# Molecular mechanisms of how black barley accumulates higher anthocyanins than blue barley following transcriptomic evaluation and expression analysis of key genes in anthocyanins biosynthesis pathway

**DOI:** 10.3389/fpls.2025.1650803

**Published:** 2025-08-29

**Authors:** Md. Mahmudul Hasan, Md. Sohel Mia, Jiazhen Yang, Yawen Zeng, Tao Yang

**Affiliations:** ^1^ Biotechnology and Germplasm Resources Institute, Yunnan Academy of Agricultural Sciences, Kunming, China; ^2^ Department of Nutrition and Food Technology, Jashore University of Science and Technology, Jashore, Bangladesh

**Keywords:** black barley (*H. vulgare*), DEGs, functional annotations, anthocyanins, qRT-PCR

## Abstract

Barley is not only a major food crop but also a medicinal plant which has considerable quantities of flavonoids. Among all flavonoids, anthocyanins play a crucial role in pigmentation, pollination, seed dispersal in plants. Anthocyanins also have antioxidant properties. Varietal differences significantly affect anthocyanins content in barley. Therefore, anthocyanins content are significantly higher in black barley than the blue one. To explore the molecular mechanisms of higher anthocyanins content in black barley, transcriptomic analysis was done to elucidate the involvement and expression of new genes in anthocyanins biosynthesis in two barley. In transcriptomic analyses, 10,579 new genes were identified, and 5,912 were functionally annotated. Twelve types of alternative splicing were found in 10,579 unigenes. Among 7,113 differentially expressed genes (DEGs), 3,235 were significantly up-regulated. The existence of the significant protein-protein interaction and involvement of many DEGs in various biological, cellular and molecular processes might reveal their significant influence on plant growth, development, yield and anthocyanins biosynthesis. Involvement of DEGs in phenylpropanoid and flavonoids biosynthesis in the black barley might be due to their great involvement in flavonoid biosynthesis, including anthocyanins. Higher expression of *ANS1, LDOX1, LDOX2*, and *LDOX3* genes of anthocyanins biosynthesis pathway in black barley than the blue one might reveal their great involvement in biosynthesis, accumulation and transformation of anthocyanins into the seed of black barley. Therefore, the current first report on DEGs in two types of barley, their expression, the unigenes and expression of major genes in anthocyanins biosynthesis pathway might guide plant biologists in reprogramming the anthocyanins biosynthesis pathway to develop barley with improved anthocyanins content by developing and transforming synthetic genetic circuits into black barley.

## 1 Introduction

Barley (*Hordeum vulgare* L.) ranks as the fourth major global cereal crop in yield and production (FAO (Food and Agricultural Organization), 2025), which is domesticated around 10000 years earlier (Van Der Leij et al., 1998). Barley was first domesticated in the eastern Mediterranean, mostly in Palaeolithic sites, and gradually disseminated to the rest world, including China. In China, barley is mostly grown in Tibet, Qinghai, Sichuan and Yunnan. In Yunnan province of China, Diqen Prefecture is the main cultivating zone of barley due to having unique climate conditions. In China, many elite cultivars of barley have already been developed and cultivated commercially (Gangopadhyay et al., 2015; Yang et al., 2018). In addition to utilizing as food, feed and brewing crop, barley is also considered a medicinal plant due to having potential bioactive compounds. Among all the bioactive compounds in barley, flavonoids possess potential quantities that play significant role in different metabolic processes. Among the flavonoids, anthocyanins are one of the major secondary metabolites and phenolic compounds that determine grain color of black and blue barley (Shi QianQian et al., 2015). The anthocyanins are mostly biosynthesized through the phenylpropane biosynthetic pathway (Wada et al., 2015). Initially, chalcone synthase (CHS) catalyzes p-Coumaroyl-CoA to generate chalcone naringenin reacting with three molecules of malonyl-CoA (Koes et al., 2005). Then, chalcone isomerase (CHI) isomerized chalcone naringenin into naringenin (Winkel-Shirley, 2001; Koes et al., 2005). Following that, flavanone 3-hydroxylase (F3H), flavonoid 3′-hydroxylase (F3′H), and flavonoid 3′5′-hydroxylase (F3′5′H) do downstream catalysis for producing various colorless intermediate flavonoids (Winkel-Shirley, 2001; Tanaka et al., 2008). After that, dihydroflavonol 4-reductase (DFR) converts these intermediates into leucoanthocyanidin, leucocyanidin, and leucodelphinidin (Koes et al., 2005). Subsequently, anthocyanidin synthase (ANS) converts these colorless precursors into pigmented anthocyanins (Albert et al., 1997, **Supplementary Figure 1**). Altered expression or function of these genes directly affect anthocyanins synthesis. Indeed, mutations of *CHS* gene restrict anthocyanins biosynthesis, consequences white petals in petunias (Spitzer et al., 2007). However, *Arabidopsis glutathione S-transferase GST* mutant tt19 transferred *lychee acyltransferase LcGST4* gene, resulted anthocyanins synthesis in the hypocotyl (Hu et al., 2016). Interestingly, transformation of antisense orientation of *CHS* gene into petunia changed the petal color from purplish red to pink or even white (van der Krol et al., 1990).

Anthocyanins consist of three aromatic rings and could be substituted by hydroxyl, sugar, acyl and methyl substitutions in various configurations depending on the plant species (Provenzano et al., 2014). Two major groups of genes guide the anthocyanins: the structural gene, which is directly involved in the formation of enzymes, and the regulatory gene that regulates the expression of the enzymatic genes (Jaakola et al., 2002). In addition to plants, anthocyanins are also beneficial to humans. Antioxidant activity is the most important physiological function of anthocyanins. Varietal differences might significantly affect anthocyanins content in barley. Anthocyanins play positive role in stress tolerance in plants. Anthocyanins regulate reactive oxygen signaling during oxidative stresses (Hatier and Gould, 2009). In addition to using as the natural colorant, the natural antioxidant anthocyanins are widely used in the food and pharmaceutical industries due to having antibacterial and anticancer effects (Flamini et al., 2013; Wang and Stoner, 2008). Anthocyanins could potentially reduce the adverse effects of N-retinylidene-N-retinylethanolamine accumulation in retinal pigment epithelium for maintaining proper eye vision (Jang et al., 2005). These metabolites could also have potential impact in reducing obesity by reducing adipose tissue (Azzini et al., 2017). Due to having above mentioned potential beneficial impact on plant and human, research on major genes involved in anthocyanins biosynthesis and its regulation is extremely important to regulate anthocyanins accumulation in barley. Indeed, content and distribution of anthocyanins in barley vary according to growth stages (Idehen et al., 2017) (Mierziak et al., 2014). Till now, no research has been published to elucidate the molecular mechanism of how higher anthocyanins are accumulated in seeds of black barley. Therefore, the current research was undertaken to explore the mechanism of higher anthocyanin content in black barley than blue one following measuring anthocyanin content in both barley seed, their transcriptome analysis, and the involvement and expression of new genes in anthocyanin biosynthesis through a series of analyses. The expression of four major genes involved in anthocyanin biosynthesis in black and blue barley were validated to elucidate the intrinsic mechanism of accumulating higher anthocyanin in black barley seed.

## 2 Materials and methods

### 2.1 Measurement of hydroxysafflor yellow A flavonoid in barley seed

Hydroxysafflor yellow A flavonoid content was measured in fully mature seeds of black (Yungongmai No. 4) and blue (Yunke No. 4) barley maintaining three independent biological replicates in each case (**Supplementary Figure 2**). Initially, HPLC-grade acetonitrile (ACN) and methanol (MeOH) were purchased from Merck (Darmstadt, Germany), formic acid from Sigma-Aldrich (St Louis, MO, USA), and all standards were obtained from MCE (China Pharmaceutical Chemical Service Company). The standard reserve solution was prepared at a concentration of 10 mmol/L using 70% methanol. All reserve solutions were stored at -20°C. Before analysis, the reserve solutions were diluted to working solutions with 70% methanol.

The black and blue barley seeds were subjected to vacuum freeze-drying and then ground in a ball mill at a frequency of 30 Hz for 1.5 min to obtain powder form, which was stored at -80°C until using it. Then, extraction was done using 20 mg seed powder and 0.5 mL 70% methanol. Following that, 10 μL internal standard (4,000 nmol/L) was added to the extract as a quantitative internal standard (IS). After ultrasonic treatment of the extract for 30 min, the solution was centrifuged at 12,000 r/min at 4 °C for 5 min. To aspirate the supernatant, the samples were filtrated through a 0.22 μm membrane, and stored in an injection bottle for LC-MS/MS analysis. The Waters ACQUITY UPLC HSST3 C181.8 μm, 2.1 mm × 100 mm chromatographic column was selected, where, ultra-pure water having 0.05% formic acid was in phase A and acetonitrile having 0.05% formic acid was in phase B. The elution gradient was: 0 min, 90:10 (A:B, v/v); 1 min, 80:20; 9 min, 30:70; 12.5 min, 5:95; 13.5 min, 90:10; 15 min, 90:10. For effective separation of the flavonoids, the flow rate was adjusted at 0.35 mL/min, maintaining the column temperature at 40 °C, and the injection volume of 2 μL. Mass spectrometry analysis was performed as previously described (Chen et al., 2013). An electrospray ion source (ESI) was used with a temperature of 550 °C, a mass spectrometry voltage of 5,500 V in positive ion mode, and the same of -4,500 V in negative ion mode. The curtain gas was 35 psi. The Analyst 1.6.3 software (Sciex) controlled Q-Trap6500+, utilized scanning and detecting of each ion pair based on optimized fragmentation voltage (DP) and collision energy (CE), for ensuring high sensitivity and specificity. An MWDB database was constructed using standards to qualitatively identify flavonoids in the sample. The multi-reaction monitoring mode (MRM) of triple quadrupole mass spectrometry was used for quantification. The target precursor ions were screened by the quadrupole, and the characteristic fragment ions were selected after collision-induced fragmentation to eliminate interference. The flavonoid content in the sample was calculated by integrating the chromatographic peak area and combining it with the standard curve. Data acquisitions were performed using Analyst 1.6.3 software (Sciex). Multiquant 3.0.3 software (Sciex) was used to quantify all compounds. The result was expressed as µg/g.

### 2.2 Measurement of cyanidin-3-O-glucoside, cyanidin 3-(6-p-caffeoyl) glucoside, and cyanidin 3-O-(6-O-para-coumaroyl) anthocyanins content in barley seed

Fully matured black (Yungongmai No. 4) and blue (Yunke No. 4) barley seeds were selected for three repeated experiments (**Supplementary Figure 2**). HPLC-grade methanol (MeOH) was purchased from Merck (Darmstadt, Germany). All standards were obtained from ISOReag (Shanghai, China). Formic acid was sourced from Sigma-Aldrich (St Louis, MO, USA). Hydrochloric acid was provided by Xinyang Chemical Reagent Factory (China). The standard solutions were prepared at a concentration of 1 mg/mL in 50% methanol. All stock solutions were stored at -20°C. Before analysis, the stock solutions were diluted with 50% methanol to prepare working solutions. The sample was subjected to vacuum freeze-drying and then ground in a ball mill at a frequency of 30 Hz for 1.5 min to form a powder, which was stored at 80°C until use. An amount of 50 mg of the powder was weighed and used for extraction with 0.5 mL of methanol/water/hydrochloric acid (500:500:1, V/V/V). The extract was vortexed for 5 min, ultrasonicated for 5 min, and centrifuged at 12,000 g for 3 min at 4°C. The residue was re-extracted under the same conditions. The supernatant was collected, and the sample was filtered through a 0.22 μm membrane. The sample was stored in a vial for LC-MS/MS analysis. An ACQUITY BEH C181.7 μm, 2.1mm × 100 mm chromatographic column was selected where ultra-pure water containing 0.1% formic acid as the A phase and methanol containing 0.1% formic acid as the B phase was used. The B phase of elution gradients was set at: 0.00 min at 5%, 6.00 min at 50%, 12.00 min at 95%, maintained this for 2 min; 14 min to decrease it to 5%, and remains balanced for 2 min. The flow rate 0.35 mL/min, and the column temperature 40 °C, and the injection volume 2 μL were maintained for achieving effective separation of anthocyanins. Mass spectrometry analysis was performed as previously described (Chen et al., 2013; Komyshev et al., 2023). The mass spectrometry conditions mainly included an electrospray ion source (ESI), with a temperature of 550 °C, a mass spectrometry voltage of 5,500 V in positive ion mode, and a curtain gas of 35 psi. The Analyst 1.6.3 software (Sciex) controlled Q-Trap6500+, utilized scanning of ion pair based on optimized fragmentation voltage (DP) and collision energy (CE) for ensuring high sensitivity and specificity in detection. Anthocyanins content were analyzed using scheduled multiple reaction monitoring (MRM). Data acquisitions were performed using Analyst 1.6.3 software (Sciex). Multiquant 3.0.3 software (Sciex) was used to quantify all compounds. The results were expressed as µg/g.

For quantitative metabolite analyses (hydroxysafflor yellow A and anthocyanins including cyanidin-3-O-glucoside, cyanidin 3-(6-p-caffeoyl) glucoside, and cyanidin 3-O-(6-O-para-coumaroyl), data from three independent biological replicates were analyzed using one-way ANOVA (Yao et al., 2022). Before ANOVA, the Shapiro-Wilk test was used to assess normality, and Levene’s test was applied to evaluate homogeneity of variances. Upon meeting ANOVA assumptions, Tukey’s HSD *post hoc* test was conducted for pairwise group comparisons. Results were reported as mean ± standard deviation (SD), and significance was set at p < 0.05 (Li et al., 2023).

### 2.3 Plant materials, RNA extraction, and library preparation

Black (Yungongmai No. 4) and blue(Yunke No. 4) barley were cultivated in the field under natural condition at Yunnan Yuxi city Experimental Station, Yunnan, Kunming, China following recommended intercultural operations. During growing period, the average daily temperature was 10-22 °C having daytime high at around 20-25°C in afternoon. The lowest temperature at night was around 8-12 °C. Significant diurnal temperature variation was observed as slightly cool at the beginning of the month, that gradually warmed up towards the end. The average relative humidity was 55-70%. At the end of the dry season, there was little precipitation, and the air tended to be dry, with occasional slight rainfall. The average diurnal sunshine duration was 6 to 8 hours. Most days were sunny or cloudy, with fewer rainy days. We have obtained the climate data of the growing region from the China Meteorological Data Service Center (CMDC; http://data.cma.cn), which is publicly accessible (Yang et al., 2022).

After harvesting the experimental tissues (immature spikelet during the grain-filling stage at 30 days after flowering) on 120 days after planting (at Mid-April), it was immediately frozen into the liquid nitrogen and stored at -80 ^0^C until using it. Following the manufacturer’s instructions, total RNA of black (Yungongmai No. 4) and blue (Yunke No. 4) barley was extracted using an RNeasy Plant Mini Kit (Qiagen, Canada). Three biological replicates from each (black: GB, and blue: GH) type of barley were subjected to RNA-Seq analysis. Before sequencing, the RNA quality and content were confirmed using an Agilent 2,100 Bioanalyzer (Agilent Technologies, Canada) and a NanoDrop 1,000 spectrophotometer (ThermoFisher Scientific, Canada). The RNA samples were shipped to Beijing Biomarker Biotechnology Co., Ltd, Beijing, China for library, preparation, RNA-Seq, and sequence analysis. In short, after completing the quality control procedures, oligo(dT) beads were used to enrich the mRNA, which was then randomly fragmented in fragmentation buffer. Random hexamers and reverse transcriptase were then used to synthesize cDNA. A custom second-strand synthesis buffer was added following first-strand synthesis to create the second strand using nick-translation. Following purification, terminal repair, A-tailing, ligation of sequencing adapters, size selection, and PCR enrichment, the final cDNA library was completed. Using a Qubit 2.0 fluorometer (Life Technologies, Carlsbad, CA, USA), the library concentration was first measured. It was then diluted to 1 ng/L before the insert size was checked on an Agilent 2,100 Bioanalyzer (Agilent Technologies, Santa Clara, CA, USA) and quantified more precisely using quantitative PCR (q-PCR). An Illumina HiSeqTM 4,000 platform was used to sequence the generated libraries, producing at least 20 million paired-end, 150 bp reads per sample (Liu et al., 2021).

### 2.4 Sequencing, assembly, and annotation

The TruSeq PE Cluster Kit v4-cBot-HS (Illumina) was used to cluster the index-coded samples on a cBot Cluster Generation System. Following cluster creation, paired-end reads were produced by sequencing the library preparations using the Illumina HiSeq X Ten platform. Low-quality, adapter-containing, and poly-N reads were eliminated from the raw data to provide clean reads. Additionally, the Q20, Q30, and GC contents in clean data were computed. With Tophat2 (parameters: -N 5 -p 30 -i 20), all downstream analyses were based on clean, high-quality data that was aligned to the reference genome: Hv_IBSC_PGSB_v2 (Elakhdar et al., 2023). The reference genome is 4.83 Gbp in length, with N50 values of 4 Mbp for contigs and 7 pseudomolecules for scaffolds, respectively (Guan et al., 2016).

In barley transcriptomic, unigenes (or unique transcripts) detection are extremely important for analyzing accurate expression of gene. More specially, it is essential when dealing with complex transcriptomes or diverse genotypes. They represent distinct gene sequences, finding to distinguish different transcripts of the same gene (isoforms) for reducing redundancy in datasets. This guide the genome biologists and synthetic biologists for critical analysis of gene function, identification of novel transcripts, for increasing the precision of gene expression analysis.

For minimizing redundancy and generating a representative transcript dataset, we have selected the longest transcript isoform per gene locus as the unigene. Following reference-guided assembly, it was done using custom in-house scripts. Based on sequence length and coverage, the longest transcript was chosen, as it represents the typical and most complete information. The mentioned method is highly consistent with the other transcriptome analysis (Long et al., 2022).

To investigate the activities of mRNA, all constructed unigenes were compared to the NCBI-non-redundant protein sequences (Nr) database using the BLAST method, which also found homologs of genes with established functions (Altschul et al., 1997). Blast2GO was used to scan the Nr database for the best gene ontology (GO) terms that were obtained (Götz et al., 2008). To identify and forecast functional classifications and molecular pathways, the assembled unigenes were also compared to the Swiss-Prot (a manually annotated and reviewed protein sequence database), NT (NCBI nucleotide sequences), Pfam (Protein family), KOG (euKaryotic Ortholog Groups)/COG (Clusters of Orthologous Groups of proteins), and KEGG (Kyoto Encyclopedia of Genes and Genomes) Orthology databases.

### 2.5 Alternative splicing event quantification

For AS analysis, AS profile (b-1.0.4) software (b-1.0.4; http://ccb.jhu.edu/software/ASprofile/) (Florea et al., 2013) was used to classified alternative splicing events into 12 types. This program explains splice types. The 12 types included alternative exon ends (AE), intron retention (IR), multi-intron retention (MIR), multi-exon skipping (MSKIP), skipped exon (SKIP), alternative 5′ first exon (TSS), alternative 3′ last exon (TTS), and approximate exons (XAE, XIR, XMIR, XMSKIP, and XSKIP).

### 2.6 Differential expression analysis

Gene expression is governed on time, tissue and condition-specific manner. Genes or transcripts having significant variation in expression pattern are called DEGs or DETs under two/multiple circumstances. Following StringTie, fragments per kilobase of transcript per million fragments mapped (FPKM) are followed in transcript reconstruction and evaluation of gene expression levels (Pertea et al., 2015). Initially each sample each sample utilizes StringTie without the -e argument to annotate fresh transcripts. After merging all genes, the gtf format files from each samples were moved into a total gtf file. The -e argument was used to calculate normalized expression values across the samples.

Differential expression analysis was performed using DEseq2 (v1.10.1) (Love et al., 2014). DEseq2 uses a negative binomial distribution model to identify digital gene expression data. In each analysis, three replicates were maintained to evaluate the treatments compared to controls. DESeq2 solves two major differential analysis problems: compensating the library size disparities and retaining the library size impact. In DESeq2, genes having 0 FPKM value was eliminated before determining the scaling factor of each sample. DEGs were estimated by comparing FPKM values and scaling factors. DEGs were screened following the Benjamini–Hochberg procedure using an absolute log2 (Fold Change) higher than one and a False Discovery Rate (FDR) less than 0.01. Fold Change is the expression ratio between samples/groups. FDR was calculated by correcting the significant difference of p-value. Differential expression analysis of transcriptome sequencing is an independent statistical hypothesis test for several gene expression evaluations, which may provide false positives. The hypothesis test p-value significance is corrected using the Benjamini-Hochberg correction procedure.

### 2.7 PPI (Protein-Protein Interaction) network analysis of the DEGs

The DEGs sequences of new genes were blasted (blastx) against the barley genomes protein data that are available in the STRING database (http://string-db.org/) to obtain protein-protein interactions for the DEGs of new genes. Then, the PPI of these DEGs of new genes was visualized using Cytoscape (Shannon et al., 2003). Here, the full STRING network, edge evidence, and the lower confidence parameter (0.150) were set (Islam et al., 2022).

### 2.8 GO functional annotation of the DEGs

Gene Ontology enrichment analysis of the DEGs was implemented by the GOseq R packages based on Wallenius non-central hyper-geometric distribution (Lee et al., 2013), which can adjust for gene length bias in DEGs.

### 2.9 KEGG pathway enrichment analysis of the DEGs

KEGG (Kanehisa et al., 2025) is a database resource for understanding the high-level functions and utilities of biological systems, such as cells, organisms, and ecosystems, from molecular-level information, especially large-scale molecular datasets generated by genome sequencing and other high-throughput experimental technologies (http://www.genome.jp/kegg/). We used KOBAS (Wu et al., 2006) software to assess the statistical enrichment of differentially expressed genes in KEGG pathways. Here, FDR-adjusted p-values were also calculated using the Benjamini–Hochberg correction to account for multiple testing (Benjamini and Hochberg, 1995).

### 2.10 Heat-map construction to visualize RNA-Seq based relative mRNA expression of major genes in anthocyanin biosynthesis pathways and evaluation of their interaction with transcription factors (TFs)

A heat-map was generated using TBtools to visualize the RNA-Seq based expression profile of key genes in anthocyanin biosynthesis (*ANS1, LDOX1, LDOX2*, and *LDOX3*). For each gene, three biological replicates from both black and blue barley were utilized. The obtained mRNA-seq log2FC values (FPKM) were utilized to construct the heat-map. To predict the interaction of these genes with transcription factors, 1,000-bp upstream promoter sequences of the above 4 genes were extracted from the barley genome and uploaded to the Plant Transcriptional Regulatory Map (PTRM) server [https://plantregmap.gao-lab.org/about.php (accessed on June 10, 2025)] maintaining p value of ≤1e-6. After obtaining the analyzed results from the server, the Cytoscape software was used to build and visualize the gene-TF network (Göös et al., 2022).

### 2.11 Construction of the weight gene co-expression network

The WGCNA (v1.47) package integrated in R package was utilized in constructing the co-expression network (Long et al., 2022). The analysis parameters were as follows: FPKM ≥ 1, CV of FPKM ≥ 0.5, an unsigned weighted network, a dynamic hybrid tree cut algorithm for hierarchical clustering, a minimum module size of 30, and a minimum merging threshold height of 0.0586. Further, we have selected soft-thresholding power (β) = 78 to achieve a signed R² = 0.8, indicating a strong fit to the scale-free topology model. The gene clustering tree was constructed according to the correlation of gene expression, and then the genes with similar expression patterns were classified into the same module, and the branches of the cluster tree were cut and distinguished to produce different modules. The WGCNA analysis was performed using Pearson correlation with two-tailed tests and Benjamini–Hochberg correction to identify trait-associated modules and significant genes (Langfelder and Horvath, 2008). The values represent the Pearson correlation coefficient. The Benjamini-Hochberg method was corrected for multiple comparisons. *p < 0.05; **p < 0.01.

### 2.12 Quantitative real-time PCR validation for expression of major genes involved in anthocyanin biosynthesis

To explore the tissue-specific expression patterns of the barley anthocyanin biosynthesis genes, barley was cultivated in pots filled with a soil and vermiculite mixture (1:1) under growth conditions of 25 ± 1°C during the day and 20 ± 1°C at night, with a relative humidity of 75%. For analyzing the expression of genes in grain filling stage in barley, immature spikelet was harvested at 30 days after flowering consequences on 120 days after planting. Similarly, for analyzing the expression of genes at physiological maturity stage in barley, spikelet was harvested at 45 days after flowering consequences on 135 days after planting. Then, the qRT-PCR experiment was performed for the selected four (*ANS1, LDOX1, LDOX2*, and *LDOX3*) anthocyanin biosynthesis genes in immature barley seeds at the grain-filling stage.

The total RNA was isolated from barley leaf tissues utilizing an E.Z.N.A. Plant RNA Kit (Omega Bio-tek, Inc., USA), by the manufacturers guidelines. The isolated RNA was subsequently reverse transcribed into first-strand cDNA with the HiScript II Q RT SuperMix for qPCR Kit (Vazyme Biotech Co., Ltd, China) in a reaction volume of 20 μL. The specific primers for quantitative PCR were developed utilizing Primer Premier (version 5.0; Premier, Canada), employing HvActin as the reference gene. The primer sequences are enumerated in **Supplementary Table 1**. Quantitative PCR was conducted with the ChamQ Universal SYBR qPCR Master Mix Kit (Vazyme Biotech Co., Ltd., China) and a fluorescence quantification kit on a CFX96 Real-Time System (Bio-Rad, USA). The relative gene expression patterns were evaluated using the 2^-ΔΔCT^ technique (Rabby et al., 2024). The expression differences between black and blue barley were assessed using the unpaired Student’s t-test (Goni et al., 2009). Normality and equal variance assumptions were verified using the Shapiro–Wilk test and F-test, respectively (Shapiro and Wilk, 1965; Tiku, 1967).

## 3 Results

### 3.1 Measurement of hydroxysafflor yellow A flavonoid content in barley seed

**Figure 1:**
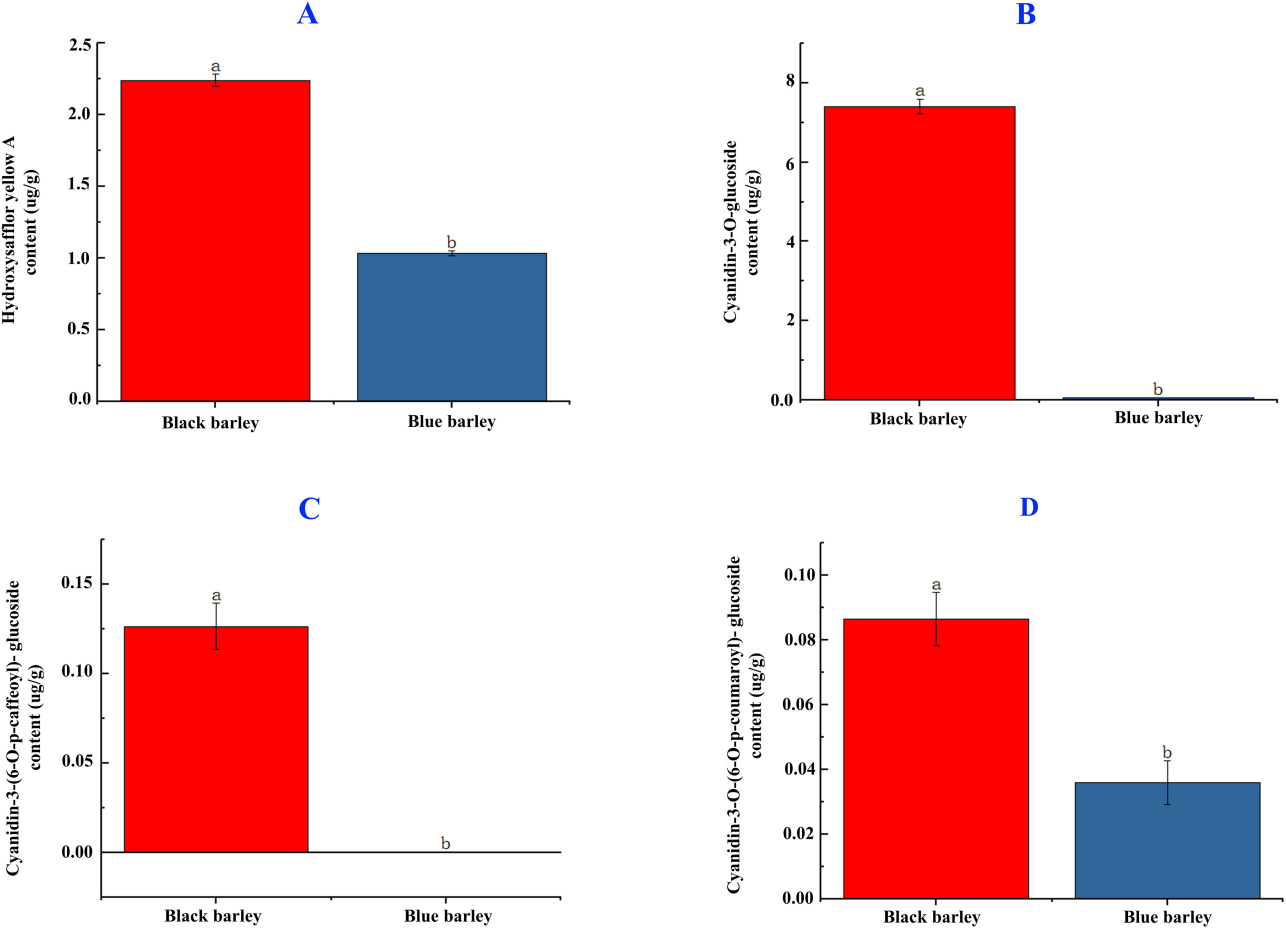
Hydroxysafflor yellow A flavonoid **(A)** and cyanidin-3-O-glucoside, cyanidin 3-(6-p-caffeoyl) glucoside, and cyanidin 3-O-(6-O-para-coumaroyl) anthocyanin content **(B-D)** in black and blue barley seed. Different letters represent significance at the 5% level of significance in the DMRT test. The bar represents the standard error value at a 5% significance level.

The hydroxysafflor yellow A flavonoid content in black barley seed is significantly higher than the blue barley (2.25 vs 1.01 μg/g). The significant higher accumulation of the mentioned flavonoid content in black barley than blue barley might be due to the differential roles of genes involved in flavonoid biosynthesis in black barley (**Figure 1A**).

### 3.2 Measurement of cyanidin-3-O-glucoside, cyanidin 3-(6-p-caffeoyl) glucoside, and cyanidin 3-O-(6-O-para-coumaroyl) anthocyanins content in barley seed

Cyanidin-3-O-glucoside anthocyanin in black barley was significantly higher than blue barley (7.40 vs 0.05μg/g) (**Figure 1B**). The same trend was observed in Cyanidin-3-(6-O-p-caffeoyl)- glucoside (0.12 vs 0 μg/g) and Cyanidin-3-O-(6-O-p-coumaroyl)- glucoside (0.09 vs 0.04 μg/g) anthocyanins (**Figures 1C, D**). The significant higher accumulation of these anthocyanins in black barley than blue barley might be due to the differential roles of genes in the anthocyanin biosynthesis pathway in black barley.

### 3.3 Overall characteristics and quality evaluation of *H. vulgare* transcriptome

The sequenced data were checked for sequencing errors and saturation to assess the adequacy of the data and ensure its suitability for subsequent analysis. Slightly high (still less than 0.05%) sequencing error rates of read 5’/3’ ends (**Supplementary Figure 3**) might suggest the good sequencing quality as expected from Illumina. In saturation of sequencing, the number of genes with different expression levels was detected, especially those with low expression, which had already reached saturation as the sequencing depth increased (**Supplementary Figure 4**). The expression distribution of different samples based on the FPKM value was also calculated, and the expression level of protein-coding genes ranged from 10–^5^ to 10^5^ (**Supplementary Figure 5**). This showed that our RNA-seq sequencing depth is reliable and that low-expression genes were properly detected (**Supplementary Figure 6**). Although there were several differences in the extreme values of expression in different samples, their distribution was nearly identical among two experimental barley. In this study, 63.30 Gb of clean data were generated, and the clean data of each sample reached 9.52 Gb. The average Q20 and Q30 values were 99.18% and 94.78%, respectively (**Supplementary Table 2**). The average GC content was 53.27% (**Supplementary Table 2**). According to the alignment results, the mapping reads in black barley ranged from 82.68% to 83.51%, and the same in blue barley ranged from 85.85% to 87.82% (**Supplementary Table 2**).

### 3.4 New gene identification and annotations in the *H. vulgare* transcriptome

The large genome size of *H. vulgare* and the considerable amount of tandem repeated sequences increase the difficulty of the assembly process, as in the case for most gymnosperms. As a result, the annotation of the selected reference genome is often not accurate enough, necessitating the optimization of the gene structure in the original annotation. RNA-seq is suitable for correcting gene structure due to its high accuracy in determining transcriptional boundaries during the mapping process. In our study, 10,579 new genes were identified, and 5,912 were functionally annotated with RNA-seq mapping data (**Supplementary Table 3**). According to the TrEMBL database results, most (5,483) of the newly identified genes were functionally annotated (**Supplementary Table 3**). The Nr database showed that most (3,524) of the new genes were highly homologous with *Triticum turgidum* species (**Supplementary Figure 7**).

The predicted unigenes were functionally annotated using KOG, GO, and KEGG databases. According to the KOG analysis, 19,856 unigenes were categorized into 25 functional groups (**Supplementary Figure 8**; **Supplementary Table 4**). Among these genes, the cluster of general function prediction (R) (3,280, 16.52%), followed by posttranslational modification, protein turnover, chaperones (O) (2,148, 10.82%), and signal transduction mechanisms (T) (1,930, 9.71%), represented the largest group. In addition, 1,012 (5.09%) unigenes were involved in secondary metabolites biosynthesis, transport and catabolism (Q) (**Supplementary Figure 8**; **Supplementary Table 4**).

### 3.5 Alternative splicing event quantification

**Figure 2:**
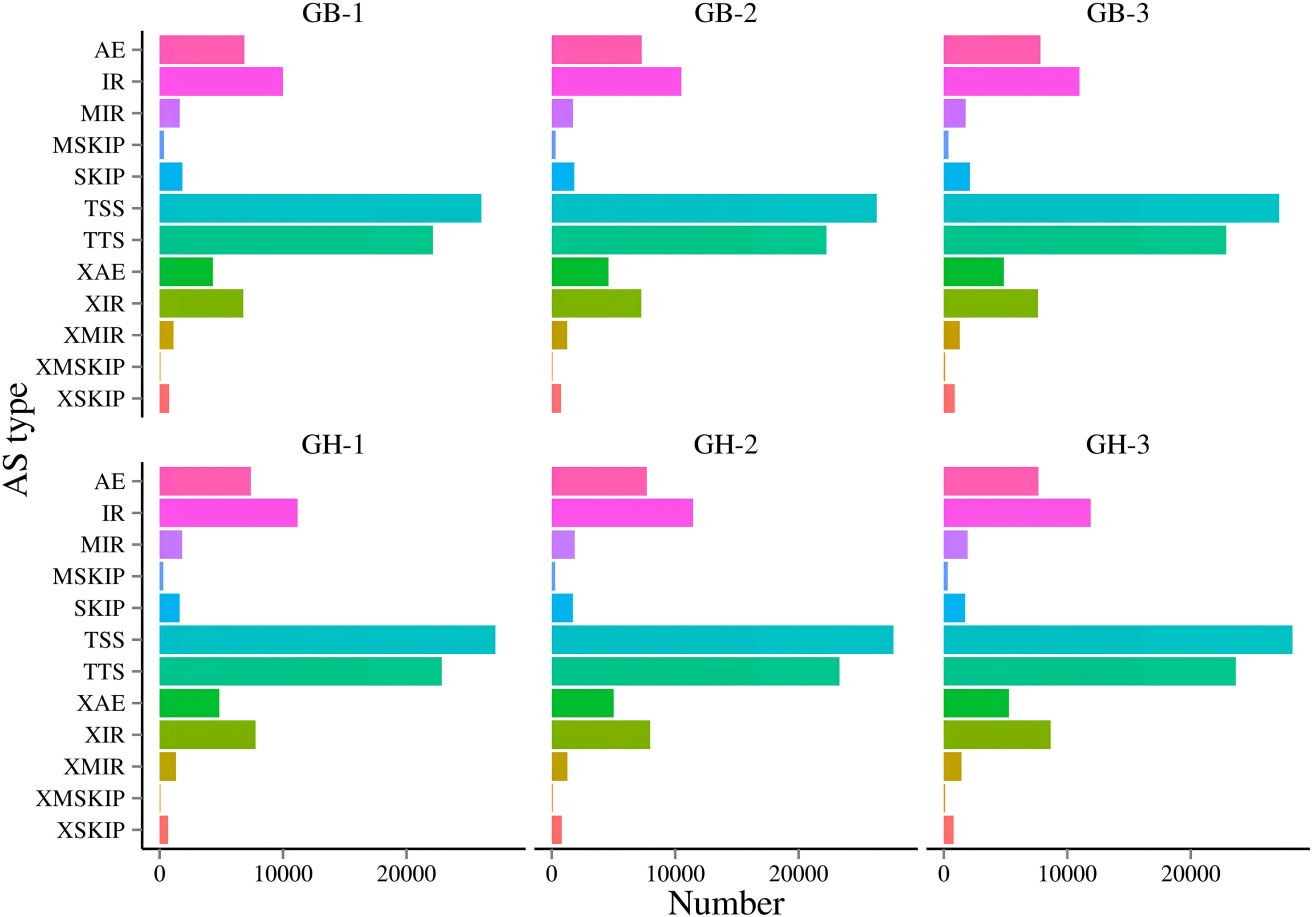
Identification of Alternative splicing (AS) events of two types of barley. Here, GB and GH represent black and blue barley respectively whereas, 1-3 represents three independent biological replicates. (1) AE: alternative exon ends (5', 3', or both); (2) IR: intron retention; (3) MIR: multi-IR; (4) MSKIP: multi-exon SKIP; (5) SKIP: skipped exon; (6) TSS: alternative 5' first exon (transcription start site); (7) TTS: alternative 3' last exon (transcription terminal site); (8) XAE: approximate AE; (9) XIR: approximate IR; (10) XMIR: approximate MIR; (11) XMSKIP: approximate MSKIP; (12) XSKIP: approximate SKIP.

In alternative splicing (AS) prediction, 12 types of AS were identified, including AE, IR, MIR, MSKIP, SKIP, TSS, TTS, XAE, XIR, XMIR, XMSKIP, and XSKIP (**Supplementary Table 5**). Notably, both black and blue barley showed difference in AS patterns. Among these, TSS-type events were the most prevalent, followed by TTS, IR, AE, XIR, XAE, SKIP, MIR, XMIR, XSKIP, MSKIP, and XMSKIP. In both transcriptomes, differences in events and quantities of alternative splicing (AS) were observed (**Figure 2**; **Supplementary Table 5**).

### 3.6 Differential expression analysis

**Figure 3:**
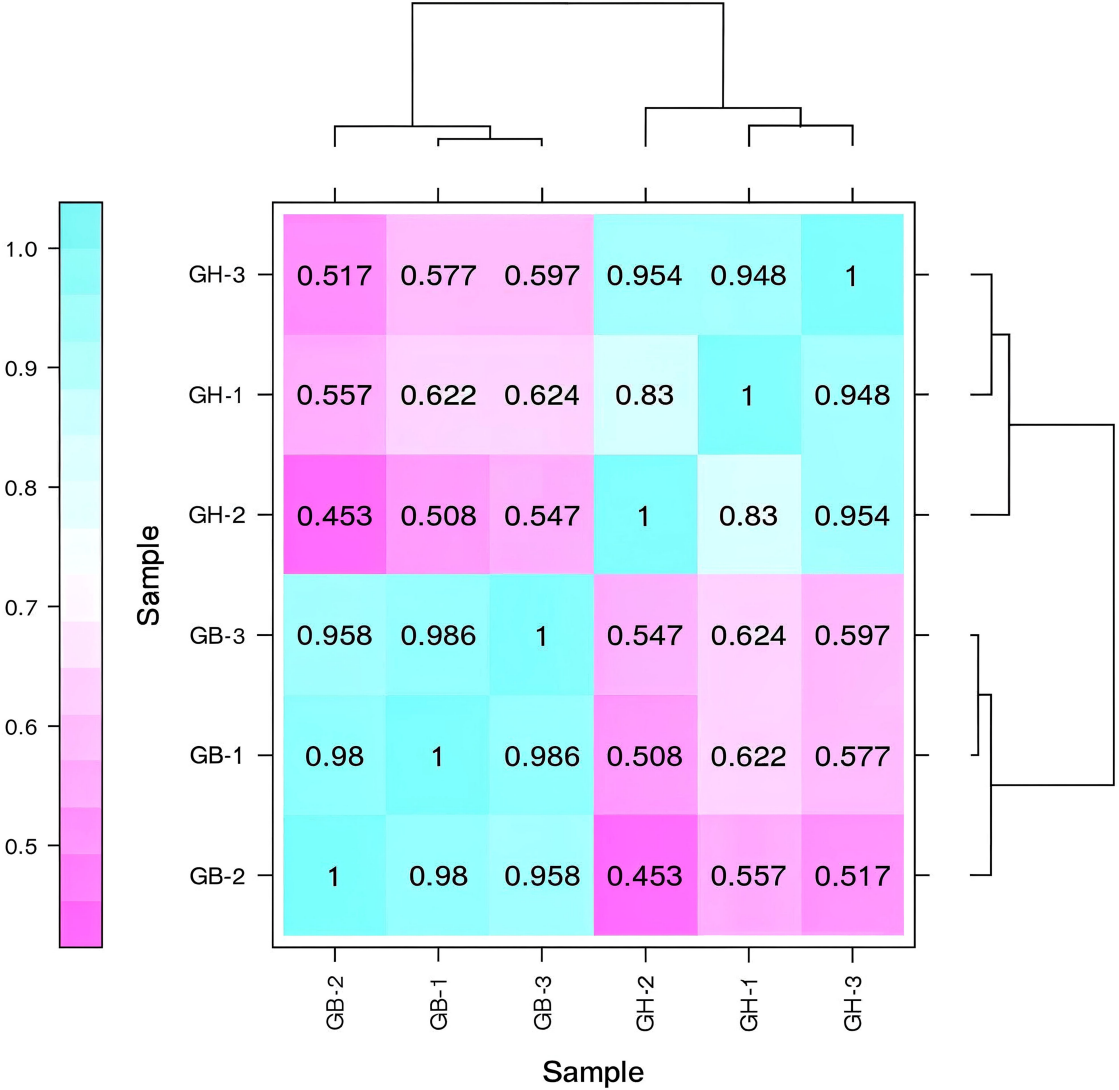
Pearson's correlation coefficient analyses among six samples from two types of barley. Here, GB and GH represent black and blue barley respectively whereas, 1-3 represents three independent biological replicates.

Pearson’s correlation coefficient (r) is used as an indicator for evaluating the correlation between biological replicates. The r value is closer to 1, the stronger the correlation between the treatments. We have ensured the highest precision in biological replication, crop microenvironment, sequencing, and analysis. To ensure that we have used all biological replicates in the same batch that were grown and extracted under the same conditions by the same person, to run sequencing in the same lane. We have also conducted a detailed analysis of abnormal samples to determine whether to repeat the experiment or exclude the abnormal samples for subsequent analysis, based on the analysis results and consensus. Statistical plot of correlation between samples were also determined (**Figure 3**). All the correlations were positive, and the strongest correlation was seen for different samples in the same cultivars (**Figure 3**). Also principal component analysis (PCA) was conducted to assess the overall transcriptomic variation and to evaluate sample clustering based on gene expression profiles. The first two principal components (PC1 and PC2) together explain 97.3% of the total variance in the dataset, with PC1 accounting for 70.8% and PC2 for 26.5% (**Supplementary Figure 9**).

**Figure 4:**
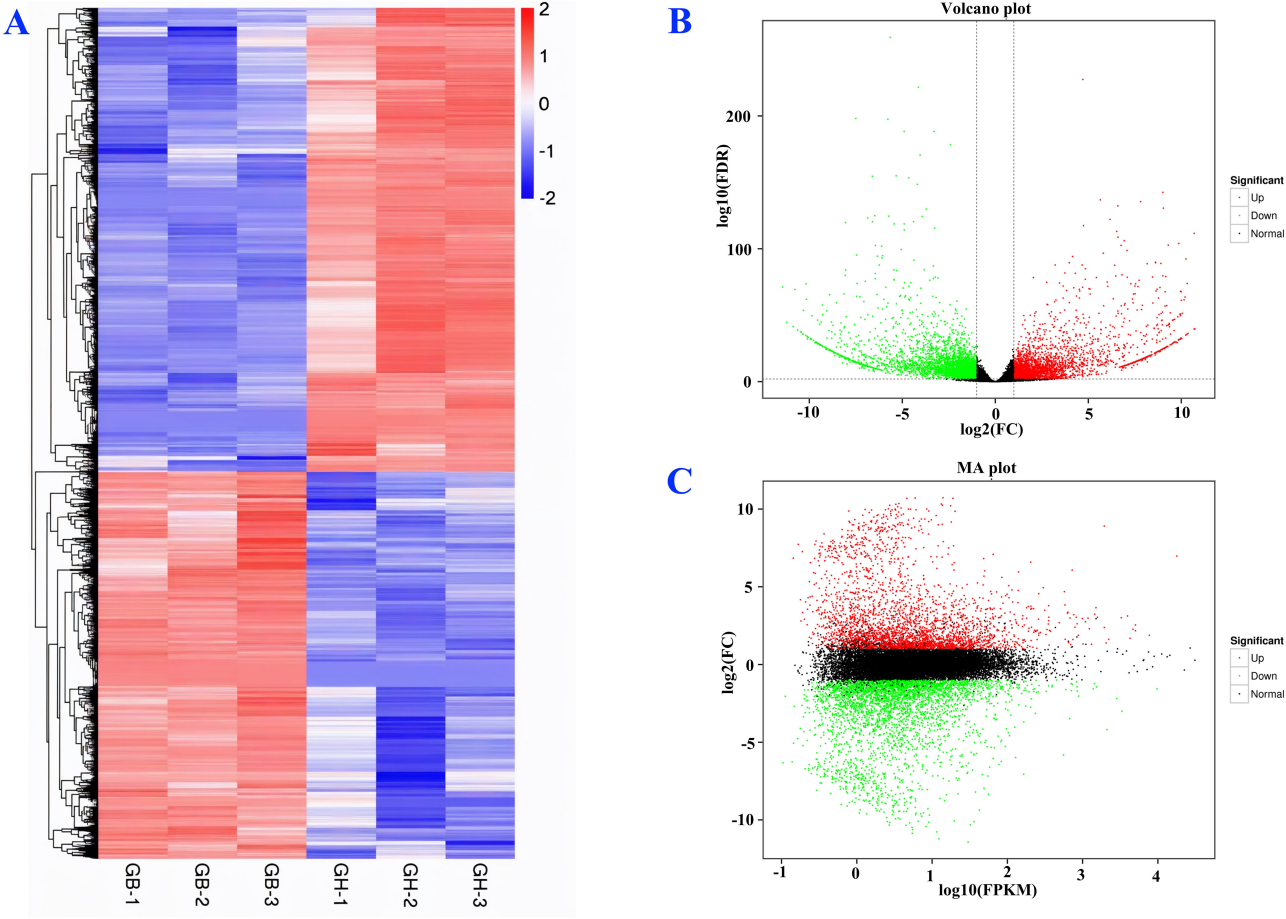
Differential expression analysis of new genes **(A)**, volcano plot **(B)**, and MA graph **(C)**. Here, GB and GH represent black and blue barley respectively whereas, 1-3 represents three independent biological replicates.

Between two types of barley, black and blue, 7,113 DEGs were identified (**Figure 4A**). Among them, 3,235 were up-regulated and 3,878 were down-regulated. A distinct expression pattern among the genes of black and blue barley was observed (**Figure 4A**). Consistent expression pattern among the biological replicates in the same barley represents the experimental validity and genetic conservation of same barley. The top ten highest DEGs were observed in phenylpropanoid biosynthesis, amino acids biosynthesis, glutathione metabolism, tyrosine metabolism, flavonoids biosynthesis, purine metabolism, ribosome biogenesis, alanine-aspartate-glutamate metabolism, glyoxylate and dicarboxylate metabolism, phenylalanine metabolism pathways (**Supplementary Figure 10A**). The notable DEGs in the above mentioned pathways among black and blue barley might regulate anthocyanin and other pigments biosynthesis interacting phenylpropanoid pathways. Indeed, these DEGs might also play potential roles in stress-responsive gene regulation interacting different stress signaling pathways.

Volcano plots reveal the log-fold change and the statistical significance (p-value) of DEGs. The volcano plot showed that the new genes were significantly expressed, where approximately 1,000 DEGs were significantly up-regulated and 1,200 were down-regulated (**Figure 4B**). The MA plots reveal the relationship between the average expression level (A) and the log-fold change (M) of DEGs. In our study, the MA plot visualizes approximately 2,500 of the DEGs that were up-regulated and 2,000 of the DEGs that were down-regulated and the majority of the DEGs that were showing no significant change (**Figure 4C**).

Based on the acceptable sequence quality, the observed significant new genes, different types of AS, and notable DEGs might play potential roles in different physiological, cellular and metabolic processes in the two barley.

### 3.7 Protein-protein interaction network analysis of the DEGs

**Figure 5:**
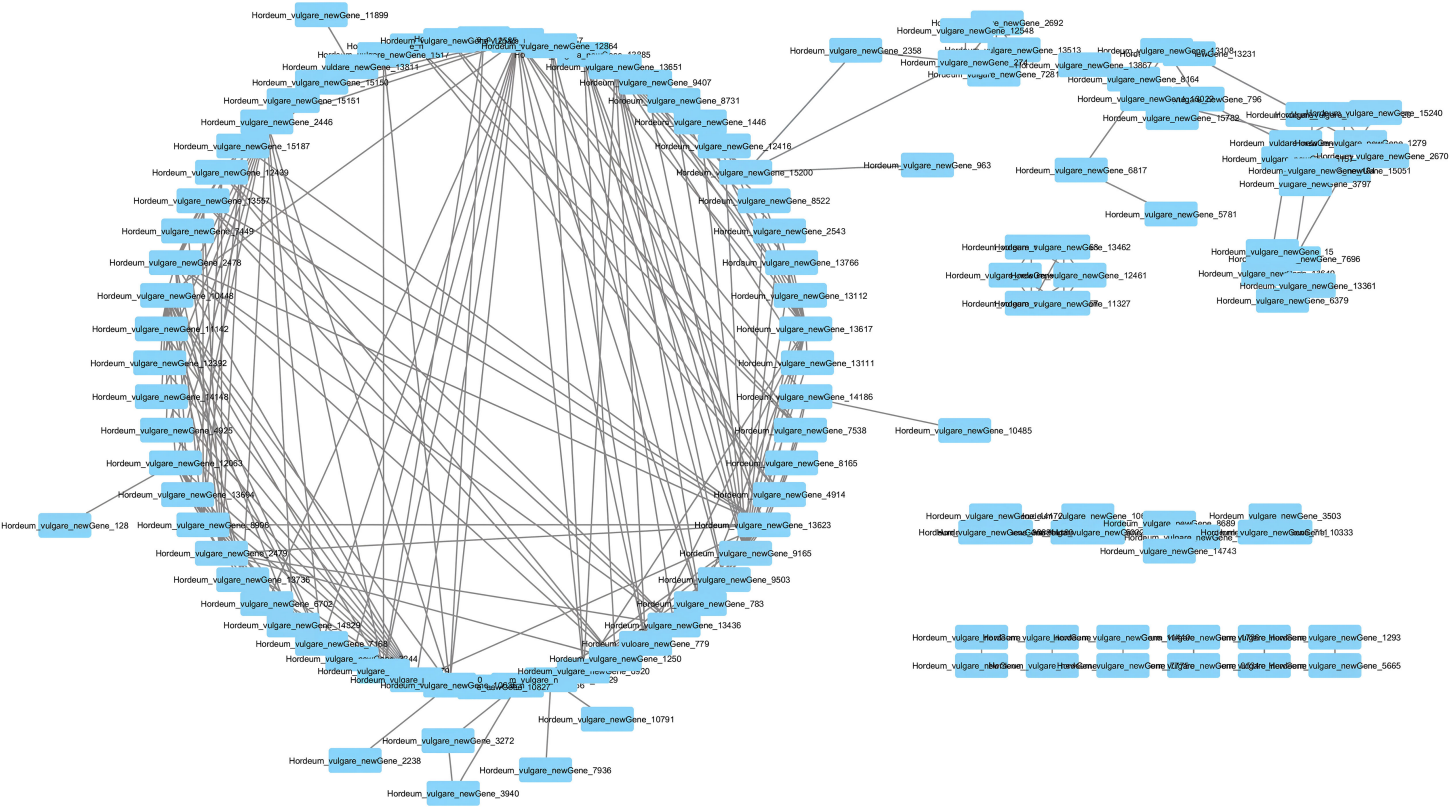
Differentially expressed genes protein-protein interaction network.

A PPI network is a graphical representation of how proteins interact with each other within a cell or organism. Each node represents a protein, and each edge (line) between nodes represents a physical or functional interaction between two proteins. In our study, the PPI network analysis of black and blue barley showed 149 significant nodes and 690 edges at medium confidence (**Figure 5**). Among the PPI of all new genes, only Hordeum_vulgare_newGene_711 and Hordeum_vulgare_newGene_8522 interacted with anthocyanin and flavonoid biosynthesis (ko00941) pathways, respectively. Significant sequence divergence in the mentioned two genes from other genes in the genome might be due to their great role in anthocyanin biosynthesis (**Supplementary Table 6**). Therefore, these genes might greatly involve in flavonoid/anthocyanin biosynthesis interacting other proteins in the mentioned pathways.

### 3.8 GO functional annotation analysis of the DEGs

**Figure 6:**
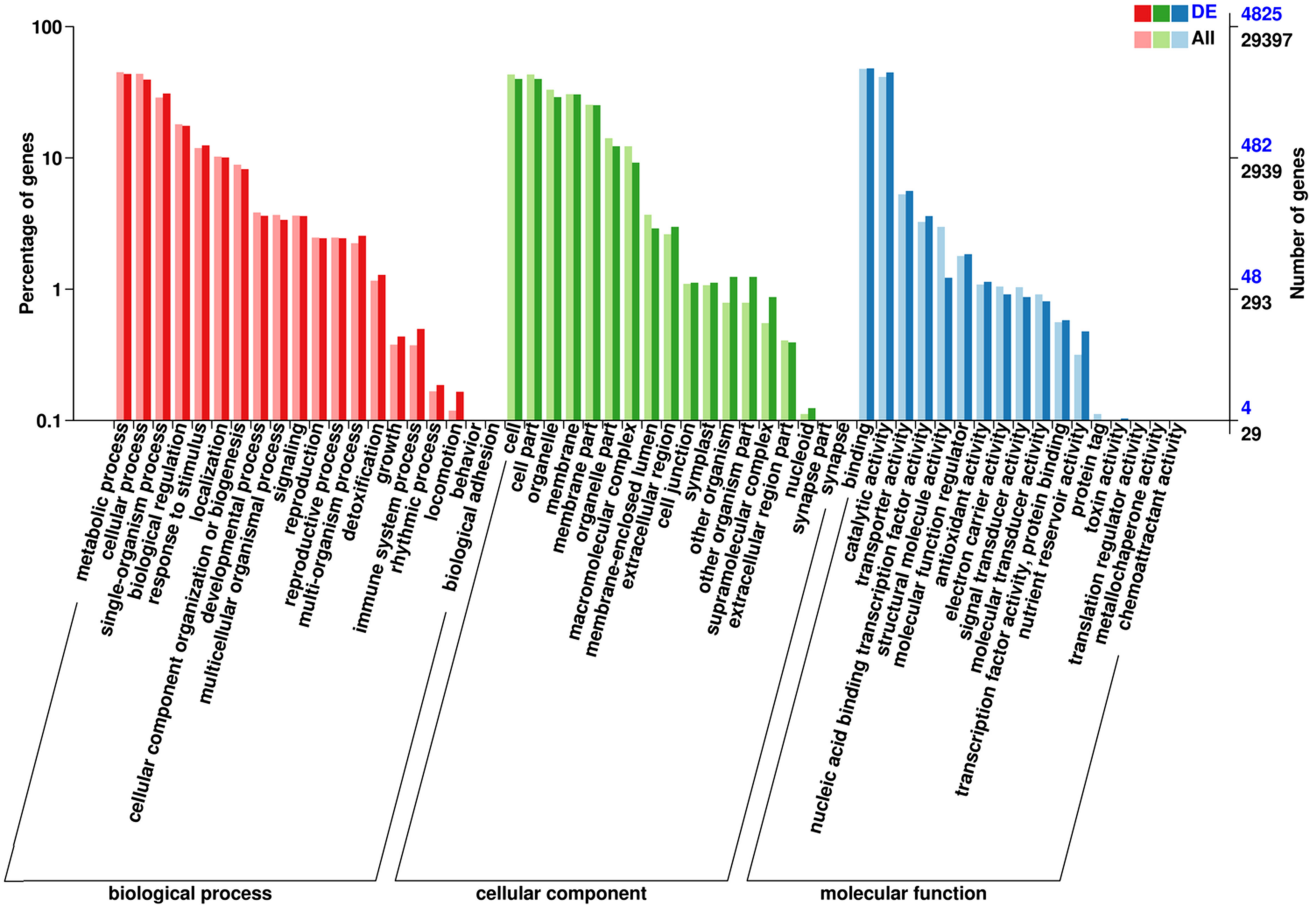
Gene ontology annotation classification statistics of differentially expressed genes of black and blue barley.

All DEGs were categorized into three GO classifications: biological process (BP), cellular component (CC), and molecular function (MF). Gene ontology annotations between blue and black barley showed 18 BP, 16 CC, and 12 MF functional subcategories (**Figure 6**). Among the various categories of BP, the top three cluster frequencies were metabolic, cellular, and single-organism processes. In the CC category, the cell, cellular organelles, and the membrane were observed as the top three clusters. Within the MF category, binding, catalytic activity, and transporter activity constituted the top three clusters (**Figure 6**, **Supplementary Table 7**).

### 3.9 KEGG pathway enrichment analysis of the DEGs

**Figure 7:**
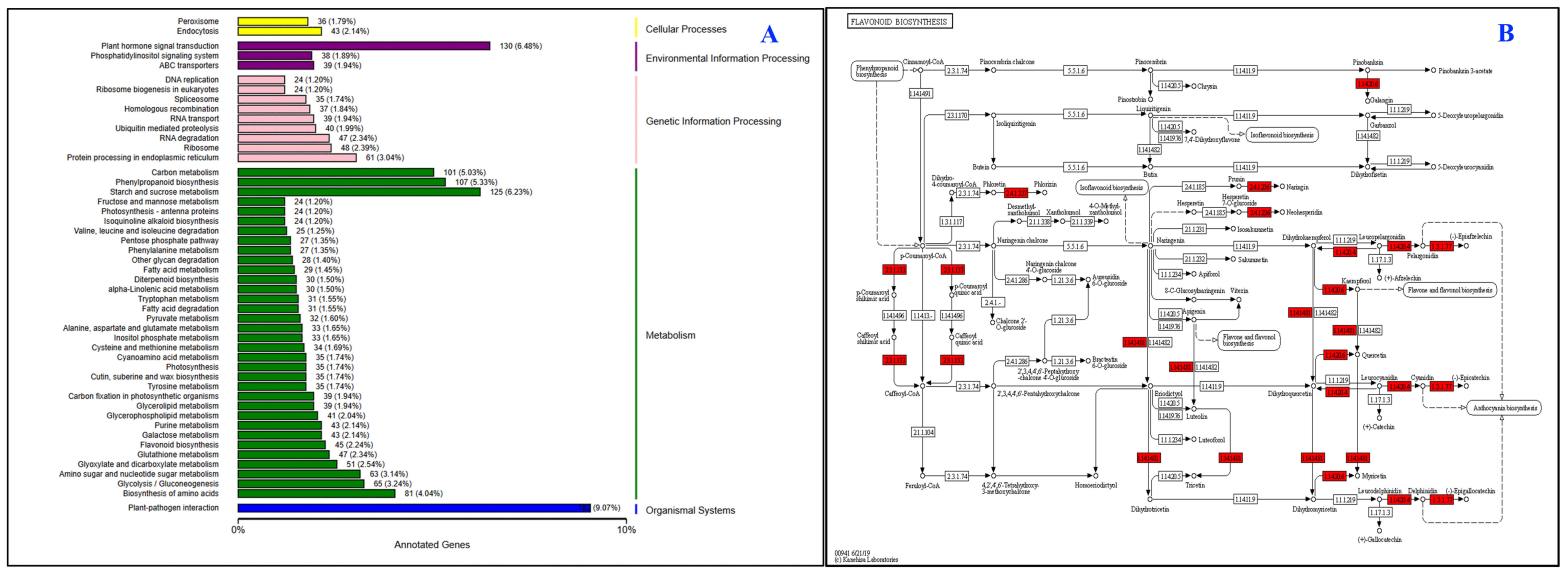
KEGG (Kyoto Encyclopedia of Genes and Genomes) classification of differentially expressed genes. Here, KEGG classification **(A)** and KEGG pathway enrichment **(B)** were shown. The vertical axis is the name of the KEGG metabolic pathway, and the horizontal axis is the number of genes annotated to the pathway and its ratio to the total number of annotated genes.

In KEGG functional enrichment analysis, most of the DEGs were found to be functionally annotated. In the KEGG findings, the most significantly enriched pathway was ‘Plant hormone signal transduction’ (q-value = 0.72022) (**Figure 7**; **Supplementary Table 8**). ‘Starch and sucrose metabolism’ (q-value = 1.40337) and ‘Phenylpropanoid biosynthesis’ (q-value = 0.02929) were the most significant pathways in KEGG analysis of the DEGs (**Figure 7**; **Supplementary Table 8**; **Supplementary Figures 11, 12**). In our study, 107 DEGs of black barley associated with the phenylpropanoid biosynthesis pathway (PATH: ko00940) and 45 DEGs were involved in the flavonoids biosynthesis pathway (PATH: ko00941). Among 107 DEGs interacted in the phenylpropanoid biosynthesis pathway, 51 were up-regulated (**Supplementary Table 9**). In this pathway, the *DFR4* (HORVU7Hr1G093360) and *DFR5* (HORVU7Hr1G093370) were up-regulated that are major enzymes responsible for flavonoids biosynthesis. Similarly, 19 genes were up-regulated in the flavonoids biosynthesis pathway (**Supplementary Table 9**). Here, *ANS1* (HORVU5Hr1G094280), *LDOX1* (HORVU5Hr1G065620), *LDOX2* (HORVU0Hr1G019590), *LDOX3* (HORVU7Hr1G009310), *LDOX4* (HORVU5Hr1G065620), and *LDOX7* (HORVU0Hr1G019590) genes are significantly up-regulated, that are core enzymes involved in anthocyanin biosynthesis (**Supplementary Table 9**). However, the *CHS1* (HORVU2Hr1G116390), *CHI2* (HORVU5Hr1G046480), *CHI6* (HORVU2Hr1G038260), *F3H3* (HORVU2Hr1G110130), *F3’H9* (HORVU1Hr1G094880) *F3’H13* (HORVU7Hr1G007580), *DFR20* (HORVU3Hr1G056560), *ANR1* (HORVU2Hr1G108110), *ANR2* (HORVU2Hr1G108260), *ANR5* (HORVU2Hr1G108180), *ANR6* (HORVU7Hr1G101860), and *LAR1* (HORVU4Hr1G061990) were down-regulated in the flavonoids biosynthesis pathway (**Supplementary Table 9**; **Supplementary Figure 10A**).

### 3.10 Heat-map construction to visualize RNA-Seq based relative mRNA expression of major genes in anthocyanin biosynthesis pathways and evaluation of their interaction with transcription factors

**Figure 8:**
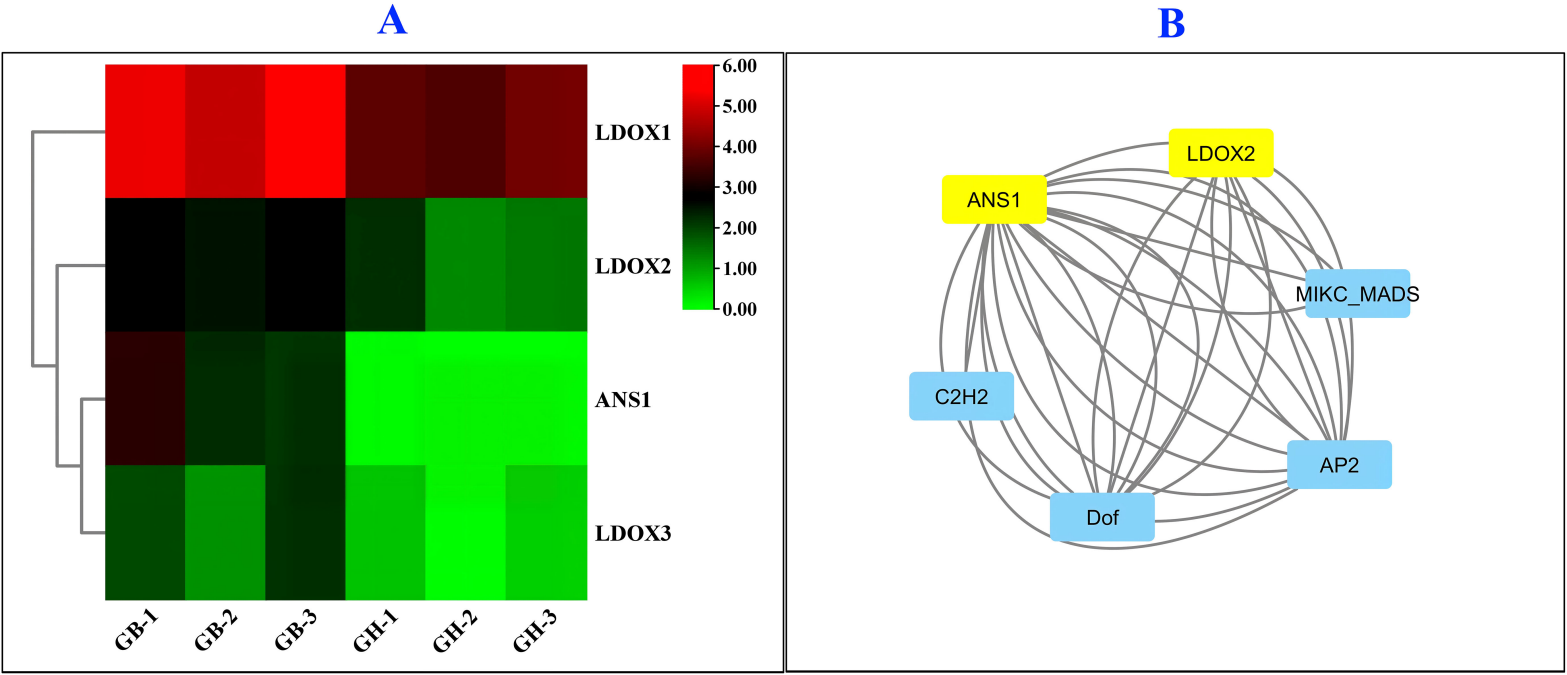
Relative mRNA expression of *ANS1*, *LDOX1*, *LDOX2*, and *LDOX3* genes in barley in RNA-Seq experiment **(A)** and transcription factor regulatory network with key genes involved in anthocyanin biosynthesis **(B)** in barley.

A significant higher expression of *ANS1, LDOX1, LDOX2*, and *LDOX3* genes were observed in black barley than blue barley in RNA-Seq based relative mRNA expression (**Figure 8A**; **Supplementary Table 10**). Among all, highest expression was observed in *LDOX1* gene in black barley. This might be due to the potential role of *LDOX1* gene in higher anthocyanin accumulation in black barley than blue barley. Although lower than the above mentioned gene, the expression of *ANS1*, *LDOX2*, and *LDOX3* genes were always higher in in black barley than blue barley (**Figure 8A**). Additionally, potential interaction of *ANS1* and *LDOX2* genes with AP2 (APETALA2), C2H2 (Cys2-His2), Dof (DNA-binding One Zinc Finger), and MIKC_MADS [MADS domain, an intervening (I) domain, a keratin-like (K) domain, and a C-terminal (C) domain] TFs might be due to great impact of these TFs on the expression of these two genes. (**Figure 8B**; **Supplementary Table 11**).

### 3.11 Construction of a weighted gene co-expression network

**Figure 9:**
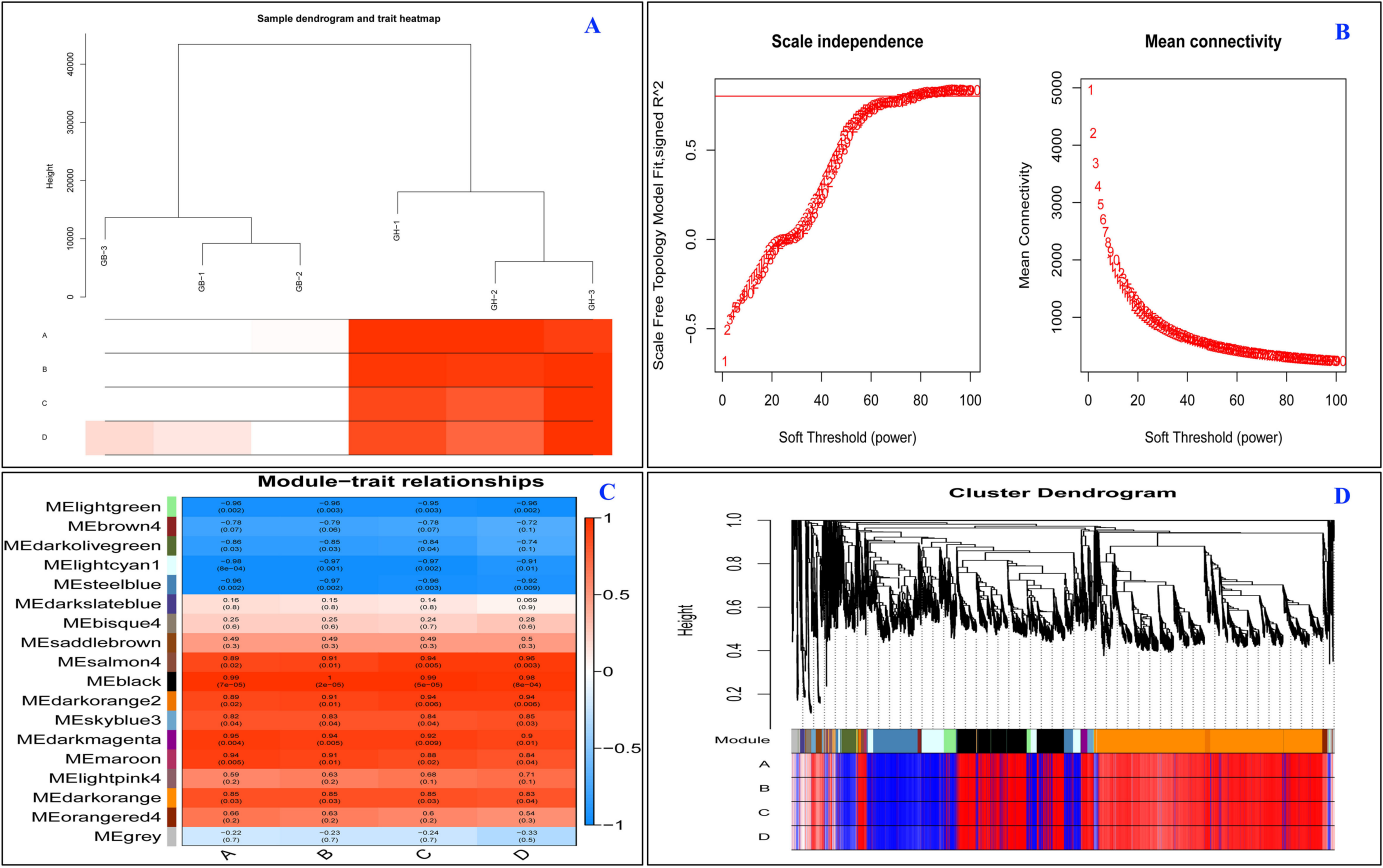
Construction of the co-expression network by WGCNA. **(A)** Sample dendogram and train heart map. **(B)** Soft power curve, the abscissa represents the power value, the ordinate (left) represents the correlation coefficient, and the ordinate (right) represents the average connectivity of genes; **(C)** Heat map of the association of gene co-expression network modules with ABCD flavonoids; **(D)** Gene cluster dendrograms and module division. Each row corresponds to a consensus module and each column to a time point. Module names;are shown on the y-axis and time points are shown n the x-axis. The table is color-coded by correlation according to the color legend. The strength and irection of the correlations are shown on the right side of the heat map (red, positive correlation; blue, negative correlation). Here, **(A–D)** represent hydroxysafflor yellow A flavonoid, cyanidin-3-O-glucoside, cyanidin 3-(6-p-caffeoyl) glucoside, and cyanidin 3-O-(6-O-para-coumaroyl) respectively.

Following WGCNA analysis, 21,049 high-quality genes were obtained (**Supplementary Table 12**). The sample dendrogram and corresponding traits of all the 21,049 genes passed the cutoff thresholds and were suitable for network analysis (**Figure 9A**). To ensure a reliable co-expression network, we carefully selected the soft-thresholding power which provided a high scale-free topology, indicating a strong network structure (**Figure 9B**). The gene co-expression network was constructed using hierarchical clustering of the calculated dissimilarities resulting in eighteen different modules (**Figures 3C, D**; **Supplementary Table 13**). Among these, the dark orange module showed the highest number (2,597) of genes. **Supplementary Table 13** contains the details of rest of genes in each module.

### 3.12 Quantitative real-time PCR validation

**Figure 10:**
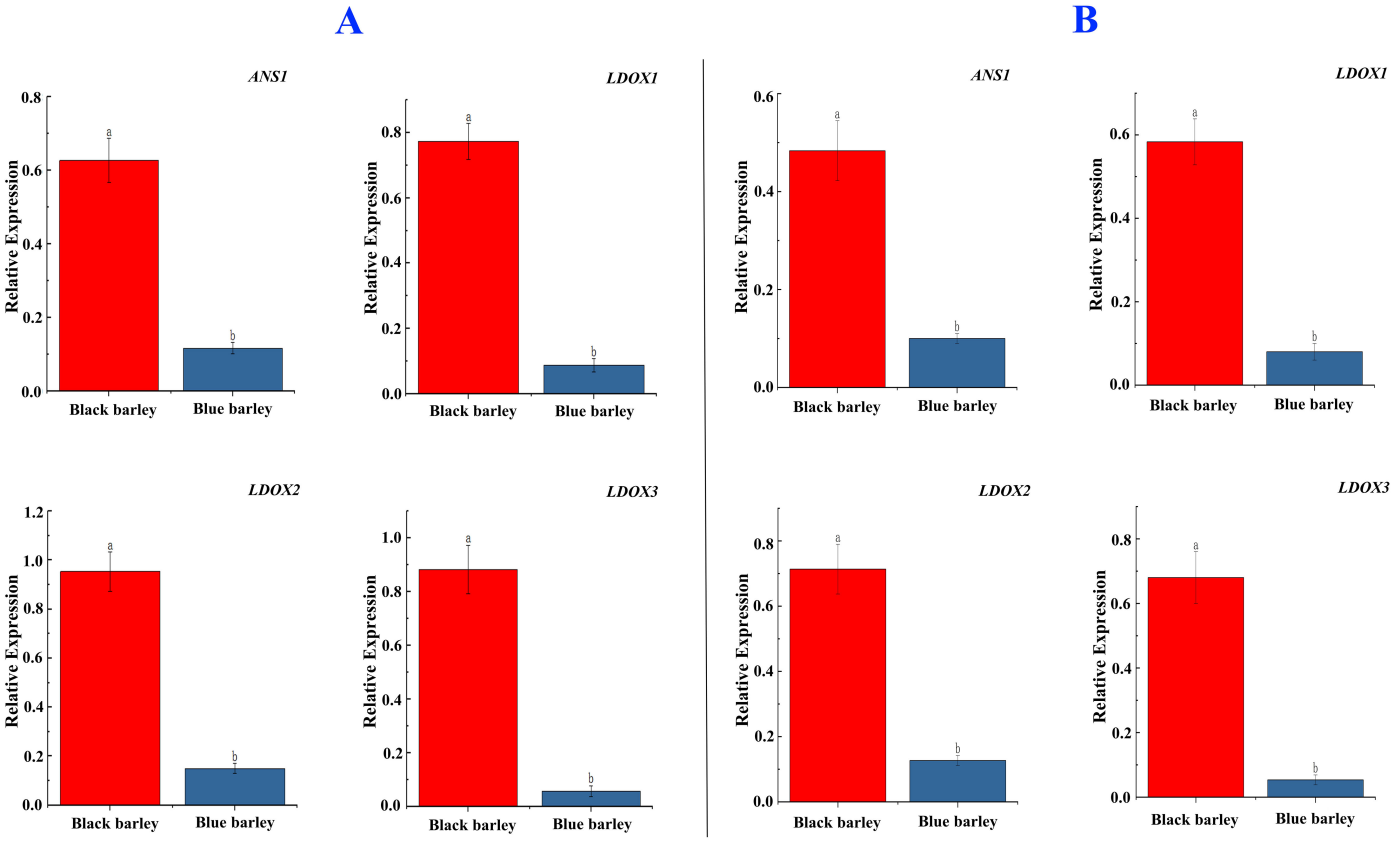
Relative mRNA expression of *ANS1*, *LDOX1*, *LDOX2*, and *LDOX3* genes (fold changes) in barley which are essential for anthocyanin biosynthesis. Here, the expression was observed in the spikelets at the grain-filling stage **(A)** and physiological maturity stage **(B)**. Different letters represent significance at the standard error value at a 5%significance level.

To explore the mechanism of higher anthocyanin production in black barley, the expression of four significant genes (*ANS1*, *LDOX1*, *LDOX2*, and *LDOX3*) involved in the anthocyanin biosynthesis pathway was evaluated at mature seed tissue in grain filling and physiological maturity stages of black and blue barley (**Figure 10**). Irrespective of two the stages, the selected four genes showed significantly higher expression in black barley than blue barley (**Figure 10**). This might be due to the great role of these genes in accumulating higher anthocyanins in black barley.

## 4 Discussion

In the current research, flavonoids and anthocyanins quantification following *de novo* transcriptomic evaluation and expression of major genes in anthocyanins biosynthesis pathway in black and blue barley were done to evaluate the molecular mechanism of anthocyanin production and accumulation in two barley (*Hordeum vulgare*). Among these two, higher accumulation of hydroxysafflor yellow A flavonoid, and cyanidin-3-O-glucoside, cyanidin-3-(6-O-p-caffeoyl)- glucoside, cyanidin-3-O-(6-O-p-coumaroyl)- glucoside anthocyanin in black barley might due to higher synthesis of these molecules in black barley (**Figure 1**). This might be either the efficient production and regulation of these anthocyanins in black barley. Anthocyanins are key secondary metabolites that possess a potential antioxidant activities, that also facilitate pollination and seed dispersal (Liu et al., 2018). Although extremely important, the transcriptome analysis of black and blue barley at the grain-filling stage, and the expression of genes involved in anthocyanins biosynthesis in barley have not yet been studied. To explore the molecular mechanism of significant higher accumulation of anthocyanins in black barley and the genes involved in the process, we have performed a series of experimental and bioinformatics analyses. Our findings provide important insights into the transcriptional regulation of the flavonoids biosynthesis pathway in black barley and major roles of potential candidate genes involved in anthocyanins biosynthesis pathway.

Most of the novel genes were aligned with the generic pathways in TrEMBL and highly homologous to *Triticum turgidum* gymnosperm sequences in the Nr database (**Supplementary Figure 7**). Following KOG analysis, we have found 1,012 novel genes involved in secondary metabolite biosynthesis, transport, and catabolism pathways, suggesting their major roles in regulating and accumulating flavonoids.

Although challenging, the considerable tandem repeated sequences in barley genome need to be critically analyzed in genomics research. Since, annotation of the reference genome is often not accurate enough, RNA-seq based transcriptomic analysis might guide the genome biologists in correcting gene structure having higher accuracy of transcriptional boundaries during the mapping and assembling. In the current research, the identified 10,579 new genes and 5,912 functionally annotated genes might guide the genome biologists in research on the roles of these genes in barley (Table 2). The TrEMBL database guided results, most (5,483) of the newly identified genes were functionally annotated (**Supplementary Table 3**). A significant number of homologs (3,524) with *Triticum turgidum* species might reveal their structural integrity and functional similarity with the genome (**Supplementary Figure 7**).

The notable unigenes (19,856) distributed into 25 functional groups might guide their functional role under different QTLs or interactions (**Supplementary Figure 8**; **Supplementary Table 4**). In addition to general function, involvement of these genes in posttranslational modification, protein turnover, chaperones, and signal transduction mechanisms might represent their potential roles in diverse biological processes. Interestingly, involvement of 1,012 (5.09%) unigenes involved in biosynthesis, transport, and catabolism of secondary metabolites might represent their major roles in regulating and accumulating flavonoids including anthocyanins (**Supplementary Figure 8**; **Supplementary Table 4**) (Biswal et al., 2021).

Alternate splicing (AS) assists in grain-filling, maturation, flowering time management, and circadian timing (Ruan et al., 2018). Variations in AS among black and blue barley and abundance of TSS and TTS type AS might guide to explore their great influence in various aspects of gene expression, including mRNA stability, localization, and translation (Ruan et al., 2018). Similar trends of AS in the potato genome might represent the nearly similar mechanisms in regulation of gene expression in the analyzed two barley (Glushkevich et al., 2022).

Almost similar expression pattern of all detected unigenes with digital gene expression analysis (Pearson correlation coefficient = 0.89) might demonstrate strong reliability of RNA-Seq data. Hence, the observed gene expression pattern in current research might guide genome and synthetic biologist in genomic and synthetic biology research (Hasan, 2024). These findings could guide wet lab research in gene function and regulation (Biswal et al., 2021).

RNA-seq data driven PCA analysis based on a small number of genomic markers, guide to evaluate the sample characteristics like gene expression (FPKM) and RNA quality (Runge et al., 2012). A significant higher variance (70.8%) in black barley than blue barley might reveal the distinct gene expression and RNA quality in black barley than the blue barley (Summers et al., 2015). The findings might guide the genome biologists in designing genomics research utilizing the advantages of synthetic biology.

Analysis of DEG expression guide to explore the mechanism of how genes are expressed in certain genome or certain condition to maintain different biological processes that utilizes transcriptome data and follows log10 (FPKM +) (Zhou et al., 2019). The 3,235 up-regulated DEGs in current research might guide their significantly regulation in biosynthesis and accumulation of different secondary metabolites, including flavonoids and anthocyanins (Yuan et al., 2022).

PPI guides to explore the mechanism of how different proteins interact in regulating physiological, morphological, and stress responsiveness in plant (Zhang et al., 2023). Significant higher interaction of black barley DEGs in PPI network might represent their potential role in regulating the cellular, biological, and metabolic processes interacting the other proteins or vice versa. Utilizing these findings, genome biologists could design a better genetic circuit to regulate and increase anthocyanins biosynthesis and accumulation in black barley.

Association of 107 DEGs with phenylpropanoid biosynthesis pathway (PATH: ko00940), including 51 up-regulated genes among those might guide their potential roles in regulating the pathway for synthesizing flavonoids including anthocyanins (Grabherr et al., 2011; Love et al., 2014; Ashburner et al., 2000). Since anthocyanins biosynthesis is the downstream pathway of phenylpropanoid biosynthesis, these genes might play significant roles in anthocyanins biosynthesis too. Not surprisingly, involvement of the identified 45 DEGs in flavonoids biosynthesis (PATH: ko00941) pathway might regulate the anthocyanins biosynthesis in barley (Grabherr et al., 2011; Love et al., 2014; Ashburner et al., 2000). In addition, upregulation of major genes in flavonoids biosynthesis, *DFR4* (HORVU7Hr1G093360) and *DFR5* (HORVU7Hr1G093370) might be due to their notable role in flavonoids biosynthesis. Regulation of these genes could significantly increase the anthocyanins biosynthesis in black barley. These notable number of genes could be critically regulated utilizing synthetic biology guided genome editing approaches.

Accordingly, 19 up-regulated genes in the flavonoids biosynthesis pathway might reveal their great involvement in flavonoids biosynthesis (**Supplementary Table 9**). Additionally, significant up-regulation of *ANS1* (HORVU5Hr1G094280), *LDOX1* (HORVU5Hr1G065620), *LDOX2* (HORVU0Hr1G019590), *LDOX3* (HORVU7Hr1G009310), *LDOX4* (HORVU5Hr1G065620), and *LDOX7* (HORVU0Hr1G019590) core enzymes involved in anthocyanins biosynthesis might reveal their potential involvement in anthocyanins biosynthesis. These genes could be critically modulated following synthetic genetic circuit enabled programming to increase anthocyanins content in barley.

Interestingly, significant higher expression of *ANS1, LDOX1, LDOX2*, and *LDOX3* genes in black barley than blue barley in RNA-Seq analysis might be due to their notable contribution in anothcyanin biosynthesis in black barley (**Figure 8A**). Following that, the consistent expression pattern of the same genes in q-RT PCR based expression might reveal the high precision in two experiments (**Figure 8A**). Therefore, these genes might play great role in anthocyanins biosynthesis black barley seed (Sreenivasulu et al., 2010).

Among four genes in anthocyanin biosynthesis pathway, potential interaction of *ANS1* and *LDOX2* genes with AP2, C2H2, Dof, and MIKC_MADS TFs might be due to great impact of these TFs on the expression of these two genes (**Figure 8B**). Overexpression and silencing of apple *MdAP2_1a TF gene resulted increased and decreased accumulation of* petal accumulation in tobacco and apple respectively (Ding et al., 2022). The overexpression of *Malus domestica* C2H2 TF *MdZAT17* TF gene improved anthocyanin accumulation, salt tolerance in apple calli and lower malondialdehyde (MDA) content, and reactive oxygen species (ROS) accumulation in *Arabidopsis* (Wang et al., 2022). Overexpression of pear Dof TF *PpCDF5* gene increased anthocyanin accumulation in pear (Yang et al., 2024). Expression of MIKC_MADS positively correlated with the anothocyanin content in purple broccoli (Liu et al., 2020). Therefore, these TFs directly affect anthocyanin biosynthesis in barly regulating the above mentioned 4 genes in anthocyanin biosynthesis pathway. Proper modulation of these TFs along with major genes in anthocyanin biosynthesis pathway might increase anthocyanin accumulation in black barley.

According to WGCNA analysis, a strong correlation between gene expression and hydroxysafflor yellow A flavonoid, cyanidin-3-O-glucoside, cyanidin 3-(6-p-caffeoyl) glucoside, and cyanidin 3-O-(6-O-para-coumaroyl) content might be due to their great involvement in regulating synthesis of the mentioned anthocyanins.

The significant higher accumulation of higher anthocyanin content in black barley might be due to the cumulative effect of the expression of major genes in anthocyanin biosynthesis pathway, PPI, GO, KEGG pathway enrichment of DEGs, co-expression network, expression of TFs and their inter-regulatory network. Therefore, proper programming considering all of the above parameters could significantly increase anthocyanin content in black barley seed. This needs synthetic biology guided reprogramming of the black barley genome.

Therefore, in current research, results of the series of transcriptional evaluation and expression analysis of major gene in anthocyanins biosynthesis pathway might guide the mechanism of how higher anthocyanins are accumulated in black barley and the genes involved in the process. This is the first transcriptome report in black barley compared to the blue one at the grain-filling stage. Integrating the above findings would guide the synthetic biologists in developing programming-based synthetic genetic circuits enabled barley having high anthocyanins content. The findings could also boost the research to increase the black barley molecular marker resources. Furthermore, the findings could forward further research related to genetic diversity studies, genetic linkage mapping, and marker-assisted selection to trigger barley breeding.

## 5 Conclusion

In the current research, significant higher accumulation of anthocyanins were observed in black barley compared to blue one. To elucidate the molecular mechanism of how anthocyanins are accumulated and the major genes involved in the process, we have done transcriptomic analyses of black and blue barley spikelets at grain-filling stage. The findings were validated through a series of statistical analyzes. Here, 10,579 new genes were identified, and 5,912 genes were functionally annotated. Additionally, 12 types of alternative splicing were found. Indeed, the observed 7,113 DEGs might involve in regulating different metabolic pathways among black and blue barley. The highest upregulated genes in black barley might be due to their involvement in regulating different cellular, biological, and metabolic processes. The significant PPI interaction among the DEGs of black barley might be due to their potential involvement in regulating different cellular, biological and metabolic processes. Involvement of many DEGs in many biological, cellular, and molecular functions reveal their great influence in plant growth, development, and yield. Irrespective of the types, the involvement of significant unigenes and DEGs in phenylpropanoid and flavonoids biosynthesis in the black barley might be due to their great involvement in flavonoids biosynthesis, including anthocyanins. Irrespective of growth stages, the higher expression of *ANS1, LDOX1, LDOX2*, and *LDOX3* genes in black barley than the blue barley might reveal their great involvement in biosynthesis, accumulation and transformation of anthocyanins into the seed of black barley. Hence, the findings of the current first research report on higher anthocyanins content in black barley, their transcriptomic regulation, and expression of major genes involved in the anthocyanins biosynthesis might add a new era to plant genome biologists to develop barley having high anthocyanins content through synthetic genetic circuit-enabled precise reprogramming.

## Data Availability

All data generated or analyzed during this study were included in the article and the supplementary files. The raw reads generated and analyzed during the current study are available on the Sequence Read Archive database of National Center for Biotechnology Information repository under the BioProject accession number PRJNA1279624 (https://www.ncbi.nlm.nih.gov/bioproject/PRJNA1279624). Scripts are available from the corresponding author on reasonable request.
