# Supplementary material for: Molecular mechanisms of how black barley accumulates higher anthocyanins than blue barley following transcriptomic evaluation and expression analysis of key genes in anthocyanins biosynthesis pathway
Source: Front Plant Sci. 2025 Aug 29;16:1650803. doi: 10.3389/fpls.2025.1650803 (PMC12427265; doi:10.3389/fpls.2025.1650803)
Supplement: Supplementary file 1 [file Supplementaryfile1.zip › Supplementary Material/Data Sheet 1.PDF]

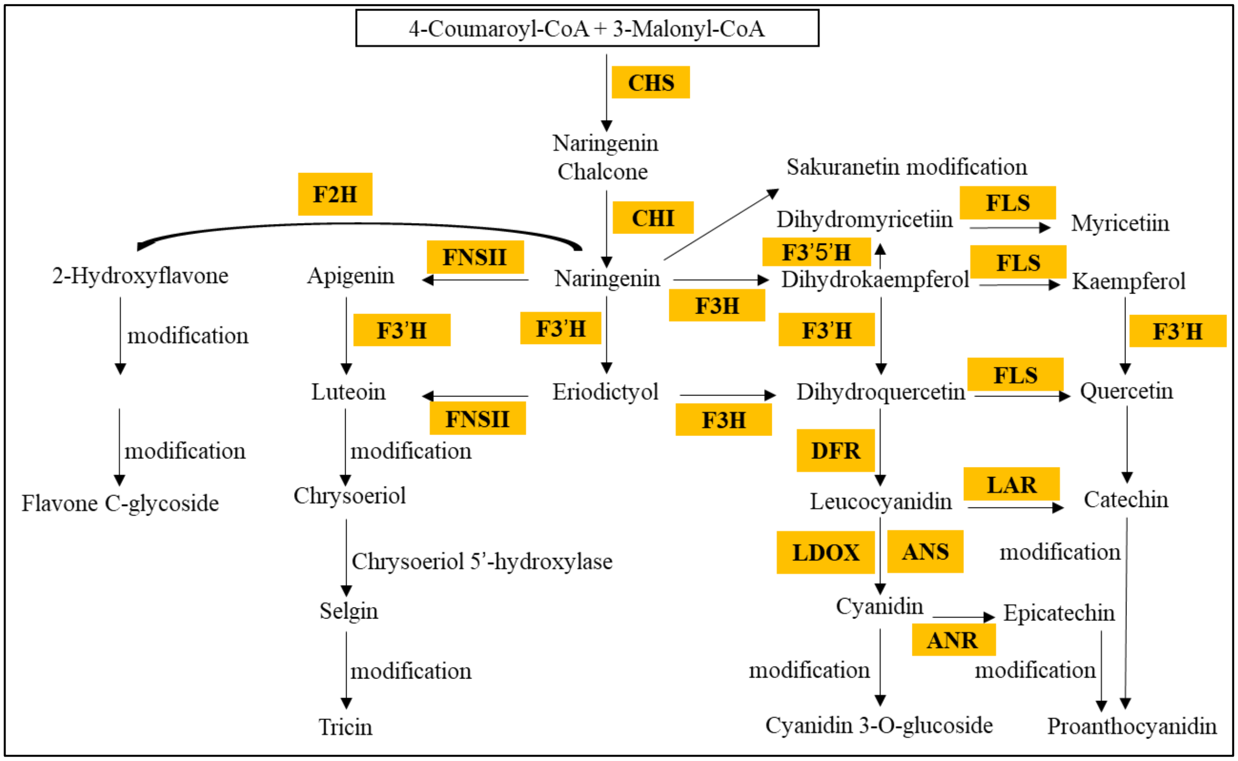

Supplementary Figure 1. Proposed flavonoid biosynthetic pathway in barley (Liu et al., 2021).

Arrows represent enzymatic steps: **CHS**, chalcone synthase; **CHI**, chalcone isomerase; **F3H**, flavanone 3-hydroxylase; **F2H**, flavanone 2-hydroxylase; **FNSII**, flavone synthase II; **F3'H**, flavonoid 3'-hydroxylase; **FLS**, flavonol synthase; **F3'5'H**, flavonoid 3'5'-hydroxylase; **DFR**, dihydroflavonol 4-reductase; **ANS**, anthocyanidin synthase; **LDOX**, leucoanthocyanidin dioxygenase; **LAR**, leucoanthocyanidin reductase; **ANR**, anthocyanidin reductase; modification means the flavonoid scaffolds modification of hydroxylation, glycosylation, and methoxylation with the aid of enzymes including glycosyltransferases, acyltransferases and methyltransferases and so on.

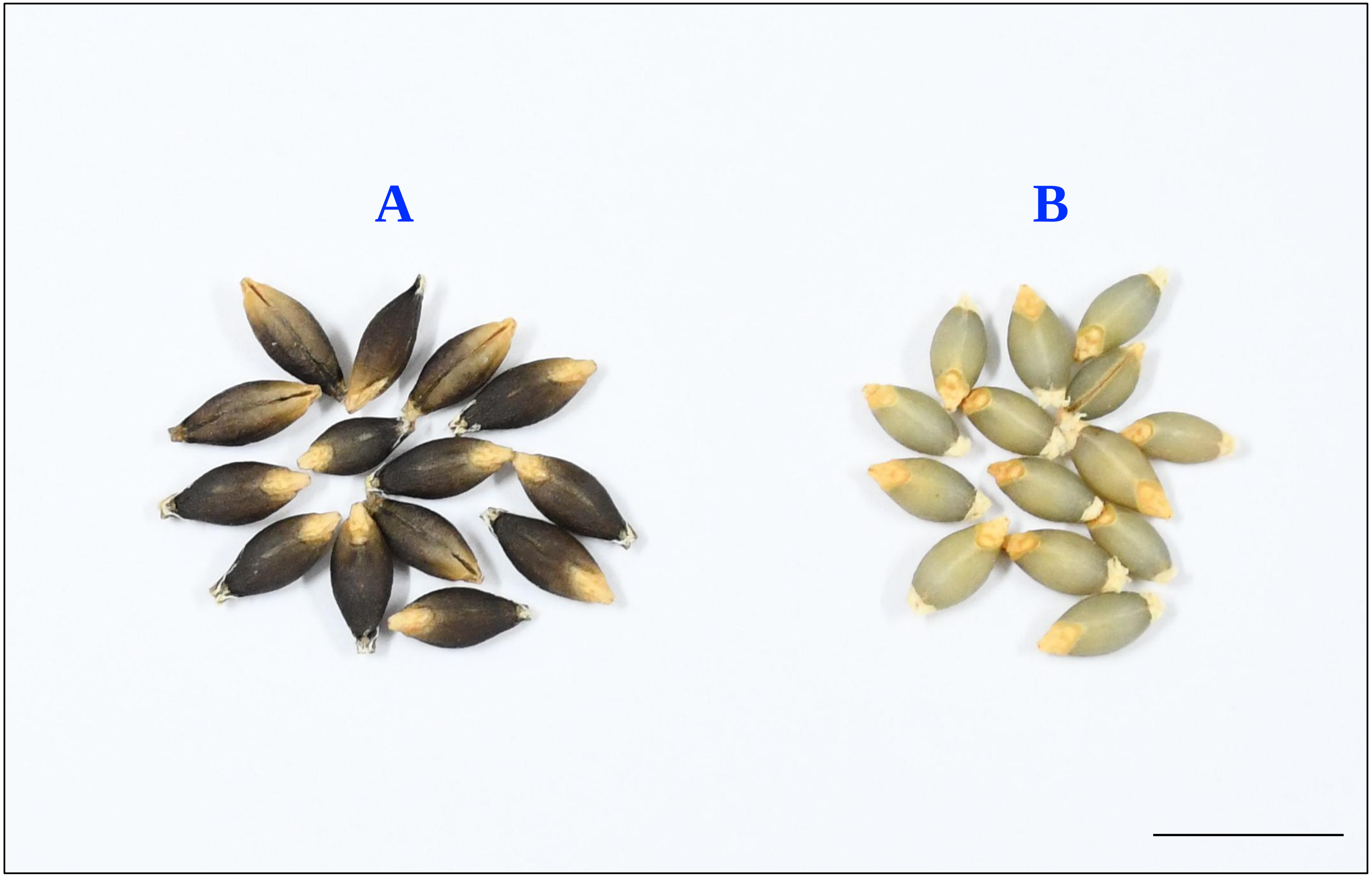

Supplementary Figure 2. Black (A) and blue (B) barley seed that are used in the experiment. Scale 1 cm.

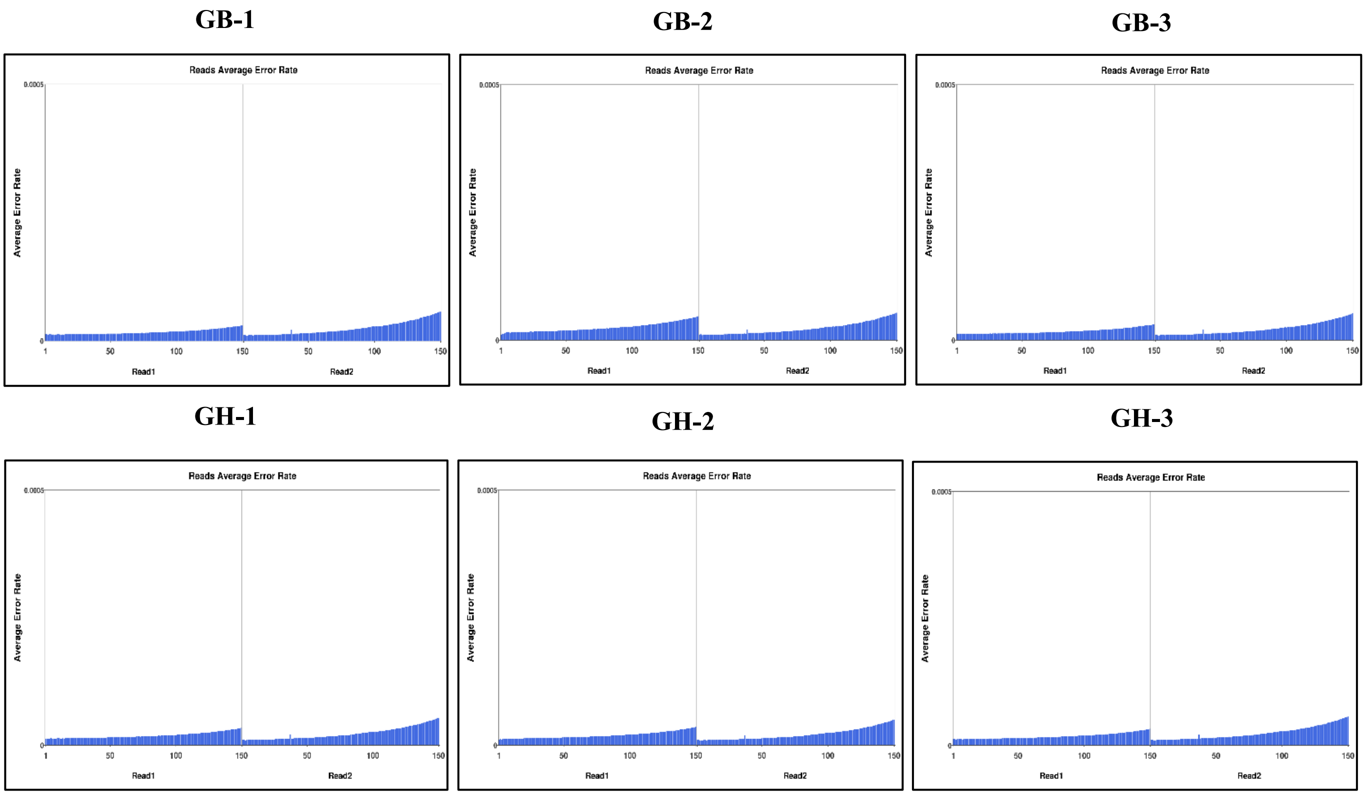

Supplementary Figure 3. Distribution of sequencing error rate. Read 1 and read 2 are paired end sequencing reads generated from Illumina platform.

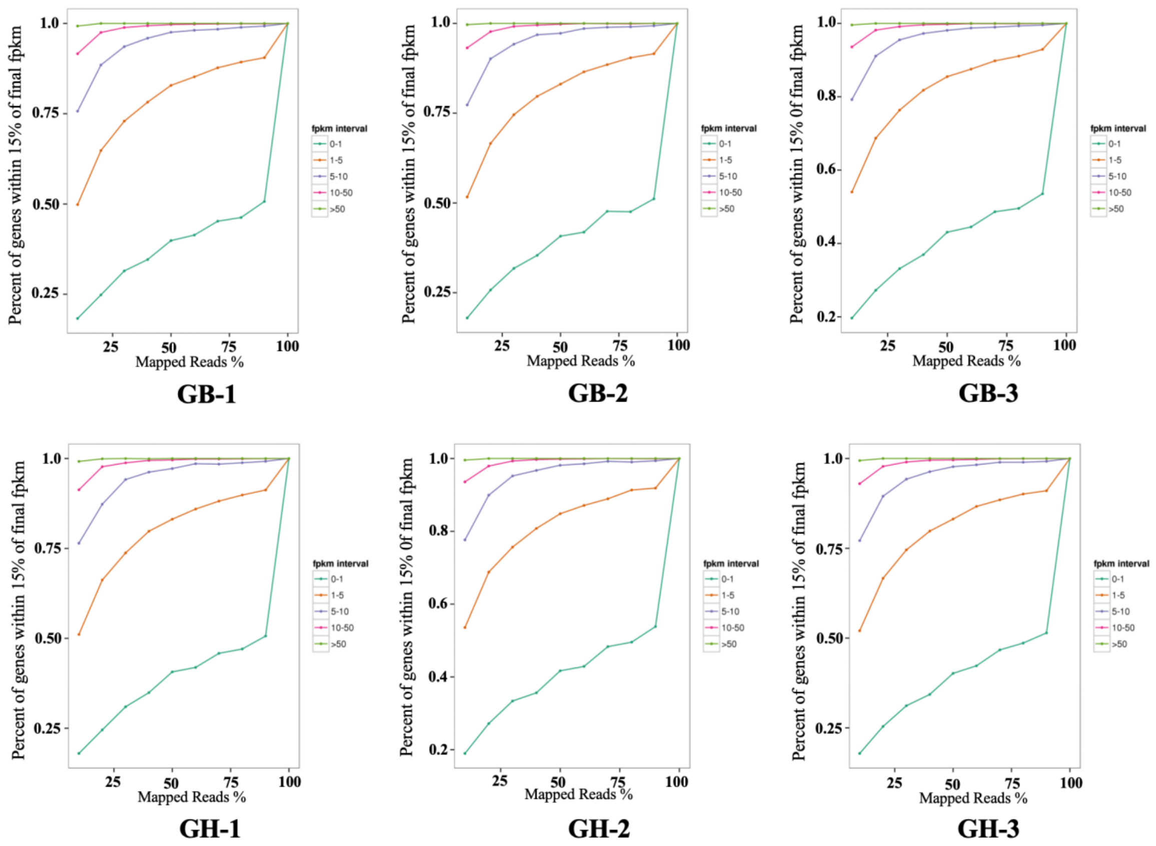

Supplementary Figure 4. Transcriptome data saturation simulation level. Here, GB and GH represent black and blue barley respectively whereas, 1-3 represents three independent biological replicates.

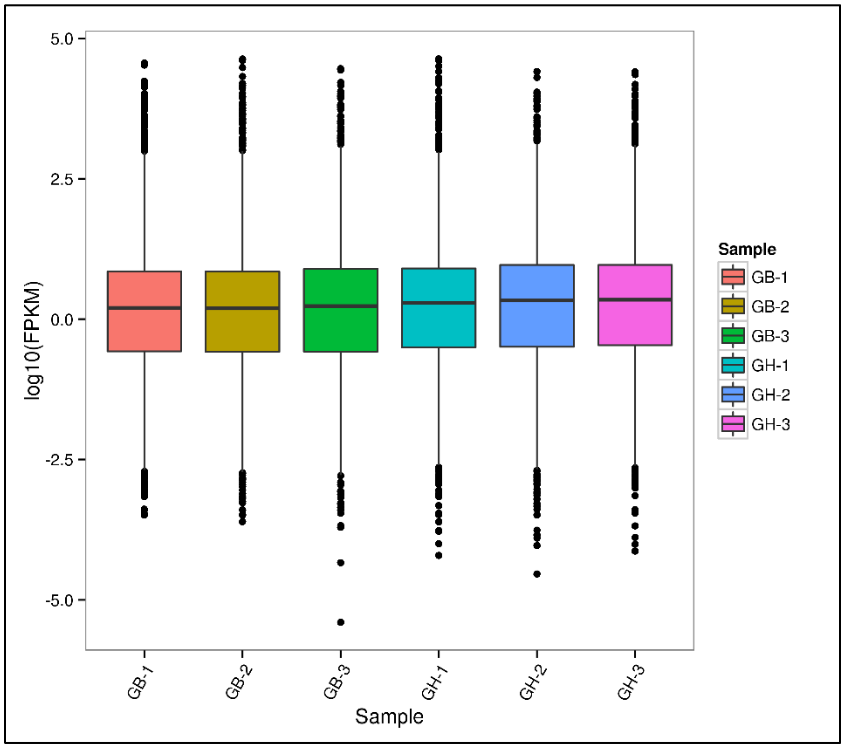

Supplementary Figure 5. Box plot of FPKM values in each sample. Box plot showing the variation in barley evaluated for the association panel. (GB1-3 = black barely having three independent replicates, and GH1-3 = blue barley having three independent replicates).

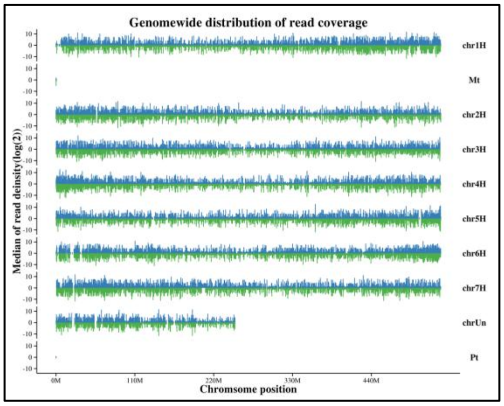

GB-1

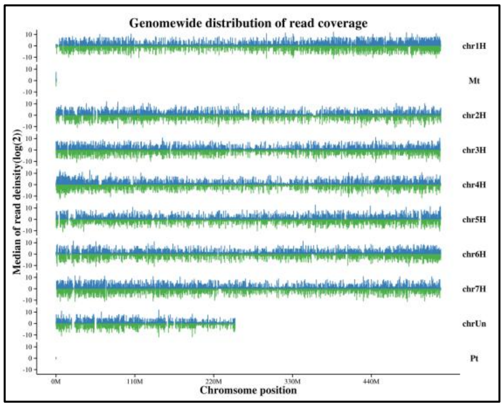

GB-2

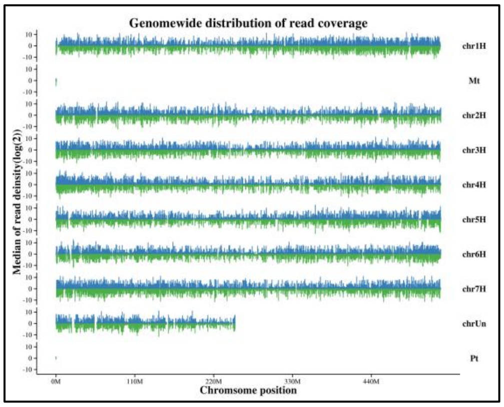

GB-3

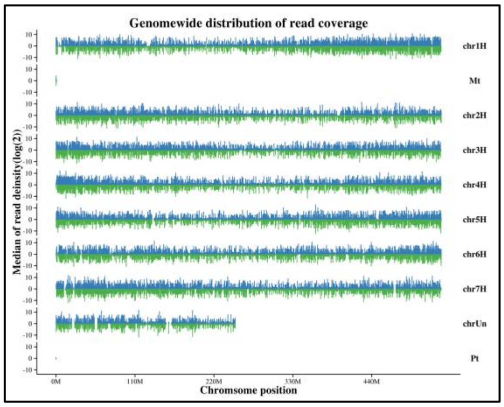

GH-1

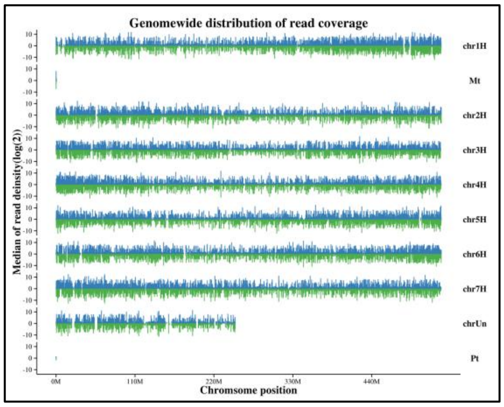

GH-2

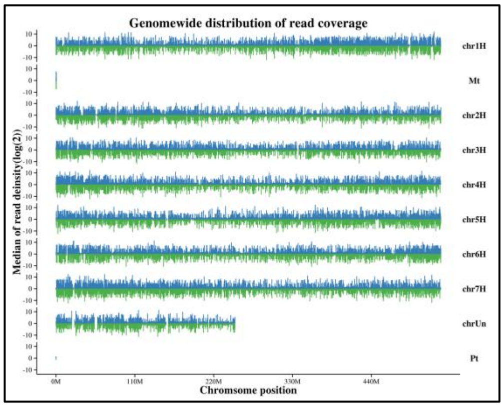

GH-3

Supplementary Figure 6. Location and coverage depth distribution of mapped reads on the reference genome. Here, GB and GH represent black and blue barley respectively whereas, 1-3 represents three independent biological replicates.

Note: The chromosome is divided into multiple small windows with 10kb as the unit length of the interval, and the mapped reads falling in each window are counted as its coverage depth. Blue color represents the positive chain and green represetns the opposite.

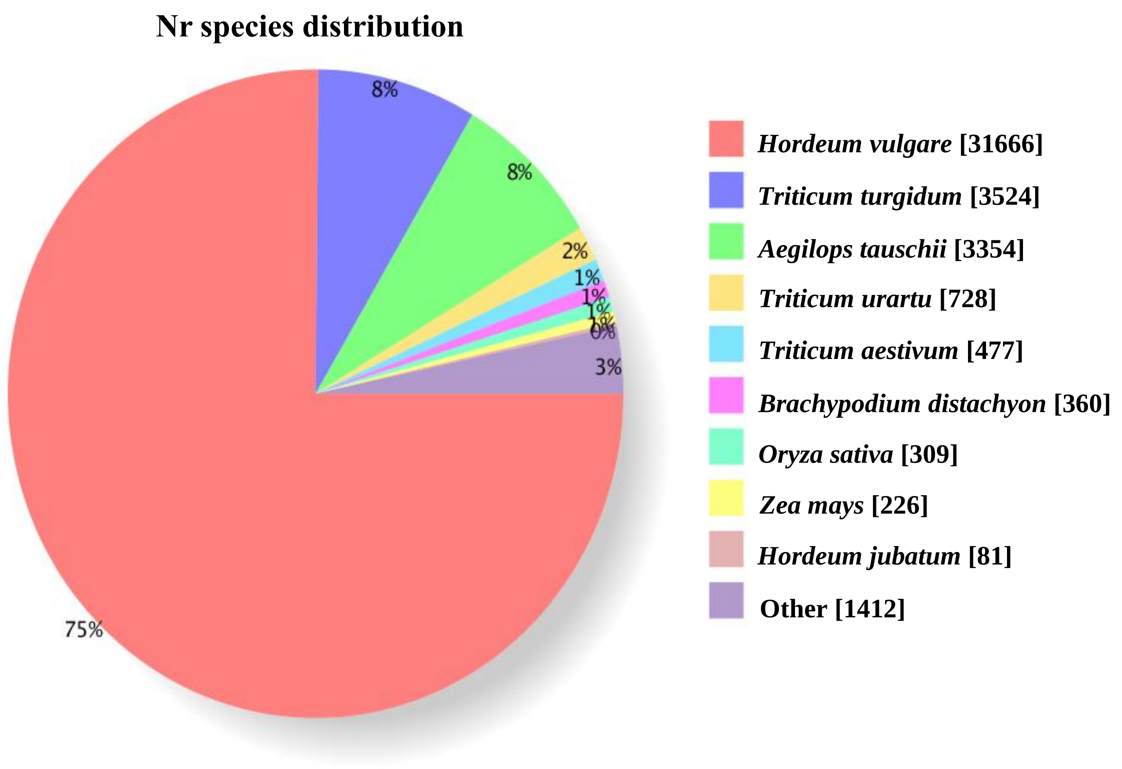

Supplementary Figure 7. Homology comparisons between *H. vulgare* (light red color) and the existing gene sets in multispecies.

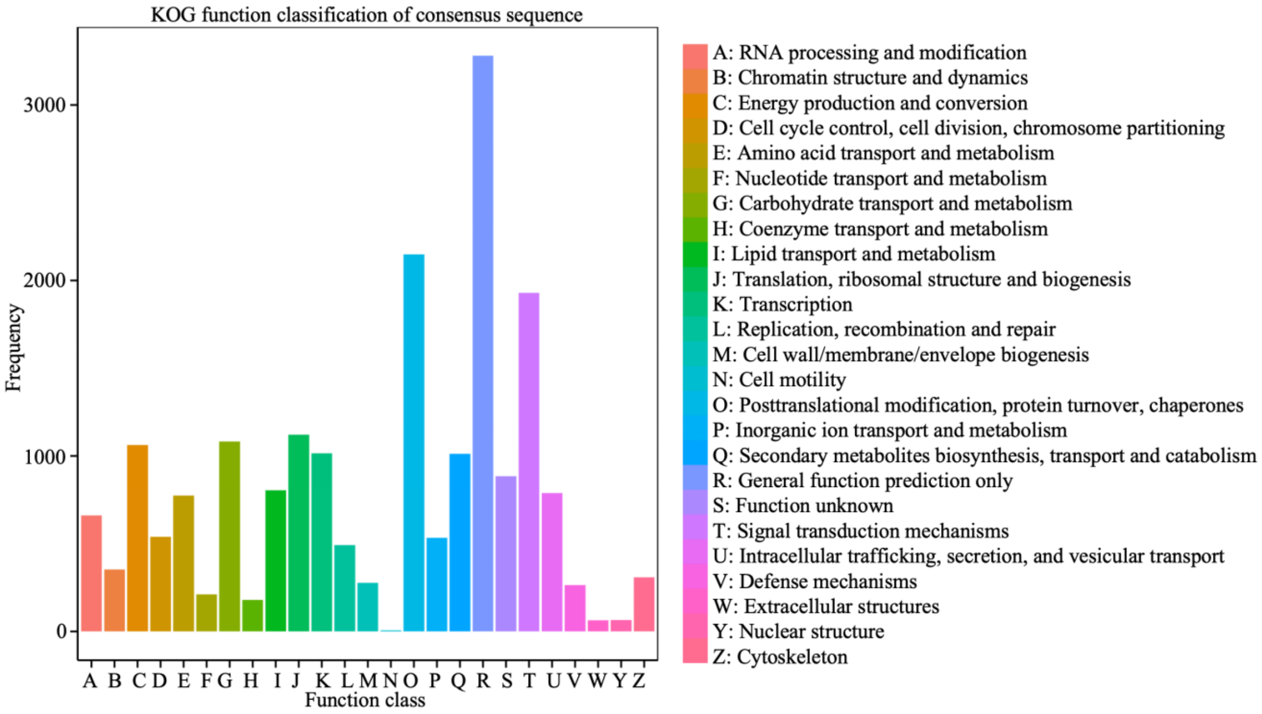

Supplementary Figure 8. KOG (EuKaryotic Orthologous Groups) annotation classification statistics of differentially expressed genes.

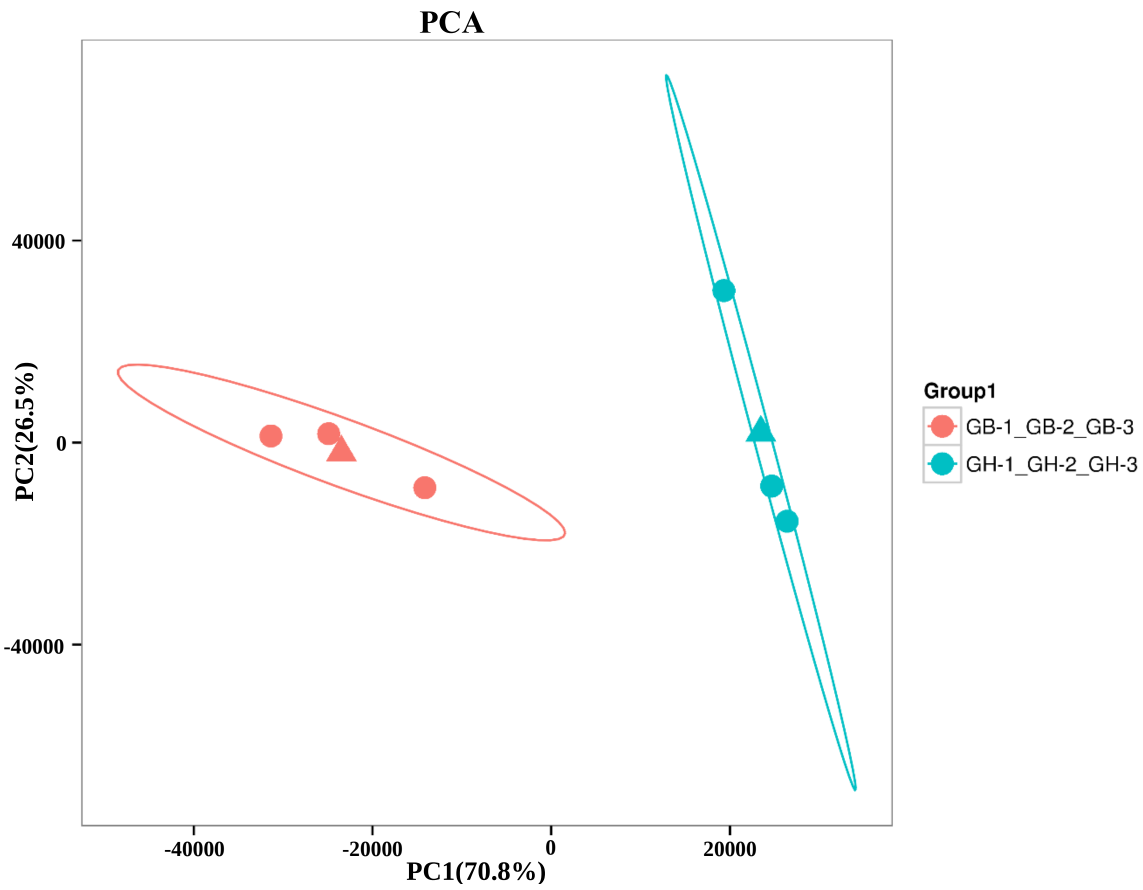

Supplementary Figure 9. Principal component analysis (PCA) of RNA-seq data of two varieties of barley.

Supplementary Figure 10. Along with flavonoid biosynthesis pathway (Figure 7B), other top 9 enriched KEGG pathways interacted upregulated DEGs.

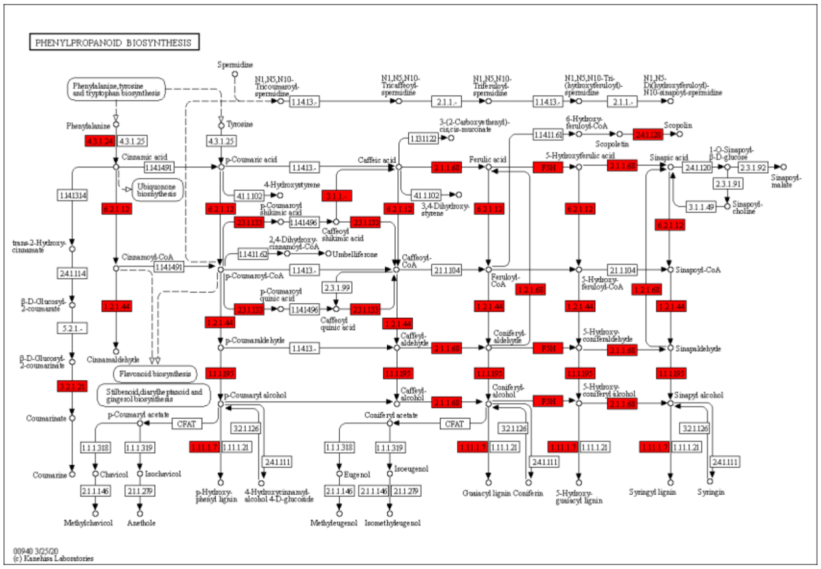

Supplementary Figure 10a. Up-regulated DEGs interacted the Phenylpropanoid biosynthesis pathway (ko00940). Red box (B) represent the point of the pathway where DEGs interacted.

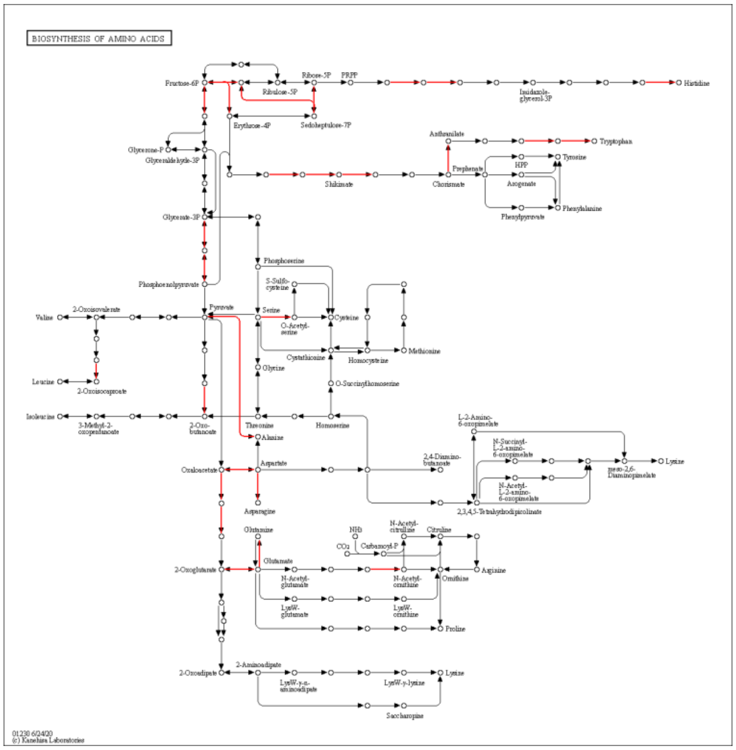

Supplementary Figure 10b. Up-regulated DEGs interacted the amino acids biosynthesis pathway (ko01230) of the up-regulated DEGs. Red box (B) represent the point of the pathway where DEGs interacted.

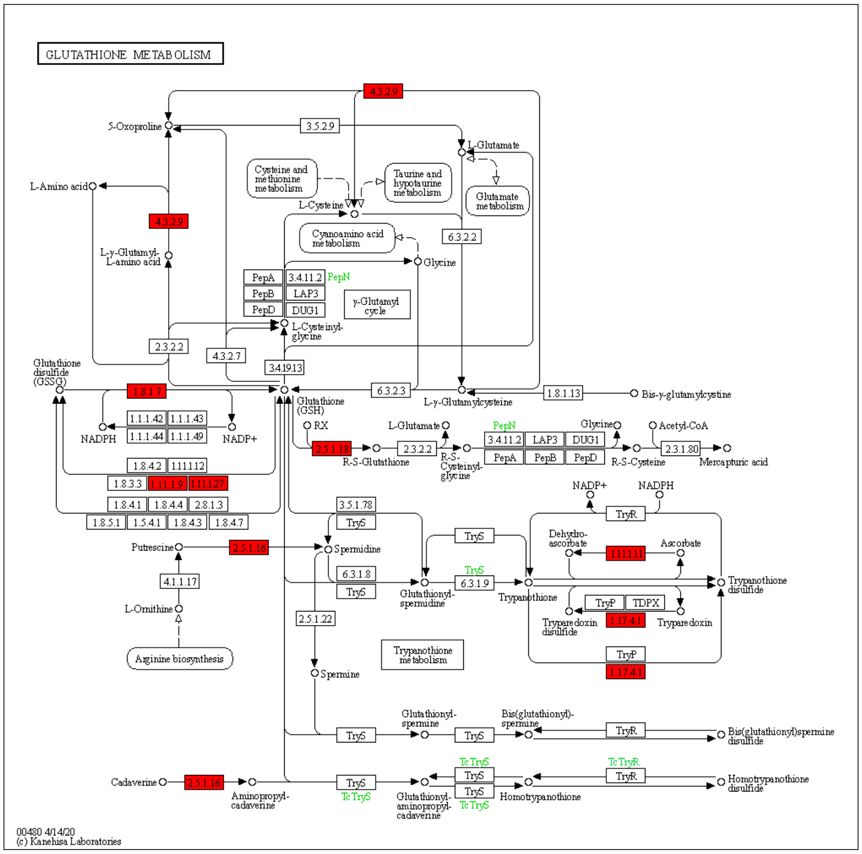

Supplementary Figure 10c. Up-regulated DEGs interacted the glutathione metabolism pathway (ko00480). Red box (B) represent the point of the pathway where DEGs interacted.

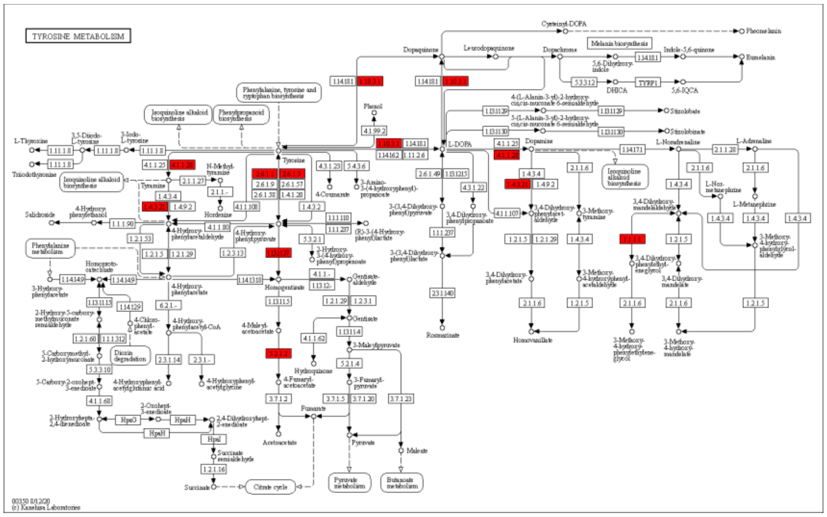

Supplementary Figure 10d. Up-regulated DEGs interacted the tyrosine metabolism pathway (ko00350). Red box (B) represent the point of the pathway where DEGs interacted.

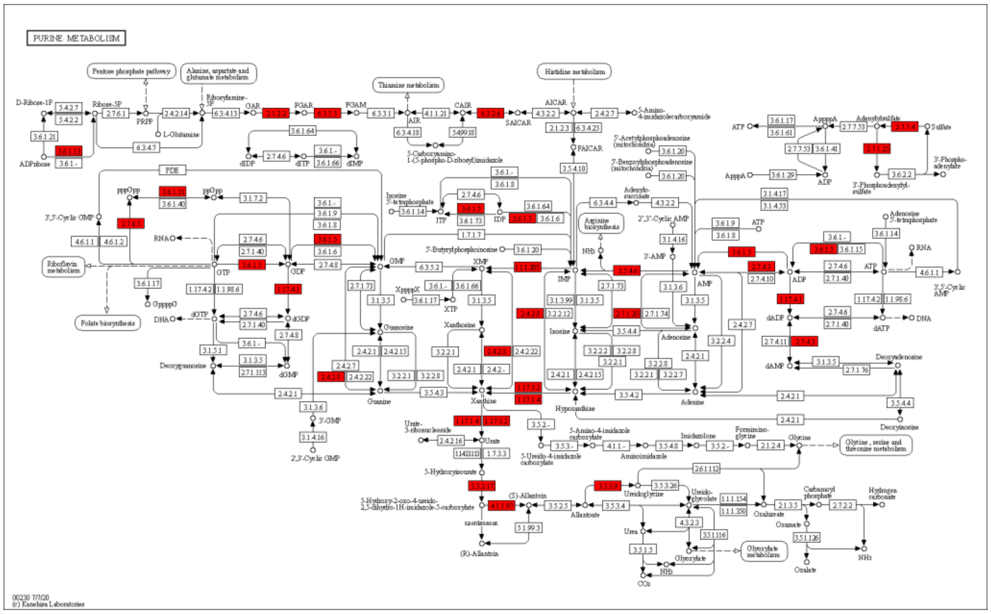

Supplementary Figure 10e. Up-regulated DEGs interacted the purine metabolism pathway (ko00230). Red box (B) represent the point of the pathway where DEGs interacted.

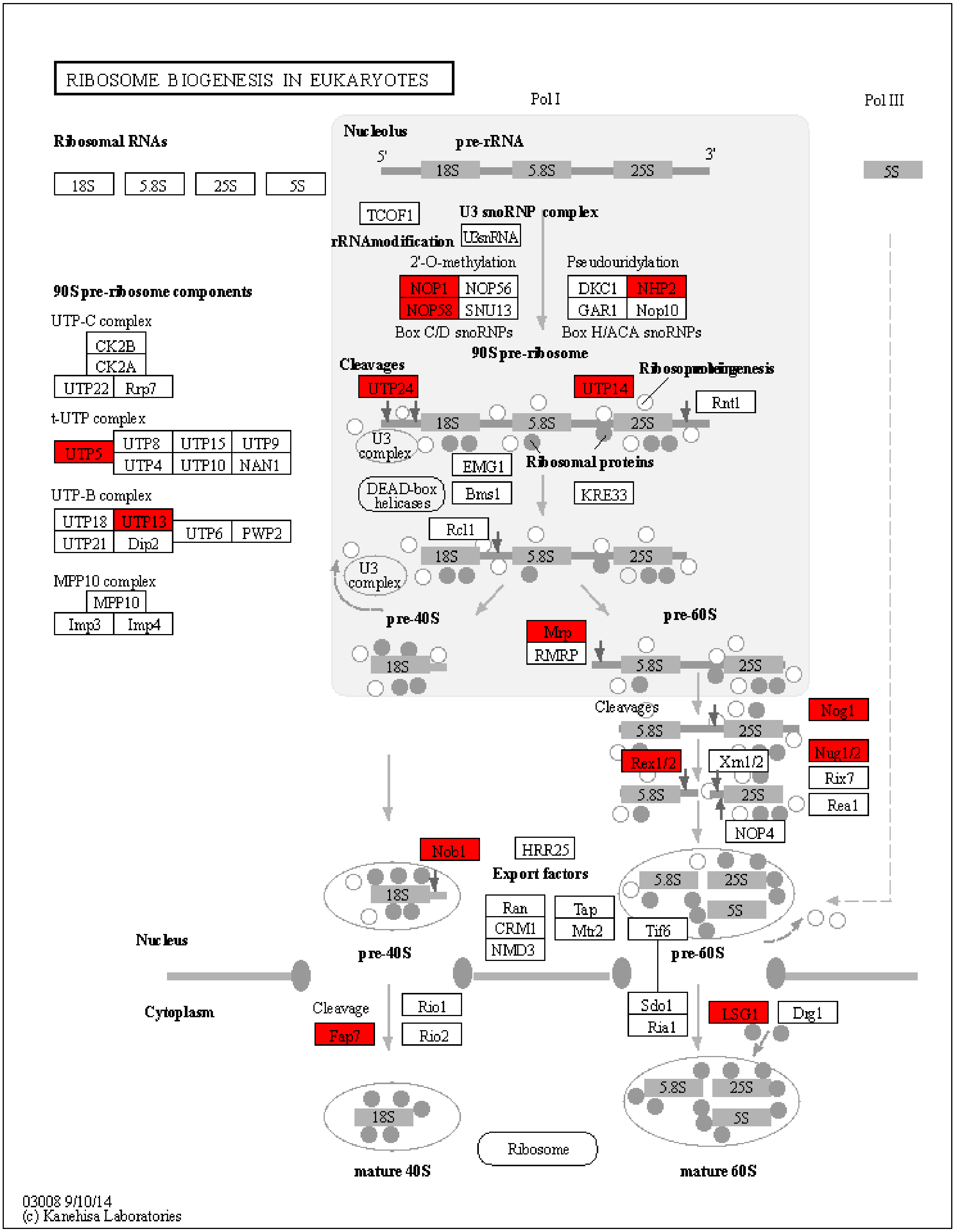

Supplementary Figure 10f. Up-regulated DEGs interacted the ribosome biogenesis pathway (ko03008). Red box (B) represent the point of the pathway where DEGs interacted.

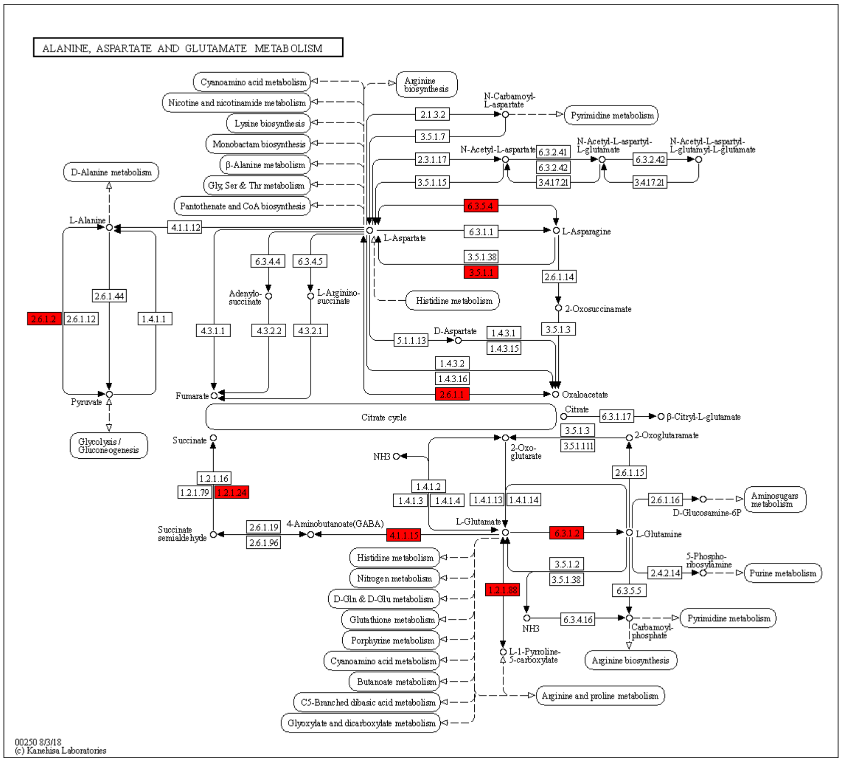

Supplementary Figure 10g. Up-regulated DEGs interacted the alanine, aspartate, and glutamate metabolism pathway (ko00250). Red box (B) represent the point of the pathway where DEGs interacted.

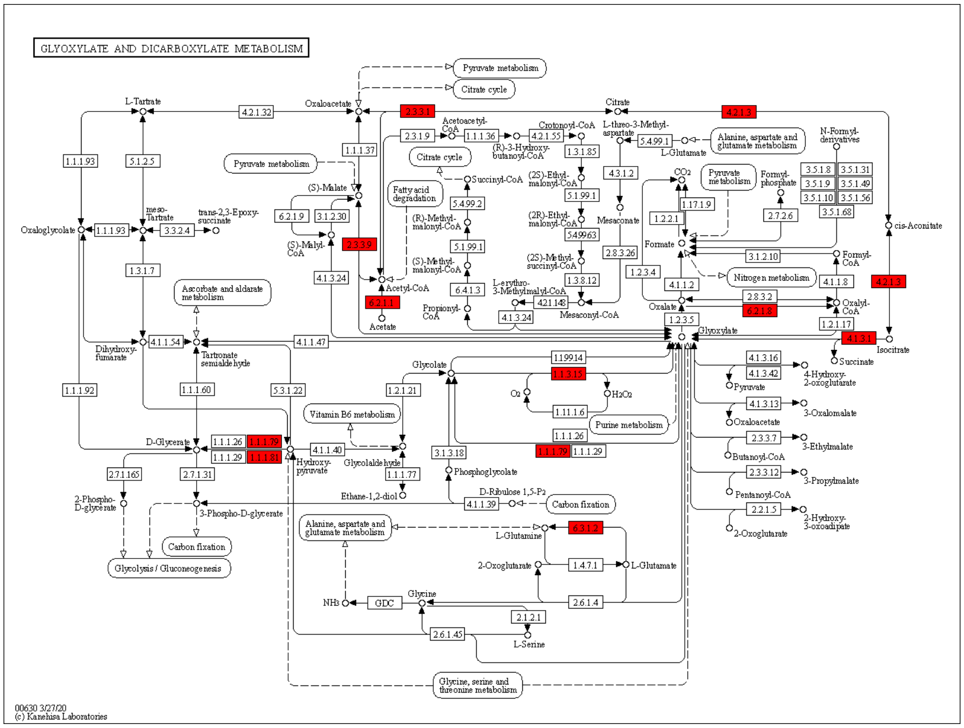

Supplementary Figure 10h. Up-regulated DEGs interacted the glyoxylate and dicarboxylate metabolism pathway (ko00630). Red box (B) represent the point of the pathway where DEGs interacted.

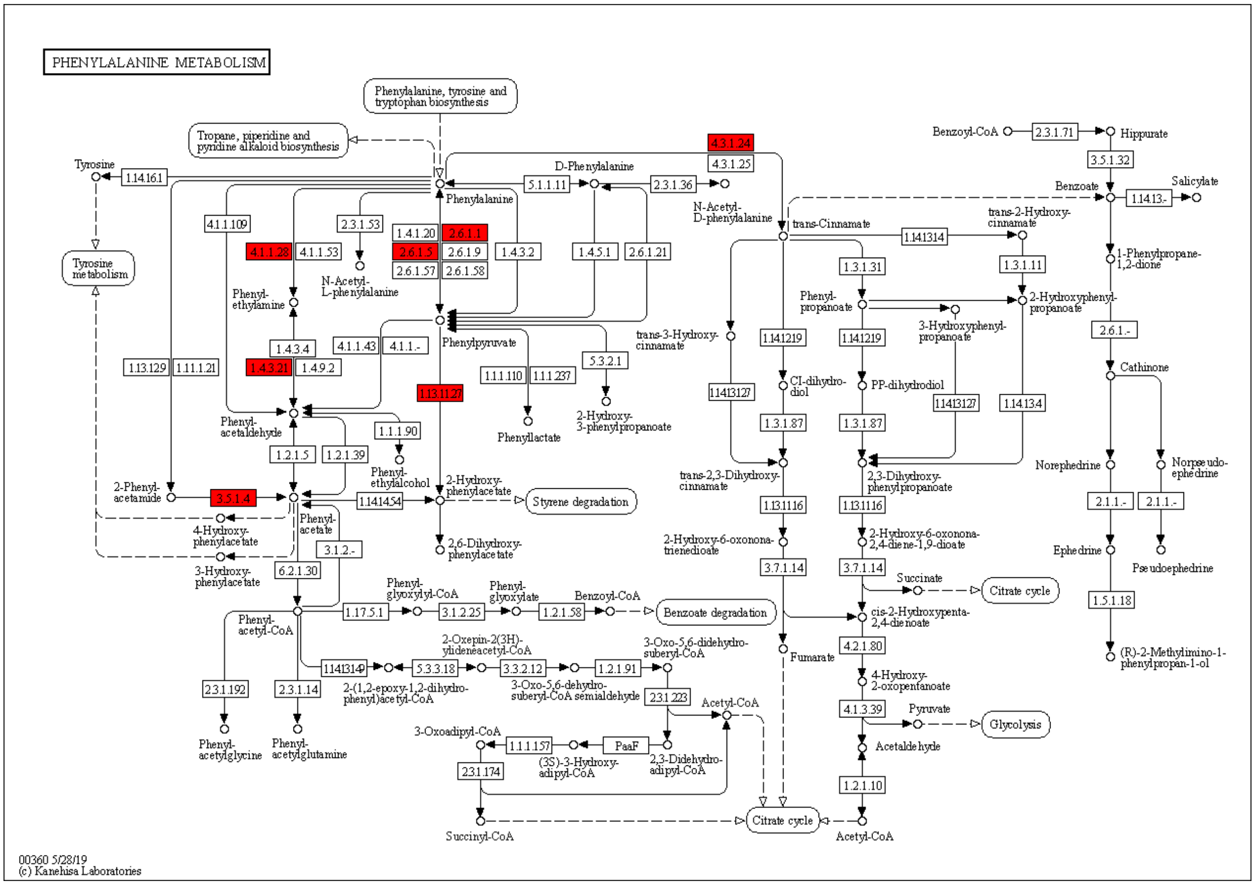

Supplementary Figure 10i. Up-regulated DEGs interacted the phenylalanine metabolism pathway (ko00360) . Red box (B) represent the point of the pathway where DEGs interacted.

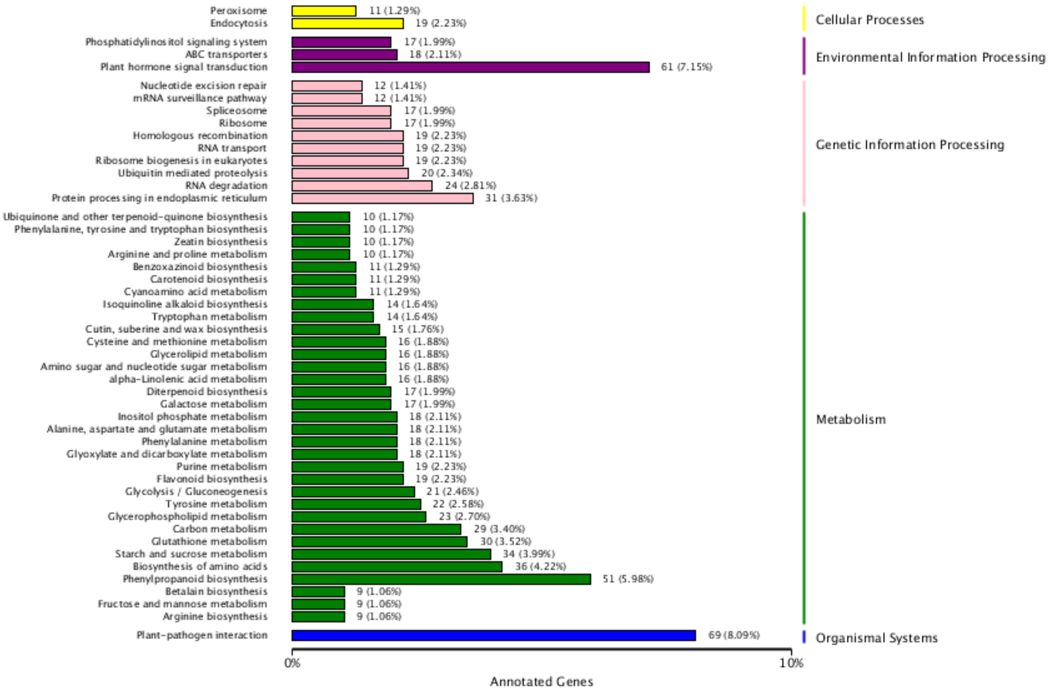

Supplementary Figure 11. All up-regulated DEGs that interacted in different pathways.

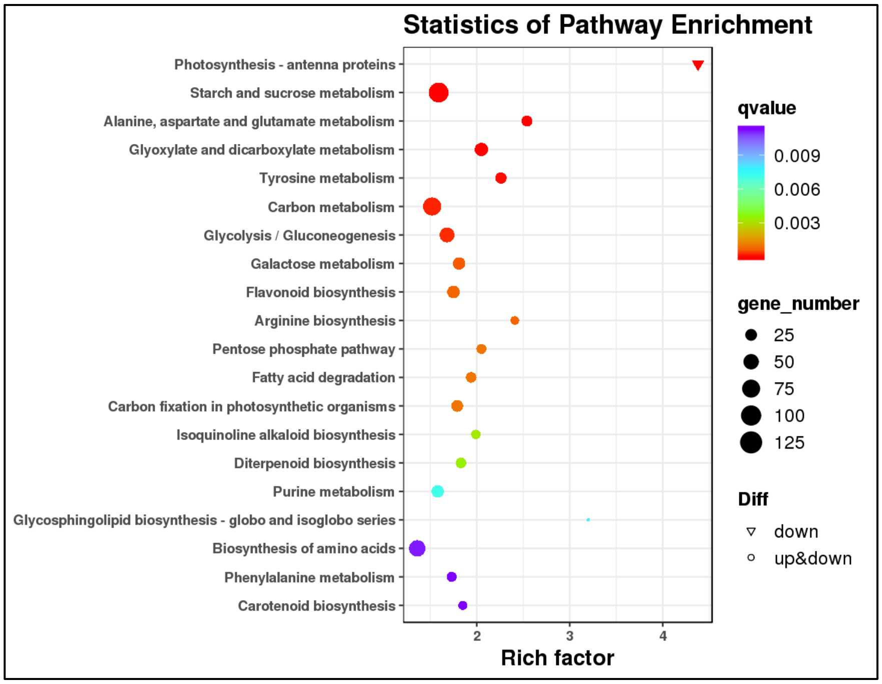

Supplementary Figure 12. Statistics of KEGG (Kyoto Encyclopedia of Genes and Genomes) classification of differentially expressed genes.
