# Supplementary material for: Molecular mechanisms of how black barley accumulates higher anthocyanins than blue barley following transcriptomic evaluation and expression analysis of key genes in anthocyanins biosynthesis pathway
Source: Front Plant Sci. 2025 Aug 29;16:1650803. doi: 10.3389/fpls.2025.1650803 (PMC12427265; doi:10.3389/fpls.2025.1650803)
Supplement: Supplementary file 1 [file Supplementaryfile1.zip › Supplementary Material/Data Sheet 10.PDF]

Supplementary Table 9a. Top 10 enriched pathways of up-regulated DEGs

ID	Pathway	GeneRatio	BgRatio	enrich_fact	pvalue
ko00940	Phenylpropanoid biosynthesis	5.98%	4.29%	1.39	0.009737
ko01230	Biosynthesis of amino acids	4.22%	2.98%	1.42	0.021623
ko00480	Glutathione metabolism	3.52%	1.57%	2.24	2.49E-05
ko00350	Tyrosine metabolism	2.58%	0.77%	3.34	4.12E-07
ko00941	Flavonoid biosynthesis	2.23%	1.28%	1.74	0.012735
ko00230	Purine metabolism	2.23%	1.36%	1.64	0.02254
ko03008	Ribosome biogenesis in eukaryotes	2.23%	1.36%	1.63	0.023772
ko00250	Alanine, aspartate and glutamate metabolism	2.11%	0.65%	3.26	6.64E-06
ko00630	Glyoxylate and dicarboxylate metabolism	2.11%	1.24%	1.7	0.018686
ko00360	Phenylalanine metabolism	2.11%	0.78%	2.71	9.33E-05

Supplementary Table 9b. KEGG enriched pathways of down-regulated DEGs

ID	Description	GeneRatio	BgRatio	enrich_fact	pvalue
ko00196	Photosynthesis - antenna proteins	2.08%	0.27%	7.63	9.90E-18
ko00500	Starch and sucrose metabolism	7.89%	3.92%	2.02	4.29E-11
ko01200	Carbon metabolism	6.24%	3.31%	1.88	8.89E-08
ko00710	Carbon fixation in photosynthetic organisms	2.69%	1.08%	2.48	1.58E-06
ko00630	Glyoxylate and dicarboxylate metabolism	2.86%	1.24%	2.31	3.97E-06
ko00010	Glycolysis / Gluconeogenesis	3.82%	1.93%	1.97	8.77E-06
ko00071	Fatty acid degradation	1.99%	0.80%	2.51	2.83E-05
ko00030	Pentose phosphate pathway	1.73%	0.66%	2.65	4.07E-05
ko00520	Amino sugar and nucleotide sugar metabolism	4.08%	2.34%	1.74	0.000115
ko00620	Pyruvate metabolism	2.34%	1.15%	2.03	0.000288
ko00903	Limonene and pinene degradation	0.52%	0.10%	5.13	0.000514
ko00052	Galactose metabolism	2.25%	1.19%	1.9	0.001016
ko00280	Valine, leucine and isoleucine degradation	1.65%	0.79%	2.09	0.001499
ko00460	Cyanoamino acid metabolism	2.08%	1.09%	1.91	0.001511
ko00603	Glycosphingolipid biosynthesis - globo and isogl	0.52%	0.12%	4.17	0.001896
ko00730	Thiamine metabolism	0.87%	0.32%	2.71	0.002804
ko00941	Flavonoid biosynthesis	2.25%	1.28%	1.76	0.003076
ko00860	Porphyrin and chlorophyll metabolism	1.47%	0.73%	2.03	0.003549
ko00061	Fatty acid biosynthesis	1.21%	0.56%	2.16	0.004398
ko01212	Fatty acid metabolism	1.91%	1.06%	1.8	0.004827

qvalue	geneID	gene_numb	Contained
0.105573	HORVU0H	51	up
0.146337	HORVU0H	36	up
0.000898	HORVU0H	30	up
4.46E-05	HORVU0H	22	up
0.115066	HORVU0H	19	up
0.146337	HORVU0H	19	up
0.146337	HORVU1H	19	up
0.00036	HORVU1H	18	up
0.146337	HORVU0H	18	up
0.00253	HORVU1H	18	up

qvalue	geneID	gene_numb	Contained
9.79E-16	HORVU1H	24	down
2.12E-09	HORVU0H	91	down
2.93E-06	HORVU0H	72	down
3.91E-05	HORVU0H	31	down
7.85E-05	HORVU0H	33	down
0.000145	HORVU0H	44	down
0.000401	HORVU1H	23	down
0.000503	HORVU0H	20	down
0.001266	HORVU0H	47	down
0.002853	HORVU0H	27	down
0.004627	HORVU2H	6	down
0.008378	HORVU0H	26	down
0.010678	HORVU1H	19	down
0.010678	HORVU0H	24	down
0.012506	HORVU0H	6	down
0.01734	HORVU1H	10	down
0.017905	HORVU1H	26	down
0.019508	HORVU0H	17	down
0.022905	HORVU1H	14	down
0.02388	HORVU1H	22	down

Supplementary Table 9c. Expression FPKM values of all DEGs.

#ID	GB-1	GB-2	GB-3	GH-1	GH-2	GH-3
HORVU7Hr1G100410	0.519567	0.083803	0.738544	1.9383	15.2213	15.53152
Hordeum_vulgare_newGene_6078	160.8344	169.4007	229.9403	43.01796	23.22261	27.95491
HORVU4Hr1G019520	0.076441	0.079138	0.204621	0.829558	1.796616	2.460529
Hordeum_vulgare_newGene_3756	0	0	0	4.18593	2.974805	3.581227
HORVU3Hr1G076940	0.349427	0.405971	0.429857	1.865517	5.211093	4.111997
Hordeum_vulgare_newGene_14186	1.112001	1.171801	2.117926	5.007684	4.67859	6.1486
Hordeum_vulgare_newGene_3759	0.748128	1.100556	1.019826	0.272618	0.304354	0.313279
Hordeum_vulgare_newGene_14180	3.243686	1.712239	2.959024	10.2215	8.752481	9.691227
HORVU6Hr1G058740	7.542984	9.238899	10.19805	4.859691	3.724931	4.642581
HORVU3Hr1G087430	0.749144	0.939607	0.86117	2.107389	3.004082	3.061704
HORVU7Hr1G008700	0	0	0.072139	2.02808	8.717706	8.785566
HORVU2Hr1G022960	1.50248	1.278798	0.873304	3.346787	3.683378	3.66532
Hordeum_vulgare_newGene_481	1.795495	2.261449	1.118561	0.071028	0.058091	0.090821
Hordeum_vulgare_newGene_486	0.161421	0.126671	0.10272	0.580963	0.796546	0.588052
HORVU3Hr1G111710	16.65565	16.56785	20.89895	12.06286	7.793631	8.703821
HORVU6Hr1G068720	10.92355	14.22175	11.88118	6.879358	7.494328	7.634486
HORVU7Hr1G035300	1.818964	1.426962	1.460725	7.289806	18.95552	13.08219
HORVU6Hr1G064430	0.085092	0.120234	0.060466	1.087194	2.365895	2.522847
Hordeum_vulgare_newGene_5759	0	0	0	4.79544	4.78694	5.778624
HORVU2Hr1G040910	0.111755	0.205384	0.28958	1.127796	1.157261	1.463227
HORVU2Hr1G004720	2.468374	1.408859	3.773045	1.027401	0.277892	0.654248
HORVU3Hr1G073940	0.3056	0.395863	0.338466	0.751149	1.520711	1.696081
HORVU4Hr1G076420	50.0227	51.72947	68.17932	2.800832	0.553854	1.536042
HORVU1Hr1G062210	2.182459	1.498114	2.753329	4.396537	7.064521	7.52983
HORVU2Hr1G073760	35.07501	30.88752	39.37736	79.24201	163.8941	158.3477
Hordeum_vulgare_newGene_1185	6.735232	5.190926	5.979586	18.0866	17.4475	18.38131
HORVU1Hr1G072430	6.686619	6.640702	6.225436	4.708837	2.982275	3.666634
HORVU3Hr1G070750	3.688617	2.879927	2.866073	1.746801	0.92071	1.199251
HORVU2Hr1G042520	0.460709	0.826768	0.748487	0.231555	0.018882	0.123011
HORVU1Hr1G076970	6.782596	6.202484	6.718065	2.561861	3.002141	3.213107
HORVU6Hr1G062820	0.679532	0.544011	0.712603	0.119756	0.416827	0.335745
HORVU5Hr1G068060	21.84419	11.62226	28.47921	9.330186	4.172189	9.103764
HORVU4Hr1G072850	11.89015	9.009773	9.808844	5.818176	3.697787	4.771887
Hordeum_vulgare_newGene_664	0.904964	1.241008	0.903988	0	0.001131	0
HORVU1Hr1G074530	2.197302	3.469146	3.43487	0.253339	0	0.094316
HORVU1Hr1G056560	6.772941	7.204537	7.075051	19.66054	22.82939	21.3044
Hordeum_vulgare_newGene_7060	0.214687	0.412055	0.235729	0.940402	0.649221	0.765997
HORVU5Hr1G012950	34.93252	31.03755	32.47427	69.12779	144.7492	125.4371
Hordeum_vulgare_newGene_6333	0	0	0	3.482359	4.646718	4.432506
HORVU0Hr1G024100	5.645631	5.777819	5.695806	0	0	0
HORVU1Hr1G088780	20.69389	19.37028	53.34999	127.1444	80.74403	69.68314
HORVU5Hr1G078630	1.035305	0.692459	1.224414	0.694883	0.356815	0.573652
HORVU6Hr1G083490	3.789156	3.766008	3.512767	0.24092	0.300151	0.408898
HORVU1Hr1G011720	0.397476	0.380545	0.447105	1.254371	8.82907	7.393462
HORVU5Hr1G106110	1.495474	1.705444	2.575272	0.560191	0.706863	0.902872

HORVU1Hr1G062970	0.575822	0.814688	0.829483	2.717665	5.90843	4.964641
HORVU5Hr1G094300	151.469	104.3379	142.9998	93.3341	74.77065	68.00931
HORVU7Hr1G069660	5.148136	5.458452	4.648616	12.75197	19.3277	17.65925
HORVU1Hr1G089780	1.038093	0.686294	1.06673	3.14699	3.061424	3.707406
HORVU3Hr1G092430	4.062932	4.186208	4.896437	24.34957	40.02198	41.61869
HORVU3Hr1G041030	2.84933	3.439118	2.608168	4.801108	6.100705	5.572236
HORVU7Hr1G057100	2.670328	4.065378	4.128111	0.119279	0.418246	0.318434
HORVU1Hr1G092670	62.68999	59.3375	86.03079	46.21898	31.89955	36.25846
HORVU2Hr1G123110	0.061024	0.059077	0.036581	0.323127	0.390017	0.402736
HORVU5Hr1G085250	6.093951	13.53534	24.76798	0.99668	1.720314	1.774596
HORVU3Hr1G009510	1.774223	2.412183	3.686761	72.1732	72.32787	91.94751
HORVU6Hr1G000090	0.079692	0.041785	0.012662	1.528757	3.921331	4.212237
HORVU6Hr1G005250	0.024394	0.089836	0.043956	0.670955	2.00562	2.183199
HORVU6Hr1G085320	0.10161	0.016297	0.045149	8.51032	11.75028	13.61881
HORVU4Hr1G057360	1.663598	1.351369	1.640623	6.212914	3.407122	4.445434
HORVU1Hr1G015610	17.06527	17.53984	19.60952	0	0	0
HORVU1Hr1G010790	1.807531	1.165903	1.563607	3.50972	5.666058	4.978509
HORVU7Hr1G085450	3.243896	3.545341	4.3519	9.200678	11.19711	15.43827
HORVU2Hr1G045810	2.607022	3.239278	4.252447	2.221636	1.405077	1.804759
HORVU6Hr1G004120	3.819853	1.356277	5.790363	0.21782	0.256429	0.359183
HORVU4Hr1G064850	0.683303	0.847386	1.032785	0.299669	0.29723	0.227477
HORVU1Hr1G016140	2.454632	2.141669	3.381992	0.969534	0.950458	1.060416
HORVU6Hr1G016710	13.87448	8.832353	12.96291	39.04999	36.45702	32.13632
HORVU5Hr1G115720	5.462039	3.719521	6.873629	1.209479	1.609517	1.860772
HORVU3Hr1G077570	0.148557	0	0.03365	1.178236	1.526367	1.937395
HORVU4Hr1G054970	12.99715	13.12851	10.33519	5.368572	3.087362	2.994306
HORVU3Hr1G064760	1.85918	2.021184	2.261595	0.090664	0.120222	0.051409
HORVU3Hr1G099740	2.205667	2.953127	3.255988	0.911148	0.748627	1.144512
HORVU4Hr1G009340	4.456398	3.535449	4.591662	9.560271	12.50654	11.75168
HORVU3Hr1G092840	1.265075	0.987344	1.394081	3.165317	3.172956	3.310953
HORVU6Hr1G013530	0.071492	0.141098	0.345469	1.118424	9.031116	6.85714
HORVU7Hr1G057650	60.36113	53.86444	38.22489	3.787646	1.78899	2.342011
Hordeum_vulgare_newGene_8143	1.297878	0.970672	1.106077	2.556925	4.521684	4.04926
Hordeum_vulgare_newGene_8140	1.387177	1.689986	1.45911	0	0	0
Hordeum_vulgare_newGene_8146	0.964746	1.549546	1.407135	0.368861	0.350651	0.312868
HORVU7Hr1G089280	2.418061	2.993935	2.252493	5.349104	22.14536	19.07077
HORVU2Hr1G122580	1.694495	1.004363	2.43502	0.41192	0.120638	0.488646
HORVU2Hr1G115600	7.68603	9.053843	9.2145	16.44795	28.32608	25.90692
HORVU2Hr1G104880	0.849838	0.815715	1.28719	0.976624	3.844547	4.488578
HORVU5Hr1G006910	12.82505	9.58352	15.08979	34.48287	43.83212	37.61452
HORVU2Hr1G073210	10.9426	7.313111	7.508002	34.41994	17.24841	21.03044
HORVU3Hr1G089420	1.999566	1.963333	4.742805	0.466791	0	0.040772
HORVU5Hr1G087930	0.485551	0.681965	0.436954	1.459867	1.874504	2.166987
HORVU4Hr1G008550	7.171636	8.646582	10.2022	2.711472	3.536268	3.923193
Hordeum_vulgare_newGene_2303	0	0	0.002462	2.224644	2.485637	2.240666
Hordeum_vulgare_newGene_2305	7.844241	7.204192	8.321982	0.004445	0	0
HORVU3Hr1G050770	1.294843	1.262482	1.177577	4.212679	5.468564	3.771865

Hordeum_vulgare_newGene_1279	1.56721	2.140591	2.164118	0.943817	0.682421	1.091135
HORVU6Hr1G012340	1.931117	1.425764	1.945995	0.618662	0.41638	0.49074
Hordeum_vulgare_newGene_9372	1.995431	2.094513	2.213673	9.027668	8.77907	8.146848
HORVU3Hr1G054100	0.092866	0.144464	0.04504	0.645419	1.275084	1.508725
HORVU1Hr1G054170	0.776939	0.494849	0.914167	0.445868	0.369571	0.308915
HORVU7Hr1G059130	5.410092	4.498657	5.103568	13.21914	19.38478	19.71555
HORVU3Hr1G011870	64.42008	42.37001	81.58424	10.82425	1.491691	7.147869
Hordeum_vulgare_newGene_8426	0.089369	0.029017	0.1656	1.280316	1.790476	1.703004
HORVU2Hr1G004550	30.03324	20.4372	38.49728	5.319626	1.071736	5.654036
HORVU3Hr1G004250	55.04682	77.48697	104.8472	2.747022	0.222555	0.394589
Hordeum_vulgare_newGene_1029	2.643342	2.964104	3.017334	0.057919	0.044685	0
HORVU0Hr1G019590	5.934137	4.635121	5.793535	3.604621	1.430911	1.766378
HORVU0Hr1G030660	9.532468	14.30292	16.12164	1.081784	0.064402	0.502135
HORVU6Hr1G020520	86.17664	79.97061	69.67044	35.7745	32.79763	31.75923
HORVU7Hr1G101500	2.703509	2.844208	2.786674	6.481604	12.83818	12.1953
HORVU1Hr1G075580	4.033792	5.876248	8.479688	0.916922	0.617737	0.733086
HORVU1Hr1G078350	1.244602	1.78228	1.951849	33.62798	68.06334	63.95725
Hordeum_vulgare_newGene_7743	0.503074	0.761963	0.670201	0	0	0
HORVU1Hr1G057880	33.86678	44.92851	50.75799	10.23969	4.732215	6.188369
HORVU4Hr1G001570	2.568188	1.960138	2.614681	0	0	0
HORVU4Hr1G073730	1.48678	1.530208	1.443179	5.23716	5.969701	5.79539
HORVU6Hr1G022500	0.576412	0.714985	1.247909	0.379159	0.025414	0.120454
HORVU4Hr1G048440	0.748016	0.708364	0.697801	3.135651	4.471059	4.628247
HORVU5Hr1G013210	1.217705	1.364524	1.498938	2.991095	9.921424	9.268975
HORVU7Hr1G056630	216.6535	197.7935	196.3881	118.0487	49.22915	61.47089
HORVU7Hr1G054980	0.130059	0.242495	0.402087	3.288019	2.336948	1.889713
HORVU6Hr1G085500	0.539101	0.601304	0.896772	1.513822	2.995413	3.284011
HORVU5Hr1G062820	0.34067	0.732646	0.409579	1.682976	1.093525	1.613829
HORVU0Hr1G035930	2.304698	1.88224	3.056104	1.078633	1.178689	0.859035
HORVU4Hr1G006530	3.027139	2.962703	3.393685	5.391183	12.97651	11.31207
HORVU7Hr1G040720	0.814876	0.484026	0.648601	2.759813	3.841145	3.204482
HORVU5Hr1G018830	0.688768	0.424328	0.847935	3.338853	6.050594	5.427663
HORVU5Hr1G075260	9.679578	9.395968	13.34183	7.646597	3.198021	6.306694
HORVU3Hr1G082460	12.71762	12.21565	15.23233	9.944491	2.448451	4.909295
HORVU5Hr1G071060	0.771074	1.099274	0.997928	1.322027	3.209835	3.605971
HORVU1Hr1G018710	19.31652	21.23274	19.29955	87.08151	121.9317	83.72511
HORVU2Hr1G085430	0.868741	0.855487	1.668918	0.338329	0.049302	0.461418
HORVU5Hr1G121600	0.707722	0.568209	0.593478	22.74617	43.58387	43.68309
HORVU5Hr1G105650	4.021574	3.855403	4.623493	15.40108	11.99604	9.65182
HORVU3Hr1G031460	8.584223	9.247656	11.70254	31.90966	57.31806	55.84853
HORVU0Hr1G007340	21.82358	15.03559	19.17027	3.387922	0.58665	1.455858
Hordeum_vulgare_newGene_12899	3.028681	3.628798	1.612149	5.185379	4.943204	5.478463
HORVU2Hr1G012550	1.081626	1.075189	0.659087	0.041246	0.138304	0.23359
HORVU0Hr1G040540	3.652533	3.852389	4.508625	23.02569	19.2143	18.20676
HORVU2Hr1G006130	1.772804	1.425566	2.253429	84.2361	1.323309	16.93777
HORVU0Hr1G024790	1.119574	1.173556	0.873138	3.95815	26.89719	42.32737
HORVU2Hr1G096130	2.063913	1.1817	2.32877	13.07387	62.94988	52.18538

HORVU1Hr1G006780	4.602324	1.715551	1.244571	1.268102	0.448321	0.531446
Hordeum_vulgare_newGene_13273	5.386025	6.277556	5.506731	2.988213	1.98145	2.560868
Hordeum_vulgare_newGene_5495	0	0	0	2.87407	4.462202	4.159362
HORVU5Hr1G016270	0.172874	0.164456	0.199256	0.934692	1.053166	1.056368
HORVU5Hr1G073010	0.737167	0.749512	0.555384	5.128226	11.53531	10.0632
Hordeum_vulgare_newGene_13856	4.50958	5.123133	4.883248	3.33619	4.065512	4.015053
Hordeum_vulgare_newGene_13857	13.39338	12.16785	11.38362	7.348937	4.524213	6.282902
Hordeum_vulgare_newGene_13859	0.702088	0.746634	0.59062	1.021477	3.442425	3.096899
HORVU7Hr1G105910	0.369231	0.461172	0.40972	2.173435	36.96904	31.04804
HORVU2Hr1G014140	3.380919	3.891994	3.199622	11.61543	11.54802	11.88771
HORVU2Hr1G090200	0.077494	0.194587	0.031924	0.730761	3.392581	2.382993
HORVU2Hr1G034540	0.625566	0.465484	0.437884	2.375884	3.688503	3.45721
HORVU2Hr1G102720	6.965954	2.323365	11.19845	0.667775	0.024154	1.165446
HORVU7Hr1G050980	0.459695	0.37594	0.453859	1.182473	2.604842	2.64914
HORVU6Hr1G036510	0.445987	0.336179	0.629463	1.559716	2.18652	1.461209
Hordeum_vulgare_newGene_7182	2.726797	1.551239	2.353956	4.392789	7.821942	7.261228
HORVU2Hr1G103890	4.431324	3.4224	6.435227	3.097887	2.150129	2.745686
Hordeum_vulgare_newGene_1822	2.262627	2.792579	1.920079	0.6336	0.383762	0.290505
HORVU3Hr1G014880	3.374576	2.666563	4.420594	0.060039	0	0
HORVU6Hr1G003170	156.0606	134.411	174.5822	111.528	81.61521	89.37232
HORVU1Hr1G029460	3.069805	3.10662	4.465367	2.144129	1.137002	1.83156
HORVU3Hr1G067760	0.646512	0.748407	1.212352	2.533326	3.331635	3.269047
HORVU7Hr1G043300	0.420458	0.362953	0.348831	2.656484	8.712784	8.13126
Hordeum_vulgare_newGene_527	0	0	0	5.918862	4.359266	3.966349
Hordeum_vulgare_newGene_529	1.100913	1.512837	1.492236	0.270061	0.300916	0.235598
HORVU4Hr1G074620	0.122132	0.129951	0.214019	3.004596	3.439992	3.623367
HORVU0Hr1G014520	0.072383	0.088636	0.054701	2.945478	118.9677	157.7692
HORVU3Hr1G093100	0.360085	0.962716	0.472114	5.446644	3.697573	9.586932
HORVU5Hr1G041400	9.086824	7.241927	9.105471	3.929047	1.536836	2.085205
HORVU5Hr1G012500	0.036141	0.183792	0.205857	5.894102	5.672198	5.824843
HORVU3Hr1G113000	11.12175	11.26946	12.88334	7.080759	6.651414	6.171501
HORVU3Hr1G117580	9.336016	5.492409	7.298362	1.689048	0.829755	3.276959
HORVU3Hr1G087940	1.03761	0.974093	1.377683	3.213011	3.323547	3.123288
HORVU2Hr1G019830	5.766245	7.466662	9.161347	2.583595	2.641389	2.825766
Hordeum_vulgare_newGene_13136	0.943102	1.50367	1.58676	0.404282	0.411874	0.510901
HORVU5Hr1G075330	0.203275	0.033876	0.058134	0.424798	0.658124	0.847152
HORVU7Hr1G060130	33.83218	29.50323	32.5213	76.93852	113.6872	111.1168
HORVU3Hr1G117630	10.5091	9.237551	11.93913	1.552151	1.644373	1.797328
HORVU0Hr1G008580	0.781642	0.820182	0.806652	3.292553	10.49792	9.554286
Hordeum_vulgare_newGene_3585	0.701723	0.873094	0.861387	0	0	0
HORVU2Hr1G005520	13.09814	11.91914	17.16977	4.581561	3.825131	5.400556
HORVU3Hr1G003060	2.27226	1.937022	2.355826	6.157319	7.111837	6.831282
HORVU2Hr1G040600	0	0	0	0.591344	1.357524	1.200154
HORVU1Hr1G055580	89.64043	93.819	65.27866	52.22179	33.10037	38.41041
HORVU3Hr1G019590	0.695324	0.743862	0.751073	2.693624	3.432616	3.643757
HORVU7Hr1G049080	2.905522	2.433329	3.031853	2.059818	0.9845	1.545508
HORVU1Hr1G073050	0.603956	0.628565	0.498393	2.003269	3.559924	3.516225

HORVU0Hr1G017230	2.407406	2.261507	2.009672	1.356318	0.944958	1.26553
HORVU4Hr1G088280	2.361336	2.769392	4.041392	0.476497	0.069441	0.273424
HORVU1Hr1G075040	10.91024	8.761778	9.355236	0.671101	0.422368	0.477043
HORVU4Hr1G005150	7.720568	8.214743	8.790012	5.329558	3.994214	3.821476
HORVU5Hr1G038370	6.519777	11.06112	8.959444	2.761366	0.063962	1.073873
HORVU7Hr1G088210	10.6311	13.17282	10.65475	4.874482	4.125009	5.154282
HORVU3Hr1G047230	0.933157	0.781804	1.08455	3.086448	12.88595	11.41027
HORVU2Hr1G009160	0.202587	0.098515	0.246837	2.221776	3.068512	3.876434
HORVU1Hr1G056270	1.707658	1.3576	1.947835	0.770889	1.155732	1.48322
HORVU1Hr1G067840	0.164267	0.229411	0.161336	0.852752	0.68719	0.754122
HORVU2Hr1G107090	0.424394	0.467074	0.48756	0.031377	0.147941	0.141044
HORVU6Hr1G069940	6.873244	7.186795	9.60771	4.494002	3.119294	3.443199
HORVU7Hr1G003010	3.434874	1.10413	2.638972	16.70909	139.7567	97.96816
HORVU1Hr1G078140	0.120945	0.328186	0.197399	2.570683	2.421576	3.762859
HORVU2Hr1G015590	6.662837	10.61367	11.04069	0.382829	0.033098	0.132018
HORVU1Hr1G089830	17.25402	14.23658	22.34624	5.563106	3.074277	5.734382
HORVU2Hr1G090950	0.075892	0.169454	0.229689	0.321764	0.278245	0.672428
HORVU4Hr1G049920	2.304672	1.810776	3.202381	4.696056	8.735882	7.1941
HORVU7Hr1G110810	6.17478	4.972788	9.121527	12.12723	21.18331	18.47935
HORVU4Hr1G025330	2.76869	2.929231	2.462435	6.342768	7.020895	8.298856
HORVU5Hr1G125500	87.48526	87.66314	125.6044	12.98171	0.728408	5.317592
HORVU7Hr1G009310	2.890128	1.309169	3.573008	0.56215	0	0.388075
HORVU7Hr1G043810	0.308765	0.407938	0.303779	0.850591	1.791915	1.549175
HORVU5Hr1G124450	5.632657	4.195019	4.659362	12.4635	10.82654	13.03614
Hordeum_vulgare_newGene_1710	0.787841	0.69246	0.716765	0.182747	0.145284	0.300219
HORVU5Hr1G056030	1324.378	1238.999	1725.563	435.0489	144.9001	207.8365
Hordeum_vulgare_newGene_14687	202.3809	277.2404	356.6377	5.88747	0.436338	1.846459
HORVU5Hr1G116910	1.035119	0.794222	1.095067	0.286814	0.199114	0.426455
HORVU7Hr1G011290	11.75511	11.63616	17.23147	7.286578	6.964237	8.696577
Hordeum_vulgare_newGene_11745	0.955071	1.302079	1.043613	7.943676	25.80082	10.38799
HORVU1Hr1G079510	0.683233	1.052443	1.248988	0.027468	0.032629	0.592849
HORVU6Hr1G050860	0	0	0.047537	0.590811	0.640817	0.681261
HORVU7Hr1G040390	3.492402	3.720175	3.604712	8.867787	12.20624	12.02318
HORVU4Hr1G051400	28.39808	24.40351	29.40508	9.52786	7.261066	8.92413
HORVU5Hr1G076770	87.79599	53.59539	74.64931	54.73504	12.31367	19.18102
HORVU3Hr1G038220	0	0	0	6.101079	5.023347	7.610142
Hordeum_vulgare_newGene_16215	1.190021	1.14573	0.905314	0.402894	0.443173	0.29766
HORVU2Hr1G057700	73.93575	57.78064	62.4465	150.6642	233.3865	227.2611
HORVU5Hr1G016780	1.33586	0.798735	1.285181	6.934563	11.12102	8.97086
HORVU7Hr1G080020	0.42479	0.164747	0.38031	1.28613	2.965374	2.337163
HORVU3Hr1G088700	1.024938	0.581067	0.918391	1.376679	4.315104	4.444196
HORVU3Hr1G023480	0.216227	0.144621	0.238567	0.538086	0.848745	0.951344
HORVU7Hr1G029620	0.340028	0.61046	0.603521	8.63641	7.788639	7.53644
HORVU4Hr1G053130	1.38322	1.332449	1.988259	4.724209	6.311209	6.50895
HORVU3Hr1G076080	120.5652	114.7353	134.5118	78.83712	66.12523	58.05535
HORVU2Hr1G060010	16.57434	14.31594	13.83428	38.32857	45.79674	48.24391
HORVU0Hr1G021280	2.004171	1.332946	1.915595	7.361509	7.787665	4.678987

HORVU7Hr1G046230	2.364694	2.97107	4.45515	1.077115	0.423329	0.912191
HORVU5Hr1G109710	17.66304	13.82604	14.76322	53.96049	82.25642	76.49728
Hordeum_vulgare_newGene_12462	1.534254	1.03061	1.191483	0.222013	0.117232	0.148474
Hordeum_vulgare_newGene_12461	0.831654	0.670923	0.723989	0.199846	0.07447	0.091911
HORVU7Hr1G002200	1.371604	1.091199	1.216155	3.889501	3.813906	3.563147
HORVU1Hr1G022530	0.050815	0.118546	0.10767	0.406053	0.570511	0.420373
HORVU2Hr1G117120	2.470075	2.525716	2.834105	0.721253	0.44017	0.860159
HORVU6Hr1G065230	22.08714	17.60355	20.39632	12.28884	12.12434	11.58836
HORVU6Hr1G056850	1.227131	1.618681	1.846012	0.455966	0.873113	1.143888
Hordeum_vulgare_newGene_10827	4.621334	1.045478	1.862391	0.158336	0.060713	0.044561
Hordeum_vulgare_newGene_10828	33.70737	25.11041	34.1622	7.995784	7.639057	6.949296
HORVU3Hr1G096280	7.242078	3.954869	9.755379	3.269121	2.16636	3.814622
HORVU5Hr1G085120	16.60535	20.92502	28.70704	8.766048	15.1444	15.40439
Hordeum_vulgare_newGene_5861	5.05062	3.903627	5.384203	2.319727	2.384958	2.287365
Hordeum_vulgare_newGene_5866	1.456636	1.854038	2.309986	0.175006	0.413066	0.22909
Hordeum_vulgare_newGene_5865	1.268008	1.118756	1.554609	0.719893	0.347102	0.530435
HORVU6Hr1G078910	1.986416	1.344197	2.011102	0.583381	0.874074	0.911021
Hordeum_vulgare_newGene_2484	0.050114	0.148925	0.139143	0.56836	0.392713	0.550841
Hordeum_vulgare_newGene_2481	0	0	0.006658	5.046153	4.787979	5.078334
HORVU3Hr1G013860	61.9968	81.24932	90.00763	43.52135	28.49076	32.53481
HORVU3Hr1G031620	15.90835	9.288516	12.72445	8.11137	6.301966	5.996488
Hordeum_vulgare_newGene_5289	0.484931	0.524678	0.459974	1.699872	2.544744	2.129693
Hordeum_vulgare_newGene_9706	23.21683	27.1012	30.94181	16.08631	15.31953	18.91172
Hordeum_vulgare_newGene_5137	3.481641	3.407291	5.492174	1.673189	1.535424	1.970997
HORVU6Hr1G016330	0.653594	0.949813	1.125263	0.205151	0.012281	0.16873
HORVU2Hr1G069490	19.52092	15.91401	19.33427	9.460156	10.0875	5.921307
HORVU3Hr1G012800	0	0.004111	0.04797	0.536645	0.979503	0.99602
HORVU7Hr1G106420	2.759436	1.996117	3.24508	1.391209	0.977829	1.384725
Hordeum_vulgare_newGene_8076	0.278597	0.221044	0.295969	2.444986	2.457988	2.990166
Hordeum_vulgare_newGene_8073	1.612641	2.208704	0.965884	0.414796	0.058208	0.258704
HORVU4Hr1G076200	2.975942	4.125685	4.57815	2.126402	1.370391	1.641139
HORVU2Hr1G028470	3.3089	2.928379	3.627814	5.484371	13.45273	11.81554
Hordeum_vulgare_newGene_14232	2.292157	2.443096	2.68941	8.70068	7.934927	8.271223
Hordeum_vulgare_newGene_3853	24.89783	21.09515	28.60109	46.41519	106.6321	91.68773
Hordeum_vulgare_newGene_7574	0	0	0	0.896375	1.228955	1.162245
HORVU5Hr1G082610	0.288437	0.101287	0.131251	0.522856	0.638566	0.723147
HORVU7Hr1G056310	0	0	0	5.652837	4.215455	5.692176
Hordeum_vulgare_newGene_12144	0.289341	0.300377	0.268238	1.549531	1.65134	0.900699
Hordeum_vulgare_newGene_12146	28.86453	24.2172	27.29351	14.97673	7.885279	9.473052
Hordeum_vulgare_newGene_6628	0.469851	0.101138	0.293768	1.157099	1.226404	1.253243
HORVU4Hr1G013370	2.107643	2.028559	1.950922	3.685576	6.481768	6.916373
HORVU5Hr1G075270	1.829805	2.024556	2.123641	6.499171	9.470958	9.222286
HORVU3Hr1G113210	0	0	0	2.739149	2.311723	3.906655
Hordeum_vulgare_newGene_2817	1.177875	1.350544	2.456229	31.42902	19.66942	27.60415
Hordeum_vulgare_newGene_2819	2.065224	2.561131	2.182802	0	0	0
HORVU1Hr1G089700	3.484824	3.21248	6.058573	0.263212	0	0.526321
HORVU5Hr1G062410	13.24229	9.890821	10.87059	35.65874	43.30037	45.89705

HORVU3Hr1G088190	0.954825	0.418833	0.888829	8.806326	8.690937	4.461746
HORVU3Hr1G082580	21.4217	17.47653	25.24689	9.897292	8.050872	9.295036
HORVU5Hr1G039960	1.123173	1.658414	1.616308	3.44549	5.112639	5.049519
HORVU2Hr1G085280	4.977337	3.701375	7.858189	26.35873	21.07122	37.1108
HORVU4Hr1G082040	15.3776	18.10612	11.80574	52.86175	68.66755	77.10108
HORVU0Hr1G017370	14.86831	12.83653	18.61686	32.56099	48.30229	51.64911
HORVU2Hr1G076060	7.832018	7.160264	9.117754	3.422043	1.536089	2.800188
HORVU4Hr1G031220	0	0	0	3.405833	2.810772	2.862864
HORVU1Hr1G079450	1.946265	1.681424	2.12798	0.540354	0.539273	0.494392
HORVU3Hr1G054240	1.200723	0.988585	1.206548	4.881062	10.81056	9.221254
HORVU4Hr1G085740	4.467202	4.21152	4.791792	2.516818	2.95338	2.482795
HORVU1Hr1G049440	0.030452	0.209851	0.143763	1.229674	1.024431	0.967659
HORVU4Hr1G012760	2.838242	4.968966	5.778819	0.453652	0.125538	0.463192
HORVU7Hr1G017620	0.299136	0.168127	0.395186	4.603034	10.2438	7.545781
HORVU4Hr1G064580	0.865315	1.275467	1.107654	4.043289	7.906944	7.069383
HORVU2Hr1G100720	1.357122	1.381965	1.42324	3.96535	9.199176	6.647812
HORVU7Hr1G110130	0.383717	0.341489	0.883631	4.81259	16.8129	17.63132
HORVU0Hr1G015840	0.976664	0.232944	0.909179	2.273263	2.848717	2.599923
HORVU3Hr1G079180	1.525336	1.245486	1.23867	4.09923	4.212358	5.075205
HORVU4Hr1G084830	2.508836	2.143502	2.302116	6.113078	12.70174	12.33626
HORVU1Hr1G039670	2.792171	2.187188	2.656506	4.586898	7.902438	7.800368
HORVU2Hr1G001600	19.25084	16.9008	21.3511	30.15657	61.33824	56.45848
HORVU7Hr1G018660	40.54533	36.93761	47.17151	17.28164	21.26741	21.052
HORVU3Hr1G074780	2.235622	3.009977	1.86751	0.379134	0.215991	0.029553
HORVU4Hr1G090160	1.46127	1.668675	1.851422	1.071185	0.855762	1.049426
Hordeum_vulgare_newGene_8522	54.57255	48.1687	51.74001	9.867384	0.254971	4.005111
HORVU5Hr1G116890	1.301892	1.187371	1.343531	0.229001	0.266648	0.275088
HORVU2Hr1G093210	0.322671	0.264164	0.199027	1.452979	1.931304	1.516267
HORVU1Hr1G081810	0.466368	0.586241	0.809656	0.156969	0.220622	0.501609
HORVU6Hr1G077150	0	0	0	0.437573	0.608462	0.782483
HORVU3Hr1G029200	0.661668	0.668807	0.70098	2.279726	2.535608	2.663961
HORVU7Hr1G030080	0.858635	0.67817	0.627766	1.895827	1.414086	1.899222
HORVU3Hr1G077250	3.839889	4.035558	5.093527	13.54109	26.84851	24.72994
HORVU0Hr1G016180	1.099264	1.012789	1.548301	0.723447	0.670811	0.788402
HORVU3Hr1G023560	0.248607	0.181463	0.176414	0.634302	0.616335	1.029617
HORVU5Hr1G076250	2.924743	1.77973	2.683643	4.853702	5.257597	8.788056
HORVU2Hr1G028670	62.27834	29.35126	54.84979	2.345957	0.752936	2.006353
HORVU3Hr1G057690	31.31265	24.48512	39.31059	7.267543	0.236802	2.47239
HORVU1Hr1G042800	1.532421	0.812326	1.285554	3.305005	5.352357	5.018411
HORVU6Hr1G062040	2.209828	2.088665	2.435394	7.131749	13.36057	12.58656
HORVU2Hr1G038940	3.814637	4.013438	2.336408	35.25893	45.50709	48.61566
Hordeum_vulgare_newGene_7801	7.10154	7.957103	7.486657	0.253451	0.109741	0.07571
Hordeum_vulgare_newGene_3389	1.05618	1.317462	1.369493	2.133907	6.183837	5.183686
HORVU5Hr1G061300	6.153422	5.702973	7.405256	2.580187	3.170869	3.492169
HORVU3Hr1G017220	3.095113	4.689912	4.439017	2.091735	2.408707	2.29077
HORVU2Hr1G103130	7.572127	8.137231	7.743203	22.61919	29.85781	30.37069
Hordeum_vulgare_newGene_13780	0.804813	0.441107	0.475343	5.255496	4.272164	5.341409

HORVU3Hr1G116550	2.090845	1.461077	4.011156	6.756697	29.57189	29.31932
Hordeum_vulgare_newGene_2382	0	0	0	1.691723	3.26457	1.921706
Hordeum_vulgare_newGene_2381	0.311652	0.121674	0.288841	1.8293	3.301715	2.41873
Hordeum_vulgare_newGene_2385	35.10052	40.49951	32.53485	14.66351	9.684687	12.607
Hordeum_vulgare_newGene_14316	0.618078	0.516881	0.711473	0.066079	0.068856	0.08294
Hordeum_vulgare_newGene_14315	0.485888	0.635107	0.579519	0.047513	0.034911	0.084644
Hordeum_vulgare_newGene_14313	2.82655	3.198923	4.012562	1.345699	1.908699	1.948757
HORVU2Hr1G027150	1.29336	1.581041	2.152306	0.385933	0.601706	0.507291
Hordeum_vulgare_newGene_6863	0.542376	0.908147	0.358134	3.857457	3.448357	5.46543
HORVU1Hr1G070450	0.895299	0.878475	0.858337	0.334582	0.031119	0.23189
Hordeum_vulgare_newGene_11923	1.358562	1.544759	1.759974	0.417584	0.630491	0.523523
HORVU7Hr1G108460	0.314376	0.287175	0.345185	4.389724	1.647385	3.961997
HORVU3Hr1G111580	52.27626	49.01556	68.56213	17.37242	2.336469	6.668888
HORVU4Hr1G070530	89.3686	93.14124	62.77719	22.20234	14.89561	19.0051
HORVU4Hr1G075140	0.693021	0.785117	0.960969	1.930935	3.019949	2.593451
HORVU1Hr1G059090	11.27202	12.22937	10.18897	4.840777	4.373678	5.132251
HORVU1Hr1G043890	567.9733	509.0375	699.4182	192.6842	42.52606	86.22847
Hordeum_vulgare_newGene_9511	7.874198	8.754026	9.478092	5.748909	4.212102	5.305038
HORVU3Hr1G042600	4.306047	5.471649	4.820688	25.31087	41.9323	40.95538
HORVU7Hr1G114050	115.1459	118.6183	88.68972	75.11191	25.64038	33.7336
HORVU3Hr1G087100	8.976765	4.926256	8.510186	1.716161	4.111207	2.367336
HORVU1Hr1G052180	1032.138	970.5813	1362.51	48.52086	1.330628	16.57175
HORVU6Hr1G031480	139.894	166.6242	190.5111	100.1204	84.03485	92.39096
HORVU4Hr1G046620	0	0	0	6.900857	6.616634	8.217867
Hordeum_vulgare_newGene_14600	1929.142	1703.11	1562.473	438.8129	125.7681	183.1519
HORVU2Hr1G008440	1.377068	1.491638	1.46307	8.054225	8.959355	11.44789
HORVU2Hr1G068880	0.933996	0.570792	0.472859	1.755735	2.06406	3.328167
HORVU6Hr1G023070	0.508248	0.506119	0.50343	1.959425	2.176841	2.44101
HORVU2Hr1G045340	36.57948	34.11241	35.41739	1.955576	0.047638	0.485826
Hordeum_vulgare_newGene_6472	0.425208	0.262907	0.541228	1.870644	10.12783	8.809697
Hordeum_vulgare_newGene_6478	0.602682	0.647209	0.672433	0.23908	0.395676	0.31817
HORVU5Hr1G013290	5.870353	4.926818	6.074229	21.50886	26.92741	24.49273
HORVU5Hr1G059890	12.02819	6.497695	13.97849	5.061423	1.688622	1.588588
HORVU3Hr1G081570	7.016309	5.520087	7.755918	3.940172	1.95316	1.89871
HORVU3Hr1G034040	1.728971	1.527572	1.754382	8.702167	10.0013	9.471847
HORVU5Hr1G012160	36.6551	39.7223	49.00139	23.19804	24.55759	27.21872
HORVU6Hr1G054770	1.214375	1.056072	1.040872	0.752067	0.309617	0.416878
HORVU6Hr1G034570	3.58953	2.687379	4.387174	2.192799	1.250686	2.020224
HORVU4Hr1G042710	0.127122	0.227869	0.164125	4.201273	8.521811	3.641457
HORVU1Hr1G020410	12.33084	5.991024	15.8469	3.692912	2.329669	3.287272
HORVU2Hr1G051030	18.18581	17.77914	14.62906	11.33691	1.758723	3.874151
HORVU3Hr1G029040	25.03214	9.733249	7.159138	4.341444	7.06606	4.177242
HORVU4Hr1G013650	0.212594	0.128545	0.233082	0.481458	1.550146	1.142
HORVU5Hr1G106960	1.525501	1.574198	1.453133	4.407501	6.101605	6.671326
HORVU2Hr1G085570	1.643758	0.972681	1.804522	6.4461	14.69112	13.29303
HORVU1Hr1G048610	0.608803	0.435577	0.583551	3.141281	3.19962	1.435914
HORVU1Hr1G013880	19.30276	18.84726	20.37227	0.018864	0.010791	0.009598

HORVU7Hr1G046040	2.603667	2.532661	2.980996	8.113063	6.159238	7.893041
HORVU5Hr1G059070	2.110888	2.201181	1.881971	6.445481	6.134323	6.120014
HORVU3Hr1G071370	118.5229	76.5212	89.1766	0.275345	0	0
Hordeum_vulgare_newGene_12819	0	0	0.031131	0.75984	1.525378	2.038488
HORVU4Hr1G069990	2.365594	2.685218	3.310085	10.99555	10.87187	11.07413
HORVU7Hr1G084420	6.419588	6.074726	7.52265	23.43782	18.68819	21.11605
HORVU3Hr1G064640	10.2023	9.102357	10.3896	6.976452	4.536087	3.382038
HORVU7Hr1G006600	1.291143	1.422111	1.274538	5.5398	6.886853	6.554507
Hordeum_vulgare_newGene_14704	1.156122	1.392309	1.155071	0.193369	0	0.046754
Hordeum_vulgare_newGene_5416	2.169677	1.579963	1.746867	0.035971	0.066534	0.026846
HORVU2Hr1G010660	0.0161	0.129769	0.430804	19.05333	14.7622	19.02225
HORVU5Hr1G117590	2.543662	2.58528	2.495763	0	0	0.011661
Hordeum_vulgare_newGene_8620	0.895231	0.708381	0.783203	1.272927	4.33568	3.118586
HORVU3Hr1G076740	0.042054	0.03354	0.093858	1.198997	0.676095	1.202175
Hordeum_vulgare_newGene_10504	0.068711	0.143736	0.106993	4.842403	2.646289	3.714732
HORVU3Hr1G073050	0.163012	0.187871	0.110332	0.411176	1.18081	1.221556
HORVU5Hr1G123530	16.60334	15.10283	13.22876	8.060689	10.27067	9.402426
HORVU3Hr1G096360	8.36992	4.831701	8.718316	0.944647	0.572593	1.047553
HORVU5Hr1G098780	3.881408	4.144804	4.890181	13.79029	15.07506	16.01812
HORVU2Hr1G090280	19.40786	17.09014	17.91693	7.6662	2.748036	6.146992
HORVU6Hr1G091840	25.54831	33.1126	39.02603	4.285658	2.749922	3.774013
HORVU2Hr1G023690	11.98447	10.98729	13.70182	7.94055	3.281756	4.206328
HORVU2Hr1G106940	0	0	0	0.616637	0.342437	0.598704
HORVU5Hr1G081520	18.94919	19.86373	20.34431	38.93699	58.20934	56.14751
HORVU3Hr1G059250	1.948075	2.707588	3.114877	0.172	0.060971	0.068598
HORVU2Hr1G091880	0.343955	0.12612	0.243008	1.338419	33.06971	25.84055
Hordeum_vulgare_newGene_5517	0	0	0	1.53631	1.263175	1.978397
Hordeum_vulgare_newGene_1884	0.837212	0.636803	0.932679	0.369318	0.224364	0.429698
HORVU3Hr1G108620	0.874831	1.018529	0.879113	2.713112	2.599412	2.691863
HORVU5Hr1G048150	8.512667	9.700254	9.359092	5.565598	3.867992	5.380765
HORVU7Hr1G009770	1.931966	1.724016	1.881633	0.828125	0.664743	0.658584
HORVU3Hr1G104790	1.260078	1.095217	1.267952	3.997242	17.16533	12.29536
HORVU6Hr1G090280	0.028359	0.049377	0	2.024356	2.459513	1.986056
HORVU1Hr1G046420	3.578658	3.592591	4.926788	0.015544	0	0
Hordeum_vulgare_newGene_3986	18.40377	16.52203	11.83047	8.49175	8.255785	8.38609
HORVU6Hr1G094170	0.041558	0.006759	0.00655	1.436104	0.924037	1.161776
HORVU5Hr1G068800	0.62361	0.500159	0.553616	2.440839	2.996017	2.447851
HORVU2Hr1G069950	2.038128	2.156026	2.451093	7.820522	10.97094	11.0866
Hordeum_vulgare_newGene_6276	0.009365	0	0.00782	0.55333	0.380365	0.368693
HORVU1Hr1G052850	4.887539	4.900425	6.142855	0.772786	1.003963	1.034422
Hordeum_vulgare_newGene_12594	2.640674	1.808176	2.580425	15.14983	31.85965	33.18554
HORVU7Hr1G035010	76.56664	58.60035	79.93854	16.2318	7.429209	9.13916
Hordeum_vulgare_newGene_13423	2.626719	2.268779	2.985651	0.225259	0.195842	0.247309
Hordeum_vulgare_newGene_14439	0.218969	0	0	3.852122	3.63864	4.062566
HORVU1Hr1G081430	0.004562	0	0.00194	4.314442	6.020483	7.414671
Hordeum_vulgare_newGene_5997	2.789209	2.719581	3.973093	8.045417	8.983738	8.670538
Hordeum_vulgare_newGene_9235	0.76924	0.652565	0.314807	0.029223	0.047035	0.091026

Hordeum_vulgare_newGene_14296	0.677066	0.480769	0.612178	1.077253	5.45137	3.021314
HORVU7Hr1G043040	0.358635	0.624915	0.693244	0.043817	0.153603	0.032154
HORVU7Hr1G107280	0.634528	0.681146	1.403473	0.190604	0.052413	0.240433
Hordeum_vulgare_newGene_8542	1.458108	0.819074	0.947614	0.380062	0.005891	0.077823
Hordeum_vulgare_newGene_8541	1.801038	1.811396	1.678227	0.889749	0.10159	0.247227
HORVU0Hr1G003280	0.252525	0.43306	0.242011	1.879273	7.471994	6.752707
HORVU2Hr1G004610	11.99078	5.987235	15.26888	2.667041	0.294329	0.718311
HORVU3Hr1G051330	1.812471	1.838061	2.2242	4.805198	8.013457	8.496548
HORVU3Hr1G115810	4.241055	3.467944	3.967573	0.787824	1.98433	1.816766
HORVU2Hr1G003340	113.2542	100.1717	97.5331	71.70479	51.90514	57.96118
HORVU5Hr1G038440	0.60175	0.465777	0.627961	0.217666	0.24842	0.189295
HORVU3Hr1G036970	11.97263	8.724021	14.77579	28.77747	36.02402	34.66867
HORVU2Hr1G088660	16.5821	15.16731	16.74349	11.75464	6.642724	8.191228
HORVU1Hr1G088870	60.2278	57.87354	80.4281	244.0532	270.4614	284.6764
Hordeum_vulgare_newGene_1698	0.16541	0.033248	0.026858	0.76666	0.759087	1.250358
Hordeum_vulgare_newGene_1699	1.33601	0.993373	1.433968	0	0	0
HORVU5Hr1G045860	5.612291	3.953455	5.130885	14.96555	24.72247	21.89053
HORVU5Hr1G105900	50.07703	57.28257	89.97537	6.362488	1.213805	3.020274
Hordeum_vulgare_newGene_4316	1.347871	1.497214	2.515635	0.169461	0.013979	0.052438
Hordeum_vulgare_newGene_4312	0.619421	0.724411	0.710258	0.377396	0.335614	0.268251
HORVU5Hr1G104050	1.292059	1.302145	1.476959	5.444107	3.958735	4.971109
HORVU0Hr1G039040	2.108573	2.302084	2.604469	4.087895	9.251698	8.277934
Hordeum_vulgare_newGene_11397	37.95224	36.33828	35.39748	22.68152	4.684057	8.459145
Hordeum_vulgare_newGene_7663	3.914973	3.456111	5.118535	10.93174	15.19068	13.51755
HORVU2Hr1G035170	0.354009	0.409346	0.467509	0.857823	4.366436	2.691749
HORVU3Hr1G001030	0.035452	0.041073	0	0.639124	0.746671	1.397362
HORVU2Hr1G008250	3.141121	3.746024	4.863203	0.741834	1.547948	1.600807
Hordeum_vulgare_newGene_12791	0.19783	0.273118	0.277571	0.883843	1.020948	1.147002
HORVU5Hr1G072120	0.124615	0.477035	0.689031	1.028095	1.791109	1.683849
HORVU5Hr1G058330	21.18523	28.90104	29.50909	4.500898	0.53662	1.531365
HORVU4Hr1G056860	7.889256	10.98402	10.69089	0	0	0
HORVU5Hr1G095540	5.417902	6.183363	8.542786	4.473183	3.61801	3.384141
Hordeum_vulgare_newGene_9606	1.452023	1.431863	1.857302	0.13875	0.038185	0.081255
HORVU5Hr1G093260	3.532568	4.836784	6.639753	6.434339	27.53324	27.06265
Hordeum_vulgare_newGene_5584	0.186048	0.164994	0.156149	1.043733	0.414486	0.570819
HORVU4Hr1G069380	3.420677	3.41998	4.454924	10.81927	9.40341	9.549363
HORVU3Hr1G078200	1.586113	1.394278	1.653842	3.479795	6.655273	8.052127
HORVU5Hr1G097940	5.803703	3.547314	5.933618	15.61728	12.79149	12.36543
HORVU3Hr1G072460	10.7775	10.94546	10.88207	6.550614	5.969093	5.460454
HORVU7Hr1G096970	0.060679	0.14457	0.07797	0.493009	0.366054	0.395549
HORVU4Hr1G023510	0.158897	0.176048	0.186106	1.695957	2.126617	1.219334
HORVU1Hr1G004580	2.596252	3.651427	7.115729	0.581431	0.035061	0.362845
HORVU7Hr1G092070	0.059472	0	0.157358	18.44412	13.43198	19.18946
HORVU6Hr1G011860	2.575273	2.197062	3.196265	0.295027	0.097814	0.43496
HORVU7Hr1G051810	6.941536	7.873271	11.273	1.713372	1.015848	1.248784
HORVU6Hr1G001160	0.493412	0.061048	0.09608	2.237693	2.019873	2.995477
HORVU6Hr1G091660	0.142438	0.342892	0	2.403342	5.867187	6.017664

HORVU3Hr1G037780	10.90142	12.1516	12.94305	3.214653	3.88999	3.468623
HORVU5Hr1G061990	0.46286	0.189657	0.272743	0.880789	1.803341	1.345529
HORVU7Hr1G057410	7.102912	6.697149	7.596263	3.749878	3.707577	3.432274
HORVU6Hr1G010400	0.035064	0.124617	0.087328	3.260879	1.513739	2.362065
HORVU6Hr1G088440	7.813722	4.168283	8.60278	0.176309	0.19605	0.19816
Hordeum_vulgare_newGene_9030	2.796476	1.441335	1.652103	0	0	0.048893
Hordeum_vulgare_newGene_9031	6.159449	4.375219	1.865898	0	0	0
Hordeum_vulgare_newGene_9034	0.909628	1.06453	1.049384	2.783989	2.611548	3.329712
Hordeum_vulgare_newGene_9037	42.79863	31.38734	38.28074	129.5202	125.4866	127.2839
HORVU2Hr1G033340	1.593398	1.945767	1.628869	0.025632	0.180107	0.055097
Hordeum_vulgare_newGene_2775	1.438591	1.194645	1.845242	0.248846	0.059758	0.178243
Hordeum_vulgare_newGene_2772	2.066913	1.489521	1.464308	0	0	0
HORVU4Hr1G021260	3.896487	3.776892	5.791279	0	0	0
HORVU2Hr1G101920	5.71821	3.967134	6.955335	1.834194	0.951124	2.066656
HORVU5Hr1G059410	0.960104	0.771727	1.093206	0.536645	0.222866	0.557593
HORVU2Hr1G060770	10.0608	8.450021	9.331051	3.877991	7.116869	7.233628
HORVU4Hr1G020130	25.65086	26.56529	28.47574	87.20487	79.73343	69.71665
HORVU2Hr1G099570	1.615706	2.110991	2.372496	0.566981	0.083641	0.337342
HORVU5Hr1G078050	4.584157	4.602651	5.371866	11.14181	12.79304	13.14959
HORVU7Hr1G003840	0.829056	0.642442	1.26051	2.956695	4.590541	4.052718
HORVU5Hr1G053320	0.326306	0.307482	0.309554	0.953116	2.847663	2.918321
HORVU7Hr1G086010	14.02047	10.01374	16.70952	7.687979	4.162544	7.236475
HORVU5Hr1G120250	3.907105	4.247778	5.090819	8.68752	16.15739	14.87865
Hordeum_vulgare_newGene_3644	0	0	0	2.512828	0.619781	2.08114
HORVU2Hr1G119430	2.877391	3.547656	2.771854	0.335137	0.225654	0.217295
HORVU5Hr1G049980	1.16286	0.966586	1.145207	3.409581	4.833937	5.550567
HORVU7Hr1G059460	4.161046	3.903011	5.46194	9.212119	15.53196	17.12671
HORVU6Hr1G010870	1.521612	1.381388	1.141582	0.080474	0.014383	0.048703
HORVU7Hr1G027370	0.52538	0.265977	0.425238	1.124859	26.86909	27.21319
Hordeum_vulgare_newGene_10714	3.167402	2.522937	4.245693	5.814806	9.918726	9.757772
Hordeum_vulgare_newGene_1458	1.230343	1.3213	1.064863	0	0	0
HORVU3Hr1G017610	8.349088	7.553771	9.688831	3.939589	3.097894	3.278714
HORVU5Hr1G067810	0.977463	1.474737	1.454198	0.574649	0.230478	0.523623
Hordeum_vulgare_newGene_4661	0	0	0	6.201936	5.559124	6.801601
HORVU1Hr1G072250	13.44733	9.287491	12.20182	36.11563	61.83012	58.24717
HORVU3Hr1G090190	365.6398	420.4736	346.9444	1037.724	1569.711	1326.774
HORVU1Hr1G075150	172.4802	155.3171	167.0572	70.25918	31.73051	36.20694
HORVU3Hr1G080100	4.508008	3.057553	4.631436	2.05436	1.627826	2.863488
HORVU6Hr1G022590	1.341459	0.658668	1.963947	0.451523	0.389269	0.712474
Hordeum_vulgare_newGene_13673	12.42684	11.55969	14.56335	8.010865	7.724198	7.843046
Hordeum_vulgare_newGene_3149	0.045987	0.208264	0.137747	0.890476	1.165131	1.621821
HORVU7Hr1G079830	0	0	0.015959	7.732756	8.807666	9.095541
HORVU3Hr1G087480	8.901705	3.98873	6.812187	21.75857	21.93821	17.49723
HORVU1Hr1G027570	9.140012	5.890405	9.70974	5.623541	4.829505	4.275639
Hordeum_vulgare_newGene_2238	13.66425	15.55524	15.8712	3.741827	4.350929	4.123425
Hordeum_vulgare_newGene_2237	1.329296	1.613614	1.729033	0.248282	0.387102	0.217288
HORVU0Hr1G005400	3.542399	4.937725	4.256168	0.81045	1.224008	1.339155

HORVU7Hr1G054530	7.664417	8.718515	11.01335	26.9109	35.39268	31.45046
HORVU5Hr1G023740	25.19552	11.42603	22.49402	3.422348	0.216961	3.90481
HORVU5Hr1G062490	62.00565	63.78369	53.65705	33.70564	19.54186	25.38825
HORVU5Hr1G061760	1.281862	1.839057	1.769839	0.442589	0.291224	0.616659
HORVU2Hr1G004790	0	0	0	2.587384	2.019773	2.524445
HORVU4Hr1G002050	0.111762	0.029267	0.20837	10.12291	13.97777	13.17422
Hordeum_vulgare_newGene_8990	32.81622	39.96676	42.44403	8.109239	4.586047	4.278336
HORVU1Hr1G021930	0.358921	0.823186	0.808317	1.054638	1.476042	1.947753
HORVU2Hr1G124270	0.172271	0.115999	0.211627	1.068927	0.551212	0.928575
HORVU1Hr1G079130	0.910355	1.037852	1.140028	1.808609	3.112508	3.834352
HORVU3Hr1G014510	0.141637	0.188948	0.281673	1.103101	3.541369	2.879315
HORVU1Hr1G058490	1.829419	1.236536	2.920438	23.98208	42.10049	39.73039
Hordeum_vulgare_newGene_1157	0	0	0	1.967113	1.749196	2.203387
HORVU2Hr1G016470	2.236412	2.416123	2.544913	0	0	0
HORVU6Hr1G085980	19.25501	21.09172	27.81318	10.80845	13.68827	13.33962
HORVU5Hr1G125280	10.5498	9.390336	10.71448	3.159006	3.99301	4.20365
HORVU4Hr1G012100	3.599363	3.338916	3.661466	1.784122	0.851261	0.91351
HORVU1Hr1G089970	4.893287	3.918163	5.795601	1.374598	1.386057	1.679083
HORVU1Hr1G090930	5.977685	3.478935	11.02859	2.873115	1.678448	1.072619
HORVU7Hr1G053260	0.426132	0.465238	0.44511	2.306537	3.065954	3.079483
HORVU4Hr1G081310	49.46605	52.91088	69.44593	10.65349	16.64642	16.58204
HORVU3Hr1G005980	12.55321	11.33816	12.65422	5.887043	6.57446	5.967048
HORVU5Hr1G124880	0.93422	0.74381	0.504537	6.060276	6.003441	4.898003
HORVU5Hr1G081770	0.132573	0.258352	0.154016	1.709501	1.764153	1.912954
HORVU3Hr1G007580	0.029119	0.044045	0.081767	1.016689	1.865983	2.650949
Hordeum_vulgare_newGene_11026	0.13972	0.111765	0.130258	0.714372	0.638186	0.71055
Hordeum_vulgare_newGene_11024	0.504287	0.274298	0.403306	2.77448	1.805816	1.698445
HORVU3Hr1G073670	0.590837	0.64994	0.6481	1.73677	2.408611	2.478473
HORVU5Hr1G111840	46.14874	38.33899	46.06824	25.84576	13.20917	14.28949
HORVU3Hr1G092620	0.69	0.683805	0.813603	0.123923	0.167101	0.238152
HORVU2Hr1G041480	1.415761	1.054936	1.086327	2.624166	3.692334	3.099013
HORVU1Hr1G045080	0.213077	0.382601	0.147884	0.602284	1.575975	1.509463
HORVU7Hr1G112900	8.903026	7.329706	8.916179	2.649985	1.662853	2.390585
HORVU3Hr1G109540	7.25535	7.388519	7.905016	4.496027	1.039576	1.758951
HORVU2Hr1G109100	6.238799	5.865468	6.963682	2.493221	0.852808	1.633557
HORVU4Hr1G014190	14.29932	21.32902	21.8895	0.263732	0.065231	0.115198
HORVU5Hr1G096800	2.860853	2.974865	3.419429	6.375267	10.1502	9.87609
HORVU2Hr1G060110	0.104355	0.107726	0.124567	0.253409	0.551917	0.404977
HORVU3Hr1G093990	1.053002	0.706932	0.610717	3.210137	4.807546	4.671108
HORVU2Hr1G097990	0.338974	0.178659	0.468183	2.915365	16.06542	10.41326
HORVU5Hr1G050990	39.22023	39.82238	32.9274	207.1349	368.527	362.5342
Hordeum_vulgare_newGene_13702	0.497221	0.48032	0.702024	2.299163	5.824797	5.815917
HORVU7Hr1G120660	2.472132	2.838742	3.002334	10.04965	11.97324	13.12252
HORVU3Hr1G086190	1.692944	1.729097	1.275091	6.31124	9.239252	9.9248
HORVU4Hr1G084600	4.029395	2.687469	4.90067	2.263797	1.107603	2.1635
Hordeum_vulgare_newGene_12705	0.430317	0.517586	0.422227	3.87797	9.419747	8.335593
Hordeum_vulgare_newGene_12704	0.971326	0.872197	1.412784	11.2452	29.37286	26.98825

HORVU5Hr1G121730	12.67299	13.59614	16.60086	4.620984	5.286835	5.050796
HORVU3Hr1G090970	1.768451	1.481348	2.050897	7.824836	15.71008	14.43182
Hordeum_vulgare_newGene_1099	9.555631	10.69851	8.753984	7.028053	4.806834	5.975356
HORVU7Hr1G077780	2.226645	2.704168	2.05767	6.420255	83.84037	78.53504
HORVU2Hr1G103710	0.420376	0.763821	0.939776	1.78441	2.592238	3.905907
HORVU2Hr1G070440	14.37936	14.30211	12.37462	10.24851	5.61678	7.873569
HORVU3Hr1G051610	2.274668	1.708142	2.053632	16.13088	11.63493	11.53784
HORVU7Hr1G078320	3.147889	2.655498	2.595158	5.12271	9.103375	9.187862
HORVU7Hr1G073440	0.817045	0.866177	0.979826	1.885611	6.306419	5.251269
HORVU5Hr1G080140	1.278131	1.45021	1.924776	0.903349	0.881388	0.721333
HORVU2Hr1G101710	0.783568	1.073181	1.169801	3.64194	4.541429	4.249761
HORVU7Hr1G078490	0.467314	0.448558	0.617831	2.053882	2.36492	2.392758
HORVU7Hr1G009930	7.869881	10.87552	9.243288	1.098066	0.964102	0.963064
HORVU5Hr1G115820	2.449158	2.834236	2.834198	7.973601	8.965995	7.758884
Hordeum_vulgare_newGene_10036	1.896862	2.509019	2.71223	0.135953	0.046285	0.094279
HORVU3Hr1G026690	1.003549	1.021353	2.220547	3.119655	3.423091	5.029656
HORVU1Hr1G000530	73.09437	78.98091	52.43355	25.24449	23.59799	26.13627
HORVU2Hr1G026200	6.070929	6.056883	9.199412	4.046052	4.395717	4.868005
Hordeum_vulgare_newGene_5070	8.143464	8.686775	8.986248	0.094388	0	0
HORVU4Hr1G067780	0.756807	0.648964	0.686866	9.38995	17.24114	16.07594
HORVU2Hr1G116390	10.54179	5.096446	5.396582	50.09695	63.65556	64.12909
HORVU4Hr1G067600	0.840004	0.962097	0.870182	2.095467	5.957514	5.459754
Hordeum_vulgare_newGene_1798	0.035436	0.040434	0.03964	0.976849	2.30282	3.142334
Hordeum_vulgare_newGene_14837	0.021558	0.035719	0.022269	8.47818	7.49634	8.439157
Hordeum_vulgare_newGene_8249	0.879116	0.995746	0.668752	0	0	0
Hordeum_vulgare_newGene_12215	0.935783	0.723282	0.661348	0.251484	0.019411	0.069904
HORVU0Hr1G029600	1.852656	2.015922	2.382305	1.232667	0.916029	1.236904
Hordeum_vulgare_newGene_7436	0.23436	0.335738	0.733952	1.972785	0.842321	1.605145
Hordeum_vulgare_newGene_7295	0	0	0	1.061546	0.38801	0.50947
Hordeum_vulgare_newGene_12068	1.420883	1.596444	2.135816	0.306628	1.172799	0.522862
Hordeum_vulgare_newGene_12063	3.849491	3.362205	3.637812	1.38781	2.583956	0.501459
HORVU5Hr1G014130	15.33852	11.59735	16.25923	0.038036	0	0
Hordeum_vulgare_newGene_15878	1.351208	1.464586	2.016568	0.450672	0.849318	1.071996
HORVU0Hr1G018190	4.291224	5.505618	5.863014	2.090565	3.334714	3.487559
HORVU6Hr1G026800	3.953635	3.206824	3.773715	7.075797	11.29873	10.48121
HORVU4Hr1G074710	80.31978	100.843	114.3935	35.42048	3.459835	17.73582
HORVU5Hr1G032650	24.95991	27.01817	38.4404	41.18345	96.60996	111.454
Hordeum_vulgare_newGene_2915	0.190786	0	0	1.3493	2.940492	1.515126
HORVU5Hr1G074780	0.571238	0.560054	0.698044	0.123771	0.124414	0.191202
HORVU5Hr1G018740	5.71504	5.318695	8.209125	3.126534	3.928874	4.33953
Hordeum_vulgare_newGene_14585	24.35772	15.61898	16.04764	43.95705	95.21497	113.0893
HORVU3Hr1G079920	0.757758	0.833041	1.058154	2.989774	4.432134	3.524003
HORVU7Hr1G107010	0.486688	0.852359	0.619458	2.872047	5.219232	2.525967
HORVU3Hr1G055260	0.129938	0.057565	0.147459	2.367946	4.515927	4.040765
HORVU3Hr1G110660	0.376709	0.32267	0.161409	0.826177	2.214763	1.526263
HORVU6Hr1G087820	515.4549	668.2174	525.7674	1437.176	2776.804	2398.947
HORVU5Hr1G065450	0.982731	1.09199	1.182267	0.727677	0.492401	0.802626

HORVU2Hr1G005630	0.736375	0.41834	0.509735	5.260888	13.57217	12.18467
HORVU3Hr1G012620	2.205085	1.39662	1.858434	0.899325	0.962716	0.635688
HORVU3Hr1G004280	66.31889	88.00965	127.4162	2.617437	0.094348	1.747525
HORVU0Hr1G018670	0	0	0	1.820236	8.627645	9.21725
HORVU5Hr1G066100	7.146626	6.590071	8.620106	2.509747	4.026294	4.11016
HORVU2Hr1G098340	0.37875	0.125371	0.853356	5.327382	40.25347	30.51802
HORVU6Hr1G011120	22.06681	12.74581	24.36875	8.998273	7.678628	10.81765
HORVU0Hr1G007690	0.126598	0.098168	0.123375	0.604561	1.294213	1.996624
HORVU6Hr1G091010	22.70512	26.08059	22.93118	16.87325	12.79885	13.89893
HORVU7Hr1G036540	3.066869	1.698858	3.324528	8.414091	14.22267	10.09666
HORVU1Hr1G048690	25.03013	21.88853	25.65061	12.14191	11.51234	12.48266
HORVU1Hr1G083160	0.501422	0.663808	0.270002	2.24761	2.440184	3.0118
HORVU6Hr1G043980	2.971627	2.182218	3.548689	0.227868	0.607217	0.457321
HORVU2Hr1G014240	2.238612	1.290936	1.650498	12.07386	22.00121	19.43743
HORVU1Hr1G064700	0.614415	0.784588	1.000704	9.42113	34.35486	30.44004
HORVU5Hr1G067760	159.394	108.1837	187.0954	36.55044	31.94248	35.86755
HORVU0Hr1G001690	0.334115	0.269355	0.441344	1.485949	2.292298	2.330252
HORVU2Hr1G002690	10.48387	10.74928	11.46468	41.24424	35.87135	42.74116
HORVU7Hr1G056060	1.139502	1.278897	1.414439	0.755515	0.161876	0.227749
HORVU0Hr1G002250	12.97215	6.142623	18.37588	2.583247	0.038973	0.371613
HORVU2Hr1G077230	1.295196	1.484823	1.775575	2.032855	4.891156	5.572675
Hordeum_vulgare_newGene_10239	0.296905	0.562531	0.435945	0.75631	1.224494	2.20912
Hordeum_vulgare_newGene_10233	1.576132	1.306418	1.813797	0.382629	0.268924	0.27098
HORVU2Hr1G115960	0.02616	0.013862	0.027013	2.248793	10.58889	8.968098
Hordeum_vulgare_newGene_10582	0.874763	1.032532	0.543513	0.076685	0.095336	0.110573
HORVU2Hr1G118360	15.19738	14.35859	19.356	5.471232	5.522737	6.427109
Hordeum_vulgare_newGene_11182	10.24533	11.23696	10.52828	0.044096	0	0.039034
Hordeum_vulgare_newGene_11183	0.017699	0	0	13.24531	15.53498	17.49214
HORVU5Hr1G122990	0	0	0	7.623988	6.096703	5.859092
HORVU7Hr1G069770	0.058594	0.084776	0.1243	8.200586	7.44794	10.51785
HORVU7Hr1G105880	205.7524	190.2874	180.7119	109.1422	99.99135	87.87653
HORVU5Hr1G046520	765.1338	481.0856	806.6072	1691.594	3148.929	2292.113
HORVU6Hr1G001270	10.83361	8.994302	11.11633	39.17379	64.4823	50.65545
HORVU4Hr1G057110	2.61943	2.443697	3.599247	1.513453	1.80693	1.180292
HORVU6Hr1G095070	9.651713	10.40544	10.3427	24.74657	30.36543	26.95234
HORVU5Hr1G119550	9.939111	13.28675	15.75021	6.321966	0.8618	4.06594
HORVU3Hr1G105600	0.224546	0.119035	0.579571	1.731241	0.873806	2.421954
HORVU3Hr1G002820	0.017097	0	0.005301	1.006908	1.891897	1.78297
HORVU2Hr1G044910	8.17133	7.167198	9.479445	5.706856	2.900542	3.989915
HORVU1Hr1G071350	1.174111	1.035101	0.968348	0.062688	0.106714	0.223954
HORVU7Hr1G038710	5.684567	4.953663	6.17287	12.34632	17.79907	17.8738
HORVU4Hr1G029700	12.08986	12.77847	12.28426	6.798425	5.954096	6.588467
HORVU5Hr1G084320	1.002708	0.644536	1.314182	0.659575	0.354652	0.398687
HORVU5Hr1G000800	4.839288	5.843176	5.042791	19.37599	14.19244	15.35899
HORVU7Hr1G077620	18.17417	16.46071	19.95459	10.72738	6.184571	6.973425
HORVU4Hr1G021570	2.343813	2.424689	2.444544	0.13926	0.108473	0.150549
HORVU7Hr1G031730	0.785613	0.537948	0.952381	0.335058	0.102897	0.371649

HORVU3Hr1G097010	11.64259	11.50076	12.05184	4.383398	2.938547	3.2194
Hordeum_vulgare_newGene_714	98.56837	135.1514	152.7857	1.159836	0.579143	1.47399
Hordeum_vulgare_newGene_711	0.282732	0.585927	0.678331	5.287012	3.832145	4.593623
Hordeum_vulgare_newGene_10914	8.2503	7.318902	8.336163	1.108861	0.141525	0.377917
Hordeum_vulgare_newGene_719	2.545997	2.232078	1.068729	8.447726	4.182415	5.813689
HORVU7Hr1G042930	0	0	0	4.079028	2.235914	2.271998
Hordeum_vulgare_newGene_8895	0.634988	0.760447	0.69857	0.242065	0.225073	0.260727
Hordeum_vulgare_newGene_5915	37.0177	52.62234	61.99332	13.58821	4.790463	9.091271
HORVU6Hr1G021620	4.021587	3.725282	3.574693	12.47993	11.86659	7.88009
HORVU1Hr1G007730	4.352816	4.055843	3.887141	2.154163	1.930039	2.663388
HORVU4Hr1G078390	17.15603	13.30299	20.42261	10.65805	6.423661	8.544709
HORVU1Hr1G000700	3311.185	3702.003	2493.376	1411.549	1261.719	1146.678
HORVU5Hr1G098110	1.375386	1.259874	4.425821	5.969705	11.85891	11.67118
HORVU0Hr1G032300	0.14737	0.124539	0.223269	2.541812	5.622018	2.749553
HORVU1Hr1G077020	2.037033	2.161706	2.369092	3.932392	7.093897	6.212585
HORVU2Hr1G021870	0.321269	0.356206	0.352538	2.198523	3.09698	2.991186
Hordeum_vulgare_newGene_9418	6.832593	6.532299	6.268735	1.109654	0.33116	0.339267
HORVU1Hr1G025530	82.45868	62.66876	77.31325	23.30791	7.758409	10.9903
HORVU5Hr1G091420	0.487247	0.354484	0.487074	1.346166	1.873092	2.132465
HORVU7Hr1G021700	5.224067	4.455734	6.000676	9.68058	19.63267	16.08035
HORVU2Hr1G014380	2.230988	4.144045	1.666743	0.060667	0.055131	0
Hordeum_vulgare_newGene_4391	5.15862	4.747896	4.584621	3.282422	2.85782	3.056144
HORVU1Hr1G043470	4.172111	3.003076	4.249347	1.276843	1.656093	1.377528
HORVU2Hr1G042490	1.720627	1.039206	1.919139	3.324516	7.254439	7.082256
HORVU3Hr1G010310	0.278972	0.162273	0.32057	1.19484	0.998363	1.111719
HORVU5Hr1G105980	2.216415	2.102729	2.729314	5.673644	7.882909	7.939775
HORVU4Hr1G076840	19.72235	15.22164	18.59297	4.605576	2.598375	3.763031
Hordeum_vulgare_newGene_4979	1.770906	1.995773	2.276769	4.693484	5.989179	6.116823
Hordeum_vulgare_newGene_4977	0.396912	0.422391	0.201751	1.09096	1.795431	1.877363
HORVU5Hr1G060030	0.514802	1.067956	0.784121	2.002612	2.132231	2.170528
HORVU5Hr1G085800	3.050949	2.603064	3.246248	2.098453	1.007589	1.569223
HORVU2Hr1G089970	2.001557	2.984421	3.392592	5.867968	12.6968	13.08241
HORVU6Hr1G008640	62.10937	45.09055	43.88735	138.1104	122.0819	178.5279
Hordeum_vulgare_newGene_15200	6.257976	4.282919	4.753634	0	0	0
HORVU4Hr1G072960	0	0	0	7.929727	15.20934	8.869328
Hordeum_vulgare_newGene_6534	2.278522	2.647083	2.548965	6.031268	6.358409	7.65958
HORVU2Hr1G114240	0.865706	1.242219	1.018388	0.179098	0.188869	0.274638
Hordeum_vulgare_newGene_15939	1.838659	2.942824	3.092453	0	0	0.011443
Hordeum_vulgare_newGene_15931	1.487636	1.980967	1.822234	4.358081	4.112494	5.389396
HORVU6Hr1G078670	2.782185	1.8367	3.463554	0.141236	0.154337	0.143051
HORVU3Hr1G041850	0	0	0	2.703755	2.719803	3.249995
HORVU2Hr1G079510	0.739599	0.861456	1.025663	0.180341	0.293501	0.441325
HORVU7Hr1G099950	1.057818	1.081952	1.264078	1.897533	8.302882	8.235463
HORVU2Hr1G125360	0.373883	0.416719	0.474909	1.018878	2.932952	2.270789
HORVU3Hr1G006800	0.07261	0.020934	0.044102	4.286872	1.430544	3.300661
HORVU7Hr1G052210	1.937268	1.993428	1.997505	3.112159	6.039615	6.852164
HORVU3Hr1G032820	1.384795	0.911598	1.370599	2.38011	4.881083	3.085144

HORVU7Hr1G118010	20.96085	15.63675	20.18509	11.56398	4.041684	5.667136
HORVU2Hr1G024570	19.30621	12.42331	18.38463	62.5454	119.0483	113.2209
Hordeum_vulgare_newGene_2090	0.89922	0.542408	0.97578	1.971539	2.665395	3.23928
HORVU5Hr1G055560	3.353671	3.578933	3.331542	13.45582	35.9273	34.0915
HORVU3Hr1G078820	0.107232	0.200661	0.212765	0.573972	0.872567	0.977288
HORVU5Hr1G079950	0.661974	0.6526	0.408189	1.856054	2.126091	2.250326
HORVU1Hr1G019500	1.886063	1.452688	2.128373	0.947849	0.812304	1.071482
HORVU6Hr1G085120	0.445922	0.806131	0.509125	2.605621	2.521141	1.788211
HORVU2Hr1G066680	18.77357	16.69989	18.14892	80.01222	104.0954	104.4938
HORVU4Hr1G063790	0.249867	0.339197	0.455815	3.548844	1.098379	2.248438
HORVU5Hr1G005290	37.94518	24.22036	55.6474	5.525278	2.223555	6.680281
Hordeum_vulgare_newGene_3687	2.209658	2.591833	5.62136	0.867142	0.648721	0.481085
HORVU3Hr1G035930	5.878538	6.435189	10.85327	3.503699	4.601286	4.41707
HORVU5Hr1G104390	11.87334	12.74593	18.04396	23.22822	48.35	43.6596
HORVU4Hr1G054110	11.06744	16.93121	20.4058	8.207094	1.277798	3.513222
HORVU2Hr1G091270	4.474535	3.683083	3.624482	11.05515	18.31919	15.82181
HORVU2Hr1G093680	0.174561	0.189786	0.891089	13.28388	32.82228	28.21261
HORVU2Hr1G036110	3.80013	3.550121	3.119405	21.66758	33.01583	32.95058
Hordeum_vulgare_newGene_13953	0.065672	0.089463	0.165495	0.670339	0.89991	1.337106
HORVU1Hr1G083100	2.007688	2.580633	2.930288	1.512269	0.834668	1.324812
HORVU4Hr1G055240	7.986556	7.838117	9.724348	14.18518	33.35145	32.98454
HORVU7Hr1G095930	0.529821	0.977208	1.074145	0.111344	0.108108	0.094588
HORVU5Hr1G053010	0.92254	1.010943	2.151947	5.108563	36.46619	42.75307
HORVU7Hr1G043600	0.031765	0.134699	0.332962	1.07907	10.64051	9.829957
HORVU1Hr1G049090	14.51694	8.172771	13.06198	7.258877	4.483371	5.009251
HORVU2Hr1G014030	0	0	0.040121	5.423322	6.40654	8.219538
HORVU3Hr1G005830	2.60304	2.185979	2.623996	3.862334	7.248925	7.904796
HORVU5Hr1G045450	7.824146	8.238957	9.46085	14.97186	25.70567	26.86291
HORVU4Hr1G000620	24.27997	25.91448	30.29247	14.24945	13.9228	16.01258
HORVU6Hr1G092010	1.975669	1.849156	1.821337	0.082393	0.196974	0.252905
HORVU6Hr1G076040	0.821434	0.633102	0.655286	4.57913	6.969854	5.71563
HORVU1Hr1G064110	41.75712	39.05671	27.30214	16.08425	6.807844	9.353201
HORVU3Hr1G062920	7.039644	5.417299	5.823422	2.49571	0.827866	1.676823
Hordeum_vulgare_newGene_5189	0	0	0	1.669296	8.36981	4.354017
HORVU4Hr1G082400	40.00701	42.94829	50.82268	24.94247	8.448372	15.45395
Hordeum_vulgare_newGene_10795	3.506344	3.978136	3.118994	0.039271	0.005542	0
Hordeum_vulgare_newGene_10792	1.355127	1.242872	1.363999	0.383692	0.017556	0.051827
Hordeum_vulgare_newGene_10793	0	0	0	0.554964	0.376779	0.300595
Hordeum_vulgare_newGene_10791	0.786697	0.738652	0.555345	0.13218	0	0.045446
HORVU2Hr1G073050	3.04977	2.165392	3.736583	1.881551	0.906711	1.360259
Hordeum_vulgare_newGene_3797	5.259576	6.32628	4.656548	20.99582	11.14785	12.65295
HORVU3Hr1G093590	2.047237	2.696331	3.415998	1.595636	1.215152	1.688451
HORVU2Hr1G122610	1.793865	0.961247	2.098482	0.141289	0	0.177396
HORVU3Hr1G104360	18.10247	18.38848	23.56361	12.63785	8.501015	10.1186
HORVU5Hr1G066780	0.455785	0.495768	0.465564	0.064826	0.009271	0.069631
HORVU6Hr1G077790	1.449761	1.983341	2.396222	0.132478	0.10133	0.138614
HORVU4Hr1G006300	0.182642	0.139307	0.175386	1.927824	0.766526	1.965024

HORVU2Hr1G036930	137.6399	128.5409	161.6915	51.00189	31.55235	32.05528
Hordeum_vulgare_newGene_179	13.4742	11.80244	15.29067	0.045856	0	0
Hordeum_vulgare_newGene_178	3.074527	2.459945	3.760643	0	0	0
HORVU3Hr1G069830	0.645905	0.42215	0.511055	2.094039	1.803984	1.791846
HORVU7Hr1G063030	0.931959	1.057861	1.089967	2.4667	3.088978	3.699493
HORVU2Hr1G011870	0.200447	0.187163	0.131988	3.02352	3.648305	3.594127
HORVU5Hr1G046480	0.215819	0.203214	0.322891	3.320687	23.36217	20.14368
HORVU5Hr1G043470	2.573729	0.766585	2.509333	4.61528	6.198801	6.296442
Hordeum_vulgare_newGene_11441	0.054428	0.14059	0.097794	0.657642	1.165521	1.507998
Hordeum_vulgare_newGene_11444	0.511333	0.46633	0.675459	0	0	0
HORVU2Hr1G083720	16.29655	20.73848	24.31445	9.80987	11.69337	11.55263
HORVU5Hr1G072770	0.831391	0.644731	0.561939	2.871019	2.898753	3.348445
HORVU0Hr1G017540	54.60076	45.01286	77.79342	29.69092	37.78629	40.65266
HORVU2Hr1G022920	12.87614	14.51963	12.93889	26.60575	54.24173	52.50852
Hordeum_vulgare_newGene_12491	9.478209	11.22076	14.74404	4.759657	4.308328	5.21817
HORVU7Hr1G035350	0.1155	0.06061	0.096011	0.768221	0.969827	0.897917
HORVU3Hr1G116790	36.25353	20.53954	24.14182	79.19886	104.4998	104.7607
HORVU1Hr1G074200	0.688299	0.730163	0.70551	0.06399	0.044657	0.011439
Hordeum_vulgare_newGene_12213	1.377051	1.014227	1.50515	0.642059	0.39026	0.771317
HORVU4Hr1G074320	0.183086	0.389252	0.296388	1.895893	2.624681	2.882521
Hordeum_vulgare_newGene_6041	0.228394	0.068976	0.091717	1.854431	3.022932	3.382884
HORVU0Hr1G005150	0.925617	0.579014	0.567992	0.259095	0.163517	0.255476
Hordeum_vulgare_newGene_7936	1.038029	1.151235	1.302045	6.919985	10.3904	9.525778
Hordeum_vulgare_newGene_7934	0	0.049261	0.095493	0.821534	2.05979	1.5979
Hordeum_vulgare_newGene_3728	1.332439	1.674298	1.487416	0	0	0
HORVU5Hr1G075810	0.616268	0.532551	0.839965	1.396133	2.235787	1.818201
Hordeum_vulgare_newGene_3727	0.541904	0.628307	0.692656	2.306695	4.569194	2.91039
Hordeum_vulgare_newGene_5899	3.153043	3.467815	2.462759	0.701645	0.424045	0.403876
Hordeum_vulgare_newGene_5897	3.566946	3.365068	2.617876	1.810317	1.563859	1.544147
HORVU7Hr1G070080	0.467054	0.68612	0.883619	0.897316	5.972393	6.847909
Hordeum_vulgare_newGene_900	2.695648	6.14456	3.602178	0.034512	0.039966	0.031098
HORVU2Hr1G119820	1.573828	1.809698	1.56154	5.183942	4.968478	5.315948
HORVU5Hr1G125810	0.00729	0.01356	0.035936	2.357963	0.436522	1.283645
HORVU1Hr1G063880	1.976328	1.627412	1.436128	1.073539	0.346876	0.559442
Hordeum_vulgare_newGene_11850	0	0	0	0.699705	0.327258	0.371311
HORVU3Hr1G016990	3.993379	5.195594	5.703317	1.026683	0.993207	1.273209
HORVU4Hr1G089450	395.4855	379.426	344.7628	166.2702	60.05418	71.06387
HORVU2Hr1G033470	1.501544	1.993186	2.760582	6.610244	23.99162	17.47339
HORVU3Hr1G071030	6.21474	6.605414	7.788758	2.274714	1.713527	2.10292
HORVU3Hr1G081940	1.631665	2.092223	2.353646	6.754932	11.18556	9.928999
HORVU5Hr1G057730	4.875363	3.670806	6.171435	1.525516	1.675249	1.13227
HORVU2Hr1G069440	5.710757	4.784446	4.289784	1.315719	1.373579	1.662014
HORVU5Hr1G078950	9.515134	9.974263	11.22434	28.12376	32.1125	32.81406
HORVU0Hr1G005970	1.356225	1.096955	1.340348	0	0.01604	0
HORVU6Hr1G094680	0.387403	0.578949	0.646651	0.888959	2.487674	2.214695
Hordeum_vulgare_newGene_7586	0.535703	0.807176	0.506319	0	0.032563	0.021283
Hordeum_vulgare_newGene_7587	9.600678	10.08951	9.047108	0.077018	0	0

HORVU1Hr1G069830	19.44512	18.90089	17.02244	68.68219	45.28541	43.8025
HORVU1Hr1G057640	0.095007	0.088371	0	0.56172	0.780339	0.763007
Hordeum_vulgare_newGene_4588	9.963075	7.007003	11.181	4.009277	5.931487	2.530185
HORVU6Hr1G081350	0.431648	0.350561	0.521049	1.106616	1.936522	1.231313
HORVU5Hr1G060480	0.04853	0.041258	0.071502	0.636302	4.695533	4.190036
HORVU0Hr1G000840	11.24694	5.784461	13.89377	0.884524	1.800015	3.34892
Hordeum_vulgare_newGene_7057	1.187604	1.735133	1.02832	0.578748	0.179252	0.335231
HORVU1Hr1G056510	4.035398	3.976843	4.384799	9.323189	14.42089	14.58624
Hordeum_vulgare_newGene_6022	6.655301	7.378869	7.721177	0	0	0
Hordeum_vulgare_newGene_1406	1.292923	1.057276	0.929878	1.939025	1.907431	2.090683
HORVU3Hr1G034860	1.067519	0.893017	1.620405	2.203	4.258446	3.90722
HORVU3Hr1G031980	0.722593	0.814343	0.727794	1.942561	3.693151	3.665886
HORVU7Hr1G093370	35.48384	37.30029	53.25448	21.44576	18.21314	23.06579
HORVU3Hr1G006100	1.450913	0.889574	1.673471	0.638931	0.128068	0.306395
HORVU7Hr1G052530	11.25666	8.012924	11.51456	21.93258	32.73902	26.22315
HORVU5Hr1G058070	2.210762	2.147957	2.563167	1.121718	1.260467	1.514376
Hordeum_vulgare_newGene_5798	2.053014	3.178751	2.451431	9.282297	7.748806	6.303563
HORVU6Hr1G029220	1.137377	0.698884	1.297376	2.117283	7.758548	8.325446
HORVU5Hr1G122960	0	0	0	6.088439	5.09801	4.984941
HORVU1Hr1G021760	1.977731	1.591134	2.146023	1.127226	0.650419	1.110802
HORVU1Hr1G078480	2.341318	2.518729	3.395198	0.834052	0.828242	0.823761
HORVU3Hr1G097830	0.210948	0.03311	0.059562	9.612537	85.69547	82.62034
HORVU7Hr1G000270	35.64158	26.86569	26.96624	10.66853	14.75117	12.31673
HORVU5Hr1G012010	2.186556	1.891944	2.603149	0.019829	0.017058	0.042592
HORVU6Hr1G038700	1.027976	0.841248	0.876532	1.293743	2.861521	3.015464
HORVU6Hr1G004980	2.768784	3.477711	3.983979	1.288124	1.226584	1.046256
HORVU2Hr1G097910	1.221325	1.591585	1.376457	4.77069	4.801636	5.07725
Hordeum_vulgare_newGene_10212	0	0	0	0.927778	1.136076	1.128321
HORVU5Hr1G011100	1.084318	0.740217	1.081672	3.942209	5.17009	5.515163
Hordeum_vulgare_newGene_10218	428.2114	491.8741	616.6581	22.99007	0.525867	9.870485
HORVU1Hr1G002170	72.06587	76.13848	100.4798	38.10297	31.85505	31.85752
HORVU4Hr1G084680	0.047133	0.065826	0.136432	0.547124	1.123416	1.008969
HORVU3Hr1G034230	11.33586	12.70884	15.2142	5.295187	6.690615	6.207234
Hordeum_vulgare_newGene_7955	3.859556	3.619026	4.017575	10.25712	21.82091	20.20947
HORVU4Hr1G012220	5.124603	4.036089	5.967935	19.33867	23.37269	23.02331
Hordeum_vulgare_newGene_4638	4.780052	4.15174	5.154964	11.18172	14.42542	13.79562
Hordeum_vulgare_newGene_2036	0.527943	0.585043	0.159132	2.63731	3.437344	4.312529
HORVU7Hr1G043410	4.041147	5.446204	6.57712	2.410077	2.377806	2.809707
HORVU3Hr1G026610	34.07551	25.97602	34.49497	16.9352	9.218002	11.36039
Hordeum_vulgare_newGene_8139	3.665292	3.966905	3.504678	0	0	0
Hordeum_vulgare_newGene_13962	0.136913	0.172866	0.301091	1.059639	0.634735	0.634935
HORVU5Hr1G052150	15.49713	6.039396	26.68473	1.516836	0.095295	1.672831
HORVU1Hr1G036200	2.977812	2.565319	2.583523	4.384392	7.321226	8.174823
HORVU5Hr1G086760	1.055233	0.956015	1.368232	2.142275	3.528226	3.233119
HORVU6Hr1G085890	68.31114	70.0669	92.4833	41.56476	43.95953	46.43352
HORVU4Hr1G076820	12.66892	9.389862	16.87196	9.046451	4.775807	6.284801
Hordeum_vulgare_newGene_1711	0.854809	0.583358	0.709921	0.107871	0.155063	0.151282

HORVU6Hr1G025460	5.216447	3.011524	4.92099	15.37071	15.69152	15.49475
HORVU1Hr1G001080	5702.466	3222.493	3376.332	25520	8074.92	12568.62
HORVU3Hr1G094820	2.655511	2.911026	2.175844	8.669497	22.59484	20.98721
HORVU0Hr1G022330	0.16361	0.160375	0.167351	0.465302	0.692194	0.624557
Hordeum_vulgare_newGene_4742	0.549189	0.651501	0.520044	0	0	0
Hordeum_vulgare_newGene_1498	0.758803	0.864756	1.097423	0	0	0
HORVU1Hr1G052350	32.8834	32.32674	29.59185	12.90625	7.038181	8.443224
Hordeum_vulgare_newGene_1492	0.913619	1.052802	0.940419	0.09001	0.168954	0.116496
HORVU6Hr1G054050	1.002469	0.533316	0.633775	1.774511	3.460591	3.715271
HORVU1Hr1G084410	1.15307	0.913578	1.602566	0.180406	0.51128	0.51656
HORVU1Hr1G030200	4.84696	5.191057	5.732203	12.98918	14.128	16.28988
HORVU2Hr1G088980	0.111875	0.031183	0.034601	0.82186	0.617776	0.745263
HORVU6Hr1G074520	2.500465	2.523424	2.423729	0.159479	0.476969	0.381718
Hordeum_vulgare_newGene_255	3.082907	3.442968	4.851519	1.920384	1.606213	1.977194
Hordeum_vulgare_newGene_13736	3.855726	2.855608	3.834775	2.441566	2.126813	1.720906
HORVU7Hr1G059850	49.13362	42.58493	48.06748	33.73925	22.49438	24.99815
HORVU1Hr1G029770	1.964264	2.117659	3.485863	6.454121	19.60048	17.92319
HORVU1Hr1G071190	0.335989	0.085955	0.177624	2.643089	13.15439	9.122224
HORVU2Hr1G027690	0.523437	0.202088	0.209724	0.845071	2.040743	1.891758
Hordeum_vulgare_newGene_2378	1.20938	0.894045	1.747225	0.583171	0.598332	0.565282
HORVU5Hr1G064580	1.893891	2.045481	1.756725	1.995446	4.707197	4.495531
Hordeum_vulgare_newGene_2376	0.860797	1.057767	0.63602	6.775144	6.465247	7.616272
HORVU3Hr1G012510	5.931804	6.17254	7.252574	0.024648	0.012536	0.048776
HORVU7Hr1G096830	0.287015	0.297783	0.477021	0.859072	2.006402	1.488426
HORVU7Hr1G054360	5.537445	3.051075	6.678024	9.118449	16.41501	13.65501
Hordeum_vulgare_newGene_15361	3.422933	3.034009	2.484304	6.483658	12.31948	8.827219
HORVU7Hr1G118770	4.353533	2.341414	5.112455	0.310766	0.111905	0.274501
HORVU1Hr1G047220	23.6275	21.94505	23.96522	9.320785	5.85909	10.6403
HORVU5Hr1G024550	96.46596	94.99058	112.8189	70.10745	54.11634	52.01602
HORVU5Hr1G097370	0.668868	0.789453	0.807204	1.974236	2.177751	2.200621
HORVU2Hr1G085740	1.204255	0.949265	1.497541	2.380597	13.77019	8.139899
HORVU2Hr1G009900	0.336676	0.317683	0.23549	3.942341	3.117347	4.018995
HORVU7Hr1G113300	16.4958	14.81119	25.47252	5.030063	1.716261	2.779159
HORVU1Hr1G093950	0.083063	0.177469	0.148393	1.33608	0.608306	0.899473
Hordeum_vulgare_newGene_1051	2.666296	3.462939	2.953786	5.022457	12.7928	11.31395
HORVU1Hr1G053260	0.275995	0.246515	0.281335	1.079218	1.004209	1.555482
HORVU7Hr1G034120	1.572448	1.518544	1.421126	13.54877	9.362925	7.836466
Hordeum_vulgare_newGene_4263	0	0	0.019704	3.888773	3.921636	4.758832
HORVU7Hr1G095760	0.491559	0.616886	0.637538	1.890678	2.196757	1.445038
HORVU6Hr1G036760	0.462774	0.3978	0.529416	1.904727	1.530387	1.294845
Hordeum_vulgare_newGene_4265	2.241836	2.5698	2.865978	0	0	0
HORVU5Hr1G021070	33.76622	29.50687	50.90569	11.40192	3.143752	6.656658
HORVU7Hr1G085660	0.47137	0.220328	0.408898	0.831576	6.456509	5.295389
HORVU1Hr1G078380	0.187998	0.024241	0	3.881154	11.43209	8.705074
Hordeum_vulgare_newGene_13643	5.975942	7.200749	7.730097	0.014746	0	0
HORVU1Hr1G090360	4.415687	5.647875	6.082101	0.363913	0.027374	0.190612
HORVU2Hr1G010590	0.292495	0.309808	0.33248	0.676322	1.051366	0.947992

HORVU5Hr1G014470	0.302746	0.481979	0.319395	1.289562	1.029669	1.658185
HORVU7Hr1G019780	640.9952	521.8188	511.759	2532.449	9351.832	6471.142
HORVU3Hr1G006310	4.331079	3.539244	4.705698	0.233546	0.125077	0.166928
HORVU7Hr1G079210	0.690996	0.465954	0.52357	1.979973	3.085795	2.036402
HORVU7Hr1G018780	1.309618	1.472232	1.148515	3.477984	3.623419	4.01106
HORVU3Hr1G070240	3.608467	3.132398	3.954904	2.080434	1.449495	1.153095
Hordeum_vulgare_newGene_11100	211.9802	279.5481	315.4369	17.49574	2.420538	8.677576
HORVU4Hr1G050510	30.16193	22.68236	39.71495	7.318924	2.293286	3.916172
HORVU7Hr1G012630	0.815058	0.706408	0.703546	0.566312	13.06461	8.739343
Hordeum_vulgare_newGene_7538	0.11508	0.086537	0.163455	0.724035	2.046253	1.890689
HORVU3Hr1G062570	1.745341	1.515109	0.952349	0.699166	0.557377	1.006843
HORVU6Hr1G046420	2.247315	2.606673	2.130659	4.484092	6.250194	7.087897
HORVU6Hr1G033600	0.510621	0.680176	0.344384	6.099081	7.822421	8.317895
HORVU1Hr1G049810	1.453383	1.361462	1.436176	0.416219	0.563977	0.51372
HORVU2Hr1G061890	6.531841	9.024848	4.753879	1.953566	3.79852	2.016834
HORVU7Hr1G076290	0.558428	0.817788	1.306842	7.665237	10.24629	7.801833
HORVU6Hr1G093190	4.611956	5.129404	5.928764	11.16745	18.00052	18.26546
HORVU2Hr1G111570	0	0	0	1.066161	2.436488	2.829083
HORVU6Hr1G090080	1.588752	1.383537	1.890153	3.450785	4.477159	4.783848
HORVU7Hr1G050030	3.396613	4.188371	3.779202	11.45425	9.898393	11.52099
HORVU4Hr1G090850	2.974164	2.990269	4.408251	0.909011	0.437427	0.595541
HORVU3Hr1G027760	1.517585	1.381104	1.512366	2.965847	4.465789	4.027998
HORVU2Hr1G018380	2.03042	1.407853	1.695526	2.51611	7.27092	6.177924
HORVU3Hr1G025720	63.8298	67.98002	92.83683	9.134382	0.701535	3.714833
Hordeum_vulgare_newGene_3227	0	0	0	0.746899	1.23293	1.594804
HORVU7Hr1G047690	0	0	0.024291	3.031897	16.26476	9.454769
HORVU1Hr1G013870	1.121802	1.074773	0.696518	0.04465	0.322561	0.148171
Hordeum_vulgare_newGene_3594	0	0	0	1.888194	0.951451	1.619042
Hordeum_vulgare_newGene_3599	0.397769	0.326114	0.53668	1.507597	1.297289	1.182854
HORVU7Hr1G028840	11.57907	6.554686	9.761244	34.10884	41.77873	48.53215
HORVU4Hr1G064670	4.376496	6.162425	4.978395	1.614441	2.839335	3.563596
HORVU4Hr1G078310	7.176014	5.503655	7.527215	3.545717	2.128359	1.951888
Hordeum_vulgare_newGene_10327	6.624724	4.526854	6.458611	0	0	0
HORVU6Hr1G073980	32.32517	34.91109	35.2791	151.6751	120.2917	108.6918
HORVU2Hr1G039880	1.890094	2.205404	1.808744	5.295725	8.761954	8.107034
Hordeum_vulgare_newGene_13244	0.610169	1.54776	0.695098	0.297351	0.256502	0.354478
HORVU2Hr1G090960	1.608665	1.715336	3.249084	13.88652	29.00977	19.79083
Hordeum_vulgare_newGene_9147	0.18186	0.090729	0.118717	0.583195	0.424578	0.510742
Hordeum_vulgare_newGene_9142	18.20672	19.88321	39.80258	12.81303	17.36983	17.01984
Hordeum_vulgare_newGene_9143	3.75513	2.902425	4.876135	26.63635	195.8851	219.3264
HORVU7Hr1G043590	0.028531	0.013942	0.373651	1.055138	10.89676	10.15009
Hordeum_vulgare_newGene_15164	0.728641	0.799963	0.901789	2.167553	1.604696	2.486458
Hordeum_vulgare_newGene_8817	18.47674	17.49857	10.12706	0.315235	0	0.133992
HORVU2Hr1G037480	10.31429	9.840875	8.426278	3.907676	1.63038	1.546103
Hordeum_vulgare_newGene_5332	12.42983	14.47596	13.05832	0.038104	0	0.031379
Hordeum_vulgare_newGene_9495	4.630996	4.600874	3.405845	11.62887	10.86333	14.35527
HORVU3Hr1G013910	57.11916	58.9274	71.79393	22.04005	10.86949	13.18385

Hordeum_vulgare_newGene_13868	6.772528	4.409983	4.595448	0	0	0
HORVU4Hr1G090070	1700.165	1701.684	1852.954	959.4144	946.4205	716.3863
HORVU3Hr1G111040	0	0	0.012601	0.212149	0.262613	0.379007
HORVU6Hr1G025780	1.044049	0.722574	0.849068	2.017012	2.746932	2.568759
HORVU1Hr1G009200	0	0	0	1.785171	1.157842	1.325242
HORVU1Hr1G073720	0	0	0	2.404233	2.244892	2.529441
HORVU3Hr1G091000	3.376838	2.838333	3.81146	9.754224	10.20322	9.501259
HORVU6Hr1G066140	0.976981	0.54472	0.560389	12.29997	19.85144	18.46944
Hordeum_vulgare_newGene_1527	5.550839	6.371141	6.300919	3.790684	3.792436	3.613922
Hordeum_vulgare_newGene_2528	2.826125	2.329599	2.885245	0	0	0
Hordeum_vulgare_newGene_1529	1.935169	0.942009	2.464476	0.278047	0.181103	0.302316
HORVU3Hr1G014140	10.0774	9.533653	9.96826	4.754956	1.341614	2.425447
HORVU3Hr1G068410	0.03745	0.033943	0.041022	0.295029	0.246219	0.468307
HORVU2Hr1G012010	0.164241	0.117	0.100947	1.121176	0.671898	0.736728
HORVU5Hr1G101680	12.55704	10.98508	13.06062	23.09576	36.79351	43.76552
Hordeum_vulgare_newGene_12650	1.995959	1.723568	2.196049	0	0	0
Hordeum_vulgare_newGene_12654	0.486675	1.128517	0.935744	0	0.021575	0.037734
HORVU6Hr1G059440	17.00297	18.14403	24.11965	10.13302	10.89452	12.23242
Hordeum_vulgare_newGene_518	1.669774	2.429311	3.228332	1.239446	1.488351	1.484477
HORVU5Hr1G064280	0	0	0	0.882618	1.576053	1.866895
Hordeum_vulgare_newGene_510	0.834466	1.235294	0.808928	0.637125	0.314231	0.352471
HORVU3Hr1G014850	0.160237	0.099535	0.22248	1.393982	3.260545	3.392232
HORVU7Hr1G118090	9.497113	8.473447	8.91691	4.563059	5.302566	4.085869
HORVU6Hr1G075240	0.247985	0.120467	0.274835	1.087094	2.28928	1.232704
HORVU3Hr1G079800	0.24919	0.316405	0.256148	9.749723	13.2608	9.079684
HORVU3Hr1G087970	0	0.126039	0.036412	0.913617	2.898111	2.621697
Hordeum_vulgare_newGene_14713	6.527189	5.584315	4.782977	0	0	0
Hordeum_vulgare_newGene_14719	0.296569	0.587593	0.449007	0.030213	0.03722	0.02897
HORVU4Hr1G084950	0.310112	0.190269	0.190478	0.778686	0.654061	0.696658
HORVU5Hr1G124650	15.74135	9.212208	19.03352	7.450055	2.415136	6.600734
HORVU3Hr1G055450	6.345028	5.983562	5.558211	3.382355	2.049433	2.400891
HORVU0Hr1G000760	23.17777	13.62267	19.9211	2.69568	0.597016	3.849432
HORVU7Hr1G054190	0.808085	0.814251	0.776902	2.273456	3.472529	3.283431
HORVU2Hr1G006720	0.9144	0.623428	0.563653	1.41876	6.808415	4.069536
HORVU5Hr1G058830	1.512926	2.081987	2.131047	0.667855	0.85878	1.32113
HORVU3Hr1G032200	1.733111	1.46433	1.214122	4.344565	6.86343	5.968316
HORVU3Hr1G087020	2.183928	1.476183	2.07923	0.870379	0.25473	0.542651
HORVU4Hr1G043930	1.521244	1.672144	1.926853	0.751778	0.746707	1.378772
HORVU3Hr1G016280	11.13115	11.54784	12.72584	4.815918	2.279539	2.623096
HORVU0Hr1G002720	1.351909	1.101945	2.803927	0.413641	0.460681	1.422131
HORVU7Hr1G117180	1.312459	1.30876	1.337254	11.20167	25.88643	21.23831
HORVU1Hr1G050450	0.612097	0.800947	0.793705	1.068856	2.185055	2.166722
HORVU3Hr1G016820	5.885169	4.58876	3.990311	0	0.023396	0.04027
HORVU3Hr1G004070	5.186191	6.665648	11.49847	1.961898	0.102749	0.780264
HORVU6Hr1G060100	1.599246	1.785406	1.735392	3.599063	5.4134	4.904629
HORVU1Hr1G067100	7.192422	8.309506	11.05359	4.446566	3.556107	3.863659
HORVU3Hr1G015730	0.3899	0.356249	0.653668	1.433607	2.146852	1.971335

HORVU5Hr1G114540	5.695655	4.360284	4.492013	21.66723	19.01085	19.78954
HORVU4Hr1G011170	1.655855	1.897769	1.521399	6.380617	8.596868	8.102591
HORVU5Hr1G114090	6.226905	6.331198	6.666037	20.46334	28.58294	28.46932
HORVU1Hr1G018370	4.345983	2.371914	5.269359	2.080026	1.366513	1.37661
HORVU5Hr1G066930	21.521	16.17071	20.00393	1.139592	4.086337	0.785822
HORVU0Hr1G013180	8.144294	4.128642	2.376556	15.531	12.42684	19.93038
HORVU4Hr1G000510	13.00414	12.17968	10.85906	7.724024	5.590293	6.875757
HORVU3Hr1G079340	0.733668	0.522657	0.733834	2.44572	1.927495	2.039716
HORVU1Hr1G056530	0.69142	0.464082	0.750303	3.327257	17.73398	16.27729
HORVU5Hr1G060570	2.285085	2.025379	2.120965	1.092264	1.253993	1.008837
HORVU7Hr1G016770	0.069967	0.089685	0.058831	1.522761	1.888303	2.59821
HORVU6Hr1G022370	13.90975	15.91801	13.22529	40.29224	42.27549	38.53698
HORVU2Hr1G109590	2.522313	1.601805	4.333025	0.78846	0.477448	1.161734
Hordeum_vulgare_newGene_10462	5.791128	6.205193	6.742593	0.34812	0.226903	0.363713
Hordeum_vulgare_newGene_10463	3.748649	4.301203	4.529651	0.280268	0.067434	0.266117
HORVU6Hr1G032050	2.337216	2.565354	3.403479	26.98048	71.28479	61.87289
HORVU7Hr1G009340	47.98628	39.86341	60.33403	7.168076	11.58875	10.10155
HORVU2Hr1G000420	0	0.023708	0.024892	1.486736	1.021861	1.339544
Hordeum_vulgare_newGene_11736	0	0	0	0.254978	0.223401	0.351998
Hordeum_vulgare_newGene_11739	9.812696	11.82596	10.35586	2.697257	1.127594	1.497122
HORVU5Hr1G042180	1.008629	1.447538	1.567501	3.393175	3.375309	3.87934
HORVU4Hr1G002800	5845.607	5551.766	5781.161	1360.334	386.774	646.7091
HORVU2Hr1G063820	2.581656	2.399122	2.860727	4.349367	11.22506	9.773486
HORVU6Hr1G085760	20.44479	27.83595	30.49549	5.949047	6.602545	6.484549
HORVU6Hr1G092780	5.078236	4.143841	5.452238	2.031791	0.68093	1.259813
HORVU6Hr1G088270	1.89756	2.11807	1.499705	0.912992	0.540604	1.105833
HORVU1Hr1G013480	0.487576	0.840401	0.91434	0.167002	0.104407	0.443711
HORVU7Hr1G041410	5.263986	5.289047	7.086306	1.812948	2.965075	3.477053
Hordeum_vulgare_newGene_12411	5.6558	6.595687	3.931148	0.963744	0.393441	0.941107
Hordeum_vulgare_newGene_12416	87.46513	116.5961	133.4077	21.76731	14.20153	14.56675
Hordeum_vulgare_newGene_12415	0.387707	0.366803	0.313868	0.065858	0	0.010071
HORVU3Hr1G116710	0.160937	0.153093	0.177925	0.372091	0.78385	0.748049
HORVU2Hr1G102930	0.116684	0.111503	0.232144	0.666091	2.191166	3.075424
HORVU5Hr1G075540	2.577066	1.266506	3.249729	0.048651	0	0.050902
Hordeum_vulgare_newGene_452	0.308276	0.157757	0.536448	1.659042	1.196728	0.968823
Hordeum_vulgare_newGene_456	0.811041	1.083952	0.539724	1.512883	2.309558	2.400148
Hordeum_vulgare_newGene_457	0.777449	0.729679	0.731281	0.014374	0.022135	0.060123
HORVU2Hr1G086170	2.691552	3.436381	4.247376	1.836813	1.916995	2.034581
Hordeum_vulgare_newGene_14157	5.833439	4.857562	6.882979	0	0	0
Hordeum_vulgare_newGene_14150	1.898315	1.437976	1.724236	0	0.133984	0.059854
HORVU2Hr1G019820	23.76954	19.74555	20.85895	45.7859	70.88899	70.64487
HORVU2Hr1G006100	0.217962	0.205441	0.214923	0.53813	0.642684	1.081544
Hordeum_vulgare_newGene_10813	0.991321	1.122287	1.092717	0.672648	0.45247	0.585606
Hordeum_vulgare_newGene_10812	0.8565	0.901725	0.831227	0	0	0
HORVU5Hr1G062720	0.314273	0.233606	0.735721	2.94404	1.281197	1.885925
Hordeum_vulgare_newGene_2450	0.075951	0.097148	0.099687	0.633917	0.461274	0.811724
Hordeum_vulgare_newGene_15281	0.949724	1.430451	1.133146	3.505764	5.429464	4.999876

HORVU5Hr1G057210	0.196897	0.305158	0.195863	0.498395	0.922607	1.045647
HORVU4Hr1G083230	0.201153	0.140344	0.148501	1.341709	1.321499	1.418192
HORVU4Hr1G076940	7.305171	7.757609	9.795372	166.7106	204.3381	188.0248
HORVU6Hr1G079030	71.22363	67.76961	88.28751	47.80499	36.62832	34.71868
HORVU7Hr1G009800	0.146532	0.133039	0.178758	0.599701	0.481872	0.889484
HORVU7Hr1G060260	12.67838	12.6549	13.37923	28.18033	33.3748	37.55291
Hordeum_vulgare_newGene_1221	1.347917	2.019495	1.26803	3.235594	5.329583	4.747703
Hordeum_vulgare_newGene_1220	1.739943	1.980112	1.995781	4.764443	7.817866	7.747708
Hordeum_vulgare_newGene_10101	10.16242	9.778526	7.067498	4.495468	2.807844	3.139381
HORVU5Hr1G007890	0.270729	0.18885	0.338059	2.143646	2.839284	2.989923
Hordeum_vulgare_newGene_5107	0.73836	0.942602	0.680722	0.080934	0.087218	0.081941
HORVU7Hr1G114610	0.063729	0.060975	0.09057	2.197913	1.456396	1.477112
HORVU5Hr1G010900	0.163161	0.250548	0.181606	0.568952	0.548257	0.434504
HORVU1Hr1G008360	42.93968	24.32901	41.93436	0.66484	0.383298	0.528349
Hordeum_vulgare_newGene_1486	31.79999	29.32552	35.80843	9.471358	4.672303	5.021204
Hordeum_vulgare_newGene_1489	4.585829	3.745827	4.272097	0.011033	0	0
Hordeum_vulgare_newGene_4051	1.181357	1.899429	1.673503	0	0	0
Hordeum_vulgare_newGene_4050	4.866401	4.264124	4.393713	0	0	0
HORVU7Hr1G101740	4.706504	4.514455	4.931756	24.64583	18.54919	16.49184
HORVU3Hr1G070700	2.549789	1.739064	3.011951	1.614117	0.846955	1.722188
HORVU2Hr1G029350	28.55163	23.51104	29.66731	54.13243	80.90602	71.91369
Hordeum_vulgare_newGene_14908	0.287596	0.281804	0.333038	0.875505	0.837154	0.830244
HORVU3Hr1G080390	0	0	0	0.643071	0.520225	0.604801
Hordeum_vulgare_newGene_7502	0	0	0	1.689611	1.449229	2.101808
HORVU1Hr1G071930	3.629059	3.752812	4.317239	2.026622	2.110377	1.954618
HORVU2Hr1G077710	217.4332	163.3992	236.916	120.0826	170.5902	90.11734
HORVU4Hr1G063660	1.42146	1.255303	1.927161	0.71625	0.471564	1.159732
HORVU2Hr1G045100	0.57389	0.402072	0.396406	1.19337	1.608226	1.721369
HORVU3Hr1G086940	57.77198	66.7599	65.26476	26.46877	20.90094	21.53189
HORVU0Hr1G001170	0.289015	0.413472	0.46076	3.886506	5.897858	5.790773
HORVU5Hr1G019030	97.3728	92.06484	95.76639	180.5372	304.6119	291.1681
Hordeum_vulgare_newGene_1557	0	0	0	3.518308	3.772372	3.799274
HORVU4Hr1G074400	34.46564	23.607	15.76918	12.30933	5.744374	10.13128
HORVU2Hr1G096510	6.319261	3.913343	5.859725	19.51217	21.38169	21.47076
Hordeum_vulgare_newGene_13436	33.16716	28.06304	22.50167	13.03177	20.49534	18.81649
HORVU6Hr1G004900	3.060766	2.411912	2.923721	0.043007	0.067247	0.019829
HORVU4Hr1G017240	2.884815	3.422833	5.010587	0.679952	0.306181	0.419619
HORVU5Hr1G122540	0	0	0	1.897789	3.28903	2.109789
HORVU1Hr1G061230	1.461017	1.783551	1.878334	7.393287	7.438268	8.43359
HORVU7Hr1G098810	0.635851	0.964189	1.018324	0.245144	0.115276	0.163148
HORVU3Hr1G029750	0.445851	0.419647	0.532183	1.09713	2.182238	2.00732
HORVU4Hr1G018430	11.43265	9.438723	6.910655	2.265293	5.174369	5.237421
HORVU7Hr1G049400	1.649675	1.329567	1.318824	0.738938	0.5896	0.944065
HORVU1Hr1G023100	87.53727	78.88799	93.17868	154.3197	537.0521	510.2295
HORVU2Hr1G080770	0.160681	0.19635	0.119087	0.818377	0.84797	1.196151
HORVU7Hr1G051750	4.442921	6.357982	4.967847	19.26355	39.95219	35.90403
HORVU6Hr1G040040	0.25795	0.378357	0.649023	9.517406	7.677933	6.88642

HORVU6Hr1G085370	0.254059	0.149448	0.208881	8.779708	11.45891	14.01549
HORVU5Hr1G106850	3.69128	1.546474	5.556507	0.796423	0.533216	0.878958
HORVU1Hr1G052620	0.745898	0.725097	1.006942	1.833684	5.347465	4.964475
HORVU4Hr1G065660	0.66862	0.538228	0.717362	1.85049	3.222365	3.329334
HORVU6Hr1G091300	24.77416	18.29023	26.54071	15.00988	14.2082	13.95945
HORVU1Hr1G043820	17.66252	16.25877	17.3238	53.78251	264.1378	240.5403
HORVU2Hr1G099100	1.454628	1.240679	1.180593	3.87424	5.40593	4.528838
HORVU5Hr1G074340	13.67948	10.39903	13.20663	41.85528	37.63276	37.65328
HORVU4Hr1G004170	0.367927	0.138901	0.193811	1.234108	6.937468	5.661502
HORVU5Hr1G125270	15.99912	15.57276	16.16419	9.825631	9.271746	9.139152
Hordeum_vulgare_newGene_10344	3.807327	4.641558	3.49131	0	0	0
Hordeum_vulgare_newGene_10347	0	0	0.006569	1.439224	1.168998	1.684091
Hordeum_vulgare_newGene_10341	24.2438	20.34279	24.36457	61.79584	109.0452	78.06347
Hordeum_vulgare_newGene_10343	0.664158	0.5422	1.105424	12.79139	101.0165	82.9831
HORVU3Hr1G109370	0.122427	0.141504	0.11002	0.778091	1.094427	0.851408
Hordeum_vulgare_newGene_10348	0	0	0	1.975102	1.379166	2.497579
HORVU3Hr1G114220	1.289004	1.778935	1.796595	4.228337	7.457868	6.14483
HORVU4Hr1G004820	48.89988	44.04359	44.30876	20.05765	9.038001	13.58745
HORVU2Hr1G019660	82.96233	92.24533	171.5368	53.67987	81.31144	72.0548
Hordeum_vulgare_newGene_10698	2.092822	1.860301	2.134432	4.945563	8.475513	7.603456
HORVU3Hr1G115170	1.243756	1.070501	1.161538	5.268008	11.63941	8.268638
HORVU7Hr1G027740	0.332827	0.5868	0.661508	2.355193	2.567652	1.939829
HORVU1Hr1G014000	10.01544	13.95021	13.82631	0.353026	0.49956	0.415677
HORVU6Hr1G093210	0.497451	0.542564	0.785101	1.47007	2.353794	3.334177
HORVU5Hr1G048240	21.39174	22.1887	23.16006	14.13239	7.629348	8.153169
HORVU7Hr1G009680	2.541378	2.091795	2.727264	0.704408	0.287247	0.87479
Hordeum_vulgare_newGene_780	0.468437	0.051507	0.461695	5.929786	5.740828	5.08186
HORVU5Hr1G099700	10.91367	11.10318	15.86805	3.896719	5.600315	5.503182
HORVU7Hr1G029580	0.89606	0.715915	1.131323	0.305274	0.407575	0.536435
HORVU2Hr1G029110	0	0.05191	0	0.933772	1.833497	3.411069
Hordeum_vulgare_newGene_1446	2.590766	2.203609	3.926949	9.029987	10.61522	9.979388
HORVU7Hr1G105190	0.520413	0.400663	0.680022	0.24154	0.076994	0.331311
HORVU3Hr1G056440	122.4932	108.0862	94.94984	22.71088	26.04339	28.65341
HORVU4Hr1G037160	0.911217	0.58259	0.744954	1.96249	3.193572	3.15806
HORVU6Hr1G011970	2.04512	1.135336	3.070448	4.798494	5.096758	6.613997
HORVU5Hr1G031870	4.364962	3.909627	4.171274	8.412105	16.11731	15.50817
Hordeum_vulgare_newGene_523	0.180955	0.175937	0.168178	1.204358	1.128472	1.105289
HORVU1Hr1G085720	0.08151	0.031184	0.200075	3.395016	1.686421	2.76892
HORVU1Hr1G008540	12.96583	13.67266	17.34044	2.572299	2.599167	2.674077
HORVU6Hr1G071500	0.195074	0.072486	0.030756	2.991603	3.733841	4.669787
HORVU1Hr1G075530	22.36445	23.58752	23.34922	17.23294	10.48227	13.12087
Hordeum_vulgare_newGene_6166	0.965399	0.943136	0.857656	3.507377	6.88967	6.782944
Hordeum_vulgare_newGene_3604	1.205539	1.216671	1.274726	0	0	0
Hordeum_vulgare_newGene_3603	0.523033	0.646397	0.609299	1.876756	1.874203	1.807696
HORVU5Hr1G028260	0.637733	0.422148	0.703296	1.526299	5.144892	4.515155
HORVU5Hr1G068350	3.344782	1.639252	3.841286	0.053321	0	0
HORVU5Hr1G074260	0.028308	0.01674	0.004747	0.734391	0.88108	1.361355

Hordeum_vulgare_newGene_8	3.8639	3.790814	3.806644	0	0	0
Hordeum_vulgare_newGene_5602	2.789734	2.761213	2.867701	0	0	0
HORVU7Hr1G043280	4.224267	4.031275	5.360272	6.435967	17.30195	13.9429
HORVU2Hr1G027160	4.124347	2.754489	5.27362	2.487009	1.583229	2.421169
HORVU3Hr1G069140	99.75956	113.3557	147.1785	15.61971	0.205543	6.522916
Hordeum_vulgare_newGene_11953	2.929458	1.514106	2.567415	0.897651	0.441755	0.410649
HORVU7Hr1G049130	2.571965	4.101863	4.806507	0.173527	0.009089	0.085033
HORVU6Hr1G064650	0.767899	0.534258	0.602373	4.067	1.649811	2.486784
HORVU5Hr1G073280	2.717433	2.811174	3.02163	1.414377	0.890929	0.993042
HORVU7Hr1G037140	0.933395	1.168994	0.97888	1.789304	12.97229	9.968855
Hordeum_vulgare_newGene_15095	0	0	0	4.770342	4.752525	8.293515
HORVU0Hr1G008640	3.583714	4.682331	5.482095	9.425286	15.61112	15.05998
HORVU6Hr1G057550	2.014151	2.7404	2.933822	4.199538	8.171679	7.708772
HORVU3Hr1G083990	2.022285	2.905673	2.408712	7.508756	13.75233	11.69239
HORVU7Hr1G067110	5.983264	6.065266	5.169611	12.71382	22.35366	21.10525
HORVU3Hr1G018550	8.285384	6.778221	6.525894	49.38966	143.0889	100.0793
HORVU7Hr1G039890	3.803836	2.741201	4.360974	1.99636	1.649228	2.059195
HORVU7Hr1G114000	196.4749	199.0226	156.4315	131.7101	54.27599	62.11837
HORVU4Hr1G008610	4.030342	6.972599	7.545248	2.90861	2.682595	2.907514
HORVU1Hr1G063700	0.795888	0.638699	1.194768	3.617584	13.68333	13.31589
Hordeum_vulgare_newGene_4155	1.245193	1.704143	1.705604	0.86393	0.737869	1.091481
HORVU3Hr1G021550	0.57073	0.228971	0.341643	6.466224	3.355185	2.970157
HORVU6Hr1G028740	4.472875	3.579809	4.75193	3.152307	1.270399	1.867517
HORVU6Hr1G081250	21.09328	21.4309	20.04073	9.428029	9.343749	9.052784
HORVU6Hr1G032220	5.24253	4.392525	5.510897	13.11323	36.23793	35.4828
Hordeum_vulgare_newGene_7710	59.89263	99.3754	141.9861	25.40372	39.00901	41.03914
Hordeum_vulgare_newGene_7719	12.33095	18.08966	22.11854	10.77882	8.245649	8.281179
HORVU1Hr1G029180	5.070913	3.548193	4.960743	21.11007	26.22487	25.10785
HORVU3Hr1G039930	8.710922	7.504847	6.8085	33.76153	64.51499	63.75953
HORVU7Hr1G025390	87.04028	80.39166	105.3416	183.6526	284.1862	256.8718
HORVU2Hr1G078730	5.126748	3.900963	3.916072	4.342341	1.773888	2.597512
HORVU5Hr1G008250	30.59729	36.11102	33.6921	18.75372	18.49737	21.9891
HORVU5Hr1G058240	0.619561	0.605579	0.603191	1.557493	1.955386	2.124671
Hordeum_vulgare_newGene_6446	0.635938	1.627041	1.749045	0.454018	0.469425	0.651932
HORVU7Hr1G055080	6.536221	6.377675	5.985494	20.38126	23.97432	27.34057
HORVU4Hr1G074230	0.386854	0.349123	0.44044	1.648901	1.453473	1.176911
HORVU1Hr1G087050	0.02755	0	0	0.616095	0.671463	0.661225
HORVU6Hr1G067880	0.242687	0.23739	0.27879	0.661527	1.381533	1.800233
HORVU0Hr1G018120	5.241058	6.693723	7.482137	2.957256	3.532002	3.770261
HORVU3Hr1G030770	40.68898	50.88973	64.48537	7.376673	4.343935	6.827011
HORVU7Hr1G055330	4287.015	4110.324	3215.866	13.05938	3.823618	6.91564
HORVU6Hr1G030880	8.186292	7.642417	8.650053	15.11283	25.59852	24.66446
HORVU5Hr1G076110	8.695241	10.47531	13.52951	5.174571	7.673049	7.699638
HORVU7Hr1G112240	0.250564	0.205306	0.196463	1.339594	1.558487	1.8553
HORVU2Hr1G017880	36.17805	36.54508	42.30657	19.72445	14.50854	20.31021
HORVU7Hr1G040080	2.352144	2.743369	2.406945	19.46339	24.30513	19.28093
HORVU1Hr1G089520	13.10833	13.7955	23.07638	0.888124	0.074545	0.825726

HORVU5Hr1G113780	5.92931	9.209668	8.340602	0.260155	0.209123	0.157015
HORVU5Hr1G122080	1.442315	1.248566	1.144971	9.532592	12.81316	10.41539
HORVU7Hr1G121470	0.045285	0.022118	0.015757	0.293346	0.193917	0.314708
HORVU7Hr1G049610	1.261134	1.131565	1.229478	0.399633	0.105249	0.162634
HORVU7Hr1G046030	38.54948	41.8255	47.0788	21.85585	5.454809	11.92235
HORVU3Hr1G071300	0.782295	0.940859	0.840429	2.609007	3.211927	4.624774
HORVU5Hr1G005180	88.46954	50.56901	116.3236	60.30116	28.50197	56.67109
HORVU1Hr1G058540	5.36813	5.964145	8.262786	2.85196	3.001322	3.267505
HORVU3Hr1G036960	2.440142	1.958865	2.340475	0.801335	1.194993	1.107438
HORVU7Hr1G047070	6.000693	5.130026	8.232494	5.463162	3.015785	3.979076
Hordeum_vulgare_newGene_13017	0.536257	0.89856	0.391495	2.790169	2.924769	3.09664
HORVU4Hr1G054200	64.52961	53.50653	66.68953	182.961	241.0486	227.9964
HORVU2Hr1G108730	22.53028	17.64782	20.36229	1.931816	3.745341	3.845742
HORVU1Hr1G011950	1.865893	2.059611	2.179591	0.171439	0	0
HORVU3Hr1G096500	1.34839	1.706669	1.528939	3.908018	4.762341	5.056268
HORVU5Hr1G093580	5.949321	7.36088	9.345167	1.345404	2.28401	1.882276
HORVU5Hr1G078960	4.587743	4.491632	6.168337	14.0461	20.58811	19.37127
HORVU4Hr1G061990	0.22767	0.17798	0.25049	1.128181	114.7094	98.54038
HORVU3Hr1G074470	0.202078	0.266664	0.199941	1.769347	1.998804	2.086288
HORVU6Hr1G014480	0.563035	0.859055	0.724879	2.341612	3.229575	3.257329
Hordeum_vulgare_newGene_10556	1.169834	1.295065	1.362655	0	0	0
HORVU5Hr1G002320	0.607363	0.419353	1.002269	0.96627	3.247841	2.955102
HORVU3Hr1G104290	0.028845	0	0.022358	1.156385	1.254172	2.108729
HORVU0Hr1G022070	0.086592	0.043644	0.061013	0.929638	0.753839	1.15156
HORVU4Hr1G077310	0.408864	0.718895	0.957715	7.54336	7.438818	9.178616
HORVU3Hr1G095360	6.51788	8.146865	10.01562	0.990533	0.558411	0.464915
HORVU1Hr1G073940	150.795	119.9377	114.8665	51.56285	38.18001	40.51075
HORVU6Hr1G020750	14.17874	11.47018	15.67353	8.13212	7.422409	7.285108
HORVU1Hr1G010810	0.66342	1.222966	1.143161	3.767503	7.120244	6.826556
HORVU4Hr1G032980	0.035538	0.021871	0.048713	13.50895	13.53975	17.11932
HORVU1Hr1G084350	10.41703	10.76513	14.02937	5.307725	8.815727	7.702086
HORVU3Hr1G094010	5.13075	4.599982	5.309139	0.301708	0.641812	0.314276
Hordeum_vulgare_newGene_599	1.674209	2.277278	1.687446	0.281776	0.07608	0.053131
Hordeum_vulgare_newGene_12301	1.621688	2.235746	1.832955	0.842577	0.382214	0.674646
Hordeum_vulgare_newGene_7155	0.746501	1.482719	1.938116	2.745219	5.185171	4.598942
HORVU6Hr1G095080	2.956686	3.093119	3.819844	8.667687	12.39473	10.5741
Hordeum_vulgare_newGene_6288	0.225262	0.248806	0.261684	0.548273	0.713802	1.026817
Hordeum_vulgare_newGene_6289	0.275825	0.284535	0.243895	0.567663	1.390035	1.27666
HORVU5Hr1G084510	6.73207	9.178247	11.61585	2.901229	3.442474	4.661879
HORVU3Hr1G113050	37.63674	30.25148	30.42259	18.11458	17.79308	16.93174
HORVU7Hr1G101860	2.488818	2.744505	2.799478	10.25676	14.17273	12.26128
HORVU3Hr1G017000	0.955751	1.225386	0.780191	4.905376	5.529126	5.151114
HORVU1Hr1G080600	1.798536	1.295001	1.256133	0.227156	0.134347	0.288708
HORVU4Hr1G074030	1.339533	1.17503	1.091388	3.432815	6.346313	5.894432
Hordeum_vulgare_newGene_14469	3.683914	3.059353	2.826898	0.226355	0.390596	0.29065
Hordeum_vulgare_newGene_14466	0	0	0	3.155768	2.655701	2.048642
HORVU3Hr1G061450	0.626546	1.281214	1.351257	0.656647	0.280265	0.59765

Hordeum_vulgare_newGene_13168	6.307444	7.699157	6.941234	0.006767	0.003007	0
Hordeum_vulgare_newGene_13161	50.24355	46.55775	33.33028	9.106679	2.495829	4.410457
Hordeum_vulgare_newGene_14797	54.76973	25.19826	42.53322	1.12916	1.311527	1.874949
HORVU5Hr1G065370	6.598546	5.337004	6.637064	3.979911	2.750101	3.953913
HORVU1Hr1G042250	2.824583	3.58072	3.691584	0.782766	0.450165	0.615432
HORVU0Hr1G005570	1.474351	2.293055	1.996646	6.340526	5.563174	6.604797
HORVU4Hr1G075070	1.518587	1.050754	1.288991	0.380729	0.312035	0.478447
HORVU2Hr1G004620	15.84843	10.99377	20.8605	1.988442	0.385946	1.906704
Hordeum_vulgare_newGene_830	11.45472	11.70196	12.24953	5.799767	1.994779	3.620821
HORVU2Hr1G075950	3.14032	3.94222	4.627973	0.938278	1.19339	1.264606
HORVU5Hr1G063690	0.597898	0.690592	0.915508	0	0	0
HORVU4Hr1G076520	11.19489	12.7113	12.42251	1.345217	0.736183	1.221456
HORVU3Hr1G018690	2.090665	2.111944	2.240531	1.375112	0.688363	1.288477
HORVU6Hr1G030600	0.907473	0.722866	0.869128	2.503518	14.26734	11.52611
HORVU7Hr1G113020	13.23131	8.694576	10.43473	2.131029	0.938413	0.978655
Hordeum_vulgare_newGene_1968	0.962739	1.22462	3.065957	0.314007	0.022687	0.196259
HORVU5Hr1G069910	1.218597	0.742496	1.341236	4.748513	7.085078	4.77076
HORVU7Hr1G010570	3.134752	4.063422	4.009089	1.080654	1.829401	1.33789
Hordeum_vulgare_newGene_1646	1.258778	1.336292	1.159524	0.509574	0.310906	0.588828
Hordeum_vulgare_newGene_1645	0.838468	0.727962	0.941624	1.662093	2.62334	2.535808
HORVU2Hr1G125430	0.361322	0.094129	0.2619	1.812878	1.988899	2.021095
HORVU3Hr1G071490	471.8783	481.8066	665.5011	195.0497	90.15669	114.2886
HORVU1Hr1G072750	4.669039	4.736707	5.860684	10.69703	16.21386	15.86155
HORVU2Hr1G080190	3.962859	3.283993	3.435344	0.293248	0.073401	0.046028
HORVU7Hr1G090460	0.422371	0.367297	0.26363	1.103194	1.230606	1.423827
Hordeum_vulgare_newGene_10097	0.234486	0.318361	0.307273	0.746231	1.014747	1.067486
HORVU1Hr1G080840	1.37932	1.905206	1.206325	3.87437	5.145454	5.488751
HORVU6Hr1G083680	0.259594	0.23841	0.252433	0.596927	0.645421	0.960987
HORVU5Hr1G125550	0.061772	0.097581	0.045523	0.365667	0.540222	0.634069
HORVU1Hr1G065130	3.205404	2.667854	4.462221	7.638216	15.21732	17.7532
HORVU7Hr1G007690	74.29261	58.11679	87.8064	6.384541	0.011419	1.768069
HORVU1Hr1G051190	0.950278	0.944164	0.98466	0.378206	0.408099	0.343207
HORVU1Hr1G094180	0.46216	0.357433	0.447302	2.952319	2.863756	3.894566
HORVU3Hr1G062430	2.171168	1.284237	1.763433	7.187924	3.73097	8.885719
HORVU5Hr1G008770	0.220893	0.207221	0.390479	1.456007	8.009475	7.30341
HORVU4Hr1G051450	4.082158	3.315461	4.325355	0.833294	0.34697	0.585224
HORVU7Hr1G083230	1.225764	0.60519	1.333149	0.250058	0.158756	0.296417
HORVU5Hr1G007480	5.12373	6.275376	7.07268	0.070008	0.011254	0.04131
HORVU7Hr1G074890	15.82137	13.35468	19.35831	38.2573	49.14975	53.6342
HORVU7Hr1G051840	3.42401	3.987794	5.368038	0.272934	0.609189	0.262724
HORVU2Hr1G063220	20.84188	20.23508	20.89914	3.273756	0.656951	1.862606
HORVU6Hr1G086480	9.844689	11.73925	12.27231	5.761751	7.356677	6.302215
HORVU4Hr1G049500	26.33492	34.61492	39.35975	8.78139	7.460604	9.936711
HORVU7Hr1G077270	3.643091	3.323165	5.211735	0.799962	0.21813	0.237576
HORVU3Hr1G092520	4.316466	3.278397	2.307381	13.07941	9.998172	11.53302
HORVU3Hr1G023230	3.516424	2.303983	4.271917	1.079132	0.271178	0.980269
HORVU3Hr1G089000	3.2168	2.774652	2.774325	6.428775	12.52615	12.73748

HORVU7Hr1G093830	6.44236	7.362943	6.165078	0.034189	0	0.034009
HORVU5Hr1G010880	58.89209	68.89705	80.5538	6.552048	0.772367	4.251762
HORVU5Hr1G041210	0.028943	0.101915	0.116087	0.376429	0.64938	0.801606
HORVU2Hr1G063510	14.56809	9.335041	12.64275	59.7635	58.1296	53.2037
HORVU3Hr1G101990	0.702738	0.500725	0.621408	3.730883	7.595078	5.446475
HORVU4Hr1G082130	5.09161	5.236824	5.033576	2.358597	3.032562	3.299159
HORVU1Hr1G015260	0.075367	0.251037	0.210921	1.109232	1.782816	1.872412
Hordeum_vulgare_newGene_13361	9.073759	11.47086	8.215452	0.051931	0	0.048134
Hordeum_vulgare_newGene_9064	0	0	0	5.939439	9.130514	10.16458
Hordeum_vulgare_newGene_2729	0.221587	0.239659	0.317517	2.035593	0.96274	0.883253
Hordeum_vulgare_newGene_2726	0.089405	0.085814	0.083149	0.401206	0.724605	0.766929
HORVU1Hr1G070690	88.31738	109.4988	142.9097	23.6914	13.60507	15.31109
Hordeum_vulgare_newGene_13984	2.741268	2.021566	3.912531	21.99278	19.89721	27.59148
Hordeum_vulgare_newGene_13985	3.866895	3.786493	4.096085	2.110468	1.092444	1.47143
Hordeum_vulgare_newGene_13980	0.657028	0.408219	0.616205	1.659642	1.943022	2.178997
HORVU3Hr1G021490	2.07153	3.209027	3.259059	0.044636	0.042417	0.053587
HORVU7Hr1G072620	12.65336	10.8257	14.09435	0.13874	0	0
Hordeum_vulgare_newGene_5188	26.25018	28.95388	38.39269	13.54599	2.732637	7.397759
HORVU7Hr1G089540	0.581496	0.255098	0.30537	3.456094	9.578941	9.900329
HORVU4Hr1G000520	1.339292	1.180931	1.471158	2.710783	4.766078	4.523093
HORVU2Hr1G023560	5.988397	5.063728	4.537307	17.77188	28.12582	30.27885
Hordeum_vulgare_newGene_10635	3.779289	3.902347	5.18005	0	0.034814	0.067492
HORVU7Hr1G081770	80.85577	87.96071	97.25822	237.3607	253.0906	234.029
HORVU6Hr1G015890	5.15377	4.317102	4.605389	2.076791	0.683679	1.127184
Hordeum_vulgare_newGene_14988	2.753393	2.110993	2.739183	0.94548	0.575102	0.549095
Hordeum_vulgare_newGene_4966	0	0	0	0.993384	0.531158	1.307946
HORVU2Hr1G026880	3.222447	5.123029	8.489375	1.992123	0.232412	1.098105
HORVU7Hr1G030810	4.890795	4.05462	5.62365	25.39054	33.2508	27.29655
HORVU2Hr1G043170	0.610324	0.973017	0.424028	1.478125	1.959124	2.390156
HORVU0Hr1G020960	2.190093	1.257024	1.280742	3.68734	10.55379	9.082496
Hordeum_vulgare_newGene_1402	0	0	0	0.786744	0.840769	0.634552
HORVU3Hr1G111830	0.185694	0.281701	0.156456	1.133905	2.153999	1.519868
HORVU1Hr1G075160	0.346499	0.198061	0.539658	3.038742	6.610257	8.469767
Hordeum_vulgare_newGene_15013	8.026796	7.594342	5.758216	1.401219	0.723893	0.74135
HORVU2Hr1G045630	2.220965	2.797768	2.829228	5.477385	9.232701	8.637435
Hordeum_vulgare_newGene_4632	3.571577	3.584054	4.041881	2.648611	1.195805	2.159689
HORVU5Hr1G014730	2.40123	2.925533	3.653921	3.633475	34.84562	35.36727
HORVU3Hr1G014950	23.89087	24.86933	23.72289	14.46261	11.88978	12.59152
HORVU6Hr1G069280	7.256457	8.821672	9.931699	3.23746	3.354177	3.282263
Hordeum_vulgare_newGene_12118	8.642522	16.70025	13.27172	0.530258	0.545223	0.650839
HORVU1Hr1G092540	3.616314	2.812714	4.049767	8.61706	19.6025	16.44123
HORVU3Hr1G085270	13.26235	12.05857	9.8545	39.72932	59.41408	61.60262
Hordeum_vulgare_newGene_257	2.655846	2.494323	2.868717	1.666538	1.369491	1.765391
Hordeum_vulgare_newGene_3115	1.175612	1.780156	1.869945	0	0	0
HORVU4Hr1G013320	0.234181	0.15632	0.240306	1.075278	3.565743	3.779409
HORVU1Hr1G026650	6.794422	5.348951	6.549394	13.60605	23.47072	21.25611
Hordeum_vulgare_newGene_3118	1.507423	1.230901	0.937915	8.27145	15.7428	17.18475

HORVU6Hr1G070740	2.64944	0.981345	3.909826	1.130181	0.78615	0.782283
Hordeum_vulgare_newGene_13649	12.13309	13.43131	10.18591	0	0	0
HORVU5Hr1G062990	120.8709	136.9959	161.5008	63.8667	5.95506	26.38049
HORVU5Hr1G107340	27.19431	22.96912	30.83866	10.82206	11.01616	10.74539
Hordeum_vulgare_newGene_14617	2.001621	2.285224	3.298909	10.94099	20.16447	15.71584
Hordeum_vulgare_newGene_13642	3.498477	2.38134	2.797508	0	0	0
HORVU5Hr1G000400	28.24764	33.17849	44.47714	2.354531	1.222403	1.511897
HORVU7Hr1G038200	3.63674	3.584227	3.620622	6.853981	19.71376	16.03303
HORVU6Hr1G082310	23.19768	22.19717	12.32659	0.571224	1.991035	1.729202
HORVU5Hr1G074170	0.188719	0.127088	0.423288	0.642118	0.84931	1.011896
HORVU7Hr1G118570	41.02916	35.15318	28.35646	4.729761	2.036481	2.707607
HORVU7Hr1G091910	3.392619	4.398141	5.885997	0.693558	0.824293	0.908611
HORVU3Hr1G068300	1.870151	1.867697	1.739416	6.809949	8.389242	8.315774
HORVU5Hr1G023730	180.3586	82.03866	241.9394	26.24835	5.378444	20.94194
HORVU5Hr1G069040	1.404198	1.850294	2.369258	6.06181	8.820946	9.792732
Hordeum_vulgare_newGene_8941	0.186182	0.198175	0.271258	0.478332	0.93475	0.532326
HORVU3Hr1G082550	0.80253	0.609603	1.002925	3.274736	6.215492	7.121939
HORVU5Hr1G061710	47.56899	46.80797	31.33487	14.63073	10.41242	13.1784
HORVU7Hr1G037780	0.387876	0.48755	0.448439	2.202997	2.589204	2.480598
HORVU3Hr1G082880	1.301937	1.314995	1.546236	0.832306	0.31985	0.479671
HORVU1Hr1G092680	34.30003	21.91934	33.48087	1.318389	1.039801	3.275405
Hordeum_vulgare_newGene_9564	0.118106	0.153329	0.251255	0.800381	1.025414	0.857977
HORVU7Hr1G092320	24.09494	22.79609	31.95679	14.82419	16.71696	16.07749
HORVU3Hr1G084480	2.700896	3.782187	3.903799	1.184348	2.122798	2.879463
HORVU3Hr1G083680	1.556437	1.013421	2.274999	5.25226	3.883938	4.627479
HORVU3Hr1G011990	1.288322	0.969071	2.104516	4.219198	2.900774	4.079219
HORVU7Hr1G036780	4.488273	4.320831	6.044183	3.108463	1.199073	2.882072
HORVU3Hr1G098150	1.423109	1.499766	1.563638	0.31176	0.331832	0.235763
HORVU4Hr1G080760	0.07161	0	0	0.92091	13.66677	12.08406
HORVU6Hr1G011090	1.614474	1.331126	1.755276	0.548727	0.331598	0.407215
HORVU2Hr1G077510	0.788928	0.820746	0.821261	3.074953	6.785751	5.326965
HORVU5Hr1G020410	2.205014	1.658896	1.838478	3.851943	5.551131	5.66631
HORVU5Hr1G120280	0.325503	0.449356	0.475511	0.665722	1.426513	1.771462
HORVU7Hr1G014410	1.100881	1.52637	1.781603	2.744067	11.45773	9.995132
HORVU2Hr1G088400	2.192674	1.176266	2.516524	4.42585	5.741818	5.359969
HORVU0Hr1G017490	6.283221	6.493536	6.42366	2.126076	3.723008	4.188255
HORVU3Hr1G009360	1.703349	0.899492	2.09238	106.2037	194.4587	195.4429
HORVU4Hr1G008180	7.580481	7.474719	8.369344	23.6699	20.40428	18.44036
HORVU2Hr1G107140	0.897025	0.843445	1.106857	0.447893	0.085883	0.2843
HORVU1Hr1G069510	2.455655	2.193257	2.763861	6.015365	6.972601	5.53683
HORVU1Hr1G057940	10.37547	13.90878	13.42253	5.746535	4.119859	4.025456
HORVU2Hr1G109650	3.544725	3.648974	3.165086	2.62664	1.233339	1.467699
HORVU5Hr1G009200	7.664004	6.248393	5.589104	17.7705	31.26962	28.8887
HORVU2Hr1G125600	0.926515	1.137924	1.165092	13.51329	10.74606	15.31038
HORVU0Hr1G015230	0.176949	0.184919	0.325828	0.744887	0.952676	1.225314
HORVU5Hr1G047530	19.77709	16.48744	23.31417	42.6424	79.26803	74.06418
HORVU4Hr1G054980	2.769565	3.583647	3.200201	0.085851	0.075141	0.030393

HORVU1Hr1G090210	225.1659	139.9839	191.4957	453.7084	782.6216	576.5759
HORVU1Hr1G012870	11.55813	13.24428	18.04024	47.06266	152.571	128.7026
HORVU3Hr1G078680	1.840063	0.097963	5.171676	17.00945	20.73931	21.80359
HORVU5Hr1G081720	1.122282	0.817053	0.917673	0.552362	0.448855	0.378369
HORVU2Hr1G108260	4.014351	3.067619	3.583682	11.50577	18.88862	19.18264
HORVU1Hr1G052420	28.3621	27.39646	24.96978	10.7294	5.51257	6.653225
HORVU7Hr1G012920	0.653139	0.635249	1.114506	3.056439	6.143288	5.893533
HORVU2Hr1G112580	67.59766	63.19467	66.98309	34.10036	10.30655	20.33545
HORVU2Hr1G016990	0.960641	0.76477	0.861722	2.696298	4.803711	4.109588
HORVU1Hr1G066630	981.8898	838.5752	924.0196	651.0134	420.5128	436.6644
HORVU7Hr1G000150	6.798833	4.781575	4.997905	13.77388	14.22173	13.31526
HORVU4Hr1G052110	0.328532	0.408981	0.430439	0.908713	1.815723	1.987732
HORVU2Hr1G102300	1.568576	1.310499	1.770006	3.543185	8.60345	8.468936
HORVU5Hr1G045820	5.634562	7.782154	7.18405	14.65002	30.74373	27.27706
HORVU7Hr1G121540	0.402893	0.388734	0.398906	0.758143	1.538592	1.133972
HORVU1Hr1G085230	0.130468	0.059808	0.07212	0.841873	7.147104	5.872144
HORVU5Hr1G082400	0	0	0	0.619355	0.506956	0.714189
Hordeum_vulgare_newGene_3357	1.032151	0.918952	1.497965	0.545397	0.377427	0.569123
HORVU4Hr1G062440	16.68975	21.06545	24.4079	7.247825	8.057524	14.32418
Hordeum_vulgare_newGene_12755	0.633104	0.900241	0.85801	0.111348	0.12797	0.129846
Hordeum_vulgare_newGene_12751	0.375512	0.504964	0.379508	0.18864	0.054566	0.123958
HORVU5Hr1G100700	241.5798	222.2719	264.3972	359.1077	859.7992	764.1139
HORVU3Hr1G069410	0.264649	0.21571	0.111423	4.138659	9.333384	7.450822
HORVU3Hr1G089210	1.025723	0.70925	0.342383	1.987442	2.222968	2.654781
Hordeum_vulgare_newGene_13089	1.129409	1.103177	2.202034	4.654445	5.824305	7.859386
HORVU6Hr1G009350	0.155921	0.187868	0.217637	5.708188	22.84272	21.28727
HORVU7Hr1G077750	2.398258	3.067618	1.90843	15.91633	30.27353	28.25323
Hordeum_vulgare_newGene_663	0.047361	0.243113	0.096897	1.440428	1.903652	2.550641
Hordeum_vulgare_newGene_665	0.353103	0.313906	0.161592	1.325955	1.679275	1.356541
HORVU4Hr1G058810	0.266196	0.124118	0.41825	9.547364	48.96917	44.75045
HORVU3Hr1G055550	54.07274	55.18807	54.33027	37.69107	29.2951	32.55564
HORVU4Hr1G015930	0.355189	0.291364	0.931273	2.905314	3.431398	3.464075
HORVU1Hr1G094230	0.552696	0.472539	0.792901	5.351058	13.08159	12.13685
HORVU2Hr1G096250	0.305336	0.163443	0.146846	2.010672	6.539341	5.336441
HORVU5Hr1G080110	0.309003	0.321935	0.295457	1.555743	6.593071	6.947262
Hordeum_vulgare_newGene_2559	1.517965	1.480619	1.472716	2.803647	5.326792	4.905889
Hordeum_vulgare_newGene_5684	93.5798	84.11966	82.09053	31.01975	27.69173	31.54636
HORVU5Hr1G099910	14.20213	14.84154	16.73844	0.483406	0.238428	0.091838
HORVU3Hr1G116580	1.067419	0.418028	0.780846	2.243657	2.334167	2.069669
Hordeum_vulgare_newGene_13976	0	0	0	2.736247	3.914782	3.933515
Hordeum_vulgare_newGene_9569	4.592375	4.109053	6.181156	21.38314	43.15253	31.09296
Hordeum_vulgare_newGene_9560	3.087348	2.927034	4.172136	1.208426	1.763445	1.983895
Hordeum_vulgare_newGene_13751	0.622405	0.624471	1.419741	2.431362	5.149242	1.793894
HORVU1Hr1G000090	2.515694	1.657431	2.000246	7.142437	6.841957	7.483458
HORVU6Hr1G059800	1.33773	0.903831	1.444891	0.882246	0.501086	0.756244
HORVU6Hr1G068150	6.680079	9.089443	13.1525	1.695286	1.573014	1.973942
HORVU7Hr1G063430	0.010086	0	0	1.678946	33.58937	25.85136

Hordeum_vulgare_newGene_8216	2.058185	2.333105	2.023833	8.344725	12.54961	10.90962
HORVU1Hr1G079650	9.607754	9.480651	10.40354	0.381802	0.474768	0.320499
HORVU1Hr1G075550	26.21995	26.30054	31.2723	16.62363	12.31327	14.75514
Hordeum_vulgare_newGene_4841	1.180671	0.860064	1.725065	0.419738	0.406016	0.200943
HORVU5Hr1G037660	8.49062	5.221429	6.981097	18.13419	14.93345	18.63079
Hordeum_vulgare_newGene_7798	3.693719	3.760088	3.361789	1.473656	0.521277	0.940518
HORVU6Hr1G062350	1.371044	0.48846	1.310879	0.317389	0.465933	0.523842
HORVU6Hr1G080690	52.60351	47.64111	53.64096	15.94807	14.06653	13.54958
HORVU3Hr1G085400	0.652627	0.270031	0.480989	1.149748	2.82829	2.002426
HORVU7Hr1G038500	0.226368	0.269274	0.183571	0.750019	0.747786	0.828413
Hordeum_vulgare_newGene_15842	2.612131	5.060329	4.104599	0	0	0
Hordeum_vulgare_newGene_15846	1.128142	1.369164	2.404993	0.314329	0.146893	0.164905
HORVU7Hr1G056570	3.32294	3.458684	3.816959	7.127473	13.19391	12.65872
HORVU1Hr1G026590	17.20132	17.26348	19.21643	8.08171	6.329764	6.44148
Hordeum_vulgare_newGene_2945	0	0	0	2.763953	3.568534	3.507884
Hordeum_vulgare_newGene_2947	0	0	0	2.368441	3.015922	3.546156
Hordeum_vulgare_newGene_2946	0	0	0	1.556999	1.845611	1.616932
HORVU3Hr1G001940	0	0	0	7.026607	6.156591	9.560797
HORVU6Hr1G003560	0.547108	0.919276	0.509094	2.631943	3.137778	2.980862
HORVU5Hr1G012190	1.687054	1.292323	1.728857	0.800899	0.332722	0.393617
HORVU3Hr1G095880	30.64019	26.59818	38.76711	25.50159	14.53177	15.94297
HORVU3Hr1G033790	1.30802	1.157996	1.036113	11.75992	15.89144	11.89827
Hordeum_vulgare_newGene_14006	2.215831	1.930252	1.839773	1.592399	1.487608	1.407957
Hordeum_vulgare_newGene_14007	4.07625	3.160775	1.526104	0	0	0
HORVU4Hr1G011910	8.252378	7.082562	8.050508	16.01244	23.13416	22.21289
HORVU1Hr1G073190	0.425441	0.254399	0.493627	1.497527	2.566993	2.12388
HORVU3Hr1G029010	182.2315	158.1483	157.3066	105.9136	68.45438	85.78391
HORVU2Hr1G007990	0.883922	1.181296	1.317095	0.454897	0.453804	0.569708
HORVU7Hr1G108370	1.280856	0.682543	0.857901	4.584711	18.96035	10.15354
HORVU1Hr1G023220	6.490311	5.264106	9.260919	37.88741	28.5003	31.57157
HORVU1Hr1G019740	10.57906	5.211001	15.45275	0.325967	0.024338	0.275011
Hordeum_vulgare_newGene_9985	0.079139	0.060776	0.279255	1.722225	5.582373	4.979474
Hordeum_vulgare_newGene_15694	0.662335	0.946778	0.978456	0.442411	0.231314	0.309428
HORVU1Hr1G066340	0.317739	0.442076	0.657225	3.23975	3.15238	3.848791
HORVU4Hr1G081600	22.49878	20.94757	24.88699	19.17491	9.893681	10.72119
HORVU3Hr1G000220	0.25873	0.386686	0.363228	0.822649	1.157904	1.317313
HORVU4Hr1G005900	65.32957	81.67776	122.3963	28.91669	48.16765	50.28031
HORVU5Hr1G009900	0	0	0	0.956356	1.453735	1.176218
HORVU7Hr1G036570	5.901906	4.942129	8.766234	3.476286	4.579666	3.081209
HORVU4Hr1G087760	0.141914	0.203259	0.056749	0.969164	4.778847	5.174659
HORVU1Hr1G066960	0.641422	0.656453	1.202525	2.311257	7.667154	9.337358
Hordeum_vulgare_newGene_11525	1.528964	1.917659	0.785495	0.606575	0.750688	0.635936
HORVU3Hr1G085890	24.55487	20.65506	26.52662	19.12579	6.382233	13.79739
HORVU3Hr1G114320	0	0.016958	0	0.686286	1.472374	0.744023
HORVU3Hr1G001570	0	0.036548	0.070373	1.144368	1.090606	1.39276
HORVU1Hr1G094430	0	0.01883	0	0.3612	1.066212	1.428563
HORVU5Hr1G101770	0.00968	0.014167	0.025442	0.235077	0.275655	0.50616

HORVU3Hr1G099690	3.242955	2.737276	3.437149	7.563815	8.964786	9.190222
Hordeum_vulgare_newGene_10264	1.816193	1.550135	1.446692	14.16247	13.71127	12.2806
HORVU4Hr1G025410	2.039449	2.774392	2.205132	6.778284	8.398539	8.245961
HORVU6Hr1G038250	11.40485	11.46228	7.081739	2.699023	1.807466	2.078403
HORVU6Hr1G076130	7.097798	5.819882	7.992789	3.462975	2.683623	3.212359
HORVU4Hr1G005380	1.657405	1.073083	1.113202	0.073741	0.03894	0.03983
HORVU3Hr1G026110	0.233821	0.108723	0.099277	0.463433	0.551558	0.557006
HORVU3Hr1G003860	0.022141	0.026445	0.158807	5.112911	4.008	3.528008
HORVU2Hr1G031410	1.252895	1.50143	1.151865	5.191157	6.248488	5.916287
HORVU2Hr1G108180	0.050502	0.022821	0.029356	5.463009	5.078475	4.868871
Hordeum_vulgare_newGene_11602	5.468095	7.025964	6.578792	0.024156	0	0
HORVU2Hr1G056510	1.326535	1.14767	1.369838	3.054641	6.069424	5.634313
HORVU7Hr1G010680	0	0.054909	0	27.35081	134.0073	132.7922
HORVU7Hr1G077170	5.423208	4.148611	5.746933	7.044201	19.16965	25.27897
HORVU3Hr1G103630	0.56864	0.658529	0.781663	2.399033	2.444095	2.937211
Hordeum_vulgare_newGene_1838	0.15618	0.179301	0.254623	0.875281	0.8455	0.652252
HORVU2Hr1G063910	0.49975	0.361375	0.306837	1.464343	5.644227	4.971667
HORVU5Hr1G051970	196.4162	155.3974	199.8591	87.99194	35.31555	65.89
Hordeum_vulgare_newGene_1833	0.561035	0.639653	0.38947	2.390956	1.830088	2.437923
HORVU2Hr1G022590	0.904569	0.991038	1.522376	3.663965	6.965932	7.010376
HORVU6Hr1G095000	0.23222	0.331477	0.277442	0.760369	0.815813	1.264661
HORVU5Hr1G070930	0.623775	0.756097	1.350859	1.966165	4.23185	4.314653
HORVU1Hr1G051300	1.433565	1.067241	2.187884	5.658128	3.14206	3.835493
HORVU3Hr1G068640	0.345577	0.469505	0.449538	1.007676	1.817653	1.835145
HORVU6Hr1G062190	0.284618	0.221452	0.245673	1.816491	14.52631	6.929004
HORVU5Hr1G071080	4.914854	4.324267	5.770667	13.08964	18.10017	16.0042
HORVU4Hr1G026680	0.136915	0.156621	0.158858	0.3245	0.55289	0.433734
HORVU7Hr1G062930	2.189684	1.790125	2.205247	4.050355	7.238258	6.199159
Hordeum_vulgare_newGene_6201	5.602679	4.778567	4.34129	1.876921	0.901009	0.695583
Hordeum_vulgare_newGene_13478	0.832882	10.14416	2.251159	0	0	0
Hordeum_vulgare_newGene_13479	0	0	0	9.048727	7.9578	10.0699
Hordeum_vulgare_newGene_13472	0.785327	0.105103	0.849837	3.363981	3.586877	3.426925
Hordeum_vulgare_newGene_13474	11.33755	8.687934	15.02752	4.974617	3.705235	4.503415
Hordeum_vulgare_newGene_13475	1.082577	0.809937	1.832194	0.32223	0.40291	0.262641
HORVU7Hr1G097000	299.8149	237.1535	328.813	125.6206	129.4404	119.5296
HORVU4Hr1G021500	0.359719	0.469907	0.337667	1.411373	2.223387	1.735585
HORVU2Hr1G032420	2.685844	2.867928	2.25465	0.091826	0	0.023951
HORVU6Hr1G012210	0.686221	1.029307	1.085722	28.45889	18.00874	21.82617
Hordeum_vulgare_newGene_15732	0.178297	0.235734	0.10234	1.018373	1.288593	1.143692
Hordeum_vulgare_newGene_5234	1.553452	1.629997	1.759524	4.409657	6.565501	5.984395
HORVU7Hr1G096750	5.351983	3.890046	4.971486	2.626195	2.425884	2.324712
HORVU7Hr1G078670	17.28421	21.29413	29.76405	2.44515	1.090181	1.597702
Hordeum_vulgare_newGene_8535	2.545919	2.551113	3.167395	0.012995	0.01458	0.019792
HORVU0Hr1G032350	0.131822	0.172654	0.158575	0.885546	1.474484	1.438346
Hordeum_vulgare_newGene_1318	0	0	0	0.511104	0.304008	0.567615
Hordeum_vulgare_newGene_1317	0	0	0	2.413853	1.461858	3.548089
HORVU2Hr1G058800	1.323117	1.074505	1.2908	2.939739	4.891218	4.777982

Hordeum_vulgare_newGene_7709	1.559029	1.010864	1.442069	0.672299	0.920659	0.731069
HORVU6Hr1G091700	3.77783	3.384693	3.613397	8.346814	8.43239	9.886287
HORVU1Hr1G062330	2.496041	2.55697	2.732483	1.441946	1.360813	1.453806
HORVU7Hr1G027430	0.655446	0.570432	1.045586	2.220583	12.65059	11.79294
HORVU6Hr1G063900	1.188538	1.450275	1.120313	0.505952	0.524528	0.449083
HORVU7Hr1G036000	26.23398	26.73662	24.82038	4.201565	2.24333	3.368151
Hordeum_vulgare_newGene_8779	2.471361	2.628525	2.875638	1.394711	1.523502	1.583642
HORVU2Hr1G046740	4.162925	3.701563	4.680052	11.36702	18.40234	18.93843
HORVU5Hr1G109640	18.89647	15.26815	21.71287	10.76663	9.364748	8.949319
HORVU3Hr1G000970	0.317592	0.432372	0.200525	8.740207	13.14266	11.69704
HORVU7Hr1G119870	8.37602	8.265372	13.32178	2.199947	4.338631	3.364241
Hordeum_vulgare_newGene_6563	0.102867	0.074236	0.093624	2.344522	0.355236	0.926366
Hordeum_vulgare_newGene_6560	3.756265	1.505819	2.229571	0.572087	0.128734	0.299071
Hordeum_vulgare_newGene_3123	0.467273	0.506066	0.507098	0.187903	0.176828	0.176768
HORVU6Hr1G002530	0.872092	0.412545	0.610143	2.134229	1.423363	2.778068
HORVU5Hr1G022550	3.177746	2.888252	5.07596	44.84941	92.71399	71.94965
HORVU6Hr1G073630	4.85572	3.733913	3.371669	0.85721	1.630924	1.026875
HORVU3Hr1G028240	0.418769	0.406697	0.446151	1.197832	1.973818	1.534916
HORVU7Hr1G094870	4.767762	4.442112	3.820053	24.02366	11.58487	16.08755
HORVU4Hr1G013700	0.797323	0.745216	0.716516	0.152433	0.070998	0.163247
HORVU0Hr1G022830	2.082414	1.900125	2.076557	4.901853	6.536258	6.67785
HORVU1Hr1G020270	0.284327	0.095028	0.150546	1.052931	1.449089	1.226289
HORVU1Hr1G041960	1.074812	1.045391	1.539577	0.196832	0.171073	0.23295
HORVU2Hr1G126150	5.190276	4.49868	7.71495	0.749478	0.21128	0.507165
HORVU3Hr1G007830	1.890947	2.691834	2.327908	0.51949	0.715291	0.640438
HORVU4Hr1G005710	0.533814	0.531707	0.625358	1.511835	1.871535	2.94132
HORVU3Hr1G056090	10.4238	9.444265	9.586525	6.704637	4.298159	6.635544
HORVU4Hr1G058940	0.651858	0.508638	0.71354	1.868747	5.58783	5.406177
HORVU1Hr1G085640	14.68941	17.20912	21.24584	1.33884	0.552756	0.764934
HORVU6Hr1G085170	67.71294	54.38556	58.56259	129.9695	166.6871	177.9769
HORVU5Hr1G111590	41.15116	44.26061	53.8477	14.85341	7.361075	11.30708
HORVU4Hr1G027970	0.233574	0.177349	0.319952	1.435741	4.210452	3.351781
HORVU2Hr1G103550	0	0	0	3.653238	109.8766	93.04417
HORVU6Hr1G000480	7.086927	5.723086	6.860664	0.026253	0	0
HORVU4Hr1G019360	2.072166	3.184696	2.193773	5.397465	7.895104	7.080975
HORVU6Hr1G006880	5.951417	6.036387	5.711847	22.96887	69.76391	66.45806
HORVU7Hr1G094010	0.599429	0.796637	0.732279	1.449409	3.397939	3.006958
HORVU2Hr1G013870	6.109244	7.233935	5.989045	14.4773	22.23244	24.84951
Hordeum_vulgare_newGene_13903	12.83435	12.95664	12.02823	7.779628	5.218424	7.455581
HORVU6Hr1G016070	1.29811	1.839614	1.1952	3.454707	4.590465	5.560012
Hordeum_vulgare_newGene_13909	0	0	0.010766	0.656127	0.932785	0.724903
HORVU5Hr1G045790	0.727085	1.008793	0.927429	0.038753	0.048629	0.013408
HORVU7Hr1G006570	46.86051	44.42237	35.17939	3.056181	5.450525	4.92087
HORVU1Hr1G083170	7.960691	7.017949	4.155114	23.84181	27.47781	19.28694
HORVU5Hr1G120110	97.40147	80.9926	99.47715	48.91726	25.40116	29.73951
HORVU2Hr1G010990	1.385672	0.932565	1.776996	7.937476	39.39933	35.12444
Hordeum_vulgare_newGene_15101	1.838119	2.369219	0.844066	0.598061	1.031755	1.184872

Hordeum_vulgare_newGene_8731	2.328021	3.219098	3.53406	0.018938	0	0
HORVU3Hr1G114350	0.985016	0.979275	1.184687	1.356511	3.95162	4.200539
Hordeum_vulgare_newGene_8737	1.566691	1.387495	2.044126	0.334835	0.06417	0.257596
Hordeum_vulgare_newGene_5275	0.941023	0.977755	1.338318	1.900532	7.57255	5.749693
HORVU6Hr1G076070	58.85736	63.03306	43.5816	17.07509	13.11467	14.42908
HORVU2Hr1G097080	5.720256	6.813095	5.642617	12.34213	22.47681	21.05857
HORVU2Hr1G118840	2.347096	3.141252	2.890822	1.329783	1.54455	1.467133
HORVU5Hr1G047480	0.148253	0.097695	0.155477	0.361339	0.940439	1.121688
HORVU5Hr1G117080	3.947676	2.533213	6.098908	0.102035	0.031958	0.048637
HORVU5Hr1G047150	1.943121	2.360405	2.728025	0.290594	0.174272	0.419855
HORVU3Hr1G095240	1.455413	1.29441	1.762999	0.859255	0.885889	0.736709
Hordeum_vulgare_newGene_11410	0.677084	0.726157	0.939747	0.35508	0.175521	0.355785
HORVU3Hr1G066580	89.79595	73.9302	89.39767	52.86449	46.00514	44.71629
HORVU7Hr1G001790	0.592162	0.358787	0.463901	1.935674	1.835319	1.406083
HORVU6Hr1G016890	0.358515	0.311297	0.4156	2.363239	5.646628	6.759569
HORVU3Hr1G080640	29.20366	28.02843	25.80862	20.44767	12.46677	16.09059
Hordeum_vulgare_newGene_12265	0	0	0	3.447357	2.437891	2.353728
HORVU3Hr1G105420	0.0602	0.002397	0.166918	2.931618	1.97899	3.693679
HORVU4Hr1G057330	1.022099	1.271445	1.387392	3.116107	3.278884	3.690663
HORVU0Hr1G004830	39.14493	36.90717	28.44606	95.47962	163.4557	164.7046
Hordeum_vulgare_newGene_6016	3.89523	2.266692	2.804707	0	0.00874	0
HORVU3Hr1G108950	2.245232	3.265051	4.470814	1.036145	0.619173	0.738987
Hordeum_vulgare_newGene_6019	2.62361	3.814463	2.952541	0.005636	0	0
Hordeum_vulgare_newGene_6018	4.352481	5.116488	4.924419	0.007928	0	0
Hordeum_vulgare_newGene_3198	2.313633	2.47126	2.680656	0	0	0
HORVU3Hr1G087450	50.51282	52.65487	69.89524	24.80987	18.37611	19.56572
HORVU2Hr1G073850	4.141814	3.607949	3.817384	8.073713	12.90909	10.91325
HORVU3Hr1G068380	30.41481	20.54035	39.09036	13.77994	2.800797	5.91006
HORVU6Hr1G062430	5.191315	3.987699	4.850404	1.272838	0.477436	0.713019
HORVU3Hr1G020400	4.970397	6.767246	7.228955	3.112186	2.915859	3.99199
HORVU2Hr1G015570	4.958749	8.201952	8.001451	0.258361	0.022219	0.051302
Hordeum_vulgare_newGene_2249	0.728236	0.584313	0.587204	0.409343	0.173716	0.219412
HORVU5Hr1G001750	14.71179	14.7762	16.87632	3.315421	1.179006	1.484027
HORVU1Hr1G020070	0.08918	0.070354	0.095374	1.681056	7.076286	6.365765
HORVU4Hr1G077140	0.37524	0.420581	0.354458	1.085478	1.386169	1.429255
HORVU3Hr1G069030	0.265561	0.559791	0.242549	2.294226	1.124737	2.453437
Hordeum_vulgare_newGene_970	2.889199	2.451395	1.37181	5.258304	22.04303	20.40316
HORVU1Hr1G062270	2.562772	2.948389	2.860498	5.231349	11.43748	11.25738
HORVU7Hr1G096270	0.317601	0.311049	1.135292	2.05219	2.861768	3.720835
Hordeum_vulgare_newGene_1293	3.548681	2.477041	3.796308	0.194938	0.028433	0.175126
HORVU5Hr1G069620	4.009742	4.17166	3.431955	19.84503	49.80547	41.95976
HORVU1Hr1G033660	1.951592	1.883775	2.088642	0	0.039187	0
HORVU1Hr1G041770	3.856731	4.227213	4.601037	10.87948	18.57496	17.61991
HORVU7Hr1G113270	2.607356	1.344895	2.713728	58.43997	123.5232	114.3782
HORVU3Hr1G052250	1.621495	1.564084	1.59118	5.745199	6.393215	7.801927
HORVU1Hr1G058420	0.26758	0.383035	0.514186	1.739496	1.731848	1.758233
HORVU1Hr1G049760	0.39608	0.296196	0.255329	1.923717	1.520248	1.72555

HORVU1Hr1G063630	1.487987	1.532979	2.093439	0.551313	0.782807	0.571649
Hordeum_vulgare_newGene_4576	0.028976	0.054091	0.032028	5.467496	4.90751	4.792584
Hordeum_vulgare_newGene_4575	0.723846	1.075612	0.85767	1.988484	2.720065	2.700143
HORVU0Hr1G031760	2.662905	3.103934	3.614341	5.985375	12.94458	12.01916
HORVU0Hr1G000810	0.483931	0.32922	0.450385	5.607998	9.714938	12.04104
Hordeum_vulgare_newGene_7004	10.87357	6.807195	12.32088	28.39042	33.82897	31.49593
HORVU5Hr1G095410	3.007676	2.55329	2.786654	6.476226	13.45495	11.42615
HORVU3Hr1G006150	1.180376	1.133066	0.857599	2.810178	3.700357	4.287462
HORVU2Hr1G077830	0.155487	0.068891	0.215857	0.942542	11.47098	10.31126
HORVU4Hr1G079600	20.1914	11.23395	26.58669	1.230093	0.882609	1.112379
HORVU3Hr1G043160	1.108986	1.667805	1.506312	5.731654	4.973198	6.61136
HORVU3Hr1G031950	2.738874	2.141631	2.258684	9.500829	5.995336	6.341758
HORVU6Hr1G029210	0.580436	0.404162	0.979451	4.525118	24.24794	24.75378
HORVU5Hr1G093150	3.994678	5.007251	5.666494	2.68632	1.642215	2.050401
HORVU2Hr1G090330	2.669535	1.893989	2.112003	11.96539	12.51207	9.116672
HORVU5Hr1G001780	1.26624	2.246714	1.708378	7.171983	7.774925	8.197026
HORVU5Hr1G122860	0	0	0.017708	1.059412	1.566447	1.457637
HORVU5Hr1G122070	9.870738	8.413014	9.781204	28.92177	40.57957	32.9947
HORVU3Hr1G028780	5.916977	5.265284	9.240364	40.32623	42.57611	42.4526
HORVU2Hr1G040780	41.36699	31.20277	34.70806	74.17873	127.3823	121.1536
HORVU3Hr1G092420	7.087975	6.530014	8.303531	15.54149	33.06196	29.31433
HORVU7Hr1G048670	0	0	0	1.632011	2.635386	2.688554
HORVU5Hr1G054420	12.13423	11.38478	7.752435	5.181413	2.497254	3.016829
HORVU2Hr1G108800	2.217978	1.620651	1.701443	0	0	0
HORVU0Hr1G016150	0.566149	0.83729	0.853943	1.831309	2.069861	2.430765
HORVU5Hr1G097100	10.64996	12.00935	17.83723	4.692865	2.957709	3.748089
HORVU2Hr1G094360	0.077309	0.233206	0.157505	1.152016	2.722997	3.323056
HORVU1Hr1G039830	0.261246	0.468779	0.357389	6.906954	19.6501	17.2659
HORVU6Hr1G000380	31.37296	27.96544	35.11559	16.1275	17.00894	15.80871
HORVU3Hr1G056110	0.956924	0.905785	1.010495	0.212017	0.200014	0.108279
HORVU1Hr1G068850	1.639529	1.930214	2.209138	4.341891	6.017461	6.221557
HORVU6Hr1G027460	0.336653	0.22855	0.464256	0.788036	1.816543	1.868374
HORVU5Hr1G119200	1.155518	1.30184	1.37169	0.588683	0.471996	0.668418
HORVU1Hr1G082180	3.333182	4.251948	3.699411	3.300907	2.122922	1.980972
HORVU6Hr1G026730	4.513731	6.125668	6.021631	2.660931	2.723767	3.313563
HORVU3Hr1G025690	0.550095	0.67331	0.788652	2.165534	2.125884	2.768235
HORVU5Hr1G115780	6.227308	5.271429	6.15167	0.826933	0.497655	0.618214
Hordeum_vulgare_newGene_2004	3.808688	4.91081	5.35936	0.021173	0	0
HORVU3Hr1G059230	6.939821	6.964003	7.539456	22.99849	47.05111	39.6414
HORVU5Hr1G053230	1.030126	1.575951	1.631434	5.72873	10.79393	9.460635
HORVU5Hr1G115870	1.811051	1.125918	2.644701	0.585865	0.286034	0.555454
HORVU5Hr1G078100	0.362146	0.405075	0.431499	1.242718	1.184153	1.472073
HORVU2Hr1G072500	54.37089	55.20374	66.16517	186.054	470.665	414.2034
HORVU2Hr1G023480	0.352689	0.322229	0.431388	5.952939	9.64549	10.55624
Hordeum_vulgare_newGene_13601	0.822441	1.728234	1.70458	3.328301	4.756764	5.187981
HORVU6Hr1G065740	1.296728	1.708415	3.006987	0.662452	0.321383	0.684837
Hordeum_vulgare_newGene_14867	6.589909	7.827991	7.939932	0.150814	0.060878	0.048259

Hordeum_vulgare_newGene_1729	2.605811	2.557305	2.7023	0.01461	0	0
HORVU2Hr1G031030	6.798623	7.042585	7.330808	15.82449	20.26449	18.27831
HORVU4Hr1G067280	44.10874	36.84109	26.40013	6.997455	6.101563	7.445778
HORVU4Hr1G063900	1.926786	1.967395	1.959069	4.256347	7.558344	6.45465
HORVU7Hr1G073190	0.299816	0.222212	0.251488	1.798113	6.565345	5.066028
HORVU3Hr1G111970	1.787204	1.380041	1.620927	0.06526	0	0
HORVU7Hr1G021050	0.882006	0.452185	0.590025	2.695327	5.183112	3.794054
HORVU6Hr1G074570	24.02354	26.29794	26.30631	12.94686	11.21194	10.70186
Hordeum_vulgare_newGene_3012	1.564101	1.480719	2.059348	0	0	0
Hordeum_vulgare_newGene_6194	0.374106	0.667643	0.668251	0.056483	0.037574	0.058483
Hordeum_vulgare_newGene_13768	5.073331	4.422269	3.542143	0	0	0
Hordeum_vulgare_newGene_13766	2.424798	2.595079	3.020439	5.276249	11.47782	10.15889
Hordeum_vulgare_newGene_13761	0	0	0	1.262059	1.327709	0.549918
Hordeum_vulgare_newGene_14086	0.364265	0.739642	0.797814	0	0.010316	0
HORVU5Hr1G075490	48.0833	47.71916	60.96025	67.62079	189.4551	180.2903
Hordeum_vulgare_newGene_15688	0.249301	0.202889	0.161359	0.658653	0.706015	0.706658
HORVU6Hr1G002290	0.67851	0.882161	0.851941	2.29552	1.78267	3.13146
HORVU4Hr1G002180	3.128497	2.963536	3.548153	1.470351	0.839691	1.421904
HORVU4Hr1G003580	2.742143	3.838025	3.840905	9.935795	12.50101	11.37544
HORVU5Hr1G103430	37.17994	45.52162	61.78166	23.88825	22.72577	24.37372
HORVU5Hr1G106400	0.402687	0.297425	0.338831	0.874552	1.448172	1.089734
HORVU1Hr1G078860	8.172816	6.750154	9.036906	2.089777	0.462511	0.506513
HORVU2Hr1G007130	8.589683	9.88914	11.4632	3.91774	2.795615	3.098821
HORVU0Hr1G003340	1.214435	1.020206	1.068777	3.931622	2.46404	2.686382
HORVU2Hr1G094160	16.69092	17.51436	19.3286	8.191285	5.275962	6.779404
HORVU2Hr1G004530	22.93878	11.64852	29.69139	7.080663	2.607566	8.987871
HORVU5Hr1G016850	1.417221	0.610108	0.748753	17.93943	20.43743	21.69081
HORVU2Hr1G047010	6.721123	8.770439	9.870931	2.854382	4.253616	4.573484
HORVU2Hr1G070620	9.051296	7.696273	7.119064	1.513107	1.267861	1.125253
Hordeum_vulgare_newGene_15526	0.190432	0.189926	0.262964	0.426445	0.751064	0.667873
HORVU4Hr1G008800	9.778764	6.14166	12.04775	23.33556	60.15301	52.79854
HORVU1Hr1G067070	1.302955	0.96074	1.814564	3.655321	3.776742	5.419348
HORVU4Hr1G018150	144.9613	131.9746	80.5259	8.055342	1.199646	3.432432
HORVU4Hr1G005320	0.026334	0	0.05225	12.62451	15.71069	12.08307
HORVU2Hr1G088520	17.42663	17.95184	25.06775	6.990774	6.067896	7.775042
HORVU2Hr1G009480	0.056257	0	0	0.879846	0.774337	1.045186
HORVU5Hr1G067890	7.882836	5.317098	7.549352	4.054149	3.399394	3.685063
HORVU6Hr1G007360	0.016726	0.237617	0	1.883024	5.713374	4.122386
HORVU4Hr1G016880	0.691996	0.395737	0.546818	3.186391	1.40675	1.438395
HORVU5Hr1G088040	3.976162	3.742318	3.319933	1.239114	0.858415	1.580084
HORVU5Hr1G096560	0.131047	0.03258	0.016068	0.906094	1.264108	1.11501
HORVU2Hr1G017860	1.900869	2.109897	1.826769	7.859162	5.950739	6.042777
HORVU2Hr1G099440	0.689179	0.900554	0.839884	1.666889	1.65054	1.923775
HORVU4Hr1G016260	1.029292	0.738476	1.379347	3.219805	3.594711	3.693087
HORVU1Hr1G012930	248.3608	252.3999	328.1755	648.2546	1633.667	1155.196
HORVU3Hr1G028080	37.00807	29.92067	28.1452	11.84541	1.903797	5.550529
HORVU6Hr1G084190	37.03775	40.89032	32.5644	20.23447	10.7358	15.36383

HORVU2Hr1G041080	0.609978	0.390542	0.3763	0.692735	6.906202	5.128592
HORVU6Hr1G006100	0.026264	0.135675	0.087389	9.183572	41.11446	33.84185
HORVU3Hr1G033450	0.250339	0.151675	0.259274	0.340452	1.613989	1.703427
Hordeum_vulgare_newGene_11133	0.090677	0.026763	0.048478	3.913518	12.33719	11.37124
HORVU7Hr1G012600	1.286875	1.002657	1.247456	4.144665	8.558009	6.168926
HORVU7Hr1G093190	4.881327	5.313109	5.221672	1.659099	1.474063	1.347848
HORVU0Hr1G012850	68.88183	55.75839	46.65571	18.86803	10.19633	11.5053
HORVU7Hr1G069230	165.2588	169.3425	228.309	90.11848	95.57763	103.2168
HORVU3Hr1G028620	4.644201	2.597024	5.754558	2.352419	1.170469	1.807279
HORVU5Hr1G077120	24.05358	23.7485	24.71899	9.332448	6.564665	8.744895
HORVU2Hr1G090100	0.220529	0.134299	0.21979	1.361206	1.673864	1.934137
HORVU5Hr1G049560	8.39395	8.729	11.26663	5.221541	5.607133	5.287794
HORVU3Hr1G026840	2.125236	1.76968	2.780206	5.161978	7.03534	6.386982
HORVU1Hr1G009430	7.840222	7.756208	8.621751	3.969941	3.006647	3.894569
HORVU3Hr1G009940	1.145364	1.626115	1.178043	2.965688	3.587767	3.646267
HORVU2Hr1G111540	1.265242	1.598501	1.119629	12.44112	22.23555	20.24595
HORVU1Hr1G005510	2.15856	0.505533	1.017124	0.083898	0	0.24073
HORVU1Hr1G067330	0.234231	0.18692	0.222647	2.30726	2.409956	3.673695
HORVU5Hr1G076380	0.613153	0.725708	0.873161	1.557089	3.028945	3.787429
HORVU6Hr1G089560	3.982225	4.132333	3.173336	0.092672	0	0.010783
Hordeum_vulgare_newGene_13891	0.500769	0.651019	0.644248	3.401142	4.729815	5.378754
HORVU3Hr1G108540	3.101162	2.082524	3.113189	8.480003	10.75733	11.32547
HORVU6Hr1G055440	9.919672	8.135072	5.584807	0.027998	0.03542	0
Hordeum_vulgare_newGene_12560	0.9703	1.392472	0.933511	0.100033	0.110593	0.028438
Hordeum_vulgare_newGene_741	0	0	0.133729	3.227236	1.972476	2.949495
Hordeum_vulgare_newGene_748	7.043738	7.811851	7.539669	3.36917	3.891344	3.785083
HORVU4Hr1G022630	1.227478	1.407675	1.835927	0.218922	0.597592	0.468205
HORVU5Hr1G075650	2.515604	3.097479	3.965738	1.336604	1.690132	1.480637
HORVU4Hr1G059260	343.5089	345.2963	356.1314	132.8353	96.58096	70.32497
HORVU7Hr1G002960	0.643123	0.400055	0.43218	0.765818	1.250307	1.437633
Hordeum_vulgare_newGene_14960	0	0	0	1.281942	1.459184	2.143406
Hordeum_vulgare_newGene_2613	0	0	0	1.636391	1.9554	2.0705
HORVU6Hr1G012290	3.297384	4.856808	3.946012	17.81563	19.47845	18.487
HORVU4Hr1G061070	0.178571	0.377841	0.165346	1.096514	7.174558	6.052259
Hordeum_vulgare_newGene_14661	0.1008	0.127866	0.167955	2.005678	2.042994	2.544189
Hordeum_vulgare_newGene_9190	0.022312	0.024116	0.013213	0.180774	1.100826	0.519939
Hordeum_vulgare_newGene_9199	0.500606	0.698458	0.584845	1.542524	1.39769	1.787729
HORVU7Hr1G102480	0.516801	0.260742	0.689244	7.457304	18.30373	13.78814
Hordeum_vulgare_newGene_9441	0.875428	0.982696	1.132326	1.472553	3.071595	3.435227
HORVU5Hr1G063340	15.4977	11.22444	13.28239	41.73784	30.89425	29.47876
HORVU4Hr1G088080	2.952221	3.572314	3.082716	8.372492	11.51357	10.10147
HORVU7Hr1G080950	1.971998	1.401542	1.749364	4.322004	5.969658	5.061889
HORVU5Hr1G081610	367.2142	360.6091	354.1078	1300.395	1006.838	844.8287
HORVU2Hr1G069600	3.291425	3.163532	3.728072	8.292354	17.97478	20.71195
HORVU4Hr1G071370	4.872666	2.79775	6.783285	0.377283	0.0954	0.371499
HORVU2Hr1G049500	5.575348	6.011363	7.831099	3.223104	4.060905	4.690157
HORVU2Hr1G081650	0.975214	1.379828	1.248927	0.923217	0.357601	0.661827

HORVU2Hr1G038030	4.006744	2.844644	4.262428	8.177072	14.10534	12.92943
HORVU0Hr1G029570	27.28485	21.37715	18.49708	1.011484	0	0.397197
HORVU3Hr1G105820	7.674356	6.210769	7.894042	22.24221	182.8323	169.6207
HORVU2Hr1G022360	1.555419	1.613925	2.213936	4.001994	4.431651	5.22885
HORVU0Hr1G026630	113.3242	109.0364	153.3421	322.0893	696.0581	580.4879
HORVU3Hr1G094600	13.0782	12.969	11.63267	5.123445	6.050566	6.825873
HORVU5Hr1G088060	0.452729	0.41784	0.296171	1.640807	1.378873	1.657644
Hordeum_vulgare_newGene_4922	0	0	0	3.356949	4.116377	5.475461
Hordeum_vulgare_newGene_4925	0.118973	0.415652	0.247206	0.818153	2.561195	2.606793
Hordeum_vulgare_newGene_4924	0	0	0	0.986886	1.696565	1.61424
HORVU0Hr1G039090	1.034014	0.943646	1.304309	3.749997	4.423782	4.673012
Hordeum_vulgare_newGene_7696	0	0	0	1.327175	0.951877	1.279833
HORVU3Hr1G088160	2.324758	2.583212	5.064157	11.16629	36.45146	30.85885
HORVU3Hr1G030340	23.13953	27.3119	33.47176	3.864279	0.991527	2.531611
HORVU2Hr1G042240	0.093371	0.07765	0.082918	0.379581	1.882039	1.530571
Hordeum_vulgare_newGene_15962	0	0	0.012621	2.27535	2.454263	3.069613
Hordeum_vulgare_newGene_15964	0.161822	0.058914	0.168445	0.643965	1.591966	1.507455
HORVU7Hr1G080740	9.195494	12.21416	12.75674	0.16042	0.096359	0
HORVU3Hr1G091920	2.07365	5.005428	6.076975	0.089572	0	0.05937
Hordeum_vulgare_newGene_12601	7.202905	6.216327	6.98377	0.025594	0.029345	0
HORVU7Hr1G031140	0.536035	0.140502	0.608572	6.359948	5.083605	5.981067
HORVU6Hr1G087220	5.393354	4.67084	5.481879	13.77166	35.04583	27.47631
Hordeum_vulgare_newGene_9830	2.280006	2.266624	2.449878	4.714857	7.87848	7.013106
HORVU2Hr1G006850	10.5799	6.515377	6.738151	2.095852	3.058854	4.863148
HORVU1Hr1G012950	146.2592	145.8347	189.224	256.7798	665.5004	711.623
HORVU3Hr1G083330	1.418458	1.481053	1.687711	9.441381	3.487763	4.796284
HORVU1Hr1G081410	17.22555	16.09174	18.2723	4.829112	1.937864	2.676933
HORVU6Hr1G035730	1.243434	1.579229	1.683397	0.363089	0.161243	0.302306
HORVU4Hr1G010250	0	0	0.064207	0.595617	0.545841	0.865806
HORVU1Hr1G092040	2.868459	4.648399	5.103223	0	0	0
HORVU1Hr1G023070	4.020327	3.96525	4.113016	9.175811	11.31568	11.33692
HORVU3Hr1G034590	0	0	0	0.98363	0.895087	1.454746
HORVU5Hr1G063620	0.495152	0.887388	0.882003	4.584249	3.0958	4.774294
HORVU6Hr1G035190	11.22215	11.35472	13.10714	25.16751	41.054	39.18243
HORVU4Hr1G059270	205.5931	177.1135	286.2883	53.41716	17.94495	31.53793
HORVU6Hr1G045180	1.802531	2.446056	1.963198	0.309417	0	0.266511
HORVU1Hr1G048290	0.468914	0.429236	0.419306	3.411033	5.207466	5.041948
HORVU5Hr1G106610	166.9535	145.143	175.3829	33.3781	97.5494	88.86061
HORVU2Hr1G007070	0.127633	0.211872	0.290941	0.729313	1.065196	1.592843
HORVU7Hr1G037090	0	0	0.004796	3.854099	3.751064	5.536282
HORVU2Hr1G098290	0.743131	0.450362	0.921119	3.124188	5.948263	3.709355
HORVU7Hr1G114400	1.253315	1.718118	1.152035	5.256501	10.50473	10.70333
HORVU5Hr1G011130	1.825782	1.096727	1.212144	0.03782	0.536343	0.636922
HORVU2Hr1G085690	0.035587	0.038279	0.052203	1.96769	10.52825	12.99672
HORVU5Hr1G067010	0.350523	0.434032	1.03011	2.161801	6.795093	4.591875
HORVU2Hr1G012980	18.22502	9.261768	19.32354	1.967141	0.532516	1.724562
HORVU1Hr1G082330	0.872198	0.637534	1.201905	4.299813	3.461687	3.928956

HORVU1Hr1G093880	4.453986	4.996764	4.824507	9.722873	18.78176	15.0569
HORVU3Hr1G047290	0.59383	0.503356	0.30009	1.080295	4.245996	4.061222
HORVU4Hr1G085480	5.414171	6.389074	6.822633	1.147689	0.079477	0.504951
HORVU7Hr1G091780	0.128716	0.057354	0.011599	1.932344	2.526678	2.125009
HORVU5Hr1G057740	12.6434	9.800129	12.47004	2.761535	1.334553	1.972271
HORVU3Hr1G009210	18.56818	16.13277	16.49723	41.7018	58.29712	60.11118
Hordeum_vulgare_newGene_6481	1.015165	1.422549	0.733781	0.078754	0.383258	0.610737
HORVU4Hr1G064440	1.945383	1.23363	1.723998	1.254153	0.640433	0.91271
HORVU4Hr1G038570	4.864399	4.311102	6.427245	14.90187	27.84003	29.06834
HORVU5Hr1G057090	0.033541	0.01773	0.106823	0.816891	1.627215	2.974731
HORVU4Hr1G055210	0	0	0	3.445267	2.554875	2.979222
HORVU4Hr1G078400	0.810996	0.871841	0.952215	2.731416	2.783105	1.685517
HORVU5Hr1G121860	0.377033	0.562083	0.80556	3.928519	9.596476	7.147694
HORVU5Hr1G045710	50.84382	47.03958	44.74177	23.64853	6.813024	7.991675
HORVU0Hr1G007170	0	0	0	5.180335	39.00599	32.46503
HORVU5Hr1G057320	1.047177	0.930212	0.90835	1.5197	4.561404	4.457771
Hordeum_vulgare_newGene_10459	0.088998	0.098699	0.061077	0.844358	1.019701	0.468655
Hordeum_vulgare_newGene_10454	0.425349	0.751424	0.42553	0.235445	0.204788	0.147773
HORVU7Hr1G090120	1.160488	0.808167	1.034857	0.320161	0.199322	0.228308
HORVU4Hr1G007530	10.33776	11.3697	17.26025	2.983202	4.207413	4.472201
HORVU7Hr1G009370	2.156055	1.67305	2.242182	0.096183	0	0.015971
HORVU7Hr1G116470	0.291732	0.427164	0.438259	0.825742	2.961333	3.053845
HORVU2Hr1G041270	15.18423	13.9336	12.03085	47.55741	63.90507	65.21267
HORVU5Hr1G093460	2.306299	2.461386	2.534838	41.44177	26.33484	29.48876
HORVU4Hr1G060630	4.156276	3.966678	3.72806	18.35744	31.82751	30.11321
HORVU6Hr1G041610	0.316273	0.441949	1.1623	5.358902	7.578824	8.487413
HORVU5Hr1G094160	0.233888	0.260083	0.283114	0.900342	1.132542	1.176093
HORVU2Hr1G022160	3.268577	4.359008	5.805179	1.985288	1.545966	1.568875
HORVU6Hr1G094980	0.918648	1.296775	1.187159	0.457266	0.421257	0.587178
HORVU4Hr1G085100	2.781767	2.837639	2.808466	9.056274	8.641715	9.369641
Hordeum_vulgare_newGene_11494	0.365837	0.408978	0.144642	2.063244	2.374823	2.446377
Hordeum_vulgare_newGene_11492	0.088437	0.010911	0.080749	5.478905	5.375399	6.884133
Hordeum_vulgare_newGene_120	0.557596	0.38766	0.485368	0	0	0.022577
Hordeum_vulgare_newGene_128	3.064337	1.928436	2.548217	0.341331	1.032126	0.846165
HORVU3Hr1G023460	1.875486	1.270471	2.437798	3.487934	12.84753	12.90792
HORVU0Hr1G028930	1.248886	2.442436	1.257856	5.927837	8.349526	4.909945
HORVU0Hr1G025870	3.523785	3.38411	4.649015	1.717835	2.717437	2.724207
HORVU2Hr1G114450	1.302219	0.768741	2.165405	0.367805	0.047989	0.423869
Hordeum_vulgare_newGene_16149	0	0	0	4.133548	4.344625	5.272391
HORVU2Hr1G114980	9.705929	8.252166	9.70402	19.88098	33.82798	30.37594
HORVU2Hr1G021530	2.431347	2.22519	3.305034	7.877971	9.348372	9.906116
HORVU2Hr1G060560	0.27863	0.492544	0.583651	1.609848	0.80011	1.609866
HORVU5Hr1G121450	0.154806	0.1105	0	2.859289	3.36181	3.827552
HORVU3Hr1G027460	0.215889	0.37945	0.420428	2.671287	15.58499	12.24547
Hordeum_vulgare_newGene_14166	0.985298	0.980423	0.926052	3.765687	5.587665	4.722621
HORVU2Hr1G100500	7.609201	6.996571	7.658433	0.551315	0.086265	0.259803
Hordeum_vulgare_newGene_3773	5.440585	5.28794	6.278745	32.74094	27.06485	31.58293

Hordeum_vulgare_newGene_3777	0	0	0	2.530535	1.639926	1.700878
Hordeum_vulgare_newGene_10841	3.085692	2.459992	3.60363	1.40728	1.516103	1.538413
HORVU5Hr1G032370	1.107416	1.484903	2.332512	3.662459	6.059603	5.59381
Hordeum_vulgare_newGene_3521	1.555566	1.981099	2.099449	0.004579	0	0
HORVU6Hr1G072050	1.688186	1.897775	2.482814	4.784687	4.947212	6.060142
HORVU1Hr1G007480	0	0	0.183624	2.527162	7.556801	7.353508
HORVU4Hr1G074840	2.887105	2.44275	2.095011	5.343581	8.706739	8.874782
Hordeum_vulgare_newGene_2422	3.397055	3.760042	4.724262	1.888754	1.90731	2.05793
Hordeum_vulgare_newGene_2427	0.864586	0.667952	0.71234	0.266845	0.441376	0.599344
HORVU1Hr1G000650	7.425556	5.248161	4.805656	2.149461	0.41353	0.883579
Hordeum_vulgare_newGene_1218	1.071774	1.134354	1.332294	2.785016	4.974284	5.4666
Hordeum_vulgare_newGene_1219	0.791777	0.745452	1.060051	2.033731	2.896176	2.889308
Hordeum_vulgare_newGene_1217	1.294324	1.166412	1.152764	2.718906	4.110003	4.407623
HORVU2Hr1G071670	1.677088	1.581136	2.308049	1.103773	0.941066	1.198887
Hordeum_vulgare_newGene_11825	2.403713	2.2091	2.649583	2.376166	11.54549	10.42464
Hordeum_vulgare_newGene_11824	0	0.073102	0.055467	1.782525	2.500645	2.527354
Hordeum_vulgare_newGene_11820	0.354635	0.438257	0.242609	2.609786	3.22491	2.965501
HORVU7Hr1G073050	22.29317	19.62937	27.13825	9.519639	6.631133	8.665853
Hordeum_vulgare_newGene_9761	0.455781	0.603251	0.567472	0.330226	0.080511	0.11337
HORVU7Hr1G086340	15.19556	11.08903	13.55173	7.216372	6.526999	6.106564
HORVU0Hr1G025460	8.734565	6.854135	8.852989	4.491255	2.747482	4.262747
HORVU6Hr1G073090	0.380681	0.490467	0.217387	22.2095	5.137409	8.137602
HORVU6Hr1G063860	3.507294	4.137901	4.538908	7.529762	15.76852	13.85848
HORVU7Hr1G039080	1.363458	1.320288	1.241657	4.503548	4.981797	4.037935
HORVU3Hr1G012860	0.076237	0.112038	0.110274	0.877923	0.521968	0.638987
HORVU4Hr1G088350	0.67971	0.944155	0.949212	0.997705	3.805717	4.432167
HORVU7Hr1G101710	1.444332	1.058261	1.043061	3.542386	6.06302	5.809919
HORVU2Hr1G059940	5.349999	4.180531	5.727411	3.162118	2.35535	2.843035
HORVU0Hr1G005640	0	0.024535	0	2.778842	3.028316	3.686233
HORVU2Hr1G075370	17.85332	12.8875	22.31124	5.237816	1.678669	2.878786
Hordeum_vulgare_newGene_4684	6.099428	7.940695	7.481353	0.012799	0	0
HORVU3Hr1G070080	2.155007	1.978063	2.83859	4.487812	7.912077	7.451846
HORVU3Hr1G000830	5.966752	7.16305	4.713036	3.279852	2.661837	2.624222
HORVU5Hr1G088210	4.643641	4.486215	5.673242	2.598098	2.451224	2.499273
Hordeum_vulgare_newGene_6681	2.30837	4.490129	2.966487	0.066892	0.019358	0.018203
Hordeum_vulgare_newGene_7088	0.03681	0.076778	0.104467	0.378896	0.74572	0.471525
HORVU4Hr1G081290	0.679217	0.672739	0.345374	2.251433	11.37011	9.504035
HORVU2Hr1G076590	6.397612	7.657936	6.178806	3.240188	2.921265	3.712726
Hordeum_vulgare_newGene_561	0.067162	0.13034	0.129443	1.349952	2.432666	3.562793
HORVU1Hr1G080640	3.487365	2.267649	3.352488	8.967485	19.35334	15.51208
HORVU6Hr1G067930	1.541266	1.917681	2.821192	4.941318	17.86986	19.60797
HORVU4Hr1G065180	0.678005	0.963408	0.702481	1.763191	3.891927	3.976332
HORVU2Hr1G056650	1.28415	1.119252	1.675861	3.117926	3.43688	3.500738
HORVU3Hr1G013380	0.452615	0.983248	0.449199	3.326355	1.284748	3.085368
HORVU4Hr1G017210	0.916833	0.504814	0.676179	1.920152	3.069812	3.20628
HORVU7Hr1G034220	0.976364	0.946136	1.069567	0	0	0
HORVU1Hr1G005760	3.791612	1.076573	1.286289	0.445249	0.09934	0.100037

HORVU7Hr1G051720	0.64369	0.900977	1.781272	3.091594	6.744376	4.620306
HORVU4Hr1G056050	81.70099	84.79095	92.2897	36.75564	28.15722	35.3837
HORVU1Hr1G090870	6.980782	4.708902	7.70608	1.670933	0.369053	0.799801
HORVU5Hr1G051760	18.21676	15.0542	14.87408	7.86386	4.607316	3.140066
HORVU5Hr1G111700	62.21108	59.77735	63.92347	38.97839	37.93531	38.56674
HORVU2Hr1G084130	56.77317	48.32355	55.15675	5.779571	1.780969	3.358431
HORVU4Hr1G062110	3.51153	2.675769	2.928269	1.479514	0.96531	1.311948
HORVU5Hr1G005340	18.37872	15.68124	17.71392	2.252869	1.579187	1.672262
HORVU2Hr1G127360	0.597207	0.706599	0.75723	1.717749	3.589456	3.552962
HORVU1Hr1G049280	45.50137	23.10556	60.67422	17.05879	21.46368	23.08357
Hordeum_vulgare_newGene_12930	7.357162	7.451324	4.354053	3.981363	2.928557	3.732672
Hordeum_vulgare_newGene_12931	0.728683	0.710711	0.60111	0.061353	0.036839	0.018596
Hordeum_vulgare_newGene_12932	0.033818	0.018046	0.024008	1.193535	0.544594	0.723961
Hordeum_vulgare_newGene_12935	4.74956	4.519859	5.32618	8.838461	18.3922	17.6302
HORVU2Hr1G061260	0.365311	0.069215	0.308858	1.483206	4.379341	4.667871
HORVU2Hr1G016720	2.16515	2.254012	2.739884	0.742721	0.210683	0.432056
HORVU1Hr1G057330	14.37922	14.11197	15.99205	37.89802	56.732	52.96902
HORVU1Hr1G082100	35.37668	37.81199	48.97111	13.37211	4.98165	5.8882
HORVU0Hr1G006750	0.631744	0.560771	0.947168	1.814567	3.796125	2.830323
HORVU5Hr1G121120	1.726777	1.912591	2.165882	0.651132	0.843117	0.897242
HORVU7Hr1G019510	0.898416	0.437437	0.699585	1.312303	1.941241	2.178892
HORVU6Hr1G012640	0.585011	0.481167	0.911717	9.335859	6.114157	3.975058
HORVU2Hr1G078270	19.11224	14.63632	19.07858	13.72101	5.420184	6.587522
HORVU1Hr1G015090	1.809146	1.293248	2.092862	0.934215	0.267741	0.852635
HORVU7Hr1G006740	0.232253	0.247788	0.951112	2.95558	40.27454	43.78703
HORVU3Hr1G099760	9.667154	5.528846	10.85595	2.186818	0.116564	0.878427
Hordeum_vulgare_newGene_8165	0.081618	0.170479	0.180959	0.650315	0.635215	0.541156
HORVU6Hr1G094780	0	0	0	6.954587	7.354752	7.945086
Hordeum_vulgare_newGene_8169	1.82948	1.295772	0.928688	0.734	0.317131	0.51685
HORVU4Hr1G076600	16.21246	15.46092	16.41445	2.507391	4.918909	3.094295
Hordeum_vulgare_newGene_1502	0.949896	1.172846	1.220315	0.162308	0.029683	0.071474
Hordeum_vulgare_newGene_10393	17.68207	18.89972	17.45555	0.02502	0	0.006849
HORVU3Hr1G109300	6.707756	6.262743	6.989339	3.513577	3.499383	3.204054
HORVU7Hr1G086580	2.431958	2.53815	3.229203	1.538611	0.872821	1.487329
Hordeum_vulgare_newGene_10645	21.43457	28.38255	33.98738	0	0	0
HORVU3Hr1G072380	0.10496	0.134851	0.18305	0.795797	10.28353	9.122038
HORVU6Hr1G076620	8.678789	6.880333	11.07217	3.410533	3.303558	2.739553
HORVU2Hr1G119700	0.320024	0.423376	0.220107	1.205928	1.459691	1.416737
HORVU1Hr1G060610	1.191546	0.898423	0.865164	4.457825	8.121756	7.422706
HORVU7Hr1G027770	0.643836	0.714625	0.935157	2.558458	2.700956	2.598391
HORVU4Hr1G063980	10.92827	12.61173	14.54374	6.650149	5.370041	5.816623
HORVU5Hr1G006930	0	0.148854	0	3.221382	4.195706	4.63942
HORVU1Hr1G004150	12.09103	17.51134	27.9215	4.783138	0.579856	2.219794
HORVU2Hr1G122070	0.096738	0.174378	0.286445	1.251115	1.058328	1.457204
HORVU5Hr1G092560	24.23208	19.36571	24.77725	7.633933	9.972465	10.54671
Hordeum_vulgare_newGene_1942	3.546599	5.12283	3.884237	0.028038	0	0
HORVU2Hr1G011960	9.101592	8.76455	10.00875	4.919177	4.74061	4.872779

HORVU1Hr1G084190	21.79492	28.8101	30.20784	3.511348	1.152782	2.010276
HORVU4Hr1G079230	111.1668	122.6262	160.9818	187.5557	469.5037	396.7577
HORVU3Hr1G058810	0.305476	0.141765	0.28817	2.409136	4.612728	3.430073
HORVU2Hr1G014410	3.465504	3.290917	3.02928	0.179184	0.031989	0
HORVU2Hr1G028340	9.506133	9.016177	10.90502	6.152758	5.034676	5.430261
Hordeum_vulgare_newGene_7244	0.657321	0.994251	0.777124	7.079848	6.830596	8.563813
Hordeum_vulgare_newGene_12094	0.990577	1.655041	1.2304	26.35251	20.30367	26.58745
Hordeum_vulgare_newGene_12091	0	0	0	3.579815	3.468713	4.617088
HORVU3Hr1G097750	0.062689	0.029305	0.07885	13.86241	62.80501	55.2799
HORVU3Hr1G081650	3.655721	4.131487	5.010787	2.106999	2.24375	2.302842
Hordeum_vulgare_newGene_3091	3.920708	4.415129	4.381081	12.53125	23.07582	20.46194
Hordeum_vulgare_newGene_3094	3.714327	4.239601	2.829582	0	0.020729	0.040753
Hordeum_vulgare_newGene_3095	1.619007	1.468941	1.993741	0	0	0
Hordeum_vulgare_newGene_3096	39.08214	37.3447	32.56337	14.90796	8.121038	9.198712
HORVU1Hr1G075910	1.671953	1.265295	1.473308	5.150762	7.635101	10.70769
HORVU3Hr1G087550	1.42628	1.180994	1.045638	2.055949	3.96299	3.683145
Hordeum_vulgare_newGene_3903	1.182074	0.953976	0.862762	3.027513	3.03526	3.440318
Hordeum_vulgare_newGene_7683	0.310385	0.89048	0.552085	0.917422	1.818371	1.538393
HORVU3Hr1G018610	2.238976	1.465329	1.365273	1.102002	0.874306	1.079792
HORVU1Hr1G008160	0	0	0	1.59992	2.669556	2.809871
Hordeum_vulgare_newGene_3635	0	0	0	6.206022	5.321818	6.397194
Hordeum_vulgare_newGene_2323	1.451562	1.736864	1.502451	0	0	0
HORVU1Hr1G070290	1.970649	2.24001	2.175658	7.519818	11.27531	12.92868
Hordeum_vulgare_newGene_10379	13.93295	12.73468	14.8896	6.988932	6.770771	7.729101
HORVU6Hr1G072390	13.11994	15.71495	17.90699	2.739008	1.077916	1.770766
Hordeum_vulgare_newGene_5678	4.692479	5.131848	5.896327	1.718685	2.817239	3.032156
Hordeum_vulgare_newGene_9352	0.332177	0.264784	0.314191	0.89615	0.926262	1.030981
HORVU2Hr1G033040	50.06277	49.83169	66.96576	29.03734	31.96289	30.76265
Hordeum_vulgare_newGene_6808	6.435276	4.559703	6.686764	1.455876	2.787509	2.711493
Hordeum_vulgare_newGene_8408	4.067356	4.301555	4.050373	0.164361	0.175728	0.275202
HORVU7Hr1G022910	1.25862	1.702103	1.816388	4.361995	4.338329	4.20619
HORVU4Hr1G005450	9.944414	8.489301	10.46335	3.758308	1.869442	2.685339
HORVU7Hr1G060480	5.415085	6.352258	6.430299	0.399344	1.647225	1.63575
HORVU7Hr1G020510	0.077985	0.077391	0.121545	1.259137	0.99255	1.406279
Hordeum_vulgare_newGene_1003	0.935391	1.299332	1.514888	3.269674	4.6628	4.827119
Hordeum_vulgare_newGene_1006	2.019153	2.126659	2.504854	10.86287	13.31416	13.93304
HORVU6Hr1G082880	24.95879	20.83451	29.39541	59.39826	79.47417	73.14975
HORVU6Hr1G020540	2.412436	1.807997	3.279304	0.025173	0	0.029406
HORVU1Hr1G085480	1.35316	1.294185	1.445771	4.047879	4.935996	4.757815
HORVU3Hr1G014080	0.575586	0.490929	0.727353	1.926681	2.838606	3.028227
Hordeum_vulgare_newGene_4234	1.200735	1.57732	2.450009	0.933535	0.590744	0.458644
HORVU6Hr1G083250	0.554791	0.481909	0.795485	1.844689	1.686227	1.93913
HORVU0Hr1G000910	0	0.046028	0.018507	6.069141	9.340391	8.575247
Hordeum_vulgare_newGene_7760	4.631583	4.314154	4.678329	0	0	0
HORVU6Hr1G008320	0	0.093865	0.057177	3.936034	4.655826	5.385456
HORVU4Hr1G072480	18.51689	11.50121	28.16428	8.493955	2.451572	10.05858
HORVU1Hr1G079680	4.142836	3.085312	3.76368	0	0	0

HORVU2Hr1G002660	6.863493	6.77078	8.003593	27.583	22.79275	26.209
HORVU5Hr1G014480	0.294415	0.370477	0.447429	1.515316	1.519551	1.725499
HORVU2Hr1G041000	0.200313	0.235119	0.168887	1.281851	3.228426	3.485427
HORVU1Hr1G064130	22.75135	31.84637	27.97663	7.726712	2.21384	3.593418
Hordeum_vulgare_newGene_3923	1.254862	1.196297	1.294563	3.860459	3.847044	4.661689
HORVU2Hr1G077970	0.164696	0.274371	0.157864	2.38496	2.024388	2.22581
HORVU6Hr1G057380	0.073584	0.08904	0.03609	1.589638	0.837879	1.537694
HORVU4Hr1G050250	0.528353	0.774119	1.118866	1.064525	2.414332	2.591261
HORVU1Hr1G023750	0	0	0	1.271883	1.100874	1.737476
HORVU3Hr1G079720	0.040565	0.060333	0.0141	3.590881	6.156611	4.131136
HORVU1Hr1G079970	1.330876	2.250076	3.36175	1.070281	1.400884	1.003659
HORVU4Hr1G052090	1.931983	1.769618	2.27196	4.197685	8.424994	7.686117
HORVU1Hr1G093480	39.68183	19.74835	57.02062	16.14172	19.05408	26.83646
HORVU5Hr1G009620	6.793788	8.918827	7.344347	14.2347	30.90126	21.83697
HORVU3Hr1G006940	4.469442	4.176414	5.777986	27.09439	18.97662	21.76607
HORVU5Hr1G112750	0.521943	0.478263	0.538141	0.707816	3.463891	2.981101
HORVU3Hr1G108360	5.634248	3.339589	5.365074	13.95478	13.60555	16.31556
HORVU0Hr1G038820	1.164088	0.958703	1.535831	0.029572	0.127637	0.117311
HORVU5Hr1G082940	0.56083	0.383092	0.46851	1.315206	2.142093	2.23984
HORVU2Hr1G112830	11.29909	8.493459	9.907658	4.98029	1.090158	5.121881
HORVU5Hr1G034820	37.18089	33.9103	24.88963	16.40433	10.45406	12.17749
HORVU0Hr1G007360	3.877316	3.293797	4.399789	10.98994	15.03623	13.87377
HORVU1Hr1G012190	0.193538	0.2931	0.150718	1.619898	1.280165	1.567113
HORVU7Hr1G093940	1.853759	2.091716	2.022186	10.59365	22.91968	19.34322
Hordeum_vulgare_newGene_12878	0.577892	0.647983	0.728489	1.670645	11.02136	7.603082
HORVU3Hr1G074100	5.71723	5.415797	6.45067	34.32031	52.96316	36.15767
HORVU7Hr1G085570	9.905254	8.047298	10.25778	4.217817	3.905006	4.561218
HORVU2Hr1G017370	1.162256	1.282609	1.294185	0.725694	0.40845	0.442587
HORVU2Hr1G066790	0.626643	1.322971	1.529514	0.456645	0.541102	0.561537
HORVU3Hr1G104090	0	0	0	5.737333	7.093224	8.475716
Hordeum_vulgare_newGene_5470	0.424079	0.467132	0.369325	1.683886	2.093208	1.996114
Hordeum_vulgare_newGene_5479	0.680111	0.52574	0.552262	3.638918	1.994231	1.550416
HORVU3Hr1G077450	13.03018	11.88542	12.17935	5.257194	3.970505	4.563024
HORVU0Hr1G010040	0.237609	0.13273	0.12611	0.675792	1.667694	1.205103
HORVU0Hr1G021850	142.8991	105.2285	167.7505	85.27717	47.35737	55.13181
HORVU4Hr1G075560	2.341573	3.484321	3.474687	1.702142	1.501272	1.795803
Hordeum_vulgare_newGene_13836	4.082773	3.941892	2.281808	0.565495	1.506297	1.755963
Hordeum_vulgare_newGene_8640	11.94241	9.865938	11.8496	27.83493	51.6229	44.76274
HORVU3Hr1G093760	2.949887	3.275715	4.459588	2.323317	1.397763	1.605572
HORVU1Hr1G022900	0.288873	0.068424	0.182448	1.915212	35.39443	37.91545
HORVU5Hr1G080630	0.946474	0.801642	1.081426	4.290006	2.419253	2.76186
HORVU7Hr1G121850	398.5731	245.3652	331.2884	126.0448	13.12632	48.58234
HORVU5Hr1G052730	3.436549	2.475529	3.388485	1.225171	1.735745	2.66172
Hordeum_vulgare_newGene_2942	2.777176	1.913366	3.991281	1.953803	1.203699	0.93319
HORVU4Hr1G048510	5.487567	5.761385	6.129332	13.14139	20.45958	20.03438
Hordeum_vulgare_newGene_10567	0.463122	0.539909	0.606456	2.402141	2.47416	2.195529
HORVU7Hr1G076850	0.172009	0.224785	0.255823	0.630124	1.011306	0.913845

Hordeum_vulgare_newGene_1574	0.204071	0.201621	0.248108	0.478238	1.174829	0.929914
HORVU6Hr1G060810	1.551666	1.732837	1.674868	6.134163	9.971317	8.602323
HORVU3Hr1G018680	0.156492	0.476961	0.598353	2.035119	1.702845	2.410302
HORVU2Hr1G015760	0.076986	0.045919	0.108025	0.31145	0.655714	0.882881
HORVU6Hr1G071860	27.64852	30.78594	33.93806	21.62073	7.821292	13.04935
HORVU7Hr1G082330	6.354164	5.596473	6.478461	15.45186	15.36922	18.08359
HORVU2Hr1G025630	0	0.017865	0	2.555769	3.73586	4.086813
Hordeum_vulgare_newGene_7165	7.309975	6.579127	10.18938	12.84128	27.95195	23.65099
Hordeum_vulgare_newGene_7166	76.65124	76.54788	97.5836	157.5101	382.994	314.1268
Hordeum_vulgare_newGene_7167	1.076584	1.142987	0.853696	4.375862	6.378935	6.184392
Hordeum_vulgare_newGene_7168	12.9024	12.67588	12.37129	8.083914	7.021123	8.581278
Hordeum_vulgare_newGene_7615	3.101196	3.029656	2.766003	8.843402	7.763347	9.097414
HORVU7Hr1G039710	0.501291	0.404438	0.816698	1.085021	2.297733	2.021
HORVU5Hr1G084520	0.225685	0.094355	0.162077	0.857678	2.806623	2.920548
HORVU1Hr1G030060	3.782958	2.926073	3.836434	15.73846	7.280002	10.27116
Hordeum_vulgare_newGene_541	0.111677	0.116058	0.12314	0.756654	1.472804	1.302602
Hordeum_vulgare_newGene_14455	1.364552	1.591061	1.541196	1.812784	1.599559	2.209262
Hordeum_vulgare_newGene_14454	0	0	0	0.765109	0.763732	1.158986
Hordeum_vulgare_newGene_14452	2.057857	2.160641	2.08054	1.058888	1.537947	1.328126
HORVU3Hr1G086030	0.09666	0.005891	0.153518	0.933739	2.460601	2.047482
Hordeum_vulgare_newGene_12683	0	0	0.161254	5.425642	6.168887	7.809114
HORVU7Hr1G025670	0.114063	0.061995	0.346599	0.798836	0.669118	1.131026
HORVU5Hr1G087690	11.83159	11.39388	12.58219	6.072043	4.519833	4.903586
HORVU0Hr1G008030	2.752979	3.546337	3.281414	6.00199	7.943108	8.535961
HORVU1Hr1G070310	624.3182	581.1111	722.5764	140.9493	3.37984	50.20824
Hordeum_vulgare_newGene_8152	0	0	0	0.62185	0.707592	0.893644
HORVU7Hr1G030670	7.335545	9.162749	10.28353	2.533666	0.785412	1.413211
HORVU6Hr1G002390	0.307554	0.271443	0.334855	1.168412	1.204172	2.280601
HORVU7Hr1G008470	0.260376	0.354053	0.512327	2.899017	10.65949	9.479142
HORVU6Hr1G082260	1.053037	1.14477	1.22688	2.581543	4.217498	3.453377
HORVU5Hr1G074000	0.222343	0.23848	0.237841	0.9649	3.625082	3.238186
HORVU6Hr1G072740	1.420704	2.061339	1.109603	13.47597	20.26894	18.8675
HORVU1Hr1G059910	69.64053	111.2953	166.9594	4.263925	0	0.796672
HORVU7Hr1G058940	1.485953	1.389778	1.209461	3.670764	7.770947	7.103776
HORVU2Hr1G040880	0.791696	0.778997	0.577799	3.408313	2.999611	2.390573
HORVU0Hr1G003260	1.860722	2.353094	2.915535	5.059085	8.357938	7.536858
HORVU7Hr1G116880	3.756534	2.639466	3.827928	1.695691	1.633123	1.908488
HORVU7Hr1G023140	2.258623	2.794027	1.874982	7.942964	11.35568	12.22641
HORVU7Hr1G090950	16.94853	13.8226	20.44121	26.85234	56.04512	54.12412
HORVU4Hr1G002730	4.169601	2.050043	1.737904	1.447613	0.361164	1.028329
HORVU5Hr1G039730	0.171459	0.099889	0.039982	1.968257	2.976638	2.134339
HORVU5Hr1G026650	3.521752	3.818672	5.795788	8.226252	20.28281	16.98199
HORVU4Hr1G080610	0.285568	0.097502	0.256335	6.276267	14.64437	16.5965
HORVU1Hr1G062380	13.89244	5.136598	11.68627	0.292075	0	0.693254
HORVU1Hr1G054250	2.595129	2.803611	3.193054	62.11631	62.4553	60.35964
HORVU7Hr1G106130	0.705511	0.963806	1.770392	7.912324	132.0068	122.9092
HORVU3Hr1G014780	2.687939	2.636022	2.467888	5.473736	8.692829	8.745338

HORVU5Hr1G062310	25.1975	24.67309	32.73853	51.77061	111.7997	93.39394
HORVU5Hr1G067360	0.543901	0.493476	0.882876	0.17674	0.019504	0.080564
Hordeum_vulgare_newGene_1637	0.10044	0.112629	0.132138	0.476208	0.391656	0.647901
HORVU2Hr1G001430	2.292507	1.382165	2.74046	35.38831	79.61037	61.25177
HORVU4Hr1G072360	6.196284	4.694623	7.137607	1.602931	0.451622	1.331897
HORVU4Hr1G081390	36.54717	39.32377	58.30293	3.584801	0.14333	1.294313
Hordeum_vulgare_newGene_2590	0	0	0	1.576933	2.03565	2.027974
HORVU7Hr1G099390	1.687873	1.753234	2.028067	1.301686	0.647678	1.162892
HORVU5Hr1G113880	10.64646	11.92382	12.74856	27.40592	57.38332	49.28138
HORVU6Hr1G013370	3.96054	3.378085	5.530686	2.45447	2.842812	2.328302
HORVU2Hr1G078910	97.42386	106.3409	179.6778	38.93427	76.1954	73.12594
HORVU7Hr1G038900	0.879463	1.16447	1.194027	3.017926	3.812137	3.542075
HORVU1Hr1G051700	3.002504	3.068251	1.866522	8.843884	16.62191	19.7498
HORVU5Hr1G045360	0.377541	0.185968	0.290594	0.616303	0.729867	1.275305
HORVU3Hr1G002010	1.361961	1.941088	2.329745	0.943164	1.159286	1.122719
HORVU2Hr1G054890	0.42759	0.228939	0.284748	1.778842	1.693108	1.646676
HORVU1Hr1G055930	6.946047	8.257089	10.24706	1.057825	0.157753	0.732188
HORVU3Hr1G095780	2.732561	2.776702	2.762946	15.06102	18.06474	17.10788
HORVU6Hr1G085730	9.550049	9.489678	13.75663	5.801902	6.601776	7.553388
HORVU7Hr1G010490	19.83995	22.42666	24.15826	10.27033	9.106522	10.20356
HORVU6Hr1G034760	13.06261	7.551456	12.43179	6.008519	2.748652	3.322993
HORVU2Hr1G019010	0.027178	0.008462	0.06079	3.804327	2.817428	3.012122
HORVU7Hr1G045290	0.136896	0.200792	0.287666	5.477224	11.46297	11.95461
HORVU5Hr1G007050	1.938153	1.947471	2.684174	7.17577	11.40144	11.36342
HORVU5Hr1G054970	0.529368	0.111723	0.247712	1.784964	1.234557	1.164345
HORVU4Hr1G005810	0.061601	0.028867	0.139366	0.490811	1.127749	1.356538
HORVU5Hr1G109250	0	0	0	1.064707	2.769989	2.550231
HORVU0Hr1G027130	532.5319	481.1016	698.4937	144.3191	164.3101	156.4169
Hordeum_vulgare_newGene_15051	5.724025	5.750841	5.564246	3.302279	3.452975	3.811652
HORVU6Hr1G090370	0	0	0.029619	25.54158	106.434	84.394
HORVU2Hr1G012200	1.309924	1.310752	1.150404	0.638878	0.568212	0.507528
HORVU1Hr1G093390	1.425573	1.191541	1.960111	6.978443	10.62487	6.95284
HORVU4Hr1G053150	1.102844	0.669238	1.355773	0.124814	0.148034	0.123503
HORVU3Hr1G084220	0.216149	0.132029	0.327739	2.170093	2.432459	4.650095
HORVU6Hr1G011510	0.524233	0.346005	0.655296	2.634836	12.13522	12.64485
HORVU2Hr1G065000	21.03168	14.02235	15.73429	5.047138	2.331331	3.265094
HORVU2Hr1G060070	6.629896	4.608854	3.47341	17.02509	17.64117	16.48657
HORVU6Hr1G054430	0.599489	1.041675	0.759405	1.455826	3.075337	2.701227
HORVU4Hr1G008950	0.67949	0.577667	0.688741	2.679884	1.783491	2.027029
HORVU7Hr1G100300	0.761233	1.096084	0.843644	0.075466	0.048455	0.060021
HORVU1Hr1G075750	5.109145	4.285688	3.770709	0	0	0
HORVU4Hr1G067180	3.938166	3.6154	4.901117	10.22196	14.22118	14.53905
HORVU1Hr1G042360	0.1226	0.132766	0.180921	0.983419	2.529884	2.187319
HORVU3Hr1G021080	0.320233	0.317336	1.147882	0.99124	1.091005	2.714989
HORVU2Hr1G036280	6.626049	6.56665	7.329595	9.491161	24.54849	22.68941
HORVU6Hr1G092990	1.251091	1.982629	1.436136	0.190464	0.214938	0.166124
HORVU5Hr1G099170	0.178615	0.574171	0.181811	4.763964	4.306576	6.772523

HORVU4Hr1G022770	0	0	0	2.431698	105.8305	63.11112
Hordeum_vulgare_newGene_3441	1.24818	1.366458	1.360864	0.535596	0.278515	0.150803
Hordeum_vulgare_newGene_3447	4.874676	6.909169	6.316462	1.7716	1.74575	2.309648
HORVU2Hr1G092530	10.6098	8.552506	10.07049	37.02628	60.56475	47.55819
HORVU4Hr1G064760	14.55636	9.245167	8.60794	15.8837	53.89906	53.39925
Hordeum_vulgare_newGene_2710	0	0	0	4.414877	8.379428	6.729455
Hordeum_vulgare_newGene_2718	71.61402	66.69168	88.45936	24.7637	4.987313	9.909307
HORVU2Hr1G126740	5.623069	2.47684	4.499816	29.5829	50.10425	52.17927
Hordeum_vulgare_newGene_5842	2.981181	2.07592	2.971342	0	0	0.053933
Hordeum_vulgare_newGene_15259	0	0	0	1.893884	3.931394	3.574496
HORVU1Hr1G003960	0.662797	0.611493	0.972719	7.045783	8.242107	8.925051
HORVU7Hr1G080850	1.58004	1.67798	2.784778	5.477701	14.76036	13.24918
HORVU4Hr1G079620	9.0568	4.618959	14.07811	1.217548	0.093107	1.829316
Hordeum_vulgare_newGene_5158	0.41257	0.280739	0.500494	5.081426	4.735043	4.688629
HORVU1Hr1G055340	12.92905	15.27662	18.33422	2.90878	1.021201	2.047087
HORVU7Hr1G037830	0.072379	0	0.015737	4.606044	3.605596	4.014874
HORVU2Hr1G076320	7.991565	5.780897	8.986521	3.034787	1.726503	2.847415
Hordeum_vulgare_newGene_8054	3.467036	4.324314	4.264964	12.16171	10.58169	11.0445
Hordeum_vulgare_newGene_8059	25.81675	31.00667	17.50473	3.326281	2.860825	3.552379
HORVU2Hr1G026850	0.042583	0.081353	0.080025	0.372509	1.137841	1.315481
HORVU7Hr1G063220	5.467455	7.306394	7.589515	9.502192	21.0379	23.41348
HORVU3Hr1G112670	0.130144	0.225249	0.157852	0.903425	0.924728	1.066102
HORVU1Hr1G073540	7.317059	8.36333	7.97162	4.006028	4.310596	4.233033
Hordeum_vulgare_newGene_4607	0	0	0.006039	4.251516	5.356397	4.973237
Hordeum_vulgare_newGene_4603	0	0	0.045651	3.245695	3.799574	5.672565
HORVU1Hr1G031270	3.828918	4.210165	3.83239	0.511626	0.796018	0.948332
HORVU2Hr1G102510	0.289168	0.524344	0.43935	2.544615	0.677652	1.482204
Hordeum_vulgare_newGene_3879	0.522586	0.240868	0.629765	2.344453	1.873221	1.663674
HORVU3Hr1G088040	4.78336	3.199705	3.272316	0.127042	0.202408	0.061335
HORVU1Hr1G063330	0.431934	0.294592	0.492294	1.198289	3.977901	5.200738
HORVU1Hr1G025060	1.279244	1.258124	1.421985	0.187233	0.467015	0.250053
HORVU2Hr1G032730	5.260264	4.126912	5.222779	9.54516	21.17961	14.73815
HORVU6Hr1G073260	42.06007	36.31969	32.55525	12.26468	6.345125	6.092105
HORVU1Hr1G011030	0.616354	0.15266	0.558663	1.763541	1.665848	1.601974
HORVU3Hr1G085980	0.767136	0.61776	0.699488	0	0	0
Hordeum_vulgare_newGene_3124	6.422686	4.531832	7.004823	2.11342	0.914356	0.763524
HORVU7Hr1G002740	7.638857	3.053233	14.06728	0.032385	0	0.085203
HORVU3Hr1G093080	0.358735	0.230646	0.219128	2.501064	30.39706	42.41828
Hordeum_vulgare_newGene_14642	4.740806	4.02315	5.234953	2.023317	0.813128	1.054575
Hordeum_vulgare_newGene_14648	0	0	0	1.228154	1.644066	1.441124
HORVU2Hr1G081490	0.133762	0.239233	0.527507	0.614467	0.723413	1.279142
HORVU3Hr1G117750	0.111481	0.200844	0.451262	1.255981	1.155807	1.575341
HORVU2Hr1G027480	4.255976	2.329488	5.736171	0.69441	0.941461	1.306987
HORVU1Hr1G047370	0.152399	0.181088	0.232069	0.701552	0.576013	0.61786
HORVU0Hr1G000450	17.02084	18.01739	14.78649	6.352407	2.747953	4.348318
HORVU3Hr1G110760	0	0	0	7.570583	4.008545	2.654083
HORVU4Hr1G003340	0.505994	0.680886	0.949633	0.115465	0	0.049886

HORVU2Hr1G043960	2.326297	2.073715	1.885662	6.292189	8.427914	7.665586
HORVU4Hr1G046810	2.953796	2.827229	4.396017	5.309918	18.42857	12.71866
Hordeum_vulgare_newGene_8933	3.697795	3.484354	3.062129	7.18502	10.42058	13.23451
HORVU3Hr1G042540	5.553418	6.595622	5.903353	29.14898	43.93145	39.94313
HORVU4Hr1G068690	1.026384	0.90736	1.233541	0.334055	0.171451	0.277513
HORVU2Hr1G015940	10.9402	9.761242	11.0986	4.903584	3.932937	4.931483
HORVU7Hr1G047500	2.718751	3.269932	2.50609	10.4153	18.06854	16.48675
HORVU5Hr1G025430	1.214603	1.292882	1.580636	3.022893	5.730603	5.050041
HORVU2Hr1G085800	11.87807	10.44732	12.29086	30.60493	46.31891	45.92241
HORVU7Hr1G084610	5.064291	3.663708	5.895564	2.155996	2.742159	3.441782
HORVU6Hr1G084070	452.9626	580.7398	720.6708	29.31871	2.653998	13.30184
Hordeum_vulgare_newGene_8251	0.076057	0.196057	0.302716	1.5371	1.63694	1.614654
HORVU7Hr1G017640	44.57529	29.2392	43.48021	25.63093	12.15198	16.39953
HORVU7Hr1G095820	1.363797	1.584595	1.848222	4.2776	7.509193	7.145551
HORVU5Hr1G011920	0.380805	0.364676	0.691736	0.143989	0.191701	0.349563
HORVU4Hr1G048110	0	0.008863	0	2.084923	2.48594	2.607711
HORVU2Hr1G080260	1.86012	1.211393	1.88002	3.603297	4.749294	6.566847
HORVU4Hr1G086300	3.640712	3.290963	6.203002	1.423309	0.491875	2.743931
HORVU1Hr1G057910	2.043762	2.222176	1.676506	1.210273	0.281809	0.444669
HORVU7Hr1G019590	0.124819	0.105263	0.178846	1.133184	3.643255	4.286395
HORVU1Hr1G078710	1.020665	0.8859	0.667442	0.240711	0.163875	0.142542
HORVU2Hr1G092180	2.052043	2.381895	2.057437	14.09233	23.99617	22.54373
HORVU3Hr1G068780	4.290923	4.817699	4.624043	8.187868	14.51152	14.84184
HORVU7Hr1G012910	0.755692	0.85823	0.956129	1.691847	2.462079	3.344047
HORVU1Hr1G080200	0.223173	0.119347	0.442917	6.471308	19.28297	15.51632
HORVU7Hr1G116220	1.579554	1.649097	1.364106	0.532724	0.386041	0.498649
HORVU1Hr1G004480	2.140594	1.909059	2.203892	4.088793	6.086203	5.813984
HORVU3Hr1G094560	1.017684	1.263955	1.027767	0.48462	0.04224	0.435227
HORVU3Hr1G092390	8.375153	9.825926	8.283401	18.33218	25.62022	25.28335
HORVU6Hr1G057500	1.522946	1.31207	1.280498	6.869247	6.782782	6.085586
HORVU7Hr1G023920	2.231357	3.211446	2.392155	1.645404	1.068674	1.249289
HORVU7Hr1G006980	0.993552	1.150716	0.943839	0.441388	0.092031	0.203935
HORVU5Hr1G049490	3.939821	4.619279	4.187252	2.659976	2.033196	2.780006
Hordeum_vulgare_newGene_15629	0.696871	0.910364	0.785116	0.378517	0.65215	0.36723
HORVU7Hr1G098320	1.904788	2.592507	3.046793	3.750122	9.258372	8.576241
HORVU7Hr1G041540	0.03738	0.094275	0.100499	0.62245	2.332655	2.438952
HORVU2Hr1G028610	0.084764	0.174251	0.223246	1.479005	1.584095	1.916289
Hordeum_vulgare_newGene_16062	1.503924	1.185136	1.760282	0.425782	0.284893	0.207711
HORVU7Hr1G001450	1.33074	0.643374	0.90262	0	0	0
HORVU3Hr1G088530	34.3696	26.45941	31.66166	15.10135	13.87255	14.21157
HORVU7Hr1G042160	6.770592	5.889987	5.723187	28.78267	59.53739	54.69323
HORVU1Hr1G012060	1.442611	1.893498	3.309039	0.193674	0	0
HORVU5Hr1G080870	1.266503	1.186684	1.859976	5.961433	10.21069	7.10559
HORVU4Hr1G090930	0.154035	0.295071	0.308194	1.199127	1.165255	1.341487
HORVU3Hr1G106090	1.343358	1.946748	2.334748	1.121406	0.693406	1.087819
HORVU6Hr1G027760	0.057503	0.07341	0.241112	0.601081	1.480216	2.400055
HORVU0Hr1G021760	25.63616	28.12697	32.29093	54.37116	133.3514	111.7413

HORVU1Hr1G082220	33.87807	35.66237	14.42867	0.167209	0.096255	0.225649
HORVU4Hr1G058840	5.457284	5.951057	5.472376	8.387869	20.72025	19.25831
Hordeum_vulgare_newGene_634	0.217684	0.168617	0.102733	2.918533	2.655124	2.127475
Hordeum_vulgare_newGene_14339	1.573276	1.605419	2.593243	7.138704	6.906333	7.500654
HORVU3Hr1G110160	4.392813	2.731805	4.652218	9.800865	15.70524	13.82693
Hordeum_vulgare_newGene_14332	0.036056	0	0	1.681605	1.972327	2.207734
Hordeum_vulgare_newGene_14333	3.622946	4.001128	4.378571	0.219937	0.053814	0.043438
HORVU5Hr1G000940	7.578803	6.897278	8.175142	1.599708	0.408978	2.297122
Hordeum_vulgare_newGene_2506	9.239454	14.23425	14.74472	0.970768	0.675666	0.538252
HORVU1Hr1G000590	14.20914	12.09382	11.65694	2.733561	1.301046	1.677776
HORVU2Hr1G077120	31.87752	40.88892	33.79223	158.6797	636.3847	516.4442
HORVU2Hr1G031990	8.981208	8.021003	11.35318	15.48463	34.55387	30.2233
HORVU1Hr1G000040	7.436662	6.104369	6.488774	22.1699	46.44427	42.08127
Hordeum_vulgare_newGene_11909	3.121195	3.936119	3.358982	1.139748	0.26788	0.543047
Hordeum_vulgare_newGene_11905	0.176862	0.352661	0.316238	0.784726	0.807038	0.940593
HORVU3Hr1G026540	5.76554	6.076304	7.268566	16.74282	41.72107	44.44587
HORVU4Hr1G060720	337.594	307.935	334.7839	21.86821	54.26731	26.81176
HORVU2Hr1G009730	0	0	0	1.161937	0.517296	1.169897
HORVU2Hr1G105140	13.58262	15.42368	21.54366	8.116495	5.755159	5.577617
HORVU0Hr1G038120	11.56544	10.28757	12.34612	24.33215	42.57736	41.6495
Hordeum_vulgare_newGene_1774	1.353046	0.872641	1.266528	0.58321	0.410435	0.566664
Hordeum_vulgare_newGene_6773	0.530964	0.370549	0.377686	0.706824	1.510745	1.209293
HORVU4Hr1G072150	2.577767	2.367374	2.763387	8.412699	12.00391	11.0162
Hordeum_vulgare_newGene_7455	0	0	0.015973	8.777846	7.451264	9.602404
Hordeum_vulgare_newGene_15883	1.218795	1.405403	1.208146	2.718684	4.762613	4.401386
Hordeum_vulgare_newGene_4819	0.784042	1.354595	0.973181	2.879879	2.844597	3.629669
HORVU3Hr1G018800	64.62316	55.1976	55.87808	26.09325	18.08493	20.74461
HORVU4Hr1G016190	0	0	0	1.695894	2.002522	1.900237
Hordeum_vulgare_newGene_6417	0.802994	0.914066	0.578262	0.039265	0	0.028316
HORVU3Hr1G034020	2.512627	1.266525	1.875508	0.789221	0.52618	1.105879
HORVU3Hr1G079940	0.256667	0.105585	0.310157	1.611054	1.7546	1.919798
HORVU1Hr1G074380	3.718501	3.416656	3.616431	6.352573	11.24234	14.2154
HORVU1Hr1G070800	0.650246	0.32187	0.376174	0.983711	1.481835	1.886816
HORVU5Hr1G089280	0.038516	0	0.048268	0.572901	0.683678	1.10639
HORVU5Hr1G009150	4.357357	5.326698	5.703892	2.695847	0.292578	1.394964
Hordeum_vulgare_newGene_9945	7.887904	9.47811	10.2996	0	0	0
HORVU2Hr1G000040	6.685266	6.986158	7.868142	16.32071	24.94122	22.25646
HORVU6Hr1G087310	0.2275	0.224414	0.16404	2.395082	1.723709	1.95987
HORVU6Hr1G084650	0.162023	0.165539	0.150254	3.459764	8.248059	7.475918
HORVU6Hr1G006480	43.64106	36.21919	46.31942	23.84716	23.15072	26.2538
HORVU2Hr1G122810	0.052384	0.096174	0.036458	0.373303	0.669422	0.620862
HORVU1Hr1G023270	3.040735	2.250722	3.541197	7.40092	11.42898	10.1013
HORVU2Hr1G051010	6.677013	6.628818	5.395277	0.265433	0.719123	1.738809
HORVU3Hr1G018390	0.08259	0.091781	0.074382	0.399893	0.366749	0.614017
HORVU2Hr1G095880	1.101018	1.358657	1.397026	6.609799	3.837755	4.836645
HORVU3Hr1G023740	4.729487	3.756666	5.159278	10.63749	14.92439	16.01684
HORVU2Hr1G094280	10.78541	11.95399	14.83362	5.05787	4.160036	5.15225

HORVU0Hr1G007050	18.14926	12.92058	19.34583	8.323256	8.28176	9.126944
HORVU5Hr1G109680	3.107598	3.058131	5.094835	6.08776	12.17449	11.18235
HORVU1Hr1G048670	0	0	0.009741	21.20963	13.13863	19.67871
HORVU7Hr1G076030	1.506003	1.367913	1.418559	4.373613	7.282907	5.867923
HORVU3Hr1G026150	3.736973	3.927727	3.360556	16.09652	34.67242	29.38391
HORVU7Hr1G088920	11.53148	14.46205	19.09283	6.622634	8.456199	7.37383
HORVU3Hr1G077980	0	0.008217	0.01444	8.461401	8.946576	10.18098
HORVU2Hr1G100660	5.754404	6.599954	6.44952	27.74675	33.09153	32.3292
HORVU1Hr1G082020	2.122129	1.643826	1.451332	0.062691	0.054191	0.033111
HORVU2Hr1G061090	2.288009	2.887145	2.690571	4.545795	14.44348	13.00499
HORVU2Hr1G108780	12.81842	10.43906	20.3788	1.70397	2.831722	3.127196
HORVU3Hr1G099390	0.377705	0.376545	0.233294	2.344476	2.892532	2.730138
HORVU4Hr1G007620	23.85792	21.19431	26.10135	49.35338	104.612	102.7779
HORVU2Hr1G048100	0.654405	0.683079	0.529623	1.917494	3.23278	3.661761
HORVU2Hr1G092390	6.489204	6.464158	9.026855	3.176281	1.253624	1.003438
HORVU2Hr1G073370	8.027568	7.224419	7.257378	24.56564	56.55009	51.63776
HORVU5Hr1G081500	24.45372	25.08862	26.5603	15.33879	7.77014	11.65327
HORVU3Hr1G104260	8.341092	8.146235	8.718006	0.470266	0.36793	0.414556
HORVU2Hr1G004820	4.249215	4.845283	2.568451	11.41819	12.70656	14.00788
HORVU1Hr1G040800	0.087563	0.204686	0.085544	1.356699	0.946494	0.889484
HORVU3Hr1G077170	2.607568	2.636661	2.843058	1.999143	1.222161	1.383282
Hordeum_vulgare_newGene_11653	1.940034	1.80667	2.193161	10.65919	9.896683	13.24348
HORVU6Hr1G024000	10.31768	10.66136	8.873952	4.990461	4.746348	4.28212
Hordeum_vulgare_newGene_5277	1.306179	1.536732	1.790741	0.046284	0	0
HORVU4Hr1G063240	0.895104	0.728894	1.01801	2.509323	2.184984	3.150703
HORVU7Hr1G080710	8.201335	8.037934	8.244514	2.974468	2.491039	2.733173
HORVU5Hr1G005740	4.79845	4.083124	5.426119	11.79518	14.38011	13.71178
HORVU3Hr1G010070	0.612361	0.503259	0.976443	2.046586	2.819108	3.031149
HORVU1Hr1G046400	0.839732	0.477084	1.293933	7.451428	40.41503	50.14784
Hordeum_vulgare_newGene_12352	0.373715	0.454988	0.500827	1.381254	1.62567	1.840504
HORVU3Hr1G107240	14.72673	17.97206	20.25605	9.091893	5.385506	5.991923
HORVU5Hr1G072650	11.62769	5.524198	17.77575	17.78079	51.87375	51.6816
HORVU2Hr1G092800	6.005135	5.784323	6.277984	18.69676	19.19193	18.05516
HORVU4Hr1G011210	4.355879	5.963871	6.719106	0.06451	1.081054	0.473793
HORVU7Hr1G030540	1.836889	2.406372	2.144542	5.911367	13.13768	10.46497
HORVU7Hr1G038770	9.595408	9.513912	8.766818	18.45487	36.61329	30.8082
HORVU1Hr1G085050	0.524538	0.400915	0.462032	1.849753	2.782412	2.868775
HORVU7Hr1G077860	494.297	527.4287	782.1662	64.10631	0.806198	20.68138
HORVU2Hr1G117290	0.055066	0.112211	0.092625	0.527153	0.531243	0.658257
Hordeum_vulgare_newGene_13403	0.945036	1.302465	1.235356	0.007382	0	0
Hordeum_vulgare_newGene_13402	0	0	0	1.888174	1.702426	1.906916
Hordeum_vulgare_newGene_13401	0.838155	0.305121	0.33342	5.295651	3.261042	4.446604
Hordeum_vulgare_newGene_13405	1.176244	1.684018	1.634541	3.878965	9.429705	7.340605
HORVU2Hr1G092510	7.127921	4.395815	7.10914	14.40389	23.64321	19.39863
HORVU3Hr1G048950	2.588434	2.250003	2.763977	7.040025	8.252683	7.722546
HORVU3Hr1G110550	0.277162	0.114276	0.140914	11.01294	12.56339	13.24065
HORVU5Hr1G069880	13.22991	9.354388	11.20322	7.126717	1.879225	3.232148

Hordeum_vulgare_newGene_15763	0	0	0.03117	9.668382	9.761772	11.29512
HORVU6Hr1G065360	1.800554	1.897195	1.688504	5.207974	10.10286	9.570909
Hordeum_vulgare_newGene_15496	2.562822	2.2828	2.717231	0.016153	0	0
Hordeum_vulgare_newGene_6920	0.23717	0.5133	0.256329	1.699799	2.162046	2.990537
Hordeum_vulgare_newGene_8565	0	0	0	2.100282	1.913534	2.372604
HORVU3Hr1G012080	0.107138	0.110852	0.249289	0.542681	0.636794	0.727417
HORVU2Hr1G088640	124.4096	101.9322	130.751	51.68365	10.47868	20.47455
HORVU5Hr1G010370	0.38086	0.254235	0.42118	1.357564	3.987129	3.889139
HORVU2Hr1G045970	0.035734	0.237123	0.236363	1.212894	2.505527	2.712367
HORVU5Hr1G061120	17.65811	15.17609	16.23458	33.17451	47.47172	46.8539
HORVU0Hr1G039020	0.024316	0.022838	0.133253	1.490895	0.976151	2.325136
Hordeum_vulgare_newGene_16156	35.02011	34.94455	49.26544	21.63992	20.83906	24.36927
HORVU6Hr1G032080	0.517094	0.478515	0.590557	2.548068	2.412569	2.086159
Hordeum_vulgare_newGene_16159	2.182347	2.720895	2.63404	0.08273	0.039862	0.016056
HORVU3Hr1G085080	2.248132	2.353803	3.85689	1.205547	0.123331	0.416199
HORVU6Hr1G072950	5.677911	6.48701	7.105461	10.73768	31.61773	28.251
HORVU2Hr1G010440	8.662336	9.091527	9.156595	23.69954	63.24487	57.82738
Hordeum_vulgare_newGene_15239	0.038334	0.041554	0.02713	0.342041	0.73177	0.872084
HORVU5Hr1G110920	0	0	0.02506	0.79105	0.411613	0.688022
HORVU1Hr1G045520	35.20602	33.50269	29.43052	23.93171	13.76004	18.348
HORVU0Hr1G000160	3.244643	3.084104	2.722851	1.969551	1.455847	1.411766
HORVU1Hr1G039540	6.628625	8.5054	8.115205	2.481147	1.252111	2.253795
HORVU4Hr1G013190	5.856635	5.444917	6.819488	15.38997	16.55287	15.74718
HORVU3Hr1G028270	26.56182	27.39549	28.41885	0	0.01096	0.007991
HORVU6Hr1G075730	0.312928	0.474621	0.56034	1.063899	2.095435	1.348088
Hordeum_vulgare_newGene_12439	0	0	0.087093	5.54764	6.247723	6.212242
HORVU7Hr1G106960	6.745219	7.172277	7.645512	23.23734	24.13566	24.84347
HORVU7Hr1G011890	18.83298	23.25588	24.15187	10.497	11.62059	12.89486
HORVU3Hr1G022620	2.070663	2.216726	3.33804	5.249222	12.29033	10.68734
HORVU2Hr1G047410	0.534052	0.59284	0.677759	1.0213	2.051384	1.764063
HORVU1Hr1G084520	2.683166	2.451668	2.138913	6.996462	11.28274	10.6604
HORVU3Hr1G106210	8.602124	8.749574	9.959427	19.02894	32.38911	28.84442
HORVU1Hr1G052110	0.423966	0.43739	0.609697	4.780273	8.420027	7.238019
HORVU5Hr1G118710	16.92116	13.87213	22.10533	8.681707	10.60489	12.01687
HORVU6Hr1G000230	1.806735	2.386707	2.258046	12.64905	30.10955	26.95053
HORVU1Hr1G013700	10.99172	10.51357	9.366142	6.151673	6.695175	6.244391
HORVU7Hr1G095080	3.49472	2.502681	4.200821	18.60977	41.90855	29.70328
HORVU2Hr1G015870	0	0	0	9.041592	6.986058	5.038074
HORVU7Hr1G088880	57.90084	42.51385	55.46439	104.2452	213.2522	171.9729
HORVU4Hr1G084410	1.194909	1.067837	0.903066	5.357932	11.48512	9.290805
HORVU5Hr1G119040	3.40202	3.843562	3.592494	9.070657	12.58972	11.5706
HORVU3Hr1G002840	0.265373	0.451089	0.391613	3.267212	3.658356	5.763688
HORVU4Hr1G058650	0.131576	0.161477	0.365027	0.575097	0.970193	0.836801
HORVU5Hr1G045290	2.019461	1.399684	2.010169	6.580198	12.11268	10.53648
HORVU5Hr1G017530	0.797999	1.325506	2.581974	4.818122	107.1288	96.52281
HORVU2Hr1G071100	1.79127	1.371947	1.71011	4.106014	6.641884	6.596992
HORVU7Hr1G082590	1.337251	1.07284	1.293246	4.60358	6.80714	6.552785

HORVU2Hr1G033320	0.162568	0.139356	0.223201	3.635081	3.254815	3.791377
Hordeum_vulgare_newGene_11808	0.771806	0.603691	0.662316	0.39345	0.256849	0.45171
HORVU5Hr1G095990	6.817269	7.814692	8.228311	24.94373	25.5581	28.25946
HORVU2Hr1G100360	0.3689	0.191479	0.251203	1.102993	0.80474	1.157649
HORVU3Hr1G006760	1.403176	1.12322	1.466901	0.307618	0.277889	0.325472
HORVU1Hr1G077420	4.624124	5.295638	6.325214	2.164445	0.665738	1.155415
HORVU2Hr1G099590	2.398398	2.510145	3.429226	7.055177	10.67014	7.77976
Hordeum_vulgare_newGene_8766	0.582029	0.332925	0.336527	0.872907	1.296486	1.689676
HORVU7Hr1G003860	5.627059	5.43004	6.285376	17.10535	21.79353	20.47282
HORVU5Hr1G073330	5.079107	5.694908	5.591481	2.102584	3.564852	3.657762
Hordeum_vulgare_newGene_5225	9.176627	6.713927	6.615855	13.57594	35.04278	32.1425
Hordeum_vulgare_newGene_5224	17.66048	12.82047	13.34354	32.26108	113.3428	126.2971
Hordeum_vulgare_newGene_5226	0.042134	0.015673	0.053216	1.826143	2.526537	2.674176
Hordeum_vulgare_newGene_5221	0	0.013286	0.004349	0.795823	0.916751	1.04157
HORVU3Hr1G075960	1.363756	0.67214	1.231281	5.635861	3.885867	4.990136
HORVU1Hr1G017830	0.52182	0.226294	0.716777	1.391052	6.927488	5.944391
Hordeum_vulgare_newGene_14177	3.776603	3.144981	2.288643	0.407307	0.367423	0.473011
HORVU3Hr1G089880	1.965953	1.828653	2.190872	3.577726	6.297064	6.878927
Hordeum_vulgare_newGene_11283	0	0	0	10.74976	12.55489	12.89047
Hordeum_vulgare_newGene_11285	36.76078	34.14207	34.94912	0.034324	0	0
HORVU3Hr1G053060	8.107412	9.350154	12.54195	5.130119	2.033764	2.176458
Hordeum_vulgare_newGene_13381	4.395149	3.964172	4.700282	11.90842	17.0827	15.87116
Hordeum_vulgare_newGene_10485	0.068748	0.131061	0.079553	0.575226	0.969706	0.818692
HORVU4Hr1G052070	0.586302	0.861207	0.694221	2.279565	3.295758	3.581482
HORVU4Hr1G023670	4.817096	5.997441	7.117258	3.321006	3.270478	4.014891
HORVU3Hr1G011260	1.737606	2.95708	2.209303	0.21052	0.284898	0.334456
HORVU2Hr1G115710	0.522669	0.793766	0.749462	4.494404	5.969607	6.561551
HORVU7Hr1G086690	14.19095	11.08854	11.15169	24.22025	48.66516	40.2671
HORVU6Hr1G017080	6.170003	5.309885	6.528623	2.927852	2.623731	3.135652
Hordeum_vulgare_newGene_10738	1.794043	0.662634	0.548072	0	0	0
HORVU7Hr1G045150	17.16749	23.68501	27.71684	4.855046	0.362517	2.988854
HORVU7Hr1G058750	16.04812	20.56457	17.43265	9.774096	6.480936	8.630456
HORVU2Hr1G114390	3.497617	2.277794	6.968773	0.299658	0	0.176469
Hordeum_vulgare_newGene_3136	43.02791	35.65467	35.82492	0.116821	0	0
Hordeum_vulgare_newGene_197	66.15965	65.75072	94.69591	24.13501	11.9469	11.83887
Hordeum_vulgare_newGene_196	0.086716	0.012199	0.014033	4.844487	4.211809	4.254045
Hordeum_vulgare_newGene_198	370.9395	372.092	472.9226	166.0834	95.84688	97.54406
Hordeum_vulgare_newGene_229	0.080366	0.076956	0.128607	0.695676	1.734025	1.144261
Hordeum_vulgare_newGene_228	0.605555	0.488382	0.43354	4.279171	2.929352	2.13117
Hordeum_vulgare_newGene_223	97.84227	109.3603	46.19711	49.50402	43.4627	31.2313
HORVU3Hr1G014980	9.822614	10.22546	10.78926	5.981049	3.996296	4.470711
HORVU4Hr1G062920	1.795745	1.266558	1.351127	4.01106	7.276977	7.923994
HORVU6Hr1G089470	14.67049	15.70517	20.35111	8.075431	11.44983	11.25195
HORVU3Hr1G017950	8.217628	8.048628	8.807134	5.897911	3.994474	5.177331
HORVU4Hr1G073650	2.207685	1.875538	2.412618	7.464902	7.435715	6.668397
HORVU2Hr1G013450	6.184483	6.679195	9.247765	2.817811	3.116894	2.493608
Hordeum_vulgare_newGene_7330	0.146519	0.018719	0.172506	0.688768	1.589217	1.334864

HORVU2Hr1G072890	0.577835	0.460175	0.366905	0.939574	1.676051	1.958791
HORVU1Hr1G059810	59.33174	68.10536	39.87145	26.65851	17.22721	23.51286
HORVU7Hr1G108280	1.249541	0.6225	0.866446	6.760797	5.577711	3.226383
HORVU6Hr1G061270	0.299507	0.244447	0.217336	12.98929	16.04587	15.22374
HORVU6Hr1G071190	0.081149	0.123065	0.105029	1.286768	2.95215	3.325193
HORVU4Hr1G075160	1.110184	0.860703	0.879977	2.251315	3.563783	4.133315
Hordeum_vulgare_newGene_5830	1.830474	2.072655	2.187135	1.127591	1.173539	1.292476
HORVU2Hr1G086220	0.312179	0.233687	0.491324	1.294909	1.835022	1.885207
HORVU2Hr1G096890	24.93155	18.55252	23.65842	7.169803	2.902801	3.909131
HORVU2Hr1G094670	0.292796	0.337153	0.551316	12.52681	27.66709	24.04885
HORVU5Hr1G051010	43.35763	44.69872	35.5656	241.9109	432.3296	422.6858
HORVU1Hr1G095430	133.6159	129.2354	148.5977	23.50774	20.01095	18.3936
HORVU1Hr1G062510	36.28477	35.78831	45.59819	22.05037	26.30879	23.73632
HORVU7Hr1G096240	47.4163	49.88311	35.01722	27.42233	19.79279	19.61016
HORVU2Hr1G013400	1.879401	2.185208	1.644522	6.310576	8.805152	9.145316
HORVU7Hr1G066630	0.165154	0.350819	0.331927	2.259363	2.224959	2.082333
HORVU4Hr1G033790	3.627816	3.675805	4.174762	0	0	0
HORVU1Hr1G058470	0.080296	0.147157	0.133004	2.827644	3.215001	4.417458
HORVU0Hr1G007730	2.869801	1.483196	1.983418	0.622801	0.67435	0.778112
HORVU2Hr1G080590	0.883309	0.552622	0.990543	3.287162	12.28783	10.44009
HORVU6Hr1G013290	0.885746	1.070764	0.801343	0.015572	0.27011	0.007123
HORVU0Hr1G001770	0.42227	0.541765	0.734204	0.371156	0.16299	0.323767
Hordeum_vulgare_newGene_4528	4.170005	4.552156	4.415417	0	0.013192	0
HORVU7Hr1G039930	95.00948	94.68708	102.184	342.4284	384.137	372.1938
HORVU1Hr1G075670	7.628741	5.141929	7.688382	5.119235	2.400811	4.20556
HORVU7Hr1G085120	1.333266	1.375019	1.705469	5.118478	13.21946	12.18567
HORVU2Hr1G120010	1.930446	2.505195	2.076434	1.137398	0.958549	1.05181
HORVU2Hr1G007490	0.024246	0	0.026951	12.88479	5.089717	4.33349
HORVU1Hr1G026320	10.05409	11.48811	10.82503	21.69168	38.20543	38.15387
HORVU7Hr1G065950	0.420274	0.410118	0.694972	0.901125	1.71253	1.665944
HORVU7Hr1G054890	35.6565	32.29127	39.78284	0.094588	0.064719	0
HORVU5Hr1G044830	1.127494	1.396926	1.244564	6.349249	6.377762	9.01611
HORVU6Hr1G080530	6.285656	4.749065	6.036534	12.85795	17.09566	18.98166
HORVU6Hr1G094790	1.302827	0.681706	1.156061	0.401436	0.259816	0.61453
Hordeum_vulgare_newGene_1132	1.681061	1.906688	1.986241	3.815262	6.010864	6.0248
HORVU4Hr1G023470	0.412678	0.242921	0.497269	0.68103	1.308614	0.954069
HORVU3Hr1G062250	0.551102	0.696975	0.737113	1.723588	2.838598	2.685528
HORVU1Hr1G016980	0.918336	0.632476	0.757203	3.964778	3.208991	4.96294
HORVU7Hr1G080320	0	0	0	0.936251	0.617217	0.683628
HORVU7Hr1G121510	0.411766	0.377704	0.447764	0.210523	0.048471	0.087805
HORVU7Hr1G090880	1.268591	1.323504	1.592624	4.291236	6.597709	6.448191
HORVU4Hr1G082600	0.238724	0.217914	0.499179	7.099617	15.02489	17.26576
HORVU1Hr1G005460	1592.335	1525.449	1195.009	302.5244	145.9375	170.9366
HORVU3Hr1G074960	11.35518	5.23252	14.78788	3.171978	0.800443	4.083337
HORVU4Hr1G085970	4.240232	4.45715	4.119967	2.03706	1.676867	1.894231
HORVU1Hr1G018600	22.87886	19.42646	20.75917	11.03639	4.167758	6.548105
Hordeum_vulgare_newGene_11835	2.093923	1.313478	2.171129	6.460828	5.324434	4.668005

HORVU4Hr1G014460	3.25544	2.532733	2.10486	14.99299	57.87645	56.36665
HORVU7Hr1G084310	1.162566	1.520513	1.847303	4.652406	6.147063	6.879068
HORVU3Hr1G089260	7.103944	6.405016	8.122454	12.62685	26.1712	22.66524
Hordeum_vulgare_newGene_3323	9.180503	8.970886	8.937501	19.59235	36.74474	33.89361
HORVU3Hr1G067990	6.192748	5.662502	9.108527	1.726725	1.237048	1.565147
HORVU4Hr1G055610	5.80356	5.175123	6.83907	2.336628	2.232993	1.889136
Hordeum_vulgare_newGene_2054	0	0	0	1.082867	0.679924	0.69937
Hordeum_vulgare_newGene_2050	0	0.033732	0	1.37824	1.179567	1.118165
Hordeum_vulgare_newGene_2058	0	0	0	2.545559	2.658728	3.123657
HORVU7Hr1G036890	0.847663	0.879456	1.222923	0.059999	0.017984	0
Hordeum_vulgare_newGene_12980	0	0	0	1.426737	1.769004	1.446702
HORVU7Hr1G073460	113.2095	82.58909	153.2591	68.50927	45.1878	70.80037
HORVU1Hr1G031490	28.77873	35.28871	52.99599	1.953668	0	0.6604
Hordeum_vulgare_newGene_9608	0.020038	0.019491	0.012062	0.558377	1.480567	0.995125
Hordeum_vulgare_newGene_15062	0.796441	0.573453	0.6779	2.037264	3.454668	3.989118
HORVU7Hr1G073790	3.871569	7.214543	8.397541	0.447412	0	0.108971
HORVU2Hr1G101730	0.033094	0.089807	0	1.390231	6.835401	4.663991
Hordeum_vulgare_newGene_2589	1.380496	0.898161	0.827418	0.072726	0.06813	0.038861
Hordeum_vulgare_newGene_2587	0.106697	0.278203	0.066926	1.179698	2.362537	2.330267
HORVU2Hr1G108010	1.160421	1.024769	1.387569	11.15604	20.78862	18.73169
Hordeum_vulgare_newGene_11989	14.01014	10.58207	17.4591	8.405167	3.270526	5.184213
HORVU7Hr1G122430	0.564922	0.655093	0.775267	0.025077	0.043567	0.062472
HORVU5Hr1G081210	0.643739	0.562717	0.623739	0.099404	0.012908	0.095208
Hordeum_vulgare_newGene_5056	0	0	0.040775	1.271807	1.180688	1.275901
HORVU7Hr1G089270	10.41103	7.383051	9.46875	1.975389	0.301153	0.805389
Hordeum_vulgare_newGene_5054	0.494998	0.567435	0.713888	0.138986	0.0271	0.060479
Hordeum_vulgare_newGene_5050	1.067764	1.239975	1.09805	0	0	0
HORVU7Hr1G007220	1.580684	1.826984	1.740102	18.91051	6.617025	13.61586
HORVU4Hr1G000280	0.055811	0.024934	0.048599	4.350935	6.126255	6.510666
HORVU2Hr1G031910	57.79128	57.33817	80.51657	46.08364	23.39527	30.99154
Hordeum_vulgare_newGene_11367	0.620192	0.582278	0.639914	1.983151	1.256599	1.948565
Hordeum_vulgare_newGene_11363	1.822654	0.62987	1.872061	21.64919	101.5676	93.08852
HORVU1Hr1G009490	16.84296	8.182198	22.33289	0.291159	0.017047	0
Hordeum_vulgare_newGene_8268	2.049724	2.397037	2.540736	0	0	0
Hordeum_vulgare_newGene_8269	5.410119	6.613448	6.78507	0.101352	0.061499	0.019764
Hordeum_vulgare_newGene_1773	0.576588	0.312231	0.592132	0.042052	0.010143	0
HORVU4Hr1G077850	12.91592	11.26976	15.3701	5.60674	2.035884	3.00945
HORVU1Hr1G005870	5.257572	2.729488	4.518505	8.098383	34.41893	31.51144
HORVU2Hr1G022250	18.55481	19.83688	26.17079	10.09347	13.20864	12.60086
HORVU1Hr1G073460	5.858977	7.248211	9.441583	2.676237	4.301731	4.631759
HORVU3Hr1G091140	0.145649	0.331992	0.129614	1.29024	2.316472	2.70803
HORVU1Hr1G039720	4.712855	3.654141	5.089846	1.802455	0.961466	1.529006
HORVU6Hr1G076520	1.273832	1.395127	1.487441	0.522296	0.266821	0.244273
HORVU7Hr1G079380	5.408789	3.418165	4.997087	11.45604	32.6037	28.42715
HORVU7Hr1G105200	3.194157	2.29068	3.131985	1.516845	1.347939	1.791961
HORVU3Hr1G104600	6.089856	7.602763	5.807789	0.010482	0	0
HORVU7Hr1G046830	7.128128	4.798821	7.433855	1.736393	0.47997	1.284001

HORVU7Hr1G058090	0.273813	0.460553	0.271712	6.305344	1.680034	3.759006
HORVU3Hr1G081590	5.418823	4.923776	6.959445	2.516401	0.989536	1.910117
Hordeum_vulgare_newGene_12047	0.786806	0.204944	0.418434	2.90745	5.503267	5.864446
HORVU3Hr1G040310	0	0	0	27.65445	20.70864	25.68922
HORVU3Hr1G108500	0	0	0	0.19123	0.185853	0.250658
HORVU6Hr1G074580	5.580185	6.46369	5.539505	2.476504	2.302894	3.09128
HORVU6Hr1G087420	1.283003	1.159888	1.579764	3.883669	4.89401	5.551361
HORVU1Hr1G026520	5.384379	4.66642	5.485649	15.11783	21.60354	23.14801
HORVU1Hr1G011660	0	0.016909	0.043108	10.44781	9.394708	10.94573
Hordeum_vulgare_newGene_3041	2.47515	2.360249	2.765603	1.268928	1.231618	1.918563
HORVU4Hr1G074770	11.80916	10.80552	12.47251	7.946228	4.524825	6.249689
Hordeum_vulgare_newGene_6496	4.594819	7.334132	3.820493	2.661501	3.231977	2.87842
HORVU6Hr1G087390	0.789204	0.720912	0.65356	0.254105	0.134419	0.187332
HORVU5Hr1G073440	1.039257	0.897324	1.279198	0.432915	0.09612	0.357497
HORVU2Hr1G117050	0	0	0	3.248628	4.206825	4.099423
HORVU3Hr1G087850	14.11305	16.0205	19.64618	6.454826	8.475912	9.132209
HORVU6Hr1G082060	10.50495	8.507518	14.19839	20.61206	35.09448	30.3602
HORVU1Hr1G070220	1.687619	1.776021	2.636613	6.119769	8.941994	8.571936
HORVU2Hr1G024940	1.251536	1.452841	1.86603	2.95473	4.121975	4.728457
HORVU5Hr1G107120	0.168146	0.126968	0.255205	1.737087	1.231416	1.64651
Hordeum_vulgare_newGene_14077	1.102983	1.353424	0.83308	0.21985	0.22457	0.233349
HORVU4Hr1G017390	14.63661	9.896576	14.06556	29.5063	42.83646	45.78766
HORVU1Hr1G056160	6.500079	6.25757	10.4421	3.795257	4.392178	4.406008
HORVU3Hr1G016010	5.60711	6.318765	6.840267	3.486422	2.829883	3.692559
HORVU3Hr1G032490	24.92796	23.51046	21.13403	54.34044	74.02408	70.65534
Hordeum_vulgare_newGene_15301	0.096061	0.070625	0.034685	0.65415	1.32424	1.981449
HORVU7Hr1G107310	3.213471	3.947347	3.97176	0.826689	1.29378	1.429902
HORVU3Hr1G082730	4.711496	3.958591	3.8938	23.34939	44.81862	49.73026
HORVU5Hr1G104580	10.39399	8.264192	12.15226	2.428599	0.660415	1.566322
HORVU5Hr1G069800	3.25412	3.346667	3.92641	1.812534	1.881629	2.041629
HORVU4Hr1G051980	0.080964	0.062412	0.099279	0.235565	1.008933	1.092284
HORVU2Hr1G084270	17.57469	17.34097	27.09446	8.525123	11.41765	11.05421
HORVU7Hr1G021660	0	0	0.064536	1.256394	10.61878	9.665977
HORVU7Hr1G115990	0	0	0	0.841423	1.16515	1.62961
HORVU3Hr1G014320	0	0.01381	0	1.513491	2.526046	2.399071
HORVU1Hr1G088920	2.007889	1.046165	1.068018	95.81943	9.694126	9.278775
Hordeum_vulgare_newGene_6089	0.699163	0.875146	0.83287	1.813425	3.0977	3.119943
HORVU5Hr1G058720	49.42878	61.74699	40.40567	50.08805	365.9541	363.4022
HORVU3Hr1G029880	0.648621	0.552221	0.707498	1.070831	2.768173	2.974035
HORVU4Hr1G084910	3.355158	3.544659	2.886321	1.084853	0.129649	0.356672
HORVU2Hr1G099490	1.24202	1.057419	1.244088	3.241043	6.166832	5.236827
HORVU5Hr1G013400	2.804938	2.828641	4.733587	1.419346	1.086291	1.023958
Hordeum_vulgare_newGene_4698	0.12055	0.124337	0.110683	6.868562	8.186208	8.010343
HORVU5Hr1G045150	17.03899	10.37824	18.86592	9.874836	7.232842	8.074278
HORVU5Hr1G009350	0.035691	0.067874	0.061884	0.522485	2.072735	3.239144
HORVU7Hr1G006370	15.23558	15.68057	15.12983	54.0597	35.18159	38.86504
HORVU3Hr1G071530	0.801967	0.642772	0.85338	8.216741	16.7954	17.89444

HORVU1Hr1G078050	91.95438	109.7602	226.3098	18.85905	0.209475	11.45595
HORVU7Hr1G084210	0.078872	0	0.057894	1.030514	2.659774	1.960653
HORVU7Hr1G018720	0.971609	1.044558	1.227785	0.617742	0.684841	0.686397
HORVU1Hr1G012960	77.79987	85.90366	105.2214	189.9339	471.804	396.8838
Hordeum_vulgare_newGene_11167	0.09768	0.074556	0.160986	9.886547	14.97837	13.93598
Hordeum_vulgare_newGene_11164	75.70952	94.3372	131.275	16.17151	3.074167	6.862639
Hordeum_vulgare_newGene_11165	1.403529	1.309022	1.151387	5.648921	14.55944	12.7298
Hordeum_vulgare_newGene_11161	6.620578	8.240027	7.481929	0.021114	0	0.002211
HORVU3Hr1G108530	0.432741	0.626554	0.656737	1.32201	1.731686	2.074024
HORVU5Hr1G077170	4.02263	4.536342	4.45122	0.249443	0.127639	0.317987
HORVU1Hr1G004670	0.790745	1.195249	2.387212	0.078068	0.020394	0.089733
HORVU7Hr1G087440	3.27419	1.960022	2.386852	9.750773	56.44135	45.42385
HORVU2Hr1G011640	0.01791	0.027577	0.039862	0.454862	1.605633	1.773273
HORVU7Hr1G045580	7.814908	9.496248	7.308679	59.89907	48.92783	48.1304
HORVU5Hr1G098960	55.21379	60.89307	62.03498	141.1704	246.3493	240.2418
HORVU2Hr1G057610	11.5843	13.60478	12.83234	4.623294	5.539771	5.820584
Hordeum_vulgare_newGene_7905	0.618293	0.458976	0.523699	0.034635	0	0
HORVU7Hr1G045630	63.5488	63.57453	74.34625	29.58411	12.90615	13.32483
HORVU6Hr1G088190	4.879115	5.558848	4.516221	3.352245	1.759188	2.109189
HORVU1Hr1G008940	0.410369	0.345019	0.368438	0.953661	1.061781	1.151461
HORVU1Hr1G084900	1.336172	1.560226	2.387374	0.156967	0.023806	0.231871
HORVU4Hr1G062510	0.326834	0.100439	0.073051	1.380582	1.034327	1.357698
HORVU0Hr1G021390	6.700229	5.81688	7.007318	14.03397	22.89971	20.24192
HORVU2Hr1G025090	9.973177	9.786543	7.955197	5.091803	2.307174	3.131395
HORVU2Hr1G021730	15.8451	10.58733	6.483057	0.633005	0.669494	0.509518
HORVU4Hr1G057790	0.033487	0.193182	0.107498	0.696373	2.473992	1.713907
HORVU2Hr1G035870	84.70452	65.84535	99.99157	50.7432	20.88819	28.03765
HORVU6Hr1G086830	2.88481	2.419401	2.926255	1.743003	1.45399	1.903909
HORVU4Hr1G065560	1.718175	1.304187	1.339763	0.540019	0.707413	0.622883
HORVU7Hr1G023820	0.009685	0.01582	0.037417	0.431154	0.506725	0.673088
HORVU0Hr1G021640	24.14134	23.77321	25.60587	13.73133	7.644741	10.36746
HORVU7Hr1G077600	0.392015	0.363473	0.332446	1.361658	1.141823	1.295576
HORVU6Hr1G078110	1.452663	1.597269	1.771447	5.579527	6.679698	7.051778
Hordeum_vulgare_newGene_779	0.072664	0.314947	0.182863	2.403746	1.955589	1.824728
Hordeum_vulgare_newGene_778	6.471299	5.503402	6.247137	0.019485	0	0
HORVU3Hr1G064070	5.685084	4.341057	5.270469	2.41789	0.812299	1.221107
Hordeum_vulgare_newGene_14270	2.166899	1.932845	2.740575	8.699257	10.81197	7.251945
HORVU6Hr1G053710	9.601401	6.893188	10.94244	1.56127	0.789816	1.004378
Hordeum_vulgare_newGene_10970	9.185183	10.3916	8.65966	0.00898	0.016842	0.003812
HORVU6Hr1G073990	36.81031	21.8559	32.04679	16.99054	10.0008	11.95356
HORVU7Hr1G042910	0.778801	0.91301	0.972062	0.133727	0.07944	0.13724
HORVU7Hr1G096700	2.530919	1.697731	2.944835	1.177591	0.872051	0.729983
Hordeum_vulgare_newGene_8878	0.102513	0.058678	0.07679	0.263947	0.300082	0.414437
Hordeum_vulgare_newGene_5939	0.810622	0.754359	0.689083	0	0	0
HORVU3Hr1G110330	0.016459	0	0	1.376122	2.740282	2.046366
HORVU3Hr1G088990	0.714569	0.825974	0.967572	1.83604	3.079299	2.334757
HORVU2Hr1G104130	5.717688	7.043123	12.4213	0.724528	0.086406	0.348813

HORVU5Hr1G063330	0.841759	0.425652	1.388101	5.023391	4.921472	5.611583
HORVU1Hr1G035100	21.82623	28.19242	29.54442	0.030445	0	0
HORVU0Hr1G010340	3.245378	3.367368	2.318841	1.306093	0.996894	1.199804
HORVU1Hr1G000720	0.473206	1.062784	0.540105	14.98624	2.72391	3.992936
HORVU1Hr1G052530	1.813089	1.430345	1.736203	6.522705	4.34649	4.757945
HORVU1Hr1G028900	1.43368	1.59086	1.414923	1.042506	0.490381	0.853339
HORVU7Hr1G027440	7.855762	7.820596	10.37161	147.2703	80.31548	148.9678
HORVU3Hr1G014120	81.59865	82.59032	88.02212	24.84631	35.8514	34.54013
HORVU0Hr1G020590	0	0.057164	0.218183	3.440345	6.141654	8.838771
HORVU1Hr1G068660	0.104362	0.116316	0.111022	0.987092	1.600095	1.269984
HORVU6Hr1G031580	2.377287	2.075848	2.784744	10.36403	16.85015	14.58646
HORVU2Hr1G077630	0.899561	1.066082	1.298098	2.509714	9.295224	9.157331
Hordeum_vulgare_newGene_4912	2.392598	1.960596	1.125297	0.677857	0.299915	1.004687
Hordeum_vulgare_newGene_4913	0.589143	0.779755	0.736054	12.7009	10.39467	10.12928
Hordeum_vulgare_newGene_4914	0.176433	0.203414	0.322669	0.702361	0.766749	1.386538
Hordeum_vulgare_newGene_57	2.196269	1.953706	1.619013	0.206569	0.14124	0.111897
HORVU5Hr1G033060	7.083841	5.286842	5.7603	22.6339	33.54104	36.46763
HORVU2Hr1G089950	7.672628	6.695485	11.78624	4.750426	3.34418	4.77157
HORVU2Hr1G071070	0.153057	0.228577	0.54293	2.49706	1.472693	2.110924
Hordeum_vulgare_newGene_15918	5.064578	7.108792	5.888255	3.261108	1.710632	3.395878
Hordeum_vulgare_newGene_15911	0	0.011247	0.030303	1.48776	0.78085	1.147853
Hordeum_vulgare_newGene_9165	0.461827	0.495203	0.400131	0.309754	0.068659	0.151526
HORVU4Hr1G032960	0.131274	0.320148	0.367296	0.685823	0.888128	0.859965
HORVU7Hr1G011050	109.9335	120.9288	72.7224	41.30646	30.1786	31.3984
HORVU1Hr1G051160	0.409175	0.486958	0.313392	3.078708	4.17284	4.402104
HORVU1Hr1G087100	46.60862	46.00786	59.96404	30.32141	35.91873	34.97301
HORVU3Hr1G086310	4.910958	4.992923	5.262312	2.403851	2.697518	2.914413
HORVU5Hr1G017640	0	0.052358	0.144247	2.519685	3.47892	3.793496
HORVU7Hr1G034020	3.0504	2.231658	3.260281	6.106517	7.975241	8.360522
HORVU2Hr1G118190	0.590921	0.547262	0.499091	1.61996	2.24489	1.801076
HORVU3Hr1G007210	0.451965	0.226524	0.360832	1.988176	1.656805	1.896287
HORVU1Hr1G091210	8.852494	7.788624	8.470411	6.549578	6.567787	6.057394
HORVU5Hr1G064130	0.405221	0.473627	0.428239	3.18807	3.680319	4.009754
HORVU5Hr1G069690	3.611252	1.491584	4.367634	10.21842	48.58	48.95672
HORVU5Hr1G034400	10.70532	11.61392	17.38925	6.973488	7.476142	8.234174
HORVU4Hr1G061680	1.580467	1.61153	2.699075	11.05055	8.002896	9.811941
HORVU6Hr1G005030	28.79536	35.67202	39.57238	12.33854	6.309498	6.743167
HORVU7Hr1G010850	0.766501	0.708131	1.562408	0.054085	0	0.064352
HORVU3Hr1G024180	0.972442	0.640398	1.388779	4.828319	8.139837	7.46288
HORVU6Hr1G021060	23.35502	18.66855	17.38326	4.574313	2.842918	2.491836
HORVU4Hr1G088140	1.035432	1.304998	1.874224	2.7607	7.055526	7.154465
HORVU5Hr1G104620	0.368064	0.606941	0.593534	1.190524	2.492961	2.578712
HORVU6Hr1G032310	3.940542	3.780264	3.299683	1.509876	1.961502	2.727165
HORVU1Hr1G082340	0	0	0	4.929973	3.938113	3.150769
HORVU5Hr1G124940	0.411542	0.543037	0.446176	1.13426	1.140744	1.042164
HORVU5Hr1G057590	0.210575	0.216019	0.264973	3.568348	3.718815	4.119691
Hordeum_vulgare_newGene_13970	3.216156	4.204992	4.809863	0	0	0

HORVU3Hr1G006450	11.29082	6.747348	11.19018	5.522329	4.067022	6.353057
HORVU4Hr1G021820	0.529963	0.360096	0.494059	1.863445	1.882867	2.290851
HORVU4Hr1G068870	0.337866	0.188842	0.510619	0.975533	1.08181	1.275108
Hordeum_vulgare_newGene_10406	1.331549	2.012149	0.688456	0.123613	0	0.036186
Hordeum_vulgare_newGene_10404	268.3654	250.3481	197.0033	97.71668	109.7299	113.8428
Hordeum_vulgare_newGene_10405	6.57439	7.927287	3.74264	1.481875	1.043243	1.124019
Hordeum_vulgare_newGene_10401	6.813795	5.688187	3.465163	0	0	0
HORVU3Hr1G065630	5.365639	3.358415	2.81781	0.504711	0.151707	0.469791
HORVU1Hr1G000120	1.096041	0.776719	1.191051	2.987168	2.13538	2.670219
HORVU3Hr1G078490	16.51502	13.35777	16.18411	7.802552	9.509518	9.483241
HORVU7Hr1G000900	9.396147	7.380397	7.461751	27.12005	45.45979	45.83384
HORVU3Hr1G095760	59.40851	39.06977	72.82432	18.02732	15.24327	14.1058
HORVU3Hr1G078140	0	0	0	0.552185	0.681021	0.433189
HORVU6Hr1G077770	8.689011	7.01729	10.416	25.36154	29.95713	27.0451
HORVU4Hr1G024500	0.024509	0.006468	0.005186	0.540975	0.686607	1.49152
HORVU7Hr1G077060	0.064362	0.060238	0.069271	1.089908	3.230805	2.369561
Hordeum_vulgare_newGene_11464	0.422542	0.583353	0.76151	4.891446	5.404634	6.134214
Hordeum_vulgare_newGene_11461	2.236379	1.967851	2.292894	0	0	0
HORVU2Hr1G060480	17.42497	15.70069	15.44688	47.23143	88.96032	87.20777
HORVU3Hr1G022340	2.077482	1.356225	1.379894	3.801397	5.952585	4.607831
HORVU6Hr1G090380	3.006447	3.301209	3.245589	6.710914	11.6952	11.23553
HORVU5Hr1G062030	2.612986	1.306598	2.330672	8.055392	8.14052	4.768647
HORVU5Hr1G071480	0.178838	0.422626	0.403331	1.813066	2.584291	2.443012
HORVU5Hr1G022940	2.194184	2.286432	1.241714	0.791963	0.876725	0.572769
HORVU6Hr1G089780	86.85086	77.40022	108.0052	51.53407	53.14842	60.29906
HORVU1Hr1G085150	12.53598	15.20799	10.31081	4.112329	5.698853	5.164652
HORVU5Hr1G037200	4.908829	4.98732	5.25781	24.44399	26.35138	21.99358
HORVU0Hr1G024560	0.953479	1.223903	0.758308	3.27425	18.21896	19.23526
HORVU7Hr1G105580	0.18937	0.257961	0.208019	1.086767	1.533496	1.099137
HORVU3Hr1G081140	3.052198	2.84048	2.392727	1.281617	1.197019	0.957164
Hordeum_vulgare_newGene_13565	1.261759	1.272382	1.091587	2.604353	3.205716	3.516774
Hordeum_vulgare_newGene_13567	0.517479	0.647521	0.507959	2.899847	13.22212	9.511845
HORVU7Hr1G100400	0.483958	1.12097	0.883109	2.144467	6.014621	7.820558
Hordeum_vulgare_newGene_7914	0	0	0	6.427857	7.394134	7.850961
HORVU1Hr1G071500	3.258777	1.912591	3.849225	26.4822	7.84113	10.67231
HORVU2Hr1G057760	0.154248	0.118478	0.237699	0.988279	1.516531	1.922106
Hordeum_vulgare_newGene_8346	4.874096	7.226021	4.068448	0	0	0
HORVU7Hr1G038120	0	0	0	32.5174	26.2721	29.11462
Hordeum_vulgare_newGene_6068	1.392567	1.480277	1.673524	0.457537	0.530306	0.695666
Hordeum_vulgare_newGene_6069	5.886055	2.929534	6.315196	22.63228	9.314087	21.00862
Hordeum_vulgare_newGene_13693	0	0	0	2.368858	1.474095	2.621185
Hordeum_vulgare_newGene_13694	3.012469	3.316972	3.60175	1.657755	1.925415	2.048821
HORVU3Hr1G052780	0	0	0	5.004249	6.937835	6.159219
Hordeum_vulgare_newGene_3747	0.37935	0.484399	0.868729	2.626113	2.836829	3.535659
HORVU5Hr1G068070	2.228578	1.953202	2.6325	1.224797	1.112429	1.280677
HORVU6Hr1G065280	14.42078	11.71025	12.72796	8.597556	7.458423	8.062198
Hordeum_vulgare_newGene_2297	3.698774	2.758014	4.342139	0.003976	0	0

HORVU1Hr1G070670	0	0	0	0.693381	0.6525	0.895878
HORVU7Hr1G078760	0	0	0.163055	2.593667	11.2685	10.6516
HORVU1Hr1G023120	1.689848	1.878751	1.481662	3.79859	6.550026	6.235448
Hordeum_vulgare_newGene_15597	6.098155	8.317492	7.873068	4.032749	2.831058	6.04938
HORVU3Hr1G033550	0.174117	0.521559	0.490925	0.79737	2.023807	1.791151
HORVU6Hr1G068730	0.681498	0.638959	0.582049	2.183377	3.021079	3.164158
HORVU7Hr1G042900	0.836744	1.229559	1.217306	0.163593	0.059427	0.180643
HORVU2Hr1G072020	80.78956	82.19728	71.15051	61.49017	28.01098	36.79893
HORVU0Hr1G009120	4.693234	3.366079	3.512923	11.05676	14.18774	14.18023
Hordeum_vulgare_newGene_5747	5.154306	4.351029	5.181052	11.40007	14.72929	17.73313
Hordeum_vulgare_newGene_5742	2.81263	2.157775	2.480999	0	0	0
HORVU3Hr1G079230	1.462556	1.527044	1.388574	0.352879	0.538536	0.354697
HORVU6Hr1G068080	4.396284	5.906826	6.534003	1.274948	2.107273	2.86912
HORVU1Hr1G053050	3.028878	3.889781	4.618584	1.318032	1.08701	1.356422
HORVU5Hr1G103890	56.97952	54.56481	57.70549	23.1658	28.29203	27.61002
HORVU1Hr1G059180	0.154545	0.080921	0.647973	1.416785	6.209481	5.643675
HORVU7Hr1G060530	5.534221	6.138817	6.386568	11.74914	16.0389	18.43802
Hordeum_vulgare_newGene_1243	15.09853	9.739768	19.21584	10.28311	6.102987	10.40725
HORVU3Hr1G081960	1.892155	3.207599	3.22134	1.501159	1.180456	1.741638
HORVU5Hr1G096760	21.01203	33.41322	34.17123	9.087858	5.490975	8.843948
HORVU6Hr1G075850	0.193748	0.280391	0.063934	7.017443	2.559282	3.847622
HORVU5Hr1G076910	0.604093	0.570087	1.322702	1.599135	2.419725	2.544447
HORVU2Hr1G032360	0.725277	0.462915	0.910817	4.104278	3.142187	3.668719
HORVU2Hr1G088180	5.800426	5.293068	5.137878	25.23446	45.4036	26.69609
HORVU5Hr1G026140	3.83403	4.327129	5.044495	8.717681	14.63165	15.57273
HORVU6Hr1G028680	5.261113	5.581575	6.502688	2.884116	3.267672	3.445675
HORVU1Hr1G068490	19.55909	16.96185	25.43803	0.388727	0.954404	0.891653
HORVU5Hr1G056400	8.094128	9.544943	12.01933	4.925882	5.649808	5.64546
HORVU0Hr1G018910	0.17756	0.254255	0.447888	4.673278	76.73794	66.84777
HORVU7Hr1G056410	15.77625	12.24154	18.56691	6.625347	6.991384	7.326672
HORVU1Hr1G056570	134.6915	126.1527	163.0697	5.077225	0.097813	3.043464
HORVU2Hr1G001150	0.106769	0.121157	0.165858	0.608107	0.514934	1.068029
HORVU6Hr1G029240	1.489272	1.269657	1.187159	0.688303	0.297449	0.646318
HORVU3Hr1G086430	17.26114	14.81166	12.43066	7.606201	8.903313	7.434204
HORVU6Hr1G075070	7.883796	9.116065	13.5332	5.218189	6.067476	5.702501
HORVU2Hr1G000280	3.035886	2.123983	4.174773	8.927398	7.779239	12.15276
HORVU3Hr1G030650	63.21161	102.8446	129.0791	2.200788	1.575389	1.356693
HORVU2Hr1G061930	14.13927	14.81959	13.76392	6.322332	5.492908	7.907475
Hordeum_vulgare_newGene_14434	1.306578	3.942829	1.953735	1.446752	0.062919	0.165531
HORVU6Hr1G087000	1.088109	0.838441	1.4832	2.547798	7.621326	5.772836
HORVU2Hr1G081440	2.120572	2.551326	2.537452	1.2395	1.535676	1.341444
HORVU6Hr1G093030	1.654231	1.707709	2.368539	9.628196	26.1356	21.8343
HORVU2Hr1G122920	13.99891	15.91816	8.566521	5.612815	6.946595	5.827033
HORVU2Hr1G085210	0.213578	0.563542	0.574048	1.174961	1.877784	1.67343
HORVU5Hr1G097150	1.000335	1.17964	1.537566	2.969388	8.726543	9.962723
HORVU7Hr1G046660	2936.549	2541.109	3024.416	1895.417	866.2228	1073.598
HORVU6Hr1G000370	1.716506	1.454105	2.16992	0	0	0

HORVU0Hr1G001280	0.33933	0.544976	0.307294	1.374534	1.618453	2.140615
Hordeum_vulgare_newGene_242	1.934796	1.674421	2.044826	0.832162	1.09544	1.307866
HORVU1Hr1G092920	2.07324	1.64192	1.728747	0	0	0
HORVU6Hr1G010700	0.828447	1.211498	1.181751	3.086694	4.76201	4.404735
HORVU4Hr1G066370	3.668657	3.433502	5.067058	9.308013	10.92162	11.06952
HORVU3Hr1G009560	1.930424	1.374147	2.940594	117.0558	94.98235	105.0214
HORVU3Hr1G031680	7.51616	9.113018	5.381777	1.009174	0.787883	1.501533
HORVU1Hr1G035130	0.452341	0.380305	1.187884	5.177581	42.02717	36.77224
HORVU7Hr1G114880	0.59868	0.422608	0.663656	1.423195	1.12857	1.731316
HORVU2Hr1G095460	8.661908	4.132344	9.379904	0.084351	0.088178	0.165627
HORVU4Hr1G082200	14.15881	16.36544	23.70996	4.344138	2.472613	2.675828
HORVU1Hr1G048700	25.94698	22.87097	26.04351	12.42138	13.0401	12.96548
HORVU1Hr1G013990	7.748222	9.283152	10.76648	0.125254	0	0.215207
HORVU1Hr1G058330	5.143344	5.015967	5.859384	2.708473	3.26355	3.883533
Hordeum_vulgare_newGene_15745	0	0	0	4.10909	1.622328	1.76921
HORVU1Hr1G049250	1.156868	1.491843	1.545818	0.409521	0.30042	0.882146
HORVU1Hr1G042280	5.552472	6.988915	7.24645	0	0	0
HORVU3Hr1G028990	57.92464	88.44444	142.2889	1.417485	0.304148	0.7022
HORVU2Hr1G003460	5.28946	4.523017	5.691867	11.02496	12.44943	14.09955
HORVU5Hr1G057410	3.152137	3.911412	3.048328	0	0	0
Hordeum_vulgare_newGene_12908	0	0	0	4.45174	2.961947	2.871362
HORVU7Hr1G047200	0.65211	0.593371	0.767537	0.498401	0.319739	0.652769
HORVU3Hr1G067910	0.049849	0.31561	0.07701	2.700848	1.766143	2.55437
Hordeum_vulgare_newGene_12907	0	0	0.001964	1.658063	4.200057	4.057198
HORVU7Hr1G084390	4.427171	4.506128	4.850647	2.03836	0.839783	1.290174
HORVU6Hr1G012270	0.166229	0.048607	0.16142	4.513354	6.582161	4.949217
HORVU2Hr1G093700	15.21513	10.26882	9.033879	4.276361	2.786611	1.737283
HORVU7Hr1G019520	1.034427	0.820472	0.963022	0.459615	0.317849	0.476187
HORVU0Hr1G010170	5.46936	6.004368	8.312928	2.548575	4.462903	4.745279
HORVU4Hr1G016940	1.209929	1.345513	1.677476	11.58832	26.78521	25.36736
HORVU3Hr1G064750	2.045562	1.52625	1.81909	0.059318	0.090374	0.083622
HORVU7Hr1G003940	8.395696	10.77203	7.83259	3.55003	2.257086	3.283028
HORVU5Hr1G095160	64.18643	66.87615	85.41721	19.85563	10.74021	9.489961
HORVU2Hr1G020920	0.270345	0.150089	0.554199	6.248466	5.397888	6.282923
HORVU5Hr1G117770	4.49263	5.179698	5.878525	2.452651	3.353067	3.354599
Hordeum_vulgare_newGene_8150	1.085053	0.705806	0.898019	0.210069	0.340179	0.338762
Hordeum_vulgare_newGene_8153	0	0.017849	0	1.321053	0.466666	0.827872
HORVU2Hr1G007840	2.698383	2.161929	1.975048	9.509051	22.16194	21.29858
HORVU3Hr1G059880	0.676392	0.674885	0.575215	1.661562	2.445091	2.334738
HORVU2Hr1G021320	0.247534	0.167734	0.127804	4.603694	7.058538	7.1721
HORVU7Hr1G027010	36.11311	27.49766	38.82394	8.200717	8.182183	7.788458
Hordeum_vulgare_newGene_1996	0.133406	0.501858	0.229634	4.062158	4.0073	3.37291
HORVU7Hr1G021310	0.915177	1.257214	2.049808	1.964156	31.72676	21.8247
HORVU1Hr1G087570	3.273206	2.631059	3.565237	16.13109	21.2807	20.10992
HORVU7Hr1G009620	0.93345	1.35233	0.766428	0.853228	0.70012	0.581986
Hordeum_vulgare_newGene_796	0.703685	1.185533	1.410411	5.26124	4.415617	6.607592
HORVU3Hr1G063000	2.911146	4.076823	3.49346	1.869799	1.255734	2.138199

HORVU3Hr1G105790	0.403945	0.442718	0.409306	1.272317	5.706058	4.029637
HORVU3Hr1G097760	0.060177	0.040948	0.217184	10.37371	69.39703	55.97339
HORVU4Hr1G073460	9.512821	6.29371	11.55639	37.10637	48.4907	52.11979
HORVU5Hr1G000330	8.74833	10.56609	16.31224	2.89067	0.119618	0.92815
Hordeum_vulgare_newGene_2319	0.46731	0.50477	0.519889	1.217219	2.094639	1.831979
HORVU2Hr1G079920	17.11747	13.18529	16.20702	56.63395	104.6731	99.36185
Hordeum_vulgare_newGene_14565	1.992184	3.509514	3.654359	0.331831	0.310672	0.31474
HORVU3Hr1G097180	47.7589	33.271	44.95411	16.63828	6.756117	8.144727
HORVU3Hr1G052420	20.35259	18.67825	19.4136	7.983497	0.940513	3.378207
HORVU3Hr1G019510	27.29531	30.24982	37.67424	10.9116	3.622464	6.213437
Hordeum_vulgare_newGene_9361	3675.525	3581.499	2494.765	1231.98	218.9173	461.7927
Hordeum_vulgare_newGene_15384	0	0.035145	0.012769	0.488628	1.228104	1.473499
HORVU7Hr1G034470	0.939042	0.989442	1.273692	2.910318	16.00658	15.13881
HORVU4Hr1G011320	5.207936	4.140821	6.364729	19.39873	18.90551	23.83318
HORVU1Hr1G059020	11.19642	9.769244	11.31634	7.271726	4.345838	5.010684
HORVU6Hr1G081080	1.059393	1.006338	1.247528	3.51534	4.390695	4.297735
HORVU3Hr1G023370	3.205314	2.962558	2.097874	10.41569	20.62254	17.75618
HORVU4Hr1G005480	0.154519	0.462046	0.167744	4.904619	4.594001	3.444245
Hordeum_vulgare_newGene_12675	3.310832	5.138124	5.660742	2.24559	2.196193	2.489692
HORVU3Hr1G117590	14.50614	8.472204	12.20986	1.298215	1.283378	1.343398
HORVU0Hr1G040410	0.238791	0.139835	0.157377	0.654358	0.792792	0.509839
HORVU5Hr1G021050	2.290764	2.718415	3.573414	1.553019	0.881681	1.1673
HORVU0Hr1G002480	2.31637	3.005361	4.720583	1.933673	1.248351	1.196365
Hordeum_vulgare_newGene_4202	0	0	0	4.499309	6.473358	7.368142
HORVU6Hr1G020530	1.644255	0.912101	1.572823	8.415405	15.69406	10.58089
HORVU1Hr1G043710	84.00881	76.28962	45.97598	13.21713	10.00777	10.40571
HORVU3Hr1G047180	2.236644	1.389782	2.415748	1.254954	1.064735	0.889307
HORVU5Hr1G060980	1.046098	1.18752	1.047313	3.819807	3.987962	4.562359
HORVU2Hr1G046600	129.9852	168.1229	201.5078	5.223211	0.45286	1.579836
HORVU5Hr1G059530	4.705834	4.154437	4.144713	8.054856	15.88502	14.35966
HORVU5Hr1G013480	0.395427	0.370927	0.354087	1.540163	0.948676	1.171451
HORVU5Hr1G013220	1.496822	1.920949	2.001382	4.038182	13.59213	12.65916
HORVU4Hr1G007190	4.490798	3.210534	3.934221	2.156005	1.136817	1.73947
HORVU2Hr1G023390	0.959417	1.409586	1.020064	0	0	0.053403
HORVU5Hr1G042390	0.431938	0.380454	0.775341	1.444398	1.86956	1.934967
HORVU2Hr1G000090	2.988415	3.621619	4.368893	0.586616	0.039328	0.276125
HORVU5Hr1G086500	15.62461	13.07528	11.23808	27.53388	42.86313	39.81582
HORVU5Hr1G062830	0.215221	0.1747	0.182835	0.637933	0.456347	0.74611
HORVU6Hr1G005400	3.56364	2.324016	4.586677	1.14875	0.446841	0.89604
HORVU5Hr1G052440	0.824058	0.594439	0.54151	2.750076	3.240944	3.260439
HORVU1Hr1G012090	3011.245	2478.244	2294.248	491.4127	167.6308	237.3562
HORVU2Hr1G048500	3.822004	4.470078	2.968095	7.311783	14.09742	13.24828
HORVU7Hr1G121410	0.185512	0.149136	0.34637	0.717358	1.537457	0.898232
HORVU5Hr1G018820	0.40837	0.554734	0.637497	0.836904	2.538855	2.491066
HORVU5Hr1G017830	1.006121	1.397055	1.383936	2.588309	5.729133	4.474033
HORVU5Hr1G071070	0.251519	0.225655	0.296415	0.821627	1.543788	1.597493
HORVU1Hr1G046370	1.785975	1.181041	1.59269	24.12882	12.21279	16.02877

HORVU5Hr1G121610	10.62718	10.77668	14.86482	23.60731	45.28126	54.47616
HORVU1Hr1G084980	5.95708	4.687272	6.856213	3.05485	1.384846	1.551049
HORVU3Hr1G067030	21.30796	23.3533	25.44969	13.8293	8.851082	11.20688
HORVU1Hr1G082670	2.858402	2.369684	2.216459	1.133247	0.827888	1.903653
HORVU2Hr1G045250	117.6334	119.73	204.425	85.31821	72.62749	82.6148
HORVU5Hr1G082990	1.657502	1.475118	1.483434	4.32212	5.803451	5.365745
Hordeum_vulgare_newGene_2155	0	0	0	11.08694	9.456748	12.4208
HORVU4Hr1G026300	5.002202	5.016611	5.776303	12.20309	22.12709	19.05322
HORVU0Hr1G022570	1.621249	1.559113	2.066394	0.104334	0.021915	0.099042
HORVU2Hr1G110860	6.301277	5.391432	6.097855	0.02274	0	0
HORVU3Hr1G061130	0.828933	0.490827	0.544857	4.639745	6.067943	3.974162
HORVU5Hr1G085070	38.18998	45.95764	43.59507	17.81576	10.26908	12.63957
HORVU2Hr1G090980	9.847755	15.28987	21.51964	4.517772	4.62375	5.953187
Hordeum_vulgare_newGene_15141	4.089805	4.42561	4.275147	2.562656	2.086659	2.395772
HORVU7Hr1G122680	437.0465	379.982	352.9666	147.3952	120.6968	126.7727
HORVU5Hr1G069380	2.052591	1.370144	1.666607	0.492915	0.66106	0.883754
Hordeum_vulgare_newGene_5310	0	0	0	1.593892	3.205638	2.591212
Hordeum_vulgare_newGene_5314	5.504019	9.386869	6.459674	1.162179	0.025305	0.449455
HORVU7Hr1G071020	2.461389	2.869215	2.677996	6.954177	9.098154	9.393632
HORVU4Hr1G060370	25.50177	17.62832	18.51404	11.09331	8.294579	8.603005
Hordeum_vulgare_newGene_13848	2.019779	3.964209	5.248344	0	0	0
Hordeum_vulgare_newGene_13847	4.589681	5.456891	4.749436	0	0	0
HORVU4Hr1G090090	14.00319	11.5741	14.14381	7.732602	2.147773	3.777405
HORVU2Hr1G121250	0.550336	0.419868	0.386019	1.526521	1.879958	1.764207
HORVU0Hr1G023760	4.091302	3.253342	4.705734	0.974899	0.83474	1.425114
Hordeum_vulgare_newGene_8349	2.763047	3.39005	3.092285	0	0	0
HORVU1Hr1G058750	1.431821	1.091773	1.127143	4.472742	4.160069	6.985529
HORVU3Hr1G105850	138.9339	119.5917	153.955	77.51773	55.30428	53.81313
HORVU1Hr1G073700	0.339281	0.309498	0.268172	0.90026	0.768056	0.754211
HORVU2Hr1G090210	1.015941	1.144476	1.37587	2.591368	11.90056	10.42464
Hordeum_vulgare_newGene_4445	1.073083	1.195272	1.165256	0.660957	0.516796	0.450622
Hordeum_vulgare_newGene_1548	1.968766	2.948262	2.368134	0	0	0
HORVU3Hr1G095300	2.21346	1.983382	2.118055	5.294662	7.737849	7.791624
Hordeum_vulgare_newGene_3940	30.34402	24.88421	28.46253	13.33909	8.698493	9.937967
HORVU2Hr1G102280	0	0	0	10.39347	12.28105	13.20785
HORVU6Hr1G073570	0.03163	0.01334	0.01288	0.659152	0.935876	1.212904
HORVU3Hr1G091680	4.537584	4.987082	5.851473	1.930971	0.581953	1.326533
Hordeum_vulgare_newGene_4999	0	0	0	0.367367	0.652709	0.783055
HORVU2Hr1G103880	10.61662	10.97657	13.12717	33.19958	48.70393	48.44629
Hordeum_vulgare_newGene_4991	0	0	0.030682	6.637783	9.222038	9.051376
HORVU0Hr1G006830	5.938606	4.55408	6.455533	3.960624	0.726507	1.989217
HORVU6Hr1G003160	0.178641	0.1237	0.218226	32.13565	62.5343	36.26693
HORVU7Hr1G100130	317.4005	135.1803	265.3436	187.8861	85.63896	106.7606
HORVU1Hr1G056920	0.223324	0.32561	0.128085	1.393126	2.061579	0.517506
HORVU0Hr1G021050	4.748939	6.616292	8.179562	1.910709	3.539112	4.031978
Hordeum_vulgare_newGene_14480	1.798539	2.200174	2.485278	1.108517	1.035371	1.306072
Hordeum_vulgare_newGene_14481	0	0	0	3.954771	4.571535	4.208457

HORVU5Hr1G116780	0	0	0	1.872596	1.709707	2.285167
HORVU1Hr1G072720	28.72744	22.60221	28.31792	5.637846	1.884656	4.064585
HORVU7Hr1G002050	1.642729	1.765631	1.470734	2.759974	6.929446	6.408362
HORVU1Hr1G010670	1.616651	1.379432	2.886342	6.009519	12.41608	11.20868
Hordeum_vulgare_newGene_13108	4.421675	4.811451	5.225782	2.514721	3.034311	1.482363
Hordeum_vulgare_newGene_14730	4.408467	4.006939	2.810006	0	0	0
Hordeum_vulgare_newGene_13101	6.623782	8.84859	6.305505	3.987859	4.326023	3.66121
HORVU0Hr1G008590	0	0.002704	0	6.713907	6.952442	7.54135
HORVU6Hr1G082230	10.93308	10.11534	11.13732	1.529355	1.904452	1.23437
HORVU7Hr1G118610	11.06392	9.056547	13.57192	5.61983	3.071622	4.753571
HORVU3Hr1G003050	69.72142	70.56155	97.28324	51.21978	35.57822	44.76587
HORVU5Hr1G061580	2.455316	2.465043	3.515025	1.680595	0.747124	1.423172
HORVU7Hr1G037510	1.815468	2.392534	2.741569	5.209769	5.455577	6.989024
Hordeum_vulgare_newGene_15462	0.18773	0.128588	0.321222	1.987264	1.597872	1.539552
Hordeum_vulgare_newGene_15464	1.305069	1.699998	1.321934	1.199819	0.688635	0.817347
HORVU6Hr1G076870	4.075214	4.478956	4.93589	3.339745	2.635333	2.409283
HORVU4Hr1G074530	0.485702	0.471992	0.297896	2.684549	6.212866	4.620292
HORVU4Hr1G003450	3.634857	3.103387	4.535286	3.081885	1.596636	1.503622
HORVU3Hr1G078880	0.018978	0.016649	0	1.183102	5.374827	7.672821
HORVU3Hr1G019580	23.15776	20.28339	25.95472	13.36982	11.15706	13.89017
HORVU5Hr1G063670	0.274693	0.198059	0.18581	2.364432	1.127079	2.525166
HORVU3Hr1G003580	0.38323	0.305303	0.459822	1.11359	1.106173	1.115882
HORVU7Hr1G017670	4.476577	4.437593	5.909209	10.86305	13.00754	15.48475
HORVU6Hr1G069440	1.337442	0.806662	1.00673	0.601404	0.211907	0.315088
HORVU3Hr1G016800	39.83741	34.43307	26.74909	4.607772	1.62368	1.826924
HORVU5Hr1G069960	0.024414	0.038725	0.229361	0.969452	1.62628	1.624707
HORVU6Hr1G011620	0.550024	0.473133	0.851012	1.403269	5.469825	8.507469
HORVU1Hr1G082790	0.141287	0.164007	0.100637	2.107912	12.26805	12.4766
HORVU5Hr1G108690	1.864523	1.495744	1.480421	0.765246	0.501347	0.561598
HORVU0Hr1G017220	35.96367	36.60043	24.66285	0.3899	0.16297	0.207545
HORVU2Hr1G016250	6.836207	7.803775	7.845057	4.20793	3.690494	4.506546
HORVU6Hr1G066400	0.031199	0.028036	0.303662	1.739707	10.72282	8.432118
HORVU3Hr1G047220	0.013293	0	0	1.404445	0.495901	0.834691
HORVU2Hr1G113320	1.209972	1.322083	1.789929	0.483897	0.614765	0.777875
HORVU3Hr1G000770	3.581962	3.608632	3.583368	10.44035	18.32127	15.88354
HORVU2Hr1G084590	0.16426	0.194951	0.085131	1.483278	2.217679	2.372536
HORVU3Hr1G030850	3.175898	1.920071	1.989986	9.619689	8.806856	8.965594
HORVU3Hr1G071470	4.518272	2.526246	6.669602	1.928828	0.55303	2.215596
HORVU7Hr1G110280	0.404336	0.250187	0.719082	1.544964	9.381605	8.257223
HORVU2Hr1G001790	7.468454	8.165386	10.2228	4.4489	4.809799	5.311932
HORVU1Hr1G069470	0.128186	0.17293	0.085909	1.769312	6.875457	6.650836
HORVU3Hr1G076840	2.605563	4.653822	3.910661	0	0	0
HORVU3Hr1G042330	1.597307	1.520961	1.68395	4.349209	5.753077	5.90321
HORVU7Hr1G006580	545.049	791.3867	520.344	58.27205	112.6468	101.9709
HORVU3Hr1G098160	38.45606	41.18575	44.03097	13.23694	8.821695	8.907976
HORVU1Hr1G093350	1.38785	1.7308	1.348524	3.334085	4.963384	5.138534
HORVU1Hr1G011260	0.014141	0.057995	0	0.962268	1.635219	1.762909

HORVU4Hr1G073070	0.211441	0.30307	0.199238	1.917457	1.715168	1.878665
HORVU2Hr1G101360	10.77876	9.930692	10.44701	24.59478	31.20717	31.33993
HORVU5Hr1G056390	6.596089	6.877141	9.772908	14.13803	30.07482	30.03027
HORVU6Hr1G083620	13.09759	11.04733	13.58693	32.81413	34.54265	32.06932
HORVU3Hr1G062490	3.802049	3.675106	4.159724	14.82533	33.71159	30.66969
HORVU4Hr1G024050	39.21092	37.82781	34.362	21.61239	20.13097	20.32807
HORVU7Hr1G049860	0.998916	1.199077	1.359501	4.410636	20.85349	20.55218
HORVU7Hr1G043800	0.245484	0.124171	0.169047	0.75847	0.935649	0.806243
HORVU1Hr1G065150	673.5662	462.524	670.4615	276.139	36.3577	133.9106
HORVU5Hr1G094400	3.468722	3.358168	3.488026	7.752865	9.030013	9.876631
HORVU7Hr1G037690	13.08978	14.84045	16.50892	46.67011	265.6282	310.8791
HORVU3Hr1G109760	0.138778	0.177606	0.335962	1.089776	3.452551	2.818274
HORVU7Hr1G010750	0.683396	1.07642	6.516579	0.076348	0	0.048833
HORVU3Hr1G077030	8.775165	13.84132	18.4303	6.020377	6.95807	8.496196
HORVU4Hr1G023580	3.67922	4.369229	3.893845	15.61287	13.92841	15.173
HORVU2Hr1G040170	4.705978	5.845404	6.413848	0.033834	0.047666	0.053365
HORVU2Hr1G118670	2.647756	1.846078	2.6866	4.495113	6.95289	7.457341
HORVU7Hr1G046970	100.7415	93.5719	112.9158	53.41932	54.74969	49.31912
Hordeum_vulgare_newGene_16197	1.111905	1.157107	1.982262	0.112521	0.251083	0.422186
HORVU7Hr1G073860	0.165925	0.145267	0.103087	0.941335	1.125791	1.255761
HORVU3Hr1G089060	1.654525	2.172144	2.050047	1.168089	1.266262	1.346145
HORVU1Hr1G012770	27.1492	28.92403	41.6737	216.8459	208.3245	166.245
HORVU7Hr1G002210	0.790519	0.904637	0.626582	7.114006	16.5649	16.77909
Hordeum_vulgare_newGene_438	0	0	0	0.85733	0.844672	1.194784
HORVU1Hr1G009800	6.394725	3.949191	6.387259	3.632265	0.621846	1.128149
HORVU3Hr1G077850	0.522032	0.290603	0.289332	3.055896	2.389311	3.213371
Hordeum_vulgare_newGene_8290	9.206327	9.524037	13.33269	5.311007	4.419414	5.224609
HORVU4Hr1G058970	20.26328	18.52078	18.63171	57.94902	87.2999	86.69085
HORVU7Hr1G043230	0.635258	0.904103	1.135808	3.213149	5.795165	5.668008
HORVU1Hr1G044890	0.510013	0.734861	0.77567	1.30481	7.260701	9.068097
HORVU3Hr1G020780	141.754	139.4527	114.951	57.13976	47.36665	53.78572
Hordeum_vulgare_newGene_2479	0.369488	0.295901	0.337054	0.917641	3.532368	2.196546
Hordeum_vulgare_newGene_2478	0.305976	0.252862	0.198751	0.793853	0.987165	1.997433
HORVU5Hr1G116590	9.528609	10.13443	11.37459	44.98499	189.7559	147.756
Hordeum_vulgare_newGene_5294	1.084794	0.852585	1.112195	4.386345	6.09707	8.193178
HORVU5Hr1G063470	1.406592	2.025695	2.475818	3.037591	6.30214	6.199102
HORVU1Hr1G004980	21.5768	16.98759	25.65894	10.57869	2.610957	4.167054
Hordeum_vulgare_newGene_3678	2.317734	1.885494	2.636525	0.021872	0	0
HORVU7Hr1G027520	5.927326	6.478684	7.17061	0.238758	0.269327	0.229431
HORVU3Hr1G083830	1.084036	1.716174	2.252836	4.86638	13.32086	11.50349
HORVU4Hr1G061120	24.28256	15.16378	38.17022	9.416977	8.981541	11.7021
HORVU7Hr1G105990	2.012517	1.026143	1.478292	9.094361	127.1269	113.0264
HORVU5Hr1G103450	2.456267	2.72602	4.307561	1.045915	1.422302	1.683043
HORVU1Hr1G073510	8.615741	9.218205	11.55102	25.75079	62.8166	48.18344
Hordeum_vulgare_newGene_16225	1.548778	1.460507	1.460386	3.779929	6.288177	5.507989
HORVU3Hr1G011560	10.03268	12.74916	16.84191	5.508395	4.003737	3.272547
HORVU1Hr1G071910	0.067396	0.126122	0.131735	1.999953	3.242093	2.710929

HORVU6Hr1G020960	4.389036	4.586348	3.598898	3.059425	2.102936	2.533124
HORVU7Hr1G052800	2.127384	3.060085	3.364085	1.625422	1.671154	1.654602
HORVU3Hr1G018110	2.928214	1.994838	2.943661	6.616453	8.23183	7.870348
HORVU5Hr1G087210	0.054862	0.036002	0.013713	0.59462	0.69744	0.605087
HORVU2Hr1G114340	0.870174	0.681789	1.206757	4.416742	3.98191	4.600193
HORVU6Hr1G074030	214.8945	150.6762	232.7486	142.2432	89.64886	127.4321
HORVU5Hr1G067880	11.80178	10.18676	14.22279	24.38087	42.30459	48.25431
Hordeum_vulgare_newGene_7387	0	0	0.019973	1.057182	1.470153	1.749414
Hordeum_vulgare_newGene_7380	8.268624	6.646458	8.944829	16.75444	39.32369	35.33292
HORVU3Hr1G039290	1.886668	1.445201	1.399328	0.112308	0.03406	0.017367
HORVU5Hr1G100670	0	0	0	1.864211	2.807574	2.672924
HORVU6Hr1G082160	37.63919	34.70097	36.00996	19.20753	17.14881	20.01728
HORVU5Hr1G084740	13.19203	10.463	11.55874	5.072952	4.715416	4.985925
HORVU1Hr1G044680	12.10334	8.766574	7.978781	21.56796	32.51155	33.18627
HORVU6Hr1G032540	0.222357	0.165171	0.157103	1.250773	0.901309	1.177809
HORVU6Hr1G074690	6.200207	4.691513	6.354505	0.38526	0.089542	0.282968
HORVU4Hr1G017260	20.33088	34.95009	43.50058	0.158771	0.192563	0.158309
HORVU4Hr1G056500	15.20932	5.312586	19.73947	1.346662	0.865317	1.868677
HORVU3Hr1G013680	1.465428	1.184353	1.933508	10.4582	32.72504	31.73777
HORVU6Hr1G065690	2.512048	2.595373	3.097128	5.534115	9.185611	8.57898
HORVU5Hr1G023460	2.912937	2.236277	3.357556	6.075203	11.64056	11.17813
HORVU2Hr1G040400	3.500878	2.083951	2.860709	5.328157	9.397595	9.39184
HORVU6Hr1G010780	0.189077	0.055145	0.215251	0.751149	0.975119	1.306392
HORVU7Hr1G037720	0.579395	0.576577	0.868908	3.070501	42.02952	49.92501
HORVU2Hr1G082410	22.3382	19.34567	24.45156	10.01814	8.020017	8.404629
HORVU4Hr1G002510	26.08134	28.22725	38.45823	13.26365	8.309795	11.8706
HORVU2Hr1G113460	341.4677	323.3326	361.8774	108.683	40.52461	80.73473
HORVU5Hr1G092220	2.732309	5.261791	5.03958	0.992449	1.059282	0.995035
HORVU4Hr1G019490	0.958263	0.793735	1.015161	4.430895	2.945385	3.979025
HORVU4Hr1G090650	0.805406	0.718402	0.779962	1.232867	5.9499	4.891144
HORVU4Hr1G088620	0.519584	0.289281	0.546426	1.051806	1.367482	1.688265
HORVU7Hr1G036450	7.06508	10.08418	10.88113	3.053281	1.541781	2.749239
HORVU2Hr1G081140	1.388022	2.045959	1.941838	3.964887	6.764419	6.800453
HORVU3Hr1G084530	2.042333	1.726033	2.298359	9.539582	15.80637	12.65701
HORVU3Hr1G030950	1.831023	1.341634	1.78169	0.812577	0.555342	0.749387
HORVU4Hr1G009520	87.65114	81.63466	82.39834	276.833	440.245	380.6787
Hordeum_vulgare_newGene_9553	0.470447	0.722508	0.774494	0.201431	0.194995	0.234672
Hordeum_vulgare_newGene_2917	0.460737	0.38635	0.313716	1.336852	3.82923	4.185023
HORVU0Hr1G001780	0	0	0.014649	2.096102	1.084308	2.065803
HORVU7Hr1G044270	0.556068	0.435354	0.480731	1.34428	1.555549	1.973636
HORVU3Hr1G099400	1.336546	1.234058	1.859297	0.796064	0.530851	0.692854
HORVU2Hr1G017420	54.38843	29.49968	55.76748	9.442678	2.458378	4.394886
HORVU5Hr1G095250	0.382273	0.678079	0.556479	1.231716	2.851424	3.616899
HORVU2Hr1G099870	159.1025	186.3031	225.6856	55.97953	24.16711	33.5699
HORVU2Hr1G115690	0.605999	0.460687	0.46071	1.284441	1.452821	1.53378
HORVU2Hr1G019680	5.846288	8.157857	10.00622	1.654314	0.196444	0.750632
HORVU5Hr1G042000	0.627038	0.512396	0.752804	0.434015	0.19692	0.343056

HORVU2Hr1G000560	0.303302	0.384748	0.393666	0.785383	1.345362	0.898295
HORVU3Hr1G078620	0.921019	0.401724	0.563671	7.722719	1.633365	2.349192
HORVU4Hr1G040520	1.396515	2.385816	2.968491	11.01042	44.37214	32.15748
HORVU1Hr1G083700	0.926267	0.802601	1.221098	0.178761	0.137051	0.292788
HORVU6Hr1G089000	0.138531	0.181122	0.177631	0.556522	1.881061	2.914994
Hordeum_vulgare_newGene_11543	14.04092	10.56361	16.40355	4.671992	2.634495	4.236765
Hordeum_vulgare_newGene_11545	0	0.015512	0	8.539339	8.892131	10.89885
HORVU5Hr1G092510	15.64075	13.9983	16.89367	7.450972	9.034157	8.494591
HORVU5Hr1G081840	9.365268	9.257136	11.66029	4.13682	2.345058	3.620214
HORVU1Hr1G084120	0.632342	0.665883	0.768377	2.280938	4.915876	4.941987
HORVU2Hr1G028660	11.22114	6.881483	8.65614	0.131952	0.030266	0.412583
HORVU7Hr1G051430	2.987987	1.916803	3.062913	0.71045	1.008112	1.177205
HORVU6Hr1G011990	1.378999	1.295153	1.721352	5.291909	11.1923	9.631508
HORVU3Hr1G026900	3.790904	3.51169	3.980709	9.890718	12.11532	12.2694
Hordeum_vulgare_newGene_16018	3.366879	4.408224	4.449875	2.776179	1.60287	2.359972
HORVU3Hr1G024500	4.984791	3.700148	5.517482	1.071319	0.009104	0.068063
Hordeum_vulgare_newGene_3017	41.78924	52.08714	21.93024	1.021012	0.153067	0.379301
HORVU0Hr1G021480	3.792365	2.589561	4.506634	2.287801	1.014766	2.149222
HORVU5Hr1G087980	12.78834	13.02303	8.500562	4.986846	6.044754	6.268934
HORVU5Hr1G071140	10.31125	8.058901	10.96961	0.491543	0.881244	1.480651
HORVU2Hr1G039000	0.937455	0.997898	1.177621	2.233136	3.470965	2.5376
Hordeum_vulgare_newGene_7817	2.962112	2.083293	2.552471	8.843987	9.910291	8.062119
Hordeum_vulgare_newGene_13793	0.326711	0.443376	0.39383	1.292426	5.991101	8.201419
Hordeum_vulgare_newGene_3667	10.26347	10.60061	12.40276	0.203789	0.166956	0
HORVU2Hr1G100450	1.67621	1.476678	2.109525	6.978862	4.84845	6.057926
HORVU6Hr1G078470	0.171674	0.118076	0.086551	2.904638	4.859292	3.858677
HORVU2Hr1G086380	23.70842	22.68581	27.5828	11.03231	8.766268	9.56373
Hordeum_vulgare_newGene_6025	2.526671	2.889702	2.114613	0.894532	1.596348	1.180897
HORVU5Hr1G115270	0.153906	0.185239	0.241834	0.639341	0.7749	0.662711
HORVU1Hr1G077820	63.17046	73.46418	75.71093	54.79822	24.26718	39.34183
HORVU2Hr1G063740	14.59835	13.04378	13.54518	58.52276	71.53561	71.43888
Hordeum_vulgare_newGene_2535	0	0	0	0.856981	2.352714	2.222496
Hordeum_vulgare_newGene_2531	0.264902	0.291611	0.189586	0.899741	1.216642	1.048468
Hordeum_vulgare_newGene_11938	0	0	0	1.21999	1.084325	1.683227
HORVU7Hr1G028670	9.863853	9.492506	10.54369	23.81209	27.48513	27.76333
HORVU7Hr1G000320	1.995608	3.337819	3.305901	0.045283	0.089499	0.01183
Hordeum_vulgare_newGene_5626	20.94848	14.32311	18.11268	7.780972	6.065192	5.8948
HORVU1Hr1G000030	1.132098	1.523881	1.914755	3.126684	4.541889	4.836378
HORVU0Hr1G022110	2.93023	3.074074	3.732654	4.080927	11.6907	10.56795
Hordeum_vulgare_newGene_9503	6.863953	8.323819	8.698557	4.958004	3.939882	5.465896
HORVU7Hr1G114020	5.75341	7.622635	9.295037	0.854798	0.871119	0.737584
HORVU1Hr1G073490	0.81318	0.776643	0.66581	7.618722	32.23202	22.34835
HORVU3Hr1G049730	34.45988	25.67876	26.47113	6.270157	6.424438	5.451755
Hordeum_vulgare_newGene_4175	0.63774	1.076608	1.087293	0	0	0
Hordeum_vulgare_newGene_4178	0.976948	0.947834	1.288404	2.417665	3.824756	3.790226
HORVU4Hr1G089990	0.068543	0.175342	0.053345	0.80796	1.379751	1.747639
Hordeum_vulgare_newGene_14888	42.4281	44.98122	57.84597	10.07159	3.357408	8.0706

Hordeum_vulgare_newGene_4283	0.824955	0.919781	0.688843	2.638827	2.685276	3.166702
HORVU3Hr1G047670	0.099972	0.181217	0.135566	2.752328	2.10738	2.697262
HORVU6Hr1G023040	1.262962	1.34192	1.537852	6.473654	10.04428	7.815945
HORVU1Hr1G078320	28.05417	24.83753	25.18198	6.993732	10.68223	10.02494
Hordeum_vulgare_newGene_4828	0	0	0	1.156566	1.168359	1.392099
HORVU7Hr1G098280	0.968022	0.534977	1.756407	8.142202	41.57582	40.13914
HORVU5Hr1G101710	0.218207	0.208989	0.163243	13.66946	1.064623	4.975473
Hordeum_vulgare_newGene_7735	0.130946	0.231846	0.196911	1.243922	4.293835	5.21451
HORVU1Hr1G021390	0.480561	0.756476	0.355384	2.452192	2.386383	2.733181
HORVU6Hr1G005950	1.074327	0.644047	1.215255	2.451125	12.75075	10.41281
HORVU4Hr1G074780	34.82208	30.82762	34.96285	16.52184	9.560769	13.36879
Hordeum_vulgare_newGene_6465	14.20728	15.5399	28.26889	1.690399	0.676876	0.669033
HORVU3Hr1G030750	1.760248	2.054105	2.261356	4.300853	6.040365	6.78478
HORVU0Hr1G040070	2.404009	2.479211	3.210629	5.540961	8.765037	7.577247
HORVU4Hr1G074250	1.192384	1.442696	1.257563	5.614823	4.941156	6.560504
HORVU2Hr1G082340	3.754736	2.439084	1.539579	0.898219	0.216184	0.355232
HORVU0Hr1G018100	0	0	0	2.815755	2.483926	2.824101
HORVU5Hr1G088130	4.445874	3.20455	4.040892	1.830749	1.257868	1.351145
HORVU5Hr1G012170	0.956459	0.580208	0.742479	3.20511	4.437795	4.111928
HORVU4Hr1G000830	4.578137	4.572903	4.834901	18.71929	12.5891	15.05613
HORVU5Hr1G124010	43.09436	38.2981	38.44755	73.05008	123.7427	114.9029
HORVU3Hr1G039790	0.65123	0.621748	0.602654	4.825242	9.637947	8.46436
HORVU1Hr1G065630	0.688042	0.786287	0.850113	13.24092	4.199235	5.531108
HORVU3Hr1G013160	1.461997	1.148505	1.482131	0.848529	0.347142	0.653997
HORVU7Hr1G069420	6.567759	9.032566	7.713393	0.007767	0	0
HORVU4Hr1G013640	10.49574	11.98797	16.10569	5.483407	7.738949	9.951237
HORVU1Hr1G050050	9.816231	9.616172	10.20503	17.64443	40.90606	23.85965
HORVU7Hr1G049580	0.415313	0.339903	0.315183	0.221105	0.09422	0.164076
HORVU6Hr1G010230	0.272975	0.2511	0.499797	1.490516	1.259338	1.649674
HORVU1Hr1G010210	96.88226	104.2424	70.28442	19.33528	8.113751	10.90237
HORVU1Hr1G067670	5.770349	4.600921	6.897534	1.596966	1.633209	1.420966
HORVU5Hr1G029060	0.627134	0.526964	0.540714	2.062436	1.477656	1.714356
HORVU4Hr1G087780	71.27714	107.5161	138.1678	10.74147	2.542072	6.504748
HORVU2Hr1G111840	2.448059	1.56168	1.673094	6.931447	9.372679	9.32335
HORVU1Hr1G090490	93.64232	97.12907	114.171	167.3989	402.6453	390.7414
HORVU6Hr1G085210	0.161418	0	0.112517	1.538317	21.99329	20.80011
HORVU6Hr1G054890	15.45494	19.41273	24.20638	2.072882	0.571042	1.205098
HORVU5Hr1G093850	1.484214	1.732642	1.851638	2.878621	5.30365	5.069662
HORVU6Hr1G088880	0.856224	0.660408	0.87635	0.364992	0.239769	0.26092
HORVU2Hr1G100690	4.683056	6.944202	9.077606	4.296742	3.691096	3.551151
HORVU2Hr1G061040	39.58652	40.96841	48.81726	27.91936	19.01516	23.15419
HORVU4Hr1G065380	24.5224	23.53264	25.50109	47.7363	76.16089	69.30928
HORVU1Hr1G056170	0.539206	0.509437	0.66013	1.348477	9.158613	6.85829
HORVU6Hr1G012530	3.985332	4.028592	5.976903	1.770781	1.778166	2.002397
HORVU7Hr1G074530	31.58385	30.19099	31.82003	17.03821	17.30253	19.54034
HORVU5Hr1G115470	39.50597	31.66661	39.92396	21.1645	17.02489	18.00167
HORVU4Hr1G054880	5.850793	7.3543	9.024644	3.580861	4.126747	4.680328

HORVU2Hr1G038570	0.502475	0.507371	0.673234	3.634829	5.240231	4.340744
HORVU3Hr1G021660	2.538656	2.696333	3.050756	6.721397	11.47648	11.10076
HORVU2Hr1G010670	0.050409	0	0.037686	2.324416	13.69478	11.37845
HORVU2Hr1G019180	5.399088	3.912519	6.114129	17.4412	33.49151	27.42438
HORVU3Hr1G110990	3.672202	2.983595	4.884065	1.67784	1.933941	1.759809
Hordeum_vulgare_newGene_8615	0	0	0	6.272756	6.028324	7.826457
HORVU4Hr1G020060	1.820346	2.12273	2.792581	0.767729	0.475627	0.976345
HORVU5Hr1G123520	3.46737	3.089324	3.645628	0.328956	0.466884	0.471775
HORVU5Hr1G086620	16.6797	8.514981	16.99724	21.46101	48.85934	53.726
HORVU2Hr1G048680	7.455216	7.319593	7.264038	16.80654	20.68507	20.77284
HORVU3Hr1G094960	0.567061	0.371481	0.420336	0.766563	1.541432	1.86447
HORVU2Hr1G011690	2.831218	2.293234	3.115033	1.788969	1.08377	0.907068
Hordeum_vulgare_newGene_1898	1.77187	1.472964	1.786391	6.158011	5.656291	5.816269
Hordeum_vulgare_newGene_1891	0	0	0	1.734791	2.119351	2.023898
HORVU7Hr1G009700	2.30587	1.952146	2.410309	5.676264	10.15256	11.57791
HORVU3Hr1G075470	4.336977	4.051183	6.275217	2.330822	2.004927	1.918938
HORVU6Hr1G063510	23.89965	28.18711	18.95911	6.067755	1.727527	3.956913
HORVU3Hr1G089550	0.493615	0.502153	0.76729	4.347655	9.65866	8.214071
HORVU2Hr1G071890	195.6133	242.8653	176.3991	11.42989	0.242692	4.50727
Hordeum_vulgare_newGene_7131	2.197197	1.290296	1.63288	0.76568	0.552093	1.423966
HORVU2Hr1G112080	0.083392	0.140794	0.085163	1.719348	4.357449	3.594265
Hordeum_vulgare_newGene_2957	13.18862	11.77204	17.0066	0.044904	0	0
Hordeum_vulgare_newGene_6265	2.124971	1.649145	1.982848	0	0	0
HORVU7Hr1G003880	1.359801	1.611098	1.817621	3.144356	6.709989	5.892486
Hordeum_vulgare_newGene_12585	1.90822	1.223293	2.239201	4.433165	4.725947	4.363269
HORVU7Hr1G042300	4.807792	4.931975	6.913433	1.332046	0.509309	0.886014
Hordeum_vulgare_newGene_9203	0.720844	0.629694	0.706326	0.174301	0.135339	0.059128
Hordeum_vulgare_newGene_9208	0.30777	0.30902	0.248464	1.06915	0.869849	0.961469
HORVU5Hr1G068100	0.58537	0.986088	1.245365	0.337312	0.029182	0.270894
HORVU5Hr1G065350	25.59506	22.84362	25.76354	10.93538	6.884053	8.415484
Hordeum_vulgare_newGene_5980	1.20204	1.042547	1.151088	0	0	0
Hordeum_vulgare_newGene_5981	2.443163	2.377782	3.465246	0.51227	0.386605	0.744076
HORVU2Hr1G018750	0.045049	0.050391	0.133403	2.052887	36.44221	25.0455
HORVU4Hr1G071570	8.615673	8.900559	8.281482	0.581521	0.335898	0.369448
HORVU1Hr1G054110	0.588647	0.875993	1.023644	8.879144	15.07569	17.99175
Hordeum_vulgare_newGene_815	3.61527	5.267454	5.882135	1.47826	0.492715	1.161245
HORVU7Hr1G034640	2.004373	1.548343	1.607945	0.913655	1.160313	0.994141
HORVU2Hr1G004600	12.84716	8.17977	16.54476	0.913184	0.23347	1.190159
HORVU4Hr1G089540	1.720976	1.451496	2.096603	4.261512	7.930563	7.232972
HORVU5Hr1G050610	10.34315	10.65765	12.50045	23.21216	38.92715	32.66244
Hordeum_vulgare_newGene_1668	0	0	0	4.157913	2.589756	3.019831
HORVU7Hr1G088260	55.54477	51.74954	56.43314	42.98793	8.804087	18.59399
HORVU2Hr1G017120	1.498508	1.361904	2.50221	3.459478	4.758716	5.260179
HORVU7Hr1G019680	0.162962	0.241482	0.195345	2.365446	6.994953	6.593908
Hordeum_vulgare_newGene_7671	0.532649	0.53941	0.539415	3.46438	8.178654	7.424758
HORVU3Hr1G001020	0.050502	0.037423	0.060151	4.269591	7.236625	14.76498
HORVU7Hr1G098670	0.181423	0.242587	0.510888	2.370442	2.032408	1.380485

HORVU6Hr1G074180	0.101096	0.179239	0.184445	1.295556	2.808736	2.969719
HORVU7Hr1G056700	0.625416	0.590002	0.694669	2.119343	3.142636	4.17661
HORVU3Hr1G086500	31.60578	34.30724	35.93944	70.98085	112.765	73.15778
HORVU7Hr1G025160	0.245367	0.174746	0.487838	4.805574	23.72172	22.38062
HORVU4Hr1G056870	30.34944	46.42831	44.44346	0	0	0
HORVU3Hr1G038290	4.273769	3.187399	3.862079	9.115084	11.21745	9.830232
HORVU1Hr1G055320	0	0	0.008682	25.58744	19.35622	24.26986
HORVU5Hr1G093270	3.353389	3.902876	4.498059	1.803253	1.288186	1.45351
HORVU5Hr1G086390	0.089717	0.070817	0.021811	0.61815	0.863638	1.171269
HORVU0Hr1G022170	0.28846	0.187965	0.157986	5.893945	88.9747	87.07952
HORVU2Hr1G115240	0.106454	0.040526	0.050676	3.619226	1.206494	1.276877
HORVU2Hr1G017900	26.22798	25.11604	33.98128	14.34809	11.17905	11.45585
HORVU7Hr1G048020	0.472101	0.574026	0.645642	1.857996	2.047919	2.329731
HORVU6Hr1G084430	9.469721	9.45753	9.857491	7.194563	4.748001	5.050635
HORVU5Hr1G122100	1.305557	1.246526	1.099317	0.662461	0.544233	0.936277
HORVU7Hr1G051860	12.66247	12.80026	10.22226	8.336182	7.073668	8.065083
HORVU4Hr1G049150	136.455	163.3646	250.708	70.09817	96.25635	97.57623
HORVU5Hr1G011730	31.01361	28.20011	17.42675	6.727332	5.034314	4.634407
HORVU2Hr1G085360	0.465742	0.712184	0.923106	1.131194	3.964823	3.721586
HORVU7Hr1G040560	0.538085	0.553732	0.40525	0.087733	0.088092	0.13246
HORVU4Hr1G050980	2.45306	2.216934	2.922227	5.64557	7.528848	7.714735
HORVU7Hr1G119200	0.023619	0.149436	0.316563	1.307816	1.246322	1.429431
HORVU4Hr1G057450	2.19472	1.75344	2.5483	4.370122	5.701795	7.196118
HORVU4Hr1G066750	1.502497	1.177396	1.253217	0.181798	0.498323	0.397603
HORVU3Hr1G031220	1.361743	1.769378	1.793488	2.782881	5.659444	4.629329
HORVU5Hr1G111640	0.564724	0.527705	0.609569	2.966586	5.069628	5.176612
HORVU3Hr1G024150	2.159866	2.855406	2.960907	5.339723	7.776364	8.655411
HORVU5Hr1G057520	8.779172	12.16171	16.76923	4.158329	5.229371	5.838536
HORVU1Hr1G057410	40.02107	31.80775	32.19605	13.44468	10.31095	10.05995
Hordeum_vulgare_newGene_13342	16.09798	24.1338	32.17321	0.975148	0.067011	0.346401
Hordeum_vulgare_newGene_13344	398.9678	456.2056	550.774	97.6134	4.170382	44.8921
Hordeum_vulgare_newGene_9041	0.340419	0.493845	0.387326	0.063738	0.023823	0.018233
Hordeum_vulgare_newGene_2747	3.723009	3.259415	2.615709	12.35806	11.74563	13.39498
HORVU4Hr1G085460	1.601664	1.849941	2.02203	0.227331	0.257809	0.228314
HORVU3Hr1G050430	0.476938	0.859585	1.136266	3.152249	11.14626	8.741098
Hordeum_vulgare_newGene_5213	0.428077	0.703769	0.533595	0	0	0
HORVU5Hr1G092910	0.825766	1.300741	0.926183	4.826757	16.84854	17.15988
HORVU7Hr1G119100	0.14395	0.160461	0.123126	0.491308	0.52572	0.567363
HORVU1Hr1G000680	7879.225	9247.93	6646.441	2594.697	2070.654	1800.417
Hordeum_vulgare_newGene_8797	3.966254	4.257173	2.849029	1.617231	0.850798	0.793324
HORVU2Hr1G116970	0.750857	0.630632	1.076822	0.353925	0.101403	0.361125
HORVU3Hr1G093500	0.830511	0.626249	0.876266	1.632747	2.873608	2.568959
HORVU2Hr1G023540	0.850569	0.691266	0.715652	4.009808	3.337256	7.848485
HORVU3Hr1G095480	0.098619	0.102269	0.065314	0.374191	0.721143	0.988047
HORVU3Hr1G042440	1.396622	1.043214	1.431064	4.319066	3.97092	4.44591
Hordeum_vulgare_newGene_10708	0.103135	0.057401	0.181044	0.434018	0.500241	0.41323
HORVU3Hr1G115530	1.662349	2.052163	1.781957	0.542119	1.003787	1.011338

Hordeum_vulgare_newGene_3825	0.992527	0.281624	0.221702	4.506269	3.196456	3.756766
HORVU7Hr1G066450	16.69823	17.2236	16.9347	0.020961	0	0
HORVU7Hr1G101140	1.785046	1.00894	1.116995	7.786658	5.019777	7.01375
Hordeum_vulgare_newGene_4657	1.138812	1.26598	1.754878	0.946512	0.826258	0.787104
Hordeum_vulgare_newGene_4655	0	0	0	2.370097	2.265032	2.734136
HORVU2Hr1G024120	0.090344	0.06812	0.049429	1.127763	0.947683	0.937029
HORVU7Hr1G099870	19.77919	19.21517	21.60385	9.942282	17.85713	14.03008
HORVU2Hr1G044560	0.872512	1.710841	1.698944	3.232825	6.367431	4.396171
HORVU5Hr1G100180	1.88187	1.00722	2.226835	0.222688	0.025891	0.065771
HORVU4Hr1G037380	1.337607	1.239135	1.579907	0.590564	0.644459	1.090096
Hordeum_vulgare_newGene_2880	0	0	0	1.643716	1.580203	1.833893
HORVU3Hr1G091830	10.70652	9.870017	14.62484	5.790403	6.183153	5.630824
HORVU5Hr1G041530	15.75468	15.30763	15.90462	18.47345	54.67702	54.60096
HORVU6Hr1G078510	1.939431	1.481576	2.418507	1.35247	0.381295	0.348807
HORVU2Hr1G117610	0.025671	0.056818	0.027511	0.836703	1.519215	1.559214
HORVU3Hr1G097800	0.226431	0.21533	0.031708	31.19769	166.3609	137.8274
HORVU6Hr1G032890	0.501522	0.445682	0.685086	2.071721	3.280671	3.347051
HORVU5Hr1G075200	66.30645	44.4432	49.21087	28.40021	23.51189	24.75831
HORVU3Hr1G053860	6.354136	4.854363	6.33885	11.15439	21.8633	19.23396
HORVU5Hr1G087830	6.702097	4.438291	7.942546	23.12242	50.49274	41.22696
HORVU7Hr1G038220	0	0	0.042986	1.21698	1.58361	1.820725
Hordeum_vulgare_newGene_2225	1.005082	1.298101	2.218983	2.265438	2.924974	2.622069
Hordeum_vulgare_newGene_10936	5.678397	4.540841	6.370612	0.122576	0.134492	0.182198
Hordeum_vulgare_newGene_15548	0.083075	0.175834	0.1259	1.098157	1.571799	0.935594
HORVU0Hr1G031850	4.789363	3.800116	4.788649	9.707493	13.49426	12.97735
HORVU6Hr1G061450	1.716505	1.150037	1.657535	4.433387	4.114598	6.070212
HORVU4Hr1G075150	2.98171	3.416368	4.196344	2.157187	1.270892	2.072748
HORVU2Hr1G062330	8.685551	9.105688	9.098604	2.227739	2.118507	2.588126
HORVU4Hr1G043910	697.206	622.3131	771.8423	1083.976	2907.139	2526.239
HORVU2Hr1G089440	15.47893	11.47455	21.20987	5.064668	8.420705	12.21494
HORVU7Hr1G049200	6.081786	6.144107	6.878073	3.705227	3.61038	3.560486
HORVU1Hr1G053080	5.166975	5.509636	5.794445	11.79606	18.67107	19.20827
HORVU7Hr1G113210	1.384861	1.274975	1.590154	5.028287	7.453967	6.544922
HORVU3Hr1G031020	2.01823	1.302165	1.654287	0.361167	0.066719	0.204533
HORVU5Hr1G107380	0.814612	0.982399	0.606619	2.766515	6.360057	6.58626
HORVU0Hr1G007700	1.733607	1.743739	1.832616	0.498378	0.969153	0.156516
Hordeum_vulgare_newGene_1143	0.598941	0.394863	0.962822	0	0	0
HORVU6Hr1G080260	13.65016	7.436686	9.099276	5.352431	1.027533	1.46911
HORVU6Hr1G007020	14.85271	16.07111	24.81914	6.558146	7.78763	7.513701
HORVU7Hr1G095890	0.16677	0.234464	0.452312	1.404871	3.829032	4.283264
Hordeum_vulgare_newGene_4516	1.319936	0.830274	1.097397	0.319141	0.375886	0.218157
Hordeum_vulgare_newGene_4517	1.436885	1.051041	0.84526	0.056953	0.182868	0.147844
HORVU3Hr1G076960	2.313741	2.238593	2.591181	6.634822	12.6698	11.51832
HORVU1Hr1G078740	2.606271	2.869303	3.541127	37.20838	41.75963	36.1098
HORVU7Hr1G055560	52.7782	51.5278	71.21967	25.8996	30.52932	34.82866
Hordeum_vulgare_newGene_9874	0.784063	1.13541	1.163025	0	0	0
HORVU4Hr1G051390	5.45516	6.877983	7.538041	0	0	0

HORVU5Hr1G125430	1.392846	1.52758	1.088102	1.161206	0.697003	0.702285
HORVU4Hr1G050660	40.30707	35.47318	31.28218	18.76467	15.55842	18.40387
HORVU7Hr1G043920	0.23195	0.582492	0.475586	4.001216	4.989952	5.654039
HORVU5Hr1G124520	0	0	0	0.776798	1.431767	1.385678
HORVU3Hr1G100350	43.69971	29.32304	34.08351	16.38416	6.977373	7.553174
HORVU5Hr1G042080	1.961413	1.439796	2.144419	5.651978	8.798221	9.159555
HORVU5Hr1G030460	1.607564	1.750358	1.83148	1.109462	0.962256	1.119786
HORVU7Hr1G051120	5.169434	3.463542	4.016674	0.839695	1.407363	1.155058
HORVU2Hr1G011400	0.682617	0.879945	0.82438	2.08718	1.938874	2.10265
HORVU2Hr1G107980	4.902539	3.725537	6.089178	13.76846	18.55577	20.28185
Hordeum_vulgare_newGene_16094	0.701483	1.075484	0.879119	0	0	0
Hordeum_vulgare_newGene_16099	0	0.095188	0.156817	1.602665	4.354323	3.662796
Hordeum_vulgare_newGene_16098	40.34445	34.0916	30.77971	0.927664	6.965797	5.876883
HORVU6Hr1G093080	18.61365	19.50384	17.79518	7.028606	9.434977	9.132595
HORVU3Hr1G066450	1.004688	0.478339	0.90296	1.288547	2.547225	3.215909
Hordeum_vulgare_newGene_12733	4.329305	3.800836	4.268369	2.0101	2.195368	2.979462
HORVU3Hr1G025670	0.855054	0.716831	0.785304	0.632554	0.349005	0.356767
HORVU3Hr1G060310	2.875686	2.427502	2.075273	3.450259	8.572849	8.875864
Hordeum_vulgare_newGene_13069	0.165613	0.237575	0.228568	0.642227	0.580847	0.664025
HORVU1Hr1G017230	7.434379	6.902384	5.701935	3.680001	3.927865	4.743626
Hordeum_vulgare_newGene_939	0.558721	0.673707	0.742109	0.036186	0.047098	0
HORVU7Hr1G007580	0.079124	0.069953	0.053201	0.892014	0.712968	1.070864
HORVU1Hr1G056120	1.722815	1.393977	1.211016	3.823373	4.392018	4.163348
HORVU5Hr1G070230	16.88704	15.69189	18.32181	36.39681	42.79601	43.26053
HORVU4Hr1G064890	2.034369	2.887755	2.574962	1.371859	1.006613	1.475776
HORVU1Hr1G031990	0.037004	0.013473	0.020797	0.379334	0.329313	0.395281
HORVU1Hr1G070730	1.569217	1.37221	2.340898	0.488808	0.33516	0.834545
HORVU4Hr1G082700	25.88078	23.5673	21.51078	69.02917	94.89952	100.8443
HORVU2Hr1G123760	4.789638	6.290604	5.228141	9.058395	22.70725	20.80261
HORVU7Hr1G073450	0.644023	0.859084	0.857501	1.819687	5.302654	4.733071
HORVU5Hr1G080170	63.94307	88.38197	124.7354	23.3666	36.1481	36.91647
HORVU6Hr1G013350	0	0.013469	0	2.534622	2.966994	5.418885
HORVU3Hr1G065230	1.531491	1.489505	1.932364	1.154344	0.851566	1.142272
HORVU1Hr1G000520	81.14265	76.46343	62.81683	22.99656	28.00226	28.57552
HORVU3Hr1G058740	0.190766	0.198729	0.14718	0.597037	0.495729	0.914486
HORVU5Hr1G116720	2.214206	2.737208	2.502215	1.423614	1.725721	1.5299
Hordeum_vulgare_newGene_5002	0	0	0	2.44087	2.581266	3.229324
HORVU7Hr1G089240	6.87989	5.952053	6.958465	4.639453	3.248808	3.597454
HORVU1Hr1G077170	1.06746	0.96316	1.227327	1.839529	4.597899	3.466491
HORVU7Hr1G044280	0.172483	0.089631	0.087176	1.114901	1.858681	2.091519
HORVU4Hr1G089910	1.420571	1.211579	1.73125	6.035412	8.456331	7.618974
HORVU7Hr1G106510	0.466985	0.593499	0.4616	2.156319	2.425109	2.662757
HORVU1Hr1G035290	0.431853	0.314045	0.556682	2.142844	3.428842	2.717492
HORVU5Hr1G104240	116.4598	76.61402	94.30656	3.230555	1.277007	1.553603
Hordeum_vulgare_newGene_13677	0.243046	0.374918	0.341579	1.410846	5.526828	5.702343
HORVU7Hr1G036210	1.133403	1.043882	1.014018	2.18258	2.68753	3.596597
HORVU2Hr1G035310	45.23888	55.28503	48.68529	18.18974	20.26274	21.38086

HORVU6Hr1G077440	3.239068	4.42798	5.64849	9.634171	34.42878	26.72435
Hordeum_vulgare_newGene_1786	0	0	0.034966	17.00216	16.21094	19.69174
Hordeum_vulgare_newGene_1787	0.192652	0.30348	0.276273	0.604256	1.009626	1.126842
Hordeum_vulgare_newGene_8237	0.693085	0.78695	0.836726	0.455494	0.434328	0.606287
HORVU2Hr1G048510	2.475666	2.832596	1.866661	6.422925	12.60508	11.21049
HORVU0Hr1G039620	3.156443	4.476988	4.31012	2.584691	1.649934	2.463117
HORVU1Hr1G024550	0	0	0	8.216133	11.61908	12.08934
Hordeum_vulgare_newGene_13657	0.873583	0.870189	1.179621	0.624565	0.343924	0.221229
HORVU7Hr1G025330	0.041857	0	0.197355	1.859904	7.189423	7.78031
HORVU2Hr1G004890	2.019648	0.906649	1.333979	4.980584	5.667729	6.721208
HORVU7Hr1G053070	4.104478	4.552274	6.248348	2.213175	2.169251	2.339089
HORVU5Hr1G087800	1.238463	1.13366	2.656065	0.570996	0.675036	0.593636
Hordeum_vulgare_newGene_6756	0.425522	0.606573	0.312038	2.051272	6.355086	5.099942
Hordeum_vulgare_newGene_6751	0.52852	0.532644	0.610192	1.642695	4.894056	4.377597
Hordeum_vulgare_newGene_15869	2.00811	3.242414	3.512445	1.478311	1.421728	1.419362
Hordeum_vulgare_newGene_15867	0	0	0	1.998305	1.545901	2.581289
Hordeum_vulgare_newGene_15863	0.513864	0.781953	0.56195	2.403509	5.425021	3.50781
HORVU1Hr1G011650	0.01797	0.016544	0	1.059146	1.340371	1.906001
HORVU7Hr1G052030	0.216864	0.159415	0.381898	1.974564	2.762608	2.506984
Hordeum_vulgare_newGene_2929	7.351923	5.976782	8.186833	0	0.050042	0
HORVU5Hr1G089780	41.89365	42.90734	53.70617	24.02151	18.84746	21.26516
HORVU4Hr1G013530	1.520361	1.894443	1.698539	5.344904	7.89779	7.615985
HORVU5Hr1G023090	5.776041	5.344987	7.174453	2.452401	2.116157	2.312326
HORVU6Hr1G070130	2.152059	1.217509	1.776804	0.305858	0.080608	0.035299
HORVU3Hr1G013250	0.436171	0.73465	0.958107	0.340379	0.317553	0.319684
HORVU7Hr1G118180	0	0	0	1.007346	2.226639	1.702369
HORVU4Hr1G017340	2.998606	3.347394	3.055968	8.20236	11.11555	11.04028
HORVU3Hr1G085290	20.6034	17.75108	23.03762	10.66343	7.813393	8.470083
HORVU1Hr1G082090	0	0	0.017283	61.65756	53.75425	65.35355
HORVU3Hr1G004290	26.37873	33.58035	41.47518	4.215867	0.028292	2.449573
HORVU4Hr1G002650	230.4417	150.5716	208.9793	30.66355	12.27263	22.80996
HORVU2Hr1G126540	5.806838	5.449023	3.872552	3.348345	2.280893	3.509477
HORVU5Hr1G066130	3.378014	2.464826	4.286821	0.865462	0.96472	1.709334
Hordeum_vulgare_newGene_13112	0.621369	0.831894	0.742051	3.367477	10.68757	8.592102
HORVU4Hr1G032660	0	0.011907	0	4.002132	2.946643	4.014416
HORVU0Hr1G017530	1.003399	1.016227	1.117174	2.786599	5.576025	5.446647
HORVU5Hr1G054400	0.887247	1.722148	1.288892	0.737604	0.503424	0.811916
HORVU5Hr1G050180	0.756915	0.966293	0.833038	2.901258	2.838784	3.376451
HORVU2Hr1G108420	0.303472	0.173392	0.626542	3.909495	2.727303	3.70994
HORVU3Hr1G041440	0	0	0	5.268004	6.004014	6.722212
HORVU3Hr1G056830	0.417721	0.628115	0.764949	0.085257	0.112481	0.155918
HORVU7Hr1G088350	107.9548	71.81407	67.569	30.72454	13.67365	13.61866
HORVU7Hr1G071530	16.94509	18.87148	18.81247	16.74498	9.066645	13.74756
HORVU5Hr1G093530	0.798249	0.514562	0.631086	1.077643	2.090727	2.11967
HORVU5Hr1G010120	3.012953	4.66706	3.264308	10.42806	5.960024	13.11146
HORVU3Hr1G099360	0.158975	0.174611	0.186995	4.952518	5.979608	6.24178
HORVU7Hr1G085180	22.7194	19.59544	25.37253	48.78079	89.83211	73.33493

HORVU2Hr1G113260	16.4401	14.12013	15.20667	11.95682	4.264235	5.371665
Hordeum_vulgare_newGene_10249	0	0	0	3.405019	4.633302	3.558126
Hordeum_vulgare_newGene_10247	0.555972	0.536968	0.797014	1.862257	2.065476	1.965236
Hordeum_vulgare_newGene_2116	3.584089	5.323445	6.675653	2.089979	1.832863	2.459256
HORVU3Hr1G028020	9.883241	5.335927	9.821723	3.587281	1.01803	1.789193
HORVU1Hr1G004080	0	0	0	2.108773	1.662262	2.662841
HORVU5Hr1G119500	3.765966	3.979934	5.073255	0	0.041865	0.111713
HORVU6Hr1G048850	0.459416	1.020701	0.988633	1.948086	4.125938	4.316829
HORVU6Hr1G020310	35.69024	33.54608	51.09923	16.81614	12.04742	15.02016
HORVU6Hr1G077600	0.639898	0.85874	1.201239	0.334814	0.147538	0.197427
HORVU4Hr1G024470	8.047196	6.358061	7.839894	14.06625	32.57206	33.28189
HORVU3Hr1G094300	0.227742	0.315792	0.228844	1.109899	0.934444	0.774062
Hordeum_vulgare_newGene_1819	6.60478	7.848365	7.208993	0.478119	0.221911	0.216888
Hordeum_vulgare_newGene_5462	1.440567	1.461059	1.007891	0	0.011526	0.025698
HORVU7Hr1G045600	8.481222	6.43285	6.449351	2.651525	0.229329	2.608156
HORVU1Hr1G012170	1284.557	1390.594	1060.752	249.4874	8.348733	82.7942
HORVU0Hr1G021670	0.581664	0.59343	0.682673	3.273918	7.275393	6.757258
HORVU3Hr1G017570	1.104941	1.03014	1.07696	2.776785	9.061752	7.320877
HORVU5Hr1G073350	20.4808	20.61939	20.78268	54.14752	91.01876	79.14354
Hordeum_vulgare_newGene_3504	4.332663	3.63941	2.961302	10.69952	48.80093	41.26321
Hordeum_vulgare_newGene_3503	3.563594	2.608943	3.393136	1.589653	0.749366	0.882358
Hordeum_vulgare_newGene_9496	0.398198	0.584262	0.528927	0.755046	1.625559	2.361593
HORVU3Hr1G044150	1.087321	1.236373	1.583182	8.423767	3.986062	4.890508
HORVU7Hr1G003090	7.592749	6.737676	9.104289	22.54845	54.73947	52.48638
HORVU2Hr1G094870	2.17763	1.3689	1.392615	0.286421	0.132962	0.343085
Hordeum_vulgare_newGene_2670	0.635327	0.658769	0.277574	2.495972	3.201578	3.890245
HORVU7Hr1G078690	0	0	0.044992	0.951724	1.881622	2.067703
Hordeum_vulgare_newGene_5902	0	0	0	0.281675	0.763228	0.713971
HORVU4Hr1G077360	3.319616	2.882965	3.618599	9.765409	10.6056	10.89074
HORVU5Hr1G072980	0.086412	0.1873	0.279765	1.152684	0.77318	1.178884
Hordeum_vulgare_newGene_9597	3.2053	2.167168	4.429516	1.733909	1.79598	1.264156
HORVU3Hr1G117600	13.58193	9.171975	12.02299	2.869359	1.489366	6.854753
HORVU4Hr1G078650	28.21639	24.31062	22.85763	11.54904	11.67664	12.38644
HORVU7Hr1G062240	0.864974	0.854527	1.19614	1.741695	3.691303	3.220104
HORVU6Hr1G051070	0.632219	0.449051	0.703628	2.055492	2.911288	2.711077
Hordeum_vulgare_newGene_9422	16.95073	9.58228	13.84818	5.123435	6.217122	7.264865
HORVU3Hr1G010980	0.426931	0.539063	0.385898	1.931528	2.162957	2.704259
HORVU6Hr1G067200	2.41515	2.129344	2.788337	5.36301	16.60485	11.21977
HORVU7Hr1G027410	3.458616	1.790317	2.600563	0.652426	0	0.441215
Hordeum_vulgare_newGene_12566	1.537159	1.234383	0.833667	0.47884	0.483465	0.601871
HORVU1Hr1G079570	0.217882	0.178768	0.345649	1.118106	1.566181	1.292241
HORVU5Hr1G108870	0.551811	0.566451	0.415081	3.684109	6.036229	5.756155
HORVU7Hr1G019600	0	0.031872	0.050084	3.174424	3.805657	3.816464
Hordeum_vulgare_newGene_4946	6.612353	7.591629	7.083525	4.860866	2.90615	3.139206
Hordeum_vulgare_newGene_4941	0.707066	0.876613	0.513591	0.130485	0	0.092728
Hordeum_vulgare_newGene_3994	10.68594	9.107053	13.15031	38.66305	31.0596	25.63153
HORVU3Hr1G000910	10.17748	9.080981	12.66186	46.6477	80.42121	85.25585

Hordeum_vulgare_newGene_1849	2.180565	3.002567	2.01903	0	0	0
Hordeum_vulgare_newGene_6549	16.30005	17.27164	11.00653	6.073493	1.657762	4.463809
HORVU7Hr1G043360	1.399792	1.822281	1.239579	3.248735	4.482736	4.303188
HORVU7Hr1G109380	0.752129	0.77914	0.643242	3.130547	4.300669	5.121108
HORVU3Hr1G090050	0.655561	0.553416	0.595219	1.727878	2.656649	1.017994
HORVU2Hr1G061910	2.987218	2.400572	2.382136	0.942905	0.784194	1.109306
HORVU7Hr1G067620	79.96028	74.32196	82.2165	289.9767	1990.926	1532.659
Hordeum_vulgare_newGene_14241	0	0	0	1.470013	1.849471	1.648199
HORVU4Hr1G051740	0.780501	0.94448	1.120301	1.876182	2.95781	3.271772
HORVU7Hr1G069500	2.446127	2.810364	2.41696	6.081906	6.892188	6.549486
HORVU1Hr1G056500	2.206322	2.866618	3.243381	9.552577	10.74095	11.48456
Hordeum_vulgare_newGene_9101	8.179466	8.722201	9.902699	17.57905	23.38963	29.85487
HORVU2Hr1G027010	1.776812	1.647819	3.069217	4.883199	7.643921	5.314139
HORVU5Hr1G122180	0.556	0.525883	0.698343	3.238018	3.204553	3.049585
HORVU5Hr1G094840	8.438275	8.938776	10.03485	4.712574	4.819411	5.500945
HORVU0Hr1G012390	0.062391	0.050797	0.079266	1.096118	7.742251	7.052784
HORVU6Hr1G073080	3.014081	3.31695	4.168977	8.132506	10.18484	10.45499
HORVU5Hr1G010860	0.067352	0.057106	0.150917	10.81626	21.5607	16.41941
Hordeum_vulgare_newGene_1340	3.782568	3.365323	3.426182	2.410949	1.471607	1.845552
HORVU6Hr1G090560	31.56839	30.3527	30.81014	13.98835	8.806177	18.06031
HORVU4Hr1G063780	21.22944	10.72744	12.86575	0.024961	0.02169	0.059661
HORVU4Hr1G003640	12.28441	11.30659	12.84387	5.185896	2.861168	2.855971
HORVU7Hr1G076120	1.300471	1.900505	1.142612	0.366898	0.057874	0.168554
HORVU3Hr1G084510	0.466915	0.573961	0.666083	0.215381	0.268636	0.386227
HORVU0Hr1G017760	5.297562	6.221766	8.671714	2.418681	0.922602	1.337731
HORVU4Hr1G087110	0.091783	0.070226	0.103021	0.907987	3.342211	7.695911
HORVU2Hr1G103570	0.459879	0.439135	0.512585	1.011292	1.303623	1.436631
HORVU7Hr1G088780	8.97588	4.277628	4.564526	1.281078	0.150738	0.507701
HORVU7Hr1G075760	0.982941	0.557862	0.712719	1.719304	9.805024	10.38571
HORVU4Hr1G012400	2.222593	1.452629	2.367354	6.533187	4.776928	4.523478
HORVU6Hr1G004040	0	0.004842	0.010334	0.306384	0.359832	0.307147
HORVU6Hr1G018520	23.19866	25.89954	30.82522	15.44072	14.08027	17.90965
HORVU7Hr1G071470	0	0	0	13.02204	12.95923	16.30275
HORVU5Hr1G013880	0.169564	0.221747	0.394337	1.327967	1.201838	1.415231
HORVU5Hr1G046330	1.498994	1.027296	1.545225	5.536052	8.610543	7.319215
HORVU4Hr1G081070	2.625018	2.657582	2.817396	4.126685	9.739401	10.43784
HORVU3Hr1G099530	3.321419	3.336641	3.45998	1.348348	0.699202	1.413876
HORVU5Hr1G080500	39.69304	46.69802	54.34984	27.52355	21.71068	22.74842
HORVU2Hr1G001760	0.61712	0.746259	0.961161	0.202267	0.029861	0.203137
HORVU4Hr1G000610	0.752209	0.589001	0.742467	0.840393	11.45144	12.00355
HORVU5Hr1G123440	0.871222	1.150198	0.571178	2.462893	3.406081	3.030173
HORVU0Hr1G010800	4.542221	4.679717	5.474491	9.666677	22.42395	19.12369
HORVU3Hr1G098660	38.78662	41.62869	45.27819	20.58541	15.98533	19.0256
HORVU1Hr1G040150	1.368393	1.041364	0.657368	9.444738	28.04437	21.774
HORVU2Hr1G111690	0	0	0	3.605765	3.446487	2.910746
HORVU3Hr1G074660	23.55261	23.35803	26.93026	15.11114	15.93307	14.6396
HORVU6Hr1G050710	3.176448	3.483536	4.430704	7.316961	9.884727	10.42923

HORVU3Hr1G066340	41.61288	41.94111	57.5993	16.21823	5.966866	10.12059
HORVU7Hr1G021550	0.053876	0.205402	0.198926	0.716435	0.849384	0.975263
HORVU0Hr1G027030	1021.402	1258.621	897.5894	521.384	319.7362	446.8465
Hordeum_vulgare_newGene_11781	0.026929	0	0	2.047663	1.560591	1.793329
Hordeum_vulgare_newGene_146	2.556048	2.526959	4.240779	0.412611	0.085472	0.111842
HORVU7Hr1G063020	2.130262	2.415275	1.962419	5.689398	11.12293	11.62898
HORVU5Hr1G046490	27.66707	29.03688	30.12614	53.56772	85.65427	85.25101
Hordeum_vulgare_newGene_11434	7.743879	10.94108	9.423735	1.589006	0.789223	1.084818
Hordeum_vulgare_newGene_11430	0.719133	0.621438	0.610452	0	0	0
Hordeum_vulgare_newGene_11431	0.851901	0.716045	0.786429	0.325896	0.316724	0.479519
HORVU6Hr1G089130	9.984445	11.11599	7.843329	7.707606	5.450425	6.563598
Hordeum_vulgare_newGene_11438	4.611739	5.011281	7.141071	3.116714	2.632156	2.810761
HORVU2Hr1G072870	1.515764	2.058998	2.418047	7.02969	6.235017	8.849426
HORVU7Hr1G122580	0.049474	0.030536	0.034921	1.303452	0.878922	1.428457
HORVU2Hr1G087630	1.943069	2.457403	1.811128	4.373824	6.473871	7.02904
HORVU5Hr1G080990	0.350914	0.350379	0.347341	0.0762	0.22864	0.05498
Hordeum_vulgare_newGene_13554	0.013743	0	0	3.250847	7.29413	6.732881
Hordeum_vulgare_newGene_13557	0.76149	0.538904	0.584125	1.888354	3.086157	2.967959
HORVU2Hr1G114920	4.265616	4.459731	5.086244	9.789418	14.50962	15.48475
HORVU4Hr1G022280	6.957969	5.357007	7.127651	38.09077	24.00378	20.63065
HORVU0Hr1G005140	5.968129	8.04911	12.05078	2.35483	0.570386	1.30017
Hordeum_vulgare_newGene_6030	0.04891	0.094973	0.055837	7.015996	6.102561	7.777973
Hordeum_vulgare_newGene_7927	2.335842	2.030084	1.932403	0.69046	0.459565	0.283295
HORVU6Hr1G079640	1.276428	0.848483	0.627448	1.102029	5.237185	5.03122
HORVU4Hr1G073880	3.777769	4.135718	4.050348	2.442472	2.218151	2.479681
Hordeum_vulgare_newGene_5345	0	0	0	1.700433	1.053407	1.649331
HORVU6Hr1G061220	0.079998	0.032083	0	1.977756	9.672614	10.84636
HORVU1Hr1G020050	0.07693	0.070818	0.142419	2.170314	11.26987	9.590675
Hordeum_vulgare_newGene_914	2.904593	1.950877	2.501522	0.14017	0.095361	0.061299
HORVU3Hr1G013530	0.03649	0	0.105376	2.685692	14.05974	12.60489
HORVU3Hr1G044720	0.698727	1.431283	1.102255	3.734102	4.392554	5.708793
HORVU5Hr1G062240	2.89354	2.071004	1.603002	6.917741	7.521118	9.181936
HORVU3Hr1G004420	2.987496	4.757764	6.499774	2.281264	1.551655	3.246249
HORVU1Hr1G054380	3.717614	3.72108	4.537767	9.00335	11.39286	12.03877
HORVU5Hr1G034290	1.134043	1.07314	1.387972	0.633016	0.136468	0.287553
HORVU4Hr1G043990	5.956212	7.079638	6.481866	1.671809	0.798248	2.191061
HORVU1Hr1G073300	0.950847	0.868392	1.142819	5.140902	6.400873	7.126732
HORVU7Hr1G113290	38.90219	34.9053	47.38606	16.53726	8.170909	8.551589
HORVU1Hr1G018930	0.785195	0.677206	0.913455	2.278522	3.719275	3.34354
HORVU5Hr1G021340	1.582139	1.414307	1.722407	4.20891	6.376659	5.599488
HORVU4Hr1G019140	10.43849	14.68544	11.35984	29.49241	42.64038	45.42586
HORVU7Hr1G121830	1.415824	1.03476	1.051744	4.062973	4.150802	4.004138
HORVU5Hr1G092240	1.2111	1.602227	1.535103	3.375405	8.561199	8.565732
HORVU7Hr1G101260	8.083518	6.168578	10.14101	3.896088	4.731647	5.265703
HORVU1Hr1G057650	0.806461	0.729739	0.749276	0.061834	0.108502	0.078344
HORVU0Hr1G015520	2.571269	2.202582	3.116922	1.581895	0.99389	1.676358
HORVU7Hr1G093360	1.159994	1.307611	1.409764	0.263271	0.091579	0.180669

HORVU1Hr1G029540	0.568066	0.325175	0.234542	0.807883	1.930528	2.547085
HORVU2Hr1G124740	1.022487	1.758017	1.828507	0.180514	0.048165	0.048189
HORVU5Hr1G123680	5.594724	5.970012	9.697502	3.781418	4.447221	4.418331
HORVU4Hr1G052200	8.330895	7.269865	9.65109	42.37729	48.53987	47.09656
HORVU6Hr1G083180	2.840304	4.565699	3.930167	0.172954	0.302582	0.276368
HORVU7Hr1G025750	1.394213	0.997685	1.143603	3.624801	3.847917	5.380037
HORVU1Hr1G069310	1.630296	1.387337	1.965756	0.109738	0.0703	0.205777
HORVU5Hr1G036330	17.04767	18.39995	19.37643	11.96569	10.95982	10.11023
HORVU5Hr1G116860	1.69696	1.904101	1.719624	0.64702	0.975925	0.870332
HORVU7Hr1G121250	0.353843	0.13017	0.32643	1.347493	2.349631	1.167098
HORVU1Hr1G087530	0.259303	0.518551	0.416013	1.453823	7.7502	6.223966
HORVU1Hr1G081240	17.20009	13.50557	20.76007	5.588326	4.41129	6.30295
HORVU4Hr1G023390	3.561682	3.044682	4.228432	1.930507	1.457494	2.333255
HORVU0Hr1G016170	66.18811	56.08604	70.87624	39.08956	20.0229	20.81004
HORVU4Hr1G063600	1.613894	1.392103	1.836236	2.593768	5.248965	5.211347
HORVU7Hr1G096760	0.841826	0.512835	0.727039	2.792973	2.023717	2.01075
HORVU4Hr1G009310	1.374319	1.117735	1.277224	2.519297	5.19086	4.965327
HORVU4Hr1G063590	2.192577	1.405678	2.261221	5.844813	4.362729	6.37968
HORVU1Hr1G068830	16.97274	15.10233	18.60979	40.21554	57.69843	51.72568
HORVU2Hr1G076000	9.980508	12.04963	9.999305	23.71274	34.36204	29.84097
HORVU6Hr1G092840	6.041893	6.389655	8.435854	2.788627	0.800336	1.351346
HORVU7Hr1G109250	1.079275	1.358657	1.239567	3.436746	5.796288	5.375382
Hordeum_vulgare_newGene_12957	4.290841	3.556217	6.769576	11.55597	30.13774	29.66908
Hordeum_vulgare_newGene_12952	0.522073	0.577778	0.589832	0.294157	0.482487	0.245392
HORVU7Hr1G082990	0.544524	0.523934	0.405454	1.317626	1.8244	2.42123
HORVU5Hr1G119220	0.908532	1.215485	1.838615	0.613468	0.926814	0.722769
HORVU1Hr1G043500	0	0.021153	0	3.182764	4.852011	5.355645
Hordeum_vulgare_newGene_5516	121.932	97.47786	133.141	77.94006	59.17915	72.67371
HORVU2Hr1G107480	3.025309	1.298032	3.169697	20.10366	53.61451	56.3963
Hordeum_vulgare_newGene_2021	0.08725	0.110521	0.153146	0.385078	0.588736	0.626229
HORVU2Hr1G006170	1.421255	0.501451	1.49077	67.30919	0.89188	12.61716
HORVU3Hr1G081030	0.001913	0.001446	0.000211	0.455991	0.565773	0.742177
HORVU7Hr1G006180	36.87244	33.38071	37.20041	10.06572	11.07589	11.45465
Hordeum_vulgare_newGene_5553	1.07223	1.295777	1.429603	0	0.00538	0.053357
HORVU2Hr1G036710	11.754	13.62841	10.65967	6.464955	6.309137	8.373175
HORVU7Hr1G008050	0	0.088775	0.050798	0.373083	0.424654	0.764015
HORVU3Hr1G077530	0.208953	0.023622	0	2.988722	3.794657	4.160983
HORVU5Hr1G078160	2.846287	2.432169	3.188198	13.88551	37.05693	29.07168
HORVU4Hr1G085250	2.008464	1.682755	2.687885	8.322786	3.95506	6.586632
HORVU2Hr1G121090	18.96081	17.99515	21.30104	4.909069	1.144604	1.943136
HORVU4Hr1G060970	2.158311	1.857482	1.863187	5.507956	7.494752	7.519253
HORVU2Hr1G018920	0	0	0.015854	1.916536	1.456408	1.153714
Hordeum_vulgare_newGene_8106	0.491489	0.468308	0.639145	2.464786	2.086974	2.220226
Hordeum_vulgare_newGene_8100	1.832578	1.137625	1.576574	0.451634	0.769556	0.952781
HORVU2Hr1G072520	0.340228	0.675183	0.419041	1.899265	1.539242	1.647091
Hordeum_vulgare_newGene_5088	2.004467	2.114676	1.531711	0	0	0
HORVU2Hr1G025710	58.37769	52.86449	58.25552	40.75365	26.58072	29.41007

HORVU5Hr1G078830	0.058451	0.150182	0.129883	1.402841	2.084939	1.854358
HORVU5Hr1G100890	4.004047	4.239958	2.525178	0.718117	1.190441	1.616372
HORVU1Hr1G004130	9.001693	7.575674	18.70218	0	0	0
HORVU3Hr1G106880	0.463913	0.482462	0.572398	1.251009	2.692233	2.513917
Hordeum_vulgare_newGene_10624	40.89964	40.29427	49.92774	21.62199	19.30861	17.01819
Hordeum_vulgare_newGene_1709	11.97967	10.73301	13.97544	27.61613	38.62267	40.5206
HORVU2Hr1G096570	0	0	0	0.899288	0.977301	1.371786
HORVU5Hr1G015110	2.077692	2.598815	2.693987	5.349351	6.982279	6.960536
HORVU7Hr1G073170	2.574719	3.188214	3.995013	11.26467	26.14541	24.74768
HORVU3Hr1G069980	1.867651	2.031784	2.394176	4.426019	7.58321	8.342229
HORVU1Hr1G062590	2.030608	1.91893	2.543588	0.48594	0.715882	0.43411
HORVU1Hr1G025280	2.877424	1.431968	1.603504	1.580188	15.26989	13.38506
Hordeum_vulgare_newGene_7221	0.095602	0.214768	0.058008	1.147516	0.849951	1.135635
Hordeum_vulgare_newGene_7228	0.418402	0.335764	0.439675	0.983892	1.367927	1.507684
HORVU3Hr1G010460	0.548972	0.235945	0.471062	1.205351	2.539197	2.733627
Hordeum_vulgare_newGene_13744	3.838775	3.994741	5.269188	2.448495	1.985274	3.09429
Hordeum_vulgare_newGene_14537	0	0	0.008027	4.057116	3.157543	3.940655
Hordeum_vulgare_newGene_14534	0	0	0	2.573815	2.339604	2.40354
Hordeum_vulgare_newGene_13742	21.04694	18.30912	21.44903	40.81521	92.63596	90.44643
Hordeum_vulgare_newGene_14531	162.1867	106.7455	119.012	0.716532	0.023605	0.19148
Hordeum_vulgare_newGene_3038	1.037918	1.186008	0.738067	0.258757	0.218549	0.393007
Hordeum_vulgare_newGene_3033	1.257534	1.66365	1.784905	0.124142	0.131788	0.148706
HORVU7Hr1G030480	3.965278	2.518818	4.207465	1.428112	1.499452	2.121456
HORVU7Hr1G038300	0.924537	0.747122	0.803434	1.576093	1.843258	2.283351
HORVU5Hr1G085530	18.02644	20.35225	28.17496	12.93564	14.11683	13.70977
HORVU1Hr1G045110	24.29977	26.83209	24.68831	9.394115	13.36457	11.79875
HORVU3Hr1G068200	0.050053	0.037099	0.069671	1.492056	0.958351	0.97111
Hordeum_vulgare_newGene_9991	0.084172	0.086953	0.116872	0.796684	2.93799	2.663748
HORVU5Hr1G074740	1.71207	1.300902	2.777625	0.842842	0.573022	0.82387
HORVU3Hr1G052470	1.083594	0.793967	1.267086	0.633886	0.558195	0.718206
Hordeum_vulgare_newGene_15373	0.500916	1.870221	2.061331	8.851161	10.16974	8.037693
Hordeum_vulgare_newGene_2576	1.372984	1.618193	1.515074	0.01883	0	0
HORVU2Hr1G004510	0.730504	0.515272	0.75035	0.090953	0.021193	0.118737
HORVU3Hr1G015510	0.121461	0.087743	0.088637	0.577893	2.132404	1.89663
HORVU3Hr1G114200	4.700401	8.169562	8.956973	0.075664	0.130725	0
HORVU7Hr1G106010	0.269609	0.168331	0.131991	1.527694	24.1349	19.02987
HORVU7Hr1G096310	0.538719	0.872573	0.740937	2.039041	12.20584	10.5283
HORVU4Hr1G005300	14.53654	13.13842	10.84406	5.301802	7.659874	5.714096
HORVU3Hr1G031340	0.296219	0.351812	0.190179	1.128732	1.278047	1.297162
Hordeum_vulgare_newGene_1067	17.16317	15.34062	21.88423	25.45413	61.58352	63.10813
Hordeum_vulgare_newGene_4259	0	0	0	1.617359	3.396557	2.615755
HORVU4Hr1G015560	0.995243	1.047369	0.725799	0.112376	0	0.065797
HORVU1Hr1G072640	18.50591	21.42192	30.79289	10.59065	11.49588	10.5908
HORVU0Hr1G013010	0.529674	0.323549	0.331801	2.351023	2.735367	3.578226
HORVU0Hr1G014040	164.2646	152.6965	108.2322	31.00839	10.25731	18.02073
HORVU5Hr1G111350	34.20364	38.39754	50.58951	5.362555	0.103184	2.688591
HORVU4Hr1G004470	34.48836	33.05303	32.59523	25.74827	6.974334	9.916778

HORVU2Hr1G002600	18.47779	16.96095	21.15732	50.26735	73.80149	63.09657
HORVU1Hr1G068760	0.231498	0.114678	1.928359	6.240638	6.408284	6.422863
HORVU7Hr1G111310	0.758912	0.433068	0.428484	3.801048	2.155408	1.722702
HORVU6Hr1G006120	0.304843	0.154912	0.580492	3.94945	35.37183	35.94046
HORVU1Hr1G012910	1.132569	0.669315	1.58869	3.572639	18.54208	13.07115
HORVU3Hr1G078780	0.830314	0.546278	1.394722	4.011322	6.472817	6.618851
HORVU3Hr1G077180	1.04669	0.371953	0.598096	2.085729	3.385162	3.246688
HORVU1Hr1G080320	40.50752	44.78314	60.95738	27.87591	28.7182	33.3245
HORVU2Hr1G090120	0.317964	0.182532	0.318142	1.691543	6.387806	5.505883
HORVU7Hr1G091860	5.397146	3.572048	7.636725	0.391166	0.016885	0.531667
HORVU4Hr1G023210	4.715882	2.915611	3.425417	1.687967	2.506243	1.723263
HORVU2Hr1G125280	20.45264	14.19445	18.91211	46.52534	66.6712	56.49534
HORVU1Hr1G019000	0.158301	0.04664	0.062691	0.697359	0.33497	0.545646
HORVU7Hr1G040760	1.698848	1.694115	2.157097	0.135552	0.050466	0.077476
HORVU5Hr1G096930	12.19261	8.803013	13.45418	2.247888	1.857008	2.00432
HORVU7Hr1G045680	2.405388	3.1058	2.951055	6.447547	10.79817	9.884656
HORVU2Hr1G023820	0.434171	0.360892	0.132123	6.283453	56.43892	43.00178
HORVU1Hr1G038220	3.092306	2.031063	2.53212	1.935267	1.054258	1.602319
HORVU4Hr1G062890	1.41113	1.481479	1.21421	0.496158	0.954776	0.810007
HORVU1Hr1G018750	28.98383	29.4029	47.48691	14.73113	16.29097	18.33744
HORVU2Hr1G012340	1.28963	0.815692	1.514078	0.063854	0.0554	0.144774
HORVU4Hr1G090820	3.226631	5.366521	2.43135	56.36562	124.4481	92.03663
HORVU4Hr1G067970	1.68217	1.966147	2.355949	4.56366	12.97664	11.64307
HORVU6Hr1G011470	1.857857	2.081468	1.91487	0.830689	0.840367	1.096912
HORVU4Hr1G084730	19.1033	17.41027	28.14878	9.737578	12.87204	12.3188
HORVU4Hr1G018670	0	0.092814	0.047285	0.898177	0.957788	0.89476
HORVU4Hr1G079940	2.43495	1.840853	2.577265	0.898611	0.791329	0.652787
HORVU7Hr1G071770	0.277729	0.158308	0.352312	1.007182	1.329551	1.188452
HORVU4Hr1G015040	0.203967	0.194529	0.586234	2.529334	0.643939	1.195928
Hordeum_vulgare_newGene_3239	3.295742	2.701654	4.023552	6.873847	12.96944	11.35087
HORVU2Hr1G070310	39.45335	44.04267	56.58944	16.56542	1.780684	8.436344
HORVU0Hr1G039000	0.143766	0.537827	0.277095	5.991785	3.520825	5.673219
HORVU6Hr1G056910	1.223978	0.844994	1.141607	11.90789	31.98573	29.52396
HORVU7Hr1G028850	1.679297	1.394041	1.48204	7.627258	16.93422	15.28568
HORVU4Hr1G059240	0.897208	1.072172	1.217964	2.147441	3.395211	3.023971
HORVU7Hr1G002940	0	0.009131	0.004756	4.216535	3.810293	4.469132
HORVU5Hr1G083320	2911.136	2405.716	3419.647	211.3991	1.057262	83.82347
Hordeum_vulgare_newGene_13234	0.710811	1.030787	1.169759	11.9389	47.20323	36.39275
Hordeum_vulgare_newGene_13231	2.070481	2.101564	1.973398	1.058025	0.751352	1.376731
HORVU5Hr1G041830	1.656479	2.194362	1.888889	4.197566	9.55177	8.419375
HORVU2Hr1G036520	0.627866	0.564768	0.604807	0.201941	0.093502	0.070101
Hordeum_vulgare_newGene_15172	40.32	34.8437	31.10746	126.877	189.3304	188.5261
Hordeum_vulgare_newGene_15174	6.509893	7.775479	8.569821	3.861851	2.898899	3.33419
HORVU3Hr1G050320	3.745426	3.522162	5.468204	1.690287	1.946439	2.56652
HORVU2Hr1G116800	56.99367	47.2024	84.27082	26.48526	14.7693	23.50021
HORVU4Hr1G061900	3.176602	2.809214	5.562032	0.399378	0.743292	0.757795
HORVU7Hr1G026540	0.362686	0.30179	0.412273	0.904103	2.41959	2.436253

HORVU6Hr1G077520	1.164959	1.170329	1.372246	3.131933	5.657358	5.208976
HORVU1Hr1G001420	35596.82	42828.22	27697.91	474.793	169.9527	238.262
HORVU6Hr1G060250	0.232755	0.153465	0.253532	1.057071	1.265683	1.284318
Hordeum_vulgare_newGene_8376	5.995469	7.579045	7.58006	0	0	0
HORVU7Hr1G087750	0.88466	0.930992	0.999202	3.921907	3.814923	4.255432
Hordeum_vulgare_newGene_1517	0	0	0	0.466444	1.708312	1.94858
HORVU4Hr1G072620	1.035704	0.90507	1.529807	2.569805	6.41834	9.790562
HORVU3Hr1G066050	0.019277	0.121841	0.008594	0.775217	0.669225	0.580823
HORVU2Hr1G071860	8.520478	8.082068	10.80692	18.09697	31.6988	31.91021
HORVU4Hr1G066860	35.90151	29.9391	32.98529	72.82776	104.9344	103.8218
HORVU7Hr1G058630	22.30298	26.13368	23.74347	0.04405	0	0
HORVU7Hr1G001570	0.850313	0.920105	1.090092	4.501271	10.0389	9.132257
Hordeum_vulgare_newGene_15940	1.086314	1.143231	1.877439	0	0.11771	0.086746
HORVU6Hr1G070860	0.60207	0.926601	0.82861	7.860907	8.244194	9.588326
HORVU0Hr1G040130	1.693408	1.400601	1.003858	0.018069	0	0.040135
HORVU2Hr1G006830	0.617687	0.707908	0.61391	3.386238	4.978104	7.205738
HORVU1Hr1G015810	1.265719	1.102607	1.082943	0.803547	0.352079	0.509638
HORVU6Hr1G075270	27.05138	34.23327	55.41225	0.688699	0.051193	0.310079
HORVU7Hr1G034070	286.8922	365.4527	268.2407	267.0662	150.2997	142.6414
HORVU3Hr1G086360	0.557517	0.504703	0.649438	1.458867	2.606247	2.963499
Hordeum_vulgare_newGene_9819	1.943209	1.905405	2.016904	0.758771	0.327769	0.411798
HORVU7Hr1G116310	41.37866	33.07811	50.09745	26.02254	18.59248	23.03516
HORVU1Hr1G061010	33.65251	33.36512	24.15277	17.2443	18.30828	20.65187
HORVU3Hr1G055420	2.034978	2.03858	1.954019	4.560713	5.734273	5.81575
HORVU6Hr1G064140	19.21063	14.37496	20.24567	84.56504	176.5717	131.376
HORVU6Hr1G039610	0.633493	0.904993	1.480834	1.875209	8.553596	7.269232
HORVU6Hr1G071380	0.101767	0.022398	0	3.058195	2.744091	2.783873
HORVU6Hr1G081950	7.23842	6.300933	7.238564	3.785567	2.656935	3.470843
HORVU3Hr1G087010	0.176427	0.187303	0.28073	19.25911	26.00741	25.37136
Hordeum_vulgare_newGene_11033	0.097714	0.057291	0.035632	0.549155	0.212639	0.340854
HORVU6Hr1G071030	18.65465	19.23143	34.55024	0.084914	0	0
HORVU1Hr1G066240	7.137742	4.080555	8.740222	16.47884	19.39792	21.62199
HORVU7Hr1G097760	0.718277	0.688517	0.924183	3.130575	3.438993	3.885715
HORVU5Hr1G063600	0	0	0.060999	0.815724	0.90416	0.916924
HORVU5Hr1G109570	2.279755	1.311009	2.60141	8.712734	12.74152	12.75255
HORVU4Hr1G005800	1242.488	1205.837	1660.169	882.4343	537.4406	556.5245
HORVU6Hr1G031340	8.024799	5.539958	9.524959	20.75983	42.07457	35.8041
HORVU5Hr1G067380	0.598842	0.332288	0.820645	3.212269	16.77473	12.49503
HORVU2Hr1G098140	13.82035	11.74968	15.63145	0.463563	0.414538	0.266171
HORVU4Hr1G069860	5.602753	5.22476	7.300114	2.106959	0.204961	0.60701
HORVU0Hr1G013190	1.11231	0.878368	0.888208	9.561812	18.64575	16.41172
HORVU0Hr1G013220	5.808461	5.659602	6.224097	10.79878	23.19393	22.87718
HORVU5Hr1G080580	1.656931	2.140778	1.607008	5.838524	7.275207	7.606112
HORVU1Hr1G080750	17.08665	14.98731	17.1823	9.878181	9.509655	8.739593
Hordeum_vulgare_newGene_10601	1.689114	2.327147	2.336095	0.005407	0	0
HORVU7Hr1G110850	0.703676	1.124571	0.906829	0.242935	0.140881	0.231981
HORVU3Hr1G040230	0.248283	0.152044	0.203239	0.667368	2.749681	2.008754

HORVU7Hr1G012780	0.106574	0.330668	0.026097	2.515391	4.423957	3.797109
Hordeum_vulgare_newGene_10478	72.84698	84.52963	48.50163	36.36157	17.2946	23.82721
HORVU7Hr1G090100	26.57704	29.77577	28.04332	56.59682	73.73839	86.1422
HORVU3Hr1G078420	0.821083	0.611296	0.935592	2.85559	2.682221	2.26664
HORVU3Hr1G100190	35.21047	17.24921	44.10307	1.97119	0.26535	1.885149
Hordeum_vulgare_newGene_11705	5.293506	6.72308	7.746608	0	0	0
HORVU1Hr1G081770	0	0	0	0.968889	3.599872	4.450748
HORVU4Hr1G067340	1.387969	1.041838	1.3382	21.22825	27.716	24.62797
HORVU3Hr1G115020	4.379296	5.814121	7.003954	2.308407	3.452988	3.834688
HORVU3Hr1G107640	0.364081	0.235451	0.068345	0.862413	0.974626	1.297562
HORVU6Hr1G085750	1.23896	2.099345	2.127016	0.084782	0.047344	0.182183
HORVU7Hr1G121090	2.813754	1.218864	4.446173	0.184051	0	0.25376
HORVU5Hr1G072780	5.946761	4.000887	6.120186	1.415352	0.411212	0.525533
Hordeum_vulgare_newGene_3900	5.648305	6.370511	7.788209	13.43747	33.3352	28.1254
Hordeum_vulgare_newGene_16169	20.82026	28.52573	29.75719	7.834005	1.401534	3.919679
HORVU3Hr1G117390	9.724322	6.936517	13.14687	2.277426	0.943689	3.068535
HORVU2Hr1G065060	3.479215	4.68524	5.431329	0	0	0
Hordeum_vulgare_newGene_12423	22.9154	26.20856	26.78439	20.24491	4.506408	9.887296
HORVU7Hr1G105620	0.03289	0.01182	0.070308	2.13825	2.566413	2.05982
HORVU4Hr1G065470	0.421732	0.641333	0.53995	2.657387	7.474466	6.560342
HORVU5Hr1G084210	2.5678	1.370457	1.593844	0.051731	0.091056	0.047311
HORVU3Hr1G077800	69.77214	61.505	74.38222	32.90926	15.79249	23.60066
Hordeum_vulgare_newGene_14148	0.542062	0.561891	0.482698	2.00573	4.355828	3.372403
HORVU2Hr1G039930	1.308756	1.12703	1.506836	0.431274	0.355499	0.511496
Hordeum_vulgare_newGene_3792	0.163848	0.080583	0.225044	2.221458	1.004905	1.297264
HORVU7Hr1G072890	14.83428	10.82134	11.88847	6.868599	6.232724	6.042481
Hordeum_vulgare_newGene_446	1.349486	1.246276	1.505184	0.04048	0.025224	0.030315
Hordeum_vulgare_newGene_449	0.464904	0.6704	0.707511	1.888228	3.130475	3.130736
Hordeum_vulgare_newGene_10862	0	0	0	1.427517	1.315356	1.630613
Hordeum_vulgare_newGene_5829	1.578448	1.32647	1.76903	0.053553	0	0.025395
Hordeum_vulgare_newGene_2446	0.499078	0.53475	0.669324	1.417452	2.471228	2.316463
HORVU4Hr1G076970	0.790551	0.772657	1.435986	3.210287	2.695279	4.413924
Hordeum_vulgare_newGene_5796	6.357546	6.645283	2.866424	0.005767	0	0
HORVU0Hr1G002530	5.180278	4.884318	6.803095	2.315087	1.978872	2.67351
HORVU3Hr1G096120	1.029258	1.050577	1.313327	3.264811	8.619494	8.82457
HORVU7Hr1G100690	9.644236	8.202702	11.89251	19.08144	38.40686	37.00658
HORVU6Hr1G058000	1.029513	1.085881	1.505029	2.297545	3.662823	5.333215
HORVU4Hr1G077730	1.872318	1.930001	2.333609	0.44287	0.861193	1.409708
Hordeum_vulgare_newGene_1490	7.99856	8.318161	9.249734	0.149269	0.263165	0.202656
HORVU2Hr1G035220	1.943411	1.838917	2.109293	5.588145	9.79441	9.618212
HORVU1Hr1G072290	3.68442	2.95209	3.756757	7.645419	11.29179	9.664054
Hordeum_vulgare_newGene_7536	501.1295	357.5631	481.5068	1133.028	1285.416	1236.862
Hordeum_vulgare_newGene_7539	0.898182	0.970707	1.102738	0.297225	0.232255	0.304004
HORVU7Hr1G052580	46.23826	39.36336	50.0598	21.64398	17.87936	17.5023
HORVU3Hr1G086970	12.80261	12.24577	15.93131	10.52305	4.653618	7.359535
HORVU5Hr1G125120	1.241482	0.931063	0.82535	4.531609	5.799391	5.314116
HORVU7Hr1G025200	0.031711	0.044236	0.308591	1.903532	13.32935	10.40964

HORVU2Hr1G087370	0.096251	0.176854	0.200499	0.91636	0.859336	0.955144
HORVU4Hr1G001900	0.294003	0.313362	0.522708	1.970173	2.121819	2.042576
Hordeum_vulgare_newGene_6379	0.658765	0.78101	1.098683	1.297666	4.818008	4.912933
HORVU0Hr1G005260	1.942307	1.815628	1.567186	2.654425	6.735614	5.525224
Hordeum_vulgare_newGene_1026	1.564866	1.467981	1.591817	0.479149	0.406276	0.19289
HORVU1Hr1G080190	1.510761	0.909093	1.883666	8.394332	31.9673	32.12314
HORVU2Hr1G106880	0.6345	0.77296	0.773432	3.013157	4.820613	4.950311
HORVU4Hr1G069230	10.57087	9.937961	10.83925	23.65594	35.72599	33.0129
HORVU1Hr1G094250	2.620369	2.49108	4.28955	6.811041	10.68588	8.88573
HORVU5Hr1G105790	1.842814	2.621574	2.910245	13.78869	10.38252	13.19412
HORVU1Hr1G017720	32.47501	42.97479	37.03041	18.06362	11.89718	15.04191
HORVU6Hr1G085360	1.018022	1.241737	1.12979	7.306045	8.104512	7.927752
HORVU3Hr1G067620	11.98867	13.90304	9.619344	5.720427	5.612739	5.719962
HORVU3Hr1G084410	1.037834	1.402866	1.033254	3.008806	4.817171	3.625638
HORVU4Hr1G006840	0.510744	0.687266	0.694779	0.170032	0.140287	0.147299
HORVU2Hr1G111930	0.93255	0.708896	0.245748	2.634453	2.691307	3.039361
HORVU6Hr1G005290	2.081436	2.903076	2.074529	3.397935	7.452029	6.353037
HORVU4Hr1G008440	0.014863	0.08504	0	0.954866	0.826007	0.898563
HORVU4Hr1G015490	11.2829	9.963311	11.91293	7.496695	4.086281	4.662021
Hordeum_vulgare_newGene_11937	0	0	0.021244	2.977842	2.654164	3.127541
HORVU1Hr1G042450	0.690264	0.531992	0.702475	1.387813	1.807615	2.000897
HORVU1Hr1G043580	51.07557	32.60567	46.49478	114.9598	110.3451	111.8739
HORVU6Hr1G084010	13.68897	19.69822	27.07347	0.297085	0.0698	0.257506
Hordeum_vulgare_newGene_8245	5.725936	6.164958	4.458935	39.1046	62.52832	56.54102
HORVU3Hr1G047040	2.109918	2.457782	2.67659	5.183524	7.842047	8.796137
HORVU0Hr1G014170	5.566771	3.44141	4.263564	14.06818	17.64566	15.96015
HORVU7Hr1G108490	0.165949	0.095381	0.11916	1.074367	0.727135	1.618333
HORVU0Hr1G013200	1.151619	0.628129	0.713014	2.120199	2.887626	2.6662
HORVU7Hr1G095840	23.42492	23.64348	16.89881	3.892239	7.041018	5.192084
HORVU6Hr1G092150	0.079684	0.068516	0.158614	0.40825	0.677504	0.708666
HORVU7Hr1G081320	2.697403	2.502057	3.563361	0.194649	0.082581	0.15964
HORVU6Hr1G021260	0.134949	0.115526	0.098936	1.430411	9.777663	6.961062
HORVU7Hr1G026000	384.6098	363.2963	332.2006	244.0309	69.57467	99.17238
HORVU4Hr1G084390	44.47511	56.96735	73.84015	10.96475	11.073	11.92069
HORVU7Hr1G006280	240.4181	281.0885	247.6731	19.82417	23.00043	17.31944
HORVU7Hr1G077970	2.744113	2.41266	2.815905	1.175577	1.365604	1.670491
HORVU2Hr1G097180	4.693479	1.95273	2.510777	294.6074	223.3608	246.6664
HORVU3Hr1G074290	22.61724	21.63006	24.14851	10.29025	1.678516	3.539879
HORVU3Hr1G115180	0.068541	0.11257	0.033234	0.619831	11.37933	5.715116
HORVU1Hr1G000340	0.998241	0.494968	1.629436	10.33102	1.070306	6.038114
HORVU3Hr1G078670	6.340976	4.269848	10.52076	19.16975	29.20772	27.31301
HORVU6Hr1G056610	5.913885	5.660164	10.06881	2.313119	4.869488	4.611496
HORVU7Hr1G082670	1.176568	1.111364	1.112808	2.259245	3.803721	3.25185
HORVU1Hr1G073230	18.19461	17.87244	25.17248	12.15179	9.93571	12.23653
HORVU4Hr1G020950	2.904277	3.062221	2.709824	12.25501	13.55525	13.89152
HORVU2Hr1G122090	0	0	0	1.195367	1.195766	1.347442
HORVU2Hr1G036800	38.81948	33.72207	39.89524	13.47623	17.87694	18.57571

HORVU6Hr1G041020	5.976375	6.642733	7.609581	17.80681	35.0939	33.58879
Hordeum_vulgare_newGene_1961	0	0	0	1.042095	0.466449	0.530797
HORVU7Hr1G040250	0.108843	0.185439	0.160405	1.016541	5.724107	5.113415
Hordeum_vulgare_newGene_11573	0.98509	1.060764	1.251722	0	0	0
HORVU5Hr1G082230	0	0	0	0.436641	0.504796	1.052548
HORVU3Hr1G075500	2.929317	2.891914	5.437245	2.225581	1.851238	1.678195
HORVU5Hr1G105080	0.382232	0.383368	0.567843	1.125517	1.362675	0.906012
HORVU3Hr1G063050	6.907349	6.408509	6.18661	13.49021	24.6412	25.11348
HORVU7Hr1G098330	0.670159	0.641721	1.005288	3.885848	8.007701	7.942889
HORVU3Hr1G105720	0.14905	0.205594	0.37811	1.197963	1.088172	1.399604
HORVU2Hr1G018680	9.037036	5.601788	4.577461	0.975498	0.357317	0.584837
HORVU0Hr1G005060	17.28462	16.37986	15.93185	49.08476	47.30554	45.10269
HORVU6Hr1G079720	35.91763	27.82585	35.48767	21.55731	16.57653	19.25614
HORVU3Hr1G096860	29.44692	16.94989	29.74959	14.74811	4.889923	15.66718
Hordeum_vulgare_newGene_3611	12.79665	12.19209	7.956319	4.047477	1.481378	2.121196
Hordeum_vulgare_newGene_3614	1.721838	1.678465	2.409267	0.565895	0.957664	0.926324
Hordeum_vulgare_newGene_3615	0.440424	0.263032	0.36346	0.778001	0.942187	1.12891
HORVU7Hr1G028960	3.290494	2.588681	3.099709	1.464348	1.639373	1.600492
HORVU1Hr1G071760	0.112475	0.085302	0.328252	1.447624	7.524407	6.155355
HORVU2Hr1G120410	82.8441	78.98367	75.16788	43.31054	16.32654	21.07413
HORVU6Hr1G072370	40.49283	36.46954	48.62746	25.28291	25.52507	27.21154
Hordeum_vulgare_newGene_15666	0.754231	1.459664	1.163935	0.117314	0.0437	0.026429
HORVU0Hr1G009030	5.331959	4.755897	7.475087	2.272962	2.721141	3.447352
Hordeum_vulgare_newGene_8462	9.226962	9.205414	10.15428	3.081722	3.341561	3.399117
HORVU3Hr1G058440	7.721168	9.192144	8.267868	4.953109	4.578279	4.803828
HORVU6Hr1G064640	0.325894	0.389028	0.400183	2.025012	0.815028	1.294375
HORVU5Hr1G038630	34.19484	32.21401	28.81052	169.8278	299.6107	287.2537
HORVU7Hr1G030380	32.01027	24.98696	35.93543	16.93194	15.16277	17.72133
HORVU6Hr1G062360	59.00196	55.4351	98.42658	33.27447	43.69336	40.87613
Hordeum_vulgare_newGene_15087	1.410832	0.872352	1.349096	2.902568	4.911959	4.429013
HORVU5Hr1G096010	1.143757	1.271729	1.598769	3.527882	3.736472	4.41183
HORVU0Hr1G002090	3.655465	2.097044	3.627182	4.802275	12.26203	9.972025
HORVU2Hr1G080370	5.11939	6.624677	9.751509	2.058399	2.582199	2.903505
HORVU5Hr1G066750	0.288517	0.217177	0.268995	0.621148	1.96869	1.413963
HORVU6Hr1G066640	14.22922	12.64587	12.48607	6.647256	6.216923	7.599337
HORVU0Hr1G005840	1.404662	1.147486	1.818381	2.532163	5.689049	4.766122
HORVU7Hr1G021620	0	0	0	1.036761	1.172748	2.009506
HORVU4Hr1G072130	2.46514	2.633181	2.446329	8.383821	9.651717	10.03679
HORVU0Hr1G031170	1.786761	1.45566	1.859829	15.91598	8.819262	11.45292
HORVU1Hr1G078310	1.010268	1.458426	2.169086	0.42249	0.720478	0.629913
HORVU4Hr1G014220	0.916151	1.852308	1.909636	0.23011	0.431241	0.530065
HORVU2Hr1G059320	18.01267	17.60486	22.8241	9.446667	5.795341	7.450239
HORVU0Hr1G004060	0.639589	0.792939	0.992238	2.167052	2.640714	1.916024
HORVU4Hr1G039870	2.509593	3.461928	3.626094	9.970605	26.52775	26.91086
HORVU4Hr1G016280	2.942495	3.690777	5.123343	15.94815	13.93917	17.56087
HORVU6Hr1G005960	0.144057	0.175426	0.446966	3.741259	17.53037	18.38216
HORVU6Hr1G033540	10.96078	10.95385	10.06944	29.25132	43.76711	40.86555

HORVU7Hr1G097250	0.810366	0.866918	0.986916	3.701136	7.734884	6.624134
HORVU5Hr1G012120	1.351239	0.976076	1.372926	0.476182	0.157159	0.474345
HORVU1Hr1G065600	15.47115	8.411351	13.2702	7.493412	1.942949	2.904748
HORVU4Hr1G083200	7.906081	8.022616	10.63185	6.879768	4.200837	4.576734
HORVU7Hr1G040090	3.912805	3.792824	4.085712	11.36716	8.931677	9.872331
HORVU1Hr1G089530	7.649691	6.201521	8.095421	2.343117	0.145317	1.157713
HORVU5Hr1G113790	6.642155	1.583435	9.225501	0.215495	0.200905	0.273707
HORVU7Hr1G071800	16.91341	19.21116	17.81154	9.822398	10.65607	11.11547
HORVU4Hr1G089060	1.887522	2.549327	1.831377	7.665921	6.912142	6.283308
HORVU4Hr1G083000	127.8373	129.9767	106.2502	79.83729	62.07689	70.12008
HORVU5Hr1G109040	1.26565	1.41434	1.352907	29.62708	35.28474	31.68437
HORVU0Hr1G017100	2.648267	1.964233	2.535616	5.104882	9.797659	8.468391
HORVU7Hr1G077320	0.563332	0.412655	0.810654	1.134985	2.103437	2.515444
HORVU5Hr1G110680	3.21811	2.307118	2.691574	6.969456	31.85847	27.13661
HORVU4Hr1G009210	2.185081	2.219684	2.805588	9.029444	7.874467	9.651148
HORVU6Hr1G035370	11.0698	9.797006	10.91951	6.48395	3.612218	4.913366
HORVU5Hr1G056620	2.740679	2.561104	2.591421	0.832132	0.822761	1.55406
HORVU6Hr1G074830	0.236401	0.739946	0.401219	6.869466	7.832078	10.75675
HORVU3Hr1G033740	7.911524	6.158672	10.57063	4.443369	1.89327	4.341207
HORVU5Hr1G111480	1.557864	1.507949	2.291675	3.508114	4.94045	5.501457
HORVU5Hr1G095910	19.07075	16.70046	25.38319	47.1903	57.76677	54.03867
HORVU7Hr1G085510	0	0	0.056719	0.914884	1.269612	0.955603
HORVU7Hr1G025990	8.657168	8.158282	11.43895	22.65747	46.91903	42.13815
Hordeum_vulgare_newGene_2105	1.111833	1.305969	1.747086	4.533029	5.131151	5.913356
Hordeum_vulgare_newGene_2107	0.090516	0.070252	0.040032	0.59427	0.789543	0.6928
Hordeum_vulgare_newGene_2109	1.023913	0.743028	0.856445	0.020648	0.079846	0.072254
HORVU2Hr1G017310	12.08603	9.85398	9.355243	5.704757	3.910398	4.42023
HORVU6Hr1G037610	11.93349	11.35679	16.44792	7.416501	4.968281	4.944011
Hordeum_vulgare_newGene_9133	0.076846	0.045096	0.16593	0.516967	1.672412	1.090888
Hordeum_vulgare_newGene_9135	1459.593	1684.238	1065.126	170.9951	89.54838	95.9025
Hordeum_vulgare_newGene_5459	3.120852	3.101465	2.773485	0.455686	0.256731	0.263124
HORVU2Hr1G117820	1.802554	2.510153	1.815924	0.465945	0.235478	0.479117
HORVU2Hr1G002520	33.66979	41.0635	65.93125	7.838958	0.252707	4.097804
HORVU7Hr1G006530	0.572982	0.394979	0.371377	1.764432	5.194412	3.710263
HORVU5Hr1G019420	1.320097	1.779416	1.267456	0.053512	0	0
Hordeum_vulgare_newGene_13811	0.499761	0.398293	0.301804	0.921826	1.087586	1.694896
HORVU5Hr1G098290	1.524407	1.723502	3.272034	1.121285	0.397715	0.416765
HORVU5Hr1G046020	0.478425	0.227828	0.46624	1.594914	3.275917	3.416648
HORVU2Hr1G116880	0.170546	0.169552	0.282998	2.452189	2.206798	2.796676
HORVU4Hr1G020030	2.022091	2.539602	2.229029	88.84834	140.471	129.1126
HORVU2Hr1G092080	136.9465	128.1223	118.9279	305.1623	464.2601	430.05
HORVU5Hr1G073050	1.256769	0.734098	1.602131	2.858227	3.985151	3.296493
HORVU5Hr1G053480	0.838056	0.660663	1.035991	4.288934	6.405121	6.817115
HORVU2Hr1G036980	0.462357	0.618703	0.634903	1.106272	2.861389	2.775669
HORVU3Hr1G105880	2.471565	2.507872	2.424165	11.12261	39.08155	33.01051
HORVU2Hr1G119680	0	0	0	0.651961	0.351924	0.552252
HORVU3Hr1G068970	0.089195	0.098474	0.199067	0.457748	0.85727	0.677565

HORVU2Hr1G035020	5.763653	3.746434	6.785571	23.79788	30.91961	27.56475
HORVU6Hr1G025730	3.939836	3.914731	3.478463	10.22834	14.27875	14.20308
HORVU3Hr1G057880	4.362189	5.91646	7.400396	2.817625	2.954404	2.678611
Hordeum_vulgare_newGene_10545	0.755304	0.56787	0.667852	3.317166	1.812493	2.496362
HORVU3Hr1G088270	54.57237	42.21919	58.79545	124.1475	258.4976	184.0912
HORVU7Hr1G082350	0.41451	0.336217	0.485413	1.218889	2.188009	2.012097
HORVU3Hr1G094000	0.085447	0.149285	0.079914	0.708595	0.802366	0.720204
Hordeum_vulgare_newGene_569	0	0	0	12.93262	11.75189	14.6332
Hordeum_vulgare_newGene_9921	0	0	0	0.704169	0.803359	1.551893
HORVU5Hr1G070630	7.379346	7.138005	9.244845	4.882473	1.971715	2.710053
Hordeum_vulgare_newGene_14471	0.516077	0.609173	0.496571	0.034809	0.040936	0.051967
Hordeum_vulgare_newGene_14477	0.113511	0.125447	0.056691	0.949233	1.37375	1.423596
HORVU3Hr1G117540	3.26668	3.802732	3.695415	1.903837	2.168724	2.173849
Hordeum_vulgare_newGene_9892	0	0	0	0.785771	0.764456	0.695156
Hordeum_vulgare_newGene_9891	0.14469	0.028812	0.080176	1.569823	1.426765	1.524458
Hordeum_vulgare_newGene_15782	0.06467	0.422676	0.590486	41.12068	45.92429	51.95782
Hordeum_vulgare_newGene_15783	0.010151	0.009219	0.020322	3.807987	3.703182	3.769775
HORVU3Hr1G032540	1.764214	2.112578	2.335513	4.751601	6.038541	5.497044
HORVU4Hr1G021020	9.329668	11.86287	10.42024	5.573109	3.373499	4.16259
HORVU5Hr1G001170	0	0	0.05378	1.049389	1.313199	1.980855
Hordeum_vulgare_newGene_13178	2.046694	1.937148	2.084642	1.18782	1.148759	1.481894
Hordeum_vulgare_newGene_13175	2.767494	2.779788	2.408251	1.412661	1.067982	2.044238
HORVU7Hr1G087860	0.953172	0.743002	1.188598	2.803263	2.472641	3.095889
Hordeum_vulgare_newGene_9270	0.98379	0.623007	0.700355	0	0	0.133602
HORVU7Hr1G048710	1.482076	1.607327	1.284493	2.955128	5.708851	4.406088
HORVU5Hr1G052010	2.157068	1.646968	2.964383	5.189624	7.684374	8.148199
Hordeum_vulgare_newGene_826	0	0	0	0.866251	0.757604	1.205067
HORVU6Hr1G035210	0.200502	0.133388	0.26836	0.460254	1.481596	1.27238
HORVU4Hr1G089510	6896.114	9038.683	5359.943	1528.636	280.5358	592.1299
HORVU5Hr1G069480	6.714025	6.402766	7.286287	4.072816	3.321304	3.658543
HORVU3Hr1G085930	20.21876	18.0213	22.99232	12.39169	9.393384	12.25821
HORVU5Hr1G000150	6.70904	7.177968	9.054821	20.34326	35.02716	32.68618
Hordeum_vulgare_newGene_1383	0.270283	0.216499	0.21611	1.305935	2.59682	2.654895
HORVU5Hr1G105360	0	0	0	2.229833	2.545593	2.412785
Hordeum_vulgare_newGene_4352	0.131392	0.094638	0.162689	0.482076	0.505184	0.422788
Hordeum_vulgare_newGene_4350	0.279473	0.378521	0.182358	0.972733	2.007704	2.468657
HORVU1Hr1G048550	0.337095	0.33755	0.282749	1.120361	1.383718	1.171298
HORVU6Hr1G082900	0.874465	0.886303	1.04646	1.829713	2.700561	2.173891
HORVU0Hr1G001490	17.69416	16.29533	15.63494	40.03136	65.3648	73.60724
HORVU1Hr1G090030	14.49561	14.89143	14.57805	9.337178	5.718666	5.861778
Hordeum_vulgare_newGene_11386	13.36877	11.70164	13.44987	6.86862	8.446039	8.160851
HORVU3Hr1G034070	0	0	0.016316	3.52238	15.94838	4.560087
HORVU6Hr1G018830	3.859879	3.486524	5.135427	11.537	20.2308	18.46128
Hordeum_vulgare_newGene_16140	0.70108	0.268671	0.964024	2.247167	2.704785	2.232985
HORVU7Hr1G024240	4.517679	2.04003	3.757505	11.2332	17.298	10.87602
HORVU5Hr1G014500	0.43926	0.160923	0.469083	2.177974	8.750884	5.676329
HORVU2Hr1G083090	0.824609	0.582314	0.905568	0.283246	0.454363	0.458931

HORVU6Hr1G091650	0.106951	0	0	2.047462	4.386578	4.806382
HORVU3Hr1G006220	2.043187	1.553639	2.083991	4.828238	6.385706	5.742252
HORVU2Hr1G086910	0.190819	0.40788	0.347457	1.192537	0.903713	1.048195
HORVU7Hr1G049830	0.140975	0.137272	0.126412	1.19247	1.662688	1.747169
HORVU5Hr1G094450	1.186435	1.125105	1.00638	0.445852	0.148942	0.244398
HORVU7Hr1G059910	2.922621	3.255464	3.264383	0	0	0
HORVU1Hr1G021600	0	0	0.013534	4.539133	4.790506	5.189551
HORVU6Hr1G080410	3.322969	2.89507	4.806148	1.114718	1.277817	1.438254
HORVU5Hr1G016210	6.668687	7.917147	7.437945	38.19333	33.89094	44.08128
HORVU0Hr1G022230	1.304389	1.769528	1.588028	0.249187	0.671261	0.436828
HORVU5Hr1G007340	0.151163	0.121819	0.117995	0.44559	0.961515	1.178913
HORVU5Hr1G112630	0	0	0	4.564594	2.642145	4.220831
HORVU7Hr1G040680	0.461629	0.313368	0.677695	1.873446	1.919395	2.461886
HORVU5Hr1G049370	3.668075	2.662722	2.888972	1.246288	1.456891	2.268076
HORVU2Hr1G061610	17.1051	15.48814	25.49058	46.44765	63.68169	60.81954
HORVU1Hr1G018140	1206.225	1198.388	1488.555	459.8895	165.1964	228.1303
HORVU6Hr1G089750	0.215018	0.058539	0.06146	5.631138	7.650641	6.936388
HORVU3Hr1G027430	0.37882	0.292441	0.303507	3.37644	8.727001	8.889828
HORVU1Hr1G068940	4.28818	5.393544	6.013829	2.524213	2.077577	0.911098
HORVU4Hr1G052490	0.229009	0.201132	0.257654	0.689941	1.077598	0.82544
HORVU3Hr1G060150	1.651259	1.942467	2.299971	1.141533	1.102468	1.206386
Hordeum_vulgare_newGene_3464	2.572475	2.935595	3.126889	0.500926	0.450093	0.481814
Hordeum_vulgare_newGene_3468	1.056556	0.923201	0.466762	1.652044	4.738173	4.115011
HORVU2Hr1G019850	5.7744	6.512303	7.80255	0.011484	0	0
HORVU7Hr1G103180	1.083752	1.459817	1.102014	0.745054	0.641326	0.612598
Hordeum_vulgare_newGene_13371	1.198538	0.984394	0.695161	0	0	0
Hordeum_vulgare_newGene_13374	0.985576	0.63773	1.399965	0.483926	0.33642	0.52218
Hordeum_vulgare_newGene_15233	0	0	0.008845	1.68179	1.57897	2.222488
HORVU1Hr1G057180	0.045037	0.025292	0.017755	0.267249	0.4602	0.419809
HORVU2Hr1G101650	0.258243	0.201599	0.316513	1.307369	1.148498	2.196214
HORVU2Hr1G060730	0.025284	0.363002	0.130662	4.188776	14.06289	13.8357
Hordeum_vulgare_newGene_13994	9.9652	8.901343	9.526864	4.144192	4.505218	4.967672
HORVU4Hr1G014600	3.817704	4.624054	5.412323	1.613889	0.862781	1.186759
HORVU4Hr1G018180	8.881537	9.478545	5.67004	0.4336	0.043996	0.073824
HORVU2Hr1G067330	1.69243	2.048902	2.010714	18.3689	10.98446	14.91068
HORVU5Hr1G096390	7.999419	6.884984	8.903628	45.92753	188.1319	160.5299
HORVU2Hr1G060880	22.75523	18.84498	18.71717	49.71775	79.4047	76.90753
HORVU6Hr1G017390	11.75768	8.484629	13.87699	3.326195	1.266	2.770454
HORVU3Hr1G022900	1.769651	1.637936	2.62623	4.819068	4.440067	4.330541
Hordeum_vulgare_newGene_8036	0	0	0	5.60563	6.337034	8.329655
Hordeum_vulgare_newGene_14169	16.96881	13.76103	15.09735	11.45042	7.300513	6.862874
HORVU6Hr1G066210	2.077146	1.97328	2.536152	4.389427	10.90874	9.228747
Hordeum_vulgare_newGene_1412	6.2322	6.720968	5.292933	12.76104	16.81306	17.84545
Hordeum_vulgare_newGene_1415	1.802307	1.16047	2.490998	3.658519	8.224895	5.865057
HORVU3Hr1G014210	0.398866	0.298173	0.356241	1.106121	2.195472	1.365528
HORVU1Hr1G031250	0.476734	0.798135	0.455157	1.240449	1.659004	2.017704
HORVU2Hr1G021060	26.0962	23.86955	26.9995	13.54048	11.61627	13.21083

HORVU1Hr1G038330	6.371742	5.792863	6.803944	17.5737	31.08894	32.78755
HORVU1Hr1G025040	0.262608	0.302045	0.322748	0.032584	0.058562	0.108046
HORVU3Hr1G025400	0.307299	0.144478	0.245958	1.193384	1.191966	1.413626
HORVU7Hr1G056770	34.5784	34.1854	34.9516	89.57992	85.14	91.92469
HORVU2Hr1G044590	1.934028	2.208861	2.929556	0.647396	0.326942	0.642188
HORVU3Hr1G096710	1.601985	1.351529	1.186635	0.9115	0.589826	1.092282
Hordeum_vulgare_newGene_6668	0.601081	0.054803	0.332061	1.59767	2.839308	3.6892
HORVU5Hr1G019040	22.85604	24.50632	28.93833	12.99905	4.336608	5.698523
HORVU3Hr1G014960	0.225793	0.316364	0.256662	3.083288	6.048575	4.992133
HORVU2Hr1G081920	0.379024	0.433919	0.753104	2.630391	5.183424	5.404145
HORVU4Hr1G080810	1.148145	0.900159	0.844344	3.20479	5.046716	4.828257
HORVU1Hr1G063250	0.705428	0.621176	0.721617	2.240594	1.730947	2.260472
HORVU3Hr1G067110	38.23433	36.17597	33.11323	103.1237	127.3485	145.7944
HORVU3Hr1G069300	7.327271	7.404445	9.658277	0.034772	0	0
HORVU1Hr1G087630	0.625558	0.509942	0.8113	0.255027	0.114467	0.204504
HORVU4Hr1G013480	7873.012	8708.045	6102.377	3261.892	790.5816	1267.763
HORVU7Hr1G118560	3.522661	2.167672	3.483463	0.861255	0.519182	0.662672
HORVU2Hr1G043900	44.03361	34.43823	52.4123	8.394596	0.2733	2.755232
HORVU4Hr1G089230	0	0	0	1.173311	1.224593	1.064536
HORVU7Hr1G034860	11.6369	8.981365	14.5774	7.425953	6.660231	7.491387
HORVU1Hr1G003300	9.457995	9.664675	10.36376	43.66045	40.16742	39.42864
HORVU6Hr1G081140	12.50632	11.54676	13.70149	6.878744	6.406446	7.232682
HORVU7Hr1G092330	0.037319	0.023177	0.019558	0.306661	1.076022	1.421885
HORVU0Hr1G013830	1.461486	1.34948	0.956815	3.470426	3.011256	3.280116
HORVU7Hr1G028720	5.780216	6.212737	6.155467	1.127618	0.726783	1.084365
HORVU5Hr1G114470	0.395427	0.317738	0.513729	1.21043	2.898233	2.301172
HORVU4Hr1G088540	3.306833	4.445917	4.221958	1.496909	1.133942	1.335226
HORVU1Hr1G085960	0.003766	0	0.003605	1.153197	1.029265	1.602345
HORVU0Hr1G017330	0.417981	0.588949	0.821506	3.166706	11.76456	11.054
HORVU7Hr1G115500	1.132575	0.844643	0.786126	1.821234	6.090383	4.063151
HORVU6Hr1G080230	0.367462	0	0.385055	5.769456	6.952045	7.342795
HORVU3Hr1G009370	0.634526	0.668117	1.202177	85.01587	118.9015	144.0396
HORVU3Hr1G099470	2.4503	3.467705	4.205587	0.977102	1.246998	1.514132
HORVU2Hr1G080240	0.508556	0.5064	0.666788	0.554445	2.533735	4.167375
HORVU3Hr1G113120	329.9952	193.0342	298.5235	54.81056	24.36062	48.49359
HORVU7Hr1G110170	0.104541	0.063021	0.551506	4.777	16.5262	13.61586
HORVU5Hr1G113900	24.48095	22.09171	19.94853	51.34256	76.50446	77.38541
HORVU4Hr1G081800	2.180583	1.76179	1.904551	2.776977	70.63028	50.51658
HORVU5Hr1G045070	6.240418	5.530971	6.295994	12.29716	17.69393	17.33784
HORVU7Hr1G016130	0	0	0	1.699109	3.863705	4.183744
HORVU6Hr1G019350	0.555939	0.73172	0.545516	1.49753	1.764826	2.073183
HORVU3Hr1G030010	0.43024	0.538199	0.383645	1.243053	2.176599	1.792792
Hordeum_vulgare_newGene_10333	2.627808	1.893494	3.417432	0	0.051669	0
Hordeum_vulgare_newGene_10335	0	0	0	8.908936	8.514646	4.763036
Hordeum_vulgare_newGene_10336	1.960748	2.264352	2.603332	0.296676	0.366356	0.395623
HORVU1Hr1G090200	0.84502	0.544877	0.777825	1.543694	2.588799	2.614923
HORVU2Hr1G097670	0.024037	0.32205	0.087936	2.734028	4.367316	4.12862

HORVU6Hr1G038120	1.291615	0.987538	1.043922	2.144638	3.604888	3.907869
HORVU2Hr1G104080	51.89335	70.79362	103.749	8.984246	2.102143	6.746041
HORVU3Hr1G073630	1.962819	2.238147	1.953468	6.833448	8.49392	9.04043
HORVU0Hr1G012940	8.894773	7.91747	9.366433	4.632864	4.196982	3.116069
HORVU3Hr1G077740	1.822504	2.36918	2.89043	0.102906	0.079368	0.054756
HORVU6Hr1G094880	9.515111	8.658051	10.95821	2.872825	1.835864	2.601339
Hordeum_vulgare_newGene_4612	14.19958	15.75101	16.21591	0.009936	0.074608	0.167419
HORVU5Hr1G072350	2.887642	2.624825	3.905458	1.84939	1.274667	1.632217
HORVU2Hr1G110680	19.68233	22.06532	27.53796	12.32257	11.8817	13.98679
HORVU7Hr1G000140	0.511346	0.847623	0.819209	0.333586	0.212814	0.433531
HORVU3Hr1G112450	0.274534	0.203933	0.435001	0.911424	1.468061	1.594807
HORVU1Hr1G040670	4.534912	5.405161	5.22036	2.520574	2.801894	3.218307
HORVU1Hr1G061140	0.840709	0.701461	1.248821	0.14754	0.036389	0.16484
HORVU2Hr1G107970	0.023706	0.028459	0.05605	0.622724	0.58056	0.819867
HORVU0Hr1G009860	136.8891	125.0524	87.06975	18.32341	6.284533	9.110543
HORVU6Hr1G090250	1.594546	1.347972	1.533998	3.739917	5.201829	4.496629
HORVU3Hr1G037370	2.159653	2.830214	3.271865	0.033697	0.038652	0.029979
HORVU1Hr1G046630	1.394257	1.048976	1.498362	3.46627	2.267626	3.500643
HORVU3Hr1G030150	2.868531	3.368533	3.697495	14.1493	16.16344	15.44823
Hordeum_vulgare_newGene_16042	2.554869	2.041237	2.910672	0	0	0
Hordeum_vulgare_newGene_16045	24.38257	38.32677	13.32411	1.000567	0.254532	1.219183
HORVU1Hr1G038130	34.91725	26.51343	31.4553	13.0919	9.41202	11.06854
HORVU1Hr1G059870	373.6321	627.4566	1066.353	46.96448	0.272268	10.58529
HORVU5Hr1G076290	0.582	0.611277	0.74801	0.11289	0.326465	0.354026
HORVU2Hr1G012180	6.839466	6.43948	7.253123	3.774081	3.883619	4.150998
HORVU1Hr1G085500	0.798846	0.896545	0.833544	3.101238	1.611449	2.375994
HORVU5Hr1G005930	28.038	34.42818	38.99928	6.253967	0.897766	2.217909
HORVU6Hr1G088540	24.49525	17.69849	18.16104	5.871128	1.360786	1.861933
HORVU3Hr1G026950	0.806975	1.326863	1.819745	6.902489	6.541203	5.805714
HORVU2Hr1G068380	4.821735	4.135641	4.483365	9.419827	23.5018	17.52354
Hordeum_vulgare_newGene_12745	1.241701	0.330543	0.214644	3.693622	5.498202	4.60465
HORVU5Hr1G050950	124.86	128.4519	194.3076	24.53704	4.029646	9.656927
HORVU3Hr1G061560	5.007202	4.638415	4.521726	2.500324	2.095218	2.421729
HORVU7Hr1G038270	0.048377	0.1632	0.068809	4.242239	5.125329	5.605597
Hordeum_vulgare_newGene_3344	0.194645	0.151419	0.213604	1.163498	0.666107	1.093047
HORVU4Hr1G073920	0.984238	0.881213	0.802624	2.571666	4.982179	4.243138
Hordeum_vulgare_newGene_7847	1.90084	3.251493	0.793134	0	0	0
HORVU2Hr1G112860	1.51721	1.851278	3.329649	0.315735	0.334141	0.421624
HORVU4Hr1G054500	15.74745	12.5751	20.21151	8.34509	8.969357	7.612015
Hordeum_vulgare_newGene_13639	2.550967	1.507239	1.704465	0.144965	0.358391	0.335331
HORVU2Hr1G084750	7.941956	6.056418	7.269291	17.83185	22.98206	21.84867
Hordeum_vulgare_newGene_3690	27.5812	18.35918	36.24069	16.6277	6.805558	18.41592
HORVU2Hr1G019950	0.39027	0.574822	1.386033	3.649543	12.88344	8.939656
HORVU7Hr1G077740	23.31421	20.97139	20.72212	69.10218	116.5354	108.791
HORVU5Hr1G070240	1.446545	1.340652	1.474426	0.743589	0.510895	0.789013
Hordeum_vulgare_newGene_658	93.08111	86.62735	76.45526	28.33193	12.46884	17.99157
Hordeum_vulgare_newGene_654	11.58397	9.453248	15.24775	5.875462	4.667346	8.072051

Hordeum_vulgare_newGene_656	4.585257	4.233272	5.544919	13.95033	22.96768	20.33961
HORVU2Hr1G070400	0.012537	0.022541	0.010469	1.362126	2.801561	3.001586
HORVU2Hr1G063710	1.690958	1.482996	2.530648	0.700769	1.037207	1.348336
HORVU4Hr1G078010	0.060887	0.084673	0.21033	2.319072	4.647296	5.097229
HORVU3Hr1G050600	238.6552	221.3213	206.3814	134.5981	79.15684	89.85724
HORVU2Hr1G101750	0.377895	0.540028	0.302342	1.123951	3.655514	3.682886
HORVU6Hr1G079100	1.79731	2.145093	2.168773	1.119193	1.580566	0.598665
HORVU1Hr1G044780	27.30023	28.98036	32.42158	9.63412	2.972	4.559531
HORVU6Hr1G021780	0.212749	0.159594	0.097111	0.641038	0.642839	0.925099
HORVU6Hr1G015760	0.437026	0.374301	0.570328	6.8411	21.20153	20.10077
Hordeum_vulgare_newGene_5694	0.107395	0	0.279542	0.810607	2.730734	1.859494
Hordeum_vulgare_newGene_10073	0.823143	0.7352	1.022499	2.807235	2.720324	3.247138
Hordeum_vulgare_newGene_10072	0.906989	1.182233	1.264325	1.012891	0.570459	0.526022
Hordeum_vulgare_newGene_15009	0.131237	0.232143	0.147693	0.499293	0.593091	0.807769
HORVU3Hr1G093270	19.63261	20.16685	32.54826	2.544292	1.275591	1.783992
HORVU5Hr1G116770	0.091383	0.070898	0.103236	1.13538	0.825933	0.945838
HORVU6Hr1G053090	0.18586	0.104967	0.329161	1.305839	0.998183	0.994345
HORVU1Hr1G081570	2.023347	1.175202	1.583368	2.435525	6.406123	5.964708
Hordeum_vulgare_newGene_5039	3.491043	4.066656	3.507461	6.933984	10.24059	11.19754
HORVU6Hr1G075900	40.61963	36.02734	41.89821	109.4151	137.3261	151.5969
HORVU7Hr1G107670	2.761576	2.144409	3.070495	1.022396	0.213436	0.548883
HORVU4Hr1G047240	12.12188	10.87299	12.32239	6.084358	4.92307	5.57585
Hordeum_vulgare_newGene_4120	7.282142	6.518713	3.585915	0	0.01498	0
HORVU5Hr1G084900	1.258943	1.714381	2.487942	9.838619	15.23703	18.7505
HORVU6Hr1G077410	0.726171	0.611445	1.062884	3.937118	3.769809	3.53328
HORVU1Hr1G068010	2.876676	2.316995	2.36191	8.099749	12.6875	10.69955
Hordeum_vulgare_newGene_8200	1.03978	1.580815	1.288297	0.495216	0.086704	0.37931
Hordeum_vulgare_newGene_4784	1.752281	2.632652	1.992685	0	0	0
Hordeum_vulgare_newGene_4781	18.17073	20.43272	23.1786	2.434121	0.610252	1.067592
HORVU6Hr1G009690	1.290864	0.855269	0.888014	0.447742	0.539813	0.348929
Hordeum_vulgare_newGene_7478	4.292475	5.550981	4.64875	0	0	0
HORVU1Hr1G071840	4.561782	4.330645	5.972451	2.712254	1.117093	2.552858
Hordeum_vulgare_newGene_4872	0.136147	0.312936	0.318404	6.249616	4.968446	6.806355
Hordeum_vulgare_newGene_4874	0	0	0.018467	0.95368	0.955126	1.414008
Hordeum_vulgare_newGene_6789	2.173599	3.636552	3.023167	1.060453	0.723037	0.970562
HORVU6Hr1G029640	2.601707	3.00096	2.920659	13.51442	16.54142	15.16218
HORVU2Hr1G007980	14.19873	12.75891	14.10779	32.42198	54.85207	44.27061
HORVU1Hr1G086020	0.339352	0.243831	0.399427	0.740778	1.173294	1.203894
HORVU1Hr1G078530	10.46529	10.76226	15.5298	25.75351	71.81692	70.15881
HORVU3Hr1G030250	0.858847	0.888011	0.894121	5.001844	7.377596	7.957095
HORVU7Hr1G079190	2.767152	3.14997	3.043895	12.31656	24.48422	23.81694
HORVU3Hr1G017700	36.61774	35.04728	55.25598	25.54793	25.46212	27.95948
HORVU7Hr1G038510	1.947915	2.260994	2.020824	0.906255	0.592436	0.916463
HORVU7Hr1G098250	3.645553	3.317097	3.523382	0.262563	0.110863	0.168023
HORVU5Hr1G040000	2.984275	2.657939	3.053373	7.338267	12.17693	12.85379
HORVU3Hr1G081530	1.766664	2.710253	2.466809	0	0.020559	0
HORVU5Hr1G048660	0.096286	0.162374	0.16746	0.344861	0.612801	0.864995

HORVU6Hr1G078770	1.681258	1.658118	1.642143	4.558225	5.86933	5.781677
HORVU6Hr1G026840	2.146716	2.49676	4.529566	1.220381	1.68383	1.823589
HORVU1Hr1G051330	12.64382	10.04263	10.81441	29.29925	40.46197	38.74958
Hordeum_vulgare_newGene_2951	10.3126	9.320454	10.97769	17.475	51.97931	46.42019
HORVU3Hr1G116650	2.745319	3.134483	3.525173	7.237973	8.508866	9.307182
HORVU4Hr1G017370	3.980484	4.972387	4.392121	10.62111	14.1917	13.66235
HORVU7Hr1G116000	0	0	0	0.982209	1.343183	1.656587
HORVU3Hr1G037570	1.823581	2.088339	1.576768	0	0	0
HORVU5Hr1G088690	0.730621	0.889589	0.794231	0.501963	0.519704	0.728881
HORVU2Hr1G085160	47.13376	40.25333	45.78382	23.35728	15.49129	18.70954
HORVU3Hr1G012660	0.773611	0.687127	0.610219	1.317555	2.893302	2.295457
HORVU4Hr1G015330	1.657017	2.613209	1.840022	8.934457	11.12606	11.80921
HORVU1Hr1G058000	1.993929	1.656121	1.910646	4.9581	5.671805	5.852335
HORVU4Hr1G001680	1.186998	0.880637	1.508092	3.188921	20.92531	20.46157
HORVU5Hr1G066490	2.776383	2.477145	3.05568	12.62419	15.31251	17.17357
HORVU0Hr1G006380	1.199978	1.287841	1.671684	0.375752	0.083387	0.212833
HORVU3Hr1G077960	242.2651	212.7656	313.4381	150.342	137.8046	138.0594
HORVU1Hr1G042710	0.398738	0.540536	0.331719	0.715294	2.786026	2.304709
HORVU0Hr1G017690	2.432025	1.335228	1.66709	0	0	0
HORVU3Hr1G067020	3.083082	3.102209	3.399184	8.323583	18.51272	17.14738
Hordeum_vulgare_newGene_13740	0.02352	0	0	2.63581	5.672853	5.14068
HORVU4Hr1G085590	73.89411	72.10374	88.66663	46.51901	37.25673	39.2712
HORVU4Hr1G016810	266.5478	250.084	335.3196	178.0323	119.2952	161.0413
HORVU1Hr1G094420	0.043968	0	0	2.009046	1.572169	3.294588
HORVU2Hr1G017530	8.049451	6.050027	6.648432	0.600712	0.563161	0.417121
HORVU6Hr1G072650	15.3063	16.58104	32.46007	6.419208	15.96184	14.68648
Hordeum_vulgare_newGene_13892	0.048524	0.088845	0.176821	0.460058	1.958004	2.654796
Hordeum_vulgare_newGene_13893	4.012585	4.483622	5.281589	0.898492	1.287628	1.49035
Hordeum_vulgare_newGene_13898	0.819265	1.023952	0.743702	1.870837	2.330359	3.068184
HORVU4Hr1G052060	0.164162	0.149743	0.105682	11.61316	18.26945	14.71643
Hordeum_vulgare_newGene_10276	0.182125	0.242503	0.095881	3.155251	1.734621	1.509235
Hordeum_vulgare_newGene_10279	0	0	0	5.049405	3.986327	5.167809
HORVU1Hr1G095410	106.9901	114.8206	104.6702	55.77916	54.11126	56.0946
HORVU7Hr1G055180	0.145798	0.149499	0.105229	1.130785	1.560495	1.339247
HORVU5Hr1G042370	5.038553	3.378063	5.075598	1.750878	1.028845	0.857022
Hordeum_vulgare_newGene_5425	1.726589	2.493393	2.208943	1.453702	0.566956	0.946305
HORVU1Hr1G080510	0.401115	0.432414	0.338573	1.710622	1.194647	1.431365
HORVU2Hr1G119600	3.886027	2.739771	2.812094	9.338768	35.82847	30.60774
HORVU5Hr1G006850	2.887986	2.297521	2.332581	5.267941	7.408283	7.154173
HORVU5Hr1G099670	2.092288	1.962689	2.374224	0.760654	0.292768	0.76904
HORVU4Hr1G066900	0.703008	0.909407	0.668807	3.644881	6.384505	6.562734
HORVU6Hr1G063250	3.457646	4.985596	4.678938	10.18086	11.04795	11.78453
HORVU5Hr1G097460	0	0	0.991604	68.82827	22.73581	28.50967
HORVU7Hr1G083080	0.476948	0.55146	0.639183	2.269683	4.63535	3.995926
HORVU7Hr1G001600	1.192064	1.164762	1.305747	17.77568	22.68699	24.57997
HORVU2Hr1G114720	0.94389	1.398457	1.319047	4.464135	6.789593	5.597923
HORVU3Hr1G093850	35.91633	31.43358	36.89342	22.8924	19.88997	18.49963

HORVU3Hr1G068650	0.133541	0.187625	0.040141	0.341416	4.568054	2.590173
HORVU3Hr1G100360	8.491461	7.532074	10.73929	4.500429	1.8315	2.425014
HORVU7Hr1G042120	0.705794	0.666751	0.759243	6.539962	6.447331	11.19042
HORVU1Hr1G075830	2.48238	2.444406	3.311329	0.877159	1.012659	0.945523
Hordeum_vulgare_newGene_6236	2.720363	1.578679	2.146855	1.528468	0.400321	0.755229
Hordeum_vulgare_newGene_13469	1.924995	2.339497	2.527759	1.560516	1.054128	0.897935
Hordeum_vulgare_newGene_13462	0.821209	0.963219	0.861407	3.333386	3.5877	3.232978
Hordeum_vulgare_newGene_13466	0.476715	0.531711	0.390742	9.144319	7.600208	7.982005
Hordeum_vulgare_newGene_3555	0.510953	0.239739	0.355597	44.12431	50.91604	50.5968
Hordeum_vulgare_newGene_3554	1.634717	1.670962	1.555934	1.228434	0.757387	0.490461
HORVU6Hr1G027620	7.719891	7.546509	11.22963	25.9355	47.11208	50.94122
HORVU7Hr1G027960	17.43998	17.14585	11.78582	0.673131	1.017218	0.984305
HORVU3Hr1G113620	111.0885	93.11264	122.3353	59.40536	23.79199	57.41203
HORVU1Hr1G043900	2.158336	1.518927	1.349838	7.386111	12.09954	9.951747
HORVU5Hr1G115360	2.875432	2.781143	3.601868	9.564424	12.87952	11.86901
HORVU3Hr1G069210	56.40402	47.62071	84.78027	49.12154	21.75277	23.64243
HORVU5Hr1G023020	32.69467	30.92527	33.30961	16.79236	14.17736	16.27852
HORVU7Hr1G078660	2.031302	1.996098	2.161085	1.130942	1.044446	1.339397
HORVU3Hr1G059610	1.307224	1.479831	1.336716	3.053321	4.107274	3.853761
Hordeum_vulgare_newGene_6903	0.229467	0.37742	0.241979	1.306655	1.06758	1.026683
Hordeum_vulgare_newGene_8503	0	0	0.061212	4.041769	14.36101	10.88014
Hordeum_vulgare_newGene_8500	0	0.029537	0	1.276073	5.087981	7.629359
HORVU3Hr1G111080	5.77411	10.20596	6.114169	29.67635	394.7747	306.321
HORVU0Hr1G019690	3.019114	2.054482	3.295866	1.503937	1.422409	1.359784
HORVU1Hr1G073680	0.056922	0.010937	0.14653	2.328882	9.642426	8.53206
Hordeum_vulgare_newGene_6354	1.154544	0.691839	0.736558	0.190456	0.427367	0.333638
HORVU7Hr1G114750	0.540317	0.109545	0.376579	1.76963	1.722604	1.666249
HORVU2Hr1G080430	1.831903	2.352975	2.35752	0.800497	0.935885	1.086825
Hordeum_vulgare_newGene_6358	0.732845	0.641112	0.84059	0.516755	0.428257	0.388114
HORVU2Hr1G089310	7.000377	6.759777	10.16403	5.883478	3.254573	4.857247
HORVU3Hr1G019140	0.532404	0.654785	0.747043	2.730221	6.352913	5.966818
HORVU5Hr1G018160	3.981412	3.899964	4.724823	9.869671	14.02568	14.81471
HORVU5Hr1G010350	1.378771	1.534296	2.057043	6.716135	12.23739	10.38595
HORVU2Hr1G032220	4.171656	2.839211	4.196135	4.052859	26.23478	12.21216
HORVU5Hr1G034810	6.861407	5.162452	5.709493	9.272623	21.68621	22.56909
HORVU3Hr1G070660	11.71809	11.80176	13.95505	4.790857	4.916054	5.325358
HORVU4Hr1G073380	0.787572	1.322364	1.487324	2.061915	4.639768	4.93283
HORVU2Hr1G120200	0.552895	0.720465	0.521713	0.318973	0.074823	0.193197
HORVU6Hr1G019700	0.653127	1.012288	1.128447	2.450638	4.502749	4.858684
HORVU5Hr1G097270	3.616944	1.975021	3.533777	0.395142	0.139313	0.243573
HORVU7Hr1G088510	13.27777	15.50055	14.46311	4.606176	7.870083	8.751709
HORVU4Hr1G088860	7.159433	7.312794	7.969713	8.50786	32.75894	26.51976
HORVU7Hr1G018580	9.159267	10.4893	14.64583	3.161249	3.039497	3.8062
HORVU5Hr1G014320	0.924084	0.930864	0.978451	1.991385	3.67218	3.818597
Hordeum_vulgare_newGene_6572	0	0.015776	0.081035	0.550495	0.829119	0.585467
HORVU6Hr1G073620	7.506573	8.305285	10.32836	39.28776	59.08603	46.39964
HORVU3Hr1G020830	7.036515	4.717465	6.15853	4.312516	1.86044	2.190006

HORVU5Hr1G012240	0.519833	0.346912	0.456341	1.089733	0.937131	2.46952
HORVU1Hr1G055370	3.300785	3.744675	3.714592	8.18631	13.86002	13.97122
HORVU5Hr1G123770	38.3468	38.73931	40.02734	18.04882	15.6618	16.54334
HORVU5Hr1G002110	0.450293	0.746173	0.775467	5.760065	5.404691	5.931311
HORVU7Hr1G011250	15.31894	17.77457	14.87513	12.27246	4.80293	8.266682
HORVU3Hr1G117550	7.451132	5.549939	6.719184	1.916003	0.737904	2.160537
HORVU2Hr1G091590	26.67739	23.1112	21.74726	13.71257	7.62737	8.291084
HORVU3Hr1G082380	1.855347	1.473469	1.615984	4.285578	4.69651	5.027196
HORVU3Hr1G078860	0.512676	0.274135	0.465804	0.99935	1.138422	1.181949
HORVU1Hr1G020240	1.766405	1.823348	1.626527	9.295461	12.46339	12.51085
HORVU3Hr1G016240	3.787926	4.369625	4.319222	7.971907	13.48622	12.63949
HORVU3Hr1G029610	1.017765	1.062488	0.960615	0.26922	0.3773	0.786431
HORVU4Hr1G068560	0.461824	0.458734	0.48883	0.812824	2.206471	1.518717
HORVU5Hr1G069360	215.4836	201.4757	302.1937	145.5821	61.27162	83.2192
HORVU1Hr1G008650	0.807924	1.216302	1.563066	0.510275	0.057963	0.177746
HORVU6Hr1G052420	13.53966	11.09821	14.37512	27.25529	46.03109	38.65383
HORVU5Hr1G066350	0.341002	0.300043	0.218889	0.622646	0.959668	1.113064
HORVU0Hr1G016840	0.371374	0.362484	0.324361	0.750725	1.451964	1.316029
HORVU3Hr1G102340	0	0	0	0.578304	0.849373	0.759769
HORVU1Hr1G090670	14.0014	9.407172	12.23646	5.688209	2.378415	3.005834
HORVU7Hr1G076150	9.615464	8.098483	9.394417	35.64974	128.7928	127.8208
Hordeum_vulgare_newGene_9484	0	0	0	1.378959	2.209894	3.612416
HORVU7Hr1G029170	1.831998	1.48772	2.008397	3.648241	5.006939	5.619701
Hordeum_vulgare_newGene_9481	0.629021	0.918053	0.899775	0.487017	0.403158	0.617582
HORVU7Hr1G042390	0.618606	0.318095	0.941839	42.88122	38.2679	40.38078
HORVU2Hr1G108600	6.069498	6.441129	10.30235	0.190517	0.235929	0.115073
HORVU7Hr1G082380	10.92333	12.50301	16.21026	4.472347	3.239384	3.747342
HORVU6Hr1G021460	0.503278	0.789726	0.530086	2.482402	2.640697	3.168712
Hordeum_vulgare_newGene_13915	0.058055	0.092998	0.293012	2.205248	9.445185	5.933243
Hordeum_vulgare_newGene_13911	2.034883	2.258837	2.583907	0.664373	0.696327	0.71449
Hordeum_vulgare_newGene_13913	30.7353	31.47899	25.70707	12.52331	9.109298	10.01347
HORVU2Hr1G118210	0.194341	0.146409	0.115695	1.78399	1.782834	2.232547
HORVU5Hr1G117610	0.405963	0.391544	0.414091	1.414931	1.909642	1.53152
Hordeum_vulgare_newGene_8741	3.167788	2.167342	3.549096	1.481776	0.489911	0.656088
HORVU5Hr1G073310	3.67534	3.556176	3.881615	1.803171	1.095253	1.192458
HORVU5Hr1G072920	20.87582	17.35026	18.06795	11.57134	6.349123	8.093795
HORVU7Hr1G081250	0.174185	0.326835	0.191578	1.298009	0.871294	1.548035
HORVU5Hr1G093700	8.689037	12.91925	15.10418	53.75076	464.8806	436.3623
HORVU7Hr1G011750	1.776148	1.754875	1.876074	0.633701	0.950147	1.035242
HORVU2Hr1G108380	0.888278	0.670709	0.990141	2.937783	2.058846	2.156026
Hordeum_vulgare_newGene_14214	0.758498	0.434763	0.88785	3.937829	2.84836	5.704885
HORVU0Hr1G016430	1.889436	1.309021	1.638694	25.24997	44.23107	39.99208
HORVU3Hr1G054810	3.010119	2.849896	4.452686	7.239124	14.47454	11.41125
HORVU3Hr1G010190	7.603583	6.050126	10.14352	4.750842	0.813763	1.920199
HORVU1Hr1G038620	0.524924	0.384114	0.638939	0.935006	1.468203	1.762518
Hordeum_vulgare_newGene_11407	2.081655	2.305206	2.005621	0	0	0
Hordeum_vulgare_newGene_11406	0.629019	0.690589	0.596827	0	0	0

HORVU3Hr1G075210	0.272796	0.237256	0.495401	7.064623	6.634956	7.65863
HORVU6Hr1G054110	1.227421	0.898684	1.662331	2.253915	3.884627	4.085639
HORVU3Hr1G105430	5.13559	3.189472	4.304793	10.53389	10.61927	11.80289
Hordeum_vulgare_newGene_13584	2.991164	2.863375	3.150792	8.522071	13.51873	12.08749
Hordeum_vulgare_newGene_13589	2.114652	2.224037	2.232799	0.258617	0.278396	0.282954
HORVU2Hr1G114910	43.69108	29.22924	61.39103	12.96296	7.449313	6.82252
Hordeum_vulgare_newGene_12189	2.297343	2.78426	2.359698	4.173979	11.11667	11.95551
Hordeum_vulgare_newGene_12188	2.338649	1.883384	1.820992	4.235774	10.68844	10.11746
Hordeum_vulgare_newGene_6000	5.673484	4.418269	4.799017	0.024663	0	0
Hordeum_vulgare_newGene_6009	33.36972	20.72864	27.81827	12.46724	5.759283	3.493675
HORVU6Hr1G064350	0.175086	0.242784	0.674583	1.547918	2.332613	1.928386
HORVU7Hr1G025460	6.404282	7.529718	8.719901	2.189945	3.546431	2.312124
HORVU1Hr1G071560	0.466506	0.592674	0.547483	1.217826	1.692236	2.006754
HORVU7Hr1G108110	0	0.00686	0.008321	2.586446	2.699748	3.46724
Hordeum_vulgare_newGene_2273	0	0	0	5.654665	6.36692	7.221462
Hordeum_vulgare_newGene_2278	2.38845	3.531612	3.144714	0.00982	0	0
HORVU2Hr1G073840	2.784166	2.760297	3.534696	0.57674	1.134805	0.747026
Hordeum_vulgare_newGene_13635	1.411575	1.510051	1.418204	5.195366	8.112419	8.182911
Hordeum_vulgare_newGene_13637	0.094679	0.17351	0.169195	3.221285	2.870213	2.940387
HORVU2Hr1G116510	0.007203	0.03813	0.045111	34.66131	129.9995	89.64554
Hordeum_vulgare_newGene_7978	7.135801	7.257251	4.595117	3.278641	3.837235	4.336068
Hordeum_vulgare_newGene_7972	5.590544	5.943265	6.169949	0.688624	0.142259	0.379565
HORVU5Hr1G103560	2.921647	2.924567	2.79898	1.944393	1.434939	1.398313
HORVU2Hr1G046550	18.13978	12.41739	10.81461	2.546816	1.932873	2.324435
HORVU1Hr1G020000	0.886053	1.033578	1.138522	3.42281	6.284699	6.926484
Hordeum_vulgare_newGene_5727	0	0	0	0.517144	0.675399	0.689133
HORVU2Hr1G075850	7.007071	7.19641	7.638342	4.785657	2.916438	3.588162
Hordeum_vulgare_newGene_949	1.036525	0.806515	0.796391	2.922567	3.393304	3.174589
Hordeum_vulgare_newGene_941	0.287222	0.595091	0.455805	1.471226	1.517541	1.670804
HORVU1Hr1G091540	0.577828	0.505447	0.367567	1.152375	2.299934	2.023431
HORVU4Hr1G089410	1.551129	1.249343	1.418514	3.521706	4.830227	3.903202
HORVU4Hr1G002010	34.7835	35.58572	35.28525	17.18676	22.23859	23.11305
HORVU3Hr1G015680	0.057816	0.030468	0.146556	0.670316	1.025951	1.438984
HORVU7Hr1G107780	3.501759	3.448236	4.041604	12.19577	15.04218	14.65014
HORVU7Hr1G025700	0.300595	0.159416	0.236885	4.029255	5.419605	5.94998
HORVU1Hr1G052960	0.46226	0.555087	0.60811	1.466221	1.623489	1.48565
Hordeum_vulgare_newGene_6261	0.989024	2.712189	2.179928	0.255367	0.275645	0.249604
HORVU2Hr1G031130	0.914288	0.499069	0.739288	4.061472	10.1871	10.83423
HORVU7Hr1G057760	3.695466	3.597028	3.700023	8.226587	16.04644	14.47375
Hordeum_vulgare_newGene_4099	9.20337	9.041578	12.74407	0	0	0
Hordeum_vulgare_newGene_1115	0	0	0	0.907463	1.514504	1.365658
HORVU2Hr1G080570	0.592259	0.509451	0.510492	0.14859	0.318974	0.342767
HORVU1Hr1G063620	1.831865	1.480824	2.103743	15.23749	12.51615	14.38176
HORVU5Hr1G105840	94.67505	83.3679	123.6937	42.25416	30.73178	24.19785
HORVU7Hr1G094640	232.8048	216.1044	281.6699	137.1361	129.7568	122.9569
HORVU4Hr1G057210	7.73692	5.786682	9.433281	15.8805	34.48789	33.81618
HORVU3Hr1G005410	0.492344	0.60065	0.8549	1.138288	2.590261	2.472649

HORVU1Hr1G083380	12.41783	9.608669	14.94323	4.164373	3.40259	3.769001
HORVU4Hr1G076350	1.50745	1.827001	1.546643	4.944192	7.24487	7.295394
HORVU7Hr1G098490	18.34417	17.1045	22.59609	6.04414	3.063046	4.373275
HORVU2Hr1G041110	0.355673	0.387912	0.546635	0.960411	1.3848	1.369399
HORVU7Hr1G019390	1.958723	1.296002	2.076433	0.764451	0.38507	1.183979
HORVU6Hr1G006090	0.354614	0.229129	0.56095	3.846634	16.68731	16.72121
HORVU5Hr1G125460	156.9445	180.253	236.3555	51.32926	3.058841	25.63485
HORVU3Hr1G031940	1.131101	1.090314	1.158244	12.47642	12.33371	16.50311
HORVU5Hr1G002090	1.406479	2.133308	3.159377	0.405395	0.019602	0.069428
HORVU1Hr1G090820	23.93076	23.27786	25.19109	7.976773	3.025361	4.681549
HORVU5Hr1G065920	45.60505	37.05935	42.06534	6.17353	3.069939	3.500118
HORVU4Hr1G006720	2.255815	3.407294	3.356693	2.065045	1.650749	2.128989
HORVU1Hr1G080120	0	0	0	0.572503	0.614433	0.548915
HORVU2Hr1G050410	0	0.131908	0	1.733992	5.880748	4.18005
HORVU6Hr1G034620	0.603516	0.330562	0.747877	1.860159	5.905181	7.628534
HORVU5Hr1G116850	0.097343	0	0	4.778791	5.971051	5.704961
HORVU3Hr1G035650	3.207327	1.999511	2.637022	12.48984	17.86112	16.32258
HORVU3Hr1G018740	1.671668	1.453711	1.778695	0.614018	0.353074	0.82061
HORVU5Hr1G009510	1.971751	1.873029	1.35681	0.471163	0.495693	0.538973
HORVU3Hr1G037040	12.10013	11.94261	18.21058	5.981471	6.460528	5.972678
HORVU4Hr1G002350	0.355455	0.341141	0.481525	2.555896	4.889418	5.373452
Hordeum_vulgare_newGene_13513	2.618874	2.603485	2.933958	1.51	1.499577	1.600866
HORVU0Hr1G016140	0.264958	0.202258	0.546593	0.798909	1.516029	1.231706
HORVU2Hr1G113300	4.200274	3.159966	6.526911	1.4825	1.333233	1.454887
HORVU6Hr1G093050	61.4703	58.19308	63.31641	41.59064	35.10545	36.41214
HORVU2Hr1G085270	15.24921	19.98892	25.62793	2.356758	5.011158	6.55649
HORVU2Hr1G037700	0.616165	0.516296	1.04063	3.686999	3.367272	4.354281
HORVU1Hr1G091060	0.005727	0.016998	0.009686	0.57084	0.246873	0.58814
HORVU1Hr1G068860	2.571845	2.648228	3.30591	1.954869	1.424618	1.849558
HORVU6Hr1G091560	9.022877	6.107535	9.78519	0.008275	0.009415	0
HORVU1Hr1G013680	448.1612	390.0703	264.1422	159.57	41.29256	70.61941
HORVU2Hr1G063460	0.073942	0.153204	0.05236	1.888307	2.698279	3.416728
HORVU3Hr1G025680	2.712565	2.527513	3.157928	6.535061	12.5713	13.42237
HORVU1Hr1G049230	9.538179	6.923818	12.75705	1.681295	0.628419	1.057338
Hordeum_vulgare_newGene_15751	1.043686	1.967177	1.366055	0.603895	0.18114	0.443635
Hordeum_vulgare_newGene_10320	0	0	0.102708	0.911514	3.945915	4.214644
HORVU2Hr1G064160	10.71711	7.668055	14.0841	3.275409	1.30787	4.681445
HORVU3Hr1G005350	9.37791	8.849092	7.776491	0.222111	0	0.048054
HORVU2Hr1G100480	6.893096	6.677926	7.051481	3.824528	2.501019	3.138494
HORVU3Hr1G074010	5.532515	3.693643	5.68463	2.791705	1.800685	2.192088
HORVU7Hr1G008420	0.690635	0.504562	0.737663	0.246777	0.070236	0.078682
Hordeum_vulgare_newGene_2072	0.32095	0.255895	0.171999	0.580983	0.889114	0.747607
HORVU6Hr1G021160	0.58716	0.35219	0.598359	2.986223	14.97232	12.96423
HORVU7Hr1G089210	0.277975	0.375127	0.48621	1.174065	9.448617	16.44558
HORVU3Hr1G065240	31.21319	34.24769	26.14738	17.11159	3.326339	7.475814
HORVU3Hr1G026650	8.429833	7.04906	8.788674	19.23795	20.76379	21.42857
HORVU4Hr1G076690	5.373002	4.320278	4.11678	3.538407	1.825167	1.983132

Hordeum_vulgare_newGene_11304	1.768309	2.210248	1.905516	0	0.009459	0
HORVU3Hr1G062730	0.190213	0.16522	0.305838	0.62073	0.826043	0.93965
Hordeum_vulgare_newGene_9207	0.24643	0.152313	0.161915	0.527386	0.972218	0.874837
Hordeum_vulgare_newGene_8283	9.706773	5.647631	11.70813	4.975278	2.023254	6.027757
HORVU7Hr1G106540	3.821797	3.965673	4.707505	10.47234	25.03616	25.76178
HORVU7Hr1G021950	0.981241	1.057819	1.028914	4.392041	6.967278	6.409337
HORVU1Hr1G013020	22.44617	22.37757	34.2117	48.64346	180.6928	144.0181
Hordeum_vulgare_newGene_4708	0.469806	0.490031	0.322753	0.017135	0.034962	0.016922
Hordeum_vulgare_newGene_12022	1.009931	1.666708	1.583188	2.523031	3.253137	4.307637
Hordeum_vulgare_newGene_12023	1.079194	1.066249	0.793165	0.214468	0.013738	0.022955
HORVU6Hr1G062320	0.906537	0.656294	0.883837	2.15296	2.557314	2.701177
HORVU6Hr1G058890	0.302804	0.295498	0.184499	0.934427	1.357304	1.357199
Hordeum_vulgare_newGene_6702	58.49821	74.25543	61.69701	45.94292	23.85558	30.67081
HORVU5Hr1G014170	5.136822	4.801272	4.036227	13.46509	15.75119	15.21913
HORVU6Hr1G074560	8.199395	6.569004	7.884947	0.030147	0	0
HORVU6Hr1G003620	0.379342	0.321435	0.448326	0.062825	0	0.041323
HORVU5Hr1G084610	0.229647	0.1742	0.199732	0.66523	2.236763	1.805591
HORVU1Hr1G056820	7.747986	6.799824	4.644069	0.755063	0.331494	0.316728
HORVU7Hr1G052770	2.431154	1.858084	1.95068	15.63757	20.05637	19.89145
HORVU2Hr1G124800	6.183052	6.181629	6.868612	15.36045	19.18892	21.82477
HORVU7Hr1G038330	32.22686	21.52693	34.73309	15.56071	4.068622	6.123715
Hordeum_vulgare_newGene_14095	3.005815	2.267548	2.439335	0.011187	0.021387	0.01724
HORVU1Hr1G070200	2.979728	2.023951	2.567881	7.675415	12.8433	12.42498
Hordeum_vulgare_newGene_14099	10.17929	13.01655	13.64333	0.010914	0	0.012436
HORVU2Hr1G091360	3.390529	3.174793	4.173185	5.718927	11.54518	11.50871
Hordeum_vulgare_newGene_504	1.728516	2.138899	2.294856	0	0	0
HORVU4Hr1G070320	35.74005	39.17165	41.83422	11.3902	10.97652	11.37815
HORVU2Hr1G005670	1.522238	2.033218	1.651341	0.428005	0.361101	0.537957
HORVU2Hr1G116300	0.776251	0.56172	0.765361	16.06452	29.43366	25.97025
HORVU6Hr1G061000	0.066978	0.086916	0	1.620286	2.987742	1.804164
HORVU6Hr1G081020	0.092954	0.103275	0.2532	1.493934	1.44929	2.191028
HORVU4Hr1G087590	0.114871	0.295771	0.278257	2.106605	2.53626	2.755886
HORVU7Hr1G112470	4.616818	6.434348	7.673244	6.56082	28.45763	28.37969
HORVU3Hr1G084750	1.265982	1.431293	1.555197	2.768244	6.338324	5.630277
HORVU6Hr1G060750	2.666175	1.856896	3.295458	1.194203	0.932058	1.429578
HORVU2Hr1G009940	3.627905	3.311786	5.026624	15.07701	28.95504	10.29587
HORVU6Hr1G000620	11.96582	10.25568	10.88344	41.92507	56.03303	55.90875
HORVU5Hr1G069860	18.23216	15.82173	16.38983	9.47376	7.288737	8.812253
HORVU6Hr1G068180	3.34972	3.477676	4.276088	0	0	0
HORVU5Hr1G109660	3.516992	2.896484	2.998114	0.436441	1.396062	0.926199
HORVU2Hr1G088530	1.61013	1.869703	2.4838	0.725633	0.998315	1.249077
HORVU4Hr1G015820	3.335337	2.505627	4.25418	9.058528	10.4686	11.03988
HORVU0Hr1G019500	6.582296	6.72054	7.135389	3.341687	5.320234	2.63726
HORVU0Hr1G017560	4.639904	3.579512	3.10808	4.629391	13.68789	17.07065
HORVU1Hr1G092960	16.5154	13.23156	9.015527	0.495063	0.690081	0.487653
HORVU7Hr1G117640	6.374917	6.54922	7.448506	20.8122	43.95828	38.08456
HORVU7Hr1G115640	0.225604	0.13115	0.22707	0.796458	1.357636	1.775213

Hordeum_vulgare_newGene_5249	1.973583	2.64359	1.969315	0.027167	0	0
HORVU4Hr1G010810	3.702587	3.43822	3.351317	0	0	0
HORVU4Hr1G039880	3.371308	4.543153	4.718289	12.87091	34.15382	34.48998
HORVU4Hr1G059930	0.018599	0	0	0.481947	0.934156	0.977645
HORVU1Hr1G012940	148.8166	171.5079	198.4528	320.7673	815.4641	694.6667
HORVU3Hr1G086380	7.805353	12.10914	14.1865	2.503105	2.93071	3.176174
Hordeum_vulgare_newGene_13117	97.83479	82.14167	57.35775	22.75471	18.14884	21.65962
Hordeum_vulgare_newGene_13116	13.53982	14.31426	16.01486	30.19909	71.72191	71.87273
Hordeum_vulgare_newGene_13111	1.614172	1.128623	2.420192	3.881189	10.09196	9.078254
HORVU3Hr1G070200	2.492025	2.210756	3.089385	5.9807	7.894048	7.251627
HORVU4Hr1G007680	2.968196	2.533424	2.573712	15.3316	50.45717	49.06297
HORVU7Hr1G010500	2.306524	3.232776	3.381742	0.843325	1.006204	1.694707
Hordeum_vulgare_newGene_11149	0	0	0	0.852442	1.332578	1.007969
Hordeum_vulgare_newGene_11142	6.955056	7.290489	9.35945	3.46068	4.208648	5.049406
Hordeum_vulgare_newGene_5534	1.727334	1.764668	0.821925	0	0	0
HORVU1Hr1G065030	103.5167	87.4625	88.24978	40.44632	22.51688	28.40821
HORVU7Hr1G007590	28.55918	37.91022	49.00424	4.458125	5.847344	6.648978
HORVU0Hr1G012860	0.839117	0.566946	0.483649	4.904646	8.968221	9.050019
HORVU2Hr1G122280	919.3782	897.8264	673.1512	4756.705	5413.956	4383.88
HORVU6Hr1G092280	0.103076	0.038416	0.24763	1.782835	10.87324	10.1038
HORVU2Hr1G123480	1.772571	0.78188	1.716893	2.613129	5.746861	5.876544
HORVU2Hr1G105980	1.024513	1.294134	0.833916	3.076048	4.375936	3.33774
HORVU1Hr1G084690	5.705275	5.548144	9.820477	3.261415	4.766218	4.16316
HORVU5Hr1G106370	1.747169	1.066522	1.523383	0.861512	0.438108	0.654984
HORVU3Hr1G094370	1.521315	0.685802	2.365723	0.087372	0	0.26727
HORVU2Hr1G111530	0	0	0	1.527831	3.796843	4.146915
HORVU3Hr1G057440	0.793423	1.036145	0.978409	1.969205	3.336685	3.011023
HORVU5Hr1G071010	1.920955	1.417978	1.845041	17.71804	36.85381	18.62997
HORVU1Hr1G067300	11.09298	10.54246	11.12591	27.57287	57.79564	57.23048
HORVU5Hr1G119510	13.82854	13.10007	19.70175	5.138788	10.70012	8.720129
HORVU6Hr1G089590	3.019681	3.275248	2.832004	0.094288	0	0.010971
Hordeum_vulgare_newGene_8164	0.076372	0.242347	0.082076	0.583676	1.316832	1.41838
HORVU0Hr1G017000	11.77383	7.095507	11.47888	23.20126	30.83187	24.54253
HORVU7Hr1G035500	45.23614	38.91502	38.03606	25.15878	23.91926	20.916
HORVU4Hr1G090810	0.024593	0	0.028483	2.871303	4.206869	2.989273
HORVU4Hr1G067940	0.2212	0.102559	0.344772	1.721703	12.32533	11.09295
HORVU5Hr1G099350	0.424026	0.42747	1.060433	4.183876	23.27794	18.81677
HORVU3Hr1G061730	6.33871	8.440534	12.85792	4.940629	5.412446	5.433671
HORVU0Hr1G030280	5.197382	4.474174	6.101806	1.854226	1.112123	1.642115
HORVU2Hr1G064640	0.144755	0.064676	0.239049	0.819805	0.784322	1.013817
HORVU6Hr1G088310	3.359336	2.095519	3.394856	1.54828	0.581415	1.173735
HORVU2Hr1G070360	136.7869	144.7412	198.3417	72.44763	59.40071	55.81119
Hordeum_vulgare_newGene_4326	0.127578	0.046595	0.260752	3.034486	7.128553	6.316862
Hordeum_vulgare_newGene_15124	6.990513	6.887373	4.680876	1.189212	1.84335	1.553997
HORVU7Hr1G074660	22.96371	12.40617	26.84522	10.23769	4.118548	10.55249
HORVU6Hr1G095380	1.052427	1.076116	0.94458	16.8932	16.82447	21.13477
HORVU3Hr1G050310	18.43463	18.81714	19.78558	10.43878	8.136199	7.932553

HORVU4Hr1G071300	1.335278	1.662337	2.1181	3.733513	6.286314	9.783023
HORVU7Hr1G106660	0.742375	1.020174	1.246914	2.706707	3.08922	4.555388
HORVU0Hr1G031500	6.94417	8.979856	9.283212	4.168181	5.181897	5.862645
HORVU1Hr1G073760	2.376384	2.160105	3.189459	5.024842	10.46096	12.12977
HORVU5Hr1G045800	2.174154	3.730609	3.092252	7.305131	13.71341	12.77833
Hordeum_vulgare_newGene_8326	1.378038	0.945913	2.215239	5.237992	15.54788	16.61508
HORVU7Hr1G036070	14.30756	7.121329	17.06929	5.221236	2.951398	5.199537
HORVU7Hr1G087700	4.304225	4.649676	6.671619	0.986844	1.046325	0.931928
HORVU6Hr1G020790	0.24903	0.418544	0.349605	0.800395	0.966312	1.394808
HORVU3Hr1G014100	5.806313	5.206578	3.896221	0.948232	1.277658	1.477907
HORVU1Hr1G024400	3.35059	3.974287	3.594545	7.385761	10.87765	13.39135
HORVU3Hr1G011620	0.145294	0.071131	0.054621	0.723972	1.179235	1.239645
HORVU2Hr1G077650	4.197793	4.85415	4.169877	18.56218	25.94014	26.59132
Hordeum_vulgare_newGene_38	6.244387	5.613332	10.48124	1.418094	3.476528	1.701061
HORVU6Hr1G062070	8.369424	9.554083	11.75823	1.59831	2.209008	1.665279
Hordeum_vulgare_newGene_34	0.348337	0.44551	0.788175	2.569417	3.163562	3.4142
HORVU0Hr1G039080	10.39321	9.35358	10.83994	2.942252	3.065931	3.01143
Hordeum_vulgare_newGene_32	0	0.572475	0.731659	7.151556	6.706931	8.151154
Hordeum_vulgare_newGene_3924	23.65448	31.60155	44.99869	9.859675	16.78246	16.20932
Hordeum_vulgare_newGene_15972	0	0	0	1.356963	1.923132	1.715652
HORVU5Hr1G060070	46.81704	47.36457	43.65385	25.65033	17.97166	21.19861
HORVU0Hr1G022360	0.140693	0.152245	0.175719	0.608002	1.208301	0.93402
HORVU2Hr1G032400	26.33572	32.2367	38.45816	18.12349	10.28965	12.34189
HORVU7Hr1G100150	42.36275	44.52721	60.95109	11.34675	1.249947	3.943336
Hordeum_vulgare_newGene_12616	1.11068	0.690204	1.353878	0.61526	0.422172	0.373578
Hordeum_vulgare_newGene_12611	4.678174	4.25219	3.939621	0.880983	0.904848	1.166316
Hordeum_vulgare_newGene_12610	2.095096	2.322686	2.372461	0.304913	0.16121	0.304546
HORVU4Hr1G074650	0.574017	0.836406	0.954275	1.149447	3.501201	3.417773
HORVU2Hr1G041520	2.951727	2.371483	2.237005	1.05346	0.786643	0.735723
HORVU2Hr1G000140	1.172193	0.968542	1.733955	5.469017	4.029049	5.236771
Hordeum_vulgare_newGene_14757	2.591889	2.629404	2.612426	0.492234	0.720115	0.824881
Hordeum_vulgare_newGene_14753	1.31169	1.253251	1.155871	0.553046	0.567003	0.48271
Hordeum_vulgare_newGene_14750	1.055417	1.427037	0.95361	9.520662	10.55211	11.24207
HORVU6Hr1G004860	1.541907	1.789275	1.643086	5.365454	14.23975	12.21554
HORVU0Hr1G003980	5.838709	5.844824	7.170609	2.478652	1.291778	1.874369
HORVU3Hr1G029120	0.775711	0.889472	0.915074	0.444563	0.441618	0.44648
HORVU6Hr1G068410	13.02167	12.36411	15.53921	13.94924	52.54284	53.34929
HORVU6Hr1G072260	1.036616	0.796157	0.846382	3.646877	8.768189	8.107994
HORVU1Hr1G091230	8.533708	9.793875	13.34257	1.063607	1.018621	1.062481
HORVU1Hr1G081400	0.138709	0.136127	0.189343	0.794601	1.011653	1.062714
HORVU6Hr1G002320	2.598366	1.298333	2.428786	6.374485	49.61435	33.36076
HORVU4Hr1G056240	0.578664	0.491956	0.867723	0.86824	4.412683	9.162718
HORVU7Hr1G008090	4.666842	5.534065	6.822155	3.24313	3.070872	3.252599
HORVU1Hr1G055510	0.196324	0.288415	0.476347	0.790747	3.042543	2.999441
HORVU2Hr1G085000	5.084211	5.742066	5.790333	15.68081	20.55274	19.55892
HORVU2Hr1G098800	49.36306	42.53806	48.49786	16.27352	12.13196	13.95237
HORVU3Hr1G016830	3.234	2.571834	2.362189	0.068445	0.039967	0

HORVU7Hr1G050670	44.47287	34.16356	44.84827	16.68669	3.265794	8.022646
HORVU4Hr1G065800	13.06342	9.781105	14.66892	5.280899	4.382753	4.456799
HORVU7Hr1G116810	1.708067	0.918576	1.807407	0.633438	0.452264	0.656752
HORVU1Hr1G052890	12.37718	12.10586	14.97246	31.99529	40.27641	37.31204
HORVU4Hr1G062730	0.198549	0.191937	0.169364	0.991159	0.718466	0.697916
Hordeum_vulgare_newGene_1948	0.58586	0.50314	0.583876	0.048677	0.02306	0.077651
HORVU1Hr1G057110	5.055187	4.100134	5.681468	3.359658	2.069009	2.321225
HORVU1Hr1G082320	0	0.018709	0	3.946468	3.047826	2.839725
HORVU1Hr1G047390	6.163836	5.41622	7.657062	3.118275	3.855363	4.304922
Hordeum_vulgare_newGene_6840	6.791212	4.296042	10.17961	0.339194	0.654893	0.729533
HORVU4Hr1G014530	0.35213	0.414866	0.450637	2.197006	6.268775	5.85795
HORVU7Hr1G095680	11.94532	9.379601	13.72996	19.94697	34.16659	35.31249
HORVU3Hr1G005780	8.178142	7.658193	9.804786	2.968252	3.970567	4.62083
HORVU7Hr1G071390	0.797385	0.834164	0.668704	1.58365	4.501629	3.821007
HORVU4Hr1G053980	4.265527	2.836636	5.390052	11.51912	7.817509	13.22326
HORVU7Hr1G099070	38.27409	33.9157	43.06639	26.28653	23.47479	23.62064
HORVU7Hr1G094080	0.262113	0.259295	0.143848	2.250964	7.39866	8.784094
HORVU3Hr1G065610	1.259275	1.251775	1.031366	0.362654	0.426119	0.364647
HORVU4Hr1G055970	2.824919	2.148742	3.054961	1.69448	0.526666	0.632462
HORVU7Hr1G086670	0.258665	0.47356	0.452909	0.475106	1.543669	1.315569
HORVU5Hr1G052090	16.8263	13.58153	19.95857	33.67313	69.97167	66.91828
HORVU2Hr1G023590	2895.309	3462.851	2176.362	639.3228	33.51153	242.1749
HORVU1Hr1G040700	6.895821	3.948308	5.562785	0	0	0
HORVU1Hr1G041250	371.6521	290.4879	395.8721	838.0687	1278.268	1196.682
Hordeum_vulgare_newGene_132	8.012126	8.621829	8.843106	0.04644	0	0
HORVU5Hr1G092430	5.520926	4.783262	5.521416	11.47713	18.77992	19.40175
HORVU1Hr1G013190	4.066052	4.168924	4.852726	8.16395	13.32999	12.03542
HORVU6Hr1G092740	47.17936	35.15695	50.68057	16.26071	7.978953	10.28429
HORVU3Hr1G057760	15.55635	17.94856	30.01525	3.868378	0.922805	2.101664
HORVU2Hr1G087660	6.849492	8.264622	6.528003	3.948288	3.275102	3.908101
HORVU3Hr1G110170	2.179461	1.349548	2.627275	5.14758	30.82113	25.11372
HORVU1Hr1G012710	86.95602	91.98424	135.6986	31.89952	4.317528	11.74088
HORVU2Hr1G103040	83.35978	89.27833	94.06683	41.9757	19.20836	25.13763
HORVU4Hr1G054610	0.173316	0.217462	0.389692	1.519231	2.643188	2.736038
Hordeum_vulgare_newGene_14877	1.46947	2.329397	1.559474	0.627886	0.293903	0.360154
Hordeum_vulgare_newGene_14193	1.009501	1.268319	0.979843	9.379558	6.564488	7.127437
Hordeum_vulgare_newGene_10855	50.13256	50.58444	57.68282	1.541149	0	0.423765
HORVU7Hr1G027070	0.103094	0	0.12187	0.646419	0.530153	0.655641
HORVU1Hr1G000660	4.560054	4.962611	3.801344	0.024324	0.010681	0
HORVU4Hr1G024950	1.870905	1.936265	1.781955	4.75282	5.873194	6.943924
HORVU4Hr1G075180	0.205602	0.091848	0.270712	0.502636	2.944354	2.723562
HORVU0Hr1G009140	0.933334	0.852784	0.863994	2.652867	4.28111	4.058857
HORVU6Hr1G078960	71.09224	61.49299	71.85876	32.61333	11.8366	14.00286
Hordeum_vulgare_newGene_12093	0.738814	0.514036	0.473221	1.00609	1.496053	1.957445
HORVU6Hr1G013720	5.499327	6.491129	8.977303	3.783676	4.382615	4.412768
HORVU5Hr1G103870	0.223243	0.315692	0.502442	1.254305	1.640015	1.174998
HORVU2Hr1G066200	11.5044	8.777157	11.67088	4.97775	1.780917	1.886808

HORVU6Hr1G067170	4.493709	3.220558	3.767216	8.992196	22.28041	17.2358
HORVU4Hr1G075720	4.775866	5.165271	7.071651	0.050098	0.024105	0.044623
HORVU2Hr1G103150	6.673163	8.219334	11.96694	1.847759	1.130002	1.438721
HORVU7Hr1G036130	0.535276	1.052767	1.017225	2.297503	1.74942	1.504116
Hordeum_vulgare_newGene_4019	24.87023	36.21969	40.46751	15.4995	3.710602	8.949746
HORVU6Hr1G081310	3.45827	3.511542	4.696362	1.607724	1.756516	2.164918
HORVU3Hr1G105290	0	0.051012	0.0616	5.392119	2.644383	6.147767
Hordeum_vulgare_newGene_14949	1.403898	2.047274	1.952898	1.071336	0.758134	1.360265
Hordeum_vulgare_newGene_14940	2.149359	2.402031	1.38585	0.397019	0.314439	0.458721
Hordeum_vulgare_newGene_14946	2.74093	3.870408	2.843193	0.767708	0.80957	0.54858
HORVU5Hr1G056420	3.253603	2.352081	3.806814	1.23382	0.414498	1.277021
HORVU3Hr1G034820	1.315336	1.571234	2.272841	1.134817	0.516268	0.90938
HORVU7Hr1G052860	0.149261	0.495329	0.349934	1.051894	2.392974	1.479086
HORVU5Hr1G076400	26.82566	21.6973	25.24197	67.66891	79.34837	76.52987
Hordeum_vulgare_newGene_7097	0.830696	0.250268	0.408678	2.567167	3.114405	2.204768
HORVU7Hr1G006240	24.65047	23.13376	22.98356	1.209608	1.956183	1.008425
HORVU3Hr1G085720	2.750436	3.351566	3.235664	1.056199	1.295875	1.305122
HORVU7Hr1G038420	66.85206	62.95652	76.63504	137.6002	381.4215	336.3954
HORVU3Hr1G034310	2.601142	2.758179	4.566868	10.53999	14.92813	29.58487
HORVU3Hr1G078360	4.211324	3.388004	3.60826	0.131231	0.335492	0.424602
HORVU1Hr1G011730	1.635724	0.996632	1.471337	4.812192	4.036417	5.230341
HORVU5Hr1G106100	1.157072	1.43804	2.479587	2.349148	2.979945	5.800915
HORVU2Hr1G061950	5.31079	6.623194	6.460295	2.86186	3.492593	3.215068
HORVU5Hr1G027980	3.747239	2.288374	2.82667	0.40355	0.250845	0.415513
HORVU5Hr1G017660	1.468237	1.154089	1.817284	5.870905	5.492092	5.622323
HORVU3Hr1G016360	4.577176	6.040827	6.005393	3.152099	2.017112	2.917901
HORVU1Hr1G065990	0.106492	0.06786	0.08769	1.992525	3.331192	2.965352
HORVU7Hr1G121200	0.131549	0.291338	0.306738	0.574478	2.236261	3.339932
HORVU5Hr1G113620	3.753455	4.040854	4.930459	2.574097	1.545456	2.267117
HORVU7Hr1G051710	12.24903	17.88316	15.54594	52.79366	125.2986	115.5502
HORVU5Hr1G107220	10.0602	8.80915	10.06737	2.316569	1.012143	1.471892
HORVU5Hr1G122060	0.123741	0.218753	0.171196	7.296955	15.59757	12.02022
HORVU5Hr1G021130	0.416703	0.800441	0.465722	0.042522	0.011755	0.03683
HORVU6Hr1G085330	1.307432	1.296385	1.451374	3.532322	3.368558	3.334462
HORVU7Hr1G095060	4.914591	4.912378	6.22068	21.95463	33.1926	33.16332
HORVU5Hr1G096260	13.56533	13.9217	12.43076	0.971244	0.803708	0.704337
HORVU4Hr1G019780	2.248015	2.09103	2.565028	5.663279	15.94522	12.98027
HORVU1Hr1G058310	2.240116	2.640415	1.043084	0.042596	0	0.049753
HORVU2Hr1G009520	0.039337	0.051425	0	0.768003	1.033284	1.71414
HORVU5Hr1G009860	0.287879	0.23406	0.774878	1.142349	2.227217	1.953582
Hordeum_vulgare_newGene_4270	1.660649	2.018435	2.488234	7.102709	5.40784	7.008555
Hordeum_vulgare_newGene_12928	0.006985	0	0	0.865956	0.397543	0.512124
HORVU7Hr1G092450	3.331958	3.696829	2.778038	30.64496	33.65401	34.928
HORVU6Hr1G011050	109.2972	105.3663	137.4095	6.406662	0.412191	1.906376
HORVU4Hr1G065110	4.600127	4.835353	5.442275	2.957721	1.970725	2.232667
HORVU2Hr1G107180	0.004086	0.025353	0.01315	0.532929	0.412512	0.650827
HORVU0Hr1G006720	1.816814	1.545128	2.386308	0.581669	0.504001	0.640682

HORVU3Hr1G077540	33.16302	30.53824	32.31464	19.37496	13.23834	15.39283
HORVU2Hr1G023170	3.228894	2.976251	3.36329	8.037091	9.498156	9.584649
HORVU5Hr1G044640	16.11397	18.33201	18.85841	11.16489	9.692815	10.75165
HORVU6Hr1G021250	0.513126	0.498782	0.414239	2.618099	19.98337	11.74177
HORVU4Hr1G020230	3.068333	1.129393	3.332956	0.499491	0.151347	0.424722
HORVU1Hr1G094880	1.590008	1.836937	1.422872	1.519663	9.783437	10.03123
HORVU6Hr1G090990	119.4386	104.7729	112.7351	70.58493	53.57127	60.00179
HORVU7Hr1G089160	5.220607	4.463957	6.359245	0.381542	0.364276	0.733628
HORVU4Hr1G076610	7.188916	8.387378	6.862609	22.56502	33.20121	29.74952
HORVU4Hr1G061740	0.076551	0.137142	0.200068	1.308527	2.23445	1.845302
HORVU7Hr1G089290	20.0095	18.87187	24.30002	25.91439	75.77928	62.97452
HORVU2Hr1G111290	0.05254	0.054071	0.130537	1.898261	2.246852	1.519226
HORVU2Hr1G048010	0.06092	0.148658	0.101147	0.573629	3.278951	4.320079
HORVU5Hr1G100910	53.63689	60.80329	80.15244	30.00916	27.28935	30.93996
HORVU2Hr1G090400	0.056611	0.148348	0.09884	1.022365	1.616106	1.460275
HORVU3Hr1G098400	5.087922	4.143902	4.810182	3.261568	2.089461	2.595023
HORVU2Hr1G122590	11.31368	8.602527	11.99946	7.815907	3.979181	5.793761
Hordeum_vulgare_newGene_11381	0	0	0	2.350157	2.952615	1.726854
HORVU3Hr1G074770	32.6931	32.0719	28.38284	22.18266	14.30188	16.54867
HORVU5Hr1G077680	2.697582	2.806736	3.537653	10.54429	10.23642	10.90583
HORVU0Hr1G022370	0.081819	0.297558	0.143936	1.443677	1.158838	0.982974
HORVU2Hr1G025810	3.892	2.389588	4.417105	12.16923	11.15525	9.384054
HORVU2Hr1G118060	0.951978	0.722622	1.50767	2.499731	2.594012	4.648557
HORVU3Hr1G011470	5.923597	5.23397	6.287846	2.15771	2.861845	2.733999
HORVU7Hr1G033430	53.22384	45.77481	56.56865	35.53559	15.1159	12.05181
HORVU1Hr1G025250	0.227394	0.10009	0.154848	0.815259	1.010383	1.298392
Hordeum_vulgare_newGene_11522	16.05851	19.77424	18.26726	0.030248	0	0.023576
HORVU7Hr1G035870	2.375509	1.650844	1.800138	0.649168	0.631188	0.840151
HORVU2Hr1G123310	1.265891	0.953094	1.029288	5.42781	9.2108	8.644777
HORVU2Hr1G029480	6.954438	6.386135	7.068603	10.35375	38.11501	31.00583
HORVU1Hr1G084450	2.3044	2.518993	2.886334	4.938718	9.164059	10.172
HORVU5Hr1G082190	0.055812	0.004354	0.081588	1.334609	1.494305	1.173173
HORVU2Hr1G117560	0	0	0	10.53819	9.981381	16.29503
HORVU3Hr1G056400	1.940851	1.126691	1.101902	3.144565	5.138334	5.574741
HORVU6Hr1G087400	1.355861	1.385649	3.091514	0.51316	0.12373	0.471235
HORVU1Hr1G042870	1.09968	1.260495	1.456166	3.283986	3.092132	4.379217
HORVU3Hr1G063680	14.48828	14.37007	10.30598	4.549801	1.516386	2.682791
Hordeum_vulgare_newGene_7775	0.280647	0.406285	0.544375	8.785561	11.307	10.04009
Hordeum_vulgare_newGene_6121	0	0	0.010034	9.544535	7.366621	10.42749
HORVU2Hr1G015140	14.61366	9.750782	13.26729	1.875703	0.381843	0.672766
HORVU1Hr1G075900	1.829864	1.203436	2.271207	8.914859	28.47207	23.1882
HORVU6Hr1G075650	0.224454	0.18882	0.224624	0.546008	0.880931	1.090369
HORVU6Hr1G034630	2.632446	2.757736	3.279267	16.12577	36.62516	34.14379
HORVU2Hr1G079900	2.186805	2.403431	3.167137	0.015149	0.01335	0
HORVU5Hr1G107230	14.59858	13.03558	14.56953	3.754134	2.274408	2.368898
HORVU4Hr1G026160	2.012063	1.870557	2.653639	1.036127	1.643921	1.432442
HORVU6Hr1G053680	16.34704	17.07883	16.37163	37.43727	55.38074	70.44215

HORVU1Hr1G068020	2.00103	0.961882	1.516286	0.410661	0.609492	0.685124
HORVU2Hr1G033070	11.55313	10.02816	11.41083	26.74461	54.0098	47.06958
HORVU4Hr1G054000	2.142868	2.606714	2.852546	1.78012	1.577469	1.754159
Hordeum_vulgare_newGene_2339	1.889857	1.567593	1.726727	0.835768	0.481579	0.441187
HORVU5Hr1G074220	0.893159	0.718878	1.197452	3.201997	2.633714	3.616215
Hordeum_vulgare_newGene_2334	0.399907	0.345132	0.290046	1.033731	1.52185	1.364689
HORVU1Hr1G032150	1.256101	0.747055	0.989311	0.711145	0.18596	0.169823
HORVU3Hr1G114920	1.247887	1.205093	1.175723	3.343757	4.363394	4.269757
HORVU3Hr1G096830	11.46272	10.75848	10.3992	11.25576	52.15378	50.17245
HORVU0Hr1G018320	0.357029	0.320012	0.322198	45.51005	31.34485	30.74337
HORVU2Hr1G062700	0.83642	0.616589	0.840924	1.875161	3.270763	2.627947
HORVU1Hr1G077770	8.975471	12.3792	8.239591	5.026959	2.769989	3.92816
HORVU2Hr1G030520	2.919906	3.726332	4.616542	7.714571	11.44459	11.28671
HORVU2Hr1G070700	6.471395	4.81888	5.923473	0.559723	0.159756	0.264718
HORVU4Hr1G083750	3.752356	5.607689	4.865101	1.914391	3.469819	3.478359
HORVU4Hr1G052730	53.51999	59.49138	62.25644	35.90485	31.33248	34.69302
HORVU7Hr1G096690	1.530406	1.289424	2.052391	0.321876	0.432049	0.595294
HORVU2Hr1G036570	8.95384	6.691725	7.800936	2.159219	0.860088	1.754988
HORVU2Hr1G004540	10.68448	7.541188	14.34337	4.205226	1.055939	4.334418
HORVU6Hr1G068100	2.661327	3.451368	5.7207	1.685274	0.648902	1.341726
HORVU0Hr1G002950	0.468735	0.165228	0.253455	1.695764	3.130736	3.090741
HORVU5Hr1G102310	1.627845	1.409194	1.974021	0.373006	0.077803	0.469146
HORVU7Hr1G020300	0.402671	0.43157	0.589993	1.306275	1.106426	1.933328
HORVU6Hr1G020080	252.8211	321.0363	382.3534	78.13657	12.70525	38.1151
HORVU5Hr1G066720	4.438929	2.80049	5.890277	0.150685	0.036564	0.299484
HORVU7Hr1G006520	2.492981	3.402482	3.490778	13.31207	16.07606	18.64496
HORVU2Hr1G098380	1.088313	0.92919	1.156038	2.844032	5.885463	6.522659
HORVU6Hr1G066610	6.886337	9.508479	11.58084	0.068187	0.979949	0.856185
HORVU1Hr1G079030	0.224692	0.246253	0.169194	1.093624	0.801659	1.236097
HORVU1Hr1G064870	8.514631	8.727958	9.574589	34.9862	61.36089	68.19064
HORVU6Hr1G062970	4.171824	4.180311	4.493897	0.959703	0.866654	1.106034
Hordeum_vulgare_newGene_4228	3.571933	5.495114	5.160017	1.908791	1.504611	1.426645
Hordeum_vulgare_newGene_4229	0	0	0	2.258672	2.035583	2.117781
HORVU6Hr1G081290	0.185366	0.220745	0.267587	0.971709	2.076861	1.973052
HORVU1Hr1G064290	1.418631	1.782194	1.347474	4.391713	6.065469	6.315035
HORVU0Hr1G000960	1.553772	1.116686	1.484359	5.080571	2.72343	4.599653
HORVU6Hr1G008330	0.081694	0.136259	0.058323	1.170697	1.484868	1.56298
HORVU6Hr1G085600	2.139277	1.957269	2.960158	8.582771	9.723973	12.22098
HORVU5Hr1G100200	215.8195	233.2561	291.3374	627.9231	1162.937	1063.265
HORVU5Hr1G042400	1.109457	1.29759	0.892903	2.315717	3.400582	3.170101
HORVU4Hr1G056950	13.2841	11.59685	14.88771	9.702891	4.076917	4.6194
HORVU5Hr1G086520	6.191579	6.865402	11.21008	0.958052	0.719636	2.384202
HORVU2Hr1G090170	0.489152	0.453799	0.953054	0.115699	0.012697	0.045953
HORVU5Hr1G017580	0.171431	0.210865	0.178722	0.524531	1.083108	1.208974
HORVU5Hr1G009650	3.382058	4.592323	5.60788	8.49364	13.55875	14.44958
HORVU5Hr1G117990	1.631089	1.201303	1.318708	3.782193	5.155923	4.377001
HORVU6Hr1G004770	4.336368	3.178728	4.612623	0.005599	0.008028	0

HORVU7Hr1G050340	0.898478	1.110649	1.011112	3.052384	4.790101	5.130735
HORVU3Hr1G086610	5.429754	5.841933	9.059854	11.49513	51.86584	40.57566
HORVU1Hr1G091650	1.01639	1.139715	1.356162	1.39251	3.249741	3.887706
HORVU7Hr1G084230	0.201242	0.455639	0.603226	1.72376	3.182497	2.252092
Hordeum_vulgare_newGene_12888	15.06136	4.856049	11.76547	0.770116	1.485	2.983205
Hordeum_vulgare_newGene_12883	0.23095	0.24022	0.483848	1.173101	1.726274	3.000114
HORVU4Hr1G087230	0.303581	0.098254	0.207966	7.365876	12.93602	12.1695
HORVU4Hr1G019570	38.31184	34.36021	33.28296	105.4101	175.3928	175.3902
HORVU1Hr1G058500	0.931049	0.846259	1.333438	7.37048	21.79228	18.70211
HORVU4Hr1G078350	5.570692	6.087279	6.828716	3.29517	3.748021	3.366647
HORVU3Hr1G005540	2.452347	1.878419	3.036624	0.039286	0.027745	0.030089
Hordeum_vulgare_newGene_2138	1.676494	1.098372	1.067001	0.090446	0.046182	0.063811
Hordeum_vulgare_newGene_2139	0.063079	0.054137	0	15.66206	16.81876	19.35563
HORVU2Hr1G019120	0.189983	0.488217	0.681994	1.659579	1.9892	2.330263
HORVU7Hr1G008320	22.78482	17.33164	25.66309	37.96385	185.1633	136.1308
HORVU1Hr1G000430	1.365691	1.431785	1.285557	2.967498	3.47294	3.990188
HORVU4Hr1G020000	0.257112	0.234787	0.176458	0.851051	1.167853	0.983955
HORVU3Hr1G065320	3.043916	2.543584	5.217073	2.107615	1.361377	1.737372
Hordeum_vulgare_newGene_13348	0	0	0.009057	1.796951	1.98233	2.470721
HORVU2Hr1G105240	0.941798	0.897091	1.496746	0.415596	0.296809	0.730224
HORVU5Hr1G084860	0.462357	0.583051	0.974281	2.127248	2.882879	3.719965
HORVU5Hr1G098770	0.837949	0.823073	0.917348	5.272532	13.19287	10.24393
HORVU3Hr1G061800	12.16872	9.9066	14.87102	7.937116	5.764821	6.713013
HORVU2Hr1G119500	4.791383	2.163532	5.469644	0.60604	0.213778	0.458583
HORVU7Hr1G082090	0.43706	0.387358	0.353407	2.063041	3.740148	3.408449
HORVU6Hr1G077590	0.809826	1.119845	0.808253	0.413915	0.252269	0.259785
HORVU1Hr1G067960	0.721617	1.03031	1.389419	0.207373	0.095124	0.230614
HORVU2Hr1G073380	0.669382	0.618036	0.723416	1.189703	2.26595	2.089754
HORVU7Hr1G080780	2.156547	1.610562	1.949248	0.772015	0.749444	0.655036
HORVU6Hr1G064070	0.014449	0.006718	0	2.18092	2.507556	2.322899
Hordeum_vulgare_newGene_4424	16.62156	23.46427	23.29549	13.57524	9.193081	11.69377
HORVU7Hr1G001030	0.525845	0.747927	1.084132	10.77514	11.02414	18.94837
HORVU4Hr1G077060	0.627721	0.353949	0.409872	0.350857	3.019074	3.330759
HORVU6Hr1G089250	0.127418	0.115796	0.152502	2.118727	10.36338	10.58309
HORVU2Hr1G045730	0.087073	0.121233	0.127651	0.393973	0.533903	0.561967
HORVU7Hr1G031280	1.5399	1.401387	1.314443	2.90352	4.41932	4.255509
HORVU7Hr1G039700	199.282	167.7666	157.1461	87.21765	79.40823	70.70287
HORVU3Hr1G039480	3.043861	3.227642	4.144715	0	0	0
HORVU3Hr1G067750	0.995152	1.285915	1.865767	3.729804	6.302969	7.38977
Hordeum_vulgare_newGene_2968	2.236271	2.394057	3.186807	0	0	0
HORVU2Hr1G020640	0.972665	0.928791	0.905483	0.479883	0.497084	0.709111
Hordeum_vulgare_newGene_554	14.68348	11.00883	18.10771	5.645569	3.766413	6.376024
HORVU3Hr1G033620	1.279469	0.953184	1.419147	9.115531	18.52464	16.32028
HORVU3Hr1G081050	1.342321	1.631757	1.310366	4.02512	6.94925	5.753675
HORVU7Hr1G028290	1.617811	1.400496	1.172144	25.02715	14.5368	17.23759
HORVU2Hr1G075030	2.572504	2.282227	2.754728	6.562486	10.23678	9.656608
HORVU0Hr1G022270	21.18446	20.70154	20.71898	11.57167	10.12	11.01646

Hordeum_vulgare_newGene_13122	8.580052	10.65701	9.840936	0.019484	0	0
HORVU4Hr1G026640	0.066411	0.05936	0.212928	0.415087	0.773375	0.434495
HORVU7Hr1G030660	8.079454	5.591332	10.35609	3.703922	3.548278	2.6856
HORVU0Hr1G003900	27.14349	22.33733	30.1363	21.18299	11.85202	11.01704
HORVU4Hr1G070240	0.21946	0.322385	0.354592	5.831079	22.01456	20.19652
HORVU2Hr1G005530	0.906572	0.916795	0.648014	5.31666	6.960515	6.862497
HORVU5Hr1G007870	1.863482	1.481035	1.743279	0.022688	0.034559	0.033461
HORVU5Hr1G092160	37.64619	46.08971	58.16726	12.82766	0.753841	6.210945
HORVU1Hr1G059900	814.7919	1049.111	1744.941	121.0314	0.511416	42.1556
HORVU5Hr1G065330	0.261405	0.133043	0.267252	0.85631	1.278974	1.712974
HORVU5Hr1G094890	0.675554	0.790052	0.841118	2.127114	3.011077	2.870032
HORVU6Hr1G064130	0.091983	0.082956	0.094672	0.47688	0.480636	0.486211
HORVU1Hr1G022400	2.923166	1.865325	3.944359	9.091656	25.28342	26.23245
HORVU0Hr1G003270	39.13344	36.97619	36.92109	89.01356	136.1519	134.209
Hordeum_vulgare_newGene_9954	1.616015	0.871688	1.047448	0	0	0
HORVU3Hr1G016860	6.888567	4.062244	7.47577	1.242276	0.715129	1.743956
HORVU3Hr1G014790	0.166089	0.155943	0.094857	1.908173	3.586711	3.569518
Hordeum_vulgare_newGene_7986	0.702767	0.621685	1.016067	1.876468	2.607886	2.631608
HORVU2Hr1G085990	7.211893	8.460628	5.964422	20.69357	24.51351	20.83178
HORVU5Hr1G108670	39.12813	39.24241	40.05481	25.22626	4.787618	8.938265
HORVU2Hr1G043330	0.029412	0.078495	0.198065	0.715499	3.249672	3.065897
HORVU2Hr1G080480	2.578851	1.158061	2.212453	6.275659	9.722667	9.723622
Hordeum_vulgare_newGene_1600	0.505654	0.512151	0.439745	1.481945	1.782003	1.996621
HORVU7Hr1G099560	3.278388	2.76179	3.677612	2.161643	0.871135	1.768364
Hordeum_vulgare_newGene_12405	61.57043	69.76566	87.84683	18.80732	12.34268	14.25702
HORVU2Hr1G001400	1.969482	2.97881	4.26071	1.475144	2.101681	1.582108
HORVU4Hr1G019000	2.244859	2.452924	1.627115	0.450551	1.041418	0.617556
HORVU5Hr1G056950	14.8359	13.97775	14.74965	28.71148	44.85517	45.93323
Hordeum_vulgare_newGene_8799	1.495022	2.40382	2.278102	0.722935	0.453881	0.657244
HORVU4Hr1G068890	12.90608	10.427	11.34396	8.207507	4.232282	4.940085
HORVU1Hr1G089840	0	0	0	0.828814	1.796854	2.23855
HORVU2Hr1G126510	0.03494	0.094472	0.153806	0.23211	0.364138	0.461634
HORVU7Hr1G024270	4.04019	4.342765	3.472964	15.02777	14.33186	12.48641
HORVU6Hr1G081730	1.243681	1.613111	1.231446	3.895006	11.10802	11.43769
HORVU3Hr1G019070	1.761676	1.612558	2.156025	6.11738	11.23842	10.22882
Hordeum_vulgare_newGene_11449	1.304227	1.37223	1.119564	3.704142	4.266524	4.974974
HORVU3Hr1G100270	1.903192	1.335638	1.964431	5.290158	9.273995	8.974154
Hordeum_vulgare_newGene_11770	1.304836	2.019686	1.991276	0.996393	0.601781	0.720208
HORVU5Hr1G077010	1.441156	1.138377	0.745563	2.742199	3.114532	3.905966
HORVU5Hr1G094280	8.67385	3.938973	3.453281	0.067627	0.019683	0.031336
HORVU2Hr1G090030	3.359729	4.201279	6.949565	0.17255	0.56126	0.096049
HORVU5Hr1G076760	0	0	0	0.954893	1.000677	1.454106
HORVU6Hr1G049050	5.727282	6.210277	6.759455	15.98709	28.28669	27.72629
HORVU3Hr1G038230	0	0	0	7.457713	6.415011	7.200843
HORVU6Hr1G092430	1.990482	1.060654	1.411081	7.041171	11.50327	9.625716
HORVU1Hr1G065820	10.30062	10.11421	12.20968	6.498006	1.929665	4.127329
HORVU3Hr1G057090	61.23663	60.71077	58.9873	85.78536	226.8035	199.7133

Hordeum_vulgare_newGene_16202	3.105991	4.732018	2.152582	0.219406	0.027029	0.057477
HORVU0Hr1G016380	1060.987	1374.337	1004.618	191.7879	75.71902	120.1405
HORVU3Hr1G084990	1.918625	1.347881	1.648248	7.731121	47.65143	32.39925
HORVU1Hr1G084800	47.39442	40.30166	52.07797	23.33815	17.38376	18.89551
HORVU2Hr1G093610	0.388137	0.053892	0.317642	1.579023	3.378053	3.619373
HORVU6Hr1G068230	16.20116	14.35686	18.35003	10.38427	6.704697	5.306083
HORVU3Hr1G063430	6.134985	5.901114	9.033319	2.258599	2.462378	2.483836
Hordeum_vulgare_newGene_12457	4.76793	5.318431	5.313495	0.009327	0	0
HORVU2Hr1G068270	19.54426	18.67842	21.86258	33.75371	90.31211	84.10614
HORVU1Hr1G071210	0.965744	0.611924	1.05979	3.002206	21.24287	15.17553
HORVU5Hr1G103460	87.82837	116.4351	152.0599	19.91757	0.442222	8.379071
Hordeum_vulgare_newGene_410	2.89361	4.294036	4.589669	7.47311	18.28135	17.37752
Hordeum_vulgare_newGene_14112	0.100332	0.095393	0.086508	4.606674	4.418377	4.613516
Hordeum_vulgare_newGene_3457	1.802696	1.195986	1.500486	0.22163	0.15178	0.203583
HORVU5Hr1G115000	4.501573	4.021896	5.298559	16.24073	25.16487	25.86152
HORVU2Hr1G101990	19.91167	12.12042	19.70905	2.302027	0.969798	2.535326
Hordeum_vulgare_newGene_14435	5.191965	5.914083	3.561693	1.999722	1.668233	1.530763
Hordeum_vulgare_newGene_15245	0.097617	0.038685	0.298856	1.485392	1.461551	1.18006
Hordeum_vulgare_newGene_4722	0	0	0	1.672786	1.08768	1.563403
Hordeum_vulgare_newGene_2494	0.730124	0.823616	0.561373	0.112768	0.527382	0.153789
HORVU6Hr1G079070	0.324461	0.190827	0.233191	1.072523	1.667526	0.87928
Hordeum_vulgare_newGene_5809	0.252244	0.166185	0.206074	2.314896	2.87329	2.891426
Hordeum_vulgare_newGene_11899	0.60411	0.832728	0.744936	2.93695	4.853212	4.455441
HORVU3Hr1G096190	0	0	0	0.54823	0.298844	0.534322
HORVU3Hr1G050450	0.56759	0.43609	1.025583	1.528439	2.498835	2.732436
Hordeum_vulgare_newGene_5147	0.158506	0.004199	0.186536	0.478412	1.063778	0.908346
Hordeum_vulgare_newGene_5148	0.960318	1.191422	1.097086	0	0	0
Hordeum_vulgare_newGene_5149	0.211777	0.123928	0.158732	2.343377	2.667904	3.240661
HORVU4Hr1G071020	24.47897	20.14919	30.45134	12.56354	4.21638	13.1799
HORVU7Hr1G109950	36.51108	37.40085	64.83212	20.75844	24.44126	25.14057
HORVU2Hr1G118550	270.9883	219.3986	184.9062	74.98001	17.81152	28.44643
HORVU4Hr1G067450	22.41515	11.65438	27.74168	6.221148	1.848888	6.149217
HORVU5Hr1G094080	3.549955	5.071003	3.009689	17.82714	40.84507	37.6211
HORVU3Hr1G089830	10.89615	9.903655	14.75054	42.96175	44.97559	59.78806
HORVU5Hr1G030290	1.003223	15.29499	2.879665	0	0	0
HORVU1Hr1G076470	0.502728	0.349354	0.582764	3.211715	8.030017	9.401341
HORVU7Hr1G021820	0.833603	0.750024	0.383513	3.763583	5.803669	5.292503
Hordeum_vulgare_newGene_2463	6.349698	6.384709	5.956422	0	0.029284	0.051598
HORVU6Hr1G081750	1.757577	1.39792	1.626007	4.03918	3.269128	5.298765
HORVU3Hr1G086690	79.7205	63.18977	65.30473	17.28986	4.207293	7.842349
Hordeum_vulgare_newGene_1467	0.768697	0.45066	1.486295	0.285804	0.233098	0.504585
Hordeum_vulgare_newGene_3843	0	0	0	4.121275	4.278665	4.197808
HORVU3Hr1G088050	0.527104	0.766913	0.543449	1.27301	1.826537	1.708444
HORVU3Hr1G052030	1.642459	1.494345	1.591878	3.802284	7.163078	5.920302
Hordeum_vulgare_newGene_13547	7.083684	4.171586	6.5674	1.700949	3.863485	2.353461
HORVU3Hr1G034640	1.416479	1.13616	1.084373	4.291534	4.023489	4.869965
HORVU2Hr1G079610	5.004259	6.096595	6.805703	1.253207	1.659333	1.391484

HORVU3Hr1G097810	0.670357	0.25738	0.504147	6.674232	6.593347	9.88133
HORVU2Hr1G032130	5.992707	5.516724	4.987078	17.71746	28.59261	23.01474
Hordeum_vulgare_newGene_2826	1.877074	2.22448	2.28684	0	0	0
Hordeum_vulgare_newGene_2824	0.429341	0.506546	0.507019	1.815952	3.507837	3.285577
HORVU1Hr1G089730	67.04691	76.50826	98.60797	5.560397	4.032785	5.388093
HORVU6Hr1G078060	13.30758	11.67981	19.38266	8.5357	4.102215	5.65179
HORVU6Hr1G029190	0.538621	0.382559	1.035532	4.261924	13.86943	12.73099
HORVU1Hr1G093220	0.068445	0.061253	0.044715	0.649062	0.804794	0.718099
HORVU6Hr1G081800	0.103401	0.283459	0.094638	4.692914	6.166144	6.194331
HORVU3Hr1G038430	0.784289	0.649265	0.744208	2.756984	10.58951	12.43042
Hordeum_vulgare_newGene_8906	6.928688	6.581005	5.618157	12.9078	41.05046	32.75248
HORVU3Hr1G082590	1.315715	1.191062	0.905317	4.835216	8.222755	7.81784
HORVU5Hr1G023480	0.14602	0.099617	0.219872	0.742059	0.53942	0.458083
HORVU6Hr1G034140	0.52177	1.19448	1.035724	1.783411	2.539545	3.484047
HORVU2Hr1G026450	0.768158	0.331516	0.701915	5.413484	3.472634	2.932834
HORVU5Hr1G106720	1.552439	1.694126	1.43251	0.337876	0.207148	0.29953
HORVU7Hr1G041610	0.854057	1.469963	1.702997	0.481905	0.632617	0.735801
HORVU5Hr1G067800	6.78533	5.665255	8.404713	4.509739	3.408557	4.132652
HORVU4Hr1G065620	0.780667	0.638458	0.866138	1.536297	2.303739	2.276929
HORVU1Hr1G069550	11.6861	9.281346	10.04985	0.368768	0.240641	0.384747
HORVU4Hr1G068910	0	0	0.091557	16.62729	32.33643	27.86507
HORVU6Hr1G090910	2.348579	2.569628	2.809223	0	0	0
HORVU7Hr1G012380	332.6264	307.3644	342.9613	1160.072	1599.795	1354.183
HORVU2Hr1G096550	0.733086	1.012751	0.994095	3.469356	4.016149	4.054787
HORVU7Hr1G098460	0.021997	0.006599	0.035608	8.465602	6.762634	8.105374
HORVU5Hr1G013510	0.28467	0.365363	0.314193	23.9869	3.229991	11.88421
HORVU2Hr1G017440	4.648197	2.951603	3.776189	5.072691	63.36477	51.2855
HORVU5Hr1G081070	6.231198	5.877959	7.418077	4.196521	2.588197	2.797324
Hordeum_vulgare_newGene_6499	6.012152	7.725397	5.23223	1.535614	2.025063	1.430873
HORVU2Hr1G099850	3.115116	3.945494	3.957767	2.388498	2.058405	1.789088
Hordeum_vulgare_newGene_10300	1.217674	0.50101	0.999555	3.439316	4.003767	3.738134
HORVU5Hr1G095180	91.38464	63.37488	85.74273	13.26068	4.732757	6.733677
HORVU1Hr1G054200	0.357689	0.326181	0.580592	36.35737	3.663305	14.44052
HORVU4Hr1G090170	1.85033	3.361574	2.819361	0.740491	0.971612	1.082122
HORVU3Hr1G106850	79.14536	44.07515	94.27611	48.48405	35.30132	35.80588
HORVU2Hr1G050260	1.809077	1.985776	2.311889	1.0661	0.746162	0.752243
HORVU7Hr1G099650	0.83103	0.748423	1.445621	7.30686	11.3261	9.371835
HORVU6Hr1G088840	3.361864	3.949116	5.185145	1.878607	2.164467	2.18139
HORVU3Hr1G077790	5.161685	3.915743	5.236413	1.221991	0.096883	0.518121
HORVU3Hr1G099990	0.218715	0.063187	0.142481	31.29987	3.469049	16.37757
HORVU4Hr1G056640	4.03761	4.726596	3.998586	1.611326	2.559168	2.97248
HORVU7Hr1G051180	0.439377	0.314715	0.472705	1.793454	4.427548	6.624299
HORVU3Hr1G029210	5.062204	5.052718	5.573543	7.856746	22.76421	16.96599
HORVU3Hr1G085210	23.05601	25.21139	32.40584	10.80495	11.44288	13.17997
HORVU0Hr1G010640	6.527097	6.163627	5.683543	2.991481	1.278398	1.200436
HORVU2Hr1G022720	1.246172	1.081072	0.788316	1.366116	6.20337	6.167242
HORVU1Hr1G004720	0.964092	0.419275	0.763988	4.48755	3.582827	3.603769

Hordeum_vulgare_newGene_1931	2.849594	2.890249	2.836673	0	0	0.00619
Hordeum_vulgare_newGene_1933	0.945082	1.098929	1.193533	0.025477	0.179117	0.058118
Hordeum_vulgare_newGene_274	1.748466	1.062087	1.796633	0.657574	0.320267	0.496557
HORVU7Hr1G092680	1.615856	2.160797	4.225348	1.033813	0.706152	0.716017
HORVU6Hr1G062050	0.389002	0.225883	0.803628	2.59113	3.849735	3.786127
HORVU7Hr1G084920	59.12038	38.28637	56.44806	9.191257	8.487234	14.51386
HORVU2Hr1G018670	50.06302	45.09942	45.13839	9.94715	1.139694	3.135186
HORVU2Hr1G073920	7.154506	6.933165	7.9231	3.888979	3.384803	3.557762
HORVU2Hr1G116650	2.134904	2.296309	2.095429	0.879337	0.655556	1.100518
HORVU5Hr1G000680	6307.31	5727.22	6427.183	2410.478	2704.947	1988.841
HORVU7Hr1G019400	1.285555	0.967979	1.465196	0.400114	0.127421	0.51144
HORVU4Hr1G090920	0.015884	0.005303	0.003983	1.152603	0.656398	1.211523
Hordeum_vulgare_newGene_3648	0.24336	0.335049	0.221913	7.035162	8.845873	11.26162
Hordeum_vulgare_newGene_3643	2.804777	4.216768	3.706077	1.789414	1.601124	2.172602
HORVU7Hr1G077710	21.7156	22.77717	15.87393	8.249249	8.468606	8.408314
HORVU6Hr1G078260	4.794705	4.181894	4.58789	1.994374	2.380976	2.653672
HORVU3Hr1G105190	0.212942	0.195261	0.139639	1.432904	1.182335	2.428346
Hordeum_vulgare_newGene_14321	4.311469	4.795742	5.250716	1.929693	2.690254	2.654507
Hordeum_vulgare_newGene_14325	0.430166	0.397977	0.205172	2.581933	1.626833	2.215146
HORVU3Hr1G110190	0.894157	0.323822	0.197054	3.593025	5.032988	4.974938
HORVU6Hr1G072300	1.136676	0.860424	1.351172	3.285947	2.781454	3.363087
Hordeum_vulgare_newGene_625	6.524886	7.51324	7.150979	3.272131	3.125202	4.502209
Hordeum_vulgare_newGene_626	12.42315	11.21964	10.28357	5.659159	4.117832	4.111866
HORVU3Hr1G065830	13.36668	12.24519	13.91588	7.896165	6.922078	7.598022
Hordeum_vulgare_newGene_2512	1.489739	1.929063	1.494838	4.455045	9.412413	9.281048
HORVU1Hr1G070460	15.2037	13.15915	14.09566	4.848942	3.134145	3.772803
HORVU7Hr1G096610	0.133661	0.118405	0.063392	2.234643	3.262281	2.023386
HORVU7Hr1G026940	57.71444	55.28981	59.34171	17.27448	5.690718	11.48435
HORVU3Hr1G054070	80.3985	72.74029	67.97424	44.45684	36.63778	38.35762
HORVU0Hr1G009060	12.46714	10.43726	11.82908	8.317739	6.662477	7.326813
HORVU5Hr1G073710	5.856651	2.602799	10.44675	3.549459	1.899815	2.048837
HORVU7Hr1G043790	1.154664	1.622326	1.183465	3.879683	8.125042	7.210649
HORVU2Hr1G033610	2.433443	2.000528	2.279258	0.863352	0.635491	0.496267
Hordeum_vulgare_newGene_9697	0.302243	0.182515	0.371664	0.968671	2.741412	1.784001
HORVU4Hr1G044890	1.176981	1.20232	1.289683	2.494006	4.187905	3.477678
Hordeum_vulgare_newGene_9523	0.807046	1.366944	1.434781	0.827471	0.52483	0.428127
HORVU5Hr1G083000	7.297843	6.893146	9.254256	4.250819	3.952125	4.610071
HORVU1Hr1G032470	1.581276	1.44525	0.790975	2.780364	4.685943	3.008108
HORVU5Hr1G077110	13.11793	8.473234	13.67491	36.29864	74.41199	68.3365
Hordeum_vulgare_newGene_4112	7.913631	6.41827	5.923534	1.598263	1.571768	1.000899
HORVU6Hr1G028250	5.942904	5.685568	6.403051	3.299221	3.336009	1.930793
HORVU5Hr1G018050	0.201921	0.153409	0.238195	0.688999	2.206236	2.055989
HORVU7Hr1G070970	3.833514	2.837034	2.447811	8.527671	15.05114	13.45312
HORVU1Hr1G063740	32.22043	31.04592	37.83268	10.2303	8.692116	10.43561
HORVU7Hr1G101590	44.55844	38.18867	52.462	11.24358	8.710489	14.09061
HORVU1Hr1G079610	9.206159	7.690471	10.29049	0.380575	0.3229	0.532302
HORVU2Hr1G087880	3.71677	3.141708	3.464135	0.912833	1.119023	0.748979

HORVU7Hr1G098260	0.234255	0.190842	0.3816	0.834659	2.341113	3.119246
HORVU2Hr1G087260	0.397207	0.341519	0.397395	0	0	0
Hordeum_vulgare_newGene_6405	3.842116	4.255299	5.830322	0.1812	0.295369	0.340598
HORVU2Hr1G027470	6.101943	4.762464	6.973851	4.209404	2.015663	2.85298
HORVU1Hr1G036340	5.02941	6.084143	5.119059	2.96803	2.016889	2.799275
HORVU4Hr1G019380	0.043567	0.112589	0.060191	0.64281	0.807685	0.646133
Hordeum_vulgare_newGene_7519	0.771535	1.455981	1.761746	2.519308	4.677451	4.329054
HORVU5Hr1G065420	1.361434	1.126866	1.011222	0.515594	0.233854	0.578229
HORVU6Hr1G087300	0.422244	0.309023	0.338263	6.221369	16.95661	13.92096
HORVU7Hr1G040040	0.206048	0.207813	0.149995	1.440262	1.422648	1.210914
HORVU7Hr1G054020	1.082683	1.345787	1.565494	0.507537	0.435634	0.711823
HORVU1Hr1G066300	4.570198	4.308897	5.35224	0	0	0
HORVU3Hr1G023750	5.866856	4.937478	4.554892	3.650192	1.903371	1.590495
HORVU1Hr1G005030	337.9413	321.0297	239.8937	105.6813	67.38455	74.6288
HORVU1Hr1G010230	1.245529	2.011047	1.063926	4.003396	135.9548	94.62184
HORVU2Hr1G123150	5.11338	4.130711	4.801649	9.900735	15.02287	14.26728
HORVU4Hr1G049770	0.110032	0.359352	0.12554	0.493575	2.28772	1.851958
HORVU6Hr1G085270	0.954806	1.28301	1.205594	8.103557	8.115788	7.994742
Hordeum_vulgare_newGene_5360	2.288948	2.223375	1.808855	0.784706	1.794037	1.216125
HORVU3Hr1G071340	0.379627	0.281444	0.410138	1.132012	2.524035	3.442733
HORVU7Hr1G037410	5.495385	3.975309	4.852976	13.0115	14.48333	13.5818
HORVU4Hr1G080190	0.539183	0.445821	0.571102	2.455477	2.227257	2.827667
HORVU4Hr1G054240	3.586556	2.184394	3.684691	7.01452	15.84734	11.93955
HORVU7Hr1G110310	8.616115	6.697701	10.98711	10.14506	34.16082	34.04316
HORVU5Hr1G045650	1.398241	0.946326	1.277904	4.381856	3.810767	7.559045
Hordeum_vulgare_newGene_5401	23.76214	30.23496	27.71975	0.052175	0.060463	0.038692
HORVU5Hr1G115980	0	0	0.025483	3.083424	3.03151	3.253341
HORVU5Hr1G121900	25.23561	25.75734	24.3462	12.88139	12.76715	14.67309
HORVU7Hr1G121820	0.098616	0.126221	0.210367	0.747587	4.125469	4.611625
HORVU2Hr1G030870	4.613805	4.619915	6.248905	38.91895	1534.594	1763.72
HORVU5Hr1G098260	0.915733	1.483612	2.279234	0.326608	0.154562	0.124848
HORVU4Hr1G082680	3.89307	5.646928	5.788502	0.917009	0.157841	0.78686
HORVU5Hr1G093090	0.111106	0.096925	0.095045	0.622199	2.197728	1.419841
HORVU3Hr1G104250	7.706865	8.733971	10.59891	4.232245	5.735015	5.151245
Hordeum_vulgare_newGene_1875	0.78636	0.670201	0.838117	2.587927	2.11504	2.462659
Hordeum_vulgare_newGene_1871	7.222611	7.927874	7.458178	0.093419	0.203869	0.116103
HORVU2Hr1G105420	0.138452	0.117218	0.21779	0.70553	2.153455	2.139348
HORVU4Hr1G027260	93.00028	89.69855	53.12656	146.4044	166.5448	197.5673
HORVU6Hr1G035260	5.738056	5.482306	6.045388	13.67004	19.8484	20.4069
HORVU3Hr1G056560	2.609212	0.986008	1.321715	160.3435	428.8413	395.1532
Hordeum_vulgare_newGene_12347	0	0.11399	0.098409	3.586758	4.665543	4.845968
HORVU2Hr1G092810	5.924326	6.510111	4.787356	17.77153	13.32936	14.45224
Hordeum_vulgare_newGene_6242	0.623104	0.815839	0.841158	0	0	0
HORVU4Hr1G046270	0.123748	0.09153	0.059008	12.06116	14.60815	17.77369
HORVU3Hr1G081760	2.174036	1.965878	2.731022	5.275151	6.280031	7.768579
Hordeum_vulgare_newGene_2692	0.450902	0.595912	0.651749	2.57103	2.837978	2.417961
Hordeum_vulgare_newGene_14420	1.483202	1.429805	1.468743	0.220855	0.183833	0.25938

Hordeum_vulgare_newGene_14423	1.237774	1.501692	1.715555	0.056597	0.051145	0.059144
Hordeum_vulgare_newGene_2696	0.099171	0.04088	0.123303	0.383046	0.357319	0.345439
HORVU2Hr1G068610	1.591737	1.557141	1.059213	3.350645	5.206253	4.782062
HORVU5Hr1G033540	28.24104	19.28613	7.379044	117.4808	198.2894	196.9915
Hordeum_vulgare_newGene_3561	0.950319	0.922531	0.700594	0	0	0
Hordeum_vulgare_newGene_15771	2.077647	2.157076	1.873002	0.691664	0.616419	0.520424
Hordeum_vulgare_newGene_15776	0.669827	0.408777	0.589966	1.774452	2.068435	1.754359
Hordeum_vulgare_newGene_15774	0	0	0	9.852174	9.994969	10.43828
HORVU3Hr1G061410	0.604057	0.387236	0.420216	1.307472	2.170297	1.715111
Hordeum_vulgare_newGene_3269	2.529434	3.491035	3.794602	0	0	0
Hordeum_vulgare_newGene_783	0.247307	0.306004	0.192442	5.603202	4.726167	6.689538
Hordeum_vulgare_newGene_784	0.582354	0.842117	0.445191	13.21065	10.14247	13.40413
Hordeum_vulgare_newGene_788	2.457158	2.50705	2.699159	0	0	0
HORVU5Hr1G063960	4.030547	4.148975	5.573057	11.80098	23.70008	20.21141
Hordeum_vulgare_newGene_15486	0.576568	0.56248	0.556723	1.654676	1.281789	1.886239
Hordeum_vulgare_newGene_8572	0.740776	0.676007	0.757196	3.173402	2.602377	3.054116
HORVU4Hr1G028110	0.027842	0	0	6.440852	6.217307	6.720296
HORVU3Hr1G004520	68.43717	76.09737	64.20551	42.83359	44.00049	44.55884
HORVU3Hr1G018650	7.64636	6.665312	7.286514	16.25712	22.86136	21.86388
HORVU4Hr1G035380	31.57897	30.88849	45.99124	18.75757	16.23525	17.67785
HORVU0Hr1G032640	2.571503	4.3975	1.274678	9.948519	83.94389	85.27399
HORVU5Hr1G063380	0.152106	0.162446	0.19839	1.043716	1.8544	1.715477
HORVU7Hr1G039330	0.453384	0.647494	1.077137	1.794952	2.253484	2.166687
Hordeum_vulgare_newGene_1689	9.66657	7.799794	11.61036	6.187826	4.566574	5.345325
HORVU3Hr1G049900	3.860538	3.761201	4.753589	13.80239	26.03617	21.74257
Hordeum_vulgare_newGene_10395	3.527632	3.127861	4.889909	0	0	0
Hordeum_vulgare_newGene_4304	2.314217	3.045563	1.886002	1.587039	0.786653	1.331103
HORVU0Hr1G006640	1.474115	1.118894	1.947631	6.249462	7.915837	8.222698
HORVU7Hr1G019830	235.9458	187.8113	207.2835	136.3359	76.02704	81.96671
Hordeum_vulgare_newGene_7658	19.82966	16.14106	16.18917	10.4089	6.237619	6.512892
HORVU3Hr1G001000	106.7827	97.99442	165.0948	59.93417	46.55067	44.56619
HORVU6Hr1G081400	4.302243	4.433727	4.715602	16.01098	14.47996	15.10829
HORVU3Hr1G035680	5.654621	2.223299	6.334851	0.143147	0.053269	0.082251
HORVU0Hr1G015420	0.630395	0.783717	0.731783	0.712808	4.151121	4.202112
HORVU7Hr1G093020	35.48233	30.09603	40.99462	13.39438	13.6313	12.18059
HORVU1Hr1G045530	44.45757	42.05206	46.31326	21.37602	16.42298	15.21543
HORVU2Hr1G079580	42.51262	39.39062	34.0633	4.253272	1.782211	3.426474
HORVU5Hr1G120210	1.649993	1.243542	1.833158	4.327095	7.151099	5.986092
HORVU3Hr1G006250	142.3749	116.3664	151.1833	77.25488	57.32586	56.7913
HORVU4Hr1G082910	6.678791	6.824169	6.405647	19.47125	18.35642	18.56279
HORVU4Hr1G010430	2.080323	2.045761	2.040094	6.059139	10.14874	9.191223
Hordeum_vulgare_newGene_14257	5.810069	4.902792	4.969429	0.637532	0.573157	0.229986
HORVU3Hr1G078210	5.137871	7.079177	9.465103	3.23072	2.251171	2.540561
HORVU5Hr1G009460	2.097624	1.992129	1.83249	12.73743	6.730438	8.736968
Hordeum_vulgare_newGene_14259	15.00617	8.498634	18.78276	0.440261	0.092681	1.336139
HORVU4Hr1G011740	222.9908	186.0448	261.3521	59.37338	23.73785	32.39656
HORVU6Hr1G086440	0.069811	0.011518	0.115215	0.6885	0.560403	1.210663

HORVU1Hr1G084880	1.137285	0.734162	0.863316	0	0.026778	0
HORVU7Hr1G088890	76.9867	54.46307	76.45125	141.2226	287.2632	226.3882
HORVU7Hr1G041230	0.836619	0.692941	1.226208	0.199753	0.209543	0.340856
HORVU3Hr1G105300	0.035816	0.039208	0.028397	1.987709	1.511438	1.701447
HORVU1Hr1G068910	26.1401	18.99239	25.39373	10.98641	8.606182	10.68793
HORVU4Hr1G084400	5.31059	5.871602	6.007781	6.019969	21.15252	30.57414
HORVU2Hr1G065090	1.237697	1.112179	0.84991	3.115128	6.148339	5.901395
HORVU7Hr1G043370	0.070365	0.079406	0.134307	0.235614	0.564618	1.04622
HORVU3Hr1G098340	5.690944	2.332957	7.925089	2.69519	2.403849	2.619777
HORVU7Hr1G108240	0.876485	1.320804	1.532605	2.734826	6.033288	5.311444
HORVU1Hr1G054530	1.546194	1.441867	2.100405	0.80356	0.827802	0.77274
HORVU7Hr1G003140	0.105844	0.04547	0	1.688849	47.91591	44.41026
Hordeum_vulgare_newGene_9029	1.212549	0.903317	0.584627	0.227223	0.024791	0.100606
HORVU0Hr1G022780	3.197332	3.017081	4.370815	9.271917	14.28261	14.31634
Hordeum_vulgare_newGene_2767	0.263491	0.527982	0.343376	13.09776	11.93591	11.39117
Hordeum_vulgare_newGene_2769	0.381458	0.225814	0.316592	4.460325	4.826955	4.967592
HORVU2Hr1G091280	2.184323	1.554091	1.954874	3.078886	7.242077	6.072994
HORVU7Hr1G027810	8.048363	5.086423	7.777548	1.723452	0.93322	1.411011
Hordeum_vulgare_newGene_5231	0	0	0	1.461659	2.099421	2.249957
HORVU5Hr1G079910	1.040051	0.841422	1.084561	0.146803	0.040607	0.02516
Hordeum_vulgare_newGene_9799	1.368615	1.428115	2.841197	5.011506	5.617127	5.42518
Hordeum_vulgare_newGene_5238	0	0	0	2.071929	1.595001	2.1769
HORVU4Hr1G090440	27.75119	25.66034	28.28235	119.5942	243.1091	199.1813
HORVU7Hr1G008170	0.308066	0.255676	0.294855	0	0	0
Hordeum_vulgare_newGene_8774	0.650807	0.47098	0.529564	15.26489	65.32352	68.03099
Hordeum_vulgare_newGene_8778	1.753776	2.147453	1.799667	0.994899	0.683781	0.19651
HORVU3Hr1G110830	2.108683	1.852973	1.609558	1.012521	0.878042	0.820663
HORVU5Hr1G053330	0.939186	1.083419	1.12668	2.07825	5.193274	5.453401
HORVU5Hr1G080560	6.636042	9.755869	12.51828	5.075017	4.26458	5.053621
HORVU3Hr1G095930	2.483282	2.707073	3.734941	0.054512	0.089204	0.031705
HORVU1Hr1G017800	1.029897	0.720363	1.399786	9.18075	33.71595	35.60981
HORVU1Hr1G040130	0.748297	0.788466	0.644365	24.36441	28.11761	34.75201
Hordeum_vulgare_newGene_10490	0	0	0	3.513037	1.27162	2.10152
Hordeum_vulgare_newGene_10492	2.708674	3.380221	2.037693	0.071508	0	0.025877
HORVU3Hr1G026180	7.328826	7.704832	10.77943	3.489101	2.526482	2.027927
HORVU7Hr1G028780	0.13365	0	0	1.019077	1.384537	0.920679
HORVU3Hr1G074600	0.658102	0.190133	0.494304	3.027176	3.512781	3.500676
Hordeum_vulgare_newGene_1444	0.28576	0.223622	0.262762	4.05913	3.35056	3.589151
Hordeum_vulgare_newGene_10721	2.057529	2.149154	1.562656	0	0	0
Hordeum_vulgare_newGene_10725	2.943203	3.195354	2.434722	1.649149	1.283084	1.135442
HORVU3Hr1G095200	2.038615	1.878793	2.077646	0.274015	0.681162	0.290965
HORVU4Hr1G029420	0.250262	0.409932	0.245095	2.661748	3.821296	2.752994
HORVU7Hr1G080510	36.61309	36.93029	33.8284	5.372752	1.065077	2.660963
HORVU7Hr1G033500	4.557137	4.455917	3.467888	11.81801	22.14108	23.57508
HORVU6Hr1G020980	4.075889	3.633676	3.154108	2.398895	1.204479	1.289822
Hordeum_vulgare_newGene_4679	7.981008	9.099386	9.442943	0.048671	0	0.040314
HORVU1Hr1G036630	3.777091	2.960492	3.516378	1.524868	2.150248	2.692128

Hordeum_vulgare_newGene_7320	1.326376	1.129167	1.320947	0	0	0
HORVU6Hr1G003020	0	0.029951	0	3.182791	7.630812	3.091117
Hordeum_vulgare_newGene_215	29.1483	23.80285	36.95786	22.83354	13.57739	17.09726
HORVU3Hr1G017940	8.055448	6.30372	7.277114	3.539084	1.967983	2.757154
Hordeum_vulgare_newGene_212	0.189665	0.146766	0.12784	0.661933	0.720376	0.869022
HORVU6Hr1G067660	12.26874	12.49173	18.38433	35.4142	59.84647	39.26063
HORVU3Hr1G095330	3.857134	3.709788	3.926001	10.49105	17.21233	17.55425
HORVU5Hr1G095580	0.516632	1.019108	0.288042	22.2703	1.637978	11.47878
HORVU4Hr1G058340	0.72512	0.419334	0.8582	1.754849	1.662706	2.078224
Hordeum_vulgare_newGene_13600	15.29407	15.20788	14.51757	36.04911	64.39185	58.05852
HORVU6Hr1G088710	2.968036	4.698071	4.207259	1.863239	1.552805	1.692355
HORVU6Hr1G078390	24.89812	21.39734	22.53678	0.355229	0.083012	0.187867
HORVU2Hr1G116540	4.238536	5.424467	7.202333	2.331336	2.424659	2.944808
Hordeum_vulgare_newGene_2363	0.232231	0.209511	0.385837	2.111538	5.740289	4.411856
HORVU6Hr1G009770	0.045311	0.138867	0.132239	0.641077	0.774772	0.736022
Hordeum_vulgare_newGene_9451	0.08918	0.079728	0.058192	0.582789	0.919668	0.73103
HORVU0Hr1G031830	1.617276	1.230273	1.275262	7.636856	5.10322	5.950725
HORVU3Hr1G003130	3.393111	4.007308	4.667968	1.35644	1.876112	0.862893
HORVU6Hr1G034110	0.365857	0.360325	0.745885	0.64447	2.152131	2.779157
HORVU3Hr1G096910	0.383554	0.4712	0.429637	5.668015	8.451061	8.117142
HORVU7Hr1G048570	12.07178	10.56781	12.64167	44.74308	45.12236	46.4559
HORVU1Hr1G021920	1.105381	1.981983	1.24446	7.477368	11.3803	7.669464
HORVU5Hr1G111040	1.333796	1.362202	1.756556	0.171907	0.012119	0.138303
HORVU4Hr1G080720	0.392878	0.347252	0.325341	3.582851	4.109783	3.542066
HORVU3Hr1G012280	14.32853	14.65617	17.6107	7.545701	7.657742	8.386378
HORVU5Hr1G040440	21.30663	26.8326	31.21773	3.437984	0.285548	1.155204
HORVU7Hr1G088560	23.21064	22.10455	25.71961	10.0769	5.19719	8.85503
Hordeum_vulgare_newGene_1166	0	0	0	2.204498	2.640988	2.088552
HORVU1Hr1G073670	2.999686	2.397053	2.619087	10.88298	41.39064	42.45311
HORVU5Hr1G096480	1.335618	1.376575	1.505801	3.631946	4.417214	3.47006
HORVU7Hr1G091180	1.567266	2.634085	2.788078	0.612819	0.290082	0.440261
Hordeum_vulgare_newGene_4538	3.255929	3.545389	3.670194	1.555812	2.330106	2.688711
Hordeum_vulgare_newGene_4530	0.835389	0.809189	0.695096	0.029993	0	0
HORVU3Hr1G031000	1.891568	1.305825	1.238644	0.152059	0.762207	0.807312
HORVU6Hr1G035930	0.789423	0.631748	0.889185	0.135845	0.112353	0.08527
HORVU7Hr1G114170	1.42191	1.476735	1.561172	8.286576	18.10565	13.68208
HORVU4Hr1G008270	15.76901	16.25237	17.61863	36.10357	58.91036	55.82242
HORVU7Hr1G022330	0.635581	0.542925	0.79138	1.703845	1.775967	2.452368
HORVU4Hr1G074750	88.11466	116.7694	129.3429	14.14853	1.478443	8.095736
HORVU1Hr1G056580	0	0	0	4.745669	3.585837	4.492761
HORVU0Hr1G001180	1.730272	1.907229	2.227172	20.71706	24.87084	23.92723
HORVU2Hr1G003210	9.820489	6.149372	13.47907	2.257723	0.503413	2.394377
HORVU5Hr1G022140	0.351346	0.196058	0.229081	4.123525	6.484403	8.880962
HORVU1Hr1G055800	1.115442	1.442777	1.536994	2.202626	6.265044	4.381138
HORVU6Hr1G032680	0.205773	0.313308	0.332053	1.457748	1.341576	2.188277
HORVU1Hr1G001490	0	0	0	12.59064	0.26059	0.306429
HORVU4Hr1G060310	6.225991	5.077884	5.978655	2.405289	1.876977	2.358542

Hordeum_vulgare_newGene_11032	0.761957	0.574957	0.754776	0.129713	0	0.011621
Hordeum_vulgare_newGene_11035	0	0	0.008475	2.669058	1.184415	1.94573
HORVU3Hr1G078390	10.76566	10.57926	16.6116	1.253104	0.030747	0.395324
HORVU5Hr1G116800	1.027702	1.199791	1.465105	3.658201	5.209569	4.233411
HORVU5Hr1G008670	1.24522	0.49011	0.893447	2.118314	2.759964	3.223132
HORVU0Hr1G027670	14.79381	12.80327	15.99692	6.881688	7.543784	9.534917
HORVU5Hr1G122820	18.49179	17.73261	17.98026	40.96212	67.06888	64.00568
HORVU2Hr1G112070	1.007174	0.827902	1.46376	1.8179	5.231848	4.072918
HORVU1Hr1G056390	33.26094	26.84692	31.23964	18.06554	17.66621	18.16507
HORVU3Hr1G075620	0.163385	0.504663	0.333157	1.614851	1.801127	1.783024
HORVU7Hr1G121500	0.234816	0.120361	0.117332	0.732998	1.18087	1.118279
HORVU3Hr1G023590	0.146177	0.084914	0.125221	0.983583	1.059362	0.861619
HORVU4Hr1G012700	1.286194	0.682666	2.243296	10.17399	21.46853	16.6394
HORVU4Hr1G085960	9.561114	8.739707	12.30362	27.8956	29.42154	28.07056
HORVU1Hr1G047930	19.69197	17.24707	20.52276	61.86363	92.42665	78.1015
HORVU1Hr1G042210	0.673333	0.858592	0.708225	0	0.015678	0
Hordeum_vulgare_newGene_12718	0.320899	0.236214	0.269539	0.639218	0.948035	1.071416
HORVU5Hr1G111870	31.11529	23.59724	36.15083	82.00191	76.55483	87.5651
HORVU3Hr1G089250	2.064784	3.27629	3.70638	0.192352	0.088367	0.118042
Hordeum_vulgare_newGene_3318	1.122383	1.42393	1.041908	0.69946	0.436431	0.612062
HORVU4Hr1G021190	13.8979	10.11907	11.77219	26.50977	39.3859	36.37479
Hordeum_vulgare_newGene_2048	2.641837	1.98614	1.625962	0	0.005627	0
HORVU2Hr1G048290	4.161128	3.719692	4.223523	0.019495	0	0
Hordeum_vulgare_newGene_13485	5.610287	5.191189	4.804944	2.189173	2.390467	2.004121
HORVU6Hr1G015790	0.077931	0.101102	0.121581	2.089527	7.241749	6.274503
HORVU6Hr1G093840	0.790606	1.019221	0.864927	2.801989	2.784091	3.523288
HORVU5Hr1G053270	1.892867	1.811007	1.777854	4.537343	6.744933	5.542853
Hordeum_vulgare_newGene_857	1158.527	910.4783	1155.827	103.0867	75.05529	89.45389
Hordeum_vulgare_newGene_15053	1.526325	1.889391	2.064133	0.701765	0.756555	0.369873
Hordeum_vulgare_newGene_10022	4.425917	3.21769	1.80612	0.441742	0.084796	0.223529
Hordeum_vulgare_newGene_10027	33.61194	38.67514	41.27845	0.039573	0	0.02152
Hordeum_vulgare_newGene_11995	0	0	0	0.433711	0.870042	0.946749
HORVU4Hr1G079710	9.71203	12.30111	8.980568	25.20694	29.18964	34.20097
HORVU5Hr1G005310	0	0	0	1.057755	2.185107	2.406735
HORVU7Hr1G089260	0.576104	0.31988	0.456101	0.860721	19.11338	14.23626
HORVU4Hr1G034860	1.022898	1.059182	1.584494	4.041879	3.570228	4.542908
HORVU4Hr1G083930	10.53704	11.15861	8.916698	76.47897	56.95601	55.15675
HORVU2Hr1G108340	25.78754	40.48501	49.0193	0.307364	0.126832	0.101195
HORVU6Hr1G070920	1.374031	2.253393	2.155566	0.06078	0	0
HORVU2Hr1G104860	32.29143	34.11059	23.16169	16.24978	9.50087	11.47884
Hordeum_vulgare_newGene_14828	0.691545	0.451402	0.586397	1.818869	2.893082	3.07712
Hordeum_vulgare_newGene_14829	14.23268	14.1603	16.42096	8.88502	8.617498	9.231445
HORVU7Hr1G106570	9.18278	10.69146	12.86569	5.752732	5.144508	5.973581
Hordeum_vulgare_newGene_8256	13.79304	11.59599	21.57618	2.775501	0.324152	1.980196
HORVU2Hr1G077410	1.0199	0.968577	0.622669	2.118434	8.468796	4.255173
Hordeum_vulgare_newGene_4734	0	0	0	5.632756	5.587023	6.928682
Hordeum_vulgare_newGene_4736	0.729577	0.8565	0.666782	0.025621	0.03481	0

Hordeum_vulgare_newGene_7424	23.94709	21.49859	22.57567	61.71468	99.1657	101.3068
Hordeum_vulgare_newGene_4882	0.298714	0.274319	0.270865	1.091716	1.319248	2.037627
Hordeum_vulgare_newGene_4889	0.192517	0.326691	0.355556	0.902974	0.846609	1.030565
HORVU4Hr1G006480	0.473172	0.466209	0.971617	1.08177	3.793745	4.955934
Hordeum_vulgare_newGene_7281	4.603646	5.127275	5.109306	3.167285	2.880971	3.171157
HORVU7Hr1G081980	0.557489	1.080455	0.805313	0.2713	0.19472	0.363301
Hordeum_vulgare_newGene_12057	0	0.046158	0	0.589687	1.21596	1.037204
HORVU6Hr1G053810	4.446266	5.129729	3.011123	146.7385	186.373	157.567
Hordeum_vulgare_newGene_6775	0.103028	0.090051	0.063913	0.546833	0.523014	0.685534
HORVU5Hr1G014120	44.21873	44.51968	59.93153	18.11877	7.349912	11.46844
Hordeum_vulgare_newGene_15889	2.057699	2.381669	1.859566	8.016134	16.52343	13.97956
Hordeum_vulgare_newGene_3053	2.447306	3.530947	3.432758	0	0	0
HORVU5Hr1G076550	12.46902	10.2452	12.25061	7.251726	6.824554	6.972046
Hordeum_vulgare_newGene_14594	2.822381	2.904498	2.526735	0.277228	0.114818	0.095596
Hordeum_vulgare_newGene_14595	8.318534	8.524498	3.964453	4.059633	1.133824	1.401126
Hordeum_vulgare_newGene_14597	2.530673	2.195295	1.5481	0.614214	0.172887	0.228485
Hordeum_vulgare_newGene_14590	15.38067	13.67091	10.28442	8.648825	4.014413	4.709742
HORVU6Hr1G074820	0.260998	0.121892	0.197906	6.250729	6.898159	7.785983
HORVU6Hr1G087380	0.311005	0.226505	0.297788	17.44467	89.47556	67.0659
HORVU3Hr1G013270	0.623542	0.517278	0.567979	0.312678	0.407996	0.422962
HORVU2Hr1G024950	0.994688	1.056236	1.164346	3.819501	3.22334	3.960821
HORVU7Hr1G096880	1.812123	1.999681	3.266754	1.264749	0.870264	1.007535
HORVU4Hr1G003270	0.878424	0.895217	0.938333	2.263982	3.074139	3.942117
Hordeum_vulgare_newGene_9935	61.78471	51.74106	62.91953	20.61831	8.931005	8.032327
HORVU6Hr1G033310	0.165364	0.150564	0.120223	0.584892	0.964392	1.460933
HORVU2Hr1G005620	3.680043	2.834959	4.330979	1.587198	1.256278	1.213868
HORVU7Hr1G119250	3.722011	4.04868	3.018695	0.259423	0.227694	0.302955
HORVU4Hr1G076850	0.870921	0.81873	0.77569	0.203459	0.067803	0.130435
Hordeum_vulgare_newGene_3430	0.30383	0.33918	0.383935	0.92879	0.881114	1.243829
HORVU5Hr1G113740	2.349428	3.377257	4.593566	1.267272	1.729141	1.725806
HORVU7Hr1G041710	2.064342	2.045323	2.796158	9.308339	18.89805	19.58447
HORVU5Hr1G109610	39.43142	30.64247	31.72987	3.040483	2.424619	2.981527
HORVU6Hr1G030150	0.392663	0.246285	0.314958	0.750484	0.995234	1.302166
HORVU7Hr1G092550	4.631752	3.699613	5.783724	16.93354	36.84461	35.1264
HORVU1Hr1G052700	35.52845	28.84025	37.82969	23.28021	18.1836	18.17749
HORVU4Hr1G051990	0.629542	0.672786	1.213383	1.091686	2.848279	3.401851
HORVU2Hr1G124010	1.025054	0.76522	1.091203	2.529846	6.430302	6.350763
HORVU5Hr1G112900	6.320052	4.945393	5.299747	0.227156	0.096421	0.043841
HORVU3Hr1G077930	3.537889	3.921146	4.654231	2.935806	1.086266	1.824578
HORVU1Hr1G079310	0.441861	0.629431	0.437343	0.877716	1.250258	1.152976
HORVU0Hr1G015950	2.195437	3.504343	4.234363	9.424709	6.785518	9.69256
HORVU2Hr1G010690	2.801033	3.382174	1.92541	65.85577	169.8878	159.5885
HORVU2Hr1G099480	1.569072	2.102198	2.017368	15.60668	22.85559	21.56132
HORVU3Hr1G064320	1.707269	0.908303	1.400616	0.749884	0.473309	0.690363
HORVU2Hr1G019250	80.01298	42.92003	54.47913	149.8454	178.2272	162.1672
HORVU7Hr1G074370	0	0	0	8.012687	13.33027	11.35569
Hordeum_vulgare_newGene_10225	2.011103	1.698535	2.407389	0.020294	0	0

HORVU3Hr1G073580	7.773995	5.964207	5.561841	2.289393	3.648121	2.67615
HORVU1Hr1G083610	2.559623	1.420356	2.458897	0.718326	0.946077	0.532878
HORVU2Hr1G097410	1.503692	1.238319	1.576802	0.402124	0.746931	0.839987
HORVU3Hr1G085680	9.289464	10.63457	6.949946	4.117427	4.577136	3.610071
HORVU1Hr1G040540	3.662878	4.960212	3.82484	1.887687	2.772569	2.675815
HORVU3Hr1G065580	2.115416	1.72392	2.087016	6.789969	11.28123	10.43729
HORVU4Hr1G023850	4.95009	5.600912	6.277912	13.13316	20.93633	19.00017
HORVU7Hr1G091800	313.052	223.1356	333.4691	1402.469	1629.192	1418.886
HORVU3Hr1G037260	2.06293	4.272973	9.010133	0.814567	0.227209	0.415699
HORVU1Hr1G066020	0.667019	0.753888	0.61757	3.852685	7.340051	6.12374
HORVU7Hr1G051310	0.013941	0.083533	0	4.355467	2.830161	5.069064
HORVU2Hr1G039100	2.883663	2.214131	2.578137	6.399876	10.08023	8.22127
HORVU7Hr1G045620	823.586	957.2052	698.1874	195.8666	83.91312	181.1061
HORVU6Hr1G067470	3.984664	2.725895	4.5458	12.03634	12.56803	8.129763
HORVU2Hr1G013090	2.961159	3.638612	5.102162	3.061553	1.390921	1.615194
Hordeum_vulgare_newGene_3292	3.676013	4.104762	4.918698	0.871004	0.614435	0.64132
HORVU1Hr1G075860	1.011096	1.34024	1.59249	0.976528	0.107263	0.456117
Hordeum_vulgare_newGene_5561	19.03581	19.56327	16.99591	13.50629	9.243657	10.05238
HORVU3Hr1G052560	0.243495	0.853058	0.832027	2.6184	2.095822	3.071972
HORVU6Hr1G027650	65.36151	54.92063	64.68364	28.51615	20.87485	24.44511
HORVU2Hr1G083020	5.540834	4.545786	5.079793	1.335742	0.384866	0.616913
HORVU2Hr1G086080	46.50759	43.90122	34.894	21.85385	18.62233	21.316
HORVU7Hr1G025800	0.789003	0.731439	0.592283	6.320054	5.416717	5.329347
HORVU5Hr1G065620	37.84762	27.98217	45.00646	13.09446	11.81215	15.48516
HORVU5Hr1G115310	3.257805	3.098863	4.26655	0.788071	1.031405	0.47054
Hordeum_vulgare_newGene_10962	0.508384	0.865492	0.510717	0	0	0
HORVU5Hr1G080050	9.910118	9.987295	12.89517	4.578101	5.719249	5.208181
Hordeum_vulgare_newGene_8864	7.747294	6.598348	7.02944	4.62155	3.750108	3.639429
HORVU2Hr1G096400	39.71174	38.00102	38.65257	2.967815	2.205999	2.84622
HORVU3Hr1G025020	8.373715	6.049977	8.369246	5.084076	4.322131	4.196011
HORVU3Hr1G075690	273.3918	377.6212	421.1875	45.87359	4.124617	18.33399
HORVU2Hr1G104120	56.98317	99.77839	114.7097	23.54737	2.103901	9.851764
HORVU0Hr1G022670	0.596851	0.814915	0.49236	5.058765	5.414032	4.767302
Hordeum_vulgare_newGene_1350	64.83419	64.04125	84.02603	25.69038	12.1148	14.06231
HORVU2Hr1G031710	5.699739	6.110558	5.294351	2.102683	0.935562	2.178757
HORVU2Hr1G026020	1.090965	1.446252	2.029678	0.384761	0	0.17457
Hordeum_vulgare_newGene_9407	1.394538	1.083987	1.093638	0	0	0
HORVU5Hr1G103900	0.643492	0.640548	0.800122	1.892759	3.100397	2.267971
HORVU3Hr1G057860	0.294025	0.113164	0.18931	4.47885	3.442684	3.136493
HORVU6Hr1G087960	1.345852	1.087835	1.445898	2.764756	6.71258	6.328
HORVU2Hr1G105740	191.6906	272.2441	424.68	58.59065	4.285436	11.68401
HORVU7Hr1G096070	1.153323	1.08573	0.902912	0.022069	0.064278	0.022353
HORVU1Hr1G009920	0.209622	0.227134	0.22072	12.61617	8.478135	9.715347
Hordeum_vulgare_newGene_45	104.9978	95.3854	104.9534	21.19647	12.83768	16.02218
HORVU5Hr1G043200	8.094377	9.157892	6.985641	4.184907	1.942117	2.041853
HORVU3Hr1G015880	6.252687	7.182331	7.716854	3.909472	3.758419	4.219795
Hordeum_vulgare_newGene_4964	0.784619	1.117699	1.216022	0.16719	0.074749	0.071846

HORVU2Hr1G020900	0.981692	2.061366	2.904887	23.28166	19.46306	22.35011
Hordeum_vulgare_newGene_4961	0.158325	0.130045	0.05933	2.928868	2.489499	3.210771
Hordeum_vulgare_newGene_4969	0.572234	1.05642	0.718121	0.330142	0.169549	0.169647
HORVU7Hr1G032340	0.630838	0.414498	0.663365	6.17845	7.97101	6.429154
HORVU6Hr1G080750	0.215857	0.548718	0.284992	1.224062	1.399831	1.112317
HORVU7Hr1G052190	3.471352	3.756591	4.479068	9.57095	11.6099	11.43131
HORVU5Hr1G064230	0.537976	0.598176	0.483762	1.218584	1.635939	2.045143
HORVU3Hr1G006830	4.625267	3.444241	4.289896	1.969562	1.973443	3.11263
HORVU1Hr1G015820	3.301715	4.044404	3.725291	0.040286	0.076016	0.136907
Hordeum_vulgare_newGene_9879	0	0	0.010191	3.928124	4.257434	4.468034
Hordeum_vulgare_newGene_9877	1.325367	1.174178	1.381955	0.866216	0.340882	0.731351
HORVU1Hr1G087110	22.25413	20.21055	21.78642	3.321929	0.420214	0.955555
HORVU5Hr1G023640	0.012913	0.010222	0.006746	0.427115	2.417598	3.183825
HORVU6Hr1G084720	2.821152	3.145632	2.507756	5.582335	9.743343	9.390321
HORVU1Hr1G015560	154.1596	146.081	142.7121	24.40655	18.1897	16.3649
HORVU6Hr1G072250	1.547777	1.440042	1.440701	13.23298	12.0635	13.16251
HORVU3Hr1G078830	3.398108	4.203098	3.908411	6.983397	11.03698	10.85681
HORVU5Hr1G122470	0.344988	0.263841	0.191274	0.660296	1.550678	1.214181
HORVU0Hr1G007370	0.274139	0.4048	0.371968	1.620457	14.64284	17.27028
HORVU2Hr1G056710	0.722065	0.812912	0.822758	2.38798	2.195574	2.56195
HORVU7Hr1G048080	1.97886	2.31561	2.137649	5.302196	9.385349	9.358364
HORVU5Hr1G069310	0.066802	0	0.040915	6.077199	6.801356	7.982941
HORVU5Hr1G111550	47.7323	39.1518	46.61467	22.14132	24.27551	24.76994
HORVU4Hr1G081670	34.46221	30.48378	38.12447	24.53544	17.03427	21.33685
HORVU2Hr1G066690	33.42274	32.7357	35.99112	21.7197	18.42928	18.9904
HORVU7Hr1G078330	1.696474	2.261644	2.702643	0.519704	0	0.149358
HORVU7Hr1G050680	62.77119	53.88192	61.8048	20.93726	4.640471	10.5742
HORVU7Hr1G120020	1.652428	2.142383	1.944562	3.920185	4.585803	4.86556
HORVU1Hr1G058150	0	0.027061	0.036757	0.897019	5.45843	3.780882
HORVU3Hr1G071220	0.144659	0.058314	0.109008	2.368165	2.54638	2.17815
HORVU1Hr1G012420	2.387924	2.354502	3.341949	5.089235	7.449461	8.044624
HORVU5Hr1G124710	0.26492	0.363587	0.421588	0.04378	0.038306	0.076369
HORVU2Hr1G081230	0.15191	0	0.696267	23.57834	317.7046	220.7487
HORVU1Hr1G089180	1.022356	0.564248	0.494597	48.76073	7.099836	7.147287
HORVU4Hr1G027930	1.374886	0.992434	1.860731	2.714329	5.064861	5.309512
HORVU5Hr1G121350	3.931908	4.328553	4.617918	0.202172	0.078905	0.098777
HORVU2Hr1G080490	3.033069	3.322521	3.893976	1.955698	1.362618	1.791681
HORVU2Hr1G107030	0.166227	0.318741	0.272827	0.736032	1.313037	1.436915
HORVU5Hr1G056670	0.447067	0.63044	0.718811	2.197219	3.069589	4.040935
HORVU0Hr1G001470	0.313952	0.128781	0.319204	0.907369	2.319853	2.570493
HORVU7Hr1G095900	2.506711	2.179195	2.337731	0.612679	0.443315	0.64462
HORVU5Hr1G074810	0.909616	1.034655	1.170402	0.355032	0.129443	0.290117
HORVU5Hr1G125300	15.59905	14.26863	16.90265	5.147755	5.265632	6.001223
HORVU5Hr1G079990	26.06849	23.12556	21.21275	13.41105	11.38917	11.76189
HORVU5Hr1G098340	1.013099	0.691246	0.981378	3.071673	5.814507	6.09465
HORVU4Hr1G000630	0.027221	0.0462	0.046259	1.63891	1.155703	1.394662
HORVU6Hr1G013450	2.367026	3.497104	3.690353	1.081396	1.383384	2.017409

HORVU4Hr1G060670	0.127857	0.194222	0.119281	15.41019	1.859906	5.952237
HORVU1Hr1G060060	1.517287	1.222511	1.707499	0.788087	0.201795	0.389658
HORVU3Hr1G098640	1.098957	0.962826	0.936485	2.95622	4.236308	4.998454
HORVU1Hr1G065250	0.619683	0.557996	0.570674	2.923974	2.185424	3.215152
HORVU0Hr1G040200	2.012408	2.045498	1.469954	4.784472	5.487642	5.114815
HORVU2Hr1G012790	88.0115	90.14346	108.1511	14.37347	1.634659	6.011482
Hordeum_vulgare_newGene_160	1.293425	2.715639	3.496089	41.99805	53.18006	53.83988
HORVU3Hr1G081610	0.719151	0.545315	0.894296	2.64696	3.385602	4.18903
HORVU4Hr1G043680	21.74156	19.41509	21.07073	47.78092	59.43983	57.70643
HORVU2Hr1G109080	0.312854	0.274185	0.167774	0.802214	0.824777	0.98973
HORVU2Hr1G072850	8.307502	8.451635	11.25788	19.44217	79.51726	91.19272
HORVU5Hr1G072760	8.763929	8.235621	8.096111	2.032112	2.035436	1.973891
HORVU5Hr1G041590	0.537607	0.535816	0.517787	1.421542	1.213661	1.990258
HORVU5Hr1G070720	55.52875	54.96861	62.59732	83.82052	233.9887	188.1015
HORVU5Hr1G113580	45.76381	52.42496	48.59397	161.8953	147.0052	130.9621
HORVU5Hr1G112390	10.78409	13.81899	18.38515	3.179857	5.818924	5.222974
Hordeum_vulgare_newGene_12487	7.754729	6.35757	7.857697	29.42458	39.69684	33.05331
HORVU2Hr1G103010	252.9156	194.4095	244.4774	92.36829	86.75196	88.89232
Hordeum_vulgare_newGene_13579	3.580317	3.111388	3.306291	2.213939	1.094012	1.778299
Hordeum_vulgare_newGene_3486	0.035184	0	0.035321	2.81837	2.730517	3.454691
Hordeum_vulgare_newGene_3485	5.902467	4.701342	6.391025	4.024872	3.616964	2.518506
HORVU5Hr1G085760	11.58352	11.46756	11.09022	8.343344	5.758947	7.735206
Hordeum_vulgare_newGene_6053	0	0	0.005139	5.148375	5.621052	5.680993
Hordeum_vulgare_newGene_13685	14.86854	14.10321	18.56896	29.93514	75.24263	75.40183
Hordeum_vulgare_newGene_13684	81.53481	68.90854	46.22179	18.21538	13.53348	14.02572
Hordeum_vulgare_newGene_5881	1.449993	0.058409	1.58402	5.056979	7.018313	6.090722
HORVU2Hr1G005470	1.754382	1.589737	1.715049	6.452731	11.80223	11.00341
HORVU6Hr1G032260	0.316277	0.638893	0.494843	0.730282	2.556966	2.607155
HORVU7Hr1G078770	2.915286	3.107081	4.014136	6.654873	13.64017	13.50492
HORVU4Hr1G061260	7.761657	7.181788	9.471839	3.318121	4.509254	4.650731
HORVU4Hr1G011500	29.11418	27.00072	28.04649	86.77551	131.9436	134.9675
HORVU2Hr1G027230	1.397447	2.409492	2.831174	0.615821	0.574927	0.91792
HORVU3Hr1G069070	1.216457	1.772834	1.631064	2.062435	7.618039	5.285968
HORVU2Hr1G077000	0.292835	0.146646	0.33369	0.734914	1.223019	1.426533
Hordeum_vulgare_newGene_11863	7.39464	8.558166	4.533512	0.705633	0.160846	0.506822
Hordeum_vulgare_newGene_11867	0.120683	0.11279	0.320598	1.641853	0.887948	0.845352
HORVU2Hr1G051180	1.112489	0.405252	1.1069	2.911096	3.333205	3.819616
HORVU3Hr1G058300	2.559759	2.081732	2.681139	1.372033	0.433586	1.013004
Hordeum_vulgare_newGene_1250	0	0	0	0.872128	0.692943	1.267476
HORVU3Hr1G014580	0.886144	1.026842	1.257933	1.8055	4.023707	4.410751
HORVU7Hr1G106480	1.180943	1.02646	1.495835	4.229757	4.715143	4.582689
Hordeum_vulgare_newGene_4024	116.0966	160.3855	178.1456	58.45396	15.54069	29.39181
Hordeum_vulgare_newGene_4025	0.24342	0.431334	0.373625	1.104112	1.569237	1.489815
Hordeum_vulgare_newGene_4026	0.669361	0.881108	1.131951	0	0	0
Hordeum_vulgare_newGene_4022	5.331226	4.280957	6.024327	1.329687	0.131477	0.432783
Hordeum_vulgare_newGene_4023	6.361042	5.367045	10.98251	0.787261	0.780888	2.344816
HORVU0Hr1G005900	0.469455	0.205955	0.327847	1.085385	1.255491	1.292071

Hordeum_vulgare_newGene_7592	0.457783	0.694657	0.775397	4.551349	4.666028	5.783162
HORVU1Hr1G069800	1.131487	1.667973	1.552298	10.88974	7.189045	11.58012
HORVU1Hr1G076950	1.375392	1.596571	1.552466	0.599389	0.921191	0.667895
HORVU0Hr1G000850	5.328503	3.870836	5.591878	0	0.02514	0.013331
HORVU5Hr1G027110	13.88664	14.49399	8.789166	0.803383	0.642054	0.924267
HORVU4Hr1G073120	313.3222	356.0598	485.8	58.25667	20.04231	27.34774
Hordeum_vulgare_newGene_5472	0.701973	0.665794	1.143164	0	0	0
HORVU3Hr1G035470	115.5633	144.1268	216.9632	12.30918	6.254481	8.344612
HORVU5Hr1G093400	5.684961	5.021487	5.28272	14.21527	21.63285	19.08256
HORVU1Hr1G074550	3.741328	2.505494	3.519069	1.773892	1.036451	1.968002
Hordeum_vulgare_newGene_11352	1.69482	1.549904	1.325881	0	0	0
Hordeum_vulgare_newGene_10166	27.64494	21.40602	32.44775	0.131259	0	0
HORVU4Hr1G074470	0.036185	0.038148	0.042841	0.777189	0.933594	1.475224
HORVU1Hr1G080680	3.088107	1.246906	4.642656	0.667991	0.110738	0.842244
Hordeum_vulgare_newGene_6310	1.085502	0.912117	0.604583	0.262928	0.383599	0.626417
HORVU1Hr1G021750	97.51536	112.9335	158.3681	32.82372	1.631948	11.53195
HORVU4Hr1G007260	2.642459	2.2402	3.317764	7.522638	9.012919	10.05453
HORVU7Hr1G000260	2.549633	2.191649	3.204763	19.5989	16.34103	17.88732
HORVU2Hr1G061920	6.434709	6.17967	6.892816	3.099424	1.838247	3.386337
HORVU3Hr1G109880	1.703337	2.357902	3.015823	3.911293	10.265	9.91565
HORVU5Hr1G106280	0.890383	0.734347	0.868519	0.123222	0.146877	0.084078
HORVU7Hr1G020580	1.991093	1.942363	2.189933	3.324282	32.82734	33.95944
HORVU1Hr1G091010	99.4419	97.5669	132.0206	39.84423	8.921203	16.61644
HORVU2Hr1G098660	40.67228	40.14005	41.14026	17.8543	11.59444	13.16274
HORVU6Hr1G000340	25.9484	29.54448	45.71153	1.549754	0.387281	0.969812
HORVU3Hr1G029470	0.388864	0.353201	0.477204	3.965095	1.914259	2.664844
HORVU1Hr1G065160	2.418603	1.948638	2.98159	7.919883	15.87819	17.01101
HORVU5Hr1G109100	0.189622	0.209411	0.228191	9.567031	9.173363	6.981811
HORVU2Hr1G015980	25.7288	27.93621	23.25977	67.01902	106.6	91.46935
HORVU7Hr1G114890	0.392255	0.39035	0.272515	5.153944	10.21462	9.037797
HORVU6Hr1G094080	0.023176	0	0.049055	0.833696	1.413629	1.099295
HORVU6Hr1G019510	4.347285	3.068131	4.010334	11.03608	17.76565	19.9618
HORVU5Hr1G035980	0.344552	0.294461	0.396712	0.778286	1.389866	1.418329
HORVU7Hr1G082970	2.802115	1.538729	1.676175	0.305892	1.135776	1.345474
HORVU4Hr1G058500	1.570883	1.860187	1.967417	1.171892	1.120286	0.933821
Hordeum_vulgare_newGene_11940	0.028612	0.018778	0.006243	0.327981	1.033003	0.763474
HORVU3Hr1G028540	6.583752	7.818103	7.811542	27.58254	24.21719	25.09776
HORVU7Hr1G093680	1.225089	0.941995	1.82014	2.338851	8.914997	8.990531
HORVU3Hr1G099490	1.691687	2.510679	3.697111	0.993313	0.967007	0.961436
Hordeum_vulgare_newGene_13739	0.118014	0.281434	0.381863	43.44859	40.82188	43.89149
HORVU3Hr1G092860	3.069655	1.618629	2.53455	4.375007	9.247157	6.667005
HORVU7Hr1G100090	232.0724	206.1294	213.4031	30.08943	27.42643	29.37872
HORVU6Hr1G014600	13.44347	9.688711	19.69915	2.533186	1.407188	3.697519
HORVU2Hr1G104570	7.69097	5.895811	8.365422	4.526594	3.609183	3.675108
HORVU5Hr1G073170	3.341837	3.694991	4.360995	2.282224	1.05572	1.612151
HORVU2Hr1G109330	0.231122	0.2842	0.212604	3.138406	0.665085	2.39518
Hordeum_vulgare_newGene_10605	1.755552	1.694639	1.650662	0.742718	0.39493	0.673258

Hordeum_vulgare_newGene_10602	2.153207	2.916578	2.900686	0	0	0
HORVU1Hr1G040920	0	0.042565	0.032991	10.8285	11.21967	15.58591
Hordeum_vulgare_newGene_1988	6.141619	7.977539	7.606974	1.155125	0.442533	0.743591
HORVU3Hr1G108120	0.238022	0.180574	0.232783	1.468018	2.754594	1.50013
HORVU0Hr1G016250	0.883724	0.738974	0.72684	2.085107	2.618605	2.666411
HORVU3Hr1G069650	2.13154	3.171796	4.63652	49.32152	31.96761	44.42965
Hordeum_vulgare_newGene_316	1.192502	0.922654	1.289618	0.605725	0.324386	0.488822
HORVU7Hr1G039140	0	0.007965	0.180682	0.848724	2.030697	2.394215
Hordeum_vulgare_newGene_7401	1.067391	0.876981	0.61014	0.020586	0.00662	0.018614
HORVU3Hr1G067590	5.052271	4.14813	6.704628	2.674111	2.344183	2.562426
Hordeum_vulgare_newGene_2379	9.751519	13.91994	13.57552	1.038058	0.349082	0.751243
Hordeum_vulgare_newGene_2986	5.39192	4.255778	5.854653	0	0	0
Hordeum_vulgare_newGene_7208	5.92961	5.86333	5.354147	1.413068	2.1178	2.229914
HORVU7Hr1G079160	2.172277	1.514361	1.950551	0.450438	0.205355	0.248757
HORVU2Hr1G075230	3.668832	3.406411	3.4475	2.821505	1.026856	2.022482
Hordeum_vulgare_newGene_13724	0.475363	0.26112	0.489834	2.6947	0.952194	1.279212
HORVU7Hr1G109730	0.134812	0.144513	0.047727	2.144112	4.193953	3.485244
HORVU6Hr1G078290	2.307223	2.284358	3.762587	6.581557	14.93307	14.76392
HORVU2Hr1G027680	13.48513	10.17131	13.6509	63.37518	38.43786	47.65575
HORVU7Hr1G103550	23.98575	17.87153	25.8547	10.87413	9.603068	12.2438
HORVU4Hr1G026150	0.860186	1.189879	1.027793	5.93427	64.8816	62.36951
HORVU5Hr1G061410	5.794245	6.89195	7.015948	0.048296	0.031395	0
HORVU2Hr1G080870	7.880054	8.659775	12.95331	4.844729	5.574224	5.817888
Hordeum_vulgare_newGene_9314	3.141876	3.774036	3.633098	0.050886	0.030779	0.01236
Hordeum_vulgare_newGene_9310	6.374624	6.654405	7.568284	2.448365	1.431993	1.003027
HORVU1Hr1G020370	1.323388	0.982208	0.842702	5.212541	4.465982	6.221592
Hordeum_vulgare_newGene_15394	2.609754	2.434255	3.546916	0	0.569579	0
HORVU3Hr1G003760	0.141421	0.262916	0.233811	0.944007	1.280948	1.217508
HORVU7Hr1G044210	0.025689	0.05261	0.06527	0.545607	0.999239	1.004557
HORVU2Hr1G092540	4.961279	5.244665	5.49964	18.69125	28.27198	26.16542
HORVU3Hr1G004230	18.36391	27.35208	34.03981	3.281546	0.022778	1.302799
HORVU3Hr1G011850	49.26153	29.98167	66.52804	2.785633	1.644632	9.329204
HORVU5Hr1G020710	1.273531	1.267445	1.984578	1.248828	1.211377	2.185259
HORVU4Hr1G005490	0.107298	0.034258	0	2.293959	2.173542	1.193382
Hordeum_vulgare_newGene_10425	1.124598	1.942301	1.21027	0.436255	0.375018	0.542826
HORVU5Hr1G064040	1.384629	1.142038	2.634239	0.270594	0.285434	0.35435
HORVU3Hr1G083450	0.305793	0.155769	0.337882	3.384766	1.420164	1.385168
HORVU2Hr1G077210	3.479184	2.065375	2.650296	2.188242	18.85045	15.74043
HORVU7Hr1G017190	50.2834	44.94442	57.87903	26.29686	25.33765	25.64738
Hordeum_vulgare_newGene_1047	1.143825	0.735213	0.814808	5.841578	3.887848	5.065557
Hordeum_vulgare_newGene_1043	0.899848	1.032046	0.783426	0.47724	0.21071	0.29514
Hordeum_vulgare_newGene_1049	0.500244	0.600841	0.704	1.547407	1.501016	1.699132
HORVU1Hr1G073130	3.104303	2.878239	4.399672	1.976887	2.237312	1.895704
HORVU1Hr1G060030	1.366627	1.333205	2.253973	7.086461	5.356433	4.232219
HORVU7Hr1G091060	11.44439	9.070576	10.65721	5.096224	2.137972	2.83888
Hordeum_vulgare_newGene_4276	0.078937	0.084857	0.115873	1.436984	0.631732	0.788804
Hordeum_vulgare_newGene_4273	6.073151	6.997934	6.540385	0.022602	0.008562	0

HORVU3Hr1G031320	0.142987	0.15611	0.071333	0.336929	0.899643	1.025071
HORVU1Hr1G058630	1.40367	1.103134	1.083878	4.159122	3.927324	3.599053
HORVU7Hr1G039800	3.009788	2.923343	3.258686	1.755934	1.446892	1.467712
HORVU2Hr1G041040	0.22265	0.24305	0.383903	2.022901	4.722349	4.160265
HORVU0Hr1G019840	0.675297	0.372814	0.719948	3.127658	6.270071	5.800987
HORVU2Hr1G100960	19.33251	18.59807	18.22884	8.779867	5.447412	5.154907
HORVU7Hr1G054610	0.759751	0.946514	1.181979	0.131991	0.02815	0.097882
HORVU3Hr1G002150	2.443873	2.55852	3.407632	0.889152	0.61669	0.831703
HORVU4Hr1G067850	24.23209	17.90159	25.53718	7.549732	6.482221	4.15351
HORVU3Hr1G082190	8.718869	8.875316	10.67449	5.607794	4.940458	5.757607
HORVU5Hr1G112710	0.507986	1.079807	0.723298	3.614912	5.361234	4.823878
HORVU7Hr1G040740	6.091837	2.831313	4.867654	1.112397	0.112993	0.557016
HORVU2Hr1G105930	9.269322	7.760936	7.821261	3.679149	5.728456	4.982594
HORVU6Hr1G084690	1.670105	1.463181	1.927183	4.613503	5.23939	6.096774
HORVU2Hr1G094240	1.208932	1.427141	1.119529	2.37872	4.823325	3.66699
HORVU3Hr1G009980	1.429756	1.632804	2.227117	16.24262	40.33504	34.491
HORVU7Hr1G071750	0.73135	1.061415	0.74359	1.293599	4.674733	4.352745
HORVU1Hr1G067370	31.68086	36.78511	58.38212	6.127261	3.662095	4.94376
HORVU4Hr1G018650	4.703737	4.73776	5.093067	7.515409	29.16053	28.12416
HORVU0Hr1G028350	36.74026	38.57864	31.37444	21.14111	9.606234	15.19463
HORVU2Hr1G110230	15.32279	19.36864	21.66577	5.104172	2.188139	3.264149
HORVU6Hr1G089730	0.058962	0.184559	0.113285	5.668427	7.140771	5.772467
Hordeum_vulgare_newGene_3219	0.795892	0.780814	1.419359	0.360234	0.527343	0.620614
HORVU1Hr1G047100	0.576381	0.38981	0.747773	2.054051	5.683414	5.74349
Hordeum_vulgare_newGene_2166	1.87072	1.867388	2.134568	4.303308	6.308328	6.513549
Hordeum_vulgare_newGene_2165	22.89504	19.95805	25.10265	0.026564	0	0
HORVU3Hr1G024220	7.330757	5.284046	7.84671	4.485946	3.850137	3.334399
HORVU5Hr1G079130	0.588481	0.23334	0.654836	3.063543	15.15029	12.96549
HORVU6Hr1G009670	11.62536	10.20263	11.33215	29.49989	27.79415	29.14985
Hordeum_vulgare_newGene_15151	2.243896	3.975841	4.406301	0.725533	1.024313	0.901136
Hordeum_vulgare_newGene_15150	5.307966	4.378515	5.146807	1.13217	1.079172	1.283125
HORVU0Hr1G002390	0.078378	0.0922	0.080375	0.489725	0.859303	0.634267
HORVU5Hr1G092800	12.96301	14.84671	18.05954	6.870317	8.215236	7.954295
Hordeum_vulgare_newGene_13878	5.594826	8.416259	7.237477	0.03569	0	0
Hordeum_vulgare_newGene_13879	17.51432	13.08374	18.68727	11.56154	5.257713	6.538229
HORVU5Hr1G098720	16.27635	14.65516	20.72473	13.18995	6.946229	7.201588
Hordeum_vulgare_newGene_8689	0.2654	0.435498	0.118077	3.919361	5.14273	4.421423
Hordeum_vulgare_newGene_8353	3.026303	2.591616	2.439551	6.99212	11.92225	11.96742
HORVU3Hr1G105860	8.886518	8.986387	9.371697	4.79106	3.027844	4.183996
HORVU5Hr1G049880	7.638118	8.700017	9.468356	5.457457	4.165044	4.165111
Hordeum_vulgare_newGene_4476	0.027612	0.039814	0.023149	0.451134	0.436435	0.430165
Hordeum_vulgare_newGene_4471	0	0	0	2.310217	1.511029	1.458231
HORVU2Hr1G011570	0.078223	0.049585	0.049582	3.278196	3.350213	3.870958
Hordeum_vulgare_newGene_1534	0.590397	0.556628	0.931528	7.919203	3.298208	3.369283
Hordeum_vulgare_newGene_1533	17.2939	13.63435	20.91875	5.535774	0.831508	1.682663
Hordeum_vulgare_newGene_3971	0	0	0	1.802738	2.456753	2.33934
Hordeum_vulgare_newGene_3976	0.304965	0.261868	0.254603	0.890765	1.492493	1.116787

HORVU3Hr1G023910	0.449171	0.309157	0.335608	1.389592	1.808949	1.350088
HORVU6Hr1G073540	143.9164	161.4699	243.3366	13.71289	1.923642	5.381627
Hordeum_vulgare_newGene_12391	1.832567	1.703934	1.645981	0	0	0
HORVU1Hr1G003990	17.25204	15.10268	16.35093	8.950081	9.225112	8.55664
HORVU3Hr1G080730	8.160783	8.52678	7.619423	5.442479	4.353194	4.51168
HORVU7Hr1G100100	4.839947	5.290318	4.821654	5.631104	25.82456	20.3919
Hordeum_vulgare_newGene_12645	0	0	0	5.140624	3.632408	4.095944
HORVU3Hr1G085520	0.365029	0.317006	0.178594	0.968043	1.691664	1.427405
HORVU4Hr1G037290	12.64677	14.37966	15.61033	31.98166	39.50696	44.47909
HORVU7Hr1G024890	13.22961	14.29648	22.05025	8.227228	7.203221	8.960232
HORVU4Hr1G074600	2.987128	3.031179	3.560151	1.600056	1.745144	1.710924
Hordeum_vulgare_newGene_500	1.782977	2.364527	2.894207	1.311006	1.244052	1.676279
HORVU3Hr1G089650	2.134096	2.266941	2.300946	9.773595	15.80781	13.94166
Hordeum_vulgare_newGene_14494	17.68154	15.00164	12.72324	8.099035	6.493872	5.668129
HORVU6Hr1G083570	0.306796	0.188523	0.135331	0.907198	1.098323	1.570347
HORVU3Hr1G113020	11.54453	6.844811	11.27934	1.582415	0.634986	1.216043
HORVU7Hr1G018470	0.530456	0.248758	0.300604	4.025033	3.259211	4.019944
HORVU0Hr1G000280	6.836832	6.537986	7.41846	3.689309	3.757603	3.569717
HORVU7Hr1G111860	0.530185	0.528814	0.411974	0.161158	0.248491	0.373217
HORVU5Hr1G075510	0.411244	0.375893	0.495942	4.371357	5.12602	4.168071
Hordeum_vulgare_newGene_13118	3.658512	3.295022	3.646132	0.880326	0.968185	1.163096
HORVU5Hr1G079670	6.932477	6.607808	9.115359	1.321635	0.353728	0.769115
HORVU0Hr1G001880	0.010446	0.011528	0.010177	0.38147	0.624705	0.971186
HORVU5Hr1G087340	0.173054	0	0.157952	2.528734	11.02105	10.55555
HORVU6Hr1G078430	0.624845	0.384002	0.314151	1.090368	0.860849	1.039962
HORVU3Hr1G003040	8.496596	7.629497	8.556773	15.24066	36.41895	29.86955
HORVU4Hr1G050210	279.7795	272.7172	325.8335	87.21632	5.237911	38.72206
HORVU6Hr1G009880	0.565901	0.643704	0.778032	0.141884	0.344771	0.353466
Hordeum_vulgare_newGene_15456	0	0	0.069139	52.35675	39.96975	49.13676
HORVU3Hr1G087030	3.112726	4.294821	3.032165	1.462581	0.425271	0.318824
HORVU6Hr1G028150	1.520584	1.139234	1.286759	0.366257	0.0405	0.502747
HORVU5Hr1G113020	2.28052	2.330895	2.151965	0.790609	0.239278	0.322769
HORVU3Hr1G054710	6.771665	6.625943	12.24107	2.488454	6.256164	5.51061
HORVU7Hr1G086570	1.322325	0.946341	1.436203	2.294517	4.479495	4.487564
HORVU4Hr1G011160	12.03205	12.9083	11.66928	38.43743	70.56	69.03645
HORVU2Hr1G096910	0.140152	0.10796	0.156034	3.88477	9.574589	8.44873
HORVU5Hr1G109880	0.875151	1.021193	1.366492	2.734655	3.651908	5.250518
HORVU7Hr1G095630	0.093554	0.0942	0.204385	1.925156	6.278392	5.203479
HORVU6Hr1G083080	0.061423	0	0	2.23343	1.452326	1.545482
HORVU3Hr1G030840	3.061581	2.338716	2.313313	11.01731	9.567875	9.600248
HORVU3Hr1G099590	0.156433	0.080998	0.103735	1.202006	0.558075	1.057338
HORVU5Hr1G080210	1.560898	1.413389	2.184878	0.38434	0.320174	0.700283
HORVU2Hr1G080100	2.866735	3.121004	3.031308	6.331349	13.75144	14.42632
HORVU3Hr1G046780	0	0	0	2.59113	2.291939	3.068828
HORVU1Hr1G056210	8.443389	7.85286	6.559061	6.214886	2.79955	4.334277
HORVU4Hr1G015550	1.007037	1.02108	1.333075	3.484352	5.34469	3.723827
HORVU2Hr1G001780	4.361939	2.435251	5.474634	1.539583	0.209958	1.352127

HORVU2Hr1G099610	35.50969	37.93358	67.08852	13.69472	25.66668	23.37768
HORVU2Hr1G096640	14.80477	17.07484	15.28156	5.679611	3.919667	2.514007
HORVU5Hr1G045750	3.684765	4.129201	5.551057	1.72312	0.733381	1.516757
HORVU3Hr1G074570	1.626892	1.655539	1.516629	5.062384	13.15865	12.2626
HORVU7Hr1G055440	18.48418	17.22621	18.43122	8.157713	9.679165	10.21493
HORVU7Hr1G098370	2.031314	1.752423	2.720157	4.29518	7.671259	11.70725
HORVU2Hr1G079020	6.856772	6.665627	7.431578	19.06961	18.06612	20.59754
HORVU4Hr1G007570	0.692067	0.762606	1.078872	0.448785	0.293006	0.286235
HORVU3Hr1G002050	1.303525	1.038425	0.897669	0.263405	0.12544	0.511782
HORVU1Hr1G051290	19.20386	15.31556	21.34661	59.14825	72.1708	63.19013
HORVU1Hr1G055970	0.670601	0.7444	0.889962	5.24459	6.432576	8.17586
HORVU5Hr1G124470	0.056123	0.062332	0.038468	2.223128	194.2115	132.9774
HORVU2Hr1G009850	0.517244	0.821025	0.308321	1.888206	1.703813	1.993943
HORVU7Hr1G037600	8.190733	11.40144	13.96175	5.005509	5.129452	4.830841
HORVU2Hr1G108350	0.205225	0.201096	0.194674	0.677884	0.68924	0.809327
HORVU3Hr1G073710	9.652142	9.766532	8.422714	4.574064	5.872112	5.85026
HORVU7Hr1G012850	23.52444	30.44458	41.11028	10.64336	15.25352	14.2083
HORVU5Hr1G117040	0	0	0	7.854235	6.572773	8.032438
HORVU4Hr1G067320	2.374258	2.597101	3.307279	4.858639	9.241374	9.044683
HORVU4Hr1G043650	3.675795	3.066228	4.253088	8.298243	12.43147	11.1824
HORVU2Hr1G013730	7.844992	7.414082	8.536693	4.19882	2.929303	3.5815
HORVU6Hr1G090330	0.312546	0.390604	0.395782	0.097324	0.054512	0.06179
HORVU2Hr1G115160	2.120763	3.099064	3.931853	4.067899	13.52467	10.95293
HORVU5Hr1G092480	1.799511	1.04649	1.516988	0.165623	0.200056	0.245156
Hordeum_vulgare_newGene_16187	0	0	0	2.189183	4.026986	5.17602
HORVU3Hr1G022510	3.338768	2.740589	3.786465	5.991122	52.60588	57.08061
HORVU7Hr1G109960	13.74642	13.19628	17.1351	7.88696	8.387177	5.819855
HORVU7Hr1G050160	1.283667	1.399825	1.762798	7.727962	24.03923	19.70081
HORVU2Hr1G120530	1.80911	1.283517	2.491949	36.34422	71.79903	52.95292
HORVU7Hr1G001750	11.58435	5.576825	8.156237	0.429703	1.714647	1.032597
HORVU3Hr1G089090	1.600661	1.604285	1.81002	4.825301	3.905336	5.843951
Hordeum_vulgare_newGene_3402	0	0	0	3.528676	3.419922	4.137387
HORVU7Hr1G109340	0	0	0	2.211486	1.734946	2.612604
HORVU4Hr1G057910	6.643569	7.074248	11.99971	21.61004	28.67904	32.30188
HORVU5Hr1G063420	0.579171	0.510056	0.756138	1.503902	1.524426	1.980275
Hordeum_vulgare_newGene_3408	33.81064	39.22233	37.29365	1.577751	1.708086	1.822192
HORVU1Hr1G062190	1.707916	2.174929	2.480546	4.911092	9.669674	8.501081
HORVU2Hr1G076620	2.341749	2.573854	2.550538	1.638108	1.367529	1.641436
Hordeum_vulgare_newGene_14129	5.214302	5.945109	6.705267	0	0	0.094302
HORVU1Hr1G070190	0.472432	0.742209	0.363931	1.398613	2.176088	2.176064
Hordeum_vulgare_newGene_14123	1.734253	1.224343	1.517939	7.121352	13.73657	13.59292
Hordeum_vulgare_newGene_10809	3.418	3.08388	5.20755	0.738649	0.568637	0.543869
Hordeum_vulgare_newGene_15898	8.061575	8.437061	8.624666	0.040186	0	0
Hordeum_vulgare_newGene_10802	2.036638	2.875667	1.942732	0	0	0
HORVU3Hr1G092940	2.684089	2.141793	4.153413	1.06428	0.925812	1.201532
HORVU1Hr1G015210	0.045386	0.01069	0.06845	0.499637	0.263016	0.511655
HORVU4Hr1G055510	8.96072	12.26105	11.85734	3.745234	3.305109	4.416478

HORVU2Hr1G033450	17.05912	13.25001	17.0095	9.335941	7.779726	9.587679
HORVU7Hr1G027860	80.39945	93.12777	84.34038	29.90267	19.03868	22.74516
HORVU3Hr1G013880	38.48793	35.03332	40.75058	19.39821	18.15354	19.83629
Hordeum_vulgare_newGene_10569	1.018677	1.199046	1.238176	0.814679	0.532118	0.597158
HORVU4Hr1G020480	34.21493	34.83194	28.3369	12.88632	10.6681	14.01893
HORVU2Hr1G104040	10.74499	12.51535	14.12449	6.521587	1.728106	3.332083
Hordeum_vulgare_newGene_5150	2.662736	2.486732	2.799055	7.94576	9.959679	10.55919
HORVU3Hr1G093310	25.29616	23.91355	23.3707	14.78064	12.08543	12.68694
HORVU4Hr1G071070	5.903745	5.774923	7.352349	2.953032	5.865174	5.458271
HORVU4Hr1G015810	0.171446	0.236886	0.398525	1.113315	1.072734	1.317578
Hordeum_vulgare_newGene_5116	0	0	0	2.006662	1.02381	1.473391
HORVU2Hr1G105390	1.150335	1.020784	1.138424	9.17252	18.08361	17.07215
HORVU7Hr1G030860	0.574528	0.740989	0.783437	0.440427	0.365239	0.390063
HORVU1Hr1G063100	36.33977	30.90334	42.11123	10.63416	3.583203	4.486185
HORVU2Hr1G026810	760.8978	716.7925	1052.759	403.4067	317.1803	272.2092
HORVU2Hr1G088110	32.34897	26.18966	32.72068	11.98174	12.0214	11.04494
HORVU5Hr1G015050	0.817899	1.002151	0.931757	2.540104	2.857324	3.351915
Hordeum_vulgare_newGene_14919	0.115049	0.160062	0.057499	0.838797	0.625697	0.505147
HORVU1Hr1G024770	4.120154	3.705461	4.005052	0	0	0
Hordeum_vulgare_newGene_14911	0	0	0	1.365357	2.180859	1.973816
HORVU4Hr1G048210	2.146233	2.017174	2.311108	11.72879	9.679056	12.63283
HORVU5Hr1G060340	0.165582	0.194971	0.185831	1.820252	1.534882	2.212516
HORVU4Hr1G072830	41.37616	43.74201	58.34073	19.78735	2.057487	7.960977
HORVU3Hr1G017990	5.804764	6.431816	5.305582	1.459086	1.946667	2.136668
HORVU3Hr1G084880	5.710117	3.895596	3.956561	11.88929	15.80855	14.07648
Hordeum_vulgare_newGene_10374	1.618961	1.801183	1.49913	0	0	0
HORVU1Hr1G086120	0.028736	0.0291	0.06102	0.377471	0.408894	0.415967
HORVU5Hr1G013040	1.562179	1.59414	1.82832	0.045115	0	0
HORVU2Hr1G079620	0.624719	0.637753	0.406472	1.996848	1.781647	3.085029
HORVU3Hr1G086220	28.18572	23.56156	34.54201	11.84058	7.519991	8.859247
HORVU1Hr1G089760	1.10896	1.029868	1.124786	5.409468	7.489629	7.880881
HORVU2Hr1G037040	7.691811	7.298565	6.076998	0.737224	0.237645	0.297595
HORVU5Hr1G100140	14.40641	12.59257	11.03421	55.6989	89.11615	93.7522
HORVU1Hr1G093250	1.058596	1.344122	1.421985	2.662123	4.041796	4.941051
HORVU1Hr1G087380	137.8498	122.4807	106.7658	18.67482	4.754916	9.423997
HORVU6Hr1G074910	0	0	0	2.214939	2.872178	2.775136
HORVU7Hr1G054220	39.11178	31.23554	36.50417	131.5476	206.5722	185.6233
HORVU5Hr1G122530	0	0	0	1.484013	2.362165	2.856564
HORVU4Hr1G010150	10.38759	10.45901	14.56674	2.395252	3.124598	4.18076
HORVU7Hr1G051760	0.611082	0.629284	0.656674	1.973853	4.276543	4.105628
HORVU7Hr1G113460	2.95581	2.231053	2.345755	1.091568	0.90866	1.088789
HORVU3Hr1G000350	2.095409	1.622869	1.97153	3.324364	7.05846	6.805904
HORVU3Hr1G008400	1.798388	1.553936	1.245454	0.28594	0.169904	0.193381
Hordeum_vulgare_newGene_7376	0	0.027121	0	0.724403	0.731142	0.81773
HORVU2Hr1G009570	0.240575	0.194177	0.076235	1.312488	2.843717	2.430308
HORVU5Hr1G009830	18.00257	20.50668	26.48585	8.266214	15.16706	16.00858
HORVU6Hr1G059500	4.020945	2.000567	3.603019	0.057196	0.078424	0.01203

HORVU1Hr1G080080	0.367445	0.614167	0.519629	1.783091	2.105919	2.28088
HORVU1Hr1G049420	1.473139	0.942556	0.945484	3.476707	2.666169	2.836141
HORVU7Hr1G099700	2.100705	2.075492	2.826485	1.249377	1.403167	1.090408
HORVU5Hr1G044360	0.589918	0.712231	0.809845	0.344998	0.473897	0.486201
HORVU0Hr1G014150	3.940409	4.452492	4.528961	16.8569	36.77879	32.29023
HORVU5Hr1G010240	0.290951	0.130054	0.296485	1.493362	1.706434	0.807955
HORVU4Hr1G084240	1.799742	1.484883	4.185912	1.173333	1.190724	1.092113
HORVU0Hr1G004850	3.909999	3.489171	4.088672	14.57289	28.67396	25.63744
HORVU4Hr1G054910	2.074065	2.200731	2.742127	5.330597	8.431554	6.798622
Hordeum_vulgare_newGene_6568	0.645877	0.672718	1.12543	1.601225	2.028476	2.772672
HORVU4Hr1G070250	0.554825	0.644094	1.357498	7.443098	25.15904	23.70275
Hordeum_vulgare_newGene_10355	1.503445	1.705863	1.695367	0.060013	0	0
Hordeum_vulgare_newGene_10353	1.391532	2.174495	2.309132	0.022616	0	0
HORVU3Hr1G059810	2.779575	2.81206	2.516013	10.82922	18.72005	20.79228
HORVU5Hr1G053810	1.92262	1.115865	1.278707	3.39222	11.52942	8.72383
HORVU6Hr1G076660	1.875261	1.767441	1.40638	5.300626	3.992585	4.900771
HORVU4Hr1G051360	13.05567	10.84054	12.78888	6.618304	4.465024	5.334414
HORVU1Hr1G012880	3.363539	2.309206	4.036175	30.35708	237.3665	214.3672
HORVU1Hr1G014010	6.242202	8.640043	9.828507	4.111835	3.831351	4.95295
Hordeum_vulgare_newGene_11089	1.681478	1.375432	1.993979	6.137244	9.635787	8.913767
Hordeum_vulgare_newGene_11088	0.532366	0.560334	0.576618	2.048525	4.441866	5.07222
Hordeum_vulgare_newGene_11559	0.57226	0.431413	0.833692	0.011156	0	0
Hordeum_vulgare_newGene_1900	3.84073	5.246592	4.018645	0.027502	0	0
Hordeum_vulgare_newGene_1903	0.071184	0.047948	0.065797	1.385951	0.827732	1.301797
Hordeum_vulgare_newGene_1905	1.808268	1.538174	1.835944	0	0.016159	0
HORVU5Hr1G043650	78.72554	76.37294	73.59341	29.31398	38.77437	37.21652
HORVU2Hr1G062910	2.859506	3.958538	2.604988	1.761847	0.856781	1.030477
HORVU6Hr1G060990	167.4511	170.5326	217.4296	63.20568	58.66641	58.11621
HORVU4Hr1G084000	42.94029	36.4131	46.03169	80.99109	126.1084	117.655
Hordeum_vulgare_newGene_16022	0.270042	0.332354	0.16845	1.334007	1.185034	1.53585
HORVU3Hr1G024530	61.79864	67.62948	84.33825	39.23518	30.51506	36.05927
HORVU2Hr1G028380	6.555333	6.621251	7.737716	3.115707	2.788629	3.752993
HORVU2Hr1G116660	0.311385	0.278537	0.436242	4.911084	5.267003	4.854135
HORVU0Hr1G021720	3.743225	3.710647	8.405396	12.20788	19.73153	16.18004
HORVU6Hr1G078210	8.037074	9.128072	11.74438	6.725857	5.941551	5.230815
Hordeum_vulgare_newGene_6157	0.22268	0.241888	0.177514	1.836844	1.067538	1.364104
Hordeum_vulgare_newGene_6150	0.076933	0.12428	0.257748	13.55966	2.109141	6.285101
HORVU3Hr1G090440	0.414539	0.316568	0.479444	1.195569	1.005917	1.259151
HORVU3Hr1G114970	2.077103	1.96296	2.334486	0.60786	0.940506	1.308245
HORVU5Hr1G068320	37.17976	35.7305	36.83265	24.01101	20.56384	18.5029
HORVU3Hr1G059480	1.33725	0.601261	0.946065	12.40968	5.878183	15.83138
HORVU2Hr1G005330	1.509102	2.441472	2.450468	1.021339	0.248752	0.82126
HORVU6Hr1G012320	926.4667	756.3854	1033.092	596.6567	263.2951	331.6649
Hordeum_vulgare_newGene_15642	0.01954	0.105422	0.01214	0.40141	0.950347	1.13622
Hordeum_vulgare_newGene_11944	0.719741	0.939299	1.300842	0	0	0
Hordeum_vulgare_newGene_11942	7.012557	5.673281	8.328072	0.067488	0.092873	0.046197
HORVU4Hr1G055300	1.742034	2.113834	3.272883	1.440934	0.860199	1.256781

Hordeum_vulgare_newGene_5638	0.695761	0.441961	0.826219	2.623732	3.802963	3.923976
Hordeum_vulgare_newGene_6842	1.927588	0.475695	1.216887	3.087483	5.374953	4.908666
Hordeum_vulgare_newGene_6844	0.306388	0.164977	0.266929	0.674233	0.69559	0.757945
HORVU0Hr1G019020	0.420843	0.554696	0.55497	10.42369	3.150048	6.312009
Hordeum_vulgare_newGene_8449	1.678691	1.859565	2.446676	8.044237	13.01046	11.4035
Hordeum_vulgare_newGene_8443	0	0	0.014534	9.412593	12.00678	14.80909
HORVU0Hr1G032400	3.519696	4.244103	5.924505	1.916968	2.088546	2.670795
HORVU5Hr1G077910	0.536172	0.52295	0.561789	2.311468	6.076406	5.792255
HORVU2Hr1G089160	6.412601	5.154234	7.423184	13.58049	31.03619	28.64522
HORVU7Hr1G023600	16.30336	16.37939	18.47551	11.12363	8.792271	10.4344
Hordeum_vulgare_newGene_15504	0.534904	0.265033	0.285632	0.124832	0.124889	0.153274
Hordeum_vulgare_newGene_7495	1.9317	2.990251	3.140129	0.912506	0.036789	0.47845
HORVU5Hr1G067420	1.289349	0.858642	0.88171	0.070456	0.045296	0.082105
Hordeum_vulgare_newGene_14892	46.49262	47.46151	62.37142	11.99953	5.023073	9.941564
HORVU1Hr1G076690	0.2784	0.267549	0.331068	1.457661	7.010556	6.013288
HORVU7Hr1G039260	2.271732	2.407687	2.268903	7.558756	15.99523	14.08385
HORVU3Hr1G035730	0	0.041855	0	2.238047	5.311267	4.017616
Hordeum_vulgare_newGene_6458	0.51232	0.349821	0.354987	1.440816	1.761339	1.078156
HORVU6Hr1G068990	57.81873	47.15866	62.48893	30.56197	21.46279	25.98627
HORVU1Hr1G074340	1.661335	0.859951	1.045015	8.637715	35.1969	29.78208
HORVU4Hr1G017860	2.67039	3.223591	3.421814	7.275723	13.71483	9.861637
HORVU5Hr1G095710	0.465468	0.513797	0.300362	4.681853	4.218595	6.202378
HORVU3Hr1G034060	0	0	0.010925	3.187743	13.6536	5.321177
HORVU3Hr1G030760	0.357026	0.253434	0.453954	3.899548	20.09947	14.90923
HORVU5Hr1G077140	1.345593	1.50106	1.993512	0.276153	0.154885	0.308492
HORVU4Hr1G010580	2.003231	1.081886	1.602053	0.873476	0.196593	0.258754
HORVU7Hr1G118130	0.830229	1.230896	1.218151	2.03244	3.907796	3.028199
HORVU2Hr1G041950	3.219167	4.06655	3.386564	2.045798	1.680142	1.753489
HORVU4Hr1G069400	42.57611	39.13882	37.42199	18.25122	13.49134	14.39468
HORVU5Hr1G018850	3.684453	5.125032	5.827538	1.962462	2.47831	1.877898
HORVU4Hr1G011670	95.73973	70.34312	44.83879	28.17078	12.08962	17.8469
HORVU3Hr1G027200	1.192897	1.831728	2.948261	0.061431	0.166878	0.259274
HORVU4Hr1G083020	1.796864	1.529972	1.532486	5.124209	4.540403	3.847397
HORVU4Hr1G063160	0.315397	0.241187	0.313189	0.674377	3.319033	2.978867
HORVU3Hr1G022780	3.089949	1.994288	2.489141	1.082529	0.427049	0.877041
HORVU2Hr1G083960	2.351356	3.741899	3.174279	0.911447	1.305723	1.137651
HORVU1Hr1G067660	11.01393	13.98091	10.39915	7.368863	2.619807	3.657885
HORVU6Hr1G064500	6.730076	6.489754	8.251647	1.697486	0.348834	0.850423
HORVU7Hr1G117000	53.85045	54.89928	57.39901	56.67747	138.5779	88.20312
HORVU5Hr1G109390	0.691177	0.768857	0.991262	0.317247	0.526324	0.487918
HORVU1Hr1G067080	0.556659	1.207125	0.675373	5.42963	6.119613	7.307349
HORVU5Hr1G096370	17.5315	12.72363	19.22134	43.76212	45.49684	49.1303
HORVU6Hr1G010580	0	0	0	12.02122	10.58516	13.29604
HORVU6Hr1G088610	38.74151	38.11782	37.36266	148.4778	76.14043	79.03402
HORVU5Hr1G114700	4.42976	2.951235	4.058901	1.49462	0.450139	0.958022
HORVU7Hr1G056990	0.946613	1.313017	1.120259	4.687842	4.580764	5.635841
HORVU6Hr1G011180	47.26406	39.60695	46.09152	24.79819	21.54385	23.11048

HORVU7Hr1G047080	4.775162	5.486548	6.931577	3.145721	2.901614	3.46806
HORVU1Hr1G010780	1.145101	1.24874	1.487891	3.127759	2.952657	3.74935
HORVU6Hr1G026030	0.172353	0.178807	0.157163	0.422511	0.436845	0.666963
HORVU0Hr1G014580	3.758183	1.937881	2.187568	0.800744	0.729509	0.906715
HORVU1Hr1G016200	15.9609	13.33025	16.74826	29.1296	68.55325	57.62574
HORVU2Hr1G062590	0.293694	0.154635	0.258813	0.789626	0.856524	0.801072
HORVU1Hr1G094480	4.185993	3.730737	4.987826	8.86721	12.79373	13.19084
HORVU7Hr1G040850	2.843333	1.474374	2.908954	0.052347	0.129696	0.363842
HORVU3Hr1G059130	118.3933	120.0009	72.04631	19.7473	10.61832	11.3647
HORVU3Hr1G065390	6.907574	4.97737	7.155159	1.724975	1.258426	2.072285
Hordeum_vulgare_newGene_10933	1.779248	1.979103	1.951108	0	0	0
HORVU1Hr1G045760	3.097427	2.521182	3.484697	9.432299	14.89812	15.92469
HORVU7Hr1G028540	10.84316	9.653031	11.12464	25.50946	26.42519	28.33391
HORVU2Hr1G104740	7.867281	8.320103	8.809208	0	0	0
HORVU4Hr1G078460	15.41492	11.85187	14.58521	9.7895	3.176178	4.409682
HORVU6Hr1G091860	23.4708	33.49815	45.32947	12.37756	14.67691	19.38173
HORVU5Hr1G118800	16.43777	10.5887	20.89254	0.036485	0	0
HORVU5Hr1G073960	20.28678	23.03342	31.53471	10.37528	19.11854	15.70962
HORVU1Hr1G025380	5.949811	5.406686	6.666021	0.495367	0.559782	0.599724
HORVU7Hr1G083390	0.847023	0.785625	1.349415	0.510814	0.2637	0.482362
HORVU2Hr1G097980	5.4989	3.904799	3.969719	1.530034	0.757574	0.767761
Hordeum_vulgare_newGene_12310	0	0	0	3.018907	3.483221	3.938774
HORVU2Hr1G020610	0.108576	0	0	1.946635	1.86216	1.848414
Hordeum_vulgare_newGene_5387	9.824335	6.994343	7.040058	0.016289	0	0.007606
HORVU4Hr1G022020	3.285704	2.738188	2.987511	8.893721	11.73003	10.30668
HORVU5Hr1G068820	1.670651	2.25445	2.565921	0.20063	0.185936	0.351238
HORVU5Hr1G068790	1.149345	1.349513	1.978383	0.034298	0.039586	0.054313
HORVU4Hr1G058450	0.081277	0.025564	0.029848	0.54211	0.68259	0.588432
Hordeum_vulgare_newGene_14410	1.313556	1.346049	1.103378	9.66913	14.34639	12.66033
Hordeum_vulgare_newGene_14412	12.32925	10.66976	11.1978	2.762593	1.857515	1.983353
Hordeum_vulgare_newGene_14415	73.50775	67.63497	77.5886	224.5426	367.8485	351.9346
HORVU2Hr1G100870	6.934245	5.230612	7.360433	3.379247	2.210229	2.553174
HORVU7Hr1G008430	0.461109	0.224272	0.483989	1.280774	4.183514	4.227407
Hordeum_vulgare_newGene_13190	7.178724	8.094691	6.509643	5.426891	4.312109	4.544245
Hordeum_vulgare_newGene_13198	0.161656	0.088023	0.04558	0.328863	0.619981	0.585828
HORVU5Hr1G103730	0.699243	0.388752	0.831899	2.104798	2.666799	2.54932
HORVU3Hr1G013790	10.83378	9.927781	13.68178	41.25219	58.37902	46.57647
HORVU5Hr1G021430	0.189016	0.067372	0.124661	0.473073	1.345526	1.453282
Hordeum_vulgare_newGene_803	0.756163	0.89747	0.662888	0	0	0
HORVU5Hr1G048810	94.12843	90.52959	95.36709	46.65825	42.95949	44.72057
HORVU5Hr1G066630	3.655237	3.616471	4.209292	2.280061	2.149703	2.001783
HORVU2Hr1G098250	0.809167	0.754487	1.225311	0.376789	0.515366	0.28492
Hordeum_vulgare_newGene_4337	9.920441	9.944296	9.508909	2.159815	1.881889	2.07707
HORVU1Hr1G058770	16.8301	16.45113	30.97534	8.863234	11.42574	12.28412
Hordeum_vulgare_newGene_1676	0.57821	0.769706	0.339933	0.108973	0.060767	0.193865
HORVU1Hr1G049690	21.99768	20.61265	22.71737	16.872	10.74801	11.73434
HORVU2Hr1G032230	0.135476	0.157519	0.214538	0.567583	2.007188	0.796752

HORVU2Hr1G028920	6.443234	8.307283	10.7782	0.832955	0.733486	0.409141
HORVU3Hr1G000980	2.916446	2.452072	2.305214	71.29704	116.9874	114.3044
HORVU7Hr1G003020	1.395596	0.810445	1.191282	5.594737	98.5127	82.61796
Hordeum_vulgare_newGene_4559	3.950013	2.742616	3.151699	1.587525	1.079071	1.637139
HORVU7Hr1G024220	3.676836	2.463736	2.749147	47.95646	92.96347	83.55225
HORVU7Hr1G098660	1.81145	1.163208	1.925108	3.316335	5.200118	4.381418
HORVU5Hr1G064800	0.444177	0.647318	0.158841	1.676744	12.30572	12.10664
HORVU6Hr1G003210	0.878974	0.730235	0.84052	0.046699	0.016318	0.040683
HORVU3Hr1G003980	11.05639	11.64749	20.04082	3.486207	0.304076	1.582858
HORVU1Hr1G065120	1.309523	1.071933	1.009623	15.26125	28.06861	27.01187
HORVU6Hr1G080430	22.38162	20.15862	22.2919	10.91594	11.35089	11.59651
HORVU4Hr1G060810	33.33248	24.32481	32.7431	16.40055	3.231439	8.914581
HORVU1Hr1G081790	15.64639	13.538	20.38164	9.218761	10.38211	9.996868
HORVU3Hr1G029670	0.657926	0.722	0.867509	1.533303	1.894882	1.995625
HORVU5Hr1G112610	1622.1	1576.681	1119.564	29.31056	2.368716	11.71979
HORVU2Hr1G113940	11.27665	8.374557	8.189304	3.127137	3.113001	3.250878
HORVU3Hr1G082320	0.12699	0.030088	0.182854	7.921983	9.630032	10.95048
HORVU0Hr1G013950	36.45239	35.99778	38.53983	22.19105	14.86272	14.5356
HORVU4Hr1G002230	16.96097	14.73016	19.23619	10.6071	9.130286	9.147785
Hordeum_vulgare_newGene_12509	21.69516	36.71482	41.07792	13.96113	9.995319	10.57383
HORVU7Hr1G058120	4.031395	3.159379	4.156999	24.97492	62.3252	52.76762
HORVU4Hr1G063730	0.108925	0.244653	0.077771	2.289491	7.602043	6.108776
HORVU7Hr1G120560	2.893716	3.269382	3.877878	1.533718	2.637871	1.586048
HORVU3Hr1G050350	3.744587	4.674501	4.501962	0	0.004411	0
HORVU4Hr1G008990	131.295	154.5509	207.8156	20.40459	2.783072	8.908362
HORVU6Hr1G010420	0.161591	0.160311	0.262436	0.886396	1.966612	2.05582
HORVU7Hr1G095370	2.073017	2.489176	2.382934	9.260375	21.80429	14.83446
HORVU7Hr1G088730	1.023579	1.216874	1.910283	2.972436	4.915903	5.029275
HORVU0Hr1G014460	8.691965	7.84467	7.945	19.84471	30.58712	29.01891
Hordeum_vulgare_newGene_13354	0.306821	0.158487	0.166994	0.935533	0.648449	0.927484
HORVU1Hr1G070110	0.61125	0.537111	0.935476	3.23028	12.1236	12.42701
Hordeum_vulgare_newGene_13359	10.57882	12.22948	11.2285	0.02175	0	0
HORVU7Hr1G003170	135.1519	192.1732	293.4129	4.537118	0.039438	1.349316
HORVU4Hr1G005690	46.3115	59.61016	113.4487	4.271666	0.045509	2.175796
Hordeum_vulgare_newGene_15210	13.86653	1.644034	1.986631	0	0	0.022029
Hordeum_vulgare_newGene_15214	1.018074	1.2974	0.907461	0.2935	0.03479	0.175169
Hordeum_vulgare_newGene_15215	0.534629	0.445547	0.448008	3.492748	4.746292	4.170583
Hordeum_vulgare_newGene_15218	1.045973	0.804027	1.293712	0	0	0
HORVU2Hr1G099550	3.209678	2.791477	3.784764	11.03468	17.41391	14.99175
HORVU5Hr1G014300	2.401417	2.410081	3.834778	8.274108	12.32846	12.46186
HORVU5Hr1G116500	3.05721	2.66563	2.687663	1.19435	1.230103	1.038945
HORVU4Hr1G069260	9.281276	7.982091	6.072627	3.379425	3.826924	2.561285
HORVU4Hr1G075200	76.30929	62.34088	58.23278	139.1827	255.0877	257.8198
HORVU5Hr1G073370	0.409707	0.35625	0.115748	9.139457	7.254446	7.776752
HORVU7Hr1G008140	0.87872	0.739476	1.025183	0.20829	0.067775	0.200295
HORVU6Hr1G013240	16.35949	14.74654	12.30374	7.809751	5.172702	6.486446
HORVU5Hr1G078030	1.121723	1.451663	1.038314	0.465526	0.338304	0.569626

HORVU1Hr1G000690	1813.541	2429.969	1666.821	596.7852	453.6355	420.0302
HORVU2Hr1G097010	5.371241	3.672867	3.492699	17.28642	16.56782	15.4965
Hordeum_vulgare_newGene_5193	0.844787	1.418781	0.920245	3.24697	2.805677	3.664532
Hordeum_vulgare_newGene_5192	0.933461	1.002736	1.339659	3.366197	2.611489	3.205267
Hordeum_vulgare_newGene_13867	2.271704	1.858021	2.710716	4.418816	8.425281	7.315955
Hordeum_vulgare_newGene_10194	0	0.023596	0.016412	2.140865	2.294641	2.217341
Hordeum_vulgare_newGene_10190	0	0	0	1.76551	1.81368	2.085853
Hordeum_vulgare_newGene_10191	0.056134	0.038901	0	0.891841	0.838014	1.004801
HORVU3Hr1G095940	1.272494	1.172881	1.288375	0.376669	0.321611	0.427632
Hordeum_vulgare_newGene_14991	0.643763	0.752712	0.993709	3.062039	7.093064	6.55221
Hordeum_vulgare_newGene_14992	2.670277	1.76051	2.553465	10.87427	13.47818	11.10481
Hordeum_vulgare_newGene_14994	0.921487	0.994318	0.777518	3.392005	4.657141	4.193787
HORVU2Hr1G026890	1.498119	1.067747	3.824099	0.435984	0.063161	0.2663
HORVU7Hr1G037870	14.47193	15.19777	18.61452	6.107187	6.745636	7.395021
HORVU7Hr1G048310	0.714643	1.053358	1.058237	5.372337	7.684109	10.84424
Hordeum_vulgare_newGene_12292	1.581083	2.591745	1.846761	0	0	0
Hordeum_vulgare_newGene_12295	1.267002	1.783816	1.650919	0	0	0
Hordeum_vulgare_newGene_4640	2.703517	2.913789	3.119404	0	0	0
HORVU7Hr1G029400	0.248937	0.509532	0.383647	1.515007	2.172375	2.155329
HORVU5Hr1G103080	7.757614	8.902192	11.4346	4.927726	3.885985	4.417473
HORVU3Hr1G091800	1.247673	2.1621	2.570639	0.077198	0.05381	0
Hordeum_vulgare_newGene_6646	0.032648	0.020535	0.021872	0.587252	0.385381	0.606521
Hordeum_vulgare_newGene_6647	0.058587	0	0.030721	1.414241	5.09481	4.051667
HORVU6Hr1G074620	0.43685	0.360072	0.329862	1.319061	1.710962	1.652342
HORVU3Hr1G081180	0.204481	0.232186	0.077215	6.421646	5.000804	5.769797
Hordeum_vulgare_newGene_12128	0	0	0	30.43407	37.7428	50.24083
Hordeum_vulgare_newGene_13659	3.020403	3.51003	5.754602	1.498234	0.438404	1.809211
Hordeum_vulgare_newGene_8457	3.454324	2.290591	3.554261	8.50759	8.034477	7.316352
HORVU5Hr1G036460	4.254404	3.162142	4.313801	6.458924	12.23012	12.32886
Hordeum_vulgare_newGene_13603	41.82968	28.76743	37.67584	15.3934	12.66079	13.79806
HORVU5Hr1G023720	96.08589	42.88845	109.7795	10.63692	2.234801	8.393285
HORVU2Hr1G082480	11.51348	11.8393	12.78807	8.807191	4.802582	5.277842
Hordeum_vulgare_newGene_8976	1.238059	1.314963	1.208594	0.656654	0.603544	0.682839
HORVU6Hr1G069710	3.829636	4.30652	4.217319	2.980605	0.839358	1.789267
HORVU7Hr1G097550	0.276982	0.4259	0.420704	4.87999	3.532558	4.164138
Hordeum_vulgare_newGene_8885	1.109454	1.058655	1.048876	0.521707	0.166549	0.564124
HORVU1Hr1G018360	3.883134	3.663553	5.874973	0.300896	0.319484	0.415504
HORVU5Hr1G111830	28.94684	23.35875	26.18254	18.13779	9.60815	10.52317
HORVU5Hr1G066280	16.72819	14.50203	15.87163	44.85002	74.94291	77.00538
HORVU0Hr1G006240	39.5079	43.22524	46.32914	11.7843	17.90968	17.29465
HORVU1Hr1G079440	6.774448	3.931846	9.595674	13.56258	31.47912	27.40319
HORVU5Hr1G109760	0.669965	0.407771	0.476334	2.787271	3.892386	3.311645
Hordeum_vulgare_newGene_5903	15.33108	15.92309	14.86609	0.02722	0	0
HORVU1Hr1G053090	1.870708	1.320305	1.723096	2.884568	5.24907	5.700034
Hordeum_vulgare_newGene_9286	0	0	0	1.441589	1.519099	1.873987
HORVU0Hr1G012490	0.999144	0.942952	1.115258	2.140347	3.655897	3.617832
HORVU5Hr1G011960	1.077773	0.692237	1.473099	2.034672	3.608	3.750669

HORVU1Hr1G047570	10.93077	5.491884	10.87089	6.84983	3.535832	4.776766
HORVU4Hr1G006730	2.836462	2.370254	2.358589	8.72467	14.63605	13.94579
Hordeum_vulgare_newGene_4466	0.435032	0.705271	0.248039	0	0	0.018799
HORVU4Hr1G084850	0.066777	0.062853	0.166115	0.561548	1.306305	1.564285
HORVU2Hr1G094390	1.761694	1.391686	1.331144	4.863991	19.9708	22.58166
HORVU2Hr1G015430	1.008708	1.513902	1.208306	4.765612	8.224181	8.28781
HORVU3Hr1G030070	0	0	0	1.667793	1.767916	1.582479
HORVU1Hr1G012800	17.99732	13.67196	22.37491	11.12721	152.2317	123.8402
HORVU1Hr1G069540	9.385867	7.765041	13.72247	1.944587	0.303565	0.411111
HORVU7Hr1G043930	0	0	0.039814	2.084017	2.812105	3.735776
Hordeum_vulgare_newGene_11000	0	0	0	8.193421	13.53406	9.741772
HORVU2Hr1G090380	1.163147	0.757556	0.934698	2.88911	4.900183	4.520856
HORVU3Hr1G028710	0.368967	0.270902	0.33698	1.928387	3.933495	2.508031
HORVU4Hr1G056630	0	0	0	0.514241	0.760252	0.906269
HORVU1Hr1G061160	104.457	80.21295	106.0524	394.6653	429.285	432.2009
HORVU2Hr1G011430	0	0.011575	0	1.015278	25.04524	18.73196
HORVU4Hr1G023300	1.839995	2.135824	1.893363	4.992963	7.531407	6.78416
HORVU7Hr1G073640	0.057554	0.022057	0.053695	0.600556	0.656474	0.914971
HORVU5Hr1G076100	175.4764	170.9446	148.6102	94.49318	76.45744	78.86345
HORVU6Hr1G090270	4.93704	4.300534	5.600282	2.536699	2.755245	2.933669
HORVU3Hr1G026970	0.572589	0.91248	0.529114	4.832025	4.491106	3.593969
HORVU5Hr1G049180	0.309477	0.496988	0.36736	1.390036	4.6619	5.834771
HORVU5Hr1G106250	2.307793	2.403733	2.205995	0.579703	0.38172	0.212864
HORVU5Hr1G005910	335.9542	350.6852	483.3883	44.63364	2.972243	17.74543
HORVU5Hr1G109400	2.133326	1.794277	2.124749	10.01447	16.89773	13.28773
HORVU4Hr1G083190	39.90994	38.76802	38.77282	24.12191	13.19464	17.56333
Hordeum_vulgare_newGene_3367	0	0	0.01159	3.887135	3.578394	4.248619
Hordeum_vulgare_newGene_3364	1.177009	1.226165	1.568374	0.817569	0.300888	0.527161
HORVU1Hr1G059950	25.59525	46.47951	70.51832	3.362435	0.1365	1.628774
HORVU6Hr1G089980	1.271254	1.246803	1.402042	14.36138	15.27058	26.12734
HORVU2Hr1G112800	5.132966	5.421522	5.875247	0.049196	0.015978	0.00913
Hordeum_vulgare_newGene_13098	0.481781	0.343377	0.598992	1.214516	1.439094	1.363306
HORVU5Hr1G000630	12.86073	12.62054	18.81477	4.559062	2.673645	3.07066
Hordeum_vulgare_newGene_14377	0	0	0	2.725329	66.51862	56.87565
Hordeum_vulgare_newGene_14379	0	0	0.014549	7.050797	0.862811	2.008109
HORVU1Hr1G070700	0.164559	0.13816	0.315246	1.25803	6.67548	5.534571
HORVU3Hr1G044660	1.334117	1.06871	1.124695	10.60917	18.90555	12.92493
HORVU2Hr1G100420	2.596972	1.299729	1.514495	0.416003	1.376299	0.376225
HORVU4Hr1G082710	5.155826	5.698064	5.747552	22.78288	14.16751	16.22323
HORVU5Hr1G006670	0.87423	0.678318	0.813876	0.019708	0.019194	0
Hordeum_vulgare_newGene_2543	1.763588	1.828517	2.048517	1.226279	0.261625	0.742182
HORVU7Hr1G082450	2.970799	4.551755	4.553637	2.354007	0.697869	1.08428
Hordeum_vulgare_newGene_15022	0.915263	0.954233	0.953313	1.817615	4.891799	5.438525
HORVU5Hr1G077990	2.225172	1.76138	2.521709	6.463004	6.865129	6.703674
Hordeum_vulgare_newGene_10018	5.163962	4.267108	3.796444	1.373125	1.925402	1.077117
Hordeum_vulgare_newGene_15028	0	0	0	27.44974	3.977727	10.31064
Hordeum_vulgare_newGene_10638	0.049955	0.024763	0.011038	0.871205	0.268878	0.360457

HORVU3Hr1G063270	0.312961	0.122096	0.137475	1.702453	6.121157	5.338991
HORVU5Hr1G007980	6.876923	7.261477	5.913704	14.74475	17.77888	19.3998
Hordeum_vulgare_newGene_9578	1.536933	1.885782	1.948166	0.17595	0.193558	0.416056
Hordeum_vulgare_newGene_5013	1.0724	1.799549	1.847017	0.581642	0.877726	1.107876
HORVU4Hr1G079740	0.61722	0.851295	0.737017	1.622243	2.175958	2.221685
HORVU6Hr1G014500	1.852392	1.843991	2.736946	6.915725	9.771043	9.911214
Hordeum_vulgare_newGene_8198	0.054418	0	0	0.525443	1.010476	0.886159
Hordeum_vulgare_newGene_8192	1.974918	1.774415	1.459992	0	0	0
HORVU5Hr1G087880	8.159143	7.318716	8.962643	5.049861	1.005317	2.644977
Hordeum_vulgare_newGene_7412	1.176343	0.87919	1.308865	4.714434	12.77117	10.16513
HORVU1Hr1G024540	1.115893	0.938305	1.44301	2.062284	3.446097	3.665294
HORVU7Hr1G036970	109.207	75.33934	103.2813	16.08306	0.671747	4.335407
Hordeum_vulgare_newGene_4852	2.429324	2.497759	1.954052	0.14418	0.00439	0.123819
Hordeum_vulgare_newGene_4857	2.111925	1.951879	2.132437	0.119352	0	0.100055
HORVU2Hr1G114090	1.494241	1.400565	1.290166	0	0	0
HORVU7Hr1G032270	0.155177	0	0.111044	1.23002	7.244527	6.326969
Hordeum_vulgare_newGene_15851	0.115992	0.204774	0.312839	0.287383	0.747119	1.118483
HORVU7Hr1G058500	14.1054	12.78308	11.97758	6.128362	5.719732	6.356667
HORVU6Hr1G083770	33.40133	28.12513	29.68449	20.54496	16.81491	19.07197
HORVU1Hr1G051310	2.93014	2.703895	3.375901	4.377003	14.50692	12.96017
Hordeum_vulgare_newGene_9902	7.912451	9.679971	7.379587	0	0	0
Hordeum_vulgare_newGene_9903	3.341002	2.890332	3.30627	1.113593	0.400666	0.630166
Hordeum_vulgare_newGene_9904	7.36142	7.301918	8.412892	23.57693	71.3939	49.78231
HORVU5Hr1G065470	0.177553	0.135338	0.175877	0.744952	1.75147	1.251115
HORVU3Hr1G079900	23.61449	23.57491	24.19976	9.292536	4.104863	5.22261
HORVU7Hr1G064170	1.979296	2.295074	2.604834	0.378076	0.111548	0.144642
HORVU4Hr1G017350	419.8281	355.7264	459.1781	176.2624	16.97499	70.36045
HORVU5Hr1G012180	3.560135	3.199554	2.962562	0.367722	0.402055	0.373244
HORVU7Hr1G116060	3.840516	3.430452	1.90771	0.591048	0.34933	0.460332
HORVU1Hr1G019750	10.21558	5.016157	17.29469	0.447161	0.024338	1.52973
HORVU7Hr1G107350	5.020769	4.561705	7.618805	3.816572	3.299209	3.0713
HORVU3Hr1G018350	0.287458	0.18384	0.343248	3.823286	2.764365	2.167638
HORVU7Hr1G049510	8.52143	7.137689	11.23203	4.5126	5.31674	6.074197
HORVU2Hr1G119780	0.52698	0.498332	0.667531	0.015718	0	0.029132
HORVU2Hr1G089640	0.136247	0.419007	0.777604	1.06781	6.856931	5.795979
HORVU4Hr1G005910	1.354812	1.333342	1.315282	5.015925	4.215076	4.878015
HORVU7Hr1G050510	1.897201	2.566054	2.661624	0	0	0
Hordeum_vulgare_newGene_60	2.038915	2.200956	2.341072	0	0	0
HORVU7Hr1G088960	53.22169	47.44978	48.6737	23.2728	13.07154	15.43907
HORVU6Hr1G088870	2.030662	2.572278	3.115979	1.176862	1.686123	1.65916
HORVU5Hr1G010130	0.530938	0.527061	0.375986	21.60318	13.23378	24.25334
HORVU1Hr1G094400	0.243292	0.174385	0.150968	9.283481	9.760542	17.00849
HORVU3Hr1G099680	1.714556	1.122956	1.254597	5.153014	6.249168	6.81903
HORVU0Hr1G001500	1.209614	0.980822	1.146429	3.342125	3.832101	4.588265
HORVU7Hr1G111010	1.105295	0.599995	1.260334	6.108947	8.120152	6.388019
HORVU3Hr1G021610	8.048346	6.371783	7.249602	0.079271	0.076133	0.115109
HORVU7Hr1G085310	8.997956	7.051055	9.905069	21.1146	33.77779	30.76109

HORVU1Hr1G094160	0.107724	0.034708	0	0.963818	1.385312	1.273504
HORVU7Hr1G012690	0.885921	0.803761	0.734991	2.378517	4.236127	3.887131
HORVU1Hr1G060110	1.493868	1.933087	1.766876	0.669361	0.350972	0.706863
HORVU6Hr1G093300	1.863194	3.949971	2.082086	1.526754	1.530189	1.864949
HORVU7Hr1G082040	144.7602	190.1167	240.519	11.87856	1.041375	5.646898
HORVU4Hr1G090310	3.613495	2.378155	2.872526	9.134506	26.67575	19.3049
HORVU1Hr1G076870	7.242312	8.110036	8.619984	19.2412	28.96284	24.23557
HORVU7Hr1G096030	0.085002	0.213979	0.067732	0.529519	0.559875	0.976863
Hordeum_vulgare_newGene_11618	0	0	0	3.261518	1.991657	1.63805
Hordeum_vulgare_newGene_11612	9.958344	13.6447	12.55445	4.230346	1.600553	2.410444
Hordeum_vulgare_newGene_11617	0	0	0	23.72733	15.45116	16.0966
HORVU6Hr1G077670	1.515835	1.601148	2.452027	0.709893	1.040997	0.94434
HORVU2Hr1G038260	2.135217	2.055841	2.653281	7.679847	14.01841	11.0061
HORVU6Hr1G089280	77.82673	75.29802	68.06988	33.89889	24.59093	26.99288
HORVU2Hr1G020560	1.11933	1.227802	1.009293	0.673137	0.627481	0.611604
Hordeum_vulgare_newGene_1823	12.85423	11.66046	14.86236	3.828584	3.16955	4.039602
HORVU6Hr1G088530	5.388026	3.903905	4.599575	0.353403	0.00815	0.144734
HORVU3Hr1G037350	97.29272	80.46062	96.878	73.6529	41.34074	44.23957
HORVU2Hr1G020690	21.23638	22.76703	34.72142	10.75366	14.0234	15.04952
HORVU6Hr1G063580	9.151232	7.54524	8.028087	6.016655	3.324612	4.160945
HORVU6Hr1G066740	5.73329	5.499347	8.060477	2.928277	2.781885	3.384155
HORVU4Hr1G057170	10.51898	7.677179	8.964575	18.22973	41.28382	40.66646
HORVU3Hr1G024470	0.648042	0.727708	0.588598	4.08062	6.893454	6.929265
HORVU4Hr1G066070	1.408323	1.761641	1.971032	4.485222	5.111329	4.923687
HORVU7Hr1G030500	19.1352	11.8993	22.67201	10.78646	6.473006	9.102883
HORVU3Hr1G093830	0.827438	1.28069	1.329154	0.055364	0.086131	0.077541
HORVU6Hr1G054580	0.602023	0.628219	0.850132	1.827009	1.961252	1.750927
HORVU2Hr1G116700	10.64865	9.168883	10.65601	8.073091	3.838183	5.376623
HORVU3Hr1G116470	0.390878	0.231632	0.402876	5.611554	27.70527	22.09811
HORVU3Hr1G090560	1.420194	1.492222	1.704958	4.471085	4.559281	5.275225
HORVU5Hr1G071340	0.384827	0.475761	0.161043	0.536489	0.836521	0.709573
Hordeum_vulgare_newGene_3534	3.63624	5.109965	5.460427	0	0	0
Hordeum_vulgare_newGene_3539	0.615439	0.510588	0.696551	2.977063	5.426965	6.274304
HORVU7Hr1G025850	0.806604	0.831546	0.648914	0.222567	0.200943	0.285988
HORVU6Hr1G018060	3.454451	3.742431	5.490432	1.746272	2.256759	2.788442
HORVU3Hr1G061750	17.03255	18.83308	24.75853	10.80132	13.43429	12.25202
HORVU7Hr1G038080	0.192184	0.211534	0.344844	1.786692	2.396557	2.867794
HORVU3Hr1G089380	2.441137	2.575185	2.652429	0.363113	0.537286	0.307614
HORVU0Hr1G005670	7.462193	7.953901	6.785597	15.14618	28.39592	21.18535
HORVU7Hr1G029110	34.42217	30.98984	35.50137	19.22321	16.8525	18.4865
HORVU5Hr1G115340	1.751189	1.656144	1.750738	5.49356	12.03769	10.6294
Hordeum_vulgare_newGene_731	0.047168	0.068442	0.065462	0.542644	0.517608	0.444015
Hordeum_vulgare_newGene_737	2.222138	2.446819	2.660816	0	0	0
Hordeum_vulgare_newGene_735	2.019706	2.205292	2.22745	0	0	0
Hordeum_vulgare_newGene_734	0.769773	0.92108	0.951283	0	0	0.013576
HORVU1Hr1G070570	1.697417	1.608319	1.822101	0.623901	0.224125	0.110056
Hordeum_vulgare_newGene_2663	3.642193	4.926689	3.661777	2.181945	0.591829	0.67594

HORVU7Hr1G096740	1.914131	1.475119	2.589493	1.192487	0.798251	0.650512
HORVU6Hr1G076880	0.191758	0.384689	0.136099	8.088132	33.46882	31.18889
HORVU5Hr1G098170	0.543364	0.723125	0.773712	1.329172	2.705544	2.752636
HORVU0Hr1G032360	2.708584	3.247155	3.633346	2.063038	1.530205	1.587123
Hordeum_vulgare_newGene_15187	2.207165	2.135439	0.908442	0	0.309503	0.423884
HORVU7Hr1G026350	19.44129	21.01173	26.099	35.65001	242.6049	229.2922
HORVU7Hr1G073650	4.001314	4.003856	3.789397	9.752391	17.96359	15.31259
HORVU3Hr1G074000	1.258571	2.471627	2.532268	0.369132	0.282562	0.432615
HORVU1Hr1G043450	3.223904	2.18065	2.38722	0.783036	0.879824	1.120228
HORVU3Hr1G010990	0.196927	0.238357	0.237333	1.517142	1.161863	2.074621
HORVU4Hr1G008700	9.375905	6.261329	9.954227	28.53304	21.10934	32.6888
HORVU5Hr1G084880	0.483795	0.591388	0.370528	1.038724	2.395643	2.199076
HORVU6Hr1G063910	6.127642	6.160055	6.067095	12.78487	24.81389	23.17549
Hordeum_vulgare_newGene_16065	1.931572	2.064658	1.800456	1.109419	0.767183	1.026714
HORVU7Hr1G013470	64.19754	75.39519	51.21071	347.1455	139.4816	186.5503
Hordeum_vulgare_newGene_11382	0	0	0	0.576961	0.41586	0.497899
HORVU4Hr1G041810	4.3142	5.086059	6.319195	2.359722	2.496189	2.96283
Hordeum_vulgare_newGene_96	1.705192	2.197263	1.552323	0.049284	0.094857	0.231091
HORVU5Hr1G010330	226.6388	230.6644	326.8095	75.58555	42.7322	53.46655
HORVU4Hr1G010940	1.672612	1.214977	1.45433	0.237816	0.154306	0.234298
HORVU7Hr1G079650	2.509599	1.620588	1.810456	1.465082	0.483719	0.781785
Hordeum_vulgare_newGene_4959	0.095721	0.076397	0.103813	0.4633	0.345773	0.686827
HORVU7Hr1G056240	0.496645	0.624959	0.690485	2.709739	4.966583	5.460778
HORVU3Hr1G000900	0.614302	0.302489	0.515969	1.072523	2.339272	1.85618
Hordeum_vulgare_newGene_3982	3.828972	2.669053	5.386671	1.981603	2.182335	1.657175
Hordeum_vulgare_newGene_7600	8.591908	8.616505	10.14369	5.560371	5.633781	5.3496
Hordeum_vulgare_newGene_7605	1.016306	0.823283	1.00156	0.408127	0.308253	0.719052
Hordeum_vulgare_newGene_7606	1.857448	1.584777	1.263685	4.913482	5.756657	6.13991
Hordeum_vulgare_newGene_1553	0	0	0	13.66351	14.66554	15.33918
HORVU2Hr1G045200	4.858975	3.627787	7.179915	2.988663	2.668323	3.384534
Hordeum_vulgare_newGene_6551	3.53778	1.784295	1.953612	9.562796	11.17892	8.666126
HORVU4Hr1G016720	1.054995	1.118615	0.916324	0.528487	0.34428	0.5583
HORVU1Hr1G070960	1.294864	0.788653	0.660296	4.798123	6.31457	5.422743
HORVU2Hr1G041580	1.280313	0.716919	1.296587	0.343977	0.103743	0.168398
HORVU5Hr1G018480	0.407749	0.328492	0.558966	2.692485	5.253389	6.963572
HORVU4Hr1G080450	10.74212	9.34601	10.24938	5.469022	6.429678	7.007618
HORVU3Hr1G002670	0.936457	1.085154	0.823935	4.994995	8.137475	7.085926
HORVU5Hr1G112340	15.74341	15.05263	16.58315	6.044047	2.874081	4.594798
HORVU7Hr1G054130	1.261161	0.814041	1.564627	0.288626	0.409343	0.181603
HORVU3Hr1G078840	9.362709	8.335436	10.58287	2.994184	2.343715	2.784957
HORVU1Hr1G089380	0.01153	0	0	8.195584	4.119687	3.752077
HORVU5Hr1G092150	4.753731	5.465058	8.530825	0.177141	0	0.233918
HORVU5Hr1G118060	0.206982	0.151456	0.22946	1.473255	2.740658	2.313975
HORVU7Hr1G051560	0.089955	0.158241	0.080018	0.61748	5.023281	4.485072
HORVU5Hr1G110180	56.41438	49.95367	49.78224	28.56995	21.67856	35.9691
HORVU3Hr1G056710	3.950576	8.799023	9.65351	0.58933	0.496575	0.320282
HORVU5Hr1G113560	0.651329	0.583783	0.688134	2.982438	3.598966	3.485257

HORVU5Hr1G109290	23.68238	23.34384	32.37737	6.538771	1.91543	3.139205
HORVU4Hr1G052470	4.675256	4.313968	5.054706	12.6339	15.33276	16.46476
HORVU4Hr1G031510	13.03819	13.45219	14.26795	3.694471	2.846327	3.955364
HORVU1Hr1G013740	0.86408	0.762389	0.812716	1.826332	4.748373	4.55249
HORVU5Hr1G066370	0.105269	0.045268	0.069501	1.87997	1.699644	1.961591
HORVU2Hr1G015830	3.804048	2.934237	3.068226	8.965556	8.235221	7.151865
HORVU4Hr1G089670	15.70195	12.06233	17.7865	32.78565	48.70058	46.16144
Hordeum_vulgare_newGene_7826	1.556312	1.58081	1.643237	0	0	0
HORVU4Hr1G068540	1.685478	1.73813	2.061175	3.356598	9.158918	8.033261
HORVU1Hr1G058180	74.45799	66.29485	81.75729	29.26559	32.17791	35.05958
HORVU5Hr1G109870	0	0	0	0.755435	0.830175	1.201936
HORVU3Hr1G071210	1.555306	1.686399	2.761532	5.885843	9.061764	9.580643
HORVU7Hr1G076680	0.152193	0.096796	0.085549	0.371694	0.645796	0.732499
HORVU6Hr1G000490	2.402136	3.926926	4.279522	0	0	0
HORVU5Hr1G060580	1.035785	0.853207	0.953946	0.477767	0.130828	0.228095
HORVU4Hr1G001450	10.24979	12.13472	12.01807	2.427244	0.759297	1.568455
HORVU1Hr1G057460	43.01022	38.97576	53.51659	12.05711	19.11617	18.26894
HORVU2Hr1G099000	0.956918	1.078987	1.244146	0.329668	0.058326	0.092039
Hordeum_vulgare_newGene_13934	1.577142	2.382936	3.360615	0.958445	0.717303	0.840605
HORVU5Hr1G115630	1.809676	1.083407	1.414029	4.779966	3.690058	4.151311
HORVU7Hr1G043620	0.434484	0.410847	0.469771	1.866605	1.571443	2.339355
HORVU1Hr1G047440	129.0793	104.648	155.8232	88.64116	74.00738	69.17893
HORVU2Hr1G066930	7.253651	7.020184	7.05229	0.06706	0.02833	0
Hordeum_vulgare_newGene_8728	0.77201	0.720478	0.664941	4.381845	2.891019	3.247817
HORVU4Hr1G007580	0.347674	0.483826	1.01284	1.313126	4.595788	3.814641
HORVU5Hr1G011160	0.532685	0.51866	0.617489	4.732998	6.438212	5.430512
Hordeum_vulgare_newGene_5263	0	0	0	1.357627	2.259779	2.185208
HORVU3Hr1G076880	1.91862	2.307349	3.048449	3.463429	9.854146	9.354388
HORVU5Hr1G112350	18.88198	19.66365	21.71122	10.17401	13.90377	11.84526
Hordeum_vulgare_newGene_8095	0	0	0	1.238812	1.442036	1.626128
HORVU3Hr1G062900	5.48137	4.785404	4.452757	3.314195	0.683121	1.783835
HORVU1Hr1G040140	1.225533	1.481337	1.504232	78.85297	167.0517	137.9266
HORVU6Hr1G091980	6.96953	6.629596	7.381891	0.046184	0	0
HORVU5Hr1G052030	2.10578	1.694502	3.288098	5.505264	8.267795	7.722027
HORVU5Hr1G081660	0.726671	0.857911	0.87416	1.524543	5.389956	4.580438
HORVU7Hr1G021560	0.383066	0.09753	0.189427	3.691781	4.95676	4.153387
Hordeum_vulgare_newGene_11795	0	0	0	1.786628	1.190429	2.099752
Hordeum_vulgare_newGene_153	0	0	0	4.514549	4.845742	5.911448
Hordeum_vulgare_newGene_157	0	0	0.036389	75.26079	70.60096	80.49504
HORVU2Hr1G110130	9.623075	4.482418	5.521516	180.6324	217.5057	224.2085
Hordeum_vulgare_newGene_11427	9.459993	9.083419	12.66435	194.7053	82.22398	108.027
HORVU3Hr1G112010	2.667121	1.270416	1.948317	17.52844	18.72509	29.61936
HORVU6Hr1G016880	0.749822	0.50798	0.607197	3.361217	3.815392	4.891788
HORVU5Hr1G065400	1.764898	1.415948	1.710247	0	0.098128	0.167667
HORVU2Hr1G072880	0.498244	0.423377	0.77913	1.913401	4.469762	3.540018
HORVU4Hr1G028720	0.73929	0.725473	2.380553	3.765752	14.92905	10.94043
HORVU3Hr1G080110	1.3534	1.686755	1.749277	0.433712	0.347098	0.925659

Hordeum_vulgare_newGene_7370	0	0	0	1.48082	2.695888	2.165826
Hordeum_vulgare_newGene_7378	0.024377	0	0	2.093936	3.015531	2.879529
HORVU2Hr1G032970	8.754157	7.548467	7.278678	19.46691	23.71932	21.99362
Hordeum_vulgare_newGene_5370	0.357018	0.37203	0.293188	0.023187	0	0.026048
HORVU0Hr1G004800	1.199074	1.420552	1.514672	0.73214	0.671468	1.099126
HORVU1Hr1G021590	27.56376	32.14017	22.66458	66.08969	111.0444	98.75097
HORVU5Hr1G061250	3.839167	4.061305	3.969998	8.297682	12.12488	11.56267
HORVU4Hr1G061770	0.12766	0.113862	0.358538	0.662133	0.755878	0.865541
HORVU2Hr1G060510	1.031161	1.026907	0.976391	5.978071	9.757051	10.10918
HORVU3Hr1G110410	0.509696	0.663987	0.576999	0.222255	0.461553	0.379785
HORVU3Hr1G068390	4.314938	3.929602	6.644265	1.657373	0.24291	0.88656
HORVU4Hr1G021720	8.583487	11.38176	12.55875	3.649141	4.046276	4.342252
HORVU0Hr1G000490	2.703756	2.507086	2.723826	7.647967	11.7308	12.93063
Hordeum_vulgare_newGene_5700	10.75537	7.244418	9.677177	108.5056	151.6817	147.7488
Hordeum_vulgare_newGene_963	0.20023	0.237151	0.336506	3.002566	2.803835	2.934231
Hordeum_vulgare_newGene_960	2.792905	1.733415	1.533095	0.157847	0	0.078244
Hordeum_vulgare_newGene_966	12.96078	12.71409	11.17753	3.5268	5.526976	5.691946
Hordeum_vulgare_newGene_964	0	0	0	0.71281	1.460092	1.432904
Hordeum_vulgare_newGene_965	0.022262	0.022957	0.039989	1.45412	1.110308	0.857657
HORVU6Hr1G064460	6.131964	5.119412	8.005063	18.10918	24.33518	21.67126
HORVU3Hr1G107350	5.515001	3.380637	5.048644	2.431739	0.788793	1.084569
HORVU7Hr1G026250	11.19343	12.64966	10.1109	6.771686	5.355671	5.099943
HORVU2Hr1G075870	0.496534	0.40153	0.726427	0.853596	2.222487	2.71651
HORVU5Hr1G063780	0.793661	1.108668	1.416813	0.023963	0	0.016289
Hordeum_vulgare_newGene_1283	1.46798	2.064537	1.921168	4.505187	4.696156	5.211563
Hordeum_vulgare_newGene_1282	11.17164	7.274679	7.77426	3.159	3.63382	3.131598
Hordeum_vulgare_newGene_1286	14.71977	21.77599	12.75583	0.736423	0.026531	0.179946
Hordeum_vulgare_newGene_1284	1.374136	1.216896	2.234889	0.217477	0.486612	0.453493
HORVU2Hr1G004480	6.188045	3.829501	7.043506	1.029355	0.145458	1.041702
HORVU7Hr1G106230	8.772307	11.15559	14.88109	4.081244	2.721193	4.173111
HORVU3Hr1G018430	1.481551	1.322459	1.09535	0.36398	0.1486	0.219694
HORVU4Hr1G055910	1.262272	1.071952	0.927152	2.170847	3.506852	4.636998
HORVU2Hr1G088760	1013.015	815.5583	939.8235	588.454	415.2602	385.4736
Hordeum_vulgare_newGene_1135	11.32396	10.99411	16.68092	5.313186	5.357474	5.518195
Hordeum_vulgare_newGene_1136	5.425307	4.715932	4.29363	36.58036	12.03726	25.40529
HORVU5Hr1G021750	60.76605	73.5616	63.47325	47.51432	23.07463	31.32158
HORVU1Hr1G067980	110.2502	81.6198	134.5803	13.52172	9.240789	10.82703
HORVU1Hr1G079150	3.008859	2.820422	3.595028	1.310295	0.917562	1.162599
HORVU2Hr1G005990	6.12911	4.795707	8.44337	4.336134	1.931964	3.168836
HORVU6Hr1G044080	11.58229	11.48071	13.55453	7.540669	6.419285	7.04204
HORVU1Hr1G092980	0.64746	0.300588	0.740305	1.49889	2.697978	2.146757
HORVU0Hr1G006420	0	0	0.007432	1.961698	7.085776	18.7259
Hordeum_vulgare_newGene_4566	0	0	0	1.518874	1.96346	1.569492
HORVU7Hr1G090280	0.911943	0.794336	1.046105	10.89217	13.31139	11.23654
HORVU4Hr1G073150	0.269043	0.457868	0.726308	8.935253	38.01434	31.54127
Hordeum_vulgare_newGene_7033	1.661266	2.172075	1.747036	0.87426	0.056183	0.201507
Hordeum_vulgare_newGene_7034	3.2386	4.605003	6.246281	0.316473	0.015223	0.143705

HORVU3Hr1G035420	13.2367	14.82205	16.33245	8.96643	5.888058	7.244698
HORVU5Hr1G012570	5.308983	4.855778	6.10632	1.857872	2.539492	2.099603
HORVU3Hr1G039700	1.577433	1.885139	2.494179	0.726398	0.878978	0.715661
HORVU6Hr1G029200	0.509225	0.384114	0.948194	3.804208	12.53447	11.39068
HORVU2Hr1G058390	3.007325	2.368517	3.691175	0.082013	0.054257	0.063624
HORVU1Hr1G050970	0.450447	0.242727	0.601504	1.143704	2.775598	2.373491
HORVU2Hr1G082170	0.585494	0.463802	0.428494	1.112185	3.824119	5.557029
HORVU5Hr1G116870	46.12382	47.07504	57.96569	24.22749	21.90517	24.53565
HORVU7Hr1G000250	0.326141	0.386203	0.396722	3.66982	5.247631	5.521454
HORVU7Hr1G041850	17.69148	16.351	19.43342	40.479	60.27195	67.53989
HORVU1Hr1G093570	0	0	0	1.0061	17.57127	12.54942
HORVU2Hr1G123270	76.71251	63.76994	99.29927	46.88578	21.34925	24.44768
HORVU4Hr1G023430	15.69667	14.51468	16.83006	36.89348	49.11498	48.13198
HORVU5Hr1G092290	5.451431	3.874688	5.366652	3.098787	2.255762	2.871634
HORVU5Hr1G011640	0.06584	0.129566	0.417354	1.280006	1.037806	0.559908
HORVU3Hr1G056100	17.28983	12.36829	19.09448	4.881968	1.360895	2.653092
HORVU7Hr1G095230	1.587169	0.908052	2.177062	0.367274	0.55802	0.665863
HORVU1Hr1G049210	3.895235	3.093181	4.841425	1.081013	1.179557	1.370039
HORVU5Hr1G083340	25.39469	20.50788	30.02417	6.409508	3.625326	4.159761
HORVU7Hr1G082980	2.42521	2.483255	1.672242	0.389251	1.247035	1.284768
HORVU4Hr1G082790	498.2702	416.3604	508.7295	314.7688	203.5297	210.134
HORVU5Hr1G120800	15.32378	14.17743	13.12874	4.797211	4.96549	7.853189
HORVU0Hr1G022470	4.748754	4.806289	5.491902	14.8073	27.71701	26.08203
Hordeum_vulgare_newGene_3420	0.222957	0.35558	0.257684	1.466615	1.261451	1.207984
Hordeum_vulgare_newGene_2019	0	0	0	0.780779	1.082695	1.295703
HORVU1Hr1G016110	2.902038	2.507323	3.409172	1.454034	2.189878	1.854067
Hordeum_vulgare_newGene_2015	7.854198	10.60931	10.35437	0.1385	0.023744	0.062801
HORVU7Hr1G042740	1.221162	1.490112	1.477978	4.458309	10.21842	8.723576
HORVU3Hr1G021320	0.514713	0.323489	0.41772	1.131531	2.61558	1.63164
HORVU1Hr1G044560	2.238218	1.996987	2.575348	1.327721	1.102592	1.334363
HORVU0Hr1G016350	8.828604	5.962611	7.547864	4.79146	3.581916	3.954414
HORVU6Hr1G013320	0.986807	1.11521	0.759799	0.009168	0	0.004552
Hordeum_vulgare_newGene_5093	0	0	0	2.4827	2.547074	3.627222
HORVU5Hr1G081250	0.224153	0.383042	0.222375	1.657112	1.058615	1.343369
Hordeum_vulgare_newGene_10098	0.740718	0.659067	0.825706	0.529269	0.304703	0.489535
Hordeum_vulgare_newGene_10096	6.765056	3.950577	7.638453	2.80532	2.920912	4.412035
HORVU4Hr1G083940	1.865539	0.835125	2.399048	6.122679	6.953885	8.315948
Hordeum_vulgare_newGene_8118	3.021462	2.803515	2.350648	1.596938	1.539336	1.711558
Hordeum_vulgare_newGene_11325	2.483913	2.615408	2.091715	0.968387	0.149963	0.385153
Hordeum_vulgare_newGene_11327	6.068437	4.649383	5.468875	11.78605	53.27768	48.37183
HORVU5Hr1G073140	2.759332	3.634988	4.191951	1.869469	0.706174	1.268094
HORVU5Hr1G080700	1.346764	1.617574	1.468328	9.09916	7.957527	8.319833
HORVU3Hr1G022800	2.618549	2.147861	2.690451	17.89406	49.22937	37.67076
HORVU6Hr1G013580	1.704298	1.277565	1.255974	0.103354	0.24089	0.321502
HORVU7Hr1G045830	0.705606	0.675716	0.769552	2.033737	2.708064	2.054815
HORVU1Hr1G004120	11.10928	9.445499	7.442278	0.701967	0.284025	0.447228
HORVU2Hr1G111140	1.080597	1.251042	1.034675	3.380373	7.520141	7.452774

HORVU3Hr1G094800	4.914366	3.429081	4.376492	2.453839	0.557934	1.945099
HORVU2Hr1G069110	1.565093	1.891201	2.298432	4.279534	5.652846	6.067416
HORVU1Hr1G072370	0.082289	0.17033	0.261742	0.715431	2.221451	3.490378
Hordeum_vulgare_newGene_374	5.427197	6.167673	7.972041	3.024302	3.498281	4.288994
HORVU4Hr1G077230	19.54387	16.96113	16.29272	39.29208	65.13286	57.35308
HORVU3Hr1G090270	0.058509	0.101034	0.108807	0.671676	0.779375	0.813027
Hordeum_vulgare_newGene_5648	0.279187	0.34327	0.586292	1.213018	1.431324	1.525923
HORVU7Hr1G033760	3.016007	3.958758	2.349495	0	0.060349	0.007082
Hordeum_vulgare_newGene_5644	0	0	0	4.579547	3.14498	2.428931
Hordeum_vulgare_newGene_6727	1.724388	2.518646	0.614862	0.099432	0.043906	0.030035
HORVU3Hr1G089490	0.656102	0.481954	0.311207	1.598331	1.733346	1.613933
HORVU3Hr1G010450	1.6432	1.818213	1.379859	0	0	0
Hordeum_vulgare_newGene_3004	0.060719	0.144933	0.261299	3.248686	47.02688	55.47268
HORVU3Hr1G103960	1.339077	1.139729	2.072119	4.060881	5.129597	3.069379
Hordeum_vulgare_newGene_6182	0	0.004262	0	1.485908	4.00789	2.117166
Hordeum_vulgare_newGene_14525	2.296015	1.441378	1.39041	3.174387	4.603537	4.464578
HORVU2Hr1G085350	1.810849	1.432717	1.491485	0.618134	0.549482	0.753628
HORVU2Hr1G079960	4.698599	3.578003	3.612786	9.019928	11.16925	9.99376
Hordeum_vulgare_newGene_13757	6.143793	8.524469	6.53636	0	0.012533	0.035677
Hordeum_vulgare_newGene_2358	8.431371	8.939508	8.979794	35.00639	38.54018	41.71089
HORVU5Hr1G095190	3.836691	2.34014	3.862946	1.501797	2.479072	2.181192
HORVU4Hr1G017600	0.125948	0.197686	0.217925	0.673193	4.704603	3.899515
HORVU5Hr1G079700	1.96031	1.205028	1.96864	5.03966	4.217755	5.566748
HORVU7Hr1G030120	5.745708	5.095827	5.994299	20.3731	27.0315	26.93386
HORVU4Hr1G010050	0.3693	0.184042	0.173765	1.294631	2.063894	2.145967
HORVU7Hr1G096850	0.377526	0.437814	0.27973	1.902665	4.612639	4.105736
HORVU4Hr1G003220	0.490128	1.123917	0.634622	0.061481	0.172337	0.152025
HORVU3Hr1G096890	3.48998	2.561254	3.25028	0.086815	0.038024	0.171471
HORVU2Hr1G075930	80.68661	101.2634	117.6305	26.94499	6.29532	16.42547
HORVU5Hr1G103420	12.42126	15.64976	17.58768	3.828308	1.037182	1.09208
HORVU3Hr1G036600	0.098447	0.244621	0.109409	3.496124	5.77993	5.216072
HORVU3Hr1G083420	0.250386	0.127687	0.272183	2.821222	1.207114	1.316203
HORVU7Hr1G113320	3.114768	3.02453	3.228757	1.759493	1.484595	2.135469
HORVU1Hr1G079050	10.73602	7.220538	11.08184	17.20603	31.6348	30.90515
HORVU6Hr1G009000	12.35269	14.28773	17.37778	8.206009	4.608845	5.712614
HORVU1Hr1G021160	7.69886	5.619417	7.838484	2.472841	0.88891	1.634748
Hordeum_vulgare_newGene_4246	5.319349	5.903521	5.667709	0	0	0
Hordeum_vulgare_newGene_4247	6.076599	7.335053	5.993193	0	0	0
HORVU4Hr1G015570	13.50796	12.99518	11.00043	50.43638	61.73982	60.10013
HORVU6Hr1G093260	479.6904	301.4955	430.6656	5.108722	5.956115	3.717343
HORVU2Hr1G017040	0.445591	1.142209	0.475374	1.142697	25.98469	19.71747
HORVU1Hr1G056180	0.289966	0.305778	0.386463	1.594538	0.89586	1.735186
HORVU1Hr1G090300	8.374445	10.65907	10.3317	2.490077	5.869304	5.271858
HORVU3Hr1G005640	29.97315	28.89941	34.0154	16.53388	20.63697	16.53618
HORVU6Hr1G006130	2.157065	1.562955	3.010489	10.46002	40.2519	37.79479
HORVU5Hr1G012710	1.779488	2.008523	2.113675	0.207781	0.28832	0.072059
HORVU2Hr1G108110	1.338088	1.510924	1.362033	8.868487	6.742383	7.178149

HORVU1Hr1G083660	19.00508	19.78258	22.89677	7.272235	7.045243	7.239374
HORVU1Hr1G045490	4.018336	3.247636	4.755101	1.463654	2.027688	2.05754
HORVU2Hr1G123710	4.652362	3.73827	5.362582	18.28823	68.49464	53.61874
HORVU5Hr1G051980	4.606318	3.633222	4.762729	0.12364	0	0.186312
HORVU5Hr1G093290	0	0.087212	0.106296	2.790471	0.775008	1.505877
HORVU7Hr1G000040	3.532185	3.373121	4.114003	0.027718	0.018627	0.034752
HORVU5Hr1G106350	26.14597	18.04035	25.24133	16.67824	13.63225	13.70514
HORVU1Hr1G016770	2.281336	2.133819	1.738824	10.13988	9.242486	7.762203
HORVU1Hr1G085090	17.24192	14.81195	18.78775	8.291095	0.989809	3.153482
HORVU6Hr1G089570	3.487536	4.330726	6.639107	1.531189	0.031989	0.471194
HORVU2Hr1G095600	1.089954	1.016013	1.034328	0.114	0.016941	0.023785
HORVU4Hr1G063420	0.329913	0.246199	0.365701	5.718832	9.10272	7.677531
HORVU7Hr1G071760	9.984807	9.866666	11.25374	36.62704	89.36035	80.77376
HORVU7Hr1G046320	60.90079	53.61734	62.87889	172.2399	226.1406	232.0687
HORVU7Hr1G046990	0	0	0	2.992363	1.579195	1.367005
Hordeum_vulgare_newGene_3240	2.112103	1.448318	2.793394	3.859568	7.463405	6.277819
Hordeum_vulgare_newGene_13228	0.057755	0.574335	0.036461	3.290789	3.503244	2.961517
HORVU6Hr1G015390	20.51085	20.56625	12.70602	4.202427	4.132186	4.001909
HORVU1Hr1G046740	0.094336	0.36521	0.42185	1.255993	2.281394	2.628914
HORVU4Hr1G061060	0.156803	0.083599	0.185148	0.474872	2.091996	2.111557
HORVU7Hr1G030000	0.024732	0.111614	0.03005	0.310196	0.702961	0.593307
HORVU1Hr1G077590	0.061108	0.013114	0.119478	0.283965	2.057367	2.019651
HORVU7Hr1G026680	24.30709	16.70931	24.58754	51.85817	56.62111	66.21983
HORVU3Hr1G013970	2.11932	2.071175	2.898581	33.66062	96.65081	70.69963
HORVU2Hr1G049510	0.218547	0.148739	0.246965	0.64003	0.570164	0.711294
HORVU2Hr1G072490	11.53268	12.31391	13.78026	39.18543	102.5253	90.38275
HORVU4Hr1G078710	2.693091	3.187332	3.20074	5.199073	9.443215	9.17141
HORVU3Hr1G057810	21.61712	28.38428	47.26308	6.222594	1.553496	3.294073
HORVU7Hr1G081610	1.851267	1.390981	1.187534	0	0	0
HORVU3Hr1G087800	0.699275	0.447545	0.662429	2.005249	2.711549	2.8177
Hordeum_vulgare_newGene_8303	9.902268	10.38025	13.66861	0.262389	0	0.05896
Hordeum_vulgare_newGene_8302	3.430227	3.273405	4.503624	0.68376	0.845777	0.52042
Hordeum_vulgare_newGene_8309	8.919594	9.823425	9.503988	3.432053	4.58167	5.532418
Hordeum_vulgare_newGene_4485	1.527743	1.899479	1.712354	0	0	0
HORVU1Hr1G076710	3.484078	2.711431	2.471262	0.311951	0.352634	0.439814
Hordeum_vulgare_newGene_4481	0.062011	0	0.022234	0.546345	0.935862	1.292986
HORVU2Hr1G102790	0.203716	0.179067	0.175357	0.580843	0.779721	0.97072
HORVU1Hr1G024460	1.685572	1.413905	1.850881	6.33939	9.651937	9.177438
HORVU0Hr1G032870	0.517412	0.742822	0.917877	2.034497	1.43908	2.550378
Hordeum_vulgare_newGene_15	0.38548	0.223011	0.527145	3.157768	20.34725	9.008778
HORVU2Hr1G102240	0	0	0	9.159032	7.825	9.093612
HORVU4Hr1G002820	0.602851	0.979174	0.888056	1.204727	3.061984	2.729463
HORVU7Hr1G032330	0.885592	0.607755	1.200546	3.484177	3.642829	4.16851
Hordeum_vulgare_newGene_3909	3.837035	3.348053	4.468796	10.03974	11.04498	11.1657
HORVU7Hr1G078960	11.72358	10.40762	21.3093	0.599297	1.76587	2.004444
HORVU3Hr1G080740	2.194359	2.237215	2.491986	7.915195	14.92198	12.01773
HORVU4Hr1G016050	0.612724	0.767841	0.814258	0.284773	0.391675	0.463632

HORVU6Hr1G068910	1.275144	1.794199	1.845282	6.272055	4.876176	4.89678
HORVU7Hr1G001540	2.435107	2.914832	2.831258	1.030059	1.088126	1.044876
Hordeum_vulgare_newGene_15956	2.185124	3.657218	2.92511	8.804359	8.726741	6.691757
Hordeum_vulgare_newGene_15955	0.943853	0.863503	0.985483	0	0	0
Hordeum_vulgare_newGene_15953	0.421499	0.37544	0.320146	0.158483	0.274047	0.207907
Hordeum_vulgare_newGene_15952	0.761969	0.548066	0.473583	0.202331	0.41469	0.286655
HORVU7Hr1G039760	0.392278	0.389863	0.717619	1.429578	1.908047	2.154489
HORVU7Hr1G100170	0	0	0	0.87172	0.960547	0.999387
HORVU4Hr1G073580	1.661212	1.720089	2.645701	10.57021	15.29488	14.30168
HORVU1Hr1G011100	0.013852	0.022685	0	0.775096	1.286103	1.104842
Hordeum_vulgare_newGene_12637	0.084328	0.107874	0.105202	2.146186	5.178493	2.781033
HORVU5Hr1G012550	23.52568	20.62115	23.07059	10.1405	11.74806	10.21072
Hordeum_vulgare_newGene_2974	2.366952	3.401339	3.770311	1.596949	1.84009	2.053261
HORVU6Hr1G087250	2.276377	1.846171	1.734235	28.01796	20.1854	22.58061
Hordeum_vulgare_newGene_9822	12.9106	14.05821	18.26189	3.4639	4.519122	4.327494
Hordeum_vulgare_newGene_9825	0.113038	0.607226	0.359591	1.3342	1.779691	1.748458
Hordeum_vulgare_newGene_9828	1.775134	1.968027	1.34377	0	0	0
HORVU7Hr1G002010	0.645579	0.492216	0.520936	0.014016	0	0.010274
HORVU5Hr1G124160	15.26692	13.98944	11.9736	52.75132	152.2905	149.9704
HORVU7Hr1G078030	0.374251	0.397153	0.784668	1.757241	2.21674	2.368319
Hordeum_vulgare_newGene_4227	17.08275	17.87482	17.23807	6.084652	5.539547	7.425684
HORVU0Hr1G019300	8.607686	6.766105	8.80043	1.200845	0.757396	1.688976
Hordeum_vulgare_newGene_13398	0	0	0.004964	1.474129	0.264823	0.745889
HORVU6Hr1G064150	2.210022	2.540267	2.848456	4.969984	9.038868	8.214041
HORVU5Hr1G023610	0	0	0	1.646956	1.684641	1.688205
HORVU4Hr1G056260	3.384966	2.55954	1.327971	0.480389	0.865872	0.613768
HORVU6Hr1G073880	0.656001	0.934578	1.19141	0.474985	0.526921	0.391519
HORVU3Hr1G003540	0.779801	0.849058	1.268008	8.253173	20.27168	21.01079
HORVU1Hr1G045360	27.31986	22.24824	31.89605	4.329739	3.386355	3.305476
HORVU1Hr1G022410	6.231883	4.848174	6.889878	13.78332	24.59674	24.8027
HORVU1Hr1G066250	0.238342	0.531047	0.474488	1.437314	1.373863	1.358611
HORVU7Hr1G108550	2.804382	3.223824	2.494497	9.087345	15.8299	14.49748
HORVU5Hr1G063630	0	0	0.019596	1.977717	1.668426	2.101882
HORVU7Hr1G037080	19.72983	15.71877	20.45131	27.82399	62.89549	55.39383
HORVU2Hr1G012440	60.99065	53.86131	54.92535	23.33565	22.12436	20.73316
HORVU5Hr1G094000	0.301419	0.254143	0.164791	0.800067	1.126695	1.283739
HORVU5Hr1G112850	9.981074	9.187447	12.01716	16.50958	46.20423	40.95312
HORVU1Hr1G088880	83.10825	57.83418	82.36071	247.9387	276.9617	284.4604
HORVU2Hr1G093950	0.82582	1.012269	1.075176	1.886631	11.27236	9.998036
HORVU7Hr1G110610	13.78487	10.02545	13.42867	6.811654	4.675096	5.412878
HORVU5Hr1G104080	4.751051	4.686936	5.250693	3.793015	1.149423	1.916643
HORVU3Hr1G082280	1.835511	2.080237	1.991194	4.693123	6.506169	6.10946
HORVU5Hr1G018720	1.708054	1.301535	1.341912	0.308012	0.046922	0.16339
HORVU4Hr1G012060	21.37803	26.12653	36.21324	1.336285	0.993369	1.519791
HORVU4Hr1G048060	0	0	0	2.866383	3.431038	3.221966
HORVU2Hr1G017650	0.892344	0.931038	0.862554	2.58445	8.491178	9.407321
HORVU3Hr1G071430	14.76904	14.8849	15.12121	9.766069	6.549837	6.981829

HORVU4Hr1G025360	5.098476	4.987363	6.517482	1.887504	0.746426	1.278824
HORVU5Hr1G045700	0.439292	0.347225	0.462886	0.526152	3.729461	3.490054
HORVU5Hr1G095310	3.67463	4.490453	6.667153	1.979569	2.711447	2.700092
HORVU2Hr1G037620	0.623678	0.467713	0.735593	1.809092	2.077855	1.131453
HORVU5Hr1G047410	1.073557	0.917718	0.840769	4.765473	13.50959	10.31722
Hordeum_vulgare_newGene_10448	4.972152	5.230182	6.008788	2.634914	3.480858	3.530257
Hordeum_vulgare_newGene_10443	32.93354	40.07112	36.07097	13.42489	18.31503	19.59959
Hordeum_vulgare_newGene_10441	0.286176	0.133818	0.219916	2.375926	35.3997	34.72789
HORVU1Hr1G081300	15.59348	12.17955	13.66782	1.672819	0.247055	0.822646
HORVU4Hr1G090210	1.597044	1.875671	2.211246	1.040484	1.127133	1.182437
HORVU4Hr1G060600	2.803003	2.447057	2.990885	7.435568	13.42509	12.58724
HORVU6Hr1G077730	1.108704	1.857822	2.90833	0.842265	1.229503	0.993555
HORVU7Hr1G010790	1.381559	1.350605	1.432213	0.583536	0.4779	0.611088
HORVU6Hr1G010890	3.820984	3.414372	8.06728	1.366769	1.303306	1.748074
HORVU3Hr1G103540	1.503461	0.686556	2.148787	2.635966	6.512862	6.858666
HORVU2Hr1G063800	1.000468	0.90195	1.403224	5.838902	6.790098	9.72671
HORVU2Hr1G090090	1.108799	1.267884	1.613138	6.228645	9.960696	9.245469
HORVU2Hr1G088540	107.9973	104.9243	139.5742	94.19576	54.06569	56.3493
HORVU7Hr1G041430	0.538143	0.622461	0.484427	0.215034	0.352111	0.323928
HORVU5Hr1G040140	1.951371	1.399667	1.80549	0.824927	1.06976	1.353043
Hordeum_vulgare_newGene_12435	3.113644	2.916588	3.915812	0.91244	1.280437	1.045061
HORVU7Hr1G035480	1.871625	1.198862	1.734156	0.864105	0.261791	0.790211
HORVU1Hr1G072890	8.595904	8.301115	11.40079	3.786329	4.45743	5.242705
HORVU2Hr1G118500	0.961111	0.689158	0.885835	0	0	0
HORVU0Hr1G040500	0.224446	0.235034	0.249977	1.042686	0.718641	0.709953
Hordeum_vulgare_newGene_12202	0.476496	1.443594	1.732382	0.071087	0.044698	0.192273
HORVU7Hr1G077560	0.944037	1.134818	1.500298	4.161483	5.972497	5.718526
HORVU4Hr1G052450	39.52961	42.97862	54.23368	7.122671	6.244946	5.211337
HORVU5Hr1G121440	3.078061	2.861549	3.073483	45.48943	53.73003	54.82717
Hordeum_vulgare_newGene_14172	1.095762	0.800697	0.915495	4.816523	3.375124	4.741051
Hordeum_vulgare_newGene_14171	26.80997	19.41678	23.24447	17.08451	11.42225	11.22496
Hordeum_vulgare_newGene_13388	0.040507	0.025593	0.031455	0.132651	0.185227	0.257477
Hordeum_vulgare_newGene_10873	0	0	0.025305	2.8755	1.321656	1.625322
Hordeum_vulgare_newGene_10870	0.339108	0.279286	0.412188	0.816751	0.876003	0.808343
Hordeum_vulgare_newGene_477	1.296143	1.641306	1.670654	0.128502	0.095928	0.080522
HORVU4Hr1G058930	5.283817	6.273769	5.817346	2.019341	2.717023	3.311353
Hordeum_vulgare_newGene_2786	0.150114	0.199876	0.214544	0.643536	0.451442	0.474322
HORVU3Hr1G110230	1.471369	0.683375	0.966724	0	0	0
Hordeum_vulgare_newGene_5836	0	0.032295	0	2.584312	2.959531	2.632397
Hordeum_vulgare_newGene_5832	2.018077	2.254081	2.370837	0.579127	0.808076	0.888127
Hordeum_vulgare_newGene_5838	0	0	0	0.731909	0.471663	1.193911
HORVU3Hr1G055980	0.255349	0.254931	0.349712	0.723855	1.243533	0.837385
HORVU5Hr1G078080	4.123133	4.224528	4.588207	8.143554	14.47824	12.43191
HORVU2Hr1G037540	0.195888	0.258569	0.180783	1.019339	1.876893	1.130123
HORVU5Hr1G006290	1.562713	1.739658	3.056673	0.303961	0	0.285414
HORVU5Hr1G032980	20.29281	17.81669	17.13261	78.50481	104.4528	107.3813
Hordeum_vulgare_newGene_11831	0.26304	0.268013	0.182713	1.904226	2.314376	1.525296

Hordeum_vulgare_newGene_11832	0.21031	0.171056	0.103661	0.931647	1.820865	1.287351
Hordeum_vulgare_newGene_9777	0.362704	0.120486	0.289828	1.807596	4.777285	4.044428
HORVU7Hr1G030210	0	0	0.02296	1.592336	1.338176	1.902238
Hordeum_vulgare_newGene_9771	0.279427	0.129769	0.265697	34.62051	20.23836	20.98108
HORVU7Hr1G073040	0	0	0	2.07357	3.193529	2.441099
HORVU7Hr1G023440	8.162586	8.629594	7.023773	4.122697	0.318656	2.257593
HORVU2Hr1G073730	6.416561	9.509965	7.284277	0.529388	0	0.052254
HORVU3Hr1G014280	1.287174	0.943988	1.367225	0.198851	0.04063	0.074329
Hordeum_vulgare_newGene_4072	6.776343	2.685662	7.428183	2.665743	1.891221	2.321204
Hordeum_vulgare_newGene_14920	1.56762	2.304587	2.620823	0.331544	0.131848	0.186821
Hordeum_vulgare_newGene_14922	11.88487	11.22036	7.590104	0.590701	0	0.284808
Hordeum_vulgare_newGene_14924	0.861492	0.248652	0.653769	2.178395	3.660723	3.384431
Hordeum_vulgare_newGene_4699	3.515352	4.007774	3.841076	0	0	0
HORVU6Hr1G076750	7.675964	8.758863	10.45876	4.330557	4.349306	4.763955
Hordeum_vulgare_newGene_7521	30.39816	27.91066	31.07676	102.9534	132.5579	101.8959
HORVU7Hr1G099250	0.480135	0.652855	0.495849	2.93926	3.781524	3.218335
Hordeum_vulgare_newGene_7528	0.62584	0.587734	0.607189	1.579713	2.094727	2.658342
HORVU5Hr1G010560	7.012038	4.976373	5.931236	1.114401	0.303225	0.703137
Hordeum_vulgare_newGene_1137	0	0.014646	0.013152	0.47504	0.499423	0.687981
HORVU5Hr1G087250	15.34258	14.12364	12.25005	75.67178	114.8852	118.9528
Hordeum_vulgare_newGene_3699	4.967424	5.871248	7.172759	2.634322	3.05115	3.4621
HORVU2Hr1G045120	1.51858	1.814914	1.310437	0.520146	0.603482	0.652006
HORVU5Hr1G125110	1.223969	1.180989	1.038327	3.320651	4.529003	4.639591
HORVU2Hr1G006320	0.789579	0.695068	1.106382	0.414553	0.461043	0.52513
HORVU6Hr1G075380	18.0244	17.83875	17.19188	0.415431	0	0.247841
HORVU3Hr1G090160	0.002409	0	0.023125	0.376818	0.462673	0.323207
HORVU2Hr1G074280	0.892174	0.830504	1.014898	2.307007	4.212576	4.005204
HORVU7Hr1G055100	16.5827	16.343	15.9264	10.14184	6.999013	8.958297
Hordeum_vulgare_newGene_1222	1.277922	1.169761	1.275632	2.362613	4.704724	4.431209
HORVU3Hr1G032920	10.04521	10.02721	12.80875	5.834792	6.420236	5.973443
HORVU4Hr1G051010	60.25206	64.14729	75.25227	19.94343	7.632819	9.721829
HORVU3Hr1G013390	0.130778	0.106211	0.086618	0.962053	0.978246	1.603858
HORVU2Hr1G005140	6.310249	4.782979	6.918292	2.680458	3.666316	3.333885
HORVU3Hr1G019130	0.493653	0.548721	0.681148	0.239836	0.306135	0.728016
HORVU5Hr1G035610	1.98522	2.119826	2.172254	5.112123	9.260604	7.768387
HORVU2Hr1G004230	26.12554	15.30748	36.05937	10.62646	6.72426	11.73945
HORVU2Hr1G019270	10.68542	14.66484	14.318	35.06579	62.07396	59.0142
HORVU4Hr1G010160	0.036639	0.137337	0.013466	1.536182	2.46728	2.402404
HORVU3Hr1G076790	2.604371	2.430691	2.303312	4.486707	10.35375	8.114368
HORVU2Hr1G123070	23.4706	23.96754	26.77902	14.20329	14.62488	15.76065
HORVU3Hr1G009520	6.109976	6.255751	9.876831	168.1132	218.8438	207.8673
HORVU5Hr1G066230	29.97924	13.13095	27.47606	0.141234	1.377911	0.383759
HORVU4Hr1G009380	97.24579	70.63874	92.99437	28.91975	19.67111	22.08756
HORVU2Hr1G065430	2.03281	1.593623	1.32225	4.855453	5.573672	6.295379
HORVU3Hr1G084420	7.075476	6.50257	10.52156	1.570721	1.101867	1.430716
HORVU4Hr1G015260	38.21108	32.65692	31.35633	95.94523	146.5496	151.9056
HORVU3Hr1G088000	0.377795	0.355372	0.309088	3.888505	4.638273	4.895039

HORVU1Hr1G015620	4.639004	5.473271	6.217294	0.054279	0.0495	0
HORVU3Hr1G000580	9.74901	9.309847	10.46627	4.592204	3.615399	4.352426
HORVU5Hr1G083920	0.150631	0.067593	0.255172	0.391106	1.460313	2.260765
HORVU2Hr1G107250	0.513221	0.398145	0.539844	1.410798	0.766984	1.197096
HORVU7Hr1G071230	8.317103	12.72544	14.73398	0.023047	0.041642	0.267087
HORVU7Hr1G093450	34.66518	27.79825	32.53757	19.2151	13.41735	14.33546
HORVU7Hr1G110470	0.261763	0.496428	0.459235	0.942877	2.429994	2.603824
HORVU3Hr1G006600	0	0	0	8.834447	11.8309	12.13866
HORVU4Hr1G055030	0.01905	0.129611	0.060861	0.861286	0.958729	0.833308
HORVU4Hr1G059050	0.180593	0.186805	0.237202	6.127949	7.041676	8.510718
HORVU3Hr1G099770	31.72287	32.16661	45.52045	10.68073	5.572494	5.817335
Hordeum_vulgare_newGene_12947	1.555768	2.2951	2.970672	5.751507	8.749606	7.974193
HORVU2Hr1G101150	60.39566	54.92524	58.365	6.557566	1.128676	4.931042
HORVU3Hr1G114240	1.723288	1.89957	3.091197	0.563346	0.670333	1.230126
HORVU5Hr1G095120	0.036869	0.027528	0.079604	0.344633	0.699152	0.618969
HORVU2Hr1G104500	11.98398	10.29828	9.610348	3.071428	4.347557	4.358122
HORVU2Hr1G067350	2.4757	1.96182	2.784513	0.383444	0.225064	0.344966
HORVU0Hr1G011480	0.581681	0.691274	0.539836	0.112872	0.06359	0.036141
Hordeum_vulgare_newGene_1953	0	0	0.098797	3.591623	2.323272	3.996952
Hordeum_vulgare_newGene_11504	4.598096	5.226281	4.44477	13.27427	20.81734	20.34763
HORVU3Hr1G073930	0	0.010088	0.005654	4.046778	2.967343	4.055447
Hordeum_vulgare_newGene_904	0.228111	0.53084	0.492588	0.701388	4.507579	4.124143
HORVU2Hr1G025290	0.240237	0.279845	0.060104	1.046134	1.004188	1.149211
HORVU2Hr1G081800	21.46344	16.55736	20.81682	13.5258	8.71765	10.79036
HORVU6Hr1G090150	3.080123	4.21396	5.926756	2.221463	1.864731	2.185773
HORVU5Hr1G014090	7.91623	6.746462	9.827641	0	0	0
Hordeum_vulgare_newGene_12080	3.36502	4.598719	5.202019	0.235504	0.085156	0.181427
Hordeum_vulgare_newGene_12088	1.976911	1.425852	2.232791	0.663159	1.632562	1.23472
HORVU4Hr1G079860	0.998722	1.235624	2.076576	0	0.069925	0.04513
HORVU1Hr1G081980	1.213944	1.267428	1.128408	5.434149	8.547186	8.129747
HORVU7Hr1G104590	4.384569	7.022275	6.779921	11.56768	17.48625	17.79997
HORVU5Hr1G075150	4.141641	2.596019	2.54405	0.38627	1.197627	1.247372
HORVU6Hr1G071520	0.620539	0.719861	0.75956	2.887703	6.781453	5.847949
HORVU7Hr1G038390	0.19894	0.048686	0.560027	6.07841	27.29332	21.09056
Hordeum_vulgare_newGene_6104	3.129636	2.549643	3.963734	9.802335	10.18116	10.80321
Hordeum_vulgare_newGene_6107	3.52251	3.193756	3.433898	0.009121	0.018666	0.024505
HORVU3Hr1G090410	6.192269	4.287738	8.638225	3.063255	0.565721	1.979279
Hordeum_vulgare_newGene_6101	0.107991	0.110497	0.240865	2.670066	10.65937	10.56168
Hordeum_vulgare_newGene_3163	2.180316	2.939371	3.13313	1.20983	1.544639	1.690154
HORVU4Hr1G045830	0.214866	0.0558	0	0.671123	2.470271	1.646016
Hordeum_vulgare_newGene_5665	0.48095	0.273149	0.354865	0.234683	4.079096	2.654708
Hordeum_vulgare_newGene_5668	34.06438	34.40938	49.73375	23.48883	25.37746	24.26328
HORVU3Hr1G013400	0.745517	0.773561	0.853596	3.955267	7.248525	7.083213
HORVU5Hr1G062110	1.989449	2.428274	2.396528	5.880331	13.75136	13.93223
HORVU6Hr1G078500	0.14565	0.157551	0.116281	0.654813	0.917379	0.776686
Hordeum_vulgare_newGene_11974	1.908036	1.556976	1.758587	0.503673	0.488292	0.639876
HORVU0Hr1G003020	2.36286	1.945502	2.221621	5.684818	6.156672	8.422304

HORVU2Hr1G070720	2.201588	1.382633	1.652325	0.750598	0.612335	1.017254
HORVU5Hr1G024390	0.160874	0.312014	0.190264	1.491002	2.785195	2.823338
Hordeum_vulgare_newGene_6817	0.525193	0.563973	0.749724	0.024083	0.060679	0.065479
Hordeum_vulgare_newGene_6814	0.180797	0.123596	0.269718	0.50568	0.549473	0.706944
HORVU4Hr1G043860	0.221797	0.267382	0.215544	1.931089	1.915861	2.398534
HORVU6Hr1G073100	0	0	0	1.220766	2.692566	1.745304
HORVU2Hr1G080340	34.56931	33.33848	61.23625	17.68895	26.34334	24.75551
HORVU7Hr1G026240	6.704476	6.636937	4.537116	3.061896	2.623924	2.349073
HORVU3Hr1G011110	5.446154	7.709148	6.903579	0.007495	0.011965	0.009306
HORVU2Hr1G069780	0.424877	0.298645	0.551827	2.092114	5.508526	4.511448
Hordeum_vulgare_newGene_14609	25.77722	19.82988	22.87209	0.327987	0.26522	0.38112
HORVU5Hr1G104230	63.36592	60.927	51.35468	9.276492	11.55297	10.61907
HORVU1Hr1G022430	1.852634	0.678059	1.751677	4.065138	3.459188	4.411776
HORVU3Hr1G014090	470.4514	421.7897	400.0932	202.6477	64.28977	93.27419
HORVU0Hr1G018850	0.750314	0.479973	0.654076	0.871792	5.597801	6.322242
HORVU1Hr1G093170	66.52535	62.7343	80.75611	34.80968	35.47332	32.33101
Hordeum_vulgare_newGene_13654	0.681292	0.513297	0.414348	8.923192	2.90908	3.893729
HORVU4Hr1G072120	0.202534	0.126271	0.206869	1.398171	1.52464	1.350144
HORVU6Hr1G070450	96.13053	78.01023	99.30921	30.05359	12.05711	12.04371
HORVU6Hr1G081270	0.220232	0.170057	0.195349	2.67724	2.73605	3.239607
Hordeum_vulgare_newGene_13651	0.146226	0.191526	0.276598	1.183809	1.131455	1.814253
HORVU6Hr1G009620	0.011989	0	0	2.262856	2.579656	2.552436
HORVU2Hr1G083100	20.89796	21.27331	27.7697	12.2708	10.41047	11.6305
HORVU7Hr1G110320	0.306073	0.11184	0.536681	4.645055	18.79827	16.54314
Hordeum_vulgare_newGene_7777	5.806874	6.583973	4.808816	1.418156	3.811329	1.851378
HORVU4Hr1G052030	0	0	0	0.766578	2.291447	1.111495
HORVU3Hr1G039660	9.693562	9.089921	11.94565	6.342234	5.986024	6.11193
HORVU5Hr1G046550	10278.38	6642.44	9458.149	2911.306	14.59161	904.972
HORVU5Hr1G008270	2.387501	1.041104	2.785767	0.383423	0.369649	0.235542
HORVU7Hr1G115040	2.663937	1.909643	2.531768	9.279957	17.60754	16.87748
HORVU1Hr1G053000	2.789953	2.91353	2.576398	5.989116	6.981976	8.523963
HORVU1Hr1G061530	7.220063	8.522571	11.3511	5.257574	5.908758	5.621565
HORVU7Hr1G093120	24.09788	21.36989	22.21738	16.56805	9.783954	13.21951
HORVU5Hr1G013260	2.169215	1.94061	2.380337	4.039891	8.815743	8.239842
HORVU2Hr1G044640	1.70337	1.060673	1.309093	3.536915	2.155483	3.928207
HORVU0Hr1G017080	2.560055	2.574105	2.880055	5.537466	9.504856	9.119916
HORVU4Hr1G007150	1.230001	2.071407	1.663281	0	0	0
HORVU3Hr1G022060	1.556018	1.735426	1.444101	0	0	0
HORVU2Hr1G041960	11.39899	10.85267	11.45121	5.894903	5.024146	6.297944
HORVU0Hr1G005420	6.055743	4.122344	8.280814	2.848131	2.363809	3.589738
HORVU1Hr1G011520	0.969915	1.213582	1.094583	2.207322	3.835852	3.015936
HORVU4Hr1G011600	0.445637	0.540927	0.323638	10.83044	20.4763	17.28731
HORVU6Hr1G093680	1.792574	2.275296	2.23546	0.964243	1.25486	1.660428
HORVU5Hr1G097010	0.854682	0.618441	1.051133	1.940549	1.677363	1.692454
HORVU5Hr1G095010	1.827393	1.484846	1.603908	0.834321	0.113517	0.368854
HORVU1Hr1G094380	0.052015	0.02871	0.065467	3.898499	3.333584	5.912922
HORVU4Hr1G014070	3.761848	3.285495	4.114111	8.956709	13.55762	12.29625

HORVU2Hr1G002750	0.004423	0.004315	0	0.343062	0.265659	0.535374
HORVU3Hr1G056660	8.410291	6.326849	6.274432	0	0	0
HORVU4Hr1G052500	0.061294	0.08472	0.050614	0.934762	0.441574	0.899952
Hordeum_vulgare_newGene_12864	0.358878	0.512876	0.472948	0.089492	0.332066	0.06052
HORVU4Hr1G026340	17.52503	16.55262	20.63264	10.27886	6.00167	9.401154
HORVU3Hr1G087170	3.524049	5.503658	5.959378	14.32949	37.39093	30.03807
HORVU1Hr1G069900	4.572371	3.39268	4.973143	8.674494	14.09774	11.21223
HORVU6Hr1G055230	11.36641	8.596325	9.411778	4.643766	3.079881	3.539506
HORVU7Hr1G094730	6.482968	4.792339	4.872577	0.12048	0.247447	0.085209
HORVU3Hr1G005560	0.16102	0.178291	0.315139	0.512402	1.081217	0.941766
Hordeum_vulgare_newGene_13801	0.585022	0.658135	1.688748	2.99031	11.99052	8.701951
HORVU4Hr1G079460	0.11671	0.06855	0.070695	0.550169	0.442728	0.614137
HORVU6Hr1G008450	0	0	0	1.249393	0.771917	1.185718
HORVU7Hr1G043530	18.7546	18.1273	15.2724	1.486756	1.888021	1.780465
HORVU2Hr1G036590	17.05135	10.94731	18.16129	4.006602	1.253295	3.849627
Hordeum_vulgare_newGene_8655	0.377154	0.45615	0.620865	1.888541	1.608339	1.587381
HORVU2Hr1G092090	24.25691	15.00211	16.48916	21.7591	171.9753	136.3244
HORVU7Hr1G012550	0.303911	0.09267	0.408495	1.935111	2.342352	1.896647
HORVU2Hr1G109440	2.057304	1.695411	2.115278	0.113412	0.016426	0.137406
HORVU4Hr1G021950	4.242645	3.769814	4.454165	12.51988	18.27478	19.186
HORVU7Hr1G020760	0.61354	1.459185	1.231031	68.0412	70.31309	73.83775
Hordeum_vulgare_newGene_10576	1.811637	2.313775	1.971123	7.26649	8.595267	10.16758
HORVU3Hr1G057890	4.050184	5.627257	7.095429	2.68819	2.822841	2.58409
Hordeum_vulgare_newGene_10571	2.787284	1.72745	3.762348	0.161282	0.083666	0.22673
HORVU2Hr1G102710	4.665316	1.657867	7.357029	0.475751	0.023725	0.815887
HORVU2Hr1G090250	1.471981	1.033857	1.286741	0.349152	0.123933	0.21764
HORVU1Hr1G035720	0.45844	0.757504	0.666453	6.30578	26.02159	30.39971
HORVU3Hr1G054050	11.62053	11.80992	13.59564	30.08789	36.95917	39.66641
HORVU3Hr1G011350	12.17672	11.91196	17.46508	5.316785	5.351674	4.746331
HORVU2Hr1G021280	13.34993	11.79792	14.16489	31.94124	40.57031	41.02688
HORVU3Hr1G095340	0.698168	0.522775	0.462244	1.407306	1.659647	2.050724
Hordeum_vulgare_newGene_4401	16.47373	22.61978	23.68559	4.624755	1.808759	2.990638
Hordeum_vulgare_newGene_4409	21.10621	18.30802	23.26818	46.10625	74.59938	66.48766
Hordeum_vulgare_newGene_6045	10.76084	13.86025	25.0624	0.750726	0.788849	0.799246
Hordeum_vulgare_newGene_7170	0.843942	0.946908	0.469102	0.130329	0.057443	0.096133
Hordeum_vulgare_newGene_577	32.94119	28.80048	42.05778	11.41938	22.68145	22.64518
Hordeum_vulgare_newGene_12327	8.352026	7.610397	9.407615	6.046135	5.801428	5.329286
Hordeum_vulgare_newGene_13491	0.549168	0.230574	0.345328	3.619579	7.910296	9.141221
HORVU1Hr1G074130	39.65957	41.72835	54.07497	21.72764	19.2969	19.29506
HORVU3Hr1G060700	0.128227	0.273385	0.216931	1.711516	1.268128	1.354435
HORVU2Hr1G068050	0.888508	0.406433	1.228241	2.484011	4.642952	4.334522
HORVU5Hr1G075050	2.404633	1.962571	2.491452	7.883846	35.05134	31.61846
HORVU5Hr1G037430	0.711937	0.474596	0.630537	1.291899	3.807383	2.447581
HORVU7Hr1G052140	43.54363	43.4551	37.62886	23.96	18.29338	21.50234
HORVU6Hr1G064720	56.85506	63.79772	88.49556	32.19915	24.20707	32.65117
HORVU1Hr1G012130	2213.413	2096.816	1885.395	510.4823	147.2864	224.0447
HORVU1Hr1G075880	1.348778	1.552611	1.234787	0.572956	1.119854	1.017035

HORVU2Hr1G087730	0.113188	0.431644	0.152726	4.126592	4.007536	4.828658
HORVU7Hr1G110900	64.23356	63.56972	46.8979	18.74374	21.66775	22.7738
HORVU4Hr1G070260	3.452742	2.666972	3.723154	23.11168	5.190857	9.509332
HORVU1Hr1G055600	10.5071	8.326413	10.77015	25.36421	27.82886	28.37113
HORVU1Hr1G059920	32.41766	53.64071	78.91367	1.568947	0	0.485313
HORVU7Hr1G037550	0.036776	0.054045	0.074393	0.388898	0.307088	0.345464
Hordeum_vulgare_newGene_4591	1.237345	1.711983	1.362572	0	0	0
HORVU0Hr1G000780	35.0172	18.26273	36.67534	3.776787	4.281404	4.399354
HORVU2Hr1G005510	3.300275	2.414573	2.723854	11.06421	21.16073	13.90988
HORVU6Hr1G061300	0.868968	1.011938	1.315869	4.453593	15.36157	18.66295
HORVU7Hr1G112710	0.389256	0.579501	0.695768	6.630883	3.651199	4.893772
HORVU2Hr1G004640	13.86658	8.31083	18.32161	2.852636	0.468256	1.067827
HORVU1Hr1G025830	1.241379	0.698156	1.509439	0.087013	0.018973	0.06082
HORVU6Hr1G030080	1.793579	3.488331	3.265154	0.840926	0.354124	0.600946
HORVU7Hr1G049070	1.805421	1.551015	2.567621	19.62575	8.693466	14.12009
HORVU0Hr1G002120	1.122067	0.887115	0.998329	1.740915	3.25275	3.164321
HORVU5Hr1G001180	0	0.038856	0.016564	5.408551	18.25318	15.52272
HORVU1Hr1G076210	0.909575	1.12152	1.540017	0.747606	0.6736	0.750681
Hordeum_vulgare_newGene_1625	37.13957	35.36806	64.5098	1.884395	2.76137	4.405071
HORVU2Hr1G043310	1.273693	1.733566	2.388877	3.836668	5.18234	4.574756
Hordeum_vulgare_newGene_1628	6.415823	11.50878	11.71681	0.87143	0.240521	0.473453
HORVU0Hr1G008870	26.48168	28.38144	29.09088	18.63582	15.37955	15.03464
HORVU4Hr1G086500	0.143704	0.099651	0.364029	1.541005	0.601967	1.107461
HORVU1Hr1G029090	3.158676	5.016618	7.175767	15.18783	41.38739	34.23777
HORVU2Hr1G082950	3.967054	4.581331	4.449071	2.185542	2.80321	2.767679
HORVU3Hr1G031140	0.611482	0.589186	0.441844	1.117864	4.384199	3.513038
HORVU7Hr1G048880	2.900411	3.132596	4.7348	5.28338	11.64893	10.95165
HORVU7Hr1G024250	0.016212	0	0.026981	1.907028	40.69446	29.03668
HORVU3Hr1G001060	0.294973	0.234058	0.286119	1.150859	1.270682	2.971579
HORVU5Hr1G014510	0.244285	0.11482	0.203993	3.357249	14.3158	10.83403
HORVU4Hr1G003800	8.444714	7.696701	8.959639	19.57042	22.51505	22.78993
HORVU4Hr1G081990	6.910626	8.560055	9.673264	4.845481	2.475881	4.016016
HORVU2Hr1G099660	10.51609	10.28229	19.72447	4.813366	6.363059	7.153524
HORVU3Hr1G070300	9.81186	10.16038	13.86342	7.153912	4.581401	6.198223
HORVU6Hr1G074410	0.705854	0.591689	0.602473	3.193242	2.311266	2.323755
HORVU3Hr1G002000	5.53706	3.187321	8.746502	0.34554	0.075932	0.117201
HORVU7Hr1G093080	16.40042	14.39038	18.91327	14.09928	8.160238	7.560744
HORVU4Hr1G052320	6.347239	3.053709	7.134206	1.636846	0.819422	1.091353
HORVU4Hr1G051780	201.3533	236.6762	288.8035	73.07365	5.101343	35.00189
HORVU3Hr1G028550	5.851232	7.58002	6.467757	15.25859	19.75634	18.74703
HORVU4Hr1G081330	0.043802	0.049103	0	1.925359	1.410242	1.610127
HORVU5Hr1G097930	1.573783	1.135207	1.955321	12.81497	6.088622	7.464816
HORVU3Hr1G072410	35.73829	33.82654	33.54608	19.01361	14.83535	17.83327
HORVU7Hr1G040690	0.2318	0.373971	0.356477	1.577119	2.416777	2.025927
Hordeum_vulgare_newGene_16229	0.068742	0	0.033661	0.601632	1.319141	0.909861
Hordeum_vulgare_newGene_16220	1.564165	2.264895	2.387649	0.197532	0.071876	0.084456
HORVU0Hr1G013900	25.36688	22.40309	20.04457	84.77275	94.70655	103.9753

HORVU5Hr1G109240	65.29718	55.01732	57.35148	156.4709	191.1733	137.1253
HORVU7Hr1G050110	0.384252	0.399286	0.685178	0.717225	2.733593	2.176605
HORVU2Hr1G009830	9.842114	12.59773	15.25354	4.256557	0.834226	2.692005
HORVU1Hr1G038060	17.07446	20.199	22.7518	13.55223	9.353856	12.04576
HORVU3Hr1G117370	7.633372	6.365371	10.1254	1.324071	0.599934	2.388586
HORVU6Hr1G093490	0.143354	0.189392	0.24414	0.775025	1.496973	2.001547
HORVU5Hr1G058060	7.968525	5.644046	6.371127	0.542904	0.014273	0.132459
Hordeum_vulgare_newGene_3474	20.6557	14.94904	17.48892	12.45246	4.276265	5.943619
Hordeum_vulgare_newGene_3470	0.307325	0.259312	0.407488	0.565794	1.362635	1.071719
Hordeum_vulgare_newGene_9082	2.908961	3.070642	3.307624	1.272923	0.794977	0.508444
Hordeum_vulgare_newGene_9085	3.240734	2.711414	3.197367	0.007918	0	0
Hordeum_vulgare_newGene_9084	2091.552	2067.006	1405.925	556.2512	173.5831	258.4333
Hordeum_vulgare_newGene_15229	9.491987	10.23999	9.447094	0.015351	0	0
HORVU3Hr1G088850	1.280737	1.539524	2.401365	0.647318	0.602391	0.760635
HORVU3Hr1G104100	0.018339	0.062664	0.01716	1.988071	1.931403	2.470578
HORVU5Hr1G063430	1.939381	1.438518	2.125238	0.554433	0.222883	0.637335
HORVU0Hr1G010220	4.390182	4.419263	5.289117	3.462872	2.043692	2.534045
HORVU7Hr1G027870	5.682282	6.503046	6.904919	2.095279	1.638037	2.228538
HORVU3Hr1G095090	0.544992	0.413376	0.882617	1.726371	1.457041	0.93308
HORVU2Hr1G097060	2.974021	4.906388	3.584046	8.467306	15.60805	14.47714
HORVU4Hr1G071000	21.2304	24.90329	19.05821	43.10206	59.77725	75.04806
HORVU2Hr1G112230	1.229685	0.902176	1.10636	3.186282	3.976387	3.129388
HORVU2Hr1G119220	0.446506	0.326289	0.243945	0.640096	1.169424	1.261251
HORVU5Hr1G117680	0.091633	0.229411	0.223649	4.982263	12.15559	10.4319
HORVU2Hr1G049410	2.187177	1.457277	1.901312	2.900588	6.633095	5.983147
HORVU2Hr1G111590	0.788916	0.637749	0.465152	1.545489	1.906973	1.821392
HORVU2Hr1G118570	113.1442	85.97543	77.0276	29.12978	7.170227	12.51041
HORVU5Hr1G072160	66.34818	68.97865	88.41889	9.467303	0.599285	3.814989
HORVU2Hr1G092900	6.50263	4.979875	6.271089	3.90505	2.51242	2.835601
Hordeum_vulgare_newGene_1426	1.127907	1.082342	0.751763	0.011625	0.010034	0
HORVU2Hr1G011200	2.177435	1.927895	3.311128	5.118346	8.007569	7.55267
HORVU7Hr1G021840	9.725108	8.412696	10.64977	24.35717	55.30259	55.3471
Hordeum_vulgare_newGene_3867	8.529072	7.899771	7.500926	43.51095	67.34712	56.78618
Hordeum_vulgare_newGene_3866	5.533894	5.044569	5.539799	2.681712	3.165907	3.681392
HORVU7Hr1G001190	1.663553	1.044579	3.212897	0	0	0
HORVU7Hr1G100850	11.62814	4.304527	12.18021	1.439453	0.771895	0.898655
HORVU7Hr1G021260	0.581494	0.522876	0.675264	0.326451	0.104485	0.309216
HORVU5Hr1G082620	14.28237	10.33967	13.31314	4.590004	2.888146	2.203422
HORVU5Hr1G060310	1.180973	1.148258	1.512888	1.816066	5.408512	5.636081
HORVU7Hr1G001220	0.014896	0	0	0.735213	0.778873	1.150858
Hordeum_vulgare_newGene_6618	0	0	0	0.715784	0.578532	0.490606
HORVU4Hr1G002530	0.025122	0.105433	0.120855	0.497971	1.942808	2.628934
HORVU5Hr1G084700	2.977982	1.558787	2.675348	0.934156	0.709604	0.714242
Hordeum_vulgare_newGene_14635	0.971422	1.437571	1.532047	5.501632	6.380838	7.161863
HORVU7Hr1G078170	4.29508	4.323505	4.33838	1.935981	1.823243	2.2717
HORVU5Hr1G064100	0.654556	0.561334	0.961719	6.156192	5.971297	6.316015
HORVU5Hr1G020350	0.717932	11.23079	1.311634	0	0	0

HORVU3Hr1G089690	0.108391	0.134841	0.127351	0.347305	0.329982	0.472128
HORVU3Hr1G012010	1.981251	1.940626	2.019419	5.974909	6.543945	6.869722
HORVU3Hr1G087780	6.122381	6.468986	7.718729	3.664181	4.16646	4.197638
HORVU5Hr1G023710	0.745892	0.851104	0.742813	5.528548	2.817852	3.391852
Hordeum_vulgare_newGene_8922	0.055893	0.209085	0.325625	0.175626	0.868506	0.663328
HORVU1Hr1G092150	5.805882	4.170397	5.540608	12.60448	18.2686	20.20257
HORVU1Hr1G061830	1.824339	1.724018	1.688087	4.110537	7.625811	7.242345
HORVU5Hr1G111240	1.923399	0.936586	2.411177	0.396183	0.281197	0.358338
HORVU1Hr1G044000	71.85217	52.43432	74.1292	6.965187	3.106206	5.113893
HORVU5Hr1G069680	1.262465	0.18384	1.331093	6.06512	37.10506	26.98038
HORVU7Hr1G010990	0.70893	0.566855	1.436902	0.100607	0	0.051238
HORVU0Hr1G012190	0.614678	0.609539	0.691249	1.792112	1.834431	2.237654
HORVU4Hr1G011250	40.76106	33.54779	53.01401	24.95891	12.61273	15.63146
Hordeum_vulgare_newGene_2954	0.756694	2.126177	2.876234	4.974051	14.07757	10.81925
HORVU2Hr1G114280	0.054098	0.082066	0.069814	0.53625	0.387713	0.471308
HORVU2Hr1G009580	24.237	32.04219	38.3933	10.7933	2.709922	7.49761
HORVU5Hr1G020430	48.44262	43.02647	46.87056	13.7495	7.309779	10.18803
HORVU3Hr1G001450	10.75672	9.647235	11.66219	5.481296	5.147777	5.725834
HORVU1Hr1G015000	0.594754	0.464394	0.499179	1.217877	3.146879	2.854238
HORVU3Hr1G047030	0.101191	0.088831	0.146768	30.81276	29.50729	40.07474
HORVU1Hr1G092930	0.735165	0.493613	0.82704	0	0	0
HORVU3Hr1G002900	0.988424	1.052992	1.612162	0.233484	0	0.124416
HORVU7Hr1G000860	23.37592	27.12999	28.8459	0.993767	2.233803	4.053427
Hordeum_vulgare_newGene_10324	0.773603	0.964543	0.645334	3.254515	3.970603	3.594472
HORVU7Hr1G043960	0	0	0	2.124329	2.839484	3.707271
Hordeum_vulgare_newGene_10329	0.431214	0.446834	0.361302	0.160642	0.01622	0.141472
HORVU7Hr1G116780	1.635916	1.455869	2.067676	0.917922	0.640428	0.71701
HORVU5Hr1G022120	0.663856	0.540348	0.411842	2.214671	7.925902	6.818721
HORVU1Hr1G012850	11.57257	8.370565	12.31289	36.10173	70.45068	66.78825
Hordeum_vulgare_newGene_11056	0.069855	0.077014	0.05289	4.86344	5.189624	5.347759
Hordeum_vulgare_newGene_11058	0	0	0	10.25011	10.08202	12.21793
HORVU3Hr1G092650	13.67747	15.98651	11.75944	8.092633	5.490124	6.238754
HORVU3Hr1G095600	8.896451	9.002638	10.14241	34.62365	37.96051	35.86098
HORVU3Hr1G014890	1.1172	1.001783	1.175478	5.438173	5.941414	6.932145
Hordeum_vulgare_newGene_11587	0	0	0	15.40485	16.07343	18.17092
HORVU2Hr1G112060	5.221498	3.452958	7.351753	0.249755	0.061157	0.081916
HORVU3Hr1G037360	97.47306	80.24349	112.2136	74.19277	42.59861	45.23584
HORVU7Hr1G045470	18.21739	23.55186	21.59271	9.338479	1.757042	3.93234
HORVU2Hr1G013590	0.06868	0	0.046642	3.847378	8.005479	5.439429
Hordeum_vulgare_newGene_16052	4.66265	2.919037	3.792691	0	0	0
HORVU5Hr1G082420	48.89469	42.45351	44.09621	107.9374	203.7628	196.5681
HORVU6Hr1G048170	2.139659	1.604916	1.670968	3.931156	4.620075	5.470505
HORVU6Hr1G024930	0	0.196107	0.251955	6.178445	7.318427	6.536694
HORVU7Hr1G037910	4.520677	2.257123	7.074099	1.082667	0.720651	1.536147
HORVU3Hr1G063620	0.027062	0.059684	0.005704	0.548277	2.12933	3.489063
HORVU2Hr1G062940	5.626168	4.9905	5.750315	0.055669	0.027163	0.044383
HORVU5Hr1G040970	1319.045	1330.152	1294.06	549.1714	360.29	420.6213

Hordeum_vulgare_newGene_647	1.179931	1.495852	1.033549	7.862555	6.839467	8.765776
HORVU7Hr1G042790	62.39595	56.04885	78.21779	14.72049	14.64274	11.28631
HORVU0Hr1G020420	38.83926	26.23596	40.78991	21.15293	23.37047	16.8163
Hordeum_vulgare_newGene_14341	2.20477	2.03748	2.920827	6.359346	7.020068	5.916852
Hordeum_vulgare_newGene_14340	0	0	0	2.078369	2.022719	2.54517
Hordeum_vulgare_newGene_14343	2.547537	2.164076	2.451145	0	0	0
Hordeum_vulgare_newGene_14342	0.505828	0.637253	0.463101	3.008167	2.78972	3.438195
Hordeum_vulgare_newGene_14344	10.41929	9.985046	12.62153	2.652771	0.222844	0.839602
HORVU3Hr1G059470	12.66195	15.46843	13.53227	26.56171	50.75177	42.58247
HORVU2Hr1G020330	6.166895	6.481002	6.763978	2.038046	2.446144	2.785884
HORVU2Hr1G096270	6.349305	6.51261	7.140965	3.838857	2.237145	2.709215
Hordeum_vulgare_newGene_2575	4.649162	8.004896	5.720476	0	0	0
HORVU7Hr1G103380	36.30016	32.02485	35.59917	7.913419	7.438676	7.896877
HORVU1Hr1G070480	0.611424	0.409728	0.331071	4.975821	5.118835	4.911893
HORVU7Hr1G096630	1.456539	0.560061	0.890875	10.51999	15.14228	11.33302
HORVU5Hr1G094680	360.75	393.2561	507.8816	54.35436	2.792549	19.87388
HORVU7Hr1G073490	0.214328	0.157966	0.183894	1.041614	6.252048	5.192441
HORVU5Hr1G063570	0.580416	0.74227	0.769773	1.572096	2.253168	3.085997
Hordeum_vulgare_newGene_10040	5.106769	5.661022	6.634061	18.9484	26.00642	24.84787
Hordeum_vulgare_newGene_13617	0.133369	0.206838	0.238051	1.144963	1.071499	1.750329
Hordeum_vulgare_newGene_8564	0	0	0	1.453851	0.977261	1.974179
HORVU3Hr1G026550	1.096518	1.327302	1.526163	73.35319	76.77562	79.47147
HORVU6Hr1G073180	2.563167	1.987779	1.548231	15.14794	19.61618	17.82978
Hordeum_vulgare_newGene_8499	0.089635	0.079163	0.206166	0.543586	1.775303	1.671518
HORVU7Hr1G102030	0	0.222376	0	2.652691	2.314472	2.502735
HORVU6Hr1G067360	2.761581	2.540553	3.069424	1.588251	1.758983	1.74764
HORVU0Hr1G026480	3.2615	2.439904	3.630451	6.158588	8.785846	8.995744
HORVU2Hr1G105130	1.916658	1.455696	1.967135	0.660438	0.872494	0.966226
Hordeum_vulgare_newGene_4130	5.552173	8.912163	8.306981	0.123248	0.106803	0.121829
Hordeum_vulgare_newGene_4131	0	0	0	0.726145	0.771051	0.795148
HORVU5Hr1G043090	3.469817	3.040616	3.269068	9.603202	12.8733	11.957
HORVU5Hr1G030330	0.565676	0.452188	0.62706	1.357049	1.397346	1.76311
Hordeum_vulgare_newGene_4796	14.7961	11.5384	12.55981	3.65841	3.416099	2.395886
Hordeum_vulgare_newGene_7443	2.781975	3.916554	3.189988	1.155318	0.99231	1.112902
Hordeum_vulgare_newGene_13125	1.285739	1.589974	1.293308	0	0	0
Hordeum_vulgare_newGene_7449	0.340841	0.406119	0.36822	2.428326	10.90593	10.22631
Hordeum_vulgare_newGene_4863	0.840171	0.962068	0.829218	0.180109	0.242959	0.150743
Hordeum_vulgare_newGene_6790	0.597027	0.797731	0.567568	0	0	0
HORVU6Hr1G058820	16.14456	10.99854	18.16671	26.47383	51.9387	58.3169
HORVU5Hr1G125010	0.745205	0.699646	0.943113	7.055034	10.56795	10.12421
HORVU3Hr1G018810	0.007742	0.04159	0.014968	0.458718	1.389281	1.353987
HORVU5Hr1G104980	1.176354	1.75511	1.236208	8.625806	9.773964	11.8581
Hordeum_vulgare_newGene_15822	1.188846	1.193126	0.68346	0.423284	0.196023	0.239046
Hordeum_vulgare_newGene_15825	1.501155	1.456428	0.862991	0.441657	0.194936	0.395568
HORVU7Hr1G052070	0.166894	0.08394	0.060362	0.686272	0.690336	0.664928
HORVU3Hr1G001920	0.168184	0.190826	0.202346	6.038656	74.40584	71.18563
Hordeum_vulgare_newGene_2962	2.00237	1.790221	1.433767	0	0	0

Hordeum_vulgare_newGene_9958	1.567777	1.644983	1.53581	1.046241	0.929077	1.015703
HORVU4Hr1G070970	486.0231	514.14	507.4331	276.4561	37.50987	132.1698
HORVU2Hr1G041610	10.2656	9.821801	9.348041	35.67606	55.32888	56.67903
HORVU5Hr1G089740	1.355319	1.028745	1.080015	7.142379	6.476669	5.693611
HORVU7Hr1G020410	0.324956	0.338889	0.353216	2.95772	2.376185	3.039508
HORVU1Hr1G065670	12.33272	5.203557	12.38246	0.955276	1.621991	3.154669
Hordeum_vulgare_newGene_5780	0.31894	0.588474	0.472401	0.037107	0.011084	0.011765
Hordeum_vulgare_newGene_5781	4.861892	4.721595	5.082545	10.49587	15.37338	12.50922
HORVU5Hr1G069180	2.864573	3.263227	4.286559	1.36367	2.022816	1.882996
HORVU5Hr1G120810	9.025956	7.175763	16.27801	4.354686	4.710254	5.064758
HORVU5Hr1G021610	0.678036	0.666769	0.687494	4.62628	5.673401	3.938228
HORVU6Hr1G085250	1.546726	1.68782	1.561082	5.68186	5.493277	5.570437
HORVU2Hr1G089670	2.176506	2.475652	3.000178	5.525475	6.225486	6.136657
HORVU2Hr1G109120	91.46356	86.43276	94.5285	43.54506	12.30431	26.44137
HORVU5Hr1G059040	0.143416	0.108072	0.137793	0.524583	0.989014	0.705785
HORVU1Hr1G085540	0.085087	0.145136	0.171863	1.183349	2.078497	2.498489
HORVU5Hr1G066460	11.42192	10.38151	11.11699	1.294399	0.509985	0.838191
HORVU4Hr1G087740	3.03795	3.942441	5.42781	5.459975	14.45646	14.95304
HORVU2Hr1G094410	1.508983	1.630749	1.088655	3.508026	3.408701	3.723427
HORVU2Hr1G061080	11.93108	11.03501	13.52269	6.931571	4.542738	6.004806
Hordeum_vulgare_newGene_12674	0.881098	0.888192	1.128747	0.48756	0.170555	0.364453
HORVU0Hr1G006020	8.149384	9.133624	13.93227	5.245175	6.283549	6.877781
HORVU1Hr1G081540	0.13765	0.068635	0.137044	0.750009	0.503317	0.588965
HORVU3Hr1G090900	16.26308	15.19032	18.81766	10.39015	8.463144	8.300129
HORVU3Hr1G064340	1.617534	1.73946	1.511459	4.797519	3.636865	4.254355
HORVU1Hr1G051470	1.816281	0.781666	0.932207	3.06582	24.68723	20.35402
HORVU5Hr1G088880	4.243322	4.23444	3.421357	15.24933	24.53481	21.52838
Hordeum_vulgare_newGene_13887	0.149348	0.142154	0.177157	1.024823	0.805977	1.404376
Hordeum_vulgare_newGene_13885	27.28565	23.64503	21.6106	13.60996	7.693218	10.13204
HORVU1Hr1G011930	2.440019	3.084852	3.112692	0.212931	0.020038	0.09372
HORVU2Hr1G010630	0.034272	0.212905	0.063048	2.406661	14.05914	12.29607
Hordeum_vulgare_newGene_10288	0	0	0	0.998896	1.058246	2.299292
HORVU6Hr1G017530	3.257749	2.399453	2.74489	8.06138	27.65113	26.72496
HORVU2Hr1G096700	0.658978	0.55974	0.589711	2.256413	3.330827	2.438746
HORVU7Hr1G093240	0.233854	0.240366	0.21792	0.770741	1.273773	1.017711
HORVU7Hr1G111060	0.736581	0.897089	1.269901	0.530124	0.111203	0.334542
HORVU1Hr1G016630	23.9591	21.77089	25.49621	12.12763	12.38901	13.19793
HORVU4Hr1G007610	135.2428	129.2257	79.5344	30.86127	33.99952	30.06808
HORVU3Hr1G073290	3.288851	2.736957	2.440103	14.76377	8.274697	10.64098
HORVU2Hr1G106970	4.986939	4.669078	4.346289	9.522237	23.28455	20.44033
HORVU5Hr1G048100	2.182552	3.402437	4.071575	1.539538	1.355745	2.591462
HORVU5Hr1G077450	0.006733	0.10726	0.040979	1.553366	0.637904	1.056187
HORVU4Hr1G069130	1.89978	1.738431	2.739313	4.137463	5.867878	6.402185
Hordeum_vulgare_newGene_11620	66.40518	64.57469	54.78125	21.33459	17.04113	19.12998
HORVU7Hr1G077110	0.131703	0.28116	0.336673	0.515475	0.607923	0.884655
HORVU2Hr1G028780	13.32341	3.251064	7.025871	0.588337	0.296322	0.510496
HORVU2Hr1G004660	0.720706	1.02879	1.080995	0.310053	0.055747	0.33157

HORVU7Hr1G105700	0.395352	0.72671	0.281272	1.812979	2.64084	2.252051
HORVU3Hr1G089510	3.703294	3.722512	4.036398	15.9991	17.56129	16.46064
HORVU0Hr1G021630	14.29313	13.7071	15.17901	56.55788	82.57212	82.44192
Hordeum_vulgare_newGene_6228	9.14789	11.44901	11.81565	2.153443	2.429337	1.953927
HORVU7Hr1G003630	0.065278	0.03022	0.097977	0.746911	0.441199	0.618967
HORVU1Hr1G019480	3.03718	2.543371	2.059254	7.290126	15.15968	17.80812
HORVU2Hr1G071270	8.428433	6.254064	7.235729	2.155517	1.126044	1.991905
HORVU2Hr1G116480	0	0	0	0.887025	0.850602	0.879986
HORVU2Hr1G093220	0.654461	0.753751	0.746055	2.567263	3.851822	4.130221
HORVU4Hr1G059280	36.12157	30.4078	51.55788	7.988581	1.766248	3.441623
Hordeum_vulgare_newGene_9853	0.608708	0.890214	0.513796	0	0.012416	0.013732
HORVU5Hr1G074360	43.66986	41.98245	52.09062	8.816417	3.138308	4.260622
HORVU3Hr1G052500	8.262766	10.1386	10.53271	0.004977	0.01728	0.010883
Hordeum_vulgare_newGene_3363	4.80446	4.576638	5.605172	11.55297	24.02376	22.00253
Hordeum_vulgare_newGene_15717	1.079891	1.10052	1.262033	2.277975	5.378354	4.015548
HORVU2Hr1G005590	8.097122	7.762844	10.83717	5.247129	4.730751	5.743488
HORVU1Hr1G074860	0.264759	0.202697	0.53379	2.168915	4.444228	4.304018
HORVU2Hr1G030460	4.422945	3.384749	3.913454	7.822649	11.41862	13.64453
Hordeum_vulgare_newGene_858	6.548177	10.29682	14.37502	0	0	0
HORVU3Hr1G058570	45.88252	45.43248	65.42605	11.06142	0.68822	4.433464
HORVU6Hr1G052560	25.1276	26.40454	44.12696	14.80739	15.21411	14.08888
HORVU0Hr1G019680	9.338894	9.067031	12.85555	5.715812	6.132207	5.240664
HORVU2Hr1G047890	2.802054	4.57586	4.633208	2.603642	1.763296	2.023512
HORVU4Hr1G005270	10.87757	9.755797	8.819624	4.419441	5.728989	4.957444
HORVU1Hr1G067140	1.5536	1.266066	1.724887	2.633562	5.511461	6.170172
HORVU5Hr1G067530	0.311838	0.605384	0.67474	1.123008	5.198288	6.013042
HORVU4Hr1G008080	0	0	0	2.07216	4.460177	4.352134
HORVU5Hr1G010360	0.221824	0.182593	0.168476	1.309979	2.719568	2.701819
HORVU5Hr1G060620	10.21429	9.220154	13.8697	7.864708	7.786974	10.50083
HORVU7Hr1G116350	0.312895	0.430146	0.328538	1.167477	1.038118	1.473082
HORVU7Hr1G033470	57.24639	71.99914	101.126	22.23907	46.77189	30.74916
HORVU7Hr1G099320	7.201779	6.361993	9.484257	5.296945	3.801087	4.083105
HORVU3Hr1G075870	10.99886	9.920421	10.05828	28.32393	38.55891	33.7495
HORVU2Hr1G041880	12.24025	12.97822	9.608239	4.576262	5.901694	6.617593
HORVU5Hr1G064250	1.33694	1.298593	1.482517	0	0	0
HORVU1Hr1G045510	130.3815	126.02	133.4504	71.84151	46.49875	47.44771
HORVU4Hr1G000350	0.269223	0.313532	0.418498	0	0	0
HORVU4Hr1G016770	31.94472	16.20626	35.44796	62.24055	73.38494	96.37153
HORVU5Hr1G022570	0.470497	0.391426	0.326151	1.949253	2.756891	3.25442
HORVU4Hr1G013180	0.980172	0.93781	0.887838	2.428753	4.132464	3.793224
HORVU3Hr1G002080	0.611764	0.403248	0.730354	0.083609	0.142553	0.311455
HORVU2Hr1G102380	4.180931	3.220167	3.209495	1.520061	0.913868	1.068826
HORVU5Hr1G027890	4.894973	5.598866	6.68755	11.36835	23.86929	20.52989
HORVU5Hr1G094220	0.789521	0.929335	1.690634	11.78715	18.26667	20.50911
HORVU4Hr1G051700	0.056175	0.06683	0.073433	0.613142	1.339798	1.429456
HORVU1Hr1G004200	4.652266	6.095721	6.90567	1.660715	2.296291	2.779667
Hordeum_vulgare_newGene_2093	0	0	0	2.522445	1.755741	2.587783

HORVU6Hr1G084740	5.683094	3.792779	8.456079	0.407194	1.320104	1.348171
HORVU5Hr1G122490	0.462474	0.466578	0.527069	1.071507	1.450492	2.066464
HORVU1Hr1G005150	13401.22	12851.82	9135.943	6756.756	1234.247	2849.644
Hordeum_vulgare_newGene_2095	1.98122	2.097215	1.641172	10.40245	12.90903	11.10997
Hordeum_vulgare_newGene_13710	0	0	0	0.553527	0.954731	0.939883
HORVU4Hr1G080460	2.186266	1.252045	3.166982	1.800589	2.515944	2.387975
HORVU4Hr1G011760	1.52711	1.180774	2.046373	0.246796	0	0.072958
HORVU5Hr1G092100	5.294539	7.130802	9.92257	0.218451	0	0.202306
HORVU5Hr1G122410	5.800413	2.83099	8.603697	0	0	0
HORVU1Hr1G010890	0.185649	0.334486	0.275045	0.814804	0.813381	0.770952
HORVU1Hr1G067480	1.086572	1.182627	1.531385	2.719906	4.886039	4.750705
HORVU6Hr1G011580	12.82485	11.22429	12.74441	3.033548	4.275587	4.625087
HORVU0Hr1G014980	0	0	0	0.43046	0.595941	0.959019
HORVU1Hr1G068930	3.11422	2.033958	1.348024	0.978322	0.591531	0.6267
HORVU5Hr1G055920	3.475724	3.346505	4.205465	9.268197	15.47825	13.03783
HORVU3Hr1G098360	12.71601	9.388605	13.96086	2.976322	2.491205	2.64836
HORVU6Hr1G088470	12.54245	7.763282	8.932903	1.113319	0.696879	0.74694
HORVU3Hr1G070850	224.9174	202.0174	232.0741	24.16484	9.786117	12.37976
HORVU6Hr1G064230	16.73038	14.33454	19.84996	30.68531	58.00555	49.35384
Hordeum_vulgare_newGene_9009	3.639257	3.202338	2.747061	8.176291	8.593799	9.599519
HORVU6Hr1G004000	0.007411	0.013924	0	1.148375	0.829724	1.4496
HORVU4Hr1G064790	12.66921	10.1591	14.15742	3.309663	0.839072	1.578131
HORVU7Hr1G094320	5.122624	4.255461	6.856823	2.671687	1.401634	2.713981
HORVU5Hr1G013840	6.948581	5.829102	5.787534	13.84985	27.80043	24.10607
HORVU2Hr1G093670	4.424312	3.736209	3.18653	1.240243	0.700314	0.785843
HORVU6Hr1G019290	0.439126	0.427178	0.372283	4.041966	12.01692	9.574206
HORVU2Hr1G097350	1.111072	0.5759	0.77947	2.809618	3.700331	3.621209
HORVU2Hr1G030660	3.706	4.24825	5.929123	1.550383	0.987561	1.222719
Hordeum_vulgare_newGene_8751	6.865517	9.053337	7.387859	0.446933	0.19333	0.25975
HORVU7Hr1G003870	11.39517	9.800385	10.12867	4.814172	6.733042	6.904542
HORVU4Hr1G090460	28.00561	31.96874	35.33307	7.834584	0.837033	3.053678
HORVU4Hr1G071730	193.6476	181.8786	228.1877	139.1336	107.4926	108.7553
HORVU1Hr1G036060	1.976988	1.850085	1.96844	5.572138	4.852613	5.298411
Hordeum_vulgare_newGene_1705	1.849756	0.857549	2.320342	26.23566	20.50224	20.98986
HORVU2Hr1G115890	5.196605	4.227687	4.472073	2.783987	1.599242	2.363559
Hordeum_vulgare_newGene_10622	4.470493	5.315084	6.871498	3.535865	4.309976	2.559419
Hordeum_vulgare_newGene_10620	4.202166	7.131645	8.565649	0.471757	0.300918	1.426764
Hordeum_vulgare_newGene_11294	0.559744	0.58935	0.434215	0.018049	0	0
Hordeum_vulgare_newGene_11295	0	0	0	1.384898	1.245357	1.192398
HORVU5Hr1G073870	5.941446	2.805631	5.387534	8.645095	19.65801	16.26302
Hordeum_vulgare_newGene_10011	6.854583	4.926802	9.18093	1.011527	0.377679	0.355411
Hordeum_vulgare_newGene_184	3.886482	3.657048	5.111548	9.975243	27.38617	23.93104
Hordeum_vulgare_newGene_181	0.624681	0.59239	0.677496	1.620003	2.400543	3.020242
HORVU2Hr1G036960	44.10928	41.08929	35.81817	122.7002	188.6651	182.6711
Hordeum_vulgare_newGene_12240	0.188849	0.183189	0.03334	0.973478	0.671941	0.68004
HORVU6Hr1G089460	5.902834	3.607858	7.252198	0.34579	0.180411	0.544457
HORVU2Hr1G069820	9.815198	14.21707	13.55256	7.326413	5.895846	8.053067

HORVU2Hr1G114960	0.370621	0.686005	0.373537	3.608413	3.406387	3.63539
Hordeum_vulgare_newGene_7340	0.021202	0.05237	0	2.403225	3.395905	3.41436
HORVU5Hr1G087780	4.243757	2.722032	3.387043	5.505244	18.0274	13.74602
HORVU4Hr1G058360	8.389636	9.71014	13.06357	4.976174	6.73604	7.662939
HORVU0Hr1G005180	32.82763	26.59059	31.21277	16.13117	3.245206	7.148188
HORVU5Hr1G083170	28.27038	26.41847	33.71605	18.76592	16.38562	16.27814
HORVU7Hr1G025450	9.38537	13.60322	13.44357	6.510456	7.547454	5.782969
HORVU5Hr1G116710	43.76073	36.28758	46.21493	77.07103	147.5944	140.0901
Hordeum_vulgare_newGene_13626	2.925906	4.650762	5.697272	1.636558	0.79499	0.867656
Hordeum_vulgare_newGene_13623	0.150813	0.112653	0.244494	0.736989	1.920509	2.65335
HORVU6Hr1G079600	13.96882	15.83995	10.29706	1.146747	1.355245	1.200807
Hordeum_vulgare_newGene_7960	0.071789	0.082517	0.108739	0.818705	0.454568	0.543582
Hordeum_vulgare_newGene_6161	1.305346	0.851827	0.709867	0.407229	0.17357	0.283844
HORVU6Hr1G058290	0.580836	0.468657	0.669951	0.263818	0.072906	0.268731
HORVU1Hr1G087320	0.719509	0.621705	0.626158	3.930302	10.00762	7.874403
Hordeum_vulgare_newGene_959	1.307847	1.587072	1.446127	0	0	0
HORVU7Hr1G064800	0	0	0	2.452745	2.786002	2.987388
HORVU7Hr1G108830	3.406082	3.308948	4.683864	8.618123	12.97177	9.58329
HORVU3Hr1G019750	0.352698	0.446139	0.263848	1.124014	7.404736	4.52109
Hordeum_vulgare_newGene_9576	8.560564	10.9492	10.3484	30.62224	39.62022	35.75771
HORVU2Hr1G094660	0.091052	0.167931	0.15754	8.514602	8.889153	8.75084
HORVU2Hr1G124200	0.016819	0	0.012454	0.899321	1.498566	1.040867
HORVU3Hr1G049060	20.1616	15.93435	18.70172	35.64968	64.52167	65.19637
HORVU5Hr1G105250	3.129969	3.100105	3.463604	5.616143	10.9918	9.906119
HORVU7Hr1G096250	6.626632	5.418486	4.974171	17.93935	17.12597	20.38881
Hordeum_vulgare_newGene_4081	0.191309	0.189522	0.216985	1.190511	1.818564	2.284313
Hordeum_vulgare_newGene_1103	9.818322	13.6131	13.29908	0.022198	0	0
HORVU6Hr1G025630	1.741146	2.592397	1.410249	5.266623	5.385911	6.044281
HORVU5Hr1G091840	0.034547	0.125817	0.023208	4.96808	6.693706	10.12882
Hordeum_vulgare_newGene_4551	0.393661	0.183134	0.374752	1.677001	4.868662	2.167652
Hordeum_vulgare_newGene_4553	1.034279	1.221624	0.924606	0	0	0
Hordeum_vulgare_newGene_4558	2.406828	2.936197	3.243393	0.009463	0	0
HORVU7Hr1G101220	0.328609	0.394357	0.232563	3.428416	6.415376	5.163257
HORVU2Hr1G034420	5.639803	4.307801	6.796576	13.59088	12.98931	13.15097
HORVU5Hr1G104120	1.788709	2.370439	1.381824	1.021902	0.565484	1.030131
HORVU5Hr1G113990	0.284641	0.310578	0.256493	1.605495	1.466771	1.310862
HORVU1Hr1G048900	1.560872	0.902266	2.211129	0.310766	0.484847	0.533331
Hordeum_vulgare_newGene_3	4.841757	4.623959	4.237199	1.734085	0.437023	0.718278
HORVU5Hr1G056490	0.046759	0.065347	0.080419	0.285134	0.340589	0.379265
HORVU5Hr1G056140	3.69273	3.778642	5.021705	6.446952	15.37515	15.82837
HORVU6Hr1G006080	0.358334	0.373545	0.784067	3.432466	26.47408	22.07381
HORVU3Hr1G079420	1.127268	1.041397	0.806487	2.743833	6.12708	5.894594
HORVU0Hr1G012930	8.990242	7.425957	10.13343	2.938887	0.885397	1.648141
HORVU4Hr1G052240	86.30694	76.88316	75.93584	46.59269	29.60435	31.79359
HORVU6Hr1G057060	0.701493	0.981761	1.277127	0.457339	0.043589	0.400142
HORVU7Hr1G028260	0.825115	0.832211	0.88955	2.165092	2.481185	2.88443
HORVU7Hr1G097930	0.121051	0.455339	0.330094	0.714635	0.916851	0.919558

HORVU2Hr1G005820	0	0.016461	0.011633	1.10874	1.253745	1.053882
HORVU7Hr1G051160	1.362543	1.097412	1.152992	3.070487	5.601775	5.48131
Hordeum_vulgare_newGene_5603	0.991522	1.729958	1.936573	2.878462	5.449734	5.247134
Hordeum_vulgare_newGene_9383	20.04774	24.16385	28.44686	4.049538	4.680331	4.407807
HORVU5Hr1G097870	0.176834	0.258762	0.161944	2.895832	8.576858	6.707243
HORVU5Hr1G049420	0.030682	0.202925	0.18213	0.548337	3.042489	3.59485
HORVU6Hr1G090220	1.760726	0.950016	0.82961	4.383109	8.347453	5.613347
HORVU5Hr1G106220	0.065913	0.133848	0.093755	1.887511	2.051077	1.492019
HORVU2Hr1G060120	1.668536	1.989955	1.89783	4.246833	5.769803	6.275367
HORVU7Hr1G077220	2.717978	2.734267	4.327106	5.368163	14.78115	22.10797
HORVU5Hr1G094460	1.756779	1.399625	1.501905	15.47972	20.1988	20.39507
HORVU5Hr1G118530	2.190031	2.826993	3.594672	0.98877	0.654686	0.92184
HORVU2Hr1G109840	1.799542	1.48875	1.881175	0.760725	1.06962	0.781952
HORVU1Hr1G013690	484.8062	431.2258	281.9769	175.5004	44.51288	78.03093
HORVU5Hr1G115100	30.00878	31.12642	35.11196	10.34827	5.04092	6.088683
HORVU3Hr1G108100	16.21119	29.52559	17.13507	1.089973	1.303831	2.450652
HORVU4Hr1G058560	4.258552	5.095306	6.808686	2.339429	0.858733	1.394243
HORVU6Hr1G027440	1.695947	1.894875	1.51219	15.89456	26.34321	25.08593
Hordeum_vulgare_newGene_3338	0	0.22279	0.095477	2.687187	2.9333	2.608058
HORVU7Hr1G026860	2.362333	2.302864	3.206248	0.587348	0.274593	0.573757
HORVU1Hr1G094290	8.089689	7.725031	7.605505	1.832723	3.516877	3.82233
Hordeum_vulgare_newGene_13022	0.419294	0.376281	0.441511	0.807962	1.241989	1.396374
Hordeum_vulgare_newGene_13025	39.91519	33.00826	41.87982	14.45765	1.178814	4.941071
HORVU4Hr1G082740	0.632761	0.349305	0.747976	1.304847	2.518967	1.452139
Hordeum_vulgare_newGene_8228	0.962312	0.951216	0.413164	0.057644	0	0
HORVU7Hr1G102300	0.085473	0.01735	0.112207	0.560821	2.11044	1.766055
Hordeum_vulgare_newGene_15076	0.358861	0.182964	0.144323	1.106848	0.754638	0.950604
HORVU2Hr1G060460	1.987608	2.148241	1.853931	10.27287	17.27102	16.12378
Hordeum_vulgare_newGene_13741	0	0	0	1.548089	3.529278	3.030576
Hordeum_vulgare_newGene_13743	142.2753	135.3496	83.49869	32.28329	34.74134	30.14973
HORVU6Hr1G021150	14.22701	16.43599	16.61892	31.32237	47.87584	44.55584
HORVU4Hr1G060200	0.202993	0.229059	0.178852	0.922141	1.519851	1.779503
Hordeum_vulgare_newGene_8279	0.332531	0.06028	0.328228	0.867728	1.52973	1.255995
HORVU6Hr1G025430	33.98625	43.85983	56.6275	10.45128	1.117842	4.210362
HORVU0Hr1G011720	12.58259	4.094718	10.42589	1.4442	0.26196	1.774832
HORVU1Hr1G005800	6.93158	6.351619	6.754475	0.636392	0.717266	0.917629
HORVU4Hr1G060840	20.09118	19.19834	22.93282	12.80556	12.52811	12.49656
HORVU2Hr1G076180	12.87716	13.44751	14.71769	86.63371	95.41223	99.12884
HORVU5Hr1G087970	0.416072	0.773661	0.527303	2.544607	2.744486	3.328526
HORVU6Hr1G020600	0.137227	0	0.229142	22.55581	27.94396	29.49477
Hordeum_vulgare_newGene_4719	0.619443	0.605873	0.652529	0.26542	0.233164	0.398989
HORVU0Hr1G022380	0.439631	0.374897	0.515179	4.412927	4.592345	5.600208
HORVU6Hr1G076530	36.03796	38.8417	51.79015	5.071947	1.803804	2.625278
HORVU3Hr1G102720	0	0.453428	0.180765	35.41938	35.21186	39.45221
Hordeum_vulgare_newGene_370	0.896005	1.251843	0.673063	0.239917	0.25433	0.226339
HORVU0Hr1G005750	1.090065	0.760167	0.753976	3.352562	4.075051	3.434749
HORVU0Hr1G000500	4.288305	5.861773	5.429062	0.093003	0.217243	0.147252

HORVU7Hr1G064100	6.298427	4.683813	6.154859	15.0085	17.98863	18.12001
HORVU7Hr1G057230	0.891262	1.181391	1.249151	0.452241	0.456172	0.346483
HORVU1Hr1G010120	2.302768	1.313833	4.98011	0	0.012191	0.127373
HORVU1Hr1G070230	17.4518	19.36402	19.98598	11.76628	8.739863	11.98612
Hordeum_vulgare_newGene_14065	0.2666	0.525308	0.456795	1.177614	1.756269	1.697842
HORVU4Hr1G010000	2.727866	2.796106	2.683175	7.837287	9.787987	10.69539
HORVU5Hr1G021560	1.564179	0.498683	2.214137	8.305888	14.01483	12.82849
HORVU2Hr1G005600	1.835008	1.901609	1.731458	5.867007	7.380798	8.735316
HORVU5Hr1G101990	1.646619	1.647486	2.206815	9.122313	11.5097	12.22801
HORVU0Hr1G018390	0.167776	0.221304	0.288205	1.237134	1.19185	1.877226
HORVU3Hr1G082740	5.929452	4.75347	4.959246	28.91325	59.56428	62.5398
HORVU5Hr1G074840	1.263269	1.778156	1.062213	11.9728	33.43038	19.36331
HORVU4Hr1G011060	0.217188	0.843926	0.613904	1.7396	3.750173	3.587428
HORVU7Hr1G092570	13.63588	11.60754	14.4916	9.588349	5.909506	6.673725
HORVU4Hr1G009190	0	0	0	6.321826	5.128115	8.004955
HORVU1Hr1G076500	3.582606	4.828483	4.005315	0.053229	0	0
HORVU5Hr1G060790	87.53014	89.49655	78.78454	23.78914	19.981	19.63244
HORVU7Hr1G040880	0.062434	0.115423	0.138648	0.998516	0.877259	1.25949
HORVU4Hr1G008310	20.19973	19.67636	18.95923	44.12732	90.54425	89.07036
HORVU3Hr1G074320	0.420119	0.244338	0.219068	1.499595	0.91285	1.09154
Hordeum_vulgare_newGene_10207	41.05271	37.59808	45.21129	24.42608	22.9202	24.53863
Hordeum_vulgare_newGene_10209	0.940296	1.393888	1.356292	3.217922	6.064143	4.940381
HORVU7Hr1G111350	2.028896	1.920945	1.386346	4.986487	5.410954	5.3716
HORVU3Hr1G086870	0.231453	0.233161	0.21453	1.952693	2.282884	1.82512
HORVU1Hr1G090330	1.186737	1.235081	1.333395	0.24786	0.207746	0.237951
HORVU5Hr1G095080	4.321743	4.135987	4.204863	4.59853	39.55717	41.3632
HORVU3Hr1G098580	3.357438	2.423578	3.64994	1.651852	0.720929	0.962812
HORVU5Hr1G081060	4.816064	5.812671	7.777933	0.336793	0.567324	0.677447
HORVU2Hr1G112170	0.715355	0.544073	1.001669	0.051473	0.01344	0.065583
HORVU2Hr1G065960	5.233013	5.540853	8.314034	2.96086	2.168314	2.602584
HORVU5Hr1G120650	1.910637	1.278277	1.673831	4.900721	6.993977	6.682001
HORVU5Hr1G048630	6.082866	5.588944	6.068159	12.60719	16.67276	16.53069
HORVU5Hr1G042130	0	0	0	54.8785	16.45381	21.87791
HORVU4Hr1G082880	3.371536	3.725798	4.091347	6.708246	16.49682	15.31517
HORVU3Hr1G026120	55.53193	55.19455	79.10635	13.00788	0.965027	5.189503
HORVU1Hr1G000810	2.197179	2.205862	1.944671	0.796272	0.219279	0.484483
HORVU5Hr1G077100	4.349427	4.516074	4.787883	2.73557	2.169704	2.880696
HORVU1Hr1G001140	141.0837	229.9292	134.6535	4235.382	4005.638	3885.246
HORVU1Hr1G049840	0.315396	0.257481	0.160163	0.88208	1.260095	1.022904
HORVU7Hr1G010620	0.032913	0	0	0.4921	1.481324	1.542959
HORVU7Hr1G044990	18.55397	16.48936	21.32196	10.35953	5.199203	5.493827
HORVU3Hr1G038540	16.20591	15.22073	24.16942	7.564566	9.06297	8.690866
HORVU2Hr1G011650	0.086783	0.073885	0.058814	0.389494	1.314208	1.637894
HORVU4Hr1G085820	18.83397	14.43318	20.60337	61.80242	46.6645	51.67169
HORVU0Hr1G027480	9.341889	14.68945	16.09811	1.108593	0.057119	0.255427
HORVU3Hr1G094340	3.332721	2.626854	4.151675	1.697339	0.501185	1.634406
HORVU7Hr1G021630	1.841549	2.878285	2.660427	7.156588	15.16727	13.23914

HORVU7Hr1G045640	12.41442	11.92588	14.39569	20.41371	58.88571	43.05928
HORVU1Hr1G016050	1.07964	1.3833	0.475441	3.904133	4.905847	5.057307
HORVU4Hr1G062500	0.577783	0.214887	0.80597	1.148616	2.590386	1.971798
HORVU3Hr1G056580	9.97018	8.471806	10.42239	23.36889	24.06058	26.74596
HORVU1Hr1G090770	27.73366	33.01897	25.64467	21.62304	13.70634	16.61363
HORVU2Hr1G015410	9.056954	6.700693	8.471542	5.673737	3.339296	4.532778
Hordeum_vulgare_newGene_3271	9.662568	6.837533	8.514607	3.925814	2.548535	2.801644
Hordeum_vulgare_newGene_3272	24.83429	22.34691	20.98984	14.47586	11.52775	12.87364
HORVU7Hr1G105780	5.254101	3.499988	7.055285	0.992984	0.439747	0.574237
HORVU7Hr1G071730	8.336432	8.179564	11.67143	30.98084	75.72455	69.58651
HORVU6Hr1G050750	14.88936	14.67297	16.60596	32.65345	48.97966	48.24639
HORVU3Hr1G089330	45.89227	44.5603	40.99507	30.29368	23.49971	27.35235
Hordeum_vulgare_newGene_12548	0.603357	0.953341	1.267287	0.132795	0	0.044389
HORVU5Hr1G121530	0.625739	0.423581	0.524307	1.373664	1.456121	2.532366
Hordeum_vulgare_newGene_14266	2.761688	2.683045	1.751219	8.164299	9.043071	10.01036
Hordeum_vulgare_newGene_14267	1.192102	1.117317	1.054764	0.443799	0.473355	0.49041
Hordeum_vulgare_newGene_14265	9.394238	10.32325	14.08082	4.802344	5.467203	5.177346
HORVU5Hr1G070040	0.437551	0.591447	0.608818	1.867643	7.13909	7.756025
Hordeum_vulgare_newGene_2635	0.04287	0	0.09567	1.225015	0.654421	1.169813
Hordeum_vulgare_newGene_8847	0.551845	1.316059	0.385832	0.019194	0	0.003974
Hordeum_vulgare_newGene_5940	2.513741	2.767971	2.650589	0	0	0
Hordeum_vulgare_newGene_5944	0	0.012606	0.020389	11.05351	9.536293	10.06909
Hordeum_vulgare_newGene_15135	1.064025	1.005877	1.254617	0.458935	0.675614	0.652
Hordeum_vulgare_newGene_15139	0	0.021079	0	1.657869	2.886273	2.413169
HORVU4Hr1G025130	1.017474	1.549407	1.29931	0.638431	0.878751	0.56715
HORVU3Hr1G110340	0.219841	0.124493	0.505037	4.532855	42.09704	37.04273
HORVU7Hr1G019700	12.38086	10.31059	11.64398	4.038447	1.237557	2.381518
Hordeum_vulgare_newGene_5363	0.685893	0.615274	0.952788	3.440172	15.90563	17.99649
HORVU5Hr1G063320	0.572938	0.39893	0.629214	4.734014	8.617468	8.014031
HORVU7Hr1G005040	0.908821	1.347969	2.564776	0	0.023064	0.031245
Hordeum_vulgare_newGene_8591	1.29543	1.216116	1.323038	0	0	0
Hordeum_vulgare_newGene_9460	0.164063	0.122785	0.129495	0.563872	0.246796	0.491586
Hordeum_vulgare_newGene_9469	1.811569	1.839074	2.111789	0	0	0
HORVU7Hr1G102170	10.29084	8.840365	8.700908	26.52603	38.67289	40.27452
HORVU3Hr1G057840	3.641433	4.402157	3.58502	8.520347	17.40437	8.260507
HORVU2Hr1G069590	7.621085	6.502557	11.0958	19.2942	49.01288	53.61733
HORVU2Hr1G105760	182.0599	208.0112	276.1355	15.10441	2.636905	5.971698
HORVU5Hr1G033050	4.803749	3.364355	3.532199	15.38252	21.63322	22.98001
Hordeum_vulgare_newGene_13324	0.026898	0.086078	0.022462	2.863676	15.52499	14.61937
HORVU6Hr1G074200	156.0263	135.5595	147.952	56.4892	40.79874	46.54874
HORVU7Hr1G026150	241.2913	237.4066	223.7271	162.1285	49.24595	67.5334
HORVU3Hr1G113080	17.71919	18.08242	26.19823	3.165286	4.728609	4.942496
HORVU6Hr1G078640	12.33929	10.06906	11.89777	5.663837	1.984418	2.953931
Hordeum_vulgare_newGene_6501	0.913865	1.306659	1.436482	0.301899	0.673532	0.305539
HORVU2Hr1G019400	1.636328	1.207807	1.29748	3.920291	4.844625	3.654425
HORVU5Hr1G015560	0.013258	0.021583	0.017838	11.59178	9.855698	11.55888
HORVU3Hr1G034280	0.164062	0.067915	0.305121	2.167494	1.74426	2.000602

HORVU1Hr1G087130	11.52412	14.55442	17.26633	0.049664	0	0.071675
Hordeum_vulgare_newGene_14743	43.91113	34.39229	36.42189	91.4312	154.1507	153.7547
Hordeum_vulgare_newGene_14742	4.058953	4.244719	3.967579	0.065657	0.139158	0.127268
HORVU0Hr1G000730	22.00908	11.66812	19.3845	10.72581	3.163466	5.195344
HORVU5Hr1G062510	19.4215	19.93694	23.30404	44.4561	56.87724	67.59872
HORVU5Hr1G110220	55.50798	47.53212	48.71351	24.11664	18.86068	30.53852
HORVU5Hr1G069850	6.651852	6.09335	8.137204	15.92731	41.20863	40.18818
HORVU0Hr1G004430	0.429284	0.058222	0.764414	1.950571	2.328741	2.085408
HORVU4Hr1G003770	22.12914	23.1991	25.1549	14.45988	13.43921	15.30008
HORVU3Hr1G093530	0.327184	0.238857	0.361694	0.795884	3.131344	2.75536
HORVU3Hr1G016230	2.101193	3.369525	3.746619	0.262389	0.162532	0.455449
HORVU5Hr1G118010	3.734713	5.699197	6.19953	2.693969	1.483004	2.324084
HORVU4Hr1G089330	0	0	0	6.033643	5.737295	7.076799
HORVU1Hr1G019530	0.234542	0.209452	0.081329	0.602335	1.087056	0.933111
HORVU3Hr1G032270	32.6949	23.28418	23.22716	5.919101	0.510619	1.248446
HORVU6Hr1G068370	3.111746	2.875653	2.909438	1.217872	1.245567	1.241513
HORVU7Hr1G050660	50.35959	43.63984	53.12793	14.03513	3.700773	8.766543
HORVU5Hr1G050840	0.332586	0.463631	0.412216	0.230931	0.23135	0.190999
HORVU5Hr1G096090	6.741922	4.565176	7.149401	9.43789	35.60079	33.52791
HORVU1Hr1G058280	0.925866	1.219376	0.875444	0.726564	0.376853	0.588572
HORVU3Hr1G009490	1.116496	0.867594	2.228354	128.5418	176.2902	154.9434
HORVU0Hr1G014900	4.815532	6.095796	6.267784	2.340745	2.158074	2.400052
HORVU6Hr1G091250	16.2308	13.80746	17.95764	9.318775	8.886918	9.127458
HORVU1Hr1G082350	0	0.01262	0.011333	1.171577	1.336936	2.309243
HORVU2Hr1G053610	0.602144	0.245063	0.422218	1.246067	0.850099	0.905859
HORVU3Hr1G029910	0.578673	0.501065	0.513472	0.872668	1.781276	1.742444
HORVU2Hr1G091220	14.35462	9.936321	8.456801	5.936653	5.554043	6.392758
HORVU1Hr1G058820	4.427979	4.075996	5.006285	3.364113	1.387456	2.737192
HORVU1Hr1G061980	19.43403	17.84238	17.8343	4.273961	2.49261	2.616573
Hordeum_vulgare_newGene_13963	3.261786	2.954424	2.795105	0	0.074645	0.045266
HORVU5Hr1G046370	1.551005	1.641619	1.92856	0.997643	0.762048	0.756162
Hordeum_vulgare_newGene_13968	12.25161	11.39246	10.19541	6.462646	3.42904	4.376285
HORVU5Hr1G053020	15.61284	18.02323	24.57524	4.148482	3.170163	2.473093
HORVU1Hr1G082820	319.9258	418.0351	403.8003	56.65051	4.92814	25.09328
Hordeum_vulgare_newGene_15240	11.17779	12.31038	14.6013	21.82385	57.28457	46.4399
HORVU6Hr1G014540	0.514484	0.462139	0.446071	1.30439	1.502576	1.525946
HORVU7Hr1G068080	2.076478	1.312311	1.947228	0.234865	0.211204	0.483785
HORVU2Hr1G092280	1.667755	1.204503	1.0966	2.818046	4.845006	4.347526
Hordeum_vulgare_newGene_10437	1.168853	1.16865	1.107124	0.454049	0.103594	0.183229
HORVU5Hr1G053680	35.30049	34.66151	45.55415	20.78396	25.64334	24.66253
HORVU2Hr1G111300	0.598771	0.678218	0.857526	5.327168	15.98637	14.50122
HORVU2Hr1G039700	3.512745	3.827964	3.372837	6.45955	13.03482	12.92781
HORVU1Hr1G080460	23.98126	25.76154	21.90565	10.58235	5.664893	6.421232
HORVU3Hr1G113810	2.89836	2.6775	2.62114	1.813323	1.359227	1.459704
HORVU7Hr1G000910	19.59243	19.20026	20.41063	9.163645	11.30504	10.90574
HORVU4Hr1G055900	0.243558	0.042626	0.254713	1.106828	1.835797	2.118933
HORVU1Hr1G065270	0.326877	0.234358	0.321487	1.722091	1.687293	2.242595

HORVU3Hr1G062370	1.33738	1.460126	1.54663	3.677595	6.819102	4.917602
HORVU4Hr1G077430	0.371026	0.196503	0.409909	1.440084	8.189604	9.755813
HORVU4Hr1G069070	0.268991	0.101646	0.300578	1.311662	4.381522	4.808055
HORVU2Hr1G038150	2.082334	3.229385	4.420545	1.627869	1.461167	1.627409
Hordeum_vulgare_newGene_11476	1.299221	1.865828	1.508934	0.937804	0.661384	0.554124
HORVU2Hr1G118350	95.43052	105.1519	70.12764	22.74655	68.3355	67.04274
HORVU5Hr1G001730	0.324874	0.224552	0.152852	1.280686	1.599901	1.25187
Hordeum_vulgare_newGene_103	8.196244	4.991307	8.045203	3.617734	1.356192	1.690473
HORVU5Hr1G071920	13.35639	11.86628	12.0132	26.63366	48.21726	43.54041
HORVU6Hr1G090390	0.01983	0	0.009123	22.67261	95.37548	75.15418
HORVU5Hr1G000170	3.985063	3.772562	4.176904	8.592528	10.8065	11.67685
HORVU3Hr1G076000	0.905221	0.897372	0.682437	7.487662	15.84758	12.42258
HORVU0Hr1G025850	18.68363	14.62919	19.0405	2.988178	3.885537	3.483863
HORVU4Hr1G000850	13.84024	7.447112	12.62268	32.78828	33.2287	36.17089
HORVU3Hr1G097860	9.894164	8.184672	18.6116	0.450412	0	0.31316
HORVU3Hr1G081170	24.69879	22.66587	51.9754	42.16164	263.1224	268.2629
HORVU5Hr1G064010	9.505368	9.961364	10.28029	4.302942	5.301509	5.875031
HORVU5Hr1G107410	4.112734	3.201987	4.249698	10.47244	12.5028	11.79172
