# Supplementary material for: Molecular mechanisms of how black barley accumulates higher anthocyanins than blue barley following transcriptomic evaluation and expression analysis of key genes in anthocyanins biosynthesis pathway
Source: Front Plant Sci. 2025 Aug 29;16:1650803. doi: 10.3389/fpls.2025.1650803 (PMC12427265; doi:10.3389/fpls.2025.1650803)
Supplement: Supplementary file 1 [file Supplementaryfile1.zip › Supplementary Material/Data Sheet 11.PDF]

Supplementary Table 10: Transcriptome expression FPKM values of *ANS1, LDOX1, LDOX2,* and *LDOX3* ge

**Gene Nam**	**GB-1**	**GB-2**	**GB-3**	**GH-1**	**GH-2**	**GH-3**
ANS1	8.67385	3.938973	3.453281	0.067627	0.019683	0.031336
LDOX1	37.84762	27.98217	45.00646	13.09446	11.81215	15.48516
LDOX2	5.934137	4.635121	5.793535	3.604621	1.430911	1.766378
LDOX3	2.890128	1.309169	3.573008	0.56215	0	0.388075

nes.
