# Supplementary material for: Molecular mechanisms of how black barley accumulates higher anthocyanins than blue barley following transcriptomic evaluation and expression analysis of key genes in anthocyanins biosynthesis pathway
Source: Front Plant Sci. 2025 Aug 29;16:1650803. doi: 10.3389/fpls.2025.1650803 (PMC12427265; doi:10.3389/fpls.2025.1650803)
Supplement: Supplementary file 1 [file Supplementaryfile1.zip › Supplementary Material/Data Sheet 12.PDF]

**Supplementary Table 11.** Transcription Factors (TFs) with flavonoid gene of barley

| Family | sequence name | start | stop | strand | score | p-value | q-value | matched se |
|---|---|---|---|---|---|---|---|---|
| AP2 | LDOX2 | 186 | 205 | + | 24.5513 | 1.26E-10 | 2.49E-05 | aaaaaaaaaa |
| AP2 | LDOX2 | 188 | 207 | + | 23.8462 | 4.5E-10 | 4.45E-05 | aaaaaaaaaa |
| AP2 | LDOX2 | 187 | 206 | + | 23.5385 | 7.72E-10 | 5.09E-05 | aaaaaaaaaa |
| AP2 | ANS1 | 307 | 326 | - | 22.1154 | 6.03E-09 | 0.000199 | CAGAAA |
| AP2 | ANS1 | 301 | 320 | - | 21.6154 | 1.13E-08 | 0.00028 | AAGAAA |
| Dof | LDOX2 | 186 | 206 | - | 20.9394 | 1.64E-08 | 0.00115 | CTTTTTT |
| AP2 | ANS1 | 707 | 726 | - | 21.2179 | 1.82E-08 | 0.000401 | AAGGAA |
| Dof | LDOX2 | 188 | 208 | - | 20.8485 | 1.82E-08 | 0.00115 | TTCTTTT |
| Dof | LDOX2 | 187 | 207 | - | 20.6212 | 2.36E-08 | 0.00115 | TCTTTTT |
| AP2 | LDOX2 | 190 | 209 | + | 20.4744 | 4.19E-08 | 0.000691 | aaaaaaaaaa |
| Dof | LDOX2 | 189 | 209 | - | 19.6212 | 6.89E-08 | 0.0018 | GTTCTTT |
| MIKC_M. | ANS1 | 312 | 330 | - | 20.3788 | 0.000000089 | 0.00887 | TAACCA |
| AP2 | ANS1 | 710 | 729 | - | 19.6538 | 9.76E-08 | 0.000965 | AAAAAG |
| Dof | ANS1 | 306 | 326 | + | 19.1818 | 0.000000107 | 0.00194 | atttcttttttc |
| C2H2 | ANS1 | 971 | 989 | - | 19.6825 | 0.000000107 | 0.00283 | TCTTTTC |
| MIKC_M. | ANS1 | 312 | 330 | + | 18.5634 | 0.000000112 | 0.0123 | ttttttcttttct |
| AP2 | ANS1 | 285 | 304 | - | 19.3462 | 0.000000131 | 0.00124 | GAAACA |
| AP2 | ANS1 | 303 | 322 | - | 19.0128 | 0.00000018 | 0.00143 | AAAAGA |
| Dof | ANS1 | 312 | 332 | - | 17.6212 | 0.000000279 | 0.00961 | AATAAC |
| Dof | ANS1 | 712 | 732 | - | 17.5909 | 0.000000289 | 0.00961 | CACAAA |
| MIKC_M. | ANS1 | 314 | 327 | - | 17.7424 | 0.000000339 | 0.0549 | CCAGAA |
| AP2 | ANS1 | 708 | 727 | - | 18.3077 | 0.000000339 | 0.00249 | AAAGGA |
| Dof | ANS1 | 707 | 727 | + | 17.697 | 0.000000434 | 0.00651 | ttttttttttcctt |
| AP2 | ANS1 | 703 | 722 | - | 17.9872 | 0.000000446 | 0.00294 | AAAAGG |
| Dof | ANS1 | 304 | 324 | - | 16.7576 | 0.000000708 | 0.0141 | GAAAAA |
| AP2 | ANS1 | 709 | 728 | - | 17.2821 | 0.000000796 | 0.00412 | AAAAGG |
| Dof | ANS1 | 314 | 334 | + | 16.8939 | 0.000000866 | 0.0113 | ttttcttttctg |
| AP2 | ANS1 | 313 | 332 | - | 17.1154 | 0.000000908 | 0.00438 | AATAAC |

equence
aaaaaaaaaaa
aaaaaaaaaga
aaaaaaaaaag
AAGAAAAAAAGAAA
AAAAGAAATAGAAA
TTTTTTTTTTTTTT
AAGGAAAAAAAAAA
TTTTTTTTTTTTTT
TTTTTTTTTTTTTT
aaaaaaagaac
TTTTTTTTTTTTTTT
GAAAAAGAAAAAA
GAAAAGGAAAAAAA
tttttctg
TTCTTCCTCCTC
ggtta
AAATAAAAGAGAAA
AAAAAAGAAATAGA
CAGAAAAAGAAAAAA
AAGGAAAAGGAAAAA
AAAGAAAA
AAAGGAAAAAAAAA
ttccttt
AAAAAAAAAACAGA
GAAAAAAAGAAATAG
AAAAGGAAAAAAAA
gttattta
CAGAAAAAGAAAAA
