# Supplementary material for: Molecular mechanisms of how black barley accumulates higher anthocyanins than blue barley following transcriptomic evaluation and expression analysis of key genes in anthocyanins biosynthesis pathway
Source: Front Plant Sci. 2025 Aug 29;16:1650803. doi: 10.3389/fpls.2025.1650803 (PMC12427265; doi:10.3389/fpls.2025.1650803)
Supplement: Supplementary file 1 [file Supplementaryfile1.zip › Supplementary Material/Data Sheet 13.PDF]

Supplementary Table 12: FPKM values of high-quality genes for WGCNA

GeneID	GB-1	GB-2	GB-3	GH-1	GH-2	GH-3
HORVU6Hr1G091110	6.608033	6.146078	6.323264	9.519673	14.54639	12.87836
HORVU6Hr1G051930	28.64349	30.93409	48.39074	29.71161	54.78149	55.51243
HORVU6Hr1G027570	3.83795	3.253358	4.854335	4.489164	6.163588	6.199794
HORVU7Hr1G100410	0.519567	0.083803	0.738544	1.9383	15.2213	15.53152
HORVU4Hr1G073840	0.054213	0.107397	0.064089	0.288266	5.025421	4.957932
HORVU0Hr1G024890	1.044371	1.576551	0.496401	3.311741	35.75906	25.72634
Hordeum_vulgare_newGene_6071	14.57864	13.54982	16.22153	12.88234	15.85234	15.20591
Hordeum_vulgare_newGene_6079	1.419042	1.604405	1.343168	1.021811	1.550989	1.203455
Hordeum_vulgare_newGene_6078	160.8344	169.4007	229.9403	43.01796	23.22261	27.95491
HORVU5Hr1G001220	4.373323	4.143809	4.214315	5.032358	5.6504	5.665106
Hordeum_vulgare_newGene_3752	1.530614	1.35435	1.823182	2.270265	3.044484	2.749758
Hordeum_vulgare_newGene_3751	9.987119	9.949352	9.298052	9.565136	9.845445	8.400636
Hordeum_vulgare_newGene_3750	3.960471	3.270686	4.554807	5.412283	5.495213	4.180575
Hordeum_vulgare_newGene_3757	0.311491	0.77812	1.177112	0.40318	8.590154	14.19565
Hordeum_vulgare_newGene_3756	0	0	0	4.18593	2.974805	3.581227
HORVU3Hr1G076940	0.349427	0.405971	0.429857	1.865517	5.211093	4.111997
Hordeum_vulgare_newGene_14186	1.112001	1.171801	2.117926	5.007684	4.67859	6.1486
Hordeum_vulgare_newGene_14180	3.243686	1.712239	2.959024	10.2215	8.752481	9.691227
HORVU6Hr1G058740	7.542984	9.238899	10.19805	4.859691	3.724931	4.642581
HORVU4Hr1G059010	3.789961	4.145114	3.591303	3.763162	5.521379	5.584015
HORVU6Hr1G018600	0.63284	0.881485	0.675037	0.933309	1.965248	2.645364
HORVU3Hr1G087430	0.749144	0.939607	0.86117	2.107389	3.004082	3.061704
HORVU7Hr1G008700	0	0	0.072139	2.02808	8.717706	8.785566
Hordeum_vulgare_newGene_488	1.714849	1.986639	2.169915	1.484673	2.388498	2.020587
HORVU2Hr1G022960	1.50248	1.278798	0.873304	3.346787	3.683378	3.66532
Hordeum_vulgare_newGene_485	12.58904	9.803878	11.64177	8.253122	13.16281	11.64284
HORVU1Hr1G074940	7.193648	7.653112	8.420928	5.792683	6.9995	7.755805
HORVU1Hr1G070640	3.856101	3.383002	3.760211	6.662054	7.411079	6.992737
HORVU7Hr1G078750	19.45361	18.0546	23.03745	23.6009	25.8871	24.93569
HORVU6Hr1G072070	5.997445	7.173635	8.805641	5.597493	8.750547	8.264776
HORVU0Hr1G009280	2.803178	1.933984	2.870692	2.170054	8.011448	5.533892
HORVU5Hr1G115520	3.32755	3.03401	3.528902	3.558793	3.90626	4.358978
HORVU1Hr1G077910	88.14641	81.48899	103.2792	58.60691	92.49691	91.05569
HORVU3Hr1G111710	16.65565	16.56785	20.89895	12.06286	7.793631	8.703821
HORVU6Hr1G068720	10.92355	14.22175	11.88118	6.879358	7.494328	7.634486
Hordeum_vulgare_newGene_2405	19.09048	19.97071	19.04333	17.51922	18.22566	18.86339
HORVU7Hr1G035300	1.818964	1.426962	1.460725	7.289806	18.95552	13.08219
HORVU3Hr1G021730	2.715939	3.086053	3.084406	4.710684	4.538839	5.739756
HORVU6Hr1G064430	0.085092	0.120234	0.060466	1.087194	2.365895	2.522847
HORVU6Hr1G030270	34.05435	31.16673	31.64627	30.33137	26.73891	27.66231
HORVU3Hr1G096160	18.86948	16.80751	21.28466	11.32252	11.75523	11.42954
Hordeum_vulgare_newGene_5759	0	0	0	4.79544	4.78694	5.778624
Hordeum_vulgare_newGene_11891	3.675189	3.810328	4.474415	3.450101	5.300147	5.122641
HORVU3Hr1G016430	8.233371	7.628331	8.486167	7.058134	3.794492	4.744776
HORVU2Hr1G004720	2.468374	1.408859	3.773045	1.027401	0.277892	0.654248

HORVU4Hr1G061850	1.608681	1.286251	1.684592	3.777825	3.173498	3.497621
HORVU4Hr1G076420	50.0227	51.72947	68.17932	2.800832	0.553854	1.536042
HORVU1Hr1G028890	17.74787	19.44616	19.86254	16.10535	15.17622	16.36184
HORVU3Hr1G058320	15.53576	17.27825	16.81055	15.09848	10.12777	10.54618
HORVU1Hr1G062210	2.182459	1.498114	2.753329	4.396537	7.064521	7.52983
HORVU7Hr1G050300	14.86504	14.31734	15.16636	15.81468	20.75445	20.38797
HORVU7Hr1G037270	2.002639	2.076544	2.449091	1.862835	2.124218	2.147097
Hordeum_vulgare_newGene_1189	34.93521	35.00079	37.13588	34.29046	67.91355	62.65897
HORVU2Hr1G073760	35.07501	30.88752	39.37736	79.24201	163.8941	158.3477
HORVU6Hr1G067160	4.768668	4.203771	4.795617	6.374236	9.704763	8.331553
HORVU7Hr1G114660	18.3444	11.96254	25.33088	9.801142	15.46005	17.99997
Hordeum_vulgare_newGene_1185	6.735232	5.190926	5.979586	18.0866	17.4475	18.38131
HORVU3Hr1G071000	17.42	17.03979	19.87517	15.47505	17.13938	19.85035
HORVU4Hr1G031700	1.346589	1.191512	0.988758	1.519891	1.008828	0.965267
HORVU4Hr1G005530	10.4495	8.366547	8.659858	12.11509	5.106975	7.252682
HORVU1Hr1G016790	7.745179	8.187744	7.736585	10.0182	13.57009	13.98566
HORVU0Hr1G029140	2.72717	3.272356	3.73073	2.486642	1.568443	2.079831
HORVU2Hr1G034130	3.135306	4.012512	3.80156	6.01686	10.08459	8.864112
HORVU3Hr1G084310	41.80932	46.85989	69.96346	44.05341	91.55622	88.50947
HORVU4Hr1G008160	2.890786	3.311905	3.336657	2.62795	3.281766	3.350965
Hordeum_vulgare_newGene_4005	3.204012	2.879847	4.306551	3.344166	3.814357	3.23688
HORVU2Hr1G036950	45.01016	42.14895	55.80531	36.73098	50.54057	54.97086
HORVU5Hr1G029410	0	0	0	2.286423	1.811313	2.732341
HORVU1Hr1G076390	1.685702	1.429358	1.749328	1.941747	1.569303	2.120577
HORVU1Hr1G072430	6.686619	6.640702	6.225436	4.708837	2.982275	3.666634
Hordeum_vulgare_newGene_14184	3.445178	3.405835	3.017121	3.46596	4.978228	5.019749
HORVU5Hr1G108970	2.305351	2.532763	3.666903	3.228691	5.808516	5.663029
HORVU3Hr1G070750	3.688617	2.879927	2.866073	1.746801	0.92071	1.199251
HORVU4Hr1G046500	0.747323	1.538375	1.17373	1.950615	2.261377	2.521961
HORVU3Hr1G022660	36.11628	33.79761	31.82587	43.17745	28.55289	28.53729
Hordeum_vulgare_newGene_14972	101.7363	87.52084	109.4799	121.267	144.6383	139.2664
HORVU2Hr1G075680	7.276038	8.166317	9.666188	7.858643	8.87031	8.090689
HORVU6Hr1G028690	40.80501	41.7308	41.45493	65.69502	93.88388	69.74342
HORVU6Hr1G070560	21.7967	26.00465	26.98965	19.83637	25.47562	27.13944
HORVU1Hr1G076970	6.782596	6.202484	6.718065	2.561861	3.002141	3.213107
HORVU3Hr1G035180	5.435023	4.577192	4.898805	8.004335	11.81335	9.904666
HORVU1Hr1G078250	4.813768	3.897287	3.810332	11.75264	7.343346	9.17714
HORVU2Hr1G035770	2.47307	2.081616	4.238789	3.227208	4.187008	5.486349
HORVU7Hr1G119950	23.01069	23.35563	25.75499	19.95881	20.41275	21.61338
HORVU5Hr1G068060	21.84419	11.62226	28.47921	9.330186	4.172189	9.103764
HORVU1Hr1G016280	1.098968	1.145043	1.187732	1.758746	2.810885	2.51654
HORVU4Hr1G072850	11.89015	9.009773	9.808844	5.818176	3.697787	4.771887
HORVU2Hr1G045170	21.97903	21.59234	22.015	17.70775	16.89459	18.23651
HORVU3Hr1G035450	7.876924	8.471733	9.868128	5.149193	7.491153	7.575795
HORVU5Hr1G104870	56.52388	58.84199	69.02184	46.71927	43.26956	45.59514
HORVU1Hr1G074530	2.197302	3.469146	3.43487	0.253339	0	0.094316
HORVU5Hr1G059990	3.685966	4.179043	4.830544	5.167617	6.245256	6.364966

Hordeum_vulgare_newGene_7068	5.519966	5.092663	5.727883	5.013517	2.573628	3.809896
HORVU1Hr1G056560	6.772941	7.204537	7.075051	19.66054	22.82939	21.3044
HORVU1Hr1G083310	7.363562	5.829427	6.169084	10.59726	14.25263	11.94023
Hordeum_vulgare_newGene_7064	91.51212	92.42184	138.1896	78.93575	149.1107	145.2403
HORVU0Hr1G001120	14.7519	18.70021	14.76706	18.98771	25.71401	24.07694
HORVU5Hr1G012950	34.93252	31.03755	32.47427	69.12779	144.7492	125.4371
HORVU4Hr1G044610	2.488153	3.571259	4.07202	2.693889	4.960981	6.022128
HORVU7Hr1G018300	5.315608	4.624724	5.824087	5.717432	7.550073	8.318007
HORVU4Hr1G074450	5.415722	5.498338	7.324333	5.545334	10.18492	8.888784
HORVU1Hr1G027350	0.657965	0.763582	0.627776	0.384441	2.738584	1.461922
Hordeum_vulgare_newGene_6333	0	0	0	3.482359	4.646718	4.432506
Hordeum_vulgare_newGene_6331	0.505134	3.433233	0.693704	0.155726	0.642824	0.762764
Hordeum_vulgare_newGene_6336	3.809182	4.439809	5.852673	4.205616	5.895568	6.766458
HORVU0Hr1G024100	5.645631	5.777819	5.695806	0	0	0
HORVU5Hr1G103060	1.528776	1.84273	2.628362	1.194292	0.014408	0.420762
HORVU5Hr1G048510	1.85619	2.691023	3.220691	2.135803	2.696934	3.141028
HORVU1Hr1G050920	12.3833	13.52875	15.30971	9.775921	13.25081	15.36979
HORVU0Hr1G040320	5.375202	4.928686	6.314064	3.970975	4.744341	4.846053
HORVU1Hr1G088780	20.69389	19.37028	53.34999	127.1444	80.74403	69.68314
HORVU1Hr1G065470	11.96238	12.74195	12.69083	15.37087	13.2877	15.19572
HORVU5Hr1G022740	13.03504	12.12089	12.76534	8.732612	12.65757	9.747414
HORVU7Hr1G111430	11.35378	11.19175	11.77099	12.02608	13.91956	12.909
HORVU5Hr1G097030	40.69535	36.92495	47.9341	68.6666	80.85157	77.19362
HORVU6Hr1G083490	3.789156	3.766008	3.512767	0.24092	0.300151	0.408898
HORVU7Hr1G011100	11.92467	13.28155	14.94754	10.39814	13.4447	12.97427
HORVU1Hr1G011720	0.397476	0.380545	0.447105	1.254371	8.82907	7.393462
HORVU5Hr1G106110	1.495474	1.705444	2.575272	0.560191	0.706863	0.902872
HORVU2Hr1G104900	5.928544	6.822665	7.010567	6.728869	6.281527	7.77144
HORVU3Hr1G002540	2.538257	3.292812	4.295042	2.640616	0.575395	1.613961
HORVU1Hr1G062970	0.575822	0.814688	0.829483	2.717665	5.90843	4.964641
HORVU2Hr1G035420	1.649257	1.04292	1.204148	2.345329	4.040453	3.461852
HORVU3Hr1G077630	0.067615	0	0	0	18.69841	17.71297
HORVU5Hr1G094300	151.469	104.3379	142.9998	93.3341	74.77065	68.00931
HORVU7Hr1G069660	5.148136	5.458452	4.648616	12.75197	19.3277	17.65925
HORVU5Hr1G058760	24.79508	22.18855	24.81082	28.11978	36.64764	35.89949
HORVU5Hr1G017670	1.828383	0.744889	0.172034	0.856416	0.605494	1.859984
HORVU7Hr1G117870	53.75752	44.07325	50.45732	76.88812	17.42424	33.07877
HORVU1Hr1G089780	1.038093	0.686294	1.06673	3.14699	3.061424	3.707406
HORVU3Hr1G078990	3.27992	8.095519	8.995371	4.985807	4.045852	6.52143
HORVU4Hr1G042610	9.294204	10.51503	10.78963	15.97574	14.51896	16.70253
HORVU1Hr1G089430	1.304202	1.108575	1.721824	2.268915	2.237705	2.609953
HORVU2Hr1G015340	3.667504	4.913342	4.307388	6.538856	5.945531	5.738986
HORVU2Hr1G059140	4.367059	4.745727	5.111947	6.74545	6.66134	6.976353
HORVU7Hr1G119080	3.717767	5.129923	3.879074	5.207955	6.176139	5.607357
HORVU3Hr1G092430	4.062932	4.186208	4.896437	24.34957	40.02198	41.61869
HORVU3Hr1G013630	0.366128	0.996358	0.424587	1.127629	2.039902	1.794614
HORVU7Hr1G121210	4.77769	3.882606	3.873026	3.42399	3.89603	3.655235

HORVU3Hr1G041030	2.84933	3.439118	2.608168	4.801108	6.100705	5.572236
HORVU2Hr1G047720	1.446083	1.674929	2.279081	1.511544	2.174614	2.07647
HORVU5Hr1G106580	176.5027	174.6508	270.5951	114.5331	262.258	223.6401
HORVU7Hr1G057100	2.670328	4.065378	4.128111	0.119279	0.418246	0.318434
HORVU3Hr1G082850	2.719966	2.91815	3.604378	4.1766	7.747279	8.305703
HORVU1Hr1G092670	62.68999	59.3375	86.03079	46.21898	31.89955	36.25846
HORVU5Hr1G085250	6.093951	13.53534	24.76798	0.99668	1.720314	1.774596
HORVU3Hr1G027540	6.162243	5.438155	6.471509	4.717546	6.327684	6.25652
HORVU5Hr1G038070	2.816618	3.468638	3.740897	5.302803	11.41059	11.58404
HORVU7Hr1G071960	50.87335	68.08855	42.41846	38.0927	31.64077	34.08612
HORVU2Hr1G094380	18.71374	13.43748	17.0742	19.50212	18.36393	21.27147
HORVU2Hr1G098600	3.720443	4.068955	4.422222	6.272983	7.404804	7.835461
HORVU5Hr1G109160	49.44539	44.84257	51.11675	39.74813	37.60851	32.8898
HORVU1Hr1G072850	2.213043	2.357145	2.649124	4.374989	3.637505	4.315887
HORVU5Hr1G046830	4.820162	3.352304	4.751401	9.299619	11.99934	9.641573
HORVU5Hr1G114920	2.630024	1.919541	2.557665	1.999787	1.962538	2.208986
HORVU3Hr1G009510	1.774223	2.412183	3.686761	72.1732	72.32787	91.94751
HORVU3Hr1G037940	13.26659	13.6485	13.51602	13.5118	14.4742	15.36873
HORVU5Hr1G066260	3.926745	2.659647	5.066777	2.727345	1.363583	2.830281
HORVU4Hr1G005670	7.498982	6.837293	7.033067	13.51333	11.40826	12.06527
HORVU3Hr1G056170	12.08263	11.01474	13.13332	17.88757	27.24969	23.96782
HORVU3Hr1G023390	5.517909	5.476297	7.906036	8.022823	10.25298	10.20827
Hordeum_vulgare_newGene_985	5.418685	5.912253	7.151865	4.169263	5.454476	5.638969
HORVU2Hr1G084150	2.511572	2.626702	2.644786	2.741345	2.445492	3.297307
HORVU4Hr1G027850	18.47867	16.40785	17.97758	16.17317	17.71851	17.59119
HORVU2Hr1G127340	3.992402	3.995076	4.048702	5.125477	7.739611	7.707189
HORVU3Hr1G084450	1.29301	1.120154	1.290242	1.815691	1.285154	1.662977
HORVU6Hr1G000090	0.079692	0.041785	0.012662	1.528757	3.921331	4.212237
HORVU1Hr1G090740	5.886396	4.508237	5.3295	11.0083	9.560587	9.635559
HORVU6Hr1G085320	0.10161	0.016297	0.045149	8.51032	11.75028	13.61881
HORVU5Hr1G120850	17.48905	15.73837	15.67571	33.3329	29.60517	32.24402
HORVU4Hr1G057360	1.663598	1.351369	1.640623	6.212914	3.407122	4.445434
Hordeum_vulgare_newGene_12917	8.110089	8.205479	9.656098	7.794909	13.66547	13.48723
HORVU5Hr1G025900	49.82569	46.74955	73.60746	38.8513	43.27779	43.30009
HORVU1Hr1G015610	17.06527	17.53984	19.60952	0	0	0
HORVU7Hr1G088620	6.355491	5.773349	6.836628	6.742162	4.106941	4.219605
HORVU6Hr1G055030	8.848701	9.84382	9.113759	11.61661	18.08864	16.44529
HORVU3Hr1G035840	0.494915	0.843174	0.685292	0.793721	2.211025	2.841837
HORVU5Hr1G108780	8.58035	10.54043	8.634501	6.896574	9.234812	8.034245
HORVU1Hr1G016490	37.04962	31.9708	39.82399	36.4311	42.55721	42.18709
HORVU1Hr1G010790	1.807531	1.165903	1.563607	3.50972	5.666058	4.978509
HORVU1Hr1G080090	2.52065	2.898181	3.413987	5.765791	7.473962	7.708833
HORVU4Hr1G065100	83.44582	81.4095	137.7714	77.27996	139.6457	145.7311
HORVU7Hr1G085450	3.243896	3.545341	4.3519	9.200678	11.19711	15.43827
HORVU7Hr1G082480	2.647993	3.212951	3.471246	4.361915	7.585813	7.436217
HORVU2Hr1G045810	2.607022	3.239278	4.252447	2.221636	1.405077	1.804759
HORVU4Hr1G087360	13.47161	15.31681	10.85424	12.96039	16.37275	14.37629

HORVU6Hr1G004120	3.819853	1.356277	5.790363	0.21782	0.256429	0.359183
HORVU1Hr1G016140	2.454632	2.141669	3.381992	0.969534	0.950458	1.060416
HORVU7Hr1G019530	2.001607	1.922243	2.127365	3.056348	4.033339	2.906233
HORVU6Hr1G016710	13.87448	8.832353	12.96291	39.04999	36.45702	32.13632
HORVU1Hr1G037580	8.576798	8.152305	11.47614	6.706457	10.90037	9.470165
HORVU7Hr1G006490	58.16219	67.01121	82.34848	63.03601	101.5391	96.68081
HORVU4Hr1G086010	2.787908	2.767235	3.52638	4.549706	6.056727	5.966505
HORVU5Hr1G079830	4.246282	3.453694	4.919609	3.000605	3.125115	3.47641
HORVU5Hr1G115720	5.462039	3.719521	6.873629	1.209479	1.609517	1.860772
HORVU2Hr1G072010	12.72912	11.26114	16.94149	17.00667	19.05435	19.90196
HORVU7Hr1G040970	2.198201	3.157737	2.661345	2.0661	2.409979	2.504972
HORVU5Hr1G117760	21.6782	17.1566	20.93428	20.89534	19.31378	19.13502
HORVU4Hr1G054970	12.99715	13.12851	10.33519	5.368572	3.087362	2.994306
HORVU3Hr1G064760	1.85918	2.021184	2.261595	0.090664	0.120222	0.051409
HORVU7Hr1G006720	0	0	0	0.595438	23.25479	19.1301
HORVU0Hr1G021970	1.562926	1.572287	1.558401	1.612246	2.09577	0.676185
HORVU3Hr1G114270	17.0715	14.99381	16.07791	16.15579	18.7948	18.37568
HORVU7Hr1G077930	6.456846	6.805515	7.438029	5.818431	6.489558	6.212015
Hordeum_vulgare_newGene_5518	6.050713	6.252213	4.919466	6.341277	7.276774	7.320449
HORVU4Hr1G004230	9.346539	11.50362	10.52485	12.32664	14.00129	13.85365
HORVU4Hr1G000750	15.52817	28.22055	37.72863	11.446	0.150997	5.601831
HORVU3Hr1G099740	2.205667	2.953127	3.255988	0.911148	0.748627	1.144512
HORVU3Hr1G117890	2.502159	2.63941	3.757288	5.590688	4.408266	5.554393
HORVU4Hr1G009340	4.456398	3.535449	4.591662	9.560271	12.50654	11.75168
HORVU7Hr1G026040	23.9206	24.70566	20.89997	15.21455	11.88997	12.12316
HORVU3Hr1G026620	27.23926	23.07485	23.97332	22.13427	15.54419	14.70714
HORVU3Hr1G092840	1.265075	0.987344	1.394081	3.165317	3.172956	3.310953
HORVU3Hr1G092590	20.20029	20.43111	15.92762	23.43379	25.42255	27.09436
HORVU6Hr1G042080	10.94248	9.663146	13.08377	9.510963	11.02897	11.34971
HORVU6Hr1G013530	0.071492	0.141098	0.345469	1.118424	9.031116	6.85714
HORVU2Hr1G101100	8.234696	7.346918	10.33035	8.857483	11.4394	11.81207
HORVU2Hr1G039960	4.895284	5.300989	5.76132	5.90085	14.40369	12.52901
HORVU7Hr1G057650	60.36113	53.86444	38.22489	3.787646	1.78899	2.342011
Hordeum_vulgare_newGene_8143	1.297878	0.970672	1.106077	2.556925	4.521684	4.04926
Hordeum_vulgare_newGene_8142	100.0457	86.23325	89.82546	76.37508	51.68367	54.40786
Hordeum_vulgare_newGene_8145	1.044472	1.014887	1.246278	1.996721	1.908834	2.686646
HORVU2Hr1G037000	1.676928	1.947236	2.616161	1.727539	1.805407	1.784584
HORVU6Hr1G014620	2.507609	1.876383	2.887672	2.152539	2.879037	2.108981
HORVU2Hr1G104550	1.248507	1.485172	1.468399	1.633594	3.439493	3.207541
HORVU7Hr1G089280	2.418061	2.993935	2.252493	5.349104	22.14536	19.07077
HORVU6Hr1G033670	0.808728	0.698776	0.771353	0.524312	2.111472	1.856388
HORVU3Hr1G026080	1.295005	1.176355	1.504492	2.325302	3.001126	3.304106
HORVU6Hr1G076600	3.966443	4.74863	4.664821	4.992953	5.436485	6.487738
HORVU6Hr1G042550	14.70526	14.98827	15.50344	12.3807	16.93863	17.58929
HORVU2Hr1G090410	1.488718	0.945545	1.930513	0.955971	1.593161	1.470109
HORVU1Hr1G024660	10.91496	10.13595	11.28191	8.740171	13.9126	11.45699
HORVU2Hr1G122580	1.694495	1.004363	2.43502	0.41192	0.120638	0.488646

HORVU4Hr1G034750	3.173926	4.218071	3.849916	3.954362	7.594697	8.140542
Hordeum_vulgare_newGene_11391	14.11166	13.96499	15.38335	17.07358	17.41144	19.24824
HORVU3Hr1G027970	2.454402	1.913727	2.171928	5.717275	3.628377	3.873074
HORVU2Hr1G115600	7.68603	9.053843	9.2145	16.44795	28.32608	25.90692
Hordeum_vulgare_newGene_10660	4.106128	2.974621	3.495401	2.493934	2.142822	2.544046
Hordeum_vulgare_newGene_10666	0.691529	0.752349	0.975061	1.33259	1.458717	1.709361
HORVU2Hr1G104880	0.849838	0.815715	1.28719	0.976624	3.844547	4.488578
HORVU2Hr1G111110	2.906037	1.882971	2.954271	4.324297	3.372736	2.90373
HORVU5Hr1G077690	13.74367	14.17478	16.21781	8.706142	9.79249	10.40371
HORVU0Hr1G022300	30.62433	29.03182	36.8215	34.47705	39.3617	38.95055
HORVU5Hr1G006910	12.82505	9.58352	15.08979	34.48287	43.83212	37.61452
HORVU2Hr1G073210	10.9426	7.313111	7.508002	34.41994	17.24841	21.03044
HORVU3Hr1G092330	2.92452	2.898414	3.061096	1.881537	1.351777	1.879327
HORVU5Hr1G081400	3.200581	2.405683	3.15514	5.538954	6.462918	5.400082
HORVU7Hr1G082100	17.2931	16.1976	18.48974	15.08524	15.98349	14.83214
HORVU5Hr1G040540	6.113397	3.493168	5.052595	4.114327	8.632184	6.908444
HORVU2Hr1G022440	4.367564	4.276945	4.735112	6.917049	9.956332	8.499328
HORVU2Hr1G042720	1.147025	1.225042	1.654375	1.352638	1.800127	1.658787
HORVU2Hr1G036840	12.85606	10.78281	15.65365	21.50384	37.36222	33.47453
Hordeum_vulgare_newGene_11533	2.459927	3.104571	3.302617	2.351126	2.968536	2.561839
HORVU3Hr1G094270	2.032047	2.406493	2.831136	2.102549	2.774644	3.315291
HORVU3Hr1G084020	2.216007	1.978784	2.191099	2.447264	2.89204	2.563857
HORVU6Hr1G054020	6.090828	5.007113	7.928506	9.027162	16.09953	12.59142
HORVU1Hr1G007010	9.70834	7.797445	9.407076	8.547691	7.024461	7.520001
HORVU3Hr1G056430	7.097888	4.665467	9.304681	7.975583	18.26029	25.41237
HORVU6Hr1G070600	150.1032	122.2544	150.9719	112.3578	82.13338	79.45953
HORVU3Hr1G089420	1.999566	1.963333	4.742805	0.466791	0	0.040772
HORVU5Hr1G037060	8.233848	8.189329	8.484058	11.66285	16.03151	15.77054
Hordeum_vulgare_newGene_7263	7.099025	7.774319	7.606172	13.28666	14.09406	15.69193
Hordeum_vulgare_newGene_7260	10.07124	12.56446	8.321079	7.433975	6.838649	8.047076
HORVU5Hr1G100770	43.8442	36.55445	51.92321	70.61492	99.2898	102.3144
HORVU5Hr1G087930	0.485551	0.681965	0.436954	1.459867	1.874504	2.166987
Hordeum_vulgare_newGene_3841	6.892235	6.955866	8.786808	6.177637	9.425376	9.338658
HORVU3Hr1G097770	6.116198	3.890261	6.040807	6.491585	5.092134	6.246706
Hordeum_vulgare_newGene_3840	2.597682	2.073588	2.395294	2.392657	3.818595	4.647668
HORVU7Hr1G038690	34.2322	35.47102	50.3967	34.05243	37.72107	40.89269
HORVU6Hr1G055770	1.210973	0.993254	0.27121	0.197659	1.916045	1.739809
Hordeum_vulgare_newGene_6138	3.335933	2.491621	2.824112	2.120488	1.542738	2.058464
Hordeum_vulgare_newGene_6139	0.765831	0.764837	0.771018	1.379347	1.896165	2.012307
HORVU2Hr1G052430	8.580594	9.511232	11.09513	12.70405	19.29985	18.44552
HORVU3Hr1G086620	0.995583	0.509799	1.264383	0.338878	10.2973	1.884656
HORVU4Hr1G030950	12.15538	12.98304	12.56228	15.37791	16.42564	18.65435
Hordeum_vulgare_newGene_2211	1.358894	1.46457	1.494251	1.88771	1.529072	1.694527
HORVU1Hr1G071490	29.33947	24.39641	31.69247	32.0797	32.95398	32.6135
HORVU4Hr1G008550	7.171636	8.646582	10.2022	2.711472	3.536268	3.923193
HORVU2Hr1G011030	5.874248	5.59168	7.032969	6.914425	8.594947	7.107689
HORVU6Hr1G075640	8.782195	8.031728	10.5423	11.85092	14.64909	13.63959

HORVU3Hr1G017150	17.70063	16.02668	16.78545	16.33848	16.04694	16.45708
Hordeum_vulgare_newGene_2303	0	0	0.002462	2.224644	2.485637	2.240666
Hordeum_vulgare_newGene_2302	1.070567	0.717573	0.48714	2.156229	1.928875	1.88912
HORVU7Hr1G038340	0.862025	0.713013	1.398932	1.013258	1.008834	1.233529
Hordeum_vulgare_newGene_2305	7.844241	7.204192	8.321982	0.004445	0	0
HORVU4Hr1G074120	22.29204	23.39243	26.6447	25.714	34.90881	33.04502
HORVU7Hr1G042180	0.015755	0.140953	0.166859	0.346441	3.718167	2.804757
Hordeum_vulgare_newGene_13706	3.24667	3.616881	2.654709	2.799837	3.567242	4.506982
Hordeum_vulgare_newGene_2218	9.746535	7.279389	7.957571	9.459729	11.55245	10.9498
HORVU4Hr1G026170	1.372352	1.477332	0.650716	1.358654	1.985853	1.549855
HORVU1Hr1G008530	38.48982	35.50107	42.38407	32.80037	39.41301	36.434
HORVU3Hr1G050770	1.294843	1.262482	1.177577	4.212679	5.468564	3.771865
Hordeum_vulgare_newGene_1276	14.54988	12.81518	12.29285	16.26137	20.90153	18.46805
HORVU1Hr1G021230	3.844787	3.957087	4.369805	5.365174	7.920995	5.935602
Hordeum_vulgare_newGene_15624	1.410623	1.917025	2.242172	3.165057	4.400791	4.670451
HORVU3Hr1G032420	22.87049	23.39133	29.31511	20.64326	26.67326	27.56959
Hordeum_vulgare_newGene_1279	1.56721	2.140591	2.164118	0.943817	0.682421	1.091135
HORVU3Hr1G096820	33.06431	29.39108	34.02945	24.39149	25.58689	25.26531
HORVU6Hr1G012340	1.931117	1.425764	1.945995	0.618662	0.41638	0.49074
Hordeum_vulgare_newGene_9372	1.995431	2.094513	2.213673	9.027668	8.77907	8.146848
HORVU1Hr1G071670	21.94717	19.01107	22.33592	45.39881	13.10629	20.23605
Hordeum_vulgare_newGene_9379	4.846575	4.240716	4.849037	4.711424	4.430231	4.666602
HORVU4Hr1G017160	20.35426	19.69234	19.53598	22.92456	19.62049	21.27785
HORVU0Hr1G004040	2.273017	4.46775	3.913559	1.373577	1.260742	2.190381
HORVU1Hr1G057240	14.12307	13.22035	12.1638	19.60701	14.09776	14.84112
HORVU1Hr1G077760	3.359761	3.260808	4.683365	2.158103	2.667239	2.488979
HORVU3Hr1G045480	21.56374	16.38989	24.03282	21.59545	19.24561	20.63703
HORVU4Hr1G070350	1.884131	2.722232	4.135845	2.282759	4.282569	4.51301
HORVU4Hr1G029350	3.049043	4.02006	4.33211	2.606995	2.594649	2.841378
HORVU5Hr1G024500	4.053491	4.462819	5.37078	7.749382	8.968677	10.69199
HORVU5Hr1G097320	2.62531	1.183466	2.327624	2.779724	0.296759	0.910501
HORVU4Hr1G089740	40.30195	34.51944	42.27894	28.46572	50.36323	47.92068
HORVU7Hr1G059130	5.410092	4.498657	5.103568	13.21914	19.38478	19.71555
HORVU7Hr1G034990	2.725039	2.483587	3.12486	4.203427	1.8022	2.993738
HORVU3Hr1G044530	12.37662	11.82813	17.33184	24.03749	30.62146	31.3975
HORVU3Hr1G011870	64.42008	42.37001	81.58424	10.82425	1.491691	7.147869
HORVU2Hr1G072600	7.825469	7.899729	8.71597	11.33057	7.859093	9.603457
Hordeum_vulgare_newGene_8424	4.394501	5.170656	6.97125	7.900739	12.63087	11.20076
Hordeum_vulgare_newGene_8427	1.666474	4.043358	4.636313	2.544711	3.64627	3.18218
Hordeum_vulgare_newGene_8422	3.423193	3.507143	3.441406	2.616456	4.164698	3.642721
HORVU1Hr1G066980	40.15024	30.61403	46.52024	37.19992	18.34426	19.92813
HORVU2Hr1G004550	30.03324	20.4372	38.49728	5.319626	1.071736	5.654036
HORVU3Hr1G004250	55.04682	77.48697	104.8472	2.747022	0.222555	0.394589
HORVU6Hr1G068110	1.52323	1.729376	2.401235	2.079495	2.167198	1.812187
HORVU3Hr1G083470	62.58991	55.72175	67.21698	82.38314	116.6195	99.50685
HORVU5Hr1G031130	1.883503	3.285394	2.684346	2.905174	4.862016	4.913626
HORVU5Hr1G088770	2.585119	2.129259	2.650171	2.66812	2.758931	3.437262

HORVU7Hr1G113350	5.072423	4.846187	8.802735	4.315272	5.408858	6.106216
HORVU4Hr1G035290	23.48233	26.23792	24.84432	20.3562	21.57234	22.6786
Hordeum_vulgare_newGene_1023	0.824203	1.107542	1.065562	1.209114	2.293458	2.468442
Hordeum_vulgare_newGene_1022	12.17573	13.07394	15.3229	8.85439	9.59152	10.38305
Hordeum_vulgare_newGene_1021	11.79161	13.44143	12.51148	21.44752	27.59679	23.99961
Hordeum_vulgare_newGene_1029	2.643342	2.964104	3.017334	0.057919	0.044685	0
HORVU7Hr1G076060	3.396699	3.715787	4.604945	2.775921	2.985427	3.169331
HORVU0Hr1G019590	5.934137	4.635121	5.793535	3.604621	1.430911	1.766378
HORVU0Hr1G030660	9.532468	14.30292	16.12164	1.081784	0.064402	0.502135
HORVU2Hr1G084420	2.159437	2.803215	4.00106	3.334437	4.014102	4.791058
Hordeum_vulgare_newGene_4212	2.442288	2.484592	2.558948	2.76604	4.250307	3.800696
HORVU2Hr1G047960	15.10825	14.79404	16.50204	13.56764	17.01846	19.00755
HORVU6Hr1G020520	86.17664	79.97061	69.67044	35.7745	32.79763	31.75923
Hordeum_vulgare_newGene_4218	6.543472	8.151037	9.572603	6.325496	5.535604	7.284398
HORVU7Hr1G101500	2.703509	2.844208	2.786674	6.481604	12.83818	12.1953
HORVU5Hr1G050140	13.11893	13.39119	15.97198	20.79676	28.13039	24.06365
HORVU3Hr1G020250	93.13239	85.05684	87.4293	116.5269	159.3755	155.4446
HORVU7Hr1G048970	27.69856	22.97998	37.71785	36.25091	31.67543	37.49882
HORVU3Hr1G049810	0.879759	1.036662	0.761698	1.330445	1.377939	1.836798
HORVU4Hr1G008390	0.398659	0.778273	0.617624	1.102376	1.542488	1.973591
HORVU1Hr1G075580	4.033792	5.876248	8.479688	0.916922	0.617737	0.733086
HORVU4Hr1G053740	34.49259	37.12909	33.90738	41.09641	40.45006	43.40607
HORVU1Hr1G078350	1.244602	1.78228	1.951849	33.62798	68.06334	63.95725
HORVU0Hr1G025440	2.041314	1.742781	1.637155	0.809298	1.125644	1.598493
HORVU6Hr1G028710	1.510548	1.468538	1.335389	2.924752	3.049026	3.695529
HORVU7Hr1G024090	2.072432	0.975637	2.385893	0.194468	0.35039	0.653717
HORVU5Hr1G059540	1.662927	0.99984	1.489173	0.585725	3.999152	2.794814
HORVU2Hr1G079270	3.289657	2.575459	2.39997	3.878566	4.809315	3.837027
HORVU1Hr1G048860	7.339616	3.621067	6.181724	11.22042	6.198198	9.714085
HORVU1Hr1G075050	20.83021	20.59564	21.29642	28.53401	27.2825	28.00273
HORVU2Hr1G087810	17.1049	18.89749	14.70983	22.50513	31.23723	28.86146
HORVU2Hr1G082860	20.60175	17.81394	21.35047	26.57864	38.38384	36.61941
HORVU1Hr1G057880	33.86678	44.92851	50.75799	10.23969	4.732215	6.188369
HORVU2Hr1G087380	12.66575	10.98867	15.58153	16.25499	18.23429	17.17975
HORVU4Hr1G001570	2.568188	1.960138	2.614681	0	0	0
HORVU6Hr1G032270	4.172907	4.149961	4.06216	4.042422	5.94307	6.057099
HORVU3Hr1G070270	5.756259	5.432586	6.365996	6.650205	3.786984	4.372887
HORVU4Hr1G019910	5.369943	6.18597	6.724858	6.795416	9.190064	9.409757
HORVU4Hr1G073730	1.48678	1.530208	1.443179	5.23716	5.969701	5.79539
HORVU4Hr1G048440	0.748016	0.708364	0.697801	3.135651	4.471059	4.628247
HORVU1Hr1G079910	5.126344	4.957356	6.441929	7.615815	14.1842	11.49794
HORVU5Hr1G081090	3.861562	4.853552	5.636093	5.983911	6.293663	7.403277
HORVU5Hr1G013210	1.217705	1.364524	1.498938	2.991095	9.921424	9.268975
HORVU7Hr1G056630	216.6535	197.7935	196.3881	118.0487	49.22915	61.47089
HORVU3Hr1G033430	5.548641	6.294353	5.641808	4.371437	4.365831	4.710542
HORVU7Hr1G054980	0.130059	0.242495	0.402087	3.288019	2.336948	1.889713
HORVU5Hr1G120520	81.23302	66.39717	87.52479	76.76405	74.05226	74.90781

HORVU4Hr1G000220	5.625752	6.06656	8.491417	4.686522	7.263612	6.809093
HORVU6Hr1G033580	9.036835	8.376813	9.479883	10.03336	12.08339	12.10305
HORVU3Hr1G071910	6.109954	5.730703	7.134698	7.455811	7.753079	7.857517
HORVU3Hr1G038010	33.48664	33.28355	43.13917	37.85003	53.73616	53.04877
HORVU0Hr1G038620	3.208894	3.191236	4.100211	4.174122	4.16068	5.648408
HORVU2Hr1G058170	29.46771	28.87926	28.60217	26.03754	33.95373	31.92432
HORVU6Hr1G085500	0.539101	0.601304	0.896772	1.513822	2.995413	3.284011
HORVU4Hr1G055850	14.88616	16.01302	21.49244	16.74272	30.58552	32.28822
HORVU7Hr1G054670	146.4636	164.7986	258.9246	103.7847	239.3487	216.1907
HORVU0Hr1G035930	2.304698	1.88224	3.056104	1.078633	1.178689	0.859035
HORVU2Hr1G074220	6.417526	7.511638	10.22425	11.53807	17.31552	16.76464
Hordeum_vulgare_newGene_10455	1.101974	1.056111	1.414013	1.981882	2.19946	2.179396
HORVU1Hr1G081900	4.099525	4.09797	4.836571	6.572559	7.70707	8.076103
HORVU6Hr1G033630	22.33399	27.51342	27.5822	25.49974	25.04638	29.72085
HORVU7Hr1G116590	12.96144	12.94017	15.08437	10.19288	10.12365	11.19555
HORVU1Hr1G088620	5.398549	4.101494	21.78016	56.33771	3.270789	4.541959
HORVU6Hr1G050490	5.916356	5.816582	7.362491	8.515666	11.20902	11.69611
HORVU3Hr1G032740	2.366752	2.358464	2.604137	1.920922	2.998872	3.034878
HORVU5Hr1G016190	8.007542	8.027435	8.070081	9.247863	11.97305	11.52082
HORVU1Hr1G080360	14.41608	13.74057	19.08234	15.55253	27.77351	23.99634
HORVU4Hr1G006530	3.027139	2.962703	3.393685	5.391183	12.97651	11.31207
HORVU7Hr1G011310	5.869381	6.612023	10.39617	4.597873	5.65284	7.085862
HORVU1Hr1G066510	1.113336	1.242362	1.484312	1.54867	1.155019	1.268776
HORVU6Hr1G036940	1.21808	0.964683	1.876203	1.371303	0.54586	0.462099
HORVU7Hr1G048110	15.40412	14.41383	20.80831	11.38577	11.8004	12.77664
HORVU1Hr1G093710	3.249886	1.630099	2.461182	4.653997	5.890393	3.685658
HORVU3Hr1G029510	8.796407	7.652187	12.67259	7.023005	14.66156	15.03197
HORVU7Hr1G040720	0.814876	0.484026	0.648601	2.759813	3.841145	3.204482
HORVU5Hr1G018830	0.688768	0.424328	0.847935	3.338853	6.050594	5.427663
HORVU1Hr1G089570	0.227321	0.953153	0.227137	0.541581	2.325199	2.431654
HORVU5Hr1G075260	9.679578	9.395968	13.34183	7.646597	3.198021	6.306694
HORVU3Hr1G082460	12.71762	12.21565	15.23233	9.944491	2.448451	4.909295
HORVU3Hr1G075380	4.451097	4.555934	6.0682	5.34924	4.971933	5.476817
HORVU4Hr1G063180	10.04485	10.60653	10.4269	16.26033	23.12347	22.69515
HORVU2Hr1G054080	2.396217	2.141962	2.955112	1.580679	1.414448	1.891837
HORVU5Hr1G071060	0.771074	1.099274	0.997928	1.322027	3.209835	3.605971
HORVU2Hr1G065270	5.229225	4.731037	5.689103	6.000253	5.233868	7.02855
HORVU5Hr1G046880	1.633232	0.108394	0.01288	0.072374	4.484826	1.168306
HORVU1Hr1G018710	19.31652	21.23274	19.29955	87.08151	121.9317	83.72511
HORVU5Hr1G110390	0	0.42158	1.307405	2.755987	2.246933	2.761497
HORVU1Hr1G082660	2.556567	2.051131	2.395673	4.449615	5.249673	6.789003
HORVU2Hr1G106290	0.755665	0.875612	1.101278	1.725375	1.30769	2.376848
HORVU5Hr1G121600	0.707722	0.568209	0.593478	22.74617	43.58387	43.68309
HORVU4Hr1G068400	1.230064	1.546052	2.576485	1.442558	1.663371	2.65
HORVU2Hr1G083340	27.48761	25.43679	25.41858	23.49841	24.26465	25.9786
HORVU2Hr1G109960	2.500549	1.301844	1.482911	2.951478	3.868736	2.953164
HORVU5Hr1G105650	4.021574	3.855403	4.623493	15.40108	11.99604	9.65182

HORVU4Hr1G062580	13.41581	11.86018	14.00892	9.388717	9.52599	9.019633
HORVU3Hr1G067000	5.468986	3.920956	7.316051	5.284976	5.702511	5.50469
HORVU0Hr1G016920	11.1309	11.29664	12.18972	15.83704	27.9891	25.0957
HORVU1Hr1G002000	0	0	0	12.08431	0	0
HORVU2Hr1G012920	4.347926	4.082395	4.749913	5.706954	6.914572	6.297168
HORVU5Hr1G050420	5.245035	5.053738	5.410953	6.4414	7.957243	8.562586
HORVU3Hr1G031460	8.584223	9.247656	11.70254	31.90966	57.31806	55.84853
HORVU2Hr1G018370	1.085586	1.554915	0.125722	0.139771	1.581855	2.144445
HORVU5Hr1G047820	6.130929	7.410415	6.69504	10.58615	11.4437	13.85866
HORVU0Hr1G007340	21.82358	15.03559	19.17027	3.387922	0.58665	1.455858
HORVU2Hr1G065180	5.979588	7.867445	8.249364	4.968751	4.48251	6.030478
HORVU7Hr1G109550	30.28626	24.76192	29.32863	23.15113	21.60177	20.90611
HORVU2Hr1G016000	3.164899	2.729844	4.258338	2.349305	2.484118	3.292199
HORVU4Hr1G057700	34.57815	32.9743	47.40621	23.08671	26.63607	27.3481
HORVU3Hr1G062000	6.872206	7.20711	8.875859	13.64433	14.10823	13.56858
HORVU0Hr1G017070	2.180975	1.891969	2.331209	1.80059	1.960924	4.3695
HORVU4Hr1G082390	460.8067	481.2383	363.1341	784.2341	117.3106	290.1852
HORVU4Hr1G082020	6.151705	7.22381	7.373229	4.933293	6.595175	7.303483
Hordeum_vulgare_newGene_12899	3.028681	3.628798	1.612149	5.185379	4.943204	5.478463
Hordeum_vulgare_newGene_12897	23.8542	25.91923	14.33523	16.81519	7.505814	9.152395
Hordeum_vulgare_newGene_12896	2.836116	1.800749	1.645763	0.651031	0.00837	0.155743
HORVU6Hr1G052250	2.343203	2.019826	2.518374	2.992442	4.77491	4.572062
HORVU5Hr1G111110	19.80774	21.28368	22.66124	23.87354	26.75677	29.14125
Hordeum_vulgare_newGene_2820	8.171761	7.931193	8.421919	7.269919	6.128895	5.995765
HORVU0Hr1G040540	3.652533	3.852389	4.508625	23.02569	19.2143	18.20676
HORVU3Hr1G024200	2.740821	3.018881	3.22128	3.447312	4.304097	4.182681
HORVU2Hr1G006130	1.772804	1.425566	2.253429	84.2361	1.323309	16.93777
HORVU6Hr1G056950	11.46069	11.08648	12.41805	14.27645	12.40464	12.46157
HORVU0Hr1G024790	1.119574	1.173556	0.873138	3.95815	26.89719	42.32737
HORVU6Hr1G074940	6.912788	7.419446	11.10379	18.56236	18.65996	20.73594
HORVU3Hr1G067040	9.620724	9.095249	13.3796	9.484378	9.126419	11.05755
HORVU2Hr1G096130	2.063913	1.1817	2.32877	13.07387	62.94988	52.18538
HORVU1Hr1G006780	4.602324	1.715551	1.244571	1.268102	0.448321	0.531446
Hordeum_vulgare_newGene_13273	5.386025	6.277556	5.506731	2.988213	1.98145	2.560868
Hordeum_vulgare_newGene_13276	0.71247	2.603811	1.439532	0.298707	1.750666	1.069306
Hordeum_vulgare_newGene_13277	2.538311	2.601486	3.001245	1.458561	2.409797	2.257694
Hordeum_vulgare_newGene_2144	2.497707	2.447052	2.581848	1.844518	1.544139	1.364239
Hordeum_vulgare_newGene_2146	5.202998	5.890143	4.900961	5.641562	4.340003	5.555188
HORVU2Hr1G021960	9.020219	9.29482	10.84358	9.103848	12.10097	12.75074
HORVU5Hr1G026060	5.94831	5.991999	7.163817	5.908799	8.023306	9.310517
HORVU2Hr1G116150	6.853689	7.627993	8.434997	18.471	22.90878	22.24563
HORVU6Hr1G035250	1.994235	1.52105	2.68136	3.505801	5.777646	5.891391
HORVU5Hr1G041870	1.637938	1.151725	1.828987	0.979135	0.955108	1.242145
HORVU1Hr1G094970	13.89834	15.95967	14.2237	14.24104	20.96822	20.11919
Hordeum_vulgare_newGene_5495	0	0	0	2.87407	4.462202	4.159362
HORVU2Hr1G060630	2.637667	1.497575	4.734277	3.714952	3.180384	1.639173
HORVU7Hr1G122690	0.608715	0.891551	0.904707	1.317716	1.203395	1.792479

HORVU4Hr1G075540	15.96111	13.42565	12.68378	16.09307	22.20088	19.79623
HORVU3Hr1G054470	1.188836	1.290417	1.085454	1.388645	1.378591	1.804867
HORVU5Hr1G073010	0.737167	0.749512	0.555384	5.128226	11.53531	10.0632
HORVU6Hr1G094650	10.8548	9.801868	12.36234	12.98558	13.14482	14.66798
HORVU5Hr1G092820	18.83889	19.96906	20.42313	15.66107	17.22954	16.91805
HORVU6Hr1G013180	4.545411	3.93201	5.775248	3.248104	4.228307	4.876986
Hordeum_vulgare_newGene_13854	15.08335	12.60334	17.09533	12.89829	12.70696	11.3453
Hordeum_vulgare_newGene_13856	4.50958	5.123133	4.883248	3.33619	4.065512	4.015053
Hordeum_vulgare_newGene_13857	13.39338	12.16785	11.38362	7.348937	4.524213	6.282902
Hordeum_vulgare_newGene_13851	0.783978	0.688947	0.64534	1.72929	0.915187	1.677626
Hordeum_vulgare_newGene_13852	0.987349	1.292628	0.848362	2.678333	1.48713	2.308383
Hordeum_vulgare_newGene_13859	0.702088	0.746634	0.59062	1.021477	3.442425	3.096899
HORVU6Hr1G061870	0.901349	1.594283	1.476815	0.884796	0.867287	0.90237
HORVU3Hr1G073240	10.15746	8.299354	10.41787	22.48569	27.02164	20.27719
HORVU3Hr1G059120	18.38321	19.09551	16.15054	17.1254	18.90069	19.22956
HORVU7Hr1G023200	1.087302	1.34062	1.719949	1.637479	2.311725	2.255397
HORVU1Hr1G001750	7.643374	19.01352	9.512444	32.95304	19.58736	18.65436
HORVU4Hr1G021960	16.88601	16.24893	14.34975	15.91004	23.66116	20.83997
HORVU7Hr1G105910	0.369231	0.461172	0.40972	2.173435	36.96904	31.04804
HORVU5Hr1G052710	44.19727	36.91241	46.813	42.18666	45.36071	47.394
HORVU2Hr1G014140	3.380919	3.891994	3.199622	11.61543	11.54802	11.88771
HORVU1Hr1G040200	1.252017	2.739214	2.035375	2.497119	2.173246	2.701768
HORVU7Hr1G106610	0.633518	0.879888	1.767358	2.240069	1.268981	1.125987
HORVU1Hr1G005920	3.217698	1.916347	2.884227	5.427394	6.043852	6.231108
HORVU2Hr1G090200	0.077494	0.194587	0.031924	0.730761	3.392581	2.382993
HORVU5Hr1G015700	1.142596	1.719463	0.984386	1.050669	5.381408	5.71706
HORVU1Hr1G028330	4.236527	4.044622	4.55047	3.381421	6.95754	6.512306
HORVU1Hr1G041150	8.565201	8.421935	9.767677	12.5963	17.38293	16.1872
HORVU7Hr1G027230	4.040297	4.42726	5.012215	5.641963	6.701443	7.2012
HORVU6Hr1G020720	23.9729	20.73487	23.38717	24.59881	25.02936	23.18276
HORVU5Hr1G118860	16.57572	13.74275	18.58663	12.21282	17.5773	15.48109
HORVU4Hr1G009620	4.196242	3.298075	4.480227	6.388206	6.415986	6.597582
HORVU2Hr1G034540	0.625566	0.465484	0.437884	2.375884	3.688503	3.45721
HORVU2Hr1G102720	6.965954	2.323365	11.19845	0.667775	0.024154	1.165446
HORVU7Hr1G050980	0.459695	0.37594	0.453859	1.182473	2.604842	2.64914
HORVU6Hr1G071840	4.189724	3.643978	3.885952	3.681715	3.140926	3.322865
HORVU6Hr1G036510	0.445987	0.336179	0.629463	1.559716	2.18652	1.461209
Hordeum_vulgare_newGene_4455	6.567945	5.498408	7.354142	9.179538	13.1959	10.87213
Hordeum_vulgare_newGene_4456	5.3128	5.232823	4.589125	9.454195	7.666158	9.581386
Hordeum_vulgare_newGene_4450	2.887087	2.934096	3.201977	3.988328	5.342244	5.663714
Hordeum_vulgare_newGene_4453	12.35914	14.08724	14.67054	14.15835	13.63493	16.08782
HORVU7Hr1G058180	11.30563	13.81413	13.599	10.13709	13.86093	15.46436
HORVU7Hr1G073300	0.582565	0.911165	0.593256	0.727334	2.126658	1.576447
HORVU2Hr1G114140	1.236291	2.217999	2.404416	3.121259	5.806211	5.375418
HORVU5Hr1G037920	1.039996	1.169149	1.02667	1.550579	1.574485	1.864092
HORVU3Hr1G090360	27.79032	26.39404	31.8665	37.22	57.54272	53.46065
Hordeum_vulgare_newGene_15988	8.410644	8.510289	11.53402	13.13017	16.52437	18.11346

HORVU7Hr1G021170	1.056766	1.662911	2.02785	1.570074	3.307061	5.250263
Hordeum_vulgare_newGene_7188	2.107018	1.680414	2.252272	2.634145	3.537751	3.329689
Hordeum_vulgare_newGene_7182	2.726797	1.551239	2.353956	4.392789	7.821942	7.261228
HORVU1Hr1G075210	27.12345	18.8886	22.47934	17.79758	14.0306	16.95382
Hordeum_vulgare_newGene_4988	14.73126	18.05542	15.77037	16.45449	11.11477	14.42162
HORVU2Hr1G103890	4.431324	3.4224	6.435227	3.097887	2.150129	2.745686
HORVU6Hr1G053970	9.614713	9.269506	14.30932	7.238866	10.96046	10.88724
Hordeum_vulgare_newGene_4985	5.83804	13.62158	13.22491	12.11778	0.069336	0.065821
Hordeum_vulgare_newGene_1822	2.262627	2.792579	1.920079	0.6336	0.383762	0.290505
Hordeum_vulgare_newGene_8679	2.185976	3.324928	3.647855	0	0	0
HORVU4Hr1G029570	5.633944	5.224642	6.956572	4.74376	5.468568	7.111468
HORVU3Hr1G090090	2.743472	2.70736	2.856961	3.150256	3.974104	4.731402
HORVU3Hr1G014880	3.374576	2.666563	4.420594	0.060039	0	0
HORVU6Hr1G003170	156.0606	134.411	174.5822	111.528	81.61521	89.37232
HORVU7Hr1G100120	13.8675	15.85877	26.30766	0.632012	32.13169	18.85611
HORVU4Hr1G022080	2.855226	3.178608	3.157387	3.634256	4.930484	5.537718
HORVU1Hr1G029460	3.069805	3.10662	4.465367	2.144129	1.137002	1.83156
HORVU5Hr1G008300	11.51433	9.074237	14.02167	12.21929	18.41936	18.93707
HORVU3Hr1G063220	32.01481	32.80784	41.52226	25.11057	32.67938	31.36827
HORVU3Hr1G067760	0.646512	0.748407	1.212352	2.533326	3.331635	3.269047
HORVU7Hr1G043300	0.420458	0.362953	0.348831	2.656484	8.712784	8.13126
HORVU1Hr1G030040	2.480025	2.452055	2.373615	2.792302	2.908213	3.14391
Hordeum_vulgare_newGene_6580	7.078024	2.063433	7.611249	8.426135	8.362648	8.812149
HORVU7Hr1G035630	4.314198	4.41242	4.7383	4.412698	6.748109	7.18364
Hordeum_vulgare_newGene_527	0	0	0	5.918862	4.359266	3.966349
Hordeum_vulgare_newGene_525	1.922079	1.675323	1.56728	3.998352	3.652594	3.955029
HORVU4Hr1G074620	0.122132	0.129951	0.214019	3.004596	3.439992	3.623367
HORVU0Hr1G014520	0.072383	0.088636	0.054701	2.945478	118.9677	157.7692
HORVU5Hr1G000260	18.83312	16.06329	25.59803	18.58844	15.78254	17.59194
Hordeum_vulgare_newGene_12661	0.051308	0	0	0.134095	5.454883	8.853501
HORVU3Hr1G093100	0.360085	0.962716	0.472114	5.446644	3.697573	9.586932
HORVU5Hr1G041400	9.086824	7.241927	9.105471	3.929047	1.536836	2.085205
HORVU5Hr1G012500	0.036141	0.183792	0.205857	5.894102	5.672198	5.824843
HORVU3Hr1G113000	11.12175	11.26946	12.88334	7.080759	6.651414	6.171501
HORVU3Hr1G117580	9.336016	5.492409	7.298362	1.689048	0.829755	3.276959
HORVU7Hr1G101830	2.162136	2.37983	2.222809	3.298476	3.451064	3.88333
HORVU3Hr1G087940	1.03761	0.974093	1.377683	3.213011	3.323547	3.123288
HORVU2Hr1G019830	5.766245	7.466662	9.161347	2.583595	2.641389	2.825766
HORVU2Hr1G084680	2.67481	2.90218	5.177274	4.910241	8.914635	7.88391
HORVU2Hr1G074950	2.09522	2.729075	3.261089	2.34116	3.453586	2.95954
Hordeum_vulgare_newGene_13135	6.274316	6.431698	6.458661	5.964422	7.000065	7.438999
HORVU7Hr1G012570	0.828752	0.718944	1.053636	1.318182	1.641502	1.571597
HORVU4Hr1G075970	2.669929	3.409564	3.452534	3.098655	4.098848	3.462214
HORVU5Hr1G068770	1.306391	0.90819	1.47117	1.399244	0.837149	1.207115
HORVU7Hr1G060130	33.83218	29.50323	32.5213	76.93852	113.6872	111.1168
HORVU3Hr1G117630	10.5091	9.237551	11.93913	1.552151	1.644373	1.797328
HORVU2Hr1G087740	0.57511	1.390456	1.989156	2.407381	3.082255	2.690943

HORVU0Hr1G003930	5.453312	5.346016	5.735819	7.463338	5.924502	5.383053
HORVU5Hr1G058960	10.63528	9.215615	12.0237	10.85802	17.99163	18.49131
HORVU5Hr1G085320	3.040864	3.497224	4.113673	5.150752	7.173715	7.765553
HORVU0Hr1G008580	0.781642	0.820182	0.806652	3.292553	10.49792	9.554286
HORVU2Hr1G080910	14.17912	12.69959	13.48856	12.9072	14.02377	14.1353
HORVU3Hr1G055460	0.954174	0.601382	1.027663	1.075587	1.971349	1.733982
HORVU5Hr1G015990	0.73903	0.521622	0.388087	0.45572	1.86602	2.357858
HORVU6Hr1G090020	1.962512	1.136593	1.859251	1.106076	0.937443	1.048826
HORVU2Hr1G005520	13.09814	11.91914	17.16977	4.581561	3.825131	5.400556
HORVU3Hr1G003060	2.27226	1.937022	2.355826	6.157319	7.111837	6.831282
Hordeum_vulgare_newGene_7063	7.322398	4.746929	6.039923	5.627694	2.606458	4.441593
HORVU5Hr1G066820	1.308522	1.354046	1.866616	1.275115	1.229498	1.841765
Hordeum_vulgare_newGene_15476	3.004176	3.554861	2.999489	3.78277	5.815179	5.2013
HORVU7Hr1G037560	0.988464	1.324475	1.494676	1.148607	1.580714	1.687833
HORVU5Hr1G068040	4.415474	0.537461	0.404312	0.700288	0.782381	0.992471
Hordeum_vulgare_newGene_15472	1.509437	1.609361	1.772212	1.708766	1.622283	1.805519
Hordeum_vulgare_newGene_15479	12.14508	11.20062	11.95496	13.10984	17.89741	17.92106
HORVU3Hr1G087050	15.79396	11.55937	19.19399	22.11072	20.82437	22.18148
HORVU6Hr1G076860	1.535392	1.281024	1.188691	2.331028	2.071909	2.243855
HORVU1Hr1G055580	89.64043	93.819	65.27866	52.22179	33.10037	38.41041
HORVU4Hr1G003440	2.787303	3.114486	2.612375	5.007812	4.129174	5.499634
HORVU5Hr1G094910	6.260769	5.912528	7.951538	7.221595	7.951562	9.100625
HORVU2Hr1G125950	3.348182	2.858584	3.061197	6.386246	6.501526	6.590634
HORVU5Hr1G103790	3.256205	2.564306	3.81383	6.074176	7.725639	7.478001
HORVU6Hr1G058450	16.18333	17.30003	16.61041	28.46916	44.96639	34.99981
HORVU7Hr1G108580	6.662273	4.658948	7.164609	7.45995	11.53504	11.84793
HORVU1Hr1G054180	4.39847	4.290865	4.134147	5.415533	6.06682	6.124448
HORVU3Hr1G019590	0.695324	0.743862	0.751073	2.693624	3.432616	3.643757
HORVU3Hr1G054730	5.806436	6.414262	8.763149	4.444011	5.578593	6.387073
HORVU1Hr1G003130	0.996915	12.36181	1.395405	13.57344	0.621095	0.214058
HORVU4Hr1G031010	28.15185	26.82531	32.00447	49.52458	60.8956	59.46747
HORVU1Hr1G048230	26.35141	22.84856	23.95418	23.54616	21.92762	24.17076
HORVU1Hr1G023670	22.5699	18.21593	21.47898	30.29378	35.16826	34.37262
HORVU7Hr1G049080	2.905522	2.433329	3.031853	2.059818	0.9845	1.545508
HORVU4Hr1G083360	23.97676	26.11992	35.91239	16.59707	22.14036	20.2838
HORVU7Hr1G106150	114.0915	127.3245	164.076	83.2239	162.3623	146.4687
HORVU6Hr1G029950	0.90097	1.602438	1.401029	1.581042	3.682564	4.002214
HORVU6Hr1G060150	4.754103	4.983526	5.395977	6.265929	8.527788	7.809658
HORVU1Hr1G018260	6.864006	6.670396	7.591777	5.708858	7.645767	7.596715
HORVU2Hr1G085980	2.168224	1.757181	2.116117	2.40304	2.258103	2.421535
HORVU1Hr1G093860	6.643424	5.394486	5.751735	5.46333	6.315873	7.342415
Hordeum_vulgare_newGene_6460	2.436132	1.049847	1.753087	1.348879	4.218931	5.627886
HORVU1Hr1G073050	0.603956	0.628565	0.498393	2.003269	3.559924	3.516225
HORVU4Hr1G011850	19.46911	18.46841	18.41502	19.76434	33.14653	33.78571
HORVU0Hr1G017230	2.407406	2.261507	2.009672	1.356318	0.944958	1.26553
HORVU7Hr1G057350	2.614976	2.404868	2.91963	3.605722	5.016073	4.906395
HORVU2Hr1G006760	1.786552	2.031898	1.68424	3.24383	2.055311	3.136122

HORVU7Hr1G108270	6.607664	6.838202	8.290426	8.503503	14.27361	12.9563
HORVU7Hr1G036380	3.387291	1.889225	3.378906	2.648953	2.901228	2.81068
HORVU6Hr1G026540	3.904663	3.691343	2.889952	4.827115	5.877089	4.776978
HORVU4Hr1G088280	2.361336	2.769392	4.041392	0.476497	0.069441	0.273424
HORVU1Hr1G075040	10.91024	8.761778	9.355236	0.671101	0.422368	0.477043
HORVU5Hr1G066960	65.94335	66.79666	70.05811	81.43815	154.8248	151.2602
HORVU4Hr1G005150	7.720568	8.214743	8.790012	5.329558	3.994214	3.821476
HORVU5Hr1G038370	6.519777	11.06112	8.959444	2.761366	0.063962	1.073873
HORVU2Hr1G064030	2.077316	2.17449	2.162441	2.712554	2.564909	2.824007
HORVU7Hr1G088210	10.6311	13.17282	10.65475	4.874482	4.125009	5.154282
HORVU3Hr1G047230	0.933157	0.781804	1.08455	3.086448	12.88595	11.41027
Hordeum_vulgare_newGene_1619	6.102184	4.685717	6.529877	2.721871	4.914957	5.483253
HORVU7Hr1G091760	17.38735	16.00284	19.87567	15.62411	20.0956	18.90588
HORVU2Hr1G045440	4.844283	4.40947	4.991567	6.96382	7.539154	6.590255
HORVU2Hr1G014080	8.715627	8.522387	9.181725	7.615915	8.325094	7.807495
HORVU1Hr1G064630	29.36763	26.38423	29.55595	27.53155	38.82213	38.9063
HORVU2Hr1G009160	0.202587	0.098515	0.246837	2.221776	3.068512	3.876434
HORVU1Hr1G056270	1.707658	1.3576	1.947835	0.770889	1.155732	1.48322
HORVU6Hr1G069940	6.873244	7.186795	9.60771	4.494002	3.119294	3.443199
HORVU5Hr1G111050	0	0	0	0.870229	296.2568	217.5879
HORVU7Hr1G003010	3.434874	1.10413	2.638972	16.70909	139.7567	97.96816
HORVU1Hr1G078140	0.120945	0.328186	0.197399	2.570683	2.421576	3.762859
HORVU2Hr1G015590	6.662837	10.61367	11.04069	0.382829	0.033098	0.132018
HORVU1Hr1G069440	2.225289	2.204982	1.720219	2.06394	2.753641	3.039322
HORVU1Hr1G089830	17.25402	14.23658	22.34624	5.563106	3.074277	5.734382
HORVU7Hr1G053300	2.265107	1.464039	4.164784	2.348286	2.349606	2.913938
HORVU4Hr1G001430	6.367959	5.922938	6.483193	7.399219	13.20462	11.41382
Hordeum_vulgare_newGene_6957	2.095933	1.680352	1.791934	1.881491	1.272115	1.631407
HORVU4Hr1G049920	2.304672	1.810776	3.202381	4.696056	8.735882	7.1941
HORVU1Hr1G086380	2.977425	3.759408	4.046674	2.728749	4.079853	3.967536
HORVU1Hr1G056490	14.71941	12.90146	17.00798	20.06942	30.09905	28.01923
HORVU7Hr1G110810	6.17478	4.972788	9.121527	12.12723	21.18331	18.47935
HORVU2Hr1G025050	1.909261	1.977975	1.875618	3.198285	3.472601	3.595596
HORVU3Hr1G040270	38.04843	35.29494	37.77144	38.19712	56.93349	57.75138
HORVU0Hr1G040430	18.92042	20.56826	17.28318	16.09276	11.61457	13.59302
HORVU2Hr1G010350	10.60914	9.301431	17.33097	12.80218	24.05933	22.01927
HORVU1Hr1G080970	1.826476	2.692842	2.150737	4.632831	4.021618	4.748906
HORVU4Hr1G025330	2.76869	2.929231	2.462435	6.342768	7.020895	8.298856
HORVU7Hr1G090140	18.51173	17.16913	21.98332	31.25807	35.52959	37.3476
HORVU4Hr1G007550	12.71986	11.93227	11.34064	10.34273	16.12455	16.11978
HORVU4Hr1G013170	6.902448	7.975465	6.824428	9.058273	6.687327	7.365047
HORVU6Hr1G083650	49.14949	49.64877	60.27278	40.0848	50.75905	51.16598
HORVU5Hr1G125500	87.48526	87.66314	125.6044	12.98171	0.728408	5.317592
HORVU5Hr1G061140	6.299816	5.912378	5.879916	8.029412	8.484872	9.584669
HORVU2Hr1G097570	3.613998	4.180447	3.862805	5.383916	6.695219	6.609584
HORVU3Hr1G002070	1.447341	1.696276	1.982894	3.436176	3.395478	4.153245
HORVU7Hr1G009310	2.890128	1.309169	3.573008	0.56215	0	0.388075

HORVU7Hr1G049870	35.2807	31.95237	30.64508	85.4504	79.49765	91.18034
HORVU2Hr1G124490	1.986337	1.379828	1.994095	1.427158	3.740798	3.993213
HORVU5Hr1G094410	90.9122	76.36038	88.68814	77.42778	93.52739	89.6615
HORVU5Hr1G124450	5.632657	4.195019	4.659362	12.4635	10.82654	13.03614
HORVU5Hr1G056030	1324.378	1238.999	1725.563	435.0489	144.9001	207.8365
Hordeum_vulgare_newGene_14687	202.3809	277.2404	356.6377	5.88747	0.436338	1.846459
HORVU3Hr1G073730	7.280042	7.619328	10.84923	6.641487	10.37885	10.53879
Hordeum_vulgare_newGene_11748	0.630241	0.735539	0.851501	1.319534	0.9593	1.511008
HORVU7Hr1G011290	11.75511	11.63616	17.23147	7.286578	6.964237	8.696577
Hordeum_vulgare_newGene_11743	21.81991	25.75483	24.55293	23.3737	20.73138	24.53121
Hordeum_vulgare_newGene_11746	6.11424	4.556871	8.813066	4.227386	5.504128	6.098799
Hordeum_vulgare_newGene_11745	0.955071	1.302079	1.043613	7.943676	25.80082	10.38799
HORVU3Hr1G038190	38.8389	36.80179	46.38617	32.52457	31.21225	33.01043
HORVU1Hr1G049960	16.66414	14.1617	19.58227	17.3317	14.9734	16.56985
HORVU2Hr1G022100	2.128304	1.850789	2.558666	4.035672	5.752571	5.846798
HORVU7Hr1G040390	3.492402	3.720175	3.604712	8.867787	12.20624	12.02318
HORVU5Hr1G123220	2.290764	2.38364	3.485122	3.198522	3.190474	3.350708
HORVU0Hr1G027010	3.61282	4.289914	4.699048	3.91357	4.963305	4.108878
HORVU4Hr1G051400	28.39808	24.40351	29.40508	9.52786	7.261066	8.92413
HORVU5Hr1G076770	87.79599	53.59539	74.64931	54.73504	12.31367	19.18102
HORVU7Hr1G087550	14.40133	12.14985	14.97931	14.83918	16.795	17.94644
HORVU1Hr1G050590	1.933384	1.275433	1.280881	1.703276	0.475427	0.677425
HORVU1Hr1G081730	0.244385	0	0	0.045837	16.84026	18.55493
HORVU5Hr1G077790	21.56034	15.29633	18.62533	39.27793	21.17732	25.22672
HORVU1Hr1G088010	0.164623	0.062487	16.37981	46.00712	0.464065	0.639796
HORVU3Hr1G038220	0	0	0	6.101079	5.023347	7.610142
HORVU7Hr1G076320	27.55607	24.08671	27.1612	29.3537	41.30778	38.73313
HORVU2Hr1G057700	73.93575	57.78064	62.4465	150.6642	233.3865	227.2611
HORVU4Hr1G068300	25.62129	26.83185	21.27105	18.05294	21.78083	21.48127
HORVU5Hr1G016780	1.33586	0.798735	1.285181	6.934563	11.12102	8.97086
HORVU2Hr1G109020	1.199643	2.7257	3.398911	3.301775	5.439779	6.345523
HORVU5Hr1G077430	1.518828	0.956517	1.080258	2.699667	2.317211	2.710345
HORVU1Hr1G001950	0.438879	8.553511	0.99457	15.47682	0.581607	0.583421
HORVU5Hr1G032550	13.50003	11.27941	11.70929	16.13432	16.87354	19.04636
HORVU7Hr1G080020	0.42479	0.164747	0.38031	1.28613	2.965374	2.337163
HORVU4Hr1G007240	14.67715	14.45245	16.94217	18.02407	19.0215	19.72518
HORVU3Hr1G074820	0.951707	1.004822	1.160146	1.490604	1.264684	1.433063
HORVU3Hr1G088700	1.024938	0.581067	0.918391	1.376679	4.315104	4.444196
HORVU2Hr1G095940	9.459775	9.111217	10.26234	11.87099	20.70223	17.08551
HORVU3Hr1G037470	7.831913	9.225906	10.88665	13.44108	30.50909	29.64697
HORVU7Hr1G050140	0.468852	0.492674	0.751312	0.725229	1.869989	2.429558
HORVU7Hr1G029620	0.340028	0.61046	0.603521	8.63641	7.788639	7.53644
HORVU7Hr1G041440	40.9072	52.2854	30.98948	33.88473	42.93666	42.70549
HORVU4Hr1G053130	1.38322	1.332449	1.988259	4.724209	6.311209	6.50895
HORVU3Hr1G076080	120.5652	114.7353	134.5118	78.83712	66.12523	58.05535
HORVU6Hr1G011570	3.97098	4.018355	4.761986	3.1879	3.345095	3.412503
HORVU6Hr1G067630	0.925415	0.686745	1.533761	0.740912	1.163766	1.479642

HORVU2Hr1G060010	16.57434	14.31594	13.83428	38.32857	45.79674	48.24391
HORVU0Hr1G017170	27.81985	27.72897	24.74438	24.66535	28.05542	27.93488
HORVU0Hr1G021280	2.004171	1.332946	1.915595	7.361509	7.787665	4.678987
HORVU6Hr1G048080	11.3371	9.453503	12.49808	7.901872	13.05887	14.15623
HORVU6Hr1G086960	37.30546	30.46976	44.68736	27.97565	39.97953	38.0362
HORVU4Hr1G027740	10.62251	8.289882	10.1266	13.39622	14.90611	16.36323
HORVU2Hr1G097890	10.23139	10.07021	10.19679	12.30831	12.2051	13.3332
HORVU6Hr1G093460	1.073422	0.918627	0.887924	1.158446	1.596739	1.301342
HORVU3Hr1G027470	2.614388	2.739411	3.102306	3.629138	4.822867	4.679922
HORVU7Hr1G035430	14.6205	10.24493	12.06356	29.75071	27.17148	30.71547
HORVU7Hr1G007950	2.161671	1.937906	2.306669	4.599671	4.475369	3.214834
HORVU7Hr1G046230	2.364694	2.97107	4.45515	1.077115	0.423329	0.912191
Hordeum_vulgare_newGene_5321	6.563118	7.369911	6.024241	13.96543	0.191312	3.227021
HORVU7Hr1G100490	57.81226	49.79793	60.98415	59.92597	69.94071	66.57808
HORVU5Hr1G109710	17.66304	13.82604	14.76322	53.96049	82.25642	76.49728
HORVU7Hr1G047720	10.03194	9.646373	10.65063	12.20025	13.79773	14.07629
HORVU5Hr1G041240	3.014669	2.616631	4.210911	4.11612	5.919177	5.5614
HORVU7Hr1G002200	1.371604	1.091199	1.216155	3.889501	3.813906	3.563147
HORVU2Hr1G117120	2.470075	2.525716	2.834105	0.721253	0.44017	0.860159
Hordeum_vulgare_newGene_405	17.27224	20.07174	17.04564	17.45651	15.4594	17.40347
Hordeum_vulgare_newGene_406	51.04277	49.48542	46.16109	49.48301	45.68125	60.25268
HORVU6Hr1G065230	22.08714	17.60355	20.39632	12.28884	12.12434	11.58836
Hordeum_vulgare_newGene_13334	3.4525	5.797112	8.024041	1.733699	0	0.568425
Hordeum_vulgare_newGene_13337	12.59003	11.74073	13.158	16.71988	23.46586	20.68474
Hordeum_vulgare_newGene_13333	8.324161	6.792975	9.103539	11.06035	10.99545	11.38546
Hordeum_vulgare_newGene_13332	0.619525	2.074893	2.536626	1.923076	2.269355	3.012289
HORVU6Hr1G094110	1.35231	1.514811	2.167571	1.67311	1.992393	1.772244
HORVU6Hr1G056850	1.227131	1.618681	1.846012	0.455966	0.873113	1.143888
Hordeum_vulgare_newGene_3426	12.64271	12.67678	9.453009	18.64412	18.47056	17.64498
HORVU2Hr1G019810	8.291739	8.034638	11.04341	11.39185	19.89967	19.11943
Hordeum_vulgare_newGene_10827	4.621334	1.045478	1.862391	0.158336	0.060713	0.044561
Hordeum_vulgare_newGene_10828	33.70737	25.11041	34.1622	7.995784	7.639057	6.949296
HORVU3Hr1G097300	1.660323	1.17611	0.548701	1.338285	0.940171	1.461142
HORVU5Hr1G115030	6.585434	7.640718	7.18803	7.075868	10.88881	10.95761
HORVU5Hr1G070160	7.596971	10.09462	11.70883	6.860459	7.894516	9.02129
HORVU1Hr1G037250	3.175421	3.273248	2.214741	2.136309	1.008388	2.149144
HORVU2Hr1G103610	9.35825	8.915085	11.94602	5.629951	6.935309	6.535669
HORVU2Hr1G020240	1.694678	3.1402	2.51675	3.440503	2.551284	3.385948
HORVU3Hr1G096280	7.242078	3.954869	9.755379	3.269121	2.16636	3.814622
HORVU5Hr1G085120	16.60535	20.92502	28.70704	8.766048	15.1444	15.40439
HORVU4Hr1G070090	2.086119	2.246643	2.93958	2.152229	2.617577	2.86112
HORVU5Hr1G074660	2.513918	2.144752	2.973319	3.732536	6.42397	6.139455
HORVU7Hr1G042800	19.18194	18.24073	22.5398	21.74318	20.75751	21.75491
HORVU2Hr1G090810	21.22501	22.76434	18.74844	17.4833	17.48547	15.82057
HORVU4Hr1G020710	17.87896	15.5331	16.07842	39.73021	37.68325	42.94021
HORVU4Hr1G059090	38.74709	32.75864	38.47283	39.37416	51.41577	47.11687
Hordeum_vulgare_newGene_5861	5.05062	3.903627	5.384203	2.319727	2.384958	2.287365

Hordeum_vulgare_newGene_5867	1.455618	1.775768	1.895914	3.061627	5.192038	4.499394
Hordeum_vulgare_newGene_5866	1.456636	1.854038	2.309986	0.175006	0.413066	0.22909
Hordeum_vulgare_newGene_5864	1.3077	2.463237	1.755362	2.708671	2.746241	1.908038
HORVU2Hr1G104060	36.9346	33.76172	41.62628	50.36839	53.53634	52.61204
Hordeum_vulgare_newGene_15274	2.130021	1.543949	1.426163	2.624863	4.20151	4.55215
Hordeum_vulgare_newGene_15272	0.998051	1.070148	1.275041	1.583689	1.600102	1.804895
HORVU6Hr1G078910	1.986416	1.344197	2.011102	0.583381	0.874074	0.911021
HORVU2Hr1G096300	1.735956	1.835944	2.187318	3.235499	4.792791	4.612292
Hordeum_vulgare_newGene_15278	2.352049	2.789368	1.833074	3.747173	3.559217	4.903744
Hordeum_vulgare_newGene_2487	9.125991	10.4149	7.808695	7.920102	2.341415	4.759226
Hordeum_vulgare_newGene_2481	0	0	0.006658	5.046153	4.787979	5.078334
HORVU2Hr1G101610	4.059352	4.286984	4.826019	3.138045	3.937574	4.437212
HORVU3Hr1G013860	61.9968	81.24932	90.00763	43.52135	28.49076	32.53481
Hordeum_vulgare_newGene_2489	5.70783	6.279423	6.135996	3.515145	3.918638	4.084377
HORVU3Hr1G021220	9.42123	8.443087	10.92718	7.531397	7.803159	8.552906
HORVU3Hr1G031620	15.90835	9.288516	12.72445	8.11137	6.301966	5.996488
HORVU1Hr1G003940	6.009504	6.130301	9.247842	4.82177	5.989155	7.026439
Hordeum_vulgare_newGene_10177	3.868471	3.175069	3.414116	2.582281	2.688539	2.597799
Hordeum_vulgare_newGene_5289	0.484931	0.524678	0.459974	1.699872	2.544744	2.129693
Hordeum_vulgare_newGene_9706	23.21683	27.1012	30.94181	16.08631	15.31953	18.91172
Hordeum_vulgare_newGene_9707	1.260739	1.200562	1.235454	1.501548	1.356925	1.348586
Hordeum_vulgare_newGene_5285	3.722888	4.519166	4.70848	5.394767	6.277818	6.121851
HORVU2Hr1G015470	11.44458	9.28498	12.09823	7.793599	9.484389	8.433635
Hordeum_vulgare_newGene_9700	1.539188	1.709461	1.950147	3.073065	2.760399	3.606486
HORVU5Hr1G063400	13.43261	13.45704	15.079	20.86641	20.55388	18.4564
HORVU3Hr1G111130	6.92998	5.079564	7.728665	3.892833	3.997051	5.646545
HORVU6Hr1G016080	9.25885	8.39441	9.439335	5.506462	5.532422	6.624618
HORVU7Hr1G080870	14.19971	14.16665	17.89172	17.99519	16.42879	18.56792
HORVU6Hr1G061440	6.492808	6.434792	8.39553	10.2224	13.10288	12.53627
HORVU3Hr1G093370	0.757064	1.013998	0.787264	1.575163	1.464447	1.887728
HORVU3Hr1G050440	44.34275	45.48318	48.27257	34.27145	38.26562	39.53721
HORVU2Hr1G062320	4.517902	4.647292	6.951514	5.446714	10.84729	9.819746
Hordeum_vulgare_newGene_5137	3.481641	3.407291	5.492174	1.673189	1.535424	1.970997
Hordeum_vulgare_newGene_5133	2.32862	2.516248	2.444777	1.570236	2.293276	2.377401
HORVU4Hr1G071050	5.568716	4.36309	4.787488	7.766515	11.57505	11.83932
HORVU7Hr1G089510	4.805856	5.048354	6.755857	3.220176	5.228895	6.617134
HORVU7Hr1G086320	2.533503	2.471926	3.420897	2.978678	3.637239	3.799322
HORVU1Hr1G001540	0.109943	8.692222	1.121293	13.22523	1.296106	1.417647
HORVU7Hr1G061180	26.95936	26.91819	32.4366	36.90168	47.33329	47.99213
HORVU1Hr1G030660	19.2062	21.87832	25.05522	34.7692	36.34064	42.66292
HORVU6Hr1G055180	2.111514	2.197022	2.799063	1.981107	2.405217	2.595451
HORVU2Hr1G069490	19.52092	15.91401	19.33427	9.460156	10.0875	5.921307
Hordeum_vulgare_newGene_11220	0.888107	0.988498	1.435305	1.503827	1.061174	1.365644
Hordeum_vulgare_newGene_11222	7.050478	6.796748	7.675993	8.548355	7.320727	9.381065
HORVU7Hr1G106420	2.759436	1.996117	3.24508	1.391209	0.977829	1.384725
HORVU2Hr1G097730	4.756232	3.783348	3.447879	3.312061	5.405256	5.575555
HORVU5Hr1G027250	11.13486	20.94111	14.33907	12.83643	19.61444	19.77243

HORVU3Hr1G089820	2.993844	2.716436	3.844697	4.583261	6.616674	5.406929
HORVU0Hr1G026710	0.530563	0.722045	0.765011	0.978999	1.592848	2.071487
Hordeum_vulgare_newGene_8076	0.278597	0.221044	0.295969	2.444986	2.457988	2.990166
HORVU7Hr1G087050	298.7221	270.2681	371.3789	449.8667	691.8764	633.8332
HORVU1Hr1G008130	31.48335	27.40677	30.03096	23.13724	42.85744	58.90931
HORVU2Hr1G026830	3.624433	3.264379	4.406971	2.280389	2.889032	2.211792
HORVU1Hr1G014230	0.268722	0.1377	0.278805	0.53333	4.241745	3.460059
HORVU5Hr1G124860	13.95109	11.29795	16.38427	19.20636	21.19863	20.28309
HORVU5Hr1G028460	1.635759	0.833689	1.406774	0.957608	0.623604	0.868632
HORVU0Hr1G038350	1.890745	1.415129	2.347872	2.317016	3.243653	1.968086
HORVU3Hr1G014250	64.26488	61.32883	63.57842	48.77604	39.37331	40.78171
HORVU4Hr1G029760	24.22821	21.93266	22.25088	17.74608	10.51621	12.8285
HORVU4Hr1G076200	2.975942	4.125685	4.57815	2.126402	1.370391	1.641139
HORVU1Hr1G071960	42.09987	36.39955	43.18637	38.90731	38.53228	40.63976
HORVU2Hr1G077760	23.7371	23.27984	35.57235	28.39778	58.35712	50.39857
HORVU4Hr1G088910	12.08044	14.65933	14.96085	19.80853	23.60758	23.40159
HORVU6Hr1G020910	56.85035	50.02819	63.42811	59.40227	82.0118	74.11117
Hordeum_vulgare_newGene_14236	10.00589	7.194871	7.601777	4.705078	3.233167	4.922314
HORVU7Hr1G115280	19.31955	21.54533	21.94552	22.97989	38.75331	37.53312
HORVU2Hr1G028470	3.3089	2.928379	3.627814	5.484371	13.45273	11.81554
HORVU0Hr1G039170	0.7323	1.021747	1.243022	1.21492	0.815423	1.379513
Hordeum_vulgare_newGene_14232	2.292157	2.443096	2.68941	8.70068	7.934927	8.271223
HORVU7Hr1G066320	3.151747	2.859888	3.276719	0.687265	4.203264	4.323298
Hordeum_vulgare_newGene_3854	0	0.541758	0.870938	1.495663	1.744899	1.699725
Hordeum_vulgare_newGene_3853	24.89783	21.09515	28.60109	46.41519	106.6321	91.68773
Hordeum_vulgare_newGene_3858	42.91239	39.02023	52.53124	49.14563	77.23972	72.93706
HORVU7Hr1G056310	0	0	0	5.652837	4.215455	5.692176
HORVU2Hr1G114330	2.812398	3.350605	3.353793	3.016481	3.89095	4.116436
HORVU6Hr1G074020	10.36519	10.41513	11.00398	11.78213	14.39888	13.09513
HORVU3Hr1G086930	85.19573	93.17518	130.5728	98.40051	232.8068	199.4056
HORVU5Hr1G033640	16.09722	15.31452	18.48906	24.41738	31.08574	32.17803
Hordeum_vulgare_newGene_7390	1.547568	1.222539	1.542478	2.122521	3.289096	3.260071
HORVU3Hr1G030550	1.973813	1.69843	2.51646	1.414659	1.508959	1.309009
Hordeum_vulgare_newGene_12147	5.495491	4.929784	5.894836	4.23008	7.411005	6.916855
Hordeum_vulgare_newGene_12146	28.86453	24.2172	27.29351	14.97673	7.885279	9.473052
Hordeum_vulgare_newGene_6625	1.89278	0.263507	1.342858	2.609369	0.199418	0.553713
HORVU6Hr1G029760	3.644596	2.944971	4.663844	3.074633	3.981379	4.453667
HORVU5Hr1G019000	5.70572	7.329177	6.648737	9.654489	9.544786	10.6435
HORVU4Hr1G038960	109.765	89.26418	108.2406	149.1148	228.5373	235.3427
HORVU5Hr1G059910	1.061291	1.057183	1.164965	1.059792	1.172343	1.707554
HORVU2Hr1G032280	8.171506	7.751467	12.22914	9.998431	14.26649	13.13052
HORVU7Hr1G039500	2.228313	2.029046	1.849916	2.209725	2.077861	2.076655
HORVU4Hr1G013370	2.107643	2.028559	1.950922	3.685576	6.481768	6.916373
HORVU5Hr1G075270	1.829805	2.024556	2.123641	6.499171	9.470958	9.222286
HORVU4Hr1G030640	4.207863	5.177149	5.591607	4.475725	4.367084	4.202018
HORVU3Hr1G113640	1.4073	1.831272	1.985949	2.137101	1.806194	2.297241
HORVU3Hr1G113210	0	0	0	2.739149	2.311723	3.906655

Hordeum_vulgare_newGene_2811	1.713839	1.805809	1.838306	1.862446	2.341695	2.530997
Hordeum_vulgare_newGene_2810	3.674803	4.532795	4.30798	4.28079	5.337943	4.789024
Hordeum_vulgare_newGene_2817	1.177875	1.350544	2.456229	31.42902	19.66942	27.60415
HORVU2Hr1G033880	9.070662	7.355197	6.789884	7.701831	1.593426	3.350524
Hordeum_vulgare_newGene_2819	2.065224	2.561131	2.182802	0	0	0
HORVU5Hr1G124610	12.86462	11.94895	14.6977	12.50528	15.06933	18.82307
Hordeum_vulgare_newGene_14662	14.74736	16.48274	14.49497	13.61601	13.64353	13.80142
HORVU6Hr1G030950	5.286498	4.441554	5.544795	8.1832	7.939801	5.058886
HORVU1Hr1G026990	1.812416	2.465374	1.369865	1.179374	6.245733	6.184233
HORVU1Hr1G089700	3.484824	3.21248	6.058573	0.263212	0	0.526321
HORVU6Hr1G029180	4.170155	3.59436	3.562784	3.499593	3.100671	3.876448
HORVU1Hr1G093230	17.36532	19.23487	20.21299	18.22954	25.65911	24.00489
HORVU5Hr1G060810	19.85736	20.0824	15.17704	14.58439	11.11844	11.97413
HORVU6Hr1G074970	7.791949	7.925523	6.514945	13.70286	10.75993	14.69342
HORVU3Hr1G033390	19.33888	17.455	24.66069	18.98515	19.51292	16.96366
HORVU5Hr1G062410	13.24229	9.890821	10.87059	35.65874	43.30037	45.89705
HORVU6Hr1G034180	0.538172	0.942866	1.507886	1.606436	1.884975	1.535769
HORVU5Hr1G052820	4.257239	3.843198	4.82828	5.537052	10.41892	9.064828
HORVU1Hr1G081530	1.07452	1.063637	1.262647	1.768081	1.942809	2.458142
HORVU6Hr1G033200	8.285267	9.110079	9.930542	8.235275	8.0961	9.541838
HORVU5Hr1G122510	1.739219	1.224492	2.130981	2.542058	1.255463	2.578159
HORVU3Hr1G088190	0.954825	0.418833	0.888829	8.806326	8.690937	4.461746
HORVU1Hr1G023480	16.99217	17.43966	18.42993	26.48026	27.83234	27.16994
HORVU2Hr1G027710	8.587527	7.82159	9.319199	7.735299	10.82636	9.531808
HORVU2Hr1G043940	0.696733	0.596621	0.219328	1.375146	9.075363	9.619523
HORVU6Hr1G059370	5.907137	6.781963	7.432936	5.651033	8.512205	8.579903
HORVU3Hr1G038420	12.12988	9.700227	11.42816	10.43003	12.61512	11.19855
HORVU3Hr1G029760	3.335197	3.137343	4.110388	4.167422	3.89821	4.562903
HORVU3Hr1G047860	5.5595	5.714923	7.075794	7.745989	10.27496	11.92854
HORVU1Hr1G055420	12.6755	12.80998	14.29072	9.73351	10.71463	10.19016
HORVU3Hr1G083180	19.35619	15.92222	18.23961	20.63762	26.76662	27.0598
HORVU7Hr1G091490	2.534291	2.693954	3.148601	2.95926	3.153698	2.988439
Hordeum_vulgare_newGene_8919	42.67381	37.00207	45.63481	39.50313	37.94355	38.43615
Hordeum_vulgare_newGene_8915	3.585486	2.381917	4.418499	1.744874	4.849658	5.226474
Hordeum_vulgare_newGene_8910	161.3062	101.6252	162.5724	29.91818	0.202072	9.177511
HORVU3Hr1G082580	21.4217	17.47653	25.24689	9.897292	8.050872	9.295036
HORVU1Hr1G020590	6.651311	8.721288	9.196729	4.802233	5.672448	5.481178
HORVU1Hr1G081280	608.1056	574.5193	555.0747	643.4291	1140.795	982.383
HORVU2Hr1G040410	8.548328	10.6642	10.04927	13.4895	21.72158	20.30441
HORVU4Hr1G089270	9.228895	8.049363	8.065954	6.95638	7.664063	7.174744
HORVU7Hr1G037480	2.557926	3.746441	3.375958	2.234172	3.810402	3.105171
HORVU5Hr1G069030	1.570542	1.779528	1.775079	1.032417	1.437641	1.572561
HORVU5Hr1G021180	40.50699	40.16565	45.17731	39.30899	48.78042	45.19699
HORVU5Hr1G039960	1.123173	1.658414	1.616308	3.44549	5.112639	5.049519
HORVU6Hr1G001670	41.3467	35.60354	43.27843	52.98054	87.31316	73.25608
HORVU1Hr1G067290	3.854815	4.332675	4.531739	2.037519	3.195957	3.171375
HORVU3Hr1G015640	12.29945	16.69851	19.5999	10.07988	10.26163	13.04514

HORVU3Hr1G018230	13.93864	13.62543	14.40437	9.623447	15.13552	12.87602
HORVU5Hr1G008810	2.373675	2.896769	2.703542	4.01032	3.842718	4.931708
HORVU7Hr1G049470	149.1728	150.7865	154.8023	198.7571	152.2864	146.6814
HORVU5Hr1G106730	43.6129	35.70035	40.95405	42.72471	127.7633	122.1018
HORVU4Hr1G009540	7.688633	8.99558	9.121329	8.881551	15.67742	12.85693
HORVU2Hr1G085280	4.977337	3.701375	7.858189	26.35873	21.07122	37.1108
HORVU6Hr1G009850	5.572589	5.398126	6.070489	10.35136	8.911922	9.47199
HORVU3Hr1G079260	1.64163	1.833627	2.003153	2.569455	4.973143	4.737054
HORVU4Hr1G002500	9.981944	9.195766	16.35525	7.054203	9.356537	10.33616
HORVU4Hr1G082040	15.3776	18.10612	11.80574	52.86175	68.66755	77.10108
HORVU2Hr1G126610	16.82063	16.24326	17.08885	22.27993	26.47925	27.4209
HORVU0Hr1G017370	14.86831	12.83653	18.61686	32.56099	48.30229	51.64911
HORVU2Hr1G076060	7.832018	7.160264	9.117754	3.422043	1.536089	2.800188
HORVU4Hr1G014720	2.81175	2.461991	4.395218	3.00915	3.519122	3.13285
HORVU4Hr1G031220	0	0	0	3.405833	2.810772	2.862864
HORVU7Hr1G020800	43.6334	47.2757	51.04189	61.59085	54.72629	60.92024
HORVU0Hr1G018730	10.96393	8.409799	8.271498	15.37108	5.868599	6.364429
HORVU4Hr1G011260	18.58494	18.95155	20.0527	18.62553	19.89559	20.92989
HORVU7Hr1G092370	6.91073	7.539514	9.533655	13.92637	19.10886	19.52308
HORVU2Hr1G098020	14.91655	18.56523	21.40885	13.72103	20.81094	19.37181
HORVU1Hr1G079450	1.946265	1.681424	2.12798	0.540354	0.539273	0.494392
Hordeum_vulgare_newGene_1066	0.395168	0	0.25387	0.766284	2.505733	2.448639
HORVU7Hr1G059270	14.07358	11.63063	15.42834	11.80276	13.0223	14.74706
HORVU1Hr1G042140	11.84683	11.67916	10.51244	10.05492	9.608952	9.539348
HORVU5Hr1G057950	1.343587	1.605222	1.949227	1.511784	1.94705	1.920715
HORVU4Hr1G018440	8.763982	6.731145	7.328451	7.111659	5.277317	7.707874
HORVU5Hr1G119150	1.811671	1.926986	1.623148	1.126001	1.355979	1.304174
HORVU4Hr1G009590	12.81939	13.16991	12.39994	12.34581	14.88674	15.88863
HORVU4Hr1G080000	6.034032	6.120933	7.025988	8.081865	12.56693	14.2838
HORVU5Hr1G097740	5.454206	5.369651	6.038779	4.391413	3.848171	4.010889
HORVU3Hr1G054240	1.200723	0.988585	1.206548	4.881062	10.81056	9.221254
HORVU5Hr1G073640	2.971412	1.282843	7.797307	2.471719	1.183244	1.283202
Hordeum_vulgare_newGene_1064	0	0	0	0	9.824663	11.10116
HORVU5Hr1G091740	0.759267	0.771442	0.886109	1.060203	1.224315	1.545317
HORVU1Hr1G058940	8.976512	6.565345	10.33043	8.513566	1.538769	4.154586
HORVU4Hr1G085740	4.467202	4.21152	4.791792	2.516818	2.95338	2.482795
HORVU1Hr1G042490	0.880966	1.225739	1.609949	3.324087	2.80994	2.796824
HORVU7Hr1G013710	28.53285	22.70866	35.52581	24.79068	25.57872	33.46168
HORVU2Hr1G120610	4.48325	4.070706	5.690733	8.828067	12.45133	13.4884
HORVU4Hr1G012760	2.838242	4.968966	5.778819	0.453652	0.125538	0.463192
HORVU1Hr1G074110	7.599688	6.016536	8.508155	5.676162	5.866831	6.694596
HORVU7Hr1G017620	0.299136	0.168127	0.395186	4.603034	10.2438	7.545781
HORVU1Hr1G082160	18.24184	16.73371	22.88452	17.96673	21.33683	22.77285
HORVU4Hr1G064580	0.865315	1.275467	1.107654	4.043289	7.906944	7.069383
Hordeum_vulgare_newGene_6964	2.156873	0.853246	3.014658	2.816198	5.313069	3.616741
HORVU5Hr1G027580	1.37507	1.248496	2.096356	1.181292	1.190339	0.584555
HORVU2Hr1G017780	38.64219	36.1707	51.56869	44.05005	68.23424	72.25883

HORVU5Hr1G046230	2.423682	2.618653	2.7272	2.980937	5.946173	5.70624
HORVU2Hr1G100720	1.357122	1.381965	1.42324	3.96535	9.199176	6.647812
HORVU7Hr1G110130	0.383717	0.341489	0.883631	4.81259	16.8129	17.63132
HORVU0Hr1G015840	0.976664	0.232944	0.909179	2.273263	2.848717	2.599923
HORVU7Hr1G052560	4.842644	4.82713	4.99212	9.015992	10.0183	12.2152
HORVU1Hr1G094740	24.98275	27.7408	24.04533	21.40851	28.51537	30.20475
HORVU3Hr1G099430	2.407813	3.736423	4.90548	3.980075	6.316725	7.298308
HORVU3Hr1G079180	1.525336	1.245486	1.23867	4.09923	4.212358	5.075205
HORVU4Hr1G084830	2.508836	2.143502	2.302116	6.113078	12.70174	12.33626
HORVU2Hr1G121050	5.873949	6.417328	6.568068	11.26348	8.882392	10.56779
HORVU1Hr1G039670	2.792171	2.187188	2.656506	4.586898	7.902438	7.800368
HORVU3Hr1G064450	1.388227	0.670446	1.662608	0.793154	1.112251	1.598945
HORVU2Hr1G113350	43.65247	38.08193	41.878	57.93179	60.07818	54.81232
HORVU4Hr1G026890	6.04944	5.517322	6.841427	8.66647	10.98511	10.79478
HORVU0Hr1G001240	7.705256	7.032341	6.569484	5.915182	6.76125	4.610056
HORVU5Hr1G093160	4.141121	4.818013	5.90245	5.18395	9.511753	8.237267
HORVU2Hr1G001600	19.25084	16.9008	21.3511	30.15657	61.33824	56.45848
HORVU4Hr1G082570	34.05841	30.09069	43.05741	47.51596	83.2729	76.63703
HORVU5Hr1G047540	5.736169	6.61756	5.233218	6.927938	9.739596	8.106426
HORVU7Hr1G018660	40.54533	36.93761	47.17151	17.28164	21.26741	21.052
Hordeum_vulgare_newGene_10378	1.780448	1.824038	1.578744	1.12741	1.078914	1.151978
HORVU2Hr1G090340	3.278197	2.568959	3.126037	4.43178	3.909257	4.906607
HORVU1Hr1G080290	8.519726	9.531459	8.717291	8.730317	6.380731	8.301228
HORVU1Hr1G051570	6.231387	6.840718	8.160053	8.244619	11.43482	11.21241
Hordeum_vulgare_newGene_10377	33.52531	27.68567	35.4749	26.32661	2.060221	9.645288
HORVU7Hr1G090260	5.527884	6.260295	6.522593	4.902806	5.902526	5.90034
HORVU6Hr1G057580	6.135812	2.705876	9.036762	4.889343	9.228338	13.21888
HORVU4Hr1G007450	8.792866	10.87328	8.815568	12.25881	18.15364	18.66563
HORVU3Hr1G074250	9.531299	7.368457	9.695632	9.576087	19.42496	18.5624
HORVU3Hr1G074780	2.235622	3.009977	1.86751	0.379134	0.215991	0.029553
HORVU4Hr1G090160	1.46127	1.668675	1.851422	1.071185	0.855762	1.049426
HORVU3Hr1G059830	106.6057	94.02183	131.3241	88.15787	115.8156	112.4931
HORVU7Hr1G086520	4.53433	5.502717	5.46276	7.470652	9.242661	9.039999
HORVU5Hr1G017680	8.470503	7.424406	10.46222	5.026882	7.570896	8.160498
HORVU6Hr1G032610	7.971096	7.397841	9.781352	13.54549	18.32178	19.96874
Hordeum_vulgare_newGene_8522	54.57255	48.1687	51.74001	9.867384	0.254971	4.005111
HORVU3Hr1G007500	461.5494	388.351	878.979	1219.303	1703.912	1225.875
HORVU3Hr1G028150	1.582738	1.725484	2.407371	1.46973	0.978109	1.510055
HORVU7Hr1G027710	11.7225	12.76241	14.17867	7.798295	11.29014	9.035355
HORVU1Hr1G044350	2.944424	3.33621	3.549896	5.731588	7.537588	8.332443
HORVU4Hr1G013330	1.366142	1.060391	1.429212	2.032218	0.760783	1.283228
HORVU2Hr1G090490	0.812495	1.49965	1.624496	1.149108	4.305	3.862006
HORVU3Hr1G077780	3.966094	3.57406	4.606974	2.895597	4.566378	6.880437
HORVU5Hr1G044480	4.57711	1.308919	4.453573	6.324938	8.688018	8.966977
HORVU7Hr1G087690	3.200669	4.41237	3.209449	8.002353	7.706067	2.618902
HORVU6Hr1G048780	24.37221	23.46037	29.4052	26.92961	38.2754	36.32334
HORVU6Hr1G041060	3.793826	3.955987	4.167711	4.55805	4.708038	5.707758

HORVU3Hr1G065960	7.668606	8.326161	11.70588	14.98522	14.54435	15.1653
HORVU1Hr1G040980	24.93255	26.82522	28.18524	26.81887	31.1316	31.77904
HORVU2Hr1G011490	0	0	0	0.891668	22.07163	15.26775
HORVU3Hr1G029200	0.661668	0.668807	0.70098	2.279726	2.535608	2.663961
HORVU7Hr1G040210	1.506612	1.737336	2.058975	3.319663	3.521539	2.420661
HORVU7Hr1G030080	0.858635	0.67817	0.627766	1.895827	1.414086	1.899222
HORVU3Hr1G077250	3.839889	4.035558	5.093527	13.54109	26.84851	24.72994
HORVU6Hr1G057520	2.509545	2.429967	2.373028	4.951327	5.318867	5.170254
HORVU7Hr1G083580	5.639752	5.342032	5.042027	6.798559	4.387466	5.542586
HORVU3Hr1G067530	12.4583	13.14124	13.82023	12.79147	15.15967	15.56386
HORVU6Hr1G090290	2.303397	2.01059	2.216831	2.469174	3.824422	3.363522
Hordeum_vulgare_newGene_1926	1.548017	1.555121	1.015105	1.291824	1.141961	1.845525
Hordeum_vulgare_newGene_1922	3.408916	3.835928	3.683375	3.671848	3.726024	4.527303
HORVU5Hr1G016060	3.587309	4.458953	2.236255	2.129764	20.09432	15.49667
HORVU1Hr1G046670	7.828225	6.877297	8.775042	8.016089	13.87282	12.92252
Hordeum_vulgare_newGene_1929	6.816691	8.881717	10.54519	7.31696	5.601737	8.180292
HORVU5Hr1G098820	2.441983	2.398736	2.520112	2.297824	2.44022	3.005708
HORVU5Hr1G076250	2.924743	1.77973	2.683643	4.853702	5.257597	8.788056
HORVU2Hr1G028670	62.27834	29.35126	54.84979	2.345957	0.752936	2.006353
HORVU3Hr1G057690	31.31265	24.48512	39.31059	7.267543	0.236802	2.47239
Hordeum_vulgare_newGene_16005	2.861262	2.533727	2.642875	3.477963	4.049298	4.188377
Hordeum_vulgare_newGene_16006	9.286506	11.01618	8.492638	9.163404	11.20582	11.8461
HORVU5Hr1G045850	8.890345	8.904699	8.906784	9.525069	4.279195	8.122668
HORVU1Hr1G042800	1.532421	0.812326	1.285554	3.305005	5.352357	5.018411
Hordeum_vulgare_newGene_16008	6.524851	6.233975	6.295675	7.052942	9.497283	9.722827
HORVU5Hr1G049160	8.878516	9.88674	8.948058	15.36474	15.16227	16.21848
HORVU1Hr1G092950	17.47915	19.79401	23.39876	14.53163	18.23307	19.97914
HORVU3Hr1G068710	2.078256	1.637337	1.753639	1.239327	1.363712	2.007849
HORVU6Hr1G062040	2.209828	2.088665	2.435394	7.131749	13.36057	12.58656
Hordeum_vulgare_newGene_1365	18.7879	18.7884	20.97563	15.02789	14.67078	14.80222
HORVU2Hr1G038940	3.814637	4.013438	2.336408	35.25893	45.50709	48.61566
HORVU4Hr1G002770	2.04619	1.159457	2.494669	1.462016	3.353873	3.761847
HORVU7Hr1G105490	7.530972	7.113915	9.614165	10.85582	10.52102	12.76119
HORVU4Hr1G079830	9.331659	11.20432	8.973404	19.08761	14.92459	16.53489
HORVU4Hr1G060040	15.28533	15.12244	19.0666	18.00322	19.01491	15.18801
HORVU3Hr1G105760	0	4.018503	4.994771	1.453121	0	0.191458
HORVU2Hr1G025170	3.121412	2.860909	4.215115	3.967356	4.519116	4.317248
HORVU2Hr1G114640	1.928098	1.997502	3.183163	2.472501	2.9859	3.061183
Hordeum_vulgare_newGene_3380	2.514303	2.686345	1.727126	4.185319	2.415462	2.823457
Hordeum_vulgare_newGene_7801	7.10154	7.957103	7.486657	0.253451	0.109741	0.07571
Hordeum_vulgare_newGene_3389	1.05618	1.317462	1.369493	2.133907	6.183837	5.183686
HORVU4Hr1G073960	5.800739	7.855699	7.723458	9.43312	7.222915	8.671572
HORVU1Hr1G064530	2.560147	2.577004	2.645215	2.15429	2.626155	2.54232
HORVU5Hr1G028180	5.507164	5.722869	8.255893	11.46598	12.9349	13.58581
HORVU0Hr1G021740	1.034171	1.432203	1.640404	1.000786	0.398388	0.838745
HORVU5Hr1G061300	6.153422	5.702973	7.405256	2.580187	3.170869	3.492169
HORVU3Hr1G017220	3.095113	4.689912	4.439017	2.091735	2.408707	2.29077

HORVU2Hr1G103130	7.572127	8.137231	7.743203	22.61919	29.85781	30.37069
HORVU7Hr1G038610	27.28205	21.56483	27.6219	18.13846	13.58467	15.51238
HORVU4Hr1G037680	7.76219	8.522631	8.982617	10.85422	13.28543	14.86194
Hordeum_vulgare_newGene_13780	0.804813	0.441107	0.475343	5.255496	4.272164	5.341409
Hordeum_vulgare_newGene_13784	33.30651	27.69433	34.86332	23.26226	28.37518	22.04942
HORVU3Hr1G116550	2.090845	1.461077	4.011156	6.756697	29.57189	29.31932
HORVU3Hr1G090460	20.11738	21.14629	23.90721	11.13716	16.69221	16.12442
Hordeum_vulgare_newGene_3659	1.494775	1.277195	1.958812	1.142694	1.471167	1.948716
HORVU7Hr1G035190	4.653495	4.145056	4.3204	2.925737	2.55033	3.960491
Hordeum_vulgare_newGene_2389	2.701752	1.966086	3.031202	2.567708	4.301756	3.47281
Hordeum_vulgare_newGene_2388	2.489108	2.442447	2.814007	2.994264	5.111491	4.971013
Hordeum_vulgare_newGene_2382	0	0	0	1.691723	3.26457	1.921706
Hordeum_vulgare_newGene_2381	0.311652	0.121674	0.288841	1.8293	3.301715	2.41873
Hordeum_vulgare_newGene_2387	6.603254	7.119668	7.911	6.40461	7.820498	8.495543
HORVU0Hr1G005790	1.217719	1.364356	2.300662	0.292582	0.505873	1.122587
Hordeum_vulgare_newGene_2385	35.10052	40.49951	32.53485	14.66351	9.684687	12.607
Hordeum_vulgare_newGene_14313	2.82655	3.198923	4.012562	1.345699	1.908699	1.948757
HORVU7Hr1G078410	11.76741	12.3264	15.39076	10.99858	15.16733	15.16622
HORVU2Hr1G027150	1.29336	1.581041	2.152306	0.385933	0.601706	0.507291
Hordeum_vulgare_newGene_616	7.862267	9.880464	11.33047	5.076931	0	2.959956
Hordeum_vulgare_newGene_617	1.507431	1.402224	1.879949	1.331424	1.014274	1.510963
HORVU5Hr1G094580	6.063871	6.93208	9.49153	8.847555	11.53527	10.97641
HORVU3Hr1G087220	5.732862	3.752129	5.322766	5.675195	5.23951	5.146768
HORVU3Hr1G069190	14.70824	14.00847	16.73453	21.17303	24.78591	25.86674
HORVU4Hr1G070680	2.358621	2.208569	2.450122	2.404573	1.8077	2.056511
Hordeum_vulgare_newGene_6866	1.445948	1.274854	1.310623	1.953978	4.331566	3.608628
Hordeum_vulgare_newGene_6863	0.542376	0.908147	0.358134	3.857457	3.448357	5.46543
Hordeum_vulgare_newGene_11922	1.408722	1.292997	1.647046	2.825067	3.988903	3.048823
Hordeum_vulgare_newGene_11923	1.358562	1.544759	1.759974	0.417584	0.630491	0.523523
HORVU2Hr1G077100	2.363438	1.776801	2.256502	1.966964	3.143253	3.184951
HORVU2Hr1G054210	3.28236	3.768613	3.602836	4.618493	4.995476	6.178292
Hordeum_vulgare_newGene_9437	2.031814	2.235547	2.499069	2.320115	2.772949	2.23716
HORVU7Hr1G108460	0.314376	0.287175	0.345185	4.389724	1.647385	3.961997
HORVU5Hr1G073700	7.377499	1.022181	8.162404	3.099315	1.49237	0.907605
HORVU7Hr1G119720	2.906668	2.628282	3.948214	2.993918	3.333745	2.861293
HORVU7Hr1G043760	1.405205	1.643342	1.881571	0.653512	1.093997	1.411947
HORVU4Hr1G078050	26.55352	26.71039	26.32018	22.0078	34.46991	31.84657
HORVU3Hr1G111580	52.27626	49.01556	68.56213	17.37242	2.336469	6.668888
HORVU4Hr1G070530	89.3686	93.14124	62.77719	22.20234	14.89561	19.0051
HORVU7Hr1G022970	23.48082	19.02963	27.38067	28.38431	42.81972	42.48974
HORVU1Hr1G000020	33.09578	27.75826	34.41842	25.71935	29.57374	32.11552
HORVU4Hr1G075140	0.693021	0.785117	0.960969	1.930935	3.019949	2.593451
HORVU7Hr1G027910	8.368703	11.75431	9.839146	8.375495	6.28248	5.053034
HORVU2Hr1G030550	1.750386	1.679218	2.057437	2.056856	2.160194	2.37049
HORVU0Hr1G023970	1.243094	1.369366	0.778662	0.638776	4.226272	2.706358
HORVU1Hr1G059090	11.27202	12.22937	10.18897	4.840777	4.373678	5.132251
HORVU7Hr1G037130	1.175108	1.436759	1.701566	0.821773	1.133253	0.961678

HORVU7Hr1G073770	5.972602	6.558998	5.696131	5.991418	8.683642	8.333444
Hordeum_vulgare_newGene_12915	3.873343	1.757183	2.627013	0.989097	1.566614	0.971204
HORVU1Hr1G077100	38.98539	34.10645	42.75665	75.49973	80.28235	80.29747
HORVU0Hr1G008670	1.440052	2.381968	3.066669	2.574712	12.03837	4.539576
HORVU1Hr1G035200	2.669393	2.699852	2.544846	4.598204	6.387679	6.63882
HORVU1Hr1G043890	567.9733	509.0375	699.4182	192.6842	42.52606	86.22847
Hordeum_vulgare_newGene_9519	9.278426	10.914	11.8519	18.47464	23.72095	29.03926
Hordeum_vulgare_newGene_9516	4.982139	5.441975	6.251303	6.499098	7.204009	7.857215
HORVU7Hr1G060790	5.188461	5.730304	6.173113	7.856948	8.253221	10.09915
Hordeum_vulgare_newGene_9511	7.874198	8.754026	9.478092	5.748909	4.212102	5.305038
HORVU5Hr1G075920	5.790731	6.021694	4.655769	6.639151	9.072638	7.259512
HORVU2Hr1G034380	26.93764	25.93931	29.01151	18.0863	23.2534	20.82035
HORVU2Hr1G073150	63.61249	66.98727	98.61398	54.34829	99.94452	97.8771
HORVU3Hr1G042600	4.306047	5.471649	4.820688	25.31087	41.9323	40.95538
HORVU7Hr1G114050	115.1459	118.6183	88.68972	75.11191	25.64038	33.7336
HORVU4Hr1G032190	3.922986	3.866624	5.690078	5.497912	6.185644	6.413891
HORVU7Hr1G021990	2.081268	1.785517	3.19338	2.551131	1.981859	2.090931
HORVU2Hr1G009710	4.819924	4.843075	5.224185	5.435582	3.854998	4.442639
HORVU2Hr1G029260	6.396962	7.242079	8.189332	6.022122	6.485939	8.03324
HORVU3Hr1G087100	8.976765	4.926256	8.510186	1.716161	4.111207	2.367336
HORVU3Hr1G049720	12.67689	12.46631	11.18089	20.95815	24.75888	25.78642
HORVU4Hr1G035210	48.60054	43.96147	53.99188	56.16534	66.85221	72.83718
HORVU1Hr1G072570	9.656348	9.154653	13.42705	10.3763	11.9296	10.74408
HORVU1Hr1G072680	13.76799	14.08843	12.71803	28.12471	19.71541	19.07082
HORVU6Hr1G009380	18.35082	15.8028	20.61376	15.46592	25.08354	23.04899
HORVU1Hr1G009250	1.684248	1.645646	1.913148	2.509356	1.750986	2.365893
HORVU1Hr1G052180	1032.138	970.5813	1362.51	48.52086	1.330628	16.57175
HORVU6Hr1G031480	139.894	166.6242	190.5111	100.1204	84.03485	92.39096
HORVU5Hr1G018040	1.374106	1.872114	1.827379	2.877136	3.595701	3.456828
HORVU4Hr1G046620	0	0	0	6.900857	6.616634	8.217867
Hordeum_vulgare_newGene_14600	1929.142	1703.11	1562.473	438.8129	125.7681	183.1519
HORVU7Hr1G101580	8.079868	6.874644	9.099928	10.771	4.648293	5.978258
Hordeum_vulgare_newGene_4294	8.315417	8.03563	9.552874	14.43967	13.85594	13.56917
HORVU2Hr1G008440	1.377068	1.491638	1.46307	8.054225	8.959355	11.44789
HORVU7Hr1G019730	0.780654	0.681133	0.962856	0.92563	1.418407	1.746826
HORVU7Hr1G099120	3.747178	2.994418	4.411651	3.277668	2.598828	3.593073
HORVU2Hr1G068880	0.933996	0.570792	0.472859	1.755735	2.06406	3.328167
HORVU1Hr1G023730	2.362986	1.840774	2.317691	3.970675	5.233515	5.381048
Hordeum_vulgare_newGene_4837	5.237686	6.035011	5.26449	5.062714	5.058502	5.942688
HORVU4Hr1G019990	83.07552	83.18908	91.54528	139.0854	76.37471	84.6672
HORVU6Hr1G023070	0.508248	0.506119	0.50343	1.959425	2.176841	2.44101
HORVU1Hr1G056600	2.432763	2.469174	2.333716	2.038718	2.743718	2.893613
HORVU6Hr1G059100	5.113312	5.524635	6.273574	5.688035	4.17663	6.840867
HORVU5Hr1G088460	2.307397	2.828563	3.624539	3.065808	3.305391	2.712461
HORVU2Hr1G045340	36.57948	34.11241	35.41739	1.955576	0.047638	0.485826
HORVU5Hr1G058270	24.62414	25.04692	19.96475	23.43771	29.13538	29.95773
Hordeum_vulgare_newGene_6474	4.43515	4.086107	4.814645	6.012097	6.332564	5.611077

Hordeum_vulgare_newGene_6471	0.590294	0	0.97706	0.779987	18.24121	14.10002
Hordeum_vulgare_newGene_6470	0.300501	5.258248	0.990869	0	0	0
Hordeum_vulgare_newGene_6472	0.425208	0.262907	0.541228	1.870644	10.12783	8.809697
HORVU4Hr1G074790	1.271639	1.30839	1.359472	0.901936	1.048864	0.939878
HORVU1Hr1G051370	14.32601	12.57475	13.20294	9.483222	9.000738	8.75131
HORVU5Hr1G013290	5.870353	4.926818	6.074229	21.50886	26.92741	24.49273
HORVU5Hr1G059890	12.02819	6.497695	13.97849	5.061423	1.688622	1.588588
HORVU3Hr1G033920	11.95477	9.674934	14.54347	16.84577	22.35488	22.04456
HORVU3Hr1G081570	7.016309	5.520087	7.755918	3.940172	1.95316	1.89871
HORVU5Hr1G103380	8.344377	7.534226	8.241345	10.02642	14.22896	14.63873
HORVU3Hr1G018860	1.366998	1.522822	1.639076	2.282042	1.918914	2.042061
HORVU1Hr1G030000	10.50704	10.15713	16.9632	7.080857	8.853516	9.454906
HORVU3Hr1G034040	1.728971	1.527572	1.754382	8.702167	10.0013	9.471847
HORVU4Hr1G016460	0	0	0.03266	4.914446	0.710354	1.684984
HORVU5Hr1G111970	1.463117	1.332725	2.081734	3.599222	2.647062	3.064889
HORVU3Hr1G082170	17.58681	13.44946	16.35046	21.88264	22.69573	22.87315
HORVU7Hr1G097210	1.653481	1.432055	1.452397	3.007947	0.80432	1.148722
HORVU5Hr1G012160	36.6551	39.7223	49.00139	23.19804	24.55759	27.21872
HORVU5Hr1G058850	7.267277	6.993087	7.820288	10.5069	10.6104	11.7835
HORVU5Hr1G094020	10.25047	9.830257	13.47522	7.736193	8.173934	8.029043
HORVU7Hr1G022500	4.029933	3.331109	3.899378	6.957983	8.364537	8.968963
HORVU6Hr1G087330	6.081517	4.983724	6.37591	9.654631	6.059699	7.825977
HORVU3Hr1G035380	97.11094	84.75908	102.1794	74.75066	60.82505	62.36259
HORVU6Hr1G034570	3.58953	2.687379	4.387174	2.192799	1.250686	2.020224
HORVU5Hr1G020280	15.1545	16.27519	16.45352	24.97071	36.43303	31.57653
HORVU4Hr1G042710	0.127122	0.227869	0.164125	4.201273	8.521811	3.641457
HORVU5Hr1G106380	30.96341	34.90198	40.88542	28.34991	42.03545	43.83252
HORVU5Hr1G023530	3.896882	3.005826	3.983507	4.169372	5.35234	5.214203
HORVU1Hr1G020410	12.33084	5.991024	15.8469	3.692912	2.329669	3.287272
HORVU1Hr1G032610	3.70512	4.265064	4.417411	6.155574	8.770432	8.336534
HORVU4Hr1G053780	2.388598	2.007953	2.81622	1.944728	4.117456	3.221404
HORVU2Hr1G051030	18.18581	17.77914	14.62906	11.33691	1.758723	3.874151
HORVU3Hr1G029040	25.03214	9.733249	7.159138	4.341444	7.06606	4.177242
HORVU2Hr1G118790	3.840816	2.594835	3.147866	7.767353	2.806905	5.051287
HORVU4Hr1G069420	13.35993	11.23434	19.12099	14.48459	20.71759	18.49797
HORVU7Hr1G054010	57.52808	57.43488	88.49903	57.28865	103.3352	92.79853
HORVU6Hr1G085590	2.213177	2.682582	2.52939	3.616444	4.630585	3.797692
HORVU1Hr1G018790	8.018577	7.163373	7.994565	9.507306	9.963597	10.8399
HORVU2Hr1G085120	27.58043	19.76055	28.02223	25.22493	30.10468	32.28075
HORVU5Hr1G118160	10.7442	7.822213	9.800298	7.019683	7.873489	7.004932
HORVU6Hr1G066810	14.95851	13.0013	16.89532	19.1151	20.29646	21.09352
HORVU1Hr1G093790	3.178768	2.567774	4.265216	3.68729	3.742472	3.954529
HORVU1Hr1G010220	1.167814	0.832915	1.265554	1.753827	1.165945	1.45697
HORVU2Hr1G083980	26.30426	26.57105	21.94451	25.01077	24.31081	24.68728
HORVU4Hr1G072010	84.9941	76.40752	88.2364	97.11805	109.3107	112.778
HORVU3Hr1G003400	3.303988	4.371212	10.36617	2.572463	2.806364	8.878483
HORVU1Hr1G053440	89.54028	62.73265	87.1775	133.0839	102.8679	105.9251

HORVU6Hr1G040130	0.889414	1.064562	1.226709	1.302245	1.198232	1.300084
HORVU6Hr1G090720	25.57899	25.26283	29.63044	25.36507	41.98831	44.30966
Hordeum_vulgare_newGene_14721	3.67465	3.338535	5.017105	3.032241	3.681964	3.362096
HORVU5Hr1G106960	1.525501	1.574198	1.453133	4.407501	6.101605	6.671326
HORVU2Hr1G085570	1.643758	0.972681	1.804522	6.4461	14.69112	13.29303
HORVU1Hr1G092440	14.05064	11.02793	13.36206	24.56903	33.58632	30.86802
HORVU5Hr1G088920	31.94839	29.20385	39.06593	19.51717	27.33599	25.07645
HORVU2Hr1G046990	1.907303	1.922253	3.385114	1.938015	2.543597	2.732165
HORVU4Hr1G010300	52.41589	47.4761	67.16778	59.37051	46.38183	43.13989
HORVU3Hr1G036190	1.618081	1.514858	1.401106	2.194371	2.81292	3.26949
Hordeum_vulgare_newGene_832	8.450407	7.949412	9.917833	8.372482	6.229186	7.069255
Hordeum_vulgare_newGene_833	0.668309	1.025211	0.85614	1.190349	1.068385	1.833267
HORVU6Hr1G001820	2.805038	5.594753	4.00106	6.573873	4.07862	5.67939
HORVU2Hr1G107830	0.934939	1.184584	0.819633	1.117702	1.244258	1.248912
HORVU1Hr1G048610	0.608803	0.435577	0.583551	3.141281	3.19962	1.435914
HORVU1Hr1G013880	19.30276	18.84726	20.37227	0.018864	0.010791	0.009598
HORVU7Hr1G095100	2.963659	2.518995	3.247405	4.852616	7.502443	6.442109
HORVU7Hr1G046040	2.603667	2.532661	2.980996	8.113063	6.159238	7.893041
HORVU3Hr1G066930	11.33106	11.38108	15.21603	22.81036	32.11838	33.32172
HORVU5Hr1G059070	2.110888	2.201181	1.881971	6.445481	6.134323	6.120014
HORVU1Hr1G085570	3.318152	3.469445	3.866103	5.030845	7.227384	7.25201
HORVU5Hr1G109910	64.83364	72.0536	102.359	43.78774	101.5246	84.3774
HORVU3Hr1G071370	118.5229	76.5212	89.1766	0.275345	0	0
Hordeum_vulgare_newGene_12812	39.8867	33.70092	47.75952	43.69811	67.48543	71.84303
HORVU4Hr1G012680	0.898556	0.669824	0.974134	2.386226	0.713007	1.726503
HORVU2Hr1G083070	4.170304	4.164277	6.094074	5.145192	9.432371	10.8023
HORVU2Hr1G041760	18.94128	21.17785	16.5189	19.01985	25.81127	25.68786
Hordeum_vulgare_newGene_99	1.563768	1.854089	1.74081	1.348901	1.919821	1.71177
HORVU1Hr1G044960	18.72779	16.01152	21.47729	14.00367	15.40012	12.4208
HORVU4Hr1G069990	2.365594	2.685218	3.310085	10.99555	10.87187	11.07413
HORVU6Hr1G016610	8.540272	8.1906	8.554065	12.40986	15.16301	14.89844
HORVU0Hr1G010020	2.525733	2.303862	2.289722	2.325189	2.449179	2.60496
HORVU3Hr1G064390	13.80557	16.31255	17.08939	10.69214	9.99197	10.73887
HORVU6Hr1G012500	2.222957	3.602941	3.411662	3.554037	5.142788	4.549375
HORVU1Hr1G011900	8.639868	9.694182	8.867173	6.372254	6.688105	7.502049
HORVU4Hr1G009040	64.80327	66.04343	86.81516	29.16387	72.51922	60.54652
HORVU1Hr1G094460	13.73901	11.28879	15.52657	21.94357	17.30522	19.39965
HORVU3Hr1G020500	279.9226	245.642	294.8141	375.292	541.0837	366.8078
HORVU7Hr1G074520	5.442685	6.641287	6.864042	5.615898	5.772175	5.976585
HORVU5Hr1G115440	1.270543	1.094301	1.323896	1.645489	0.724065	0.730509
HORVU7Hr1G084420	6.419588	6.074726	7.52265	23.43782	18.68819	21.11605
HORVU2Hr1G091530	15.16067	11.13779	11.85373	16.76538	15.70213	13.7618
HORVU2Hr1G018840	10.16712	13.71123	18.80562	8.733042	24.77542	21.48494
HORVU3Hr1G094570	12.65621	11.20296	13.82909	9.909052	19.64559	17.49021
HORVU3Hr1G064640	10.2023	9.102357	10.3896	6.976452	4.536087	3.382038
HORVU5Hr1G045640	0.788903	0.494813	0.899548	0.579511	2.440846	2.263257
HORVU7Hr1G006600	1.291143	1.422111	1.274538	5.5398	6.886853	6.554507

HORVU5Hr1G090000	11.21114	13.09089	14.11523	10.80684	12.5293	13.1326
HORVU2Hr1G078400	4.286166	5.04292	4.271683	3.789241	4.670686	5.263675
Hordeum_vulgare_newGene_5411	1.752077	2.20834	2.176053	2.376489	2.735196	2.687759
HORVU4Hr1G022540	27.3305	27.04031	26.01457	23.14149	29.45883	28.19451
HORVU2Hr1G010660	0.0161	0.129769	0.430804	19.05333	14.7622	19.02225
HORVU1Hr1G003750	11.84046	10.76848	10.06068	14.15139	13.30694	14.16476
HORVU3Hr1G067370	31.98257	31.14807	35.89962	25.63018	33.92258	32.88251
HORVU5Hr1G117590	2.543662	2.58528	2.495763	0	0	0.011661
HORVU2Hr1G076530	1.982681	2.188907	2.67904	2.342249	2.282212	2.956931
Hordeum_vulgare_newGene_8627	4.127424	1.979058	5.019941	5.105915	3.790746	4.450546
Hordeum_vulgare_newGene_8623	29.97053	24.92751	21.86598	25.79181	25.64991	23.96078
Hordeum_vulgare_newGene_8620	0.895231	0.708381	0.783203	1.272927	4.33568	3.118586
HORVU5Hr1G081380	24.11863	20.02444	26.93972	21.00894	23.33751	22.17966
HORVU6Hr1G040900	17.28844	14.11916	23.50847	17.03764	32.37821	27.80326
HORVU6Hr1G035160	3.209515	3.430767	3.45733	4.772925	4.914653	5.136579
HORVU4Hr1G020070	1.302676	1.447746	1.572048	1.169137	1.485663	1.536687
HORVU7Hr1G074380	8.515403	8.161343	8.3233	5.806671	7.290526	6.483295
HORVU2Hr1G113570	1.775714	1.295294	2.121223	3.175556	5.172955	3.475008
HORVU1Hr1G036370	0.897334	0.815627	1.050072	0.763738	1.223385	1.254704
HORVU3Hr1G061870	4.555751	4.534124	4.985774	4.194853	6.106746	6.039662
HORVU3Hr1G065510	9.431317	9.857157	9.442617	8.6171	8.364994	10.13764
HORVU2Hr1G011040	37.16017	44.76399	33.48745	44.07444	60.41032	59.06908
Hordeum_vulgare_newGene_10504	0.068711	0.143736	0.106993	4.842403	2.646289	3.714732
HORVU4Hr1G025750	2.24167	3.43347	3.354342	2.142908	1.559125	2.794822
HORVU5Hr1G123530	16.60334	15.10283	13.22876	8.060689	10.27067	9.402426
HORVU3Hr1G098530	22.15435	18.6263	22.90811	19.93632	23.49189	21.86838
HORVU3Hr1G096360	8.36992	4.831701	8.718316	0.944647	0.572593	1.047553
HORVU3Hr1G096550	2.659125	2.648935	2.90336	3.275602	4.266536	5.23825
HORVU5Hr1G098780	3.881408	4.144804	4.890181	13.79029	15.07506	16.01812
HORVU4Hr1G067630	1.059071	0.675324	0.986228	1.077599	1.090908	1.719171
HORVU0Hr1G022020	1.540285	0.863835	2.371095	2.865498	2.00258	2.327824
HORVU3Hr1G106650	4.887909	4.66187	5.69181	6.146232	6.199878	7.590644
HORVU2Hr1G073310	1.615289	1.169482	2.098819	1.825992	0.98715	0.956675
HORVU2Hr1G090280	19.40786	17.09014	17.91693	7.6662	2.748036	6.146992
HORVU2Hr1G031190	2.248339	2.139183	1.917622	1.075753	1.721577	1.520227
HORVU0Hr1G023990	0.497482	1.176582	0.605826	0.394158	2.705844	1.767231
HORVU2Hr1G093390	2.171026	2.299717	3.084123	2.43557	4.467247	4.052184
HORVU6Hr1G091840	25.54831	33.1126	39.02603	4.285658	2.749922	3.774013
HORVU5Hr1G052790	2.298221	1.954873	2.416028	2.756701	5.664242	4.626586
HORVU5Hr1G002310	3.561183	4.558669	5.88336	3.429146	3.876736	4.039429
HORVU7Hr1G087240	7.757479	5.089709	6.069033	13.48643	12.65444	12.15557
HORVU2Hr1G023690	11.98447	10.98729	13.70182	7.94055	3.281756	4.206328
HORVU5Hr1G081520	18.94919	19.86373	20.34431	38.93699	58.20934	56.14751
HORVU3Hr1G112860	0.924917	0.631663	1.067799	3.435877	0.1542	0.882813
HORVU3Hr1G059250	1.948075	2.707588	3.114877	0.172	0.060971	0.068598
HORVU4Hr1G024400	6.518672	7.139501	8.905479	9.727398	8.767346	11.03292
Hordeum_vulgare_newGene_9815	3.236381	2.998999	2.909518	6.903927	5.409921	6.638226

HORVU2Hr1G091880	0.343955	0.12612	0.243008	1.338419	33.06971	25.84055
Hordeum_vulgare_newGene_9813	1.702143	2.117887	2.461464	1.987783	1.815625	2.375925
Hordeum_vulgare_newGene_1880	15.25152	16.80174	16.11719	23.37867	18.21588	23.19565
HORVU7Hr1G021460	30.60539	33.29028	27.50503	83.92211	42.9643	47.09544
HORVU5Hr1G029910	11.2032	16.89177	13.25249	14.47091	14.91008	14.43955
HORVU2Hr1G093020	5.834412	4.963341	6.268951	9.155174	13.95282	13.47717
HORVU5Hr1G006000	47.72824	47.45313	46.59351	42.23948	39.97667	42.6622
HORVU7Hr1G033360	33.90715	26.61443	33.1308	33.3505	29.00624	32.4848
HORVU3Hr1G108620	0.874831	1.018529	0.879113	2.713112	2.599412	2.691863
HORVU5Hr1G048150	8.512667	9.700254	9.359092	5.565598	3.867992	5.380765
HORVU7Hr1G009770	1.931966	1.724016	1.881633	0.828125	0.664743	0.658584
HORVU3Hr1G104790	1.260078	1.095217	1.267952	3.997242	17.16533	12.29536
HORVU3Hr1G075460	1.301808	1.692426	3.12964	1.398465	1.49383	1.453581
HORVU6Hr1G077340	73.48291	60.93569	83.10063	67.9519	103.0657	107.9602
HORVU7Hr1G031210	3.185412	3.791109	2.695532	6.764951	5.236928	6.133074
HORVU6Hr1G090280	0.028359	0.049377	0	2.024356	2.459513	1.986056
HORVU1Hr1G046420	3.578658	3.592591	4.926788	0.015544	0	0
HORVU0Hr1G016190	16.63129	17.64489	16.57625	22.04219	24.07862	27.26321
HORVU2Hr1G035630	9.414103	7.617721	9.17204	10.64524	10.38373	10.41037
HORVU4Hr1G027270	15.92134	21.60771	21.77394	20.96422	22.27442	26.2409
HORVU2Hr1G100970	87.92652	95.34121	145.261	61.19306	108.5791	109.7071
HORVU4Hr1G027180	7.76523	5.463503	6.57462	7.075207	13.60518	12.68971
Hordeum_vulgare_newGene_3986	18.40377	16.52203	11.83047	8.49175	8.255785	8.38609
HORVU3Hr1G089540	1.141475	1.245137	0.528285	1.432809	2.291653	1.975585
Hordeum_vulgare_newGene_7108	5.897225	4.128684	5.356547	8.676007	10.54225	9.547421
Hordeum_vulgare_newGene_12376	1.588356	0.614055	1.520074	1.765915	4.054494	4.884761
HORVU3Hr1G080790	216.2679	190.2054	219.598	147.7888	190.2662	184.4408
HORVU5Hr1G068800	0.62361	0.500159	0.553616	2.440839	2.996017	2.447851
HORVU3Hr1G017580	5.570522	5.83231	5.880748	6.559473	8.164579	9.173009
HORVU2Hr1G069950	2.038128	2.156026	2.451093	7.820522	10.97094	11.0866
Hordeum_vulgare_newGene_6273	13.70723	14.15657	16.10642	15.76222	25.20669	26.22448
HORVU2Hr1G066560	32.50553	31.24537	34.51503	33.65605	54.29141	52.43894
HORVU1Hr1G037810	11.1953	11.23327	13.46603	13.42274	12.26649	13.68253
HORVU2Hr1G039190	0.469711	0.977607	0.762784	0.469502	3.641853	2.512445
HORVU6Hr1G054690	1.129779	1.046778	0.831038	1.184866	1.335865	1.595215
HORVU5Hr1G084540	12.61638	12.29365	14.16592	13.76155	20.09007	20.88816
HORVU1Hr1G052850	4.887539	4.900425	6.142855	0.772786	1.003963	1.034422
HORVU4Hr1G026580	3.088323	5.062217	3.983218	2.367806	2.145513	3.46484
HORVU2Hr1G013370	2.484868	2.346179	2.795864	5.52489	6.511425	6.938865
HORVU3Hr1G088490	8.463071	8.476431	8.699487	8.99601	9.837165	10.31134
Hordeum_vulgare_newGene_1938	1.254674	0.632888	1.238208	2.624554	0.997503	0.971185
HORVU7Hr1G028220	0.759019	1.365501	1.121918	0.92101	0.921956	1.243346
Hordeum_vulgare_newGene_12594	2.640674	1.808176	2.580425	15.14983	31.85965	33.18554
Hordeum_vulgare_newGene_12595	3.787681	3.258054	4.707791	3.63692	5.140132	5.207384
Hordeum_vulgare_newGene_12593	0.747724	0.608013	1.830455	1.503047	1.89767	1.770682
HORVU7Hr1G035010	76.56664	58.60035	79.93854	16.2318	7.429209	9.13916
HORVU6Hr1G002620	0.084151	2.291485	2.389859	3.582659	2.699162	3.401744

HORVU0Hr1G008050	1.201941	1.261281	1.378545	1.070168	1.446806	1.047871
Hordeum_vulgare_newGene_13427	1.806375	0.448244	1.664392	1.70352	1.356072	1.412973
Hordeum_vulgare_newGene_13426	2.467809	1.923546	2.77316	3.303143	2.691549	4.295662
Hordeum_vulgare_newGene_13421	20.89393	18.79889	19.83565	19.48971	23.74228	23.66191
Hordeum_vulgare_newGene_13423	2.626719	2.268779	2.985651	0.225259	0.195842	0.247309
Hordeum_vulgare_newGene_14430	12.8641	13.04886	12.74667	14.34925	15.05663	14.9676
Hordeum_vulgare_newGene_13429	3.648023	4.294535	3.882252	2.353396	3.667068	4.740082
Hordeum_vulgare_newGene_14439	0.218969	0	0	3.852122	3.63864	4.062566
HORVU6Hr1G023890	5.896157	3.133198	5.981051	3.723317	2.428774	2.797932
HORVU4Hr1G026630	2.642457	2.783261	3.850418	3.035255	3.328645	3.730012
HORVU2Hr1G071200	8.878455	9.209168	9.939222	8.193055	9.955182	10.1399
HORVU0Hr1G030920	3.693449	4.791587	5.636003	3.907151	3.707637	4.312137
HORVU5Hr1G080090	68.09637	58.93154	86.1205	73.5305	28.97498	38.62941
HORVU1Hr1G059970	47.39482	46.00226	68.53002	46.90324	63.36934	64.01433
HORVU5Hr1G061530	4.91773	4.971	6.09809	5.190051	6.111129	6.047227
HORVU3Hr1G020260	2.739156	3.8564	3.511061	3.27895	3.622755	3.771796
Hordeum_vulgare_newGene_13149	3.003397	4.290653	5.041395	2.994178	4.733649	3.977475
Hordeum_vulgare_newGene_10998	3.534269	2.685949	3.302943	7.085115	4.755863	5.991226
HORVU5Hr1G074350	17.61033	15.9255	21.60275	15.78135	15.20977	17.05342
HORVU6Hr1G012260	6.517251	6.476991	7.715536	5.144381	7.303935	7.869082
HORVU6Hr1G069120	2.360235	3.192945	3.140676	6.600677	6.600468	6.22176
HORVU1Hr1G081430	0.004562	0	0.00194	4.314442	6.020483	7.414671
Hordeum_vulgare_newGene_5998	5.953148	6.932958	6.560571	12.41091	13.83782	10.27053
Hordeum_vulgare_newGene_5997	2.789209	2.719581	3.973093	8.045417	8.983738	8.670538
Hordeum_vulgare_newGene_5993	2.145969	4.647987	0	5.884585	9.071188	6.196743
Hordeum_vulgare_newGene_9231	2.490872	2.134152	2.091197	2.935743	3.810441	3.715432
HORVU3Hr1G013770	2.325557	2.380867	2.770039	4.790154	6.378818	7.105059
Hordeum_vulgare_newGene_791	9.041144	8.478116	9.745337	9.097811	12.82686	14.16178
Hordeum_vulgare_newGene_790	6.550211	7.154788	6.729644	9.266412	12.77244	11.62855
Hordeum_vulgare_newGene_792	13.32444	12.99318	17.0473	14.39867	18.64363	17.61284
Hordeum_vulgare_newGene_14293	4.601968	4.277793	4.371797	3.241141	2.965526	3.476035
Hordeum_vulgare_newGene_14292	2.209492	2.917561	2.316379	2.130787	1.5211	2.235057
Hordeum_vulgare_newGene_14296	0.677066	0.480769	0.612178	1.077253	5.45137	3.021314
Hordeum_vulgare_newGene_14294	1.183456	1.55732	1.514014	1.42979	1.333113	1.724991
HORVU1Hr1G070920	15.40848	14.45784	14.23005	16.84863	22.81736	23.69462
HORVU1Hr1G050480	3.031332	3.027253	3.26977	5.336278	6.174202	6.946049
HORVU1Hr1G054100	22.41476	21.28618	20.6063	20.34241	11.5366	13.68629
HORVU5Hr1G097200	0.564162	0.678742	0.769422	1.70073	1.470436	1.898617
HORVU5Hr1G073510	12.39783	11.38518	13.04696	10.54314	10.0651	9.629052
HORVU3Hr1G051080	3.361641	3.306866	5.202267	4.280972	6.036392	6.397049
Hordeum_vulgare_newGene_869	2.310112	1.628996	2.759943	1.910307	1.923131	1.865292
HORVU7Hr1G022830	3.22063	3.552204	3.030142	4.394489	4.007025	5.228658
HORVU7Hr1G109190	3.022837	2.452966	3.157066	1.903578	2.804979	2.977657
HORVU7Hr1G096010	2.584711	2.795583	2.162724	4.025655	4.427643	4.603323
Hordeum_vulgare_newGene_8541	1.801038	1.811396	1.678227	0.889749	0.10159	0.247227
HORVU0Hr1G003280	0.252525	0.43306	0.242011	1.879273	7.471994	6.752707
HORVU2Hr1G004610	11.99078	5.987235	15.26888	2.667041	0.294329	0.718311

HORVU3Hr1G004510	15.79208	14.00176	17.09848	16.24012	19.99418	20.59854
HORVU3Hr1G051330	1.812471	1.838061	2.2242	4.805198	8.013457	8.496548
HORVU3Hr1G115810	4.241055	3.467944	3.967573	0.787824	1.98433	1.816766
HORVU5Hr1G102040	2.943116	1.923715	1.625521	1.966252	0.223828	0.656448
HORVU2Hr1G119720	7.989959	7.973183	10.25078	7.154121	7.606302	7.749154
HORVU3Hr1G041560	6.235412	6.251434	6.797344	9.55388	16.22407	15.13073
HORVU2Hr1G003340	113.2542	100.1717	97.5331	71.70479	51.90514	57.96118
HORVU5Hr1G069990	5.231136	4.676563	6.231156	4.670594	3.79617	3.819107
HORVU1Hr1G049100	1.159011	0.775579	1.980265	2.289669	0.618907	1.558924
HORVU2Hr1G058850	3.831748	3.201802	4.268106	6.141259	7.423722	8.024398
HORVU3Hr1G036970	11.97263	8.724021	14.77579	28.77747	36.02402	34.66867
HORVU7Hr1G010230	5.108174	3.841459	5.748015	4.958532	6.887165	6.40884
HORVU1Hr1G063090	11.1551	12.06555	13.72749	11.92687	10.16162	11.74224
HORVU2Hr1G084500	2.382927	2.521932	2.856993	3.047837	3.662563	3.330973
HORVU2Hr1G047840	3.88086	4.315504	4.459961	4.075354	4.414773	2.734289
HORVU4Hr1G066510	17.85762	19.77857	18.25677	37.56524	34.16388	37.52041
HORVU2Hr1G088660	16.5821	15.16731	16.74349	11.75464	6.642724	8.191228
HORVU1Hr1G088870	60.2278	57.87354	80.4281	244.0532	270.4614	284.6764
HORVU0Hr1G008880	2.599642	2.579934	2.916851	2.793566	4.79403	4.744155
HORVU6Hr1G031520	1.035515	1.2255	1.070486	1.732134	1.847223	2.472051
HORVU7Hr1G048830	9.777664	9.293744	11.66967	7.508358	13.80078	12.54921
HORVU5Hr1G045860	5.612291	3.953455	5.130885	14.96555	24.72247	21.89053
HORVU3Hr1G049910	16.14429	15.92695	18.7711	11.76092	11.35	13.4982
HORVU5Hr1G021270	11.13298	10.25745	12.68345	9.944785	9.048404	8.922002
HORVU5Hr1G105900	50.07703	57.28257	89.97537	6.362488	1.213805	3.020274
HORVU2Hr1G028940	15.37864	16.79754	12.83513	22.72575	34.95027	35.62455
HORVU6Hr1G088370	30.30743	27.31598	28.01748	34.32164	42.76825	39.71876
HORVU5Hr1G104050	1.292059	1.302145	1.476959	5.444107	3.958735	4.971109
HORVU0Hr1G039040	2.108573	2.302084	2.604469	4.087895	9.251698	8.277934
HORVU1Hr1G019390	12.84291	11.2459	13.58139	13.90503	13.87263	14.65628
Hordeum_vulgare_newGene_11397	37.95224	36.33828	35.39748	22.68152	4.684057	8.459145
Hordeum_vulgare_newGene_7664	1.739168	1.807676	1.622258	3.124041	4.01863	4.588741
Hordeum_vulgare_newGene_7663	3.914973	3.456111	5.118535	10.93174	15.19068	13.51755
HORVU2Hr1G035170	0.354009	0.409346	0.467509	0.857823	4.366436	2.691749
HORVU4Hr1G012330	8.812004	8.988669	8.459877	25.94371	12.07264	14.12226
HORVU0Hr1G037040	3.46168	2.69441	1.62724	1.716733	20.27055	8.469364
HORVU7Hr1G098640	0.721598	0.647665	0.956068	0.837809	2.061888	1.631814
HORVU7Hr1G064620	3.024052	2.53493	2.459381	3.385253	2.386769	2.976564
HORVU0Hr1G015410	1.022934	1.672104	1.400587	1.179419	0.898567	1.166748
HORVU5Hr1G110900	6.12583	4.615261	6.558826	7.145941	7.395782	7.324948
HORVU2Hr1G008250	3.141121	3.746024	4.863203	0.741834	1.547948	1.600807
HORVU1Hr1G051460	4.379448	4.430535	4.505271	5.084914	7.466531	7.572447
HORVU6Hr1G074440	9.880701	9.924293	12.43886	15.55622	14.74105	15.36103
HORVU3Hr1G086510	13.59153	10.00485	12.67183	19.29045	25.70157	22.95493
Hordeum_vulgare_newGene_15327	1.059386	0.928145	0.960232	1.493973	3.03987	2.94137
HORVU7Hr1G025150	0.125056	0.261283	0.35158	1.515091	8.250855	10.72502
HORVU5Hr1G058330	21.18523	28.90104	29.50909	4.500898	0.53662	1.531365

HORVU4Hr1G056860	7.889256	10.98402	10.69089	0	0	0
HORVU1Hr1G061790	2.992756	2.833999	3.012355	3.837919	4.014359	4.638269
HORVU1Hr1G056470	5.59802	6.684322	6.973717	4.257362	7.929213	7.445602
HORVU3Hr1G007130	8.771096	9.106973	9.873119	9.475596	11.65476	12.79266
HORVU6Hr1G057770	2.733391	2.785085	2.926662	3.515913	9.527689	4.763871
HORVU5Hr1G095540	5.417902	6.183363	8.542786	4.473183	3.61801	3.384141
HORVU1Hr1G050810	7.947996	6.282233	8.287446	8.659612	10.10942	10.23188
HORVU7Hr1G018230	1.60365	2.524112	2.67903	3.537065	6.435959	5.633347
Hordeum_vulgare_newGene_5580	2.691168	2.817813	4.044562	2.856135	1.239676	1.623222
HORVU1Hr1G027770	20.98612	19.88711	20.50845	32.57324	27.33516	28.36672
HORVU5Hr1G093260	3.532568	4.836784	6.639753	6.434339	27.53324	27.06265
HORVU4Hr1G069380	3.420677	3.41998	4.454924	10.81927	9.40341	9.549363
HORVU4Hr1G040900	7.603905	6.931626	9.113074	8.424463	9.751758	10.05895
HORVU4Hr1G007330	5.711453	5.835789	5.784856	4.688442	4.664221	5.786322
HORVU7Hr1G054730	7.803702	7.789799	10.31206	12.62399	16.93239	16.99286
HORVU1Hr1G004230	7.435719	7.576649	7.693001	16.11691	14.56815	15.53338
Hordeum_vulgare_newGene_9601	2.552061	2.204226	1.754423	2.631185	2.611924	2.604531
HORVU3Hr1G078200	1.586113	1.394278	1.653842	3.479795	6.655273	8.052127
HORVU5Hr1G002150	890.1505	758.7244	1110.72	794.6967	840.2877	761.9182
HORVU7Hr1G011210	1.707319	2.159187	1.815471	3.194989	4.658188	3.392785
HORVU4Hr1G039020	20.1644	23.47585	25.35419	23.46764	27.61302	27.30971
HORVU7Hr1G068990	6.804689	7.542878	9.04515	7.791113	13.13091	14.14291
HORVU1Hr1G066410	1.014861	1.667299	1.696889	1.168949	2.677658	3.184464
HORVU5Hr1G097940	5.803703	3.547314	5.933618	15.61728	12.79149	12.36543
HORVU4Hr1G068380	11.51127	13.149	11.38811	11.3288	7.651666	10.54055
HORVU1Hr1G005180	2.613754	0.47996	0.816196	3.045041	2.755142	2.631361
HORVU3Hr1G057020	3.968138	3.55353	4.527549	6.148066	5.999728	6.962186
HORVU3Hr1G072460	10.7775	10.94546	10.88207	6.550614	5.969093	5.460454
HORVU3Hr1G022600	2.474214	2.573276	3.588563	2.02666	3.669824	2.712503
HORVU5Hr1G112670	6.377264	5.149251	6.753564	5.95374	12.45624	12.96255
HORVU6Hr1G087100	4.737561	5.879945	8.094255	4.684663	6.690895	7.232343
HORVU1Hr1G004580	2.596252	3.651427	7.115729	0.581431	0.035061	0.362845
HORVU0Hr1G016330	105.566	86.11279	109.5669	83.76352	67.73338	81.56086
HORVU5Hr1G106020	0.058752	0	0.11317	4.749259	0.381993	2.866815
HORVU7Hr1G092070	0.059472	0	0.157358	18.44412	13.43198	19.18946
HORVU4Hr1G011750	10.44616	10.21439	13.43599	9.68328	14.33258	13.89239
Hordeum_vulgare_newGene_3393	0.851412	0.919329	0.624832	1.355366	1.45681	1.264069
HORVU5Hr1G122170	33.16194	49.37202	61.6405	38.28568	88.79357	85.16214
Hordeum_vulgare_newGene_3390	112.8498	90.92347	91.57723	85.93501	103.7027	98.65638
HORVU6Hr1G011860	2.575273	2.197062	3.196265	0.295027	0.097814	0.43496
HORVU6Hr1G035470	3.452012	2.985287	4.282097	8.07349	6.172932	5.369961
HORVU7Hr1G051810	6.941536	7.873271	11.273	1.713372	1.015848	1.248784
HORVU4Hr1G049680	32.30372	32.42025	27.39992	26.46213	27.02952	28.97714
HORVU6Hr1G001160	0.493412	0.061048	0.09608	2.237693	2.019873	2.995477
HORVU6Hr1G091660	0.142438	0.342892	0	2.403342	5.867187	6.017664
HORVU1Hr1G053890	2.304672	1.879673	2.474778	2.274124	2.429471	3.137881
HORVU5Hr1G084160	2.954446	3.031597	3.882287	3.177948	3.206419	3.343234

HORVU3Hr1G037780	10.90142	12.1516	12.94305	3.214653	3.88999	3.468623
HORVU5Hr1G110120	57.47736	50.04442	60.62434	39.85768	35.82214	36.81113
HORVU4Hr1G063710	1.68163	1.922591	1.960119	3.170911	3.851881	4.220457
HORVU2Hr1G126100	6.891486	5.146471	5.726472	10.10058	8.653024	9.719473
HORVU1Hr1G063930	7.022388	7.050009	7.991187	7.589209	14.29592	13.06825
HORVU7Hr1G091520	6.670144	6.546027	6.738544	5.663093	5.384459	7.179325
HORVU5Hr1G114630	2.58231	3.832058	3.669512	2.499618	2.637452	2.690905
HORVU0Hr1G021200	2.392145	2.600557	3.064642	1.379576	1.679592	2.140498
HORVU1Hr1G002320	40.08941	39.59894	51.86404	33.12875	30.53152	28.61664
HORVU3Hr1G023260	17.62081	15.93125	21.33651	17.15841	23.98828	23.54867
HORVU5Hr1G005210	4.111261	1.697734	5.299114	0.971591	0.749762	1.047992
Hordeum_vulgare_newGene_11980	2.834002	3.080699	3.471059	4.488662	3.682832	4.571502
HORVU4Hr1G065920	15.79445	13.58226	17.97367	31.00836	37.93949	30.81588
HORVU6Hr1G091440	1.49226	0.713781	1.537376	1.01563	0.700984	0.580764
HORVU5Hr1G105040	0.745963	0.952044	1.35105	1.72253	2.239009	2.347309
HORVU7Hr1G057410	7.102912	6.697149	7.596263	3.749878	3.707577	3.432274
HORVU2Hr1G013170	31.05114	30.97604	35.73945	17.13014	22.08074	23.53969
HORVU6Hr1G010400	0.035064	0.124617	0.087328	3.260879	1.513739	2.362065
HORVU4Hr1G066290	3.291181	4.168982	4.957697	3.893405	5.576909	5.84043
HORVU5Hr1G119060	8.180766	8.153051	7.46533	8.51965	5.950289	6.714604
HORVU4Hr1G062040	1.659043	1.662965	2.134992	1.04818	3.870336	3.85146
HORVU1Hr1G094050	12.82688	12.1208	15.66493	22.83773	29.92087	28.59519
HORVU1Hr1G015720	1.263031	1.342061	1.595834	1.406107	1.130739	1.668354
HORVU0Hr1G014400	0.087321	1.445297	1.038479	1.580636	5.241328	5.085896
HORVU1Hr1G058250	15.26514	15.52004	13.25253	10.06782	8.813891	9.382371
HORVU5Hr1G002130	473.9954	404.6082	585.1418	360.4085	401.276	357.2491
HORVU6Hr1G088440	7.813722	4.168283	8.60278	0.176309	0.19605	0.19816
HORVU4Hr1G026030	27.42576	23.94386	26.47851	22.00337	33.15639	32.01953
HORVU1Hr1G017380	33.1113	28.03719	28.0643	19.13018	19.61955	18.98174
HORVU7Hr1G113500	63.55397	56.33042	83.93553	81.00381	100.0288	95.0482
HORVU6Hr1G094190	2.249659	2.361415	1.862216	2.955831	3.186912	2.783432
HORVU2Hr1G037820	2.37796	1.662038	1.841249	3.183286	2.690597	2.750732
HORVU2Hr1G002710	9.775533	8.589003	8.452357	9.266258	9.63521	10.24759
HORVU1Hr1G064080	14136.26	13875.37	10244.68	15443	6325.132	7413.604
HORVU2Hr1G096070	2.34415	3.46497	3.040719	2.320833	12.48285	11.93954
HORVU5Hr1G002970	41.21112	33.9153	39.70657	33.33741	34.96675	37.31378
HORVU2Hr1G126750	9.120929	8.317217	6.066337	10.05936	18.69622	16.35273
HORVU2Hr1G113190	3.856142	5.165811	4.634467	6.340658	10.10604	8.978293
HORVU3Hr1G088800	1.000217	0.722508	1.33508	1.03121	1.39664	1.768007
HORVU0Hr1G015750	26.29854	26.31141	36.69841	25.65609	32.06082	31.68031
Hordeum_vulgare_newGene_9031	6.159449	4.375219	1.865898	0	0	0
HORVU0Hr1G022790	26.69981	34.34214	29.43319	53.93797	82.44828	73.5319
Hordeum_vulgare_newGene_9034	0.909628	1.06453	1.049384	2.783989	2.611548	3.329712
HORVU6Hr1G078990	7.390746	8.111921	7.245753	13.05203	12.15375	12.89661
Hordeum_vulgare_newGene_9037	42.79863	31.38734	38.28074	129.5202	125.4866	127.2839
Hordeum_vulgare_newGene_2773	2.594354	3.344403	2.777758	2.660077	6.083205	4.303547
HORVU4Hr1G021260	3.896487	3.776892	5.791279	0	0	0

HORVU4Hr1G001170	113.6077	114.3762	223.3231	95.11259	192.0002	186.8326
HORVU2Hr1G101920	5.71821	3.967134	6.955335	1.834194	0.951124	2.066656
Hordeum_vulgare_newGene_2779	7.278933	8.098419	8.889163	7.375786	9.720107	9.635601
HORVU5Hr1G030040	14.41781	12.99381	17.59407	16.98074	22.30551	21.21171
HORVU7Hr1G042880	65.01407	56.93732	66.80411	116.0138	160.9551	167.8793
HORVU1Hr1G004910	2.549981	3.053789	3.105803	1.580422	3.522445	3.117893
HORVU2Hr1G060770	10.0608	8.450021	9.331051	3.877991	7.116869	7.233628
HORVU5Hr1G007840	1.028978	1.127247	1.382684	0.985397	0.585328	1.172464
HORVU6Hr1G012710	9.175671	8.55575	10.97409	7.787193	10.38587	10.47047
HORVU6Hr1G056140	0.522113	0.719225	0.656698	1.410543	2.236741	2.671666
Hordeum_vulgare_newGene_9782	3.388401	4.004334	3.092909	8.382122	6.152947	5.882812
HORVU4Hr1G020130	25.65086	26.56529	28.47574	87.20487	79.73343	69.71665
HORVU2Hr1G099570	1.615706	2.110991	2.372496	0.566981	0.083641	0.337342
HORVU2Hr1G096380	1.744484	1.760748	3.062393	1.58046	1.358461	2.078479
HORVU2Hr1G036440	1.513657	2.167692	5.367247	4.328546	11.51443	10.88819
HORVU7Hr1G083990	3.467607	3.907432	4.005091	4.712132	5.407698	6.864763
HORVU5Hr1G078050	4.584157	4.602651	5.371866	11.14181	12.79304	13.14959
Hordeum_vulgare_newGene_8789	4.604968	5.401059	4.987829	8.001853	6.303705	7.593271
HORVU7Hr1G003840	0.829056	0.642442	1.26051	2.956695	4.590541	4.052718
HORVU3Hr1G096290	3.652792	3.006331	3.846263	7.552158	6.085769	7.738542
HORVU5Hr1G116380	0.765967	0.978556	1.067372	1.074981	2.053746	2.521994
HORVU7Hr1G072610	0.706043	0.55462	1.026705	1.138206	1.2769	1.700324
HORVU5Hr1G098420	1.660485	1.934744	1.339273	0.705382	0.518689	1.051707
HORVU5Hr1G053320	0.326306	0.307482	0.309554	0.953116	2.847663	2.918321
HORVU7Hr1G086010	14.02047	10.01374	16.70952	7.687979	4.162544	7.236475
HORVU7Hr1G104350	3.961047	2.610733	5.581823	5.150208	3.843468	5.435722
HORVU7Hr1G081290	74.81117	76.90148	90.76399	71.15475	85.86503	81.27718
HORVU4Hr1G060190	9.262592	9.551918	11.26829	9.2035	17.44875	17.11912
HORVU5Hr1G074880	1.923176	2.655397	1.122447	1.284518	11.89658	8.195464
HORVU2Hr1G031870	7.297102	7.903945	7.183246	13.92045	15.2082	16.53749
HORVU6Hr1G070810	44.57845	43.88407	42.85072	29.15777	28.90801	28.89851
HORVU3Hr1G073470	3.355618	3.687194	3.048047	3.818182	3.852713	3.932246
HORVU2Hr1G092230	16.00635	14.15136	14.36539	11.59966	12.63808	13.58504
HORVU5Hr1G120250	3.907105	4.247778	5.090819	8.68752	16.15739	14.87865
HORVU3Hr1G061930	9.076459	7.243298	10.94911	8.330946	10.1462	8.64198
HORVU2Hr1G119430	2.877391	3.547656	2.771854	0.335137	0.225654	0.217295
HORVU5Hr1G049980	1.16286	0.966586	1.145207	3.409581	4.833937	5.550567
HORVU7Hr1G059460	4.161046	3.903011	5.46194	9.212119	15.53196	17.12671
HORVU1Hr1G065770	5.141794	4.621744	5.65559	7.785711	10.48386	10.62378
HORVU7Hr1G027370	0.52538	0.265977	0.425238	1.124859	26.86909	27.21319
HORVU2Hr1G115730	19.40147	18.76319	23.91587	23.46038	23.27819	26.87818
HORVU2Hr1G111600	129.1016	105.7208	127.1817	125.0734	128.868	124.7864
Hordeum_vulgare_newGene_1453	2.232375	1.693586	2.604817	2.873568	5.970591	5.354135
Hordeum_vulgare_newGene_10719	1.322138	1.375037	1.036717	1.353655	0.584317	1.301147
Hordeum_vulgare_newGene_10717	18.11738	15.6846	21.20107	26.55089	37.36673	33.47787
Hordeum_vulgare_newGene_10714	3.167402	2.522937	4.245693	5.814806	9.918726	9.757772
Hordeum_vulgare_newGene_10715	19.19276	17.26288	17.55398	36.55925	31.88297	31.29831

HORVU2Hr1G039400	1.655884	1.856021	1.082251	1.126484	8.519524	7.800135
Hordeum_vulgare_newGene_10713	38.05399	39.10668	53.83558	44.22341	65.91972	70.24608
HORVU5Hr1G049670	11.07785	12.45301	13.17562	8.666951	9.018585	10.3858
HORVU5Hr1G033310	11.19659	10.69923	14.91612	17.4342	28.7605	26.09865
HORVU6Hr1G071960	0	0.021175	0.042567	0.412992	3.413733	3.170385
HORVU3Hr1G017610	8.349088	7.553771	9.688831	3.939589	3.097894	3.278714
HORVU1Hr1G041740	22.97665	20.76046	17.86902	14.99052	13.927	15.56814
Hordeum_vulgare_newGene_13821	14.89771	14.91289	13.89701	11.78086	14.31695	14.33899
HORVU5Hr1G073820	2.978338	2.875556	3.045957	4.37624	5.865343	5.9761
HORVU4Hr1G077480	1.881161	2.22461	1.909632	1.034342	2.462046	2.42905
HORVU2Hr1G021020	11.1258	8.768204	7.645861	11.39509	10.54948	11.38266
HORVU7Hr1G029420	1.184918	1.083267	1.6029	1.419012	1.927463	1.482313
HORVU7Hr1G021230	9.25564	7.717546	9.572607	11.44558	16.24038	16.02593
HORVU2Hr1G029560	0.953594	0.830944	0.825381	2.316085	0.889467	1.271695
HORVU7Hr1G057390	10.74413	8.406174	13.13831	16.55358	31.36402	30.25723
Hordeum_vulgare_newGene_4667	1.797014	1.228386	1.189537	1.036199	1.516119	1.143763
Hordeum_vulgare_newGene_4662	2.220369	2.498823	2.850983	3.04655	3.982086	3.167378
Hordeum_vulgare_newGene_4660	9.727392	9.316396	8.990544	10.38717	14.21883	12.01576
Hordeum_vulgare_newGene_4661	0	0	0	6.201936	5.559124	6.801601
Hordeum_vulgare_newGene_4669	1.256693	1.443071	1.78028	1.279935	1.905655	1.840682
HORVU1Hr1G072250	13.44733	9.287491	12.20182	36.11563	61.83012	58.24717
HORVU3Hr1G066680	0.665028	0.611657	0.921199	1.156354	1.647364	1.286608
HORVU7Hr1G058730	13.81435	10.84453	15.9567	14.04199	17.24873	18.82118
HORVU5Hr1G019080	9.79321	8.397059	10.14004	7.821566	6.941321	7.71018
HORVU7Hr1G031000	4.402146	4.453728	4.577411	0.070522	0.266964	0.083359
HORVU3Hr1G090190	365.6398	420.4736	346.9444	1037.724	1569.711	1326.774
HORVU3Hr1G010620	8.666463	8.748345	9.376863	8.637938	7.291039	7.829818
Hordeum_vulgare_newGene_7316	10.41001	10.47153	9.20109	17.66183	19.2622	14.46959
HORVU1Hr1G075150	172.4802	155.3171	167.0572	70.25918	31.73051	36.20694
HORVU1Hr1G038370	8.973408	9.877185	7.627848	9.265267	10.69218	10.63355
HORVU3Hr1G063300	7.15682	7.507473	8.52898	10.87417	16.27683	16.57229
HORVU2Hr1G035400	30.59643	26.68294	31.06736	25.3465	29.75502	29.10681
Hordeum_vulgare_newGene_207	20.91773	21.05535	30.27941	14.92945	15.90667	16.77312
Hordeum_vulgare_newGene_204	2.833662	3.677014	3.590396	4.161924	3.208647	3.809181
Hordeum_vulgare_newGene_201	3.469131	2.82488	3.162986	2.300604	0.618228	1.250832
HORVU3Hr1G080100	4.508008	3.057553	4.631436	2.05436	1.627826	2.863488
HORVU3Hr1G105470	2.347477	1.829731	1.947777	3.444233	2.528977	3.562683
Hordeum_vulgare_newGene_2899	6.458112	6.694072	6.588949	4.72989	5.545999	5.607296
HORVU2Hr1G028210	51.41842	38.89651	47.86157	46.39533	57.35588	55.57001
Hordeum_vulgare_newGene_2896	3.079745	5.524674	2.978875	9.037084	0.959029	3.097266
Hordeum_vulgare_newGene_2895	1.917121	2.625048	2.058734	2.37792	2.28101	3.144343
HORVU3Hr1G086750	7.325704	6.104329	7.96003	10.11882	11.38736	12.58621
HORVU3Hr1G039200	7.336602	5.666411	8.706941	8.553977	8.149586	8.189708
HORVU3Hr1G034630	4.993578	5.728015	6.578452	5.424333	6.208574	5.988415
HORVU5Hr1G041520	10.06971	10.42101	12.21441	11.65649	11.79192	11.47483
HORVU7Hr1G038180	20.56804	20.69016	25.16465	20.02136	26.81373	26.67448
Hordeum_vulgare_newGene_13674	3.730105	3.639683	3.455452	2.942266	4.388658	4.555937

Hordeum_vulgare_newGene_13675	1.242833	1.248223	2.111524	0.703867	1.795667	0.483331
Hordeum_vulgare_newGene_13673	12.42684	11.55969	14.56335	8.010865	7.724198	7.843046
HORVU5Hr1G062940	358.9642	338.3846	432.0437	418.4957	472.0896	422.4422
HORVU3Hr1G081250	1.050085	0.854927	1.590934	1.909695	1.480175	1.530525
HORVU5Hr1G068630	22.90598	20.10257	26.59555	22.92665	27.16281	28.77381
HORVU7Hr1G079830	0	0	0.015959	7.732756	8.807666	9.095541
HORVU3Hr1G087480	8.901705	3.98873	6.812187	21.75857	21.93821	17.49723
HORVU0Hr1G040440	2.244376	1.892259	2.140134	2.518819	2.889202	2.876347
HORVU1Hr1G027570	9.140012	5.890405	9.70974	5.623541	4.829505	4.275639
Hordeum_vulgare_newGene_2238	13.66425	15.55524	15.8712	3.741827	4.350929	4.123425
HORVU0Hr1G005400	3.542399	4.937725	4.256168	0.81045	1.224008	1.339155
Hordeum_vulgare_newGene_2233	1.69163	2.257862	1.943479	4.280671	5.161879	6.501655
Hordeum_vulgare_newGene_2231	1.951721	2.126995	1.878546	3.228271	4.428663	4.504893
HORVU7Hr1G054530	7.664417	8.718515	11.01335	26.9109	35.39268	31.45046
HORVU4Hr1G017140	11.64177	10.97928	11.66476	13.97166	23.49141	23.34224
HORVU5Hr1G079040	0.207978	0.193057	0.224484	5.568961	0.043782	1.524411
HORVU5Hr1G023740	25.19552	11.42603	22.49402	3.422348	0.216961	3.90481
HORVU7Hr1G108150	1.095559	0.470362	1.791377	1.120817	0.869146	0.891773
HORVU3Hr1G012410	17.20578	17.35114	20.21655	28.70913	40.67769	39.85996
HORVU7Hr1G028610	18.91446	16.38134	19.64866	21.43277	31.5764	29.44876
HORVU3Hr1G003140	9.72833	10.32242	10.43667	18.91964	27.25157	25.63634
HORVU2Hr1G024840	6.65452	8.739645	9.590274	7.301477	10.47815	11.70512
HORVU5Hr1G062490	62.00565	63.78369	53.65705	33.70564	19.54186	25.38825
HORVU2Hr1G030860	17.35839	14.88384	16.6205	13.94987	12.89058	13.4216
HORVU6Hr1G058790	3.106511	3.94579	2.343888	4.357276	8.158675	9.076107
HORVU4Hr1G065840	5.004819	5.804935	6.168714	6.629557	6.937537	7.280723
HORVU1Hr1G059780	1.447139	2.400597	1.157592	1.821536	2.797567	3.027285
HORVU5Hr1G061760	1.281862	1.839057	1.769839	0.442589	0.291224	0.616659
HORVU4Hr1G011590	6.862159	5.949576	7.696672	5.631	6.925901	6.869824
HORVU7Hr1G097570	5.425218	6.906312	7.598415	4.399996	4.074444	5.339943
HORVU7Hr1G087130	2.996511	3.060865	4.134296	3.475388	2.779256	2.575358
HORVU2Hr1G004790	0	0	0	2.587384	2.019773	2.524445
HORVU2Hr1G075810	22.95902	21.77891	24.78864	28.47866	38.92565	35.82224
HORVU5Hr1G059200	9.324244	9.090473	10.12502	14.43203	11.63737	15.80965
HORVU4Hr1G002050	0.111762	0.029267	0.20837	10.12291	13.97777	13.17422
Hordeum_vulgare_newGene_8995	40.6728	33.22164	45.29046	34.9996	54.9436	54.63804
Hordeum_vulgare_newGene_8990	32.81622	39.96676	42.44403	8.109239	4.586047	4.278336
Hordeum_vulgare_newGene_8992	1.936137	1.434869	1.163812	1.705717	1.980288	1.47919
HORVU1Hr1G021930	0.358921	0.823186	0.808317	1.054638	1.476042	1.947753
Hordeum_vulgare_newGene_8999	1.830789	2.861785	3.030837	3.279387	2.351564	3.446884
HORVU6Hr1G058240	2.364457	2.020978	2.85212	5.279733	6.189736	4.942048
HORVU2Hr1G094650	6.467166	7.742982	4.458274	12.06362	18.71977	13.02619
HORVU6Hr1G060610	28.53711	22.93241	30.6983	30.19467	27.64706	23.92151
Hordeum_vulgare_newGene_2513	1.342195	1.046735	1.547905	1.767691	1.524625	2.115476
HORVU7Hr1G020880	53.98864	43.98434	58.46849	84.36041	94.52778	95.18445
HORVU1Hr1G018340	0.44265	0.972506	2.320447	1.272566	1.358074	1.439656
HORVU1Hr1G062530	1.540698	1.532206	1.826011	1.899333	2.599242	2.985324

HORVU2Hr1G088780	5.065756	5.357597	6.185932	6.838128	7.418709	8.114773
HORVU4Hr1G012480	0.949181	0.896606	1.49259	0.871267	1.463479	1.61502
HORVU2Hr1G006480	2.346994	1.982418	2.545155	2.930584	3.106785	3.348738
HORVU4Hr1G018260	13.37081	10.63661	14.70155	11.08974	11.40489	13.59861
HORVU7Hr1G095550	9.376588	8.869882	9.369519	22.44275	17.33198	19.77089
HORVU6Hr1G066510	4.166071	4.273946	5.163853	4.302037	5.418087	5.623308
HORVU6Hr1G036990	33.49362	28.66046	39.77251	35.86516	36.52229	36.17834
HORVU1Hr1G079130	0.910355	1.037852	1.140028	1.808609	3.112508	3.834352
HORVU3Hr1G014510	0.141637	0.188948	0.281673	1.103101	3.541369	2.879315
HORVU1Hr1G058490	1.829419	1.236536	2.920438	23.98208	42.10049	39.73039
HORVU0Hr1G008960	0	1.688718	0.024207	1.426996	4.534922	6.718813
Hordeum_vulgare_newGene_1151	14.42087	14.58972	15.30254	21.9857	27.62582	27.11692
HORVU1Hr1G073640	119.4372	104.4216	151.4922	106.1823	155.9305	149.2082
HORVU2Hr1G016470	2.236412	2.416123	2.544913	0	0	0
HORVU1Hr1G053340	16.43415	12.99834	16.65238	17.27081	16.79234	16.55925
HORVU2Hr1G078290	39.14047	34.57167	37.19427	42.58206	93.86454	81.64156
Hordeum_vulgare_newGene_4504	0.805941	0.729507	0.911317	2.593619	0.099893	1.182
Hordeum_vulgare_newGene_4501	3.307716	3.186795	2.612877	4.640175	4.039178	4.787085
HORVU4Hr1G015450	18.55257	19.47749	14.93072	24.18952	19.16711	22.63782
HORVU6Hr1G085980	19.25501	21.09172	27.81318	10.80845	13.68827	13.33962
HORVU2Hr1G125580	1.209844	1.34185	1.28588	2.978777	2.516336	3.228047
HORVU2Hr1G125630	3.101505	2.890682	3.253802	2.893206	5.665504	4.710742
HORVU5Hr1G125280	10.5498	9.390336	10.71448	3.159006	3.99301	4.20365
HORVU1Hr1G007600	21.61155	19.65916	25.72538	22.14563	23.41337	25.7461
HORVU0Hr1G028710	12.64702	13.32181	10.48039	14.55476	13.2776	12.87075
HORVU4Hr1G012100	3.599363	3.338916	3.661466	1.784122	0.851261	0.91351
HORVU1Hr1G017050	139.8932	108.7832	155.8346	84.32695	83.29887	86.48918
HORVU1Hr1G089970	4.893287	3.918163	5.795601	1.374598	1.386057	1.679083
HORVU1Hr1G090930	5.977685	3.478935	11.02859	2.873115	1.678448	1.072619
HORVU2Hr1G002480	9.122831	9.007738	10.02447	8.962019	7.928037	9.470685
HORVU0Hr1G013340	1.693839	1.80057	2.056702	2.547014	3.752613	3.004659
HORVU7Hr1G053260	0.426132	0.465238	0.44511	2.306537	3.065954	3.079483
HORVU4Hr1G001770	6.480999	3.418442	6.230659	5.759382	22.59605	26.66882
HORVU6Hr1G087720	12.58542	10.34471	11.79468	20.01575	28.47429	28.17958
HORVU5Hr1G018350	12.42936	12.36235	12.92934	18.14533	12.12803	18.26788
HORVU3Hr1G046970	6.079136	5.179148	6.741596	8.08327	10.4052	10.69908
HORVU1Hr1G044020	5.565488	5.737558	7.188974	5.996429	7.8446	8.350598
HORVU4Hr1G081310	49.46605	52.91088	69.44593	10.65349	16.64642	16.58204
HORVU4Hr1G016410	29.57969	26.44157	38.92558	16.15802	36.62415	37.92903
HORVU2Hr1G099770	45.22743	42.68493	45.90452	55.21775	68.85324	61.99598
HORVU3Hr1G005980	12.55321	11.33816	12.65422	5.887043	6.57446	5.967048
HORVU6Hr1G081600	1.989129	2.065017	3.005422	3.605176	5.445244	4.229645
HORVU1Hr1G068430	2.590201	1.552769	2.257546	2.411083	3.034742	1.942745
HORVU5Hr1G124880	0.93422	0.74381	0.504537	6.060276	6.003441	4.898003
HORVU6Hr1G006050	24.37077	27.55578	20.08301	20.98462	23.93906	22.91186
HORVU5Hr1G125420	11.79701	15.85813	15.81455	10.14315	9.89967	9.937476
HORVU1Hr1G055810	5.720663	7.895945	6.656625	6.492293	5.404448	5.609173

HORVU2Hr1G112000	20.22529	20.44997	25.29469	17.57337	26.45246	26.55007
HORVU5Hr1G056170	4.220729	4.534174	3.913201	3.21517	8.90338	7.953872
HORVU3Hr1G033500	3.320143	2.614887	3.30465	6.068485	5.758404	4.332834
HORVU2Hr1G028880	0.728458	1.247601	1.287099	0.403373	1.766099	1.639145
Hordeum_vulgare_newGene_11024	0.504287	0.274298	0.403306	2.77448	1.805816	1.698445
HORVU3Hr1G073670	0.590837	0.64994	0.6481	1.73677	2.408611	2.478473
HORVU5Hr1G111840	46.14874	38.33899	46.06824	25.84576	13.20917	14.28949
HORVU3Hr1G072260	6.55363	5.818988	7.469132	6.612863	8.32022	8.859803
HORVU2Hr1G041480	1.415761	1.054936	1.086327	2.624166	3.692334	3.099013
HORVU1Hr1G080160	1.907172	1.722137	2.345041	3.161188	2.420738	2.561777
HORVU7Hr1G040290	5.036047	5.14802	5.46672	7.499885	11.71684	12.42742
HORVU4Hr1G023490	1.092153	0.875772	1.51656	1.463053	4.631567	5.189579
HORVU6Hr1G034660	1.280983	1.130049	1.265179	1.59164	2.064168	2.120041
HORVU7Hr1G087610	1.513819	1.047298	1.042993	2.243167	1.542145	2.341243
HORVU3Hr1G037080	12.30181	11.24943	14.38712	9.600662	11.26998	11.30061
HORVU5Hr1G122810	21.23701	19.04795	23.22697	13.24381	13.67858	14.77332
HORVU7Hr1G112900	8.903026	7.329706	8.916179	2.649985	1.662853	2.390585
HORVU4Hr1G026140	2.38899	2.013481	3.438313	2.266015	5.64569	5.379395
HORVU7Hr1G018000	2.597424	2.764033	2.818542	2.819166	5.692879	4.641199
HORVU3Hr1G109540	7.25535	7.388519	7.905016	4.496027	1.039576	1.758951
HORVU3Hr1G038300	17.69454	13.96684	13.83525	24.11908	23.2375	25.17048
HORVU5Hr1G016350	15.5741	13.1356	14.70101	31.60393	40.72753	38.39505
HORVU2Hr1G064900	1.364235	2.087416	2.228163	1.269098	2.829659	2.291275
HORVU1Hr1G065950	0.809168	1.157779	0.852203	2.12429	1.974955	3.158335
HORVU2Hr1G109100	6.238799	5.865468	6.963682	2.493221	0.852808	1.633557
HORVU3Hr1G082260	3.890726	3.718716	3.99416	5.939787	8.368431	7.303459
HORVU1Hr1G062980	21.35583	20.17738	22.05959	23.4758	25.17878	23.84186
Hordeum_vulgare_newGene_2306	2.374125	2.727621	2.574624	3.05012	2.07383	2.941581
HORVU7Hr1G074920	1.003706	0.870205	1.070608	1.575277	1.912635	1.406147
HORVU3Hr1G026990	4.69104	4.469372	4.292226	9.81803	7.271193	8.562659
HORVU7Hr1G040430	1209.693	1196.548	1022.367	1046.982	955.8191	962.2473
HORVU1Hr1G043280	3.06534	3.367263	3.546903	4.300942	5.163008	5.300446
HORVU1Hr1G067550	1.99719	1.282369	2.283742	1.721234	1.551419	2.289924
HORVU4Hr1G014190	14.29932	21.32902	21.8895	0.263732	0.065231	0.115198
HORVU5Hr1G096800	2.860853	2.974865	3.419429	6.375267	10.1502	9.87609
HORVU3Hr1G037330	1.166867	0.818657	0.754986	1.488403	1.437074	1.793369
HORVU1Hr1G002820	0.058361	8.247181	0.708891	16.533	0.065908	0.08348
HORVU6Hr1G093090	9.269153	8.199506	9.212866	7.485841	7.539308	8.149273
HORVU7Hr1G041520	22.2162	20.90094	24.84143	20.72	22.73922	22.44452
HORVU4Hr1G053670	47.01719	46.68319	47.18714	45.05041	64.88697	54.62235
HORVU3Hr1G066440	12.12223	10.05561	13.58696	17.36995	19.94729	16.7338
HORVU4Hr1G063690	9.098346	7.472365	11.64342	14.6868	11.78073	11.94906
HORVU3Hr1G093990	1.053002	0.706932	0.610717	3.210137	4.807546	4.671108
HORVU5Hr1G109420	3.663292	2.84518	3.545262	4.622869	5.378016	5.329952
HORVU4Hr1G014440	0.772688	0.798972	0.708708	1.093647	1.36945	1.43522
Hordeum_vulgare_newGene_11534	2.840221	2.443796	3.191981	1.956748	2.55454	2.855004
HORVU1Hr1G002790	0	0	0	12.13377	0.034718	0

HORVU2Hr1G097990	0.338974	0.178659	0.468183	2.915365	16.06542	10.41326
HORVU6Hr1G093540	6.207624	6.136419	5.876407	5.280168	9.389557	8.903396
HORVU3Hr1G075090	0.686255	0.745544	1.100511	0.639759	4.314047	6.360323
HORVU5Hr1G050990	39.22023	39.82238	32.9274	207.1349	368.527	362.5342
Hordeum_vulgare_newGene_13702	0.497221	0.48032	0.702024	2.299163	5.824797	5.815917
HORVU7Hr1G120660	2.472132	2.838742	3.002334	10.04965	11.97324	13.12252
Hordeum_vulgare_newGene_13703	19.40784	18.11798	23.16881	12.82948	14.3991	15.2482
Hordeum_vulgare_newGene_13704	1.494474	1.54293	1.696129	2.330391	3.335731	2.327108
HORVU3Hr1G086190	1.692944	1.729097	1.275091	6.31124	9.239252	9.9248
HORVU4Hr1G084600	4.029395	2.687469	4.90067	2.263797	1.107603	2.1635
HORVU2Hr1G102840	57.26059	50.16177	52.53538	41.026	41.57328	43.67421
HORVU3Hr1G024590	10.78664	7.227277	10.61638	9.880453	7.46363	10.38889
Hordeum_vulgare_newGene_12705	0.430317	0.517586	0.422227	3.87797	9.419747	8.335593
Hordeum_vulgare_newGene_12704	0.971326	0.872197	1.412784	11.2452	29.37286	26.98825
Hordeum_vulgare_newGene_12701	5.926283	7.409921	4.485911	5.775928	1.46018	2.270776
HORVU7Hr1G047800	2.563285	3.35419	4.000352	6.411837	4.859701	6.207697
HORVU3Hr1G063670	78.00672	79.07462	106.5165	75.89178	154.8175	159.8588
HORVU5Hr1G025310	6.114735	6.122123	7.010338	10.1952	17.31976	16.07212
HORVU5Hr1G121730	12.67299	13.59614	16.60086	4.620984	5.286835	5.050796
Hordeum_vulgare_newGene_13077	1.139818	1.959814	1.571298	1.863926	1.77782	2.172517
Hordeum_vulgare_newGene_13075	12.5555	11.5779	17.0906	13.69101	28.17712	27.09678
Hordeum_vulgare_newGene_13072	7.605308	9.755481	9.305883	5.786511	6.744904	7.539953
HORVU3Hr1G116880	13.77066	13.19463	14.88791	12.04372	13.36689	13.46809
Hordeum_vulgare_newGene_13071	15.20698	16.94421	16.01152	12.61239	12.89991	13.30518
HORVU3Hr1G090970	1.768451	1.481348	2.050897	7.824836	15.71008	14.43182
Hordeum_vulgare_newGene_1099	9.555631	10.69851	8.753984	7.028053	4.806834	5.975356
Hordeum_vulgare_newGene_13079	4.837443	6.204528	5.769692	6.992448	11.70966	11.28405
Hordeum_vulgare_newGene_3308	22.89511	25.61045	26.20656	35.4423	28.06523	32.53577
HORVU4Hr1G022450	5.057221	5.510993	5.558782	5.197611	6.525268	7.004327
HORVU1Hr1G017220	2.221256	2.194194	2.135437	1.65901	2.256676	2.328587
Hordeum_vulgare_newGene_7881	9.629525	9.408574	9.923929	8.173914	6.512263	7.366077
HORVU7Hr1G077780	2.226645	2.704168	2.05767	6.420255	83.84037	78.53504
HORVU5Hr1G115170	5.539558	6.343871	6.866072	4.615947	4.579964	4.767891
HORVU2Hr1G103710	0.420376	0.763821	0.939776	1.78441	2.592238	3.905907
HORVU2Hr1G070440	14.37936	14.30211	12.37462	10.24851	5.61678	7.873569
HORVU3Hr1G051610	2.274668	1.708142	2.053632	16.13088	11.63493	11.53784
HORVU7Hr1G076780	37.77334	41.35758	40.77576	37.76245	44.72467	52.24294
HORVU4Hr1G086600	1.294425	0.927479	1.088669	1.067071	1.083788	1.120172
HORVU7Hr1G078320	3.147889	2.655498	2.595158	5.12271	9.103375	9.187862
HORVU4Hr1G026200	2.684028	3.348727	3.596073	8.509148	6.8958	5.944886
HORVU1Hr1G038820	8.441756	8.526078	6.992525	7.844002	8.826539	9.32044
HORVU1Hr1G053840	32.17422	27.13875	37.47596	32.87516	35.3168	37.30012
HORVU0Hr1G009560	1.248501	1.244797	1.591874	1.458696	1.573079	1.739924
HORVU3Hr1G020690	12.83085	10.72784	12.04712	17.0746	23.99775	20.2796
HORVU6Hr1G078810	1.251156	0.851001	1.177701	0.997552	0.869995	1.053097
HORVU7Hr1G074490	3.488484	2.634429	3.774056	2.393209	2.1493	2.530359
HORVU7Hr1G073440	0.817045	0.866177	0.979826	1.885611	6.306419	5.251269

HORVU0Hr1G027290	4.423363	5.15261	6.204095	2.82652	3.902383	4.023879
HORVU5Hr1G080140	1.278131	1.45021	1.924776	0.903349	0.881388	0.721333
HORVU2Hr1G101710	0.783568	1.073181	1.169801	3.64194	4.541429	4.249761
HORVU7Hr1G078490	0.467314	0.448558	0.617831	2.053882	2.36492	2.392758
HORVU4Hr1G047270	0.982939	0.494554	0.902612	1.674259	1.270622	0.932454
HORVU7Hr1G009930	7.869881	10.87552	9.243288	1.098066	0.964102	0.963064
HORVU5Hr1G098090	3.023436	2.357085	3.0006	4.499765	3.914935	4.780384
HORVU3Hr1G059280	8.947108	8.066856	9.667397	11.22809	11.44114	13.85934
HORVU5Hr1G115820	2.449158	2.834236	2.834198	7.973601	8.965995	7.758884
HORVU3Hr1G045150	29.01446	23.38165	33.50225	41.26038	56.75956	56.10794
Hordeum_vulgare_newGene_10036	1.896862	2.509019	2.71223	0.135953	0.046285	0.094279
HORVU3Hr1G026690	1.003549	1.021353	2.220547	3.119655	3.423091	5.029656
HORVU1Hr1G000530	73.09437	78.98091	52.43355	25.24449	23.59799	26.13627
HORVU6Hr1G012690	9.261792	9.722522	9.262051	10.36068	16.2451	16.31947
HORVU2Hr1G071710	2.786828	4.011049	4.84274	5.969884	5.982711	6.845251
HORVU2Hr1G026200	6.070929	6.056883	9.199412	4.046052	4.395717	4.868005
Hordeum_vulgare_newGene_5070	8.143464	8.686775	8.986248	0.094388	0	0
Hordeum_vulgare_newGene_9596	6.714323	7.070318	7.775739	4.57063	12.15202	12.33382
Hordeum_vulgare_newGene_9593	2.646567	1.792244	2.608994	12.49444	2.342803	3.746184
HORVU4Hr1G090640	3.438972	3.492197	4.948949	2.2879	2.906752	2.719305
Hordeum_vulgare_newGene_9598	13.08103	14.87564	12.20815	15.0188	17.04255	17.83583
HORVU7Hr1G026740	3.287397	3.061052	3.74041	2.559887	1.864496	2.234725
HORVU0Hr1G021980	12.18114	11.15673	10.5932	16.8124	21.21909	20.08194
HORVU2Hr1G017920	28.87733	22.39368	32.75131	28.42795	33.30115	32.27341
HORVU3Hr1G096460	29.50512	27.99364	25.54201	33.97062	29.7648	29.77147
HORVU7Hr1G072420	30.40179	24.47715	38.33399	30.13401	46.49869	43.86677
Hordeum_vulgare_newGene_11348	0.968562	0.937992	0.446347	0.397941	1.98176	2.206073
Hordeum_vulgare_newGene_11346	3.05654	1.879565	2.24181	0.486803	1.015788	0.710736
Hordeum_vulgare_newGene_11343	0.778055	1.094929	1.183302	1.205998	1.849344	1.611356
HORVU4Hr1G067780	0.756807	0.648964	0.686866	9.38995	17.24114	16.07594
HORVU3Hr1G026520	53.43631	50.88458	52.87426	43.43635	30.97218	28.34041
HORVU7Hr1G045890	23.74446	20.01234	20.52053	25.56408	20.39974	20.81845
HORVU1Hr1G035280	0.915405	0.998209	1.122537	1.202581	1.530077	1.711375
HORVU2Hr1G031680	2.245502	2.131557	2.640399	2.37124	4.310638	4.232018
HORVU1Hr1G028680	5.805262	5.235381	6.267086	6.424495	8.511849	8.993373
HORVU6Hr1G061980	8.115154	6.666091	9.020269	8.823978	9.188922	9.760653
HORVU2Hr1G035300	1.60882	1.898452	1.994551	2.057737	1.155336	1.406574
HORVU4Hr1G087100	2.081567	2.862284	4.674771	2.331701	2.922529	3.582711
HORVU2Hr1G116390	10.54179	5.096446	5.396582	50.09695	63.65556	64.12909
HORVU3Hr1G112990	1.463609	1.525448	1.556066	1.170931	2.416514	2.117655
HORVU6Hr1G070480	43.87723	35.80728	56.81075	38.22189	64.66262	62.99643
HORVU4Hr1G067600	0.840004	0.962097	0.870182	2.095467	5.957514	5.459754
Hordeum_vulgare_newGene_1799	1.199607	0.039843	0.706345	1.603641	2.16483	2.550364
Hordeum_vulgare_newGene_1798	0.035436	0.040434	0.03964	0.976849	2.30282	3.142334
HORVU2Hr1G011350	1.193229	1.46366	1.506999	1.013491	2.654297	2.681598
Hordeum_vulgare_newGene_1793	3.770571	6.248156	4.05303	3.470995	6.355081	5.642487
Hordeum_vulgare_newGene_1795	3.526386	3.038174	3.57501	3.575123	3.637191	3.790016

HORVU7Hr1G021910	2.766501	2.168248	3.097128	4.841409	7.833963	6.877358
Hordeum_vulgare_newGene_14837	0.021558	0.035719	0.022269	8.47818	7.49634	8.439157
Hordeum_vulgare_newGene_8246	11.35865	10.45024	10.58583	20.99044	25.9757	25.6441
HORVU7Hr1G024610	9.875693	5.37772	9.038759	2.127807	0.009149	2.546327
HORVU0Hr1G029600	1.852656	2.015922	2.382305	1.232667	0.916029	1.236904
Hordeum_vulgare_newGene_15894	0	2.366638	0.042662	0	3.002952	3.718351
Hordeum_vulgare_newGene_7433	3.30071	4.234491	3.936621	4.960548	4.019272	4.017853
HORVU2Hr1G006010	12.85591	13.71589	29.201	25.98151	16.2272	34.5233
HORVU1Hr1G011780	4.069701	3.935876	5.882222	3.906336	5.516875	6.320933
HORVU6Hr1G043390	10.7498	11.12653	11.64691	16.43469	16.32758	16.84092
HORVU2Hr1G028530	20.46428	18.84044	24.05673	25.95458	33.93665	35.91563
HORVU7Hr1G001330	19.47169	18.16405	21.04179	19.52782	20.41257	21.7774
HORVU3Hr1G013360	0.482593	0.461895	0.377379	0.367375	2.519218	2.709253
HORVU1Hr1G074650	11.20167	11.81242	13.59109	8.096856	13.73874	13.25648
HORVU3Hr1G010290	15.84123	15.82211	18.9328	14.95471	14.16426	16.60148
HORVU4Hr1G050710	9.821697	12.43006	14.54112	10.50335	14.59518	14.69326
HORVU7Hr1G066170	7.571193	8.749469	7.09815	9.5762	8.694023	10.25995
HORVU3Hr1G081860	3.818418	4.981363	5.508122	3.894084	3.543138	3.528084
HORVU7Hr1G078860	2.079142	2.13884	1.344157	4.045354	4.53558	4.649352
Hordeum_vulgare_newGene_7293	3.436424	3.491103	3.567482	3.311855	3.553341	4.089445
HORVU0Hr1G030450	4.256756	4.658096	5.134971	4.424823	4.639492	4.544051
Hordeum_vulgare_newGene_7297	3.730563	3.961317	3.928151	5.755947	8.768674	8.89826
Hordeum_vulgare_newGene_7294	2.034278	2.099197	2.190562	3.923136	3.936631	4.69356
HORVU0Hr1G003010	3.060688	3.253483	3.788836	3.641834	6.733067	6.757502
Hordeum_vulgare_newGene_12068	1.420883	1.596444	2.135816	0.306628	1.172799	0.522862
Hordeum_vulgare_newGene_12062	2.081754	2.803772	2.542109	1.915545	3.102958	4.201966
Hordeum_vulgare_newGene_12063	3.849491	3.362205	3.637812	1.38781	2.583956	0.501459
Hordeum_vulgare_newGene_6741	1.412332	1.746255	1.592745	1.526967	1.78968	1.779742
HORVU7Hr1G098210	2.938222	1.840483	2.448488	6.754595	3.714261	5.327979
HORVU5Hr1G014130	15.33852	11.59735	16.25923	0.038036	0	0
HORVU5Hr1G040040	12.04498	11.21699	13.39938	19.0051	24.76023	23.14254
Hordeum_vulgare_newGene_15878	1.351208	1.464586	2.016568	0.450672	0.849318	1.071996
HORVU1Hr1G074670	7.728841	6.430849	7.857602	10.26667	8.55233	8.712047
HORVU7Hr1G039420	2.144321	1.946685	1.646914	2.492503	2.483914	2.743597
HORVU6Hr1G067850	1.146185	1.134806	2.066778	3.845368	3.301553	2.102202
HORVU5Hr1G084630	3.60971	2.843325	4.010638	3.079595	4.01313	3.738759
HORVU0Hr1G018190	4.291224	5.505618	5.863014	2.090565	3.334714	3.487559
HORVU6Hr1G059720	1.456251	1.221554	1.666845	2.346061	3.424395	2.378291
HORVU5Hr1G100450	1.681426	1.403567	2.643774	2.474321	3.839487	3.836494
HORVU4Hr1G027610	1.347139	1.956733	1.926543	1.942151	1.72348	2.452617
HORVU6Hr1G022380	52.2737	48.4086	45.9684	43.73487	22.64576	24.5437
HORVU6Hr1G026800	3.953635	3.206824	3.773715	7.075797	11.29873	10.48121
HORVU7Hr1G024980	7.1124	8.179828	8.078802	7.992177	8.678925	9.364754
HORVU4Hr1G074710	80.31978	100.843	114.3935	35.42048	3.459835	17.73582
HORVU5Hr1G032650	24.95991	27.01817	38.4404	41.18345	96.60996	111.454
Hordeum_vulgare_newGene_2919	10.38369	10.58057	11.96664	9.536599	14.30463	12.65987
HORVU3Hr1G116690	4.398528	3.525898	3.802454	3.778089	3.757383	4.123415

HORVU2Hr1G088900	0.42743	0.460385	0.865234	0.921145	1.986634	2.49367
Hordeum_vulgare_newGene_14583	20.49865	11.73495	15.32993	14.47826	16.73774	17.42684
Hordeum_vulgare_newGene_694	9.824704	9.403359	11.33591	8.787189	9.753852	10.41298
Hordeum_vulgare_newGene_14581	20.5779	26.93441	21.49185	24.39887	27.25741	25.22673
HORVU5Hr1G018740	5.71504	5.318695	8.209125	3.126534	3.928874	4.33953
Hordeum_vulgare_newGene_14585	24.35772	15.61898	16.04764	43.95705	95.21497	113.0893
HORVU5Hr1G020200	20.89539	23.11719	23.98139	25.5409	27.46581	29.63229
HORVU5Hr1G075420	99.59045	95.38484	158.2284	92.62458	181.8507	166.4383
HORVU3Hr1G079920	0.757758	0.833041	1.058154	2.989774	4.432134	3.524003
HORVU7Hr1G107010	0.486688	0.852359	0.619458	2.872047	5.219232	2.525967
HORVU5Hr1G064500	9.445991	9.129997	11.87043	20.74186	23.18668	23.25369
HORVU3Hr1G055260	0.129938	0.057565	0.147459	2.367946	4.515927	4.040765
HORVU6Hr1G087820	515.4549	668.2174	525.7674	1437.176	2776.804	2398.947
HORVU5Hr1G025850	1.08137	1.368079	1.867977	1.678077	2.262476	2.694229
HORVU5Hr1G028030	7.229174	4.733687	8.329111	5.899958	4.872079	6.42117
Hordeum_vulgare_newGene_9926	5.974847	5.977215	6.558567	10.23557	11.84553	12.26077
HORVU6Hr1G033320	4.514499	5.123765	6.507566	4.884226	5.622008	5.763808
HORVU7Hr1G023980	6.304968	5.581406	7.066206	6.076281	9.249346	8.510807
HORVU2Hr1G051740	46.52286	48.75368	54.14831	71.15556	108.2364	105.6812
HORVU2Hr1G043860	22.4256	22.75739	29.63505	26.13552	65.43936	61.08004
HORVU1Hr1G051840	43.27785	36.50287	39.26905	40.18387	50.09318	44.7
HORVU2Hr1G005630	0.736375	0.41834	0.509735	5.260888	13.57217	12.18467
HORVU5Hr1G029780	1.481683	1.622368	1.501585	1.135853	1.077918	1.379429
HORVU7Hr1G054090	72.87839	56.82031	70.88283	87.4049	57.11324	58.3397
HORVU1Hr1G059660	9.829719	6.822945	7.936229	6.580142	6.832473	8.845537
HORVU1Hr1G020490	1.427969	1.391336	1.940214	2.22081	5.248659	3.922771
HORVU7Hr1G065280	14.77113	13.21243	12.42273	14.26367	17.40433	17.16548
HORVU3Hr1G004860	1.419111	0.679823	1.859538	0.775003	1.332245	1.122376
HORVU6Hr1G066890	23.22759	18.50199	21.52905	23.78572	27.5383	28.68511
HORVU6Hr1G002240	31.41534	35.44362	58.07893	44.12636	72.14799	60.4627
HORVU7Hr1G077650	0.80597	1.17077	1.078026	1.460975	1.96477	2.287239
HORVU3Hr1G003480	27.68572	20.1528	19.48623	37.92403	20.888	21.71248
HORVU5Hr1G106470	8.091717	8.403558	10.60853	6.340405	9.771633	8.159846
HORVU1Hr1G078890	1.122896	1.250316	1.110866	2.251292	2.033779	2.542378
HORVU3Hr1G012620	2.205085	1.39662	1.858434	0.899325	0.962716	0.635688
HORVU4Hr1G068770	8.076079	7.780875	9.975832	8.430273	15.35253	13.79407
HORVU3Hr1G032160	5.052101	4.570642	7.031575	7.715433	7.996953	7.836313
HORVU3Hr1G004280	66.31889	88.00965	127.4162	2.617437	0.094348	1.747525
HORVU7Hr1G090800	1.817427	2.038105	2.144633	2.673595	3.071172	3.41733
HORVU1Hr1G088360	1.105184	0.778026	20.32275	59.36243	0.374658	0.244462
HORVU5Hr1G109620	55.34169	48.36988	61.54591	47.17193	82.66371	87.61197
HORVU1Hr1G067020	1.273902	1.691013	1.356655	1.798815	2.793626	2.684373
HORVU1Hr1G048340	7.402066	5.624772	7.24694	4.598577	5.001704	6.015595
HORVU0Hr1G018670	0	0	0	1.820236	8.627645	9.21725
HORVU5Hr1G066100	7.146626	6.590071	8.620106	2.509747	4.026294	4.11016
HORVU5Hr1G014950	2.488681	3.006363	2.430429	2.227608	2.244632	2.697462
HORVU7Hr1G041090	5.900503	7.144289	8.534228	5.566015	5.577041	6.952495

HORVU2Hr1G094190	2.475949	1.939651	2.556956	4.846078	5.276092	5.09855
HORVU7Hr1G092520	2.358209	2.091073	3.410814	2.040206	0.996638	1.564041
HORVU2Hr1G098340	0.37875	0.125371	0.853356	5.327382	40.25347	30.51802
HORVU6Hr1G000660	0	0	0	56.27704	0	63.68158
HORVU6Hr1G011120	22.06681	12.74581	24.36875	8.998273	7.678628	10.81765
HORVU7Hr1G114550	2.68354	2.390497	3.543469	3.066919	1.01165	1.54272
HORVU1Hr1G042060	8.765343	8.431757	7.864565	10.34231	13.11984	15.25273
HORVU6Hr1G091010	22.70512	26.08059	22.93118	16.87325	12.79885	13.89893
HORVU7Hr1G036540	3.066869	1.698858	3.324528	8.414091	14.22267	10.09666
HORVU4Hr1G062630	5.606498	4.317844	5.599913	7.421124	8.840014	10.19794
HORVU1Hr1G048690	25.03013	21.88853	25.65061	12.14191	11.51234	12.48266
HORVU7Hr1G049390	2.257904	2.039834	2.256347	5.124173	7.642852	6.738638
HORVU5Hr1G112930	10.83162	7.792229	12.67462	13.25112	3.780586	4.604945
HORVU6Hr1G043490	0.636739	0.919371	0.925727	2.458829	2.400284	1.561857
HORVU2Hr1G046000	22.88448	20.88485	25.68968	27.36182	43.51002	42.83663
HORVU1Hr1G083160	0.501422	0.663808	0.270002	2.24761	2.440184	3.0118
HORVU4Hr1G052880	0.527841	0.736884	0.953711	0.383602	2.321535	4.091749
HORVU2Hr1G081360	5.030508	5.497498	6.209673	5.389092	8.799609	8.119226
HORVU5Hr1G054010	0.74082	0.49997	0.483304	1.444409	1.751063	1.323561
HORVU5Hr1G058740	0.411043	1.003598	0.867196	1.88684	2.302629	1.522575
HORVU1Hr1G057290	0.309144	0.5103	0.321108	0.653074	2.281173	2.355137
HORVU6Hr1G043980	2.971627	2.182218	3.548689	0.227868	0.607217	0.457321
HORVU7Hr1G115600	4.735309	4.443631	4.516324	6.228398	10.89653	10.6773
HORVU2Hr1G014240	2.238612	1.290936	1.650498	12.07386	22.00121	19.43743
HORVU5Hr1G050240	7.143578	5.959699	7.610282	8.046159	5.659299	7.486395
HORVU1Hr1G064700	0.614415	0.784588	1.000704	9.42113	34.35486	30.44004
HORVU5Hr1G067760	159.394	108.1837	187.0954	36.55044	31.94248	35.86755
HORVU7Hr1G094270	4.491659	4.525736	3.892081	5.765418	13.39889	13.02565
HORVU7Hr1G096680	13.80053	15.32811	11.70597	25.58583	34.29667	32.35709
HORVU0Hr1G001690	0.334115	0.269355	0.441344	1.485949	2.292298	2.330252
HORVU2Hr1G100600	0.939405	1.025906	1.109689	1.212074	1.107439	1.580113
HORVU0Hr1G015960	10.59224	10.37316	13.2084	10.37336	10.63331	11.09391
HORVU2Hr1G109760	15.46626	12.8932	19.7507	12.46765	13.21334	12.37736
HORVU2Hr1G002690	10.48387	10.74928	11.46468	41.24424	35.87135	42.74116
HORVU0Hr1G002250	12.97215	6.142623	18.37588	2.583247	0.038973	0.371613
HORVU2Hr1G077230	1.295196	1.484823	1.775575	2.032855	4.891156	5.572675
HORVU7Hr1G091380	10.12224	11.11928	11.46233	11.40586	8.084111	8.57748
HORVU3Hr1G034980	8.606279	8.662706	7.097598	6.098765	3.622441	4.76891
HORVU7Hr1G055860	64.75645	55.84016	74.53937	57.27211	60.98517	64.86537
HORVU0Hr1G015380	3.608967	3.475151	4.383887	4.73509	4.360453	4.429914
HORVU2Hr1G014050	8.303342	8.795523	9.413516	7.906633	8.606068	8.34887
HORVU7Hr1G113680	1.26516	0.867138	1.287546	2.052911	3.250233	2.463299
HORVU7Hr1G074300	27.58259	23.23482	25.69931	28.96067	30.15488	31.59161
HORVU5Hr1G121970	1.720418	1.449558	1.644744	2.739488	1.876233	1.797412
HORVU0Hr1G017120	9.274416	9.301305	10.73955	11.44771	14.40444	14.71857
HORVU7Hr1G090380	46.76271	46.51896	55.26786	50.93802	69.021	73.26879
HORVU5Hr1G105030	3.250844	3.705928	4.000691	3.003529	2.757524	3.050563

HORVU6Hr1G056510	1.90502	1.840405	2.126644	3.796745	0.225577	3.597261
HORVU0Hr1G015030	1.082241	1.174569	1.563072	1.339806	0.819822	1.352382
HORVU2Hr1G115960	0.02616	0.013862	0.027013	2.248793	10.58889	8.968098
HORVU2Hr1G019790	57.07926	46.2036	52.97505	80.71087	92.68356	95.91243
HORVU2Hr1G118360	15.19738	14.35859	19.356	5.471232	5.522737	6.427109
HORVU4Hr1G025420	1.197935	1.626672	0.99337	0.989231	8.404167	6.377944
HORVU7Hr1G122340	6.324297	6.669239	6.919403	4.453006	5.994502	6.008342
HORVU2Hr1G097400	0.676137	1.964444	2.4304	2.139137	3.10251	3.116198
Hordeum_vulgare_newGene_11181	2.68869	2.402569	2.017558	5.414478	4.053216	3.721864
Hordeum_vulgare_newGene_11182	10.24533	11.23696	10.52828	0.044096	0	0.039034
Hordeum_vulgare_newGene_11183	0.017699	0	0	13.24531	15.53498	17.49214
Hordeum_vulgare_newGene_11187	0.320204	0.421223	1.769885	0.221939	3.782179	3.343411
HORVU6Hr1G037500	29.00794	33.12609	37.54219	31.77081	37.27048	41.41658
HORVU5Hr1G122990	0	0	0	7.623988	6.096703	5.859092
HORVU5Hr1G077150	4.011528	3.212483	2.842652	3.045685	3.804628	4.433973
HORVU3Hr1G092780	28.40742	26.05626	26.81215	20.60365	22.47175	21.30789
HORVU5Hr1G093570	1.674405	1.376612	1.448933	0.867177	1.159618	1.683653
HORVU4Hr1G060540	3.072008	3.821064	5.851431	2.319122	2.844389	2.886189
HORVU4Hr1G023860	5.386166	5.741693	7.033514	4.823076	6.150233	6.981278
HORVU6Hr1G077610	3.049063	2.466771	3.009605	4.256265	4.70629	5.131921
HORVU2Hr1G118110	30.50928	26.06667	30.10226	39.132	23.03944	28.61577
HORVU1Hr1G094060	3.41862	3.404357	3.0614	4.145902	6.460134	6.198748
HORVU5Hr1G122660	1.2653	1.36303	1.415744	0.422551	0.870393	1.094229
HORVU2Hr1G091800	1.415717	1.47648	1.024677	2.463652	2.23721	2.88983
HORVU4Hr1G006510	0.657481	0.851598	0.593631	1.639886	1.486723	1.677611
HORVU1Hr1G065560	10.704	9.422745	8.042902	15.05342	16.14335	15.85973
HORVU3Hr1G029360	8.055244	5.65056	4.999103	6.707627	10.08211	8.049812
HORVU5Hr1G112160	21.10218	23.38358	20.53686	24.1085	22.29149	23.83002
HORVU7Hr1G069770	0.058594	0.084776	0.1243	8.200586	7.44794	10.51785
HORVU3Hr1G077110	1.498197	1.431511	1.732727	1.380116	1.150697	1.9156
HORVU6Hr1G057640	25.93064	26.47754	34.67154	27.29075	49.77268	51.63607
HORVU0Hr1G023910	4.127155	4.867234	2.541385	2.713407	17.92528	14.39076
HORVU7Hr1G091810	16.30313	14.48958	15.49455	22.787	23.84094	25.51404
HORVU1Hr1G004610	5.446091	4.421205	5.401018	7.164374	8.40586	7.060738
HORVU4Hr1G061550	5.645424	4.787129	5.769475	6.858755	7.907851	8.276958
HORVU7Hr1G087420	6.925625	6.496698	9.40048	5.898762	7.823969	5.019171
HORVU3Hr1G037270	10.2684	9.652532	9.040268	5.952033	6.655496	7.654186
Hordeum_vulgare_newGene_5262	5.089329	3.772148	4.193669	3.43788	2.921586	4.093727
HORVU1Hr1G066030	5.182799	5.260213	7.249226	3.592427	4.236538	4.534197
HORVU6Hr1G063210	9.93885	10.59079	10.71767	9.527225	10.35847	11.48093
HORVU3Hr1G094330	5.510042	5.242455	7.662515	3.961651	4.134255	3.750507
HORVU7Hr1G105880	205.7524	190.2874	180.7119	109.1422	99.99135	87.87653
Hordeum_vulgare_newGene_3209	11.35032	10.03907	14.49314	14.0199	15.78187	15.28388
HORVU5Hr1G046520	765.1338	481.0856	806.6072	1691.594	3148.929	2292.113
HORVU6Hr1G001270	10.83361	8.994302	11.11633	39.17379	64.4823	50.65545
HORVU4Hr1G057110	2.61943	2.443697	3.599247	1.513453	1.80693	1.180292
HORVU1Hr1G013210	15.57923	13.39742	14.17682	11.33992	12.21719	11.74019

HORVU6Hr1G090000	9.260957	9.433956	10.89799	14.60034	24.40981	22.74417
HORVU6Hr1G095070	9.651713	10.40544	10.3427	24.74657	30.36543	26.95234
HORVU2Hr1G102140	1.602319	3.057432	2.476064	1.375005	1.992002	2.287754
HORVU4Hr1G001850	13.0018	13.13881	13.66967	13.81138	14.23006	14.62735
HORVU6Hr1G013950	17.51922	19.32053	27.65004	20.94131	35.24539	34.03912
HORVU7Hr1G121700	110.4217	98.40746	116.6333	96.16739	153.2719	144.2784
HORVU2Hr1G023850	67.42611	55.36822	65.15604	40.40612	41.94283	40.27988
HORVU5Hr1G119550	9.939111	13.28675	15.75021	6.321966	0.8618	4.06594
HORVU4Hr1G062570	8.124722	7.281551	9.534204	7.61618	8.630811	8.975627
HORVU1Hr1G042350	9.362806	8.733672	8.667531	8.607818	7.971376	8.38722
HORVU6Hr1G093660	19.03081	20.30051	21.43377	20.85174	21.28134	22.04614
HORVU3Hr1G034170	4.929281	5.719143	4.639432	6.067717	7.253511	7.183513
HORVU2Hr1G102780	81.92635	67.76927	89.19359	53.89528	62.69957	65.21742
HORVU7Hr1G100250	1.538255	1.783647	1.705339	1.703849	1.235679	1.688136
Hordeum_vulgare_newGene_12517	90.1506	71.64743	80.90588	64.65711	50.7794	58.87636
Hordeum_vulgare_newGene_12512	2.812412	3.667758	3.928006	2.93705	2.248607	2.660233
HORVU2Hr1G044910	8.17133	7.167198	9.479445	5.706856	2.900542	3.989915
HORVU7Hr1G038710	5.684567	4.953663	6.17287	12.34632	17.79907	17.8738
HORVU2Hr1G063260	10.90641	12.05534	11.44052	11.5826	14.08702	15.01904
HORVU3Hr1G116450	11.30837	11.52444	11.05869	14.06707	13.90852	17.7559
HORVU3Hr1G090540	168.013	145.1949	162.2839	93.57747	104.7888	106.9945
HORVU4Hr1G029700	12.08986	12.77847	12.28426	6.798425	5.954096	6.588467
HORVU2Hr1G119180	5.133804	5.092519	5.675206	9.014186	8.986391	9.837341
HORVU3Hr1G052570	4.117206	4.469447	3.610727	3.917638	5.457741	5.145958
HORVU6Hr1G027660	2.306178	3.002057	2.475079	2.789512	3.671224	4.389368
Hordeum_vulgare_newGene_3511	1.056491	0.932918	1.003517	1.702934	2.140473	2.356167
Hordeum_vulgare_newGene_3513	3.265535	3.498599	3.802351	4.418599	4.664956	5.85876
Hordeum_vulgare_newGene_3512	0.933197	1.42323	1.262949	1.975308	3.146955	2.295839
Hordeum_vulgare_newGene_3517	7.685725	6.060209	6.758387	5.995917	8.602873	8.414683
HORVU5Hr1G000800	4.839288	5.843176	5.042791	19.37599	14.19244	15.35899
Hordeum_vulgare_newGene_8091	2.390332	1.76733	3.012245	1.584402	2.332897	2.236795
HORVU7Hr1G077620	18.17417	16.46071	19.95459	10.72738	6.184571	6.973425
HORVU6Hr1G078130	16.55902	14.86803	18.79865	16.70077	26.82095	26.81582
Hordeum_vulgare_newGene_8093	15.07802	15.34116	15.63376	19.00573	25.44699	23.82097
HORVU1Hr1G043940	19.17095	17.2726	19.76843	22.42427	30.57418	29.45732
HORVU4Hr1G021570	2.343813	2.424689	2.444544	0.13926	0.108473	0.150549
HORVU4Hr1G025850	2.050029	1.3034	2.092728	1.882181	2.547988	2.50768
HORVU0Hr1G023280	14.65261	15.12459	24.02201	4.403432	5.248299	6.039102
HORVU6Hr1G065300	2.583725	2.599127	3.44504	2.182564	3.592744	4.66042
Hordeum_vulgare_newGene_14210	14.26073	14.19831	17.44351	13.94166	15.92599	16.50636
HORVU7Hr1G075170	28.55049	32.54421	31.75953	29.59625	27.83635	36.57349
Hordeum_vulgare_newGene_14219	11.42726	15.33953	15.47712	19.9314	58.83341	56.99158
HORVU4Hr1G061080	1.319153	1.676596	1.765937	2.588185	2.750957	2.606107
HORVU0Hr1G010810	0.247597	0.773565	0.789817	1.542827	1.318823	1.667963
HORVU3Hr1G097010	11.64259	11.50076	12.05184	4.383398	2.938547	3.2194
Hordeum_vulgare_newGene_714	98.56837	135.1514	152.7857	1.159836	0.579143	1.47399
Hordeum_vulgare_newGene_711	0.282732	0.585927	0.678331	5.287012	3.832145	4.593623

Hordeum_vulgare_newGene_710	0.769703	0.850131	0.975459	1.780671	2.306088	2.404752
Hordeum_vulgare_newGene_10914	8.2503	7.318902	8.336163	1.108861	0.141525	0.377917
Hordeum_vulgare_newGene_719	2.545997	2.232078	1.068729	8.447726	4.182415	5.813689
HORVU2Hr1G119980	5.621348	6.106611	5.843909	8.018735	14.23231	14.46934
Hordeum_vulgare_newGene_6945	1.1048	1.525519	1.658386	1.381942	1.50901	3.149652
HORVU7Hr1G042930	0	0	0	4.079028	2.235914	2.271998
HORVU3Hr1G048310	19.10887	20.05649	30.91839	16.59764	21.26538	21.45734
HORVU5Hr1G062040	8.658119	7.109801	11.47909	12.81169	13.35243	16.24672
Hordeum_vulgare_newGene_8897	1.616272	1.296736	1.662414	2.528657	2.624406	2.741913
Hordeum_vulgare_newGene_5916	2.163595	2.1709	2.512301	2.627125	2.696051	3.231649
Hordeum_vulgare_newGene_5915	37.0177	52.62234	61.99332	13.58821	4.790463	9.091271
HORVU6Hr1G021620	4.021587	3.725282	3.574693	12.47993	11.86659	7.88009
HORVU6Hr1G015440	1.915229	1.975192	2.934256	2.548179	3.559981	3.342906
Hordeum_vulgare_newGene_5910	5.438874	4.103443	5.484661	9.250211	13.29848	12.01039
HORVU4Hr1G071870	27.33731	23.64001	30.5249	30.89565	42.92935	44.21105
HORVU1Hr1G007730	4.352816	4.055843	3.887141	2.154163	1.930039	2.663388
Hordeum_vulgare_newGene_5919	18.95131	15.43439	22.7829	9.988713	16.07173	14.34738
HORVU5Hr1G073660	15.80963	19.45346	22.52314	13.32392	16.08965	14.20268
HORVU4Hr1G078390	17.15603	13.30299	20.42261	10.65805	6.423661	8.544709
HORVU1Hr1G090760	128.806	110.1257	158.144	199.2919	279.7269	284.1501
HORVU2Hr1G096410	9.644226	8.831862	10.69662	14.79804	17.74576	19.59628
HORVU6Hr1G022330	15.48308	16.99193	17.81149	18.42529	22.9616	23.26232
HORVU4Hr1G074990	8.79615	9.23162	10.39317	6.917675	5.480835	6.862613
HORVU4Hr1G070470	1.681338	2.793726	2.23043	2.519726	2.6854	2.229015
HORVU1Hr1G000700	3311.185	3702.003	2493.376	1411.549	1261.719	1146.678
HORVU1Hr1G053600	34.30735	27.99337	38.86269	31.3252	58.4058	55.59359
HORVU6Hr1G088580	9.194022	8.048848	9.548395	18.82078	18.90352	18.56685
HORVU5Hr1G098110	1.375386	1.259874	4.425821	5.969705	11.85891	11.67118
Hordeum_vulgare_newGene_16167	2.906005	2.061351	2.32452	4.034185	3.332846	3.432983
HORVU0Hr1G032300	0.14737	0.124539	0.223269	2.541812	5.622018	2.749553
HORVU3Hr1G097900	4.703404	2.567161	4.360002	4.954179	9.820417	6.260892
HORVU1Hr1G077020	2.037033	2.161706	2.369092	3.932392	7.093897	6.212585
HORVU7Hr1G019870	35.98593	36.82212	38.56043	23.90783	24.92117	27.70382
HORVU5Hr1G047490	1.057917	1.090466	1.597317	1.619381	3.199656	2.048307
HORVU2Hr1G060680	6.358651	6.641972	6.867008	10.12224	10.13175	11.26872
HORVU3Hr1G096050	92.32799	74.18195	101.7915	81.00111	107.6706	121.3183
HORVU2Hr1G021870	0.321269	0.356206	0.352538	2.198523	3.09698	2.991186
Hordeum_vulgare_newGene_9418	6.832593	6.532299	6.268735	1.109654	0.33116	0.339267
Hordeum_vulgare_newGene_9415	5.186879	5.682588	4.949678	7.64195	9.540353	9.249531
HORVU7Hr1G023700	5.796388	4.992254	4.609424	3.289861	3.344679	3.273403
HORVU5Hr1G006040	2.009683	1.620144	2.02825	1.351325	1.734589	1.425666
HORVU4Hr1G047430	2.077626	1.686462	2.014075	1.362717	2.165001	3.705767
HORVU5Hr1G014810	1.131526	2.34002	1.424008	1.080505	1.375547	1.430865
HORVU2Hr1G102620	278.1928	243.2698	274.8597	239.6695	146.36	164.7445
HORVU1Hr1G025530	82.45868	62.66876	77.31325	23.30791	7.758409	10.9903
HORVU5Hr1G091420	0.487247	0.354484	0.487074	1.346166	1.873092	2.132465
HORVU5Hr1G018120	2.483788	2.619182	3.047989	2.039452	2.925913	2.970685

HORVU7Hr1G021700	5.224067	4.455734	6.000676	9.68058	19.63267	16.08035
HORVU1Hr1G063010	4.434666	4.770312	4.436728	4.966501	3.979098	4.365454
HORVU2Hr1G014380	2.230988	4.144045	1.666743	0.060667	0.055131	0
HORVU2Hr1G105750	0	0	0	0.111554	16.85349	14.43724
HORVU7Hr1G076910	9.359144	8.69166	10.73057	9.132092	14.72822	14.12117
Hordeum_vulgare_newGene_4396	1.649178	1.060802	0.975162	2.049721	1.611426	1.79847
Hordeum_vulgare_newGene_4394	1.016421	1.024587	1.004665	1.711381	2.555807	2.703503
Hordeum_vulgare_newGene_4392	16.43515	13.68664	16.66111	13.50503	13.34196	13.07698
Hordeum_vulgare_newGene_4391	5.15862	4.747896	4.584621	3.282422	2.85782	3.056144
HORVU1Hr1G043470	4.172111	3.003076	4.249347	1.276843	1.656093	1.377528
HORVU2Hr1G042490	1.720627	1.039206	1.919139	3.324516	7.254439	7.082256
HORVU2Hr1G007570	7.671345	4.033472	8.976358	20.58621	9.088769	15.85734
Hordeum_vulgare_newGene_77	9.430355	11.21131	11.5092	11.4273	14.0634	13.76013
Hordeum_vulgare_newGene_79	6.072164	6.243852	7.036736	7.006707	7.09601	7.614028
HORVU3Hr1G070620	0.734503	0.642756	0.753317	1.663817	1.717495	1.772055
HORVU4Hr1G053660	7.542906	6.621256	8.222633	13.10466	17.78093	17.64718
HORVU5Hr1G105980	2.216415	2.102729	2.729314	5.673644	7.882909	7.939775
HORVU4Hr1G076840	19.72235	15.22164	18.59297	4.605576	2.598375	3.763031
HORVU3Hr1G042680	13.21117	15.26898	16.85065	12.09919	13.37961	16.08353
HORVU6Hr1G020820	11.46906	10.0764	12.35674	10.58071	11.39239	11.72035
HORVU7Hr1G119820	11.11608	9.642664	11.18894	17.06625	20.20854	20.99834
Hordeum_vulgare_newGene_4979	1.770906	1.995773	2.276769	4.693484	5.989179	6.116823
HORVU1Hr1G079890	15.8355	15.69542	16.9187	12.87101	14.15412	14.78578
HORVU5Hr1G060030	0.514802	1.067956	0.784121	2.002612	2.132231	2.170528
HORVU5Hr1G101600	29.94615	29.31394	51.45935	23.31572	55.72647	47.72681
HORVU2Hr1G126950	3.402431	3.332773	4.608467	4.754181	7.131549	6.787143
HORVU3Hr1G093570	1.227166	1.490164	1.94461	1.375147	2.318339	2.060403
HORVU5Hr1G085800	3.050949	2.603064	3.246248	2.098453	1.007589	1.569223
HORVU4Hr1G073340	3.040223	2.978802	3.10407	5.989122	8.768142	8.494459
HORVU2Hr1G089970	2.001557	2.984421	3.392592	5.867968	12.6968	13.08241
HORVU6Hr1G069560	29.37448	30.15878	26.82849	22.86278	21.73995	22.78365
HORVU1Hr1G033210	30.0547	36.71403	30.68308	36.01045	28.92191	38.63948
HORVU3Hr1G018940	9.478672	9.44859	9.21806	11.19071	11.67728	9.820969
HORVU3Hr1G030390	3.203465	4.473377	2.787761	6.54455	8.452422	6.69441
HORVU6Hr1G008640	62.10937	45.09055	43.88735	138.1104	122.0819	178.5279
Hordeum_vulgare_newGene_15200	6.257976	4.282919	4.753634	0	0	0
HORVU4Hr1G072960	0	0	0	7.929727	15.20934	8.869328
Hordeum_vulgare_newGene_6530	14.8159	6.59569	15.85451	7.789419	4.764353	7.331039
Hordeum_vulgare_newGene_6534	2.278522	2.647083	2.548965	6.031268	6.358409	7.65958
HORVU7Hr1G098020	26.19424	25.20205	27.10776	28.48695	21.27622	21.7567
Hordeum_vulgare_newGene_15939	1.838659	2.942824	3.092453	0	0	0.011443
Hordeum_vulgare_newGene_15931	1.487636	1.980967	1.822234	4.358081	4.112494	5.389396
Hordeum_vulgare_newGene_15209	1.211942	0.747729	1.003907	2.923089	0.347123	1.152916
HORVU1Hr1G021530	15.9455	15.43341	18.00281	10.11086	12.46632	13.45221
HORVU2Hr1G084800	6.270092	4.915776	5.856204	6.558451	3.786157	4.985548
HORVU3Hr1G085640	4.262374	4.57191	5.407537	6.521517	9.295598	9.492658
HORVU6Hr1G078670	2.782185	1.8367	3.463554	0.141236	0.154337	0.143051

HORVU2Hr1G082090	4.659573	4.413067	5.151576	7.807463	7.306003	7.476576
HORVU7Hr1G053560	1.826267	2.129718	1.327943	1.139816	10.23207	7.251479
HORVU3Hr1G116150	6.397045	7.358098	6.889652	16.42741	15.83484	14.45524
HORVU3Hr1G041850	0	0	0	2.703755	2.719803	3.249995
HORVU6Hr1G073660	9.024405	8.305404	7.665524	16.41371	13.58642	16.36768
HORVU7Hr1G099950	1.057818	1.081952	1.264078	1.897533	8.302882	8.235463
HORVU3Hr1G028210	31.16437	28.52953	34.07495	29.0722	36.33098	32.89201
HORVU5Hr1G001410	2.768018	2.41546	3.346406	2.457499	2.731223	4.134603
HORVU2Hr1G125360	0.373883	0.416719	0.474909	1.018878	2.932952	2.270789
HORVU3Hr1G006800	0.07261	0.020934	0.044102	4.286872	1.430544	3.300661
HORVU5Hr1G012200	213.5689	209.8669	213.1965	274.9414	171.7764	193.3561
HORVU7Hr1G052210	1.937268	1.993428	1.997505	3.112159	6.039615	6.852164
HORVU3Hr1G032820	1.384795	0.911598	1.370599	2.38011	4.881083	3.085144
HORVU7Hr1G118010	20.96085	15.63675	20.18509	11.56398	4.041684	5.667136
HORVU2Hr1G024570	19.30621	12.42331	18.38463	62.5454	119.0483	113.2209
HORVU5Hr1G023670	2.020224	3.721531	4.242094	6.96848	3.91107	6.266479
HORVU4Hr1G029940	4.171184	5.163161	5.06746	4.772733	5.961664	5.873558
Hordeum_vulgare_newGene_2090	0.89922	0.542408	0.97578	1.971539	2.665395	3.23928
HORVU3Hr1G029160	13.51527	13.99604	15.45345	20.35804	31.30864	26.86371
HORVU5Hr1G039100	10.55538	13.15895	11.41838	18.4007	23.17867	21.29031
HORVU1Hr1G022370	17.34404	12.8005	17.08831	16.37797	16.62153	16.85273
HORVU6Hr1G045820	2.092556	3.73879	3.715091	3.738617	4.528801	4.490799
HORVU3Hr1G072760	1.103036	1.030318	1.183543	1.988963	2.479346	2.925654
HORVU5Hr1G055560	3.353671	3.578933	3.331542	13.45582	35.9273	34.0915
HORVU6Hr1G039660	12.29646	9.559547	15.5012	19.75578	23.5532	22.73571
HORVU3Hr1G082690	30.3062	30.66121	33.80081	31.77497	52.62777	46.6937
HORVU4Hr1G083270	9.551755	8.807628	9.077288	9.96617	9.930116	10.06443
HORVU4Hr1G056200	7.604086	7.834756	9.116943	15.56752	17.661	19.00604
HORVU1Hr1G005100	25.88975	32.4854	32.24443	27.73961	25.22402	21.72052
HORVU5Hr1G079950	0.661974	0.6526	0.408189	1.856054	2.126091	2.250326
HORVU2Hr1G123200	6.505984	6.179891	5.958832	9.132458	14.20477	13.83749
HORVU1Hr1G021880	33.65338	31.26444	26.92773	34.09684	58.81722	51.66941
HORVU6Hr1G040270	8.03508	10.30245	14.58868	9.534961	14.64946	14.07011
HORVU5Hr1G118040	4.010315	3.408902	4.858266	4.921788	6.923424	7.490567
HORVU5Hr1G088840	1.246251	1.295814	1.519358	1.269788	1.621492	1.686618
HORVU4Hr1G063020	9.675746	9.79398	11.44633	12.54132	14.17715	15.21639
HORVU1Hr1G019500	1.886063	1.452688	2.128373	0.947849	0.812304	1.071482
HORVU2Hr1G080630	64.01628	43.54456	82.69276	81.06302	210.0324	154.8932
HORVU6Hr1G085120	0.445922	0.806131	0.509125	2.605621	2.521141	1.788211
HORVU6Hr1G001700	5.605174	6.232087	7.035292	5.521333	11.74843	11.99358
HORVU2Hr1G066680	18.77357	16.69989	18.14892	80.01222	104.0954	104.4938
HORVU5Hr1G119790	4.739143	4.858916	5.682235	8.273212	14.31029	13.2233
HORVU1Hr1G048360	5.587196	5.453633	6.802408	9.146711	16.81025	17.77368
HORVU4Hr1G031530	1.247206	1.411375	1.968642	1.199771	1.221513	1.473149
HORVU4Hr1G066210	3.441425	3.618038	3.787273	3.157934	1.056734	2.177156
HORVU4Hr1G062770	2.713514	3.895249	3.339959	2.085494	2.681424	2.515321
HORVU2Hr1G102920	1.636748	1.481595	1.201654	5.986261	2.35202	3.315328

HORVU5Hr1G034460	1.191603	1.239219	1.371751	2.168509	2.529617	3.842149
HORVU4Hr1G063790	0.249867	0.339197	0.455815	3.548844	1.098379	2.248438
HORVU1Hr1G085630	11.13321	12.09691	11.91234	14.69044	20.94529	20.03031
HORVU6Hr1G005010	14.93381	13.67519	12.09707	15.93435	21.17795	20.05391
HORVU5Hr1G055970	7.325765	6.553037	8.266986	9.865737	11.2289	10.60199
HORVU0Hr1G016880	3.432593	3.664967	4.830792	3.933791	3.574895	3.624119
HORVU4Hr1G005740	5.688723	4.672679	5.050881	5.822823	7.139857	7.608136
HORVU4Hr1G039720	16.36184	16.2377	16.97929	12.44776	15.78433	16.39229
HORVU2Hr1G012840	22.9502	23.77384	26.82218	35.4755	26.1302	24.51686
HORVU1Hr1G080040	5.164345	5.546725	6.548835	3.85011	3.272486	3.878701
HORVU5Hr1G005290	37.94518	24.22036	55.6474	5.525278	2.223555	6.680281
HORVU2Hr1G094760	1.249682	1.051466	0.992906	2.851469	1.687451	2.169473
HORVU0Hr1G014950	1.258584	0.86531	1.753675	0.776751	0.541331	1.047806
HORVU3Hr1G084520	9.286514	9.886584	12.42144	11.5093	17.27894	15.27031
HORVU2Hr1G118320	21.64833	19.65495	24.7555	16.34037	17.44666	18.98463
HORVU7Hr1G031850	31.87541	33.77605	49.44196	25.73228	52.27085	52.46668
Hordeum_vulgare_newGene_3687	2.209658	2.591833	5.62136	0.867142	0.648721	0.481085
HORVU7Hr1G022510	0.522938	0.534054	0.741122	1.509264	1.961594	2.173105
HORVU2Hr1G103560	4.355757	4.543049	6.255034	3.79798	4.167154	4.689557
HORVU1Hr1G087900	251.0232	239.4741	224.3711	261.9022	177.6928	172.9778
HORVU2Hr1G053620	0.943694	1.709756	0.749939	1.108087	8.39972	5.972856
HORVU3Hr1G035930	5.878538	6.435189	10.85327	3.503699	4.601286	4.41707
HORVU5Hr1G104390	11.87334	12.74593	18.04396	23.22822	48.35	43.6596
HORVU5Hr1G124920	1.428432	1.662636	1.976929	1.91092	2.362967	2.804779
HORVU4Hr1G054110	11.06744	16.93121	20.4058	8.207094	1.277798	3.513222
HORVU1Hr1G068320	3.615579	4.762337	3.867029	4.223264	6.239775	6.06485
HORVU2Hr1G091270	4.474535	3.683083	3.624482	11.05515	18.31919	15.82181
HORVU3Hr1G070540	54.91156	48.28169	51.30764	68.2065	34.08319	44.43486
HORVU7Hr1G075770	6.856922	7.546396	8.764034	8.97261	9.451923	10.29372
HORVU2Hr1G093680	0.174561	0.189786	0.891089	13.28388	32.82228	28.21261
HORVU1Hr1G004920	1.129646	1.219387	1.172913	1.517032	1.394779	1.39598
HORVU3Hr1G064290	23.43312	22.61781	27.38153	28.64736	33.66538	37.02297
HORVU1Hr1G056280	0.089168	0.127469	0.230623	0.190394	3.598744	3.546863
HORVU5Hr1G044410	2.076827	0.875574	1.006838	3.813766	3.995887	1.453256
HORVU7Hr1G088080	26.81579	25.787	33.46425	34.11453	51.2417	53.70259
HORVU2Hr1G124610	54.68207	51.06772	56.27601	47.90086	44.60194	42.48042
HORVU6Hr1G018530	9.619777	9.135499	10.26715	11.79371	13.23129	12.39602
HORVU3Hr1G093380	2.783278	3.550738	3.648604	3.868949	4.953813	4.471594
HORVU2Hr1G036110	3.80013	3.550121	3.119405	21.66758	33.01583	32.95058
HORVU5Hr1G027740	1.041853	0.475704	0.443911	0.940442	2.144106	1.781112
HORVU7Hr1G083910	3.562436	2.649154	2.166198	0.298801	8.657143	8.947513
HORVU1Hr1G083100	2.007688	2.580633	2.930288	1.512269	0.834668	1.324812
HORVU7Hr1G091220	26.51791	25.26996	23.87496	18.43115	18.66924	19.21115
HORVU4Hr1G000590	3.169364	2.571571	3.388138	2.420357	2.850405	3.742481
HORVU4Hr1G055240	7.986556	7.838117	9.724348	14.18518	33.35145	32.98454
HORVU5Hr1G053010	0.92254	1.010943	2.151947	5.108563	36.46619	42.75307
HORVU7Hr1G043600	0.031765	0.134699	0.332962	1.07907	10.64051	9.829957

HORVU1Hr1G049090	14.51694	8.172771	13.06198	7.258877	4.483371	5.009251
HORVU2Hr1G014030	0	0	0.040121	5.423322	6.40654	8.219538
HORVU3Hr1G005830	2.60304	2.185979	2.623996	3.862334	7.248925	7.904796
HORVU5Hr1G117650	9.69296	9.102566	10.04777	6.936033	7.257819	7.764611
Hordeum_vulgare_newGene_8701	1.304775	1.053923	0.637359	1.167269	1.038891	1.172174
HORVU2Hr1G066910	16.05975	17.04339	18.81848	20.83656	26.01668	27.64923
Hordeum_vulgare_newGene_8702	2.35162	2.51139	2.127737	3.384621	5.755805	3.963848
HORVU5Hr1G074800	28.13584	28.85347	33.32196	29.43281	33.03356	31.90363
Hordeum_vulgare_newGene_8704	11.80366	12.83655	11.31205	10.10052	12.04894	12.11744
Hordeum_vulgare_newGene_8706	0.987718	1.646092	1.177794	2.616007	1.175756	1.447689
HORVU1Hr1G000140	7.062592	5.813236	7.183536	9.581434	14.85504	13.91797
HORVU5Hr1G045450	7.824146	8.238957	9.46085	14.97186	25.70567	26.86291
HORVU4Hr1G014660	4.639976	5.055567	5.600925	5.619271	5.620762	6.675342
HORVU4Hr1G000620	24.27997	25.91448	30.29247	14.24945	13.9228	16.01258
HORVU7Hr1G089040	6.189982	5.917973	8.18434	8.93946	11.59874	12.4857
HORVU5Hr1G006170	8.14864	7.427806	8.493072	9.417642	15.55657	14.01455
HORVU4Hr1G064160	15.66802	15.58214	18.35083	18.71449	30.56448	24.49037
HORVU6Hr1G092010	1.975669	1.849156	1.821337	0.082393	0.196974	0.252905
Hordeum_vulgare_newGene_14142	2.059722	1.756855	2.485769	3.137043	4.670776	4.027327
HORVU3Hr1G065650	3.45463	1.323189	3.463607	1.388908	3.692394	3.083211
HORVU2Hr1G011120	1.530259	1.363223	1.479136	1.427969	0.979749	1.034255
HORVU6Hr1G076040	0.821434	0.633102	0.655286	4.57913	6.969854	5.71563
HORVU1Hr1G064110	41.75712	39.05671	27.30214	16.08425	6.807844	9.353201
HORVU3Hr1G098670	3.158625	3.085005	3.232931	3.187537	4.728951	3.923519
HORVU5Hr1G095660	2.538738	3.17043	3.04858	2.225638	2.09207	2.44825
HORVU0Hr1G035530	0.695843	0.836013	0.339239	0.614797	3.432599	2.41045
HORVU3Hr1G096210	82.64367	66.81409	84.11714	112.6175	84.0254	77.97345
HORVU1Hr1G016360	1.942528	1.961292	1.644186	3.547965	3.782827	4.456115
HORVU3Hr1G062920	7.039644	5.417299	5.823422	2.49571	0.827866	1.676823
Hordeum_vulgare_newGene_5189	0	0	0	1.669296	8.36981	4.354017
HORVU6Hr1G056490	115.4016	115.3632	100.9832	105.7149	129.2944	127.2188
HORVU4Hr1G024380	11.29017	9.958484	10.48969	14.16379	15.27443	16.15336
HORVU4Hr1G082400	40.00701	42.94829	50.82268	24.94247	8.448372	15.45395
HORVU2Hr1G104640	1.931529	1.711465	1.731376	2.186982	2.145028	2.644234
Hordeum_vulgare_newGene_10795	3.506344	3.978136	3.118994	0.039271	0.005542	0
HORVU2Hr1G111680	0	0	0	1.244031	6.63435	4.435377
HORVU2Hr1G073050	3.04977	2.165392	3.736583	1.881551	0.906711	1.360259
HORVU2Hr1G112420	13.57106	15.3607	15.07122	18.10181	20.2441	21.13148
Hordeum_vulgare_newGene_3797	5.259576	6.32628	4.656548	20.99582	11.14785	12.65295
HORVU4Hr1G051230	3.26459	3.537873	3.70363	2.973785	2.881018	3.153049
HORVU3Hr1G093590	2.047237	2.696331	3.415998	1.595636	1.215152	1.688451
HORVU6Hr1G056720	2.450243	2.567205	2.924146	3.087719	3.128895	3.365962
HORVU3Hr1G104360	18.10247	18.38848	23.56361	12.63785	8.501015	10.1186
HORVU6Hr1G077790	1.449761	1.983341	2.396222	0.132478	0.10133	0.138614
HORVU7Hr1G080670	2.99445	2.980774	3.760544	4.12912	5.385697	5.100343
HORVU0Hr1G023890	28.13877	29.40153	13.37692	21.02318	47.53123	45.76256
HORVU3Hr1G073780	48.98402	51.03045	71.03243	51.8801	59.7256	67.81582

Hordeum_vulgare_newGene_175	14.556	14.77428	15.35466	16.04772	11.30959	9.855599
HORVU5Hr1G048360	7.865681	8.480274	9.230722	8.88309	11.17398	10.30318
HORVU2Hr1G036930	137.6399	128.5409	161.6915	51.00189	31.55235	32.05528
Hordeum_vulgare_newGene_179	13.4742	11.80244	15.29067	0.045856	0	0
Hordeum_vulgare_newGene_178	3.074527	2.459945	3.760643	0	0	0
HORVU3Hr1G069830	0.645905	0.42215	0.511055	2.094039	1.803984	1.791846
HORVU7Hr1G063030	0.931959	1.057861	1.089967	2.4667	3.088978	3.699493
HORVU2Hr1G011870	0.200447	0.187163	0.131988	3.02352	3.648305	3.594127
HORVU0Hr1G029340	0.813069	0.860687	0.592918	0.494606	2.616748	1.501265
HORVU2Hr1G022600	1.145819	0.849911	1.639206	1.418806	1.399311	1.590744
HORVU5Hr1G046480	0.215819	0.203214	0.322891	3.320687	23.36217	20.14368
HORVU5Hr1G043470	2.573729	0.766585	2.509333	4.61528	6.198801	6.296442
HORVU1Hr1G084220	13.41948	11.31551	18.01389	13.89341	17.67124	17.15608
Hordeum_vulgare_newGene_11445	0.842233	1.085411	0.933175	1.359587	1.498423	1.740365
HORVU2Hr1G038160	1.641732	1.940556	1.29442	3.097565	3.782383	3.396594
HORVU1Hr1G064890	2.648776	3.789541	5.424042	2.423729	0.405255	1.60936
Hordeum_vulgare_newGene_11273	4.693441	5.404517	5.550601	6.490108	7.502992	8.757739
HORVU3Hr1G075250	14.00491	14.88761	13.7119	18.30161	25.68134	24.73094
Hordeum_vulgare_newGene_11270	0.886333	2.714674	2.264883	2.633893	2.983306	3.846874
HORVU2Hr1G083720	16.29655	20.73848	24.31445	9.80987	11.69337	11.55263
Hordeum_vulgare_newGene_284	14.2026	12.23366	17.37637	21.10962	25.74042	23.72647
HORVU7Hr1G080580	16.96889	15.48297	17.16671	24.39507	28.21252	29.04614
HORVU2Hr1G071980	19.19461	17.53589	13.05007	21.06602	19.23719	18.54476
HORVU7Hr1G083160	12.45797	14.34444	12.23062	14.37747	19.09961	18.36887
HORVU5Hr1G070120	5.137275	5.302174	6.842005	7.804621	7.563676	9.198652
Hordeum_vulgare_newGene_12232	7.096413	5.670636	6.957404	12.59711	15.11604	15.85825
Hordeum_vulgare_newGene_12230	28.82502	23.17178	25.40284	28.82908	37.23838	38.48626
HORVU5Hr1G072770	0.831391	0.644731	0.561939	2.871019	2.898753	3.348445
HORVU3Hr1G067400	36.20338	36.12064	28.04217	30.95664	31.28301	26.8243
HORVU5Hr1G087730	1.829392	22.4311	30.6351	2.086015	4.850479	4.570321
HORVU6Hr1G024850	2.952197	2.592798	2.565284	2.487446	1.139964	2.062966
HORVU2Hr1G062800	8.491227	9.165885	7.336343	9.560809	10.25355	10.36024
Hordeum_vulgare_newGene_8001	2.81176	3.977066	2.543834	4.021296	3.254009	3.561973
HORVU1Hr1G085170	84.06779	74.12015	128.2394	73.1482	144.6224	126.1772
HORVU1Hr1G032900	11.95046	11.84549	12.89425	14.19881	16.73151	15.28072
HORVU7Hr1G039670	19.66855	24.33106	18.70909	8.360541	30.24753	23.90848
HORVU0Hr1G017540	54.60076	45.01286	77.79342	29.69092	37.78629	40.65266
HORVU2Hr1G022920	12.87614	14.51963	12.93889	26.60575	54.24173	52.50852
Hordeum_vulgare_newGene_12491	9.478209	11.22076	14.74404	4.759657	4.308328	5.21817
HORVU2Hr1G103000	9.17784	8.056496	6.823583	29.48666	12.84431	17.43681
HORVU6Hr1G065800	1.080526	1.096477	1.408854	0.560046	1.221384	0.878716
Hordeum_vulgare_newGene_13542	0.675697	0.752874	0.599299	1.614623	1.818295	1.297141
HORVU3Hr1G116790	36.25353	20.53954	24.14182	79.19886	104.4998	104.7607
HORVU3Hr1G094130	5.373037	6.777911	8.832675	7.832286	7.406505	7.897688
Hordeum_vulgare_newGene_13549	15.13781	12.72477	17.55675	11.99509	16.45423	15.65673
HORVU5Hr1G107440	12.66522	13.57331	14.74011	21.15611	35.44081	29.35359
HORVU3Hr1G068000	0.896116	1.910341	1.95929	2.163905	1.532443	2.316493

HORVU1Hr1G023260	15.37149	13.35685	18.97149	29.69247	47.63526	40.27768
Hordeum_vulgare_newGene_6048	2.359915	12.68969	2.982427	12.05291	0	0.103233
HORVU4Hr1G074320	0.183086	0.389252	0.296388	1.895893	2.624681	2.882521
HORVU2Hr1G018260	9.474499	8.856854	9.6434	12.11248	17.01429	21.37627
HORVU4Hr1G054650	3.549802	2.701586	3.735667	2.48428	2.39381	3.26791
Hordeum_vulgare_newGene_6040	6.691748	7.016001	6.496369	6.972719	8.586435	9.646565
Hordeum_vulgare_newGene_6041	0.228394	0.068976	0.091717	1.854431	3.022932	3.382884
HORVU6Hr1G032800	30.39328	25.37034	29.77134	31.8717	42.49147	43.17992
HORVU2Hr1G074850	33.64248	27.66558	33.20022	29.23149	39.06245	40.63556
Hordeum_vulgare_newGene_7936	1.038029	1.151235	1.302045	6.919985	10.3904	9.525778
Hordeum_vulgare_newGene_7935	2.66348	2.653923	2.40491	3.126474	3.602241	3.144129
HORVU3Hr1G110430	3.024387	4.110167	3.867848	5.443253	8.155096	8.160349
Hordeum_vulgare_newGene_3720	3.795118	4.885393	5.99389	5.207865	5.989734	6.150553
Hordeum_vulgare_newGene_3721	1.294547	2.402697	0.220945	1.029058	1.456358	0.21735
HORVU5Hr1G075810	0.616268	0.532551	0.839965	1.396133	2.235787	1.818201
Hordeum_vulgare_newGene_3727	0.541904	0.628307	0.692656	2.306695	4.569194	2.91039
HORVU6Hr1G021980	6.989657	6.659575	7.492866	6.611621	6.268839	7.47365
HORVU6Hr1G058710	5.328732	4.846788	6.07981	4.37249	4.001498	4.640056
Hordeum_vulgare_newGene_5899	3.153043	3.467815	2.462759	0.701645	0.424045	0.403876
Hordeum_vulgare_newGene_5893	0.921399	0.508336	0.668624	0.665557	1.753843	1.776787
Hordeum_vulgare_newGene_5897	3.566946	3.365068	2.617876	1.810317	1.563859	1.544147
HORVU1Hr1G034880	22.49266	23.71186	25.22869	27.9747	24.53243	25.36191
HORVU7Hr1G070080	0.467054	0.68612	0.883619	0.897316	5.972393	6.847909
HORVU5Hr1G057440	2.800786	3.858162	3.676544	5.583059	6.935739	6.637843
HORVU7Hr1G110990	3.916132	4.53062	4.384377	6.532349	5.082916	5.961072
Hordeum_vulgare_newGene_5760	7.96305	7.372913	10.06692	8.84667	10.16235	10.74376
HORVU4Hr1G011510	1.253574	1.487544	1.211779	2.34772	3.456951	3.375712
HORVU3Hr1G016440	1.028668	0.964343	2.358231	1.062417	1.26886	0.881852
HORVU5Hr1G115510	10.8297	10.33146	10.54414	12.76029	14.8934	13.02242
Hordeum_vulgare_newGene_900	2.695648	6.14456	3.602178	0.034512	0.039966	0.031098
Hordeum_vulgare_newGene_902	1.47241	1.513091	1.399598	1.267013	1.476339	1.906186
HORVU3Hr1G111740	95.77992	77.44586	116.9392	74.82526	141.1449	129.4793
Hordeum_vulgare_newGene_908	18.72891	15.39555	27.07124	16.51862	37.49487	34.30105
HORVU2Hr1G119820	1.573828	1.809698	1.56154	5.183942	4.968478	5.315948
HORVU2Hr1G031260	49.97637	45.62528	52.38986	48.82875	67.33287	62.61742
HORVU6Hr1G064440	3.368772	3.538222	3.045882	4.345865	7.323647	6.66223
HORVU1Hr1G063880	1.976328	1.627412	1.436128	1.073539	0.346876	0.559442
HORVU0Hr1G004350	4.317333	5.146666	4.360042	7.02615	5.372274	6.763716
HORVU7Hr1G119610	11.09524	10.40273	13.60048	7.89886	10.03422	9.10716
HORVU4Hr1G074810	19.57167	16.47978	21.28387	34.05125	44.03426	46.22208
HORVU6Hr1G068750	34.63717	29.31802	37.30899	36.88728	62.69808	57.99164
HORVU0Hr1G019750	3.244478	3.13814	3.756343	3.86791	4.568019	5.041767
HORVU1Hr1G032960	2.944396	3.93292	5.123636	3.097365	3.669161	4.639786
HORVU7Hr1G113110	29.29401	24.94799	30.06478	62.00331	70.35615	64.81085
HORVU3Hr1G016990	3.993379	5.195594	5.703317	1.026683	0.993207	1.273209
HORVU4Hr1G089450	395.4855	379.426	344.7628	166.2702	60.05418	71.06387
HORVU6Hr1G060690	11.86684	13.21274	14.53593	10.8891	10.6965	13.33225

HORVU0Hr1G008470	11.76198	11.30065	11.31243	18.20055	22.22803	23.50426
HORVU6Hr1G073040	2.981745	2.918822	2.916389	4.551076	6.113041	4.113645
HORVU1Hr1G027810	22.10094	23.87149	22.06748	25.02423	19.6255	24.13669
HORVU2Hr1G033470	1.501544	1.993186	2.760582	6.610244	23.99162	17.47339
HORVU3Hr1G071030	6.21474	6.605414	7.788758	2.274714	1.713527	2.10292
HORVU2Hr1G095160	3.732244	3.424745	3.535714	4.37305	4.773704	4.249261
HORVU6Hr1G020320	2.466699	2.556191	4.193087	3.282206	4.14145	4.231937
HORVU4Hr1G005500	2.566472	1.793333	3.091498	2.690099	3.01879	4.141113
HORVU1Hr1G061690	4.520431	4.909328	6.100455	5.440372	7.538059	7.682401
HORVU3Hr1G014590	2.614559	2.829063	2.627673	4.236077	4.592429	5.97093
HORVU3Hr1G081940	1.631665	2.092223	2.353646	6.754932	11.18556	9.928999
HORVU3Hr1G042770	3.749663	4.960319	5.002518	6.060203	6.381085	6.972364
HORVU6Hr1G031640	6.1821	5.337986	7.583382	5.444294	1.902952	3.671657
HORVU2Hr1G104940	6.862735	7.088247	7.727636	9.556275	14.84548	14.13372
HORVU3Hr1G046600	2.867852	3.316312	3.334656	4.481229	6.249446	5.959168
HORVU4Hr1G008150	11.46951	12.1349	13.09836	9.754098	10.48243	10.88857
HORVU4Hr1G018800	5.745259	8.602758	5.932386	2.501303	5.255431	4.965739
HORVU5Hr1G057730	4.875363	3.670806	6.171435	1.525516	1.675249	1.13227
HORVU2Hr1G069440	5.710757	4.784446	4.289784	1.315719	1.373579	1.662014
HORVU7Hr1G115870	23.59751	27.24883	29.04996	20.8894	49.47374	40.22081
HORVU5Hr1G078950	9.515134	9.974263	11.22434	28.12376	32.1125	32.81406
HORVU1Hr1G064970	2.247655	2.100079	2.166088	2.238815	4.605733	4.964683
HORVU6Hr1G009490	10.43266	7.40219	12.01629	14.96215	15.56409	12.44803
HORVU3Hr1G070780	1.493595	0.919043	1.585219	2.003533	2.83743	3.14149
HORVU7Hr1G090510	0.989308	0.936696	1.25916	1.531979	0.833477	1.082533
HORVU6Hr1G094680	0.387403	0.578949	0.646651	0.888959	2.487674	2.214695
Hordeum_vulgare_newGene_7589	7.524182	4.849018	7.025956	5.643811	5.228333	4.630222
HORVU3Hr1G000880	21.4663	22.5009	24.99185	19.50273	19.74599	23.62435
Hordeum_vulgare_newGene_7582	45.98329	46.21309	55.65308	62.37855	76.33753	73.02832
HORVU6Hr1G062870	1.46744	1.766575	1.329265	1.300966	1.885	2.304854
Hordeum_vulgare_newGene_7587	9.600678	10.08951	9.047108	0.077018	0	0
Hordeum_vulgare_newGene_4587	19.33065	23.24079	25.97006	20.78043	19.0153	24.56977
Hordeum_vulgare_newGene_4585	5.626085	5.34078	6.367304	9.210668	8.701484	10.25039
Hordeum_vulgare_newGene_4582	9.818379	10.14427	13.1026	12.2874	15.84905	16.78717
HORVU1Hr1G069830	19.44512	18.90089	17.02244	68.68219	45.28541	43.8025
Hordeum_vulgare_newGene_4588	9.963075	7.007003	11.181	4.009277	5.931487	2.530185
HORVU6Hr1G028660	1.94726	1.8986	1.977368	1.342923	2.487024	1.620866
HORVU0Hr1G006440	1.883898	0.473265	1.804084	2.092249	0.964903	1.862072
HORVU0Hr1G031730	11.5873	11.66529	9.608034	13.16477	9.515597	11.22795
HORVU5Hr1G060480	0.04853	0.041258	0.071502	0.636302	4.695533	4.190036
HORVU1Hr1G087820	16865.48	14523.3	16373.99	17676.07	10855.66	10194.16
HORVU0Hr1G000840	11.24694	5.784461	13.89377	0.884524	1.800015	3.34892
HORVU0Hr1G039250	20.96975	23.9863	24.34855	24.86079	38.8116	36.4104
Hordeum_vulgare_newGene_7054	2.214518	2.518862	2.491671	2.703112	3.581017	3.53363
HORVU4Hr1G073130	11.54461	10.38391	14.22199	14.48396	13.88142	14.28017
HORVU1Hr1G056510	4.035398	3.976843	4.384799	9.323189	14.42089	14.58624
HORVU3Hr1G091270	8.3193	8.095056	8.836372	10.31001	12.32575	13.2094

Hordeum_vulgare_newGene_6022	6.655301	7.378869	7.721177	0	0	0
HORVU1Hr1G075610	0.682133	0.758625	0.700488	1.782944	0.926344	2.112744
Hordeum_vulgare_newGene_1406	1.292923	1.057276	0.929878	1.939025	1.907431	2.090683
HORVU7Hr1G013170	15.8745	12.08061	16.17827	14.30299	4.689708	9.619242
HORVU3Hr1G034860	1.067519	0.893017	1.620405	2.203	4.258446	3.90722
HORVU0Hr1G015510	1.668692	1.660574	2.105785	1.246062	0.802328	1.05131
HORVU3Hr1G031980	0.722593	0.814343	0.727794	1.942561	3.693151	3.665886
HORVU3Hr1G035400	18.94424	15.22884	19.82246	12.66983	18.26099	16.6086
HORVU1Hr1G055890	27.89395	29.3948	27.88768	24.24527	28.04218	28.02162
HORVU7Hr1G093370	35.48384	37.30029	53.25448	21.44576	18.21314	23.06579
HORVU7Hr1G056430	4.095914	5.632565	5.903175	5.22763	4.341473	5.484221
HORVU3Hr1G086410	26.93956	24.19441	37.43053	23.62973	37.6433	33.54244
HORVU3Hr1G039760	1.787177	2.031729	2.179554	2.58967	3.839027	4.278878
HORVU5Hr1G012900	16.51165	16.59371	12.18327	16.15783	15.7959	16.73296
HORVU7Hr1G052530	11.25666	8.012924	11.51456	21.93258	32.73902	26.22315
HORVU4Hr1G000020	62.68244	53.40673	76.1865	62.80812	124.1722	106.6727
HORVU3Hr1G075540	23.46925	24.00804	23.54614	33.47963	46.23926	41.82198
HORVU5Hr1G058070	2.210762	2.147957	2.563167	1.121718	1.260467	1.514376
Hordeum_vulgare_newGene_5798	2.053014	3.178751	2.451431	9.282297	7.748806	6.303563
HORVU2Hr1G051850	7.793419	7.06492	7.931292	10.83029	11.96388	12.51884
Hordeum_vulgare_newGene_8551	6.16979	6.440599	6.806497	8.233449	8.662655	9.744671
HORVU4Hr1G081220	21.93421	21.90342	24.25592	36.5048	65.27003	64.14245
HORVU6Hr1G029220	1.137377	0.698884	1.297376	2.117283	7.758548	8.325446
HORVU5Hr1G122960	0	0	0	6.088439	5.09801	4.984941
HORVU5Hr1G123670	3.049509	3.525431	4.656747	4.541432	3.322993	2.797118
HORVU5Hr1G006280	1.334131	0.736524	0.845879	1.436484	0.866161	1.348389
HORVU5Hr1G095460	3.441645	2.472526	3.245231	3.149821	1.855707	2.255726
HORVU1Hr1G021760	1.977731	1.591134	2.146023	1.127226	0.650419	1.110802
HORVU1Hr1G078480	2.341318	2.518729	3.395198	0.834052	0.828242	0.823761
HORVU6Hr1G080550	13.35218	11.65834	14.59358	9.180185	11.15269	10.38252
HORVU3Hr1G030580	8.897643	10.16557	8.663922	12.30746	10.54129	13.96307
HORVU5Hr1G065960	9.090698	7.903598	7.97107	15.98179	18.5372	19.73221
HORVU6Hr1G033430	7.200579	8.552404	8.958959	11.10929	14.92658	13.31541
HORVU3Hr1G097830	0.210948	0.03311	0.059562	9.612537	85.69547	82.62034
HORVU7Hr1G112980	6.254348	8.916674	8.31022	9.638537	18.21449	15.54176
HORVU7Hr1G000270	35.64158	26.86569	26.96624	10.66853	14.75117	12.31673
HORVU5Hr1G012010	2.186556	1.891944	2.603149	0.019829	0.017058	0.042592
HORVU6Hr1G038700	1.027976	0.841248	0.876532	1.293743	2.861521	3.015464
HORVU1Hr1G093550	2.352027	2.389584	2.48482	3.460023	3.988793	3.53067
HORVU3Hr1G072540	11.4984	12.90602	10.4879	13.68218	18.03295	18.29926
HORVU5Hr1G112530	9.418743	9.996002	11.00017	16.13414	17.00801	18.26085
HORVU7Hr1G022210	11.5518	13.64584	10.75775	14.62347	20.24141	18.46057
HORVU5Hr1G029890	3.181518	2.260125	3.354094	3.263432	5.339413	4.921929
HORVU1Hr1G026920	6.313147	7.96892	4.481186	4.170062	32.62252	24.30222
HORVU7Hr1G094980	3.995127	4.552616	7.182856	3.543117	1.096162	2.679072
HORVU4Hr1G080570	20.60503	19.63529	17.87746	18.04462	13.27618	13.46471
HORVU6Hr1G004980	2.768784	3.477711	3.983979	1.288124	1.226584	1.046256

HORVU5Hr1G106140	2.437758	3.175445	3.64755	5.184461	7.353754	7.224233
HORVU1Hr1G062900	34.35046	30.81823	46.53532	37.02756	73.53647	73.87641
HORVU5Hr1G028410	3.730473	3.342406	3.487114	3.26635	6.094515	5.30464
HORVU3Hr1G019190	33.27296	27.78706	38.73984	43.99495	20.87525	27.46066
HORVU5Hr1G092270	5.842253	5.683024	5.760203	5.15651	5.939794	6.25021
HORVU7Hr1G045330	1.754501	2.001442	2.690794	4.565534	4.211458	4.842112
HORVU4Hr1G007890	18.38807	20.73641	20.02286	17.85146	23.27904	24.00752
HORVU2Hr1G057860	8.998736	9.793458	9.093219	12.30462	17.57003	19.18967
HORVU5Hr1G110000	70.4898	75.62652	74.57756	72.30316	83.37381	75.22126
HORVU2Hr1G097910	1.221325	1.591585	1.376457	4.77069	4.801636	5.07725
HORVU5Hr1G097170	4.754351	4.853509	6.778146	3.437176	4.487309	4.325729
HORVU7Hr1G120190	2.415099	2.333818	3.016147	4.761485	7.219057	7.163772
HORVU2Hr1G098670	1.738615	0.943913	2.106031	0.374046	0.428523	0.586107
HORVU5Hr1G003010	27.61872	23.36931	25.03683	27.90363	29.11963	27.53346
HORVU5Hr1G109130	28.3739	23.97393	22.77045	31.8809	80.97403	34.07357
HORVU4Hr1G014110	0.970633	0.685618	0.732117	1.583234	2.671388	2.215088
HORVU7Hr1G091440	6.10893	3.984689	3.961488	6.529379	4.608634	4.595762
HORVU2Hr1G047260	0.937672	1.156693	1.101977	1.413116	3.108343	3.455156
HORVU7Hr1G077470	3.592986	3.797841	3.524924	3.584765	2.813716	3.137398
HORVU4Hr1G009360	2.578664	2.903894	3.396461	3.232263	4.349983	5.203048
HORVU5Hr1G011100	1.084318	0.740217	1.081672	3.942209	5.17009	5.515163
HORVU6Hr1G091520	4.530925	4.303944	4.926906	6.327245	9.593848	9.818187
HORVU4Hr1G052790	2.501827	2.711981	2.817745	1.011261	2.863539	1.911857
Hordeum_vulgare_newGene_10218	428.2114	491.8741	616.6581	22.99007	0.525867	9.870485
HORVU5Hr1G119180	11.11948	9.494389	9.639118	16.6351	16.74775	18.69811
HORVU7Hr1G095210	5.453955	4.982607	6.42127	3.216127	4.971817	4.409073
HORVU1Hr1G002170	72.06587	76.13848	100.4798	38.10297	31.85505	31.85752
HORVU2Hr1G103240	0.835156	0.889783	1.032495	1.552585	1.597929	1.720359
HORVU2Hr1G063420	57.41373	56.6802	69.11096	58.47709	83.79571	82.43437
HORVU6Hr1G092870	3.89855	3.702411	3.669715	3.555255	4.152548	4.24765
HORVU1Hr1G063220	4.436603	4.743912	5.335141	9.125544	8.737852	9.0218
HORVU0Hr1G000320	2.7817	3.809713	4.110862	4.29535	3.770201	4.34858
HORVU1Hr1G049270	1.033878	1.143176	1.5593	1.068244	0.432625	1.576421
Hordeum_vulgare_newGene_12804	9.55356	11.17564	8.110152	10.35788	13.41429	12.93029
HORVU7Hr1G057280	2.828823	2.823507	2.979572	4.217916	3.061625	4.111554
Hordeum_vulgare_newGene_12961	1.243229	4.201388	1.728557	2.340602	1.128829	2.441936
Hordeum_vulgare_newGene_12966	1.935588	2.429018	2.753055	1.683022	2.613658	3.028771
Hordeum_vulgare_newGene_12969	2.722081	3.031736	3.012572	6.275677	3.45482	4.496397
Hordeum_vulgare_newGene_12809	3.438967	3.133786	2.336611	4.245325	3.671332	4.844672
HORVU3Hr1G034230	11.33586	12.70884	15.2142	5.295187	6.690615	6.207234
Hordeum_vulgare_newGene_7955	3.859556	3.619026	4.017575	10.25712	21.82091	20.20947
HORVU5Hr1G108770	0.952286	1.142163	1.223552	1.453005	1.865485	1.272065
HORVU5Hr1G104450	30.93468	27.46917	29.84198	23.04388	24.65861	26.51351
HORVU4Hr1G051850	3.919313	3.985551	5.288407	3.197162	2.472339	3.501804
HORVU5Hr1G116400	1.55974	1.422946	1.788886	2.111244	3.410664	3.727857
HORVU2Hr1G013900	6.58023	7.23656	6.799551	7.850723	11.22927	11.02903
HORVU6Hr1G079670	0.934517	0.521283	1.600439	1.050331	1.665377	2.238402

HORVU0Hr1G015890	5.07166	6.311403	6.403017	4.648426	4.093002	5.942679
HORVU4Hr1G059660	38.93922	34.77962	36.59306	34.93166	39.16466	39.01658
HORVU4Hr1G012220	5.124603	4.036089	5.967935	19.33867	23.37269	23.02331
Hordeum_vulgare_newGene_4638	4.780052	4.15174	5.154964	11.18172	14.42542	13.79562
Hordeum_vulgare_newGene_2032	6.399619	5.823546	6.144722	6.332409	6.337413	6.291821
Hordeum_vulgare_newGene_2035	7.249621	6.972618	6.096688	12.22449	14.75636	15.56854
Hordeum_vulgare_newGene_2036	0.527943	0.585043	0.159132	2.63731	3.437344	4.312529
HORVU1Hr1G016170	10.97938	10.29235	12.25535	8.257426	5.903284	6.779995
HORVU1Hr1G046940	9.748795	8.613197	9.499672	14.52517	23.65867	19.98571
HORVU2Hr1G108070	2.342009	1.843401	1.999251	3.490264	2.678548	2.92097
HORVU5Hr1G007920	19.93493	20.51342	21.58229	18.66172	21.37623	23.52098
Hordeum_vulgare_newGene_9662	4.314199	3.856578	1.600908	5.575701	5.171541	5.650438
HORVU2Hr1G099230	125.625	124.5547	191.4929	177.2708	254.5919	215.1349
HORVU2Hr1G101790	20.40923	18.20101	20.81806	26.85229	27.53319	28.2624
HORVU2Hr1G055830	3.946803	4.418967	3.83181	4.249435	4.075766	4.628939
HORVU3Hr1G064730	8.718616	9.415292	9.324576	5.888669	12.98822	15.1785
HORVU1Hr1G032310	12.55017	13.14081	12.2016	15.88804	20.82601	20.17541
HORVU7Hr1G072710	3.037627	3.368306	5.127444	3.720732	3.56077	4.464845
HORVU4Hr1G061700	109.5326	88.12239	124.2334	82.76287	84.50434	81.3766
HORVU7Hr1G043410	4.041147	5.446204	6.57712	2.410077	2.377806	2.809707
HORVU3Hr1G065280	1.173921	1.039647	0.743216	2.346546	1.698065	2.468757
HORVU5Hr1G042600	10.64757	13.62902	13.20543	13.68276	15.92334	13.82573
HORVU3Hr1G026610	34.07551	25.97602	34.49497	16.9352	9.218002	11.36039
HORVU5Hr1G046110	1.313227	0.801422	1.005527	0.964114	1.468983	1.22294
Hordeum_vulgare_newGene_8139	3.665292	3.966905	3.504678	0	0	0
HORVU4Hr1G031740	15.53036	16.48557	19.32374	10.22601	14.44234	15.44381
HORVU2Hr1G072530	11.44584	10.87537	14.76129	11.15427	13.74767	13.01166
HORVU5Hr1G052150	15.49713	6.039396	26.68473	1.516836	0.095295	1.672831
HORVU6Hr1G014610	14.8882	16.7681	21.90994	19.04804	27.01986	25.67256
HORVU1Hr1G036200	2.977812	2.565319	2.583523	4.384392	7.321226	8.174823
HORVU1Hr1G001660	1.474278	8.829568	2.374689	10.50469	0.377476	0.603049
HORVU5Hr1G093660	1.438876	1.994837	2.129508	2.390454	1.73792	2.022381
Hordeum_vulgare_newGene_11279	1.064299	1.946728	2.120698	0.457229	2.143318	0.602294
HORVU4Hr1G036670	8.474803	8.606003	9.211841	8.383083	9.549172	9.997437
HORVU5Hr1G086760	1.055233	0.956015	1.368232	2.142275	3.528226	3.233119
HORVU1Hr1G051410	10.77953	10.78939	13.36244	9.109621	15.76348	14.2622
HORVU1Hr1G014370	16.71542	18.63648	18.59267	13.49156	18.00641	20.40016
HORVU3Hr1G074730	2.159251	1.754747	1.729737	1.703907	2.190422	1.843357
HORVU6Hr1G085890	68.31114	70.0669	92.4833	41.56476	43.95953	46.43352
HORVU4Hr1G076820	12.66892	9.389862	16.87196	9.046451	4.775807	6.284801
HORVU6Hr1G025460	5.216447	3.011524	4.92099	15.37071	15.69152	15.49475
Hordeum_vulgare_newGene_10612	14.34926	13.25427	12.89268	14.80079	13.96405	15.35272
Hordeum_vulgare_newGene_10611	14.56789	15.46291	17.1082	15.96657	15.80254	16.41633
HORVU1Hr1G031660	6.991355	8.585812	8.461582	16.24231	15.05612	19.37676
HORVU1Hr1G001080	5702.466	3222.493	3376.332	25520	8074.92	12568.62
HORVU5Hr1G049730	7.167772	7.07357	7.924495	8.799995	9.227048	8.979335
HORVU2Hr1G122000	6.11081	6.10924	6.897269	6.001386	6.272582	6.934544

Hordeum_vulgare_newGene_13967	34.45117	33.35749	32.65388	38.63451	43.44708	40.23663
HORVU4Hr1G022830	0.944721	0.991527	0.914249	1.42947	0.885325	1.303523
HORVU3Hr1G094820	2.655511	2.911026	2.175844	8.669497	22.59484	20.98721
HORVU2Hr1G022410	1.980633	1.625587	2.030687	2.302789	3.85896	4.013825
HORVU2Hr1G029440	28.21516	22.21965	19.15957	65.59208	34.25385	38.20581
HORVU5Hr1G067910	5.873012	6.452532	6.799391	5.320332	5.122105	5.003928
HORVU7Hr1G101050	1.027475	1.152151	1.560994	1.679502	2.359796	2.184142
HORVU2Hr1G036870	7.203136	7.712643	6.544958	9.616024	7.760222	9.938685
HORVU7Hr1G046890	2.847249	2.687566	3.610774	2.961886	3.912083	4.826953
HORVU1Hr1G052350	32.8834	32.32674	29.59185	12.90625	7.038181	8.443224
HORVU2Hr1G108150	2.353656	2.443717	2.540386	1.539457	2.205235	2.513172
HORVU7Hr1G031320	44.14246	46.30529	46.01576	56.52473	73.8949	57.18941
HORVU6Hr1G054050	1.002469	0.533316	0.633775	1.774511	3.460591	3.715271
HORVU3Hr1G080150	6.794735	7.577581	10.2901	4.763618	7.548638	7.42831
HORVU1Hr1G030200	4.84696	5.191057	5.732203	12.98918	14.128	16.28988
Hordeum_vulgare_newGene_322	1.588932	1.853772	1.329693	1.633989	4.236176	3.817091
Hordeum_vulgare_newGene_328	9.84917	7.063928	9.712452	15.39894	9.227318	11.75583
Hordeum_vulgare_newGene_329	10.80828	9.8957	11.77868	20.24908	21.90652	21.92297
HORVU2Hr1G033960	7.760886	8.156655	10.57381	8.225221	8.508409	9.188592
Hordeum_vulgare_newGene_2992	5.639558	2.037243	3.707007	3.355935	2.165368	3.540219
Hordeum_vulgare_newGene_2991	0.04162	3.126189	3.598066	2.681897	1.549262	0.638428
Hordeum_vulgare_newGene_2998	7.445225	7.192045	7.881025	10.27655	14.81191	13.7467
Hordeum_vulgare_newGene_2999	3.355316	3.49146	2.526972	2.921073	3.390206	3.854513
HORVU6Hr1G074520	2.500465	2.523424	2.423729	0.159479	0.476969	0.381718
HORVU3Hr1G062590	57.90349	55.42288	91.95174	59.89236	142.4042	135.3007
HORVU2Hr1G013570	6.177115	5.828497	7.073845	10.98717	12.56008	11.59192
HORVU3Hr1G107310	2.858954	3.323909	3.592552	3.1691	3.09896	3.749573
Hordeum_vulgare_newGene_255	3.082907	3.442968	4.851519	1.920384	1.606213	1.977194
HORVU7Hr1G052730	2.635435	2.870296	2.688561	1.351319	3.089679	2.995135
HORVU7Hr1G002600	0.619054	0.808831	0.879302	0.826275	1.676346	1.681877
HORVU2Hr1G020120	1.67482	1.593703	1.293447	0.807193	1.912354	1.519405
HORVU2Hr1G075220	3.454991	3.479785	4.578584	3.938	6.686903	7.289317
HORVU6Hr1G070180	10.50557	8.496426	10.60651	9.294261	10.70329	9.745317
Hordeum_vulgare_newGene_13735	1.075625	1.168255	1.191265	1.430259	0.915404	1.262998
Hordeum_vulgare_newGene_13736	3.855726	2.855608	3.834775	2.441566	2.126813	1.720906
HORVU7Hr1G059850	49.13362	42.58493	48.06748	33.73925	22.49438	24.99815
Hordeum_vulgare_newGene_14508	4.116353	3.530355	4.140648	4.408155	5.670563	5.651881
HORVU3Hr1G060040	8.06678	7.383389	10.21238	14.13488	24.32251	23.73745
HORVU1Hr1G029770	1.964264	2.117659	3.485863	6.454121	19.60048	17.92319
HORVU4Hr1G057530	7.194068	7.093319	8.793627	6.173178	8.0775	6.285554
HORVU1Hr1G071190	0.335989	0.085955	0.177624	2.643089	13.15439	9.122224
HORVU4Hr1G003090	3.999382	5.615993	4.373223	5.289437	5.715851	5.82315
HORVU4Hr1G045700	1.298017	1.632196	1.660089	1.713376	1.676781	2.363425
HORVU3Hr1G017120	1.955925	1.871306	2.110068	2.558182	4.124995	4.444334
HORVU2Hr1G048870	2.413154	2.350195	3.636636	3.105234	8.727187	7.054878
HORVU7Hr1G038370	2.919002	2.919903	3.79156	2.156839	2.982189	4.321451
HORVU6Hr1G002130	9.299953	8.576456	8.161627	10.29803	10.73117	10.43712

HORVU5Hr1G085520	26.26522	23.74454	33.75576	19.66209	15.52105	20.30541
HORVU5Hr1G064580	1.893891	2.045481	1.756725	1.995446	4.707197	4.495531
Hordeum_vulgare_newGene_2373	1.712356	1.795464	1.824344	3.534583	2.809729	3.089656
Hordeum_vulgare_newGene_2376	0.860797	1.057767	0.63602	6.775144	6.465247	7.616272
HORVU2Hr1G032180	0.606464	0.653871	0.637509	0.810387	3.765286	2.317414
HORVU3Hr1G068270	1.359385	1.316294	0.669406	2.356945	1.390502	2.363166
HORVU4Hr1G075840	15.80253	15.08183	19.14276	23.43015	21.64306	20.86774
HORVU3Hr1G012510	5.931804	6.17254	7.252574	0.024648	0.012536	0.048776
HORVU7Hr1G065200	4.490444	5.315209	6.013777	5.050343	6.030002	6.4026
HORVU2Hr1G081930	8.163646	7.8359	9.219263	9.35469	14.61751	13.54653
HORVU3Hr1G087210	11.42068	12.71667	11.16094	28.37281	27.51043	27.96681
HORVU3Hr1G048620	25.0039	21.006	28.69439	31.91989	43.52033	40.62872
HORVU7Hr1G054360	5.537445	3.051075	6.678024	9.118449	16.41501	13.65501
HORVU2Hr1G000360	4.868089	4.588931	5.980463	6.863629	7.168994	7.466679
HORVU6Hr1G053310	22.27926	21.96485	23.05504	28.54766	68.69775	55.81689
Hordeum_vulgare_newGene_9308	1.898834	2.192277	1.909029	3.504306	3.324307	3.896003
HORVU1Hr1G020360	7.890017	7.133218	7.837422	16.18675	18.36127	19.27059
HORVU5Hr1G065230	18.48651	16.33458	20.13746	17.836	25.86271	23.19029
Hordeum_vulgare_newGene_9300	0.873364	1.129203	1.244693	1.207155	1.680533	1.248985
Hordeum_vulgare_newGene_9307	3.40031	3.566597	3.616291	5.669928	6.225724	6.578246
Hordeum_vulgare_newGene_9305	9.287038	9.513931	9.057175	8.866162	6.057497	8.682112
Hordeum_vulgare_newGene_15369	3.39587	3.581227	3.001779	5.582426	6.127844	5.469335
HORVU1Hr1G077730	5.831995	5.570398	6.688296	7.497876	9.094776	8.983037
HORVU2Hr1G075910	4.622944	5.122865	6.989521	5.179025	7.676962	8.02695
Hordeum_vulgare_newGene_15361	3.422933	3.034009	2.484304	6.483658	12.31948	8.827219
HORVU5Hr1G074770	11.72907	10.24395	13.54953	12.08678	12.92547	13.24104
HORVU7Hr1G118770	4.353533	2.341414	5.112455	0.310766	0.111905	0.274501
HORVU1Hr1G047220	23.6275	21.94505	23.96522	9.320785	5.85909	10.6403
HORVU5Hr1G024550	96.46596	94.99058	112.8189	70.10745	54.11634	52.01602
HORVU5Hr1G097370	0.668868	0.789453	0.807204	1.974236	2.177751	2.200621
HORVU2Hr1G094110	18.73951	16.02149	18.05866	16.0543	15.14627	14.39461
HORVU2Hr1G098470	1.954927	3.312573	2.023421	0.448143	0.421555	0.57038
HORVU4Hr1G043800	10.7186	10.44642	10.82711	15.34124	19.49275	18.33327
HORVU7Hr1G106390	10.11324	7.556049	12.37476	4.97576	7.012099	6.769101
HORVU5Hr1G035300	1.096062	2.108607	1.964574	2.598323	3.862675	4.561687
HORVU3Hr1G023790	15.21193	12.2634	15.21967	17.41773	16.57374	18.24974
HORVU2Hr1G085740	1.204255	0.949265	1.497541	2.380597	13.77019	8.139899
HORVU2Hr1G009900	0.336676	0.317683	0.23549	3.942341	3.117347	4.018995
HORVU7Hr1G015690	1.666058	1.657525	1.277123	2.159938	3.809695	3.30354
HORVU7Hr1G096300	27.15214	24.2383	24.13144	33.89894	44.54326	46.53111
HORVU2Hr1G077200	8.559486	7.659619	10.68153	6.363984	7.693745	7.604782
HORVU0Hr1G002020	4.28916	1.158175	3.086785	6.553071	7.595872	7.376801
Hordeum_vulgare_newGene_5440	3.946409	3.383233	3.406289	2.941048	2.235273	2.767799
HORVU7Hr1G113300	16.4958	14.81119	25.47252	5.030063	1.716261	2.779159
HORVU3Hr1G085840	14.16213	13.47387	13.38055	16.43236	15.2412	14.1847
HORVU4Hr1G005310	17.63	14.9813	21.7996	20.56715	25.63815	26.5883
HORVU1Hr1G079070	460.5827	480.0851	412.6177	362.0414	350.8203	346.5059

HORVU7Hr1G106020	0.249445	0.132359	0	1.285313	21.88177	24.23755
Hordeum_vulgare_newGene_1050	1.719769	1.736191	1.696809	2.882246	4.031703	3.410583
Hordeum_vulgare_newGene_1051	2.666296	3.462939	2.953786	5.022457	12.7928	11.31395
Hordeum_vulgare_newGene_1055	6.056177	5.659041	5.997835	9.520775	8.845392	9.602212
HORVU1Hr1G049070	8.899119	6.000075	9.208093	6.1864	3.689789	5.22059
HORVU6Hr1G068270	9.813242	10.38389	11.57797	12.5081	16.5272	14.86398
HORVU5Hr1G057670	3.378111	3.020472	3.531383	3.58723	3.97775	4.205167
HORVU2Hr1G095390	1.612949	1.459913	2.125223	2.531892	2.397637	3.124563
HORVU7Hr1G034120	1.572448	1.518544	1.421126	13.54877	9.362925	7.836466
HORVU7Hr1G101550	0.647555	0.412432	0.320986	0.395776	2.736653	2.798565
Hordeum_vulgare_newGene_4263	0	0	0.019704	3.888773	3.921636	4.758832
HORVU7Hr1G095760	0.491559	0.616886	0.637538	1.890678	2.196757	1.445038
HORVU6Hr1G036760	0.462774	0.3978	0.529416	1.904727	1.530387	1.294845
Hordeum_vulgare_newGene_4265	2.241836	2.5698	2.865978	0	0	0
HORVU0Hr1G029320	1.495598	1.905306	1.120932	1.12012	4.828164	5.092402
HORVU1Hr1G064830	19.24595	17.34692	18.07903	19.42521	17.07969	17.69377
HORVU3Hr1G010800	23.79882	23.595	41.51881	29.34754	36.59124	36.96346
HORVU5Hr1G021070	33.76622	29.50687	50.90569	11.40192	3.143752	6.656658
Hordeum_vulgare_newGene_13648	31.92394	30.1372	40.28239	30.5506	56.51904	50.93789
HORVU3Hr1G049820	33.61217	32.48058	38.27063	41.27122	48.44275	50.76272
HORVU7Hr1G085660	0.47137	0.220328	0.408898	0.831576	6.456509	5.295389
HORVU1Hr1G078380	0.187998	0.024241	0	3.881154	11.43209	8.705074
HORVU3Hr1G039130	7.433497	8.126499	12.50392	10.42034	23.61344	21.85248
HORVU4Hr1G072450	30.01233	27.93308	33.38515	24.14767	27.29017	24.29469
HORVU1Hr1G072650	49.86929	43.38009	43.9244	47.37029	50.15602	48.55741
HORVU1Hr1G043730	3.396526	4.237602	4.506611	3.871459	4.324313	4.678332
HORVU0Hr1G014070	6.095656	6.408377	6.84281	13.14555	13.38314	15.64268
HORVU1Hr1G064780	6.455874	5.221292	7.207965	11.58583	12.20208	13.99773
Hordeum_vulgare_newGene_13643	5.975942	7.200749	7.730097	0.014746	0	0
HORVU7Hr1G048940	3.805371	3.860199	5.349469	2.941927	5.106241	4.755713
HORVU7Hr1G114330	38.35423	38.06233	37.96273	27.13525	35.50625	30.33577
HORVU1Hr1G090360	4.415687	5.647875	6.082101	0.363913	0.027374	0.190612
HORVU3Hr1G098020	29.57322	27.34781	30.53102	24.13613	26.40013	27.02202
HORVU5Hr1G111340	10.72263	9.325871	16.52409	6.668122	10.58312	8.684478
HORVU1Hr1G075000	7.502462	8.210942	10.02053	5.1629	5.628839	7.180316
HORVU1Hr1G078030	6.070524	6.572731	8.525585	6.274819	7.819642	8.489716
HORVU4Hr1G004440	14.41094	11.50351	14.13293	13.83896	16.14944	14.38725
HORVU7Hr1G088980	3.077867	2.764608	3.832033	3.156837	4.307497	4.368457
HORVU6Hr1G021510	5.815206	4.677326	4.801336	5.157706	8.554267	6.486599
HORVU7Hr1G019780	640.9952	521.8188	511.759	2532.449	9351.832	6471.142
HORVU2Hr1G079200	10.11387	7.634508	10.00999	9.358808	9.739282	10.04001
HORVU6Hr1G003300	0.966268	1.116114	1.014505	1.379242	1.058241	1.567431
HORVU4Hr1G003940	5.792321	6.061803	7.497594	11.63342	12.0512	13.36657
HORVU2Hr1G065930	6.225076	6.714862	6.94453	9.072961	16.74005	17.56114
HORVU6Hr1G086060	5.126462	4.675999	5.783421	4.71482	4.140711	5.500101
HORVU3Hr1G006310	4.331079	3.539244	4.705698	0.233546	0.125077	0.166928
HORVU7Hr1G079210	0.690996	0.465954	0.52357	1.979973	3.085795	2.036402

HORVU7Hr1G018780	1.309618	1.472232	1.148515	3.477984	3.623419	4.01106
HORVU4Hr1G015000	20.05895	15.19179	7.763926	30.9577	2.820309	41.74735
HORVU7Hr1G007000	79.13988	83.09239	73.60112	58.84947	64.52535	64.88518
HORVU2Hr1G048110	9.302608	10.0312	11.66355	9.556989	11.19685	12.38438
HORVU6Hr1G000960	20.14537	19.46009	29.92309	17.44894	0.512669	7.974071
HORVU3Hr1G070240	3.608467	3.132398	3.954904	2.080434	1.449495	1.153095
HORVU7Hr1G056600	2.298435	1.997504	2.483282	3.932243	3.131301	3.18032
HORVU4Hr1G040290	41.1518	41.28569	38.82394	68.48384	83.50996	88.02406
HORVU6Hr1G057170	4.637174	3.219961	4.488102	2.928517	3.199724	4.280492
HORVU5Hr1G006330	2.045867	1.067515	2.346192	1.034687	0.309356	1.193956
Hordeum_vulgare_newGene_11100	211.9802	279.5481	315.4369	17.49574	2.420538	8.677576
Hordeum_vulgare_newGene_11101	0	0	0	2.479085	3.054544	4.000642
Hordeum_vulgare_newGene_11102	1.828889	1.734705	1.889112	2.481439	2.256852	2.743863
Hordeum_vulgare_newGene_11104	16.72724	13.21214	16.12685	12.14149	17.43981	15.92422
HORVU3Hr1G021810	5.092818	4.419382	5.255501	3.068941	6.921943	7.185698
Hordeum_vulgare_newGene_7532	4.253577	3.901094	4.794557	4.568056	4.091166	4.488243
HORVU7Hr1G092810	16.29431	13.44002	21.64003	14.13314	14.47548	17.55504
HORVU4Hr1G004990	3.934451	3.272146	4.928865	4.846353	7.513286	7.105203
HORVU4Hr1G050510	30.16193	22.68236	39.71495	7.318924	2.293286	3.916172
HORVU7Hr1G116650	1.402873	1.333532	2.264353	0.854314	0.130458	0.813422
Hordeum_vulgare_newGene_7530	1.078213	1.080019	0.982158	1.848104	1.32816	1.511702
HORVU7Hr1G089410	8.128419	7.970347	10.16611	13.21591	17.02354	18.78571
HORVU7Hr1G012630	0.815058	0.706408	0.703546	0.566312	13.06461	8.739343
HORVU1Hr1G065070	1.745441	2.898924	2.694065	2.034317	4.500938	4.175339
HORVU1Hr1G061550	10.50965	9.272749	15.01861	10.48989	12.07766	12.41549
HORVU5Hr1G094780	2.47439	2.402655	2.733384	3.735682	4.098344	5.220604
HORVU2Hr1G123730	2.117555	1.560195	1.781665	2.936322	4.088404	4.238265
HORVU5Hr1G086560	2.419503	3.20473	3.540687	2.800411	2.081	2.363266
HORVU3Hr1G062570	1.745341	1.515109	0.952349	0.699166	0.557377	1.006843
HORVU6Hr1G046420	2.247315	2.606673	2.130659	4.484092	6.250194	7.087897
HORVU6Hr1G041430	10.41586	8.131059	9.850435	9.118587	8.902328	8.80601
HORVU6Hr1G033600	0.510621	0.680176	0.344384	6.099081	7.822421	8.317895
HORVU2Hr1G014740	1.191731	0.897276	1.271942	1.558175	1.077657	1.032428
HORVU1Hr1G080330	1.612188	1.859628	2.679608	2.280174	3.157251	3.771739
HORVU5Hr1G093310	1.157771	1.309685	1.435896	1.530105	1.354238	1.897025
HORVU5Hr1G123350	6.6236	8.374623	6.139086	8.684115	9.709604	10.31663
HORVU4Hr1G069490	12.31289	10.24636	13.13281	14.90498	16.10009	16.54707
HORVU4Hr1G023200	10.48622	9.328524	10.82654	10.41746	23.683	24.00418
HORVU6Hr1G034050	3.179688	3.183313	4.306888	5.063368	9.699808	9.72073
HORVU1Hr1G004690	1.738315	2.34475	2.789745	3.238008	2.327613	2.521194
HORVU5Hr1G007270	8.486588	7.324571	7.077592	13.92819	16.46197	17.79406
HORVU1Hr1G066520	13.93231	10.86944	15.28899	15.26173	24.10529	17.1664
HORVU6Hr1G054720	9.337528	9.667379	11.38943	14.37119	19.01526	19.34691
HORVU2Hr1G061890	6.531841	9.024848	4.753879	1.953566	3.79852	2.016834
HORVU1Hr1G038950	2.018449	1.7331	2.157686	1.911848	1.298794	1.578832
HORVU3Hr1G026890	10.28602	8.044982	8.665073	18.14298	13.47115	14.02699
HORVU7Hr1G076290	0.558428	0.817788	1.306842	7.665237	10.24629	7.801833

HORVU5Hr1G049280	9.643876	11.04116	9.584219	6.666481	5.34528	7.141952
HORVU5Hr1G076600	33.83263	27.25151	44.68629	28.88722	35.01871	38.53464
HORVU2Hr1G067390	2.451319	3.204846	3.277121	3.221244	3.344825	4.234296
HORVU6Hr1G093190	4.611956	5.129404	5.928764	11.16745	18.00052	18.26546
HORVU5Hr1G054860	1.318232	0.8945	1.060836	1.292144	1.111601	0.656395
HORVU5Hr1G122000	3.80011	4.131307	3.377071	4.501474	6.059172	5.539067
HORVU2Hr1G111570	0	0	0	1.066161	2.436488	2.829083
HORVU5Hr1G051300	9.232186	10.42739	10.60231	7.809299	9.930708	10.54329
HORVU5Hr1G109300	63.48135	65.29927	61.71998	137.1232	129.7928	114.852
HORVU1Hr1G018740	13.00296	12.71566	15.00255	13.00975	15.66518	15.85305
HORVU6Hr1G090080	1.588752	1.383537	1.890153	3.450785	4.477159	4.783848
HORVU7Hr1G050030	3.396613	4.188371	3.779202	11.45425	9.898393	11.52099
HORVU3Hr1G023060	0.796798	0.845102	1.138777	0.978169	2.572749	2.804358
HORVU7Hr1G071740	0.675862	0.91622	0.94494	1.638878	1.753328	1.57166
HORVU7Hr1G120780	7.599792	7.48086	6.838824	8.199064	11.33807	11.89294
HORVU4Hr1G090850	2.974164	2.990269	4.408251	0.909011	0.437427	0.595541
HORVU6Hr1G014450	199.7614	179.1524	188.7729	227.4994	260.1034	229.6627
HORVU4Hr1G054420	3.203003	2.455925	2.834216	1.84538	1.521534	1.38036
HORVU6Hr1G029720	5.754149	5.289178	6.196507	4.953759	5.675103	7.243308
HORVU2Hr1G109910	6.131682	6.046051	7.391476	3.896174	6.69985	7.254795
HORVU2Hr1G060380	23.42942	21.91869	23.35325	28.62889	31.6109	34.90731
HORVU6Hr1G054520	986.6797	659.1664	1045.494	1106.9	1315.785	1401.123
HORVU7Hr1G047920	3.546514	2.461306	3.542108	4.825813	3.96437	4.31882
HORVU3Hr1G099130	6.425261	5.041369	7.060598	5.006673	5.36403	5.668026
HORVU1Hr1G082650	4.8751	3.855176	5.165607	2.885356	3.163647	3.280047
HORVU3Hr1G027760	1.517585	1.381104	1.512366	2.965847	4.465789	4.027998
HORVU3Hr1G105020	13.13782	14.48402	17.97425	11.11422	12.05488	13.61751
HORVU2Hr1G018380	2.03042	1.407853	1.695526	2.51611	7.27092	6.177924
HORVU3Hr1G025720	63.8298	67.98002	92.83683	9.134382	0.701535	3.714833
HORVU3Hr1G089360	17.9414	16.61179	15.9659	18.96174	27.88104	24.99895
HORVU7Hr1G075240	16.62067	14.78716	16.13806	17.76352	22.50612	24.32244
HORVU6Hr1G027370	7.881225	8.089339	11.82868	7.260674	9.822033	8.499807
HORVU6Hr1G088680	3.58238	3.890546	4.138006	1.738814	4.581771	4.228457
Hordeum_vulgare_newGene_3228	2.922726	2.649915	2.631343	3.374314	4.12054	3.939031
HORVU6Hr1G080290	1.49034	1.478714	2.299393	3.054782	3.022064	3.004555
HORVU7Hr1G047690	0	0	0.024291	3.031897	16.26476	9.454769
HORVU4Hr1G066580	1.194133	1.096806	2.059838	1.831456	1.391244	1.73392
HORVU7Hr1G095400	28.46739	28.60628	35.31986	23.47157	29.44769	28.54522
HORVU6Hr1G017720	2.366911	2.576479	3.865638	1.984397	2.380352	2.865126
HORVU2Hr1G070320	1.620242	1.487311	1.70873	1.779575	1.691791	1.665404
Hordeum_vulgare_newGene_2174	24.46822	29.16313	28.63526	24.67167	28.07278	30.2841
Hordeum_vulgare_newGene_2176	5.217465	5.738186	6.38009	5.30472	6.546099	6.745188
HORVU6Hr1G065380	32.28303	31.17785	31.72605	24.67379	21.55766	24.66343
Hordeum_vulgare_newGene_3593	0.894131	1.48357	1.306597	1.945221	0.942342	1.514901
Hordeum_vulgare_newGene_3595	1.176302	1.420513	1.188962	1.671185	0.348314	0.584899
HORVU7Hr1G028840	11.57907	6.554686	9.761244	34.10884	41.77873	48.53215
HORVU4Hr1G082070	6.157775	2.007841	6.59263	6.089668	5.095908	0.041241

HORVU2Hr1G107520	50.88353	49.85979	49.15317	75.02982	98.03898	86.03874
HORVU0Hr1G022550	1.502463	2.547422	1.402526	2.033551	1.233438	1.950886
HORVU7Hr1G074590	84.53084	76.81882	86.41802	57.3828	47.94528	49.93897
HORVU4Hr1G064670	4.376496	6.162425	4.978395	1.614441	2.839335	3.563596
HORVU4Hr1G078310	7.176014	5.503655	7.527215	3.545717	2.128359	1.951888
HORVU3Hr1G005500	8.957199	7.806735	7.54202	19.40722	14.21313	17.96606
Hordeum_vulgare_newGene_10327	6.624724	4.526854	6.458611	0	0	0
HORVU6Hr1G073980	32.32517	34.91109	35.2791	151.6751	120.2917	108.6918
HORVU0Hr1G001160	2.566906	2.448817	3.579476	3.113735	3.224663	3.457504
Hordeum_vulgare_newGene_13249	3.137538	0.649721	2.406107	2.367283	5.472486	0.788785
Hordeum_vulgare_newGene_13248	1.272539	1.108216	1.574896	1.546155	1.245908	1.505571
HORVU2Hr1G039880	1.890094	2.205404	1.808744	5.295725	8.761954	8.107034
Hordeum_vulgare_newGene_13240	1.1051	1.135023	1.243115	1.061484	0.770763	1.297111
Hordeum_vulgare_newGene_13242	4.810994	5.551642	4.921504	4.81818	9.884504	6.455848
HORVU5Hr1G026050	0.133999	0.267938	0.329714	0.511369	5.756369	4.640111
Hordeum_vulgare_newGene_13247	18.43767	21.17509	22.98754	14.566	24.03875	22.78145
HORVU2Hr1G116100	5.22881	4.212712	4.58648	5.785939	6.677174	7.836635
HORVU2Hr1G090960	1.608665	1.715336	3.249084	13.88652	29.00977	19.79083
Hordeum_vulgare_newGene_9146	4.109775	4.404416	4.786766	4.935204	7.019848	7.189884
HORVU5Hr1G098190	28.48557	28.96882	35.36611	36.65115	28.8079	33.14701
Hordeum_vulgare_newGene_9142	18.20672	19.88321	39.80258	12.81303	17.36983	17.01984
Hordeum_vulgare_newGene_9143	3.75513	2.902425	4.876135	26.63635	195.8851	219.3264
HORVU2Hr1G019160	76.0024	74.0303	124.7137	76.77179	127.1354	119.8679
HORVU7Hr1G074620	40.02912	37.16643	43.63684	37.37859	26.9166	26.26957
HORVU7Hr1G043590	0.028531	0.013942	0.373651	1.055138	10.89676	10.15009
HORVU3Hr1G045610	1.580666	3.592598	3.760801	1.873016	4.339784	2.734444
HORVU3Hr1G065360	0.643951	1.235572	0.908962	1.290748	1.356809	1.417587
Hordeum_vulgare_newGene_15167	2.551416	2.569488	3.34857	5.410523	6.984375	6.379809
Hordeum_vulgare_newGene_15164	0.728641	0.799963	0.901789	2.167553	1.604696	2.486458
Hordeum_vulgare_newGene_8810	9.172223	11.71796	11.10777	12.14647	13.16441	15.54968
Hordeum_vulgare_newGene_8817	18.47674	17.49857	10.12706	0.315235	0	0.133992
Hordeum_vulgare_newGene_8818	3.298838	1.862435	2.140057	0	0	0
HORVU3Hr1G031260	0.491579	1.451902	2.22267	1.285557	0.864994	0.877501
HORVU2Hr1G037480	10.31429	9.840875	8.426278	3.907676	1.63038	1.546103
Hordeum_vulgare_newGene_5332	12.42983	14.47596	13.05832	0.038104	0	0.031379
Hordeum_vulgare_newGene_5330	1.37721	2.188655	2.571613	4.015362	4.63138	4.96338
Hordeum_vulgare_newGene_9497	27.55838	31.94219	33.75947	26.56188	15.91169	25.33909
Hordeum_vulgare_newGene_9495	4.630996	4.600874	3.405845	11.62887	10.86333	14.35527
Hordeum_vulgare_newGene_9498	1.307377	1.720885	1.953632	2.678282	2.381937	2.836062
HORVU4Hr1G071690	24.62964	23.08918	26.0007	20.91873	25.60381	26.33831
HORVU1Hr1G025360	15.66172	12.94021	15.83435	16.06475	24.08477	25.48212
HORVU7Hr1G023250	1.785303	2.334877	2.406148	5.390268	5.372839	5.13578
HORVU3Hr1G093710	233.4194	193.5343	267.6	230.3259	261.936	242.8123
HORVU4Hr1G025140	7.997683	9.574108	10.0641	8.976184	8.49956	10.17101
HORVU4Hr1G083840	109.2368	105.4865	164.099	120.3262	288.4163	239.086
HORVU3Hr1G013910	57.11916	58.9274	71.79393	22.04005	10.86949	13.18385
HORVU4Hr1G060350	15.23346	11.67351	16.02225	14.86243	16.44496	16.51934

HORVU5Hr1G045930	8.274477	7.395444	10.08765	8.784672	12.03337	11.70832
Hordeum_vulgare_newGene_13868	6.772528	4.409983	4.595448	0	0	0
HORVU7Hr1G080980	7.751601	7.157689	8.88998	6.814252	7.329983	7.669281
Hordeum_vulgare_newGene_13864	5.913528	5.70729	7.15341	3.534278	3.912121	4.49229
HORVU6Hr1G012800	14.08942	12.70335	11.23093	20.2295	14.43671	14.93847
HORVU7Hr1G061070	9.65743	10.19569	10.18396	20.75494	20.24522	19.65522
HORVU4Hr1G079190	11.98135	12.6207	17.11928	9.884641	14.34716	13.62993
HORVU4Hr1G090070	1700.165	1701.684	1852.954	959.4144	946.4205	716.3863
HORVU3Hr1G061120	4.319886	6.282708	5.756133	5.196893	2.367486	3.995836
HORVU6Hr1G076280	0.80245	1.759124	1.765207	0.349696	1.496383	1.447364
HORVU4Hr1G071960	104.5578	92.50487	101.2724	116.0077	162.074	161.0867
HORVU7Hr1G020780	9.184782	7.237638	9.892793	23.88011	15.56089	19.40625
HORVU2Hr1G118410	31.03476	24.89173	28.72047	22.04179	27.58979	26.54197
HORVU6Hr1G025780	1.044049	0.722574	0.849068	2.017012	2.746932	2.568759
HORVU4Hr1G077970	15.49657	13.52958	18.07796	16.34877	15.86199	15.77392
HORVU4Hr1G067620	3.44162	4.04735	3.453128	3.741521	2.913506	3.458298
HORVU1Hr1G073720	0	0	0	2.404233	2.244892	2.529441
HORVU5Hr1G043140	3.527872	4.418529	5.061257	5.747383	7.705863	8.428875
HORVU1Hr1G024190	21.88559	18.76922	20.65719	18.09171	16.6688	19.68459
HORVU5Hr1G001670	0.936111	0.506611	0.830587	1.033851	1.758961	1.674387
HORVU7Hr1G028060	1.170798	1.379042	1.373457	1.680679	2.082761	2.497922
HORVU3Hr1G091000	3.376838	2.838333	3.81146	9.754224	10.20322	9.501259
HORVU3Hr1G015810	38.91518	34.68544	43.86183	35.24223	44.3558	45.17992
HORVU7Hr1G101390	13.3488	12.28795	13.5097	9.934672	11.09587	12.69125
HORVU2Hr1G075590	3.708608	4.256205	4.412089	5.40362	5.606064	6.333226
HORVU3Hr1G112760	21.99947	19.79058	28.47751	14.6277	14.66028	15.35672
HORVU6Hr1G066140	0.976981	0.54472	0.560389	12.29997	19.85144	18.46944
HORVU4Hr1G063870	3.732767	3.204277	3.435404	3.706288	5.772293	5.514005
Hordeum_vulgare_newGene_1527	5.550839	6.371141	6.300919	3.790684	3.792436	3.613922
Hordeum_vulgare_newGene_2528	2.826125	2.329599	2.885245	0	0	0
Hordeum_vulgare_newGene_1529	1.935169	0.942009	2.464476	0.278047	0.181103	0.302316
HORVU3Hr1G014140	10.0774	9.533653	9.96826	4.754956	1.341614	2.425447
HORVU0Hr1G020840	2.072123	3.596777	8.870519	4.878165	5.760513	4.377217
HORVU2Hr1G029760	5.261417	5.278944	4.047408	3.785769	3.448063	4.035143
HORVU7Hr1G033620	67.00803	41.73158	70.60345	59.58881	26.18094	42.06467
HORVU5Hr1G082050	4.609375	4.226387	4.97428	6.154699	4.406214	6.217647
HORVU5Hr1G088030	6.955677	7.02678	8.475633	9.720803	13.16407	14.52545
HORVU7Hr1G080210	3.260934	3.898225	3.439087	4.077583	5.111781	5.372739
HORVU7Hr1G021120	8.72614	8.904481	9.317178	9.531239	14.35113	10.89547
Hordeum_vulgare_newGene_12382	270.3395	266.1325	358.8968	317.0697	523.5837	494.0413
HORVU3Hr1G030310	1.099261	1.460313	1.798178	0.61295	1.441316	1.006146
Hordeum_vulgare_newGene_12385	2.108133	1.867529	2.036372	2.79993	3.6043	3.312303
HORVU0Hr1G006810	8.983606	6.977793	10.19381	9.044858	6.702348	6.545234
HORVU7Hr1G024570	11.31442	10.15179	15.97718	6.017539	8.442617	8.924251
HORVU5Hr1G101680	12.55704	10.98508	13.06062	23.09576	36.79351	43.76552
HORVU7Hr1G001560	12.29587	9.853723	13.79656	14.76458	18.18981	19.46466
HORVU7Hr1G029330	4.74495	4.073148	4.884278	6.426437	8.729585	8.277388

HORVU3Hr1G090350	7.009852	6.233634	7.004751	12.64679	11.8731	11.65875
HORVU6Hr1G059440	17.00297	18.14403	24.11965	10.13302	10.89452	12.23242
HORVU5Hr1G103190	503.2736	459.0415	474.0987	338.1473	419.6806	388.3097
HORVU3Hr1G091910	1.742535	1.741786	2.265059	4.051372	3.873814	4.457548
HORVU1Hr1G071010	0.192801	0.797652	0.636871	3.034171	0.573016	0.822399
HORVU4Hr1G027150	39.85893	34.121	50.46291	39.03384	80.64862	73.44117
HORVU2Hr1G042270	40.68774	35.05694	42.48432	29.70764	28.59073	27.20577
Hordeum_vulgare_newGene_518	1.669774	2.429311	3.228332	1.239446	1.488351	1.484477
HORVU6Hr1G032710	4.219591	3.925144	7.485303	3.382746	5.716657	6.511281
Hordeum_vulgare_newGene_515	7.146576	6.581059	6.840621	5.781894	6.170293	5.620854
HORVU3Hr1G014850	0.160237	0.099535	0.22248	1.393982	3.260545	3.392232
HORVU3Hr1G089640	3.402245	3.620468	4.208509	3.153398	3.882374	3.84201
HORVU7Hr1G118090	9.497113	8.473447	8.91691	4.563059	5.302566	4.085869
HORVU3Hr1G033660	2.020728	2.035091	2.607508	2.486734	3.655719	3.176393
HORVU3Hr1G117910	1.023631	0.919342	1.676794	0.928509	1.700776	1.611358
HORVU4Hr1G017780	1.03723	1.462627	2.174834	1.711454	2.494718	2.794473
HORVU1Hr1G026750	6.673159	6.370269	6.319195	6.526095	6.310176	5.68067
HORVU7Hr1G090530	12.14709	9.063857	9.964038	11.62582	26.49173	24.52154
HORVU0Hr1G040120	1.164082	1.865281	1.674267	2.012145	2.644139	1.797063
HORVU5Hr1G041450	3.168769	3.073104	2.916396	5.2199	7.393594	6.17878
HORVU7Hr1G042000	4.055613	2.863723	4.771313	2.907629	1.770916	2.541407
HORVU3Hr1G079800	0.24919	0.316405	0.256148	9.749723	13.2608	9.079684
Hordeum_vulgare_newGene_5481	12.68933	13.52739	11.19612	31.37656	26.82328	25.60291
HORVU3Hr1G087970	0	0.126039	0.036412	0.913617	2.898111	2.621697
HORVU2Hr1G065170	0.310013	0	0	2.402959	5.401609	5.731556
Hordeum_vulgare_newGene_14713	6.527189	5.584315	4.782977	0	0	0
HORVU3Hr1G110580	1.476364	0.416484	1.998278	1.046981	0.328408	1.00582
HORVU7Hr1G054440	74.22164	63.5325	91.06392	90.18786	139.8232	140.6754
HORVU6Hr1G027390	1.08472	0.998671	1.081131	1.226891	2.029436	3.128492
Hordeum_vulgare_newGene_9800	4.803497	6.40078	6.815656	5.225101	5.506195	5.934018
HORVU5Hr1G058950	3.174835	3.448032	4.089323	4.430698	9.11287	8.348022
HORVU5Hr1G085310	9.920193	6.74172	10.95193	23.2568	15.50025	22.7658
HORVU5Hr1G124650	15.74135	9.212208	19.03352	7.450055	2.415136	6.600734
HORVU3Hr1G055450	6.345028	5.983562	5.558211	3.382355	2.049433	2.400891
HORVU0Hr1G000760	23.17777	13.62267	19.9211	2.69568	0.597016	3.849432
HORVU1Hr1G010380	8.021298	7.986914	8.981438	12.52829	21.72403	21.32766
HORVU1Hr1G022790	7.87247	7.689004	9.32842	10.1114	11.44655	11.65394
HORVU7Hr1G054190	0.808085	0.814251	0.776902	2.273456	3.472529	3.283431
HORVU2Hr1G006720	0.9144	0.623428	0.563653	1.41876	6.808415	4.069536
HORVU5Hr1G058830	1.512926	2.081987	2.131047	0.667855	0.85878	1.32113
HORVU2Hr1G059780	7.92711	6.89757	7.081702	4.779343	4.03576	4.1339
HORVU7Hr1G085160	1.840773	1.564741	2.583258	2.458077	5.027228	4.352352
HORVU0Hr1G003490	3.459655	3.148043	3.997186	3.632261	5.72584	4.756081
HORVU2Hr1G091890	2.965201	3.610471	4.338266	3.825158	0.410658	3.287843
HORVU2Hr1G027080	4.688394	3.975551	4.105625	4.536509	3.163002	3.354477
HORVU3Hr1G032200	1.733111	1.46433	1.214122	4.344565	6.86343	5.968316
Hordeum_vulgare_newGene_15448	4.450786	3.975393	5.121117	3.132067	2.496478	3.168077

HORVU3Hr1G087020	2.183928	1.476183	2.07923	0.870379	0.25473	0.542651
HORVU7Hr1G048760	3.058641	2.977403	3.06764	3.779996	6.269433	6.013024
Hordeum_vulgare_newGene_15446	0.589539	0.780078	0.924823	1.09733	1.995449	1.273403
HORVU4Hr1G043930	1.521244	1.672144	1.926853	0.751778	0.746707	1.378772
HORVU5Hr1G029130	16.66987	19.25467	18.73553	13.44759	8.896784	12.03047
HORVU6Hr1G043680	10.17626	10.14586	11.81603	12.5923	19.28404	19.84905
HORVU7Hr1G108570	5.749231	4.188222	6.980135	4.952511	3.426332	6.36768
HORVU3Hr1G016280	11.13115	11.54784	12.72584	4.815918	2.279539	2.623096
HORVU1Hr1G025600	6.36627	6.240266	8.023342	5.38942	6.475105	6.810726
HORVU4Hr1G068030	2.687251	2.855924	4.719091	2.559837	3.632619	3.430012
HORVU0Hr1G003230	0.970214	1.015035	1.398443	0.888321	1.757449	1.960958
HORVU2Hr1G094090	1.275599	1.113178	1.51788	3.5053	1.834714	2.357491
HORVU7Hr1G090920	4.549763	4.873381	4.735336	6.567843	5.009651	5.092806
HORVU0Hr1G002720	1.351909	1.101945	2.803927	0.413641	0.460681	1.422131
HORVU7Hr1G117180	1.312459	1.30876	1.337254	11.20167	25.88643	21.23831
HORVU2Hr1G057430	45.91568	52.24617	66.8536	37.92943	64.85249	69.57864
HORVU4Hr1G005870	64.80966	55.39614	61.55783	44.01904	37.75992	38.9633
HORVU1Hr1G062080	12.90229	12.28365	13.66485	15.50917	18.18918	18.22524
HORVU1Hr1G050450	0.612097	0.800947	0.793705	1.068856	2.185055	2.166722
HORVU3Hr1G016820	5.885169	4.58876	3.990311	0	0.023396	0.04027
HORVU5Hr1G097500	11.90975	10.95522	12.89077	15.52425	7.719648	11.18799
HORVU4Hr1G067920	11.84535	11.26312	13.84287	11.73803	9.17927	9.974154
HORVU3Hr1G004070	5.186191	6.665648	11.49847	1.961898	0.102749	0.780264
HORVU6Hr1G060100	1.599246	1.785406	1.735392	3.599063	5.4134	4.904629
HORVU6Hr1G031370	2.845454	2.193606	2.12167	5.612027	4.395509	5.116067
HORVU1Hr1G067100	7.192422	8.309506	11.05359	4.446566	3.556107	3.863659
HORVU3Hr1G015730	0.3899	0.356249	0.653668	1.433607	2.146852	1.971335
HORVU5Hr1G114540	5.695655	4.360284	4.492013	21.66723	19.01085	19.78954
HORVU4Hr1G080640	33.8838	36.53247	39.66972	21.70299	27.65214	31.57723
HORVU4Hr1G011170	1.655855	1.897769	1.521399	6.380617	8.596868	8.102591
HORVU1Hr1G062620	3.471061	3.635394	4.516768	4.583011	7.562766	7.571733
HORVU2Hr1G085950	4.870872	3.300651	4.287541	4.033414	4.430337	5.116405
HORVU4Hr1G066970	0.655705	0.736983	1.153206	0.942788	1.468983	1.224377
HORVU2Hr1G046160	4.56816	4.854242	4.070304	7.781603	12.55611	11.448
HORVU3Hr1G036430	23.62062	21.39576	28.37954	18.68594	19.76662	19.57773
HORVU2Hr1G126760	1.453379	1.396415	2.409848	1.115004	0.478147	0.847812
HORVU0Hr1G026500	15.32614	12.16997	21.04205	10.90021	17.79228	15.87883
HORVU0Hr1G017200	0.271315	3.01582	1.948653	2.39714	5.148516	3.684885
HORVU2Hr1G089090	33.42136	31.09805	49.64716	28.5685	50.4764	52.95558
HORVU5Hr1G114090	6.226905	6.331198	6.666037	20.46334	28.58294	28.46932
HORVU6Hr1G064820	2.681165	1.990795	2.495109	3.117037	0.990064	1.496394
HORVU1Hr1G018370	4.345983	2.371914	5.269359	2.080026	1.366513	1.37661
HORVU5Hr1G020510	8.272086	7.397302	9.154672	9.669458	13.85346	14.51754
HORVU5Hr1G025740	11.43525	9.784589	12.37747	19.56968	26.45872	26.88169
HORVU5Hr1G058170	2.206478	1.405684	2.126072	3.141759	3.075631	3.287936
HORVU5Hr1G066930	21.521	16.17071	20.00393	1.139592	4.086337	0.785822
HORVU7Hr1G115760	18.53459	16.81359	19.76704	12.18038	12.76245	13.27876

HORVU1Hr1G047350	0.824827	0.663282	0.675551	1.358592	1.787051	1.690648
HORVU0Hr1G013180	8.144294	4.128642	2.376556	15.531	12.42684	19.93038
HORVU1Hr1G042650	16.11447	17.54681	16.12805	15.06012	18.10969	18.40153
HORVU7Hr1G099520	0.853911	1.025176	1.291722	1.195085	1.062881	1.061361
HORVU2Hr1G107750	17.27147	15.50348	17.59119	15.35985	9.99554	11.31982
HORVU2Hr1G095050	5.219491	5.231303	5.002975	3.249922	6.037156	6.013249
HORVU3Hr1G009240	1.441385	1.206813	1.802425	2.510247	3.892865	4.379085
HORVU6Hr1G027640	0.847237	1.494482	1.594926	1.433278	1.621346	1.321371
HORVU4Hr1G000510	13.00414	12.17968	10.85906	7.724024	5.590293	6.875757
HORVU4Hr1G079300	0.9714	1.227649	1.317597	1.894669	2.500717	3.2349
HORVU3Hr1G079340	0.733668	0.522657	0.733834	2.44572	1.927495	2.039716
HORVU1Hr1G056530	0.69142	0.464082	0.750303	3.327257	17.73398	16.27729
HORVU5Hr1G060570	2.285085	2.025379	2.120965	1.092264	1.253993	1.008837
HORVU7Hr1G093640	0.809076	0.682787	1.141144	1.966884	1.361906	1.875335
HORVU2Hr1G017630	5.050442	5.05542	6.173981	10.36972	12.67457	12.06376
HORVU6Hr1G003800	4.461826	3.709908	6.541516	5.232873	4.393688	3.997451
HORVU7Hr1G006250	81.22549	390.4138	105.2996	69.18795	30.04094	17.01227
HORVU7Hr1G020830	219.6827	188.7865	225.3626	183.0357	137.1848	147.7952
HORVU7Hr1G016770	0.069967	0.089685	0.058831	1.522761	1.888303	2.59821
HORVU0Hr1G001400	22.06378	20.87232	22.37544	27.31289	31.84148	32.78287
HORVU3Hr1G071410	1.377463	1.12512	1.563557	1.286466	3.078554	2.775887
Hordeum_vulgare_newGene_13850	2.586107	2.507588	2.990032	4.180378	4.605713	4.472547
HORVU3Hr1G006430	0.589363	0.746828	0.781677	1.560249	1.637394	1.908512
HORVU6Hr1G022370	13.90975	15.91801	13.22529	40.29224	42.27549	38.53698
HORVU2Hr1G005150	79.10028	77.41949	82.72015	97.1408	86.30855	96.52151
HORVU1Hr1G078170	44.04512	41.06992	41.0772	38.37043	48.35426	46.42129
HORVU5Hr1G113830	13.29467	11.80533	13.51696	13.86362	12.92058	12.87195
HORVU2Hr1G019360	107.8489	67.95567	75.51061	92.33971	83.87825	79.81879
HORVU2Hr1G036030	65.56696	53.54136	67.48588	70.01048	70.23534	70.10219
HORVU5Hr1G050250	22.50941	23.71571	32.42333	24.94265	30.17621	30.74338
HORVU2Hr1G109590	2.522313	1.601805	4.333025	0.78846	0.477448	1.161734
HORVU1Hr1G090080	15.36159	15.2903	15.52895	14.63158	17.12325	16.82156
HORVU3Hr1G006290	17.04589	14.03736	21.02521	22.5331	16.12582	23.22909
Hordeum_vulgare_newGene_12531	2.555794	2.619107	3.314519	4.27558	3.750236	4.457287
Hordeum_vulgare_newGene_10460	19.3081	19.62652	19.21603	16.47162	25.78768	23.58459
Hordeum_vulgare_newGene_10462	5.791128	6.205193	6.742593	0.34812	0.226903	0.363713
Hordeum_vulgare_newGene_10463	3.748649	4.301203	4.529651	0.280268	0.067434	0.266117
Hordeum_vulgare_newGene_10464	14.01413	14.66354	18.28761	16.34628	19.74633	19.70821
HORVU5Hr1G047470	6.652801	6.603373	6.944513	7.505633	8.219991	8.861329
HORVU1Hr1G080980	67.73723	68.20357	85.98689	92.0754	157.9486	146.0287
HORVU6Hr1G032050	2.337216	2.565354	3.403479	26.98048	71.28479	61.87289
HORVU4Hr1G007560	24.86725	22.11828	28.98086	20.4718	23.57666	24.43065
HORVU5Hr1G052320	7.312745	7.156705	8.907165	11.23489	13.43331	14.05093
HORVU6Hr1G019840	19.2127	20.88117	23.32009	20.7366	30.45835	31.66533
HORVU7Hr1G009340	47.98628	39.86341	60.33403	7.168076	11.58875	10.10155
HORVU4Hr1G090270	1.820492	1.485308	2.150116	1.826701	2.096166	2.365082
HORVU3Hr1G059980	9.687054	10.71463	11.26713	10.92252	9.511437	11.73418

HORVU1Hr1G094870	13.60101	15.35419	12.85597	11.89355	10.68009	12.06325
HORVU6Hr1G050810	1.656787	1.694764	1.638552	2.616518	2.414504	2.758631
HORVU5Hr1G094420	9.404014	10.78424	7.720698	9.815556	16.26551	13.44724
Hordeum_vulgare_newGene_11730	7.120145	6.868708	6.296531	7.766247	10.64687	10.66765
Hordeum_vulgare_newGene_11737	12.04406	14.72652	16.20738	11.97681	10.91645	15.67432
HORVU5Hr1G044920	7.644564	5.76244	7.767743	5.314722	6.268962	6.14084
Hordeum_vulgare_newGene_11739	9.812696	11.82596	10.35586	2.697257	1.127594	1.497122
HORVU6Hr1G077710	43.30787	42.73761	75.80252	49.83972	57.37292	40.04115
HORVU5Hr1G092740	13.70783	11.05371	13.52828	25.43477	35.42307	33.33175
HORVU6Hr1G014170	8.000952	8.135518	6.151269	6.443558	7.200183	7.060198
HORVU3Hr1G109780	11.39731	9.169678	13.43615	4.199112	10.7868	10.05932
HORVU5Hr1G042180	1.008629	1.447538	1.567501	3.393175	3.375309	3.87934
HORVU3Hr1G016330	5.135541	6.053419	6.038446	4.956531	5.322749	5.948598
HORVU5Hr1G117030	31.19649	29.65014	32.51758	44.61964	58.99265	56.21758
HORVU3Hr1G077010	6.04315	5.524603	7.06845	4.474381	3.784151	4.202884
HORVU6Hr1G039140	29.18686	29.60506	37.10955	34.62629	43.03929	41.97067
HORVU5Hr1G072260	17.22004	18.30549	23.053	16.9357	21.42776	22.24172
HORVU1Hr1G066770	3.20804	3.098084	2.902214	3.717371	3.887895	4.453776
HORVU4Hr1G043660	8.684326	7.610872	11.01377	6.931031	7.777454	7.943082
HORVU4Hr1G002800	5845.607	5551.766	5781.161	1360.334	386.774	646.7091
HORVU2Hr1G063820	2.581656	2.399122	2.860727	4.349367	11.22506	9.773486
HORVU6Hr1G085760	20.44479	27.83595	30.49549	5.949047	6.602545	6.484549
HORVU2Hr1G022680	9.286064	7.872229	7.613311	11.86719	14.49077	16.20291
HORVU1Hr1G017240	1.471224	1.76811	1.36019	2.391699	2.34635	2.584455
HORVU6Hr1G095130	2.383157	2.395797	2.247927	4.557314	5.239942	4.982985
HORVU5Hr1G058630	1.533643	1.509719	1.785825	2.833207	5.091955	3.577954
HORVU5Hr1G068230	29.96258	32.11036	29.59498	30.54547	29.22369	28.64198
HORVU6Hr1G092780	5.078236	4.143841	5.452238	2.031791	0.68093	1.259813
HORVU3Hr1G022500	12.78738	13.57977	14.93053	15.1447	17.16853	19.61205
HORVU2Hr1G039250	4.582482	6.232551	3.215397	3.302111	24.7577	18.44756
HORVU7Hr1G045710	10.90076	8.981189	12.22898	14.88854	15.98702	14.64852
HORVU6Hr1G088270	1.89756	2.11807	1.499705	0.912992	0.540604	1.105833
Hordeum_vulgare_newGene_16173	6.710686	6.723674	6.788365	4.464748	4.466091	4.512103
HORVU7Hr1G050170	37.90659	43.48717	69.9488	32.42955	58.3927	56.69023
HORVU1Hr1G064040	5.32068	6.32437	7.966388	9.516141	11.33022	11.63472
HORVU7Hr1G041410	5.263986	5.289047	7.086306	1.812948	2.965075	3.477053
HORVU2Hr1G028160	3.913367	4.024119	4.587892	2.66685	5.3859	4.522335
HORVU7Hr1G074810	0.738019	0.860661	1.730641	0.766749	1.75325	1.40115
HORVU2Hr1G111430	0.836986	0.942327	0.896268	1.079555	2.403432	1.624573
HORVU5Hr1G118500	2.927845	2.196076	3.022192	3.905347	4.812741	5.424415
HORVU3Hr1G057720	8.342787	10.12096	9.80966	13.85677	12.05678	14.74409
HORVU5Hr1G086650	1.300698	0.219549	0	2.787702	0.759389	1.592117
HORVU1Hr1G003140	0	0	0	12.08225	0	0.008486
HORVU6Hr1G051470	83.58156	76.57304	84.23459	51.03248	75.97549	66.44656
Hordeum_vulgare_newGene_12412	0.600869	0.882396	0.817226	0.851832	1.14885	2.036591
Hordeum_vulgare_newGene_12411	5.6558	6.595687	3.931148	0.963744	0.393441	0.941107
Hordeum_vulgare_newGene_12416	87.46513	116.5961	133.4077	21.76731	14.20153	14.56675

Hordeum_vulgare_newGene_12419	21.44628	15.41142	18.43688	15.24774	11.36455	14.35673
HORVU5Hr1G011200	45.03055	33.1189	57.60748	119.8517	38.95672	52.22529
HORVU3Hr1G017420	2.525076	2.019587	3.035723	4.090642	4.281585	4.650293
HORVU6Hr1G055570	0.97899	0.969844	1.661747	1.565228	1.460616	1.275233
HORVU2Hr1G091030	6.054671	7.442478	7.113336	10.94485	10.95306	13.08619
HORVU0Hr1G008950	5.048314	5.117912	6.106383	5.384942	0.641359	2.294857
HORVU2Hr1G102930	0.116684	0.111503	0.232144	0.666091	2.191166	3.075424
HORVU6Hr1G091140	4.413332	4.581817	5.819265	4.242665	6.261878	7.740948
HORVU6Hr1G027500	1.406092	1.259343	1.254317	0.993912	1.077148	1.185559
HORVU7Hr1G109370	0.175272	0.148566	0.241653	0.669473	14.09216	11.1451
HORVU5Hr1G075540	2.577066	1.266506	3.249729	0.048651	0	0.050902
HORVU2Hr1G018190	4.674611	4.276286	3.741499	6.27775	8.950601	10.07078
Hordeum_vulgare_newGene_453	166.3215	124.1982	179.05	176.3373	173.9308	175.135
Hordeum_vulgare_newGene_456	0.811041	1.083952	0.539724	1.512883	2.309558	2.400148
Hordeum_vulgare_newGene_455	1.332155	1.081907	1.422593	2.026925	2.215778	3.015228
HORVU2Hr1G086170	2.691552	3.436381	4.247376	1.836813	1.916995	2.034581
Hordeum_vulgare_newGene_14157	5.833439	4.857562	6.882979	0	0	0
HORVU2Hr1G019820	23.76954	19.74555	20.85895	45.7859	70.88899	70.64487
HORVU3Hr1G113740	14.09579	14.51903	14.40006	34.11971	26.10501	31.91321
HORVU3Hr1G020100	8.073283	8.856714	8.887734	10.18447	11.20644	11.32515
Hordeum_vulgare_newGene_10818	0.413702	1.006729	0.648525	1.25897	2.04415	3.465045
HORVU6Hr1G018670	0	0	0	0.207561	81.13638	61.75726
HORVU6Hr1G022950	3.274864	3.141501	3.421506	5.742606	8.972651	8.456641
Hordeum_vulgare_newGene_984	5.475179	5.960547	6.425305	5.410005	5.229474	5.969438
HORVU4Hr1G058910	1.071049	1.587682	2.359276	2.526826	3.28492	3.194367
HORVU1Hr1G070610	13.79174	15.28074	20.37551	10.08143	12.09271	12.95522
HORVU3Hr1G048210	4.713724	6.113882	8.301929	5.233243	5.901051	6.634998
HORVU5Hr1G062720	0.314273	0.233606	0.735721	2.94404	1.281197	1.885925
Hordeum_vulgare_newGene_5816	21.92592	21.07933	22.14414	21.89688	23.31664	23.43366
HORVU4Hr1G020740	5.028655	4.934052	5.876014	6.945998	10.93042	11.00019
HORVU3Hr1G109170	9.517024	7.698112	10.41244	21.03414	1.605824	4.818243
HORVU5Hr1G006720	1.78137	3.408412	7.889194	16.80226	0.046173	0.116923
HORVU2Hr1G012520	3.056435	2.451321	2.933401	3.188121	5.021518	4.58496
HORVU3Hr1G097870	1.658372	0.998739	1.860866	1.780696	2.871312	2.922278
Hordeum_vulgare_newGene_15285	2.032505	1.88482	2.608941	1.987527	2.618085	3.430377
Hordeum_vulgare_newGene_15281	0.949724	1.430451	1.133146	3.505764	5.429464	4.999876
HORVU7Hr1G029900	2.756774	3.458849	3.005139	5.116117	9.547142	6.42746
HORVU4Hr1G076940	7.305171	7.757609	9.795372	166.7106	204.3381	188.0248
HORVU1Hr1G000620	23.26401	20.68092	18.98529	21.15295	19.50877	19.6112
HORVU6Hr1G079030	71.22363	67.76961	88.28751	47.80499	36.62832	34.71868
HORVU2Hr1G037560	11.35226	11.13129	10.63766	13.06442	18.75073	19.90633
HORVU1Hr1G050560	11.72261	10.65608	11.6696	14.03491	14.85918	16.72163
HORVU7Hr1G060260	12.67838	12.6549	13.37923	28.18033	33.3748	37.55291
Hordeum_vulgare_newGene_1221	1.347917	2.019495	1.26803	3.235594	5.329583	4.747703
Hordeum_vulgare_newGene_1220	1.739943	1.980112	1.995781	4.764443	7.817866	7.747708
Hordeum_vulgare_newGene_1229	7.625537	7.133755	9.652938	9.176045	14.94566	12.28368
Hordeum_vulgare_newGene_1228	2.847107	2.656238	3.858994	4.717546	1.559445	2.339121

Hordeum_vulgare_newGene_10101	10.16242	9.778526	7.067498	4.495468	2.807844	3.139381
Hordeum_vulgare_newGene_10100	6.694168	6.278082	6.92439	8.56763	10.5221	8.666059
Hordeum_vulgare_newGene_10107	1.544931	0	2.696319	2.136085	1.299455	1.026965
HORVU6Hr1G013760	12.89166	10.7742	16.02801	10.31403	7.434386	8.437433
HORVU4Hr1G060140	2.488017	2.25144	1.568238	1.616416	0.972087	1.543877
HORVU7Hr1G080820	1.986476	1.577947	1.863695	2.635982	2.98708	3.509219
HORVU3Hr1G053610	2.252977	2.333804	3.166918	2.305419	2.471894	2.236478
HORVU0Hr1G002520	2.107609	1.795275	2.300287	2.045686	4.442759	2.709665
HORVU5Hr1G007890	0.270729	0.18885	0.338059	2.143646	2.839284	2.989923
HORVU3Hr1G050490	5.183363	6.381773	7.311175	5.488197	7.111671	7.307498
Hordeum_vulgare_newGene_9750	9.243336	7.753455	7.262255	5.679977	5.874236	6.949489
Hordeum_vulgare_newGene_9753	2.788588	1.698584	2.34982	2.234594	3.868826	3.63157
HORVU4Hr1G071060	2.150075	2.182105	2.126581	3.016631	4.374961	4.039832
Hordeum_vulgare_newGene_5105	1.47432	1.792003	1.492407	2.925376	2.816896	3.5112
HORVU1Hr1G001570	0.029242	9.068978	0.538459	18.07495	5.81809	10.93213
HORVU3Hr1G026230	16.14538	13.21877	16.70467	14.10581	11.13555	12.65863
HORVU6Hr1G067130	19.47294	18.88115	22.41312	13.4456	12.80716	13.0116
HORVU7Hr1G107740	98.58043	93.94199	102.1651	165.1286	128.7534	153.2821
HORVU0Hr1G033170	0	0.094798	0.190207	2.774565	3.045694	3.220729
HORVU6Hr1G030590	28.43397	31.27322	23.03985	28.24359	35.54107	37.02122
HORVU5Hr1G089950	11.70579	13.23772	10.94024	11.63758	16.87258	15.76271
HORVU7Hr1G027500	7.794547	7.688311	8.661946	5.497418	6.570152	7.934791
HORVU1Hr1G008360	42.93968	24.32901	41.93436	0.66484	0.383298	0.528349
HORVU6Hr1G063830	67.00912	61.30651	72.40008	105.9574	170.4105	167.973
HORVU2Hr1G018420	6.853195	3.429219	1.965212	0.09106	0	0
HORVU3Hr1G012850	6.537508	7.342791	7.58266	10.37868	21.45975	20.38524
Hordeum_vulgare_newGene_1485	3.580049	5.079708	6.977946	5.347743	13.1118	12.34743
Hordeum_vulgare_newGene_1486	31.79999	29.32552	35.80843	9.471358	4.672303	5.021204
Hordeum_vulgare_newGene_1489	4.585829	3.745827	4.272097	0.011033	0	0
HORVU7Hr1G070780	17.07994	15.34969	17.11946	13.38573	16.03263	15.2454
Hordeum_vulgare_newGene_4050	4.866401	4.264124	4.393713	0	0	0
HORVU7Hr1G036170	4.585139	4.443418	4.763349	4.753235	6.286861	6.124323
HORVU7Hr1G101740	4.706504	4.514455	4.931756	24.64583	18.54919	16.49184
HORVU2Hr1G026800	3.37315	2.712887	3.502258	6.685491	7.788824	8.52191
HORVU6Hr1G009410	7.093389	7.162237	7.953816	14.33727	5.68013	5.648739
HORVU1Hr1G064460	1.402368	0.59811	1.063974	1.618301	1.045975	1.061565
HORVU3Hr1G070700	2.549789	1.739064	3.011951	1.614117	0.846955	1.722188
HORVU2Hr1G029350	28.55163	23.51104	29.66731	54.13243	80.90602	71.91369
HORVU7Hr1G070870	21.43416	23.63591	24.49928	46.68291	43.29561	52.91385
HORVU5Hr1G030250	2.508649	1.486618	3.830514	9.602922	2.031563	1.967407
Hordeum_vulgare_newGene_14907	0.723312	0.843717	1.047932	0.945532	1.455178	1.607223
HORVU2Hr1G035230	3.042954	2.671563	3.626863	4.179913	3.462256	4.311403
HORVU6Hr1G063160	20.69481	16.09577	19.59912	20.86189	20.71862	22.43374
HORVU2Hr1G014440	26.60342	29.05285	24.17002	17.81563	24.13126	23.27169
HORVU3Hr1G071750	10.94102	7.88561	11.51127	10.03889	11.61587	10.26556
HORVU7Hr1G053190	5.733827	3.644973	5.444764	7.359463	8.387197	8.309409
HORVU1Hr1G087330	1.368043	0.840586	1.343464	1.821803	2.495729	2.340166

Hordeum_vulgare_newGene_7500	12.86966	12.84865	13.47603	8.514428	9.4189	10.9961
HORVU5Hr1G085920	1.054527	0.836116	1.704604	1.522486	1.584859	1.972742
Hordeum_vulgare_newGene_7506	4.005566	3.263344	5.172233	2.622548	3.962375	3.248457
Hordeum_vulgare_newGene_7507	6.116738	6.02623	9.425123	7.106206	7.212201	8.287094
HORVU5Hr1G096440	8.75379	9.630264	9.419561	14.97382	16.15369	14.70008
HORVU7Hr1G100830	7.948376	7.425593	7.830031	9.243478	7.996332	9.495485
HORVU6Hr1G056160	4.170058	4.016711	4.485416	7.118052	5.84132	5.002394
HORVU6Hr1G083600	13.70324	8.951232	16.90298	12.92737	21.9513	20.9971
HORVU1Hr1G071930	3.629059	3.752812	4.317239	2.026622	2.110377	1.954618
HORVU2Hr1G077710	217.4332	163.3992	236.916	120.0826	170.5902	90.11734
HORVU6Hr1G080000	7.824811	5.383118	7.001358	6.266637	7.681805	7.546378
HORVU1Hr1G069650	10.11676	8.970932	10.77294	12.94431	13.43485	13.64891
HORVU4Hr1G063660	1.42146	1.255303	1.927161	0.71625	0.471564	1.159732
HORVU5Hr1G037700	4.294124	4.523527	5.527881	7.228983	9.843191	12.83814
HORVU3Hr1G086940	57.77198	66.7599	65.26476	26.46877	20.90094	21.53189
HORVU1Hr1G086110	6.468994	6.925528	7.182375	7.080912	7.392839	7.415908
HORVU6Hr1G071660	13.17344	8.594499	13.47947	19.26594	29.27569	26.52001
HORVU0Hr1G001170	0.289015	0.413472	0.46076	3.886506	5.897858	5.790773
HORVU1Hr1G029370	2.730478	2.251711	3.041695	2.366835	2.942989	3.078113
HORVU3Hr1G085760	10.49585	10.32185	12.27173	19.36133	18.87354	24.23162
HORVU1Hr1G069380	11.0769	12.38208	17.032	8.27401	9.728421	10.03599
HORVU2Hr1G039320	4.473605	4.368809	3.227634	2.455774	18.94978	17.66258
HORVU5Hr1G019030	97.3728	92.06484	95.76639	180.5372	304.6119	291.1681
HORVU7Hr1G098100	10.8824	9.767768	9.689723	7.084403	9.713352	9.095743
HORVU7Hr1G058410	6.024836	6.097595	6.733527	6.852701	7.661122	7.703227
HORVU7Hr1G053440	0.725659	1.18864	1.011612	0.85479	1.131086	1.201044
HORVU3Hr1G116050	2.888763	3.60719	3.807675	5.310559	5.806337	6.019957
Hordeum_vulgare_newGene_1557	0	0	0	3.518308	3.772372	3.799274
HORVU5Hr1G059960	3.579048	4.411815	4.936236	5.274329	5.761457	5.970715
HORVU6Hr1G073740	3.787932	3.052611	3.632115	7.013757	6.004552	5.517391
HORVU0Hr1G030520	0.561611	0.601946	1.174029	1.160869	1.855853	1.958821
HORVU2Hr1G044270	17.11572	15.0111	17.29314	18.39489	27.60883	27.34946
HORVU0Hr1G040350	5.458655	5.41254	6.160322	6.689799	7.343875	8.346042
HORVU6Hr1G037460	2.217466	1.421525	2.437534	2.960143	4.988276	4.821714
HORVU7Hr1G103870	0.988795	0.583686	1.039957	0.923649	2.237579	2.424326
HORVU4Hr1G074400	34.46564	23.607	15.76918	12.30933	5.744374	10.13128
Hordeum_vulgare_newGene_2866	2.497261	3.628399	3.966306	2.692899	3.006063	3.004698
Hordeum_vulgare_newGene_2864	4.305799	4.340302	4.916241	3.777464	2.818191	2.819176
HORVU2Hr1G096510	6.319261	3.913343	5.859725	19.51217	21.38169	21.47076
Hordeum_vulgare_newGene_13436	33.16716	28.06304	22.50167	13.03177	20.49534	18.81649
HORVU6Hr1G004900	3.060766	2.411912	2.923721	0.043007	0.067247	0.019829
HORVU5Hr1G012090	5.547362	6.384938	7.498046	4.652178	5.943541	6.366021
Hordeum_vulgare_newGene_14426	0	0	0.070733	3.479526	2.171732	3.053176
HORVU4Hr1G040660	58.71732	57.96696	74.05525	73.42537	106.0252	102.5522
HORVU4Hr1G017240	2.884815	3.422833	5.010587	0.679952	0.306181	0.419619
HORVU5Hr1G036940	9.304473	7.822564	9.786939	12.40437	18.01286	18.29836
HORVU2Hr1G038040	2.119331	2.147663	2.296401	4.791915	5.437034	5.75974

HORVU5Hr1G122540	0	0	0	1.897789	3.28903	2.109789
HORVU3Hr1G045960	12.60157	11.01615	14.03616	11.17534	13.55193	14.5406
HORVU2Hr1G027760	5.02636	5.609394	7.129756	3.980001	4.180816	4.062824
HORVU4Hr1G006140	32.2338	26.14478	26.8371	19.12117	18.66576	17.15136
HORVU1Hr1G061230	1.461017	1.783551	1.878334	7.393287	7.438268	8.43359
HORVU3Hr1G029750	0.445851	0.419647	0.532183	1.09713	2.182238	2.00732
HORVU7Hr1G040120	2.342036	1.616204	2.698301	1.296218	1.239816	1.583835
HORVU1Hr1G010020	0.949212	1.012949	1.233096	1.488526	3.254593	2.899999
HORVU5Hr1G105410	7.733638	9.415691	9.473698	5.770814	5.41707	5.908498
HORVU7Hr1G096410	2.907854	1.973383	3.064483	3.104386	4.025245	3.128236
HORVU4Hr1G018430	11.43265	9.438723	6.910655	2.265293	5.174369	5.237421
HORVU6Hr1G010050	6.179111	5.356643	7.505074	8.821594	6.555308	6.412578
HORVU6Hr1G064320	1.801967	1.517168	1.846089	2.407436	2.334989	3.041993
HORVU4Hr1G050810	62.90239	61.06912	70.33617	44.07821	40.73905	41.18992
HORVU5Hr1G110080	3.203718	3.337114	3.277557	3.555676	6.547257	5.765961
HORVU7Hr1G049400	1.649675	1.329567	1.318824	0.738938	0.5896	0.944065
HORVU3Hr1G082800	2.596444	2.734651	3.959912	4.117064	6.145805	6.729806
HORVU4Hr1G068170	17.79372	18.594	15.43796	18.43163	19.27896	18.82308
HORVU1Hr1G023100	87.53727	78.88799	93.17868	154.3197	537.0521	510.2295
HORVU1Hr1G064440	3.456113	3.292274	4.598963	7.129835	6.909964	7.044365
HORVU7Hr1G051750	4.442921	6.357982	4.967847	19.26355	39.95219	35.90403
HORVU1Hr1G086930	10.393	9.344371	8.390995	13.16349	10.84342	12.01633
HORVU6Hr1G045030	3.360222	3.815333	3.6182	3.997348	5.486199	5.85004
HORVU6Hr1G040040	0.25795	0.378357	0.649023	9.517406	7.677933	6.88642
HORVU4Hr1G066310	1.796712	1.277377	1.893639	1.633229	2.441997	2.165252
Hordeum_vulgare_newGene_3565	0.155313	0	0	0.06132	13.48632	10.41313
HORVU6Hr1G067670	14.10467	12.9567	18.95365	15.1848	23.51255	21.33815
HORVU6Hr1G085370	0.254059	0.149448	0.208881	8.779708	11.45891	14.01549
HORVU7Hr1G001020	2.437678	4.642343	4.116304	9.448738	11.73211	15.36366
HORVU3Hr1G024040	1.86049	1.88775	3.318177	3.249242	1.576588	1.733498
HORVU5Hr1G106850	3.69128	1.546474	5.556507	0.796423	0.533216	0.878958
HORVU1Hr1G002240	13.72828	24.12855	14.8754	27.88484	9.270086	9.250779
HORVU3Hr1G040870	9.752063	8.097228	9.679404	12.00154	10.70673	11.52392
HORVU1Hr1G085470	19.97063	21.94119	27.7915	29.44588	38.66918	32.30794
HORVU7Hr1G041380	2.839661	1.962554	2.784263	2.527993	2.626574	3.316605
HORVU2Hr1G094680	0.010736	0.005087	0	0	15.85389	10.01635
HORVU7Hr1G090710	5.040688	4.594883	5.215109	5.594837	9.104865	9.508411
HORVU4Hr1G006850	5.056208	5.262208	7.059616	11.6087	12.67856	15.66183
HORVU1Hr1G052620	0.745898	0.725097	1.006942	1.833684	5.347465	4.964475
HORVU4Hr1G065660	0.66862	0.538228	0.717362	1.85049	3.222365	3.329334
HORVU6Hr1G091300	24.77416	18.29023	26.54071	15.00988	14.2082	13.95945
HORVU3Hr1G046320	1.692174	0.021879	0.076679	0	8.906093	0
HORVU4Hr1G008450	4.184571	5.576733	5.945521	4.55474	5.034241	5.116616
HORVU2Hr1G023000	26.42787	26.96015	28.31028	41.81696	44.96689	37.65941
HORVU4Hr1G062120	9.871006	7.517199	13.25335	9.093334	14.29088	13.07873
HORVU1Hr1G048760	13.33352	11.26688	15.05179	11.21457	11.99848	12.43046
HORVU4Hr1G080050	9.637304	7.700104	12.01511	14.61554	15.5472	17.60846

HORVU7Hr1G004350	27.2138	29.19409	32.99373	35.17955	43.61949	50.60797
HORVU5Hr1G097710	8.654558	8.351663	9.884467	9.193326	9.520638	11.33303
HORVU1Hr1G043820	17.66252	16.25877	17.3238	53.78251	264.1378	240.5403
HORVU3Hr1G031590	4.648338	6.534068	6.841493	9.202094	8.159098	9.402431
HORVU2Hr1G083450	0.793611	1.182232	1.11095	1.6617	2.415708	2.781046
Hordeum_vulgare_newGene_15980	53.59217	45.93983	57.27429	38.29715	38.40138	36.03732
HORVU6Hr1G085950	5.33271	4.346251	7.472673	7.110128	7.893303	7.308671
HORVU2Hr1G016750	6.249745	4.734955	5.482967	7.036548	10.45893	9.047201
HORVU3Hr1G005770	3.708609	4.11139	3.894654	4.134275	7.681043	7.066739
HORVU4Hr1G009500	2.654408	2.835627	4.187049	4.567163	8.89547	8.627301
HORVU2Hr1G099100	1.454628	1.240679	1.180593	3.87424	5.40593	4.528838
HORVU3Hr1G001430	16.84843	17.08375	18.09135	13.27612	12.5806	12.81371
HORVU5Hr1G096780	3.874143	4.500438	3.96387	5.93675	10.14119	8.956807
HORVU1Hr1G079410	15.58452	11.41196	16.00859	13.62631	13.54885	13.32058
HORVU5Hr1G074340	13.67948	10.39903	13.20663	41.85528	37.63276	37.65328
HORVU3Hr1G077580	1.03432	0.566771	1.709341	1.313051	0.710497	0.955773
HORVU6Hr1G032930	6.491049	5.489596	6.016755	5.509796	6.000626	6.258205
HORVU7Hr1G019540	5.951433	5.476843	7.318527	6.180367	7.032918	8.120477
HORVU6Hr1G084500	30.7742	26.66011	30.02386	34.93867	39.17662	40.8931
HORVU0Hr1G015810	4.938969	4.969847	5.424157	9.12518	13.09283	14.27704
HORVU4Hr1G004170	0.367927	0.138901	0.193811	1.234108	6.937468	5.661502
HORVU5Hr1G110500	2.871689	3.164005	3.257211	3.858767	3.543363	4.031836
HORVU5Hr1G115750	5.029027	1.0392	4.507112	0.070755	0.102258	0.25568
HORVU3Hr1G030980	4.56386	3.924869	5.450007	5.565393	7.265516	13.07995
HORVU7Hr1G084550	60.97262	54.63638	65.86996	67.94635	93.48055	87.11969
HORVU2Hr1G121060	59.08931	54.91707	66.40662	76.1561	66.20165	75.08363
HORVU7Hr1G081310	1.640515	2.002513	2.690526	3.378534	3.884201	3.70191
HORVU2Hr1G017400	7.807661	8.272773	8.236441	7.32444	5.627841	7.706415
HORVU5Hr1G074920	12.70105	11.62445	19.20738	10.29278	14.56064	16.55214
HORVU5Hr1G125270	15.99912	15.57276	16.16419	9.825631	9.271746	9.139152
HORVU5Hr1G113970	1.8608	2.456371	2.988202	2.867043	3.941972	4.276842
HORVU4Hr1G050020	1.389107	2.429664	2.88435	2.658477	4.430782	3.535232
HORVU4Hr1G068950	16.56335	16.59054	13.69932	12.84145	14.71165	12.65341
HORVU4Hr1G055050	23.75914	19.69051	29.5797	19.02753	25.53327	24.35496
Hordeum_vulgare_newGene_10344	3.807327	4.641558	3.49131	0	0	0
Hordeum_vulgare_newGene_10346	1.475049	0.882771	1.195615	2.194185	0.907654	1.157688
Hordeum_vulgare_newGene_10341	24.2438	20.34279	24.36457	61.79584	109.0452	78.06347
Hordeum_vulgare_newGene_10343	0.664158	0.5422	1.105424	12.79139	101.0165	82.9831
HORVU2Hr1G099810	11.03411	11.59222	11.36373	9.351241	12.95517	11.92814
HORVU2Hr1G109380	18.44015	18.14125	22.34475	29.41479	32.0043	31.26626
HORVU7Hr1G007790	40.25271	31.19225	7.563416	5.218122	0.012073	1.500565
HORVU5Hr1G095490	93.3469	76.28667	25.36945	185.254	53.05627	201.6556
HORVU3Hr1G114220	1.289004	1.778935	1.796595	4.228337	7.457868	6.14483
HORVU3Hr1G093140	2.368416	2.831004	2.756692	8.582811	4.88977	3.964544
HORVU4Hr1G004820	48.89988	44.04359	44.30876	20.05765	9.038001	13.58745
HORVU2Hr1G019660	82.96233	92.24533	171.5368	53.67987	81.31144	72.0548
Hordeum_vulgare_newGene_10690	7.455601	7.57508	7.583614	9.069516	9.988895	9.03041

Hordeum_vulgare_newGene_10695	0.983507	4.332811	14.90743	1.84083	14.15606	8.26602
Hordeum_vulgare_newGene_10699	5.073863	4.249904	5.997881	6.946417	7.855059	7.852254
Hordeum_vulgare_newGene_10698	2.092822	1.860301	2.134432	4.945563	8.475513	7.603456
HORVU2Hr1G097640	1.675394	0.952564	1.326284	0.765356	1.6185	1.594466
HORVU3Hr1G115170	1.243756	1.070501	1.161538	5.268008	11.63941	8.268638
HORVU5Hr1G094670	23.06059	20.37714	24.2391	28.53565	38.15768	35.52381
HORVU1Hr1G000350	5.908051	7.784511	12.42674	8.390221	2.168294	6.29205
HORVU3Hr1G058150	2.386152	2.532547	2.660986	2.553635	2.667339	3.134713
HORVU7Hr1G027740	0.332827	0.5868	0.661508	2.355193	2.567652	1.939829
HORVU2Hr1G023450	9.464482	6.791554	8.640158	11.7827	4.381882	8.478242
HORVU1Hr1G014000	10.01544	13.95021	13.82631	0.353026	0.49956	0.415677
HORVU6Hr1G093210	0.497451	0.542564	0.785101	1.47007	2.353794	3.334177
HORVU4Hr1G077050	1.362406	1.51279	1.51397	2.838019	3.431081	3.673405
HORVU5Hr1G068120	2.852221	1.896159	8.066087	6.024626	4.581654	6.898425
HORVU7Hr1G089870	1.147519	0.931255	1.688755	1.00711	1.84203	2.281539
HORVU3Hr1G109590	39.28321	34.21576	32.31572	50.85177	67.75048	63.31536
HORVU2Hr1G073260	11.29478	12.8753	11.8367	14.80644	15.96483	16.88936
HORVU2Hr1G105650	13.77148	10.8121	15.94859	24.07621	14.95137	15.73882
HORVU5Hr1G048240	21.39174	22.1887	23.16006	14.13239	7.629348	8.153169
HORVU7Hr1G009680	2.541378	2.091795	2.727264	0.704408	0.287247	0.87479
HORVU6Hr1G077100	31.23189	27.99936	29.77447	24.48394	25.08962	27.68712
Hordeum_vulgare_newGene_780	0.468437	0.051507	0.461695	5.929786	5.740828	5.08186
HORVU5Hr1G099700	10.91367	11.10318	15.86805	3.896719	5.600315	5.503182
HORVU3Hr1G069970	101.7493	83.0746	103.8758	132.3172	174.0329	157.4815
HORVU5Hr1G077570	7.904511	8.204488	9.99262	7.74885	10.49068	10.03674
HORVU2Hr1G127720	2.884966	3.928453	4.004878	2.369254	2.387095	2.641922
HORVU2Hr1G029110	0	0.05191	0	0.933772	1.833497	3.411069
Hordeum_vulgare_newGene_11565	2.350153	2.149447	2.158996	1.384996	1.667323	1.354185
HORVU1Hr1G054240	65.31033	40.83987	55.18877	52.00207	52.802	64.29508
HORVU3Hr1G073910	1.415023	0.711485	1.605153	1.753022	1.511978	1.711896
HORVU2Hr1G110610	16.6212	16.49691	19.71205	10.9419	13.43532	14.41026
HORVU2Hr1G038330	10.06667	8.474826	7.592029	5.333414	7.883378	8.287397
HORVU4Hr1G079260	6.082156	7.212352	7.245028	14.71346	12.42287	13.05796
HORVU2Hr1G020430	5.643769	2.924039	4.075634	1.911898	0.036087	0.598477
HORVU3Hr1G075510	0.75742	0.700969	3.192771	1.397537	0.477243	0.28723
HORVU3Hr1G057390	6.204355	5.454676	7.040702	8.404318	11.33738	11.86703
Hordeum_vulgare_newGene_1978	2.300556	2.424455	2.390046	2.919412	2.231646	2.689886
HORVU6Hr1G005920	10.46737	10.06581	10.80284	12.25345	10.98106	11.68608
Hordeum_vulgare_newGene_1976	25.31651	25.32805	39.21919	22.9449	57.74986	55.65926
HORVU2Hr1G123350	2.762688	2.766903	3.792703	2.022811	1.684752	2.2993
Hordeum_vulgare_newGene_1973	1.67388	0.031881	0.17969	2.194033	2.978351	3.367385
HORVU5Hr1G043640	16.13199	13.76436	15.29748	17.11731	16.91805	18.40163
Hordeum_vulgare_newGene_1446	2.590766	2.203609	3.926949	9.029987	10.61522	9.979388
HORVU5Hr1G000060	12.73818	12.88779	13.3712	15.21871	19.56994	18.02945
HORVU1Hr1G033390	6.185078	4.890084	6.843374	6.512862	8.78487	9.106191
HORVU1Hr1G013360	3.677743	4.859651	4.839778	5.38296	6.133858	7.320438
HORVU6Hr1G090170	61.58144	62.20368	100.2026	51.47436	114.1502	114.5186

HORVU3Hr1G056440	122.4932	108.0862	94.94984	22.71088	26.04339	28.65341
HORVU4Hr1G037160	0.911217	0.58259	0.744954	1.96249	3.193572	3.15806
HORVU2Hr1G025140	10.93861	13.17434	8.976801	14.81131	23.78674	21.87395
HORVU6Hr1G011970	2.04512	1.135336	3.070448	4.798494	5.096758	6.613997
HORVU5Hr1G037540	6.450321	6.435751	7.674748	10.5347	11.48816	13.63244
HORVU2Hr1G049830	3.911376	3.843026	5.618376	5.274635	5.721473	6.024088
Hordeum_vulgare_newGene_16039	6.250516	8.154232	7.648604	5.450973	8.109515	8.048465
Hordeum_vulgare_newGene_16038	4.39596	5.078837	5.055721	4.067971	4.988608	5.498035
HORVU5Hr1G049110	15.69487	14.55467	17.1792	16.60352	18.89892	19.78561
HORVU5Hr1G068970	4.281286	4.32484	4.758124	5.662711	10.786	9.043687
HORVU5Hr1G031870	4.364962	3.909627	4.171274	8.412105	16.11731	15.50817
HORVU7Hr1G038660	32.14089	30.79634	43.42664	27.60619	31.61421	33.30768
HORVU6Hr1G063560	29.64208	25.69875	34.96737	44.90366	51.94195	57.53729
HORVU1Hr1G085720	0.08151	0.031184	0.200075	3.395016	1.686421	2.76892
HORVU2Hr1G114610	12.1622	10.22842	12.78053	13.51109	13.87461	15.42729
HORVU3Hr1G081600	1.181498	1.001734	0.982935	1.844167	0.737217	1.265491
HORVU3Hr1G068760	0.900745	0.671966	1.621391	1.359013	2.083267	1.377697
HORVU1Hr1G008540	12.96583	13.67266	17.34044	2.572299	2.599167	2.674077
HORVU4Hr1G082290	19.57017	17.12552	25.9899	12.26483	15.54754	15.10916
HORVU5Hr1G033730	2.212762	1.57264	3.860434	2.928181	6.213694	5.241671
HORVU6Hr1G071500	0.195074	0.072486	0.030756	2.991603	3.733841	4.669787
HORVU1Hr1G075530	22.36445	23.58752	23.34922	17.23294	10.48227	13.12087
HORVU0Hr1G010790	9.364103	9.94774	13.60757	6.174744	11.25555	11.45377
Hordeum_vulgare_newGene_6162	1.07869	1.082897	1.993696	3.424501	3.666697	3.962645
Hordeum_vulgare_newGene_6166	0.965399	0.943136	0.857656	3.507377	6.88967	6.782944
HORVU3Hr1G052370	18.5881	18.60253	26.93348	21.16332	44.63206	40.23203
Hordeum_vulgare_newGene_3600	21.88295	25.25809	22.92176	23.48959	21.40969	25.42886
Hordeum_vulgare_newGene_3603	0.523033	0.646397	0.609299	1.876756	1.874203	1.807696
HORVU6Hr1G058610	3.198397	3.112651	3.297576	3.236478	3.50084	3.622296
HORVU5Hr1G028260	0.637733	0.422148	0.703296	1.526299	5.144892	4.515155
HORVU5Hr1G068350	3.344782	1.639252	3.841286	0.053321	0	0
HORVU5Hr1G061480	16.01804	17.91572	13.90113	9.448069	17.699	15.33129
HORVU6Hr1G014800	5.373513	5.277578	6.019547	5.258386	7.171144	8.095216
HORVU2Hr1G007080	0.554253	1.032775	0.550232	1.199679	0.952954	1.905222
HORVU7Hr1G118440	2.227991	1.946244	2.079967	4.151224	3.16205	3.602554
HORVU2Hr1G005320	72.87446	58.11259	76.55639	60.55338	39.40007	41.4567
HORVU7Hr1G042480	9.171835	9.445521	8.255584	9.69551	16.41182	18.00087
HORVU2Hr1G049160	8.467224	11.03497	11.09691	8.531201	9.086397	11.28505
Hordeum_vulgare_newGene_8	3.8639	3.790814	3.806644	0	0	0
HORVU5Hr1G062680	0.763808	0.969811	1.320282	1.069417	1.119105	1.250531
HORVU2Hr1G032920	4.570515	4.588229	5.358821	9.64797	10.54971	9.858178
Hordeum_vulgare_newGene_3758	1.079181	1.13937	1.274531	2.845725	2.825762	2.322013
HORVU1Hr1G077630	17.50534	16.73628	16.26214	12.88088	13.59374	13.59361
Hordeum_vulgare_newGene_5602	2.789734	2.761213	2.867701	0	0	0
Hordeum_vulgare_newGene_9380	1.412756	2.082966	1.869062	1.052842	1.294024	1.610723
HORVU5Hr1G081860	19.71766	21.58334	20.48235	25.06077	16.6579	21.97524
Hordeum_vulgare_newGene_9389	2.824291	2.676748	3.325711	2.686389	3.618259	4.397665

HORVU5Hr1G115250	7.196609	4.465069	6.021021	6.774036	5.326021	5.864066
HORVU7Hr1G043280	4.224267	4.031275	5.360272	6.435967	17.30195	13.9429
HORVU2Hr1G027160	4.124347	2.754489	5.27362	2.487009	1.583229	2.421169
Hordeum_vulgare_newGene_15673	3.227974	2.865086	3.873888	4.002253	4.554259	3.90081
HORVU3Hr1G069140	99.75956	113.3557	147.1785	15.61971	0.205543	6.522916
HORVU7Hr1G070210	12.55452	12.47025	12.48922	11.43533	9.55563	11.32498
HORVU1Hr1G070420	1.574958	1.073271	1.64275	2.199989	1.689635	1.382089
HORVU7Hr1G096650	20.01379	19.49809	22.3528	22.84626	30.21819	32.23314
Hordeum_vulgare_newGene_11953	2.929458	1.514106	2.567415	0.897651	0.441755	0.410649
Hordeum_vulgare_newGene_11954	5.357466	2.796908	6.27694	3.661431	3.560921	4.462959
HORVU1Hr1G007640	1.130339	1.344831	1.315363	1.397649	1.714407	2.130984
HORVU3Hr1G059720	1.948393	1.529904	2.084872	1.128017	1.442514	1.170974
HORVU7Hr1G049130	2.571965	4.101863	4.806507	0.173527	0.009089	0.085033
HORVU0Hr1G003040	4.421523	4.99663	5.350069	7.298584	9.752076	8.062623
HORVU7Hr1G043750	4.876371	5.165421	4.652766	7.25203	9.097029	9.959519
Hordeum_vulgare_newGene_6830	47.15574	48.12825	74.91051	26.38476	55.07015	53.10665
HORVU5Hr1G117170	1.401868	2.279061	1.806529	0.805845	1.298609	1.393117
HORVU4Hr1G086050	2.990572	3.043365	2.610671	5.498661	4.548827	5.459145
Hordeum_vulgare_newGene_8471	0.131062	0	0	0	8.850692	10.5726
HORVU7Hr1G022940	2.617448	2.967173	2.868132	2.143054	5.298777	4.133041
HORVU1Hr1G000010	11.18888	10.65521	12.61252	13.6932	20.08106	19.19402
HORVU2Hr1G006560	12.59186	10.65227	11.45458	12.24003	11.75182	13.01282
HORVU3Hr1G053400	34.1364	26.98794	37.34977	33.10906	53.8977	48.7565
HORVU6Hr1G064650	0.767899	0.534258	0.602373	4.067	1.649811	2.486784
HORVU7Hr1G117570	0.734648	0.595804	1.107771	1.591225	1.394649	1.62864
HORVU7Hr1G113650	1.862303	1.580891	2.043764	3.17466	5.361366	4.446226
HORVU0Hr1G013470	2.897009	1.920162	2.483548	0.97119	0.014026	0.657216
HORVU5Hr1G073280	2.717433	2.811174	3.02163	1.414377	0.890929	0.993042
HORVU7Hr1G037140	0.933395	1.168994	0.97888	1.789304	12.97229	9.968855
HORVU4Hr1G028050	1.39999	1.494078	1.603514	2.713125	2.573383	3.079171
Hordeum_vulgare_newGene_15097	2.523232	3.033051	3.055338	5.540673	7.302979	7.566048
Hordeum_vulgare_newGene_15095	0	0	0	4.770342	4.752525	8.293515
HORVU0Hr1G008640	3.583714	4.682331	5.482095	9.425286	15.61112	15.05998
HORVU6Hr1G057550	2.014151	2.7404	2.933822	4.199538	8.171679	7.708772
HORVU0Hr1G030300	1.979108	2.665061	2.61451	2.089095	1.493631	1.554339
HORVU3Hr1G083990	2.022285	2.905673	2.408712	7.508756	13.75233	11.69239
HORVU7Hr1G067110	5.983264	6.065266	5.169611	12.71382	22.35366	21.10525
HORVU1Hr1G074410	14.46119	14.11984	15.40178	12.72991	13.70108	14.54322
HORVU2Hr1G006690	2.138858	2.711242	2.290383	3.799563	3.522463	4.618046
HORVU3Hr1G018550	8.285384	6.778221	6.525894	49.38966	143.0889	100.0793
HORVU1Hr1G062470	17.52847	18.33678	21.86987	14.3947	16.16252	18.85464
HORVU5Hr1G019650	3.140056	3.034263	3.913578	2.738707	2.967507	2.983584
HORVU6Hr1G075970	1.364894	2.090943	2.094886	1.341787	1.320574	1.818773
HORVU2Hr1G073100	1.166928	1.333417	1.617891	1.486633	1.462349	1.730806
HORVU6Hr1G067300	15.5174	14.58701	14.4989	16.60341	16.2532	17.37131
HORVU7Hr1G039890	3.803836	2.741201	4.360974	1.99636	1.649228	2.059195
HORVU7Hr1G114000	196.4749	199.0226	156.4315	131.7101	54.27599	62.11837

HORVU2Hr1G029250	0.626833	0.899592	0.828144	1.318952	1.190637	1.29611
HORVU4Hr1G008610	4.030342	6.972599	7.545248	2.90861	2.682595	2.907514
HORVU1Hr1G063700	0.795888	0.638699	1.194768	3.617584	13.68333	13.31589
HORVU3Hr1G071660	8.241273	6.275327	8.522877	10.28103	11.50877	11.79583
Hordeum_vulgare_newGene_4155	1.245193	1.704143	1.705604	0.86393	0.737869	1.091481
Hordeum_vulgare_newGene_4152	5.574612	4.5991	8.03819	5.156004	7.053169	7.543086
HORVU6Hr1G036810	20.7157	21.07975	28.43048	19.71058	23.50032	27.0768
HORVU6Hr1G028290	22.48998	20.18883	23.61188	18.50685	17.62162	19.01472
HORVU1Hr1G076060	3.099495	3.878802	5.418259	2.29907	4.674096	5.812008
HORVU3Hr1G021550	0.57073	0.228971	0.341643	6.466224	3.355185	2.970157
HORVU6Hr1G031450	1.226642	1.905603	1.900765	1.94664	3.701935	3.146232
HORVU5Hr1G020960	6.322504	6.232524	6.853018	11.54042	8.938723	9.501867
HORVU2Hr1G069270	4.988849	5.344735	5.642795	5.372043	5.107908	7.897606
HORVU6Hr1G028740	4.472875	3.579809	4.75193	3.152307	1.270399	1.867517
HORVU6Hr1G081250	21.09328	21.4309	20.04073	9.428029	9.343749	9.052784
HORVU6Hr1G032220	5.24253	4.392525	5.510897	13.11323	36.23793	35.4828
HORVU2Hr1G046660	52.78851	50.83938	49.15445	40.05592	33.36905	35.26916
Hordeum_vulgare_newGene_7710	59.89263	99.3754	141.9861	25.40372	39.00901	41.03914
Hordeum_vulgare_newGene_7719	12.33095	18.08966	22.11854	10.77882	8.245649	8.281179
HORVU1Hr1G029180	5.070913	3.548193	4.960743	21.11007	26.22487	25.10785
HORVU7Hr1G026300	3.817777	3.974867	5.748051	4.67206	4.464143	5.855615
HORVU3Hr1G080210	25.31532	30.793	24.10065	23.86249	35.5893	35.75938
HORVU4Hr1G044910	3.160374	3.607634	3.134956	5.564151	9.551219	8.535999
HORVU2Hr1G034350	2.617381	2.605807	2.369141	2.956111	3.114305	3.36975
HORVU3Hr1G039930	8.710922	7.504847	6.8085	33.76153	64.51499	63.75953
HORVU3Hr1G035720	13.69203	13.84105	15.52456	15.40729	22.58052	20.65929
HORVU2Hr1G092430	25.94252	20.68839	24.90791	18.23892	20.59913	20.24386
HORVU1Hr1G064160	60.34948	49.77219	61.17207	44.17497	50.3755	50.01451
HORVU4Hr1G019940	10.17359	9.292249	12.61972	14.54384	22.52707	21.68748
Hordeum_vulgare_newGene_13132	0	0.079272	0.081622	0.986706	3.30105	3.136513
HORVU4Hr1G056600	11.66708	11.87244	12.79525	14.31035	19.64317	18.06571
Hordeum_vulgare_newGene_4915	2.209661	1.884297	2.041835	2.861383	3.897633	3.731526
Hordeum_vulgare_newGene_13137	2.376702	3.030881	2.55076	2.2394	2.215304	3.07469
HORVU7Hr1G025390	87.04028	80.39166	105.3416	183.6526	284.1862	256.8718
HORVU3Hr1G039600	22.41078	27.05651	23.0521	52.50316	59.1526	57.61899
HORVU2Hr1G078730	5.126748	3.900963	3.916072	4.342341	1.773888	2.597512
HORVU2Hr1G004870	0.672667	0.71062	1.19507	1.412173	1.636247	2.231805
HORVU3Hr1G079710	4.989907	4.008881	4.76544	5.775559	6.357128	6.652727
HORVU5Hr1G008250	30.59729	36.11102	33.6921	18.75372	18.49737	21.9891
HORVU5Hr1G058240	0.619561	0.605579	0.603191	1.557493	1.955386	2.124671
HORVU2Hr1G124560	8.287554	7.760792	9.810385	5.332333	5.182121	5.863568
HORVU1Hr1G051380	35.49741	34.56368	32.65953	35.12566	34.21296	36.73515
Hordeum_vulgare_newGene_6440	9.18656	1.876598	9.085219	10.50991	12.23581	11.63764
Hordeum_vulgare_newGene_6441	13.10338	12.05415	13.7958	13.65893	13.01683	13.69022
Hordeum_vulgare_newGene_6442	9.730549	10.27163	10.59947	10.8395	9.502388	11.38571
Hordeum_vulgare_newGene_6443	26.37403	27.2912	26.86959	23.36012	17.06689	21.37458
HORVU2Hr1G001340	1.260118	1.316957	1.230911	1.013081	0.773238	1.231227

HORVU1Hr1G017570	23.41284	17.16321	21.72508	29.7932	21.33572	23.45217
HORVU7Hr1G055080	6.536221	6.377675	5.985494	20.38126	23.97432	27.34057
HORVU3Hr1G054920	0.752417	0.7244	0.352832	1.987638	2.875287	2.671251
HORVU5Hr1G124030	2.560036	1.718761	2.711839	2.091485	6.758624	7.923383
HORVU4Hr1G007130	3.584293	4.044715	3.797218	3.358497	4.700969	4.356324
HORVU1Hr1G060420	8.600111	8.229926	9.692489	7.240185	7.476557	8.695048
HORVU0Hr1G018120	5.241058	6.693723	7.482137	2.957256	3.532002	3.770261
HORVU5Hr1G111920	1377.296	1465.793	1037.149	783.5092	53.27335	318.7098
HORVU5Hr1G103600	15.09051	15.77024	16.39616	16.46428	21.8174	20.32093
HORVU3Hr1G030770	40.68898	50.88973	64.48537	7.376673	4.343935	6.827011
HORVU7Hr1G000530	1.561471	1.023645	1.353684	1.258248	0.359948	1.199396
HORVU7Hr1G097240	40.83164	34.6756	40.31442	76.7752	58.98273	63.36348
HORVU5Hr1G012110	13.61911	15.77672	21.66998	8.798242	11.5294	11.82288
HORVU7Hr1G055330	4287.015	4110.324	3215.866	13.05938	3.823618	6.91564
HORVU6Hr1G038620	2.389221	3.242756	4.904871	5.065222	3.978946	3.852986
HORVU6Hr1G050440	6.357297	5.33723	6.329943	11.54693	11.00045	12.01096
HORVU3Hr1G032730	10.15687	11.08235	9.036634	13.06855	20.77549	20.45485
HORVU4Hr1G006260	24.44049	22.53162	24.49395	29.94486	33.02323	34.03639
HORVU6Hr1G087340	14.60226	13.45363	17.96444	14.98883	23.65386	22.30265
HORVU6Hr1G030880	8.186292	7.642417	8.650053	15.11283	25.59852	24.66446
HORVU1Hr1G026840	73.49412	68.93731	69.97788	123.394	158.2687	128.0056
HORVU4Hr1G064230	13.79452	14.5062	16.09431	17.6671	18.81532	20.05537
HORVU3Hr1G006930	9.309459	8.194933	12.92434	8.739147	5.838572	8.82145
HORVU1Hr1G023320	4.530308	3.796746	4.684603	10.24048	8.645077	9.973723
HORVU3Hr1G019090	8.787465	8.851561	10.65241	10.90499	14.18416	13.41275
HORVU5Hr1G076110	8.695241	10.47531	13.52951	5.174571	7.673049	7.699638
HORVU2Hr1G017880	36.17805	36.54508	42.30657	19.72445	14.50854	20.31021
HORVU2Hr1G003350	1.821204	1.867066	3.630169	2.511214	3.247842	3.43032
HORVU5Hr1G039210	9.145739	7.851874	8.884473	13.02824	15.55865	15.36994
HORVU7Hr1G040080	2.352144	2.743369	2.406945	19.46339	24.30513	19.28093
HORVU7Hr1G034350	7.721213	6.162274	7.82217	10.26094	12.60112	12.59211
HORVU5Hr1G018840	133.2332	117.7447	129.8842	111.0557	93.02645	88.09536
HORVU5Hr1G017890	1.151552	1.522313	1.788137	2.911516	3.344228	4.150932
HORVU1Hr1G089520	13.10833	13.7955	23.07638	0.888124	0.074545	0.825726
HORVU5Hr1G080790	56.20658	45.46799	58.25033	65.91731	47.71126	43.55743
HORVU3Hr1G027270	17.97216	15.96321	18.50697	29.31098	36.70348	35.53683
HORVU3Hr1G019320	5.015666	4.480534	4.565029	2.797533	3.16752	1.933309
HORVU5Hr1G113780	5.92931	9.209668	8.340602	0.260155	0.209123	0.157015
HORVU7Hr1G071870	0.806482	1.274351	1.055673	1.394263	1.416511	2.290223
HORVU5Hr1G122080	1.442315	1.248566	1.144971	9.532592	12.81316	10.41539
HORVU4Hr1G063150	3.455215	4.140555	6.231998	5.130064	8.451835	6.790708
HORVU1Hr1G093760	14.36676	9.987852	14.71538	18.05928	27.47288	25.99659
HORVU4Hr1G014010	3.018128	3.524355	3.522053	5.07338	6.194332	7.08762
Hordeum_vulgare_newGene_12945	14.18972	13.91625	16.09719	27.25684	43.83568	39.44143
HORVU1Hr1G062130	5.470597	5.321248	4.431474	5.630394	4.098957	5.432554
Hordeum_vulgare_newGene_12941	1.849526	1.208064	1.457038	3.763027	1.584123	3.65326
HORVU7Hr1G046030	38.54948	41.8255	47.0788	21.85585	5.454809	11.92235

HORVU7Hr1G020620	4.642244	4.216923	4.797439	4.610394	6.682902	6.831634
HORVU1Hr1G038880	2.566008	2.977217	3.246578	4.00139	5.373835	5.183778
HORVU5Hr1G109380	18.53034	16.65058	22.53572	20.29551	32.84765	32.5543
HORVU6Hr1G094460	26.56857	24.75251	25.73671	37.24589	79.57232	72.28224
HORVU5Hr1G114780	0.099227	0.257661	0.055141	0.259241	1.385049	4.172931
HORVU5Hr1G050470	3.682116	3.158855	3.366896	7.286191	8.270453	7.344143
HORVU1Hr1G058090	6.936779	5.148316	7.278043	8.282485	8.832103	9.15007
HORVU2Hr1G042210	1.479621	1.61116	1.858669	1.782928	2.329493	2.878668
HORVU2Hr1G123800	0.472802	0.658072	0.643127	5.391965	0.867534	6.020511
HORVU1Hr1G083960	9.443874	9.289257	13.49382	7.998961	16.82083	20.5752
HORVU3Hr1G071300	0.782295	0.940859	0.840429	2.609007	3.211927	4.624774
Hordeum_vulgare_newGene_12848	2.932008	2.560879	3.929384	4.299896	3.983587	5.853001
Hordeum_vulgare_newGene_12845	34.44062	35.86307	36.34175	61.53323	58.58492	70.58003
Hordeum_vulgare_newGene_12846	4.440775	6.123575	5.098692	10.2629	8.7453	11.20626
HORVU7Hr1G095480	4.935816	4.726241	4.6779	3.114117	3.962969	4.023143
Hordeum_vulgare_newGene_12843	0.095759	0.029036	0	0.502925	6.888302	6.385886
HORVU5Hr1G005180	88.46954	50.56901	116.3236	60.30116	28.50197	56.67109
HORVU4Hr1G019530	22.81952	21.64683	20.60551	31.96729	37.52987	36.67353
HORVU1Hr1G058540	5.36813	5.964145	8.262786	2.85196	3.001322	3.267505
HORVU3Hr1G098260	7.913605	7.442954	7.608654	7.844885	10.74897	9.761435
HORVU5Hr1G111140	13.94754	13.66396	15.33008	14.53122	17.13816	17.12304
HORVU3Hr1G036960	2.440142	1.958865	2.340475	0.801335	1.194993	1.107438
HORVU0Hr1G017620	19.05816	18.51121	18.08559	21.55859	19.66663	20.21165
HORVU7Hr1G085500	3.69125	5.180883	5.66055	4.00808	6.274311	6.619075
HORVU7Hr1G047070	6.000693	5.130026	8.232494	5.463162	3.015785	3.979076
HORVU4Hr1G012500	2.22604	2.303051	2.494545	2.277098	2.836999	3.282624
HORVU6Hr1G055250	27.35097	20.36383	32.23185	25.34929	22.01554	27.47369
Hordeum_vulgare_newGene_13017	0.536257	0.89856	0.391495	2.790169	2.924769	3.09664
HORVU2Hr1G061060	2.919498	2.738455	2.786142	2.947726	4.152514	3.971702
HORVU4Hr1G054200	64.52961	53.50653	66.68953	182.961	241.0486	227.9964
Hordeum_vulgare_newGene_15912	2.297746	2.247576	2.864892	1.736817	2.298186	2.950712
HORVU4Hr1G085690	4.415686	3.842985	5.110789	5.850177	6.062671	4.998446
HORVU2Hr1G108730	22.53028	17.64782	20.36229	1.931816	3.745341	3.845742
HORVU3Hr1G064110	1.635868	0.958853	1.250707	1.767615	1.664824	2.41988
HORVU2Hr1G100100	22.65773	24.26803	27.80488	27.20719	21.91165	23.50805
HORVU1Hr1G011950	1.865893	2.059611	2.179591	0.171439	0	0
HORVU6Hr1G021550	15.48787	17.718	11.72635	15.04864	18.26138	17.99988
HORVU5Hr1G115490	23.32671	20.90213	24.73138	16.88447	16.73733	17.11285
HORVU2Hr1G049200	5.538519	4.892122	4.965055	6.046982	8.552493	9.582961
HORVU0Hr1G021860	2.45757	1.426181	1.658143	1.438398	2.469546	2.773874
HORVU5Hr1G095060	4.526784	5.620351	7.126187	7.001602	27.62694	29.749
HORVU6Hr1G066710	3.703945	2.705248	4.45061	3.692227	6.032113	6.710442
HORVU1Hr1G094920	51.5084	46.34955	49.58331	39.83633	44.93765	44.66232
HORVU3Hr1G079050	4.345129	5.533925	6.216577	4.915647	4.524597	5.151422
HORVU3Hr1G020880	1.0593	1.226997	1.488507	1.122182	1.317062	1.476868
HORVU1Hr1G077530	11.26516	15.44649	10.75181	6.283309	18.25798	18.72313
HORVU1Hr1G047730	11.44163	11.71032	12.60046	16.6668	20.67264	21.54055

HORVU0Hr1G027080	1.491276	1.146559	1.453737	1.699224	1.735796	1.639563
HORVU6Hr1G055870	3.13911	3.355917	3.596045	3.796302	5.860542	6.32145
HORVU7Hr1G121860	905.2222	608.1927	746.6499	250.8765	8.708881	89.16792
HORVU6Hr1G013680	4.174879	2.971277	3.281093	5.74921	9.183927	9.338753
HORVU7Hr1G045910	0.417049	0.829787	1.186103	1.745778	2.404143	1.949782
HORVU2Hr1G066860	3.053994	3.724296	4.308028	3.521308	5.339095	4.289437
HORVU6Hr1G088220	1.488798	2.4221	1.161893	3.78492	2.42029	3.447858
HORVU2Hr1G072470	9.532471	7.958043	9.465884	8.131894	8.346624	8.14748
HORVU3Hr1G093750	4.251947	3.478761	4.061356	4.182897	3.559907	2.498078
HORVU3Hr1G096500	1.34839	1.706669	1.528939	3.908018	4.762341	5.056268
HORVU6Hr1G017590	10.74881	10.12911	12.01523	9.920622	17.22323	16.88675
HORVU4Hr1G064010	23.8611	22.626	18.73845	16.4235	11.60295	16.9808
HORVU2Hr1G109460	26.59511	28.87389	35.12899	36.76003	41.13731	38.24258
HORVU5Hr1G093580	5.949321	7.36088	9.345167	1.345404	2.28401	1.882276
HORVU5Hr1G078960	4.587743	4.491632	6.168337	14.0461	20.58811	19.37127
HORVU0Hr1G010900	14.70441	11.35101	14.5343	10.87114	18.86509	18.11441
HORVU7Hr1G068410	11.26398	11.11449	14.95167	20.83001	20.50522	21.25026
HORVU4Hr1G061990	0.22767	0.17798	0.25049	1.128181	114.7094	98.54038
HORVU3Hr1G074470	0.202078	0.266664	0.199941	1.769347	1.998804	2.086288
HORVU2Hr1G092340	5.763606	6.0736	6.113859	4.763385	5.809324	6.579049
HORVU6Hr1G014480	0.563035	0.859055	0.724879	2.341612	3.229575	3.257329
Hordeum_vulgare_newGene_10554	0.9941	1.540721	1.577353	0.810752	1.077047	1.285451
Hordeum_vulgare_newGene_10553	1.565729	0.892813	1.932527	0.896914	1.506054	0.602624
HORVU3Hr1G061840	1.62647	1.595894	2.201158	1.942639	0.795909	0.814939
HORVU4Hr1G078470	2.776841	1.969466	3.279478	4.200296	1.613674	2.863975
HORVU2Hr1G119540	8.184856	8.67234	10.73656	7.661764	10.47917	10.92757
HORVU5Hr1G002320	0.607363	0.419353	1.002269	0.96627	3.247841	2.955102
HORVU7Hr1G087210	10.10866	12.48676	14.21672	11.97848	13.3893	12.97189
HORVU1Hr1G040850	5.662992	5.463794	6.604551	6.845156	8.388028	10.44001
HORVU7Hr1G058130	4.270181	4.678236	5.322431	6.006123	7.314962	7.668789
HORVU4Hr1G077310	0.408864	0.718895	0.957715	7.54336	7.438818	9.178616
HORVU3Hr1G115450	1.165698	1.298467	1.995058	1.695934	2.09018	2.350428
HORVU3Hr1G095360	6.51788	8.146865	10.01562	0.990533	0.558411	0.464915
HORVU1Hr1G026130	3.11785	3.380257	4.429765	4.369249	7.590787	7.604205
HORVU1Hr1G073940	150.795	119.9377	114.8665	51.56285	38.18001	40.51075
HORVU7Hr1G072650	10.70759	10.07761	11.82123	14.36348	18.68025	20.26423
HORVU5Hr1G064220	6.800699	5.409597	6.426318	5.493292	6.052385	6.381757
HORVU6Hr1G020750	14.17874	11.47018	15.67353	8.13212	7.422409	7.285108
HORVU7Hr1G101310	29.39788	23.82014	27.17555	18.13299	22.56666	21.53572
HORVU3Hr1G023960	22.97022	22.14504	31.61852	18.98045	28.03746	28.43747
HORVU1Hr1G025390	11.15978	13.32607	10.51679	23.4667	27.27093	31.01013
HORVU1Hr1G010810	0.66342	1.222966	1.143161	3.767503	7.120244	6.826556
HORVU7Hr1G080290	19.7282	20.35202	17.5813	21.89597	30.2459	30.53578
HORVU4Hr1G032980	0.035538	0.021871	0.048713	13.50895	13.53975	17.11932
HORVU1Hr1G084350	10.41703	10.76513	14.02937	5.307725	8.815727	7.702086
HORVU2Hr1G103820	4.335536	4.086234	3.95821	2.406848	5.490866	4.054454
HORVU3Hr1G094010	5.13075	4.599982	5.309139	0.301708	0.641812	0.314276

HORVU6Hr1G047910	3.72028	2.816376	3.8303	3.867869	4.633239	4.884119
HORVU2Hr1G028050	10.05274	9.225317	11.17184	12.12633	16.93302	14.44921
Hordeum_vulgare_newGene_599	1.674209	2.277278	1.687446	0.281776	0.07608	0.053131
Hordeum_vulgare_newGene_598	1.170457	1.078598	0.874687	1.214908	0.916768	1.44955
HORVU0Hr1G022340	12.48406	9.930681	9.710267	12.13249	11.60597	11.18901
HORVU6Hr1G054640	4.539084	3.91696	4.785047	4.910992	5.547409	6.325684
Hordeum_vulgare_newGene_12302	1.348763	1.51182	1.053268	2.24805	2.411636	2.971731
Hordeum_vulgare_newGene_12301	1.621688	2.235746	1.832955	0.842577	0.382214	0.674646
Hordeum_vulgare_newGene_12306	1.59722	1.635378	1.464498	1.71181	2.501262	1.945742
Hordeum_vulgare_newGene_12305	0.661494	1.445144	0.450718	5.176585	0.227631	2.241523
Hordeum_vulgare_newGene_7155	0.746501	1.482719	1.938116	2.745219	5.185171	4.598942
Hordeum_vulgare_newGene_7154	1.53982	1.45469	1.495064	0.133462	4.239989	3.564932
Hordeum_vulgare_newGene_7151	4.051585	3.08331	5.319634	5.198302	4.609549	5.065449
Hordeum_vulgare_newGene_7150	48.84561	47.95073	48.53757	51.44115	48.03195	50.30262
HORVU6Hr1G095080	2.956686	3.093119	3.819844	8.667687	12.39473	10.5741
HORVU2Hr1G070160	347.5203	355.8431	302.2428	472.9656	572.2792	553.5335
HORVU6Hr1G070060	7.361576	5.273022	7.117505	10.71674	10.6453	10.29459
HORVU5Hr1G102530	0	0	0.014626	0	12.68427	8.001614
Hordeum_vulgare_newGene_6281	6.780836	6.937812	6.523459	5.273333	6.724416	8.744293
HORVU3Hr1G089950	12.99175	12.13002	17.18497	11.77239	15.11995	17.24696
HORVU5Hr1G084510	6.73207	9.178247	11.61585	2.901229	3.442474	4.661879
HORVU6Hr1G088160	25.78972	23.71574	29.35734	26.16497	23.40723	23.66129
HORVU5Hr1G055430	5.555198	5.35171	5.932429	7.055394	8.424033	8.088969
HORVU7Hr1G025620	9.773086	10.20264	10.19562	12.72187	16.10875	13.78234
HORVU3Hr1G113050	37.63674	30.25148	30.42259	18.11458	17.79308	16.93174
HORVU7Hr1G101860	2.488818	2.744505	2.799478	10.25676	14.17273	12.26128
HORVU3Hr1G117680	4.10945	2.941994	4.101653	5.759514	5.758878	5.465047
HORVU3Hr1G017000	0.955751	1.225386	0.780191	4.905376	5.529126	5.151114
HORVU7Hr1G103420	1.999867	2.60918	3.077236	2.171186	2.082791	2.722703
HORVU4Hr1G074030	1.339533	1.17503	1.091388	3.432815	6.346313	5.894432
HORVU2Hr1G058700	44.37212	47.29743	46.17417	53.07618	73.93873	69.78907
Hordeum_vulgare_newGene_14469	3.683914	3.059353	2.826898	0.226355	0.390596	0.29065
Hordeum_vulgare_newGene_14464	1.629576	2.706739	2.047337	5.139567	5.854676	3.038988
Hordeum_vulgare_newGene_14466	0	0	0	3.155768	2.655701	2.048642
HORVU4Hr1G022580	13.25034	9.585549	13.00469	13.2431	16.07677	14.47212
Hordeum_vulgare_newGene_14463	0.12677	0.822384	0.841367	1.426917	1.688387	1.229728
HORVU3Hr1G050880	9.360649	8.767849	9.435396	14.95284	16.71919	17.67519
Hordeum_vulgare_newGene_5547	1.317359	1.395632	1.215467	1.697147	1.067954	0.893811
HORVU0Hr1G040270	3.257972	1.597826	1.644593	3.198513	1.657202	1.534668
Hordeum_vulgare_newGene_5542	1.650521	1.85937	1.816355	2.954171	4.56216	3.68758
HORVU5Hr1G088510	5.228689	5.500135	5.94339	5.541976	5.361271	5.903558
HORVU6Hr1G075420	21.1135	16.10416	19.79455	21.3436	19.54064	18.28714
Hordeum_vulgare_newGene_9866	1.100062	1.507799	1.803389	1.260402	1.941449	2.113267
Hordeum_vulgare_newGene_13168	6.307444	7.699157	6.941234	0.006767	0.003007	0
HORVU0Hr1G000270	6.132745	5.558443	7.347295	7.290088	6.223367	6.933105
Hordeum_vulgare_newGene_13160	1.985424	2.617743	2.629192	3.363245	3.825111	4.231389
Hordeum_vulgare_newGene_13161	50.24355	46.55775	33.33028	9.106679	2.495829	4.410457

Hordeum_vulgare_newGene_14797	54.76973	25.19826	42.53322	1.12916	1.311527	1.874949
Hordeum_vulgare_newGene_9266	2.77203	2.890401	3.297518	5.066602	4.924868	5.339128
HORVU5Hr1G103720	14.27971	12.8956	18.38527	20.18039	26.15883	28.24673
HORVU1Hr1G078910	29.90336	31.86839	29.41546	27.81104	28.93026	28.69992
HORVU5Hr1G065370	6.598546	5.337004	6.637064	3.979911	2.750101	3.953913
HORVU0Hr1G016720	3.546618	3.470483	3.843227	4.273091	8.279832	8.057107
HORVU3Hr1G069260	47.51792	44.72739	42.01907	29.2671	29.04592	28.3933
HORVU1Hr1G042250	2.824583	3.58072	3.691584	0.782766	0.450165	0.615432
HORVU0Hr1G005570	1.474351	2.293055	1.996646	6.340526	5.563174	6.604797
HORVU4Hr1G029200	6.540247	7.155999	6.754769	8.51184	13.96418	12.98255
HORVU6Hr1G091620	1.751737	1.724896	1.85429	1.571868	2.277053	2.427668
HORVU5Hr1G030970	22.18665	14.08653	19.21225	23.80646	26.28182	28.90321
HORVU2Hr1G086040	4.550352	4.00175	4.934976	7.48511	8.09897	8.196628
Hordeum_vulgare_newGene_7872	23.93444	26.79952	27.96414	23.22472	24.20837	29.12878
HORVU7Hr1G100540	3.356769	3.391449	3.256487	3.417903	3.293244	3.141651
HORVU4Hr1G011460	14.47175	14.65714	17.65729	19.7342	24.41578	24.88647
HORVU1Hr1G059360	2.636448	2.438895	2.684917	3.255453	4.066572	4.526456
HORVU5Hr1G097230	47.15155	42.54269	42.27522	32.26835	36.97614	36.19359
HORVU5Hr1G073560	15.23271	11.892	15.25129	10.90537	18.30983	16.28062
HORVU2Hr1G004620	15.84843	10.99377	20.8605	1.988442	0.385946	1.906704
HORVU6Hr1G076810	24.35246	19.76806	24.78905	33.04376	47.64772	45.05002
Hordeum_vulgare_newGene_830	11.45472	11.70196	12.24953	5.799767	1.994779	3.620821
HORVU5Hr1G035220	24.76017	27.59711	26.12885	22.87924	20.65306	24.13234
Hordeum_vulgare_newGene_834	0.412676	1.211246	0.611908	1.279169	1.292746	1.578862
Hordeum_vulgare_newGene_837	2.579323	2.737838	2.950782	3.768512	3.593608	4.085808
HORVU3Hr1G080830	29.93086	23.38252	31.18553	19.30583	29.4669	33.5145
HORVU6Hr1G061580	4.375072	5.823095	5.509908	5.140043	5.955145	6.136678
HORVU7Hr1G036660	14.13261	13.6512	15.75691	28.72271	28.78349	28.40924
HORVU2Hr1G050640	20.3788	18.9035	17.74806	15.61114	15.35933	16.45458
HORVU7Hr1G049050	8.204376	7.889114	6.356698	13.04065	15.45015	16.22454
HORVU2Hr1G075950	3.14032	3.94222	4.627973	0.938278	1.19339	1.264606
HORVU4Hr1G089520	4.063309	3.483727	4.227017	5.061801	3.301918	4.119137
HORVU3Hr1G115860	3.019076	2.458463	2.317168	1.971518	2.078131	2.242361
HORVU1Hr1G034650	2.405128	2.3599	3.204193	0.929627	1.6311	2.465413
HORVU3Hr1G060920	17.77229	15.77369	21.9443	28.62741	29.12849	24.60244
HORVU1Hr1G040940	3.437264	3.486871	3.44541	5.030691	7.294561	9.001798
HORVU3Hr1G083560	64.26807	56.5329	62.50619	66.72437	60.02054	62.21032
HORVU2Hr1G089540	1.764135	0.924252	2.710952	1.477624	2.451458	3.511261
HORVU7Hr1G096020	1.70395	1.765748	1.367217	2.264665	5.478484	5.063563
HORVU4Hr1G076520	11.19489	12.7113	12.42251	1.345217	0.736183	1.221456
HORVU3Hr1G018690	2.090665	2.111944	2.240531	1.375112	0.688363	1.288477
HORVU6Hr1G030600	0.907473	0.722866	0.869128	2.503518	14.26734	11.52611
HORVU2Hr1G120850	7.35846	7.880635	9.288368	7.57132	9.750227	8.156022
HORVU7Hr1G113020	13.23131	8.694576	10.43473	2.131029	0.938413	0.978655
HORVU1Hr1G076180	24.42358	21.25191	25.62699	22.05785	22.17411	24.1257
HORVU2Hr1G098220	1.977641	1.300076	1.572108	3.053448	3.14843	2.696615
Hordeum_vulgare_newGene_1392	4.035026	3.923145	4.87103	5.0542	6.084502	5.54636

HORVU5Hr1G069910	1.218597	0.742496	1.341236	4.748513	7.085078	4.77076
HORVU7Hr1G010570	3.134752	4.063422	4.009089	1.080654	1.829401	1.33789
HORVU3Hr1G071140	3.52257	4.019124	4.477179	6.189039	7.820688	8.609434
HORVU2Hr1G047870	5.813277	7.077707	6.60394	5.824877	7.657718	8.44048
Hordeum_vulgare_newGene_4344	9.300021	8.681793	9.948224	8.082466	8.89127	8.710082
Hordeum_vulgare_newGene_4342	19.20255	19.86505	18.32598	26.39589	33.48675	36.60742
Hordeum_vulgare_newGene_4349	2.549317	3.086396	3.00727	2.806419	5.883294	6.243498
Hordeum_vulgare_newGene_1645	0.838468	0.727962	0.941624	1.662093	2.62334	2.535808
HORVU5Hr1G108630	5.026753	5.667544	5.977367	3.973418	5.03825	5.459307
Hordeum_vulgare_newGene_1649	10.39333	10.59838	11.05385	9.125954	8.165874	8.255161
Hordeum_vulgare_newGene_1648	0.817201	1.033685	0.922107	0.726077	1.697349	1.692681
HORVU2Hr1G083620	4.423937	4.792492	5.946768	6.198539	7.310123	6.412756
HORVU1Hr1G058700	8.153476	7.401425	9.401186	7.04231	9.216927	9.13034
HORVU0Hr1G008850	10.80471	8.453454	13.23848	8.982826	7.123012	7.406112
HORVU3Hr1G049940	8.82599	5.864568	8.786079	9.100623	8.084129	8.043923
HORVU5Hr1G021240	5.564397	5.934418	7.314375	5.849507	7.863286	8.810206
HORVU5Hr1G067590	1.045639	1.586996	1.360379	0.747941	0.95247	1.417519
HORVU5Hr1G105950	11.04494	12.06885	13.42868	19.29275	31.02671	30.01654
HORVU2Hr1G125430	0.361322	0.094129	0.2619	1.812878	1.988899	2.021095
HORVU3Hr1G071490	471.8783	481.8066	665.5011	195.0497	90.15669	114.2886
HORVU4Hr1G012340	6.643499	7.828088	12.41491	7.590998	9.83784	8.578456
HORVU4Hr1G072310	1.854284	1.761388	1.59551	2.995659	3.034106	4.458385
HORVU1Hr1G072750	4.669039	4.736707	5.860684	10.69703	16.21386	15.86155
HORVU5Hr1G104020	16.05499	13.87136	15.31427	17.486	22.47616	20.20749
HORVU4Hr1G019040	15.94224	13.33714	13.69853	16.22237	15.81263	15.00814
HORVU2Hr1G080190	3.962859	3.283993	3.435344	0.293248	0.073401	0.046028
HORVU5Hr1G018220	3.55394	1.873179	1.609622	3.179437	4.882848	4.425655
HORVU7Hr1G094510	6.9905	6.160034	6.550385	7.086582	7.896607	8.26393
HORVU1Hr1G092890	29.01959	23.45255	24.17544	27.56021	48.64717	47.38926
HORVU1Hr1G048580	9.806976	11.33417	14.73934	7.548478	7.446539	8.023407
HORVU3Hr1G001040	0.947283	0.690765	1.063418	0.929649	1.582993	2.031081
HORVU7Hr1G098610	1.340431	1.450222	1.998882	1.447259	3.508355	2.214424
HORVU4Hr1G053940	39.78429	34.33732	16.75406	58.47658	61.22303	64.86173
HORVU2Hr1G083080	7.127398	6.415755	5.441203	6.876109	7.572812	8.268253
HORVU6Hr1G044440	7.731628	7.726088	9.576355	6.768793	9.815816	9.036033
HORVU4Hr1G003820	22.76484	22.86581	23.6254	21.96042	24.62248	22.34176
HORVU1Hr1G080840	1.37932	1.905206	1.206325	3.87437	5.145454	5.488751
Hordeum_vulgare_newGene_13085	6.835581	7.207358	8.650578	4.938751	5.979441	5.594415
HORVU0Hr1G005210	22.78367	14.60907	22.84924	24.27861	24.98707	19.47619
HORVU3Hr1G029140	17.693	17.48828	20.98915	30.14403	35.41724	34.74521
HORVU7Hr1G038950	2.363271	2.730343	3.27117	2.057647	2.62425	2.101331
Hordeum_vulgare_newGene_9287	30.36292	30.38886	34.79319	45.82958	55.85397	54.93613
HORVU1Hr1G026100	4.478014	2.838537	1.605586	0.733505	1.441127	1.221364
HORVU5Hr1G027160	15.01958	14.72949	13.60134	10.46201	10.5337	11.39841
HORVU0Hr1G036340	0	0	0.03266	4.085519	0.646948	1.591538
HORVU1Hr1G080900	0.931106	1.300994	1.244522	1.934423	2.781879	2.872965
HORVU5Hr1G013160	12.41465	9.881406	13.60111	13.95683	15.9774	15.67642

HORVU2Hr1G086900	2.499456	2.520493	2.786529	3.497516	5.71241	5.012787
HORVU3Hr1G021910	3.124099	3.435632	3.921214	6.641301	8.332627	9.409571
HORVU7Hr1G052400	11.2775	9.727461	10.32397	13.5802	15.71219	16.60428
HORVU2Hr1G078630	2.265019	1.610797	2.13895	2.31378	3.351212	4.258249
HORVU4Hr1G048620	1.835396	1.791682	1.808678	3.653379	4.785644	4.652604
HORVU1Hr1G065130	3.205404	2.667854	4.462221	7.638216	15.21732	17.7532
HORVU7Hr1G007690	74.29261	58.11679	87.8064	6.384541	0.011419	1.768069
HORVU1Hr1G010510	7.978075	6.069207	8.245189	8.207465	12.92287	13.64116
Hordeum_vulgare_newGene_9284	1.219346	0.938559	1.254155	1.434577	1.075938	1.221597
HORVU7Hr1G054760	55.01503	53.85334	58.09294	38.56227	32.62015	33.73262
HORVU1Hr1G094180	0.46216	0.357433	0.447302	2.952319	2.863756	3.894566
HORVU3Hr1G062430	2.171168	1.284237	1.763433	7.187924	3.73097	8.885719
HORVU6Hr1G046540	14.27718	16.07627	17.12278	22.25437	27.67254	26.96221
HORVU5Hr1G008770	0.220893	0.207221	0.390479	1.456007	8.009475	7.30341
HORVU1Hr1G088590	0.566719	0.621175	27.87127	71.32177	2.749115	3.351818
HORVU3Hr1G038140	10.89926	10.71444	11.13073	12.23118	16.59946	15.39617
HORVU4Hr1G002880	3.177152	3.813766	3.411667	4.98069	7.603434	6.708272
HORVU5Hr1G027830	13.86021	13.11265	16.78711	13.09893	15.49485	16.12615
HORVU6Hr1G082150	14.16259	16.90705	13.80736	17.79485	20.69132	19.87558
HORVU5Hr1G093210	3.215428	2.883685	3.82383	2.401376	2.931596	3.777362
HORVU4Hr1G015170	18.34112	17.40966	20.33174	22.14301	44.91387	20.96653
HORVU2Hr1G090070	1.386864	1.214937	2.372002	1.394516	3.309842	3.12608
HORVU6Hr1G045890	1.444108	1.836912	1.925908	2.195416	1.981766	1.576263
HORVU4Hr1G051450	4.082158	3.315461	4.325355	0.833294	0.34697	0.585224
HORVU5Hr1G036250	44.41129	51.99725	51.41717	46.8402	58.49933	60.8548
HORVU1Hr1G029480	2.624219	2.788382	3.03744	3.960918	5.150057	4.766622
HORVU4Hr1G016640	48.19276	45.64035	40.50075	52.72123	65.16	66.27494
HORVU1Hr1G019330	15.85127	14.9108	17.36572	23.08324	22.17256	21.58884
HORVU1Hr1G088040	69.21306	75.61715	135.0028	94.45797	79.54759	81.44711
HORVU3Hr1G076320	1.888159	1.840179	2.134382	2.268871	2.233938	2.830953
HORVU7Hr1G048040	8.283244	8.601001	9.549843	9.173521	11.00131	12.14796
HORVU5Hr1G039400	22.11551	16.18115	26.02544	19.76536	18.31366	16.61472
HORVU2Hr1G113970	40.21881	35.06107	40.19805	29.08874	23.16828	25.03053
HORVU7Hr1G045240	18.23545	19.08108	18.05723	18.48274	23.75562	23.60008
Hordeum_vulgare_newGene_16245	2.378506	2.120722	2.571116	2.790372	3.33839	3.680424
HORVU1Hr1G095080	3.623976	4.052016	4.958777	3.282581	4.524123	4.891061
HORVU5Hr1G007480	5.12373	6.275376	7.07268	0.070008	0.011254	0.04131
HORVU0Hr1G016340	1.470724	0.945018	0.093742	0.094443	0.126551	3.893301
HORVU3Hr1G082310	30.66027	31.01832	31.4894	39.77822	38.36851	38.32338
HORVU2Hr1G067290	0.987725	1.132122	1.409574	1.544417	3.324438	4.211148
Hordeum_vulgare_newGene_11326	6.147511	5.894768	0	2.179052	0.540155	0.954033
HORVU2Hr1G061600	15.52488	12.94383	22.19851	30.60421	56.34514	39.14513
HORVU7Hr1G074890	15.82137	13.35468	19.35831	38.2573	49.14975	53.6342
HORVU4Hr1G056100	3.635057	2.959648	3.787778	5.495551	5.561537	6.361973
HORVU7Hr1G051840	3.42401	3.987794	5.368038	0.272934	0.609189	0.262724
HORVU2Hr1G063220	20.84188	20.23508	20.89914	3.273756	0.656951	1.862606
HORVU5Hr1G109220	5.593548	6.486618	7.112733	5.567114	7.883823	8.088965

HORVU1Hr1G089310	8.399337	3.152329	11.32092	50.49815	9.491497	8.503733
HORVU6Hr1G086480	9.844689	11.73925	12.27231	5.761751	7.356677	6.302215
HORVU1Hr1G002950	0	0	0	12.09353	0.025952	0.096198
Hordeum_vulgare_newGene_15757	20.64284	22.43745	19.33234	23.94084	30.01344	28.8127
HORVU4Hr1G049500	26.33492	34.61492	39.35975	8.78139	7.460604	9.936711
HORVU5Hr1G114660	0	0.051725	1.106909	1.604378	2.420767	1.574673
HORVU3Hr1G065370	9.774512	9.469193	9.362655	9.526559	9.118307	10.96455
HORVU0Hr1G021230	30.02329	28.18219	36.54583	18.67168	31.84488	32.25945
HORVU7Hr1G077270	3.643091	3.323165	5.211735	0.799962	0.21813	0.237576
HORVU5Hr1G110170	18.72803	16.72496	24.28274	13.99656	13.02874	14.02367
HORVU5Hr1G011280	19.15859	19.94042	25.105	29.37145	54.77342	42.30726
HORVU3Hr1G092520	4.316466	3.278397	2.307381	13.07941	9.998172	11.53302
HORVU3Hr1G023230	3.516424	2.303983	4.271917	1.079132	0.271178	0.980269
HORVU1Hr1G068950	7.003068	6.55324	7.003658	8.287551	10.59989	10.5814
HORVU3Hr1G089000	3.2168	2.774652	2.774325	6.428775	12.52615	12.73748
HORVU7Hr1G093830	6.44236	7.362943	6.165078	0.034189	0	0.034009
HORVU5Hr1G010880	58.89209	68.89705	80.5538	6.552048	0.772367	4.251762
HORVU2Hr1G066650	12.01921	12.54399	14.86583	18.59415	19.86202	18.41426
HORVU5Hr1G079230	25.77308	25.13881	33.25813	52.70318	76.62912	67.20592
HORVU7Hr1G047790	0.900899	1.54922	1.007479	4.003373	1.43314	1.572053
HORVU5Hr1G044190	1.483082	1.621028	1.361989	1.990847	3.229815	2.301773
HORVU7Hr1G087800	3.377646	3.193519	3.368748	8.838611	3.365754	4.503805
HORVU7Hr1G088720	32.25639	35.31027	28.04376	63.54888	66.90812	75.74241
HORVU2Hr1G063510	14.56809	9.335041	12.64275	59.7635	58.1296	53.2037
HORVU3Hr1G031200	35.27345	33.44021	45.26606	44.30739	52.19179	55.53806
HORVU0Hr1G020180	7.158325	6.056879	6.412548	6.547733	7.962402	6.512477
HORVU3Hr1G024170	16.32358	17.44562	24.10275	18.5195	22.99426	22.90165
HORVU3Hr1G101990	0.702738	0.500725	0.621408	3.730883	7.595078	5.446475
HORVU5Hr1G000760	6.271983	6.593773	5.860544	7.026291	8.063513	8.800096
HORVU4Hr1G082130	5.09161	5.236824	5.033576	2.358597	3.032562	3.299159
HORVU7Hr1G047130	3.356328	2.79344	4.09654	4.913671	5.678364	5.34385
HORVU3Hr1G067840	3.964724	3.43812	4.088576	6.233021	5.034671	4.924844
HORVU3Hr1G117250	0.673097	1.114341	0.983443	1.212363	1.63587	1.432966
HORVU5Hr1G085150	4.036851	3.545882	4.151068	3.095841	5.947415	6.155931
Hordeum_vulgare_newGene_13368	5.474753	2.786552	6.040816	10.12101	8.113811	6.498584
HORVU1Hr1G070120	2.396407	3.531566	5.10909	2.982939	6.62338	5.156859
Hordeum_vulgare_newGene_13361	9.073759	11.47086	8.215452	0.051931	0	0.048134
Hordeum_vulgare_newGene_13366	1.402392	1.11576	0.85629	0.903879	1.033961	0.800057
HORVU7Hr1G003100	21.40593	16.57938	20.95028	28.0023	35.35051	32.35572
HORVU2Hr1G116220	1.845231	1.867566	2.910887	2.3184	0	1.045489
HORVU7Hr1G082540	120.8763	90.53438	138.7882	142.1912	195.9226	188.4003
Hordeum_vulgare_newGene_9064	0	0	0	5.939439	9.130514	10.16458
HORVU6Hr1G053290	6.572622	7.644492	11.15396	5.026318	5.732487	6.715315
Hordeum_vulgare_newGene_9060	129.6799	101.1378	107.1512	107.7952	111.7418	108.357
Hordeum_vulgare_newGene_15207	0	0	0	1.017039	2.56779	3.542055
Hordeum_vulgare_newGene_15205	1.175915	1.205912	1.070214	0.972305	1.159351	1.219776
HORVU7Hr1G073570	6.696106	8.435367	10.11414	7.599302	9.91361	8.957024

HORVU1Hr1G077470	31.57038	26.87833	32.17406	23.78476	34.7038	33.08096
Hordeum_vulgare_newGene_4484	6.836852	7.493065	7.716718	7.334817	8.370709	10.3217
HORVU4Hr1G001140	2.173572	2.245603	3.78614	3.443111	3.466731	4.512583
Hordeum_vulgare_newGene_2727	3.966888	3.616683	4.640074	5.070683	7.152145	7.656071
HORVU1Hr1G070690	88.31738	109.4988	142.9097	23.6914	13.60507	15.31109
HORVU3Hr1G037720	10.62903	9.562619	13.19404	9.89576	14.02984	16.14856
HORVU6Hr1G056110	33.47634	40.23159	37.19181	35.75933	35.87867	34.9396
HORVU6Hr1G021320	2.337962	2.490297	3.745448	2.178225	1.311824	2.652699
HORVU3Hr1G013810	1.030842	0.829568	1.170113	0.973854	1.108772	1.460313
HORVU5Hr1G078020	1.779013	2.389759	2.832496	0.428022	0.366592	0.441976
Hordeum_vulgare_newGene_13988	4.348871	4.005352	5.165889	4.000223	3.91889	3.614398
Hordeum_vulgare_newGene_13986	1.308695	1.053217	1.166362	1.722256	0.745182	1.195207
Hordeum_vulgare_newGene_13984	2.741268	2.021566	3.912531	21.99278	19.89721	27.59148
Hordeum_vulgare_newGene_13985	3.866895	3.786493	4.096085	2.110468	1.092444	1.47143
Hordeum_vulgare_newGene_13980	0.657028	0.408219	0.616205	1.659642	1.943022	2.178997
HORVU2Hr1G038720	21.65278	23.37018	24.28007	24.42691	34.44798	35.19412
HORVU3Hr1G021490	2.07153	3.209027	3.259059	0.044636	0.042417	0.053587
HORVU7Hr1G027580	1.026557	1.097328	1.263352	0.65044	0.709824	1.304335
HORVU7Hr1G072620	12.65336	10.8257	14.09435	0.13874	0	0
HORVU2Hr1G082280	4.499496	4.902387	5.308117	7.682838	9.937531	10.997
HORVU4Hr1G061810	1.053971	1.536382	1.487889	1.550421	1.369013	1.280853
HORVU2Hr1G030130	7.889849	7.467486	8.759963	8.17801	7.588992	6.666144
HORVU5Hr1G078180	36.88104	32.65252	39.24061	28.15585	26.66587	25.90952
Hordeum_vulgare_newGene_5188	26.25018	28.95388	38.39269	13.54599	2.732637	7.397759
HORVU7Hr1G089540	0.581496	0.255098	0.30537	3.456094	9.578941	9.900329
HORVU3Hr1G075910	8.397469	7.05176	9.38091	6.359012	11.54329	10.82281
HORVU1Hr1G031680	1.841384	2.027648	1.215406	1.045546	1.76457	2.511204
HORVU7Hr1G070700	21.49256	18.74443	22.92715	39.15273	36.09565	38.23855
HORVU1Hr1G024090	4.460261	4.377292	5.338506	5.340567	4.888471	5.45947
Hordeum_vulgare_newGene_11277	6.062166	6.197241	5.686552	5.763758	6.499182	5.943328
Hordeum_vulgare_newGene_8004	5.725013	4.606285	7.827595	10.71074	11.22396	10.10621
HORVU4Hr1G000520	1.339292	1.180931	1.471158	2.710783	4.766078	4.523093
HORVU2Hr1G023560	5.988397	5.063728	4.537307	17.77188	28.12582	30.27885
Hordeum_vulgare_newGene_10635	3.779289	3.902347	5.18005	0	0.034814	0.067492
HORVU5Hr1G048550	1.62999	2.302305	1.609354	2.186464	2.301595	2.431252
HORVU2Hr1G039540	4.338613	4.187878	3.915784	6.239358	7.287881	8.210531
HORVU7Hr1G081770	80.85577	87.96071	97.25822	237.3607	253.0906	234.029
HORVU0Hr1G023510	3.076383	3.975293	4.755341	4.809639	5.372392	5.177618
Hordeum_vulgare_newGene_14987	2.146987	1.774854	2.268079	3.002473	2.843661	3.359884
HORVU6Hr1G015890	5.15377	4.317102	4.605389	2.076791	0.683679	1.127184
Hordeum_vulgare_newGene_15947	5.170362	4.744504	5.710237	6.302491	7.254636	7.529652
Hordeum_vulgare_newGene_14988	2.753393	2.110993	2.739183	0.94548	0.575102	0.549095
HORVU2Hr1G026880	3.222447	5.123029	8.489375	1.992123	0.232412	1.098105
HORVU2Hr1G084890	44.23266	37.69719	45.63675	55.74035	65.7197	54.57877
HORVU7Hr1G030810	4.890795	4.05462	5.62365	25.39054	33.2508	27.29655
HORVU7Hr1G037840	2.616982	3.714866	3.886368	6.218332	6.958946	6.479579
HORVU2Hr1G043170	0.610324	0.973017	0.424028	1.478125	1.959124	2.390156

HORVU0Hr1G020960	2.190093	1.257024	1.280742	3.68734	10.55379	9.082496
Hordeum_vulgare_newGene_1409	3.124344	2.150787	2.752445	2.758314	1.95262	2.52229
Hordeum_vulgare_newGene_1408	4.590968	3.988849	4.386588	0.455606	0	0
Hordeum_vulgare_newGene_1405	10.50302	9.384561	8.943453	7.879923	6.562983	7.468601
HORVU7Hr1G021860	1.094486	0.880529	0.83201	1.121182	1.799278	1.845369
HORVU5Hr1G097020	5.956174	5.737223	7.835645	8.858073	13.90361	15.41693
HORVU6Hr1G027910	11.81909	11.7356	10.92959	12.39503	11.11416	12.42637
Hordeum_vulgare_newGene_3801	0	0	0	0	3.088542	4.006878
Hordeum_vulgare_newGene_3800	0	0	0.146549	1.596487	14.80446	15.34318
HORVU6Hr1G071910	11.12051	12.3119	12.16955	9.982511	16.82335	17.42441
HORVU3Hr1G094730	2.565728	1.780865	1.777766	2.920309	3.185727	4.085853
HORVU7Hr1G120960	4.817835	6.820234	8.846599	9.053381	18.91279	18.93273
HORVU1Hr1G039090	4.695363	6.146086	6.352599	7.465195	8.992011	10.75863
HORVU2Hr1G034720	8.616302	8.306458	10.02089	10.88062	13.47942	14.0839
Hordeum_vulgare_newGene_10065	2.042961	1.752733	1.752474	2.119471	1.394783	1.139758
HORVU2Hr1G029530	1.34438	1.354586	1.343139	1.485125	1.649319	1.71403
HORVU2Hr1G071930	12.40829	14.99831	10.54545	17.58114	17.03476	20.97666
HORVU1Hr1G075160	0.346499	0.198061	0.539658	3.038742	6.610257	8.469767
Hordeum_vulgare_newGene_15013	8.026796	7.594342	5.758216	1.401219	0.723893	0.74135
HORVU2Hr1G045630	2.220965	2.797768	2.829228	5.477385	9.232701	8.637435
Hordeum_vulgare_newGene_4635	4.121066	3.601102	3.43671	4.000152	5.169597	5.434059
Hordeum_vulgare_newGene_341	6.359022	7.957172	8.098039	7.877535	15.8406	15.61947
HORVU1Hr1G072220	315.1646	317.1019	314.8583	715.4561	935.2398	660.243
Hordeum_vulgare_newGene_4632	3.571577	3.584054	4.041881	2.648611	1.195805	2.159689
HORVU5Hr1G014730	2.40123	2.925533	3.653921	3.633475	34.84562	35.36727
HORVU5Hr1G114840	13.2417	14.01692	21.56959	11.61672	24.35279	25.23408
HORVU3Hr1G052070	11.13391	12.22005	12.84444	10.65003	11.88253	12.05014
HORVU6Hr1G016810	8.681904	8.379431	10.32782	7.026195	9.280484	9.297596
HORVU3Hr1G014950	23.89087	24.86933	23.72289	14.46261	11.88978	12.59152
HORVU5Hr1G084490	2.022623	1.860149	1.893288	4.718664	5.175939	4.599307
Hordeum_vulgare_newGene_12698	1.231778	1.766463	1.441063	2.481037	3.010877	3.617732
HORVU2Hr1G068170	10.78574	9.500201	15.53716	7.64012	8.915565	8.655776
HORVU6Hr1G069280	7.256457	8.821672	9.931699	3.23746	3.354177	3.282263
Hordeum_vulgare_newGene_12118	8.642522	16.70025	13.27172	0.530258	0.545223	0.650839
HORVU1Hr1G092540	3.616314	2.812714	4.049767	8.61706	19.6025	16.44123
HORVU3Hr1G085270	13.26235	12.05857	9.8545	39.72932	59.41408	61.60262
HORVU4Hr1G057580	1.672558	0.560501	1.326033	1.192474	1.635393	1.375562
HORVU7Hr1G105350	3.765326	4.755304	5.082194	4.04692	3.785315	5.432348
HORVU4Hr1G072880	2.875212	3.180444	9.865041	2.963612	10.54061	13.74508
Hordeum_vulgare_newGene_258	13.69668	12.99556	14.12665	20.12512	35.04118	31.67235
Hordeum_vulgare_newGene_6672	1.099882	0.229725	0.966503	1.766704	2.981513	2.872306
HORVU7Hr1G058490	23.34262	20.39443	31.07498	26.43526	50.16269	48.67362
Hordeum_vulgare_newGene_254	1.249702	0.740599	0.774369	0.425474	4.791289	1.604084
Hordeum_vulgare_newGene_6676	3.687532	2.560288	3.913498	4.39717	4.726661	4.175593
Hordeum_vulgare_newGene_257	2.655846	2.494323	2.868717	1.666538	1.369491	1.765391
HORVU0Hr1G005290	0.860337	1.240603	1.218002	1.974191	2.668946	2.717875
HORVU1Hr1G022720	6.093897	6.5099	8.960019	4.418377	6.542066	7.49243

Hordeum_vulgare_newGene_3117	7.049424	1.863317	2.166958	4.132384	4.601338	2.820976
Hordeum_vulgare_newGene_3113	2.795542	3.997272	2.606828	4.300103	4.822193	4.957025
HORVU4Hr1G013320	0.234181	0.15632	0.240306	1.075278	3.565743	3.779409
HORVU1Hr1G026650	6.794422	5.348951	6.549394	13.60605	23.47072	21.25611
Hordeum_vulgare_newGene_3118	1.507423	1.230901	0.937915	8.27145	15.7428	17.18475
HORVU6Hr1G063700	1.203218	1.0659	0.79482	0.876027	1.286585	1.150964
HORVU6Hr1G070740	2.64944	0.981345	3.909826	1.130181	0.78615	0.782283
HORVU2Hr1G034670	11.94217	9.945369	11.25163	15.58259	22.56727	16.29743
HORVU4Hr1G074480	4.173308	5.330678	7.713919	4.721543	4.743084	3.536309
Hordeum_vulgare_newGene_6099	1.885053	2.53907	2.924795	2.920454	2.85638	2.945105
HORVU3Hr1G013370	5.893635	7.149457	7.706475	8.562022	11.15383	12.4479
Hordeum_vulgare_newGene_6093	0.890207	0	1.16614	2.996322	4.46897	4.768412
HORVU7Hr1G064240	4.304135	3.53585	3.963566	5.671285	7.333682	8.081226
Hordeum_vulgare_newGene_13649	12.13309	13.43131	10.18591	0	0	0
HORVU5Hr1G062990	120.8709	136.9959	161.5008	63.8667	5.95506	26.38049
HORVU5Hr1G107340	27.19431	22.96912	30.83866	10.82206	11.01616	10.74539
Hordeum_vulgare_newGene_14618	0.016243	0.822259	2.366589	6.578238	8.249638	8.603329
Hordeum_vulgare_newGene_14617	2.001621	2.285224	3.298909	10.94099	20.16447	15.71584
Hordeum_vulgare_newGene_14615	0	0.610148	1.503034	6.161	7.14756	5.365471
Hordeum_vulgare_newGene_13640	21.92038	15.95336	26.71732	13.93449	33.28703	30.72154
HORVU1Hr1G060850	11.21094	13.31793	13.27717	11.06921	9.450968	10.89619
Hordeum_vulgare_newGene_13642	3.498477	2.38134	2.797508	0	0	0
HORVU5Hr1G000400	28.24764	33.17849	44.47714	2.354531	1.222403	1.511897
HORVU7Hr1G052390	2.735303	2.602688	3.517704	2.955065	3.946736	3.504238
HORVU2Hr1G124930	1.822984	0.826221	3.811187	3.79084	1.391811	6.219981
HORVU6Hr1G070290	8.969976	6.956059	12.30469	6.419532	9.217125	8.240621
HORVU7Hr1G038200	3.63674	3.584227	3.620622	6.853981	19.71376	16.03303
HORVU1Hr1G027520	0.443078	0.773687	1.257911	0.942613	1.369233	1.833728
HORVU6Hr1G082310	23.19768	22.19717	12.32659	0.571224	1.991035	1.729202
HORVU7Hr1G118570	41.02916	35.15318	28.35646	4.729761	2.036481	2.707607
HORVU7Hr1G091910	3.392619	4.398141	5.885997	0.693558	0.824293	0.908611
HORVU3Hr1G068300	1.870151	1.867697	1.739416	6.809949	8.389242	8.315774
HORVU5Hr1G036490	5.690173	6.849165	7.338777	3.621971	4.774599	4.73316
HORVU5Hr1G023730	180.3586	82.03866	241.9394	26.24835	5.378444	20.94194
HORVU7Hr1G108180	0.927057	1.327065	1.580326	1.636485	1.710801	1.109067
HORVU5Hr1G069040	1.404198	1.850294	2.369258	6.06181	8.820946	9.792732
Hordeum_vulgare_newGene_15563	20.26117	19.51603	21.79617	25.18411	25.4055	28.17045
Hordeum_vulgare_newGene_15560	1.760001	2.076907	0.815464	1.601209	0.863458	2.362261
Hordeum_vulgare_newGene_8943	1.125572	0.778275	1.198022	0.764042	1.303013	1.242783
HORVU5Hr1G094950	24.30794	25.48463	30.37255	27.62869	34.03458	29.81899
HORVU6Hr1G034150	8.756875	6.943132	8.804963	7.359284	9.438587	10.34339
HORVU6Hr1G072580	0.123424	0.546979	0.285949	1.688402	7.637815	7.186339
HORVU3Hr1G078940	78.87135	80.51847	116.3468	115.6189	146.401	131.7937
HORVU3Hr1G082550	0.80253	0.609603	1.002925	3.274736	6.215492	7.121939
HORVU1Hr1G092130	2.635206	1.653287	2.554412	3.169345	1.577034	1.510244
HORVU7Hr1G108230	10.7503	8.10923	11.86949	24.4037	24.21797	23.67896
HORVU5Hr1G061710	47.56899	46.80797	31.33487	14.63073	10.41242	13.1784

HORVU1Hr1G054580	1.026086	0.795728	1.05638	1.096258	1.210043	1.626902
HORVU3Hr1G012420	0.663329	1.679909	2.034172	1.388192	1.533334	1.226146
HORVU5Hr1G067930	10.84082	11.00012	12.82642	10.06712	10.05419	10.2223
HORVU4Hr1G089220	1.302388	1.132258	1.153559	2.337315	1.128728	1.022744
HORVU7Hr1G037470	6.050065	6.114063	7.998317	4.445817	6.18394	5.288996
HORVU7Hr1G037780	0.387876	0.48755	0.448439	2.202997	2.589204	2.480598
HORVU3Hr1G032090	2.938634	3.002824	3.564037	5.778595	9.073032	9.846162
HORVU1Hr1G061810	4.612115	4.02674	6.654497	4.245275	4.687864	4.297709
HORVU7Hr1G054160	9.753275	9.530856	12.4036	7.070389	10.12547	8.963596
HORVU6Hr1G089180	1.079044	2.271514	1.164342	1.751386	4.674259	1.65834
HORVU6Hr1G058230	11.99358	11.19162	12.0079	15.10452	18.94595	20.8587
HORVU1Hr1G092680	34.30003	21.91934	33.48087	1.318389	1.039801	3.275405
HORVU7Hr1G092320	24.09494	22.79609	31.95679	14.82419	16.71696	16.07749
HORVU0Hr1G013800	3.741956	2.381044	3.446902	2.868505	1.865737	2.592729
HORVU2Hr1G098580	11.72162	8.018257	10.45801	10.06132	12.36089	11.09704
HORVU3Hr1G084480	2.700896	3.782187	3.903799	1.184348	2.122798	2.879463
HORVU3Hr1G004330	4.38939	4.203119	4.832928	5.055482	7.273443	7.369136
HORVU7Hr1G090790	2.163356	1.547301	2.425063	2.695501	3.134322	2.523694
HORVU4Hr1G032490	0.985798	1.110585	1.024481	0.804765	1.489569	1.368136
HORVU3Hr1G083680	1.556437	1.013421	2.274999	5.25226	3.883938	4.627479
HORVU3Hr1G011990	1.288322	0.969071	2.104516	4.219198	2.900774	4.079219
HORVU4Hr1G088570	6.551713	6.733116	6.972137	5.78409	7.143832	6.313926
HORVU7Hr1G036780	4.488273	4.320831	6.044183	3.108463	1.199073	2.882072
HORVU4Hr1G047030	17.89092	17.67215	16.10766	18.33869	21.35822	21.71948
HORVU1Hr1G048450	4.197677	3.788972	7.198642	1.640077	4.240789	3.693156
HORVU4Hr1G080760	0.07161	0	0	0.92091	13.66677	12.08406
HORVU5Hr1G054250	8.157995	7.079128	10.17808	6.404869	7.05381	7.270386
HORVU3Hr1G019850	9.062615	9.528019	9.419069	6.152353	11.83695	10.1188
HORVU2Hr1G126640	3.302682	2.100701	1.871419	1.922351	2.012313	1.776181
HORVU0Hr1G017320	10.10679	9.897001	8.929907	9.708125	12.30186	11.14226
HORVU2Hr1G077510	0.788928	0.820746	0.821261	3.074953	6.785751	5.326965
HORVU6Hr1G064940	9.69669	8.837413	10.22369	9.695892	16.03083	15.11995
HORVU3Hr1G043660	0.708017	0.885243	0.945268	0.929204	1.433668	1.947541
HORVU5Hr1G020410	2.205014	1.658896	1.838478	3.851943	5.551131	5.66631
HORVU7Hr1G014410	1.100881	1.52637	1.781603	2.744067	11.45773	9.995132
HORVU2Hr1G127510	2.810092	3.395468	2.886333	2.35125	2.328309	2.662752
HORVU2Hr1G088400	2.192674	1.176266	2.516524	4.42585	5.741818	5.359969
HORVU0Hr1G017490	6.283221	6.493536	6.42366	2.126076	3.723008	4.188255
HORVU3Hr1G009360	1.703349	0.899492	2.09238	106.2037	194.4587	195.4429
HORVU4Hr1G048120	9.400032	10.70439	10.88378	6.84143	7.481833	6.875677
HORVU1Hr1G015060	8.942208	9.984448	13.91526	5.856784	7.739987	7.301236
HORVU1Hr1G043640	5.362773	6.550685	6.968445	6.229449	8.709229	8.637723
HORVU3Hr1G035210	2.369494	1.063105	2.241034	1.183444	3.513561	3.153678
HORVU1Hr1G049490	2.373769	2.263541	2.954587	2.918388	3.768898	5.305789
HORVU4Hr1G008180	7.580481	7.474719	8.369344	23.6699	20.40428	18.44036
HORVU1Hr1G067910	53.0367	52.30479	51.48437	55.71114	76.67277	78.59457
HORVU5Hr1G057780	4.379712	1.57832	4.89991	2.157278	3.750715	4.093633

HORVU2Hr1G023150	1.993633	1.190577	0.611297	0.857109	0.489075	0.927063
HORVU2Hr1G099630	7.553332	8.32454	9.81853	7.395835	7.527825	8.24345
HORVU3Hr1G108030	0	0	0	0	2.139234	4.338712
HORVU1Hr1G069510	2.455655	2.193257	2.763861	6.015365	6.972601	5.53683
HORVU1Hr1G057940	10.37547	13.90878	13.42253	5.746535	4.119859	4.025456
HORVU4Hr1G084840	10.73506	11.95137	12.12838	11.02551	8.718926	9.185086
HORVU2Hr1G109650	3.544725	3.648974	3.165086	2.62664	1.233339	1.467699
HORVU5Hr1G009200	7.664004	6.248393	5.589104	17.7705	31.26962	28.8887
HORVU1Hr1G056090	2.553953	1.559851	2.412276	2.002221	4.907929	3.715235
HORVU2Hr1G125600	0.926515	1.137924	1.165092	13.51329	10.74606	15.31038
HORVU5Hr1G010600	9.042616	9.562553	8.662368	11.34806	10.65737	10.21051
HORVU0Hr1G012520	1.696155	1.630797	1.977579	2.405555	2.631376	2.973404
HORVU3Hr1G074220	13.27427	11.10405	13.57186	16.22677	17.5089	18.90505
HORVU4Hr1G059800	26.19414	24.00315	26.19982	22.67866	15.47343	17.13407
HORVU5Hr1G047530	19.77709	16.48744	23.31417	42.6424	79.26803	74.06418
HORVU7Hr1G074270	11.14369	10.39878	12.60662	18.32849	25.03789	17.36496
HORVU6Hr1G081630	1.119274	1.465002	0.375945	2.660716	3.398171	3.094641
HORVU3Hr1G091400	6.842282	6.592369	7.697768	10.19808	13.40934	13.16077
HORVU5Hr1G013550	2.506421	2.737253	3.425008	2.415995	2.094458	2.947926
HORVU4Hr1G054980	2.769565	3.583647	3.200201	0.085851	0.075141	0.030393
HORVU4Hr1G007400	13.5455	12.10378	12.39238	11.47579	13.25774	14.14672
HORVU1Hr1G090210	225.1659	139.9839	191.4957	453.7084	782.6216	576.5759
HORVU1Hr1G012870	11.55813	13.24428	18.04024	47.06266	152.571	128.7026
HORVU3Hr1G078680	1.840063	0.097963	5.171676	17.00945	20.73931	21.80359
HORVU6Hr1G083160	19.49078	20.43573	19.04204	30.33134	43.10273	42.51408
HORVU1Hr1G014080	7.379522	7.25638	9.658336	8.969761	13.47863	12.68069
HORVU5Hr1G012600	5.597378	6.692418	5.157942	11.53123	11.62935	13.18437
HORVU2Hr1G010130	18.25947	18.21467	20.19778	16.96655	19.64695	17.46185
HORVU2Hr1G099890	36.26493	31.88547	39.84275	40.25107	23.40554	20.55355
HORVU5Hr1G022100	47.73181	44.92186	53.6928	58.14219	66.77968	62.32714
HORVU5Hr1G094540	4.076004	4.113121	4.329324	7.487154	7.835002	9.589839
HORVU4Hr1G025590	0.925705	1.14073	0.674116	0.615458	5.017726	4.444434
HORVU5Hr1G124500	12.47969	14.65257	13.08576	12.31555	12.07963	12.86048
HORVU5Hr1G056120	3.310223	3.632966	3.833149	3.451639	4.948746	4.979723
HORVU0Hr1G010770	6.51121	1.286086	4.428135	4.223708	4.083011	2.730244
HORVU2Hr1G108260	4.014351	3.067619	3.583682	11.50577	18.88862	19.18264
HORVU2Hr1G093400	2.1784	2.014026	3.044105	2.791994	4.507167	4.682692
HORVU1Hr1G052420	28.3621	27.39646	24.96978	10.7294	5.51257	6.653225
Hordeum_vulgare_newGene_11073	1.791718	2.2535	2.158846	1.81715	1.963484	2.522967
HORVU7Hr1G089700	16.33676	15.40764	15.44277	14.93629	15.89412	18.49236
HORVU7Hr1G012920	0.653139	0.635249	1.114506	3.056439	6.143288	5.893533
HORVU4Hr1G010880	1.619077	1.012766	1.294975	1.770721	2.624158	2.231534
HORVU2Hr1G112580	67.59766	63.19467	66.98309	34.10036	10.30655	20.33545
HORVU6Hr1G077210	17.26164	21.36446	18.32248	30.78145	27.07765	26.72982
HORVU2Hr1G016990	0.960641	0.76477	0.861722	2.696298	4.803711	4.109588
HORVU3Hr1G077280	1.534265	2.064202	1.243357	1.677695	1.684232	1.382344
HORVU6Hr1G094740	2.209323	0.582804	5.384958	2.333608	1.896618	3.839056

HORVU1Hr1G066630	981.8898	838.5752	924.0196	651.0134	420.5128	436.6644
HORVU7Hr1G000150	6.798833	4.781575	4.997905	13.77388	14.22173	13.31526
HORVU1Hr1G040660	3.737287	4.358916	4.495689	3.671677	3.957239	5.554319
HORVU2Hr1G028330	5.482567	3.233253	6.352342	9.757872	6.411633	8.008066
HORVU5Hr1G049440	11.50126	9.061066	12.34729	7.777751	7.441403	7.72419
HORVU7Hr1G083280	4.635898	4.85281	6.514609	4.839075	4.008999	4.223095
HORVU4Hr1G020540	10.0792	8.975755	10.73595	11.22886	13.59531	13.38763
HORVU1Hr1G041530	3.966256	5.067743	6.524705	5.063421	7.131495	7.181484
HORVU0Hr1G009850	1.467069	1.164193	1.295417	1.316034	0.804076	1.189995
HORVU1Hr1G039250	0.433088	1.05581	0.520833	0.970118	1.936106	2.508421
HORVU3Hr1G076480	37.19342	27.05978	40.87823	31.38685	25.56851	26.95236
HORVU4Hr1G078820	23.28174	20.30908	28.44058	13.27409	23.95973	22.48785
HORVU2Hr1G102300	1.568576	1.310499	1.770006	3.543185	8.60345	8.468936
HORVU4Hr1G058010	8.302425	8.61441	12.21345	10.12365	17.29053	17.81295
HORVU1Hr1G046620	4.617655	3.360461	4.660133	3.741231	4.22172	3.789302
HORVU5Hr1G045820	5.634562	7.782154	7.18405	14.65002	30.74373	27.27706
HORVU3Hr1G022420	2.08636	1.29013	1.411109	1.018826	1.607492	1.257089
HORVU2Hr1G052170	6.702828	9.805468	8.505968	10.08929	13.53299	13.68548
HORVU7Hr1G045450	10.59362	11.34364	11.62142	13.56225	18.09285	17.98333
HORVU6Hr1G088330	2.773695	2.300225	3.028675	2.653811	2.723084	2.548169
HORVU2Hr1G066620	27.60556	22.41769	32.51964	24.79728	35.20349	34.78987
HORVU1Hr1G085230	0.130468	0.059808	0.07212	0.841873	7.147104	5.872144
Hordeum_vulgare_newGene_3006	14.52566	12.91798	16.44191	17.57706	13.38036	12.70494
HORVU3Hr1G064830	12.26281	12.30964	13.65977	13.58981	13.46759	17.12965
HORVU2Hr1G060140	7.448987	5.824865	7.24569	6.222866	9.909653	8.918619
HORVU5Hr1G004340	3.054049	3.113244	4.764035	2.767516	3.50034	3.468396
HORVU6Hr1G051590	1.391944	1.479048	1.655122	2.842754	3.277071	3.48754
HORVU7Hr1G077230	12.48954	10.79521	14.78773	17.88791	26.25545	22.03711
HORVU6Hr1G086690	16.18075	12.89018	16.8676	12.32772	13.81435	12.72074
HORVU6Hr1G093570	31.79035	27.80082	35.31038	26.25848	26.46411	28.07879
HORVU5Hr1G050940	15.37597	6.504	12.67079	12.91485	1.07817	4.797281
HORVU5Hr1G040370	3.594339	1.148184	3.122203	5.890577	5.279979	7.118055
HORVU7Hr1G042150	0.785293	1.537642	0.600515	2.31473	2.738409	3.408189
HORVU5Hr1G084120	25.80141	19.88209	33.83113	27.55857	62.29329	63.00483
Hordeum_vulgare_newGene_3352	3.120948	3.415081	3.437396	3.07778	4.590584	3.943786
HORVU4Hr1G062440	16.68975	21.06545	24.4079	7.247825	8.057524	14.32418
Hordeum_vulgare_newGene_12752	5.118415	6.021509	3.430831	8.468469	4.812889	4.786323
HORVU3Hr1G063590	5.471456	5.676938	6.424558	5.372875	12.07098	12.38911
HORVU3Hr1G027610	29.8634	26.11679	26.57714	34.85095	36.00522	32.80752
HORVU5Hr1G100700	241.5798	222.2719	264.3972	359.1077	859.7992	764.1139
HORVU3Hr1G069410	0.264649	0.21571	0.111423	4.138659	9.333384	7.450822
HORVU6Hr1G065540	0.546765	2.021536	1.617316	2.644173	1.188878	2.380726
HORVU3Hr1G025610	2.486288	2.04386	3.083539	2.809402	3.025126	3.718713
HORVU3Hr1G089210	1.025723	0.70925	0.342383	1.987442	2.222968	2.654781
HORVU1Hr1G037340	6.137298	6.318821	9.008992	4.160073	5.779939	5.929387
HORVU1Hr1G032190	23.09764	22.46926	28.00028	22.44581	38.16311	34.48785
Hordeum_vulgare_newGene_13089	1.129409	1.103177	2.202034	4.654445	5.824305	7.859386

Hordeum_vulgare_newGene_13080	1.153808	0.981498	0.850318	1.803505	3.013296	2.060527
HORVU3Hr1G011460	6.554646	8.430935	10.84416	4.504456	5.888888	7.438865
HORVU6Hr1G009350	0.155921	0.187868	0.217637	5.708188	22.84272	21.28727
HORVU7Hr1G077750	2.398258	3.067618	1.90843	15.91633	30.27353	28.25323
HORVU7Hr1G072980	2.482717	2.346351	3.425182	5.165198	6.138179	6.37148
Hordeum_vulgare_newGene_660	0.654823	0.975942	0.788711	1.135445	1.595255	1.190462
Hordeum_vulgare_newGene_663	0.047361	0.243113	0.096897	1.440428	1.903652	2.550641
Hordeum_vulgare_newGene_669	3.682075	4.305774	5.487393	6.457675	8.295759	8.479717
HORVU4Hr1G058810	0.266196	0.124118	0.41825	9.547364	48.96917	44.75045
HORVU3Hr1G055550	54.07274	55.18807	54.33027	37.69107	29.2951	32.55564
Hordeum_vulgare_newGene_14364	11.33756	12.47811	13.33427	12.96811	14.4277	14.80813
HORVU1Hr1G070710	11.53351	10.61212	11.6876	11.14198	15.13451	14.43206
Hordeum_vulgare_newGene_14368	4.759947	4.800388	5.622966	11.71699	10.38764	10.8399
HORVU6Hr1G012060	2.693286	2.715768	2.578273	4.964509	4.705618	4.835547
Hordeum_vulgare_newGene_3689	64.52778	42.25865	83.9777	219.0322	83.65957	190.5584
HORVU5Hr1G075750	1.298645	1.520669	2.409624	1.441279	1.582541	1.83971
Hordeum_vulgare_newGene_3684	3.652092	4.281484	7.333548	4.055128	7.517375	6.524645
Hordeum_vulgare_newGene_3686	2.947993	0.964091	4.542325	1.154714	0.727197	6.076093
HORVU4Hr1G015930	0.355189	0.291364	0.931273	2.905314	3.431398	3.464075
HORVU1Hr1G094230	0.552696	0.472539	0.792901	5.351058	13.08159	12.13685
HORVU3Hr1G020390	20.60358	16.96872	24.45931	23.31896	18.78883	12.38631
HORVU5Hr1G006640	3.138714	3.722267	3.810409	3.608089	3.634237	3.821418
HORVU2Hr1G020310	40.60056	31.44018	37.5854	34.23586	33.91774	29.01202
HORVU2Hr1G096250	0.305336	0.163443	0.146846	2.010672	6.539341	5.336441
HORVU5Hr1G080110	0.309003	0.321935	0.295457	1.555743	6.593071	6.947262
Hordeum_vulgare_newGene_2559	1.517965	1.480619	1.472716	2.803647	5.326792	4.905889
HORVU6Hr1G059920	10.32899	8.113048	10.77259	9.373353	15.36788	15.32018
HORVU3Hr1G110150	8.775046	7.608122	8.871078	7.464918	8.929114	9.029616
HORVU4Hr1G061130	4.309698	4.031486	5.481649	4.858658	4.580006	4.929217
HORVU1Hr1G059510	7.366141	7.128257	7.33176	8.12483	8.406017	10.69596
Hordeum_vulgare_newGene_5682	5.993761	3.510761	4.516164	6.41252	4.412445	4.944778
Hordeum_vulgare_newGene_5684	93.5798	84.11966	82.09053	31.01975	27.69173	31.54636
HORVU3Hr1G054680	5.7121	6.787527	7.91689	9.003293	9.76346	9.43513
HORVU3Hr1G117870	1.053249	0.922321	1.134362	1.706594	2.411947	2.573592
Hordeum_vulgare_newGene_10061	1.768849	2.014367	2.064207	2.873446	3.202539	3.324149
HORVU5Hr1G099910	14.20213	14.84154	16.73844	0.483406	0.238428	0.091838
HORVU3Hr1G116580	1.067419	0.418028	0.780846	2.243657	2.334167	2.069669
Hordeum_vulgare_newGene_13976	0	0	0	2.736247	3.914782	3.933515
HORVU5Hr1G077020	47.13391	52.2593	65.02959	44.03711	62.38307	66.97749
Hordeum_vulgare_newGene_9569	4.592375	4.109053	6.181156	21.38314	43.15253	31.09296
Hordeum_vulgare_newGene_5026	4.2896	3.504189	3.592794	3.31086	3.315427	2.776433
Hordeum_vulgare_newGene_9563	2.91215	3.774556	3.218551	3.046373	2.914444	3.370745
Hordeum_vulgare_newGene_9560	3.087348	2.927034	4.172136	1.208426	1.763445	1.983895
Hordeum_vulgare_newGene_13751	0.622405	0.624471	1.419741	2.431362	5.149242	1.793894
HORVU1Hr1G000090	2.515694	1.657431	2.000246	7.142437	6.841957	7.483458
Hordeum_vulgare_newGene_13756	0.548668	0.748618	0.069069	2.756089	1.5621	2.759721
HORVU7Hr1G030990	2.170059	2.407946	2.11319	0	0.006661	0.062871

HORVU7Hr1G110200	12.33905	12.56054	13.44543	10.43653	25.76173	23.85004
HORVU4Hr1G060770	7.295126	6.956087	7.644869	8.223253	9.96482	10.10052
HORVU6Hr1G063650	4.266542	4.396816	4.779526	7.377703	7.8434	7.661389
HORVU2Hr1G072580	3.546204	3.464423	4.018976	1.923965	3.622595	2.986774
Hordeum_vulgare_newGene_13632	6.240976	4.397284	4.910596	5.327712	7.191022	7.088091
HORVU5Hr1G050570	58.32729	53.79119	55.2987	52.58871	38.58006	42.01554
HORVU6Hr1G077460	6.611672	6.894234	8.015734	7.627079	12.41583	12.47695
HORVU6Hr1G068150	6.680079	9.089443	13.1525	1.695286	1.573014	1.973942
HORVU7Hr1G063430	0.010086	0	0	1.678946	33.58937	25.85136
HORVU7Hr1G064420	13.39644	14.45649	12.90607	0.150573	0	0
HORVU1Hr1G025480	2.924777	4.113006	4.625646	3.458769	4.221013	4.378253
HORVU6Hr1G052890	10.56658	9.721263	13.15496	13.52901	16.59739	15.80056
HORVU5Hr1G102930	17.24379	15.20248	16.3655	12.5946	17.71221	17.42949
Hordeum_vulgare_newGene_8216	2.058185	2.333105	2.023833	8.344725	12.54961	10.90962
HORVU2Hr1G120400	1.497104	1.076044	1.782932	1.972426	1.046494	1.909893
HORVU6Hr1G063000	1.839844	1.213965	1.894765	1.691817	1.652212	2.036981
HORVU1Hr1G079650	9.607754	9.480651	10.40354	0.381802	0.474768	0.320499
HORVU4Hr1G078580	8.928181	10.67322	15.20945	7.088225	8.969704	8.642885
HORVU0Hr1G039600	4.776329	5.846739	5.215557	5.003106	6.846779	6.275842
HORVU3Hr1G068520	5.245051	5.387675	6.549903	5.171959	4.843267	6.517418
HORVU5Hr1G043560	21.51494	18.73805	19.58013	14.81241	23.89535	21.26592
HORVU1Hr1G075550	26.21995	26.30054	31.2723	16.62363	12.31327	14.75514
Hordeum_vulgare_newGene_4842	1.028615	1.472767	1.155017	1.784282	2.531273	2.966916
HORVU4Hr1G048490	8.917873	8.629866	9.238123	11.91061	14.67728	15.39675
HORVU1Hr1G064250	30.47166	30.26606	35.97516	34.26424	41.42128	40.68284
HORVU4Hr1G009990	1.043182	0.959997	0.942054	1.501851	1.633385	1.078164
HORVU5Hr1G037660	8.49062	5.221429	6.981097	18.13419	14.93345	18.63079
HORVU2Hr1G032680	1.509906	1.702794	1.481701	2.057051	2.967199	3.081915
Hordeum_vulgare_newGene_7798	3.693719	3.760088	3.361789	1.473656	0.521277	0.940518
HORVU6Hr1G080690	52.60351	47.64111	53.64096	15.94807	14.06653	13.54958
HORVU5Hr1G100790	8.850593	4.734132	9.787226	6.59102	14.35938	7.409707
HORVU3Hr1G085400	0.652627	0.270031	0.480989	1.149748	2.82829	2.002426
Hordeum_vulgare_newGene_15842	2.612131	5.060329	4.104599	0	0	0
HORVU7Hr1G098220	19.59251	12.38529	19.6251	48.84425	26.58172	37.80578
Hordeum_vulgare_newGene_15844	0.036578	3.745096	2.604508	0.889214	1.918866	2.309641
HORVU5Hr1G040030	10.44352	11.29879	11.76758	12.5607	14.17593	17.20105
Hordeum_vulgare_newGene_15848	12.96001	13.08462	14.6409	13.52152	17.92218	20.92878
HORVU7Hr1G056570	3.32294	3.458684	3.816959	7.127473	13.19391	12.65872
HORVU1Hr1G074600	3.339995	3.24459	3.111268	5.536007	11.0449	5.975655
HORVU5Hr1G059840	1.090056	0.899979	1.078695	1.281129	3.374092	3.348379
HORVU5Hr1G077970	1.832015	1.860737	3.724034	2.002582	3.549602	2.47535
HORVU1Hr1G026590	17.20132	17.26348	19.21643	8.08171	6.329764	6.44148
HORVU5Hr1G103680	124.3253	105.8939	126.4393	223.7624	295.5678	213.7355
HORVU6Hr1G028980	4.642518	4.258949	3.943732	6.721002	6.212641	7.177517
HORVU7Hr1G106860	6.285417	6.063234	6.732384	6.880723	8.831967	8.315895
HORVU3Hr1G039020	0.648155	0.267394	0.599477	0.811313	0.4538	3.422137
HORVU3Hr1G113340	2.937816	2.979287	3.637608	2.923854	4.668646	5.670013

Hordeum_vulgare_newGene_2945	0	0	0	2.763953	3.568534	3.507884
Hordeum_vulgare_newGene_2947	0	0	0	2.368441	3.015922	3.546156
HORVU3Hr1G001940	0	0	0	7.026607	6.156591	9.560797
HORVU4Hr1G074720	1.74487	1.945707	1.614343	2.42631	3.257809	3.588439
HORVU2Hr1G058230	4.580011	6.385094	6.4684	6.661562	7.717593	8.800479
HORVU6Hr1G032400	8.140662	7.869627	8.788542	11.24092	13.1981	13.51365
HORVU7Hr1G036900	3.472691	3.372489	5.334318	3.56813	4.453486	3.542728
HORVU6Hr1G003560	0.547108	0.919276	0.509094	2.631943	3.137778	2.980862
HORVU6Hr1G030800	20.02085	14.44326	21.89664	16.89998	19.56605	20.3389
HORVU5Hr1G095960	5.275758	6.179389	7.661152	7.856094	8.748875	8.645648
Hordeum_vulgare_newGene_9973	11.47736	8.642779	9.966054	14.58057	20.91992	14.82982
HORVU5Hr1G028060	1.954794	2.062971	2.382572	2.327184	2.75044	2.763922
HORVU5Hr1G012190	1.687054	1.292323	1.728857	0.800899	0.332722	0.393617
HORVU3Hr1G095880	30.64019	26.59818	38.76711	25.50159	14.53177	15.94297
HORVU2Hr1G124870	3.546994	2.542628	4.469602	5.638521	5.628402	4.418052
Hordeum_vulgare_newGene_13173	14.99655	14.59005	12.88144	10.26027	9.244113	10.9541
HORVU5Hr1G022690	1.795046	0.813593	1.930327	2.81468	1.993978	1.749268
HORVU4Hr1G045510	11.96919	13.41349	16.87039	10.59731	10.90157	12.2431
HORVU2Hr1G050790	16.4841	14.88037	17.7647	20.532	20.50405	20.14053
HORVU3Hr1G033790	1.30802	1.157996	1.036113	11.75992	15.89144	11.89827
Hordeum_vulgare_newGene_14006	2.215831	1.930252	1.839773	1.592399	1.487608	1.407957
Hordeum_vulgare_newGene_14007	4.07625	3.160775	1.526104	0	0	0
Hordeum_vulgare_newGene_14002	3.233593	3.6418	4.430655	3.050039	2.16648	0.492864
HORVU5Hr1G020180	3.016253	2.331998	2.107117	2.415754	2.143808	2.710536
HORVU4Hr1G011910	8.252378	7.082562	8.050508	16.01244	23.13416	22.21289
HORVU3Hr1G097200	30.62265	36.26934	34.83308	20.86471	31.70376	29.52962
HORVU2Hr1G027640	9.935984	10.28252	12.82832	8.136143	10.57924	8.846617
HORVU1Hr1G073190	0.425441	0.254399	0.493627	1.497527	2.566993	2.12388
HORVU3Hr1G029010	182.2315	158.1483	157.3066	105.9136	68.45438	85.78391
HORVU7Hr1G054060	26.32355	22.67605	28.28596	34.49029	50.27283	51.24464
HORVU3Hr1G037540	4.188706	3.89038	4.787632	5.932926	4.644819	5.661011
HORVU7Hr1G108370	1.280856	0.682543	0.857901	4.584711	18.96035	10.15354
HORVU3Hr1G019010	24.88993	22.78002	32.96418	15.42566	21.74654	19.7265
Hordeum_vulgare_newGene_11468	4.249686	5.175073	4.79855	3.27889	2.908185	2.753758
HORVU5Hr1G088680	66.93628	56.67109	69.81688	88.84799	126.3839	123.6582
HORVU1Hr1G023220	6.490311	5.264106	9.260919	37.88741	28.5003	31.57157
HORVU1Hr1G019740	10.57906	5.211001	15.45275	0.325967	0.024338	0.275011
Hordeum_vulgare_newGene_9985	0.079139	0.060776	0.279255	1.722225	5.582373	4.979474
HORVU5Hr1G021580	18.87308	17.44581	17.51843	17.32397	20.05106	20.58191
HORVU5Hr1G039870	7.91308	8.758581	8.520339	9.134243	11.28296	11.17928
HORVU3Hr1G003450	13.62869	12.35602	19.12279	16.71038	20.02077	22.78408
HORVU1Hr1G048390	16.7163	15.06825	16.95141	14.23068	17.31722	18.37723
HORVU5Hr1G106480	12.23505	10.75626	12.33265	10.44241	7.33858	8.530918
HORVU1Hr1G066340	0.317739	0.442076	0.657225	3.23975	3.15238	3.848791
HORVU0Hr1G032110	0.774465	1.076863	1.625909	0.347443	1.478599	1.971827
Hordeum_vulgare_newGene_15693	4.125466	3.883951	3.1865	4.956195	5.018476	5.262129
HORVU5Hr1G088970	11.96971	10.74852	11.22612	12.07373	18.80515	18.31606

HORVU3Hr1G032110	7.993534	8.878796	10.29268	6.561897	7.302397	7.785642
HORVU4Hr1G081600	22.49878	20.94757	24.88699	19.17491	9.893681	10.72119
HORVU4Hr1G084230	9.149602	12.29395	14.53662	5.836304	9.000794	9.288708
HORVU6Hr1G005650	3.421712	4.028104	3.918371	3.166892	3.567256	4.076681
HORVU4Hr1G002670	2.441912	1.607428	2.743414	3.579136	5.503598	3.819786
HORVU1Hr1G061360	1.471296	1.430891	1.757474	1.445668	1.581447	1.168632
HORVU1Hr1G090430	5.260785	4.126477	5.430951	5.569307	4.157973	5.852776
HORVU5Hr1G111410	28.70086	22.17543	32.04969	32.29619	45.29175	39.68634
HORVU2Hr1G089650	22.66566	24.2565	14.948	17.75288	9.51069	12.60125
HORVU4Hr1G014630	10.92092	9.471514	10.13531	12.71445	15.68208	12.8815
Hordeum_vulgare_newGene_10409	3.326981	3.634687	4.153683	4.283352	4.896792	5.471342
HORVU4Hr1G088720	8.028585	5.913305	8.06736	6.349651	3.886074	5.719439
Hordeum_vulgare_newGene_522	44.26714	43.71145	46.07169	45.30312	55.2045	60.0447
HORVU4Hr1G005900	65.32957	81.67776	122.3963	28.91669	48.16765	50.28031
HORVU6Hr1G064040	1.565849	1.49128	2.141545	1.821208	2.818799	2.923947
HORVU2Hr1G115180	0.83566	1.313464	1.592497	1.202863	0.745561	1.329826
HORVU1Hr1G016540	2.549564	2.909828	3.32938	2.280961	2.436167	3.073699
HORVU6Hr1G067020	1.184031	0.732628	0.806761	1.493096	1.884088	2.492199
HORVU5Hr1G067280	11.01758	8.188172	9.907367	10.51765	11.97747	12.06922
HORVU0Hr1G017510	11.19168	9.69243	10.37574	11.28294	14.68537	13.63912
HORVU1Hr1G095140	45.63302	50.62912	72.50649	51.17011	82.7538	84.50414
HORVU7Hr1G036570	5.901906	4.942129	8.766234	3.476286	4.579666	3.081209
HORVU2Hr1G081080	46.27817	35.96805	45.56455	43.54228	50.49719	52.50687
Hordeum_vulgare_newGene_12313	38.73629	35.40695	50.52906	34.58591	36.66	38.14843
HORVU4Hr1G087760	0.141914	0.203259	0.056749	0.969164	4.778847	5.174659
HORVU4Hr1G039650	11.22581	10.30373	11.04956	9.839057	10.6722	11.55557
HORVU1Hr1G066960	0.641422	0.656453	1.202525	2.311257	7.667154	9.337358
Hordeum_vulgare_newGene_11525	1.528964	1.917659	0.785495	0.606575	0.750688	0.635936
HORVU7Hr1G092510	3.003798	1.702704	4.354497	3.24437	0.675472	3.243846
HORVU3Hr1G007410	2.188202	2.123725	3.339486	4.167923	4.055227	4.221801
HORVU4Hr1G069920	16.44124	18.4908	17.29917	18.41377	17.22367	14.89702
HORVU3Hr1G107820	9.749642	8.894155	10.62146	11.53403	12.74438	12.73124
HORVU0Hr1G007930	4.817671	5.592684	5.267885	5.754338	6.240135	6.293774
HORVU1Hr1G049510	1.348695	1.526641	2.059499	1.419571	1.981408	1.422192
HORVU5Hr1G067750	1.850394	1.572504	1.920288	1.211314	2.518066	2.302718
HORVU3Hr1G085890	24.55487	20.65506	26.52662	19.12579	6.382233	13.79739
HORVU6Hr1G008550	0.864827	0.846573	0.995363	1.687041	0.634319	1.871921
HORVU5Hr1G008050	2.748756	2.749787	2.889016	4.560723	1.844903	0.708136
HORVU4Hr1G009420	3.799999	3.562718	6.204658	2.131125	2.927961	3.640972
HORVU2Hr1G124050	1.198667	1.139216	1.675498	2.212618	1.171961	1.854316
HORVU2Hr1G116320	9.454669	8.735333	8.862455	10.85232	12.03567	14.26195
HORVU7Hr1G088370	82.53278	84.24014	86.69957	72.64826	66.11416	70.84742
HORVU2Hr1G016650	17.98262	19.70908	27.75278	12.41324	16.76517	17.91312
HORVU1Hr1G044930	13.66455	11.03741	14.78433	11.33403	7.870697	8.357221
HORVU6Hr1G039890	31.92697	30.41641	37.64725	41.02038	48.56737	48.94316
HORVU2Hr1G067040	1.373463	1.894669	1.978829	1.154826	1.780564	2.070134
HORVU4Hr1G012580	1.249022	1.027189	1.113711	1.535936	1.558079	1.52393

HORVU2Hr1G109750	3.176498	2.42757	3.92796	3.948878	3.467067	3.221061
Hordeum_vulgare_newGene_6748	43.20998	49.45905	51.65683	36.41905	39.76961	49.02511
HORVU5Hr1G060140	40.19972	26.51559	43.66742	63.98142	50.46473	36.07819
HORVU7Hr1G083860	47.47133	40.71022	57.89337	38.01131	59.22306	61.40095
HORVU1Hr1G056110	88.9895	71.50271	88.19659	58.3002	56.77619	53.94289
HORVU0Hr1G001570	83.22424	74.64953	84.88253	77.02457	86.95187	82.29896
HORVU7Hr1G091350	37.33688	34.4386	33.6028	27.25645	19.59102	21.5581
HORVU3Hr1G099690	3.242955	2.737276	3.437149	7.563815	8.964786	9.190222
Hordeum_vulgare_newGene_10267	0	0.05475	0.144262	0.380626	7.021039	5.723662
Hordeum_vulgare_newGene_10264	1.816193	1.550135	1.446692	14.16247	13.71127	12.2806
Hordeum_vulgare_newGene_10263	18.57092	16.09768	17.74289	21.06391	20.85153	19.07424
HORVU7Hr1G074330	0.806854	0.755955	1.207778	0.597924	1.672133	2.122818
HORVU4Hr1G064090	1.62162	1.770138	1.715646	1.933824	2.410522	2.166692
HORVU5Hr1G121940	2.315565	2.598591	2.059479	5.238629	2.350478	2.410226
HORVU7Hr1G093260	3.455323	3.282112	4.760268	3.471284	9.133664	11.92414
HORVU4Hr1G025410	2.039449	2.774392	2.205132	6.778284	8.398539	8.245961
HORVU7Hr1G111000	3.223513	3.502135	3.41724	4.380172	5.575227	4.21082
HORVU6Hr1G038250	11.40485	11.46228	7.081739	2.699023	1.807466	2.078403
HORVU5Hr1G120090	6.763121	6.121652	6.696749	7.02046	6.817073	7.007123
HORVU5Hr1G098220	3.909026	2.738748	4.315608	4.850926	2.965319	2.91433
HORVU2Hr1G039640	5.747788	5.338442	4.918486	4.186087	1.005984	1.935303
HORVU2Hr1G112620	0.855683	0.79349	0.888951	3.414475	1.226116	2.230091
HORVU6Hr1G076130	7.097798	5.819882	7.992789	3.462975	2.683623	3.212359
HORVU7Hr1G012820	285.5175	265.8195	310.7153	260.4076	334.924	302.8431
HORVU2Hr1G079700	47.35466	43.50465	46.0028	63.62616	40.49004	46.62868
HORVU0Hr1G010630	0.171743	0.580909	2.232705	0.117427	0.276307	3.668536
HORVU1Hr1G060100	2.353368	1.638067	2.46061	3.335149	3.75795	4.527449
HORVU7Hr1G122370	14.2178	16.85038	12.08782	11.28506	13.10435	13.4065
HORVU6Hr1G093310	6.243257	5.786836	8.060963	9.750118	9.053985	9.534652
HORVU1Hr1G040620	1.838044	1.155561	1.707674	1.225693	0.774239	0.897996
HORVU4Hr1G090300	5.70942	5.077026	4.494575	9.053377	9.569382	9.10187
HORVU6Hr1G014370	1.433441	1.884521	2.119247	1.461635	2.375039	2.664457
HORVU7Hr1G086740	1.86079	1.872735	2.194742	1.666897	1.649017	1.961407
HORVU1Hr1G001120	17209.67	20878.4	14435.08	39858.67	20043.32	22566.54
HORVU1Hr1G065550	2.182416	2.148817	2.445676	1.962151	1.587201	1.558485
HORVU3Hr1G003860	0.022141	0.026445	0.158807	5.112911	4.008	3.528008
HORVU2Hr1G031410	1.252895	1.50143	1.151865	5.191157	6.248488	5.916287
HORVU2Hr1G108180	0.050502	0.022821	0.029356	5.463009	5.078475	4.868871
HORVU7Hr1G011080	76.81562	77.49233	80.96179	72.09966	61.84642	64.17994
HORVU2Hr1G012540	5.15939	5.918807	5.860512	11.58419	9.185205	10.59558
Hordeum_vulgare_newGene_11602	5.468095	7.025964	6.578792	0.024156	0	0
HORVU6Hr1G077660	17.42383	16.26912	16.01289	19.56081	25.11841	22.37328
HORVU2Hr1G056510	1.326535	1.14767	1.369838	3.054641	6.069424	5.634313
HORVU3Hr1G029350	3.634711	3.76409	5.70089	4.029542	4.392679	4.479841
HORVU7Hr1G010680	0	0.054909	0	27.35081	134.0073	132.7922
HORVU4Hr1G050580	3.001583	1.97669	3.231319	2.768878	3.273147	3.572317
HORVU7Hr1G069720	2.30998	2.070853	1.949218	2.948515	4.358065	4.685412

HORVU2Hr1G093070	1.028059	1.179458	1.194733	1.271233	0.995575	1.32793
HORVU2Hr1G105460	1.549181	1.338922	1.466074	1.203616	1.027657	1.463078
HORVU7Hr1G077170	5.423208	4.148611	5.746933	7.044201	19.16965	25.27897
HORVU3Hr1G103630	0.56864	0.658529	0.781663	2.399033	2.444095	2.937211
HORVU2Hr1G020570	46.1958	36.75469	38.82129	29.37464	30.59504	31.40675
HORVU6Hr1G063240	0.510541	0.527923	0.577485	2.147589	1.478127	1.7661
HORVU2Hr1G118270	0.604825	1.015016	0.976307	1.672896	2.104399	1.908439
HORVU5Hr1G118270	56.48977	56.90779	58.32368	104.353	156.966	139.7079
HORVU2Hr1G028760	2.555175	3.134787	5.264204	0.346264	0.165092	0.299908
HORVU1Hr1G034470	21.03126	22.1711	23.00325	23.02066	22.18574	25.40321
HORVU2Hr1G063910	0.49975	0.361375	0.306837	1.464343	5.644227	4.971667
HORVU5Hr1G051970	196.4162	155.3974	199.8591	87.99194	35.31555	65.89
Hordeum_vulgare_newGene_1833	0.561035	0.639653	0.38947	2.390956	1.830088	2.437923
HORVU2Hr1G022590	0.904569	0.991038	1.522376	3.663965	6.965932	7.010376
HORVU4Hr1G023100	12.64745	11.86437	14.58389	8.698434	10.02094	10.2053
HORVU6Hr1G015130	0.326706	1.024117	0.500749	1.125909	1.967899	2.077856
HORVU2Hr1G020680	14.67981	12.13478	12.38666	10.23545	12.03028	12.35804
HORVU5Hr1G070930	0.623775	0.756097	1.350859	1.966165	4.23185	4.314653
HORVU3Hr1G094090	7.170631	7.766439	7.116267	6.229346	11.04309	8.9756
HORVU2Hr1G013630	0	0	0	0.920669	3.062711	2.074847
HORVU1Hr1G051300	1.433565	1.067241	2.187884	5.658128	3.14206	3.835493
HORVU3Hr1G107290	7.031941	6.457104	7.451539	9.645261	9.119205	9.170535
HORVU6Hr1G062190	0.284618	0.221452	0.245673	1.816491	14.52631	6.929004
HORVU5Hr1G014290	0.551728	1.19764	1.334807	1.105831	1.211415	1.377644
HORVU5Hr1G017510	0.902084	1.114872	0.842295	1.553144	1.321977	1.935005
HORVU5Hr1G071080	4.914854	4.324267	5.770667	13.08964	18.10017	16.0042
HORVU2Hr1G032330	1.994476	2.071095	2.271541	4.733183	2.328024	3.613449
HORVU4Hr1G026570	2.567031	2.91821	2.867705	4.358682	2.963377	5.126264
HORVU3Hr1G050800	3.544963	4.119788	4.27154	6.364642	9.939709	10.15646
HORVU7Hr1G062930	2.189684	1.790125	2.205247	4.050355	7.238258	6.199159
HORVU1Hr1G075820	6.715173	6.535815	6.180762	6.679965	8.050435	7.72567
HORVU6Hr1G022170	14.52507	13.60178	15.51708	13.96486	22.43811	24.23928
Hordeum_vulgare_newGene_6200	1.198403	1.099256	1.077255	1.433317	0.778314	1.609059
Hordeum_vulgare_newGene_6201	5.602679	4.778567	4.34129	1.876921	0.901009	0.695583
HORVU6Hr1G050740	0.563466	0.804811	1.385676	2.035095	2.786971	2.482175
Hordeum_vulgare_newGene_6209	8.749633	9.132889	8.778194	10.40345	13.05604	12.67427
Hordeum_vulgare_newGene_13478	0.832882	10.14416	2.251159	0	0	0
Hordeum_vulgare_newGene_13479	0	0	0	9.048727	7.9578	10.0699
Hordeum_vulgare_newGene_13472	0.785327	0.105103	0.849837	3.363981	3.586877	3.426925
Hordeum_vulgare_newGene_13473	2.778939	1.641458	2.049494	1.932484	1.615096	1.87013
Hordeum_vulgare_newGene_13471	1.072686	1.403073	1.006237	0.799768	2.224412	2.325254
Hordeum_vulgare_newGene_13477	1.530848	1.954428	1.842524	2.185592	1.967595	2.500092
Hordeum_vulgare_newGene_13474	11.33755	8.687934	15.02752	4.974617	3.705235	4.503415
HORVU7Hr1G097000	299.8149	237.1535	328.813	125.6206	129.4404	119.5296
HORVU6Hr1G018070	17.75827	15.95536	18.64566	19.23433	2.505815	7.884656
HORVU1Hr1G043930	10.07973	8.487245	10.86317	7.402033	8.781995	7.355128
HORVU4Hr1G021500	0.359719	0.469907	0.337667	1.411373	2.223387	1.735585

HORVU3Hr1G069590	5.48661	4.990667	5.266517	3.671683	4.014504	2.559189
HORVU7Hr1G029120	1.347373	1.31208	2.401886	2.436707	6.456428	5.851498
HORVU4Hr1G029280	6.922695	7.339911	7.704817	8.76894	12.56185	11.19367
HORVU2Hr1G032420	2.685844	2.867928	2.25465	0.091826	0	0.023951
Hordeum_vulgare_newGene_13295	4.122015	8.243709	6.71559	5.32795	5.16033	4.991368
HORVU6Hr1G012210	0.686221	1.029307	1.085722	28.45889	18.00874	21.82617
HORVU6Hr1G021610	0.98422	1.265064	1.801591	0.746033	0.875372	1.086227
Hordeum_vulgare_newGene_15736	2.582562	3.074292	3.004487	1.244549	3.059448	3.684494
Hordeum_vulgare_newGene_9792	5.413806	5.545115	8.554166	5.49746	7.883917	7.84918
HORVU2Hr1G030680	1.892668	1.904208	2.455951	2.404815	3.517244	3.436533
Hordeum_vulgare_newGene_5234	1.553452	1.629997	1.759524	4.409657	6.565501	5.984395
HORVU7Hr1G096750	5.351983	3.890046	4.971486	2.626195	2.425884	2.324712
HORVU7Hr1G078670	17.28421	21.29413	29.76405	2.44515	1.090181	1.597702
HORVU4Hr1G055450	3.114846	2.553291	3.804469	5.410896	5.478738	6.364883
HORVU3Hr1G059600	2.171725	2.542337	2.541336	0	0	0
HORVU5Hr1G006430	4.882856	5.602244	5.29199	3.112654	3.838452	3.672534
HORVU4Hr1G074960	1.350625	1.614548	1.662244	2.633098	4.125376	3.289654
HORVU6Hr1G068620	19.85559	18.80917	18.67334	15.07549	23.97058	22.35001
HORVU6Hr1G034870	14.74773	13.17784	14.29921	11.99097	14.87626	15.09248
Hordeum_vulgare_newGene_8535	2.545919	2.551113	3.167395	0.012995	0.01458	0.019792
HORVU7Hr1G108600	7.60864	7.00716	7.391111	7.333211	8.18765	7.545436
HORVU2Hr1G040830	2.800174	2.365753	2.857761	3.850692	2.289927	2.414851
HORVU7Hr1G060970	12.28171	12.66201	10.45085	11.30682	11.99068	13.49037
HORVU7Hr1G073660	4.540179	5.210936	6.795913	4.375203	4.884515	5.874363
HORVU6Hr1G076270	1.913777	1.691305	1.519885	0.848828	1.509642	2.359814
Hordeum_vulgare_newGene_1314	4.003671	3.944886	4.392222	3.809225	5.674521	7.454473
Hordeum_vulgare_newGene_1317	0	0	0	2.413853	1.461858	3.548089
HORVU2Hr1G058800	1.323117	1.074505	1.2908	2.939739	4.891218	4.777982
Hordeum_vulgare_newGene_7709	1.559029	1.010864	1.442069	0.672299	0.920659	0.731069
HORVU5Hr1G096100	14.5001	12.96588	14.91471	17.04008	15.87315	16.82994
HORVU6Hr1G091700	3.77783	3.384693	3.613397	8.346814	8.43239	9.886287
HORVU1Hr1G030410	0.622709	0.590132	1.564328	0.580693	1.10011	1.545513
HORVU1Hr1G062330	2.496041	2.55697	2.732483	1.441946	1.360813	1.453806
HORVU2Hr1G104470	12.85347	11.71933	14.60293	9.036708	8.248667	9.906012
HORVU3Hr1G054490	20.99969	23.86058	24.78545	27.23515	32.12787	34.6506
HORVU7Hr1G014130	0.118946	1.198944	0.741889	1.724432	3.598749	0.075522
HORVU7Hr1G060640	6.465146	5.147055	7.895747	10.15131	16.66664	14.44741
HORVU5Hr1G015550	4.016524	5.240274	2.937266	2.657948	18.41604	14.39768
HORVU5Hr1G063070	1.457345	1.899008	2.301394	2.163018	2.365458	1.68998
HORVU2Hr1G089320	4.456547	4.537732	5.267911	7.960186	6.630386	8.142635
HORVU7Hr1G027430	0.655446	0.570432	1.045586	2.220583	12.65059	11.79294
HORVU1Hr1G081120	3.597155	3.966828	4.212901	4.236605	4.828808	5.406544
HORVU5Hr1G066640	2.820881	1.364458	2.166199	1.183673	0.313229	0.600565
HORVU1Hr1G076100	34.47639	43.1352	35.9346	65.69003	95.40179	68.77808
HORVU6Hr1G087920	16.0435	15.72638	17.55945	25.34365	38.85596	37.31925
HORVU7Hr1G021770	2.980508	3.018788	3.579674	2.715276	4.528363	5.055006
HORVU7Hr1G036000	26.23398	26.73662	24.82038	4.201565	2.24333	3.368151

HORVU4Hr1G047790	4.908291	4.937233	6.925829	6.347095	11.6657	12.39091
HORVU4Hr1G072060	2.979624	2.564626	3.931575	3.505875	2.636972	3.180298
HORVU2Hr1G045920	44.52131	41.3394	65.55152	34.50222	61.88879	58.54386
HORVU6Hr1G058100	16.39583	15.27264	22.05702	14.22227	19.64365	18.91943
Hordeum_vulgare_newGene_8786	4.613176	5.653484	3.783864	12.67499	10.63942	14.12281
HORVU1Hr1G034730	18.61235	16.51518	18.744	22.3268	25.49643	27.31715
HORVU3Hr1G115580	3.37741	2.784699	4.476837	3.764019	8.201707	8.40915
Hordeum_vulgare_newGene_8779	2.471361	2.628525	2.875638	1.394711	1.523502	1.583642
HORVU2Hr1G046740	4.162925	3.701563	4.680052	11.36702	18.40234	18.93843
Hordeum_vulgare_newGene_81	0.631771	0.704367	1.049469	1.883375	1.968082	2.09882
HORVU6Hr1G072420	3.676714	4.415104	4.134224	8.273783	8.61912	9.354961
Hordeum_vulgare_newGene_85	22.59774	21.54847	34.41859	16.48753	24.46522	24.37388
HORVU0Hr1G012460	57.24981	49.50626	59.43991	54.40614	44.03126	49.14555
HORVU2Hr1G120230	71.38421	62.7635	79.65822	51.96743	51.37246	48.96371
HORVU6Hr1G036020	2.333267	2.542682	2.873928	2.963739	4.728437	4.282172
HORVU5Hr1G109640	18.89647	15.26815	21.71287	10.76663	9.364748	8.949319
HORVU7Hr1G053680	3.029824	4.20496	4.295853	5.055876	6.140401	7.954011
HORVU3Hr1G000970	0.317592	0.432372	0.200525	8.740207	13.14266	11.69704
HORVU7Hr1G099340	5.618157	4.387223	6.397858	3.82208	1.859073	4.002311
Hordeum_vulgare_newGene_7614	16.81534	15.18598	18.82552	25.42784	32.23533	30.536
Hordeum_vulgare_newGene_7612	2.182066	1.958627	2.749914	1.779235	1.059362	1.514095
Hordeum_vulgare_newGene_7613	21.94361	19.57043	27.75483	13.97781	17.25695	19.47018
HORVU4Hr1G088870	8.856065	8.351948	8.682737	13.27548	12.88667	13.29212
HORVU7Hr1G119870	8.37602	8.265372	13.32178	2.199947	4.338631	3.364241
HORVU7Hr1G055900	16.83807	19.22674	14.36966	18.77487	19.35087	17.96982
HORVU2Hr1G004930	21.38267	22.01973	29.1002	19.42635	21.01014	21.7926
HORVU1Hr1G084200	1.881536	2.494874	2.90408	1.4523	2.569947	2.061901
HORVU5Hr1G014310	9.448636	7.778742	10.39355	12.09149	15.92333	14.36712
HORVU7Hr1G093060	25.05752	26.17949	23.89874	35.87598	43.2957	43.99436
Hordeum_vulgare_newGene_6562	5.619922	5.528607	6.102058	4.8337	4.843052	4.955891
Hordeum_vulgare_newGene_6560	3.756265	1.505819	2.229571	0.572087	0.128734	0.299071
HORVU1Hr1G026140	12.02702	9.732701	13.34681	12.62234	17.94925	18.68603
HORVU5Hr1G125620	9.713053	9.856405	10.83488	15.50092	27.88437	26.06006
HORVU5Hr1G012870	1.421721	1.336545	1.945297	1.289445	1.728258	2.244099
HORVU7Hr1G075000	31.64819	27.31293	33.44081	28.27579	26.81966	24.1532
HORVU1Hr1G088510	150.0774	147.7033	193.9623	244.1759	177.7589	169.5646
HORVU6Hr1G002530	0.872092	0.412545	0.610143	2.134229	1.423363	2.778068
HORVU5Hr1G022550	3.177746	2.888252	5.07596	44.84941	92.71399	71.94965
HORVU6Hr1G073630	4.85572	3.733913	3.371669	0.85721	1.630924	1.026875
HORVU2Hr1G086980	45.7864	51.41793	45.20486	29.47755	28.99639	32.27182
HORVU1Hr1G060740	55.87251	53.2099	47.93108	37.99876	33.86025	30.25033
HORVU1Hr1G021690	21.16236	17.45203	23.6437	27.88751	35.14071	35.66848
HORVU0Hr1G005320	23.06497	18.04317	20.56334	33.66965	42.49862	36.69824
HORVU5Hr1G123700	6.749191	7.610631	7.907673	9.23197	11.07754	11.15089
HORVU7Hr1G022450	8.174904	11.62813	10.3938	10.59576	13.92894	13.16825
HORVU2Hr1G000130	1.101959	1.526771	1.350666	1.496038	1.248915	1.553267
HORVU7Hr1G110660	27.75844	29.70207	43.53671	21.93588	40.78764	38.85163

HORVU4Hr1G051720	14.698	14.45798	20.64242	14.92137	27.27272	28.5482
HORVU5Hr1G002120	3.707648	2.957031	4.368381	3.32114	0.537791	0.56862
HORVU4Hr1G039070	49.10284	47.94132	44.73825	35.75565	37.64229	40.37583
HORVU7Hr1G094870	4.767762	4.442112	3.820053	24.02366	11.58487	16.08755
HORVU5Hr1G000710	2623.596	2487.943	2405.607	1636.762	2237.702	1350.354
HORVU7Hr1G092080	8.958771	9.259436	9.844978	10.23598	13.08625	10.53048
HORVU1Hr1G071590	18.38434	19.07395	16.85031	20.53577	26.1007	25.06518
HORVU2Hr1G056750	11.57511	13.54192	12.44401	7.635237	13.26688	13.91965
HORVU3Hr1G109960	15.49148	17.71226	15.47241	15.19698	18.05671	18.7554
HORVU7Hr1G069520	4.062625	3.527148	3.353045	8.317345	8.898118	9.082086
HORVU6Hr1G087150	18.12454	17.38769	17.72832	20.50999	25.15573	24.47584
HORVU0Hr1G022830	2.082414	1.900125	2.076557	4.901853	6.536258	6.67785
HORVU1Hr1G054430	0.660867	0.877915	0.728475	1.407126	1.549252	1.371238
HORVU4Hr1G063050	0.862559	1.193076	1.259921	1.08703	3.597683	2.678323
HORVU7Hr1G051590	3.220163	2.877558	3.848507	4.972444	3.754215	4.428891
HORVU2Hr1G083850	14.58647	12.98426	16.14813	14.70905	16.96996	17.16979
HORVU5Hr1G006710	10.55864	7.543585	8.761628	7.137073	9.342764	10.76204
HORVU3Hr1G082930	0.838735	1.03373	1.61526	1.215497	3.539091	3.653229
HORVU3Hr1G075160	17.24102	14.82985	17.29996	16.42616	13.94318	10.99932
HORVU2Hr1G056280	1.564871	2.144831	2.226184	3.146226	4.527749	6.032701
HORVU3Hr1G038850	0.939881	0.97496	1.100496	1.641324	1.766572	2.002372
HORVU5Hr1G069350	6.447152	7.570784	6.471877	6.56515	5.392121	5.562222
HORVU5Hr1G016400	7.74685	4.223524	10.62203	15.84665	23.5158	23.49014
HORVU1Hr1G005680	4.340449	2.047478	2.700977	2.339113	4.089535	3.805149
HORVU4Hr1G049670	151.1156	149.0227	273.6522	127.3317	240.023	215.0301
HORVU2Hr1G126150	5.190276	4.49868	7.71495	0.749478	0.21128	0.507165
HORVU3Hr1G007830	1.890947	2.691834	2.327908	0.51949	0.715291	0.640438
HORVU7Hr1G091570	0.025595	0	1.991463	3.7355	4.414498	4.206511
HORVU1Hr1G048560	5.911371	5.433689	5.858227	6.552813	11.25729	10.00951
HORVU1Hr1G013730	6.737619	7.29687	7.416414	8.625184	9.532817	9.306129
HORVU5Hr1G082830	3.344223	2.147171	4.563311	4.275535	0.957779	1.460438
HORVU5Hr1G066360	1.778173	1.599232	2.078951	1.960915	2.066564	3.856204
HORVU4Hr1G005710	0.533814	0.531707	0.625358	1.511835	1.871535	2.94132
HORVU7Hr1G058580	4.588095	4.872062	3.670681	7.075927	4.224381	6.171488
HORVU3Hr1G056090	10.4238	9.444265	9.586525	6.704637	4.298159	6.635544
HORVU2Hr1G082720	1.206315	0.772314	1.075462	0.823961	1.642631	1.630779
HORVU4Hr1G058940	0.651858	0.508638	0.71354	1.868747	5.58783	5.406177
HORVU1Hr1G085640	14.68941	17.20912	21.24584	1.33884	0.552756	0.764934
HORVU6Hr1G085170	67.71294	54.38556	58.56259	129.9695	166.6871	177.9769
HORVU5Hr1G111590	41.15116	44.26061	53.8477	14.85341	7.361075	11.30708
HORVU5Hr1G111620	39.3065	33.68371	43.78525	70.1786	86.87979	93.04795
HORVU0Hr1G014960	1.513755	1.3912	1.642405	1.829684	1.773968	2.277444
HORVU0Hr1G039970	2.378449	2.447122	2.526159	5.754399	3.102258	5.372638
HORVU2Hr1G044650	7.169393	5.94461	7.458507	11.43093	11.50636	13.15313
HORVU4Hr1G027970	0.233574	0.177349	0.319952	1.435741	4.210452	3.351781
HORVU2Hr1G103550	0	0	0	3.653238	109.8766	93.04417
HORVU1Hr1G015770	6.743277	5.683653	6.343204	4.510037	5.350286	4.451295

HORVU6Hr1G000480	7.086927	5.723086	6.860664	0.026253	0	0
HORVU4Hr1G019360	2.072166	3.184696	2.193773	5.397465	7.895104	7.080975
HORVU6Hr1G006880	5.951417	6.036387	5.711847	22.96887	69.76391	66.45806
HORVU7Hr1G094010	0.599429	0.796637	0.732279	1.449409	3.397939	3.006958
HORVU2Hr1G013870	6.109244	7.233935	5.989045	14.4773	22.23244	24.84951
HORVU0Hr1G015700	7.486306	7.196844	6.442463	6.899841	7.925425	7.167899
HORVU6Hr1G018500	7.162955	7.524457	8.68516	13.27573	9.788309	10.64806
HORVU2Hr1G099030	3.625509	2.878914	4.89053	3.711274	4.608826	3.891863
HORVU3Hr1G061210	3.100272	3.484379	3.617554	5.03509	6.313148	7.154598
HORVU2Hr1G101950	6.155439	6.908756	6.24045	7.919329	6.158887	9.430003
Hordeum_vulgare_newGene_13904	9.223341	10.47855	10.67748	13.99003	13.74828	15.85448
Hordeum_vulgare_newGene_13903	12.83435	12.95664	12.02823	7.779628	5.218424	7.455581
Hordeum_vulgare_newGene_13900	20.56768	16.61857	20.76564	28.94313	29.57447	29.27619
HORVU3Hr1G107960	0.878506	0.980265	1.318718	1.345406	0.862786	1.124042
HORVU6Hr1G016070	1.29811	1.839614	1.1952	3.454707	4.590465	5.560012
HORVU0Hr1G010280	1.229534	1.16734	1.601242	1.219121	2.473155	2.488564
HORVU7Hr1G006570	46.86051	44.42237	35.17939	3.056181	5.450525	4.92087
HORVU2Hr1G038410	15.95627	19.327	15.62566	19.38415	25.13351	25.80548
HORVU1Hr1G083170	7.960691	7.017949	4.155114	23.84181	27.47781	19.28694
HORVU5Hr1G120110	97.40147	80.9926	99.47715	48.91726	25.40116	29.73951
HORVU2Hr1G010990	1.385672	0.932565	1.776996	7.937476	39.39933	35.12444
HORVU3Hr1G099550	2.527016	2.741399	4.640297	2.110384	0.11501	1.216428
Hordeum_vulgare_newGene_15101	1.838119	2.369219	0.844066	0.598061	1.031755	1.184872
HORVU5Hr1G041930	2.924568	1.917281	3.176432	7.890247	3.377259	5.027787
HORVU5Hr1G074850	5.965858	7.398274	10.76628	5.180426	7.23835	8.00526
Hordeum_vulgare_newGene_8738	2.124225	2.653328	2.549759	3.098178	2.150659	1.620465
Hordeum_vulgare_newGene_8739	4.34657	4.439547	4.175831	5.495385	3.749822	3.409077
Hordeum_vulgare_newGene_8731	2.328021	3.219098	3.53406	0.018938	0	0
HORVU3Hr1G110870	36.89404	31.7137	30.94263	34.58146	29.97052	30.69229
HORVU3Hr1G114350	0.985016	0.979275	1.184687	1.356511	3.95162	4.200539
Hordeum_vulgare_newGene_8735	3.885833	5.033305	3.898723	8.234886	7.597101	7.087281
HORVU2Hr1G030640	50.7273	42.65987	60.67853	53.24134	69.01661	64.07806
Hordeum_vulgare_newGene_5275	0.941023	0.977755	1.338318	1.900532	7.57255	5.749693
HORVU4Hr1G020160	18.5021	14.51977	21.96675	20.6174	41.44057	36.63345
HORVU4Hr1G004590	12.34463	12.32444	12.58448	9.638534	8.118845	8.142066
HORVU4Hr1G000670	18.4105	21.45069	24.19108	21.42262	21.48237	24.42388
HORVU3Hr1G079640	2.320419	2.128766	2.42745	5.136894	5.320008	5.400515
HORVU4Hr1G064110	14.47966	14.29003	17.39894	16.62777	28.92375	30.01979
HORVU2Hr1G096680	12.00582	12.2983	17.25942	7.969365	8.636062	9.611418
HORVU3Hr1G075990	3.260435	4.254862	4.691195	4.429967	6.55871	6.947897
EPlHVUG00000039856	0.74453	0.484806	1.937897	0.371799	1.507488	1.249857
HORVU3Hr1G022990	2.504834	4.201434	3.158997	3.506626	2.816623	4.069173
HORVU6Hr1G076070	58.85736	63.03306	43.5816	17.07509	13.11467	14.42908
HORVU2Hr1G101040	16.31545	17.6614	21.37463	14.45823	8.756418	12.58302
Hordeum_vulgare_newGene_8086	6.220139	6.294943	8.094992	4.581036	5.157929	5.238325
HORVU6Hr1G057240	5.792026	4.595321	4.981391	11.1105	5.376065	10.50756
HORVU2Hr1G055140	24.83645	22.93121	26.94388	24.04753	29.46658	28.59177

Hordeum_vulgare_newGene_8083	0.945913	0.90593	0.964598	1.301525	1.458514	1.656831
HORVU4Hr1G007020	51.44156	45.99864	49.81163	41.65148	35.81319	36.92713
HORVU4Hr1G076720	20.05793	20.70909	22.66742	25.72935	25.00794	24.938
HORVU2Hr1G097080	5.720256	6.813095	5.642617	12.34213	22.47681	21.05857
HORVU2Hr1G118840	2.347096	3.141252	2.890822	1.329783	1.54455	1.467133
HORVU2Hr1G092260	1.22748	1.731594	1.232334	1.441876	2.022259	2.224761
HORVU1Hr1G040170	2.880307	0.154127	3.270588	0.705686	4.280544	0.224745
HORVU3Hr1G095790	1.375318	0.970219	1.839602	0.478962	2.507304	3.635734
HORVU5Hr1G117080	3.947676	2.533213	6.098908	0.102035	0.031958	0.048637
HORVU3Hr1G074640	13.32488	11.10228	12.04278	19.07249	24.09084	21.19369
HORVU2Hr1G118590	1.217232	1.696357	2.625439	1.952246	1.0155	1.04852
HORVU4Hr1G067490	3.805292	5.243479	3.816977	2.965013	2.546504	2.83922
HORVU5Hr1G047150	1.943121	2.360405	2.728025	0.290594	0.174272	0.419855
HORVU3Hr1G106700	1.443717	1.987876	2.088214	1.95816	1.95152	2.562093
HORVU0Hr1G022240	3.335652	3.700554	3.656343	4.789917	6.819371	6.033159
HORVU4Hr1G077450	12.89333	12.71152	11.18788	18.31119	19.16895	24.41848
HORVU3Hr1G095240	1.455413	1.29441	1.762999	0.859255	0.885889	0.736709
HORVU2Hr1G029600	8.128364	7.941752	9.831665	15.33705	21.97607	21.73561
HORVU1Hr1G041220	3.681557	4.315291	5.328692	4.901437	7.136972	7.06452
HORVU7Hr1G082240	79.77712	65.4521	88.45316	73.31222	95.401	96.10419
HORVU3Hr1G112680	6.835519	7.070322	6.312468	5.082105	6.158678	6.655004
HORVU2Hr1G025920	1.120289	1.359044	1.469344	1.344273	1.956278	2.26169
HORVU5Hr1G099410	2.913348	2.099483	4.77794	3.746507	2.169488	1.974405
HORVU7Hr1G058760	12.96085	13.57826	15.24954	20.06178	21.45314	21.43388
HORVU3Hr1G011580	1.515599	1.021416	0.888573	1.523611	2.544358	2.010763
HORVU5Hr1G082310	4.065155	2.758109	4.575214	6.82648	6.973126	6.840824
HORVU3Hr1G075200	0.999789	0.721064	1.72468	1.075452	2.628421	2.365189
HORVU3Hr1G066580	89.79595	73.9302	89.39767	52.86449	46.00514	44.71629
HORVU7Hr1G001790	0.592162	0.358787	0.463901	1.935674	1.835319	1.406083
HORVU6Hr1G016890	0.358515	0.311297	0.4156	2.363239	5.646628	6.759569
HORVU2Hr1G049900	4.42762	3.523284	4.791986	5.85825	8.309202	8.513638
HORVU3Hr1G024890	2.686806	1.678027	2.754961	2.679415	1.858934	2.040329
HORVU4Hr1G062970	4.417844	3.743328	4.760424	5.194695	4.22598	6.453088
HORVU5Hr1G072720	8.017148	7.716323	7.383852	11.14827	13.37079	13.36907
HORVU3Hr1G080640	29.20366	28.02843	25.80862	20.44767	12.46677	16.09059
Hordeum_vulgare_newGene_12265	0	0	0	3.447357	2.437891	2.353728
HORVU7Hr1G072120	2.755352	2.614008	3.511785	4.339515	4.372458	5.19879
HORVU7Hr1G079310	6.06907	5.67262	8.178846	8.182463	13.35374	12.50766
HORVU1Hr1G030190	5.57809	4.800585	5.330249	10.40251	7.649427	7.255235
HORVU2Hr1G070000	35.53685	30.87606	47.72014	33.50926	59.09136	50.55321
HORVU6Hr1G024860	0.617011	0.636829	0.651198	0.932757	1.725933	2.053154
HORVU2Hr1G033850	15.53742	9.968757	10.86942	12.0429	0.775539	3.924489
HORVU3Hr1G105420	0.0602	0.002397	0.166918	2.931618	1.97899	3.693679
HORVU2Hr1G028260	17.23533	17.69695	19.68713	21.12349	26.4059	26.33974
HORVU5Hr1G064070	1.04377	0.885344	1.160652	2.649935	2.124886	2.485554
HORVU6Hr1G065940	6.079256	7.442677	7.396896	11.23348	16.86255	16.41088
HORVU7Hr1G001860	2.462076	2.07124	2.823938	3.525437	3.564474	2.880235

Hordeum_vulgare_newGene_10175	2.405239	3.205964	3.231536	3.116428	4.797049	4.404888
HORVU4Hr1G057330	1.022099	1.271445	1.387392	3.116107	3.278884	3.690663
HORVU6Hr1G059520	3.875471	3.177172	3.918826	4.783202	3.56664	4.31259
Hordeum_vulgare_newGene_12192	10.01164	10.09115	11.553	12.78063	16.01742	17.74352
Hordeum_vulgare_newGene_12190	1.130982	2.064871	2.666943	1.697703	1.530275	1.853735
Hordeum_vulgare_newGene_12191	1.151137	1.284467	1.311725	1.829972	1.689624	2.26671
Hordeum_vulgare_newGene_5284	2.138498	2.656025	2.251371	1.91489	1.865934	1.921361
HORVU0Hr1G004830	39.14493	36.90717	28.44606	95.47962	163.4557	164.7046
HORVU7Hr1G035360	32.08411	33.2608	30.4973	29.84054	33.93636	33.67581
Hordeum_vulgare_newGene_6016	3.89523	2.266692	2.804707	0	0.00874	0
HORVU6Hr1G052600	141.6492	158.4301	237.8077	97.53962	171.5423	166.9244
HORVU6Hr1G029150	5.516806	5.969737	6.179137	8.905444	11.39307	11.75828
HORVU3Hr1G108950	2.245232	3.265051	4.470814	1.036145	0.619173	0.738987
Hordeum_vulgare_newGene_6019	2.62361	3.814463	2.952541	0.005636	0	0
Hordeum_vulgare_newGene_6018	4.352481	5.116488	4.924419	0.007928	0	0
HORVU5Hr1G037160	9.218706	7.692597	11.89397	12.3266	23.59132	23.3407
HORVU3Hr1G081110	2.804492	2.722219	3.643793	1.973722	2.385768	2.527479
HORVU7Hr1G061990	1.517865	1.492818	1.466687	1.245987	1.142192	1.251012
HORVU4Hr1G026720	22.02635	19.50248	21.51713	40.33402	48.03501	50.34451
HORVU2Hr1G071350	92.33899	81.37504	114.5405	82.76698	133.0031	134.5156
HORVU0Hr1G030830	0.511981	0.235152	0.473618	4.239712	0.884892	3.711379
Hordeum_vulgare_newGene_3198	2.313633	2.47126	2.680656	0	0	0
HORVU1Hr1G071570	79.70703	80.90327	119.0491	56.98428	128.3297	119.4767
HORVU1Hr1G059860	35.82562	33.40415	40.94375	37.58879	39.23249	40.12826
HORVU3Hr1G087450	50.51282	52.65487	69.89524	24.80987	18.37611	19.56572
HORVU2Hr1G073850	4.141814	3.607949	3.817384	8.073713	12.90909	10.91325
HORVU3Hr1G052280	2.450506	2.403663	2.764368	2.326579	4.092014	4.690462
HORVU2Hr1G079830	16.68105	14.99259	19.65822	19.00198	29.42272	28.63789
Hordeum_vulgare_newGene_14699	6.151452	3.667203	4.861718	5.239681	0.526165	1.640024
Hordeum_vulgare_newGene_7948	21.73209	22.90231	25.41318	24.21704	30.48473	26.92769
HORVU6Hr1G079660	3.516267	4.406255	4.895765	3.342876	5.649469	4.982572
HORVU2Hr1G116500	4.280507	7.09083	4.683939	6.518434	10.42476	8.882871
HORVU3Hr1G068380	30.41481	20.54035	39.09036	13.77994	2.800797	5.91006
HORVU6Hr1G062430	5.191315	3.987699	4.850404	1.272838	0.477436	0.713019
Hordeum_vulgare_newGene_7946	6.092156	5.867876	5.674077	8.353035	14.927	12.97573
HORVU6Hr1G053550	8.805534	7.647792	9.367666	14.13359	12.26844	11.2811
HORVU7Hr1G097630	8.30373	9.417361	8.71034	11.89153	13.03149	15.85684
HORVU3Hr1G020400	4.970397	6.767246	7.228955	3.112186	2.915859	3.99199
HORVU2Hr1G015570	4.958749	8.201952	8.001451	0.258361	0.022219	0.051302
HORVU4Hr1G078590	42.94349	40.32795	51.68202	50.52907	63.24667	58.77925
Hordeum_vulgare_newGene_2246	3.382989	3.7649	4.8598	6.470414	4.64104	5.335736
Hordeum_vulgare_newGene_2245	3.613435	3.454685	3.48919	4.296978	4.050523	3.577984
HORVU5Hr1G001750	14.71179	14.7762	16.87632	3.315421	1.179006	1.484027
HORVU6Hr1G061200	9.693367	8.541711	9.823454	11.74699	10.82064	12.37461
HORVU7Hr1G078730	9.080574	9.028376	13.5397	19.78664	29.02085	24.24335
HORVU6Hr1G012400	11.78402	9.557579	12.89055	9.929205	7.861189	7.724356
HORVU2Hr1G003870	3.127387	3.136748	3.508607	2.036529	4.221122	3.324067

Hordeum_vulgare_newGene_13115	2.537917	1.96494	2.381111	2.965136	4.577039	6.06229
HORVU3Hr1G053620	35.09536	34.91139	37.21953	44.87197	43.33894	41.75785
HORVU1Hr1G020070	0.08918	0.070354	0.095374	1.681056	7.076286	6.365765
HORVU3Hr1G016490	1.920993	1.670707	1.538541	2.523033	1.714429	2.044836
HORVU7Hr1G119620	7.583638	7.987624	8.485994	11.59717	11.21176	10.78027
HORVU3Hr1G113720	3.357697	3.5174	3.779268	5.07677	4.879962	5.266391
HORVU3Hr1G069030	0.265561	0.559791	0.242549	2.294226	1.124737	2.453437
Hordeum_vulgare_newGene_976	5.642486	6.346983	7.576572	4.159357	5.651285	5.520676
Hordeum_vulgare_newGene_970	2.889199	2.451395	1.37181	5.258304	22.04303	20.40316
Hordeum_vulgare_newGene_973	1.189654	1.988913	1.48879	2.033457	2.421715	2.382896
HORVU5Hr1G024240	13.11706	14.73345	12.73094	13.38815	13.67603	13.20598
HORVU3Hr1G011910	7.075597	8.219099	8.241632	13.59247	14.40286	17.33868
HORVU5Hr1G100900	85.77321	73.50157	86.3444	84.25851	66.83962	66.97649
HORVU2Hr1G053160	4.635516	4.838529	6.56234	5.914656	8.542554	7.136367
HORVU1Hr1G062270	2.562772	2.948389	2.860498	5.231349	11.43748	11.25738
HORVU2Hr1G085500	1.362154	1.498535	1.793141	1.252614	0	0.529477
HORVU3Hr1G019770	3.074505	3.130354	3.297331	3.759045	4.22138	4.469599
HORVU3Hr1G004440	1.431585	1.062567	1.921697	1.210945	1.748797	2.165468
HORVU3Hr1G051220	15.92768	14.86694	22.30474	12.10928	14.98553	15.63635
HORVU3Hr1G115940	12.07025	9.079283	12.84878	11.19761	7.220867	8.145758
HORVU4Hr1G002020	3.262738	2.739098	2.108464	3.855972	5.822735	5.981528
HORVU1Hr1G095400	37.58263	34.44506	45.26876	46.20789	68.62685	69.30056
HORVU7Hr1G107790	5.355444	6.493799	5.761228	8.330223	9.88546	7.946995
HORVU7Hr1G096270	0.317601	0.311049	1.135292	2.05219	2.861768	3.720835
HORVU0Hr1G002220	79.30013	63.33485	71.02862	80.59143	97.7984	100.6986
HORVU2Hr1G003290	1.153009	2.232803	2.604677	3.016272	1.800166	2.893627
HORVU7Hr1G049260	1.526672	1.809878	1.979506	2.667952	6.413201	4.969085
HORVU7Hr1G030280	4.8763	4.526831	7.22443	3.900791	5.102229	5.231187
Hordeum_vulgare_newGene_1292	152.8585	123.3236	135.0493	121.769	202.2104	202.469
Hordeum_vulgare_newGene_1293	3.548681	2.477041	3.796308	0.194938	0.028433	0.175126
Hordeum_vulgare_newGene_1294	3.248666	2.719595	3.819395	3.783021	4.773631	5.996276
Hordeum_vulgare_newGene_1296	1.266904	0.889261	0.935203	2.125706	2.957677	2.930496
Hordeum_vulgare_newGene_1297	14.08024	16.98198	16.75191	11.45946	10.75527	12.81909
HORVU5Hr1G069620	4.009742	4.17166	3.431955	19.84503	49.80547	41.95976
HORVU6Hr1G014790	3.592153	3.560064	3.821592	3.109821	6.036332	5.973954
HORVU0Hr1G019700	5.99813	7.02116	6.267208	7.620972	12.47139	12.79814
HORVU5Hr1G102220	9.021768	8.723457	10.02801	9.742792	11.7313	11.57886
HORVU0Hr1G030010	1.202665	1.101683	1.192515	1.39924	1.726761	1.799206
HORVU2Hr1G084670	21.02442	22.08559	23.47896	16.9261	23.30058	24.50222
HORVU1Hr1G041770	3.856731	4.227213	4.601037	10.87948	18.57496	17.61991
Hordeum_vulgare_newGene_13692	4.072963	3.97127	4.944238	4.64183	6.341289	2.80967
HORVU7Hr1G113270	2.607356	1.344895	2.713728	58.43997	123.5232	114.3782
HORVU4Hr1G047610	5.411117	6.12368	3.753633	2.272819	6.533666	6.389367
HORVU3Hr1G052250	1.621495	1.564084	1.59118	5.745199	6.393215	7.801927
Hordeum_vulgare_newGene_13696	19.15055	17.52303	20.45823	28.13246	38.03499	42.24382
HORVU1Hr1G058420	0.26758	0.383035	0.514186	1.739496	1.731848	1.758233
HORVU6Hr1G089190	3.561469	3.907612	3.528965	8.084706	11.39299	10.79546

HORVU6Hr1G073010	1.020409	0.755948	0.972514	1.776576	1.2336	2.070851
HORVU1Hr1G049760	0.39608	0.296196	0.255329	1.923717	1.520248	1.72555
HORVU5Hr1G021360	7.639588	8.396808	8.387302	6.091867	8.181229	8.532189
HORVU1Hr1G067990	2.427739	2.802437	2.94733	1.900568	1.912619	1.740795
HORVU1Hr1G063630	1.487987	1.532979	2.093439	0.551313	0.782807	0.571649
HORVU2Hr1G012710	32.95324	28.34142	28.79781	57.21793	63.04306	57.57195
HORVU1Hr1G069840	7.607335	9.279928	8.42937	8.185804	8.951422	11.01202
HORVU7Hr1G101240	57.88979	42.61442	69.73185	43.47504	56.84977	56.49624
HORVU2Hr1G069470	15.80004	16.96118	17.0889	10.03829	9.596123	12.70594
HORVU4Hr1G005080	0.939683	1.433121	0.930315	0.649289	1.821561	1.750798
Hordeum_vulgare_newGene_13362	0.054785	0.050516	3.47891	2.92809	3.664163	5.255583
HORVU1Hr1G072490	67.09951	61.92576	61.94074	49.34591	64.10596	72.26935
HORVU1Hr1G079140	0	0.038689	0.046224	9.235077	0.429628	4.639092
HORVU2Hr1G034440	2.57454	2.441497	2.867472	2.476311	3.465247	4.42907
HORVU2Hr1G088480	5.897146	7.341508	7.297767	5.320219	6.775153	7.986234
HORVU6Hr1G035970	16.49509	17.98921	20.201	16.50991	8.464634	9.67788
HORVU7Hr1G090540	1.506404	1.924542	2.064829	2.567487	2.237378	3.352329
HORVU5Hr1G018300	2.010732	2.404793	1.993272	1.409147	2.072932	2.287459
HORVU2Hr1G125680	1.737161	2.194768	1.950149	1.511189	2.155665	2.345299
HORVU1Hr1G075640	6.12765	5.265027	7.20933	10.66132	11.43503	13.17223
HORVU2Hr1G120060	3.031517	3.24966	2.949991	3.011204	4.428287	4.185913
HORVU4Hr1G072210	7.199521	7.149271	7.092844	6.209699	9.30559	9.9115
Hordeum_vulgare_newGene_4576	0.028976	0.054091	0.032028	5.467496	4.90751	4.792584
Hordeum_vulgare_newGene_4575	0.723846	1.075612	0.85767	1.988484	2.720065	2.700143
HORVU0Hr1G031760	2.662905	3.103934	3.614341	5.985375	12.94458	12.01916
HORVU2Hr1G017770	82.47661	38.54533	39.57968	61.59372	116.8492	99.79473
HORVU1Hr1G052040	30.55751	35.2528	41.7553	42.25948	52.53038	57.04877
HORVU3Hr1G042290	12.76989	13.02425	13.31597	14.60684	14.08724	14.51266
HORVU0Hr1G000810	0.483931	0.32922	0.450385	5.607998	9.714938	12.04104
HORVU1Hr1G083370	6.69953	7.197149	8.021781	7.44992	9.09447	9.133391
Hordeum_vulgare_newGene_7004	10.87357	6.807195	12.32088	28.39042	33.82897	31.49593
HORVU7Hr1G110720	3.583992	3.934978	1.688652	1.323147	7.453686	9.037307
HORVU3Hr1G085130	9.350063	9.287099	9.527462	11.55786	11.90738	13.21336
HORVU7Hr1G079450	15.85788	15.47679	20.50604	16.12699	21.00329	20.29899
HORVU1Hr1G091870	19.18382	19.66151	19.59142	27.25573	27.4833	29.86607
HORVU4Hr1G059000	1.645896	2.143901	2.422722	3.647119	4.441559	4.782443
HORVU4Hr1G053250	0.106463	0.151181	0.307164	3.708799	0.328821	1.624076
HORVU2Hr1G008380	4.512474	4.086753	4.609443	6.911184	8.928333	9.829159
HORVU7Hr1G055570	2.110405	2.530549	2.451756	4.308908	7.063734	6.326061
HORVU1Hr1G068460	1.729853	1.38846	1.994925	2.776903	4.122793	3.961676
HORVU7Hr1G093380	17.94661	14.95751	22.31727	9.890976	11.47309	16.0213
HORVU2Hr1G000580	3.900386	3.688974	4.616074	3.113204	4.132477	4.61829
HORVU5Hr1G095410	3.007676	2.55329	2.786654	6.476226	13.45495	11.42615
HORVU3Hr1G006150	1.180376	1.133066	0.857599	2.810178	3.700357	4.287462
HORVU2Hr1G077830	0.155487	0.068891	0.215857	0.942542	11.47098	10.31126
HORVU3Hr1G034520	0.036555	0	0.099254	0.328474	30.14764	24.38993
HORVU4Hr1G079600	20.1914	11.23395	26.58669	1.230093	0.882609	1.112379

HORVU4Hr1G004750	8.158142	8.523155	9.09443	8.752014	9.277975	8.507284
HORVU4Hr1G000070	1.383217	1.439145	2.266129	1.536227	2.413369	1.903559
HORVU4Hr1G016380	3.365623	3.588995	4.000379	2.948913	3.424511	4.607056
HORVU3Hr1G043160	1.108986	1.667805	1.506312	5.731654	4.973198	6.61136
HORVU3Hr1G079400	4.553989	3.233691	5.361774	3.421693	4.242792	2.483202
HORVU4Hr1G048700	16.14369	13.4676	16.06348	16.36596	13.12459	15.15982
HORVU3Hr1G031950	2.738874	2.141631	2.258684	9.500829	5.995336	6.341758
HORVU6Hr1G029210	0.580436	0.404162	0.979451	4.525118	24.24794	24.75378
HORVU3Hr1G007280	91.87436	82.53806	127.008	72.56095	55.53172	56.15531
HORVU2Hr1G055380	74.15419	88.24517	78.39229	70.38076	77.75501	81.84574
HORVU4Hr1G007220	2.415759	2.266325	4.36022	4.130077	0.75135	2.855505
HORVU4Hr1G051080	18.51895	13.74274	17.2409	17.86984	25.52135	28.15764
HORVU5Hr1G125450	2.175893	2.031188	2.918882	6.5554	0.271279	2.297275
HORVU6Hr1G080500	45.08822	33.34817	42.16136	26.5349	28.48929	29.13942
HORVU4Hr1G081210	0.775981	1.271568	1.284455	0.935031	1.017303	1.12083
HORVU2Hr1G014450	17.99318	15.66081	19.21217	24.00779	20.25957	19.52617
HORVU1Hr1G027000	2.115357	1.261438	1.564852	1.003714	10.31186	8.688635
HORVU2Hr1G041780	4.83159	6.484333	6.743617	10.47698	12.3748	14.43891
HORVU5Hr1G093150	3.994678	5.007251	5.666494	2.68632	1.642215	2.050401
HORVU2Hr1G090330	2.669535	1.893989	2.112003	11.96539	12.51207	9.116672
HORVU5Hr1G097000	6.632356	6.640469	9.339228	8.368893	10.47703	10.72047
HORVU0Hr1G022260	1.651242	1.24886	1.717595	2.0727	1.702986	1.544286
HORVU5Hr1G001780	1.26624	2.246714	1.708378	7.171983	7.774925	8.197026
HORVU5Hr1G111810	1.613632	2.056791	3.103604	0.935116	0.927317	1.27515
HORVU3Hr1G030600	38.46891	36.62505	35.60171	27.05854	22.04068	24.26002
HORVU1Hr1G081840	3.148944	3.073961	4.285713	4.48313	7.292009	6.469081
HORVU0Hr1G003890	87.86318	54.90483	83.81541	121.3217	75.29008	96.52881
HORVU5Hr1G122070	9.870738	8.413014	9.781204	28.92177	40.57957	32.9947
HORVU7Hr1G116180	3.821261	4.128494	4.85149	3.339324	5.211614	5.16559
HORVU1Hr1G043440	6.045372	7.045747	6.926729	9.78971	13.63643	13.1306
HORVU7Hr1G121270	113.2852	196.9585	97.58953	96.99917	14.08657	34.10096
HORVU2Hr1G113830	2.245127	2.221894	2.719715	3.890147	6.734289	5.629349
HORVU4Hr1G051550	5.229495	7.163461	7.943879	7.050885	5.605115	7.43526
HORVU3Hr1G028780	5.916977	5.265284	9.240364	40.32623	42.57611	42.4526
HORVU3Hr1G082210	3.930738	4.933863	5.074876	5.980118	6.463216	6.497141
HORVU2Hr1G020760	1.599886	1.824086	2.817764	4.285906	4.039753	3.528654
HORVU2Hr1G040780	41.36699	31.20277	34.70806	74.17873	127.3823	121.1536
HORVU4Hr1G085300	9.3934	6.040176	8.61207	9.87821	14.46308	14.90721
HORVU1Hr1G081260	0.802477	1.321268	1.405367	0.403378	1.332232	1.309316
HORVU3Hr1G092420	7.087975	6.530014	8.303531	15.54149	33.06196	29.31433
HORVU6Hr1G084510	3.395402	3.187007	3.531656	6.977531	4.230004	5.228577
HORVU7Hr1G048670	0	0	0	1.632011	2.635386	2.688554
HORVU5Hr1G071610	1.357126	1.102609	1.402411	1.214491	1.296055	1.653836
HORVU7Hr1G080310	34.01369	36.1048	42.55317	43.14144	84.71643	70.93818
HORVU7Hr1G040460	27.6014	32.52049	34.89671	24.94759	32.06716	28.16574
HORVU5Hr1G054420	12.13423	11.38478	7.752435	5.181413	2.497254	3.016829
HORVU2Hr1G105830	3.833562	4.547484	5.153453	3.389833	3.672064	4.453805

HORVU0Hr1G016150	0.566149	0.83729	0.853943	1.831309	2.069861	2.430765
HORVU1Hr1G002850	0.010827	13.24607	0.884017	24.10592	0.021114	0
HORVU6Hr1G093060	11.60392	13.17954	12.38243	13.5567	11.05677	12.86433
HORVU4Hr1G069790	3.776853	4.156172	4.718571	2.55224	3.576966	3.653436
HORVU5Hr1G097100	10.64996	12.00935	17.83723	4.692865	2.957709	3.748089
HORVU7Hr1G071900	4.742103	5.283487	5.07935	4.794126	6.8037	7.147413
HORVU2Hr1G094360	0.077309	0.233206	0.157505	1.152016	2.722997	3.323056
HORVU1Hr1G039830	0.261246	0.468779	0.357389	6.906954	19.6501	17.2659
HORVU6Hr1G000380	31.37296	27.96544	35.11559	16.1275	17.00894	15.80871
HORVU7Hr1G114850	0.989355	0.871917	0.755146	1.637331	0.958506	1.344152
HORVU3Hr1G083160	7.436976	4.663431	6.100133	6.293867	8.385469	7.620322
HORVU1Hr1G068850	1.639529	1.930214	2.209138	4.341891	6.017461	6.221557
HORVU3Hr1G053080	1.383282	1.126745	1.852914	1.154411	1.046216	1.364648
HORVU3Hr1G025920	21.11191	20.24255	24.03581	12.6628	13.16342	16.44979
HORVU6Hr1G091510	0.377175	0.747246	1.306512	1.001771	1.635493	1.991742
HORVU5Hr1G115530	5.876115	4.833894	5.846666	3.564108	4.529711	6.151719
HORVU5Hr1G105770	4.280429	4.113004	3.775622	3.91411	5.301906	3.648438
HORVU5Hr1G053750	1.836243	2.071889	2.594852	3.355787	5.014876	5.352598
HORVU2Hr1G114940	10.37593	8.997443	9.694027	11.4063	11.67604	11.10114
HORVU1Hr1G094100	24.48584	24.10918	37.11679	42.94956	69.27366	78.38943
HORVU3Hr1G090690	6.315097	5.734254	6.575005	6.98844	10.10143	10.26166
HORVU6Hr1G055090	0.191801	0.297754	0.209158	1.08046	4.135242	3.655433
HORVU3Hr1G031560	24.20091	22.7642	19.57798	23.29766	19.05022	18.41969
HORVU2Hr1G076970	9.74465	4.894895	7.196214	5.964358	6.677198	4.34279
HORVU2Hr1G030200	24.69086	20.90326	31.38807	33.66719	65.82845	55.67133
HORVU3Hr1G000390	7.66921	6.147321	8.850742	10.85184	10.93608	12.09866
HORVU5Hr1G083350	29.17353	27.81691	34.10079	30.17479	36.33417	38.16779
HORVU5Hr1G000640	3.115014	1.501069	0.861376	1.636615	3.03225	3.093122
HORVU4Hr1G066430	1.579752	1.403479	1.521993	0.888272	1.060344	1.136105
HORVU1Hr1G082180	3.333182	4.251948	3.699411	3.300907	2.122922	1.980972
HORVU6Hr1G026730	4.513731	6.125668	6.021631	2.660931	2.723767	3.313563
HORVU3Hr1G025690	0.550095	0.67331	0.788652	2.165534	2.125884	2.768235
Hordeum_vulgare_newGene_13004	9.445367	11.96289	12.95273	11.86326	13.10396	15.51189
Hordeum_vulgare_newGene_13001	1.976333	2.142802	3.081964	1.45317	1.491726	1.346319
HORVU7Hr1G075370	7.658704	9.142051	7.340714	8.147647	9.096303	9.564084
HORVU5Hr1G093900	1.993243	2.526024	2.593287	4.940067	3.411172	1.900096
HORVU4Hr1G086650	16.74355	16.48727	17.24851	17.16119	17.34487	19.29873
HORVU0Hr1G022460	6.499487	7.147138	6.768252	10.31534	11.31457	12.8044
HORVU7Hr1G074420	2.156823	2.461739	2.148145	4.55026	2.286842	2.728475
HORVU5Hr1G115780	6.227308	5.271429	6.15167	0.826933	0.497655	0.618214
HORVU2Hr1G099200	9.351102	9.24181	8.310451	16.80831	14.97988	16.0066
Hordeum_vulgare_newGene_2000	5.470682	6.241986	6.391024	8.176372	9.368992	9.757109
Hordeum_vulgare_newGene_2004	3.808688	4.91081	5.35936	0.021173	0	0
Hordeum_vulgare_newGene_5237	1.101029	0.984146	0.961832	1.863329	2.194721	2.459913
HORVU7Hr1G076480	1.743898	2.122165	1.161852	3.237496	4.477964	4.926057
HORVU4Hr1G026870	8.324161	8.921712	8.237168	8.816217	8.545358	10.78943
HORVU5Hr1G007910	7.534257	6.575427	7.967615	7.824149	8.671606	9.198513

HORVU4Hr1G004250	2.565797	2.195715	2.991829	1.483365	0.821269	1.848872
HORVU3Hr1G059230	6.939821	6.964003	7.539456	22.99849	47.05111	39.6414
HORVU3Hr1G054600	4.80989	6.062891	6.052691	7.9044	10.33707	11.88043
HORVU5Hr1G053230	1.030126	1.575951	1.631434	5.72873	10.79393	9.460635
HORVU5Hr1G115870	1.811051	1.125918	2.644701	0.585865	0.286034	0.555454
HORVU2Hr1G033520	7.017265	4.841935	6.705367	1.780522	13.2299	6.569425
HORVU7Hr1G071280	2.441218	1.917143	3.15768	4.328271	5.655683	5.485732
HORVU7Hr1G008070	16.47564	19.35796	18.25403	28.78802	40.77129	35.03273
HORVU2Hr1G077010	4.021229	6.244247	4.582568	10.22225	7.803519	9.363902
HORVU3Hr1G021700	26.10639	24.14067	22.50464	23.51167	24.87537	26.64262
HORVU1Hr1G000540	10.36508	10.98119	5.740046	5.038473	5.820015	7.661979
HORVU2Hr1G060440	2.85125	2.637631	2.378857	4.629016	8.722398	5.621716
HORVU6Hr1G021170	17.89248	18.07315	21.63194	17.14977	21.62187	21.79283
HORVU7Hr1G012370	43.91764	41.17599	61.50757	41.39891	55.03683	56.91438
HORVU3Hr1G083770	1.392905	1.707573	1.677441	2.955569	8.372423	6.688777
HORVU7Hr1G083770	18.77625	21.32225	23.63893	21.36074	34.36394	33.86769
HORVU2Hr1G072500	54.37089	55.20374	66.16517	186.054	470.665	414.2034
HORVU2Hr1G105190	3.282535	2.69805	3.547797	3.323803	1.889414	2.331564
HORVU2Hr1G023480	0.352689	0.322229	0.431388	5.952939	9.64549	10.55624
HORVU3Hr1G062700	3.652513	3.429999	4.931426	6.120872	6.152	6.229252
HORVU2Hr1G030380	0	0.277376	0.095823	0.656664	11.13454	5.475865
HORVU5Hr1G098640	1.251569	0.929516	1.102713	1.849347	1.47731	2.491098
HORVU3Hr1G059060	106.7513	82.28318	110.1853	129.1607	185.4656	196.8702
Hordeum_vulgare_newGene_13601	0.822441	1.728234	1.70458	3.328301	4.756764	5.187981
Hordeum_vulgare_newGene_5239	1.231361	1.462938	1.245337	1.785547	1.073229	1.321565
HORVU1Hr1G001630	0.006716	8.246095	0.532584	12.13259	0.03002	0.070389
HORVU6Hr1G065740	1.296728	1.708415	3.006987	0.662452	0.321383	0.684837
HORVU3Hr1G092530	8.129464	9.421618	9.017144	9.466206	8.68078	9.291597
HORVU4Hr1G001590	5.81371	4.053247	6.867599	5.727409	17.77065	29.79323
HORVU5Hr1G078810	18.57116	17.65476	22.2405	18.53002	31.92822	31.65226
Hordeum_vulgare_newGene_8293	8.780453	6.624771	12.53875	13.0529	23.01013	21.99036
Hordeum_vulgare_newGene_8292	19.84015	15.34332	22.12678	16.97075	19.88171	20.5352
Hordeum_vulgare_newGene_14866	5.630287	5.697901	6.15547	6.5522	7.548014	7.877775
Hordeum_vulgare_newGene_14867	6.589909	7.827991	7.939932	0.150814	0.060878	0.048259
Hordeum_vulgare_newGene_14868	81.03856	89.35844	122.8805	73.99342	157.086	165.3106
HORVU3Hr1G103120	2.992388	2.880021	3.237972	4.341631	6.819599	7.384038
Hordeum_vulgare_newGene_8754	17.58191	15.10835	20.861	20.40648	25.80468	26.91223
HORVU3Hr1G012950	63.8596	60.19622	62.79779	45.01396	53.53227	50.25484
HORVU2Hr1G056820	3.727689	3.411859	3.828153	5.477953	5.652817	5.751408
HORVU6Hr1G025410	3.844927	4.819825	5.764156	5.201986	8.512288	9.024225
Hordeum_vulgare_newGene_1729	2.605811	2.557305	2.7023	0.01461	0	0
HORVU2Hr1G043030	1.471825	1.597585	1.455904	2.97183	2.771084	2.677604
HORVU2Hr1G011300	1.674951	1.682298	1.679228	2.128351	2.161836	2.261112
Hordeum_vulgare_newGene_1724	1.940966	1.64782	2.102331	3.800145	4.900981	5.249548
HORVU2Hr1G031030	6.798623	7.042585	7.330808	15.82449	20.26449	18.27831
HORVU6Hr1G060500	4.061402	2.84061	3.907922	8.595352	8.644132	7.023011
HORVU4Hr1G066880	2.890204	2.172583	2.606734	2.216421	2.152141	2.508906

HORVU4Hr1G067280	44.10874	36.84109	26.40013	6.997455	6.101563	7.445778
HORVU4Hr1G063900	1.926786	1.967395	1.959069	4.256347	7.558344	6.45465
HORVU3Hr1G066280	1.181386	0.568313	0.864966	1.030141	1.230873	1.291362
HORVU7Hr1G073190	0.299816	0.222212	0.251488	1.798113	6.565345	5.066028
HORVU4Hr1G078500	1.939045	1.938144	1.989271	1.923601	1.580776	1.45154
HORVU2Hr1G043780	26.49734	22.46823	23.28539	24.26991	22.43306	21.27214
HORVU3Hr1G057320	4.210572	4.115963	4.478895	5.457232	9.045849	10.04758
HORVU6Hr1G071780	23.29532	18.72999	23.7818	19.69923	20.35776	21.74744
HORVU0Hr1G019770	5.964413	5.097758	6.44963	7.606559	10.33074	10.59885
HORVU7Hr1G039180	2.559605	1.268862	2.206345	0.81222	0	0.337622
HORVU7Hr1G021050	0.882006	0.452185	0.590025	2.695327	5.183112	3.794054
HORVU5Hr1G099200	24.42678	19.88364	23.15267	20.77779	18.59284	21.89121
HORVU6Hr1G022660	8.984248	8.637881	8.172996	9.385676	9.240502	10.62136
Hordeum_vulgare_newGene_355	8.004238	8.616673	8.225459	6.721623	5.463689	5.161673
Hordeum_vulgare_newGene_350	1.906358	1.870624	1.591225	0.372427	0.475839	0.782757
HORVU3Hr1G089480	19.44199	21.09839	19.72599	25.60718	26.79198	25.91961
HORVU6Hr1G074570	24.02354	26.29794	26.30631	12.94686	11.21194	10.70186
Hordeum_vulgare_newGene_3016	1.031962	1.429356	1.363149	4.136352	2.733801	2.599824
Hordeum_vulgare_newGene_3019	1.139565	2.09968	2.753844	3.553978	1.685133	2.689152
HORVU0Hr1G021100	12.75269	12.37908	12.7178	10.34123	11.47987	11.18296
Hordeum_vulgare_newGene_3856	26.42748	20.67103	25.76718	44.63808	50.64352	55.74644
Hordeum_vulgare_newGene_6199	0.958539	1.485	1.216861	0.944498	0.550052	0.900173
Hordeum_vulgare_newGene_13768	5.073331	4.422269	3.542143	0	0	0
Hordeum_vulgare_newGene_13766	2.424798	2.595079	3.020439	5.276249	11.47782	10.15889
HORVU3Hr1G081300	1.858949	1.804444	2.005008	1.952092	2.584673	2.499771
Hordeum_vulgare_newGene_13765	4.070619	6.75467	9.531192	0.026429	14.44636	12.78757
Hordeum_vulgare_newGene_13760	6.682281	0	7.190204	0.043957	3.93503	0.0214
HORVU7Hr1G101910	1.103903	1.439222	1.758573	1.923257	2.043773	2.15779
HORVU6Hr1G078780	0.224767	0.219758	0.384009	0.377716	3.78001	3.018004
HORVU2Hr1G048840	17.05944	15.24054	16.97225	18.33401	20.77035	20.28088
HORVU4Hr1G041320	15.2172	12.85354	16.67533	13.90128	19.89033	18.98402
HORVU5Hr1G085550	1.868616	1.952852	1.663938	1.956624	1.484338	1.964158
HORVU7Hr1G103510	2.883983	2.904223	3.551763	5.217494	5.042874	5.250047
HORVU3Hr1G048610	48.35736	40.22398	52.64766	63.98705	157.4236	126.4856
HORVU7Hr1G096840	4.121063	3.786309	5.767841	5.133832	5.377184	5.181456
HORVU5Hr1G075490	48.0833	47.71916	60.96025	67.62079	189.4551	180.2903
HORVU5Hr1G079770	0.03727	0.146057	0.057635	0.298373	5.119167	5.390486
HORVU4Hr1G075810	4.370123	5.306134	5.036786	6.075464	10.43785	11.52872
HORVU3Hr1G117190	8.167439	5.157331	6.265465	10.41646	8.825048	10.87732
HORVU2Hr1G087400	15.95694	14.41038	18.85628	20.14437	39.3794	34.7177
HORVU6Hr1G033350	9.645088	8.279373	9.986364	13.33453	17.23987	17.96836
Hordeum_vulgare_newGene_15689	13.8706	13.55322	13.68562	12.49384	12.26487	14.04046
HORVU4Hr1G034360	17.80602	19.04744	20.20179	20.52514	22.38415	24.60267
HORVU3Hr1G032440	3.78943	3.672271	2.941639	7.165892	7.73514	9.607885
HORVU6Hr1G002290	0.67851	0.882161	0.851941	2.29552	1.78267	3.13146
Hordeum_vulgare_newGene_15687	7.262559	9.297467	9.413206	9.445516	9.658281	11.11264
Hordeum_vulgare_newGene_15685	1.006447	0.981706	1.308725	0.719168	1.022967	1.019175

HORVU4Hr1G070330	8.675491	8.274474	11.84513	9.253982	9.215348	10.01943
HORVU1Hr1G055710	0.869232	0.412115	0.873577	1.86833	1.424534	2.059777
HORVU5Hr1G039560	0	0	0.697215	2.667807	3.658735	3.709631
HORVU7Hr1G118740	1.763454	1.73517	2.18494	2.901126	1.843539	3.30125
HORVU6Hr1G071200	1.802317	2.038964	2.19169	3.500115	4.883318	4.352396
HORVU7Hr1G058860	2.723327	2.134573	2.246698	4.501142	4.587724	4.780768
HORVU3Hr1G111560	5.779972	4.155281	2.359106	4.045659	1.050516	2.191334
HORVU7Hr1G048470	10.78245	7.261831	10.94709	6.775068	4.374839	7.716185
HORVU4Hr1G002180	3.128497	2.963536	3.548153	1.470351	0.839691	1.421904
HORVU1Hr1G061910	0	0	0	0.027453	62.43767	57.69853
HORVU4Hr1G012070	3.0923	3.236563	4.654086	3.427205	7.025161	8.375724
HORVU4Hr1G003580	2.742143	3.838025	3.840905	9.935795	12.50101	11.37544
HORVU5Hr1G103430	37.17994	45.52162	61.78166	23.88825	22.72577	24.37372
HORVU6Hr1G058570	6.429141	4.753716	6.13564	10.57253	13.04827	12.65799
HORVU1Hr1G078860	8.172816	6.750154	9.036906	2.089777	0.462511	0.506513
HORVU1Hr1G050290	4.443454	4.279461	5.414676	5.580909	7.529804	7.633581
HORVU2Hr1G007130	8.589683	9.88914	11.4632	3.91774	2.795615	3.098821
HORVU1Hr1G054040	1.131885	1.742625	1.95697	1.334943	0.399712	1.330431
HORVU5Hr1G014980	65.88742	58.80175	69.58857	55.26053	58.77907	54.91028
HORVU0Hr1G003340	1.214435	1.020206	1.068777	3.931622	2.46404	2.686382
HORVU2Hr1G094160	16.69092	17.51436	19.3286	8.191285	5.275962	6.779404
HORVU2Hr1G004530	22.93878	11.64852	29.69139	7.080663	2.607566	8.987871
HORVU5Hr1G016850	1.417221	0.610108	0.748753	17.93943	20.43743	21.69081
Hordeum_vulgare_newGene_9487	4.298652	4.944783	4.51987	6.119822	5.508491	6.194837
Hordeum_vulgare_newGene_5178	4.64938	4.351888	4.425292	7.708441	9.381115	10.17546
HORVU5Hr1G029080	6.270951	11.24432	8.315521	6.981802	7.410718	9.779209
HORVU2Hr1G047010	6.721123	8.770439	9.870931	2.854382	4.253616	4.573484
HORVU3Hr1G080940	12.41733	15.33994	18.1545	12.54487	24.25333	19.68587
HORVU0Hr1G002690	11.21959	12.87517	11.25315	12.25164	14.76201	14.7255
HORVU2Hr1G070620	9.051296	7.696273	7.119064	1.513107	1.267861	1.125253
HORVU1Hr1G018030	2.312178	2.027299	2.175212	3.297103	2.451792	3.25697
HORVU3Hr1G019980	1.924346	1.895354	2.03504	1.37826	1.861566	1.659536
HORVU3Hr1G036630	3.597086	4.43136	7.266321	6.930116	8.386901	8.32165
HORVU4Hr1G008800	9.778764	6.14166	12.04775	23.33556	60.15301	52.79854
HORVU1Hr1G067070	1.302955	0.96074	1.814564	3.655321	3.776742	5.419348
HORVU1Hr1G095230	6.537521	7.253019	8.533541	4.829875	6.866521	7.294725
HORVU4Hr1G018150	144.9613	131.9746	80.5259	8.055342	1.199646	3.432432
HORVU0Hr1G038290	1.241486	1.730352	1.597873	1.975322	3.263582	3.258024
HORVU4Hr1G035180	10.73142	11.61805	9.615859	13.17183	17.95982	17.12966
HORVU3Hr1G085810	4.462727	4.529536	5.275493	3.331054	3.847393	5.808414
HORVU4Hr1G087350	1.831626	2.751294	2.419659	4.927389	4.647133	5.932894
HORVU4Hr1G005320	0.026334	0	0.05225	12.62451	15.71069	12.08307
HORVU4Hr1G070430	2.896982	3.126621	3.417361	4.426053	4.464747	5.354121
HORVU7Hr1G084100	27.30687	25.21493	33.84579	38.82334	53.88225	50.40249
HORVU3Hr1G031360	5.911008	5.991546	7.244576	10.1989	13.46366	12.37202
HORVU2Hr1G088520	17.42663	17.95184	25.06775	6.990774	6.067896	7.775042
HORVU0Hr1G008690	39.14087	38.78276	39.05995	51.22593	69.45754	70.56051

HORVU1Hr1G073170	1.464484	1.607564	1.604527	2.810859	3.665716	3.925207
HORVU0Hr1G019570	0	0	0	0.087246	4.133897	4.54403
HORVU3Hr1G046280	9.485748	9.312003	10.89108	11.5937	14.38363	14.11798
HORVU2Hr1G016300	6.647625	7.094102	6.037061	6.111982	3.942279	4.832099
HORVU5Hr1G067890	7.882836	5.317098	7.549352	4.054149	3.399394	3.685063
HORVU6Hr1G007360	0.016726	0.237617	0	1.883024	5.713374	4.122386
HORVU4Hr1G016880	0.691996	0.395737	0.546818	3.186391	1.40675	1.438395
HORVU7Hr1G095730	72.63997	60.21663	106.9804	68.08439	108.5073	100.4514
HORVU5Hr1G011850	5.053404	6.236907	7.150224	4.899351	6.50778	7.038932
HORVU2Hr1G083710	4.722681	4.255207	4.64952	6.443066	8.472992	7.467791
HORVU4Hr1G019280	1.151834	1.221392	1.295141	1.124475	1.098197	1.27033
HORVU7Hr1G019930	48.86702	48.58097	53.10247	48.50061	63.44561	63.71353
HORVU5Hr1G088040	3.976162	3.742318	3.319933	1.239114	0.858415	1.580084
HORVU6Hr1G047650	12.2296	14.83713	10.21793	15.29513	19.57191	18.68511
HORVU1Hr1G049590	12.17804	11.98262	12.55934	17.08271	16.07172	17.8626
HORVU3Hr1G000400	5.144462	4.785904	5.991937	4.360864	7.087613	7.601326
Hordeum_vulgare_newGene_7394	1.115256	1.205938	1.699026	2.835976	1.736975	2.397607
HORVU2Hr1G017860	1.900869	2.109897	1.826769	7.859162	5.950739	6.042777
HORVU3Hr1G002800	15.02393	17.23532	16.87846	14.54339	14.2726	14.4753
HORVU2Hr1G045500	16.06259	15.35384	26.33931	13.80548	19.12289	17.91335
HORVU4Hr1G064360	10.24735	4.108735	12.78726	9.088717	10.26162	10.29687
HORVU2Hr1G099440	0.689179	0.900554	0.839884	1.666889	1.65054	1.923775
HORVU1Hr1G064750	4.229431	4.482011	4.316891	4.935087	5.272983	5.754083
HORVU5Hr1G056860	80.19892	71.03406	78.78585	52.87832	59.17406	55.30286
HORVU3Hr1G046860	3.21952	2.513638	4.330458	3.165734	2.514406	2.215217
HORVU2Hr1G113200	0.955341	1.024364	1.079407	1.821593	1.764952	1.481233
HORVU4Hr1G039890	19.93733	17.96185	18.6702	20.77406	27.5591	28.9747
HORVU1Hr1G078000	14.38072	14.66559	13.30121	13.51161	15.17933	16.60812
HORVU4Hr1G016260	1.029292	0.738476	1.379347	3.219805	3.594711	3.693087
HORVU3Hr1G028000	2.162556	2.537547	3.477873	4.05865	5.098858	5.697417
HORVU0Hr1G000990	5.771434	6.42495	5.114972	7.804226	2.030478	4.492237
HORVU1Hr1G006930	4.944672	5.298715	6.660197	4.72566	4.626787	2.487953
HORVU6Hr1G044040	2.527746	1.65653	2.736465	1.380244	2.39086	2.954659
HORVU1Hr1G012930	248.3608	252.3999	328.1755	648.2546	1633.667	1155.196
HORVU3Hr1G028080	37.00807	29.92067	28.1452	11.84541	1.903797	5.550529
HORVU1Hr1G011370	22.41455	18.80491	24.67685	14.97473	16.18349	17.06467
HORVU2Hr1G015670	19.89897	20.45508	15.89353	14.33188	16.66119	15.99076
HORVU4Hr1G050520	1.342432	1.22533	1.354317	0.966456	0.518797	0.932336
HORVU7Hr1G116680	2.824823	3.340133	2.646525	2.641825	2.917629	2.871335
Hordeum_vulgare_newGene_3676	3.021348	3.726618	4.488885	0	0.038216	0
HORVU6Hr1G084190	37.03775	40.89032	32.5644	20.23447	10.7358	15.36383
HORVU5Hr1G022060	19.89544	17.41583	23.89613	20.87953	37.33863	35.48596
HORVU2Hr1G041080	0.609978	0.390542	0.3763	0.692735	6.906202	5.128592
HORVU2Hr1G079780	12.83544	12.75076	20.09671	14.75866	25.97025	26.95172
HORVU6Hr1G006100	0.026264	0.135675	0.087389	9.183572	41.11446	33.84185
HORVU2Hr1G106990	1.28417	1.424663	1.421449	0.788914	2.026468	1.708932
HORVU3Hr1G062540	1.479738	1.306045	2.175694	2.47964	2.320048	3.483057

Hordeum_vulgare_newGene_11133	0.090677	0.026763	0.048478	3.913518	12.33719	11.37124
HORVU7Hr1G000580	46.68517	41.5017	47.79346	32.62857	42.91453	44.15341
HORVU7Hr1G009230	21.84926	22.83433	23.89815	23.05065	29.58164	25.68475
Hordeum_vulgare_newGene_11137	1.364055	2.271305	1.3317	1.650259	0.319757	0.634835
HORVU5Hr1G053740	4.663273	4.132251	4.200408	5.862116	6.643738	6.699557
HORVU7Hr1G012600	1.286875	1.002657	1.247456	4.144665	8.558009	6.168926
HORVU1Hr1G065000	6.691303	6.228407	7.309173	9.366922	11.57493	10.80283
HORVU1Hr1G061580	2.233571	2.348468	2.289458	2.626303	2.814421	2.591282
HORVU7Hr1G093190	4.881327	5.313109	5.221672	1.659099	1.474063	1.347848
HORVU0Hr1G012850	68.88183	55.75839	46.65571	18.86803	10.19633	11.5053
HORVU7Hr1G104300	7.364479	7.510665	10.93444	7.210844	10.64011	11.1021
HORVU7Hr1G069230	165.2588	169.3425	228.309	90.11848	95.57763	103.2168
HORVU4Hr1G069190	0.952653	1.133989	1.662852	1.09715	0.761874	1.194154
HORVU4Hr1G052010	2.220489	1.72255	2.749224	2.795346	2.097284	2.796662
HORVU2Hr1G024150	13.29557	13.00885	13.95298	13.5843	13.81316	15.03104
HORVU3Hr1G028620	4.644201	2.597024	5.754558	2.352419	1.170469	1.807279
Hordeum_vulgare_newGene_11683	6.715307	7.306337	7.15019	5.916127	6.774029	7.640741
Hordeum_vulgare_newGene_11681	5.946185	5.698059	5.091508	5.414954	3.821997	5.694531
Hordeum_vulgare_newGene_11680	6.621795	7.522712	8.26729	6.276434	4.731779	6.534329
HORVU3Hr1G062090	1.593396	1.875186	3.006708	2.132191	2.465045	1.934147
HORVU7Hr1G112850	3.586608	3.769535	4.299112	5.266468	5.638441	6.832182
HORVU2Hr1G108490	1.798578	1.453641	1.341856	1.736264	1.798919	1.632893
HORVU5Hr1G077120	24.05358	23.7485	24.71899	9.332448	6.564665	8.744895
HORVU1Hr1G061630	5.5895	4.877321	5.714156	4.485001	4.000362	3.791769
HORVU1Hr1G049860	1.889339	2.08915	2.88321	2.618996	2.34863	2.832007
HORVU5Hr1G055740	2.188941	1.176261	1.913689	3.429309	4.485605	3.320372
HORVU7Hr1G010600	2.578334	2.934933	3.638218	5.44352	5.741526	5.809449
HORVU1Hr1G080300	2.307719	0.954478	1.919549	2.126276	0.49735	1.495094
HORVU5Hr1G049560	8.39395	8.729	11.26663	5.221541	5.607133	5.287794
HORVU2Hr1G055640	25.06988	22.06808	24.88839	23.12788	25.12064	25.83025
HORVU7Hr1G083360	19.6249	16.18225	20.46232	19.90821	15.59959	17.92645
HORVU2Hr1G091850	7.089886	7.621733	9.01845	10.83504	12.42382	12.22426
HORVU2Hr1G081960	37.61799	35.37479	40.79123	31.43637	42.33901	41.2201
HORVU3Hr1G026840	2.125236	1.76968	2.780206	5.161978	7.03534	6.386982
HORVU1Hr1G009430	7.840222	7.756208	8.621751	3.969941	3.006647	3.894569
HORVU5Hr1G117900	28.62485	26.06398	27.28836	27.39212	34.11829	34.99744
HORVU2Hr1G052030	5.578551	5.934734	6.879886	9.342396	13.8186	13.58924
HORVU3Hr1G024990	7.820838	7.694683	8.298772	15.4658	22.90083	20.49036
HORVU5Hr1G096950	1.420119	1.247131	1.690874	2.123769	1.918613	2.478425
HORVU1Hr1G038240	9.447571	9.636763	9.959281	10.75704	12.4748	11.65044
HORVU3Hr1G009940	1.145364	1.626115	1.178043	2.965688	3.587767	3.646267
HORVU7Hr1G110340	7.729322	7.28489	7.334394	8.133019	10.41906	9.621685
HORVU3Hr1G112270	25.60305	18.44575	25.43953	35.14756	27.37426	24.56834
HORVU5Hr1G113260	0.577285	0.952975	0.922271	0.72738	1.695358	1.686262
HORVU4Hr1G053760	1.089625	1.299241	1.225311	1.023288	1.95694	1.322985
HORVU2Hr1G111540	1.265242	1.598501	1.119629	12.44112	22.23555	20.24595
HORVU1Hr1G067330	0.234231	0.18692	0.222647	2.30726	2.409956	3.673695

HORVU4Hr1G053860	2.148682	2.072024	2.884754	1.666891	2.334227	2.155708
HORVU1Hr1G084970	30.45242	26.52098	30.13202	34.16971	41.42021	44.54269
HORVU1Hr1G018770	1.705882	0.382881	1.086945	0.84524	1.555766	0.919005
HORVU5Hr1G076380	0.613153	0.725708	0.873161	1.557089	3.028945	3.787429
HORVU6Hr1G089560	3.982225	4.132333	3.173336	0.092672	0	0.010783
HORVU7Hr1G050000	5.865451	5.169969	5.688481	5.551807	6.1723	5.976696
HORVU6Hr1G084300	4.211782	4.428309	4.635027	8.840542	6.873546	8.993854
HORVU1Hr1G003710	1.490407	1.238886	1.225422	1.496022	1.106366	1.31118
Hordeum_vulgare_newGene_13891	0.500769	0.651019	0.644248	3.401142	4.729815	5.378754
HORVU3Hr1G070760	3.848729	4.012137	3.049438	3.198053	1.026006	1.726407
HORVU2Hr1G065120	6.271523	4.69164	6.524096	8.757538	9.060461	12.16325
HORVU6Hr1G044590	19.0496	22.3829	20.5186	31.7784	30.42061	36.75737
HORVU2Hr1G052690	5.237831	6.234089	6.51532	6.594296	5.455216	7.373785
HORVU6Hr1G051760	4.465494	4.479462	4.751795	7.628107	11.20137	10.34542
HORVU6Hr1G027340	1.968658	2.21959	2.296693	3.251441	4.919913	5.563399
HORVU3Hr1G108540	3.101162	2.082524	3.113189	8.480003	10.75733	11.32547
HORVU3Hr1G001670	5.427036	5.310426	6.238974	4.869019	5.337161	7.049405
HORVU6Hr1G055440	9.919672	8.135072	5.584807	0.027998	0.03542	0
HORVU4Hr1G090800	60.52819	52.93372	71.32831	57.01207	66.33211	62.71785
HORVU4Hr1G067950	31.06213	30.67112	27.07481	49.21987	40.78566	38.23899
HORVU2Hr1G049080	6.68049	8.195465	7.824982	11.21864	11.61832	11.34866
HORVU2Hr1G052960	7.505167	6.945444	8.349367	10.38519	15.18114	15.68086
HORVU7Hr1G033820	7.273792	9.357974	7.131342	14.69314	17.72829	16.34005
Hordeum_vulgare_newGene_12565	1.176436	1.044077	1.272433	1.278136	1.326943	1.39041
HORVU3Hr1G062010	1.616421	2.485015	2.182094	4.375894	4.981153	6.243457
Hordeum_vulgare_newGene_12568	0.978241	1.0623	1.652541	2.760124	0	0.438917
HORVU5Hr1G041100	16.7294	14.18358	16.47837	14.12309	14.04245	15.03958
HORVU5Hr1G025570	3.011866	2.849834	3.280253	3.530426	3.635934	4.544138
HORVU2Hr1G064650	1.387679	1.712098	1.347635	1.894451	1.169126	1.340078
Hordeum_vulgare_newGene_741	0	0	0.133729	3.227236	1.972476	2.949495
HORVU6Hr1G032550	2.358402	0	0	0	0	3.740059
Hordeum_vulgare_newGene_748	7.043738	7.811851	7.539669	3.36917	3.891344	3.785083
Hordeum_vulgare_newGene_749	5.277752	9.296803	6.048829	6.828851	6.469694	6.356976
Hordeum_vulgare_newGene_13216	1.853736	1.006235	2.059225	3.14745	1.67266	2.848062
Hordeum_vulgare_newGene_13215	0	0.051499	0	0.381303	11.35354	6.998295
HORVU0Hr1G023180	1.753834	1.710411	2.406308	1.725923	1.391331	1.593123
Hordeum_vulgare_newGene_13213	1.044327	1.407701	1.420097	1.329692	1.936822	1.348498
Hordeum_vulgare_newGene_14246	4.239406	3.802726	5.205325	4.630431	4.152911	4.094114
HORVU2Hr1G021980	1.215153	1.683573	1.583513	2.027248	2.148028	3.129803
HORVU2Hr1G039850	3.889973	3.410822	5.702116	4.067632	3.631957	4.245879
HORVU2Hr1G107540	5.382459	7.147756	4.370404	9.19525	12.76277	10.93715
HORVU5Hr1G075650	2.515604	3.097479	3.965738	1.336604	1.690132	1.480637
Hordeum_vulgare_newGene_13897	3.20968	2.464923	3.199128	4.497651	5.347509	5.311578
HORVU4Hr1G059260	343.5089	345.2963	356.1314	132.8353	96.58096	70.32497
HORVU0Hr1G009470	1.579112	0.488274	1.955396	1.695092	0.769116	1.67038
HORVU4Hr1G065350	1.483819	1.901451	1.765136	2.887073	2.953775	3.083064
HORVU6Hr1G017710	45.09807	38.56158	46.69575	60.07643	35.40501	42.2968

HORVU5Hr1G085020	1.008072	1.054176	1.008401	2.07827	2.50559	2.754186
Hordeum_vulgare_newGene_2611	9.153411	8.576913	8.818945	6.259623	5.776688	6.325329
Hordeum_vulgare_newGene_2610	3.068648	3.068782	3.403915	1.987768	1.791198	2.568495
HORVU5Hr1G074520	3.466446	4.782802	3.905404	3.630764	4.884466	4.8943
Hordeum_vulgare_newGene_2615	5.33795	7.992304	5.388896	7.814044	5.154103	7.182786
Hordeum_vulgare_newGene_2617	1.499989	1.506119	2.2705	1.614753	1.383775	1.438506
Hordeum_vulgare_newGene_2616	2.068749	9.629358	2.630418	9.424543	3.972158	8.330019
HORVU2Hr1G032880	4.679389	5.705225	5.91529	8.611304	10.27209	11.20174
HORVU5Hr1G026000	1.796052	3.707119	1.569894	1.902229	1.845625	2.043457
HORVU2Hr1G116130	5.409844	4.283631	4.281241	10.75958	10.44157	11.94411
HORVU2Hr1G090930	6.238312	8.374003	9.00936	5.242559	6.920372	5.304412
HORVU6Hr1G012290	3.297384	4.856808	3.946012	17.81563	19.47845	18.487
HORVU4Hr1G061070	0.178571	0.377841	0.165346	1.096514	7.174558	6.052259
HORVU2Hr1G030950	8.000388	7.657227	7.508407	10.65422	19.54468	15.99937
Hordeum_vulgare_newGene_5964	9.666889	8.869102	13.19557	9.639649	13.2194	11.43897
HORVU6Hr1G021690	3.663304	3.927744	3.656601	6.879313	10.65771	9.152856
Hordeum_vulgare_newGene_5963	0.75127	0.842524	0.674158	1.690734	1.385018	2.378024
HORVU3Hr1G064600	7.335119	7.167882	8.261126	8.384032	7.413259	7.349234
Hordeum_vulgare_newGene_15117	8.784224	8.650505	9.754545	9.924712	12.57744	12.95192
Hordeum_vulgare_newGene_15115	4.623373	4.622175	4.758793	5.507581	7.093816	6.925857
HORVU7Hr1G060310	19.18917	18.54165	19.30508	22.57804	31.22615	32.35332
HORVU2Hr1G086460	4.616111	4.302215	4.914458	7.772208	8.851918	8.627655
HORVU3Hr1G065350	1.713712	1.42137	2.38237	2.145804	2.865265	3.121748
Hordeum_vulgare_newGene_14661	0.1008	0.127866	0.167955	2.005678	2.042994	2.544189
HORVU4Hr1G060380	87.01653	68.7932	88.32512	70.84952	121.6232	123.1821
HORVU1Hr1G070580	1.220611	1.653978	1.844369	1.823398	1.750599	1.613242
HORVU3Hr1G110360	1.076502	1.479357	2.156859	1.576283	2.151461	2.083091
Hordeum_vulgare_newGene_8825	9.187564	8.648257	9.741597	6.239134	5.359334	6.937952
Hordeum_vulgare_newGene_8826	7.833793	7.95513	8.912611	10.05266	8.405402	10.09478
HORVU1Hr1G008690	1.666458	1.45087	1.423924	2.098554	3.635095	3.062277
Hordeum_vulgare_newGene_9197	7.67149	9.699604	8.20696	5.994201	9.862921	10.41432
HORVU2Hr1G030480	5.638397	4.483701	5.898051	7.751107	13.47987	13.9093
Hordeum_vulgare_newGene_9199	0.500606	0.698458	0.584845	1.542524	1.39769	1.787729
HORVU7Hr1G102480	0.516801	0.260742	0.689244	7.457304	18.30373	13.78814
HORVU7Hr1G102150	4.161145	3.812327	4.585963	4.281676	4.518868	4.556727
Hordeum_vulgare_newGene_9441	0.875428	0.982696	1.132326	1.472553	3.071595	3.435227
Hordeum_vulgare_newGene_9447	0.842607	0.738684	1.279732	2.272901	1.166968	1.004419
Hordeum_vulgare_newGene_9446	23.71073	22.14032	23.36554	21.36822	22.96726	25.38329
HORVU5Hr1G063340	15.4977	11.22444	13.28239	41.73784	30.89425	29.47876
HORVU3Hr1G026430	0.873115	1.136245	1.238752	0.971918	1.156748	1.4929
HORVU4Hr1G088080	2.952221	3.572314	3.082716	8.372492	11.51357	10.10147
HORVU7Hr1G080950	1.971998	1.401542	1.749364	4.322004	5.969658	5.061889
HORVU5Hr1G081610	367.2142	360.6091	354.1078	1300.395	1006.838	844.8287
HORVU2Hr1G026060	30.21341	28.26648	40.5136	37.51158	71.73674	67.93881
HORVU2Hr1G030220	22.79605	20.14381	22.69252	24.16579	33.43785	30.89108
HORVU2Hr1G069600	3.291425	3.163532	3.728072	8.292354	17.97478	20.71195
HORVU4Hr1G071370	4.872666	2.79775	6.783285	0.377283	0.0954	0.371499

HORVU1Hr1G001400	0	0	0.678062	12.08247	0	0.011631
HORVU2Hr1G049500	5.575348	6.011363	7.831099	3.223104	4.060905	4.690157
HORVU2Hr1G038030	4.006744	2.844644	4.262428	8.177072	14.10534	12.92943
HORVU6Hr1G077500	11.1118	11.3474	15.24892	13.82619	21.08071	19.21668
HORVU2Hr1G118420	2.789795	3.123776	4.17053	3.032174	6.005339	6.181818
HORVU0Hr1G029570	27.28485	21.37715	18.49708	1.011484	0	0.397197
HORVU6Hr1G060230	15.18885	14.01618	13.78413	16.09076	21.46871	21.03041
HORVU3Hr1G105820	7.674356	6.210769	7.894042	22.24221	182.8323	169.6207
HORVU2Hr1G022360	1.555419	1.613925	2.213936	4.001994	4.431651	5.22885
HORVU0Hr1G026630	113.3242	109.0364	153.3421	322.0893	696.0581	580.4879
HORVU7Hr1G059290	17.44516	18.00818	25.59679	24.65513	71.35683	52.92547
Hordeum_vulgare_newGene_8319	1.823	0.298782	1.529784	1.298012	1.61283	1.724726
Hordeum_vulgare_newGene_8312	4.17931	3.54303	3.696214	4.158555	5.206458	5.70942
Hordeum_vulgare_newGene_8310	0.718876	0.601005	1.416825	0.331252	2.112558	2.752549
HORVU2Hr1G105780	0.242269	0.070783	0.745261	0.973179	2.221918	1.894884
HORVU3Hr1G094600	13.0782	12.969	11.63267	5.123445	6.050566	6.825873
Hordeum_vulgare_newGene_12574	4.19278	2.632189	3.384319	3.216312	0.478774	1.303843
HORVU4Hr1G046730	2.750859	2.817045	3.2868	2.285857	2.377485	2.117144
Hordeum_vulgare_newGene_12579	18.83151	18.84298	20.86523	25.33202	47.20625	47.12343
HORVU7Hr1G028050	31.99684	28.19566	48.83548	22.15731	37.23722	38.11571
HORVU4Hr1G044460	9.958297	8.336531	11.23288	12.24066	9.972449	11.88088
Hordeum_vulgare_newGene_4922	0	0	0	3.356949	4.116377	5.475461
Hordeum_vulgare_newGene_4925	0.118973	0.415652	0.247206	0.818153	2.561195	2.606793
HORVU4Hr1G033020	2.312269	3.005553	2.800896	2.113004	3.086175	3.616552
HORVU7Hr1G001080	4.157927	4.401054	5.05498	4.583081	4.779992	5.24056
HORVU2Hr1G088050	4.216944	3.926098	4.755272	8.772901	14.42532	9.044837
HORVU0Hr1G039090	1.034014	0.943646	1.304309	3.749997	4.423782	4.673012
HORVU3Hr1G088160	2.324758	2.583212	5.064157	11.16629	36.45146	30.85885
HORVU1Hr1G021560	3.072699	3.362337	3.703807	3.756405	4.306599	4.559463
HORVU3Hr1G030340	23.13953	27.3119	33.47176	3.864279	0.991527	2.531611
Hordeum_vulgare_newGene_15962	0	0	0.012621	2.27535	2.454263	3.069613
Hordeum_vulgare_newGene_15960	0.759534	0.729361	1.002402	1.888782	1.162968	1.735146
Hordeum_vulgare_newGene_15966	4.616852	4.029302	4.116493	4.15162	3.866987	4.351687
HORVU5Hr1G060060	3.248889	2.941386	3.239796	4.930192	4.58377	5.215596
Hordeum_vulgare_newGene_15969	2.326828	2.220995	2.461824	2.383029	1.074502	1.809338
HORVU7Hr1G080740	9.195494	12.21416	12.75674	0.16042	0.096359	0
HORVU6Hr1G051240	4.113565	3.961358	5.793438	3.13136	2.289781	3.783253
HORVU7Hr1G100140	4.781114	3.508427	3.977402	7.269732	8.11847	9.548708
HORVU4Hr1G073590	1.273998	1.836888	1.983511	1.09737	1.594575	1.539176
HORVU5Hr1G036120	1.705724	1.341911	1.97853	2.104756	3.876184	3.945957
HORVU3Hr1G091920	2.07365	5.005428	6.076975	0.089572	0	0.05937
HORVU3Hr1G096510	18.11423	21.84119	19.68058	21.40585	20.89431	22.27116
Hordeum_vulgare_newGene_12609	1.29886	1.295667	1.440493	2.644489	2.330346	2.636847
HORVU3Hr1G085690	12.65576	8.161876	12.54738	12.72357	18.01587	17.63172
Hordeum_vulgare_newGene_12601	7.202905	6.216327	6.98377	0.025594	0.029345	0
HORVU6Hr1G022430	9.24312	11.91379	9.800336	9.968852	10.22502	12.46375
HORVU5Hr1G022260	9.685257	5.102211	13.04969	9.471869	11.46398	12.55772

HORVU5Hr1G019140	1.233219	1.48732	1.803169	1.964125	2.375643	2.879696
HORVU7Hr1G031140	0.536035	0.140502	0.608572	6.359948	5.083605	5.981067
HORVU3Hr1G090030	3.263822	2.861185	2.84561	3.3434	2.941763	3.139168
HORVU1Hr1G074490	2.156759	2.467087	2.974314	1.996611	1.528212	1.611603
HORVU6Hr1G087220	5.393354	4.67084	5.481879	13.77166	35.04583	27.47631
HORVU6Hr1G003440	9.723314	10.85295	12.66013	9.197036	10.86866	11.79463
HORVU5Hr1G084030	3.10316	2.856845	3.67431	2.907617	9.143872	7.848115
Hordeum_vulgare_newGene_9830	2.280006	2.266624	2.449878	4.714857	7.87848	7.013106
HORVU4Hr1G000100	10.76193	11.08845	11.18188	19.77116	21.5689	22.84121
Hordeum_vulgare_newGene_9834	27.9219	25.49547	41.53005	27.24364	46.57387	45.41142
Hordeum_vulgare_newGene_9836	5.334359	5.75629	5.630226	5.166467	6.13229	6.647417
HORVU2Hr1G006850	10.5799	6.515377	6.738151	2.095852	3.058854	4.863148
HORVU4Hr1G075990	6.362375	6.459156	7.320889	4.364159	5.214316	4.321236
HORVU5Hr1G065500	16.94882	14.76721	19.14228	15.877	18.61062	18.22733
HORVU2Hr1G117740	89.08649	79.54943	117.7718	61.48193	80.80326	76.55366
HORVU6Hr1G075210	3.3622	3.370948	4.651353	2.976602	4.214829	4.550049
Hordeum_vulgare_newGene_13222	0.98294	1.859549	1.027252	0.803965	2.698293	2.028324
Hordeum_vulgare_newGene_14769	0.025255	0	1.645951	2.805889	0	4.332013
HORVU1Hr1G012950	146.2592	145.8347	189.224	256.7798	665.5004	711.623
Hordeum_vulgare_newGene_14761	2.248544	1.560027	0.972574	2.94974	2.919779	3.240745
HORVU4Hr1G017000	0.867764	0.945921	0.878297	1.138544	2.110741	1.648613
HORVU4Hr1G000950	0.789738	0.61868	0.796824	1.394673	1.297887	1.604073
HORVU6Hr1G033090	1.22893	1.158599	2.055223	1.406699	1.651248	2.208576
HORVU7Hr1G116330	603.894	596.8306	779.293	598.3734	962.6951	656.0733
HORVU3Hr1G083330	1.418458	1.481053	1.687711	9.441381	3.487763	4.796284
HORVU7Hr1G054140	3.315115	2.898299	3.066873	4.115427	5.51247	5.273482
HORVU1Hr1G059990	4.08419	3.329133	4.428227	4.221293	4.828011	4.735239
HORVU1Hr1G081410	17.22555	16.09174	18.2723	4.829112	1.937864	2.676933
HORVU5Hr1G122430	44.94022	40.48217	52.3114	28.68636	32.01794	32.34861
HORVU5Hr1G125150	13.82834	12.02278	13.929	11.78449	14.12937	12.73589
HORVU1Hr1G055520	59.76662	50.84489	62.25983	45.5524	44.56376	47.64773
HORVU1Hr1G024860	3.724897	3.189933	3.835614	3.722907	3.529747	2.590175
HORVU7Hr1G081830	1.681299	1.573325	2.067028	2.03993	2.829402	3.54952
HORVU1Hr1G092040	2.868459	4.648399	5.103223	0	0	0
HORVU2Hr1G085030	26.55714	26.36146	28.15235	16.98135	20.62929	21.63034
HORVU3Hr1G019280	0.111655	0.173581	0.161177	3.275289	3.408678	0.176077
HORVU5Hr1G113060	1.173998	1.295441	1.700467	0.654195	1.094627	1.717581
HORVU4Hr1G089310	32.02268	33.63062	32.33822	32.93207	42.31922	41.44041
HORVU5Hr1G088830	9.376573	9.289392	11.44011	11.34478	15.63426	15.64443
HORVU1Hr1G023070	4.020327	3.96525	4.113016	9.175811	11.31568	11.33692
HORVU5Hr1G113010	141.2959	123.4859	145.3223	174.1747	232.1207	232.3538
HORVU5Hr1G063620	0.495152	0.887388	0.882003	4.584249	3.0958	4.774294
HORVU1Hr1G088270	4.344875	3.997023	35.42382	72.69338	2.306664	2.232447
HORVU6Hr1G035190	11.22215	11.35472	13.10714	25.16751	41.054	39.18243
HORVU4Hr1G059270	205.5931	177.1135	286.2883	53.41716	17.94495	31.53793
HORVU5Hr1G111510	15.86855	15.70766	26.34882	9.511445	16.9436	15.405
HORVU3Hr1G015700	13.02545	11.40937	14.95375	12.52953	10.86044	12.02322

HORVU6Hr1G045180	1.802531	2.446056	1.963198	0.309417	0	0.266511
HORVU1Hr1G048290	0.468914	0.429236	0.419306	3.411033	5.207466	5.041948
HORVU2Hr1G057400	2.902311	3.40301	2.741204	6.214774	4.90755	5.972382
HORVU6Hr1G069410	3.408693	2.743039	4.339172	2.314181	3.152491	3.120776
HORVU5Hr1G106610	166.9535	145.143	175.3829	33.3781	97.5494	88.86061
HORVU1Hr1G002400	0.420505	8.598687	0.876767	12.19055	0	0.041142
HORVU6Hr1G046980	3.489158	3.741985	4.718205	5.000485	5.610296	6.352368
HORVU5Hr1G050820	34.88822	33.18301	36.68875	29.74101	35.64297	34.90456
HORVU7Hr1G037090	0	0	0.004796	3.854099	3.751064	5.536282
HORVU2Hr1G098290	0.743131	0.450362	0.921119	3.124188	5.948263	3.709355
HORVU5Hr1G059690	10.5183	9.140195	9.684301	8.705036	11.61207	10.11399
HORVU7Hr1G114400	1.253315	1.718118	1.152035	5.256501	10.50473	10.70333
HORVU5Hr1G051440	14.27957	12.46194	16.97221	13.05382	12.64904	13.50575
HORVU2Hr1G126730	5.221363	4.473519	5.027178	2.201437	6.306798	6.159553
HORVU5Hr1G050760	20.09043	19.58285	19.68015	20.8562	23.64803	25.46266
HORVU0Hr1G017250	0.900924	1.669933	0.953995	0.936411	1.209612	2.246389
Hordeum_vulgare_newGene_2655	1.137802	1.311486	1.111664	2.03798	2.759056	2.941866
HORVU4Hr1G011120	0.981167	1.333761	2.155717	1.517887	1.948066	1.269847
HORVU2Hr1G085690	0.035587	0.038279	0.052203	1.96769	10.52825	12.99672
HORVU2Hr1G094730	3.25068	2.333704	2.138142	3.327381	1.783829	2.715507
HORVU1Hr1G087970	0.186034	0.221942	16.60779	44.46703	0.121666	0.37488
HORVU2Hr1G061380	2.68984	2.504042	3.503189	2.896921	4.050793	4.176196
HORVU2Hr1G093960	12.19107	7.325535	14.41286	20.67991	15.46973	15.45569
HORVU1Hr1G012460	0.81055	0.879101	1.515007	1.645187	2.599944	2.934694
HORVU5Hr1G067010	0.350523	0.434032	1.03011	2.161801	6.795093	4.591875
HORVU4Hr1G057140	20.60995	18.6679	16.98761	20.79568	16.44251	18.409
HORVU2Hr1G012980	18.22502	9.261768	19.32354	1.967141	0.532516	1.724562
HORVU4Hr1G088190	20.85087	23.169	25.25939	27.12006	25.57844	27.75868
HORVU7Hr1G047420	15.7112	16.58574	22.27314	12.09568	13.50573	14.13236
HORVU1Hr1G082330	0.872198	0.637534	1.201905	4.299813	3.461687	3.928956
HORVU2Hr1G085960	20.89283	17.63332	22.92509	24.23311	41.00943	39.23702
HORVU5Hr1G121310	31.03212	32.224	27.56772	24.21898	29.605	31.05929
HORVU1Hr1G093880	4.453986	4.996764	4.824507	9.722873	18.78176	15.0569
HORVU5Hr1G050310	3.546825	3.657967	3.55105	3.672137	7.619752	6.557297
HORVU3Hr1G047290	0.59383	0.503356	0.30009	1.080295	4.245996	4.061222
HORVU5Hr1G027730	13.93704	15.21129	13.60155	19.22506	34.93797	36.03524
HORVU4Hr1G019090	11.96875	13.8234	15.97306	19.06468	21.97652	25.90035
HORVU7Hr1G075700	240.8495	250.9471	314.805	250.5537	281.2212	199.2801
HORVU4Hr1G085480	5.414171	6.389074	6.822633	1.147689	0.079477	0.504951
HORVU2Hr1G016510	6.897543	6.985442	7.929773	8.053475	8.239525	7.702478
HORVU7Hr1G091780	0.128716	0.057354	0.011599	1.932344	2.526678	2.125009
HORVU5Hr1G057740	12.6434	9.800129	12.47004	2.761535	1.334553	1.972271
HORVU3Hr1G009210	18.56818	16.13277	16.49723	41.7018	58.29712	60.11118
HORVU5Hr1G044440	4.203921	5.718309	6.431589	7.237183	8.753127	8.937501
HORVU7Hr1G028250	11.12308	10.05995	14.07164	17.02577	11.59393	14.7816
HORVU4Hr1G064440	1.945383	1.23363	1.723998	1.254153	0.640433	0.91271
HORVU4Hr1G038570	4.864399	4.311102	6.427245	14.90187	27.84003	29.06834

HORVU7Hr1G088140	12.33657	10.38644	13.02778	12.36436	16.92216	14.25324
HORVU5Hr1G046350	9.405563	10.0253	10.55226	12.0149	11.17638	11.33857
HORVU0Hr1G001430	2.66471	1.386957	3.460438	4.507959	1.74106	4.190107
HORVU7Hr1G091250	2.048744	1.267584	1.528652	3.560101	4.280978	4.22978
HORVU2Hr1G078330	2.220517	2.496466	3.216839	3.37211	2.632572	3.088584
HORVU0Hr1G015780	1.048135	1.347364	1.167135	1.88988	1.942125	2.032524
HORVU4Hr1G055210	0	0	0	3.445267	2.554875	2.979222
HORVU4Hr1G050190	0.809394	0.924575	1.3485	1.397776	2.522645	2.27907
HORVU4Hr1G068820	14.7654	16.33454	19.4119	15.90352	20.29129	22.32959
HORVU4Hr1G078400	0.810996	0.871841	0.952215	2.731416	2.783105	1.685517
HORVU4Hr1G064190	1.237785	0.850971	1.050362	1.796606	1.976214	2.09971
HORVU5Hr1G121860	0.377033	0.562083	0.80556	3.928519	9.596476	7.147694
HORVU7Hr1G111120	14.68647	14.33325	16.12623	12.0657	20.94884	23.29422
HORVU5Hr1G045710	50.84382	47.03958	44.74177	23.64853	6.813024	7.991675
HORVU0Hr1G007170	0	0	0	5.180335	39.00599	32.46503
HORVU2Hr1G125110	0.67366	1.12692	1.599728	2.636309	0.067651	1.304358
HORVU5Hr1G120190	1.887195	1.728597	1.703874	1.098067	1.388077	0.814271
HORVU5Hr1G057320	1.047177	0.930212	0.90835	1.5197	4.561404	4.457771
HORVU5Hr1G113800	3.028521	2.705759	3.86709	3.213443	2.623219	1.743601
HORVU5Hr1G098300	2.901692	2.605876	3.18623	3.460019	2.809741	3.581721
Hordeum_vulgare_newGene_10450	2.428333	2.321705	2.647951	2.649657	3.705119	3.452791
Hordeum_vulgare_newGene_10457	5.664135	5.38437	6.32514	7.602172	6.297977	7.085447
HORVU4Hr1G007530	10.33776	11.3697	17.26025	2.983202	4.207413	4.472201
HORVU4Hr1G003890	19.71372	15.96387	19.68155	19.82902	17.42815	21.66807
HORVU1Hr1G000110	2.247042	2.988927	3.340599	3.822749	4.76166	4.57697
HORVU0Hr1G035560	1.063915	1.824007	1.122336	1.934037	1.399404	2.945728
HORVU2Hr1G023210	4.26684	4.056646	5.080487	3.776626	6.085764	5.732544
HORVU1Hr1G044120	2.114035	2.725094	3.457995	3.294872	5.363003	5.314287
HORVU3Hr1G062970	9.933034	10.06005	11.42574	8.616459	8.128606	8.870347
HORVU5Hr1G061150	2.686943	2.180589	3.165403	3.055459	4.242837	3.803902
HORVU2Hr1G106810	94.70242	84.26588	103.1544	95.245	149.9446	147.3567
HORVU7Hr1G082710	13.44709	11.33847	15.96593	11.03299	10.82068	13.17327
HORVU7Hr1G009370	2.156055	1.67305	2.242182	0.096183	0	0.015971
HORVU4Hr1G090220	0.845083	1.730879	1.71471	2.153466	2.851204	2.968099
HORVU5Hr1G095170	3.899777	3.119569	1.978696	4.25013	7.545497	5.498932
HORVU7Hr1G116470	0.291732	0.427164	0.438259	0.825742	2.961333	3.053845
HORVU5Hr1G053950	9.074665	10.2745	11.6262	14.23341	25.25708	25.51672
HORVU7Hr1G012810	4.708426	4.148597	3.567028	2.934415	3.91598	3.7717
HORVU2Hr1G041270	15.18423	13.9336	12.03085	47.55741	63.90507	65.21267
HORVU5Hr1G093460	2.306299	2.461386	2.534838	41.44177	26.33484	29.48876
HORVU5Hr1G123420	6.396752	5.753598	6.091779	3.916515	4.981727	5.18357
HORVU4Hr1G060630	4.156276	3.966678	3.72806	18.35744	31.82751	30.11321
HORVU3Hr1G003940	22.09537	16.13622	16.16139	14.84935	16.21325	15.03046
HORVU5Hr1G116970	2.357656	3.202433	3.702752	1.844432	1.993256	1.888543
HORVU6Hr1G041610	0.316273	0.441949	1.1623	5.358902	7.578824	8.487413
HORVU4Hr1G067360	1.500755	1.212508	1.182916	1.412724	1.628025	2.255026
HORVU3Hr1G115000	4.587441	3.383464	5.341561	6.699653	4.755147	4.647737

HORVU6Hr1G047760	5.448678	5.606597	7.74783	7.433634	7.181192	8.05803
HORVU2Hr1G022160	3.268577	4.359008	5.805179	1.985288	1.545966	1.568875
HORVU4Hr1G085100	2.781767	2.837639	2.808466	9.056274	8.641715	9.369641
Hordeum_vulgare_newGene_11494	0.365837	0.408978	0.144642	2.063244	2.374823	2.446377
Hordeum_vulgare_newGene_11492	0.088437	0.010911	0.080749	5.478905	5.375399	6.884133
Hordeum_vulgare_newGene_11499	5.968517	9.271749	9.18867	9.774206	10.22521	10.86482
Hordeum_vulgare_newGene_122	15.94118	13.14757	16.2028	16.48919	9.392117	12.07913
HORVU3Hr1G094440	1.500678	1.024571	1.368899	0.979217	1.800342	1.904088
HORVU5Hr1G099490	5.816284	6.204316	5.968424	3.680738	4.472663	4.722779
Hordeum_vulgare_newGene_128	3.064337	1.928436	2.548217	0.341331	1.032126	0.846165
HORVU6Hr1G025840	3.518687	3.781492	6.530531	2.485093	4.209803	4.395911
HORVU4Hr1G056730	13.01814	10.00492	17.05032	12.90331	20.36614	22.90078
HORVU7Hr1G051250	19.63924	18.61997	24.76137	11.7173	16.20868	13.89686
HORVU1Hr1G055210	17.84208	14.21786	16.40619	15.52254	13.86569	12.51054
HORVU5Hr1G079680	1.776546	1.992681	2.045867	1.381299	5.725204	4.207068
HORVU6Hr1G048390	16.01307	16.57409	22.76315	18.46358	30.62295	30.0839
HORVU3Hr1G023460	1.875486	1.270471	2.437798	3.487934	12.84753	12.90792
HORVU1Hr1G015940	2.773444	3.096924	2.98123	4.734101	4.647213	6.730266
HORVU3Hr1G004360	8.648237	7.904671	9.123829	8.121084	9.532084	11.11159
HORVU2Hr1G013480	1.728684	1.748992	2.363934	2.909022	3.328418	3.678679
HORVU5Hr1G040460	12.48286	10.06514	12.12639	16.62896	22.67283	22.27804
HORVU7Hr1G028110	0.84616	1.050913	0.979203	0.906099	1.06303	1.255565
HORVU0Hr1G028930	1.248886	2.442436	1.257856	5.927837	8.349526	4.909945
HORVU2Hr1G062850	8.601773	9.299299	10.79244	10.95226	12.89724	12.9997
HORVU0Hr1G025870	3.523785	3.38411	4.649015	1.717835	2.717437	2.724207
HORVU7Hr1G074820	1.797454	1.646974	1.957668	1.959478	2.659789	2.778598
HORVU1Hr1G085120	6.399107	6.230329	4.598781	7.52044	13.35409	8.44051
HORVU6Hr1G067640	6.069116	6.471283	9.006553	6.832174	5.867743	5.989972
Hordeum_vulgare_newGene_16149	0	0	0	4.133548	4.344625	5.272391
HORVU6Hr1G088200	10.71483	13.70193	14.64801	12.69764	12.85097	14.31298
HORVU7Hr1G003710	24.34567	20.11667	25.92855	23.40551	28.05582	27.22248
HORVU3Hr1G050920	27.20781	27.51918	27.04148	28.66122	31.4628	33.79602
HORVU1Hr1G071280	1.238767	1.42443	1.317633	2.397278	1.394879	1.653092
HORVU2Hr1G018700	83.43936	85.49279	111.1631	83.68501	131.7634	127.3267
HORVU2Hr1G114980	9.705929	8.252166	9.70402	19.88098	33.82798	30.37594
HORVU5Hr1G071270	6.363191	6.632554	5.605351	5.478468	2.113875	3.200791
HORVU2Hr1G021530	2.431347	2.22519	3.305034	7.877971	9.348372	9.906116
HORVU6Hr1G091130	247.3728	201.5944	254.0174	152.8954	149.5058	171.5547
HORVU6Hr1G065970	0.885206	1.07934	0.981179	1.348229	1.876956	1.826457
Hordeum_vulgare_newGene_5356	1.195455	2.845807	1.124697	1.487667	0.805709	0.500694
HORVU5Hr1G068590	2.706889	2.847568	5.810982	2.572133	3.211692	3.70333
HORVU1Hr1G082470	2.889001	2.62498	2.433587	4.506452	2.996704	2.902109
HORVU5Hr1G121450	0.154806	0.1105	0	2.859289	3.36181	3.827552
HORVU4Hr1G054600	2.93231	2.679395	2.238642	3.636956	2.606795	2.656554
HORVU3Hr1G027460	0.215889	0.37945	0.420428	2.671287	15.58499	12.24547
Hordeum_vulgare_newGene_14164	16.96264	19.54424	13.87346	14.40154	15.14011	14.35588
Hordeum_vulgare_newGene_14166	0.985298	0.980423	0.926052	3.765687	5.587665	4.722621

Hordeum_vulgare_newGene_14162	49.22272	58.63968	45.92878	79.79893	74.71724	78.14825
Hordeum_vulgare_newGene_13397	8.154719	8.884045	9.315325	13.77092	14.20306	15.13627
Hordeum_vulgare_newGene_13394	0.175048	3.227595	1.998727	1.965102	1.271946	2.205994
HORVU2Hr1G086120	2.729787	2.668964	3.381788	3.036491	2.602695	2.420108
HORVU2Hr1G100500	7.609201	6.996571	7.658433	0.551315	0.086265	0.259803
HORVU1Hr1G044820	9.845101	11.21186	10.47568	9.823511	14.42349	13.4577
Hordeum_vulgare_newGene_3773	5.440585	5.28794	6.278745	32.74094	27.06485	31.58293
HORVU7Hr1G027270	3.686373	3.666303	4.514581	4.138091	4.696578	5.45577
HORVU5Hr1G065740	5.687105	4.466845	5.091091	3.640449	5.287573	4.817216
Hordeum_vulgare_newGene_10841	3.085692	2.459992	3.60363	1.40728	1.516103	1.538413
HORVU6Hr1G018620	35.83661	44.78358	76.11685	28.95238	0.281562	11.67679
HORVU3Hr1G045900	1.149879	1.111103	1.860774	2.452893	3.427884	3.843812
HORVU7Hr1G008760	7.381919	5.806978	8.787087	7.586492	7.831544	6.910395
Hordeum_vulgare_newGene_466	3.696774	3.291668	3.555774	2.884662	3.431727	3.094691
HORVU2Hr1G076660	12.19538	14.54879	14.45046	11.69686	13.14406	13.82521
HORVU5Hr1G032370	1.107416	1.484903	2.332512	3.662459	6.059603	5.59381
Hordeum_vulgare_newGene_2799	3.458944	3.134687	3.885639	3.486864	4.179227	4.130966
Hordeum_vulgare_newGene_2792	0.624305	0.340893	0.680753	0.715901	2.699146	1.743109
HORVU6Hr1G072050	1.688186	1.897775	2.482814	4.784687	4.947212	6.060142
HORVU4Hr1G006120	17.79015	12.6624	24.24776	22.70846	5.276897	4.518003
HORVU1Hr1G007480	0	0	0.183624	2.527162	7.556801	7.353508
HORVU3Hr1G054330	1.53952	1.823186	2.086796	1.766507	2.944913	3.484379
HORVU4Hr1G074840	2.887105	2.44275	2.095011	5.343581	8.706739	8.874782
Hordeum_vulgare_newGene_2422	3.397055	3.760042	4.724262	1.888754	1.90731	2.05793
Hordeum_vulgare_newGene_2424	3.407473	3.059399	3.436588	4.035323	5.548458	6.175508
Hordeum_vulgare_newGene_2425	2.691038	2.260287	2.773558	2.637436	2.180217	2.567961
HORVU6Hr1G059810	8.978029	9.733777	10.0034	13.19157	18.94286	18.54316
HORVU1Hr1G000650	7.425556	5.248161	4.805656	2.149461	0.41353	0.883579
HORVU7Hr1G096140	1.594998	1.648301	1.693546	2.435307	3.932437	3.66638
HORVU6Hr1G061420	8.612388	4.151706	8.798128	4.571301	8.712152	6.82905
HORVU2Hr1G037550	7.892996	7.359158	7.191459	8.639786	12.86799	13.39801
HORVU1Hr1G054630	17.28545	13.77399	19.59206	21.5258	35.2109	32.54291
HORVU5Hr1G024880	2.311074	2.160356	3.123877	1.835813	2.291961	2.253962
Hordeum_vulgare_newGene_1218	1.071774	1.134354	1.332294	2.785016	4.974284	5.4666
Hordeum_vulgare_newGene_1219	0.791777	0.745452	1.060051	2.033731	2.896176	2.889308
HORVU6Hr1G021480	11.22989	12.41521	14.04893	19.92262	11.96243	13.71638
Hordeum_vulgare_newGene_1217	1.294324	1.166412	1.152764	2.718906	4.110003	4.407623
HORVU2Hr1G031890	35.17162	31.86459	46.29946	39.59039	49.94158	44.75402
HORVU2Hr1G078860	15.44882	13.51744	17.80785	10.95362	14.84699	14.79551
HORVU3Hr1G058340	1.861307	2.075875	1.7225	2.400937	1.995925	2.749615
HORVU2Hr1G071670	1.677088	1.581136	2.308049	1.103773	0.941066	1.198887
Hordeum_vulgare_newGene_11828	0.861947	0.999251	0.914093	1.106489	1.748782	1.826498
Hordeum_vulgare_newGene_11825	2.403713	2.2091	2.649583	2.376166	11.54549	10.42464
Hordeum_vulgare_newGene_11824	0	0.073102	0.055467	1.782525	2.500645	2.527354
Hordeum_vulgare_newGene_11823	5.870272	6.389031	7.482032	7.045172	10.18557	10.94983
Hordeum_vulgare_newGene_11820	0.354635	0.438257	0.242609	2.609786	3.22491	2.965501
HORVU7Hr1G108890	6.927083	6.160355	6.786616	4.761355	3.108364	4.197257

Hordeum_vulgare_newGene_9768	7.650575	7.889077	9.679262	6.785735	10.64152	10.56633
Hordeum_vulgare_newGene_9766	26.64974	28.04683	26.02901	30.58774	18.62329	20.81181
HORVU7Hr1G073050	22.29317	19.62937	27.13825	9.519639	6.631133	8.665853
HORVU7Hr1G086340	15.19556	11.08903	13.55173	7.216372	6.526999	6.106564
HORVU0Hr1G025460	8.734565	6.854135	8.852989	4.491255	2.747482	4.262747
HORVU6Hr1G075800	9.454506	9.216465	10.45068	12.54913	13.53812	14.9578
HORVU2Hr1G073740	3.860489	3.415619	3.358688	3.103455	2.049582	2.888149
HORVU3Hr1G095450	12.90246	12.15003	11.75096	16.8404	25.92872	27.55215
HORVU6Hr1G073090	0.380681	0.490467	0.217387	22.2095	5.137409	8.137602
HORVU5Hr1G089980	10.82377	10.52927	14.10594	9.688204	11.41482	12.75354
HORVU4Hr1G047340	27.02504	22.80545	35.88125	26.55588	31.07919	30.4478
HORVU1Hr1G008330	22.38214	20.58866	20.43626	22.38612	29.93653	28.28182
HORVU6Hr1G063860	3.507294	4.137901	4.538908	7.529762	15.76852	13.85848
HORVU4Hr1G075710	179.157	205.6464	266.6713	159.0865	338.7231	311.8167
HORVU7Hr1G039080	1.363458	1.320288	1.241657	4.503548	4.981797	4.037935
HORVU1Hr1G072410	2.324558	2.185725	3.125287	1.537138	1.528039	1.831834
HORVU6Hr1G087800	5.572284	6.325198	6.132769	5.09345	4.678051	5.581348
HORVU6Hr1G031610	1.629468	1.587357	1.757537	1.97928	2.445927	2.868703
HORVU2Hr1G105060	2.725091	2.607142	3.637212	6.691933	6.652092	5.200614
HORVU4Hr1G088350	0.67971	0.944155	0.949212	0.997705	3.805717	4.432167
HORVU4Hr1G047690	12.59625	13.13886	16.7574	12.45623	14.99606	15.11135
HORVU7Hr1G101710	1.444332	1.058261	1.043061	3.542386	6.06302	5.809919
Hordeum_vulgare_newGene_4061	0.72403	0.676479	0.862022	1.04443	1.85253	1.899039
Hordeum_vulgare_newGene_4064	1.870573	1.470236	1.256545	1.864433	2.908846	1.991536
HORVU4Hr1G041690	22.69136	23.67059	24.28039	34.64977	51.87161	55.66208
HORVU2Hr1G059940	5.349999	4.180531	5.727411	3.162118	2.35535	2.843035
HORVU0Hr1G005640	0	0.024535	0	2.778842	3.028316	3.686233
HORVU7Hr1G063720	0.827451	0.795777	1.028259	1.593298	2.254429	2.311625
HORVU1Hr1G068170	33.54923	35.51252	50.32777	46.23237	80.72641	73.56129
HORVU1Hr1G064490	47.36762	44.77939	54.94695	58.14181	61.31926	64.85031
HORVU0Hr1G039110	2.828454	2.677047	3.307291	3.09514	3.60286	3.921454
Hordeum_vulgare_newGene_14950	9.38504	5.903055	7.401814	6.151219	9.903098	10.08123
HORVU2Hr1G075370	17.85332	12.8875	22.31124	5.237816	1.678669	2.878786
Hordeum_vulgare_newGene_4684	6.099428	7.940695	7.481353	0.012799	0	0
Hordeum_vulgare_newGene_4682	19.02844	17.97613	23.55302	23.35538	29.67255	29.60472
HORVU3Hr1G070080	2.155007	1.978063	2.83859	4.487812	7.912077	7.451846
HORVU6Hr1G074040	4.455924	4.317637	6.230286	5.588738	7.589101	8.52357
HORVU3Hr1G000830	5.966752	7.16305	4.713036	3.279852	2.661837	2.624222
HORVU7Hr1G099260	1.196487	0.953274	1.872297	0.646836	0.612481	0.749776
HORVU1Hr1G089280	15.44138	16.44826	17.04625	61.44162	16.89952	17.94187
HORVU7Hr1G052870	3.362563	2.164593	2.604514	1.571975	5.411968	4.780454
HORVU5Hr1G088210	4.643641	4.486215	5.673242	2.598098	2.451224	2.499273
HORVU1Hr1G023650	2.843184	1.882515	1.796235	1.237448	3.151494	2.923906
HORVU2Hr1G045150	2.920202	3.465963	3.352053	2.524164	2.336765	3.050337
HORVU5Hr1G058000	1.399267	1.041754	1.975463	3.803933	2.866088	3.067016
Hordeum_vulgare_newGene_6688	4.454038	0.811037	6.14634	5.081479	5.82045	6.141774
HORVU7Hr1G093300	7.165491	7.193545	6.751072	7.688945	5.762545	6.952956

Hordeum_vulgare_newGene_6681	2.30837	4.490129	2.966487	0.066892	0.019358	0.018203
Hordeum_vulgare_newGene_7087	4.92653	5.545533	7.373657	8.940234	10.2765	10.91109
Hordeum_vulgare_newGene_7084	7.520049	8.616328	9.904638	8.006663	10.89265	11.37319
Hordeum_vulgare_newGene_7083	1.079079	1.281183	0.906175	1.805684	2.832794	2.12604
HORVU6Hr1G059040	27.20273	25.38491	29.10174	21.48671	22.34837	20.93274
HORVU2Hr1G006330	22.73084	20.46599	30.90936	18.34418	19.60886	22.24171
HORVU3Hr1G085710	24.26062	17.14193	27.96718	23.27634	24.19377	20.17709
HORVU7Hr1G018320	9.29792	5.326301	8.722215	8.031452	8.816442	8.462641
HORVU4Hr1G081290	0.679217	0.672739	0.345374	2.251433	11.37011	9.504035
Hordeum_vulgare_newGene_6357	2.309275	1.74067	2.734971	2.445738	2.635937	2.589453
HORVU6Hr1G029290	15.71081	19.62576	16.03537	15.9134	16.81335	19.07066
Hordeum_vulgare_newGene_6359	6.979015	1.547314	7.361582	8.146686	2.165974	1.938625
HORVU1Hr1G087690	26.45103	40.38845	72.03801	20.71249	44.80073	78.3548
HORVU3Hr1G090170	5.449634	4.377031	5.455559	4.655987	1.527567	2.202964
HORVU3Hr1G041900	2.350955	2.897024	3.2674	3.754151	3.862617	3.943027
HORVU0Hr1G035760	0.700134	0.68549	0.72596	1.472073	2.002115	1.89558
HORVU5Hr1G103040	2.50775	2.29936	3.140907	3.282288	2.904623	3.317861
HORVU4Hr1G039130	1.157318	1.612462	1.348204	1.87662	2.581758	2.262177
HORVU2Hr1G076590	6.397612	7.657936	6.178806	3.240188	2.921265	3.712726
HORVU3Hr1G032930	3.484341	3.860497	4.351721	5.780972	5.493873	6.520782
Hordeum_vulgare_newGene_561	0.067162	0.13034	0.129443	1.349952	2.432666	3.562793
Hordeum_vulgare_newGene_10920	22.44114	22.78319	24.15118	33.27752	49.42959	43.03543
HORVU1Hr1G080640	3.487365	2.267649	3.352488	8.967485	19.35334	15.51208
HORVU3Hr1G007200	2.886975	2.963442	3.370939	5.159063	6.0817	6.15721
HORVU6Hr1G067930	1.541266	1.917681	2.821192	4.941318	17.86986	19.60797
Hordeum_vulgare_newGene_9527	5.310322	7.356162	7.831158	6.917587	5.987002	9.064943
HORVU5Hr1G009580	20.23737	18.28273	17.38692	14.09671	10.28892	10.41524
HORVU6Hr1G004930	0.563191	0.854248	1.281355	1.592083	1.370071	1.660674
HORVU1Hr1G062950	3.360942	3.142768	3.665138	3.150768	1.419854	2.146925
HORVU6Hr1G038750	61.51088	70.9923	62.03906	84.32889	85.72052	97.72944
HORVU4Hr1G065180	0.678005	0.963408	0.702481	1.763191	3.891927	3.976332
HORVU2Hr1G056650	1.28415	1.119252	1.675861	3.117926	3.43688	3.500738
HORVU5Hr1G022760	4.074007	4.362901	3.861961	6.576772	8.271948	9.320208
HORVU1Hr1G087340	30.43457	31.9024	29.66444	23.5243	21.78336	22.78075
HORVU3Hr1G013380	0.452615	0.983248	0.449199	3.326355	1.284748	3.085368
HORVU5Hr1G094360	2.20093	2.556526	2.697382	1.807665	2.983411	2.950486
HORVU5Hr1G029820	5.789329	1.121992	1.288511	6.662121	7.695918	9.468592
HORVU6Hr1G030930	4.742039	3.764509	4.938094	0.106132	1.444584	4.305835
HORVU1Hr1G087520	1.553217	1.97363	2.218564	2.278982	3.664809	3.586371
HORVU4Hr1G017210	0.916833	0.504814	0.676179	1.920152	3.069812	3.20628
HORVU1Hr1G018500	11.51806	9.816234	9.996281	12.4185	11.01253	11.22749
HORVU1Hr1G020530	76.41394	58.37796	83.47026	131.9177	155.3545	137.5235
HORVU3Hr1G016350	9.344824	9.477102	9.454064	7.130351	6.765546	7.5871
HORVU6Hr1G084590	18.40072	17.36469	22.27424	18.37588	18.29677	20.28721
HORVU4Hr1G089290	11.78375	12.66718	11.04845	9.764812	9.231825	10.7242
HORVU1Hr1G061200	5.14358	3.113033	6.283899	2.540324	3.388285	3.282052
HORVU3Hr1G013650	9.888656	9.150162	9.165904	10.28984	5.260659	6.262282

HORVU7Hr1G010250	62.93751	48.25372	53.62657	41.4458	38.39555	41.42524
HORVU2Hr1G047700	2.537495	2.572124	2.837732	3.902691	4.473972	4.818268
HORVU0Hr1G038980	0.606351	1.113857	1.761521	2.460234	2.688999	2.896073
HORVU4Hr1G050840	12.87003	13.33974	13.8825	14.32093	18.72167	19.14219
HORVU5Hr1G106790	0.906896	0.628124	0.750188	1.210078	1.473793	1.656096
Hordeum_vulgare_newGene_12339	4.527959	3.719333	6.287982	9.088396	8.037076	8.596883
HORVU5Hr1G113630	49.97283	49.31422	57.71561	44.38139	81.15051	73.39204
HORVU5Hr1G069090	0.424605	1.748825	3.530619	2.364538	5.436192	0.174685
HORVU1Hr1G005760	3.791612	1.076573	1.286289	0.445249	0.09934	0.100037
HORVU7Hr1G051720	0.64369	0.900977	1.781272	3.091594	6.744376	4.620306
HORVU4Hr1G052720	10.02339	9.914453	10.70342	10.80501	17.33734	16.71664
HORVU5Hr1G029310	2.173064	1.574568	2.278098	1.372805	1.886623	1.941843
HORVU4Hr1G056050	81.70099	84.79095	92.2897	36.75564	28.15722	35.3837
HORVU1Hr1G048480	2.818873	2.57625	3.297642	2.153631	2.94446	3.205813
HORVU2Hr1G057210	6.724973	7.118826	10.64506	9.501965	22.97074	20.9802
HORVU5Hr1G034570	9.380532	12.334	12.30234	9.814732	12.8621	13.19809
HORVU4Hr1G089120	2.635293	2.536615	2.45961	2.34513	6.242359	4.936968
HORVU2Hr1G082440	171.8784	200.4052	148.3026	169.9922	229.0346	165.6343
HORVU1Hr1G090870	6.980782	4.708902	7.70608	1.670933	0.369053	0.799801
HORVU2Hr1G104280	6.804087	8.6164	8.6155	7.192863	11.73162	11.43705
HORVU5Hr1G051760	18.21676	15.0542	14.87408	7.86386	4.607316	3.140066
HORVU5Hr1G111700	62.21108	59.77735	63.92347	38.97839	37.93531	38.56674
HORVU2Hr1G084130	56.77317	48.32355	55.15675	5.779571	1.780969	3.358431
HORVU2Hr1G069910	1.020032	1.250088	1.223524	1.276615	1.082054	1.549333
HORVU4Hr1G062110	3.51153	2.675769	2.928269	1.479514	0.96531	1.311948
HORVU5Hr1G054190	2.894006	2.21208	3.630016	2.073152	1.89499	1.995513
HORVU5Hr1G005340	18.37872	15.68124	17.71392	2.252869	1.579187	1.672262
HORVU2Hr1G127360	0.597207	0.706599	0.75723	1.717749	3.589456	3.552962
HORVU1Hr1G085450	0	0	0	0	11.63623	14.04551
HORVU1Hr1G049280	45.50137	23.10556	60.67422	17.05879	21.46368	23.08357
HORVU1Hr1G090720	6.083	6.162772	6.129793	5.302764	9.0184	6.875045
HORVU3Hr1G072810	3.389618	2.096106	2.824303	6.324983	2.232904	2.372229
HORVU6Hr1G091330	7.228442	5.491418	6.392997	3.651473	4.253974	6.179787
HORVU5Hr1G093980	15.1476	15.02097	16.69138	17.4957	16.26415	13.92265
HORVU1Hr1G059490	1.175164	1.336273	1.313293	0.980612	1.306064	1.059938
Hordeum_vulgare_newGene_12930	7.357162	7.451324	4.354053	3.981363	2.928557	3.732672
Hordeum_vulgare_newGene_12933	0.264214	1.543835	2.881279	0.708262	0.678521	0.98208
Hordeum_vulgare_newGene_12935	4.74956	4.519859	5.32618	8.838461	18.3922	17.6302
HORVU7Hr1G082930	139.1355	115.9171	139.2113	106.8932	165.7005	149.1726
HORVU2Hr1G061260	0.365311	0.069215	0.308858	1.483206	4.379341	4.667871
HORVU5Hr1G114910	10.35565	9.909191	12.47457	15.2143	23.04744	21.63241
HORVU1Hr1G043870	42.17905	37.11541	35.25533	26.36843	24.16149	21.46364
HORVU6Hr1G092820	13.98876	13.59655	15.91384	12.86413	17.12485	16.133
HORVU6Hr1G011020	1.294823	1.123818	1.85875	1.475406	3.455368	3.641462
HORVU3Hr1G089630	6.963923	5.586614	9.823071	6.36326	9.987456	10.86293
HORVU1Hr1G046990	11.33789	11.54585	13.4297	14.92901	15.73432	15.46499
HORVU7Hr1G094110	2.493056	2.197628	2.636103	2.46664	1.627291	2.146073

HORVU2Hr1G016720	2.16515	2.254012	2.739884	0.742721	0.210683	0.432056
HORVU2Hr1G100740	14.1101	16.55724	21.49349	11.31699	10.17699	10.61318
HORVU7Hr1G110190	0.309781	0.124396	0.208791	0.744271	2.877653	1.909489
HORVU2Hr1G070660	31.55348	27.45708	37.28634	27.23564	21.94732	22.60633
HORVU1Hr1G057330	14.37922	14.11197	15.99205	37.89802	56.732	52.96902
HORVU1Hr1G082100	35.37668	37.81199	48.97111	13.37211	4.98165	5.8882
HORVU6Hr1G018420	73.18317	70.39134	77.64963	88.4993	176.9365	147.5572
HORVU0Hr1G006750	0.631744	0.560771	0.947168	1.814567	3.796125	2.830323
HORVU4Hr1G064870	1.061714	1.292855	1.476166	1.49052	1.620059	2.139572
HORVU5Hr1G121120	1.726777	1.912591	2.165882	0.651132	0.843117	0.897242
HORVU5Hr1G054730	13.35327	14.05674	15.23341	16.61057	23.06914	21.27057
HORVU2Hr1G061190	7.045889	6.500958	8.679773	10.50317	12.16056	13.71051
HORVU5Hr1G046250	11.95904	13.10258	10.19747	26.97075	35.61077	35.68429
HORVU7Hr1G019510	0.898416	0.437437	0.699585	1.312303	1.941241	2.178892
HORVU6Hr1G012640	0.585011	0.481167	0.911717	9.335859	6.114157	3.975058
HORVU2Hr1G100290	19.49138	19.05684	19.3671	17.85278	24.66383	21.31407
HORVU7Hr1G110440	5.0099	4.280889	3.910547	3.954143	3.514403	3.168071
HORVU2Hr1G078270	19.11224	14.63632	19.07858	13.72101	5.420184	6.587522
HORVU2Hr1G010890	47.42448	46.51552	67.23908	59.46779	120.4161	110.5936
HORVU3Hr1G079290	5.154313	5.546613	7.049706	3.560101	6.954449	9.516022
HORVU3Hr1G020640	2.309482	1.825727	2.780689	3.468041	3.90137	1.574759
HORVU2Hr1G107110	8.787086	8.324414	11.20255	7.386447	10.95672	12.15977
HORVU1Hr1G015090	1.809146	1.293248	2.092862	0.934215	0.267741	0.852635
HORVU4Hr1G076440	6.693228	5.196453	7.305529	4.919367	5.63039	5.796059
HORVU3Hr1G100500	0	0	0	0.0854	7.146092	6.226826
HORVU7Hr1G006740	0.232253	0.247788	0.951112	2.95558	40.27454	43.78703
HORVU0Hr1G021950	8.766054	7.54298	9.562553	13.38976	18.42224	15.17957
HORVU3Hr1G114250	9.187945	6.142218	12.46534	6.15879	4.895794	6.035876
HORVU1Hr1G044590	12.64659	13.98997	13.94289	12.0011	14.2574	14.75166
Hordeum_vulgare_newGene_5536	7.54349	8.744224	9.907874	7.949551	12.29403	8.927549
HORVU1Hr1G007310	24.41371	22.22982	25.38021	28.58517	31.4045	34.21621
HORVU3Hr1G099760	9.667154	5.528846	10.85595	2.186818	0.116564	0.878427
HORVU7Hr1G026020	10.54425	12.14035	13.79697	7.742463	8.792345	8.802246
HORVU4Hr1G084890	4.387815	6.025123	5.747931	5.170602	7.366835	7.982395
HORVU2Hr1G121030	51.91725	51.59591	64.82154	42.26626	70.79958	60.48336
HORVU1Hr1G047640	2.024941	2.113829	1.780365	3.412736	3.822417	4.040946
HORVU2Hr1G109370	26.37705	26.64845	33.54254	34.83288	50.41431	60.90088
HORVU1Hr1G045640	42.67132	36.28052	40.69542	33.28771	35.51567	33.42658
HORVU6Hr1G094780	0	0	0	6.954587	7.354752	7.945086
HORVU4Hr1G076600	16.21246	15.46092	16.41445	2.507391	4.918909	3.094295
HORVU3Hr1G096340	3.673477	3.980358	2.943787	2.810534	3.862761	4.65844
HORVU7Hr1G002080	0.836547	0.596438	1.035047	1.351824	2.286002	2.45973
Hordeum_vulgare_newGene_10393	17.68207	18.89972	17.45555	0.02502	0	0.006849
HORVU2Hr1G092100	2.033008	2.45458	2.427599	2.291088	5.919744	5.76219
HORVU3Hr1G079270	35.07691	31.27024	40.79438	37.35612	42.54333	41.013
HORVU2Hr1G104530	9.508929	10.32758	10.22953	8.341933	9.445696	9.138305
HORVU3Hr1G109300	6.707756	6.262743	6.989339	3.513577	3.499383	3.204054

HORVU7Hr1G086580	2.431958	2.53815	3.229203	1.538611	0.872821	1.487329
Hordeum_vulgare_newGene_10641	9.57465	10.71347	8.721474	11.75836	11.39425	10.4956
Hordeum_vulgare_newGene_10642	3.51553	4.219065	5.940531	2.757277	3.723438	4.194849
Hordeum_vulgare_newGene_10645	21.43457	28.38255	33.98738	0	0	0
HORVU3Hr1G072380	0.10496	0.134851	0.18305	0.795797	10.28353	9.122038
HORVU6Hr1G076620	8.678789	6.880333	11.07217	3.410533	3.303558	2.739553
HORVU1Hr1G060610	1.191546	0.898423	0.865164	4.457825	8.121756	7.422706
HORVU3Hr1G058100	16.98845	13.85722	15.70684	20.3518	19.23609	18.86465
HORVU3Hr1G078650	33.82587	31.22285	32.34396	34.93037	34.34162	39.7537
HORVU5Hr1G052100	62.92459	61.95888	64.68977	70.38388	88.01503	91.28247
HORVU7Hr1G027770	0.643836	0.714625	0.935157	2.558458	2.700956	2.598391
HORVU5Hr1G081790	8.384225	9.024911	9.108076	12.55984	15.87985	15.71427
HORVU2Hr1G115620	5.795477	5.473406	6.459844	4.966753	4.071821	4.529238
HORVU2Hr1G025800	8.866634	7.645041	9.041117	9.774233	1.576986	4.846669
HORVU7Hr1G038820	12.87924	12.82537	15.47606	13.26677	16.07174	14.36089
HORVU4Hr1G063980	10.92827	12.61173	14.54374	6.650149	5.370041	5.816623
HORVU5Hr1G006930	0	0.148854	0	3.221382	4.195706	4.63942
HORVU2Hr1G073230	1.242113	1.436048	2.027176	1.42095	1.774991	2.218501
HORVU1Hr1G028500	3.016421	3.295213	3.043691	2.663284	3.51083	3.373783
HORVU1Hr1G004150	12.09103	17.51134	27.9215	4.783138	0.579856	2.219794
HORVU5Hr1G081420	11.59212	13.45225	14.96217	9.44517	10.30005	10.09777
HORVU3Hr1G094870	8.554361	10.46025	9.882785	9.794694	10.10447	13.39789
HORVU5Hr1G092560	24.23208	19.36571	24.77725	7.633933	9.972465	10.54671
Hordeum_vulgare_newGene_1945	4.233146	5.896848	5.470609	6.389436	6.07573	6.750544
Hordeum_vulgare_newGene_1942	3.546599	5.12283	3.884237	0.028038	0	0
HORVU2Hr1G011960	9.101592	8.76455	10.00875	4.919177	4.74061	4.872779
HORVU1Hr1G084190	21.79492	28.8101	30.20784	3.511348	1.152782	2.010276
HORVU4Hr1G066800	7.180575	5.711838	8.05522	19.16365	10.83199	15.77855
Hordeum_vulgare_newGene_11519	8.17757	7.848633	7.465267	8.743458	7.174359	7.786482
HORVU2Hr1G042700	32.53238	29.23219	31.3331	35.64839	46.03611	47.99932
HORVU4Hr1G079230	111.1668	122.6262	160.9818	187.5557	469.5037	396.7577
HORVU6Hr1G077280	2.709557	2.896583	3.463058	4.358711	7.410234	8.057339
HORVU3Hr1G094250	11.96267	6.805654	15.15419	15.96609	11.29946	12.77451
HORVU3Hr1G086050	97.26179	100.1402	159.8584	79.30716	153.9173	137.5496
HORVU3Hr1G088350	42.8448	37.89575	39.76212	30.14766	24.55777	26.21011
HORVU3Hr1G058810	0.305476	0.141765	0.28817	2.409136	4.612728	3.430073
HORVU2Hr1G014410	3.465504	3.290917	3.02928	0.179184	0.031989	0
HORVU7Hr1G072240	18.60203	20.44418	22.72552	12.90699	13.36989	13.97428
HORVU2Hr1G028340	9.506133	9.016177	10.90502	6.152758	5.034676	5.430261
Hordeum_vulgare_newGene_7241	2.966601	2.758605	2.931432	4.725122	6.087092	5.883577
Hordeum_vulgare_newGene_7243	2.755358	2.600814	2.796148	4.436316	5.714629	5.447834
Hordeum_vulgare_newGene_7244	0.657321	0.994251	0.777124	7.079848	6.830596	8.563813
Hordeum_vulgare_newGene_7246	11.98413	12.69693	15.32607	7.308843	9.877247	9.655599
HORVU7Hr1G109880	4.031396	4.313593	2.801306	4.266384	3.560053	3.809927
HORVU1Hr1G003320	4.599778	3.63401	4.897164	19.26791	7.764604	8.426159
HORVU3Hr1G107360	3.290598	3.657379	3.691062	3.509624	4.246395	3.548246
Hordeum_vulgare_newGene_12094	0.990577	1.655041	1.2304	26.35251	20.30367	26.58745

Hordeum_vulgare_newGene_12091	0	0	0	3.579815	3.468713	4.617088
HORVU3Hr1G063690	5.291013	5.878298	6.940117	4.871912	4.573431	5.589869
HORVU5Hr1G068940	4.522389	4.94332	5.725344	6.23351	5.899995	7.203338
HORVU3Hr1G097750	0.062689	0.029305	0.07885	13.86241	62.80501	55.2799
Hordeum_vulgare_newGene_6112	3.18617	5.049587	4.417106	3.30296	3.964543	4.210758
Hordeum_vulgare_newGene_6111	1.342011	1.149408	1.320363	1.646563	2.257582	2.601155
HORVU6Hr1G055750	5.692944	4.821532	5.731256	8.697278	8.733384	9.861248
HORVU6Hr1G052520	5.087799	5.275275	5.248148	8.194186	10.99914	9.922124
HORVU6Hr1G029050	7.398549	5.328739	8.284837	9.101031	13.9318	13.22332
HORVU3Hr1G081650	3.655721	4.131487	5.010787	2.106999	2.24375	2.302842
HORVU7Hr1G109770	71.08707	64.69657	86.87215	82.47372	116.3288	117.0077
HORVU2Hr1G092650	23.24153	21.90256	22.86997	19.2399	27.00385	27.24977
Hordeum_vulgare_newGene_3091	3.920708	4.415129	4.381081	12.53125	23.07582	20.46194
Hordeum_vulgare_newGene_3094	3.714327	4.239601	2.829582	0	0.020729	0.040753
Hordeum_vulgare_newGene_3096	39.08214	37.3447	32.56337	14.90796	8.121038	9.198712
HORVU6Hr1G071530	5.73433	5.785987	8.764523	6.091545	6.900777	8.198223
HORVU1Hr1G075910	1.671953	1.265295	1.473308	5.150762	7.635101	10.70769
HORVU7Hr1G108010	0.786524	0.742095	0.973096	1.279982	1.35511	1.317904
HORVU5Hr1G061360	9.850004	9.715814	11.56031	9.185528	13.57347	11.97971
HORVU7Hr1G035130	4.264551	4.59789	4.877826	5.229246	4.923422	5.524229
HORVU2Hr1G043890	499.784	305.192	367.3079	175.8781	14.36535	67.03828
HORVU3Hr1G087550	1.42628	1.180994	1.045638	2.055949	3.96299	3.683145
HORVU7Hr1G008680	2.239743	3.087095	2.82255	2.558609	4.521742	3.836629
HORVU5Hr1G064620	1.440488	1.674666	1.848233	0.992867	1.51379	2.513558
Hordeum_vulgare_newGene_3903	1.182074	0.953976	0.862762	3.027513	3.03526	3.440318
HORVU1Hr1G074060	0.780132	0.995056	1.060334	1.418941	1.310608	1.414945
Hordeum_vulgare_newGene_7683	0.310385	0.89048	0.552085	0.917422	1.818371	1.538393
HORVU3Hr1G018610	2.238976	1.465329	1.365273	1.102002	0.874306	1.079792
HORVU3Hr1G081380	5.011459	3.457523	5.002345	5.825014	4.876359	5.201606
HORVU2Hr1G116620	3.158014	1.986351	3.840112	1.57566	2.62801	2.397966
HORVU1Hr1G008510	1.518027	1.725288	1.84435	0.674344	1.461512	1.287574
HORVU1Hr1G008160	0	0	0	1.59992	2.669556	2.809871
Hordeum_vulgare_newGene_3635	0	0	0	6.206022	5.321818	6.397194
HORVU5Hr1G068360	20.33457	23.28033	19.48113	41.74876	30.05957	25.58409
Hordeum_vulgare_newGene_15607	0.624375	0.865084	0.849561	0.923266	1.611257	1.390513
HORVU4Hr1G074140	14.25499	14.4051	17.70594	11.87625	14.2525	14.3176
HORVU5Hr1G074230	16.36939	14.41398	18.31138	15.47746	14.70317	15.38106
HORVU1Hr1G070290	1.970649	2.24001	2.175658	7.519818	11.27531	12.92868
HORVU3Hr1G110690	7.307967	6.395475	9.792204	8.533311	11.49725	10.64472
Hordeum_vulgare_newGene_10379	13.93295	12.73468	14.8896	6.988932	6.770771	7.729101
HORVU6Hr1G072390	13.11994	15.71495	17.90699	2.739008	1.077916	1.770766
HORVU1Hr1G003200	0.026055	8.267878	0	0	0	0
Hordeum_vulgare_newGene_5678	4.692479	5.131848	5.896327	1.718685	2.817239	3.032156
HORVU1Hr1G003530	1.554257	1.536931	1.889773	1.368673	2.023483	1.783475
HORVU2Hr1G059360	1.951481	2.734797	3.935749	4.139924	9.770506	9.024166
HORVU6Hr1G033930	1.774307	1.394072	1.964605	1.392989	2.540864	2.291315
HORVU3Hr1G065840	2.673095	4.027565	4.654661	3.576888	3.954902	4.876625

HORVU2Hr1G033040	50.06277	49.83169	66.96576	29.03734	31.96289	30.76265
HORVU2Hr1G119050	0.032483	0	0.035291	0.699943	6.868887	5.739448
HORVU4Hr1G035450	73.59028	68.96098	77.28894	67.70477	93.70267	96.15882
HORVU4Hr1G011370	6.392322	6.70248	6.28876	6.755291	8.023081	8.134099
HORVU1Hr1G083000	2.554192	3.094896	3.597977	3.96599	6.382755	6.417737
Hordeum_vulgare_newGene_6808	6.435276	4.559703	6.686764	1.455876	2.787509	2.711493
HORVU3Hr1G055870	15.13797	16.76126	13.98776	27.07077	29.12401	27.3676
HORVU5Hr1G035350	2.287119	0.473017	2.133926	4.136284	5.083404	5.083069
Hordeum_vulgare_newGene_6805	24.54838	26.29928	45.40019	22.13722	49.73691	42.25755
Hordeum_vulgare_newGene_6802	3.42896	3.903465	2.892499	5.055182	6.442257	7.575031
HORVU3Hr1G044550	4.143336	3.806207	4.494193	4.242566	4.904405	5.385362
Hordeum_vulgare_newGene_8408	4.067356	4.301555	4.050373	0.164361	0.175728	0.275202
HORVU7Hr1G022910	1.25862	1.702103	1.816388	4.361995	4.338329	4.20619
Hordeum_vulgare_newGene_8400	2.546366	2.959269	2.947591	3.18747	5.02316	6.243422
Hordeum_vulgare_newGene_8404	1.306497	1.60754	2.234265	1.161735	1.130758	1.147775
Hordeum_vulgare_newGene_8405	1.994275	2.096534	1.980354	2.080122	1.279399	1.472135
HORVU4Hr1G031480	18.52485	20.85226	19.71807	22.06358	30.89683	24.92015
HORVU7Hr1G065590	21.45713	21.57672	31.13689	21.09086	28.51175	29.69404
HORVU4Hr1G005450	9.944414	8.489301	10.46335	3.758308	1.869442	2.685339
HORVU2Hr1G104270	2.063911	2.350322	2.793554	4.008639	8.512015	6.239538
HORVU7Hr1G060480	5.415085	6.352258	6.430299	0.399344	1.647225	1.63575
HORVU4Hr1G089720	9.073289	9.140318	10.86457	7.452979	10.52013	11.25517
HORVU7Hr1G106340	19.23507	15.72875	21.60816	18.24727	25.3253	20.60274
HORVU5Hr1G069700	0.838603	0.646559	1.150477	1.01952	1.941091	2.013223
HORVU2Hr1G042250	1.150748	0.879901	1.265422	1.645683	2.943221	2.266135
HORVU3Hr1G049120	59.28712	51.11096	70.09758	82.24929	81.04739	79.12678
HORVU1Hr1G015660	51.77687	44.39732	41.29658	35.30629	27.2421	25.68834
HORVU1Hr1G062420	2.458257	1.564924	2.376816	2.626979	3.576333	3.358392
HORVU2Hr1G098390	1.619198	1.137566	2.051149	1.652949	1.422412	2.020964
HORVU6Hr1G025110	2.69703	2.58339	3.23076	5.13979	7.390303	6.769956
HORVU3Hr1G014660	1.315332	1.091447	1.280836	0.899525	1.367264	1.424004
Hordeum_vulgare_newGene_1003	0.935391	1.299332	1.514888	3.269674	4.6628	4.827119
Hordeum_vulgare_newGene_1006	2.019153	2.126659	2.504854	10.86287	13.31416	13.93304
HORVU3Hr1G049780	26.7458	23.99219	29.06177	44.09588	47.85951	37.04782
HORVU6Hr1G082880	24.95879	20.83451	29.39541	59.39826	79.47417	73.14975
HORVU3Hr1G046570	2.525917	2.619343	2.727494	3.770209	4.761354	5.144958
HORVU6Hr1G093240	11.49201	9.685507	11.69971	14.91713	14.14607	14.90956
HORVU5Hr1G124750	1.656497	1.316807	1.668297	1.438734	0.831963	0.899034
Hordeum_vulgare_newGene_9279	3.953706	4.64897	2.867776	2.48223	3.474421	4.043284
HORVU2Hr1G084440	18.31831	19.31172	21.42398	23.28546	17.52955	20.02739
HORVU5Hr1G023890	0.960172	1.25057	1.314797	4.475657	4.24461	4.726992
HORVU6Hr1G020540	2.412436	1.807997	3.279304	0.025173	0	0.029406
HORVU5Hr1G050160	29.54644	29.14798	41.71284	29.07925	42.05335	44.1286
HORVU2Hr1G059060	4.476923	4.269259	4.499367	6.212077	7.408951	8.591062
HORVU0Hr1G005860	5.826664	7.859128	8.027678	16.26647	12.7843	17.47906
HORVU1Hr1G064860	7.477217	7.730134	8.838959	9.680934	17.12212	16.57385
HORVU2Hr1G074700	9.590734	9.396416	12.94152	12.85686	13.97859	16.68447

HORVU1Hr1G085480	1.35316	1.294185	1.445771	4.047879	4.935996	4.757815
HORVU3Hr1G014080	0.575586	0.490929	0.727353	1.926681	2.838606	3.028227
HORVU6Hr1G044600	3.022845	4.504213	3.687737	5.231593	5.327542	5.757755
HORVU1Hr1G078370	3.520625	3.674433	4.768377	4.32269	4.899991	5.115371
HORVU1Hr1G057530	37.02068	29.18943	45.90576	51.0545	90.46837	81.67285
HORVU4Hr1G032920	6.730116	7.574689	7.193275	6.391151	5.134076	5.559106
Hordeum_vulgare_newGene_4233	11.25303	10.28091	5.086433	4.073188	0.034697	1.823685
Hordeum_vulgare_newGene_4232	2.685213	10.90985	4.731314	13.78883	1.377942	1.135552
Hordeum_vulgare_newGene_4234	1.200735	1.57732	2.450009	0.933535	0.590744	0.458644
HORVU6Hr1G083250	0.554791	0.481909	0.795485	1.844689	1.686227	1.93913
HORVU1Hr1G052160	15.94988	14.6886	15.79651	24.51118	34.21649	35.59667
HORVU0Hr1G000910	0	0.046028	0.018507	6.069141	9.340391	8.575247
HORVU5Hr1G015530	2.409924	3.166412	1.806347	1.622291	16.60905	12.04622
Hordeum_vulgare_newGene_7760	4.631583	4.314154	4.678329	0	0	0
Hordeum_vulgare_newGene_7765	8.081952	8.558085	8.663578	8.209971	7.922095	7.376371
HORVU6Hr1G008320	0	0.093865	0.057177	3.936034	4.655826	5.385456
HORVU7Hr1G079790	0.184914	0.037681	0.095367	0.882466	3.652232	2.537544
HORVU4Hr1G072480	18.51689	11.50121	28.16428	8.493955	2.451572	10.05858
HORVU1Hr1G079680	4.142836	3.085312	3.76368	0	0	0
HORVU2Hr1G002660	6.863493	6.77078	8.003593	27.583	22.79275	26.209
HORVU5Hr1G124550	8.28798	8.271852	8.186908	9.969568	11.60765	11.49206
HORVU3Hr1G031850	118.7921	95.28022	114.0101	98.02628	78.44201	83.33869
HORVU5Hr1G104910	10.18172	11.82405	8.874662	15.18846	7.546124	6.908446
HORVU2Hr1G041000	0.200313	0.235119	0.168887	1.281851	3.228426	3.485427
HORVU1Hr1G064130	22.75135	31.84637	27.97663	7.726712	2.21384	3.593418
HORVU7Hr1G029720	4.441515	5.134156	6.336177	4.199043	4.112669	4.830568
Hordeum_vulgare_newGene_3923	1.254862	1.196297	1.294563	3.860459	3.847044	4.661689
HORVU2Hr1G050270	29.69401	25.87399	33.74898	32.85678	43.82984	43.36227
HORVU2Hr1G077970	0.164696	0.274371	0.157864	2.38496	2.024388	2.22581
HORVU2Hr1G059470	4.710282	6.926534	6.831027	4.233669	6.028551	6.804749
HORVU4Hr1G004410	28.59194	28.65854	35.08825	32.12713	41.38145	42.00264
HORVU4Hr1G050250	0.528353	0.774119	1.118866	1.064525	2.414332	2.591261
HORVU3Hr1G079720	0.040565	0.060333	0.0141	3.590881	6.156611	4.131136
HORVU1Hr1G079970	1.330876	2.250076	3.36175	1.070281	1.400884	1.003659
HORVU2Hr1G124530	8.57646	10.62235	9.750571	23.68277	16.08801	14.88237
HORVU6Hr1G029550	20.75996	19.9174	17.25671	14.90709	14.85428	15.50776
HORVU1Hr1G061500	165.8853	144.4453	167.7154	147.2735	182.9379	179.2576
HORVU4Hr1G056070	18.27401	15.49457	17.78347	22.30268	28.70211	19.6646
HORVU1Hr1G083520	1.348598	1.740186	2.12099	0.581035	1.357254	1.774945
HORVU5Hr1G125710	16.16582	17.49012	14.27999	22.04049	21.67463	25.41001
HORVU2Hr1G001310	3.247559	3.882801	4.446329	3.364543	5.096277	5.474438
HORVU3Hr1G018880	6.437905	5.745056	5.347178	5.557554	4.133996	5.392749
HORVU7Hr1G116600	1.415875	1.816237	1.806609	1.831922	2.476863	3.06587
HORVU7Hr1G098010	59.48073	43.98543	67.96332	70.28268	65.57253	80.5339
HORVU1Hr1G088600	15.34103	15.21448	51.36501	108.9852	12.00171	12.30113
HORVU1Hr1G027120	0.299296	0.733051	0.698802	0.798102	3.810495	3.680978
HORVU6Hr1G085560	1.801827	1.86707	1.961873	1.849102	2.788826	2.724828

HORVU4Hr1G052090	1.931983	1.769618	2.27196	4.197685	8.424994	7.686117
HORVU2Hr1G055530	4.624665	3.799755	6.614757	4.233625	5.447043	5.433888
HORVU3Hr1G072320	10.24278	11.39334	13.11643	6.696133	8.042114	8.162751
HORVU1Hr1G014070	1.723968	1.713996	2.114863	2.591738	2.277873	3.46083
HORVU6Hr1G037680	1.178293	1.10423	1.499166	1.3223	1.924235	1.82632
HORVU3Hr1G076520	6.837118	6.919996	7.929072	9.208542	10.32049	10.87795
HORVU3Hr1G032760	5.204724	4.403637	5.81501	6.075088	6.224764	6.339023
HORVU6Hr1G091550	53.27852	40.75724	54.27124	45.52564	46.27563	48.87933
HORVU4Hr1G010540	4.635282	5.523014	8.393474	11.95423	7.600904	9.12026
HORVU1Hr1G093480	39.68183	19.74835	57.02062	16.14172	19.05408	26.83646
HORVU1Hr1G080380	1.6335	1.797531	1.042917	0.926806	0.818485	1.380818
HORVU5Hr1G009620	6.793788	8.918827	7.344347	14.2347	30.90126	21.83697
HORVU3Hr1G006940	4.469442	4.176414	5.777986	27.09439	18.97662	21.76607
HORVU1Hr1G020470	2.99752	3.360144	3.222022	3.830632	4.185644	4.736834
HORVU5Hr1G112750	0.521943	0.478263	0.538141	0.707816	3.463891	2.981101
HORVU5Hr1G020150	10.47031	14.53925	12.00016	14.47556	21.56241	20.1394
HORVU3Hr1G108360	5.634248	3.339589	5.365074	13.95478	13.60555	16.31556
HORVU0Hr1G016050	3.721184	3.745039	4.439042	3.730053	3.508884	4.695545
HORVU7Hr1G050080	2.052772	2.625172	2.62522	2.983056	5.043931	4.73991
HORVU3Hr1G027240	3.340178	1.700487	3.904801	6.408483	2.800121	3.581061
HORVU1Hr1G081030	2.903158	3.024049	3.931808	4.002798	8.408193	8.212334
HORVU4Hr1G083060	4.340755	3.865753	4.646049	3.592737	2.980846	2.930311
HORVU4Hr1G084280	14.34494	12.29069	15.74757	13.55262	13.2985	14.32891
HORVU5Hr1G020840	1.87959	2.116997	2.444255	1.705738	1.274448	1.553704
HORVU5Hr1G046860	2.832823	2.2338	3.179325	2.98162	4.066528	3.334106
HORVU6Hr1G048290	7.941127	7.73526	10.13792	12.94793	18.63916	18.71455
HORVU6Hr1G090780	2.15891	1.769948	2.768716	1.942388	1.750026	2.821054
HORVU4Hr1G009270	26.68475	22.06561	33.44377	24.90713	34.08326	30.09417
HORVU3Hr1G056650	4.93296	5.49997	7.414471	7.242024	12.10088	12.87715
HORVU5Hr1G121620	2.530515	2.71224	3.733461	4.392013	4.50273	5.338684
HORVU7Hr1G071790	50.96161	39.35697	47.98353	97.50862	90.79032	97.93148
HORVU1Hr1G046320	4.66153	4.408776	4.88417	5.925772	11.02092	10.66714
HORVU3Hr1G083750	13.20499	9.88609	13.97653	12.68563	16.39897	15.19135
HORVU6Hr1G040190	2.725889	2.76615	3.122289	3.687693	4.925408	5.573209
HORVU5Hr1G119580	1.005994	1.505783	2.533464	0.999206	0.146054	0.868138
HORVU1Hr1G041890	4.838383	3.319495	3.779546	4.509204	3.683172	3.630373
HORVU1Hr1G013820	42.08139	37.33918	37.48453	78.21423	55.56836	55.36774
HORVU5Hr1G082940	0.56083	0.383092	0.46851	1.315206	2.142093	2.23984
HORVU4Hr1G040550	7.695683	8.011392	11.13733	9.020143	18.51831	17.72646
HORVU2Hr1G112830	11.29909	8.493459	9.907658	4.98029	1.090158	5.121881
HORVU6Hr1G011490	0.499713	0.617059	1.178157	0.889855	4.346012	5.919163
HORVU5Hr1G034820	37.18089	33.9103	24.88963	16.40433	10.45406	12.17749
HORVU0Hr1G007360	3.877316	3.293797	4.399789	10.98994	15.03623	13.87377
HORVU7Hr1G093940	1.853759	2.091716	2.022186	10.59365	22.91968	19.34322
HORVU6Hr1G091780	1.312968	1.285345	1.268676	1.261311	1.07683	1.294931
HORVU0Hr1G017090	1.289663	2.194541	2.334835	1.447061	1.722237	1.756112
HORVU6Hr1G010540	0.483251	0.413514	0.328296	0.039718	38.2991	26.37295

Hordeum_vulgare_newGene_12873	37.83029	34.00971	40.06577	41.68483	51.26408	51.24408
Hordeum_vulgare_newGene_12872	0.052119	0.033673	1.476464	1.974795	2.300718	2.302967
Hordeum_vulgare_newGene_12879	4.015759	3.953794	5.639533	3.30644	3.982773	3.685415
Hordeum_vulgare_newGene_12878	0.577892	0.647983	0.728489	1.670645	11.02136	7.603082
HORVU5Hr1G121080	1.091587	0.807343	1.622161	2.712159	1.47971	2.171975
HORVU2Hr1G063680	4.259242	5.825156	6.71089	6.282424	12.80613	10.88702
HORVU3Hr1G098230	13.46729	13.30258	12.5215	9.282723	9.677051	9.18229
HORVU5Hr1G111130	21.94644	18.10415	22.84582	25.5056	13.28876	15.50035
HORVU3Hr1G074100	5.71723	5.415797	6.45067	34.32031	52.96316	36.15767
HORVU7Hr1G085570	9.905254	8.047298	10.25778	4.217817	3.905006	4.561218
HORVU6Hr1G015490	8.644789	7.548907	10.23944	8.184998	10.20098	10.35425
HORVU0Hr1G022500	39.66704	32.42969	39.33532	30.43388	41.28528	42.2516
HORVU3Hr1G020560	8.598016	8.907563	10.44007	4.53587	7.221654	7.732974
HORVU2Hr1G099320	1.315711	1.463218	1.642767	2.326382	2.4097	2.599472
HORVU0Hr1G014540	0	0	0	0.076147	3.671341	5.120923
Hordeum_vulgare_newGene_2123	4.564815	6.147306	6.051222	5.713642	6.152587	7.333593
Hordeum_vulgare_newGene_2126	1.309633	1.258994	1.643305	1.316636	1.493179	1.888329
HORVU4Hr1G065080	28.50721	30.58383	37.81068	31.64708	59.83328	53.85068
HORVU3Hr1G064080	9.514195	9.902414	10.34405	6.302562	7.218613	8.19897
HORVU3Hr1G104090	0	0	0	5.737333	7.093224	8.475716
Hordeum_vulgare_newGene_5470	0.424079	0.467132	0.369325	1.683886	2.093208	1.996114
Hordeum_vulgare_newGene_5479	0.680111	0.52574	0.552262	3.638918	1.994231	1.550416
Hordeum_vulgare_newGene_2690	41.14843	34.59342	45.4903	31.64761	37.18996	34.39522
HORVU2Hr1G002500	20.07709	25.7037	33.70492	5.284771	0.044062	2.75679
Hordeum_vulgare_newGene_2697	0.661991	0.782074	0.801465	1.461373	1.696152	1.74954
HORVU3Hr1G077450	13.03018	11.88542	12.17935	5.257194	3.970505	4.563024
HORVU6Hr1G016670	7.851688	8.356331	7.645208	9.108692	7.972627	8.988981
HORVU2Hr1G060650	3.56771	4.982257	3.80156	3.108909	3.447727	5.625877
HORVU0Hr1G021850	142.8991	105.2285	167.7505	85.27717	47.35737	55.13181
HORVU3Hr1G006000	62.26654	64.59653	73.79366	40.20241	54.12304	56.95424
HORVU7Hr1G034060	2.171897	2.418785	2.28355	1.635392	2.327943	2.092979
HORVU4Hr1G075560	2.341573	3.484321	3.474687	1.702142	1.501272	1.795803
HORVU5Hr1G092840	25.64332	28.72414	29.88578	23.49315	31.35264	30.95988
Hordeum_vulgare_newGene_13832	0.648991	0.85546	1.013593	1.531737	1.904352	1.694596
Hordeum_vulgare_newGene_13836	4.082773	3.941892	2.281808	0.565495	1.506297	1.755963
Hordeum_vulgare_newGene_13838	3.132491	3.459008	4.265253	2.454743	3.582365	3.451584
Hordeum_vulgare_newGene_8646	1.081924	1.177963	1.091252	1.754234	1.708284	1.887347
Hordeum_vulgare_newGene_8640	11.94241	9.865938	11.8496	27.83493	51.6229	44.76274
Hordeum_vulgare_newGene_8643	1.451668	1.345048	1.538522	1.227427	1.654629	1.567593
HORVU2Hr1G072420	128.8917	102.8672	142.3909	127.0374	248.2212	217.2388
HORVU3Hr1G073220	872.7646	589.0508	858.9424	701.4789	1044.768	1056.61
HORVU3Hr1G093760	2.949887	3.275715	4.459588	2.323317	1.397763	1.605572
HORVU1Hr1G022900	0.288873	0.068424	0.182448	1.915212	35.39443	37.91545
Hordeum_vulgare_newGene_8961	2.266795	3.243467	2.751033	2.429821	2.765681	2.998132
HORVU5Hr1G073070	1.913883	1.849279	2.21251	2.316261	3.035853	3.295194
HORVU7Hr1G086170	12.00106	9.103852	11.11977	18.71944	22.14548	21.73872
HORVU1Hr1G001770	0.051121	8.272525	0.596244	12.12245	0.123459	0.254265

HORVU5Hr1G080630	0.946474	0.801642	1.081426	4.290006	2.419253	2.76186
HORVU7Hr1G121850	398.5731	245.3652	331.2884	126.0448	13.12632	48.58234
HORVU2Hr1G076550	7.494994	6.341252	9.077899	6.297744	7.942879	8.242202
HORVU3Hr1G114280	7.47773	4.902583	7.258663	7.799212	9.989015	10.0917
HORVU5Hr1G052730	3.436549	2.475529	3.388485	1.225171	1.735745	2.66172
HORVU4Hr1G067650	6.876749	7.541878	10.13581	10.76131	26.96245	24.70434
Hordeum_vulgare_newGene_2942	2.777176	1.913366	3.991281	1.953803	1.203699	0.93319
HORVU2Hr1G111070	59.68197	49.43876	64.13076	55.96279	75.32444	76.43622
HORVU4Hr1G048510	5.487567	5.761385	6.129332	13.14139	20.45958	20.03438
HORVU1Hr1G001480	0	8.268515	0	0	0	0
HORVU7Hr1G008800	5.839827	5.384	5.750429	6.301818	7.644028	8.701932
Hordeum_vulgare_newGene_10567	0.463122	0.539909	0.606456	2.402141	2.47416	2.195529
Hordeum_vulgare_newGene_10560	2.578376	2.40783	1.946818	2.097173	2.912166	2.585265
HORVU6Hr1G076450	0.118294	0.282786	0.243961	0.328735	9.717063	5.600645
HORVU7Hr1G062400	3.49468	5.323504	5.974351	9.755742	9.773303	11.97887
HORVU7Hr1G052270	2.888123	3.224671	3.754051	3.827083	10.35109	8.750784
HORVU1Hr1G009900	8.570917	9.134338	10.42471	9.520955	11.62263	12.1368
HORVU4Hr1G000940	4.83774	4.653263	5.903487	3.625118	4.862995	4.139025
Hordeum_vulgare_newGene_13041	3.442151	5.573712	5.856469	7.621545	9.19763	9.469234
HORVU4Hr1G063820	70.61597	59.86989	80.38842	69.01078	86.96846	88.30093
Hordeum_vulgare_newGene_13040	2.432087	3.171998	2.88567	3.456601	5.968174	5.339164
Hordeum_vulgare_newGene_1577	5.44674	5.113177	4.555144	13.78509	8.872077	9.931999
HORVU3Hr1G057240	4.301681	4.071135	3.207019	10.52605	8.084494	9.330447
HORVU6Hr1G060810	1.551666	1.732837	1.674868	6.134163	9.971317	8.602323
HORVU7Hr1G080790	21.7505	23.35191	22.80867	23.64311	21.29569	24.24295
HORVU3Hr1G018680	0.156492	0.476961	0.598353	2.035119	1.702845	2.410302
HORVU5Hr1G043790	2.245599	2.095583	2.739904	1.529621	2.406426	2.883503
HORVU6Hr1G071860	27.64852	30.78594	33.93806	21.62073	7.821292	13.04935
HORVU3Hr1G011690	0.696474	0.784048	0.36986	1.069703	39.25122	31.19274
HORVU5Hr1G030630	1.315377	1.964088	1.83529	3.278139	3.772775	4.741972
HORVU7Hr1G082330	6.354164	5.596473	6.478461	15.45186	15.36922	18.08359
Hordeum_vulgare_newGene_4430	29.917	24.71679	31.54103	31.12706	36.21877	34.10261
Hordeum_vulgare_newGene_4437	2.979541	3.226828	2.924302	3.442721	2.411949	3.212778
HORVU2Hr1G025630	0	0.017865	0	2.555769	3.73586	4.086813
HORVU1Hr1G047610	11.30548	12.89227	10.87466	12.80472	9.178898	12.25363
HORVU1Hr1G025690	4.334075	4.158113	6.184019	8.432016	9.641558	8.468357
HORVU4Hr1G077070	15.29864	14.44551	11.22965	7.146042	14.81313	13.59705
HORVU5Hr1G071560	15.04276	11.954	14.9993	16.38156	23.20784	21.14321
Hordeum_vulgare_newGene_7165	7.309975	6.579127	10.18938	12.84128	27.95195	23.65099
Hordeum_vulgare_newGene_7166	76.65124	76.54788	97.5836	157.5101	382.994	314.1268
Hordeum_vulgare_newGene_7167	1.076584	1.142987	0.853696	4.375862	6.378935	6.184392
Hordeum_vulgare_newGene_7161	1.377019	1.274572	1.619515	1.782267	2.738126	2.159708
Hordeum_vulgare_newGene_7168	12.9024	12.67588	12.37129	8.083914	7.021123	8.581278
Hordeum_vulgare_newGene_7169	4.379378	5.336949	6.665385	3.923689	5.704383	4.959722
HORVU2Hr1G012040	3.424158	2.132283	4.902294	2.785446	2.618725	2.895251
HORVU6Hr1G077320	13.58115	13.29366	17.57484	10.79619	10.38929	11.37507
HORVU2Hr1G028060	4.028744	4.460638	5.304488	2.988186	3.846887	4.323003

Hordeum_vulgare_newGene_7615	3.101196	3.029656	2.766003	8.843402	7.763347	9.097414
HORVU7Hr1G039710	0.501291	0.404438	0.816698	1.085021	2.297733	2.021
HORVU5Hr1G084520	0.225685	0.094355	0.162077	0.857678	2.806623	2.920548
HORVU1Hr1G075270	32.80322	42.36945	37.14559	58.80249	83.41429	83.23353
HORVU3Hr1G039470	5.013313	3.486828	4.909049	0	0	0
HORVU3Hr1G067740	12.82956	14.44764	14.193	15.40255	17.09484	20.89027
Hordeum_vulgare_newGene_544	4.138163	4.381294	4.915342	3.749643	3.852409	4.824883
HORVU7Hr1G079000	4.571168	5.342126	5.891677	4.279469	4.704567	5.511594
HORVU1Hr1G030060	3.782958	2.926073	3.836434	15.73846	7.280002	10.27116
HORVU6Hr1G063540	25.43802	21.92484	28.25912	39.42633	43.23189	48.04027
Hordeum_vulgare_newGene_543	43.65958	46.97702	40.26144	53.89172	47.93514	50.25509
Hordeum_vulgare_newGene_14455	1.364552	1.591061	1.541196	1.812784	1.599559	2.209262
Hordeum_vulgare_newGene_14452	2.057857	2.160641	2.08054	1.058888	1.537947	1.328126
HORVU3Hr1G033610	1.178946	0.910281	1.8988	1.281383	0.69684	0.802327
Hordeum_vulgare_newGene_12687	4.19274	4.886773	5.392412	3.496194	2.764023	4.246844
Hordeum_vulgare_newGene_12685	4.853855	4.910358	4.878876	3.127396	4.100974	3.475708
Hordeum_vulgare_newGene_12683	0	0	0.161254	5.425642	6.168887	7.809114
Hordeum_vulgare_newGene_12681	1.992826	2.485811	1.669947	1.930066	1.311514	1.520039
HORVU5Hr1G087690	11.83159	11.39388	12.58219	6.072043	4.519833	4.903586
HORVU3Hr1G113060	12.6496	19.36507	16.68761	10.54921	12.34485	14.08861
HORVU7Hr1G002060	109.4168	116.9507	172.6475	94.88498	225.987	208.0537
HORVU4Hr1G028600	12.71115	13.11047	15.96981	7.368627	10.47365	10.77867
HORVU7Hr1G038040	13.81562	13.7351	20.13287	24.51842	22.72453	21.79603
HORVU0Hr1G008030	2.752979	3.546337	3.281414	6.00199	7.943108	8.535961
HORVU2Hr1G099160	2.292604	1.597856	1.86777	4.733806	3.618098	4.017351
HORVU0Hr1G019390	1.490683	1.960442	2.065625	1.488775	2.12999	1.911389
Hordeum_vulgare_newGene_13154	3.405789	4.264109	3.84421	4.344505	4.697439	5.221565
Hordeum_vulgare_newGene_13156	1.0521	0.99418	2.197396	1.427772	0.355441	0.721036
HORVU6Hr1G032940	6.456335	5.690199	7.249091	5.867377	7.516397	8.471914
HORVU1Hr1G070310	624.3182	581.1111	722.5764	140.9493	3.37984	50.20824
HORVU3Hr1G068140	2.626456	2.527822	2.710906	2.505886	0.209684	0.853766
HORVU5Hr1G068750	1.632048	1.515652	1.874541	2.930928	3.960131	3.595759
HORVU7Hr1G030670	7.335545	9.162749	10.28353	2.533666	0.785412	1.413211
HORVU0Hr1G003910	7.619675	7.344672	8.310527	9.541359	12.38688	11.70349
HORVU5Hr1G048950	2.438483	2.840263	3.027322	4.41558	5.049532	5.143663
HORVU1Hr1G015500	22.90429	21.8367	27.14161	20.78474	24.65938	25.11877
HORVU7Hr1G008470	0.260376	0.354053	0.512327	2.899017	10.65949	9.479142
HORVU4Hr1G021000	30.45616	31.04058	31.32689	21.74066	19.6766	21.3586
HORVU2Hr1G080970	66.19327	66.69438	75.48075	66.53548	67.18726	70.33783
HORVU6Hr1G082260	1.053037	1.14477	1.22688	2.581543	4.217498	3.453377
HORVU5Hr1G074000	0.222343	0.23848	0.237841	0.9649	3.625082	3.238186
HORVU1Hr1G014610	23.28567	20.79005	25.9745	22.15055	26.50859	27.68925
HORVU6Hr1G072740	1.420704	2.061339	1.109603	13.47597	20.26894	18.8675
HORVU7Hr1G057310	23.3841	21.67076	23.75989	17.85043	16.96531	19.67256
HORVU1Hr1G059910	69.64053	111.2953	166.9594	4.263925	0	0.796672
Hordeum_vulgare_newGene_15413	2.539395	2.961666	3.007869	3.039091	3.265122	4.01699
HORVU7Hr1G058940	1.485953	1.389778	1.209461	3.670764	7.770947	7.103776

HORVU7Hr1G048730	18.39554	16.45475	17.4674	25.96399	30.42858	26.3483
HORVU5Hr1G024480	3.894415	2.288269	3.505315	2.670757	2.923393	2.508989
HORVU0Hr1G018270	4.516216	4.855943	4.22547	2.954781	8.961123	4.627042
HORVU2Hr1G051150	20.01968	15.93897	35.37575	23.47857	46.3681	47.4827
HORVU5Hr1G103770	1.998352	0.706324	2.319576	0.60055	0.012935	0.463134
HORVU5Hr1G097260	10.19859	8.172572	11.3618	14.48583	16.89154	15.09456
HORVU2Hr1G040880	0.791696	0.778997	0.577799	3.408313	2.999611	2.390573
HORVU0Hr1G003260	1.860722	2.353094	2.915535	5.059085	8.357938	7.536858
HORVU7Hr1G116880	3.756534	2.639466	3.827928	1.695691	1.633123	1.908488
HORVU7Hr1G023140	2.258623	2.794027	1.874982	7.942964	11.35568	12.22641
HORVU7Hr1G090950	16.94853	13.8226	20.44121	26.85234	56.04512	54.12412
HORVU4Hr1G002730	4.169601	2.050043	1.737904	1.447613	0.361164	1.028329
HORVU5Hr1G039730	0.171459	0.099889	0.039982	1.968257	2.976638	2.134339
HORVU5Hr1G026650	3.521752	3.818672	5.795788	8.226252	20.28281	16.98199
HORVU4Hr1G080610	0.285568	0.097502	0.256335	6.276267	14.64437	16.5965
HORVU1Hr1G062380	13.89244	5.136598	11.68627	0.292075	0	0.693254
HORVU2Hr1G085610	0.497106	0.530122	1.229749	2.252496	2.342677	1.765467
HORVU1Hr1G054250	2.595129	2.803611	3.193054	62.11631	62.4553	60.35964
HORVU7Hr1G106130	0.705511	0.963806	1.770392	7.912324	132.0068	122.9092
HORVU3Hr1G014780	2.687939	2.636022	2.467888	5.473736	8.692829	8.745338
HORVU5Hr1G062310	25.1975	24.67309	32.73853	51.77061	111.7997	93.39394
HORVU5Hr1G114510	21.71314	20.71137	21.42954	29.02081	37.89585	37.72802
HORVU4Hr1G088440	3.858335	3.595828	3.125154	9.908311	6.63786	8.398564
HORVU5Hr1G058430	28.7606	27.06056	32.65668	32.98784	47.22296	46.56375
HORVU0Hr1G029210	55.60816	44.36358	57.56588	73.2674	83.66849	85.03038
HORVU6Hr1G066780	0.648179	0.620536	0.840825	1.364203	1.646865	1.666077
HORVU1Hr1G062650	89.68962	79.01707	103.4605	133.7108	181.432	177.8511
HORVU0Hr1G008790	10.33502	11.24427	11.24814	12.43776	15.61488	18.04075
HORVU4Hr1G018360	2.148529	1.786964	2.109558	1.512649	1.341371	1.642036
HORVU6Hr1G039910	15.86302	13.83702	16.35463	25.14636	32.07844	32.77443
HORVU4Hr1G042970	31.28458	42.79734	33.78894	35.74119	32.14416	42.77315
HORVU6Hr1G066430	4.302076	4.736666	5.279945	5.653359	7.038167	8.250661
HORVU2Hr1G083610	63.52543	45.66177	52.14084	38.34167	43.04007	40.2112
HORVU1Hr1G088810	11.42952	11.70565	29.47847	61.49655	12.30201	13.64435
HORVU0Hr1G008860	12.94707	13.80941	14.42342	12.60728	10.72165	14.31193
Hordeum_vulgare_newGene_1634	3.236139	4.183924	4.329196	3.191508	3.106738	3.565316
Hordeum_vulgare_newGene_1635	0.770924	0.82405	0.973284	1.592074	1.713636	2.023257
HORVU3Hr1G000720	5.035897	3.245415	2.939118	4.620172	11.37785	8.705454
HORVU5Hr1G067090	20.51567	21.11331	21.62989	24.74782	34.25936	32.36072
HORVU7Hr1G032750	47.66155	46.53349	49.28849	57.29293	54.1115	60.90329
HORVU4Hr1G077640	5.994695	6.250435	6.005621	4.300625	4.99011	5.36935
HORVU5Hr1G057010	5.653186	7.32868	7.276559	8.511484	8.997322	9.998719
HORVU2Hr1G001430	2.292507	1.382165	2.74046	35.38831	79.61037	61.25177
HORVU4Hr1G072360	6.196284	4.694623	7.137607	1.602931	0.451622	1.331897
Hordeum_vulgare_newGene_14507	11.61315	9.3406	6.505135	5.999617	12.25267	8.848684
HORVU4Hr1G081390	36.54717	39.32377	58.30293	3.584801	0.14333	1.294313
HORVU1Hr1G058880	16.01157	14.80391	17.89767	20.85496	26.08268	25.79874

HORVU2Hr1G080140	17.50526	12.25071	18.33561	7.366813	15.77316	19.53128
HORVU1Hr1G090050	1.045706	1.06849	1.267591	1.285947	3.19028	2.883959
HORVU7Hr1G099390	1.687873	1.753234	2.028067	1.301686	0.647678	1.162892
HORVU5Hr1G010440	6.48554	8.618652	10.9596	4.658878	5.816965	5.685243
HORVU5Hr1G113880	10.64646	11.92382	12.74856	27.40592	57.38332	49.28138
HORVU1Hr1G043150	10.12211	9.311371	11.11996	10.62825	10.52374	13.03717
HORVU7Hr1G093630	4.088274	4.254257	4.750281	3.843239	6.452418	5.151488
HORVU6Hr1G013370	3.96054	3.378085	5.530686	2.45447	2.842812	2.328302
Hordeum_vulgare_newGene_2926	13.51667	11.73013	14.8558	11.71486	14.26219	15.43631
HORVU2Hr1G098860	49.30629	54.15626	41.90374	72.15599	70.11253	68.54841
HORVU2Hr1G001290	23.01384	17.27325	25.42634	22.24285	38.60046	36.53125
HORVU5Hr1G081120	10.23325	11.02964	13.16632	8.835097	8.861367	10.08799
HORVU3Hr1G040290	3.733162	5.218648	6.018839	4.466369	4.700972	5.661691
HORVU2Hr1G078910	97.42386	106.3409	179.6778	38.93427	76.1954	73.12594
HORVU7Hr1G092940	23.05758	19.7339	19.57064	23.54994	16.96008	17.39514
HORVU7Hr1G038900	0.879463	1.16447	1.194027	3.017926	3.812137	3.542075
HORVU1Hr1G051700	3.002504	3.068251	1.866522	8.843884	16.62191	19.7498
HORVU7Hr1G006160	77.88519	63.91973	78.14341	63.07958	80.35586	91.98269
HORVU3Hr1G002010	1.361961	1.941088	2.329745	0.943164	1.159286	1.122719
HORVU2Hr1G054890	0.42759	0.228939	0.284748	1.778842	1.693108	1.646676
HORVU2Hr1G082940	5.9683	6.258828	8.553415	4.371655	5.581546	5.17989
HORVU5Hr1G053660	13.57531	12.64305	12.8563	10.40458	10.58467	11.38979
HORVU2Hr1G119460	41.49849	36.10957	44.72556	36.99824	56.41218	57.96157
HORVU2Hr1G121440	24.02265	23.42971	29.99638	25.20924	30.90008	34.78355
HORVU1Hr1G055930	6.946047	8.257089	10.24706	1.057825	0.157753	0.732188
HORVU1Hr1G080480	9.466275	8.186793	15.36894	11.89633	11.02649	18.25189
HORVU2Hr1G066320	5.732011	5.858669	9.287126	8.78269	11.2011	12.79133
HORVU3Hr1G007190	46.5421	41.13039	42.60283	51.19143	76.36939	66.29375
HORVU5Hr1G086470	3.578241	2.133896	2.93349	5.326149	8.791578	6.422183
HORVU4Hr1G051790	9.09591	7.571899	7.534722	8.571664	12.35468	12.55734
Hordeum_vulgare_newGene_11763	4.181468	3.818471	4.37695	6.101328	6.412308	6.163313
Hordeum_vulgare_newGene_11766	0.862471	0.928058	1.398198	1.629772	1.505584	1.46804
Hordeum_vulgare_newGene_11768	5.436768	4.42222	6.35366	6.57292	8.647886	7.877469
HORVU3Hr1G095780	2.732561	2.776702	2.762946	15.06102	18.06474	17.10788
HORVU6Hr1G033750	3.939419	4.664562	5.31886	6.157684	4.884051	5.118613
HORVU5Hr1G094290	99.57308	86.80225	102.2466	93.05089	142.3077	132.1966
HORVU6Hr1G085730	9.550049	9.489678	13.75663	5.801902	6.601776	7.553388
HORVU7Hr1G010490	19.83995	22.42666	24.15826	10.27033	9.106522	10.20356
HORVU5Hr1G123200	2.239728	1.599986	2.177109	2.341288	2.59501	2.654842
HORVU2Hr1G123590	4.828	4.567674	5.076797	8.375593	3.676746	7.307681
HORVU4Hr1G052680	2.085338	2.396132	1.890926	1.981685	1.839934	1.974362
HORVU6Hr1G034760	13.06261	7.551456	12.43179	6.008519	2.748652	3.322993
Hordeum_vulgare_newGene_4995	1.230343	1.14795	0.998986	1.635067	1.344731	1.314418
HORVU2Hr1G115210	236.2946	218.5219	241.585	218.7403	201.1066	207.8271
HORVU2Hr1G040140	17.9599	15.85643	17.61908	15.92393	20.71452	19.26499
HORVU4Hr1G014540	14.41352	16.02887	17.64334	18.8847	23.71339	23.94075
HORVU2Hr1G019010	0.027178	0.008462	0.06079	3.804327	2.817428	3.012122

HORVU1Hr1G065830	2.77651	3.012134	3.849228	1.907978	2.306023	2.613018
Hordeum_vulgare_newGene_16235	12.78474	14.53958	13.53542	16.60819	14.64351	17.61031
Hordeum_vulgare_newGene_16239	2.430777	2.579103	2.112006	2.126689	2.673058	2.932598
HORVU7Hr1G045290	0.136896	0.200792	0.287666	5.477224	11.46297	11.95461
HORVU5Hr1G007050	1.938153	1.947471	2.684174	7.17577	11.40144	11.36342
HORVU3Hr1G082360	2.093535	2.009808	2.959874	4.0397	4.538941	5.796925
HORVU3Hr1G075750	3.434683	4.093694	4.685151	3.594007	3.705143	4.16127
HORVU5Hr1G113300	12.07954	9.844133	11.3872	12.54837	19.11819	17.48801
HORVU0Hr1G013910	1.500687	0.775771	1.519495	1.705063	1.846212	2.0487
HORVU6Hr1G038840	2.382673	3.560112	3.847802	1.932679	1.756742	1.946159
HORVU2Hr1G025430	3.278613	3.525996	2.943287	2.614488	3.030138	3.625807
HORVU2Hr1G017950	50.97833	35.74453	46.74104	42.37592	14.63502	18.10934
HORVU3Hr1G076310	1.910338	2.209301	2.509944	3.995551	4.365719	4.133364
HORVU2Hr1G056230	13.21602	14.19208	19.5227	13.54277	20.29388	20.28672
HORVU3Hr1G022550	5.852004	5.870398	5.890085	7.612563	12.14374	11.31225
HORVU5Hr1G109250	0	0	0	1.064707	2.769989	2.550231
HORVU0Hr1G027130	532.5319	481.1016	698.4937	144.3191	164.3101	156.4169
HORVU6Hr1G001490	5.381831	5.263834	6.349123	3.783017	4.480736	4.594502
HORVU3Hr1G071830	8.812324	9.113194	10.21826	6.062464	5.368234	6.658578
HORVU3Hr1G074800	5.127947	5.591714	5.957064	6.337603	7.62699	7.351513
Hordeum_vulgare_newGene_15051	5.724025	5.750841	5.564246	3.302279	3.452975	3.811652
HORVU1Hr1G013160	2.1006	2.127204	2.855746	2.447234	3.162645	4.72118
HORVU6Hr1G090370	0	0	0.029619	25.54158	106.434	84.394
HORVU5Hr1G106550	100.7821	85.6926	98.39635	92.31876	224.8094	214.1241
HORVU1Hr1G002920	0	0	0	12.04928	0	0
HORVU7Hr1G120380	2.308278	2.564572	2.557872	3.426295	2.903644	3.430072
HORVU1Hr1G093390	1.425573	1.191541	1.960111	6.978443	10.62487	6.95284
HORVU4Hr1G083210	37.44321	24.90304	50.55631	26.91295	54.18808	54.70173
HORVU3Hr1G084220	0.216149	0.132029	0.327739	2.170093	2.432459	4.650095
HORVU6Hr1G011510	0.524233	0.346005	0.655296	2.634836	12.13522	12.64485
HORVU2Hr1G065000	21.03168	14.02235	15.73429	5.047138	2.331331	3.265094
HORVU2Hr1G060070	6.629896	4.608854	3.47341	17.02509	17.64117	16.48657
HORVU6Hr1G054430	0.599489	1.041675	0.759405	1.455826	3.075337	2.701227
HORVU3Hr1G036720	6.151303	6.962498	6.600182	8.79088	12.09469	13.931
HORVU6Hr1G027260	2.0708	1.607626	1.882005	2.099956	3.585145	1.864334
HORVU4Hr1G008950	0.67949	0.577667	0.688741	2.679884	1.783491	2.027029
HORVU5Hr1G114650	12.83778	12.92217	14.42998	12.93155	12.88824	13.52588
HORVU4Hr1G066180	19.98737	18.75982	17.86173	24.5366	22.74511	22.54536
HORVU5Hr1G082570	35.00702	33.61894	37.66538	31.51698	49.44808	46.84502
HORVU5Hr1G011270	14.8499	13.64853	14.93462	19.79639	24.16002	24.46984
HORVU1Hr1G075750	5.109145	4.285688	3.770709	0	0	0
HORVU6Hr1G093480	6.647274	6.035027	10.19046	4.17548	6.04818	6.29723
HORVU4Hr1G067180	3.938166	3.6154	4.901117	10.22196	14.22118	14.53905
Hordeum_vulgare_newGene_1990	1.539709	0.802826	2.302202	2.474909	7.175344	4.796556
HORVU1Hr1G042360	0.1226	0.132766	0.180921	0.983419	2.529884	2.187319
HORVU7Hr1G057470	81.31458	64.82147	95.85474	108.3775	230.0218	205.2374
HORVU2Hr1G068200	38.99026	39.55416	40.37432	44.03583	28.41507	43.01625

Hordeum_vulgare_newGene_12445	4.569029	4.144111	4.442361	17.17686	3.292305	5.458716
Hordeum_vulgare_newGene_12446	3.811433	3.227436	3.587316	13.04052	1.462633	4.15093
Hordeum_vulgare_newGene_12447	1.686035	1.33432	3.213886	1.357111	0	0.266987
HORVU3Hr1G021080	0.320233	0.317336	1.147882	0.99124	1.091005	2.714989
HORVU6Hr1G018330	9.028152	10.00129	11.89803	7.952836	11.21948	10.72561
HORVU2Hr1G103580	56.28166	58.88445	52.83964	90.66379	86.31764	84.48156
HORVU2Hr1G064750	1.480915	0	0.791355	1.851514	2.760784	1.136999
HORVU2Hr1G036280	6.626049	6.56665	7.329595	9.491161	24.54849	22.68941
HORVU3Hr1G092980	0.192573	0.024112	0	0	6.773956	2.660049
HORVU5Hr1G099170	0.178615	0.574171	0.181811	4.763964	4.306576	6.772523
HORVU6Hr1G065250	1.962795	1.872585	2.626308	1.343764	2.091314	2.199442
HORVU4Hr1G022770	0	0	0	2.431698	105.8305	63.11112
HORVU7Hr1G085980	1.414849	1.936396	1.554809	2.997966	2.490429	3.275323
Hordeum_vulgare_newGene_3447	4.874676	6.909169	6.316462	1.7716	1.74575	2.309648
HORVU2Hr1G092530	10.6098	8.552506	10.07049	37.02628	60.56475	47.55819
HORVU2Hr1G053290	24.19414	23.92177	38.58407	19.84015	44.90727	47.79294
HORVU4Hr1G064760	14.55636	9.245167	8.60794	15.8837	53.89906	53.39925
HORVU2Hr1G020260	6.129331	6.753551	6.54862	4.379248	4.354974	5.182095
Hordeum_vulgare_newGene_2717	1.354538	1.372165	1.07108	1.574705	2.213088	2.229896
Hordeum_vulgare_newGene_2710	0	0	0	4.414877	8.379428	6.729455
Hordeum_vulgare_newGene_2713	10.64551	13.10578	13.44524	11.91101	10.54869	13.8939
HORVU4Hr1G070070	7.23637	7.736432	6.835724	13.57536	13.50248	12.88276
HORVU6Hr1G082670	23.21271	23.06713	18.39987	16.25438	15.83167	17.43964
Hordeum_vulgare_newGene_2718	71.61402	66.69168	88.45936	24.7637	4.987313	9.909307
HORVU5Hr1G026760	3.166541	4.29287	5.487411	3.699451	0.975705	5.62193
HORVU2Hr1G126740	5.623069	2.47684	4.499816	29.5829	50.10425	52.17927
Hordeum_vulgare_newGene_5842	2.981181	2.07592	2.971342	0	0	0.053933
HORVU0Hr1G017260	30.13865	22.67675	32.87113	26.37451	47.93388	50.38113
Hordeum_vulgare_newGene_5849	25.75058	27.55073	21.97685	19.40095	23.08889	21.91296
Hordeum_vulgare_newGene_5848	7.655299	7.237388	9.323745	10.21399	12.63712	13.09188
HORVU3Hr1G106070	1.602571	1.184178	1.949172	1.686866	1.546516	1.620544
Hordeum_vulgare_newGene_9099	0.588014	0.782295	0.673687	1.770644	1.219919	1.338151
Hordeum_vulgare_newGene_9097	11.87776	9.226908	15.3362	12.6153	15.91884	15.83106
HORVU0Hr1G005020	6.327588	5.045459	5.525333	4.49829	3.254846	3.702378
HORVU7Hr1G073500	8.323745	7.778899	11.13387	7.014454	8.7909	7.847852
Hordeum_vulgare_newGene_15259	0	0	0	1.893884	3.931394	3.574496
Hordeum_vulgare_newGene_15256	3.032502	2.228509	2.567935	3.287809	6.793446	6.609005
HORVU1Hr1G081490	6.829547	6.851394	8.831875	4.995826	5.163726	5.306898
Hordeum_vulgare_newGene_15250	2.559431	2.687425	3.035767	2.239824	1.731308	2.115171
HORVU6Hr1G013200	4.901755	4.795619	7.241334	4.452147	8.032242	6.9034
HORVU3Hr1G048240	5.367682	5.464279	6.595895	6.898392	8.363008	9.351984
HORVU7Hr1G082510	15.16059	16.4658	15.69737	16.04468	18.82348	18.22895
HORVU3Hr1G088860	8.579736	8.217333	9.839578	11.26354	11.37715	10.78118
HORVU4Hr1G020190	4.384631	4.515168	5.041844	5.687422	8.278483	8.492568
HORVU1Hr1G003960	0.662797	0.611493	0.972719	7.045783	8.242107	8.925051
HORVU5Hr1G028400	1.226607	1.078745	1.322623	2.567605	2.316288	2.556366
HORVU4Hr1G053470	14.45337	15.73267	17.67539	12.06185	18.93097	20.37653

HORVU4Hr1G035890	9.214474	9.735364	10.42086	13.45523	19.1941	19.27684
HORVU3Hr1G013840	289.9933	270.4248	366.6983	224.1584	271.3485	284.7684
HORVU1Hr1G077190	3.723836	3.647698	4.133472	5.07547	6.15377	6.693392
HORVU0Hr1G010230	2.277754	1.973295	2.950048	4.16652	3.735707	3.132682
HORVU4Hr1G076910	5.931701	6.55893	5.099041	6.004304	5.69827	7.537566
HORVU7Hr1G080850	1.58004	1.67798	2.784778	5.477701	14.76036	13.24918
HORVU3Hr1G093350	206.38	165.2896	176.1517	142.1562	231.4481	233.7579
HORVU5Hr1G011140	0	0	0.040136	2.184552	36.68377	28.04375
HORVU4Hr1G079620	9.0568	4.618959	14.07811	1.217548	0.093107	1.829316
Hordeum_vulgare_newGene_5156	4.43454	5.2585	4.902399	5.376808	4.98435	4.260153
Hordeum_vulgare_newGene_5154	1.300716	1.708418	1.884027	1.378707	2.011002	1.715016
Hordeum_vulgare_newGene_5158	0.41257	0.280739	0.500494	5.081426	4.735043	4.688629
HORVU1Hr1G055340	12.92905	15.27662	18.33422	2.90878	1.021201	2.047087
HORVU7Hr1G026400	19.83702	17.26168	23.25822	13.34377	15.42234	16.24295
HORVU4Hr1G034950	2.372546	1.922696	2.368968	3.23972	4.41847	3.865609
HORVU2Hr1G038770	0.638175	0.575573	0.662805	1.424224	1.664518	1.367097
HORVU4Hr1G069350	7.512245	7.050608	8.185953	13.19809	21.50826	19.49796
Hordeum_vulgare_newGene_11201	3.355408	3.440445	4.429923	3.81819	3.427673	3.626609
Hordeum_vulgare_newGene_11200	7.718339	7.818613	7.368536	12.46077	11.56428	10.13313
Hordeum_vulgare_newGene_3657	11.60712	5.177001	8.760955	13.44207	5.891568	9.87029
HORVU7Hr1G037830	0.072379	0	0.015737	4.606044	3.605596	4.014874
HORVU3Hr1G003880	1.201378	0.953231	1.032442	3.814451	2.029613	2.436282
HORVU7Hr1G081720	3.275861	3.828723	3.415262	6.561929	7.789747	7.367405
HORVU2Hr1G076320	7.991565	5.780897	8.986521	3.034787	1.726503	2.847415
Hordeum_vulgare_newGene_8055	34.42079	32.93864	57.41406	23.19195	39.56492	41.02633
Hordeum_vulgare_newGene_8054	3.467036	4.324314	4.264964	12.16171	10.58169	11.0445
Hordeum_vulgare_newGene_8056	60.29663	58.08625	94.68974	88.89873	172.6378	169.5403
Hordeum_vulgare_newGene_8051	1.970645	2.425689	2.94011	2.021996	3.180557	3.945564
Hordeum_vulgare_newGene_8053	3.092293	2.202569	4.05842	5.18298	5.133005	5.372462
HORVU7Hr1G070730	5.026013	5.123348	7.392615	7.453287	10.54348	10.06044
Hordeum_vulgare_newGene_8059	25.81675	31.00667	17.50473	3.326281	2.860825	3.552379
HORVU5Hr1G075620	4.945668	4.551428	4.966715	6.929894	5.849857	6.884945
HORVU7Hr1G063220	5.467455	7.306394	7.589515	9.502192	21.0379	23.41348
HORVU7Hr1G115510	12.96365	11.40218	13.06054	16.48265	17.75592	17.3426
HORVU3Hr1G014230	3.403919	2.667879	3.739673	3.806976	5.969346	6.7745
HORVU1Hr1G090180	4.65932	4.383186	6.550951	0.340078	0.27946	0.166652
HORVU7Hr1G021830	1.013635	0.781065	1.018412	1.475435	0.8589	0.909846
HORVU1Hr1G073540	7.317059	8.36333	7.97162	4.006028	4.310596	4.233033
Hordeum_vulgare_newGene_4607	0	0	0.006039	4.251516	5.356397	4.973237
Hordeum_vulgare_newGene_4602	2.775434	3.666619	3.312206	2.583554	3.321971	4.06131
Hordeum_vulgare_newGene_4603	0	0	0.045651	3.245695	3.799574	5.672565
HORVU1Hr1G031270	3.828918	4.210165	3.83239	0.511626	0.796018	0.948332
Hordeum_vulgare_newGene_7550	3.499671	2.847689	2.879601	2.977107	5.852931	5.145387
HORVU7Hr1G066300	24.77734	28.25742	27.68782	26.89553	29.41159	30.13251
Hordeum_vulgare_newGene_3879	0.522586	0.240868	0.629765	2.344453	1.873221	1.663674
HORVU1Hr1G035960	2.095975	2.411553	2.530609	4.61374	5.632054	5.640418
HORVU2Hr1G071960	16.30855	15.34017	17.3638	17.00164	18.93117	19.50095

HORVU5Hr1G033660	10.01477	12.8774	11.47115	18.84101	19.51603	20.53579
HORVU3Hr1G088040	4.78336	3.199705	3.272316	0.127042	0.202408	0.061335
HORVU1Hr1G063330	0.431934	0.294592	0.492294	1.198289	3.977901	5.200738
HORVU0Hr1G006940	1.747781	3.35923	2.278555	2.915589	2.652757	2.840463
HORVU1Hr1G076990	16.28514	16.27123	17.80067	17.76518	17.45526	19.73399
HORVU7Hr1G001210	3.102626	3.330563	3.975162	3.889424	4.540326	5.006522
HORVU2Hr1G032730	5.260264	4.126912	5.222779	9.54516	21.17961	14.73815
HORVU6Hr1G073260	42.06007	36.31969	32.55525	12.26468	6.345125	6.092105
HORVU1Hr1G081140	0.55102	0.570012	1.089036	3.026831	0.832999	2.287229
HORVU4Hr1G025670	3.699724	4.837134	7.067921	3.477419	5.952066	5.628797
HORVU7Hr1G099800	5.174385	4.743502	4.180212	2.322503	4.00995	4.786194
HORVU6Hr1G062260	13.49708	12.62276	12.93644	12.69581	20.16586	19.41182
HORVU1Hr1G011030	0.616354	0.15266	0.558663	1.763541	1.665848	1.601974
HORVU1Hr1G078450	6.725098	6.224105	5.92735	13.73179	13.94713	13.29216
HORVU7Hr1G052690	0.810999	0.483757	0.91404	1.215218	2.093968	2.606101
Hordeum_vulgare_newGene_6602	11.20736	2.643246	11.22949	12.89358	11.79944	14.84479
Hordeum_vulgare_newGene_6604	5.893679	7.161502	8.264502	7.028446	8.560105	8.926809
Hordeum_vulgare_newGene_6609	3.004601	1.757071	2.065709	2.155813	0.810704	1.190229
HORVU1Hr1G074590	19.19059	16.54277	20.03551	31.13151	39.44246	36.34393
HORVU5Hr1G059930	10.94187	10.5455	12.27189	11.07626	19.2689	18.80594
Hordeum_vulgare_newGene_14067	1.415109	1.285114	1.835347	1.291045	1.700235	2.088457
Hordeum_vulgare_newGene_3124	6.422686	4.531832	7.004823	2.11342	0.914356	0.763524
HORVU7Hr1G002740	7.638857	3.053233	14.06728	0.032385	0	0.085203
HORVU2Hr1G087390	75.24227	73.20397	84.01565	83.668	133.2288	139.7643
HORVU3Hr1G092570	0.933586	1.259446	1.11452	1.245481	0.880693	1.06618
HORVU6Hr1G070790	1.601627	2.250755	2.532016	5.128577	5.880126	3.30125
Hordeum_vulgare_newGene_2831	18.11062	18.60913	17.48494	29.97697	32.62508	34.71312
Hordeum_vulgare_newGene_2835	8.294032	8.097259	9.117962	11.73436	15.48454	15.81659
HORVU6Hr1G032570	5.926621	5.223178	6.083545	5.856457	6.420254	7.078029
HORVU4Hr1G005100	151.9184	146.612	130.5782	120.912	183.2477	186.7864
Hordeum_vulgare_newGene_14646	0.237017	2.379622	5.213382	0	0.062268	0.183929
HORVU3Hr1G093080	0.358735	0.230646	0.219128	2.501064	30.39706	42.41828
Hordeum_vulgare_newGene_14642	4.740806	4.02315	5.234953	2.023317	0.813128	1.054575
HORVU3Hr1G033000	7.580729	8.763948	8.114685	7.912031	6.672579	8.297938
HORVU7Hr1G078140	43.03728	44.08479	42.23395	47.58615	56.29755	54.77133
HORVU2Hr1G027480	4.255976	2.329488	5.736171	0.69441	0.941461	1.306987
HORVU3Hr1G081100	3.611516	2.447331	4.436669	2.211223	1.765163	2.592856
HORVU3Hr1G013300	43.68752	48.07398	37.6311	37.49228	44.65079	44.54897
HORVU0Hr1G000450	17.02084	18.01739	14.78649	6.352407	2.747953	4.348318
HORVU7Hr1G091920	19.79625	19.10263	22.45853	20.30895	28.53908	27.57989
HORVU3Hr1G110760	0	0	0	7.570583	4.008545	2.654083
HORVU3Hr1G078930	3.0868	3.138818	2.599348	6.058232	4.127017	4.411983
HORVU1Hr1G059890	5.700825	5.342504	6.726163	10.29114	12.70755	13.95283
HORVU2Hr1G043960	2.326297	2.073715	1.885662	6.292189	8.427914	7.665586
HORVU4Hr1G046810	2.953796	2.827229	4.396017	5.309918	18.42857	12.71866
Hordeum_vulgare_newGene_846	1.336284	1.3365	1.726011	3.331859	0.489733	1.728872
HORVU2Hr1G000940	13.81113	15.70762	13.68416	15.11476	19.3607	17.56373

Hordeum_vulgare_newGene_8932	6.653125	6.643525	7.508064	6.712116	6.010134	7.668197
Hordeum_vulgare_newGene_8933	3.697795	3.484354	3.062129	7.18502	10.42058	13.23451
Hordeum_vulgare_newGene_611	3.176997	3.182745	2.516335	1.647142	3.370596	2.813646
HORVU1Hr1G066100	11.7867	13.10044	15.4935	18.22859	20.47729	20.45181
HORVU2Hr1G082370	10.5008	8.251356	10.09153	10.02754	7.088764	8.006259
HORVU3Hr1G042540	5.553418	6.595622	5.903353	29.14898	43.93145	39.94313
HORVU1Hr1G061820	5.49167	5.307422	6.382762	9.463351	13.78038	13.05633
HORVU0Hr1G007150	3.583533	3.420142	1.855292	4.342967	6.785793	6.060761
HORVU1Hr1G086980	20.66517	16.10523	27.12254	11.85594	18.82408	19.07412
HORVU5Hr1G053980	2.585408	3.276636	3.945905	2.803493	3.293341	4.098833
HORVU3Hr1G015620	17.839	16.01269	16.3007	19.22151	20.78042	22.31122
HORVU2Hr1G013550	4.148005	4.375884	4.069643	6.655704	8.847003	8.124632
HORVU3Hr1G012160	2.513972	3.102661	3.837719	3.181268	4.639878	3.606029
HORVU3Hr1G107330	10.03164	6.954829	11.02318	11.58976	9.850192	11.16873
Hordeum_vulgare_newGene_16150	4.559355	4.93029	3.986011	3.911289	3.994046	5.177611
HORVU0Hr1G013850	76.98031	63.63678	78.60585	67.4231	98.64388	99.27292
HORVU6Hr1G067620	1.329907	0.344619	0.864944	1.621194	1.66073	1.836216
HORVU4Hr1G035740	3.67404	3.699245	4.89169	4.336418	7.849508	8.195237
HORVU1Hr1G048400	6.820744	7.203168	11.09621	6.177381	7.760975	7.110714
HORVU5Hr1G011070	12.45422	13.57999	13.88402	14.90308	23.24392	20.06262
HORVU4Hr1G039410	3.030412	3.248387	3.227148	2.646471	3.856275	3.615819
HORVU2Hr1G015940	10.9402	9.761242	11.0986	4.903584	3.932937	4.931483
HORVU6Hr1G081160	1.681423	1.799515	2.481187	2.89743	2.683086	3.159768
HORVU1Hr1G016430	9.68221	8.834405	10.49476	9.177651	10.01145	10.0531
HORVU4Hr1G065610	6.817363	5.607345	7.378774	6.213569	6.684497	7.753451
HORVU4Hr1G014740	21.0434	23.35385	20.7941	24.49501	25.76906	26.67656
HORVU7Hr1G047500	2.718751	3.269932	2.50609	10.4153	18.06854	16.48675
HORVU4Hr1G084370	0.650598	0.664781	0.262214	1.26448	1.879753	2.11961
HORVU5Hr1G025430	1.214603	1.292882	1.580636	3.022893	5.730603	5.050041
HORVU2Hr1G085800	11.87807	10.44732	12.29086	30.60493	46.31891	45.92241
HORVU6Hr1G004180	3.698063	3.607602	4.408027	2.878136	3.4633	2.987365
HORVU7Hr1G084610	5.064291	3.663708	5.895564	2.155996	2.742159	3.441782
HORVU6Hr1G084070	452.9626	580.7398	720.6708	29.31871	2.653998	13.30184
HORVU5Hr1G067110	9.101267	8.384431	14.26361	8.734261	11.49468	11.36439
HORVU5Hr1G008160	1.52258	1.493355	1.535663	1.484855	1.840421	2.217846
HORVU7Hr1G017640	44.57529	29.2392	43.48021	25.63093	12.15198	16.39953
HORVU3Hr1G079210	27.07543	24.71354	31.10541	29.79403	46.877	47.98086
HORVU7Hr1G095820	1.363797	1.584595	1.848222	4.2776	7.509193	7.145551
HORVU4Hr1G048110	0	0.008863	0	2.084923	2.48594	2.607711
HORVU5Hr1G060460	79.01324	78.76578	56.63031	62.1636	63.9621	65.40901
HORVU0Hr1G014110	1.90117	2.353757	2.181223	3.597515	6.575194	5.593822
HORVU2Hr1G080260	1.86012	1.211393	1.88002	3.603297	4.749294	6.566847
HORVU1Hr1G049460	4.146397	4.761977	5.248275	5.000198	6.752058	6.57766
HORVU5Hr1G026160	1.265093	0.922185	1.711104	1.997578	1.808797	2.1221
HORVU4Hr1G086300	3.640712	3.290963	6.203002	1.423309	0.491875	2.743931
HORVU3Hr1G071880	1.327494	1.468366	1.841944	1.776713	1.670419	1.225072
HORVU4Hr1G004120	2.856961	2.972448	2.992671	7.714556	5.155363	5.611724

HORVU4Hr1G014970	12.63407	10.45002	11.4883	16.29018	10.18662	11.99229
HORVU6Hr1G081890	9.23031	8.583482	8.697185	7.101241	7.064277	7.017702
HORVU1Hr1G057910	2.043762	2.222176	1.676506	1.210273	0.281809	0.444669
HORVU1Hr1G036120	3.661187	2.671832	3.341379	2.568984	2.170607	2.068791
HORVU3Hr1G005920	4.764043	5.179562	6.68505	10.25086	11.72891	9.698527
HORVU7Hr1G098470	0.916162	1.065399	1.176273	1.165138	2.319894	2.272173
HORVU2Hr1G017450	323.8346	253.4412	367.6675	337.3566	848.2517	862.7424
HORVU7Hr1G019590	0.124819	0.105263	0.178846	1.133184	3.643255	4.286395
HORVU3Hr1G064470	80.91923	64.80487	112.8647	96.3584	40.70479	75.20686
HORVU4Hr1G008200	6.327053	6.423256	6.407891	6.586341	3.94061	4.69807
HORVU5Hr1G095260	7.124328	6.974795	5.491193	5.058639	6.729871	9.872614
HORVU2Hr1G037710	2.448602	2.25256	2.813806	2.799626	3.163861	3.057732
HORVU4Hr1G004780	10.98907	8.7774	11.93628	16.54389	20.79684	20.42197
HORVU2Hr1G092180	2.052043	2.381895	2.057437	14.09233	23.99617	22.54373
HORVU2Hr1G019500	31.17624	20.01412	20.56027	31.26706	26.49498	28.03405
HORVU3Hr1G076680	4.558784	4.623293	4.959068	4.225832	4.457393	4.748202
HORVU7Hr1G074240	17.33579	15.57117	18.51581	15.53137	17.68126	17.57017
Hordeum_vulgare_newGene_10311	24.37309	22.99858	26.63829	25.32868	22.62255	24.89583
HORVU7Hr1G090240	10.94739	10.82966	12.87275	11.13701	15.03036	13.90239
HORVU7Hr1G085250	2.216737	2.655442	2.776012	1.744004	2.092727	1.780335
HORVU3Hr1G068780	4.290923	4.817699	4.624043	8.187868	14.51152	14.84184
HORVU4Hr1G050630	28.32141	26.90549	33.03706	37.89295	50.65735	46.97597
HORVU7Hr1G012910	0.755692	0.85823	0.956129	1.691847	2.462079	3.344047
HORVU4Hr1G006780	1.337446	1.937763	2.071012	2.701566	4.50724	3.626781
HORVU6Hr1G092440	19.16682	20.1914	17.98601	24.05969	27.24293	29.98284
HORVU2Hr1G093580	5.282731	6.348636	6.972412	11.06072	17.7768	16.52428
HORVU1Hr1G080200	0.223173	0.119347	0.442917	6.471308	19.28297	15.51632
Hordeum_vulgare_newGene_11041	0.900953	0.972576	1.225091	2.676623	1.957198	2.948187
Hordeum_vulgare_newGene_11043	3.085494	2.444343	3.173581	2.005946	1.563785	2.3479
Hordeum_vulgare_newGene_11049	0.713457	2.938675	0.013834	0	0.74515	4.519001
Hordeum_vulgare_newGene_11048	1.416345	1.76401	1.581841	1.906311	2.905992	1.701941
HORVU1Hr1G081890	1.239925	1.167951	1.30002	1.441513	1.908951	1.592657
HORVU7Hr1G116220	1.579554	1.649097	1.364106	0.532724	0.386041	0.498649
HORVU3Hr1G026060	96.47256	99.60482	142.8043	79.48525	153.4344	148.0791
HORVU2Hr1G108210	13.12385	13.37401	11.16152	13.29505	18.01132	17.38695
HORVU1Hr1G004480	2.140594	1.909059	2.203892	4.088793	6.086203	5.813984
Hordeum_vulgare_newGene_11596	1.292918	1.462541	1.759152	1.900758	1.220104	1.536245
HORVU3Hr1G062160	12.04254	13.64747	10.72039	11.76695	13.82851	13.82094
HORVU7Hr1G000120	2.49756	2.353805	1.986795	1.30095	1.655447	1.48542
HORVU2Hr1G115000	2.053371	1.909127	3.333588	3.340457	3.99476	4.24163
HORVU3Hr1G111930	7.958817	7.100346	6.96257	8.157749	13.81314	13.93426
HORVU3Hr1G038360	14.73122	14.22543	17.86014	14.11136	21.626	22.11706
HORVU4Hr1G056670	6.401928	7.345202	8.363713	6.632368	7.792042	7.042076
HORVU3Hr1G029260	94.03079	94.69701	75.4222	86.70229	68.07688	65.79919
HORVU3Hr1G092390	8.375153	9.825926	8.283401	18.33218	25.62022	25.28335
HORVU6Hr1G076510	2.262646	1.137869	2.323792	1.571061	0.93572	1.091176
HORVU6Hr1G057500	1.522946	1.31207	1.280498	6.869247	6.782782	6.085586

HORVU7Hr1G083560	5.296764	4.512321	5.865227	6.310354	5.06242	5.886876
HORVU6Hr1G040680	6.580358	6.968832	8.20624	7.690764	7.632356	8.984846
HORVU7Hr1G023920	2.231357	3.211446	2.392155	1.645404	1.068674	1.249289
HORVU2Hr1G057880	5.360829	5.905425	6.824193	9.822587	16.71673	17.84018
HORVU4Hr1G009960	3.028656	3.653891	4.513653	4.083733	5.66711	5.812548
HORVU3Hr1G051910	25.89295	24.72301	28.21675	21.96657	27.61552	23.66835
HORVU1Hr1G005280	2.562697	1.279584	1.776478	2.39324	2.261772	2.746298
HORVU5Hr1G049490	3.939821	4.619279	4.187252	2.659976	2.033196	2.780006
Hordeum_vulgare_newGene_2316	8.933608	9.301064	7.558645	9.36894	9.619031	9.57497
HORVU5Hr1G026460	4.177039	4.121654	4.494519	5.787128	5.413077	6.649905
HORVU6Hr1G015080	6.134832	6.186576	7.997076	8.457962	14.22242	13.24217
HORVU4Hr1G071480	22.48792	23.35784	26.98969	22.61056	34.8058	37.26997
HORVU7Hr1G098320	1.904788	2.592507	3.046793	3.750122	9.258372	8.576241
HORVU6Hr1G024900	1.889922	2.169681	2.414436	2.734872	2.355136	2.661308
HORVU7Hr1G026880	0.770133	1.062403	1.527436	2.215946	1.846662	1.74268
HORVU3Hr1G066420	12.5076	13.13809	13.62463	12.97126	11.84814	12.36878
Hordeum_vulgare_newGene_16069	16.16521	13.38614	15.17187	17.91738	18.07966	17.99275
HORVU2Hr1G039030	1.917805	2.044086	1.886869	2.461104	3.665999	3.435032
HORVU3Hr1G093930	50.84131	40.56019	51.08348	53.52683	47.87981	50.77341
HORVU6Hr1G088090	5.67087	5.552473	6.524927	4.240041	8.210315	7.056799
HORVU3Hr1G024570	1.260609	0.859508	0.878463	1.072892	1.284371	1.25266
HORVU3Hr1G088530	34.3696	26.45941	31.66166	15.10135	13.87255	14.21157
HORVU5Hr1G083210	1.817217	2.593411	3.217141	3.253799	5.497826	5.026515
Hordeum_vulgare_newGene_12768	4.056991	4.272479	4.313282	6.229564	2.491527	2.45452
HORVU1Hr1G014730	8.341787	7.504005	8.44281	16.16589	10.69494	13.29528
HORVU6Hr1G093520	15.34001	15.8961	18.39023	20.64524	8.803474	7.298431
HORVU0Hr1G020200	3.082337	1.969971	3.311434	4.256736	6.56004	6.220894
HORVU2Hr1G025190	1.561351	1.707082	1.847293	1.461027	2.547605	2.416087
HORVU5Hr1G107210	41.11734	33.83829	45.08928	47.45364	61.6927	54.96259
HORVU2Hr1G114660	10.13798	7.851236	9.22228	11.94724	9.591645	11.19476
HORVU7Hr1G042160	6.770592	5.889987	5.723187	28.78267	59.53739	54.69323
HORVU1Hr1G012060	1.442611	1.893498	3.309039	0.193674	0	0
HORVU3Hr1G052320	15.43363	18.86627	21.96588	11.24491	20.74943	19.22703
Hordeum_vulgare_newGene_13713	2.839666	3.542691	2.300857	4.912985	3.735873	4.977612
Hordeum_vulgare_newGene_7828	8.865208	7.827065	8.257806	9.193696	8.658178	7.437329
HORVU7Hr1G038630	2.965608	2.356174	7.771307	1.916422	2.536279	1.576692
HORVU5Hr1G080870	1.266503	1.186684	1.859976	5.961433	10.21069	7.10559
HORVU4Hr1G083400	15.45088	13.67287	14.88128	13.4687	17.9642	15.05446
HORVU1Hr1G047990	50.87334	37.5426	62.66889	37.01433	38.45776	41.18443
HORVU3Hr1G106090	1.343358	1.946748	2.334748	1.121406	0.693406	1.087819
HORVU7Hr1G075380	11.1917	13.60582	12.0735	12.61261	10.7315	14.43571
HORVU5Hr1G001090	6.087155	5.854913	6.678215	11.52378	16.71177	17.23064
HORVU7Hr1G070150	16.46577	15.36231	22.06559	20.37554	45.79662	44.00821
HORVU6Hr1G012050	44.50203	38.14511	52.97396	45.60641	69.89034	64.55454
HORVU2Hr1G100460	1.236364	0.648972	1.188072	1.645874	1.838394	1.933845
HORVU0Hr1G021760	25.63616	28.12697	32.29093	54.37116	133.3514	111.7413
HORVU1Hr1G082220	33.87807	35.66237	14.42867	0.167209	0.096255	0.225649

HORVU5Hr1G070260	9.259176	8.489928	10.93537	15.03141	20.38303	18.67376
HORVU7Hr1G081140	7.218236	5.645062	5.921891	7.596741	11.58807	11.76749
HORVU2Hr1G084120	62.54035	56.22694	77.82934	64.36079	71.88624	67.88996
HORVU4Hr1G058840	5.457284	5.951057	5.472376	8.387869	20.72025	19.25831
HORVU7Hr1G063970	68.66491	75.13326	72.60768	93.49171	146.8213	149.2966
Hordeum_vulgare_newGene_634	0.217684	0.168617	0.102733	2.918533	2.655124	2.127475
HORVU3Hr1G064130	1.272277	1.000115	1.760212	3.036706	2.791371	3.241169
Hordeum_vulgare_newGene_14339	1.573276	1.605419	2.593243	7.138704	6.906333	7.500654
HORVU3Hr1G110160	4.392813	2.731805	4.652218	9.800865	15.70524	13.82693
HORVU6Hr1G079120	3.560971	3.819207	4.055608	7.196764	7.22541	9.462564
Hordeum_vulgare_newGene_14333	3.622946	4.001128	4.378571	0.219937	0.053814	0.043438
HORVU5Hr1G000940	7.578803	6.897278	8.175142	1.599708	0.408978	2.297122
HORVU1Hr1G003020	0	8.294106	0.544202	12.08005	0	0
HORVU4Hr1G055690	40.02818	30.33924	35.90631	46.61381	89.12968	80.21493
HORVU5Hr1G065170	74.48056	62.15477	83.34735	82.52152	110.8843	116.8778
HORVU3Hr1G059440	1.384115	1.730796	1.620308	0.755154	1.27116	1.632225
Hordeum_vulgare_newGene_2506	9.239454	14.23425	14.74472	0.970768	0.675666	0.538252
Hordeum_vulgare_newGene_2501	1.641558	1.698088	2.289973	2.325149	2.289267	2.584623
Hordeum_vulgare_newGene_2500	7.030778	7.699914	7.601659	5.357188	4.308591	4.071187
Hordeum_vulgare_newGene_6883	8.987162	9.058919	10.19734	15.2094	21.0473	19.88347
Hordeum_vulgare_newGene_6889	1.987819	0.542551	2.438393	2.773207	3.577225	3.734347
HORVU6Hr1G059910	2.635518	2.300382	2.843144	4.360003	7.22391	7.610903
HORVU1Hr1G000590	14.20914	12.09382	11.65694	2.733561	1.301046	1.677776
HORVU7Hr1G096620	0.091359	0.257174	0.09706	1.700622	3.389435	2.757268
HORVU2Hr1G077120	31.87752	40.88892	33.79223	158.6797	636.3847	516.4442
HORVU4Hr1G078030	13.39909	13.71404	14.80953	8.675227	8.774911	8.863274
HORVU1Hr1G077290	5.093949	4.386453	6.744968	9.119178	6.301979	7.532239
HORVU2Hr1G033620	1.540557	1.425265	1.831143	2.524201	2.750775	3.491153
Hordeum_vulgare_newGene_10056	7.410277	9.057668	8.825186	9.381788	14.53741	13.64562
HORVU2Hr1G031990	8.981208	8.021003	11.35318	15.48463	34.55387	30.2233
Hordeum_vulgare_newGene_8485	2.535596	3.044822	2.786644	3.901616	4.308366	4.593136
HORVU1Hr1G000040	7.436662	6.104369	6.488774	22.1699	46.44427	42.08127
Hordeum_vulgare_newGene_11909	3.121195	3.936119	3.358982	1.139748	0.26788	0.543047
HORVU5Hr1G116750	6.62276	6.537947	5.736293	5.391161	6.938116	6.36678
HORVU4Hr1G061490	2.512084	2.793448	2.946771	3.187271	4.71446	5.167274
Hordeum_vulgare_newGene_11901	11.17256	12.03139	11.03243	18.13322	13.5918	16.8034
Hordeum_vulgare_newGene_11906	5.573347	6.646314	6.905796	6.457255	9.923417	11.50489
Hordeum_vulgare_newGene_9687	2.732207	1.38565	1.867012	6.034356	4.002885	3.318869
Hordeum_vulgare_newGene_9683	5.168715	5.983415	6.724799	9.36845	10.97407	9.252074
Hordeum_vulgare_newGene_1082	2.669881	2.624798	2.9835	2.559861	2.279722	2.783542
Hordeum_vulgare_newGene_1084	8.170943	8.019272	9.922926	5.538724	6.768657	6.460125
HORVU3Hr1G026540	5.76554	6.076304	7.268566	16.74282	41.72107	44.44587
HORVU6Hr1G076340	69.76726	58.20329	67.43253	106.3324	117.1685	118.1122
HORVU4Hr1G060720	337.594	307.935	334.7839	21.86821	54.26731	26.81176
HORVU0Hr1G002340	2.428382	2.252539	3.851751	5.231118	6.588722	7.566948
HORVU7Hr1G122120	38.91512	26.19878	59.14069	50.07172	10.26588	27.73688
HORVU4Hr1G047260	1.145736	0.883501	1.098322	1.337824	1.423196	1.360471

Hordeum_vulgare_newGene_9534	23.25145	21.07422	26.07564	16.83026	24.50934	25.61928
Hordeum_vulgare_newGene_9537	3.18654	2.967101	4.475053	6.443316	8.280489	7.091329
Hordeum_vulgare_newGene_9539	15.49044	16.37965	17.42067	16.67746	18.15889	18.77177
HORVU2Hr1G074780	8.185494	7.131366	9.065993	9.94735	13.6821	14.23652
Hordeum_vulgare_newGene_15426	3.750036	4.588831	4.371204	3.37579	3.341808	4.149818
HORVU3Hr1G034760	2.005862	0.609245	2.3274	1.208435	1.423399	1.886038
HORVU2Hr1G105140	13.58262	15.42368	21.54366	8.116495	5.755159	5.577617
HORVU0Hr1G038120	11.56544	10.28757	12.34612	24.33215	42.57736	41.6495
HORVU7Hr1G020590	1.27663	1.066646	1.36089	1.520454	2.368719	2.433199
HORVU1Hr1G072740	0.949766	2.235336	1.534931	2.330353	1.25501	1.721729
HORVU6Hr1G077430	5.991908	5.012749	6.791171	7.8026	5.889178	7.750854
HORVU6Hr1G070420	118.882	137.5651	161.8032	131.8493	146.2447	172.2105
HORVU5Hr1G052300	3.113482	3.514305	4.546637	1.883449	2.618139	2.715925
Hordeum_vulgare_newGene_6771	0	0	0	1.513113	3.576345	3.61007
HORVU1Hr1G068030	4.29354	3.961849	4.432392	4.061362	5.436965	5.296835
HORVU5Hr1G116360	6.539894	7.745819	11.57979	6.069553	11.39273	11.48316
HORVU5Hr1G043080	3.922557	5.256292	5.778004	5.010429	3.465609	5.476014
HORVU4Hr1G072150	2.577767	2.367374	2.763387	8.412699	12.00391	11.0162
HORVU3Hr1G043530	5.047313	5.37512	8.032837	8.181483	8.900268	9.01797
HORVU6Hr1G066000	3.431843	3.426783	4.114669	8.338341	8.11824	11.284
HORVU3Hr1G035900	10.83139	9.09036	10.3121	14.923	15.88765	17.95529
HORVU3Hr1G035080	1.337313	1.161866	1.247338	1.778917	1.025602	1.051032
HORVU1Hr1G073480	4.794311	3.734331	5.234741	5.041618	12.58954	6.874484
HORVU1Hr1G064200	2.205085	2.973348	3.167998	4.736064	4.817626	5.193314
HORVU2Hr1G083170	19.09923	17.36482	24.49682	12.45736	16.92293	20.40434
Hordeum_vulgare_newGene_7455	0	0	0.015973	8.777846	7.451264	9.602404
HORVU5Hr1G015600	14.14995	15.28893	10.64348	8.517668	55.02505	45.72933
HORVU5Hr1G043550	1.123264	1.728354	1.431343	2.772093	3.626136	3.436352
HORVU1Hr1G075560	42.67278	31.93383	53.14867	82.1904	118.2003	119.3763
Hordeum_vulgare_newGene_4814	0.615963	0.960806	1.304266	0.92506	1.258011	1.774719
Hordeum_vulgare_newGene_15883	1.218795	1.405403	1.208146	2.718684	4.762613	4.401386
Hordeum_vulgare_newGene_4819	0.784042	1.354595	0.973181	2.879879	2.844597	3.629669
HORVU7Hr1G024690	5.647717	5.372017	6.12455	6.61254	8.422022	7.94692
HORVU5Hr1G060110	5.063932	4.044639	4.89424	6.078969	6.352202	6.918272
HORVU5Hr1G101740	21.07626	19.27555	24.33663	21.83274	20.62064	21.56685
HORVU6Hr1G071770	18.0619	15.83933	16.94874	27.42487	33.26151	29.38548
HORVU3Hr1G018800	64.62316	55.1976	55.87808	26.09325	18.08493	20.74461
HORVU6Hr1G005900	3.813013	2.634358	3.843734	2.585201	1.586911	2.418416
Hordeum_vulgare_newGene_6418	10.95856	9.215649	9.314928	8.714887	6.313985	9.082162
HORVU7Hr1G024960	8.401039	9.581033	10.92595	12.26877	15.73971	16.43967
HORVU4Hr1G001840	14.17697	15.04152	12.61274	15.46714	17.20605	17.46053
Hordeum_vulgare_newGene_15819	1.265302	0.996118	1.07025	0.843635	0.934684	1.18834
Hordeum_vulgare_newGene_15817	8.649845	12.53689	12.55753	9.234431	8.563479	12.03421
Hordeum_vulgare_newGene_15388	17.45404	19.13474	17.29859	26.86929	25.39693	23.41312
HORVU3Hr1G034020	2.512627	1.266525	1.875508	0.789221	0.52618	1.105879
HORVU5Hr1G012490	2.434873	2.155979	3.074757	3.224525	3.387487	3.64274
HORVU7Hr1G052060	1.488868	1.794137	2.126547	1.277311	1.380754	1.750501

HORVU4Hr1G016440	9.654366	9.410933	11.86278	8.562664	11.99946	12.8361
HORVU1Hr1G088680	0	0	0	44.15083	0	0
HORVU1Hr1G051350	6.961864	7.325528	10.37568	11.32278	16.55949	14.93693
HORVU5Hr1G124770	16.65145	17.64179	17.07227	23.39206	32.35673	32.99658
HORVU1Hr1G074380	3.718501	3.416656	3.616431	6.352573	11.24234	14.2154
HORVU5Hr1G009150	4.357357	5.326698	5.703892	2.695847	0.292578	1.394964
HORVU4Hr1G053560	11.48393	9.16344	10.83502	17.7159	22.35629	17.4679
Hordeum_vulgare_newGene_680	5.27637	3.770684	5.023776	7.131828	6.282193	7.001933
HORVU3Hr1G082150	3.048293	2.652163	3.480229	2.686805	3.4907	3.665441
Hordeum_vulgare_newGene_9944	13.44757	13.77826	13.74688	14.44649	11.50649	9.595582
Hordeum_vulgare_newGene_9945	7.887904	9.47811	10.2996	0	0	0
HORVU5Hr1G022640	3.127282	3.033291	4.097532	2.994105	3.603669	4.583406
HORVU1Hr1G080070	1.298503	1.735492	2.001681	1.60037	3.16963	3.208227
HORVU0Hr1G000080	8.611115	7.467267	8.819368	6.836455	7.145322	7.319548
HORVU1Hr1G022100	6.963966	6.62149	8.384804	9.449317	9.321823	10.54472
HORVU2Hr1G000040	6.685266	6.986158	7.868142	16.32071	24.94122	22.25646
HORVU6Hr1G087310	0.2275	0.224414	0.16404	2.395082	1.723709	1.95987
HORVU6Hr1G030870	5.026347	3.506567	5.786513	7.429701	10.44785	11.2398
HORVU7Hr1G054380	13.53734	13.75548	14.92668	22.53367	34.57895	35.96554
HORVU4Hr1G027600	8.506517	9.641401	10.48751	12.29388	16.56554	17.72502
HORVU3Hr1G016090	0.084117	1.987462	2.400268	0.97019	0.480501	1.08767
HORVU6Hr1G084650	0.162023	0.165539	0.150254	3.459764	8.248059	7.475918
HORVU6Hr1G044840	20.65595	22.61941	35.87394	15.01164	25.69145	27.98524
HORVU4Hr1G056470	6.846536	6.391206	8.837298	17.78629	12.24649	15.26985
HORVU1Hr1G093400	11.01949	12.04226	17.65157	8.456047	10.53566	11.26251
HORVU3Hr1G029060	3.265461	3.942769	2.865101	3.656207	4.791114	5.343948
HORVU5Hr1G112420	11.17895	8.77346	10.31949	8.357112	11.89606	9.370615
HORVU2Hr1G043770	4.060739	4.233007	5.943894	7.605027	9.456015	9.473264
HORVU7Hr1G040030	4.840315	2.064066	5.620637	5.901771	2.356261	2.545578
HORVU6Hr1G006480	43.64106	36.21919	46.31942	23.84716	23.15072	26.2538
HORVU5Hr1G110370	27.29948	31.94409	40.2538	24.18239	34.37512	34.33397
HORVU2Hr1G085100	7.409769	7.033799	7.428499	5.497273	6.929008	9.219893
HORVU1Hr1G023270	3.040735	2.250722	3.541197	7.40092	11.42898	10.1013
HORVU2Hr1G051010	6.677013	6.628818	5.395277	0.265433	0.719123	1.738809
HORVU3Hr1G038610	3.41007	3.457558	4.097794	4.982551	5.66638	5.833008
HORVU1Hr1G011390	2.196425	1.354296	2.970113	4.260151	4.29863	3.110859
HORVU2Hr1G093530	11.5798	8.596722	15.43637	16.33938	11.08332	11.4675
HORVU3Hr1G083200	1.718222	1.87327	1.933777	2.302522	2.578328	2.974289
HORVU6Hr1G094410	7.100036	6.667753	6.693699	8.482412	7.598556	8.317658
HORVU2Hr1G095880	1.101018	1.358657	1.397026	6.609799	3.837755	4.836645
HORVU7Hr1G031620	8.532103	4.281148	7.320039	6.704455	5.392343	6.063218
HORVU6Hr1G090700	8.119217	6.477024	6.691772	8.161192	7.16796	8.916354
Hordeum_vulgare_newGene_2903	1.995927	2.234354	2.910901	3.460333	3.478697	4.276987
HORVU0Hr1G032120	11.63393	9.753491	16.05072	7.441617	10.45484	10.34964
HORVU3Hr1G023740	4.729487	3.756666	5.159278	10.63749	14.92439	16.01684
Hordeum_vulgare_newGene_2902	4.137179	4.545133	5.705862	3.907634	4.949417	5.225502
HORVU2Hr1G094280	10.78541	11.95399	14.83362	5.05787	4.160036	5.15225

HORVU4Hr1G068710	4.210891	4.223225	6.009571	6.34309	7.918038	8.047179
HORVU6Hr1G031830	564.3218	461.4768	672.1451	564.4607	698.5569	701.4868
HORVU4Hr1G010320	23.3242	24.24388	23.50763	19.75782	27.488	26.58698
HORVU0Hr1G007050	18.14926	12.92058	19.34583	8.323256	8.28176	9.126944
HORVU5Hr1G109680	3.107598	3.058131	5.094835	6.08776	12.17449	11.18235
HORVU5Hr1G097310	2.579116	2.176906	2.828588	4.056784	0.484569	1.060996
HORVU4Hr1G052570	9.261122	9.005541	11.23185	9.090395	10.22896	9.861172
HORVU3Hr1G015010	0.925541	0.820302	1.395608	1.074442	1.233751	0.982398
HORVU1Hr1G048670	0	0	0.009741	21.20963	13.13863	19.67871
HORVU5Hr1G059050	18.61025	14.90182	21.92825	19.98871	19.58505	20.73766
HORVU1Hr1G058020	6.303918	6.246008	6.339861	5.545777	4.98481	4.681632
HORVU1Hr1G085550	5.449513	5.626343	7.509746	4.839543	7.042629	6.78825
HORVU5Hr1G051530	5.058998	6.273548	6.411805	3.696926	5.632705	6.275389
HORVU3Hr1G072910	8.061537	9.93271	14.30394	17.97321	18.57883	19.99577
HORVU7Hr1G076030	1.506003	1.367913	1.418559	4.373613	7.282907	5.867923
HORVU3Hr1G071350	17.49925	17.25984	19.45501	12.6488	13.31375	13.54252
HORVU2Hr1G103310	124.465	101.5948	125.3616	241.5286	312.3754	316.8831
HORVU3Hr1G026150	3.736973	3.927727	3.360556	16.09652	34.67242	29.38391
HORVU7Hr1G088920	11.53148	14.46205	19.09283	6.622634	8.456199	7.37383
HORVU2Hr1G094420	1.594998	1.383025	2.856963	1.840429	2.780351	2.97406
HORVU3Hr1G084260	7.151123	7.174124	8.266186	5.749961	6.518108	6.873799
HORVU3Hr1G077980	0	0.008217	0.01444	8.461401	8.946576	10.18098
HORVU6Hr1G085880	44.32253	42.1215	43.6248	44.90175	42.57043	44.30948
HORVU2Hr1G100660	5.754404	6.599954	6.44952	27.74675	33.09153	32.3292
HORVU7Hr1G110090	0.495028	0.274431	0.561625	1.024866	9.029962	9.979461
HORVU2Hr1G067870	9.929833	10.42767	12.84089	13.46587	22.9329	20.5968
HORVU6Hr1G018740	3.081858	1.841802	2.117823	2.771264	3.458175	3.946041
HORVU6Hr1G057780	1.009891	0.792387	0.757088	1.159374	3.251148	1.794465
HORVU5Hr1G112990	33.21035	32.73324	30.67234	39.67628	48.66952	46.1969
HORVU2Hr1G061090	2.288009	2.887145	2.690571	4.545795	14.44348	13.00499
HORVU3Hr1G070400	1.201967	0.022789	0.842401	2.178427	1.179591	1.924191
HORVU6Hr1G003940	5.719827	5.217615	5.51868	12.05382	10.30446	12.26334
HORVU2Hr1G108780	12.81842	10.43906	20.3788	1.70397	2.831722	3.127196
Hordeum_vulgare_newGene_7863	71.84686	60.45526	74.75432	82.06445	112.6425	101.6983
HORVU4Hr1G055290	4.828191	5.35147	5.361176	6.887121	7.274635	6.556534
HORVU1Hr1G069990	4.221978	4.101208	4.266219	2.759537	2.561894	2.560147
HORVU3Hr1G099390	0.377705	0.376545	0.233294	2.344476	2.892532	2.730138
HORVU3Hr1G053460	24.65144	21.95559	24.23923	26.73606	34.25201	35.95492
HORVU5Hr1G045620	11.28327	11.82375	16.1436	11.84299	24.85394	24.47026
HORVU7Hr1G006660	2.937016	3.196755	2.595791	2.5599	5.973329	4.264073
HORVU6Hr1G021520	9.60408	9.870351	9.601586	11.97025	12.96349	13.98325
HORVU2Hr1G014060	25.6612	27.06218	27.83165	20.44619	21.02967	26.80772
HORVU1Hr1G094990	7.301824	7.443786	10.51405	8.515419	11.72493	11.58872
HORVU5Hr1G053170	5.248321	5.844849	6.27141	4.915032	4.232446	5.679036
HORVU4Hr1G064060	12.94446	11.40982	12.0502	14.5614	17.12251	17.75776
HORVU5Hr1G121910	0.083628	0.071904	0.078014	0.427794	3.925686	3.210768
HORVU0Hr1G011310	10.09317	10.28338	12.2171	9.519549	14.10126	14.6192

HORVU2Hr1G109430	1.096287	1.089283	1.92062	0.794337	0.312033	1.545254
Hordeum_vulgare_newGene_10295	3.257937	2.130918	3.19181	2.896391	3.584693	3.021646
HORVU2Hr1G066830	22.71893	32.47927	28.76972	21.26444	23.57641	23.23693
HORVU3Hr1G007680	0.932638	1.456189	1.115589	1.613399	1.062621	1.726815
HORVU4Hr1G007620	23.85792	21.19431	26.10135	49.35338	104.612	102.7779
HORVU3Hr1G021650	2.357085	2.498324	2.933732	2.685298	4.069901	4.02128
HORVU5Hr1G043530	5.1796	3.90577	5.427046	3.621166	5.555375	4.995533
HORVU2Hr1G097240	6.223112	5.991515	6.451266	9.743953	7.032131	7.753638
Hordeum_vulgare_newGene_7866	10.02148	10.30773	11.39359	9.092297	11.14358	11.19
HORVU6Hr1G040960	3.322003	0.829638	4.216786	5.406598	7.013184	7.847069
HORVU4Hr1G082690	9.105746	9.759411	10.61382	11.47479	11.62209	12.61404
HORVU7Hr1G049370	283.5701	258.0871	274.037	424.6954	778.5991	607.6806
HORVU7Hr1G089380	6.808369	5.292276	7.030899	6.619346	8.310253	7.143601
HORVU2Hr1G048100	0.654405	0.683079	0.529623	1.917494	3.23278	3.661761
HORVU7Hr1G008880	7.003223	6.999526	11.21099	7.861127	9.414951	7.645357
HORVU4Hr1G025770	3.740092	5.028524	6.841605	3.980776	9.201206	10.96378
HORVU1Hr1G060150	11.81698	9.140153	13.0831	11.70638	17.35953	17.0251
HORVU3Hr1G114440	1.491255	1.481749	1.554669	2.136493	3.127495	3.441182
HORVU7Hr1G068440	19.53684	24.16164	21.37162	29.27272	20.46294	27.99278
HORVU2Hr1G092390	6.489204	6.464158	9.026855	3.176281	1.253624	1.003438
HORVU4Hr1G024790	6.19492	7.11052	9.094511	4.923411	5.639822	6.298944
HORVU7Hr1G089960	5.638892	5.96103	5.721909	8.567134	11.26799	12.55056
HORVU0Hr1G022000	4.185906	4.191097	3.721252	5.529046	4.122746	3.537948
HORVU2Hr1G073370	8.027568	7.224419	7.257378	24.56564	56.55009	51.63776
HORVU2Hr1G022090	210.1984	228.561	341.561	271.6336	654.3827	591.1891
HORVU7Hr1G027120	17.68759	15.54997	19.29538	17.94053	23.79901	22.94426
HORVU2Hr1G122730	3.679236	4.33169	5.012667	6.851598	9.159936	10.13184
HORVU2Hr1G106960	4.422781	3.538532	4.597028	5.943759	7.990222	8.750684
HORVU5Hr1G081500	24.45372	25.08862	26.5603	15.33879	7.77014	11.65327
HORVU3Hr1G104260	8.341092	8.146235	8.718006	0.470266	0.36793	0.414556
HORVU2Hr1G004820	4.249215	4.845283	2.568451	11.41819	12.70656	14.00788
Hordeum_vulgare_newGene_1862	0.018166	0	0.007004	0.089034	6.240592	4.356753
HORVU7Hr1G069710	22.0784	20.01452	20.37415	36.83073	37.80969	42.08566
HORVU3Hr1G077170	2.607568	2.636661	2.843058	1.999143	1.222161	1.383282
HORVU3Hr1G073840	26.2996	25.96433	29.16533	26.39611	49.25055	48.65255
Hordeum_vulgare_newGene_11653	1.940034	1.80667	2.193161	10.65919	9.896683	13.24348
HORVU2Hr1G110740	2.061	2.598036	2.627396	1.936789	1.960337	2.632655
HORVU6Hr1G024000	10.31768	10.66136	8.873952	4.990461	4.746348	4.28212
Hordeum_vulgare_newGene_11658	14.93843	15.05154	16.34685	19.01074	30.07213	30.23903
HORVU3Hr1G027160	39.47081	33.99583	37.00454	37.02181	31.60716	40.28843
HORVU2Hr1G118240	213.4334	194.9156	233.3544	285.6532	286.1272	288.2117
HORVU4Hr1G063240	0.895104	0.728894	1.01801	2.509323	2.184984	3.150703
HORVU7Hr1G080710	8.201335	8.037934	8.244514	2.974468	2.491039	2.733173
HORVU3Hr1G058910	11.21183	11.83845	11.17774	16.44829	22.39622	22.77331
HORVU2Hr1G038220	5.319155	5.031609	6.551248	6.131868	8.028719	9.450802
HORVU5Hr1G076330	4.282464	5.133335	5.896552	5.42435	4.356278	5.382867
HORVU7Hr1G072050	10.82555	10.15926	10.18021	9.887917	8.379152	9.865909

HORVU5Hr1G005740	4.79845	4.083124	5.426119	11.79518	14.38011	13.71178
Hordeum_vulgare_newGene_5279	2.432962	3.449685	4.07301	1.377565	8.392754	6.760755
HORVU3Hr1G010070	0.612361	0.503259	0.976443	2.046586	2.819108	3.031149
HORVU1Hr1G046400	0.839732	0.477084	1.293933	7.451428	40.41503	50.14784
Hordeum_vulgare_newGene_12352	0.373715	0.454988	0.500827	1.381254	1.62567	1.840504
Hordeum_vulgare_newGene_12353	7.533108	6.669312	9.837499	9.437689	9.91643	10.18153
HORVU3Hr1G107240	14.72673	17.97206	20.25605	9.091893	5.385506	5.991923
Hordeum_vulgare_newGene_12356	3.299353	2.804452	2.068714	4.848986	2.955656	3.617635
Hordeum_vulgare_newGene_12357	5.007167	3.885751	3.357989	6.844164	4.034807	5.480522
HORVU2Hr1G072960	1.42045	1.054513	0.68108	0.563192	4.130538	3.014625
HORVU5Hr1G072650	11.62769	5.524198	17.77575	17.78079	51.87375	51.6816
HORVU2Hr1G092800	6.005135	5.784323	6.277984	18.69676	19.19193	18.05516
HORVU1Hr1G036040	5.301867	5.602329	7.195801	7.536348	8.336376	10.51486
HORVU4Hr1G011210	4.355879	5.963871	6.719106	0.06451	1.081054	0.473793
HORVU7Hr1G030540	1.836889	2.406372	2.144542	5.911367	13.13768	10.46497
HORVU2Hr1G087050	2.838662	2.877023	4.223041	4.200835	5.597712	6.200792
HORVU7Hr1G038770	9.595408	9.513912	8.766818	18.45487	36.61329	30.8082
Hordeum_vulgare_newGene_14117	2.380081	2.20087	1.546933	1.999297	0.620402	1.112586
HORVU1Hr1G085050	0.524538	0.400915	0.462032	1.849753	2.782412	2.868775
HORVU7Hr1G077860	494.297	527.4287	782.1662	64.10631	0.806198	20.68138
Hordeum_vulgare_newGene_3469	7.375219	7.797815	7.672466	12.40433	17.28846	15.11196
HORVU2Hr1G013390	0.020895	0.051579	0.012061	0.653165	3.558091	2.296876
HORVU3Hr1G063860	21.48537	23.31423	26.69459	21.62455	26.55778	25.98018
HORVU3Hr1G061790	84.38084	71.01356	85.36387	87.59827	99.82616	101.299
Hordeum_vulgare_newGene_13401	0.838155	0.305121	0.33342	5.295651	3.261042	4.446604
Hordeum_vulgare_newGene_6258	15.41714	14.91257	23.93612	19.65308	33.85496	33.85283
Hordeum_vulgare_newGene_13405	1.176244	1.684018	1.634541	3.878965	9.429705	7.340605
HORVU6Hr1G051790	1.6563	2.287448	2.92917	2.305184	5.730074	5.55133
HORVU4Hr1G022680	35.18569	33.00217	29.62045	22.65216	19.4329	21.57598
Hordeum_vulgare_newGene_3575	25.59857	21.80482	26.62888	24.30172	27.50155	27.85721
HORVU5Hr1G068280	1.399944	1.605857	1.406581	2.538284	2.269043	2.09387
HORVU2Hr1G092510	7.127921	4.395815	7.10914	14.40389	23.64321	19.39863
HORVU6Hr1G026330	1.256853	1.738936	1.160808	1.586405	1.147495	2.252332
HORVU4Hr1G054770	16.72265	17.00582	18.33469	18.64032	13.60497	12.03082
HORVU5Hr1G074370	1.368285	1.849816	1.849745	1.4456	1.705769	2.441113
HORVU7Hr1G118370	35.58327	34.71339	33.14395	41.18871	60.54483	57.87341
HORVU1Hr1G070390	9.958107	8.254379	11.19276	11.11987	9.934815	9.289114
HORVU2Hr1G049070	6.764966	7.380088	8.65836	8.33708	12.14922	12.14678
HORVU3Hr1G048950	2.588434	2.250003	2.763977	7.040025	8.252683	7.722546
HORVU6Hr1G051860	8.283787	8.318023	7.269812	15.90221	19.46273	18.75618
HORVU3Hr1G110550	0.277162	0.114276	0.140914	11.01294	12.56339	13.24065
HORVU2Hr1G032830	175.4664	148.0615	211.1335	215.6511	317.3892	304.9477
HORVU5Hr1G069880	13.22991	9.354388	11.20322	7.126717	1.879225	3.232148
HORVU4Hr1G020680	9.028917	8.453702	8.987932	8.738354	8.493134	8.702693
HORVU1Hr1G003270	4.114524	14.27959	4.896025	21.47651	8.275084	8.824519
HORVU7Hr1G097780	20.91738	18.73773	23.25302	16.16648	16.62517	16.40555
HORVU7Hr1G028430	6.484804	4.773481	6.274001	8.356662	2.264503	4.678843

HORVU3Hr1G054200	10.24317	11.96259	12.04245	14.64263	14.60473	16.96615
HORVU5Hr1G073680	47.18619	49.97581	58.02921	45.18538	42.52926	42.81501
Hordeum_vulgare_newGene_15764	0.037179	1.440052	0.733581	0.672364	2.622899	3.121839
Hordeum_vulgare_newGene_15765	0.813166	0.551156	1.666883	2.147867	2.589421	3.037842
Hordeum_vulgare_newGene_15763	0	0	0.03117	9.668382	9.761772	11.29512
HORVU6Hr1G065360	1.800554	1.897195	1.688504	5.207974	10.10286	9.570909
HORVU0Hr1G023150	3.782068	2.950386	4.353146	3.811063	2.297506	2.758397
HORVU5Hr1G078500	3.748896	3.177427	3.771746	5.467166	5.835501	5.901143
HORVU5Hr1G024400	6.708924	3.165222	7.978035	9.557331	3.695384	4.293422
Hordeum_vulgare_newGene_11659	1.803731	2.347439	2.035231	2.371854	5.682853	5.382285
HORVU4Hr1G011430	11.01219	10.48863	12.93975	7.799149	9.541584	8.497458
Hordeum_vulgare_newGene_15493	1.216901	1.796212	1.450233	1.607301	1.834055	2.132027
Hordeum_vulgare_newGene_15491	13.81394	12.16016	16.08015	13.07404	14.02941	12.46992
Hordeum_vulgare_newGene_15496	2.562822	2.2828	2.717231	0.016153	0	0
Hordeum_vulgare_newGene_15497	16.57299	20.51787	24.74389	25.66557	34.26372	33.09642
HORVU3Hr1G111600	62.25254	31.37642	91.57836	141.188	69.84364	120.1265
HORVU2Hr1G119920	1.935858	2.172278	2.441059	2.370568	3.389948	3.416676
Hordeum_vulgare_newGene_6929	1.242884	0.612727	1.137833	1.383303	0.888616	1.236105
Hordeum_vulgare_newGene_840	1.801283	2.292296	2.464131	3.244688	3.324658	3.630343
Hordeum_vulgare_newGene_845	2.301084	2.455998	2.227441	4.133934	2.420446	3.013961
Hordeum_vulgare_newGene_6920	0.23717	0.5133	0.256329	1.699799	2.162046	2.990537
Hordeum_vulgare_newGene_8565	0	0	0	2.100282	1.913534	2.372604
Hordeum_vulgare_newGene_8567	1.5518	0.840835	1.716823	1.13117	1.942365	2.445184
HORVU1Hr1G059610	8.279482	6.511461	7.158862	7.602351	5.38133	5.696842
Hordeum_vulgare_newGene_2735	3.587911	4.744653	4.166058	4.512214	6.646219	7.050649
Hordeum_vulgare_newGene_2738	8.747617	7.880691	7.481184	10.37202	10.9568	9.431427
HORVU3Hr1G016700	3.976115	4.156615	4.741519	2.063584	1.858673	2.683542
HORVU4Hr1G021220	16.61341	16.56751	20.56299	21.30374	20.55091	20.89644
HORVU2Hr1G040800	37.26721	31.04232	31.62556	30.83017	16.76165	19.33952
HORVU0Hr1G004270	11.28684	11.47031	10.87944	16.97614	14.71924	20.05465
HORVU4Hr1G074930	2.116445	2.057579	3.612117	3.834592	4.364865	3.879125
HORVU4Hr1G076570	5.294074	5.517385	7.479264	4.211511	2.994164	4.149013
HORVU7Hr1G037320	1.760332	1.50125	1.796713	2.508074	4.593189	3.565835
Hordeum_vulgare_newGene_1329	1.84391	1.64028	1.53567	2.330746	3.447967	3.120104
HORVU6Hr1G090690	1.353175	1.556394	1.965055	3.338059	1.786787	2.21974
HORVU7Hr1G042100	131.9738	119.7556	123.8771	104.9397	118.9652	115.8294
HORVU4Hr1G081640	9323.106	10427.8	8752.169	6814.479	11015.34	7188.361
HORVU1Hr1G076170	2.218399	2.204817	2.495091	3.267682	5.643079	5.857022
HORVU2Hr1G034200	1.93113	2.443836	1.95912	1.949363	2.923646	3.162863
HORVU2Hr1G088640	124.4096	101.9322	130.751	51.68365	10.47868	20.47455
HORVU6Hr1G069620	1.137487	1.342391	1.135872	1.330042	1.234334	1.919958
Hordeum_vulgare_newGene_15316	2.706627	4.847727	4.786081	5.470362	7.597944	7.816231
HORVU5Hr1G091480	1.940113	1.947353	2.335834	2.182789	3.767283	4.292269
HORVU6Hr1G035880	3.195002	2.81947	3.587138	4.761793	4.631327	4.609931
HORVU7Hr1G048810	18.29897	14.09199	18.20246	20.30506	23.5295	24.86142
Hordeum_vulgare_newGene_15310	1.318272	0.95365	1.134631	1.477726	1.470013	2.163625
HORVU5Hr1G010370	0.38086	0.254235	0.42118	1.357564	3.987129	3.889139

HORVU5Hr1G093710	5.820479	5.846368	8.375143	9.689517	22.97474	10.21156
HORVU6Hr1G020220	1.685067	1.392266	1.780783	2.36277	2.578202	2.752043
HORVU2Hr1G045970	0.035734	0.237123	0.236363	1.212894	2.505527	2.712367
HORVU5Hr1G061120	17.65811	15.17609	16.23458	33.17451	47.47172	46.8539
HORVU2Hr1G083030	3.378244	4.111114	4.24955	4.584131	5.086008	4.524135
HORVU7Hr1G090430	1.060124	1.715257	1.645756	1.474972	1.580752	1.368423
Hordeum_vulgare_newGene_16156	35.02011	34.94455	49.26544	21.63992	20.83906	24.36927
HORVU5Hr1G060090	15.60364	15.42825	14.95389	13.92909	17.26706	17.39013
HORVU6Hr1G032080	0.517094	0.478515	0.590557	2.548068	2.412569	2.086159
Hordeum_vulgare_newGene_16159	2.182347	2.720895	2.63404	0.08273	0.039862	0.016056
HORVU3Hr1G040740	15.56219	12.96501	14.79738	18.53127	18.60755	20.1323
HORVU3Hr1G085080	2.248132	2.353803	3.85689	1.205547	0.123331	0.416199
HORVU6Hr1G072950	5.677911	6.48701	7.105461	10.73768	31.61773	28.251
HORVU2Hr1G010440	8.662336	9.091527	9.156595	23.69954	63.24487	57.82738
HORVU7Hr1G038980	2.182842	1.102621	2.047638	1.887985	0.194916	0.661974
HORVU1Hr1G074460	1.712809	1.756127	1.814119	0.736199	0.957314	1.857753
HORVU1Hr1G045520	35.20602	33.50269	29.43052	23.93171	13.76004	18.348
HORVU2Hr1G078990	1.907265	2.00741	1.746283	2.774331	4.666868	4.681472
Hordeum_vulgare_newGene_15234	1.074776	1.032929	0.403549	1.242102	1.630526	1.368672
HORVU5Hr1G088360	12.22078	11.7778	17.33559	16.70892	24.1091	24.16233
HORVU5Hr1G058310	21.10969	29.32571	29.39549	4.579747	0	1.50237
HORVU0Hr1G020210	3.012172	2.493253	2.939389	2.130663	2.352704	2.571447
HORVU0Hr1G018890	3.088985	2.097733	3.46948	2.492641	0.195866	0.60163
HORVU1Hr1G087180	11.76362	12.24961	9.846099	11.95975	13.52339	14.03463
HORVU0Hr1G000160	3.244643	3.084104	2.722851	1.969551	1.455847	1.411766
HORVU1Hr1G039540	6.628625	8.5054	8.115205	2.481147	1.252111	2.253795
HORVU3Hr1G007110	1.225247	0.670442	1.439576	0.942945	1.795181	0.960534
HORVU4Hr1G013190	5.856635	5.444917	6.819488	15.38997	16.55287	15.74718
HORVU1Hr1G060790	2.922493	2.250892	3.241248	1.864633	2.74356	2.655382
HORVU3Hr1G028270	26.56182	27.39549	28.41885	0	0.01096	0.007991
HORVU7Hr1G055220	2.157679	2.039036	2.535494	4.31439	5.882997	5.455731
HORVU6Hr1G038590	23.09271	24.82898	21.66905	24.78967	22.15437	23.46308
HORVU1Hr1G019340	3.253308	3.698794	4.458198	3.48072	3.478459	4.54783
HORVU3Hr1G087620	7.901027	7.112978	11.30203	7.80343	7.898084	9.09849
HORVU5Hr1G094210	4.146169	4.356887	4.180019	7.013343	8.610842	8.874118
Hordeum_vulgare_newGene_12439	0	0	0.087093	5.54764	6.247723	6.212242
HORVU5Hr1G036220	2.655322	2.843864	1.918929	1.779164	4.427811	3.491851
HORVU7Hr1G106960	6.745219	7.172277	7.645512	23.23734	24.13566	24.84347
HORVU4Hr1G053400	4.824483	5.244653	5.437829	6.563215	9.526859	9.628551
HORVU6Hr1G019190	21.7219	25.21407	20.25109	25.57005	29.6885	29.30091
HORVU0Hr1G011840	2.888905	3.059352	3.306551	3.145689	2.886825	3.973683
HORVU1Hr1G022310	1.504885	0.689072	1.016594	4.437964	4.93848	4.865559
HORVU7Hr1G096990	75.52999	75.5519	112.215	76.2189	116.3997	118.1634
HORVU3Hr1G108000	26.76911	22.79069	23.74571	28.02147	19.26972	19.67522
HORVU0Hr1G016310	1.833671	1.022391	1.961866	1.182209	0.593687	1.267774
HORVU7Hr1G011890	18.83298	23.25588	24.15187	10.497	11.62059	12.89486
HORVU5Hr1G011760	3.421815	2.955766	4.298629	3.236117	4.823305	4.359556

HORVU4Hr1G011770	7.12519	7.112548	6.999687	11.52351	15.28316	15.90967
HORVU5Hr1G122150	13.23709	11.17028	13.79738	26.6953	34.80609	34.42235
HORVU4Hr1G002270	71.48319	53.9134	75.26769	88.0721	109.1251	136.2424
HORVU3Hr1G022620	2.070663	2.216726	3.33804	5.249222	12.29033	10.68734
HORVU2Hr1G047410	0.534052	0.59284	0.677759	1.0213	2.051384	1.764063
HORVU1Hr1G084520	2.683166	2.451668	2.138913	6.996462	11.28274	10.6604
HORVU3Hr1G106210	8.602124	8.749574	9.959427	19.02894	32.38911	28.84442
HORVU1Hr1G091120	1.213748	1.458582	1.724305	2.589542	1.943127	2.466406
HORVU1Hr1G052110	0.423966	0.43739	0.609697	4.780273	8.420027	7.238019
HORVU5Hr1G008980	15.63439	16.59107	18.72122	18.72101	21.11379	21.6009
HORVU6Hr1G023820	9.661086	10.27199	9.784226	12.0631	11.28016	11.91216
HORVU5Hr1G118710	16.92116	13.87213	22.10533	8.681707	10.60489	12.01687
HORVU6Hr1G000230	1.806735	2.386707	2.258046	12.64905	30.10955	26.95053
HORVU3Hr1G066850	13.01832	12.34447	14.68746	10.58036	13.39875	11.48506
HORVU7Hr1G040590	3.143211	2.98279	3.022209	3.640634	2.125173	2.57245
HORVU7Hr1G091500	29.95276	24.53172	30.89695	20.64522	17.49866	18.67718
Hordeum_vulgare_newGene_682	1.149018	1.186643	1.438178	1.59029	2.207229	1.945387
HORVU1Hr1G013700	10.99172	10.51357	9.366142	6.151673	6.695175	6.244391
HORVU7Hr1G095080	3.49472	2.502681	4.200821	18.60977	41.90855	29.70328
HORVU1Hr1G002300	6.221811	17.84891	11.80651	15.1096	1.219219	2.43713
HORVU2Hr1G015870	0	0	0	9.041592	6.986058	5.038074
HORVU7Hr1G088880	57.90084	42.51385	55.46439	104.2452	213.2522	171.9729
HORVU7Hr1G046290	41.41562	44.2324	47.51304	26.32012	25.1249	27.28311
HORVU4Hr1G084410	1.194909	1.067837	0.903066	5.357932	11.48512	9.290805
HORVU4Hr1G065900	2.477012	2.223765	2.477717	1.599949	1.326605	1.78848
HORVU2Hr1G065080	18.46923	16.6533	17.59111	13.12726	13.03512	13.15508
HORVU2Hr1G052550	7.507358	9.956242	9.946351	9.145711	10.40017	12.4169
HORVU5Hr1G105060	0.624199	0.133779	0.677009	0.908794	2.714244	1.998524
HORVU4Hr1G052430	6.107036	5.862428	6.676651	5.916303	6.146335	6.920245
HORVU2Hr1G013150	1.713534	1.889296	2.737836	0.838704	2.220587	1.67134
HORVU4Hr1G014850	1.735444	1.765039	2.106975	2.554055	2.919882	2.79908
HORVU5Hr1G119040	3.40202	3.843562	3.592494	9.070657	12.58972	11.5706
HORVU6Hr1G017850	11.83519	11.37554	12.49479	11.54786	12.64019	12.24835
HORVU2Hr1G106750	2.097726	2.028764	2.758457	2.827698	4.714098	5.391675
HORVU3Hr1G084540	1.996739	2.403445	3.155875	1.189835	1.810935	2.123147
HORVU0Hr1G039920	4.165541	3.296536	5.230479	3.008107	4.416001	4.310809
HORVU3Hr1G002840	0.265373	0.451089	0.391613	3.267212	3.658356	5.763688
HORVU0Hr1G020130	2.078287	2.518398	2.163283	4.610434	7.110677	6.787864
HORVU2Hr1G052880	49.57268	39.3605	58.24395	50.67312	101.927	102.2498
HORVU5Hr1G111670	9.231094	8.830134	9.95658	8.953348	10.27299	11.69299
HORVU4Hr1G026010	0.95026	1.028777	1.293264	0.723258	1.41786	1.138271
HORVU5Hr1G057510	21.56914	23.411	26.38883	24.58642	18.11318	26.02024
HORVU5Hr1G025810	9.088152	8.41106	11.7377	7.538179	8.309637	8.382049
HORVU6Hr1G092910	14.10729	16.11631	12.04375	17.18732	15.51922	14.56584
HORVU5Hr1G045290	2.019461	1.399684	2.010169	6.580198	12.11268	10.53648
HORVU7Hr1G094060	0.359793	0.423724	0.230424	1.965633	5.79083	5.463424
HORVU5Hr1G017530	0.797999	1.325506	2.581974	4.818122	107.1288	96.52281

HORVU2Hr1G071100	1.79127	1.371947	1.71011	4.106014	6.641884	6.596992
Hordeum_vulgare_newGene_9016	3.734297	3.67786	4.480184	3.153321	3.898409	2.916316
HORVU7Hr1G082590	1.337251	1.07284	1.293246	4.60358	6.80714	6.552785
Hordeum_vulgare_newGene_9015	7.015033	7.773777	8.0502	15.49615	19.42819	18.54371
Hordeum_vulgare_newGene_9013	2.855361	2.878522	3.193311	4.831317	5.516194	6.16488
HORVU0Hr1G015770	9.340794	9.547252	9.876145	7.98555	7.92016	9.108155
HORVU4Hr1G004090	5.350764	6.165464	5.204569	7.515414	6.770114	7.753744
HORVU2Hr1G033320	0.162568	0.139356	0.223201	3.635081	3.254815	3.791377
HORVU6Hr1G026680	3.306795	3.477836	3.999915	6.964991	8.455377	8.486005
HORVU2Hr1G091290	3.511201	3.447014	4.223851	7.475091	7.225331	7.811368
HORVU2Hr1G101900	2.236063	2.452691	2.638408	2.864733	2.110872	3.14777
HORVU3Hr1G025270	30.75792	32.07107	30.55389	24.88583	24.75986	27.90602
HORVU5Hr1G095990	6.817269	7.814692	8.228311	24.94373	25.5581	28.25946
HORVU1Hr1G004970	12.89288	15.35967	16.86684	27.38715	37.32699	36.19377
HORVU6Hr1G019280	18.56388	20.17103	19.16729	15.79178	19.52608	19.66068
HORVU2Hr1G030670	8.262319	8.720946	10.06768	10.84859	13.98195	13.81383
HORVU4Hr1G020110	7.659419	7.516175	7.331617	12.66847	17.68554	16.49751
HORVU1Hr1G077420	4.624124	5.295638	6.325214	2.164445	0.665738	1.155415
HORVU2Hr1G099590	2.398398	2.510145	3.429226	7.055177	10.67014	7.77976
HORVU4Hr1G025090	9.75936	8.441806	13.16332	7.889581	11.47847	6.325281
HORVU2Hr1G066970	2.026071	1.392495	2.94715	3.501412	4.601483	4.105403
Hordeum_vulgare_newGene_8763	2.113567	2.516778	2.797678	1.812558	2.536079	2.910074
Hordeum_vulgare_newGene_8769	2.738645	2.98358	2.872315	3.761135	3.204082	3.50833
HORVU3Hr1G110820	1.285108	1.330797	1.668502	1.910748	2.115886	2.365932
HORVU7Hr1G003860	5.627059	5.43004	6.285376	17.10535	21.79353	20.47282
HORVU7Hr1G064770	2.491562	3.237473	3.471267	5.489981	9.431178	7.556687
HORVU2Hr1G084260	7.112182	5.984624	9.376601	9.238832	12.04143	12.80986
HORVU3Hr1G015060	1.770537	1.807431	1.547578	2.430641	2.43424	2.266647
HORVU5Hr1G073330	5.079107	5.694908	5.591481	2.102584	3.564852	3.657762
Hordeum_vulgare_newGene_5225	9.176627	6.713927	6.615855	13.57594	35.04278	32.1425
Hordeum_vulgare_newGene_5224	17.66048	12.82047	13.34354	32.26108	113.3428	126.2971
Hordeum_vulgare_newGene_5226	0.042134	0.015673	0.053216	1.826143	2.526537	2.674176
HORVU1Hr1G050130	4.148913	3.558857	5.876327	7.235539	10.43493	11.67486
HORVU3Hr1G075960	1.363756	0.67214	1.231281	5.635861	3.885867	4.990136
HORVU5Hr1G117630	1.499021	1.523825	1.328997	1.130373	1.031933	1.050956
HORVU1Hr1G017830	0.52182	0.226294	0.716777	1.391052	6.927488	5.944391
Hordeum_vulgare_newGene_14177	3.776603	3.144981	2.288643	0.407307	0.367423	0.473011
HORVU3Hr1G089880	1.965953	1.828653	2.190872	3.577726	6.297064	6.878927
Hordeum_vulgare_newGene_11283	0	0	0	10.74976	12.55489	12.89047
HORVU7Hr1G028460	29.36783	28.17354	27.51552	23.47191	18.40255	19.95809
Hordeum_vulgare_newGene_11285	36.76078	34.14207	34.94912	0.034324	0	0
HORVU3Hr1G053060	8.107412	9.350154	12.54195	5.130119	2.033764	2.176458
HORVU5Hr1G052070	5.604256	7.691802	7.781794	6.432617	6.10766	7.91025
HORVU7Hr1G087340	48.42002	36.77419	54.78101	41.46204	60.27493	60.85011
Hordeum_vulgare_newGene_13381	4.395149	3.964172	4.700282	11.90842	17.0827	15.87116
HORVU4Hr1G024320	1.701916	1.644772	1.632147	2.202211	2.734773	3.073183
Hordeum_vulgare_newGene_10486	1.101282	1.191096	0.995697	1.485599	1.190986	1.557598

Hordeum_vulgare_newGene_10487	1.467378	1.210351	1.34509	1.511912	1.754286	2.490771
Hordeum_vulgare_newGene_10483	24.33992	29.02664	15.14465	20.51219	8.482581	11.56354
HORVU4Hr1G052070	0.586302	0.861207	0.694221	2.279565	3.295758	3.581482
HORVU2Hr1G031230	6.236462	6.16108	7.259507	7.108648	9.091967	9.011378
HORVU4Hr1G023670	4.817096	5.997441	7.117258	3.321006	3.270478	4.014891
HORVU3Hr1G011260	1.737606	2.95708	2.209303	0.21052	0.284898	0.334456
HORVU2Hr1G115710	0.522669	0.793766	0.749462	4.494404	5.969607	6.561551
Hordeum_vulgare_newGene_10734	19.83295	17.20653	24.10391	19.94513	29.30928	29.67139
HORVU7Hr1G086690	14.19095	11.08854	11.15169	24.22025	48.66516	40.2671
HORVU6Hr1G017080	6.170003	5.309885	6.528623	2.927852	2.623731	3.135652
HORVU4Hr1G022850	0.942989	0.902744	0.882972	1.18282	1.214853	1.831873
HORVU7Hr1G001160	0.709549	1.487011	0.858814	1.47083	1.289407	1.084514
HORVU7Hr1G066380	3.923017	3.319697	3.344326	4.946026	6.104774	5.541357
HORVU7Hr1G045150	17.16749	23.68501	27.71684	4.855046	0.362517	2.988854
HORVU1Hr1G073890	9.371724	8.943633	10.51185	11.12869	13.50341	13.93641
HORVU7Hr1G027350	4.365133	3.863734	5.605117	3.41546	5.206696	4.890559
HORVU6Hr1G020420	5.11279	5.853389	5.91138	5.177287	7.769101	8.124509
HORVU0Hr1G016450	11.78448	11.60957	13.36392	11.33699	12.13879	13.25368
HORVU7Hr1G058750	16.04812	20.56457	17.43265	9.774096	6.480936	8.630456
HORVU7Hr1G001290	7.69927	7.506171	10.79083	5.875676	8.440175	9.822967
HORVU2Hr1G114390	3.497617	2.277794	6.968773	0.299658	0	0.176469
Hordeum_vulgare_newGene_3136	43.02791	35.65467	35.82492	0.116821	0	0
HORVU7Hr1G101170	1.111097	1.350797	2.163121	1.513289	1.402711	2.014942
Hordeum_vulgare_newGene_197	66.15965	65.75072	94.69591	24.13501	11.9469	11.83887
Hordeum_vulgare_newGene_196	0.086716	0.012199	0.014033	4.844487	4.211809	4.254045
Hordeum_vulgare_newGene_195	1.641307	1.613482	2.3765	1.343724	1.058964	2.012437
Hordeum_vulgare_newGene_192	25.0657	25.26688	23.80118	19.90811	17.43632	20.16596
Hordeum_vulgare_newGene_198	370.9395	372.092	472.9226	166.0834	95.84688	97.54406
Hordeum_vulgare_newGene_228	0.605555	0.488382	0.43354	4.279171	2.929352	2.13117
Hordeum_vulgare_newGene_11260	1.265063	1.303288	1.219564	1.68746	1.699478	1.15653
Hordeum_vulgare_newGene_223	97.84227	109.3603	46.19711	49.50402	43.4627	31.2313
Hordeum_vulgare_newGene_222	8.37817	7.542693	8.140286	10.93841	15.25394	13.41028
HORVU3Hr1G116080	2.653695	3.414467	4.871696	3.241929	5.382768	7.681383
HORVU3Hr1G014980	9.822614	10.22546	10.78926	5.981049	3.996296	4.470711
HORVU3Hr1G010600	15.2671	13.0714	18.4435	20.97745	19.87385	21.67082
HORVU7Hr1G039610	1.07897	1.235329	1.585251	2.118587	3.0976	2.574741
HORVU5Hr1G084440	19.78002	16.72536	21.2589	30.82481	40.28164	31.865
HORVU4Hr1G062920	1.795745	1.266558	1.351127	4.01106	7.276977	7.923994
HORVU5Hr1G100150	0.926214	0.791695	1.236175	1.462263	3.740793	3.052547
HORVU6Hr1G089470	14.67049	15.70517	20.35111	8.075431	11.44983	11.25195
HORVU7Hr1G057640	10.0263	10.32802	15.00799	11.18797	23.94475	24.04435
HORVU1Hr1G030140	20.52397	20.33397	22.32864	19.69435	26.2429	25.33271
HORVU3Hr1G017950	8.217628	8.048628	8.807134	5.897911	3.994474	5.177331
HORVU7Hr1G035910	18.91794	15.95231	19.88135	17.76253	19.76548	18.63901
HORVU2Hr1G033860	8.219821	5.358687	4.891543	6.353315	0.556203	1.457242
Hordeum_vulgare_newGene_476	1.667273	1.419602	1.342188	5.228099	2.749046	2.625937
HORVU2Hr1G028230	15.82498	17.44633	16.46228	16.69881	13.97436	11.76719

HORVU7Hr1G001830	8.644953	10.72761	11.62085	16.20709	17.86765	18.8463
HORVU1Hr1G074260	0.918895	0.69092	1.006557	1.392969	1.988676	2.012494
HORVU4Hr1G073650	2.207685	1.875538	2.412618	7.464902	7.435715	6.668397
HORVU2Hr1G013450	6.184483	6.679195	9.247765	2.817811	3.116894	2.493608
Hordeum_vulgare_newGene_7339	0.685994	1.121507	1.42902	1.961957	2.244578	2.645141
HORVU6Hr1G059510	36.25625	33.43884	34.71318	54.68611	36.85789	39.23891
Hordeum_vulgare_newGene_7334	4.788064	4.650578	5.07783	4.041552	5.421914	5.362031
Hordeum_vulgare_newGene_7337	10.19714	10.59685	10.47603	12.64813	16.97801	14.24886
HORVU5Hr1G041500	3.000406	2.382107	3.462366	2.252515	2.868007	3.442351
HORVU3Hr1G113100	1.524968	1.163468	1.348424	3.386256	2.786426	4.29924
HORVU2Hr1G027400	8.305962	10.27388	11.43631	7.653664	11.95978	13.3562
Hordeum_vulgare_newGene_13618	4.245484	3.94963	5.069713	4.16675	5.96383	10.33403
HORVU6Hr1G032860	2.143563	3.007199	3.102917	2.786374	2.765978	2.629061
Hordeum_vulgare_newGene_2787	1.583485	1.526999	2.37852	1.769577	1.804889	2.44665
HORVU7Hr1G025440	3.157294	4.03232	6.042	5.699271	5.783256	5.700118
HORVU7Hr1G030730	1.053929	1.32963	1.400941	0.684811	1.010622	1.003758
HORVU7Hr1G079810	13.28591	9.229772	11.81786	8.456182	16.11809	12.37495
Hordeum_vulgare_newGene_2214	79.16821	74.87985	91.00085	61.08004	85.73479	80.30193
Hordeum_vulgare_newGene_2216	2.09678	2.554407	2.929528	3.471181	3.810965	3.628304
HORVU0Hr1G008280	24.93314	23.13786	27.2542	26.32226	38.32845	36.7975
HORVU1Hr1G027550	2.984742	3.379333	3.971036	4.117196	6.198905	6.758159
HORVU6Hr1G082340	6.681569	6.744867	8.402089	5.1885	6.756548	7.359674
HORVU2Hr1G021580	2.951085	0.985468	2.577428	3.839289	6.389134	6.441928
HORVU3Hr1G081270	1.007276	1.128427	0.872886	1.633337	1.643381	2.244072
HORVU7Hr1G054510	1.604215	1.251393	1.525478	3.246032	1.510427	1.07778
HORVU6Hr1G079630	3.55362	4.423678	6.710943	2.550516	6.42587	6.620608
HORVU5Hr1G079060	19.88667	18.62431	29.15031	15.33515	21.49852	22.0626
HORVU2Hr1G063280	4.505947	3.762734	4.536109	3.81459	5.01714	5.42306
HORVU1Hr1G059810	59.33174	68.10536	39.87145	26.65851	17.22721	23.51286
HORVU7Hr1G051620	6.894985	4.902048	7.740441	11.54222	11.16274	11.49149
HORVU7Hr1G108280	1.249541	0.6225	0.866446	6.760797	5.577711	3.226383
HORVU7Hr1G037420	17.43873	18.54663	18.19353	30.88807	41.22303	39.35072
HORVU3Hr1G032350	0.089406	0.134057	0.157977	0.154901	14.77915	13.47105
Hordeum_vulgare_newGene_15519	5.027356	5.205618	5.222814	6.521744	8.640141	9.100926
Hordeum_vulgare_newGene_15514	13.52594	11.05908	14.57945	17.96156	20.1453	19.37881
HORVU5Hr1G026360	1.645215	1.621154	2.155003	2.748897	3.870875	4.912725
HORVU6Hr1G061270	0.299507	0.244447	0.217336	12.98929	16.04587	15.22374
HORVU2Hr1G026750	13.21999	13.27229	12.33789	16.99705	32.51492	29.32807
HORVU7Hr1G043340	9.469023	9.707619	11.88456	6.790587	8.709418	7.273431
HORVU6Hr1G071190	0.081149	0.123065	0.105029	1.286768	2.95215	3.325193
Hordeum_vulgare_newGene_5834	2.276632	1.8482	2.842989	5.086391	6.218464	5.197825
HORVU4Hr1G075160	1.110184	0.860703	0.879977	2.251315	3.563783	4.133315
HORVU0Hr1G004330	5.091694	5.949867	6.623468	4.997199	5.221628	6.359188
Hordeum_vulgare_newGene_5830	1.830474	2.072655	2.187135	1.127591	1.173539	1.292476
HORVU7Hr1G088330	2.199382	1.374906	2.273695	2.311634	3.438084	3.864608
HORVU3Hr1G069000	3.597847	3.388653	4.499781	5.121121	5.273215	5.882153
HORVU1Hr1G055480	37.8844	34.40228	39.09862	36.33426	46.3602	45.8971

HORVU5Hr1G078330	3.844149	3.010053	4.906923	6.201467	7.022216	7.032654
HORVU7Hr1G107100	36.17234	35.45688	41.73778	36.00768	50.60432	51.93563
HORVU2Hr1G086220	0.312179	0.233687	0.491324	1.294909	1.835022	1.885207
HORVU2Hr1G085550	15.28141	13.5984	16.96317	16.85984	23.93397	23.29267
HORVU2Hr1G096890	24.93155	18.55252	23.65842	7.169803	2.902801	3.909131
HORVU2Hr1G094670	0.292796	0.337153	0.551316	12.52681	27.66709	24.04885
HORVU2Hr1G098550	14.85234	13.7288	15.5945	11.51669	8.951486	8.745164
HORVU7Hr1G028730	1.741956	1.341182	1.426323	1.029613	0.847228	1.615243
HORVU5Hr1G051010	43.35763	44.69872	35.5656	241.9109	432.3296	422.6858
HORVU6Hr1G010610	1.426336	1.213252	1.983564	1.740972	1.595895	1.617666
HORVU1Hr1G095430	133.6159	129.2354	148.5977	23.50774	20.01095	18.3936
HORVU3Hr1G053540	4.779929	4.853595	6.039254	6.322649	7.234687	8.11148
HORVU0Hr1G002270	2.149056	1.647365	1.92522	4.401515	0.612238	1.682961
HORVU2Hr1G124210	7.973512	8.974106	8.902495	15.05917	20.80946	22.17021
HORVU1Hr1G062510	36.28477	35.78831	45.59819	22.05037	26.30879	23.73632
HORVU2Hr1G085880	16.5526	19.84618	29.51684	15.84721	19.66617	20.94378
HORVU2Hr1G127090	4.046196	4.372046	7.84061	3.94259	6.292621	6.821762
HORVU6Hr1G060630	7.179753	7.377726	7.659815	7.714738	12.36932	13.69179
HORVU4Hr1G032440	0.699684	0.781833	0.910777	1.271884	1.723519	1.447545
HORVU7Hr1G096240	47.4163	49.88311	35.01722	27.42233	19.79279	19.61016
HORVU6Hr1G088820	5.105634	3.80786	4.3315	5.115645	7.489344	7.359207
HORVU2Hr1G077540	14.86324	15.22486	17.49031	17.31085	22.70369	21.41431
HORVU3Hr1G071090	10.07276	11.09977	14.22314	8.479509	14.15846	14.19737
HORVU2Hr1G013400	1.879401	2.185208	1.644522	6.310576	8.805152	9.145316
HORVU7Hr1G066630	0.165154	0.350819	0.331927	2.259363	2.224959	2.082333
HORVU4Hr1G033790	3.627816	3.675805	4.174762	0	0	0
HORVU2Hr1G098080	6.778041	6.444671	7.344471	11.92851	15.70523	16.02995
HORVU3Hr1G084380	15.00084	13.95247	16.61574	13.09464	17.50118	16.84545
Hordeum_vulgare_newGene_1171	15.41032	15.23898	10.42093	9.912527	12.09823	9.466422
HORVU1Hr1G025960	2.185653	1.763511	2.238988	2.60189	2.16328	2.336716
HORVU1Hr1G062160	26.63766	23.63946	25.53159	19.10652	21.87386	21.43978
HORVU1Hr1G058470	0.080296	0.147157	0.133004	2.827644	3.215001	4.417458
HORVU0Hr1G007730	2.869801	1.483196	1.983418	0.622801	0.67435	0.778112
HORVU2Hr1G080590	0.883309	0.552622	0.990543	3.287162	12.28783	10.44009
HORVU5Hr1G096520	9.398474	9.729728	11.11555	13.59983	15.09647	16.06049
HORVU1Hr1G073660	33.69902	25.84532	39.10986	25.22203	31.90781	33.98372
HORVU5Hr1G105880	4.942153	4.654862	5.512078	3.885784	3.895198	4.08305
HORVU1Hr1G063640	10.38355	10.2782	10.22574	11.0294	12.68142	14.3541
HORVU5Hr1G096490	7.274861	5.58295	8.718495	15.27474	5.276866	7.805751
Hordeum_vulgare_newGene_4525	4.153454	5.016165	4.171738	1.137705	0	0.190442
Hordeum_vulgare_newGene_4526	8.749241	10.4083	8.872957	9.756451	0.879707	2.713834
HORVU7Hr1G101230	12.66015	11.88355	14.91853	17.06749	17.36879	16.92802
Hordeum_vulgare_newGene_15280	5.498944	5.65831	5.760228	9.905273	13.24552	12.06958
Hordeum_vulgare_newGene_4528	4.170005	4.552156	4.415417	0	0.013192	0
HORVU7Hr1G088570	2.810543	2.905639	3.756996	2.760197	2.612289	3.139057
HORVU3Hr1G067180	1.461875	1.944633	2.7437	1.114128	1.475409	1.78305
HORVU3Hr1G031070	3.16422	4.686717	4.126761	3.806066	3.319177	4.967339

HORVU2Hr1G127540	7.303244	7.649308	10.23666	7.801279	7.202149	8.862416
HORVU3Hr1G042790	10.4268	9.65091	13.59006	11.31348	11.88344	13.04078
HORVU2Hr1G050770	4.222928	4.928411	4.368659	4.210679	3.639338	4.774145
HORVU7Hr1G039930	95.00948	94.68708	102.184	342.4284	384.137	372.1938
HORVU5Hr1G021310	89.95811	74.16559	134.6751	111.7012	163.7664	142.1328
HORVU1Hr1G075670	7.628741	5.141929	7.688382	5.119235	2.400811	4.20556
HORVU7Hr1G085120	1.333266	1.375019	1.705469	5.118478	13.21946	12.18567
HORVU2Hr1G120010	1.930446	2.505195	2.076434	1.137398	0.958549	1.05181
HORVU4Hr1G033420	4.837686	5.153677	5.571527	5.401436	6.686527	7.031491
HORVU2Hr1G007490	0.024246	0	0.026951	12.88479	5.089717	4.33349
HORVU3Hr1G005430	1.642042	1.658473	1.591077	2.782017	3.531066	3.403014
HORVU4Hr1G051620	15.02411	15.20276	22.08618	12.31694	20.47282	19.99806
HORVU1Hr1G052010	1.671264	1.732638	1.99931	2.320717	2.300858	2.828266
HORVU5Hr1G018330	9.293988	11.57417	12.41529	12.7484	22.54173	21.95718
Hordeum_vulgare_newGene_5783	2.590998	2.073524	1.657099	1.713663	0.930621	1.11223
HORVU0Hr1G001190	1.513445	1.383613	1.648947	2.498163	3.350691	3.07506
HORVU3Hr1G034510	0.084988	0	0.148682	0.667455	40.8274	31.6933
HORVU1Hr1G089950	5.049396	4.414964	5.211569	6.13176	8.429279	7.34633
HORVU7Hr1G024450	6.209146	7.779411	10.2627	4.803721	5.091913	5.354285
HORVU4Hr1G038270	18.02176	18.60983	19.30937	20.38905	35.86014	30.1114
HORVU1Hr1G087880	0.550375	0.595187	21.01865	44.49977	0.240674	0.351201
HORVU3Hr1G070050	8.737078	11.11128	11.12315	14.06879	19.51159	20.08432
HORVU5Hr1G056480	5.954888	6.112899	5.013936	9.317807	10.74484	8.120076
HORVU2Hr1G050290	1.983663	2.541917	3.502294	2.74343	3.270102	3.621907
HORVU1Hr1G026320	10.05409	11.48811	10.82503	21.69168	38.20543	38.15387
HORVU7Hr1G098540	21.1109	20.34047	19.20966	22.0935	15.72037	15.50213
HORVU7Hr1G052550	2.931207	2.695023	4.396818	2.586957	3.378694	1.298093
HORVU4Hr1G016350	9.349822	9.704391	14.20517	10.57402	16.52046	16.57355
HORVU2Hr1G041130	18.1018	15.24247	22.00885	22.07132	20.71117	24.62981
HORVU1Hr1G045310	6.076839	6.537694	6.99667	7.944025	9.808578	9.722467
Hordeum_vulgare_newGene_15039	3.059277	3.816751	3.450991	4.40873	4.395893	5.138692
HORVU7Hr1G054890	35.6565	32.29127	39.78284	0.094588	0.064719	0
HORVU5Hr1G044830	1.127494	1.396926	1.244564	6.349249	6.377762	9.01611
HORVU5Hr1G111820	155.2397	152.018	216.6835	154.3683	357.5529	328.1996
HORVU6Hr1G080530	6.285656	4.749065	6.036534	12.85795	17.09566	18.98166
HORVU7Hr1G097920	2.735218	2.673863	3.331857	2.158204	2.59858	2.598788
HORVU6Hr1G033490	2.866018	2.845529	3.589328	4.715543	7.116502	6.880507
HORVU1Hr1G065390	15.97614	17.79273	17.50059	13.44046	14.24686	15.0283
HORVU1Hr1G061430	9.607078	7.643337	10.18477	10.97088	16.28084	13.65498
HORVU3Hr1G086720	3.20834	2.613636	4.303944	5.670779	7.470677	7.770021
HORVU2Hr1G064920	16.64774	15.89087	17.53516	15.30526	16.8455	16.48491
HORVU5Hr1G123120	3.830167	2.40836	3.42238	5.411442	1.953479	3.339376
HORVU2Hr1G123650	6.964223	6.687582	10.09713	7.929881	6.470599	7.096881
HORVU1Hr1G017580	9.480591	7.201909	9.386613	11.25471	10.03674	9.749993
Hordeum_vulgare_newGene_1132	1.681061	1.906688	1.986241	3.815262	6.010864	6.0248
HORVU4Hr1G003060	261.6403	281.8398	416.8708	169.9777	273.3829	261.9383
HORVU1Hr1G004400	4.959005	3.927252	4.46017	6.784531	9.638506	9.173887

HORVU3Hr1G062250	0.551102	0.696975	0.737113	1.723588	2.838598	2.685528
HORVU5Hr1G097840	19.86544	19.16913	19.18698	17.00342	18.83272	19.45115
HORVU2Hr1G118700	8.036459	6.375368	7.524181	11.05994	12.48219	9.685187
HORVU0Hr1G001530	7.608222	7.700081	7.614309	7.654127	8.775021	9.73841
HORVU3Hr1G072520	19.08928	19.07365	17.80373	16.4091	20.85432	22.32588
Hordeum_vulgare_newGene_1205	3.228713	4.036331	3.280704	3.470561	4.953351	4.817808
HORVU6Hr1G040600	4.811734	5.194704	5.266163	5.116302	4.38074	5.031512
HORVU5Hr1G009530	14.43491	13.3497	15.16538	10.74814	9.879689	10.65278
HORVU5Hr1G007550	48.53205	49.0162	40.63828	32.39858	30.42246	29.50493
HORVU3Hr1G082240	27.8308	28.78892	27.62454	26.72666	24.38613	22.8254
HORVU1Hr1G016980	0.918336	0.632476	0.757203	3.964778	3.208991	4.96294
HORVU7Hr1G048620	15.87129	10.86793	19.1409	33.07952	29.52016	31.11145
HORVU5Hr1G109190	4.593996	2.810234	6.114867	2.114148	2.320453	4.298684
HORVU1Hr1G067530	1.718179	2.539594	2.46213	2.184854	2.533564	2.605108
HORVU7Hr1G090880	1.268591	1.323504	1.592624	4.291236	6.597709	6.448191
HORVU2Hr1G105800	5.071102	5.566016	5.985481	4.453318	2.765602	3.772934
HORVU5Hr1G076140	26.43553	20.86423	30.24681	20.87195	19.95913	20.21686
HORVU4Hr1G082600	0.238724	0.217914	0.499179	7.099617	15.02489	17.26576
HORVU5Hr1G118690	8.680393	7.992212	10.48019	6.883282	8.691259	7.59633
HORVU4Hr1G084590	1.892611	1.722863	1.61959	2.202547	1.923627	2.266396
HORVU1Hr1G005460	1592.335	1525.449	1195.009	302.5244	145.9375	170.9366
HORVU6Hr1G091540	1.505533	1.408077	1.734353	1.017208	0.974979	0.851525
HORVU5Hr1G109440	2.933469	3.63078	4.313454	5.10341	9.784849	9.192149
HORVU3Hr1G074960	11.35518	5.23252	14.78788	3.171978	0.800443	4.083337
HORVU4Hr1G085970	4.240232	4.45715	4.119967	2.03706	1.676867	1.894231
HORVU1Hr1G018600	22.87886	19.42646	20.75917	11.03639	4.167758	6.548105
HORVU6Hr1G048180	19.04011	15.67418	17.7532	19.94093	21.69675	18.98398
HORVU1Hr1G012680	4.593679	5.665196	7.08717	6.561735	6.451407	8.829881
Hordeum_vulgare_newGene_11835	2.093923	1.313478	2.171129	6.460828	5.324434	4.668005
HORVU4Hr1G014460	3.25544	2.532733	2.10486	14.99299	57.87645	56.36665
HORVU4Hr1G062490	1.488514	1.966985	1.709762	2.159185	2.726597	3.343452
HORVU3Hr1G056270	5.51938	5.353541	4.233795	7.466372	2.010305	4.576609
HORVU7Hr1G084310	1.162566	1.520513	1.847303	4.652406	6.147063	6.879068
Hordeum_vulgare_newGene_9776	15.6624	11.34774	15.21497	15.1834	16.37015	16.79878
HORVU6Hr1G032830	4.086295	2.875279	5.125499	3.252733	1.462283	1.768066
HORVU3Hr1G089260	7.103944	6.405016	8.122454	12.62685	26.1712	22.66524
Hordeum_vulgare_newGene_3323	9.180503	8.970886	8.937501	19.59235	36.74474	33.89361
Hordeum_vulgare_newGene_3320	7.435989	6.982764	7.548862	6.367881	7.340699	8.855024
HORVU6Hr1G094070	2.11067	2.100986	2.617371	1.687643	0.696624	0.697247
HORVU4Hr1G082280	17.23072	17.92962	17.55269	19.31343	23.77633	23.98939
Hordeum_vulgare_newGene_9770	2.174648	3.381862	3.669619	3.317542	3.691118	4.5852
HORVU3Hr1G067990	6.192748	5.662502	9.108527	1.726725	1.237048	1.565147
HORVU4Hr1G055610	5.80356	5.175123	6.83907	2.336628	2.232993	1.889136
HORVU2Hr1G024720	1.026205	1.007005	1.24675	1.404886	1.32852	1.603102
HORVU2Hr1G020340	1.543586	1.167646	1.412418	1.990527	1.834842	1.641545
Hordeum_vulgare_newGene_9023	1.387213	0.793201	0.712097	1.600817	0.903159	1.229075
HORVU2Hr1G064140	4.545015	5.951637	4.133492	11.9733	5.929101	7.993203

HORVU5Hr1G085220	1.188889	1.694343	1.761447	1.269887	1.202081	1.519096
Hordeum_vulgare_newGene_2058	0	0	0	2.545559	2.658728	3.123657
Hordeum_vulgare_newGene_13058	5.80861	7.32334	9.607816	3.751055	6.17794	6.753184
HORVU5Hr1G026640	48.41158	52.10754	63.51945	51.07649	57.02073	54.50246
HORVU4Hr1G086660	6.86708	4.591891	7.975738	10.1575	6.142331	6.971305
HORVU7Hr1G078300	1.269736	1.731045	2.321213	1.530513	2.675293	2.278688
Hordeum_vulgare_newGene_12981	3.506587	3.022514	3.563212	6.062373	3.462326	4.512274
Hordeum_vulgare_newGene_12986	7.571086	10.25168	8.61263	12.87614	8.693784	13.68872
Hordeum_vulgare_newGene_12989	6.826151	6.901618	5.759818	9.328234	7.354925	8.605316
HORVU6Hr1G078830	4.946747	4.661733	6.418345	4.371034	5.896658	6.241641
HORVU7Hr1G073460	113.2095	82.58909	153.2591	68.50927	45.1878	70.80037
HORVU2Hr1G099250	1.237125	1.587144	1.702279	2.671608	2.676018	3.217455
HORVU1Hr1G031490	28.77873	35.28871	52.99599	1.953668	0	0.6604
HORVU2Hr1G033510	1.876018	3.640237	3.345902	2.160007	2.716402	3.597488
HORVU5Hr1G033420	8.453607	8.101664	9.775728	8.42704	8.393038	9.005284
HORVU1Hr1G070740	2.746818	2.418643	2.240908	2.973093	3.130249	3.488611
HORVU3Hr1G048520	30.90006	30.77664	35.81366	33.65384	29.66472	34.82945
HORVU0Hr1G020490	10.71942	10.79217	10.08905	9.669146	8.64933	9.153746
HORVU4Hr1G061140	2.993761	2.664027	2.760917	6.000151	7.386818	6.915428
HORVU2Hr1G023140	7.158243	5.547092	8.249672	6.665532	8.956286	8.381834
Hordeum_vulgare_newGene_9600	3.173379	3.0791	2.666144	2.984634	4.497097	3.972552
HORVU4Hr1G075360	162.9624	152.1498	140.7066	153.2926	151.7003	144.6515
Hordeum_vulgare_newGene_15066	6.850389	8.650446	6.096335	11.43198	16.65359	16.67757
Hordeum_vulgare_newGene_15063	1.967152	1.982681	2.517531	2.234776	2.534699	2.537183
Hordeum_vulgare_newGene_15062	0.796441	0.573453	0.6779	2.037264	3.454668	3.989118
HORVU7Hr1G073790	3.871569	7.214543	8.397541	0.447412	0	0.108971
HORVU5Hr1G019590	47.50123	54.51114	44.72312	51.89268	53.75987	58.58299
HORVU3Hr1G065260	2.602766	3.342553	3.535444	4.886736	5.195011	6.222306
Hordeum_vulgare_newGene_15069	2.292913	2.525759	3.100317	1.968084	2.663067	2.792828
HORVU1Hr1G014120	52.58693	56.35987	41.97924	43.38654	47.95828	47.84893
HORVU4Hr1G070620	1.525275	1.3978	1.477264	1.419509	2.756073	2.457155
HORVU2Hr1G101730	0.033094	0.089807	0	1.390231	6.835401	4.663991
HORVU5Hr1G074450	6.471052	5.515722	6.691709	9.924573	10.00455	9.536148
HORVU5Hr1G078130	2.525356	2.776785	3.307591	3.417369	3.973781	4.971238
Hordeum_vulgare_newGene_2587	0.106697	0.278203	0.066926	1.179698	2.362537	2.330267
Hordeum_vulgare_newGene_2580	1.551794	1.220355	2.055854	1.279291	1.817935	2.52227
HORVU2Hr1G108010	1.160421	1.024769	1.387569	11.15604	20.78862	18.73169
Hordeum_vulgare_newGene_11981	1.183674	1.517063	1.072206	0.898899	3.284366	2.410022
Hordeum_vulgare_newGene_11984	2.599211	3.396776	3.673591	2.999809	4.481703	4.994332
Hordeum_vulgare_newGene_11985	1.364869	0	0.152812	0.758527	4.347896	3.67311
Hordeum_vulgare_newGene_11989	14.01014	10.58207	17.4591	8.405167	3.270526	5.184213
HORVU7Hr1G029460	1.580388	1.033759	1.751174	1.894354	1.005311	1.627709
HORVU2Hr1G097150	2.436715	1.989844	2.90293	5.819154	4.401936	5.78037
HORVU6Hr1G034070	12.10588	10.50568	12.54603	8.646172	8.899474	10.03092
HORVU7Hr1G089270	10.41103	7.383051	9.46875	1.975389	0.301153	0.805389
HORVU7Hr1G007220	1.580684	1.826984	1.740102	18.91051	6.617025	13.61586
HORVU4Hr1G000280	0.055811	0.024934	0.048599	4.350935	6.126255	6.510666

HORVU4Hr1G060230	1.976067	2.093691	2.50786	1.049283	2.049755	1.994697
HORVU2Hr1G031910	57.79128	57.33817	80.51657	46.08364	23.39527	30.99154
HORVU3Hr1G053380	5.252087	3.845264	6.84109	7.562402	4.854507	7.233953
Hordeum_vulgare_newGene_11367	0.620192	0.582278	0.639914	1.983151	1.256599	1.948565
Hordeum_vulgare_newGene_11361	0.947536	1.171819	1.109405	1.108811	1.173572	1.15654
Hordeum_vulgare_newGene_11363	1.822654	0.62987	1.872061	21.64919	101.5676	93.08852
HORVU6Hr1G048550	21.36334	16.66327	22.99847	16.13382	21.21408	19.75772
HORVU5Hr1G031380	1.969578	1.646964	2.199125	1.982302	4.027282	4.278592
HORVU1Hr1G009490	16.84296	8.182198	22.33289	0.291159	0.017047	0
HORVU2Hr1G002040	10.13173	9.697241	12.35897	13.42924	13.29311	13.99291
Hordeum_vulgare_newGene_14858	4.477514	4.913791	5.321699	4.045431	4.552234	4.874319
HORVU4Hr1G076100	60.92644	60.92592	62.92111	63.05948	73.97006	73.06732
Hordeum_vulgare_newGene_8268	2.049724	2.397037	2.540736	0	0	0
Hordeum_vulgare_newGene_8269	5.410119	6.613448	6.78507	0.101352	0.061499	0.019764
Hordeum_vulgare_newGene_14851	1.250233	1.069105	1.341543	2.059967	2.330689	1.992079
Hordeum_vulgare_newGene_14853	3.722625	5.036223	4.892293	3.089361	2.568801	3.090374
Hordeum_vulgare_newGene_1777	0.612564	8.830245	1.20011	12.86738	0.67183	0.873795
HORVU4Hr1G077850	12.91592	11.26976	15.3701	5.60674	2.035884	3.00945
HORVU1Hr1G005870	5.257572	2.729488	4.518505	8.098383	34.41893	31.51144
HORVU2Hr1G022250	18.55481	19.83688	26.17079	10.09347	13.20864	12.60086
HORVU1Hr1G073460	5.858977	7.248211	9.441583	2.676237	4.301731	4.631759
HORVU1Hr1G024580	3.552735	4.205866	3.573943	7.016632	8.077138	8.518183
HORVU3Hr1G091140	0.145649	0.331992	0.129614	1.29024	2.316472	2.70803
HORVU7Hr1G033490	5.973984	4.574833	4.962095	8.065149	2.848852	4.738084
Hordeum_vulgare_newGene_4725	2.260841	3.007041	2.916925	1.869579	2.401785	2.46143
HORVU1Hr1G038280	15.24916	18.70488	18.87835	20.2473	20.0533	22.64015
HORVU1Hr1G039720	4.712855	3.654141	5.089846	1.802455	0.961466	1.529006
HORVU2Hr1G034630	2.236016	2.354073	3.083619	2.992579	6.28565	6.540513
HORVU7Hr1G063440	0.244007	0.074015	0.02147	1.428643	2.903591	2.354498
HORVU2Hr1G088210	6.718117	5.286885	9.565948	8.843732	11.38212	12.57344
HORVU7Hr1G100920	18.50538	18.44608	16.16213	15.01714	11.6437	13.46781
HORVU7Hr1G079380	5.408789	3.418165	4.997087	11.45604	32.6037	28.42715
HORVU3Hr1G011760	0.285298	0.278414	0.072393	0.631445	17.01127	13.2719
HORVU7Hr1G105200	3.194157	2.29068	3.131985	1.516845	1.347939	1.791961
HORVU3Hr1G104600	6.089856	7.602763	5.807789	0.010482	0	0
HORVU7Hr1G046830	7.128128	4.798821	7.433855	1.736393	0.47997	1.284001
HORVU3Hr1G035000	44.09009	34.31245	46.67787	44.77485	73.68966	68.71801
HORVU7Hr1G058090	0.273813	0.460553	0.271712	6.305344	1.680034	3.759006
HORVU7Hr1G001310	2.083336	2.200384	1.933171	2.314687	2.696912	2.7018
HORVU3Hr1G081590	5.418823	4.923776	6.959445	2.516401	0.989536	1.910117
Hordeum_vulgare_newGene_12047	0.786806	0.204944	0.418434	2.90745	5.503267	5.864446
HORVU5Hr1G103360	4.745818	4.731579	5.253842	6.434973	9.914619	9.300736
HORVU3Hr1G040310	0	0	0	27.65445	20.70864	25.68922
HORVU3Hr1G034750	53.57537	61.81469	79.6699	43.92947	56.84655	56.95221
HORVU7Hr1G038860	14.64971	13.58609	16.95135	14.34427	12.30636	13.00624
Hordeum_vulgare_newGene_4897	6.702508	8.400915	7.949115	6.740429	7.200958	8.740456
Hordeum_vulgare_newGene_4892	3.757266	3.021821	4.625299	5.53079	4.613417	3.968562

HORVU3Hr1G069620	3.866005	4.180507	3.665379	5.683629	4.609915	4.492318
HORVU5Hr1G019300	19.91992	17.20184	14.43653	12.29553	15.32433	15.88755
Hordeum_vulgare_newGene_15892	3.587743	3.911983	4.067977	6.654676	12.98687	10.07494
Hordeum_vulgare_newGene_15891	2.783206	2.933846	2.629531	4.610501	8.554216	7.702692
Hordeum_vulgare_newGene_6764	14.91721	18.47587	16.92739	17.9832	18.1639	24.34466
HORVU2Hr1G114580	58.90758	67.47179	58.37606	77.47195	107.2929	103.9249
HORVU6Hr1G074580	5.580185	6.46369	5.539505	2.476504	2.302894	3.09128
HORVU6Hr1G087420	1.283003	1.159888	1.579764	3.883669	4.89401	5.551361
HORVU6Hr1G067870	95.29744	105.8809	159.7893	86.00279	182.9494	182.1101
HORVU1Hr1G026520	5.384379	4.66642	5.485649	15.11783	21.60354	23.14801
Hordeum_vulgare_newGene_3044	0.651886	1.45364	2.457729	0.697356	0.952037	0.64335
Hordeum_vulgare_newGene_3043	1.129447	1.967162	2.132796	1.32107	1.543267	2.092649
HORVU1Hr1G011660	0	0.016909	0.043108	10.44781	9.394708	10.94573
Hordeum_vulgare_newGene_3041	2.47515	2.360249	2.765603	1.268928	1.231618	1.918563
HORVU6Hr1G008580	1.299661	1.084029	1.207399	2.002297	1.410144	2.418828
HORVU0Hr1G038850	7.095644	5.659182	5.955948	8.281137	9.917434	10.51222
HORVU4Hr1G074770	11.80916	10.80552	12.47251	7.946228	4.524825	6.249689
Hordeum_vulgare_newGene_6496	4.594819	7.334132	3.820493	2.661501	3.231977	2.87842
Hordeum_vulgare_newGene_6495	0.484591	0.244195	0.373059	2.694507	1.050014	1.192427
HORVU3Hr1G059030	145.6305	137.9871	210.3736	38.94238	0.457706	13.64584
HORVU3Hr1G033760	5.822782	6.868498	10.02093	5.128603	4.977905	8.470828
HORVU5Hr1G107290	18.76243	15.73741	18.12137	26.73843	26.08047	26.70949
HORVU1Hr1G048000	7.951755	7.532476	8.156322	6.569388	9.045053	8.31076
HORVU5Hr1G095930	3.972982	2.498989	4.469819	3.981326	5.814579	4.926522
HORVU3Hr1G039050	135.8599	166.7532	197.8536	41.34232	1.144532	17.89955
HORVU2Hr1G117050	0	0	0	3.248628	4.206825	4.099423
HORVU6Hr1G075180	10.28349	7.279505	10.44162	10.00301	10.73038	11.36819
HORVU2Hr1G075280	17.76929	16.04169	22.57102	20.32494	44.27729	36.49623
HORVU3Hr1G087850	14.11305	16.0205	19.64618	6.454826	8.475912	9.132209
HORVU6Hr1G029080	16.08902	12.70839	15.2216	12.97909	10.74198	10.9325
HORVU1Hr1G027690	80.55826	70.19321	92.0455	63.98203	75.61462	73.28082
HORVU6Hr1G082060	10.50495	8.507518	14.19839	20.61206	35.09448	30.3602
HORVU5Hr1G035540	7.92797	7.638213	10.02345	9.903354	13.19396	14.26768
HORVU1Hr1G070220	1.687619	1.776021	2.636613	6.119769	8.941994	8.571936
HORVU2Hr1G024940	1.251536	1.452841	1.86603	2.95473	4.121975	4.728457
HORVU4Hr1G017390	14.63661	9.896576	14.06556	29.5063	42.83646	45.78766
HORVU1Hr1G056160	6.500079	6.25757	10.4421	3.795257	4.392178	4.406008
Hordeum_vulgare_newGene_974	8.303229	8.749139	9.419136	11.4495	20.82337	21.96226
HORVU2Hr1G043840	76.20855	70.08286	90.9417	62.14746	58.72719	60.82665
HORVU5Hr1G069110	6.430184	2.334141	3.194719	4.266439	3.068666	4.613793
HORVU0Hr1G000660	4.566107	4.550857	5.162675	4.499448	3.631244	3.449304
HORVU6Hr1G061020	6.509515	6.154338	6.649116	5.832733	7.884285	8.690998
HORVU4Hr1G011940	20.91019	18.6205	21.44281	22.26331	27.21175	24.85528
HORVU3Hr1G016010	5.60711	6.318765	6.840267	3.486422	2.829883	3.692559
HORVU2Hr1G040570	39.38517	32.68441	41.86792	32.97961	55.40836	57.80818
HORVU5Hr1G088630	18.23768	14.044	16.64059	10.84482	10.33477	11.01874
Hordeum_vulgare_newGene_3105	3.046725	2.238125	3.030769	2.919333	2.266708	2.71394

HORVU3Hr1G032490	24.92796	23.51046	21.13403	54.34044	74.02408	70.65534
HORVU2Hr1G082520	3.408409	2.563893	2.377949	0.640522	2.277191	1.732226
Hordeum_vulgare_newGene_15304	5.170759	3.626579	4.066258	5.724868	2.887525	3.960549
Hordeum_vulgare_newGene_15302	9.988665	9.812537	13.23417	10.71426	11.01388	10.99276
Hordeum_vulgare_newGene_15300	1.474494	1.688257	2.034082	1.893241	3.279788	2.954877
HORVU7Hr1G107310	3.213471	3.947347	3.97176	0.826689	1.29378	1.429902
HORVU6Hr1G064010	6.428779	6.055165	7.213203	6.573399	10.38361	8.090054
HORVU7Hr1G113890	1.513804	1.31078	1.375071	0.905379	0.936401	1.040808
HORVU3Hr1G037510	1.739355	2.112967	3.281953	1.625395	1.89871	2.14438
HORVU6Hr1G028020	1.800508	1.56698	1.712051	2.52084	2.785775	2.904381
HORVU3Hr1G082730	4.711496	3.958591	3.8938	23.34939	44.81862	49.73026
HORVU4Hr1G089080	17.89692	15.87465	17.81531	29.64279	40.32192	35.00139
HORVU7Hr1G112410	119.0424	116.3729	126.6156	54.31646	67.83456	72.67575
HORVU7Hr1G090820	1.171556	1.577524	1.381444	2.006232	2.557118	2.58313
HORVU5Hr1G051600	1.87078	2.525545	2.939684	2.779048	3.817136	3.609143
HORVU6Hr1G083890	2.390144	2.985443	3.229764	2.897393	3.243723	4.63771
HORVU5Hr1G066160	21.0171	17.8384	20.32331	24.66063	32.49146	28.57147
HORVU4Hr1G005950	7.45041	8.724312	6.92374	5.57553	4.720745	5.13289
HORVU6Hr1G081000	41.21967	27.86823	48.87016	44.18994	39.61628	47.86622
HORVU2Hr1G098320	0.090631	0.020689	0.068399	0.150122	5.547099	6.126575
HORVU5Hr1G104580	10.39399	8.264192	12.15226	2.428599	0.660415	1.566322
HORVU6Hr1G000640	273.6011	238.4691	311.8393	237.6147	271.7104	281.1781
HORVU7Hr1G106040	0.039207	0	0	0.216996	26.02697	26.4239
HORVU1Hr1G052730	2.531831	2.22802	2.42572	2.272852	1.973688	2.091326
HORVU5Hr1G069800	3.25412	3.346667	3.92641	1.812534	1.881629	2.041629
HORVU6Hr1G011140	8.500135	8.071523	9.263293	5.662928	7.151611	7.573307
HORVU5Hr1G109600	2.234956	2.170152	2.421514	2.652145	2.834721	3.038561
HORVU2Hr1G084270	17.57469	17.34097	27.09446	8.525123	11.41765	11.05421
HORVU0Hr1G005430	0.346994	2.379447	0.377327	2.065911	0.659452	2.077338
HORVU5Hr1G114200	1.451986	1.833271	2.041533	2.740017	4.593446	5.556749
HORVU7Hr1G021660	0	0	0.064536	1.256394	10.61878	9.665977
HORVU2Hr1G002050	10.80347	10.97838	14.01829	10.42121	12.26501	11.23518
HORVU6Hr1G009040	0.725481	0.657787	1.327909	1.513045	1.541451	1.393951
HORVU2Hr1G088550	4.387544	5.100226	5.304421	4.221015	4.628171	4.674978
HORVU4Hr1G069970	3.263683	4.183363	3.275535	4.008911	2.28247	2.357898
HORVU4Hr1G015530	5.158	5.055821	6.108684	5.476043	9.432114	8.994338
HORVU3Hr1G014320	0	0.01381	0	1.513491	2.526046	2.399071
HORVU1Hr1G088920	2.007889	1.046165	1.068018	95.81943	9.694126	9.278775
Hordeum_vulgare_newGene_6089	0.699163	0.875146	0.83287	1.813425	3.0977	3.119943
HORVU5Hr1G086730	2.201883	2.952356	1.436262	3.655654	4.525763	2.865767
HORVU2Hr1G118100	1.453967	1.099875	1.040593	1.792339	1.622442	3.061797
HORVU0Hr1G012710	0.573752	0.736663	0.467307	0.894957	1.902204	1.486596
HORVU4Hr1G008580	13.2343	11.89273	13.85838	10.37894	9.61657	11.62625
HORVU1Hr1G095190	1.328663	1.1283	0.870917	2.660571	3.063184	2.761802
HORVU3Hr1G085820	9.088834	9.852893	8.088105	10.52215	10.87435	10.60643
HORVU5Hr1G044200	1.422697	2.206663	1.911239	1.736877	2.236309	2.393736
HORVU5Hr1G058720	49.42878	61.74699	40.40567	50.08805	365.9541	363.4022

HORVU4Hr1G042800	13.4663	15.58133	18.204	14.33012	17.58764	18.98296
HORVU3Hr1G029880	0.648621	0.552221	0.707498	1.070831	2.768173	2.974035
HORVU0Hr1G013040	3.34719	3.826696	4.950288	2.264414	2.873296	3.075077
HORVU2Hr1G017080	1.964009	1.639251	0.853396	1.335106	2.180507	2.374777
HORVU5Hr1G023860	0.771758	0.746914	0.875331	0.80399	1.783923	1.036669
HORVU4Hr1G084910	3.355158	3.544659	2.886321	1.084853	0.129649	0.356672
HORVU2Hr1G099490	1.24202	1.057419	1.244088	3.241043	6.166832	5.236827
HORVU5Hr1G060250	24.21948	23.39487	24.706	26.3675	22.86397	24.77824
HORVU5Hr1G013400	2.804938	2.828641	4.733587	1.419346	1.086291	1.023958
HORVU6Hr1G072640	3.509488	4.234367	5.634064	3.34226	4.014142	4.777847
HORVU6Hr1G063100	3.957916	3.661218	3.459802	2.630281	3.111981	3.378196
Hordeum_vulgare_newGene_4698	0.12055	0.124337	0.110683	6.868562	8.186208	8.010343
HORVU5Hr1G045150	17.03899	10.37824	18.86592	9.874836	7.232842	8.074278
HORVU7Hr1G006370	15.23558	15.68057	15.12983	54.0597	35.18159	38.86504
HORVU3Hr1G071530	0.801967	0.642772	0.85338	8.216741	16.7954	17.89444
HORVU1Hr1G078050	91.95438	109.7602	226.3098	18.85905	0.209475	11.45595
HORVU3Hr1G074330	13.89382	14.39831	14.40517	11.42059	12.33913	13.91244
HORVU6Hr1G056280	11.673	11.85259	12.5529	16.83344	18.49556	17.65586
HORVU4Hr1G059950	5.007245	4.827831	6.302549	9.067637	10.94932	8.872927
Hordeum_vulgare_newGene_10219	1.503141	40.58003	77.80347	0.41527	0.005889	0.111275
Hordeum_vulgare_newGene_5413	2.509314	4.148998	3.722155	2.596514	2.683267	2.843705
HORVU7Hr1G056040	21.39316	20.95576	23.39862	16.61947	21.14182	19.17869
HORVU4Hr1G054850	10.3162	9.032747	12.21742	14.1015	24.59117	20.08736
HORVU7Hr1G109640	47.00971	39.49169	47.39466	34.9762	26.53141	26.59507
HORVU3Hr1G074480	24.36966	24.14602	26.32325	21.75044	25.22644	23.28705
HORVU6Hr1G014630	2.199528	1.420462	3.035945	2.535887	2.31041	3.740094
HORVU4Hr1G000290	1.699074	1.848566	1.918975	3.232987	4.017276	2.180933
HORVU5Hr1G047790	50.90109	48.9317	53.40579	58.12425	59.06837	59.10868
HORVU7Hr1G089470	6.544137	6.643724	9.050346	5.00378	8.116035	8.405529
HORVU5Hr1G022050	9.774495	13.39929	12.38087	10.07106	10.4383	11.7237
HORVU6Hr1G032170	1.595865	1.13531	1.660891	2.521944	3.082488	3.46071
HORVU0Hr1G010660	0.564914	0.883322	0.605916	0.766103	2.290618	1.946362
Hordeum_vulgare_newGene_14199	0.120186	0.284958	0.142104	1.201757	3.383541	2.319041
HORVU1Hr1G012960	77.79987	85.90366	105.2214	189.9339	471.804	396.8838
Hordeum_vulgare_newGene_11166	10.90549	11.27228	13.78604	8.848653	10.03725	11.55528
Hordeum_vulgare_newGene_11167	0.09768	0.074556	0.160986	9.886547	14.97837	13.93598
Hordeum_vulgare_newGene_11164	75.70952	94.3372	131.275	16.17151	3.074167	6.862639
Hordeum_vulgare_newGene_11165	1.403529	1.309022	1.151387	5.648921	14.55944	12.7298
HORVU3Hr1G073590	7.536043	7.63947	9.415703	7.693593	10.793	9.517597
Hordeum_vulgare_newGene_11161	6.620578	8.240027	7.481929	0.021114	0	0.002211
HORVU3Hr1G108530	0.432741	0.626554	0.656737	1.32201	1.731686	2.074024
HORVU2Hr1G097420	0.960787	0.952282	1.307888	0.942229	1.642085	1.525163
HORVU1Hr1G081990	11.3746	11.22348	11.74171	8.328776	7.98869	8.951805
HORVU4Hr1G024710	16.19976	18.21843	16.82915	24.99458	40.66317	42.91071
HORVU5Hr1G042310	29.1544	28.39546	30.103	52.9282	67.63557	74.09947
HORVU6Hr1G055820	4.985972	4.640197	5.167638	5.378529	5.407276	5.820763
HORVU5Hr1G123590	7.952827	6.971927	9.81015	15.36288	20.76216	21.3009

HORVU2Hr1G093330	5.157627	5.83203	6.967745	7.607214	8.317033	8.818905
HORVU5Hr1G079850	2.699894	2.217463	1.841756	4.319547	4.799126	5.824697
HORVU3Hr1G062040	24.41571	23.24151	35.50298	31.76692	52.99368	62.02054
HORVU4Hr1G009670	4.863439	4.327002	4.963796	6.002967	5.191427	7.944667
HORVU5Hr1G077170	4.02263	4.536342	4.45122	0.249443	0.127639	0.317987
HORVU1Hr1G001150	0	0	1.0302	12.32213	0	0.050528
HORVU1Hr1G061600	9.205138	11.18671	7.832823	13.58021	9.490733	10.38845
HORVU3Hr1G029300	1.06875	1.260093	2.12969	1.492397	2.108203	2.445245
HORVU4Hr1G052040	24.54313	23.9706	25.22398	34.33315	31.20202	28.79886
HORVU0Hr1G023930	32.1945	49.95772	53.42642	18.55226	217.183	380.1143
HORVU3Hr1G058990	1.538502	1.145096	0.963255	1.958988	2.576919	3.946489
HORVU5Hr1G002510	36.94326	42.08493	36.393	32.69913	38.34772	38.92513
HORVU7Hr1G087440	3.27419	1.960022	2.386852	9.750773	56.44135	45.42385
HORVU3Hr1G108390	9.729677	9.686566	10.99505	13.26671	23.40715	29.23653
HORVU2Hr1G098500	4.433499	2.484191	3.734113	2.954438	0.07329	1.084713
HORVU5Hr1G122640	26.81354	30.2311	33.80446	26.07128	30.63515	32.8637
HORVU2Hr1G035780	4.765968	4.460959	5.084663	5.691227	7.394425	8.070582
HORVU4Hr1G067070	1.665824	2.975501	3.463144	1.499013	2.484314	3.365733
HORVU5Hr1G016120	1.57214	1.327307	1.320921	0.905845	7.156359	8.043215
HORVU4Hr1G067270	10.35526	12.67471	11.9093	10.49801	12.7525	12.85525
HORVU4Hr1G052920	6.393951	6.305695	7.550379	9.749149	15.75987	15.41609
HORVU7Hr1G045580	7.814908	9.496248	7.308679	59.89907	48.92783	48.1304
HORVU4Hr1G085830	3.18554	3.217339	5.20597	5.918645	9.569692	9.744726
HORVU5Hr1G098960	55.21379	60.89307	62.03498	141.1704	246.3493	240.2418
HORVU2Hr1G057610	11.5843	13.60478	12.83234	4.623294	5.539771	5.820584
HORVU5Hr1G082770	7.785231	7.911142	12.01947	6.03065	8.238197	8.912554
HORVU3Hr1G026870	17.418	18.511	17.97506	28.16088	26.13207	24.58979
HORVU2Hr1G039170	8.413598	9.146068	11.50267	12.78841	15.28134	15.57024
HORVU7Hr1G045630	63.5488	63.57453	74.34625	29.58411	12.90615	13.32483
HORVU6Hr1G088190	4.879115	5.558848	4.516221	3.352245	1.759188	2.109189
HORVU3Hr1G088470	1.068717	1.20558	1.374716	0.840103	0.683004	1.240198
HORVU7Hr1G047980	9.272404	11.0894	13.58247	8.290485	11.51059	11.05186
HORVU2Hr1G092880	9.060669	6.777099	7.786352	14.45415	6.771198	8.456947
HORVU3Hr1G063710	0.436455	0.617956	0.745867	0.766322	1.566303	1.886803
HORVU0Hr1G021390	6.700229	5.81688	7.007318	14.03397	22.89971	20.24192
HORVU2Hr1G064620	32.90861	32.09984	40.69788	30.09021	46.82266	47.45917
HORVU2Hr1G025090	9.973177	9.786543	7.955197	5.091803	2.307174	3.131395
HORVU2Hr1G111510	3.37618	1.959899	3.541873	2.699846	0.360909	0.932277
HORVU2Hr1G021730	15.8451	10.58733	6.483057	0.633005	0.669494	0.509518
HORVU1Hr1G020620	4.648838	5.266698	6.126307	4.410046	5.629907	6.316392
HORVU0Hr1G017020	26.3994	25.51615	32.83288	36.66704	60.13774	57.39954
Hordeum_vulgare_newGene_3281	14.56922	14.37196	13.80494	15.61827	19.87284	18.99684
HORVU4Hr1G076330	9.140876	10.41465	8.307213	9.410639	10.72205	11.04891
Hordeum_vulgare_newGene_3286	2.445321	2.368788	2.387831	2.168323	1.799864	1.850242
HORVU2Hr1G035870	84.70452	65.84535	99.99157	50.7432	20.88819	28.03765
HORVU6Hr1G086830	2.88481	2.419401	2.926255	1.743003	1.45399	1.903909
HORVU1Hr1G050110	2.032375	1.734043	2.558264	2.998028	4.300428	4.490299

HORVU4Hr1G065560	1.718175	1.304187	1.339763	0.540019	0.707413	0.622883
HORVU0Hr1G021640	24.14134	23.77321	25.60587	13.73133	7.644741	10.36746
HORVU6Hr1G078110	1.452663	1.597269	1.771447	5.579527	6.679698	7.051778
Hordeum_vulgare_newGene_12533	1.537088	1.620465	1.236207	2.21246	1.908048	2.145036
HORVU3Hr1G056330	1.058102	0.996989	1.21559	1.686133	1.905609	1.48902
Hordeum_vulgare_newGene_779	0.072664	0.314947	0.182863	2.403746	1.955589	1.824728
Hordeum_vulgare_newGene_778	6.471299	5.503402	6.247137	0.019485	0	0
Hordeum_vulgare_newGene_777	1.698105	1.59179	3.199818	1.22996	1.738167	1.670963
Hordeum_vulgare_newGene_776	9.621156	8.406603	10.1772	18.30604	25.06892	28.01037
Hordeum_vulgare_newGene_772	7.704567	8.656892	9.052227	6.532212	6.053353	7.414468
Hordeum_vulgare_newGene_770	6.643743	8.511153	9.254327	5.811347	5.939289	7.040754
Hordeum_vulgare_newGene_14279	1.7495	1.160068	1.958309	0.270798	1.678472	1.054986
HORVU3Hr1G064070	5.685084	4.341057	5.270469	2.41789	0.812299	1.221107
Hordeum_vulgare_newGene_14270	2.166899	1.932845	2.740575	8.699257	10.81197	7.251945
HORVU4Hr1G086700	48.50049	41.90226	45.58086	48.00703	38.12327	40.94027
HORVU7Hr1G078530	21.80675	18.05941	21.06125	28.63678	23.6167	24.81339
HORVU5Hr1G079180	8.946179	9.315822	8.858966	9.872627	15.31189	14.16574
HORVU6Hr1G053710	9.601401	6.893188	10.94244	1.56127	0.789816	1.004378
HORVU5Hr1G068200	12.70064	10.88919	16.99359	21.22565	21.45873	15.95543
HORVU5Hr1G065630	12.23566	10.47393	10.4018	12.05548	14.22923	14.47186
Hordeum_vulgare_newGene_10978	15.39907	15.9319	18.01902	14.89746	18.1025	17.62745
HORVU6Hr1G017740	16.77554	18.61905	15.30027	14.44413	16.23296	17.61586
HORVU3Hr1G097070	4.414334	5.721082	7.246249	4.029508	5.510291	5.802607
Hordeum_vulgare_newGene_10970	9.185183	10.3916	8.65966	0.00898	0.016842	0.003812
HORVU2Hr1G063630	11.65627	10.66781	12.82373	9.622463	12.89347	12.14285
Hordeum_vulgare_newGene_2623	8.455639	8.572746	8.113561	8.661421	6.44664	8.424259
Hordeum_vulgare_newGene_2627	11.32797	12.73866	10.43387	18.77938	19.94201	18.52801
HORVU6Hr1G073990	36.81031	21.8559	32.04679	16.99054	10.0008	11.95356
HORVU4Hr1G070180	14.18708	13.42061	13.90886	14.46085	15.80945	16.87489
HORVU3Hr1G055730	11.92263	10.9687	12.2549	20.1772	31.11702	31.87316
HORVU7Hr1G059090	75.95569	78.38398	131.4848	64.08441	208.695	172.795
Hordeum_vulgare_newGene_8872	1.085903	0.735777	1.30884	1.881973	2.176071	2.109485
Hordeum_vulgare_newGene_8871	1.115061	1.101678	1.190652	1.461038	1.126653	1.434949
HORVU7Hr1G096700	2.530919	1.697731	2.944835	1.177591	0.872051	0.729983
Hordeum_vulgare_newGene_8953	2.270972	2.577365	2.98865	2.542295	1.810812	1.923007
HORVU5Hr1G073600	10.6767	9.533455	9.898129	8.671802	10.3783	10.29552
Hordeum_vulgare_newGene_5937	3.57875	2.917231	3.486118	2.706196	2.255385	2.540686
Hordeum_vulgare_newGene_5931	6.4711	6.227151	9.960031	10.00825	8.105749	9.8512
HORVU2Hr1G070690	25.55456	21.66433	25.38099	32.33896	47.25361	44.70573
Hordeum_vulgare_newGene_5932	3.621503	3.203892	4.096675	7.498243	6.211332	6.373897
HORVU7Hr1G074640	4.282963	3.056375	5.731217	3.94523	1.983602	3.596814
HORVU4Hr1G028180	9.580817	12.77595	10.82686	17.63207	19.9777	20.8472
HORVU7Hr1G073610	2.889322	3.075989	2.855672	3.56948	4.775677	5.161854
HORVU2Hr1G096430	1.315703	1.575941	1.401378	1.404689	2.060509	2.096201
HORVU3Hr1G110330	0.016459	0	0	1.376122	2.740282	2.046366
HORVU5Hr1G116650	38.48798	34.87064	50.151	33.90605	51.65494	53.47118
HORVU3Hr1G088990	0.714569	0.825974	0.967572	1.83604	3.079299	2.334757

HORVU3Hr1G012000	0.231577	0.586345	0.636573	3.387639	2.957081	2.131079
HORVU2Hr1G104130	5.717688	7.043123	12.4213	0.724528	0.086406	0.348813
HORVU7Hr1G060360	11.78693	9.219709	12.24011	14.11199	18.58601	19.0781
HORVU7Hr1G086180	1.179944	1.400447	1.484504	1.23985	1.704744	1.73572
HORVU5Hr1G063330	0.841759	0.425652	1.388101	5.023391	4.921472	5.611583
HORVU6Hr1G076220	23.63128	24.08061	20.51143	20.16363	19.12382	20.66326
Hordeum_vulgare_newGene_1127	1.637139	1.859039	2.084028	1.856478	1.741092	1.957654
HORVU4Hr1G060000	1.598925	1.381346	2.097049	2.455105	2.964234	2.621215
HORVU1Hr1G035100	21.82623	28.19242	29.54442	0.030445	0	0
HORVU0Hr1G010340	3.245378	3.367368	2.318841	1.306093	0.996894	1.199804
HORVU7Hr1G107550	21.01624	20.15656	24.42966	24.02443	24.41191	23.95978
HORVU1Hr1G000720	0.473206	1.062784	0.540105	14.98624	2.72391	3.992936
HORVU4Hr1G046610	5.724981	3.77525	6.010081	10.73831	8.14051	9.681548
HORVU7Hr1G122600	25.97898	25.47726	24.02832	18.20308	16.12441	17.76773
HORVU5Hr1G038970	12.86782	14.19226	16.42496	13.30857	10.76056	16.6606
HORVU7Hr1G023760	25.53373	23.91064	29.98081	26.8764	33.11165	30.25259
HORVU7Hr1G118860	0.90513	0.795299	0.501698	1.795721	0.878771	1.690754
HORVU1Hr1G052530	1.813089	1.430345	1.736203	6.522705	4.34649	4.757945
HORVU3Hr1G052820	4.094552	3.724684	4.38559	5.75628	11.29818	9.325447
HORVU1Hr1G028900	1.43368	1.59086	1.414923	1.042506	0.490381	0.853339
HORVU7Hr1G027440	7.855762	7.820596	10.37161	147.2703	80.31548	148.9678
HORVU3Hr1G050080	13.642	12.22757	15.97828	13.63026	10.99689	11.51251
HORVU6Hr1G077570	5.513681	6.302803	7.301602	5.334758	8.150237	8.084537
HORVU1Hr1G072780	137.4299	140.0572	103.7721	117.7514	94.42129	100.696
HORVU6Hr1G009280	1.400695	1.192088	1.375976	2.007487	2.824703	3.357567
HORVU3Hr1G014120	81.59865	82.59032	88.02212	24.84631	35.8514	34.54013
HORVU0Hr1G039750	7.313979	4.096948	7.438151	9.518276	13.71762	10.71883
HORVU2Hr1G022350	7.930055	7.435401	9.05201	12.03589	19.97486	15.40266
HORVU1Hr1G063030	14.24503	15.79981	15.6037	18.90391	23.74653	21.84433
HORVU2Hr1G105770	0.014908	0	0	0.497138	123.7006	82.45307
HORVU6Hr1G066120	2.201782	0.19786	2.092328	0.959247	1.048801	1.542712
HORVU7Hr1G115460	5.724491	6.97211	8.658478	5.943942	7.57191	8.913089
HORVU0Hr1G020590	0	0.057164	0.218183	3.440345	6.141654	8.838771
HORVU0Hr1G031690	3.533638	1.256382	0.489617	2.977184	3.968966	4.688709
HORVU1Hr1G064560	5.782347	5.695871	7.584098	5.07304	5.922237	7.442608
HORVU6Hr1G031580	2.377287	2.075848	2.784744	10.36403	16.85015	14.58646
HORVU7Hr1G021720	1.674309	1.605697	1.997566	2.451099	2.313925	2.620629
HORVU2Hr1G068980	1.305766	1.401124	1.212062	1.137091	1.171411	1.351223
HORVU1Hr1G024420	3.778302	5.246586	4.667171	7.996724	8.555513	9.821557
HORVU2Hr1G120260	2.863835	2.92225	2.599206	4.411134	4.402386	5.027671
HORVU2Hr1G077630	0.899561	1.066082	1.298098	2.509714	9.295224	9.157331
Hordeum_vulgare_newGene_4912	2.392598	1.960596	1.125297	0.677857	0.299915	1.004687
Hordeum_vulgare_newGene_4913	0.589143	0.779755	0.736054	12.7009	10.39467	10.12928
HORVU0Hr1G004960	11.83559	10.43951	12.62081	10.6166	13.60859	14.30909
HORVU6Hr1G043230	1.070192	0.803233	1.658031	1.010799	1.053135	1.422316
HORVU1Hr1G088110	11.94046	11.17998	43.79527	101.8546	21.11141	21.8572
HORVU4Hr1G038060	8.769092	6.542069	8.582231	13.50671	20.22302	19.34495

HORVU5Hr1G037990	2.427902	3.039128	2.562366	4.468951	5.527173	5.847869
Hordeum_vulgare_newGene_52	33.78483	28.22186	32.89373	24.06381	20.93798	22.8792
Hordeum_vulgare_newGene_50	5.165613	4.308808	6.916897	3.531178	5.627876	6.406317
Hordeum_vulgare_newGene_57	2.196269	1.953706	1.619013	0.206569	0.14124	0.111897
Hordeum_vulgare_newGene_56	1.960087	2.949445	2.701729	2.924997	4.753486	4.139154
Hordeum_vulgare_newGene_54	5.424788	5.256306	5.207147	7.217856	8.547814	8.481731
HORVU5Hr1G033060	7.083841	5.286842	5.7603	22.6339	33.54104	36.46763
HORVU2Hr1G089950	7.672628	6.695485	11.78624	4.750426	3.34418	4.77157
HORVU0Hr1G001300	12.22269	14.22454	19.51029	8.909137	10.5745	10.55279
HORVU1Hr1G021550	18.95148	19.69142	25.67944	19.56643	32.57474	29.06167
HORVU2Hr1G071070	0.153057	0.228577	0.54293	2.49706	1.472693	2.110924
HORVU4Hr1G072940	214.8037	223.6235	239.7284	309.717	437.0231	384.6712
HORVU5Hr1G040740	25.33308	25.16391	28.57552	16.31489	16.67658	18.33441
Hordeum_vulgare_newGene_15918	5.064578	7.108792	5.888255	3.261108	1.710632	3.395878
HORVU2Hr1G114260	10.89314	8.447126	11.71566	7.573914	7.403321	7.797492
Hordeum_vulgare_newGene_15917	11.49913	14.94734	12.67083	9.011453	8.396189	11.12476
HORVU5Hr1G103280	11.18914	11.23386	11.22185	12.06637	11.09581	12.87605
HORVU5Hr1G103130	1.379942	1.302073	4.856336	1.846702	1.638058	1.261297
HORVU6Hr1G078650	2.270183	1.585092	2.758614	2.875167	1.792789	3.102692
HORVU5Hr1G039780	2.153039	2.344808	2.707586	1.826531	4.394949	3.072153
HORVU5Hr1G019110	7.444895	7.307302	7.42219	9.611519	8.3951	12.30844
HORVU5Hr1G014340	42.60512	40.92583	45.05456	55.31472	64.15076	58.4977
HORVU3Hr1G090000	4.976428	6.122617	6.595276	11.13204	16.94164	17.48791
HORVU5Hr1G009740	12.34309	12.22061	13.71387	12.62251	16.96405	17.05018
HORVU7Hr1G011050	109.9335	120.9288	72.7224	41.30646	30.1786	31.3984
HORVU3Hr1G034270	7.651255	6.732116	6.917151	7.452711	7.757479	8.214617
HORVU3Hr1G019360	3.999401	4.309732	3.375322	5.934264	7.251615	6.487615
HORVU1Hr1G051160	0.409175	0.486958	0.313392	3.078708	4.17284	4.402104
HORVU1Hr1G087100	46.60862	46.00786	59.96404	30.32141	35.91873	34.97301
HORVU6Hr1G047440	6.976354	7.861652	7.14582	7.007051	6.822645	7.423636
HORVU2Hr1G041540	6.818255	8.354588	7.253252	8.498993	11.82883	11.39897
HORVU0Hr1G027060	1.684096	1.896726	2.14301	1.752763	1.903993	2.166066
HORVU6Hr1G003470	24.33322	22.091	26.14735	22.8602	35.294	35.07312
HORVU5Hr1G089010	8.087593	8.036484	10.88835	6.494038	5.722304	6.512621
HORVU3Hr1G086310	4.910958	4.992923	5.262312	2.403851	2.697518	2.914413
HORVU5Hr1G017640	0	0.052358	0.144247	2.519685	3.47892	3.793496
HORVU6Hr1G070330	12.35904	13.53564	14.6476	13.90322	18.3784	18.98163
HORVU7Hr1G034020	3.0504	2.231658	3.260281	6.106517	7.975241	8.360522
HORVU4Hr1G069320	34.12817	30.99325	34.876	44.05072	25.06419	28.09788
HORVU1Hr1G022390	6.625475	3.30015	9.512374	3.261816	9.382244	7.844155
HORVU2Hr1G118190	0.590921	0.547262	0.499091	1.61996	2.24489	1.801076
HORVU6Hr1G006560	38.43575	34.83072	40.88065	36.51519	34.46453	35.96239
HORVU3Hr1G007210	0.451965	0.226524	0.360832	1.988176	1.656805	1.896287
HORVU1Hr1G091210	8.852494	7.788624	8.470411	6.549578	6.567787	6.057394
HORVU7Hr1G011810	0.302296	0.567091	0.924052	1.017935	1.476889	1.770537
HORVU5Hr1G064130	0.405221	0.473627	0.428239	3.18807	3.680319	4.009754
HORVU2Hr1G027020	0.732035	0.961363	0.862559	1.277234	2.436854	2.263431

HORVU7Hr1G051520	4.815618	5.185325	4.755638	6.261036	12.82103	10.42037
HORVU5Hr1G015900	0.38302	0.842395	0.299438	0.683276	2.471729	2.424376
HORVU3Hr1G083090	20.12263	21.87218	23.08702	29.32848	42.9752	46.28593
HORVU4Hr1G043920	22.95719	19.72871	23.06368	26.3348	30.67392	30.76505
HORVU7Hr1G054850	5.863799	5.462022	6.584026	7.480918	8.418505	10.28413
HORVU7Hr1G091580	6.055994	5.253569	0	0	0	0
HORVU6Hr1G064220	1.419048	1.224429	1.499143	1.72766	1.235168	1.417119
HORVU1Hr1G041910	4.17149	5.191372	4.859931	4.199601	3.431607	3.771839
HORVU3Hr1G021190	49.20094	41.32309	49.74125	47.23213	59.82002	60.58766
HORVU5Hr1G066080	9.069146	11.8647	17.86723	6.84268	10.26547	9.698411
HORVU2Hr1G085060	22.09107	20.76396	21.70841	31.46014	38.59513	40.05375
HORVU1Hr1G025670	7.896908	7.612631	9.567058	13.22112	12.01461	11.72178
HORVU5Hr1G088860	33.48774	27.26564	31.95429	46.42841	37.34446	39.00477
HORVU2Hr1G098820	5.052644	5.494848	6.973771	4.046761	4.684698	5.337043
HORVU4Hr1G034100	6.184678	7.570476	6.145577	7.008539	13.47644	11.49261
HORVU4Hr1G063000	34.94663	34.99971	40.00464	28.78485	28.07782	28.71662
HORVU3Hr1G032260	41.61889	28.97758	28.51578	2.923391	0	0.999881
HORVU5Hr1G069690	3.611252	1.491584	4.367634	10.21842	48.58	48.95672
HORVU5Hr1G016450	1.86713	2.824454	2.925976	2.170519	2.245456	2.227952
HORVU4Hr1G010200	18.30349	19.84594	18.39747	40.00725	23.17025	26.88346
HORVU4Hr1G041610	5.464684	4.958655	5.003324	6.224267	9.784238	10.418
HORVU5Hr1G111540	35.93795	34.24917	36.19013	30.63532	38.3787	35.99283
Hordeum_vulgare_newGene_1881	5.022803	5.761672	5.849638	7.018881	7.155857	8.23763
HORVU3Hr1G024630	3.233654	3.135977	3.778053	3.573459	2.769962	3.21989
HORVU4Hr1G088490	17.03837	14.61723	18.58134	16.32937	22.54658	21.82184
Hordeum_vulgare_newGene_4571	0.774568	0.67095	0.729596	0.990066	1.717021	2.394307
HORVU1Hr1G048530	2.659581	2.309414	3.653704	3.133267	2.60558	2.758395
HORVU5Hr1G050870	7.144889	6.657105	7.94158	9.124675	9.427149	10.27924
HORVU5Hr1G034400	10.70532	11.61392	17.38925	6.973488	7.476142	8.234174
HORVU7Hr1G120030	15.6951	17.60239	17.51705	19.94541	35.76921	36.68927
HORVU3Hr1G008680	7.233108	9.889155	8.339937	12.87547	10.13911	12.46773
HORVU4Hr1G061680	1.580467	1.61153	2.699075	11.05055	8.002896	9.811941
HORVU1Hr1G058140	4.127631	4.230155	5.212834	4.86638	7.731505	8.558859
HORVU6Hr1G005030	28.79536	35.67202	39.57238	12.33854	6.309498	6.743167
HORVU4Hr1G049080	2.746239	2.985465	2.56683	4.986526	2.295601	1.532278
HORVU3Hr1G024180	0.972442	0.640398	1.388779	4.828319	8.139837	7.46288
HORVU6Hr1G021060	23.35502	18.66855	17.38326	4.574313	2.842918	2.491836
HORVU5Hr1G114070	1.489377	1.291169	2.130438	2.176914	3.198888	3.87254
HORVU4Hr1G088140	1.035432	1.304998	1.874224	2.7607	7.055526	7.154465
HORVU2Hr1G012860	54.93754	53.63964	56.52901	67.87959	84.49293	76.04952
HORVU4Hr1G048970	0.791292	0.876978	0.565841	1.279959	1.901911	1.888299
HORVU5Hr1G097560	10.51475	10.58299	10.30015	6.21889	8.436526	7.32682
HORVU2Hr1G127250	2.980116	4.018533	3.63838	4.728768	6.36948	7.335905
HORVU5Hr1G104620	0.368064	0.606941	0.593534	1.190524	2.492961	2.578712
HORVU4Hr1G015180	4.610118	4.709319	4.505825	7.93048	5.964715	6.800601
HORVU7Hr1G090670	17.64453	14.78119	18.75175	9.533932	11.25693	14.08168
HORVU1Hr1G016320	3.944746	3.929702	2.852036	5.034393	5.54465	5.99128

HORVU1Hr1G022800	2.757734	2.420959	3.779864	2.966728	5.731193	4.892357
HORVU6Hr1G032310	3.940542	3.780264	3.299683	1.509876	1.961502	2.727165
HORVU1Hr1G049660	1.890738	1.665237	2.572906	2.529542	4.766916	3.790756
HORVU6Hr1G091240	2.078322	2.167167	3.152688	1.86281	2.603037	2.115348
HORVU7Hr1G099580	38.63444	35.91088	34.97298	26.37377	26.4439	28.46186
HORVU7Hr1G020840	61.94958	63.58852	78.48312	76.49522	54.34541	55.62538
HORVU7Hr1G047470	7.334074	7.71162	7.441218	8.956663	13.62964	12.30167
HORVU5Hr1G025720	12.28733	11.65736	11.8375	14.12114	14.60243	13.67916
HORVU1Hr1G082340	0	0	0	4.929973	3.938113	3.150769
HORVU1Hr1G087920	0.096846	0.167936	16.3814	0	0	0
Hordeum_vulgare_newGene_11677	0.611024	0.763377	1.098245	1.688474	1.056324	1.708562
HORVU5Hr1G057590	0.210575	0.216019	0.264973	3.568348	3.718815	4.119691
HORVU7Hr1G082390	2.448078	1.365739	2.867328	2.764265	0.5824	1.087908
HORVU5Hr1G044430	0.832791	0.276502	0.37213	1.970391	1.503929	2.233983
HORVU5Hr1G025890	11.10905	9.795354	11.62948	8.39676	13.038	13.74227
Hordeum_vulgare_newGene_11670	1.894241	2.363641	1.860997	1.964795	3.312737	3.107714
HORVU4Hr1G083860	3.026815	3.932005	3.592604	3.471757	5.048438	5.009419
HORVU5Hr1G080280	34.63845	26.85507	29.89231	23.7692	25.03645	24.39769
Hordeum_vulgare_newGene_13970	3.216156	4.204992	4.809863	0	0	0
HORVU5Hr1G046070	5.282283	5.697004	4.534789	5.560339	5.010221	5.800876
HORVU0Hr1G001460	8.089911	7.279502	7.896543	11.43059	14.5366	14.17358
HORVU1Hr1G011800	1.804146	1.869737	2.624783	2.786771	3.386034	3.395767
HORVU3Hr1G006450	11.29082	6.747348	11.19018	5.522329	4.067022	6.353057
HORVU2Hr1G054370	2.333424	2.275592	2.897766	2.688025	3.419348	3.826012
HORVU7Hr1G022270	14.49637	13.97211	14.55311	19.37887	19.10107	20.42406
HORVU4Hr1G055220	2.848032	1.985572	1.962149	1.916167	2.056594	2.01969
HORVU2Hr1G051930	12.59337	11.92882	11.4416	12.1544	9.961764	11.92122
HORVU4Hr1G021820	0.529963	0.360096	0.494059	1.863445	1.882867	2.290851
HORVU5Hr1G074820	22.85448	27.03462	17.91512	31.52058	75.28573	58.63603
HORVU1Hr1G056770	9.0212	9.683891	9.995057	10.23001	15.00172	14.07771
HORVU4Hr1G077250	32.66936	33.23232	44.29999	42.81659	70.02947	67.84989
Hordeum_vulgare_newGene_13845	0.640887	0.649434	0.842714	1.128764	1.391572	1.529276
Hordeum_vulgare_newGene_13844	0.808961	0.963742	0.787445	1.603829	1.19045	1.080087
Hordeum_vulgare_newGene_10404	268.3654	250.3481	197.0033	97.71668	109.7299	113.8428
Hordeum_vulgare_newGene_10405	6.57439	7.927287	3.74264	1.481875	1.043243	1.124019
Hordeum_vulgare_newGene_10402	6.546442	6.01154	6.221293	8.043998	6.713006	9.963907
Hordeum_vulgare_newGene_10401	6.813795	5.688187	3.465163	0	0	0
HORVU6Hr1G017370	10.9727	9.52692	12.73992	8.429118	8.394176	8.839395
HORVU3Hr1G065630	5.365639	3.358415	2.81781	0.504711	0.151707	0.469791
HORVU5Hr1G042290	8.005512	6.743149	8.054601	8.115848	0.912277	3.594976
HORVU1Hr1G051200	10.2989	8.211694	7.861889	12.07067	19.7679	21.74854
HORVU2Hr1G039710	5.786869	5.982091	7.153745	5.538353	7.918273	5.969511
HORVU1Hr1G080470	9.096466	7.317412	7.120355	16.97332	15.38184	16.02272
HORVU6Hr1G076020	12.88745	9.698201	9.815084	3.719946	10.8194	8.277706
HORVU2Hr1G101090	1.515516	1.410568	1.99887	1.937632	0.80468	0.995636
HORVU5Hr1G123410	1.126436	0.571738	1.10279	1.259352	1.676084	1.409055
HORVU1Hr1G000120	1.096041	0.776719	1.191051	2.987168	2.13538	2.670219

HORVU0Hr1G007650	14.85086	14.05536	17.15921	20.819	29.53565	27.72939
HORVU3Hr1G078490	16.51502	13.35777	16.18411	7.802552	9.509518	9.483241
HORVU5Hr1G052340	10.70812	11.38455	11.8607	9.783454	11.70732	12.52164
HORVU5Hr1G116320	6.953675	7.404242	9.836356	5.707421	9.368207	9.451956
HORVU7Hr1G068320	5.204709	5.656837	6.17198	6.524287	7.538637	7.584089
HORVU1Hr1G044170	2.568796	1.787501	3.527497	4.037543	2.893082	3.802747
HORVU3Hr1G062940	3.140304	3.467767	3.326192	2.506387	0.463466	1.016893
HORVU7Hr1G000900	9.396147	7.380397	7.461751	27.12005	45.45979	45.83384
HORVU1Hr1G017700	99.4987	89.46557	113.0528	107.0069	64.71271	74.28189
HORVU3Hr1G072230	8.588478	9.18456	11.8288	7.286176	10.5877	10.76507
HORVU3Hr1G095760	59.40851	39.06977	72.82432	18.02732	15.24327	14.1058
HORVU1Hr1G087870	0.418813	0.403969	17.42865	45.69343	0.821105	0.979133
HORVU4Hr1G007090	6.277175	5.616412	7.574193	8.281349	9.278771	9.547724
HORVU4Hr1G051210	5.664729	5.749403	6.200648	6.382349	9.938145	10.09978
HORVU7Hr1G027600	7.090393	8.929779	8.521928	7.071365	9.080877	8.340994
HORVU5Hr1G081620	21.44556	19.26704	19.95162	22.20111	23.47805	24.0656
HORVU4Hr1G014760	4.30696	3.730668	4.582874	5.631361	7.4348	7.55184
HORVU6Hr1G077770	8.689011	7.01729	10.416	25.36154	29.95713	27.0451
HORVU6Hr1G077860	1.253269	1.032039	1.51691	0.772469	1.123156	2.087279
HORVU2Hr1G056440	1.407863	2.088888	2.220647	1.661298	2.756805	3.302583
HORVU4Hr1G006320	22.77842	23.02253	25.83193	25.63035	23.42577	28.65424
HORVU4Hr1G077420	12.68232	13.28742	15.97708	11.01428	17.13986	14.26382
HORVU2Hr1G021080	28.85658	21.7239	31.28831	46.36261	76.16338	70.20702
HORVU5Hr1G049960	15.32109	13.04533	15.3584	19.5961	26.69127	26.89239
HORVU0Hr1G023870	2.224411	2.743911	1.570148	1.081443	10.52564	8.548283
HORVU2Hr1G093160	4.291172	0.893867	0.559746	0.120392	4.495384	4.352255
HORVU7Hr1G077060	0.064362	0.060238	0.069271	1.089908	3.230805	2.369561
HORVU4Hr1G079030	3.65363	5.074289	6.715034	4.723313	10.00791	8.371775
HORVU5Hr1G048380	4.703479	4.278305	5.790084	6.308951	6.562187	4.993541
Hordeum_vulgare_newGene_11464	0.422542	0.583353	0.76151	4.891446	5.404634	6.134214
Hordeum_vulgare_newGene_11463	12.98751	11.48758	11.81333	15.97547	13.06517	14.17182
Hordeum_vulgare_newGene_11461	2.236379	1.967851	2.292894	0	0	0
HORVU2Hr1G060480	17.42497	15.70069	15.44688	47.23143	88.96032	87.20777
Hordeum_vulgare_newGene_111	6.427046	5.791735	6.982886	6.808484	6.529774	5.865293
Hordeum_vulgare_newGene_110	3.771833	4.055929	4.594273	5.948737	8.035182	8.468578
HORVU3Hr1G022340	2.077482	1.356225	1.379894	3.801397	5.952585	4.607831
HORVU7Hr1G083490	11.32132	9.990864	12.515	11.9825	17.74825	16.25772
HORVU3Hr1G107690	6.254585	6.257986	6.41182	4.739672	7.098315	6.828926
HORVU3Hr1G064040	1.388226	1.066359	1.276563	0.855419	0.928347	0.807646
HORVU4Hr1G079310	5.958183	5.824369	6.265706	6.136601	11.12365	13.19377
Hordeum_vulgare_newGene_14209	3.272045	5.014421	6.433766	2.775435	3.186136	3.422665
HORVU6Hr1G090380	3.006447	3.301209	3.245589	6.710914	11.6952	11.23553
HORVU5Hr1G062030	2.612986	1.306598	2.330672	8.055392	8.14052	4.768647
HORVU4Hr1G027080	1.925951	2.037318	1.919729	2.952702	3.312401	3.064819
HORVU7Hr1G046460	26.68597	34.61102	27.51651	34.89984	28.4413	33.50845
HORVU4Hr1G009570	8.758659	7.353529	10.10204	10.28925	12.36908	12.63563
HORVU5Hr1G071480	0.178838	0.422626	0.403331	1.813066	2.584291	2.443012

HORVU6Hr1G054240	2.779956	1.05283	1.68039	2.949537	3.754559	11.48264
HORVU5Hr1G070980	5.021363	5.977328	7.959041	6.919485	12.66493	9.973934
HORVU5Hr1G022940	2.194184	2.286432	1.241714	0.791963	0.876725	0.572769
HORVU6Hr1G089780	86.85086	77.40022	108.0052	51.53407	53.14842	60.29906
HORVU0Hr1G025820	1.698844	1.940423	1.952567	2.235551	2.65248	2.854707
HORVU7Hr1G074870	3.379558	3.324171	3.988865	3.418812	6.336557	6.212398
HORVU1Hr1G085150	12.53598	15.20799	10.31081	4.112329	5.698853	5.164652
HORVU2Hr1G114460	4.370363	2.800363	4.550871	3.362829	7.392783	5.453876
HORVU5Hr1G037200	4.908829	4.98732	5.25781	24.44399	26.35138	21.99358
HORVU4Hr1G059220	6.36587	5.102586	7.420296	7.084132	6.101157	5.533858
Hordeum_vulgare_newGene_16119	0.85903	1.1314	1.380617	2.624635	1.54101	3.429963
HORVU5Hr1G049030	2.24969	1.998351	2.10419	4.274471	3.374878	4.087033
Hordeum_vulgare_newGene_16113	2.446166	2.917185	3.279418	1.989705	2.277976	2.801064
Hordeum_vulgare_newGene_16110	6.945427	7.629335	7.164663	9.971981	12.34533	13.62801
HORVU5Hr1G104190	23.06331	21.59166	27.44934	22.02525	27.60445	29.82775
HORVU5Hr1G079570	26.06258	28.24176	24.83979	25.96893	31.31035	29.62323
HORVU0Hr1G024560	0.953479	1.223903	0.758308	3.27425	18.21896	19.23526
HORVU5Hr1G064020	44.17427	29.37992	46.80542	30.21622	25.58392	35.99096
Hordeum_vulgare_newGene_13569	18.38067	14.24863	18.98899	22.23797	29.92236	31.34092
HORVU3Hr1G081140	3.052198	2.84048	2.392727	1.281617	1.197019	0.957164
Hordeum_vulgare_newGene_13565	1.261759	1.272382	1.091587	2.604353	3.205716	3.516774
Hordeum_vulgare_newGene_13566	2.448652	1.743024	2.662833	3.604975	4.025954	3.681106
Hordeum_vulgare_newGene_13567	0.517479	0.647521	0.507959	2.899847	13.22212	9.511845
Hordeum_vulgare_newGene_13563	0.952552	1.711539	1.413203	2.742896	2.671726	2.957526
HORVU7Hr1G100400	0.483958	1.12097	0.883109	2.144467	6.014621	7.820558
HORVU7Hr1G104180	2.458046	2.355099	2.634958	1.835484	2.434421	3.26285
Hordeum_vulgare_newGene_7915	26.39701	26.40539	29.14761	25.27341	29.48616	29.18884
Hordeum_vulgare_newGene_7914	0	0	0	6.427857	7.394134	7.850961
HORVU5Hr1G000780	22.74567	23.98544	24.55279	38.55128	39.876	37.32117
HORVU1Hr1G071500	3.258777	1.912591	3.849225	26.4822	7.84113	10.67231
HORVU6Hr1G051110	5.509149	6.002218	7.592788	6.400248	6.79659	7.478692
Hordeum_vulgare_newGene_8346	4.874096	7.226021	4.068448	0	0	0
HORVU6Hr1G078320	6.534174	7.247342	6.064327	8.605786	9.282056	8.062727
HORVU7Hr1G038120	0	0	0	32.5174	26.2721	29.11462
Hordeum_vulgare_newGene_6064	1.007881	1.069958	1.083523	1.767898	1.681746	1.682799
HORVU5Hr1G085710	2.080379	0.682941	2.048967	0.886813	0.640822	1.144576
HORVU5Hr1G009890	2.40834	2.418148	2.217969	3.449474	4.448823	4.875366
HORVU4Hr1G054630	0.993473	1.041533	1.634208	0.603095	0.76901	1.032704
Hordeum_vulgare_newGene_6068	1.392567	1.480277	1.673524	0.457537	0.530306	0.695666
Hordeum_vulgare_newGene_6069	5.886055	2.929534	6.315196	22.63228	9.314087	21.00862
Hordeum_vulgare_newGene_13698	12.99472	16.02262	12.5303	16.60423	22.25849	21.15795
HORVU7Hr1G118230	29.96723	27.09539	32.2893	36.09075	64.67929	65.39302
Hordeum_vulgare_newGene_13693	0	0	0	2.368858	1.474095	2.621185
Hordeum_vulgare_newGene_13691	2.27901	3.047858	2.102612	3.785218	4.5649	4.744607
HORVU2Hr1G060550	3.515041	3.82305	4.817133	6.377021	9.449662	8.156321
Hordeum_vulgare_newGene_13694	3.012469	3.316972	3.60175	1.657755	1.925415	2.048821
HORVU3Hr1G052780	0	0	0	5.004249	6.937835	6.159219

Hordeum_vulgare_newGene_3745	9.660368	9.799563	15.87056	10.34213	12.17566	11.89004
Hordeum_vulgare_newGene_3747	0.37935	0.484399	0.868729	2.626113	2.836829	3.535659
HORVU1Hr1G044810	17.00112	16.65146	16.63475	23.26455	26.99397	26.99916
HORVU5Hr1G068070	2.228578	1.953202	2.6325	1.224797	1.112429	1.280677
HORVU6Hr1G018610	6.643142	7.354892	8.791069	11.66443	18.36451	16.59518
HORVU6Hr1G065280	14.42078	11.71025	12.72796	8.597556	7.458423	8.062198
Hordeum_vulgare_newGene_2297	3.698774	2.758014	4.342139	0.003976	0	0
Hordeum_vulgare_newGene_2290	6.631963	9.081872	8.537337	12.62684	9.812903	13.57148
HORVU5Hr1G062740	3.688845	4.841061	4.441282	7.148531	9.107473	10.90602
HORVU7Hr1G078760	0	0	0.163055	2.593667	11.2685	10.6516
HORVU1Hr1G023120	1.689848	1.878751	1.481662	3.79859	6.550026	6.235448
Hordeum_vulgare_newGene_15597	6.098155	8.317492	7.873068	4.032749	2.831058	6.04938
Hordeum_vulgare_newGene_15596	1.102295	3.397157	2.159879	1.826922	1.362307	2.567755
HORVU3Hr1G020470	50.74756	43.03574	53.83253	54.91441	63.04139	66.65403
Hordeum_vulgare_newGene_15593	1.692437	1.466051	1.866137	2.947748	4.029701	4.660318
Hordeum_vulgare_newGene_15592	1.099737	1.770213	1.428139	1.266628	0.643523	0.433645
HORVU5Hr1G006780	95.5331	96.64409	112.9141	87.81185	122.4178	140.5341
HORVU1Hr1G077900	26.34829	25.59898	25.46152	26.33733	30.83688	27.62411
HORVU7Hr1G043140	226.1492	208.3002	194.088	139.6396	134.8216	133.1618
Hordeum_vulgare_newGene_15599	0.93804	1.780678	1.888145	0.873215	0.520435	0.936758
HORVU3Hr1G111760	135.2842	144.76	215.8814	112.0732	249.8867	237.2137
HORVU7Hr1G103170	32.77438	42.10894	31.11512	39.38595	30.25006	38.81818
HORVU6Hr1G068730	0.681498	0.638959	0.582049	2.183377	3.021079	3.164158
HORVU5Hr1G062290	31.7036	16.98415	46.46891	24.74776	4.133446	11.45438
HORVU2Hr1G072020	80.78956	82.19728	71.15051	61.49017	28.01098	36.79893
HORVU7Hr1G117320	1.178522	0.861466	1.028683	2.03927	2.746157	2.617157
HORVU3Hr1G016420	28.88503	28.46755	29.40192	30.2628	29.02921	30.09888
HORVU0Hr1G009120	4.693234	3.366079	3.512923	11.05676	14.18774	14.18023
HORVU3Hr1G054090	3.290692	2.736818	3.185667	6.187355	6.800953	7.399585
Hordeum_vulgare_newGene_5747	5.154306	4.351029	5.181052	11.40007	14.72929	17.73313
Hordeum_vulgare_newGene_5742	2.81263	2.157775	2.480999	0	0	0
HORVU2Hr1G004730	1.672844	2.109536	2.046962	2.914859	2.654124	2.421214
HORVU7Hr1G066670	12.19559	11.57862	12.09637	12.69905	14.95549	15.78209
HORVU2Hr1G029990	44.20081	42.06488	60.88201	41.02101	57.39921	61.06151
HORVU6Hr1G068080	4.396284	5.906826	6.534003	1.274948	2.107273	2.86912
HORVU1Hr1G053050	3.028878	3.889781	4.618584	1.318032	1.08701	1.356422
HORVU3Hr1G015190	87.26106	91.11387	127.3947	77.31703	148.4143	142.4046
HORVU6Hr1G045310	8.696942	8.548212	9.987064	14.9211	21.45171	21.08088
HORVU5Hr1G103890	56.97952	54.56481	57.70549	23.1658	28.29203	27.61002
HORVU7Hr1G025730	4.6061	6.555998	6.599053	5.521315	5.189524	6.457303
HORVU1Hr1G059180	0.154545	0.080921	0.647973	1.416785	6.209481	5.643675
HORVU1Hr1G033800	10.07457	10.80542	9.268288	9.575221	12.49213	11.29174
HORVU2Hr1G104300	1.639695	1.061389	1.319153	1.724323	4.488793	2.85313
HORVU7Hr1G060530	5.534221	6.138817	6.386568	11.74914	16.0389	18.43802
Hordeum_vulgare_newGene_1243	15.09853	9.739768	19.21584	10.28311	6.102987	10.40725
Hordeum_vulgare_newGene_1242	15.0949	10.2858	15.22481	16.27909	21.16247	22.3496
Hordeum_vulgare_newGene_1249	50.32461	44.03559	77.85332	105.5192	2.81374	24.72138

HORVU3Hr1G005490	4.861348	4.165268	5.617449	4.876971	5.111987	5.178805
HORVU3Hr1G081960	1.892155	3.207599	3.22134	1.501159	1.180456	1.741638
HORVU0Hr1G008980	19.45296	18.88674	19.54255	15.84643	19.65869	22.39911
HORVU7Hr1G114670	2.431308	2.911411	1.333211	1.018412	1.012424	1.260555
HORVU3Hr1G068840	8.337948	8.546372	10.09083	7.729503	9.911284	11.6218
HORVU2Hr1G084600	0.931727	1.109184	1.239038	1.265767	0.875811	1.351148
HORVU5Hr1G096760	21.01203	33.41322	34.17123	9.087858	5.490975	8.843948
HORVU6Hr1G063850	1.513242	1.401094	1.317405	1.667313	1.666783	1.76061
HORVU2Hr1G066740	3.188055	3.177034	4.636003	2.784044	5.276425	4.991644
HORVU7Hr1G050900	1.485656	1.701714	2.526802	1.530263	1.538444	1.726113
HORVU5Hr1G019730	1.047526	1.014393	1.183066	1.742156	3.398496	2.734436
HORVU6Hr1G075850	0.193748	0.280391	0.063934	7.017443	2.559282	3.847622
HORVU2Hr1G029330	2.415606	3.694939	3.50852	3.258204	3.991146	4.117859
HORVU5Hr1G076910	0.604093	0.570087	1.322702	1.599135	2.419725	2.544447
Hordeum_vulgare_newGene_4442	13.89985	14.65245	13.17517	16.37704	16.02912	18.81411
HORVU2Hr1G032360	0.725277	0.462915	0.910817	4.104278	3.142187	3.668719
HORVU2Hr1G092920	25.32735	27.4154	26.2465	27.22547	28.64355	30.67549
HORVU4Hr1G089350	2.027659	2.801146	3.036464	2.177185	2.326307	2.902151
Hordeum_vulgare_newGene_4447	7.818526	5.396709	5.92757	9.004354	11.76699	12.32151
HORVU6Hr1G081370	8.062172	8.583499	7.359438	12.25927	18.34566	18.25264
HORVU0Hr1G031710	3.452761	2.969704	3.353526	2.664058	3.934592	4.061169
HORVU2Hr1G088180	5.800426	5.293068	5.137878	25.23446	45.4036	26.69609
HORVU6Hr1G087780	11.63592	12.33025	18.36868	11.40074	21.51582	19.60014
HORVU5Hr1G026140	3.83403	4.327129	5.044495	8.717681	14.63165	15.57273
HORVU1Hr1G047030	3.168991	3.139249	4.438892	2.517319	2.02441	3.470635
Hordeum_vulgare_newGene_14967	2.223656	2.2462	3.001097	3.596046	5.584326	5.308933
Hordeum_vulgare_newGene_14963	3.343949	1.766476	2.351099	2.506254	0.216904	0.511949
HORVU6Hr1G028680	5.261113	5.581575	6.502688	2.884116	3.267672	3.445675
HORVU6Hr1G070510	5.353538	4.193639	7.498115	7.355523	7.214664	6.039069
HORVU7Hr1G051140	4.187266	3.454776	4.346696	6.321047	9.060756	8.839461
HORVU7Hr1G024100	1.703145	1.778946	1.714411	2.247311	4.009158	3.141933
HORVU4Hr1G038380	27.12381	28.27192	34.76673	33.20712	40.90318	42.24101
HORVU3Hr1G047530	4.704732	5.554925	5.204486	6.363304	10.0234	9.41311
HORVU1Hr1G087800	33320.8	30210.63	28909.33	42902.87	25582.66	25368.49
HORVU1Hr1G068490	19.55909	16.96185	25.43803	0.388727	0.954404	0.891653
HORVU5Hr1G056400	8.094128	9.544943	12.01933	4.925882	5.649808	5.64546
HORVU0Hr1G039270	12.36343	15.03052	13.22636	12.64587	14.10954	13.93325
HORVU1Hr1G039600	2.453537	1.982335	2.755822	4.172214	3.545272	3.845555
HORVU0Hr1G018910	0.17756	0.254255	0.447888	4.673278	76.73794	66.84777
HORVU3Hr1G091250	11.8955	10.93201	14.28585	12.61338	6.621613	8.319977
HORVU7Hr1G013190	34.85687	28.50608	40.73616	28.32783	27.26803	26.38959
HORVU3Hr1G080370	14.6916	16.31694	11.41969	11.45149	11.0761	9.973161
HORVU7Hr1G079400	11.70921	10.05384	11.56568	14.46064	15.0241	14.78045
HORVU1Hr1G091820	9.487082	9.518688	12.08332	11.2282	11.93109	13.7996
HORVU3Hr1G043440	19.86831	18.26734	22.07275	18.69294	21.12844	20.31145
HORVU0Hr1G029500	4.158786	3.250889	3.056488	0.167046	1.635207	2.130661
HORVU5Hr1G058050	0.965204	0.785407	1.023883	1.539305	2.479214	2.597621

HORVU5Hr1G104860	33.85359	33.6674	32.45372	22.81254	22.60688	25.36874
HORVU7Hr1G056410	15.77625	12.24154	18.56691	6.625347	6.991384	7.326672
HORVU1Hr1G056570	134.6915	126.1527	163.0697	5.077225	0.097813	3.043464
HORVU6Hr1G083200	1.545558	1.039434	1.300636	3.145988	3.746365	3.306142
HORVU2Hr1G078890	4.362103	3.541911	4.299512	3.290405	5.866015	4.580225
HORVU5Hr1G012960	2.661636	2.739723	3.493073	4.857886	4.095208	4.793427
HORVU1Hr1G023660	95.15081	109.4359	153.9692	91.52226	172.0997	172.1209
HORVU7Hr1G018310	4.031875	4.41246	6.147628	3.380745	4.396953	4.110692
HORVU1Hr1G088440	0.324587	0.359209	16.55919	44.64967	0.280097	0.413946
HORVU4Hr1G074460	0	0	0.057388	0.195034	6.445642	4.992912
HORVU4Hr1G089090	25.62379	26.37038	30.94358	58.65102	56.13159	50.15666
HORVU3Hr1G081420	1.955509	2.532865	2.646589	2.858517	4.773644	4.799787
HORVU3Hr1G086430	17.26114	14.81166	12.43066	7.606201	8.903313	7.434204
HORVU2Hr1G059280	1.773009	1.134375	0.958106	2.24728	1.950555	1.773291
HORVU5Hr1G095440	15.62573	11.97102	16.92326	12.10052	14.46179	14.40671
HORVU5Hr1G103070	53.2935	49.68203	56.84279	36.97119	39.42838	40.92123
HORVU1Hr1G069360	64.48716	49.69101	75.81863	51.14122	69.87904	75.35398
HORVU0Hr1G040330	11.23825	13.00972	13.04107	10.91421	14.45185	14.77888
HORVU6Hr1G075070	7.883796	9.116065	13.5332	5.218189	6.067476	5.702501
HORVU5Hr1G055850	18.63712	13.98686	19.17021	30.58833	25.62215	32.18
HORVU3Hr1G087740	18.20348	14.99916	19.08383	22.13916	12.43273	16.11243
HORVU5Hr1G022730	3.929873	4.82768	5.053907	8.769785	9.937959	12.4729
HORVU2Hr1G109640	2.170997	1.191375	2.439311	2.68927	0.518535	2.987929
HORVU2Hr1G000280	3.035886	2.123983	4.174773	8.927398	7.779239	12.15276
HORVU4Hr1G017480	2.278651	2.945055	3.601028	3.996151	5.900857	5.262948
HORVU3Hr1G078300	2.841463	2.839125	2.868773	3.508037	4.656289	4.415323
HORVU5Hr1G002030	22.00301	25.05294	19.01285	17.58935	19.24948	19.67731
HORVU3Hr1G030650	63.21161	102.8446	129.0791	2.200788	1.575389	1.356693
HORVU7Hr1G041810	23.40694	16.85722	18.36648	20.99797	27.99093	23.43939
HORVU2Hr1G061930	14.13927	14.81959	13.76392	6.322332	5.492908	7.907475
Hordeum_vulgare_newGene_14434	1.306578	3.942829	1.953735	1.446752	0.062919	0.165531
HORVU7Hr1G048330	4.861202	7.189998	8.2865	5.215206	5.450966	6.691373
HORVU1Hr1G093530	4.539064	3.711129	3.66605	5.809537	5.906227	5.814491
HORVU5Hr1G124350	2.098251	1.382028	2.082476	3.173246	4.159375	3.768297
HORVU4Hr1G069280	27.75409	38.57652	30.78946	49.61085	56.16262	62.24617
HORVU6Hr1G087000	1.088109	0.838441	1.4832	2.547798	7.621326	5.772836
HORVU2Hr1G081440	2.120572	2.551326	2.537452	1.2395	1.535676	1.341444
HORVU6Hr1G054540	1.261926	1.26558	1.700914	1.347443	1.175034	1.606752
HORVU1Hr1G020500	20.26536	20.51907	19.07184	16.87191	10.35898	13.26431
HORVU6Hr1G093030	1.654231	1.707709	2.368539	9.628196	26.1356	21.8343
HORVU5Hr1G092250	9.878864	9.428319	9.215278	13.10954	20.50283	19.66231
HORVU2Hr1G051300	16.86753	17.92788	18.25347	16.09162	16.74205	17.09082
HORVU0Hr1G032830	4.094259	4.148996	5.134034	4.043894	5.600973	4.243003
HORVU7Hr1G121220	1.504568	0.969152	0.466098	1.879081	1.503939	1.590626
Hordeum_vulgare_newGene_2503	1.630953	1.745069	1.995017	1.566677	0.485365	0.906489
HORVU5Hr1G052890	16.00882	13.91634	16.98619	15.70928	18.63728	19.46459
HORVU2Hr1G122920	13.99891	15.91816	8.566521	5.612815	6.946595	5.827033

HORVU2Hr1G085210	0.213578	0.563542	0.574048	1.174961	1.877784	1.67343
HORVU3Hr1G027550	12.25287	11.93702	11.25582	13.83488	12.67753	13.6961
HORVU5Hr1G097150	1.000335	1.17964	1.537566	2.969388	8.726543	9.962723
HORVU7Hr1G046660	2936.549	2541.109	3024.416	1895.417	866.2228	1073.598
HORVU4Hr1G002330	1.067607	1.128332	0.730284	1.359883	1.675041	1.823122
HORVU0Hr1G001280	0.33933	0.544976	0.307294	1.374534	1.618453	2.140615
Hordeum_vulgare_newGene_242	1.934796	1.674421	2.044826	0.832162	1.09544	1.307866
HORVU2Hr1G126000	0.099732	0	0	0.324667	14.42484	19.1784
HORVU2Hr1G123030	16.81238	10.17759	16.63945	17.08711	10.5793	12.69807
HORVU3Hr1G083110	58.94031	53.08581	63.11491	37.82732	36.31939	37.1457
HORVU6Hr1G010700	0.828447	1.211498	1.181751	3.086694	4.76201	4.404735
HORVU4Hr1G066370	3.668657	3.433502	5.067058	9.308013	10.92162	11.06952
HORVU3Hr1G009560	1.930424	1.374147	2.940594	117.0558	94.98235	105.0214
HORVU5Hr1G066270	1.985347	1.007838	2.336041	2.943734	4.415449	3.971004
HORVU3Hr1G066020	18.10508	15.7563	21.95638	23.04839	29.26207	26.97852
HORVU3Hr1G031680	7.51616	9.113018	5.381777	1.009174	0.787883	1.501533
HORVU1Hr1G035130	0.452341	0.380305	1.187884	5.177581	42.02717	36.77224
HORVU4Hr1G084510	2.450969	1.721772	2.304024	4.288339	3.998876	5.266476
HORVU6Hr1G001680	15.76971	15.37617	14.50529	14.2633	18.31863	17.87908
HORVU2Hr1G013210	3.875452	3.900918	4.045808	5.810824	9.489088	8.95172
HORVU6Hr1G094560	49.87704	45.75567	52.40474	37.01088	45.12769	45.61717
HORVU4Hr1G014790	5.988874	5.481747	6.671033	5.377863	5.376264	4.81575
HORVU5Hr1G096200	12.23609	9.751884	16.62471	19.97539	23.5391	24.6853
HORVU2Hr1G095460	8.661908	4.132344	9.379904	0.084351	0.088178	0.165627
HORVU4Hr1G082200	14.15881	16.36544	23.70996	4.344138	2.472613	2.675828
HORVU1Hr1G048700	25.94698	22.87097	26.04351	12.42138	13.0401	12.96548
HORVU1Hr1G013990	7.748222	9.283152	10.76648	0.125254	0	0.215207
HORVU6Hr1G017950	1.986636	1.907052	1.781008	2.113455	2.096991	2.090812
HORVU5Hr1G037910	4.576734	4.11985	6.665276	3.483562	4.737067	4.606212
HORVU1Hr1G058330	5.143344	5.015967	5.859384	2.708473	3.26355	3.883533
HORVU6Hr1G005260	2.29151	3.040523	1.634851	3.730373	5.415582	5.105443
Hordeum_vulgare_newGene_15745	0	0	0	4.10909	1.622328	1.76921
HORVU1Hr1G042280	5.552472	6.988915	7.24645	0	0	0
HORVU3Hr1G028990	57.92464	88.44444	142.2889	1.417485	0.304148	0.7022
HORVU7Hr1G109260	0.2139	0.201755	0.079833	0.184129	4.322516	4.546481
HORVU5Hr1G009840	2.140584	2.087521	2.921252	4.078933	4.167838	5.891151
HORVU2Hr1G003460	5.28946	4.523017	5.691867	11.02496	12.44943	14.09955
HORVU3Hr1G079490	9.27555	8.194207	10.46001	9.609335	13.20531	13.24355
HORVU1Hr1G008120	168.6038	156.2458	200.8782	255.3006	389.4612	378.7458
HORVU5Hr1G057410	3.152137	3.911412	3.048328	0	0	0
HORVU3Hr1G099950	26.07814	26.88738	28.29785	21.01884	28.75468	26.75167
Hordeum_vulgare_newGene_12908	0	0	0	4.45174	2.961947	2.871362
HORVU3Hr1G067910	0.049849	0.31561	0.07701	2.700848	1.766143	2.55437
Hordeum_vulgare_newGene_12902	1.128166	0.875619	0.862266	0.730142	1.029065	1.697677
Hordeum_vulgare_newGene_12905	0.872478	0.870036	0.896147	1.496946	1.913455	2.053334
Hordeum_vulgare_newGene_12904	27.26043	19.26718	23.20375	23.1353	33.13222	32.80337
Hordeum_vulgare_newGene_12907	0	0	0.001964	1.658063	4.200057	4.057198

Hordeum_vulgare_newGene_12906	0.376024	0.895965	0.844991	0.766376	1.867439	1.591568
HORVU7Hr1G084390	4.427171	4.506128	4.850647	2.03836	0.839783	1.290174
HORVU7Hr1G088630	1.774831	1.780082	2.629502	4.248792	1.25353	2.915935
HORVU3Hr1G061400	1.833499	1.413309	1.724727	1.355884	1.184753	1.095736
HORVU1Hr1G016480	8.084257	7.804319	10.15645	10.76154	17.18407	14.01736
HORVU4Hr1G019410	6.331624	5.278547	7.07897	11.6898	10.44515	11.7337
HORVU2Hr1G033100	203.1264	163.0277	234.072	148.6436	112.4681	120.963
HORVU3Hr1G072860	4.562826	5.755191	8.536847	6.330123	10.72472	10.61676
HORVU1Hr1G042420	24.002	19.71613	28.64447	19.6092	22.9962	26.91841
HORVU7Hr1G082490	6.395798	8.110778	6.378253	5.338955	5.904931	5.514025
HORVU4Hr1G012240	3.630794	2.858199	4.126905	5.318531	4.944495	5.215007
HORVU1Hr1G015590	34.73856	33.77631	43.32503	25.89179	32.81504	33.7121
HORVU4Hr1G003180	3.291624	4.213876	4.052424	4.901502	4.090301	5.121864
HORVU6Hr1G012270	0.166229	0.048607	0.16142	4.513354	6.582161	4.949217
HORVU2Hr1G093700	15.21513	10.26882	9.033879	4.276361	2.786611	1.737283
HORVU6Hr1G016700	1.87928	2.197286	1.667075	3.351582	4.093879	4.86257
HORVU1Hr1G037570	0.495726	0.781588	1.25138	1.865607	2.24486	2.129452
HORVU0Hr1G010170	5.46936	6.004368	8.312928	2.548575	4.462903	4.745279
HORVU4Hr1G086000	93.86307	87.02162	89.842	117.1374	74.911	88.7415
HORVU3Hr1G006640	1.79942	1.304557	1.859566	5.819491	1.960389	5.01311
HORVU4Hr1G016940	1.209929	1.345513	1.677476	11.58832	26.78521	25.36736
HORVU7Hr1G074470	1.834118	0.472643	1.2549	1.173831	0.815414	1.051038
HORVU0Hr1G027230	3.176558	3.307962	3.331411	5.74732	6.942951	10.16159
HORVU3Hr1G001140	36.37954	35.36773	54.6391	20.56533	39.40043	39.78858
HORVU2Hr1G034290	2.710891	0.107056	2.824039	0.412323	0.209487	0
HORVU3Hr1G100570	0.286545	0.116003	0.063629	0	3.051759	3.660567
HORVU7Hr1G003940	8.395696	10.77203	7.83259	3.55003	2.257086	3.283028
HORVU5Hr1G095160	64.18643	66.87615	85.41721	19.85563	10.74021	9.489961
HORVU3Hr1G078540	7.6305	7.01549	10.26266	6.250471	7.899649	8.1337
HORVU3Hr1G099730	50.2013	43.20687	47.23184	82.09887	83.84193	84.71445
Hordeum_vulgare_newGene_5504	28.21421	26.76164	30.60637	28.13084	45.45964	43.65118
Hordeum_vulgare_newGene_5506	2.424811	2.181254	1.481536	3.647066	4.932091	4.770944
HORVU2Hr1G020920	0.270345	0.150089	0.554199	6.248466	5.397888	6.282923
HORVU3Hr1G092870	10.71414	9.982625	12.02999	7.79647	7.045339	7.562332
HORVU5Hr1G117770	4.49263	5.179698	5.878525	2.452651	3.353067	3.354599
HORVU4Hr1G025570	1.118574	1.343229	1.02483	0.560603	5.015059	4.544184
HORVU5Hr1G117480	22.79153	19.5272	18.97873	13.13607	28.60742	17.01515
HORVU1Hr1G045610	10.77456	9.228466	11.77675	17.32365	32.00285	33.1546
HORVU6Hr1G083150	79.71022	72.5259	93.2777	64.55112	85.07378	79.4103
HORVU4Hr1G076630	12.42063	12.53025	13.67479	14.25054	17.28047	16.97986
Hordeum_vulgare_newGene_8155	2.197835	2.225604	2.569418	3.632184	6.81285	6.138304
HORVU5Hr1G081290	22.68681	27.67209	21.62912	20.44534	22.49851	21.99999
HORVU2Hr1G007840	2.698383	2.161929	1.975048	9.509051	22.16194	21.29858
HORVU7Hr1G074290	1.273738	1.328286	1.321699	1.893393	1.793115	1.777097
HORVU3Hr1G036380	17.69771	14.96666	16.76838	14.54312	14.20949	14.38034
HORVU0Hr1G018210	16.90924	20.42873	17.0843	22.54519	12.70025	14.78157
HORVU2Hr1G048070	10.45309	9.552783	12.9413	12.95404	17.48173	17.63736

HORVU2Hr1G039500	1.656158	1.32863	2.317124	2.16341	2.797074	2.834085
HORVU5Hr1G093640	1.294642	1.108755	2.394003	1.136928	0.221834	0.717128
HORVU1Hr1G024670	4.304665	3.81737	4.788516	4.595413	5.885164	5.482071
HORVU5Hr1G098690	64.35652	54.51512	69.53169	62.48834	105.388	103.6815
HORVU3Hr1G059880	0.676392	0.674885	0.575215	1.661562	2.445091	2.334738
HORVU3Hr1G109680	1.734206	1.739419	1.984977	3.221097	4.879377	4.486782
HORVU5Hr1G006900	74.76794	72.42327	80.33438	57.43431	45.22894	52.65322
HORVU2Hr1G021320	0.247534	0.167734	0.127804	4.603694	7.058538	7.1721
HORVU2Hr1G022780	14.79623	13.13068	19.85643	16.32456	12.30426	16.68725
Hordeum_vulgare_newGene_9237	5.750913	6.035952	6.093155	8.292064	7.348624	8.919077
HORVU7Hr1G027010	36.11311	27.49766	38.82394	8.200717	8.182183	7.788458
HORVU3Hr1G108480	3.629705	3.992879	5.319864	5.14275	4.907413	4.875041
HORVU7Hr1G020040	5.338401	9.896583	4.872237	2.086567	24.60737	23.62531
HORVU2Hr1G122020	3.30572	3.910418	5.324621	2.32143	4.385102	3.604902
HORVU3Hr1G112930	1.852834	1.671156	1.602926	2.776451	2.719359	3.107004
HORVU1Hr1G077600	10.17781	11.47706	9.420595	9.702109	11.94274	11.85055
HORVU0Hr1G022310	45.93236	36.25674	38.86605	50.39	62.23555	59.66318
Hordeum_vulgare_newGene_1993	2.568465	2.895958	3.334707	4.355785	5.43529	4.99152
HORVU1Hr1G040930	28.60865	24.92946	29.07816	24.74951	24.81688	26.92996
Hordeum_vulgare_newGene_1996	0.133406	0.501858	0.229634	4.062158	4.0073	3.37291
HORVU3Hr1G057370	13.09892	11.89674	12.58677	21.12577	20.85835	20.42789
HORVU7Hr1G021310	0.915177	1.257214	2.049808	1.964156	31.72676	21.8247
HORVU1Hr1G087570	3.273206	2.631059	3.565237	16.13109	21.2807	20.10992
HORVU4Hr1G025580	1.208779	1.561717	0.946062	0.901432	4.83658	3.788785
HORVU2Hr1G025780	30.34713	25.41677	38.4991	31.16446	45.9529	48.50813
HORVU3Hr1G094260	13.504	13.82558	15.88677	14.3346	18.89613	18.63277
Hordeum_vulgare_newGene_796	0.703685	1.185533	1.410411	5.26124	4.415617	6.607592
Hordeum_vulgare_newGene_309	0.046549	0	0.038872	0.261191	8.373329	9.938689
Hordeum_vulgare_newGene_304	5.93204	6.834481	6.469124	6.373513	6.453519	7.230674
Hordeum_vulgare_newGene_300	1.511182	1.664933	1.505111	1.906021	1.98625	1.995674
HORVU6Hr1G011910	33.74193	30.65791	33.72568	29.02726	41.28479	41.58789
HORVU6Hr1G051000	4.613001	4.794771	4.033685	7.050359	4.953905	7.108287
HORVU3Hr1G088360	2.047266	1.514443	1.446263	2.565456	2.193015	2.705128
HORVU2Hr1G050230	31.96649	27.01096	36.08337	28.92398	27.43888	30.07482
HORVU3Hr1G063000	2.911146	4.076823	3.49346	1.869799	1.255734	2.138199
HORVU3Hr1G067770	1.391861	1.583357	1.44236	1.369915	1.03219	1.257272
HORVU3Hr1G051430	7.520832	8.343742	6.861328	10.11826	13.77746	13.50856
HORVU3Hr1G105790	0.403945	0.442718	0.409306	1.272317	5.706058	4.029637
HORVU6Hr1G070610	3.577566	2.395362	2.771108	2.155816	1.45044	1.890719
HORVU2Hr1G028370	6.521618	6.303638	8.36795	8.725373	10.56426	10.78043
HORVU4Hr1G083100	30.51564	20.01822	29.60275	57.33439	52.42661	54.3288
HORVU4Hr1G057080	13.28443	12.72834	16.6359	13.92295	14.56797	12.85138
HORVU6Hr1G062380	1.506275	0.908381	2.097795	1.372107	0.436054	1.069102
Hordeum_vulgare_newGene_7270	11.8913	13.82044	13.93439	10.08024	10.58567	12.90978
HORVU3Hr1G039500	5.71047	6.136872	5.257816	8.228815	9.190607	9.971499
HORVU3Hr1G097760	0.060177	0.040948	0.217184	10.37371	69.39703	55.97339
HORVU4Hr1G033990	24.26574	25.04391	33.14709	22.8859	25.19636	27.26091

HORVU3Hr1G105240	14.93773	14.39965	14.69275	13.58442	13.63054	14.82829
HORVU4Hr1G058250	8.999807	11.45602	10.12518	13.83495	19.29758	17.61597
HORVU7Hr1G059870	2.56627	2.535347	2.43431	4.457892	4.166753	4.724059
HORVU7Hr1G039480	26.82527	26.09854	25.70176	28.90572	32.35772	24.5596
HORVU3Hr1G086630	11.88925	11.29495	16.49467	6.197991	9.056265	10.37691
HORVU2Hr1G086830	3.291727	3.547626	3.435226	4.366511	6.514904	7.434614
HORVU4Hr1G073460	9.512821	6.29371	11.55639	37.10637	48.4907	52.11979
HORVU4Hr1G057550	11.5915	13.01221	13.38492	13.50564	23.59433	21.94947
HORVU2Hr1G044360	16.66068	8.380632	13.25761	3.549554	0	2.269917
HORVU5Hr1G075110	5.629254	5.414055	6.529463	6.564159	8.925299	8.776343
HORVU5Hr1G000330	8.74833	10.56609	16.31224	2.89067	0.119618	0.92815
Hordeum_vulgare_newGene_539	12.05065	12.32033	12.99606	10.47769	9.130048	9.959965
HORVU7Hr1G030160	194.0273	158.6305	202.4887	203.2089	116.6285	98.67723
Hordeum_vulgare_newGene_5582	0	0.828794	0.929616	2.176086	3.946419	3.769904
HORVU1Hr1G023500	32.5729	27.85226	35.24354	41.84982	59.45863	56.35054
Hordeum_vulgare_newGene_2311	2.479522	1.440934	2.559806	2.617992	2.353964	2.722051
HORVU6Hr1G026200	230.5618	167.0586	212.4923	252.9438	226.4453	220.178
Hordeum_vulgare_newGene_2319	0.46731	0.50477	0.519889	1.217219	2.094639	1.831979
HORVU3Hr1G060060	7.332486	12.79159	13.97254	8.883838	21.31591	18.53881
HORVU2Hr1G079920	17.11747	13.18529	16.20702	56.63395	104.6731	99.36185
Hordeum_vulgare_newGene_14561	4.334628	5.412163	6.918858	6.15296	9.314117	9.113614
Hordeum_vulgare_newGene_13716	0.878905	1.042387	1.113784	0.89	1.124194	1.276003
Hordeum_vulgare_newGene_14565	1.992184	3.509514	3.654359	0.331831	0.310672	0.31474
Hordeum_vulgare_newGene_7898	3.354724	3.731483	2.740619	2.648803	0.294924	0.833974
Hordeum_vulgare_newGene_7895	1.730372	1.597874	2.016266	0.080172	1.535983	1.758689
Hordeum_vulgare_newGene_7890	3.12985	3.908967	4.741319	3.242612	5.285758	4.556007
Hordeum_vulgare_newGene_7893	1.502153	2.043357	1.941853	1.631252	2.716745	3.350301
HORVU1Hr1G071710	2.342656	1.926176	2.53103	1.791392	1.72745	2.241434
HORVU3Hr1G012530	13.28299	13.69039	13.91567	16.01389	35.15915	29.93119
HORVU3Hr1G113910	9.384769	8.796105	10.27786	10.86843	12.07802	12.96421
HORVU3Hr1G097180	47.7589	33.271	44.95411	16.63828	6.756117	8.144727
HORVU2Hr1G005340	3.083893	4.298514	3.481785	3.080149	5.226969	4.169394
HORVU7Hr1G042420	1.163931	1.275939	1.901369	1.457723	2.271432	3.02763
HORVU3Hr1G052420	20.35259	18.67825	19.4136	7.983497	0.940513	3.378207
HORVU3Hr1G019510	27.29531	30.24982	37.67424	10.9116	3.622464	6.213437
Hordeum_vulgare_newGene_9361	3675.525	3581.499	2494.765	1231.98	218.9173	461.7927
HORVU0Hr1G018300	83.85999	72.69515	92.67325	103.0206	130.9722	128.5009
HORVU6Hr1G053330	3.354148	3.99912	3.245472	4.795743	5.578185	7.067919
HORVU1Hr1G020340	4.496361	4.738905	7.32655	2.948225	6.466101	7.145794
Hordeum_vulgare_newGene_15387	11.78712	10.36646	12.56883	14.49213	25.24011	25.82912
HORVU1Hr1G077710	7.931933	5.591896	7.543263	5.362995	9.665015	8.913976
HORVU2Hr1G075970	11.535	12.96216	18.04489	13.47197	18.44968	17.33103
HORVU5Hr1G063820	0.957134	1.076146	1.125957	1.488305	1.359662	1.869473
HORVU7Hr1G099670	3.892112	4.343951	6.17844	4.083222	4.758098	4.161182
Hordeum_vulgare_newGene_683	0.805625	0.929427	1.240432	1.609822	3.278299	2.327099
HORVU7Hr1G031480	0.261972	0.228302	0.770189	1.813051	1.438567	2.305426
Hordeum_vulgare_newGene_681	4.411256	4.518886	4.160226	5.985875	8.831536	8.235595

HORVU7Hr1G034470	0.939042	0.989442	1.273692	2.910318	16.00658	15.13881
HORVU2Hr1G005690	4.393398	5.399281	6.295624	5.433094	9.379116	8.793437
HORVU4Hr1G072020	20.4716	13.90668	20.79148	19.98358	22.97522	23.93648
Hordeum_vulgare_newGene_14387	6.853106	6.267945	7.391098	5.0816	6.337371	5.879124
HORVU7Hr1G077420	46.00395	36.33122	59.58028	34.21674	46.04715	44.93269
HORVU6Hr1G083810	1.322699	1.759035	1.944472	3.078341	2.690153	3.442168
HORVU4Hr1G011320	5.207936	4.140821	6.364729	19.39873	18.90551	23.83318
HORVU1Hr1G059020	11.19642	9.769244	11.31634	7.271726	4.345838	5.010684
HORVU6Hr1G081080	1.059393	1.006338	1.247528	3.51534	4.390695	4.297735
HORVU5Hr1G073220	1.527756	2.030215	1.6258	3.613735	2.117951	3.152121
HORVU7Hr1G023070	3.905856	4.050867	5.275654	4.39954	5.594004	5.464264
HORVU3Hr1G015540	3.192328	3.024787	3.098849	3.950551	5.247621	4.854983
HORVU2Hr1G077220	6.63223	4.898919	7.582711	4.709948	3.223469	4.375751
HORVU5Hr1G070560	2.906481	3.282843	3.333898	6.323166	9.210402	8.508802
HORVU7Hr1G117510	5.143456	4.537105	6.666656	6.145663	7.781522	7.352028
HORVU3Hr1G023370	3.205314	2.962558	2.097874	10.41569	20.62254	17.75618
HORVU7Hr1G017210	1.132498	1.866243	1.109882	1.680225	2.559933	1.914771
HORVU4Hr1G005480	0.154519	0.462046	0.167744	4.904619	4.594001	3.444245
HORVU5Hr1G069730	1.6869	1.71336	1.832347	1.548467	4.21477	3.759378
HORVU3Hr1G095850	25.00021	20.91101	26.19201	23.33455	25.62394	27.12469
Hordeum_vulgare_newGene_12675	3.310832	5.138124	5.660742	2.24559	2.196193	2.489692
HORVU7Hr1G096360	0.910568	1.107356	1.075702	2.784072	2.017565	2.384349
HORVU1Hr1G021120	5.526912	5.897147	8.878594	6.283662	12.97494	13.03345
HORVU3Hr1G117590	14.50614	8.472204	12.20986	1.298215	1.283378	1.343398
HORVU2Hr1G120970	43.92631	37.71518	45.53685	49.52656	51.17981	50.037
HORVU5Hr1G062370	10.17537	11.00115	10.89529	14.2539	19.34322	19.05049
HORVU2Hr1G127190	6.59109	6.060316	8.174887	8.791449	10.96794	10.70218
HORVU3Hr1G014630	1.286497	1.267515	1.45143	0.996721	1.307258	1.410531
HORVU2Hr1G058940	5.540958	5.419794	5.771216	9.188095	11.83403	12.9986
HORVU5Hr1G021050	2.290764	2.718415	3.573414	1.553019	0.881681	1.1673
HORVU0Hr1G002480	2.31637	3.005361	4.720583	1.933673	1.248351	1.196365
HORVU0Hr1G001630	3.833949	3.869492	3.648743	6.110911	5.026095	5.143257
Hordeum_vulgare_newGene_4202	0	0	0	4.499309	6.473358	7.368142
HORVU6Hr1G020530	1.644255	0.912101	1.572823	8.415405	15.69406	10.58089
HORVU3Hr1G031330	23.03754	21.98335	27.69217	25.56674	43.24519	42.46101
HORVU0Hr1G013770	5.235736	5.07748	6.102555	5.650161	5.195713	6.563585
HORVU2Hr1G034370	3.205322	2.585435	3.041566	2.257037	2.668391	2.983479
HORVU0Hr1G039590	1.817017	1.880152	2.174749	1.860498	3.51132	3.967174
HORVU2Hr1G009780	2.746272	3.392643	3.167942	2.624902	3.462585	3.550237
HORVU1Hr1G043710	84.00881	76.28962	45.97598	13.21713	10.00777	10.40571
HORVU3Hr1G047180	2.236644	1.389782	2.415748	1.254954	1.064735	0.889307
HORVU5Hr1G060700	25.82303	23.44766	29.66058	18.9148	29.5222	30.62328
HORVU5Hr1G060980	1.046098	1.18752	1.047313	3.819807	3.987962	4.562359
HORVU1Hr1G022470	0.84171	0.983306	1.142497	1.465036	1.574825	1.943899
HORVU2Hr1G046600	129.9852	168.1229	201.5078	5.223211	0.45286	1.579836
HORVU5Hr1G059530	4.705834	4.154437	4.144713	8.054856	15.88502	14.35966
HORVU5Hr1G035420	15.08776	14.0396	16.29887	19.48371	24.99311	21.44556

HORVU7Hr1G110630	1.086005	0.923901	1.364738	1.467338	1.934038	2.712167
HORVU6Hr1G056200	9.852864	8.216789	11.63355	8.19729	7.248883	6.70612
HORVU2Hr1G082810	7.44147	5.947883	7.517441	5.618011	4.041715	4.376978
HORVU2Hr1G099410	26.33608	22.76088	29.97636	25.16954	23.67743	23.27425
HORVU7Hr1G024310	2.30313	2.992582	3.290801	2.21036	1.935188	2.642555
HORVU0Hr1G000940	7.322682	6.031236	8.418185	4.338651	6.469669	6.124799
HORVU7Hr1G056590	10.81557	10.52941	12.8357	22.16195	23.97397	21.62541
HORVU6Hr1G074260	11.98182	11.29525	14.24059	13.68302	14.50852	16.16215
HORVU5Hr1G056210	0.046822	0.076637	0.008081	1.085212	4.759634	2.971832
Hordeum_vulgare_newGene_13107	10.30687	11.48748	13.88245	11.87507	19.98017	15.66363
HORVU4Hr1G050280	73.79692	65.74154	74.57315	87.50007	97.89685	103.0924
HORVU1Hr1G079900	7.260003	7.044572	8.998807	7.598096	12.58593	13.06779
HORVU3Hr1G031800	17.71542	16.89602	18.75655	20.96164	26.30878	28.15358
HORVU5Hr1G013220	1.496822	1.920949	2.001382	4.038182	13.59213	12.65916
HORVU3Hr1G051850	21.42426	20.99633	20.75142	15.33394	18.6099	18.92136
HORVU2Hr1G016840	8.48184	10.48917	14.42739	6.209549	6.910064	8.84301
HORVU4Hr1G007190	4.490798	3.210534	3.934221	2.156005	1.136817	1.73947
HORVU1Hr1G060480	21.91416	22.4643	20.18333	19.94799	22.00658	21.92004
HORVU3Hr1G006330	10.86984	11.06985	14.58659	11.71389	20.56041	20.6809
HORVU5Hr1G041720	2.14012	1.653274	2.47669	3.415583	6.047062	6.576354
HORVU6Hr1G080670	11.04596	9.821199	10.78431	14.15443	10.77222	10.52976
HORVU5Hr1G042390	0.431938	0.380454	0.775341	1.444398	1.86956	1.934967
HORVU2Hr1G000090	2.988415	3.621619	4.368893	0.586616	0.039328	0.276125
HORVU5Hr1G086500	15.62461	13.07528	11.23808	27.53388	42.86313	39.81582
HORVU7Hr1G054660	2.27111	2.060896	2.746836	6.509935	5.243931	4.851434
HORVU3Hr1G062510	2.200044	2.358697	3.479162	2.226913	2.371972	2.571536
HORVU7Hr1G000550	1.417796	1.806536	1.971856	1.880381	2.629599	2.553814
HORVU4Hr1G082870	2.881757	2.722034	3.677295	3.443249	4.702522	5.327209
HORVU7Hr1G018100	6.847963	4.50803	7.502591	6.367234	7.043111	8.878502
HORVU3Hr1G038000	1.696266	1.179937	1.597065	1.986437	1.300843	1.349849
HORVU6Hr1G005400	3.56364	2.324016	4.586677	1.14875	0.446841	0.89604
HORVU3Hr1G032750	3.871463	3.49552	3.503814	3.214506	3.28196	3.494146
HORVU3Hr1G072640	4.720941	5.365108	5.507643	3.807106	3.004106	3.408633
HORVU5Hr1G123370	25.32754	23.75658	29.7126	16.40681	15.36116	15.68669
HORVU5Hr1G052440	0.824058	0.594439	0.54151	2.750076	3.240944	3.260439
HORVU7Hr1G011300	5.800976	4.6741	7.381407	8.100188	10.82872	10.39479
HORVU1Hr1G012090	3011.245	2478.244	2294.248	491.4127	167.6308	237.3562
HORVU2Hr1G094890	3.24994	2.562909	2.708377	2.575027	2.274776	2.394639
HORVU2Hr1G048500	3.822004	4.470078	2.968095	7.311783	14.09742	13.24828
HORVU2Hr1G017820	4.545461	4.852822	5.856447	5.606784	5.111687	6.776456
HORVU1Hr1G005090	2.90956	2.442491	1.544737	0.8913	2.401888	1.990119
HORVU1Hr1G093700	3.509366	2.49868	3.827967	5.162789	6.368289	5.736296
HORVU5Hr1G007680	11.13527	9.762755	12.17599	13.24411	8.92139	8.873612
HORVU3Hr1G022710	11.59195	11.07445	10.93669	10.02005	16.29638	16.22374
HORVU4Hr1G069740	2.474775	2.800356	3.828353	3.15049	3.118635	3.592209
HORVU5Hr1G018820	0.40837	0.554734	0.637497	0.836904	2.538855	2.491066
HORVU5Hr1G017830	1.006121	1.397055	1.383936	2.588309	5.729133	4.474033

HORVU0Hr1G016000	4.618938	5.345234	4.32547	8.301614	12.90426	11.79298
HORVU5Hr1G106310	4.012185	3.702363	5.183229	6.685757	8.094861	7.303443
HORVU3Hr1G019340	1.629799	1.588499	1.985194	1.919456	2.090549	2.188386
HORVU1Hr1G046370	1.785975	1.181041	1.59269	24.12882	12.21279	16.02877
HORVU5Hr1G046890	26.237	23.2662	27.90217	27.10879	26.95897	28.69188
HORVU3Hr1G024920	0.56491	0.960535	0.741277	0.538829	2.904893	2.562255
HORVU5Hr1G114890	3.577238	4.087673	5.231131	2.877687	2.842337	3.530239
HORVU1Hr1G038210	15.72524	17.21651	17.07415	13.01793	11.1956	14.72353
HORVU1Hr1G018720	22.99957	22.40531	21.87345	35.33815	25.60528	23.57736
HORVU5Hr1G110380	0.988259	1.245384	1.907012	1.215955	1.669652	2.196094
HORVU2Hr1G092400	5.138977	4.873265	4.420337	6.067418	5.865067	3.634954
HORVU5Hr1G121610	10.62718	10.77668	14.86482	23.60731	45.28126	54.47616
HORVU4Hr1G029410	42.40202	47.41426	47.72065	69.33842	73.19377	83.65783
HORVU4Hr1G018640	13.09402	7.89468	10.99489	13.01168	17.11038	14.36299
HORVU1Hr1G084980	5.95708	4.687272	6.856213	3.05485	1.384846	1.551049
HORVU3Hr1G067030	21.30796	23.3533	25.44969	13.8293	8.851082	11.20688
HORVU3Hr1G097950	87.45128	60.42602	91.71133	68.49104	135.6267	158.8494
HORVU1Hr1G082670	2.858402	2.369684	2.216459	1.133247	0.827888	1.903653
HORVU2Hr1G102330	20.21437	18.45471	31.75039	28.15234	43.37855	37.77369
HORVU2Hr1G064590	17.80016	15.93961	21.71861	17.63855	20.95669	21.80632
HORVU4Hr1G015070	1.552222	2.98899	3.394131	2.644128	4.14462	4.37161
HORVU7Hr1G104710	7.879354	8.631188	8.38058	6.067046	6.101173	5.505035
HORVU2Hr1G045250	117.6334	119.73	204.425	85.31821	72.62749	82.6148
HORVU5Hr1G082990	1.657502	1.475118	1.483434	4.32212	5.803451	5.365745
Hordeum_vulgare_newGene_2151	5.365467	4.633469	6.207363	6.431289	5.140242	5.545432
Hordeum_vulgare_newGene_2157	3.344082	3.179181	3.671916	3.038976	4.414917	4.082509
Hordeum_vulgare_newGene_2155	0	0	0	11.08694	9.456748	12.4208
HORVU6Hr1G088300	20.94422	21.20955	30.88353	22.0153	24.35988	26.12098
HORVU4Hr1G026300	5.002202	5.016611	5.776303	12.20309	22.12709	19.05322
HORVU3Hr1G024210	4.767473	4.80028	6.111843	6.391938	5.913494	7.908843
HORVU5Hr1G079100	7.226735	8.046122	7.096788	11.101	15.7856	15.65776
HORVU5Hr1G053840	0.78396	0.631432	0.82022	1.211364	1.806127	2.008017
HORVU4Hr1G082010	16.12021	15.78949	18.18532	9.941646	13.29286	11.83228
Hordeum_vulgare_newGene_13006	2.913893	4.541313	5.141846	2.628226	2.42506	3.3068
HORVU2Hr1G110860	6.301277	5.391432	6.097855	0.02274	0	0
HORVU2Hr1G099350	7.932734	6.120035	11.54783	3.535663	6.916008	7.421426
HORVU3Hr1G061130	0.828933	0.490827	0.544857	4.639745	6.067943	3.974162
HORVU5Hr1G085070	38.18998	45.95764	43.59507	17.81576	10.26908	12.63957
Hordeum_vulgare_newGene_13263	2.899893	3.519223	2.986139	4.178909	4.437333	4.351505
HORVU5Hr1G001660	1.47973	2.039307	2.547973	2.598733	2.432395	2.602233
HORVU2Hr1G021950	2.704834	3.511585	4.224684	2.533773	3.137262	3.309274
HORVU7Hr1G003040	0.134966	0.069931	0.068571	0.278673	5.636597	4.945098
HORVU2Hr1G090980	9.847755	15.28987	21.51964	4.517772	4.62375	5.953187
HORVU1Hr1G044440	4.58341	5.636633	6.057064	7.896352	10.05883	10.69763
HORVU3Hr1G088910	6.16596	5.653826	5.275211	7.923875	8.610946	8.83617
Hordeum_vulgare_newGene_15141	4.089805	4.42561	4.275147	2.562656	2.086659	2.395772
HORVU0Hr1G020730	15.11644	15.25676	20.386	17.45723	27.54252	26.62869

HORVU5Hr1G053180	13.60579	15.4527	15.4836	18.81516	18.30158	21.21943
HORVU3Hr1G045630	1.840083	0.779812	0.884488	1.738634	1.237464	1.659051
HORVU7Hr1G122680	437.0465	379.982	352.9666	147.3952	120.6968	126.7727
HORVU6Hr1G012820	2.027565	1.775426	2.592634	1.403377	2.293708	2.519398
HORVU4Hr1G079420	15.6143	20.38286	16.80001	16.18481	21.74152	20.62432
HORVU5Hr1G069380	2.052591	1.370144	1.666607	0.492915	0.66106	0.883754
Hordeum_vulgare_newGene_5319	2.111096	1.832584	1.803314	3.535338	0	0.900775
Hordeum_vulgare_newGene_5310	0	0	0	1.593892	3.205638	2.591212
Hordeum_vulgare_newGene_5312	6.180803	7.980761	8.215281	7.770416	8.322723	8.331321
Hordeum_vulgare_newGene_5314	5.504019	9.386869	6.459674	1.162179	0.025305	0.449455
HORVU7Hr1G071020	2.461389	2.869215	2.677996	6.954177	9.098154	9.393632
HORVU4Hr1G060370	25.50177	17.62832	18.51404	11.09331	8.294579	8.603005
Hordeum_vulgare_newGene_13848	2.019779	3.964209	5.248344	0	0	0
Hordeum_vulgare_newGene_13847	4.589681	5.456891	4.749436	0	0	0
HORVU2Hr1G060620	1.573581	1.847362	1.771225	1.118905	2.08331	3.015532
HORVU0Hr1G021880	13.18043	12.71679	13.88812	10.21266	11.8297	10.74621
HORVU2Hr1G030250	5.191885	4.789379	5.253665	6.933072	11.40999	10.00084
HORVU4Hr1G090090	14.00319	11.5741	14.14381	7.732602	2.147773	3.777405
HORVU4Hr1G067680	11.52498	9.71464	9.171892	22.56599	19.4387	27.97448
HORVU1Hr1G077080	0.808582	0.596378	0.612492	1.459825	1.510787	1.706405
HORVU2Hr1G121250	0.550336	0.419868	0.386019	1.526521	1.879958	1.764207
Hordeum_vulgare_newGene_8692	1.731657	2.128823	1.954366	1.390458	0.914832	1.536479
Hordeum_vulgare_newGene_1697	5.957009	7.888466	6.498	11.62113	9.4205	10.24133
Hordeum_vulgare_newGene_8695	2.824812	2.782155	3.132649	2.680845	4.28649	4.123072
HORVU0Hr1G023760	4.091302	3.253342	4.705734	0.974899	0.83474	1.425114
Hordeum_vulgare_newGene_1690	3.464628	3.166886	3.44307	3.59472	4.310369	4.459417
Hordeum_vulgare_newGene_8349	2.763047	3.39005	3.092285	0	0	0
HORVU3Hr1G096650	5.1444	5.957214	5.954138	9.572028	12.38572	13.14919
HORVU2Hr1G070850	12.17366	10.91298	17.29701	10.79249	20.68884	20.87151
HORVU1Hr1G058750	1.431821	1.091773	1.127143	4.472742	4.160069	6.985529
HORVU3Hr1G078660	7.069137	5.511969	8.293327	8.125157	7.620889	7.955174
HORVU3Hr1G109160	22.66175	21.65192	20.83635	28.22499	26.23604	28.31884
HORVU2Hr1G022820	0.154672	0.235042	0.184245	0.467466	2.422873	2.886346
HORVU7Hr1G106600	3.483358	2.119495	2.328522	3.213671	2.529044	2.581679
HORVU3Hr1G105850	138.9339	119.5917	153.955	77.51773	55.30428	53.81313
HORVU5Hr1G051090	2.037073	2.028512	2.959196	2.483872	2.377374	2.408771
HORVU2Hr1G090210	1.015941	1.144476	1.37587	2.591368	11.90056	10.42464
HORVU3Hr1G068430	3.311832	2.975314	3.602657	4.118442	6.256868	6.091548
HORVU7Hr1G122210	6.313821	7.755226	8.390156	11.04792	11.28616	10.63745
Hordeum_vulgare_newGene_4443	1.069064	1.481608	0.945153	2.096616	1.249811	1.796467
HORVU7Hr1G027220	27.67932	24.42235	51.84819	28.15796	42.51697	39.62853
HORVU5Hr1G118870	3.327292	3.268586	3.978281	2.229856	2.379319	2.87767
HORVU7Hr1G063520	12.83596	12.42447	11.94634	13.96684	16.28241	15.32815
Hordeum_vulgare_newGene_1548	1.968766	2.948262	2.368134	0	0	0
HORVU2Hr1G011560	0.994021	1.414019	1.173572	1.947079	1.792028	2.394235
HORVU0Hr1G020860	12.14925	11.43765	11.69928	15.65001	9.37051	11.3193
Hordeum_vulgare_newGene_1543	4.193184	4.498579	4.193483	3.458788	3.91628	3.879778

Hordeum_vulgare_newGene_1547	3.439083	5.171952	3.016582	2.875509	1.8771	2.213359
HORVU3Hr1G095300	2.21346	1.983382	2.118055	5.294662	7.737849	7.791624
Hordeum_vulgare_newGene_3940	30.34402	24.88421	28.46253	13.33909	8.698493	9.937967
HORVU5Hr1G014350	44.25181	41.32975	48.5816	48.81532	50.70892	49.39465
HORVU1Hr1G016120	5.840309	8.507106	8.231056	6.652746	6.313312	1.776489
HORVU3Hr1G011640	2.193436	1.232813	1.361706	3.445712	2.776691	3.002215
HORVU5Hr1G030640	2.659916	2.028256	2.072356	5.476219	5.110551	5.50313
HORVU4Hr1G088880	19.33506	20.56776	14.77737	4.295526	6.644072	8.099062
HORVU3Hr1G094650	0.881933	1.759572	1.528218	3.355466	2.660299	3.528647
HORVU1Hr1G072070	78.32317	73.69605	77.77226	63.29171	88.62357	86.52122
HORVU5Hr1G113030	3.150054	2.349879	2.923212	3.4313	2.80445	2.56035
HORVU2Hr1G059520	5.137571	5.076311	7.282109	5.492976	9.94151	8.926787
HORVU2Hr1G102280	0	0	0	10.39347	12.28105	13.20785
HORVU1Hr1G010800	8.262483	9.417552	7.719536	6.945374	8.064367	7.702459
Hordeum_vulgare_newGene_7075	1.946406	1.840815	2.255498	2.690711	1.909143	2.038504
HORVU3Hr1G088130	3.546525	3.147582	3.490199	3.377941	6.537083	6.206881
HORVU3Hr1G091680	4.537584	4.987082	5.851473	1.930971	0.581953	1.326533
HORVU2Hr1G103880	10.61662	10.97657	13.12717	33.19958	48.70393	48.44629
Hordeum_vulgare_newGene_4991	0	0	0.030682	6.637783	9.222038	9.051376
HORVU0Hr1G006830	5.938606	4.55408	6.455533	3.960624	0.726507	1.989217
HORVU6Hr1G022770	39.84501	35.95938	35.35328	31.06078	49.04385	44.78443
Hordeum_vulgare_newGene_4997	0.776244	1.198694	1.359852	1.776152	1.495717	1.542105
Hordeum_vulgare_newGene_15993	0.907652	1.205522	1.22243	3.131974	1.83605	1.261013
HORVU1Hr1G034490	2.051333	1.335462	1.69413	0.546239	1.887559	1.395417
Hordeum_vulgare_newGene_15996	0.633524	1.102279	1.58826	1.066852	2.024893	2.08202
Hordeum_vulgare_newGene_15998	25.96376	23.85864	28.6604	34.18968	34.35495	33.94257
HORVU1Hr1G025370	2.836549	2.907934	2.666001	2.510476	0.506523	1.077755
HORVU3Hr1G090080	12.96575	14.40381	18.39309	12.7444	19.28794	20.25465
HORVU2Hr1G074360	29.35712	28.71965	31.70544	27.59066	32.29423	31.7044
HORVU6Hr1G003160	0.178641	0.1237	0.218226	32.13565	62.5343	36.26693
HORVU6Hr1G051210	29.50783	23.36076	32.31743	27.90429	41.10466	40.1895
HORVU6Hr1G027050	5.14425	6.399016	7.114406	3.326897	4.575849	4.508819
HORVU7Hr1G100130	317.4005	135.1803	265.3436	187.8861	85.63896	106.7606
HORVU1Hr1G029490	12.73372	12.31248	12.80047	15.70488	18.70254	19.97156
HORVU6Hr1G059420	8.840732	7.409909	8.504433	9.392023	7.907785	11.76922
HORVU0Hr1G021050	4.748939	6.616292	8.179562	1.910709	3.539112	4.031978
HORVU7Hr1G090900	3.56211	3.455534	3.637443	3.976898	3.599775	4.477587
HORVU7Hr1G079030	21.10723	22.86881	28.10573	29.21461	44.71238	39.47968
HORVU1Hr1G030030	32.90347	30.13802	35.29745	40.82189	48.27969	48.25022
HORVU4Hr1G016010	2.249033	2.369515	3.039457	3.01124	4.106604	3.794055
Hordeum_vulgare_newGene_6597	1.809248	3.840918	2.67638	1.905775	1.839187	2.452157
Hordeum_vulgare_newGene_6595	2.523945	2.926103	3.482266	5.615669	1.673179	1.683657
Hordeum_vulgare_newGene_6590	0.300856	7.669157	1.030577	0	0	0
HORVU0Hr1G018000	2.135375	3.129824	2.855617	3.420894	4.19519	3.539867
Hordeum_vulgare_newGene_14480	1.798539	2.200174	2.485278	1.108517	1.035371	1.306072
Hordeum_vulgare_newGene_14481	0	0	0	3.954771	4.571535	4.208457
Hordeum_vulgare_newGene_14487	3.646697	3.630095	5.022914	4.368946	6.857048	4.823241

Hordeum_vulgare_newGene_14489	1.884055	1.94705	2.177914	2.176586	1.780275	1.575629
HORVU1Hr1G072720	28.72744	22.60221	28.31792	5.637846	1.884656	4.064585
Hordeum_vulgare_newGene_12677	8.991292	11.32053	7.017589	9.286709	7.324408	7.278178
HORVU5Hr1G012510	15.0409	17.3504	14.12822	30.70033	27.55694	34.04246
HORVU7Hr1G002050	1.642729	1.765631	1.470734	2.759974	6.929446	6.408362
Hordeum_vulgare_newGene_12671	1.799753	1.806977	2.014088	1.865351	1.572476	1.896975
HORVU3Hr1G097680	35.88518	31.83948	30.92878	24.97991	27.47451	31.77908
HORVU1Hr1G039880	5.408699	5.38955	5.891196	6.435616	9.286229	8.87242
Hordeum_vulgare_newGene_459	3.43756	4.312929	6.900996	2.564176	6.994304	5.478168
HORVU4Hr1G037400	5.676288	5.367637	5.679884	8.228588	12.1034	12.7783
HORVU4Hr1G074090	4.544236	6.494792	6.057677	9.374335	11.5824	10.76767
HORVU1Hr1G010670	1.616651	1.379432	2.886342	6.009519	12.41608	11.20868
Hordeum_vulgare_newGene_13108	4.421675	4.811451	5.225782	2.514721	3.034311	1.482363
Hordeum_vulgare_newGene_13109	9.061524	8.591603	8.595453	10.53973	9.756829	9.192712
Hordeum_vulgare_newGene_14730	4.408467	4.006939	2.810006	0	0	0
HORVU5Hr1G107000	20.66947	17.56538	22.87711	18.78369	21.46244	15.65363
Hordeum_vulgare_newGene_13101	6.623782	8.84859	6.305505	3.987859	4.326023	3.66121
HORVU5Hr1G058970	1.186072	1.378213	1.168462	1.228729	1.264126	1.753383
HORVU2Hr1G075010	10.98789	7.728511	10.52373	6.734673	6.14975	6.416528
HORVU0Hr1G008590	0	0.002704	0	6.713907	6.952442	7.54135
HORVU1Hr1G027420	49.55152	46.42056	53.39818	74.59869	92.6264	88.27013
HORVU6Hr1G082230	10.93308	10.11534	11.13732	1.529355	1.904452	1.23437
HORVU5Hr1G015980	0.782027	1.244294	1.124712	1.373564	3.660242	3.554455
HORVU7Hr1G118610	11.06392	9.056547	13.57192	5.61983	3.071622	4.753571
HORVU3Hr1G003050	69.72142	70.56155	97.28324	51.21978	35.57822	44.76587
HORVU6Hr1G038500	5.65219	8.120469	8.115469	6.056905	7.198744	8.212467
HORVU6Hr1G075780	9.825044	9.270698	10.6189	13.81112	16.36621	18.20612
HORVU2Hr1G046710	1.013423	1.181499	0.98459	1.322152	1.841386	1.75628
HORVU5Hr1G061580	2.455316	2.465043	3.515025	1.680595	0.747124	1.423172
HORVU5Hr1G088570	7.227812	6.72713	9.968334	6.488043	11.06934	8.940542
HORVU4Hr1G034270	3.034263	6.134998	4.641234	4.452921	6.360509	7.205605
HORVU0Hr1G018510	91.3228	82.8667	107.9091	99.79545	180.4333	179.2278
HORVU7Hr1G037510	1.815468	2.392534	2.741569	5.209769	5.455577	6.989024
Hordeum_vulgare_newGene_15460	3.680233	3.208822	3.22309	4.46737	6.668472	7.130934
HORVU3Hr1G087040	5.636074	4.795224	5.267703	6.283371	0.553793	1.72236
Hordeum_vulgare_newGene_15464	1.305069	1.699998	1.321934	1.199819	0.688635	0.817347
HORVU6Hr1G076870	4.075214	4.478956	4.93589	3.339745	2.635333	2.409283
HORVU1Hr1G055570	1.780878	1.852176	1.613994	2.248792	3.113008	3.05733
HORVU4Hr1G074530	0.485702	0.471992	0.297896	2.684549	6.212866	4.620292
HORVU4Hr1G003450	3.634857	3.103387	4.535286	3.081885	1.596636	1.503622
HORVU3Hr1G078880	0.018978	0.016649	0	1.183102	5.374827	7.672821
HORVU6Hr1G069190	17.79674	16.01098	27.70281	36.14598	18.7668	19.01567
HORVU3Hr1G082670	7.030676	8.709477	7.995662	7.075464	9.567992	8.837515
HORVU5Hr1G034770	89.32639	92.42086	129.8068	161.0418	290.0813	272.1585
HORVU3Hr1G019580	23.15776	20.28339	25.95472	13.36982	11.15706	13.89017
HORVU0Hr1G003210	4.152375	3.994466	4.533083	3.637106	2.973341	3.256931
HORVU5Hr1G063670	0.274693	0.198059	0.18581	2.364432	1.127079	2.525166

HORVU5Hr1G111860	127.7813	137.6974	100.6992	629.3873	21.98869	227.6615
HORVU5Hr1G059310	2.108888	2.578656	2.218809	1.384476	2.89334	1.673769
HORVU7Hr1G114960	5.146533	4.386827	4.852041	7.222127	5.173824	7.290216
HORVU5Hr1G051160	13.84453	7.093761	16.63111	11.51857	7.666569	5.882358
HORVU1Hr1G024810	24.46485	23.15675	27.84238	14.76007	18.79874	19.12846
HORVU5Hr1G029150	0.957706	1.349681	1.475685	2.093755	1.206982	1.694756
HORVU1Hr1G081910	4.516868	4.226507	4.48009	10.98321	3.866759	5.492249
HORVU7Hr1G017670	4.476577	4.437593	5.909209	10.86305	13.00754	15.48475
HORVU3Hr1G016800	39.83741	34.43307	26.74909	4.607772	1.62368	1.826924
HORVU7Hr1G106160	2.606247	2.125561	1.911922	2.144543	1.341216	2.57025
HORVU6Hr1G011620	0.550024	0.473133	0.851012	1.403269	5.469825	8.507469
HORVU5Hr1G109520	16.81168	14.55782	16.21387	16.87849	13.07565	14.64362
HORVU4Hr1G088410	9.07154	6.895691	7.502875	6.020103	10.60415	9.272778
HORVU1Hr1G082790	0.141287	0.164007	0.100637	2.107912	12.26805	12.4766
HORVU4Hr1G080660	1.737533	1.449204	2.128401	1.45104	1.598505	1.71751
HORVU0Hr1G006190	3.411749	6.574612	8.022027	3.23683	4.685737	5.197975
HORVU5Hr1G050690	6.078859	5.27174	7.734118	4.557337	4.392812	4.807697
HORVU5Hr1G108690	1.864523	1.495744	1.480421	0.765246	0.501347	0.561598
HORVU4Hr1G015670	5.295396	4.919838	4.414878	4.521761	3.749817	3.565808
HORVU1Hr1G073060	9.390743	10.30013	10.91293	13.40566	18.52274	17.86464
HORVU0Hr1G017220	35.96367	36.60043	24.66285	0.3899	0.16297	0.207545
HORVU2Hr1G016250	6.836207	7.803775	7.845057	4.20793	3.690494	4.506546
HORVU4Hr1G018330	36.02522	35.20824	39.80598	41.17753	53.72439	54.92846
HORVU2Hr1G120830	3.453697	3.199875	2.953102	3.00274	4.083104	3.675767
HORVU6Hr1G039940	85.08327	69.60451	67.48464	69.72653	75.81048	71.34132
HORVU1Hr1G091730	71.98178	66.56932	75.27062	58.84608	61.98193	59.95783
HORVU5Hr1G058190	82.36542	83.43296	109.249	28.81069	1.214791	12.54278
HORVU6Hr1G066400	0.031199	0.028036	0.303662	1.739707	10.72282	8.432118
HORVU5Hr1G110710	0.383991	0.129133	0.06993	0.04447	33.59928	28.98252
HORVU1Hr1G079200	40.13852	37.93704	47.72531	51.40785	48.93511	48.68013
HORVU1Hr1G068380	34.96737	29.12271	37.31417	4.07969	25.36249	24.53777
HORVU1Hr1G064600	1.43837	1.650593	2.048216	1.189693	1.641909	2.07724
HORVU1Hr1G085870	6.464988	5.952428	9.887755	5.669812	10.25329	10.47963
HORVU0Hr1G008830	1.791351	0.753837	1.985143	0.867596	0.628007	1.811457
HORVU2Hr1G113320	1.209972	1.322083	1.789929	0.483897	0.614765	0.777875
HORVU3Hr1G000770	3.581962	3.608632	3.583368	10.44035	18.32127	15.88354
HORVU4Hr1G086540	14.79263	12.80434	16.40757	13.44536	11.97184	12.93619
HORVU2Hr1G084590	0.16426	0.194951	0.085131	1.483278	2.217679	2.372536
HORVU3Hr1G041680	0.980485	1.389454	1.113773	1.48053	2.133908	1.461632
HORVU3Hr1G030850	3.175898	1.920071	1.989986	9.619689	8.806856	8.965594
HORVU2Hr1G078070	8.892277	8.212684	10.73555	7.291843	8.696444	7.917467
HORVU1Hr1G079370	65.5502	64.70349	100.4655	58.28325	162.2603	143.1892
HORVU2Hr1G078380	3.137339	3.507865	3.417374	3.89771	2.495907	3.074808
HORVU2Hr1G082990	4.764722	5.256677	6.12898	6.251242	10.58581	8.113951
HORVU6Hr1G055960	114.5174	91.63885	93.58797	231.8955	214.1331	205.7567
HORVU4Hr1G066810	11.5037	10.11902	11.96265	31.45949	19.35269	28.61807
HORVU2Hr1G125490	2.070801	2.462094	3.060494	3.251878	3.517941	3.506874

HORVU3Hr1G071470	4.518272	2.526246	6.669602	1.928828	0.55303	2.215596
HORVU7Hr1G110280	0.404336	0.250187	0.719082	1.544964	9.381605	8.257223
Hordeum_vulgare_newGene_4512	16.36738	13.60142	16.87635	15.1203	24.0192	23.45857
HORVU7Hr1G085010	4.971792	7.252631	8.485406	3.950465	5.851489	7.117392
HORVU2Hr1G001790	7.468454	8.165386	10.2228	4.4489	4.809799	5.311932
HORVU1Hr1G069470	0.128186	0.17293	0.085909	1.769312	6.875457	6.650836
HORVU3Hr1G076840	2.605563	4.653822	3.910661	0	0	0
HORVU1Hr1G089820	2.498767	2.790276	2.845697	6.31637	5.50264	6.186432
HORVU3Hr1G042330	1.597307	1.520961	1.68395	4.349209	5.753077	5.90321
HORVU5Hr1G018240	9.68292	10.8148	12.32417	9.23145	11.58872	12.15409
HORVU7Hr1G006580	545.049	791.3867	520.344	58.27205	112.6468	101.9709
HORVU3Hr1G098160	38.45606	41.18575	44.03097	13.23694	8.821695	8.907976
HORVU1Hr1G093350	1.38785	1.7308	1.348524	3.334085	4.963384	5.138534
HORVU0Hr1G040420	10.08989	7.574333	8.012814	11.91314	12.03629	10.77202
HORVU5Hr1G012890	6.442809	7.271607	10.06739	5.906276	8.167318	9.4136
HORVU1Hr1G055380	0.504721	1.682831	1.47727	0.757697	1.722026	1.496107
HORVU4Hr1G073070	0.211441	0.30307	0.199238	1.917457	1.715168	1.878665
HORVU4Hr1G083970	7.612659	8.311123	11.69396	5.877113	7.646321	9.002435
HORVU1Hr1G080960	2.806266	2.854966	4.314509	5.147216	5.381716	4.59567
HORVU4Hr1G025320	5.420392	5.071991	8.017608	3.563725	4.820904	8.031634
HORVU2Hr1G101360	10.77876	9.930692	10.44701	24.59478	31.20717	31.33993
HORVU5Hr1G056390	6.596089	6.877141	9.772908	14.13803	30.07482	30.03027
HORVU1Hr1G062600	2.691466	1.994778	2.864963	0.261414	0.527591	0.334436
HORVU4Hr1G007540	9.575235	11.25259	11.16183	12.19339	17.89616	16.05327
HORVU4Hr1G003840	8.571866	10.27963	8.610636	14.50751	17.74542	17.54403
HORVU4Hr1G051290	29.17756	27.97761	29.46916	60.94017	45.56637	47.05421
HORVU5Hr1G046160	1.183084	0.807974	1.212138	3.729049	1.821934	2.110112
HORVU3Hr1G021970	18.12488	18.92218	15.24475	20.50499	15.17858	19.5175
HORVU6Hr1G083620	13.09759	11.04733	13.58693	32.81413	34.54265	32.06932
HORVU2Hr1G105090	12.883	12.32896	13.65237	12.73024	7.014659	7.857203
HORVU3Hr1G062490	3.802049	3.675106	4.159724	14.82533	33.71159	30.66969
HORVU1Hr1G050880	13.83345	13.36077	15.65386	18.19201	17.9456	19.48309
HORVU4Hr1G024050	39.21092	37.82781	34.362	21.61239	20.13097	20.32807
HORVU7Hr1G116420	45.73461	45.35472	47.93761	37.02056	43.87508	43.64873
HORVU7Hr1G049860	0.998916	1.199077	1.359501	4.410636	20.85349	20.55218
HORVU1Hr1G065150	673.5662	462.524	670.4615	276.139	36.3577	133.9106
HORVU1Hr1G051280	39.27446	32.51632	52.62735	30.52825	50.78305	50.2609
HORVU1Hr1G087460	2.179749	3.070885	2.702496	4.530196	5.330128	5.678588
HORVU5Hr1G094400	3.468722	3.358168	3.488026	7.752865	9.030013	9.876631
HORVU5Hr1G068470	3.642737	4.721247	2.49497	3.224497	2.446378	2.150268
HORVU5Hr1G123490	5.205232	5.794374	7.419226	3.837172	5.817903	5.844773
HORVU4Hr1G085150	1.713142	1.41127	1.282299	1.489963	2.155583	1.873167
HORVU7Hr1G037690	13.08978	14.84045	16.50892	46.67011	265.6282	310.8791
HORVU5Hr1G028360	41.47335	35.96032	42.10563	46.89652	80.40771	74.92035
Hordeum_vulgare_newGene_11754	1.025621	1.867067	1.097165	0.828486	1.29913	1.773333
HORVU1Hr1G081700	22.79678	22.32237	23.29427	23.44633	34.09173	30.66787
HORVU5Hr1G008710	5.241395	7.705551	11.60109	5.831265	7.234722	6.841958

HORVU7Hr1G089640	9.107313	8.999332	12.04832	8.283046	17.56832	15.93324
HORVU3Hr1G109760	0.138778	0.177606	0.335962	1.089776	3.452551	2.818274
HORVU1Hr1G093000	1.51335	2.124675	1.514169	1.440149	1.78732	1.878886
HORVU5Hr1G055610	7.567883	4.495444	10.52196	4.404095	5.28568	4.861664
HORVU7Hr1G010750	0.683396	1.07642	6.516579	0.076348	0	0.048833
HORVU1Hr1G045220	81.3669	89.95788	86.07741	77.49657	47.39967	58.79371
HORVU3Hr1G077030	8.775165	13.84132	18.4303	6.020377	6.95807	8.496196
HORVU7Hr1G083410	4.649247	5.316373	5.207696	4.729351	3.138147	4.604283
HORVU4Hr1G023580	3.67922	4.369229	3.893845	15.61287	13.92841	15.173
HORVU5Hr1G076780	22.6788	16.67153	21.85923	31.05459	37.08536	38.39828
HORVU2Hr1G040170	4.705978	5.845404	6.413848	0.033834	0.047666	0.053365
HORVU2Hr1G118670	2.647756	1.846078	2.6866	4.495113	6.95289	7.457341
HORVU4Hr1G056740	88.31467	95.37708	116.8376	116.0124	279.1199	248.0025
HORVU2Hr1G063840	12.99261	12.0657	13.038	10.12877	11.92703	11.98778
HORVU7Hr1G040380	0	0	0	0.492877	5.02606	2.698817
Hordeum_vulgare_newGene_11339	69.25221	73.40434	107.037	34.23505	70.47578	68.04919
Hordeum_vulgare_newGene_11337	1.05921	0.684744	1.080651	1.697045	1.789872	1.829462
HORVU4Hr1G023050	2.117792	2.180479	2.443456	2.901107	3.919016	4.651043
HORVU1Hr1G053160	1.615367	1.427267	1.461827	2.587988	2.334875	1.66869
HORVU5Hr1G092490	2.430675	3.044287	4.394557	3.646762	3.039809	3.297401
HORVU7Hr1G046970	100.7415	93.5719	112.9158	53.41932	54.74969	49.31912
HORVU1Hr1G039370	1.970254	2.32077	2.405105	0.896847	1.85707	1.847089
HORVU5Hr1G016790	3.215897	3.611735	4.931844	7.775003	7.191425	6.406953
HORVU4Hr1G078920	2.652014	2.802575	3.164852	2.494917	3.355197	3.32987
HORVU2Hr1G102040	1.743759	2.389281	2.842838	3.0453	2.805376	3.206841
Hordeum_vulgare_newGene_16195	8.365602	7.746262	8.988941	10.06168	16.29297	14.77501
Hordeum_vulgare_newGene_16190	1.476317	1.501228	1.256266	1.620013	2.956315	2.705134
HORVU2Hr1G052290	1.604587	2.796159	2.711429	3.316042	3.795499	3.992999
HORVU7Hr1G045770	30.88607	34.48233	48.10904	21.95819	33.84391	31.16376
HORVU7Hr1G109970	1.222241	1.284314	1.161282	1.436421	1.848324	1.880099
HORVU7Hr1G036390	6.980848	4.07387	7.904162	5.010765	3.941377	4.941478
HORVU1Hr1G084860	3.803876	4.543366	6.085376	5.473844	6.645214	6.69104
HORVU2Hr1G012230	24.06495	20.63422	26.03731	19.19446	21.72601	22.27353
Hordeum_vulgare_newGene_14358	1.06071	0.533685	0.732458	0.603125	3.760002	3.705479
HORVU7Hr1G041470	4.616087	4.963793	4.596528	7.023874	6.383331	6.844339
HORVU2Hr1G060000	21.6147	18.53842	21.76373	23.03205	24.3658	23.60581
HORVU2Hr1G073450	11.39963	7.329251	10.8115	7.826868	6.074597	8.12857
HORVU3Hr1G036710	5.984566	6.034182	7.867933	5.093891	6.704866	6.743068
HORVU6Hr1G051450	20.34595	21.7302	37.14211	16.2784	29.66834	27.53609
HORVU2Hr1G071380	8.445708	7.632683	9.679895	7.913887	7.756704	7.613484
HORVU3Hr1G063450	10.5115	9.672414	16.09699	11.8067	16.82568	16.57473
HORVU2Hr1G032990	2.961019	3.90427	3.158252	2.094324	1.955388	2.633222
HORVU6Hr1G086970	7.180028	6.612798	8.372849	11.16212	15.44426	13.67773
HORVU6Hr1G089700	20.32807	21.06389	21.42692	40.31069	43.74697	41.48523
HORVU3Hr1G075180	3.299215	2.561257	3.118755	3.81478	7.147299	6.827686
HORVU7Hr1G035440	5.736696	3.954021	6.241258	14.74429	6.516651	7.625268
HORVU3Hr1G110140	2.13766	2.705131	3.573741	2.037673	2.016126	2.683562

HORVU6Hr1G000710	36.79876	72.76917	84.34595	36.83821	0.586495	16.35974
HORVU3Hr1G089060	1.654525	2.172144	2.050047	1.168089	1.266262	1.346145
HORVU2Hr1G102950	2.261838	1.419308	1.457462	7.29188	3.052739	4.152351
Hordeum_vulgare_newGene_7991	9.259536	8.170919	11.21584	18.40067	20.86521	18.35707
Hordeum_vulgare_newGene_3433	51.92717	49.77275	59.30962	38.19345	43.72881	41.76715
HORVU1Hr1G012770	27.1492	28.92403	41.6737	216.8459	208.3245	166.245
Hordeum_vulgare_newGene_3436	2.290819	2.096787	2.522661	5.380295	5.662951	4.92048
HORVU7Hr1G002210	0.790519	0.904637	0.626582	7.114006	16.5649	16.77909
HORVU2Hr1G090470	5.658613	6.331588	6.353637	7.977786	10.45586	9.632683
HORVU3Hr1G092970	87.9431	68.38036	95.85154	78.95488	129.0787	190.5394
HORVU1Hr1G009800	6.394725	3.949191	6.387259	3.632265	0.621846	1.128149
HORVU3Hr1G077850	0.522032	0.290603	0.289332	3.055896	2.389311	3.213371
Hordeum_vulgare_newGene_14862	4.572741	4.640519	4.064496	4.146146	3.367826	4.106109
HORVU7Hr1G019810	2.067521	2.610474	2.598107	3.854084	4.304817	5.321207
HORVU3Hr1G031100	4.218212	5.585873	3.876106	3.729312	4.868499	4.52318
HORVU2Hr1G031380	1.508716	1.710961	0.971067	1.178883	1.188708	0.922819
HORVU3Hr1G101930	11.68453	8.237351	11.77328	14.59748	26.9564	22.53478
HORVU5Hr1G000700	9.223996	8.389606	10.37106	7.316467	6.354903	7.40602
HORVU7Hr1G025910	7.427748	6.629414	8.92162	9.05632	9.226168	8.856849
Hordeum_vulgare_newGene_8290	9.206327	9.524037	13.33269	5.311007	4.419414	5.224609
HORVU4Hr1G058970	20.26328	18.52078	18.63171	57.94902	87.2999	86.69085
HORVU3Hr1G055670	3.664141	3.7834	4.133104	6.960101	10.52558	10.91601
HORVU7Hr1G043230	0.635258	0.904103	1.135808	3.213149	5.795165	5.668008
HORVU1Hr1G044890	0.510013	0.734861	0.77567	1.30481	7.260701	9.068097
HORVU4Hr1G055500	24.42931	20.79652	27.24464	24.96947	36.22527	35.86152
HORVU3Hr1G020780	141.754	139.4527	114.951	57.13976	47.36665	53.78572
HORVU7Hr1G074760	4.116757	4.284926	4.977013	3.306858	3.681331	4.257406
HORVU2Hr1G096370	4.037894	4.509471	3.787389	6.043607	6.777198	7.416185
Hordeum_vulgare_newGene_2479	0.369488	0.295901	0.337054	0.917641	3.532368	2.196546
Hordeum_vulgare_newGene_2471	3.902886	4.242497	3.442349	5.449634	7.152504	6.733962
Hordeum_vulgare_newGene_2470	10.56182	9.996959	7.912442	15.84225	15.44703	19.4859
Hordeum_vulgare_newGene_2472	8.909685	9.873289	10.04216	14.06826	23.01065	20.94831
HORVU7Hr1G029960	5.031511	5.130065	6.475839	4.360833	3.70955	3.698877
HORVU5Hr1G116590	9.528609	10.13443	11.37459	44.98499	189.7559	147.756
HORVU4Hr1G017450	32.44616	26.7828	33.5014	24.65356	14.11912	19.44062
HORVU2Hr1G037500	1.919088	2.482127	2.952077	2.656551	3.925988	3.331759
HORVU6Hr1G015670	3.112864	3.01949	3.698922	5.972757	8.895789	8.67254
HORVU4Hr1G020490	3.651452	4.683863	4.214172	4.281225	3.873912	4.14616
Hordeum_vulgare_newGene_10161	0	0	0.057478	0.320837	12.66942	17.38163
Hordeum_vulgare_newGene_10160	0.08296	0.049577	0.071528	0.788703	2.914044	2.194668
HORVU3Hr1G059390	41.0794	50.77788	42.18002	46.41087	37.08844	46.05073
Hordeum_vulgare_newGene_10165	46.90424	44.26416	51.72817	58.15551	99.25733	90.4642
Hordeum_vulgare_newGene_5294	1.084794	0.852585	1.112195	4.386345	6.09707	8.193178
Hordeum_vulgare_newGene_5293	7.197726	7.044326	11.05184	5.9422	8.026427	9.42512
HORVU5Hr1G063470	1.406592	2.025695	2.475818	3.037591	6.30214	6.199102
HORVU0Hr1G010260	5.757119	7.036949	8.624524	9.494147	10.81727	11.44033
HORVU1Hr1G000600	49.32654	41.29805	36.42708	38.75996	33.14287	32.72356

HORVU1Hr1G004980	21.5768	16.98759	25.65894	10.57869	2.610957	4.167054
HORVU7Hr1G080800	161.5534	132.9403	147.319	153.7547	128.4596	131.6
Hordeum_vulgare_newGene_9733	9.458937	10.28961	11.14903	7.707455	7.290701	8.753407
Hordeum_vulgare_newGene_9737	2.350749	2.510156	1.384221	3.988777	2.92896	3.911047
Hordeum_vulgare_newGene_9739	2.088739	2.556099	2.349619	2.209776	3.323231	3.562052
Hordeum_vulgare_newGene_3678	2.317734	1.885494	2.636525	0.021872	0	0
HORVU4Hr1G075740	2.376365	2.736156	2.995837	4.777774	5.727109	5.911706
Hordeum_vulgare_newGene_5127	2.419956	2.642351	2.400964	3.683979	3.120194	4.231774
HORVU7Hr1G026430	9.670377	11.07153	10.56129	10.16904	13.1426	13.17551
HORVU3Hr1G095400	2.603271	2.376426	2.949067	2.049312	2.478973	2.571198
HORVU7Hr1G107760	1.198038	1.120609	1.089181	1.921291	2.116608	2.295717
HORVU1Hr1G028820	69.84055	68.19722	119.4948	58.63333	108.0618	106.2813
HORVU2Hr1G105380	10.92997	10.11759	13.63103	8.971401	14.90729	12.27912
HORVU2Hr1G038740	56.47331	57.86589	63.24233	126.7675	154.3538	152.4387
HORVU7Hr1G027520	5.927326	6.478684	7.17061	0.238758	0.269327	0.229431
HORVU2Hr1G104920	2.525061	3.145954	2.708954	3.978679	7.175372	6.757256
HORVU1Hr1G052360	1.000919	1.328371	1.189164	1.55632	0.997681	1.65371
HORVU3Hr1G014240	2.059448	1.996632	2.210495	2.836788	4.366571	4.435386
HORVU4Hr1G027580	8.683454	8.624328	8.796636	10.19514	11.4831	10.63576
HORVU1Hr1G035390	22.08466	22.79226	23.82111	24.09203	31.21185	32.20619
HORVU3Hr1G083830	1.084036	1.716174	2.252836	4.86638	13.32086	11.50349
HORVU4Hr1G061120	24.28256	15.16378	38.17022	9.416977	8.981541	11.7021
HORVU2Hr1G049760	7.439084	8.59948	11.03173	7.364808	10.15709	10.53748
Hordeum_vulgare_newGene_8069	1.021188	1.241545	0.85732	1.982721	1.372323	1.944864
HORVU3Hr1G053090	9.239699	6.835661	9.113454	7.136608	3.438031	4.602691
HORVU6Hr1G020380	21.37901	23.19527	19.18681	22.21536	40.50391	34.32116
HORVU2Hr1G026820	2.337673	2.062998	2.564361	1.407188	1.275698	1.842223
HORVU7Hr1G105990	2.012517	1.026143	1.478292	9.094361	127.1269	113.0264
HORVU6Hr1G009430	10.99065	11.91594	14.30073	16.23749	31.01996	30.52475
HORVU6Hr1G052970	14.46375	12.80438	16.90197	16.68253	21.54479	18.21485
HORVU5Hr1G103450	2.456267	2.72602	4.307561	1.045915	1.422302	1.683043
HORVU1Hr1G009030	7.162992	6.142267	8.581938	6.656599	5.641251	5.729118
HORVU1Hr1G073510	8.615741	9.218205	11.55102	25.75079	62.8166	48.18344
Hordeum_vulgare_newGene_16225	1.548778	1.460507	1.460386	3.779929	6.288177	5.507989
HORVU1Hr1G024740	16.94822	18.25606	19.82793	13.22048	15.68753	15.86742
HORVU3Hr1G011560	10.03268	12.74916	16.84191	5.508395	4.003737	3.272547
HORVU1Hr1G071910	0.067396	0.126122	0.131735	1.999953	3.242093	2.710929
HORVU6Hr1G020960	4.389036	4.586348	3.598898	3.059425	2.102936	2.533124
HORVU7Hr1G052800	2.127384	3.060085	3.364085	1.625422	1.671154	1.654602
HORVU2Hr1G045690	41.20776	36.73969	47.05897	28.00341	51.97311	53.12508
HORVU5Hr1G041860	15.13193	13.90598	14.6092	15.73421	16.95393	16.6605
HORVU1Hr1G031220	5.671391	6.416113	7.737369	8.237223	12.34142	12.92336
HORVU5Hr1G085900	1.153065	0.702443	0.589784	1.479455	1.325568	1.622696
HORVU3Hr1G018110	2.928214	1.994838	2.943661	6.616453	8.23183	7.870348
HORVU7Hr1G100810	41.02332	30.47927	43.44443	38.07715	16.70748	22.661
HORVU1Hr1G035930	3.070638	3.815661	4.569754	3.374788	5.636033	5.012334
HORVU5Hr1G033380	1.864861	2.344657	2.620884	3.069327	3.177808	3.566816

Hordeum_vulgare_newGene_7565	6.914618	5.245884	6.412969	12.01786	9.787893	10.11196
HORVU6Hr1G059090	3.308732	2.860273	3.655959	4.155928	3.859165	3.966674
HORVU2Hr1G118340	2.063584	1.906672	2.763019	1.589974	2.966797	3.296718
HORVU7Hr1G105330	11.12473	9.834882	12.54284	13.33164	17.20406	16.80118
HORVU3Hr1G081660	14.8881	14.51447	14.10878	9.44813	10.32044	10.65216
HORVU3Hr1G035170	77.42841	75.35983	73.62	99.01045	98.72295	98.46518
HORVU5Hr1G014790	3.427917	4.405859	3.549131	5.400199	6.795024	8.880073
HORVU7Hr1G056320	0	0	0	3.876827	2.151748	2.556966
HORVU2Hr1G114340	0.870174	0.681789	1.206757	4.416742	3.98191	4.600193
HORVU6Hr1G074030	214.8945	150.6762	232.7486	142.2432	89.64886	127.4321
HORVU5Hr1G037760	7.576425	6.618633	6.092043	10.86086	11.64133	12.68552
Hordeum_vulgare_newGene_8180	1.110872	0.634838	1.212699	3.42287	1.029329	1.55503
HORVU5Hr1G067880	11.80178	10.18676	14.22279	24.38087	42.30459	48.25431
HORVU1Hr1G008810	17.11235	14.10178	16.46485	26.77244	10.20771	15.17113
HORVU6Hr1G062230	4.645723	5.213223	7.433072	5.669898	9.187249	9.21331
Hordeum_vulgare_newGene_7389	0.394891	0.225836	0.575528	1.215262	25.47559	14.48086
Hordeum_vulgare_newGene_7386	3.999713	3.505205	3.956941	3.504064	4.390241	4.349957
Hordeum_vulgare_newGene_7380	8.268624	6.646458	8.944829	16.75444	39.32369	35.33292
HORVU3Hr1G030520	1.324789	1.661014	1.7531	2.919621	2.911755	4.040645
HORVU5Hr1G100670	0	0	0	1.864211	2.807574	2.672924
HORVU4Hr1G016600	0.758454	0.675271	0.687451	2.486072	0.838053	1.124748
HORVU7Hr1G056490	13.01441	12.52466	13.08822	15.85686	33.66107	32.47947
HORVU6Hr1G082160	37.63919	34.70097	36.00996	19.20753	17.14881	20.01728
HORVU5Hr1G084740	13.19203	10.463	11.55874	5.072952	4.715416	4.985925
HORVU7Hr1G025490	2.641462	3.206161	3.081458	1.116729	1.851156	3.571853
HORVU3Hr1G002280	23.67291	25.0158	27.94794	27.53178	32.69531	34.14047
HORVU3Hr1G039120	3.486249	3.384887	4.027421	4.253077	7.252488	6.949381
HORVU3Hr1G113200	0.672207	0.310216	1.537616	1.390585	1.073342	1.24237
HORVU1Hr1G044680	12.10334	8.766574	7.978781	21.56796	32.51155	33.18627
HORVU7Hr1G038440	2.769505	2.679945	2.802845	2.805321	2.850046	2.437368
Hordeum_vulgare_newGene_2806	13.2745	10.24286	13.1861	7.73316	9.363185	9.984715
HORVU5Hr1G022400	1.459027	1.924142	2.081912	1.109017	2.756925	2.871641
HORVU1Hr1G087660	58.58367	67.72131	74.88879	63.75994	62.69725	69.51898
HORVU6Hr1G074690	6.200207	4.691513	6.354505	0.38526	0.089542	0.282968
HORVU4Hr1G045760	17.23634	18.6026	16.89879	20.23286	18.37415	19.8119
HORVU5Hr1G024800	2.557967	2.702797	2.497347	3.393068	5.060059	4.365139
Hordeum_vulgare_newGene_14678	0	3.109703	4.337694	0	0	0
HORVU3Hr1G086210	2.074627	2.037489	2.650763	1.985827	2.037214	2.401228
Hordeum_vulgare_newGene_14674	1.997993	0.757373	0.00868	0.966306	1.092691	1.94222
HORVU6Hr1G030940	39.49744	31.76659	44.26081	34.24702	51.99093	49.53394
Hordeum_vulgare_newGene_14672	3.094714	3.215633	3.323098	3.657116	4.917963	5.618868
HORVU1Hr1G089710	4.717287	4.155789	5.149211	7.810653	6.44398	7.842639
HORVU2Hr1G075190	0.87673	1.029581	1.440692	0.852415	0.960019	1.543057
HORVU3Hr1G032960	4.515523	5.655392	3.678177	7.489592	7.438787	8.197127
HORVU6Hr1G070270	22.31626	19.7357	27.96193	38.6329	63.12379	62.33546
HORVU1Hr1G093240	10.25354	11.84036	15.99932	5.3659	14.41699	12.55768
HORVU6Hr1G068570	4.855896	5.692601	5.365513	3.989347	5.017056	5.633662

HORVU7Hr1G034140	28.8357	30.37458	37.98255	26.7661	39.93345	44.85804
HORVU6Hr1G074960	0.575035	0.317339	0.732063	0.666142	2.563225	3.614559
HORVU4Hr1G069200	28.71547	23.70244	25.48316	26.18301	37.35267	34.67795
HORVU4Hr1G002400	7.030431	7.347137	7.48285	9.210091	9.144675	8.500328
HORVU2Hr1G047750	3.387335	1.894169	2.502276	2.446706	2.353863	2.248402
HORVU7Hr1G054230	9.687962	11.87379	10.21696	20.15413	27.47481	25.87255
HORVU4Hr1G017260	20.33088	34.95009	43.50058	0.158771	0.192563	0.158309
HORVU1Hr1G011430	13.81534	10.65187	13.71834	20.2176	13.74805	13.87212
HORVU4Hr1G056500	15.20932	5.312586	19.73947	1.346662	0.865317	1.868677
HORVU3Hr1G013680	1.465428	1.184353	1.933508	10.4582	32.72504	31.73777
HORVU2Hr1G126080	0.989569	1.406507	1.245002	1.804408	2.62824	3.010976
HORVU5Hr1G105430	3.871669	4.836866	4.362792	5.554618	4.111696	5.401819
HORVU4Hr1G013540	12.87368	16.85333	23.27807	17.07802	16.31948	13.33936
HORVU7Hr1G096470	2.423627	2.040209	4.069203	4.698409	7.401406	5.491381
HORVU6Hr1G065690	2.512048	2.595373	3.097128	5.534115	9.185611	8.57898
HORVU5Hr1G023460	2.912937	2.236277	3.357556	6.075203	11.64056	11.17813
HORVU2Hr1G040400	3.500878	2.083951	2.860709	5.328157	9.397595	9.39184
HORVU4Hr1G010120	4.686891	4.315936	4.602551	7.227224	7.291123	8.436295
HORVU7Hr1G051770	0.5128	1.059824	0.487827	1.760678	1.53874	2.841375
HORVU1Hr1G051950	5.905986	5.496923	5.955177	12.73919	12.09966	12.98235
HORVU7Hr1G107400	5.113563	4.113237	3.514253	2.413603	5.110335	4.425645
HORVU3Hr1G018220	12.63009	13.08059	13.29711	12.1895	19.11306	18.06532
HORVU3Hr1G037600	18.77446	17.71157	22.22435	19.50586	26.98771	23.94857
HORVU3Hr1G012150	5.682093	6.507375	7.18538	6.366995	7.093994	7.071447
HORVU7Hr1G037720	0.579395	0.576577	0.868908	3.070501	42.02952	49.92501
HORVU2Hr1G082410	22.3382	19.34567	24.45156	10.01814	8.020017	8.404629
HORVU1Hr1G088140	12.1938	13.37519	42.33494	76.16431	12.08444	13.37021
HORVU6Hr1G011780	1.117266	0.719971	0.996949	0.924503	3.228375	3.837417
HORVU4Hr1G002510	26.08134	28.22725	38.45823	13.26365	8.309795	11.8706
HORVU5Hr1G055890	1.926565	1.909264	2.416277	3.740171	3.666014	4.256935
HORVU0Hr1G017380	1.744534	1.105626	1.909855	3.370944	2.704282	3.695235
HORVU2Hr1G089770	2.07442	0.732088	2.536789	1.182508	1.675822	0.902947
HORVU5Hr1G114330	11.40765	6.164828	9.236815	11.75885	21.11987	20.09212
HORVU7Hr1G020810	1.655912	1.441228	1.747068	1.581563	1.246061	1.786108
HORVU5Hr1G011020	1.570695	1.447343	1.656478	2.615118	2.824394	3.669354
HORVU1Hr1G002260	42.78594	33.13505	39.36667	44.30309	40.50255	31.33852
HORVU2Hr1G015930	3.115699	3.120381	2.749887	0.916101	2.715674	2.845538
HORVU2Hr1G113460	341.4677	323.3326	361.8774	108.683	40.52461	80.73473
HORVU7Hr1G092380	0.840664	0.870398	0.867897	1.090754	1.399612	1.444678
HORVU5Hr1G092220	2.732309	5.261791	5.03958	0.992449	1.059282	0.995035
HORVU4Hr1G019490	0.958263	0.793735	1.015161	4.430895	2.945385	3.979025
HORVU6Hr1G044360	3.229319	3.316744	4.646447	3.46845	5.828282	4.839653
HORVU4Hr1G090650	0.805406	0.718402	0.779962	1.232867	5.9499	4.891144
Hordeum_vulgare_newGene_4777	0.93939	1.197679	0.876338	1.551222	1.154923	1.645747
HORVU4Hr1G052990	3.227427	3.623684	3.234122	6.43949	7.103464	8.800636
HORVU7Hr1G036450	7.06508	10.08418	10.88113	3.053281	1.541781	2.749239
HORVU2Hr1G081140	1.388022	2.045959	1.941838	3.964887	6.764419	6.800453

HORVU2Hr1G085870	5.669917	7.035584	9.789269	5.132886	8.389698	8.441343
HORVU5Hr1G097730	21.7307	18.40539	21.58153	25.2191	12.134	13.63295
Hordeum_vulgare_newGene_217	3.176221	4.536211	4.025451	4.488438	4.369666	5.218063
HORVU3Hr1G084530	2.042333	1.726033	2.298359	9.539582	15.80637	12.65701
HORVU2Hr1G083430	7.985917	5.313594	8.287373	8.142551	5.455154	5.419038
HORVU6Hr1G058670	12.8776	10.03119	16.43542	12.16319	10.33436	9.08429
HORVU1Hr1G056380	31.74076	27.56933	28.92948	21.82408	20.08505	20.35429
HORVU3Hr1G030950	1.831023	1.341634	1.78169	0.812577	0.555342	0.749387
HORVU2Hr1G045860	3.053168	3.447368	3.385915	3.844631	4.40259	4.796298
HORVU4Hr1G009520	87.65114	81.63466	82.39834	276.833	440.245	380.6787
HORVU4Hr1G064590	16.9081	17.13953	19.93792	18.07179	29.44573	23.76842
HORVU4Hr1G048140	1.130065	1.486856	1.379954	0.827925	3.07128	2.038911
HORVU1Hr1G079430	34.56084	30.06447	40.08658	25.2843	30.31973	28.93172
HORVU5Hr1G027590	0.754088	0.723723	0.934274	1.399543	1.39965	1.710444
Hordeum_vulgare_newGene_2917	0.460737	0.38635	0.313716	1.336852	3.82923	4.185023
Hordeum_vulgare_newGene_9552	9.352336	8.005834	8.116063	10.45022	7.246724	8.823905
HORVU7Hr1G044270	0.556068	0.435354	0.480731	1.34428	1.555549	1.973636
HORVU0Hr1G015830	0.062994	1.228869	0.905465	2.409576	3.258923	3.129384
HORVU5Hr1G073460	2.623472	3.028336	2.635454	1.880491	0.955856	1.557326
HORVU1Hr1G091500	1.183142	1.17637	1.188726	3.319569	1.556283	1.921652
HORVU3Hr1G099400	1.336546	1.234058	1.859297	0.796064	0.530851	0.692854
HORVU7Hr1G095810	62.72065	58.2259	67.84275	53.03629	46.2902	45.88812
HORVU3Hr1G050210	3.048274	2.606276	4.126755	3.953559	6.663284	6.812717
HORVU1Hr1G047690	2.328753	2.361103	2.852815	3.756953	5.550982	5.451078
HORVU7Hr1G098440	16.86202	12.61926	21.65623	32.88137	4.092262	9.917381
HORVU2Hr1G017420	54.38843	29.49968	55.76748	9.442678	2.458378	4.394886
HORVU5Hr1G074900	45.72112	34.73963	43.2689	46.56496	35.04739	38.04935
HORVU1Hr1G086270	7.716973	7.604902	7.99528	12.0681	12.08983	14.3184
HORVU6Hr1G091230	0.920326	0.760558	1.068114	1.23034	2.203191	1.619511
HORVU5Hr1G095250	0.382273	0.678079	0.556479	1.231716	2.851424	3.616899
HORVU6Hr1G019320	3.312099	3.430047	4.232911	3.005881	4.702061	4.343348
HORVU1Hr1G019290	19.15973	19.26076	19.73174	14.35601	13.48259	13.79687
HORVU5Hr1G113950	2.536205	2.047338	2.262351	2.614097	3.908139	3.873357
HORVU7Hr1G055880	1.129396	1.230635	1.668874	0.329148	0.855249	1.336987
HORVU4Hr1G055070	2.104329	1.665233	1.971669	2.632576	2.331996	2.559126
HORVU0Hr1G017740	16.47374	15.3172	20.99769	14.78273	19.24453	18.11953
Hordeum_vulgare_newGene_10362	0.695691	0.964548	1.239569	1.404911	0.797561	1.565405
HORVU5Hr1G047550	0.681854	1.007765	0.931952	0.356765	1.920444	1.704246
HORVU6Hr1G049910	4.772246	4.932146	6.342476	6.538533	7.195055	7.766148
HORVU2Hr1G099870	159.1025	186.3031	225.6856	55.97953	24.16711	33.5699
HORVU5Hr1G028510	1.724	1.834324	1.498163	2.92484	1.386201	2.416021
HORVU6Hr1G047040	14.10917	15.14334	15.61179	23.09284	29.33037	25.75027
HORVU3Hr1G019720	38.50475	25.20469	35.72355	28.58569	17.26993	20.45249
HORVU2Hr1G101190	22.90024	23.90457	25.07884	29.1878	34.97283	31.48254
HORVU1Hr1G010940	6.403053	5.879996	6.703892	9.741502	12.18649	11.67096
HORVU2Hr1G079170	3.752992	3.680664	4.519406	4.670884	3.593146	4.529708
HORVU3Hr1G073020	41.46831	52.84061	47.05491	88.2604	119.8263	97.03211

HORVU2Hr1G015480	19.73277	16.52226	20.50969	12.57124	15.25005	13.38853
HORVU7Hr1G000820	4.732697	5.248669	4.572803	4.912231	6.242642	5.961006
HORVU7Hr1G082640	1.266606	0.773208	0.86108	0.766286	2.365744	1.583935
HORVU4Hr1G004800	11.36985	12.55742	14.22546	11.34316	9.313698	11.2557
HORVU2Hr1G019680	5.846288	8.157857	10.00622	1.654314	0.196444	0.750632
HORVU7Hr1G012940	19.60888	16.59627	20.04176	29.2323	33.75877	32.24086
HORVU3Hr1G092690	13.4179	16.8333	18.81238	9.221568	13.01813	11.98422
HORVU1Hr1G012890	0.031855	0	0.148613	0.520898	5.094869	5.847876
HORVU3Hr1G078620	0.921019	0.401724	0.563671	7.722719	1.633365	2.349192
HORVU5Hr1G073540	1.797507	2.675301	2.878326	1.528693	1.531766	1.868242
HORVU7Hr1G089850	2.654729	2.843039	3.191541	5.561093	6.76528	7.072965
HORVU1Hr1G001020	8890.279	7092.061	5454.794	16522.28	5435.715	7639.873
HORVU4Hr1G060450	35.64593	32.45415	33.51314	29.53366	25.59201	28.25344
HORVU4Hr1G040520	1.396515	2.385816	2.968491	11.01042	44.37214	32.15748
Hordeum_vulgare_newGene_11099	0	0	0	2.769866	2.308505	2.67675
Hordeum_vulgare_newGene_11540	2.63689	2.750073	2.635686	1.748242	2.567947	2.839468
Hordeum_vulgare_newGene_11541	51.97323	45.17499	56.74738	50.41827	63.41498	63.43892
Hordeum_vulgare_newGene_11542	12.76881	11.94655	13.66953	11.04299	16.73641	16.49553
Hordeum_vulgare_newGene_11543	14.04092	10.56361	16.40355	4.671992	2.634495	4.236765
Hordeum_vulgare_newGene_11544	2.724096	2.719499	2.87124	6.567802	5.914423	6.935814
Hordeum_vulgare_newGene_11545	0	0.015512	0	8.539339	8.892131	10.89885
Hordeum_vulgare_newGene_11546	23.72434	24.76742	33.89525	37.64745	48.36621	51.40351
Hordeum_vulgare_newGene_11547	6.311147	4.475153	5.143561	6.398672	6.071824	6.36628
HORVU5Hr1G092510	15.64075	13.9983	16.89367	7.450972	9.034157	8.494591
HORVU2Hr1G094960	1.789215	1.278646	1.648978	3.398295	3.911367	3.270394
HORVU1Hr1G039230	6.463352	6.63861	6.877162	8.700121	7.370877	8.762078
HORVU5Hr1G081840	9.365268	9.257136	11.66029	4.13682	2.345058	3.620214
Hordeum_vulgare_newGene_5667	0.864724	1.302732	1.803409	1.01951	1.048158	2.42883
HORVU6Hr1G094720	4.345314	4.271867	5.389077	6.03982	11.47765	10.519
Hordeum_vulgare_newGene_6738	3.34613	3.769359	4.457876	3.491261	1.491168	1.382479
HORVU1Hr1G084120	0.632342	0.665883	0.768377	2.280938	4.915876	4.941987
HORVU5Hr1G007630	1.150314	0.761071	1.2158	0.57617	4.650602	3.357416
Hordeum_vulgare_newGene_385	1.949657	3.257715	3.200288	2.205893	2.149378	3.475189
HORVU5Hr1G005980	139.2272	118.8073	149.6907	78.71389	97.3939	109.3148
HORVU2Hr1G028660	11.22114	6.881483	8.65614	0.131952	0.030266	0.412583
HORVU5Hr1G016050	0.894694	0.784855	0.423894	0.310279	2.711037	1.77068
HORVU7Hr1G051430	2.987987	1.916803	3.062913	0.71045	1.008112	1.177205
Hordeum_vulgare_newGene_1913	3.188808	3.312868	3.463216	4.813875	5.717564	6.456089
HORVU2Hr1G123370	7.22378	6.651024	9.035044	6.957877	6.611854	5.486588
HORVU5Hr1G086120	3.1797	1.318354	1.18135	3.137498	3.369792	3.800109
HORVU6Hr1G015050	7.648079	7.501623	8.953414	8.998087	15.59914	14.38401
HORVU1Hr1G038180	3.646802	5.119532	5.418962	3.687911	4.746341	4.836046
HORVU4Hr1G035250	22.62052	25.25158	23.76163	26.35935	31.43662	33.74387
HORVU4Hr1G028840	1.849279	1.795149	2.063187	1.608311	1.380194	1.162728
HORVU5Hr1G070810	4.87242	4.567723	4.899462	4.803847	3.859653	4.942655
HORVU7Hr1G084900	3.275114	2.360676	3.303918	7.468967	6.916979	7.033556
HORVU6Hr1G011990	1.378999	1.295153	1.721352	5.291909	11.1923	9.631508

HORVU3Hr1G010760	3.454071	3.97774	3.067897	3.620168	2.871826	3.332439
HORVU3Hr1G026900	3.790904	3.51169	3.980709	9.890718	12.11532	12.2694
HORVU1Hr1G042810	8.401946	5.825449	6.557769	6.937784	1.502952	2.085325
Hordeum_vulgare_newGene_16018	3.366879	4.408224	4.449875	2.776179	1.60287	2.359972
HORVU4Hr1G057000	9.68436	8.508532	13.3764	13.20001	7.069816	6.135089
HORVU6Hr1G051560	23.88473	21.66488	27.32455	24.7192	22.22897	24.39641
HORVU3Hr1G068700	9.490584	6.175611	13.71163	7.020159	6.878488	6.193625
HORVU3Hr1G024500	4.984791	3.700148	5.517482	1.071319	0.009104	0.068063
HORVU3Hr1G088500	260.2657	200.9715	250.2912	291.6903	401.4258	422.8244
HORVU1Hr1G055900	41.29828	35.86006	27.44685	42.11355	29.81377	29.93615
Hordeum_vulgare_newGene_3017	41.78924	52.08714	21.93024	1.021012	0.153067	0.379301
HORVU7Hr1G028370	20.72212	22.99621	20.32989	48.04945	32.6524	41.73935
HORVU0Hr1G021480	3.792365	2.589561	4.506634	2.287801	1.014766	2.149222
HORVU5Hr1G087980	12.78834	13.02303	8.500562	4.986846	6.044754	6.268934
HORVU4Hr1G079820	2.042426	2.365048	2.301586	2.346464	2.873948	2.853229
HORVU2Hr1G025160	3.834822	4.755455	4.447645	5.389069	6.142595	5.657536
HORVU3Hr1G089450	21.23291	22.24483	21.01886	22.29283	19.72928	17.75463
HORVU1Hr1G085740	1.826723	2.853474	2.287963	2.522929	2.748425	3.010131
HORVU5Hr1G071140	10.31125	8.058901	10.96961	0.491543	0.881244	1.480651
HORVU3Hr1G060570	1.718303	0.913057	3.077507	2.854407	2.250983	1.492246
HORVU2Hr1G039000	0.937455	0.997898	1.177621	2.233136	3.470965	2.5376
HORVU7Hr1G109780	13.66517	14.63507	14.33491	14.76087	19.42545	20.99503
Hordeum_vulgare_newGene_7817	2.962112	2.083293	2.552471	8.843987	9.910291	8.062119
Hordeum_vulgare_newGene_7811	1.792653	1.736942	0.569975	3.69126	3.559416	4.406599
HORVU5Hr1G033710	3.130481	1.95096	3.36588	3.669337	5.037101	4.471896
HORVU0Hr1G021730	2.769373	2.195434	2.735709	1.646372	1.338156	1.026764
Hordeum_vulgare_newGene_6149	4.572394	4.45976	3.854982	6.12361	6.8762	6.735238
Hordeum_vulgare_newGene_13795	1.820222	1.549596	2.001624	2.136496	0.899875	1.065685
Hordeum_vulgare_newGene_13793	0.326711	0.443376	0.39383	1.292426	5.991101	8.201419
HORVU7Hr1G042130	6.480096	4.85976	6.778127	4.149502	4.633018	4.185903
Hordeum_vulgare_newGene_13798	0.630965	0.766939	0.975543	1.821455	1.322123	1.079948
HORVU1Hr1G037320	2.564787	2.502346	1.88721	1.280733	2.302802	2.380313
Hordeum_vulgare_newGene_3660	4.719398	4.743145	4.018307	6.208407	6.664635	4.644116
Hordeum_vulgare_newGene_3667	10.26347	10.60061	12.40276	0.203789	0.166956	0
HORVU3Hr1G096850	29.30501	26.25906	35.58265	24.81462	16.34487	20.17197
HORVU2Hr1G100450	1.67621	1.476678	2.109525	6.978862	4.84845	6.057926
HORVU6Hr1G078470	0.171674	0.118076	0.086551	2.904638	4.859292	3.858677
HORVU7Hr1G028950	5.937616	7.03315	6.940138	11.00676	14.7472	14.40374
HORVU5Hr1G068330	3.275536	2.856472	3.511909	4.048645	8.342544	7.06043
HORVU6Hr1G018150	0	0.058883	0	0.481304	6.329819	7.921945
HORVU3Hr1G061620	0.972273	1.132643	1.268407	1.18161	1.636949	1.160852
HORVU3Hr1G087500	58.52491	55.88685	56.80411	58.47939	27.76669	35.37743
HORVU6Hr1G026280	152.7079	113.097	134.4589	130.6185	87.53876	107.4956
HORVU6Hr1G014860	32.66699	31.93963	45.31973	26.71803	37.31165	35.96732
HORVU1Hr1G034980	45.46586	45.93516	45.8616	55.08524	70.0841	62.50339
Hordeum_vulgare_newGene_2392	10.88841	10.83684	8.323302	9.852589	12.18126	12.44595
HORVU4Hr1G029030	43.88612	52.25555	50.37498	51.10392	61.76583	70.18053

HORVU7Hr1G070230	5.32617	6.412331	6.928016	5.69968	5.992876	7.415216
HORVU2Hr1G086380	23.70842	22.68581	27.5828	11.03231	8.766268	9.56373
HORVU7Hr1G078400	0.039978	2.572025	0.156116	2.592217	2.521545	3.559633
HORVU2Hr1G116090	4.070913	3.141722	4.089919	8.92369	11.51129	12.46709
HORVU6Hr1G072320	44.61303	45.5312	49.39172	45.42756	49.96019	54.50887
HORVU4Hr1G061190	29.6004	23.69563	31.61238	43.11722	70.87316	52.84925
Hordeum_vulgare_newGene_6025	2.526671	2.889702	2.114613	0.894532	1.596348	1.180897
Hordeum_vulgare_newGene_15650	0.808678	0.489621	0.77101	0.730189	2.063524	2.232613
HORVU1Hr1G077820	63.17046	73.46418	75.71093	54.79822	24.26718	39.34183
HORVU2Hr1G063740	14.59835	13.04378	13.54518	58.52276	71.53561	71.43888
Hordeum_vulgare_newGene_600	23.91966	27.11495	34.65929	28.16126	43.32279	43.38574
HORVU0Hr1G031960	18.16109	16.35197	20.27187	23.07721	24.47309	22.28413
HORVU0Hr1G023640	10.6335	9.838713	9.4861	6.158372	6.947534	6.489733
Hordeum_vulgare_newGene_2539	5.28917	5.442235	7.948623	4.229618	4.898627	5.865131
HORVU1Hr1G070440	4.35577	4.851548	5.618847	3.296404	5.824691	4.443366
Hordeum_vulgare_newGene_2532	13.66082	12.88143	13.02992	24.51692	36.34298	39.22668
HORVU7Hr1G096670	14.51538	11.90379	15.14362	23.04047	28.88795	27.02226
HORVU2Hr1G077110	1.800857	1.122225	1.457017	1.35542	0.846122	1.228393
HORVU5Hr1G048070	5.933729	4.914686	5.724066	5.845446	6.241948	6.891986
Hordeum_vulgare_newGene_5629	17.08356	14.28663	17.90988	16.27367	17.60805	15.71398
HORVU7Hr1G028670	9.863853	9.492506	10.54369	23.81209	27.48513	27.76333
Hordeum_vulgare_newGene_5621	0.919315	1.004853	1.374086	0.724056	1.476928	1.378847
HORVU7Hr1G000320	1.995608	3.337819	3.305901	0.045283	0.089499	0.01183
Hordeum_vulgare_newGene_5626	20.94848	14.32311	18.11268	7.780972	6.065192	5.8948
Hordeum_vulgare_newGene_6853	4.119753	1.573159	2.066187	5.187317	5.24077	0.396891
HORVU1Hr1G077790	2.460316	2.645033	2.677544	2.541073	3.064932	3.106237
Hordeum_vulgare_newGene_6858	2.873713	2.159515	2.326466	2.384469	3.283169	2.656724
HORVU1Hr1G080180	5.874704	5.414895	8.040738	7.087488	9.721536	9.929221
HORVU4Hr1G070520	1.08055	0.524299	1.443515	3.307016	2.726021	1.984666
HORVU2Hr1G029890	34.45426	36.79122	49.51873	33.07873	53.38118	55.8298
HORVU1Hr1G000030	1.132098	1.523881	1.914755	3.126684	4.541889	4.836378
HORVU3Hr1G058470	3.946997	4.409085	4.87801	4.91434	4.95117	5.705538
HORVU6Hr1G064670	0.855302	1.171579	1.438811	1.733503	3.674082	3.272925
Hordeum_vulgare_newGene_8207	4.994933	5.192109	5.47985	5.09876	5.660861	5.817718
HORVU5Hr1G098000	8.642141	7.523649	9.48443	9.816569	7.118131	8.787712
HORVU0Hr1G022110	2.93023	3.074074	3.732654	4.080927	11.6907	10.56795
HORVU5Hr1G077920	53.15117	56.44697	64.28761	64.33319	28.54821	40.7266
HORVU4Hr1G035700	8.319149	7.67206	6.954701	7.551997	2.5578	4.14678
HORVU6Hr1G076310	18.90492	17.73472	22.98688	17.13024	28.06119	25.54924
HORVU1Hr1G035210	6.783632	8.607213	10.90149	9.058144	11.39505	11.5274
Hordeum_vulgare_newGene_13767	1.586627	2.904331	3.100708	3.509076	4.683116	4.674289
HORVU2Hr1G092600	78.77375	63.54395	92.45318	74.57769	68.98466	75.47396
HORVU3Hr1G050240	8.0162	7.887583	9.868462	8.096155	9.609451	11.82885
Hordeum_vulgare_newGene_9509	1.28	1.675137	1.679909	1.60161	2.303675	2.032862
HORVU7Hr1G058210	4.321429	4.889146	4.273067	3.25583	3.768346	3.574112
Hordeum_vulgare_newGene_9506	0.671285	0.421197	0.766279	1.210949	1.678966	1.432656
HORVU2Hr1G034040	43.48555	39.26148	40.4218	36.76249	41.54728	44.39553

HORVU6Hr1G075950	3.843152	4.775941	5.276132	6.27919	7.287653	7.818974
Hordeum_vulgare_newGene_9503	6.863953	8.323819	8.698557	4.958004	3.939882	5.465896
HORVU7Hr1G023610	16.52877	12.11059	10.95457	10.95502	10.47329	10.53228
HORVU7Hr1G114020	5.75341	7.622635	9.295037	0.854798	0.871119	0.737584
HORVU1Hr1G073490	0.81318	0.776643	0.66581	7.618722	32.23202	22.34835
HORVU3Hr1G049730	34.45988	25.67876	26.47113	6.270157	6.424438	5.451755
Hordeum_vulgare_newGene_4173	17.09299	12.99493	21.49346	16.97911	22.59122	22.42921
Hordeum_vulgare_newGene_4174	2.063411	1.961812	2.141244	2.304348	2.613706	2.770439
Hordeum_vulgare_newGene_4178	0.976948	0.947834	1.288404	2.417665	3.824756	3.790226
HORVU7Hr1G024060	2.450922	1.989161	2.499495	0.358217	1.942126	1.278958
HORVU7Hr1G063490	0	2.209933	2.60676	2.748463	7.521868	6.599044
HORVU1Hr1G063720	8.685394	8.158324	7.749828	5.650918	5.828094	6.120578
Hordeum_vulgare_newGene_14888	42.4281	44.98122	57.84597	10.07159	3.357408	8.0706
Hordeum_vulgare_newGene_14880	9.005363	10.14012	13.23578	9.108132	14.30899	11.79844
HORVU5Hr1G020900	9.635153	10.95916	10.78139	9.042683	8.754737	9.459149
Hordeum_vulgare_newGene_4283	0.824955	0.919781	0.688843	2.638827	2.685276	3.166702
HORVU6Hr1G054850	1.664422	1.15782	1.469739	1.183841	1.10586	0.92232
HORVU3Hr1G047670	0.099972	0.181217	0.135566	2.752328	2.10738	2.697262
HORVU5Hr1G056520	1.704808	1.640123	2.151418	2.342834	3.823426	3.433766
HORVU2Hr1G046680	3.331035	3.449312	4.551193	2.913433	3.173225	3.576144
HORVU6Hr1G023040	1.262962	1.34192	1.537852	6.473654	10.04428	7.815945
HORVU1Hr1G012270	4.000237	4.28434	4.350391	3.309595	5.311411	6.656096
HORVU1Hr1G071890	12.55261	12.5343	13.49165	15.1354	14.13118	14.08737
HORVU1Hr1G078320	28.05417	24.83753	25.18198	6.993732	10.68223	10.02494
HORVU7Hr1G017730	9.271426	8.172657	9.585624	13.71736	15.61414	14.87564
Hordeum_vulgare_newGene_4821	9.933489	8.20748	9.172153	6.321115	6.251368	4.851063
HORVU7Hr1G098280	0.968022	0.534977	1.756407	8.142202	41.57582	40.13914
HORVU5Hr1G101710	0.218207	0.208989	0.163243	13.66946	1.064623	4.975473
HORVU1Hr1G056630	1.786271	1.713153	1.73755	2.824166	2.8804	3.312297
HORVU1Hr1G086070	1.355988	1.407066	3.078998	1.954142	2.015152	2.041431
Hordeum_vulgare_newGene_7732	7.358488	5.927514	8.469123	8.063374	7.893364	8.264605
Hordeum_vulgare_newGene_7735	0.130946	0.231846	0.196911	1.243922	4.293835	5.21451
HORVU5Hr1G125050	3.731051	4.637835	4.887563	5.089631	8.911642	7.115958
HORVU1Hr1G021390	0.480561	0.756476	0.355384	2.452192	2.386383	2.733181
HORVU5Hr1G061870	12.43015	13.30135	15.56033	13.23277	24.26336	24.57477
HORVU7Hr1G065420	20.45587	18.77309	21.02887	22.56159	36.6196	37.11426
HORVU6Hr1G005950	1.074327	0.644047	1.215255	2.451125	12.75075	10.41281
HORVU6Hr1G029520	3.601913	3.516084	4.284062	3.762874	5.126188	4.89824
HORVU4Hr1G074780	34.82208	30.82762	34.96285	16.52184	9.560769	13.36879
HORVU1Hr1G051360	0.787987	1.045205	1.022901	0.76517	1.799048	1.266718
Hordeum_vulgare_newGene_6465	14.20728	15.5399	28.26889	1.690399	0.676876	0.669033
HORVU3Hr1G081540	16.79017	15.59086	18.80658	30.45219	37.1553	31.56581
HORVU6Hr1G073440	13.15317	15.80649	14.53054	15.05596	19.43018	20.83028
HORVU2Hr1G044680	4.385808	3.425018	4.54667	4.448324	3.083838	4.465435
HORVU2Hr1G001360	11.51541	11.85285	13.85189	12.00956	15.02827	16.57931
HORVU3Hr1G018850	11.38901	10.47814	11.63315	8.773535	9.353815	10.22219
HORVU3Hr1G030750	1.760248	2.054105	2.261356	4.300853	6.040365	6.78478

HORVU0Hr1G040070	2.404009	2.479211	3.210629	5.540961	8.765037	7.577247
HORVU4Hr1G016470	10.87656	12.04876	11.30813	15.60443	15.47651	16.21889
HORVU6Hr1G005480	4.571697	5.423376	5.02235	4.465571	5.615555	5.157052
HORVU2Hr1G077990	16.95251	14.4635	18.01229	17.60052	10.81129	13.06474
HORVU4Hr1G074250	1.192384	1.442696	1.257563	5.614823	4.941156	6.560504
HORVU3Hr1G090710	65.78108	59.17902	64.79152	44.53092	41.2981	38.98118
HORVU2Hr1G082340	3.754736	2.439084	1.539579	0.898219	0.216184	0.355232
HORVU4Hr1G007110	17.24622	21.25605	22.6966	15.87413	12.31092	14.30944
HORVU0Hr1G018100	0	0	0	2.815755	2.483926	2.824101
HORVU5Hr1G111940	4.91953	4.45988	4.552089	3.899018	4.70512	5.478289
HORVU5Hr1G103620	2.684085	1.818661	2.604332	2.223373	2.563467	2.284517
HORVU5Hr1G088130	4.445874	3.20455	4.040892	1.830749	1.257868	1.351145
HORVU5Hr1G012170	0.956459	0.580208	0.742479	3.20511	4.437795	4.111928
HORVU4Hr1G000830	4.578137	4.572903	4.834901	18.71929	12.5891	15.05613
HORVU5Hr1G058840	2.880811	3.687769	3.65813	1.737522	3.020409	3.345592
HORVU5Hr1G094050	6.666146	5.740927	7.200669	13.10596	18.20111	15.87207
HORVU5Hr1G124010	43.09436	38.2981	38.44755	73.05008	123.7427	114.9029
HORVU2Hr1G000010	6.689234	7.121198	7.753771	9.991327	10.14325	12.11708
HORVU6Hr1G003500	12.0669	11.34795	10.6986	10.12388	12.47474	11.93962
HORVU1Hr1G026860	0.514279	0.444742	0.099672	0.397456	2.307887	2.28472
HORVU3Hr1G039790	0.65123	0.621748	0.602654	4.825242	9.637947	8.46436
HORVU1Hr1G089500	1.104567	1.101148	1.12844	0.427088	1.434297	1.955934
HORVU5Hr1G106390	7.330453	8.196884	10.71244	7.896576	8.41895	10.80304
HORVU2Hr1G058900	3.069015	4.01978	3.33461	6.076613	7.54422	8.630837
HORVU4Hr1G053790	0.270854	0.110295	0.048282	0.117616	3.952806	5.048856
HORVU4Hr1G038160	7.646826	7.820668	10.47023	13.10825	26.29135	24.70877
HORVU2Hr1G024650	0.798982	0.499018	0.687213	1.285876	1.369341	1.674593
HORVU1Hr1G065630	0.688042	0.786287	0.850113	13.24092	4.199235	5.531108
HORVU3Hr1G022020	37.22859	31.97219	37.23509	25.05394	24.47369	24.99487
HORVU7Hr1G069420	6.567759	9.032566	7.713393	0.007767	0	0
HORVU6Hr1G045970	10.42919	11.48212	14.99432	15.1252	21.3535	20.05498
HORVU4Hr1G013640	10.49574	11.98797	16.10569	5.483407	7.738949	9.951237
HORVU4Hr1G090550	2.78596	2.404354	3.176362	2.228124	4.025509	3.607243
HORVU1Hr1G050050	9.816231	9.616172	10.20503	17.64443	40.90606	23.85965
HORVU3Hr1G082780	5.552128	5.109103	7.457506	6.493505	9.380633	9.216158
HORVU1Hr1G010210	96.88226	104.2424	70.28442	19.33528	8.113751	10.90237
HORVU2Hr1G083970	9.037455	3.130349	5.582275	4.072445	3.75222	6.0453
HORVU1Hr1G067670	5.770349	4.600921	6.897534	1.596966	1.633209	1.420966
HORVU5Hr1G029060	0.627134	0.526964	0.540714	2.062436	1.477656	1.714356
HORVU6Hr1G094440	2.014529	2.174061	1.92338	1.34357	1.751917	2.16274
HORVU7Hr1G065250	6.011508	7.314884	7.196548	5.240909	5.058262	5.958766
HORVU4Hr1G088060	5.195682	4.759875	5.795145	8.040576	8.522887	8.610898
HORVU5Hr1G106970	11.14889	10.29366	15.63536	13.87783	16.83154	16.69681
HORVU1Hr1G002540	0	0	0	12.05931	0	0
HORVU2Hr1G082590	97.79218	99.28092	96.44424	83.39048	120.1386	117.055
HORVU4Hr1G038940	1.089156	1.736867	1.908459	1.507606	0.537781	1.595299
HORVU7Hr1G051980	1.517853	2.887954	2.117931	3.701961	4.108511	4.839191

HORVU4Hr1G049710	13.90704	18.29822	15.23045	17.01019	19.73106	20.11589
HORVU3Hr1G083780	18.76364	16.22403	20.74264	19.06136	24.73304	25.35712
HORVU1Hr1G048620	2.897072	2.46146	3.135984	3.404215	4.64155	3.914352
HORVU4Hr1G087780	71.27714	107.5161	138.1678	10.74147	2.542072	6.504748
HORVU1Hr1G002090	0.021814	0	0.538068	12.05024	0.053263	0.019755
HORVU5Hr1G118480	4.736199	5.168586	5.924184	6.794543	7.128682	6.991711
HORVU7Hr1G046050	2.32516	4.239175	3.191449	8.292304	7.092902	8.536998
HORVU4Hr1G006930	29.08544	29.47277	30.40038	31.70415	31.93859	38.34407
HORVU2Hr1G111840	2.448059	1.56168	1.673094	6.931447	9.372679	9.32335
HORVU1Hr1G090490	93.64232	97.12907	114.171	167.3989	402.6453	390.7414
HORVU6Hr1G085210	0.161418	0	0.112517	1.538317	21.99329	20.80011
HORVU5Hr1G051500	7.604036	9.20012	7.165625	10.10128	14.93116	14.15702
HORVU6Hr1G054890	15.45494	19.41273	24.20638	2.072882	0.571042	1.205098
HORVU5Hr1G093850	1.484214	1.732642	1.851638	2.878621	5.30365	5.069662
HORVU4Hr1G014960	23.25138	23.74119	23.47385	36.52659	48.00898	43.09939
Hordeum_vulgare_newGene_12823	3.655165	3.425109	3.754255	4.902629	5.479524	5.692402
Hordeum_vulgare_newGene_12820	6.022443	7.062804	6.644221	6.24426	2.653846	3.189288
Hordeum_vulgare_newGene_12826	4.718802	4.845805	6.169955	5.571493	3.949285	4.792096
HORVU1Hr1G057220	1.909571	1.274129	2.284611	1.79702	0.794424	1.274629
HORVU7Hr1G084290	4.254577	3.807592	4.494972	6.784025	6.486736	6.993389
Hordeum_vulgare_newGene_4908	0.664647	1.035491	0.760769	1.895107	1.663677	2.659667
HORVU7Hr1G056980	8.657206	7.696589	9.915999	9.868633	14.00059	13.08213
HORVU2Hr1G100690	4.683056	6.944202	9.077606	4.296742	3.691096	3.551151
HORVU6Hr1G004270	2.459537	2.142451	2.49971	2.928142	4.662318	4.451597
HORVU6Hr1G018790	5.740267	6.682335	5.259167	6.716094	9.615887	9.202066
HORVU1Hr1G015490	15.90989	15.08549	19.11447	13.85415	12.5053	12.48266
HORVU3Hr1G073170	12.1671	10.83723	12.46822	9.905561	10.39252	10.10037
HORVU6Hr1G055270	2.090045	2.704719	4.427487	3.152695	7.061881	9.031322
HORVU7Hr1G036920	27.9709	26.03964	27.65068	26.84882	29.43538	29.24566
HORVU2Hr1G061040	39.58652	40.96841	48.81726	27.91936	19.01516	23.15419
HORVU4Hr1G065380	24.5224	23.53264	25.50109	47.7363	76.16089	69.30928
HORVU3Hr1G060800	157.8117	166.5088	235.404	174.9346	334.9544	294.9469
HORVU1Hr1G056170	0.539206	0.509437	0.66013	1.348477	9.158613	6.85829
HORVU6Hr1G012530	3.985332	4.028592	5.976903	1.770781	1.778166	2.002397
HORVU7Hr1G110370	3.3819	2.625522	2.875883	3.639632	7.137837	6.059337
HORVU1Hr1G094490	43.31245	39.31199	41.19078	34.61961	55.2166	57.982
HORVU7Hr1G074530	31.58385	30.19099	31.82003	17.03821	17.30253	19.54034
HORVU5Hr1G115470	39.50597	31.66661	39.92396	21.1645	17.02489	18.00167
HORVU3Hr1G061240	3.339904	3.648904	3.365882	4.240623	5.807236	6.192272
HORVU4Hr1G054880	5.850793	7.3543	9.024644	3.580861	4.126747	4.680328
HORVU2Hr1G038570	0.502475	0.507371	0.673234	3.634829	5.240231	4.340744
HORVU3Hr1G021660	2.538656	2.696333	3.050756	6.721397	11.47648	11.10076
HORVU6Hr1G021570	20.6247	22.1105	22.55033	15.0866	16.82707	19.10041
HORVU2Hr1G010670	0.050409	0	0.037686	2.324416	13.69478	11.37845
HORVU2Hr1G019180	5.399088	3.912519	6.114129	17.4412	33.49151	27.42438
Hordeum_vulgare_newGene_5426	7.075847	6.700781	7.376552	6.846895	8.479455	8.680475
HORVU4Hr1G064380	25.37652	21.67783	30.78829	26.099	22.75892	25.03074

HORVU1Hr1G047710	1.931684	1.779193	2.179621	2.654323	1.18482	0.892055
HORVU7Hr1G093200	11.22844	12.55476	13.58637	10.32633	12.16964	12.62979
HORVU6Hr1G013660	3.678937	3.04838	3.470506	3.857551	3.876715	4.029081
HORVU2Hr1G076520	1.696301	1.520703	1.73753	2.357074	3.637887	3.345275
HORVU3Hr1G110990	3.672202	2.983595	4.884065	1.67784	1.933941	1.759809
Hordeum_vulgare_newGene_8615	0	0	0	6.272756	6.028324	7.826457
Hordeum_vulgare_newGene_5397	2.090215	1.842859	2.007542	2.975515	6.556501	5.409643
HORVU4Hr1G020060	1.820346	2.12273	2.792581	0.767729	0.475627	0.976345
HORVU3Hr1G076750	15.65315	14.21088	19.58435	17.08064	16.02834	18.31012
HORVU6Hr1G017570	28.25519	25.41731	26.42343	40.98819	54.92217	51.933
HORVU3Hr1G061860	8.617615	10.16201	8.431497	13.52774	14.11214	13.81046
HORVU2Hr1G063350	233.5176	202.3579	293.496	246.1301	328.0821	303.3295
HORVU6Hr1G042200	19.95504	21.06816	22.54835	16.33797	17.24546	22.21887
HORVU5Hr1G123520	3.46737	3.089324	3.645628	0.328956	0.466884	0.471775
HORVU5Hr1G086620	16.6797	8.514981	16.99724	21.46101	48.85934	53.726
HORVU1Hr1G082970	5.214883	4.318864	7.114166	4.522867	6.526101	4.900503
HORVU1Hr1G028450	12.3816	14.18334	15.66346	19.60577	21.95276	19.14049
HORVU2Hr1G020030	3.032641	0.958049	1.441212	1.479637	1.340076	1.818976
HORVU4Hr1G024290	3.301415	2.979811	4.913615	2.779954	2.590994	3.806239
Hordeum_vulgare_newGene_10530	0	0	0	0	0.096952	6.026935
HORVU0Hr1G022050	8.252798	7.624195	6.43269	7.189578	8.994674	9.469365
HORVU2Hr1G048680	7.455216	7.319593	7.264038	16.80654	20.68507	20.77284
HORVU2Hr1G073320	34.10752	41.12634	57.01098	27.87912	64.19754	64.82959
HORVU2Hr1G029070	0.110405	0.041574	0.137163	0.466394	14.03979	17.85466
HORVU5Hr1G049810	5.096041	6.732953	6.905394	6.340848	6.447093	6.193395
HORVU7Hr1G087270	6.517737	6.682221	6.972195	6.319103	6.587717	7.668538
HORVU7Hr1G020720	10.25636	8.973859	14.16061	8.003368	9.489055	9.718275
HORVU5Hr1G073970	20.48395	17.98518	22.17446	18.23366	20.43571	21.17983
HORVU6Hr1G025980	16.75196	16.34352	17.05005	20.34839	25.97491	25.72558
HORVU7Hr1G073370	1.220813	1.221116	1.14097	0.952898	1.002855	1.482348
HORVU1Hr1G025660	8.135109	8.218105	10.49942	11.16608	12.20278	13.24981
HORVU2Hr1G011690	2.831218	2.293234	3.115033	1.788969	1.08377	0.907068
Hordeum_vulgare_newGene_1898	1.77187	1.472964	1.786391	6.158011	5.656291	5.816269
Hordeum_vulgare_newGene_1895	4.576392	4.027177	4.726497	2.521732	3.518413	4.172682
Hordeum_vulgare_newGene_1890	2.161397	0.933632	1.582601	2.287339	1.90094	3.075336
HORVU3Hr1G108610	14.70029	17.31139	16.88789	26.50647	25.01915	25.79439
HORVU7Hr1G009700	2.30587	1.952146	2.410309	5.676264	10.15256	11.57791
HORVU2Hr1G069050	3.597321	2.998249	3.612556	3.619712	5.288342	5.437834
Hordeum_vulgare_newGene_11662	10.70637	13.46068	12.14593	17.12504	17.59959	21.51348
HORVU3Hr1G075470	4.336977	4.051183	6.275217	2.330822	2.004927	1.918938
HORVU1Hr1G052150	3.619544	3.301586	3.886107	4.378343	8.204664	7.462956
HORVU7Hr1G063300	3.522421	3.294787	3.909618	6.098474	6.031335	5.384496
HORVU6Hr1G080190	4.234021	3.45999	4.729125	3.606014	4.262235	4.608939
HORVU7Hr1G032080	3.080403	3.445347	4.495175	2.624192	2.370309	2.381165
HORVU5Hr1G046590	9.397076	7.330287	7.258547	8.380499	6.198082	9.073509
HORVU3Hr1G088200	6.010106	4.515065	6.896096	4.614315	2.726891	4.65667
HORVU1Hr1G084370	7.437544	7.091168	9.242482	10.08063	23.4792	23.4542

HORVU7Hr1G087490	7.740863	7.940519	8.789114	6.191123	6.236511	6.292258
HORVU2Hr1G103800	0.996396	1.595596	1.470373	1.413871	1.983905	1.734015
HORVU6Hr1G063510	23.89965	28.18711	18.95911	6.067755	1.727527	3.956913
HORVU7Hr1G035680	7.133208	5.701525	8.002026	5.628408	5.896951	5.940621
HORVU7Hr1G078500	17.4989	15.95729	19.02334	14.69879	20.63356	20.01514
HORVU7Hr1G031190	1.611292	2.00614	2.497394	2.349609	3.669284	4.983151
HORVU3Hr1G089550	0.493615	0.502153	0.76729	4.347655	9.65866	8.214071
HORVU7Hr1G021180	4.123634	3.511463	4.485897	3.797155	5.944252	6.069408
HORVU2Hr1G071890	195.6133	242.8653	176.3991	11.42989	0.242692	4.50727
HORVU7Hr1G083030	13.0354	12.33075	18.30813	8.656826	10.70096	10.41987
Hordeum_vulgare_newGene_7131	2.197197	1.290296	1.63288	0.76568	0.552093	1.423966
Hordeum_vulgare_newGene_2771	162.3497	202.7624	144.5207	250.7498	184.4266	215.1375
Hordeum_vulgare_newGene_7136	5.345416	5.394732	6.968132	7.66137	7.705816	9.810103
Hordeum_vulgare_newGene_12364	1.922329	2.191421	1.650807	2.761679	3.043344	3.168472
HORVU2Hr1G117790	1.407749	1.005531	1.327936	1.435218	1.021696	1.451686
HORVU2Hr1G112080	0.083392	0.140794	0.085163	1.719348	4.357449	3.594265
Hordeum_vulgare_newGene_2956	4.320511	4.5484	4.105636	5.195644	7.256674	6.856147
Hordeum_vulgare_newGene_2957	13.18862	11.77204	17.0066	0.044904	0	0
HORVU2Hr1G018440	4.149269	7.542136	12.52955	4.234462	0	2.097787
HORVU1Hr1G050740	20.94017	18.69092	26.53354	22.14774	40.77673	40.7238
HORVU1Hr1G045320	1.105019	0.822424	1.164539	1.134761	0.898149	1.468596
HORVU2Hr1G068090	3.049199	3.516234	3.461351	2.999975	2.419386	3.943474
HORVU7Hr1G003880	1.359801	1.611098	1.817621	3.144356	6.709989	5.892486
HORVU5Hr1G068810	18.36166	20.01266	20.2454	20.93571	33.31902	30.14339
Hordeum_vulgare_newGene_12585	1.90822	1.223293	2.239201	4.433165	4.725947	4.363269
HORVU3Hr1G017060	1.889936	1.497387	1.587626	1.699443	2.324158	2.597389
HORVU7Hr1G035020	6021.25	6773.847	5865.008	6406.072	7509.646	5744.404
HORVU4Hr1G037480	0.612066	0.833985	1.544026	1.132784	2.030484	2.231042
Hordeum_vulgare_newGene_14409	0.568734	0.960839	0.91511	2.810866	3.364448	2.271649
HORVU1Hr1G059960	61.21255	57.05753	75.81407	73.66327	108.5116	106.691
HORVU5Hr1G065680	15.44078	13.62203	17.95682	20.65036	26.22141	24.13734
Hordeum_vulgare_newGene_15754	0.890237	1.704706	1.062287	2.30668	1.838532	1.654954
HORVU6Hr1G075440	7.138918	8.450072	9.124374	7.396648	8.352986	9.080262
Hordeum_vulgare_newGene_10985	69.62277	59.88094	78.17053	38.31783	50.54242	44.2733
Hordeum_vulgare_newGene_13186	8.680633	9.020972	9.15156	8.519409	12.78619	13.90168
Hordeum_vulgare_newGene_13187	1.552016	1.740285	1.526991	1.924602	1.948011	1.902169
HORVU7Hr1G118340	21.38305	17.79102	20.30016	25.13868	18.05724	21.2413
HORVU7Hr1G042300	4.807792	4.931975	6.913433	1.332046	0.509309	0.886014
Hordeum_vulgare_newGene_13189	7.349161	2.092065	6.179057	5.485076	5.008142	5.076512
HORVU5Hr1G084390	9.953848	7.215464	10.62038	9.183299	10.60541	10.59856
HORVU7Hr1G027800	0.584898	0.68766	0.919457	1.258979	1.463394	1.465537
HORVU6Hr1G069110	5.01286	5.407735	7.343814	3.56753	6.460407	7.346773
HORVU2Hr1G126290	0.679999	0.775389	0.584927	1.449423	1.271846	1.850818
HORVU1Hr1G050340	5.654568	5.656539	6.462239	7.060096	8.455176	9.467248
HORVU5Hr1G065350	25.59506	22.84362	25.76354	10.93538	6.884053	8.415484
Hordeum_vulgare_newGene_5988	29.9143	34.27787	27.64187	34.13102	34.36992	39.06117
HORVU2Hr1G091010	4.711475	3.867112	5.333562	4.340306	4.826673	4.345107

Hordeum_vulgare_newGene_5984	1.068687	1.107567	1.076038	1.423919	1.944978	1.651671
Hordeum_vulgare_newGene_5981	2.443163	2.377782	3.465246	0.51227	0.386605	0.744076
Hordeum_vulgare_newGene_5982	7.30616	7.080571	8.02223	9.930138	14.78961	13.57842
HORVU7Hr1G118690	4.290091	3.326539	4.394894	4.55408	6.866683	6.060321
HORVU2Hr1G018750	0.045049	0.050391	0.133403	2.052887	36.44221	25.0455
HORVU2Hr1G086020	1.531245	2.362798	2.900388	2.416193	3.301292	3.341683
HORVU5Hr1G089620	6.088557	5.775802	8.111821	7.65799	8.260583	10.29654
Hordeum_vulgare_newGene_5200	2.733616	2.820859	2.558661	2.793348	3.288251	4.373271
HORVU4Hr1G071570	8.615673	8.900559	8.281482	0.581521	0.335898	0.369448
HORVU1Hr1G054110	0.588647	0.875993	1.023644	8.879144	15.07569	17.99175
Hordeum_vulgare_newGene_814	64.66519	54.63802	71.19385	82.05766	78.17436	79.34582
Hordeum_vulgare_newGene_815	3.61527	5.267454	5.882135	1.47826	0.492715	1.161245
HORVU2Hr1G119970	3.784199	2.572145	3.607115	4.614868	7.075635	7.068784
HORVU6Hr1G032200	14.92118	12.69539	14.88311	14.94592	11.44119	9.952981
HORVU7Hr1G034640	2.004373	1.548343	1.607945	0.913655	1.160313	0.994141
HORVU7Hr1G033230	591.325	464.1262	600.7881	398.2791	1487.882	1459.453
HORVU0Hr1G027310	0.078778	0.244258	0.18612	1.71152	3.237032	2.992069
HORVU2Hr1G004600	12.84716	8.17977	16.54476	0.913184	0.23347	1.190159
HORVU5Hr1G092900	18.97611	19.69985	21.71881	23.39346	24.21658	25.74639
HORVU6Hr1G068640	8.949501	9.742789	10.51889	6.802261	9.560727	8.856976
HORVU5Hr1G102070	4.245589	2.763678	3.957234	0.344343	8.620128	1.556014
HORVU3Hr1G053250	5.380254	5.513597	6.6605	9.798866	9.889593	10.53161
HORVU7Hr1G117780	13.38348	13.80151	15.09356	15.54977	18.21282	17.61852
HORVU2Hr1G003370	1.37881	1.140081	0.824805	0.78886	2.053494	2.165482
HORVU7Hr1G060620	5.81002	6.715056	6.712637	6.975396	9.396149	8.649379
HORVU4Hr1G089540	1.720976	1.451496	2.096603	4.261512	7.930563	7.232972
HORVU3Hr1G014770	3.418922	3.44733	3.499884	4.49569	4.269309	4.540603
HORVU2Hr1G058860	22.15262	21.22842	24.47435	18.78995	23.40951	25.96196
HORVU2Hr1G047850	14.68124	13.452	15.51722	15.42741	19.40295	22.28209
Hordeum_vulgare_newGene_4320	9.991163	11.05412	12.22787	7.597593	8.270523	9.121052
HORVU5Hr1G054350	11.0118	11.19597	14.63721	14.66741	17.1358	17.81936
Hordeum_vulgare_newGene_4324	3.245219	3.532994	1.622176	1.627707	2.131429	1.711556
Hordeum_vulgare_newGene_4328	5.537846	6.485755	5.484929	6.4645	5.517507	6.295176
HORVU7Hr1G050870	5.478769	8.046632	10.66932	8.407487	10.55266	10.82522
HORVU5Hr1G050610	10.34315	10.65765	12.50045	23.21216	38.92715	32.66244
HORVU2Hr1G127700	2.194278	2.036718	1.691615	3.123591	4.070123	3.497256
HORVU2Hr1G034230	2.789171	1.674705	0.617853	1.895683	2.658265	2.478519
HORVU5Hr1G108610	25.80748	19.84006	28.03775	29.43408	36.13711	37.04974
Hordeum_vulgare_newGene_1668	0	0	0	4.157913	2.589756	3.019831
HORVU5Hr1G063010	1.199134	2.02989	1.412424	1.230394	1.432331	2.092689
HORVU2Hr1G046180	1.410948	1.320438	1.429689	1.127202	1.217536	1.795862
Hordeum_vulgare_newGene_1661	2.572521	3.217862	3.870079	3.410591	3.280255	4.238483
HORVU6Hr1G003550	1.511546	1.348755	1.892484	1.400194	1.03838	1.315349
HORVU2Hr1G080460	16.49416	16.00158	18.3583	21.0066	41.86199	31.19934
HORVU6Hr1G031530	2.844751	2.320572	4.619824	1.917698	2.316054	2.547621
HORVU7Hr1G114250	0.841569	0.527883	0.959898	1.293469	1.927016	1.548281
HORVU3Hr1G049960	63.98517	60.89447	67.42685	62.58367	75.3878	77.14709

HORVU3Hr1G033250	4.592877	4.866058	3.981495	5.868783	6.585685	6.518311
Hordeum_vulgare_newGene_6105	9.326786	7.445315	7.712117	7.915973	10.49737	9.354208
Hordeum_vulgare_newGene_11019	2.677859	2.71889	2.752594	2.955023	3.201466	3.487112
HORVU7Hr1G115780	9.531333	9.984527	12.47143	7.311018	12.76993	14.23617
HORVU1Hr1G072730	30.79962	28.48615	32.79226	26.63042	26.53963	26.97089
HORVU7Hr1G088260	55.54477	51.74954	56.43314	42.98793	8.804087	18.59399
HORVU2Hr1G017120	1.498508	1.361904	2.50221	3.459478	4.758716	5.260179
HORVU5Hr1G059470	8.902555	11.63164	7.865214	7.128608	6.756165	7.174981
HORVU7Hr1G019680	0.162962	0.241482	0.195345	2.365446	6.994953	6.593908
HORVU2Hr1G079300	10.38954	10.35835	11.16886	14.05875	12.93075	13.30803
HORVU4Hr1G049990	39.63168	38.33068	37.94231	52.22617	25.50407	29.58625
Hordeum_vulgare_newGene_7678	1.923195	1.736452	2.229833	1.256805	2.451009	1.204848
Hordeum_vulgare_newGene_7674	4.80873	4.409911	4.001639	4.303543	4.111886	4.106513
Hordeum_vulgare_newGene_7671	0.532649	0.53941	0.539415	3.46438	8.178654	7.424758
HORVU1Hr1G073820	2.214673	1.994028	2.149926	2.639593	0.982194	0.904257
HORVU7Hr1G024210	1.824667	1.509218	1.506262	2.97789	3.419491	3.33489
HORVU3Hr1G001020	0.050502	0.037423	0.060151	4.269591	7.236625	14.76498
HORVU7Hr1G098670	0.181423	0.242587	0.510888	2.370442	2.032408	1.380485
HORVU3Hr1G070690	7.288772	9.152063	8.403313	10.45263	19.42648	14.2191
HORVU1Hr1G064020	0.754119	1.01516	2.281504	4.017223	1.064738	2.138774
HORVU3Hr1G070340	22.81205	18.57229	22.24386	26.83663	24.33248	25.02065
HORVU6Hr1G074180	0.101096	0.179239	0.184445	1.295556	2.808736	2.969719
HORVU4Hr1G073060	10.54317	10.3082	10.28833	10.6798	8.382225	10.27898
HORVU3Hr1G039980	26.50402	27.96372	31.58767	35.74461	50.70286	50.93089
HORVU3Hr1G040710	6.553385	7.229229	9.078585	9.778472	14.79481	15.1004
HORVU7Hr1G115120	15.76503	18.34034	18.43467	15.08429	19.9519	19.24996
HORVU1Hr1G079860	6.83879	6.805898	7.393157	5.533514	4.028694	4.801945
HORVU7Hr1G056700	0.625416	0.590002	0.694669	2.119343	3.142636	4.17661
HORVU3Hr1G086500	31.60578	34.30724	35.93944	70.98085	112.765	73.15778
HORVU2Hr1G086920	1.552057	1.779192	2.046896	1.133394	1.660895	1.403777
HORVU7Hr1G025160	0.245367	0.174746	0.487838	4.805574	23.72172	22.38062
HORVU3Hr1G006270	5.322246	4.970394	6.017637	10.66009	10.54174	10.83222
HORVU3Hr1G079540	33.96585	29.36785	47.80064	52.81676	22.80808	24.96912
HORVU6Hr1G002550	1.113501	1.773711	1.848843	3.475125	2.364229	3.198758
HORVU4Hr1G056870	30.34944	46.42831	44.44346	0	0	0
HORVU1Hr1G080780	2.514235	2.392981	3.382748	3.572936	1.28934	2.238893
HORVU6Hr1G094950	1.384944	1.768556	1.422723	2.303623	3.958001	2.679764
HORVU5Hr1G095530	10.31419	8.938396	8.256594	7.331936	6.493773	6.821799
HORVU5Hr1G125570	3.205474	3.073216	2.768473	0.111874	0	0.103678
HORVU7Hr1G068630	44.86364	56.34753	88.54251	37.61586	64.32572	83.35976
HORVU1Hr1G069210	14.98896	14.36661	17.97235	18.04336	22.30504	22.18428
HORVU7Hr1G018220	2.452971	3.092596	3.469539	3.865694	4.583225	4.45528
HORVU1Hr1G088060	1.390506	1.031755	17.76306	45.63612	0.234176	0.538179
HORVU3Hr1G038290	4.273769	3.187399	3.862079	9.115084	11.21745	9.830232
HORVU7Hr1G051280	19.10589	16.81734	19.20928	18.30039	18.30103	18.02967
HORVU1Hr1G055320	0	0	0.008682	25.58744	19.35622	24.26986
HORVU5Hr1G093270	3.353389	3.902876	4.498059	1.803253	1.288186	1.45351

HORVU5Hr1G002140	0.538235	1.351581	1.208365	1.72435	3.355227	3.438185
HORVU7Hr1G011220	35.62328	32.32721	44.22342	33.32012	57.47971	53.16507
HORVU0Hr1G022170	0.28846	0.187965	0.157986	5.893945	88.9747	87.07952
HORVU3Hr1G082080	5.936347	6.406456	6.429416	15.60508	13.56211	13.03476
HORVU1Hr1G066460	6.808148	5.739709	7.88703	6.452604	12.55496	9.920506
HORVU7Hr1G120340	3.141228	2.576613	2.289854	6.328231	3.613624	5.329033
HORVU2Hr1G115240	0.106454	0.040526	0.050676	3.619226	1.206494	1.276877
HORVU1Hr1G071580	11.84988	11.47879	10.15236	14.12689	15.91825	16.49553
HORVU2Hr1G078710	11.84608	13.22741	12.92151	15.83029	23.81025	23.62707
HORVU4Hr1G068390	1.678062	2.20286	1.766611	3.325818	1.654739	2.021374
HORVU2Hr1G017900	26.22798	25.11604	33.98128	14.34809	11.17905	11.45585
HORVU7Hr1G048020	0.472101	0.574026	0.645642	1.857996	2.047919	2.329731
HORVU1Hr1G093620	0	0	0	0	26.06364	14.32404
HORVU7Hr1G121350	66.3617	85.90693	59.26227	52.42968	8.764524	20.20012
HORVU4Hr1G069660	14.28844	12.92091	15.0474	19.36707	22.80168	22.19684
HORVU6Hr1G034730	5.634934	0.902911	7.225031	4.073093	1.58152	1.561004
HORVU5Hr1G076700	68.52254	73.10129	99.70475	69.88965	144.8719	149.7075
HORVU7Hr1G094890	2.134753	2.844238	3.429119	1.939959	0.36663	1.172924
HORVU4Hr1G080420	7.904294	7.160666	7.554448	10.53108	10.5245	10.32352
HORVU1Hr1G024060	6.762602	11.66225	12.58297	5.8127	8.145421	7.299593
HORVU6Hr1G084430	9.469721	9.45753	9.857491	7.194563	4.748001	5.050635
HORVU4Hr1G053180	21.23592	18.37308	23.12232	13.83367	14.07532	14.85096
HORVU3Hr1G038830	19.46197	22.96203	25.12149	32.92251	50.78021	49.96455
HORVU6Hr1G011890	2.159715	1.957946	2.112044	2.408488	2.65139	2.538998
HORVU7Hr1G051860	12.66247	12.80026	10.22226	8.336182	7.073668	8.065083
HORVU3Hr1G057780	4.354078	3.766831	3.185548	1.770121	0	0.197382
HORVU5Hr1G051200	4.327998	4.296406	4.650005	7.122077	9.017143	9.339878
HORVU4Hr1G049150	136.455	163.3646	250.708	70.09817	96.25635	97.57623
HORVU5Hr1G011730	31.01361	28.20011	17.42675	6.727332	5.034314	4.634407
HORVU6Hr1G023100	0.867089	1.043382	1.088612	1.204736	2.247347	1.817033
HORVU2Hr1G097800	6.79598	8.017811	11.91805	21.19734	20.98173	18.32911
HORVU2Hr1G085360	0.465742	0.712184	0.923106	1.131194	3.964823	3.721586
HORVU3Hr1G075100	1.16731	1.749304	2.121979	1.503347	3.187847	3.248558
HORVU4Hr1G068530	16.56376	15.13914	23.67103	15.60631	16.14499	16.65727
HORVU3Hr1G066880	1.786303	1.368248	1.717112	2.217197	1.6581	1.467497
HORVU4Hr1G084420	105.8613	112.9272	182.2358	98.47485	216.3431	194.328
HORVU5Hr1G109200	2.561691	2.469137	3.202611	3.6677	5.203295	5.403409
HORVU1Hr1G063940	95.16578	79.94565	112.2554	61.1166	67.46068	70.55425
HORVU3Hr1G074850	1.540423	1.619298	1.48859	1.440819	1.993494	1.911114
HORVU5Hr1G114600	2.349594	2.991192	2.086044	3.193636	1.546244	1.548738
HORVU4Hr1G050980	2.45306	2.216934	2.922227	5.64557	7.528848	7.714735
HORVU3Hr1G099010	2.198873	2.409091	1.998715	3.400734	3.390828	3.427526
HORVU5Hr1G082890	21.04984	17.41376	22.86498	20.91653	14.59771	23.18986
HORVU6Hr1G080360	7.744981	5.147375	7.555835	10.84407	9.532161	11.36087
HORVU6Hr1G068940	2.299057	2.264321	2.080049	3.400438	2.532747	3.080779
HORVU3Hr1G025820	7.600642	7.837285	11.37056	4.948845	8.552766	8.373093
HORVU5Hr1G105050	2.787587	2.304169	2.950468	6.422233	6.923113	6.830119

HORVU4Hr1G057450	2.19472	1.75344	2.5483	4.370122	5.701795	7.196118
HORVU0Hr1G003000	3.18356	2.354173	3.655358	4.002604	2.234982	4.480786
HORVU3Hr1G034540	7.913228	8.898635	12.1473	10.52232	10.98316	12.46056
HORVU6Hr1G092920	24.88408	25.97269	19.87226	18.30901	17.44781	19.29579
HORVU3Hr1G031220	1.361743	1.769378	1.793488	2.782881	5.659444	4.629329
HORVU6Hr1G057110	3.095951	3.248216	3.334007	3.738873	5.502782	5.2869
HORVU2Hr1G095340	1.209812	1.693931	2.13555	1.626861	1.509598	1.724972
HORVU0Hr1G039910	2.749912	1.854329	2.431293	2.182581	1.624597	2.366381
Hordeum_vulgare_newGene_14492	0.578482	0.20822	0.956161	3.800842	0.374176	1.460554
HORVU5Hr1G013230	10.89221	11.15103	10.68567	9.222095	8.778454	8.769168
HORVU5Hr1G111640	0.564724	0.527705	0.609569	2.966586	5.069628	5.176612
HORVU3Hr1G024150	2.159866	2.855406	2.960907	5.339723	7.776364	8.655411
HORVU5Hr1G057520	8.779172	12.16171	16.76923	4.158329	5.229371	5.838536
HORVU2Hr1G037850	1.994386	2.375776	2.372463	2.119843	3.074953	2.513603
HORVU1Hr1G057410	40.02107	31.80775	32.19605	13.44468	10.31095	10.05995
HORVU4Hr1G078230	2.087762	2.117903	1.747417	4.266326	4.027371	4.280463
HORVU4Hr1G058600	28.30851	23.79996	32.29641	27.1583	24.83205	24.35906
Hordeum_vulgare_newGene_13342	16.09798	24.1338	32.17321	0.975148	0.067011	0.346401
Hordeum_vulgare_newGene_13344	398.9678	456.2056	550.774	97.6134	4.170382	44.8921
HORVU7Hr1G019890	22.3988	14.45349	27.08054	19.09138	10.85737	14.47857
HORVU3Hr1G090820	21.43193	19.69539	23.43507	48.69224	50.85772	44.34141
HORVU7Hr1G042540	1.171048	1.299108	1.522264	1.435126	1.017419	1.395193
HORVU6Hr1G091290	19.61505	16.27255	21.07382	16.4936	17.43251	17.03605
HORVU5Hr1G026730	17.66284	18.95967	22.47639	23.24398	32.55095	33.82913
HORVU2Hr1G116200	1.728896	0.344189	3.01118	2.865932	10.32892	9.158628
HORVU2Hr1G100080	9.387791	10.87838	8.984281	12.61032	13.28054	13.62225
HORVU3Hr1G088810	3.186296	2.134356	3.582165	2.499959	3.689776	3.686496
HORVU5Hr1G044710	3.324892	2.314311	3.565744	3.262103	3.662189	2.033316
HORVU7Hr1G073550	4.483343	3.8153	4.90749	4.889984	5.641418	6.258662
Hordeum_vulgare_newGene_2748	2.710429	2.245506	3.454879	2.541008	1.879884	2.373155
HORVU4Hr1G021270	52.47667	47.16856	53.48389	63.06458	158.0423	127.8075
Hordeum_vulgare_newGene_2742	1.480797	1.266064	1.565053	1.231018	1.328888	1.372861
Hordeum_vulgare_newGene_2747	3.723009	3.259415	2.615709	12.35806	11.74563	13.39498
HORVU4Hr1G085460	1.601664	1.849941	2.02203	0.227331	0.257809	0.228314
HORVU6Hr1G012700	17.63842	18.21559	22.81812	14.51945	21.00202	22.12203
HORVU2Hr1G100330	2.423035	2.543742	2.86174	2.420588	3.53804	3.582545
HORVU3Hr1G050430	0.476938	0.859585	1.136266	3.152249	11.14626	8.741098
Hordeum_vulgare_newGene_5214	0	0	0	12.09283	0.118204	0
HORVU5Hr1G053310	3.416017	3.715095	3.872606	6.361674	10.29494	10.19552
HORVU5Hr1G092910	0.825766	1.300741	0.926183	4.826757	16.84854	17.15988
HORVU6Hr1G010650	41.91014	34.27247	50.21825	37.0322	26.01191	31.5004
HORVU1Hr1G000680	7879.225	9247.93	6646.441	2594.697	2070.654	1800.417
Hordeum_vulgare_newGene_8797	3.966254	4.257173	2.849029	1.617231	0.850798	0.793324
Hordeum_vulgare_newGene_8790	9.174192	10.93152	11.87631	9.856228	7.940724	10.33893
HORVU6Hr1G014700	6.373447	6.775411	8.37394	7.746836	9.735851	9.858655
HORVU3Hr1G111140	5.721499	8.17553	6.998744	6.717386	6.395723	7.336869
HORVU1Hr1G024070	9.312267	8.526845	9.155271	11.64043	15.5411	14.13931

HORVU5Hr1G025320	11.91258	12.28659	15.15834	13.06671	16.3096	17.43898
HORVU3Hr1G093500	0.830511	0.626249	0.876266	1.632747	2.873608	2.568959
HORVU2Hr1G023540	0.850569	0.691266	0.715652	4.009808	3.337256	7.848485
Hordeum_vulgare_newGene_11250	3.041715	3.290505	3.760792	2.742362	2.33146	3.103697
HORVU7Hr1G062770	3.891521	4.62397	5.939571	3.346864	4.807564	4.933787
HORVU2Hr1G098920	34.55063	31.65337	45.4078	30.94847	33.46322	34.95878
HORVU6Hr1G057800	6.435904	5.175995	7.038211	7.724502	11.63712	12.00518
HORVU2Hr1G111610	2.882655	3.705119	4.683356	3.369134	1.230518	2.085661
HORVU3Hr1G042440	1.396622	1.043214	1.431064	4.319066	3.97092	4.44591
Hordeum_vulgare_newGene_1464	4.605884	4.639859	4.189604	10.98191	9.411514	11.67965
HORVU3Hr1G115530	1.662349	2.052163	1.781957	0.542119	1.003787	1.011338
Hordeum_vulgare_newGene_3825	0.992527	0.281624	0.221702	4.506269	3.196456	3.756766
Hordeum_vulgare_newGene_3824	4.985738	5.142803	5.004868	8.198377	10.47744	9.274385
Hordeum_vulgare_newGene_3827	3.738322	4.216279	4.512317	5.983283	6.956134	8.281896
HORVU6Hr1G027970	7.657502	8.890376	9.238443	7.30915	8.165115	9.391697
HORVU5Hr1G049660	2.258953	0.268099	1.759413	0.41613	0.811642	0.842495
Hordeum_vulgare_newGene_10716	2.416262	2.166939	1.595474	1.524595	1.342372	1.208897
HORVU7Hr1G033520	2.318721	1.631614	2.505725	2.77477	2.019521	3.198471
HORVU7Hr1G082220	14.1954	11.61264	17.07149	12.94344	21.47607	21.32836
HORVU2Hr1G069390	8.746306	8.051581	9.100181	11.95201	15.22623	16.22394
HORVU2Hr1G034740	11.59514	8.844646	13.75007	10.82943	12.44224	11.85206
HORVU7Hr1G066450	16.69823	17.2236	16.9347	0.020961	0	0
HORVU2Hr1G032740	3.867346	4.337262	4.578579	5.895635	8.875035	9.700176
Hordeum_vulgare_newGene_12288	14.24365	13.27336	13.39631	10.73305	10.0723	10.92587
Hordeum_vulgare_newGene_12289	6.713004	5.764318	8.985613	6.499761	4.115942	3.811754
HORVU4Hr1G023340	9.173908	6.459402	9.593627	6.883981	6.507864	5.777631
Hordeum_vulgare_newGene_4658	0.978798	1.736372	2.398337	0.411822	2.053392	1.861308
HORVU7Hr1G101140	1.785046	1.00894	1.116995	7.786658	5.019777	7.01375
Hordeum_vulgare_newGene_4650	5.131551	4.912749	4.392447	3.409267	3.691664	4.934445
Hordeum_vulgare_newGene_4657	1.138812	1.26598	1.754878	0.946512	0.826258	0.787104
Hordeum_vulgare_newGene_4655	0	0	0	2.370097	2.265032	2.734136
HORVU4Hr1G084920	50.53341	39.53897	39.51888	54.27143	57.24805	46.45319
HORVU5Hr1G099470	1.483248	0.985608	2.333152	1.034002	0.396312	0.913369
HORVU4Hr1G041570	3.760791	4.22929	5.024846	4.351674	6.455761	7.464525
HORVU6Hr1G048410	21.91292	22.32354	28.20196	18.21701	22.26802	25.12788
HORVU7Hr1G053130	1.74244	2.480196	3.011034	3.287649	3.909476	3.554663
HORVU4Hr1G047800	2.2373	2.810336	2.365848	2.313276	3.475244	4.374413
HORVU2Hr1G091870	4.680964	4.345755	5.288994	6.725747	8.108008	7.883855
HORVU7Hr1G099870	19.77919	19.21517	21.60385	9.942282	17.85713	14.03008
HORVU2Hr1G044560	0.872512	1.710841	1.698944	3.232825	6.367431	4.396171
HORVU1Hr1G081950	15.47198	14.28762	15.24558	21.51459	20.88045	20.44627
HORVU4Hr1G027030	5.717462	7.961798	10.23762	7.574522	21.85069	19.93039
HORVU6Hr1G063490	52.49762	43.12532	60.32119	63.64756	80.62028	88.72168
Hordeum_vulgare_newGene_6653	0.140263	1.297628	0.474427	0.781891	2.09329	2.252673
HORVU4Hr1G037380	1.337607	1.239135	1.579907	0.590564	0.644459	1.090096
Hordeum_vulgare_newGene_6659	2.360182	2.938409	3.625365	1.486204	2.311272	1.61625
Hordeum_vulgare_newGene_13578	1.990618	2.877876	3.381009	2.835225	4.278525	4.313662

Hordeum_vulgare_newGene_3171	3.969032	3.269643	4.306521	4.608187	4.789803	4.351252
Hordeum_vulgare_newGene_3174	3.346445	2.940559	3.487377	4.004826	4.466376	5.89985
Hordeum_vulgare_newGene_12138	4.233789	3.515753	3.907169	6.407003	8.799953	8.477303
HORVU3Hr1G091830	10.70652	9.870017	14.62484	5.790403	6.183153	5.630824
HORVU5Hr1G041530	15.75468	15.30763	15.90462	18.47345	54.67702	54.60096
Hordeum_vulgare_newGene_12135	5.595786	55.36641	44.19783	24.45162	6.042398	14.49145
HORVU6Hr1G078510	1.939431	1.481576	2.418507	1.35247	0.381295	0.348807
HORVU3Hr1G097800	0.226431	0.21533	0.031708	31.19769	166.3609	137.8274
HORVU2Hr1G032140	2.68809	4.427166	4.636003	6.159989	8.00085	5.996209
HORVU7Hr1G033530	47.17205	26.21953	94.92348	36.52942	14.60794	24.76406
HORVU4Hr1G033860	12.95017	9.485613	15.02834	10.97866	10.37135	12.20064
HORVU6Hr1G052660	9.683841	14.65594	11.80457	15.33197	14.73161	16.84008
HORVU6Hr1G065760	4.139556	2.883003	5.549455	4.832297	3.587031	4.644143
HORVU3Hr1G013350	475.483	386.2066	525.5418	663.5836	985.5888	954.1317
HORVU6Hr1G032890	0.501522	0.445682	0.685086	2.071721	3.280671	3.347051
HORVU7Hr1G111930	0	0	0	0	3.115804	5.076731
HORVU2Hr1G024780	4.784605	5.766735	6.25703	2.804867	4.442957	5.825436
Hordeum_vulgare_newGene_13661	0.192332	0.308467	0.283285	0.234451	51.349	86.52663
HORVU5Hr1G062970	15.72167	12.91309	16.79421	14.11479	14.52531	13.60929
HORVU3Hr1G110480	6.858581	8.845806	10.03737	10.42247	11.68204	11.37519
HORVU5Hr1G075200	66.30645	44.4432	49.21087	28.40021	23.51189	24.75831
HORVU3Hr1G053860	6.354136	4.854363	6.33885	11.15439	21.8633	19.23396
HORVU5Hr1G087830	6.702097	4.438291	7.942546	23.12242	50.49274	41.22696
HORVU2Hr1G075110	1.912834	2.024437	2.313256	2.732712	1.977725	2.907861
HORVU4Hr1G010650	206.2866	193.4681	232.1953	235.0761	244.923	225.526
HORVU2Hr1G058530	18.24133	17.15587	17.53636	20.10032	21.83796	21.97018
Hordeum_vulgare_newGene_2225	1.005082	1.298101	2.218983	2.265438	2.924974	2.622069
HORVU4Hr1G045980	38.36412	36.55027	34.43756	43.43638	62.45038	62.82056
HORVU3Hr1G003150	20.97994	23.28163	25.32339	18.99317	22.31016	23.45242
Hordeum_vulgare_newGene_10936	5.678397	4.540841	6.370612	0.122576	0.134492	0.182198
HORVU2Hr1G087540	3.288291	4.02617	3.722277	2.505888	2.983382	2.747099
HORVU3Hr1G113820	19.25546	16.4653	20.73143	28.77102	37.21921	38.18327
Hordeum_vulgare_newGene_15546	2.358398	0.065647	0.626644	1.6519	2.531544	1.380836
HORVU1Hr1G044750	12.5986	12.9415	13.87668	11.04591	14.60267	13.82705
HORVU3Hr1G032300	2.370116	2.148817	2.079728	2.256272	1.716451	2.669338
Hordeum_vulgare_newGene_15549	11.48321	11.71553	11.55921	11.17267	11.81645	12.26458
HORVU0Hr1G031850	4.789363	3.800116	4.788649	9.707493	13.49426	12.97735
HORVU6Hr1G034130	1.895533	2.135054	2.265989	2.708873	4.875275	3.977787
HORVU3Hr1G078960	3.199673	2.980189	2.492304	3.532338	4.927816	4.09647
HORVU6Hr1G058780	19.10197	19.7799	22.52754	24.9679	19.55935	24.50854
HORVU1Hr1G092110	1.764639	1.503866	1.9421	0.990138	2.151058	2.225863
HORVU5Hr1G061770	31.50697	28.29185	35.65989	46.76039	34.78148	51.87344
HORVU6Hr1G061450	1.716505	1.150037	1.657535	4.433387	4.114598	6.070212
HORVU4Hr1G075150	2.98171	3.416368	4.196344	2.157187	1.270892	2.072748
HORVU6Hr1G033850	12.80883	12.86759	13.57826	11.97027	13.50123	13.85148
HORVU6Hr1G009820	1.720577	1.710422	1.397312	2.81758	4.267023	3.829226
Hordeum_vulgare_newGene_8965	2.567366	0.957335	2.631794	2.324911	2.064959	2.251509

HORVU6Hr1G058250	2.611797	2.5369	1.219752	1.842708	3.575312	3.126582
HORVU4Hr1G080740	0.03295	0	0	0.180399	12.03613	20.11306
HORVU2Hr1G062330	8.685551	9.105688	9.098604	2.227739	2.118507	2.588126
HORVU2Hr1G085560	1.76443	1.416818	2.513999	0.815119	2.023825	2.03461
HORVU1Hr1G054300	24.56184	24.82751	19.78706	22.20513	24.64363	25.35324
HORVU5Hr1G097400	11.17752	9.314404	11.69446	11.2845	17.9515	17.96296
HORVU7Hr1G092300	14.56939	14.70535	17.94315	11.82376	18.64321	17.79694
HORVU4Hr1G043910	697.206	622.3131	771.8423	1083.976	2907.139	2526.239
HORVU7Hr1G106280	5.642554	6.568021	7.621149	4.404572	13.90726	14.37664
HORVU1Hr1G052930	1.476453	0.898317	1.284467	1.694738	2.120619	1.960332
HORVU6Hr1G060600	21.20617	16.63311	21.67579	22.2047	20.48189	17.70068
HORVU7Hr1G034850	47.19957	42.65087	43.22884	47.11822	53.10611	51.29695
HORVU1Hr1G067200	2.792296	3.62991	4.457075	5.316107	6.136722	5.865533
HORVU2Hr1G089440	15.47893	11.47455	21.20987	5.064668	8.420705	12.21494
HORVU2Hr1G081670	14.68874	13.05441	14.75523	11.12456	19.21571	16.01334
HORVU3Hr1G043640	7.156654	7.936247	9.383763	8.995473	13.82885	13.61451
HORVU2Hr1G067370	327.9409	362.1359	605.4716	234.3116	504.1457	463.9148
HORVU7Hr1G049200	6.081786	6.144107	6.878073	3.705227	3.61038	3.560486
HORVU0Hr1G006250	9.362965	6.682794	9.289407	9.045329	13.13294	14.68662
Hordeum_vulgare_newGene_6056	35.8979	37.23212	64.16925	27.49539	43.27788	44.1114
HORVU4Hr1G049320	1.703574	1.764539	1.948977	1.68685	3.004123	2.725652
HORVU4Hr1G071040	4.455628	4.723043	3.924345	4.540827	2.201406	2.604942
HORVU1Hr1G053080	5.166975	5.509636	5.794445	11.79606	18.67107	19.20827
HORVU4Hr1G018270	1.114938	0.904326	1.723473	1.413999	0.523777	1.148693
HORVU6Hr1G064960	2.122931	2.277091	3.979265	0.67587	2.382383	2.111385
HORVU7Hr1G113210	1.384861	1.274975	1.590154	5.028287	7.453967	6.544922
HORVU5Hr1G066810	3.489157	3.371177	3.573481	3.526978	4.53829	4.490897
HORVU1Hr1G076350	44.17784	49.06275	42.27114	29.99081	41.27803	36.37143
HORVU5Hr1G107380	0.814612	0.982399	0.606619	2.766515	6.360057	6.58626
HORVU6Hr1G009170	0.982324	0.814145	0.792598	1.190474	1.759109	1.762297
HORVU1Hr1G058480	9.193073	9.839844	11.38335	8.145678	12.83016	10.68948
HORVU1Hr1G085930	2.307878	2.687112	2.074941	3.998301	4.608339	4.897655
HORVU0Hr1G007700	1.733607	1.743739	1.832616	0.498378	0.969153	0.156516
Hordeum_vulgare_newGene_1140	0	0	0	0.258339	66.26367	45.48081
HORVU2Hr1G016440	1.44918	1.644036	1.370673	4.015945	1.616896	2.102572
HORVU6Hr1G080260	13.65016	7.436686	9.099276	5.352431	1.027533	1.46911
HORVU6Hr1G007020	14.85271	16.07111	24.81914	6.558146	7.78763	7.513701
Hordeum_vulgare_newGene_13593	4.64684	4.746718	5.413843	2.877957	4.870708	4.248451
HORVU7Hr1G095890	0.16677	0.234464	0.452312	1.404871	3.829032	4.283264
Hordeum_vulgare_newGene_4515	7.20435	6.594592	7.26147	12.39554	16.87248	17.12028
HORVU1Hr1G015040	4.721683	5.016374	6.617418	6.301846	9.752784	8.831126
HORVU2Hr1G088420	3.500857	2.370547	3.237161	6.309159	2.672039	3.710038
HORVU7Hr1G104220	166.175	180.7169	274.9956	94.20106	176.1823	171.7791
HORVU7Hr1G039900	14.19675	8.86084	14.95638	9.508762	11.90401	14.1171
HORVU3Hr1G000540	46.48247	45.5076	43.53141	45.13555	51.64733	50.88027
HORVU5Hr1G125290	13.60255	14.0736	14.04379	9.877312	8.585241	8.356092
HORVU7Hr1G085110	0.110459	0.398219	6.07059	6.272824	13.32525	13.49808

HORVU2Hr1G001690	2.291729	2.344463	2.877269	2.750855	3.068327	3.521487
HORVU1Hr1G069530	47.32107	36.82945	42.10355	47.31752	55.58971	56.2461
HORVU2Hr1G087950	23.53372	20.79716	25.22174	26.6108	38.30754	37.81394
HORVU3Hr1G076960	2.313741	2.238593	2.591181	6.634822	12.6698	11.51832
HORVU5Hr1G045020	4.350007	4.636077	4.435841	6.338981	7.33793	8.149185
HORVU5Hr1G010620	9.851282	8.517259	14.49959	8.385058	10.6022	14.07268
HORVU5Hr1G111250	1.222104	1.700352	1.278676	2.758417	2.285735	2.372153
HORVU1Hr1G078740	2.606271	2.869303	3.541127	37.20838	41.75963	36.1098
HORVU2Hr1G078840	17.34209	18.29997	19.36835	37.78929	36.67249	40.4325
HORVU5Hr1G012990	1.690279	2.658095	1.135124	1.353575	9.491651	6.045716
HORVU3Hr1G110890	63.51387	56.75717	53.4176	84.12251	73.66098	75.46852
HORVU7Hr1G024400	14.92583	15.65605	16.74258	12.93508	13.3882	14.76689
HORVU3Hr1G005990	5.150109	5.119105	5.681408	4.539264	3.147782	2.426484
HORVU4Hr1G015590	2.133842	2.326371	2.239677	2.065242	2.71201	3.447041
HORVU7Hr1G055560	52.7782	51.5278	71.21967	25.8996	30.52932	34.82866
HORVU7Hr1G090210	5.172989	5.193129	6.131167	3.90915	6.333253	5.350482
HORVU4Hr1G007420	3.468324	3.475508	2.767812	6.292823	7.949441	8.417035
HORVU4Hr1G051390	5.45516	6.877983	7.538041	0	0	0
HORVU1Hr1G083420	11.92538	12.3066	11.20374	15.61178	13.28632	12.81256
HORVU5Hr1G125430	1.392846	1.52758	1.088102	1.161206	0.697003	0.702285
HORVU5Hr1G120400	2.059529	2.193509	2.359135	1.779304	2.118713	1.893384
HORVU7Hr1G097970	5.343295	6.30544	5.445617	6.981985	5.753218	7.233543
HORVU5Hr1G012660	10.8883	11.33495	10.83295	7.665502	6.941916	8.282956
HORVU4Hr1G090110	32.75537	25.59565	37.02792	57.25244	87.64378	84.50208
HORVU4Hr1G050660	40.30707	35.47318	31.28218	18.76467	15.55842	18.40387
HORVU5Hr1G053550	3.076192	4.27574	4.822378	2.708071	3.44683	3.726744
HORVU7Hr1G043920	0.23195	0.582492	0.475586	4.001216	4.989952	5.654039
HORVU3Hr1G065770	33.38139	26.92333	37.43709	33.64973	43.52419	41.47703
HORVU3Hr1G100350	43.69971	29.32304	34.08351	16.38416	6.977373	7.553174
HORVU6Hr1G047300	7.154309	6.861908	9.586969	9.245922	13.33856	14.10006
Hordeum_vulgare_newGene_11013	12.87057	8.695094	12.61161	16.93274	33.25991	31.53174
HORVU6Hr1G088850	8.449757	7.628409	9.694789	11.31926	12.84341	13.88446
HORVU6Hr1G032120	0.940631	1.733178	1.427787	1.437882	0.861507	0.740683
HORVU5Hr1G042080	1.961413	1.439796	2.144419	5.651978	8.798221	9.159555
Hordeum_vulgare_newGene_113	2.432616	1.419898	3.226723	3.773751	2.709305	3.544652
HORVU0Hr1G012970	1.992165	0.012541	2.819358	2.852037	5.564342	5.220714
HORVU1Hr1G045070	5.771982	5.021543	6.508593	4.84997	7.441336	7.193336
HORVU3Hr1G057990	1.56282	1.800356	1.962548	1.907164	2.527854	2.432523
HORVU5Hr1G030460	1.607564	1.750358	1.83148	1.109462	0.962256	1.119786
HORVU3Hr1G028720	2.050692	2.392561	2.732812	3.37566	4.249695	4.711141
HORVU7Hr1G087600	1.221341	0.875107	1.047149	1.503147	1.005806	0.771702
HORVU3Hr1G109530	0	0	0	1.873238	2.248269	2.109797
HORVU2Hr1G098990	1.003894	1.138032	1.252543	2.16124	2.812409	3.005433
HORVU7Hr1G051120	5.169434	3.463542	4.016674	0.839695	1.407363	1.155058
HORVU2Hr1G011400	0.682617	0.879945	0.82438	2.08718	1.938874	2.10265
HORVU2Hr1G107980	4.902539	3.725537	6.089178	13.76846	18.55577	20.28185
Hordeum_vulgare_newGene_14011	1.663132	2.344383	2.564427	0	0	0

HORVU4Hr1G078800	21.87647	17.75152	24.96034	28.16878	31.98581	30.75523
HORVU3Hr1G026980	5.461176	5.434322	6.589036	4.587784	7.130976	6.378084
Hordeum_vulgare_newGene_16099	0	0.095188	0.156817	1.602665	4.354323	3.662796
Hordeum_vulgare_newGene_16098	40.34445	34.0916	30.77971	0.927664	6.965797	5.876883
HORVU7Hr1G045430	23.96367	29.51661	28.07427	27.80255	34.22878	32.66845
HORVU2Hr1G123080	13.87846	11.54156	14.96129	20.46668	25.97521	26.0861
HORVU1Hr1G093990	1.202182	0.93333	1.505537	1.632508	1.665469	2.02761
HORVU4Hr1G085940	0.649486	1.043771	1.241919	1.83715	2.447372	2.443063
HORVU4Hr1G009930	0.739244	2.64851	1.289368	1.362105	2.078032	1.426265
HORVU1Hr1G002830	1.208447	14.90715	2.835485	22.19896	3.231361	3.392117
HORVU6Hr1G093080	18.61365	19.50384	17.79518	7.028606	9.434977	9.132595
HORVU3Hr1G066450	1.004688	0.478339	0.90296	1.288547	2.547225	3.215909
HORVU3Hr1G093980	4.193659	3.302651	4.175034	4.704668	4.630587	5.437326
HORVU5Hr1G046850	6.704072	5.159317	5.648374	7.25675	3.710514	3.759947
HORVU5Hr1G109430	20.41212	19.49736	21.15792	31.36246	44.27861	42.83823
HORVU2Hr1G023940	4.20036	4.567397	5.269419	3.910688	3.701153	3.602777
HORVU5Hr1G082460	6.327783	5.500558	7.758509	10.76844	21.04662	17.61257
Hordeum_vulgare_newGene_11135	5.459188	4.161347	5.464105	7.995739	14.45578	13.48176
HORVU2Hr1G067750	282.5607	261.9644	272.7521	419.319	448.4725	311.5559
HORVU3Hr1G027580	4.258487	4.117521	3.838998	7.120468	6.673021	7.048135
HORVU4Hr1G083470	9.059159	6.035258	9.360614	10.85026	15.17414	15.66661
HORVU1Hr1G042270	45.3302	48.29063	81.58888	42.57802	84.25482	81.00629
HORVU5Hr1G083330	2933.908	2237.073	3320.432	413.6938	1.686556	145.933
HORVU6Hr1G023980	1.163013	1.169454	1.182977	0.996096	0.563439	1.217114
HORVU7Hr1G104660	1.158244	1.586475	1.63	0.792907	6.214085	5.423813
Hordeum_vulgare_newGene_12733	4.329305	3.800836	4.268369	2.0101	2.195368	2.979462
HORVU3Hr1G063660	2.563379	1.579894	2.317228	1.539558	3.010156	2.284849
Hordeum_vulgare_newGene_12735	2.357905	2.900244	2.318062	3.193697	3.617764	3.379686
HORVU7Hr1G002150	7.919967	7.141039	7.835568	7.877547	7.797911	7.852323
HORVU3Hr1G027630	2.022586	1.355711	1.749382	1.913618	0.451626	0.903501
HORVU7Hr1G084320	4.222924	3.825288	4.824298	3.179046	3.045362	2.46791
HORVU2Hr1G076910	30.15765	32.73094	44.48795	21.24696	28.82088	27.65922
Hordeum_vulgare_newGene_13062	6.091418	5.793343	5.755798	3.205891	4.037409	3.946281
HORVU3Hr1G060310	2.875686	2.427502	2.075273	3.450259	8.572849	8.875864
Hordeum_vulgare_newGene_13068	10.03583	12.09429	11.77023	7.636227	10.31672	10.634
HORVU1Hr1G017230	7.434379	6.902384	5.701935	3.680001	3.927865	4.743626
Hordeum_vulgare_newGene_3379	20.33056	18.39144	18.58662	21.93929	18.5848	19.69014
Hordeum_vulgare_newGene_3378	3.070581	3.343025	3.575521	5.907891	6.558966	7.039251
Hordeum_vulgare_newGene_3371	0.160544	0	0.213099	0.172265	12.83442	12.83836
Hordeum_vulgare_newGene_3370	0.15235	0	0.148793	0.106749	9.84921	10.10669
Hordeum_vulgare_newGene_3372	0.195518	0	0.210677	0.169244	13.01827	13.5234
HORVU2Hr1G019960	8.996779	8.221619	11.36278	7.322307	12.80378	11.76411
HORVU7Hr1G087980	15.92439	17.39213	17.61971	14.04621	14.89991	14.47371
HORVU1Hr1G056120	1.722815	1.393977	1.211016	3.823373	4.392018	4.163348
HORVU5Hr1G070230	16.88704	15.69189	18.32181	36.39681	42.79601	43.26053
HORVU4Hr1G064890	2.034369	2.887755	2.574962	1.371859	1.006613	1.475776
HORVU2Hr1G070450	12.9676	14.02295	13.08858	12.4072	11.7291	13.44954

HORVU1Hr1G047000	2.134005	1.623124	2.4855	2.735904	4.407175	3.671288
HORVU4Hr1G054390	4.501328	2.006663	2.983342	3.938164	0.049995	0.460981
HORVU7Hr1G075680	0.077408	0	0	0.249877	6.274131	5.678781
HORVU1Hr1G070730	1.569217	1.37221	2.340898	0.488808	0.33516	0.834545
HORVU5Hr1G093960	0.931602	0.765489	1.305326	1.399719	1.104315	1.513961
HORVU4Hr1G086630	6.855235	6.41893	7.633935	6.962932	10.43379	9.933697
HORVU4Hr1G022990	43.45493	45.19405	42.2179	50.03108	33.31128	37.96406
HORVU7Hr1G115540	123.7244	124.5914	108.1498	116.5918	160.2532	136.8478
HORVU5Hr1G075770	10.35597	10.83315	13.50901	13.18519	10.29175	11.60251
HORVU4Hr1G082700	25.88078	23.5673	21.51078	69.02917	94.89952	100.8443
HORVU1Hr1G003070	1.140323	9.05017	0.903643	12.44301	2.684208	1.524383
HORVU0Hr1G009550	6.789019	6.042352	7.366652	8.416604	8.199573	8.370402
HORVU0Hr1G004700	56.25236	56.12381	89.49245	39.09788	87.07775	76.59951
HORVU0Hr1G009080	6.620628	6.853962	6.530602	7.989329	7.45736	7.059662
HORVU2Hr1G123760	4.789638	6.290604	5.228141	9.058395	22.70725	20.80261
HORVU7Hr1G073450	0.644023	0.859084	0.857501	1.819687	5.302654	4.733071
HORVU2Hr1G096230	1.006916	0.498719	1.116595	1.531616	2.993347	2.142982
HORVU3Hr1G051190	8.419629	7.941081	7.304621	12.32305	16.84518	16.65099
HORVU5Hr1G080170	63.94307	88.38197	124.7354	23.3666	36.1481	36.91647
HORVU7Hr1G066930	18.50727	20.20571	19.72695	21.60881	29.11221	29.16952
HORVU6Hr1G013350	0	0.013469	0	2.534622	2.966994	5.418885
HORVU2Hr1G101760	5.397984	4.865731	6.184638	6.368821	8.794098	8.019151
HORVU6Hr1G079150	33.97591	31.06064	48.36748	83.68792	75.54931	67.73509
HORVU2Hr1G084050	1.347484	2.051092	1.674322	1.82964	2.757881	2.518551
HORVU7Hr1G108410	2.15212	1.192929	1.871519	3.646807	2.768103	4.061385
Hordeum_vulgare_newGene_10003	1.764906	0.654669	1.850068	1.230716	3.461052	1.347885
HORVU4Hr1G075390	6.395094	6.653338	8.375448	6.524415	7.156631	7.377613
HORVU3Hr1G059290	13.30616	12.75035	24.51408	28.63099	29.77842	31.81098
Hordeum_vulgare_newGene_10005	3.081159	2.23694	3.112523	0.696611	8.087608	5.590343
HORVU3Hr1G011950	2.664376	2.412999	4.373126	3.669361	5.618675	5.395645
HORVU3Hr1G065230	1.531491	1.489505	1.932364	1.154344	0.851566	1.142272
Hordeum_vulgare_newGene_15035	0.910921	1.099697	1.572803	1.278281	1.48188	1.437821
Hordeum_vulgare_newGene_15036	20.14973	18.71098	21.52514	19.49946	23.84553	24.04251
Hordeum_vulgare_newGene_15031	0.323866	1.416759	1.446797	1.942107	2.171514	2.293088
HORVU1Hr1G000520	81.14265	76.46343	62.81683	22.99656	28.00226	28.57552
HORVU5Hr1G116720	2.214206	2.737208	2.502215	1.423614	1.725721	1.5299
Hordeum_vulgare_newGene_5006	2.595527	2.856005	2.71298	2.439023	2.202364	2.691692
Hordeum_vulgare_newGene_5002	0	0	0	2.44087	2.581266	3.229324
HORVU7Hr1G089240	6.87989	5.952053	6.958465	4.639453	3.248808	3.597454
HORVU7Hr1G026770	15.14996	13.06934	16.81709	10.13117	7.186343	8.252939
HORVU1Hr1G077170	1.06746	0.96316	1.227327	1.839529	4.597899	3.466491
HORVU6Hr1G060040	3.626687	3.498612	4.669781	3.66109	5.528427	5.419892
HORVU6Hr1G016200	4.223121	3.67564	4.299821	5.448917	6.676788	6.381438
Hordeum_vulgare_newGene_11689	1.271254	2.360446	1.823994	0.779965	1.195097	1.431974
HORVU1Hr1G030710	4.534235	4.07038	4.953984	9.81168	8.435811	8.617365
HORVU6Hr1G058310	6.278089	6.281152	6.853838	6.265858	6.831526	7.167302
HORVU6Hr1G014510	5.053208	5.054235	5.174437	7.441689	8.66996	9.107933

HORVU4Hr1G089910	1.420571	1.211579	1.73125	6.035412	8.456331	7.618974
HORVU7Hr1G106510	0.466985	0.593499	0.4616	2.156319	2.425109	2.662757
Hordeum_vulgare_newGene_13890	1.888031	1.989306	1.735357	2.74318	3.431025	3.676008
HORVU5Hr1G117450	13.252	12.48617	15.73946	10.47984	9.992996	10.3669
HORVU1Hr1G035290	0.431853	0.314045	0.556682	2.142844	3.428842	2.717492
HORVU5Hr1G104240	116.4598	76.61402	94.30656	3.230555	1.277007	1.553603
Hordeum_vulgare_newGene_13677	0.243046	0.374918	0.341579	1.410846	5.526828	5.702343
HORVU2Hr1G049640	3.916479	6.289253	6.496165	7.109792	12.82259	13.20357
HORVU7Hr1G036210	1.133403	1.043882	1.014018	2.18258	2.68753	3.596597
HORVU2Hr1G035310	45.23888	55.28503	48.68529	18.18974	20.26274	21.38086
HORVU6Hr1G077440	3.239068	4.42798	5.64849	9.634171	34.42878	26.72435
HORVU3Hr1G094890	8.094065	8.273909	8.249186	7.011569	7.016776	8.088389
Hordeum_vulgare_newGene_1786	0	0	0.034966	17.00216	16.21094	19.69174
HORVU2Hr1G022260	11.27292	11.44686	17.32188	14.3072	10.96919	10.83472
HORVU4Hr1G076150	26.58233	23.29978	29.36264	22.19547	27.42951	25.92741
HORVU5Hr1G107330	3.420718	3.026591	3.120544	4.292	3.932894	4.076462
HORVU1Hr1G028030	40.28861	35.7902	40.54639	54.05378	25.3833	35.1112
HORVU1Hr1G071810	4.029068	3.219561	3.367912	5.065894	6.926028	6.726318
HORVU7Hr1G115370	24.84416	24.35241	26.02597	26.27459	29.79568	23.32925
HORVU1Hr1G076620	6.776494	6.663827	8.851556	6.131007	8.008223	7.780836
HORVU2Hr1G028500	0.594888	0.473211	0.588944	0.490869	4.708338	5.086864
HORVU2Hr1G048510	2.475666	2.832596	1.866661	6.422925	12.60508	11.21049
HORVU0Hr1G039620	3.156443	4.476988	4.31012	2.584691	1.649934	2.463117
HORVU3Hr1G068500	19.18933	18.66853	27.20222	19.07676	27.84338	30.42123
Hordeum_vulgare_newGene_7403	0.62571	1.281599	1.49512	0.825633	0.752534	1.304216
HORVU5Hr1G015670	5.42074	4.055977	3.244249	2.682052	15.98471	10.0498
Hordeum_vulgare_newGene_7405	6.599948	6.497038	10.53095	8.101679	10.9353	14.40709
HORVU1Hr1G024550	0	0	0	8.216133	11.61908	12.08934
HORVU7Hr1G025330	0.041857	0	0.197355	1.859904	7.189423	7.78031
HORVU7Hr1G105270	1.559156	1.563702	1.528694	1.359766	1.294981	1.684963
HORVU2Hr1G004890	2.019648	0.906649	1.333979	4.980584	5.667729	6.721208
Hordeum_vulgare_newGene_15163	1.61247	1.257146	1.273623	2.822685	3.796677	3.781288
HORVU7Hr1G053070	4.104478	4.552274	6.248348	2.213175	2.169251	2.339089
HORVU7Hr1G001340	7.781746	8.03861	8.386493	7.811193	8.153332	8.809962
HORVU3Hr1G010280	0	0	0	0.347918	10.76117	8.812929
HORVU5Hr1G037600	15.3697	15.64965	16.90874	27.01454	32.41242	30.82503
HORVU7Hr1G032240	8.378161	8.985839	9.705942	11.61401	15.35354	14.69725
HORVU6Hr1G073370	56.24283	45.57026	60.58728	36.99762	31.91698	36.91844
HORVU5Hr1G033110	1.92728	2.035347	1.847567	3.270059	2.844937	3.834987
Hordeum_vulgare_newGene_13655	11.26209	10.47457	10.92051	9.611852	8.481322	9.517447
HORVU5Hr1G087800	1.238463	1.13366	2.656065	0.570996	0.675036	0.593636
HORVU0Hr1G030460	13.67166	11.04955	14.63062	8.800115	10.05829	10.75549
HORVU5Hr1G103330	125.2132	123.8381	198.839	100.7664	202.8394	201.3051
HORVU3Hr1G034780	18.16828	19.35438	18.8687	21.74201	29.25675	30.3502
HORVU3Hr1G080410	0.66888	0.398456	0.510354	0.967831	2.194448	1.445475
Hordeum_vulgare_newGene_12074	1.874968	1.719887	1.95667	3.012482	2.767957	3.641216
HORVU1Hr1G069080	7.329624	3.416193	7.818084	6.062157	10.92349	10.00376

Hordeum_vulgare_newGene_12076	3.181654	2.562029	3.173041	2.355512	2.138131	2.447478
Hordeum_vulgare_newGene_12071	80.30471	66.23459	80.81549	61.72656	96.18588	87.89863
Hordeum_vulgare_newGene_6756	0.425522	0.606573	0.312038	2.051272	6.355086	5.099942
Hordeum_vulgare_newGene_6757	0.507232	0.657092	0.772154	1.505334	1.448428	1.742101
Hordeum_vulgare_newGene_6751	0.52852	0.532644	0.610192	1.642695	4.894056	4.377597
Hordeum_vulgare_newGene_6753	1.651581	1.26128	1.698021	2.309212	3.628995	4.025585
HORVU7Hr1G098200	4.555237	4.452672	5.391876	6.603914	7.23328	8.067368
HORVU1Hr1G074660	85.74558	70.34248	95.63773	70.73109	90.65799	87.85328
Hordeum_vulgare_newGene_15869	2.00811	3.242414	3.512445	1.478311	1.421728	1.419362
Hordeum_vulgare_newGene_15867	0	0	0	1.998305	1.545901	2.581289
Hordeum_vulgare_newGene_15863	0.513864	0.781953	0.56195	2.403509	5.425021	3.50781
HORVU6Hr1G051500	1.169717	1.098025	1.17658	1.319557	1.498687	1.521638
HORVU6Hr1G059730	15.87723	14.91582	17.1695	26.06807	32.82636	25.91818
HORVU7Hr1G052030	0.216864	0.159415	0.381898	1.974564	2.762608	2.506984
Hordeum_vulgare_newGene_2921	2.480092	1.879671	3.047193	4.414209	3.940495	4.430084
HORVU7Hr1G035730	0.363548	0.188332	0.32549	0.152672	4.247306	2.323436
HORVU7Hr1G038520	54.4133	51.76006	72.80591	43.59908	59.5069	52.81336
Hordeum_vulgare_newGene_2929	7.351923	5.976782	8.186833	0	0.050042	0
HORVU3Hr1G001960	9.787126	6.769039	9.555574	14.56726	23.09312	22.95809
HORVU5Hr1G022360	14.66434	12.72144	13.74335	12.25696	10.34277	10.201
HORVU1Hr1G022660	6.028166	6.376169	7.950683	12.50258	15.00969	16.51033
HORVU1Hr1G074080	2.544643	0.942165	2.210415	1.023653	1.640593	1.19494
HORVU5Hr1G089780	41.89365	42.90734	53.70617	24.02151	18.84746	21.26516
HORVU4Hr1G013530	1.520361	1.894443	1.698539	5.344904	7.89779	7.615985
HORVU1Hr1G029870	29.84322	29.48277	43.18818	32.19037	71.563	66.214
Hordeum_vulgare_newGene_7560	1.075153	0	1.956136	1.365096	2.407393	1.161851
Hordeum_vulgare_newGene_9910	58.34978	50.46951	64.20045	41.89798	58.44743	56.3977
Hordeum_vulgare_newGene_9916	12.20393	10.76062	11.82827	8.674826	11.85859	8.985092
HORVU5Hr1G075430	12.09619	12.09172	12.10207	20.09126	27.8823	24.50857
HORVU3Hr1G072350	25.72313	20.22902	32.10659	36.20314	31.21086	29.00881
HORVU5Hr1G023090	5.776041	5.344987	7.174453	2.452401	2.116157	2.312326
HORVU7Hr1G017950	2.284245	2.16412	2.278116	3.182818	2.269961	2.550496
HORVU5Hr1G124090	4.106453	4.505511	5.279838	8.243365	10.63267	11.9427
Hordeum_vulgare_newGene_14024	4.020192	5.245371	19.01124	48.03001	3.465424	3.153022
Hordeum_vulgare_newGene_14026	12.87664	11.86135	34.48489	72.09098	7.915374	8.726082
Hordeum_vulgare_newGene_14027	0.535518	1.176976	5.542765	28.03962	0.763684	1.768604
Hordeum_vulgare_newGene_14020	7.672623	8.903464	27.74224	63.86079	5.386577	4.677445
Hordeum_vulgare_newGene_14021	13.27789	11.539	35.90968	76.35373	8.630901	8.751666
HORVU6Hr1G082360	80.15356	74.01687	97.47394	81.54382	28.9584	34.91173
Hordeum_vulgare_newGene_14028	1.536961	1.432132	14.60331	25.37889	0.570241	0.150247
Hordeum_vulgare_newGene_14029	1.073392	0.935944	0.920374	1.883363	1.352075	1.42143
HORVU4Hr1G003290	1.123118	1.46203	1.482367	1.385942	1.379781	2.078908
HORVU6Hr1G079450	2.134875	2.313779	2.700871	6.066964	4.435807	4.817573
HORVU4Hr1G017340	2.998606	3.347394	3.055968	8.20236	11.11555	11.04028
HORVU7Hr1G023990	12.64931	11.88233	13.93218	14.25157	15.16376	15.49172
HORVU4Hr1G046940	18.15027	23.76601	30.71469	20.97884	28.63837	27.89384
HORVU0Hr1G000630	24.20718	23.57914	21.98622	31.04526	50.72327	53.85897

HORVU6Hr1G039550	2.403905	1.968509	2.632701	2.306299	2.507376	3.300625
HORVU3Hr1G085290	20.6034	17.75108	23.03762	10.66343	7.813393	8.470083
HORVU2Hr1G059690	10.80061	13.11954	11.70714	14.65308	11.49562	13.20043
HORVU0Hr1G035340	0.561611	0.686362	1.3108	1.173828	1.926926	1.248778
HORVU6Hr1G002230	36.32228	33.37851	36.64221	41.21835	38.50363	37.97447
HORVU5Hr1G069140	2.129488	3.647359	5.266124	5.681145	4.20929	6.181437
HORVU1Hr1G082090	0	0	0.017283	61.65756	53.75425	65.35355
HORVU0Hr1G007000	1.967397	1.068294	0.965989	1.81538	1.252316	2.228347
HORVU5Hr1G039810	8.854556	22.5492	15.77439	15.79247	358.169	8.771812
HORVU6Hr1G067180	1.226527	1.011329	1.510379	1.593201	3.204539	2.619947
HORVU3Hr1G003470	2.891696	2.083403	2.955506	2.931604	3.547148	4.863369
HORVU3Hr1G015590	58.80704	55.94502	57.72465	65.00403	52.79923	55.99212
HORVU6Hr1G030170	1.093918	1.077572	1.123261	1.533763	1.582067	2.070575
HORVU3Hr1G037520	3.005735	2.865124	3.953219	3.64527	5.060151	4.065826
HORVU5Hr1G103490	0.888743	2.032382	1.476428	1.562659	1.158029	1.68301
HORVU5Hr1G106460	12.7266	10.17549	16.06104	18.50748	27.50816	26.53575
HORVU4Hr1G046380	13.97295	13.11314	15.16935	17.77165	18.36333	20.33924
HORVU1Hr1G078880	19.19508	17.4813	19.21317	15.7235	14.99671	16.23098
HORVU2Hr1G001070	1.425912	1.124317	1.625366	1.994261	0.928792	0.999941
HORVU3Hr1G032170	4.144856	3.7451	3.837191	3.636964	4.073341	3.797417
HORVU3Hr1G004290	26.37873	33.58035	41.47518	4.215867	0.028292	2.449573
HORVU3Hr1G084210	30.71604	25.40537	36.4239	35.23358	10.90744	9.748911
HORVU4Hr1G002650	230.4417	150.5716	208.9793	30.66355	12.27263	22.80996
HORVU5Hr1G101500	11.65531	10.92848	20.58181	24.43421	53.38452	40.76297
HORVU1Hr1G090410	6.691999	5.987649	7.555377	6.712729	8.760196	7.977387
HORVU2Hr1G126540	5.806838	5.449023	3.872552	3.348345	2.280893	3.509477
HORVU3Hr1G108970	2.739862	1.56158	3.104073	3.676076	2.622007	4.583708
HORVU5Hr1G066130	3.378014	2.464826	4.286821	0.865462	0.96472	1.709334
HORVU4Hr1G005920	111.2479	141.3257	151.8369	64.45096	87.53827	107.1063
HORVU1Hr1G050720	3.27541	3.207224	3.842661	3.10258	3.93435	3.102294
Hordeum_vulgare_newGene_13112	0.621369	0.831894	0.742051	3.367477	10.68757	8.592102
HORVU5Hr1G014920	4.486338	5.016264	4.841104	3.238408	3.986148	3.990849
HORVU7Hr1G041080	1.986354	2.173966	2.056937	1.904724	2.854419	3.111214
HORVU2Hr1G094180	1.625979	1.485865	1.431012	4.655948	2.937806	3.821963
HORVU2Hr1G094450	10.67997	9.041281	12.22613	10.6006	15.29968	13.98686
HORVU1Hr1G052760	7.912845	7.382691	8.587324	4.479941	5.437074	6.148191
HORVU4Hr1G032660	0	0.011907	0	4.002132	2.946643	4.014416
HORVU0Hr1G017530	1.003399	1.016227	1.117174	2.786599	5.576025	5.446647
HORVU4Hr1G088700	8.319032	10.08694	9.658312	12.41885	20.31014	18.69409
Hordeum_vulgare_newGene_15536	2.269682	1.762597	1.544348	2.42532	2.186363	2.085618
HORVU4Hr1G087700	2.192414	2.037404	2.217375	2.590531	1.402236	1.909242
HORVU4Hr1G011020	37.14016	34.2592	50.28098	40.91707	53.61323	51.09131
HORVU2Hr1G106110	4.654532	5.038724	6.719564	3.137192	3.599876	3.900685
HORVU5Hr1G097670	0.80587	0.791698	0.930486	1.489454	1.162517	1.38825
HORVU7Hr1G092530	3.825372	2.952616	3.737259	6.568953	5.90697	6.138265
HORVU5Hr1G050180	0.756915	0.966293	0.833038	2.901258	2.838784	3.376451
HORVU4Hr1G001350	80.22921	77.41082	95.26332	82.30486	103.0874	108.0719

HORVU3Hr1G077950	3.320364	2.570493	5.855693	3.708953	1.061508	4.685988
HORVU7Hr1G019990	4.933361	5.362942	6.682624	5.016405	7.225168	7.533687
HORVU2Hr1G108420	0.303472	0.173392	0.626542	3.909495	2.727303	3.70994
HORVU4Hr1G052890	5.058296	4.265882	6.386033	5.814334	11.3775	11.17808
HORVU3Hr1G041440	0	0	0	5.268004	6.004014	6.722212
HORVU6Hr1G008920	4.59804	4.584862	6.06026	9.878218	12.35571	10.29944
HORVU6Hr1G018710	62.72864	63.74586	50.91525	60.0528	41.56839	44.13996
HORVU7Hr1G088350	107.9548	71.81407	67.569	30.72454	13.67365	13.61866
HORVU7Hr1G071530	16.94509	18.87148	18.81247	16.74498	9.066645	13.74756
HORVU4Hr1G042670	5.389098	6.602132	7.288622	11.38537	10.40939	11.15253
HORVU2Hr1G112650	0.855343	1.560207	2.11451	2.566446	3.714185	2.260196
HORVU6Hr1G003910	5.068005	4.685454	6.530873	3.526647	4.154526	5.488082
HORVU5Hr1G093530	0.798249	0.514562	0.631086	1.077643	2.090727	2.11967
HORVU6Hr1G088800	19.15376	23.48152	21.09577	21.76143	24.0296	22.15314
HORVU5Hr1G010120	3.012953	4.66706	3.264308	10.42806	5.960024	13.11146
HORVU3Hr1G099360	0.158975	0.174611	0.186995	4.952518	5.979608	6.24178
HORVU4Hr1G064300	1.243629	1.589152	1.257889	1.420428	1.225424	1.299262
HORVU7Hr1G085180	22.7194	19.59544	25.37253	48.78079	89.83211	73.33493
HORVU4Hr1G053850	0.110008	0.068503	0.06391	0.295964	7.896416	13.23171
HORVU1Hr1G006950	11.46483	10.50389	13.94357	10.01278	10.26897	10.98355
HORVU3Hr1G064300	14.98979	14.76298	15.58829	17.58478	23.25625	19.93306
HORVU5Hr1G045180	16.41911	14.69449	16.91206	18.28087	21.50002	19.33442
HORVU2Hr1G113260	16.4401	14.12013	15.20667	11.95682	4.264235	5.371665
HORVU0Hr1G001510	26.05447	23.23908	34.48525	19.97714	21.09849	21.44442
HORVU3Hr1G071500	8.033639	5.898787	7.622983	5.612039	7.568382	6.457231
HORVU0Hr1G015390	2.852866	2.298234	2.25502	1.914989	5.831094	4.609499
HORVU4Hr1G082620	9.607088	9.111107	9.813641	5.55698	6.022568	6.863059
HORVU5Hr1G044540	3.513139	3.41093	3.700684	2.828449	4.290875	3.816932
Hordeum_vulgare_newGene_10249	0	0	0	3.405019	4.633302	3.558126
Hordeum_vulgare_newGene_10244	3.22168	3.06245	3.104419	4.426684	3.409425	3.824867
HORVU1Hr1G051440	5.343882	3.636497	6.587483	6.786783	6.722087	7.228355
Hordeum_vulgare_newGene_10247	0.555972	0.536968	0.797014	1.862257	2.065476	1.965236
HORVU2Hr1G089070	26.617	23.75786	39.57906	21.89825	40.56552	41.01309
HORVU3Hr1G086830	23.41561	20.7805	26.969	20.72011	27.76241	27.35726
Hordeum_vulgare_newGene_2116	3.584089	5.323445	6.675653	2.089979	1.832863	2.459256
HORVU6Hr1G093330	6.694626	8.253007	10.11681	3.746653	6.679792	6.992285
HORVU1Hr1G045790	0.705178	0.593029	0.53386	0.948359	2.151142	1.509784
HORVU7Hr1G090020	10.95947	9.987564	11.30111	11.51858	13.67252	14.17207
HORVU3Hr1G028020	9.883241	5.335927	9.821723	3.587281	1.01803	1.789193
HORVU2Hr1G122470	4.120496	3.188746	3.681541	2.676044	6.171502	5.152579
Hordeum_vulgare_newGene_11199	15.99655	14.54613	16.96499	16.91282	24.45545	22.4351
HORVU2Hr1G054930	5.549588	4.808094	5.328388	9.042101	12.09837	11.79728
HORVU3Hr1G104230	4.145042	2.219726	3.886286	2.535169	2.959532	2.56711
Hordeum_vulgare_newGene_11191	1.724185	1.686669	1.817421	1.219503	1.138648	1.699924
Hordeum_vulgare_newGene_11190	34.88925	37.18146	30.38755	33.84905	37.6932	40.24703
HORVU6Hr1G037510	1.802654	2.138239	1.906251	3.165932	3.460614	4.244238
HORVU4Hr1G055860	1.00406	1.164108	1.694881	2.376935	3.606721	3.596377

HORVU1Hr1G040560	10.69834	12.09489	12.214	17.50524	27.89659	26.99339
HORVU2Hr1G112600	9.197318	9.438183	12.33636	9.655231	14.47704	12.43508
HORVU2Hr1G090740	0.865442	0.446607	1.099385	0.649307	1.315328	1.810724
HORVU3Hr1G007380	365.7611	305.9178	488.5069	783.339	846.023	652.9808
HORVU1Hr1G004080	0	0	0	2.108773	1.662262	2.662841
HORVU5Hr1G119500	3.765966	3.979934	5.073255	0	0.041865	0.111713
HORVU6Hr1G048850	0.459416	1.020701	0.988633	1.948086	4.125938	4.316829
HORVU6Hr1G020310	35.69024	33.54608	51.09923	16.81614	12.04742	15.02016
HORVU4Hr1G024470	8.047196	6.358061	7.839894	14.06625	32.57206	33.28189
HORVU2Hr1G094840	3.002271	2.857635	3.489395	3.058783	3.710775	4.15673
HORVU4Hr1G067020	47.12042	41.2267	52.42729	42.96502	11.90487	18.44083
HORVU1Hr1G065570	1.998289	2.568564	2.252229	3.035742	4.74759	4.549641
HORVU3Hr1G029370	1.529492	1.119526	1.232364	1.370654	0.695169	1.105918
HORVU3Hr1G095070	10.9555	7.784466	8.901819	8.701553	7.41374	7.385151
HORVU7Hr1G069740	1.678511	2.232875	2.689365	3.198011	4.640602	5.158824
HORVU1Hr1G045130	34.15215	33.05293	51.73633	23.25233	31.90927	31.32824
HORVU5Hr1G029960	10.99843	4.527391	11.89576	13.33605	10.11782	13.71115
HORVU5Hr1G007770	4.771094	6.504044	6.896753	14.68156	11.99557	15.91303
HORVU4Hr1G009870	2.673885	3.291422	3.773027	2.670956	3.189013	3.329838
Hordeum_vulgare_newGene_1812	28.34459	23.45324	28.92797	24.73535	33.10104	32.31646
Hordeum_vulgare_newGene_1813	11.32261	9.189788	15.19845	18.1216	25.79691	25.09349
HORVU7Hr1G051370	1.067075	0.952279	1.170078	1.577308	1.849984	1.475618
HORVU2Hr1G011610	25.85539	31.19502	23.06968	47.3068	46.95478	38.43036
Hordeum_vulgare_newGene_1819	6.60478	7.848365	7.208993	0.478119	0.221911	0.216888
HORVU2Hr1G080580	4.664278	4.747427	3.749053	7.018839	9.471791	8.93401
HORVU5Hr1G046510	2.668892	2.58722	3.129614	2.380852	2.951418	1.054895
HORVU7Hr1G031580	1.051518	0.977151	1.100783	1.583913	1.468963	1.473996
HORVU2Hr1G072930	195.6185	200.9858	142.2552	153.0754	627.9716	708.2182
HORVU6Hr1G090010	42.75563	36.0977	44.83431	46.7967	57.66357	48.3474
HORVU6Hr1G063590	4.448542	4.208247	5.482258	3.715929	6.800334	6.566325
HORVU5Hr1G070910	7.949735	6.742866	8.433407	10.29771	14.57062	14.67984
Hordeum_vulgare_newGene_5467	120.9433	95.35169	119.5469	131.4909	231.1117	145.3879
HORVU4Hr1G084110	16.11914	15.34887	17.17946	21.38367	20.07869	18.87345
HORVU2Hr1G102130	16.38122	13.27683	22.12768	15.43887	14.28163	15.18333
HORVU5Hr1G045960	6.384903	6.106282	7.518151	11.05465	9.419406	11.01492
HORVU2Hr1G052050	11.54794	11.12704	11.18822	12.39364	14.63709	15.101
HORVU7Hr1G045600	8.481222	6.43285	6.449351	2.651525	0.229329	2.608156
HORVU0Hr1G015600	2.262278	2.560982	2.946991	3.082666	1.648674	2.476439
HORVU3Hr1G068620	0.926305	1.020916	1.273156	1.60385	1.220643	1.524518
HORVU2Hr1G037990	41.7793	37.24294	35.41476	33.77218	38.04836	36.59628
HORVU7Hr1G030530	7.995288	7.515597	8.531243	6.837412	7.23145	6.157001
HORVU5Hr1G040210	10.89902	8.755017	8.806468	8.376708	5.790167	6.829497
HORVU1Hr1G001810	0.547519	9.953193	0.974925	14.72349	1.698952	2.189429
HORVU3Hr1G059140	75.59167	64.49276	78.16347	91.49623	44.86507	56.78761
HORVU3Hr1G060430	25.12656	23.26917	27.22409	21.04179	20.60135	23.29135
Hordeum_vulgare_newGene_9129	1.794809	1.447811	1.769867	2.081157	1.306033	1.385019
HORVU1Hr1G012170	1284.557	1390.594	1060.752	249.4874	8.348733	82.7942

Hordeum_vulgare_newGene_2802	2.88027	2.766759	3.112866	4.428108	7.496303	7.896156
HORVU3Hr1G050820	2.904163	2.379535	2.336453	5.375701	5.66799	6.278709
Hordeum_vulgare_newGene_12500	14.30106	17.95345	19.52877	11.32941	14.67204	15.0479
HORVU7Hr1G047600	0.860648	2.520464	1.27271	2.913694	1.885427	2.876741
HORVU1Hr1G075840	15.3441	15.17691	19.09973	12.47324	14.00731	14.78189
HORVU0Hr1G021670	0.581664	0.59343	0.682673	3.273918	7.275393	6.757258
HORVU2Hr1G106250	20.94311	16.38958	23.05189	10.36192	14.81502	12.75476
HORVU3Hr1G017570	1.104941	1.03014	1.07696	2.776785	9.061752	7.320877
Hordeum_vulgare_newGene_13455	57.84957	86.4738	90.44986	31.96021	0.368898	12.58839
Hordeum_vulgare_newGene_3254	12.32942	11.49407	12.53252	15.31264	19.53659	19.75605
HORVU3Hr1G060290	4.338463	4.37365	4.885041	5.674046	5.30118	6.009029
HORVU5Hr1G073350	20.4808	20.61939	20.78268	54.14752	91.01876	79.14354
Hordeum_vulgare_newGene_3255	0.365126	0.582393	0.620665	1.113887	2.119739	2.153465
HORVU7Hr1G100790	5.035289	4.015149	4.822117	6.248022	4.962247	5.976653
HORVU2Hr1G068670	52.8494	47.70767	59.92209	48.60328	72.51006	74.92578
HORVU5Hr1G075630	1.297494	1.17463	4.84335	2.123546	3.499074	2.299358
Hordeum_vulgare_newGene_3504	4.332663	3.63941	2.961302	10.69952	48.80093	41.26321
Hordeum_vulgare_newGene_3503	3.563594	2.608943	3.393136	1.589653	0.749366	0.882358
HORVU6Hr1G018050	12.90464	13.43075	12.55023	18.41096	9.931188	14.78596
Hordeum_vulgare_newGene_9496	0.398198	0.584262	0.528927	0.755046	1.625559	2.361593
HORVU5Hr1G121570	8.251366	5.673121	8.81458	7.04044	8.351925	9.663352
HORVU0Hr1G023290	82.93091	97.13271	141.2652	44.53592	79.98051	107.6626
HORVU2Hr1G092680	8.542217	8.931113	9.151155	7.802048	12.02055	10.92169
HORVU7Hr1G031720	3.125233	3.79553	3.15578	4.989812	5.518901	4.885625
HORVU3Hr1G044150	1.087321	1.236373	1.583182	8.423767	3.986062	4.890508
HORVU7Hr1G003090	7.592749	6.737676	9.104289	22.54845	54.73947	52.48638
HORVU6Hr1G081180	4.585508	5.039149	4.944277	8.432052	8.981785	10.54579
HORVU2Hr1G116190	21.98175	20.47555	25.33301	16.27094	24.54003	21.13917
HORVU2Hr1G050120	2.630425	2.416722	2.600367	3.09076	2.951442	3.251557
Hordeum_vulgare_newGene_720	1.082649	0.811924	1.444073	0.774451	1.27303	0.621319
Hordeum_vulgare_newGene_724	1.354595	1.20658	1.241969	1.279322	2.110073	1.897965
Hordeum_vulgare_newGene_726	0.965658	1.224283	0.988732	1.030748	2.249336	1.901859
HORVU3Hr1G055700	6.053231	5.488515	7.247105	7.433572	11.4396	11.27351
Hordeum_vulgare_newGene_6955	17.29335	14.45887	16.12613	33.3337	39.43457	38.5226
Hordeum_vulgare_newGene_2670	0.635327	0.658769	0.277574	2.495972	3.201578	3.890245
HORVU5Hr1G062050	19.04001	11.76479	16.8144	15.45688	7.289229	6.295757
HORVU3Hr1G021140	31.63891	32.59738	32.06954	47.0495	44.06643	44.48495
HORVU4Hr1G027400	2.822059	4.096659	3.215594	5.570509	7.481465	7.374539
Hordeum_vulgare_newGene_9281	3.237437	2.5673	3.462508	3.311989	5.167997	5.759664
Hordeum_vulgare_newGene_5908	13.17202	8.494809	15.13267	14.72742	11.02395	8.70209
HORVU3Hr1G054250	7.583141	6.971619	7.084628	9.151544	12.31129	12.65042
Hordeum_vulgare_newGene_9285	12.01601	11.7941	12.59399	21.29107	26.79852	27.77981
HORVU4Hr1G077360	3.319616	2.882965	3.618599	9.765409	10.6056	10.89074
HORVU7Hr1G043030	8.477154	9.262943	12.26139	7.60697	6.031997	6.107626
HORVU3Hr1G051010	11.04994	10.18862	12.20526	13.65581	21.72235	21.3687
HORVU3Hr1G111650	22.24958	19.92326	23.51495	24.59016	30.06363	38.0403
HORVU2Hr1G033730	2.140658	3.06191	1.350731	4.774777	4.144568	4.945144

HORVU3Hr1G115880	6.743589	7.315161	8.378268	8.300908	9.320002	9.078976
HORVU4Hr1G074980	5.74536	5.316038	6.73302	8.587049	12.977	12.07443
HORVU4Hr1G070400	1.787375	1.827305	2.614432	2.614602	3.715707	3.511184
HORVU7Hr1G071710	46.65323	46.00002	41.65975	33.88597	37.79638	37.07282
HORVU4Hr1G061320	15.28475	14.91843	17.61135	14.78677	17.59123	18.48384
HORVU1Hr1G072180	2.90966	2.832731	2.757345	4.281979	3.257017	3.587176
Hordeum_vulgare_newGene_9597	3.2053	2.167168	4.429516	1.733909	1.79598	1.264156
HORVU3Hr1G117600	13.58193	9.171975	12.02299	2.869359	1.489366	6.854753
HORVU5Hr1G073580	1.776126	2.275946	2.595031	1.402542	1.444681	1.678256
HORVU7Hr1G030090	6.017695	5.524678	6.237388	11.94455	13.48506	11.49239
HORVU4Hr1G034450	4.137924	5.139489	5.146799	8.219119	8.283886	10.48214
HORVU4Hr1G083310	19.24313	20.13592	30.94686	16.52561	17.81335	16.17573
HORVU4Hr1G078650	28.21639	24.31062	22.85763	11.54904	11.67664	12.38644
HORVU6Hr1G076210	9.813913	8.486227	11.25312	17.08055	25.7031	20.35956
HORVU0Hr1G008740	49.2375	40.48659	58.92378	32.21588	35.00471	37.66132
Hordeum_vulgare_newGene_1375	1.959928	2.320037	2.165759	1.468769	2.1398	2.195781
HORVU0Hr1G030200	5.81834	6.321134	5.519631	4.709748	3.620669	4.097217
HORVU7Hr1G062240	0.864974	0.854527	1.19614	1.741695	3.691303	3.220104
HORVU3Hr1G096040	17.73845	13.98998	20.01877	24.15672	24.12708	25.87824
HORVU7Hr1G122650	24.46158	20.22916	24.82795	29.53504	33.68531	35.29365
HORVU4Hr1G075580	4.482933	5.54011	4.490076	6.511795	9.892577	9.720915
Hordeum_vulgare_newGene_9429	3.755838	4.461171	4.529908	4.17807	3.680594	4.325597
HORVU3Hr1G084800	0.163255	0.046919	0.033999	0.410671	3.770554	3.611803
Hordeum_vulgare_newGene_9427	4.935679	6.32338	9.796945	6.006755	8.316178	7.935419
HORVU6Hr1G051070	0.632219	0.449051	0.703628	2.055492	2.911288	2.711077
HORVU5Hr1G034150	5.890346	6.123371	5.428392	7.73998	9.121315	6.913639
HORVU7Hr1G058310	8.464108	8.430285	11.25157	7.843555	13.27832	12.3288
Hordeum_vulgare_newGene_9422	16.95073	9.58228	13.84818	5.123435	6.217122	7.264865
Hordeum_vulgare_newGene_9421	1.992554	1.175805	1.84587	2.883304	5.091438	4.588009
HORVU1Hr1G052560	11.92691	11.58005	14.28122	13.01233	16.57204	17.36624
HORVU3Hr1G010980	0.426931	0.539063	0.385898	1.931528	2.162957	2.704259
HORVU6Hr1G067200	2.41515	2.129344	2.788337	5.36301	16.60485	11.21977
HORVU3Hr1G049470	15.44214	13.28496	14.73535	14.60465	16.43679	17.58217
HORVU1Hr1G017460	11.01893	11.26735	11.66079	11.07752	12.7593	14.41257
HORVU5Hr1G056470	45.34583	33.46241	50.2835	29.37413	28.96774	26.0651
HORVU7Hr1G027410	3.458616	1.790317	2.600563	0.652426	0	0.441215
HORVU7Hr1G113080	1.619659	1.003709	1.90571	2.306327	0.800494	1.794283
HORVU6Hr1G020140	87.78246	73.44458	86.79868	95.60203	99.75897	117.8465
HORVU5Hr1G014800	9.068871	11.61918	9.639367	10.32583	10.48757	11.05784
HORVU5Hr1G092850	1.151866	2.084292	0.492527	2.129297	1.558113	1.485114
Hordeum_vulgare_newGene_1591	1.690957	1.920442	1.918354	2.3378	2.823981	3.233579
HORVU2Hr1G089340	0.299803	0.611313	0.968875	2.201908	1.849895	1.979434
HORVU4Hr1G088270	12.01659	12.82111	13.5083	11.69411	11.83733	12.62242
HORVU6Hr1G024710	9.150789	7.682174	9.715053	7.701572	8.219422	8.311774
HORVU2Hr1G045900	19.58883	13.63242	22.62322	21.91595	33.96348	38.36083
HORVU6Hr1G081460	89.18787	87.97644	133.0717	104.5755	133.6617	122.9649
HORVU5Hr1G108870	0.551811	0.566451	0.415081	3.684109	6.036229	5.756155

Hordeum_vulgare_newGene_67	3.22538	2.593958	2.501898	2.310281	2.589543	3.731054
HORVU3Hr1G070610	1.521445	1.073382	1.766427	1.58765	1.745852	1.686896
HORVU7Hr1G019600	0	0.031872	0.050084	3.174424	3.805657	3.816464
HORVU5Hr1G067570	41.82648	37.35368	48.89373	53.93285	99.2396	86.31157
HORVU4Hr1G018990	8.13915	9.086105	9.108713	7.301289	9.531083	9.926228
HORVU7Hr1G079640	60.40429	61.57197	71.09873	50.72414	70.86518	68.07266
Hordeum_vulgare_newGene_4948	1.093967	1.304704	1.324867	1.173589	1.323565	1.049883
Hordeum_vulgare_newGene_4946	6.612353	7.591629	7.083525	4.860866	2.90615	3.139206
HORVU3Hr1G047440	10.71442	10.42412	13.26613	12.75788	20.11943	19.86585
Hordeum_vulgare_newGene_3994	10.68594	9.107053	13.15031	38.66305	31.0596	25.63153
HORVU3Hr1G000910	10.17748	9.080981	12.66186	46.6477	80.42121	85.25585
HORVU0Hr1G001350	5.269812	2.800062	0.08828	1.041613	0.130296	0.14047
HORVU3Hr1G091320	116.519	99.72598	119.6513	85.93415	69.80091	71.54177
HORVU4Hr1G088850	6.763275	6.833817	8.088322	7.289062	8.304012	8.174809
HORVU5Hr1G061930	2.817387	2.778797	2.643027	3.584626	1.900408	2.661512
HORVU1Hr1G025940	1.931721	1.493933	1.94694	3.23142	3.585403	3.469738
HORVU7Hr1G119810	7.636762	8.409435	11.48912	10.44887	13.05539	12.55543
HORVU5Hr1G088330	3.043764	3.220296	4.072549	4.093979	5.100345	5.522148
HORVU4Hr1G072910	15.61007	10.17456	12.83763	10.1203	9.586893	12.95421
Hordeum_vulgare_newGene_1849	2.180565	3.002567	2.01903	0	0	0
HORVU3Hr1G035590	1.55213	1.286209	1.629467	1.959424	1.22401	1.784084
Hordeum_vulgare_newGene_6549	16.30005	17.27164	11.00653	6.073493	1.657762	4.463809
HORVU7Hr1G053550	5.344165	5.723896	3.542387	4.259524	20.41409	16.85226
HORVU7Hr1G043360	1.399792	1.822281	1.239579	3.248735	4.482736	4.303188
HORVU3Hr1G085630	4.418617	4.509412	5.041187	4.872568	6.173478	6.085439
HORVU2Hr1G082080	5.043053	4.932854	5.630032	8.529505	8.987404	10.19022
HORVU4Hr1G016730	3.52665	3.80972	4.601883	2.317084	5.770921	5.308835
HORVU3Hr1G079890	2.001577	2.376623	3.381732	3.614725	3.788121	4.00539
HORVU2Hr1G126690	3.136719	4.266702	4.022629	4.476456	5.937003	5.977473
HORVU7Hr1G109380	0.752129	0.77914	0.643242	3.130547	4.300669	5.121108
HORVU7Hr1G058850	26.58759	26.08824	30.70589	36.92994	66.06166	71.46304
HORVU5Hr1G022530	1.300076	1.45455	1.97957	1.261721	2.083344	1.763231
HORVU3Hr1G090050	0.655561	0.553416	0.595219	1.727878	2.656649	1.017994
HORVU1Hr1G010820	6.510392	6.458764	7.249466	5.302902	6.714339	6.734304
HORVU3Hr1G086580	0.150502	0.155215	0.665724	0.710045	6.754894	8.506946
HORVU6Hr1G073650	3.597476	4.403467	4.515677	4.345515	6.915414	7.466829
HORVU2Hr1G061910	2.987218	2.400572	2.382136	0.942905	0.784194	1.109306
HORVU7Hr1G067620	79.96028	74.32196	82.2165	289.9767	1990.926	1532.659
HORVU0Hr1G040240	0	0	0.117838	0	34.40644	30.97215
HORVU6Hr1G038520	2.289612	3.208337	2.737053	4.06085	4.129133	5.153227
HORVU6Hr1G075760	2.487079	2.299426	2.967597	2.763944	2.174871	2.89527
HORVU4Hr1G070870	1.177335	1.397955	1.114993	1.465357	1.064784	0.958446
HORVU1Hr1G080700	1.10037	1.222546	1.202953	0.975299	1.826667	1.650301
HORVU2Hr1G041590	9.272633	7.794905	10.10759	13.40518	15.81017	15.46871
HORVU0Hr1G005300	1.246151	1.679835	1.670268	5.003573	2.237786	5.559058
Hordeum_vulgare_newGene_14167	32.78288	31.08851	44.20409	31.25836	30.97742	32.85236
Hordeum_vulgare_newGene_2952	1.440855	0.939373	1.392789	0.895433	1.337435	1.258455

HORVU4Hr1G051740	0.780501	0.94448	1.120301	1.876182	2.95781	3.271772
Hordeum_vulgare_newGene_13212	2.07249	3.06818	3.009871	2.997886	2.21636	2.330969
HORVU3Hr1G078270	9.528244	7.382413	9.79919	15.98415	21.95541	21.92133
HORVU5Hr1G023660	1.853979	0.529541	1.51688	0.659008	0.366381	1.139112
HORVU6Hr1G084700	2.457436	2.423625	2.087803	2.257835	4.584763	4.418124
HORVU6Hr1G070300	4.066458	3.974279	5.257614	3.997024	6.587653	5.841777
HORVU1Hr1G061090	1.931092	2.084515	2.093832	3.681337	1.952436	3.656168
HORVU3Hr1G053300	13.92854	13.295	15.83	17.00136	22.16273	22.44097
HORVU0Hr1G036310	0	0.075894	0.157986	0.062711	55.14114	54.77175
HORVU7Hr1G069500	2.446127	2.810364	2.41696	6.081906	6.892188	6.549486
HORVU3Hr1G003390	2.834111	2.655738	3.583799	2.162749	1.946898	2.520536
HORVU3Hr1G033140	2.699185	1.577944	1.249382	3.112525	3.120155	2.872678
HORVU6Hr1G072230	11.28665	9.866359	11.06405	12.99271	24.36663	20.68848
HORVU1Hr1G056500	2.206322	2.866618	3.243381	9.552577	10.74095	11.48456
Hordeum_vulgare_newGene_9101	8.179466	8.722201	9.902699	17.57905	23.38963	29.85487
HORVU3Hr1G078850	1.633025	2.364842	2.94696	2.71776	2.193543	2.24149
HORVU1Hr1G083330	5.343431	5.36151	5.953443	8.25577	7.306832	7.321232
HORVU2Hr1G013080	0.849845	0.765034	0.630063	0.59011	2.791174	2.689424
HORVU2Hr1G027010	1.776812	1.647819	3.069217	4.883199	7.643921	5.314139
HORVU6Hr1G066920	20.23179	16.14371	22.60922	21.29359	22.598	24.1252
HORVU4Hr1G056210	0.917366	0.929238	1.100364	2.013636	2.313366	2.61617
HORVU1Hr1G005110	690.7315	525.7563	800.393	835.8835	742.0151	790.5952
HORVU7Hr1G051570	1.188694	1.141838	1.03357	2.40889	1.831196	2.717218
Hordeum_vulgare_newGene_2776	3.038306	3.524668	2.993351	2.490249	5.339628	4.323212
HORVU5Hr1G112680	2.913948	2.755542	3.464339	5.129534	7.787543	6.819567
HORVU5Hr1G015950	0.854491	0.825601	0.458069	0.429579	3.006506	2.137417
HORVU7Hr1G034560	10.73945	13.24568	16.96256	17.25279	29.77563	27.96247
HORVU3Hr1G083350	29.97648	21.32575	29.73082	45.25805	100.2788	91.62537
HORVU4Hr1G064040	0.261017	0.4044	0	4.261092	3.15773	2.72667
HORVU1Hr1G002460	1.174127	9.8853	1.945873	15.37055	0.590136	0.962814
HORVU6Hr1G032870	2.801611	3.338979	3.361548	2.324332	1.625379	2.361876
HORVU5Hr1G122180	0.556	0.525883	0.698343	3.238018	3.204553	3.049585
HORVU6Hr1G031920	4.028734	3.230992	3.592621	3.274061	3.225059	3.723548
HORVU5Hr1G094840	8.438275	8.938776	10.03485	4.712574	4.819411	5.500945
HORVU0Hr1G012390	0.062391	0.050797	0.079266	1.096118	7.742251	7.052784
HORVU6Hr1G073080	3.014081	3.31695	4.168977	8.132506	10.18484	10.45499
HORVU5Hr1G010860	0.067352	0.057106	0.150917	10.81626	21.5607	16.41941
HORVU3Hr1G018130	3.874614	4.580294	5.90157	3.162003	5.110066	5.719549
HORVU6Hr1G010490	5.394982	5.967439	6.74884	4.167999	5.122249	4.593021
Hordeum_vulgare_newGene_1340	3.782568	3.365323	3.426182	2.410949	1.471607	1.845552
HORVU1Hr1G013750	2.281261	3.582092	3.357202	3.878691	4.738893	4.714132
HORVU6Hr1G090560	31.56839	30.3527	30.81014	13.98835	8.806177	18.06031
HORVU5Hr1G066300	3.169216	3.667462	3.747743	5.515458	6.071681	6.944637
HORVU0Hr1G016890	3.507968	3.600584	3.816452	5.546525	4.715261	5.148522
HORVU4Hr1G005770	4.44364	5.137679	4.705468	6.584578	7.893101	7.676142
HORVU5Hr1G113550	19.70422	17.21969	23.19514	17.75308	18.61356	17.53477
HORVU1Hr1G024170	13.5212	13.88535	17.30721	12.7947	17.35146	18.48653

HORVU4Hr1G063780	21.22944	10.72744	12.86575	0.024961	0.02169	0.059661
HORVU3Hr1G066800	50.00672	45.97662	53.78088	58.62124	70.70004	74.42466
HORVU5Hr1G061540	0.989939	1.137438	1.464159	1.495428	1.471663	1.543725
HORVU1Hr1G085620	3.683402	4.092989	4.752107	6.497357	8.789135	6.383498
HORVU4Hr1G003640	12.28441	11.30659	12.84387	5.185896	2.861168	2.855971
HORVU3Hr1G046180	4.694145	4.77317	5.236523	5.334626	6.869047	6.984052
HORVU4Hr1G014800	45.15312	37.94656	50.87464	33.63555	31.29659	36.61626
HORVU5Hr1G114020	2.195666	2.275671	2.389016	3.993414	4.66885	5.266161
HORVU4Hr1G082190	5.608088	6.993313	5.169739	5.201472	6.545584	5.919307
Hordeum_vulgare_newGene_1150	4.373841	4.524041	4.052535	5.920552	9.670865	9.603054
HORVU3Hr1G031730	21.56083	16.45851	20.106	21.26445	27.55922	23.69395
HORVU0Hr1G014940	2.131592	1.976158	0.920245	1.214306	1.428468	1.795556
HORVU6Hr1G005390	34.48773	39.6388	44.79126	52.69068	159.0308	125.2417
HORVU5Hr1G080010	13.68288	14.58845	17.85017	11.10134	11.43762	12.29808
HORVU6Hr1G091210	3.516825	3.607923	4.726352	3.679763	4.915211	4.589312
HORVU0Hr1G017760	5.297562	6.221766	8.671714	2.418681	0.922602	1.337731
HORVU5Hr1G082950	2.468553	2.81114	3.757545	2.652663	4.359989	4.771856
HORVU5Hr1G020580	2.937887	2.94686	4.024636	4.775221	5.113822	6.154148
HORVU4Hr1G087110	0.091783	0.070226	0.103021	0.907987	3.342211	7.695911
HORVU7Hr1G088780	8.97588	4.277628	4.564526	1.281078	0.150738	0.507701
HORVU1Hr1G087910	0.067647	0.117795	16.44386	0	0	0
HORVU5Hr1G098980	13.16021	12.20925	9.621471	16.95628	25.06502	23.57368
HORVU3Hr1G070550	99.94745	88.26753	98.46152	70.73802	77.11137	84.12603
HORVU7Hr1G075760	0.982941	0.557862	0.712719	1.719304	9.805024	10.38571
HORVU1Hr1G042550	10.1467	10.66931	12.29333	9.180275	10.57879	11.10109
HORVU1Hr1G014800	5.773529	6.140782	7.695047	4.56507	6.265749	8.079377
HORVU4Hr1G012400	2.222593	1.452629	2.367354	6.533187	4.776928	4.523478
HORVU1Hr1G057490	6.513547	7.92393	6.336679	7.642955	10.28325	12.05846
HORVU2Hr1G110900	36.26863	35.56936	32.30537	82.54436	71.98058	68.41102
HORVU6Hr1G017200	1.403506	1.605024	1.75312	0.776552	0.546532	1.017252
HORVU6Hr1G018520	23.19866	25.89954	30.82522	15.44072	14.08027	17.90965
HORVU3Hr1G029950	0.892793	0.804331	1.78248	0.995399	1.184256	1.533484
HORVU4Hr1G066010	2.667452	3.289985	4.173086	3.602256	3.523843	4.035776
HORVU7Hr1G071470	0	0	0	13.02204	12.95923	16.30275
HORVU5Hr1G046330	1.498994	1.027296	1.545225	5.536052	8.610543	7.319215
HORVU4Hr1G081070	2.625018	2.657582	2.817396	4.126685	9.739401	10.43784
Hordeum_vulgare_newGene_13927	33.03284	18.17299	37.86661	83.69494	21.54956	25.79732
HORVU7Hr1G091230	13.40553	12.81439	20.51774	11.98764	15.00315	14.8511
HORVU2Hr1G078350	12.86711	14.1419	17.28866	9.334614	13.72348	14.24353
HORVU4Hr1G055270	4.751251	3.759545	6.912932	6.017889	4.52949	5.406996
HORVU3Hr1G099530	3.321419	3.336641	3.45998	1.348348	0.699202	1.413876
HORVU7Hr1G095920	4.102369	3.496323	4.624617	9.748571	7.754376	6.341736
HORVU6Hr1G076190	14.56856	15.43187	19.71915	12.38797	17.93549	17.24867
HORVU5Hr1G092990	1.127305	1.459966	1.496975	2.121881	1.571343	1.867977
HORVU5Hr1G080500	39.69304	46.69802	54.34984	27.52355	21.71068	22.74842
HORVU2Hr1G066920	54.93335	42.45828	66.14971	28.34962	45.33729	39.31078
HORVU3Hr1G033800	11.4342	12.91232	9.656555	32.61573	22.00558	21.97163

Hordeum_vulgare_newGene_8713	2.571691	1.443651	2.005736	0.924059	2.037664	1.142043
HORVU7Hr1G057330	4.096343	2.976617	3.937466	4.772163	3.660347	2.948094
HORVU4Hr1G007590	3.119967	8.274777	11.27002	5.867803	9.308935	0
HORVU5Hr1G045440	47.44296	40.54804	63.44656	45.25026	35.97676	41.2388
Hordeum_vulgare_newGene_8718	1.629286	1.457998	1.692066	1.79331	1.394834	1.356953
Hordeum_vulgare_newGene_8719	1.857617	1.989736	2.5414	2.153377	2.489996	2.66487
HORVU4Hr1G050360	9.814166	8.976287	10.33581	11.02136	17.64006	15.68909
HORVU4Hr1G000610	0.752209	0.589001	0.742467	0.840393	11.45144	12.00355
HORVU3Hr1G076890	2.458711	1.678853	1.359095	1.036928	1.592503	1.681459
HORVU6Hr1G015410	12.24935	10.44293	11.27039	11.85225	11.26768	11.37497
HORVU2Hr1G063250	2.812359	3.504454	2.937386	3.446479	4.278657	5.312679
HORVU4Hr1G025660	3.955359	4.392655	4.132281	6.709855	5.772335	6.567597
HORVU5Hr1G123440	0.871222	1.150198	0.571178	2.462893	3.406081	3.030173
HORVU2Hr1G055160	6.931192	7.095429	7.831947	7.742784	8.495799	9.375547
HORVU0Hr1G010800	4.542221	4.679717	5.474491	9.666677	22.42395	19.12369
HORVU3Hr1G098660	38.78662	41.62869	45.27819	20.58541	15.98533	19.0256
HORVU3Hr1G114370	1.272717	1.904944	1.787656	1.374319	1.810209	1.843964
HORVU2Hr1G118820	1.647044	0.954964	1.384307	2.315881	0.71461	1.420931
HORVU0Hr1G022580	5.842746	5.934197	8.179525	4.460611	9.293365	8.93344
HORVU1Hr1G040150	1.368393	1.041364	0.657368	9.444738	28.04437	21.774
Hordeum_vulgare_newGene_10786	2.14534	2.77937	3.181413	4.408379	7.766399	7.082976
HORVU3Hr1G106720	9.98415	10.57028	12.73615	5.543631	7.292537	9.089574
HORVU2Hr1G111690	0	0	0	3.605765	3.446487	2.910746
HORVU7Hr1G076900	25.49022	24.96974	26.42952	31.48843	27.98782	32.80167
HORVU4Hr1G016520	7.871299	6.268056	7.935599	7.207751	6.474771	7.912608
HORVU3Hr1G074660	23.55261	23.35803	26.93026	15.11114	15.93307	14.6396
HORVU4Hr1G090280	14.34108	15.37453	20.13649	11.23044	11.32237	13.41196
HORVU4Hr1G024530	12.40546	8.455403	7.933473	15.21307	11.80093	17.64831
HORVU2Hr1G025940	3.900177	4.136931	6.193867	2.622309	3.486989	3.85607
HORVU5Hr1G008860	1.361879	1.678214	1.824678	1.346399	1.525404	1.982037
HORVU6Hr1G050710	3.176448	3.483536	4.430704	7.316961	9.884727	10.42923
HORVU3Hr1G066340	41.61288	41.94111	57.5993	16.21823	5.966866	10.12059
HORVU2Hr1G011790	0.771755	0.717373	1.31901	1.884538	2.100575	1.626693
HORVU0Hr1G027030	1021.402	1258.621	897.5894	521.384	319.7362	446.8465
HORVU2Hr1G105520	3.12397	4.106838	5.764917	3.140308	5.092058	3.568293
Hordeum_vulgare_newGene_11786	1.147839	1.301412	1.517154	2.685881	3.168093	3.70912
HORVU6Hr1G077010	3.716951	2.848885	3.986333	2.9271	3.686589	3.507301
Hordeum_vulgare_newGene_146	2.556048	2.526959	4.240779	0.412611	0.085472	0.111842
HORVU2Hr1G071240	27.88263	28.87829	27.97759	33.38229	54.26996	49.8424
HORVU7Hr1G063020	2.130262	2.415275	1.962419	5.689398	11.12293	11.62898
HORVU2Hr1G123570	0.509628	0.968593	0.476961	0.497233	3.276627	2.675389
HORVU2Hr1G029620	20.60008	18.29575	26.01326	20.43293	30.50293	27.90733
HORVU5Hr1G046490	27.66707	29.03688	30.12614	53.56772	85.65427	85.25101
HORVU5Hr1G043460	1.652426	0.750595	1.931076	2.321331	1.844096	2.389024
Hordeum_vulgare_newGene_11434	7.743879	10.94108	9.423735	1.589006	0.789223	1.084818
HORVU7Hr1G087590	13.75038	14.05306	13.11311	19.8411	27.35658	25.93143
HORVU2Hr1G110120	87.14586	74.79826	94.57528	104.168	111.3505	134.9625

HORVU6Hr1G089130	9.984445	11.11599	7.843329	7.707606	5.450425	6.563598
Hordeum_vulgare_newGene_11438	4.611739	5.011281	7.141071	3.116714	2.632156	2.810761
HORVU3Hr1G075220	24.36703	25.30426	24.58413	29.30579	38.07304	37.67024
HORVU2Hr1G013790	13.24117	15.10738	12.62256	13.51478	15.44548	17.42081
HORVU2Hr1G072870	1.515764	2.058998	2.418047	7.02969	6.235017	8.849426
Hordeum_vulgare_newGene_12200	2.841124	3.284924	3.601414	2.719558	2.743626	3.805163
HORVU0Hr1G028990	9.976933	9.155744	10.28322	9.745186	9.363139	8.689607
HORVU6Hr1G095230	15.55683	15.81548	18.04071	29.51884	33.65888	34.19201
HORVU4Hr1G006870	21.35672	19.1784	19.62768	29.59034	51.13518	45.91453
HORVU2Hr1G049960	2.793819	2.290684	4.144509	2.922256	5.573231	4.650933
HORVU7Hr1G039640	57.35936	54.56363	60.92154	48.88139	43.91971	48.16806
HORVU2Hr1G087630	1.943069	2.457403	1.811128	4.373824	6.473871	7.02904
HORVU3Hr1G017300	9.834867	9.600062	11.24998	6.480253	8.95711	10.16615
HORVU2Hr1G090000	4.980151	3.409666	4.787885	4.477505	3.727907	4.182596
HORVU7Hr1G035340	7.691412	6.182382	8.63381	15.15321	13.31853	14.5831
HORVU3Hr1G106260	9.148326	7.74543	9.225963	5.760527	5.687424	6.050961
Hordeum_vulgare_newGene_13554	0.013743	0	0	3.250847	7.29413	6.732881
Hordeum_vulgare_newGene_13557	0.76149	0.538904	0.584125	1.888354	3.086157	2.967959
HORVU2Hr1G114920	4.265616	4.459731	5.086244	9.789418	14.50962	15.48475
Hordeum_vulgare_newGene_13558	0.961377	1.258166	1.237979	1.838199	1.47487	1.559379
HORVU4Hr1G022280	6.957969	5.357007	7.127651	38.09077	24.00378	20.63065
HORVU3Hr1G068010	1.145328	1.797773	1.708845	1.809628	1.607511	2.656588
HORVU0Hr1G030810	3.016377	2.817968	2.974554	4.66929	4.072443	5.011102
HORVU5Hr1G064120	3.043073	2.402539	3.044419	2.435274	1.927131	2.244982
HORVU1Hr1G036920	37.73728	28.98279	46.85144	37.90808	18.78687	28.53242
HORVU3Hr1G055000	26.83595	28.48525	29.58757	17.59018	18.68009	24.18081
HORVU0Hr1G005140	5.968129	8.04911	12.05078	2.35483	0.570386	1.30017
HORVU6Hr1G032810	1.795693	0.917171	2.933835	1.187984	0.63616	0.860932
Hordeum_vulgare_newGene_6030	0.04891	0.094973	0.055837	7.015996	6.102561	7.777973
Hordeum_vulgare_newGene_6033	4.520495	4.276734	4.852473	5.171833	5.014846	5.606686
Hordeum_vulgare_newGene_7922	0.983805	0.72101	1.399178	3.990245	1.109833	1.906666
Hordeum_vulgare_newGene_7927	2.335842	2.030084	1.932403	0.69046	0.459565	0.283295
HORVU6Hr1G079640	1.276428	0.848483	0.627448	1.102029	5.237185	5.03122
HORVU4Hr1G073880	3.777769	4.135718	4.050348	2.442472	2.218151	2.479681
HORVU4Hr1G053570	8.832402	7.664022	9.057507	10.82486	13.12036	10.83006
HORVU3Hr1G087470	6.48936	5.232493	7.393876	9.794925	10.41298	12.05328
HORVU6Hr1G014910	34.64699	30.90259	34.14292	32.01609	36.90242	35.59813
HORVU6Hr1G061220	0.079998	0.032083	0	1.977756	9.672614	10.84636
HORVU6Hr1G072030	10.81589	11.34993	14.84479	10.86254	13.87429	13.52062
Hordeum_vulgare_newGene_5716	2.856325	3.439529	3.405715	2.895784	2.7734	3.53909
Hordeum_vulgare_newGene_5713	3.00244	3.821976	3.525965	2.400109	3.768202	4.069052
HORVU7Hr1G108780	17.16809	17.66992	24.32991	15.03005	23.17164	20.87009
HORVU1Hr1G020050	0.07693	0.070818	0.142419	2.170314	11.26987	9.590675
Hordeum_vulgare_newGene_914	2.904593	1.950877	2.501522	0.14017	0.095361	0.061299
Hordeum_vulgare_newGene_913	3.619133	3.019963	4.884505	5.089919	7.351417	6.905738
Hordeum_vulgare_newGene_910	2.298132	2.913127	2.766609	2.902786	2.063405	2.558944
HORVU3Hr1G013530	0.03649	0	0.105376	2.685692	14.05974	12.60489

HORVU3Hr1G019790	11.51163	11.45316	16.43179	10.01934	17.04536	18.15431
HORVU1Hr1G074900	11.15188	8.963457	10.4722	8.20229	12.16339	9.30036
HORVU3Hr1G044720	0.698727	1.431283	1.102255	3.734102	4.392554	5.708793
HORVU5Hr1G062240	2.89354	2.071004	1.603002	6.917741	7.521118	9.181936
HORVU6Hr1G064470	12.26416	11.99688	11.15136	7.764862	12.00898	11.11149
Hordeum_vulgare_newGene_11849	530.0896	469.4025	577.2352	352.2527	328.1534	321.1214
Hordeum_vulgare_newGene_11848	1.621227	1.619868	2.938737	1.81699	1.825554	2.329288
HORVU5Hr1G073930	22.88242	19.98342	31.10127	23.76728	32.87567	31.92587
HORVU1Hr1G062250	2.714251	2.052107	3.219455	4.187656	2.620002	4.199711
HORVU3Hr1G004420	2.987496	4.757764	6.499774	2.281264	1.551655	3.246249
HORVU3Hr1G055910	2.654735	2.636265	2.570871	1.525168	3.042057	3.47562
HORVU6Hr1G068760	12.38538	21.04857	27.2283	6.818764	20.66633	24.4485
HORVU6Hr1G045360	9.405789	10.05166	10.00312	15.48387	24.59737	19.9125
HORVU5Hr1G066540	2.365256	2.330137	3.225597	2.810929	3.340665	3.070266
HORVU1Hr1G054380	3.717614	3.72108	4.537767	9.00335	11.39286	12.03877
HORVU4Hr1G043990	5.956212	7.079638	6.481866	1.671809	0.798248	2.191061
HORVU5Hr1G091880	5.016749	8.108457	5.735857	4.243957	4.382256	4.128002
HORVU1Hr1G073300	0.950847	0.868392	1.142819	5.140902	6.400873	7.126732
HORVU1Hr1G028720	34.51957	31.0418	40.00114	42.00807	80.89213	88.02416
HORVU2Hr1G095170	4.749772	3.630206	4.802178	5.094946	5.553074	5.577231
HORVU7Hr1G113290	38.90219	34.9053	47.38606	16.53726	8.170909	8.551589
HORVU6Hr1G020330	0.82448	0.882841	0.887394	1.887385	1.12826	1.222394
HORVU1Hr1G018930	0.785195	0.677206	0.913455	2.278522	3.719275	3.34354
HORVU3Hr1G084350	7.002473	7.542573	8.489725	7.673949	8.825473	9.714667
HORVU6Hr1G025670	9.961046	10.10253	9.965318	7.857661	10.20605	10.18391
HORVU1Hr1G058400	113.2267	82.73672	107.1445	164.855	46.68049	83.588
HORVU6Hr1G035950	5.055102	5.630422	5.694367	5.688986	4.884133	6.081674
HORVU7Hr1G039980	1.959604	1.209249	1.676001	2.549358	2.907022	3.178297
HORVU6Hr1G091360	24.2995	23.3681	25.85489	28.26778	24.43695	32.2737
HORVU5Hr1G021340	1.582139	1.414307	1.722407	4.20891	6.376659	5.599488
HORVU4Hr1G008120	3.537447	3.271894	4.620963	5.537764	12.66388	10.63463
HORVU1Hr1G063610	1.572376	1.098898	1.635531	1.830598	1.40156	1.614714
HORVU2Hr1G120590	18.78504	17.93895	17.66105	12.89349	12.98424	13.07382
HORVU7Hr1G050270	30.63827	24.12599	24.01635	19.57417	14.10461	13.68983
Hordeum_vulgare_newGene_1892	94.71084	85.25818	92.88239	69.08598	49.13059	50.67451
HORVU0Hr1G005960	19.0479	17.58873	19.08869	21.07246	28.15957	23.90628
HORVU5Hr1G104160	7.068467	7.093877	9.706463	9.54545	11.94209	11.60642
HORVU4Hr1G019140	10.43849	14.68544	11.35984	29.49241	42.64038	45.42586
HORVU7Hr1G121830	1.415824	1.03476	1.051744	4.062973	4.150802	4.004138
HORVU5Hr1G092240	1.2111	1.602227	1.535103	3.375405	8.561199	8.565732
HORVU6Hr1G062860	3.508696	2.806561	3.23657	4.12058	4.757687	5.184701
HORVU1Hr1G075620	2.120229	1.647469	3.181044	2.148078	1.492465	1.940107
Hordeum_vulgare_newGene_4596	1.405829	1.541589	1.929079	1.964158	2.356135	2.558488
HORVU6Hr1G024100	36.66836	37.44847	45.45871	34.00265	47.2094	47.14269
HORVU7Hr1G101260	8.083518	6.168578	10.14101	3.896088	4.731647	5.265703
HORVU2Hr1G042560	6.897992	7.568027	9.908266	6.022182	6.944974	7.678409
Hordeum_vulgare_newGene_4598	22.7664	24.83311	27.81227	18.79716	15.85683	17.78734

Hordeum_vulgare_newGene_4599	1.85743	2.369305	2.095564	1.450863	1.560914	1.715779
HORVU6Hr1G081340	21.52645	22.18643	18.15398	22.57485	33.51422	34.32307
HORVU7Hr1G090290	8.717786	8.9566	10.75601	14.55998	24.06886	21.0703
HORVU7Hr1G039320	6.625171	5.868889	7.765255	4.953311	5.022984	4.83631
Hordeum_vulgare_newGene_7027	3.268005	3.207401	3.52645	7.267761	7.913355	6.423541
Hordeum_vulgare_newGene_7026	41.54763	37.23638	49.70023	52.38683	50.46016	55.68648
Hordeum_vulgare_newGene_7020	7.819558	7.771023	10.01248	6.726472	6.269464	6.585067
HORVU4Hr1G073140	2.445568	2.52948	2.279306	2.897639	3.725656	3.925794
Hordeum_vulgare_newGene_7029	4.226221	3.263551	4.356735	4.515594	12.61874	9.918918
HORVU6Hr1G083270	19.5017	22.13816	20.25481	20.23174	22.84292	23.19886
HORVU0Hr1G015520	2.571269	2.202582	3.116922	1.581895	0.99389	1.676358
HORVU7Hr1G079430	13.40679	10.76373	14.53054	25.79813	23.21521	25.36198
HORVU1Hr1G091850	3.141527	1.997956	2.861823	3.996133	2.053926	2.606267
HORVU6Hr1G035520	9.826027	9.226482	11.19569	16.00018	27.35764	30.0088
HORVU7Hr1G056420	46.04041	36.57393	45.81892	38.22125	42.53856	44.72833
HORVU4Hr1G019850	4.28286	3.736868	4.547189	6.184061	7.70933	7.85473
HORVU1Hr1G029540	0.568066	0.325175	0.234542	0.807883	1.930528	2.547085
HORVU7Hr1G052500	7.315871	6.182877	6.939441	7.165101	3.478736	4.493751
HORVU5Hr1G088270	16.58901	15.36599	15.79327	16.12415	20.88726	19.94785
HORVU4Hr1G000050	1.514603	1.267091	2.208615	4.463435	1.375708	3.057476
HORVU1Hr1G023630	33.16155	31.48996	37.77941	27.96763	43.19635	46.10111
HORVU6Hr1G043010	39.17855	35.56791	31.27621	39.26469	40.8026	42.20825
HORVU5Hr1G008340	5.098386	3.352872	4.118314	5.039901	5.838375	4.134323
HORVU7Hr1G049900	10.00129	7.472323	9.416043	9.308827	12.08622	10.15961
HORVU5Hr1G123680	5.594724	5.970012	9.697502	3.781418	4.447221	4.418331
HORVU4Hr1G052200	8.330895	7.269865	9.65109	42.37729	48.53987	47.09656
HORVU1Hr1G055220	7.687476	11.19191	10.02686	6.29307	8.313416	13.17778
HORVU6Hr1G083180	2.840304	4.565699	3.930167	0.172954	0.302582	0.276368
HORVU5Hr1G120480	4.271607	4.822814	4.433008	3.37945	2.794901	3.3643
HORVU7Hr1G025750	1.394213	0.997685	1.143603	3.624801	3.847917	5.380037
HORVU1Hr1G050960	86.49665	90.69032	88.99602	68.93198	62.79472	68.44747
HORVU6Hr1G033420	3.516609	3.285721	3.564895	4.445807	5.577929	5.382107
HORVU4Hr1G059740	6.810616	6.458577	9.725096	5.369305	5.250093	5.665595
HORVU4Hr1G016500	0	0.044008	0	0.265684	46.87319	56.62543
HORVU3Hr1G032990	1.779006	1.233146	3.21376	1.33512	2.203288	3.622099
HORVU4Hr1G002980	27.47298	22.51025	25.36438	38.74677	37.44886	38.32493
HORVU5Hr1G027950	47.80905	43.08922	49.09106	48.88325	47.75247	49.7276
HORVU1Hr1G090810	41.19567	35.31987	43.05085	36.12714	44.5829	46.08871
HORVU3Hr1G078330	3.893642	3.374102	4.670653	3.920452	5.070153	6.428363
HORVU5Hr1G052600	24.22706	27.12787	29.25312	29.09096	23.17097	28.04336
HORVU5Hr1G036330	17.04767	18.39995	19.37643	11.96569	10.95982	10.11023
HORVU5Hr1G116860	1.69696	1.904101	1.719624	0.64702	0.975925	0.870332
HORVU3Hr1G030620	3.728151	4.113981	4.119121	3.405365	3.037954	2.657259
HORVU4Hr1G053550	16.46461	15.02576	16.77649	11.54829	13.70081	13.31283
HORVU1Hr1G093540	3.451287	3.64105	2.987298	4.137685	4.911689	5.031459
HORVU6Hr1G087030	9.161896	7.848856	10.48858	10.2706	10.24173	10.82412
HORVU5Hr1G017630	1.469742	0.418773	0.581142	1.951521	3.043175	2.674259

HORVU6Hr1G030990	5.268237	5.86027	5.160049	7.002192	10.35905	10.11443
HORVU1Hr1G087530	0.259303	0.518551	0.416013	1.453823	7.7502	6.223966
HORVU5Hr1G009560	8.062668	8.745623	7.678945	6.382014	4.917314	4.894248
HORVU7Hr1G094990	6.354262	5.583768	4.263931	6.871109	10.25476	7.382467
HORVU6Hr1G004990	18.0505	18.11839	18.48206	12.89174	15.74454	16.22675
HORVU2Hr1G122350	5.197825	3.802792	5.570949	7.327349	5.427369	7.439012
HORVU5Hr1G094510	16.512	13.95676	16.21023	15.53883	18.98207	17.56929
HORVU4Hr1G011880	4.713991	4.428194	4.596904	8.661064	9.324264	8.668211
HORVU6Hr1G093000	10.06653	9.41893	11.32932	17.62277	24.03602	22.86315
HORVU1Hr1G081240	17.20009	13.50557	20.76007	5.588326	4.41129	6.30295
HORVU5Hr1G092260	2.55294	3.37403	3.169616	2.588908	3.310954	3.24998
HORVU7Hr1G048690	4.447108	4.380177	5.21774	4.168763	3.277219	4.138469
HORVU4Hr1G002360	2.868571	2.455037	2.365651	2.939015	2.596954	2.859514
HORVU4Hr1G023390	3.561682	3.044682	4.228432	1.930507	1.457494	2.333255
HORVU0Hr1G016170	66.18811	56.08604	70.87624	39.08956	20.0229	20.81004
HORVU3Hr1G027500	10.62294	13.55343	13.8655	4.78557	9.423384	9.793478
HORVU2Hr1G094340	4.260471	5.30953	5.165675	4.07055	3.900267	3.161078
HORVU6Hr1G028790	3.658871	3.165818	4.511512	3.860502	3.325644	5.41761
HORVU5Hr1G003000	29.96738	22.29329	32.56408	31.17146	33.45975	31.95345
HORVU4Hr1G063600	1.613894	1.392103	1.836236	2.593768	5.248965	5.211347
HORVU5Hr1G109120	16.38582	13.53101	15.83851	26.80976	6.935099	25.71168
HORVU4Hr1G014100	9.330996	9.884067	12.05543	10.49292	11.69301	11.49888
HORVU7Hr1G091450	2.188342	2.350084	3.135244	1.50353	2.952894	3.070345
HORVU7Hr1G096760	0.841826	0.512835	0.727039	2.792973	2.023717	2.01075
HORVU4Hr1G009310	1.374319	1.117735	1.277224	2.519297	5.19086	4.965327
HORVU2Hr1G112890	7.417367	7.299439	9.568847	6.261673	6.082475	6.586775
HORVU4Hr1G063590	2.192577	1.405678	2.261221	5.844813	4.362729	6.37968
HORVU1Hr1G068830	16.97274	15.10233	18.60979	40.21554	57.69843	51.72568
HORVU1Hr1G090860	3.798134	1.478482	4.937692	1.578055	0.823624	1.527233
HORVU7Hr1G114870	0.981924	1.016479	1.175116	2.019555	1.077642	1.148344
HORVU4Hr1G035770	4.06581	4.007576	3.758069	4.895735	4.605385	5.215394
HORVU2Hr1G076000	9.980508	12.04963	9.999305	23.71274	34.36204	29.84097
HORVU2Hr1G092420	5.181519	5.763676	5.316427	4.340352	4.940891	4.552129
HORVU7Hr1G087900	17.04013	20.13	22.16156	12.45739	12.15271	13.45223
HORVU7Hr1G095220	1.805093	1.646079	1.833004	1.913272	2.391777	2.279135
HORVU6Hr1G017900	54.56425	65.26954	64.65008	65.50326	57.88936	58.13986
HORVU1Hr1G002160	0	10.90715	0.912273	15.58263	0.299176	0.874947
HORVU6Hr1G092840	6.041893	6.389655	8.435854	2.788627	0.800336	1.351346
HORVU4Hr1G084670	43.02632	39.47913	42.70546	47.26383	52.99981	53.26594
HORVU0Hr1G007420	2.092788	2.710617	2.246419	2.468936	2.113524	4.402966
Hordeum_vulgare_newGene_8318	4.569777	5.754773	6.827526	5.226276	6.633347	7.247486
HORVU7Hr1G109250	1.079275	1.358657	1.239567	3.436746	5.796288	5.375382
HORVU2Hr1G066480	2.537104	2.553751	3.030503	2.578659	2.272435	3.485001
HORVU7Hr1G071270	35.79462	35.36176	38.25586	38.20683	48.63797	49.67807
Hordeum_vulgare_newGene_12957	4.290841	3.556217	6.769576	11.55597	30.13774	29.66908
Hordeum_vulgare_newGene_12955	4.42442	4.984218	4.801975	5.849896	5.47015	6.838406
HORVU7Hr1G082990	0.544524	0.523934	0.405454	1.317626	1.8244	2.42123

HORVU5Hr1G119220	0.908532	1.215485	1.838615	0.613468	0.926814	0.722769
Hordeum_vulgare_newGene_12951	1.912311	1.133907	1.710464	0.427914	0.357846	0.572762
HORVU1Hr1G038890	115.3772	118.0062	223.6885	97.65002	248.3849	221.6611
HORVU6Hr1G018480	27.21958	18.68414	29.57816	39.4914	40.13505	39.20981
HORVU4Hr1G064810	3.581849	3.996827	4.081003	5.637339	5.911818	7.184239
HORVU1Hr1G043500	0	0.021153	0	3.182764	4.852011	5.355645
HORVU3Hr1G070960	4.264676	3.470598	3.902587	6.294151	8.890962	8.739817
HORVU4Hr1G019460	2.080633	1.767354	1.969185	2.593313	2.177996	2.464731
HORVU7Hr1G075350	4.226335	5.549024	4.441756	4.650944	6.845453	5.921056
Hordeum_vulgare_newGene_8317	13.05901	13.60813	13.33554	16.31652	23.3908	24.37794
Hordeum_vulgare_newGene_5516	121.932	97.47786	133.141	77.94006	59.17915	72.67371
HORVU1Hr1G083830	4.840596	6.179882	5.746593	7.781773	9.091756	8.009994
HORVU2Hr1G107480	3.025309	1.298032	3.169697	20.10366	53.61451	56.3963
HORVU5Hr1G044610	23.42877	12.91363	24.26523	24.6321	52.29931	56.18758
HORVU0Hr1G022440	0.013395	1.434382	0.599509	1.793927	1.587095	2.187181
Hordeum_vulgare_newGene_2026	7.620313	9.564322	8.123973	10.67461	14.9522	14.0573
HORVU2Hr1G006170	1.421255	0.501451	1.49077	67.30919	0.89188	12.61716
Hordeum_vulgare_newGene_2028	2.155331	2.285147	2.491593	2.220399	1.434159	1.81212
HORVU7Hr1G006180	36.87244	33.38071	37.20041	10.06572	11.07589	11.45465
HORVU1Hr1G044570	7.42475	7.382869	7.001268	5.171469	7.075911	6.756202
Hordeum_vulgare_newGene_9674	1.004545	2.169229	2.418711	1.262837	1.187959	1.255203
HORVU2Hr1G013300	1.334279	0.956139	0.862274	0.917345	0.906201	1.040962
HORVU4Hr1G004270	0.789193	1.062602	0.854976	0.892194	1.487111	1.424173
HORVU4Hr1G075310	41.76025	32.71774	43.62237	38.51907	36.50605	40.06013
Hordeum_vulgare_newGene_9679	6.05499	6.624399	7.173326	5.744227	5.590165	6.792894
HORVU2Hr1G099220	27.33655	27.73907	29.89972	40.17419	47.1592	42.51506
HORVU2Hr1G036710	11.754	13.62841	10.65967	6.464955	6.309137	8.373175
HORVU2Hr1G033540	2.153339	2.347121	1.904273	1.823361	1.057588	1.144166
HORVU3Hr1G077530	0.208953	0.023622	0	2.988722	3.794657	4.160983
HORVU5Hr1G078160	2.846287	2.432169	3.188198	13.88551	37.05693	29.07168
HORVU6Hr1G016750	16.74554	18.22243	14.25554	32.45581	29.70908	30.07324
HORVU4Hr1G085250	2.008464	1.682755	2.687885	8.322786	3.95506	6.586632
Hordeum_vulgare_newGene_9657	1.231786	0.788276	1.37656	3.961771	1.326665	1.608009
HORVU0Hr1G021930	2.706222	2.738764	3.879514	2.929598	2.074937	2.189282
Hordeum_vulgare_newGene_3206	6.043906	5.049772	5.835773	5.322407	9.86168	9.26932
HORVU1Hr1G030790	137.9566	138.8088	119.4199	166.6066	277.2035	213.2149
HORVU5Hr1G048960	10.85724	11.75106	10.78347	14.17048	17.02053	17.22478
HORVU1Hr1G077240	7.615853	7.267496	8.139632	8.453861	11.50845	9.771943
HORVU2Hr1G121090	18.96081	17.99515	21.30104	4.909069	1.144604	1.943136
Hordeum_vulgare_newGene_10086	2.181625	2.93411	2.698613	1.934184	1.566374	1.907257
HORVU4Hr1G060970	2.158311	1.857482	1.863187	5.507956	7.494752	7.519253
HORVU2Hr1G112330	1.100071	1.073742	0.850808	0.846276	2.600688	2.229923
HORVU4Hr1G083950	25.39689	24.40567	26.10388	42.49742	34.03918	39.44263
HORVU2Hr1G031940	23.7112	30.15053	35.97781	33.99439	31.54898	41.57213
Hordeum_vulgare_newGene_8106	0.491489	0.468308	0.639145	2.464786	2.086974	2.220226
Hordeum_vulgare_newGene_8100	1.832578	1.137625	1.576574	0.451634	0.769556	0.952781
HORVU2Hr1G072520	0.340228	0.675183	0.419041	1.899265	1.539242	1.647091

Hordeum_vulgare_newGene_8108	13.48423	13.01454	16.03846	11.33322	12.3514	9.430364
HORVU3Hr1G053350	5.412423	2.881337	5.919661	5.23189	6.454272	6.993783
HORVU3Hr1G096410	17.18585	17.20636	19.97521	20.0778	17.85163	20.494
HORVU7Hr1G101800	8.38362	10.45799	8.344054	3.870251	6.273652	6.254524
HORVU1Hr1G040050	5.530088	5.414404	6.475571	8.755153	11.86275	12.49317
Hordeum_vulgare_newGene_5086	1.513069	1.039339	1.510471	1.34799	2.029094	1.562308
HORVU6Hr1G017480	1.019667	0.822481	1.007839	1.663317	2.315436	1.775295
HORVU7Hr1G023560	9.404558	12.02601	11.93862	9.750304	6.444276	7.821995
HORVU4Hr1G029960	51.87614	39.9841	57.79981	49.48717	71.10596	69.56631
HORVU4Hr1G003040	1.446182	1.75275	1.62101	2.175325	1.869536	2.551109
HORVU2Hr1G025710	58.37769	52.86449	58.25552	40.75365	26.58072	29.41007
HORVU7Hr1G045800	4.386975	4.309738	6.222349	5.177509	8.981616	9.468002
HORVU2Hr1G119760	1.204569	1.141701	0.831547	1.060358	1.415817	1.367292
HORVU5Hr1G100890	4.004047	4.239958	2.525178	0.718117	1.190441	1.616372
HORVU1Hr1G004130	9.001693	7.575674	18.70218	0	0	0
HORVU2Hr1G115640	5.967493	5.901401	6.626688	8.59796	9.182434	9.487832
HORVU3Hr1G012970	3480.542	3160.485	3351.033	3045.849	2268.221	1818.918
HORVU1Hr1G039440	3.813047	5.690822	8.76196	5.002753	9.353349	7.260642
HORVU7Hr1G030930	2.755897	3.27498	3.741945	5.43955	6.648218	7.201079
HORVU3Hr1G106880	0.463913	0.482462	0.572398	1.251009	2.692233	2.513917
HORVU2Hr1G111150	0.616611	0.929105	1.27301	1.623624	2.978921	2.435252
Hordeum_vulgare_newGene_10624	40.89964	40.29427	49.92774	21.62199	19.30861	17.01819
Hordeum_vulgare_newGene_10623	37.57605	32.48014	40.62825	28.9638	27.86242	20.61161
Hordeum_vulgare_newGene_1709	11.97967	10.73301	13.97544	27.61613	38.62267	40.5206
HORVU7Hr1G066250	0.92906	0.927215	0.93685	1.205786	1.329258	1.350508
HORVU5Hr1G015110	2.077692	2.598815	2.693987	5.349351	6.982279	6.960536
HORVU5Hr1G049700	7.573955	8.767454	13.91571	6.272396	7.488581	8.642329
HORVU1Hr1G017740	2.973051	3.184736	3.015645	4.579616	4.2944	5.647004
HORVU7Hr1G069070	24.05881	20.98925	21.67836	16.03634	12.25872	14.24565
HORVU2Hr1G034660	5.947177	7.488245	7.346344	6.318461	5.483334	5.905676
HORVU7Hr1G073170	2.574719	3.188214	3.995013	11.26467	26.14541	24.74768
HORVU2Hr1G048590	12.39454	14.91945	15.23707	9.813368	10.93074	12.80211
HORVU2Hr1G084170	4.776528	3.165447	4.149699	6.639393	11.37625	8.72918
HORVU2Hr1G114060	3.737685	2.247604	2.685727	2.668822	4.525806	4.607809
HORVU2Hr1G099460	21.88444	20.20695	26.38909	28.62607	43.89339	43.08605
Hordeum_vulgare_newGene_4752	32.7656	31.42158	55.3	21.63733	42.85255	38.30331
HORVU6Hr1G003750	1.08471	0.830544	1.683837	0.766019	2.38605	3.455738
Hordeum_vulgare_newGene_4750	12.06515	11.52262	9.513255	15.57279	11.54592	9.372336
HORVU2Hr1G012630	6.075279	6.519588	6.9703	4.010711	3.911069	4.614537
HORVU1Hr1G072360	2.31314	1.787951	2.86107	4.496175	2.759036	3.232414
HORVU3Hr1G069980	1.867651	2.031784	2.394176	4.426019	7.58321	8.342229
HORVU1Hr1G060810	11.30879	10.82899	12.78144	11.39874	11.00931	12.41561
HORVU1Hr1G062590	2.030608	1.91893	2.543588	0.48594	0.715882	0.43411
HORVU1Hr1G025280	2.877424	1.431968	1.603504	1.580188	15.26989	13.38506
Hordeum_vulgare_newGene_7227	0.690207	1.112972	0.981472	1.177438	1.967178	1.843482
HORVU6Hr1G054060	9.013651	8.89268	8.662431	9.826099	14.83962	13.93762
HORVU7Hr1G033770	11.11767	11.48473	15.44032	14.86893	25.74339	25.80776

Hordeum_vulgare_newGene_334	17.60987	19.55042	18.41486	19.9332	18.93891	21.0945
Hordeum_vulgare_newGene_330	1.725286	1.46822	1.864613	3.543875	2.319673	2.618649
HORVU3Hr1G069670	2.516905	2.855731	3.046797	2.815583	4.813081	4.866625
HORVU2Hr1G114680	0.031479	0.08949	0.173428	0.320921	3.616264	2.790641
HORVU3Hr1G010460	0.548972	0.235945	0.471062	1.205351	2.539197	2.733627
HORVU5Hr1G037020	10.44687	11.03931	12.6184	10.85011	15.22515	15.14847
HORVU7Hr1G032420	50.96917	53.01765	48.36957	54.76387	57.72585	54.59952
HORVU5Hr1G057230	23.29107	22.53871	24.97817	21.42523	21.1531	21.12757
HORVU7Hr1G052740	4.752811	3.512725	3.41687	5.335971	4.874491	6.285237
HORVU4Hr1G027660	150.6413	94.08039	150.8619	111.4358	74.63892	97.22135
HORVU2Hr1G033970	5.591078	6.65256	7.210443	7.902265	11.92434	12.68681
Hordeum_vulgare_newGene_13744	3.838775	3.994741	5.269188	2.448495	1.985274	3.09429
Hordeum_vulgare_newGene_14537	0	0	0.008027	4.057116	3.157543	3.940655
Hordeum_vulgare_newGene_14534	0	0	0	2.573815	2.339604	2.40354
Hordeum_vulgare_newGene_13742	21.04694	18.30912	21.44903	40.81521	92.63596	90.44643
Hordeum_vulgare_newGene_14531	162.1867	106.7455	119.012	0.716532	0.023605	0.19148
Hordeum_vulgare_newGene_13748	7.462123	9.149616	9.024494	8.462259	10.3437	8.860239
Hordeum_vulgare_newGene_3039	3.238301	2.673915	2.70941	4.03972	4.930974	4.980027
Hordeum_vulgare_newGene_3037	2.645761	2.349105	3.088659	3.664285	4.689343	5.099456
HORVU7Hr1G030480	3.965278	2.518818	4.207465	1.428112	1.499452	2.121456
HORVU7Hr1G038300	0.924537	0.747122	0.803434	1.576093	1.843258	2.283351
HORVU5Hr1G085530	18.02644	20.35225	28.17496	12.93564	14.11683	13.70977
HORVU1Hr1G027620	56.95759	55.64935	56.83691	47.62887	50.22046	51.65518
HORVU1Hr1G070270	4.930313	4.112196	5.899996	6.310108	6.428937	7.031022
HORVU1Hr1G045110	24.29977	26.83209	24.68831	9.394115	13.36457	11.79875
HORVU4Hr1G017610	4.525345	3.848925	5.002889	4.534989	4.096976	3.508007
Hordeum_vulgare_newGene_7694	8.441601	13.00224	10.82042	15.54699	15.73793	16.33224
HORVU5Hr1G023010	31.8843	26.00408	35.80582	34.44119	70.41817	67.84395
Hordeum_vulgare_newGene_9991	0.084172	0.086953	0.116872	0.796684	2.93799	2.663748
Hordeum_vulgare_newGene_9996	14.93499	14.17745	18.9561	14.23811	14.31097	14.39255
HORVU3Hr1G113940	25.11284	22.4058	32.05151	18.05341	21.03199	21.12059
HORVU6Hr1G047770	10.65194	10.31574	14.49096	14.33357	16.53224	14.61285
HORVU4Hr1G044260	1.307515	1.297791	1.364212	2.491491	3.312035	3.48705
Hordeum_vulgare_newGene_7691	0	0	0.035871	0.581879	2.967187	4.039385
HORVU4Hr1G070310	2.078949	2.375981	2.567709	2.41496	1.533653	2.197587
HORVU5Hr1G039540	9.09329	7.836809	9.584398	7.277909	12.68432	12.57187
HORVU5Hr1G074740	1.71207	1.300902	2.777625	0.842842	0.573022	0.82387
HORVU7Hr1G042410	10.59472	11.00033	9.381198	10.50341	12.43744	11.68422
HORVU5Hr1G107170	26.46051	21.46461	29.95608	32.53883	40.65572	37.78199
HORVU3Hr1G114990	6.00491	2.973919	6.312838	11.76297	0.288038	2.924671
HORVU1Hr1G068650	1.937229	2.384905	2.163201	2.104132	3.236899	3.146064
Hordeum_vulgare_newGene_3938	0.913445	1.138476	0.630993	1.873313	1.739377	2.213353
HORVU1Hr1G020390	39.40189	42.41503	46.29425	36.22857	47.48109	49.944
Hordeum_vulgare_newGene_9339	2.773734	2.900165	3.376688	2.831073	2.473268	3.365451
Hordeum_vulgare_newGene_9337	1.236731	1.266688	1.405755	1.217165	0.99729	1.921191
Hordeum_vulgare_newGene_15377	0.94858	1.117554	1.923779	2.647723	2.271828	2.701832
Hordeum_vulgare_newGene_15375	0.501864	0.729253	1.180273	1.192558	1.435408	1.85859

HORVU4Hr1G002160	0.715936	1.031118	1.350943	1.914634	2.085174	2.514492
Hordeum_vulgare_newGene_15373	0.500916	1.870221	2.061331	8.851161	10.16974	8.037693
HORVU7Hr1G118760	3.59841	3.154187	3.714054	3.06819	1.20479	2.090046
HORVU7Hr1G107050	2.912813	3.216482	4.191548	5.301096	5.588004	5.680878
HORVU1Hr1G053900	24.35371	22.13497	27.93014	20.14499	16.50865	18.87874
HORVU6Hr1G083840	63.02615	57.30987	67.90336	50.22216	71.82594	65.33615
HORVU4Hr1G039540	8.386403	9.876484	9.920997	13.76146	15.44038	16.75794
HORVU6Hr1G060720	12.83536	11.56404	9.879932	22.18377	19.00502	21.02305
HORVU5Hr1G062160	3.857931	5.178898	4.908614	5.72433	10.25408	9.386042
HORVU2Hr1G081020	0	2.597339	3.395536	3.953763	4.95329	4.196747
HORVU3Hr1G114200	4.700401	8.169562	8.956973	0.075664	0.130725	0
HORVU2Hr1G085730	17.35225	17.09735	20.15889	14.32624	14.55014	14.36012
HORVU5Hr1G025960	15.17324	12.04847	15.70436	24.52676	33.74594	31.78186
HORVU7Hr1G106010	0.269609	0.168331	0.131991	1.527694	24.1349	19.02987
HORVU2Hr1G029840	1.588233	1.433871	2.054894	2.835342	3.466892	2.976663
HORVU0Hr1G012050	3.73285	3.84912	3.925185	6.672753	8.398177	7.151155
HORVU7Hr1G096310	0.538719	0.872573	0.740937	2.039041	12.20584	10.5283
Hordeum_vulgare_newGene_12973	2.672735	3.041482	3.425093	4.46875	3.150428	4.276413
HORVU4Hr1G088780	30.66423	24.97357	28.38848	72.27989	34.92818	40.33054
HORVU1Hr1G050420	2.751286	3.213322	4.430197	2.792635	2.741726	3.634118
HORVU4Hr1G005300	14.53654	13.13842	10.84406	5.301802	7.659874	5.714096
HORVU1Hr1G079040	1.111944	1.553595	1.352162	2.954345	2.145656	2.366588
HORVU0Hr1G029310	1.703514	1.565824	1.078533	1.143975	6.03408	5.118858
Hordeum_vulgare_newGene_1068	0.873291	1.203919	1.077187	1.509179	1.742633	1.742056
Hordeum_vulgare_newGene_1067	17.16317	15.34062	21.88423	25.45413	61.58352	63.10813
HORVU6Hr1G029800	3.666843	3.955787	5.014326	3.717042	3.444401	2.780953
HORVU4Hr1G052810	4.252172	4.964141	6.519424	6.030539	7.454446	8.826408
HORVU1Hr1G063790	1.739522	2.164436	2.457461	2.874431	2.777506	3.037143
HORVU7Hr1G053600	0.572986	0.744064	0.480471	0.490508	2.622643	2.453339
HORVU7Hr1G091040	5.310688	4.663825	5.782084	10.53512	4.850054	6.140591
Hordeum_vulgare_newGene_4259	0	0	0	1.617359	3.396557	2.615755
Hordeum_vulgare_newGene_4255	13.93623	17.55038	17.2008	13.13221	9.526228	13.12996
Hordeum_vulgare_newGene_4250	8.817399	8.244504	8.823616	9.670158	8.789115	9.220783
HORVU6Hr1G009030	1.326682	1.053687	1.400131	2.045057	2.355706	2.736284
HORVU1Hr1G063050	14.75585	13.08281	18.38094	25.73692	16.32056	17.21618
HORVU7Hr1G114090	1.490434	2.569091	1.137109	1.02153	0.350825	0.32463
HORVU6Hr1G085870	22.98495	20.10078	20.62414	27.65666	44.90393	44.61786
HORVU4Hr1G008680	1.56257	1.94237	2.221	2.310272	4.24096	3.788708
HORVU7Hr1G085650	1.865628	1.302985	1.799059	2.140786	5.279444	4.574689
HORVU1Hr1G078390	8.922227	8.240204	11.13311	7.901083	8.924522	8.251394
HORVU2Hr1G045560	3.07608	4.9699	5.35933	6.504745	0.085092	3.065474
HORVU1Hr1G072640	18.50591	21.42192	30.79289	10.59065	11.49588	10.5908
HORVU0Hr1G013010	0.529674	0.323549	0.331801	2.351023	2.735367	3.578226
HORVU0Hr1G014040	164.2646	152.6965	108.2322	31.00839	10.25731	18.02073
HORVU6Hr1G087650	79.38697	68.8036	88.38516	69.41485	83.81891	84.88384
HORVU5Hr1G018080	0.60897	0.895644	1.567769	0.533987	2.17085	1.78858
HORVU5Hr1G111350	34.20364	38.39754	50.58951	5.362555	0.103184	2.688591

HORVU6Hr1G056230	7.757264	6.634995	7.278558	9.221152	14.49311	13.456
HORVU4Hr1G004470	34.48836	33.05303	32.59523	25.74827	6.974334	9.916778
HORVU4Hr1G016200	2.257622	2.637909	2.727357	3.0334	3.182772	3.325395
HORVU2Hr1G002600	18.47779	16.96095	21.15732	50.26735	73.80149	63.09657
HORVU1Hr1G068760	0.231498	0.114678	1.928359	6.240638	6.408284	6.422863
HORVU7Hr1G111310	0.758912	0.433068	0.428484	3.801048	2.155408	1.722702
HORVU2Hr1G079230	8.996646	8.701692	10.08701	13.63548	13.76317	14.83401
HORVU6Hr1G006120	0.304843	0.154912	0.580492	3.94945	35.37183	35.94046
HORVU1Hr1G012910	1.132569	0.669315	1.58869	3.572639	18.54208	13.07115
HORVU3Hr1G078780	0.830314	0.546278	1.394722	4.011322	6.472817	6.618851
HORVU2Hr1G015690	31.43143	32.84671	27.30939	22.84802	24.03483	25.38994
HORVU7Hr1G092800	54.01364	33.5471	66.33427	26.8936	29.09796	35.73174
HORVU3Hr1G079780	1.530028	1.72899	2.165058	1.750387	3.121843	2.764965
HORVU6Hr1G047360	27.80063	25.90182	32.5696	36.4098	50.80572	48.98864
HORVU4Hr1G040280	42.37007	40.11047	51.93769	57.34965	77.99249	75.9621
HORVU4Hr1G085310	2.836142	3.856262	4.987554	2.441843	1.99426	2.918745
HORVU1Hr1G090990	7.653298	8.290983	6.771952	19.4235	16.51311	17.78328
Hordeum_vulgare_newGene_11113	1.237033	1.285962	1.564188	1.170472	1.073691	0.796869
HORVU6Hr1G038010	12.12703	11.69818	15.28208	17.29909	29.50615	29.57632
HORVU3Hr1G077180	1.04669	0.371953	0.598096	2.085729	3.385162	3.246688
HORVU7Hr1G027720	2.345717	3.59146	3.386414	1.589178	2.566347	2.322802
HORVU5Hr1G007260	73.79483	60.26213	69.52189	121.648	139.9456	145.7454
HORVU2Hr1G036080	10.41619	8.775871	10.70062	14.74309	19.21628	17.12105
HORVU5Hr1G023350	1.7824	2.253356	2.08022	3.366797	1.587213	2.223849
HORVU4Hr1G082840	5.827513	4.634187	7.166213	4.666199	5.076228	5.74743
HORVU2Hr1G015150	1.723201	1.531517	1.502208	1.968757	1.474454	1.205879
HORVU0Hr1G011560	11.95587	10.77833	15.2336	12.47825	20.73765	19.55425
HORVU2Hr1G014770	58.96098	53.71482	66.86675	57.50151	67.63895	73.46556
HORVU1Hr1G080320	40.50752	44.78314	60.95738	27.87591	28.7182	33.3245
HORVU2Hr1G090120	0.317964	0.182532	0.318142	1.691543	6.387806	5.505883
HORVU7Hr1G091860	5.397146	3.572048	7.636725	0.391166	0.016885	0.531667
HORVU4Hr1G023210	4.715882	2.915611	3.425417	1.687967	2.506243	1.723263
HORVU2Hr1G125280	20.45264	14.19445	18.91211	46.52534	66.6712	56.49534
HORVU2Hr1G040000	3.311398	2.604112	3.379081	6.051644	4.045569	5.0918
HORVU3Hr1G038580	17.52375	17.48531	23.08311	16.69348	19.89207	19.66499
HORVU7Hr1G048150	14.34976	14.2926	15.57933	14.74382	17.96948	17.80817
HORVU3Hr1G057140	11.92167	12.40475	10.59458	11.69313	8.898358	9.48331
HORVU5Hr1G096930	12.19261	8.803013	13.45418	2.247888	1.857008	2.00432
HORVU4Hr1G085860	9.993881	10.29734	9.93401	13.9609	24.1344	22.32204
HORVU1Hr1G041650	1.566163	2.7605	2.687539	1.930002	1.693744	2.417276
HORVU5Hr1G113240	4.669165	3.979082	4.669404	7.444684	9.288868	9.212414
HORVU2Hr1G061710	18.34896	17.71999	19.22811	23.03091	34.15998	29.29504
HORVU1Hr1G001890	9.015238	26.3796	11.90332	32.12492	10.04279	11.23637
HORVU5Hr1G071020	4.667355	5.038162	4.576924	6.127826	7.761502	7.862487
HORVU5Hr1G051330	54.92491	51.79829	63.45161	44.71931	41.38412	43.33736
HORVU3Hr1G036150	10.47689	11.03235	13.27034	12.25154	22.70222	23.14617
HORVU7Hr1G045680	2.405388	3.1058	2.951055	6.447547	10.79817	9.884656

HORVU2Hr1G023820	0.434171	0.360892	0.132123	6.283453	56.43892	43.00178
HORVU4Hr1G014310	6.251462	6.179156	7.186612	10.63788	10.65428	12.37273
HORVU1Hr1G038220	3.092306	2.031063	2.53212	1.935267	1.054258	1.602319
HORVU4Hr1G062890	1.41113	1.481479	1.21421	0.496158	0.954776	0.810007
HORVU1Hr1G018750	28.98383	29.4029	47.48691	14.73113	16.29097	18.33744
HORVU5Hr1G082700	2.220876	2.661196	3.656236	2.59645	4.998185	4.34226
HORVU7Hr1G050770	7.582075	6.413595	8.428843	6.088525	5.626067	6.082332
HORVU6Hr1G089540	1.96461	1.738911	1.899405	3.116191	2.20305	3.47441
HORVU5Hr1G082560	28.30663	27.46085	31.51297	31.56922	43.18308	44.42061
HORVU4Hr1G083080	27.56613	22.16679	23.29895	48.23987	50.23697	53.50961
HORVU4Hr1G090820	3.226631	5.366521	2.43135	56.36562	124.4481	92.03663
HORVU7Hr1G046080	7.137089	9.975574	9.264651	7.550624	7.345797	6.756584
HORVU4Hr1G067970	1.68217	1.966147	2.355949	4.56366	12.97664	11.64307
HORVU0Hr1G006630	9.558728	8.04283	10.18238	16.93163	17.75772	16.96837
HORVU6Hr1G011470	1.857857	2.081468	1.91487	0.830689	0.840367	1.096912
HORVU4Hr1G084730	19.1033	17.41027	28.14878	9.737578	12.87204	12.3188
HORVU2Hr1G060390	3.938701	4.513823	3.775108	6.032507	9.538839	8.130644
HORVU6Hr1G051740	26.3643	25.82206	36.99348	15.52747	20.45337	22.66638
HORVU6Hr1G027360	9.817918	5.965996	10.98645	12.13171	7.952748	11.89204
HORVU7Hr1G109590	9.195796	9.188475	11.08039	8.124584	12.23916	11.30149
HORVU2Hr1G038830	14.78507	13.13944	17.23257	22.19506	48.61506	40.38098
HORVU3Hr1G099120	3.653611	4.235377	3.690758	5.044544	3.509046	3.767692
HORVU4Hr1G079940	2.43495	1.840853	2.577265	0.898611	0.791329	0.652787
HORVU4Hr1G025820	12.95249	13.28187	12.34234	17.15217	21.85244	21.80839
HORVU2Hr1G076810	14.79438	13.31668	16.81374	10.36029	12.22416	11.58429
HORVU3Hr1G089370	2.785907	1.858492	2.840928	2.323057	0.906296	1.45958
HORVU7Hr1G075250	71.6017	75.5957	123.1278	51.99234	105.3464	104.7058
Hordeum_vulgare_newGene_3239	3.295742	2.701654	4.023552	6.873847	12.96944	11.35087
HORVU2Hr1G016040	9.152626	11.4691	11.04894	16.06162	27.63065	26.12028
Hordeum_vulgare_newGene_3233	5.180021	5.447328	6.364157	8.741762	13.60091	12.7109
Hordeum_vulgare_newGene_3237	4.897506	4.399657	5.185675	4.929297	4.196782	5.832797
HORVU2Hr1G010500	0.970247	1.214821	1.16651	2.147719	2.249532	2.337186
HORVU4Hr1G004210	2.785696	2.283942	2.815087	3.68082	4.253378	4.965668
HORVU4Hr1G064990	7.163032	8.197644	7.006136	6.538693	11.61949	12.74195
HORVU2Hr1G070310	39.45335	44.04267	56.58944	16.56542	1.780684	8.436344
HORVU4Hr1G054290	1.105856	2.108832	1.946387	3.443135	3.033869	1.893665
HORVU5Hr1G093880	0.982856	0.189799	0.801707	1.469194	2.709994	3.123833
HORVU4Hr1G026330	23.42629	26.24592	34.7063	17.15723	21.72354	23.97621
HORVU2Hr1G107560	1.401888	1.409877	1.496789	2.049759	3.467759	3.135774
HORVU0Hr1G039000	0.143766	0.537827	0.277095	5.991785	3.520825	5.673219
Hordeum_vulgare_newGene_253	5236.121	4709.679	4052.031	5482.01	1283.441	2211.243
HORVU6Hr1G056910	1.223978	0.844994	1.141607	11.90789	31.98573	29.52396
HORVU7Hr1G028850	1.679297	1.394041	1.48204	7.627258	16.93422	15.28568
HORVU4Hr1G059240	0.897208	1.072172	1.217964	2.147441	3.395211	3.023971
HORVU2Hr1G110580	14.34928	14.07945	18.6781	15.96005	16.3282	17.47534
HORVU2Hr1G096290	8.309003	8.521547	10.88264	6.479704	10.15782	8.609257
HORVU7Hr1G002940	0	0.009131	0.004756	4.216535	3.810293	4.469132

HORVU4Hr1G064660	5.893715	5.93888	6.63092	6.026474	8.037056	8.282625
HORVU7Hr1G008390	3.941078	4.511844	6.429966	3.052595	5.301705	5.059301
HORVU4Hr1G070150	10.3763	8.576114	8.742161	10.72453	24.05529	23.96241
HORVU5Hr1G083320	2911.136	2405.716	3419.647	211.3991	1.057262	83.82347
Hordeum_vulgare_newGene_13239	1.220438	0.62994	1.875124	1.742328	2.240161	2.172986
Hordeum_vulgare_newGene_13234	0.710811	1.030787	1.169759	11.9389	47.20323	36.39275
HORVU5Hr1G026020	19.28941	12.39922	16.02403	21.84154	17.34253	23.10098
Hordeum_vulgare_newGene_13237	1.256168	0.454659	0.960917	2.010699	1.723828	0.339679
Hordeum_vulgare_newGene_13231	2.070481	2.101564	1.973398	1.058025	0.751352	1.376731
HORVU2Hr1G030930	6.289768	6.209944	7.109348	13.05485	17.80601	16.60411
HORVU5Hr1G041830	1.656479	2.194362	1.888889	4.197566	9.55177	8.419375
HORVU3Hr1G020810	4.518832	4.890049	7.904636	5.709721	7.578377	6.6689
HORVU5Hr1G077880	31.39712	40.48546	52.38199	18.25144	0.469343	9.293497
Hordeum_vulgare_newGene_15172	40.32	34.8437	31.10746	126.877	189.3304	188.5261
Hordeum_vulgare_newGene_15174	6.509893	7.775479	8.569821	3.861851	2.898899	3.33419
Hordeum_vulgare_newGene_8805	2.434243	2.671092	2.728716	2.944869	4.498277	4.177101
HORVU1Hr1G000440	1.837668	1.644088	3.526411	18.54759	1.417193	12.49671
HORVU2Hr1G072220	12.28776	11.49193	14.56856	14.41404	21.08798	22.68404
Hordeum_vulgare_newGene_8809	6.415013	7.752072	8.319579	8.437687	9.096122	11.48197
HORVU3Hr1G104070	1.987688	2.850798	4.07952	4.024261	4.740652	5.132857
HORVU3Hr1G050320	3.745426	3.522162	5.468204	1.690287	1.946439	2.56652
HORVU7Hr1G034010	2.405795	0.940923	1.902221	3.298978	3.026581	2.551408
Hordeum_vulgare_newGene_5328	4.037407	2.993006	4.363605	2.735076	3.82224	3.886898
HORVU4Hr1G067100	17.63152	17.98886	16.45548	11.66419	10.04206	11.85316
HORVU6Hr1G016120	6.767231	7.632894	9.796899	6.505715	9.10666	9.152497
HORVU6Hr1G012810	8.292661	8.179319	9.948414	6.568463	11.03542	9.842414
HORVU2Hr1G116800	56.99367	47.2024	84.27082	26.48526	14.7693	23.50021
HORVU7Hr1G027490	81.67865	79.59596	98.82846	52.73585	58.00891	55.35939
HORVU4Hr1G061900	3.176602	2.809214	5.562032	0.399378	0.743292	0.757795
HORVU4Hr1G002720	4.079808	5.369489	6.060373	3.322608	4.256478	4.196845
HORVU4Hr1G024220	3.056348	3.179712	3.046401	3.786481	4.589563	4.596381
HORVU5Hr1G038920	128.8132	146.6022	236.4545	111.7722	243.3973	225.1973
HORVU7Hr1G026540	0.362686	0.30179	0.412273	0.904103	2.41959	2.436253
HORVU7Hr1G023790	3.91702	2.492851	2.944265	2.862512	3.604655	2.915073
HORVU7Hr1G081590	8.562684	9.186352	10.22729	9.636639	10.16282	8.656208
HORVU4Hr1G013780	0.740489	0.826054	1.065414	0.91638	1.295486	1.606149
HORVU1Hr1G032510	31.27521	30.82779	33.35123	37.00063	40.49582	43.13853
HORVU3Hr1G110920	17.91713	17.84842	20.03707	12.24727	14.74331	15.04035
HORVU3Hr1G096620	10.55427	5.622756	12.55282	5.174529	9.537605	7.344293
HORVU6Hr1G077520	1.164959	1.170329	1.372246	3.131933	5.657358	5.208976
HORVU3Hr1G094990	25.15333	25.29491	20.66164	39.95742	47.25523	45.52924
HORVU1Hr1G001420	35596.82	42828.22	27697.91	474.793	169.9527	238.262
HORVU2Hr1G073680	1.407856	0.981903	1.815604	1.7437	0.182502	0.853216
Hordeum_vulgare_newGene_8371	3.702787	3.391558	5.544564	3.399442	5.255017	5.122886
Hordeum_vulgare_newGene_8373	0.845051	0.87045	0.944112	1.754417	1.656752	1.543879
Hordeum_vulgare_newGene_8376	5.995469	7.579045	7.58006	0	0	0
Hordeum_vulgare_newGene_8377	0.827695	0.747897	0.7662	2.723509	1.844766	1.808985

HORVU3Hr1G091010	29.58369	27.60412	28.63623	27.53676	29.21509	27.37668
HORVU7Hr1G087750	0.88466	0.930992	0.999202	3.921907	3.814923	4.255432
HORVU2Hr1G076920	17.57557	17.70615	15.44021	18.16664	21.88926	23.69041
Hordeum_vulgare_newGene_1516	10.00363	11.40217	11.95493	12.07505	14.26873	15.24387
HORVU2Hr1G034580	4.565576	4.156263	4.92248	6.600714	8.170012	13.91735
HORVU6Hr1G093230	75.01608	62.82083	97.77165	71.90636	47.25659	44.83253
HORVU7Hr1G063510	5.073074	4.135821	5.551846	8.248581	14.69398	12.38447
HORVU7Hr1G066590	47.58932	55.19749	44.26854	43.31467	51.98031	54.32389
HORVU6Hr1G076430	20.99506	26.15319	22.95652	25.98379	34.96887	32.0025
HORVU0Hr1G023360	0.85761	0.976328	1.068418	1.592839	2.145368	2.312203
HORVU2Hr1G068970	6.187852	5.912778	5.891939	6.748415	9.030335	8.853893
HORVU6Hr1G020850	27.89266	31.8145	28.50164	20.29959	25.1697	25.41314
HORVU4Hr1G072620	1.035704	0.90507	1.529807	2.569805	6.41834	9.790562
HORVU6Hr1G060870	6.070575	6.448105	7.739749	12.13412	14.13716	14.13454
HORVU2Hr1G071860	8.520478	8.082068	10.80692	18.09697	31.6988	31.91021
HORVU4Hr1G066860	35.90151	29.9391	32.98529	72.82776	104.9344	103.8218
HORVU1Hr1G080890	5.671484	5.669831	6.473744	7.03828	13.92327	13.7143
HORVU1Hr1G069160	0.612013	0.730491	0.638122	0.274059	4.235692	2.680264
HORVU0Hr1G006800	17.43475	12.08585	18.7745	16.24918	17.4042	18.31344
HORVU7Hr1G058630	22.30298	26.13368	23.74347	0.04405	0	0
Hordeum_vulgare_newGene_15949	0.603243	0.900357	1.397145	0.983492	1.462217	1.072668
HORVU7Hr1G001570	0.850313	0.920105	1.090092	4.501271	10.0389	9.132257
Hordeum_vulgare_newGene_15945	0.963924	1.487782	1.169758	1.6992	1.013685	0.990001
HORVU6Hr1G070860	0.60207	0.926601	0.82861	7.860907	8.244194	9.588326
HORVU5Hr1G084580	0.683535	0.886443	1.432255	1.028308	0.963994	1.219125
HORVU7Hr1G100160	13.40302	14.32989	12.8913	12.69149	17.72461	17.63677
HORVU6Hr1G069390	4.709637	5.063578	5.438352	4.414847	5.857444	5.958738
Hordeum_vulgare_newGene_12626	2.170169	2.595118	2.153871	1.078807	2.267101	2.442248
HORVU5Hr1G103180	10.28721	8.674337	11.22115	10.21565	17.2324	15.71695
Hordeum_vulgare_newGene_12622	0.90168	1.047298	1.22587	2.826744	1.882293	1.78703
Hordeum_vulgare_newGene_12623	0.876095	1.335974	1.827166	0.841365	0.989529	1.076144
HORVU1Hr1G071060	1.570594	1.461752	2.957551	2.854271	3.058267	3.729167
HORVU3Hr1G030490	1.286748	1.507138	1.900805	1.752472	1.991289	2.762818
HORVU4Hr1G027140	1.217058	1.231508	1.515222	1.649045	2.175434	1.814458
HORVU3Hr1G017630	6.098956	5.996152	5.611206	6.520404	4.858738	6.397484
HORVU0Hr1G000710	8.932216	9.464203	11.14169	14.60066	20.1338	21.93343
HORVU7Hr1G035670	15.21032	10.10491	16.00736	18.00063	31.14346	22.72346
HORVU5Hr1G018410	15.12804	17.4636	13.79823	24.04914	26.74685	26.64119
HORVU1Hr1G060180	5.100818	5.634675	6.387366	7.179296	10.63399	10.77732
HORVU2Hr1G006830	0.617687	0.707908	0.61391	3.386238	4.978104	7.205738
HORVU6Hr1G075270	27.05138	34.23327	55.41225	0.688699	0.051193	0.310079
HORVU5Hr1G114250	31.95047	27.1243	35.87267	44.6606	53.80053	53.21909
Hordeum_vulgare_newGene_14707	8.56731	10.61034	9.715842	10.82411	11.19326	12.45124
HORVU7Hr1G034070	286.8922	365.4527	268.2407	267.0662	150.2997	142.6414
HORVU5Hr1G107030	1.78213	2.01882	2.180648	2.294204	2.250484	2.231518
HORVU3Hr1G086360	0.557517	0.504703	0.649438	1.458867	2.606247	2.963499
HORVU7Hr1G078000	11.08163	9.18027	11.1467	9.064402	9.492629	9.42888

HORVU6Hr1G029930	1.539896	2.309504	3.151453	2.661987	6.274241	7.003036
HORVU5Hr1G023190	40.07858	41.55458	47.90462	46.23004	61.74029	58.90368
HORVU3Hr1G053910	2.159814	1.954444	2.019807	2.643982	3.154575	3.457766
HORVU5Hr1G065520	4.207672	3.092407	4.797977	4.931138	4.80207	4.79213
Hordeum_vulgare_newGene_9819	1.943209	1.905405	2.016904	0.758771	0.327769	0.411798
HORVU3Hr1G117670	6.706021	7.405074	7.914586	10.18467	8.111297	8.210569
Hordeum_vulgare_newGene_9814	11.37806	10.45512	10.54146	14.61216	18.84497	17.57019
HORVU6Hr1G033070	9.068683	10.10093	10.96575	8.896682	8.041534	9.905984
HORVU7Hr1G116310	41.37866	33.07811	50.09745	26.02254	18.59248	23.03516
HORVU1Hr1G047010	1.344293	1.061449	1.594662	3.736357	2.444395	2.131067
HORVU1Hr1G061010	33.65251	33.36512	24.15277	17.2443	18.30828	20.65187
HORVU3Hr1G055420	2.034978	2.03858	1.954019	4.560713	5.734273	5.81575
HORVU6Hr1G064140	19.21063	14.37496	20.24567	84.56504	176.5717	131.376
HORVU6Hr1G039610	0.633493	0.904993	1.480834	1.875209	8.553596	7.269232
HORVU6Hr1G071380	0.101767	0.022398	0	3.058195	2.744091	2.783873
HORVU6Hr1G081950	7.23842	6.300933	7.238564	3.785567	2.656935	3.470843
HORVU2Hr1G040640	9.996144	10.09241	10.97476	14.4987	16.21057	16.99546
HORVU0Hr1G004470	0.889488	1.634798	1.768082	1.266816	2.093385	1.912559
HORVU5Hr1G088580	6.992801	5.450893	6.967135	8.512559	10.34623	8.748172
HORVU2Hr1G051140	1.373627	1.50049	0.539896	1.500568	2.33072	2.813954
HORVU3Hr1G087010	0.176427	0.187303	0.28073	19.25911	26.00741	25.37136
HORVU4Hr1G081100	17.55354	14.69245	20.10674	23.12744	26.37299	26.72554
HORVU5Hr1G024350	25.26941	25.49897	23.78989	25.65485	26.00844	26.74077
HORVU6Hr1G071030	18.65465	19.23143	34.55024	0.084914	0	0
HORVU3Hr1G082620	0.581243	0.792089	1.103629	1.217842	1.878378	1.666605
HORVU1Hr1G066240	7.137742	4.080555	8.740222	16.47884	19.39792	21.62199
HORVU7Hr1G108540	28.20287	26.50095	24.53335	26.4925	21.04927	21.0402
HORVU7Hr1G097760	0.718277	0.688517	0.924183	3.130575	3.438993	3.885715
HORVU5Hr1G113000	8.372505	6.816066	9.548278	8.685375	9.395631	9.442756
HORVU1Hr1G019570	4.980271	5.09247	5.466385	5.290982	7.497773	7.214349
HORVU3Hr1G032230	1.276999	2.233237	1.761518	1.698501	6.476044	5.928923
HORVU6Hr1G009910	1.40896	1.698751	1.787753	0.856916	1.144601	1.629379
HORVU5Hr1G093970	1.808115	1.648824	2.137232	1.749445	1.973731	1.826115
HORVU5Hr1G111530	54.83186	53.93726	50.5361	39.01864	40.15183	41.97285
HORVU5Hr1G109570	2.279755	1.311009	2.60141	8.712734	12.74152	12.75255
HORVU0Hr1G002730	27.74613	23.09191	32.07988	29.20369	39.74157	35.06237
HORVU7Hr1G025480	1.250724	2.488979	0.292812	0.584229	3.731951	4.136066
HORVU5Hr1G008950	8.166019	8.268699	11.04058	9.790401	13.42313	10.307
HORVU4Hr1G005800	1242.488	1205.837	1660.169	882.4343	537.4406	556.5245
HORVU5Hr1G050800	48.0482	47.49781	47.32227	40.53624	53.94333	49.92469
HORVU4Hr1G089590	37.42479	34.38352	55.89715	45.25137	65.48567	66.7985
HORVU6Hr1G011670	1.353637	1.845198	1.61747	3.281842	2.361373	1.058319
HORVU4Hr1G002460	19.64591	18.08471	18.18042	20.11589	25.44553	25.10251
HORVU6Hr1G031340	8.024799	5.539958	9.524959	20.75983	42.07457	35.8041
HORVU2Hr1G065700	17.57139	15.94759	15.96972	15.33098	11.02186	12.76221
HORVU5Hr1G067380	0.598842	0.332288	0.820645	3.212269	16.77473	12.49503
HORVU7Hr1G036690	7.905632	8.568679	9.08821	7.161297	5.927393	7.312216

HORVU2Hr1G081780	4.611243	3.159104	5.613038	4.124964	3.696816	4.651839
HORVU5Hr1G078800	2.617432	1.334578	1.850703	2.943533	2.259074	3.186906
HORVU4Hr1G011100	41.96566	35.06182	41.34892	45.17779	36.29648	36.05395
HORVU2Hr1G106010	10.5551	9.752205	11.4872	15.96774	16.13847	17.42605
HORVU5Hr1G097530	9.690875	10.02001	9.805414	9.771316	13.07755	12.40182
HORVU2Hr1G034270	17.65051	13.60622	17.72341	26.10625	38.48727	34.34035
HORVU2Hr1G085940	1.069195	1.259296	1.684165	1.440329	2.033018	1.844546
HORVU4Hr1G082730	25.75161	21.3841	26.12305	22.90164	34.20083	38.13571
HORVU1Hr1G087990	0.507893	0.501382	16.44785	44.96184	1.100193	0.544405
HORVU5Hr1G112840	3.09127	3.636389	6.213387	2.093877	4.638898	4.912058
HORVU2Hr1G098140	13.82035	11.74968	15.63145	0.463563	0.414538	0.266171
HORVU4Hr1G069860	5.602753	5.22476	7.300114	2.106959	0.204961	0.60701
HORVU3Hr1G070880	7.516504	9.21778	8.862754	7.688588	7.673301	8.832348
HORVU1Hr1G058790	3.561326	4.273138	3.232504	7.981552	9.499577	10.68638
HORVU1Hr1G063320	43.07187	37.26517	52.04303	44.50156	50.39664	45.16131
HORVU5Hr1G058490	7.329271	7.934981	8.371108	11.16122	13.73765	15.07884
HORVU1Hr1G057140	3.718536	4.2967	4.902387	6.37712	8.6711	9.117406
HORVU2Hr1G064070	4.336653	4.994935	6.123355	6.092066	7.254365	6.899907
HORVU0Hr1G013190	1.11231	0.878368	0.888208	9.561812	18.64575	16.41172
HORVU5Hr1G104090	183.4844	149.0518	180.5676	251.4439	308.6164	273.1988
HORVU5Hr1G056920	32.59246	29.07016	29.35151	23.99198	27.3511	26.38975
HORVU1Hr1G058860	9.833097	9.4283	10.55144	13.10819	18.82761	18.9644
HORVU2Hr1G107760	0.642711	0.872885	1.483928	2.437393	1.64113	2.264274
HORVU5Hr1G044460	3.873073	4.130207	4.470992	5.019481	7.022287	7.699986
HORVU7Hr1G012120	16.40221	16.7886	26.92229	12.70258	22.23783	19.54089
HORVU7Hr1G088160	1.702089	3.163726	3.24025	3.745477	3.830718	4.681857
HORVU0Hr1G013220	5.808461	5.659602	6.224097	10.79878	23.19393	22.87718
HORVU7Hr1G006260	1245.978	187.533	1415.662	40.24501	531.1409	465.5988
HORVU1Hr1G069400	1.109189	0.824876	1.208859	1.460799	1.84743	1.929733
HORVU2Hr1G019370	0.899588	1.03171	1.030014	1.21305	1.030159	1.194543
HORVU5Hr1G105970	5.048416	4.887682	6.839206	4.444959	6.467907	6.80118
HORVU3Hr1G065170	0.209466	0	0	0	3.310192	3.103859
HORVU5Hr1G080580	1.656931	2.140778	1.607008	5.838524	7.275207	7.606112
HORVU1Hr1G080750	17.08665	14.98731	17.1823	9.878181	9.509655	8.739593
HORVU5Hr1G001920	17.27932	13.58815	15.70984	25.4863	12.06728	19.42925
HORVU4Hr1G005940	6.277028	5.439773	8.112006	8.859647	12.64507	13.16023
HORVU3Hr1G064590	11.70567	6.115026	7.179914	7.497899	3.328689	6.954948
HORVU2Hr1G113450	2.89626	3.147478	5.141199	5.0044	10.90361	8.92514
HORVU7Hr1G085020	2.394974	3.102997	6.284282	2.4236	4.548002	4.539492
Hordeum_vulgare_newGene_10601	1.689114	2.327147	2.336095	0.005407	0	0
HORVU3Hr1G077690	0.113458	0.09841	0.040911	0.223644	8.888414	8.576329
HORVU3Hr1G040230	0.248283	0.152044	0.203239	0.667368	2.749681	2.008754
Hordeum_vulgare_newGene_10477	4.041444	5.887067	3.730015	0.326543	5.068232	3.657669
Hordeum_vulgare_newGene_10475	3.043782	2.846154	1.160531	0	2.14303	2.075503
Hordeum_vulgare_newGene_10474	17.04237	18.61362	18.68105	24.61038	30.56039	30.64328
Hordeum_vulgare_newGene_10473	22.42593	22.37142	22.15934	31.09231	35.46503	37.22544
Hordeum_vulgare_newGene_10471	2.112806	2.298205	2.674992	4.86175	5.505981	4.926919

HORVU7Hr1G012780	0.106574	0.330668	0.026097	2.515391	4.423957	3.797109
HORVU2Hr1G111340	25.04301	22.90859	23.25562	35.15777	35.48591	35.22406
Hordeum_vulgare_newGene_10478	72.84698	84.52963	48.50163	36.36157	17.2946	23.82721
HORVU0Hr1G028170	0.134207	0.208069	1.41507	1.518758	1.954702	2.500426
HORVU7Hr1G090100	26.57704	29.77577	28.04332	56.59682	73.73839	86.1422
HORVU4Hr1G007510	2.500816	2.821766	3.315863	4.340952	5.971853	5.074305
HORVU3Hr1G078420	0.821083	0.611296	0.935592	2.85559	2.682221	2.26664
HORVU1Hr1G083730	43.15414	42.17527	39.95721	40.97352	42.96899	44.53693
HORVU7Hr1G082730	29.6017	25.48194	31.48169	30.40183	49.7586	52.71595
HORVU6Hr1G079140	7.168944	7.897882	10.988	8.610792	11.99636	12.44138
HORVU4Hr1G055940	2.073473	2.710996	2.454445	1.536756	2.378985	1.668388
HORVU7Hr1G089610	20.82667	20.38647	23.33767	16.29958	19.14094	19.73319
HORVU6Hr1G047130	1.220073	0.951763	0.862298	1.158465	2.525425	1.443014
HORVU2Hr1G119110	7.571778	5.809505	7.223909	14.1527	13.16705	11.84604
HORVU5Hr1G124410	52.60129	44.82743	48.92048	53.78028	43.24236	44.20027
HORVU3Hr1G003920	3.59725	2.755066	4.862888	3.489081	1.390815	2.824455
HORVU3Hr1G100190	35.21047	17.24921	44.10307	1.97119	0.26535	1.885149
HORVU3Hr1G072070	4.026642	2.788443	3.093358	4.57839	8.116792	6.275446
Hordeum_vulgare_newGene_11707	8.68552	7.797706	9.532962	9.307223	10.80757	10.77772
Hordeum_vulgare_newGene_11705	5.293506	6.72308	7.746608	0	0	0
HORVU7Hr1G000370	26.97565	29.39195	25.24068	24.1156	22.21841	21.78474
HORVU1Hr1G081770	0	0	0	0.968889	3.599872	4.450748
HORVU2Hr1G118640	10.29125	11.80795	13.76221	9.799628	8.449388	8.346687
HORVU4Hr1G067340	1.387969	1.041838	1.3382	21.22825	27.716	24.62797
HORVU1Hr1G001220	2.607165	12.57905	4.679352	24.86394	0.007671	0.367695
HORVU3Hr1G115020	4.379296	5.814121	7.003954	2.308407	3.452988	3.834688
HORVU3Hr1G095730	4.933397	2.895739	6.791381	3.371258	0.607477	3.21018
HORVU2Hr1G022140	17.66855	21.19001	27.76701	15.81363	17.94109	21.44251
HORVU7Hr1G075860	5.03313	5.707979	6.292007	7.075591	7.643322	8.258671
HORVU7Hr1G044850	35.6091	41.05211	41.45186	28.98158	23.10443	29.27401
HORVU3Hr1G034210	1.538593	1.599583	1.522888	3.759786	3.705885	3.985112
HORVU1Hr1G066760	4.226466	4.564669	5.431695	6.898924	7.025679	8.498391
HORVU2Hr1G118390	1.196863	1.651715	1.496916	0.779478	1.014299	1.457289
HORVU3Hr1G038260	3.556515	7.783116	8.168453	10.84069	9.479782	10.42077
HORVU4Hr1G002870	37.05271	33.12393	36.7814	39.15729	36.44701	38.01378
HORVU6Hr1G018280	2.253745	1.706409	2.473409	4.085557	5.030706	4.396971
HORVU7Hr1G121090	2.813754	1.218864	4.446173	0.184051	0	0.25376
HORVU6Hr1G040540	3.747656	3.946133	4.23785	4.803625	4.194957	5.328306
HORVU5Hr1G072780	5.946761	4.000887	6.120186	1.415352	0.411212	0.525533
HORVU6Hr1G095100	18.18461	14.74246	15.8241	30.38807	28.41842	30.13655
HORVU7Hr1G046980	6.120198	5.943499	8.022847	7.557257	11.02242	12.28876
HORVU1Hr1G019630	9.682756	9.889128	16.77409	9.47433	16.30168	14.52263
Hordeum_vulgare_newGene_3900	5.648305	6.370511	7.788209	13.43747	33.3352	28.1254
HORVU7Hr1G076360	9.9026	12.5616	10.54718	11.10077	12.66973	13.78498
HORVU7Hr1G121630	4.672682	3.762041	3.249892	2.490702	2.913332	3.049715
EPlHVUG00000039881	1.184228	1.974038	2.712579	0.394379	0.204983	1.002291
HORVU7Hr1G109940	4.709998	5.418097	4.548884	4.885045	4.739024	5.001168

Hordeum_vulgare_newGene_16169	20.82026	28.52573	29.75719	7.834005	1.401534	3.919679
HORVU5Hr1G049080	2.15183	2.753618	2.818678	3.735026	5.006018	5.809552
Hordeum_vulgare_newGene_16165	2.797497	2.461667	2.5568	2.425868	4.770426	3.796463
Hordeum_vulgare_newGene_16162	4.000608	4.624338	3.316783	6.341611	6.780702	7.782087
Hordeum_vulgare_newGene_16161	4.160061	4.938522	4.206562	6.402584	7.651781	8.522225
Hordeum_vulgare_newGene_16160	1.091906	1.169977	0.937863	1.510446	1.788755	1.910309
HORVU1Hr1G033060	4.616793	3.504004	5.207582	6.809843	8.80456	8.849465
HORVU1Hr1G013490	2.863556	2.881222	3.179013	2.448433	2.249204	2.638225
HORVU7Hr1G081910	16.94991	19.23957	28.46591	12.2519	27.97662	28.99759
HORVU3Hr1G117390	9.724322	6.936517	13.14687	2.277426	0.943689	3.068535
HORVU1Hr1G001910	78.42104	82.88732	73.15169	75.33837	55.47783	59.49241
HORVU3Hr1G057750	12.35616	11.7222	17.34148	14.15696	11.20872	10.55543
HORVU5Hr1G071780	5.581189	4.598811	5.298732	4.251489	3.319792	3.938761
HORVU4Hr1G071250	5.114913	3.125183	5.867811	3.500966	11.08545	9.413854
HORVU5Hr1G071250	6.52957	6.874768	7.330365	11.02383	14.85819	13.05785
HORVU2Hr1G065060	3.479215	4.68524	5.431329	0	0	0
HORVU7Hr1G100360	0.886664	1.201297	1.161886	0.665828	1.579911	1.118964
Hordeum_vulgare_newGene_12423	22.9154	26.20856	26.78439	20.24491	4.506408	9.887296
HORVU7Hr1G047760	30.74193	26.56193	30.1426	43.74202	58.03722	52.18368
HORVU1Hr1G071260	23.46444	17.36186	20.44976	17.4437	27.55814	26.1935
Hordeum_vulgare_newGene_12429	2.771445	2.077091	3.100381	4.584057	4.931412	5.948383
HORVU7Hr1G105620	0.03289	0.01182	0.070308	2.13825	2.566413	2.05982
HORVU3Hr1G066240	2.180675	2.271661	2.56449	2.954398	2.495432	3.131579
HORVU1Hr1G047820	2.315322	2.122229	2.417015	4.651353	4.718484	5.531112
HORVU4Hr1G037030	32.02979	33.28992	32.8358	41.28864	53.90278	55.92016
HORVU3Hr1G116700	1.875208	1.208766	2.560511	0.80826	1.373825	1.375185
HORVU4Hr1G065470	0.421732	0.641333	0.53995	2.657387	7.474466	6.560342
HORVU6Hr1G088480	2.566231	2.06065	1.836779	1.109416	0.063674	0.429034
HORVU5Hr1G075570	7.089195	7.684167	8.65964	11.80691	24.51435	19.68343
HORVU2Hr1G053110	6.773131	5.679188	7.284513	9.220187	10.96755	12.72051
HORVU3Hr1G077800	69.77214	61.505	74.38222	32.90926	15.79249	23.60066
Hordeum_vulgare_newGene_14144	0	0	0	1.026317	38.79242	30.85314
HORVU7Hr1G075040	1.344476	1.404648	1.772472	1.505311	1.470023	1.49991
Hordeum_vulgare_newGene_14148	0.542062	0.561891	0.482698	2.00573	4.355828	3.372403
Hordeum_vulgare_newGene_3794	6.177368	6.837553	8.336704	7.009382	10.34216	10.45786
HORVU2Hr1G086140	745.3263	669.0493	852.3519	932.6971	976.8272	1054.698
Hordeum_vulgare_newGene_3799	0	0	0	0.42941	30.04596	29.31823
HORVU1Hr1G094090	2.265171	0.110223	0.570772	2.035886	5.4559	1.775997
HORVU3Hr1G113750	9.328211	10.38178	12.63613	9.067136	13.99404	11.98737
HORVU7Hr1G072890	14.83428	10.82134	11.88847	6.868599	6.232724	6.042481
HORVU2Hr1G065720	18.14463	16.21304	15.3831	15.28033	10.99276	11.88431
Hordeum_vulgare_newGene_449	0.464904	0.6704	0.707511	1.888228	3.130475	3.130736
Hordeum_vulgare_newGene_10861	1.787238	2.515781	2.456838	1.987705	11.45285	8.769983
Hordeum_vulgare_newGene_996	1.264641	1.568276	1.956782	1.577131	2.724877	2.990326
HORVU3Hr1G055620	2.344576	2.00013	2.096642	2.580198	0.972133	1.31569
Hordeum_vulgare_newGene_9592	0.251013	0.127088	0.8482	3.530905	0.787105	0.956875
Hordeum_vulgare_newGene_5825	15.19251	12.65948	16.09035	18.78025	23.99739	22.72399

Hordeum_vulgare_newGene_5822	2.527079	3.055336	3.162088	0	0	0
HORVU6Hr1G049800	0	0	12.17671	12.16134	14.98008	18.70574
HORVU1Hr1G095060	6.738027	5.977017	7.988247	6.410831	8.280449	8.364777
Hordeum_vulgare_newGene_2440	3.261527	5.012206	4.48178	6.516009	7.677432	8.186754
Hordeum_vulgare_newGene_2442	6.14734	6.915025	7.355265	5.094397	8.896834	8.770561
Hordeum_vulgare_newGene_2443	3.359909	4.01949	4.5688	4.679515	4.779231	4.83336
Hordeum_vulgare_newGene_13791	1.930995	1.525864	2.00407	1.953766	1.522578	2.296365
Hordeum_vulgare_newGene_2446	0.499078	0.53475	0.669324	1.417452	2.471228	2.316463
Hordeum_vulgare_newGene_2447	16.69475	15.17753	14.99919	20.05719	26.62622	25.59162
Hordeum_vulgare_newGene_2448	1.576415	2.112243	2.161944	2.614995	3.889038	3.896816
HORVU4Hr1G070720	4.512443	4.224798	4.878997	6.181397	8.1583	7.842046
HORVU3Hr1G055990	31.45882	29.95809	31.27803	32.35024	36.74248	36.62603
HORVU5Hr1G078090	5.140853	4.914312	6.857703	4.924423	6.568273	7.656388
HORVU6Hr1G084770	1.291749	1.137835	1.38081	3.028543	1.151124	1.758595
HORVU4Hr1G076970	0.790551	0.772657	1.435986	3.210287	2.695279	4.413924
HORVU2Hr1G037570	4.626224	4.691946	4.121854	5.715835	7.812229	8.516895
HORVU1Hr1G059220	14.94797	17.75729	15.16109	20.46869	25.4565	24.13034
HORVU0Hr1G009170	17.17871	10.51538	17.15209	9.773536	14.80675	14.45414
Hordeum_vulgare_newGene_5795	2.205787	1.674026	2.469821	3.699854	4.363915	5.471056
HORVU7Hr1G119680	1.282022	1.415507	1.514498	2.292923	2.672966	2.633363
Hordeum_vulgare_newGene_5796	6.357546	6.645283	2.866424	0.005767	0	0
Hordeum_vulgare_newGene_5790	0	0	0.032983	0.525955	3.224112	3.578103
Hordeum_vulgare_newGene_1236	59.60931	42.87273	66.03221	82.36661	53.98399	67.35632
Hordeum_vulgare_newGene_1233	8.805518	5.609615	10.95334	6.863308	3.297585	7.14531
Hordeum_vulgare_newGene_1231	11.56879	12.08131	13.91789	9.207193	17.28406	16.10141
HORVU5Hr1G063440	1.427844	1.159756	1.450996	1.850918	1.653574	1.824891
Hordeum_vulgare_newGene_10132	9.055053	8.659855	10.27754	7.432761	8.050049	8.684276
Hordeum_vulgare_newGene_10133	2.84156	2.4876	2.874545	1.58218	2.247637	1.792729
Hordeum_vulgare_newGene_1238	5.493814	3.618777	6.22313	7.312578	4.196699	6.253706
HORVU3Hr1G042890	30.75695	30.97071	31.49011	46.53157	46.49892	47.09412
HORVU6Hr1G013770	3.995825	4.043411	3.996224	5.045597	6.544122	6.124054
HORVU3Hr1G060390	9.557964	9.118478	7.661967	16.68947	23.71249	23.86586
HORVU4Hr1G060150	14.53138	14.31409	19.86439	15.8539	10.5792	7.997069
HORVU3Hr1G093330	12.26789	11.43574	13.35443	11.56118	12.02521	13.42064
HORVU0Hr1G002530	5.180278	4.884318	6.803095	2.315087	1.978872	2.67351
HORVU3Hr1G096120	1.029258	1.050577	1.313327	3.264811	8.619494	8.82457
HORVU7Hr1G122750	1.498422	2.073658	2.268618	2.456173	3.187748	3.587369
HORVU0Hr1G011860	1.34509	1.630943	1.946137	1.995342	1.637472	2.315128
HORVU7Hr1G030220	14.17219	12.93408	14.69389	13.98952	14.46954	16.32438
HORVU7Hr1G100690	9.644236	8.202702	11.89251	19.08144	38.40686	37.00658
HORVU7Hr1G114620	83.7128	80.59666	85.36693	124.8848	177.8681	182.1408
HORVU1Hr1G008350	14.58551	15.99511	18.87773	10.8499	13.00808	12.04164
HORVU6Hr1G058000	1.029513	1.085881	1.505029	2.297545	3.662823	5.333215
Hordeum_vulgare_newGene_709	1.837854	0.66767	0.914431	1.885018	2.221204	2.060275
HORVU4Hr1G077730	1.872318	1.930001	2.333609	0.44287	0.861193	1.409708
HORVU4Hr1G045210	2.663553	1.977498	1.761766	2.421826	2.973646	3.044029
HORVU3Hr1G014290	16.52696	12.1241	16.09508	18.3194	24.92318	27.44729

HORVU2Hr1G046370	6.679067	5.585492	7.328713	9.446214	3.599783	5.098701
Hordeum_vulgare_newGene_1490	7.99856	8.318161	9.249734	0.149269	0.263165	0.202656
Hordeum_vulgare_newGene_1497	0.687825	3.9866	9.383177	1.651419	0	0
HORVU3Hr1G083800	1.04717	1.115238	0.896665	1.027205	0.965991	1.035511
HORVU2Hr1G078960	3.708107	2.250063	4.166546	6.495936	5.651718	6.427378
HORVU4Hr1G059120	9.360091	8.398108	9.743592	14.35075	16.04978	15.789
HORVU1Hr1G016370	7.810606	8.358174	7.982838	11.52536	16.21941	16.34513
HORVU7Hr1G031330	2.836261	3.717884	3.105727	2.934963	3.633058	3.466168
HORVU3Hr1G116350	4.931587	3.978107	5.229574	4.385638	6.753172	6.38718
HORVU1Hr1G068110	0	0	0	1.043053	2.648355	2.509956
HORVU3Hr1G049640	3.173141	2.475856	3.45051	3.832256	3.679365	5.164762
HORVU5Hr1G067630	4.875026	5.048305	5.216713	5.916708	8.940855	8.230433
HORVU5Hr1G105870	65.79351	65.24001	86.5859	58.32575	87.26571	88.13165
HORVU0Hr1G028270	3.014115	2.272286	2.95024	4.573658	4.905519	5.961453
HORVU2Hr1G035220	1.943411	1.838917	2.109293	5.588145	9.79441	9.618212
HORVU6Hr1G024180	425.9778	398.3756	332.3726	240.6521	1241.354	1163.275
HORVU7Hr1G052850	0.902814	0.604605	1.088127	2.204395	2.843376	1.603092
HORVU5Hr1G085580	5.350047	4.956038	7.39836	5.158824	7.13933	6.310151
HORVU1Hr1G072290	3.68442	2.95209	3.756757	7.645419	11.29179	9.664054
HORVU7Hr1G008670	5.486957	3.720602	4.488351	4.335271	3.975037	4.636006
HORVU7Hr1G053180	1.176397	0.643226	1.16047	1.458717	1.09185	0.875582
HORVU6Hr1G032640	8.350243	7.701553	7.671649	9.509962	12.93871	12.02364
Hordeum_vulgare_newGene_7536	501.1295	357.5631	481.5068	1133.028	1285.416	1236.862
Hordeum_vulgare_newGene_3893	23.26898	24.39513	29.7774	17.28247	29.39179	29.54557
HORVU4Hr1G046540	6.747379	6.550251	8.442136	7.719173	10.64584	11.37628
Hordeum_vulgare_newGene_7531	1.545262	1.562629	1.47443	3.018987	2.0227	3.385685
HORVU3Hr1G091200	88.79616	74.25379	100.978	113.5018	104.1033	105.784
HORVU2Hr1G077890	16.46943	14.76105	18.42018	17.08924	17.9928	18.92825
HORVU7Hr1G052580	46.23826	39.36336	50.0598	21.64398	17.87936	17.5023
HORVU2Hr1G045130	40.60197	35.92557	39.88597	33.10384	40.7409	37.79844
HORVU7Hr1G098110	0	0	0	0.61395	6.023651	6.042391
HORVU3Hr1G086970	12.80261	12.24577	15.93131	10.52305	4.653618	7.359535
HORVU6Hr1G073280	7.787384	7.555765	9.500341	4.643094	6.05438	5.695172
HORVU5Hr1G092170	7.32361	7.712527	7.653856	7.379255	6.434736	7.499105
HORVU5Hr1G125120	1.241482	0.931063	0.82535	4.531609	5.799391	5.314116
HORVU7Hr1G025200	0.031711	0.044236	0.308591	1.903532	13.32935	10.40964
HORVU2Hr1G084930	4.112673	4.913317	4.845824	5.390367	7.285327	8.219985
HORVU7Hr1G018340	14.14363	12.55256	13.08315	10.6694	11.76943	12.64678
HORVU3Hr1G116060	33.17077	19.61471	34.98508	44.18216	45.1507	62.9868
HORVU4Hr1G001900	0.294003	0.313362	0.522708	1.970173	2.121819	2.042576
HORVU2Hr1G032240	2.045004	2.760811	3.077769	2.400009	15.64965	5.571015
HORVU7Hr1G051580	40.29731	33.96985	42.07243	50.86971	68.66647	57.3276
HORVU7Hr1G035820	0.103343	0.46658	0.747146	0.370654	4.240008	5.967849
Hordeum_vulgare_newGene_6379	0.658765	0.78101	1.098683	1.297666	4.818008	4.912933
HORVU0Hr1G005260	1.942307	1.815628	1.567186	2.654425	6.735614	5.525224
HORVU6Hr1G032510	30.90477	25.23189	30.74948	34.17503	34.34908	33.02166
Hordeum_vulgare_newGene_6371	4.114413	3.746051	4.740071	3.096195	3.27915	3.804845

HORVU1Hr1G022130	44.34829	39.06735	42.99827	40.66987	41.9988	40.6589
Hordeum_vulgare_newGene_2981	13.56032	17.69324	17.27625	14.10078	11.21838	15.26955
Hordeum_vulgare_newGene_2980	2.202072	2.54799	2.645228	0.414258	2.588384	0.445582
HORVU6Hr1G004910	0	0.057355	0.205572	0.272661	5.383902	2.681301
HORVU6Hr1G081850	42.73954	35.46368	35.00179	31.95373	50.75102	56.71805
HORVU2Hr1G083860	7.263885	6.155251	9.211716	8.227907	10.61028	9.829847
HORVU6Hr1G038770	11.4742	12.75018	14.55018	13.71441	18.30506	16.49118
HORVU1Hr1G080190	1.510761	0.909093	1.883666	8.394332	31.9673	32.12314
HORVU2Hr1G106880	0.6345	0.77296	0.773432	3.013157	4.820613	4.950311
HORVU5Hr1G102770	9.537182	7.828767	10.02526	14.46853	20.46684	18.12681
HORVU5Hr1G124380	6.21068	6.598913	7.199902	15.48121	13.98315	12.42112
HORVU6Hr1G074930	9.208105	8.618311	13.64233	9.9503	13.45273	13.5874
HORVU4Hr1G069230	10.57087	9.937961	10.83925	23.65594	35.72599	33.0129
HORVU5Hr1G062450	1.218648	1.189149	1.703161	1.035455	0.930501	0.944758
HORVU1Hr1G094250	2.620369	2.49108	4.28955	6.811041	10.68588	8.88573
HORVU2Hr1G027750	2.005735	2.078031	2.116597	1.147123	3.894881	2.285303
HORVU3Hr1G038460	55.89884	50.10962	70.47405	55.95782	89.50453	83.72929
HORVU4Hr1G056550	1.823424	1.857873	2.258208	4.153172	4.572755	4.026813
HORVU7Hr1G001940	1.524116	1.371099	1.726609	2.472106	4.082377	3.970583
HORVU5Hr1G015850	0.728541	1.30071	0.846167	0.7574	5.598889	4.333535
HORVU7Hr1G040110	6.212343	5.543726	6.203079	5.288169	8.064021	7.681482
HORVU3Hr1G033350	4.099753	3.420501	4.676289	5.944149	8.417016	8.32159
HORVU5Hr1G114960	10.18349	9.658631	11.19664	10.03201	10.18006	10.28158
HORVU3Hr1G037630	1.371496	1.473015	2.085014	0.801697	1.014101	1.384163
HORVU4Hr1G043300	23.94633	29.64474	40.2244	19.13647	27.06568	30.5552
HORVU5Hr1G105790	1.842814	2.621574	2.910245	13.78869	10.38252	13.19412
HORVU1Hr1G017720	32.47501	42.97479	37.03041	18.06362	11.89718	15.04191
HORVU6Hr1G094530	0	0	0	0	7.705706	8.446242
HORVU7Hr1G113400	10.34502	9.855317	10.8673	10.47105	10.74159	10.25267
HORVU2Hr1G092930	20.22492	21.22793	23.75478	20.46547	21.71979	22.19252
HORVU1Hr1G018380	1.448933	1.794714	2.067649	2.420625	4.496548	5.209472
HORVU3Hr1G012100	1.374776	1.33883	1.531659	1.641278	1.421165	1.699854
HORVU7Hr1G120120	5.792522	3.510325	5.592476	5.151131	4.479226	5.787495
HORVU2Hr1G082420	206.8145	212.9921	174.6938	219.2777	242.4993	231.2398
HORVU1Hr1G088190	0	0.056252	0	44.96709	0.082047	0.143615
HORVU5Hr1G021140	30.55963	27.56757	43.95147	23.96954	44.9807	45.08427
HORVU5Hr1G055840	103.7459	94.25451	136.4181	92.59145	111.1857	101.6176
HORVU6Hr1G085360	1.018022	1.241737	1.12979	7.306045	8.104512	7.927752
HORVU6Hr1G001980	27.46954	26.18538	25.28797	25.25307	25.11029	29.43191
HORVU2Hr1G084110	0.78508	0.768004	0.789865	1.341923	1.054244	1.266323
HORVU5Hr1G038720	16.82986	13.33535	16.92405	16.44299	23.69749	23.76471
HORVU4Hr1G088670	0	0	0	0.396962	7.042669	3.928637
HORVU7Hr1G056020	5.53	5.97339	7.245657	4.975226	3.419235	6.139575
HORVU4Hr1G009390	20.759	24.92465	22.17519	15.46257	15.79762	16.61927
HORVU5Hr1G106840	7.721909	6.745688	15.41929	11.37043	14.90529	14.05583
HORVU3Hr1G067620	11.98867	13.90304	9.619344	5.720427	5.612739	5.719962
HORVU2Hr1G094690	2.707767	2.66886	2.543728	4.105552	2.82149	4.567729

HORVU3Hr1G084410	1.037834	1.402866	1.033254	3.008806	4.817171	3.625638
HORVU3Hr1G008420	60.26234	61.29703	50.74536	52.94513	53.49534	53.89912
HORVU7Hr1G090760	17.03838	13.42804	17.61346	18.27649	19.70056	19.55175
HORVU2Hr1G111930	0.93255	0.708896	0.245748	2.634453	2.691307	3.039361
HORVU6Hr1G005290	2.081436	2.903076	2.074529	3.397935	7.452029	6.353037
HORVU7Hr1G106500	11.31527	8.947715	10.40629	6.622611	8.750442	7.89122
HORVU6Hr1G091310	30.94762	31.00973	26.79327	21.86918	20.48907	20.91204
HORVU5Hr1G067170	1.853548	1.353438	2.067471	2.376935	3.388059	2.974286
HORVU7Hr1G082910	8.237632	9.276785	11.15966	9.379787	14.56692	14.83772
HORVU2Hr1G017290	7.195941	6.603707	6.758105	10.41985	11.24911	11.71301
HORVU4Hr1G015490	11.2829	9.963311	11.91293	7.496695	4.086281	4.662021
Hordeum_vulgare_newGene_11937	0	0	0.021244	2.977842	2.654164	3.127541
HORVU1Hr1G050220	33.68396	34.04814	55.14218	23.17342	25.62547	23.3228
HORVU1Hr1G042450	0.690264	0.531992	0.702475	1.387813	1.807615	2.000897
Hordeum_vulgare_newGene_1796	2.322045	3.730351	3.052313	2.744907	4.639042	5.326576
HORVU2Hr1G016740	39.17229	32.91095	40.37882	34.89277	50.50754	49.53574
Hordeum_vulgare_newGene_8242	13.86534	13.74424	12.95913	16.08701	18.93729	19.37388
HORVU2Hr1G099110	3.278012	2.844683	2.344898	2.882508	3.664104	3.886822
HORVU1Hr1G043580	51.07557	32.60567	46.49478	114.9598	110.3451	111.8739
HORVU5Hr1G031300	18.26825	21.85511	20.23176	21.27398	22.98758	23.81455
HORVU6Hr1G084010	13.68897	19.69822	27.07347	0.297085	0.0698	0.257506
Hordeum_vulgare_newGene_8245	5.725936	6.164958	4.458935	39.1046	62.52832	56.54102
HORVU3Hr1G047040	2.109918	2.457782	2.67659	5.183524	7.842047	8.796137
HORVU0Hr1G014170	5.566771	3.44141	4.263564	14.06818	17.64566	15.96015
Hordeum_vulgare_newGene_5628	1.643228	1.417103	1.62602	1.994985	2.457614	2.326941
HORVU3Hr1G064410	2.952275	3.226112	3.7422	3.133528	4.024241	5.670379
HORVU1Hr1G056000	0.968896	0.641385	0.8756	1.546483	3.346296	2.202452
HORVU3Hr1G071890	2.903245	3.245285	3.721596	6.111964	7.512646	8.194631
HORVU0Hr1G013200	1.151619	0.628129	0.713014	2.120199	2.887626	2.6662
HORVU7Hr1G091170	12.5422	12.31454	14.04716	14.24758	14.27636	12.88362
HORVU2Hr1G078250	19.9893	18.88221	19.46284	21.77954	24.34558	25.4856
HORVU7Hr1G028630	5.280914	3.32452	3.171718	2.836743	5.227863	4.789927
HORVU4Hr1G059610	12.24625	11.03799	12.25482	11.35647	11.65413	13.23056
HORVU7Hr1G095840	23.42492	23.64348	16.89881	3.892239	7.041018	5.192084
HORVU2Hr1G014670	6.624421	5.724516	5.793038	8.659816	9.221155	6.953527
HORVU7Hr1G081320	2.697403	2.502057	3.563361	0.194649	0.082581	0.15964
HORVU7Hr1G093440	5.567774	5.439134	5.665972	6.4275	8.308942	8.275014
HORVU5Hr1G117720	16.8234	17.825	15.27481	20.22501	23.94403	24.05591
HORVU6Hr1G045130	15.8374	15.2035	17.32028	15.57849	18.85132	19.67602
HORVU7Hr1G083790	4.252704	3.752749	4.668086	5.604449	5.415063	5.626566
HORVU2Hr1G089830	2.185774	3.308857	3.644571	3.64628	3.878889	4.079333
HORVU2Hr1G037770	32.52231	28.9337	31.62624	24.4481	26.13218	25.39727
HORVU2Hr1G001640	14.36036	14.34306	12.79089	12.41517	15.83051	14.65858
HORVU6Hr1G021260	0.134949	0.115526	0.098936	1.430411	9.777663	6.961062
HORVU2Hr1G092690	17.17563	17.73247	16.3261	16.67461	22.37237	23.25204
HORVU4Hr1G068960	4.012132	4.114095	3.984308	3.410381	6.665231	7.055306
HORVU4Hr1G055040	14.9721	15.1252	18.1344	14.69509	23.38847	21.61564

HORVU7Hr1G026000	384.6098	363.2963	332.2006	244.0309	69.57467	99.17238
HORVU7Hr1G018620	9.344631	6.794089	9.405961	10.63061	12.94698	12.93949
HORVU4Hr1G006670	15.54932	13.26748	15.15319	14.00626	13.32951	15.75793
HORVU7Hr1G081490	13.61676	16.0631	15.72474	12.11114	12.20246	14.58466
HORVU2Hr1G119090	0	0	0	0.325368	5.02764	3.644249
HORVU1Hr1G010930	2.711066	2.524088	3.113076	3.228722	3.859902	3.759515
HORVU4Hr1G084390	44.47511	56.96735	73.84015	10.96475	11.073	11.92069
HORVU3Hr1G007540	2.151503	1.737176	2.731044	1.727036	1.034146	1.809002
HORVU7Hr1G006280	240.4181	281.0885	247.6731	19.82417	23.00043	17.31944
HORVU5Hr1G095130	23.5435	19.83631	23.7287	17.63434	30.74225	29.68885
HORVU7Hr1G077970	2.744113	2.41266	2.815905	1.175577	1.365604	1.670491
HORVU5Hr1G021730	167.2245	175.0508	247.4206	152.898	302.8338	294.2137
HORVU2Hr1G097180	4.693479	1.95273	2.510777	294.6074	223.3608	246.6664
HORVU3Hr1G074290	22.61724	21.63006	24.14851	10.29025	1.678516	3.539879
HORVU5Hr1G028810	4.536646	4.291902	5.503927	5.188944	7.849938	10.70091
HORVU2Hr1G104510	16.12927	16.55418	24.23851	19.45514	24.30322	22.34043
HORVU5Hr1G087990	7.575586	6.96447	8.503368	9.195351	10.3039	11.01873
HORVU5Hr1G047630	0.011029	0.027454	0.015802	0.121438	3.846959	2.891424
HORVU5Hr1G042030	8.94162	7.064918	8.887431	8.391766	9.453365	8.54099
HORVU5Hr1G027810	26.04317	27.36501	28.50539	34.56538	59.95331	62.198
HORVU3Hr1G115180	0.068541	0.11257	0.033234	0.619831	11.37933	5.715116
HORVU5Hr1G094660	8.640234	8.269656	8.333807	7.872471	10.71527	10.8297
HORVU1Hr1G000340	0.998241	0.494968	1.629436	10.33102	1.070306	6.038114
HORVU6Hr1G013060	1.499011	1.512553	1.872258	0.975951	1.431466	1.494073
HORVU3Hr1G078670	6.340976	4.269848	10.52076	19.16975	29.20772	27.31301
HORVU6Hr1G056610	5.913885	5.660164	10.06881	2.313119	4.869488	4.611496
HORVU7Hr1G082670	1.176568	1.111364	1.112808	2.259245	3.803721	3.25185
HORVU1Hr1G073230	18.19461	17.87244	25.17248	12.15179	9.93571	12.23653
HORVU2Hr1G019650	7.265341	7.505903	10.76701	9.920839	15.45514	14.89236
HORVU4Hr1G020950	2.904277	3.062221	2.709824	12.25501	13.55525	13.89152
HORVU6Hr1G038430	14.10787	15.72571	14.15836	14.09221	22.51574	17.15956
HORVU4Hr1G040510	0	0	0	0.412909	6.84568	3.198837
HORVU2Hr1G050500	8.361311	9.479897	7.517649	8.43597	9.343609	9.530972
HORVU1Hr1G030540	2.232115	2.462367	3.043681	2.28003	1.166676	1.72965
HORVU2Hr1G036800	38.81948	33.72207	39.89524	13.47623	17.87694	18.57571
HORVU6Hr1G041020	5.976375	6.642733	7.609581	17.80681	35.0939	33.58879
HORVU1Hr1G085140	1.319757	0.479536	0.907588	0.428006	1.909682	1.909166
HORVU4Hr1G077280	9.803198	7.723596	9.940953	11.81316	13.60283	14.434
Hordeum_vulgare_newGene_1969	4.129964	4.068998	3.56507	5.628683	11.35099	9.542097
HORVU1Hr1G005260	18.43941	17.66378	23.78052	18.3554	24.32206	23.69478
HORVU3Hr1G057380	6.71744	7.201279	7.404884	8.559338	12.26376	11.28091
HORVU7Hr1G040250	0.108843	0.185439	0.160405	1.016541	5.724107	5.113415
HORVU2Hr1G029100	43.98144	37.39032	44.19414	42.56874	45.34123	44.57979
HORVU3Hr1G077210	2.082407	1.789545	2.799524	1.83422	2.343088	2.164687
HORVU6Hr1G034990	19.41588	16.71316	18.49589	34.28371	57.12703	44.76825
HORVU1Hr1G000960	30.58478	29.48794	33.62978	45.57552	48.49967	37.51712
HORVU2Hr1G110600	2.630436	2.197062	2.76846	2.255047	3.547331	2.923316

HORVU4Hr1G079250	13.45313	13.02078	16.20177	10.05901	15.69011	14.02827
HORVU3Hr1G075500	2.929317	2.891914	5.437245	2.225581	1.851238	1.678195
HORVU7Hr1G031350	2.760423	2.379447	2.276614	2.560082	3.870139	3.034246
HORVU7Hr1G021340	8.286507	8.397263	9.132415	10.25799	14.54434	13.6841
HORVU2Hr1G123320	24.82614	23.53118	27.16068	29.38928	30.20489	33.38655
Hordeum_vulgare_newGene_2367	6.150961	6.532504	7.286365	11.31054	17.03446	15.34063
HORVU5Hr1G015660	0.688585	0.56865	0.279429	0.277087	2.140162	2.247378
HORVU3Hr1G058830	9.049481	8.383032	8.117693	17.27749	23.12025	19.85395
HORVU5Hr1G072420	301.9683	358.1119	493.4097	222.0591	259.6971	266.0571
HORVU3Hr1G063050	6.907349	6.408509	6.18661	13.49021	24.6412	25.11348
HORVU7Hr1G098330	0.670159	0.641721	1.005288	3.885848	8.007701	7.942889
HORVU6Hr1G090160	7.766013	6.211583	7.285353	14.03505	15.88272	15.74824
HORVU7Hr1G084950	2.861773	3.962604	3.806163	3.603053	3.593939	4.070395
HORVU1Hr1G027600	25.45948	22.81522	27.92807	33.41158	35.82349	36.52816
HORVU2Hr1G018680	9.037036	5.601788	4.577461	0.975498	0.357317	0.584837
HORVU3Hr1G071270	14.2671	16.27761	21.31162	12.94277	18.3718	19.13551
HORVU2Hr1G101930	26.45877	21.66053	22.42243	31.72918	23.6332	25.21261
HORVU2Hr1G013500	1.925656	1.750497	1.844348	1.897093	2.681242	2.861548
HORVU5Hr1G079460	6.005443	5.154646	6.515024	8.812131	9.64256	9.358341
HORVU7Hr1G025320	2.055997	1.630312	1.50891	0.339207	1.956785	1.783446
HORVU4Hr1G027590	27.14278	26.26569	26.27421	27.52187	29.34091	30.65118
HORVU7Hr1G038650	12.5099	12.36782	13.37257	14.03139	13.07913	13.72025
HORVU6Hr1G052500	7.692662	6.974328	8.40183	12.2759	10.98216	11.90078
HORVU1Hr1G085730	14.98895	16.44649	20.84071	10.41691	15.06678	14.56764
HORVU3Hr1G060500	2.07362	2.229164	2.442254	2.289941	1.492365	2.05496
HORVU3Hr1G085380	7.617139	9.739736	10.80461	13.58748	17.10823	16.20154
HORVU2Hr1G068470	22.54201	20.9584	24.15343	23.6207	25.3141	26.40761
HORVU7Hr1G072910	2.958821	2.243563	2.77224	2.831873	4.387371	4.045217
HORVU6Hr1G075600	9.637598	9.615978	7.315689	8.169566	10.57151	8.022037
HORVU3Hr1G017190	2.702408	2.242282	3.951592	2.407536	1.637903	1.681778
HORVU4Hr1G054580	4.420896	4.850995	4.713373	8.747768	7.059056	6.24558
HORVU0Hr1G005060	17.28462	16.37986	15.93185	49.08476	47.30554	45.10269
HORVU5Hr1G001380	1.566938	1.162527	1.784738	1.314583	1.226441	1.463586
HORVU3Hr1G052380	6.248525	6.871503	8.376841	10.13578	13.37843	13.70665
HORVU6Hr1G079720	35.91763	27.82585	35.48767	21.55731	16.57653	19.25614
HORVU2Hr1G116600	10.05577	8.907387	11.85803	14.58703	19.39947	19.62687
HORVU3Hr1G096860	29.44692	16.94989	29.74959	14.74811	4.889923	15.66718
HORVU1Hr1G092860	36.15419	33.37751	37.97116	32.37364	30.64511	30.95935
Hordeum_vulgare_newGene_3610	1.350025	1.684343	2.344252	2.962161	3.748041	2.921328
Hordeum_vulgare_newGene_3611	12.79665	12.19209	7.956319	4.047477	1.481378	2.121196
Hordeum_vulgare_newGene_3614	1.721838	1.678465	2.409267	0.565895	0.957664	0.926324
HORVU7Hr1G028960	3.290494	2.588681	3.099709	1.464348	1.639373	1.600492
HORVU1Hr1G071760	0.112475	0.085302	0.328252	1.447624	7.524407	6.155355
HORVU7Hr1G121390	166.9789	134.0491	179.5211	233.8117	354.1227	348.1031
HORVU3Hr1G020300	3.389422	2.409312	2.967459	4.423277	6.026419	5.85661
HORVU3Hr1G087530	20.79994	19.22034	21.47531	16.08134	15.1042	15.58661
HORVU5Hr1G099090	5.090008	5.724489	6.961628	6.478512	9.754734	9.006194

HORVU1Hr1G055000	60.11847	68.51187	61.50863	45.12159	58.61299	49.79373
HORVU2Hr1G120410	82.8441	78.98367	75.16788	43.31054	16.32654	21.07413
HORVU7Hr1G042490	4.072273	3.191548	5.109549	4.205044	3.978846	4.811086
HORVU2Hr1G032910	3.47197	3.368222	4.44289	4.260374	8.355752	8.306822
HORVU6Hr1G072370	40.49283	36.46954	48.62746	25.28291	25.52507	27.21154
Hordeum_vulgare_newGene_5614	1.091428	0.846653	1.223187	0.974312	2.034269	1.331407
HORVU6Hr1G021700	44.77626	36.50608	42.803	60.18442	74.12457	74.62741
Hordeum_vulgare_newGene_5619	5.304955	6.206354	5.37013	5.209508	4.843751	5.969211
HORVU3Hr1G059710	5.656935	4.686786	8.563251	4.444485	6.416242	6.229015
Hordeum_vulgare_newGene_1873	1.394544	1.483337	1.674901	1.15808	0.912069	0.891116
Hordeum_vulgare_newGene_15661	1.305332	1.18161	1.535762	2.052102	2.221005	1.965416
Hordeum_vulgare_newGene_15664	2.906531	3.591733	2.987623	5.276325	4.285273	4.943044
HORVU6Hr1G065020	2.236765	2.53947	2.490704	3.345333	6.384431	6.034518
HORVU7Hr1G063910	3.003073	3.12576	4.493259	2.914498	3.328051	3.960714
HORVU7Hr1G034400	15.67634	12.27471	14.77492	15.88794	22.23119	21.11599
HORVU7Hr1G096640	4.065247	6.050687	5.935411	8.786023	9.99047	7.885578
HORVU2Hr1G077140	3.204134	3.778819	4.021683	3.191978	2.896472	3.550027
Hordeum_vulgare_newGene_16191	24.85098	23.72343	24.69927	18.50701	20.9373	21.77412
Hordeum_vulgare_newGene_11966	1.380278	1.106188	1.115371	0.704678	5.118046	2.833156
Hordeum_vulgare_newGene_11965	4.938887	3.473726	4.338562	8.878509	8.775809	8.565628
HORVU0Hr1G009030	5.331959	4.755897	7.475087	2.272962	2.721141	3.447352
HORVU6Hr1G076980	5.627216	5.505568	7.745955	8.401962	7.464099	10.28167
Hordeum_vulgare_newGene_6823	7.849532	9.501156	8.7107	10.44446	11.7958	12.72678
HORVU5Hr1G035370	8.440778	11.71543	9.983125	12.61107	13.93515	13.66416
Hordeum_vulgare_newGene_6824	2.991006	3.34934	2.422409	3.812062	2.81119	3.351668
HORVU6Hr1G023380	6.171497	8.535656	7.732721	12.57821	12.3407	13.83143
Hordeum_vulgare_newGene_8462	9.226962	9.205414	10.15428	3.081722	3.341561	3.399117
Hordeum_vulgare_newGene_8464	0.853929	0.934895	0.9505	1.776552	1.387348	1.098607
HORVU3Hr1G058440	7.721168	9.192144	8.267868	4.953109	4.578279	4.803828
HORVU7Hr1G117540	1.698666	1.544515	2.09404	3.937652	5.366382	4.970958
HORVU1Hr1G059050	8.410397	8.517413	8.279187	8.042743	7.196338	7.995306
HORVU5Hr1G038630	34.19484	32.21401	28.81052	169.8278	299.6107	287.2537
HORVU7Hr1G030380	32.01027	24.98696	35.93543	16.93194	15.16277	17.72133
HORVU6Hr1G025280	1.133565	1.226643	1.553868	2.183452	2.714116	2.998028
HORVU6Hr1G062360	59.00196	55.4351	98.42658	33.27447	43.69336	40.87613
Hordeum_vulgare_newGene_15087	1.410832	0.872352	1.349096	2.902568	4.911959	4.429013
Hordeum_vulgare_newGene_15083	0	6.475869	0	0	0	0
HORVU7Hr1G055410	24.96509	23.19497	29.29303	19.17237	17.34685	19.5013
HORVU6Hr1G076320	46.51117	41.20355	52.63119	53.1318	33.5919	35.15871
HORVU6Hr1G029880	7.731106	9.111822	9.040725	5.513774	6.346198	7.550353
HORVU4Hr1G036960	19.7318	19.07687	22.01797	27.87088	36.89855	38.09014
HORVU5Hr1G096010	1.143757	1.271729	1.598769	3.527882	3.736472	4.41183
HORVU0Hr1G002090	3.655465	2.097044	3.627182	4.802275	12.26203	9.972025
HORVU2Hr1G118930	30.73406	25.53942	31.41078	27.9701	32.38977	33.11426
Hordeum_vulgare_newGene_9502	10.64068	8.835769	12.63212	10.55495	11.57372	11.23502
HORVU3Hr1G045220	0.261102	0.208002	0.304579	0.941683	4.630083	6.998264
HORVU2Hr1G073110	2.649411	2.579782	2.673144	2.448308	3.483067	3.84394

HORVU7Hr1G114010	1.888809	2.651037	1.67722	1.250813	0.13895	0.235363
HORVU2Hr1G080370	5.11939	6.624677	9.751509	2.058399	2.582199	2.903505
HORVU3Hr1G036870	3.098653	2.709214	3.072516	3.355724	3.249505	3.488902
HORVU5Hr1G057600	0.233995	0.235854	0.337325	0.916909	13.25756	7.0418
HORVU0Hr1G038490	28.99108	26.95238	25.8323	28.62695	25.57291	33.36177
HORVU6Hr1G066640	14.22922	12.64587	12.48607	6.647256	6.216923	7.599337
HORVU1Hr1G076010	3.222159	3.692228	4.72771	1.946909	3.153567	3.294531
HORVU0Hr1G005840	1.404662	1.147486	1.818381	2.532163	5.689049	4.766122
HORVU5Hr1G104200	5.735855	6.618877	7.878933	8.734773	12.54177	13.3447
HORVU1Hr1G022420	25.46369	21.07711	27.52052	22.03703	26.95858	30.52957
HORVU6Hr1G031440	66.83606	56.92211	58.78826	62.43256	54.82121	56.75512
HORVU5Hr1G067400	3.546076	3.19684	3.353437	4.962072	3.856192	3.8647
HORVU2Hr1G120490	3.407898	2.641742	2.90741	5.487235	4.459446	5.318728
HORVU6Hr1G020560	0.828369	0.832502	0.511617	1.118713	1.565685	1.652389
HORVU4Hr1G072130	2.46514	2.633181	2.446329	8.383821	9.651717	10.03679
HORVU1Hr1G057510	2.167435	2.299274	3.070285	2.580773	2.859884	2.614594
HORVU6Hr1G081260	3.172672	2.826151	3.946695	4.877609	7.229915	8.34471
HORVU0Hr1G031170	1.786761	1.45566	1.859829	15.91598	8.819262	11.45292
HORVU0Hr1G018370	7.784503	8.052771	11.28438	6.208567	7.522736	7.436425
Hordeum_vulgare_newGene_7700	23.95529	19.55312	28.50817	30.54864	50.68255	48.33079
Hordeum_vulgare_newGene_7704	1.945258	2.265226	3.265044	1.801901	2.554908	3.431033
Hordeum_vulgare_newGene_7707	8.114216	6.885407	8.803967	5.71241	8.643517	7.755863
HORVU6Hr1G028050	5.665459	4.93182	6.133963	8.13834	10.58207	12.06091
HORVU7Hr1G063280	59.84502	61.80851	88.84469	67.99821	137.9904	131.506
HORVU1Hr1G078310	1.010268	1.458426	2.169086	0.42249	0.720478	0.629913
HORVU4Hr1G048400	3.071415	3.485198	3.166484	3.654635	4.443911	4.427688
HORVU2Hr1G059320	18.01267	17.60486	22.8241	9.446667	5.795341	7.450239
HORVU3Hr1G035750	9.102458	9.34482	9.714118	13.0041	20.2746	20.33387
HORVU2Hr1G041020	6.222631	4.96099	6.377323	7.847794	11.75988	13.20577
HORVU6Hr1G074210	2.06971	2.004449	2.43006	2.225269	1.133605	1.927622
HORVU0Hr1G004060	0.639589	0.792939	0.992238	2.167052	2.640714	1.916024
HORVU6Hr1G077450	1.064838	1.124248	1.432798	0.641202	1.033039	1.04722
HORVU4Hr1G039870	2.509593	3.461928	3.626094	9.970605	26.52775	26.91086
HORVU4Hr1G050230	3.658749	3.068105	2.852484	5.64309	5.417919	5.533103
HORVU4Hr1G016280	2.942495	3.690777	5.123343	15.94815	13.93917	17.56087
HORVU3Hr1G079700	219.0895	183.3965	227.6854	208.2757	241.0361	229.6214
HORVU7Hr1G115050	12.52054	12.80764	15.17227	14.12818	17.29534	17.87157
HORVU6Hr1G005960	0.144057	0.175426	0.446966	3.741259	17.53037	18.38216
HORVU6Hr1G068970	8.404338	9.643256	10.38307	12.6225	11.43331	12.12052
HORVU6Hr1G032490	3.797731	3.669533	3.30516	6.490198	6.134681	8.459421
HORVU1Hr1G017900	1.639449	1.64621	1.90784	1.97545	3.611253	3.109684
HORVU6Hr1G057100	8.093919	6.583972	9.365033	8.627839	16.60074	15.1693
HORVU1Hr1G029220	44.51125	38.38175	45.86947	94.7817	100.0749	94.9372
HORVU0Hr1G035460	1.256522	1.496597	0.76938	0.86156	6.28705	5.224195
HORVU6Hr1G080600	5.591919	6.184587	6.133483	4.836211	8.023943	7.392589
HORVU5Hr1G012780	483.6885	367.5547	420.1551	532.0421	455.4335	486.0594
HORVU5Hr1G088420	0.843081	1.614883	1.665447	1.733873	3.990582	4.138652

HORVU6Hr1G033540	10.96078	10.95385	10.06944	29.25132	43.76711	40.86555
HORVU7Hr1G055090	29.92187	27.47395	32.82015	35.24125	42.71515	41.80998
HORVU3Hr1G038050	1.013923	1.161526	1.553441	2.569074	2.487458	3.074395
HORVU4Hr1G074200	0.852628	1.078296	1.144908	1.11129	1.807712	1.666453
HORVU4Hr1G090760	5.388163	5.535543	5.328096	6.390433	6.329553	7.371357
HORVU1Hr1G087040	1.655829	2.364632	4.828511	0.937254	0	0.404501
HORVU6Hr1G067890	11.92891	12.43828	10.83434	18.01776	18.18656	20.03573
HORVU1Hr1G090910	1.6607	1.869625	2.096197	2.508134	4.100105	3.458443
HORVU1Hr1G058580	2.625693	1.800078	4.297461	1.295123	2.961767	2.882038
HORVU7Hr1G110260	18.20887	16.63931	20.02891	17.60053	21.34141	23.14057
HORVU7Hr1G097250	0.810366	0.866918	0.986916	3.701136	7.734884	6.624134
HORVU1Hr1G065600	15.47115	8.411351	13.2702	7.493412	1.942949	2.904748
HORVU3Hr1G029080	12.13193	12.25915	13.25526	15.50773	21.41576	23.09997
HORVU4Hr1G083200	7.906081	8.022616	10.63185	6.879768	4.200837	4.576734
HORVU5Hr1G018780	13.87844	8.629983	12.95665	16.28764	8.93138	11.63462
HORVU5Hr1G017570	1.947844	1.848027	1.674024	3.346871	5.559856	5.457763
HORVU1Hr1G004330	6.418877	6.557269	5.390128	5.539002	4.41621	4.713388
HORVU7Hr1G117970	30.5112	23.69036	29.98961	34.70883	37.39669	35.25702
HORVU5Hr1G009600	46.3523	45.08049	69.25506	39.02632	68.35491	62.80683
HORVU3Hr1G082130	0.915567	0.948712	0.787203	0.976307	1.295435	1.795086
HORVU1Hr1G020450	20.50386	11.51656	22.30919	24.85903	7.524372	17.05223
HORVU2Hr1G061790	5.384441	5.802907	7.600933	5.822971	8.383818	8.228738
HORVU2Hr1G035440	0.690734	0.836056	0.92679	1.386997	1.707914	1.973567
HORVU7Hr1G040090	3.912805	3.792824	4.085712	11.36716	8.931677	9.872331
HORVU5Hr1G018870	3.536597	4.372956	4.626531	6.139008	7.59441	6.680389
HORVU4Hr1G013610	8.675197	7.517696	9.142266	6.983383	8.331896	9.039581
HORVU5Hr1G020170	4.510334	7.218221	6.733533	7.604274	13.67475	10.2103
HORVU1Hr1G089530	7.649691	6.201521	8.095421	2.343117	0.145317	1.157713
HORVU3Hr1G037280	22.1942	20.8304	27.88919	14.62262	13.24318	15.64159
HORVU4Hr1G011610	9.55589	9.22357	8.476273	12.59385	19.94479	18.57001
HORVU5Hr1G113790	6.642155	1.583435	9.225501	0.215495	0.200905	0.273707
HORVU7Hr1G071800	16.91341	19.21116	17.81154	9.822398	10.65607	11.11547
HORVU4Hr1G089060	1.887522	2.549327	1.831377	7.665921	6.912142	6.283308
HORVU4Hr1G083000	127.8373	129.9767	106.2502	79.83729	62.07689	70.12008
HORVU4Hr1G063140	4.772507	4.692183	5.195607	5.642481	5.384222	5.48925
HORVU5Hr1G055140	11.28584	6.394175	11.15987	10.07508	14.23915	12.82131
HORVU5Hr1G035030	26.86002	26.11851	25.26648	21.30359	24.34944	26.26973
HORVU7Hr1G121440	0.000689	0.002417	0.006235	0.071101	22.4559	4.688018
HORVU5Hr1G109040	1.26565	1.41434	1.352907	29.62708	35.28474	31.68437
HORVU2Hr1G083940	28.58342	25.0141	34.80036	47.56633	63.34592	79.87994
HORVU5Hr1G119830	1.368333	0.969245	1.183222	0.946788	1.852189	1.562364
HORVU0Hr1G017100	2.648267	1.964233	2.535616	5.104882	9.797659	8.468391
HORVU7Hr1G117020	12.20598	12.47872	16.69879	10.87481	17.64487	17.49515
HORVU1Hr1G091660	1.1946	1.125144	2.288925	1.725788	1.184275	1.525857
HORVU7Hr1G077320	0.563332	0.412655	0.810654	1.134985	2.103437	2.515444
HORVU5Hr1G110680	3.21811	2.307118	2.691574	6.969456	31.85847	27.13661
HORVU4Hr1G009210	2.185081	2.219684	2.805588	9.029444	7.874467	9.651148

HORVU1Hr1G002590	1.689009	13.13428	2.115922	22.6555	2.242828	4.10854
HORVU3Hr1G081720	6.57816	6.223694	7.195383	6.207469	10.66668	12.30014
Hordeum_vulgare_newGene_2914	3.110518	3.398203	3.657124	6.594615	8.536993	7.728984
HORVU4Hr1G071420	2.565895	2.31058	3.076983	3.831249	5.806811	6.053617
HORVU6Hr1G035370	11.0698	9.797006	10.91951	6.48395	3.612218	4.913366
HORVU6Hr1G094470	3.921715	3.569379	3.924188	4.290543	2.932485	3.24714
HORVU5Hr1G056620	2.740679	2.561104	2.591421	0.832132	0.822761	1.55406
HORVU6Hr1G074830	0.236401	0.739946	0.401219	6.869466	7.832078	10.75675
HORVU0Hr1G016960	12.29594	13.46711	13.54834	9.426141	9.760782	10.2809
HORVU7Hr1G023060	3.679098	3.244654	4.207938	3.364174	5.393273	4.804821
HORVU3Hr1G033740	7.911524	6.158672	10.57063	4.443369	1.89327	4.341207
HORVU5Hr1G111480	1.557864	1.507949	2.291675	3.508114	4.94045	5.501457
HORVU6Hr1G088630	10.31922	12.17033	12.1562	11.56747	11.23932	14.07136
HORVU4Hr1G057740	2.199318	1.087917	2.415252	2.677545	4.675203	3.44599
Hordeum_vulgare_newGene_12850	6.714531	7.70994	6.73225	7.003633	6.79122	8.465175
HORVU3Hr1G074120	4.770592	4.180035	5.648881	3.940918	4.88328	4.489728
Hordeum_vulgare_newGene_12859	18.57053	20.47116	24.26949	30.12779	35.78507	39.25017
Hordeum_vulgare_newGene_12858	1.978752	2.259967	2.904917	2.531525	3.012187	3.683159
HORVU1Hr1G038990	7.079456	8.189448	8.534066	7.302712	12.03187	12.19495
HORVU4Hr1G087200	1.902731	1.965803	2.463896	3.056776	1.009434	1.590002
HORVU5Hr1G040870	23.5024	21.0627	25.78544	19.24507	19.99953	21.67225
HORVU3Hr1G072970	5.833803	5.351849	6.019547	5.526172	5.205105	5.043986
HORVU7Hr1G071600	14.27456	16.43411	15.6342	17.92285	23.16548	32.30475
HORVU7Hr1G076580	0.08083	0.053145	0.126951	0.209929	4.944856	3.66525
HORVU5Hr1G095910	19.07075	16.70046	25.38319	47.1903	57.76677	54.03867
HORVU7Hr1G025990	8.657168	8.158282	11.43895	22.65747	46.91903	42.13815
Hordeum_vulgare_newGene_2100	2.673379	2.998221	3.902002	5.770431	7.208509	7.92884
Hordeum_vulgare_newGene_2105	1.111833	1.305969	1.747086	4.533029	5.131151	5.913356
HORVU7Hr1G120510	6.087341	6.478184	5.443968	6.597547	8.361894	9.108749
HORVU2Hr1G017310	12.08603	9.85398	9.355243	5.704757	3.910398	4.42023
HORVU4Hr1G054210	20.31926	19.04384	25.02068	24.68252	24.71476	25.02352
HORVU2Hr1G021920	1.952594	1.526332	1.896323	3.192367	4.133667	4.813194
HORVU1Hr1G046870	52.21139	58.17432	63.05302	61.27523	50.05335	61.2251
HORVU6Hr1G037610	11.93349	11.35679	16.44792	7.416501	4.968281	4.944011
HORVU7Hr1G122520	5.097401	4.485572	4.665308	3.143409	3.167447	3.533799
HORVU6Hr1G012540	6.242614	4.253584	4.336816	4.325929	4.612907	4.986572
Hordeum_vulgare_newGene_9135	1459.593	1684.238	1065.126	170.9951	89.54838	95.9025
HORVU6Hr1G021540	3.317451	3.752313	4.564517	5.725085	6.082608	6.88171
Hordeum_vulgare_newGene_5459	3.120852	3.101465	2.773485	0.455686	0.256731	0.263124
HORVU2Hr1G117820	1.802554	2.510153	1.815924	0.465945	0.235478	0.479117
HORVU2Hr1G002520	33.66979	41.0635	65.93125	7.838958	0.252707	4.097804
HORVU7Hr1G008310	14.91618	12.96843	16.21944	25.92301	38.50943	38.45756
HORVU7Hr1G006530	0.572982	0.394979	0.371377	1.764432	5.194412	3.710263
HORVU1Hr1G037460	20.47337	21.12366	17.09693	20.27025	32.02233	30.09364
Hordeum_vulgare_newGene_13812	2.412825	1.773433	2.139942	2.724701	2.039625	2.194278
HORVU2Hr1G113560	5.46968	5.269851	3.973845	9.920353	12.03031	11.93649
HORVU5Hr1G098290	1.524407	1.723502	3.272034	1.121285	0.397715	0.416765

HORVU7Hr1G074690	407.4312	347.161	478.68	648.0586	1257.859	1173.085
HORVU1Hr1G077520	2.033524	2.334909	2.603659	4.210804	6.324146	5.752886
HORVU7Hr1G043520	29.85918	23.25367	30.1656	30.2013	32.64547	31.58186
HORVU2Hr1G091680	11.64959	13.36692	11.36166	23.1245	27.61303	29.30884
HORVU2Hr1G018800	0.734003	2.272777	2.926173	1.632932	3.061449	1.859405
HORVU5Hr1G046020	0.478425	0.227828	0.46624	1.594914	3.275917	3.416648
Hordeum_vulgare_newGene_8663	6.833919	6.168231	9.706369	6.698021	8.614799	7.877402
Hordeum_vulgare_newGene_8668	4.643218	5.417412	4.514679	3.990521	5.302435	5.50945
HORVU2Hr1G072400	0.104996	0.29304	0.250203	0.083021	16.98721	11.25053
HORVU5Hr1G052280	159.9229	169.6341	252.0288	150.286	410.8459	351.1976
HORVU2Hr1G116880	0.170546	0.169552	0.282998	2.452189	2.206798	2.796676
HORVU3Hr1G062600	5.897264	4.984187	7.297529	4.024555	4.201271	4.877867
HORVU7Hr1G018750	2.996111	2.21385	2.214748	2.97172	3.444872	4.010897
HORVU4Hr1G020030	2.022091	2.539602	2.229029	88.84834	140.471	129.1126
HORVU2Hr1G092080	136.9465	128.1223	118.9279	305.1623	464.2601	430.05
HORVU5Hr1G073050	1.256769	0.734098	1.602131	2.858227	3.985151	3.296493
HORVU5Hr1G053480	0.838056	0.660663	1.035991	4.288934	6.405121	6.817115
HORVU4Hr1G064000	8.133694	10.09237	14.54657	11.55282	11.26454	7.51222
HORVU1Hr1G001710	0	0	0	12.26216	0.052469	0.015948
HORVU5Hr1G080610	6.417758	6.053322	7.169928	9.285735	9.974927	9.471189
HORVU2Hr1G036980	0.462357	0.618703	0.634903	1.106272	2.861389	2.775669
HORVU3Hr1G105880	2.471565	2.507872	2.424165	11.12261	39.08155	33.01051
HORVU2Hr1G030280	2.341337	4.25843	2.753657	2.562435	2.78558	2.969009
HORVU2Hr1G124850	5.024558	4.304482	5.728755	8.408507	8.055453	9.555722
HORVU6Hr1G014490	4.859284	5.502866	6.125585	4.838717	5.593121	5.787791
HORVU2Hr1G035020	5.763653	3.746434	6.785571	23.79788	30.91961	27.56475
Hordeum_vulgare_newGene_10548	44.41361	41.35036	41.17009	47.19372	49.23901	54.30505
HORVU2Hr1G111050	3.880402	4.583995	3.748011	4.11641	4.412349	4.570638
HORVU6Hr1G025730	3.939836	3.914731	3.478463	10.22834	14.27875	14.20308
HORVU3Hr1G057880	4.362189	5.91646	7.400396	2.817625	2.954404	2.678611
Hordeum_vulgare_newGene_10545	0.755304	0.56787	0.667852	3.317166	1.812493	2.496362
HORVU2Hr1G039360	0.945023	1.101892	0.522684	0.725348	6.095751	4.52752
HORVU7Hr1G066510	29.37302	27.79661	29.11877	21.28514	22.15458	25.68784
HORVU7Hr1G079720	27.5338	23.98078	31.28349	20.4489	20.43687	20.79435
HORVU7Hr1G020770	11.93404	9.997629	10.3927	18.77268	14.21045	16.55125
HORVU2Hr1G070800	13.87216	15.19272	16.58095	12.1383	16.11507	16.39849
HORVU3Hr1G095820	1.077794	1.025951	1.187989	2.502565	2.312043	2.643007
HORVU0Hr1G022060	7.406273	7.282776	7.095873	7.713847	9.59413	11.1255
HORVU7Hr1G058140	4.977836	4.276866	4.690156	5.058799	5.933999	7.129435
Hordeum_vulgare_newGene_8745	1.083853	0.792789	0.999553	2.078549	1.221242	2.300651
HORVU2Hr1G035750	3.530769	3.771006	4.947317	4.592366	4.284682	7.387392
HORVU3Hr1G088270	54.57237	42.21919	58.79545	124.1475	258.4976	184.0912
Hordeum_vulgare_newGene_4415	1.416787	1.336083	1.456246	2.075827	1.927639	2.26263
Hordeum_vulgare_newGene_4414	5.064019	12.88861	5.434672	17.97242	4.515299	5.751649
HORVU7Hr1G101300	9.395149	8.118901	8.910934	6.027982	6.139926	6.780086
HORVU7Hr1G082350	0.41451	0.336217	0.485413	1.218889	2.188009	2.012097
HORVU3Hr1G023970	16.58536	15.82128	22.39275	17.42377	23.77002	24.2705

Hordeum_vulgare_newGene_7142	4.560049	5.030712	4.372995	4.666993	5.446987	5.623924
Hordeum_vulgare_newGene_7146	4.684922	3.289551	4.630973	8.127932	9.817351	9.412611
Hordeum_vulgare_newGene_7145	2.906306	3.697406	2.36856	5.233517	6.78639	6.318856
Hordeum_vulgare_newGene_7148	1.376563	1.225785	1.5908	1.342732	0.839498	1.229019
Hordeum_vulgare_newGene_7149	12.85139	11.63425	16.22825	23.12728	37.59555	29.12298
HORVU1Hr1G053780	7.634536	7.616217	7.534424	9.261146	12.56476	13.86597
HORVU6Hr1G077300	75.58013	61.67323	77.57213	63.86707	56.3994	53.93629
Hordeum_vulgare_newGene_569	0	0	0	12.93262	11.75189	14.6332
HORVU3Hr1G089580	8.154472	4.585901	8.434572	9.522116	15.39428	13.64153
HORVU1Hr1G064180	3.818895	2.437847	1.519908	4.136519	4.152712	4.439514
Hordeum_vulgare_newGene_12336	43.76133	41.9138	47.14173	38.88131	37.77092	36.54992
HORVU0Hr1G021000	16.23672	15.28977	21.47743	16.94158	25.99104	22.31086
HORVU2Hr1G117450	5.192069	4.427435	7.857376	5.787877	8.946168	7.846706
HORVU2Hr1G070110	2.241529	2.135744	2.569615	4.289872	4.236609	4.802268
HORVU5Hr1G070630	7.379346	7.138005	9.244845	4.882473	1.971715	2.710053
HORVU6Hr1G029370	15.89201	13.81971	16.811	31.50687	38.11749	34.74693
HORVU3Hr1G090580	25.36283	30.4871	31.45915	29.00267	46.48056	38.65927
Hordeum_vulgare_newGene_14478	3.877503	4.11838	4.051859	3.575345	2.472502	3.226332
HORVU7Hr1G025650	3.298023	3.983576	3.914219	3.701257	4.365813	5.006005
Hordeum_vulgare_newGene_7730	1.122734	1.312681	1.424331	0.943598	1.00933	1.491477
HORVU3Hr1G117540	3.26668	3.802732	3.695415	1.903837	2.168724	2.173849
HORVU3Hr1G097580	47.28127	44.43848	46.97511	42.88658	41.88917	39.02217
HORVU6Hr1G032960	7.839166	7.587487	6.704242	8.094164	19.57254	18.86012
HORVU3Hr1G068120	11.53592	11.0135	13.24698	18.07204	17.44593	14.60391
Hordeum_vulgare_newGene_9893	1.178207	1.254589	1.257996	1.277216	1.237894	1.788114
HORVU3Hr1G053990	21.04298	21.76973	24.43423	22.48673	28.52159	30.26417
Hordeum_vulgare_newGene_15782	0.06467	0.422676	0.590486	41.12068	45.92429	51.95782
Hordeum_vulgare_newGene_15783	0.010151	0.009219	0.020322	3.807987	3.703182	3.769775
HORVU3Hr1G032540	1.764214	2.112578	2.335513	4.751601	6.038541	5.497044
HORVU4Hr1G021020	9.329668	11.86287	10.42024	5.573109	3.373499	4.16259
HORVU1Hr1G055630	3.085348	3.421185	3.900599	8.523726	7.838979	9.578859
Hordeum_vulgare_newGene_14788	7.606454	5.618464	6.902328	6.966118	6.026362	6.001928
HORVU1Hr1G070330	7.611346	7.340337	7.592577	6.550696	8.069486	8.274946
HORVU2Hr1G074910	8.075908	8.917152	9.239774	6.078908	7.353191	8.00455
Hordeum_vulgare_newGene_13178	2.046694	1.937148	2.084642	1.18782	1.148759	1.481894
Hordeum_vulgare_newGene_14781	1.429167	0.832434	1.339079	2.291949	2.416039	1.868532
Hordeum_vulgare_newGene_13175	2.767494	2.779788	2.408251	1.412661	1.067982	2.044238
HORVU3Hr1G110530	8.955209	8.964801	11.718	5.960424	6.005	9.285792
HORVU7Hr1G122060	5.026859	4.519074	3.325353	3.62219	2.78574	3.14998
HORVU7Hr1G087860	0.953172	0.743002	1.188598	2.803263	2.472641	3.095889
Hordeum_vulgare_newGene_9278	0.799892	0.865837	0.497298	0.75634	2.157861	1.68969
Hordeum_vulgare_newGene_9271	1.68622	2.986081	2.995982	3.609093	3.646807	4.152802
Hordeum_vulgare_newGene_9277	1.412703	1.893569	1.374551	2.342713	2.521431	2.334103
Hordeum_vulgare_newGene_15431	9.325086	9.744391	9.490995	11.15477	12.83932	14.66336
HORVU3Hr1G087090	22.38257	17.64527	23.63193	19.82446	30.13976	92.15163
HORVU7Hr1G048710	1.482076	1.607327	1.284493	2.955128	5.708851	4.406088
HORVU6Hr1G076820	4.472652	4.032748	5.13685	3.915034	3.765416	3.890375

HORVU1Hr1G046710	6.697729	6.388037	7.409377	7.059172	7.550959	7.611873
HORVU3Hr1G003020	1.440831	1.148732	2.122972	1.357411	1.81309	1.41713
HORVU7Hr1G061220	24.69539	21.762	23.10336	26.34771	30.33119	32.49075
HORVU5Hr1G052010	2.157068	1.646968	2.964383	5.189624	7.684374	8.148199
HORVU1Hr1G027980	5.084865	5.540582	3.83198	2.395443	3.810528	4.630723
HORVU4Hr1G064710	24.06128	23.91282	27.64145	34.60253	47.90563	50.88233
HORVU4Hr1G011450	8.684977	8.680571	8.105054	14.19775	21.00529	21.184
HORVU3Hr1G067380	8.338772	8.736656	9.283575	14.44916	12.78543	14.15425
HORVU1Hr1G020120	4.869007	5.115176	6.39354	6.311251	10.0493	9.461989
HORVU5Hr1G073550	11.88286	10.91573	12.08707	9.659471	6.46027	9.187371
HORVU7Hr1G119510	1.563984	1.441749	1.595754	1.272335	1.621011	1.436436
HORVU2Hr1G094020	1.935733	2.094036	2.327415	2.225268	1.597691	2.467964
HORVU2Hr1G096080	5.612692	5.345535	5.76672	5.8596	6.168633	6.292221
HORVU3Hr1G043800	2.015607	2.948835	3.249209	2.601382	4.18315	4.211044
HORVU4Hr1G009050	4.561834	3.06708	5.258852	4.125712	4.794803	4.437278
HORVU3Hr1G019880	12.21755	11.27392	10.8359	9.481664	6.655681	8.39556
HORVU5Hr1G034180	30.59223	11.2315	35.06761	16.44823	11.79336	15.67594
HORVU4Hr1G089510	6896.114	9038.683	5359.943	1528.636	280.5358	592.1299
HORVU5Hr1G069480	6.714025	6.402766	7.286287	4.072816	3.321304	3.658543
HORVU6Hr1G068690	0.803193	0.781465	0.692621	2.011856	1.098226	1.453938
HORVU5Hr1G096190	1.970754	2.249079	2.151574	3.209536	4.934809	4.489857
HORVU5Hr1G022850	4.088789	4.284476	3.857793	3.863912	5.074996	4.749691
HORVU3Hr1G085930	20.21876	18.0213	22.99232	12.39169	9.393384	12.25821
HORVU5Hr1G000150	6.70904	7.177968	9.054821	20.34326	35.02716	32.68618
HORVU2Hr1G124370	36.39731	34.95482	38.63376	35.17261	32.0948	33.02761
HORVU1Hr1G076190	102.6353	110.9132	102.3833	199.7485	239.6802	182.2171
Hordeum_vulgare_newGene_1388	1.211952	1.621267	1.477818	1.664771	1.99928	1.722635
Hordeum_vulgare_newGene_1383	0.270283	0.216499	0.21611	1.305935	2.59682	2.654895
Hordeum_vulgare_newGene_1386	1.405143	1.567952	1.409377	2.304869	2.697606	2.725688
HORVU3Hr1G070560	29.73504	22.92555	25.8173	38.16494	21.35431	24.93594
Hordeum_vulgare_newGene_1385	53.27798	54.83169	76.81556	46.23017	88.416	89.37813
HORVU1Hr1G073010	6.461617	7.733514	7.812051	13.46484	14.1835	14.03675
HORVU0Hr1G019610	1.280579	1.626157	1.805886	1.664289	2.257951	3.055364
HORVU5Hr1G105360	0	0	0	2.229833	2.545593	2.412785
HORVU0Hr1G038750	8.546355	6.381208	8.3577	10.24861	12.67025	13.31293
HORVU7Hr1G091720	146.1565	116.0066	158.7859	196.4402	273.2135	274.6437
Hordeum_vulgare_newGene_4350	0.279473	0.378521	0.182358	0.972733	2.007704	2.468657
HORVU2Hr1G060210	7.557973	6.433474	8.365045	11.65044	10.66388	11.43267
Hordeum_vulgare_newGene_1652	5.583112	6.584732	5.37738	5.031055	4.975328	6.182625
Hordeum_vulgare_newGene_1655	0.992301	0.99756	1.168531	1.127558	1.568012	1.520151
Hordeum_vulgare_newGene_1657	1.0144	0.708341	0.847625	0.970476	1.114465	1.572134
HORVU2Hr1G088620	5.172599	4.197885	4.823009	4.112292	0.609499	1.755786
HORVU5Hr1G091970	14.96109	16.81153	17.02169	23.95079	25.4139	25.33517
HORVU7Hr1G095770	14.12782	13.05571	16.90059	17.91657	18.32725	19.14404
HORVU6Hr1G082900	0.874465	0.886303	1.04646	1.829713	2.700561	2.173891
HORVU7Hr1G099550	3.361162	3.640414	3.749609	1.655737	3.267671	2.712465
HORVU5Hr1G021230	1.569608	1.468105	1.857833	1.876635	3.862548	3.426217

HORVU5Hr1G050120	6.777013	6.188332	7.794401	10.34825	14.14088	11.24341
HORVU1Hr1G067880	5.179782	4.959057	5.821477	4.767107	3.934109	4.957187
HORVU0Hr1G001490	17.69416	16.29533	15.63494	40.03136	65.3648	73.60724
HORVU7Hr1G085750	6.041719	4.99261	6.585304	8.621673	8.984639	10.45924
HORVU7Hr1G088250	16.72854	16.62035	17.21333	14.23799	16.72243	15.34834
HORVU1Hr1G052260	84.96659	72.36407	80.51147	72.55468	89.50439	84.69955
HORVU6Hr1G087990	5.185335	5.523256	5.723428	8.835042	11.01514	10.01354
HORVU6Hr1G035820	9.465981	9.030055	16.33702	7.443274	10.15218	11.42975
HORVU6Hr1G003890	41.23265	37.95927	41.07489	33.3379	36.94267	37.11822
HORVU1Hr1G090030	14.49561	14.89143	14.57805	9.337178	5.718666	5.861778
HORVU5Hr1G010420	0.97969	1.199405	0.821295	2.588471	1.729109	2.548054
HORVU5Hr1G111010	6.828469	6.404188	7.679587	9.901895	13.13251	13.15107
Hordeum_vulgare_newGene_11386	13.36877	11.70164	13.44987	6.86862	8.446039	8.160851
HORVU3Hr1G034070	0	0	0.016316	3.52238	15.94838	4.560087
HORVU3Hr1G040720	0.485113	1.06902	1.021131	1.590141	1.480026	1.891797
HORVU2Hr1G078680	10.04574	11.75023	11.03353	13.00182	16.47544	17.5294
HORVU0Hr1G015450	17.46441	14.27093	21.06373	11.16133	18.59048	18.08248
HORVU6Hr1G018830	3.859879	3.486524	5.135427	11.537	20.2308	18.46128
Hordeum_vulgare_newGene_16140	0.70108	0.268671	0.964024	2.247167	2.704785	2.232985
HORVU7Hr1G024240	4.517679	2.04003	3.757505	11.2332	17.298	10.87602
HORVU5Hr1G014500	0.43926	0.160923	0.469083	2.177974	8.750884	5.676329
HORVU5Hr1G018210	4.456843	4.493673	5.912248	3.896281	3.586479	3.734606
HORVU2Hr1G079080	3.423403	3.347111	1.742045	3.283039	5.518569	5.524401
HORVU6Hr1G075130	34.0263	27.9217	36.96912	71.70287	68.75879	64.22338
HORVU2Hr1G065800	2.681094	3.15753	3.547194	3.509103	3.32401	4.425435
HORVU6Hr1G091650	0.106951	0	0	2.047462	4.386578	4.806382
HORVU3Hr1G006220	2.043187	1.553639	2.083991	4.828238	6.385706	5.742252
HORVU3Hr1G043380	2.370082	2.599988	3.558224	3.852672	6.578592	5.668976
HORVU1Hr1G068530	5.97786	4.693846	5.009874	5.405559	3.42657	3.131487
HORVU3Hr1G070370	5.431193	6.044632	6.747058	5.538592	6.529044	6.82195
HORVU5Hr1G124490	16.67453	17.10063	17.44628	21.49343	19.56843	22.5382
HORVU6Hr1G083690	2.277506	2.148451	2.306348	3.534206	2.245279	2.732468
HORVU5Hr1G120790	2.022095	2.222866	2.956159	2.313789	1.652966	1.686683
HORVU0Hr1G015180	2.339886	2.437236	2.793196	2.182006	1.843625	2.477182
HORVU7Hr1G012700	34.99586	31.93448	33.16182	44.76011	55.08585	56.43033
HORVU1Hr1G065100	2.303819	2.785745	4.15301	3.230023	1.035577	2.454445
HORVU1Hr1G067820	7.236457	9.198397	6.860687	9.550518	12.29115	11.89263
HORVU7Hr1G059910	2.922621	3.255464	3.264383	0	0	0
HORVU1Hr1G051180	1.090213	1.135789	1.564853	1.836742	2.48928	2.288395
HORVU5Hr1G003820	17.38378	17.63743	16.87017	22.85974	28.0908	27.80509
HORVU4Hr1G052330	32.45263	29.32251	33.38515	22.64139	21.26019	22.56898
HORVU3Hr1G073770	17.84192	10.52081	21.54421	34.10646	29.1111	37.63679
HORVU5Hr1G052510	17.51445	19.11633	17.10863	23.6943	35.25274	34.54601
HORVU1Hr1G021600	0	0	0.013534	4.539133	4.790506	5.189551
HORVU6Hr1G080410	3.322969	2.89507	4.806148	1.114718	1.277817	1.438254
HORVU7Hr1G000480	4.318364	4.071247	5.471639	5.42133	7.742513	7.186942
HORVU1Hr1G088580	0.299847	0.258798	40.11689	56.84013	0.271507	0.58772

HORVU0Hr1G011600	6.432641	7.559234	6.585554	11.11698	14.65717	15.02687
HORVU4Hr1G056820	39.361	27.6379	44.34267	60.24575	88.83028	92.11887
HORVU1Hr1G049920	0	0.027838	0.018027	0.539877	2.733676	4.436633
HORVU5Hr1G016210	6.668687	7.917147	7.437945	38.19333	33.89094	44.08128
HORVU4Hr1G010440	3.39988	3.446106	5.679862	4.602334	9.372228	9.012844
HORVU0Hr1G022230	1.304389	1.769528	1.588028	0.249187	0.671261	0.436828
HORVU6Hr1G082120	16.35737	15.71687	16.79087	14.13549	18.2925	19.08975
HORVU4Hr1G060870	19.49552	13.18014	19.17234	16.61029	23.29078	23.02095
HORVU6Hr1G064770	5.101122	5.568343	5.20748	4.20409	6.094956	6.067536
HORVU7Hr1G096930	0.896907	1.176295	1.01788	1.347736	1.463897	1.557044
HORVU4Hr1G023550	14.97796	14.6588	18.07236	22.04911	33.30353	30.14975
HORVU4Hr1G060950	5.329207	6.429362	5.222716	4.964847	5.710144	5.957976
HORVU1Hr1G066450	1.300054	0.967155	1.266683	1.999169	2.664004	2.233967
HORVU1Hr1G019320	22.92297	21.57085	19.81211	21.23707	19.8109	22.25053
HORVU1Hr1G051420	7.408869	10.63937	9.56562	5.298182	7.456843	6.793966
HORVU1Hr1G058040	28.51473	23.46004	24.57866	46.31865	48.69257	41.97473
HORVU5Hr1G112630	0	0	0	4.564594	2.642145	4.220831
HORVU7Hr1G040680	0.461629	0.313368	0.677695	1.873446	1.919395	2.461886
HORVU5Hr1G049370	3.668075	2.662722	2.888972	1.246288	1.456891	2.268076
HORVU2Hr1G061610	17.1051	15.48814	25.49058	46.44765	63.68169	60.81954
HORVU1Hr1G001990	1.05713	9.53981	1.220893	12.21787	0.102421	0
HORVU4Hr1G058070	2.269477	1.374609	1.879173	1.667261	8.3957	5.281572
HORVU3Hr1G036290	9.797787	9.980872	11.0625	12.93025	13.46581	14.64608
HORVU5Hr1G076080	51.6797	45.15644	49.40926	45.94051	30.46259	33.82818
HORVU6Hr1G030140	4.155273	3.78038	4.049301	7.415688	8.927844	11.1945
HORVU1Hr1G038070	2.113755	1.912424	2.851647	3.433743	2.924355	2.753819
HORVU1Hr1G018140	1206.225	1198.388	1488.555	459.8895	165.1964	228.1303
HORVU6Hr1G089750	0.215018	0.058539	0.06146	5.631138	7.650641	6.936388
HORVU6Hr1G020400	6.335816	5.353991	6.808945	5.440332	5.377083	4.965595
HORVU3Hr1G027430	0.37882	0.292441	0.303507	3.37644	8.727001	8.889828
HORVU6Hr1G080180	1.318	1.899552	1.853184	1.717486	0.110039	0.770696
HORVU5Hr1G054000	6.040438	7.939265	8.984889	4.955308	4.209697	5.581225
HORVU2Hr1G053190	4.735834	4.690087	5.228983	6.444873	7.703168	7.569055
HORVU7Hr1G046270	21.40698	22.07067	24.09335	24.80969	32.10823	33.2952
HORVU1Hr1G068940	4.28818	5.393544	6.013829	2.524213	2.077577	0.911098
HORVU5Hr1G047980	105.8071	111.7982	188.1347	103.7229	201.1996	175.273
HORVU3Hr1G089030	2.269582	2.183731	3.28426	2.166625	5.301058	6.186791
HORVU2Hr1G013130	3.159049	2.793081	3.425493	4.278279	5.644121	5.446399
HORVU4Hr1G018020	6.504779	6.057235	6.968985	4.753192	5.745449	5.723777
HORVU2Hr1G033090	9.428883	3.882542	10.39116	12.71382	14.91849	15.23671
HORVU5Hr1G115070	6.021696	6.241966	6.044763	7.256881	5.624512	7.894634
HORVU2Hr1G064730	5.740245	4.352618	6.749584	6.473074	13.20922	10.68506
HORVU0Hr1G027970	2.940307	1.759059	1.947226	1.751745	2.580736	1.113375
HORVU3Hr1G060150	1.651259	1.942467	2.299971	1.141533	1.102468	1.206386
Hordeum_vulgare_newGene_3464	2.572475	2.935595	3.126889	0.500926	0.450093	0.481814
HORVU6Hr1G088400	1.595055	0.915972	1.978268	1.587292	1.13895	1.596365
Hordeum_vulgare_newGene_3468	1.056556	0.923201	0.466762	1.652044	4.738173	4.115011

HORVU7Hr1G031870	19.4301	21.45924	19.75441	17.69617	19.80064	21.24402
HORVU5Hr1G000750	0.082359	0	0	0.678281	4.69922	5.98661
HORVU4Hr1G082140	13.01404	12.50962	14.33281	7.499194	9.669845	10.76561
HORVU2Hr1G019850	5.7744	6.512303	7.80255	0.011484	0	0
HORVU1Hr1G015270	1.456334	1.766544	1.976532	2.524325	2.296259	3.042492
Hordeum_vulgare_newGene_2739	7.698716	6.381143	7.842712	7.178921	8.382506	8.394105
HORVU3Hr1G090870	5.636256	4.943561	5.579517	6.994458	9.005865	9.074664
HORVU7Hr1G042590	2.058889	2.397101	2.99783	2.217623	2.709339	3.116137
Hordeum_vulgare_newGene_13370	19.80578	19.45151	19.23477	15.42184	13.17916	14.50621
Hordeum_vulgare_newGene_13373	10.60205	10.53361	12.42411	13.41521	18.33978	19.1172
Hordeum_vulgare_newGene_9077	7.160617	6.39617	7.579662	6.551425	6.764185	7.129371
HORVU2Hr1G099530	1.981241	1.350489	3.001247	3.207124	4.141955	4.935494
Hordeum_vulgare_newGene_15230	3.414211	3.396222	3.16955	4.911709	3.599568	4.401009
Hordeum_vulgare_newGene_15231	5.036034	5.038037	4.432736	7.845403	4.633384	6.223487
Hordeum_vulgare_newGene_1414	1.147456	1.18882	2.298071	1.852543	1.456877	2.538137
HORVU5Hr1G007830	42.99611	35.22099	58.07717	31.91452	34.08381	34.65583
HORVU5Hr1G001400	8.902354	8.182498	10.47333	8.905048	10.38314	10.43818
HORVU4Hr1G061280	2.355098	2.485024	3.018364	0	0	0
HORVU3Hr1G050400	6.026277	5.376875	7.819322	7.063746	8.266917	8.303283
HORVU2Hr1G091720	23.8972	21.18647	30.32118	24.61876	19.28214	23.29982
HORVU3Hr1G013820	1.935334	2.142992	2.101775	2.569937	1.867593	2.596525
HORVU2Hr1G060730	0.025284	0.363002	0.130662	4.188776	14.06289	13.8357
Hordeum_vulgare_newGene_13994	9.9652	8.901343	9.526864	4.144192	4.505218	4.967672
Hordeum_vulgare_newGene_13992	4.71772	6.289274	6.130615	6.483324	7.762199	8.54586
HORVU7Hr1G027590	6.205644	6.621763	8.438778	6.280635	10.20765	8.879631
HORVU4Hr1G014600	3.817704	4.624054	5.412323	1.613889	0.862781	1.186759
HORVU4Hr1G018180	8.881537	9.478545	5.67004	0.4336	0.043996	0.073824
HORVU2Hr1G067330	1.69243	2.048902	2.010714	18.3689	10.98446	14.91068
HORVU0Hr1G000950	2.090911	1.734434	2.404217	2.864942	2.230306	2.24352
HORVU5Hr1G096390	7.999419	6.884984	8.903628	45.92753	188.1319	160.5299
HORVU7Hr1G089550	8.415469	8.079131	9.023499	9.67256	8.978668	9.675251
HORVU5Hr1G117690	3.201781	3.660883	3.65811	2.251939	3.624351	3.899579
HORVU2Hr1G031830	1.354187	1.370196	1.367815	1.327455	2.131861	1.881118
HORVU2Hr1G060880	22.75523	18.84498	18.71717	49.71775	79.4047	76.90753
HORVU1Hr1G032780	5.062557	4.838865	6.715011	6.685507	7.617205	7.728963
Hordeum_vulgare_newGene_11269	3.561486	3.559412	5.194991	6.67689	7.033581	9.331678
HORVU7Hr1G075060	0.805686	0.91113	1.485491	1.477157	2.130998	1.339842
HORVU2Hr1G118560	7.282068	5.797342	7.697056	5.745102	6.438817	5.870856
HORVU6Hr1G017390	11.75768	8.484629	13.87699	3.326195	1.266	2.770454
HORVU7Hr1G081700	2.13524	1.48513	2.364634	1.926557	0.503608	2.278911
HORVU3Hr1G022900	1.769651	1.637936	2.62623	4.819068	4.440067	4.330541
Hordeum_vulgare_newGene_8033	1.406766	1.906524	1.771621	1.434669	2.873467	2.393401
Hordeum_vulgare_newGene_8036	0	0	0	5.60563	6.337034	8.329655
Hordeum_vulgare_newGene_8035	4.397525	4.443737	4.47884	5.02215	6.490297	6.097207
HORVU7Hr1G059420	9.002826	10.0846	10.00039	12.84429	16.0834	17.25846
HORVU1Hr1G024710	236.9432	225.6928	319.3228	233.3125	430.932	395.9606
Hordeum_vulgare_newGene_14169	16.96881	13.76103	15.09735	11.45042	7.300513	6.862874

HORVU2Hr1G026870	22.08616	18.79615	26.73876	26.53982	32.68391	31.92746
HORVU6Hr1G066210	2.077146	1.97328	2.536152	4.389427	10.90874	9.228747
Hordeum_vulgare_newGene_1412	6.2322	6.720968	5.292933	12.76104	16.81306	17.84545
Hordeum_vulgare_newGene_1413	0.805547	1.252116	1.460514	1.73377	1.996893	2.136506
HORVU7Hr1G030800	0.659888	0.723218	0.827662	2.226235	1.425912	1.14456
Hordeum_vulgare_newGene_1415	1.802307	1.16047	2.490998	3.658519	8.224895	5.865057
HORVU6Hr1G076770	0.563863	0.705699	0.576188	0.481033	3.39412	3.447918
HORVU5Hr1G049630	34.56712	31.16007	31.50132	26.87704	20.5447	23.23419
HORVU6Hr1G071920	14.46741	11.30476	19.168	7.600006	35.53103	33.04779
Hordeum_vulgare_newGene_3815	0	0	0	0	2.849265	4.153496
Hordeum_vulgare_newGene_4628	1.519691	2.076491	1.828165	1.058322	1.07642	1.544736
HORVU2Hr1G025990	1.176155	1.375343	1.762088	1.3992	5.303274	5.217923
HORVU1Hr1G076420	19.82106	16.98038	23.58189	16.06885	17.59287	13.38212
HORVU1Hr1G031250	0.476734	0.798135	0.455157	1.240449	1.659004	2.017704
HORVU2Hr1G021060	26.0962	23.86955	26.9995	13.54048	11.61627	13.21083
HORVU2Hr1G032710	1.344032	1.284221	1.520098	0.99904	2.154511	2.134341
HORVU2Hr1G071940	1.209539	0.746088	1.666882	2.560785	1.980073	4.362053
HORVU3Hr1G088020	8.109954	6.970627	9.312481	7.895593	4.790612	6.914939
HORVU6Hr1G071690	54.07706	48.23785	59.333	49.8693	57.62057	51.96012
HORVU1Hr1G038330	6.371742	5.792863	6.803944	17.5737	31.08894	32.78755
HORVU6Hr1G008730	10.75646	5.762273	5.652898	3.925174	14.92622	23.65141
HORVU4Hr1G041540	8.372613	9.115995	8.969837	8.727561	11.4181	13.01394
HORVU1Hr1G072210	44.80725	48.426	47.75183	67.97376	72.64876	54.55625
HORVU7Hr1G053100	1.515504	1.889348	2.331916	0.436832	0.377551	1.362284
HORVU7Hr1G001230	4.123626	4.732634	4.723494	7.46376	8.015803	7.594439
HORVU7Hr1G056770	34.5784	34.1854	34.9516	89.57992	85.14	91.92469
HORVU2Hr1G064130	5.458556	5.154008	5.986022	7.319627	11.51935	10.69204
HORVU2Hr1G044590	1.934028	2.208861	2.929556	0.647396	0.326942	0.642188
HORVU2Hr1G068140	3.517218	2.289137	2.896374	2.536895	2.643562	3.195972
Hordeum_vulgare_newGene_12107	45.19534	44.12055	51.51597	32.0263	34.57442	33.38507
HORVU7Hr1G105340	8.618326	9.413522	9.761074	12.59828	13.94885	14.52933
HORVU3Hr1G096710	1.601985	1.351529	1.186635	0.9115	0.589826	1.092282
Hordeum_vulgare_newGene_6668	0.601081	0.054803	0.332061	1.59767	2.839308	3.6892
HORVU5Hr1G019040	22.85604	24.50632	28.93833	12.99905	4.336608	5.698523
Hordeum_vulgare_newGene_6661	13.07914	13.06621	10.59954	13.49051	14.12489	15.98876
Hordeum_vulgare_newGene_241	3.345081	3.02046	2.512652	3.721765	5.362099	5.669323
HORVU3Hr1G014960	0.225793	0.316364	0.256662	3.083288	6.048575	4.992133
HORVU2Hr1G081920	0.379024	0.433919	0.753104	2.630391	5.183424	5.404145
Hordeum_vulgare_newGene_3100	3.592661	3.156208	5.390397	5.348548	6.44659	5.020269
HORVU4Hr1G080810	1.148145	0.900159	0.844344	3.20479	5.046716	4.828257
HORVU1Hr1G063250	0.705428	0.621176	0.721617	2.240594	1.730947	2.260472
HORVU3Hr1G067110	38.23433	36.17597	33.11323	103.1237	127.3485	145.7944
HORVU3Hr1G034670	7.236885	7.284024	8.680768	8.390032	10.98305	10.77576
HORVU3Hr1G080560	17.2064	17.13369	19.39165	15.94033	22.75998	22.38234
HORVU3Hr1G069300	7.327271	7.404445	9.658277	0.034772	0	0
HORVU5Hr1G022470	28.81393	24.31083	39.48439	26.08262	31.71428	30.706
Hordeum_vulgare_newGene_14623	0.920332	1.288383	1.33432	1.207459	1.222416	1.703044

Hordeum_vulgare_newGene_14620	12.1378	9.144047	13.04005	11.44444	13.14768	9.6264
Hordeum_vulgare_newGene_6088	5.065781	4.986878	6.465632	3.936786	5.354177	4.254683
HORVU7Hr1G034130	10.58452	10.21836	14.80796	7.756611	11.39106	11.5897
Hordeum_vulgare_newGene_6080	6.21433	6.830804	6.563796	7.497803	6.73168	7.318651
HORVU4Hr1G013480	7873.012	8708.045	6102.377	3261.892	790.5816	1267.763
HORVU4Hr1G017100	29.64474	26.05043	27.62432	35.4892	28.71376	30.31245
HORVU5Hr1G061180	4.082967	3.69248	4.289984	4.785476	5.280961	4.565648
HORVU3Hr1G117730	14.74366	15.64223	11.47285	15.68174	19.72555	19.51699
HORVU1Hr1G070620	16.99981	16.09898	14.73756	14.11243	15.32722	16.00447
HORVU6Hr1G082320	4.048809	5.985781	7.324062	3.792382	6.049387	5.449908
HORVU7Hr1G118560	3.522661	2.167672	3.483463	0.861255	0.519182	0.662672
HORVU7Hr1G002450	8.139985	6.268261	10.388	5.77797	18.10014	13.74255
HORVU2Hr1G005480	3.610367	3.832971	4.318434	5.332532	5.184543	4.664287
HORVU7Hr1G091900	7.131709	6.355546	6.795475	8.70228	13.68033	13.50273
HORVU7Hr1G108190	2.372922	2.017158	2.967076	2.319726	3.400381	2.54248
HORVU7Hr1G051630	13.90509	13.81682	16.51205	12.32572	11.64351	12.81993
HORVU2Hr1G087510	1.800364	1.743816	1.339162	1.33356	1.722523	1.545459
HORVU6Hr1G075590	55.96429	46.0985	58.12699	46.10141	33.98666	36.24507
HORVU2Hr1G043900	44.03361	34.43823	52.4123	8.394596	0.2733	2.755232
HORVU6Hr1G002030	5.327627	6.440476	8.852908	4.78264	6.474632	6.017161
HORVU3Hr1G087110	13.20153	10.07368	12.99839	9.823408	17.76782	18.73521
Hordeum_vulgare_newGene_15575	6.618145	6.308186	6.485064	7.071886	9.369554	8.451368
Hordeum_vulgare_newGene_15574	2.527021	2.723742	0.664245	2.308427	3.235541	1.41649
Hordeum_vulgare_newGene_15577	3.699703	4.5549	4.520823	4.97164	6.483231	6.972882
Hordeum_vulgare_newGene_15576	2.688824	3.333186	3.454996	4.074125	4.604823	6.569554
Hordeum_vulgare_newGene_8950	4.215821	4.656316	4.415903	3.802817	3.57864	4.475765
Hordeum_vulgare_newGene_8951	2.927978	2.399619	2.79933	2.480088	2.127615	2.697309
Hordeum_vulgare_newGene_8957	1.744225	2.833892	2.802506	2.07465	1.802006	2.771267
HORVU7Hr1G069840	11.94379	13.00892	12.55156	10.50878	12.05307	13.96479
HORVU7Hr1G096480	8.948746	7.879298	10.14851	6.205979	5.568304	6.750481
HORVU3Hr1G051550	11.93431	8.183441	12.06817	11.92008	12.14934	11.89227
HORVU3Hr1G078950	4.955936	4.433927	5.49656	4.818652	7.667681	6.752358
HORVU2Hr1G030820	4.757148	5.036918	6.004919	6.916996	8.748983	9.403868
HORVU1Hr1G054590	4.62414	6.054098	5.581682	7.083456	9.472843	9.255822
HORVU2Hr1G004280	0.648391	0.802554	0.914737	0.933981	2.428901	1.844505
HORVU7Hr1G034860	11.6369	8.981365	14.5774	7.425953	6.660231	7.491387
HORVU1Hr1G003300	9.457995	9.664675	10.36376	43.66045	40.16742	39.42864
Hordeum_vulgare_newGene_13587	1.591101	2.294646	1.677448	1.182525	1.136528	1.349759
HORVU6Hr1G081140	12.50632	11.54676	13.70149	6.878744	6.406446	7.232682
HORVU4Hr1G089490	0	0	0.142058	0.024411	152.2631	105.9583
HORVU0Hr1G013830	1.461486	1.34948	0.956815	3.470426	3.011256	3.280116
HORVU7Hr1G028720	5.780216	6.212737	6.155467	1.127618	0.726783	1.084365
HORVU4Hr1G002540	1.018612	1.186676	1.795226	3.10412	3.188641	3.715508
HORVU5Hr1G024160	88.73152	70.34908	91.63136	59.46832	53.05028	51.14695
HORVU3Hr1G015600	8.184885	8.979444	10.10319	13.11596	15.27955	14.58698
HORVU5Hr1G114470	0.395427	0.317738	0.513729	1.21043	2.898233	2.301172
HORVU4Hr1G088540	3.306833	4.445917	4.221958	1.496909	1.133942	1.335226

Hordeum_vulgare_newGene_9351	11.0625	9.732849	13.78598	12.79815	21.60296	19.30365
HORVU1Hr1G050650	4.82278	4.90745	4.765534	5.165868	7.600858	6.310378
HORVU2Hr1G085820	11.14838	9.93647	14.35469	8.260593	11.50884	11.72674
HORVU6Hr1G000500	3.681932	4.090895	3.285657	4.232008	4.998483	5.197495
HORVU7Hr1G109990	29.33163	30.97162	32.03776	22.77051	20.28504	20.78939
HORVU6Hr1G068060	18.28177	15.82917	17.76359	18.67342	17.3729	17.18609
HORVU2Hr1G126650	1.751896	1.954675	2.138233	0.564969	1.099204	0.973075
HORVU0Hr1G017330	0.417981	0.588949	0.821506	3.166706	11.76456	11.054
HORVU2Hr1G084190	3.310346	3.815032	3.62825	5.375293	6.71752	7.339851
HORVU1Hr1G089240	0	0.056405	0	44.1861	0.03291	0.057066
HORVU6Hr1G066550	6.103367	4.694323	6.824597	4.664131	6.739384	7.379028
HORVU7Hr1G084630	20.67553	20.00818	28.50072	20.05197	25.42443	25.79156
HORVU2Hr1G127520	16.60906	15.46088	17.35525	17.01693	18.49553	18.48173
HORVU1Hr1G062110	18.60207	16.59779	22.00865	21.80831	39.71709	32.63237
HORVU0Hr1G039470	14.89031	14.44223	15.94704	19.72736	33.31335	35.88623
HORVU1Hr1G073600	1.811421	2.008386	2.37448	1.28159	3.949651	2.673198
HORVU7Hr1G115500	1.132575	0.844643	0.786126	1.821234	6.090383	4.063151
HORVU6Hr1G080230	0.367462	0	0.385055	5.769456	6.952045	7.342795
HORVU3Hr1G030900	8.773217	8.221696	8.984616	12.02276	15.56312	15.589
Hordeum_vulgare_newGene_11635	5.346711	7.808493	7.083817	9.013988	8.590167	10.45863
HORVU3Hr1G009370	0.634526	0.668117	1.202177	85.01587	118.9015	144.0396
HORVU2Hr1G010870	108.0279	122.4368	181.2511	122.8754	297.8025	242.3947
HORVU1Hr1G091550	5.277455	5.648327	8.577871	9.533438	11.521	9.956863
HORVU1Hr1G057680	34.25706	40.85904	45.49378	54.42034	32.97463	34.87135
HORVU3Hr1G099470	2.4503	3.467705	4.205587	0.977102	1.246998	1.514132
HORVU5Hr1G011940	14.92321	14.85957	16.77166	17.70942	17.99065	20.32713
HORVU2Hr1G008640	0.463518	0.657362	0.359327	2.492671	1.449497	2.824858
HORVU2Hr1G080240	0.508556	0.5064	0.666788	0.554445	2.533735	4.167375
HORVU1Hr1G049480	0.889685	0.981955	1.386471	1.751987	2.367241	2.529734
HORVU7Hr1G106450	2.982627	2.625197	4.073353	3.706976	5.296229	5.720971
HORVU3Hr1G113120	329.9952	193.0342	298.5235	54.81056	24.36062	48.49359
HORVU3Hr1G071740	9.417066	8.165827	13.43281	12.39712	19.39075	15.82208
HORVU7Hr1G110170	0.104541	0.063021	0.551506	4.777	16.5262	13.61586
HORVU5Hr1G113900	24.48095	22.09171	19.94853	51.34256	76.50446	77.38541
HORVU1Hr1G069560	5.623087	4.902514	5.949521	6.336883	7.791397	7.913689
HORVU3Hr1G076910	9.597548	8.827682	9.387754	9.686508	4.098178	4.047301
HORVU1Hr1G057970	79.70159	72.91493	104.7437	79.49707	152.9101	140.4198
HORVU1Hr1G089930	1.045605	1.017146	1.666017	0.921685	1.381958	1.262529
HORVU4Hr1G081800	2.180583	1.76179	1.904551	2.776977	70.63028	50.51658
HORVU7Hr1G098410	1.551293	1.077569	1.428441	2.096844	5.611973	5.033562
HORVU5Hr1G056790	10.23558	11.11726	17.05009	8.556677	11.46667	12.55766
HORVU5Hr1G045070	6.240418	5.530971	6.295994	12.29716	17.69393	17.33784
HORVU7Hr1G016130	0	0	0	1.699109	3.863705	4.183744
HORVU5Hr1G095200	9.465062	10.32218	12.07163	7.844386	9.569505	10.20784
HORVU6Hr1G019350	0.555939	0.73172	0.545516	1.49753	1.764826	2.073183
HORVU2Hr1G028430	17.51159	12.85695	21.71391	19.89532	39.53925	40.4098
HORVU5Hr1G057260	5.463394	5.807941	6.07699	7.049226	10.08598	8.949439

HORVU7Hr1G085270	7.519582	6.45037	7.860429	7.600705	7.24821	7.68749
HORVU3Hr1G074210	5.35808	5.705382	7.729433	7.068398	8.822102	8.540494
HORVU3Hr1G030010	0.43024	0.538199	0.383645	1.243053	2.176599	1.792792
HORVU7Hr1G000850	4.302067	3.952965	4.393534	5.746582	6.888429	7.321703
HORVU2Hr1G019520	9.248217	3.264691	1.217899	7.667513	2.615395	3.000377
Hordeum_vulgare_newGene_10333	2.627808	1.893494	3.417432	0	0.051669	0
Hordeum_vulgare_newGene_10335	0	0	0	8.908936	8.514646	4.763036
Hordeum_vulgare_newGene_10336	1.960748	2.264352	2.603332	0.296676	0.366356	0.395623
HORVU5Hr1G053560	10.30528	8.879738	10.15292	11.09759	10.84379	10.65746
HORVU2Hr1G017470	2.548705	2.157953	3.133748	7.631301	4.81693	6.628349
HORVU1Hr1G090200	0.84502	0.544877	0.777825	1.543694	2.588799	2.614923
HORVU1Hr1G081920	8.822106	5.300148	8.873581	6.42894	8.247084	8.085616
HORVU2Hr1G097670	0.024037	0.32205	0.087936	2.734028	4.367316	4.12862
HORVU6Hr1G038120	1.291615	0.987538	1.043922	2.144638	3.604888	3.907869
HORVU2Hr1G104080	51.89335	70.79362	103.749	8.984246	2.102143	6.746041
HORVU5Hr1G022130	10.90294	10.59078	13.85809	7.207603	18.66583	16.97283
HORVU5Hr1G056130	0.856154	0.848171	1.426683	1.904234	2.525095	2.554685
Hordeum_vulgare_newGene_3865	74.50227	64.48059	84.07665	85.64014	69.95571	66.40729
HORVU3Hr1G073630	1.962819	2.238147	1.953468	6.833448	8.49392	9.04043
HORVU5Hr1G048700	75.2061	75.21295	81.2495	88.03868	71.41167	71.26598
Hordeum_vulgare_newGene_3862	23.82769	21.52513	23.52256	26.40232	33.56366	34.02582
HORVU4Hr1G010890	1.448039	0.718069	2.779769	0.964114	0.741315	1.236305
HORVU3Hr1G092660	32.62704	32.10662	34.66504	39.72435	66.39621	66.88484
HORVU0Hr1G012940	8.894773	7.91747	9.366433	4.632864	4.196982	3.116069
HORVU3Hr1G077740	1.822504	2.36918	2.89043	0.102906	0.079368	0.054756
HORVU6Hr1G094880	9.515111	8.658051	10.95821	2.872825	1.835864	2.601339
Hordeum_vulgare_newGene_4612	14.19958	15.75101	16.21591	0.009936	0.074608	0.167419
HORVU5Hr1G072350	2.887642	2.624825	3.905458	1.84939	1.274667	1.632217
HORVU2Hr1G110680	19.68233	22.06532	27.53796	12.32257	11.8817	13.98679
HORVU1Hr1G040670	4.534912	5.405161	5.22036	2.520574	2.801894	3.218307
HORVU4Hr1G056610	92.77777	77.94886	85.35745	105.5015	89.45178	92.03972
HORVU7Hr1G110380	0.689719	0.173994	1.221655	3.2937	1.275544	2.019738
HORVU4Hr1G052100	8.128279	7.569293	11.27827	7.473179	11.33596	11.32595
HORVU6Hr1G057560	1.656645	1.897564	2.267358	1.249901	1.666027	2.072184
HORVU7Hr1G120720	4.706537	5.593524	5.615695	4.683388	4.857081	5.865831
HORVU3Hr1G107070	4.913811	5.608032	7.123657	4.077951	3.251505	3.713701
HORVU6Hr1G039740	12.09406	11.23962	11.46946	9.644577	10.31424	11.08964
HORVU0Hr1G009860	136.8891	125.0524	87.06975	18.32341	6.284533	9.110543
HORVU6Hr1G090250	1.594546	1.347972	1.533998	3.739917	5.201829	4.496629
HORVU3Hr1G037370	2.159653	2.830214	3.271865	0.033697	0.038652	0.029979
HORVU2Hr1G006970	1.509615	0.994065	1.034271	0.680208	1.196964	1.462344
HORVU7Hr1G061850	37.606	35.14442	39.57895	48.94462	59.60252	62.29408
HORVU1Hr1G039260	25.33283	23.73287	36.84982	19.60882	30.5936	32.97998
HORVU1Hr1G046630	1.394257	1.048976	1.498362	3.46627	2.267626	3.500643
HORVU2Hr1G113880	10.00934	10.91769	11.68453	11.7971	9.617792	11.04332
HORVU3Hr1G030150	2.868531	3.368533	3.697495	14.1493	16.16344	15.44823
Hordeum_vulgare_newGene_16042	2.554869	2.041237	2.910672	0	0	0

Hordeum_vulgare_newGene_16045	24.38257	38.32677	13.32411	1.000567	0.254532	1.219183
HORVU2Hr1G088140	8.951459	8.565442	11.33733	9.634464	10.65241	11.68325
HORVU5Hr1G096840	5.961857	5.216088	4.625912	3.326812	3.579585	3.171386
HORVU1Hr1G038130	34.91725	26.51343	31.4553	13.0919	9.41202	11.06854
HORVU1Hr1G059870	373.6321	627.4566	1066.353	46.96448	0.272268	10.58529
HORVU1Hr1G033300	1.098571	0.981144	0.991572	1.180536	1.610311	1.518888
HORVU2Hr1G012180	6.839466	6.43948	7.253123	3.774081	3.883619	4.150998
HORVU3Hr1G075580	12.79955	13.55028	19.90413	7.432443	10.92053	11.97702
HORVU1Hr1G085500	0.798846	0.896545	0.833544	3.101238	1.611449	2.375994
HORVU5Hr1G005930	28.038	34.42818	38.99928	6.253967	0.897766	2.217909
HORVU6Hr1G088540	24.49525	17.69849	18.16104	5.871128	1.360786	1.861933
HORVU3Hr1G026950	0.806975	1.326863	1.819745	6.902489	6.541203	5.805714
HORVU2Hr1G039050	4.187818	1.828632	4.470147	2.772398	1.698253	2.012935
Hordeum_vulgare_newGene_12741	2.329676	2.153333	1.557345	4.433653	1.985857	2.715833
Hordeum_vulgare_newGene_12740	1.8328	2.493774	1.415604	3.560758	2.413985	2.827279
HORVU2Hr1G068380	4.821735	4.135641	4.483365	9.419827	23.5018	17.52354
Hordeum_vulgare_newGene_12745	1.241701	0.330543	0.214644	3.693622	5.498202	4.60465
Hordeum_vulgare_newGene_12744	0	0	0	0.306259	15.21897	14.94824
Hordeum_vulgare_newGene_12749	4.34073	3.960469	4.396386	5.339848	4.049574	4.231876
HORVU7Hr1G018700	2.6924	2.266518	2.072845	2.496825	3.293875	3.439269
HORVU6Hr1G024920	45.40934	47.10011	48.41681	39.66944	48.97081	39.63074
HORVU1Hr1G014710	23.81415	18.21063	30.79338	26.42774	42.79861	37.26075
HORVU5Hr1G050950	124.86	128.4519	194.3076	24.53704	4.029646	9.656927
HORVU3Hr1G061560	5.007202	4.638415	4.521726	2.500324	2.095218	2.421729
HORVU5Hr1G040360	0	0.123585	0.156663	0.138987	2.682107	3.353297
HORVU7Hr1G038270	0.048377	0.1632	0.068809	4.242239	5.125329	5.605597
HORVU5Hr1G103860	5.440072	6.015006	6.631837	12.58019	8.338426	7.76085
HORVU2Hr1G102800	11.00822	10.71877	12.53631	12.52641	14.7002	13.13783
HORVU2Hr1G065650	9.031602	8.960257	10.50965	13.25923	14.7274	15.14589
Hordeum_vulgare_newGene_3340	1.358153	1.256883	1.517627	0.826815	1.231461	2.01755
HORVU4Hr1G073920	0.984238	0.881213	0.802624	2.571666	4.982179	4.243138
Hordeum_vulgare_newGene_7846	9.829473	8.178186	11.05915	6.856057	9.606607	8.987244
HORVU2Hr1G040020	16.58064	17.2215	17.2532	20.30305	31.09673	31.70342
HORVU2Hr1G112860	1.51721	1.851278	3.329649	0.315735	0.334141	0.421624
HORVU4Hr1G054500	15.74745	12.5751	20.21151	8.34509	8.969357	7.612015
HORVU3Hr1G069400	12.84756	12.64801	16.56679	16.15388	19.18812	18.40831
Hordeum_vulgare_newGene_13639	2.550967	1.507239	1.704465	0.144965	0.358391	0.335331
HORVU2Hr1G084750	7.941956	6.056418	7.269291	17.83185	22.98206	21.84867
HORVU7Hr1G070170	2.907124	2.968583	2.559928	3.028128	3.018257	3.03702
Hordeum_vulgare_newGene_3690	27.5812	18.35918	36.24069	16.6277	6.805558	18.41592
HORVU6Hr1G027780	1.54135	1.793054	2.425951	1.541415	0.387883	1.187312
HORVU6Hr1G012070	2.458059	3.098959	3.43032	3.518618	3.021158	3.156435
HORVU2Hr1G019950	0.39027	0.574822	1.386033	3.649543	12.88344	8.939656
Hordeum_vulgare_newGene_9760	4.54211	4.229928	4.55198	4.954137	5.784867	5.739839
HORVU7Hr1G077740	23.31421	20.97139	20.72212	69.10218	116.5354	108.791
HORVU5Hr1G070240	1.446545	1.340652	1.474426	0.743589	0.510895	0.789013
Hordeum_vulgare_newGene_658	93.08111	86.62735	76.45526	28.33193	12.46884	17.99157

Hordeum_vulgare_newGene_654	11.58397	9.453248	15.24775	5.875462	4.667346	8.072051
Hordeum_vulgare_newGene_656	4.585257	4.233272	5.544919	13.95033	22.96768	20.33961
Hordeum_vulgare_newGene_652	3.276784	3.091322	4.478223	5.544627	4.846004	5.233299
HORVU7Hr1G075670	3.158223	4.280653	3.82118	6.158515	6.04775	7.210241
HORVU3Hr1G044640	5.046041	5.338261	7.5189	4.051593	4.430154	4.27375
Hordeum_vulgare_newGene_14353	2.479919	1.590094	0.83139	6.696701	4.855747	3.981419
HORVU3Hr1G021380	6.559216	6.225021	7.234832	7.664421	10.58209	10.91907
HORVU2Hr1G054780	13.37157	11.3889	11.56858	12.48701	13.42387	14.15794
HORVU1Hr1G007580	20.79845	21.3225	23.84657	25.93169	27.77531	29.73414
HORVU3Hr1G059460	5.971642	5.475576	6.288964	6.995315	11.24491	10.57059
HORVU0Hr1G004730	1.183131	1.154585	0.791936	1.049706	1.840256	2.299549
HORVU2Hr1G070400	0.012537	0.022541	0.010469	1.362126	2.801561	3.001586
HORVU3Hr1G051650	46.26388	54.87242	51.26928	72.7823	75.17222	86.35858
HORVU2Hr1G063710	1.690958	1.482996	2.530648	0.700769	1.037207	1.348336
HORVU4Hr1G078010	0.060887	0.084673	0.21033	2.319072	4.647296	5.097229
HORVU3Hr1G050600	238.6552	221.3213	206.3814	134.5981	79.15684	89.85724
HORVU2Hr1G101750	0.377895	0.540028	0.302342	1.123951	3.655514	3.682886
HORVU0Hr1G006930	24.76892	26.41108	29.70977	18.1471	20.73404	20.88566
HORVU5Hr1G036400	8.116758	7.684761	10.43996	8.013716	8.965702	9.627576
HORVU3Hr1G048090	123.373	99.04676	131.6748	114.173	137.7176	131.8433
HORVU2Hr1G113050	18.72152	17.36066	20.48689	20.56911	36.56705	28.55722
HORVU6Hr1G079100	1.79731	2.145093	2.168773	1.119193	1.580566	0.598665
HORVU5Hr1G033940	63.90438	56.61606	44.85139	57.85385	56.31559	49.51038
HORVU1Hr1G044780	27.30023	28.98036	32.42158	9.63412	2.972	4.559531
HORVU6Hr1G015760	0.437026	0.374301	0.570328	6.8411	21.20153	20.10077
Hordeum_vulgare_newGene_5698	0.058485	0.083206	0.140518	0.767407	2.723207	2.807966
Hordeum_vulgare_newGene_5696	3.336964	3.267416	5.051067	1.709095	3.49751	2.794776
Hordeum_vulgare_newGene_5697	4.304097	3.239822	4.684786	6.270703	3.143177	3.912157
HORVU3Hr1G117860	0.145594	0.175476	0.048632	0.053923	8.903131	7.749749
HORVU7Hr1G030300	29.56617	33.40699	37.04161	29.80538	53.50336	50.0424
Hordeum_vulgare_newGene_15000	0.805876	1.142369	1.956512	1.082417	0.967549	1.048242
Hordeum_vulgare_newGene_10073	0.823143	0.7352	1.022499	2.807235	2.720324	3.247138
Hordeum_vulgare_newGene_15008	7.458121	6.537017	6.236749	6.519083	7.061323	6.278167
HORVU3Hr1G093270	19.63261	20.16685	32.54826	2.544292	1.275591	1.783992
HORVU2Hr1G062440	2.511977	2.859545	4.043649	4.165989	4.120491	4.140931
HORVU1Hr1G081570	2.023347	1.175202	1.583368	2.435525	6.406123	5.964708
HORVU3Hr1G012340	1.85668	1.442341	1.613979	1.324338	2.375506	2.576027
HORVU0Hr1G022140	0.035927	0	0	0.097126	3.961695	2.722808
Hordeum_vulgare_newGene_5039	3.491043	4.066656	3.507461	6.933984	10.24059	11.19754
Hordeum_vulgare_newGene_5030	19.77215	20.49372	16.54792	15.00784	8.802454	11.15126
HORVU6Hr1G075900	40.61963	36.02734	41.89821	109.4151	137.3261	151.5969
Hordeum_vulgare_newGene_5032	11.09343	11.312	13.44997	10.72469	10.6115	12.81139
HORVU3Hr1G026560	72.52373	64.18213	71.56576	83.64878	80.8909	83.37431
HORVU4Hr1G060740	8.518461	9.690931	10.54842	18.13078	30.23232	30.08182
HORVU6Hr1G016230	3.652356	4.715866	4.588221	5.138436	5.565789	5.637109
HORVU7Hr1G107670	2.761576	2.144409	3.070495	1.022396	0.213436	0.548883
HORVU1Hr1G000060	10.05113	12.46351	11.7058	14.65334	15.62071	18.37997

HORVU3Hr1G053490	11.55606	10.9001	14.83413	9.712648	14.56106	14.36704
HORVU4Hr1G047240	12.12188	10.87299	12.32239	6.084358	4.92307	5.57585
HORVU1Hr1G008250	28.56388	34.16049	27.25119	23.98665	19.14349	23.56994
HORVU1Hr1G030740	5.162201	4.392301	6.005846	6.878171	9.269864	7.799671
Hordeum_vulgare_newGene_9554	7.743493	8.672745	11.39148	6.861244	9.079279	9.835256
Hordeum_vulgare_newGene_9558	2.228624	2.57839	3.426697	2.342669	3.454169	3.450715
HORVU2Hr1G069710	63.9028	71.03755	83.07805	36.98402	58.2894	45.28049
HORVU6Hr1G028200	3.957931	4.717487	4.165551	1.914467	0	1.682003
HORVU5Hr1G106320	13.51297	13.33671	17.74117	10.06978	11.32625	12.5086
HORVU3Hr1G073100	4.668624	5.518841	7.520228	8.008427	4.132651	5.301975
HORVU3Hr1G089980	168.2514	158.7623	129.5199	117.804	145.2341	132.3707
Hordeum_vulgare_newGene_4120	7.282142	6.518713	3.585915	0	0.01498	0
HORVU5Hr1G084900	1.258943	1.714381	2.487942	9.838619	15.23703	18.7505
HORVU6Hr1G079810	0.937125	0.837894	1.018283	1.204532	1.002665	1.291766
HORVU7Hr1G036220	0.972771	1.144792	1.056325	2.058546	1.975201	1.992863
Hordeum_vulgare_newGene_4127	1.616506	1.866848	2.214612	1.860718	2.080415	2.241455
HORVU6Hr1G042160	6.746135	8.608458	8.41541	10.06006	14.40255	15.33302
HORVU6Hr1G077410	0.726171	0.611445	1.062884	3.937118	3.769809	3.53328
HORVU4Hr1G041970	12.90154	13.22737	12.89159	19.08408	13.15746	17.677
HORVU2Hr1G034690	10.56266	11.11924	13.26127	10.79535	14.33026	15.71093
HORVU1Hr1G068010	2.876676	2.316995	2.36191	8.099749	12.6875	10.69955
HORVU2Hr1G088270	3.55078	4.135446	4.295748	6.029704	7.358396	7.990267
HORVU2Hr1G029220	25.68036	25.26372	25.38609	21.82638	19.0759	19.27082
HORVU4Hr1G076160	16.68712	16.55568	15.92268	22.96177	21.64688	24.36448
Hordeum_vulgare_newGene_8205	1.621361	0.51758	1.005248	1.688579	1.841464	1.73555
Hordeum_vulgare_newGene_4785	1.37941	1.576577	1.619216	0.88122	1.531313	1.48521
Hordeum_vulgare_newGene_4784	1.752281	2.632652	1.992685	0	0	0
Hordeum_vulgare_newGene_4787	2.809036	3.254558	2.986687	3.567234	8.417308	7.621703
Hordeum_vulgare_newGene_4781	18.17073	20.43272	23.1786	2.434121	0.610252	1.067592
HORVU3Hr1G043510	18.43624	17.02599	17.20947	16.33605	16.6602	18.09397
HORVU2Hr1G076540	0	0	0	0	4.362648	3.077118
Hordeum_vulgare_newGene_4788	1.376102	2.200408	2.161567	2.481711	5.245614	5.879631
HORVU4Hr1G033280	15.53464	14.16917	16.26434	13.24385	13.30684	15.24709
HORVU4Hr1G048330	1.573492	1.337084	2.142816	1.977142	0.111123	1.002418
HORVU0Hr1G039670	2.963951	1.735261	1.772367	2.281205	2.165795	2.535345
Hordeum_vulgare_newGene_7476	0.826905	0.228437	1.099784	1.582695	2.284256	2.560505
Hordeum_vulgare_newGene_7477	9.102454	10.13818	14.1279	9.468797	13.12457	12.55686
HORVU0Hr1G010600	2.549691	2.354753	2.151746	4.68141	3.084579	4.0941
Hordeum_vulgare_newGene_7478	4.292475	5.550981	4.64875	0	0	0
Hordeum_vulgare_newGene_7479	7.121724	7.857317	7.370331	8.309047	8.275844	9.066345
HORVU1Hr1G075540	23.89141	27.22035	36.87317	26.94366	35.79434	35.7754
HORVU1Hr1G071840	4.561782	4.330645	5.972451	2.712254	1.117093	2.552858
HORVU3Hr1G011700	0.213545	0.17798	0.128017	0.697066	15.94107	12.18852
Hordeum_vulgare_newGene_4872	0.136147	0.312936	0.318404	6.249616	4.968446	6.806355
Hordeum_vulgare_newGene_4871	0.544884	8.607585	0.931138	12.06714	0	0.025543
HORVU7Hr1G060990	18.43106	17.00799	19.554	15.64644	17.5891	19.67847
HORVU7Hr1G065740	11.80866	10.97348	14.36193	13.14577	15.69511	16.93354

Hordeum_vulgare_newGene_6782	8.530956	9.731144	9.806439	14.3806	17.17866	17.4936
Hordeum_vulgare_newGene_6780	0.778229	1.524253	3.31138	0	0	2.508013
Hordeum_vulgare_newGene_6787	10.35202	7.728537	13.36168	16.00699	16.51667	16.76216
Hordeum_vulgare_newGene_6784	0.552062	0.611828	0.909068	1.925639	1.412047	1.345342
Hordeum_vulgare_newGene_6789	2.173599	3.636552	3.023167	1.060453	0.723037	0.970562
HORVU6Hr1G029640	2.601707	3.00096	2.920659	13.51442	16.54142	15.16218
HORVU2Hr1G007980	14.19873	12.75891	14.10779	32.42198	54.85207	44.27061
HORVU5Hr1G052240	197.6492	196.3974	270.958	223.8247	439.2408	391.0661
HORVU7Hr1G001370	40.18319	44.5477	33.87897	30.42165	29.25143	28.91349
HORVU2Hr1G032690	18.92361	19.30814	22.40099	13.77975	17.78399	15.42427
HORVU1Hr1G029110	1.878908	2.01247	2.690797	2.883948	7.203252	6.744225
Hordeum_vulgare_newGene_7780	1.172481	1.325301	0.539852	1.298523	2.245768	1.985711
HORVU6Hr1G071710	13.41018	10.16286	12.84955	22.13734	22.80036	24.41333
HORVU3Hr1G035340	3.766023	3.770933	3.968957	3.109936	3.771907	4.002162
HORVU1Hr1G078530	10.46529	10.76226	15.5298	25.75351	71.81692	70.15881
HORVU3Hr1G018820	1.005661	0.719475	1.385736	3.26025	1.941976	2.3684
HORVU3Hr1G030250	0.858847	0.888011	0.894121	5.001844	7.377596	7.957095
HORVU7Hr1G079190	2.767152	3.14997	3.043895	12.31656	24.48422	23.81694
HORVU3Hr1G017700	36.61774	35.04728	55.25598	25.54793	25.46212	27.95948
HORVU2Hr1G045380	6.0809	7.134151	7.216565	8.720795	5.481672	7.635429
HORVU7Hr1G038510	1.947915	2.260994	2.020824	0.906255	0.592436	0.916463
HORVU6Hr1G022340	11.97193	10.33803	11.40249	7.009526	7.047576	7.536784
HORVU7Hr1G098250	3.645553	3.317097	3.523382	0.262563	0.110863	0.168023
HORVU5Hr1G040000	2.984275	2.657939	3.053373	7.338267	12.17693	12.85379
HORVU3Hr1G081530	1.766664	2.710253	2.466809	0	0.020559	0
HORVU6Hr1G083790	4.36213	4.316583	4.357341	6.00107	5.855195	6.253927
HORVU0Hr1G040000	5.565257	5.975436	5.34139	7.938201	8.699794	10.32468
HORVU6Hr1G078770	1.681258	1.658118	1.642143	4.558225	5.86933	5.781677
HORVU3Hr1G079960	16.17642	13.2745	19.02126	18.12174	22.94474	27.5748
HORVU6Hr1G026840	2.146716	2.49676	4.529566	1.220381	1.68383	1.823589
Hordeum_vulgare_newGene_2959	1.540327	1.84113	1.498249	2.446355	2.044774	1.993421
HORVU1Hr1G051330	12.64382	10.04263	10.81441	29.29925	40.46197	38.74958
Hordeum_vulgare_newGene_2951	10.3126	9.320454	10.97769	17.475	51.97931	46.42019
HORVU3Hr1G116650	2.745319	3.134483	3.525173	7.237973	8.508866	9.307182
HORVU6Hr1G032410	1.445509	1.865079	1.538921	1.422461	0.958655	0.893418
HORVU3Hr1G025640	12.41079	12.44814	16.59052	12.92442	15.66988	15.19109
HORVU6Hr1G038330	21.77875	19.96758	21.73619	16.70563	19.07285	19.20662
HORVU5Hr1G018700	19.05816	19.22924	19.43328	17.28255	16.61218	17.28467
HORVU1Hr1G029800	16.28457	16.9205	16.38446	18.43906	23.12715	22.17339
HORVU5Hr1G020240	1.812934	0.72124	1.290804	1.211425	1.735997	1.534428
HORVU3Hr1G022360	0.841721	0.810376	1.198929	1.256488	2.183369	2.094962
Hordeum_vulgare_newGene_5057	2.202228	2.069683	2.767182	2.470173	4.005973	3.863378
Hordeum_vulgare_newGene_9969	10.26153	10.0755	9.539572	7.784684	8.760103	8.833389
Hordeum_vulgare_newGene_9963	4.421692	2.555272	3.323509	4.469474	5.249578	4.185598
HORVU2Hr1G124880	3.883173	3.451248	4.120652	5.999138	9.555063	8.71719
HORVU4Hr1G074280	7.766834	6.327806	7.703674	9.141465	3.96437	7.138082
HORVU4Hr1G070900	1.430635	1.301731	1.233278	2.957219	2.531936	2.566748

HORVU3Hr1G013280	2.35751	2.84276	2.753674	2.916778	2.706112	3.149642
HORVU2Hr1G041600	1.62545	1.425502	1.377018	2.185839	3.834209	3.142969
HORVU4Hr1G045500	3.161754	3.099164	3.501054	2.433348	2.912398	3.065095
Hordeum_vulgare_newGene_5723	1.08659	1.312499	1.163872	0.708115	1.076493	0.854803
HORVU3Hr1G041130	1.580394	0.98824	0.508967	0.969001	2.131112	1.881572
HORVU4Hr1G017370	3.980484	4.972387	4.392121	10.62111	14.1917	13.66235
Hordeum_vulgare_newGene_14010	1.718867	2.84426	4.432797	0	0	0
Hordeum_vulgare_newGene_14012	3.223439	2.608544	3.516907	2.296879	3.761706	4.118476
HORVU5Hr1G036690	9.906998	9.585215	12.61596	13.09289	15.88243	16.30935
HORVU3Hr1G002790	2.45488	3.477416	2.921318	3.780296	5.076748	4.790355
HORVU5Hr1G094700	0.910487	0.726649	1.944727	1.427447	0.846625	2.961586
HORVU3Hr1G097210	6.357913	4.870616	7.253237	7.634659	4.999327	4.293459
HORVU5Hr1G058810	3.874048	4.569062	5.814882	3.686807	4.692601	5.283176
HORVU4Hr1G006210	41.72889	32.67501	40.44259	29.56977	26.32225	27.33962
Hordeum_vulgare_newGene_4065	6.420111	6.285394	7.037902	5.658006	5.535611	6.373593
HORVU3Hr1G047980	3.445266	3.327265	3.437816	3.679113	3.181561	3.30238
HORVU5Hr1G112440	1.736832	2.386615	2.519123	2.721052	2.002005	2.715567
HORVU0Hr1G000600	10.35618	10.35028	11.16174	10.1395	10.70579	13.76276
HORVU3Hr1G083260	1.520091	1.980617	2.232893	3.046097	3.723405	4.282624
HORVU7Hr1G096560	7.108439	5.896372	9.428952	6.060664	5.111431	6.302095
HORVU7Hr1G115590	30.67346	31.31496	33.88205	47.25572	52.28109	51.78831
HORVU2Hr1G090750	4129.139	3734.897	3179.034	3935.114	915.6368	1587.944
HORVU7Hr1G064830	9.371426	7.766131	10.68656	13.86639	15.96518	14.07076
HORVU1Hr1G081320	19.38809	17.67539	25.87134	15.01232	15.47414	16.53312
HORVU5Hr1G122340	11.49232	10.45401	14.39284	8.399328	9.554984	9.793385
HORVU3Hr1G004820	4.784323	4.304023	5.056125	3.543629	3.91921	4.177381
HORVU6Hr1G066850	2.412215	2.934417	2.88191	3.379653	5.588765	5.196622
HORVU1Hr1G034070	10.15311	10.0037	10.65731	12.96274	16.90897	15.6512
Hordeum_vulgare_newGene_3112	7.341551	7.183699	5.39696	10.24771	10.84181	13.51776
HORVU1Hr1G051800	1.270273	1.204765	1.480591	2.322343	2.024943	2.14752
HORVU1Hr1G053400	3.316587	3.881129	4.565291	5.391591	2.526114	3.554135
HORVU4Hr1G011690	1.121903	1.406915	0.809629	1.514088	0.778936	0.89827
HORVU2Hr1G085160	47.13376	40.25333	45.78382	23.35728	15.49129	18.70954
HORVU3Hr1G012660	0.773611	0.687127	0.610219	1.317555	2.893302	2.295457
HORVU3Hr1G032120	2.131686	2.1229	2.077178	2.473007	2.930256	3.239201
HORVU2Hr1G117870	2.340931	2.229788	2.201633	2.257047	1.583142	2.704394
HORVU0Hr1G007380	10.00172	12.26355	13.32651	8.541545	10.30083	10.72874
HORVU3Hr1G000230	35.82095	32.57891	36.54765	41.17379	50.78643	48.94154
HORVU2Hr1G089660	2.237595	2.009736	2.171264	2.975747	3.108287	3.759246
HORVU5Hr1G119650	121.5189	143.9422	103.7662	112.6456	333.0829	309.939
HORVU5Hr1G106920	2.080395	2.209814	2.105602	0	0	0
HORVU1Hr1G002510	7.648967	20.91432	10.83875	27.18363	9.238432	8.741891
HORVU7Hr1G050530	17.63945	16.82048	15.82065	13.43766	12.79626	12.39675
HORVU2Hr1G082670	6.066341	5.612301	0.617926	0.573355	0.69592	6.483499
HORVU4Hr1G015330	1.657017	2.613209	1.840022	8.934457	11.12606	11.80921
HORVU1Hr1G058000	1.993929	1.656121	1.910646	4.9581	5.671805	5.852335
HORVU7Hr1G114510	2.611869	2.840285	2.969201	1.986568	2.239294	1.994097

HORVU3Hr1G098290	1.115134	1.78387	1.740647	1.075757	1.097237	1.338928
HORVU4Hr1G008520	2.27805	1.443027	3.265613	0	0	0
HORVU2Hr1G084780	11.97917	7.227223	9.613043	14.2601	8.040682	10.79245
HORVU5Hr1G114150	11.98415	11.55316	14.01548	7.833899	9.046088	10.57192
HORVU4Hr1G001680	1.186998	0.880637	1.508092	3.188921	20.92531	20.46157
HORVU5Hr1G066490	2.776383	2.477145	3.05568	12.62419	15.31251	17.17357
HORVU4Hr1G087750	4.386242	4.786011	4.843935	5.827477	6.90215	5.329201
HORVU2Hr1G085780	3.590015	4.493418	4.27082	2.917314	3.277714	2.79249
HORVU2Hr1G094400	180.1552	172.2132	227.4218	192.0133	194.6471	175.3408
HORVU3Hr1G077400	0.823608	1.045897	1.286222	0.960396	1.131336	1.094928
HORVU2Hr1G083780	7.691048	9.378172	9.679713	5.6703	13.498	11.88836
HORVU3Hr1G077960	242.2651	212.7656	313.4381	150.342	137.8046	138.0594
HORVU1Hr1G049520	35.94217	31.21523	35.91521	31.30078	41.15387	39.3011
HORVU1Hr1G042710	0.398738	0.540536	0.331719	0.715294	2.786026	2.304709
HORVU3Hr1G000490	15.26331	13.16178	15.34999	15.14444	18.58129	18.06307
HORVU5Hr1G067720	3.926934	2.441776	3.310482	3.45717	2.966662	3.07133
HORVU2Hr1G016660	3.417861	3.662875	3.236582	5.133338	7.008399	6.96145
HORVU2Hr1G081320	2.705036	2.359872	2.453816	5.281036	6.038632	5.219968
HORVU4Hr1G067010	1.196534	1.515639	1.764896	1.009331	1.477966	1.587545
HORVU5Hr1G121060	6.801557	6.295069	6.8633	7.706468	7.905382	7.33738
HORVU3Hr1G067020	3.083082	3.102209	3.399184	8.323583	18.51272	17.14738
HORVU3Hr1G005040	12.92693	13.78337	13.52228	12.81849	19.40972	17.28459
HORVU3Hr1G047160	5.559177	5.022999	6.309383	8.431159	9.679185	10.13869
HORVU6Hr1G064170	2.25137	1.682654	1.706641	1.443289	4.538154	3.147933
Hordeum_vulgare_newGene_13740	0.02352	0	0	2.63581	5.672853	5.14068
HORVU4Hr1G085590	73.89411	72.10374	88.66663	46.51901	37.25673	39.2712
HORVU1Hr1G095170	1.938104	2.224842	2.590639	3.131643	5.352652	5.579567
HORVU2Hr1G100640	25.55913	21.61505	28.69996	20.17429	22.74835	21.01182
HORVU4Hr1G016810	266.5478	250.084	335.3196	178.0323	119.2952	161.0413
HORVU1Hr1G094420	0.043968	0	0	2.009046	1.572169	3.294588
HORVU2Hr1G099380	15.50979	16.04325	15.92318	15.55129	29.87965	38.41047
HORVU2Hr1G121110	5.860811	6.329182	8.684503	5.997449	9.589595	9.756604
HORVU2Hr1G014130	18.68677	18.27462	22.0573	14.68894	14.07186	15.7369
HORVU0Hr1G021800	2.678515	3.050584	4.173164	2.363462	5.395895	4.421152
HORVU2Hr1G017530	8.049451	6.050027	6.648432	0.600712	0.563161	0.417121
HORVU6Hr1G072650	15.3063	16.58104	32.46007	6.419208	15.96184	14.68648
Hordeum_vulgare_newGene_13893	4.012585	4.483622	5.281589	0.898492	1.287628	1.49035
Hordeum_vulgare_newGene_13896	1.506321	1.327122	1.594563	2.288544	2.993759	3.055518
Hordeum_vulgare_newGene_13898	0.819265	1.023952	0.743702	1.870837	2.330359	3.068184
HORVU4Hr1G086150	4.118749	3.200887	5.822446	5.935911	3.985743	4.53725
HORVU3Hr1G006530	243.5917	159.2645	257.6393	345.3034	597.3399	347.2197
HORVU2Hr1G054010	1.965478	2.425584	0.899413	0.785576	0.108056	0.248524
HORVU4Hr1G052060	0.164162	0.149743	0.105682	11.61316	18.26945	14.71643
HORVU4Hr1G055160	4.103998	4.373828	5.917773	3.266973	4.6671	4.370544
HORVU5Hr1G053150	74.17985	62.75968	82.37055	58.98194	48.64829	52.33022
Hordeum_vulgare_newGene_10275	0.081263	0	0.072907	0.910671	3.378694	4.73597
Hordeum_vulgare_newGene_10276	0.182125	0.242503	0.095881	3.155251	1.734621	1.509235

Hordeum_vulgare_newGene_10271	2.256139	1.42606	0.913602	2.005374	4.830792	3.890661
HORVU4Hr1G064080	35.19618	30.16641	40.58817	36.69215	49.76828	45.93046
Hordeum_vulgare_newGene_10279	0	0	0	5.049405	3.986327	5.167809
HORVU5Hr1G080690	35.06012	33.95679	41.0373	29.80892	27.26681	26.77887
HORVU5Hr1G027080	5.896291	6.528939	7.290371	5.703839	6.822807	7.090961
HORVU6Hr1G013630	10.80036	12.23507	13.314	10.54213	9.696058	9.062449
HORVU1Hr1G095410	106.9901	114.8206	104.6702	55.77916	54.11126	56.0946
HORVU3Hr1G064680	162.7795	141.3128	127.3839	125.5857	171.7176	156.2844
HORVU5Hr1G111380	22.65896	16.48815	21.98563	14.14276	22.91446	24.84441
HORVU3Hr1G074390	1.614824	0.868764	1.739028	0.752998	0.849697	0.345026
HORVU2Hr1G019280	1.791803	1.293882	1.237297	1.753911	1.27574	1.789205
HORVU2Hr1G018390	3.711121	2.419395	2.910056	4.817986	3.98315	4.322753
HORVU2Hr1G019750	6.682787	5.108074	5.651409	5.555439	2.37315	3.480899
HORVU5Hr1G042370	5.038553	3.378063	5.075598	1.750878	1.028845	0.857022
Hordeum_vulgare_newGene_5425	1.726589	2.493393	2.208943	1.453702	0.566956	0.946305
HORVU2Hr1G119600	3.886027	2.739771	2.812094	9.338768	35.82847	30.60774
HORVU1Hr1G060170	4.975654	5.943557	4.951197	5.404784	5.549496	5.218291
HORVU5Hr1G052200	2.105702	2.289048	2.276484	2.676148	3.154758	3.237489
HORVU7Hr1G044070	35.71928	36.42509	47.08748	36.75909	59.204	52.74628
HORVU6Hr1G093360	6.742122	7.300991	7.717625	9.047335	13.6174	13.20091
HORVU2Hr1G115920	3.738886	3.725844	4.135994	6.350768	6.066225	6.264258
HORVU6Hr1G014360	10.38489	10.2982	11.7988	12.48851	13.00968	12.13587
HORVU4Hr1G063880	6.958765	6.958386	7.716264	6.557651	7.413699	7.810504
HORVU5Hr1G077190	13.59253	13.6148	16.03224	13.62934	11.82333	13.07814
HORVU5Hr1G006850	2.887986	2.297521	2.332581	5.267941	7.408283	7.154173
HORVU4Hr1G060500	13.0781	13.17103	12.97547	15.10167	17.70724	16.62166
HORVU3Hr1G003850	11.21224	11.28887	12.14066	7.541447	7.167911	8.343261
HORVU2Hr1G031400	6.847025	7.735623	8.024691	6.498721	3.059771	4.462963
HORVU5Hr1G017230	4.246054	3.784755	4.829079	4.962303	8.793037	7.412946
HORVU6Hr1G091800	70.59533	63.49544	66.53963	100.9488	77.29124	76.87749
HORVU7Hr1G011090	1.104982	1.389292	0.872727	1.160909	1.753892	1.531482
HORVU0Hr1G016640	1.643806	1.59002	1.807836	1.978414	1.670534	1.604653
HORVU5Hr1G099670	2.092288	1.962689	2.374224	0.760654	0.292768	0.76904
HORVU1Hr1G039180	0	0	0	0	9.180105	11.752
HORVU5Hr1G077440	33.23887	28.94094	36.04778	19.27386	20.47825	22.30685
HORVU7Hr1G029650	50.62682	54.50563	47.13456	41.57233	51.89207	49.90205
HORVU4Hr1G069100	4.712742	2.968431	3.215702	8.270607	2.080958	4.578948
HORVU5Hr1G029950	14.32989	14.76651	13.786	18.35569	20.39428	21.66381
Hordeum_vulgare_newGene_11632	2.272199	2.377121	3.13339	2.24056	2.214435	2.468155
Hordeum_vulgare_newGene_11636	16.54093	20.38463	18.75698	22.47614	20.1998	25.2506
Hordeum_vulgare_newGene_11634	1.217596	0.527201	1.013166	1.208159	0.953139	1.165771
HORVU4Hr1G066900	0.703008	0.909407	0.668807	3.644881	6.384505	6.562734
Hordeum_vulgare_newGene_11639	10.27082	10.48622	10.17955	11.07594	13.22847	13.04302
HORVU6Hr1G063250	3.457646	4.985596	4.678938	10.18086	11.04795	11.78453
HORVU2Hr1G118260	7.824655	9.522847	9.205282	8.06012	9.301506	9.890291
HORVU4Hr1G063260	2.500193	2.878584	3.139807	1.932573	4.580055	4.565512
HORVU5Hr1G055260	0.974486	1.876231	1.648243	1.329218	1.707981	1.427473

HORVU5Hr1G051960	0.014133	0	0.05579	1.855018	6.920315	7.543767
HORVU7Hr1G080770	31.19191	32.15543	34.51259	21.9816	35.12827	33.17029
Hordeum_vulgare_newGene_1847	2.293764	3.101337	3.121193	2.048108	2.20561	3.325727
HORVU2Hr1G084700	1.617968	2.670609	3.148746	1.422111	7.433808	4.851348
HORVU5Hr1G072670	8.436931	4.595774	8.026637	5.669818	6.297294	6.03784
HORVU5Hr1G097460	0	0	0.991604	68.82827	22.73581	28.50967
HORVU5Hr1G041730	229.6508	208.6191	234.2969	216.0025	295.8794	290.612
HORVU4Hr1G028910	4.309028	4.346707	4.697952	6.566198	8.00537	8.857764
HORVU4Hr1G047580	18.98693	21.27227	35.93787	12.14834	19.70322	23.45865
HORVU6Hr1G061800	25.91439	27.103	28.16825	37.04143	40.92659	42.57396
HORVU6Hr1G013990	4.950526	4.653241	6.088333	4.492104	3.404621	3.649417
HORVU5Hr1G093390	10.66532	11.12222	13.26165	17.96729	15.68592	19.48205
HORVU5Hr1G049240	2.202109	2.098763	1.980834	2.227613	1.705024	2.848125
HORVU7Hr1G083080	0.476948	0.55146	0.639183	2.269683	4.63535	3.995926
HORVU6Hr1G059300	6.794676	6.95247	7.233853	10.75678	14.45173	14.35425
HORVU6Hr1G015100	4.958711	5.408956	5.02234	5.691631	3.81835	4.880313
HORVU1Hr1G033110	2.817338	3.119963	3.008428	3.0317	3.258661	3.708054
HORVU2Hr1G087030	1.866084	1.685057	2.239562	2.521539	1.619688	1.885513
HORVU7Hr1G001600	1.192064	1.164762	1.305747	17.77568	22.68699	24.57997
HORVU2Hr1G114720	0.94389	1.398457	1.319047	4.464135	6.789593	5.597923
HORVU2Hr1G032320	3.029698	1.417872	2.852051	2.386633	1.529688	1.547091
HORVU3Hr1G093850	35.91633	31.43358	36.89342	22.8924	19.88997	18.49963
HORVU3Hr1G068650	0.133541	0.187625	0.040141	0.341416	4.568054	2.590173
HORVU2Hr1G037940	13.01762	11.86998	16.43529	12.28812	21.21169	20.99374
HORVU3Hr1G100360	8.491461	7.532074	10.73929	4.500429	1.8315	2.425014
HORVU5Hr1G079690	2.880833	3.094247	3.258011	5.843198	9.539884	6.713505
HORVU7Hr1G042120	0.705794	0.666751	0.759243	6.539962	6.447331	11.19042
HORVU1Hr1G075830	2.48238	2.444406	3.311329	0.877159	1.012659	0.945523
HORVU1Hr1G071390	1.855484	1.498511	3.096842	1.782825	0.104291	0.883028
HORVU3Hr1G063840	4.152913	2.66782	3.805758	2.431382	2.483737	3.308797
HORVU7Hr1G030690	17.79823	22.11873	36.83401	6.711078	0.066297	3.797702
HORVU7Hr1G053470	3.652781	3.243803	3.555404	3.312033	4.016703	3.495842
HORVU6Hr1G065960	11.25872	8.459794	12.53541	10.84812	12.44892	14.50259
Hordeum_vulgare_newGene_6236	2.720363	1.578679	2.146855	1.528468	0.400321	0.755229
Hordeum_vulgare_newGene_13469	1.924995	2.339497	2.527759	1.560516	1.054128	0.897935
HORVU5Hr1G107520	3.975133	4.407537	6.143496	4.568536	6.213159	5.237604
Hordeum_vulgare_newGene_13462	0.821209	0.963219	0.861407	3.333386	3.5877	3.232978
Hordeum_vulgare_newGene_13465	2.302013	2.88022	2.822034	2.099988	3.46548	3.581114
Hordeum_vulgare_newGene_13464	3.385937	4.463594	4.088233	3.854497	3.359749	3.902597
Hordeum_vulgare_newGene_13467	1.048343	1.286698	1.418006	1.924232	2.221733	3.327594
Hordeum_vulgare_newGene_13466	0.476715	0.531711	0.390742	9.144319	7.600208	7.982005
Hordeum_vulgare_newGene_3555	0.510953	0.239739	0.355597	44.12431	50.91604	50.5968
Hordeum_vulgare_newGene_3554	1.634717	1.670962	1.555934	1.228434	0.757387	0.490461
HORVU6Hr1G027620	7.719891	7.546509	11.22963	25.9355	47.11208	50.94122
HORVU7Hr1G027960	17.43998	17.14585	11.78582	0.673131	1.017218	0.984305
HORVU7Hr1G028880	1.644804	0.970511	1.152068	1.942012	2.602179	2.156792
HORVU7Hr1G097010	11.29188	9.970308	11.79801	18.62474	21.40297	23.69986

HORVU5Hr1G065650	6.221925	5.302215	5.290109	6.266653	8.305128	8.663612
HORVU3Hr1G113620	111.0885	93.11264	122.3353	59.40536	23.79199	57.41203
HORVU3Hr1G020220	6.700918	6.190766	6.534423	5.474715	5.706988	5.893141
HORVU5Hr1G121540	3.571744	3.961245	3.892066	5.593945	7.636199	8.17188
HORVU7Hr1G035050	1.614829	1.885453	2.531674	1.680828	5.43029	4.15008
HORVU1Hr1G043900	2.158336	1.518927	1.349838	7.386111	12.09954	9.951747
HORVU4Hr1G074040	4.810485	7.311123	4.223232	8.674405	7.006741	9.17146
HORVU1Hr1G006020	19.03461	22.94093	21.31336	21.87148	20.94358	28.79166
HORVU4Hr1G029740	1.214298	1.698015	0.898088	1.719232	0.96926	0.912267
HORVU2Hr1G074990	2.698282	1.483991	2.619375	2.031805	1.474311	1.757641
HORVU5Hr1G082790	23.48842	21.82745	28.91575	20.30456	19.48228	20.52455
Hordeum_vulgare_newGene_13288	8.706813	8.4492	9.275381	8.765078	6.940236	7.717049
Hordeum_vulgare_newGene_13284	1.447674	1.005636	2.257653	2.785981	3.326117	3.439442
Hordeum_vulgare_newGene_13282	3.946599	4.487507	3.910267	3.881974	2.206753	3.700102
HORVU1Hr1G003210	0.144943	8.32813	0.605488	0	0	0
HORVU5Hr1G115360	2.875432	2.781143	3.601868	9.564424	12.87952	11.86901
HORVU3Hr1G087360	33.21894	30.99081	40.48592	32.47566	46.5642	43.27838
Hordeum_vulgare_newGene_15705	1.3039	1.14321	1.023013	1.504458	0.576024	1.535626
HORVU2Hr1G022850	2.642007	2.777335	2.87458	2.432024	4.94428	4.153229
HORVU3Hr1G069210	56.40402	47.62071	84.78027	49.12154	21.75277	23.64243
HORVU3Hr1G013730	6.609507	4.374343	7.61155	6.800492	5.754046	6.473295
HORVU5Hr1G023020	32.69467	30.92527	33.30961	16.79236	14.17736	16.27852
HORVU5Hr1G062000	3.023792	2.717206	3.130554	2.580387	2.94207	2.977153
HORVU7Hr1G078660	2.031302	1.996098	2.161085	1.130942	1.044446	1.339397
HORVU6Hr1G072140	47.54974	33.18711	52.57526	49.5686	37.08882	30.08815
Hordeum_vulgare_newGene_1116	19.13495	18.92311	19.62437	32.25421	36.91566	33.81682
HORVU3Hr1G059610	1.307224	1.479831	1.336716	3.053321	4.107274	3.853761
HORVU7Hr1G119040	3.996353	3.763283	3.642388	3.918132	4.845598	4.22087
HORVU2Hr1G070670	23.17275	22.70625	30.20509	12.3268	18.59062	17.94258
Hordeum_vulgare_newGene_6901	3.81885	4.437739	4.657362	5.203	5.908959	5.486363
Hordeum_vulgare_newGene_6902	7.302123	5.44541	9.431973	7.802593	12.43311	11.07296
Hordeum_vulgare_newGene_6909	3.565589	4.780312	3.630564	4.2473	3.822018	4.780148
Hordeum_vulgare_newGene_8503	0	0	0.061212	4.041769	14.36101	10.88014
Hordeum_vulgare_newGene_8500	0	0.029537	0	1.276073	5.087981	7.629359
HORVU2Hr1G072120	3.79703	2.040104	2.886049	3.486846	3.578509	2.739735
HORVU6Hr1G034840	14.72799	14.81292	16.14281	19.16447	22.05839	20.69284
HORVU7Hr1G113760	7.795577	7.968138	9.097322	6.693689	9.894263	11.2979
HORVU1Hr1G059310	80.08703	70.57974	83.68151	69.77724	68.32103	68.60231
HORVU2Hr1G104190	3.80978	4.564732	4.032118	6.8331	7.492851	8.840369
HORVU7Hr1G119590	54.34409	50.77453	56.61676	66.99038	83.26123	87.37327
HORVU7Hr1G026370	7.129977	7.319901	9.58444	8.941095	7.818083	10.02248
HORVU4Hr1G034400	15.06676	14.5416	17.19011	21.86608	27.12388	26.72647
HORVU3Hr1G051370	0.879178	1.16017	0.942911	1.03641	1.380633	0.982201
HORVU6Hr1G060440	5.668776	4.257934	3.233515	3.239027	3.890084	3.972937
HORVU3Hr1G111080	5.77411	10.20596	6.114169	29.67635	394.7747	306.321
HORVU2Hr1G058810	6.465516	6.857638	7.748881	14.98216	19.81922	16.71058
HORVU0Hr1G019690	3.019114	2.054482	3.295866	1.503937	1.422409	1.359784

HORVU1Hr1G024220	1.802721	2.348734	2.803252	2.473168	3.071645	3.237265
HORVU5Hr1G096110	4.505247	4.199513	4.665491	5.32521	5.17324	5.034177
HORVU1Hr1G073680	0.056922	0.010937	0.14653	2.328882	9.642426	8.53206
HORVU5Hr1G019980	18.5259	16.13919	18.46761	22.37888	30.77905	31.74662
HORVU5Hr1G034100	7.014598	7.339477	8.186134	11.39897	22.16489	16.27493
Hordeum_vulgare_newGene_1301	0.921765	0.758529	0.71548	1.281094	2.404042	1.86925
Hordeum_vulgare_newGene_1309	20.4318	17.50358	19.85727	24.44216	31.2581	28.7628
HORVU3Hr1G014720	7.790052	8.133026	8.363947	7.285004	9.066621	10.00155
HORVU3Hr1G052880	4.72361	2.856571	4.210982	3.717291	3.181906	3.450015
HORVU7Hr1G114750	0.540317	0.109545	0.376579	1.76963	1.722604	1.666249
HORVU2Hr1G080430	1.831903	2.352975	2.35752	0.800497	0.935885	1.086825
HORVU3Hr1G049480	28.57765	30.28693	32.05575	34.89954	32.59882	37.28123
HORVU3Hr1G036930	10.15229	8.627231	10.94381	8.648896	12.79088	11.99824
HORVU2Hr1G089310	7.000377	6.759777	10.16403	5.883478	3.254573	4.857247
HORVU3Hr1G019140	0.532404	0.654785	0.747043	2.730221	6.352913	5.966818
HORVU4Hr1G078120	2.452947	2.357504	2.700051	4.355469	5.678524	5.784863
HORVU1Hr1G076110	3.449144	3.558698	3.402014	5.348636	6.879068	7.131643
HORVU2Hr1G127770	1.480655	1.655459	1.478852	2.273379	2.472658	2.381143
HORVU7Hr1G118880	0.202866	0.140947	0.419185	0.403377	6.597868	7.679547
HORVU7Hr1G037960	4.954328	6.043815	6.38324	4.552155	4.848304	5.7684
HORVU6Hr1G044700	15.77584	13.76156	17.31972	15.57339	18.36195	21.4413
HORVU5Hr1G018160	3.981412	3.899964	4.724823	9.869671	14.02568	14.81471
HORVU5Hr1G010350	1.378771	1.534296	2.057043	6.716135	12.23739	10.38595
HORVU2Hr1G032220	4.171656	2.839211	4.196135	4.052859	26.23478	12.21216
HORVU5Hr1G034810	6.861407	5.162452	5.709493	9.272623	21.68621	22.56909
HORVU6Hr1G081450	1.092532	1.115849	1.716404	1.468409	1.828952	1.271791
HORVU3Hr1G070660	11.71809	11.80176	13.95505	4.790857	4.916054	5.325358
Hordeum_vulgare_newGene_7622	2.041184	1.626799	2.276005	2.505221	3.354997	2.792861
Hordeum_vulgare_newGene_7621	7.639623	7.299474	8.354858	8.419041	10.7766	10.88444
Hordeum_vulgare_newGene_7625	2.518789	2.635821	2.963048	3.114832	4.302796	3.848732
HORVU5Hr1G018290	1.733488	2.105606	2.583457	1.446334	1.358104	1.357108
HORVU4Hr1G073380	0.787572	1.322364	1.487324	2.061915	4.639768	4.93283
HORVU2Hr1G045480	10.55044	7.945156	8.295903	16.13901	10.53215	11.60406
HORVU7Hr1G115480	27.71777	16.54324	34.1706	24.47899	32.99138	32.87104
HORVU6Hr1G019700	0.653127	1.012288	1.128447	2.450638	4.502749	4.858684
HORVU1Hr1G076800	1.31355	0.954355	1.24642	1.615777	3.166469	2.102468
HORVU5Hr1G097270	3.616944	1.975021	3.533777	0.395142	0.139313	0.243573
HORVU7Hr1G053690	4.650144	5.336148	4.336081	7.184399	7.327463	8.101438
HORVU7Hr1G099330	0.933622	1.759528	2.592181	0.955431	0.597369	0.700981
HORVU7Hr1G088510	13.27777	15.50055	14.46311	4.606176	7.870083	8.751709
HORVU4Hr1G088860	7.159433	7.312794	7.969713	8.50786	32.75894	26.51976
HORVU5Hr1G061960	6.144826	6.769577	8.273944	5.528742	5.07817	5.934674
HORVU7Hr1G055930	1.321056	1.141853	1.493687	1.583028	1.585896	1.395392
HORVU3Hr1G043300	5.588678	5.233942	6.222509	13.02546	18.52711	18.49896
HORVU4Hr1G072920	7.794617	7.676311	9.246006	10.9234	13.03651	13.5642
HORVU2Hr1G045220	3.008006	3.54999	4.703008	3.214488	3.392198	3.503516
HORVU7Hr1G018580	9.159267	10.4893	14.64583	3.161249	3.039497	3.8062

Hordeum_vulgare_newGene_9156	1.180357	1.375005	0.840002	0.661499	1.397386	0.660564
HORVU5Hr1G014320	0.924084	0.930864	0.978451	1.991385	3.67218	3.818597
Hordeum_vulgare_newGene_6575	6.972838	13.20479	7.985712	10.42659	7.862103	9.978921
HORVU6Hr1G073590	0.966525	1.353225	1.314315	2.109054	2.851114	2.653781
HORVU1Hr1G086610	7.39397	4.0804	6.940051	9.714306	10.38319	12.72483
HORVU5Hr1G125630	7.836207	8.61481	8.219127	11.52992	15.12118	15.27514
Hordeum_vulgare_newGene_15445	6.645104	5.828189	8.733589	9.495245	11.21342	11.12315
HORVU0Hr1G040300	4.455376	4.280062	5.621832	5.382449	5.348536	5.799438
HORVU5Hr1G088300	45.2228	38.88214	54.4107	53.76757	83.34876	75.08476
HORVU6Hr1G042410	7.298877	8.797972	9.215427	9.048488	12.39379	12.0751
HORVU3Hr1G041810	2.999496	3.334171	4.089611	3.161168	4.220618	3.974852
HORVU6Hr1G073620	7.506573	8.305285	10.32836	39.28776	59.08603	46.39964
HORVU2Hr1G086990	10.28342	10.90893	11.45456	10.62855	18.62751	18.38352
HORVU1Hr1G060770	26.1487	22.24372	28.07838	45.27177	32.44919	28.97963
HORVU3Hr1G020830	7.036515	4.717465	6.15853	4.312516	1.86044	2.190006
HORVU0Hr1G004130	75.90092	82.85821	121.0605	62.88151	123.7083	117.1992
HORVU6Hr1G080490	4.243392	3.486567	2.417778	3.167653	2.898648	3.580757
HORVU3Hr1G030420	2.903296	2.625791	3.230658	4.074852	5.770461	5.595957
HORVU1Hr1G050850	1.654661	1.398022	1.963377	1.515774	1.588241	1.730438
HORVU0Hr1G040210	5.100066	4.594925	3.329228	8.261264	8.373129	8.358941
HORVU6Hr1G075750	6.767097	6.083065	7.477271	8.431868	7.701848	8.29465
HORVU3Hr1G087600	8.517713	7.841878	9.466125	7.001816	10.07158	8.928449
HORVU1Hr1G055370	3.300785	3.744675	3.714592	8.18631	13.86002	13.97122
HORVU5Hr1G123770	38.3468	38.73931	40.02734	18.04882	15.6618	16.54334
HORVU7Hr1G022460	0.482271	1.349849	0.903988	0.826318	1.439119	1.195093
HORVU4Hr1G007370	154.7928	133.1141	168.4541	212.4112	295.2804	305.9653
HORVU5Hr1G002110	0.450293	0.746173	0.775467	5.760065	5.404691	5.931311
HORVU7Hr1G011250	15.31894	17.77457	14.87513	12.27246	4.80293	8.266682
HORVU3Hr1G117550	7.451132	5.549939	6.719184	1.916003	0.737904	2.160537
HORVU2Hr1G122110	2.626547	4.096959	3.677099	4.057758	1.492158	2.503465
HORVU3Hr1G082070	10.19146	7.997933	9.992455	10.59038	11.24689	11.72912
HORVU2Hr1G091590	26.67739	23.1112	21.74726	13.71257	7.62737	8.291084
HORVU3Hr1G082380	1.855347	1.473469	1.615984	4.285578	4.69651	5.027196
HORVU6Hr1G041140	8.168913	7.58858	8.816397	16.34461	20.73151	20.41594
HORVU4Hr1G053460	2.049207	2.692589	3.293705	2.059741	3.003114	3.005234
HORVU4Hr1G006020	1.136161	1.037347	1.034503	1.936932	1.519993	1.71156
HORVU5Hr1G094230	4.323725	4.450545	5.869264	8.02453	8.552815	9.444073
HORVU7Hr1G034080	26.71947	26.06927	27.40097	17.32968	21.06049	20.22834
HORVU6Hr1G087140	11.26552	14.06499	17.49656	8.571229	12.18342	13.22001
HORVU5Hr1G018930	1.332908	1.546731	2.129285	2.171671	3.648069	3.421111
HORVU4Hr1G071670	10.7158	9.84512	10.79717	17.83618	20.41329	21.76826
HORVU7Hr1G094860	2.309989	1.913346	2.047503	3.180335	5.885525	5.575153
HORVU1Hr1G089360	298.3505	280.7592	342.3419	476.6804	405.4626	427.7889
HORVU6Hr1G086430	2.410034	2.163278	2.756678	2.395212	2.466272	2.094038
HORVU1Hr1G020240	1.766405	1.823348	1.626527	9.295461	12.46339	12.51085
HORVU3Hr1G016240	3.787926	4.369625	4.319222	7.971907	13.48622	12.63949
HORVU3Hr1G027340	0.62293	0.718002	1.120769	1.948399	0.523099	1.215949

HORVU5Hr1G118080	43.42772	33.65132	42.47006	44.54411	33.25002	33.00212
HORVU2Hr1G098880	40.59905	43.32735	40.25816	32.73216	32.75033	35.53074
HORVU4Hr1G063060	1.127462	1.388634	1.364231	0.804346	0.972823	1.26848
HORVU1Hr1G005140	8.403526	7.468722	5.623961	6.111894	7.128377	7.008223
HORVU3Hr1G040640	3.929971	4.752377	5.350359	9.124845	10.33313	11.95788
HORVU5Hr1G055060	16.80284	13.24989	17.66239	19.55702	13.22128	15.3411
HORVU7Hr1G045230	25.36996	24.34146	24.93083	17.61713	16.15015	16.70027
HORVU3Hr1G083380	6.010664	5.929073	6.463971	9.68922	11.6258	10.94533
HORVU2Hr1G108990	2.698056	2.077664	3.167037	4.708744	6.524177	5.627693
HORVU4Hr1G003700	39.57857	38.62151	44.40744	32.93077	23.88114	24.66333
HORVU1Hr1G041970	21.11696	17.71675	29.07226	25.60914	45.61826	40.84739
HORVU7Hr1G077200	35.10396	33.18335	42.74684	45.97602	67.34814	55.80957
HORVU7Hr1G007990	2.029001	1.924769	2.550563	3.930172	4.314772	3.7339
HORVU5Hr1G069360	215.4836	201.4757	302.1937	145.5821	61.27162	83.2192
HORVU4Hr1G002250	3.60281	4.04084	4.317124	2.921078	5.139001	4.929968
HORVU4Hr1G053950	8.627986	8.899997	6.830045	6.06465	8.85271	9.657134
HORVU4Hr1G049640	13.01389	13.39275	14.79521	23.9135	27.07762	28.91633
HORVU5Hr1G002000	2.659461	2.614901	3.402128	2.297881	2.704298	3.220704
HORVU1Hr1G067490	17.40943	22.00213	11.18441	21.06448	9.703935	11.77357
HORVU6Hr1G010440	7.822914	8.203143	10.09469	6.75048	10.47912	10.72173
HORVU6Hr1G052420	13.53966	11.09821	14.37512	27.25529	46.03109	38.65383
HORVU6Hr1G090530	2.88469	2.388069	3.019897	2.619579	4.550478	3.925999
HORVU2Hr1G015850	7.224203	6.360773	8.002447	20.80941	21.24196	14.96276
HORVU5Hr1G054080	111.4516	90.85512	119.6845	121.8984	137.2526	131.5612
HORVU6Hr1G000210	3.122286	1.942266	2.95347	2.501371	2.920265	3.594524
HORVU1Hr1G088280	15.43968	10.87129	34.39489	53.41607	5.283513	6.219564
HORVU6Hr1G085160	6.725621	7.097922	7.286946	13.8156	13.68102	15.83727
HORVU4Hr1G062000	37.88926	35.78737	37.47424	27.76152	39.80869	38.11877
HORVU1Hr1G052030	18.06456	13.66375	23.72811	25.5116	30.90776	28.05434
HORVU4Hr1G087120	3.59079	3.575358	3.719063	3.197984	3.002069	2.732974
HORVU6Hr1G005360	4.549984	4.613964	4.423152	4.695555	2.977387	4.323611
HORVU7Hr1G090550	1.391637	1.096203	1.515207	1.8992	2.307702	1.79225
HORVU1Hr1G090670	14.0014	9.407172	12.23646	5.688209	2.378415	3.005834
HORVU0Hr1G020110	23.66148	23.22332	28.15468	24.52506	35.14378	31.86812
HORVU7Hr1G076150	9.615464	8.098483	9.394417	35.64974	128.7928	127.8208
HORVU1Hr1G042690	2.552946	2.435919	2.887672	3.422486	4.069853	3.977116
HORVU5Hr1G026780	9.009765	7.098706	8.94901	6.201162	7.479957	6.735754
HORVU5Hr1G019950	0.324764	0.260563	0.389233	1.065377	5.552824	8.714008
HORVU5Hr1G057570	1.407275	1.091626	1.668443	2.465836	3.181095	3.99601
HORVU1Hr1G018310	36.05965	33.1618	28.69936	21.2367	21.31441	20.80369
Hordeum_vulgare_newGene_9484	0	0	0	1.378959	2.209894	3.612416
HORVU7Hr1G029170	1.831998	1.48772	2.008397	3.648241	5.006939	5.619701
HORVU4Hr1G053530	4.594931	4.445454	5.105528	8.071245	8.040755	9.428343
HORVU7Hr1G092710	2.57988	2.116886	2.789954	2.907354	1.480241	1.492332
HORVU6Hr1G092970	36.73827	35.50677	42.67494	43.81889	40.99355	44.16093
HORVU7Hr1G042390	0.618606	0.318095	0.941839	42.88122	38.2679	40.38078
HORVU2Hr1G108600	6.069498	6.441129	10.30235	0.190517	0.235929	0.115073

HORVU1Hr1G042520	2.165498	3.245003	3.367622	3.990397	6.973489	7.829913
HORVU7Hr1G082380	10.92333	12.50301	16.21026	4.472347	3.239384	3.747342
HORVU2Hr1G062670	3.362198	2.978082	3.046838	3.258495	3.743936	3.935202
HORVU6Hr1G021460	0.503278	0.789726	0.530086	2.482402	2.640697	3.168712
HORVU0Hr1G015710	1.653793	1.14324	1.488899	1.454028	1.364502	1.297407
HORVU2Hr1G121990	128.6257	118.6764	144.0466	208.9245	332.7951	320.6626
HORVU1Hr1G050640	2.701015	2.900732	2.831913	5.670789	7.224095	7.582628
HORVU4Hr1G001130	0.40275	0.620012	0.877947	1.360712	1.993489	1.826976
Hordeum_vulgare_newGene_13915	0.058055	0.092998	0.293012	2.205248	9.445185	5.933243
Hordeum_vulgare_newGene_13911	2.034883	2.258837	2.583907	0.664373	0.696327	0.71449
HORVU3Hr1G107990	8.005116	7.715397	10.91637	19.31388	13.3065	12.41805
Hordeum_vulgare_newGene_13913	30.7353	31.47899	25.70707	12.52331	9.109298	10.01347
HORVU5Hr1G019580	1.364476	0.757284	0.832673	1.847871	1.811154	1.760038
HORVU3Hr1G064520	1.903596	4.549764	5.45647	5.534673	6.205021	5.445655
HORVU2Hr1G118210	0.194341	0.146409	0.115695	1.78399	1.782834	2.232547
HORVU2Hr1G100380	1.788323	1.826595	2.13909	1.304649	2.244471	1.793587
HORVU2Hr1G037680	25.21276	22.04767	24.78605	26.94697	26.22177	25.52955
HORVU6Hr1G056180	2.812841	2.907835	3.688973	2.850347	4.660034	4.692102
HORVU2Hr1G010980	26.82474	25.16334	23.1118	19.78127	20.87875	21.67766
HORVU2Hr1G023120	7.131636	8.302289	9.644034	6.66731	10.03928	10.49123
Hordeum_vulgare_newGene_3516	1.012372	1.360445	1.607643	0.950577	0.594649	0.851409
HORVU5Hr1G117610	0.405963	0.391544	0.414091	1.414931	1.909642	1.53152
HORVU2Hr1G066950	5.492719	3.262789	5.003593	4.129099	5.328066	4.287
HORVU5Hr1G025500	8.452859	7.450936	8.200074	6.706047	5.451056	7.20932
HORVU3Hr1G114340	2.42122	2.236274	3.152269	3.897317	5.880574	5.270975
HORVU3Hr1G096250	2.56036	1.995491	2.88155	4.314427	6.022199	6.329497
Hordeum_vulgare_newGene_8741	3.167788	2.167342	3.549096	1.481776	0.489911	0.656088
Hordeum_vulgare_newGene_8740	6.916056	7.057194	10.63432	6.472676	6.584256	7.059468
HORVU5Hr1G098390	3.939386	3.538538	3.707967	3.751852	3.021962	3.36627
HORVU6Hr1G047170	1.428501	0.955842	0.734793	2.399321	2.347565	1.227538
HORVU7Hr1G031260	107.0357	103.0749	90.77343	56.13409	85.7487	73.59763
HORVU5Hr1G073310	3.67534	3.556176	3.881615	1.803171	1.095253	1.192458
HORVU5Hr1G072920	20.87582	17.35026	18.06795	11.57134	6.349123	8.093795
HORVU4Hr1G064120	13.91813	13.18916	14.16051	14.86435	17.49077	16.88243
HORVU5Hr1G080550	2.436641	3.020375	4.134245	2.443874	2.734544	2.859794
HORVU6Hr1G057270	2.605958	0	0.797945	1.876258	1.017283	0.815348
HORVU0Hr1G010870	24.29964	32.95443	25.3926	27.88475	37.52072	37.02957
HORVU3Hr1G093550	1.364912	1.359061	1.415317	2.831458	1.369272	2.291511
HORVU6Hr1G014750	15.17819	17.00554	22.26054	11.38266	14.18884	15.32245
HORVU1Hr1G040160	4.181866	5.340352	4.266916	4.472171	3.568149	6.19793
HORVU5Hr1G093700	8.689037	12.91925	15.10418	53.75076	464.8806	436.3623
HORVU3Hr1G096780	12.57614	12.71357	13.38999	12.79199	17.11038	18.40878
HORVU7Hr1G011750	1.776148	1.754875	1.876074	0.633701	0.950147	1.035242
HORVU7Hr1G064050	13.19134	9.9143	12.67264	16.52334	33.97989	33.68904
Hordeum_vulgare_newGene_10759	2.690541	2.02811	2.763027	4.036066	2.899663	3.40329
HORVU2Hr1G111640	167.876	143.4909	164.6556	176.5108	189.4324	222.015
HORVU6Hr1G050720	6.99815	5.081113	5.545318	6.488719	8.291998	8.441989

HORVU1Hr1G054570	1.863365	1.347746	2.302961	2.664696	4.105746	3.236167
HORVU7Hr1G029750	18.05969	24.43059	30.33035	10.98852	22.30371	21.26348
Hordeum_vulgare_newGene_14211	36.45854	33.42437	40.73351	47.04528	49.76217	55.42592
HORVU3Hr1G115560	5.5657	5.349192	7.031261	3.896332	6.992234	7.311382
HORVU3Hr1G092200	16.4832	15.215	20.30223	18.00602	19.36051	17.12034
HORVU2Hr1G022640	2.920195	1.879744	1.775402	1.868499	0	0.497927
HORVU5Hr1G086610	0.187173	0.558239	0.891392	3.194712	0.720937	1.751913
HORVU5Hr1G033350	4.374435	1.065767	2.67579	3.248955	2.657965	2.40224
HORVU2Hr1G108380	0.888278	0.670709	0.990141	2.937783	2.058846	2.156026
Hordeum_vulgare_newGene_14214	0.758498	0.434763	0.88785	3.937829	2.84836	5.704885
HORVU0Hr1G016430	1.889436	1.309021	1.638694	25.24997	44.23107	39.99208
HORVU3Hr1G112690	6.663221	6.632342	7.367039	6.576702	6.761391	6.926364
HORVU3Hr1G054810	3.010119	2.849896	4.452686	7.239124	14.47454	11.41125
HORVU7Hr1G058770	1.746222	1.735353	1.96148	1.862228	0.756734	1.806291
HORVU3Hr1G010190	7.603583	6.050126	10.14352	4.750842	0.813763	1.920199
HORVU1Hr1G084260	11.57904	10.82196	11.76406	8.982396	8.707994	9.742835
HORVU2Hr1G110110	28.11574	26.85868	33.28061	31.07546	34.41545	42.00956
HORVU4Hr1G079090	1.878526	2.543107	2.408484	4.04632	4.230314	3.806083
Hordeum_vulgare_newGene_11407	2.081655	2.305206	2.005621	0	0	0
Hordeum_vulgare_newGene_11400	6.757035	6.937409	8.117283	6.483217	6.886527	7.267875
HORVU3Hr1G075210	0.272796	0.237256	0.495401	7.064623	6.634956	7.65863
HORVU2Hr1G070140	248.1143	250.3596	222.1594	349.3108	418.6845	396.4103
HORVU6Hr1G054110	1.227421	0.898684	1.662331	2.253915	3.884627	4.085639
HORVU7Hr1G083120	4.520829	4.361945	6.113799	4.36836	4.936966	4.92825
Hordeum_vulgare_newGene_12277	4.732144	3.833962	4.629662	4.712175	6.302111	6.167388
Hordeum_vulgare_newGene_12275	7.398111	7.917463	9.191185	8.426019	9.504777	9.92366
HORVU7Hr1G040170	1.753174	1.479384	1.017428	4.441571	1.77639	2.955482
HORVU1Hr1G075110	11.18994	8.748658	13.14756	26.72815	16.0542	19.90108
HORVU4Hr1G062940	11.87891	8.838283	11.2914	13.55529	6.511486	10.38479
HORVU2Hr1G020730	8.127647	6.776333	9.103768	5.892167	7.607081	8.079966
HORVU3Hr1G017970	16.71163	15.28302	18.18129	16.63467	19.46605	17.60919
HORVU6Hr1G063770	29.86779	29.50431	36.01675	26.69307	33.01424	33.83329
HORVU3Hr1G105430	5.13559	3.189472	4.304793	10.53389	10.61927	11.80289
HORVU3Hr1G034530	0	0	0	0.073823	8.871887	7.394436
Hordeum_vulgare_newGene_13583	117.7906	98.5982	140.902	153.9059	209.2003	187.5911
Hordeum_vulgare_newGene_13580	4.829945	5.455348	4.861866	4.832568	4.595903	5.212716
Hordeum_vulgare_newGene_13586	1.457134	2.046667	0.891866	0.866994	0.549068	1.060519
Hordeum_vulgare_newGene_13584	2.991164	2.863375	3.150792	8.522071	13.51873	12.08749
Hordeum_vulgare_newGene_13589	2.114652	2.224037	2.232799	0.258617	0.278396	0.282954
HORVU2Hr1G114910	43.69108	29.22924	61.39103	12.96296	7.449313	6.82252
HORVU4Hr1G073630	151.122	136.5943	158.5778	169.5205	242.9064	249.2922
Hordeum_vulgare_newGene_7352	9.207002	7.653221	10.11063	16.95204	22.70513	24.35987
Hordeum_vulgare_newGene_7357	0.927919	1.136573	1.078227	2.426938	2.722907	3.609457
Hordeum_vulgare_newGene_7356	1.364796	1.23157	0.961741	1.984603	1.160845	1.506004
HORVU6Hr1G059530	2.679065	2.900472	3.904455	3.631863	4.765182	5.507459
Hordeum_vulgare_newGene_7354	0.729444	1.035387	1.103559	1.222253	1.156878	1.882441
Hordeum_vulgare_newGene_12189	2.297343	2.78426	2.359698	4.173979	11.11667	11.95551

Hordeum_vulgare_newGene_12188	2.338649	1.883384	1.820992	4.235774	10.68844	10.11746
Hordeum_vulgare_newGene_12183	17.35374	19.17242	18.21448	18.19428	22.30815	25.16924
Hordeum_vulgare_newGene_12182	24.84845	27.13665	24.01769	25.44275	29.50961	32.80367
Hordeum_vulgare_newGene_12184	5.699248	7.798198	7.404265	6.75433	5.256976	7.231807
Hordeum_vulgare_newGene_12187	4.941941	3.321406	4.397312	5.204324	7.741173	6.271953
HORVU7Hr1G026910	1.515508	1.957359	2.299344	2.413504	2.002372	2.107197
Hordeum_vulgare_newGene_6000	5.673484	4.418269	4.799017	0.024663	0	0
HORVU4Hr1G074360	20.85205	25.46247	21.27153	25.50175	34.66838	32.56539
Hordeum_vulgare_newGene_6009	33.36972	20.72864	27.81827	12.46724	5.759283	3.493675
HORVU6Hr1G064350	0.175086	0.242784	0.674583	1.547918	2.332613	1.928386
Hordeum_vulgare_newGene_3189	1.163708	1.747645	1.676401	1.108466	1.047893	1.277545
HORVU5Hr1G070010	24.31458	21.8153	28.70828	34.23356	41.12674	38.18695
Hordeum_vulgare_newGene_10918	0.562379	0.591644	0.914571	0.695936	1.843087	2.194831
HORVU7Hr1G025460	6.404282	7.529718	8.719901	2.189945	3.546431	2.312124
HORVU1Hr1G071560	0.466506	0.592674	0.547483	1.217826	1.692236	2.006754
HORVU7Hr1G108110	0	0.00686	0.008321	2.586446	2.699748	3.46724
HORVU3Hr1G113780	1.307992	1.060023	1.380847	2.029718	1.573349	2.743668
Hordeum_vulgare_newGene_2273	0	0	0	5.654665	6.36692	7.221462
Hordeum_vulgare_newGene_2272	0.947906	0.764005	0.727122	1.135124	1.376426	1.089724
Hordeum_vulgare_newGene_2270	36.42471	28.55404	36.77809	23.29247	31.50652	30.0316
Hordeum_vulgare_newGene_2277	7.708941	7.414125	8.720122	10.65549	10.26261	13.32757
Hordeum_vulgare_newGene_2278	2.38845	3.531612	3.144714	0.00982	0	0
HORVU4Hr1G021700	14.86644	13.03678	16.87148	19.49554	26.2852	27.02794
HORVU0Hr1G000340	13.44676	5.840716	5.911487	4.216131	12.10173	8.860329
HORVU7Hr1G118290	0.014567	0.113951	0	0.267298	4.04346	4.09094
HORVU2Hr1G073840	2.784166	2.760297	3.534696	0.57674	1.134805	0.747026
HORVU2Hr1G079820	19.25449	14.99817	19.81672	15.6369	20.38051	20.62154
Hordeum_vulgare_newGene_13635	1.411575	1.510051	1.418204	5.195366	8.112419	8.182911
HORVU3Hr1G110470	50.84673	52.56306	54.73685	46.70427	58.92114	57.84641
Hordeum_vulgare_newGene_13637	0.094679	0.17351	0.169195	3.221285	2.870213	2.940387
HORVU2Hr1G116510	0.007203	0.03813	0.045111	34.66131	129.9995	89.64554
Hordeum_vulgare_newGene_7978	7.135801	7.257251	4.595117	3.278641	3.837235	4.336068
Hordeum_vulgare_newGene_7976	2.383163	2.152196	2.011441	1.804	3.986862	3.889323
Hordeum_vulgare_newGene_7975	8.387832	8.416338	11.63846	8.567459	11.62091	11.57451
Hordeum_vulgare_newGene_7972	5.590544	5.943265	6.169949	0.688624	0.142259	0.379565
Hordeum_vulgare_newGene_7970	6.145558	5.42474	5.944046	7.556602	8.328697	7.583403
HORVU4Hr1G021390	5.742648	4.716416	4.977638	6.614664	9.402208	9.005321
Hordeum_vulgare_newGene_2259	4.950833	3.56997	4.63081	5.499872	5.363953	6.645652
HORVU3Hr1G087190	9.472305	10.33933	11.32488	12.61715	14.15138	14.48003
HORVU5Hr1G103560	2.921647	2.924567	2.79898	1.944393	1.434939	1.398313
HORVU2Hr1G046550	18.13978	12.41739	10.81461	2.546816	1.932873	2.324435
HORVU1Hr1G059290	3.175778	2.713104	5.925445	4.526939	4.978117	4.226789
HORVU1Hr1G020000	0.886053	1.033578	1.138522	3.42281	6.284699	6.926484
HORVU3Hr1G059000	1.720296	2.053936	2.530327	2.17808	3.207829	2.94638
HORVU3Hr1G054030	1.24094	1.487477	1.351736	2.023249	0.957806	1.653973
HORVU5Hr1G073450	31.67988	35.59974	39.67829	23.87166	40.42932	37.95107
HORVU1Hr1G021150	1.534442	1.608027	3.277968	1.415639	1.619602	1.756695

Hordeum_vulgare_newGene_5721	5.137276	4.373255	5.728308	3.602957	3.546852	3.415109
Hordeum_vulgare_newGene_5722	6.535752	6.547449	6.363894	5.372534	6.327853	6.26545
HORVU2Hr1G075850	7.007071	7.19641	7.638342	4.785657	2.916438	3.588162
Hordeum_vulgare_newGene_949	1.036525	0.806515	0.796391	2.922567	3.393304	3.174589
HORVU6Hr1G073910	45.88007	39.37495	52.3242	33.92111	43.64573	44.81002
Hordeum_vulgare_newGene_945	5.091248	5.982989	8.002448	4.100158	5.36263	4.351051
Hordeum_vulgare_newGene_947	9.254683	11.68249	9.008251	16.89398	29.37699	24.17223
HORVU5Hr1G024250	11.8075	10.84157	14.09865	16.71325	27.50388	24.86539
HORVU2Hr1G086240	44.41335	43.63439	52.39525	51.05612	62.58464	66.44776
HORVU0Hr1G002870	1.022731	0.890409	1.629152	1.405468	0.755449	0.682252
HORVU6Hr1G064480	5.54732	6.197738	7.454404	5.33753	7.896596	8.382332
HORVU6Hr1G083930	20.80298	19.01673	19.82522	29.42416	29.55568	27.56331
HORVU1Hr1G091540	0.577828	0.505447	0.367567	1.152375	2.299934	2.023431
HORVU4Hr1G089410	1.551129	1.249343	1.418514	3.521706	4.830227	3.903202
HORVU4Hr1G002010	34.7835	35.58572	35.28525	17.18676	22.23859	23.11305
HORVU7Hr1G030370	2.099941	1.763113	2.332986	5.172516	4.785667	5.06506
HORVU3Hr1G055920	59.0466	54.85016	69.53754	81.33461	100.5704	98.58154
HORVU6Hr1G068790	1.816843	2.081966	2.076458	1.771957	1.157722	1.592685
HORVU7Hr1G107780	3.501759	3.448236	4.041604	12.19577	15.04218	14.65014
HORVU2Hr1G077560	15.07081	15.71776	18.92696	19.36217	14.52108	17.76608
HORVU3Hr1G043690	0.210426	1.829348	1.164637	0.073167	2.400348	1.594755
HORVU7Hr1G025700	0.300595	0.159416	0.236885	4.029255	5.419605	5.94998
HORVU7Hr1G049250	18.58033	17.21524	19.70863	12.62356	13.81381	15.16402
HORVU4Hr1G005540	661.0609	537.9559	641.2728	542.0057	185.6491	265.2919
HORVU2Hr1G004460	32.31285	22.00278	21.88578	21.27567	20.98402	25.09406
Hordeum_vulgare_newGene_5911	4.17496	4.379043	5.204964	3.720622	5.029047	4.912898
HORVU1Hr1G052960	0.46226	0.555087	0.60811	1.466221	1.623489	1.48565
HORVU5Hr1G082840	4.403421	2.116572	3.759615	7.389994	4.376761	4.667358
HORVU0Hr1G019710	3.181481	3.675952	3.684585	5.187686	6.882849	8.489315
HORVU7Hr1G100750	7.250926	8.492686	11.2941	18.32388	14.52644	12.82148
Hordeum_vulgare_newGene_6261	0.989024	2.712189	2.179928	0.255367	0.275645	0.249604
HORVU2Hr1G031130	0.914288	0.499069	0.739288	4.061472	10.1871	10.83423
HORVU1Hr1G059460	1.173455	2.94457	2.938032	3.521484	3.164976	3.90894
HORVU7Hr1G091600	47.7613	42.98116	46.38358	35.54888	36.3488	38.82146
HORVU4Hr1G031840	2.901248	3.473822	3.818273	5.168152	5.968261	5.886734
Hordeum_vulgare_newGene_4092	15.17199	15.53328	16.05055	11.39635	14.70774	11.41132
HORVU7Hr1G057760	3.695466	3.597028	3.700023	8.226587	16.04644	14.47375
Hordeum_vulgare_newGene_4099	9.20337	9.041578	12.74407	0	0	0
Hordeum_vulgare_newGene_4098	3.622113	3.282995	3.878655	4.610814	4.962549	5.386898
HORVU3Hr1G084360	77.62395	77.46461	81.09439	39.54312	63.73454	59.872
Hordeum_vulgare_newGene_1119	3.975031	3.42518	4.29519	4.53349	6.355282	5.612231
HORVU7Hr1G106780	4.837945	4.964132	4.96651	3.891863	3.889861	4.306568
HORVU1Hr1G027850	7.782467	8.364685	6.576588	8.220862	8.465225	8.778271
HORVU1Hr1G063620	1.831865	1.480824	2.103743	15.23749	12.51615	14.38176
HORVU0Hr1G001750	2.663072	3.607728	2.139965	3.203444	4.991351	4.265241
HORVU5Hr1G105840	94.67505	83.3679	123.6937	42.25416	30.73178	24.19785
HORVU4Hr1G072220	1.55139	2.231664	2.192617	2.957174	4.955404	4.130551

HORVU6Hr1G028590	1.5941	1.315233	2.145307	2.766271	1.831433	1.694553
HORVU7Hr1G115830	0.462633	0.721477	0.926763	1.509443	1.189174	1.960082
HORVU1Hr1G079170	5.996836	5.649541	5.736465	6.997001	3.075531	5.556718
HORVU1Hr1G056340	30.82306	25.43264	31.08052	17.12221	26.72855	26.47711
HORVU7Hr1G094640	232.8048	216.1044	281.6699	137.1361	129.7568	122.9569
HORVU4Hr1G086330	20.85395	18.99468	24.01137	12.48561	14.18225	15.29399
HORVU4Hr1G057210	7.73692	5.786682	9.433281	15.8805	34.48789	33.81618
HORVU3Hr1G085100	4.67364	5.169523	5.203679	2.659478	5.256994	6.030224
HORVU3Hr1G005410	0.492344	0.60065	0.8549	1.138288	2.590261	2.472649
HORVU2Hr1G002440	114.088	144.2575	223.9639	45.02144	1.734	23.22152
HORVU5Hr1G091160	3.820187	4.134858	4.337178	4.928667	5.184698	5.915981
HORVU1Hr1G083380	12.41783	9.608669	14.94323	4.164373	3.40259	3.769001
Hordeum_vulgare_newGene_7017	19.53223	21.41335	17.38408	20.82328	25.83392	25.20451
HORVU7Hr1G079460	3.46997	2.64908	4.423357	4.327634	2.103262	3.325292
HORVU1Hr1G091880	5.443711	4.489785	4.779533	11.38525	12.99477	12.43644
HORVU2Hr1G008390	4.132213	3.132436	3.628453	9.29642	5.640791	8.890274
HORVU4Hr1G076350	1.50745	1.827001	1.546643	4.944192	7.24487	7.295394
HORVU7Hr1G098490	18.34417	17.1045	22.59609	6.04414	3.063046	4.373275
HORVU5Hr1G104800	0.985042	0.47827	1.040287	1.113401	1.189373	1.999753
HORVU7Hr1G056470	23.88492	21.76359	25.32352	33.3151	40.74758	43.91219
HORVU6Hr1G074360	18.0099	17.20168	16.76988	26.31922	31.47223	28.83747
HORVU2Hr1G000590	18.02507	17.73224	21.04229	17.42841	16.11911	18.34272
HORVU7Hr1G019390	1.958723	1.296002	2.076433	0.764451	0.38507	1.183979
HORVU3Hr1G041990	2.97219	2.914082	3.347951	5.093591	5.644615	5.448899
HORVU6Hr1G006090	0.354614	0.229129	0.56095	3.846634	16.68731	16.72121
HORVU6Hr1G019930	4.86418	5.726034	6.077794	6.45579	9.804412	9.786908
HORVU5Hr1G125460	156.9445	180.253	236.3555	51.32926	3.058841	25.63485
HORVU3Hr1G030090	2.063581	2.306909	2.816111	1.665088	1.81389	1.572259
HORVU7Hr1G052570	11.57926	12.05428	14.49662	10.15201	10.58665	11.38533
HORVU3Hr1G079410	4.027192	2.631133	3.97133	2.521239	3.694332	3.349647
HORVU2Hr1G039300	1.395586	1.891318	1.138031	1.397981	6.386451	5.324521
HORVU2Hr1G124790	13.17337	13.36182	21.2052	15.74711	24.13154	23.54528
HORVU3Hr1G031940	1.131101	1.090314	1.158244	12.47642	12.33371	16.50311
HORVU5Hr1G002090	1.406479	2.133308	3.159377	0.405395	0.019602	0.069428
HORVU3Hr1G100380	2.097521	2.633408	2.676994	0	0	0.556994
HORVU1Hr1G090820	23.93076	23.27786	25.19109	7.976773	3.025361	4.681549
HORVU1Hr1G021720	234.713	246.9606	374.5481	201.7765	480.5229	447.1412
HORVU1Hr1G050950	0.970928	0.926998	1.070713	1.992217	2.588177	2.861383
HORVU5Hr1G065920	45.60505	37.05935	42.06534	6.17353	3.069939	3.500118
HORVU4Hr1G006720	2.255815	3.407294	3.356693	2.065045	1.650749	2.128989
HORVU6Hr1G005540	74.16823	85.75832	80.80553	92.97288	83.45894	80.18756
HORVU6Hr1G092320	11.66945	7.345866	10.53744	11.9884	11.43931	13.36223
HORVU2Hr1G050410	0	0.131908	0	1.733992	5.880748	4.18005
HORVU5Hr1G086240	1.35918	1.641203	1.878325	1.779035	2.16885	2.00187
HORVU6Hr1G034620	0.603516	0.330562	0.747877	1.860159	5.905181	7.628534
HORVU5Hr1G036360	8.641167	8.52109	9.192181	6.905007	10.55259	11.96908
HORVU5Hr1G116850	0.097343	0	0	4.778791	5.971051	5.704961

HORVU7Hr1G067800	14.86167	13.36821	14.66958	16.92387	22.13927	23.97803
HORVU3Hr1G035650	3.207327	1.999511	2.637022	12.48984	17.86112	16.32258
HORVU5Hr1G097860	7.151642	7.087379	8.892911	9.975862	14.2824	13.83329
HORVU6Hr1G033160	27.93849	23.50486	24.59236	84.07005	80.17654	72.73157
HORVU4Hr1G043530	7.512463	7.150571	8.271343	7.498397	6.505082	8.346555
HORVU1Hr1G065910	4.138343	4.546175	4.413984	4.246264	4.282275	4.37381
HORVU2Hr1G109140	5.366512	5.137186	8.666299	3.860712	3.960628	4.611724
HORVU3Hr1G029790	13.32159	12.53536	12.99503	12.3845	15.45388	16.16732
HORVU5Hr1G055380	7.297459	7.385126	8.847639	6.839214	7.449196	7.725556
HORVU7Hr1G045370	1.750284	0.540183	0.491237	0.974987	6.646616	0.576201
HORVU3Hr1G018740	1.671668	1.453711	1.778695	0.614018	0.353074	0.82061
HORVU5Hr1G049430	8.14767	7.893936	11.53566	7.445787	8.00049	6.621826
HORVU5Hr1G009510	1.971751	1.873029	1.35681	0.471163	0.495693	0.538973
HORVU3Hr1G099940	1.74339	2.108542	2.314972	1.212328	3.320985	2.850517
HORVU3Hr1G037040	12.10013	11.94261	18.21058	5.981471	6.460528	5.972678
HORVU4Hr1G088650	2.286843	2.56781	2.405417	6.270315	4.758956	4.771464
HORVU2Hr1G040790	39.79161	29.46398	31.36152	30.70875	18.11344	19.13426
HORVU4Hr1G053580	1.270424	1.270243	1.464669	1.530709	1.034725	1.153403
HORVU4Hr1G053630	7.965179	6.249502	6.967725	4.536652	7.090798	5.63241
HORVU5Hr1G047000	186.5776	156.6373	170.8186	115.1207	126.5865	118.3489
HORVU7Hr1G074960	1.107261	0.823507	1.081971	1.280134	1.635452	1.621803
HORVU4Hr1G002350	0.355455	0.341141	0.481525	2.555896	4.889418	5.373452
Hordeum_vulgare_newGene_13513	2.618874	2.603485	2.933958	1.51	1.499577	1.600866
HORVU2Hr1G108810	4.567223	4.391097	6.380988	3.404573	4.138905	4.443797
HORVU5Hr1G076160	1.441372	1.122529	1.200607	1.891706	2.950739	2.159711
HORVU1Hr1G089440	12.58524	14.52899	18.54771	8.696426	12.61486	14.23675
HORVU2Hr1G113300	4.200274	3.159966	6.526911	1.4825	1.333233	1.454887
HORVU6Hr1G093050	61.4703	58.19308	63.31641	41.59064	35.10545	36.41214
HORVU2Hr1G085270	15.24921	19.98892	25.62793	2.356758	5.011158	6.55649
HORVU6Hr1G076200	25.76316	24.39007	28.39621	18.36205	18.53851	17.99248
HORVU4Hr1G049430	9.618997	9.263833	9.330512	5.837746	13.58057	14.4778
HORVU1Hr1G046010	7.269101	8.079905	8.182741	5.729342	6.721283	6.380174
HORVU2Hr1G105430	15.91808	12.303	14.46121	41.96384	19.29066	24.84729
HORVU2Hr1G037700	0.616165	0.516296	1.04063	3.686999	3.367272	4.354281
HORVU6Hr1G086600	3.725587	3.875493	3.111316	2.15401	2.515723	3.791712
HORVU7Hr1G049110	10.01166	10.43228	10.42004	9.817649	12.92597	12.93621
HORVU3Hr1G084430	7.764448	8.349927	7.557341	5.035907	5.9427	5.536325
HORVU1Hr1G068860	2.571845	2.648228	3.30591	1.954869	1.424618	1.849558
HORVU2Hr1G109850	4.977542	5.134406	5.738265	9.845786	9.79251	10.47721
HORVU6Hr1G091560	9.022877	6.107535	9.78519	0.008275	0.009415	0
HORVU5Hr1G105760	32.74584	27.97266	25.99025	29.99184	39.39762	38.36865
HORVU1Hr1G013680	448.1612	390.0703	264.1422	159.57	41.29256	70.61941
HORVU3Hr1G067350	9.793366	9.146919	9.994183	14.45099	15.12733	16.701
HORVU7Hr1G080900	5.746324	6.059019	7.149083	6.12495	8.499979	8.84199
HORVU6Hr1G017930	1.578121	1.628914	3.655611	2.021484	2.980073	2.689972
HORVU2Hr1G103280	2.266324	2.226135	2.697382	2.000027	2.40621	1.917613
HORVU2Hr1G063460	0.073942	0.153204	0.05236	1.888307	2.698279	3.416728

HORVU1Hr1G009950	111.2106	111.667	113.9397	128.9326	158.7697	142.001
HORVU6Hr1G065530	10.38803	10.43209	10.65688	11.08705	15.86984	16.17307
HORVU3Hr1G025680	2.712565	2.527513	3.157928	6.535061	12.5713	13.42237
HORVU0Hr1G007410	1.632602	1.750931	1.307918	0.389767	0.697365	0.761557
HORVU2Hr1G102880	16.01708	13.00552	15.1959	19.25596	7.433046	11.40979
HORVU1Hr1G049230	9.538179	6.923818	12.75705	1.681295	0.628419	1.057338
HORVU1Hr1G042220	54.58532	50.95707	72.4665	51.38187	121.8335	110.5453
HORVU5Hr1G083360	17.04271	17.92405	18.46606	14.62199	13.277	13.4363
HORVU1Hr1G013970	9.135141	10.12335	9.324608	10.7987	8.963115	9.819021
Hordeum_vulgare_newGene_10320	0	0	0.102708	0.911514	3.945915	4.214644
HORVU6Hr1G017680	1.900871	1.818678	2.173797	3.597677	3.207097	3.598646
HORVU2Hr1G064160	10.71711	7.668055	14.0841	3.275409	1.30787	4.681445
HORVU3Hr1G005350	9.37791	8.849092	7.776491	0.222111	0	0.048054
HORVU6Hr1G052360	1.897943	2.922591	2.17166	1.765957	1.864958	2.292855
HORVU4Hr1G021140	4.723641	3.551462	3.974213	7.495343	7.193769	7.962425
Hordeum_vulgare_newGene_13039	1.823169	2.279058	1.887085	3.604433	4.730513	4.612135
HORVU7Hr1G075360	124.8226	121.7731	171.7438	128.057	293.9738	285.4521
HORVU6Hr1G023290	1.168224	0.891258	0.792468	2.020867	1.554107	1.974224
HORVU3Hr1G072880	2.336112	3.265523	4.075404	2.631694	5.088979	6.115224
HORVU5Hr1G093930	10.86529	8.834896	12.34733	8.096742	4.553065	6.128713
HORVU6Hr1G054940	31.13589	25.59615	34.22302	32.16475	24.47267	32.24728
HORVU4Hr1G086640	3.626319	3.75806	3.320458	4.560434	5.81378	6.403591
HORVU2Hr1G100480	6.893096	6.677926	7.051481	3.824528	2.501019	3.138494
HORVU3Hr1G074010	5.532515	3.693643	5.68463	2.791705	1.800685	2.192088
HORVU2Hr1G099270	1.283469	1.362745	1.798822	1.306281	1.071502	1.321164
HORVU3Hr1G061010	39.67227	39.68522	37.47929	36.29262	37.88826	41.29158
Hordeum_vulgare_newGene_2079	2.708456	1.622329	2.489098	3.283388	3.312353	4.159433
Hordeum_vulgare_newGene_2078	0.67857	0.977612	0.798129	0.575456	1.89485	1.942586
HORVU2Hr1G016100	3.030551	3.207216	3.856822	1.845879	2.469633	2.280489
Hordeum_vulgare_newGene_2077	2.383534	2.562567	2.38653	1.886973	2.02996	2.515154
HORVU5Hr1G083980	1.143864	1.46253	0.650814	0.742042	5.41096	4.257921
HORVU2Hr1G116060	2.682507	3.30629	3.570892	4.015845	6.261098	6.275668
HORVU5Hr1G007960	9.526587	7.584105	11.69801	13.33067	15.27358	15.17148
HORVU2Hr1G100220	2.839935	1.772717	2.49306	1.433595	1.431607	1.1497
HORVU2Hr1G071750	3.888171	4.120278	4.238835	4.956677	4.563224	5.423468
HORVU1Hr1G044500	3.193738	3.974671	3.924603	2.324924	2.72348	3.341671
Hordeum_vulgare_newGene_12937	0.396509	1.546919	2.357584	0.349182	0.698341	0.785123
HORVU4Hr1G004240	138.4422	116.5675	141.6359	168.0759	269.3376	247.8083
Hordeum_vulgare_newGene_9629	1.073301	1.058781	1.212092	1.552333	2.309381	1.558428
HORVU1Hr1G094800	28.06803	25.07171	30.20876	31.7331	42.17198	43.34552
Hordeum_vulgare_newGene_9624	1.112193	1.46593	0.970052	1.114698	1.324788	1.27964
HORVU1Hr1G077230	1.815542	1.970109	2.458758	2.570736	2.692754	2.974396
Hordeum_vulgare_newGene_9622	1.784548	1.228214	1.872319	2.386723	3.544072	4.01071
HORVU5Hr1G027570	16.61964	15.30596	17.80607	17.34705	27.63726	28.63447
HORVU4Hr1G083690	0.824284	0.955617	1.281433	0.907421	1.099385	1.009304
HORVU5Hr1G078110	7.517882	7.15341	9.640005	10.44864	11.90014	10.53822
HORVU7Hr1G003920	2.38601	3.087893	3.286921	2.81534	3.779411	3.963283

HORVU7Hr1G072750	3.833744	3.946483	4.777931	5.499332	7.214771	6.295797
HORVU3Hr1G050290	20.42191	19.66488	16.80278	25.75433	21.56745	22.52862
HORVU5Hr1G081270	3.839723	3.221866	4.055262	4.554076	0.148055	5.577926
HORVU6Hr1G021160	0.58716	0.35219	0.598359	2.986223	14.97232	12.96423
HORVU7Hr1G089210	0.277975	0.375127	0.48621	1.174065	9.448617	16.44558
HORVU3Hr1G065240	31.21319	34.24769	26.14738	17.11159	3.326339	7.475814
HORVU3Hr1G075820	3.163165	3.292466	3.812636	6.392618	7.027082	7.66366
HORVU3Hr1G111230	2.018916	2.380313	3.438861	2.522036	4.157159	4.337046
HORVU3Hr1G026650	8.429833	7.04906	8.788674	19.23795	20.76379	21.42857
HORVU2Hr1G112360	36.05549	25.11562	32.92302	56.01762	68.25635	64.67212
HORVU4Hr1G083960	15.37392	14.20976	17.59228	11.65188	12.11677	13.25866
HORVU4Hr1G076690	5.373002	4.320278	4.11678	3.538407	1.825167	1.983132
HORVU7Hr1G081420	10.51917	9.787558	10.94348	14.26789	17.56018	16.36158
HORVU6Hr1G060010	10.15116	10.392	12.32167	13.99218	12.13944	14.58669
Hordeum_vulgare_newGene_14874	2.320338	2.455104	2.568739	3.30243	4.182509	4.344018
Hordeum_vulgare_newGene_8283	9.706773	5.647631	11.70813	4.975278	2.023254	6.027757
Hordeum_vulgare_newGene_14878	1.752594	2.396028	2.421732	1.963659	1.721329	1.669679
HORVU4Hr1G029510	3.390056	3.469423	3.633161	3.047443	3.388495	4.157059
HORVU6Hr1G017100	0.879021	1.275629	0.906131	1.252379	2.572613	2.71876
HORVU7Hr1G106540	3.821797	3.965673	4.707505	10.47234	25.03616	25.76178
Hordeum_vulgare_newGene_1759	4.161214	4.86766	4.362798	5.528143	5.986237	6.153973
HORVU7Hr1G021950	0.981241	1.057819	1.028914	4.392041	6.967278	6.409337
Hordeum_vulgare_newGene_1752	2.225996	3.169357	3.326295	3.294949	3.730616	4.673775
HORVU7Hr1G062510	32.04362	29.99711	36.50137	33.0913	44.24019	46.40177
HORVU1Hr1G013020	22.44617	22.37757	34.2117	48.64346	180.6928	144.0181
HORVU1Hr1G041060	10.88976	13.24389	14.60664	18.69921	32.31081	23.03656
HORVU2Hr1G025890	15.34502	13.41042	13.16149	19.30043	23.22141	22.9056
HORVU7Hr1G080460	2.533205	2.487149	3.321774	2.624958	4.362499	4.412919
HORVU6Hr1G071790	8.201443	8.290751	9.112222	17.55717	19.15948	20.54499
HORVU3Hr1G011780	0.552277	0.344464	0.366137	0.105856	24.61198	21.63679
HORVU5Hr1G030500	6.329424	5.775225	6.920789	5.801911	3.818094	5.415922
HORVU2Hr1G042790	4.425927	4.587808	4.126049	4.609587	4.956617	5.105312
HORVU4Hr1G033200	485.3973	498.7174	801.9622	346.582	615.7305	583.6203
Hordeum_vulgare_newGene_1887	1.379956	0.978754	1.874324	0.655116	0.757207	1.652329
HORVU3Hr1G010780	43.90962	36.82545	42.98026	48.08367	59.5252	54.41322
HORVU2Hr1G074130	2.606968	1.725295	2.901273	1.563875	1.535325	1.631086
Hordeum_vulgare_newGene_12022	1.009931	1.666708	1.583188	2.523031	3.253137	4.307637
Hordeum_vulgare_newGene_12026	1.706847	1.739768	2.273733	2.219711	2.7698	2.657037
HORVU6Hr1G062320	0.906537	0.656294	0.883837	2.15296	2.557314	2.701177
HORVU3Hr1G088690	2.429595	2.862547	5.172184	3.738743	6.848969	6.520813
HORVU7Hr1G105220	6.980401	6.610211	9.5325	5.446701	5.118221	6.478038
HORVU4Hr1G072750	16.67978	15.89129	18.59168	16.56213	18.27555	18.98851
HORVU7Hr1G024670	77.55228	63.1565	66.78783	166.0786	144.8347	149.612
Hordeum_vulgare_newGene_6702	58.49821	74.25543	61.69701	45.94292	23.85558	30.67081
HORVU5Hr1G014170	5.136822	4.801272	4.036227	13.46509	15.75119	15.21913
Hordeum_vulgare_newGene_6706	11.07998	11.69628	13.17479	12.76951	20.05108	19.63888
Hordeum_vulgare_newGene_366	5.253275	4.878788	6.121955	6.785895	7.597466	5.26583

Hordeum_vulgare_newGene_367	3.09963	4.042094	3.779225	3.4125	4.30384	4.652483
HORVU2Hr1G088940	2.448736	1.827773	2.994034	2.847371	5.782623	4.40096
HORVU6Hr1G074560	8.199395	6.569004	7.884947	0.030147	0	0
HORVU7Hr1G039460	2.862955	2.868836	3.317543	2.488797	3.825415	3.446381
HORVU7Hr1G072090	1.287104	0.818786	0.957488	1.116189	1.50911	1.286409
HORVU5Hr1G089260	2.003655	2.319815	2.291385	2.781405	3.626373	3.918534
HORVU4Hr1G073400	0.803315	0.994987	0.937204	1.253786	2.457344	3.356394
HORVU1Hr1G056820	7.747986	6.799824	4.644069	0.755063	0.331494	0.316728
HORVU2Hr1G044300	1.048707	1.103289	0.794696	1.544451	0.898073	1.202796
HORVU7Hr1G052770	2.431154	1.858084	1.95068	15.63757	20.05637	19.89145
HORVU6Hr1G078440	12.47567	15.93654	15.75497	13.82715	13.37429	14.64501
HORVU6Hr1G070670	111.2114	117.8437	186.0289	91.56778	186.3559	165.9572
HORVU2Hr1G106590	0.485718	0.390881	0.840721	1.089993	2.833376	3.52299
HORVU4Hr1G070980	16.43772	17.16039	19.58871	24.02145	35.66619	35.94914
HORVU7Hr1G064110	5.176458	3.344199	3.476906	6.515472	11.41213	11.37787
HORVU6Hr1G079790	8.828532	7.588107	9.848306	4.760838	7.281385	6.735356
HORVU6Hr1G028910	1.890806	2.002942	2.693462	2.521458	3.66541	3.303237
HORVU1Hr1G071150	1.020889	0.731819	0.846292	1.054466	1.387754	1.826187
Hordeum_vulgare_newGene_509	3.185654	3.251698	3.762301	2.822494	3.634876	3.652481
HORVU2Hr1G124800	6.183052	6.181629	6.868612	15.36045	19.18892	21.82477
HORVU7Hr1G038330	32.22686	21.52693	34.73309	15.56071	4.068622	6.123715
HORVU5Hr1G085560	39.1484	28.17953	39.34884	25.02394	23.90999	31.34808
HORVU5Hr1G022220	3.415606	2.630184	3.577697	4.744122	7.155398	7.365732
Hordeum_vulgare_newGene_14095	3.005815	2.267548	2.439335	0.011187	0.021387	0.01724
HORVU1Hr1G070200	2.979728	2.023951	2.567881	7.675415	12.8433	12.42498
HORVU3Hr1G048660	10.31194	11.45432	10.30053	10.44915	12.04123	13.04721
Hordeum_vulgare_newGene_14099	10.17929	13.01655	13.64333	0.010914	0	0.012436
Hordeum_vulgare_newGene_14098	1.570693	1.765684	2.099232	2.637999	2.479673	3.162638
HORVU2Hr1G091360	3.390529	3.174793	4.173185	5.718927	11.54518	11.50871
Hordeum_vulgare_newGene_504	1.728516	2.138899	2.294856	0	0	0
HORVU4Hr1G075800	18.11709	16.55565	22.67024	21.57422	36.2796	38.28983
HORVU7Hr1G023940	0.311428	0.407544	0.515069	1.941212	2.302773	1.358795
HORVU3Hr1G087250	6.729729	8.018301	8.775795	7.177287	11.86893	12.84678
HORVU5Hr1G069130	2.129611	2.585367	2.168449	3.287275	3.035473	3.154398
HORVU1Hr1G020150	1.069491	0.598447	1.194724	1.838659	1.7773	1.863365
HORVU4Hr1G010030	3.978431	3.371066	4.367416	2.952069	3.093626	3.892493
HORVU4Hr1G070320	35.74005	39.17165	41.83422	11.3902	10.97652	11.37815
HORVU0Hr1G031980	21.07636	18.70365	19.7765	20.04138	23.05611	23.54228
HORVU2Hr1G005670	1.522238	2.033218	1.651341	0.428005	0.361101	0.537957
HORVU7Hr1G107080	2.848454	2.352134	4.291913	3.751021	6.686641	6.869449
HORVU2Hr1G116300	0.776251	0.56172	0.765361	16.06452	29.43366	25.97025
HORVU7Hr1G065240	12.40894	13.26449	13.86669	10.27951	9.948791	10.92689
Hordeum_vulgare_newGene_15320	1.406777	1.883727	2.558765	2.471903	2.183958	2.885213
Hordeum_vulgare_newGene_15323	2.410082	3.023269	1.94673	5.360564	5.459783	5.995862
HORVU3Hr1G045410	30.97977	30.52497	36.34297	34.12146	47.77897	46.26642
HORVU7Hr1G015380	2.105251	2.767868	3.777374	3.607412	7.951525	7.332159
Hordeum_vulgare_newGene_817	1.664243	1.780792	1.817045	2.190249	4.140536	4.314355

HORVU6Hr1G061000	0.066978	0.086916	0	1.620286	2.987742	1.804164
HORVU0Hr1G002930	1.302589	1.083572	1.822401	2.008605	1.308187	1.785514
HORVU6Hr1G058560	19.98866	20.79119	33.02872	14.17326	30.76148	29.5218
HORVU3Hr1G082750	19.91821	18.13484	27.59085	13.04119	14.91587	14.38535
Hordeum_vulgare_newGene_5522	5.352807	4.170748	7.379798	9.370117	5.75338	6.240541
HORVU7Hr1G097470	6.676212	6.938755	9.797153	15.03995	12.00889	12.2131
HORVU4Hr1G087590	0.114871	0.295771	0.278257	2.106605	2.53626	2.755886
Hordeum_vulgare_newGene_3767	1.010767	0.314823	0.724326	0.978648	6.979538	7.069313
HORVU7Hr1G112470	4.616818	6.434348	7.673244	6.56082	28.45763	28.37969
HORVU3Hr1G084750	1.265982	1.431293	1.555197	2.768244	6.338324	5.630277
HORVU6Hr1G060750	2.666175	1.856896	3.295458	1.194203	0.932058	1.429578
HORVU5Hr1G016840	12.25357	12.05206	10.71531	14.20595	14.5346	14.24742
HORVU2Hr1G009940	3.627905	3.311786	5.026624	15.07701	28.95504	10.29587
HORVU0Hr1G002680	21.40831	18.57903	26.46474	35.20706	57.63812	56.47466
HORVU7Hr1G092560	10.54617	11.37131	13.58772	9.785039	15.9262	16.81556
HORVU6Hr1G000620	11.96582	10.25568	10.88344	41.92507	56.03303	55.90875
HORVU5Hr1G069860	18.23216	15.82173	16.38983	9.47376	7.288737	8.812253
HORVU6Hr1G011160	5.256889	4.152128	4.811373	4.081982	5.907777	5.988941
HORVU2Hr1G029870	161.565	135.9079	146.583	111.6754	81.71359	101.531
HORVU6Hr1G068180	3.34972	3.477676	4.276088	0	0	0
HORVU5Hr1G109660	3.516992	2.896484	2.998114	0.436441	1.396062	0.926199
HORVU7Hr1G036500	4.514336	4.54836	6.694623	2.161096	9.59175	6.319616
HORVU7Hr1G063630	14.68097	16.64752	18.30677	19.04043	24.93391	24.35925
HORVU4Hr1G005350	7.078592	5.310353	8.452009	7.007228	5.3456	3.730183
HORVU1Hr1G068640	33.62662	37.15648	58.72412	43.80078	119.8007	92.25246
HORVU1Hr1G062740	2.404058	2.12997	3.138249	4.433148	4.941331	5.395448
HORVU2Hr1G127640	0.25933	0.238091	0.392108	1.220528	3.280314	3.480909
HORVU2Hr1G088530	1.61013	1.869703	2.4838	0.725633	0.998315	1.249077
HORVU4Hr1G015820	3.335337	2.505627	4.25418	9.058528	10.4686	11.03988
HORVU1Hr1G042020	3.88026	4.440291	5.940974	5.7327	9.397008	10.76506
HORVU0Hr1G019500	6.582296	6.72054	7.135389	3.341687	5.320234	2.63726
HORVU5Hr1G067230	1.575246	1.465375	1.16452	1.809608	1.928125	2.59548
HORVU0Hr1G017560	4.639904	3.579512	3.10808	4.629391	13.68789	17.07065
HORVU4Hr1G052840	1.583871	1.748977	1.706493	1.32192	2.539866	2.198407
HORVU1Hr1G021140	1.826448	1.667883	1.991135	1.425952	0.991657	1.475949
HORVU1Hr1G092960	16.5154	13.23156	9.015527	0.495063	0.690081	0.487653
HORVU7Hr1G117640	6.374917	6.54922	7.448506	20.8122	43.95828	38.08456
HORVU3Hr1G085800	2.691821	2.629159	4.445	1.561183	1.721017	2.211945
HORVU7Hr1G095720	91.00606	76.5265	108.9922	95.80409	138.9841	127.0366
HORVU6Hr1G036720	73.64785	60.78847	88.5716	80.3666	109.5513	103.6976
HORVU1Hr1G076530	24.69294	22.52541	28.85568	23.97977	36.70935	35.82604
HORVU0Hr1G014030	1.605525	1.187346	1.312692	0.728602	1.15715	0.881292
HORVU7Hr1G039850	35.64719	44.54159	47.2005	27.28431	26.07348	32.32486
Hordeum_vulgare_newGene_5249	1.973583	2.64359	1.969315	0.027167	0	0
HORVU5Hr1G023800	14.28777	12.34551	11.70554	24.26254	26.17527	23.81997
HORVU4Hr1G010810	3.702587	3.43822	3.351317	0	0	0
HORVU4Hr1G084930	4.506083	4.305853	6.057458	3.540001	5.279167	4.572737

HORVU2Hr1G058790	3.117023	2.711409	3.161086	3.101434	1.639426	2.496191
HORVU3Hr1G042140	1.970468	1.814506	2.153324	1.961047	3.136982	3.408603
HORVU2Hr1G080050	37.90036	30.5035	39.38318	33.7185	26.55825	40.03397
HORVU4Hr1G013910	4.740911	5.186233	5.806864	14.29878	5.468476	7.099089
HORVU4Hr1G008300	16.68845	14.39765	17.87594	21.01829	19.30586	21.63127
HORVU3Hr1G071550	8.523916	7.123095	8.318212	7.399696	6.479698	6.024046
HORVU5Hr1G010730	4.21814	4.026585	3.833207	5.448011	6.667397	6.146987
HORVU4Hr1G039880	3.371308	4.543153	4.718289	12.87091	34.15382	34.48998
HORVU1Hr1G044740	5.750493	6.807517	6.435291	11.25929	11.86241	12.61034
HORVU1Hr1G078070	0.778703	2.385853	2.096394	0.661735	0	0.2106
HORVU3Hr1G074310	1.004155	1.267362	1.409764	1.240313	2.088031	2.385731
HORVU6Hr1G056260	15.91581	14.957	19.89782	19.52251	31.43106	28.66249
HORVU5Hr1G014430	2.460973	2.414022	3.168668	3.888584	8.22677	6.588693
HORVU1Hr1G012940	148.8166	171.5079	198.4528	320.7673	815.4641	694.6667
HORVU5Hr1G107010	13.40858	13.23696	15.72583	11.17943	12.04957	12.96397
HORVU6Hr1G083050	154.3971	143.6094	123.5868	150.4333	96.69491	113.5123
HORVU4Hr1G006070	4.125669	3.762046	4.250001	7.06158	5.444338	5.695534
Hordeum_vulgare_newGene_4978	5.341142	6.011288	6.362442	7.143719	6.499277	7.317952
HORVU3Hr1G086380	7.805353	12.10914	14.1865	2.503105	2.93071	3.176174
HORVU7Hr1G097860	18.03974	18.90362	20.28525	13.43655	15.34815	16.77556
HORVU3Hr1G110590	12.06414	10.38414	11.62675	21.53139	17.21653	18.20442
HORVU7Hr1G116690	14.15388	14.69959	14.40245	9.16363	10.12588	10.68158
Hordeum_vulgare_newGene_13117	97.83479	82.14167	57.35775	22.75471	18.14884	21.65962
Hordeum_vulgare_newGene_13116	13.53982	14.31426	16.01486	30.19909	71.72191	71.87273
HORVU3Hr1G065000	16.23477	13.57949	18.04945	15.04802	16.62812	16.32543
HORVU5Hr1G022070	69.44929	76.02588	60.27682	100.5115	103.5006	115.6767
Hordeum_vulgare_newGene_13111	1.614172	1.128623	2.420192	3.881189	10.09196	9.078254
HORVU3Hr1G070200	2.492025	2.210756	3.089385	5.9807	7.894048	7.251627
HORVU5Hr1G056270	4.221207	2.883993	3.134518	3.089735	3.576738	3.505042
HORVU1Hr1G060960	2.140772	2.083684	2.112013	4.088283	3.276453	4.029581
HORVU2Hr1G079790	0.218114	0.211289	0.250393	0.104964	5.898349	3.944835
HORVU3Hr1G093190	1.827312	1.874473	1.925949	2.640046	1.262335	1.8902
HORVU4Hr1G007680	2.968196	2.533424	2.573712	15.3316	50.45717	49.06297
HORVU7Hr1G010500	2.306524	3.232776	3.381742	0.843325	1.006204	1.694707
HORVU4Hr1G080940	2.19332	2.010802	2.729001	2.883107	3.788509	3.550689
Hordeum_vulgare_newGene_11140	1.092145	1.829967	1.36802	2.565786	1.613376	3.643076
Hordeum_vulgare_newGene_11142	6.955056	7.290489	9.35945	3.46068	4.208648	5.049406
HORVU0Hr1G026030	14.82625	17.09419	23.03221	21.86975	37.87622	32.95634
Hordeum_vulgare_newGene_5537	5.258474	7.661712	7.107812	4.062583	4.005197	4.869568
HORVU1Hr1G065030	103.5167	87.4625	88.24978	40.44632	22.51688	28.40821
HORVU7Hr1G007590	28.55918	37.91022	49.00424	4.458125	5.847344	6.648978
HORVU0Hr1G012860	0.839117	0.566946	0.483649	4.904646	8.968221	9.050019
HORVU3Hr1G077660	2.47255	1.663522	2.707727	4.145323	3.190664	3.154586
HORVU3Hr1G007080	7.071495	5.699877	8.099486	8.025595	8.858497	9.080389
HORVU2Hr1G122280	919.3782	897.8264	673.1512	4756.705	5413.956	4383.88
HORVU4Hr1G026660	3.040017	3.72999	4.192095	3.190551	4.004541	3.750562
HORVU6Hr1G092280	0.103076	0.038416	0.24763	1.782835	10.87324	10.1038

HORVU6Hr1G042780	0.338109	1.089486	0.679173	2.366047	0.82776	0.768408
HORVU2Hr1G123770	10.76179	9.752512	14.46608	14.12266	6.509324	9.155974
HORVU1Hr1G045160	34.21115	29.27267	35.5682	30.64963	32.2078	32.89875
HORVU2Hr1G123480	1.772571	0.78188	1.716893	2.613129	5.746861	5.876544
HORVU5Hr1G046560	3.845528	4.459659	4.248562	2.738691	3.217149	4.091276
HORVU6Hr1G040780	1.936719	1.95099	2.299557	2.207956	2.835964	2.570366
HORVU2Hr1G105980	1.024513	1.294134	0.833916	3.076048	4.375936	3.33774
HORVU5Hr1G072540	1.175982	1.345425	1.21843	1.390262	1.468949	1.617434
HORVU5Hr1G092660	9.250707	6.223747	8.7435	8.992295	8.48889	7.438595
HORVU2Hr1G091840	1.970061	2.4829	3.27287	1.676531	1.326169	2.144495
HORVU6Hr1G091690	49.72971	50.3353	73.26782	43.48095	50.83491	51.50471
HORVU3Hr1G022770	20.13369	14.8917	19.49353	27.53135	21.23797	25.84025
HORVU5Hr1G117910	123.2217	88.46646	170.9042	81.0138	187.1077	162.6398
HORVU6Hr1G013910	0.716574	0.597187	0.649324	1.235745	1.977698	1.529168
HORVU1Hr1G047110	5.633543	6.3218	6.912069	8.025762	14.69686	14.20368
HORVU1Hr1G084690	5.705275	5.548144	9.820477	3.261415	4.766218	4.16316
HORVU1Hr1G038270	15.68351	14.23108	23.79143	16.83504	18.89906	12.89694
HORVU2Hr1G057670	12.28106	9.223073	11.23964	11.43229	12.18646	12.59608
HORVU5Hr1G106370	1.747169	1.066522	1.523383	0.861512	0.438108	0.654984
HORVU6Hr1G071890	60.04241	55.18946	57.7673	84.84231	75.83717	76.95914
HORVU3Hr1G066720	6.923012	6.320954	7.094463	8.096512	13.05064	12.34502
HORVU2Hr1G111530	0	0	0	1.527831	3.796843	4.146915
HORVU3Hr1G057440	0.793423	1.036145	0.978409	1.969205	3.336685	3.011023
HORVU5Hr1G071010	1.920955	1.417978	1.845041	17.71804	36.85381	18.62997
HORVU7Hr1G121740	1.064118	1.058535	1.224099	1.398968	1.574815	1.700807
HORVU5Hr1G109340	71.45912	67.93457	68.9687	95.51579	116.794	120.4893
HORVU1Hr1G067300	11.09298	10.54246	11.12591	27.57287	57.79564	57.23048
HORVU4Hr1G014360	15.82272	16.45952	19.25482	9.641465	12.15172	13.95084
HORVU3Hr1G088450	9.473897	9.870732	10.28392	10.13397	11.90651	12.29186
HORVU5Hr1G119510	13.82854	13.10007	19.70175	5.138788	10.70012	8.720129
HORVU0Hr1G005500	5.68781	6.341193	7.318547	10.52466	13.32449	14.90443
HORVU6Hr1G089590	3.019681	3.275248	2.832004	0.094288	0	0.010971
HORVU2Hr1G012310	1.188429	0.89978	1.317717	1.140554	2.956535	2.633506
HORVU5Hr1G092080	5.586048	4.277166	5.261465	4.635135	4.407775	5.519376
HORVU5Hr1G047210	3.011633	3.648064	3.681709	3.822367	3.165067	4.144063
HORVU4Hr1G054460	2.068783	2.171459	3.189677	1.408754	1.992743	2.368631
HORVU2Hr1G065110	4.640657	3.920356	4.609586	6.314027	7.195999	8.368792
HORVU6Hr1G091710	13.92214	14.21827	20.33225	11.77431	9.895961	9.875965
Hordeum_vulgare_newGene_3264	8.548539	10.7678	9.754621	8.742058	12.60161	13.3517
HORVU0Hr1G017000	11.77383	7.095507	11.47888	23.20126	30.83187	24.54253
HORVU2Hr1G068530	11.77035	9.620641	11.46712	11.97617	15.99433	15.90037
HORVU5Hr1G057840	10.3985	9.335753	8.742974	19.46211	15.75198	17.3226
HORVU4Hr1G066090	17.36221	18.11699	21.14567	16.30687	9.611655	11.246
HORVU1Hr1G005290	1.891777	0.361475	1.115205	0.882284	0.886367	1.02464
HORVU1Hr1G082690	26.73537	26.72924	24.83142	19.72907	25.2476	22.08646
HORVU7Hr1G035500	45.23614	38.91502	38.03606	25.15878	23.91926	20.916
HORVU7Hr1G071700	16.16852	12.88181	20.14217	20.66212	33.68946	29.3535

HORVU2Hr1G068120	126.2473	145.8005	213.3564	131.8676	336.4234	295.1488
HORVU4Hr1G090810	0.024593	0	0.028483	2.871303	4.206869	2.989273
HORVU4Hr1G067940	0.2212	0.102559	0.344772	1.721703	12.32533	11.09295
HORVU5Hr1G099350	0.424026	0.42747	1.060433	4.183876	23.27794	18.81677
HORVU4Hr1G054790	4.398377	3.781589	4.570513	6.670271	11.90226	10.16305
Hordeum_vulgare_newGene_12558	3.540896	3.988646	3.879918	4.933098	6.543345	6.466069
HORVU5Hr1G000590	5.005011	4.2743	5.687045	7.670417	9.627011	12.09433
HORVU1Hr1G082520	4.20208	3.771618	5.50586	3.137607	3.750578	3.777901
HORVU2Hr1G117270	2.326544	1.688131	2.882168	2.055744	2.977607	2.191525
HORVU3Hr1G061730	6.33871	8.440534	12.85792	4.940629	5.412446	5.433671
HORVU0Hr1G030280	5.197382	4.474174	6.101806	1.854226	1.112123	1.642115
HORVU2Hr1G036390	18.68303	16.37024	20.16075	23.2834	29.48923	32.52031
HORVU6Hr1G055290	4.222754	4.619821	7.102222	4.054615	5.475672	5.345482
HORVU3Hr1G069290	3.581487	3.891257	4.120068	7.625222	7.355496	8.285932
Hordeum_vulgare_newGene_13207	0.538574	0.663038	0.350344	0.530559	3.724426	3.467103
Hordeum_vulgare_newGene_13201	0.522167	0.741408	0.384587	0.394462	3.507752	2.398521
HORVU6Hr1G088310	3.359336	2.095519	3.394856	1.54828	0.581415	1.173735
HORVU2Hr1G107550	40.27558	39.85243	36.48594	43.34945	58.33915	64.36601
HORVU5Hr1G075660	0	0	0.01534	0.081044	8.404364	0.268107
HORVU5Hr1G079160	4.068308	4.532695	5.030536	4.227239	7.372373	8.559
HORVU6Hr1G053730	8.273796	9.198225	7.944328	13.35634	7.357391	8.056386
HORVU6Hr1G017760	7.395226	6.60612	11.83946	6.349532	7.772198	7.739601
HORVU4Hr1G057040	1.498916	1.733393	1.637136	1.551585	1.312831	1.366325
HORVU2Hr1G070360	136.7869	144.7412	198.3417	72.44763	59.40071	55.81119
HORVU5Hr1G080060	18.59245	20.52308	19.79057	19.12365	12.9667	10.51609
HORVU6Hr1G022890	13.09959	15.148	11.83829	20.67211	32.32523	31.49882
Hordeum_vulgare_newGene_6989	2.214324	0.247047	1.993278	6.014748	6.315528	4.244772
Hordeum_vulgare_newGene_2602	1.039142	0.857427	0.90064	1.478381	2.421418	1.745428
Hordeum_vulgare_newGene_6983	2.251884	1.543905	2.759002	2.155267	2.791154	3.667029
HORVU4Hr1G061040	12.08663	12.03497	12.87499	13.15524	10.31359	13.84934
HORVU2Hr1G030940	72.08893	65.71392	93.1126	47.18786	61.49047	64.92548
Hordeum_vulgare_newGene_5958	1.657555	2.158547	2.439811	2.201783	2.183445	2.512742
HORVU4Hr1G020660	10.65389	12.26964	12.34604	13.49357	20.43477	19.10565
Hordeum_vulgare_newGene_5953	2.909611	1.994625	2.507427	0.878635	0	0.304951
Hordeum_vulgare_newGene_4326	0.127578	0.046595	0.260752	3.034486	7.128553	6.316862
Hordeum_vulgare_newGene_15126	1.357318	0.561046	1.279862	1.15684	2.163865	1.682934
HORVU2Hr1G104110	19.80124	16.62973	23.31542	26.26783	21.20879	24.44013
Hordeum_vulgare_newGene_15124	6.990513	6.887373	4.680876	1.189212	1.84335	1.553997
HORVU3Hr1G020840	2.249422	2.311401	2.921003	2.253277	3.21522	2.577184
HORVU7Hr1G030020	1.061762	1.124958	1.028006	1.528128	1.540638	1.439193
HORVU7Hr1G074660	22.96371	12.40617	26.84522	10.23769	4.118548	10.55249
HORVU5Hr1G099820	60.23457	63.04596	68.61873	45.10475	34.05834	41.91264
HORVU7Hr1G031990	1.950354	1.67652	2.035647	2.972264	3.297979	3.402708
HORVU2Hr1G072210	12.05143	8.370551	14.75345	17.9493	7.645726	13.13739
HORVU7Hr1G122590	18.52679	22.47588	19.66278	20.41779	25.15619	24.7954
HORVU6Hr1G095380	1.052427	1.076116	0.94458	16.8932	16.82447	21.13477
Hordeum_vulgare_newGene_9180	2.480501	3.045243	1.675465	2.740492	3.530317	3.766259

Hordeum_vulgare_newGene_9181	1.313875	1.776822	1.731903	1.716153	1.408597	1.791238
Hordeum_vulgare_newGene_9182	1.528475	1.844439	1.804701	1.385241	1.373232	1.555725
Hordeum_vulgare_newGene_9185	1.975414	2.618262	2.089529	3.745035	5.367381	6.099404
Hordeum_vulgare_newGene_9188	2.894908	3.544053	4.10761	2.975305	2.83115	3.398333
HORVU4Hr1G049830	1.001709	1.027038	1.222089	0.778608	1.16139	1.32874
HORVU7Hr1G108670	7.082892	3.234662	12.33679	5.323209	2.61171	1.421113
HORVU4Hr1G020080	2.437131	2.571729	1.930546	2.945639	4.332103	5.0585
Hordeum_vulgare_newGene_9458	1.687926	2.026319	2.020238	1.851163	1.906119	2.274837
HORVU7Hr1G029860	44.00298	42.16134	63.21309	42.25224	76.85326	70.98113
HORVU2Hr1G071540	17.67376	18.25395	19.03068	30.92026	37.10891	41.72866
HORVU3Hr1G050310	18.43463	18.81714	19.78558	10.43878	8.136199	7.932553
HORVU2Hr1G069610	4.425206	4.356044	4.898251	4.539888	4.994346	5.800282
HORVU4Hr1G090030	3.090133	2.491718	3.001103	2.284055	2.656952	3.062378
HORVU2Hr1G035970	5.781234	4.947493	5.129816	8.753019	9.194752	9.093791
HORVU4Hr1G071300	1.335278	1.662337	2.1181	3.733513	6.286314	9.783023
HORVU7Hr1G106660	0.742375	1.020174	1.246914	2.706707	3.08922	4.555388
HORVU6Hr1G060220	0.14468	2.124744	10.62954	8.550662	3.416077	9.089396
HORVU1Hr1G035160	6.791534	7.450291	6.713558	10.03727	9.302268	11.86542
HORVU1Hr1G028920	22.5892	19.18187	20.84871	18.01659	10.00428	11.84888
HORVU6Hr1G058170	0.1596	1.436907	0.48894	2.510211	1.261454	2.006871
HORVU0Hr1G031500	6.94417	8.979856	9.283212	4.168181	5.181897	5.862645
HORVU1Hr1G073760	2.376384	2.160105	3.189459	5.024842	10.46096	12.12977
HORVU5Hr1G045800	2.174154	3.730609	3.092252	7.305131	13.71341	12.77833
HORVU1Hr1G032670	4.723189	6.40986	6.166233	8.716523	10.91174	11.84842
HORVU2Hr1G105790	0	0	0	0.032063	38.44707	23.06623
Hordeum_vulgare_newGene_8326	1.378038	0.945913	2.215239	5.237992	15.54788	16.61508
HORVU7Hr1G036070	14.30756	7.121329	17.06929	5.221236	2.951398	5.199537
HORVU5Hr1G072020	4.779784	4.839125	5.96776	5.377577	8.579692	9.071166
HORVU5Hr1G051590	0.532769	0.348931	1.075445	2.22937	2.507502	4.224914
HORVU7Hr1G087700	4.304225	4.649676	6.671619	0.986844	1.046325	0.931928
HORVU3Hr1G112720	34.81354	32.40479	37.78142	24.79647	27.32698	26.64792
HORVU3Hr1G014100	5.806313	5.206578	3.896221	0.948232	1.277658	1.477907
HORVU4Hr1G046700	15.60966	15.8355	14.64358	17.69804	15.95063	18.82245
HORVU1Hr1G024400	3.35059	3.974287	3.594545	7.385761	10.87765	13.39135
HORVU1Hr1G016130	21.57691	22.62578	20.47941	22.43402	26.46497	27.97354
HORVU2Hr1G077650	4.197793	4.85415	4.169877	18.56218	25.94014	26.59132
HORVU6Hr1G020860	9.405988	7.592937	7.743585	9.986692	11.80788	10.89685
HORVU2Hr1G042180	18.01437	16.55195	20.20869	22.43213	30.05061	29.3785
Hordeum_vulgare_newGene_4939	2.153356	2.065241	2.123555	4.201352	4.726054	5.159326
Hordeum_vulgare_newGene_4936	0.801392	0.764444	0.785398	0.912379	2.266356	1.702873
Hordeum_vulgare_newGene_4934	3.284533	3.376359	3.554341	4.460918	5.890919	5.91119
HORVU1Hr1G076880	1.523941	2.375754	3.952812	2.991716	2.543304	3.468966
Hordeum_vulgare_newGene_38	6.244387	5.613332	10.48124	1.418094	3.476528	1.701061
HORVU6Hr1G062070	8.369424	9.554083	11.75823	1.59831	2.209008	1.665279
Hordeum_vulgare_newGene_34	0.348337	0.44551	0.788175	2.569417	3.163562	3.4142
HORVU0Hr1G039080	10.39321	9.35358	10.83994	2.942252	3.065931	3.01143
HORVU7Hr1G032310	79.15201	66.28972	79.26273	69.00683	65.26068	66.18342

HORVU3Hr1G024540	6.083149	4.416619	5.384877	7.356636	8.794817	8.509189
Hordeum_vulgare_newGene_32	0	0.572475	0.731659	7.151556	6.706931	8.151154
Hordeum_vulgare_newGene_3924	23.65448	31.60155	44.99869	9.859675	16.78246	16.20932
Hordeum_vulgare_newGene_3927	61.22177	56.57121	58.85643	50.1209	41.1769	44.0796
Hordeum_vulgare_newGene_3928	1.798141	1.542849	1.260312	0.932698	0.841138	1.326763
Hordeum_vulgare_newGene_3929	0.264568	0.296205	0.371245	1.326335	2.445531	2.296753
HORVU3Hr1G088150	1.829456	1.709858	1.569085	2.001679	0.435784	0.887399
HORVU3Hr1G030350	33.58354	29.58573	41.81759	25.6016	30.86929	30.38216
HORVU5Hr1G060070	46.81704	47.36457	43.65385	25.65033	17.97166	21.19861
Hordeum_vulgare_newGene_15974	7.003869	9.835109	8.854314	6.849426	4.105176	6.178152
Hordeum_vulgare_newGene_15979	36.52856	34.63519	40.99915	35.60051	49.1753	50.87008
HORVU2Hr1G032400	26.33572	32.2367	38.45816	18.12349	10.28965	12.34189
HORVU7Hr1G100150	42.36275	44.52721	60.95109	11.34675	1.249947	3.943336
HORVU7Hr1G099910	1.057666	1.115913	1.391639	2.392931	2.107303	2.790529
HORVU3Hr1G113090	23.88329	21.60903	26.28558	23.7982	25.47813	25.42893
Hordeum_vulgare_newGene_12611	4.678174	4.25219	3.939621	0.880983	0.904848	1.166316
Hordeum_vulgare_newGene_12610	2.095096	2.322686	2.372461	0.304913	0.16121	0.304546
Hordeum_vulgare_newGene_12613	3.139536	3.532572	4.10259	5.798438	7.281472	7.202757
HORVU4Hr1G037240	1.494375	1.662592	1.698105	1.675589	1.590806	1.942411
HORVU4Hr1G074650	0.574017	0.836406	0.954275	1.149447	3.501201	3.417773
HORVU6Hr1G032750	3.149083	2.810261	0.080934	3.007233	6.832594	5.229166
HORVU2Hr1G079550	39.85502	38.09835	44.54666	34.05036	52.29791	66.77039
HORVU5Hr1G089380	26.76821	24.12442	32.12729	24.97012	35.76115	32.95461
HORVU3Hr1G086080	24.55295	18.96704	25.06758	29.23783	18.25488	19.61596
Hordeum_vulgare_newGene_9847	1.199982	1.156348	1.660085	0.953664	1.268386	1.304646
HORVU2Hr1G001810	1.827019	2.124168	1.738355	3.628711	4.780003	5.250023
HORVU7Hr1G017880	1.158997	1.761145	1.892319	2.222979	2.173592	2.958597
HORVU6Hr1G002580	18.99292	21.26532	20.63957	29.91674	44.93696	35.63602
HORVU3Hr1G087930	1.588041	1.473689	1.618013	2.132332	2.292924	2.759673
HORVU4Hr1G010910	16.13782	19.74453	23.0628	12.39371	19.50821	16.26206
HORVU2Hr1G041520	2.951727	2.371483	2.237005	1.05346	0.786643	0.735723
HORVU0Hr1G005350	3.607455	3.043923	4.02678	8.406818	1.065168	2.722596
HORVU2Hr1G000140	1.172193	0.968542	1.733955	5.469017	4.029049	5.236771
Hordeum_vulgare_newGene_14757	2.591889	2.629404	2.612426	0.492234	0.720115	0.824881
Hordeum_vulgare_newGene_14750	1.055417	1.427037	0.95361	9.520662	10.55211	11.24207
HORVU5Hr1G036790	6.363668	5.315816	6.152077	7.718951	5.535497	5.656274
HORVU6Hr1G004860	1.541907	1.789275	1.643086	5.365454	14.23975	12.21554
HORVU0Hr1G003980	5.838709	5.844824	7.170609	2.478652	1.291778	1.874369
HORVU5Hr1G058910	0.560961	0.538769	0.480974	0.442723	2.122699	2.810338
HORVU3Hr1G087680	4.28282	4.014484	4.716281	3.647355	2.741867	4.192224
HORVU6Hr1G070350	21.81682	20.77182	20.62463	13.38562	14.62174	14.28675
HORVU5Hr1G060920	30.86309	31.38868	34.6789	35.74953	60.59934	61.08234
HORVU6Hr1G068410	13.02167	12.36411	15.53921	13.94924	52.54284	53.34929
HORVU5Hr1G035440	4.981788	6.10751	6.992054	5.402468	10.86236	10.90369
HORVU3Hr1G055410	2.872517	2.690866	4.319215	3.3285	4.218134	4.858623
HORVU3Hr1G083300	16.02582	12.76556	16.26491	12.19889	17.17947	17.74124
HORVU0Hr1G039070	3.449524	1.880445	2.373956	2.25872	2.77578	2.549509

HORVU6Hr1G072260	1.036616	0.796157	0.846382	3.646877	8.768189	8.107994
HORVU1Hr1G091230	8.533708	9.793875	13.34257	1.063607	1.018621	1.062481
HORVU2Hr1G026340	340.7493	263.3262	340.8565	428.5136	352.2482	355.472
HORVU7Hr1G011830	9.035972	9.466467	13.47302	7.447427	15.63961	12.83647
HORVU0Hr1G004400	6.805532	6.792535	8.095805	9.406039	11.75246	10.91246
HORVU2Hr1G027040	6.43628	6.912263	6.869232	13.57542	15.40924	17.84167
HORVU6Hr1G066950	1.487831	1.283419	1.826243	2.257405	5.242998	4.346255
HORVU6Hr1G002320	2.598366	1.298333	2.428786	6.374485	49.61435	33.36076
Hordeum_vulgare_newGene_9623	16.53828	16.22351	23.62619	11.93531	13.14352	14.96844
HORVU4Hr1G056240	0.578664	0.491956	0.867723	0.86824	4.412683	9.162718
HORVU7Hr1G008090	4.666842	5.534065	6.822155	3.24313	3.070872	3.252599
HORVU7Hr1G071290	10.57228	9.332905	11.11588	9.28524	7.883441	8.149998
HORVU1Hr1G055510	0.196324	0.288415	0.476347	0.790747	3.042543	2.999441
HORVU5Hr1G094810	8.499424	8.628467	8.595355	6.594089	8.881953	8.332017
HORVU4Hr1G011790	5.238054	7.040026	8.796536	5.883728	7.620376	8.592922
HORVU2Hr1G091440	6.551023	5.107832	7.57233	2.870798	4.777421	4.053239
HORVU2Hr1G085000	5.084211	5.742066	5.790333	15.68081	20.55274	19.55892
HORVU2Hr1G098800	49.36306	42.53806	48.49786	16.27352	12.13196	13.95237
HORVU1Hr1G023060	18.36303	17.11316	21.83268	28.99807	47.69506	50.57902
HORVU4Hr1G081150	2.08778	2.792033	2.554258	1.370637	0.0931	0.654652
HORVU5Hr1G051180	7.02277	6.577946	7.49233	8.403308	12.42582	11.30507
HORVU3Hr1G016830	3.234	2.571834	2.362189	0.068445	0.039967	0
HORVU3Hr1G024610	27.07074	28.05618	34.42361	27.73808	31.86824	31.89643
HORVU1Hr1G002410	0.092547	8.591535	0.82851	12.0963	0.030599	0.025521
HORVU7Hr1G050670	44.47287	34.16356	44.84827	16.68669	3.265794	8.022646
HORVU4Hr1G065800	13.06342	9.781105	14.66892	5.280899	4.382753	4.456799
HORVU5Hr1G050850	0.838362	1.089635	1.399073	1.091345	0.873402	1.231265
HORVU7Hr1G116810	1.708067	0.918576	1.807407	0.633438	0.452264	0.656752
HORVU1Hr1G052890	12.37718	12.10586	14.97246	31.99529	40.27641	37.31204
HORVU7Hr1G090650	10.65113	13.64061	12.04955	10.17265	9.798476	10.79277
HORVU6Hr1G011640	2.801785	1.941044	3.617041	1.91688	2.163715	3.124531
HORVU2Hr1G065750	41.49041	30.84039	47.53372	24.28732	34.95738	32.96881
HORVU3Hr1G049360	2.899362	3.696227	2.983504	6.000704	6.482765	6.096992
HORVU2Hr1G089050	24.36086	22.48759	27.47575	20.55641	13.6303	17.10539
HORVU7Hr1G095020	33.0724	27.55994	30.7669	35.90533	39.28403	36.84427
HORVU4Hr1G005780	15.8913	14.90301	16.63637	17.926	20.64522	20.82784
HORVU2Hr1G106040	7.59341	5.941584	9.509418	8.983378	9.478485	9.124273
HORVU3Hr1G031740	39.68477	39.1443	39.07217	21.34363	34.2342	33.73095
HORVU2Hr1G098110	4.12361	4.8055	6.519584	3.379927	3.697946	2.911708
HORVU5Hr1G124960	20.04775	19.28726	21.89333	23.81937	26.32259	27.01672
HORVU2Hr1G081540	3.606833	3.306053	3.926198	13.03967	3.66485	6.771988
HORVU6Hr1G011390	1.492479	1.690344	1.626985	0.780228	1.04363	2.485285
HORVU4Hr1G062080	8.266264	5.478392	9.662999	5.060239	6.937611	6.785352
HORVU1Hr1G057110	5.055187	4.100134	5.681468	3.359658	2.069009	2.321225
HORVU1Hr1G082320	0	0.018709	0	3.946468	3.047826	2.839725
HORVU2Hr1G124380	4.667816	4.576814	5.713732	4.946289	5.02132	5.997823
HORVU2Hr1G085910	12.90454	13.42147	12.10807	27.94736	28.33809	27.73812

HORVU1Hr1G093890	12.23167	11.35476	16.45529	12.68705	5.367722	9.83625
HORVU1Hr1G087940	0.421807	0.415107	17.07268	44.43752	0.159067	0.231722
HORVU1Hr1G047390	6.163836	5.41622	7.657062	3.118275	3.855363	4.304922
HORVU7Hr1G088280	12.73081	15.23633	12.61468	24.24461	8.42157	10.03433
HORVU5Hr1G060590	1.764676	0.902529	0.910705	0.790413	0.690446	0.960675
HORVU2Hr1G091230	7.185214	6.91997	8.132085	6.441173	6.761226	6.552042
Hordeum_vulgare_newGene_6840	6.791212	4.296042	10.17961	0.339194	0.654893	0.729533
HORVU4Hr1G014530	0.35213	0.414866	0.450637	2.197006	6.268775	5.85795
HORVU2Hr1G078090	6.360912	6.756599	8.6841	5.656807	6.911506	7.076243
HORVU7Hr1G095680	11.94532	9.379601	13.72996	19.94697	34.16659	35.31249
HORVU1Hr1G036780	0.784325	1.152859	0.728503	0.813128	1.363924	1.476494
HORVU4Hr1G048000	3.352463	3.06436	4.483454	2.952047	4.041813	3.913509
HORVU3Hr1G005780	8.178142	7.658193	9.804786	2.968252	3.970567	4.62083
HORVU3Hr1G047750	10.41921	9.384968	10.00301	9.232124	10.4406	11.0534
HORVU7Hr1G071390	0.797385	0.834164	0.668704	1.58365	4.501629	3.821007
HORVU4Hr1G053980	4.265527	2.836636	5.390052	11.51912	7.817509	13.22326
HORVU5Hr1G021280	11.99008	10.96435	13.80337	10.95951	9.744135	12.67363
HORVU7Hr1G099070	38.27409	33.9157	43.06639	26.28653	23.47479	23.62064
HORVU7Hr1G094080	0.262113	0.259295	0.143848	2.250964	7.39866	8.784094
HORVU0Hr1G001440	9.68315	10.52353	12.74393	10.369	11.30181	12.73974
HORVU7Hr1G091260	35.13736	29.7486	44.49049	34.65094	62.59558	63.76172
Hordeum_vulgare_newGene_12149	35.0946	35.37068	44.50146	29.45873	40.55003	37.65753
HORVU5Hr1G092630	138.0875	145.1465	198.6249	123.3842	248.5551	242.1935
HORVU0Hr1G027160	3.572778	3.414197	4.117005	5.906848	8.307192	10.64919
HORVU0Hr1G037150	0	0.376961	1.420783	2.625775	2.862826	3.2234
HORVU2Hr1G101300	5.774897	5.933738	5.722594	4.674165	5.07625	4.845769
HORVU7Hr1G111130	16.2639	14.13919	17.57805	25.58729	26.04439	25.75309
HORVU2Hr1G019410	88.63491	84.72943	96.9036	72.92886	85.12263	90.14834
HORVU1Hr1G051220	1.356338	1.619167	1.741664	2.568359	2.208483	2.768283
HORVU4Hr1G007520	19.84729	19.87087	18.08792	15.87615	19.30013	19.25385
HORVU4Hr1G017950	5.799281	8.278644	8.800706	8.355596	9.169212	12.19847
HORVU5Hr1G052360	9.266168	9.518049	10.10753	8.015529	9.438094	10.73419
HORVU0Hr1G035570	0.767909	1.055546	0.965429	0.88364	3.130505	2.621832
HORVU5Hr1G116340	10.51334	9.798305	10.08597	6.798555	7.402388	8.007411
HORVU4Hr1G055970	2.824919	2.148742	3.054961	1.69448	0.526666	0.632462
HORVU2Hr1G090610	12.61289	11.75275	15.04492	17.25695	22.58007	21.37945
HORVU3Hr1G003950	10.98872	9.946627	11.98673	8.551336	10.06894	11.75885
HORVU5Hr1G052090	16.8263	13.58153	19.95857	33.67313	69.97167	66.91828
HORVU2Hr1G023590	2895.309	3462.851	2176.362	639.3228	33.51153	242.1749
HORVU2Hr1G106820	12.23077	10.45808	10.24817	11.41636	13.88235	13.03544
HORVU7Hr1G070450	24.4825	20.64979	26.80415	15.08073	20.77885	19.65961
HORVU5Hr1G048010	35.56151	39.06008	35.08068	40.53216	65.28768	66.84647
HORVU6Hr1G077750	1.559248	1.162988	2.204524	5.238672	4.875034	2.525322
HORVU4Hr1G024560	1.027213	1.210476	0.850321	1.858533	2.285113	2.064521
HORVU4Hr1G067390	15.27313	14.72815	21.71174	14.30765	18.72145	17.94452
HORVU1Hr1G040700	6.895821	3.948308	5.562785	0	0	0
HORVU3Hr1G095700	23.81942	22.17789	22.55674	25.56641	29.75505	43.65858

HORVU4Hr1G069060	5.844234	6.594526	5.914333	8.504992	10.82183	9.649039
HORVU1Hr1G041250	371.6521	290.4879	395.8721	838.0687	1278.268	1196.682
HORVU7Hr1G077000	3.764359	4.725043	4.454926	7.756592	10.58894	11.02183
Hordeum_vulgare_newGene_11484	6.423079	7.864704	7.056743	6.360631	6.530993	8.326174
Hordeum_vulgare_newGene_11486	2.97539	2.702743	3.476428	4.314239	5.835171	6.370578
HORVU2Hr1G038120	1.599742	1.187328	1.979883	1.580125	1.750494	1.980161
HORVU4Hr1G038090	16.10411	12.14218	18.71824	20.56728	22.47051	24.42724
HORVU1Hr1G059830	2.480198	2.792423	1.850164	1.383639	0.597708	0.981099
Hordeum_vulgare_newGene_132	8.012126	8.621829	8.843106	0.04644	0	0
HORVU5Hr1G092430	5.520926	4.783262	5.521416	11.47713	18.77992	19.40175
HORVU7Hr1G051260	6.504025	6.263396	8.842422	3.031196	6.992464	5.72165
HORVU7Hr1G080630	29.80295	28.45217	44.10416	20.84505	34.10383	37.11214
HORVU0Hr1G017210	0.414201	6.913879	5.154841	6.019397	8.387619	7.403678
HORVU4Hr1G052610	12.71374	11.58799	14.65261	8.301045	9.045315	8.97304
HORVU5Hr1G086370	4.615763	2.035707	2.264893	1.981695	4.456004	3.904578
HORVU4Hr1G023030	6.375281	5.665656	5.857294	6.163303	7.998865	7.132444
HORVU1Hr1G013190	4.066052	4.168924	4.852726	8.16395	13.32999	12.03542
HORVU3Hr1G067440	12.52037	12.63779	13.42126	14.67519	15.35106	15.31959
HORVU0Hr1G009910	3.38039	3.720047	4.436477	5.963649	11.58683	10.20995
HORVU1Hr1G002990	0.069309	8.246414	0.552444	12.09663	0	0.026967
HORVU2Hr1G070090	22.34587	20.67384	23.36257	28.35429	36.53604	45.3098
HORVU5Hr1G016730	1.313661	1.122026	1.343719	1.912591	2.081971	2.151045
HORVU6Hr1G092740	47.17936	35.15695	50.68057	16.26071	7.978953	10.28429
HORVU5Hr1G000140	8.568455	9.456017	9.178614	8.507173	6.451765	7.548977
HORVU4Hr1G028780	1.568582	1.710706	1.450761	1.751729	1.665251	2.076346
HORVU2Hr1G062840	3.390073	3.026183	3.521628	2.611903	4.550958	4.607919
HORVU2Hr1G028120	1.449766	2.736124	2.422293	1.958806	2.914563	3.682301
HORVU0Hr1G022810	832.9747	999.0641	941.4143	952.0918	267.2475	384.9994
HORVU5Hr1G040160	4.846224	2.053304	3.97422	6.037055	5.81627	5.950024
HORVU7Hr1G074850	27.38678	25.13561	24.61129	44.43529	49.97721	48.00434
HORVU3Hr1G057760	15.55635	17.94856	30.01525	3.868378	0.922805	2.101664
Hordeum_vulgare_newGene_16136	5.174196	4.323327	5.580065	5.708897	6.432344	5.94207
Hordeum_vulgare_newGene_16132	1.245162	1.604155	2.188105	3.429472	2.650399	3.141006
HORVU5Hr1G049050	6.078962	5.623209	5.893859	6.895043	13.55667	11.90402
HORVU3Hr1G064100	3.051983	3.32619	4.63203	4.622978	5.573654	4.846765
HORVU2Hr1G087660	6.849492	8.264622	6.528003	3.948288	3.275102	3.908101
HORVU3Hr1G110170	2.179461	1.349548	2.627275	5.14758	30.82113	25.11372
HORVU6Hr1G022220	6.766064	6.914796	9.536179	6.791101	11.17403	13.81006
HORVU2Hr1G018730	0.858097	0.895699	1.250023	2.014016	3.032744	2.824307
HORVU5Hr1G064000	5.755292	5.808358	4.824405	15.59217	11.36053	11.13504
Hordeum_vulgare_newGene_13508	17.00259	15.46249	20.76735	13.6834	18.23364	18.11956
HORVU2Hr1G032170	17.63636	16.28805	17.47895	18.21887	19.80921	18.66763
Hordeum_vulgare_newGene_13504	4.148618	3.970487	4.281859	3.547111	2.418894	3.473669
HORVU1Hr1G012710	86.95602	91.98424	135.6986	31.89952	4.317528	11.74088
HORVU7Hr1G003490	1.596074	1.023249	1.46489	1.890647	0	0.27538
HORVU4Hr1G022780	61.11475	64.67658	123.3871	85.47723	42.19504	78.10934
HORVU4Hr1G059390	85.61319	54.55157	78.08509	176.742	176.3668	169.9027

HORVU2Hr1G103040	83.35978	89.27833	94.06683	41.9757	19.20836	25.13763
HORVU7Hr1G038140	2.25434	1.93024	3.241483	3.382375	3.984213	3.483662
HORVU4Hr1G054610	0.173316	0.217462	0.389692	1.519231	2.643188	2.736038
HORVU4Hr1G029660	5.527032	5.371548	5.776287	5.056975	5.694099	4.795341
Hordeum_vulgare_newGene_14877	1.46947	2.329397	1.559474	0.627886	0.293903	0.360154
Hordeum_vulgare_newGene_14190	22.08374	19.78615	21.49828	20.52959	23.04661	23.95852
Hordeum_vulgare_newGene_14191	3.636436	3.44343	5.47594	3.35334	5.174683	4.605176
Hordeum_vulgare_newGene_14192	40.65355	36.26373	45.77519	35.8493	53.10855	49.86447
Hordeum_vulgare_newGene_14193	1.009501	1.268319	0.979843	9.379558	6.564488	7.127437
Hordeum_vulgare_newGene_14195	0	0	0	0	5.180775	6.176503
Hordeum_vulgare_newGene_3768	1.594969	1.733352	1.851663	2.55736	3.90793	3.231669
Hordeum_vulgare_newGene_3766	1.087925	1.40123	1.437096	0.361374	1.046111	1.209195
HORVU5Hr1G043060	7.455092	8.582203	9.866978	10.78755	20.46263	18.71876
Hordeum_vulgare_newGene_10855	50.13256	50.58444	57.68282	1.541149	0	0.423765
Hordeum_vulgare_newGene_10851	0.547426	0	0.238173	0.276735	4.644816	3.973731
HORVU1Hr1G036990	1.410682	1.211244	1.611549	1.322087	1.538316	2.583151
HORVU2Hr1G036210	2.853593	2.669594	3.098894	3.434101	3.061847	3.474986
Hordeum_vulgare_newGene_10858	65.0601	68.1139	72.44444	111.2895	163.8761	158.7475
Hordeum_vulgare_newGene_494	0.846026	1.006524	1.362345	1.005615	1.824365	1.855745
Hordeum_vulgare_newGene_492	1.095361	0.928765	1.591019	1.202536	1.928013	1.791283
Hordeum_vulgare_newGene_491	0.555299	0.722587	0.979885	1.172039	1.504565	1.898633
HORVU6Hr1G054000	7.231954	4.226263	6.405671	7.235159	22.09756	13.55443
HORVU1Hr1G094530	25.57039	30.29043	30.76195	25.44965	33.6022	31.71136
HORVU7Hr1G043120	10.22882	9.521817	9.534137	5.775854	5.973179	5.884678
HORVU7Hr1G103150	16.52907	20.47292	17.95819	17.46035	9.58258	12.93139
HORVU3Hr1G061080	0.737414	0.819359	1.199067	1.784441	2.149299	2.164702
Hordeum_vulgare_newGene_2417	19.71226	15.09955	18.57287	20.33479	32.73245	33.54448
Hordeum_vulgare_newGene_2416	4.501313	4.088078	5.022042	4.917932	5.363607	3.998059
Hordeum_vulgare_newGene_2414	7.035789	7.656925	8.354249	4.647035	6.095072	6.572907
Hordeum_vulgare_newGene_2413	17.59771	14.15865	19.79704	17.51919	26.95351	26.13975
Hordeum_vulgare_newGene_11813	3.630828	4.111385	3.615829	2.806753	3.117183	3.991372
HORVU1Hr1G000660	4.560054	4.962611	3.801344	0.024324	0.010681	0
HORVU7Hr1G096150	0.872542	0.789284	0.823719	1.501468	1.060443	1.04649
Hordeum_vulgare_newGene_11815	4.025426	3.858679	3.606311	3.899251	6.003414	4.792727
HORVU6Hr1G030260	0.686051	0.437391	0.396824	1.411047	1.119824	2.169113
HORVU3Hr1G096110	6.965655	7.714095	8.726685	10.78298	10.28122	10.72382
HORVU7Hr1G117300	1.465565	1.377219	1.156057	1.934903	3.032671	3.470731
HORVU6Hr1G026420	2.018279	1.598675	1.348821	3.114054	1.62148	1.930844
HORVU4Hr1G024950	1.870905	1.936265	1.781955	4.75282	5.873194	6.943924
HORVU4Hr1G075180	0.205602	0.091848	0.270712	0.502636	2.944354	2.723562
HORVU7Hr1G108730	6.906099	5.622833	7.201824	6.426882	9.247058	10.18981
HORVU1Hr1G054620	2.367853	2.486021	3.855564	2.003492	2.418845	2.139147
HORVU0Hr1G032260	2.351959	3.07393	1.842933	0.869558	1.512043	1.153457
HORVU0Hr1G009140	0.933334	0.852784	0.863994	2.652867	4.28111	4.058857
HORVU2Hr1G040900	3.832571	3.528766	6.414457	6.090301	5.894588	4.841702
HORVU6Hr1G078960	71.09224	61.49299	71.85876	32.61333	11.8366	14.00286
Hordeum_vulgare_newGene_12093	0.738814	0.514036	0.473221	1.00609	1.496053	1.957445

HORVU6Hr1G013720	5.499327	6.491129	8.977303	3.783676	4.382615	4.412768
HORVU2Hr1G031880	9.60773	10.41921	11.619	12.16154	14.98469	15.32785
HORVU4Hr1G082110	4.148072	4.848651	5.857062	3.861796	3.981555	4.358633
HORVU7Hr1G107700	9.955287	10.65669	10.64909	12.19132	15.32171	15.30465
HORVU1Hr1G053070	1.224503	1.177119	1.503166	1.214447	0.935425	1.457995
HORVU3Hr1G058350	8.970687	11.70279	9.467601	12.18812	12.64516	15.01922
HORVU5Hr1G077350	31.90613	25.59123	32.44143	24.2711	21.71558	22.48103
HORVU7Hr1G044470	2.062063	1.982695	1.956793	1.792506	1.27732	1.59476
HORVU2Hr1G066200	11.5044	8.777157	11.67088	4.97775	1.780917	1.886808
HORVU7Hr1G073020	3.370917	3.798696	3.831168	3.98385	4.863041	4.944276
HORVU2Hr1G073750	2.893018	2.686795	2.903292	2.627918	3.566294	3.383035
HORVU6Hr1G067170	4.493709	3.220558	3.767216	8.992196	22.28041	17.2358
HORVU7Hr1G114650	17.57442	17.26614	22.4227	17.14147	10.52043	16.3833
HORVU4Hr1G084340	26.42207	21.49089	27.3435	41.63263	33.45922	32.8831
HORVU4Hr1G038850	33.62689	36.24674	39.08632	35.36888	40.8848	42.09046
HORVU7Hr1G087560	2.362492	3.297432	2.957218	2.115985	1.622268	1.949617
HORVU4Hr1G075720	4.775866	5.165271	7.071651	0.050098	0.024105	0.044623
HORVU2Hr1G034100	3.501911	2.687951	3.251283	3.000679	4.32021	3.369035
HORVU3Hr1G049610	14.90689	17.33669	17.2718	18.94184	39.84601	39.15564
HORVU2Hr1G049700	35.34671	33.69871	39.45963	39.40293	39.77676	44.20969
HORVU2Hr1G103150	6.673163	8.219334	11.96694	1.847759	1.130002	1.438721
HORVU2Hr1G105050	5.76059	4.450943	6.160594	9.84382	3.844439	6.275752
HORVU0Hr1G038650	7.556356	8.120651	6.309176	9.445446	10.71484	10.61309
HORVU7Hr1G061640	4.684282	5.148897	5.479017	6.595718	8.840228	9.977042
HORVU7Hr1G036130	0.535276	1.052767	1.017225	2.297503	1.74942	1.504116
HORVU7Hr1G035280	66.60602	66.85348	66.47715	62.87864	71.7181	69.28497
Hordeum_vulgare_newGene_4019	24.87023	36.21969	40.46751	15.4995	3.710602	8.949746
HORVU6Hr1G036950	16.67633	14.75691	23.84683	20.04746	23.71147	19.64299
Hordeum_vulgare_newGene_4015	2.775263	2.703916	3.363105	1.811175	2.416821	2.843004
Hordeum_vulgare_newGene_4014	12.4116	9.854583	10.80574	23.67078	21.45169	26.33998
Hordeum_vulgare_newGene_4017	15.51916	14.98067	17.52913	20.44159	24.94994	28.78319
Hordeum_vulgare_newGene_4011	8.411159	5.986038	10.64324	3.623235	9.023911	9.683371
HORVU1Hr1G072400	14.37524	9.413189	14.25514	11.41071	7.416965	6.604417
Hordeum_vulgare_newGene_4012	36.68107	30.84894	43.4667	38.24201	37.76246	43.96958
HORVU6Hr1G081310	3.45827	3.511542	4.696362	1.607724	1.756516	2.164918
HORVU1Hr1G068160	0.083303	0.034908	0.06459	0.50215	1.829356	3.502361
Hordeum_vulgare_newGene_11760	2.51688	1.152346	1.682086	1.562318	2.013441	1.929691
HORVU3Hr1G105290	0	0.051012	0.0616	5.392119	2.644383	6.147767
HORVU5Hr1G091250	18.01886	15.48114	17.31477	11.31064	12.00218	14.7388
HORVU2Hr1G046410	0.413582	0.883147	1.537476	2.07408	3.617281	1.755145
Hordeum_vulgare_newGene_14949	1.403898	2.047274	1.952898	1.071336	0.758134	1.360265
Hordeum_vulgare_newGene_14942	197.0012	159.3409	229.6644	145.0862	109.0803	117.0765
Hordeum_vulgare_newGene_14941	0.428947	0.307774	0.387643	1.163402	3.066777	2.901202
Hordeum_vulgare_newGene_14940	2.149359	2.402031	1.38585	0.397019	0.314439	0.458721
Hordeum_vulgare_newGene_14946	2.74093	3.870408	2.843193	0.767708	0.80957	0.54858
Hordeum_vulgare_newGene_14945	1.162356	1.40819	1.768707	0.886263	0.124867	0.993353
HORVU3Hr1G047510	25.88299	19.75356	32.8802	25.01918	36.86624	36.75387

HORVU5Hr1G056420	3.253603	2.352081	3.806814	1.23382	0.414498	1.277021
HORVU6Hr1G062830	3.461648	3.375489	4.144764	3.981207	4.002756	4.921906
HORVU0Hr1G018970	1.195935	0.971405	1.170607	2.754375	2.221055	2.577003
HORVU3Hr1G091230	11.65017	9.151337	9.472962	8.601478	9.490595	7.819976
HORVU3Hr1G034820	1.315336	1.571234	2.272841	1.134817	0.516268	0.90938
HORVU3Hr1G085180	2.090514	1.769721	2.92122	3.344072	3.416249	2.81946
HORVU5Hr1G076400	26.82566	21.6973	25.24197	67.66891	79.34837	76.52987
HORVU2Hr1G045140	7.980634	8.300318	9.457815	5.96176	7.334431	7.287967
Hordeum_vulgare_newGene_7097	0.830696	0.250268	0.408678	2.567167	3.114405	2.204768
HORVU7Hr1G006240	24.65047	23.13376	22.98356	1.209608	1.956183	1.008425
HORVU1Hr1G083300	5.458863	3.986805	5.548771	5.63046	4.197508	4.265906
HORVU3Hr1G085720	2.750436	3.351566	3.235664	1.056199	1.295875	1.305122
HORVU1Hr1G069340	31.00154	22.22541	28.2007	23.32559	36.89348	32.13859
HORVU2Hr1G051810	4.561341	5.923174	7.005228	6.386682	9.934587	9.634251
HORVU7Hr1G038420	66.85206	62.95652	76.63504	137.6002	381.4215	336.3954
HORVU1Hr1G088460	1.050395	1.066117	17.55383	44.87153	0.734876	0.740477
Hordeum_vulgare_newGene_6322	0.837565	1.077924	0.916435	1.105371	1.356846	1.500492
HORVU1Hr1G027340	0.608758	0.811883	0.586025	0.942622	4.198079	2.55927
HORVU3Hr1G081400	18.7921	14.84453	15.79741	14.27176	8.309238	8.242176
HORVU1Hr1G086480	5.20848	5.77489	5.39603	7.747032	8.286795	7.915093
HORVU3Hr1G030670	1.051873	1.504368	2.060559	3.44383	4.261861	3.332134
HORVU3Hr1G034310	2.601142	2.758179	4.566868	10.53999	14.92813	29.58487
HORVU6Hr1G075010	19.95371	21.75457	25.27508	26.25198	17.33467	26.45411
HORVU1Hr1G065440	34.38229	30.1229	34.31245	18.18189	20.85982	22.97219
HORVU5Hr1G123630	0.695275	0.797083	1.088791	2.013993	2.291969	2.233762
HORVU2Hr1G055310	3.412999	4.164408	6.348158	2.904202	5.596466	6.550623
HORVU4Hr1G051070	1.933861	1.5294	1.65075	1.59987	10.08122	6.715882
HORVU3Hr1G078360	4.211324	3.388004	3.60826	0.131231	0.335492	0.424602
HORVU5Hr1G002010	66.86718	58.39242	75.15747	69.87656	94.55743	89.40262
HORVU1Hr1G011730	1.635724	0.996632	1.471337	4.812192	4.036417	5.230341
HORVU5Hr1G106100	1.157072	1.43804	2.479587	2.349148	2.979945	5.800915
HORVU1Hr1G062940	23.62213	19.40795	30.67999	15.03802	12.02269	14.43262
HORVU6Hr1G033290	11.00869	10.06652	10.34042	6.726173	7.980217	7.854811
HORVU6Hr1G038740	5.03669	4.593102	5.73661	4.660132	5.777452	6.478055
HORVU2Hr1G061950	5.31079	6.623194	6.460295	2.86186	3.492593	3.215068
HORVU5Hr1G027980	3.747239	2.288374	2.82667	0.40355	0.250845	0.415513
HORVU5Hr1G094370	63.50334	51.35524	74.86196	53.15213	61.89029	69.86312
HORVU7Hr1G022250	1.47949	1.582277	1.947925	3.224413	2.100227	2.312498
HORVU5Hr1G017660	1.468237	1.154089	1.817284	5.870905	5.492092	5.622323
HORVU2Hr1G081460	5.176933	4.618195	5.640419	7.956796	11.84628	11.15734
HORVU1Hr1G026960	1.887469	2.614676	1.749756	2.019474	10.65969	7.409561
HORVU4Hr1G017200	0.693551	1.309288	0.923859	2.052284	2.03232	1.651068
HORVU2Hr1G059170	20.05126	21.81484	19.1556	31.23982	30.89621	34.68336
HORVU3Hr1G016360	4.577176	6.040827	6.005393	3.152099	2.017112	2.917901
HORVU3Hr1G061690	15.59953	13.76779	19.73407	19.98639	18.69943	19.14542
HORVU4Hr1G006100	40.63759	39.52601	39.73004	42.15758	32.44327	36.65898
HORVU1Hr1G065990	0.106492	0.06786	0.08769	1.992525	3.331192	2.965352

HORVU5Hr1G055300	8.23949	9.447935	9.204642	8.981226	6.672874	8.316903
HORVU7Hr1G121200	0.131549	0.291338	0.306738	0.574478	2.236261	3.339932
HORVU3Hr1G019480	3.002891	3.206557	2.784055	4.231179	3.180339	3.567996
HORVU5Hr1G113620	3.753455	4.040854	4.930459	2.574097	1.545456	2.267117
HORVU7Hr1G051710	12.24903	17.88316	15.54594	52.79366	125.2986	115.5502
HORVU5Hr1G094990	4.761207	6.35983	6.437948	7.297405	6.404333	7.011065
HORVU3Hr1G036310	24.94613	32.53682	30.94277	19.8734	18.90542	22.2699
HORVU6Hr1G045070	3.215618	3.794627	3.487244	3.834748	5.485723	5.598681
HORVU1Hr1G013600	16.09624	16.47461	16.88395	17.17555	19.75906	18.72529
HORVU5Hr1G066250	80.22511	76.2539	77.25225	101.2986	93.83171	106.6757
HORVU7Hr1G049190	16.45359	14.97178	17.0657	16.25648	23.42195	23.63623
HORVU2Hr1G061620	2.094586	1.888559	2.615218	3.225799	2.218783	2.200234
HORVU2Hr1G082470	4.021864	4.052081	4.059541	6.54956	8.664711	8.859468
HORVU4Hr1G063650	6.392506	7.563985	5.823511	3.70326	0.299821	1.158959
HORVU5Hr1G107220	10.0602	8.80915	10.06737	2.316569	1.012143	1.471892
HORVU5Hr1G122060	0.123741	0.218753	0.171196	7.296955	15.59757	12.02022
HORVU0Hr1G011630	3.488614	1.563371	2.418989	1.609666	2.377203	2.397008
HORVU6Hr1G085330	1.307432	1.296385	1.451374	3.532322	3.368558	3.334462
HORVU6Hr1G001950	1.694432	1.21528	0.982527	1.846496	3.47733	2.953671
HORVU7Hr1G095060	4.914591	4.912378	6.22068	21.95463	33.1926	33.16332
HORVU5Hr1G096260	13.56533	13.9217	12.43076	0.971244	0.803708	0.704337
HORVU2Hr1G095440	1.127325	1.157905	1.805313	1.031505	1.273603	0.797279
HORVU1Hr1G048720	5.452808	6.131158	5.656911	7.899283	9.646663	10.85479
HORVU2Hr1G127370	1.53197	1.457345	1.96031	2.898268	3.787848	4.299306
HORVU4Hr1G019780	2.248015	2.09103	2.565028	5.663279	15.94522	12.98027
HORVU5Hr1G059140	9.584204	7.873526	7.66441	8.218729	7.829287	8.243324
HORVU1Hr1G058310	2.240116	2.640415	1.043084	0.042596	0	0.049753
HORVU6Hr1G005240	2.889317	3.296333	3.784474	2.147491	3.977985	4.049567
Hordeum_vulgare_newGene_4707	3.200574	4.409635	4.026478	2.092183	2.410436	2.766269
HORVU3Hr1G072800	40.69898	38.91917	55.27939	35.60318	37.26017	35.54325
HORVU5Hr1G009860	0.287879	0.23406	0.774878	1.142349	2.227217	1.953582
HORVU0Hr1G017450	4.95378	4.534751	5.447139	6.373454	9.562577	9.277653
HORVU1Hr1G083840	71.81461	71.72075	83.4982	82.29382	138.0604	142.4838
HORVU6Hr1G019520	21.91424	21.34676	32.64993	27.17259	26.05086	24.16897
Hordeum_vulgare_newGene_12925	4.172516	4.082648	5.235036	3.697927	5.436915	3.646985
Hordeum_vulgare_newGene_12924	6.046019	5.871613	4.368267	4.885099	5.966413	8.406517
Hordeum_vulgare_newGene_12923	4.014242	3.933133	5.108508	4.019379	5.076129	4.943042
Hordeum_vulgare_newGene_4270	1.660649	2.018435	2.488234	7.102709	5.40784	7.008555
HORVU2Hr1G064470	33.93329	31.86256	33.91878	33.44081	43.75258	45.86878
HORVU7Hr1G092450	3.331958	3.696829	2.778038	30.64496	33.65401	34.928
HORVU5Hr1G104410	1.861695	2.084652	2.46209	3.938649	3.315874	3.578271
HORVU6Hr1G011050	109.2972	105.3663	137.4095	6.406662	0.412191	1.906376
HORVU4Hr1G051810	1.733295	1.632225	2.052352	2.146678	2.595103	3.354537
HORVU4Hr1G065110	4.600127	4.835353	5.442275	2.957721	1.970725	2.232667
HORVU1Hr1G014950	14.75594	11.67655	14.13766	10.80895	8.309005	7.821392
HORVU2Hr1G062730	1.773899	0.996332	1.589142	2.215584	2.323207	2.656017
HORVU4Hr1G059170	3.369834	2.703438	6.417459	2.962554	1.847111	3.126348

HORVU0Hr1G006720	1.816814	1.545128	2.386308	0.581669	0.504001	0.640682
HORVU4Hr1G064860	17.1348	15.75732	16.44753	17.53685	21.15781	20.79303
HORVU2Hr1G036050	12.82017	12.72139	14.87839	20.76358	23.50785	23.66107
HORVU2Hr1G091370	3.743102	4.606016	4.938732	4.232061	3.968895	4.288974
HORVU3Hr1G077540	33.16302	30.53824	32.31464	19.37496	13.23834	15.39283
HORVU2Hr1G055870	28.54033	31.86578	27.84518	34.25193	37.77531	38.74975
HORVU1Hr1G037590	25.04111	21.63852	24.96936	20.60385	19.78989	16.89864
HORVU6Hr1G019380	15.52519	15.2166	14.31696	19.72036	11.44052	13.83258
HORVU2Hr1G023170	3.228894	2.976251	3.36329	8.037091	9.498156	9.584649
HORVU3Hr1G006620	10.35477	8.923871	13.64302	20.17345	5.242754	15.74763
HORVU5Hr1G044640	16.11397	18.33201	18.85841	11.16489	9.692815	10.75165
HORVU5Hr1G115710	4.526696	4.400532	4.779334	3.133854	3.635322	3.782351
HORVU4Hr1G065740	4.291869	4.093902	5.917679	5.330205	5.831501	6.226369
HORVU0Hr1G021940	14.42663	13.06104	13.29337	11.54474	10.13747	11.94632
HORVU3Hr1G073040	11.73441	11.88365	12.97905	12.43487	15.30821	13.9428
HORVU7Hr1G077900	8.591943	7.628568	9.70377	9.657965	12.78131	10.94385
Hordeum_vulgare_newGene_5526	8.305846	10.26357	11.82675	10.89359	11.44831	11.42857
HORVU6Hr1G021250	0.513126	0.498782	0.414239	2.618099	19.98337	11.74177
HORVU4Hr1G020230	3.068333	1.129393	3.332956	0.499491	0.151347	0.424722
HORVU4Hr1G050060	1.024257	2.157272	1.342385	2.045497	3.317627	3.416432
HORVU1Hr1G094880	1.590008	1.836937	1.422872	1.519663	9.783437	10.03123
HORVU6Hr1G090990	119.4386	104.7729	112.7351	70.58493	53.57127	60.00179
HORVU7Hr1G089160	5.220607	4.463957	6.359245	0.381542	0.364276	0.733628
HORVU7Hr1G026030	2.547994	2.5111	3.592297	6.055639	5.887209	8.910916
HORVU7Hr1G012300	1.024603	0.846387	1.083354	0.670623	1.191012	1.288948
HORVU2Hr1G121020	19.48757	17.3278	18.22368	13.53423	14.07921	13.46768
HORVU2Hr1G109690	1.594138	1.330791	1.432145	1.02919	0.953742	1.39203
HORVU7Hr1G081350	21.33436	18.08221	19.9137	26.82414	25.54559	28.96146
HORVU3Hr1G092580	18.15098	13.2272	18.53498	20.09588	19.2491	23.05086
HORVU4Hr1G053480	0.869699	0.739077	1.142695	0.67409	1.750087	1.54708
HORVU1Hr1G045670	4.134519	4.43309	3.80771	3.445817	2.447003	2.285231
HORVU3Hr1G019260	8.484483	8.67129	9.309362	9.898359	12.74514	13.33274
HORVU4Hr1G076610	7.188916	8.387378	6.862609	22.56502	33.20121	29.74952
HORVU2Hr1G072570	8.650692	7.959636	10.8186	12.91857	8.849744	10.20685
Hordeum_vulgare_newGene_8179	0.712859	0.661744	1.082552	1.478249	1.40207	1.967667
HORVU6Hr1G028850	0.638283	0.592669	0.69467	1.299544	1.317964	1.473681
HORVU2Hr1G030040	2.370773	2.347882	2.615074	2.950123	5.688263	5.152094
HORVU2Hr1G019590	2.604749	4.359963	6.239192	9.979456	8.157418	8.335395
Hordeum_vulgare_newGene_10380	1.358726	1.209	2.247341	1.194715	1.041306	1.074627
HORVU7Hr1G089290	20.0095	18.87187	24.30002	25.91439	75.77928	62.97452
Hordeum_vulgare_newGene_10388	43.62013	49.0424	45.89527	40.72665	50.3539	50.36684
HORVU2Hr1G048010	0.06092	0.148658	0.101147	0.573629	3.278951	4.320079
HORVU5Hr1G100910	53.63689	60.80329	80.15244	30.00916	27.28935	30.93996
Hordeum_vulgare_newGene_10653	6.498984	6.409599	10.81396	5.708861	7.976934	8.160955
Hordeum_vulgare_newGene_10652	8.180504	12.30881	10.69786	6.690193	7.956591	9.741843
Hordeum_vulgare_newGene_10650	6.115854	5.385133	6.381872	7.149922	7.151213	6.710943
HORVU2Hr1G031310	66.31521	48.43032	72.3894	66.13838	48.64019	59.68016

HORVU3Hr1G098400	5.087922	4.143902	4.810182	3.261568	2.089461	2.595023
HORVU2Hr1G122590	11.31368	8.602527	11.99946	7.815907	3.979181	5.793761
Hordeum_vulgare_newGene_11380	16.90042	10.71116	13.12425	23.74856	26.38882	28.60735
Hordeum_vulgare_newGene_11381	0	0	0	2.350157	2.952615	1.726854
HORVU3Hr1G074770	32.6931	32.0719	28.38284	22.18266	14.30188	16.54867
HORVU3Hr1G085570	1.194791	1.245815	1.374731	0.953931	2.166413	1.370989
HORVU2Hr1G115630	2.401003	2.9856	2.047771	2.203384	3.730798	3.28062
HORVU2Hr1G019620	1.252141	1.658283	1.16224	1.017797	1.10294	1.343819
HORVU4Hr1G067740	24.18054	27.3146	22.11847	38.68924	29.64065	37.27004
HORVU5Hr1G077680	2.697582	2.806736	3.537653	10.54429	10.23642	10.90583
HORVU7Hr1G073100	6.293807	5.591485	5.131047	4.779068	7.528443	6.244284
HORVU1Hr1G028570	15.10291	13.96076	10.65823	14.82836	15.47676	20.11104
HORVU7Hr1G087190	35.95535	32.69261	38.66191	40.34225	59.15782	60.4308
HORVU2Hr1G122040	32.03334	36.01539	38.94798	3.431592	0	1.312687
HORVU7Hr1G082110	13.765	11.65248	13.48464	12.9291	12.17759	13.02285
HORVU3Hr1G094860	8.208854	7.721369	8.548773	9.016532	10.39269	9.62634
HORVU2Hr1G025810	3.892	2.389588	4.417105	12.16923	11.15525	9.384054
HORVU2Hr1G118060	0.951978	0.722622	1.50767	2.499731	2.594012	4.648557
HORVU6Hr1G050960	1.970954	0.66691	3.287518	3.990167	4.887596	4.579733
Hordeum_vulgare_newGene_6701	5.1698	4.357909	6.369681	5.071371	11.04566	12.52818
HORVU2Hr1G022450	6.116122	8.867151	7.008694	6.270928	6.908923	7.235553
HORVU3Hr1G011470	5.923597	5.23397	6.287846	2.15771	2.861845	2.733999
HORVU7Hr1G033430	53.22384	45.77481	56.56865	35.53559	15.1159	12.05181
HORVU3Hr1G116300	0.476803	0.623757	1.556166	0.873983	1.531621	1.982519
Hordeum_vulgare_newGene_11522	16.05851	19.77424	18.26726	0.030248	0	0.023576
Hordeum_vulgare_newGene_11520	2.785622	3.432074	3.598404	5.245101	4.708336	5.514019
HORVU3Hr1G075550	21.30084	17.33173	22.51748	23.26583	23.1234	28.38328
HORVU7Hr1G035870	2.375509	1.650844	1.800138	0.649168	0.631188	0.840151
HORVU6Hr1G077270	0.07151	0.177044	0.203014	0.052818	5.880109	4.677398
HORVU3Hr1G094240	0.699248	0.95942	1.15764	2.116601	1.454313	1.886155
Hordeum_vulgare_newGene_5672	25.2251	21.90183	28.35025	27.56051	10.08776	11.77007
HORVU5Hr1G037580	12.81645	10.44021	19.64327	15.12116	30.72903	25.40137
HORVU2Hr1G123310	1.265891	0.953094	1.029288	5.42781	9.2108	8.644777
HORVU2Hr1G029480	6.954438	6.386135	7.068603	10.35375	38.11501	31.00583
HORVU5Hr1G043600	5.502329	5.245576	5.353111	10.5471	9.803608	10.05431
HORVU1Hr1G084450	2.3044	2.518993	2.886334	4.938718	9.164059	10.172
HORVU2Hr1G117560	0	0	0	10.53819	9.981381	16.29503
HORVU3Hr1G056400	1.940851	1.126691	1.101902	3.144565	5.138334	5.574741
HORVU6Hr1G087400	1.355861	1.385649	3.091514	0.51316	0.12373	0.471235
HORVU1Hr1G042870	1.09968	1.260495	1.456166	3.283986	3.092132	4.379217
Hordeum_vulgare_newGene_7257	1.00517	1.166015	0.91585	1.557658	1.806397	2.252227
HORVU6Hr1G054010	3.746924	2.763295	2.924972	3.465133	2.719677	2.77897
Hordeum_vulgare_newGene_7255	1.082745	1.277346	0.572436	1.732419	1.569277	1.230244
Hordeum_vulgare_newGene_7253	2.068877	1.139515	2.497377	1.335316	2.922559	1.737975
Hordeum_vulgare_newGene_7251	1.206505	1.162521	1.331125	1.75385	2.223012	2.375593
HORVU0Hr1G024130	1.976928	2.349503	1.687692	1.218183	7.92176	6.69036
HORVU1Hr1G042560	3.649742	3.562198	4.223892	3.424738	5.574752	5.937701

HORVU6Hr1G095320	5.39611	6.095354	6.446893	2.741221	3.825415	3.744847
HORVU3Hr1G063680	14.48828	14.37007	10.30598	4.549801	1.516386	2.682791
Hordeum_vulgare_newGene_7775	0.280647	0.406285	0.544375	8.785561	11.307	10.04009
HORVU6Hr1G055760	2.424052	2.64288	1.331617	1.12262	7.847086	6.514063
Hordeum_vulgare_newGene_6123	6.481033	6.79067	5.560833	13.23835	11.88755	14.84703
Hordeum_vulgare_newGene_6121	0	0	0.010034	9.544535	7.366621	10.42749
HORVU2Hr1G015140	14.61366	9.750782	13.26729	1.875703	0.381843	0.672766
HORVU3Hr1G081640	0.717375	1.117108	1.499631	3.481126	2.215709	2.73121
HORVU5Hr1G084690	1.340824	1.029449	1.068384	1.47819	1.975908	2.015805
HORVU2Hr1G086810	3.25906	2.797704	2.97619	5.174943	7.3823	6.153899
HORVU6Hr1G059760	6.402172	3.800808	6.669641	9.000264	11.72998	12.30454
HORVU1Hr1G075900	1.829864	1.203436	2.271207	8.914859	28.47207	23.1882
HORVU2Hr1G087490	2.267956	1.947443	2.516818	3.247477	2.728314	2.5655
HORVU3Hr1G017160	15.04107	8.764255	12.09495	9.764603	11.41361	10.19822
HORVU6Hr1G034630	2.632446	2.757736	3.279267	16.12577	36.62516	34.14379
HORVU7Hr1G008690	75.51285	73.61585	68.13581	86.87355	70.64055	78.87665
HORVU3Hr1G060080	15.75034	15.55813	12.87783	10.03781	11.11183	11.83803
HORVU0Hr1G020260	5.349306	6.022623	6.220765	12.00847	11.82177	11.73896
HORVU2Hr1G079900	2.186805	2.403431	3.167137	0.015149	0.01335	0
HORVU5Hr1G107230	14.59858	13.03558	14.56953	3.754134	2.274408	2.368898
HORVU4Hr1G026160	2.012063	1.870557	2.653639	1.036127	1.643921	1.432442
HORVU3Hr1G050760	5.494865	6.125519	7.340675	5.19208	10.68522	11.92393
HORVU6Hr1G053680	16.34704	17.07883	16.37163	37.43727	55.38074	70.44215
HORVU1Hr1G068020	2.00103	0.961882	1.516286	0.410661	0.609492	0.685124
HORVU2Hr1G033070	11.55313	10.02816	11.41083	26.74461	54.0098	47.06958
HORVU4Hr1G054000	2.142868	2.606714	2.852546	1.78012	1.577469	1.754159
Hordeum_vulgare_newGene_2339	1.889857	1.567593	1.726727	0.835768	0.481579	0.441187
HORVU5Hr1G074220	0.893159	0.718878	1.197452	3.201997	2.633714	3.616215
HORVU3Hr1G052400	15.85423	14.51741	12.96113	24.81011	27.63893	26.03326
HORVU2Hr1G046530	7.58656	9.040024	11.70916	13.79468	22.90354	26.50183
HORVU3Hr1G114920	1.247887	1.205093	1.175723	3.343757	4.363394	4.269757
HORVU3Hr1G096830	11.46272	10.75848	10.3992	11.25576	52.15378	50.17245
HORVU7Hr1G078460	2.296323	0.93737	2.205936	2.642393	1.449753	1.253846
HORVU0Hr1G018320	0.357029	0.320012	0.322198	45.51005	31.34485	30.74337
HORVU3Hr1G005290	1.43665	1.150352	1.629109	2.727713	2.838329	3.060705
Hordeum_vulgare_newGene_9344	1.906455	1.670977	1.408987	1.683259	1.340177	1.60617
HORVU2Hr1G062700	0.83642	0.616589	0.840924	1.875161	3.270763	2.627947
HORVU1Hr1G077770	8.975471	12.3792	8.239591	5.026959	2.769989	3.92816
HORVU5Hr1G078410	0.987187	0.782576	1.311625	1.299282	2.282024	2.652797
HORVU2Hr1G086360	106.466	102.3192	142.8362	127.4072	266.4551	233.0812
HORVU3Hr1G080930	23.22415	17.67841	18.15166	17.78919	26.83923	33.92069
HORVU2Hr1G030520	2.919906	3.726332	4.616542	7.714571	11.44459	11.28671
HORVU4Hr1G011340	3.346999	3.725334	3.980649	5.365667	7.24639	6.642884
HORVU7Hr1G108960	168.939	151.7996	180.4854	180.5788	132.8423	142.6912
HORVU3Hr1G016610	2.171449	3.068059	2.234259	4.165347	6.612507	5.947164
HORVU2Hr1G070700	6.471395	4.81888	5.923473	0.559723	0.159756	0.264718
Hordeum_vulgare_newGene_630	51.07829	49.68127	47.92018	35.18708	38.54152	34.02763

HORVU4Hr1G083750	3.752356	5.607689	4.865101	1.914391	3.469819	3.478359
HORVU4Hr1G052730	53.51999	59.49138	62.25644	35.90485	31.33248	34.69302
HORVU1Hr1G061960	29.21131	23.43564	34.41702	22.72475	38.5242	37.23738
HORVU7Hr1G096690	1.530406	1.289424	2.052391	0.321876	0.432049	0.595294
HORVU6Hr1G064610	2.171018	1.569211	2.573955	2.460737	2.060691	2.183333
Hordeum_vulgare_newGene_8433	5.371893	6.269759	6.255612	4.103195	3.800051	3.857079
Hordeum_vulgare_newGene_8432	9.988283	8.059645	10.75276	6.609234	6.747338	6.112571
HORVU2Hr1G036570	8.95384	6.691725	7.800936	2.159219	0.860088	1.754988
HORVU4Hr1G005460	10.54283	8.275493	13.44405	6.792662	9.547584	9.006091
HORVU7Hr1G037180	3.486655	3.905663	3.030735	7.118023	4.680957	5.625096
HORVU2Hr1G004540	10.68448	7.541188	14.34337	4.205226	1.055939	4.334418
HORVU4Hr1G043840	2.043364	2.420977	2.398528	4.14413	5.795467	5.62563
HORVU5Hr1G069710	24.96434	29.2771	25.60485	30.52981	25.07741	27.81097
HORVU3Hr1G033700	1.762142	2.153638	1.994192	2.604104	2.655501	2.248924
HORVU6Hr1G068100	2.661327	3.451368	5.7207	1.685274	0.648902	1.341726
HORVU0Hr1G002950	0.468735	0.165228	0.253455	1.695764	3.130736	3.090741
HORVU5Hr1G035960	12.13186	15.29021	13.94845	11.8138	11.46504	11.6866
Hordeum_vulgare_newGene_4195	6.578193	7.66523	7.395182	7.723358	9.443847	9.913331
HORVU6Hr1G020080	252.8211	321.0363	382.3534	78.13657	12.70525	38.1151
Hordeum_vulgare_newGene_4198	0.272746	8.584736	0.910875	12.2113	0.081537	0.121146
HORVU7Hr1G026140	1.353583	1.221917	1.607836	2.643004	4.028587	3.870967
HORVU5Hr1G058780	30.77524	27.88187	26.08832	33.46015	13.89557	17.6722
HORVU5Hr1G066720	4.438929	2.80049	5.890277	0.150685	0.036564	0.299484
HORVU7Hr1G006520	2.492981	3.402482	3.490778	13.31207	16.07606	18.64496
HORVU2Hr1G098380	1.088313	0.92919	1.156038	2.844032	5.885463	6.522659
HORVU5Hr1G043380	12.91291	14.71657	15.29486	17.40983	20.97685	21.78778
Hordeum_vulgare_newGene_1019	3.718335	3.277563	4.886178	2.585914	4.002681	3.503542
Hordeum_vulgare_newGene_1016	2.89164	2.321115	2.202714	1.940132	0.569474	1.076459
HORVU2Hr1G068550	11.76107	12.94219	15.36857	13.61032	17.62763	18.38697
HORVU3Hr1G049790	18.02909	17.84825	24.64164	21.52662	26.77335	28.76746
HORVU5Hr1G023880	9.15019	9.975401	10.02052	16.7186	20.53138	22.82732
HORVU6Hr1G066610	6.886337	9.508479	11.58084	0.068187	0.979949	0.856185
HORVU1Hr1G064870	8.514631	8.727958	9.574589	34.9862	61.36089	68.19064
HORVU4Hr1G019230	4.39433	5.626427	7.402614	3.308865	4.139911	4.775811
HORVU2Hr1G046590	15.22409	13.6406	15.8312	15.86989	25.05574	24.39318
HORVU7Hr1G048900	41.55694	33.88206	44.01844	45.12201	35.72607	33.89683
HORVU6Hr1G062970	4.171824	4.180311	4.493897	0.959703	0.866654	1.106034
HORVU1Hr1G063470	4.423838	3.948761	4.256976	4.239472	2.22921	3.013059
HORVU1Hr1G078340	31.38957	28.84759	32.53364	38.4516	38.87113	35.91777
HORVU0Hr1G028010	3.662428	4.37469	4.397545	3.173283	3.736687	3.778338
HORVU1Hr1G057520	3.999082	3.561679	4.88849	4.840551	7.540735	7.10873
Hordeum_vulgare_newGene_4228	3.571933	5.495114	5.160017	1.908791	1.504611	1.426645
Hordeum_vulgare_newGene_4229	0	0	0	2.258672	2.035583	2.117781
HORVU0Hr1G006520	0.950021	1.508773	1.221714	1.774777	3.041313	2.391757
HORVU4Hr1G038750	6.724561	6.446806	6.403175	5.135754	8.826104	10.34391
Hordeum_vulgare_newGene_4221	0.903916	1.284988	1.533463	1.11712	2.105761	1.941637
HORVU1Hr1G064290	1.418631	1.782194	1.347474	4.391713	6.065469	6.315035

HORVU0Hr1G000960	1.553772	1.116686	1.484359	5.080571	2.72343	4.599653
HORVU5Hr1G059550	16.96961	16.17025	14.50222	15.90337	16.09747	17.48335
HORVU2Hr1G079240	4.648732	3.56953	6.287589	4.91913	5.406379	5.55099
HORVU6Hr1G064620	99.80028	75.76227	88.7794	208.9508	141.7573	155.5282
HORVU2Hr1G082870	1.193458	1.568977	1.732253	1.524105	1.925887	1.669272
Hordeum_vulgare_newGene_7440	0.960552	0.981386	1.593006	1.571497	2.52955	2.133142
Hordeum_vulgare_newGene_6439	1.167679	1.241375	1.432352	1.869555	2.155458	2.187102
HORVU6Hr1G085600	2.139277	1.957269	2.960158	8.582771	9.723973	12.22098
HORVU4Hr1G008980	1.385233	0.932656	1.409479	2.54422	2.803218	2.667617
Hordeum_vulgare_newGene_7448	4.359433	4.554457	5.320945	4.484386	3.041721	4.144153
HORVU2Hr1G077940	2.255569	2.129014	2.481071	3.022125	3.604996	3.845338
HORVU5Hr1G100200	215.8195	233.2561	291.3374	627.9231	1162.937	1063.265
HORVU7Hr1G049340	157.7339	92.10035	173.4601	232.9912	275.3562	358.5474
HORVU1Hr1G079960	1.078427	1.140736	1.778565	0.438722	0.578178	1.353249
HORVU5Hr1G042400	1.109457	1.29759	0.892903	2.315717	3.400582	3.170101
HORVU6Hr1G029540	15.95417	13.33132	18.2054	23.77469	25.07822	20.19076
HORVU2Hr1G091910	5.187443	3.433561	4.309043	7.929249	2.922505	3.71789
HORVU4Hr1G007170	2.433178	3.359701	4.031781	3.285218	3.428245	3.823189
HORVU5Hr1G120550	16.78297	19.55838	21.3471	17.02876	13.27044	13.03355
HORVU1Hr1G069060	17.01084	18.95094	19.96448	21.92655	27.6471	27.72125
HORVU4Hr1G000230	1.42366	1.150429	0.993332	1.826515	2.617056	2.19911
HORVU1Hr1G033470	9.250357	8.602199	10.54833	8.150565	7.893712	8.585558
HORVU3Hr1G038060	1.712221	1.737518	1.79163	3.94174	3.038119	4.361709
Hordeum_vulgare_newGene_8707	2.400803	2.469148	1.60976	2.417	4.293181	3.741107
HORVU4Hr1G056950	13.2841	11.59685	14.88771	9.702891	4.076917	4.6194
HORVU0Hr1G000030	17.6654	12.80509	18.4933	13.72967	14.41422	12.74719
HORVU1Hr1G017480	21.63219	20.72524	26.62846	18.40915	15.95033	17.91211
HORVU5Hr1G086520	6.191579	6.865402	11.21008	0.958052	0.719636	2.384202
HORVU0Hr1G035920	0.160679	0.284395	1.477071	1.467865	2.001887	3.159832
HORVU5Hr1G088120	1.292915	1.662516	1.598868	1.850157	3.069669	2.932458
HORVU1Hr1G093490	3.314574	2.885928	2.670661	3.066115	4.825202	4.427674
HORVU1Hr1G080370	29.01617	26.08229	40.88807	30.86298	61.43659	59.85723
HORVU5Hr1G093350	10.44149	10.31991	9.750099	8.871048	7.208225	8.730889
HORVU5Hr1G123390	5.209698	6.706679	5.387037	7.389636	8.458625	8.60459
HORVU5Hr1G009650	3.382058	4.592323	5.60788	8.49364	13.55875	14.44958
HORVU7Hr1G011320	1.921795	4.177178	2.101869	2.867004	3.674954	4.857179
HORVU2Hr1G030610	3.723655	2.725875	4.35837	2.85525	1.941904	2.217399
Hordeum_vulgare_newGene_6798	11.80333	8.782937	15.01575	7.668474	11.50427	12.11425
HORVU1Hr1G093720	67.46326	59.73375	77.89497	43.62395	49.57683	53.51659
HORVU5Hr1G045990	5.712969	6.831192	8.78537	3.842371	5.016507	4.79843
HORVU5Hr1G055110	1.53983	0.182441	0.028014	1.532663	1.142495	1.671436
HORVU5Hr1G117990	1.631089	1.201303	1.318708	3.782193	5.155923	4.377001
HORVU7Hr1G040730	1.371502	0.345709	1.05942	4.85301	0.459191	3.0043
HORVU6Hr1G004770	4.336368	3.178728	4.612623	0.005599	0.008028	0
HORVU7Hr1G050340	0.898478	1.110649	1.011112	3.052384	4.790101	5.130735
HORVU4Hr1G011620	3.42428	3.189404	4.400281	3.32776	3.746201	4.081952
HORVU1Hr1G081020	2.703571	2.744118	3.338932	1.631052	3.238832	3.337146

HORVU6Hr1G084390	2.390896	2.46456	2.485888	2.867258	4.411157	4.051794
HORVU5Hr1G122040	0.065279	0.10937	0.048455	0.74803	6.75345	4.987371
HORVU4Hr1G083070	16.45291	11.00469	16.62631	11.78347	10.46095	10.59827
HORVU4Hr1G063190	10.90658	9.868726	13.64449	24.10957	24.93598	23.12593
HORVU2Hr1G056360	83.76271	63.03551	96.30562	89.12191	148.9206	136.8034
HORVU4Hr1G069760	18.41368	20.74115	17.97163	16.56824	19.9429	18.41153
HORVU2Hr1G047500	7.711801	7.093999	7.353079	4.938768	5.963727	5.885096
HORVU5Hr1G058890	3.865665	3.55843	4.189626	4.441506	5.917188	5.9084
HORVU1Hr1G018700	2.378425	3.067307	4.100096	3.069916	3.497135	3.601013
HORVU6Hr1G087200	1.423498	1.349004	2.064118	4.183804	3.327082	4.495063
HORVU6Hr1G020090	2.316464	2.893256	2.975949	2.993611	3.944188	3.88743
HORVU3Hr1G066940	1.696343	1.226218	1.316907	1.240379	0.504586	0.336577
HORVU4Hr1G084760	50.12462	45.88395	62.77348	46.42187	79.03916	75.75271
HORVU2Hr1G066080	8.821559	7.256363	8.653935	9.179398	8.761236	10.49496
HORVU5Hr1G114740	0.531414	0.645597	1.792005	2.442683	1.653593	0.123403
HORVU3Hr1G086610	5.429754	5.841933	9.059854	11.49513	51.86584	40.57566
HORVU1Hr1G091650	1.01639	1.139715	1.356162	1.39251	3.249741	3.887706
HORVU2Hr1G038860	2.528185	2.336076	2.597472	3.365246	3.991328	4.34333
HORVU7Hr1G095190	1.335812	1.374005	1.363136	1.090965	1.383222	1.497902
HORVU2Hr1G012930	5.007399	5.541908	6.027036	7.886737	8.722971	9.027687
HORVU7Hr1G084230	0.201242	0.455639	0.603226	1.72376	3.182497	2.252092
HORVU2Hr1G080820	4.85212	4.437886	3.411946	3.756066	6.431607	5.50002
HORVU7Hr1G046380	2.890944	2.767402	2.681172	1.866974	2.104873	1.377081
HORVU0Hr1G001050	7.702149	7.906576	10.5257	5.636309	6.884887	6.840806
HORVU2Hr1G018340	1.372474	1.206396	2.136493	1.064506	1.80231	2.720911
HORVU7Hr1G109560	2.964901	2.1906	2.413292	3.166823	3.819133	3.469005
HORVU2Hr1G016030	1.540181	1.895933	3.237241	0.614018	1.688347	1.848349
Hordeum_vulgare_newGene_12888	15.06136	4.856049	11.76547	0.770116	1.485	2.983205
HORVU4Hr1G062310	10.70679	9.315967	10.99552	23.84165	22.53676	24.36548
Hordeum_vulgare_newGene_12883	0.23095	0.24022	0.483848	1.173101	1.726274	3.000114
Hordeum_vulgare_newGene_12884	7.904399	6.811567	7.955828	14.93029	20.5446	20.73799
Hordeum_vulgare_newGene_12885	0.568529	1.16989	2.001981	0.899598	0.720314	1.028169
Hordeum_vulgare_newGene_12887	19.74575	20.53444	19.214	28.77937	45.00479	47.30205
HORVU4Hr1G087230	0.303581	0.098254	0.207966	7.365876	12.93602	12.1695
HORVU2Hr1G106420	0.920299	0.831272	0.865027	1.288852	2.694086	2.377181
HORVU6Hr1G026060	2.428913	1.938835	2.173284	2.369033	2.601808	3.490233
HORVU0Hr1G014570	2.716832	3.011447	0.987904	0.009189	0.076469	0
HORVU4Hr1G019570	38.31184	34.36021	33.28296	105.4101	175.3928	175.3902
HORVU1Hr1G058500	0.931049	0.846259	1.333438	7.37048	21.79228	18.70211
HORVU5Hr1G024590	3.781403	3.631476	4.434102	5.136982	7.487849	7.687356
HORVU1Hr1G043660	27.19884	26.96954	28.14814	22.86489	27.40997	27.89394
HORVU6Hr1G015480	4.97928	8.074025	8.195733	4.742877	5.24962	6.662962
HORVU0Hr1G022510	41.44473	33.80745	37.97718	26.61675	20.92814	21.91187
HORVU2Hr1G117810	2.103057	2.454872	2.152304	2.660207	3.229028	2.676587
HORVU2Hr1G099330	5.950183	4.684805	5.519639	8.172812	7.317009	8.673829
HORVU4Hr1G078350	5.570692	6.087279	6.828716	3.29517	3.748021	3.366647
HORVU2Hr1G096100	6.381139	6.469156	6.491266	4.110641	5.676307	6.137077

HORVU3Hr1G005540	2.452347	1.878419	3.036624	0.039286	0.027745	0.030089
Hordeum_vulgare_newGene_2139	0.063079	0.054137	0	15.66206	16.81876	19.35563
HORVU1Hr1G008620	38.28489	37.51934	40.02504	42.02471	48.63879	50.05484
HORVU6Hr1G053110	6.334088	4.351837	9.735977	3.281798	4.883235	4.568004
HORVU2Hr1G019120	0.189983	0.488217	0.681994	1.659579	1.9892	2.330263
Hordeum_vulgare_newGene_2687	7.816063	7.697471	8.249204	5.482508	5.363367	5.534811
Hordeum_vulgare_newGene_2684	16.84033	18.41352	17.51826	21.46551	18.43563	20.28497
HORVU7Hr1G008320	22.78482	17.33164	25.66309	37.96385	185.1633	136.1308
HORVU4Hr1G070120	2.632122	2.13388	2.85937	2.774779	2.364245	2.384016
Hordeum_vulgare_newGene_2689	38.23729	33.04007	41.1311	29.15915	34.34173	32.83487
HORVU0Hr1G010050	1.211273	1.200692	1.308022	1.488523	1.750216	1.476375
HORVU1Hr1G000430	1.365691	1.431785	1.285557	2.967498	3.47294	3.990188
HORVU2Hr1G049240	2.37297	1.754783	2.487234	4.328598	4.694079	5.151996
HORVU2Hr1G060640	3.270374	3.789041	4.848968	3.31832	4.483053	4.994945
HORVU5Hr1G013770	0.223063	1.425254	0.846059	2.111224	1.936322	2.133463
HORVU4Hr1G079400	14.91486	13.85351	14.81232	22.89266	25.99818	26.89108
HORVU6Hr1G021040	1.383586	1.40558	1.659473	1.414975	1.655216	2.487721
HORVU4Hr1G014270	11.96259	9.881625	13.68825	9.913234	7.744696	7.746209
HORVU3Hr1G065320	3.043916	2.543584	5.217073	2.107615	1.361377	1.737372
HORVU4Hr1G083800	0.813194	0.513651	0.442736	1.303122	1.703078	1.38115
HORVU2Hr1G018830	18.43251	9.397774	7.817414	45.11979	15.88887	17.93201
Hordeum_vulgare_newGene_13348	0	0	0.009057	1.796951	1.98233	2.470721
Hordeum_vulgare_newGene_13827	2.877882	2.94257	2.281733	2.731037	1.490638	3.23267
HORVU1Hr1G000290	53.14962	51.70502	48.19392	40.37201	45.16826	46.97064
HORVU3Hr1G073230	50.91322	60.69087	74.15343	39.70513	50.56456	53.91916
HORVU5Hr1G084860	0.462357	0.583051	0.974281	2.127248	2.882879	3.719965
HORVU6Hr1G033980	27.5248	28.43502	37.25292	23.06563	35.85229	38.47224
HORVU2Hr1G097760	4.283705	4.851918	4.294526	4.301513	4.950199	4.745703
HORVU5Hr1G098770	0.837949	0.823073	0.917348	5.272532	13.19287	10.24393
HORVU4Hr1G024270	3.38691	3.042445	3.815384	5.660408	9.340209	8.785436
HORVU5Hr1G053470	17.0107	16.64965	17.29761	20.18869	25.61534	29.13212
HORVU7Hr1G012530	18.7032	17.70467	21.61177	22.67994	26.72111	29.44459
HORVU2Hr1G082540	11.26623	10.15702	11.26197	12.10584	14.9425	15.11016
HORVU7Hr1G059220	2.396686	2.304678	2.527281	2.824075	7.870614	7.245378
HORVU7Hr1G028530	2.600813	2.312621	3.388586	4.203887	5.758791	5.036463
HORVU1Hr1G014580	22.27296	20.12632	23.66052	33.90929	28.96922	28.60563
HORVU4Hr1G071380	9.747256	9.366916	14.31853	8.626474	12.57544	11.99693
HORVU6Hr1G017220	2.865748	3.323067	3.714906	3.034336	4.553436	5.540035
HORVU3Hr1G061800	12.16872	9.9066	14.87102	7.937116	5.764821	6.713013
HORVU2Hr1G119500	4.791383	2.163532	5.469644	0.60604	0.213778	0.458583
HORVU5Hr1G076560	4.047565	3.395715	5.483754	5.124772	10.84552	9.634485
HORVU7Hr1G027240	23.13461	21.91344	31.23267	17.09148	17.96559	18.06244
HORVU7Hr1G082090	0.43706	0.387358	0.353407	2.063041	3.740148	3.408449
HORVU4Hr1G063830	8.344438	5.481985	6.836643	11.53447	11.85854	12.73352
HORVU1Hr1G031010	2.947999	3.374729	3.920732	4.804426	6.394403	7.182336
HORVU2Hr1G073380	0.669382	0.618036	0.723416	1.189703	2.26595	2.089754
HORVU2Hr1G102730	2.460147	2.185827	2.426529	2.559058	2.65103	3.173202

HORVU0Hr1G020800	1.071599	1.060156	1.633558	1.187185	2.08177	1.713183
Hordeum_vulgare_newGene_1569	4.981524	6.930799	5.55017	6.276765	3.890402	5.859908
HORVU7Hr1G080780	2.156547	1.610562	1.949248	0.772015	0.749444	0.655036
HORVU2Hr1G029720	3.190308	3.346972	3.724729	5.055059	6.559078	6.91042
HORVU6Hr1G071850	39.32756	33.0202	39.00027	29.02093	25.1609	26.61337
HORVU7Hr1G073870	5.200167	6.897998	7.990034	11.59926	15.88235	13.68597
Hordeum_vulgare_newGene_4429	8.391146	9.769703	9.87362	10.42291	7.234946	9.993551
Hordeum_vulgare_newGene_4422	1.07046	1.02916	0.858695	1.922714	1.6716	1.459316
HORVU6Hr1G064070	0.014449	0.006718	0	2.18092	2.507556	2.322899
Hordeum_vulgare_newGene_4424	16.62156	23.46427	23.29549	13.57524	9.193081	11.69377
HORVU3Hr1G113360	9.755835	9.65598	11.98591	6.178817	6.091408	6.472561
HORVU7Hr1G001030	0.525845	0.747927	1.084132	10.77514	11.02414	18.94837
HORVU2Hr1G021110	145.3673	107.7021	138.5404	175.3082	279.1977	250.2755
HORVU4Hr1G077060	0.627721	0.353949	0.409872	0.350857	3.019074	3.330759
HORVU2Hr1G071830	8.393111	7.983258	9.113152	9.507039	11.90468	12.30205
Hordeum_vulgare_newGene_7190	1.895295	1.34672	1.763632	2.09561	1.447568	1.491926
HORVU2Hr1G035680	9.440031	8.900352	11.01781	11.8586	4.593207	5.038176
HORVU5Hr1G087060	8.677941	7.611449	8.572617	8.57606	11.10747	11.82092
HORVU6Hr1G089250	0.127418	0.115796	0.152502	2.118727	10.36338	10.58309
Hordeum_vulgare_newGene_9040	2.159083	1.851679	1.955243	5.465343	4.532444	5.196417
HORVU5Hr1G099550	4.175188	3.918308	5.129702	5.634008	10.42372	8.376787
HORVU5Hr1G071840	10.9554	10.98259	12.76958	12.61846	17.37378	15.44878
HORVU7Hr1G031280	1.5399	1.401387	1.314443	2.90352	4.41932	4.255509
HORVU7Hr1G031130	73.23979	60.32204	76.4668	56.88606	61.91023	60.42185
HORVU3Hr1G010570	27.77534	22.68019	26.71686	17.72991	17.52608	18.95666
HORVU5Hr1G092200	12.38009	10.68149	12.1654	11.43818	14.97113	11.87742
HORVU7Hr1G039700	199.282	167.7666	157.1461	87.21765	79.40823	70.70287
HORVU6Hr1G054600	14.02173	13.29421	13.70165	12.21224	6.597474	8.835093
HORVU4Hr1G047940	6.439221	5.944223	7.14418	5.712407	7.302199	8.648441
HORVU1Hr1G017500	1.025279	0.713921	1.141347	1.635524	1.664339	1.213393
HORVU3Hr1G039480	3.043861	3.227642	4.144715	0	0	0
HORVU3Hr1G067750	0.995152	1.285915	1.865767	3.729804	6.302969	7.38977
Hordeum_vulgare_newGene_2968	2.236271	2.394057	3.186807	0	0	0
Hordeum_vulgare_newGene_556	4.871935	5.896558	7.041516	9.164845	12.78111	14.39897
Hordeum_vulgare_newGene_555	5.810146	6.987979	7.30221	4.522593	5.985519	6.287943
Hordeum_vulgare_newGene_554	14.68348	11.00883	18.10771	5.645569	3.766413	6.376024
Hordeum_vulgare_newGene_550	3.543261	3.570402	3.805789	2.249213	2.726744	2.610199
HORVU5Hr1G074610	8.023112	6.027645	9.4615	9.00202	6.774763	7.425274
HORVU3Hr1G033620	1.279469	0.953184	1.419147	9.115531	18.52464	16.32028
HORVU3Hr1G081050	1.342321	1.631757	1.310366	4.02512	6.94925	5.753675
Hordeum_vulgare_newGene_12695	1.146349	1.136718	0.856532	1.700317	1.790232	2.147875
Hordeum_vulgare_newGene_12696	2.513285	3.479285	1.573661	2.53524	1.474742	1.997182
Hordeum_vulgare_newGene_12691	2.450758	2.546736	1.995983	2.600173	2.080185	3.36921
HORVU7Hr1G028290	1.617811	1.400496	1.172144	25.02715	14.5368	17.23759
HORVU5Hr1G087680	3.8074	4.886383	5.288109	4.060595	5.339207	5.188223
HORVU7Hr1G017800	3.41342	4.398367	4.873416	1.779071	2.933933	3.345915
HORVU2Hr1G075030	2.572504	2.282227	2.754728	6.562486	10.23678	9.656608

HORVU0Hr1G022270	21.18446	20.70154	20.71898	11.57167	10.12	11.01646
Hordeum_vulgare_newGene_13122	8.580052	10.65701	9.840936	0.019484	0	0
HORVU1Hr1G070360	18.87063	16.50843	19.75309	22.94746	26.59566	26.76505
Hordeum_vulgare_newGene_13128	2.84562	2.818661	3.156236	5.072114	4.507183	4.123307
HORVU3Hr1G068170	7.836428	9.86237	7.7749	5.732552	16.0924	12.62402
HORVU5Hr1G023140	77.08431	61.47259	88.3543	92.43273	132.7555	126.4289
HORVU2Hr1G113180	41.55632	31.1925	54.79638	22.24404	32.27256	43.84519
HORVU7Hr1G030660	8.079454	5.591332	10.35609	3.703922	3.548278	2.6856
Hordeum_vulgare_newGene_4230	2.915162	3.568396	1.848394	2.272621	0	1.10221
HORVU0Hr1G003900	27.14349	22.33733	30.1363	21.18299	11.85202	11.01704
HORVU1Hr1G023350	1.088508	1.483362	1.146451	1.326361	2.473671	2.987549
HORVU3Hr1G032570	35.46399	27.25323	34.24087	27.04634	25.74709	24.13919
HORVU4Hr1G021010	9.851157	8.818456	9.277794	7.044553	5.586927	5.377315
HORVU4Hr1G070240	0.21946	0.322385	0.354592	5.831079	22.01456	20.19652
HORVU7Hr1G035450	7.501322	6.692758	8.002672	7.667043	10.19753	11.36161
HORVU2Hr1G005530	0.906572	0.916795	0.648014	5.31666	6.960515	6.862497
HORVU5Hr1G092160	37.64619	46.08971	58.16726	12.82766	0.753841	6.210945
HORVU5Hr1G095940	10.21598	9.390873	13.96886	8.817015	8.63571	8.130951
HORVU1Hr1G059900	814.7919	1049.111	1744.941	121.0314	0.511416	42.1556
HORVU0Hr1G004480	28.86446	33.7969	55.73633	32.65869	106.577	90.17315
HORVU7Hr1G048720	4.524863	4.259344	5.206859	6.542945	9.054451	8.170105
HORVU4Hr1G015800	4.183438	7.351258	4.199441	5.693503	6.816361	6.386598
HORVU5Hr1G094890	0.675554	0.790052	0.841118	2.127114	3.011077	2.870032
HORVU1Hr1G022400	2.923166	1.865325	3.944359	9.091656	25.28342	26.23245
HORVU7Hr1G097730	1.446549	0.466186	0.944684	0.969711	0.991942	1.184212
HORVU6Hr1G046910	16.46302	15.20597	16.80864	19.19736	22.79428	22.93088
HORVU0Hr1G003270	39.13344	36.97619	36.92109	89.01356	136.1519	134.209
HORVU5Hr1G063650	1.985969	2.375107	2.968516	4.304915	5.331337	5.754651
HORVU4Hr1G002700	1.734429	1.625894	2.279635	2.359097	3.244472	4.057735
HORVU1Hr1G021840	17.41703	19.81406	16.08158	36.03813	50.87055	47.76108
HORVU1Hr1G018020	1.720297	2.896495	1.793376	2.232991	2.099158	2.465437
HORVU1Hr1G009140	77.58074	84.40356	124.3493	9.169891	0	3.282547
HORVU3Hr1G016860	6.888567	4.062244	7.47577	1.242276	0.715129	1.743956
HORVU3Hr1G014790	0.166089	0.155943	0.094857	1.908173	3.586711	3.569518
Hordeum_vulgare_newGene_7986	0.702767	0.621685	1.016067	1.876468	2.607886	2.631608
HORVU5Hr1G051100	7.343112	8.049122	6.502952	8.830395	9.868496	11.29219
HORVU5Hr1G062300	1.967673	1.377313	3.572617	0.760973	0.504477	1.115375
HORVU0Hr1G033330	1.404699	1.175025	1.134712	1.723714	2.017136	2.074114
HORVU5Hr1G114500	7.932338	6.646304	8.140366	10.12486	10.46948	12.11805
HORVU4Hr1G088470	3.11394	3.146082	3.309716	3.509614	3.208311	3.522264
HORVU1Hr1G080060	31.12623	27.99983	35.90582	53.19491	75.34913	65.7966
HORVU2Hr1G085990	7.211893	8.460628	5.964422	20.69357	24.51351	20.83178
HORVU5Hr1G108670	39.12813	39.24241	40.05481	25.22626	4.787618	8.938265
Hordeum_vulgare_newGene_170	1.160578	1.60248	1.647038	2.37938	2.93649	3.216767
HORVU6Hr1G031330	14.26229	12.76322	16.55044	20.88741	14.53352	15.06889
HORVU1Hr1G073040	5.80601	3.866993	4.566067	7.853548	9.89725	10.47859
HORVU0Hr1G012630	3.792003	4.282901	4.714513	4.005806	4.102029	4.811917

HORVU1Hr1G063390	5.98066	5.284901	7.058881	12.93418	16.47736	16.25418
HORVU1Hr1G000450	1.450446	1.540176	2.749012	6.161118	0.986553	4.188888
HORVU1Hr1G022110	1.463458	0.821769	1.451445	1.786807	1.003682	1.901117
HORVU7Hr1G091770	1.050164	0.807673	0.889779	1.350169	1.972455	1.710417
HORVU5Hr1G092570	120.5484	123.7183	132.9904	100.8757	102.4164	99.3988
HORVU1Hr1G091710	3.027018	2.919139	2.996993	4.338412	3.837904	4.683527
HORVU6Hr1G036640	25.72026	24.18753	26.52176	29.2888	46.24059	44.73834
HORVU2Hr1G064000	13.56741	14.6589	12.67654	12.89863	11.62739	11.90607
HORVU2Hr1G043330	0.029412	0.078495	0.198065	0.715499	3.249672	3.065897
Hordeum_vulgare_newGene_1606	25.88294	21.1007	39.12743	21.16335	22.90478	14.50864
Hordeum_vulgare_newGene_1605	0.75384	0.591501	0.608156	1.546499	1.253092	1.363973
HORVU2Hr1G080480	2.578851	1.158061	2.212453	6.275659	9.722667	9.723622
Hordeum_vulgare_newGene_1602	5.558434	6.733199	5.070952	8.369259	2.622851	3.144547
Hordeum_vulgare_newGene_1600	0.505654	0.512151	0.439745	1.481945	1.782003	1.996621
HORVU7Hr1G099560	3.278388	2.76179	3.677612	2.161643	0.871135	1.768364
HORVU5Hr1G015220	0.980486	0.849664	1.267753	1.133513	1.206379	1.616622
Hordeum_vulgare_newGene_12405	61.57043	69.76566	87.84683	18.80732	12.34268	14.25702
HORVU2Hr1G016580	26.2099	22.17148	26.3675	34.77113	29.60058	32.47876
HORVU7Hr1G002220	73.85795	57.02962	76.30254	80.68009	112.2403	106.4632
HORVU2Hr1G001400	1.969482	2.97881	4.26071	1.475144	2.101681	1.582108
HORVU4Hr1G019000	2.244859	2.452924	1.627115	0.450551	1.041418	0.617556
HORVU5Hr1G056950	14.8359	13.97775	14.74965	28.71148	44.85517	45.93323
Hordeum_vulgare_newGene_8799	1.495022	2.40382	2.278102	0.722935	0.453881	0.657244
HORVU3Hr1G073190	2.412057	1.425365	2.053762	0.7683	1.704222	2.447702
HORVU1Hr1G067850	0.979341	1.836825	4.404004	0.579187	0.598232	0.730372
HORVU7Hr1G099380	9.194387	8.360815	9.399514	6.972332	9.770787	8.436051
HORVU1Hr1G086390	30.06888	29.46884	30.59281	32.62811	33.99523	33.83656
Hordeum_vulgare_newGene_8792	3.452562	3.737385	3.509482	4.152171	3.369023	4.884841
HORVU4Hr1G068890	12.90608	10.427	11.34396	8.207507	4.232282	4.940085
HORVU7Hr1G024270	4.04019	4.342765	3.472964	15.02777	14.33186	12.48641
HORVU2Hr1G053800	3.000837	3.80765	4.848031	6.357596	6.440782	7.283858
HORVU7Hr1G053310	6.845436	7.495139	7.281497	7.536382	8.192747	8.448891
HORVU4Hr1G001420	3.301232	2.922655	3.83458	2.943831	12.24336	7.694439
HORVU2Hr1G059620	120.6866	106.1164	132.3242	83.99289	87.61038	86.85824
HORVU3Hr1G078470	8.046822	8.031065	10.60051	6.059232	8.529341	8.86956
HORVU0Hr1G040400	1.886749	1.353066	2.286658	2.43341	0.272304	0.531071
HORVU2Hr1G022900	12.55419	15.83791	24.71902	9.00228	15.0692	13.83997
HORVU2Hr1G099640	2.622009	1.681457	3.362382	2.856519	3.569975	5.672111
HORVU6Hr1G081730	1.243681	1.613111	1.231446	3.895006	11.10802	11.43769
HORVU3Hr1G019070	1.761676	1.612558	2.156025	6.11738	11.23842	10.22882
HORVU7Hr1G105960	0	0.01741	0.04897	0.078906	6.203875	4.039676
HORVU2Hr1G093410	2.228317	1.849455	2.939291	3.11032	3.584911	4.50153
HORVU3Hr1G021950	9.300702	10.03072	7.437159	10.47935	16.73157	16.10109
Hordeum_vulgare_newGene_11449	1.304227	1.37223	1.119564	3.704142	4.266524	4.974974
HORVU2Hr1G027660	1.891782	1.756906	2.153513	2.284595	4.703801	3.465661
HORVU2Hr1G004190	2.554865	1.540301	2.980539	3.300409	3.009579	2.474608
HORVU6Hr1G047180	3.89976	4.726521	3.509356	8.57207	9.578868	8.51784

HORVU5Hr1G056040	61.54816	41.22967	82.05943	60.33462	20.35417	43.44428
Hordeum_vulgare_newGene_9817	3.868532	4.899855	5.85289	4.45969	4.988612	5.162678
HORVU2Hr1G050380	4.807057	5.609342	6.737373	7.461129	8.182933	9.119023
HORVU2Hr1G066330	6.126024	5.654131	8.018388	7.724466	8.612561	8.97741
HORVU3Hr1G100270	1.903192	1.335638	1.964431	5.290158	9.273995	8.974154
Hordeum_vulgare_newGene_11775	1.110486	1.406225	2.066137	1.301235	1.167774	1.562231
Hordeum_vulgare_newGene_11770	1.304836	2.019686	1.991276	0.996393	0.601781	0.720208
HORVU7Hr1G116400	7.318434	7.702594	7.759454	10.16974	8.778843	8.996552
Hordeum_vulgare_newGene_9470	2.088955	2.455297	1.745754	0.708617	3.596254	2.40357
HORVU5Hr1G008730	43.6297	49.65571	72.72773	39.74205	92.28938	84.41083
HORVU5Hr1G077010	1.441156	1.138377	0.745563	2.742199	3.114532	3.905966
HORVU0Hr1G040280	5.926255	7.295012	6.792616	7.330903	7.210795	7.811275
HORVU3Hr1G092170	29.31631	21.0124	32.79382	29.53594	44.74185	46.89724
HORVU6Hr1G085490	1.306301	1.5734	1.92831	2.757617	4.705255	3.823344
HORVU5Hr1G094280	8.67385	3.938973	3.453281	0.067627	0.019683	0.031336
HORVU6Hr1G085720	13.57932	13.35648	12.454	9.724867	11.78909	12.28075
HORVU5Hr1G123210	7.127885	5.489104	10.65348	5.803549	7.405284	8.323302
HORVU4Hr1G039810	8.058741	8.448027	11.54205	9.088608	14.50419	14.88775
HORVU2Hr1G090030	3.359729	4.201279	6.949565	0.17255	0.56126	0.096049
HORVU5Hr1G028340	7.889416	7.460671	7.705526	5.673703	5.662801	6.568475
HORVU5Hr1G092700	2.642696	2.160364	3.428036	7.371032	5.18927	6.3109
HORVU4Hr1G043620	2.734929	2.701308	2.7668	4.190355	5.683652	5.514039
HORVU2Hr1G078700	9.021216	9.477959	8.407429	8.9867	12.71378	12.62722
HORVU6Hr1G049050	5.727282	6.210277	6.759455	15.98709	28.28669	27.72629
HORVU3Hr1G038230	0	0	0	7.457713	6.415011	7.200843
HORVU6Hr1G092430	1.990482	1.060654	1.411081	7.041171	11.50327	9.625716
HORVU1Hr1G065820	10.30062	10.11421	12.20968	6.498006	1.929665	4.127329
HORVU3Hr1G057090	61.23663	60.71077	58.9873	85.78536	226.8035	199.7133
Hordeum_vulgare_newGene_16205	9.517547	10.71958	7.867219	8.022478	6.069998	7.34159
Hordeum_vulgare_newGene_16202	3.105991	4.732018	2.152582	0.219406	0.027029	0.057477
HORVU2Hr1G113930	44.98336	40.65466	45.79051	35.5734	27.5663	30.91843
Hordeum_vulgare_newGene_16209	4.395349	5.662045	6.108433	5.074934	4.630553	5.109666
HORVU2Hr1G010560	7.584013	7.586089	9.290377	15.90264	11.99111	16.31306
HORVU0Hr1G016380	1060.987	1374.337	1004.618	191.7879	75.71902	120.1405
HORVU0Hr1G038890	25.42369	22.5016	22.86433	20.24648	22.76133	23.22955
HORVU7Hr1G014140	24.68279	23.95509	40.06558	25.71808	59.66419	60.31594
HORVU3Hr1G022540	11.85278	10.2061	12.49647	15.24645	20.38218	20.24529
HORVU2Hr1G039210	8.800397	9.381933	5.624743	5.791161	28.79217	25.17791
HORVU3Hr1G098920	43.99661	36.15445	53.03268	34.42672	54.12595	51.34382
HORVU3Hr1G084990	1.918625	1.347881	1.648248	7.731121	47.65143	32.39925
HORVU5Hr1G109260	22.85302	13.1685	15.52648	15.39913	17.95238	26.28442
HORVU1Hr1G015050	14.52401	9.855595	16.84249	26.65971	12.68733	18.09511
HORVU1Hr1G067460	7.376242	5.534033	6.935618	9.309487	11.44113	8.575691
HORVU1Hr1G084800	47.39442	40.30166	52.07797	23.33815	17.38376	18.89551
Hordeum_vulgare_newGene_11258	0.853737	0.554797	0.731193	1.599056	1.196193	1.465561
HORVU5Hr1G106520	2.445075	2.888743	2.280268	1.599827	2.326669	2.030359
HORVU2Hr1G093610	0.388137	0.053892	0.317642	1.579023	3.378053	3.619373

HORVU7Hr1G041450	26.28263	31.97552	20.18142	24.84749	36.79157	31.47336
HORVU6Hr1G068230	16.20116	14.35686	18.35003	10.38427	6.704697	5.306083
HORVU6Hr1G050140	5.5154	5.31261	5.717936	6.065458	7.367183	8.154729
HORVU4Hr1G063760	3.026987	2.323125	1.938025	1.416548	0	0.546217
HORVU5Hr1G071280	9.859342	9.781606	12.36763	11.227	13.86611	14.26933
HORVU0Hr1G017160	6.034122	5.172674	5.637163	5.002812	4.899177	4.293544
HORVU7Hr1G100310	70.01511	64.61619	65.54286	110.826	74.54455	80.17891
HORVU3Hr1G063430	6.134985	5.901114	9.033319	2.258599	2.462378	2.483836
HORVU6Hr1G048090	1.545061	1.674497	0.762983	1.947561	2.151502	2.343308
HORVU5Hr1G025190	0.574618	0.813658	0.425563	0.429475	2.008912	2.042254
HORVU4Hr1G027750	1.707853	1.429134	2.199494	4.341105	1.916162	2.718223
HORVU7Hr1G035420	2.330417	2.333909	2.116856	1.790891	2.924537	2.663927
HORVU7Hr1G007940	19.71847	20.05385	26.07334	15.32738	17.11437	16.90187
HORVU3Hr1G105380	40.48446	37.4407	31.09742	63.7195	75.45573	59.16053
HORVU0Hr1G019800	1.253865	1.222803	1.370338	6.082738	1.972482	3.421168
HORVU3Hr1G089040	2.738436	3.851942	3.84785	4.403813	5.51058	5.6336
HORVU1Hr1G012790	11.53471	9.279284	16.23105	7.949928	92.98183	75.75272
HORVU5Hr1G084240	8.703505	7.785097	8.881777	13.29279	16.27432	16.06209
Hordeum_vulgare_newGene_12457	4.76793	5.318431	5.313495	0.009327	0	0
HORVU2Hr1G068270	19.54426	18.67842	21.86258	33.75371	90.31211	84.10614
HORVU1Hr1G071210	0.965744	0.611924	1.05979	3.002206	21.24287	15.17553
HORVU5Hr1G041250	0.909142	0.639288	0.938636	1.299272	2.028652	2.122335
Hordeum_vulgare_newGene_12239	2.034913	2.156852	1.472517	1.68889	0.338991	0.957752
HORVU7Hr1G035390	113.6499	100.4906	127.0731	99.41828	105.7217	102.4644
HORVU5Hr1G103460	87.82837	116.4351	152.0599	19.91757	0.442222	8.379071
Hordeum_vulgare_newGene_415	12.23009	13.99846	12.7774	12.7126	14.54182	15.35888
Hordeum_vulgare_newGene_410	2.89361	4.294036	4.589669	7.47311	18.28135	17.37752
Hordeum_vulgare_newGene_411	39.99603	42.03855	35.78828	43.37876	44.08415	48.25042
HORVU5Hr1G099160	2.862576	2.755693	3.463403	4.353098	7.399047	7.200598
Hordeum_vulgare_newGene_14112	0.100332	0.095393	0.086508	4.606674	4.418377	4.613516
HORVU5Hr1G075500	53.29531	60.9545	89.79208	64.40185	155.3387	138.5808
HORVU4Hr1G066730	2.919382	3.638579	3.813405	3.09712	4.628819	4.728584
HORVU4Hr1G010080	6.392921	8.849733	10.43466	4.997233	6.247572	7.054844
HORVU7Hr1G051670	2.514523	3.122308	3.181424	3.980188	4.328221	3.689177
HORVU3Hr1G020140	3.537321	1.866258	4.322297	1.713314	0.1736	1.628778
HORVU5Hr1G115000	4.501573	4.021896	5.298559	16.24073	25.16487	25.86152
HORVU7Hr1G002890	2.49927	2.664148	3.2333	3.508939	3.601122	3.905199
HORVU2Hr1G020250	1.011973	1.526554	1.502957	2.076785	1.713633	1.987563
HORVU2Hr1G101990	19.91167	12.12042	19.70905	2.302027	0.969798	2.535326
HORVU3Hr1G055650	44.047	38.61422	41.8124	50.97357	40.37197	46.27317
HORVU5Hr1G074670	2.45603	2.881494	3.478029	1.553312	2.011916	1.964473
HORVU7Hr1G078210	4.78543	4.931971	5.374814	6.322712	8.809838	9.606592
HORVU2Hr1G090800	4.512507	4.799527	6.028981	4.315439	5.733157	6.000292
HORVU3Hr1G059550	5.053186	5.080156	4.984702	5.900638	5.454846	6.121788
Hordeum_vulgare_newGene_5859	1.399195	1.142302	1.823252	2.056327	3.389854	2.907063
Hordeum_vulgare_newGene_15243	10.63846	10.29151	11.08199	8.526434	7.358603	9.153884
Hordeum_vulgare_newGene_14435	5.191965	5.914083	3.561693	1.999722	1.668233	1.530763

Hordeum_vulgare_newGene_15244	3.511187	3.175706	3.713129	5.847771	2.588247	3.400485
HORVU6Hr1G080620	4.197804	5.691898	6.902424	2.486894	5.435542	5.27533
HORVU3Hr1G045750	5.56713	4.785668	6.289849	6.341435	11.63444	11.44545
HORVU6Hr1G013230	8.865194	9.68891	13.77917	10.26093	12.48523	14.98414
HORVU7Hr1G082500	3.970493	4.28665	4.308785	3.461656	2.393782	1.00658
Hordeum_vulgare_newGene_5809	0.252244	0.166185	0.206074	2.314896	2.87329	2.891426
HORVU6Hr1G015690	0.963244	1.253765	1.372918	1.705887	1.969572	2.140731
Hordeum_vulgare_newGene_9713	8.815306	10.65627	10.27672	8.29292	9.749227	11.39482
HORVU7Hr1G030270	10.3561	8.397815	10.62901	8.633988	7.307226	7.872331
HORVU5Hr1G006180	3.135631	4.355834	4.397611	7.093801	5.04789	7.204946
Hordeum_vulgare_newGene_10147	0.228718	1.863747	4.278137	0	0.286197	0
Hordeum_vulgare_newGene_10146	11.06961	10.16019	15.10976	20.04069	26.92529	28.06014
Hordeum_vulgare_newGene_10144	10.55574	11.6999	10.25237	17.14973	20.11662	18.7864
Hordeum_vulgare_newGene_11899	0.60411	0.832728	0.744936	2.93695	4.853212	4.455441
Hordeum_vulgare_newGene_11892	2.224818	2.160337	3.339067	2.656164	5.210325	4.495102
Hordeum_vulgare_newGene_11890	2.029866	2.445824	3.064527	2.434393	3.187845	3.243972
HORVU3Hr1G050450	0.56759	0.43609	1.025583	1.528439	2.498835	2.732436
Hordeum_vulgare_newGene_5149	0.211777	0.123928	0.158732	2.343377	2.667904	3.240661
HORVU4Hr1G071020	24.47897	20.14919	30.45134	12.56354	4.21638	13.1799
HORVU7Hr1G089500	0.791739	0.911171	0.865342	1.334415	1.118622	1.523375
HORVU7Hr1G086330	1.421848	1.220939	0.900191	1.28187	1.316253	1.170461
HORVU5Hr1G013250	5.183211	5.192755	7.00659	5.901548	7.582528	7.354469
HORVU7Hr1G109950	36.51108	37.40085	64.83212	20.75844	24.44126	25.14057
HORVU2Hr1G038760	9.215937	7.510954	9.502989	6.200612	7.074816	6.24948
Hordeum_vulgare_newGene_11238	8.256411	4.683827	4.90889	3.867368	3.063618	4.266315
HORVU2Hr1G118550	270.9883	219.3986	184.9062	74.98001	17.81152	28.44643
HORVU4Hr1G067450	22.41515	11.65438	27.74168	6.221148	1.848888	6.149217
HORVU3Hr1G095420	17.51714	18.68588	16.65412	18.94031	23.07935	23.76507
HORVU5Hr1G094080	3.549955	5.071003	3.009689	17.82714	40.84507	37.6211
HORVU3Hr1G089830	10.89615	9.903655	14.75054	42.96175	44.97559	59.78806
HORVU0Hr1G026700	14.13551	11.75338	14.1596	19.783	26.63317	27.77455
Hordeum_vulgare_newGene_8047	0.64941	0.604467	1.500955	0.907511	0.949893	1.921785
HORVU5Hr1G030290	1.003223	15.29499	2.879665	0	0	0
HORVU2Hr1G026840	5.303225	5.871129	6.994356	3.211812	4.317676	4.615286
HORVU2Hr1G075610	3.215898	2.684731	3.718723	4.349517	4.431052	4.686033
HORVU7Hr1G115520	40.5018	32.67383	43.54307	37.08582	52.08319	40.3322
HORVU1Hr1G076470	0.502728	0.349354	0.582764	3.211715	8.030017	9.401341
HORVU1Hr1G039050	16.26105	18.35502	20.79414	18.97533	23.87002	26.36215
HORVU7Hr1G021820	0.833603	0.750024	0.383513	3.763583	5.803669	5.292503
Hordeum_vulgare_newGene_2463	6.349698	6.384709	5.956422	0	0.029284	0.051598
HORVU6Hr1G081750	1.757577	1.39792	1.626007	4.03918	3.269128	5.298765
HORVU3Hr1G011540	10.79978	9.330941	10.2908	13.78135	27.24886	25.00242
HORVU4Hr1G088900	26.18635	28.86008	31.91102	23.13429	31.61377	31.47497
HORVU4Hr1G072590	18.12384	15.27362	21.12873	20.98285	19.76759	19.89095
Hordeum_vulgare_newGene_10704	1.984103	0.783957	1.853649	1.304387	1.324374	2.051351
HORVU3Hr1G086690	79.7205	63.18977	65.30473	17.28986	4.207293	7.842349
HORVU7Hr1G115290	5.025821	5.192077	6.026418	5.575885	6.738442	7.591183

HORVU0Hr1G006950	1.400349	2.661801	2.984897	2.236952	3.039137	3.307348
HORVU0Hr1G022280	13.84769	13.7991	12.55294	16.55032	16.93768	16.34594
HORVU6Hr1G019130	7.750029	8.930223	9.796143	9.027622	14.1539	12.93638
HORVU2Hr1G102500	4.367286	4.874416	4.922922	4.99663	5.165642	4.917286
Hordeum_vulgare_newGene_3847	7.864827	9.209439	8.109115	15.00174	15.73226	19.00583
Hordeum_vulgare_newGene_3843	0	0	0	4.121275	4.278665	4.197808
Hordeum_vulgare_newGene_3848	3.354417	2.330179	2.577302	3.313828	2.92915	3.09728
HORVU3Hr1G088050	0.527104	0.766913	0.543449	1.27301	1.826537	1.708444
HORVU3Hr1G052030	1.642459	1.494345	1.591878	3.802284	7.163078	5.920302
HORVU2Hr1G032720	1.555001	1.153034	1.424022	1.243336	1.767123	1.342351
HORVU3Hr1G026930	6.144501	5.502258	6.23127	7.546248	8.639773	9.346343
Hordeum_vulgare_newGene_13547	7.083684	4.171586	6.5674	1.700949	3.863485	2.353461
HORVU3Hr1G091850	6.658668	7.138447	7.673835	9.338699	10.89824	11.14808
HORVU3Hr1G030540	6.238829	7.921132	9.019127	8.679442	7.79792	9.248514
HORVU3Hr1G034640	1.416479	1.13616	1.084373	4.291534	4.023489	4.869965
HORVU3Hr1G080530	94.86269	84.24622	97.60545	74.27583	115.0001	116.3394
Hordeum_vulgare_newGene_12153	2.338604	2.414522	2.25113	2.100523	2.272724	2.67968
Hordeum_vulgare_newGene_6633	1.556597	0.295823	0.99599	2.198359	1.990682	2.575681
HORVU7Hr1G058340	10.15058	10.16328	8.498451	10.73947	17.32606	14.81333
HORVU7Hr1G053480	2.341208	2.418102	2.474909	1.688873	2.185659	2.012244
HORVU2Hr1G079610	5.004259	6.096595	6.805703	1.253207	1.659333	1.391484
HORVU7Hr1G039510	8.308808	7.864878	8.508347	9.458389	16.43262	13.44381
HORVU2Hr1G044230	90.81469	81.13842	88.66767	78.7004	74.70089	76.74153
HORVU6Hr1G059610	1.326452	1.644637	2.190089	2.134799	2.151417	2.67422
HORVU4Hr1G080820	3.786171	5.380664	4.03254	10.2091	8.201973	7.157198
HORVU6Hr1G028820	9.392019	7.94229	11.25313	6.366835	7.551906	7.246468
HORVU4Hr1G016620	10.15376	10.77196	13.14941	14.2818	19.00242	18.44606
HORVU3Hr1G092230	6.584211	4.824406	6.197416	9.94903	7.249409	6.62279
HORVU3Hr1G097810	0.670357	0.25738	0.504147	6.674232	6.593347	9.88133
HORVU2Hr1G032130	5.992707	5.516724	4.987078	17.71746	28.59261	23.01474
HORVU6Hr1G070780	165.6544	178.3856	242.9012	108.6602	211.0844	220.608
HORVU1Hr1G087600	4.802994	4.930892	7.62006	7.61742	7.488351	6.337295
Hordeum_vulgare_newGene_2826	1.877074	2.22448	2.28684	0	0	0
Hordeum_vulgare_newGene_2824	0.429341	0.506546	0.507019	1.815952	3.507837	3.285577
HORVU7Hr1G034160	10.60815	11.92455	10.7375	11.07625	12.25431	12.54523
Hordeum_vulgare_newGene_14653	3.210474	3.596868	3.525168	3.101933	3.015231	3.046082
Hordeum_vulgare_newGene_14652	7.978847	7.879351	6.08265	6.168155	5.264984	5.998252
HORVU3Hr1G086270	15.19093	13.30854	13.56591	10.81742	14.10611	12.39702
HORVU5Hr1G020370	4.042836	3.889554	4.402601	4.905491	7.953755	8.622762
HORVU1Hr1G089730	67.04691	76.50826	98.60797	5.560397	4.032785	5.388093
HORVU7Hr1G052350	83.45559	92.02397	101.6664	65.42287	82.55253	85.85974
HORVU6Hr1G037420	2.418396	2.351045	2.257583	3.877058	3.435116	4.191159
HORVU6Hr1G078060	13.30758	11.67981	19.38266	8.5357	4.102215	5.65179
HORVU6Hr1G029190	0.538621	0.382559	1.035532	4.261924	13.86943	12.73099
HORVU5Hr1G060800	5.146187	5.209947	5.861662	7.096173	17.20723	18.08594
HORVU2Hr1G041770	14.69096	16.51168	12.93697	14.71587	20.25981	20.41196
HORVU5Hr1G102720	4.120888	4.991492	5.693949	8.595994	9.419755	10.42247

HORVU3Hr1G047870	1.668305	2.034545	2.270365	3.348408	4.780946	4.441963
HORVU3Hr1G055350	13.43642	10.96677	13.49672	20.85767	19.06044	21.62541
HORVU7Hr1G034290	2.277346	1.033914	2.818735	1.779066	1.621965	2.446831
HORVU4Hr1G007850	47.84806	45.07081	54.29561	43.94368	59.10189	59.1448
HORVU3Hr1G078900	11.50396	10.59694	11.85544	11.9182	8.531891	9.909367
HORVU6Hr1G081800	0.103401	0.283459	0.094638	4.692914	6.166144	6.194331
HORVU3Hr1G038430	0.784289	0.649265	0.744208	2.756984	10.58951	12.43042
HORVU4Hr1G056520	15.59838	19.27134	17.88939	24.46388	23.6465	29.64233
HORVU1Hr1G051970	2.557721	2.653678	2.805348	3.951234	5.863508	4.963801
HORVU3Hr1G007980	3.643592	3.849641	4.126191	4.992423	5.723407	5.697355
HORVU3Hr1G003600	1.587968	1.183658	1.643418	4.270072	1.282561	2.264748
HORVU7Hr1G091480	9.747562	6.804682	9.919372	6.96171	7.023597	7.141866
Hordeum_vulgare_newGene_8907	1.345428	0.874938	1.330246	1.486318	1.095597	1.017034
Hordeum_vulgare_newGene_8906	6.928688	6.581005	5.618157	12.9078	41.05046	32.75248
Hordeum_vulgare_newGene_8903	0.476209	1.003609	0.913701	0.84562	1.277145	1.662363
HORVU4Hr1G051610	3.223455	2.967572	3.201214	2.522334	2.438552	2.64017
HORVU6Hr1G071120	8.144245	7.739855	11.23168	12.06453	18.77116	16.22646
HORVU3Hr1G082590	1.315715	1.191062	0.905317	4.835216	8.222755	7.81784
HORVU4Hr1G071920	1.535065	1.704828	2.603943	1.836037	2.33312	2.110115
HORVU2Hr1G019200	11.70955	11.57716	13.75351	12.12177	12.10823	13.19649
HORVU6Hr1G034140	0.52177	1.19448	1.035724	1.783411	2.539545	3.484047
HORVU2Hr1G046860	158.9972	127.9212	162.1272	136.7133	156.6484	148.5814
Hordeum_vulgare_newGene_10897	1.284863	2.088709	2.227412	1.741311	1.049459	0.716755
HORVU6Hr1G005710	1.976532	1.939057	2.447771	2.261383	3.034654	2.961093
HORVU7Hr1G108810	1.474698	2.35369	2.317573	1.624592	1.75782	1.672091
HORVU6Hr1G001660	23.32752	25.86539	27.95002	26.43977	31.64254	32.57801
HORVU3Hr1G018200	1.709927	1.853381	2.201001	3.150006	3.708925	3.765216
HORVU2Hr1G026450	0.768158	0.331516	0.701915	5.413484	3.472634	2.932834
HORVU3Hr1G032050	2.237669	2.736014	3.207471	2.197357	3.876168	2.929831
HORVU4Hr1G043970	14.59968	17.23919	18.00434	15.76903	18.98729	19.57593
HORVU1Hr1G060250	9.618462	8.696692	9.833832	8.592001	7.98888	7.57967
HORVU5Hr1G051710	11.73084	12.27238	11.91859	15.33039	16.12133	15.07283
Hordeum_vulgare_newGene_9729	107.7382	88.81427	111.0553	74.05344	90.79028	81.77455
HORVU0Hr1G017360	67.80233	68.01901	118.9977	52.34294	121.6904	107.5376
HORVU2Hr1G089710	36.7383	39.23892	25.1775	28.00655	16.10084	22.44785
HORVU7Hr1G113450	1.853264	2.336812	2.738676	4.580623	6.162496	6.624717
HORVU4Hr1G047070	8.648059	9.597384	9.514656	13.30249	12.96801	14.02284
HORVU1Hr1G048410	8.814505	9.252605	11.02793	7.924256	8.06944	7.51053
HORVU5Hr1G109940	44.39005	33.94752	53.64171	33.04184	22.93947	30.24389
HORVU5Hr1G106890	0.800066	0.868049	1.37919	1.038967	1.592192	1.278164
HORVU1Hr1G050620	0.546333	0.750037	1.077906	0.950197	2.966167	3.348935
HORVU5Hr1G067800	6.78533	5.665255	8.404713	4.509739	3.408557	4.132652
HORVU7Hr1G092360	2.787875	1.616377	2.840947	3.485913	2.960299	3.112402
Hordeum_vulgare_newGene_12284	2.24906	2.743455	3.211721	3.041818	2.23288	3.015279
HORVU2Hr1G098010	4.160749	2.401105	3.922052	1.641185	2.247032	3.067165
HORVU1Hr1G052660	0.934188	0.746353	1.162732	1.275944	1.358169	1.646823
HORVU6Hr1G044300	5.961331	6.065433	7.280499	7.758304	6.949153	8.249701

HORVU4Hr1G065620	0.780667	0.638458	0.866138	1.536297	2.303739	2.276929
HORVU5Hr1G067120	2.588837	3.147765	2.864806	3.190348	2.030841	2.627809
HORVU7Hr1G036470	14.16348	9.372859	18.63081	6.016962	29.50225	19.20993
HORVU5Hr1G025420	10.6748	11.0084	11.21487	14.49653	23.10927	21.02406
HORVU3Hr1G048870	6.836244	6.141356	7.70264	9.618913	11.18892	9.37844
HORVU2Hr1G083410	5.623722	4.182669	6.804472	4.003069	3.310554	3.6696
HORVU5Hr1G091750	6.31201	6.712533	6.850872	8.984445	8.753143	10.00241
HORVU4Hr1G085750	11.7359	9.622273	15.41237	17.13204	22.80632	25.74622
HORVU4Hr1G012770	12.99695	13.31659	20.29576	9.747331	18.68063	17.06223
HORVU5Hr1G058670	5.083844	4.910653	6.75176	6.801294	8.259333	7.766718
HORVU7Hr1G017650	5.928341	3.752114	5.868813	6.948106	9.010817	8.513665
HORVU2Hr1G124110	23.75184	21.10801	25.23423	20.19755	29.55277	28.19995
HORVU7Hr1G095830	2.654273	2.088393	2.331039	3.435778	2.780299	2.856377
HORVU4Hr1G048160	1.269718	1.264036	1.843359	1.640443	1.097293	1.241431
HORVU7Hr1G084590	9.077816	9.17182	7.677954	4.974897	6.193199	7.103257
HORVU1Hr1G043680	14.70045	16.17477	16.64599	13.17949	17.55983	18.98691
HORVU0Hr1G014100	0.714803	1.070739	0.988705	1.552801	3.537948	2.565994
HORVU5Hr1G046220	5.282219	4.341415	6.012322	4.571241	8.04198	7.280753
HORVU5Hr1G097630	50.20196	42.95804	54.56915	68.32375	74.5557	69.97537
HORVU2Hr1G107100	3.666398	2.688015	4.664326	6.130664	1.347899	2.593187
HORVU2Hr1G095220	2.433061	2.608947	5.358869	3.287746	5.31368	5.996285
HORVU4Hr1G004130	2.809567	2.294486	3.265241	1.750903	2.376058	1.85146
HORVU1Hr1G069550	11.6861	9.281346	10.04985	0.368768	0.240641	0.384747
HORVU4Hr1G068910	0	0	0.091557	16.62729	32.33643	27.86507
HORVU7Hr1G085220	18.20962	14.26016	19.24619	24.05985	42.92248	45.45468
HORVU6Hr1G090910	2.348579	2.569628	2.809223	0	0	0
Hordeum_vulgare_newGene_3726	2.024987	3.076926	3.12368	2.135469	2.429913	2.950418
HORVU7Hr1G012380	332.6264	307.3644	342.9613	1160.072	1599.795	1354.183
HORVU2Hr1G096550	0.733086	1.012751	0.994095	3.469356	4.016149	4.054787
HORVU3Hr1G005930	7.949518	9.091245	9.706505	9.670492	8.251213	7.762728
HORVU5Hr1G080450	3.069477	2.36712	2.906466	4.657496	1.405263	2.26323
HORVU7Hr1G098460	0.021997	0.006599	0.035608	8.465602	6.762634	8.105374
HORVU5Hr1G013510	0.28467	0.365363	0.314193	23.9869	3.229991	11.88421
HORVU2Hr1G017440	4.648197	2.951603	3.776189	5.072691	63.36477	51.2855
HORVU5Hr1G081070	6.231198	5.877959	7.418077	4.196521	2.588197	2.797324
HORVU5Hr1G045040	17.6914	14.86956	22.72996	19.79981	27.35292	30.67098
HORVU7Hr1G107750	6.566565	8.442797	7.857841	9.680439	13.49466	12.36254
HORVU1Hr1G086210	1.691767	1.238408	1.867253	1.575697	1.660056	1.455491
HORVU6Hr1G019300	4.748355	3.567681	5.388423	5.741517	11.69611	12.90846
HORVU0Hr1G001270	76.69581	69.89128	86.2368	112.7155	141.7833	139.2865
Hordeum_vulgare_newGene_6499	6.012152	7.725397	5.23223	1.535614	2.025063	1.430873
HORVU3Hr1G002920	12.8302	9.452656	12.804	20.67095	25.27572	26.8857
HORVU4Hr1G059060	38.56423	33.66251	45.35078	41.61578	38.8596	36.14622
HORVU4Hr1G059840	45.68113	38.98017	46.39929	45.64181	48.28682	47.2443
HORVU7Hr1G018670	10.41502	9.914949	16.01207	9.55413	8.930323	10.64597
HORVU2Hr1G099850	3.115116	3.945494	3.957767	2.388498	2.058405	1.789088
Hordeum_vulgare_newGene_10300	1.217674	0.50101	0.999555	3.439316	4.003767	3.738134

HORVU1Hr1G051500	3.167637	2.871132	3.506164	3.833385	3.742884	3.573811
HORVU4Hr1G007440	16.53576	14.65837	15.73879	20.162	21.42866	19.66236
HORVU5Hr1G095180	91.38464	63.37488	85.74273	13.26068	4.732757	6.733677
HORVU2Hr1G037090	2.381117	2.259546	2.126046	2.169428	2.569399	2.892676
HORVU1Hr1G054200	0.357689	0.326181	0.580592	36.35737	3.663305	14.44052
HORVU4Hr1G090170	1.85033	3.361574	2.819361	0.740491	0.971612	1.082122
HORVU3Hr1G106850	79.14536	44.07515	94.27611	48.48405	35.30132	35.80588
HORVU4Hr1G009890	13.13804	14.25189	15.54953	11.75785	16.91609	15.40271
HORVU4Hr1G010840	2.769215	2.868512	3.761234	3.562683	3.329787	3.273042
HORVU1Hr1G051250	4.413908	3.33414	6.054269	4.605729	7.301277	6.714552
HORVU2Hr1G050260	1.809077	1.985776	2.311889	1.0661	0.746162	0.752243
HORVU7Hr1G099650	0.83103	0.748423	1.445621	7.30686	11.3261	9.371835
HORVU6Hr1G088840	3.361864	3.949116	5.185145	1.878607	2.164467	2.18139
HORVU6Hr1G049790	1.775436	2.591478	1.607618	2.339564	2.860459	3.353096
HORVU2Hr1G041430	2.186105	2.480697	3.282148	1.732422	2.04107	2.403127
HORVU3Hr1G077790	5.161685	3.915743	5.236413	1.221991	0.096883	0.518121
HORVU5Hr1G097060	11.41265	10.38208	14.09409	10.22987	9.418708	9.13763
HORVU1Hr1G004490	14.76959	12.76054	16.86572	17.68628	24.42347	25.19691
HORVU3Hr1G099990	0.218715	0.063187	0.142481	31.29987	3.469049	16.37757
HORVU5Hr1G048280	1.435178	1.447719	1.395146	1.31458	1.574599	1.690054
HORVU6Hr1G041090	4.240264	3.165707	2.786773	3.025509	2.64313	2.952996
HORVU4Hr1G056640	4.03761	4.726596	3.998586	1.611326	2.559168	2.97248
HORVU1Hr1G001040	0	0	0	7.086504	0	0
HORVU7Hr1G051180	0.439377	0.314715	0.472705	1.793454	4.427548	6.624299
HORVU3Hr1G029210	5.062204	5.052718	5.573543	7.856746	22.76421	16.96599
HORVU3Hr1G085210	23.05601	25.21139	32.40584	10.80495	11.44288	13.17997
HORVU0Hr1G010640	6.527097	6.163627	5.683543	2.991481	1.278398	1.200436
HORVU2Hr1G022720	1.246172	1.081072	0.788316	1.366116	6.20337	6.167242
HORVU1Hr1G000930	4074.761	4329.824	3193.729	4852.036	1529.561	2487.601
HORVU3Hr1G107020	1.219753	2.17144	2.640336	1.903276	2.075261	2.969207
HORVU1Hr1G004720	0.964092	0.419275	0.763988	4.48755	3.582827	3.603769
HORVU1Hr1G008900	2.209231	1.408545	1.931406	2.614836	2.259587	2.381815
HORVU4Hr1G009950	2.281627	2.374075	3.350473	1.374219	2.557023	2.406116
HORVU1Hr1G002890	0.536032	8.611369	0.837648	12.09076	0	0.086771
HORVU6Hr1G063320	1.937965	1.816369	2.600665	1.570542	1.557265	1.519353
Hordeum_vulgare_newGene_1931	2.849594	2.890249	2.836673	0	0	0.00619
HORVU3Hr1G048890	23.22211	19.37808	30.55048	35.81654	53.73797	52.49286
Hordeum_vulgare_newGene_274	1.748466	1.062087	1.796633	0.657574	0.320267	0.496557
Hordeum_vulgare_newGene_6656	2.283078	1.247798	3.08477	3.77987	4.500099	4.498935
HORVU7Hr1G092680	1.615856	2.160797	4.225348	1.033813	0.706152	0.716017
HORVU4Hr1G061270	35.47384	34.88241	43.05292	30.57785	34.61362	34.40431
HORVU2Hr1G028600	30.00947	28.89061	36.93116	28.86096	32.06263	31.3927
HORVU4Hr1G063350	114.359	112.3062	135.2222	152.1305	316.6606	171.054
HORVU3Hr1G066430	86.72424	87.11769	148.9892	60.58133	94.17679	99.25655
HORVU0Hr1G027660	3.058025	2.780123	2.897191	4.582431	4.95566	5.027024
HORVU3Hr1G026920	153.4736	102.15	183.0515	349.6535	217.3111	228.0947
HORVU7Hr1G062090	24.24133	29.52988	34.10674	23.9793	24.38989	30.02112

HORVU7Hr1G109810	6.176082	5.751376	8.705663	9.488352	9.140387	9.294288
HORVU6Hr1G062050	0.389002	0.225883	0.803628	2.59113	3.849735	3.786127
HORVU3Hr1G024560	8.002992	4.681099	7.34903	5.57353	3.52851	5.504202
Hordeum_vulgare_newGene_12798	34.44738	30.73837	33.04131	26.45493	29.1102	27.8274
Hordeum_vulgare_newGene_12799	6.403081	5.071036	6.496452	5.810931	6.638096	6.313649
HORVU2Hr1G038950	2.087772	2.485954	2.759036	2.62779	2.506701	2.334869
Hordeum_vulgare_newGene_12793	36.96525	28.88216	37.09519	47.6861	40.40659	41.74011
Hordeum_vulgare_newGene_12794	1.996402	1.32222	1.917191	3.898582	3.653521	3.71534
HORVU7Hr1G084920	59.12038	38.28637	56.44806	9.191257	8.487234	14.51386
HORVU2Hr1G025180	5.796005	6.098158	6.639177	4.890002	6.127125	6.526769
HORVU2Hr1G018670	50.06302	45.09942	45.13839	9.94715	1.139694	3.135186
HORVU3Hr1G057530	32.0993	35.21698	26.99489	55.00326	19.38045	26.88862
HORVU2Hr1G073920	7.154506	6.933165	7.9231	3.888979	3.384803	3.557762
HORVU7Hr1G100500	117.9621	109.4609	117.7319	77.51624	75.50677	74.79962
HORVU2Hr1G116650	2.134904	2.296309	2.095429	0.879337	0.655556	1.100518
HORVU4Hr1G057680	10.65029	10.53636	11.63647	13.27472	16.16939	17.1283
HORVU7Hr1G043160	3.427941	3.609707	3.774255	2.00484	2.697714	2.599756
HORVU5Hr1G000680	6307.31	5727.22	6427.183	2410.478	2704.947	1988.841
HORVU5Hr1G080840	13.7345	11.01468	14.3692	11.6941	13.50918	9.674068
HORVU4Hr1G083410	5.170455	5.038587	6.935772	3.926044	5.861804	5.955685
Hordeum_vulgare_newGene_3170	0.923043	0.807842	0.97526	1.74187	1.831315	2.155051
HORVU5Hr1G078760	21.09788	20.05466	21.15316	17.89866	19.06363	18.96172
HORVU7Hr1G033900	12.24727	10.09275	8.030391	21.77092	42.91136	27.2259
Hordeum_vulgare_newGene_3648	0.24336	0.335049	0.221913	7.035162	8.845873	11.26162
HORVU4Hr1G022930	1.372141	1.60983	1.657814	1.762778	4.024488	3.535502
HORVU2Hr1G100470	3.125424	3.095145	3.507863	6.025862	6.811111	8.045534
Hordeum_vulgare_newGene_3643	2.804777	4.216768	3.706077	1.789414	1.601124	2.172602
HORVU7Hr1G077710	21.7156	22.77717	15.87393	8.249249	8.468606	8.408314
HORVU7Hr1G072940	8.454216	8.791816	10.79696	12.74245	15.0531	15.60464
HORVU6Hr1G078260	4.794705	4.181894	4.58789	1.994374	2.380976	2.653672
HORVU5Hr1G121250	4.185325	5.078676	6.460554	4.153661	5.935662	5.960011
HORVU1Hr1G009980	0.371816	0.140472	1.163262	4.14463	0.204398	0.393992
Hordeum_vulgare_newGene_14323	1.288301	2.530839	1.662718	1.251947	1.014141	1.47058
Hordeum_vulgare_newGene_14321	4.311469	4.795742	5.250716	1.929693	2.690254	2.654507
HORVU0Hr1G023660	1.496891	1.573666	1.41606	1.814632	3.646573	3.597569
Hordeum_vulgare_newGene_14325	0.430166	0.397977	0.205172	2.581933	1.626833	2.215146
HORVU5Hr1G078490	3.405505	3.055824	4.659074	5.784608	3.938368	3.882912
HORVU3Hr1G064120	0.837014	0.963125	1.281728	1.319051	3.143911	1.899963
HORVU3Hr1G110190	0.894157	0.323822	0.197054	3.593025	5.032988	4.974938
HORVU6Hr1G072300	1.136676	0.860424	1.351172	3.285947	2.781454	3.363087
HORVU2Hr1G118680	8.604756	8.173319	8.340892	11.06002	8.424755	11.89888
Hordeum_vulgare_newGene_625	6.524886	7.51324	7.150979	3.272131	3.125202	4.502209
Hordeum_vulgare_newGene_624	2.759199	2.169763	3.155888	3.127165	3.488541	3.281386
Hordeum_vulgare_newGene_627	2.122339	2.030211	2.444154	2.062764	1.842933	3.172578
Hordeum_vulgare_newGene_626	12.42315	11.21964	10.28357	5.659159	4.117832	4.111866
HORVU3Hr1G065830	13.36668	12.24519	13.91588	7.896165	6.922078	7.598022
HORVU6Hr1G026400	7.228032	8.220084	7.155415	10.37252	14.70849	13.61861

Hordeum_vulgare_newGene_2518	11.82051	10.83107	14.70651	13.74758	17.02361	19.23839
Hordeum_vulgare_newGene_2519	4.722507	4.422043	5.163403	8.087223	10.72913	10.26904
Hordeum_vulgare_newGene_2512	1.489739	1.929063	1.494838	4.455045	9.412413	9.281048
Hordeum_vulgare_newGene_2511	1.322542	1.155858	1.434708	2.753672	2.759836	2.880237
HORVU1Hr1G070460	15.2037	13.15915	14.09566	4.848942	3.134145	3.772803
HORVU7Hr1G096610	0.133661	0.118405	0.063392	2.234643	3.262281	2.023386
HORVU7Hr1G026940	57.71444	55.28981	59.34171	17.27448	5.690718	11.48435
HORVU3Hr1G054070	80.3985	72.74029	67.97424	44.45684	36.63778	38.35762
HORVU6Hr1G021750	126.1532	111.6329	128.0903	105.1413	111.3425	112.381
HORVU4Hr1G055350	6.890051	6.992992	7.898885	7.857407	8.369874	9.222939
HORVU0Hr1G009060	12.46714	10.43726	11.82908	8.317739	6.662477	7.326813
HORVU5Hr1G073710	5.856651	2.602799	10.44675	3.549459	1.899815	2.048837
HORVU7Hr1G043790	1.154664	1.622326	1.183465	3.879683	8.125042	7.210649
HORVU1Hr1G031460	2.11479	2.487502	3.406363	4.497719	6.157548	6.934656
HORVU2Hr1G033610	2.433443	2.000528	2.279258	0.863352	0.635491	0.496267
HORVU6Hr1G013000	2.01481	1.885912	2.335324	1.132206	1.800212	1.443896
HORVU3Hr1G058410	2.187543	2.571798	2.013402	3.147668	3.045278	3.752802
HORVU5Hr1G116740	5.811824	7.865843	5.701587	5.813553	8.083897	8.362091
Hordeum_vulgare_newGene_11910	3.119112	3.769826	4.214264	2.60617	2.880689	3.27359
HORVU4Hr1G071490	1.231118	0.754517	0.768904	1.306997	0.728276	1.653265
HORVU7Hr1G060410	6.299345	5.116229	6.081841	6.6806	10.56384	10.82981
Hordeum_vulgare_newGene_9697	0.302243	0.182515	0.371664	0.968671	2.741412	1.784001
HORVU4Hr1G044890	1.176981	1.20232	1.289683	2.494006	4.187905	3.477678
HORVU6Hr1G025270	26.083	26.98299	36.13403	29.20501	43.25008	42.53518
Hordeum_vulgare_newGene_1092	2.064153	1.550372	2.405751	1.85137	0.972503	0.709053
HORVU3Hr1G045270	16.85121	17.0816	20.27785	16.53815	30.99618	28.39278
Hordeum_vulgare_newGene_9528	12.34379	14.97435	16.18436	13.12999	11.84735	14.97793
HORVU5Hr1G019610	3.786871	2.152306	5.701602	5.117354	7.001968	5.903828
Hordeum_vulgare_newGene_9525	9.932181	11.98283	11.75945	9.811587	12.59103	14.27344
HORVU1Hr1G072560	2.146117	2.361571	2.593112	2.725212	3.044044	3.161984
HORVU5Hr1G073410	14.57772	12.09348	11.49884	10.30946	20.13161	18.90948
HORVU7Hr1G021980	4.622291	4.66853	5.293252	5.81923	8.330129	8.128757
HORVU5Hr1G083000	7.297843	6.893146	9.254256	4.250819	3.952125	4.610071
HORVU1Hr1G024390	6.401942	6.309779	6.818493	8.660486	8.282863	9.595857
HORVU1Hr1G032470	1.581276	1.44525	0.790975	2.780364	4.685943	3.008108
HORVU5Hr1G077110	13.11793	8.473234	13.67491	36.29864	74.41199	68.3365
HORVU0Hr1G000390	11.47851	12.54056	7.93243	10.89932	10.27461	12.15464
Hordeum_vulgare_newGene_4112	7.913631	6.41827	5.923534	1.598263	1.571768	1.000899
HORVU6Hr1G028250	5.942904	5.685568	6.403051	3.299221	3.336009	1.930793
Hordeum_vulgare_newGene_4114	4.621062	3.658525	3.844661	5.748626	2.940753	6.065019
Hordeum_vulgare_newGene_4115	61.6236	52.84615	59.69278	66.86736	56.08889	54.91858
HORVU6Hr1G081210	4.781162	5.403852	6.062102	5.224565	2.71653	3.555163
HORVU1Hr1G025440	5.772736	4.968142	6.239901	2.050254	7.53701	6.346784
HORVU0Hr1G039640	4.656435	5.080507	5.462915	5.606102	7.145587	7.284394
Hordeum_vulgare_newGene_13660	2.85331	2.333233	2.472176	3.799906	4.801159	4.829493
HORVU7Hr1G070970	3.833514	2.837034	2.447811	8.527671	15.05114	13.45312
HORVU1Hr1G063740	32.22043	31.04592	37.83268	10.2303	8.692116	10.43561

HORVU7Hr1G033170	1.566146	1.450933	1.166869	1.832143	3.175248	2.975966
Hordeum_vulgare_newGene_13665	2.348281	2.506083	3.53078	2.458156	2.978305	4.106543
HORVU7Hr1G101590	44.55844	38.18867	52.462	11.24358	8.710489	14.09061
Hordeum_vulgare_newGene_13888	4.029605	4.335392	5.519918	5.053109	4.920028	5.184622
HORVU2Hr1G075790	49.31262	38.72645	57.43873	44.12293	94.25967	89.60146
HORVU3Hr1G029320	176.8091	145.6137	189.3399	192.1868	273.569	266.7126
HORVU2Hr1G075420	3.823507	4.30585	4.697292	2.848876	3.222497	3.483709
HORVU1Hr1G079610	9.206159	7.690471	10.29049	0.380575	0.3229	0.532302
HORVU5Hr1G101480	2.788813	2.52502	3.197956	2.276884	2.984616	2.838923
HORVU1Hr1G064210	53.24011	62.458	82.83271	40.2314	58.66613	80.77738
HORVU5Hr1G043520	11.79059	10.55629	13.06978	12.68838	15.20707	16.13199
HORVU0Hr1G018830	3.033887	2.78185	3.165445	3.18009	6.073449	6.873339
HORVU2Hr1G087880	3.71677	3.141708	3.464135	0.912833	1.119023	0.748979
Hordeum_vulgare_newGene_4805	2.02524	2.31758	3.103236	1.721087	2.101832	2.443021
HORVU7Hr1G098260	0.234255	0.190842	0.3816	0.834659	2.341113	3.119246
HORVU7Hr1G053720	14.46646	14.0311	14.29983	16.84988	18.05626	11.93498
HORVU7Hr1G056530	13.76323	12.56063	12.13541	59.41153	14.60295	23.2036
HORVU4Hr1G083340	6.692442	7.796659	10.21042	5.142207	5.527796	5.829546
HORVU4Hr1G073230	154.819	128.0615	126.6163	108.1614	109.1802	104.1602
HORVU1Hr1G056610	1.811337	1.746698	2.721173	3.711734	2.958075	4.083347
HORVU6Hr1G071740	35.48227	30.80424	45.41552	68.8184	73.18834	68.8144
HORVU3Hr1G091460	2.128316	2.009991	2.524253	1.559477	3.141198	2.660815
Hordeum_vulgare_newGene_10066	1.599859	1.762673	1.910496	0.91896	2.016682	2.076191
HORVU6Hr1G080160	26.06726	20.14001	33.46026	32.98484	43.55534	44.51678
HORVU7Hr1G083370	29.89092	29.46012	30.64223	42.90892	25.51639	32.83246
Hordeum_vulgare_newGene_6403	14.29721	10.44321	15.09166	18.13435	17.24457	21.98842
Hordeum_vulgare_newGene_6405	3.842116	4.255299	5.830322	0.1812	0.295369	0.340598
Hordeum_vulgare_newGene_6406	6.143615	5.61862	7.688531	10.53034	13.10236	12.92997
HORVU5Hr1G022380	1.14453	0.871809	0.7111	0.364333	1.05641	1.888548
HORVU4Hr1G019980	55.75596	57.29309	78.50606	55.25277	102.143	96.20584
Hordeum_vulgare_newGene_15805	1.317156	1.281836	1.708789	1.63161	2.667925	2.714351
HORVU2Hr1G032570	21.18871	21.6201	25.9545	19.31695	23.89077	22.89172
Hordeum_vulgare_newGene_15803	20.97813	20.8597	24.96428	19.61923	23.27248	23.52549
HORVU2Hr1G027470	6.101943	4.762464	6.973851	4.209404	2.015663	2.85298
HORVU1Hr1G082900	8.750197	7.26551	6.405501	10.03062	9.328134	11.13436
HORVU6Hr1G078740	7.692335	6.699162	10.1143	10.54259	13.81368	11.54016
HORVU6Hr1G075110	13.24156	13.46429	15.29896	15.48197	17.97847	20.1199
HORVU1Hr1G036340	5.02941	6.084143	5.119059	2.96803	2.016889	2.799275
HORVU3Hr1G090730	13.7396	12.96418	18.49141	9.415104	11.55219	11.50852
HORVU4Hr1G051130	2.321472	2.833033	2.75407	3.584927	6.215405	5.890839
Hordeum_vulgare_newGene_7519	0.771535	1.455981	1.761746	2.519308	4.677451	4.329054
HORVU1Hr1G062820	3.901812	3.210414	3.66616	5.318147	7.568493	6.442032
HORVU7Hr1G097200	21.7265	22.8401	21.83632	24.11064	25.51407	23.76307
HORVU4Hr1G000810	3.509413	2.565825	3.012249	3.278101	1.472638	2.407081
HORVU4Hr1G074270	27.15522	28.73735	38.091	21.82563	33.73397	32.56533
HORVU4Hr1G070950	6.487849	7.319176	9.26533	6.275716	7.278826	6.915874
HORVU2Hr1G041630	21.39849	20.78835	24.92872	19.64178	23.48941	26.57308

HORVU2Hr1G000030	2.344888	2.734047	2.961726	3.370333	3.980697	4.174119
HORVU6Hr1G087300	0.422244	0.309023	0.338263	6.221369	16.95661	13.92096
HORVU5Hr1G018730	8.023704	6.572048	8.467813	7.055507	11.81419	10.47111
Hordeum_vulgare_newGene_2228	5.121732	4.441404	7.221148	5.343216	6.603455	6.483184
HORVU4Hr1G051680	12.37683	12.22234	15.23019	11.90631	14.81185	16.10281
HORVU4Hr1G013660	1.718092	1.939738	2.048989	1.728145	1.876964	1.993712
HORVU5Hr1G023520	3.511434	4.788718	5.073548	3.742203	6.404118	6.671692
HORVU1Hr1G020400	54.44655	52.03337	54.38284	47.80268	50.56223	50.13594
HORVU1Hr1G081310	35.97286	37.10806	42.87259	27.13012	24.49881	27.55507
HORVU6Hr1G084640	14.37683	10.4348	16.49969	20.75095	27.75041	26.83357
Hordeum_vulgare_newGene_2220	0.782035	1.136863	1.495937	1.618539	2.747785	2.641201
HORVU3Hr1G022000	11.68459	13.29598	11.09775	9.915949	7.319378	7.768286
HORVU4Hr1G069450	8.252623	6.34142	9.563751	5.956292	4.574778	4.844598
HORVU3Hr1G041160	5.212481	5.895106	6.055711	6.800754	6.844108	8.298194
HORVU1Hr1G018780	10.51976	11.7201	11.90425	13.17917	13.32811	13.78955
HORVU3Hr1G037580	3.969054	3.150914	1.999909	0	0	0
HORVU1Hr1G066300	4.570198	4.308897	5.35224	0	0	0
HORVU3Hr1G075310	5.247693	5.481764	5.667348	5.111787	5.190577	5.633214
HORVU3Hr1G023750	5.866856	4.937478	4.554892	3.650192	1.903371	1.590495
HORVU5Hr1G095000	27.4219	22.62888	32.84938	48.77344	22.22385	26.7533
HORVU1Hr1G057270	0.208092	0	0.381447	2.938185	0	3.341563
HORVU1Hr1G005030	337.9413	321.0297	239.8937	105.6813	67.38455	74.6288
HORVU7Hr1G010050	40.82659	43.62996	45.25329	43.53109	42.61896	40.18044
HORVU1Hr1G010230	1.245529	2.011047	1.063926	4.003396	135.9548	94.62184
HORVU2Hr1G123150	5.11338	4.130711	4.801649	9.900735	15.02287	14.26728
HORVU2Hr1G106410	12.34686	13.6278	14.78513	21.71342	27.11413	24.56546
HORVU5Hr1G055190	5.191762	5.060844	5.974089	4.642309	8.625868	8.906042
HORVU5Hr1G066190	18.77975	13.81555	17.5952	20.30333	29.3075	24.14856
HORVU2Hr1G094290	8.525633	8.0568	9.53526	10.19888	11.17379	10.95241
HORVU2Hr1G046980	6.52617	6.262629	6.535298	7.175188	6.917563	7.878349
HORVU3Hr1G000260	8.260673	8.887114	8.685466	11.95479	14.01436	13.59389
HORVU6Hr1G085270	0.954806	1.28301	1.205594	8.103557	8.115788	7.994742
HORVU2Hr1G107800	22.53955	22.38178	19.65799	21.90811	21.98057	25.81169
HORVU6Hr1G000180	20.73256	18.66239	27.32103	23.92139	43.31112	46.07969
HORVU7Hr1G090890	25.88307	25.66468	27.79123	21.45049	23.18752	24.27735
Hordeum_vulgare_newGene_5360	2.288948	2.223375	1.808855	0.784706	1.794037	1.216125
HORVU1Hr1G012690	14.4944	14.50567	17.38532	21.0298	26.40442	28.14905
HORVU3Hr1G072920	2.59817	3.022142	3.189616	4.543304	4.285345	4.402642
HORVU5Hr1G111180	5.245611	4.926031	5.790706	8.806703	7.85633	9.941386
HORVU1Hr1G095100	31.44254	32.19722	41.84526	23.06897	30.71809	31.29239
Hordeum_vulgare_newGene_12801	0	0	0	0	4.072364	4.529336
HORVU3Hr1G071340	0.379627	0.281444	0.410138	1.132012	2.524035	3.442733
HORVU3Hr1G074190	2.029227	2.041505	3.276385	2.224557	3.378034	2.459716
Hordeum_vulgare_newGene_12805	6.351903	8.209245	5.887568	7.92139	9.562783	8.890924
Hordeum_vulgare_newGene_12808	83.7879	115.5179	118.1973	53.6492	0.454579	21.17758
HORVU7Hr1G037410	5.495385	3.975309	4.852976	13.0115	14.48333	13.5818
HORVU4Hr1G080190	0.539183	0.445821	0.571102	2.455477	2.227257	2.827667

HORVU2Hr1G082320	6.325063	4.475408	5.58719	5.229015	5.317308	5.085175
HORVU6Hr1G011260	16.96723	17.16111	17.9675	26.0394	38.6024	39.10024
HORVU6Hr1G018770	16.55054	14.42878	16.58846	9.038351	14.56155	13.57618
HORVU4Hr1G054240	3.586556	2.184394	3.684691	7.01452	15.84734	11.93955
HORVU2Hr1G119610	34.94143	32.36262	38.23936	21.92414	29.27892	28.78097
HORVU6Hr1G012510	23.62298	18.79589	28.69767	24.66853	44.5961	36.96406
HORVU7Hr1G110310	8.616115	6.697701	10.98711	10.14506	34.16082	34.04316
HORVU5Hr1G045650	1.398241	0.946326	1.277904	4.381856	3.810767	7.559045
Hordeum_vulgare_newGene_5401	23.76214	30.23496	27.71975	0.052175	0.060463	0.038692
HORVU1Hr1G003740	2.470641	2.078397	2.85546	1.318834	2.040869	2.313796
HORVU3Hr1G079010	3.346189	4.305712	4.276203	3.698089	2.578836	2.600241
HORVU5Hr1G115980	0	0	0.025483	3.083424	3.03151	3.253341
HORVU7Hr1G043550	101.0381	83.05016	101.9432	111.6643	127.534	122.9242
HORVU1Hr1G047770	0.95037	1.049133	1.556566	1.331848	0.80248	1.710639
HORVU6Hr1G055830	31.11537	23.33314	36.52361	23.32338	27.91805	27.10803
HORVU5Hr1G121900	25.23561	25.75734	24.3462	12.88139	12.76715	14.67309
HORVU7Hr1G081540	1.288891	2.233789	1.186172	2.138698	1.66284	2.249405
HORVU7Hr1G121820	0.098616	0.126221	0.210367	0.747587	4.125469	4.611625
HORVU7Hr1G111040	12.58774	12.26118	15.6251	16.06813	18.97596	16.72801
HORVU2Hr1G066820	2.470342	1.565168	2.706519	2.129937	2.257058	2.757367
Hordeum_vulgare_newGene_8632	2.049345	1.987931	1.933325	1.306139	1.391584	1.774645
Hordeum_vulgare_newGene_8637	2.709493	3.425537	3.601115	3.745377	5.548965	4.857996
Hordeum_vulgare_newGene_8638	3.458414	3.561915	4.764256	2.725663	2.436172	2.432913
HORVU5Hr1G090010	4.182099	5.978992	4.07178	4.626805	3.872987	4.44232
HORVU2Hr1G030870	4.613805	4.619915	6.248905	38.91895	1534.594	1763.72
HORVU4Hr1G082680	3.89307	5.646928	5.788502	0.917009	0.157841	0.78686
HORVU3Hr1G065500	31.98279	27.85888	37.97808	22.41428	22.6739	23.51125
HORVU1Hr1G080520	8.486514	7.589075	7.940686	11.24459	8.101065	9.71649
HORVU4Hr1G025760	4.798736	5.985693	6.053121	5.035091	4.328009	6.093341
HORVU4Hr1G060530	51.32681	44.21109	48.47799	36.82833	36.2914	38.55252
HORVU3Hr1G068900	1.221075	1.357638	2.838949	1.35138	1.082194	1.838159
HORVU2Hr1G031450	174.475	175.1099	252.6029	229.6796	467.4709	406.4465
HORVU1Hr1G060140	4.535381	5.723668	7.145618	2.511106	5.006437	5.268102
HORVU3Hr1G078760	1.155203	0.942223	0.939438	2.202104	1.959995	1.852806
HORVU1Hr1G016240	4.394129	6.817948	8.940414	6.696034	10.01007	10.40303
HORVU2Hr1G023350	56.34391	56.85784	53.09078	56.20614	80.24019	76.73641
HORVU7Hr1G068450	1.793356	1.806772	1.181598	2.251218	2.195091	1.725925
HORVU7Hr1G000630	1.005175	0.945038	1.491384	2.209282	2.530829	3.68817
HORVU2Hr1G092380	13.39472	13.83287	9.206328	10.52388	12.40659	14.97486
HORVU4Hr1G055800	1575.336	1645.684	1517.438	1609.603	1909.108	1472.577
HORVU2Hr1G073300	40.36668	38.46553	38.64673	47.60831	49.19902	52.85361
HORVU3Hr1G072100	8.039451	6.178787	8.868876	11.03462	9.465016	10.66302
HORVU2Hr1G022080	42.70684	41.73674	51.08674	37.79745	49.83304	48.14386
HORVU7Hr1G087250	2.144121	2.442188	3.192471	1.026618	1.953847	1.972678
HORVU3Hr1G104250	7.706865	8.733971	10.59891	4.232245	5.735015	5.151245
HORVU6Hr1G041750	26.76218	27.37997	39.07183	31.77064	59.51887	58.66828
HORVU5Hr1G073990	56.80401	54.00711	48.91663	59.25719	76.02595	73.1531

Hordeum_vulgare_newGene_1875	0.78636	0.670201	0.838117	2.587927	2.11504	2.462659
Hordeum_vulgare_newGene_1876	0.21131	0.056211	0	4.788407	5.323842	2.826004
Hordeum_vulgare_newGene_1877	1.554202	1.844971	1.541056	1.408331	1.87853	2.321994
Hordeum_vulgare_newGene_1871	7.222611	7.927874	7.458178	0.093419	0.203869	0.116103
HORVU1Hr1G005370	4.188916	2.040714	3.797099	1.926313	2.892615	2.953962
HORVU2Hr1G021190	10.72586	11.94016	8.884955	6.928364	6.819891	6.955517
HORVU2Hr1G063950	17.11996	15.01419	17.72057	23.76983	36.17775	34.66689
HORVU7Hr1G121170	2.124023	2.446033	2.325197	3.784554	5.003811	4.305385
HORVU2Hr1G031180	6.073674	8.402452	7.780887	6.843793	9.866358	10.17371
HORVU2Hr1G093030	54.56275	32.60891	57.31902	70.30534	104.8457	111.724
HORVU3Hr1G011330	13.58582	12.40182	14.72168	23.06773	30.31585	28.52761
HORVU3Hr1G073850	1.387041	0.866373	1.641249	1.541413	1.518281	1.633489
HORVU7Hr1G033370	19.32234	17.60441	17.47749	23.54293	35.68513	34.81669
HORVU5Hr1G048120	4.413219	5.049459	5.181146	9.730416	10.27857	9.58244
HORVU4Hr1G027260	93.00028	89.69855	53.12656	146.4044	166.5448	197.5673
HORVU6Hr1G063280	2.202069	2.094123	2.689567	2.405739	7.026623	5.20287
HORVU2Hr1G118230	6.562944	5.462523	7.02783	7.446727	6.612768	5.88812
HORVU6Hr1G035260	5.738056	5.482306	6.045388	13.67004	19.8484	20.4069
HORVU3Hr1G088220	4.864335	4.646901	3.554243	7.26203	11.48136	6.341326
HORVU1Hr1G084310	1.712237	1.616143	2.735705	0	0	0
HORVU2Hr1G038230	1.954655	2.357034	3.1091	2.414239	2.540421	2.751441
HORVU3Hr1G080010	18.74512	14.30717	20.31922	19.98755	27.45488	30.44023
HORVU6Hr1G090070	87.7993	84.77591	155.0641	58.45258	110.6814	108.4742
HORVU3Hr1G056560	2.609212	0.986008	1.321715	160.3435	428.8413	395.1532
HORVU3Hr1G094050	83.78627	87.54326	94.17777	73.09884	78.56248	79.35391
HORVU2Hr1G025020	1.258057	0.961682	1.315052	1.673195	2.267732	1.68211
Hordeum_vulgare_newGene_7113	9.422084	8.265843	8.183975	15.62845	13.62991	16.18583
Hordeum_vulgare_newGene_7114	17.88691	19.76911	21.66063	28.47592	37.27534	39.6889
HORVU6Hr1G053100	4.842435	4.297307	4.676051	8.054191	8.615931	9.010914
Hordeum_vulgare_newGene_12347	0	0.11399	0.098409	3.586758	4.665543	4.845968
Hordeum_vulgare_newGene_12345	2.375505	3.917631	2.816312	4.188222	1.557559	2.190569
Hordeum_vulgare_newGene_12342	4.939139	4.860468	5.051746	5.416276	5.606477	5.776914
Hordeum_vulgare_newGene_12341	6.318228	7.087434	7.675075	4.700779	5.100122	5.364686
HORVU2Hr1G072950	0	1.253637	1.382135	0.043358	42.91116	4.167226
HORVU5Hr1G072640	6.557177	6.48249	8.323666	7.656128	5.759834	5.030181
HORVU3Hr1G039400	13.3024	13.40141	15.71164	21.13111	28.69194	30.31031
HORVU2Hr1G092810	5.924326	6.510111	4.787356	17.77153	13.32936	14.45224
HORVU5Hr1G068870	2.957275	2.878155	4.618121	3.933279	3.203284	3.760466
HORVU5Hr1G041490	11.77374	8.772951	10.53877	15.0762	17.35828	17.92469
HORVU3Hr1G097620	2.272924	2.424707	2.736456	2.308938	2.300545	2.818908
HORVU3Hr1G017590	5.240683	7.769403	6.823452	6.079816	7.28242	6.421117
HORVU2Hr1G069960	4.779169	4.194391	5.97379	4.025134	4.58927	4.80503
HORVU3Hr1G059170	19.71704	22.1072	20.27105	25.27378	21.84952	22.97213
HORVU1Hr1G037800	42.65721	37.11286	39.54032	47.74522	69.8874	64.12046
HORVU4Hr1G046270	0.123748	0.09153	0.059008	12.06116	14.60815	17.77369
HORVU7Hr1G039780	32.35007	25.32946	32.88545	25.3904	21.26276	20.40866
HORVU3Hr1G081760	2.174036	1.965878	2.731022	5.275151	6.280031	7.768579

HORVU3Hr1G088480	4.56347	4.780832	4.890415	4.460479	5.377861	6.086739
Hordeum_vulgare_newGene_2692	0.450902	0.595912	0.651749	2.57103	2.837978	2.417961
HORVU6Hr1G026300	1.679783	1.982806	3.122651	1.626442	2.09136	1.940976
Hordeum_vulgare_newGene_13432	2.852479	4.707751	2.866504	2.758551	2.314188	3.201147
Hordeum_vulgare_newGene_13430	2.953607	3.720888	3.822324	5.619368	6.392686	6.935247
HORVU3Hr1G090530	1.97193	2.343303	2.958471	1.659969	2.294831	2.096299
Hordeum_vulgare_newGene_13438	4.76544	2.710276	4.528867	2.72108	1.878338	1.375814
HORVU0Hr1G020300	0.836372	1.06652	0.886828	1.151355	1.594318	1.638473
HORVU2Hr1G068610	1.591737	1.557141	1.059213	3.350645	5.206253	4.782062
HORVU5Hr1G033540	28.24104	19.28613	7.379044	117.4808	198.2894	196.9915
HORVU7Hr1G082830	3.453364	3.664774	4.465744	6.034349	8.827825	7.232254
Hordeum_vulgare_newGene_3564	0.145589	0	0	0.223192	8.940639	6.863758
Hordeum_vulgare_newGene_3008	13.77285	11.60349	14.80607	11.93129	11.8366	12.66912
Hordeum_vulgare_newGene_15771	2.077647	2.157076	1.873002	0.691664	0.616419	0.520424
Hordeum_vulgare_newGene_15776	0.669827	0.408777	0.589966	1.774452	2.068435	1.754359
Hordeum_vulgare_newGene_15774	0	0	0	9.852174	9.994969	10.43828
Hordeum_vulgare_newGene_15778	2.010403	2.25067	2.688746	1.678704	2.26902	2.346371
HORVU3Hr1G061410	0.604057	0.387236	0.420216	1.307472	2.170297	1.715111
HORVU5Hr1G124670	20.51991	19.40597	19.34616	20.66644	18.81027	19.93497
Hordeum_vulgare_newGene_3269	2.529434	3.491035	3.794602	0	0	0
HORVU3Hr1G110540	1.745461	2.37575	2.194682	0.069329	0.106697	0.300877
HORVU5Hr1G075960	6.890003	7.096927	7.522242	6.816047	8.03849	7.881898
HORVU0Hr1G018200	26.38574	21.7685	26.2211	24.07082	28.76323	28.99642
HORVU1Hr1G003260	6.254728	25.39213	6.47815	21.79893	0.471368	0.319105
HORVU5Hr1G073690	5.058525	0.988491	5.860141	5.482268	2.513185	3.060634
HORVU2Hr1G036320	20.54271	21.10675	24.7642	34.1031	39.07348	39.10989
Hordeum_vulgare_newGene_783	0.247307	0.306004	0.192442	5.603202	4.726167	6.689538
HORVU2Hr1G033130	1.633748	1.732662	1.961541	1.604699	5.056607	3.873769
Hordeum_vulgare_newGene_784	0.582354	0.842117	0.445191	13.21065	10.14247	13.40413
HORVU4Hr1G021090	1.805547	1.613204	1.924351	1.327795	6.994369	7.519737
Hordeum_vulgare_newGene_788	2.457158	2.50705	2.699159	0	0	0
HORVU2Hr1G035810	2.344702	1.131035	1.859155	2.355002	0.114331	0.631854
HORVU3Hr1G013740	1.986426	1.895959	1.831692	2.214449	3.986297	4.538015
HORVU0Hr1G023140	5.422917	5.030397	5.802342	6.631689	8.31431	8.178094
Hordeum_vulgare_newGene_14287	10.24814	7.851631	11.23185	12.03146	20.46925	18.47481
HORVU5Hr1G024410	8.122007	6.970702	8.916615	12.32133	15.96054	13.3238
Hordeum_vulgare_newGene_14288	37.29143	33.24595	37.48816	26.63793	36.06535	39.62903
HORVU2Hr1G003970	5.966355	7.67874	6.483097	11.01394	17.35498	14.79847
HORVU4Hr1G027410	46.0367	49.76581	47.35949	48.62481	59.39747	60.08366
HORVU4Hr1G011420	1.49788	1.638296	1.617929	2.540925	3.059172	3.461064
HORVU1Hr1G020170	1.156543	1.098464	0.859981	1.939855	0.908946	1.39869
HORVU1Hr1G054130	9.164875	12.9019	12.79443	9.666844	10.41368	11.26549
HORVU7Hr1G043050	4.419519	4.642076	5.193088	10.18164	6.699861	8.135522
Hordeum_vulgare_newGene_872	5.070189	5.247245	4.133842	2.335794	5.283875	3.88817
HORVU5Hr1G063960	4.030547	4.148975	5.573057	11.80098	23.70008	20.21141
Hordeum_vulgare_newGene_15481	2.84385	2.747758	2.52444	3.768792	2.143466	2.317421
Hordeum_vulgare_newGene_878	4.202508	3.716939	4.341605	4.705308	6.532807	6.51611

Hordeum_vulgare_newGene_15486	0.576568	0.56248	0.556723	1.654676	1.281789	1.886239
Hordeum_vulgare_newGene_6939	3.816423	3.528833	3.965015	3.816605	5.645561	5.477862
HORVU5Hr1G106800	27.82742	24.20608	32.00167	30.26574	44.08162	40.51455
HORVU7Hr1G059060	4.661691	6.529294	7.101098	3.911556	4.047142	4.412837
HORVU1Hr1G074840	4.728214	3.362057	4.624219	3.520936	2.87201	3.23842
Hordeum_vulgare_newGene_8572	0.740776	0.676007	0.757196	3.173402	2.602377	3.054116
Hordeum_vulgare_newGene_8570	6.642438	6.315849	8.47877	6.470195	7.286916	8.57714
HORVU4Hr1G028110	0.027842	0	0	6.440852	6.217307	6.720296
HORVU3Hr1G004520	68.43717	76.09737	64.20551	42.83359	44.00049	44.55884
HORVU3Hr1G051340	11.9592	14.10544	13.74897	11.3763	12.4452	15.73297
HORVU3Hr1G115820	62.41577	59.65738	106.6788	73.36475	146.4348	139.8811
HORVU6Hr1G068660	4.319685	3.850248	3.377857	5.516872	6.082382	5.538888
HORVU1Hr1G024210	24.40141	19.11627	27.82385	28.40512	43.20403	40.92795
HORVU3Hr1G018650	7.64636	6.665312	7.286514	16.25712	22.86136	21.86388
HORVU4Hr1G031600	6.959995	7.867719	7.520604	7.769389	7.501352	8.831724
HORVU4Hr1G035380	31.57897	30.88849	45.99124	18.75757	16.23525	17.67785
HORVU0Hr1G032640	2.571503	4.3975	1.274678	9.948519	83.94389	85.27399
HORVU5Hr1G038450	5.825191	3.502636	4.04719	4.922806	4.105526	4.243762
HORVU7Hr1G060600	7.791725	9.454145	9.357919	7.567535	8.948297	9.281479
HORVU4Hr1G089560	2.189774	1.961976	1.823129	2.915634	5.971348	5.417955
HORVU3Hr1G069520	5.866053	5.813485	6.501231	10.2006	14.75252	13.91115
HORVU5Hr1G037290	9.696792	10.201	12.67109	11.59932	14.34979	14.97973
HORVU4Hr1G032550	1.364765	1.375151	1.922196	2.407111	2.628561	3.581412
HORVU6Hr1G082970	2.334001	1.937996	2.725096	3.384849	3.734545	4.253119
Hordeum_vulgare_newGene_11626	7.336969	6.409991	7.35572	5.754096	8.718265	7.703887
HORVU4Hr1G008750	16.51401	20.15109	15.80894	19.50988	32.04691	33.25148
HORVU4Hr1G088210	30.066	33.63483	22.93245	31.86204	45.74435	42.51587
Hordeum_vulgare_newGene_1685	1.172224	1.173846	1.204048	3.130589	2.4488	2.246912
HORVU7Hr1G039330	0.453384	0.647494	1.077137	1.794952	2.253484	2.166687
Hordeum_vulgare_newGene_1683	1.398449	1.56255	1.512661	2.467239	3.167125	3.90366
Hordeum_vulgare_newGene_1681	3.840997	4.435586	4.747651	4.131527	3.193614	3.496768
Hordeum_vulgare_newGene_1689	9.66657	7.799794	11.61036	6.187826	4.566574	5.345325
HORVU7Hr1G048820	0.668751	4.472567	5.635473	3.385642	3.318371	2.603185
HORVU5Hr1G018150	3.923128	5.011713	9.000698	6.358494	7.082471	7.254362
HORVU3Hr1G049900	3.860538	3.761201	4.753589	13.80239	26.03617	21.74257
Hordeum_vulgare_newGene_10395	3.527632	3.127861	4.889909	0	0	0
HORVU7Hr1G101410	2.781119	3.29226	3.133727	4.90557	6.617721	6.664565
Hordeum_vulgare_newGene_4304	2.314217	3.045563	1.886002	1.587039	0.786653	1.331103
HORVU0Hr1G006640	1.474115	1.118894	1.947631	6.249462	7.915837	8.222698
HORVU1Hr1G072710	7.086382	5.841737	8.652656	10.25578	10.68151	7.819401
HORVU2Hr1G018820	41.62607	43.19683	66.91548	36.38147	87.29551	76.42333
HORVU7Hr1G019830	235.9458	187.8113	207.2835	136.3359	76.02704	81.96671
HORVU1Hr1G052230	6.502536	4.735427	7.075422	7.924486	11.88422	11.93466
HORVU7Hr1G053390	4.173042	2.805639	3.710734	5.030897	5.611976	6.780376
HORVU7Hr1G064610	3.625759	3.245703	3.020495	3.462376	2.584763	3.60684
HORVU0Hr1G039050	13.80236	11.82707	14.98476	23.47838	23.24101	20.85442
HORVU2Hr1G079320	3.614172	3.013674	1.963801	3.663224	1.683594	3.267248

Hordeum_vulgare_newGene_7658	19.82966	16.14106	16.18917	10.4089	6.237619	6.512892
Hordeum_vulgare_newGene_7656	35.39044	26.64278	38.02694	24.89463	31.6729	32.40908
HORVU2Hr1G035160	1.232971	1.355237	2.475545	1.412073	1.8817	1.576921
HORVU2Hr1G042460	23.88865	24.30941	24.01261	31.40772	37.04981	32.38133
HORVU3Hr1G001000	106.7827	97.99442	165.0948	59.93417	46.55067	44.56619
HORVU6Hr1G081400	4.302243	4.433727	4.715602	16.01098	14.47996	15.10829
HORVU3Hr1G035680	5.654621	2.223299	6.334851	0.143147	0.053269	0.082251
HORVU3Hr1G084920	1.796606	1.98771	2.000797	2.302007	4.673885	5.118573
Hordeum_vulgare_newGene_8645	36.59902	39.74307	34.31254	28.17524	35.63725	34.06289
HORVU1Hr1G068580	8.180788	6.830362	9.548966	7.818413	9.805683	9.74326
HORVU5Hr1G061950	3.997368	2.765468	2.980217	3.446762	1.94362	2.813551
HORVU0Hr1G024320	2.080609	2.391082	2.381533	2.021475	1.519812	2.326389
HORVU0Hr1G015420	0.630395	0.783717	0.731783	0.712808	4.151121	4.202112
HORVU6Hr1G072940	7.903348	9.050558	11.61126	7.296819	10.57472	10.22514
HORVU7Hr1G079510	3.69865	3.401057	4.835892	4.800805	3.730013	4.113112
HORVU7Hr1G038990	5.846796	5.878982	6.163401	4.459279	4.827747	5.513146
HORVU7Hr1G115100	2.628294	2.838391	3.594495	3.302326	5.690719	5.312448
HORVU2Hr1G124400	6.251654	6.934797	8.467781	8.15356	16.68693	16.23637
HORVU7Hr1G093020	35.48233	30.09603	40.99462	13.39438	13.6313	12.18059
HORVU0Hr1G036550	1.0928	1.376558	2.642076	1.068079	1.227233	0.7974
HORVU1Hr1G074450	3.765725	3.388707	3.001499	6.681658	4.450028	4.522136
HORVU1Hr1G045530	44.45757	42.05206	46.31326	21.37602	16.42298	15.21543
HORVU3Hr1G041820	3.056566	3.083258	3.148783	2.750479	2.41584	1.873848
HORVU2Hr1G079580	42.51262	39.39062	34.0633	4.253272	1.782211	3.426474
HORVU1Hr1G083270	1.349316	0.89441	1.170355	0.869068	2.249633	2.417679
HORVU5Hr1G120210	1.649993	1.243542	1.833158	4.327095	7.151099	5.986092
HORVU3Hr1G006250	142.3749	116.3664	151.1833	77.25488	57.32586	56.7913
HORVU0Hr1G040480	0.118589	0.158641	0.025744	0	4.567154	7.42626
HORVU1Hr1G069180	10.35735	8.701348	10.44102	9.451581	7.48149	9.929015
HORVU1Hr1G061780	12.38157	11.52097	14.60992	7.885433	11.33393	13.37976
HORVU6Hr1G085410	6.996763	6.113885	7.714042	9.924988	8.513852	9.25942
Hordeum_vulgare_newGene_5246	1.868719	1.978265	1.604598	0.461134	0.006852	0.311132
HORVU1Hr1G045280	1.958598	2.124472	2.762467	3.979506	5.161252	5.065082
HORVU3Hr1G007120	0.503182	0.665264	1.14635	1.004734	1.379005	1.658781
HORVU3Hr1G028280	1.739174	1.053881	1.931039	2.561361	1.247259	2.024196
HORVU5Hr1G095550	2.67465	2.943617	2.768236	2.375298	2.084005	1.504146
HORVU4Hr1G082910	6.678791	6.824169	6.405647	19.47125	18.35642	18.56279
HORVU4Hr1G010430	2.080323	2.045761	2.040094	6.059139	10.14874	9.191223
HORVU1Hr1G055300	17.17036	15.94634	19.72963	19.45215	20.71796	23.85079
HORVU5Hr1G124240	1.895332	1.901627	2.190115	0.747083	1.573537	1.740058
HORVU4Hr1G069390	4.089388	4.529143	4.65136	6.504387	7.319634	7.969248
Hordeum_vulgare_newGene_14257	5.810069	4.902792	4.969429	0.637532	0.573157	0.229986
HORVU4Hr1G007340	7.793537	4.507606	7.847791	6.915535	5.676752	4.597244
HORVU3Hr1G078210	5.137871	7.079177	9.465103	3.23072	2.251171	2.540561
HORVU7Hr1G000380	8.979752	9.013249	9.690984	8.913939	13.73396	13.93784
Hordeum_vulgare_newGene_13200	2.657071	2.659114	3.167053	3.02495	4.575707	4.581456
HORVU7Hr1G092040	0.024527	0	0.024992	0.458291	5.014194	3.917018

HORVU3Hr1G057010	1.337354	1.329879	1.365029	2.11846	3.276184	3.461049
HORVU1Hr1G004590	10.24	10.16981	11.08379	12.51527	11.7463	11.21773
HORVU5Hr1G009460	2.097624	1.992129	1.83249	12.73743	6.730438	8.736968
Hordeum_vulgare_newGene_14259	15.00617	8.498634	18.78276	0.440261	0.092681	1.336139
HORVU1Hr1G089680	4.771312	4.073405	4.996678	7.557808	8.701385	8.909127
HORVU3Hr1G037170	3.322512	3.88662	4.200599	4.683577	6.799567	6.042697
HORVU4Hr1G011740	222.9908	186.0448	261.3521	59.37338	23.73785	32.39656
HORVU5Hr1G092120	51.34617	57.47506	83.7829	17.25612	0.405442	8.038272
HORVU4Hr1G056140	46.46519	38.18119	45.98995	57.51585	74.60631	72.83326
HORVU5Hr1G110280	28.19619	27.51126	30.70773	32.88898	45.2258	41.87523
HORVU3Hr1G037790	31.26442	24.14039	38.50126	34.85723	44.71238	46.56514
HORVU2Hr1G085300	1.444529	1.731844	1.63876	2.68811	3.438304	3.481457
HORVU5Hr1G038710	2.147052	2.580253	3.912973	2.550772	2.711773	2.503662
HORVU3Hr1G066860	0	0	0	0	5.732639	8.919146
HORVU5Hr1G051220	15.30578	12.67836	15.94325	15.30364	21.48565	20.90788
HORVU5Hr1G114620	16.86516	17.61357	19.60215	12.15861	13.84193	13.72116
HORVU5Hr1G043130	3.425033	3.657537	4.200145	2.35267	3.130709	2.222601
HORVU1Hr1G021990	0.399846	0.722743	1.391295	0.991006	1.550748	1.604158
HORVU2Hr1G110340	4.373843	5.576521	4.000874	5.340939	10.63316	10.67336
HORVU7Hr1G088890	76.9867	54.46307	76.45125	141.2226	287.2632	226.3882
HORVU1Hr1G068910	26.1401	18.99239	25.39373	10.98641	8.606182	10.68793
HORVU4Hr1G084400	5.31059	5.871602	6.007781	6.019969	21.15252	30.57414
HORVU6Hr1G005080	2.074166	2.770973	1.740468	1.812434	2.165842	2.849245
HORVU2Hr1G009890	12.54333	13.12343	13.92892	17.45866	18.49837	20.8652
HORVU2Hr1G065090	1.237697	1.112179	0.84991	3.115128	6.148339	5.901395
HORVU0Hr1G012370	3.533133	3.57077	3.816481	3.767568	3.381212	3.360549
HORVU4Hr1G015110	1.66792	2.168228	2.557806	1.721204	2.132649	2.564078
HORVU0Hr1G020140	1.982856	1.690413	2.440614	2.93916	3.843642	4.046675
HORVU3Hr1G098340	5.690944	2.332957	7.925089	2.69519	2.403849	2.619777
HORVU7Hr1G108240	0.876485	1.320804	1.532605	2.734826	6.033288	5.311444
HORVU5Hr1G057500	7.506447	7.169224	7.929875	7.903734	7.574018	8.480231
HORVU1Hr1G054530	1.546194	1.441867	2.100405	0.80356	0.827802	0.77274
HORVU4Hr1G012460	4.803421	4.202201	5.80984	5.759382	7.789349	1.023631
HORVU6Hr1G033380	5.408305	5.462255	5.747422	4.197912	4.471838	4.619407
HORVU7Hr1G003140	0.105844	0.04547	0	1.688849	47.91591	44.41026
HORVU2Hr1G071110	4.154684	3.388311	4.4336	3.588466	5.561675	5.340259
HORVU6Hr1G053250	177.3966	172.1575	161.4605	143.4129	188.7156	185.7686
HORVU4Hr1G004080	8.044419	7.055305	8.023898	6.907399	5.697172	6.547492
Hordeum_vulgare_newGene_9020	3.92012	4.745251	3.684084	2.59167	2.850105	4.289735
HORVU0Hr1G022780	3.197332	3.017081	4.370815	9.271917	14.28261	14.31634
Hordeum_vulgare_newGene_9024	1.150938	0.848118	1.289362	2.692232	2.571182	2.744756
Hordeum_vulgare_newGene_2767	0.263491	0.527982	0.343376	13.09776	11.93591	11.39117
HORVU6Hr1G025750	1.098308	1.378247	1.509352	1.324019	2.173303	1.741983
Hordeum_vulgare_newGene_2769	0.381458	0.225814	0.316592	4.460325	4.826955	4.967592
Hordeum_vulgare_newGene_2768	2.468657	2.450156	3.023552	3.516701	3.586526	3.777482
HORVU4Hr1G021250	1.799835	0.978376	1.11904	2.31737	2.497025	2.248171
HORVU2Hr1G091280	2.184323	1.554091	1.954874	3.078886	7.242077	6.072994

Hordeum_vulgare_newGene_2603	1.028866	1.253352	1.014234	2.555242	2.616452	2.861737
Hordeum_vulgare_newGene_2600	0	0.648469	1.035883	1.366074	3.819999	1.714988
HORVU3Hr1G064570	0.718996	1.09269	2.044964	0.954077	1.378292	1.416063
HORVU1Hr1G004960	27.49226	25.85299	28.23024	46.36895	60.4855	54.75419
HORVU7Hr1G027810	8.048363	5.086423	7.777548	1.723452	0.93322	1.411011
Hordeum_vulgare_newGene_5230	6.560726	7.718661	6.853211	6.075469	7.397443	7.405695
Hordeum_vulgare_newGene_9797	2.017453	1.424783	1.45841	1.850376	2.200488	2.574016
Hordeum_vulgare_newGene_10564	2.067298	1.645554	3.789151	0.851201	0.624831	1.285205
Hordeum_vulgare_newGene_9799	1.368615	1.428115	2.841197	5.011506	5.617127	5.42518
HORVU4Hr1G090440	27.75119	25.66034	28.28235	119.5942	243.1091	199.1813
HORVU1Hr1G077430	23.09836	20.05222	25.85107	30.60345	36.14613	34.72621
HORVU7Hr1G043690	3.608619	2.559569	4.171483	3.069401	2.710407	3.654076
Hordeum_vulgare_newGene_15558	2.320314	2.790026	2.732591	3.263979	3.510256	4.580911
Hordeum_vulgare_newGene_8774	0.650807	0.47098	0.529564	15.26489	65.32352	68.03099
Hordeum_vulgare_newGene_8778	1.753776	2.147453	1.799667	0.994899	0.683781	0.19651
HORVU2Hr1G105320	7.802956	7.485529	7.901515	7.39141	3.848026	5.517511
HORVU3Hr1G110830	2.108683	1.852973	1.609558	1.012521	0.878042	0.820663
HORVU5Hr1G053330	0.939186	1.083419	1.12668	2.07825	5.193274	5.453401
HORVU2Hr1G027860	30.70974	25.69691	37.12257	30.23879	57.08187	61.46883
HORVU5Hr1G080560	6.636042	9.755869	12.51828	5.075017	4.26458	5.053621
HORVU2Hr1G112250	0.63287	0.587552	0.483281	1.012712	1.998647	2.205706
HORVU3Hr1G095930	2.483282	2.707073	3.734941	0.054512	0.089204	0.031705
HORVU1Hr1G057320	0.824609	0.950316	1.553942	1.845696	3.12841	3.58695
HORVU7Hr1G083670	1.747631	1.057093	2.188824	2.463175	3.254127	2.581921
HORVU1Hr1G017800	1.029897	0.720363	1.399786	9.18075	33.71595	35.60981
HORVU7Hr1G020660	3.538617	3.915235	4.470548	5.420118	7.02207	5.5211
Hordeum_vulgare_newGene_5957	5.261612	6.323855	3.691294	5.732894	6.526394	4.301925
HORVU1Hr1G040130	0.748297	0.788466	0.644365	24.36441	28.11761	34.75201
Hordeum_vulgare_newGene_10495	1.531314	1.274953	2.447341	1.247422	1.691047	1.374632
Hordeum_vulgare_newGene_10490	0	0	0	3.513037	1.27162	2.10152
Hordeum_vulgare_newGene_10492	2.708674	3.380221	2.037693	0.071508	0	0.025877
HORVU3Hr1G026180	7.328826	7.704832	10.77943	3.489101	2.526482	2.027927
HORVU3Hr1G074600	0.658102	0.190133	0.494304	3.027176	3.512781	3.500676
HORVU2Hr1G115700	4.673654	4.291593	5.472715	4.880872	6.751445	5.996887
HORVU1Hr1G018060	56.61482	47.29522	64.62096	48.89605	62.17117	61.47982
Hordeum_vulgare_newGene_1444	0.28576	0.223622	0.262762	4.05913	3.35056	3.589151
Hordeum_vulgare_newGene_10725	2.943203	3.195354	2.434722	1.649149	1.283084	1.135442
HORVU2Hr1G102580	1.008671	1.068457	1.109628	1.183384	1.132249	1.311708
HORVU3Hr1G072730	7.758751	5.178941	7.278719	6.458407	5.285658	4.653626
HORVU3Hr1G095200	2.038615	1.878793	2.077646	0.274015	0.681162	0.290965
HORVU1Hr1G035990	1.973406	2.512757	1.7728	0	0.058702	0
HORVU6Hr1G071950	1.111224	0.938539	1.075926	1.338293	1.641851	1.47919
HORVU1Hr1G028190	28.76289	25.08208	29.72192	30.90612	41.09735	40.38822
HORVU3Hr1G068660	43.14619	37.45615	48.72287	46.27695	65.77536	62.98915
HORVU7Hr1G082200	0.729577	0.76952	0.724869	1.643695	1.17149	1.332754
HORVU4Hr1G029420	0.250262	0.409932	0.245095	2.661748	3.821296	2.752994
HORVU7Hr1G080510	36.61309	36.93029	33.8284	5.372752	1.065077	2.660963

HORVU7Hr1G033500	4.557137	4.455917	3.467888	11.81801	22.14108	23.57508
HORVU6Hr1G048600	1.02445	1.251195	1.652618	1.189961	3.671102	3.072832
HORVU6Hr1G020980	4.075889	3.633676	3.154108	2.398895	1.204479	1.289822
Hordeum_vulgare_newGene_4679	7.981008	9.099386	9.442943	0.048671	0	0.040314
HORVU1Hr1G030480	20.30411	20.00341	18.598	22.05158	25.19817	26.6549
HORVU6Hr1G077070	1.964154	2.112507	2.285521	3.254301	3.689905	3.723436
HORVU2Hr1G028190	15.67271	14.93556	14.18262	14.54206	17.58705	14.53995
HORVU1Hr1G036630	3.777091	2.960492	3.516378	1.524868	2.150248	2.692128
Hordeum_vulgare_newGene_7322	0.181104	2.642791	3.592284	3.467117	3.904284	4.779225
Hordeum_vulgare_newGene_7326	0.729684	0.904414	0.917063	1.708791	1.521803	2.262202
HORVU6Hr1G003020	0	0.029951	0	3.182791	7.630812	3.091117
HORVU5Hr1G084450	7.747068	8.706421	8.494819	8.606495	11.78702	12.9832
HORVU1Hr1G075120	1.77764	1.658096	0.997786	2.754006	3.328431	3.463086
HORVU4Hr1G062930	1.631193	1.498767	2.229153	1.508553	2.884083	3.521029
HORVU5Hr1G082680	5.100624	6.405936	8.111846	7.384821	8.821357	8.891287
HORVU3Hr1G085230	6.038233	6.059925	6.771152	8.744365	7.759807	9.537355
Hordeum_vulgare_newGene_214	4.2246	4.911078	7.743568	6.069856	21.38163	20.92756
Hordeum_vulgare_newGene_215	29.1483	23.80285	36.95786	22.83354	13.57739	17.09726
HORVU2Hr1G070040	21.17103	21.84423	23.97047	24.4148	31.95025	35.41721
HORVU3Hr1G017940	8.055448	6.30372	7.277114	3.539084	1.967983	2.757154
HORVU7Hr1G035900	4.689874	5.105579	6.007289	7.015327	11.53542	11.61343
HORVU2Hr1G028220	25.4163	19.64036	25.21177	24.37052	36.97135	34.09819
HORVU6Hr1G067660	12.26874	12.49173	18.38433	35.4142	59.84647	39.26063
HORVU5Hr1G037120	1.297056	1.549632	1.299503	2.584198	3.734431	3.271667
HORVU3Hr1G060640	4.007771	4.317994	4.333836	4.237634	4.730504	4.51337
HORVU2Hr1G013440	3.378135	3.614541	4.340176	4.341868	3.451486	3.100997
HORVU7Hr1G011150	0.272626	0.30892	0.272464	0.882141	7.92913	5.637592
HORVU3Hr1G039230	5.040901	4.411489	6.268123	6.949648	6.034895	6.839493
HORVU6Hr1G095250	8.360534	8.209073	8.80823	13.98068	14.21061	14.20576
HORVU3Hr1G113110	17.59936	13.05576	22.04344	13.63735	19.12322	18.15734
HORVU3Hr1G095330	3.857134	3.709788	3.926001	10.49105	17.21233	17.55425
HORVU5Hr1G095580	0.516632	1.019108	0.288042	22.2703	1.637978	11.47878
HORVU6Hr1G029110	28.58622	31.61289	26.61984	32.90271	29.2941	35.51394
HORVU3Hr1G035230	8.86007	8.771608	8.853503	12.45906	13.00123	13.06156
HORVU4Hr1G058340	0.72512	0.419334	0.8582	1.754849	1.662706	2.078224
Hordeum_vulgare_newGene_13600	15.29407	15.20788	14.51757	36.04911	64.39185	58.05852
Hordeum_vulgare_newGene_13605	0.946595	0.783327	0.640313	0.460295	3.832828	4.04614
HORVU6Hr1G088710	2.968036	4.698071	4.207259	1.863239	1.552805	1.692355
Hordeum_vulgare_newGene_3151	1.195335	0.92759	0.918921	1.976051	2.380224	2.695577
Hordeum_vulgare_newGene_3157	2.064898	0.901188	2.619399	1.681722	2.358074	1.72799
HORVU5Hr1G031980	33.58245	33.49988	33.22492	40.29005	56.28147	48.8976
HORVU5Hr1G087810	57.64579	60.15086	93.83713	134.1767	158.908	195.5083
HORVU6Hr1G078390	24.89812	21.39734	22.53678	0.355229	0.083012	0.187867
HORVU3Hr1G087490	6.909115	5.927968	6.657444	7.384627	11.64387	9.355616
HORVU1Hr1G055140	16.59961	13.1064	9.669093	10.21207	13.91758	13.81968
HORVU6Hr1G082350	34.00314	34.01154	45.48225	45.09217	48.01648	45.30978
Hordeum_vulgare_newGene_2207	16.25536	16.15669	19.58165	25.93952	27.24611	28.11168

HORVU5Hr1G057970	3.913005	0.547158	0.755812	0.437748	2.555927	2.432749
HORVU7Hr1G003460	26.9357	33.18148	27.83474	36.62654	50.73194	48.10783
HORVU7Hr1G054520	3.113646	2.858216	3.942849	2.988469	2.941375	3.928827
HORVU2Hr1G116540	4.238536	5.424467	7.202333	2.331336	2.424659	2.944808
Hordeum_vulgare_newGene_2363	0.232231	0.209511	0.385837	2.111538	5.740289	4.411856
HORVU3Hr1G060780	11.96567	12.27402	11.86936	13.84326	18.40432	17.44353
Hordeum_vulgare_newGene_13419	2.425051	2.679094	3.176773	4.0663	4.937657	5.392505
Hordeum_vulgare_newGene_15529	3.306557	3.506803	3.261641	4.930909	5.865111	5.728049
HORVU3Hr1G087140	5.641171	6.877996	6.228118	5.593949	6.404106	7.512365
HORVU4Hr1G010180	2.038844	2.528077	2.612984	3.178154	3.314787	3.991724
HORVU4Hr1G013840	81.17916	57.84473	66.53171	154.6824	91.58234	111.5102
HORVU1Hr1G055490	5.832981	5.441685	6.367853	6.886807	7.353174	6.776241
HORVU0Hr1G031830	1.617276	1.230273	1.275262	7.636856	5.10322	5.950725
HORVU3Hr1G003130	3.393111	4.007308	4.667968	1.35644	1.876112	0.862893
HORVU3Hr1G048750	3.633304	3.513244	3.575788	5.055189	6.289824	5.846062
HORVU6Hr1G034110	0.365857	0.360325	0.745885	0.64447	2.152131	2.779157
HORVU2Hr1G086790	1.461652	1.102592	1.429936	2.093968	3.543903	3.243798
Hordeum_vulgare_newGene_3549	0.095661	0.078236	0.151268	0.757813	16.10056	17.50457
HORVU3Hr1G096910	0.383554	0.4712	0.429637	5.668015	8.451061	8.117142
HORVU7Hr1G048570	12.07178	10.56781	12.64167	44.74308	45.12236	46.4559
Hordeum_vulgare_newGene_8980	2.541503	3.33871	3.454197	2.54163	5.157812	6.067726
HORVU1Hr1G021920	1.105381	1.981983	1.24446	7.477368	11.3803	7.669464
Hordeum_vulgare_newGene_8988	143.0926	133.8131	205.5795	135.8128	129.6484	142.6483
HORVU0Hr1G002840	1.328538	1.507508	1.975612	1.135476	0.130984	0.356207
HORVU2Hr1G068630	0.881962	0.631112	1.006404	1.379571	2.195714	2.535929
HORVU6Hr1G083900	6.401457	5.722982	7.504635	6.828541	6.984833	7.134643
HORVU4Hr1G080720	0.392878	0.347252	0.325341	3.582851	4.109783	3.542066
HORVU7Hr1G082850	3.346508	4.787521	3.625389	4.466942	6.762738	5.381031
HORVU3Hr1G012280	14.32853	14.65617	17.6107	7.545701	7.657742	8.386378
HORVU6Hr1G016170	9.721237	8.090513	9.420094	5.994528	5.988421	6.529858
HORVU1Hr1G052910	27.741	25.02508	26.38123	19.49855	20.72245	20.29193
HORVU5Hr1G040440	21.30663	26.8326	31.21773	3.437984	0.285548	1.155204
HORVU3Hr1G030040	11.1425	11.55445	12.72037	9.048847	9.923425	9.354709
HORVU5Hr1G062220	5.213221	4.551723	6.466015	5.350724	6.759885	6.225856
HORVU2Hr1G077080	13.90822	11.57768	15.01089	16.81551	9.9863	15.46513
HORVU7Hr1G097020	10.05236	9.533682	11.46608	7.857676	11.21099	10.97795
HORVU7Hr1G049220	40.04891	33.11699	38.12878	34.58506	38.25075	39.44982
HORVU4Hr1G005590	4.760681	5.143875	4.803719	4.827098	5.275451	6.057768
HORVU2Hr1G124260	7.071576	7.087131	7.640922	8.300193	9.637292	9.424907
HORVU1Hr1G062500	9.046063	8.821137	10.09129	7.632348	14.91513	12.41721
HORVU1Hr1G025340	7.87497	6.945288	7.807633	8.868788	11.04894	10.01949
HORVU2Hr1G029900	16.40181	13.60022	18.83753	25.56148	19.39535	19.21658
HORVU5Hr1G051790	18.3386	16.22471	21.84464	17.26572	27.06145	24.48454
HORVU4Hr1G018250	2.166451	1.822904	2.348417	1.411297	1.334356	1.518668
HORVU4Hr1G088390	43.64806	44.66691	43.38913	52.92034	64.98123	68.04614
HORVU7Hr1G113230	1.245736	1.508333	1.158368	1.827474	2.950071	2.955989
HORVU5Hr1G066830	1.760603	1.420698	1.630416	1.691952	1.93576	1.703213

HORVU7Hr1G115800	72.24897	59.2008	69.00506	53.27771	64.30469	62.26393
HORVU7Hr1G088560	23.21064	22.10455	25.71961	10.0769	5.19719	8.85503
Hordeum_vulgare_newGene_1161	3.103909	2.866157	3.069678	3.427575	1.668966	1.706802
Hordeum_vulgare_newGene_1166	0	0	0	2.204498	2.640988	2.088552
Hordeum_vulgare_newGene_1165	1.223607	1.334564	1.424615	1.332488	1.609868	1.297057
HORVU1Hr1G073670	2.999686	2.397053	2.619087	10.88298	41.39064	42.45311
HORVU5Hr1G096480	1.335618	1.376575	1.505801	3.631946	4.417214	3.47006
HORVU7Hr1G091180	1.567266	2.634085	2.788078	0.612819	0.290082	0.440261
HORVU2Hr1G078280	5.016685	4.144081	7.171511	4.243413	6.036428	5.972005
Hordeum_vulgare_newGene_4538	3.255929	3.545389	3.670194	1.555812	2.330106	2.688711
Hordeum_vulgare_newGene_4539	16.60715	14.19603	15.63252	33.54657	33.214	34.44591
HORVU1Hr1G057630	2.645182	2.964886	2.601264	4.916512	7.444346	6.729576
Hordeum_vulgare_newGene_4533	1.578903	1.682521	1.31016	2.65701	3.430708	4.133541
Hordeum_vulgare_newGene_4531	11.62402	8.999039	11.33901	11.4511	22.62742	19.85064
Hordeum_vulgare_newGene_4536	12.54179	12.96991	13.90462	21.66445	28.67966	27.32182
HORVU3Hr1G031000	1.891568	1.305825	1.238644	0.152059	0.762207	0.807312
HORVU2Hr1G127550	1.72071	2.104326	1.331236	1.301957	1.519023	1.585452
HORVU0Hr1G014630	1.633044	2.206979	2.078607	2.12692	1.81894	1.750406
HORVU7Hr1G114170	1.42191	1.476735	1.561172	8.286576	18.10565	13.68208
HORVU4Hr1G008270	15.76901	16.25237	17.61863	36.10357	58.91036	55.82242
HORVU7Hr1G022330	0.635581	0.542925	0.79138	1.703845	1.775967	2.452368
HORVU7Hr1G085130	14.99086	12.58588	17.81267	23.98191	20.47526	14.47527
HORVU2Hr1G087970	38.14765	31.64426	49.76208	15.98295	42.76951	42.98842
HORVU4Hr1G074750	88.11466	116.7694	129.3429	14.14853	1.478443	8.095736
HORVU3Hr1G005420	1.905732	2.00292	1.757393	3.307643	3.636034	4.016847
HORVU1Hr1G052000	1.904189	2.179438	1.825549	2.22692	2.905333	2.91889
HORVU5Hr1G018340	3.890846	2.966468	3.120263	6.798164	4.838637	5.27001
HORVU1Hr1G090250	2.726452	1.9862	2.436069	3.60113	2.12725	2.291722
HORVU1Hr1G056580	0	0	0	4.745669	3.585837	4.492761
HORVU3Hr1G078530	8.557231	8.262457	7.843324	14.28046	15.31021	17.08108
HORVU0Hr1G001180	1.730272	1.907229	2.227172	20.71706	24.87084	23.92723
HORVU2Hr1G003210	9.820489	6.149372	13.47907	2.257723	0.503413	2.394377
HORVU7Hr1G018900	3.037285	2.695915	3.092648	3.559847	5.157503	5.053719
HORVU6Hr1G081670	1.364037	1.144576	1.244834	0.968947	1.3469	1.124502
HORVU1Hr1G068420	1.982518	1.704997	2.273538	0	0.579778	0.461786
HORVU3Hr1G070040	1.925264	2.155203	2.257255	2.288497	2.669646	3.215456
HORVU6Hr1G083120	53.73167	55.38942	92.78159	40.20325	76.95653	66.49002
HORVU4Hr1G016340	19.01042	16.93411	21.73586	28.82465	26.95311	23.82422
Hordeum_vulgare_newGene_9678	4.330862	3.594652	3.631417	3.195663	2.486646	2.667987
HORVU6Hr1G002980	5.756362	5.477204	6.038758	4.78731	5.645756	6.625935
HORVU5Hr1G022140	0.351346	0.196058	0.229081	4.123525	6.484403	8.880962
HORVU1Hr1G055800	1.115442	1.442777	1.536994	2.202626	6.265044	4.381138
HORVU5Hr1G094500	58.77947	48.26448	59.35579	51.40704	70.66356	71.32471
HORVU1Hr1G019580	2.038555	2.685034	3.233492	1.340187	1.845917	1.87923
HORVU1Hr1G001490	0	0	0	12.59064	0.26059	0.306429
HORVU3Hr1G077710	1.167065	1.649541	1.37773	1.813203	2.053634	2.378042
HORVU4Hr1G060310	6.225991	5.077884	5.978655	2.405289	1.876977	2.358542

HORVU4Hr1G017430	18.19804	17.16166	18.8383	15.18725	15.28522	17.87792
HORVU3Hr1G078390	10.76566	10.57926	16.6116	1.253104	0.030747	0.395324
HORVU5Hr1G052660	2.79805	3.161368	3.593605	3.4074	4.23607	4.837653
HORVU5Hr1G111850	5.630232	6.086393	7.332401	7.858722	7.97436	7.364102
HORVU5Hr1G116800	1.027702	1.199791	1.465105	3.658201	5.209569	4.233411
HORVU6Hr1G033480	1.92856	2.102196	2.173116	1.971153	2.628528	2.418523
HORVU4Hr1G059750	3.391331	3.819455	5.138579	3.930143	5.320508	4.57377
HORVU5Hr1G008670	1.24522	0.49011	0.893447	2.118314	2.759964	3.223132
HORVU4Hr1G006710	1.250792	1.206148	1.679867	0.803753	1.390053	1.552908
HORVU1Hr1G061420	21.51279	22.38118	24.4148	23.68191	31.32425	32.84895
HORVU3Hr1G092630	1.422749	1.604009	1.612198	2.120752	1.724793	1.849311
HORVU5Hr1G027930	6.263541	6.803203	6.430356	6.969696	8.746319	9.36787
HORVU7Hr1G040280	1.532141	1.13645	2.290921	0.946114	3.155993	2.777694
HORVU6Hr1G034650	171.7209	161.9695	248.818	163.0366	155.0812	172.7697
HORVU0Hr1G027670	14.79381	12.80327	15.99692	6.881688	7.543784	9.534917
HORVU2Hr1G040290	2.979009	2.95166	3.626354	5.731775	6.870358	7.433025
HORVU0Hr1G003850	1.054799	0.823485	1.30187	1.385424	1.133501	1.486943
HORVU5Hr1G122820	18.49179	17.73261	17.98026	40.96212	67.06888	64.00568
HORVU1Hr1G088760	19.70875	20.84949	40.70525	61.73016	31.0729	31.10506
HORVU6Hr1G005510	1.290793	1.058639	0.942509	1.930202	2.355113	2.595895
HORVU0Hr1G011460	5.857138	5.665746	7.088846	6.291135	8.902951	9.14856
HORVU5Hr1G016340	2.970434	2.634153	3.628461	3.810079	5.40286	5.113032
HORVU2Hr1G112070	1.007174	0.827902	1.46376	1.8179	5.231848	4.072918
HORVU1Hr1G056390	33.26094	26.84692	31.23964	18.06554	17.66621	18.16507
HORVU3Hr1G075620	0.163385	0.504663	0.333157	1.614851	1.801127	1.783024
HORVU2Hr1G115090	1.174647	1.136896	0.585971	3.825658	0.201526	1.989143
HORVU2Hr1G094980	3.97053	4.278017	6.788914	3.614793	1.736728	3.696906
HORVU5Hr1G071650	2.04932	2.320761	3.037209	1.578804	2.424443	2.27251
HORVU0Hr1G005720	0.461792	0.516476	0.477277	5.013113	0.611873	7.707989
HORVU7Hr1G045410	15.70904	16.4618	16.03308	23.01513	32.03587	30.50315
HORVU3Hr1G019440	5.355503	5.353813	7.210885	4.917351	7.783749	6.59957
HORVU5Hr1G076150	28.64517	30.40873	31.43248	65.71314	61.82851	62.64281
HORVU0Hr1G021060	23.59768	24.76114	23.43525	19.09132	21.18054	23.00321
HORVU2Hr1G025270	41.83315	31.91065	40.18975	34.82995	31.13827	29.00862
HORVU1Hr1G016660	19.49033	16.61069	21.22446	27.99834	21.28682	24.71984
HORVU3Hr1G076190	3.830697	4.109982	4.811815	4.397915	3.715527	3.794405
HORVU2Hr1G094320	10.03038	9.179389	12.29167	13.66152	20.38952	18.85892
HORVU2Hr1G091130	15.08878	14.11094	15.31992	10.87962	12.21398	12.4795
HORVU3Hr1G106370	3.491067	3.758237	4.616518	4.881436	5.135092	5.813738
HORVU7Hr1G022550	26.65678	28.40934	34.92001	42.05912	58.95001	63.38617
HORVU1Hr1G005450	2.763919	1.086599	1.28627	0.482684	0.690749	0.977326
HORVU4Hr1G012700	1.286194	0.682666	2.243296	10.17399	21.46853	16.6394
HORVU4Hr1G085960	9.561114	8.739707	12.30362	27.8956	29.42154	28.07056
Hordeum_vulgare_newGene_545	0.96501	0.93457	0.574403	1.566027	2.050873	2.48018
HORVU7Hr1G050760	3.078174	3.680015	4.780509	4.755761	5.295506	5.313509
Hordeum_vulgare_newGene_540	1.833115	2.26378	2.210152	4.424444	4.251957	4.827877
HORVU1Hr1G047930	19.69197	17.24707	20.52276	61.86363	92.42665	78.1015

HORVU7Hr1G060930	4.113882	4.172305	5.503175	9.845919	11.65265	12.77394
HORVU5Hr1G118510	0.806799	0.667443	0.924127	1.192467	1.416024	1.605506
HORVU2Hr1G053260	3.736681	3.275846	3.953663	5.054884	6.292804	6.670491
Hordeum_vulgare_newGene_542	2.279642	1.744598	2.990171	1.616683	1.344759	1.199703
HORVU2Hr1G109820	10.84004	9.807364	10.77771	8.31095	7.441214	7.712646
HORVU5Hr1G083310	9.493791	9.756048	13.98585	12.9921	18.37579	19.05439
Hordeum_vulgare_newGene_12710	0.760789	1.191612	1.150591	1.159264	1.333722	1.203623
Hordeum_vulgare_newGene_12714	2.894245	2.627611	2.487576	7.697135	3.302963	3.690463
Hordeum_vulgare_newGene_12717	1.554903	1.04536	1.601764	1.410118	1.379138	1.554708
HORVU3Hr1G067300	4.720899	4.726052	4.398934	6.365361	3.383172	4.912241
HORVU5Hr1G111870	31.11529	23.59724	36.15083	82.00191	76.55483	87.5651
HORVU4Hr1G079880	1.686708	1.386745	1.604891	1.16964	1.487761	1.208787
HORVU3Hr1G027650	9.445538	9.674005	9.734843	11.92844	18.23274	16.53764
HORVU1Hr1G002100	0.243079	9.03147	1.114614	33.53461	55.953	58.41645
HORVU5Hr1G115160	23.84489	28.74543	19.41274	33.7775	13.93042	18.73315
HORVU5Hr1G005030	10.56451	5.774682	8.329206	4.925834	0	1.488815
HORVU3Hr1G105110	3.729859	3.661928	4.939312	3.147448	4.333628	4.945373
Hordeum_vulgare_newGene_13043	4.245088	4.398577	4.533117	3.201259	3.513159	3.930786
Hordeum_vulgare_newGene_13042	5.960913	4.788627	8.080889	9.093905	11.81065	11.80474
HORVU4Hr1G058580	8.257766	7.721675	7.786973	11.23592	10.76457	9.621695
HORVU3Hr1G070900	3.517699	4.540428	6.013842	4.411284	6.303445	6.48005
HORVU3Hr1G089250	2.064784	3.27629	3.70638	0.192352	0.088367	0.118042
HORVU4Hr1G022420	207.3706	189.0189	189.1922	146.2661	166.8265	170.987
HORVU1Hr1G017210	2.936042	3.589461	3.615854	2.981051	3.190844	3.815196
HORVU5Hr1G046970	3.744109	3.3458	4.12663	5.336388	4.906161	6.168934
Hordeum_vulgare_newGene_3315	1.633622	1.087342	1.157028	1.192906	0.716719	1.19913
HORVU1Hr1G013900	13.92332	13.81199	15.04534	13.25063	9.423211	11.77635
HORVU2Hr1G019900	1.529843	0.689668	0.882551	1.750046	0.729162	1.228519
HORVU5Hr1G041060	5.77237	5.301625	7.795781	7.948435	11.19018	10.51702
HORVU7Hr1G077790	5.781647	7.297422	6.647093	5.771202	6.564828	7.673627
HORVU3Hr1G020020	7.587348	7.572041	9.206752	9.729061	9.301139	10.527
HORVU5Hr1G006600	23.935	24.53459	30.41822	20.30155	22.27601	22.68182
HORVU2Hr1G103700	6.678576	6.455612	6.872702	7.869635	9.707208	10.08365
HORVU1Hr1G031970	4.01575	3.640722	4.179309	6.770365	9.319802	10.20194
HORVU1Hr1G047060	13.48348	11.48612	12.93049	13.0861	13.56368	13.38814
HORVU2Hr1G053480	9.291944	9.343993	11.03593	13.90092	22.22394	21.0647
Hordeum_vulgare_newGene_2045	1.515435	1.496261	1.730396	1.760371	2.158437	3.040747
Hordeum_vulgare_newGene_2047	1.64626	2.337118	1.850725	2.698599	2.707608	3.551938
Hordeum_vulgare_newGene_2040	5.152871	6.268114	7.252989	8.205798	7.916616	8.866895
HORVU4Hr1G021190	13.8979	10.11907	11.77219	26.50977	39.3859	36.37479
Hordeum_vulgare_newGene_2042	5.190393	7.121697	8.71378	4.37164	5.082831	7.330087
Hordeum_vulgare_newGene_2048	2.641837	1.98614	1.625962	0	0.005627	0
HORVU7Hr1G109180	8.464253	8.446219	8.05164	16.69438	13.65619	14.04945
HORVU4Hr1G026210	8.037773	8.141441	10.02751	8.706863	9.884508	10.94032
HORVU2Hr1G107460	0	0	0	0.254152	5.064136	4.913455
HORVU6Hr1G012020	13.50178	16.49661	14.71062	23.96327	40.6546	36.42606
HORVU4Hr1G082720	1.758917	1.420671	1.924991	1.846651	1.909546	2.062897

HORVU1Hr1G003050	0.507924	10.0788	1.411044	14.27006	1.030652	1.172775
HORVU7Hr1G073470	2.679407	2.534266	3.187214	2.749065	2.531737	2.870882
HORVU2Hr1G099240	6.250617	7.02781	7.318107	5.617234	8.152798	6.501662
HORVU2Hr1G048290	4.161128	3.719692	4.223523	0.019495	0	0
HORVU2Hr1G096210	3.431147	3.088964	2.695444	6.9619	6.893579	7.613369
HORVU4Hr1G070630	10.83592	11.41734	14.32747	13.05539	22.71809	21.65424
HORVU2Hr1G101700	1.33291	1.189695	1.268227	2.006724	1.687296	2.119699
HORVU3Hr1G081020	7.175157	7.564536	7.368485	9.435641	20.04061	19.64427
HORVU2Hr1G113000	7.188107	6.491308	7.797444	5.91718	5.029225	5.855506
Hordeum_vulgare_newGene_13485	5.610287	5.191189	4.804944	2.189173	2.390467	2.004121
HORVU4Hr1G061170	20.31052	19.0339	20.93703	20.38506	19.23043	18.18875
HORVU7Hr1G082460	10.07633	9.692782	10.90367	9.661819	9.801164	9.866673
HORVU6Hr1G015790	0.077931	0.101102	0.121581	2.089527	7.241749	6.274503
HORVU6Hr1G093840	0.790606	1.019221	0.864927	2.801989	2.784091	3.523288
Hordeum_vulgare_newGene_9617	2.502753	1.585759	1.059427	2.454405	3.593509	3.376235
Hordeum_vulgare_newGene_9610	2.277756	1.867295	2.626828	3.399848	2.278147	2.628066
HORVU7Hr1G049150	0.976322	1.118334	1.784677	0.982359	1.261789	1.15201
HORVU3Hr1G092610	37.4449	43.6199	44.26553	29.66686	34.17086	38.4545
Hordeum_vulgare_newGene_9619	5.69507	5.402682	6.217522	9.536032	9.722376	7.762151
HORVU3Hr1G054640	23.6635	21.77701	27.46771	38.61942	68.84277	66.78077
HORVU3Hr1G117830	5.195937	5.54583	4.910259	6.351975	7.023973	8.085413
HORVU5Hr1G053270	1.892867	1.811007	1.777854	4.537343	6.744933	5.542853
HORVU7Hr1G074170	35.0473	32.24387	63.42207	24.16895	41.84039	38.43881
Hordeum_vulgare_newGene_15059	12.5805	11.34749	14.26091	7.277637	8.767012	9.738275
HORVU7Hr1G073780	3.295663	3.076089	4.188672	4.562477	5.830891	5.542686
Hordeum_vulgare_newGene_857	1158.527	910.4783	1155.827	103.0867	75.05529	89.45389
Hordeum_vulgare_newGene_15052	31.35172	28.51648	34.03045	20.67048	19.64219	20.8048
Hordeum_vulgare_newGene_15053	1.526325	1.889391	2.064133	0.701765	0.756555	0.369873
Hordeum_vulgare_newGene_10022	4.425917	3.21769	1.80612	0.441742	0.084796	0.223529
HORVU1Hr1G047430	9.130991	7.02422	12.51273	10.55124	4.6802	4.886329
Hordeum_vulgare_newGene_10027	33.61194	38.67514	41.27845	0.039573	0	0.02152
Hordeum_vulgare_newGene_6911	0.951857	0.938802	1.109534	1.198811	1.112251	1.505334
HORVU2Hr1G055880	1.494108	1.888999	1.744288	2.096686	1.845877	2.368632
HORVU2Hr1G026210	9.485796	9.959107	13.61422	7.037567	9.84931	9.930912
Hordeum_vulgare_newGene_11998	1.015037	1.176535	0.926606	1.330924	0.999067	0.724726
HORVU4Hr1G079710	9.71203	12.30111	8.980568	25.20694	29.18964	34.20097
Hordeum_vulgare_newGene_5064	1.818061	1.475199	2.476815	1.575493	0.996399	1.572616
HORVU4Hr1G090630	2.281343	3.941791	2.579499	6.468029	7.208297	8.289208
HORVU7Hr1G089260	0.576104	0.31988	0.456101	0.860721	19.11338	14.23626
HORVU4Hr1G034860	1.022898	1.059182	1.584494	4.041879	3.570228	4.542908
HORVU4Hr1G083930	10.53704	11.15861	8.916698	76.47897	56.95601	55.15675
HORVU4Hr1G004160	2.235713	2.058034	2.048424	2.589616	2.668738	3.870948
HORVU0Hr1G010470	2.908295	1.072155	2.089271	1.457868	0.339807	0.374744
HORVU5Hr1G069510	1.359542	1.455383	1.573765	1.847359	1.813744	1.82645
HORVU7Hr1G101810	2.040911	1.3167	1.570772	3.338019	3.716078	4.585226
HORVU2Hr1G059290	0.94407	1.176945	0.482871	2.165439	1.736253	2.179327
HORVU2Hr1G108340	25.78754	40.48501	49.0193	0.307364	0.126832	0.101195

HORVU5Hr1G098600	2.560704	2.481468	3.319096	4.993599	7.18886	6.883957
HORVU4Hr1G071270	6.537165	8.24987	9.258167	7.204554	10.02777	11.21108
HORVU7Hr1G095860	2.310326	0.763693	1.174751	2.184849	0.992118	1.29152
HORVU2Hr1G104860	32.29143	34.11059	23.16169	16.24978	9.50087	11.47884
HORVU2Hr1G073410	12.21759	8.968513	9.35532	11.74326	12.842	11.71148
HORVU6Hr1G060020	6.489952	4.740048	7.637224	16.05493	2.796173	3.715839
HORVU3Hr1G022850	33.47431	37.3055	36.05976	28.80254	37.96614	37.94711
HORVU5Hr1G117470	4.097627	4.781668	4.845945	6.568373	12.57072	11.42059
HORVU2Hr1G031690	3.520244	4.358812	3.911196	4.892427	7.851493	7.401181
HORVU5Hr1G100880	2.592323	1.978852	3.24996	3.709526	4.47737	5.528557
Hordeum_vulgare_newGene_14828	0.691545	0.451402	0.586397	1.818869	2.893082	3.07712
Hordeum_vulgare_newGene_14829	14.23268	14.1603	16.42096	8.88502	8.617498	9.231445
HORVU2Hr1G075710	8.443775	8.456879	9.22839	11.23565	10.27272	11.56331
HORVU7Hr1G106570	9.18278	10.69146	12.86569	5.752732	5.144508	5.973581
Hordeum_vulgare_newGene_1761	1.751107	3.325146	2.37198	4.570632	4.850464	5.150532
Hordeum_vulgare_newGene_1763	1.578969	1.533551	1.478879	1.408312	0.797001	0.912093
HORVU2Hr1G121070	16.34668	13.47853	17.84778	13.50246	10.20052	11.3478
HORVU4Hr1G046140	26.43769	27.73967	24.75317	25.55763	27.33762	28.32994
HORVU2Hr1G076160	2.888868	3.311326	3.017052	2.309745	1.990674	2.687718
HORVU2Hr1G029290	22.93515	12.07707	29.95244	20.95035	15.16145	15.51127
Hordeum_vulgare_newGene_8256	13.79304	11.59599	21.57618	2.775501	0.324152	1.980196
Hordeum_vulgare_newGene_8258	16.89744	14.53418	16.95443	19.09777	16.06544	17.31426
HORVU2Hr1G068810	3.914161	4.139487	5.545022	5.502019	9.029714	8.059622
HORVU3Hr1G091170	1.055307	0.910687	1.245073	1.279511	1.092123	1.002166
HORVU2Hr1G077410	1.0199	0.968577	0.622669	2.118434	8.468796	4.255173
Hordeum_vulgare_newGene_4734	0	0	0	5.632756	5.587023	6.928682
Hordeum_vulgare_newGene_4730	16.3939	19.22233	19.95497	19.79522	19.96624	23.79375
HORVU7Hr1G100740	1.56831	1.541737	3.044015	7.051312	2.38859	1.002766
Hordeum_vulgare_newGene_7421	1.021916	1.285493	1.49029	2.169945	2.001593	2.758061
Hordeum_vulgare_newGene_7423	11.44606	9.166476	11.60707	12.11691	13.32949	14.20794
Hordeum_vulgare_newGene_7422	11.09723	10.1326	12.34014	11.69559	14.74078	14.62979
Hordeum_vulgare_newGene_7424	23.94709	21.49859	22.57567	61.71468	99.1657	101.3068
Hordeum_vulgare_newGene_7427	64.52872	53.92566	76.75398	60.94511	89.79568	87.69685
HORVU7Hr1G100930	4.021682	4.175566	4.399515	6.128011	8.860023	8.339062
Hordeum_vulgare_newGene_13159	1.052249	1.07042	1.183921	0.935556	1.158103	1.468464
Hordeum_vulgare_newGene_4880	9.026812	10.47335	10.32946	15.77688	22.62324	21.58758
HORVU5Hr1G088140	8.615502	8.334245	9.036959	5.806332	5.366902	5.536402
HORVU7Hr1G046800	1.239953	1.095556	1.373073	0.860411	0.973292	0.71864
HORVU2Hr1G028520	3.582802	2.728994	3.133588	2.948263	4.152197	2.018096
HORVU7Hr1G058080	4.535529	4.991551	6.076636	5.875346	8.167292	8.234105
HORVU4Hr1G006480	0.473172	0.466209	0.971617	1.08177	3.793745	4.955934
HORVU1Hr1G046020	15.15203	15.90025	15.30857	16.71344	20.83256	20.57958
Hordeum_vulgare_newGene_7281	4.603646	5.127275	5.109306	3.167285	2.880971	3.171157
Hordeum_vulgare_newGene_7280	1.6978	1.411658	1.752713	0.987437	1.10346	1.147562
HORVU7Hr1G078870	6.50252	6.704565	7.228346	7.70269	8.205504	8.187893
HORVU0Hr1G030440	18.85503	16.43173	20.95791	16.06857	22.14432	20.48663
HORVU1Hr1G061190	15.8257	13.67714	19.18421	13.25668	17.6722	14.30999

Hordeum_vulgare_newGene_12050	4.106371	3.026605	4.57527	4.670374	8.958607	8.04129
HORVU6Hr1G008590	3.591799	3.757904	4.82771	8.458216	9.820863	10.87348
HORVU6Hr1G053810	4.446266	5.129729	3.011123	146.7385	186.373	157.567
Hordeum_vulgare_newGene_6778	1.481706	1.481993	0.921508	2.073628	2.908003	1.904018
HORVU5Hr1G014120	44.21873	44.51968	59.93153	18.11877	7.349912	11.46844
HORVU5Hr1G040070	66.48117	71.12465	108.8333	55.83558	124.0857	115.0815
Hordeum_vulgare_newGene_15881	1.265372	0.472338	1.551597	1.708864	1.839028	1.105115
Hordeum_vulgare_newGene_15880	3.098694	3.370185	3.540853	2.40979	3.068347	2.882337
Hordeum_vulgare_newGene_15882	1.828876	2.173152	2.387964	2.659412	2.560441	3.327671
HORVU2Hr1G114590	11.08963	10.64257	12.72767	15.94379	21.12024	18.75956
Hordeum_vulgare_newGene_15889	2.057699	2.381669	1.859566	8.016134	16.52343	13.97956
HORVU6Hr1G067840	25.79542	21.10671	29.07589	33.27561	32.39309	28.51864
HORVU7Hr1G100060	3.129319	2.887687	4.05786	2.765538	4.978168	4.544905
HORVU1Hr1G056870	13.74181	12.94178	15.35489	15.81987	15.64599	15.61935
HORVU6Hr1G083720	29.90398	31.25971	37.84143	33.75079	29.02803	23.55119
HORVU6Hr1G059710	5.233752	5.177841	5.727889	8.273523	11.95316	11.75461
Hordeum_vulgare_newGene_3056	1.208666	1.66657	1.209102	2.43328	2.762344	2.783312
HORVU6Hr1G028940	2.517182	2.767416	2.905156	4.547282	4.040444	4.534913
HORVU1Hr1G071160	2.631205	2.409813	2.752595	4.888384	6.10781	6.093411
Hordeum_vulgare_newGene_3053	2.447306	3.530947	3.432758	0	0	0
HORVU7Hr1G002670	2.453711	2.49168	2.176603	5.791218	4.929884	5.14558
HORVU3Hr1G017750	3.657375	3.511745	4.327824	4.196405	4.641621	4.912024
HORVU5Hr1G076550	12.46902	10.2452	12.25061	7.251726	6.824554	6.972046
Hordeum_vulgare_newGene_4930	1.194316	12.73	3.763031	0	0.379655	0.152756
HORVU5Hr1G032660	1.895193	1.522559	2.40821	1.588122	1.350049	1.991542
Hordeum_vulgare_newGene_2900	1.544948	1.749665	1.477609	2.282031	2.418157	2.495999
Hordeum_vulgare_newGene_6482	2.531881	2.863064	2.543637	1.386912	3.632385	2.776201
Hordeum_vulgare_newGene_14594	2.822381	2.904498	2.526735	0.277228	0.114818	0.095596
Hordeum_vulgare_newGene_14595	8.318534	8.524498	3.964453	4.059633	1.133824	1.401126
Hordeum_vulgare_newGene_14597	2.530673	2.195295	1.5481	0.614214	0.172887	0.228485
Hordeum_vulgare_newGene_14590	15.38067	13.67091	10.28442	8.648825	4.014413	4.709742
HORVU6Hr1G074820	0.260998	0.121892	0.197906	6.250729	6.898159	7.785983
HORVU6Hr1G087380	0.311005	0.226505	0.297788	17.44467	89.47556	67.0659
Hordeum_vulgare_newGene_14598	3.646942	1.727849	0.813783	3.543766	4.788014	5.476548
HORVU4Hr1G013550	13.3838	14.02016	14.98245	20.92772	26.22892	25.74434
HORVU1Hr1G029850	8.889183	8.121616	11.69325	11.73363	10.82429	10.51272
Hordeum_vulgare_newGene_39	2.597734	1.881545	2.626272	2.389406	2.378363	1.424824
HORVU3Hr1G087840	8.647973	8.947114	9.239007	7.652878	8.328972	8.603992
Hordeum_vulgare_newGene_14042	1.929122	1.365001	9.59148	40.29534	1.601698	1.669443
Hordeum_vulgare_newGene_14043	0	0.088501	0	44.52848	0.215637	0.146898
HORVU7Hr1G098950	1.97472	2.226708	1.764088	2.130879	1.972498	2.779636
Hordeum_vulgare_newGene_14041	1.159857	1.781831	11.99816	25.41543	0.718463	0.294726
Hordeum_vulgare_newGene_10354	12.161	9.526705	13.20477	15.56947	14.17215	13.83489
Hordeum_vulgare_newGene_30	5.547659	5.602355	5.757892	9.078009	12.12328	11.45278
HORVU2Hr1G024950	0.994688	1.056236	1.164346	3.819501	3.22334	3.960821
HORVU7Hr1G096880	1.812123	1.999681	3.266754	1.264749	0.870264	1.007535
HORVU4Hr1G003270	0.878424	0.895217	0.938333	2.263982	3.074139	3.942117

Hordeum_vulgare_newGene_9930	0.749987	0.708162	0.882494	0.458216	1.737841	1.943538
HORVU4Hr1G034200	10.05468	9.952758	13.0758	14.83343	22.73817	23.50494
Hordeum_vulgare_newGene_9935	61.78471	51.74106	62.91953	20.61831	8.931005	8.032327
HORVU4Hr1G056400	9.95924	11.92616	10.79755	13.092	19.39009	19.63882
HORVU0Hr1G000650	13.401	13.11228	12.85948	19.09426	21.4229	23.38937
HORVU2Hr1G005620	3.680043	2.834959	4.330979	1.587198	1.256278	1.213868
HORVU5Hr1G029770	9.366626	10.00715	11.34615	8.881512	10.66923	11.07334
HORVU1Hr1G020480	1.751066	1.5294	2.21078	2.796904	6.068721	4.749229
HORVU3Hr1G016000	8.89726	8.6044	10.10182	7.477562	8.242595	8.39601
HORVU7Hr1G119250	3.722011	4.04868	3.018695	0.259423	0.227694	0.302955
HORVU3Hr1G004850	5.50578	3.910931	5.127056	6.589962	4.320716	6.629483
HORVU3Hr1G032480	31.81006	25.83546	31.42744	20.78353	23.59437	21.90851
HORVU2Hr1G118080	1.511808	1.322694	2.542003	1.759872	0.38039	1.487539
HORVU2Hr1G046900	1.565747	1.28863	1.643453	2.413322	3.699036	3.625395
HORVU7Hr1G048430	1.151557	0.842495	1.32134	1.598548	0.794612	1.351334
HORVU4Hr1G010390	71.77786	68.50716	87.96866	122.7902	166.3526	145.2278
Hordeum_vulgare_newGene_15311	17.614	17.02769	18.57078	15.47285	21.21781	22.35024
Hordeum_vulgare_newGene_12967	0.078842	0.123433	1.832784	2.018711	1.394805	1.871905
Hordeum_vulgare_newGene_1336	3.026276	2.363058	3.405395	2.921223	5.021925	6.735706
HORVU0Hr1G005540	1.353891	2.049488	2.949079	3.05198	5.764627	4.559751
HORVU5Hr1G106440	31.92542	34.6063	48.45842	32.57576	54.63766	56.68111
HORVU5Hr1G113740	2.349428	3.377257	4.593566	1.267272	1.729141	1.725806
HORVU7Hr1G041710	2.064342	2.045323	2.796158	9.308339	18.89805	19.58447
HORVU7Hr1G053830	6.077182	5.83786	6.999996	7.223356	7.427149	7.872749
HORVU4Hr1G068760	6.807937	6.297889	7.493498	8.069283	9.376988	9.147184
HORVU1Hr1G088370	22.51522	18.67825	80.95392	161.9581	12.93897	13.53683
HORVU4Hr1G002630	1.048795	0.583133	0.853465	0.911011	2.02155	1.906404
HORVU5Hr1G109610	39.43142	30.64247	31.72987	3.040483	2.424619	2.981527
HORVU1Hr1G048350	8.980029	7.961099	10.70034	8.177695	7.909473	8.133494
HORVU7Hr1G050560	9.350716	9.284014	10.03747	8.122877	8.349771	8.002737
HORVU5Hr1G097650	4.736302	3.762985	4.878992	5.968772	6.771303	7.093694
HORVU7Hr1G092550	4.631752	3.699613	5.783724	16.93354	36.84461	35.1264
HORVU3Hr1G004140	123.2096	147.5815	255.5694	252.6011	248.3132	280.9988
HORVU1Hr1G052700	35.52845	28.84025	37.82969	23.28021	18.1836	18.17749
HORVU6Hr1G067060	3.241694	2.398013	4.499011	5.518639	4.17282	5.849807
HORVU6Hr1G031200	0.830297	1.193569	1.079251	1.642784	1.677013	1.542469
HORVU3Hr1G017660	20.78929	16.65933	28.36309	17.80664	20.77007	23.28175
HORVU0Hr1G007680	4.577196	2.261511	6.788365	5.422749	1.470918	3.532994
HORVU4Hr1G051990	0.629542	0.672786	1.213383	1.091686	2.848279	3.401851
HORVU1Hr1G042050	23.74116	24.02856	24.592	32.57244	46.45351	46.1637
HORVU4Hr1G014670	1.566767	1.156058	1.876103	1.336993	0.831185	1.422075
HORVU1Hr1G044080	2.918031	2.731234	2.682235	3.30738	1.816849	1.704468
HORVU4Hr1G080110	1.792602	1.613575	1.911251	1.28471	1.0979	1.166348
HORVU4Hr1G087720	33.63445	30.1143	33.85896	26.65147	27.81512	30.15703
HORVU2Hr1G106130	0.048686	1.538801	2.141158	3.390583	1.049063	2.705635
HORVU4Hr1G005360	36.71667	30.96388	36.71061	28.86351	26.21157	26.46674
HORVU2Hr1G124010	1.025054	0.76522	1.091203	2.529846	6.430302	6.350763

HORVU3Hr1G001530	8.758316	8.033042	10.58364	8.140421	12.86558	14.77031
HORVU5Hr1G112900	6.320052	4.945393	5.299747	0.227156	0.096421	0.043841
HORVU3Hr1G077930	3.537889	3.921146	4.654231	2.935806	1.086266	1.824578
Hordeum_vulgare_newGene_15975	8.625971	7.85581	10.17118	7.923264	8.73342	8.646154
HORVU7Hr1G013600	76.68694	70.0592	82.24485	78.90337	125.1586	131.6765
HORVU2Hr1G081370	28.27731	24.59653	30.85395	61.06759	66.28722	75.05872
Hordeum_vulgare_newGene_15978	2.536201	2.141247	3.343653	5.191802	7.025967	6.384117
HORVU4Hr1G012630	3.72725	3.478418	2.59267	3.903812	5.201851	4.683338
HORVU1Hr1G091400	5.575223	4.321012	7.491558	8.292481	7.778531	7.667025
HORVU3Hr1G099300	8.583864	7.97444	7.852523	4.072084	5.184908	6.446864
HORVU6Hr1G066330	0.84853	12.5496	17.03741	3.795562	0.873227	1.883994
HORVU3Hr1G047110	8.457535	8.141341	8.260377	10.48471	15.53002	14.46205
HORVU5Hr1G056820	3.09981	3.579681	3.492821	4.132856	4.673591	5.294592
HORVU1Hr1G049550	2.489734	2.184719	2.337073	2.359464	2.266095	2.583813
HORVU5Hr1G067790	2.822705	3.169599	3.236142	3.27575	4.474577	5.230309
HORVU7Hr1G094240	7.385824	7.148104	9.87119	6.256123	5.567659	6.498375
HORVU2Hr1G107660	3.84989	4.509893	5.175214	6.586413	6.031468	5.844974
HORVU5Hr1G023980	27.01463	28.52497	30.95474	25.97735	26.44538	27.22944
HORVU0Hr1G015950	2.195437	3.504343	4.234363	9.424709	6.785518	9.69256
Hordeum_vulgare_newGene_15418	4.133943	4.029385	5.537083	6.461717	8.452959	9.610906
HORVU2Hr1G010690	2.801033	3.382174	1.92541	65.85577	169.8878	159.5885
HORVU2Hr1G082880	3.415621	4.061976	4.396505	2.956741	4.005812	4.872627
HORVU6Hr1G090260	37.26581	39.29444	53.59723	55.80636	94.46561	94.13074
HORVU6Hr1G090810	11.78496	9.86228	13.41679	10.35451	10.51508	12.53108
HORVU1Hr1G036670	1.116835	1.131066	1.482932	2.001372	2.361492	2.438742
HORVU2Hr1G099480	1.569072	2.102198	2.017368	15.60668	22.85559	21.56132
HORVU5Hr1G060220	1.606693	1.754181	1.817446	1.411158	1.279611	1.167174
HORVU4Hr1G053830	4.547166	6.104811	4.525249	5.748768	6.476639	8.458123
HORVU1Hr1G037150	1.452497	1.069998	1.64016	2.280975	1.766272	2.102838
HORVU2Hr1G080020	12.00257	10.13252	15.75458	12.93178	11.79232	13.55142
HORVU3Hr1G071520	9.253918	10.18627	11.61307	6.659357	9.771205	9.142353
HORVU3Hr1G006540	2.29585	1.890785	2.955648	3.706088	4.076209	3.799018
HORVU2Hr1G054040	8.925706	12.35945	9.722886	11.16003	15.73288	15.60684
HORVU4Hr1G010830	3.629141	3.732153	5.299382	4.523133	4.725522	4.866139
HORVU2Hr1G092070	3.30189	2.322898	3.58578	2.361561	2.158772	2.498654
HORVU2Hr1G019250	80.01298	42.92003	54.47913	149.8454	178.2272	162.1672
HORVU7Hr1G074370	0	0	0	8.012687	13.33027	11.35569
HORVU2Hr1G096760	17.07942	13.54602	17.56235	31.70674	30.78499	33.57644
Hordeum_vulgare_newGene_10226	4.239856	4.024553	4.220295	2.947973	3.787359	3.438237
Hordeum_vulgare_newGene_10225	2.011103	1.698535	2.407389	0.020294	0	0
HORVU3Hr1G026020	29.93298	26.26783	31.55117	23.85931	35.50303	34.71197
HORVU3Hr1G092680	45.78224	33.17224	43.57858	47.3361	54.13095	60.82422
HORVU5Hr1G111330	2.415272	2.883917	2.750841	1.773706	2.967957	2.976357
HORVU7Hr1G110930	3.310388	3.778373	3.951507	4.109768	7.17349	6.513483
HORVU5Hr1G081040	10.51049	11.0471	12.06742	8.268301	9.592194	9.137639
HORVU7Hr1G055520	4.516406	3.843578	7.60745	4.782776	7.266023	7.002539
HORVU2Hr1G019780	5.952712	7.24514	9.984472	12.40262	18.88218	20.90995

HORVU5Hr1G053700	2.250982	3.700584	3.579804	2.059719	3.268498	2.50965
HORVU3Hr1G106950	2.108305	1.394295	2.638807	4.70396	3.976522	5.76862
HORVU4Hr1G025450	1.044544	0.793562	1.13369	0.709088	5.608462	4.888748
HORVU6Hr1G006140	0.743016	1.701709	2.094237	1.637723	3.612839	6.943634
HORVU2Hr1G065940	10.79842	8.77564	12.54596	8.48002	9.111163	9.502897
HORVU5Hr1G052250	13.05792	11.74814	12.78858	13.75388	19.99159	20.63364
HORVU3Hr1G073580	7.773995	5.964207	5.561841	2.289393	3.648121	2.67615
HORVU1Hr1G083610	2.559623	1.420356	2.458897	0.718326	0.946077	0.532878
HORVU6Hr1G086560	100.8016	92.50019	86.88501	86.12665	79.9381	79.29634
HORVU0Hr1G016630	15.37589	12.69681	21.30159	20.1685	13.68961	13.26211
HORVU2Hr1G097410	1.503692	1.238319	1.576802	0.402124	0.746931	0.839987
HORVU5Hr1G081590	46.41253	45.00183	66.77604	39.51818	61.80229	56.09818
HORVU6Hr1G064120	1.634845	2.568403	2.619349	1.91089	2.007715	2.192733
HORVU3Hr1G085680	9.289464	10.63457	6.949946	4.117427	4.577136	3.610071
HORVU4Hr1G055880	16.18639	16.98925	14.77193	10.92295	14.85629	14.75649
HORVU1Hr1G040540	3.662878	4.960212	3.82484	1.887687	2.772569	2.675815
HORVU3Hr1G065580	2.115416	1.72392	2.087016	6.789969	11.28123	10.43729
HORVU5Hr1G042300	2.102126	2.470548	2.448647	1.738335	2.151712	1.922718
HORVU7Hr1G007540	4.72318	4.134555	6.473487	6.403866	4.840072	5.848246
HORVU3Hr1G092770	5.570808	5.757483	8.052891	6.230858	8.719248	7.95703
HORVU3Hr1G003820	5.387855	5.022142	6.675105	7.826189	7.931666	9.121512
HORVU4Hr1G023850	4.95009	5.600912	6.277912	13.13316	20.93633	19.00017
HORVU5Hr1G006880	151.076	118.0231	157.8702	149.482	95.12417	95.63504
HORVU4Hr1G006500	23.84902	22.18522	28.82747	15.56711	17.66478	18.5407
HORVU3Hr1G029310	2.27634	2.098552	2.239118	4.062678	4.085572	4.065966
HORVU4Hr1G069150	27.00571	30.59394	41.19745	0.871427	34.71813	32.37211
HORVU6Hr1G094660	11.80668	14.22303	12.09254	13.20382	19.40522	19.11413
HORVU7Hr1G091800	313.052	223.1356	333.4691	1402.469	1629.192	1418.886
HORVU3Hr1G107700	3.954366	4.804768	4.618029	7.634463	9.774772	10.18259
HORVU1Hr1G084390	6.577583	3.939331	8.706196	7.054311	5.551813	4.197392
HORVU5Hr1G072530	45.7709	38.04196	47.79786	50.61512	57.75512	56.08032
HORVU5Hr1G007750	3.710143	3.695395	3.735935	3.462271	4.275497	4.654239
HORVU3Hr1G037260	2.06293	4.272973	9.010133	0.814567	0.227209	0.415699
HORVU1Hr1G066020	0.667019	0.753888	0.61757	3.852685	7.340051	6.12374
HORVU7Hr1G051310	0.013941	0.083533	0	4.355467	2.830161	5.069064
HORVU6Hr1G085030	2.631166	3.066122	2.909008	2.237754	2.328054	2.323792
HORVU3Hr1G019570	4.257533	4.032544	5.426855	4.982869	10.27002	11.58405
HORVU4Hr1G057100	48.0585	39.89085	42.80957	35.28245	71.2215	60.41126
HORVU7Hr1G105050	2.020904	1.211629	1.647522	2.800439	3.004578	2.352948
HORVU1Hr1G002600	1.583252	23.70061	3.480108	38.41193	2.580164	4.111523
HORVU2Hr1G094040	10.05831	8.275355	10.39907	12.02015	11.01686	11.60995
HORVU2Hr1G039100	2.883663	2.214131	2.578137	6.399876	10.08023	8.22127
HORVU7Hr1G045620	823.586	957.2052	698.1874	195.8666	83.91312	181.1061
HORVU2Hr1G023840	4.475797	3.626275	5.273994	7.010539	10.21763	17.36346
HORVU3Hr1G068600	35.17604	35.55266	33.89177	50.89733	38.12738	44.80611
HORVU6Hr1G062150	3.179197	2.199697	2.588695	4.615301	0.128167	0.838572
HORVU7Hr1G047990	29.90887	27.61763	39.19013	32.24979	37.01243	35.18496

HORVU1Hr1G033160	0.890185	0.807007	0.822309	1.766349	1.937526	2.098454
HORVU7Hr1G050740	14.20322	11.29168	14.76009	18.97101	18.38467	20.00344
HORVU2Hr1G035860	25.65907	23.89714	31.38472	27.91606	50.42143	47.99182
HORVU2Hr1G073270	3.251409	2.835264	4.073633	3.670071	4.246747	5.393439
HORVU6Hr1G067470	3.984664	2.725895	4.5458	12.03634	12.56803	8.129763
HORVU2Hr1G013090	2.961159	3.638612	5.102162	3.061553	1.390921	1.615194
Hordeum_vulgare_newGene_3298	4.121582	4.125088	4.146519	4.164469	3.907506	5.115817
Hordeum_vulgare_newGene_3297	6.048005	6.325725	7.108613	8.999892	12.07008	11.53106
HORVU3Hr1G104940	7.091985	5.292121	7.084235	4.567822	2.712076	6.289983
Hordeum_vulgare_newGene_3292	3.676013	4.104762	4.918698	0.871004	0.614435	0.64132
HORVU1Hr1G071360	11.22199	11.57202	12.29626	18.16585	28.72142	28.80136
HORVU5Hr1G075340	9.513983	12.61772	14.31359	19.34922	16.36746	18.31927
HORVU7Hr1G038700	1.548686	1.975713	2.590518	1.31797	0.871976	1.691585
HORVU0Hr1G013160	1.980684	0.661121	1.456486	3.193799	0.142904	3.16336
Hordeum_vulgare_newGene_5561	19.03581	19.56327	16.99591	13.50629	9.243657	10.05238
HORVU3Hr1G116440	0.863574	0.828167	0.735286	1.166769	1.35227	1.210455
HORVU3Hr1G025750	7.127216	8.042501	7.230966	10.54327	15.67721	15.44554
Hordeum_vulgare_newGene_5564	4.920814	4.614019	3.993561	7.857409	2.487067	3.576912
HORVU3Hr1G052560	0.243495	0.853058	0.832027	2.6184	2.095822	3.071972
HORVU2Hr1G052920	1.602871	1.178384	2.946976	0.060201	0.464703	1.291658
HORVU5Hr1G084330	0.780208	0.68728	1.281824	1.639433	0.757776	0.96785
HORVU6Hr1G027650	65.36151	54.92063	64.68364	28.51615	20.87485	24.44511
HORVU4Hr1G026390	3.380492	3.067133	4.566224	2.899546	2.556204	3.701081
HORVU5Hr1G017310	1.434366	1.039705	0.47296	1.208076	1.286235	1.1906
HORVU3Hr1G034290	48.7783	40.7672	51.90583	42.68332	45.2926	43.5509
HORVU6Hr1G018030	36.45702	35.75414	39.30597	41.64903	44.43072	43.39065
HORVU5Hr1G070390	10.96752	12.36073	16.22235	7.981179	11.91039	12.27108
HORVU4Hr1G025840	92.75918	76.75447	110.4065	99.73441	121.9614	124.5994
Hordeum_vulgare_newGene_708	15.31263	13.685	20.38911	23.61299	22.55335	22.76626
HORVU2Hr1G083020	5.540834	4.545786	5.079793	1.335742	0.384866	0.616913
Hordeum_vulgare_newGene_14208	27.51562	23.38615	32.28143	13.91275	19.94284	18.88844
Hordeum_vulgare_newGene_14205	0.754655	0.669015	0.839376	1.251857	1.347047	1.361505
HORVU2Hr1G086080	46.50759	43.90122	34.894	21.85385	18.62233	21.316
HORVU6Hr1G053760	46.95382	48.05529	49.93963	66.61082	62.19667	70.28998
HORVU5Hr1G000830	32.32486	31.33374	30.90548	31.04062	47.25111	46.21333
HORVU7Hr1G025800	0.789003	0.731439	0.592283	6.320054	5.416717	5.329347
HORVU1Hr1G003150	1.338183	17.22102	2.16547	20.62532	1.742871	1.723861
HORVU5Hr1G065620	37.84762	27.98217	45.00646	13.09446	11.81215	15.48516
HORVU5Hr1G115310	3.257805	3.098863	4.26655	0.788071	1.031405	0.47054
HORVU2Hr1G020090	0.557246	0.380141	1.083048	2.264193	1.317813	1.437657
HORVU5Hr1G080050	9.910118	9.987295	12.89517	4.578101	5.719249	5.208181
Hordeum_vulgare_newGene_2657	5.581112	6.544519	8.200338	7.01951	12.2467	11.85244
Hordeum_vulgare_newGene_2659	1.854534	2.316769	2.278198	1.655663	1.244836	2.272089
Hordeum_vulgare_newGene_2658	1.860636	2.194382	2.231312	2.870665	5.252459	5.157924
Hordeum_vulgare_newGene_8864	7.747294	6.598348	7.02944	4.62155	3.750108	3.639429
Hordeum_vulgare_newGene_8866	1.446217	0.036457	1.255619	3.215583	4.979861	5.031971
Hordeum_vulgare_newGene_8867	72.33498	49.2404	83.93166	58.5073	69.62662	64.63196

Hordeum_vulgare_newGene_8860	1.612612	1.59655	2.261262	2.344193	2.675754	3.676192
Hordeum_vulgare_newGene_8861	17.73442	14.00681	17.14597	33.4663	36.32085	37.05991
Hordeum_vulgare_newGene_8862	1.292481	1.288586	1.679089	1.846472	2.157602	2.236327
HORVU5Hr1G062070	11.9982	7.679931	12.1807	9.207148	3.14522	2.026191
HORVU5Hr1G084100	3.296957	2.301997	3.227816	5.963105	4.744668	7.730819
Hordeum_vulgare_newGene_5922	12.5546	11.65678	11.20091	12.22619	11.31737	13.2931
Hordeum_vulgare_newGene_5920	5.082984	5.040544	5.756551	4.327643	4.471414	4.167676
Hordeum_vulgare_newGene_5921	4.100545	4.092661	5.586567	6.269872	12.74018	11.26665
HORVU5Hr1G081900	6.480563	6.702927	7.378358	7.738663	8.691464	9.941337
HORVU6Hr1G015450	20.61659	19.6388	29.91309	18.81481	24.44827	25.63985
Hordeum_vulgare_newGene_5928	2.576364	0	0.502863	1.228077	7.904534	0.26318
HORVU1Hr1G059450	12.75997	11.81324	13.65576	12.14883	15.03628	15.29886
HORVU5Hr1G073610	21.5033	21.14296	27.22915	26.8762	28.41646	31.03455
HORVU2Hr1G027370	7.104267	8.760618	10.87988	4.787113	7.279956	7.572919
HORVU4Hr1G078670	1.411188	1.471547	2.192851	2.665353	3.236814	2.418201
HORVU2Hr1G096400	39.71174	38.00102	38.65257	2.967815	2.205999	2.84622
HORVU3Hr1G025020	8.373715	6.049977	8.369246	5.084076	4.322131	4.196011
HORVU3Hr1G075690	273.3918	377.6212	421.1875	45.87359	4.124617	18.33399
HORVU6Hr1G013120	1.059125	1.020864	0.700057	1.099452	1.563766	1.9978
HORVU1Hr1G000710	19.13637	20.42233	23.61921	25.81774	52.43353	57.53854
HORVU3Hr1G088980	2.878606	2.604759	3.031282	1.867762	2.572728	2.532502
HORVU7Hr1G108640	2.17527	2.412003	3.56797	3.13183	3.231106	3.341653
HORVU3Hr1G012030	2.507953	2.473524	2.453486	3.014251	3.913427	5.465798
HORVU2Hr1G104120	56.98317	99.77839	114.7097	23.54737	2.103901	9.851764
HORVU0Hr1G022670	0.596851	0.814915	0.49236	5.058765	5.414032	4.767302
HORVU1Hr1G037550	21.59536	18.81657	19.56798	15.59501	15.16734	18.32919
Hordeum_vulgare_newGene_1350	64.83419	64.04125	84.02603	25.69038	12.1148	14.06231
Hordeum_vulgare_newGene_1359	8.387367	7.694225	10.2323	9.705601	9.228183	6.675353
HORVU1Hr1G034630	30.52696	26.7647	33.00281	35.75811	41.5199	41.21858
HORVU2Hr1G073590	3.909432	3.867336	4.620315	5.048855	6.701172	6.538504
HORVU3Hr1G026470	23.32721	21.95714	25.01193	18.27686	20.61188	18.48927
HORVU5Hr1G035800	4.430003	6.112186	4.506648	7.082363	9.861525	9.087074
HORVU2Hr1G031710	5.699739	6.110558	5.294351	2.102683	0.935562	2.178757
HORVU2Hr1G071570	9.345627	4.840838	10.21677	6.826428	13.92443	21.30431
Hordeum_vulgare_newGene_9405	5.076532	7.546683	7.626966	6.111282	6.598857	6.462983
HORVU5Hr1G103900	0.643492	0.640548	0.800122	1.892759	3.100397	2.267971
HORVU7Hr1G086420	7.027504	8.403217	8.094796	6.33213	7.579661	7.926616
HORVU0Hr1G025540	4.824162	4.550083	4.499971	5.487191	7.447728	8.030034
HORVU3Hr1G057860	0.294025	0.113164	0.18931	4.47885	3.442684	3.136493
HORVU4Hr1G017030	31.56173	32.17687	31.91863	64.81497	55.9054	63.3807
HORVU5Hr1G092620	6.451269	7.226382	8.620153	5.435488	6.322393	5.655035
HORVU7Hr1G072570	3.204895	2.981521	3.304691	7.912198	5.618997	8.619441
HORVU4Hr1G079100	8.93459	9.684866	8.821245	8.278989	11.04268	11.56152
HORVU6Hr1G077540	48.53331	44.92007	51.21909	33.38335	31.25839	32.23938
HORVU6Hr1G087960	1.345852	1.087835	1.445898	2.764756	6.71258	6.328
HORVU6Hr1G031590	52.99962	54.18434	60.20485	47.43154	58.07013	50.60367
HORVU7Hr1G062190	17.58712	16.65812	19.1446	20.35631	32.52287	29.10833

HORVU2Hr1G105740	191.6906	272.2441	424.68	58.59065	4.285436	11.68401
HORVU7Hr1G033030	120.2531	109.9077	114.2071	122.5387	153.4681	136.2133
Hordeum_vulgare_newGene_4389	0.637998	0.69149	0.584799	0.815791	2.329682	2.011422
HORVU7Hr1G101490	0.82395	1.216902	1.274929	0.9409	1.113405	1.142472
HORVU5Hr1G020800	6.814092	1.716087	7.764717	4.75494	4.769521	5.580333
Hordeum_vulgare_newGene_3872	7.705629	6.673473	8.05288	6.329389	6.583329	7.56313
HORVU1Hr1G009920	0.209622	0.227134	0.22072	12.61617	8.478135	9.715347
HORVU7Hr1G055280	23.59192	23.2881	25.85446	21.64949	31.07014	30.98097
HORVU7Hr1G115450	3.531058	3.456032	3.718611	5.84062	8.403063	8.349238
Hordeum_vulgare_newGene_45	104.9978	95.3854	104.9534	21.19647	12.83768	16.02218
HORVU5Hr1G108810	1.125553	1.009641	1.640873	1.944837	2.484362	2.491935
HORVU3Hr1G070630	6.00645	4.364638	5.959052	5.502453	6.573952	6.99718
HORVU3Hr1G010300	40.8708	35.13037	39.4898	47.74441	52.2053	51.55369
HORVU5Hr1G043200	8.094377	9.157892	6.985641	4.184907	1.942117	2.041853
HORVU5Hr1G010300	3.782083	3.986754	5.065271	4.693041	8.099508	7.003472
HORVU3Hr1G015880	6.252687	7.182331	7.716854	3.909472	3.758419	4.219795
HORVU0Hr1G004970	9.454691	8.431888	14.32664	11.64821	10.10673	14.27954
HORVU2Hr1G020900	0.981692	2.061366	2.904887	23.28166	19.46306	22.35011
Hordeum_vulgare_newGene_4961	0.158325	0.130045	0.05933	2.928868	2.489499	3.210771
Hordeum_vulgare_newGene_4960	1.166859	1.60136	1.611201	2.401943	2.240026	2.905966
HORVU6Hr1G036060	1.8452	2.195175	1.955075	2.252456	2.237645	2.849081
HORVU7Hr1G115180	6.940467	8.391634	9.862574	12.80529	18.39694	17.66893
HORVU5Hr1G101670	14.63678	15.39141	8.802446	7.462484	0	1.886735
HORVU5Hr1G037980	0.878479	0.138336	0.614124	0	3.08232	2.363892
HORVU7Hr1G032340	0.630838	0.414498	0.663365	6.17845	7.97101	6.429154
HORVU4Hr1G046480	9.234935	9.902898	12.56332	13.12196	20.07102	20.38611
HORVU3Hr1G000930	0.139662	0.061141	0.031018	0.479058	24.09503	21.88495
HORVU5Hr1G015480	0.722231	0.735061	0.411414	0.420699	2.993623	2.351894
HORVU2Hr1G089940	61.53228	64.91542	72.02318	78.96158	127.5388	128.1168
HORVU2Hr1G066400	2.651077	1.852025	2.307306	3.387371	4.208276	4.08135
HORVU1Hr1G069100	5.136868	5.409679	5.310109	4.017694	4.249752	4.75161
HORVU4Hr1G072970	0.817629	1.271196	0.753119	2.180471	1.385961	1.357301
Hordeum_vulgare_newGene_6521	5.816702	6.509509	6.867158	4.75917	5.772276	5.845974
HORVU7Hr1G018530	3.200333	1.61026	3.018598	0.324875	1.899943	1.524706
Hordeum_vulgare_newGene_6525	0.977578	1.671691	1.753975	2.094728	3.14713	3.270156
HORVU4Hr1G037210	5.162834	5.617547	7.424084	6.24893	7.279797	7.647047
Hordeum_vulgare_newGene_6529	1.277752	0.375089	1.341093	1.335952	2.219044	1.409223
HORVU4Hr1G074680	24.86273	26.01394	34.1815	35.88852	60.56004	55.4346
Hordeum_vulgare_newGene_390	21.57799	20.75306	24.61481	18.74437	22.41964	21.81012
Hordeum_vulgare_newGene_15926	1.224205	1.385738	1.642977	1.301395	3.48306	3.304064
Hordeum_vulgare_newGene_15924	4.388583	1.633037	0.127244	0.292515	0.058506	0.674219
Hordeum_vulgare_newGene_392	11.87299	9.263708	12.07922	19.34724	6.990876	8.439082
HORVU3Hr1G094210	4.805415	3.616598	4.173791	7.208579	5.611663	5.975358
HORVU5Hr1G036160	2.325835	3.010344	3.432202	2.516324	3.05809	3.53569
Hordeum_vulgare_newGene_8133	6.196381	6.577784	4.131712	17.74079	6.600646	5.909046
HORVU1Hr1G093800	16.42795	17.64209	20.44068	23.25594	24.81813	25.5275
HORVU7Hr1G052190	3.471352	3.756591	4.479068	9.57095	11.6099	11.43131

HORVU3Hr1G079870	22.06033	21.25851	25.93848	18.6444	35.73015	33.87805
HORVU1Hr1G088550	0.065908	0.126396	17.34805	44.19513	0	0
HORVU5Hr1G019100	4.452509	4.47405	5.647225	3.59008	3.388839	4.642345
HORVU5Hr1G064230	0.537976	0.598176	0.483762	1.218584	1.635939	2.045143
HORVU7Hr1G053570	1.70464	1.990174	1.067069	0.95753	8.586192	6.846576
HORVU6Hr1G032760	3.319154	1.959077	2.777337	8.113854	5.691523	5.485444
HORVU7Hr1G111600	88.90083	71.66861	110.6151	65.73177	131.01	102.9362
HORVU6Hr1G069370	63.93948	52.62026	84.76014	61.99302	118.9716	121.1894
HORVU1Hr1G029950	4.537984	4.048439	6.377499	7.613989	11.26973	12.26843
HORVU3Hr1G006830	4.625267	3.444241	4.289896	1.969562	1.973443	3.11263
HORVU1Hr1G015820	3.301715	4.044404	3.725291	0.040286	0.076016	0.136907
Hordeum_vulgare_newGene_9879	0	0	0.010191	3.928124	4.257434	4.468034
HORVU4Hr1G000910	6.932232	6.280399	7.236388	6.6573	8.171669	8.892563
HORVU6Hr1G070090	1.120782	1.574323	2.287765	1.292573	0.16258	0.605924
HORVU5Hr1G022510	1.690994	1.338376	2.404813	2.96719	3.170462	3.288172
HORVU1Hr1G051150	15.96824	15.38994	16.92169	13.89318	19.56364	19.72082
HORVU1Hr1G087110	22.25413	20.21055	21.78642	3.321929	0.420214	0.955555
HORVU1Hr1G080720	13.94911	13.24627	15.23526	18.06704	23.99987	21.57656
HORVU7Hr1G022410	8.445492	7.353246	7.439603	10.09529	12.97324	11.44227
HORVU4Hr1G017040	1.175444	1.403623	1.067551	0.274487	1.449576	0.769135
HORVU7Hr1G094830	5.865756	7.044825	5.414636	7.849788	7.40525	8.978965
HORVU5Hr1G023640	0.012913	0.010222	0.006746	0.427115	2.417598	3.183825
HORVU6Hr1G084720	2.821152	3.145632	2.507756	5.582335	9.743343	9.390321
HORVU1Hr1G015560	154.1596	146.081	142.7121	24.40655	18.1897	16.3649
HORVU0Hr1G012620	0.462882	5.497959	6.112502	12.13227	11.86298	0.901572
HORVU6Hr1G087190	104.7078	110.0816	143.2013	46.98269	118.7211	97.58858
HORVU3Hr1G033120	4.506371	4.098144	4.702672	5.23508	4.184891	6.079212
HORVU5Hr1G062530	4.515713	4.444108	5.164387	2.835792	4.349022	4.455811
HORVU6Hr1G072250	1.547777	1.440042	1.440701	13.23298	12.0635	13.16251
HORVU3Hr1G078830	3.398108	4.203098	3.908411	6.983397	11.03698	10.85681
HORVU5Hr1G110200	24.86225	23.12991	24.91843	13.67873	16.11433	15.58774
HORVU1Hr1G054470	8.185057	7.09527	9.127807	10.07256	8.999558	10.71711
HORVU0Hr1G007370	0.274139	0.4048	0.371968	1.620457	14.64284	17.27028
HORVU2Hr1G056710	0.722065	0.812912	0.822758	2.38798	2.195574	2.56195
HORVU7Hr1G048080	1.97886	2.31561	2.137649	5.302196	9.385349	9.358364
HORVU1Hr1G093680	4.519501	4.593977	3.932282	1.777645	2.510605	3.84864
Hordeum_vulgare_newGene_6952	1.45407	2.537373	1.819376	3.084755	3.345623	2.762571
HORVU1Hr1G027490	5.96677	4.719317	5.636637	6.658452	9.018555	9.446223
HORVU7Hr1G040670	24.03728	22.73057	22.04638	19.67616	24.36892	26.55358
HORVU1Hr1G021890	7.612281	7.405164	7.383585	10.16232	16.93734	16.21393
HORVU4Hr1G050960	6.78974	7.213193	9.506795	7.027677	12.05323	10.10594
HORVU1Hr1G023030	13.2317	13.83782	11.77103	12.87214	10.95804	11.51195
HORVU4Hr1G063010	1.6067	1.729295	2.176543	1.716385	2.80997	2.454362
HORVU5Hr1G069310	0.066802	0	0.040915	6.077199	6.801356	7.982941
HORVU4Hr1G084330	1.736087	1.431265	1.891874	3.199723	3.664554	3.278816
HORVU7Hr1G076790	9.274052	9.794745	8.671516	12.29121	17.05119	14.37131
HORVU5Hr1G111550	47.7323	39.1518	46.61467	22.14132	24.27551	24.76994

HORVU4Hr1G081670	34.46221	30.48378	38.12447	24.53544	17.03427	21.33685
HORVU2Hr1G066690	33.42274	32.7357	35.99112	21.7197	18.42928	18.9904
HORVU3Hr1G015740	10.93848	10.85492	9.912875	8.308864	10.20973	11.43064
HORVU3Hr1G024640	5.993978	5.551027	5.831263	5.886222	2.353264	3.106721
HORVU4Hr1G088480	1.310462	1.634267	1.56328	2.151313	4.900411	3.912376
HORVU7Hr1G078330	1.696474	2.261644	2.702643	0.519704	0	0.149358
HORVU4Hr1G062760	1.672521	1.71262	1.431028	3.247303	4.070719	2.708273
HORVU1Hr1G002440	0	0	0	12.07062	0.013598	0
HORVU7Hr1G050680	62.77119	53.88192	61.8048	20.93726	4.640471	10.5742
HORVU5Hr1G038790	11.07073	11.8502	13.84318	8.31574	11.28418	7.267058
HORVU7Hr1G120020	1.652428	2.142383	1.944562	3.920185	4.585803	4.86556
HORVU6Hr1G012000	1.924118	1.58629	1.697348	1.875657	1.381556	2.109007
HORVU1Hr1G058150	0	0.027061	0.036757	0.897019	5.45843	3.780882
HORVU6Hr1G067740	1.064168	0.33713	2.317489	0.40577	0.999353	1.350598
HORVU4Hr1G049090	11.68014	11.70425	13.37741	18.57628	30.16556	27.71805
HORVU3Hr1G000090	18.72994	13.99474	17.98289	18.00681	20.58542	19.31169
HORVU3Hr1G071220	0.144659	0.058314	0.109008	2.368165	2.54638	2.17815
HORVU7Hr1G095010	6.804968	7.882631	7.351589	9.483656	6.780911	8.218115
HORVU1Hr1G047300	17.14688	14.34312	20.30546	13.70298	18.24156	18.36504
HORVU1Hr1G022810	101.7348	94.65438	127.175	96.18248	138.4807	143.7122
HORVU3Hr1G025590	84.66051	75.55324	72.35626	60.49199	64.87103	62.35753
HORVU7Hr1G051600	3.274061	3.330233	3.43539	4.238415	9.01805	8.894186
HORVU1Hr1G012420	2.387924	2.354502	3.341949	5.089235	7.449461	8.044624
HORVU0Hr1G012510	10.71441	10.20097	11.30361	10.13648	10.83615	11.25072
HORVU2Hr1G010380	38.34835	37.24332	37.42423	34.7967	46.10121	48.14858
HORVU2Hr1G081230	0.15191	0	0.696267	23.57834	317.7046	220.7487
HORVU1Hr1G024870	5.695064	3.519363	5.752374	7.590263	7.950897	9.874533
HORVU6Hr1G014970	52.33477	44.93408	49.39569	47.51962	56.42656	52.987
HORVU1Hr1G089180	1.022356	0.564248	0.494597	48.76073	7.099836	7.147287
HORVU4Hr1G027930	1.374886	0.992434	1.860731	2.714329	5.064861	5.309512
HORVU2Hr1G085920	4.857906	4.678169	5.387262	5.629587	5.720152	7.163684
HORVU5Hr1G121350	3.931908	4.328553	4.617918	0.202172	0.078905	0.098777
HORVU2Hr1G053630	6.676333	6.637316	3.983214	3.541509	14.23902	16.4274
HORVU5Hr1G104380	47.49074	54.66782	70.1793	42.0835	74.82829	72.90468
HORVU2Hr1G091200	14.00037	13.4508	15.69256	21.89604	23.36859	24.17438
HORVU2Hr1G093690	1.00481	1.762952	2.09431	3.448278	4.821733	4.370342
Hordeum_vulgare_newGene_4047	3.301012	3.293166	4.189672	5.524469	6.682744	7.454188
HORVU2Hr1G080490	3.033069	3.322521	3.893976	1.955698	1.362618	1.791681
HORVU2Hr1G117940	1.327924	0.794763	1.530052	3.285336	1.105695	2.50793
HORVU5Hr1G044400	10.06048	10.92143	9.038919	13.69317	17.37001	13.0934
HORVU6Hr1G018540	7.516193	8.961204	12.42139	4.642385	6.892833	6.970521
HORVU7Hr1G084750	2.185798	4.708316	3.150315	4.784108	4.197785	5.896636
HORVU7Hr1G071450	10.33881	8.852235	13.46483	8.836767	11.76659	11.48517
HORVU0Hr1G014210	5.882892	5.617843	6.354525	6.111197	4.733902	6.125478
HORVU5Hr1G056670	0.447067	0.63044	0.718811	2.197219	3.069589	4.040935
HORVU3Hr1G107920	7.123482	7.009583	7.012158	6.747655	6.576897	6.41851
Hordeum_vulgare_newGene_13944	29.46667	28.47056	33.89381	24.45345	25.19304	27.59679

HORVU0Hr1G001470	0.313952	0.128781	0.319204	0.907369	2.319853	2.570493
HORVU7Hr1G091210	1.32588	1.400086	1.289148	0.848587	1.481868	1.94695
HORVU4Hr1G000580	6.650149	6.96851	6.996658	7.867505	9.103914	8.971535
HORVU7Hr1G095900	2.506711	2.179195	2.337731	0.612679	0.443315	0.64462
HORVU4Hr1G081940	4.815563	4.946427	5.983346	4.104992	3.434758	4.317511
HORVU4Hr1G064150	2.171656	1.613703	2.265966	1.423379	2.053666	2.076561
HORVU2Hr1G109500	64.93758	56.72926	64.20176	48.68832	63.51501	63.42767
HORVU1Hr1G043220	5.817935	4.529355	5.149484	4.277798	5.444546	5.226743
HORVU7Hr1G081220	11.11087	11.36767	10.36455	9.29245	9.954779	8.2533
HORVU4Hr1G021830	7.000479	6.760936	5.569882	7.519993	10.63998	11.93361
HORVU6Hr1G085430	0.970569	1.132005	0.877393	1.678271	2.300052	2.492851
HORVU5Hr1G125300	15.59905	14.26863	16.90265	5.147755	5.265632	6.001223
HORVU4Hr1G061500	42.53389	40.03554	51.64954	23.31046	38.00938	33.12879
HORVU5Hr1G079990	26.06849	23.12556	21.21275	13.41105	11.38917	11.76189
HORVU5Hr1G098340	1.013099	0.691246	0.981378	3.071673	5.814507	6.09465
Hordeum_vulgare_newGene_10414	27.69215	26.85348	35.42948	38.40022	46.28725	43.85278
HORVU6Hr1G017340	5.692872	6.302124	4.755469	3.282589	3.618611	4.46605
HORVU1Hr1G051210	1.147548	1.766073	1.626708	1.210506	1.127892	1.591629
HORVU1Hr1G080440	10.12413	5.313542	9.698603	8.243006	8.401349	9.780172
HORVU6Hr1G013450	2.367026	3.497104	3.690353	1.081396	1.383384	2.017409
HORVU4Hr1G060670	0.127857	0.194222	0.119281	15.41019	1.859906	5.952237
HORVU3Hr1G098640	1.098957	0.962826	0.936485	2.95622	4.236308	4.998454
HORVU5Hr1G116310	21.02919	20.67425	21.66201	14.76376	22.80058	20.23333
HORVU1Hr1G044160	17.44621	14.6565	23.35134	11.87493	19.15509	19.12434
HORVU2Hr1G037380	4.612103	4.851905	4.969035	6.12887	6.680293	8.870902
HORVU7Hr1G041920	48.25841	49.40916	38.94156	76.46525	69.75339	78.73014
HORVU5Hr1G047190	5.596353	6.335247	7.026672	7.044202	6.727471	8.004178
HORVU1Hr1G065250	0.619683	0.557996	0.570674	2.923974	2.185424	3.215152
HORVU3Hr1G072220	1.908936	1.999565	2.329158	1.749605	2.869914	2.803965
HORVU3Hr1G095750	15.79957	11.63206	17.98064	16.61913	22.32183	21.91775
HORVU4Hr1G056060	6.220031	7.147014	6.331097	11.55171	13.49752	13.67199
HORVU3Hr1G078150	0.958959	3.501106	1.285874	5.118599	4.8773	5.30579
HORVU7Hr1G027670	60.21055	51.71039	60.92409	52.43724	34.48512	35.75542
HORVU2Hr1G106850	1.353712	1.576466	1.335926	1.694687	2.049823	2.133469
HORVU3Hr1G074680	54.18022	51.85063	64.5095	35.052	58.24782	45.46724
HORVU7Hr1G082280	53.87136	39.30926	51.52152	62.55425	63.15627	74.96298
HORVU6Hr1G041650	5.294418	4.844151	5.478432	3.800899	6.888088	5.138954
HORVU2Hr1G091990	6.058666	5.326005	7.467777	11.31192	3.616319	6.228055
HORVU6Hr1G049080	6.484996	7.35195	8.805064	11.26096	15.9267	14.78327
HORVU4Hr1G006330	9.758348	10.36521	11.31035	10.4752	9.061527	9.913383
HORVU2Hr1G021090	5.725064	2.739308	0.944851	1.755989	1.772636	1.725568
HORVU7Hr1G000410	3.945253	5.104809	4.449265	2.275237	3.572241	3.271157
HORVU3Hr1G092250	36.81331	27.62895	37.42722	30.73928	27.02202	28.53535
HORVU4Hr1G069050	0.235464	0.900773	0.678806	1.603769	2.081448	2.029718
HORVU3Hr1G077080	2.066281	2.044453	2.308001	1.700834	2.470342	3.062606
HORVU5Hr1G017450	1.435338	1.309011	2.038814	1.527304	1.973261	1.909746
HORVU2Hr1G017170	3.543967	4.340105	4.277465	4.498597	4.775506	4.709206

HORVU3Hr1G073790	2.029512	1.739742	1.740439	3.455402	4.772134	5.321574
HORVU0Hr1G040200	2.012408	2.045498	1.469954	4.784472	5.487642	5.114815
HORVU2Hr1G012790	88.0115	90.14346	108.1511	14.37347	1.634659	6.011482
HORVU3Hr1G075790	2.072127	1.699225	1.865656	3.996998	1.550437	2.736108
HORVU3Hr1G094480	6.440566	5.553254	8.184678	6.306008	7.146605	6.520999
HORVU5Hr1G027180	1.800565	1.91861	1.412482	1.614258	1.828842	1.441727
HORVU1Hr1G034570	3.364109	5.202064	4.672145	2.984966	3.144718	4.282055
Hordeum_vulgare_newGene_160	1.293425	2.715639	3.496089	41.99805	53.18006	53.83988
HORVU5Hr1G051870	15.61711	13.56243	15.56025	18.2452	11.1444	15.63696
HORVU7Hr1G021530	13.48812	14.06173	17.69555	20.59211	16.88978	19.80185
Hordeum_vulgare_newGene_10004	4.395141	4.993134	6.32416	5.738927	7.483908	7.5129
HORVU2Hr1G029640	54.03044	55.02861	65.58144	52.68945	70.15119	65.17275
HORVU7Hr1G083480	2.312808	2.276606	3.513194	2.23541	2.040739	2.463618
Hordeum_vulgare_newGene_11450	14.24105	14.75728	16.19049	23.65931	33.73767	32.66412
HORVU3Hr1G081610	0.719151	0.545315	0.894296	2.64696	3.385602	4.18903
HORVU4Hr1G043680	21.74156	19.41509	21.07073	47.78092	59.43983	57.70643
Hordeum_vulgare_newGene_292	18.69346	20.29207	22.57507	16.26462	15.55703	16.98173
HORVU7Hr1G021280	11.91421	9.198872	11.06096	17.34458	17.77774	19.52478
HORVU5Hr1G026240	4.144086	3.629478	4.842282	4.84424	4.564264	5.120864
Hordeum_vulgare_newGene_12220	3.900964	4.512742	5.680417	3.224461	2.658911	3.227743
Hordeum_vulgare_newGene_12221	1.869269	2.445479	2.091337	2.316073	2.193897	3.289276
Hordeum_vulgare_newGene_12222	1.621771	2.195615	2.194548	3.068315	3.390091	4.279869
Hordeum_vulgare_newGene_12225	2.790284	1.698341	2.901353	2.487559	3.252609	3.720425
Hordeum_vulgare_newGene_12227	6.136032	8.452724	9.013726	5.813006	7.825549	9.276301
Hordeum_vulgare_newGene_12229	19.78977	16.80504	21.26764	21.73882	22.39974	24.71524
HORVU2Hr1G072850	8.307502	8.451635	11.25788	19.44217	79.51726	91.19272
HORVU5Hr1G072760	8.763929	8.235621	8.096111	2.032112	2.035436	1.973891
HORVU1Hr1G027300	0.510532	0.516737	0.471274	0.390782	1.968961	2.473156
HORVU5Hr1G041590	0.537607	0.535816	0.517787	1.421542	1.213661	1.990258
HORVU5Hr1G070720	55.52875	54.96861	62.59732	83.82052	233.9887	188.1015
HORVU1Hr1G085160	1.086941	1.088095	1.319402	1.225131	3.424095	3.932622
HORVU5Hr1G113580	45.76381	52.42496	48.59397	161.8953	147.0052	130.9621
EPlHVUG00000039864	0.564185	0.096561	0.140442	0.102417	3.152931	2.100193
HORVU5Hr1G049020	32.66032	26.82562	36.13866	34.58279	31.32813	37.31832
HORVU5Hr1G112390	10.78409	13.81899	18.38515	3.179857	5.818924	5.222974
Hordeum_vulgare_newGene_16102	7.904926	8.764445	8.389454	13.54536	10.67797	13.09373
Hordeum_vulgare_newGene_12486	888.5273	685.7609	1122.476	663.5668	869.1788	752.4455
Hordeum_vulgare_newGene_12487	7.754729	6.35757	7.857697	29.42458	39.69684	33.05331
Hordeum_vulgare_newGene_12482	5.882053	4.679934	5.36551	3.015404	6.303635	4.381435
HORVU2Hr1G103010	252.9156	194.4095	244.4774	92.36829	86.75196	88.89232
HORVU7Hr1G035320	17.77439	19.41877	19.8629	28.03476	11.61919	18.72219
Hordeum_vulgare_newGene_13574	5.068337	5.467194	4.725115	4.813019	2.992128	3.651139
Hordeum_vulgare_newGene_13579	3.580317	3.111388	3.306291	2.213939	1.094012	1.778299
HORVU3Hr1G090670	7.885463	7.210518	8.550683	8.479496	9.78181	10.55969
Hordeum_vulgare_newGene_7908	23.23499	19.07632	25.49193	32.61983	39.90072	31.34073
Hordeum_vulgare_newGene_7906	1.340125	1.602526	1.562492	2.472878	1.971041	2.550309
Hordeum_vulgare_newGene_3486	0.035184	0	0.035321	2.81837	2.730517	3.454691

Hordeum_vulgare_newGene_3485	5.902467	4.701342	6.391025	4.024872	3.616964	2.518506
Hordeum_vulgare_newGene_7902	1.481956	1.171337	1.986748	2.100846	0.610814	0.34918
Hordeum_vulgare_newGene_10882	3.564024	4.179428	4.728906	2.745	3.012114	3.420708
Hordeum_vulgare_newGene_10881	1.389123	1.661354	2.109061	2.874535	4.876241	4.369635
HORVU5Hr1G061200	12.38249	13.31455	13.58308	13.60879	15.38477	14.23731
Hordeum_vulgare_newGene_10885	33.44426	37.65348	34.8995	43.35091	58.26377	55.12306
HORVU5Hr1G085760	11.58352	11.46756	11.09022	8.343344	5.758947	7.735206
Hordeum_vulgare_newGene_6053	0	0	0.005139	5.148375	5.621052	5.680993
Hordeum_vulgare_newGene_6057	0.768359	1.084393	1.785159	1.289924	1.452799	1.359549
HORVU0Hr1G005160	45.69667	38.06499	42.5297	56.53364	29.7131	37.33247
Hordeum_vulgare_newGene_13681	3.15827	3.011159	3.392237	3.188374	4.196915	4.123683
Hordeum_vulgare_newGene_13683	14.0961	9.119272	11.84076	8.22675	9.661499	19.03661
Hordeum_vulgare_newGene_13682	2.879261	2.117765	3.129222	3.899772	4.227259	3.974622
Hordeum_vulgare_newGene_13685	14.86854	14.10321	18.56896	29.93514	75.24263	75.40183
Hordeum_vulgare_newGene_13684	81.53481	68.90854	46.22179	18.21538	13.53348	14.02572
HORVU5Hr1G079380	0.555461	0.681593	1.038727	2.32352	1.440805	1.440314
Hordeum_vulgare_newGene_5887	1.818961	2.335266	1.72844	4.865515	2.763829	2.712421
Hordeum_vulgare_newGene_5881	1.449993	0.058409	1.58402	5.056979	7.018313	6.090722
HORVU0Hr1G004690	1.994196	2.320284	2.308125	2.140576	2.862409	3.403367
Hordeum_vulgare_newGene_2282	0.63384	0.959228	0.982476	1.124168	1.74089	1.61118
HORVU3Hr1G061510	5.612576	4.86074	5.787543	4.236329	6.837578	6.730801
HORVU2Hr1G036240	11.31651	9.990848	12.37103	11.46448	9.432007	8.815741
HORVU7Hr1G008720	4.696634	6.10962	5.253185	5.484983	8.099366	7.558143
Hordeum_vulgare_newGene_2288	1.409062	1.241849	1.316754	0.020239	0.858058	1.259176
HORVU2Hr1G005470	1.754382	1.589737	1.715049	6.452731	11.80223	11.00341
HORVU1Hr1G032060	19.83341	17.81507	22.42432	21.7578	26.13331	27.43742
HORVU6Hr1G032260	0.316277	0.638893	0.494843	0.730282	2.556966	2.607155
Hordeum_vulgare_newGene_9584	4.915568	5.880522	7.387172	3.877192	5.062158	3.644798
HORVU7Hr1G078770	2.915286	3.107081	4.014136	6.654873	13.64017	13.50492
HORVU6Hr1G012440	2.753193	3.130106	4.762047	3.368336	4.461629	4.561779
HORVU4Hr1G061260	7.761657	7.181788	9.471839	3.318121	4.509254	4.650731
HORVU4Hr1G011500	29.11418	27.00072	28.04649	86.77551	131.9436	134.9675
HORVU5Hr1G115500	1.581702	2.001154	1.362579	1.601847	1.177639	1.58434
HORVU7Hr1G043150	4.13854	4.201735	3.172825	2.779886	2.495175	2.613482
HORVU2Hr1G027230	1.397447	2.409492	2.831174	0.615821	0.574927	0.91792
HORVU1Hr1G063310	47.50879	42.834	44.68696	50.47355	65.07017	66.58203
HORVU7Hr1G103160	27.57976	21.21021	17.13473	16.20963	15.02451	16.51265
HORVU3Hr1G069070	1.216457	1.772834	1.631064	2.062435	7.618039	5.285968
HORVU5Hr1G035100	51.57753	61.67176	55.18415	66.88518	96.62921	91.56315
HORVU1Hr1G074960	24.81322	20.95206	23.67381	18.60622	26.4188	25.36725
HORVU1Hr1G041020	0.957178	0.822202	1.18456	0.639809	1.375521	1.344131
HORVU6Hr1G064450	3.09794	2.308129	3.438265	4.079698	5.602262	6.391525
HORVU3Hr1G096140	52.46432	45.97278	52.6882	73.17734	93.59893	79.35504
Hordeum_vulgare_newGene_11863	7.39464	8.558166	4.533512	0.705633	0.160846	0.506822
Hordeum_vulgare_newGene_11865	1.420328	1.805182	3.085841	1.630889	3.57289	2.298565
HORVU2Hr1G051180	1.112489	0.405252	1.1069	2.911096	3.333205	3.819616
HORVU7Hr1G024900	4.983564	4.093362	7.540791	3.538812	3.93725	4.654412

HORVU4Hr1G074800	6.699452	8.000144	9.860055	6.772168	14.71004	14.63743
HORVU6Hr1G013710	23.00666	13.86831	29.45282	30.16456	10.71665	33.7163
HORVU1Hr1G053020	20.32098	21.14728	19.99293	22.91889	29.46074	28.13586
HORVU3Hr1G015180	20.61447	20.81844	25.06045	18.15279	19.03053	19.9319
HORVU3Hr1G058300	2.559759	2.081732	2.681139	1.372033	0.433586	1.013004
Hordeum_vulgare_newGene_1254	3.667518	3.055355	3.820037	4.356636	7.181907	6.694819
Hordeum_vulgare_newGene_1255	1.851178	2.430538	2.194326	1.477952	1.676666	1.959131
HORVU7Hr1G067060	51.48367	53.42569	90.77475	30.54541	48.9245	51.5065
HORVU1Hr1G066830	5.308794	6.28748	6.838248	4.769829	6.044518	6.632594
HORVU3Hr1G018460	2.032356	1.814669	2.818553	2.720082	5.425953	3.944305
HORVU4Hr1G005510	1.030472	0.606774	1.263731	1.203611	2.81683	2.824499
HORVU5Hr1G019720	7.647315	6.491371	9.819639	7.23657	3.210182	5.734967
HORVU5Hr1G063170	1.804279	2.176442	2.115153	1.851839	1.810448	1.69371
HORVU3Hr1G014580	0.886144	1.026842	1.257933	1.8055	4.023707	4.410751
HORVU7Hr1G106480	1.180943	1.02646	1.495835	4.229757	4.715143	4.582689
HORVU6Hr1G031650	7.515074	10.92628	8.776957	19.20307	15.72048	16.73527
HORVU5Hr1G105810	16.87964	13.11361	16.76842	16.5529	17.27306	16.82113
Hordeum_vulgare_newGene_4024	116.0966	160.3855	178.1456	58.45396	15.54069	29.39181
Hordeum_vulgare_newGene_4022	5.331226	4.280957	6.024327	1.329687	0.131477	0.432783
Hordeum_vulgare_newGene_4023	6.361042	5.367045	10.98251	0.787261	0.780888	2.344816
HORVU4Hr1G059130	64.4848	55.28634	64.14453	91.01842	131.4876	138.0953
HORVU4Hr1G033760	8.680248	8.08761	8.806875	8.919049	9.446745	9.79474
HORVU4Hr1G013720	9.893923	9.325335	12.24503	16.03616	19.56168	21.26372
HORVU2Hr1G034480	1.030045	1.112652	0.715813	1.272143	1.066542	1.225947
HORVU5Hr1G108990	126.7023	132.0179	202.8111	123.5542	297.492	283.6811
HORVU6Hr1G009480	4.410845	4.614523	4.300964	10.35266	8.745626	11.16898
HORVU2Hr1G046420	1.941716	1.326406	1.736328	2.839682	2.8055	2.811157
Hordeum_vulgare_newGene_7594	2.278305	3.790006	2.867026	6.41557	6.029543	7.057589
Hordeum_vulgare_newGene_7592	0.457783	0.694657	0.775397	4.551349	4.666028	5.783162
HORVU6Hr1G062800	17.82073	17.61117	11.97358	14.27519	11.78237	11.66563
HORVU1Hr1G069800	1.131487	1.667973	1.552298	10.88974	7.189045	11.58012
HORVU6Hr1G070500	5.777441	4.532563	8.165641	10.07978	16.03209	13.45055
HORVU5Hr1G074250	3.237695	2.947315	4.557677	4.692453	2.928056	3.664181
HORVU2Hr1G002410	11.24124	10.24001	11.89602	11.60115	13.58053	13.79944
HORVU1Hr1G076950	1.375392	1.596571	1.552466	0.599389	0.921191	0.667895
Hordeum_vulgare_newGene_6852	9.135722	13.34178	13.84968	8.462846	9.250224	14.72132
HORVU0Hr1G000850	5.328503	3.870836	5.591878	0	0.02514	0.013331
HORVU5Hr1G027110	13.88664	14.49399	8.789166	0.803383	0.642054	0.924267
HORVU4Hr1G073120	313.3222	356.0598	485.8	58.25667	20.04231	27.34774
HORVU1Hr1G080950	5.361573	6.14325	7.008242	6.254548	6.567485	7.544302
HORVU3Hr1G039880	5.716742	6.318319	5.743321	7.572543	7.146409	8.835984
HORVU2Hr1G078570	14.18514	11.80672	13.29418	10.7301	8.078047	9.46285
HORVU7Hr1G079410	0.257285	1.772896	0.565362	1.966522	1.561381	5.15071
HORVU2Hr1G075330	3.3375	4.232081	4.746814	3.732335	4.301283	4.99483
HORVU2Hr1G015260	6.274695	5.516237	9.483849	13.2521	14.17623	13.60101
HORVU6Hr1G029780	1.355636	1.629203	1.682788	0.973172	1.234679	1.174281
HORVU3Hr1G035470	115.5633	144.1268	216.9632	12.30918	6.254481	8.344612

HORVU7Hr1G093340	6.980855	10.01079	9.104429	5.434867	7.334891	5.499504
HORVU5Hr1G104850	5.236269	5.05231	5.174718	6.646896	2.797441	5.144573
HORVU5Hr1G093400	5.684961	5.021487	5.28272	14.21527	21.63285	19.08256
HORVU1Hr1G074550	3.741328	2.505494	3.519069	1.773892	1.036451	1.968002
Hordeum_vulgare_newGene_10166	27.64494	21.40602	32.44775	0.131259	0	0
HORVU7Hr1G052520	19.1321	19.7014	24.67216	20.72714	25.30899	27.31104
HORVU4Hr1G044670	9.307432	8.772615	13.97003	12.38354	23.0255	21.37563
HORVU1Hr1G080680	3.088107	1.246906	4.642656	0.667991	0.110738	0.842244
HORVU0Hr1G012990	6.944516	7.423449	6.902693	14.52612	9.988903	10.75831
Hordeum_vulgare_newGene_6314	1.527802	2.16473	2.579362	2.126603	1.935715	2.418
Hordeum_vulgare_newGene_6312	1.519497	1.223846	2.074787	2.723543	3.175061	3.422744
HORVU6Hr1G073730	92.86948	84.83338	94.32604	77.30586	95.805	98.30428
HORVU1Hr1G021750	97.51536	112.9335	158.3681	32.82372	1.631948	11.53195
HORVU1Hr1G078490	36.84058	26.89928	39.40775	40.51416	32.74334	36.16361
HORVU1Hr1G050900	4.918583	5.254764	5.074616	7.166345	13.57084	14.77796
HORVU2Hr1G093420	3.067598	2.546643	3.630721	4.090987	5.639946	5.551664
Hordeum_vulgare_newGene_3804	0.097922	0	0.047726	0.44429	5.250271	6.634634
HORVU6Hr1G075060	5.367388	6.169476	6.104631	7.525071	10.05464	10.08737
HORVU6Hr1G030390	24.71833	19.5667	27.07489	16.76817	14.50281	17.84265
HORVU4Hr1G007260	2.642459	2.2402	3.317764	7.522638	9.012919	10.05453
HORVU5Hr1G002040	323.3701	283.466	366.6956	362.9176	537.2512	566.4994
HORVU7Hr1G011120	8.01311	8.528004	9.938611	7.882867	9.961026	10.1134
HORVU3Hr1G030640	3.752251	4.476877	5.816959	3.658998	2.728279	2.92731
HORVU7Hr1G000260	2.549633	2.191649	3.204763	19.5989	16.34103	17.88732
HORVU1Hr1G038700	3.789493	4.680433	4.549196	5.434552	5.612588	6.18811
Hordeum_vulgare_newGene_2997	33.29218	28.26025	36.07437	25.45463	20.19835	21.63466
HORVU6Hr1G037960	7.106045	9.038671	10.0655	9.706604	17.37494	16.72949
HORVU6Hr1G038710	113.1008	97.60038	127.0828	174.9878	235.9931	187.3853
HORVU2Hr1G061920	6.434709	6.17967	6.892816	3.099424	1.838247	3.386337
HORVU5Hr1G050320	1.769276	2.203397	2.999742	2.488628	3.261981	3.568452
HORVU3Hr1G109880	1.703337	2.357902	3.015823	3.911293	10.265	9.91565
HORVU1Hr1G093520	20.95773	25.66273	24.08472	26.99644	32.72695	32.84511
HORVU5Hr1G029490	3.73963	5.20032	6.037791	3.108463	3.272691	4.153604
HORVU2Hr1G081450	3.0514	3.483718	3.718652	3.647294	3.463803	4.439071
HORVU5Hr1G009540	0.073584	0.126596	0.064263	0.866617	10.88647	4.891857
HORVU4Hr1G080560	9.143452	5.578411	11.47089	5.511497	2.903554	4.020659
HORVU2Hr1G122370	26.73908	24.84436	29.31692	32.39144	34.97487	38.09709
HORVU5Hr1G106170	4.108575	4.444565	5.784756	3.240924	4.13744	4.528371
Hordeum_vulgare_newGene_15647	6.138602	6.134805	6.373933	6.583316	6.121763	7.13044
HORVU2Hr1G015360	25.88401	17.94343	23.82081	17.37318	14.55207	18.15194
HORVU0Hr1G004790	5.046912	3.965671	6.062893	6.135872	8.586672	8.753223
HORVU7Hr1G020580	1.991093	1.942363	2.189933	3.324282	32.82734	33.95944
HORVU4Hr1G002300	7.220015	6.27247	8.920728	8.309558	9.819697	9.596921
HORVU5Hr1G055350	73.25758	73.27786	84.74215	48.44847	79.58714	79.87547
HORVU7Hr1G121230	20.81531	23.69958	23.09001	26.86452	32.24536	34.44549
HORVU4Hr1G007880	30.4317	27.92222	35.05268	39.82901	45.07286	46.90532
HORVU2Hr1G108840	2.783695	2.932243	3.088058	4.632969	6.43512	6.145907

HORVU1Hr1G018540	223.8869	203.791	200.4741	286.1142	531.1614	542.8085
HORVU3Hr1G010470	5.229581	5.173215	7.422041	5.750029	4.307026	5.318997
HORVU1Hr1G091010	99.4419	97.5669	132.0206	39.84423	8.921203	16.61644
HORVU2Hr1G122950	10.49967	7.927087	12.50645	16.9336	30.15721	29.394
HORVU7Hr1G071940	3.572068	4.619302	3.44645	6.235791	8.341189	8.855226
HORVU2Hr1G098660	40.67228	40.14005	41.14026	17.8543	11.59444	13.16274
HORVU1Hr1G039870	15.5639	13.24436	16.56472	12.14097	15.28503	16.47745
HORVU6Hr1G000340	25.9484	29.54448	45.71153	1.549754	0.387281	0.969812
HORVU3Hr1G029470	0.388864	0.353201	0.477204	3.965095	1.914259	2.664844
HORVU1Hr1G065160	2.418603	1.948638	2.98159	7.919883	15.87819	17.01101
HORVU7Hr1G099640	15.77383	15.95669	13.87996	13.05262	12.39317	14.55813
HORVU2Hr1G126010	9.855668	11.2612	10.9889	8.90139	12.09802	11.03535
HORVU5Hr1G109100	0.189622	0.209411	0.228191	9.567031	9.173363	6.981811
HORVU5Hr1G105480	10.63306	9.147678	11.66748	11.4386	14.61554	15.80588
HORVU7Hr1G119700	0.901725	0.827583	1.065871	1.365715	1.500996	1.704013
HORVU3Hr1G037960	12.75967	11.07104	11.27883	14.73898	15.60614	16.25603
HORVU7Hr1G049140	4.174713	3.789675	4.773669	3.150208	2.548728	2.83868
HORVU2Hr1G015980	25.7288	27.93621	23.25977	67.01902	106.6	91.46935
HORVU5Hr1G118590	2.139751	1.205418	2.395089	2.026913	0.804816	2.379981
HORVU3Hr1G031690	17.62445	17.1197	18.23978	15.56318	19.80344	18.94835
HORVU4Hr1G081770	1.598244	1.281233	1.97563	2.73441	3.745857	3.932698
HORVU7Hr1G114890	0.392255	0.39035	0.272515	5.153944	10.21462	9.037797
HORVU6Hr1G001690	1.270489	1.300082	1.594742	1.643061	1.944554	1.949674
HORVU2Hr1G013200	26.71109	22.97376	30.60081	19.83445	21.63346	22.36471
HORVU4Hr1G014780	3.105064	3.614376	3.961436	3.439309	2.507707	2.214674
HORVU3Hr1G018700	1.84175	1.641605	1.918004	1.338086	0.326638	0.812541
HORVU2Hr1G095410	2.805244	3.03943	2.941524	3.547238	3.620396	3.350179
HORVU6Hr1G017960	3.637337	2.089023	3.190065	4.118133	5.702972	5.710561
HORVU5Hr1G070290	6.300922	6.900812	7.435625	3.929885	4.525103	4.588263
HORVU3Hr1G084470	3.533908	4.267107	5.186608	3.044178	3.28695	4.039096
HORVU1Hr1G058340	6.885616	6.11929	8.481511	7.874515	11.0862	9.348531
HORVU1Hr1G083810	1.208898	1.046974	0.921915	0.438221	1.208813	1.197161
HORVU6Hr1G019510	4.347285	3.068131	4.010334	11.03608	17.76565	19.9618
HORVU5Hr1G057460	1.138037	1.01269	1.335273	1.745482	2.58227	2.809532
HORVU7Hr1G082970	2.802115	1.538729	1.676175	0.305892	1.135776	1.345474
HORVU6Hr1G031960	6.004149	5.848155	6.022255	4.979917	5.039145	5.494503
HORVU4Hr1G012780	1.47621	1.640223	1.662106	3.367237	3.474353	3.873973
Hordeum_vulgare_newGene_12974	1.912327	1.401495	2.165554	3.854174	2.797345	3.742084
HORVU4Hr1G087340	3.391429	2.647674	4.518776	3.184221	2.313594	3.203317
HORVU7Hr1G092460	4.854855	5.55816	5.778205	4.786807	3.946407	4.555051
HORVU6Hr1G004090	8.797705	8.617439	8.926288	8.395991	4.990248	6.343738
HORVU4Hr1G058500	1.570883	1.860187	1.967417	1.171892	1.120286	0.933821
HORVU6Hr1G011060	0.55493	5.216732	1.063882	0.409646	0.244176	0.26911
HORVU3Hr1G028540	6.583752	7.818103	7.811542	27.58254	24.21719	25.09776
HORVU5Hr1G044970	1.250725	1.076565	1.528787	1.089004	2.217563	1.677042
HORVU2Hr1G103780	0.892738	0.742124	0.884924	1.134919	1.569145	1.961636
HORVU1Hr1G043520	8.339749	11.00773	12.0277	6.084645	7.390291	10.28769

HORVU2Hr1G017270	4.356946	5.487852	7.764466	5.423818	1.049673	2.009901
Hordeum_vulgare_newGene_14800	0.784212	1.347261	1.206825	1.534138	2.398573	3.360274
HORVU3Hr1G064850	11.46207	14.17842	11.15956	10.17307	13.19844	13.07929
HORVU7Hr1G093680	1.225089	0.941995	1.82014	2.338851	8.914997	8.990531
HORVU4Hr1G082980	19.04164	16.26948	19.20447	15.33746	22.66406	21.83683
HORVU3Hr1G099490	1.691687	2.510679	3.697111	0.993313	0.967007	0.961436
Hordeum_vulgare_newGene_13731	2.596417	2.603186	2.799507	2.256155	1.804318	3.012567
HORVU7Hr1G074460	10.02384	10.83189	10.6045	10.31442	9.950213	11.38439
Hordeum_vulgare_newGene_13730	5.306914	5.515914	6.646999	3.585404	4.656619	4.565064
HORVU3Hr1G061060	25.55954	24.30745	31.8712	21.54991	25.6747	24.63926
HORVU1Hr1G006880	5.840348	5.969973	7.902674	4.876766	7.996166	8.326641
HORVU3Hr1G100540	8.351427	8.252548	9.547777	7.055977	7.606618	7.209401
HORVU3Hr1G064740	2.790782	2.972343	3.682711	2.885219	5.501835	5.25016
HORVU2Hr1G049330	10.72562	11.5534	13.47179	13.9183	18.50136	16.69775
Hordeum_vulgare_newGene_13739	0.118014	0.281434	0.381863	43.44859	40.82188	43.89149
HORVU3Hr1G114290	20.60283	20.11977	23.81662	29.8154	33.45333	29.86375
HORVU5Hr1G095150	18.76284	19.7797	24.30046	21.48302	27.75369	25.97682
HORVU2Hr1G037020	0.705611	0.997632	0.859827	1.108888	1.164151	1.328292
HORVU4Hr1G000770	1.900545	1.737924	2.517416	3.020293	3.306291	2.978404
Hordeum_vulgare_newGene_5577	35.2451	33.10631	37.34391	29.02181	31.83167	31.20376
Hordeum_vulgare_newGene_5571	4.315417	4.767361	4.002422	3.53776	2.565785	3.437743
Hordeum_vulgare_newGene_5570	11.96722	12.5019	12.29361	12.82567	14.87853	15.08785
HORVU3Hr1G075850	3.090755	3.096562	4.307034	4.898824	5.433106	5.206334
HORVU3Hr1G092860	3.069655	1.618629	2.53455	4.375007	9.247157	6.667005
HORVU4Hr1G006660	6.91825	6.487428	6.816079	5.365736	4.116797	6.066145
HORVU7Hr1G121910	1.769862	2.254791	1.384156	1.441698	1.346019	0.787592
HORVU7Hr1G045860	19.95632	17.98224	18.79565	16.20118	17.33959	18.87178
HORVU7Hr1G100090	232.0724	206.1294	213.4031	30.08943	27.42643	29.37872
HORVU3Hr1G089970	24.98554	19.68463	33.01675	30.34201	41.49699	40.78026
HORVU7Hr1G109160	0.141729	0.255992	0.786828	1.335858	3.158412	5.107198
HORVU4Hr1G076640	1.123097	1.404853	1.413245	0.720704	1.074251	0.853131
HORVU3Hr1G093400	33.15562	36.18979	50.47391	24.19354	31.40536	35.0748
HORVU5Hr1G028870	11.07853	8.486241	14.02852	9.435137	11.07222	11.75657
HORVU4Hr1G082590	3.043362	3.234496	3.504919	2.601626	4.12412	3.302355
HORVU6Hr1G014600	13.44347	9.688711	19.69915	2.533186	1.407188	3.697519
HORVU2Hr1G104570	7.69097	5.895811	8.365422	4.526594	3.609183	3.675108
HORVU5Hr1G073170	3.341837	3.694991	4.360995	2.282224	1.05572	1.612151
HORVU2Hr1G109330	0.231122	0.2842	0.212604	3.138406	0.665085	2.39518
HORVU1Hr1G001670	0.080444	1.354656	0.645806	7.305305	0.220565	0.380235
HORVU5Hr1G080730	2.415253	2.573649	2.470495	1.673556	1.798344	2.502413
HORVU2Hr1G039510	3.281732	2.758396	4.061574	4.672956	6.085334	5.398472
HORVU6Hr1G052850	5.051014	4.55166	5.440335	4.76134	4.821821	4.927084
HORVU5Hr1G093650	4.867026	4.845224	5.599696	9.769723	14.05709	12.88472
HORVU5Hr1G079490	32.10306	25.96913	32.3826	29.86002	34.63772	33.41824
HORVU1Hr1G024640	11.78417	11.79504	15.22056	15.0354	19.46178	16.74983
HORVU3Hr1G062740	7.262094	7.109694	9.268647	9.772981	12.30036	11.19462
HORVU5Hr1G068380	2.206522	2.266557	2.464124	5.504578	6.371891	5.979156

Hordeum_vulgare_newGene_10605	1.755552	1.694639	1.650662	0.742718	0.39493	0.673258
Hordeum_vulgare_newGene_10602	2.153207	2.916578	2.900686	0	0	0
HORVU4Hr1G060850	15.58951	14.97561	16.72748	10.95389	11.48981	12.81714
HORVU4Hr1G078540	15.65416	16.69255	14.31678	19.85562	12.09969	15.27682
HORVU7Hr1G027000	11.23283	13.7826	13.20122	8.983881	11.37321	11.39346
HORVU1Hr1G039790	36.31837	32.87561	38.47102	29.28118	30.10579	32.82998
HORVU1Hr1G040920	0	0.042565	0.032991	10.8285	11.21967	15.58591
Hordeum_vulgare_newGene_1982	32.51032	37.19542	36.70709	44.30088	45.21185	53.49474
HORVU5Hr1G040560	5.412913	5.084801	5.892223	6.75176	4.807869	6.257781
HORVU4Hr1G003100	23.49364	24.44725	30.70285	32.31679	43.29878	46.15157
Hordeum_vulgare_newGene_1988	6.141619	7.977539	7.606974	1.155125	0.442533	0.743591
Hordeum_vulgare_newGene_1989	1.158668	1.39184	1.276954	1.705983	1.656314	2.052967
HORVU4Hr1G036280	26.43864	26.12156	27.48562	39.74654	48.64438	50.28364
Hordeum_vulgare_newGene_8444	2.332488	0.502521	0.975375	2.475887	2.143232	2.214783
HORVU1Hr1G041030	6.45554	7.52433	8.092578	9.279809	9.51724	12.19937
HORVU3Hr1G108120	0.238022	0.180574	0.232783	1.468018	2.754594	1.50013
HORVU0Hr1G016250	0.883724	0.738974	0.72684	2.085107	2.618605	2.666411
HORVU7Hr1G009610	7.128108	6.287696	7.638449	5.043746	5.770279	5.851973
HORVU3Hr1G069650	2.13154	3.171796	4.63652	49.32152	31.96761	44.42965
Hordeum_vulgare_newGene_311	0.037052	0.068277	0	0.431021	5.195911	5.669645
Hordeum_vulgare_newGene_310	2.687948	2.09323	2.767029	4.958868	4.730511	4.598956
HORVU3Hr1G107090	1.283846	1.23376	1.60586	1.754988	1.656775	2.073838
HORVU3Hr1G067590	5.052271	4.14813	6.704628	2.674111	2.344183	2.562426
Hordeum_vulgare_newGene_2379	9.751519	13.91994	13.57552	1.038058	0.349082	0.751243
HORVU6Hr1G053890	10.8529	12.65457	14.89701	9.486476	11.36879	13.05059
HORVU5Hr1G099240	13.73988	11.87579	17.18207	19.83699	26.00185	23.60329
HORVU5Hr1G070880	1.198611	1.358547	1.636567	1.782014	1.553705	1.727763
Hordeum_vulgare_newGene_2986	5.39192	4.255778	5.854653	0	0	0
HORVU2Hr1G013560	1.00427	1.54585	2.550247	1.529294	1.153861	1.678809
Hordeum_vulgare_newGene_7208	5.92961	5.86333	5.354147	1.413068	2.1178	2.229914
HORVU3Hr1G107320	38.77605	33.13379	45.76034	34.03043	30.2851	30.76596
Hordeum_vulgare_newGene_7205	1.392046	1.822851	2.725461	0.952785	1.998745	1.073217
Hordeum_vulgare_newGene_7200	4.423271	3.406128	4.447392	6.165277	7.939526	7.616367
Hordeum_vulgare_newGene_7203	0.79125	1.651797	1.836828	1.586328	2.51491	2.001955
HORVU7Hr1G052720	22.99163	20.77198	26.92074	16.66492	28.32579	26.96624
HORVU7Hr1G079160	2.172277	1.514361	1.950551	0.450438	0.205355	0.248757
HORVU3Hr1G097710	0.34008	0.121849	0.131949	0.647492	37.18718	42.1152
HORVU2Hr1G075230	3.668832	3.406411	3.4475	2.821505	1.026856	2.022482
Hordeum_vulgare_newGene_13723	3.280084	3.430554	3.053884	4.090624	4.467096	5.343055
HORVU6Hr1G065620	3.375886	3.837845	3.40566	4.405709	4.159749	5.282481
Hordeum_vulgare_newGene_13724	0.475363	0.26112	0.489834	2.6947	0.952194	1.279212
HORVU3Hr1G089190	4.860569	5.257489	6.695957	3.435647	5.379535	5.052541
Hordeum_vulgare_newGene_13729	1.859724	1.886053	2.053569	2.057948	2.046187	2.183378
HORVU7Hr1G109730	0.134812	0.144513	0.047727	2.144112	4.193953	3.485244
HORVU2Hr1G086820	4.563054	5.280397	5.027419	7.452383	9.278462	10.61787
HORVU4Hr1G057520	8.096433	8.481478	5.868864	8.81045	6.920167	7.3556
HORVU5Hr1G000320	27.2377	20.91889	22.62028	39.26248	40.21455	46.21865

HORVU1Hr1G086310	3.160678	2.527087	3.787532	12.56989	5.246714	5.897615
HORVU5Hr1G068900	3.04301	2.824172	4.319681	2.434675	2.46934	2.661462
HORVU7Hr1G052090	36.23231	32.50095	36.50054	33.02469	37.04474	38.97832
HORVU6Hr1G078290	2.307223	2.284358	3.762587	6.581557	14.93307	14.76392
HORVU1Hr1G023510	21.14485	16.16594	20.58008	29.88953	28.78985	27.95677
HORVU2Hr1G027680	13.48513	10.17131	13.6509	63.37518	38.43786	47.65575
HORVU5Hr1G085510	3.315473	2.536427	2.924035	4.607656	5.480876	5.262699
HORVU7Hr1G103550	23.98575	17.87153	25.8547	10.87413	9.603068	12.2438
HORVU2Hr1G079930	3.870545	3.389794	5.296538	5.200549	8.004276	8.505707
HORVU4Hr1G026150	0.860186	1.189879	1.027793	5.93427	64.8816	62.36951
HORVU1Hr1G092850	3.057786	3.237618	2.537696	2.790276	2.739219	3.288877
HORVU5Hr1G079730	6.982142	5.952115	8.38093	8.558737	8.36884	9.239732
HORVU5Hr1G061410	5.794245	6.89195	7.015948	0.048296	0.031395	0
HORVU3Hr1G042920	6.942243	5.970835	8.179844	11.07443	15.24069	14.6569
HORVU2Hr1G033000	2.459751	3.1993	3.024337	5.121893	4.99634	4.752303
HORVU2Hr1G080870	7.880054	8.659775	12.95331	4.844729	5.574224	5.817888
HORVU4Hr1G070370	185.4902	204.9731	313.626	168.1826	426.8451	374.628
HORVU4Hr1G029080	5.136839	4.32626	4.600543	4.703591	5.120593	4.919663
HORVU5Hr1G107190	6.821171	7.737353	7.897896	6.870102	8.389046	8.459982
Hordeum_vulgare_newGene_9314	3.141876	3.774036	3.633098	0.050886	0.030779	0.01236
Hordeum_vulgare_newGene_9311	4.62116	5.685307	4.280107	4.210493	3.463199	4.511027
Hordeum_vulgare_newGene_9310	6.374624	6.654405	7.568284	2.448365	1.431993	1.003027
HORVU1Hr1G020370	1.323388	0.982208	0.842702	5.212541	4.465982	6.221592
HORVU5Hr1G065240	5.352374	5.971619	5.185955	8.569378	8.416483	10.55252
HORVU3Hr1G054120	11.77521	11.43005	10.57927	11.29174	16.51148	16.40689
Hordeum_vulgare_newGene_15394	2.609754	2.434255	3.546916	0	0.569579	0
Hordeum_vulgare_newGene_15390	16.39083	15.38262	16.60809	14.86438	13.93537	15.27727
HORVU6Hr1G066250	1.725312	1.932757	2.519597	2.371278	6.593372	6.418793
HORVU7Hr1G107070	2.968632	2.488497	4.620344	6.50475	8.209972	6.039261
HORVU0Hr1G002960	3.033552	2.903703	4.160379	3.127085	3.874907	3.31265
HORVU1Hr1G050780	42.72821	41.23491	60.63187	37.43687	33.91045	33.99635
HORVU2Hr1G092540	4.961279	5.244665	5.49964	18.69125	28.27198	26.16542
HORVU3Hr1G019670	3.968714	1.907421	4.621101	7.494811	6.357965	6.254654
HORVU0Hr1G003380	6.333801	6.316104	7.327936	8.17127	8.702897	9.457854
HORVU3Hr1G004230	18.36391	27.35208	34.03981	3.281546	0.022778	1.302799
HORVU7Hr1G106380	6.731185	7.617126	8.741913	8.915029	12.05235	12.5979
HORVU1Hr1G093180	3.887295	3.634601	3.145943	4.67686	4.420712	4.826292
HORVU3Hr1G015570	30.22151	30.46031	33.42173	22.5858	20.97214	23.44635
HORVU3Hr1G011850	49.26153	29.98167	66.52804	2.785633	1.644632	9.329204
HORVU2Hr1G047050	3.722905	3.961671	4.579896	3.9868	3.385517	3.649389
HORVU5Hr1G089510	3.662325	2.716156	4.245864	3.151687	4.952509	3.928016
HORVU6Hr1G034900	10.02154	8.116844	10.05572	5.075258	6.633909	6.803536
HORVU5Hr1G020710	1.273531	1.267445	1.984578	1.248828	1.211377	2.185259
HORVU7Hr1G017260	2.739691	3.593466	4.019225	3.410262	3.710677	3.614824
HORVU5Hr1G016810	2.714206	3.198955	4.127184	5.005109	4.69572	4.619656
HORVU1Hr1G080410	7.526191	9.075894	12.1935	5.058681	9.804984	8.070462
HORVU5Hr1G064040	1.384629	1.142038	2.634239	0.270594	0.285434	0.35435

HORVU1Hr1G073280	57.8276	46.24303	57.70565	65.45118	70.61033	61.69615
HORVU2Hr1G119160	3.661353	2.584544	3.059958	6.27142	5.457748	7.140193
HORVU3Hr1G083450	0.305793	0.155769	0.337882	3.384766	1.420164	1.385168
HORVU7Hr1G046920	4.357557	3.426759	4.031292	5.530636	6.954774	6.387121
HORVU1Hr1G095270	7.083216	6.940332	7.114488	11.97597	14.00191	14.52249
HORVU2Hr1G077210	3.479184	2.065375	2.650296	2.188242	18.85045	15.74043
HORVU1Hr1G021130	19.703	19.20046	25.37493	23.31338	29.19947	29.06541
HORVU6Hr1G080320	0.972199	0.820255	1.205788	1.436904	1.0575	1.114299
HORVU7Hr1G017190	50.2834	44.94442	57.87903	26.29686	25.33765	25.64738
Hordeum_vulgare_newGene_1044	1.630668	1.309185	1.464231	1.222534	1.752389	2.002086
Hordeum_vulgare_newGene_1047	1.143825	0.735213	0.814808	5.841578	3.887848	5.065557
HORVU1Hr1G052780	3.773284	3.547265	3.284878	4.131005	5.062261	5.72646
Hordeum_vulgare_newGene_1049	0.500244	0.600841	0.704	1.547407	1.501016	1.699132
HORVU1Hr1G073130	3.104303	2.878239	4.399672	1.976887	2.237312	1.895704
HORVU5Hr1G015390	3.381011	4.244478	1.96785	2.459998	19.25651	14.47673
HORVU2Hr1G084400	22.98834	22.40734	25.38456	23.30875	13.0811	15.84372
HORVU1Hr1G060030	1.366627	1.333205	2.253973	7.086461	5.356433	4.232219
HORVU0Hr1G038470	2.045412	2.047519	2.236578	1.749015	2.336453	2.430837
HORVU7Hr1G091060	11.44439	9.070576	10.65721	5.096224	2.137972	2.83888
Hordeum_vulgare_newGene_4271	3.262503	2.979977	4.539058	4.835522	6.3813	6.259172
Hordeum_vulgare_newGene_4273	6.073151	6.997934	6.540385	0.022602	0.008562	0
HORVU1Hr1G057570	12.27035	10.61366	15.02084	9.754587	14.56809	13.45584
Hordeum_vulgare_newGene_4279	1.592039	0.995118	1.083771	1.133895	0.826673	1.776026
Hordeum_vulgare_newGene_4278	8.049974	8.096127	8.87843	8.356092	5.790599	6.471935
HORVU3Hr1G093620	32.62588	28.44117	48.85333	30.78128	36.44455	39.42527
HORVU2Hr1G074740	7.434284	6.844261	7.920183	8.447213	12.1934	12.37515
HORVU1Hr1G058630	1.40367	1.103134	1.083878	4.159122	3.927324	3.599053
HORVU6Hr1G067390	2.115397	2.725668	2.801666	6.697503	5.884027	5.913309
HORVU7Hr1G039800	3.009788	2.923343	3.258686	1.755934	1.446892	1.467712
HORVU0Hr1G030640	0.598918	0.927756	0.773378	1.303192	1.992509	2.071596
HORVU2Hr1G120340	1.106064	1.70283	1.608757	1.024529	1.341442	2.087275
HORVU2Hr1G082800	6.524298	6.187456	5.634041	9.033259	8.819787	11.5877
HORVU6Hr1G028730	22.71286	18.91343	23.57855	28.157	27.83898	30.30372
HORVU1Hr1G072660	1.582797	1.397402	1.664683	1.569825	2.388498	2.472066
HORVU5Hr1G060730	17.65838	17.38894	19.98613	26.95949	30.92036	32.06838
HORVU6Hr1G044640	13.82328	14.71639	12.93069	13.69162	17.42125	17.79162
HORVU5Hr1G040050	9.186075	6.707694	11.76611	9.170794	19.53792	19.53867
HORVU1Hr1G075030	30.97681	29.00283	31.13204	24.96171	32.42632	34.50563
HORVU1Hr1G044710	21.55406	19.19372	23.91031	28.95284	35.24192	33.57376
HORVU4Hr1G014230	31.14004	33.65702	45.57951	25.13921	25.86186	24.95594
HORVU7Hr1G090330	3.355198	3.106726	3.858559	5.84275	7.998774	6.558767
HORVU5Hr1G059820	13.73389	11.98338	20.3312	15.99351	31.8995	30.3083
HORVU1Hr1G041440	4.418999	3.964174	4.641599	5.116702	5.958214	6.433447
HORVU5Hr1G092760	4.378264	4.831742	4.450125	6.83251	6.399599	5.848605
HORVU3Hr1G006320	2.552887	2.544453	3.17143	2.876456	2.636071	2.845916
HORVU4Hr1G050290	1.614194	1.603631	2.037285	2.223677	3.112612	2.951192
HORVU3Hr1G079760	16.77554	17.39384	16.43966	17.03529	18.55378	18.72029

HORVU1Hr1G079930	15.92537	15.12686	17.05606	14.1787	12.96759	14.29251
HORVU2Hr1G041040	0.22265	0.24305	0.383903	2.022901	4.722349	4.160265
HORVU0Hr1G019840	0.675297	0.372814	0.719948	3.127658	6.270071	5.800987
HORVU3Hr1G033410	3.932529	4.668781	4.377995	4.57938	5.85279	5.995555
HORVU2Hr1G122850	41.98218	39.22684	40.21907	28.52041	24.9656	28.34381
HORVU4Hr1G007180	90.30946	66.79096	114.5355	68.94304	65.64601	76.16064
HORVU1Hr1G060490	20.83294	20.52654	22.34509	18.03639	18.753	18.19881
HORVU0Hr1G035910	5.571627	6.621535	8.633887	4.000364	6.187284	5.306115
HORVU2Hr1G100960	19.33251	18.59807	18.22884	8.779867	5.447412	5.154907
HORVU6Hr1G038030	3.323957	2.261174	3.25943	3.287328	4.424027	3.74542
HORVU7Hr1G116640	6.039716	6.410079	6.399472	9.955939	12.15054	11.21044
HORVU5Hr1G092190	2.899798	2.358049	3.391144	4.681283	4.586864	4.963427
HORVU7Hr1G067950	4.7545	5.218781	5.534544	6.180697	6.835591	7.969521
HORVU3Hr1G062500	5.064823	4.901245	6.48075	8.008646	7.230962	7.673072
HORVU3Hr1G002150	2.443873	2.55852	3.407632	0.889152	0.61669	0.831703
HORVU2Hr1G040390	6.784291	7.221703	7.19697	10.27173	17.47433	15.7877
HORVU7Hr1G018110	1.618756	2.025364	1.031989	1.714459	3.897655	2.134507
HORVU4Hr1G067850	24.23209	17.90159	25.53718	7.549732	6.482221	4.15351
HORVU5Hr1G123360	2.878616	2.880323	3.280662	3.212333	4.171022	4.833999
HORVU4Hr1G069480	11.34129	8.782459	9.006055	13.14025	10.5916	12.51829
HORVU6Hr1G040770	2.98534	2.286212	2.560216	1.839097	1.858814	2.255306
HORVU6Hr1G034040	3.788575	3.665807	4.014446	3.504957	3.67692	4.236043
HORVU3Hr1G082190	8.718869	8.875316	10.67449	5.607794	4.940458	5.757607
HORVU1Hr1G066530	22.62347	14.22046	28.10638	19.88775	14.65893	14.79309
HORVU3Hr1G026880	57.65902	53.22933	81.67925	53.39989	109.8188	99.55677
HORVU5Hr1G112710	0.507986	1.079807	0.723298	3.614912	5.361234	4.823878
HORVU7Hr1G040740	6.091837	2.831313	4.867654	1.112397	0.112993	0.557016
Hordeum_vulgare_newGene_7490	1.380619	1.836155	1.570877	1.437477	1.091195	1.425389
HORVU2Hr1G105930	9.269322	7.760936	7.821261	3.679149	5.728456	4.982594
HORVU1Hr1G041630	3.63651	3.6821	4.231782	4.705386	6.536333	6.153266
HORVU6Hr1G093180	13.40371	11.95065	14.26501	21.7132	21.21318	22.30496
HORVU6Hr1G084690	1.670105	1.463181	1.927183	4.613503	5.23939	6.096774
HORVU2Hr1G025530	26.2415	24.32015	34.41909	24.94655	47.30427	49.19523
HORVU2Hr1G094240	1.208932	1.427141	1.119529	2.37872	4.823325	3.66699
HORVU7Hr1G051930	4.868188	4.893634	5.878629	8.294185	10.62259	10.55029
HORVU5Hr1G071040	0.645342	1.309835	0.681809	1.050973	1.376929	1.346
HORVU5Hr1G051310	6.198087	4.984285	6.926083	8.124227	10.63463	10.59328
HORVU3Hr1G036130	5.206879	5.32535	5.867391	5.689987	6.261936	8.950887
HORVU3Hr1G024950	10.6613	11.42033	14.06932	12.83648	24.74059	17.61546
HORVU2Hr1G047550	6.199193	6.202208	6.625916	8.067737	9.694849	9.963685
HORVU6Hr1G040480	12.63577	13.9182	14.10684	14.62688	20.39559	22.42383
HORVU3Hr1G009980	1.429756	1.632804	2.227117	16.24262	40.33504	34.491
HORVU5Hr1G011400	3.037718	2.433275	3.172415	4.089828	4.738775	4.174382
HORVU1Hr1G002680	0.010317	0	0	12.04706	0	0
HORVU7Hr1G071750	0.73135	1.061415	0.74359	1.293599	4.674733	4.352745
HORVU4Hr1G090840	1.688354	1.599567	2.40113	2.616182	5.80988	5.500252
HORVU6Hr1G011450	0.753126	2.036341	1.476143	0.688335	1.110296	0.796161

HORVU4Hr1G084710	10.88718	11.9223	11.26159	8.009878	12.3421	12.04528
HORVU2Hr1G118690	14.03164	16.12163	21.66378	11.55357	15.98249	15.7192
HORVU1Hr1G067370	31.68086	36.78511	58.38212	6.127261	3.662095	4.94376
HORVU4Hr1G018650	4.703737	4.73776	5.093067	7.515409	29.16053	28.12416
HORVU0Hr1G028350	36.74026	38.57864	31.37444	21.14111	9.606234	15.19463
HORVU1Hr1G091600	378.4979	314.9226	448.8709	553.016	923.5768	1050.856
HORVU1Hr1G037730	3.024504	2.84767	3.662446	5.490715	9.092491	9.016323
HORVU2Hr1G110230	15.32279	19.36864	21.66577	5.104172	2.188139	3.264149
HORVU7Hr1G046350	5.867797	5.16153	6.446795	5.060213	8.413608	8.200196
HORVU4Hr1G015060	12.41814	11.49853	14.78436	16.70321	18.93523	19.89631
HORVU6Hr1G065420	0.905366	1.438349	1.226462	1.048138	1.233252	1.514379
HORVU3Hr1G089350	0.964639	0.603598	0.924763	1.888677	2.702608	1.698986
Hordeum_vulgare_newGene_3216	14.94551	13.19875	15.02023	13.66898	14.36483	14.36778
HORVU6Hr1G089730	0.058962	0.184559	0.113285	5.668427	7.140771	5.772467
HORVU2Hr1G068560	2.685768	3.291534	3.83394	3.058959	6.536406	5.912257
HORVU6Hr1G010500	18.62163	16.67819	16.1915	11.6526	11.75375	12.39384
HORVU7Hr1G043320	19.33993	18.84892	15.45442	17.08565	47.92677	53.27458
HORVU1Hr1G047100	0.576381	0.38981	0.747773	2.054051	5.683414	5.74349
Hordeum_vulgare_newGene_2166	1.87072	1.867388	2.134568	4.303308	6.308328	6.513549
Hordeum_vulgare_newGene_2165	22.89504	19.95805	25.10265	0.026564	0	0
HORVU4Hr1G057890	69.82646	48.56336	68.00401	86.50082	110.0296	96.512
HORVU3Hr1G024220	7.330757	5.284046	7.84671	4.485946	3.850137	3.334399
HORVU5Hr1G079130	0.588481	0.23334	0.654836	3.063543	15.15029	12.96549
HORVU7Hr1G047000	12.02185	10.14549	12.83429	10.56097	11.60925	12.51398
Hordeum_vulgare_newGene_9915	10.59286	12.98295	13.64608	8.497689	8.37757	9.524849
HORVU0Hr1G009380	3.388844	3.505048	4.834278	4.728608	5.347054	5.480463
HORVU7Hr1G074580	10.04404	11.29894	13.06308	13.67305	18.31135	20.82646
HORVU2Hr1G033240	10.11705	13.31043	12.91571	7.652035	7.542828	9.361102
Hordeum_vulgare_newGene_13252	1.4633	1.64426	1.76459	1.521288	2.082608	2.114479
HORVU2Hr1G039890	2.468922	0.93109	1.215951	1.554282	1.818153	1.880396
HORVU6Hr1G093960	2.354337	2.084762	2.416318	1.499989	1.837544	2.187181
HORVU6Hr1G009670	11.62536	10.20263	11.33215	29.49989	27.79415	29.14985
Hordeum_vulgare_newGene_15154	1.691022	1.462262	1.500628	1.569645	1.208562	1.2308
Hordeum_vulgare_newGene_15153	3.556208	3.769332	3.434045	5.496088	7.696571	7.583287
Hordeum_vulgare_newGene_15152	11.40054	11.21133	15.51138	9.22948	10.33526	11.36979
Hordeum_vulgare_newGene_15151	2.243896	3.975841	4.406301	0.725533	1.024313	0.901136
Hordeum_vulgare_newGene_15150	5.307966	4.378515	5.146807	1.13217	1.079172	1.283125
Hordeum_vulgare_newGene_15158	1.751729	0.793117	0.526125	4.431753	0.193566	0
HORVU4Hr1G001610	20.93878	15.06816	28.42195	30.98488	33.23974	26.4914
HORVU4Hr1G070170	9.205022	11.19444	9.372714	10.88961	12.73603	14.75688
HORVU7Hr1G005720	29.37265	27.3482	35.30689	35.2459	66.04268	56.30806
HORVU7Hr1G044670	4.343605	5.561282	3.162819	5.189774	3.370854	4.057366
HORVU3Hr1G050340	21.6366	27.54959	31.21304	18.49106	16.90489	19.84683
HORVU1Hr1G022270	19.54829	23.30978	23.36887	21.6078	36.56907	39.16574
Hordeum_vulgare_newGene_9489	2.50255	2.878582	3.996061	3.988515	4.879999	4.63874
Hordeum_vulgare_newGene_9488	1.382369	1.890177	1.987123	2.820449	4.247034	4.1014
Hordeum_vulgare_newGene_5309	13.12651	12.56144	18.35414	10.02324	16.63013	16.5611

Hordeum_vulgare_newGene_5307	0.716194	0.875265	0.773546	1.514654	1.370115	1.566247
Hordeum_vulgare_newGene_5302	1.956439	1.733425	1.836887	2.602091	2.11883	2.53182
Hordeum_vulgare_newGene_11480	0.794856	1.020027	1.053923	1.833271	1.467991	1.88486
HORVU5Hr1G092800	12.96301	14.84671	18.05954	6.870317	8.215236	7.954295
HORVU4Hr1G025150	13.36129	12.70208	13.23761	11.04091	9.001861	11.60911
HORVU6Hr1G016100	21.86532	20.10883	23.82164	21.18271	22.28185	22.38319
Hordeum_vulgare_newGene_13878	5.594826	8.416259	7.237477	0.03569	0	0
Hordeum_vulgare_newGene_13879	17.51432	13.08374	18.68727	11.56154	5.257713	6.538229
Hordeum_vulgare_newGene_13876	29.6527	28.97194	29.86445	21.42734	22.20458	24.1225
HORVU7Hr1G080990	3.23019	3.839622	3.981424	5.832665	7.212098	7.559885
Hordeum_vulgare_newGene_13871	1.503273	1.924145	1.849285	2.753429	2.104297	2.708129
HORVU2Hr1G030260	0.900762	0.952016	1.367425	1.617083	2.148213	1.853301
HORVU5Hr1G098720	16.27635	14.65516	20.72473	13.18995	6.946229	7.201588
HORVU6Hr1G077890	15.65345	11.53203	15.68523	21.10949	27.92429	26.76793
HORVU5Hr1G077200	11.59689	11.68333	14.32675	10.44276	12.01024	12.06466
HORVU2Hr1G121240	2.000065	0.589986	0.800019	1.73048	0.845774	0.851939
HORVU3Hr1G092220	14.90782	14.87896	15.6084	27.19069	20.8773	29.39397
HORVU7Hr1G045980	8.557873	10.13722	11.47115	6.818855	7.269729	7.743081
Hordeum_vulgare_newGene_7974	1.212692	2.168589	2.087529	1.090309	1.617119	1.151356
Hordeum_vulgare_newGene_8689	0.2654	0.435498	0.118077	3.919361	5.14273	4.421423
Hordeum_vulgare_newGene_134	2.942521	5.31431	5.928352	8.831874	8.959103	7.058632
Hordeum_vulgare_newGene_8358	5.749141	5.308317	6.770339	3.49779	4.214732	4.141751
Hordeum_vulgare_newGene_8359	3.662051	2.515383	3.157494	2.442151	4.164219	2.792941
Hordeum_vulgare_newGene_8356	5.894096	6.670467	8.187558	8.211088	9.627207	10.69032
Hordeum_vulgare_newGene_8353	3.026303	2.591616	2.439551	6.99212	11.92225	11.96742
HORVU7Hr1G026560	12.30772	14.98822	12.30252	14.14025	6.875302	8.424804
HORVU7Hr1G106630	7.823721	6.6508	9.653898	10.85959	11.54417	12.35315
HORVU7Hr1G109360	1.589006	2.059186	1.3505	1.149726	2.615722	3.956332
HORVU3Hr1G105860	8.886518	8.986387	9.371697	4.79106	3.027844	4.183996
HORVU5Hr1G049880	7.638118	8.700017	9.468356	5.457457	4.165044	4.165111
HORVU3Hr1G015800	11.05792	10.2172	14.05183	9.784467	11.33181	11.83927
HORVU5Hr1G072070	4.133844	3.717891	5.223864	4.454442	3.131763	4.258907
Hordeum_vulgare_newGene_4477	7.301762	5.896455	6.929395	9.04063	5.257407	5.954442
HORVU7Hr1G087770	3.82121	4.392339	5.100935	4.877985	4.539815	5.945938
HORVU2Hr1G014590	19.26349	18.47427	20.30325	13.84468	18.8397	16.87152
Hordeum_vulgare_newGene_4472	3.279654	10.4348	2.819625	14.47351	2.609962	3.477573
HORVU4Hr1G067520	7.633562	7.748995	9.385404	10.16672	17.78386	19.87294
HORVU2Hr1G011570	0.078223	0.049585	0.049582	3.278196	3.350213	3.870958
HORVU0Hr1G020850	6.557211	6.58561	7.322405	10.4329	6.384197	7.414598
Hordeum_vulgare_newGene_3359	1.159636	1.087334	1.391606	1.531687	1.160919	1.261454
Hordeum_vulgare_newGene_1535	1.764406	2.247731	2.660006	2.239054	2.354699	2.194831
Hordeum_vulgare_newGene_1534	0.590397	0.556628	0.931528	7.919203	3.298208	3.369283
Hordeum_vulgare_newGene_1536	1.265175	1.320888	1.667054	2.191216	1.416244	1.400792
Hordeum_vulgare_newGene_1533	17.2939	13.63435	20.91875	5.535774	0.831508	1.682663
Hordeum_vulgare_newGene_3973	12.6754	10.18175	11.34316	9.095214	9.446598	10.43251
Hordeum_vulgare_newGene_3971	0	0	0	1.802738	2.456753	2.33934
Hordeum_vulgare_newGene_3977	4.508103	5.719865	8.849564	4.628147	5.97977	7.381309

HORVU3Hr1G011650	13.68875	19.83341	15.77942	16.25151	17.19278	15.94167
HORVU1Hr1G038560	6.658309	6.2266	6.504623	7.45023	8.455121	6.072672
HORVU5Hr1G088020	4.005	3.754951	4.012036	2.797251	4.123434	3.680502
HORVU6Hr1G036570	0.781575	1.026808	0.903733	1.167603	0.995481	1.176232
HORVU1Hr1G072060	43.26268	43.66964	67.61405	32.5793	68.93852	67.46847
HORVU1Hr1G022250	24.67428	34.35148	30.38621	32.61723	44.40443	45.9128
HORVU1Hr1G031370	6.550833	7.90112	8.314726	5.322078	7.218825	8.364385
HORVU6Hr1G090090	1.177294	1.254235	0.697872	1.579964	2.143209	1.859417
HORVU6Hr1G073540	143.9164	161.4699	243.3366	13.71289	1.923642	5.381627
HORVU2Hr1G071800	3.660888	3.133798	3.574718	6.187261	9.546059	8.751581
HORVU3Hr1G088120	1.404872	2.088334	2.048771	1.045499	6.299113	4.094574
HORVU4Hr1G000650	8.865079	9.753571	9.544395	10.53011	10.88398	12.29368
HORVU1Hr1G003990	17.25204	15.10268	16.35093	8.950081	9.225112	8.55664
HORVU3Hr1G080730	8.160783	8.52678	7.619423	5.442479	4.353194	4.51168
HORVU1Hr1G030390	14.30866	14.61961	13.96104	12.29663	12.74271	14.34181
HORVU6Hr1G053910	14.0819	13.17399	12.41116	16.3092	19.44209	15.64321
HORVU5Hr1G040730	10.97776	9.316237	10.44607	14.10904	20.45698	19.41416
HORVU7Hr1G100100	4.839947	5.290318	4.821654	5.631104	25.82456	20.3919
Hordeum_vulgare_newGene_12645	0	0	0	5.140624	3.632408	4.095944
HORVU6Hr1G030720	0	0.271225	0.20199	2.026657	2.167473	2.006557
HORVU2Hr1G042280	26.86833	25.99727	31.66426	26.66545	36.54097	36.8444
HORVU4Hr1G037290	12.64677	14.37966	15.61033	31.98166	39.50696	44.47909
HORVU7Hr1G024890	13.22961	14.29648	22.05025	8.227228	7.203221	8.960232
HORVU4Hr1G074600	2.987128	3.031179	3.560151	1.600056	1.745144	1.710924
Hordeum_vulgare_newGene_500	1.782977	2.364527	2.894207	1.311006	1.244052	1.676279
HORVU7Hr1G031100	3.213708	3.437042	3.566762	3.073181	3.843598	4.212589
Hordeum_vulgare_newGene_14491	2.847615	3.465552	3.558944	3.498971	4.883291	5.186665
Hordeum_vulgare_newGene_14493	1.151594	1.176083	0.995145	1.183759	1.548237	1.283741
HORVU3Hr1G089650	2.134096	2.266941	2.300946	9.773595	15.80781	13.94166
Hordeum_vulgare_newGene_14494	17.68154	15.00164	12.72324	8.099035	6.493872	5.668129
HORVU3Hr1G081060	21.60018	16.48877	24.1768	20.66257	34.47414	34.4318
HORVU5Hr1G084070	31.94852	24.64655	37.25708	48.3154	45.50858	49.91417
HORVU4Hr1G013210	35.94711	41.50954	56.63865	50.31462	14.45803	30.0962
HORVU0Hr1G030980	1.130925	2.164495	2.000883	4.276852	4.245419	4.102047
HORVU3Hr1G039300	1.126162	1.531617	1.168174	1.096384	0.576197	1.074391
HORVU3Hr1G034150	4.601732	3.327332	4.965084	5.360646	9.061488	9.254886
HORVU5Hr1G065890	3.61908	3.251398	4.068062	5.835365	9.255377	9.602841
HORVU3Hr1G113020	11.54453	6.844811	11.27934	1.582415	0.634986	1.216043
HORVU7Hr1G018470	0.530456	0.248758	0.300604	4.025033	3.259211	4.019944
HORVU0Hr1G000280	6.836832	6.537986	7.41846	3.689309	3.757603	3.569717
HORVU7Hr1G034050	1.12498	1.254181	1.332058	0.852136	0.710504	0.874116
HORVU5Hr1G075510	0.411244	0.375893	0.495942	4.371357	5.12602	4.168071
Hordeum_vulgare_newGene_13118	3.658512	3.295022	3.646132	0.880326	0.968185	1.163096
Hordeum_vulgare_newGene_13114	1.490852	1.046547	1.137294	2.180021	2.437672	2.661273
Hordeum_vulgare_newGene_14722	1.037582	0.880865	0.740419	1.302093	1.349829	1.424421
Hordeum_vulgare_newGene_13113	16.53281	15.12221	19.99863	10.1859	11.27025	12.14984
HORVU5Hr1G079670	6.932477	6.607808	9.115359	1.321635	0.353728	0.769115

HORVU4Hr1G075950	23.35055	15.94523	25.50016	16.57521	20.56588	18.71455
HORVU5Hr1G087340	0.173054	0	0.157952	2.528734	11.02105	10.55555
HORVU3Hr1G087380	88.65655	94.04065	161.2896	66.53052	174.3864	146.2872
HORVU3Hr1G003040	8.496596	7.629497	8.556773	15.24066	36.41895	29.86955
HORVU4Hr1G050210	279.7795	272.7172	325.8335	87.21632	5.237911	38.72206
HORVU4Hr1G007000	1.987602	2.268474	2.694028	2.771678	3.584774	3.859262
HORVU0Hr1G003480	16.16742	11.13689	14.17259	13.39255	6.686446	7.634043
Hordeum_vulgare_newGene_15456	0	0	0.069139	52.35675	39.96975	49.13676
HORVU3Hr1G087030	3.112726	4.294821	3.032165	1.462581	0.425271	0.318824
HORVU7Hr1G048770	5.882449	7.277509	7.789919	9.023303	13.47256	11.74435
HORVU7Hr1G002880	9.493888	10.80274	12.40518	7.64462	7.338412	7.245025
HORVU4Hr1G003420	40.69867	41.54364	36.16081	88.20321	96.35782	89.05621
HORVU6Hr1G069180	1.559144	0.792933	1.036965	2.495514	0.033031	0.618005
HORVU7Hr1G097740	6.51842	7.612235	7.649304	6.706051	7.701273	7.315959
HORVU5Hr1G113020	2.28052	2.330895	2.151965	0.790609	0.239278	0.322769
HORVU3Hr1G054710	6.771665	6.625943	12.24107	2.488454	6.256164	5.51061
HORVU0Hr1G003220	14.31183	11.74811	14.35872	5.695381	11.8157	12.23548
HORVU2Hr1G094080	1.597695	1.640346	1.926137	3.132181	4.199973	4.14843
HORVU7Hr1G112740	82.75074	90.90922	129.8968	57.64818	84.08387	89.35705
HORVU3Hr1G084600	3.067207	3.251885	3.960001	2.56607	2.276825	3.268572
HORVU5Hr1G063660	0.799472	1.762515	2.068131	2.574209	1.318103	2.717175
HORVU7Hr1G086570	1.322325	0.946341	1.436203	2.294517	4.479495	4.487564
HORVU6Hr1G026600	0.034869	0.044589	0.094977	0.20984	3.620842	3.310262
HORVU6Hr1G069450	12.41168	15.2088	11.47418	12.92558	18.70152	18.79552
HORVU3Hr1G019820	92.40683	70.12218	90.96085	72.49367	62.39009	54.06809
Hordeum_vulgare_newGene_6455	1.279208	0.784812	1.406287	1.658236	1.553322	1.734166
HORVU2Hr1G084300	11.81393	10.17596	13.66734	14.2488	23.78023	21.58387
HORVU5Hr1G119700	80.53308	76.06732	115.5128	72.84601	77.22403	81.29438
HORVU4Hr1G047140	6.427944	8.393691	8.198921	8.22296	9.285681	10.60596
HORVU5Hr1G011170	15.67693	14.94521	14.65487	21.62476	28.69013	26.23264
HORVU4Hr1G011160	12.03205	12.9083	11.66928	38.43743	70.56	69.03645
HORVU6Hr1G004350	17.02758	20.12512	14.84687	9.925334	14.86126	16.07913
HORVU6Hr1G070830	3.993968	2.962144	3.849739	3.326797	4.962177	2.926494
HORVU5Hr1G054500	15.13709	15.49298	15.89343	11.81355	14.97105	14.7339
HORVU2Hr1G098160	562.2612	595.4127	908.823	575.0137	454.2881	435.2599
HORVU6Hr1G000440	7.394808	7.749827	5.936213	6.670785	7.184153	11.68973
HORVU2Hr1G096910	0.140152	0.10796	0.156034	3.88477	9.574589	8.44873
HORVU3Hr1G070860	1.854582	2.305127	2.522123	4.43298	5.54777	6.255737
HORVU3Hr1G025510	12.69438	11.36903	16.85639	13.59427	22.41488	19.06719
HORVU5Hr1G091910	3.268572	3.120995	3.506088	4.476327	5.407616	5.139496
HORVU5Hr1G109880	0.875151	1.021193	1.366492	2.734655	3.651908	5.250518
HORVU5Hr1G058160	4.77062	6.230903	7.22665	3.381029	5.19919	5.975668
HORVU3Hr1G099220	8.345247	7.697647	8.838249	13.531	24.29461	22.97381
HORVU7Hr1G095630	0.093554	0.0942	0.204385	1.925156	6.278392	5.203479
HORVU6Hr1G066470	1.372629	1.430305	1.894259	2.084189	2.755507	2.513586
HORVU5Hr1G050350	9.688005	9.173132	11.8169	12.36539	17.72697	18.4268
HORVU1Hr1G054610	2.669112	2.235511	2.77004	3.500278	3.03344	3.232362

HORVU4Hr1G046230	8.128891	8.312392	15.40494	4.895663	8.353432	9.582272
HORVU3Hr1G000760	30.80381	31.3791	38.62717	37.09117	35.85847	36.15635
HORVU2Hr1G107740	5.755548	3.696327	5.166377	4.262826	2.534497	2.592671
HORVU3Hr1G030840	3.061581	2.338716	2.313313	11.01731	9.567875	9.600248
HORVU6Hr1G018890	6.376905	6.752417	7.815171	7.698439	9.338484	9.008002
HORVU4Hr1G033300	56.74752	58.30468	56.83858	60.77101	75.99999	75.5439
HORVU1Hr1G043110	5.747375	5.633253	7.549716	6.011981	7.836739	7.371366
HORVU5Hr1G080210	1.560898	1.413389	2.184878	0.38434	0.320174	0.700283
HORVU0Hr1G013240	2.033524	2.081941	3.200041	1.623576	1.69146	1.984134
HORVU2Hr1G080100	2.866735	3.121004	3.031308	6.331349	13.75144	14.42632
HORVU6Hr1G006840	44.82401	27.86598	34.75443	15.66864	33.84814	27.00242
HORVU3Hr1G046780	0	0	0	2.59113	2.291939	3.068828
HORVU1Hr1G056210	8.443389	7.85286	6.559061	6.214886	2.79955	4.334277
HORVU4Hr1G015550	1.007037	1.02108	1.333075	3.484352	5.34469	3.723827
HORVU1Hr1G092820	27.74373	25.92063	39.69937	23.59623	24.49001	29.33216
HORVU1Hr1G078160	2.105995	2.423697	2.431067	2.212607	2.438466	2.179067
HORVU2Hr1G001780	4.361939	2.435251	5.474634	1.539583	0.209958	1.352127
HORVU2Hr1G092730	3.211441	2.62591	2.712148	2.846207	2.789304	2.920562
HORVU2Hr1G099610	35.50969	37.93358	67.08852	13.69472	25.66668	23.37768
HORVU2Hr1G096640	14.80477	17.07484	15.28156	5.679611	3.919667	2.514007
HORVU5Hr1G045750	3.684765	4.129201	5.551057	1.72312	0.733381	1.516757
HORVU3Hr1G098170	32.71308	28.4915	29.93558	20.41746	18.34808	16.05237
HORVU7Hr1G094580	1.19389	1.160305	1.351125	1.534782	1.146908	1.488288
HORVU5Hr1G125380	1.735623	3.661697	3.468398	0.097445	0.367228	0.37703
HORVU3Hr1G074570	1.626892	1.655539	1.516629	5.062384	13.15865	12.2626
HORVU7Hr1G055440	18.48418	17.22621	18.43122	8.157713	9.679165	10.21493
HORVU7Hr1G098370	2.031314	1.752423	2.720157	4.29518	7.671259	11.70725
HORVU7Hr1G093580	57.19239	46.99555	68.04305	44.9277	60.62535	45.99018
HORVU2Hr1G101370	5.096744	3.926843	4.82599	4.604997	2.544146	3.650519
HORVU1Hr1G045870	0.064985	1.796299	5.072623	3.609611	1.08192	0.364675
HORVU2Hr1G079020	6.856772	6.665627	7.431578	19.06961	18.06612	20.59754
HORVU5Hr1G026690	3.663183	2.588046	2.53157	5.688706	3.141877	4.337076
HORVU1Hr1G026400	2.261527	1.667338	2.823086	2.63541	3.512578	3.153747
HORVU6Hr1G091950	161.2006	171.6058	157.4051	110.7873	121.9157	130.8016
HORVU1Hr1G083710	26.25601	21.03235	39.16822	16.50705	97.09627	88.29246
HORVU4Hr1G090260	1.411664	1.282728	1.709881	1.224916	1.00019	1.149541
HORVU7Hr1G043870	11.088	15.48414	13.65565	9.003132	7.257646	9.439507
Hordeum_vulgare_newGene_15257	0.951659	1.349319	1.133534	2.066674	1.817325	2.23228
HORVU1Hr1G051290	19.20386	15.31556	21.34661	59.14825	72.1708	63.19013
HORVU1Hr1G055970	0.670601	0.7444	0.889962	5.24459	6.432576	8.17586
HORVU5Hr1G094430	1.703764	1.168148	1.39254	1.932889	2.17601	2.121569
HORVU5Hr1G124470	0.056123	0.062332	0.038468	2.223128	194.2115	132.9774
HORVU2Hr1G009850	0.517244	0.821025	0.308321	1.888206	1.703813	1.993943
HORVU2Hr1G101670	71.20269	59.82979	96.48674	72.64544	106.0542	101.1051
HORVU7Hr1G037600	8.190733	11.40144	13.96175	5.005509	5.129452	4.830841
HORVU3Hr1G073710	9.652142	9.766532	8.422714	4.574064	5.872112	5.85026
HORVU5Hr1G116930	16.38127	16.23159	17.43542	11.88432	16.19865	16.10917

Hordeum_vulgare_newGene_10235	37.84391	35.39235	39.37154	54.17858	74.38358	83.93691
HORVU2Hr1G110410	6.486659	8.862259	7.069069	10.50125	11.14741	12.41836
Hordeum_vulgare_newGene_11724	3.521289	4.554542	4.466559	3.007582	3.102283	4.592827
Hordeum_vulgare_newGene_11726	0.785513	0.789996	0.890274	1.066949	1.887023	2.091694
HORVU6Hr1G077700	16.87182	17.64232	19.87359	17.21162	19.07651	19.72948
HORVU4Hr1G024590	18.32695	18.64914	17.60463	20.6113	24.14757	24.51535
Hordeum_vulgare_newGene_11723	1.124033	1.24481	1.478357	0.570223	0.822938	1.107658
HORVU5Hr1G077040	0.697977	0.676209	0.927949	1.729273	1.850302	1.729486
HORVU2Hr1G056980	9.361847	8.308706	15.58839	7.738447	10.81314	10.46266
HORVU7Hr1G012850	23.52444	30.44458	41.11028	10.64336	15.25352	14.2083
HORVU0Hr1G011660	0.372049	0.301999	0.477612	1.121134	6.024247	9.295663
HORVU1Hr1G001200	1.167789	7.312605	1.285232	13.91831	1.611129	2.064978
HORVU5Hr1G117040	0	0	0	7.854235	6.572773	8.032438
HORVU2Hr1G022120	1.840752	1.253821	1.75651	2.476003	1.200904	1.194017
HORVU4Hr1G052390	26.76373	25.48511	25.18111	26.58142	34.82502	34.82626
HORVU3Hr1G077000	32.61306	30.36597	32.49098	39.39426	42.65815	41.76365
HORVU3Hr1G100240	1.744058	1.6005	1.61833	3.072962	1.812832	2.508674
HORVU4Hr1G043890	3.897973	3.494565	4.206975	5.193179	5.91582	6.593285
HORVU7Hr1G054920	21.98091	15.00255	32.66295	27.4049	54.84455	49.76241
HORVU5Hr1G046470	6.431575	6.166154	6.059187	8.886024	15.72643	15.26137
HORVU3Hr1G037180	6.717744	6.636121	7.043346	9.425239	12.26869	13.05545
HORVU4Hr1G067320	2.374258	2.597101	3.307279	4.858639	9.241374	9.044683
HORVU4Hr1G043650	3.675795	3.066228	4.253088	8.298243	12.43147	11.1824
HORVU3Hr1G038200	4.786867	4.627539	5.069199	5.351131	6.367533	6.397879
HORVU4Hr1G056770	91.94733	88.90917	76.5733	112.459	76.41239	77.43733
HORVU5Hr1G093280	14.94165	12.25202	15.41234	20.00146	19.07459	19.10061
HORVU2Hr1G013730	7.844992	7.414082	8.536693	4.19882	2.929303	3.5815
HORVU1Hr1G079380	16.97113	15.44568	19.28389	15.27679	13.85993	14.26917
HORVU6Hr1G095120	5.721722	4.929454	6.320749	5.023927	0.804959	1.649804
HORVU2Hr1G115160	2.120763	3.099064	3.931853	4.067899	13.52467	10.95293
HORVU1Hr1G019650	2.642179	2.749133	2.691993	2.403022	2.713687	3.224991
HORVU6Hr1G092790	22.58008	18.77398	24.72131	20.90408	30.62339	30.45158
HORVU7Hr1G076300	40.32076	33.15568	50.67847	35.74467	56.60137	60.3466
Hordeum_vulgare_newGene_16187	0	0	0	2.189183	4.026986	5.17602
HORVU3Hr1G022510	3.338768	2.740589	3.786465	5.991122	52.60588	57.08061
HORVU2Hr1G039220	0.638404	0.850285	1.082423	0.398646	3.861221	4.035981
HORVU7Hr1G109960	13.74642	13.19628	17.1351	7.88696	8.387177	5.819855
HORVU3Hr1G071870	53.26926	41.47995	60.06571	63.3806	110.7159	99.45042
HORVU5Hr1G109630	8.747983	8.357167	12.30613	8.361822	9.465928	8.597611
HORVU1Hr1G022450	4.310435	5.275609	4.580251	8.822987	6.54148	8.12515
HORVU7Hr1G050160	1.283667	1.399825	1.762798	7.727962	24.03923	19.70081
HORVU2Hr1G120530	1.80911	1.283517	2.491949	36.34422	71.79903	52.95292
HORVU7Hr1G081930	63.23361	57.93896	58.99846	60.44104	50.09689	48.94399
HORVU7Hr1G041460	17.49739	14.40414	14.85694	12.77391	9.906876	11.17343
HORVU3Hr1G076060	1.000036	0.619776	1.141218	1.574636	1.753483	1.953173
HORVU7Hr1G001750	11.58435	5.576825	8.156237	0.429703	1.714647	1.032597
HORVU1Hr1G001970	0.079982	8.372117	0.643443	12.11268	0.088787	0.124528

HORVU3Hr1G057730	10.27481	10.33974	15.0981	11.78179	10.405	8.899547
HORVU6Hr1G051440	12.80747	11.82942	11.43524	11.88182	12.57219	11.85814
HORVU7Hr1G047700	299.2968	239.8764	297.7976	379.8215	590.7549	566.5003
HORVU6Hr1G086940	14.90411	14.23827	13.36528	27.23744	27.58675	29.5931
HORVU5Hr1G011230	87.80546	60.69732	102.9527	163.7306	52.53096	74.82987
HORVU7Hr1G105640	1.381582	1.855719	1.519259	1.54869	2.547863	2.620252
HORVU3Hr1G017410	20.58879	25.93835	26.36648	22.1311	25.94983	26.83743
HORVU1Hr1G047800	4.896509	4.384949	4.31799	8.415555	8.127779	8.085319
Hordeum_vulgare_newGene_13507	3.154123	3.406957	3.782162	3.254664	3.197718	3.212528
HORVU4Hr1G067810	59.6954	59.9894	66.33157	82.71378	114.0409	100.4211
HORVU3Hr1G089090	1.600661	1.604285	1.81002	4.825301	3.905336	5.843951
HORVU7Hr1G029200	1.698098	1.448909	2.394364	2.553968	4.673752	4.926178
HORVU4Hr1G065410	4.797558	3.958071	3.728022	2.84126	5.141588	4.784295
Hordeum_vulgare_newGene_7987	0	0	0	4.94758	0	1.305243
Hordeum_vulgare_newGene_3404	0.786813	0.701355	1.238716	0.576972	2.757112	2.948609
HORVU6Hr1G051990	6.441609	9.226895	8.532975	5.172766	5.262243	6.100806
Hordeum_vulgare_newGene_3402	0	0	0	3.528676	3.419922	4.137387
HORVU7Hr1G109340	0	0	0	2.211486	1.734946	2.612604
HORVU4Hr1G057910	6.643569	7.074248	11.99971	21.61004	28.67904	32.30188
HORVU5Hr1G063420	0.579171	0.510056	0.756138	1.503902	1.524426	1.980275
Hordeum_vulgare_newGene_3408	33.81064	39.22233	37.29365	1.577751	1.708086	1.822192
HORVU1Hr1G062190	1.707916	2.174929	2.480546	4.911092	9.669674	8.501081
HORVU7Hr1G077550	0.844315	0.628189	0.829482	2.325765	2.545642	2.453212
HORVU1Hr1G060200	2.742949	2.287574	1.907699	2.644728	1.640578	3.153391
HORVU2Hr1G076620	2.341749	2.573854	2.550538	1.638108	1.367529	1.641436
HORVU6Hr1G065210	8.809646	7.313305	7.411473	7.740877	4.650779	5.574682
Hordeum_vulgare_newGene_426	9.670604	9.313771	8.729367	17.43018	21.45711	19.07166
HORVU3Hr1G090890	3.112574	2.171262	3.404037	1.813012	3.239432	3.036861
Hordeum_vulgare_newGene_14129	5.214302	5.945109	6.705267	0	0	0.094302
HORVU7Hr1G070010	123.9214	122.3928	115.2737	129.0395	107.9389	93.53953
HORVU1Hr1G070190	0.472432	0.742209	0.363931	1.398613	2.176088	2.176064
Hordeum_vulgare_newGene_14123	1.734253	1.224343	1.517939	7.121352	13.73657	13.59292
HORVU2Hr1G086160	6.146027	6.013333	7.223459	5.933973	4.601605	3.841307
HORVU5Hr1G075550	7.204253	6.837624	7.259107	10.03164	14.45501	11.61614
HORVU7Hr1G116940	1.477156	1.637325	1.838059	3.540204	3.912658	4.186493
HORVU4Hr1G059340	2.965278	2.835031	5.185195	1.945115	3.807503	3.739537
HORVU5Hr1G044160	2.500104	2.879074	3.226027	2.861877	4.120253	3.745618
Hordeum_vulgare_newGene_10808	3.288787	3.6604	3.317844	5.690714	4.410067	6.078725
Hordeum_vulgare_newGene_10809	3.418	3.08388	5.20755	0.738649	0.568637	0.543869
Hordeum_vulgare_newGene_15898	8.061575	8.437061	8.624666	0.040186	0	0
Hordeum_vulgare_newGene_10802	2.036638	2.875667	1.942732	0	0	0
HORVU3Hr1G092940	2.684089	2.141793	4.153413	1.06428	0.925812	1.201532
HORVU3Hr1G051770	9.711781	9.138015	10.88639	11.66833	8.849891	9.29546
HORVU3Hr1G044780	16.90557	18.18327	17.04389	13.1481	13.7918	14.37832
HORVU5Hr1G017550	16.88454	15.40957	14.42332	23.22303	20.58407	21.90284
HORVU6Hr1G053590	1.713251	1.516546	1.464558	1.994227	3.623327	3.688635
HORVU4Hr1G020770	30.66918	30.53571	26.51558	45.88229	29.76146	36.84606

HORVU4Hr1G055510	8.96072	12.26105	11.85734	3.745234	3.305109	4.416478
HORVU5Hr1G006730	41.51269	41.23848	55.71743	35.37089	41.46542	41.64649
HORVU2Hr1G020220	22.61765	23.82125	25.51779	22.72592	21.72452	24.29269
HORVU2Hr1G096360	136.5455	126.6037	157.1497	263.7879	262.7961	232.9974
HORVU2Hr1G033450	17.05912	13.25001	17.0095	9.335941	7.779726	9.587679
HORVU3Hr1G115980	2.37708	1.52353	2.41533	3.497871	4.992089	5.228185
HORVU7Hr1G027860	80.39945	93.12777	84.34038	29.90267	19.03868	22.74516
HORVU2Hr1G101630	1.324347	2.287732	2.225806	2.216713	3.912696	4.897492
HORVU3Hr1G013880	38.48793	35.03332	40.75058	19.39821	18.15354	19.83629
HORVU3Hr1G110200	0	0	0	0.369781	35.06813	53.08779
HORVU6Hr1G079040	12.07492	16.51264	15.44917	21.33847	19.15794	20.0454
HORVU5Hr1G116580	168.6347	174.1975	200.8742	236.4646	359.1853	342.9382
HORVU4Hr1G020480	34.21493	34.83194	28.3369	12.88632	10.6681	14.01893
HORVU1Hr1G059240	1.168575	1.543668	1.337632	1.27232	0.802284	1.350806
HORVU2Hr1G104040	10.74499	12.51535	14.12449	6.521587	1.728106	3.332083
Hordeum_vulgare_newGene_10110	2.60472	2.636506	2.768227	3.370849	3.535634	2.244552
HORVU6Hr1G025340	1.188777	0.863461	0.953877	1.509963	0.650291	1.274286
Hordeum_vulgare_newGene_5150	2.662736	2.486732	2.799055	7.94576	9.959679	10.55919
HORVU4Hr1G060130	6.297135	5.482672	6.689657	6.187623	6.318962	6.689973
HORVU3Hr1G053600	0.639098	2.27838	2.590202	1.559133	2.15927	2.305354
HORVU3Hr1G093310	25.29616	23.91355	23.3707	14.78064	12.08543	12.68694
HORVU2Hr1G017350	2.094655	1.606994	2.217326	1.448742	1.853951	2.318836
Hordeum_vulgare_newGene_9723	6.674421	5.69848	4.842155	9.106506	12.50907	7.559068
HORVU5Hr1G109980	52.25445	49.61025	39.63504	3.553337	0	0.979432
Hordeum_vulgare_newGene_9727	4.599171	6.418469	5.675744	8.239075	6.051607	7.159876
HORVU4Hr1G071070	5.903745	5.774923	7.352349	2.953032	5.865174	5.458271
HORVU1Hr1G001560	1.044509	10.72492	1.833894	15.05834	8.433715	10.64822
Hordeum_vulgare_newGene_887	2.662217	2.338972	2.288764	4.275511	4.808236	3.832713
HORVU3Hr1G052950	6.480127	5.725107	6.663497	9.014909	12.62294	13.33406
HORVU2Hr1G126230	3.102152	4.073748	3.938046	4.388223	7.738172	7.340276
HORVU0Hr1G033160	0.051499	0.155028	0.329269	2.732924	3.046336	2.284316
HORVU2Hr1G105390	1.150335	1.020784	1.138424	9.17252	18.08361	17.07215
HORVU2Hr1G005940	7.106419	7.008518	6.956558	6.728948	6.457478	7.371325
HORVU3Hr1G014270	0.992534	0.731151	1.805645	0.934628	0.551138	1.173585
HORVU6Hr1G044030	21.74893	23.51429	20.05035	23.36946	27.5482	27.61211
HORVU1Hr1G063100	36.33977	30.90334	42.11123	10.63416	3.583203	4.486185
HORVU1Hr1G028780	2.773302	2.940553	4.482068	4.353985	7.370056	5.930137
HORVU7Hr1G061610	5.348825	7.160703	7.092367	8.658032	11.96506	10.79231
HORVU7Hr1G036160	9.595979	8.224596	11.16125	9.900175	12.81292	14.14617
HORVU7Hr1G072320	6.499486	5.171373	6.928164	7.96655	8.215892	8.31374
HORVU2Hr1G026810	760.8978	716.7925	1052.759	403.4067	317.1803	272.2092
HORVU7Hr1G115550	7.955534	7.326092	8.235449	10.32488	10.38568	11.1668
HORVU1Hr1G076480	11.00878	10.90645	9.089454	12.98132	11.69489	11.47888
HORVU1Hr1G039000	10.24227	10.96489	12.56709	12.53683	16.75217	15.65214
HORVU6Hr1G009400	95.95603	98.43361	141.6233	93.62215	165.6161	167.7043
HORVU2Hr1G088110	32.34897	26.18966	32.72068	11.98174	12.0214	11.04494
HORVU1Hr1G073500	12.34806	11.71977	13.75305	16.49506	18.75115	19.18848

HORVU5Hr1G067610	5.751239	3.933518	5.745776	6.032183	6.169546	5.874643
HORVU5Hr1G015050	0.817899	1.002151	0.931757	2.540104	2.857324	3.351915
HORVU1Hr1G024770	4.120154	3.705461	4.005052	0	0	0
Hordeum_vulgare_newGene_14912	0.799505	1.351321	1.641886	0.976376	1.500217	1.272776
Hordeum_vulgare_newGene_14914	7.039215	7.746915	11.16151	9.58989	13.78319	12.502
Hordeum_vulgare_newGene_14915	1.783493	2.039411	1.768803	1.256591	1.124375	1.128363
HORVU4Hr1G048210	2.146233	2.017174	2.311108	11.72879	9.679056	12.63283
HORVU5Hr1G060340	0.165582	0.194971	0.185831	1.820252	1.534882	2.212516
HORVU1Hr1G031230	7.097782	7.409546	7.080706	12.04815	14.74852	18.86568
HORVU7Hr1G100800	2.695117	2.14217	2.935592	3.692322	4.202787	4.815718
HORVU6Hr1G058750	62.55056	54.41642	73.53151	80.67037	121.844	128.0773
HORVU6Hr1G071980	2.805388	2.779417	2.615896	3.027461	2.720333	3.246744
HORVU1Hr1G075680	16.98647	18.02359	23.54545	24.06891	49.6205	49.98535
Hordeum_vulgare_newGene_7512	1.830786	1.64348	1.343559	1.276974	1.625096	1.52497
Hordeum_vulgare_newGene_235	4.80316	3.490989	5.331243	4.946448	6.522387	5.326903
HORVU3Hr1G085280	21.13102	22.67958	22.35561	20.62964	24.95729	24.01761
HORVU1Hr1G023690	3.324462	3.332409	3.75213	4.339489	5.965861	6.177252
HORVU4Hr1G072830	41.37616	43.74201	58.34073	19.78735	2.057487	7.960977
HORVU3Hr1G017990	5.804764	6.431816	5.305582	1.459086	1.946667	2.136668
HORVU6Hr1G058930	4.78678	4.529985	5.757908	10.09064	9.490417	13.89782
HORVU3Hr1G084880	5.710117	3.895596	3.956561	11.88929	15.80855	14.07648
HORVU3Hr1G086950	8.777936	8.95352	9.658977	9.090156	11.00893	10.70171
HORVU6Hr1G062220	5.151494	4.338052	7.0111	7.26161	5.020559	5.539101
HORVU2Hr1G084910	29.09602	25.16365	33.59275	51.04759	54.82836	51.56381
HORVU1Hr1G078410	15.47961	16.04416	18.94698	19.3301	17.00701	18.7392
HORVU3Hr1G080500	1.643732	0.873095	1.810843	2.093665	1.21254	1.898193
HORVU7Hr1G055680	1.887968	1.687819	0.394559	0.980328	0.935404	0.343421
HORVU6Hr1G075350	20.83179	21.11882	22.05108	14.23837	19.31323	21.08891
HORVU5Hr1G059970	7.510926	6.785478	7.293973	8.272574	11.54397	10.82242
HORVU2Hr1G079620	0.624719	0.637753	0.406472	1.996848	1.781647	3.085029
HORVU3Hr1G086480	28.47204	30.11904	25.39967	22.0499	21.46613	22.68923
Hordeum_vulgare_newGene_12246	38.36374	40.50947	53.32008	36.42774	37.80793	36.73974
HORVU6Hr1G083430	26.8811	25.59276	26.92221	25.06207	30.69219	29.32413
HORVU0Hr1G040340	40.10797	34.62052	51.52785	40.93441	53.08494	56.76042
Hordeum_vulgare_newGene_2875	2.604874	1.930891	3.508668	3.350959	5.938325	5.420683
Hordeum_vulgare_newGene_2874	2.687095	3.002588	3.326173	4.575857	6.260023	6.913961
Hordeum_vulgare_newGene_6397	1.051515	1.679002	1.315658	1.23117	1.724432	1.757022
Hordeum_vulgare_newGene_2871	63.1576	51.32406	76.34632	64.98866	138.3779	125.4074
Hordeum_vulgare_newGene_2870	55.67753	49.16744	68.11398	68.85387	98.69557	92.68911
HORVU1Hr1G051050	0.027009	0	0	0.576067	2.13066	3.506689
HORVU4Hr1G003120	3.125429	3.77014	5.335789	3.713547	6.051799	6.326631
Hordeum_vulgare_newGene_490	2.358027	0.781467	1.962383	0.374605	0.272495	0.263812
HORVU6Hr1G087540	1.398057	0.613824	0.705334	0.744994	2.982911	2.641706
HORVU3Hr1G086220	28.18572	23.56156	34.54201	11.84058	7.519991	8.859247
HORVU2Hr1G086400	12.39743	10.73974	10.21366	10.38699	9.640327	10.74451
HORVU5Hr1G020300	5.502118	5.136142	6.497393	6.532721	9.168926	8.855877
HORVU1Hr1G089760	1.10896	1.029868	1.124786	5.409468	7.489629	7.880881

HORVU1Hr1G081550	48.72304	36.40668	59.51233	52.33438	56.07637	70.06212
HORVU2Hr1G037040	7.691811	7.298565	6.076998	0.737224	0.237645	0.297595
HORVU5Hr1G100140	14.40641	12.59257	11.03421	55.6989	89.11615	93.7522
HORVU1Hr1G093250	1.058596	1.344122	1.421985	2.662123	4.041796	4.941051
HORVU1Hr1G087380	137.8498	122.4807	106.7658	18.67482	4.754916	9.423997
HORVU6Hr1G074910	0	0	0	2.214939	2.872178	2.775136
HORVU4Hr1G069210	5.821157	6.305044	7.942599	6.101614	6.698538	6.666133
HORVU6Hr1G087090	5.854325	4.724382	5.403733	5.334942	5.806137	5.718375
HORVU3Hr1G033370	6.428989	7.201542	11.33302	10.7103	12.05374	13.31887
HORVU7Hr1G054220	39.11178	31.23554	36.50417	131.5476	206.5722	185.6233
HORVU5Hr1G016680	22.1844	19.8192	28.95951	19.72008	25.98549	23.62905
HORVU6Hr1G072510	13.26736	8.926391	13.23566	18.56845	16.45512	21.83811
HORVU5Hr1G122530	0	0	0	1.484013	2.362165	2.856564
HORVU2Hr1G027770	14.64679	14.06412	17.32891	18.04319	22.11112	21.73786
HORVU3Hr1G013690	10.35128	9.57562	11.33333	19.05128	23.18644	22.49799
HORVU5Hr1G112580	21.38396	22.59122	17.29084	8.004901	20.57776	17.14988
HORVU7Hr1G096460	2.649128	2.377244	3.091288	2.671183	3.02786	3.89544
HORVU6Hr1G061140	7.636849	8.376204	7.760593	6.591173	4.057399	5.287739
HORVU6Hr1G010040	0	0.062257	1.19553	3.539615	3.426654	3.496853
HORVU4Hr1G003650	0.853724	0.654183	0.9681	1.663145	1.334222	1.667144
HORVU5Hr1G070740	35.36297	36.40591	48.852	34.35371	43.63436	44.53845
HORVU1Hr1G091090	2.261568	2.151735	1.87007	3.347405	5.926093	5.933198
HORVU1Hr1G059720	6.519658	5.570984	6.773734	9.279821	13.73122	13.0436
HORVU5Hr1G028480	6.047361	6.104521	6.083209	5.008858	9.868208	8.247874
HORVU3Hr1G016310	1.744269	1.114615	1.547288	0.806049	1.127662	1.297571
HORVU5Hr1G113180	382.8996	386.4495	540.4595	360.5464	453.5678	491.5192
HORVU2Hr1G004980	3.056841	3.428496	4.509333	4.498618	5.860873	5.636892
HORVU7Hr1G065180	1.289203	1.625133	1.871196	1.535796	1.105703	1.227098
HORVU5Hr1G050230	0.93976	0.99047	0.880312	0.853554	1.999742	1.956867
HORVU4Hr1G010150	10.38759	10.45901	14.56674	2.395252	3.124598	4.18076
HORVU7Hr1G051760	0.611082	0.629284	0.656674	1.973853	4.276543	4.105628
HORVU0Hr1G007110	13.91293	11.91553	14.98304	18.21518	21.57225	21.72659
HORVU5Hr1G039900	12.94527	14.58673	13.02393	10.7125	7.677007	8.857367
HORVU6Hr1G080830	3.214244	3.267369	5.014482	3.808989	5.094923	4.147712
HORVU7Hr1G113460	2.95581	2.231053	2.345755	1.091568	0.90866	1.088789
HORVU4Hr1G066300	9.228887	9.051855	10.64432	11.60437	15.91982	16.82389
HORVU5Hr1G103580	21.13386	22.32598	21.61342	26.42393	38.0797	36.13277
HORVU5Hr1G034530	28.96897	33.31814	33.2794	31.29401	28.54838	33.50755
HORVU7Hr1G041660	14.32866	14.19562	17.83618	16.64283	26.89955	28.15736
HORVU7Hr1G053940	13.19013	12.72885	13.23944	13.78954	8.266111	10.7152
HORVU3Hr1G020490	237.7594	197.8441	278.3752	205.0094	345.4004	226.0857
HORVU3Hr1G042500	25.95614	22.92579	26.54739	18.91478	21.23932	19.89326
HORVU6Hr1G068820	2.111259	2.494105	2.382386	1.168854	2.298931	2.345402
HORVU6Hr1G011790	4.046036	3.26409	3.517537	5.748207	5.078171	5.500858
Hordeum_vulgare_newGene_13597	1.077993	2.257267	3.541646	4.491438	5.941891	4.054737
HORVU3Hr1G000350	2.095409	1.622869	1.97153	3.324364	7.05846	6.805904
HORVU4Hr1G088610	28.85683	26.99756	33.30674	25.52077	39.70114	36.40792

HORVU0Hr1G018710	1.363822	1.059583	1.600186	1.489322	1.672918	1.870832
HORVU0Hr1G013890	11.35516	11.29085	15.01783	13.63599	19.08938	18.24961
HORVU4Hr1G077840	3.59095	4.107223	4.538629	3.584195	6.971498	6.573878
HORVU5Hr1G104750	1.087836	0.533523	0.565856	0.349938	4.369464	1.625891
HORVU2Hr1G009570	0.240575	0.194177	0.076235	1.312488	2.843717	2.430308
HORVU5Hr1G009830	18.00257	20.50668	26.48585	8.266214	15.16706	16.00858
HORVU2Hr1G095490	2.949087	3.145764	3.725397	3.699725	5.535061	4.707045
HORVU5Hr1G097720	17.81402	15.99692	19.22625	20.9638	25.75762	22.12425
HORVU1Hr1G043830	5.034188	4.469282	4.937588	8.062846	3.530628	5.48406
HORVU6Hr1G059500	4.020945	2.000567	3.603019	0.057196	0.078424	0.01203
HORVU2Hr1G091320	5.044009	5.160664	7.30231	8.635414	11.59604	9.173079
HORVU1Hr1G080080	0.367445	0.614167	0.519629	1.783091	2.105919	2.28088
HORVU1Hr1G037650	1.192088	1.183837	1.596436	1.903018	2.872711	2.873928
HORVU1Hr1G049420	1.473139	0.942556	0.945484	3.476707	2.666169	2.836141
HORVU7Hr1G099700	2.100705	2.075492	2.826485	1.249377	1.403167	1.090408
HORVU6Hr1G008870	8.593132	9.547009	11.27227	9.919191	10.80246	11.18223
HORVU0Hr1G006790	6.782434	7.203152	8.635604	5.341838	5.641304	6.828573
HORVU2Hr1G036000	137.8884	119.8001	129.9317	114.0605	115.2655	113.0577
HORVU7Hr1G116040	8.604517	7.883056	12.84097	5.350294	6.808576	6.585793
HORVU6Hr1G017000	1.9237	2.015896	0.498804	0.099324	1.244875	1.309503
HORVU7Hr1G071240	43.81637	44.09034	78.67457	43.61315	116.5881	98.71953
HORVU0Hr1G014150	3.940409	4.452492	4.528961	16.8569	36.77879	32.29023
HORVU4Hr1G083640	1.435245	0.968387	1.685465	0.802177	0.825282	0.906927
Hordeum_vulgare_newGene_2412	1.32259	2.186512	2.153546	0.362446	1.279655	1.052402
HORVU7Hr1G019550	5.48288	6.932717	7.539854	5.244779	6.11401	5.34598
HORVU6Hr1G095270	6.670698	5.247941	5.0309	6.38699	7.856005	7.655673
HORVU2Hr1G016760	1.878752	2.116105	2.224792	1.713601	2.220797	2.241885
HORVU2Hr1G100700	2.802813	2.990969	3.0895	3.283248	4.732742	4.595848
HORVU0Hr1G015820	16.66112	15.98892	20.6128	18.86506	28.96603	30.1726
HORVU4Hr1G084240	1.799742	1.484883	4.185912	1.173333	1.190724	1.092113
HORVU0Hr1G004850	3.909999	3.489171	4.088672	14.57289	28.67396	25.63744
HORVU1Hr1G047680	16.39292	16.38332	21.61693	21.05436	35.12536	32.32937
HORVU7Hr1G081300	2.447338	1.486701	2.612432	1.806998	1.828869	2.767642
HORVU4Hr1G054910	2.074065	2.200731	2.742127	5.330597	8.431554	6.798622
HORVU7Hr1G111280	3.812033	4.198863	4.460015	3.767803	3.64663	4.081547
HORVU1Hr1G003080	1.210679	9.454101	1.666435	14.13591	2.742635	2.646528
HORVU1Hr1G056020	14.88195	16.25656	17.47552	16.22668	14.67415	15.01221
Hordeum_vulgare_newGene_6568	0.645877	0.672718	1.12543	1.601225	2.028476	2.772672
HORVU2Hr1G107280	28.22826	25.76349	33.35483	27.92766	34.61181	32.28374
HORVU2Hr1G037750	6.377555	5.380052	7.826882	15.80148	14.95923	16.96152
HORVU7Hr1G055890	0.71579	1.353425	2.052837	1.968264	3.250984	3.191202
HORVU4Hr1G055060	48.65981	42.87218	51.12666	47.36121	52.43146	54.18151
HORVU4Hr1G070250	0.554825	0.644094	1.357498	7.443098	25.15904	23.70275
HORVU6Hr1G090960	17.9019	13.51095	19.2797	14.30523	15.73412	16.0753
HORVU7Hr1G018600	9.439272	8.27117	9.732612	17.13425	24.15742	18.87552
HORVU3Hr1G109340	3.619799	3.456824	4.787498	5.285731	8.15357	8.161545
HORVU2Hr1G111240	3.008787	5.083015	5.532012	3.733661	3.844181	4.54031

Hordeum_vulgare_newGene_10359	95.94889	82.20921	99.3976	50.93765	63.3479	55.48623
HORVU2Hr1G119070	0.68264	0.656457	0.658979	1.367117	1.77712	1.681002
HORVU1Hr1G060380	2.927851	2.910864	3.66459	1.891049	2.83575	2.775831
HORVU3Hr1G062870	9.949089	7.998034	11.6441	9.556525	8.463789	8.679282
HORVU5Hr1G098570	1.068472	1.072295	1.43357	1.086413	1.200023	1.687788
HORVU3Hr1G059080	7.131558	7.243475	6.183184	6.080054	5.388478	5.61706
HORVU3Hr1G059810	2.779575	2.81206	2.516013	10.82922	18.72005	20.79228
HORVU5Hr1G053810	1.92262	1.115865	1.278707	3.39222	11.52942	8.72383
HORVU3Hr1G072340	5.818593	6.210952	6.816951	5.836646	15.93822	16.35283
HORVU6Hr1G076660	1.875261	1.767441	1.40638	5.300626	3.992585	4.900771
HORVU4Hr1G051360	13.05567	10.84054	12.78888	6.618304	4.465024	5.334414
HORVU1Hr1G012880	3.363539	2.309206	4.036175	30.35708	237.3665	214.3672
HORVU7Hr1G044150	11.61482	8.097758	12.3337	7.12155	7.589929	6.5744
HORVU1Hr1G014010	6.242202	8.640043	9.828507	4.111835	3.831351	4.95295
HORVU7Hr1G000780	4.353712	4.15438	5.026294	6.001753	4.973107	6.310727
HORVU5Hr1G081460	11.27761	9.529619	13.26622	5.576748	23.42609	20.19642
HORVU6Hr1G038450	10.03464	8.932846	13.43938	14.69742	23.91915	22.79271
HORVU7Hr1G089840	8.258998	7.649656	7.914402	11.07261	13.09776	14.6564
HORVU2Hr1G021350	5.292991	5.6701	6.367249	8.628317	8.205316	9.600331
HORVU4Hr1G060440	45.92998	36.86679	45.17292	45.67217	27.80856	31.56743
HORVU2Hr1G093230	1.69443	1.768468	2.147022	5.033806	3.404227	5.247718
HORVU7Hr1G051070	47.87819	35.60525	49.40342	42.91208	43.61924	44.3965
Hordeum_vulgare_newGene_11089	1.681478	1.375432	1.993979	6.137244	9.635787	8.913767
Hordeum_vulgare_newGene_11088	0.532366	0.560334	0.576618	2.048525	4.441866	5.07222
HORVU7Hr1G027080	4.731288	4.521558	5.63712	6.658912	7.460362	5.9185
HORVU2Hr1G110590	47.71035	44.32062	56.101	39.97235	45.63375	47.50596
HORVU6Hr1G089010	13.96904	13.3595	16.23528	18.60645	21.6837	21.66277
HORVU2Hr1G012690	49.62519	43.40228	53.91979	55.87752	67.10986	61.21268
Hordeum_vulgare_newGene_11552	6.197594	6.724667	8.544732	7.220552	10.81218	11.5511
Hordeum_vulgare_newGene_11550	9.337863	8.962879	9.985137	9.416071	12.40623	13.23719
HORVU5Hr1G055410	7.957126	6.703645	7.47607	5.569761	4.814954	6.405844
HORVU2Hr1G029120	2.709626	2.736931	2.427476	4.045384	7.41403	6.710349
Hordeum_vulgare_newGene_4609	1.519014	2.154578	1.087828	1.702976	2.463871	2.602225
HORVU1Hr1G000940	13.58525	12.46613	13.50959	23.08269	13.40865	13.16984
HORVU1Hr1G038730	0.058118	0	0.082841	0	56.18406	65.87297
HORVU3Hr1G073960	7.253664	6.454121	9.464883	8.053873	10.67202	11.86866
HORVU5Hr1G072400	0.933978	1.00901	0.940353	0.963156	1.467077	1.420456
HORVU7Hr1G072200	10.1592	11.44395	13.74446	10.69052	11.78176	13.09137
Hordeum_vulgare_newGene_3882	2.63516	2.390656	1.382291	2.286967	2.300728	2.142976
Hordeum_vulgare_newGene_391	2.955921	3.05697	3.270056	2.686663	3.196901	3.114836
HORVU5Hr1G005990	10.11771	9.376879	9.988491	7.111941	14.50491	11.61733
Hordeum_vulgare_newGene_396	1.561637	1.889393	1.54254	1.727634	1.382379	1.381179
HORVU7Hr1G051400	77.45663	92.33293	57.32044	56.03234	44.90697	51.59178
Hordeum_vulgare_newGene_1900	3.84073	5.246592	4.018645	0.027502	0	0
HORVU5Hr1G043650	78.72554	76.37294	73.59341	29.31398	38.77437	37.21652
HORVU3Hr1G107010	1.295694	1.38462	1.418394	1.310504	2.181974	2.314721
HORVU6Hr1G040310	45.66969	51.44682	50.95634	42.37525	53.38254	61.56695

HORVU5Hr1G000010	19.30957	20.0473	22.37615	24.27562	21.76468	24.66735
HORVU5Hr1G076230	12.93593	9.646017	15.41331	13.13545	15.93317	15.5403
HORVU2Hr1G062910	2.859506	3.958538	2.604988	1.761847	0.856781	1.030477
HORVU5Hr1G070800	14.46335	13.27359	15.68449	17.54985	18.19792	18.35261
HORVU2Hr1G025150	11.44916	8.040449	12.03037	18.71433	29.87391	27.16409
HORVU6Hr1G060990	167.4511	170.5326	217.4296	63.20568	58.66641	58.11621
HORVU4Hr1G084000	42.94029	36.4131	46.03169	80.99109	126.1084	117.655
Hordeum_vulgare_newGene_3870	11.15559	9.363413	10.63089	10.27394	9.315492	10.29192
HORVU1Hr1G042820	11.2038	12.44963	18.22366	8.68246	15.77783	14.78221
HORVU7Hr1G104830	3.448531	2.894782	3.676847	1.820206	2.5974	2.818738
HORVU3Hr1G024530	61.79864	67.62948	84.33825	39.23518	30.51506	36.05927
HORVU2Hr1G038960	2.149229	6.357343	4.957841	4.030117	0.613686	0.36546
HORVU2Hr1G052650	87.92688	99.38447	157.4547	57.97862	146.4175	125.4271
HORVU2Hr1G028380	6.555333	6.621251	7.737716	3.115707	2.788629	3.752993
HORVU2Hr1G032060	15.81424	13.62992	17.38429	15.98058	18.07185	18.25869
HORVU3Hr1G093970	7.65083	6.195682	6.725289	6.764975	11.35605	4.648258
HORVU2Hr1G116660	0.311385	0.278537	0.436242	4.911084	5.267003	4.854135
HORVU0Hr1G021720	3.743225	3.710647	8.405396	12.20788	19.73153	16.18004
Hordeum_vulgare_newGene_10245	0.54419	0.805023	0.500196	1.474641	1.814535	1.982939
HORVU1Hr1G083910	3.831073	5.95037	4.579917	8.318889	3.313081	5.682139
HORVU6Hr1G078210	8.037074	9.128072	11.74438	6.725857	5.941551	5.230815
Hordeum_vulgare_newGene_6156	0.724183	0.67913	1.318514	0.966103	1.866743	1.733239
Hordeum_vulgare_newGene_6152	4.799347	4.378611	5.355476	6.184519	5.639583	6.527469
Hordeum_vulgare_newGene_6150	0.076933	0.12428	0.257748	13.55966	2.109141	6.285101
HORVU4Hr1G025430	0.449458	0.666794	0.327504	0.502239	3.298721	2.523494
HORVU5Hr1G107260	14.55279	19.32783	21.96857	13.56053	17.87017	17.90923
HORVU3Hr1G114970	2.077103	1.96296	2.334486	0.60786	0.940506	1.308245
HORVU4Hr1G022960	4.629615	4.668452	5.590057	5.047598	7.204485	7.963114
HORVU1Hr1G071780	25.91986	28.79404	27.84788	27.97477	23.14298	25.43099
HORVU5Hr1G068320	37.17976	35.7305	36.83265	24.01101	20.56384	18.5029
HORVU3Hr1G059480	1.33725	0.601261	0.946065	12.40968	5.878183	15.83138
HORVU7Hr1G008640	2.354229	3.899717	4.574614	3.702584	3.636121	3.451367
HORVU5Hr1G074270	4.44075	3.242994	4.708207	5.398873	7.054718	5.905043
HORVU2Hr1G005330	1.509102	2.441472	2.450468	1.021339	0.248752	0.82126
HORVU1Hr1G070780	0.957642	0.442284	1.048709	1.961203	1.202369	0.944953
HORVU5Hr1G082630	33.01556	33.52952	51.83758	27.1826	25.99702	30.92605
HORVU2Hr1G086390	160.8836	146.0338	207.4687	199.3666	426.0406	365.1137
HORVU6Hr1G012320	926.4667	756.3854	1033.092	596.6567	263.2951	331.6649
HORVU3Hr1G031400	3.945842	2.294739	4.38221	4.772077	1.965848	2.381094
Hordeum_vulgare_newGene_13620	1.920475	3.182946	2.912502	4.237292	4.795218	5.982163
HORVU5Hr1G088290	4.574998	4.408586	5.641434	7.028081	6.655891	6.963681
Hordeum_vulgare_newGene_15645	12.75795	11.1938	12.64429	10.71037	10.331	11.33453
Hordeum_vulgare_newGene_15643	5.64616	4.775488	7.090651	5.288647	5.186206	3.447973
HORVU1Hr1G077830	1.157398	1.008963	1.19137	0.886837	1.055973	1.018486
HORVU2Hr1G020380	60.95207	45.90238	34.96097	55.51026	80.43672	64.91699
HORVU2Hr1G027170	2.023487	2.022892	2.432507	2.661104	3.555603	3.140485
HORVU0Hr1G031970	6.039867	4.693218	6.262678	7.810583	8.607491	10.01253

HORVU3Hr1G055830	8.651007	9.738823	7.132106	11.98042	29.22101	21.78548
HORVU6Hr1G059950	2.257317	2.291234	3.009504	3.115222	4.458189	4.064153
HORVU1Hr1G070430	5.009161	4.81163	4.820282	6.454493	6.361136	5.597418
HORVU7Hr1G096660	18.45009	17.72795	19.64983	16.2093	23.10351	20.8645
HORVU3Hr1G021050	7.974319	9.438781	7.230425	9.868055	13.67554	12.64214
Hordeum_vulgare_newGene_11942	7.012557	5.673281	8.328072	0.067488	0.092873	0.046197
Hordeum_vulgare_newGene_11949	11.56063	11.22143	12.03988	10.48758	11.63397	10.76005
HORVU4Hr1G055300	1.742034	2.113834	3.272883	1.440934	0.860199	1.256781
Hordeum_vulgare_newGene_9398	5.489104	6.768234	5.06438	6.667775	10.99037	8.572204
Hordeum_vulgare_newGene_5638	0.695761	0.441961	0.826219	2.623732	3.802963	3.923976
Hordeum_vulgare_newGene_5639	1.431143	3.143586	4.483319	2.288758	1.791191	2.127507
HORVU5Hr1G073720	1.700731	1.746461	2.602028	1.698649	2.994104	3.683731
HORVU6Hr1G033970	9.746008	9.270633	13.06761	16.38411	22.10244	23.0154
Hordeum_vulgare_newGene_9390	2.318598	3.186133	2.853277	4.93761	6.921835	7.537437
HORVU7Hr1G043740	2.011022	1.875061	2.011906	1.792092	2.304786	2.232735
HORVU2Hr1G048220	15.75044	14.93022	20.62336	10.98425	16.90151	15.47855
Hordeum_vulgare_newGene_6842	1.927588	0.475695	1.216887	3.087483	5.374953	4.908666
Hordeum_vulgare_newGene_6846	2.682813	0.713011	2.999299	4.67539	4.370635	4.865293
Hordeum_vulgare_newGene_6848	2.19749	2.379472	1.985152	3.798426	2.860331	3.07792
HORVU7Hr1G103330	69.14717	54.52518	57.87266	56.0381	59.71675	65.05336
HORVU4Hr1G069720	0.720206	1.083432	1.17707	1.240339	1.30768	1.493026
HORVU0Hr1G019020	0.420843	0.554696	0.55497	10.42369	3.150048	6.312009
HORVU2Hr1G072660	28.38552	30.44978	42.0152	36.08439	83.652	82.36779
Hordeum_vulgare_newGene_8449	1.678691	1.859565	2.446676	8.044237	13.01046	11.4035
Hordeum_vulgare_newGene_8443	0	0	0.014534	9.412593	12.00678	14.80909
HORVU5Hr1G098030	1.326833	0.768031	1.309006	2.009986	0.89534	1.101356
HORVU1Hr1G054080	24.87005	32.83332	28.15081	15.14672	15.74998	16.69876
HORVU0Hr1G032400	3.519696	4.244103	5.924505	1.916968	2.088546	2.670795
HORVU5Hr1G077910	0.536172	0.52295	0.561789	2.311468	6.076406	5.792255
HORVU7Hr1G073750	4.747748	4.945348	4.591497	5.403817	8.306102	7.103653
HORVU1Hr1G063150	0.620073	0.621096	0.912134	0.649823	2.156795	1.942936
HORVU0Hr1G008650	3.700691	3.115027	3.164414	3.023815	6.102787	3.781314
HORVU2Hr1G058930	5.067573	7.121163	6.747609	10.3047	11.16246	12.03029
HORVU2Hr1G031620	4.636842	5.240359	4.612932	3.913143	4.272722	4.668421
HORVU2Hr1G089160	6.412601	5.154234	7.423184	13.58049	31.03619	28.64522
HORVU1Hr1G008230	12.1686	10.10529	10.82953	12.02594	14.35497	13.69912
HORVU2Hr1G118950	5.549751	4.838955	5.994195	9.542527	3.880463	4.241969
HORVU1Hr1G062460	8.623249	7.928295	11.00712	10.40784	11.74299	12.54306
HORVU5Hr1G019640	8.620483	8.255665	7.515257	10.56614	14.02807	14.45655
HORVU5Hr1G034040	6.776787	6.987163	9.603879	10.40867	21.61788	19.86616
HORVU2Hr1G127120	6.286997	6.012247	9.202631	5.45875	8.969112	7.724305
HORVU7Hr1G023600	16.30336	16.37939	18.47551	11.12363	8.792271	10.4344
HORVU1Hr1G022990	26.55964	23.44095	24.72975	37.5047	57.97762	51.38821
HORVU6Hr1G067310	1.725909	2.179694	2.168111	1.519307	1.412457	1.408969
HORVU7Hr1G039880	6.417357	5.385932	7.596788	8.244479	8.903326	9.277933
HORVU7Hr1G114030	6.932447	8.297087	5.89642	6.462339	3.567948	4.998801
HORVU2Hr1G029240	4.181286	3.202156	4.994903	4.56854	4.894405	4.505355

HORVU1Hr1G092270	27.58508	26.20632	27.613	19.38717	21.41225	20.27889
HORVU7Hr1G036280	0.304108	0.631955	0.547722	1.165681	89.03895	149.3474
HORVU3Hr1G075340	6.43393	7.098588	6.851388	8.436666	14.68942	14.89874
Hordeum_vulgare_newGene_4142	1.040725	1.797717	1.467383	1.572743	0.227365	1.629995
HORVU0Hr1G005820	0.309091	0.667122	0.583257	1.838405	2.4	2.454949
Hordeum_vulgare_newGene_7495	1.9317	2.990251	3.140129	0.912506	0.036789	0.47845
HORVU2Hr1G120470	1.387133	1.18049	2.031453	1.621771	2.577227	2.893633
HORVU6Hr1G083440	4.150437	4.873364	4.66288	9.716197	13.26864	6.938254
Hordeum_vulgare_newGene_14892	46.49262	47.46151	62.37142	11.99953	5.023073	9.941564
HORVU7Hr1G024070	5.714294	5.514326	4.965177	4.912563	4.823131	2.718621
HORVU1Hr1G076690	0.2784	0.267549	0.331068	1.457661	7.010556	6.013288
HORVU7Hr1G063150	20.73915	21.43441	22.47201	19.44358	22.43777	24.86666
HORVU5Hr1G056530	14.48645	17.00007	18.25585	11.12049	12.62985	12.66067
HORVU7Hr1G039260	2.271732	2.407687	2.268903	7.558756	15.99523	14.08385
HORVU6Hr1G023050	2.645909	3.055325	3.886518	4.875308	5.234601	5.391473
HORVU1Hr1G075520	2.210631	1.143292	1.777915	3.625624	3.983975	4.161044
HORVU2Hr1G075470	21.10773	16.22847	23.27735	32.81949	30.64751	36.35944
HORVU3Hr1G031890	17.81636	16.60268	18.24875	30.58191	31.4754	34.64468
HORVU3Hr1G035730	0	0.041855	0	2.238047	5.311267	4.017616
HORVU5Hr1G056280	3.68459	3.905565	3.200051	4.267183	2.896199	3.595189
Hordeum_vulgare_newGene_7722	3.468943	4.538658	5.933909	4.149274	4.976827	4.345214
HORVU1Hr1G022750	100.9023	96.77976	116.2139	104.7272	102.8432	105.1193
HORVU2Hr1G010520	9.830645	9.438265	10.16344	8.514582	12.77268	13.63142
HORVU6Hr1G005940	7.718235	6.303265	7.663618	6.285906	8.698795	8.777889
Hordeum_vulgare_newGene_6459	2.596202	2.244071	2.737687	3.136013	3.603903	3.130642
HORVU6Hr1G068990	57.81873	47.15866	62.48893	30.56197	21.46279	25.98627
HORVU1Hr1G074340	1.661335	0.859951	1.045015	8.637715	35.1969	29.78208
HORVU2Hr1G032540	15.16353	16.01162	15.2477	26.7145	35.03273	35.58736
HORVU6Hr1G003390	1.269875	1.322984	1.520915	1.702277	1.751856	2.205855
HORVU4Hr1G017860	2.67039	3.223591	3.421814	7.275723	13.71483	9.861637
HORVU5Hr1G095710	0.465468	0.513797	0.300362	4.681853	4.218595	6.202378
HORVU7Hr1G067480	13.19494	14.57288	13.41408	15.16213	18.16857	17.58185
HORVU2Hr1G001350	36.70682	32.70923	52.06368	26.05545	32.82363	30.57113
HORVU3Hr1G018840	2.713884	2.694431	5.453496	5.008714	5.874715	7.129889
HORVU7Hr1G097890	3.928744	3.724037	4.195756	3.65246	4.610453	4.288466
HORVU3Hr1G034060	0	0	0.010925	3.187743	13.6536	5.321177
HORVU2Hr1G040310	5.766299	6.137159	6.851182	5.903834	7.881559	7.912293
HORVU6Hr1G033690	1.605069	1.613109	1.939349	2.549034	2.916312	4.227237
HORVU4Hr1G016400	38.11693	39.97079	36.93525	37.27226	23.81672	28.58926
HORVU4Hr1G090320	7.539995	5.172476	7.432821	4.090464	4.379593	4.174936
HORVU1Hr1G070840	1.061919	1.044738	1.6336	1.563643	1.445105	2.315262
HORVU3Hr1G033490	7.679928	7.227154	8.733311	8.724523	9.736038	9.521782
HORVU4Hr1G007100	1.397475	1.083662	1.360233	1.946963	1.843785	2.004067
HORVU0Hr1G004320	1.367123	2.934253	2.426314	1.95786	3.108933	3.2062
HORVU3Hr1G030760	0.357026	0.253434	0.453954	3.899548	20.09947	14.90923
HORVU3Hr1G082110	4.993687	4.619613	5.866865	3.999453	4.692891	4.612084
HORVU1Hr1G062810	9.864684	7.221713	8.116121	5.508459	4.958604	5.234947

HORVU7Hr1G097270	1.91553	1.364043	1.84273	2.243566	1.191243	1.30334
HORVU7Hr1G055340	24.85058	31.98668	39.70922	22.15132	38.58908	36.53406
HORVU5Hr1G058870	10.20612	10.018	11.57796	11.69836	11.82684	12.03994
HORVU4Hr1G010580	2.003231	1.081886	1.602053	0.873476	0.196593	0.258754
HORVU7Hr1G118130	0.830229	1.230896	1.218151	2.03244	3.907796	3.028199
HORVU5Hr1G123050	11.74228	11.09172	14.54747	8.671418	8.252397	9.431598
HORVU7Hr1G022560	2.351995	2.886739	2.325754	3.129645	3.612688	5.117362
HORVU7Hr1G054690	6.718674	6.045023	8.202764	10.26667	17.76589	15.78197
HORVU4Hr1G013580	1.442323	2.093408	1.507874	2.907068	4.008206	4.926857
HORVU4Hr1G006520	10.83239	9.520056	14.65034	13.99627	17.88513	16.37591
HORVU1Hr1G089510	0.316623	0.365543	0.304514	0.170624	3.217746	2.052079
HORVU2Hr1G067320	38.9613	36.38875	36.11735	37.61512	42.67093	47.57263
HORVU1Hr1G020430	2.527034	3.136892	4.241776	2.905015	1.391379	3.431876
HORVU3Hr1G092790	4.39471	3.925699	5.3589	4.505495	6.472065	5.541374
HORVU3Hr1G022030	17.09439	13.7451	15.05187	17.56244	18.37436	18.55657
HORVU2Hr1G041950	3.219167	4.06655	3.386564	2.045798	1.680142	1.753489
HORVU7Hr1G034340	8.229689	8.17249	10.35884	9.192447	10.9621	12.5233
HORVU4Hr1G069400	42.57611	39.13882	37.42199	18.25122	13.49134	14.39468
HORVU1Hr1G076380	28.73343	31.4217	31.49619	20.53984	17.77749	19.20735
HORVU3Hr1G033230	2.846586	3.644547	1.75053	2.606176	3.676707	4.034993
HORVU5Hr1G018850	3.684453	5.125032	5.827538	1.962462	2.47831	1.877898
Hordeum_vulgare_newGene_950	12.10172	12.95662	15.8685	10.20183	10.65727	11.71565
HORVU7Hr1G110360	5.872619	4.719404	6.222513	3.600136	4.77626	4.798432
HORVU5Hr1G110330	42.78688	41.95862	49.96736	46.37185	62.02111	56.09818
HORVU4Hr1G011670	95.73973	70.34312	44.83879	28.17078	12.08962	17.8469
HORVU1Hr1G092790	1.880695	1.388573	2.120805	3.210568	3.686718	3.66684
HORVU3Hr1G027200	1.192897	1.831728	2.948261	0.061431	0.166878	0.259274
HORVU3Hr1G019330	4.222991	4.28318	3.659388	3.365792	3.700631	4.43745
HORVU4Hr1G083020	1.796864	1.529972	1.532486	5.124209	4.540403	3.847397
HORVU4Hr1G063160	0.315397	0.241187	0.313189	0.674377	3.319033	2.978867
HORVU4Hr1G056300	1.092335	1.450221	1.520266	1.657476	4.039208	4.773683
HORVU1Hr1G093770	12.92799	13.61564	13.23919	14.87022	17.96417	18.07802
HORVU3Hr1G022780	3.089949	1.994288	2.489141	1.082529	0.427049	0.877041
HORVU5Hr1G112790	13.65618	12.94449	16.98433	14.42624	15.67646	16.84336
HORVU2Hr1G083960	2.351356	3.741899	3.174279	0.911447	1.305723	1.137651
HORVU1Hr1G067660	11.01393	13.98091	10.39915	7.368863	2.619807	3.657885
HORVU6Hr1G064500	6.730076	6.489754	8.251647	1.697486	0.348834	0.850423
HORVU7Hr1G117000	53.85045	54.89928	57.39901	56.67747	138.5779	88.20312
HORVU3Hr1G099180	0.321133	0	0.283352	1.709356	2.2001	2.290472
HORVU4Hr1G005990	5.031626	4.950362	5.310149	7.65565	6.632497	8.134223
HORVU4Hr1G015940	1.35952	2.505535	2.156461	3.442415	4.095978	3.907944
HORVU5Hr1G051390	8.457981	8.950211	11.88914	9.693265	10.92072	10.49798
HORVU3Hr1G083790	1.951467	2.232646	1.768739	2.547806	2.012916	2.542008
HORVU1Hr1G067080	0.556659	1.207125	0.675373	5.42963	6.119613	7.307349
HORVU5Hr1G096370	17.5315	12.72363	19.22134	43.76212	45.49684	49.1303
HORVU5Hr1G089430	10.65972	19.66118	5.294996	11.70004	13.86048	14.91603
HORVU6Hr1G010580	0	0	0	12.02122	10.58516	13.29604

HORVU4Hr1G066530	7.557132	8.381427	10.04024	11.43685	13.59567	13.41721
HORVU1Hr1G048630	6.585423	4.038223	6.238941	8.226344	4.601192	5.767894
HORVU4Hr1G068460	38.93617	36.8745	44.1967	42.03908	53.61975	52.19344
HORVU3Hr1G108690	1.456429	1.045478	1.551134	1.518724	3.044941	2.563666
HORVU2Hr1G109980	22.54317	21.92498	26.71667	24.01647	17.96673	21.39648
HORVU3Hr1G046900	1.699028	1.659966	1.511257	0.511206	0.450907	0.403882
HORVU6Hr1G088610	38.74151	38.11782	37.36266	148.4778	76.14043	79.03402
HORVU4Hr1G086790	45.87412	41.53781	66.13999	43.49123	86.95229	87.87577
HORVU2Hr1G107580	9.788957	10.84309	11.70572	9.128538	12.59588	12.19362
HORVU4Hr1G014910	67.63658	60.93011	69.69861	83.01088	67.53238	67.37268
Hordeum_vulgare_newGene_12839	1.022059	1.21315	1.394019	1.889245	2.288597	2.519641
Hordeum_vulgare_newGene_12838	2.148647	2.169374	3.048275	3.577847	4.263223	3.614422
HORVU4Hr1G018660	17.2229	18.60374	22.08655	13.672	14.32626	19.72424
Hordeum_vulgare_newGene_12835	0.871409	1.63265	1.163142	0.867651	0.829158	0.745125
HORVU5Hr1G114700	4.42976	2.951235	4.058901	1.49462	0.450139	0.958022
HORVU7Hr1G056990	0.946613	1.313017	1.120259	4.687842	4.580764	5.635841
HORVU5Hr1G124630	7.844747	6.312499	9.134648	7.973902	7.86233	8.149267
HORVU6Hr1G011180	47.26406	39.60695	46.09152	24.79819	21.54385	23.11048
HORVU3Hr1G072950	2.543314	2.339598	2.540955	3.714315	3.502498	4.931459
HORVU6Hr1G019780	2.604559	1.789501	2.627344	2.536554	3.162532	3.73114
HORVU7Hr1G085530	0.979144	0.88337	1.369533	1.636716	1.680501	1.139419
HORVU7Hr1G047080	4.775162	5.486548	6.931577	3.145721	2.901614	3.46806
HORVU1Hr1G010780	1.145101	1.24874	1.487891	3.127759	2.952657	3.74935
HORVU6Hr1G004260	21.03625	19.63393	25.81148	15.50892	16.94393	16.96753
HORVU1Hr1G047180	7.110834	5.542328	6.647735	8.559603	12.68246	11.50451
HORVU6Hr1G055260	45.6496	42.76957	47.30146	35.35746	40.62899	40.48675
HORVU0Hr1G014580	3.758183	1.937881	2.187568	0.800744	0.729509	0.906715
HORVU1Hr1G016200	15.9609	13.33025	16.74826	29.1296	68.55325	57.62574
HORVU6Hr1G010840	0	0.226152	0.133861	2.867751	4.065728	4.795131
HORVU0Hr1G010000	8.303253	8.828725	8.588479	7.852015	6.839837	6.547837
HORVU6Hr1G053140	1.183576	1.324885	0.593172	0.259532	4.001472	1.614348
HORVU1Hr1G094480	4.185993	3.730737	4.987826	8.86721	12.79373	13.19084
HORVU3Hr1G020520	575.5464	521.372	665.1488	527.0465	895.1108	629.5592
HORVU2Hr1G110810	11.48078	11.18116	12.54702	21.83006	36.68698	28.62345
HORVU5Hr1G110490	0.985661	1.318872	1.248763	0.959953	1.089924	1.116528
HORVU2Hr1G117840	2.647257	3.076869	2.885792	2.368702	2.124514	2.488553
HORVU7Hr1G040850	2.843333	1.474374	2.908954	0.052347	0.129696	0.363842
HORVU4Hr1G054890	2.357173	2.523276	2.611366	3.500556	4.296901	4.036497
Hordeum_vulgare_newGene_10860	1.335117	1.083753	1.547397	1.164591	8.102474	4.592182
HORVU0Hr1G021810	11.58454	10.97063	10.44422	9.427693	7.08738	7.418791
HORVU2Hr1G113540	0.833631	1.007864	1.158726	1.557765	2.913732	3.155249
HORVU5Hr1G090020	11.72084	10.11192	12.41532	12.82593	14.25407	12.89023
HORVU5Hr1G120060	4.869927	5.029967	5.164049	4.7336	5.567335	6.083511
HORVU1Hr1G030870	7.154061	5.757	5.750481	4.087869	4.38969	4.162262
Hordeum_vulgare_newGene_5439	7.819471	6.801123	10.06018	7.364034	7.484575	9.015888
HORVU3Hr1G059130	118.3933	120.0009	72.04631	19.7473	10.61832	11.3647
Hordeum_vulgare_newGene_5430	1.085045	1.018728	1.084813	1.032535	0.965308	1.161117

HORVU3Hr1G079040	6.492341	6.456905	5.814981	13.36381	15.81079	12.63883
HORVU2Hr1G014150	4.879066	4.228975	4.934999	12.48417	8.176975	10.52476
HORVU3Hr1G065390	6.907574	4.97737	7.155159	1.724975	1.258426	2.072285
HORVU6Hr1G055840	36.67677	42.66049	37.28063	58.71403	95.46833	90.85624
HORVU5Hr1G092880	2.556755	2.387194	3.208283	2.052819	2.286785	2.217866
HORVU2Hr1G076510	17.68874	14.97839	16.36412	31.16715	28.04367	27.71291
HORVU1Hr1G045760	3.097427	2.521182	3.484697	9.432299	14.89812	15.92469
HORVU2Hr1G019190	3.682148	3.681728	5.609569	8.096236	10.14686	10.46411
Hordeum_vulgare_newGene_8602	2.658251	1.623418	1.876839	1.775206	0.92441	1.027659
HORVU6Hr1G040920	9.537571	12.79965	13.23476	12.06011	10.89641	13.42654
Hordeum_vulgare_newGene_5382	6.949258	0.065637	4.654645	0.095882	0.168424	3.994851
HORVU4Hr1G064020	38.78972	43.31841	73.84733	30.04634	48.63066	52.27616
HORVU2Hr1G109470	2.194959	1.839621	4.809582	1.992756	1.205476	2.796152
HORVU2Hr1G063340	27.13439	27.20539	27.7548	28.31066	30.30212	31.71828
HORVU5Hr1G093590	10.26545	10.13802	10.09422	9.086619	12.46935	12.2861
HORVU0Hr1G023200	5.272079	5.59913	5.54381	4.556651	3.012295	4.313205
HORVU5Hr1G086630	20.26195	24.65057	37.83537	27.45668	58.2281	40.63385
HORVU7Hr1G028540	10.84316	9.653031	11.12464	25.50946	26.42519	28.33391
HORVU3Hr1G062620	5.655999	6.481085	6.710171	5.659781	4.900672	5.551377
HORVU2Hr1G019730	38.71768	36.25643	30.92647	44.17675	54.65423	52.70223
HORVU2Hr1G104740	7.867281	8.320103	8.809208	0	0	0
HORVU3Hr1G061850	4.146627	3.498074	4.445403	9.93836	11.90595	13.68826
HORVU5Hr1G006830	1.641056	2.08127	2.779405	1.495065	2.060983	2.257023
HORVU4Hr1G078460	15.41492	11.85187	14.58521	9.7895	3.176178	4.409682
HORVU2Hr1G073330	1.989292	1.918169	2.192109	1.275315	1.474999	1.278728
HORVU6Hr1G076490	6.867299	6.617813	8.439224	5.8988	5.625455	6.405452
HORVU2Hr1G090260	3.514075	3.753853	3.994357	5.650455	11.08601	10.41084
HORVU5Hr1G015710	66.96787	66.56706	52.42849	51.67279	265.9382	236.0287
HORVU6Hr1G091860	23.4708	33.49815	45.32947	12.37756	14.67691	19.38173
HORVU5Hr1G052770	3.791013	3.407075	4.771419	3.715497	4.047202	4.066879
HORVU2Hr1G066510	23.60181	26.24323	27.41658	32.59518	39.66652	36.6803
HORVU5Hr1G118800	16.43777	10.5887	20.89254	0.036485	0	0
HORVU1Hr1G059350	31.07402	26.80245	32.39977	20.99899	19.15315	17.99741
HORVU5Hr1G073960	20.28678	23.03342	31.53471	10.37528	19.11854	15.70962
HORVU5Hr1G040400	7.927712	10.12265	11.54391	11.01754	18.1914	16.22097
HORVU7Hr1G001040	4.955409	6.126987	7.055671	8.255997	5.493094	8.507952
HORVU3Hr1G057200	4.820745	4.127385	5.634985	10.31753	11.80473	13.29297
HORVU2Hr1G118290	10.3777	13.29089	12.37238	11.4373	12.80812	14.48301
HORVU3Hr1G026100	4.075827	4.034336	6.037218	3.589672	6.693717	5.470625
HORVU2Hr1G069060	15.39441	13.42585	15.4112	25.77132	26.02028	21.72871
HORVU5Hr1G024060	16.71167	19.11882	20.15277	18.58923	19.95168	19.48857
HORVU1Hr1G034400	4.233438	4.160313	6.540823	8.511061	14.18946	14.72639
HORVU1Hr1G025380	5.949811	5.406686	6.666021	0.495367	0.559782	0.599724
HORVU3Hr1G038500	2.594419	1.700901	2.380672	3.713893	3.902087	3.998356
HORVU3Hr1G088210	6.547481	6.64272	9.320096	10.68237	18.66397	19.61482
HORVU2Hr1G035650	11.43299	10.13371	11.07205	13.29937	18.19195	18.15131
HORVU4Hr1G079330	7.052411	6.690869	7.428334	13.89631	12.65493	12.66643

HORVU3Hr1G056550	3.246301	3.25143	2.388391	3.415965	2.930293	3.529125
Hordeum_vulgare_newGene_582	34.93034	39.20517	42.17421	28.74123	32.45072	34.29271
Hordeum_vulgare_newGene_587	6.768586	5.383276	5.884374	6.74084	6.391916	7.07933
Hordeum_vulgare_newGene_588	4.608609	4.31851	3.990124	3.320565	5.228298	5.225472
HORVU0Hr1G025900	14.68847	11.26176	15.48464	12.31306	14.91758	13.94566
HORVU7Hr1G031180	3.131276	3.162759	3.303105	3.660164	4.855072	4.867615
HORVU2Hr1G097980	5.4989	3.904799	3.969719	1.530034	0.757574	0.767761
HORVU5Hr1G037320	1.829625	2.741718	1.946533	1.608865	1.167612	1.57875
HORVU3Hr1G060480	2.55435	3.320299	3.182035	3.632981	5.713382	5.885992
Hordeum_vulgare_newGene_7129	1.499262	1.099524	1.41669	1.373249	0.857544	0.631143
HORVU2Hr1G071880	88.68207	77.39349	88.09592	56.58406	57.93504	63.59331
HORVU2Hr1G013680	415.2328	456.3735	611.6264	591.8731	945.9983	878.512
Hordeum_vulgare_newGene_7124	17.28618	14.62042	20.89172	17.50459	36.98407	36.49441
Hordeum_vulgare_newGene_7126	15.79947	17.17614	14.50227	27.75641	48.33774	43.42478
Hordeum_vulgare_newGene_7127	3.359598	4.126534	4.001955	3.319532	3.960594	4.351796
HORVU3Hr1G063280	7.182316	5.755388	6.295599	11.94439	13.84277	14.55622
HORVU6Hr1G095090	29.53518	32.61338	26.70843	45.47189	55.39383	58.41938
Hordeum_vulgare_newGene_12310	0	0	0	3.018907	3.483221	3.938774
HORVU2Hr1G020000	4.41407	2.417222	0.232496	0.747683	0.689115	0.988315
HORVU2Hr1G087090	17.07	13.209	15.83204	25.82267	18.6102	21.648
Hordeum_vulgare_newGene_5387	9.824335	6.994343	7.040058	0.016289	0	0.007606
HORVU6Hr1G029350	0.462027	1.864799	1.084616	1.290232	2.42034	2.865616
HORVU4Hr1G058180	5.618955	6.035754	5.671717	7.185562	9.912241	8.555762
Hordeum_vulgare_newGene_6292	1.596443	2.182316	1.665476	1.99367	2.082727	2.16185
Hordeum_vulgare_newGene_6297	2.160904	2.508044	3.137561	3.165606	3.837882	3.734056
HORVU4Hr1G022020	3.285704	2.738188	2.987511	8.893721	11.73003	10.30668
HORVU4Hr1G057200	16.52977	11.01847	16.53535	17.15208	19.13921	20.54255
HORVU5Hr1G075060	14.97041	11.25606	20.71131	16.72363	48.11985	43.16819
HORVU5Hr1G000200	22.21531	24.25258	22.17926	23.95628	15.17177	18.58858
HORVU5Hr1G068820	1.670651	2.25445	2.565921	0.20063	0.185936	0.351238
HORVU7Hr1G035030	10.04868	9.521752	12.67257	11.91433	14.8078	16.08932
HORVU0Hr1G008070	1.378141	1.919797	2.164003	3.230811	3.878994	3.978147
HORVU3Hr1G060730	4.222515	4.228968	5.959082	9.011798	13.22542	10.36643
Hordeum_vulgare_newGene_14410	1.313556	1.346049	1.103378	9.66913	14.34639	12.66033
Hordeum_vulgare_newGene_14412	12.32925	10.66976	11.1978	2.762593	1.857515	1.983353
Hordeum_vulgare_newGene_14415	73.50775	67.63497	77.5886	224.5426	367.8485	351.9346
Hordeum_vulgare_newGene_14417	1.490791	0.972971	1.679915	1.074571	2.105254	2.27067
HORVU2Hr1G116780	6.990345	11.38231	12.08172	8.292994	11.16243	7.27272
HORVU2Hr1G100870	6.934245	5.230612	7.360433	3.379247	2.210229	2.553174
HORVU7Hr1G012560	8.742412	7.095235	10.15918	6.940581	8.247524	8.149616
HORVU1Hr1G071620	10.65326	15.44449	17.04669	13.89521	21.17916	18.46177
HORVU5Hr1G061510	3.140432	3.561621	4.448931	3.787422	3.988742	4.167545
Hordeum_vulgare_newGene_3592	29.28403	21.70687	38.79056	36.44243	23.71961	39.87394
HORVU1Hr1G031860	17.1606	16.81915	14.9319	13.72125	15.76656	17.11467
HORVU7Hr1G008430	0.461109	0.224272	0.483989	1.280774	4.183514	4.227407
HORVU4Hr1G021040	3.493415	2.907161	3.275707	2.719156	2.87658	3.131155
Hordeum_vulgare_newGene_13196	1.919525	1.701901	1.634528	2.106883	2.085384	2.935461

Hordeum_vulgare_newGene_13190	7.178724	8.094691	6.509643	5.426891	4.312109	4.544245
HORVU7Hr1G042370	7.005542	7.757271	8.039457	17.84275	14.78174	20.50113
HORVU2Hr1G024610	8.087589	8.727207	9.752118	9.63365	12.91807	12.74745
HORVU7Hr1G027900	3.583651	1.848419	3.453919	4.045654	3.440488	3.116938
HORVU5Hr1G036740	6.314708	7.886631	6.978182	7.186711	9.103319	10.15846
HORVU2Hr1G026610	6.849942	4.621124	4.025665	5.248106	3.21806	3.755077
HORVU4Hr1G030120	6.968352	8.505764	6.723576	11.21792	10.81208	11.18642
HORVU5Hr1G103730	0.699243	0.388752	0.831899	2.104798	2.666799	2.54932
HORVU6Hr1G083220	10.79118	10.38107	10.9997	9.813814	14.08705	13.54936
HORVU3Hr1G069270	5.062716	4.725991	4.845296	5.602929	5.410702	6.405513
HORVU3Hr1G013790	10.83378	9.927781	13.68178	41.25219	58.37902	46.57647
HORVU5Hr1G078540	13.60611	12.53715	15.81343	19.77896	40.60629	38.92289
HORVU4Hr1G029270	1.173196	1.366736	1.11906	1.253208	2.827828	2.340867
HORVU5Hr1G024440	11.95503	14.30937	14.59777	10.03887	11.29005	12.43037
HORVU4Hr1G075040	2.293341	2.497523	2.209508	1.883083	2.577613	2.291906
HORVU7Hr1G023100	22.87343	20.07312	25.88844	23.26461	32.33793	31.15773
Hordeum_vulgare_newGene_801	9.765029	9.723326	11.1822	9.787951	11.48888	11.27852
HORVU5Hr1G039770	2.50385	3.426941	2.719039	2.69489	2.115619	3.207668
HORVU4Hr1G059250	17.92287	17.01622	15.21933	17.8429	18.88065	18.30419
HORVU7Hr1G059050	24.74629	18.42289	23.49665	23.79535	22.28509	25.54099
HORVU1Hr1G022550	4.125325	4.738808	5.74198	5.449931	4.082167	5.004772
HORVU3Hr1G080820	34.4419	33.72435	35.90604	30.82185	36.60916	37.15062
HORVU2Hr1G072140	4.600533	4.500475	5.992941	4.482736	3.690481	3.804635
HORVU5Hr1G048810	94.12843	90.52959	95.36709	46.65825	42.95949	44.72057
HORVU1Hr1G062340	1.778252	1.852942	1.52989	1.425815	1.630968	1.941205
HORVU2Hr1G085650	5.212896	4.411083	5.554277	7.787611	2.739877	5.025884
Hordeum_vulgare_newGene_65	3.922837	4.531597	4.681975	4.175849	3.640741	4.927362
HORVU5Hr1G097590	82.87148	76.25773	73.21406	58.41121	52.89063	56.95182
HORVU5Hr1G016910	2.697216	2.814785	8.674971	6.35168	7.163281	9.866366
HORVU0Hr1G007740	1.795312	1.206231	1.727538	1.505531	1.680873	1.393208
HORVU5Hr1G066630	3.655237	3.616471	4.209292	2.280061	2.149703	2.001783
HORVU1Hr1G036500	2.509824	3.285305	4.017899	3.292641	8.802421	7.582823
HORVU1Hr1G062690	5.682382	6.305627	6.781465	9.362362	12.4686	10.62566
HORVU7Hr1G060630	10.34691	10.77681	13.33342	12.00825	14.78764	14.7666
Hordeum_vulgare_newGene_6324	5.357881	4.070465	4.830621	9.94433	11.06784	12.64901
HORVU2Hr1G036680	0.576657	0.426897	0.971732	0.44265	0.803293	3.234133
HORVU6Hr1G082920	15.17974	12.14639	16.32285	21.73934	34.22254	27.68817
HORVU0Hr1G030120	99.35925	90.23641	98.60254	77.96104	109.0862	103.5938
HORVU1Hr1G059760	8.562007	8.178584	8.466645	11.37446	17.23679	18.03827
Hordeum_vulgare_newGene_4330	2.926799	2.171361	3.115697	3.721348	4.20872	4.675703
HORVU7Hr1G036090	7.129256	7.669672	6.867515	9.322407	15.1503	14.73588
Hordeum_vulgare_newGene_4337	9.920441	9.944296	9.508909	2.159815	1.881889	2.07707
HORVU4Hr1G072090	15.50237	15.17772	15.55605	12.36412	14.02562	14.12614
HORVU4Hr1G057290	6.251842	4.833748	6.486194	5.785502	5.549158	5.709469
HORVU5Hr1G063000	18.34664	17.18472	18.50778	21.80186	23.86361	25.27871
HORVU1Hr1G058770	16.8301	16.45113	30.97534	8.863234	11.42574	12.28412
HORVU2Hr1G080450	1.319523	1.793687	1.779145	1.62885	1.519179	1.92765

Hordeum_vulgare_newGene_1672	0.756782	1.182739	1.374293	1.269858	1.711366	2.047321
HORVU1Hr1G049690	21.99768	20.61265	22.71737	16.872	10.74801	11.73434
Hordeum_vulgare_newGene_1670	18.09111	18.11322	12.07884	20.19845	17.30169	21.90336
HORVU2Hr1G080180	11.43434	10.87822	11.47261	10.33682	13.23677	11.35188
HORVU5Hr1G057050	11.19601	11.43155	16.35509	13.08183	25.97494	27.09527
HORVU2Hr1G028920	6.443234	8.307283	10.7782	0.832955	0.733486	0.409141
Hordeum_vulgare_newGene_13243	1.572336	2.178015	1.937746	2.022833	2.366674	2.154594
HORVU2Hr1G079310	2.460214	2.388386	1.488806	3.087352	1.660123	1.952342
HORVU3Hr1G000980	2.916446	2.452072	2.305214	71.29704	116.9874	114.3044
HORVU3Hr1G032060	12.13941	12.12557	12.50737	7.162102	13.18444	11.61013
HORVU6Hr1G069920	28.88733	31.38142	29.70646	41.65354	62.99891	56.95892
HORVU7Hr1G003020	1.395596	0.810445	1.191282	5.594737	98.5127	82.61796
Hordeum_vulgare_newGene_4559	3.950013	2.742616	3.151699	1.587525	1.079071	1.637139
HORVU2Hr1G120150	15.94869	13.40666	14.61481	11.732	8.404522	10.60342
HORVU6Hr1G018810	1.231808	0.923657	1.301237	0.956701	1.625777	1.223944
HORVU4Hr1G041760	1.626443	2.255923	1.580985	1.683406	1.573928	1.854909
HORVU7Hr1G024220	3.676836	2.463736	2.749147	47.95646	92.96347	83.55225
HORVU2Hr1G002700	9.022829	9.367735	11.37649	6.988954	5.424823	7.310328
HORVU7Hr1G098660	1.81145	1.163208	1.925108	3.316335	5.200118	4.381418
HORVU5Hr1G064800	0.444177	0.647318	0.158841	1.676744	12.30572	12.10664
HORVU2Hr1G017680	3.846333	2.169037	0.017041	0.62282	0.920288	0.982088
HORVU4Hr1G015880	1.867488	1.543575	2.039952	2.151937	2.95445	2.854267
HORVU6Hr1G083320	2.661288	3.412549	3.205105	3.647587	3.758519	4.365634
HORVU7Hr1G076810	19.51588	20.16328	18.95501	18.57399	20.95162	18.93923
HORVU5Hr1G120260	53.93745	53.78881	66.12306	55.71396	73.31101	65.61091
HORVU2Hr1G025040	5.315144	7.492505	5.775106	8.104834	8.093941	9.530287
HORVU6Hr1G072910	28.91317	27.33813	39.18292	43.29808	57.17993	58.06974
HORVU7Hr1G055990	7.28109	8.404031	10.81754	9.315489	16.02784	15.69027
HORVU1Hr1G089890	1.620238	1.741849	2.345588	2.246793	1.672715	1.755844
HORVU5Hr1G110960	2.256859	2.768126	3.951005	3.163954	5.073139	5.320815
HORVU1Hr1G080910	3.92361	3.456819	4.170293	5.745534	5.27053	7.551007
HORVU5Hr1G013170	26.10192	22.20293	28.52364	24.03513	30.30156	30.45226
HORVU3Hr1G088970	0.791678	0.979372	1.745061	2.080397	1.759375	2.497736
HORVU3Hr1G003980	11.05639	11.64749	20.04082	3.486207	0.304076	1.582858
HORVU2Hr1G006280	16.2768	17.20725	21.10551	12.14233	12.16267	12.36714
HORVU3Hr1G006200	5.796171	5.438692	6.395029	4.675348	4.985752	5.309855
HORVU4Hr1G050400	10.69443	10.07631	9.835274	12.04531	16.52474	15.925
HORVU2Hr1G124430	5.742073	4.612333	6.574253	4.254519	2.681431	4.14139
HORVU1Hr1G065120	1.309523	1.071933	1.009623	15.26125	28.06861	27.01187
HORVU4Hr1G056800	2.160797	3.460253	3.745702	0	0	0
HORVU1Hr1G049900	9.475657	7.832117	10.50441	11.58164	9.203321	9.010357
HORVU5Hr1G041840	7.090064	8.138686	10.26982	14.88588	20.22582	19.26234
HORVU1Hr1G010500	1.171158	1.465475	2.338185	1.510732	0.331626	0.817366
HORVU6Hr1G034490	2.007612	1.598251	1.975685	2.81561	2.588531	3.365033
HORVU6Hr1G080430	22.38162	20.15862	22.2919	10.91594	11.35089	11.59651
HORVU1Hr1G050830	2.682992	2.137867	2.351979	5.315561	5.125174	5.230271
HORVU2Hr1G082030	2.717677	1.879059	2.616474	3.166186	8.875863	7.056934

HORVU3Hr1G038170	21.32397	20.80687	19.68461	22.62531	26.96546	27.4488
HORVU1Hr1G088070	0.355774	0.216411	16.61664	44.63674	1.610592	1.469379
HORVU4Hr1G002890	3.566105	3.110906	4.465154	2.001741	3.060933	3.836306
HORVU5Hr1G124210	16.08408	13.59806	13.88835	23.7952	25.57558	27.14121
HORVU5Hr1G123240	1.125481	1.404062	1.238764	2.027832	2.635517	2.872033
HORVU4Hr1G060810	33.33248	24.32481	32.7431	16.40055	3.231439	8.914581
HORVU5Hr1G007320	11.56717	12.23843	10.89135	11.82968	20.31173	20.31599
HORVU1Hr1G050580	10.3685	12.68277	9.861383	12.10195	16.84762	16.31875
HORVU1Hr1G081790	15.64639	13.538	20.38164	9.218761	10.38211	9.996868
Hordeum_vulgare_newGene_15165	1.200958	0.90458	1.267891	1.195172	1.233094	1.357841
HORVU3Hr1G042830	3.737722	3.521029	1.738548	0.155367	4.418104	3.8267
HORVU5Hr1G038090	10.14755	9.550785	11.50185	12.89348	26.75166	25.00806
HORVU3Hr1G029670	0.657926	0.722	0.867509	1.533303	1.894882	1.995625
HORVU5Hr1G112610	1622.1	1576.681	1119.564	29.31056	2.368716	11.71979
HORVU2Hr1G113940	11.27665	8.374557	8.189304	3.127137	3.113001	3.250878
HORVU7Hr1G045250	8.304481	6.011731	11.15712	9.005677	6.988019	11.56786
HORVU5Hr1G073100	10.87874	9.19302	13.62688	13.32344	21.8452	19.5181
HORVU4Hr1G023570	30.58814	34.01476	51.00973	22.48271	47.78768	49.13543
HORVU6Hr1G034720	15.68763	14.23146	18.2092	16.89904	19.71556	20.49902
HORVU5Hr1G076710	14.23055	10.46953	12.55879	16.67927	21.27674	17.99558
HORVU2Hr1G050140	27.1309	26.51893	32.05305	51.03114	78.43292	77.05636
HORVU3Hr1G082320	0.12699	0.030088	0.182854	7.921983	9.630032	10.95048
HORVU0Hr1G013950	36.45239	35.99778	38.53983	22.19105	14.86272	14.5356
HORVU6Hr1G038800	2.574563	3.419863	2.986228	5.689383	4.582424	4.715955
HORVU3Hr1G008310	5.460108	11.82848	2.774246	9.278213	4.881446	7.978992
HORVU4Hr1G002230	16.96097	14.73016	19.23619	10.6071	9.130286	9.147785
HORVU5Hr1G071720	66.1288	56.08249	72.4448	55.351	60.32488	59.3871
HORVU3Hr1G022590	2.178621	3.367845	2.521312	2.160457	1.848058	2.781221
HORVU7Hr1G121690	0.925	0.83828	1.39983	1.510545	0.828209	1.387784
HORVU7Hr1G040550	89.83586	72.9202	102.881	139.9946	220.5233	192.7229
HORVU2Hr1G047450	1.057079	1.278714	1.262137	2.091298	1.932117	2.219347
Hordeum_vulgare_newGene_12509	21.69516	36.71482	41.07792	13.96113	9.995319	10.57383
HORVU7Hr1G058120	4.031395	3.159379	4.156999	24.97492	62.3252	52.76762
HORVU5Hr1G011720	1.546842	2.181145	2.26113	2.151483	2.477588	2.63617
HORVU1Hr1G002960	3.360975	14.67508	3.730327	17.89711	3.957525	3.565965
HORVU7Hr1G029630	0.500747	0.274584	0.394117	0.548167	2.993429	1.898103
HORVU3Hr1G028440	2.330596	2.474683	3.165791	7.835629	3.694204	3.410829
HORVU1Hr1G045170	2.326914	2.874524	2.880743	2.9907	3.917105	3.785607
HORVU4Hr1G063730	0.108925	0.244653	0.077771	2.289491	7.602043	6.108776
HORVU3Hr1G066890	6.132713	6.218342	6.655444	7.495503	8.49401	9.186256
HORVU2Hr1G009840	8.325288	11.75365	8.704803	11.21373	15.12148	16.49918
HORVU2Hr1G126120	4.544534	4.046413	4.081579	6.064957	5.690134	4.750247
HORVU0Hr1G016820	5.600106	5.915637	5.306874	10.51169	15.23307	14.43404
HORVU1Hr1G002340	7.821114	23.64087	10.19754	26.48964	7.681408	10.00859
HORVU6Hr1G018370	46.3812	40.76438	52.61573	59.25414	76.56105	67.97536
HORVU5Hr1G070450	3.726374	3.985988	4.588442	3.388911	4.283721	4.069359
HORVU5Hr1G054060	21.98153	18.80806	24.96714	19.89717	20.98774	22.01618

HORVU7Hr1G120560	2.893716	3.269382	3.877878	1.533718	2.637871	1.586048
HORVU0Hr1G013390	0.839745	0.297871	3.036923	0.484124	0.501192	3.709242
HORVU1Hr1G068960	1.592684	1.556362	1.341779	2.50863	1.123164	1.802292
HORVU3Hr1G050350	3.744587	4.674501	4.501962	0	0.004411	0
HORVU5Hr1G081370	19.26113	16.91467	20.98536	19.19745	20.15223	21.18686
HORVU6Hr1G027220	6.429374	7.20122	7.267121	6.548491	5.910255	8.361129
HORVU4Hr1G008990	131.295	154.5509	207.8156	20.40459	2.783072	8.908362
HORVU5Hr1G010890	3.934542	3.572852	4.607643	4.575196	5.498051	7.039098
HORVU7Hr1G057430	35.43704	30.69964	36.96142	40.74958	56.07265	53.99599
HORVU3Hr1G106140	21.1931	11.94953	24.78245	13.80498	5.265249	12.67856
HORVU1Hr1G094070	5.597871	4.337138	6.070836	5.909184	5.01152	5.349624
HORVU7Hr1G095370	2.073017	2.489176	2.382934	9.260375	21.80429	14.83446
HORVU7Hr1G088730	1.023579	1.216874	1.910283	2.972436	4.915903	5.029275
HORVU6Hr1G016460	30.00772	25.31845	31.20002	29.9142	37.11984	33.0186
HORVU0Hr1G014460	8.691965	7.84467	7.945	19.84471	30.58712	29.01891
HORVU4Hr1G026050	14.49415	14.10399	15.98389	15.19379	16.70882	19.68219
HORVU5Hr1G019940	9.525047	10.70413	10.53998	10.24433	9.060088	8.97412
HORVU6Hr1G023860	1.781971	1.56795	1.412165	1.237707	0.866451	1.684595
HORVU5Hr1G000770	3.25265	3.023382	3.288506	2.823186	3.431311	3.69304
HORVU7Hr1G047120	2.60616	2.338465	2.911337	4.758892	6.429604	6.549645
HORVU5Hr1G053430	10.68205	11.74135	10.10861	10.43766	12.24438	11.9594
HORVU0Hr1G019870	9.339776	11.10577	5.839975	13.02319	32.23468	26.40609
HORVU2Hr1G103670	18.06747	15.09346	19.28753	13.95569	16.37916	14.86121
Hordeum_vulgare_newGene_13356	1.951666	0.862123	2.262595	0.794194	0.161922	1.440565
HORVU1Hr1G070110	0.61125	0.537111	0.935476	3.23028	12.1236	12.42701
Hordeum_vulgare_newGene_13359	10.57882	12.22948	11.2285	0.02175	0	0
HORVU7Hr1G003170	135.1519	192.1732	293.4129	4.537118	0.039438	1.349316
HORVU2Hr1G116230	10.70661	11.63909	10.74156	5.030863	0.208553	9.144663
HORVU4Hr1G005690	46.3115	59.61016	113.4487	4.271666	0.045509	2.175796
HORVU2Hr1G100090	3.884811	3.428246	3.433207	4.449554	3.911221	5.003166
HORVU2Hr1G037590	2.743521	2.72138	2.809745	3.632734	4.718275	5.427508
Hordeum_vulgare_newGene_15210	13.86653	1.644034	1.986631	0	0	0.022029
Hordeum_vulgare_newGene_15211	1.755084	1.565304	1.639084	1.393739	1.655066	2.046129
Hordeum_vulgare_newGene_15215	0.534629	0.445547	0.448008	3.492748	4.746292	4.170583
Hordeum_vulgare_newGene_15216	101.27	73.0454	117.8236	218.464	147.5233	130.5493
HORVU6Hr1G082680	2.74674	2.275939	1.847752	1.868509	0.556454	0.80023
HORVU2Hr1G099550	3.209678	2.791477	3.784764	11.03468	17.41391	14.99175
HORVU3Hr1G045700	5.624755	4.729995	6.607406	6.322516	11.02971	10.72306
HORVU2Hr1G036460	2.015045	1.042433	1.531397	3.032256	2.231613	2.117422
Hordeum_vulgare_newGene_2759	9.249593	9.410504	10.57212	7.455215	6.762271	7.004186
Hordeum_vulgare_newGene_2753	3.642715	3.594524	2.91548	3.755381	4.214926	3.53644
HORVU5Hr1G014300	2.401417	2.410081	3.834778	8.274108	12.32846	12.46186
HORVU5Hr1G116500	3.05721	2.66563	2.687663	1.19435	1.230103	1.038945
HORVU4Hr1G069260	9.281276	7.982091	6.072627	3.379425	3.826924	2.561285
HORVU1Hr1G030930	0.734785	0.809809	1.083027	1.093885	1.487372	1.92617
HORVU4Hr1G075200	76.30929	62.34088	58.23278	139.1827	255.0877	257.8198
HORVU5Hr1G073370	0.409707	0.35625	0.115748	9.139457	7.254446	7.776752

HORVU5Hr1G053300	6.339104	6.899144	8.328743	12.2617	15.937	14.39145
HORVU5Hr1G092920	14.62196	11.54379	18.79926	20.09434	27.84269	34.49125
HORVU6Hr1G013240	16.35949	14.74654	12.30374	7.809751	5.172702	6.486446
HORVU1Hr1G000690	1813.541	2429.969	1666.821	596.7852	453.6355	420.0302
HORVU2Hr1G060710	4.081152	4.424587	5.904032	8.38536	10.51472	10.6499
HORVU2Hr1G038480	31.7095	34.64916	27.05282	37.44365	46.97671	46.75582
HORVU2Hr1G049420	14.69152	16.89596	14.6982	16.94413	15.28607	19.2842
HORVU2Hr1G072780	8.494208	9.193008	12.12651	6.175139	6.497556	8.298019
HORVU3Hr1G073180	320.0765	302.5096	274.2826	364.1392	384.7797	345.9045
HORVU4Hr1G003080	10.02548	8.764735	9.918205	8.486902	6.909461	8.079531
HORVU2Hr1G097010	5.371241	3.672867	3.492699	17.28642	16.56782	15.4965
HORVU3Hr1G062980	27.93902	25.70877	33.07501	18.94944	18.44195	18.29956
Hordeum_vulgare_newGene_5193	0.844787	1.418781	0.920245	3.24697	2.805677	3.664532
Hordeum_vulgare_newGene_5192	0.933461	1.002736	1.339659	3.366197	2.611489	3.205267
HORVU5Hr1G098440	2.529936	2.923632	2.870158	4.076165	2.691071	3.622768
HORVU4Hr1G024360	4.746928	4.495397	5.132826	8.659516	12.26348	13.31281
Hordeum_vulgare_newGene_13867	2.271704	1.858021	2.710716	4.418816	8.425281	7.315955
Hordeum_vulgare_newGene_10194	0	0.023596	0.016412	2.140865	2.294641	2.217341
Hordeum_vulgare_newGene_10195	0.894336	1.210516	1.055977	0.684429	1.191226	1.425682
Hordeum_vulgare_newGene_10193	1.931664	2.488862	4.910295	3.79446	0.119327	2.317085
HORVU7Hr1G083600	24.14634	22.0023	25.52297	21.29769	18.90595	16.87688
HORVU6Hr1G016390	4.701791	4.540256	7.013304	5.248392	8.810524	8.936028
HORVU6Hr1G072540	2.500415	2.497494	2.833909	2.334739	3.742424	3.965794
Hordeum_vulgare_newGene_8015	41.46693	41.03807	78.08602	58.79309	113.9116	111.7793
HORVU3Hr1G096760	2.519555	2.512446	1.954798	2.698224	6.2226	6.219572
HORVU3Hr1G096580	15.6634	14.13826	22.55032	14.44284	11.25998	12.49122
Hordeum_vulgare_newGene_11249	24.99288	20.30649	25.34296	54.78922	49.00958	50.79344
Hordeum_vulgare_newGene_11248	56.49512	48.18472	54.92614	100.7816	89.06522	84.33908
Hordeum_vulgare_newGene_11247	46.23373	39.76844	46.58991	86.42733	79.12045	74.10033
Hordeum_vulgare_newGene_11243	1.228962	2.226883	1.779106	2.268147	2.68205	3.001936
Hordeum_vulgare_newGene_11240	2.481469	2.405501	2.023129	3.565229	3.500881	3.9659
HORVU2Hr1G011180	1.772894	1.467067	1.77666	1.521101	1.184522	1.12462
Hordeum_vulgare_newGene_14991	0.643763	0.752712	0.993709	3.062039	7.093064	6.55221
Hordeum_vulgare_newGene_14992	2.670277	1.76051	2.553465	10.87427	13.47818	11.10481
Hordeum_vulgare_newGene_14994	0.921487	0.994318	0.777518	3.392005	4.657141	4.193787
HORVU7Hr1G027310	3.945421	4.139734	3.538651	6.713648	5.584266	8.465506
HORVU7Hr1G064020	7.188308	5.64165	7.535408	10.18754	15.98285	16.80132
HORVU2Hr1G026890	1.498119	1.067747	3.824099	0.435984	0.063161	0.2663
HORVU7Hr1G037870	14.47193	15.19777	18.61452	6.107187	6.745636	7.395021
HORVU1Hr1G050040	25.08422	15.72136	24.51126	25.3668	26.52219	27.0167
HORVU4Hr1G078780	6.820721	5.711218	6.704392	7.991236	8.503752	8.946338
Hordeum_vulgare_newGene_1470	1.736255	1.802954	2.066907	2.704719	3.404024	3.752637
Hordeum_vulgare_newGene_1471	2.175422	2.028439	2.209047	4.693123	4.821727	5.518063
Hordeum_vulgare_newGene_1476	2.698878	2.749494	3.043357	3.363424	2.899754	3.35968
Hordeum_vulgare_newGene_3832	7.354725	6.902697	7.809046	10.92952	16.84501	14.29352
Hordeum_vulgare_newGene_3831	6.963692	5.602972	7.959782	16.04085	11.41347	13.72295
HORVU5Hr1G049610	1.676236	2.089405	2.340752	2.711103	3.659617	3.339043

Hordeum_vulgare_newGene_3839	1.282817	1.269972	1.851004	0.725059	1.268503	1.189417
HORVU2Hr1G089820	7.284808	2.803342	5.133377	5.438276	0.677903	10.73819
HORVU3Hr1G011510	9.70334	8.366016	8.548223	8.334189	7.503517	9.01071
HORVU1Hr1G038680	13.59396	14.50037	18.39321	15.70387	29.27959	22.27168
HORVU1Hr1G041720	5.718199	4.079178	5.648403	7.787345	6.252206	8.859659
HORVU7Hr1G082230	5.692658	6.39527	6.17252	7.093312	7.962293	8.463697
HORVU3Hr1G094720	2.393373	1.371381	2.683841	1.405638	0.260822	0.648834
HORVU3Hr1G080040	9.854531	10.29215	10.9955	10.80712	11.23388	12.06431
HORVU7Hr1G080560	11.01739	12.44196	13.87852	9.575485	18.33967	17.71083
HORVU7Hr1G048310	0.714643	1.053358	1.058237	5.372337	7.684109	10.84424
HORVU7Hr1G021250	3.107318	2.75391	3.677193	3.119274	3.269214	3.471809
HORVU6Hr1G073220	10.47524	9.89369	14.38997	11.4151	14.92828	15.39354
Hordeum_vulgare_newGene_12293	6.697901	7.982259	5.838132	9.358995	8.015793	10.18962
Hordeum_vulgare_newGene_12292	1.581083	2.591745	1.846761	0	0	0
HORVU3Hr1G063320	6.36513	7.314295	7.796516	8.348598	10.45732	13.22454
Hordeum_vulgare_newGene_4649	3.227277	4.091518	4.08337	3.371341	3.423003	4.043969
HORVU4Hr1G072520	6.970819	6.936709	7.579611	7.153321	7.026195	6.733178
HORVU3Hr1G056960	1.249126	1.130017	1.533101	1.522806	4.370708	3.533589
Hordeum_vulgare_newGene_4640	2.703517	2.913789	3.119404	0	0	0
HORVU7Hr1G029400	0.248937	0.509532	0.383647	1.515007	2.172375	2.155329
HORVU5Hr1G103080	7.757614	8.902192	11.4346	4.927726	3.885985	4.417473
HORVU3Hr1G091800	1.247673	2.1621	2.570639	0.077198	0.05381	0
HORVU5Hr1G082670	5.583395	4.613963	7.07149	5.046336	7.085657	6.792852
HORVU7Hr1G081880	12.2648	12.54488	15.76004	13.81186	14.97279	16.05975
HORVU1Hr1G030100	4.437384	4.302789	4.892472	3.179895	5.570103	4.273094
Hordeum_vulgare_newGene_6647	0.058587	0	0.030721	1.414241	5.09481	4.051667
Hordeum_vulgare_newGene_264	1.660122	1.587456	1.225385	1.903374	1.791655	1.575547
Hordeum_vulgare_newGene_267	1.549487	1.378698	1.648487	1.456681	1.932611	2.114724
Hordeum_vulgare_newGene_260	7.078349	6.340513	9.163655	5.907487	8.684774	7.924283
HORVU7Hr1G031020	20.14693	19.13981	24.2788	34.28042	29.1035	31.47599
HORVU0Hr1G020940	1.376579	1.166531	1.251976	2.272603	2.861632	3.663646
HORVU3Hr1G081180	0.204481	0.232186	0.077215	6.421646	5.000804	5.769797
Hordeum_vulgare_newGene_3164	2.95904	3.750213	3.764864	3.508298	3.115633	3.887434
Hordeum_vulgare_newGene_3169	13.32918	13.06728	12.3107	16.99369	25.18666	23.58435
HORVU4Hr1G013310	30.70759	29.36803	30.75797	22.26636	24.73855	21.779
Hordeum_vulgare_newGene_12128	0	0	0	30.43407	37.7428	50.24083
HORVU3Hr1G051530	2.173345	1.697894	2.846399	2.37032	3.074754	3.701364
HORVU2Hr1G033820	3.175049	3.153451	4.607506	4.536572	5.224049	6.855507
HORVU6Hr1G070750	17.5886	17.15991	21.72965	14.16073	13.58101	15.46966
HORVU6Hr1G065710	1.107156	1.277603	1.738795	4.146513	1.167916	2.367661
Hordeum_vulgare_newGene_14608	1.919705	1.403655	1.378617	0.931124	1.452867	1.610008
Hordeum_vulgare_newGene_13659	3.020403	3.51003	5.754602	1.498234	0.438404	1.809211
Hordeum_vulgare_newGene_14603	3.193617	1.876053	0	2.870189	4.478495	3.066909
Hordeum_vulgare_newGene_13653	4.186629	4.868481	5.773098	5.184688	9.501813	9.75671
HORVU2Hr1G006910	0.981006	0.750552	0.724807	1.8507	1.811045	1.481908
HORVU5Hr1G068610	2.061044	1.906503	2.25362	2.559657	2.954412	2.167283
HORVU7Hr1G038210	3.95213	3.925705	3.855849	3.753441	3.205475	3.958894

Hordeum_vulgare_newGene_8457	3.454324	2.290591	3.554261	8.50759	8.034477	7.316352
HORVU6Hr1G047970	6.579146	7.908034	9.461662	6.882084	6.45992	8.516966
HORVU0Hr1G000410	19.09343	18.17136	21.33635	17.75425	19.63818	21.82877
HORVU2Hr1G116570	18.93095	17.86192	23.07637	22.51325	29.99858	29.87491
HORVU5Hr1G036460	4.254404	3.162142	4.313801	6.458924	12.23012	12.32886
Hordeum_vulgare_newGene_13603	41.82968	28.76743	37.67584	15.3934	12.66079	13.79806
HORVU5Hr1G023720	96.08589	42.88845	109.7795	10.63692	2.234801	8.393285
HORVU3Hr1G107200	1.868036	1.259924	1.999848	2.333308	1.476319	0.866422
HORVU4Hr1G044040	20.45242	17.22349	18.6331	16.10678	21.39652	22.19634
Hordeum_vulgare_newGene_15559	2.32569	2.778245	2.862964	3.227703	3.451074	4.472023
Hordeum_vulgare_newGene_15550	5.599822	4.993369	5.537846	6.137342	7.009743	7.52756
HORVU5Hr1G069050	3.665096	3.220044	3.371588	5.425047	4.64467	5.495453
HORVU1Hr1G055440	38.17527	34.31407	52.55123	47.96336	130.2411	112.4695
HORVU5Hr1G094940	0.405514	0.712274	0.446876	0.929	3.696493	3.217307
HORVU3Hr1G075050	10.821	9.380716	9.364577	7.452748	8.126732	8.188929
HORVU6Hr1G079360	8.183818	8.686859	8.405767	7.005463	7.536441	7.757298
Hordeum_vulgare_newGene_6959	2.187529	2.114595	2.071997	4.159549	4.260119	4.789738
Hordeum_vulgare_newGene_6956	1.705981	1.500956	1.494772	1.631072	1.647029	1.337937
HORVU6Hr1G071150	1.256283	0.245311	0.800237	1.94077	0.793812	0.968755
HORVU1Hr1G092120	3.511929	3.470301	5.0215	4.81021	7.911548	7.514462
HORVU4Hr1G067510	8.178013	8.639544	8.328959	17.67096	18.9653	16.96055
HORVU6Hr1G033840	17.30329	14.31597	16.14245	14.76835	14.85711	16.18674
HORVU2Hr1G082480	11.51348	11.8393	12.78807	8.807191	4.802582	5.277842
HORVU4Hr1G076770	54.4007	50.5911	65.77759	51.22873	61.07672	58.32011
HORVU1Hr1G081050	3.563255	3.354218	3.623299	3.791493	4.667712	4.414633
HORVU6Hr1G069710	3.829636	4.30652	4.217319	2.980605	0.839358	1.789267
HORVU7Hr1G097550	0.276982	0.4259	0.420704	4.87999	3.532558	4.164138
HORVU5Hr1G022920	1.007241	1.020145	1.323932	1.503814	1.496967	1.198489
HORVU2Hr1G094630	1.913219	1.651026	2.338547	1.795005	0.696565	0.965776
Hordeum_vulgare_newGene_8886	2.698966	2.440371	3.408948	2.806898	2.458636	2.668617
HORVU7Hr1G090780	2.952028	3.111327	3.464128	4.165762	5.510677	4.568412
HORVU6Hr1G011710	0.058378	0.146106	0.100968	1.701105	6.004431	5.191312
HORVU4Hr1G032480	4.938695	5.825845	4.60828	7.486079	8.876583	9.294315
Hordeum_vulgare_newGene_13609	1.338707	1.874633	1.038986	0.899609	1.428003	1.248514
HORVU7Hr1G034840	0.897682	1.534881	1.587365	2.0987	1.373122	1.556876
HORVU1Hr1G067230	8.77815	5.72972	9.472655	6.100691	10.01176	8.800246
HORVU5Hr1G024180	8.362172	9.843941	7.557194	10.01348	9.217844	12.82517
Hordeum_vulgare_newGene_16077	8.692441	9.676928	10.24961	12.46396	12.93167	13.38159
HORVU4Hr1G031280	9.831875	10.53221	10.62672	9.904546	14.5117	14.3002
HORVU1Hr1G018360	3.883134	3.663553	5.874973	0.300896	0.319484	0.415504
HORVU5Hr1G111830	28.94684	23.35875	26.18254	18.13779	9.60815	10.52317
HORVU5Hr1G066280	16.72819	14.50203	15.87163	44.85002	74.94291	77.00538
HORVU5Hr1G020400	3.95678	3.082276	4.293876	7.629589	6.814386	7.745378
HORVU0Hr1G006240	39.5079	43.22524	46.32914	11.7843	17.90968	17.29465
HORVU6Hr1G021630	195.4921	172.4973	178.4981	145.5217	148.2584	159.7975
HORVU6Hr1G015470	2.652891	4.323387	3.496509	3.789441	4.290046	4.081708
HORVU2Hr1G098040	6.190804	4.922097	6.116598	5.106283	4.616164	4.082168

HORVU1Hr1G079440	6.774448	3.931846	9.595674	13.56258	31.47912	27.40319
HORVU5Hr1G091810	6.510197	9.092378	9.955263	13.783	19.00099	18.53758
HORVU0Hr1G012140	4.293159	3.846166	4.337573	6.686938	5.28035	4.676669
HORVU5Hr1G109760	0.669965	0.407771	0.476334	2.787271	3.892386	3.311645
Hordeum_vulgare_newGene_5903	15.33108	15.92309	14.86609	0.02722	0	0
HORVU1Hr1G053090	1.870708	1.320305	1.723096	2.884568	5.24907	5.700034
HORVU7Hr1G113200	22.6389	19.19028	24.02369	33.54087	30.20292	28.7614
HORVU7Hr1G095570	16.54613	17.3477	19.17423	21.27374	19.13802	19.04488
HORVU5Hr1G073670	3.242153	1.950033	3.779716	1.397803	4.697967	5.496473
HORVU3Hr1G031030	9.030241	9.029869	11.722	14.05581	14.47083	14.09068
HORVU2Hr1G127500	6.336646	6.71412	7.793633	9.026831	13.21295	11.67589
HORVU2Hr1G088410	1.374935	1.443113	1.904147	3.210551	2.068427	2.412925
HORVU6Hr1G029910	8.162272	7.250477	9.539581	5.749748	8.202205	6.158456
HORVU4Hr1G046370	6.295476	6.846689	7.923541	10.33486	10.27673	11.71142
HORVU0Hr1G012490	0.999144	0.942952	1.115258	2.140347	3.655897	3.617832
HORVU4Hr1G052900	29.08635	23.71019	39.10124	30.45583	48.22129	48.06969
HORVU1Hr1G021000	6.569331	4.622369	5.690892	5.879708	10.04106	8.72037
HORVU2Hr1G114870	20.05734	13.80921	23.01677	19.25113	28.01239	24.96851
HORVU6Hr1G007030	31.97298	30.40833	33.40413	28.93621	39.61661	39.71094
HORVU1Hr1G091570	3.715348	4.514654	4.276089	8.024702	8.391884	8.93129
HORVU5Hr1G011960	1.077773	0.692237	1.473099	2.034672	3.608	3.750669
HORVU1Hr1G047570	10.93077	5.491884	10.87089	6.84983	3.535832	4.776766
HORVU4Hr1G006730	2.836462	2.370254	2.358589	8.72467	14.63605	13.94579
HORVU7Hr1G039970	1.743234	1.612891	2.339508	5.173003	2.703811	2.285979
HORVU5Hr1G026120	13.93191	11.48794	15.70049	13.70712	16.99195	18.72089
HORVU7Hr1G067520	8.322541	7.884843	9.032162	17.87206	23.52747	23.47997
HORVU5Hr1G057790	6.972673	8.60329	7.207113	9.621122	5.89827	6.283548
HORVU1Hr1G057950	24.35158	21.31299	33.39142	20.27393	26.56097	24.82736
HORVU5Hr1G106810	7.170668	7.509233	7.629406	9.111213	10.75609	11.41603
HORVU2Hr1G099710	4.966713	3.319404	5.017246	3.8049	5.78866	11.24254
HORVU6Hr1G091020	1.783742	1.491689	1.690929	0.902838	2.226186	1.729638
HORVU4Hr1G001790	43.13845	30.63791	44.19687	38.08855	41.1753	37.83731
HORVU3Hr1G078560	9.248694	9.432273	12.5448	14.26139	31.12528	21.77967
HORVU2Hr1G094390	1.761694	1.391686	1.331144	4.863991	19.9708	22.58166
HORVU2Hr1G015430	1.008708	1.513902	1.208306	4.765612	8.224181	8.28781
HORVU3Hr1G074230	61.00124	58.22736	65.46421	34.00861	54.16746	41.89048
HORVU2Hr1G002930	1.6952	1.49006	1.385132	2.197321	3.670342	3.632616
HORVU5Hr1G027290	2.615417	3.75536	3.653872	2.270095	2.103277	2.088566
HORVU2Hr1G017490	4.571402	5.368838	5.19136	5.640512	7.106353	6.695863
HORVU7Hr1G121430	1.56297	1.642999	1.943547	1.673274	3.263449	3.141876
HORVU1Hr1G012800	17.99732	13.67196	22.37491	11.12721	152.2317	123.8402
HORVU5Hr1G095480	26.45398	21.27991	23.76611	43.82076	41.97836	43.16514
Hordeum_vulgare_newGene_15547	1.228379	0.55244	0.610022	2.066049	2.571271	1.750077
HORVU1Hr1G069540	9.385867	7.765041	13.72247	1.944587	0.303565	0.411111
HORVU5Hr1G081750	2.18082	3.371676	2.602155	6.443424	6.595991	6.915284
HORVU7Hr1G043930	0	0	0.039814	2.084017	2.812105	3.735776
HORVU5Hr1G094570	0.896561	1.060045	0.61406	2.84749	1.100965	1.34828

HORVU1Hr1G004430	4.042261	3.55629	4.344001	8.163449	10.11975	11.53421
HORVU3Hr1G114170	10.69908	9.476069	10.88652	9.558486	11.67893	11.8746
HORVU3Hr1G033520	26.31102	21.23286	28.37694	33.35447	45.75738	43.51483
HORVU5Hr1G017390	2.845515	3.125471	2.966073	4.583696	4.928507	5.366236
HORVU0Hr1G035820	8.776851	3.621023	7.022645	4.00923	5.187477	4.48415
Hordeum_vulgare_newGene_11000	0	0	0	8.193421	13.53406	9.741772
Hordeum_vulgare_newGene_11002	0.825128	0.987969	1.164581	1.125576	2.415352	2.144574
HORVU4Hr1G084810	4.416961	3.733967	5.199227	5.266284	4.095208	6.34249
HORVU4Hr1G006740	25.85487	22.48381	22.95089	17.67849	22.17303	20.78893
HORVU3Hr1G092600	32.01139	40.43512	39.9173	29.0317	34.18005	37.12789
HORVU6Hr1G057090	4.370752	4.24722	4.364576	6.366701	8.620485	8.327454
HORVU5Hr1G123160	35.92576	30.05638	38.89945	37.95608	39.31485	37.98977
HORVU2Hr1G123610	2.922582	3.333889	3.033986	2.895483	1.517493	1.058131
HORVU2Hr1G090380	1.163147	0.757556	0.934698	2.88911	4.900183	4.520856
HORVU6Hr1G094750	2.226185	2.545727	0.085923	4.062352	4.477684	2.773422
HORVU3Hr1G028710	0.368967	0.270902	0.33698	1.928387	3.933495	2.508031
HORVU3Hr1G099920	8.13008	7.932807	11.06427	7.006753	6.842961	6.851811
HORVU2Hr1G118740	14.3492	15.65071	15.01313	20.42118	31.69618	28.14841
HORVU2Hr1G051560	26.96957	26.22709	26.8909	26.91687	17.37678	19.582
HORVU0Hr1G011430	1.285401	1.123552	0.961588	1.654958	1.083488	1.066215
HORVU1Hr1G061160	104.457	80.21295	106.0524	394.6653	429.285	432.2009
HORVU2Hr1G011430	0	0.011575	0	1.015278	25.04524	18.73196
HORVU2Hr1G107990	10.2789	8.168314	11.97675	13.06131	6.946093	9.37833
HORVU4Hr1G023300	1.839995	2.135824	1.893363	4.992963	7.531407	6.78416
HORVU5Hr1G076100	175.4764	170.9446	148.6102	94.49318	76.45744	78.86345
HORVU6Hr1G090270	4.93704	4.300534	5.600282	2.536699	2.755245	2.933669
HORVU3Hr1G037310	17.51814	16.13008	22.20096	17.64125	19.10946	20.49615
HORVU2Hr1G040730	4.043958	4.403282	3.353162	5.912108	7.723688	8.029098
HORVU3Hr1G045680	7.139931	7.890183	10.41266	9.181406	14.50393	14.0558
HORVU7Hr1G047590	29.68391	28.54295	33.04509	25.5153	28.8665	27.38945
HORVU1Hr1G005790	10.09176	7.522238	9.225273	15.51299	20.36019	20.98134
HORVU3Hr1G026970	0.572589	0.91248	0.529114	4.832025	4.491106	3.593969
HORVU5Hr1G117830	5.773413	4.425991	6.478487	9.571929	13.97564	15.42257
HORVU7Hr1G045420	13.41939	11.96935	18.1851	14.15174	24.17824	23.81352
HORVU5Hr1G049180	0.309477	0.496988	0.36736	1.390036	4.6619	5.834771
HORVU4Hr1G085930	2.819199	3.400559	4.751999	6.058056	8.111028	8.102478
HORVU5Hr1G098840	14.36523	15.10835	12.15689	22.8422	33.20161	31.97518
Hordeum_vulgare_newGene_14416	5.326088	6.400408	6.648193	11.41352	12.70069	11.11725
HORVU5Hr1G106250	2.307793	2.403733	2.205995	0.579703	0.38172	0.212864
HORVU5Hr1G005910	335.9542	350.6852	483.3883	44.63364	2.972243	17.74543
HORVU4Hr1G053650	13.60481	14.10515	12.60673	25.05631	30.95411	32.41872
HORVU5Hr1G047060	2.241721	2.327742	2.518357	1.719706	1.325182	1.710497
HORVU5Hr1G109400	2.133326	1.794277	2.124749	10.01447	16.89773	13.28773
HORVU2Hr1G068360	49.03046	48.22966	68.3682	47.45401	71.9409	70.87986
HORVU3Hr1G088570	29.52636	26.91795	34.90701	44.64093	67.15531	66.97733
HORVU5Hr1G119470	12.03759	12.81685	12.70309	11.81847	7.874046	8.828546
HORVU5Hr1G011570	4.27198	3.462416	4.885336	5.915063	8.589243	7.757464

HORVU7Hr1G050750	0.965552	1.109183	1.336665	2.060105	1.662816	1.230137
HORVU3Hr1G027590	19.1138	19.10904	17.77695	35.02177	22.57489	26.78294
HORVU5Hr1G050970	29.78105	25.03841	27.00643	20.47079	16.92562	15.29355
HORVU4Hr1G083190	39.90994	38.76802	38.77282	24.12191	13.19464	17.56333
HORVU4Hr1G054560	3.626064	3.319782	3.131749	4.225829	4.11043	4.772119
HORVU5Hr1G001320	4.919019	5.037559	5.760261	3.154693	4.303627	3.966844
HORVU2Hr1G102860	13.26774	12.99764	16.32757	10.28546	12.34973	12.92382
HORVU2Hr1G065670	1.046031	1.05551	0.949391	1.712481	1.763419	2.549633
Hordeum_vulgare_newGene_3367	0	0	0.01159	3.887135	3.578394	4.248619
HORVU7Hr1G090750	5.367279	4.437555	3.954128	5.365975	7.252923	7.402425
HORVU1Hr1G059950	25.59525	46.47951	70.51832	3.362435	0.1365	1.628774
HORVU6Hr1G089980	1.271254	1.246803	1.402042	14.36138	15.27058	26.12734
HORVU4Hr1G087570	1.111651	1.343793	1.187624	1.68018	1.88312	1.945059
HORVU2Hr1G112800	5.132966	5.421522	5.875247	0.049196	0.015978	0.00913
HORVU5Hr1G099070	14.02612	11.12549	12.9204	10.25003	8.171138	9.68881
HORVU5Hr1G040920	4.562385	6.833519	8.1099	5.273713	16.09217	15.07265
HORVU0Hr1G023300	2.030124	1.728896	0.942537	1.662606	2.292344	1.901029
Hordeum_vulgare_newGene_13091	1.25697	1.790193	1.596397	2.235148	2.315687	2.866849
Hordeum_vulgare_newGene_13097	2.382461	2.350926	3.027164	1.415571	2.494004	2.454702
HORVU2Hr1G023070	3.528288	3.283955	4.343388	5.059188	7.229948	6.524787
HORVU5Hr1G000630	12.86073	12.62054	18.81477	4.559062	2.673645	3.07066
HORVU1Hr1G071470	7.269479	6.787949	6.901186	7.866736	9.44575	8.845557
HORVU5Hr1G053580	5.787949	5.065415	7.548928	7.082459	12.002	10.66021
HORVU5Hr1G041030	3.47318	3.557234	4.883887	2.498194	3.225896	3.38155
HORVU3Hr1G045890	14.36563	15.76383	16.38033	8.740323	12.73732	12.67385
HORVU5Hr1G039660	1.455469	1.99717	1.655388	2.037022	3.080314	2.718641
Hordeum_vulgare_newGene_14375	0	0.048636	0	0.902074	56.05418	40.26339
Hordeum_vulgare_newGene_14377	0	0	0	2.725329	66.51862	56.87565
Hordeum_vulgare_newGene_14379	0	0	0.014549	7.050797	0.862811	2.008109
HORVU1Hr1G070700	0.164559	0.13816	0.315246	1.25803	6.67548	5.534571
HORVU3Hr1G044660	1.334117	1.06871	1.124695	10.60917	18.90555	12.92493
HORVU3Hr1G048560	31.02357	25.48559	35.79203	39.649	44.47852	46.13824
Hordeum_vulgare_newGene_2315	7.903759	8.557666	7.75926	8.374712	9.843801	9.759434
HORVU6Hr1G012010	40.19699	34.44999	45.7365	39.38155	50.21479	50.56065
HORVU2Hr1G100420	2.596972	1.299729	1.514495	0.416003	1.376299	0.376225
HORVU5Hr1G120880	4.728002	3.80427	5.070119	4.627035	6.643229	6.451784
HORVU5Hr1G075760	59.01094	51.15798	56.02058	154.5882	96.17941	100.7333
HORVU6Hr1G089600	7.696909	6.063751	9.014116	7.503984	5.905518	8.401686
HORVU4Hr1G082710	5.155826	5.698064	5.747552	22.78288	14.16751	16.22323
HORVU1Hr1G003060	0.154699	8.364144	0.591601	12.37419	1.996732	1.344044
HORVU1Hr1G051510	3.148191	3.555575	3.546195	2.9509	2.391088	4.569635
HORVU2Hr1G117970	2.450674	2.183298	2.355654	1.656748	2.661002	3.156601
HORVU2Hr1G096240	3.226595	3.26552	2.8766	5.19665	5.499909	5.231728
HORVU5Hr1G080160	2.628475	2.367458	3.230483	3.822457	5.201467	5.057176
Hordeum_vulgare_newGene_2549	3.974861	4.914382	4.884195	3.707888	6.070229	6.056857
Hordeum_vulgare_newGene_2548	15.18227	14.90992	17.03193	16.79627	20.99398	21.51622
HORVU2Hr1G101770	5.768609	7.084187	7.669153	4.075368	3.968959	3.789547

Hordeum_vulgare_newGene_2541	1.275661	1.37556	1.524538	2.117893	2.911788	2.743614
HORVU0Hr1G000200	37.19136	34.36042	51.60087	45.79056	57.72078	58.50598
Hordeum_vulgare_newGene_2543	1.763588	1.828517	2.048517	1.226279	0.261625	0.742182
HORVU3Hr1G088110	2.895973	2.616542	2.980347	2.429972	2.689264	2.682804
HORVU3Hr1G034440	50.42584	43.39389	51.68556	51.72653	62.7496	61.39515
HORVU7Hr1G029190	0.01679	0.092904	0.143151	1.010867	3.846823	3.93753
HORVU6Hr1G019670	26.44879	24.44709	22.06327	20.74515	17.4512	17.65931
HORVU7Hr1G082450	2.970799	4.551755	4.553637	2.354007	0.697869	1.08428
HORVU2Hr1G054270	13.61388	11.76729	15.92762	15.95222	20.48357	20.4807
Hordeum_vulgare_newGene_15023	0.33777	0.6551	0.856531	1.464328	1.442555	1.379028
Hordeum_vulgare_newGene_15022	0.915263	0.954233	0.953313	1.817615	4.891799	5.438525
Hordeum_vulgare_newGene_10013	22.40491	20.80295	24.84048	21.03828	27.41709	22.06914
HORVU0Hr1G022160	3.556005	3.436006	3.976884	4.797225	6.390106	5.713437
HORVU5Hr1G077990	2.225172	1.76138	2.521709	6.463004	6.865129	6.703674
Hordeum_vulgare_newGene_10018	5.163962	4.267108	3.796444	1.373125	1.925402	1.077117
Hordeum_vulgare_newGene_15029	75.28239	63.14968	82.92366	64.5913	91.98686	80.82289
Hordeum_vulgare_newGene_15028	0	0	0	27.44974	3.977727	10.31064
HORVU4Hr1G053620	21.74182	17.84735	19.79693	19.39006	29.86381	27.08087
HORVU3Hr1G063270	0.312961	0.122096	0.137475	1.702453	6.121157	5.338991
HORVU5Hr1G045580	28.26147	22.84512	26.89714	24.56978	20.53168	22.34061
HORVU6Hr1G061700	1.662216	1.434791	1.31648	1.315585	0.704577	1.058536
HORVU5Hr1G007980	6.876923	7.261477	5.913704	14.74475	17.77888	19.3998
HORVU2Hr1G026220	77.20081	69.01433	94.92361	64.56284	65.73538	70.41545
HORVU4Hr1G077220	20.09719	18.64943	16.23306	22.22365	22.04543	23.6466
Hordeum_vulgare_newGene_9579	9.508265	9.271415	11.92235	9.866271	11.96937	10.10469
Hordeum_vulgare_newGene_9578	1.536933	1.885782	1.948166	0.17595	0.193558	0.416056
HORVU7Hr1G102060	10.15563	9.648682	10.76397	12.07645	15.9718	13.98364
Hordeum_vulgare_newGene_5019	29.77526	34.02825	32.70215	24.722	19.5815	29.2648
Hordeum_vulgare_newGene_9571	35.8353	39.3142	66.84758	28.2346	61.6672	54.05873
Hordeum_vulgare_newGene_5013	1.0724	1.799549	1.847017	0.581642	0.877726	1.107876
Hordeum_vulgare_newGene_9573	34.19305	29.02183	38.28557	28.80367	24.44619	26.55811
Hordeum_vulgare_newGene_5011	11.93874	17.0252	18.23985	13.81922	13.44226	18.84611
Hordeum_vulgare_newGene_9575	10.01052	7.047839	11.01954	9.094121	9.613247	8.485912
Hordeum_vulgare_newGene_9574	8.103931	9.102537	10.23575	9.899609	7.481173	9.262165
HORVU3Hr1G026500	2.610767	3.213497	3.845047	4.353174	3.316155	3.70689
HORVU1Hr1G000080	68.52107	61.70327	81.5343	38.639	75.24495	75.51594
HORVU2Hr1G060900	10.01285	11.80745	12.29022	14.31021	13.53997	16.0404
HORVU4Hr1G079740	0.61722	0.851295	0.737017	1.622243	2.175958	2.221685
Hordeum_vulgare_newGene_9211	5.296796	4.942023	4.857055	4.773149	8.089488	9.694871
HORVU6Hr1G028220	2.515683	2.500519	3.507147	3.239029	3.195809	2.447354
HORVU6Hr1G014500	1.852392	1.843991	2.736946	6.915725	9.771043	9.911214
HORVU1Hr1G001350	921.2834	2114.917	783.4791	3854.832	1121.99	1707.938
HORVU1Hr1G052430	66.23339	45.69501	98.89178	111.1322	56.98396	98.69334
Hordeum_vulgare_newGene_9217	6.474195	5.80449	7.408937	10.28992	9.726065	9.736466
HORVU2Hr1G072590	5.122753	5.675737	6.935464	6.737319	6.559493	8.121509
Hordeum_vulgare_newGene_8190	0.460383	0.311273	1.055608	2.043748	1.726193	1.995947
HORVU5Hr1G087880	8.159143	7.318716	8.962643	5.049861	1.005317	2.644977

HORVU2Hr1G075740	24.63772	19.96283	26.71244	32.00279	37.59734	38.99959
HORVU2Hr1G010100	9.851248	10.67576	13.204	12.71294	16.98536	16.20895
HORVU1Hr1G009100	3.99251	4.838798	4.444234	8.628758	10.5928	10.17148
HORVU3Hr1G011490	1.057476	1.165861	1.36859	2.004094	3.202313	3.181466
HORVU7Hr1G090360	14.25386	18.30025	21.62555	11.66622	13.04969	14.97422
HORVU6Hr1G066040	1.896079	2.37225	1.922659	1.949589	1.363434	2.478689
HORVU1Hr1G076610	10.11937	8.750478	12.7443	9.151507	11.34059	11.94637
HORVU2Hr1G028510	29.28014	34.29071	52.2391	18.54289	44.4172	45.75675
HORVU2Hr1G093720	2.896917	3.442405	2.566064	5.622312	4.328199	6.143309
HORVU2Hr1G048520	1.576107	1.501533	1.796746	1.150438	1.033413	1.09425
HORVU6Hr1G027880	0.962385	1.137829	1.064118	1.211502	1.266399	1.244649
Hordeum_vulgare_newGene_7419	69.01389	62.72172	91.20889	102.4092	232.6607	217.9571
HORVU5Hr1G015640	1.445145	1.299664	0.840528	0.783749	6.389077	4.983834
Hordeum_vulgare_newGene_7412	1.176343	0.87919	1.308865	4.714434	12.77117	10.16513
HORVU1Hr1G024540	1.115893	0.938305	1.44301	2.062284	3.446097	3.665294
Hordeum_vulgare_newGene_7415	3.398825	3.488424	3.730184	5.072142	3.098491	4.297831
HORVU7Hr1G025300	36.6049	29.744	37.38735	26.62477	22.80192	21.54942
HORVU7Hr1G036970	109.207	75.33934	103.2813	16.08306	0.671747	4.335407
Hordeum_vulgare_newGene_4859	1.654129	1.365352	1.168517	1.119331	2.222953	2.470143
Hordeum_vulgare_newGene_4852	2.429324	2.497759	1.954052	0.14418	0.00439	0.123819
HORVU6Hr1G058870	1.649686	1.548688	0.941165	2.67322	2.690392	2.498977
Hordeum_vulgare_newGene_4857	2.111925	1.951879	2.132437	0.119352	0	0.100055
HORVU1Hr1G064240	50.47867	44.81516	62.89725	70.11302	89.03907	71.61095
HORVU7Hr1G032270	0.155177	0	0.111044	1.23002	7.244527	6.326969
HORVU3Hr1G081880	25.85087	22.70213	27.29871	39.29861	53.32521	46.00075
HORVU6Hr1G062340	10.38165	8.602418	10.32546	8.082163	7.988014	10.04613
HORVU3Hr1G088630	2.459364	2.257558	2.450948	4.44616	6.99843	7.147816
HORVU5Hr1G103320	23.34738	16.97226	28.17835	17.88595	15.72917	18.84757
HORVU3Hr1G040350	12.87594	12.11414	13.32914	11.17176	15.03391	15.96519
HORVU3Hr1G030210	5.832785	7.67045	7.59262	6.350657	6.739535	7.397934
HORVU7Hr1G058500	14.1054	12.78308	11.97758	6.128362	5.719732	6.356667
HORVU4Hr1G083120	3.814082	4.066124	4.357004	3.347782	5.18224	5.747344
HORVU7Hr1G078800	24.05209	22.37254	21.40713	33.54854	67.42079	58.39299
HORVU4Hr1G051320	48.38506	74.56293	95.26194	81.20008	59.28736	79.33012
HORVU6Hr1G083770	33.40133	28.12513	29.68449	20.54496	16.81491	19.07197
HORVU7Hr1G095390	14.22074	13.18488	16.50997	11.75421	13.15547	14.13116
HORVU7Hr1G035720	29.38875	25.94492	28.15674	17.04321	18.34619	16.85984
Hordeum_vulgare_newGene_2934	3.395377	3.579375	4.140144	3.244807	4.214831	4.434567
HORVU6Hr1G022360	2.458578	2.850496	2.901706	2.857899	3.046409	3.823189
HORVU1Hr1G051310	2.93014	2.703895	3.375901	4.377003	14.50692	12.96017
HORVU7Hr1G031610	8.70358	7.094211	9.303603	9.392803	12.80067	11.89136
HORVU3Hr1G090760	9.061617	9.922957	11.17606	17.28372	17.90928	20.45403
HORVU6Hr1G087460	253.8423	244.3428	242.0546	235.6923	179.8264	208.2677
HORVU6Hr1G003570	2.549669	2.448135	2.487356	1.666631	2.443229	2.459375
HORVU4Hr1G017680	5.965956	7.599287	7.760309	6.831671	9.619497	9.717807
Hordeum_vulgare_newGene_9902	7.912451	9.679971	7.379587	0	0	0
Hordeum_vulgare_newGene_9903	3.341002	2.890332	3.30627	1.113593	0.400666	0.630166

Hordeum_vulgare_newGene_9904	7.36142	7.301918	8.412892	23.57693	71.3939	49.78231
Hordeum_vulgare_newGene_1595	6.154067	6.596101	6.266448	8.83798	11.30447	10.91322
HORVU3Hr1G079900	23.61449	23.57491	24.19976	9.292536	4.104863	5.22261
HORVU2Hr1G124860	0.892399	1.434024	1.433649	2.450797	2.576171	2.927727
HORVU6Hr1G070120	10.2973	7.739572	10.86378	13.50179	19.39993	20.9799
HORVU7Hr1G064170	1.979296	2.295074	2.604834	0.378076	0.111548	0.144642
Hordeum_vulgare_newGene_14032	0.152453	0.106553	16.46388	0	0	0
Hordeum_vulgare_newGene_14037	0	0.032208	16.48958	44.21115	0	0.052729
Hordeum_vulgare_newGene_14036	0	0	16.53503	44.21387	0.058113	0
Hordeum_vulgare_newGene_14035	0.542502	0.38541	18.44164	50.30865	1.28431	0.929249
HORVU4Hr1G017350	419.8281	355.7264	459.1781	176.2624	16.97499	70.36045
HORVU5Hr1G012180	3.560135	3.199554	2.962562	0.367722	0.402055	0.373244
HORVU3Hr1G097230	12.23547	11.16539	13.25713	15.66377	18.02625	20.51958
HORVU7Hr1G116060	3.840516	3.430452	1.90771	0.591048	0.34933	0.460332
HORVU1Hr1G019590	22.77227	20.32817	24.01212	28.8554	32.29367	32.39929
Hordeum_vulgare_newGene_516	2.208968	2.947957	4.295372	1.612413	2.188578	2.062651
HORVU0Hr1G008360	5.057919	3.847734	6.633381	5.434475	7.135062	6.807797
HORVU3Hr1G029020	17.04439	19.08716	16.59095	20.72985	23.1083	24.98066
HORVU5Hr1G102140	3.137895	1.766008	2.031476	1.171758	0	0.167537
HORVU5Hr1G097220	2.397901	2.369148	2.918209	1.956548	2.51511	2.54452
HORVU3Hr1G025060	3.494943	3.681657	4.529484	3.727072	3.386408	3.727536
HORVU6Hr1G061060	4.400727	4.599228	4.629341	5.495121	6.882365	7.707139
HORVU7Hr1G054070	1.613568	1.824139	2.146556	1.866203	2.298884	2.583527
HORVU1Hr1G091350	4.559716	3.551435	6.917408	6.172262	5.464532	3.078597
HORVU1Hr1G019750	10.21558	5.016157	17.29469	0.447161	0.024338	1.52973
HORVU6Hr1G035640	1.785606	1.592303	1.563373	2.877717	3.765204	3.782905
HORVU4Hr1G010360	5.130459	5.772811	7.282643	3.756744	4.069658	4.138116
HORVU4Hr1G085280	7.377423	6.000555	6.665002	7.575032	7.810055	7.866426
HORVU5Hr1G042050	15.89319	17.64616	17.78505	17.05666	28.20068	28.6887
HORVU7Hr1G107350	5.020769	4.561705	7.618805	3.816572	3.299209	3.0713
HORVU3Hr1G018350	0.287458	0.18384	0.343248	3.823286	2.764365	2.167638
HORVU2Hr1G095840	9.634232	6.954616	9.253959	9.08295	9.373089	9.090237
HORVU4Hr1G031320	3.072879	3.586477	2.782104	4.028947	5.034513	7.197417
HORVU7Hr1G049510	8.52143	7.137689	11.23203	4.5126	5.31674	6.074197
HORVU1Hr1G092710	38.52989	36.45463	41.17745	32.33018	34.94998	34.81475
HORVU3Hr1G023700	6.832745	8.452352	9.122584	13.47524	15.96941	17.06221
HORVU5Hr1G088940	3.354056	3.529132	4.268916	3.163714	3.161982	3.970266
HORVU2Hr1G098940	20.26352	18.55628	17.37378	27.89492	64.8651	54.55151
HORVU4Hr1G034660	1.18568	1.087807	1.848894	1.930746	2.919169	2.586374
HORVU1Hr1G029920	1.599291	0.976316	1.531824	1.378582	2.025852	2.400309
HORVU5Hr1G059010	24.22134	23.72004	25.27905	20.9221	18.56287	20.53393
HORVU1Hr1G088340	1.387485	1.222596	17.98167	47.38559	1.815093	1.68452
HORVU3Hr1G028860	4.984106	4.733715	4.237979	8.068431	5.549786	10.00536
HORVU5Hr1G111460	6.216246	8.11033	6.001145	5.33758	11.23713	11.06247
HORVU1Hr1G067000	9.189869	8.786769	8.573865	9.419217	11.00422	11.07094
HORVU2Hr1G089640	0.136247	0.419007	0.777604	1.06781	6.856931	5.795979
Hordeum_vulgare_newGene_802	4.291938	5.419499	5.384614	6.493941	4.806252	6.733589

HORVU3Hr1G015050	3.029404	2.569133	2.992724	6.321708	4.847573	5.966573
HORVU1Hr1G018080	9.744222	11.07917	12.22352	18.6166	17.48943	19.78831
HORVU2Hr1G057350	11.87429	10.54221	12.22567	14.60043	22.15732	22.36037
HORVU4Hr1G005910	1.354812	1.333342	1.315282	5.015925	4.215076	4.878015
HORVU7Hr1G050510	1.897201	2.566054	2.661624	0	0	0
HORVU6Hr1G000680	65.98744	59.84011	131.7436	35.22911	0.376922	8.751901
HORVU7Hr1G056910	38.56893	36.67755	36.94549	27.3714	33.41168	33.40673
HORVU1Hr1G052770	8.090225	6.874272	8.75057	7.684969	8.259276	8.291484
HORVU5Hr1G124850	21.71724	18.517	27.2188	26.58893	35.21494	33.18861
HORVU5Hr1G051570	2.556602	3.819302	3.712378	4.68881	5.821197	5.404329
HORVU6Hr1G054820	1.194313	1.377003	1.232618	1.843099	3.314208	3.603785
HORVU1Hr1G095150	25.96739	27.95668	20.82499	25.04906	27.63508	30.28422
Hordeum_vulgare_newGene_60	2.038915	2.200956	2.341072	0	0	0
HORVU1Hr1G095010	4.040574	4.110658	4.739744	3.428478	4.340443	4.170348
HORVU7Hr1G088960	53.22169	47.44978	48.6737	23.2728	13.07154	15.43907
HORVU4Hr1G016780	2.714121	1.209733	2.870288	5.714585	5.024026	4.832899
HORVU4Hr1G069930	2.462883	2.523015	1.809263	3.728207	3.123977	3.495627
HORVU1Hr1G049500	9.883003	8.958264	11.00009	18.58345	24.62791	24.27008
HORVU5Hr1G067740	16.41887	12.41385	18.91995	16.19538	18.45329	16.57631
HORVU7Hr1G088340	5.774964	4.444236	5.239122	6.315585	8.452737	8.217095
HORVU3Hr1G070440	1.333371	2.397184	2.347363	2.381883	1.385	2.282134
HORVU5Hr1G105750	33.21489	27.70714	38.77092	30.36559	26.53852	20.46694
HORVU7Hr1G075450	37.02146	37.35067	40.85923	34.33983	43.73942	43.87944
HORVU6Hr1G003900	6.32337	6.407487	8.037641	9.658143	17.1452	16.29469
HORVU6Hr1G088870	2.030662	2.572278	3.115979	1.176862	1.686123	1.65916
HORVU5Hr1G010130	0.530938	0.527061	0.375986	21.60318	13.23378	24.25334
HORVU2Hr1G100620	2.965962	2.384586	3.241021	4.239216	4.891036	4.321699
HORVU1Hr1G094400	0.243292	0.174385	0.150968	9.283481	9.760542	17.00849
HORVU3Hr1G099680	1.714556	1.122956	1.254597	5.153014	6.249168	6.81903
HORVU1Hr1G036640	19.03426	20.77358	22.55609	17.2968	21.57013	23.93475
HORVU2Hr1G109740	9.269906	8.99861	10.46487	7.418048	7.049089	7.940475
HORVU4Hr1G053840	0.707114	0.985839	1.194185	1.116915	1.613295	1.768646
HORVU1Hr1G006940	0.718084	0.677719	1.61221	1.601701	1.443141	2.111372
HORVU7Hr1G083870	3.000926	3.926425	2.386826	5.827212	6.376849	6.883622
HORVU6Hr1G068450	1.972162	1.849705	2.149968	2.231539	2.796578	4.101624
HORVU3Hr1G064370	2.875812	2.817106	3.263383	6.584648	7.230382	6.578626
HORVU1Hr1G054290	17.88842	17.88989	23.6364	16.82659	21.48613	18.5626
HORVU2Hr1G113270	89.18654	68.87603	84.78321	101.575	39.60937	48.38679
HORVU0Hr1G001500	1.209614	0.980822	1.146429	3.342125	3.832101	4.588265
HORVU7Hr1G091360	18.3964	17.10642	17.90697	13.76328	12.06003	11.64099
HORVU3Hr1G034960	0.963585	0.863759	1.919234	1.004779	1.169295	1.214416
HORVU4Hr1G055140	2.725443	1.863747	1.584279	1.199951	1.793119	2.261538
Hordeum_vulgare_newGene_10253	2.527459	2.501297	1.776576	2.701774	2.877788	3.228822
HORVU6Hr1G013610	1.452767	1.472239	2.099999	0.701978	1.371872	1.249206
HORVU7Hr1G111010	1.105295	0.599995	1.260334	6.108947	8.120152	6.388019
HORVU2Hr1G074960	1.853651	2.584024	1.702718	2.776772	1.75297	2.681637
HORVU3Hr1G086800	3.637566	2.624792	3.37898	4.818271	5.725039	5.112159

HORVU3Hr1G021610	8.048346	6.371783	7.249602	0.079271	0.076133	0.115109
HORVU3Hr1G093050	53.60835	50.62091	54.04686	58.52211	89.22858	82.81323
HORVU0Hr1G035320	0.80735	0.955554	1.157	2.571742	2.453268	2.138092
HORVU6Hr1G060420	5.826237	6.636651	6.9708	11.0623	14.15153	14.39168
HORVU7Hr1G085310	8.997956	7.051055	9.905069	21.1146	33.77779	30.76109
HORVU6Hr1G067430	1.238838	1.642403	1.642561	1.753376	3.555592	3.277299
HORVU7Hr1G012690	0.885921	0.803761	0.734991	2.378517	4.236127	3.887131
HORVU6Hr1G042680	14.28184	12.92201	14.25998	14.41862	13.69182	13.97627
HORVU7Hr1G030010	0.48574	0.875256	0.25143	0.18665	3.229442	3.204431
HORVU1Hr1G060110	1.493868	1.933087	1.766876	0.669361	0.350972	0.706863
HORVU6Hr1G093300	1.863194	3.949971	2.082086	1.526754	1.530189	1.864949
HORVU5Hr1G120600	16.69583	12.46088	18.00936	17.38902	25.19396	21.44214
HORVU0Hr1G016660	3.278985	4.079643	4.867282	3.983513	4.558759	6.094723
HORVU7Hr1G082040	144.7602	190.1167	240.519	11.87856	1.041375	5.646898
HORVU3Hr1G104220	5.359519	4.634354	6.457397	4.067585	3.824597	4.370526
HORVU4Hr1G090310	3.613495	2.378155	2.872526	9.134506	26.67575	19.3049
HORVU1Hr1G076870	7.242312	8.110036	8.619984	19.2412	28.96284	24.23557
HORVU4Hr1G060560	0.719577	0.376044	0.927316	2.03301	1.158192	1.617743
Hordeum_vulgare_newGene_1460	10.07106	10.41068	12.14198	13.88321	22.29192	21.53092
Hordeum_vulgare_newGene_11618	0	0	0	3.261518	1.991657	1.63805
Hordeum_vulgare_newGene_11619	2.961858	3.238607	3.430762	1.790957	2.588515	2.41218
HORVU0Hr1G016590	0.968375	0.721748	0.970492	1.018585	1.242272	1.348684
Hordeum_vulgare_newGene_11612	9.958344	13.6447	12.55445	4.230346	1.600553	2.410444
Hordeum_vulgare_newGene_11617	0	0	0	23.72733	15.45116	16.0966
HORVU2Hr1G115360	1.991517	2.576783	3.494942	1.634479	7.242049	6.329489
HORVU6Hr1G077670	1.515835	1.601148	2.452027	0.709893	1.040997	0.94434
HORVU6Hr1G041720	34.29225	51.42552	43.32511	73.25358	84.32506	121.4808
HORVU1Hr1G024240	66.77055	59.38818	69.45187	62.17891	55.33243	60.20657
HORVU1Hr1G039160	20.90212	22.73215	32.99354	22.55681	49.32099	45.97038
HORVU2Hr1G006700	7.406051	9.500258	9.872485	8.987989	8.932273	10.68345
HORVU1Hr1G093120	2.728475	2.838841	2.436897	2.112314	1.955742	2.700005
HORVU3Hr1G029340	3.836375	3.129569	4.097973	4.429878	7.408596	6.259291
HORVU5Hr1G112100	3.016704	3.05837	4.675865	5.68202	4.580727	3.413084
HORVU2Hr1G022050	2.323916	2.590194	2.688722	2.347887	2.284489	3.437463
HORVU6Hr1G045410	0	1.599696	1.160505	2.493997	2.855	2.755356
HORVU4Hr1G066920	7.549368	8.102641	10.13093	9.093171	10.32872	10.77854
HORVU3Hr1G073800	13.26011	12.48614	17.73707	13.308	21.7187	21.21249
HORVU2Hr1G038260	2.135217	2.055841	2.653281	7.679847	14.01841	11.0061
HORVU6Hr1G089280	77.82673	75.29802	68.06988	33.89889	24.59093	26.99288
HORVU5Hr1G054880	3.378815	3.081392	4.050836	3.798703	5.788746	6.291947
HORVU2Hr1G025580	8.129631	8.607139	10.41839	7.645776	4.879279	6.018939
Hordeum_vulgare_newGene_1828	4.881249	5.564656	5.080821	6.832469	6.870708	8.026376
HORVU3Hr1G057280	36.65381	35.06133	35.19649	53.62761	34.2996	37.44929
Hordeum_vulgare_newGene_1823	12.85423	11.66046	14.86236	3.828584	3.16955	4.039602
Hordeum_vulgare_newGene_1826	1.322078	1.465132	1.073844	2.208776	1.828863	2.100975
HORVU6Hr1G088530	5.388026	3.903905	4.599575	0.353403	0.00815	0.144734
HORVU3Hr1G037350	97.29272	80.46062	96.878	73.6529	41.34074	44.23957

HORVU3Hr1G087390	25.74251	23.80343	23.21271	20.37855	27.50888	28.42905
HORVU4Hr1G023110	1.263606	1.370529	1.734078	1.147206	0.569376	0.886454
Hordeum_vulgare_newGene_3998	0.828557	3.948946	5.420551	2.76683	5.720882	5.171623
HORVU5Hr1G078470	21.25828	19.92571	24.34291	28.03102	36.19892	37.91315
HORVU2Hr1G020690	21.23638	22.76703	34.72142	10.75366	14.0234	15.04952
HORVU6Hr1G063580	9.151232	7.54524	8.028087	6.016655	3.324612	4.160945
HORVU7Hr1G084830	4.39001	3.608684	4.529547	9.828878	7.791461	8.749066
HORVU6Hr1G066740	5.73329	5.499347	8.060477	2.928277	2.781885	3.384155
HORVU3Hr1G010030	15.77175	14.68726	15.98314	19.54212	31.12153	26.99014
HORVU2Hr1G102120	3.27058	2.64339	3.856593	3.324938	4.482779	4.914008
HORVU7Hr1G032070	9.904402	9.525121	12.3893	11.65296	16.24779	16.44282
HORVU7Hr1G076230	19.94146	23.93869	22.75803	33.80992	42.26411	44.09674
HORVU6Hr1G042090	3.65713	2.091805	2.896454	3.494481	2.518285	3.015593
HORVU5Hr1G049220	5.476187	6.937335	7.496043	8.587174	15.00226	14.39089
HORVU4Hr1G057170	10.51898	7.677179	8.964575	18.22973	41.28382	40.66646
HORVU3Hr1G068630	7.910738	6.479964	8.007809	8.678919	9.071203	8.616084
HORVU6Hr1G062180	3.414862	2.057867	2.59619	4.543425	0.11631	0.773398
HORVU3Hr1G024470	0.648042	0.727708	0.588598	4.08062	6.893454	6.929265
HORVU7Hr1G028240	4.978757	4.885317	5.374127	6.851013	5.062911	6.498565
HORVU4Hr1G066070	1.408323	1.761641	1.971032	4.485222	5.111329	4.923687
HORVU1Hr1G033130	6.225672	6.552097	7.182617	5.172426	5.58935	5.418621
HORVU7Hr1G030500	19.1352	11.8993	22.67201	10.78646	6.473006	9.102883
HORVU2Hr1G087010	59.47194	59.66785	56.88332	57.34644	35.55746	35.20736
HORVU3Hr1G017560	104.7235	88.12814	101.1382	90.21611	63.40213	59.64912
HORVU3Hr1G089520	8.047607	7.442059	10.74336	15.94178	15.98625	16.34797
HORVU1Hr1G001800	15.89094	26.40074	15.21563	24.13253	4.770017	5.838077
HORVU2Hr1G039130	12.22805	12.97651	11.45733	18.93443	21.42819	20.42845
HORVU6Hr1G054580	0.602023	0.628219	0.850132	1.827009	1.961252	1.750927
HORVU2Hr1G116700	10.64865	9.168883	10.65601	8.073091	3.838183	5.376623
Hordeum_vulgare_newGene_6212	2.078945	2.393933	2.797477	2.886201	3.206977	3.05738
Hordeum_vulgare_newGene_6215	5.859322	7.087953	7.065279	5.984408	10.02116	9.031059
Hordeum_vulgare_newGene_6214	6.961015	15.06567	7.911792	15.77191	3.66832	3.608042
Hordeum_vulgare_newGene_6217	8.190019	6.270513	6.540626	7.562808	6.960906	8.223045
HORVU4Hr1G054480	2.805851	2.305955	3.100099	4.503863	2.373236	2.738111
Hordeum_vulgare_newGene_13447	14.78791	15.06088	19.84833	12.62552	12.12696	12.72682
HORVU3Hr1G116470	0.390878	0.231632	0.402876	5.611554	27.70527	22.09811
HORVU4Hr1G003430	5.086655	6.47057	8.466991	3.879825	4.534474	5.244644
HORVU3Hr1G090560	1.420194	1.492222	1.704958	4.471085	4.559281	5.275225
Hordeum_vulgare_newGene_13448	2.109563	1.874822	1.996556	2.359961	2.530792	2.93072
HORVU4Hr1G065520	226.5494	208.7825	234.5719	250.9949	218.7751	262.4507
Hordeum_vulgare_newGene_3531	11.73444	8.041626	11.31052	13.47734	8.53969	12.89426
Hordeum_vulgare_newGene_3534	3.63624	5.109965	5.460427	0	0	0
Hordeum_vulgare_newGene_3539	0.615439	0.510588	0.696551	2.977063	5.426965	6.274304
HORVU3Hr1G096920	1.436714	1.446352	1.724574	1.931969	2.615108	2.647437
HORVU7Hr1G082860	38.00318	26.45263	27.80657	21.74765	22.41407	19.3317
HORVU1Hr1G082540	2.942402	2.567325	3.154122	4.869594	5.360643	6.269232
HORVU6Hr1G018060	3.454451	3.742431	5.490432	1.746272	2.256759	2.788442

HORVU3Hr1G061750	17.03255	18.83308	24.75853	10.80132	13.43429	12.25202
HORVU1Hr1G043920	77.3989	53.25569	83.13765	60.89579	37.39531	54.0492
HORVU7Hr1G038080	0.192184	0.211534	0.344844	1.786692	2.396557	2.867794
HORVU5Hr1G048930	1.184366	0.318677	2.436262	0.719607	0.937132	0.948421
HORVU2Hr1G064170	15.82182	15.56151	18.63296	15.36226	19.61382	19.5488
Hordeum_vulgare_newGene_2061	1.660103	1.27043	1.742445	1.345254	2.639181	2.195864
HORVU3Hr1G089380	2.441137	2.575185	2.652429	0.363113	0.537286	0.307614
HORVU0Hr1G005670	7.462193	7.953901	6.785597	15.14618	28.39592	21.18535
HORVU7Hr1G029110	34.42217	30.98984	35.50137	19.22321	16.8525	18.4865
HORVU7Hr1G075150	23.00096	23.47505	26.9116	26.57117	35.23279	35.40064
Hordeum_vulgare_newGene_14231	1.02715	0.91928	1.54988	1.125826	1.189899	0.788076
Hordeum_vulgare_newGene_14233	101.3744	83.58821	94.12813	128.5552	144.0188	142.5182
Hordeum_vulgare_newGene_14239	0.858225	1.060846	0.834716	2.145973	1.616286	1.794519
HORVU7Hr1G003080	0.151691	0.164135	0.064308	0.013184	3.92257	4.948908
HORVU5Hr1G081930	33.61539	33.81272	37.5122	38.99802	45.07149	48.29493
HORVU4Hr1G059990	4.025683	4.333618	5.282908	3.256031	3.921195	4.342937
Hordeum_vulgare_newGene_15726	10.45191	10.5149	10.60046	17.09055	20.08088	16.1834
HORVU5Hr1G115340	1.751189	1.656144	1.750738	5.49356	12.03769	10.6294
HORVU4Hr1G057070	1.07333	1.94576	1.744135	1.148615	2.426424	2.148591
Hordeum_vulgare_newGene_737	2.222138	2.446819	2.660816	0	0	0
Hordeum_vulgare_newGene_735	2.019706	2.205292	2.22745	0	0	0
HORVU2Hr1G076710	11.2437	9.90828	15.1516	15.36501	18.50872	18.77764
HORVU3Hr1G013710	18.81177	18.98174	22.60516	14.95281	16.38837	15.96231
HORVU7Hr1G075310	2.562014	3.060977	2.82222	4.473476	4.27481	4.022924
HORVU1Hr1G074890	3.514448	2.896119	3.238603	5.263223	8.291432	7.738596
HORVU1Hr1G070570	1.697417	1.608319	1.822101	0.623901	0.224125	0.110056
Hordeum_vulgare_newGene_2663	3.642193	4.926689	3.661777	2.181945	0.591829	0.67594
HORVU7Hr1G096740	1.914131	1.475119	2.589493	1.192487	0.798251	0.650512
HORVU3Hr1G021150	33.87085	34.31036	33.91182	46.08605	42.18661	43.06695
Hordeum_vulgare_newGene_9298	25.16304	22.99785	36.88873	31.25931	78.77556	68.26024
HORVU1Hr1G003230	4.450988	30.85755	6.017696	46.22964	5.438036	6.796935
Hordeum_vulgare_newGene_9293	4.163833	2.537064	2.110179	3.299059	2.505827	3.357421
Hordeum_vulgare_newGene_9294	0.960698	1.144722	1.391481	1.987843	2.564671	3.021121
HORVU7Hr1G119060	0	0	0	0.408974	1.892903	7.810733
Hordeum_vulgare_newGene_884	11.99759	10.13221	14.37401	12.80071	19.53353	19.45142
Hordeum_vulgare_newGene_880	7.2863	6.71207	5.795341	7.492843	11.89631	11.39399
HORVU6Hr1G076880	0.191758	0.384689	0.136099	8.088132	33.46882	31.18889
HORVU4Hr1G070410	8.365201	5.895815	9.884853	9.341821	11.6554	10.05546
HORVU6Hr1G068630	0.733115	1.391888	0.994496	2.016775	1.425717	1.914255
HORVU7Hr1G107590	11.96517	12.46249	12.2044	10.00685	6.984382	6.97818
Hordeum_vulgare_newGene_8523	0.944009	1.360982	1.159756	1.424709	2.235356	2.319578
HORVU3Hr1G114160	4.360609	2.777346	4.737769	0	5.238134	4.984519
HORVU6Hr1G064740	1.817257	1.980307	2.327069	1.399169	2.62683	2.237871
HORVU5Hr1G098170	0.543364	0.723125	0.773712	1.329172	2.705544	2.752636
Hordeum_vulgare_newGene_27	1.419614	1.646487	1.863596	1.456793	1.185344	1.484337
HORVU0Hr1G032360	2.708584	3.247155	3.633346	2.063038	1.530205	1.587123
Hordeum_vulgare_newGene_15185	13.13428	13.21098	13.70334	11.67811	8.753339	11.39981

HORVU5Hr1G077810	31.26419	39.8761	45.16622	15.64245	0.729522	7.500639
HORVU7Hr1G026350	19.44129	21.01173	26.099	35.65001	242.6049	229.2922
HORVU7Hr1G073650	4.001314	4.003856	3.789397	9.752391	17.96359	15.31259
HORVU4Hr1G078620	29.14246	26.38944	29.79284	40.03831	45.1473	44.39008
HORVU3Hr1G074000	1.258571	2.471627	2.532268	0.369132	0.282562	0.432615
HORVU4Hr1G036820	2.549503	2.48369	2.31311	4.430593	3.30823	4.10445
HORVU2Hr1G031740	7.330913	7.893488	8.732731	5.677874	5.456621	5.590028
HORVU1Hr1G053190	13.81346	18.49279	16.20178	10.46112	11.95606	12.30666
HORVU5Hr1G048890	22.21866	22.95757	29.8224	26.14549	34.66188	36.59239
HORVU1Hr1G043450	3.223904	2.18065	2.38722	0.783036	0.879824	1.120228
Hordeum_vulgare_newGene_9434	1.361912	1.011177	0.906869	2.110358	2.719834	2.546492
HORVU7Hr1G058360	5.885762	5.736963	7.547367	7.959355	9.584	10.89624
HORVU1Hr1G038850	17.99456	18.43598	21.67116	15.28123	17.47452	19.28766
HORVU7Hr1G086410	31.33161	27.80049	31.03594	32.4437	35.776	35.59156
HORVU2Hr1G073670	1.209877	1.24538	2.283963	1.660829	0.47862	0.817073
HORVU3Hr1G095520	9.27363	8.283932	9.517858	11.5271	14.36383	14.90747
HORVU1Hr1G035490	23.76625	22.25412	28.42097	22.89612	25.43539	28.81964
HORVU4Hr1G008700	9.375905	6.261329	9.954227	28.53304	21.10934	32.6888
HORVU5Hr1G084880	0.483795	0.591388	0.370528	1.038724	2.395643	2.199076
Hordeum_vulgare_newGene_16063	2.195539	1.964885	2.183485	2.969988	1.059265	1.623699
HORVU4Hr1G084040	6.757665	6.733948	7.008375	5.850416	6.764339	6.473996
HORVU6Hr1G063910	6.127642	6.160055	6.067095	12.78487	24.81389	23.17549
HORVU1Hr1G021800	4.714966	5.246974	6.020309	8.613797	9.090617	10.55985
Hordeum_vulgare_newGene_16065	1.931572	2.064658	1.800456	1.109419	0.767183	1.026714
Hordeum_vulgare_newGene_1588	0	0	0.070396	0.076858	12.90568	17.39284
Hordeum_vulgare_newGene_1587	0.474812	0.067273	0.344679	0.690188	8.858671	13.00566
HORVU2Hr1G089330	2.100835	2.061563	2.265754	2.113365	1.291592	1.656288
HORVU2Hr1G105730	5.537278	6.262516	6.68911	11.03061	12.36943	11.61338
HORVU7Hr1G013470	64.19754	75.39519	51.21071	347.1455	139.4816	186.5503
HORVU6Hr1G017500	27.90117	24.15356	29.4656	31.33231	40.0557	42.87379
HORVU4Hr1G041810	4.3142	5.086059	6.319195	2.359722	2.496189	2.96283
HORVU6Hr1G081470	25.14636	22.05528	25.27884	22.53918	17.25971	18.0664
HORVU3Hr1G070600	1.732147	2.102188	2.349062	1.742642	4.463951	4.419942
Hordeum_vulgare_newGene_94	0.20043	1.110276	1.61282	2.896091	3.499897	2.769189
HORVU5Hr1G010330	226.6388	230.6644	326.8095	75.58555	42.7322	53.46655
HORVU4Hr1G018980	1.552564	1.612472	2.033071	1.818478	3.960876	3.246891
HORVU2Hr1G120220	5.175027	4.466405	7.135707	4.21243	4.463583	4.668288
HORVU7Hr1G079650	2.509599	1.620588	1.810456	1.465082	0.483719	0.781785
HORVU2Hr1G090310	16.6205	12.96138	15.41947	13.08383	11.66074	14.47171
HORVU3Hr1G043320	0.927864	1.15455	0.963461	1.346689	1.515296	1.708221
HORVU6Hr1G036010	8.900008	14.08838	10.61187	10.57396	12.61115	15.91167
HORVU3Hr1G084970	1.344108	1.283562	1.989845	2.570886	2.534808	3.003765
HORVU7Hr1G056240	0.496645	0.624959	0.690485	2.709739	4.966583	5.460778
HORVU5Hr1G056360	2.632593	2.6113	3.337275	3.532716	3.750031	4.301564
HORVU3Hr1G000900	0.614302	0.302489	0.515969	1.072523	2.339272	1.85618
Hordeum_vulgare_newGene_3982	3.828972	2.669053	5.386671	1.981603	2.182335	1.657175
Hordeum_vulgare_newGene_7600	8.591908	8.616505	10.14369	5.560371	5.633781	5.3496

HORVU0Hr1G001340	39.067	37.01511	43.43343	37.08291	41.76874	43.65993
Hordeum_vulgare_newGene_7606	1.857448	1.584777	1.263685	4.913482	5.756657	6.13991
Hordeum_vulgare_newGene_1553	0	0	0	13.66351	14.66554	15.33918
HORVU4Hr1G088840	2.908702	2.769275	3.335373	4.850616	5.845277	6.361468
HORVU2Hr1G010400	10.04887	8.967335	9.379615	8.49893	11.1111	12.08544
HORVU3Hr1G085590	10.79997	8.805861	9.38244	9.235843	9.816054	9.403367
HORVU4Hr1G072900	41.05323	41.17182	33.93602	41.12541	25.37931	26.96003
HORVU2Hr1G045200	4.858975	3.627787	7.179915	2.988663	2.668323	3.384534
HORVU7Hr1G089830	2.498095	2.297246	2.617838	4.955118	5.284496	5.822076
HORVU7Hr1G093050	1.432632	0.744521	1.763651	1.130609	1.333453	0.963038
Hordeum_vulgare_newGene_6551	3.53778	1.784295	1.953612	9.562796	11.17892	8.666126
HORVU7Hr1G056790	5.879487	5.350152	6.646505	9.765625	11.9913	11.56768
HORVU5Hr1G125650	27.18572	25.04647	28.98562	31.08996	28.27916	28.18473
HORVU1Hr1G088560	1.668077	1.63087	21.73232	68.35083	9.601897	13.82222
HORVU6Hr1G002520	7.096794	6.250353	7.364065	7.540361	7.34278	8.391006
HORVU6Hr1G069310	11.75331	9.338275	12.1599	13.01672	15.85291	14.26883
HORVU5Hr1G022520	3.420285	4.095097	4.618217	3.408498	2.999625	3.755787
HORVU1Hr1G049980	8.065668	9.450645	11.0114	9.807567	15.31823	16.37215
HORVU1Hr1G051120	10.61516	9.431527	11.47442	10.34399	11.99406	12.05207
HORVU1Hr1G074420	1.640087	1.777446	2.175438	1.772254	1.931082	2.519544
HORVU1Hr1G070960	1.294864	0.788653	0.660296	4.798123	6.31457	5.422743
HORVU5Hr1G033930	26.21566	29.93671	22.12971	32.71691	47.6106	45.21471
HORVU3Hr1G082010	8.851481	8.905429	10.94514	10.89299	14.43906	15.17571
HORVU5Hr1G012220	84.05561	68.34069	91.70917	78.8487	90.58753	78.68202
HORVU2Hr1G040180	13.22255	14.3372	15.61192	15.48722	18.9092	18.81865
HORVU6Hr1G075770	4.456651	3.759289	4.53515	3.308577	2.424611	3.464383
HORVU1Hr1G044530	16.05318	14.08704	16.28764	12.77626	14.14688	15.69211
HORVU0Hr1G005330	6.105188	7.252184	5.92978	15.46994	8.770053	12.03828
HORVU7Hr1G022440	1.074258	0.944594	1.740326	1.09244	1.111995	1.092022
HORVU3Hr1G082000	1.367078	1.48169	1.995369	3.811425	0.505072	2.159567
HORVU2Hr1G024550	26.81247	21.77391	27.02963	17.32487	15.79752	15.98103
HORVU2Hr1G055230	3.982527	4.129461	6.011985	6.078226	5.774128	6.721473
HORVU5Hr1G018480	0.407749	0.328492	0.558966	2.692485	5.253389	6.963572
HORVU4Hr1G080450	10.74212	9.34601	10.24938	5.469022	6.429678	7.007618
HORVU3Hr1G034240	6.117488	6.478172	8.805193	6.225913	8.691271	8.543986
HORVU3Hr1G002670	0.936457	1.085154	0.823935	4.994995	8.137475	7.085926
HORVU5Hr1G094250	30.73324	30.59578	32.22096	28.29885	34.29764	32.93062
HORVU5Hr1G112340	15.74341	15.05263	16.58315	6.044047	2.874081	4.594798
HORVU6Hr1G045800	3.008546	3.141329	3.6638	4.128652	4.162956	4.338668
HORVU3Hr1G078840	9.362709	8.335436	10.58287	2.994184	2.343715	2.784957
HORVU1Hr1G089380	0.01153	0	0	8.195584	4.119687	3.752077
HORVU4Hr1G011730	15.82781	14.62082	12.38436	9.325604	8.422278	10.2297
HORVU5Hr1G092150	4.753731	5.465058	8.530825	0.177141	0	0.233918
HORVU5Hr1G118060	0.206982	0.151456	0.22946	1.473255	2.740658	2.313975
HORVU6Hr1G066930	28.29038	22.8756	27.99303	29.97736	28.68435	30.03576
HORVU1Hr1G005120	78.25588	64.32235	88.79671	96.04349	186.9764	148.1493
HORVU7Hr1G051560	0.089955	0.158241	0.080018	0.61748	5.023281	4.485072

HORVU2Hr1G123220	11.20803	11.05784	16.15157	9.47326	10.80701	12.79452
HORVU4Hr1G050930	1.724662	2.098175	2.439741	2.906442	3.140933	3.860165
HORVU1Hr1G041950	0.966544	1.542518	1.204788	1.004899	1.308698	1.003648
HORVU5Hr1G110180	56.41438	49.95367	49.78224	28.56995	21.67856	35.9691
HORVU3Hr1G082900	7.789564	7.951716	8.954676	9.529874	11.39102	11.8831
HORVU1Hr1G002470	2.823027	22.54163	4.369286	24.50996	0.251203	0.520557
HORVU3Hr1G056710	3.950576	8.799023	9.65351	0.58933	0.496575	0.320282
HORVU5Hr1G113560	0.651329	0.583783	0.688134	2.982438	3.598966	3.485257
HORVU2Hr1G051260	3.09796	4.214663	4.500407	3.918694	5.780389	5.444539
HORVU5Hr1G051290	12.31597	9.529178	12.3457	9.966387	5.756714	7.615273
HORVU5Hr1G109290	23.68238	23.34384	32.37737	6.538771	1.91543	3.139205
HORVU4Hr1G052470	4.675256	4.313968	5.054706	12.6339	15.33276	16.46476
HORVU6Hr1G094240	7.412498	6.640945	7.209985	5.09572	5.54955	6.786833
HORVU4Hr1G031510	13.03819	13.45219	14.26795	3.694471	2.846327	3.955364
HORVU4Hr1G066270	33.61638	46.39711	57.72475	36.02855	25.15555	41.03457
HORVU1Hr1G013740	0.86408	0.762389	0.812716	1.826332	4.748373	4.55249
HORVU2Hr1G015830	3.804048	2.934237	3.068226	8.965556	8.235221	7.151865
HORVU4Hr1G089670	15.70195	12.06233	17.7865	32.78565	48.70058	46.16144
HORVU7Hr1G046700	16.87078	17.12767	14.47305	14.42675	19.12487	18.86453
HORVU4Hr1G068540	1.685478	1.73813	2.061175	3.356598	9.158918	8.033261
HORVU3Hr1G066810	18.44195	14.46904	21.57044	14.01503	9.576754	11.40126
HORVU1Hr1G058180	74.45799	66.29485	81.75729	29.26559	32.17791	35.05958
HORVU1Hr1G071430	1.596559	1.854478	1.874375	2.457311	1.476212	2.902978
HORVU4Hr1G049590	18.10628	17.40409	22.01492	22.97606	47.02892	42.1139
HORVU7Hr1G010810	3.186922	4.241561	3.802721	2.634453	3.265246	3.762636
HORVU3Hr1G071210	1.555306	1.686399	2.761532	5.885843	9.061764	9.580643
HORVU1Hr1G001090	0.940309	1.769917	1.612791	19.16181	2.273708	2.014825
HORVU5Hr1G059600	19.86165	17.1934	20.34476	19.9964	21.74187	23.20467
HORVU0Hr1G039960	5.577387	5.398297	6.63464	6.033326	6.120163	6.880639
HORVU5Hr1G055950	127.2768	70.34558	179.842	162.7939	56.66756	96.93826
HORVU3Hr1G030890	21.1658	20.61482	20.10282	21.08137	17.58462	19.65783
HORVU1Hr1G082380	11.27902	12.26363	13.35497	17.14206	25.8602	24.24208
HORVU4Hr1G027900	1.610608	1.127557	1.676192	1.883653	1.575613	1.835024
HORVU6Hr1G000490	2.402136	3.926926	4.279522	0	0	0
HORVU4Hr1G054130	2.072556	2.482103	3.059202	2.17873	1.994082	2.618844
HORVU7Hr1G075790	39.09993	36.86738	43.41053	43.63644	50.18556	53.1433
HORVU7Hr1G099010	34.93972	31.74025	36.68155	29.87743	29.06442	28.87837
HORVU4Hr1G086270	42.52681	36.56432	44.64485	39.2812	21.47926	23.37024
HORVU7Hr1G083180	10.73117	9.473244	14.49634	14.24388	31.72322	29.75853
HORVU1Hr1G011840	0.917193	1.000177	1.312383	0.993528	1.21587	1.301875
HORVU4Hr1G001450	10.24979	12.13472	12.01807	2.427244	0.759297	1.568455
HORVU1Hr1G057460	43.01022	38.97576	53.51659	12.05711	19.11617	18.26894
HORVU2Hr1G101940	44.29445	37.94719	42.99076	61.77166	42.15267	42.93018
Hordeum_vulgare_newGene_13933	1.379933	1.733263	1.846396	1.248024	3.604867	1.829138
HORVU4Hr1G066830	32.14075	32.69234	29.8338	28.91434	28.94578	31.77527
Hordeum_vulgare_newGene_13934	1.577142	2.382936	3.360615	0.958445	0.717303	0.840605
HORVU3Hr1G107970	31.46087	25.79393	30.89969	54.48475	38.83928	40.46453

HORVU2Hr1G081790	13.64635	12.42941	12.77861	24.51107	29.76484	27.2831
HORVU5Hr1G044780	4.533143	3.702357	4.334437	5.826216	5.809378	6.985578
HORVU5Hr1G053070	39.25055	38.84062	42.18323	28.42028	33.23966	34.10426
HORVU5Hr1G115630	1.809676	1.083407	1.414029	4.779966	3.690058	4.151311
HORVU7Hr1G043620	0.434484	0.410847	0.469771	1.866605	1.571443	2.339355
HORVU1Hr1G047440	129.0793	104.648	155.8232	88.64116	74.00738	69.17893
HORVU2Hr1G066930	7.253651	7.020184	7.05229	0.06706	0.02833	0
HORVU5Hr1G074860	6.786363	6.057092	9.822981	7.00826	7.027795	6.346648
Hordeum_vulgare_newGene_8728	0.77201	0.720478	0.664941	4.381845	2.891019	3.247817
HORVU4Hr1G007580	0.347674	0.483826	1.01284	1.313126	4.595788	3.814641
Hordeum_vulgare_newGene_8722	20.27148	17.81779	23.21063	26.08529	24.16216	30.52393
HORVU5Hr1G011160	0.532685	0.51866	0.617489	4.732998	6.438212	5.430512
HORVU3Hr1G114360	105.09	89.94091	136.5818	102.9737	157.9855	161.7721
HORVU2Hr1G030630	8.779337	8.502618	8.146607	14.34962	18.26093	17.20369
Hordeum_vulgare_newGene_5267	3.463336	2.995489	3.276148	3.452878	3.799753	3.693867
HORVU4Hr1G020150	8.032971	0.724114	7.754022	7.624127	10.90533	9.4226
HORVU4Hr1G050160	7.908483	8.968725	10.44322	8.839342	11.61641	11.6983
HORVU3Hr1G076880	1.91862	2.307349	3.048449	3.463429	9.854146	9.354388
HORVU5Hr1G080530	30.7497	24.72747	29.26874	41.75616	32.19758	31.10011
HORVU3Hr1G061950	3.616328	3.330242	2.869342	4.760382	7.318817	7.870167
HORVU5Hr1G112350	18.88198	19.66365	21.71122	10.17401	13.90377	11.84526
HORVU2Hr1G011100	18.52817	17.94902	17.01611	12.89325	10.07287	11.75055
Hordeum_vulgare_newGene_8090	7.985536	6.657306	8.833983	9.186141	12.87617	13.36633
HORVU4Hr1G060680	2.753904	2.44653	1.980557	2.337282	4.199368	3.486572
HORVU3Hr1G112370	21.11735	18.0548	19.54483	19.0143	21.78231	20.89441
HORVU4Hr1G007050	44.80622	39.15846	50.5499	32.54728	27.03777	30.34838
HORVU4Hr1G061880	6.262788	6.230605	6.174407	6.324567	5.892057	5.137447
HORVU3Hr1G062900	5.48137	4.785404	4.452757	3.314195	0.683121	1.783835
HORVU2Hr1G084030	4.640577	5.18793	6.010558	7.350581	5.795208	5.73622
HORVU5Hr1G054510	5.709464	4.674356	6.010591	10.12485	8.504295	10.49853
HORVU2Hr1G092250	3.238087	3.55807	4.526673	3.361414	6.673622	6.007662
HORVU2Hr1G104660	6.402052	5.656161	6.816524	7.075165	8.684786	8.830192
HORVU1Hr1G040140	1.225533	1.481337	1.504232	78.85297	167.0517	137.9266
HORVU3Hr1G106730	37.35941	31.42378	42.21059	38.7418	30.47366	30.21759
Hordeum_vulgare_newGene_10770	44.48835	38.74527	38.48226	30.65326	22.27489	23.45355
HORVU2Hr1G112400	0.982743	1.090557	1.138783	1.151512	1.20037	0.954151
HORVU5Hr1G093720	28.36512	25.63209	27.06163	22.50445	17.30604	18.03779
HORVU1Hr1G035650	6.001377	6.385416	3.928632	3.702912	4.411777	4.248876
HORVU2Hr1G010480	15.05501	16.28185	21.46011	13.72844	16.75964	18.89687
HORVU6Hr1G091980	6.96953	6.629596	7.381891	0.046184	0	0
HORVU5Hr1G052030	2.10578	1.694502	3.288098	5.505264	8.267795	7.722027
HORVU5Hr1G081660	0.726671	0.857911	0.87416	1.524543	5.389956	4.580438
HORVU4Hr1G067480	37.04932	37.928	33.30744	34.34083	31.28905	31.39775
HORVU2Hr1G025930	6.053315	5.644275	9.980144	8.848826	18.54817	14.86268
HORVU2Hr1G063880	70.66245	78.01432	118.3944	42.54058	62.49857	65.55362
HORVU3Hr1G115540	11.56104	11.83376	10.80319	14.65919	19.72469	18.93368
HORVU7Hr1G021560	0.383066	0.09753	0.189427	3.691781	4.95676	4.153387

HORVU2Hr1G022620	1.831947	2.16248	2.430832	3.272764	2.327786	3.347744
HORVU2Hr1G029630	1.23538	1.461024	1.495417	1.376863	1.47193	0.738374
HORVU5Hr1G026200	5.033563	4.172417	4.792365	4.212817	4.478048	5.347705
HORVU6Hr1G063600	4.229129	2.821166	4.677216	2.481724	5.267296	4.760517
HORVU0Hr1G012410	34.15424	33.29679	37.7565	25.97466	26.55007	30.13645
HORVU7Hr1G033260	16.17059	15.34492	14.45968	11.52429	12.64219	13.22281
HORVU3Hr1G108720	9.870771	6.123042	13.96066	6.037414	10.17976	11.01661
Hordeum_vulgare_newGene_11790	19.49548	20.70861	20.73813	25.07162	33.94512	31.76937
Hordeum_vulgare_newGene_153	0	0	0	4.514549	4.845742	5.911448
HORVU6Hr1G077000	13.53231	14.48944	14.61035	23.59695	38.68815	36.34787
Hordeum_vulgare_newGene_157	0	0	0.036389	75.26079	70.60096	80.49504
Hordeum_vulgare_newGene_156	1.802307	2.301186	2.333228	2.931639	4.207563	4.2447
Hordeum_vulgare_newGene_155	2.563667	2.54623	2.665506	3.45715	5.225844	5.282019
HORVU2Hr1G011810	2.423002	1.816264	2.388461	2.11699	2.469419	2.000923
HORVU6Hr1G054280	22.77382	19.93666	27.53196	27.63483	31.03227	31.57449
HORVU5Hr1G049690	1.776908	2.691584	2.820638	1.442725	1.54004	2.021802
HORVU2Hr1G038180	35.35502	29.51037	41.60582	23.44687	24.81293	23.91492
HORVU2Hr1G110130	9.623075	4.482418	5.521516	180.6324	217.5057	224.2085
HORVU4Hr1G079070	0.627171	0.494778	1.025111	1.23062	2.087472	1.717764
Hordeum_vulgare_newGene_11427	9.459993	9.083419	12.66435	194.7053	82.22398	108.027
HORVU2Hr1G036910	21.06208	17.88035	19.83857	20.05085	23.36777	20.67981
HORVU3Hr1G112010	2.667121	1.270416	1.948317	17.52844	18.72509	29.61936
HORVU6Hr1G016880	0.749822	0.50798	0.607197	3.361217	3.815392	4.891788
HORVU3Hr1G088080	15.44243	14.17863	17.54578	13.75203	42.17039	26.01848
Hordeum_vulgare_newGene_12218	1.982085	3.231648	6.055739	2.655854	1.573727	0.810007
HORVU2Hr1G072880	0.498244	0.423377	0.77913	1.913401	4.469762	3.540018
HORVU5Hr1G072710	4.179444	4.442063	4.924551	4.155887	3.498612	3.674453
HORVU6Hr1G095220	0.682505	0.749936	0.878391	1.15305	1.467557	1.367338
HORVU4Hr1G028720	0.73929	0.725473	2.380553	3.765752	14.92905	10.94043
HORVU3Hr1G058460	24.50729	23.99679	28.45101	23.98338	45.64943	46.731
HORVU3Hr1G080110	1.3534	1.686755	1.749277	0.433712	0.347098	0.925659
HORVU1Hr1G072810	19.66146	19.92157	19.96373	46.62065	43.11185	45.51938
Hordeum_vulgare_newGene_7370	0	0	0	1.48082	2.695888	2.165826
HORVU1Hr1G046270	3.376842	3.422743	3.955919	6.400623	7.072699	7.817764
Hordeum_vulgare_newGene_7378	0.024377	0	0	2.093936	3.015531	2.879529
HORVU6Hr1G059550	28.22528	25.80192	31.71464	28.71601	29.41815	30.44278
HORVU7Hr1G028160	0.805698	0.632509	0.643338	0.830237	1.588676	2.335457
HORVU2Hr1G032970	8.754157	7.548467	7.278678	19.46691	23.71932	21.99362
HORVU0Hr1G004800	1.199074	1.420552	1.514672	0.73214	0.671468	1.099126
HORVU3Hr1G017330	1.380254	1.502215	1.842556	1.034093	1.634376	1.772434
HORVU2Hr1G103020	1.376005	1.222743	1.406064	1.183153	1.086292	1.143822
HORVU5Hr1G080980	4.151339	4.49566	4.59467	3.546999	8.188212	5.567006
HORVU6Hr1G029160	4.945077	5.256494	6.485669	5.993968	7.129794	7.35113
HORVU3Hr1G064990	47.36612	38.26485	43.52433	44.42743	34.42669	39.61824
HORVU7Hr1G104490	0.836359	0.688984	1.175325	0.987719	3.189702	2.708091
HORVU3Hr1G050990	45.05376	40.37133	46.99319	52.13153	74.05511	74.17475
HORVU1Hr1G021590	27.56376	32.14017	22.66458	66.08969	111.0444	98.75097

HORVU5Hr1G061250	3.839167	4.061305	3.969998	8.297682	12.12488	11.56267
HORVU7Hr1G038290	2.342975	3.474099	4.143086	5.268263	5.828608	6.554031
HORVU6Hr1G032840	2.094198	2.773386	2.505057	1.789291	2.074853	2.238418
HORVU2Hr1G060510	1.031161	1.026907	0.976391	5.978071	9.757051	10.10918
Hordeum_vulgare_newGene_7954	3.770178	3.793588	4.355575	6.733694	7.638426	7.863178
Hordeum_vulgare_newGene_7957	1.267906	0.907895	1.345595	1.362397	2.524389	1.783096
Hordeum_vulgare_newGene_7952	6.260173	5.544026	8.025801	7.71997	9.57114	7.981418
HORVU3Hr1G068390	4.314938	3.929602	6.644265	1.657373	0.24291	0.88656
HORVU6Hr1G062440	15.33867	13.75631	19.37098	13.7667	22.67645	22.86734
HORVU2Hr1G026710	41.57	26.86089	46.25831	86.70158	45.39077	44.04562
HORVU6Hr1G053540	1.483522	1.253258	2.041392	2.003978	4.331219	3.941314
Hordeum_vulgare_newGene_3706	68.63432	66.45183	96.93649	68.66102	174.549	142.1499
HORVU3Hr1G113850	15.88623	16.41114	15.78573	23.4271	32.56941	33.18584
HORVU5Hr1G099180	2.811185	1.674652	2.07007	4.122439	2.272313	3.497943
HORVU4Hr1G021720	8.583487	11.38176	12.55875	3.649141	4.046276	4.342252
HORVU0Hr1G000490	2.703756	2.507086	2.723826	7.647967	11.7308	12.93063
HORVU7Hr1G108790	7.873095	7.977633	14.40563	5.072432	0.326549	2.037317
HORVU1Hr1G020060	0.136277	0.206106	0.199466	1.732992	5.013509	4.742547
Hordeum_vulgare_newGene_5700	10.75537	7.244418	9.677177	108.5056	151.6817	147.7488
Hordeum_vulgare_newGene_963	0.20023	0.237151	0.336506	3.002566	2.803835	2.934231
Hordeum_vulgare_newGene_960	2.792905	1.733415	1.533095	0.157847	0	0.078244
Hordeum_vulgare_newGene_961	33.10662	33.50183	40.71618	55.51246	63.87141	54.57959
Hordeum_vulgare_newGene_966	12.96078	12.71409	11.17753	3.5268	5.526976	5.691946
HORVU5Hr1G078370	34.58933	28.95251	35.36018	43.39393	42.53412	40.54505
HORVU7Hr1G034770	4.56138	4.862946	4.918021	9.59021	10.00834	11.4356
HORVU7Hr1G107140	0.824114	1.005771	0.92604	1.276045	1.436654	1.325762
HORVU6Hr1G064460	6.131964	5.119412	8.005063	18.10918	24.33518	21.67126
HORVU4Hr1G011280	81.47091	82.38646	113.0965	93.65252	164.8487	150.6609
HORVU3Hr1G107350	5.515001	3.380637	5.048644	2.431739	0.788793	1.084569
HORVU1Hr1G054390	2.102054	2.195394	2.119438	3.343322	3.629408	2.883348
HORVU7Hr1G026250	11.19343	12.64966	10.1109	6.771686	5.355671	5.099943
HORVU2Hr1G075870	0.496534	0.40153	0.726427	0.853596	2.222487	2.71651
HORVU5Hr1G023960	3.137793	4.30256	4.173138	3.576842	3.321411	4.707837
HORVU6Hr1G068770	18.37373	15.14289	21.45063	14.07588	12.36095	11.53621
HORVU5Hr1G066570	28.40967	24.55452	27.28178	26.16152	24.71331	26.7001
Hordeum_vulgare_newGene_1283	1.46798	2.064537	1.921168	4.505187	4.696156	5.211563
Hordeum_vulgare_newGene_1282	11.17164	7.274679	7.77426	3.159	3.63382	3.131598
Hordeum_vulgare_newGene_1280	2.543401	2.815914	2.675689	7.030726	5.432504	6.47638
Hordeum_vulgare_newGene_1286	14.71977	21.77599	12.75583	0.736423	0.026531	0.179946
HORVU2Hr1G004480	6.188045	3.829501	7.043506	1.029355	0.145458	1.041702
HORVU7Hr1G106230	8.772307	11.15559	14.88109	4.081244	2.721193	4.173111
HORVU1Hr1G073330	4.49428	3.667217	5.010533	5.38711	2.025419	3.838906
HORVU2Hr1G084640	18.82719	20.19791	17.75031	18.76335	10.49086	12.86829
HORVU4Hr1G055910	1.262272	1.071952	0.927152	2.170847	3.506852	4.636998
Hordeum_vulgare_newGene_6869	1.913322	1.547794	1.925675	1.9123	2.189636	2.247433
HORVU5Hr1G050720	5.75301	5.683482	6.66359	5.495294	8.445123	8.177356
HORVU7Hr1G054710	22.69565	14.95382	17.64133	32.5746	38.50922	40.93967

HORVU2Hr1G088760	1013.015	815.5583	939.8235	588.454	415.2602	385.4736
HORVU2Hr1G043220	34.42852	37.77456	60.78571	31.84099	72.64612	70.39439
HORVU2Hr1G074580	0.582728	0.424623	0.641315	0.793298	7.922162	8.516645
Hordeum_vulgare_newGene_1135	11.32396	10.99411	16.68092	5.313186	5.357474	5.518195
Hordeum_vulgare_newGene_1136	5.425307	4.715932	4.29363	36.58036	12.03726	25.40529
HORVU5Hr1G021750	60.76605	73.5616	63.47325	47.51432	23.07463	31.32158
HORVU5Hr1G051280	1.569255	3.212713	2.930728	0.208417	0.908065	0.816365
HORVU4Hr1G008130	9.407922	8.825722	8.313854	10.43267	8.593602	9.849868
HORVU1Hr1G067980	110.2502	81.6198	134.5803	13.52172	9.240789	10.82703
HORVU2Hr1G120580	9.966497	9.82889	10.45124	9.878024	16.19066	15.9611
HORVU2Hr1G038990	5.046035	7.396164	9.237492	5.790339	4.250863	5.269568
HORVU6Hr1G058090	3.34112	3.456126	4.049168	3.704469	3.519536	4.107083
HORVU7Hr1G121570	6.049755	3.798815	8.055745	5.027954	0.148272	1.448892
HORVU1Hr1G079150	3.008859	2.820422	3.595028	1.310295	0.917562	1.162599
HORVU2Hr1G034470	12.56157	12.17521	12.44217	11.01077	13.56036	16.15912
HORVU1Hr1G064950	1.30633	1.534544	0.951887	1.945119	2.184125	2.208062
HORVU2Hr1G005990	6.12911	4.795707	8.44337	4.336134	1.931964	3.168836
HORVU6Hr1G044080	11.58229	11.48071	13.55453	7.540669	6.419285	7.04204
HORVU6Hr1G062850	1.602361	1.472286	1.730881	5.559647	2.483098	4.021628
HORVU1Hr1G092980	0.64746	0.300588	0.740305	1.49889	2.697978	2.146757
HORVU7Hr1G101270	7.189392	6.958472	7.570445	14.16059	15.56989	14.32137
HORVU1Hr1G057660	12.70939	13.10885	14.74078	15.42088	16.8853	17.57299
Hordeum_vulgare_newGene_4560	2.118492	2.400857	1.847836	1.48095	1.604031	1.283863
Hordeum_vulgare_newGene_4564	1.400106	1.622209	1.53857	1.06645	0.688199	1.152076
HORVU0Hr1G006420	0	0	0.007432	1.961698	7.085776	18.7259
HORVU1Hr1G072170	3.295226	3.783249	3.925298	5.286636	6.081701	5.650131
Hordeum_vulgare_newGene_6862	3.392674	3.911323	4.994853	3.048884	1.998078	2.97294
HORVU2Hr1G017760	8.105866	9.757605	8.035512	13.78975	13.916	16.47835
HORVU1Hr1G022570	118.0794	127.7431	200.3397	102.6413	193.0431	190.3771
HORVU3Hr1G084830	15.05012	13.36948	12.64138	17.17143	8.82186	9.600563
HORVU7Hr1G090280	0.911943	0.794336	1.046105	10.89217	13.31139	11.23654
HORVU2Hr1G079180	0	0.091981	0.085075	0.466099	4.560035	4.307247
Hordeum_vulgare_newGene_7038	2.623645	2.567082	2.697471	4.658978	4.367413	5.19503
HORVU4Hr1G073150	0.269043	0.457868	0.726308	8.935253	38.01434	31.54127
HORVU1Hr1G048970	10.25708	11.14159	11.81589	11.73533	15.61502	16.28081
Hordeum_vulgare_newGene_7033	1.661266	2.172075	1.747036	0.87426	0.056183	0.201507
Hordeum_vulgare_newGene_7030	5.579512	4.226163	4.621237	5.452934	7.370593	6.86947
Hordeum_vulgare_newGene_7034	3.2386	4.605003	6.246281	0.316473	0.015223	0.143705
HORVU0Hr1G015530	6.35413	6.048013	7.561116	6.811188	7.157613	6.78985
HORVU6Hr1G072810	25.61412	27.29351	32.93832	23.67995	23.50225	27.18941
HORVU1Hr1G089990	2.211577	1.664838	1.900271	3.848433	4.281682	5.029636
HORVU7Hr1G115270	10.70181	10.85872	11.67709	6.112276	7.745096	7.977338
HORVU4Hr1G066200	2.539836	1.084762	4.644089	0.503343	0.069967	0.228228
HORVU5Hr1G027210	1.931279	1.896316	2.084082	1.859678	2.193515	2.559627
HORVU3Hr1G035420	13.2367	14.82205	16.33245	8.96643	5.888058	7.244698
HORVU4Hr1G019840	82.49288	63.55748	89.23736	76.62596	96.50495	94.95258
HORVU5Hr1G082810	241.1047	201.4943	254.8978	275.0825	230.5134	266.7616

HORVU1Hr1G086460	13.66561	12.98515	13.2517	18.37374	21.28222	23.23622
HORVU5Hr1G120340	3.263944	3.452592	3.473902	4.715481	6.535098	8.118154
HORVU5Hr1G012570	5.308983	4.855778	6.10632	1.857872	2.539492	2.099603
HORVU3Hr1G039700	1.577433	1.885139	2.494179	0.726398	0.878978	0.715661
HORVU3Hr1G040590	8.833647	11.35874	10.97958	9.740509	12.87667	13.26233
HORVU1Hr1G023620	20.51538	16.77289	20.97758	41.20651	32.33697	35.64343
HORVU7Hr1G097460	17.08432	14.57351	15.71721	12.53143	8.456039	8.482134
HORVU6Hr1G070870	17.55931	15.75783	17.02125	15.61225	20.30488	16.40066
HORVU6Hr1G029200	0.509225	0.384114	0.948194	3.804208	12.53447	11.39068
HORVU1Hr1G062030	5.379312	2.649447	6.983322	7.537116	1.558731	4.073993
HORVU2Hr1G058390	3.007325	2.368517	3.691175	0.082013	0.054257	0.063624
HORVU5Hr1G123690	1.029359	1.674892	1.874423	1.446033	2.175285	1.846732
HORVU4Hr1G052210	10.77918	8.019239	12.25469	15.99559	15.78573	16.54672
HORVU3Hr1G007290	1.279322	1.034723	1.321018	1.890803	1.477351	2.007938
HORVU5Hr1G095400	18.89046	17.64087	19.32623	17.41141	19.55473	20.09598
HORVU1Hr1G021700	7.372804	8.309102	8.335582	6.496577	10.29389	10.73344
HORVU1Hr1G050970	0.450447	0.242727	0.601504	1.143704	2.775598	2.373491
HORVU2Hr1G082170	0.585494	0.463802	0.428494	1.112185	3.824119	5.557029
HORVU2Hr1G082700	3.43737	5.291201	3.521887	12.39171	6.557364	11.04044
HORVU4Hr1G002990	6.802031	7.755786	8.731222	11.80516	17.42333	16.53909
HORVU7Hr1G051150	6.844529	6.305634	7.691136	10.1715	8.668992	11.34551
HORVU1Hr1G027010	0.443742	0.593038	0.296934	0.299258	2.763338	1.912931
HORVU1Hr1G055250	10.38012	8.909529	10.80825	10.47928	15.30158	15.05568
HORVU5Hr1G093120	2.997621	3.249024	4.200329	4.897907	9.165807	7.48117
HORVU1Hr1G090800	27.49822	24.80569	20.5335	21.75915	18.75815	21.91947
HORVU5Hr1G116870	46.12382	47.07504	57.96569	24.22749	21.90517	24.53565
HORVU7Hr1G011170	1.945778	0.226351	2.106402	2.999695	3.733333	2.597921
HORVU7Hr1G067860	9.423044	10.13171	9.683007	10.23795	9.768634	11.1828
HORVU3Hr1G030610	7.581525	5.653023	6.78087	6.145754	4.931562	5.250441
HORVU7Hr1G000250	0.326141	0.386203	0.396722	3.66982	5.247631	5.521454
HORVU5Hr1G097800	4.647865	4.144032	4.861905	5.71055	7.18103	6.220825
HORVU7Hr1G041850	17.69148	16.351	19.43342	40.479	60.27195	67.53989
HORVU7Hr1G116190	18.45485	18.43449	18.85535	15.07427	11.83231	13.34712
HORVU1Hr1G093570	0	0	0	1.0061	17.57127	12.54942
HORVU5Hr1G055360	27.6094	24.87252	32.6595	38.10119	47.72897	50.21832
HORVU5Hr1G039370	27.31368	27.04128	28.8159	30.74652	31.36628	34.13372
HORVU7Hr1G045350	79.17998	74.21072	73.66394	58.61687	70.72435	63.59235
HORVU2Hr1G123270	76.71251	63.76994	99.29927	46.88578	21.34925	24.44768
HORVU4Hr1G023430	15.69667	14.51468	16.83006	36.89348	49.11498	48.13198
HORVU4Hr1G051540	0.978669	1.469252	1.361878	2.263645	2.27629	3.113526
HORVU3Hr1G023810	36.34503	30.49328	33.22224	32.01833	34.21983	33.64349
HORVU6Hr1G093070	3.23041	3.432553	3.822034	3.737318	5.487557	5.149593
HORVU5Hr1G092290	5.451431	3.874688	5.366652	3.098787	2.255762	2.871634
HORVU5Hr1G122250	7.783886	6.215526	8.40774	5.456846	4.967695	6.052881
HORVU6Hr1G050090	8.465311	7.293898	9.530069	9.258342	15.65093	16.79223
HORVU5Hr1G016590	2.988946	3.187659	3.648741	4.881031	5.248622	5.854939
HORVU6Hr1G026260	196.3224	158.1442	160.7529	208.8753	157.5779	179.2561

HORVU1Hr1G002840	105.5541	110.9586	131.0535	146.2273	168.2779	167.4082
HORVU2Hr1G067430	4.201962	4.861036	7.135765	2.500562	3.936368	3.600005
Hordeum_vulgare_newGene_9688	6.129551	3.97723	6.020825	6.355387	6.359491	6.358936
HORVU2Hr1G094370	4.838581	4.084799	5.021362	5.340323	5.515676	6.258496
HORVU2Hr1G098650	8.960884	8.347234	8.011828	12.16628	14.10984	14.32904
HORVU1Hr1G024140	8.707023	8.802086	11.74045	12.03517	13.12671	14.14229
HORVU7Hr1G114840	1.131371	0.957216	0.901006	1.194479	1.59076	1.964093
HORVU3Hr1G083150	0	0	0	0.298962	9.25988	9.55551
HORVU5Hr1G046950	3.856332	3.444152	3.215532	2.653772	4.819012	4.649352
HORVU3Hr1G056100	17.28983	12.36829	19.09448	4.881968	1.360895	2.653092
HORVU4Hr1G049410	5.900216	5.676379	5.718113	7.606981	9.814862	9.982395
HORVU6Hr1G091500	4.34011	3.220208	3.816323	3.498061	3.536979	3.430722
HORVU0Hr1G012260	4.798633	5.797449	5.468903	6.555922	5.776096	7.206543
HORVU5Hr1G109480	3.84129	3.409299	4.542176	4.114145	6.75549	6.277942
HORVU6Hr1G010740	4.929248	4.672648	3.71203	4.65459	5.114083	6.574196
HORVU7Hr1G095230	1.587169	0.908052	2.177062	0.367274	0.55802	0.665863
HORVU6Hr1G017910	3.628796	3.878613	4.065371	4.143516	4.460657	4.22871
HORVU1Hr1G002150	4.32714	22.53782	6.733603	30.18567	4.628909	4.933596
HORVU1Hr1G049210	3.895235	3.093181	4.841425	1.081013	1.179557	1.370039
HORVU5Hr1G083340	25.39469	20.50788	30.02417	6.409508	3.625326	4.159761
HORVU7Hr1G057260	1.213067	0.766417	1.283099	0.545809	4.594525	3.051196
HORVU4Hr1G057650	10.11569	9.409736	11.98721	15.81903	25.97084	25.25437
HORVU7Hr1G082980	2.42521	2.483255	1.672242	0.389251	1.247035	1.284768
HORVU2Hr1G054760	10.63277	9.073665	10.93634	11.72556	13.7055	12.4444
Hordeum_vulgare_newGene_12940	44.84845	37.05672	57.91604	31.92941	51.26318	45.1893
HORVU4Hr1G082790	498.2702	416.3604	508.7295	314.7688	203.5297	210.134
Hordeum_vulgare_newGene_12948	9.110772	2.956808	0.025821	8.981887	8.060799	10.31165
HORVU5Hr1G025950	1.228932	1.352283	1.869729	1.631195	1.604935	1.172128
HORVU4Hr1G058550	3.466233	2.652051	3.917606	6.308991	7.645857	8.936912
HORVU4Hr1G015770	5.590934	7.00051	8.017562	6.448388	4.290841	5.536281
Hordeum_vulgare_newGene_13013	3.775158	4.187548	3.124996	5.057905	3.97971	5.403785
Hordeum_vulgare_newGene_13019	2.988219	3.584328	2.893812	2.919065	4.20072	3.840844
HORVU3Hr1G090910	13.26194	16.6947	13.84149	11.63485	12.54016	13.14553
HORVU6Hr1G019540	5.003519	5.660552	7.455818	7.039875	9.611622	9.826379
HORVU5Hr1G120800	15.32378	14.17743	13.12874	4.797211	4.96549	7.853189
HORVU4Hr1G059680	7.714868	8.225418	8.634082	9.884062	11.02481	10.54329
HORVU0Hr1G022470	4.748754	4.806289	5.491902	14.8073	27.71701	26.08203
HORVU7Hr1G074430	3.162465	3.277123	5.387799	4.039594	9.907583	7.762929
HORVU6Hr1G084080	5.22269	4.064702	5.19583	5.94084	13.70005	10.26143
HORVU1Hr1G016110	2.902038	2.507323	3.409172	1.454034	2.189878	1.854067
Hordeum_vulgare_newGene_2015	7.854198	10.60931	10.35437	0.1385	0.023744	0.062801
HORVU4Hr1G065170	35.81436	35.38624	34.72231	28.18336	33.87103	33.67607
HORVU7Hr1G042740	1.221162	1.490112	1.477978	4.458309	10.21842	8.723576
HORVU2Hr1G108580	1.242373	1.42456	2.421542	0.104649	0.558407	0.281658
HORVU3Hr1G021320	0.514713	0.323489	0.41772	1.131531	2.61558	1.63164
HORVU3Hr1G093290	12.07077	9.241386	15.52733	12.66533	13.4527	13.59682
HORVU1Hr1G044560	2.238218	1.996987	2.575348	1.327721	1.102592	1.334363

HORVU6Hr1G056000	7.454446	7.036169	8.649593	9.266012	9.3579	8.760053
HORVU4Hr1G004260	69.83401	51.84716	76.67264	59.50632	47.14313	49.19582
HORVU4Hr1G075320	3.918439	3.568667	5.022611	6.379008	8.893015	8.635693
Hordeum_vulgare_newGene_9642	0.956737	1.31536	1.210601	3.384184	1.662876	1.884354
Hordeum_vulgare_newGene_5549	29.06612	30.74346	33.69787	40.12806	56.91866	56.14265
HORVU0Hr1G016350	8.828604	5.962611	7.547864	4.79146	3.581916	3.954414
HORVU5Hr1G078170	4.920329	4.952746	5.984103	4.694683	5.26546	5.789179
HORVU2Hr1G108050	10.99866	11.5859	15.29005	13.97272	22.57377	23.18319
Hordeum_vulgare_newGene_5093	0	0	0	2.4827	2.547074	3.627222
Hordeum_vulgare_newGene_5091	8.434858	8.970316	9.052069	8.959386	10.9941	12.69406
Hordeum_vulgare_newGene_5095	5.251885	4.748755	5.330298	5.598778	10.07702	8.709378
HORVU2Hr1G121080	5.228023	4.318309	4.78454	4.607084	6.538407	7.112772
HORVU3Hr1G075800	12.57247	11.89949	10.30733	19.71833	27.81474	27.35311
Hordeum_vulgare_newGene_10096	6.765056	3.950577	7.638453	2.80532	2.920912	4.412035
HORVU4Hr1G083940	1.865539	0.835125	2.399048	6.122679	6.953885	8.315948
HORVU4Hr1G060270	26.28761	28.48453	31.85168	26.19371	22.69582	29.32204
HORVU5Hr1G046170	5.028466	5.948575	6.490791	8.172591	9.67297	10.74464
HORVU5Hr1G019600	85.9578	81.40796	76.20884	83.05204	82.33786	95.20248
Hordeum_vulgare_newGene_8114	3.76858	3.722602	4.451069	4.35664	6.246367	6.921812
Hordeum_vulgare_newGene_8111	17.72417	11.92373	17.36404	16.66076	7.714548	14.82843
Hordeum_vulgare_newGene_8112	168.707	182.1567	283.6747	159.6229	342.1055	318.7715
HORVU7Hr1G073060	12.54292	9.363409	13.34854	10.2496	16.86078	15.65111
HORVU2Hr1G072510	0.15397	0.492115	0.143422	1.564696	1.84313	2.06856
Hordeum_vulgare_newGene_8118	3.021462	2.803515	2.350648	1.596938	1.539336	1.711558
HORVU3Hr1G093430	3.778945	5.564033	4.22411	5.157118	7.381642	8.059973
HORVU7Hr1G072480	5.454525	4.854785	6.565472	6.65752	8.176469	8.801423
HORVU3Hr1G062710	31.50148	28.50985	36.53148	21.67277	22.54569	21.64571
Hordeum_vulgare_newGene_11325	2.483913	2.615408	2.091715	0.968387	0.149963	0.385153
Hordeum_vulgare_newGene_11327	6.068437	4.649383	5.468875	11.78605	53.27768	48.37183
HORVU2Hr1G104580	12.16687	9.856822	11.63382	9.236855	6.794056	6.708917
HORVU5Hr1G073140	2.759332	3.634988	4.191951	1.869469	0.706174	1.268094
HORVU5Hr1G080700	1.346764	1.617574	1.468328	9.09916	7.957527	8.319833
HORVU7Hr1G081400	1.01313	0.784526	1.24066	2.469511	2.226812	2.273224
HORVU3Hr1G022800	2.618549	2.147861	2.690451	17.89406	49.22937	37.67076
HORVU7Hr1G045830	0.705606	0.675716	0.769552	2.033737	2.708064	2.054815
HORVU4Hr1G060490	67.1375	57.46561	69.44805	49.84017	53.76991	52.52411
HORVU7Hr1G059320	19.40151	21.83106	34.8037	19.49291	33.60464	31.80028
HORVU1Hr1G004120	11.10928	9.445499	7.442278	0.701967	0.284025	0.447228
HORVU7Hr1G027050	17.17894	16.5924	16.57424	19.53386	23.9158	24.58035
HORVU2Hr1G111140	1.080597	1.251042	1.034675	3.380373	7.520141	7.452774
HORVU2Hr1G043020	25.43209	25.7256	27.3336	18.11296	23.48803	23.6215
Hordeum_vulgare_newGene_10631	0.165892	0.138294	0.072417	1.023902	2.132091	2.47679
HORVU7Hr1G062530	14.0894	14.97088	18.82705	22.42733	37.97424	32.18422
HORVU3Hr1G094800	4.914366	3.429081	4.376492	2.453839	0.557934	1.945099
HORVU5Hr1G118970	0	0.005289	0.019607	0.252306	12.13301	9.997186
HORVU2Hr1G028840	5.828174	7.659791	6.462598	6.180047	6.105346	6.222654
HORVU4Hr1G063910	4.723377	4.460017	5.380084	3.310111	4.092399	4.149855

HORVU0Hr1G025120	0.071487	0.150704	0.076048	0.410014	7.544945	7.036926
HORVU7Hr1G080440	89.43339	81.18916	86.02241	68.43292	61.80983	55.8546
HORVU5Hr1G076130	33.18338	28.48217	37.55497	35.96805	42.19169	44.29675
HORVU5Hr1G033180	4.291332	5.33347	4.331394	6.772294	8.377339	8.621847
Hordeum_vulgare_newGene_372	7.234043	7.622533	7.901829	5.335882	4.17885	5.449945
HORVU3Hr1G088320	1.633887	0.132428	0.197446	1.557556	0.129501	2.824106
HORVU1Hr1G038410	3.220611	3.984743	4.303217	3.606504	4.09829	4.787393
HORVU3Hr1G108110	22.11871	26.32039	18.54424	31.6903	39.51614	36.79797
HORVU2Hr1G069110	1.565093	1.891201	2.298432	4.279534	5.652846	6.067416
Hordeum_vulgare_newGene_4765	10.37605	8.494316	9.554925	14.22296	14.69482	15.28884
HORVU1Hr1G072370	0.082289	0.17033	0.261742	0.715431	2.221451	3.490378
Hordeum_vulgare_newGene_374	5.427197	6.167673	7.972041	3.024302	3.498281	4.288994
HORVU4Hr1G077230	19.54387	16.96113	16.29272	39.29208	65.13286	57.35308
HORVU2Hr1G083540	1.003242	1.573343	1.850725	0.926058	0.632193	1.092676
HORVU3Hr1G116360	0.892865	1.153868	0.924079	1.809444	3.354909	1.983229
HORVU6Hr1G054070	3.452567	4.359944	4.099812	6.233306	7.327619	7.523434
Hordeum_vulgare_newGene_7230	1.441912	1.593456	1.777555	1.578837	1.668804	1.988729
HORVU6Hr1G059290	1.62625	1.691339	1.841345	2.370527	3.532568	3.521909
HORVU7Hr1G033760	3.016007	3.958758	2.349495	0	0.060349	0.007082
Hordeum_vulgare_newGene_5644	0	0	0	4.579547	3.14498	2.428931
HORVU3Hr1G085320	2.91647	2.564335	2.473661	3.495017	4.736982	6.214165
Hordeum_vulgare_newGene_6724	1.225351	3.054706	0.746739	0.838921	2.92751	2.027909
Hordeum_vulgare_newGene_6729	4.79111	6.6581	6.593531	4.965267	7.549251	7.364254
HORVU1Hr1G072990	4.651709	4.108794	4.928877	5.392487	7.686201	6.878787
HORVU7Hr1G031690	3.846535	3.635503	3.331339	3.42742	4.010118	4.130927
HORVU3Hr1G089490	0.656102	0.481954	0.311207	1.598331	1.733346	1.613933
HORVU6Hr1G074540	2.79107	2.99932	3.241582	4.97	5.616365	5.827915
HORVU7Hr1G039440	8.375258	7.637784	11.33067	5.959354	10.39949	7.253237
Hordeum_vulgare_newGene_3004	0.060719	0.144933	0.261299	3.248686	47.02688	55.47268
HORVU3Hr1G086670	7.820109	5.252563	7.956863	9.386234	10.57259	9.579435
HORVU2Hr1G086870	10.73525	9.675291	12.24927	6.152844	6.334201	7.576376
HORVU7Hr1G007610	51.84976	41.27612	63.16475	49.43784	85.95633	86.02475
Hordeum_vulgare_newGene_12009	0.912813	0.887008	0.872593	1.70398	0.701135	1.016829
HORVU3Hr1G039540	3.004941	2.528396	2.810971	2.116619	2.143419	2.314074
HORVU7Hr1G052750	6.236842	5.482156	5.549232	11.87735	10.70671	10.63979
HORVU2Hr1G020100	0	0	0	0.090419	3.248226	3.133194
HORVU5Hr1G106010	10.17383	6.09491	12.18562	25.05631	11.17475	22.8101
HORVU3Hr1G103960	1.339077	1.139729	2.072119	4.060881	5.129597	3.069379
HORVU5Hr1G093430	1.025862	1.392209	2.300096	1.090026	1.13897	1.152875
HORVU6Hr1G070650	3.413343	2.678521	3.72873	5.205376	6.902638	7.385714
Hordeum_vulgare_newGene_6184	6.346591	6.055057	7.031042	4.635838	4.421739	4.762058
Hordeum_vulgare_newGene_6183	1.108625	1.013001	1.357873	1.847582	1.356669	1.460959
Hordeum_vulgare_newGene_6182	0	0.004262	0	1.485908	4.00789	2.117166
Hordeum_vulgare_newGene_14525	2.296015	1.441378	1.39041	3.174387	4.603537	4.464578
Hordeum_vulgare_newGene_14527	6.658872	6.095215	7.240807	8.216143	12.09296	14.2278
HORVU2Hr1G085350	1.810849	1.432717	1.491485	0.618134	0.549482	0.753628
Hordeum_vulgare_newGene_14521	0.60609	0.503919	0.642261	2.226891	1.990588	2.234917

HORVU3Hr1G033720	18.05555	17.64408	17.06701	28.45446	36.39668	35.16627
HORVU2Hr1G079960	4.698599	3.578003	3.612786	9.019928	11.16925	9.99376
Hordeum_vulgare_newGene_13757	6.143793	8.524469	6.53636	0	0.012533	0.035677
HORVU4Hr1G057510	6.684152	7.638319	6.382107	4.094866	4.444764	5.517192
HORVU6Hr1G059740	14.37396	14.06294	14.77144	21.12442	24.2859	26.77835
HORVU7Hr1G101920	158.7202	137.327	181.6006	185.3024	303.4995	215.8783
HORVU2Hr1G075240	1.466447	1.506296	2.167093	1.734261	2.472903	1.851073
HORVU3Hr1G067640	8.068951	7.742116	9.816008	7.324966	8.618012	8.982962
HORVU7Hr1G103560	10.32412	9.354996	10.62047	7.942237	8.198094	10.18123
Hordeum_vulgare_newGene_2358	8.431371	8.939508	8.979794	35.00639	38.54018	41.71089
HORVU3Hr1G055200	2.595898	3.962656	3.299119	3.053553	3.735647	3.94182
HORVU5Hr1G095190	3.836691	2.34014	3.862946	1.501797	2.479072	2.181192
HORVU0Hr1G000510	4.1135	3.881857	4.460402	4.758908	6.207384	7.524608
HORVU3Hr1G044380	9.802002	11.55764	11.94189	21.6249	22.97049	24.66863
HORVU2Hr1G024900	7.210991	5.683555	7.361395	8.60898	10.47508	10.36987
HORVU4Hr1G017600	0.125948	0.197686	0.217925	0.673193	4.704603	3.899515
HORVU5Hr1G079700	1.96031	1.205028	1.96864	5.03966	4.217755	5.566748
HORVU5Hr1G023000	36.38516	28.36666	38.69366	55.56331	93.58331	86.43864
HORVU7Hr1G030120	5.745708	5.095827	5.994299	20.3731	27.0315	26.93386
HORVU3Hr1G117180	3.379024	4.215115	4.136914	5.428241	6.295831	6.31182
HORVU3Hr1G113950	7.477088	8.095794	9.245422	6.165869	8.549756	8.924632
Hordeum_vulgare_newGene_15698	2.611733	3.346055	2.831524	3.805258	4.174946	4.572144
Hordeum_vulgare_newGene_15699	14.05993	12.69998	14.55192	17.05052	13.21778	14.61361
HORVU4Hr1G010050	0.3693	0.184042	0.173765	1.294631	2.063894	2.145967
HORVU5Hr1G021530	9.54542	10.56343	10.13611	10.02829	11.40585	11.30337
HORVU5Hr1G074750	43.69723	39.68322	43.33836	44.14559	38.02361	37.92123
HORVU7Hr1G096850	0.377526	0.437814	0.27973	1.902665	4.612639	4.105736
HORVU3Hr1G096890	3.48998	2.561254	3.25028	0.086815	0.038024	0.171471
HORVU5Hr1G036630	70.56695	79.68061	115.6715	61.85381	144.3782	138.8375
Hordeum_vulgare_newGene_15342	28.90339	27.79005	33.69103	24.3177	24.29422	24.86057
Hordeum_vulgare_newGene_15345	1.426664	1.421113	1.514826	3.010939	3.645352	3.925399
Hordeum_vulgare_newGene_9327	3.251328	5.070293	4.750139	4.820957	5.721099	7.707544
HORVU2Hr1G075930	80.68661	101.2634	117.6305	26.94499	6.29532	16.42547
HORVU4Hr1G002170	31.48608	20.60565	33.28911	28.18764	39.57367	38.4371
HORVU2Hr1G005650	21.15782	22.40934	20.08888	23.58988	34.82682	33.5259
HORVU1Hr1G053930	2.315439	2.606328	3.08895	5.639155	5.132917	5.331481
HORVU5Hr1G103420	12.42126	15.64976	17.58768	3.828308	1.037182	1.09208
HORVU7Hr1G076840	2.208871	2.685344	2.872252	3.367268	4.009522	3.999705
HORVU4Hr1G034590	5.547326	5.503388	6.826688	6.694797	10.08157	8.494042
HORVU1Hr1G059000	34.87008	34.35371	35.50056	35.26371	36.14293	44.5654
HORVU0Hr1G018690	1.496755	1.192334	1.164076	1.039702	1.350975	1.413369
HORVU3Hr1G043730	6.567035	6.902779	8.82359	8.030687	7.933055	9.602222
HORVU5Hr1G020720	2.411425	1.582517	2.060109	3.552185	2.842822	3.523919
HORVU4Hr1G080140	42.27897	35.26853	46.94078	50.76893	93.53163	96.98486
HORVU4Hr1G009140	6.820197	8.003364	6.348315	6.046319	7.628784	7.917155
HORVU4Hr1G011090	6.046382	5.490422	5.624627	6.880302	5.571106	5.766315
HORVU7Hr1G012840	4.676845	3.783346	4.492675	6.86635	6.504095	6.704883

HORVU2Hr1G085720	16.92609	12.93642	21.24433	18.89233	18.86447	20.99877
HORVU4Hr1G045570	3.273458	3.010271	3.147108	2.776033	4.088662	4.298312
HORVU3Hr1G036600	0.098447	0.244621	0.109409	3.496124	5.77993	5.216072
HORVU5Hr1G020930	1.407769	0.965972	0.986212	1.80787	3.02219	2.960651
HORVU7Hr1G096320	8.113496	7.817848	10.30239	8.548688	6.789813	6.684938
HORVU4Hr1G088790	5.244847	6.18575	7.594107	5.757217	5.925237	5.941246
HORVU7Hr1G113320	3.114768	3.02453	3.228757	1.759493	1.484595	2.135469
HORVU3Hr1G085860	9.756497	9.363044	12.54481	13.29901	12.66516	10.86224
HORVU1Hr1G079050	10.73602	7.220538	11.08184	17.20603	31.6348	30.90515
HORVU6Hr1G009000	12.35269	14.28773	17.37778	8.206009	4.608845	5.712614
HORVU6Hr1G029810	0.932278	1.17762	1.57524	0.994561	0.8148	0.884005
HORVU0Hr1G007630	1.68071	3.043928	1.866507	1.967426	2.636666	2.571783
HORVU1Hr1G021160	7.69886	5.619417	7.838484	2.472841	0.88891	1.634748
HORVU6Hr1G080310	47.63769	43.3506	62.32972	35.62365	32.23706	35.28992
Hordeum_vulgare_newGene_4244	0.717629	0.866821	0.957014	1.891047	1.770574	2.162885
Hordeum_vulgare_newGene_4246	5.319349	5.903521	5.667709	0	0	0
Hordeum_vulgare_newGene_4247	6.076599	7.335053	5.993193	0	0	0
HORVU1Hr1G015310	2.002956	2.395754	2.622459	1.886985	1.376503	1.729666
HORVU4Hr1G015570	13.50796	12.99518	11.00043	50.43638	61.73982	60.10013
HORVU4Hr1G019290	11.46924	12.12751	11.58293	12.76553	15.77875	16.60812
HORVU7Hr1G067200	7.678079	7.40676	7.685697	8.456396	9.723199	11.05523
HORVU1Hr1G063410	13.48726	9.151644	13.2127	18.94126	17.55781	19.52382
HORVU3Hr1G071570	53.67599	51.33557	49.50797	62.08124	53.58894	52.61816
HORVU6Hr1G018960	0.676103	0.80612	0.894693	1.043928	1.338367	1.423197
HORVU6Hr1G091380	1.397466	1.525184	1.681443	2.006788	2.581048	2.726165
HORVU0Hr1G014050	4.22001	6.174831	8.148288	4.421075	5.008881	5.810763
Hordeum_vulgare_newGene_8439	0.546193	0.59229	0.955337	1.372494	1.398693	1.206923
HORVU6Hr1G093260	479.6904	301.4955	430.6656	5.108722	5.956115	3.717343
HORVU2Hr1G017040	0.445591	1.142209	0.475374	1.142697	25.98469	19.71747
HORVU2Hr1G080070	34.76806	40.25347	32.83156	43.91573	50.63517	55.32414
HORVU1Hr1G090300	8.374445	10.65907	10.3317	2.490077	5.869304	5.271858
HORVU5Hr1G111360	9.493124	7.938406	9.227263	10.86569	9.76519	11.02284
HORVU2Hr1G078790	4.573163	3.945019	3.634131	3.604209	2.328329	2.229666
HORVU4Hr1G004460	86.95603	69.49198	81.88545	158.9956	120.2322	132.1357
HORVU4Hr1G016210	0.845583	1.126111	0.715636	1.94043	1.902794	2.131874
HORVU7Hr1G024350	7.279978	5.547808	8.472247	10.6998	3.254255	5.53494
HORVU3Hr1G005640	29.97315	28.89941	34.0154	16.53388	20.63697	16.53618
HORVU2Hr1G079220	9.377062	9.113827	9.681089	11.15166	9.80406	11.4411
HORVU6Hr1G006130	2.157065	1.562955	3.010489	10.46002	40.2519	37.79479
HORVU2Hr1G065910	21.9709	22.48309	20.97893	24.6502	24.83788	26.626
HORVU5Hr1G081010	35.79485	32.44313	32.67816	27.59109	45.24976	49.49345
HORVU7Hr1G097840	15.73252	15.90438	17.35107	13.49502	11.97613	13.37395
HORVU5Hr1G012710	1.779488	2.008523	2.113675	0.207781	0.28832	0.072059
HORVU3Hr1G065020	7.091096	8.138782	8.926316	5.674027	5.819897	7.172531
HORVU4Hr1G040140	6.645671	8.826757	8.143514	9.253412	10.63464	9.281955
HORVU3Hr1G077640	0	0	0	0	14.22297	11.90247
HORVU6Hr1G082070	2.318123	2.49495	2.913111	2.425583	2.536122	3.000665

HORVU2Hr1G108110	1.338088	1.510924	1.362033	8.868487	6.742383	7.178149
HORVU1Hr1G083660	19.00508	19.78258	22.89677	7.272235	7.045243	7.239374
Hordeum_vulgare_newGene_11125	3.701359	4.692324	2.594742	4.136589	3.517927	4.872358
HORVU7Hr1G089430	2.862602	3.654913	3.46078	3.081157	4.510307	5.190359
HORVU7Hr1G012610	2.651741	1.781793	2.470141	4.542762	3.680518	4.042978
HORVU1Hr1G045490	4.018336	3.247636	4.755101	1.463654	2.027688	2.05754
HORVU5Hr1G123080	25.35722	25.78872	26.03772	21.43415	23.38304	23.79071
HORVU2Hr1G123710	4.652362	3.73827	5.362582	18.28823	68.49464	53.61874
HORVU4Hr1G052000	1.968994	1.397539	2.283277	2.712069	2.337262	2.342802
Hordeum_vulgare_newGene_11697	3.695266	4.957349	4.587887	4.842529	4.907848	6.320139
HORVU7Hr1G087810	1.092476	0.994592	1.201928	3.660256	3.110481	2.947333
HORVU4Hr1G043490	2.777399	2.706382	2.601231	2.597441	2.626188	3.029803
HORVU6Hr1G050970	36.45674	32.66409	43.53408	37.01272	51.16557	49.64421
HORVU0Hr1G011570	6.217521	4.308705	7.435813	8.832517	9.149463	10.0229
HORVU1Hr1G078940	1.547189	0.760155	1.588293	1.453114	1.452889	1.729091
HORVU5Hr1G051980	4.606318	3.633222	4.762729	0.12364	0	0.186312
HORVU2Hr1G090110	4.138477	4.240685	4.526954	5.003895	6.220592	6.332116
HORVU7Hr1G099030	1.680953	1.680711	1.864702	2.133186	1.597907	2.067508
HORVU6Hr1G034030	6.626965	7.313314	8.348059	10.20376	13.44815	14.43576
HORVU5Hr1G072560	32.48836	31.15799	39.22163	28.77065	43.2189	44.91045
HORVU7Hr1G000040	3.532185	3.373121	4.114003	0.027718	0.018627	0.034752
HORVU1Hr1G054930	11.95236	9.506396	9.881769	17.97223	22.77524	19.33477
HORVU4Hr1G060880	19.41957	16.33795	17.00492	24.94646	31.06365	32.59313
HORVU5Hr1G122680	3.353526	3.931888	3.913485	9.914643	13.0571	12.50904
HORVU4Hr1G075830	28.31239	27.54806	27.63574	48.40033	47.27388	42.35781
HORVU5Hr1G117970	22.05833	24.37911	22.42559	19.33583	23.70365	21.03008
HORVU4Hr1G069700	4.807504	4.659179	4.533112	5.144073	5.593901	6.790056
HORVU5Hr1G096940	7.760111	5.815305	8.979356	5.981156	3.220722	5.151894
HORVU5Hr1G007780	9.234961	10.03395	12.69394	14.18841	19.03638	20.7574
HORVU5Hr1G106350	26.14597	18.04035	25.24133	16.67824	13.63225	13.70514
HORVU1Hr1G066090	5.475412	3.697935	5.605818	5.363114	2.842809	4.062054
HORVU1Hr1G016770	2.281336	2.133819	1.738824	10.13988	9.242486	7.762203
HORVU1Hr1G085090	17.24192	14.81195	18.78775	8.291095	0.989809	3.153482
HORVU1Hr1G005500	4.449731	2.38524	3.000559	2.800462	4.26533	4.093485
HORVU5Hr1G109360	7.397884	6.29731	6.392257	3.140167	1.060181	7.579624
HORVU4Hr1G062880	46.77017	44.72534	49.84421	59.39714	70.76819	66.40203
HORVU4Hr1G042450	6.347759	7.778285	6.764191	7.102601	9.065539	8.677904
HORVU6Hr1G089570	3.487536	4.330726	6.639107	1.531189	0.031989	0.471194
HORVU6Hr1G093600	6.118985	5.937291	7.607184	4.396886	5.448812	5.049852
HORVU5Hr1G005780	1.521836	1.901449	1.680449	2.123308	2.758896	2.920927
HORVU4Hr1G067960	11.54074	13.00491	14.80813	11.09745	10.73429	14.3215
HORVU4Hr1G063420	0.329913	0.246199	0.365701	5.718832	9.10272	7.677531
HORVU4Hr1G054400	5.748776	6.33495	6.322848	5.907071	9.329346	10.33387
HORVU6Hr1G023870	2.476396	2.604932	2.098823	2.239743	1.33151	3.337859
HORVU7Hr1G109580	1.471903	1.772557	1.925655	2.326612	2.452039	2.711703
HORVU7Hr1G100230	5.611137	3.089491	4.890756	9.216375	4.370862	8.175295
HORVU2Hr1G035830	10.52305	5.590171	7.836569	8.9646	0.190051	1.86911

HORVU4Hr1G079950	10.03805	11.32763	12.37458	12.83037	18.88667	19.20323
HORVU3Hr1G027700	0.872649	1.2078	0.944641	1.20976	0.905009	2.032902
HORVU7Hr1G071760	9.984807	9.866666	11.25374	36.62704	89.36035	80.77376
HORVU7Hr1G046320	60.90079	53.61734	62.87889	172.2399	226.1406	232.0687
HORVU4Hr1G025830	10.69067	10.54689	12.96902	11.24273	11.92453	12.87321
Hordeum_vulgare_newGene_3240	2.112103	1.448318	2.793394	3.859568	7.463405	6.277819
HORVU7Hr1G077640	4.507048	3.110239	2.868069	2.858347	3.451993	3.33426
HORVU2Hr1G070300	19.71168	16.60007	19.36512	17.21312	15.87769	17.25493
HORVU0Hr1G027680	2.14118	3.67071	3.100142	2.906226	4.327219	4.515846
Hordeum_vulgare_newGene_13223	5.777745	6.642556	7.186297	8.847422	11.84544	12.50836
HORVU2Hr1G076790	14.82961	16.57209	18.0954	24.91864	29.11788	30.28376
Hordeum_vulgare_newGene_13221	1.95786	1.573834	2.099265	2.252699	3.599512	4.288934
Hordeum_vulgare_newGene_13228	0.057755	0.574335	0.036461	3.290789	3.503244	2.961517
HORVU5Hr1G075640	0.488097	0.355753	0.537181	0.20515	2.372862	2.138125
HORVU6Hr1G015390	20.51085	20.56625	12.70602	4.202427	4.132186	4.001909
HORVU4Hr1G082050	51.20045	47.12466	55.66987	85.49182	115.5799	109.8343
HORVU1Hr1G003100	0	0	0	14.22451	0	0.028984
HORVU1Hr1G046740	0.094336	0.36521	0.42185	1.255993	2.281394	2.628914
HORVU4Hr1G071810	1.205267	0.888802	1.310982	1.203199	1.576821	1.532393
HORVU4Hr1G064720	24.19828	22.08799	31.26592	26.98323	41.17372	38.31493
HORVU7Hr1G074600	1.729148	1.729562	2.354414	3.132926	2.239578	2.180085
HORVU2Hr1G036510	11.67924	11.18269	14.99735	9.344794	19.95343	17.24212
Hordeum_vulgare_newGene_15106	39.27123	41.82423	42.67244	96.97652	62.03334	66.22253
Hordeum_vulgare_newGene_15100	1.304531	1.199659	1.982965	1.177069	2.59375	1.783511
Hordeum_vulgare_newGene_5303	4.505226	4.522083	5.071021	4.584108	6.099237	4.116416
Hordeum_vulgare_newGene_8830	11.69774	12.60217	11.55075	14.07662	14.47214	17.47331
Hordeum_vulgare_newGene_5355	0.760216	1.219828	1.119347	1.230266	1.31193	1.003092
Hordeum_vulgare_newGene_5354	1.575445	6.578157	1.309036	1.982087	1.176404	0.584184
HORVU6Hr1G021020	36.84943	40.49998	44.49308	35.61644	45.98903	48.70173
Hordeum_vulgare_newGene_5350	4.121348	2.746357	4.16804	5.989804	8.657692	7.935365
Hordeum_vulgare_newGene_5358	1.554926	1.301359	1.637986	1.54791	3.314548	3.463512
HORVU7Hr1G026680	24.30709	16.70931	24.58754	51.85817	56.62111	66.21983
HORVU3Hr1G026420	10.69471	8.522704	11.95704	9.993987	9.037643	8.951658
HORVU3Hr1G013970	2.11932	2.071175	2.898581	33.66062	96.65081	70.69963
HORVU5Hr1G088620	6.314267	5.311271	6.064415	6.150301	8.061808	7.268223
HORVU6Hr1G016150	28.35291	23.87852	27.4804	30.42872	36.35221	37.19958
HORVU4Hr1G079170	6.809581	6.130529	7.636595	8.217752	12.66402	11.02587
HORVU4Hr1G071360	13.63841	8.940444	16.79554	13.21717	3.353882	10.33
HORVU5Hr1G053930	3.452221	2.934341	3.894857	6.651329	5.984479	4.111456
HORVU2Hr1G039990	20.09129	16.36653	18.3163	15.93817	16.97175	15.64471
HORVU1Hr1G040250	4.453616	4.609699	4.886646	5.096879	6.759517	6.77161
HORVU4Hr1G089820	15.33033	14.76104	16.16507	16.27376	17.13608	18.17779
HORVU3Hr1G063470	2.292058	1.685109	2.028211	2.76064	3.058677	2.838792
HORVU2Hr1G072490	11.53268	12.31391	13.78026	39.18543	102.5253	90.38275
HORVU2Hr1G105220	0	0	0	0.165561	6.564539	4.724041
HORVU7Hr1G027480	7.287545	5.296353	7.718782	8.266239	5.17633	6.16519
HORVU2Hr1G070890	4.577552	4.823636	4.855535	4.466249	6.492234	5.620461

HORVU4Hr1G078710	2.693091	3.187332	3.20074	5.199073	9.443215	9.17141
HORVU0Hr1G031520	5.833159	4.231152	7.265561	7.12051	9.661838	9.049074
HORVU3Hr1G057810	21.61712	28.38428	47.26308	6.222594	1.553496	3.294073
HORVU2Hr1G022310	9.55665	11.91084	9.212993	8.394783	9.015972	9.13592
Hordeum_vulgare_newGene_8305	5.440617	5.325646	6.936268	12.83487	14.57705	17.19646
HORVU3Hr1G087800	0.699275	0.447545	0.662429	2.005249	2.711549	2.8177
Hordeum_vulgare_newGene_8303	9.902268	10.38025	13.66861	0.262389	0	0.05896
Hordeum_vulgare_newGene_8302	3.430227	3.273405	4.503624	0.68376	0.845777	0.52042
HORVU7Hr1G028590	14.00695	12.42034	13.33128	26.95733	31.28196	32.48824
Hordeum_vulgare_newGene_8309	8.919594	9.823425	9.503988	3.432053	4.58167	5.532418
Hordeum_vulgare_newGene_9052	2.716141	3.18102	3.221904	3.291711	4.688483	4.842943
HORVU3Hr1G112700	1.497355	1.578791	1.516847	2.545879	2.73096	2.931923
HORVU6Hr1G066160	18.83165	15.41862	25.20052	12.46462	21.50123	18.91781
HORVU3Hr1G094610	0.309607	1.482568	1.648496	1.110989	1.430263	1.040175
HORVU1Hr1G076710	3.484078	2.711431	2.471262	0.311951	0.352634	0.439814
Hordeum_vulgare_newGene_1504	5.014641	5.109913	4.967827	4.931884	5.325309	5.705242
Hordeum_vulgare_newGene_1506	4.063645	3.630034	4.642118	2.097606	2.980053	3.938834
HORVU2Hr1G046220	4.769565	4.023704	5.042515	4.579423	4.952972	5.594029
Hordeum_vulgare_newGene_9055	11.3128	11.87546	17.20538	10.13387	22.24337	22.084
Hordeum_vulgare_newGene_1508	4.688912	3.584529	5.356881	4.139415	3.169325	3.061932
Hordeum_vulgare_newGene_1509	19.62469	17.64422	27.13694	22.30496	27.94704	23.68836
HORVU4Hr1G046720	8.698659	8.336591	12.08494	6.41024	13.59963	11.26779
Hordeum_vulgare_newGene_11314	3.876013	3.457297	2.671434	2.778569	3.104024	2.878979
HORVU1Hr1G024460	1.685572	1.413905	1.850881	6.33939	9.651937	9.177438
HORVU0Hr1G032870	0.517412	0.742822	0.917877	2.034497	1.43908	2.550378
HORVU4Hr1G072630	9.900051	9.166836	10.56578	16.72451	24.46656	23.13955
Hordeum_vulgare_newGene_15	0.38548	0.223011	0.527145	3.157768	20.34725	9.008778
Hordeum_vulgare_newGene_12	2.153094	2.586883	3.100705	1.647299	1.904598	1.966307
HORVU7Hr1G001090	2.660058	2.527937	2.915556	5.871062	6.997381	5.79652
HORVU2Hr1G114190	1.723915	1.933005	1.78309	1.829265	2.243395	2.782034
HORVU2Hr1G102240	0	0	0	9.159032	7.825	9.093612
HORVU4Hr1G002820	0.602851	0.979174	0.888056	1.204727	3.061984	2.729463
HORVU7Hr1G032330	0.885592	0.607755	1.200546	3.484177	3.642829	4.16851
Hordeum_vulgare_newGene_7684	11.02959	10.40554	10.45613	9.802775	10.42112	10.80241
Hordeum_vulgare_newGene_3909	3.837035	3.348053	4.468796	10.03974	11.04498	11.1657
HORVU4Hr1G073320	48.31721	48.06931	51.90439	84.77345	137.3014	128.4869
HORVU7Hr1G078960	11.72358	10.40762	21.3093	0.599297	1.76587	2.004444
HORVU2Hr1G071850	9.944384	8.09131	10.19139	16.19062	17.76951	18.76448
HORVU1Hr1G078650	1.574051	1.458371	1.688633	2.094474	2.88383	2.603971
HORVU1Hr1G033270	13.30065	13.66819	14.38485	21.70374	28.34798	30.71708
HORVU3Hr1G063250	3.034604	3.182026	3.457249	6.15691	7.639178	8.5246
HORVU3Hr1G030330	10.94941	11.71158	15.61063	9.253653	11.9338	13.05738
HORVU3Hr1G080740	2.194359	2.237215	2.491986	7.915195	14.92198	12.01773
HORVU3Hr1G017600	1.890863	2.392797	2.92354	1.359587	2.166059	3.12807
HORVU5Hr1G014650	1.185404	1.241967	1.531742	3.464425	1.539406	1.789017
HORVU6Hr1G068910	1.275144	1.794199	1.845282	6.272055	4.876176	4.89678
HORVU7Hr1G001540	2.435107	2.914832	2.831258	1.030059	1.088126	1.044876

HORVU4Hr1G077000	5.338363	5.15116	6.78972	10.91956	13.55515	12.17636
Hordeum_vulgare_newGene_15956	2.185124	3.657218	2.92511	8.804359	8.726741	6.691757
HORVU7Hr1G039760	0.392278	0.389863	0.717619	1.429578	1.908047	2.154489
HORVU2Hr1G058260	11.27431	10.15394	14.77766	10.30296	13.94199	12.03295
HORVU4Hr1G073580	1.661212	1.720089	2.645701	10.57021	15.29488	14.30168
HORVU6Hr1G069380	4.036746	2.655837	4.902698	5.244136	2.859545	2.857915
HORVU3Hr1G034100	1.208228	0.855884	0.741106	1.166531	0.9619	1.136827
Hordeum_vulgare_newGene_12637	0.084328	0.107874	0.105202	2.146186	5.178493	2.781033
HORVU5Hr1G012550	23.52568	20.62115	23.07059	10.1405	11.74806	10.21072
Hordeum_vulgare_newGene_2974	2.366952	3.401339	3.770311	1.596949	1.84009	2.053261
HORVU3Hr1G044880	31.21476	34.23582	31.29737	35.28122	42.47764	40.06646
HORVU2Hr1G074320	18.33064	18.4534	20.20558	23.22485	28.02938	26.95699
HORVU1Hr1G022020	0	0	0.015376	0.260299	190.6611	171.5257
HORVU6Hr1G087250	2.276377	1.846171	1.734235	28.01796	20.1854	22.58061
HORVU5Hr1G018400	84.59863	83.71742	66.128	114.4856	128.5286	149.1653
Hordeum_vulgare_newGene_9822	12.9106	14.05821	18.26189	3.4639	4.519122	4.327494
Hordeum_vulgare_newGene_9824	23.96961	17.86727	23.05503	29.58761	57.73969	42.48984
HORVU2Hr1G001830	3.127416	2.549196	3.39824	3.031249	3.143585	3.445913
HORVU1Hr1G037760	10.57177	10.13596	9.867344	14.91348	10.15787	13.38189
HORVU1Hr1G087140	2.266972	0.738874	1.90479	1.83959	0.85908	1.213423
HORVU2Hr1G120330	4.054116	4.799336	6.66898	3.368886	3.724848	3.951669
HORVU5Hr1G124160	15.26692	13.98944	11.9736	52.75132	152.2905	149.9704
Hordeum_vulgare_newGene_14776	13.86215	12.87131	13.52127	8.135275	10.56478	9.649244
Hordeum_vulgare_newGene_14777	12.16365	9.92699	10.81897	6.736487	10.91595	8.507222
HORVU7Hr1G064340	19.52931	19.56493	19.53184	15.84152	18.08927	18.23054
HORVU7Hr1G078030	0.374251	0.397153	0.784668	1.757241	2.21674	2.368319
HORVU4Hr1G017010	14.44839	15.23532	12.7192	13.34348	13.40785	14.50114
HORVU5Hr1G065530	1.902369	1.587332	0.176156	0.659362	1.852126	0.795626
HORVU6Hr1G037760	5.23668	4.608421	5.915345	5.872735	7.515138	9.64894
HORVU6Hr1G033060	4.881588	4.959297	9.602816	2.769426	15.11683	16.89818
HORVU7Hr1G116300	52.2847	45.85474	45.46414	88.88231	90.08047	82.24915
HORVU0Hr1G008080	2.576687	2.522607	3.208369	4.096197	4.846423	5.85843
Hordeum_vulgare_newGene_4227	17.08275	17.87482	17.23807	6.084652	5.539547	7.425684
HORVU0Hr1G018170	1.709255	2.187266	1.101782	2.069527	2.095492	2.298027
HORVU0Hr1G019300	8.607686	6.766105	8.80043	1.200845	0.757396	1.688976
Hordeum_vulgare_newGene_4223	48.25719	45.60714	59.18162	60.10648	111.5337	106.7323
HORVU6Hr1G061380	3.798459	3.657973	4.043696	8.078195	6.398869	7.175897
HORVU6Hr1G064150	2.210022	2.540267	2.848456	4.969984	9.038868	8.214041
HORVU6Hr1G071390	0.228129	0.161145	0.38801	1.100346	2.595007	1.906284
HORVU3Hr1G016150	0	0.729436	0.300944	3.745766	3.813457	0.967951
HORVU7Hr1G119300	1.071756	0.801492	1.367761	1.400583	1.927199	2.435641
HORVU4Hr1G083260	8.172099	7.194528	8.9967	9.08501	8.9952	8.144593
HORVU4Hr1G043120	11.29788	12.55117	9.922333	15.05694	18.4407	19.30739
HORVU6Hr1G002300	29.67132	21.6872	20.25139	46.21516	39.90641	46.53349
HORVU0Hr1G008550	20.05991	19.97602	18.70515	19.72882	17.53227	20.80861
HORVU4Hr1G056260	3.384966	2.55954	1.327971	0.480389	0.865872	0.613768
HORVU3Hr1G087000	25.78349	18.28124	24.98925	32.59072	40.79149	29.88101

HORVU1Hr1G055530	1.598882	1.397109	2.506118	2.283989	3.792101	3.808583
HORVU7Hr1G034680	5.505013	6.019607	7.715976	5.50709	5.589267	5.356264
HORVU3Hr1G003540	0.779801	0.849058	1.268008	8.253173	20.27168	21.01079
HORVU5Hr1G024340	4.474691	2.845022	3.408976	7.42007	7.724409	9.393714
HORVU5Hr1G029110	31.27739	30.55679	45.59746	29.56977	29.46837	29.6664
HORVU1Hr1G045360	27.31986	22.24824	31.89605	4.329739	3.386355	3.305476
HORVU6Hr1G030000	38.21541	33.18342	43.77091	50.30177	92.73233	91.69645
HORVU1Hr1G022410	6.231883	4.848174	6.889878	13.78332	24.59674	24.8027
HORVU7Hr1G108550	2.804382	3.223824	2.494497	9.087345	15.8299	14.49748
Hordeum_vulgare_newGene_4367	0.860741	1.201946	1.094097	0.735531	2.173314	2.218204
HORVU1Hr1G058100	7.90573	7.367733	8.535476	9.619768	11.72205	11.4724
HORVU5Hr1G111500	181.9338	138.0579	192.102	222.6943	359.7993	344.1924
HORVU6Hr1G001760	20.08442	22.61021	17.15115	19.25686	22.32131	22.13299
HORVU1Hr1G048280	6.225668	5.698935	7.118061	8.405772	12.65004	13.40939
HORVU7Hr1G090340	1.95238	1.070104	2.610144	2.322049	3.186727	3.134296
HORVU3Hr1G082980	0.719046	1.14996	1.936279	1.663022	1.997204	1.527063
HORVU7Hr1G041170	5.339999	4.253893	5.958791	6.965658	9.96571	9.025791
HORVU7Hr1G037080	19.72983	15.71877	20.45131	27.82399	62.89549	55.39383
HORVU6Hr1G011660	8.867413	7.771056	8.020477	10.53324	21.29835	18.5519
HORVU7Hr1G010890	1.624608	1.998479	4.87253	0.098826	0	0
HORVU5Hr1G067390	1.306177	1.142865	1.374896	0.36394	1.999811	2.403464
HORVU2Hr1G123990	1.673728	1.644706	1.546743	3.167923	3.253739	2.972506
HORVU2Hr1G012440	60.99065	53.86131	54.92535	23.33565	22.12436	20.73316
Hordeum_vulgare_newGene_11782	1.982302	2.535454	2.477154	1.727959	1.20848	1.709749
HORVU5Hr1G015520	1.42545	2.198694	0.575248	1.128238	9.266392	6.366179
HORVU5Hr1G112850	9.981074	9.187447	12.01716	16.50958	46.20423	40.95312
HORVU6Hr1G000410	1.969979	1.977451	2.510079	3.476378	5.241711	4.433165
HORVU4Hr1G001220	152.042	149.7306	229.6163	149.0221	303.2295	279.0133
HORVU4Hr1G069870	2.762241	2.635936	3.123495	2.901264	3.220175	2.495178
HORVU1Hr1G022840	11.73445	10.11604	13.28282	16.99618	21.49562	21.48331
HORVU1Hr1G088880	83.10825	57.83418	82.36071	247.9387	276.9617	284.4604
HORVU0Hr1G007860	16.95565	16.51195	16.92921	16.90629	21.63545	21.17884
HORVU2Hr1G093950	0.82582	1.012269	1.075176	1.886631	11.27236	9.998036
HORVU1Hr1G012470	37.96365	39.0676	61.97524	39.05932	66.49537	65.0731
HORVU2Hr1G081260	1.477437	1.52719	1.860748	2.40742	4.229417	4.339991
HORVU7Hr1G110610	13.78487	10.02545	13.42867	6.811654	4.675096	5.412878
HORVU1Hr1G057170	43.62844	38.31659	9.434517	9.011927	9.203306	8.399533
HORVU4Hr1G040770	34.94475	35.88579	47.50322	33.84993	45.78502	46.2956
HORVU5Hr1G027720	4.414453	4.24828	5.416821	7.232893	11.23954	11.9879
HORVU0Hr1G014790	15.32314	17.9489	16.14285	16.94926	15.34935	15.64369
HORVU5Hr1G104080	4.751051	4.686936	5.250693	3.793015	1.149423	1.916643
HORVU2Hr1G083530	11.92092	11.03613	16.17754	10.8751	16.11748	14.3037
HORVU5Hr1G056930	9.692025	10.0163	10.2887	11.45106	11.4645	10.97333
HORVU1Hr1G058810	106.205	87.35928	125.1951	112.7295	110.996	113.6394
HORVU3Hr1G082280	1.835511	2.080237	1.991194	4.693123	6.506169	6.10946
HORVU7Hr1G075710	1.211386	1.104094	2.37021	1.558203	2.008568	2.172038
HORVU6Hr1G082990	0	0	0	0.258468	96.57166	31.00915

HORVU7Hr1G094310	3.823364	3.779179	5.287932	5.027954	5.486251	5.115623
HORVU4Hr1G012060	21.37803	26.12653	36.21324	1.336285	0.993369	1.519791
HORVU3Hr1G041640	11.06755	9.530918	11.07365	13.77932	17.33423	17.44311
HORVU7Hr1G091790	7.781939	9.023799	8.117827	14.08644	21.87977	20.58813
HORVU2Hr1G095070	59.67164	51.93906	69.65805	59.60313	49.72479	49.69639
HORVU2Hr1G051970	18.68457	19.33487	17.15822	19.43763	12.1676	14.04968
HORVU7Hr1G012130	2.314554	3.039799	3.20317	2.296742	3.734456	3.736623
HORVU4Hr1G048060	0	0	0	2.866383	3.431038	3.221966
HORVU5Hr1G080240	2.444932	2.623765	2.923682	3.110268	4.501924	3.977426
HORVU0Hr1G014240	8.197444	8.103473	8.501836	5.628816	5.765316	5.955667
HORVU2Hr1G017650	0.892344	0.931038	0.862554	2.58445	8.491178	9.407321
HORVU1Hr1G006860	51.44378	49.33366	53.5198	64.19708	124.4721	112.2788
HORVU6Hr1G006810	2.837507	2.878585	2.284812	4.588991	6.565017	5.7015
HORVU3Hr1G064230	4.951186	3.975025	5.443267	7.693008	7.926262	6.865902
HORVU1Hr1G056220	14.39104	16.82601	13.33296	19.11089	29.97266	27.67472
HORVU0Hr1G001420	0.211013	0.345445	0.101138	1.868263	2.109148	2.578001
HORVU3Hr1G071430	14.76904	14.8849	15.12121	9.766069	6.549837	6.981829
HORVU5Hr1G057080	22.43665	23.17098	22.08135	44.06506	43.24402	40.03744
HORVU1Hr1G069430	2.357951	2.365266	2.477649	1.939475	2.956756	2.573585
HORVU4Hr1G068830	3.231618	3.091771	3.512257	6.023693	8.08996	8.060464
HORVU1Hr1G073840	10.54238	9.25462	11.85204	12.16033	13.22145	14.6185
HORVU3Hr1G001360	1.351337	1.640858	2.454631	1.684492	2.716036	3.183913
HORVU2Hr1G096670	2.592705	1.699683	1.909521	2.648915	1.966595	2.403425
HORVU4Hr1G025360	5.098476	4.987363	6.517482	1.887504	0.746426	1.278824
HORVU1Hr1G038800	6.156944	4.664865	7.661765	7.367105	8.970515	8.829372
HORVU5Hr1G045700	0.439292	0.347225	0.462886	0.526152	3.729461	3.490054
HORVU5Hr1G095310	3.67463	4.490453	6.667153	1.979569	2.711447	2.700092
HORVU5Hr1G057330	3.476324	3.482883	4.362303	2.861563	5.386866	6.230333
HORVU2Hr1G037620	0.623678	0.467713	0.735593	1.809092	2.077855	1.131453
HORVU5Hr1G113810	45.72916	41.54813	52.61536	61.50605	53.5727	45.1857
HORVU5Hr1G081190	17.65688	17.41725	20.71137	16.9674	20.22608	20.09102
HORVU5Hr1G028980	1.342049	0.681898	1.152165	0.692554	1.630099	0.944095
HORVU5Hr1G047410	1.073557	0.917718	0.840769	4.765473	13.50959	10.31722
Hordeum_vulgare_newGene_10448	4.972152	5.230182	6.008788	2.634914	3.480858	3.530257
HORVU5Hr1G104920	1.820254	2.408626	2.56577	3.268851	3.967889	4.236478
HORVU2Hr1G111370	8.969339	9.723902	9.990929	15.02058	17.15074	14.83803
Hordeum_vulgare_newGene_10443	32.93354	40.07112	36.07097	13.42489	18.31503	19.59959
Hordeum_vulgare_newGene_10441	0.286176	0.133818	0.219916	2.375926	35.3997	34.72789
HORVU6Hr1G032070	13.68723	12.91912	14.89308	15.27429	30.78516	33.25171
HORVU1Hr1G081300	15.59348	12.17955	13.66782	1.672819	0.247055	0.822646
HORVU7Hr1G090110	5.318744	5.572983	7.070163	6.528052	7.276839	6.786293
HORVU5Hr1G030720	4.14257	1.643387	5.185286	3.574775	2.754288	3.545413
HORVU4Hr1G002760	13.89408	8.321275	10.66619	12.92287	11.83029	16.86665
HORVU1Hr1G060010	14.71572	14.62664	14.68649	14.17109	14.7117	14.37187
HORVU7Hr1G044310	1.752388	2.234592	3.052748	2.002099	1.417535	1.710995
HORVU4Hr1G090210	1.597044	1.875671	2.211246	1.040484	1.127133	1.182437
HORVU4Hr1G050700	23.18844	22.18123	23.34738	43.48937	53.44268	47.55499

HORVU7Hr1G116460	9.956664	9.159487	9.547391	12.43945	11.26199	12.367
HORVU1Hr1G045630	57.85749	47.56726	62.86979	68.75476	83.70157	87.53122
HORVU6Hr1G047120	1.188825	0.515793	0.991167	0.690527	2.266291	1.068974
HORVU2Hr1G041260	70.18758	60.48057	67.47758	89.48322	75.51727	80.54278
HORVU3Hr1G075920	0.88021	0.736693	0.943967	0.79316	2.218614	2.371525
HORVU4Hr1G060600	2.803003	2.447057	2.990885	7.435568	13.42509	12.58724
HORVU5Hr1G086970	2.341174	2.534112	1.960843	3.102694	2.887003	3.894069
HORVU0Hr1G010890	2.308043	2.377547	3.106378	2.321965	3.10725	1.804648
HORVU4Hr1G051250	6.593832	7.033862	7.48074	11.49424	17.23931	18.02733
Hordeum_vulgare_newGene_11715	0.987863	1.423401	1.414433	0.407836	0.986296	1.125502
Hordeum_vulgare_newGene_11719	39.13783	28.48122	37.67856	46.03667	46.512	54.26714
HORVU5Hr1G048030	22.77505	22.37526	20.35646	19.12615	17.42811	19.31825
HORVU6Hr1G077730	1.108704	1.857822	2.90833	0.842265	1.229503	0.993555
HORVU5Hr1G122760	2.253036	2.643765	2.882653	1.87013	4.007633	8.032951
HORVU4Hr1G067370	23.34711	18.84222	24.48458	34.6482	51.78624	47.91688
HORVU1Hr1G040720	17.04578	15.4425	13.61578	35.15166	34.25688	35.41829
HORVU5Hr1G042160	1.667043	1.262086	1.363078	2.612206	3.931035	3.15249
HORVU2Hr1G112480	1.615654	1.61262	2.048805	1.618166	1.619163	1.739229
HORVU6Hr1G010890	3.820984	3.414372	8.06728	1.366769	1.303306	1.748074
HORVU2Hr1G108320	3.01878	4.048462	4.380596	2.598193	3.880405	4.082903
HORVU3Hr1G092880	1.113054	1.099549	1.893814	1.074022	0.93296	1.242325
HORVU3Hr1G103540	1.503461	0.686556	2.148787	2.635966	6.512862	6.858666
HORVU7Hr1G044860	4.080489	4.393912	4.3448	4.863914	8.325547	7.35541
HORVU2Hr1G115130	1.050877	1.281146	1.403678	0.838152	1.408689	1.759118
HORVU3Hr1G094450	5.598666	6.608077	7.549352	10.5814	17.42652	16.04674
HORVU6Hr1G025830	10.4864	8.68107	12.04118	11.39061	7.831937	7.638053
HORVU4Hr1G056700	35.20564	28.68934	37.22637	58.49573	51.6562	48.95135
HORVU2Hr1G063800	1.000468	0.90195	1.403224	5.838902	6.790098	9.72671
HORVU5Hr1G071910	8.631802	8.242836	7.396612	11.18758	17.1446	16.67951
HORVU3Hr1G022380	3.074906	2.549217	2.363807	2.483613	3.278002	3.201316
HORVU2Hr1G090090	1.108799	1.267884	1.613138	6.228645	9.960696	9.245469
HORVU5Hr1G046420	0.940725	2.889589	3.089684	1.900888	2.872185	3.55382
HORVU2Hr1G013760	1.542372	0.575645	2.178091	0.45002	0.196126	1.466397
HORVU5Hr1G076030	32.7716	26.90963	42.34969	25.37016	34.01139	32.85904
HORVU6Hr1G095110	3.229173	3.533016	4.418876	3.0878	3.02609	4.234879
HORVU2Hr1G088540	107.9973	104.9243	139.5742	94.19576	54.06569	56.3493
HORVU2Hr1G020790	15.71761	12.89413	18.26748	29.1173	31.29341	28.93484
HORVU1Hr1G019600	15.86304	19.10775	29.52313	16.41294	29.77192	28.50747
HORVU5Hr1G016750	1.513266	0.985543	1.024469	1.285542	2.582335	3.048945
HORVU2Hr1G113990	1.39749	1.36343	1.707272	1.094529	1.211026	1.097448
HORVU2Hr1G095910	2.841782	3.498964	3.429529	4.031719	5.134419	5.074266
HORVU2Hr1G063590	15.42288	13.1983	18.36424	13.21775	17.61389	16.63346
HORVU3Hr1G076030	2.270679	2.071403	2.739777	2.482752	3.358355	3.171842
HORVU5Hr1G040140	1.951371	1.399667	1.80549	0.824927	1.06976	1.353043
Hordeum_vulgare_newGene_16152	6.55576	7.281459	9.107072	7.535805	7.73761	7.862372
HORVU1Hr1G001920	29.60841	33.65759	35.70806	59.4441	46.5852	37.6748
Hordeum_vulgare_newGene_16158	1.337268	3.256934	3.04703	0.203218	0	0

HORVU2Hr1G039270	0.549852	0.846602	0.192954	0.477166	3.229734	2.259558
HORVU7Hr1G100370	68.72683	75.80875	80.32101	71.67787	81.44719	81.35843
Hordeum_vulgare_newGene_12435	3.113644	2.916588	3.915812	0.91244	1.280437	1.045061
Hordeum_vulgare_newGene_12436	11.02581	10.71708	11.30812	12.12728	10.14715	11.2154
HORVU7Hr1G047770	2.124646	2.618438	2.675018	3.61994	4.960592	5.575883
Hordeum_vulgare_newGene_12432	9.469846	8.907184	9.417489	16.74941	18.471	18.64277
HORVU7Hr1G035480	1.871625	1.198862	1.734156	0.864105	0.261791	0.790211
HORVU1Hr1G072890	8.595904	8.301115	11.40079	3.786329	4.45743	5.242705
Hordeum_vulgare_newGene_13525	5.457454	5.257079	5.646747	6.114116	7.990536	8.288797
Hordeum_vulgare_newGene_13526	6.023117	6.552353	7.34765	6.931651	9.086988	8.571383
Hordeum_vulgare_newGene_13527	9.610889	9.412567	12.05095	8.257049	8.785814	9.38659
Hordeum_vulgare_newGene_13528	2.794206	3.023797	3.974794	2.43896	3.456777	3.145821
Hordeum_vulgare_newGene_13529	0.639448	0.524612	0.79373	1.328894	1.584281	1.572329
HORVU4Hr1G065440	0.09177	0.106705	0.013193	0.411584	7.178448	5.382391
HORVU2Hr1G021540	4.27194	2.507834	2.692414	6.09468	7.729124	8.413897
HORVU1Hr1G012730	14.80562	13.99743	18.88921	10.67419	9.907685	11.19369
HORVU6Hr1G027520	0.774334	0.835442	1.330001	1.413029	1.676716	1.427575
HORVU7Hr1G077560	0.944037	1.134818	1.500298	4.161483	5.972497	5.718526
HORVU4Hr1G052450	39.52961	42.97862	54.23368	7.122671	6.244946	5.211337
HORVU5Hr1G121440	3.078061	2.861549	3.073483	45.48943	53.73003	54.82717
HORVU2Hr1G056040	20.44651	19.41225	21.21358	18.20561	19.4117	21.1604
HORVU7Hr1G038160	3.970347	3.077407	5.203307	5.36828	8.525816	6.893524
HORVU5Hr1G080900	2.179834	2.870407	2.893915	3.884291	1.648484	2.542494
HORVU3Hr1G080660	2.112045	2.486076	2.901667	2.159989	2.149479	2.215536
Hordeum_vulgare_newGene_13384	1.984147	1.972427	2.570303	1.722583	2.811146	3.196017
Hordeum_vulgare_newGene_14172	1.095762	0.800697	0.915495	4.816523	3.375124	4.741051
Hordeum_vulgare_newGene_13382	1.348379	1.378783	1.938761	2.106703	1.767978	2.088126
Hordeum_vulgare_newGene_14171	26.80997	19.41678	23.24447	17.08451	11.42225	11.22496
HORVU6Hr1G035600	1.136903	1.253088	1.260102	1.487414	2.662788	2.719536
HORVU6Hr1G014980	6.773887	3.52638	3.40499	5.304558	2.243876	4.52997
HORVU4Hr1G058930	5.283817	6.273769	5.817346	2.019341	2.717023	3.311353
HORVU3Hr1G055630	13.50129	9.716679	12.9468	13.9449	16.39334	16.00113
Hordeum_vulgare_newGene_2780	0.123343	1.393908	1.648133	0.706367	1.667647	2.181756
HORVU3Hr1G021290	1.914215	1.581433	1.772744	1.191103	4.594244	4.98515
HORVU6Hr1G072040	19.89891	15.37935	22.69922	31.99844	27.42012	28.95909
HORVU2Hr1G054470	6.803271	6.359521	6.769571	9.200105	10.3254	9.323543
Hordeum_vulgare_newGene_5836	0	0.032295	0	2.584312	2.959531	2.632397
Hordeum_vulgare_newGene_5837	34.71722	31.72285	31.32895	41.50993	28.14575	32.69852
HORVU7Hr1G102840	82.79454	74.17415	79.8499	95.92536	72.70425	80.3583
Hordeum_vulgare_newGene_5832	2.018077	2.254081	2.370837	0.579127	0.808076	0.888127
HORVU3Hr1G059530	0.806856	0.514628	0.96774	1.43277	1.31459	2.069923
Hordeum_vulgare_newGene_2435	3.499868	3.565593	4.978862	3.104352	7.782976	7.423267
Hordeum_vulgare_newGene_2433	3.551856	4.451027	3.787573	4.651755	5.716444	6.505963
Hordeum_vulgare_newGene_2439	0	0	0	0	3.214485	4.333582
HORVU5Hr1G078080	4.123133	4.224528	4.588207	8.143554	14.47824	12.43191
HORVU6Hr1G059860	5.477306	5.834095	7.976795	5.763641	4.764941	5.684332
HORVU1Hr1G000640	7436.788	6365.695	5904.268	11085.92	3390.09	4808.801

HORVU7Hr1G096170	10.72347	9.172109	13.86478	6.778154	9.232976	8.604956
HORVU5Hr1G088410	5.22112	4.21222	7.255667	4.061516	6.108441	6.39963
HORVU4Hr1G061210	26.78536	23.76596	29.13407	29.36418	27.51608	27.10266
Hordeum_vulgare_newGene_5786	4.699638	5.331432	4.712617	5.014049	6.618006	6.926392
Hordeum_vulgare_newGene_5785	1.071588	1.365986	1.13738	1.681035	1.415011	1.713606
Hordeum_vulgare_newGene_5788	10.37161	12.54745	11.37179	10.32284	11.05988	11.93681
Hordeum_vulgare_newGene_5789	2.781436	2.898308	2.547591	3.087829	2.980174	3.014171
HORVU5Hr1G006290	1.562713	1.739658	3.056673	0.303961	0	0.285414
HORVU1Hr1G077360	2.128855	2.107593	3.577166	3.453482	4.813151	4.626047
Hordeum_vulgare_newGene_10129	4.982383	3.284977	5.151239	2.835908	3.412772	3.698818
Hordeum_vulgare_newGene_10127	8.918452	8.027543	7.711739	9.766027	13.85196	12.60737
Hordeum_vulgare_newGene_10126	3.270195	3.260215	2.593755	1.917579	2.192112	2.287772
Hordeum_vulgare_newGene_10121	0	0	0	0.249437	4.469646	1.952977
HORVU5Hr1G032980	20.29281	17.81669	17.13261	78.50481	104.4528	107.3813
HORVU7Hr1G107720	7.661139	6.723841	8.541384	15.9435	13.27974	12.60873
HORVU3Hr1G093320	35.601	35.83124	35.43212	30.00204	39.64045	41.16558
HORVU7Hr1G044450	10.80251	12.84835	10.78329	12.79685	11.75603	14.12624
Hordeum_vulgare_newGene_11831	0.26304	0.268013	0.182713	1.904226	2.314376	1.525296
HORVU5Hr1G103850	29.04869	26.91727	32.2261	40.34555	59.3301	56.4051
Hordeum_vulgare_newGene_9777	0.362704	0.120486	0.289828	1.807596	4.777285	4.044428
HORVU5Hr1G019880	1.866698	1.207395	2.386376	2.376714	1.586148	2.196707
Hordeum_vulgare_newGene_9771	0.279427	0.129769	0.265697	34.62051	20.23836	20.98108
HORVU7Hr1G073040	0	0	0	2.07357	3.193529	2.441099
HORVU7Hr1G023440	8.162586	8.629594	7.023773	4.122697	0.318656	2.257593
HORVU6Hr1G075810	4.44988	4.063555	5.056588	4.681696	3.752725	4.938864
HORVU2Hr1G073730	6.416561	9.509965	7.284277	0.529388	0	0.052254
Hordeum_vulgare_newGene_15343	5.297737	4.721232	6.116813	5.151427	6.238607	7.710653
HORVU3Hr1G052980	1.926783	1.679075	1.885498	3.388326	3.713138	3.997197
HORVU6Hr1G005420	2.827471	0.913001	3.957723	1.27779	0.830333	2.518307
HORVU3Hr1G015150	81.22556	81.27662	61.46716	52.73049	51.31275	50.25145
HORVU5Hr1G089970	9.183011	8.551715	8.69619	15.14201	14.8897	15.68931
HORVU4Hr1G057310	3.033228	2.713525	3.904688	2.754148	3.978745	4.071089
HORVU4Hr1G075700	19.34208	18.31718	21.25292	19.71856	24.77225	23.03402
HORVU3Hr1G115990	398.1054	311.8207	542.4282	302.015	219.1025	313.8914
HORVU2Hr1G046380	64.19888	70.76115	100.1389	51.62759	94.5751	85.55755
HORVU5Hr1G031470	42.78722	37.30982	42.37806	67.9994	69.20952	73.80542
HORVU0Hr1G038670	2.124706	2.177097	1.718705	0.748825	1.627703	1.691589
HORVU7Hr1G061620	6.234189	6.083365	8.41581	8.479294	12.84544	12.73323
HORVU4Hr1G088340	4.090643	4.361551	4.489038	6.473416	8.772229	11.13527
HORVU7Hr1G036110	18.30611	17.78668	20.78836	14.2184	13.75449	14.29259
HORVU5Hr1G124070	10.68018	10.60833	14.04578	14.07392	16.73002	15.70707
Hordeum_vulgare_newGene_4073	7.637208	8.642858	7.025502	6.914417	4.1954	7.668732
Hordeum_vulgare_newGene_4072	6.776343	2.685662	7.428183	2.665743	1.891221	2.321204
HORVU5Hr1G117800	2.737847	2.698903	4.483122	4.85719	5.678015	5.080321
HORVU7Hr1G115580	3.631814	2.974278	3.665855	6.584959	7.253533	7.811961
HORVU1Hr1G064480	38.29616	33.58731	42.37494	52.28244	64.99665	61.30165
HORVU6Hr1G057680	5.413629	4.373837	6.060913	3.744995	3.53848	3.622364

Hordeum_vulgare_newGene_14920	1.56762	2.304587	2.620823	0.331544	0.131848	0.186821
Hordeum_vulgare_newGene_14922	11.88487	11.22036	7.590104	0.590701	0	0.284808
Hordeum_vulgare_newGene_14925	1.641107	2.254479	2.948001	1.661129	2.163058	1.891974
Hordeum_vulgare_newGene_14924	0.861492	0.248652	0.653769	2.178395	3.660723	3.384431
HORVU6Hr1G024190	21.47803	16.6992	46.26791	15.41875	85.78044	132.4527
HORVU4Hr1G072280	17.37373	18.4502	19.25544	16.78053	23.41364	22.4875
HORVU3Hr1G074040	46.6168	50.37739	66.09533	50.38546	57.33216	60.24892
Hordeum_vulgare_newGene_4699	3.515352	4.007774	3.841076	0	0	0
Hordeum_vulgare_newGene_4697	5.079652	5.475889	6.267924	2.815462	2.351683	2.977413
HORVU1Hr1G076900	19.29423	19.12328	25.83993	15.58856	20.97092	20.20376
HORVU6Hr1G076750	7.675964	8.758863	10.45876	4.330557	4.349306	4.763955
HORVU3Hr1G070090	1.948714	2.539986	4.59862	1.860726	3.612829	5.28347
Hordeum_vulgare_newGene_7521	30.39816	27.91066	31.07676	102.9534	132.5579	101.8959
Hordeum_vulgare_newGene_7522	4.58779	4.682834	5.491208	5.732864	6.819243	6.02322
Hordeum_vulgare_newGene_7523	4.408986	5.492099	5.119984	6.664376	5.351526	6.291922
HORVU7Hr1G099250	0.480135	0.652855	0.495849	2.93926	3.781524	3.218335
Hordeum_vulgare_newGene_7528	0.62584	0.587734	0.607189	1.579713	2.094727	2.658342
HORVU5Hr1G010560	7.012038	4.976373	5.931236	1.114401	0.303225	0.703137
HORVU3Hr1G091210	7.89285	6.444999	8.462144	11.02178	13.79421	10.36542
HORVU7Hr1G052840	8.486051	5.174182	10.16703	4.165234	8.358288	7.719516
HORVU5Hr1G087250	15.34258	14.12364	12.25005	75.67178	114.8852	118.9528
HORVU5Hr1G088200	0.90678	0.828082	1.105091	1.997246	2.13056	2.050024
Hordeum_vulgare_newGene_3699	4.967424	5.871248	7.172759	2.634322	3.05115	3.4621
HORVU4Hr1G064340	18.02803	13.65386	19.88101	18.9467	15.13377	16.73293
HORVU2Hr1G045120	1.51858	1.814914	1.310437	0.520146	0.603482	0.652006
Hordeum_vulgare_newGene_6696	1.361946	1.471177	1.67471	2.560267	3.928302	4.389837
HORVU7Hr1G053460	1.356668	0.819795	1.56222	1.853516	2.241594	2.222002
HORVU2Hr1G032780	0.97025	0.782777	0.944995	0.954611	1.716728	1.166149
HORVU5Hr1G125110	1.223969	1.180989	1.038327	3.320651	4.529003	4.639591
HORVU1Hr1G021780	94.64015	91.67721	116.579	131.8627	134.8354	135.0302
HORVU6Hr1G033450	6.472017	5.919394	6.669782	6.820224	13.379	12.67809
HORVU6Hr1G075380	18.0244	17.83875	17.19188	0.415431	0	0.247841
HORVU7Hr1G018350	5.810678	7.105603	8.16469	8.436331	10.4686	10.59949
HORVU7Hr1G038400	7.597795	4.435567	5.166338	2.495792	0	0.611414
Hordeum_vulgare_newGene_6344	5.024935	5.324371	5.057806	4.91412	5.53006	6.105722
Hordeum_vulgare_newGene_6348	1.391	2.724064	3.016077	3.119566	6.752838	5.530405
Hordeum_vulgare_newGene_13550	0.079404	0.027808	0.221164	0.192079	43.60619	72.33116
HORVU2Hr1G074280	0.892174	0.830504	1.014898	2.307007	4.212576	4.005204
HORVU2Hr1G032250	10.05057	10.04564	10.08336	11.27441	12.442	12.29519
HORVU6Hr1G069260	20.04656	17.35173	24.34653	20.2515	21.70273	17.66291
HORVU7Hr1G067710	1.234521	1.120851	1.716449	2.788075	3.58075	3.665802
HORVU7Hr1G055100	16.5827	16.343	15.9264	10.14184	6.999013	8.958297
Hordeum_vulgare_newGene_1222	1.277922	1.169761	1.275632	2.362613	4.704724	4.431209
HORVU3Hr1G032920	10.04521	10.02721	12.80875	5.834792	6.420236	5.973443
HORVU3Hr1G087700	39.0683	36.11366	48.57791	29.45293	28.27597	27.44806
HORVU1Hr1G077340	6.152311	5.891085	5.604623	4.88399	6.066436	6.250609
Hordeum_vulgare_newGene_2844	1.960005	1.758488	2.037833	2.180111	1.153291	1.214251

Hordeum_vulgare_newGene_2841	2.288097	2.012658	2.252921	2.357514	2.441031	2.277369
HORVU6Hr1G003770	24.02216	22.97587	23.6192	15.3709	20.87642	21.98946
HORVU4Hr1G051010	60.25206	64.14729	75.25227	19.94343	7.632819	9.721829
HORVU1Hr1G026940	2.502049	3.122178	1.594471	1.940544	14.83376	10.8371
HORVU5Hr1G106120	24.6343	21.91628	28.3835	17.76617	13.69064	16.1022
HORVU6Hr1G038760	11.9802	13.87938	12.6176	13.22523	16.15966	19.1674
HORVU5Hr1G122560	38.55466	33.90719	45.43888	28.55225	30.96959	33.03483
HORVU2Hr1G051690	5.697524	5.899998	6.103117	8.498279	8.599922	9.37525
HORVU6Hr1G070230	6.083252	7.666903	7.615377	5.169057	7.168613	6.426144
HORVU1Hr1G027090	0.476557	0.644964	0.40082	0.366801	2.604902	1.62524
HORVU5Hr1G094350	11.87256	13.03742	13.09529	10.38603	11.9097	12.71337
HORVU5Hr1G124390	1.749544	1.551413	2.24354	1.728394	1.228876	2.137149
HORVU2Hr1G005140	6.310249	4.782979	6.918292	2.680458	3.666316	3.333885
HORVU6Hr1G034680	7.204724	8.952398	6.634584	11.95792	18.80883	18.6473
HORVU2Hr1G081400	3.891899	4.697724	4.816802	5.889515	6.952104	8.429171
HORVU1Hr1G003590	11.85103	13.71443	16.68235	8.30333	9.135366	9.931775
HORVU4Hr1G042640	2.12162	1.331587	1.751261	3.888645	3.625774	4.088641
HORVU1Hr1G066190	2.477919	2.036375	2.764267	6.1202	6.168004	5.683579
HORVU1Hr1G054560	8.981606	8.202789	9.291165	10.43783	8.248113	7.891566
HORVU4Hr1G089280	7.249273	6.913482	8.093489	6.706699	8.027502	7.871474
HORVU3Hr1G038490	6.669568	6.09332	7.59032	7.046049	10.61389	10.32603
HORVU1Hr1G061210	6.492614	5.777143	8.315162	7.351072	11.28574	9.963757
HORVU3Hr1G029730	4.369891	3.363799	5.979886	5.531187	7.93913	8.382292
HORVU3Hr1G013640	6.547358	6.90665	6.939929	6.468972	6.654911	7.010463
HORVU5Hr1G035610	1.98522	2.119826	2.172254	5.112123	9.260604	7.768387
HORVU5Hr1G073730	3.564372	2.647731	4.57703	4.06986	5.400218	7.421165
HORVU5Hr1G114950	10.44048	10.21889	9.439591	10.74749	12.43912	13.00883
HORVU4Hr1G003600	84.84583	70.02003	87.48832	98.89089	84.17127	76.62761
HORVU1Hr1G088300	0.553045	0.253817	18.11986	49.1145	0.548329	0.744902
HORVU6Hr1G061470	1.418217	1.542874	2.150264	2.103493	0.927001	1.705952
HORVU2Hr1G004230	26.12554	15.30748	36.05937	10.62646	6.72426	11.73945
HORVU2Hr1G019270	10.68542	14.66484	14.318	35.06579	62.07396	59.0142
HORVU6Hr1G030220	2.177764	2.070174	2.153293	4.021785	4.829096	4.333785
HORVU4Hr1G010160	0.036639	0.137337	0.013466	1.536182	2.46728	2.402404
HORVU7Hr1G051730	1.677023	1.596836	1.459737	1.289723	0.963742	0.647455
HORVU5Hr1G021480	2.13606	1.764138	3.272892	2.843831	0.156026	1.357243
Hordeum_vulgare_newGene_1936	1.016663	0.846178	1.149595	1.174966	1.068196	1.244378
HORVU3Hr1G076790	2.604371	2.430691	2.303312	4.486707	10.35375	8.114368
HORVU2Hr1G123070	23.4706	23.96754	26.77902	14.20329	14.62488	15.76065
HORVU5Hr1G105780	2.161328	1.440845	2.236415	1.393527	1.368373	1.4587
HORVU1Hr1G017710	33.97555	36.27632	47.8395	26.87196	27.18137	28.22298
HORVU1Hr1G053510	1.256202	1.296522	1.400857	1.322948	1.272094	1.371851
HORVU6Hr1G094520	0	0	0	0	7.480793	9.647411
HORVU6Hr1G080800	11.95193	10.38947	15.66428	13.25606	23.30066	20.8596
HORVU3Hr1G009520	6.109976	6.255751	9.876831	168.1132	218.8438	207.8673
HORVU5Hr1G066230	29.97924	13.13095	27.47606	0.141234	1.377911	0.383759
HORVU4Hr1G009380	97.24579	70.63874	92.99437	28.91975	19.67111	22.08756

HORVU4Hr1G005600	10.63351	8.807424	10.17715	8.533118	9.691552	9.543628
HORVU7Hr1G046620	1.395124	1.908207	1.527419	1.73578	3.010069	3.104606
HORVU2Hr1G065430	2.03281	1.593623	1.32225	4.855453	5.573672	6.295379
HORVU5Hr1G069570	4.089481	4.035151	4.749891	5.437842	6.140994	6.890623
HORVU1Hr1G090710	41.08647	37.43901	54.511	37.46185	42.87269	44.26886
HORVU5Hr1G051770	6.552107	7.096292	5.740856	5.687416	6.663185	7.373022
HORVU5Hr1G093860	3.321995	2.531257	3.948952	7.350921	2.670637	4.82618
HORVU4Hr1G082240	44.3018	34.26064	46.01808	59.66	62.5193	59.02343
HORVU5Hr1G054180	20.94179	14.83599	23.36535	31.71761	55.15172	52.30785
HORVU3Hr1G084420	7.075476	6.50257	10.52156	1.570721	1.101867	1.430716
HORVU3Hr1G008430	6.420852	7.067231	8.564299	3.835341	6.998181	5.725627
HORVU4Hr1G015260	38.21108	32.65692	31.35633	95.94523	146.5496	151.9056
HORVU5Hr1G059160	7.078844	3.750147	7.610226	13.10323	12.76483	9.727229
Hordeum_vulgare_newGene_4435	0.911833	0.799712	0.906053	1.311189	2.686483	2.230846
HORVU3Hr1G088000	0.377795	0.355372	0.309088	3.888505	4.638273	4.895039
HORVU4Hr1G051830	31.65505	28.11471	40.49857	38.86454	61.83974	59.1156
HORVU4Hr1G008470	43.98678	45.4488	41.66988	42.14203	35.9573	35.49039
HORVU2Hr1G073830	2.604518	2.896759	3.570464	2.667273	3.321499	4.309386
HORVU4Hr1G027820	6.073178	5.671349	6.516365	6.69351	6.056472	6.542544
HORVU1Hr1G015620	4.639004	5.473271	6.217294	0.054279	0.0495	0
HORVU3Hr1G035810	3.552775	2.704777	4.709498	3.922657	4.119185	4.612276
HORVU2Hr1G094510	34.23729	32.72337	34.22385	67.04084	74.70246	66.19972
HORVU7Hr1G056820	21.43902	22.41416	20.79407	25.1154	6.03754	9.438575
Hordeum_vulgare_newGene_5106	4.709749	4.844005	5.638851	5.835798	6.841311	7.303332
HORVU4Hr1G085730	8.134781	7.619741	9.141035	8.603715	8.578683	9.488324
HORVU3Hr1G071320	2.508453	2.540273	3.331208	3.50961	5.308035	5.630482
HORVU3Hr1G000580	9.74901	9.309847	10.46627	4.592204	3.615399	4.352426
HORVU2Hr1G100750	18.09295	17.18219	20.54452	18.34917	24.38192	24.98199
HORVU1Hr1G082110	8.278052	5.870862	8.564953	7.847629	3.457598	3.304871
HORVU2Hr1G053450	5.040636	5.721663	6.724216	5.154486	6.924292	7.71044
HORVU5Hr1G050480	3.197373	3.905015	4.139308	4.62311	4.844309	5.20795
HORVU1Hr1G056070	3.29188	3.219188	4.580074	4.407789	4.717902	5.390641
HORVU2Hr1G078260	1.205385	1.218144	1.357644	1.124338	1.304364	1.331082
HORVU4Hr1G004150	1.077898	1.370669	1.420888	1.096481	1.123059	0.8622
HORVU7Hr1G095850	0.444129	0.304728	0.499609	0.42508	3.877173	3.017435
HORVU1Hr1G075920	11.13531	10.23402	10.34109	12.83235	12.4243	12.79125
HORVU2Hr1G099290	12.88863	12.30272	12.20856	10.52611	11.20511	10.82929
HORVU2Hr1G036780	11.81548	10.55918	13.84306	12.64203	19.68747	17.31681
HORVU6Hr1G069010	14.25719	15.05981	13.18823	17.4139	20.68798	20.17369
HORVU7Hr1G071230	8.317103	12.72544	14.73398	0.023047	0.041642	0.267087
HORVU3Hr1G005950	31.14821	29.0554	30.90828	21.10505	21.88086	20.54839
HORVU7Hr1G093450	34.66518	27.79825	32.53757	19.2151	13.41735	14.33546
HORVU2Hr1G112380	16.53526	11.9227	17.451	18.96635	41.71873	40.79969
HORVU4Hr1G054920	7.459084	7.863048	10.27385	11.83609	24.61133	23.8759
HORVU3Hr1G100530	16.50293	13.236	18.1479	15.04421	19.30441	19.07067
HORVU4Hr1G076490	3.942483	3.438005	4.977028	2.623044	2.467214	4.149253
HORVU3Hr1G064790	1.071812	1.393821	1.138187	1.393481	0.494959	1.080229

HORVU7Hr1G110470	0.261763	0.496428	0.459235	0.942877	2.429994	2.603824
HORVU3Hr1G006600	0	0	0	8.834447	11.8309	12.13866
HORVU3Hr1G002980	8.515574	9.423406	7.970979	34.66453	4.393356	11.0115
HORVU2Hr1G054100	4.318205	4.062448	5.776167	4.156762	3.286581	3.560628
HORVU1Hr1G025020	7.167485	6.714811	6.966154	12.23758	16.93906	18.407
HORVU4Hr1G059050	0.180593	0.186805	0.237202	6.127949	7.041676	8.510718
HORVU3Hr1G099770	31.72287	32.16661	45.52045	10.68073	5.572494	5.817335
HORVU6Hr1G049950	3.891584	4.017675	4.229006	8.08875	8.53541	9.620939
HORVU2Hr1G096820	1.45523	0.819545	1.772888	0.491181	0.697918	0.794653
Hordeum_vulgare_newGene_12947	1.555768	2.2951	2.970672	5.751507	8.749606	7.974193
HORVU2Hr1G101150	60.39566	54.92524	58.365	6.557566	1.128676	4.931042
HORVU3Hr1G114240	1.723288	1.89957	3.091197	0.563346	0.670333	1.230126
HORVU0Hr1G003660	34.6583	40.29488	77.96873	33.20241	489.7831	28.3761
HORVU5Hr1G048910	1.455111	2.862893	1.01431	2.109973	1.120041	1.061773
HORVU2Hr1G104500	11.98398	10.29828	9.610348	3.071428	4.347557	4.358122
HORVU1Hr1G031600	70.58598	59.88234	87.87285	86.52162	196.1484	199.2382
HORVU5Hr1G117190	62.40435	59.65045	50.39744	53.50082	52.58772	51.40422
HORVU5Hr1G086780	2.832685	1.120506	2.128028	2.943825	5.837974	5.389078
HORVU2Hr1G067350	2.4757	1.96182	2.784513	0.383444	0.225064	0.344966
HORVU3Hr1G058170	14.04752	13.02517	19.71032	10.19726	11.82464	14.19977
HORVU7Hr1G027760	26.09602	28.75103	44.90711	43.71912	73.92287	72.04012
HORVU7Hr1G000750	0	0	0	0.096272	5.448523	7.365258
HORVU4Hr1G025470	5.092861	5.180975	3.967177	4.046163	22.14604	17.88237
HORVU7Hr1G099840	9.213416	8.447516	11.46185	7.795535	22.07864	20.10524
HORVU2Hr1G021360	2.036268	2.526482	2.711223	3.894165	4.969776	4.925316
HORVU3Hr1G022260	9.603762	6.209311	8.8313	16.07826	11.81414	13.50271
HORVU6Hr1G015900	7.844157	4.818169	9.777914	5.696173	7.742438	6.836459
Hordeum_vulgare_newGene_1953	0	0	0.098797	3.591623	2.323272	3.996952
Hordeum_vulgare_newGene_11504	4.598096	5.226281	4.44477	13.27427	20.81734	20.34763
HORVU4Hr1G062830	2.894894	2.251652	2.465587	4.499149	6.74278	7.187829
HORVU3Hr1G073930	0	0.010088	0.005654	4.046778	2.967343	4.055447
HORVU7Hr1G033450	4.814277	4.853417	6.048287	6.74122	9.159885	9.75334
Hordeum_vulgare_newGene_11501	4.189564	5.016039	5.212812	5.84184	7.578763	7.546134
HORVU4Hr1G027040	14.34011	17.63125	21.76655	13.68226	21.1584	21.00194
Hordeum_vulgare_newGene_904	0.228111	0.53084	0.492588	0.701388	4.507579	4.124143
HORVU3Hr1G112350	121.5139	89.65354	120.7787	129.7574	25.48448	62.79038
HORVU3Hr1G094220	12.53435	9.855167	11.6771	12.62079	16.11427	14.07914
HORVU2Hr1G081800	21.46344	16.55736	20.81682	13.5258	8.71765	10.79036
HORVU2Hr1G106490	19.6045	17.9556	19.41619	15.45058	14.98124	16.19599
HORVU7Hr1G083220	12.72598	12.67183	17.96796	9.319524	13.52145	12.80471
HORVU3Hr1G080130	119.8959	142.5062	252.3638	104.3704	220.2013	218.7304
HORVU0Hr1G009740	11.54532	8.652905	13.47841	16.69428	20.57734	18.54705
HORVU6Hr1G090150	3.080123	4.21396	5.926756	2.221463	1.864731	2.185773
HORVU1Hr1G072910	1.210456	0.710121	1.846772	1.267251	1.345787	2.065117
HORVU2Hr1G052190	9.831978	11.85338	13.70214	9.618817	9.551037	11.16014
HORVU5Hr1G014090	7.91623	6.746462	9.827641	0	0	0
Hordeum_vulgare_newGene_12080	3.36502	4.598719	5.202019	0.235504	0.085156	0.181427

HORVU6Hr1G059210	5.919001	5.080396	6.028794	6.981416	9.218129	8.030001
Hordeum_vulgare_newGene_12088	1.976911	1.425852	2.232791	0.663159	1.632562	1.23472
HORVU5Hr1G098890	49.41744	44.95438	49.13787	65.75412	67.59016	73.88075
HORVU0Hr1G038830	0.512207	0.984469	1.069275	1.893541	2.064475	2.421425
HORVU1Hr1G081980	1.213944	1.267428	1.128408	5.434149	8.547186	8.129747
HORVU6Hr1G055740	6.437918	6.689292	6.995141	6.663886	6.979926	7.249559
HORVU3Hr1G060530	2.744254	2.792028	3.303346	2.867889	2.325937	2.906548
HORVU6Hr1G088020	0.953695	1.122585	1.098367	0.899182	1.987439	1.75948
HORVU7Hr1G104590	4.384569	7.022275	6.779921	11.56768	17.48625	17.79997
HORVU5Hr1G075150	4.141641	2.596019	2.54405	0.38627	1.197627	1.247372
HORVU5Hr1G079470	27.71245	23.7442	31.55897	23.87499	21.71331	24.96573
HORVU6Hr1G071520	0.620539	0.719861	0.75956	2.887703	6.781453	5.847949
HORVU6Hr1G075630	11.49292	10.19545	12.36503	14.4744	26.18317	24.04425
HORVU7Hr1G038390	0.19894	0.048686	0.560027	6.07841	27.29332	21.09056
HORVU4Hr1G058210	33.07042	40.12002	35.14409	31.43459	34.49051	35.74201
HORVU0Hr1G005070	4.9031	5.163022	4.42462	6.578867	8.238288	8.762363
Hordeum_vulgare_newGene_6104	3.129636	2.549643	3.963734	9.802335	10.18116	10.80321
Hordeum_vulgare_newGene_6107	3.52251	3.193756	3.433898	0.009121	0.018666	0.024505
HORVU3Hr1G090410	6.192269	4.287738	8.638225	3.063255	0.565721	1.979279
Hordeum_vulgare_newGene_6101	0.107991	0.110497	0.240865	2.670066	10.65937	10.56168
HORVU0Hr1G020240	1.3618	3.559166	2.087267	2.739954	3.082773	4.30327
Hordeum_vulgare_newGene_3163	2.180316	2.939371	3.13313	1.20983	1.544639	1.690154
HORVU5Hr1G107250	61.41327	59.85669	56.68719	52.97969	66.27862	67.64205
HORVU3Hr1G060850	9.479472	7.274667	8.695909	11.35565	12.96462	12.64584
HORVU6Hr1G079730	1.897175	2.478404	2.63147	2.859573	3.421134	3.701927
HORVU2Hr1G116630	4.881125	4.947666	5.875339	5.507469	7.945026	7.819423
HORVU1Hr1G008560	2.870186	3.264303	3.662726	5.118053	3.568848	5.034327
HORVU4Hr1G022950	23.47682	20.6511	27.04536	24.27387	48.4846	39.64503
Hordeum_vulgare_newGene_3629	1.810996	1.348918	1.950776	2.4535	2.605721	3.081319
Hordeum_vulgare_newGene_3628	0.407671	1.939224	2.451787	2.16894	2.929141	0.553186
Hordeum_vulgare_newGene_3627	2.515027	2.759357	2.463057	3.395213	3.471242	3.236906
HORVU4Hr1G022270	2.199142	2.910979	3.012214	3.137294	11.10849	7.960817
Hordeum_vulgare_newGene_7110	0.726455	2.15579	0.402862	2.005313	2.255483	2.321883
HORVU4Hr1G025980	14.78468	12.64357	16.73796	14.57795	9.683931	12.80838
HORVU5Hr1G074200	16.2971	15.62105	17.74684	22.39199	25.01873	26.25354
HORVU5Hr1G078780	13.38935	13.49261	12.81358	16.84974	22.6423	22.12253
HORVU2Hr1G086340	1.801474	1.856626	2.933843	1.112074	2.581567	2.230972
HORVU7Hr1G078440	6.389724	7.958014	9.185809	5.203743	7.288737	7.154088
Hordeum_vulgare_newGene_5665	0.48095	0.273149	0.354865	0.234683	4.079096	2.654708
HORVU3Hr1G077670	5.947237	6.24825	4.763629	4.348553	5.850242	5.730952
HORVU5Hr1G118460	4.567045	3.431397	4.486681	5.728431	9.1474	8.648232
HORVU3Hr1G059700	27.2627	31.76427	28.58806	25.04095	24.60769	30.27047
Hordeum_vulgare_newGene_5668	34.06438	34.40938	49.73375	23.48883	25.37746	24.26328
Hordeum_vulgare_newGene_15617	1.263377	1.282968	1.531152	2.274261	2.653929	2.702748
HORVU4Hr1G070380	1.552779	1.458731	1.882293	0.746653	2.108535	2.270685
HORVU3Hr1G013400	0.745517	0.773561	0.853596	3.955267	7.248525	7.083213
HORVU7Hr1G034430	1.620074	1.99196	2.073308	2.594763	1.975689	2.321414

HORVU7Hr1G070270	6.650387	5.429074	5.797032	6.514204	7.509148	8.777314
HORVU5Hr1G062110	1.989449	2.428274	2.396528	5.880331	13.75136	13.93223
HORVU3Hr1G011800	0.050243	0	0	0.046849	16.00682	12.86688
HORVU1Hr1G052060	10.66868	8.665772	11.58699	10.99795	10.83984	9.198885
HORVU4Hr1G085510	22.36888	26.94861	23.23747	28.69492	40.90413	39.07307
Hordeum_vulgare_newGene_11974	1.908036	1.556976	1.758587	0.503673	0.488292	0.639876
Hordeum_vulgare_newGene_12120	1.401376	1.614957	2.307754	0.127892	5.741462	0.289405
HORVU4Hr1G011360	7.47613	6.900161	7.730344	9.522091	11.44788	11.19718
HORVU4Hr1G071470	10.43082	8.539262	10.88913	17.29284	18.94081	18.27258
HORVU0Hr1G003020	2.36286	1.945502	2.221621	5.684818	6.156672	8.422304
HORVU2Hr1G070720	2.201588	1.382633	1.652325	0.750598	0.612335	1.017254
HORVU3Hr1G051150	4.304586	4.136215	4.870739	4.261321	3.348192	3.781985
HORVU5Hr1G024390	0.160874	0.312014	0.190264	1.491002	2.785195	2.823338
Hordeum_vulgare_newGene_8414	25.35807	27.39605	24.7615	26.66685	26.8158	30.27499
Hordeum_vulgare_newGene_8416	7.404759	6.596228	6.348806	11.14825	7.808727	8.798407
Hordeum_vulgare_newGene_8413	8.402362	8.459289	9.480177	6.878599	7.521786	7.73369
Hordeum_vulgare_newGene_8412	3.360713	3.601526	4.277636	5.036379	10.69337	6.464557
HORVU7Hr1G107680	3.617798	2.191663	2.11802	2.170648	0.485929	1.415211
HORVU2Hr1G072650	44.68864	37.14044	50.18253	47.37811	114.3698	120.3448
HORVU6Hr1G045250	14.92871	13.65598	17.5628	26.20188	29.44294	33.5176
HORVU6Hr1G064630	29.4751	24.78155	27.79705	40.35004	45.09717	44.5383
HORVU7Hr1G117550	4.785553	5.38141	5.897056	3.851659	5.651349	5.429056
HORVU3Hr1G016630	4.437591	5.270107	4.624673	6.93388	11.70236	9.957476
HORVU6Hr1G025290	11.29489	11.29009	13.1348	11.44569	13.76607	15.52091
HORVU4Hr1G043860	0.221797	0.267382	0.215544	1.931089	1.915861	2.398534
HORVU3Hr1G095890	53.86154	48.45378	56.1756	43.70028	70.51318	65.11769
HORVU1Hr1G053280	7.433611	7.299335	8.361623	7.201578	11.34777	10.07876
HORVU6Hr1G030780	21.88918	20.635	22.88489	15.55182	20.88811	19.53412
HORVU1Hr1G062450	7.381228	7.946508	8.407532	9.778677	9.504202	8.351107
HORVU7Hr1G065990	12.23131	12.06011	12.63267	14.96898	19.73984	20.50537
HORVU6Hr1G025120	1.678047	1.48673	1.205631	1.891912	2.954865	2.672194
Hordeum_vulgare_newGene_11842	3.444329	3.449251	4.197241	3.912213	4.945681	4.767324
HORVU0Hr1G008620	5.555618	6.661795	6.286531	11.13378	13.7868	13.44569
HORVU2Hr1G080340	34.56931	33.33848	61.23625	17.68895	26.34334	24.75551
HORVU7Hr1G026240	6.704476	6.636937	4.537116	3.061896	2.623924	2.349073
HORVU7Hr1G067280	89.58458	92.93304	133.2395	161.6417	296.5789	281.0559
HORVU3Hr1G011110	5.446154	7.709148	6.903579	0.007495	0.011965	0.009306
HORVU5Hr1G057610	3.034416	3.927725	3.280888	3.459596	4.320318	4.293654
HORVU5Hr1G092310	1.335611	1.539669	2.087921	1.057519	2.448671	2.158479
HORVU2Hr1G069780	0.424877	0.298645	0.551827	2.092114	5.508526	4.511448
Hordeum_vulgare_newGene_14609	25.77722	19.82988	22.87209	0.327987	0.26522	0.38112
HORVU6Hr1G036830	16.24854	20.05468	20.47878	20.71951	14.60857	17.11699
HORVU1Hr1G076000	4.219248	4.565633	6.057686	2.934791	7.86556	7.075053
HORVU2Hr1G034330	7.045284	6.27711	7.663685	7.645133	11.07405	11.28877
HORVU5Hr1G104230	63.36592	60.927	51.35468	9.276492	11.55297	10.61907
HORVU1Hr1G064850	1.112949	1.170737	1.48188	0.944848	0.578626	0.887965
HORVU1Hr1G022430	1.852634	0.678059	1.751677	4.065138	3.459188	4.411776

HORVU3Hr1G014090	470.4514	421.7897	400.0932	202.6477	64.28977	93.27419
HORVU6Hr1G031430	2.81667	3.598466	3.309312	4.532983	10.45965	8.818248
HORVU2Hr1G079840	18.0324	15.21217	23.0012	20.84048	19.82153	23.07993
HORVU0Hr1G018850	0.750314	0.479973	0.654076	0.871792	5.597801	6.322242
HORVU2Hr1G125790	4.594518	4.667618	4.925769	5.663927	5.723658	5.426647
HORVU1Hr1G093170	66.52535	62.7343	80.75611	34.80968	35.47332	32.33101
Hordeum_vulgare_newGene_13654	0.681292	0.513297	0.414348	8.923192	2.90908	3.893729
HORVU6Hr1G020570	2.237148	2.121872	2.309148	2.262666	3.070618	2.492526
HORVU6Hr1G070450	96.13053	78.01023	99.30921	30.05359	12.05711	12.04371
HORVU7Hr1G054550	51.35998	42.9518	54.47195	52.49122	72.81593	69.35601
HORVU0Hr1G016930	0.771891	0.758049	1.454829	1.563284	1.467595	1.381794
HORVU6Hr1G081270	0.220232	0.170057	0.195349	2.67724	2.73605	3.239607
HORVU6Hr1G009620	0.011989	0	0	2.262856	2.579656	2.552436
HORVU2Hr1G083100	20.89796	21.27331	27.7697	12.2708	10.41047	11.6305
HORVU7Hr1G110320	0.306073	0.11184	0.536681	4.645055	18.79827	16.54314
Hordeum_vulgare_newGene_7777	5.806874	6.583973	4.808816	1.418156	3.811329	1.851378
HORVU4Hr1G073290	16.83775	17.38734	14.97187	16.78463	19.12122	19.03525
Hordeum_vulgare_newGene_7779	3.48731	3.163751	2.591402	2.818185	4.819009	4.019892
Hordeum_vulgare_newGene_7778	3.496277	3.255091	2.389395	1.613638	3.078505	2.415072
HORVU2Hr1G045590	0.895219	0.719869	0.838896	1.219307	1.315518	1.488955
HORVU2Hr1G030170	2.391582	2.195949	3.644354	2.629275	2.330228	2.306169
HORVU5Hr1G102240	4.927337	5.237123	5.482429	5.229482	2.842571	3.673314
HORVU6Hr1G059150	9.700543	10.99003	10.81513	10.26383	12.96182	13.31028
HORVU5Hr1G060370	2.740771	3.524619	3.87218	4.771655	6.044025	5.641557
HORVU3Hr1G039660	9.693562	9.089921	11.94565	6.342234	5.986024	6.11193
HORVU5Hr1G046550	10278.38	6642.44	9458.149	2911.306	14.59161	904.972
HORVU7Hr1G079270	4.778135	6.216104	5.867214	7.116153	4.574343	4.640817
HORVU3Hr1G043210	3.047802	3.873143	3.123531	0.122398	0	0
HORVU5Hr1G008270	2.387501	1.041104	2.785767	0.383423	0.369649	0.235542
HORVU7Hr1G115040	2.663937	1.909643	2.531768	9.279957	17.60754	16.87748
HORVU3Hr1G079000	7.789331	7.768071	8.808918	13.60462	12.34016	11.57137
HORVU1Hr1G053000	2.789953	2.91353	2.576398	5.989116	6.981976	8.523963
HORVU2Hr1G124540	116.431	106.8416	117.1936	103.7193	90.27285	89.04777
HORVU1Hr1G061530	7.220063	8.522571	11.3511	5.257574	5.908758	5.621565
HORVU7Hr1G093120	24.09788	21.36989	22.21738	16.56805	9.783954	13.21951
HORVU5Hr1G013260	2.169215	1.94061	2.380337	4.039891	8.815743	8.239842
HORVU7Hr1G056660	21.58603	18.84375	23.34619	20.33351	23.21062	25.48273
Hordeum_vulgare_newGene_3880	4.006381	2.957906	4.47107	3.141854	4.847344	4.280377
HORVU1Hr1G074330	19.12891	15.79004	18.44414	10.92991	10.85899	9.979563
HORVU2Hr1G016800	1.47015	1.880482	1.893385	1.514595	2.283563	2.028651
HORVU2Hr1G044640	1.70337	1.060673	1.309093	3.536915	2.155483	3.928207
HORVU1Hr1G083550	2.877696	3.214051	4.112733	3.910425	4.965276	5.372243
HORVU5Hr1G120570	6.1905	6.642186	7.380869	7.560268	6.608198	8.301504
HORVU7Hr1G025000	3.470951	5.367769	7.195168	12.13519	10.87476	13.83863
HORVU5Hr1G012790	6.175332	5.69151	5.079544	5.746051	8.636675	8.296515
HORVU5Hr1G088430	2.551874	2.594929	3.171122	2.228834	1.646382	1.276103
HORVU6Hr1G054560	10.63127	10.8861	9.950271	11.8522	12.73632	11.29873

HORVU6Hr1G075170	10.40352	9.4904	11.39559	12.28234	13.58022	14.00576
HORVU1Hr1G087070	55.70887	47.73752	62.12601	65.51048	55.39441	47.73534
HORVU0Hr1G017080	2.560055	2.574105	2.880055	5.537466	9.504856	9.119916
HORVU4Hr1G052080	13.66177	13.27251	17.82781	14.65525	21.00559	20.55398
HORVU3Hr1G028340	0.918263	1.473623	1.184303	2.179117	1.823355	1.773461
HORVU7Hr1G014870	1.510558	1.574812	1.225066	2.590433	2.708955	4.726007
HORVU5Hr1G092610	112.038	122.0464	202.8308	85.67436	106.4353	103.4812
HORVU2Hr1G041960	11.39899	10.85267	11.45121	5.894903	5.024146	6.297944
HORVU5Hr1G093370	13.29394	10.98357	13.73806	11.16218	11.53337	12.25448
HORVU5Hr1G018790	4.286878	3.386302	4.69783	2.883055	5.356837	5.092524
HORVU0Hr1G005420	6.055743	4.122344	8.280814	2.848131	2.363809	3.589738
HORVU1Hr1G011520	0.969915	1.213582	1.094583	2.207322	3.835852	3.015936
HORVU2Hr1G122260	3163.093	2992.712	2652.563	5837.979	5419.003	4771.579
HORVU1Hr1G045720	5.647056	4.563193	5.638811	6.472668	7.400014	7.459494
HORVU1Hr1G092290	1.881444	0.98471	1.570103	1.283409	0.745614	1.311448
HORVU5Hr1G028770	15.8947	11.42386	18.52891	12.61257	14.98488	15.87335
HORVU2Hr1G011700	13.59764	14.30962	16.32648	12.04744	13.38595	13.05437
HORVU6Hr1G041230	7.889402	7.286793	7.253756	9.490164	7.850215	9.799719
HORVU3Hr1G057130	2.722089	2.849369	2.803737	3.01727	3.936626	3.313901
HORVU4Hr1G023260	11.30707	10.57101	9.306885	10.01701	6.957686	9.515871
HORVU2Hr1G057960	7.032027	8.296464	6.804234	7.907932	6.373002	6.981575
HORVU4Hr1G011600	0.445637	0.540927	0.323638	10.83044	20.4763	17.28731
HORVU6Hr1G093680	1.792574	2.275296	2.23546	0.964243	1.25486	1.660428
HORVU5Hr1G097010	0.854682	0.618441	1.051133	1.940549	1.677363	1.692454
HORVU5Hr1G095010	1.827393	1.484846	1.603908	0.834321	0.113517	0.368854
HORVU1Hr1G094380	0.052015	0.02871	0.065467	3.898499	3.333584	5.912922
HORVU2Hr1G094230	3.980096	2.610137	4.313148	3.330759	0.799215	2.186181
HORVU4Hr1G083010	5.301502	5.109589	5.788568	6.504065	9.375476	10.47338
HORVU4Hr1G084290	32.71463	33.60553	30.91556	27.17468	30.35825	25.96312
HORVU5Hr1G055130	4.308256	2.115518	0.677145	6.150809	6.551255	6.736573
HORVU3Hr1G083290	7.209508	7.039691	7.716314	5.531103	6.915707	7.304754
HORVU4Hr1G014070	3.761848	3.285495	4.114111	8.956709	13.55762	12.29625
HORVU4Hr1G061650	10.61956	12.56715	11.7806	14.65691	19.18294	20.07206
HORVU1Hr1G013580	3.130992	3.457145	3.300781	3.595523	4.472295	4.603597
HORVU3Hr1G056660	8.410291	6.326849	6.274432	0	0	0
HORVU3Hr1G066960	1.029736	0.839265	0.757377	2.036312	1.361652	1.648215
HORVU3Hr1G083740	5.65421	3.893652	6.982109	6.268995	3.935831	4.153087
HORVU6Hr1G040180	6.030304	6.943556	5.972939	6.77934	8.869614	8.311141
HORVU3Hr1G076760	4.910719	5.635756	6.188904	5.740727	7.537653	7.219892
HORVU2Hr1G064550	7.792369	8.589289	12.35076	12.3452	19.79215	20.96923
HORVU7Hr1G120490	66.32626	53.2262	71.33703	56.03532	62.33812	62.50095
HORVU4Hr1G084740	3.690928	2.754356	3.735616	4.3434	3.550958	3.997712
HORVU1Hr1G091490	7.589704	7.014341	7.038145	9.199175	13.82562	12.61606
HORVU1Hr1G049310	1.771609	1.769032	2.554441	1.247426	2.074103	1.184828
HORVU7Hr1G023800	12.27263	11.28779	12.53005	7.656783	7.286754	6.078564
HORVU3Hr1G018440	0.490428	1.161749	1.816666	1.425492	1.549655	0.879207
HORVU5Hr1G120960	20.81246	17.45363	19.2368	18.89168	28.59414	28.01449

Hordeum_vulgare_newGene_12863	1.410671	1.37817	0.843108	2.52863	1.065587	1.475155
Hordeum_vulgare_newGene_12861	0.978683	1.26437	1.218177	1.062296	1.363685	1.30302
HORVU7Hr1G095460	2.592921	2.113929	2.553662	1.389409	2.713095	2.538016
HORVU1Hr1G090100	1.343941	1.319872	2.130006	2.356185	4.919099	4.948442
HORVU3Hr1G000130	15.0597	14.62233	20.20992	10.6636	13.20725	12.88425
HORVU5Hr1G111120	43.88264	36.65919	46.98313	50.53179	28.94612	32.70002
HORVU4Hr1G026340	17.52503	16.55262	20.63264	10.27886	6.00167	9.401154
HORVU1Hr1G003250	0.11033	8.279582	0.572566	17.0287	5.69281	6.72163
HORVU3Hr1G087170	3.524049	5.503658	5.959378	14.32949	37.39093	30.03807
HORVU1Hr1G005980	8.88384	6.847701	8.638022	9.167082	13.94982	16.45381
HORVU1Hr1G069900	4.572371	3.39268	4.973143	8.674494	14.09774	11.21223
HORVU6Hr1G055230	11.36641	8.596325	9.411778	4.643766	3.079881	3.539506
Hordeum_vulgare_newGene_2114	0.588966	0.915835	0.613015	1.879529	1.531219	1.815984
Hordeum_vulgare_newGene_2113	1.998689	1.468593	1.759164	1.425517	0.264012	0.550311
HORVU2Hr1G017360	1.680347	1.091359	1.174369	0.441119	1.273745	1.194053
HORVU1Hr1G006770	16.86126	13.5825	13.90702	16.74073	22.61119	22.43124
HORVU5Hr1G125130	61.29817	51.41239	69.08425	78.2287	178.1527	143.6873
HORVU5Hr1G094760	3.100042	4.939119	4.355626	6.932631	8.417703	8.068113
HORVU7Hr1G076590	1.307366	1.34208	1.917781	1.647441	2.36088	2.351715
HORVU7Hr1G094730	6.482968	4.792339	4.872577	0.12048	0.247447	0.085209
HORVU6Hr1G012570	235.8143	190.4068	271.8548	189.2269	283.1162	299.485
Hordeum_vulgare_newGene_9126	7.519745	8.76942	6.049152	6.356334	19.18298	22.73238
Hordeum_vulgare_newGene_9120	19.46281	19.17075	19.18717	26.89407	44.29714	44.73004
HORVU1Hr1G094900	20.42156	18.94209	20.65795	21.65753	29.57592	28.73716
HORVU3Hr1G077440	1.39386	1.499097	1.828829	1.311157	1.895714	1.736882
Hordeum_vulgare_newGene_13808	13.70587	12.57952	12.83826	12.84819	14.21196	15.24928
Hordeum_vulgare_newGene_13802	3.85353	3.606951	3.462729	3.24063	2.045741	2.734747
Hordeum_vulgare_newGene_13801	0.585022	0.658135	1.688748	2.99031	11.99052	8.701951
HORVU5Hr1G098280	1.058599	1.393543	2.143779	1.175757	0.151345	0.450949
HORVU3Hr1G099630	2.105048	1.051389	1.497861	5.230742	1.539831	2.523224
HORVU5Hr1G006390	6.529944	6.66606	5.568504	6.674486	7.306717	7.418709
HORVU1Hr1G077510	28.80821	24.57152	28.17063	27.75924	23.74844	24.8647
HORVU7Hr1G043530	18.7546	18.1273	15.2724	1.486756	1.888021	1.780465
HORVU2Hr1G036590	17.05135	10.94731	18.16129	4.006602	1.253295	3.849627
HORVU7Hr1G071060	4.585296	5.182314	5.038108	6.445882	8.36032	8.672208
HORVU5Hr1G003910	5.305923	5.648057	5.976104	7.902575	11.4935	11.37079
HORVU2Hr1G018810	0.016241	5.73677	10.2939	4.486129	4.014137	3.644393
HORVU4Hr1G060330	11.42655	11.26244	16.96695	15.66733	15.38983	17.48265
HORVU4Hr1G054060	15.56311	13.52977	19.98339	13.88801	18.47815	20.66412
Hordeum_vulgare_newGene_8655	0.377154	0.45615	0.620865	1.888541	1.608339	1.587381
Hordeum_vulgare_newGene_8654	1.820817	2.170233	2.402732	1.710006	1.346936	1.492096
HORVU6Hr1G091420	2.436414	2.357728	2.639312	4.301585	5.435975	5.212181
HORVU7Hr1G074550	8.618641	7.726258	9.927921	13.0328	13.73392	13.4722
HORVU2Hr1G092090	24.25691	15.00211	16.48916	21.7591	171.9753	136.3244
HORVU7Hr1G012550	0.303911	0.09267	0.408495	1.935111	2.342352	1.896647
HORVU2Hr1G109440	2.057304	1.695411	2.115278	0.113412	0.016426	0.137406
HORVU1Hr1G001760	24.18726	34.8946	19.97105	32.53901	6.290505	8.923293

HORVU6Hr1G056570	3.127839	5.168082	3.370028	11.30865	6.476892	8.508724
HORVU3Hr1G098870	17.03809	19.87501	23.41149	14.27201	23.1733	22.16268
HORVU4Hr1G021950	4.242645	3.769814	4.454165	12.51988	18.27478	19.186
HORVU5Hr1G086660	50.97815	45.12618	48.58365	53.61637	57.9198	49.83382
HORVU0Hr1G010920	6.498088	6.368112	8.323967	10.26783	14.81722	14.88734
HORVU3Hr1G053150	1.883247	2.463535	2.284507	1.498625	2.057158	2.279523
HORVU3Hr1G096690	3.363697	2.963943	3.608474	2.528826	3.667398	4.020767
HORVU7Hr1G020760	0.61354	1.459185	1.231031	68.0412	70.31309	73.83775
HORVU2Hr1G023660	8.79906	11.08323	8.208415	11.57663	12.62618	15.20493
HORVU3Hr1G093660	4.734732	3.123439	3.889507	3.711352	3.303317	2.553018
Hordeum_vulgare_newGene_10579	2.282549	2.436709	2.294222	1.495559	1.888453	1.435336
Hordeum_vulgare_newGene_10576	1.811637	2.313775	1.971123	7.26649	8.595267	10.16758
HORVU3Hr1G057890	4.050184	5.627257	7.095429	2.68819	2.822841	2.58409
Hordeum_vulgare_newGene_10575	3.080243	2.943406	2.980042	5.201521	4.924143	6.335876
Hordeum_vulgare_newGene_10573	4.836609	4.40221	3.899827	5.731896	4.811771	6.189004
Hordeum_vulgare_newGene_10571	2.787284	1.72745	3.762348	0.161282	0.083666	0.22673
HORVU2Hr1G102710	4.665316	1.657867	7.357029	0.475751	0.023725	0.815887
HORVU2Hr1G039370	4.231423	4.949201	2.605044	2.73794	22.91369	19.60632
HORVU6Hr1G076440	21.11269	20.06285	17.94224	27.40586	17.4833	20.90305
HORVU3Hr1G088460	5.901025	6.233601	6.274582	6.153378	5.932853	6.208575
HORVU1Hr1G035720	0.45844	0.757504	0.666453	6.30578	26.02159	30.39971
HORVU0Hr1G017050	3.708974	3.030077	4.145921	4.016717	6.264466	6.260309
HORVU3Hr1G054050	11.62053	11.80992	13.59564	30.08789	36.95917	39.66641
HORVU3Hr1G015870	7.051374	8.285202	7.850143	8.834485	10.83751	12.74658
HORVU3Hr1G011350	12.17672	11.91196	17.46508	5.316785	5.351674	4.746331
HORVU4Hr1G063810	13.66041	11.08742	12.35522	21.64853	18.96323	19.3815
HORVU4Hr1G078410	2.645047	2.187156	2.660501	4.759696	6.097081	6.378751
HORVU2Hr1G021280	13.34993	11.79792	14.16489	31.94124	40.57031	41.02688
HORVU7Hr1G001010	35.23601	22.82234	38.41237	39.85669	64.88148	69.02221
HORVU2Hr1G114110	12.69963	12.5875	12.63819	12.22356	13.57318	13.93758
HORVU3Hr1G057270	9.654101	9.175215	9.94855	11.61232	12.2912	13.23486
HORVU3Hr1G095340	0.698168	0.522775	0.462244	1.407306	1.659647	2.050724
HORVU5Hr1G043780	4.083064	5.055527	5.108163	4.306715	4.883717	5.625782
HORVU1Hr1G002620	0.489641	8.652237	0.851132	12.25601	0.164229	0.18498
Hordeum_vulgare_newGene_4407	12.45587	9.548103	14.54036	10.31345	12.79294	12.76917
Hordeum_vulgare_newGene_4401	16.47373	22.61978	23.68559	4.624755	1.808759	2.990638
HORVU4Hr1G066480	13.01113	11.5594	14.6939	15.67446	19.82154	18.23596
Hordeum_vulgare_newGene_4408	19.06941	15.06362	22.86865	16.53594	19.62435	17.69896
Hordeum_vulgare_newGene_4409	21.10621	18.30802	23.26818	46.10625	74.59938	66.48766
Hordeum_vulgare_newGene_6045	10.76084	13.86025	25.0624	0.750726	0.788849	0.799246
HORVU3Hr1G116200	16.25236	14.72887	23.70342	19.07491	36.33111	30.22356
HORVU3Hr1G102030	6.631121	5.411941	6.90909	5.709827	13.32299	11.62878
HORVU6Hr1G089230	0.100387	0.052252	0	0.302953	15.91001	15.47164
HORVU2Hr1G042120	13.07109	11.94168	10.05692	10.2116	9.982647	9.339308
HORVU2Hr1G028070	5.422459	5.119745	5.132344	9.34556	13.66453	13.9451
Hordeum_vulgare_newGene_571	2.59304	2.692766	2.861029	2.849254	4.013277	3.674799
Hordeum_vulgare_newGene_572	11.97741	9.746577	12.5867	11.14206	17.56399	15.40298

HORVU3Hr1G089590	2.028861	2.477801	2.280082	2.703029	2.954915	3.016846
Hordeum_vulgare_newGene_577	32.94119	28.80048	42.05778	11.41938	22.68145	22.64518
Hordeum_vulgare_newGene_576	3.340087	3.956009	2.698028	1.920198	3.935685	1.749394
Hordeum_vulgare_newGene_12325	2.033654	1.905279	1.56905	1.669395	1.707253	2.031116
Hordeum_vulgare_newGene_12327	8.352026	7.610397	9.407615	6.046135	5.801428	5.329286
Hordeum_vulgare_newGene_12322	14.0852	16.35499	17.87315	9.892576	8.221549	11.28538
HORVU5Hr1G041780	2.157957	1.978125	2.574513	2.345062	2.19608	2.199866
HORVU3Hr1G105560	1.04958	0.655235	0.993553	2.483965	1.002484	2.045156
HORVU3Hr1G070790	101.882	110.2649	87.66485	93.31735	56.38695	60.78963
Hordeum_vulgare_newGene_14446	8.744868	7.309547	8.095164	9.114889	4.517094	5.165485
HORVU5Hr1G041880	152.7623	137.4007	147.6841	119.7807	180.9984	161.9694
HORVU3Hr1G089620	0	0	0.04722	1.102009	6.34985	8.936245
Hordeum_vulgare_newGene_13491	0.549168	0.230574	0.345328	3.619579	7.910296	9.141221
HORVU1Hr1G074130	39.65957	41.72835	54.07497	21.72764	19.2969	19.29506
HORVU4Hr1G073500	15.81832	15.01131	17.99295	15.53096	28.75692	28.55353
HORVU2Hr1G068050	0.888508	0.406433	1.228241	2.484011	4.642952	4.334522
HORVU5Hr1G075050	2.404633	1.962571	2.491452	7.883846	35.05134	31.61846
HORVU5Hr1G037430	0.711937	0.474596	0.630537	1.291899	3.807383	2.447581
HORVU2Hr1G127060	3.006142	2.267584	2.925274	3.912139	5.25764	5.859098
HORVU7Hr1G052140	43.54363	43.4551	37.62886	23.96	18.29338	21.50234
HORVU7Hr1G002090	13.04301	11.95348	12.60239	13.37369	12.54972	12.56712
HORVU4Hr1G028610	4.239653	4.677075	5.01405	5.788671	8.6604	8.074698
Hordeum_vulgare_newGene_5486	4.177677	4.809027	5.739975	3.526285	4.11996	4.260553
HORVU6Hr1G064720	56.85506	63.79772	88.49556	32.19915	24.20707	32.65117
HORVU3Hr1G017020	1.923323	1.742919	2.110786	2.865237	1.784724	2.86343
HORVU2Hr1G048950	3.141988	3.291052	4.137405	2.36839	2.208462	2.807292
HORVU1Hr1G012130	2213.413	2096.816	1885.395	510.4823	147.2864	224.0447
Hordeum_vulgare_newGene_13141	2.195921	2.568801	2.059116	2.920158	1.836272	3.312166
Hordeum_vulgare_newGene_13148	1.661202	0.943449	0.940005	1.078406	1.371934	1.443992
HORVU0Hr1G000250	3.96115	4.750914	5.930236	4.152669	2.988409	3.986372
HORVU3Hr1G086020	10.60805	10.828	12.70081	12.2753	21.48126	19.38029
HORVU3Hr1G068150	0.587825	0.305487	0.493903	1.276703	5.412648	6.119123
HORVU1Hr1G075880	1.348778	1.552611	1.234787	0.572956	1.119854	1.017035
HORVU2Hr1G087730	0.113188	0.431644	0.152726	4.126592	4.007536	4.828658
Hordeum_vulgare_newGene_15791	1.859792	0.896445	2.595445	2.859814	0.592152	1.021217
Hordeum_vulgare_newGene_15790	11.50646	11.6551	11.58301	13.32283	17.14258	17.22128
Hordeum_vulgare_newGene_15793	2.439109	3.120071	2.13562	0.715818	0.545942	1.247671
HORVU1Hr1G060330	2.34466	2.659752	2.499576	2.206816	2.70953	2.574205
HORVU5Hr1G088530	3.39298	3.082613	3.212326	5.506419	6.359384	6.975913
HORVU6Hr1G075400	15.33952	15.07854	14.86201	18.56675	23.10079	24.23795
Hordeum_vulgare_newGene_2867	2.28006	2.368675	2.796897	3.973468	4.103918	5.391045
HORVU7Hr1G110900	64.23356	63.56972	46.8979	18.74374	21.66775	22.7738
HORVU4Hr1G070260	3.452742	2.666972	3.723154	23.11168	5.190857	9.509332
HORVU1Hr1G055600	10.5071	8.326413	10.77015	25.36421	27.82886	28.37113
HORVU2Hr1G085590	9.578616	11.43452	10.55959	9.175732	7.497087	8.247153
HORVU3Hr1G110520	8.754419	8.106549	8.974206	11.86948	11.39603	13.21522
HORVU5Hr1G023690	8.96843	3.080515	10.69177	0.721105	0.365338	1.122617

HORVU1Hr1G059920	32.41766	53.64071	78.91367	1.568947	0	0.485313
HORVU1Hr1G078930	7.163048	7.382484	6.740487	5.592246	6.031614	6.561403
HORVU1Hr1G050380	4.302185	4.892847	4.958732	4.589065	4.370712	3.844562
HORVU2Hr1G027390	1.354263	1.206291	1.358459	2.409252	2.396675	2.602633
Hordeum_vulgare_newGene_15425	4.414692	4.642498	4.634188	5.953116	6.254705	8.163032
HORVU0Hr1G000780	35.0172	18.26273	36.67534	3.776787	4.281404	4.399354
HORVU7Hr1G118650	30.17896	30.03947	32.65586	22.85501	21.98325	27.56004
HORVU2Hr1G005510	3.300275	2.414573	2.723854	11.06421	21.16073	13.90988
HORVU5Hr1G024470	1.698487	1.945739	2.303067	3.554728	5.123138	5.16423
HORVU6Hr1G061300	0.868968	1.011938	1.315869	4.453593	15.36157	18.66295
HORVU6Hr1G069150	14.31556	13.84198	19.1798	13.76422	15.53782	14.20259
HORVU6Hr1G069480	6.03538	7.151368	9.345705	9.000319	20.25538	16.54878
HORVU4Hr1G011440	1.896056	2.186768	2.487105	2.202024	2.290204	2.814051
HORVU4Hr1G075010	0.756772	1.165483	1.331893	0.781686	1.073977	1.445764
HORVU3Hr1G019540	42.88806	37.87723	46.65847	45.70801	59.86029	63.28258
HORVU0Hr1G004200	1.712288	1.791716	1.769377	1.475168	1.745509	2.130362
HORVU7Hr1G119520	1.387166	1.887957	1.877961	3.326315	3.533884	3.65897
HORVU2Hr1G094030	5.31259	4.820584	7.199124	5.479648	8.978679	9.158642
HORVU7Hr1G112710	0.389256	0.579501	0.695768	6.630883	3.651199	4.893772
HORVU2Hr1G004640	13.86658	8.31083	18.32161	2.852636	0.468256	1.067827
HORVU5Hr1G039740	5.585759	5.893859	6.492868	5.495248	7.175322	6.982022
HORVU4Hr1G041630	0.611926	0.668669	0.481685	1.103139	1.834038	1.647131
HORVU5Hr1G062090	129.6385	122.6754	133.6032	175.5132	185.7305	162.2306
HORVU6Hr1G030080	1.793579	3.488331	3.265154	0.840926	0.354124	0.600946
HORVU7Hr1G049070	1.805421	1.551015	2.567621	19.62575	8.693466	14.12009
HORVU1Hr1G054260	4.056425	4.560158	4.188872	6.951155	6.13137	8.478843
HORVU4Hr1G089500	264.6553	166.1731	241.8124	559.3768	270.4099	324.067
HORVU3Hr1G115840	3.07767	2.859901	3.720976	3.806067	5.599477	5.893479
HORVU4Hr1G058150	3.711365	2.917862	3.618242	4.097894	4.555288	4.696941
HORVU0Hr1G002120	1.122067	0.887115	0.998329	1.740915	3.25275	3.164321
HORVU3Hr1G085900	3.518699	3.377961	3.907536	5.564593	7.871876	7.069434
HORVU5Hr1G058400	0.862674	0.904696	1.075499	0.923773	2.167449	1.875763
HORVU1Hr1G093830	3.771612	4.036165	2.420056	2.370357	3.059027	2.916086
HORVU0Hr1G008780	31.72639	42.09216	39.90967	26.45046	21.37267	22.15778
HORVU7Hr1G057300	51.03056	43.7631	34.51752	21.26507	32.22102	32.02777
HORVU7Hr1G020860	66.68387	53.02835	66.38363	89.01113	98.08031	107.3006
HORVU5Hr1G001180	0	0.038856	0.016564	5.408551	18.25318	15.52272
HORVU4Hr1G038670	12.30566	13.24839	17.36151	14.05202	20.15268	21.45974
Hordeum_vulgare_newGene_1625	37.13957	35.36806	64.5098	1.884395	2.76137	4.405071
Hordeum_vulgare_newGene_1623	2.526013	1.913585	1.368638	2.262871	2.36482	2.238097
HORVU2Hr1G043310	1.273693	1.733566	2.388877	3.836668	5.18234	4.574756
Hordeum_vulgare_newGene_1628	6.415823	11.50878	11.71681	0.87143	0.240521	0.473453
HORVU0Hr1G008870	26.48168	28.38144	29.09088	18.63582	15.37955	15.03464
HORVU1Hr1G067870	16.09033	13.29181	22.41128	45.57744	33.21885	37.85565
HORVU1Hr1G063510	46.58131	39.7002	51.02493	34.70649	42.54067	39.86601
HORVU5Hr1G023070	5.715027	4.340434	6.031217	3.807794	4.211078	4.28079
HORVU1Hr1G029090	3.158676	5.016618	7.175767	15.18783	41.38739	34.23777

HORVU2Hr1G082950	3.967054	4.581331	4.449071	2.185542	2.80321	2.767679
HORVU3Hr1G031140	0.611482	0.589186	0.441844	1.117864	4.384199	3.513038
HORVU5Hr1G104000	3.159298	2.74145	5.088398	5.086177	4.86347	4.266108
HORVU1Hr1G086180	3.415178	2.453132	3.252827	2.179405	3.419096	3.410937
HORVU2Hr1G088380	4.156311	3.440824	5.002099	9.792191	4.411088	6.131121
HORVU7Hr1G048880	2.900411	3.132596	4.7348	5.28338	11.64893	10.95165
Hordeum_vulgare_newGene_13824	0	0	0	1.874329	2.52885	2.234216
HORVU1Hr1G090020	15.58907	13.38572	18.21945	14.96921	19.76221	20.85975
HORVU5Hr1G111000	2.731252	2.220734	2.625295	3.712109	2.990501	2.89548
HORVU1Hr1G078110	1.21657	1.268538	2.073233	2.655867	3.369993	2.439409
HORVU0Hr1G015440	0.883942	0.580856	1.121558	1.193914	1.537306	1.393866
HORVU1Hr1G091990	2.905566	2.039008	3.253758	1.227878	0	0.169933
HORVU6Hr1G018840	3.566817	4.241778	3.75939	4.855748	6.063963	6.129476
HORVU7Hr1G024250	0.016212	0	0.026981	1.907028	40.69446	29.03668
HORVU3Hr1G001060	0.294973	0.234058	0.286119	1.150859	1.270682	2.971579
HORVU5Hr1G014510	0.244285	0.11482	0.203993	3.357249	14.3158	10.83403
HORVU7Hr1G093620	10.24414	13.3022	12.4838	7.752435	7.866602	8.810136
HORVU7Hr1G053370	1.462213	1.387533	1.658851	1.179209	0.932166	1.644938
HORVU5Hr1G093300	11.48686	11.70128	15.40095	19.92347	24.272	21.85045
HORVU5Hr1G041120	106.7193	102.6446	114.3038	103.5494	186.6677	171.3237
HORVU4Hr1G003800	8.444714	7.696701	8.959639	19.57042	22.51505	22.78993
HORVU1Hr1G060090	32.47883	32.76662	39.26184	28.2067	39.34727	38.98663
HORVU7Hr1G110820	2.605335	2.558512	2.578666	1.82253	4.667353	3.440351
HORVU3Hr1G040280	11.4159	10.86342	14.68968	13.78964	20.33645	20.7408
HORVU3Hr1G034460	1.919536	1.66794	2.294337	1.477571	2.053294	2.952661
HORVU3Hr1G043390	16.7775	16.74465	17.00966	25.92315	37.64789	38.83392
HORVU4Hr1G081990	6.910626	8.560055	9.673264	4.845481	2.475881	4.016016
HORVU2Hr1G099660	10.51609	10.28229	19.72447	4.813366	6.363059	7.153524
HORVU2Hr1G002880	1.124329	1.250492	1.120399	1.171808	3.192503	3.171153
HORVU5Hr1G027100	7.921009	6.389067	9.548639	8.193324	7.939729	9.405773
HORVU1Hr1G080920	7.092075	7.895351	6.877879	6.189669	5.926303	6.261188
HORVU1Hr1G068520	2.705455	3.368964	2.750695	3.842359	4.87423	4.685556
HORVU3Hr1G070300	9.81186	10.16038	13.86342	7.153912	4.581401	6.198223
HORVU7Hr1G111190	2.085168	2.170137	2.058896	3.387358	4.020897	4.5095
HORVU2Hr1G000770	1.324184	1.680994	1.657107	1.543	1.800011	2.102271
HORVU6Hr1G074410	0.705854	0.591689	0.602473	3.193242	2.311266	2.323755
HORVU1Hr1G045590	17.74948	14.83895	19.66387	11.62139	24.8214	21.73036
HORVU6Hr1G072500	32.72272	31.32197	36.43941	42.6703	61.74222	54.35387
HORVU6Hr1G083660	3.778261	3.833452	5.018224	2.995759	7.483341	7.272079
HORVU3Hr1G002000	5.53706	3.187321	8.746502	0.34554	0.075932	0.117201
HORVU3Hr1G079500	5.03917	4.985317	5.628321	9.689076	14.38554	13.8245
HORVU7Hr1G093080	16.40042	14.39038	18.91327	14.09928	8.160238	7.560744
HORVU7Hr1G069090	6.921365	7.11352	8.705291	10.62187	10.09689	11.67409
HORVU4Hr1G052320	6.347239	3.053709	7.134206	1.636846	0.819422	1.091353
HORVU3Hr1G077070	2.346531	1.39954	2.85993	3.527402	1.510117	1.437721
HORVU4Hr1G051780	201.3533	236.6762	288.8035	73.07365	5.101343	35.00189
HORVU3Hr1G028550	5.851232	7.58002	6.467757	15.25859	19.75634	18.74703

HORVU5Hr1G002180	3.520732	4.161077	5.849491	2.958295	4.977199	6.338508
HORVU6Hr1G080400	3.10781	3.352374	3.973864	2.511067	3.015795	2.817254
HORVU4Hr1G050780	8.83981	8.376449	11.36131	12.64094	20.26827	20.14753
HORVU4Hr1G056830	119.3716	143.0465	142.488	187.1463	273.0725	287.2642
HORVU3Hr1G016320	11.44432	11.62604	11.91573	13.91448	12.23408	13.15815
HORVU1Hr1G027700	2.235786	0.711886	2.195614	1.237611	0.299818	1.119429
HORVU2Hr1G123580	0.561307	0.946353	0.572615	0.585994	2.810691	2.315516
HORVU4Hr1G040950	11.48166	8.858424	13.77803	16.13126	17.98339	19.39347
HORVU4Hr1G003110	9.539222	9.264593	10.79935	16.47027	18.9169	17.94563
HORVU5Hr1G097930	1.573783	1.135207	1.955321	12.81497	6.088622	7.464816
HORVU6Hr1G041680	8.19918	21.1621	12.80803	16.32286	16.75775	13.09191
HORVU4Hr1G006090	1.324776	1.42291	1.629783	4.249362	3.066778	3.534782
HORVU3Hr1G072410	35.73829	33.82654	33.54608	19.01361	14.83535	17.83327
HORVU7Hr1G040690	0.2318	0.373971	0.356477	1.577119	2.416777	2.025927
Hordeum_vulgare_newGene_16228	1.200238	1.180483	0.849473	1.390249	1.505019	1.667199
Hordeum_vulgare_newGene_16224	8.196909	6.067679	8.851418	13.48301	7.534938	8.798106
Hordeum_vulgare_newGene_16226	0.800214	1.027505	1.110091	1.003835	2.201987	2.155368
Hordeum_vulgare_newGene_16220	1.564165	2.264895	2.387649	0.197532	0.071876	0.084456
HORVU0Hr1G016360	6.914448	6.686516	7.582494	9.692195	12.94877	9.267118
HORVU0Hr1G013900	25.36688	22.40309	20.04457	84.77275	94.70655	103.9753
HORVU6Hr1G038870	5.376134	6.895686	8.213694	5.117886	5.766939	5.22704
HORVU6Hr1G050160	10.166	8.777031	10.6702	12.74907	14.47087	16.97329
HORVU1Hr1G085190	1.342938	1.254647	1.059298	0.562035	4.874117	5.444888
HORVU5Hr1G051240	3.708925	3.903165	4.283597	7.090905	6.778757	6.385934
HORVU7Hr1G080070	24.12156	30.2013	31.41162	19.18019	15.71619	20.86712
HORVU5Hr1G109240	65.29718	55.01732	57.35148	156.4709	191.1733	137.1253
HORVU6Hr1G001480	2.515961	1.338921	2.002252	3.978889	1.652811	2.622014
HORVU3Hr1G071820	22.69245	21.7785	24.845	21.79289	26.08011	27.24644
HORVU1Hr1G018480	5.634635	2.767296	5.247386	2.764394	2.683044	3.391615
HORVU1Hr1G002930	0.005863	8.251803	0.567603	0	0	0
HORVU7Hr1G050110	0.384252	0.399286	0.685178	0.717225	2.733593	2.176605
HORVU5Hr1G092180	5.724581	6.659472	7.493433	7.247395	9.736908	10.48379
HORVU4Hr1G067840	1.974977	1.512513	2.986547	1.983481	3.854238	5.419484
HORVU6Hr1G000280	9.75438	10.71719	13.83719	12.55337	19.79351	18.25641
HORVU1Hr1G003170	3.14958	11.52621	4.276223	17.7243	6.009385	6.582078
HORVU2Hr1G009830	9.842114	12.59773	15.25354	4.256557	0.834226	2.692005
HORVU6Hr1G091430	25.39093	24.03555	25.78228	26.38911	23.74053	29.23011
HORVU4Hr1G008940	23.39364	20.45685	22.40238	24.34909	28.99477	28.19373
HORVU5Hr1G114640	1.892206	1.970423	1.509776	3.0067	3.371807	4.319145
HORVU1Hr1G038060	17.07446	20.199	22.7518	13.55223	9.353856	12.04576
HORVU1Hr1G018150	15.5775	16.05048	21.61146	8.367577	15.77799	14.04541
HORVU3Hr1G117370	7.633372	6.365371	10.1254	1.324071	0.599934	2.388586
HORVU7Hr1G007920	3.048457	3.01247	3.3836	3.554995	2.844422	2.977443
HORVU3Hr1G105360	3.287785	3.448408	4.073336	8.110747	1.14405	4.442564
HORVU5Hr1G058060	7.968525	5.644046	6.371127	0.542904	0.014273	0.132459
HORVU0Hr1G008940	2.558999	2.320851	2.992517	2.808603	3.062029	3.101482
HORVU3Hr1G089020	3.120553	2.474307	4.188506	3.185391	2.269867	2.072506

HORVU1Hr1G042370	1.540529	1.128759	1.466625	2.511647	1.031451	2.259439
HORVU5Hr1G041230	6.062425	6.964083	5.875073	7.35968	9.97929	9.534821
Hordeum_vulgare_newGene_2633	4.789643	6.157635	5.541725	4.912781	5.296921	7.353451
HORVU3Hr1G086040	1.171599	0.751397	1.028513	1.506427	2.690356	3.072625
HORVU6Hr1G052140	6.044172	5.745186	7.730726	6.072743	6.101266	6.387788
HORVU0Hr1G014450	0.385238	0.261068	0.303918	1.227567	2.401423	3.593541
HORVU3Hr1G077890	1.599073	2.273006	2.427559	3.143699	1.143399	1.435818
HORVU7Hr1G019850	21.01663	21.66548	22.49041	17.38048	15.93397	18.95499
HORVU3Hr1G090860	0.645982	1.133685	0.871804	2.724102	1.360613	1.983668
Hordeum_vulgare_newGene_3474	20.6557	14.94904	17.48892	12.45246	4.276265	5.943619
Hordeum_vulgare_newGene_3473	15.71177	16.54203	13.04643	13.61161	5.618234	9.683862
HORVU4Hr1G026060	6.219368	6.614776	10.49682	4.79241	6.005907	6.821694
Hordeum_vulgare_newGene_3478	2.932482	2.96602	4.394701	3.236301	2.568497	3.418837
HORVU6Hr1G056860	3.954797	3.369902	3.365092	3.835373	4.053201	4.787857
HORVU7Hr1G057140	4.906452	4.676242	5.36152	7.175151	6.986696	6.583336
HORVU5Hr1G068520	18.75718	11.45812	22.11659	12.37248	9.052005	11.79085
Hordeum_vulgare_newGene_2639	3.543502	5.010828	5.536007	5.147967	6.905106	8.351699
HORVU3Hr1G067860	4.933342	3.973922	5.828418	7.590827	8.537749	7.683791
HORVU1Hr1G053320	14.77629	12.91204	16.70614	19.15149	34.19135	30.77152
HORVU2Hr1G020270	83.78585	86.02864	108.6104	114.8175	137.5141	136.3827
HORVU1Hr1G015240	1.284912	1.316624	1.843895	1.486732	1.980323	1.590566
Hordeum_vulgare_newGene_2703	4.798776	5.440559	5.646385	5.490633	7.129484	8.057626
Hordeum_vulgare_newGene_2702	4.790285	3.847428	4.950109	4.712499	3.45165	4.074662
Hordeum_vulgare_newGene_2708	6.301186	6.431023	7.147781	6.757091	5.830471	5.502876
HORVU5Hr1G026770	12.96688	12.88881	13.89501	15.52947	18.77764	19.57267
Hordeum_vulgare_newGene_13307	0.469521	0.343781	0.475111	4.20937	0.589357	0.748445
Hordeum_vulgare_newGene_13300	2.279505	2.651812	2.307407	1.349673	2.187855	1.980931
Hordeum_vulgare_newGene_13302	2.506723	3.497266	1.749356	3.024242	1.632572	2.499843
Hordeum_vulgare_newGene_13303	5.777842	4.389363	5.448024	4.626877	6.258895	5.471524
HORVU2Hr1G037890	5.280699	5.944567	8.199277	6.599739	9.757196	10.54427
HORVU3Hr1G055740	2.803617	2.725941	3.481811	2.553672	0.827773	1.381979
HORVU6Hr1G015500	17.10783	12.9981	14.3652	12.00577	7.647375	11.87477
Hordeum_vulgare_newGene_9082	2.908961	3.070642	3.307624	1.272923	0.794977	0.508444
Hordeum_vulgare_newGene_9085	3.240734	2.711414	3.197367	0.007918	0	0
Hordeum_vulgare_newGene_9084	2091.552	2067.006	1405.925	556.2512	173.5831	258.4333
Hordeum_vulgare_newGene_9086	4.138733	3.471565	4.710541	5.033425	7.348158	7.182435
Hordeum_vulgare_newGene_15229	9.491987	10.23999	9.447094	0.015351	0	0
Hordeum_vulgare_newGene_15225	7.457616	5.680433	6.133847	7.712446	7.1929	8.671514
Hordeum_vulgare_newGene_15227	28.3902	26.27241	29.68638	24.42012	27.20892	29.52868
Hordeum_vulgare_newGene_15226	12.58197	12.40837	11.31185	11.60327	11.79762	13.78643
HORVU2Hr1G071170	5.1612	4.510969	7.178007	7.193541	9.445971	10.02312
HORVU7Hr1G082560	1.716685	1.493872	2.14796	2.256947	2.045911	2.575741
HORVU3Hr1G088850	1.280737	1.539524	2.401365	0.647318	0.602391	0.760635
HORVU3Hr1G104100	0.018339	0.062664	0.01716	1.988071	1.931403	2.470578
HORVU5Hr1G079970	5.881764	1.350022	1.285887	1.152134	0	0.346077
HORVU4Hr1G010140	7.294091	6.155655	6.773768	8.126752	9.008082	9.471087
HORVU6Hr1G021340	11.17668	11.50918	12.76336	10.80604	15.86451	16.70011

HORVU4Hr1G075250	4.501184	5.023614	4.937235	8.338231	6.771634	8.818806
HORVU5Hr1G073380	7.691725	6.505989	7.38002	7.589958	6.566335	6.639384
HORVU5Hr1G063430	1.939381	1.438518	2.125238	0.554433	0.222883	0.637335
HORVU2Hr1G103620	23.08181	22.17619	23.51525	22.54586	24.06463	28.27743
HORVU5Hr1G078000	81.45915	65.67387	84.46724	101.1552	90.85781	87.76794
HORVU0Hr1G010220	4.390182	4.419263	5.289117	3.462872	2.043692	2.534045
HORVU7Hr1G027870	5.682282	6.503046	6.904919	2.095279	1.638037	2.228538
HORVU2Hr1G097060	2.974021	4.906388	3.584046	8.467306	15.60805	14.47714
HORVU2Hr1G030150	15.28979	13.69979	15.29037	16.84064	12.67218	13.313
HORVU4Hr1G071000	21.2304	24.90329	19.05821	43.10206	59.77725	75.04806
Hordeum_vulgare_newGene_5165	11.82238	11.50413	12.31405	16.6371	22.7442	23.19886
HORVU2Hr1G112230	1.229685	0.902176	1.10636	3.186282	3.976387	3.129388
HORVU5Hr1G117680	0.091633	0.229411	0.223649	4.982263	12.15559	10.4319
HORVU6Hr1G016360	4.796551	4.548448	8.074175	3.853394	4.988106	5.132292
HORVU2Hr1G049410	2.187177	1.457277	1.901312	2.900588	6.633095	5.983147
Hordeum_vulgare_newGene_11215	0.863214	0.825256	0.801798	0.983145	1.622767	1.34796
Hordeum_vulgare_newGene_11210	2.539193	3.37008	3.652745	3.25498	4.155643	4.472775
Hordeum_vulgare_newGene_11212	11.07177	12.04465	11.54698	9.382067	8.604574	10.53753
HORVU2Hr1G111590	0.788916	0.637749	0.465152	1.545489	1.906973	1.821392
HORVU2Hr1G118570	113.1442	85.97543	77.0276	29.12978	7.170227	12.51041
HORVU2Hr1G104960	8.067581	7.762935	8.404724	14.07384	13.72883	13.52346
HORVU1Hr1G001510	0	9.908692	0.530906	0	0.016032	0.0109
Hordeum_vulgare_newGene_8023	0.570732	0.730639	0.713245	1.57308	1.237738	1.693807
HORVU1Hr1G024700	35.76943	30.57236	35.35488	39.85456	48.97751	47.2106
HORVU1Hr1G028280	36.59305	31.54503	43.14952	40.85878	54.14509	48.8338
HORVU7Hr1G030050	1.250644	1.478315	1.655714	2.136197	1.893622	2.69799
HORVU5Hr1G072160	66.34818	68.97865	88.41889	9.467303	0.599285	3.814989
HORVU7Hr1G087000	3.976216	4.152414	3.967549	5.252157	5.591446	7.001246
HORVU2Hr1G026860	2.743504	2.617828	2.866551	4.331537	4.411899	5.728246
Hordeum_vulgare_newGene_15136	1.393982	1.497699	2.631362	0.190593	1.036864	1.234607
HORVU3Hr1G081750	6.693607	4.498658	8.747124	6.531052	8.578678	7.647741
HORVU2Hr1G075670	9.278544	6.831216	9.69995	10.57908	8.955263	10.16225
HORVU2Hr1G092900	6.50263	4.979875	6.271089	3.90505	2.51242	2.835601
Hordeum_vulgare_newGene_1421	8.40062	8.588758	11.88681	7.057415	6.521157	7.980932
HORVU2Hr1G011200	2.177435	1.927895	3.311128	5.118346	8.007569	7.55267
Hordeum_vulgare_newGene_1429	1.781839	2.157147	2.196447	2.218893	1.761248	2.010702
HORVU6Hr1G076760	3.306061	2.31757	3.899004	3.654519	6.78768	6.658072
HORVU4Hr1G078690	13.45841	12.66241	14.61256	8.929932	14.02194	15.15587
HORVU7Hr1G021840	9.725108	8.412696	10.64977	24.35717	55.30259	55.3471
HORVU6Hr1G071930	16.33722	15.59231	17.80317	16.01586	25.55269	24.4738
Hordeum_vulgare_newGene_3864	67.84344	60.32799	73.03615	50.92671	60.29135	64.38864
Hordeum_vulgare_newGene_3867	8.529072	7.899771	7.500926	43.51095	67.34712	56.78618
Hordeum_vulgare_newGene_3866	5.533894	5.044569	5.539799	2.681712	3.165907	3.681392
HORVU1Hr1G071950	25.13594	18.75553	24.2704	16.81891	20.99966	22.14785
Hordeum_vulgare_newGene_3863	4.591874	6.0705	6.327962	5.010639	3.934572	4.474322
HORVU5Hr1G016320	7.86601	6.813587	8.732763	9.346633	10.03112	12.34196
HORVU1Hr1G041710	11.00765	9.989406	14.93027	10.82935	11.22365	10.35732

HORVU6Hr1G020920	10.88491	9.576491	14.91606	9.419542	13.38348	14.64893
Hordeum_vulgare_newGene_4619	8.300307	7.82787	7.967269	9.196846	10.18047	10.93041
Hordeum_vulgare_newGene_4618	1.00494	0.856267	0.953956	1.059349	1.627013	1.817987
HORVU2Hr1G025980	15.80224	12.10559	14.05435	19.06332	24.36234	22.34595
HORVU3Hr1G066380	3.854246	2.873905	3.431556	6.337166	6.954743	5.84845
HORVU2Hr1G021070	41.6899	34.1012	47.90398	44.62071	88.965	87.9965
HORVU7Hr1G100850	11.62814	4.304527	12.18021	1.439453	0.771895	0.898655
HORVU5Hr1G082620	14.28237	10.33967	13.31314	4.590004	2.888146	2.203422
HORVU7Hr1G105370	3.420126	4.302667	5.409025	3.597617	4.010996	5.370555
HORVU4Hr1G033170	12.49372	12.28933	17.05272	12.32104	17.08409	18.24084
HORVU1Hr1G072200	79.39146	68.46785	91.15299	82.75828	105.3077	97.97894
HORVU3Hr1G066650	1.647309	1.433748	2.175095	2.484563	1.354325	2.567308
HORVU5Hr1G060310	1.180973	1.148258	1.512888	1.816066	5.408512	5.636081
HORVU7Hr1G053170	112.1872	106.1146	137.664	155.5016	311.6113	257.4681
HORVU3Hr1G052010	1.248656	0.700818	1.006392	1.105087	1.424426	1.882639
HORVU2Hr1G024160	2.075975	1.69631	1.798381	2.5376	1.611856	2.882505
HORVU2Hr1G068150	4.640647	2.951823	3.89821	4.637838	3.464543	3.28951
Hordeum_vulgare_newGene_12175	12.48633	15.23893	15.55658	16.80565	16.70758	19.67199
Hordeum_vulgare_newGene_12178	1.878241	1.534696	1.787797	7.443023	1.631135	3.062205
HORVU3Hr1G040040	84.94829	78.5304	101.1055	94.0463	151.4135	148.5709
HORVU4Hr1G027070	7.966989	7.57028	9.410025	4.177414	5.809931	6.309127
HORVU2Hr1G011580	5.443689	6.124454	6.788	8.946762	13.44605	12.90193
HORVU2Hr1G039350	3.158932	4.183963	2.213083	1.234166	12.46351	10.02133
Hordeum_vulgare_newGene_6614	2.706786	4.522816	4.846807	4.152769	4.296197	5.584108
HORVU1Hr1G074580	0	0.103829	0.380603	8.917956	0.240737	3.491985
HORVU1Hr1G022700	9.680463	7.782816	10.49029	6.845327	5.731799	6.597148
HORVU2Hr1G071080	104.3723	104.2231	109.0524	117.38	128.4555	135.2603
HORVU5Hr1G084700	2.977982	1.558787	2.675348	0.934156	0.709604	0.714242
Hordeum_vulgare_newGene_3137	2.147183	2.511548	1.095293	5.336326	6.314332	6.59462
HORVU4Hr1G030610	3.682212	4.296012	4.251397	9.165578	8.749971	8.694551
Hordeum_vulgare_newGene_3130	0.348692	0.366716	0.380606	2.175184	2.202766	1.894948
HORVU5Hr1G041570	0.771972	0.861863	1.003165	1.38494	1.308771	1.434852
HORVU3Hr1G085780	8.95989	9.965972	12.85923	10.12169	7.67393	10.50142
HORVU6Hr1G075300	0.739038	0.882005	1.001695	0.78736	1.901028	1.827967
HORVU1Hr1G036160	0.856296	0.997098	0.962691	1.156295	0.839857	1.434967
HORVU1Hr1G087620	6.040656	5.055774	7.485088	4.049681	4.718489	5.033852
Hordeum_vulgare_newGene_14635	0.971422	1.437571	1.532047	5.501632	6.380838	7.161863
HORVU5Hr1G107360	3.837102	3.283931	4.095581	3.624655	4.068578	4.198509
HORVU7Hr1G078170	4.29508	4.323505	4.33838	1.935981	1.823243	2.2717
HORVU5Hr1G064100	0.654556	0.561334	0.961719	6.156192	5.971297	6.316015
HORVU5Hr1G020350	0.717932	11.23079	1.311634	0	0	0
HORVU3Hr1G039160	1.952376	3.002628	3.611948	2.328787	1.821967	2.44497
HORVU3Hr1G117740	1.80926	4.525821	4.652299	3.601713	7.949725	5.696805
HORVU3Hr1G012010	1.981251	1.940626	2.019419	5.974909	6.543945	6.869722
HORVU3Hr1G087780	6.122381	6.468986	7.718729	3.664181	4.16646	4.197638
HORVU5Hr1G023710	0.745892	0.851104	0.742813	5.528548	2.817852	3.391852
HORVU4Hr1G044090	3.517273	2.492474	3.484858	4.129924	4.352485	5.018676

HORVU2Hr1G043970	6.392129	6.242123	9.133262	6.003976	7.156395	8.563999
HORVU1Hr1G055470	7.833188	6.364456	11.06572	6.932544	2.528666	2.66218
Hordeum_vulgare_newGene_8927	2.926694	2.991039	3.922164	3.875316	4.841164	5.413536
HORVU7Hr1G064880	17.9855	20.38142	19.7908	22.02026	17.10157	20.42779
Hordeum_vulgare_newGene_8929	26.90848	25.59302	23.57116	31.65675	29.888	30.38858
HORVU5Hr1G036920	8.634953	6.585977	9.151285	6.110045	11.31665	11.03752
HORVU7Hr1G021200	1.211567	1.86185	1.527328	1.473935	2.037893	2.420663
HORVU1Hr1G092150	5.805882	4.170397	5.540608	12.60448	18.2686	20.20257
HORVU5Hr1G088700	6.372461	6.250434	6.637471	4.955545	5.217172	4.714366
HORVU5Hr1G063770	33.1761	35.02695	41.53835	30.88243	41.78524	44.38705
HORVU3Hr1G098720	48.21124	42.98558	44.57233	53.0737	45.47648	46.71169
HORVU6Hr1G005730	17.27008	19.78357	20.06376	20.43978	22.85415	24.71031
HORVU1Hr1G061830	1.824339	1.724018	1.688087	4.110537	7.625811	7.242345
HORVU5Hr1G039950	1.858223	2.275953	2.894735	1.708727	1.195943	1.188789
HORVU5Hr1G111240	1.923399	0.936586	2.411177	0.396183	0.281197	0.358338
HORVU1Hr1G067240	4.886711	3.806298	5.002439	4.870853	6.787031	6.815903
HORVU5Hr1G008820	4.418646	4.145607	5.064886	3.153021	3.97599	4.76364
HORVU6Hr1G058210	39.06586	34.97868	45.03733	44.46205	75.05205	81.73549
HORVU1Hr1G044000	71.85217	52.43432	74.1292	6.965187	3.106206	5.113893
HORVU4Hr1G043950	9.115542	10.95955	10.3875	7.805397	8.654591	8.944848
HORVU5Hr1G069680	1.262465	0.18384	1.331093	6.06512	37.10506	26.98038
HORVU0Hr1G012190	0.614678	0.609539	0.691249	1.792112	1.834431	2.237654
HORVU3Hr1G015610	19.92933	16.63309	21.98617	15.86875	17.00329	17.89988
HORVU7Hr1G073310	14.70669	15.62964	16.34469	13.63232	29.69376	23.15078
HORVU1Hr1G048430	5.287478	4.378688	5.961113	3.945691	2.738139	3.482548
HORVU4Hr1G080780	17.34748	14.40503	17.22474	16.04026	18.08107	16.08729
HORVU4Hr1G011250	40.76106	33.54779	53.01401	24.95891	12.61273	15.63146
HORVU2Hr1G085830	5.351772	5.600613	5.701749	4.136102	4.040658	4.86274
HORVU5Hr1G097770	0	0	0.051371	0.450293	21.51779	16.74934
HORVU7Hr1G106710	6.658558	5.298606	7.355325	9.49097	10.96997	12.16511
Hordeum_vulgare_newGene_2954	0.756694	2.126177	2.876234	4.974051	14.07757	10.81925
HORVU2Hr1G050980	15.22147	14.51739	19.99231	15.97993	20.55229	20.18578
HORVU2Hr1G009580	24.237	32.04219	38.3933	10.7933	2.709922	7.49761
HORVU5Hr1G109730	10.17268	9.903791	9.399273	8.525084	8.429214	9.154922
HORVU1Hr1G095310	34.98603	31.31092	33.40606	20.16389	20.5518	17.77645
HORVU4Hr1G014750	6.832381	6.761524	8.111665	9.665442	15.83098	15.68532
HORVU4Hr1G018230	1.522	1.520525	1.612072	3.115975	3.678504	4.450826
HORVU5Hr1G020430	48.44262	43.02647	46.87056	13.7495	7.309779	10.18803
HORVU4Hr1G009560	4.668628	4.907732	4.302101	6.178577	3.593957	5.919784
HORVU7Hr1G088580	138.5669	139.7174	200.1836	151.5175	403.2718	357.1641
HORVU3Hr1G001450	10.75672	9.647235	11.66219	5.481296	5.147777	5.725834
HORVU0Hr1G039460	3.656325	3.044691	4.273855	5.355395	6.937883	6.208319
HORVU5Hr1G103920	3.015086	3.229865	3.276392	2.208266	3.865528	2.525018
HORVU1Hr1G073610	3.943138	4.283088	5.255329	3.164646	7.067512	6.281311
HORVU5Hr1G009880	15.84422	16.2335	17.98707	17.1928	20.22987	20.98224
HORVU3Hr1G041760	11.10819	10.80816	11.51617	9.903325	9.200974	9.690271
HORVU5Hr1G096790	0.501465	0.363487	1.729492	1.725636	2.421794	0.482567

HORVU3Hr1G030910	3.587588	3.540505	3.950218	5.335806	8.264939	8.107057
HORVU2Hr1G120620	6.094767	6.449063	6.078451	3.009627	5.158215	4.000115
HORVU1Hr1G015000	0.594754	0.464394	0.499179	1.217877	3.146879	2.854238
HORVU3Hr1G005480	2.050158	2.579497	3.019162	2.391099	1.898878	2.046083
HORVU3Hr1G047030	0.101191	0.088831	0.146768	30.81276	29.50729	40.07474
HORVU5Hr1G060450	2.498927	2.132624	2.304624	3.913291	4.544204	4.039659
HORVU5Hr1G026170	14.63049	13.82268	16.83797	24.48556	29.3838	30.80588
HORVU5Hr1G010080	7.539293	6.984497	9.716733	7.157561	6.22052	7.294284
HORVU2Hr1G107120	5.376403	4.723403	5.218589	6.803699	7.218444	6.778181
HORVU3Hr1G071770	2.166699	3.323399	1.49322	1.666796	2.508865	3.295919
HORVU5Hr1G096420	6.59677	3.257022	4.168617	13.79332	9.087573	8.288599
HORVU2Hr1G005230	1.420734	0.837443	1.204648	2.027136	2.113639	2.22737
HORVU1Hr1G069570	3.366161	2.283798	2.934031	5.996185	1.785544	2.250331
HORVU4Hr1G050080	3.518275	4.698425	3.524791	4.734465	5.218964	6.324615
HORVU5Hr1G082010	29.05285	25.5794	40.57133	22.06428	42.57423	36.80834
HORVU5Hr1G080470	1.421261	1.003155	1.986777	2.377263	1.974096	1.722291
HORVU5Hr1G009220	1.581497	1.016628	1.58317	3.420841	3.579922	2.863096
HORVU4Hr1G008210	2.571755	2.878822	2.677203	3.689215	7.807692	8.055587
HORVU7Hr1G000860	23.37592	27.12999	28.8459	0.993767	2.233803	4.053427
HORVU2Hr1G092190	110.7565	96.94526	112.3912	184.2448	283.8608	216.6289
HORVU4Hr1G059860	49.90468	34.5773	53.81994	61.22312	38.79334	48.60482
Hordeum_vulgare_newGene_10324	0.773603	0.964543	0.645334	3.254515	3.970603	3.594472
HORVU5Hr1G047510	39.17956	25.58761	32.809	8.496193	0.058111	2.366219
HORVU4Hr1G056000	2.759851	3.122679	3.532242	8.121964	9.86735	10.88039
HORVU7Hr1G043960	0	0	0	2.124329	2.839484	3.707271
HORVU1Hr1G045960	2.933734	3.394965	3.442293	3.335983	3.663772	4.880729
HORVU4Hr1G090150	1.715073	1.914602	1.407039	1.682951	2.250714	2.638654
HORVU7Hr1G116780	1.635916	1.455869	2.067676	0.917922	0.640428	0.71701
HORVU3Hr1G059840	2.804044	2.53179	2.349444	5.802551	7.338535	6.159407
HORVU7Hr1G089720	4.536641	5.485217	6.057003	3.700147	5.144578	4.503341
HORVU5Hr1G022120	0.663856	0.540348	0.411842	2.214671	7.925902	6.818721
HORVU1Hr1G055860	22.85586	22.88094	30.75844	31.32369	72.99102	70.78072
HORVU2Hr1G041180	2.017836	1.820995	2.316462	1.816007	3.059197	2.977933
HORVU5Hr1G124560	9.438409	8.907285	9.486835	14.17575	15.08745	14.07487
HORVU7Hr1G111780	9.606576	7.785545	10.18934	11.31524	20.18784	15.26478
HORVU0Hr1G010750	7.133841	6.19084	8.130311	6.349113	7.322784	7.446337
HORVU2Hr1G093570	8.241216	7.607214	10.28754	6.042351	5.897454	7.795887
HORVU1Hr1G012850	11.57257	8.370565	12.31289	36.10173	70.45068	66.78825
Hordeum_vulgare_newGene_11056	0.069855	0.077014	0.05289	4.86344	5.189624	5.347759
Hordeum_vulgare_newGene_11053	1.105872	1.151305	2.891847	1.418812	1.434885	1.147169
Hordeum_vulgare_newGene_11058	0	0	0	10.25011	10.08202	12.21793
HORVU7Hr1G000280	1.231505	1.517064	1.392743	1.449075	1.20585	1.559221
HORVU5Hr1G068130	12.22946	12.95466	9.990671	7.315387	10.33464	8.958509
HORVU7Hr1G007480	0	0	0	3.365812	0.760432	2.927597
HORVU3Hr1G026050	5.198824	3.978513	6.251519	7.046377	3.183881	5.796204
HORVU3Hr1G092650	13.67747	15.98651	11.75944	8.092633	5.490124	6.238754
HORVU3Hr1G095600	8.896451	9.002638	10.14241	34.62365	37.96051	35.86098

HORVU3Hr1G014890	1.1172	1.001783	1.175478	5.438173	5.941414	6.932145
HORVU1Hr1G084160	64.68766	51.86384	66.94344	42.70788	48.81238	48.04847
Hordeum_vulgare_newGene_12800	17.71458	18.09149	21.3365	16.86303	20.22572	21.62019
Hordeum_vulgare_newGene_11588	6.868796	7.674269	7.41785	8.839595	14.19192	14.93455
HORVU1Hr1G066650	15780.59	15768.26	11287.59	19757.88	7758.363	9301.196
HORVU7Hr1G000130	1.934038	1.408663	2.295388	1.891352	3.331609	3.194211
Hordeum_vulgare_newGene_11587	0	0	0	15.40485	16.07343	18.17092
HORVU2Hr1G115070	68.68533	62.47964	60.49879	72.81355	86.34449	79.38718
HORVU3Hr1G094550	19.1836	17.7074	25.74343	21.27401	29.67133	32.48685
HORVU3Hr1G038350	4.051841	4.131286	4.248492	5.556935	5.742489	6.4835
HORVU4Hr1G056660	2.715208	3.471879	3.012922	3.77024	5.247486	5.19079
HORVU6Hr1G092390	0.116818	0	0.222739	1.186224	7.111657	5.792146
HORVU7Hr1G040220	4.165755	4.175457	4.728396	3.711952	3.751671	3.891162
HORVU4Hr1G052170	6.206482	4.780855	5.564053	11.08359	9.77101	9.334992
HORVU6Hr1G057570	7.382302	8.800265	11.34496	6.675751	9.106121	9.480327
HORVU7Hr1G083550	4.900973	2.145819	4.838226	6.579296	3.010731	9.223503
HORVU3Hr1G107040	30.51094	30.09259	35.28076	48.56236	41.74607	49.55565
HORVU0Hr1G009870	3.21991	2.280638	4.054111	2.514719	2.637665	2.317169
HORVU2Hr1G112060	5.221498	3.452958	7.351753	0.249755	0.061157	0.081916
HORVU3Hr1G037360	97.47306	80.24349	112.2136	74.19277	42.59861	45.23584
HORVU4Hr1G078840	11.94711	14.08125	13.66008	25.82184	25.39068	28.18198
HORVU1Hr1G046600	1.756228	1.568634	1.932278	2.320769	2.410578	2.519235
HORVU7Hr1G006990	3.451735	3.972297	3.117299	3.400108	2.735303	3.123667
HORVU0Hr1G017800	2.705007	2.689728	2.583812	3.536109	4.578364	4.64595
HORVU7Hr1G121560	4.316082	2.94388	7.024885	7.368597	0.657692	2.345672
HORVU7Hr1G045470	18.21739	23.55186	21.59271	9.338479	1.757042	3.93234
HORVU6Hr1G013800	16.70766	16.96914	22.92834	21.28429	30.09848	32.08607
HORVU2Hr1G013590	0.06868	0	0.046642	3.847378	8.005479	5.439429
Hordeum_vulgare_newGene_16052	4.66265	2.919037	3.792691	0	0	0
HORVU5Hr1G082420	48.89469	42.45351	44.09621	107.9374	203.7628	196.5681
HORVU6Hr1G048170	2.139659	1.604916	1.670968	3.931156	4.620075	5.470505
HORVU6Hr1G024930	0	0.196107	0.251955	6.178445	7.318427	6.536694
HORVU7Hr1G041570	10.74417	8.705763	11.91299	12.03959	16.45573	14.66831
HORVU2Hr1G025210	8.036799	7.34051	9.759351	6.486598	8.428481	10.79183
HORVU3Hr1G066410	4.887509	4.671224	9.71788	3.932265	4.341472	3.967994
HORVU7Hr1G001420	1.173282	1.036124	1.223776	1.164625	0.594053	1.232609
HORVU7Hr1G037910	4.520677	2.257123	7.074099	1.082667	0.720651	1.536147
HORVU3Hr1G088540	347.0097	259.0183	336.1619	418.1042	568.3897	612.0822
HORVU4Hr1G062460	2.68504	3.258081	3.749405	2.715201	3.932629	4.519712
HORVU3Hr1G063620	0.027062	0.059684	0.005704	0.548277	2.12933	3.489063
Hordeum_vulgare_newGene_12779	4.052169	2.777278	2.87723	18.20201	3.265302	5.150895
HORVU5Hr1G011540	13.71365	12.11377	12.70728	11.34638	7.498566	9.035531
HORVU1Hr1G002740	1.689932	10.84346	2.884808	18.19222	7.465441	6.896066
HORVU2Hr1G062940	5.626168	4.9905	5.750315	0.055669	0.027163	0.044383
HORVU6Hr1G093510	42.12979	40.80211	48.30764	26.17872	28.99068	29.11483
HORVU1Hr1G052290	3.581198	3.806245	4.353732	1.916641	3.169788	2.379472
HORVU6Hr1G063570	36.22339	31.81557	42.6771	54.57222	65.23322	69.42134

HORVU3Hr1G057550	3.912155	3.784762	5.043492	5.067528	6.596196	7.585489
HORVU5Hr1G001310	18.87684	18.53726	19.51194	16.22297	18.57719	18.50688
HORVU2Hr1G102810	28.4306	23.32035	31.24008	27.75675	34.05723	32.51878
HORVU5Hr1G084140	8.787945	8.02851	8.44435	10.27045	13.74574	13.81648
HORVU7Hr1G109290	1.297258	1.228175	1.778985	0.509389	1.306014	1.727608
HORVU7Hr1G100520	3.110955	3.402909	2.98538	6.184099	6.697638	6.806029
HORVU4Hr1G073930	9.082754	9.433804	10.16484	8.222135	9.712054	10.79618
Hordeum_vulgare_newGene_7853	20.62959	19.20567	22.29804	50.2845	31.67478	36.97106
HORVU7Hr1G022570	173.0354	132.519	191.1622	212.3478	276.8293	263.9817
HORVU2Hr1G106310	4.158219	3.537225	4.981029	2.805542	2.387261	3.131978
HORVU5Hr1G080860	35.06633	29.22659	38.54519	25.61809	17.54012	20.88253
HORVU3Hr1G069470	3.087463	2.684612	4.542649	5.216467	8.966887	8.31886
HORVU5Hr1G040970	1319.045	1330.152	1294.06	549.1714	360.29	420.6213
HORVU7Hr1G029060	2.380284	2.0771	2.543265	1.689624	1.871987	2.00121
HORVU7Hr1G070140	2.018137	2.293329	3.311331	2.561518	3.465316	2.591592
HORVU1Hr1G003460	10.56102	9.044973	9.815236	16.39308	20.79162	19.31119
HORVU0Hr1G020790	8.495561	8.85398	9.725795	7.04492	7.449593	8.726661
HORVU6Hr1G012040	1.296862	1.640513	1.439421	1.273173	1.178358	1.298123
HORVU2Hr1G100410	0.932766	0.923275	0.94926	2.224535	1.752921	2.342815
HORVU5Hr1G068480	1.792238	1.137377	1.419759	1.114549	1.013512	1.163443
HORVU3Hr1G056200	30.95234	29.28772	37.35247	22.4902	21.53995	23.09816
HORVU5Hr1G070270	7.42644	6.016135	7.564378	6.281783	8.96485	8.317611
HORVU3Hr1G061660	7.315361	8.88177	7.522769	10.50671	12.73996	12.87777
HORVU4Hr1G083650	21.73982	17.29648	19.48719	15.7415	9.33896	12.04996
Hordeum_vulgare_newGene_647	1.179931	1.495852	1.033549	7.862555	6.839467	8.765776
Hordeum_vulgare_newGene_645	21.64741	19.70584	22.66875	23.92473	27.09416	27.63153
Hordeum_vulgare_newGene_643	7.94142	6.074302	7.386216	23.54382	9.765847	13.64436
HORVU7Hr1G042790	62.39595	56.04885	78.21779	14.72049	14.64274	11.28631
HORVU0Hr1G020420	38.83926	26.23596	40.78991	21.15293	23.37047	16.8163
Hordeum_vulgare_newGene_14341	2.20477	2.03748	2.920827	6.359346	7.020068	5.916852
Hordeum_vulgare_newGene_14340	0	0	0	2.078369	2.022719	2.54517
Hordeum_vulgare_newGene_14343	2.547537	2.164076	2.451145	0	0	0
Hordeum_vulgare_newGene_14342	0.505828	0.637253	0.463101	3.008167	2.78972	3.438195
Hordeum_vulgare_newGene_14344	10.41929	9.985046	12.62153	2.652771	0.222844	0.839602
HORVU3Hr1G059470	12.66195	15.46843	13.53227	26.56171	50.75177	42.58247
HORVU3Hr1G097140	18.79902	17.70189	23.33581	12.64466	13.69281	13.6701
HORVU5Hr1G115230	83.18997	68.57023	89.79448	132.7148	142.4227	152.7875
HORVU1Hr1G077860	5.28336	5.209188	7.648334	4.789718	7.452446	7.430155
HORVU2Hr1G020330	6.166895	6.481002	6.763978	2.038046	2.446144	2.785884
HORVU2Hr1G096270	6.349305	6.51261	7.140965	3.838857	2.237145	2.709215
Hordeum_vulgare_newGene_2571	1.50712	2.207986	1.993706	2.269114	2.665869	3.543671
Hordeum_vulgare_newGene_2575	4.649162	8.004896	5.720476	0	0	0
HORVU7Hr1G103380	36.30016	32.02485	35.59917	7.913419	7.438676	7.896877
HORVU1Hr1G070480	0.611424	0.409728	0.331071	4.975821	5.118835	4.911893
HORVU7Hr1G096630	1.456539	0.560061	0.890875	10.51999	15.14228	11.33302
HORVU7Hr1G009940	5.139575	3.662261	3.350159	3.653246	3.269333	3.673509
HORVU6Hr1G015770	18.5836	15.15342	19.51382	42.77034	45.83066	17.29181

HORVU1Hr1G059570	75.36545	69.08525	126.5878	58.29205	131.927	114.4536
HORVU0Hr1G009040	4.077129	4.195237	4.873246	3.57142	3.929901	4.095511
HORVU0Hr1G022480	0.747823	0.8071	1.352612	0.870022	1.236187	1.475307
HORVU5Hr1G094680	360.75	393.2561	507.8816	54.35436	2.792549	19.87388
HORVU7Hr1G073490	0.214328	0.157966	0.183894	1.041614	6.252048	5.192441
HORVU4Hr1G078020	23.58371	24.39008	33.08744	16.18883	21.23607	21.25599
HORVU7Hr1G073720	178.3854	205.9867	223.2264	87.12552	222.9611	218.7742
HORVU5Hr1G063570	0.580416	0.74227	0.769773	1.572096	2.253168	3.085997
Hordeum_vulgare_newGene_10043	1.22557	1.007758	1.429932	1.801514	1.489482	2.247166
Hordeum_vulgare_newGene_10040	5.106769	5.661022	6.634061	18.9484	26.00642	24.84787
HORVU6Hr1G013020	2.795814	4.000379	2.951126	3.881237	5.558273	5.126992
HORVU4Hr1G060280	2.171756	1.360801	2.392954	1.384973	0.343985	1.020328
Hordeum_vulgare_newGene_12690	3.80729	4.339502	3.324785	4.986317	4.682903	4.754212
HORVU6Hr1G061750	21.27156	21.85377	18.24931	28.26551	18.70054	28.97759
HORVU3Hr1G093240	6.45292	5.623556	6.593689	4.652701	7.093944	6.570877
HORVU5Hr1G098040	5.621191	5.563316	7.101418	7.145783	8.547652	8.85076
HORVU7Hr1G030310	2.671077	1.904646	3.200985	1.845115	3.31056	3.067519
HORVU7Hr1G058250	4.86555	6.413193	5.069278	10.78963	7.799688	9.570791
HORVU7Hr1G086250	3.06481	2.981794	3.616308	3.441539	4.005455	3.610069
HORVU4Hr1G060990	18.64847	18.93331	18.84266	25.45217	17.45683	17.77602
HORVU3Hr1G026550	1.096518	1.327302	1.526163	73.35319	76.77562	79.47147
HORVU6Hr1G073180	2.563167	1.987779	1.548231	15.14794	19.61618	17.82978
HORVU7Hr1G044240	8.386765	6.665585	8.236259	14.41006	18.84923	16.82088
Hordeum_vulgare_newGene_9543	9.097508	7.200345	8.79547	9.434702	13.67245	12.33732
Hordeum_vulgare_newGene_9545	2.415897	2.755559	3.184195	2.7229	2.013382	2.524591
Hordeum_vulgare_newGene_9547	5.541236	5.882603	6.995972	3.909668	4.00739	4.507252
HORVU7Hr1G102030	0	0.222376	0	2.652691	2.314472	2.502735
HORVU2Hr1G118980	7.120093	6.387043	7.806054	14.09613	5.579886	8.201676
HORVU4Hr1G071210	6.2091	4.265206	6.74161	5.212425	1.864365	2.360975
HORVU1Hr1G072500	2.122132	1.030925	2.030817	0.62268	4.111963	2.993536
Hordeum_vulgare_newGene_8569	58.2439	41.68019	50.81256	28.88135	34.86851	34.2148
HORVU6Hr1G067360	2.761581	2.540553	3.069424	1.588251	1.758983	1.74764
HORVU6Hr1G031700	3.860181	3.052435	3.180067	1.380225	3.422538	2.887958
HORVU3Hr1G089990	27.76668	23.25651	18.63513	17.55574	23.87738	22.00652
HORVU0Hr1G026480	3.2615	2.439904	3.630451	6.158588	8.785846	8.995744
HORVU2Hr1G105130	1.916658	1.455696	1.967135	0.660438	0.872494	0.966226
Hordeum_vulgare_newGene_4133	158.1281	170.9232	286.158	107.5835	163.3524	141.3192
Hordeum_vulgare_newGene_4130	5.552173	8.912163	8.306981	0.123248	0.106803	0.121829
HORVU6Hr1G077400	5.111859	6.0725	6.055307	9.639272	18.78708	15.86301
HORVU7Hr1G064400	0.617739	0.943315	1.029782	1.663671	1.836472	2.072108
HORVU2Hr1G076100	2.060517	1.699933	2.811869	3.431139	3.780108	4.201922
HORVU5Hr1G043090	3.469817	3.040616	3.269068	9.603202	12.8733	11.957
HORVU5Hr1G030330	0.565676	0.452188	0.62706	1.357049	1.397346	1.76311
Hordeum_vulgare_newGene_4798	3.115228	4.165724	4.438566	6.270955	6.818905	8.236327
Hordeum_vulgare_newGene_4796	14.7961	11.5384	12.55981	3.65841	3.416099	2.395886
HORVU2Hr1G028540	13.77414	14.02113	15.16374	16.36327	13.12899	15.27614
HORVU1Hr1G068790	179.8036	168.3494	257.5153	421.738	466.5876	463.0385

Hordeum_vulgare_newGene_7445	0.691632	0.812531	0.955162	1.944502	0.73961	1.071077
Hordeum_vulgare_newGene_7443	2.781975	3.916554	3.189988	1.155318	0.99231	1.112902
Hordeum_vulgare_newGene_7441	1.275801	0.948303	1.386813	1.584632	2.26315	2.136063
Hordeum_vulgare_newGene_7449	0.340841	0.406119	0.36822	2.428326	10.90593	10.22631
HORVU1Hr1G024510	4.218872	3.929173	5.189125	5.024952	5.276295	4.68265
HORVU2Hr1G120390	14.40772	13.89076	15.1853	18.51315	19.04631	21.64101
Hordeum_vulgare_newGene_4860	3.150439	2.49784	4.765887	4.84555	5.013629	5.409042
Hordeum_vulgare_newGene_4868	12.26254	14.71491	13.51757	9.384925	10.84862	14.08095
Hordeum_vulgare_newGene_4869	10.71698	10.37824	11.24709	16.72603	14.9506	13.62451
HORVU5Hr1G046190	2.641566	2.539552	3.903641	1.178393	2.142071	4.074729
HORVU6Hr1G058820	16.14456	10.99854	18.16671	26.47383	51.9387	58.3169
Hordeum_vulgare_newGene_6795	20.47084	19.69726	17.89165	21.08146	24.2624	24.53463
HORVU3Hr1G010240	4.739205	3.501811	5.8982	3.626534	0.16386	1.12375
HORVU4Hr1G052350	4.376875	5.030181	5.890643	3.773963	5.392727	7.004139
Hordeum_vulgare_newGene_13129	1.703127	2.085732	1.999275	2.51166	4.409971	3.899374
HORVU5Hr1G125010	0.745205	0.699646	0.943113	7.055034	10.56795	10.12421
HORVU3Hr1G091480	5.907046	4.29838	5.747661	5.65488	8.144295	6.964514
HORVU2Hr1G006040	5.138883	5.959051	6.473335	6.094582	7.195401	7.249904
HORVU3Hr1G040360	11.51827	10.31838	12.3657	17.38758	25.51164	26.59937
HORVU3Hr1G085420	11.69822	11.67874	16.18617	22.07708	24.12967	24.53501
HORVU2Hr1G045370	1.477325	1.295607	1.271276	1.889399	2.531326	3.054559
HORVU5Hr1G104980	1.176354	1.75511	1.236208	8.625806	9.773964	11.8581
HORVU0Hr1G001230	3.194421	3.495507	3.00386	3.712777	3.875362	4.804858
HORVU5Hr1G059860	2.219239	2.219883	3.24689	2.823646	4.122915	4.234223
HORVU1Hr1G029290	3.921443	3.008567	2.23962	4.776915	4.029288	5.06483
HORVU0Hr1G018140	5.298604	4.49654	7.19032	6.741799	9.383893	8.824937
HORVU3Hr1G030790	2.306702	2.436897	3.003407	2.937391	2.741818	3.119066
HORVU6Hr1G078760	7.55477	6.225984	7.927669	8.687293	10.20593	9.719429
HORVU3Hr1G079950	7.271353	3.999557	6.249046	7.011708	3.790689	4.871709
HORVU3Hr1G001920	0.168184	0.190826	0.202346	6.038656	74.40584	71.18563
Hordeum_vulgare_newGene_6427	1.781604	2.07686	2.243843	2.135499	2.84547	3.620721
Hordeum_vulgare_newGene_6425	4.672906	1.490137	5.680867	4.539268	5.256706	6.313664
Hordeum_vulgare_newGene_6421	17.21645	16.86829	12.28558	17.81235	13.40299	13.96577
HORVU5Hr1G089290	0.617946	1.040537	1.032065	2.033325	2.514499	2.368802
HORVU2Hr1G001900	26.37966	27.1804	24.73963	37.26613	77.44496	67.62109
Hordeum_vulgare_newGene_9958	1.567777	1.644983	1.53581	1.046241	0.929077	1.015703
Hordeum_vulgare_newGene_9957	1.213262	2.223388	1.554926	1.298129	1.107394	1.080174
Hordeum_vulgare_newGene_9950	2.193407	0.697632	1.745876	2.955247	1.143955	1.394593
HORVU6Hr1G070170	2.014235	1.861612	1.650054	2.718817	2.37748	2.602217
HORVU4Hr1G070970	486.0231	514.14	507.4331	276.4561	37.50987	132.1698
HORVU5Hr1G094010	10.86267	10.59131	12.66772	9.437715	10.35942	11.86015
HORVU2Hr1G041610	10.2656	9.821801	9.348041	35.67606	55.32888	56.67903
HORVU7Hr1G089120	28.06801	25.86282	35.7893	23.23631	33.47823	31.40514
HORVU7Hr1G064180	3.416427	6.880398	11.362	7.021993	5.27332	2.808692
HORVU2Hr1G005020	2.34515	2.118652	2.509235	4.953145	5.671331	5.321858
HORVU4Hr1G040760	19.71675	18.50621	18.04457	16.17988	13.40035	16.65299
HORVU5Hr1G089740	1.355319	1.028745	1.080015	7.142379	6.476669	5.693611

HORVU4Hr1G013680	2.212458	2.343583	2.80855	3.206544	2.670612	3.246294
HORVU0Hr1G011940	21.00432	17.29771	27.1337	20.4391	45.70223	40.04856
HORVU3Hr1G002780	61.04788	50.59504	69.66389	76.17328	90.57576	99.4793
HORVU1Hr1G013040	3.749408	3.411317	4.183002	4.350349	5.758077	5.781773
HORVU3Hr1G032790	10.22349	10.34048	10.81106	15.70233	13.96446	18.38202
HORVU7Hr1G020410	0.324956	0.338889	0.353216	2.95772	2.376185	3.039508
HORVU4Hr1G006200	5.419075	6.07142	6.798632	9.510291	11.94351	12.48978
HORVU1Hr1G065670	12.33272	5.203557	12.38246	0.955276	1.621991	3.154669
HORVU5Hr1G102130	0.984078	0.980799	0.920708	2.099141	1.625189	1.881978
HORVU6Hr1G088570	52.72983	50.15343	56.78289	32.36106	34.74905	33.77535
HORVU7Hr1G069460	49.28638	49.98272	77.47005	68.82697	101.3547	105.622
HORVU5Hr1G114850	1.823054	2.042612	2.699991	3.569013	3.094103	4.131629
HORVU6Hr1G039510	2.739155	2.805824	2.876788	1.756643	2.085097	2.139199
HORVU3Hr1G037560	12.2968	15.93889	14.69593	14.61589	13.70467	15.86704
Hordeum_vulgare_newGene_5781	4.861892	4.721595	5.082545	10.49587	15.37338	12.50922
HORVU3Hr1G089760	18.52198	15.14094	20.0105	13.81078	16.78473	17.39035
HORVU0Hr1G003540	6.123462	5.622605	7.323866	7.14858	11.04304	10.30381
HORVU5Hr1G122350	17.82541	18.97723	25.53629	13.79327	20.41644	22.5559
HORVU6Hr1G066840	6.297283	4.439557	3.802093	3.302123	4.433091	6.02922
HORVU5Hr1G069180	2.864573	3.263227	4.286559	1.36367	2.022816	1.882996
HORVU6Hr1G035610	22.16937	18.95581	22.01153	15.82357	13.77271	15.31905
HORVU2Hr1G080890	23.92265	18.07707	31.83445	13.38261	19.82093	20.96536
HORVU1Hr1G010250	2.090336	2.48248	5.332835	1.444166	6.306239	5.365335
HORVU5Hr1G039850	45.11363	41.51885	53.99024	83.13948	117.0778	114.7618
HORVU5Hr1G120810	9.025956	7.175763	16.27801	4.354686	4.710254	5.064758
HORVU6Hr1G001560	0.96841	0.763823	0.934667	1.70745	1.558338	1.745638
HORVU6Hr1G094400	4.31322	5.347933	5.503409	4.826169	7.343929	7.82102
HORVU6Hr1G080920	15.46282	16.67096	15.72091	17.20469	14.27003	14.553
HORVU2Hr1G095890	119.8722	113.9451	161.0214	68.13467	108.5268	111.5221
HORVU1Hr1G041820	3.984687	3.131371	4.968636	7.531091	9.113242	7.954493
HORVU3Hr1G108930	91.1717	81.11505	109.1559	61.1861	62.43993	62.44456
HORVU4Hr1G034650	1.89142	1.940781	1.769918	2.652979	3.418277	3.526055
HORVU5Hr1G021610	0.678036	0.666769	0.687494	4.62628	5.673401	3.938228
HORVU6Hr1G085250	1.546726	1.68782	1.561082	5.68186	5.493277	5.570437
HORVU2Hr1G089670	2.176506	2.475652	3.000178	5.525475	6.225486	6.136657
HORVU2Hr1G109120	91.46356	86.43276	94.5285	43.54506	12.30431	26.44137
HORVU1Hr1G002500	35.69612	39.41966	39.1099	63.54253	58.1338	61.16234
HORVU2Hr1G082660	36.55711	35.63402	35.87669	28.51966	34.99466	31.72137
HORVU4Hr1G081590	3.239809	2.603709	3.373993	3.080397	3.073398	3.773333
HORVU1Hr1G085540	0.085087	0.145136	0.171863	1.183349	2.078497	2.498489
HORVU3Hr1G072900	65.07992	50.59422	70.70305	73.30018	128.9612	105.7716
HORVU4Hr1G008530	12.07816	11.63735	11.55272	11.16075	11.92522	12.72856
HORVU1Hr1G063200	1.199037	1.314959	1.685244	1.598422	2.312959	2.361619
HORVU2Hr1G084790	5.513611	5.236248	6.615132	6.404372	7.211829	7.177397
HORVU7Hr1G085870	9.817693	8.482802	10.20781	8.840822	10.05736	10.13477
HORVU5Hr1G114140	2.985133	1.773807	3.21414	3.293143	2.818983	3.142665
HORVU5Hr1G066460	11.42192	10.38151	11.11699	1.294399	0.509985	0.838191

HORVU4Hr1G087740	3.03795	3.942441	5.42781	5.459975	14.45646	14.95304
HORVU4Hr1G048800	4.623412	4.667329	6.176209	5.440128	5.107919	5.516356
HORVU3Hr1G019920	15.91404	17.95821	15.20084	16.43008	20.08885	22.32462
HORVU2Hr1G094410	1.508983	1.630749	1.088655	3.508026	3.408701	3.723427
HORVU5Hr1G047890	1.438388	1.124982	0.836505	1.835649	2.207771	1.678983
HORVU2Hr1G061080	11.93108	11.03501	13.52269	6.931571	4.542738	6.004806
Hordeum_vulgare_newGene_15906	4.586461	4.60342	4.861692	3.359166	4.480067	4.378046
HORVU0Hr1G017680	3.947874	2.782724	3.201338	4.954912	5.361675	5.604369
HORVU4Hr1G008740	2.93423	3.082979	2.989326	4.789063	5.828671	6.219596
HORVU4Hr1G012650	4.571023	4.594584	5.634546	4.345665	6.176499	7.284185
HORVU1Hr1G082010	53.4116	46.90772	74.60452	54.34115	77.8745	75.23683
HORVU0Hr1G006020	8.149384	9.133624	13.93227	5.245175	6.283549	6.877781
HORVU7Hr1G040800	4.143318	3.998876	5.658087	3.771123	4.441307	4.100645
HORVU4Hr1G083530	21.66543	22.77802	21.88392	21.11972	31.93959	28.73734
Hordeum_vulgare_newGene_10691	1.470145	1.697559	2.096363	2.497323	3.110236	3.670905
HORVU3Hr1G090900	16.26308	15.19032	18.81766	10.39015	8.463144	8.300129
HORVU3Hr1G064340	1.617534	1.73946	1.511459	4.797519	3.636865	4.254355
HORVU5Hr1G023990	6.706399	6.98563	7.763386	7.017078	16.18272	12.63739
HORVU4Hr1G086160	10.9664	10.36962	11.5785	11.00414	15.0367	14.19793
HORVU1Hr1G051470	1.816281	0.781666	0.932207	3.06582	24.68723	20.35402
HORVU7Hr1G058950	7.286622	6.625234	7.922676	8.842192	10.18497	11.60774
HORVU5Hr1G088880	4.243322	4.23444	3.421357	15.24933	24.53481	21.52838
Hordeum_vulgare_newGene_13885	27.28565	23.64503	21.6106	13.60996	7.693218	10.13204
Hordeum_vulgare_newGene_13889	1.467848	1.489535	1.556089	2.299479	2.705987	3.210736
HORVU5Hr1G045630	54.29086	48.60208	64.61084	55.68999	69.06985	72.17719
HORVU6Hr1G019080	3.530696	4.006246	3.715497	5.104135	4.908612	5.562478
HORVU1Hr1G011930	2.440019	3.084852	3.112692	0.212931	0.020038	0.09372
HORVU2Hr1G010630	0.034272	0.212905	0.063048	2.406661	14.05914	12.29607
HORVU1Hr1G094980	7.581601	8.229546	7.358879	8.271439	8.888572	9.557721
HORVU6Hr1G017530	3.257749	2.399453	2.74489	8.06138	27.65113	26.72496
HORVU2Hr1G096700	0.658978	0.55974	0.589711	2.256413	3.330827	2.438746
Hordeum_vulgare_newGene_10283	8.644716	10.09597	10.6722	12.97558	17.59374	17.14847
HORVU6Hr1G013620	7.03795	6.818205	7.218617	7.960754	8.026128	8.456749
HORVU2Hr1G091830	7.003771	8.368003	9.463011	18.54804	21.00074	21.77869
HORVU5Hr1G021780	15.3439	12.302	15.73403	20.84871	23.02204	21.42132
HORVU1Hr1G016630	23.9591	21.77089	25.49621	12.12763	12.38901	13.19793
HORVU2Hr1G112670	1.633214	1.413675	1.649518	2.738122	2.856016	1.792565
HORVU5Hr1G094750	0.306102	0.442509	0.963948	3.346456	0.666829	1.253588
HORVU6Hr1G076110	2.482177	2.006018	2.154094	2.291631	3.311693	4.404411
HORVU4Hr1G060510	3.417378	3.96807	3.725008	3.693206	3.782675	4.547662
HORVU4Hr1G007610	135.2428	129.2257	79.5344	30.86127	33.99952	30.06808
HORVU1Hr1G000270	9.322178	7.259353	10.39113	9.371638	11.51162	9.985493
HORVU3Hr1G028060	31.50146	24.61071	30.55125	58.13128	67.69082	69.69116
HORVU3Hr1G073290	3.288851	2.736957	2.440103	14.76377	8.274697	10.64098
HORVU5Hr1G052230	21.54661	23.99052	35.57379	27.52792	23.23378	25.87394
HORVU2Hr1G067130	3.188794	3.786794	3.536407	3.460991	4.47499	4.853649
HORVU4Hr1G055820	7.960365	8.668257	8.339617	18.02169	18.0924	16.2894

HORVU7Hr1G089910	6.446841	4.86526	7.516681	4.529657	4.638663	4.93502
HORVU2Hr1G031470	22.62005	20.39011	20.15888	14.11705	11.73623	12.56738
HORVU2Hr1G106970	4.986939	4.669078	4.346289	9.522237	23.28455	20.44033
HORVU3Hr1G062030	5.195103	6.078665	6.711615	7.463808	11.93847	10.64884
HORVU5Hr1G048100	2.182552	3.402437	4.071575	1.539538	1.355745	2.591462
HORVU2Hr1G096350	5.602448	6.174049	5.739383	6.860737	7.553821	8.05892
Hordeum_vulgare_newGene_1855	0.93571	0.660699	0.49889	0.968863	1.76848	1.796256
Hordeum_vulgare_newGene_1858	7.394568	6.983706	8.161984	8.083607	9.542012	10.17126
Hordeum_vulgare_newGene_1859	0.033673	0.035949	0	0.362976	16.41537	12.11885
HORVU4Hr1G069130	1.89978	1.738431	2.739313	4.137463	5.867878	6.402185
HORVU7Hr1G075980	0.334106	0.344122	0.70709	2.467147	36.30928	34.62296
HORVU2Hr1G093050	10.15772	9.64708	10.76659	13.71068	16.23998	15.26771
Hordeum_vulgare_newGene_11621	46.79547	39.17745	42.99334	46.43659	48.45111	46.79161
Hordeum_vulgare_newGene_11620	66.40518	64.57469	54.78125	21.33459	17.04113	19.12998
Hordeum_vulgare_newGene_11623	105.8965	116.6687	188.1288	108.2195	253.5479	220.8748
HORVU3Hr1G073870	1.858897	2.131597	2.973897	3.252181	3.504165	3.255104
HORVU7Hr1G053590	0.961238	1.110615	0.498084	0.56217	2.714862	2.338949
HORVU2Hr1G028780	13.32341	3.251064	7.025871	0.588337	0.296322	0.510496
HORVU4Hr1G063270	12.14141	10.79665	11.52294	19.05442	24.32486	23.41556
HORVU3Hr1G069780	3.265574	3.038297	3.319164	7.313263	3.892884	4.666003
HORVU6Hr1G092560	4.127777	4.48732	5.195267	5.80783	6.639562	6.284988
HORVU5Hr1G055270	5.63957	2.190299	4.6051	8.043215	7.035784	9.192927
HORVU2Hr1G029780	24.8925	24.65918	27.68173	36.06695	45.31825	46.72646
HORVU5Hr1G043700	4.936195	7.824058	3.544329	3.941778	4.102627	4.226753
HORVU3Hr1G112160	0.535488	1.265932	1.538389	0.619432	1.075497	1.481639
HORVU5Hr1G005770	2.535137	3.691727	4.453748	2.526865	3.969408	4.214848
HORVU7Hr1G032040	2.352609	3.182096	2.513365	0	0	0
HORVU5Hr1G049270	3.3513	3.106941	4.444181	4.758004	4.573515	4.736566
HORVU7Hr1G083070	12.95076	16.15299	20.03789	10.84839	13.22071	14.44855
HORVU3Hr1G086090	0.817429	1.193636	1.841237	0.712557	2.328872	1.472211
HORVU3Hr1G063780	6.035361	6.013045	7.191574	4.383731	5.621437	5.053641
HORVU2Hr1G087040	2.62192	3.490909	2.723578	3.46848	4.302015	5.226922
HORVU7Hr1G105700	0.395352	0.72671	0.281272	1.812979	2.64084	2.252051
HORVU3Hr1G089510	3.703294	3.722512	4.036398	15.9991	17.56129	16.46064
HORVU1Hr1G032850	12.47792	13.24772	12.75271	18.33983	26.48848	26.17948
HORVU3Hr1G093860	12.75371	8.420654	10.10075	10.08142	9.424376	9.544505
Hordeum_vulgare_newGene_13552	11.00923	12.01781	17.28963	15.37406	28.19329	24.71326
HORVU0Hr1G021630	14.29313	13.7071	15.17901	56.55788	82.57212	82.44192
HORVU6Hr1G078160	0.850846	1.180441	0.911936	1.69749	2.008879	1.996868
Hordeum_vulgare_newGene_6228	9.14789	11.44901	11.81565	2.153443	2.429337	1.953927
Hordeum_vulgare_newGene_6223	2.532755	3.33921	3.054983	4.303114	5.822058	5.841946
HORVU2Hr1G099210	2.766082	2.991071	3.624594	1.445897	3.271505	4.618223
Hordeum_vulgare_newGene_13418	2.328272	2.017913	2.502825	1.729619	1.935554	1.890659
HORVU0Hr1G020320	5.415871	3.591115	5.355886	7.26625	4.419522	5.15926
HORVU2Hr1G060350	60.86426	54.31728	56.21395	45.19517	49.79916	53.75678
HORVU5Hr1G107510	114.6906	93.88023	133.1235	97.36067	106.7091	106.4381
Hordeum_vulgare_newGene_9672	2.928379	2.656834	2.110942	3.684764	5.014797	3.71831

HORVU1Hr1G019480	3.03718	2.543371	2.059254	7.290126	15.15968	17.80812
Hordeum_vulgare_newGene_13411	4.818663	5.760231	5.413928	5.704755	5.016483	6.056758
HORVU2Hr1G071270	8.428433	6.254064	7.235729	2.155517	1.126044	1.991905
Hordeum_vulgare_newGene_3548	10.7759	10.13179	11.03488	13.85092	14.37975	14.84226
HORVU2Hr1G093220	0.654461	0.753751	0.746055	2.567263	3.851822	4.130221
Hordeum_vulgare_newGene_3547	0	0	0.062293	0.15868	8.247631	10.06125
HORVU4Hr1G059280	36.12157	30.4078	51.55788	7.988581	1.766248	3.441623
HORVU3Hr1G020230	27.11851	20.52619	29.52407	31.73384	45.23041	39.35104
HORVU1Hr1G043910	22.69744	16.88138	35.28113	51.55667	28.21313	70.55471
HORVU4Hr1G058480	9.203044	9.611556	11.12625	12.02931	11.84154	12.98692
HORVU5Hr1G074360	43.66986	41.98245	52.09062	8.816417	3.138308	4.260622
HORVU7Hr1G118300	26.2436	27.06467	26.24861	21.16358	25.98593	23.29738
HORVU7Hr1G055290	4.455831	5.448898	4.811954	4.850471	5.274306	5.417395
HORVU2Hr1G024660	1.37573	0.646842	0.974608	1.071724	1.27942	1.066281
HORVU3Hr1G052500	8.262766	10.1386	10.53271	0.004977	0.01728	0.010883
HORVU6Hr1G021670	1.102319	1.088514	1.436329	1.030533	0.983732	1.119048
HORVU5Hr1G068140	1.09525	1.106123	1.322641	1.630561	1.540868	1.883834
HORVU7Hr1G097790	10.70146	10.53155	10.08036	10.29479	12.60103	13.59125
HORVU3Hr1G059620	2.142799	2.217857	2.376827	5.311982	6.182956	7.021339
Hordeum_vulgare_newGene_3363	4.80446	4.576638	5.605172	11.55297	24.02376	22.00253
Hordeum_vulgare_newGene_15713	1.353595	1.860439	1.845124	2.371946	2.763244	2.844908
Hordeum_vulgare_newGene_15717	1.079891	1.10052	1.262033	2.277975	5.378354	4.015548
HORVU3Hr1G069200	6.015718	3.737716	5.850638	6.910357	8.09753	6.869975
HORVU3Hr1G013720	5.992633	5.418051	7.08643	5.550152	5.752717	5.771719
HORVU7Hr1G070330	9.312429	7.840597	9.841974	11.59843	23.52768	23.30078
HORVU2Hr1G005590	8.097122	7.762844	10.83717	5.247129	4.730751	5.743488
HORVU6Hr1G091630	7.151867	5.419912	7.462496	13.65715	17.4236	17.08908
HORVU1Hr1G074860	0.264759	0.202697	0.53379	2.168915	4.444228	4.304018
HORVU3Hr1G044440	27.01172	26.81076	26.92164	27.03854	25.40448	24.29342
HORVU3Hr1G048340	12.1171	11.56417	13.07343	14.82974	15.81094	16.03193
HORVU5Hr1G062010	10.78121	12.65853	13.88855	9.377866	9.900619	8.941093
HORVU2Hr1G030460	4.422945	3.384749	3.913454	7.822649	11.41862	13.64453
HORVU2Hr1G004170	2.827802	1.61004	1.376653	0.237078	2.359238	2.749962
HORVU5Hr1G063940	5.892555	5.040867	4.998846	6.658539	4.706656	4.994632
HORVU7Hr1G094540	1.320189	0.803882	0.95231	1.215337	1.551252	1.625727
Hordeum_vulgare_newGene_858	6.548177	10.29682	14.37502	0	0	0
Hordeum_vulgare_newGene_6912	2.110746	0.29226	2.354516	1.948962	2.379199	2.210193
Hordeum_vulgare_newGene_6913	127.4996	122.7786	124.8834	84.90174	75.05366	76.37917
Hordeum_vulgare_newGene_8515	0.532326	0.51255	0.720516	1.159749	1.710844	1.806931
Hordeum_vulgare_newGene_8517	28.46607	23.21669	30.88166	23.83873	29.80422	29.63466
HORVU3Hr1G058570	45.88252	45.43248	65.42605	11.06142	0.68822	4.433464
HORVU1Hr1G059300	11.24697	9.726671	12.36074	17.83366	19.88046	16.99486
HORVU6Hr1G052560	25.1276	26.40454	44.12696	14.80739	15.21411	14.08888
HORVU3Hr1G012090	1.415235	1.734969	2.138964	1.301484	1.745141	1.815026
HORVU2Hr1G040810	1.769128	1.737915	2.237997	1.973895	0.569557	2.594488
HORVU4Hr1G078610	10.95428	9.084985	12.44322	16.37251	15.70584	16.68679
HORVU0Hr1G008700	52.97797	47.3554	52.99169	51.80002	53.21231	54.93406

HORVU2Hr1G058820	1.290508	0.918335	1.402879	1.922701	1.007041	1.324034
HORVU0Hr1G019680	9.338894	9.067031	12.85555	5.715812	6.132207	5.240664
HORVU7Hr1G107830	1.774579	1.946136	2.075703	1.44243	0.613267	2.728387
Hordeum_vulgare_newGene_12727	4.409254	4.667041	3.257676	2.076198	3.410519	3.037895
HORVU4Hr1G031620	7.483117	7.726785	9.892235	10.17273	12.32811	13.31929
HORVU2Hr1G104450	12.95298	14.40517	12.83428	15.84223	14.59314	17.02479
HORVU7Hr1G014150	6.722579	6.555178	8.029486	3.19625	4.364992	5.069831
HORVU7Hr1G037330	18.3632	13.95668	21.25241	19.83187	36.65383	34.04274
Hordeum_vulgare_newGene_1334	9.371265	9.531096	10.4484	13.54725	10.7633	13.28818
Hordeum_vulgare_newGene_1333	9190.188	8701.269	8582.307	8007.131	6000.732	4583.861
Hordeum_vulgare_newGene_1332	5.137111	5.681637	5.811871	5.050405	5.487044	5.921896
HORVU3Hr1G014730	82.55189	73.19439	93.09601	109.3583	196.3893	195.5325
HORVU1Hr1G035440	13.09659	13.74949	14.96146	13.45473	16.06498	15.6434
HORVU3Hr1G071120	9.874825	10.76728	11.01773	23.56977	26.48684	16.45222
HORVU5Hr1G080830	11.74543	9.545623	11.62219	11.92776	8.269838	8.169164
HORVU2Hr1G047890	2.802054	4.57586	4.633208	2.603642	1.763296	2.023512
HORVU3Hr1G050010	15.19968	16.82561	15.12741	19.57236	28.93214	29.54679
HORVU4Hr1G005270	10.87757	9.755797	8.819624	4.419441	5.728989	4.957444
HORVU2Hr1G127740	19.30774	19.30801	21.7443	32.84308	59.19499	59.00719
HORVU0Hr1G025520	0	0.119893	0.076286	0.632967	112.7933	103.6371
HORVU3Hr1G014190	25.82244	26.20691	27.87615	27.42966	24.4868	26.88911
HORVU1Hr1G067140	1.5536	1.266066	1.724887	2.633562	5.511461	6.170172
HORVU5Hr1G067530	0.311838	0.605384	0.67474	1.123008	5.198288	6.013042
HORVU4Hr1G008080	0	0	0	2.07216	4.460177	4.352134
HORVU5Hr1G010360	0.221824	0.182593	0.168476	1.309979	2.719568	2.701819
HORVU1Hr1G063590	105.2893	97.18784	113.3151	75.83569	78.42869	78.84939
HORVU4Hr1G047770	3.665736	4.742169	4.247616	4.85895	5.698735	6.278108
HORVU0Hr1G006620	0.879025	1.199648	1.120205	2.708903	2.562484	2.6042
HORVU5Hr1G060620	10.21429	9.220154	13.8697	7.864708	7.786974	10.50083
HORVU1Hr1G068590	6.200392	4.731475	3.263714	9.805205	0.243008	3.324867
Hordeum_vulgare_newGene_7635	2.8036	2.252285	2.570152	6.139545	3.086966	3.559737
HORVU4Hr1G046420	25.53107	25.38304	24.40117	19.68409	25.34054	25.23656
Hordeum_vulgare_newGene_7637	1.324086	1.405586	1.234345	2.36851	2.691782	3.599764
HORVU7Hr1G033470	57.24639	71.99914	101.126	22.23907	46.77189	30.74916
HORVU7Hr1G099320	7.201779	6.361993	9.484257	5.296945	3.801087	4.083105
HORVU5Hr1G015460	0.539874	0.769823	0.472882	0.463238	3.218225	2.656408
HORVU2Hr1G045490	36.18704	37.06718	52.34626	27.88313	40.39502	36.61595
Hordeum_vulgare_newGene_13711	2.180563	1.920943	1.940893	2.066339	2.773788	2.750256
HORVU3Hr1G075870	10.99886	9.920421	10.05828	28.32393	38.55891	33.7495
HORVU5Hr1G056350	0.821163	0.809422	0.884987	1.86708	1.835205	1.716035
HORVU0Hr1G001390	4.085996	4.854537	5.265276	7.254134	8.39571	9.539376
HORVU3Hr1G091360	2.590182	2.934077	2.540861	3.83356	5.194705	5.344693
HORVU5Hr1G061970	19.71233	18.77041	22.89675	29.29931	50.375	46.00365
HORVU7Hr1G079530	19.67741	16.75627	19.2716	22.05773	29.80029	31.05517
HORVU3Hr1G043310	0.956794	0.993678	1.473611	2.099084	2.067132	2.479205
HORVU2Hr1G045230	3.285102	1.235866	4.468374	1.95421	1.917892	3.477669
HORVU5Hr1G058300	12.70732	9.418214	11.55884	16.59032	12.12442	12.43181

HORVU4Hr1G048680	0.547793	0.368139	0.039342	2.231009	3.2768	0.396218
HORVU2Hr1G041880	12.24025	12.97822	9.608239	4.576262	5.901694	6.617593
HORVU3Hr1G091350	7.680684	6.425774	8.588754	8.578117	8.251746	8.632255
HORVU1Hr1G045510	130.3815	126.02	133.4504	71.84151	46.49875	47.44771
HORVU3Hr1G027070	6.156858	7.263064	8.794263	6.970129	9.628491	9.69645
HORVU3Hr1G044830	7.858988	7.986273	8.743245	10.09624	13.25147	14.12453
HORVU4Hr1G073020	10.41564	10.08831	8.644728	14.50883	4.235661	6.581612
HORVU5Hr1G120230	20.22807	11.41065	14.54924	10.13634	14.85268	15.74581
HORVU7Hr1G065540	0.838623	0.559661	0.536588	1.009431	1.447322	1.715534
HORVU6Hr1G043150	1.65779	2.167811	2.391016	1.252321	1.310828	1.671457
HORVU4Hr1G012850	5.970348	5.078813	6.678948	6.22215	7.780285	8.864744
HORVU4Hr1G016770	31.94472	16.20626	35.44796	62.24055	73.38494	96.37153
HORVU4Hr1G010980	1.930736	2.145638	2.015151	2.515147	0.944822	1.801822
HORVU5Hr1G022570	0.470497	0.391426	0.326151	1.949253	2.756891	3.25442
Hordeum_vulgare_newGene_9303	7.888811	9.125615	9.728816	12.64841	16.61001	16.84797
HORVU3Hr1G086540	54.28866	52.99114	61.73066	58.24865	70.26669	67.67274
HORVU4Hr1G013180	0.980172	0.93781	0.887838	2.428753	4.132464	3.793224
HORVU3Hr1G028260	3.062726	1.971812	2.451774	1.944454	0.4298	1.119141
HORVU5Hr1G095570	21.49617	17.56554	21.29225	13.4627	13.78678	13.57439
HORVU2Hr1G004960	6.911165	7.173383	9.069173	5.791361	6.439021	6.238315
HORVU6Hr1G080480	90.61611	86.22476	119.8618	61.30556	97.03314	104.8828
HORVU1Hr1G069250	15.1676	11.00554	15.58574	27.7944	34.76399	37.20959
HORVU5Hr1G061020	1.978345	1.463645	1.863661	1.219931	1.928293	2.155596
HORVU6Hr1G075720	20.58462	19.75945	24.18543	22.38106	25.54931	29.67377
HORVU2Hr1G102380	4.180931	3.220167	3.209495	1.520061	0.913868	1.068826
HORVU2Hr1G061110	2.799046	3.307777	3.324495	2.839268	6.093259	6.611938
HORVU5Hr1G027890	4.894973	5.598866	6.68755	11.36835	23.86929	20.52989
HORVU5Hr1G094220	0.789521	0.929335	1.690634	11.78715	18.26667	20.50911
HORVU2Hr1G041800	10.9044	9.896092	10.28219	15.15906	16.82872	18.95116
HORVU3Hr1G007100	0.895028	1.040389	1.338295	1.062278	0.823691	1.001548
HORVU4Hr1G007360	4.713464	5.060289	5.836057	4.756272	2.632339	3.323209
HORVU1Hr1G004200	4.652266	6.095721	6.90567	1.660715	2.296291	2.779667
HORVU1Hr1G026780	18.6566	16.46768	16.82414	22.71163	22.05501	20.40338
Hordeum_vulgare_newGene_1974	1.781134	1.524438	2.026168	2.640854	1.083813	1.305372
HORVU1Hr1G011400	1.844097	1.754851	1.721385	2.650354	3.300689	2.899966
Hordeum_vulgare_newGene_2093	0	0	0	2.522445	1.755741	2.587783
HORVU2Hr1G022490	5.724715	6.588271	9.130904	5.614057	7.67283	6.903484
HORVU2Hr1G059750	5.2378	5.480659	5.538984	6.636793	9.361737	9.242601
HORVU6Hr1G084740	5.683094	3.792779	8.456079	0.407194	1.320104	1.348171
HORVU6Hr1G037860	3.119342	2.915691	4.024409	3.399528	3.65579	4.059326
HORVU5Hr1G122490	0.462474	0.466578	0.527069	1.071507	1.450492	2.066464
Hordeum_vulgare_newGene_675	19.5989	16.95133	23.49895	20.04912	21.97003	22.87252
HORVU1Hr1G005150	13401.22	12851.82	9135.943	6756.756	1234.247	2849.644
Hordeum_vulgare_newGene_2095	1.98122	2.097215	1.641172	10.40245	12.90903	11.10997
HORVU4Hr1G003190	1.284603	1.539001	1.724587	0.929837	1.51659	1.920287
HORVU4Hr1G080460	2.186266	1.252045	3.166982	1.800589	2.515944	2.387975
HORVU3Hr1G108010	60.27606	52.64088	56.07883	49.01412	71.40779	67.89148

HORVU1Hr1G014570	0.98756	1.375318	1.303269	1.368071	6.022463	5.588743
HORVU7Hr1G058810	1.932274	2.347584	3.35598	0	0	0
HORVU5Hr1G092100	5.294539	7.130802	9.92257	0.218451	0	0.202306
HORVU5Hr1G118090	2.946416	2.955997	2.963005	2.587729	2.039866	2.536799
HORVU4Hr1G014130	56.43449	55.19878	78.7757	44.52634	129.8731	109.2336
HORVU3Hr1G038870	8.634475	7.342729	8.037147	12.32595	5.208459	8.39516
HORVU5Hr1G055070	15.12646	12.69447	15.29614	15.83248	20.84532	20.09198
HORVU5Hr1G035760	19.88466	20.40929	26.27711	15.96943	17.63132	18.52609
HORVU5Hr1G122410	5.800413	2.83099	8.603697	0	0	0
HORVU2Hr1G083870	4.073224	3.711072	4.685363	3.145549	4.828837	4.473591
HORVU6Hr1G001150	4265.57	4280.708	4137.527	8653.221	3854.25	3784.025
Hordeum_vulgare_newGene_5085	1.333857	1.339883	1.219588	1.307832	1.902128	1.666793
HORVU7Hr1G071680	6.156302	5.547341	5.414738	5.685669	6.827981	5.99629
HORVU6Hr1G035420	91.91245	65.92066	95.58757	143.9617	98.60858	118.8283
HORVU2Hr1G024790	0.939473	1.211316	1.438424	0.691355	1.11397	0.891185
HORVU3Hr1G083020	1.690591	1.778949	2.192391	1.408538	1.450298	1.595073
HORVU1Hr1G067480	1.086572	1.182627	1.531385	2.719906	4.886039	4.750705
HORVU5Hr1G082480	10.8618	10.12931	11.71486	9.955744	9.771039	8.260953
HORVU3Hr1G018170	9.241968	11.79315	10.08126	10.17845	14.86689	15.01138
HORVU4Hr1G066240	2.159335	0.804645	2.764893	0.321328	0.064191	0.169351
HORVU6Hr1G090520	3.195147	2.305958	3.138685	3.319844	2.783137	3.873739
HORVU4Hr1G005730	10.42668	9.119128	12.1145	7.888535	7.654541	7.762883
HORVU1Hr1G002310	9.038459	18.01706	13.35954	18.61173	8.748054	8.150343
HORVU5Hr1G050510	3.326428	4.625563	4.529211	6.204075	9.076845	10.58744
HORVU6Hr1G011580	12.82485	11.22429	12.74441	3.033548	4.275587	4.625087
HORVU1Hr1G085660	1.969358	2.981383	2.799344	2.833376	2.812896	2.995059
HORVU7Hr1G109420	3.883852	3.939774	3.822555	4.519902	4.290643	5.584647
HORVU4Hr1G057410	7.098039	7.694459	7.473239	8.86562	7.229133	9.051203
HORVU3Hr1G071240	7.775767	7.457832	8.582325	9.283849	7.129598	8.076846
Hordeum_vulgare_newGene_7856	2.272954	2.678551	2.275402	3.723668	3.163872	3.571344
HORVU6Hr1G017860	2.4405	2.339813	2.23517	2.06118	3.218404	3.113426
HORVU2Hr1G127220	10.01246	8.781464	11.03274	10.25302	16.23954	16.41756
HORVU7Hr1G046280	35.74025	26.95866	41.26608	27.7351	25.75293	26.45164
HORVU1Hr1G068930	3.11422	2.033958	1.348024	0.978322	0.591531	0.6267
HORVU0Hr1G040490	1.484605	1.021117	1.203922	1.761875	1.300112	1.650375
HORVU6Hr1G005350	3.703636	4.185376	4.360834	4.619844	5.394858	5.898529
HORVU5Hr1G055920	3.475724	3.346505	4.205465	9.268197	15.47825	13.03783
HORVU3Hr1G098360	12.71601	9.388605	13.96086	2.976322	2.491205	2.64836
HORVU7Hr1G076160	12.25672	12.09834	14.35009	11.04438	12.96871	13.78017
HORVU6Hr1G088470	12.54245	7.763282	8.932903	1.113319	0.696879	0.74694
HORVU4Hr1G086820	19.72609	20.15511	19.71671	24.81282	30.93962	30.57557
HORVU3Hr1G070850	224.9174	202.0174	232.0741	24.16484	9.786117	12.37976
HORVU5Hr1G057560	14.53469	10.43943	12.50779	16.30623	16.88883	15.68628
HORVU7Hr1G047150	1.479894	1.961315	1.819395	1.823141	3.253505	3.082973
HORVU4Hr1G012440	9.976179	9.924167	10.83718	11.04553	15.51321	14.76495
HORVU6Hr1G092960	42.16024	46.53986	62.14262	36.41805	47.41999	57.64024
HORVU6Hr1G026120	3.151827	3.067783	3.510783	2.572493	2.266561	3.245675

HORVU6Hr1G064230	16.73038	14.33454	19.84996	30.68531	58.00555	49.35384
HORVU7Hr1G094070	0.317513	0.403239	0.12741	1.589024	6.742447	7.373738
HORVU2Hr1G013850	5.888917	5.994076	6.596288	7.131148	9.688133	11.29549
Hordeum_vulgare_newGene_9002	5.689082	5.14716	4.376085	4.63792	5.981458	6.343405
Hordeum_vulgare_newGene_9005	1.829659	2.088647	2.487018	1.504206	2.063618	2.487484
Hordeum_vulgare_newGene_9006	23.90702	28.63435	28.057	20.757	19.08289	23.29528
Hordeum_vulgare_newGene_9009	3.639257	3.202338	2.747061	8.176291	8.593799	9.599519
HORVU7Hr1G120540	9.467359	9.846159	11.11064	12.49986	12.69722	13.67608
HORVU4Hr1G064790	12.66921	10.1591	14.15742	3.309663	0.839072	1.578131
HORVU7Hr1G094320	5.122624	4.255461	6.856823	2.671687	1.401634	2.713981
HORVU6Hr1G041920	19.1453	21.60165	23.07187	20.84481	20.27571	23.13565
HORVU5Hr1G013840	6.948581	5.829102	5.787534	13.84985	27.80043	24.10607
HORVU3Hr1G025240	2.866331	5.082682	2.147072	3.794563	3.422683	4.273918
HORVU2Hr1G093670	4.424312	3.736209	3.18653	1.240243	0.700314	0.785843
HORVU3Hr1G107980	0.640486	0.817719	0.693007	2.071723	1.913963	1.495447
HORVU3Hr1G064550	51.54651	50.59529	44.64184	47.76299	55.0043	44.84758
HORVU4Hr1G057020	5.17458	4.234521	6.417502	10.79731	9.077991	9.731644
HORVU6Hr1G019290	0.439126	0.427178	0.372283	4.041966	12.01692	9.574206
HORVU2Hr1G097350	1.111072	0.5759	0.77947	2.809618	3.700331	3.621209
HORVU2Hr1G030660	3.706	4.24825	5.929123	1.550383	0.987561	1.222719
HORVU1Hr1G054450	8.466481	6.743019	9.534854	7.093186	7.760978	8.512079
HORVU2Hr1G036490	2.011571	2.188345	2.377943	2.05638	3.085592	3.454446
HORVU7Hr1G071300	2.381611	3.15346	3.385408	2.443243	2.438518	3.361739
HORVU4Hr1G025080	6.473444	7.5172	7.794509	11.57926	12.40306	12.157
Hordeum_vulgare_newGene_8752	0.911613	0.736725	0.817766	1.118948	1.455599	1.441814
Hordeum_vulgare_newGene_8751	6.865517	9.053337	7.387859	0.446933	0.19333	0.25975
HORVU4Hr1G059980	8.42799	10.2845	11.12146	6.548619	7.910208	8.132296
HORVU3Hr1G110850	97.26637	94.62624	91.30863	145.9101	127.7178	132.3834
HORVU7Hr1G003870	11.39517	9.800385	10.12867	4.814172	6.733042	6.904542
HORVU5Hr1G095630	10.83159	11.34736	12.24843	23.13605	32.3771	31.78492
Hordeum_vulgare_newGene_11664	1.288958	1.475293	1.394272	1.829765	2.969496	3.484305
HORVU4Hr1G090460	28.00561	31.96874	35.33307	7.834584	0.837033	3.053678
HORVU4Hr1G050130	15.23736	15.64306	23.02997	13.14761	26.11388	24.27828
HORVU4Hr1G071730	193.6476	181.8786	228.1877	139.1336	107.4926	108.7553
HORVU3Hr1G079620	24.73286	21.00009	28.33444	20.49526	24.87819	29.58658
Hordeum_vulgare_newGene_5257	5.413521	2.529782	2.090521	3.304229	3.151977	3.494079
HORVU1Hr1G036060	1.976988	1.850085	1.96844	5.572138	4.852613	5.298411
Hordeum_vulgare_newGene_1705	1.849756	0.857549	2.320342	26.23566	20.50224	20.98986
HORVU5Hr1G080540	27.70785	12.24975	14.76434	22.09329	25.41133	22.98849
HORVU4Hr1G025620	1.581631	1.680739	1.902182	1.422504	2.425312	2.133575
HORVU6Hr1G057260	4.545523	4.722652	6.070002	6.723894	7.547439	7.629473
HORVU0Hr1G010840	0.7821	0.762007	0.975745	1.210893	1.678723	1.987373
HORVU5Hr1G052000	5.241296	5.645651	7.540444	5.069673	5.66956	4.900898
HORVU5Hr1G098410	7.485907	8.098851	8.801641	4.084395	8.519275	6.630244
HORVU2Hr1G115890	5.196605	4.227687	4.472073	2.783987	1.599242	2.363559
Hordeum_vulgare_newGene_10622	4.470493	5.315084	6.871498	3.535865	4.309976	2.559419
HORVU1Hr1G040110	2.448094	2.7534	2.754096	2.576581	3.861829	4.220025

Hordeum_vulgare_newGene_10620	4.202166	7.131645	8.565649	0.471757	0.300918	1.426764
HORVU3Hr1G061900	6.240161	6.483445	8.235127	7.520747	9.621969	10.21407
HORVU1Hr1G001590	0.23639	8.271729	0.734987	14.00372	2.855921	1.357349
HORVU2Hr1G063210	346.3563	324.6405	438.767	306.7156	485.7545	478.9781
HORVU4Hr1G078750	20.7332	19.53052	21.80031	18.76757	24.48028	24.73051
HORVU2Hr1G102610	47.7253	51.09779	72.65947	47.12732	121.461	105.3894
HORVU5Hr1G093770	0.012785	0.084789	0.021883	0.114848	5.197343	5.646668
HORVU2Hr1G061100	0.406824	1.672249	1.385369	0.475814	1.19562	1.225353
Hordeum_vulgare_newGene_5663	17.99855	15.75971	17.94098	11.82142	11.19323	12.62945
HORVU1Hr1G035600	13.41439	15.43039	17.90145	16.50868	23.89215	20.77168
HORVU2Hr1G031200	2.710566	3.134063	3.745068	2.442681	2.283674	2.457309
HORVU5Hr1G033960	9.147754	8.943435	8.597394	9.26939	10.54376	11.03001
HORVU4Hr1G023660	9.585492	6.885453	10.16652	6.95865	9.159586	7.373171
HORVU1Hr1G028200	13.78117	12.08029	14.94875	11.11805	15.55462	13.7793
HORVU3Hr1G074620	5.610088	5.009561	6.384125	7.137459	8.726839	9.182616
Hordeum_vulgare_newGene_11293	35.45847	34.68489	40.4535	25.1702	26.45401	26.51785
Hordeum_vulgare_newGene_10744	5.669695	6.42711	5.943483	5.168909	5.064184	5.671747
Hordeum_vulgare_newGene_10747	23.45632	22.33097	29.61102	31.3301	53.87649	48.34102
HORVU7Hr1G045140	4.785758	5.309499	8.485605	4.585949	7.274241	7.867105
HORVU5Hr1G033340	3.393825	1.0625	3.369463	4.565996	3.895483	5.162497
HORVU2Hr1G031050	7.857243	7.14979	9.757777	6.089912	8.54135	8.069659
HORVU5Hr1G073870	5.941446	2.805631	5.387534	8.645095	19.65801	16.26302
Hordeum_vulgare_newGene_10011	6.854583	4.926802	9.18093	1.011527	0.377679	0.355411
HORVU2Hr1G114380	15.11604	14.24482	14.55683	22.79624	18.07764	19.43387
HORVU3Hr1G117840	19.655	19.55539	19.12719	20.20003	22.36981	22.73132
HORVU1Hr1G041790	7.639244	7.727043	8.583038	10.00876	14.72706	14.52847
Hordeum_vulgare_newGene_184	3.886482	3.657048	5.111548	9.975243	27.38617	23.93104
Hordeum_vulgare_newGene_185	2.511808	2.514491	1.951416	2.658067	3.931042	4.837937
Hordeum_vulgare_newGene_187	4.662786	4.250372	3.581064	5.956668	11.29814	9.514289
Hordeum_vulgare_newGene_180	0.491109	0.74963	0.647537	1.096415	1.872491	2.4796
Hordeum_vulgare_newGene_181	0.624681	0.59239	0.677496	1.620003	2.400543	3.020242
HORVU2Hr1G036960	44.10928	41.08929	35.81817	122.7002	188.6651	182.6711
Hordeum_vulgare_newGene_5669	1.077304	0.899655	1.609931	0.649967	0.988477	1.169516
Hordeum_vulgare_newGene_237	3.184487	3.561308	2.94436	11.25834	2.427472	3.752652
HORVU6Hr1G054100	9.885687	10.74864	15.50884	10.97923	13.71127	14.19632
HORVU6Hr1G003000	1.874817	1.520628	1.690414	1.303311	0.543643	0.57724
HORVU7Hr1G083130	5.497913	5.133529	7.2135	5.603844	6.112829	5.769638
Hordeum_vulgare_newGene_12241	1.912627	2.100696	1.654234	2.544933	1.028955	0.925927
Hordeum_vulgare_newGene_5709	2.476801	3.084026	2.762579	3.671167	8.018103	5.956951
HORVU4Hr1G062950	6.166767	6.164229	8.229228	6.161369	7.198855	7.400917
Hordeum_vulgare_newGene_12248	3.958896	5.649711	4.87086	9.971833	12.00272	12.61479
HORVU6Hr1G089460	5.902834	3.607858	7.252198	0.34579	0.180411	0.544457
HORVU3Hr1G051540	9.091792	7.23445	8.549972	16.45802	14.71861	16.0336
HORVU2Hr1G069820	9.815198	14.21707	13.55256	7.326413	5.895846	8.053067
HORVU4Hr1G073990	1.498963	1.342869	1.231812	3.165144	3.110543	4.180277
HORVU5Hr1G064090	8.74401	9.358695	9.840548	11.65198	20.42957	17.78443
HORVU7Hr1G001800	2.302487	2.164429	2.315472	2.861625	4.278543	3.711213

Hordeum_vulgare_newGene_13599	2.777785	2.482189	3.447424	2.464682	3.509283	4.755085
HORVU2Hr1G114960	0.370621	0.686005	0.373537	3.608413	3.406387	3.63539
HORVU3Hr1G060620	2.694372	2.933891	2.45485	3.184158	3.972968	4.137029
Hordeum_vulgare_newGene_7340	0.021202	0.05237	0	2.403225	3.395905	3.41436
HORVU5Hr1G112380	8.132791	6.801925	8.331602	5.673124	8.009507	6.794123
HORVU5Hr1G087780	4.243757	2.722032	3.387043	5.505244	18.0274	13.74602
HORVU3Hr1G113130	28.07173	30.16401	24.13549	19.49768	17.37141	18.76845
HORVU7Hr1G105510	9.427508	10.91521	13.51011	7.418974	11.09617	8.715493
HORVU2Hr1G027430	110.5465	101.0999	124.3843	135.8796	96.99646	102.6847
HORVU6Hr1G052620	27.88994	27.69964	26.74894	17.62064	16.99092	18.05424
HORVU4Hr1G058360	8.389636	9.71014	13.06357	4.976174	6.73604	7.662939
HORVU0Hr1G005180	32.82763	26.59059	31.21277	16.13117	3.245206	7.148188
HORVU7Hr1G059900	11.34799	9.840664	9.294688	11.88228	9.108081	9.941385
HORVU5Hr1G083170	28.27038	26.41847	33.71605	18.76592	16.38562	16.27814
HORVU4Hr1G026740	8.436364	7.397616	12.65136	7.575622	11.62732	12.40765
HORVU2Hr1G044290	8.801987	7.323047	10.77054	6.396109	9.799447	8.495519
HORVU7Hr1G025450	9.38537	13.60322	13.44357	6.510456	7.547454	5.782969
HORVU5Hr1G087870	0.911833	1.304157	1.266149	0.700528	1.019403	0.962147
HORVU3Hr1G113790	4.185643	4.401668	4.30258	4.604958	4.072017	4.43505
Hordeum_vulgare_newGene_2267	2.740865	2.861218	3.872897	3.86286	5.317987	4.284685
Hordeum_vulgare_newGene_2269	3.138805	3.246019	3.063001	2.886359	2.320272	2.895182
HORVU5Hr1G116710	43.76073	36.28758	46.21493	77.07103	147.5944	140.0901
HORVU3Hr1G081280	3.884301	2.939381	4.928831	8.535053	6.446142	9.04094
Hordeum_vulgare_newGene_13626	2.925906	4.650762	5.697272	1.636558	0.79499	0.867656
HORVU7Hr1G054500	8.130969	7.408632	9.217861	5.517529	5.330757	5.766762
HORVU6Hr1G079600	13.96882	15.83995	10.29706	1.146747	1.355245	1.200807
HORVU2Hr1G116520	3.554601	2.825273	3.57363	8.225856	8.054232	8.348646
HORVU3Hr1G068360	74.48808	70.91747	102.5194	57.74667	82.63064	80.86665
HORVU6Hr1G062410	3.180401	2.189402	3.279783	3.889754	4.505548	4.4619
Hordeum_vulgare_newGene_7968	100.6275	105.0506	156.9203	90.97858	182.8911	174.4751
Hordeum_vulgare_newGene_7969	7.710869	7.425094	7.095845	9.460366	9.682072	9.695584
Hordeum_vulgare_newGene_7967	4.234721	4.262533	4.843782	6.107793	6.794514	6.768517
HORVU1Hr1G077990	39.32214	35.27581	28.16046	65.23836	53.01644	71.48245
HORVU2Hr1G027290	5.267882	6.639275	7.861116	5.841059	5.824066	6.481207
HORVU3Hr1G032340	8.324467	9.032281	12.44109	5.480584	7.015482	7.731372
Hordeum_vulgare_newGene_15503	4.557511	5.951958	5.517391	6.866111	7.525456	8.474911
Hordeum_vulgare_newGene_15506	6.742884	6.50574	6.960952	10.3901	12.33087	12.30526
HORVU2Hr1G086180	2.885094	3.277559	3.734288	4.702437	6.33499	6.320644
HORVU2Hr1G026740	2.535833	3.143727	2.765437	3.089158	2.437847	3.000432
HORVU4Hr1G053900	26.13228	24.52231	28.36011	39.30099	46.23083	43.13975
HORVU4Hr1G075110	29.30613	23.20151	38.13393	38.35607	22.38075	23.13094
HORVU1Hr1G087320	0.719509	0.621705	0.626158	3.930302	10.00762	7.874403
HORVU3Hr1G059010	10.65617	9.377066	11.49056	12.41389	14.32351	15.23776
HORVU4Hr1G090660	29.05395	33.13666	28.94562	76.81615	89.81486	63.96773
HORVU0Hr1G031810	6.495013	6.263741	5.957224	4.167059	4.18296	8.360673
Hordeum_vulgare_newGene_951	0.154302	0.114797	0.353873	0.646441	4.122568	2.032777
HORVU7Hr1G064800	0	0	0	2.452745	2.786002	2.987388

Hordeum_vulgare_newGene_957	12.27684	10.4901	12.60536	12.16643	14.96645	15.03494
Hordeum_vulgare_newGene_956	2.526516	1.752617	2.132965	4.323588	1.091404	2.931734
Hordeum_vulgare_newGene_955	1.826408	1.200884	1.284546	1.963236	0.910231	1.209217
Hordeum_vulgare_newGene_954	0.970593	0.302828	1.128697	3.674916	0.648267	1.754569
HORVU3Hr1G044760	12.04768	13.14755	12.16376	8.40934	9.106573	9.938601
HORVU2Hr1G086230	3.914403	3.571439	4.559682	4.654792	7.736961	7.050798
HORVU4Hr1G031120	1.99004	2.80325	2.605994	3.712976	3.353253	5.069301
HORVU4Hr1G062740	8.78585	9.260768	10.1865	6.735244	7.927749	7.848722
HORVU3Hr1G043930	4.333948	4.561192	4.664062	6.537797	8.536434	8.493516
HORVU1Hr1G062290	1.762556	1.297089	1.657064	2.434644	3.477978	3.656709
HORVU7Hr1G108830	3.406082	3.308948	4.683864	8.618123	12.97177	9.58329
HORVU1Hr1G054340	2.265372	2.52883	3.132401	2.17911	2.57858	3.485351
HORVU3Hr1G019750	0.352698	0.446139	0.263848	1.124014	7.404736	4.52109
Hordeum_vulgare_newGene_9576	8.560564	10.9492	10.3484	30.62224	39.62022	35.75771
HORVU2Hr1G094660	0.091052	0.167931	0.15754	8.514602	8.889153	8.75084
HORVU4Hr1G067610	5.380801	5.787969	7.961211	5.864894	8.051977	8.301691
HORVU3Hr1G083620	3.81903	3.227968	4.692019	5.028782	8.773688	8.17773
HORVU5Hr1G062200	0.747449	1.13786	1.243294	1.142791	1.214372	1.30613
HORVU3Hr1G015690	1.380704	1.388375	1.170441	1.660405	1.830008	2.312215
HORVU2Hr1G081180	9.231175	7.440223	9.484283	11.74447	10.78229	11.23635
HORVU7Hr1G049240	12.49131	12.16027	15.77144	17.35293	29.36026	28.98147
HORVU2Hr1G105010	15.30594	15.2343	18.24282	17.36072	23.63404	22.12158
HORVU4Hr1G022970	3.056721	3.183376	5.029472	2.761185	7.278556	6.79528
HORVU5Hr1G069600	18.08319	16.28161	16.43229	22.1498	27.39764	27.88121
HORVU3Hr1G049060	20.1616	15.93435	18.70172	35.64968	64.52167	65.19637
HORVU5Hr1G105250	3.129969	3.100105	3.463604	5.616143	10.9918	9.906119
HORVU1Hr1G095390	7.995892	9.536031	10.98488	7.824208	15.50836	15.70552
HORVU7Hr1G096250	6.626632	5.418486	4.974171	17.93935	17.12597	20.38881
HORVU2Hr1G006160	3.997916	3.556517	5.404575	16.72246	0.267854	7.025553
HORVU4Hr1G031870	11.525	9.594613	11.50558	13.75818	23.97748	24.80657
HORVU5Hr1G058690	0.372535	0.550297	0.369054	0.38586	2.523994	2.1832
HORVU3Hr1G025630	1.139956	0.390888	0.873747	2.354772	1.765445	1.54545
Hordeum_vulgare_newGene_1103	9.818322	13.6131	13.29908	0.022198	0	0
HORVU6Hr1G025630	1.741146	2.592397	1.410249	5.266623	5.385911	6.044281
HORVU5Hr1G091840	0.034547	0.125817	0.023208	4.96808	6.693706	10.12882
Hordeum_vulgare_newGene_158	82.99687	78.21953	86.13712	81.65795	48.09742	46.96331
HORVU6Hr1G073030	6.516694	7.154994	7.648996	7.628741	5.298742	6.90322
HORVU4Hr1G032780	1.668478	2.424003	2.751865	4.135979	3.708936	3.767948
HORVU1Hr1G049740	2.782248	3.102204	3.643781	3.460095	2.978603	3.800015
HORVU1Hr1G027840	14.24325	14.07442	18.36596	15.87852	22.59066	22.94443
HORVU5Hr1G021300	22.85786	85.18842	101.0886	27.2427	36.71572	36.15984
HORVU5Hr1G057760	2.221029	2.558393	3.277188	1.679448	2.474818	2.536267
HORVU4Hr1G023530	16.36269	25.18898	27.69266	13.69941	26.7527	24.69426
Hordeum_vulgare_newGene_4550	10.90835	12.7173	12.79472	11.14623	12.38799	15.40359
Hordeum_vulgare_newGene_4551	0.393661	0.183134	0.374752	1.677001	4.868662	2.167652
Hordeum_vulgare_newGene_4558	2.406828	2.936197	3.243393	0.009463	0	0
HORVU7Hr1G101220	0.328609	0.394357	0.232563	3.428416	6.415376	5.163257

HORVU1Hr1G076310	59.562	43.57882	67.0247	82.84872	63.42147	68.20732
HORVU0Hr1G029490	5.09823	5.194106	4.175257	3.28467	6.995985	6.772732
HORVU2Hr1G034420	5.639803	4.307801	6.796576	13.59088	12.98931	13.15097
HORVU5Hr1G104120	1.788709	2.370439	1.381824	1.021902	0.565484	1.030131
Hordeum_vulgare_newGene_8549	3.327091	2.496315	1.684092	0.068631	0	0.161574
HORVU7Hr1G085150	361.1495	325.5532	417.8008	391.4746	959.7313	802.4039
HORVU4Hr1G080410	1.63732	1.761225	1.951353	2.118377	2.962752	3.455733
HORVU0Hr1G015560	3.186456	3.550427	3.770463	6.176884	8.976293	8.466965
HORVU6Hr1G028610	23.91812	21.22535	26.57084	26.18139	25.90111	25.71082
HORVU3Hr1G005400	1.04083	0.766378	0.660408	1.976425	1.757101	1.880106
HORVU6Hr1G081380	6.575779	6.789883	7.592289	15.44492	19.14256	17.6516
HORVU7Hr1G053230	5.219608	5.323125	6.685917	3.826638	3.77502	4.14307
HORVU5Hr1G099080	2.237803	1.946182	2.65163	2.120546	1.837524	1.608943
HORVU1Hr1G048900	1.560872	0.902266	2.211129	0.310766	0.484847	0.533331
HORVU1Hr1G083390	4.499134	3.986159	5.335039	4.878444	7.05413	7.826858
HORVU5Hr1G120370	4.01395	4.315287	4.803168	5.143329	7.131207	7.112096
HORVU3Hr1G006170	5.614532	5.732773	7.359006	4.381716	4.870193	5.742707
HORVU3Hr1G030080	2.905068	2.179315	2.268289	1.402663	1.906524	2.075366
HORVU2Hr1G078800	2.944527	3.130889	3.673463	2.293604	3.484092	3.572118
HORVU4Hr1G012130	1.820718	1.686759	1.450904	1.184178	1.226428	2.199209
HORVU1Hr1G091890	24.52063	21.20518	25.34606	20.49952	25.11857	23.53798
HORVU1Hr1G051610	75.88808	70.81538	80.75714	104.7945	164.1067	140.4548
Hordeum_vulgare_newGene_3	4.841757	4.623959	4.237199	1.734085	0.437023	0.718278
HORVU3Hr1G070020	95.29054	62.44697	75.75019	115.2227	42.19426	82.46891
HORVU7Hr1G056460	7.925599	7.518527	8.144816	7.191118	10.02818	9.558116
HORVU5Hr1G056140	3.69273	3.778642	5.021705	6.446952	15.37515	15.82837
HORVU2Hr1G050280	5.839598	6.115194	5.559712	9.718419	14.75169	12.47795
HORVU6Hr1G006080	0.358334	0.373545	0.784067	3.432466	26.47408	22.07381
HORVU1Hr1G026330	2.126416	3.980374	2.252455	3.366546	6.1332	6.763956
HORVU6Hr1G019900	14.28464	11.93721	16.58839	9.395626	9.084508	8.394031
HORVU4Hr1G000090	17.12712	17.09598	15.97245	13.14908	11.54694	13.12825
HORVU3Hr1G079420	1.127268	1.041397	0.806487	2.743833	6.12708	5.894594
HORVU6Hr1G047080	0.948429	0.98749	0.600941	1.390289	4.813853	2.911871
HORVU3Hr1G032400	7.082525	6.996696	8.815923	14.84491	11.18377	12.29958
HORVU0Hr1G012930	8.990242	7.425957	10.13343	2.938887	0.885397	1.648141
HORVU4Hr1G052240	86.30694	76.88316	75.93584	46.59269	29.60435	31.79359
HORVU3Hr1G077730	10.46623	11.06246	13.80965	9.554783	13.08513	12.82958
HORVU7Hr1G028260	0.825115	0.832211	0.88955	2.165092	2.481185	2.88443
HORVU1Hr1G061400	1.874658	1.628732	1.903715	1.300746	1.392905	2.271815
HORVU5Hr1G016360	2.346107	2.655793	3.130463	3.643067	4.772104	4.828433
HORVU7Hr1G051160	1.362543	1.097412	1.152992	3.070487	5.601775	5.48131
HORVU5Hr1G027910	17.55374	22.21866	24.61729	20.81192	24.82536	26.70846
HORVU5Hr1G094380	19.06401	22.66078	21.68903	18.69367	36.56507	15.49432
HORVU4Hr1G060940	36.08136	35.08806	42.59714	26.3475	22.36247	25.58205
HORVU1Hr1G000990	36170.84	40401.6	27206.08	31773.28	10472.42	15071.27
HORVU6Hr1G039200	4.35655	4.167569	4.133439	6.810018	8.831981	9.298858
HORVU4Hr1G060760	99.62424	101.9885	133.4691	180.9945	262.8075	157.4181

Hordeum_vulgare_newGene_5603	0.991522	1.729958	1.936573	2.878462	5.449734	5.247134
Hordeum_vulgare_newGene_9383	20.04774	24.16385	28.44686	4.049538	4.680331	4.407807
HORVU5Hr1G097870	0.176834	0.258762	0.161944	2.895832	8.576858	6.707243
HORVU0Hr1G037780	2.536912	3.022554	2.052542	2.402701	1.433089	1.427489
HORVU0Hr1G003870	1.961519	2.285627	2.093964	2.497258	2.898546	2.94641
HORVU7Hr1G112930	3.441721	4.622272	4.266544	6.078208	9.043486	10.58324
Hordeum_vulgare_newGene_9387	0.229254	1.184036	0.987074	1.032079	2.25386	1.113486
HORVU5Hr1G016090	1.273744	1.673372	0.678664	0.671469	7.167662	5.336681
HORVU1Hr1G065960	17.87716	16.54961	20.66299	30.29054	42.48398	43.12527
HORVU2Hr1G109150	1.119244	1.316586	1.138832	2.351275	3.271403	3.169951
Hordeum_vulgare_newGene_5604	2.078672	2.293408	2.465896	1.901556	1.725467	2.707961
HORVU5Hr1G112560	18.68665	16.91473	19.29042	13.57276	20.55656	15.30141
HORVU5Hr1G049420	0.030682	0.202925	0.18213	0.548337	3.042489	3.59485
HORVU3Hr1G037050	16.26919	18.12067	24.73548	27.61862	32.86329	29.82768
HORVU2Hr1G122310	7.410173	6.851397	9.739195	7.888478	10.96228	11.687
HORVU3Hr1G082230	8.11937	7.449552	10.02072	5.019977	8.285243	8.1118
HORVU2Hr1G105100	25.88269	22.82722	34.82756	18.60446	16.28359	18.43981
HORVU1Hr1G016640	10.78402	11.15261	11.86292	12.24255	13.60119	14.46756
HORVU1Hr1G067520	4.642994	4.098821	5.617682	6.651856	6.295573	7.266833
HORVU5Hr1G076170	4.158567	3.168947	4.996723	5.069693	5.200543	4.546381
HORVU6Hr1G090220	1.760726	0.950016	0.82961	4.383109	8.347453	5.613347
HORVU6Hr1G020050	1.895324	2.073747	2.208756	4.237811	3.659011	3.729034
Hordeum_vulgare_newGene_15672	7.624093	6.499517	8.670053	8.479701	10.26425	8.567274
HORVU6Hr1G084530	9.525379	8.449661	11.35023	8.188158	10.8854	10.13216
HORVU5Hr1G118680	0.514457	2.534708	3.42914	2.849053	0.780941	3.247035
HORVU3Hr1G060580	5.274313	5.542766	5.737695	5.38864	4.037319	5.515989
HORVU1Hr1G041990	3.728785	4.07802	4.534558	4.306467	6.61022	6.490412
HORVU2Hr1G065330	92.10325	88.36597	103.0741	216.4209	169.2059	165.246
HORVU2Hr1G060120	1.668536	1.989955	1.89783	4.246833	5.769803	6.275367
HORVU2Hr1G047230	4.633987	4.513062	4.834671	6.274505	7.180592	8.006438
HORVU1Hr1G084700	7.325791	7.976364	10.21725	10.52696	16.47285	16.34931
HORVU7Hr1G077220	2.717978	2.734267	4.327106	5.368163	14.78115	22.10797
HORVU1Hr1G055920	1.709281	1.25271	1.35217	2.192565	1.82548	1.508646
HORVU5Hr1G094460	1.756779	1.399625	1.501905	15.47972	20.1988	20.39507
HORVU2Hr1G067790	1.030824	1.398471	0.962347	2.081345	2.185034	3.173388
HORVU5Hr1G118530	2.190031	2.826993	3.594672	0.98877	0.654686	0.92184
HORVU2Hr1G053240	5.284566	5.934616	7.616093	6.957456	10.06722	10.51381
HORVU1Hr1G068870	8.590295	6.064093	7.718447	13.89911	15.7205	16.21857
HORVU2Hr1G083490	2.226841	0.973193	1.807922	0.407135	0.475161	0.280243
HORVU2Hr1G109840	1.799542	1.48875	1.881175	0.760725	1.06962	0.781952
HORVU5Hr1G109470	2.141021	2.118393	1.83079	4.316878	4.940646	5.40192
HORVU4Hr1G014490	12.31168	12.85389	15.64462	18.23746	26.92456	23.92312
HORVU1Hr1G013690	484.8062	431.2258	281.9769	175.5004	44.51288	78.03093
HORVU7Hr1G002110	24.81144	21.57184	24.36893	29.46961	64.40075	57.40328
HORVU7Hr1G087940	15.7261	15.33951	16.92916	20.96416	25.14724	25.074
HORVU5Hr1G115100	30.00878	31.12642	35.11196	10.34827	5.04092	6.088683
HORVU3Hr1G108100	16.21119	29.52559	17.13507	1.089973	1.303831	2.450652

HORVU6Hr1G066340	1.41385	1.709638	0.860654	0.969155	1.961033	2.551558
HORVU4Hr1G058560	4.258552	5.095306	6.808686	2.339429	0.858733	1.394243
HORVU3Hr1G070920	29.13709	27.52039	28.58162	28.48261	29.28487	28.92918
Hordeum_vulgare_newGene_3330	0.959379	1.098981	0.984673	0.953044	1.368102	1.004419
Hordeum_vulgare_newGene_3337	2.916635	2.770112	4.43415	3.506949	3.509279	4.530941
HORVU6Hr1G027440	1.695947	1.894875	1.51219	15.89456	26.34321	25.08593
Hordeum_vulgare_newGene_3338	0	0.22279	0.095477	2.687187	2.9333	2.608058
HORVU7Hr1G026860	2.362333	2.302864	3.206248	0.587348	0.274593	0.573757
HORVU5Hr1G000660	5172.423	5150.712	6185.933	11561.14	7844.038	7182.984
HORVU2Hr1G019920	1.827541	1.33546	1.669903	1.612412	1.640914	1.929343
HORVU1Hr1G094290	8.089689	7.725031	7.605505	1.832723	3.516877	3.82233
Hordeum_vulgare_newGene_2062	17.96975	19.22271	21.37823	17.4428	22.52202	22.65719
HORVU1Hr1G031950	23.40803	23.52246	26.97923	21.31115	22.33082	23.21349
Hordeum_vulgare_newGene_2060	1.173213	1.38481	1.450308	1.379935	1.207402	1.368925
Hordeum_vulgare_newGene_2065	1.533302	1.800601	2.159675	0.966851	2.207974	1.890301
Hordeum_vulgare_newGene_13029	0.54765	2.323815	2.802229	0.431249	0.873804	0.361634
Hordeum_vulgare_newGene_13028	0	2.886607	0.090519	4.223582	5.950715	5.373433
Hordeum_vulgare_newGene_13025	39.91519	33.00826	41.87982	14.45765	1.178814	4.941071
Hordeum_vulgare_newGene_13024	5.406123	7.042622	7.293358	4.56458	6.132346	5.750124
HORVU4Hr1G026230	2.250792	1.885932	2.000124	2.324193	2.392866	2.442868
Hordeum_vulgare_newGene_12994	4.597272	4.983562	4.735591	3.985036	5.177126	4.883029
HORVU7Hr1G084730	1.238579	1.16801	1.448232	0.826165	0.861496	1.051399
HORVU4Hr1G082740	0.632761	0.349305	0.747976	1.304847	2.518967	1.452139
HORVU6Hr1G078820	1.95137	1.354491	2.353914	2.265869	1.924247	1.52586
HORVU7Hr1G073410	5.19184	6.056168	6.573517	5.023538	7.120733	7.662814
HORVU3Hr1G061000	9.401892	6.62288	11.44243	9.13113	7.314249	8.013605
HORVU2Hr1G036750	14.51605	17.85365	20.42361	14.33325	15.35099	16.66081
HORVU7Hr1G066970	18.40415	24.35839	18.04371	25.27094	23.11922	24.91779
HORVU5Hr1G045300	7.840144	5.092092	7.118277	6.527607	7.567363	7.47722
HORVU3Hr1G021310	23.8823	22.12478	23.15838	14.51486	17.10724	18.41018
HORVU4Hr1G061150	2.929026	2.337639	2.70789	6.217357	6.782485	7.402836
HORVU2Hr1G062780	1.435162	1.949832	2.078223	1.09048	1.424087	1.337712
Hordeum_vulgare_newGene_5590	69.51811	67.07717	60.0431	49.89077	105.3502	106.27
Hordeum_vulgare_newGene_5597	4.406421	4.02527	4.118668	5.578583	6.162036	6.39323
HORVU6Hr1G053000	3.071193	2.485502	3.059246	5.804039	4.532038	4.331431
HORVU0Hr1G014270	5.921305	4.982034	8.405208	4.011062	4.243539	6.149915
HORVU2Hr1G019070	16.30567	18.33836	17.3552	16.7745	16.42873	18.65355
HORVU4Hr1G070650	1.173157	1.358518	2.063558	1.882029	2.37971	2.41945
HORVU2Hr1G101720	2.557866	3.341449	5.994096	3.5305	4.155613	4.170969
HORVU5Hr1G078120	29.99296	25.95695	33.88318	53.94317	71.02554	69.34122
HORVU3Hr1G089170	1.414695	1.390329	1.755845	1.409851	1.234315	1.157695
HORVU2Hr1G060460	1.987608	2.148241	1.853931	10.27287	17.27102	16.12378
HORVU7Hr1G089660	5.019832	4.74519	6.661893	4.393221	7.72211	7.909544
Hordeum_vulgare_newGene_13741	0	0	0	1.548089	3.529278	3.030576
Hordeum_vulgare_newGene_13743	142.2753	135.3496	83.49869	32.28329	34.74134	30.14973
HORVU5Hr1G081260	5.533093	4.333292	4.972871	5.487312	13.47798	7.011732
HORVU6Hr1G021150	14.22701	16.43599	16.61892	31.32237	47.87584	44.55584

Hordeum_vulgare_newGene_5049	2.117182	2.149646	2.007203	1.915776	2.533273	3.14332
HORVU3Hr1G109740	3.812256	3.915479	4.719077	6.538458	8.310834	9.037854
Hordeum_vulgare_newGene_5043	4.977617	5.596663	4.366518	4.742343	2.045568	3.637729
Hordeum_vulgare_newGene_5047	2.259713	18.15437	12.32913	14.89785	0.27916	0.465399
HORVU3Hr1G075830	2.849328	2.950333	3.502872	4.957084	5.016794	5.6414
HORVU2Hr1G112370	16.51204	14.35629	23.54179	21.52055	48.34769	48.2953
HORVU4Hr1G083910	4.661205	5.223606	6.725038	3.966237	3.726749	5.147409
Hordeum_vulgare_newGene_12547	1.002391	2.637148	3.164354	2.146847	4.20219	4.745274
HORVU6Hr1G016240	7.994215	7.840515	7.857445	8.837774	9.331218	10.16366
Hordeum_vulgare_newGene_11310	2.054723	1.239612	1.733348	1.490157	1.838257	2.486418
HORVU3Hr1G053390	14.73575	10.95471	15.97794	16.74122	22.96946	22.06841
HORVU3Hr1G073360	2.713149	2.824212	2.915333	3.684893	4.397941	4.435278
HORVU3Hr1G096450	21.08846	20.8268	23.31403	18.97968	24.08949	24.23868
HORVU7Hr1G005270	12.50355	12.40259	12.54351	25.49729	24.31719	22.65193
HORVU6Hr1G070900	2.213087	2.532801	2.761563	3.992188	7.183009	6.750189
HORVU0Hr1G016860	16.78953	16.23313	21.34831	16.85865	11.06736	10.3995
Hordeum_vulgare_newGene_14848	2.517097	2.847345	3.425029	4.423915	6.925652	6.304244
Hordeum_vulgare_newGene_14842	20.17	16.9533	19.78599	19.92071	28.51062	26.49327
Hordeum_vulgare_newGene_14843	0.805008	1.166993	0.671233	1.222635	1.078922	1.154203
Hordeum_vulgare_newGene_14840	2.07054	2.351465	2.458923	2.083692	1.694845	1.993591
Hordeum_vulgare_newGene_14841	3.580705	2.814474	3.029002	1.919168	3.280674	2.739649
Hordeum_vulgare_newGene_8275	5.173929	4.997077	5.19584	7.841177	7.047509	7.035881
HORVU6Hr1G028920	5.182262	6.967836	5.466747	7.915157	8.81781	9.319024
HORVU7Hr1G027060	4.622375	4.513203	3.936539	5.986748	5.933754	4.632943
HORVU2Hr1G023710	49.50354	48.84528	51.54913	39.15316	48.57414	46.05086
HORVU5Hr1G050560	98.00396	101.465	141.1271	64.76131	78.19158	76.8558
Hordeum_vulgare_newGene_1742	9.482695	9.486377	10.37018	10.52697	11.28167	11.56106
HORVU6Hr1G025430	33.98625	43.85983	56.6275	10.45128	1.117842	4.210362
HORVU0Hr1G011720	12.58259	4.094718	10.42589	1.4442	0.26196	1.774832
HORVU1Hr1G005800	6.93158	6.351619	6.754475	0.636392	0.717266	0.917629
HORVU2Hr1G039570	4.10142	3.667225	4.068016	6.503816	7.316591	7.585267
HORVU4Hr1G060840	20.09118	19.19834	22.93282	12.80556	12.52811	12.49656
HORVU2Hr1G076180	12.87716	13.44751	14.71769	86.63371	95.41223	99.12884
HORVU5Hr1G087970	0.416072	0.773661	0.527303	2.544607	2.744486	3.328526
HORVU3Hr1G091150	12.45995	12.3653	11.93332	8.021964	8.389635	8.687807
Hordeum_vulgare_newGene_4716	2.426511	1.824431	3.343752	1.663364	1.545995	4.70087
HORVU6Hr1G020600	0.137227	0	0.229142	22.55581	27.94396	29.49477
HORVU1Hr1G053770	6.008646	4.497915	5.677709	3.774565	3.778308	3.931928
HORVU0Hr1G022380	0.439631	0.374897	0.515179	4.412927	4.592345	5.600208
HORVU2Hr1G034620	1.226929	1.633696	1.909012	2.743856	3.285771	3.19558
HORVU6Hr1G009330	11.31378	8.676278	12.94531	6.477932	8.221791	7.47675
HORVU2Hr1G102400	6.504113	5.305472	7.091785	5.799828	7.250848	7.975303
HORVU3Hr1G111910	12.03766	10.26288	13.95219	12.32635	10.81906	12.39012
HORVU6Hr1G076530	36.03796	38.8417	51.79015	5.071947	1.803804	2.625278
Hordeum_vulgare_newGene_2730	4.498729	3.333603	5.107563	5.26627	4.226034	5.016349
HORVU1Hr1G004510	6.772278	6.53768	8.881028	5.083728	7.664179	7.551591
HORVU3Hr1G011770	0.696474	1.02144	0.36986	1.084205	37.13473	29.24832

HORVU3Hr1G085350	6.145417	7.11691	5.902234	9.757331	12.30635	13.64601
HORVU2Hr1G025750	1.354615	1.363221	1.949975	0.827357	1.009317	1.339693
HORVU7Hr1G046820	0.786817	1.026469	1.178593	1.359297	0.934275	1.664585
HORVU4Hr1G077240	58.55545	67.24207	91.54465	66.09079	159.2787	154.5861
HORVU2Hr1G032590	4.690834	4.863954	4.91073	5.091691	6.934518	7.778076
HORVU3Hr1G102720	0	0.453428	0.180765	35.41938	35.21186	39.45221
HORVU7Hr1G078850	32.96649	31.42413	26.03457	25.80679	25.55381	28.55906
HORVU3Hr1G088660	3.738472	4.435183	4.106386	5.08173	3.616637	4.508499
Hordeum_vulgare_newGene_12037	4.489778	4.927954	4.858151	5.685211	10.83977	9.482357
HORVU6Hr1G053830	12.64963	10.1775	13.67588	19.67141	18.76015	15.53046
HORVU5Hr1G019310	1.890495	2.223655	1.673004	2.142126	3.214386	3.089424
HORVU5Hr1G040090	3.054769	3.819892	4.585334	2.692247	3.992774	4.260539
HORVU7Hr1G053780	5.037399	5.16978	5.548902	5.909727	6.689036	6.574416
Hordeum_vulgare_newGene_6714	2.267751	3.185222	3.290269	3.103078	3.330422	4.660659
Hordeum_vulgare_newGene_6715	7.51225	1.952153	7.259544	9.315326	9.436713	10.862
Hordeum_vulgare_newGene_6717	1.13502	1.241324	1.476195	1.824181	2.457984	4.191661
HORVU0Hr1G005750	1.090065	0.760167	0.753976	3.352562	4.075051	3.434749
Hordeum_vulgare_newGene_3075	2.388542	1.881752	2.550063	1.938467	1.078181	1.411261
HORVU0Hr1G000500	4.288305	5.861773	5.429062	0.093003	0.217243	0.147252
HORVU2Hr1G006030	33.30823	28.67322	36.92591	43.35831	55.87307	56.14876
HORVU1Hr1G063300	2.912247	3.48543	4.54047	3.337122	5.221142	5.59987
HORVU4Hr1G074740	1.151593	1.531449	1.276849	6.050316	1.070915	2.721643
HORVU3Hr1G089130	6.925389	7.237792	7.686711	14.67516	14.0964	13.15509
HORVU7Hr1G064100	6.298427	4.683813	6.154859	15.0085	17.98863	18.12001
HORVU1Hr1G022190	10.04408	10.43584	9.749504	12.96433	12.38558	13.22902
HORVU3Hr1G039040	0.977694	1.404984	1.560026	1.229348	2.260728	2.234575
HORVU2Hr1G113410	2.267535	2.901372	0.491243	4.143281	6.20312	7.832098
HORVU3Hr1G087860	19.76523	18.80166	25.05375	23.25108	28.76818	30.07143
HORVU3Hr1G022640	4.641854	4.546327	5.344364	3.546805	7.796167	6.45759
HORVU6Hr1G052010	6.905471	6.786935	8.051449	8.095817	11.39301	11.0293
HORVU2Hr1G109030	11.18879	10.71612	10.18978	7.427552	6.032161	6.830336
HORVU1Hr1G027660	0.967642	1.121697	1.223969	1.56085	2.078996	2.243262
HORVU1Hr1G010120	2.302768	1.313833	4.98011	0	0.012191	0.127373
HORVU1Hr1G070230	17.4518	19.36402	19.98598	11.76628	8.739863	11.98612
HORVU7Hr1G085060	4.470682	5.564783	5.122774	10.54009	12.81006	12.36815
HORVU5Hr1G075470	5.34293	5.026962	6.625253	4.657639	7.255163	8.836279
HORVU5Hr1G079750	6.113202	5.881416	6.201917	6.66415	8.312485	7.996995
HORVU5Hr1G055090	1.851964	2.292841	2.254255	2.676994	3.401566	3.694334
HORVU5Hr1G028000	41.59028	34.30975	56.07109	37.31016	68.06095	57.13405
HORVU7Hr1G030110	5.814769	4.336225	6.500185	4.750655	4.618249	9.21637
HORVU7Hr1G088770	0	0.590927	0.875967	3.14142	4.816697	3.571204
HORVU6Hr1G002270	9.505448	4.355212	7.101991	4.909254	5.384216	4.286938
HORVU4Hr1G010000	2.727866	2.796106	2.683175	7.837287	9.787987	10.69539
HORVU5Hr1G021560	1.564179	0.498683	2.214137	8.305888	14.01483	12.82849
HORVU0Hr1G000670	20.93972	19.85082	18.77527	28.7021	30.98238	33.10051
HORVU7Hr1G118720	12.82695	11.17286	15.45315	15.02269	23.60659	18.87301
HORVU2Hr1G005600	1.835008	1.901609	1.731458	5.867007	7.380798	8.735316

HORVU1Hr1G059630	3.893485	4.416887	6.554297	4.27806	4.972491	4.64972
HORVU5Hr1G101990	1.646619	1.647486	2.206815	9.122313	11.5097	12.22801
HORVU4Hr1G034300	29.09342	27.28393	32.01886	35.46049	42.88558	41.7905
Hordeum_vulgare_newGene_10432	6.093392	5.434517	5.733169	7.399032	5.977384	6.356796
Hordeum_vulgare_newGene_15337	12.12063	15.23869	15.03472	26.80502	27.58852	32.01528
HORVU4Hr1G081660	3738.139	4475.601	3418.903	6191.959	8376.934	5734.614
HORVU5Hr1G021690	5.918061	4.850178	6.534279	8.489068	11.74077	11.87645
HORVU7Hr1G002140	13.97863	14.8961	11.98985	9.486429	8.240197	10.68442
HORVU6Hr1G061010	106.4183	98.1894	118.6422	114.401	128.4011	121.3346
HORVU3Hr1G082740	5.929452	4.75347	4.959246	28.91325	59.56428	62.5398
HORVU2Hr1G059380	0.765515	0.817951	1.196453	1.288773	2.743268	3.295149
HORVU1Hr1G054060	2.739571	1.902479	2.637415	2.712303	0.577967	1.487929
HORVU3Hr1G084740	3.456874	4.74347	4.546711	5.446101	8.862199	10.87638
HORVU6Hr1G060760	4.840976	4.958675	6.299473	4.996876	2.798363	3.938026
HORVU1Hr1G090450	8.354484	8.14174	11.50019	10.08209	12.54319	10.86681
HORVU5Hr1G074840	1.263269	1.778156	1.062213	11.9728	33.43038	19.36331
HORVU6Hr1G069570	49.68602	49.00587	53.137	32.34499	39.23178	38.9083
HORVU4Hr1G005960	8.932533	6.296176	8.315967	7.362602	9.206702	9.341623
HORVU7Hr1G050540	19.10238	16.22736	18.8295	14.25543	12.23773	12.31008
HORVU1Hr1G050760	5.866346	5.368914	6.754855	4.69058	5.404051	5.701766
HORVU4Hr1G011060	0.217188	0.843926	0.613904	1.7396	3.750173	3.587428
HORVU1Hr1G062480	37.99653	28.50615	40.66755	29.36098	33.18469	25.34768
HORVU7Hr1G092570	13.63588	11.60754	14.4916	9.588349	5.909506	6.673725
HORVU2Hr1G094490	0.348612	2.127825	2.105397	1.41581	1.571093	1.31979
HORVU2Hr1G098330	4.776445	2.95431	6.152343	5.902968	2.42339	3.348655
HORVU6Hr1G000650	9.138792	7.11306	7.154662	16.34176	15.2924	16.72964
HORVU5Hr1G094490	48.57089	42.53427	46.19401	58.66883	35.15225	24.76222
HORVU6Hr1G068190	2.980331	3.186681	2.899827	4.770964	4.320905	5.176199
HORVU3Hr1G036650	6.142273	8.451379	6.897718	7.773021	11.16923	11.09647
HORVU4Hr1G008820	6.846291	7.223197	7.824438	7.296741	8.770418	8.898351
HORVU1Hr1G095210	18.37033	16.02837	17.89436	21.10509	18.62181	17.2306
HORVU5Hr1G114230	1.162808	0.980375	1.37445	0.980805	1.368999	0.97686
HORVU2Hr1G017960	7.519981	8.174641	5.556015	12.35842	13.72026	12.42334
Hordeum_vulgare_newGene_9153	4.963677	5.519019	5.995459	5.364079	5.855817	5.895923
HORVU4Hr1G009190	0	0	0	6.321826	5.128115	8.004955
HORVU5Hr1G066790	3.527573	2.994389	4.181515	3.197312	7.502291	6.704441
HORVU7Hr1G115980	26.19248	41.89646	31.67708	20.95246	30.81929	29.80526
HORVU1Hr1G079080	4.645019	4.348268	5.2955	3.397445	3.864061	4.564686
HORVU6Hr1G009070	1.635759	2.548218	0.741033	4.803511	5.735853	5.54653
HORVU4Hr1G069940	1.06228	1.100777	0.997624	1.885702	1.590557	1.554586
HORVU1Hr1G088930	0	0	0	44.2166	0.179165	0
HORVU1Hr1G042030	28.0316	25.03284	31.24815	25.34216	18.98253	21.17284
HORVU3Hr1G030270	1.921821	1.883909	2.577093	4.132464	4.844418	4.406801
HORVU0Hr1G017570	18.27988	21.09689	21.74434	31.82034	27.93307	40.03164
HORVU7Hr1G057010	6.791676	7.742432	7.88691	7.687663	10.43826	10.69243
HORVU6Hr1G080340	19.4275	17.39959	19.26025	22.90395	32.80574	32.85855
HORVU3Hr1G085830	5.300167	3.805218	6.108021	7.846178	11.16317	10.0665

HORVU1Hr1G091460	30.50066	25.46978	35.98071	46.54978	64.9304	61.19829
HORVU6Hr1G066350	2.564304	2.868997	3.045541	2.456278	3.721275	3.349576
HORVU5Hr1G110440	2.387021	1.918371	2.216222	3.283274	5.27612	4.461308
HORVU7Hr1G115670	1.853402	0.889002	1.16962	1.405998	0.463221	0.755006
HORVU1Hr1G076500	3.582606	4.828483	4.005315	0.053229	0	0
HORVU5Hr1G060790	87.53014	89.49655	78.78454	23.78914	19.981	19.63244
HORVU1Hr1G064770	0.717497	0.884307	0.907574	1.081125	1.412304	1.59121
HORVU3Hr1G000420	5.242207	6.253241	4.996938	3.423594	3.966269	4.195055
HORVU6Hr1G085830	1.204594	1.18925	2.52038	2.124348	3.790225	4.910891
HORVU5Hr1G015370	0.584106	0.572901	0.456231	0.434673	2.612785	2.219313
HORVU2Hr1G095360	16.48068	15.14554	18.88232	18.61019	24.99369	25.8269
HORVU1Hr1G057800	16.16121	17.44345	15.76649	13.76524	18.04132	15.67674
HORVU4Hr1G013960	7.408736	7.297771	9.2392	6.082314	6.387822	7.372817
HORVU4Hr1G008310	20.19973	19.67636	18.95923	44.12732	90.54425	89.07036
HORVU3Hr1G071540	5.920727	5.665602	7.567058	5.231842	4.836761	4.129557
Hordeum_vulgare_newGene_10201	3.638088	3.965321	3.810528	3.874709	4.303306	4.950195
Hordeum_vulgare_newGene_10207	41.05271	37.59808	45.21129	24.42608	22.9202	24.53863
Hordeum_vulgare_newGene_10209	0.940296	1.393888	1.356292	3.217922	6.064143	4.940381
HORVU7Hr1G093710	9.843779	12.03106	10.10561	17.802	12.62479	14.95539
HORVU5Hr1G065220	15.49749	13.00959	17.5798	14.57429	22.69261	20.38985
HORVU7Hr1G111350	2.028896	1.920945	1.386346	4.986487	5.410954	5.3716
Hordeum_vulgare_newGene_9338	0.996469	0.487665	1.028519	0.87152	1.631131	1.528138
HORVU3Hr1G086870	0.231453	0.233161	0.21453	1.952693	2.282884	1.82512
HORVU5Hr1G095080	4.321743	4.135987	4.204863	4.59853	39.55717	41.3632
HORVU3Hr1G098580	3.357438	2.423578	3.64994	1.651852	0.720929	0.962812
HORVU7Hr1G011570	0.81942	1.231661	0.895264	1.367532	1.384793	2.616935
HORVU3Hr1G074490	4.937963	4.495992	5.976431	4.915335	5.774329	5.544014
HORVU5Hr1G081060	4.816064	5.812671	7.777933	0.336793	0.567324	0.677447
HORVU5Hr1G012740	3.965613	2.808622	3.47428	3.451727	1.888817	1.900367
HORVU6Hr1G038050	1.644989	2.268804	1.954424	0.43957	0.324254	0.421115
HORVU5Hr1G053720	26.093	25.39812	24.28337	41.09032	30.39583	35.06614
HORVU4Hr1G003930	10.14701	10.31157	10.52815	11.76051	15.30599	15.40444
HORVU2Hr1G065960	5.233013	5.540853	8.314034	2.96086	2.168314	2.602584
HORVU4Hr1G013030	9.66038	8.183758	8.298327	7.05589	6.218389	7.541452
HORVU3Hr1G073560	21.86282	18.52227	23.47618	22.71341	25.85471	27.85887
HORVU5Hr1G120650	1.910637	1.278277	1.673831	4.900721	6.993977	6.682001
HORVU5Hr1G048630	6.082866	5.588944	6.068159	12.60719	16.67276	16.53069
HORVU5Hr1G042130	0	0	0	54.8785	16.45381	21.87791
HORVU4Hr1G082880	3.371536	3.725798	4.091347	6.708246	16.49682	15.31517
HORVU3Hr1G092750	3.075623	3.29909	3.588586	3.574028	3.964848	2.829222
HORVU3Hr1G026120	55.53193	55.19455	79.10635	13.00788	0.965027	5.189503
HORVU2Hr1G000390	7.477107	9.862047	8.888862	9.992462	11.46879	12.22848
HORVU1Hr1G000810	2.197179	2.205862	1.944671	0.796272	0.219279	0.484483
HORVU5Hr1G048180	1.726077	1.349488	2.292396	1.364066	3.653944	3.68799
HORVU5Hr1G077100	4.349427	4.516074	4.787883	2.73557	2.169704	2.880696
HORVU1Hr1G001140	141.0837	229.9292	134.6535	4235.382	4005.638	3885.246
HORVU3Hr1G084170	3.294189	3.217977	4.13711	1.764535	2.024538	2.471286

HORVU6Hr1G092290	16.00322	14.36311	16.97411	13.80915	12.30947	13.20559
HORVU6Hr1G057630	9.655201	9.7722	12.29277	7.111234	17.78487	16.06818
HORVU7Hr1G083340	3.544871	4.725809	4.412373	5.002731	7.947981	7.411517
HORVU7Hr1G044990	18.55397	16.48936	21.32196	10.35953	5.199203	5.493827
HORVU2Hr1G118120	1.125456	1.275578	0.623397	3.070913	1.348363	2.067951
HORVU4Hr1G067060	9.422373	9.640556	9.467311	11.66562	10.53678	11.55543
HORVU3Hr1G038540	16.20591	15.22073	24.16942	7.564566	9.06297	8.690866
HORVU1Hr1G005390	2.622196	0.979403	1.636098	1.169014	0.717197	1.02551
HORVU3Hr1G004300	25.91997	38.20913	47.79753	4.793792	0	1.5859
HORVU5Hr1G117920	14.1644	13.6583	14.88269	16.97649	17.88186	17.17649
HORVU4Hr1G085820	18.83397	14.43318	20.60337	61.80242	46.6645	51.67169
HORVU5Hr1G098910	57.12102	55.24711	80.96119	53.78015	82.24861	87.62894
HORVU3Hr1G051140	2.876991	3.29536	4.698449	4.145636	5.400481	6.022987
HORVU0Hr1G027480	9.341889	14.68945	16.09811	1.108593	0.057119	0.255427
HORVU3Hr1G094340	3.332721	2.626854	4.151675	1.697339	0.501185	1.634406
HORVU7Hr1G021630	1.841549	2.878285	2.660427	7.156588	15.16727	13.23914
HORVU5Hr1G103410	1.212049	1.477551	1.712126	0.744928	1.540563	1.332995
HORVU5Hr1G022350	14.69479	14.682	12.75285	12.79326	11.55785	11.62802
HORVU3Hr1G102520	31.23626	29.29057	36.09751	36.56362	40.33673	39.17755
HORVU7Hr1G045640	12.41442	11.92588	14.39569	20.41371	58.88571	43.05928
HORVU4Hr1G008920	10.95256	10.8153	15.36825	8.303849	8.962028	10.56822
HORVU1Hr1G016050	1.07964	1.3833	0.475441	3.904133	4.905847	5.057307
HORVU4Hr1G062500	0.577783	0.214887	0.80597	1.148616	2.590386	1.971798
HORVU5Hr1G011460	11.77983	13.59589	17.35788	8.216085	9.992023	12.42241
Hordeum_vulgare_newGene_557	50.63135	50.73711	66.57401	71.7885	85.89333	90.05513
HORVU2Hr1G067610	3.190734	2.916342	3.483339	3.124035	4.660297	4.385752
HORVU3Hr1G056580	9.97018	8.471806	10.42239	23.36889	24.06058	26.74596
HORVU1Hr1G090770	27.73366	33.01897	25.64467	21.62304	13.70634	16.61363
HORVU2Hr1G111520	10.06774	9.671151	12.72888	15.89473	18.16828	18.15859
HORVU1Hr1G001850	0	0	0	12.06461	0	0
HORVU6Hr1G016420	1.952402	1.969062	1.69227	2.127068	2.041089	2.77887
HORVU5Hr1G092790	2.601596	2.054148	3.841245	2.395535	2.791464	2.803012
HORVU2Hr1G021700	5.237875	7.340709	7.959384	11.46285	18.07468	18.47691
HORVU2Hr1G065100	10.60035	11.97033	10.07757	13.69899	15.78699	18.07839
HORVU2Hr1G015410	9.056954	6.700693	8.471542	5.673737	3.339296	4.532778
HORVU6Hr1G091720	63.90828	57.41572	70.46287	57.84092	63.81636	56.13464
Hordeum_vulgare_newGene_3270	72.76457	60.74392	76.55637	76.62686	112.0975	115.2859
Hordeum_vulgare_newGene_3271	9.662568	6.837533	8.514607	3.925814	2.548535	2.801644
Hordeum_vulgare_newGene_3272	24.83429	22.34691	20.98984	14.47586	11.52775	12.87364
Hordeum_vulgare_newGene_3279	9.304874	9.99047	10.41457	8.934067	8.09164	9.1612
HORVU6Hr1G086800	6.975587	6.376669	5.655082	5.915013	8.209234	7.815046
HORVU7Hr1G105780	5.254101	3.499988	7.055285	0.992984	0.439747	0.574237
HORVU4Hr1G079900	8.458985	10.96379	12.59448	8.061855	11.75515	11.67938
HORVU2Hr1G106210	4.719345	1.333334	2.975154	2.755901	2.384563	3.44374
HORVU3Hr1G027730	24.43601	22.47645	25.78602	25.84516	35.96795	37.08089
HORVU7Hr1G071730	8.336432	8.179564	11.67143	30.98084	75.72455	69.58651
HORVU6Hr1G055460	14.72531	13.41238	16.51144	18.16923	22.25521	21.43629

HORVU4Hr1G067930	65.6859	58.54883	61.7762	48.19157	37.75956	36.65843
HORVU4Hr1G054470	7.302377	8.321338	9.752409	8.238332	2.055431	2.390848
HORVU6Hr1G050750	14.88936	14.67297	16.60596	32.65345	48.97966	48.24639
HORVU3Hr1G089330	45.89227	44.5603	40.99507	30.29368	23.49971	27.35235
HORVU6Hr1G086910	2.338133	1.952481	1.77974	2.496944	1.880912	1.496178
HORVU7Hr1G075290	60.509	59.56709	88.35884	83.51571	186.1858	162.1266
HORVU2Hr1G086710	34.21147	32.51455	30.6679	25.52972	28.20225	29.52645
Hordeum_vulgare_newGene_13305	0	1.186457	0.307615	0.350561	3.836316	3.607747
HORVU5Hr1G000580	79.6777	64.13747	84.56411	48.94292	87.67932	80.40951
Hordeum_vulgare_newGene_12541	4.802251	3.551541	4.759652	5.410557	6.508235	7.113001
HORVU5Hr1G033480	23.24742	25.06117	28.19461	25.41511	43.53523	46.44692
HORVU1Hr1G082510	2.480097	1.648578	1.969958	3.331285	1.600609	2.459819
HORVU5Hr1G121530	0.625739	0.423581	0.524307	1.373664	1.456121	2.532366
HORVU3Hr1G061700	5.071342	4.496588	5.41816	6.420047	6.317322	7.586984
HORVU2Hr1G119480	1.034331	0.818333	0.919637	1.964216	2.152701	2.833653
Hordeum_vulgare_newGene_764	2.910991	3.165838	2.82387	4.481722	5.712382	6.352067
Hordeum_vulgare_newGene_765	4.630394	5.65393	5.271204	6.618048	9.422123	11.21799
Hordeum_vulgare_newGene_761	2.929745	3.585178	3.390216	4.718114	5.310725	5.142116
Hordeum_vulgare_newGene_763	2.073496	2.660866	2.273387	3.621258	4.840112	5.292032
HORVU0Hr1G020500	2.070127	2.406011	2.410803	0	0	0
Hordeum_vulgare_newGene_14266	2.761688	2.683045	1.751219	8.164299	9.043071	10.01036
Hordeum_vulgare_newGene_14264	3.096634	2.021434	2.557994	4.907461	4.326959	6.400052
Hordeum_vulgare_newGene_14265	9.394238	10.32325	14.08082	4.802344	5.467203	5.177346
HORVU5Hr1G079190	50.61209	49.55012	46.36219	36.88671	37.53897	38.52543
HORVU5Hr1G095970	7.227252	6.451555	7.50949	14.55227	15.45037	14.16498
Hordeum_vulgare_newGene_10941	0.96366	0.603348	1.122655	1.217142	1.150294	1.51544
HORVU2Hr1G020070	0	0.08591	0.193919	0.087212	5.684868	4.752207
HORVU5Hr1G070040	0.437551	0.591447	0.608818	1.867643	7.13909	7.756025
HORVU2Hr1G036380	3.347476	3.957424	4.260254	5.014459	6.307857	5.782902
HORVU3Hr1G051050	0.817483	1.404272	1.655845	1.440013	1.99017	1.818694
Hordeum_vulgare_newGene_2634	5.526844	4.742054	5.662977	7.233191	8.018066	7.634576
Hordeum_vulgare_newGene_8842	21.50148	16.85687	20.6111	22.82745	26.92481	26.68965
Hordeum_vulgare_newGene_8846	2.280184	2.81416	2.894484	3.38082	4.341685	4.923465
HORVU4Hr1G061050	12.36488	9.392784	11.47031	10.18258	23.87502	23.20611
HORVU1Hr1G003280	0.121695	0.120687	0.619888	12.78766	0.255952	0.253656
Hordeum_vulgare_newGene_5940	2.513741	2.767971	2.650589	0	0	0
HORVU5Hr1G073630	3.744023	3.457397	3.422559	1.212149	5.07932	4.413863
Hordeum_vulgare_newGene_5943	0	0	0	1.863354	0.173145	4.959356
Hordeum_vulgare_newGene_5944	0	0.012606	0.020389	11.05351	9.536293	10.06909
Hordeum_vulgare_newGene_5947	1.68791	1.002203	1.336247	1.523398	1.350306	1.04257
Hordeum_vulgare_newGene_15134	5.01362	6.209961	5.639234	5.596817	5.302114	6.214218
HORVU7Hr1G074650	1.123708	0.871037	1.598423	1.200548	1.04666	0.810917
Hordeum_vulgare_newGene_15132	1.16058	1.45893	1.4473	2.006785	3.212574	2.328856
Hordeum_vulgare_newGene_15139	0	0.021079	0	1.657869	2.886273	2.413169
HORVU3Hr1G110340	0.219841	0.124493	0.505037	4.532855	42.09704	37.04273
HORVU2Hr1G037470	17.36541	19.74167	16.51821	22.15463	38.31614	33.17117
HORVU7Hr1G019700	12.38086	10.31059	11.64398	4.038447	1.237557	2.381518

HORVU0Hr1G022610	0	0	0	0.148939	5.452398	3.31645
Hordeum_vulgare_newGene_5367	1.401133	1.78701	1.313328	0.944084	2.444069	1.744231
Hordeum_vulgare_newGene_5363	0.685893	0.615274	0.952788	3.440172	15.90563	17.99649
HORVU5Hr1G063320	0.572938	0.39893	0.629214	4.734014	8.617468	8.014031
HORVU2Hr1G119310	3.222637	3.82777	4.75911	2.876044	3.631541	3.109415
HORVU5Hr1G092960	43.28118	40.61013	36.65544	34.4123	21.2983	24.46547
HORVU7Hr1G107520	12.19106	13.17541	15.53351	14.64946	14.15782	14.74838
HORVU2Hr1G064610	19.06764	19.31825	23.79993	35.2096	51.77364	53.24348
HORVU2Hr1G026040	2.492209	2.477881	2.92122	3.251819	3.540661	4.199886
Hordeum_vulgare_newGene_9466	3.927808	3.27519	3.94123	5.868354	7.310766	7.638068
HORVU7Hr1G102170	10.29084	8.840365	8.700908	26.52603	38.67289	40.27452
HORVU6Hr1G028330	37.85754	34.06169	43.027	45.28544	59.13256	56.94632
HORVU4Hr1G075670	7.889337	6.126462	7.926165	8.310114	10.38915	9.521202
HORVU1Hr1G025890	3.103999	3.408398	2.66661	4.185047	2.124127	4.059598
HORVU1Hr1G001460	0.010892	8.258896	0.533797	12.04836	0.02204	0.015263
HORVU3Hr1G057840	3.641433	4.402157	3.58502	8.520347	17.40437	8.260507
HORVU5Hr1G035820	3.925597	4.399702	5.25154	3.486161	4.514692	5.73243
HORVU7Hr1G031800	1.350945	0.909813	0.996111	1.751816	2.449819	2.137131
HORVU4Hr1G035360	11.29596	13.43744	11.60568	17.00645	19.621	19.39457
HORVU2Hr1G069590	7.621085	6.502557	11.0958	19.2942	49.01288	53.61733
HORVU2Hr1G022340	61.0816	61.25664	78.40218	62.04878	69.464	65.0234
HORVU5Hr1G043190	37.93792	36.68125	36.5428	51.05868	90.26441	86.59532
HORVU3Hr1G059320	52.86251	42.78324	60.87294	50.26233	77.71777	77.79352
HORVU2Hr1G105760	182.0599	208.0112	276.1355	15.10441	2.636905	5.971698
HORVU4Hr1G035980	3.261982	3.786667	3.744544	3.138823	4.097153	4.188706
HORVU7Hr1G079600	3.80474	1.908928	4.656717	2.347525	1.16032	2.82716
HORVU5Hr1G034880	6.998663	7.414169	7.527452	10.38394	10.35451	12.39591
HORVU2Hr1G046270	2.766399	1.127974	4.813877	0.176642	4.687735	4.258389
Hordeum_vulgare_newGene_4907	4.270388	3.348222	3.749748	4.418838	4.206129	5.268368
Hordeum_vulgare_newGene_4906	1.213805	0.998743	1.851725	1.703391	2.756414	2.625743
Hordeum_vulgare_newGene_4904	0.981526	1.028514	0.989564	2.096775	1.39894	2.215529
HORVU0Hr1G004990	31.81486	27.74916	38.15939	43.07758	90.91229	82.6938
HORVU1Hr1G031310	14.31557	15.07356	16.66107	20.66269	26.39936	24.80923
HORVU2Hr1G102230	1.728966	2.601127	3.277901	2.373134	2.547552	2.190559
Hordeum_vulgare_newGene_29	12.02927	9.447022	11.81793	7.010743	5.222729	7.235755
Hordeum_vulgare_newGene_20	1.10936	1.195683	2.350029	0.939187	0.988382	1.44399
HORVU2Hr1G120780	0	0	0	0	3.322756	2.912336
HORVU5Hr1G033050	4.803749	3.364355	3.532199	15.38252	21.63322	22.98001
HORVU3Hr1G088140	1.664747	1.394954	1.387909	2.012404	0.381592	0.830135
HORVU1Hr1G021540	18.52968	20.20791	20.21392	18.41417	21.59177	23.06391
HORVU6Hr1G080770	32.32082	25.67548	34.35169	44.58081	63.52438	58.8128
HORVU3Hr1G030360	2.015915	1.280477	1.873648	1.326371	1.324952	1.102812
HORVU4Hr1G016080	2.915891	2.514809	2.692144	3.987964	3.347653	3.506964
HORVU5Hr1G060000	9.214135	7.918376	9.357421	11.25099	14.49783	13.02109
HORVU5Hr1G101650	32.55434	30.9336	52.10794	27.95501	51.3649	50.72209
Hordeum_vulgare_newGene_13324	0.026898	0.086078	0.022462	2.863676	15.52499	14.61937
HORVU6Hr1G074200	156.0263	135.5595	147.952	56.4892	40.79874	46.54874

HORVU7Hr1G026150	241.2913	237.4066	223.7271	162.1285	49.24595	67.5334
HORVU6Hr1G069350	6.013363	6.506318	6.226978	6.924307	9.181697	9.249574
HORVU3Hr1G113080	17.71919	18.08242	26.19823	3.165286	4.728609	4.942496
HORVU6Hr1G078640	12.33929	10.06906	11.89777	5.663837	1.984418	2.953931
Hordeum_vulgare_newGene_6500	2.068083	3.457457	2.587719	1.470721	1.500636	1.791883
HORVU5Hr1G013180	30.02708	26.80314	32.30726	29.43864	35.45989	33.68556
Hordeum_vulgare_newGene_6502	1.599759	1.686265	1.423662	1.592147	2.176729	1.624273
HORVU5Hr1G042740	2.15099	2.278493	3.261799	3.522538	4.149009	4.272353
Hordeum_vulgare_newGene_6507	3.764229	3.791414	4.428296	5.568051	7.604473	9.317814
Hordeum_vulgare_newGene_6506	0.952783	1.436816	1.954125	2.957315	0	1.885452
HORVU2Hr1G019400	1.636328	1.207807	1.29748	3.920291	4.844625	3.654425
HORVU2Hr1G079560	92.77842	87.42649	114.6935	109.9329	167.1815	160.0043
HORVU5Hr1G084010	25.09065	25.01052	27.52179	29.50551	36.73632	36.24573
HORVU5Hr1G015560	0.013258	0.021583	0.017838	11.59178	9.855698	11.55888
HORVU3Hr1G034280	0.164062	0.067915	0.305121	2.167494	1.74426	2.000602
HORVU5Hr1G065560	2.934076	2.080742	2.794359	2.915091	3.076417	3.199881
HORVU4Hr1G000930	13.39406	10.28117	12.80302	18.90959	31.63987	31.78765
HORVU1Hr1G087130	11.52412	14.55442	17.26633	0.049664	0	0.071675
HORVU2Hr1G041550	9.793513	9.366992	10.95814	14.88326	22.35579	21.91211
HORVU7Hr1G118020	4.468943	3.624648	5.019103	6.682424	5.572869	8.974087
Hordeum_vulgare_newGene_14743	43.91113	34.39229	36.42189	91.4312	154.1507	153.7547
Hordeum_vulgare_newGene_14742	4.058953	4.244719	3.967579	0.065657	0.139158	0.127268
HORVU2Hr1G092520	11.09679	13.65391	13.50813	14.25813	13.45825	15.91239
HORVU0Hr1G019370	1.474299	1.386227	1.76109	1.472102	1.6424	1.690201
HORVU0Hr1G000730	22.00908	11.66812	19.3845	10.72581	3.163466	5.195344
HORVU3Hr1G083310	10.48582	8.585747	14.29682	15.9449	26.38506	25.6379
HORVU5Hr1G062510	19.4215	19.93694	23.30404	44.4561	56.87724	67.59872
HORVU5Hr1G110220	55.50798	47.53212	48.71351	24.11664	18.86068	30.53852
HORVU5Hr1G069850	6.651852	6.09335	8.137204	15.92731	41.20863	40.18818
HORVU1Hr1G081470	15.23281	17.19304	16.83691	10.52759	12.28126	11.22881
HORVU0Hr1G004430	0.429284	0.058222	0.764414	1.950571	2.328741	2.085408
HORVU2Hr1G027050	2.158265	1.824994	2.232143	1.992087	1.910483	1.90185
HORVU6Hr1G066960	19.66465	18.82784	16.34504	20.54296	16.01786	20.24188
HORVU6Hr1G002330	28.64253	24.14373	30.37787	28.84951	30.92892	29.03661
HORVU6Hr1G040220	30.33802	30.79407	32.46448	27.59219	35.37783	35.28082
HORVU4Hr1G003770	22.12914	23.1991	25.1549	14.45988	13.43921	15.30008
HORVU3Hr1G093530	0.327184	0.238857	0.361694	0.795884	3.131344	2.75536
HORVU3Hr1G016230	2.101193	3.369525	3.746619	0.262389	0.162532	0.455449
HORVU5Hr1G113040	0.725543	1.143809	1.144035	1.183847	1.85322	2.041335
HORVU5Hr1G118010	3.734713	5.699197	6.19953	2.693969	1.483004	2.324084
HORVU4Hr1G089330	0	0	0	6.033643	5.737295	7.076799
HORVU4Hr1G034110	6.878908	7.610227	7.215612	6.480903	7.993581	8.785467
HORVU3Hr1G032270	32.6949	23.28418	23.22716	5.919101	0.510619	1.248446
HORVU6Hr1G068370	3.111746	2.875653	2.909438	1.217872	1.245567	1.241513
HORVU4Hr1G010270	4.787323	4.589175	5.906137	8.553852	9.774843	10.74625
HORVU6Hr1G005020	1.344987	1.346952	1.391468	2.440728	3.198661	1.590475
HORVU6Hr1G085110	0	0	0	0.051882	22.40994	19.93509

HORVU3Hr1G015760	9.291094	10.5887	9.178013	7.287436	7.802435	8.061036
HORVU1Hr1G002420	0	16.48294	0	0	0	0
HORVU7Hr1G050660	50.35959	43.63984	53.12793	14.03513	3.700773	8.766543
HORVU3Hr1G023160	1.611859	1.895912	2.42163	2.205621	3.160462	2.723668
HORVU7Hr1G037070	21.0338	22.35777	25.51429	16.65729	19.26877	20.40197
HORVU2Hr1G082740	9.56692	8.973896	7.008309	20.19322	10.98192	14.60592
HORVU5Hr1G096090	6.741922	4.565176	7.149401	9.43789	35.60079	33.52791
HORVU1Hr1G058170	10.24092	9.503582	13.23016	6.571066	8.255533	8.337387
HORVU5Hr1G013240	7.821157	7.459412	11.24119	7.745201	9.351469	10.92503
HORVU4Hr1G019610	2.40029	3.33057	2.883003	2.750822	4.757199	3.936886
HORVU5Hr1G111680	5.837903	6.998034	7.954047	7.246847	8.791785	9.294429
HORVU2Hr1G089020	1.76382	0.990121	2.23391	1.376315	0.814729	0.979964
HORVU5Hr1G096540	1.30571	0.758071	2.221147	1.162379	1.710171	1.17045
HORVU3Hr1G009490	1.116496	0.867594	2.228354	128.5418	176.2902	154.9434
HORVU1Hr1G074350	163.309	163.7838	195.0283	242.0138	260.6854	261.7442
HORVU7Hr1G028130	0.983929	1.82126	2.121807	1.48723	1.365649	1.04906
HORVU1Hr1G002390	15.25199	25.20383	14.71368	25.93347	14.7842	15.3653
HORVU4Hr1G048960	2.733638	3.314892	3.217186	4.786126	5.560746	6.01514
HORVU3Hr1G019840	2.272916	2.383937	2.704951	6.252421	3.845507	4.19002
HORVU0Hr1G014900	4.815532	6.095796	6.267784	2.340745	2.158074	2.400052
HORVU5Hr1G104630	1388.692	1546.269	1842.97	1291.928	42.09866	532.9968
HORVU2Hr1G098100	1.528937	1.696475	2.098908	1.637864	1.746488	1.788157
HORVU1Hr1G085880	11.26082	9.579641	12.07019	11.67611	13.9175	15.63407
HORVU1Hr1G012440	1.041273	1.230207	0.941623	1.127907	1.892858	2.429372
HORVU6Hr1G091250	16.2308	13.80746	17.95764	9.318775	8.886918	9.127458
HORVU6Hr1G088960	1.437277	0.798498	1.204818	0.686926	0.830529	1.16721
HORVU2Hr1G011140	6.550392	6.9764	8.415206	11.67447	14.59264	13.62371
HORVU7Hr1G047400	10.14521	11.96228	10.15319	16.58817	15.44243	16.563
HORVU4Hr1G009760	4.963786	5.218351	5.680405	4.891283	3.235671	3.99837
HORVU5Hr1G112210	14.91605	13.3865	14.82283	12.13267	6.472918	8.490634
HORVU1Hr1G087950	0.336763	0.458251	16.67852	44.50647	0.121356	0.124972
HORVU5Hr1G050330	5.696554	6.530804	6.740728	6.951963	11.42942	10.89615
HORVU5Hr1G027710	4.704653	5.21777	6.846232	4.478275	4.756225	5.980066
HORVU2Hr1G091220	14.35462	9.936321	8.456801	5.936653	5.554043	6.392758
HORVU7Hr1G095750	4.504623	4.772333	3.541719	3.490593	4.003813	4.984953
HORVU1Hr1G058820	4.427979	4.075996	5.006285	3.364113	1.387456	2.737192
HORVU1Hr1G061980	19.43403	17.84238	17.8343	4.273961	2.49261	2.616573
HORVU7Hr1G075720	3.432644	3.555817	5.773228	3.39926	1.903295	2.550323
Hordeum_vulgare_newGene_5857	3.369976	3.412592	2.779462	2.634013	1.496284	1.739501
HORVU2Hr1G110940	7.298149	7.45538	8.705356	7.368806	7.284957	7.547207
HORVU5Hr1G054650	43.41656	37.62058	34.25571	29.48001	26.07526	29.73661
HORVU2Hr1G014000	18.08401	18.33316	21.63683	16.10506	16.99849	18.525
HORVU6Hr1G055910	22.73495	23.24406	30.73476	26.7757	30.60291	29.36517
HORVU1Hr1G006890	3.859888	4.254149	4.871654	3.966808	4.745335	4.16994
Hordeum_vulgare_newGene_13963	3.261786	2.954424	2.795105	0	0.074645	0.045266
HORVU5Hr1G046370	1.551005	1.641619	1.92856	0.997643	0.762048	0.756162
Hordeum_vulgare_newGene_13968	12.25161	11.39246	10.19541	6.462646	3.42904	4.376285

HORVU0Hr1G001450	4.188042	3.110898	5.517814	5.255362	11.00162	10.13078
HORVU3Hr1G006440	18.99435	12.05357	19.01996	25.48708	20.14376	24.17986
HORVU7Hr1G091270	47.6715	40.34167	58.72084	48.60529	85.47391	85.70184
HORVU4Hr1G055230	204.9435	217.5975	337.5736	188.8864	469.0172	431.5182
HORVU7Hr1G095960	6.446746	5.380697	8.256275	11.18619	11.68111	14.39924
HORVU5Hr1G053020	15.61284	18.02323	24.57524	4.148482	3.170163	2.473093
HORVU5Hr1G029010	1.956137	2.447904	3.195042	1.917956	2.850223	3.184581
HORVU1Hr1G082820	319.9258	418.0351	403.8003	56.65051	4.92814	25.09328
Hordeum_vulgare_newGene_15240	11.17779	12.31038	14.6013	21.82385	57.28457	46.4399
HORVU7Hr1G111140	14.37149	13.3392	13.69587	12.86266	14.69935	16.62287
HORVU7Hr1G060220	1.854359	2.375553	2.18734	2.074238	1.330883	1.828117
HORVU7Hr1G068080	2.076478	1.312311	1.947228	0.234865	0.211204	0.483785
HORVU5Hr1G098320	3.618866	5.13968	5.073452	3.604299	4.468845	3.869044
HORVU2Hr1G092750	1.637392	2.06141	1.795102	1.525125	2.894916	3.015152
HORVU2Hr1G092280	1.667755	1.204503	1.0966	2.818046	4.845006	4.347526
Hordeum_vulgare_newGene_10435	18.17624	15.24616	16.78412	3.298704	22.55429	25.44516
HORVU5Hr1G047420	11.83023	12.67412	12.99286	7.882963	9.243348	9.949111
HORVU5Hr1G053680	35.30049	34.66151	45.55415	20.78396	25.64334	24.66253
HORVU2Hr1G111300	0.598771	0.678218	0.857526	5.327168	15.98637	14.50122
HORVU3Hr1G061980	27.17813	27.2498	28.61569	25.5248	12.00906	12.96659
HORVU2Hr1G039700	3.512745	3.827964	3.372837	6.45955	13.03482	12.92781
HORVU1Hr1G080460	23.98126	25.76154	21.90565	10.58235	5.664893	6.421232
HORVU0Hr1G010590	30.80911	25.20222	26.4996	34.41583	38.7214	36.65615
HORVU3Hr1G113810	2.89836	2.6775	2.62114	1.813323	1.359227	1.459704
HORVU7Hr1G000910	19.59243	19.20026	20.41063	9.163645	11.30504	10.90574
HORVU1Hr1G065270	0.326877	0.234358	0.321487	1.722091	1.687293	2.242595
HORVU0Hr1G020980	1.914783	2.061692	3.540191	2.129741	1.539021	2.371773
HORVU3Hr1G007420	3.384702	3.290486	4.507376	3.966487	3.911439	3.616181
HORVU2Hr1G031530	0.198484	4.200007	2.826222	0.061264	0.142709	0.35221
HORVU5Hr1G047100	46.43935	44.60393	54.88449	42.2482	37.04042	40.32471
HORVU3Hr1G062370	1.33738	1.460126	1.54663	3.677595	6.819102	4.917602
Hordeum_vulgare_newGene_10732	0.726145	0.651662	1.073808	1.17331	1.210421	1.639416
HORVU4Hr1G024570	13.84772	12.24098	16.84331	19.84594	30.48612	28.63071
HORVU4Hr1G077430	0.371026	0.196503	0.409909	1.440084	8.189604	9.755813
HORVU3Hr1G076220	14.28095	13.47886	13.08187	12.45412	20.0005	19.95648
HORVU2Hr1G022180	6.151071	5.071337	7.004318	8.417386	11.79267	11.47116
HORVU4Hr1G069070	0.268991	0.101646	0.300578	1.311662	4.381522	4.808055
HORVU2Hr1G093150	4.682496	6.776175	7.560394	6.118629	5.00395	5.776329
HORVU6Hr1G070690	3.6485	3.65543	4.586514	2.671597	1.840058	3.42079
HORVU5Hr1G044990	22.46957	18.83957	20.70604	21.30744	18.14308	18.63883
HORVU7Hr1G005730	2.273838	1.912095	2.643849	2.404032	2.650088	2.456328
HORVU2Hr1G038150	2.082334	3.229385	4.420545	1.627869	1.461167	1.627409
Hordeum_vulgare_newGene_11472	60.46003	53.48111	58.97388	56.71425	77.80296	77.29439
Hordeum_vulgare_newGene_11474	1.209821	1.075912	1.549876	1.949551	2.517835	0.13441
Hordeum_vulgare_newGene_11475	3.683013	4.569979	5.25628	3.86511	12.82074	11.44041
Hordeum_vulgare_newGene_11476	1.299221	1.865828	1.508934	0.937804	0.661384	0.554124
HORVU3Hr1G112590	4.245427	5.407781	6.764485	5.83353	7.425386	8.417971

HORVU2Hr1G118350	95.43052	105.1519	70.12764	22.74655	68.3355	67.04274
Hordeum_vulgare_newGene_109	4.656676	3.513835	4.120507	6.361333	4.126139	5.922593
Hordeum_vulgare_newGene_103	8.196244	4.991307	8.045203	3.617734	1.356192	1.690473
HORVU2Hr1G014580	14.40799	14.84357	17.98149	13.94975	16.69608	19.65465
HORVU6Hr1G092480	21.14647	18.64395	19.94135	17.99175	21.21372	22.16638
HORVU5Hr1G071920	13.35639	11.86628	12.0132	26.63366	48.21726	43.54041
HORVU3Hr1G022350	21.5305	25.33746	28.07662	25.64454	34.54937	35.7968
HORVU5Hr1G051850	63.45137	56.76606	61.3918	75.30383	90.21502	93.5117
HORVU7Hr1G010470	2.916983	2.604568	3.35027	4.594773	4.51569	5.246216
HORVU7Hr1G021510	174.6783	161.5018	138.5457	109.2091	134.5848	130.1383
HORVU2Hr1G029660	45.3554	43.898	46.98539	43.96914	44.0092	46.84949
HORVU1Hr1G038650	0.703935	0.959406	0.906325	1.814347	2.493922	1.516512
HORVU2Hr1G072830	11.5784	11.0073	13.6662	10.52914	21.4086	20.12489
HORVU3Hr1G073700	2.193447	1.919298	2.04863	0.911077	1.73952	0.247421
HORVU6Hr1G090390	0.01983	0	0.009123	22.67261	95.37548	75.15418
HORVU7Hr1G072140	1.394303	1.070185	1.824216	1.575581	1.59689	2.21615
HORVU2Hr1G025320	8.33261	7.455451	11.55777	7.485272	11.11036	9.343415
HORVU2Hr1G022660	14.69795	13.6115	15.16896	11.3019	12.76135	12.75054
HORVU2Hr1G018720	21.38208	23.16886	16.91714	16.19549	14.32899	14.6037
HORVU5Hr1G000170	3.985063	3.772562	4.176904	8.592528	10.8065	11.67685
HORVU6Hr1G024800	1.625069	1.564403	1.514692	1.507212	1.596002	1.953863
HORVU6Hr1G055580	7.77597	7.129878	8.142659	7.465249	10.6346	8.131726
HORVU3Hr1G076000	0.905221	0.897372	0.682437	7.487662	15.84758	12.42258
HORVU0Hr1G025850	18.68363	14.62919	19.0405	2.988178	3.885537	3.483863
HORVU5Hr1G040190	1.754649	1.57018	2.405192	2.477204	2.861873	3.17907
HORVU7Hr1G074840	18.46179	16.35936	18.81584	21.66334	28.75301	26.78391
Hordeum_vulgare_newGene_13518	2.317718	2.334619	2.824707	3.951807	3.902926	1.757978
HORVU2Hr1G111480	3.880065	4.073447	4.789664	3.235624	5.328252	4.685186
HORVU1Hr1G042920	108.6811	81.66766	120.0113	123.314	177.5382	151.3626
Hordeum_vulgare_newGene_16128	2.386175	2.965805	2.957542	2.669885	1.473164	1.902146
HORVU4Hr1G000850	13.84024	7.447112	12.62268	32.78828	33.2287	36.17089
HORVU2Hr1G071330	5.901045	4.703652	5.977251	10.02664	9.687178	11.342
HORVU7Hr1G062810	2.202868	2.523535	3.79945	2.277762	3.559599	3.261832
HORVU3Hr1G067290	13.85996	10.4898	14.67202	14.05962	9.799598	12.75955
HORVU3Hr1G097860	9.894164	8.184672	18.6116	0.450412	0	0.31316
HORVU3Hr1G081170	24.69879	22.66587	51.9754	42.16164	263.1224	268.2629
HORVU4Hr1G058050	9.755086	9.699815	11.21987	10.47225	12.01741	12.84734
HORVU5Hr1G064010	9.505368	9.961364	10.28029	4.302942	5.301509	5.875031
HORVU7Hr1G001880	40.38682	37.23438	44.69489	28.95234	32.23262	34.03615
HORVU5Hr1G107410	4.112734	3.201987	4.249698	10.47244	12.5028	11.79172
Hordeum_vulgare_newGene_13512	5.982419	5.985219	9.292842	8.999416	9.08997	8.392128
