# Supplementary material for: Molecular mechanisms of how black barley accumulates higher anthocyanins than blue barley following transcriptomic evaluation and expression analysis of key genes in anthocyanins biosynthesis pathway
Source: Front Plant Sci. 2025 Aug 29;16:1650803. doi: 10.3389/fpls.2025.1650803 (PMC12427265; doi:10.3389/fpls.2025.1650803)
Supplement: Supplementary file 1 [file Supplementaryfile1.zip › Supplementary Material/Data Sheet 14.PDF]

Supplementary Table 13: Eighteen WGCNA modules and involved genes

geneID	Modules
HORVU7Hr1G100410	darkorange
HORVU4Hr1G073840	darkorange
HORVU0Hr1G024890	darkorange
Hordeum_vulgare_newGene_3757	darkorange
HORVU3Hr1G076940	darkorange
HORVU6Hr1G018600	darkorange
HORVU3Hr1G087430	darkorange
HORVU7Hr1G008700	darkorange
HORVU0Hr1G009280	darkorange
HORVU7Hr1G035300	darkorange
HORVU6Hr1G064430	darkorange
HORVU1Hr1G062210	darkorange
HORVU2Hr1G073760	darkorange
HORVU5Hr1G012950	darkorange
HORVU1Hr1G011720	darkorange
HORVU1Hr1G062970	darkorange
HORVU2Hr1G035420	darkorange
HORVU3Hr1G077630	darkorange
HORVU7Hr1G069660	darkorange
HORVU3Hr1G092430	darkorange
HORVU3Hr1G082850	darkorange
HORVU5Hr1G038070	darkorange
HORVU6Hr1G000090	darkorange
HORVU3Hr1G035840	darkorange
HORVU1Hr1G010790	darkorange
HORVU7Hr1G006720	darkorange
HORVU6Hr1G013530	darkorange
HORVU2Hr1G039960	darkorange
Hordeum_vulgare_newGene_8143	darkorange
HORVU7Hr1G089280	darkorange
HORVU6Hr1G033670	darkorange
HORVU2Hr1G115600	darkorange
HORVU2Hr1G104880	darkorange
HORVU2Hr1G036840	darkorange
HORVU3Hr1G056430	darkorange
HORVU7Hr1G042180	darkorange
HORVU7Hr1G059130	darkorange
HORVU7Hr1G101500	darkorange
HORVU1Hr1G078350	darkorange
HORVU5Hr1G059540	darkorange
HORVU4Hr1G048440	darkorange
HORVU5Hr1G013210	darkorange
HORVU6Hr1G085500	darkorange
HORVU4Hr1G006530	darkorange
HORVU5Hr1G018830	darkorange

HORVU1Hr1G089570	darkorange
HORVU5Hr1G071060	darkorange
HORVU5Hr1G121600	darkorange
HORVU3Hr1G031460	darkorange
HORVU0Hr1G024790	darkorange
HORVU2Hr1G096130	darkorange
HORVU6Hr1G035250	darkorange
HORVU5Hr1G073010	darkorange
Hordeum_vulgare_newGene_13859	darkorange
HORVU7Hr1G105910	darkorange
HORVU2Hr1G090200	darkorange
HORVU5Hr1G015700	darkorange
HORVU2Hr1G034540	darkorange
HORVU7Hr1G050980	darkorange
HORVU7Hr1G073300	darkorange
HORVU2Hr1G114140	darkorange
HORVU7Hr1G021170	darkorange
Hordeum_vulgare_newGene_7182	darkorange
HORVU7Hr1G043300	darkorange
HORVU0Hr1G014520	darkorange
Hordeum_vulgare_newGene_12661	darkorange
HORVU7Hr1G060130	darkorange
HORVU0Hr1G008580	darkorange
HORVU5Hr1G015990	darkorange
HORVU6Hr1G029950	darkorange
Hordeum_vulgare_newGene_6460	darkorange
HORVU1Hr1G073050	darkorange
HORVU3Hr1G047230	darkorange
HORVU5Hr1G111050	darkorange
HORVU7Hr1G003010	darkorange
HORVU4Hr1G049920	darkorange
HORVU7Hr1G110810	darkorange
HORVU7Hr1G040390	darkorange
HORVU1Hr1G081730	darkorange
HORVU2Hr1G057700	darkorange
HORVU5Hr1G016780	darkorange
HORVU2Hr1G109020	darkorange
HORVU7Hr1G080020	darkorange
HORVU3Hr1G088700	darkorange
HORVU3Hr1G037470	darkorange
HORVU7Hr1G050140	darkorange
HORVU4Hr1G053130	darkorange
HORVU5Hr1G109710	darkorange
Hordeum_vulgare_newGene_5867	darkorange
Hordeum_vulgare_newGene_5289	darkorange
HORVU0Hr1G026710	darkorange
HORVU1Hr1G014230	darkorange

HORVU2Hr1G028470	darkorange
Hordeum_vulgare_newGene_3853	darkorange
HORVU4Hr1G013370	darkorange
HORVU5Hr1G075270	darkorange
HORVU1Hr1G026990	darkorange
HORVU2Hr1G043940	darkorange
HORVU5Hr1G039960	darkorange
HORVU5Hr1G106730	darkorange
HORVU3Hr1G079260	darkorange
HORVU0Hr1G017370	darkorange
Hordeum_vulgare_newGene_1066	darkorange
HORVU3Hr1G054240	darkorange
Hordeum_vulgare_newGene_1064	darkorange
HORVU2Hr1G120610	darkorange
HORVU7Hr1G017620	darkorange
HORVU4Hr1G064580	darkorange
Hordeum_vulgare_newGene_6964	darkorange
HORVU2Hr1G100720	darkorange
HORVU7Hr1G110130	darkorange
HORVU0Hr1G015840	darkorange
HORVU4Hr1G084830	darkorange
HORVU1Hr1G039670	darkorange
HORVU2Hr1G001600	darkorange
HORVU2Hr1G090490	darkorange
HORVU5Hr1G044480	darkorange
HORVU2Hr1G011490	darkorange
HORVU3Hr1G077250	darkorange
HORVU5Hr1G016060	darkorange
HORVU1Hr1G042800	darkorange
HORVU6Hr1G062040	darkorange
Hordeum_vulgare_newGene_3389	darkorange
HORVU3Hr1G116550	darkorange
Hordeum_vulgare_newGene_2382	darkorange
Hordeum_vulgare_newGene_2381	darkorange
Hordeum_vulgare_newGene_6866	darkorange
HORVU4Hr1G075140	darkorange
HORVU0Hr1G023970	darkorange
HORVU3Hr1G042600	darkorange
Hordeum_vulgare_newGene_6471	darkorange
Hordeum_vulgare_newGene_6472	darkorange
HORVU4Hr1G042710	darkorange
HORVU5Hr1G106960	darkorange
HORVU2Hr1G085570	darkorange
HORVU5Hr1G045640	darkorange
Hordeum_vulgare_newGene_8620	darkorange
HORVU5Hr1G081520	darkorange
HORVU2Hr1G091880	darkorange

HORVU3Hr1G104790	darkorange
Hordeum_vulgare_newGene_12376	darkorange
HORVU2Hr1G069950	darkorange
HORVU2Hr1G039190	darkorange
Hordeum_vulgare_newGene_12594	darkorange
HORVU0Hr1G003280	darkorange
HORVU3Hr1G051330	darkorange
HORVU5Hr1G045860	darkorange
HORVU0Hr1G039040	darkorange
HORVU2Hr1G035170	darkorange
HORVU7Hr1G098640	darkorange
Hordeum_vulgare_newGene_15327	darkorange
HORVU7Hr1G025150	darkorange
HORVU7Hr1G018230	darkorange
HORVU5Hr1G093260	darkorange
HORVU3Hr1G078200	darkorange
HORVU6Hr1G091660	darkorange
HORVU4Hr1G062040	darkorange
HORVU0Hr1G014400	darkorange
HORVU2Hr1G096070	darkorange
HORVU6Hr1G056140	darkorange
HORVU2Hr1G036440	darkorange
HORVU7Hr1G003840	darkorange
HORVU5Hr1G053320	darkorange
HORVU5Hr1G074880	darkorange
HORVU5Hr1G120250	darkorange
HORVU5Hr1G049980	darkorange
HORVU7Hr1G059460	darkorange
HORVU7Hr1G027370	darkorange
Hordeum_vulgare_newGene_1453	darkorange
Hordeum_vulgare_newGene_10714	darkorange
HORVU2Hr1G039400	darkorange
HORVU6Hr1G071960	darkorange
HORVU7Hr1G057390	darkorange
HORVU1Hr1G072250	darkorange
HORVU3Hr1G090190	darkorange
HORVU6Hr1G058790	darkorange
HORVU1Hr1G021930	darkorange
HORVU2Hr1G094650	darkorange
HORVU1Hr1G079130	darkorange
HORVU3Hr1G014510	darkorange
HORVU1Hr1G058490	darkorange
HORVU0Hr1G008960	darkorange
HORVU4Hr1G001770	darkorange
HORVU3Hr1G073670	darkorange
HORVU2Hr1G041480	darkorange
HORVU4Hr1G023490	darkorange

HORVU5Hr1G096800	darkorange
HORVU3Hr1G093990	darkorange
HORVU2Hr1G097990	darkorange
HORVU3Hr1G075090	darkorange
HORVU5Hr1G050990	darkorange
Hordeum_vulgare_newGene_13702	darkorange
HORVU3Hr1G086190	darkorange
Hordeum_vulgare_newGene_12705	darkorange
Hordeum_vulgare_newGene_12704	darkorange
HORVU3Hr1G090970	darkorange
HORVU7Hr1G077780	darkorange
HORVU7Hr1G078320	darkorange
HORVU7Hr1G073440	darkorange
Hordeum_vulgare_newGene_11348	darkorange
HORVU4Hr1G067780	darkorange
HORVU4Hr1G067600	darkorange
Hordeum_vulgare_newGene_1799	darkorange
Hordeum_vulgare_newGene_1798	darkorange
HORVU7Hr1G021910	darkorange
HORVU3Hr1G013360	darkorange
HORVU6Hr1G026800	darkorange
HORVU5Hr1G032650	darkorange
HORVU2Hr1G088900	darkorange
Hordeum_vulgare_newGene_14585	darkorange
HORVU7Hr1G107010	darkorange
HORVU3Hr1G055260	darkorange
HORVU6Hr1G087820	darkorange
HORVU2Hr1G043860	darkorange
HORVU2Hr1G005630	darkorange
HORVU1Hr1G020490	darkorange
HORVU0Hr1G018670	darkorange
HORVU2Hr1G098340	darkorange
HORVU7Hr1G036540	darkorange
HORVU7Hr1G049390	darkorange
HORVU4Hr1G052880	darkorange
HORVU1Hr1G057290	darkorange
HORVU2Hr1G014240	darkorange
HORVU1Hr1G064700	darkorange
HORVU7Hr1G094270	darkorange
HORVU0Hr1G001690	darkorange
HORVU2Hr1G077230	darkorange
HORVU2Hr1G115960	darkorange
HORVU4Hr1G025420	darkorange
Hordeum_vulgare_newGene_11187	darkorange
HORVU0Hr1G023910	darkorange
HORVU5Hr1G046520	darkorange
HORVU6Hr1G001270	darkorange

HORVU7Hr1G038710	darkorange
Hordeum_vulgare_newGene_14219	darkorange
HORVU5Hr1G098110	darkorange
HORVU0Hr1G032300	darkorange
HORVU1Hr1G077020	darkorange
HORVU5Hr1G091420	darkorange
HORVU7Hr1G021700	darkorange
HORVU2Hr1G105750	darkorange
HORVU2Hr1G042490	darkorange
HORVU5Hr1G105980	darkorange
HORVU4Hr1G073340	darkorange
HORVU2Hr1G089970	darkorange
HORVU4Hr1G072960	darkorange
HORVU7Hr1G053560	darkorange
HORVU7Hr1G099950	darkorange
HORVU2Hr1G125360	darkorange
HORVU7Hr1G052210	darkorange
HORVU3Hr1G032820	darkorange
HORVU2Hr1G024570	darkorange
HORVU5Hr1G055560	darkorange
HORVU2Hr1G080630	darkorange
HORVU5Hr1G119790	darkorange
HORVU1Hr1G048360	darkorange
HORVU7Hr1G022510	darkorange
HORVU2Hr1G053620	darkorange
HORVU5Hr1G104390	darkorange
HORVU2Hr1G091270	darkorange
HORVU2Hr1G093680	darkorange
HORVU1Hr1G056280	darkorange
HORVU2Hr1G036110	darkorange
HORVU5Hr1G027740	darkorange
HORVU7Hr1G083910	darkorange
HORVU4Hr1G055240	darkorange
HORVU5Hr1G053010	darkorange
HORVU7Hr1G043600	darkorange
HORVU3Hr1G005830	darkorange
HORVU5Hr1G045450	darkorange
HORVU0Hr1G035530	darkorange
Hordeum_vulgare_newGene_5189	darkorange
HORVU2Hr1G111680	darkorange
HORVU5Hr1G046480	darkorange
HORVU5Hr1G043470	darkorange
HORVU2Hr1G022920	darkorange
HORVU3Hr1G116790	darkorange
HORVU1Hr1G023260	darkorange
Hordeum_vulgare_newGene_6041	darkorange
Hordeum_vulgare_newGene_7936	darkorange

HORVU5Hr1G075810	darkorange
Hordeum_vulgare_newGene_3727	darkorange
Hordeum_vulgare_newGene_5893	darkorange
HORVU7Hr1G070080	darkorange
HORVU2Hr1G033470	darkorange
HORVU3Hr1G081940	darkorange
HORVU6Hr1G094680	darkorange
HORVU5Hr1G060480	darkorange
HORVU1Hr1G056510	darkorange
HORVU3Hr1G034860	darkorange
HORVU3Hr1G031980	darkorange
HORVU7Hr1G052530	darkorange
HORVU4Hr1G081220	darkorange
HORVU6Hr1G029220	darkorange
HORVU3Hr1G097830	darkorange
HORVU6Hr1G038700	darkorange
HORVU1Hr1G026920	darkorange
HORVU7Hr1G120190	darkorange
HORVU4Hr1G014110	darkorange
HORVU2Hr1G047260	darkorange
Hordeum_vulgare_newGene_7955	darkorange
HORVU1Hr1G036200	darkorange
HORVU5Hr1G086760	darkorange
HORVU3Hr1G094820	darkorange
HORVU6Hr1G054050	darkorange
Hordeum_vulgare_newGene_322	darkorange
HORVU3Hr1G060040	darkorange
HORVU1Hr1G029770	darkorange
HORVU1Hr1G071190	darkorange
HORVU2Hr1G048870	darkorange
HORVU7Hr1G054360	darkorange
HORVU6Hr1G053310	darkorange
Hordeum_vulgare_newGene_15361	darkorange
HORVU2Hr1G085740	darkorange
HORVU7Hr1G106020	darkorange
Hordeum_vulgare_newGene_1051	darkorange
HORVU7Hr1G101550	darkorange
HORVU0Hr1G029320	darkorange
HORVU7Hr1G085660	darkorange
HORVU1Hr1G078380	darkorange
HORVU3Hr1G039130	darkorange
HORVU7Hr1G019780	darkorange
HORVU7Hr1G079210	darkorange
HORVU7Hr1G012630	darkorange
HORVU6Hr1G046420	darkorange
HORVU6Hr1G034050	darkorange
HORVU6Hr1G093190	darkorange

HORVU2Hr1G111570	darkorange
HORVU6Hr1G090080	darkorange
HORVU3Hr1G023060	darkorange
HORVU3Hr1G027760	darkorange
HORVU2Hr1G018380	darkorange
HORVU7Hr1G047690	darkorange
HORVU2Hr1G039880	darkorange
HORVU5Hr1G026050	darkorange
HORVU2Hr1G090960	darkorange
Hordeum_vulgare_newGene_9143	darkorange
HORVU7Hr1G043590	darkorange
HORVU6Hr1G025780	darkorange
HORVU6Hr1G066140	darkorange
HORVU5Hr1G101680	darkorange
HORVU3Hr1G014850	darkorange
HORVU3Hr1G087970	darkorange
HORVU2Hr1G065170	darkorange
HORVU6Hr1G027390	darkorange
HORVU7Hr1G054190	darkorange
HORVU2Hr1G006720	darkorange
HORVU3Hr1G032200	darkorange
HORVU7Hr1G117180	darkorange
HORVU1Hr1G050450	darkorange
HORVU6Hr1G060100	darkorange
HORVU3Hr1G015730	darkorange
HORVU0Hr1G017200	darkorange
HORVU5Hr1G114090	darkorange
HORVU3Hr1G009240	darkorange
HORVU1Hr1G056530	darkorange
HORVU6Hr1G032050	darkorange
HORVU2Hr1G063820	darkorange
HORVU5Hr1G058630	darkorange
HORVU2Hr1G039250	darkorange
HORVU2Hr1G102930	darkorange
HORVU7Hr1G109370	darkorange
Hordeum_vulgare_newGene_456	darkorange
HORVU2Hr1G019820	darkorange
Hordeum_vulgare_newGene_10818	darkorange
HORVU6Hr1G018670	darkorange
Hordeum_vulgare_newGene_15281	darkorange
HORVU7Hr1G029900	darkorange
Hordeum_vulgare_newGene_1221	darkorange
Hordeum_vulgare_newGene_1220	darkorange
HORVU3Hr1G012850	darkorange
Hordeum_vulgare_newGene_1485	darkorange
HORVU2Hr1G029350	darkorange
HORVU0Hr1G001170	darkorange

HORVU2Hr1G039320	darkorange
HORVU5Hr1G019030	darkorange
HORVU7Hr1G103870	darkorange
HORVU3Hr1G029750	darkorange
HORVU1Hr1G010020	darkorange
HORVU1Hr1G023100	darkorange
HORVU7Hr1G051750	darkorange
Hordeum_vulgare_newGene_3565	darkorange
HORVU2Hr1G094680	darkorange
HORVU1Hr1G052620	darkorange
HORVU4Hr1G065660	darkorange
HORVU1Hr1G043820	darkorange
HORVU4Hr1G009500	darkorange
HORVU4Hr1G004170	darkorange
Hordeum_vulgare_newGene_10341	darkorange
Hordeum_vulgare_newGene_10343	darkorange
HORVU3Hr1G114220	darkorange
Hordeum_vulgare_newGene_10698	darkorange
HORVU3Hr1G115170	darkorange
HORVU2Hr1G029110	darkorange
HORVU4Hr1G037160	darkorange
HORVU5Hr1G031870	darkorange
Hordeum_vulgare_newGene_6166	darkorange
HORVU5Hr1G028260	darkorange
HORVU7Hr1G043280	darkorange
Hordeum_vulgare_newGene_8471	darkorange
HORVU7Hr1G113650	darkorange
HORVU7Hr1G037140	darkorange
HORVU0Hr1G008640	darkorange
HORVU6Hr1G057550	darkorange
HORVU3Hr1G083990	darkorange
HORVU7Hr1G067110	darkorange
HORVU3Hr1G018550	darkorange
HORVU1Hr1G063700	darkorange
HORVU6Hr1G032220	darkorange
HORVU4Hr1G044910	darkorange
HORVU3Hr1G039930	darkorange
Hordeum_vulgare_newGene_13132	darkorange
HORVU7Hr1G025390	darkorange
HORVU3Hr1G054920	darkorange
HORVU5Hr1G124030	darkorange
HORVU6Hr1G030880	darkorange
Hordeum_vulgare_newGene_12945	darkorange
HORVU6Hr1G094460	darkorange
Hordeum_vulgare_newGene_12843	darkorange
HORVU5Hr1G095060	darkorange
HORVU7Hr1G045910	darkorange

HORVU5Hr1G078960	darkorange
HORVU4Hr1G061990	darkorange
HORVU6Hr1G014480	darkorange
HORVU5Hr1G002320	darkorange
HORVU1Hr1G010810	darkorange
Hordeum_vulgare_newGene_7155	darkorange
Hordeum_vulgare_newGene_7154	darkorange
HORVU6Hr1G095080	darkorange
HORVU5Hr1G102530	darkorange
HORVU4Hr1G074030	darkorange
HORVU7Hr1G096020	darkorange
HORVU6Hr1G030600	darkorange
Hordeum_vulgare_newGene_1645	darkorange
HORVU1Hr1G072750	darkorange
HORVU1Hr1G080840	darkorange
HORVU1Hr1G065130	darkorange
HORVU5Hr1G008770	darkorange
HORVU2Hr1G067290	darkorange
HORVU2Hr1G061600	darkorange
HORVU7Hr1G074890	darkorange
HORVU3Hr1G089000	darkorange
HORVU3Hr1G101990	darkorange
Hordeum_vulgare_newGene_9064	darkorange
Hordeum_vulgare_newGene_15207	darkorange
HORVU7Hr1G089540	darkorange
HORVU4Hr1G000520	darkorange
HORVU2Hr1G023560	darkorange
HORVU2Hr1G043170	darkorange
HORVU0Hr1G020960	darkorange
Hordeum_vulgare_newGene_3801	darkorange
Hordeum_vulgare_newGene_3800	darkorange
HORVU7Hr1G120960	darkorange
HORVU1Hr1G075160	darkorange
HORVU2Hr1G045630	darkorange
HORVU5Hr1G014730	darkorange
HORVU1Hr1G092540	darkorange
HORVU3Hr1G085270	darkorange
Hordeum_vulgare_newGene_6672	darkorange
HORVU4Hr1G013320	darkorange
HORVU1Hr1G026650	darkorange
Hordeum_vulgare_newGene_3118	darkorange
Hordeum_vulgare_newGene_6093	darkorange
Hordeum_vulgare_newGene_14617	darkorange
HORVU7Hr1G038200	darkorange
HORVU5Hr1G069040	darkorange
HORVU6Hr1G072580	darkorange
HORVU3Hr1G082550	darkorange

HORVU3Hr1G032090	darkorange
HORVU4Hr1G080760	darkorange
HORVU2Hr1G077510	darkorange
HORVU5Hr1G020410	darkorange
HORVU7Hr1G014410	darkorange
HORVU2Hr1G088400	darkorange
HORVU3Hr1G009360	darkorange
HORVU3Hr1G108030	darkorange
HORVU5Hr1G009200	darkorange
HORVU5Hr1G047530	darkorange
HORVU1Hr1G090210	darkorange
HORVU1Hr1G012870	darkorange
HORVU4Hr1G025590	darkorange
HORVU2Hr1G108260	darkorange
HORVU7Hr1G012920	darkorange
HORVU2Hr1G016990	darkorange
HORVU1Hr1G039250	darkorange
HORVU2Hr1G102300	darkorange
HORVU5Hr1G045820	darkorange
HORVU1Hr1G085230	darkorange
HORVU5Hr1G100700	darkorange
HORVU3Hr1G069410	darkorange
Hordeum_vulgare_newGene_13080	darkorange
HORVU6Hr1G009350	darkorange
HORVU7Hr1G077750	darkorange
HORVU4Hr1G058810	darkorange
HORVU1Hr1G094230	darkorange
HORVU2Hr1G096250	darkorange
HORVU5Hr1G080110	darkorange
Hordeum_vulgare_newGene_2559	darkorange
Hordeum_vulgare_newGene_9569	darkorange
Hordeum_vulgare_newGene_13751	darkorange
HORVU7Hr1G063430	darkorange
Hordeum_vulgare_newGene_8216	darkorange
HORVU3Hr1G085400	darkorange
HORVU7Hr1G056570	darkorange
HORVU5Hr1G059840	darkorange
HORVU4Hr1G011910	darkorange
HORVU1Hr1G073190	darkorange
HORVU7Hr1G108370	darkorange
Hordeum_vulgare_newGene_9985	darkorange
HORVU4Hr1G087760	darkorange
HORVU1Hr1G066960	darkorange
Hordeum_vulgare_newGene_10267	darkorange
HORVU7Hr1G093260	darkorange
HORVU2Hr1G056510	darkorange
HORVU7Hr1G010680	darkorange

HORVU7Hr1G077170	darkorange
HORVU2Hr1G063910	darkorange
HORVU2Hr1G022590	darkorange
HORVU6Hr1G015130	darkorange
HORVU5Hr1G070930	darkorange
HORVU2Hr1G013630	darkorange
HORVU7Hr1G062930	darkorange
HORVU4Hr1G021500	darkorange
HORVU7Hr1G029120	darkorange
Hordeum_vulgare_newGene_5234	darkorange
HORVU2Hr1G058800	darkorange
HORVU5Hr1G015550	darkorange
HORVU7Hr1G027430	darkorange
HORVU2Hr1G046740	darkorange
HORVU5Hr1G022550	darkorange
HORVU0Hr1G022830	darkorange
HORVU4Hr1G063050	darkorange
HORVU3Hr1G082930	darkorange
HORVU2Hr1G056280	darkorange
HORVU5Hr1G016400	darkorange
HORVU4Hr1G058940	darkorange
HORVU6Hr1G085170	darkorange
HORVU4Hr1G027970	darkorange
HORVU2Hr1G103550	darkorange
HORVU4Hr1G019360	darkorange
HORVU6Hr1G006880	darkorange
HORVU7Hr1G094010	darkorange
HORVU2Hr1G013870	darkorange
HORVU2Hr1G010990	darkorange
HORVU3Hr1G114350	darkorange
Hordeum_vulgare_newGene_5275	darkorange
HORVU2Hr1G097080	darkorange
HORVU6Hr1G016890	darkorange
HORVU6Hr1G024860	darkorange
HORVU0Hr1G004830	darkorange
HORVU2Hr1G073850	darkorange
HORVU1Hr1G020070	darkorange
Hordeum_vulgare_newGene_970	darkorange
HORVU1Hr1G062270	darkorange
HORVU7Hr1G049260	darkorange
Hordeum_vulgare_newGene_1296	darkorange
HORVU5Hr1G069620	darkorange
HORVU1Hr1G041770	darkorange
HORVU7Hr1G113270	darkorange
HORVU6Hr1G089190	darkorange
Hordeum_vulgare_newGene_4575	darkorange
HORVU0Hr1G031760	darkorange

HORVU7Hr1G110720	darkorange
HORVU7Hr1G055570	darkorange
HORVU5Hr1G095410	darkorange
HORVU3Hr1G006150	darkorange
HORVU2Hr1G077830	darkorange
HORVU3Hr1G034520	darkorange
HORVU6Hr1G029210	darkorange
HORVU1Hr1G027000	darkorange
HORVU2Hr1G040780	darkorange
HORVU3Hr1G092420	darkorange
HORVU7Hr1G048670	darkorange
HORVU2Hr1G094360	darkorange
HORVU1Hr1G039830	darkorange
HORVU1Hr1G068850	darkorange
HORVU6Hr1G091510	darkorange
HORVU6Hr1G055090	darkorange
HORVU7Hr1G076480	darkorange
HORVU3Hr1G059230	darkorange
HORVU5Hr1G053230	darkorange
HORVU2Hr1G060440	darkorange
HORVU3Hr1G083770	darkorange
HORVU2Hr1G072500	darkorange
HORVU2Hr1G023480	darkorange
Hordeum_vulgare_newGene_13601	darkorange
HORVU4Hr1G001590	darkorange
HORVU4Hr1G063900	darkorange
HORVU7Hr1G073190	darkorange
HORVU7Hr1G021050	darkorange
Hordeum_vulgare_newGene_13766	darkorange
HORVU6Hr1G078780	darkorange
HORVU3Hr1G048610	darkorange
HORVU5Hr1G075490	darkorange
HORVU5Hr1G079770	darkorange
HORVU1Hr1G061910	darkorange
HORVU4Hr1G008800	darkorange
HORVU0Hr1G019570	darkorange
HORVU6Hr1G007360	darkorange
HORVU1Hr1G012930	darkorange
HORVU2Hr1G041080	darkorange
HORVU6Hr1G006100	darkorange
Hordeum_vulgare_newGene_11133	darkorange
HORVU7Hr1G012600	darkorange
HORVU3Hr1G026840	darkorange
HORVU2Hr1G111540	darkorange
HORVU5Hr1G076380	darkorange
HORVU4Hr1G061070	darkorange
HORVU7Hr1G102480	darkorange

Hordeum_vulgare_newGene_9441	darkorange
HORVU2Hr1G069600	darkorange
HORVU2Hr1G038030	darkorange
HORVU3Hr1G105820	darkorange
HORVU0Hr1G026630	darkorange
HORVU7Hr1G059290	darkorange
HORVU2Hr1G105780	darkorange
Hordeum_vulgare_newGene_4925	darkorange
HORVU2Hr1G088050	darkorange
HORVU3Hr1G088160	darkorange
HORVU6Hr1G087220	darkorange
HORVU5Hr1G084030	darkorange
Hordeum_vulgare_newGene_9830	darkorange
HORVU1Hr1G012950	darkorange
HORVU6Hr1G035190	darkorange
HORVU1Hr1G048290	darkorange
HORVU2Hr1G098290	darkorange
HORVU7Hr1G114400	darkorange
HORVU2Hr1G085690	darkorange
HORVU1Hr1G012460	darkorange
HORVU5Hr1G067010	darkorange
HORVU1Hr1G093880	darkorange
HORVU3Hr1G047290	darkorange
HORVU3Hr1G009210	darkorange
HORVU4Hr1G038570	darkorange
HORVU5Hr1G121860	darkorange
HORVU0Hr1G007170	darkorange
HORVU5Hr1G057320	darkorange
HORVU7Hr1G116470	darkorange
HORVU2Hr1G041270	darkorange
HORVU4Hr1G060630	darkorange
HORVU5Hr1G079680	darkorange
HORVU3Hr1G023460	darkorange
HORVU2Hr1G114980	darkorange
HORVU3Hr1G027460	darkorange
HORVU5Hr1G032370	darkorange
Hordeum_vulgare_newGene_2792	darkorange
HORVU1Hr1G007480	darkorange
HORVU4Hr1G074840	darkorange
Hordeum_vulgare_newGene_1218	darkorange
Hordeum_vulgare_newGene_1219	darkorange
Hordeum_vulgare_newGene_1217	darkorange
Hordeum_vulgare_newGene_11825	darkorange
HORVU6Hr1G063860	darkorange
HORVU4Hr1G088350	darkorange
HORVU7Hr1G101710	darkorange
HORVU3Hr1G070080	darkorange

HORVU4Hr1G081290	darkorange
HORVU1Hr1G080640	darkorange
HORVU6Hr1G067930	darkorange
HORVU4Hr1G065180	darkorange
HORVU4Hr1G017210	darkorange
HORVU7Hr1G051720	darkorange
HORVU2Hr1G057210	darkorange
HORVU2Hr1G127360	darkorange
HORVU1Hr1G085450	darkorange
Hordeum_vulgare_newGene_12935	darkorange
HORVU2Hr1G061260	darkorange
HORVU6Hr1G011020	darkorange
HORVU7Hr1G110190	darkorange
HORVU1Hr1G057330	darkorange
HORVU0Hr1G006750	darkorange
HORVU5Hr1G046250	darkorange
HORVU7Hr1G019510	darkorange
HORVU3Hr1G100500	darkorange
HORVU7Hr1G006740	darkorange
HORVU7Hr1G002080	darkorange
HORVU2Hr1G092100	darkorange
HORVU3Hr1G072380	darkorange
HORVU1Hr1G060610	darkorange
HORVU4Hr1G079230	darkorange
HORVU3Hr1G058810	darkorange
HORVU3Hr1G097750	darkorange
Hordeum_vulgare_newGene_3091	darkorange
HORVU3Hr1G087550	darkorange
Hordeum_vulgare_newGene_7683	darkorange
HORVU1Hr1G008160	darkorange
HORVU1Hr1G070290	darkorange
HORVU2Hr1G059360	darkorange
HORVU2Hr1G119050	darkorange
HORVU5Hr1G035350	darkorange
HORVU2Hr1G104270	darkorange
Hordeum_vulgare_newGene_1003	darkorange
HORVU3Hr1G014080	darkorange
HORVU5Hr1G015530	darkorange
HORVU7Hr1G079790	darkorange
HORVU2Hr1G041000	darkorange
HORVU4Hr1G050250	darkorange
HORVU1Hr1G027120	darkorange
HORVU4Hr1G052090	darkorange
HORVU5Hr1G009620	darkorange
HORVU5Hr1G112750	darkorange
HORVU1Hr1G081030	darkorange
HORVU5Hr1G082940	darkorange

HORVU6Hr1G011490	darkorange
HORVU7Hr1G093940	darkorange
HORVU6Hr1G010540	darkorange
Hordeum_vulgare_newGene_12878	darkorange
HORVU0Hr1G014540	darkorange
Hordeum_vulgare_newGene_8640	darkorange
HORVU1Hr1G022900	darkorange
HORVU4Hr1G067650	darkorange
HORVU4Hr1G048510	darkorange
HORVU7Hr1G052270	darkorange
HORVU6Hr1G060810	darkorange
HORVU3Hr1G011690	darkorange
Hordeum_vulgare_newGene_7165	darkorange
Hordeum_vulgare_newGene_7166	darkorange
Hordeum_vulgare_newGene_7167	darkorange
HORVU7Hr1G039710	darkorange
HORVU5Hr1G084520	darkorange
HORVU7Hr1G008470	darkorange
HORVU6Hr1G082260	darkorange
HORVU5Hr1G074000	darkorange
HORVU7Hr1G058940	darkorange
HORVU0Hr1G003260	darkorange
HORVU7Hr1G023140	darkorange
HORVU7Hr1G090950	darkorange
HORVU5Hr1G026650	darkorange
HORVU4Hr1G080610	darkorange
HORVU7Hr1G106130	darkorange
HORVU3Hr1G014780	darkorange
HORVU5Hr1G062310	darkorange
HORVU3Hr1G000720	darkorange
HORVU2Hr1G001430	darkorange
HORVU1Hr1G090050	darkorange
HORVU5Hr1G113880	darkorange
HORVU1Hr1G051700	darkorange
HORVU5Hr1G086470	darkorange
HORVU7Hr1G045290	darkorange
HORVU5Hr1G007050	darkorange
HORVU5Hr1G109250	darkorange
HORVU6Hr1G090370	darkorange
HORVU6Hr1G011510	darkorange
HORVU6Hr1G054430	darkorange
HORVU4Hr1G067180	darkorange
Hordeum_vulgare_newGene_1990	darkorange
HORVU1Hr1G042360	darkorange
HORVU7Hr1G057470	darkorange
HORVU2Hr1G036280	darkorange
HORVU2Hr1G092530	darkorange

HORVU4Hr1G064760	darkorange
Hordeum_vulgare_newGene_2710	darkorange
HORVU2Hr1G126740	darkorange
Hordeum_vulgare_newGene_15259	darkorange
Hordeum_vulgare_newGene_15256	darkorange
HORVU7Hr1G080850	darkorange
HORVU5Hr1G011140	darkorange
HORVU7Hr1G063220	darkorange
HORVU1Hr1G063330	darkorange
HORVU2Hr1G032730	darkorange
HORVU7Hr1G052690	darkorange
HORVU3Hr1G093080	darkorange
HORVU4Hr1G046810	darkorange
HORVU3Hr1G042540	darkorange
HORVU7Hr1G047500	darkorange
HORVU4Hr1G084370	darkorange
HORVU5Hr1G025430	darkorange
HORVU2Hr1G085800	darkorange
HORVU7Hr1G095820	darkorange
HORVU0Hr1G014110	darkorange
HORVU2Hr1G017450	darkorange
HORVU7Hr1G019590	darkorange
HORVU2Hr1G092180	darkorange
HORVU3Hr1G068780	darkorange
HORVU2Hr1G093580	darkorange
HORVU1Hr1G080200	darkorange
HORVU1Hr1G004480	darkorange
HORVU3Hr1G092390	darkorange
HORVU2Hr1G057880	darkorange
HORVU7Hr1G098320	darkorange
HORVU7Hr1G042160	darkorange
HORVU5Hr1G080870	darkorange
HORVU7Hr1G070150	darkorange
HORVU0Hr1G021760	darkorange
HORVU4Hr1G058840	darkorange
HORVU3Hr1G110160	darkorange
HORVU6Hr1G059910	darkorange
HORVU7Hr1G096620	darkorange
HORVU2Hr1G077120	darkorange
HORVU2Hr1G031990	darkorange
HORVU1Hr1G000040	darkorange
HORVU3Hr1G026540	darkorange
HORVU0Hr1G038120	darkorange
Hordeum_vulgare_newGene_6771	darkorange
HORVU4Hr1G072150	darkorange
HORVU5Hr1G015600	darkorange
HORVU1Hr1G075560	darkorange

Hordeum_vulgare_newGene_15883	darkorange
HORVU1Hr1G074380	darkorange
HORVU2Hr1G000040	darkorange
HORVU6Hr1G084650	darkorange
HORVU1Hr1G023270	darkorange
HORVU3Hr1G023740	darkorange
HORVU5Hr1G109680	darkorange
HORVU7Hr1G076030	darkorange
HORVU3Hr1G026150	darkorange
HORVU7Hr1G110090	darkorange
HORVU2Hr1G061090	darkorange
HORVU5Hr1G121910	darkorange
HORVU4Hr1G007620	darkorange
HORVU6Hr1G040960	darkorange
HORVU2Hr1G048100	darkorange
HORVU2Hr1G073370	darkorange
HORVU2Hr1G022090	darkorange
Hordeum_vulgare_newGene_1862	darkorange
Hordeum_vulgare_newGene_5279	darkorange
HORVU3Hr1G010070	darkorange
HORVU1Hr1G046400	darkorange
HORVU2Hr1G072960	darkorange
HORVU5Hr1G072650	darkorange
HORVU7Hr1G030540	darkorange
HORVU7Hr1G038770	darkorange
HORVU1Hr1G085050	darkorange
HORVU2Hr1G013390	darkorange
Hordeum_vulgare_newGene_13405	darkorange
HORVU6Hr1G051790	darkorange
HORVU2Hr1G092510	darkorange
Hordeum_vulgare_newGene_15764	darkorange
HORVU6Hr1G065360	darkorange
Hordeum_vulgare_newGene_11659	darkorange
HORVU5Hr1G010370	darkorange
HORVU2Hr1G045970	darkorange
HORVU5Hr1G061120	darkorange
HORVU6Hr1G072950	darkorange
HORVU2Hr1G010440	darkorange
HORVU3Hr1G022620	darkorange
HORVU2Hr1G047410	darkorange
HORVU1Hr1G084520	darkorange
HORVU3Hr1G106210	darkorange
HORVU1Hr1G052110	darkorange
HORVU6Hr1G000230	darkorange
HORVU7Hr1G095080	darkorange
HORVU7Hr1G088880	darkorange
HORVU4Hr1G084410	darkorange

HORVU5Hr1G105060	darkorange
HORVU5Hr1G119040	darkorange
HORVU0Hr1G020130	darkorange
HORVU5Hr1G045290	darkorange
HORVU7Hr1G094060	darkorange
HORVU5Hr1G017530	darkorange
HORVU2Hr1G071100	darkorange
HORVU7Hr1G082590	darkorange
HORVU2Hr1G099590	darkorange
HORVU7Hr1G064770	darkorange
Hordeum_vulgare_newGene_5225	darkorange
Hordeum_vulgare_newGene_5224	darkorange
HORVU1Hr1G017830	darkorange
HORVU3Hr1G089880	darkorange
Hordeum_vulgare_newGene_13381	darkorange
HORVU4Hr1G052070	darkorange
HORVU7Hr1G086690	darkorange
HORVU4Hr1G062920	darkorange
HORVU5Hr1G100150	darkorange
HORVU2Hr1G021580	darkorange
HORVU3Hr1G032350	darkorange
HORVU6Hr1G071190	darkorange
HORVU4Hr1G075160	darkorange
HORVU2Hr1G086220	darkorange
HORVU2Hr1G094670	darkorange
HORVU5Hr1G051010	darkorange
HORVU2Hr1G013400	darkorange
HORVU2Hr1G080590	darkorange
HORVU7Hr1G085120	darkorange
HORVU3Hr1G034510	darkorange
HORVU1Hr1G026320	darkorange
HORVU6Hr1G080530	darkorange
Hordeum_vulgare_newGene_1132	darkorange
HORVU3Hr1G062250	darkorange
HORVU7Hr1G090880	darkorange
HORVU4Hr1G082600	darkorange
HORVU5Hr1G109440	darkorange
HORVU4Hr1G014460	darkorange
HORVU7Hr1G084310	darkorange
HORVU3Hr1G089260	darkorange
Hordeum_vulgare_newGene_3323	darkorange
Hordeum_vulgare_newGene_15062	darkorange
HORVU2Hr1G101730	darkorange
Hordeum_vulgare_newGene_2587	darkorange
HORVU2Hr1G108010	darkorange
Hordeum_vulgare_newGene_11981	darkorange
Hordeum_vulgare_newGene_11985	darkorange

Hordeum_vulgare_newGene_11363	darkorange
HORVU1Hr1G005870	darkorange
HORVU3Hr1G091140	darkorange
HORVU2Hr1G034630	darkorange
HORVU7Hr1G063440	darkorange
HORVU7Hr1G079380	darkorange
HORVU3Hr1G011760	darkorange
Hordeum_vulgare_newGene_12047	darkorange
Hordeum_vulgare_newGene_15892	darkorange
Hordeum_vulgare_newGene_15891	darkorange
HORVU1Hr1G026520	darkorange
HORVU6Hr1G082060	darkorange
HORVU1Hr1G070220	darkorange
HORVU2Hr1G024940	darkorange
HORVU4Hr1G017390	darkorange
HORVU3Hr1G082730	darkorange
HORVU2Hr1G098320	darkorange
HORVU7Hr1G106040	darkorange
HORVU5Hr1G114200	darkorange
HORVU7Hr1G021660	darkorange
HORVU3Hr1G014320	darkorange
Hordeum_vulgare_newGene_6089	darkorange
HORVU0Hr1G012710	darkorange
HORVU5Hr1G058720	darkorange
HORVU3Hr1G029880	darkorange
HORVU2Hr1G099490	darkorange
HORVU3Hr1G071530	darkorange
HORVU0Hr1G010660	darkorange
Hordeum_vulgare_newGene_14199	darkorange
HORVU1Hr1G012960	darkorange
Hordeum_vulgare_newGene_11165	darkorange
HORVU0Hr1G023930	darkorange
HORVU7Hr1G087440	darkorange
HORVU3Hr1G108390	darkorange
HORVU5Hr1G016120	darkorange
HORVU5Hr1G098960	darkorange
HORVU3Hr1G063710	darkorange
HORVU0Hr1G021390	darkorange
Hordeum_vulgare_newGene_776	darkorange
HORVU3Hr1G110330	darkorange
HORVU3Hr1G088990	darkorange
HORVU2Hr1G105770	darkorange
HORVU6Hr1G031580	darkorange
HORVU2Hr1G077630	darkorange
HORVU5Hr1G033060	darkorange
HORVU3Hr1G090000	darkorange
HORVU7Hr1G034020	darkorange

HORVU7Hr1G011810	darkorange
HORVU2Hr1G027020	darkorange
HORVU5Hr1G015900	darkorange
HORVU5Hr1G069690	darkorange
Hordeum_vulgare_newGene_4571	darkorange
HORVU3Hr1G024180	darkorange
HORVU4Hr1G088140	darkorange
HORVU5Hr1G104620	darkorange
HORVU5Hr1G074820	darkorange
HORVU7Hr1G000900	darkorange
HORVU0Hr1G023870	darkorange
HORVU7Hr1G077060	darkorange
HORVU2Hr1G060480	darkorange
HORVU3Hr1G022340	darkorange
HORVU6Hr1G090380	darkorange
HORVU0Hr1G024560	darkorange
Hordeum_vulgare_newGene_13567	darkorange
HORVU7Hr1G100400	darkorange
HORVU7Hr1G078760	darkorange
HORVU1Hr1G023120	darkorange
HORVU6Hr1G068730	darkorange
HORVU1Hr1G059180	darkorange
HORVU5Hr1G019730	darkorange
HORVU5Hr1G076910	darkorange
HORVU2Hr1G088180	darkorange
HORVU5Hr1G026140	darkorange
HORVU0Hr1G018910	darkorange
HORVU5Hr1G058050	darkorange
HORVU4Hr1G074460	darkorange
HORVU6Hr1G087000	darkorange
HORVU6Hr1G093030	darkorange
HORVU2Hr1G085210	darkorange
HORVU5Hr1G097150	darkorange
HORVU2Hr1G126000	darkorange
HORVU6Hr1G010700	darkorange
HORVU1Hr1G035130	darkorange
HORVU7Hr1G109260	darkorange
Hordeum_vulgare_newGene_12907	darkorange
Hordeum_vulgare_newGene_12906	darkorange
HORVU4Hr1G016940	darkorange
HORVU3Hr1G100570	darkorange
HORVU4Hr1G025570	darkorange
HORVU1Hr1G045610	darkorange
Hordeum_vulgare_newGene_8155	darkorange
HORVU2Hr1G007840	darkorange
HORVU3Hr1G059880	darkorange
HORVU2Hr1G021320	darkorange

HORVU7Hr1G020040	darkorange
HORVU7Hr1G021310	darkorange
HORVU4Hr1G025580	darkorange
Hordeum_vulgare_newGene_309	darkorange
HORVU3Hr1G105790	darkorange
HORVU3Hr1G097760	darkorange
Hordeum_vulgare_newGene_5582	darkorange
Hordeum_vulgare_newGene_2319	darkorange
HORVU2Hr1G079920	darkorange
Hordeum_vulgare_newGene_683	darkorange
HORVU7Hr1G034470	darkorange
HORVU5Hr1G070560	darkorange
HORVU3Hr1G023370	darkorange
HORVU6Hr1G020530	darkorange
HORVU5Hr1G059530	darkorange
HORVU5Hr1G056210	darkorange
HORVU5Hr1G013220	darkorange
HORVU5Hr1G041720	darkorange
HORVU5Hr1G086500	darkorange
HORVU2Hr1G048500	darkorange
HORVU5Hr1G018820	darkorange
HORVU5Hr1G017830	darkorange
HORVU3Hr1G024920	darkorange
HORVU5Hr1G121610	darkorange
HORVU4Hr1G026300	darkorange
HORVU7Hr1G003040	darkorange
Hordeum_vulgare_newGene_5310	darkorange
HORVU7Hr1G071020	darkorange
HORVU2Hr1G022820	darkorange
HORVU2Hr1G090210	darkorange
HORVU3Hr1G095300	darkorange
HORVU2Hr1G103880	darkorange
HORVU6Hr1G003160	darkorange
HORVU7Hr1G002050	darkorange
HORVU1Hr1G010670	darkorange
HORVU5Hr1G015980	darkorange
HORVU4Hr1G074530	darkorange
HORVU3Hr1G078880	darkorange
HORVU5Hr1G034770	darkorange
HORVU6Hr1G011620	darkorange
HORVU1Hr1G082790	darkorange
HORVU6Hr1G066400	darkorange
HORVU5Hr1G110710	darkorange
HORVU3Hr1G000770	darkorange
HORVU2Hr1G084590	darkorange
HORVU7Hr1G110280	darkorange
HORVU1Hr1G069470	darkorange

HORVU1Hr1G093350	darkorange
HORVU5Hr1G056390	darkorange
HORVU3Hr1G062490	darkorange
HORVU7Hr1G049860	darkorange
HORVU7Hr1G037690	darkorange
HORVU3Hr1G109760	darkorange
HORVU2Hr1G118670	darkorange
HORVU4Hr1G056740	darkorange
Hordeum_vulgare_newGene_14358	darkorange
HORVU7Hr1G002210	darkorange
HORVU4Hr1G058970	darkorange
HORVU3Hr1G055670	darkorange
HORVU7Hr1G043230	darkorange
HORVU1Hr1G044890	darkorange
Hordeum_vulgare_newGene_2479	darkorange
HORVU5Hr1G116590	darkorange
Hordeum_vulgare_newGene_10161	darkorange
Hordeum_vulgare_newGene_10160	darkorange
HORVU5Hr1G063470	darkorange
HORVU3Hr1G083830	darkorange
HORVU7Hr1G105990	darkorange
HORVU1Hr1G073510	darkorange
Hordeum_vulgare_newGene_16225	darkorange
HORVU1Hr1G071910	darkorange
HORVU5Hr1G067880	darkorange
Hordeum_vulgare_newGene_7389	darkorange
Hordeum_vulgare_newGene_7380	darkorange
HORVU7Hr1G056490	darkorange
HORVU1Hr1G044680	darkorange
HORVU6Hr1G074960	darkorange
HORVU3Hr1G013680	darkorange
HORVU6Hr1G065690	darkorange
HORVU5Hr1G023460	darkorange
HORVU2Hr1G040400	darkorange
HORVU7Hr1G037720	darkorange
HORVU6Hr1G011780	darkorange
HORVU4Hr1G090650	darkorange
HORVU2Hr1G081140	darkorange
HORVU3Hr1G084530	darkorange
HORVU4Hr1G009520	darkorange
Hordeum_vulgare_newGene_2917	darkorange
HORVU5Hr1G095250	darkorange
HORVU5Hr1G047550	darkorange
HORVU1Hr1G012890	darkorange
HORVU4Hr1G040520	darkorange
HORVU1Hr1G084120	darkorange
HORVU5Hr1G007630	darkorange

HORVU5Hr1G016050	darkorange
HORVU6Hr1G011990	darkorange
HORVU2Hr1G039000	darkorange
Hordeum_vulgare_newGene_13793	darkorange
HORVU6Hr1G078470	darkorange
HORVU6Hr1G018150	darkorange
HORVU2Hr1G116090	darkorange
Hordeum_vulgare_newGene_15650	darkorange
Hordeum_vulgare_newGene_2532	darkorange
HORVU1Hr1G000030	darkorange
HORVU6Hr1G064670	darkorange
HORVU0Hr1G022110	darkorange
HORVU1Hr1G073490	darkorange
Hordeum_vulgare_newGene_4178	darkorange
HORVU7Hr1G063490	darkorange
HORVU6Hr1G023040	darkorange
HORVU7Hr1G098280	darkorange
Hordeum_vulgare_newGene_7735	darkorange
HORVU6Hr1G005950	darkorange
HORVU3Hr1G030750	darkorange
HORVU0Hr1G040070	darkorange
HORVU5Hr1G124010	darkorange
HORVU1Hr1G026860	darkorange
HORVU3Hr1G039790	darkorange
HORVU4Hr1G053790	darkorange
HORVU4Hr1G038160	darkorange
HORVU2Hr1G111840	darkorange
HORVU1Hr1G090490	darkorange
HORVU6Hr1G085210	darkorange
HORVU5Hr1G093850	darkorange
HORVU6Hr1G055270	darkorange
HORVU4Hr1G065380	darkorange
HORVU1Hr1G056170	darkorange
HORVU3Hr1G021660	darkorange
HORVU2Hr1G010670	darkorange
HORVU2Hr1G019180	darkorange
Hordeum_vulgare_newGene_5397	darkorange
HORVU5Hr1G086620	darkorange
HORVU2Hr1G029070	darkorange
HORVU7Hr1G009700	darkorange
HORVU1Hr1G084370	darkorange
HORVU3Hr1G089550	darkorange
HORVU2Hr1G112080	darkorange
HORVU7Hr1G003880	darkorange
HORVU2Hr1G018750	darkorange
HORVU7Hr1G033230	darkorange
HORVU0Hr1G027310	darkorange

HORVU4Hr1G089540	darkorange
HORVU5Hr1G050610	darkorange
HORVU2Hr1G017120	darkorange
HORVU7Hr1G019680	darkorange
Hordeum_vulgare_newGene_7671	darkorange
HORVU6Hr1G074180	darkorange
HORVU3Hr1G086500	darkorange
HORVU7Hr1G025160	darkorange
HORVU5Hr1G002140	darkorange
HORVU0Hr1G022170	darkorange
HORVU2Hr1G085360	darkorange
HORVU4Hr1G050980	darkorange
HORVU3Hr1G031220	darkorange
HORVU5Hr1G111640	darkorange
HORVU3Hr1G024150	darkorange
HORVU2Hr1G116200	darkorange
HORVU4Hr1G021270	darkorange
HORVU3Hr1G050430	darkorange
HORVU5Hr1G053310	darkorange
HORVU5Hr1G092910	darkorange
HORVU3Hr1G093500	darkorange
HORVU2Hr1G044560	darkorange
HORVU4Hr1G027030	darkorange
Hordeum_vulgare_newGene_6653	darkorange
HORVU5Hr1G041530	darkorange
HORVU3Hr1G097800	darkorange
HORVU6Hr1G032890	darkorange
HORVU7Hr1G111930	darkorange
Hordeum_vulgare_newGene_13661	darkorange
HORVU3Hr1G053860	darkorange
HORVU5Hr1G087830	darkorange
HORVU0Hr1G031850	darkorange
HORVU4Hr1G080740	darkorange
HORVU4Hr1G043910	darkorange
HORVU1Hr1G053080	darkorange
HORVU7Hr1G113210	darkorange
HORVU5Hr1G107380	darkorange
Hordeum_vulgare_newGene_1140	darkorange
HORVU7Hr1G095890	darkorange
HORVU7Hr1G085110	darkorange
HORVU3Hr1G076960	darkorange
HORVU5Hr1G012990	darkorange
Hordeum_vulgare_newGene_11013	darkorange
HORVU5Hr1G042080	darkorange
HORVU0Hr1G012970	darkorange
HORVU2Hr1G107980	darkorange
Hordeum_vulgare_newGene_16099	darkorange

HORVU3Hr1G066450	darkorange
HORVU5Hr1G082460	darkorange
Hordeum_vulgare_newGene_11135	darkorange
HORVU7Hr1G104660	darkorange
HORVU3Hr1G060310	darkorange
Hordeum_vulgare_newGene_3371	darkorange
Hordeum_vulgare_newGene_3370	darkorange
Hordeum_vulgare_newGene_3372	darkorange
HORVU7Hr1G075680	darkorange
HORVU4Hr1G082700	darkorange
HORVU2Hr1G123760	darkorange
HORVU7Hr1G073450	darkorange
HORVU2Hr1G096230	darkorange
Hordeum_vulgare_newGene_10005	darkorange
HORVU1Hr1G077170	darkorange
HORVU1Hr1G035290	darkorange
Hordeum_vulgare_newGene_13677	darkorange
HORVU6Hr1G077440	darkorange
HORVU2Hr1G028500	darkorange
HORVU2Hr1G048510	darkorange
HORVU5Hr1G015670	darkorange
HORVU7Hr1G025330	darkorange
HORVU3Hr1G010280	darkorange
Hordeum_vulgare_newGene_6756	darkorange
Hordeum_vulgare_newGene_6751	darkorange
Hordeum_vulgare_newGene_15863	darkorange
HORVU4Hr1G013530	darkorange
HORVU4Hr1G017340	darkorange
HORVU5Hr1G101500	darkorange
Hordeum_vulgare_newGene_13112	darkorange
HORVU0Hr1G017530	darkorange
HORVU5Hr1G093530	darkorange
HORVU7Hr1G085180	darkorange
HORVU4Hr1G053850	darkorange
HORVU1Hr1G045790	darkorange
HORVU4Hr1G055860	darkorange
HORVU6Hr1G048850	darkorange
HORVU4Hr1G024470	darkorange
HORVU2Hr1G072930	darkorange
HORVU0Hr1G021670	darkorange
HORVU3Hr1G017570	darkorange
HORVU5Hr1G073350	darkorange
Hordeum_vulgare_newGene_3255	darkorange
Hordeum_vulgare_newGene_3504	darkorange
Hordeum_vulgare_newGene_9496	darkorange
HORVU7Hr1G003090	darkorange
HORVU7Hr1G062240	darkorange

HORVU3Hr1G084800	darkorange
Hordeum_vulgare_newGene_9421	darkorange
HORVU6Hr1G067200	darkorange
HORVU5Hr1G108870	darkorange
HORVU3Hr1G000910	darkorange
HORVU7Hr1G053550	darkorange
HORVU7Hr1G043360	darkorange
HORVU3Hr1G090050	darkorange
HORVU3Hr1G086580	darkorange
HORVU7Hr1G067620	darkorange
HORVU0Hr1G040240	darkorange
HORVU4Hr1G051740	darkorange
HORVU0Hr1G036310	darkorange
HORVU2Hr1G013080	darkorange
HORVU2Hr1G027010	darkorange
HORVU5Hr1G015950	darkorange
HORVU3Hr1G083350	darkorange
HORVU0Hr1G012390	darkorange
HORVU5Hr1G010860	darkorange
HORVU6Hr1G005390	darkorange
HORVU7Hr1G075760	darkorange
HORVU5Hr1G046330	darkorange
HORVU4Hr1G081070	darkorange
HORVU4Hr1G000610	darkorange
HORVU0Hr1G010800	darkorange
HORVU1Hr1G040150	darkorange
Hordeum_vulgare_newGene_10786	darkorange
HORVU7Hr1G063020	darkorange
HORVU2Hr1G123570	darkorange
HORVU5Hr1G046490	darkorange
HORVU2Hr1G087630	darkorange
Hordeum_vulgare_newGene_13554	darkorange
Hordeum_vulgare_newGene_13557	darkorange
HORVU2Hr1G114920	darkorange
HORVU6Hr1G079640	darkorange
HORVU6Hr1G061220	darkorange
HORVU1Hr1G020050	darkorange
HORVU3Hr1G013530	darkorange
HORVU1Hr1G018930	darkorange
HORVU5Hr1G021340	darkorange
HORVU4Hr1G008120	darkorange
HORVU4Hr1G019140	darkorange
HORVU5Hr1G092240	darkorange
Hordeum_vulgare_newGene_7029	darkorange
HORVU6Hr1G035520	darkorange
HORVU1Hr1G029540	darkorange
HORVU4Hr1G016500	darkorange

HORVU5Hr1G017630	darkorange
HORVU1Hr1G087530	darkorange
HORVU4Hr1G063600	darkorange
HORVU4Hr1G009310	darkorange
HORVU1Hr1G068830	darkorange
HORVU2Hr1G076000	darkorange
HORVU7Hr1G109250	darkorange
Hordeum_vulgare_newGene_12957	darkorange
HORVU1Hr1G043500	darkorange
HORVU2Hr1G107480	darkorange
HORVU5Hr1G044610	darkorange
HORVU5Hr1G078160	darkorange
HORVU4Hr1G060970	darkorange
HORVU2Hr1G112330	darkorange
HORVU3Hr1G106880	darkorange
HORVU2Hr1G111150	darkorange
Hordeum_vulgare_newGene_1709	darkorange
HORVU5Hr1G015110	darkorange
HORVU7Hr1G073170	darkorange
HORVU3Hr1G069980	darkorange
HORVU1Hr1G025280	darkorange
HORVU2Hr1G114680	darkorange
HORVU3Hr1G010460	darkorange
Hordeum_vulgare_newGene_13742	darkorange
Hordeum_vulgare_newGene_9991	darkorange
Hordeum_vulgare_newGene_7691	darkorange
HORVU7Hr1G106010	darkorange
HORVU7Hr1G096310	darkorange
HORVU0Hr1G029310	darkorange
Hordeum_vulgare_newGene_1067	darkorange
HORVU7Hr1G053600	darkorange
Hordeum_vulgare_newGene_4259	darkorange
HORVU7Hr1G085650	darkorange
HORVU2Hr1G002600	darkorange
HORVU6Hr1G006120	darkorange
HORVU1Hr1G012910	darkorange
HORVU3Hr1G078780	darkorange
HORVU3Hr1G077180	darkorange
HORVU2Hr1G090120	darkorange
HORVU2Hr1G125280	darkorange
HORVU7Hr1G045680	darkorange
HORVU2Hr1G023820	darkorange
HORVU4Hr1G090820	darkorange
HORVU4Hr1G067970	darkorange
HORVU2Hr1G038830	darkorange
Hordeum_vulgare_newGene_3239	darkorange
HORVU5Hr1G093880	darkorange

HORVU6Hr1G056910	darkorange
HORVU7Hr1G028850	darkorange
HORVU4Hr1G059240	darkorange
HORVU4Hr1G070150	darkorange
Hordeum_vulgare_newGene_13234	darkorange
HORVU5Hr1G041830	darkorange
Hordeum_vulgare_newGene_15172	darkorange
HORVU7Hr1G026540	darkorange
HORVU6Hr1G077520	darkorange
HORVU7Hr1G063510	darkorange
HORVU4Hr1G072620	darkorange
HORVU2Hr1G071860	darkorange
HORVU4Hr1G066860	darkorange
HORVU1Hr1G069160	darkorange
HORVU7Hr1G001570	darkorange
HORVU3Hr1G086360	darkorange
HORVU6Hr1G029930	darkorange
HORVU6Hr1G064140	darkorange
HORVU6Hr1G039610	darkorange
HORVU3Hr1G032230	darkorange
HORVU5Hr1G109570	darkorange
HORVU7Hr1G025480	darkorange
HORVU6Hr1G031340	darkorange
HORVU5Hr1G067380	darkorange
HORVU0Hr1G013190	darkorange
HORVU0Hr1G013220	darkorange
HORVU3Hr1G065170	darkorange
HORVU2Hr1G113450	darkorange
HORVU3Hr1G077690	darkorange
HORVU3Hr1G040230	darkorange
HORVU7Hr1G012780	darkorange
HORVU1Hr1G081770	darkorange
Hordeum_vulgare_newGene_3900	darkorange
HORVU4Hr1G071250	darkorange
HORVU4Hr1G065470	darkorange
HORVU5Hr1G075570	darkorange
Hordeum_vulgare_newGene_14144	darkorange
Hordeum_vulgare_newGene_14148	darkorange
Hordeum_vulgare_newGene_3799	darkorange
Hordeum_vulgare_newGene_449	darkorange
Hordeum_vulgare_newGene_10861	darkorange
Hordeum_vulgare_newGene_2446	darkorange
Hordeum_vulgare_newGene_5790	darkorange
HORVU3Hr1G096120	darkorange
HORVU7Hr1G100690	darkorange
HORVU6Hr1G058000	darkorange
HORVU1Hr1G068110	darkorange

HORVU2Hr1G035220	darkorange
HORVU6Hr1G024180	darkorange
HORVU1Hr1G072290	darkorange
HORVU7Hr1G098110	darkorange
HORVU7Hr1G025200	darkorange
HORVU7Hr1G035820	darkorange
Hordeum_vulgare_newGene_6379	darkorange
HORVU0Hr1G005260	darkorange
HORVU1Hr1G080190	darkorange
HORVU2Hr1G106880	darkorange
HORVU4Hr1G069230	darkorange
HORVU1Hr1G094250	darkorange
HORVU5Hr1G015850	darkorange
HORVU6Hr1G094530	darkorange
HORVU1Hr1G018380	darkorange
HORVU3Hr1G084410	darkorange
HORVU6Hr1G005290	darkorange
HORVU1Hr1G042450	darkorange
Hordeum_vulgare_newGene_8245	darkorange
HORVU3Hr1G047040	darkorange
HORVU0Hr1G013200	darkorange
HORVU6Hr1G021260	darkorange
HORVU2Hr1G119090	darkorange
HORVU5Hr1G047630	darkorange
HORVU3Hr1G078670	darkorange
HORVU7Hr1G082670	darkorange
HORVU6Hr1G041020	darkorange
HORVU1Hr1G085140	darkorange
Hordeum_vulgare_newGene_1969	darkorange
HORVU7Hr1G040250	darkorange
HORVU6Hr1G034990	darkorange
HORVU5Hr1G015660	darkorange
HORVU3Hr1G063050	darkorange
HORVU7Hr1G098330	darkorange
HORVU1Hr1G071760	darkorange
HORVU7Hr1G117540	darkorange
HORVU5Hr1G038630	darkorange
Hordeum_vulgare_newGene_15087	darkorange
HORVU0Hr1G002090	darkorange
HORVU3Hr1G045220	darkorange
HORVU0Hr1G005840	darkorange
HORVU4Hr1G039870	darkorange
HORVU6Hr1G005960	darkorange
HORVU0Hr1G035460	darkorange
HORVU5Hr1G088420	darkorange
HORVU6Hr1G033540	darkorange
HORVU7Hr1G097250	darkorange

HORVU5Hr1G017570	darkorange
HORVU0Hr1G017100	darkorange
HORVU7Hr1G077320	darkorange
HORVU5Hr1G110680	darkorange
HORVU5Hr1G111480	darkorange
HORVU7Hr1G076580	darkorange
HORVU7Hr1G025990	darkorange
HORVU7Hr1G006530	darkorange
HORVU7Hr1G074690	darkorange
HORVU5Hr1G046020	darkorange
HORVU2Hr1G072400	darkorange
HORVU4Hr1G020030	darkorange
HORVU2Hr1G092080	darkorange
HORVU5Hr1G073050	darkorange
HORVU5Hr1G053480	darkorange
HORVU2Hr1G036980	darkorange
HORVU3Hr1G105880	darkorange
HORVU6Hr1G025730	darkorange
HORVU2Hr1G039360	darkorange
HORVU3Hr1G088270	darkorange
HORVU7Hr1G082350	darkorange
HORVU6Hr1G032960	darkorange
Hordeum_vulgare_newGene_9278	darkorange
HORVU7Hr1G048710	darkorange
HORVU5Hr1G052010	darkorange
HORVU5Hr1G000150	darkorange
Hordeum_vulgare_newGene_1383	darkorange
Hordeum_vulgare_newGene_4350	darkorange
HORVU0Hr1G001490	darkorange
HORVU6Hr1G018830	darkorange
HORVU7Hr1G024240	darkorange
HORVU5Hr1G014500	darkorange
HORVU6Hr1G091650	darkorange
HORVU1Hr1G049920	darkorange
HORVU2Hr1G061610	darkorange
HORVU3Hr1G027430	darkorange
Hordeum_vulgare_newGene_3468	darkorange
HORVU5Hr1G000750	darkorange
HORVU2Hr1G060730	darkorange
HORVU5Hr1G096390	darkorange
HORVU2Hr1G060880	darkorange
HORVU6Hr1G066210	darkorange
Hordeum_vulgare_newGene_1412	darkorange
Hordeum_vulgare_newGene_1415	darkorange
HORVU6Hr1G076770	darkorange
HORVU6Hr1G071920	darkorange
Hordeum_vulgare_newGene_3815	darkorange

HORVU2Hr1G025990	darkorange
HORVU1Hr1G038330	darkorange
Hordeum_vulgare_newGene_6668	darkorange
HORVU3Hr1G014960	darkorange
HORVU2Hr1G081920	darkorange
HORVU4Hr1G080810	darkorange
HORVU2Hr1G004280	darkorange
HORVU4Hr1G089490	darkorange
HORVU5Hr1G114470	darkorange
HORVU0Hr1G017330	darkorange
HORVU7Hr1G115500	darkorange
HORVU2Hr1G080240	darkorange
HORVU7Hr1G110170	darkorange
HORVU5Hr1G113900	darkorange
HORVU4Hr1G081800	darkorange
HORVU7Hr1G098410	darkorange
HORVU5Hr1G045070	darkorange
HORVU7Hr1G016130	darkorange
HORVU3Hr1G030010	darkorange
HORVU1Hr1G090200	darkorange
HORVU6Hr1G038120	darkorange
HORVU2Hr1G068380	darkorange
Hordeum_vulgare_newGene_12745	darkorange
Hordeum_vulgare_newGene_12744	darkorange
HORVU5Hr1G040360	darkorange
HORVU4Hr1G073920	darkorange
HORVU2Hr1G019950	darkorange
HORVU7Hr1G077740	darkorange
Hordeum_vulgare_newGene_656	darkorange
HORVU2Hr1G070400	darkorange
HORVU4Hr1G078010	darkorange
HORVU2Hr1G101750	darkorange
HORVU6Hr1G015760	darkorange
Hordeum_vulgare_newGene_5698	darkorange
HORVU3Hr1G117860	darkorange
HORVU1Hr1G081570	darkorange
HORVU0Hr1G022140	darkorange
Hordeum_vulgare_newGene_5039	darkorange
HORVU4Hr1G060740	darkorange
HORVU1Hr1G068010	darkorange
Hordeum_vulgare_newGene_4787	darkorange
HORVU2Hr1G076540	darkorange
Hordeum_vulgare_newGene_4788	darkorange
Hordeum_vulgare_newGene_7476	darkorange
HORVU3Hr1G011700	darkorange
HORVU2Hr1G007980	darkorange
HORVU1Hr1G029110	darkorange

HORVU1Hr1G078530	darkorange
HORVU3Hr1G030250	darkorange
HORVU7Hr1G079190	darkorange
HORVU5Hr1G040000	darkorange
HORVU1Hr1G051330	darkorange
Hordeum_vulgare_newGene_2951	darkorange
HORVU3Hr1G012660	darkorange
HORVU5Hr1G119650	darkorange
HORVU4Hr1G001680	darkorange
HORVU1Hr1G042710	darkorange
HORVU3Hr1G067020	darkorange
Hordeum_vulgare_newGene_13740	darkorange
Hordeum_vulgare_newGene_10275	darkorange
Hordeum_vulgare_newGene_10271	darkorange
HORVU2Hr1G119600	darkorange
HORVU5Hr1G006850	darkorange
HORVU1Hr1G039180	darkorange
HORVU4Hr1G066900	darkorange
HORVU5Hr1G051960	darkorange
HORVU7Hr1G083080	darkorange
HORVU2Hr1G114720	darkorange
HORVU5Hr1G079690	darkorange
HORVU6Hr1G027620	darkorange
HORVU7Hr1G035050	darkorange
HORVU1Hr1G043900	darkorange
Hordeum_vulgare_newGene_8503	darkorange
Hordeum_vulgare_newGene_8500	darkorange
HORVU3Hr1G111080	darkorange
HORVU1Hr1G073680	darkorange
HORVU5Hr1G034100	darkorange
Hordeum_vulgare_newGene_1301	darkorange
HORVU3Hr1G019140	darkorange
HORVU7Hr1G118880	darkorange
HORVU5Hr1G018160	darkorange
HORVU5Hr1G010350	darkorange
HORVU5Hr1G034810	darkorange
HORVU4Hr1G073380	darkorange
HORVU6Hr1G019700	darkorange
HORVU4Hr1G088860	darkorange
HORVU3Hr1G043300	darkorange
HORVU5Hr1G014320	darkorange
HORVU1Hr1G055370	darkorange
HORVU7Hr1G094860	darkorange
HORVU3Hr1G016240	darkorange
HORVU6Hr1G052420	darkorange
HORVU7Hr1G076150	darkorange
HORVU5Hr1G019950	darkorange

HORVU7Hr1G029170	darkorange
HORVU4Hr1G001130	darkorange
Hordeum_vulgare_newGene_13915	darkorange
HORVU5Hr1G093700	darkorange
HORVU7Hr1G064050	darkorange
HORVU0Hr1G016430	darkorange
HORVU3Hr1G054810	darkorange
HORVU6Hr1G054110	darkorange
HORVU3Hr1G034530	darkorange
Hordeum_vulgare_newGene_13584	darkorange
Hordeum_vulgare_newGene_12189	darkorange
Hordeum_vulgare_newGene_12188	darkorange
HORVU6Hr1G064350	darkorange
Hordeum_vulgare_newGene_10918	darkorange
HORVU7Hr1G118290	darkorange
Hordeum_vulgare_newGene_13635	darkorange
HORVU2Hr1G116510	darkorange
HORVU1Hr1G020000	darkorange
Hordeum_vulgare_newGene_947	darkorange
HORVU1Hr1G091540	darkorange
HORVU2Hr1G031130	darkorange
HORVU7Hr1G057760	darkorange
HORVU4Hr1G057210	darkorange
HORVU3Hr1G005410	darkorange
HORVU4Hr1G076350	darkorange
HORVU6Hr1G006090	darkorange
HORVU2Hr1G039300	darkorange
HORVU2Hr1G050410	darkorange
HORVU6Hr1G034620	darkorange
HORVU4Hr1G002350	darkorange
HORVU3Hr1G025680	darkorange
Hordeum_vulgare_newGene_10320	darkorange
Hordeum_vulgare_newGene_2078	darkorange
HORVU5Hr1G083980	darkorange
HORVU6Hr1G021160	darkorange
HORVU7Hr1G089210	darkorange
HORVU6Hr1G017100	darkorange
HORVU7Hr1G106540	darkorange
HORVU7Hr1G021950	darkorange
HORVU1Hr1G013020	darkorange
HORVU3Hr1G011780	darkorange
HORVU4Hr1G073400	darkorange
HORVU2Hr1G106590	darkorange
HORVU7Hr1G064110	darkorange
HORVU1Hr1G070200	darkorange
HORVU2Hr1G091360	darkorange
HORVU2Hr1G116300	darkorange

HORVU7Hr1G015380	darkorange
HORVU6Hr1G061000	darkorange
Hordeum_vulgare_newGene_3767	darkorange
HORVU7Hr1G112470	darkorange
HORVU3Hr1G084750	darkorange
HORVU2Hr1G009940	darkorange
HORVU1Hr1G068640	darkorange
HORVU2Hr1G127640	darkorange
HORVU0Hr1G017560	darkorange
HORVU7Hr1G117640	darkorange
HORVU4Hr1G039880	darkorange
HORVU5Hr1G014430	darkorange
HORVU1Hr1G012940	darkorange
Hordeum_vulgare_newGene_13116	darkorange
Hordeum_vulgare_newGene_13111	darkorange
HORVU2Hr1G079790	darkorange
HORVU4Hr1G007680	darkorange
HORVU0Hr1G012860	darkorange
HORVU6Hr1G092280	darkorange
HORVU2Hr1G123480	darkorange
HORVU6Hr1G013910	darkorange
HORVU2Hr1G111530	darkorange
HORVU3Hr1G057440	darkorange
HORVU5Hr1G071010	darkorange
HORVU1Hr1G067300	darkorange
HORVU2Hr1G012310	darkorange
HORVU4Hr1G067940	darkorange
HORVU5Hr1G099350	darkorange
Hordeum_vulgare_newGene_13207	darkorange
Hordeum_vulgare_newGene_13201	darkorange
Hordeum_vulgare_newGene_4326	darkorange
HORVU4Hr1G071300	darkorange
HORVU1Hr1G073760	darkorange
HORVU5Hr1G045800	darkorange
HORVU2Hr1G105790	darkorange
Hordeum_vulgare_newGene_8326	darkorange
HORVU1Hr1G024400	darkorange
HORVU2Hr1G077650	darkorange
Hordeum_vulgare_newGene_4936	darkorange
Hordeum_vulgare_newGene_3929	darkorange
HORVU4Hr1G074650	darkorange
HORVU6Hr1G004860	darkorange
HORVU5Hr1G058910	darkorange
HORVU6Hr1G068410	darkorange
HORVU6Hr1G072260	darkorange
HORVU6Hr1G066950	darkorange
HORVU6Hr1G002320	darkorange

HORVU4Hr1G056240	darkorange
HORVU1Hr1G055510	darkorange
HORVU4Hr1G014530	darkorange
HORVU7Hr1G095680	darkorange
HORVU7Hr1G071390	darkorange
HORVU7Hr1G094080	darkorange
HORVU0Hr1G035570	darkorange
HORVU5Hr1G052090	darkorange
HORVU1Hr1G041250	darkorange
HORVU5Hr1G092430	darkorange
HORVU1Hr1G013190	darkorange
HORVU0Hr1G009910	darkorange
HORVU3Hr1G110170	darkorange
HORVU2Hr1G018730	darkorange
HORVU4Hr1G054610	darkorange
Hordeum_vulgare_newGene_14195	darkorange
Hordeum_vulgare_newGene_10851	darkorange
HORVU4Hr1G075180	darkorange
HORVU0Hr1G009140	darkorange
HORVU6Hr1G067170	darkorange
HORVU1Hr1G068160	darkorange
HORVU2Hr1G046410	darkorange
Hordeum_vulgare_newGene_14941	darkorange
HORVU7Hr1G038420	darkorange
HORVU4Hr1G051070	darkorange
HORVU1Hr1G026960	darkorange
HORVU1Hr1G065990	darkorange
HORVU7Hr1G121200	darkorange
HORVU7Hr1G051710	darkorange
HORVU5Hr1G122060	darkorange
HORVU7Hr1G095060	darkorange
HORVU4Hr1G019780	darkorange
HORVU5Hr1G009860	darkorange
HORVU6Hr1G021250	darkorange
HORVU1Hr1G094880	darkorange
HORVU4Hr1G076610	darkorange
HORVU7Hr1G089290	darkorange
HORVU2Hr1G048010	darkorange
HORVU6Hr1G077270	darkorange
HORVU2Hr1G123310	darkorange
HORVU2Hr1G029480	darkorange
HORVU1Hr1G084450	darkorange
HORVU3Hr1G056400	darkorange
HORVU0Hr1G024130	darkorange
HORVU6Hr1G055760	darkorange
HORVU1Hr1G075900	darkorange
HORVU6Hr1G034630	darkorange

HORVU2Hr1G033070	darkorange
HORVU2Hr1G046530	darkorange
HORVU3Hr1G096830	darkorange
HORVU2Hr1G062700	darkorange
HORVU2Hr1G030520	darkorange
HORVU0Hr1G002950	darkorange
HORVU7Hr1G026140	darkorange
HORVU2Hr1G098380	darkorange
HORVU1Hr1G064870	darkorange
HORVU1Hr1G064290	darkorange
HORVU5Hr1G100200	darkorange
HORVU5Hr1G042400	darkorange
HORVU5Hr1G009650	darkorange
HORVU7Hr1G050340	darkorange
HORVU5Hr1G122040	darkorange
HORVU3Hr1G086610	darkorange
HORVU1Hr1G091650	darkorange
HORVU7Hr1G084230	darkorange
HORVU4Hr1G087230	darkorange
HORVU2Hr1G106420	darkorange
HORVU4Hr1G019570	darkorange
HORVU1Hr1G058500	darkorange
HORVU7Hr1G008320	darkorange
HORVU5Hr1G098770	darkorange
HORVU7Hr1G059220	darkorange
HORVU7Hr1G082090	darkorange
HORVU2Hr1G073380	darkorange
HORVU4Hr1G077060	darkorange
HORVU6Hr1G089250	darkorange
HORVU7Hr1G031280	darkorange
HORVU3Hr1G067750	darkorange
HORVU3Hr1G033620	darkorange
HORVU3Hr1G081050	darkorange
HORVU2Hr1G075030	darkorange
HORVU4Hr1G070240	darkorange
HORVU0Hr1G004480	darkorange
HORVU5Hr1G094890	darkorange
HORVU1Hr1G022400	darkorange
HORVU0Hr1G003270	darkorange
HORVU1Hr1G021840	darkorange
HORVU3Hr1G014790	darkorange
Hordeum_vulgare_newGene_7986	darkorange
HORVU2Hr1G043330	darkorange
HORVU2Hr1G080480	darkorange
HORVU5Hr1G056950	darkorange
HORVU6Hr1G081730	darkorange
HORVU3Hr1G019070	darkorange

HORVU7Hr1G105960	darkorange
HORVU3Hr1G100270	darkorange
HORVU6Hr1G085490	darkorange
HORVU6Hr1G049050	darkorange
HORVU6Hr1G092430	darkorange
HORVU3Hr1G057090	darkorange
HORVU2Hr1G039210	darkorange
HORVU3Hr1G084990	darkorange
HORVU2Hr1G093610	darkorange
HORVU5Hr1G025190	darkorange
HORVU1Hr1G012790	darkorange
HORVU2Hr1G068270	darkorange
HORVU1Hr1G071210	darkorange
Hordeum_vulgare_newGene_410	darkorange
HORVU5Hr1G115000	darkorange
Hordeum_vulgare_newGene_11899	darkorange
HORVU3Hr1G050450	darkorange
HORVU5Hr1G094080	darkorange
HORVU1Hr1G076470	darkorange
HORVU3Hr1G088050	darkorange
HORVU3Hr1G052030	darkorange
HORVU2Hr1G032130	darkorange
Hordeum_vulgare_newGene_2824	darkorange
HORVU6Hr1G029190	darkorange
HORVU5Hr1G060800	darkorange
HORVU3Hr1G038430	darkorange
Hordeum_vulgare_newGene_8906	darkorange
HORVU3Hr1G082590	darkorange
HORVU7Hr1G113450	darkorange
HORVU1Hr1G050620	darkorange
HORVU4Hr1G065620	darkorange
HORVU0Hr1G014100	darkorange
HORVU4Hr1G068910	darkorange
HORVU2Hr1G017440	darkorange
HORVU6Hr1G019300	darkorange
HORVU7Hr1G051180	darkorange
HORVU3Hr1G029210	darkorange
HORVU2Hr1G022720	darkorange
HORVU6Hr1G062050	darkorange
HORVU7Hr1G033900	darkorange
HORVU3Hr1G110190	darkorange
Hordeum_vulgare_newGene_2512	darkorange
HORVU7Hr1G043790	darkorange
Hordeum_vulgare_newGene_9697	darkorange
HORVU4Hr1G044890	darkorange
HORVU1Hr1G032470	darkorange
HORVU5Hr1G077110	darkorange

HORVU7Hr1G070970	darkorange
HORVU7Hr1G098260	darkorange
Hordeum_vulgare_newGene_7519	darkorange
HORVU6Hr1G087300	darkorange
HORVU1Hr1G010230	darkorange
HORVU2Hr1G123150	darkorange
Hordeum_vulgare_newGene_12801	darkorange
HORVU3Hr1G071340	darkorange
HORVU4Hr1G054240	darkorange
HORVU7Hr1G110310	darkorange
HORVU7Hr1G121820	darkorange
HORVU2Hr1G030870	darkorange
HORVU6Hr1G063280	darkorange
HORVU6Hr1G035260	darkorange
HORVU3Hr1G056560	darkorange
HORVU2Hr1G068610	darkorange
HORVU5Hr1G033540	darkorange
Hordeum_vulgare_newGene_3564	darkorange
HORVU3Hr1G061410	darkorange
HORVU2Hr1G033130	darkorange
HORVU4Hr1G021090	darkorange
HORVU5Hr1G063960	darkorange
HORVU3Hr1G018650	darkorange
HORVU0Hr1G032640	darkorange
HORVU4Hr1G089560	darkorange
HORVU3Hr1G049900	darkorange
HORVU3Hr1G084920	darkorange
HORVU0Hr1G015420	darkorange
HORVU5Hr1G120210	darkorange
HORVU0Hr1G040480	darkorange
HORVU4Hr1G010430	darkorange
HORVU7Hr1G092040	darkorange
HORVU3Hr1G066860	darkorange
HORVU7Hr1G088890	darkorange
HORVU4Hr1G084400	darkorange
HORVU2Hr1G065090	darkorange
HORVU7Hr1G108240	darkorange
HORVU7Hr1G003140	darkorange
HORVU0Hr1G022780	darkorange
HORVU2Hr1G091280	darkorange
HORVU4Hr1G090440	darkorange
Hordeum_vulgare_newGene_8774	darkorange
HORVU5Hr1G053330	darkorange
HORVU2Hr1G112250	darkorange
HORVU1Hr1G057320	darkorange
HORVU1Hr1G017800	darkorange
HORVU7Hr1G033500	darkorange

HORVU6Hr1G048600	darkorange
HORVU6Hr1G003020	darkorange
Hordeum_vulgare_newGene_214	darkorange
HORVU6Hr1G067660	darkorange
HORVU7Hr1G011150	darkorange
HORVU3Hr1G095330	darkorange
Hordeum_vulgare_newGene_13600	darkorange
Hordeum_vulgare_newGene_13605	darkorange
Hordeum_vulgare_newGene_2363	darkorange
HORVU6Hr1G034110	darkorange
Hordeum_vulgare_newGene_3549	darkorange
HORVU2Hr1G068630	darkorange
HORVU1Hr1G073670	darkorange
HORVU7Hr1G114170	darkorange
HORVU4Hr1G008270	darkorange
HORVU1Hr1G055800	darkorange
HORVU5Hr1G122820	darkorange
HORVU2Hr1G112070	darkorange
HORVU4Hr1G012700	darkorange
HORVU1Hr1G047930	darkorange
HORVU1Hr1G002100	darkorange
HORVU4Hr1G021190	darkorange
HORVU2Hr1G107460	darkorange
HORVU3Hr1G081020	darkorange
HORVU6Hr1G015790	darkorange
HORVU3Hr1G054640	darkorange
HORVU5Hr1G053270	darkorange
HORVU7Hr1G089260	darkorange
Hordeum_vulgare_newGene_14828	darkorange
Hordeum_vulgare_newGene_7424	darkorange
HORVU4Hr1G006480	darkorange
Hordeum_vulgare_newGene_15889	darkorange
HORVU6Hr1G087380	darkorange
Hordeum_vulgare_newGene_9930	darkorange
HORVU1Hr1G020480	darkorange
HORVU7Hr1G041710	darkorange
HORVU7Hr1G092550	darkorange
HORVU4Hr1G051990	darkorange
HORVU2Hr1G124010	darkorange
HORVU2Hr1G010690	darkorange
HORVU7Hr1G074370	darkorange
HORVU4Hr1G025450	darkorange
HORVU3Hr1G065580	darkorange
HORVU4Hr1G023850	darkorange
HORVU1Hr1G066020	darkorange
HORVU2Hr1G039100	darkorange
Hordeum_vulgare_newGene_8866	darkorange

Hordeum_vulgare_newGene_5921	darkorange
HORVU1Hr1G000710	darkorange
HORVU5Hr1G103900	darkorange
HORVU6Hr1G087960	darkorange
Hordeum_vulgare_newGene_4389	darkorange
HORVU5Hr1G037980	darkorange
HORVU3Hr1G000930	darkorange
HORVU5Hr1G015480	darkorange
Hordeum_vulgare_newGene_15926	darkorange
HORVU7Hr1G053570	darkorange
HORVU5Hr1G023640	darkorange
HORVU6Hr1G084720	darkorange
HORVU3Hr1G078830	darkorange
HORVU0Hr1G007370	darkorange
HORVU7Hr1G048080	darkorange
HORVU4Hr1G088480	darkorange
HORVU1Hr1G058150	darkorange
HORVU7Hr1G051600	darkorange
HORVU1Hr1G012420	darkorange
HORVU2Hr1G081230	darkorange
HORVU4Hr1G027930	darkorange
HORVU2Hr1G053630	darkorange
HORVU2Hr1G093690	darkorange
HORVU0Hr1G001470	darkorange
HORVU5Hr1G098340	darkorange
HORVU3Hr1G073790	darkorange
HORVU2Hr1G072850	darkorange
HORVU1Hr1G027300	darkorange
HORVU5Hr1G070720	darkorange
HORVU1Hr1G085160	darkorange
EPlHVUG00000039864	darkorange
Hordeum_vulgare_newGene_10881	darkorange
Hordeum_vulgare_newGene_13685	darkorange
HORVU2Hr1G005470	darkorange
HORVU6Hr1G032260	darkorange
HORVU7Hr1G078770	darkorange
HORVU4Hr1G011500	darkorange
HORVU3Hr1G069070	darkorange
HORVU4Hr1G005510	darkorange
HORVU3Hr1G014580	darkorange
HORVU5Hr1G093400	darkorange
HORVU1Hr1G050900	darkorange
Hordeum_vulgare_newGene_3804	darkorange
HORVU3Hr1G109880	darkorange
HORVU7Hr1G020580	darkorange
HORVU2Hr1G122950	darkorange
HORVU1Hr1G065160	darkorange

HORVU2Hr1G015980	darkorange
HORVU7Hr1G114890	darkorange
HORVU6Hr1G019510	darkorange
Hordeum_vulgare_newGene_14800	darkorange
HORVU7Hr1G093680	darkorange
HORVU3Hr1G092860	darkorange
HORVU7Hr1G109160	darkorange
HORVU3Hr1G108120	darkorange
Hordeum_vulgare_newGene_311	darkorange
HORVU3Hr1G097710	darkorange
HORVU7Hr1G109730	darkorange
HORVU6Hr1G078290	darkorange
HORVU4Hr1G026150	darkorange
HORVU6Hr1G066250	darkorange
HORVU2Hr1G092540	darkorange
HORVU2Hr1G077210	darkorange
HORVU5Hr1G015390	darkorange
HORVU2Hr1G041040	darkorange
HORVU0Hr1G019840	darkorange
HORVU2Hr1G094240	darkorange
HORVU3Hr1G009980	darkorange
HORVU7Hr1G071750	darkorange
HORVU4Hr1G090840	darkorange
HORVU4Hr1G018650	darkorange
HORVU1Hr1G037730	darkorange
HORVU3Hr1G089350	darkorange
HORVU7Hr1G043320	darkorange
HORVU1Hr1G047100	darkorange
Hordeum_vulgare_newGene_2166	darkorange
HORVU5Hr1G079130	darkorange
Hordeum_vulgare_newGene_8353	darkorange
HORVU3Hr1G088120	darkorange
HORVU7Hr1G100100	darkorange
HORVU3Hr1G089650	darkorange
HORVU5Hr1G087340	darkorange
HORVU3Hr1G003040	darkorange
HORVU7Hr1G086570	darkorange
HORVU6Hr1G026600	darkorange
HORVU4Hr1G011160	darkorange
HORVU2Hr1G096910	darkorange
HORVU3Hr1G099220	darkorange
HORVU7Hr1G095630	darkorange
HORVU2Hr1G080100	darkorange
HORVU4Hr1G015550	darkorange
HORVU3Hr1G074570	darkorange
HORVU7Hr1G098370	darkorange
HORVU1Hr1G083710	darkorange

HORVU5Hr1G124470	darkorange
HORVU0Hr1G011660	darkorange
HORVU4Hr1G067320	darkorange
HORVU4Hr1G043650	darkorange
HORVU2Hr1G115160	darkorange
HORVU3Hr1G022510	darkorange
HORVU2Hr1G039220	darkorange
HORVU7Hr1G050160	darkorange
HORVU2Hr1G120530	darkorange
HORVU7Hr1G029200	darkorange
Hordeum_vulgare_newGene_3404	darkorange
HORVU4Hr1G057910	darkorange
HORVU1Hr1G062190	darkorange
HORVU1Hr1G070190	darkorange
Hordeum_vulgare_newGene_14123	darkorange
HORVU3Hr1G110200	darkorange
HORVU2Hr1G105390	darkorange
HORVU1Hr1G075680	darkorange
HORVU1Hr1G051050	darkorange
HORVU6Hr1G087540	darkorange
HORVU5Hr1G100140	darkorange
HORVU1Hr1G093250	darkorange
HORVU7Hr1G054220	darkorange
HORVU1Hr1G091090	darkorange
HORVU7Hr1G051760	darkorange
HORVU3Hr1G000350	darkorange
HORVU2Hr1G009570	darkorange
HORVU0Hr1G014150	darkorange
HORVU0Hr1G004850	darkorange
HORVU4Hr1G054910	darkorange
HORVU4Hr1G070250	darkorange
HORVU3Hr1G059810	darkorange
HORVU5Hr1G053810	darkorange
HORVU3Hr1G072340	darkorange
HORVU1Hr1G012880	darkorange
Hordeum_vulgare_newGene_11089	darkorange
Hordeum_vulgare_newGene_11088	darkorange
HORVU2Hr1G029120	darkorange
HORVU1Hr1G038730	darkorange
HORVU2Hr1G025150	darkorange
HORVU4Hr1G084000	darkorange
HORVU0Hr1G021720	darkorange
HORVU4Hr1G025430	darkorange
HORVU3Hr1G055830	darkorange
Hordeum_vulgare_newGene_5638	darkorange
Hordeum_vulgare_newGene_6842	darkorange
HORVU2Hr1G072660	darkorange

Hordeum_vulgare_newGene_8449	darkorange
HORVU5Hr1G077910	darkorange
HORVU1Hr1G063150	darkorange
HORVU2Hr1G089160	darkorange
HORVU5Hr1G034040	darkorange
HORVU7Hr1G036280	darkorange
HORVU1Hr1G076690	darkorange
HORVU7Hr1G039260	darkorange
HORVU3Hr1G035730	darkorange
HORVU1Hr1G074340	darkorange
HORVU4Hr1G017860	darkorange
HORVU3Hr1G030760	darkorange
HORVU7Hr1G118130	darkorange
HORVU1Hr1G089510	darkorange
HORVU4Hr1G063160	darkorange
HORVU4Hr1G056300	darkorange
HORVU1Hr1G016200	darkorange
HORVU1Hr1G094480	darkorange
HORVU2Hr1G110810	darkorange
HORVU2Hr1G113540	darkorange
HORVU1Hr1G045760	darkorange
HORVU2Hr1G090260	darkorange
HORVU5Hr1G015710	darkorange
HORVU1Hr1G034400	darkorange
Hordeum_vulgare_newGene_7126	darkorange
HORVU6Hr1G029350	darkorange
HORVU5Hr1G075060	darkorange
Hordeum_vulgare_newGene_14415	darkorange
HORVU7Hr1G008430	darkorange
HORVU5Hr1G078540	darkorange
HORVU1Hr1G036500	darkorange
HORVU3Hr1G000980	darkorange
HORVU7Hr1G003020	darkorange
HORVU7Hr1G024220	darkorange
HORVU7Hr1G098660	darkorange
HORVU5Hr1G064800	darkorange
HORVU1Hr1G065120	darkorange
HORVU2Hr1G082030	darkorange
HORVU7Hr1G058120	darkorange
HORVU7Hr1G029630	darkorange
HORVU4Hr1G063730	darkorange
HORVU7Hr1G095370	darkorange
HORVU7Hr1G088730	darkorange
HORVU0Hr1G014460	darkorange
HORVU0Hr1G019870	darkorange
HORVU1Hr1G070110	darkorange
HORVU2Hr1G099550	darkorange

HORVU5Hr1G014300	darkorange
HORVU4Hr1G075200	darkorange
Hordeum_vulgare_newGene_13867	darkorange
HORVU3Hr1G096760	darkorange
Hordeum_vulgare_newGene_14991	darkorange
HORVU3Hr1G056960	darkorange
HORVU7Hr1G029400	darkorange
Hordeum_vulgare_newGene_6647	darkorange
HORVU5Hr1G036460	darkorange
HORVU1Hr1G055440	darkorange
HORVU5Hr1G094940	darkorange
HORVU6Hr1G011710	darkorange
HORVU5Hr1G066280	darkorange
HORVU1Hr1G079440	darkorange
HORVU1Hr1G053090	darkorange
HORVU0Hr1G012490	darkorange
HORVU5Hr1G011960	darkorange
HORVU4Hr1G006730	darkorange
HORVU7Hr1G067520	darkorange
HORVU3Hr1G078560	darkorange
HORVU2Hr1G094390	darkorange
HORVU2Hr1G015430	darkorange
HORVU1Hr1G012800	darkorange
HORVU2Hr1G090380	darkorange
HORVU3Hr1G028710	darkorange
HORVU2Hr1G011430	darkorange
HORVU4Hr1G023300	darkorange
HORVU5Hr1G049180	darkorange
HORVU5Hr1G109400	darkorange
HORVU5Hr1G040920	darkorange
Hordeum_vulgare_newGene_14375	darkorange
Hordeum_vulgare_newGene_14377	darkorange
HORVU1Hr1G070700	darkorange
HORVU3Hr1G044660	darkorange
HORVU7Hr1G029190	darkorange
Hordeum_vulgare_newGene_15022	darkorange
HORVU3Hr1G063270	darkorange
HORVU4Hr1G079740	darkorange
HORVU6Hr1G014500	darkorange
Hordeum_vulgare_newGene_7419	darkorange
HORVU5Hr1G015640	darkorange
Hordeum_vulgare_newGene_7412	darkorange
HORVU1Hr1G024540	darkorange
HORVU7Hr1G032270	darkorange
HORVU3Hr1G088630	darkorange
HORVU7Hr1G078800	darkorange
HORVU1Hr1G051310	darkorange

Hordeum_vulgare_newGene_9904	darkorange
HORVU2Hr1G098940	darkorange
HORVU2Hr1G089640	darkorange
HORVU6Hr1G054820	darkorange
HORVU7Hr1G085310	darkorange
HORVU7Hr1G012690	darkorange
HORVU7Hr1G030010	darkorange
HORVU4Hr1G090310	darkorange
HORVU1Hr1G076870	darkorange
HORVU2Hr1G115360	darkorange
HORVU2Hr1G038260	darkorange
HORVU4Hr1G057170	darkorange
HORVU3Hr1G024470	darkorange
HORVU3Hr1G116470	darkorange
Hordeum_vulgare_newGene_3539	darkorange
HORVU0Hr1G005670	darkorange
HORVU7Hr1G003080	darkorange
HORVU5Hr1G115340	darkorange
Hordeum_vulgare_newGene_9298	darkorange
HORVU6Hr1G076880	darkorange
HORVU5Hr1G098170	darkorange
HORVU7Hr1G026350	darkorange
HORVU7Hr1G073650	darkorange
HORVU5Hr1G084880	darkorange
HORVU6Hr1G063910	darkorange
Hordeum_vulgare_newGene_1588	darkorange
Hordeum_vulgare_newGene_1587	darkorange
HORVU7Hr1G056240	darkorange
HORVU3Hr1G000900	darkorange
HORVU3Hr1G002670	darkorange
HORVU5Hr1G118060	darkorange
HORVU7Hr1G051560	darkorange
HORVU1Hr1G013740	darkorange
HORVU4Hr1G089670	darkorange
HORVU4Hr1G068540	darkorange
HORVU3Hr1G071210	darkorange
HORVU7Hr1G083180	darkorange
HORVU4Hr1G007580	darkorange
HORVU3Hr1G076880	darkorange
HORVU1Hr1G040140	darkorange
HORVU5Hr1G052030	darkorange
HORVU5Hr1G081660	darkorange
HORVU4Hr1G079070	darkorange
HORVU2Hr1G072880	darkorange
HORVU4Hr1G028720	darkorange
Hordeum_vulgare_newGene_7370	darkorange
HORVU7Hr1G028160	darkorange

HORVU7Hr1G104490	darkorange
HORVU1Hr1G021590	darkorange
HORVU5Hr1G061250	darkorange
HORVU2Hr1G060510	darkorange
HORVU6Hr1G053540	darkorange
HORVU0Hr1G000490	darkorange
HORVU1Hr1G020060	darkorange
HORVU2Hr1G075870	darkorange
HORVU2Hr1G074580	darkorange
HORVU1Hr1G092980	darkorange
HORVU2Hr1G079180	darkorange
HORVU4Hr1G073150	darkorange
HORVU6Hr1G029200	darkorange
HORVU1Hr1G050970	darkorange
HORVU2Hr1G082170	darkorange
HORVU1Hr1G027010	darkorange
HORVU1Hr1G093570	darkorange
HORVU4Hr1G023430	darkorange
HORVU3Hr1G083150	darkorange
HORVU7Hr1G057260	darkorange
HORVU0Hr1G022470	darkorange
HORVU6Hr1G084080	darkorange
HORVU7Hr1G042740	darkorange
HORVU3Hr1G021320	darkorange
Hordeum_vulgare_newGene_11327	darkorange
HORVU3Hr1G022800	darkorange
HORVU2Hr1G111140	darkorange
Hordeum_vulgare_newGene_10631	darkorange
HORVU5Hr1G118970	darkorange
HORVU0Hr1G025120	darkorange
HORVU2Hr1G069110	darkorange
HORVU1Hr1G072370	darkorange
HORVU4Hr1G077230	darkorange
HORVU3Hr1G116360	darkorange
Hordeum_vulgare_newGene_3004	darkorange
HORVU2Hr1G020100	darkorange
Hordeum_vulgare_newGene_6182	darkorange
HORVU4Hr1G017600	darkorange
HORVU4Hr1G010050	darkorange
HORVU7Hr1G096850	darkorange
HORVU3Hr1G036600	darkorange
HORVU5Hr1G020930	darkorange
HORVU1Hr1G079050	darkorange
HORVU2Hr1G017040	darkorange
HORVU6Hr1G006130	darkorange
HORVU3Hr1G077640	darkorange
HORVU2Hr1G123710	darkorange

HORVU4Hr1G063420	darkorange
HORVU7Hr1G071760	darkorange
Hordeum_vulgare_newGene_3240	darkorange
HORVU5Hr1G075640	darkorange
HORVU1Hr1G046740	darkorange
HORVU3Hr1G013970	darkorange
HORVU2Hr1G072490	darkorange
HORVU2Hr1G105220	darkorange
HORVU4Hr1G078710	darkorange
HORVU3Hr1G087800	darkorange
HORVU1Hr1G024460	darkorange
HORVU4Hr1G002820	darkorange
HORVU3Hr1G080740	darkorange
HORVU7Hr1G039760	darkorange
Hordeum_vulgare_newGene_12637	darkorange
HORVU1Hr1G022020	darkorange
HORVU5Hr1G124160	darkorange
HORVU6Hr1G064150	darkorange
HORVU6Hr1G071390	darkorange
HORVU3Hr1G003540	darkorange
HORVU1Hr1G022410	darkorange
HORVU7Hr1G108550	darkorange
HORVU7Hr1G037080	darkorange
HORVU5Hr1G015520	darkorange
HORVU5Hr1G112850	darkorange
HORVU2Hr1G093950	darkorange
HORVU3Hr1G082280	darkorange
HORVU2Hr1G017650	darkorange
HORVU5Hr1G045700	darkorange
HORVU5Hr1G047410	darkorange
Hordeum_vulgare_newGene_10441	darkorange
HORVU3Hr1G075920	darkorange
HORVU4Hr1G060600	darkorange
HORVU3Hr1G103540	darkorange
HORVU2Hr1G090090	darkorange
HORVU2Hr1G039270	darkorange
HORVU4Hr1G065440	darkorange
HORVU7Hr1G077560	darkorange
HORVU3Hr1G021290	darkorange
Hordeum_vulgare_newGene_2439	darkorange
HORVU5Hr1G078080	darkorange
Hordeum_vulgare_newGene_9777	darkorange
Hordeum_vulgare_newGene_14924	darkorange
HORVU5Hr1G087250	darkorange
Hordeum_vulgare_newGene_6696	darkorange
HORVU5Hr1G125110	darkorange
Hordeum_vulgare_newGene_6348	darkorange

Hordeum_vulgare_newGene_13550	darkorange
HORVU2Hr1G074280	darkorange
Hordeum_vulgare_newGene_1222	darkorange
HORVU1Hr1G026940	darkorange
HORVU1Hr1G027090	darkorange
HORVU5Hr1G035610	darkorange
HORVU2Hr1G019270	darkorange
HORVU4Hr1G010160	darkorange
HORVU3Hr1G076790	darkorange
HORVU6Hr1G094520	darkorange
HORVU5Hr1G054180	darkorange
HORVU4Hr1G015260	darkorange
Hordeum_vulgare_newGene_4435	darkorange
HORVU7Hr1G095850	darkorange
HORVU2Hr1G112380	darkorange
HORVU4Hr1G054920	darkorange
HORVU7Hr1G110470	darkorange
Hordeum_vulgare_newGene_12947	darkorange
HORVU1Hr1G031600	darkorange
HORVU5Hr1G086780	darkorange
HORVU7Hr1G000750	darkorange
HORVU4Hr1G025470	darkorange
Hordeum_vulgare_newGene_11504	darkorange
HORVU4Hr1G062830	darkorange
Hordeum_vulgare_newGene_904	darkorange
HORVU1Hr1G081980	darkorange
HORVU7Hr1G104590	darkorange
HORVU6Hr1G071520	darkorange
HORVU7Hr1G038390	darkorange
Hordeum_vulgare_newGene_6101	darkorange
HORVU4Hr1G022270	darkorange
Hordeum_vulgare_newGene_5665	darkorange
HORVU3Hr1G013400	darkorange
HORVU5Hr1G062110	darkorange
HORVU3Hr1G011800	darkorange
HORVU5Hr1G024390	darkorange
HORVU2Hr1G072650	darkorange
HORVU7Hr1G067280	darkorange
HORVU2Hr1G069780	darkorange
HORVU6Hr1G031430	darkorange
HORVU0Hr1G018850	darkorange
HORVU7Hr1G110320	darkorange
HORVU7Hr1G115040	darkorange
HORVU5Hr1G013260	darkorange
HORVU0Hr1G017080	darkorange
HORVU1Hr1G011520	darkorange
HORVU4Hr1G011600	darkorange

HORVU4Hr1G014070	darkorange
HORVU1Hr1G090100	darkorange
HORVU3Hr1G087170	darkorange
HORVU1Hr1G069900	darkorange
HORVU5Hr1G125130	darkorange
Hordeum_vulgare_newGene_9126	darkorange
Hordeum_vulgare_newGene_13801	darkorange
HORVU2Hr1G092090	darkorange
HORVU4Hr1G021950	darkorange
HORVU2Hr1G039370	darkorange
HORVU1Hr1G035720	darkorange
Hordeum_vulgare_newGene_4409	darkorange
HORVU6Hr1G089230	darkorange
HORVU3Hr1G089620	darkorange
Hordeum_vulgare_newGene_13491	darkorange
HORVU2Hr1G068050	darkorange
HORVU5Hr1G075050	darkorange
HORVU5Hr1G037430	darkorange
HORVU3Hr1G068150	darkorange
HORVU2Hr1G005510	darkorange
HORVU6Hr1G061300	darkorange
HORVU4Hr1G041630	darkorange
HORVU0Hr1G002120	darkorange
HORVU5Hr1G001180	darkorange
HORVU2Hr1G043310	darkorange
HORVU1Hr1G029090	darkorange
HORVU3Hr1G031140	darkorange
HORVU7Hr1G048880	darkorange
HORVU7Hr1G024250	darkorange
HORVU5Hr1G014510	darkorange
HORVU2Hr1G002880	darkorange
HORVU2Hr1G123580	darkorange
HORVU1Hr1G085190	darkorange
HORVU7Hr1G050110	darkorange
HORVU3Hr1G086040	darkorange
HORVU0Hr1G014450	darkorange
HORVU2Hr1G097060	darkorange
HORVU5Hr1G117680	darkorange
HORVU2Hr1G049410	darkorange
HORVU2Hr1G011200	darkorange
HORVU7Hr1G021840	darkorange
Hordeum_vulgare_newGene_3867	darkorange
HORVU5Hr1G060310	darkorange
HORVU2Hr1G039350	darkorange
HORVU1Hr1G092150	darkorange
HORVU1Hr1G061830	darkorange
HORVU5Hr1G069680	darkorange

HORVU5Hr1G097770	darkorange
Hordeum_vulgare_newGene_2954	darkorange
HORVU7Hr1G088580	darkorange
HORVU1Hr1G015000	darkorange
HORVU4Hr1G008210	darkorange
HORVU5Hr1G022120	darkorange
HORVU1Hr1G055860	darkorange
HORVU1Hr1G012850	darkorange
HORVU6Hr1G092390	darkorange
HORVU2Hr1G013590	darkorange
HORVU5Hr1G082420	darkorange
HORVU3Hr1G063620	darkorange
HORVU3Hr1G059470	darkorange
HORVU7Hr1G073490	darkorange
HORVU1Hr1G072500	darkorange
HORVU0Hr1G026480	darkorange
HORVU6Hr1G077400	darkorange
Hordeum_vulgare_newGene_7449	darkorange
HORVU6Hr1G058820	darkorange
HORVU5Hr1G125010	darkorange
HORVU3Hr1G001920	darkorange
HORVU2Hr1G001900	darkorange
HORVU2Hr1G041610	darkorange
Hordeum_vulgare_newGene_5781	darkorange
HORVU4Hr1G087740	darkorange
HORVU1Hr1G051470	darkorange
HORVU5Hr1G088880	darkorange
HORVU2Hr1G010630	darkorange
HORVU6Hr1G017530	darkorange
HORVU2Hr1G106970	darkorange
Hordeum_vulgare_newGene_1855	darkorange
Hordeum_vulgare_newGene_1859	darkorange
HORVU4Hr1G069130	darkorange
HORVU7Hr1G075980	darkorange
HORVU7Hr1G053590	darkorange
HORVU0Hr1G021630	darkorange
HORVU1Hr1G019480	darkorange
HORVU2Hr1G093220	darkorange
Hordeum_vulgare_newGene_3547	darkorange
Hordeum_vulgare_newGene_3363	darkorange
Hordeum_vulgare_newGene_15717	darkorange
HORVU7Hr1G070330	darkorange
HORVU1Hr1G074860	darkorange
HORVU2Hr1G030460	darkorange
Hordeum_vulgare_newGene_8515	darkorange
HORVU2Hr1G127740	darkorange
HORVU0Hr1G025520	darkorange

HORVU1Hr1G067140	darkorange
HORVU5Hr1G067530	darkorange
HORVU4Hr1G008080	darkorange
HORVU5Hr1G010360	darkorange
HORVU5Hr1G015460	darkorange
HORVU5Hr1G022570	darkorange
HORVU4Hr1G013180	darkorange
HORVU5Hr1G027890	darkorange
HORVU5Hr1G094220	darkorange
HORVU1Hr1G014570	darkorange
HORVU1Hr1G067480	darkorange
HORVU5Hr1G055920	darkorange
HORVU6Hr1G064230	darkorange
HORVU7Hr1G094070	darkorange
HORVU5Hr1G013840	darkorange
HORVU6Hr1G019290	darkorange
HORVU5Hr1G095630	darkorange
HORVU5Hr1G093770	darkorange
HORVU5Hr1G073870	darkorange
Hordeum_vulgare_newGene_184	darkorange
Hordeum_vulgare_newGene_180	darkorange
HORVU2Hr1G036960	darkorange
Hordeum_vulgare_newGene_5709	darkorange
HORVU5Hr1G087780	darkorange
HORVU5Hr1G116710	darkorange
HORVU1Hr1G087320	darkorange
HORVU7Hr1G108830	darkorange
HORVU3Hr1G019750	darkorange
HORVU3Hr1G049060	darkorange
HORVU5Hr1G105250	darkorange
HORVU5Hr1G058690	darkorange
HORVU7Hr1G101220	darkorange
HORVU5Hr1G056140	darkorange
HORVU6Hr1G006080	darkorange
HORVU3Hr1G079420	darkorange
HORVU6Hr1G047080	darkorange
HORVU7Hr1G051160	darkorange
Hordeum_vulgare_newGene_5603	darkorange
HORVU5Hr1G097870	darkorange
HORVU5Hr1G016090	darkorange
HORVU5Hr1G049420	darkorange
HORVU6Hr1G090220	darkorange
HORVU2Hr1G060120	darkorange
HORVU7Hr1G077220	darkorange
HORVU7Hr1G002110	darkorange
HORVU6Hr1G027440	darkorange
HORVU4Hr1G082740	darkorange

HORVU2Hr1G060460	darkorange
Hordeum_vulgare_newGene_13741	darkorange
HORVU6Hr1G021150	darkorange
HORVU2Hr1G112370	darkorange
HORVU6Hr1G070900	darkorange
HORVU3Hr1G011770	darkorange
HORVU2Hr1G113410	darkorange
HORVU5Hr1G021560	darkorange
HORVU3Hr1G082740	darkorange
HORVU2Hr1G059380	darkorange
HORVU5Hr1G074840	darkorange
HORVU4Hr1G011060	darkorange
HORVU6Hr1G085830	darkorange
HORVU5Hr1G015370	darkorange
HORVU4Hr1G008310	darkorange
Hordeum_vulgare_newGene_10209	darkorange
HORVU5Hr1G095080	darkorange
HORVU5Hr1G120650	darkorange
HORVU5Hr1G048630	darkorange
HORVU4Hr1G082880	darkorange
HORVU7Hr1G021630	darkorange
HORVU7Hr1G045640	darkorange
HORVU4Hr1G062500	darkorange
HORVU7Hr1G071730	darkorange
HORVU6Hr1G050750	darkorange
HORVU7Hr1G075290	darkorange
Hordeum_vulgare_newGene_13305	darkorange
HORVU2Hr1G020070	darkorange
HORVU5Hr1G070040	darkorange
Hordeum_vulgare_newGene_15139	darkorange
HORVU3Hr1G110340	darkorange
Hordeum_vulgare_newGene_5363	darkorange
HORVU5Hr1G063320	darkorange
HORVU7Hr1G102170	darkorange
HORVU2Hr1G069590	darkorange
HORVU0Hr1G004990	darkorange
HORVU2Hr1G120780	darkorange
HORVU5Hr1G033050	darkorange
Hordeum_vulgare_newGene_13324	darkorange
Hordeum_vulgare_newGene_14743	darkorange
HORVU5Hr1G069850	darkorange
HORVU3Hr1G093530	darkorange
HORVU6Hr1G085110	darkorange
HORVU5Hr1G096090	darkorange
Hordeum_vulgare_newGene_15240	darkorange
HORVU2Hr1G092280	darkorange
HORVU2Hr1G111300	darkorange

HORVU2Hr1G039700	darkorange
HORVU3Hr1G062370	darkorange
HORVU4Hr1G077430	darkorange
HORVU4Hr1G069070	darkorange
Hordeum_vulgare_newGene_11475	darkorange
HORVU5Hr1G071920	darkorange
HORVU6Hr1G090390	darkorange
HORVU3Hr1G076000	darkorange
HORVU3Hr1G081170	darkorange
Hordeum_vulgare_newGene_6078	steelblue
HORVU4Hr1G076420	steelblue
HORVU1Hr1G074530	steelblue
HORVU5Hr1G103060	steelblue
HORVU3Hr1G002540	steelblue
HORVU7Hr1G057100	steelblue
HORVU1Hr1G015610	steelblue
HORVU1Hr1G016140	steelblue
HORVU3Hr1G064760	steelblue
HORVU4Hr1G000750	steelblue
HORVU3Hr1G099740	steelblue
HORVU4Hr1G008550	steelblue
HORVU0Hr1G004040	steelblue
HORVU3Hr1G004250	steelblue
HORVU0Hr1G030660	steelblue
HORVU1Hr1G075580	steelblue
HORVU1Hr1G057880	steelblue
Hordeum_vulgare_newGene_8679	steelblue
HORVU2Hr1G019830	steelblue
HORVU2Hr1G005520	steelblue
HORVU4Hr1G088280	steelblue
HORVU5Hr1G038370	steelblue
HORVU2Hr1G015590	steelblue
HORVU5Hr1G125500	steelblue
Hordeum_vulgare_newGene_14687	steelblue
HORVU7Hr1G046230	steelblue
Hordeum_vulgare_newGene_13334	steelblue
Hordeum_vulgare_newGene_5866	steelblue
Hordeum_vulgare_newGene_5137	steelblue
Hordeum_vulgare_newGene_2819	steelblue
HORVU1Hr1G089700	steelblue
HORVU4Hr1G012760	steelblue
Hordeum_vulgare_newGene_7801	steelblue
HORVU0Hr1G005790	steelblue
Hordeum_vulgare_newGene_11923	steelblue
HORVU1Hr1G052180	steelblue
HORVU6Hr1G091840	steelblue
HORVU3Hr1G059250	steelblue

HORVU1Hr1G046420	steelblue
HORVU1Hr1G052850	steelblue
HORVU5Hr1G105900	steelblue
HORVU2Hr1G008250	steelblue
HORVU5Hr1G058330	steelblue
HORVU4Hr1G056860	steelblue
HORVU1Hr1G004580	steelblue
HORVU7Hr1G051810	steelblue
HORVU3Hr1G037780	steelblue
HORVU4Hr1G021260	steelblue
HORVU2Hr1G099570	steelblue
HORVU2Hr1G119430	steelblue
HORVU0Hr1G005400	steelblue
HORVU5Hr1G061760	steelblue
Hordeum_vulgare_newGene_8990	steelblue
HORVU4Hr1G081310	steelblue
HORVU4Hr1G014190	steelblue
HORVU5Hr1G121730	steelblue
HORVU7Hr1G009930	steelblue
Hordeum_vulgare_newGene_10036	steelblue
Hordeum_vulgare_newGene_12068	steelblue
HORVU4Hr1G074710	steelblue
HORVU3Hr1G004280	steelblue
HORVU2Hr1G118360	steelblue
HORVU5Hr1G119550	steelblue
HORVU0Hr1G023280	steelblue
Hordeum_vulgare_newGene_714	steelblue
Hordeum_vulgare_newGene_5915	steelblue
Hordeum_vulgare_newGene_15939	steelblue
HORVU4Hr1G054110	steelblue
Hordeum_vulgare_newGene_10795	steelblue
HORVU6Hr1G077790	steelblue
HORVU1Hr1G064890	steelblue
Hordeum_vulgare_newGene_12491	steelblue
Hordeum_vulgare_newGene_900	steelblue
HORVU3Hr1G016990	steelblue
HORVU3Hr1G071030	steelblue
HORVU1Hr1G078480	steelblue
HORVU7Hr1G094980	steelblue
HORVU6Hr1G004980	steelblue
Hordeum_vulgare_newGene_10218	steelblue
HORVU3Hr1G012510	steelblue
HORVU2Hr1G098470	steelblue
HORVU7Hr1G113300	steelblue
Hordeum_vulgare_newGene_4265	steelblue
HORVU5Hr1G021070	steelblue
Hordeum_vulgare_newGene_13643	steelblue

HORVU1Hr1G090360	steelblue
HORVU6Hr1G000960	steelblue
Hordeum_vulgare_newGene_11100	steelblue
HORVU4Hr1G090850	steelblue
HORVU3Hr1G025720	steelblue
Hordeum_vulgare_newGene_5332	steelblue
HORVU0Hr1G002720	steelblue
HORVU3Hr1G004070	steelblue
HORVU2Hr1G126760	steelblue
Hordeum_vulgare_newGene_10462	steelblue
Hordeum_vulgare_newGene_10463	steelblue
Hordeum_vulgare_newGene_11739	steelblue
HORVU6Hr1G085760	steelblue
Hordeum_vulgare_newGene_12416	steelblue
HORVU3Hr1G086940	steelblue
HORVU4Hr1G017240	steelblue
Hordeum_vulgare_newGene_10344	steelblue
HORVU1Hr1G014000	steelblue
HORVU5Hr1G099700	steelblue
HORVU1Hr1G008540	steelblue
HORVU3Hr1G069140	steelblue
HORVU7Hr1G049130	steelblue
HORVU3Hr1G030770	steelblue
HORVU1Hr1G089520	steelblue
HORVU5Hr1G113780	steelblue
HORVU1Hr1G011950	steelblue
HORVU5Hr1G093580	steelblue
HORVU3Hr1G095360	steelblue
Hordeum_vulgare_newGene_599	steelblue
Hordeum_vulgare_newGene_13168	steelblue
HORVU1Hr1G042250	steelblue
HORVU2Hr1G075950	steelblue
HORVU4Hr1G076520	steelblue
HORVU7Hr1G010570	steelblue
HORVU3Hr1G071490	steelblue
HORVU5Hr1G007480	steelblue
HORVU7Hr1G051840	steelblue
HORVU4Hr1G049500	steelblue
HORVU7Hr1G077270	steelblue
HORVU7Hr1G093830	steelblue
HORVU5Hr1G010880	steelblue
Hordeum_vulgare_newGene_13361	steelblue
HORVU1Hr1G070690	steelblue
HORVU5Hr1G078020	steelblue
HORVU3Hr1G021490	steelblue
Hordeum_vulgare_newGene_10635	steelblue
HORVU2Hr1G026880	steelblue

HORVU6Hr1G069280	steelblue
Hordeum_vulgare_newGene_12118	steelblue
HORVU5Hr1G000400	steelblue
HORVU7Hr1G091910	steelblue
HORVU1Hr1G057940	steelblue
HORVU4Hr1G054980	steelblue
HORVU5Hr1G099910	steelblue
HORVU6Hr1G068150	steelblue
Hordeum_vulgare_newGene_15842	steelblue
Hordeum_vulgare_newGene_11602	steelblue
HORVU2Hr1G028760	steelblue
HORVU7Hr1G078670	steelblue
HORVU3Hr1G059600	steelblue
Hordeum_vulgare_newGene_8535	steelblue
HORVU7Hr1G119870	steelblue
HORVU2Hr1G126150	steelblue
HORVU3Hr1G007830	steelblue
HORVU1Hr1G085640	steelblue
HORVU5Hr1G111590	steelblue
HORVU3Hr1G099550	steelblue
Hordeum_vulgare_newGene_8731	steelblue
HORVU5Hr1G047150	steelblue
HORVU3Hr1G108950	steelblue
Hordeum_vulgare_newGene_6019	steelblue
Hordeum_vulgare_newGene_6018	steelblue
Hordeum_vulgare_newGene_3198	steelblue
HORVU3Hr1G087450	steelblue
HORVU2Hr1G015570	steelblue
HORVU1Hr1G063630	steelblue
HORVU5Hr1G097100	steelblue
Hordeum_vulgare_newGene_2004	steelblue
Hordeum_vulgare_newGene_14867	steelblue
HORVU2Hr1G007130	steelblue
HORVU2Hr1G088520	steelblue
Hordeum_vulgare_newGene_3676	steelblue
HORVU7Hr1G093190	steelblue
HORVU3Hr1G030340	steelblue
HORVU7Hr1G080740	steelblue
HORVU3Hr1G091920	steelblue
HORVU1Hr1G092040	steelblue
HORVU4Hr1G059270	steelblue
HORVU6Hr1G045180	steelblue
HORVU4Hr1G085480	steelblue
HORVU4Hr1G007530	steelblue
HORVU2Hr1G022160	steelblue
HORVU6Hr1G018620	steelblue
HORVU7Hr1G073050	steelblue

Hordeum_vulgare_newGene_4684	steelblue
Hordeum_vulgare_newGene_6681	steelblue
Hordeum_vulgare_newGene_10645	steelblue
HORVU1Hr1G004150	steelblue
Hordeum_vulgare_newGene_1942	steelblue
HORVU1Hr1G084190	steelblue
HORVU6Hr1G072390	steelblue
Hordeum_vulgare_newGene_4234	steelblue
HORVU1Hr1G064130	steelblue
HORVU5Hr1G119580	steelblue
HORVU2Hr1G002500	steelblue
HORVU7Hr1G030670	steelblue
HORVU1Hr1G059910	steelblue
HORVU4Hr1G081390	steelblue
HORVU1Hr1G055930	steelblue
HORVU0Hr1G027130	steelblue
Hordeum_vulgare_newGene_12447	steelblue
Hordeum_vulgare_newGene_3447	steelblue
HORVU1Hr1G055340	steelblue
HORVU1Hr1G090180	steelblue
HORVU6Hr1G084070	steelblue
HORVU4Hr1G086300	steelblue
HORVU1Hr1G012060	steelblue
Hordeum_vulgare_newGene_14333	steelblue
Hordeum_vulgare_newGene_2506	steelblue
HORVU2Hr1G105140	steelblue
Hordeum_vulgare_newGene_9945	steelblue
HORVU2Hr1G094280	steelblue
HORVU2Hr1G108780	steelblue
HORVU3Hr1G107240	steelblue
HORVU4Hr1G011210	steelblue
HORVU7Hr1G077860	steelblue
Hordeum_vulgare_newGene_16159	steelblue
HORVU3Hr1G085080	steelblue
HORVU5Hr1G058310	steelblue
HORVU1Hr1G039540	steelblue
HORVU1Hr1G077420	steelblue
HORVU3Hr1G011260	steelblue
HORVU7Hr1G045150	steelblue
Hordeum_vulgare_newGene_197	steelblue
HORVU2Hr1G013450	steelblue
HORVU4Hr1G033790	steelblue
HORVU3Hr1G067990	steelblue
HORVU1Hr1G031490	steelblue
HORVU7Hr1G073790	steelblue
Hordeum_vulgare_newGene_8268	steelblue
Hordeum_vulgare_newGene_8269	steelblue

HORVU3Hr1G104600	steelblue
HORVU3Hr1G059030	steelblue
HORVU3Hr1G039050	steelblue
HORVU7Hr1G107310	steelblue
HORVU5Hr1G013400	steelblue
Hordeum_vulgare_newGene_11164	steelblue
Hordeum_vulgare_newGene_11161	steelblue
Hordeum_vulgare_newGene_10970	steelblue
HORVU2Hr1G104130	steelblue
HORVU1Hr1G035100	steelblue
HORVU6Hr1G005030	steelblue
Hordeum_vulgare_newGene_13970	steelblue
Hordeum_vulgare_newGene_8346	steelblue
Hordeum_vulgare_newGene_6068	steelblue
HORVU6Hr1G068080	steelblue
HORVU1Hr1G053050	steelblue
HORVU5Hr1G096760	steelblue
HORVU3Hr1G030650	steelblue
HORVU4Hr1G082200	steelblue
HORVU1Hr1G013990	steelblue
HORVU1Hr1G042280	steelblue
HORVU3Hr1G028990	steelblue
HORVU5Hr1G057410	steelblue
HORVU5Hr1G095160	steelblue
HORVU5Hr1G093640	steelblue
HORVU5Hr1G000330	steelblue
Hordeum_vulgare_newGene_14565	steelblue
HORVU5Hr1G021050	steelblue
HORVU2Hr1G046600	steelblue
HORVU2Hr1G000090	steelblue
HORVU5Hr1G085070	steelblue
Hordeum_vulgare_newGene_5314	steelblue
Hordeum_vulgare_newGene_13848	steelblue
Hordeum_vulgare_newGene_13847	steelblue
Hordeum_vulgare_newGene_8349	steelblue
Hordeum_vulgare_newGene_1548	steelblue
HORVU3Hr1G076840	steelblue
HORVU7Hr1G006580	steelblue
HORVU3Hr1G098160	steelblue
HORVU2Hr1G040170	steelblue
HORVU6Hr1G000710	steelblue
HORVU7Hr1G027520	steelblue
HORVU3Hr1G011560	steelblue
HORVU4Hr1G017260	steelblue
HORVU4Hr1G002510	steelblue
HORVU5Hr1G092220	steelblue
HORVU7Hr1G036450	steelblue

HORVU2Hr1G099870	steelblue
HORVU2Hr1G019680	steelblue
Hordeum_vulgare_newGene_3667	steelblue
HORVU7Hr1G000320	steelblue
HORVU7Hr1G114020	steelblue
Hordeum_vulgare_newGene_14888	steelblue
Hordeum_vulgare_newGene_6465	steelblue
HORVU7Hr1G069420	steelblue
HORVU4Hr1G087780	steelblue
HORVU6Hr1G054890	steelblue
HORVU6Hr1G012530	steelblue
HORVU4Hr1G020060	steelblue
HORVU2Hr1G071890	steelblue
HORVU2Hr1G018440	steelblue
HORVU7Hr1G042300	steelblue
Hordeum_vulgare_newGene_5981	steelblue
Hordeum_vulgare_newGene_815	steelblue
HORVU4Hr1G056870	steelblue
HORVU5Hr1G093270	steelblue
HORVU7Hr1G094890	steelblue
Hordeum_vulgare_newGene_13342	steelblue
Hordeum_vulgare_newGene_13344	steelblue
HORVU4Hr1G085460	steelblue
HORVU6Hr1G007020	steelblue
HORVU4Hr1G051390	steelblue
Hordeum_vulgare_newGene_14011	steelblue
HORVU1Hr1G070730	steelblue
HORVU5Hr1G023090	steelblue
HORVU3Hr1G004290	steelblue
HORVU5Hr1G119500	steelblue
HORVU6Hr1G020310	steelblue
Hordeum_vulgare_newGene_1819	steelblue
Hordeum_vulgare_newGene_13455	steelblue
Hordeum_vulgare_newGene_1849	steelblue
HORVU0Hr1G017760	steelblue
HORVU3Hr1G066340	steelblue
Hordeum_vulgare_newGene_146	steelblue
Hordeum_vulgare_newGene_11434	steelblue
HORVU0Hr1G005140	steelblue
HORVU6Hr1G083180	steelblue
HORVU1Hr1G004130	steelblue
HORVU3Hr1G114200	steelblue
HORVU5Hr1G111350	steelblue
HORVU5Hr1G077880	steelblue
HORVU4Hr1G061900	steelblue
Hordeum_vulgare_newGene_8376	steelblue
HORVU7Hr1G058630	steelblue

HORVU6Hr1G075270	steelblue
HORVU6Hr1G071030	steelblue
Hordeum_vulgare_newGene_10601	steelblue
Hordeum_vulgare_newGene_11705	steelblue
Hordeum_vulgare_newGene_16169	steelblue
HORVU2Hr1G065060	steelblue
Hordeum_vulgare_newGene_5822	steelblue
Hordeum_vulgare_newGene_1490	steelblue
HORVU6Hr1G084010	steelblue
HORVU7Hr1G081320	steelblue
HORVU4Hr1G084390	steelblue
HORVU7Hr1G006280	steelblue
Hordeum_vulgare_newGene_8462	steelblue
HORVU2Hr1G117820	steelblue
HORVU2Hr1G002520	steelblue
HORVU6Hr1G080410	steelblue
HORVU1Hr1G068940	steelblue
Hordeum_vulgare_newGene_3464	steelblue
HORVU2Hr1G019850	steelblue
HORVU4Hr1G061280	steelblue
HORVU4Hr1G014600	steelblue
HORVU7Hr1G053100	steelblue
HORVU2Hr1G044590	steelblue
HORVU3Hr1G069300	steelblue
HORVU4Hr1G088540	steelblue
HORVU3Hr1G099470	steelblue
Hordeum_vulgare_newGene_10336	steelblue
HORVU2Hr1G104080	steelblue
HORVU3Hr1G077740	steelblue
HORVU3Hr1G037370	steelblue
HORVU1Hr1G059870	steelblue
HORVU5Hr1G005930	steelblue
HORVU5Hr1G050950	steelblue
HORVU3Hr1G093270	steelblue
Hordeum_vulgare_newGene_4784	steelblue
Hordeum_vulgare_newGene_4781	steelblue
Hordeum_vulgare_newGene_7478	steelblue
Hordeum_vulgare_newGene_6789	steelblue
HORVU3Hr1G081530	steelblue
Hordeum_vulgare_newGene_14010	steelblue
Hordeum_vulgare_newGene_13893	steelblue
HORVU1Hr1G075830	steelblue
HORVU1Hr1G071390	steelblue
HORVU7Hr1G030690	steelblue
HORVU7Hr1G099330	steelblue
HORVU7Hr1G018580	steelblue
HORVU2Hr1G108600	steelblue

HORVU7Hr1G082380	steelblue
Hordeum_vulgare_newGene_13911	steelblue
Hordeum_vulgare_newGene_11407	steelblue
HORVU7Hr1G025460	steelblue
Hordeum_vulgare_newGene_2278	steelblue
HORVU2Hr1G073840	steelblue
Hordeum_vulgare_newGene_6261	steelblue
Hordeum_vulgare_newGene_4099	steelblue
HORVU5Hr1G105840	steelblue
HORVU2Hr1G002440	steelblue
HORVU7Hr1G098490	steelblue
HORVU5Hr1G125460	steelblue
HORVU5Hr1G002090	steelblue
HORVU3Hr1G100380	steelblue
HORVU2Hr1G085270	steelblue
Hordeum_vulgare_newGene_14099	steelblue
Hordeum_vulgare_newGene_504	steelblue
HORVU4Hr1G070320	steelblue
HORVU2Hr1G005670	steelblue
HORVU6Hr1G068180	steelblue
Hordeum_vulgare_newGene_5249	steelblue
HORVU1Hr1G078070	steelblue
HORVU3Hr1G086380	steelblue
HORVU7Hr1G010500	steelblue
HORVU7Hr1G007590	steelblue
HORVU6Hr1G089590	steelblue
HORVU2Hr1G070360	steelblue
HORVU7Hr1G087700	steelblue
Hordeum_vulgare_newGene_38	steelblue
HORVU6Hr1G062070	steelblue
HORVU7Hr1G100150	steelblue
HORVU1Hr1G091230	steelblue
HORVU3Hr1G057760	steelblue
HORVU1Hr1G012710	steelblue
Hordeum_vulgare_newGene_10855	steelblue
HORVU4Hr1G075720	steelblue
HORVU2Hr1G103150	steelblue
Hordeum_vulgare_newGene_4019	steelblue
Hordeum_vulgare_newGene_14946	steelblue
Hordeum_vulgare_newGene_14945	steelblue
HORVU0Hr1G006720	steelblue
HORVU2Hr1G122040	steelblue
Hordeum_vulgare_newGene_11522	steelblue
HORVU2Hr1G079900	steelblue
HORVU7Hr1G096690	steelblue
HORVU6Hr1G068100	steelblue
HORVU6Hr1G020080	steelblue

HORVU6Hr1G066610	steelblue
Hordeum_vulgare_newGene_4228	steelblue
HORVU5Hr1G086520	steelblue
HORVU3Hr1G039480	steelblue
Hordeum_vulgare_newGene_2968	steelblue
Hordeum_vulgare_newGene_13122	steelblue
HORVU5Hr1G092160	steelblue
HORVU1Hr1G059900	steelblue
HORVU1Hr1G009140	steelblue
Hordeum_vulgare_newGene_12405	steelblue
Hordeum_vulgare_newGene_8799	steelblue
HORVU2Hr1G090030	steelblue
HORVU0Hr1G016380	steelblue
HORVU3Hr1G063430	steelblue
HORVU5Hr1G103460	steelblue
HORVU2Hr1G079610	steelblue
HORVU1Hr1G089730	steelblue
HORVU6Hr1G078060	steelblue
HORVU6Hr1G090910	steelblue
HORVU4Hr1G090170	steelblue
Hordeum_vulgare_newGene_6405	steelblue
Hordeum_vulgare_newGene_5401	steelblue
HORVU4Hr1G082680	steelblue
HORVU1Hr1G084310	steelblue
Hordeum_vulgare_newGene_3269	steelblue
HORVU3Hr1G110540	steelblue
Hordeum_vulgare_newGene_10395	steelblue
HORVU3Hr1G001000	steelblue
HORVU3Hr1G078210	steelblue
HORVU5Hr1G092120	steelblue
HORVU3Hr1G095930	steelblue
HORVU3Hr1G026180	steelblue
HORVU1Hr1G035990	steelblue
HORVU3Hr1G003130	steelblue
HORVU5Hr1G040440	steelblue
HORVU7Hr1G091180	steelblue
HORVU4Hr1G074750	steelblue
HORVU3Hr1G078390	steelblue
HORVU3Hr1G089250	steelblue
Hordeum_vulgare_newGene_15053	steelblue
Hordeum_vulgare_newGene_10027	steelblue
HORVU2Hr1G108340	steelblue
Hordeum_vulgare_newGene_8256	steelblue
HORVU5Hr1G014120	steelblue
Hordeum_vulgare_newGene_3053	steelblue
HORVU3Hr1G037260	steelblue
Hordeum_vulgare_newGene_3292	steelblue

HORVU2Hr1G052920	steelblue
HORVU5Hr1G115310	steelblue
HORVU3Hr1G075690	steelblue
HORVU2Hr1G104120	steelblue
HORVU2Hr1G105740	steelblue
HORVU6Hr1G070090	steelblue
HORVU7Hr1G078330	steelblue
HORVU5Hr1G121350	steelblue
Hordeum_vulgare_newGene_4024	steelblue
Hordeum_vulgare_newGene_4023	steelblue
HORVU4Hr1G073120	steelblue
HORVU3Hr1G035470	steelblue
HORVU1Hr1G021750	steelblue
HORVU6Hr1G000340	steelblue
HORVU2Hr1G017270	steelblue
Hordeum_vulgare_newGene_10602	steelblue
Hordeum_vulgare_newGene_1988	steelblue
Hordeum_vulgare_newGene_2379	steelblue
HORVU5Hr1G061410	steelblue
Hordeum_vulgare_newGene_9314	steelblue
Hordeum_vulgare_newGene_15394	steelblue
HORVU3Hr1G004230	steelblue
HORVU5Hr1G064040	steelblue
Hordeum_vulgare_newGene_4273	steelblue
HORVU3Hr1G002150	steelblue
HORVU1Hr1G067370	steelblue
HORVU2Hr1G110230	steelblue
Hordeum_vulgare_newGene_15151	steelblue
Hordeum_vulgare_newGene_13878	steelblue
HORVU6Hr1G073540	steelblue
HORVU5Hr1G079670	steelblue
HORVU5Hr1G080210	steelblue
HORVU5Hr1G045750	steelblue
HORVU5Hr1G125380	steelblue
Hordeum_vulgare_newGene_14129	steelblue
Hordeum_vulgare_newGene_10809	steelblue
Hordeum_vulgare_newGene_10802	steelblue
HORVU4Hr1G055510	steelblue
HORVU7Hr1G027860	steelblue
HORVU2Hr1G026810	steelblue
HORVU3Hr1G017990	steelblue
HORVU4Hr1G010150	steelblue
Hordeum_vulgare_newGene_1900	steelblue
HORVU6Hr1G060990	steelblue
HORVU2Hr1G005330	steelblue
Hordeum_vulgare_newGene_7495	steelblue
Hordeum_vulgare_newGene_14892	steelblue

HORVU2Hr1G083960	steelblue
HORVU5Hr1G068820	steelblue
HORVU2Hr1G028920	steelblue
HORVU3Hr1G003980	steelblue
HORVU4Hr1G056800	steelblue
HORVU1Hr1G010500	steelblue
Hordeum_vulgare_newGene_12509	steelblue
HORVU3Hr1G050350	steelblue
HORVU4Hr1G008990	steelblue
Hordeum_vulgare_newGene_13359	steelblue
HORVU7Hr1G003170	steelblue
HORVU4Hr1G005690	steelblue
HORVU1Hr1G000690	steelblue
Hordeum_vulgare_newGene_12292	steelblue
HORVU3Hr1G091800	steelblue
Hordeum_vulgare_newGene_13659	steelblue
HORVU1Hr1G018360	steelblue
HORVU0Hr1G006240	steelblue
HORVU1Hr1G069540	steelblue
HORVU5Hr1G005910	steelblue
HORVU1Hr1G059950	steelblue
HORVU5Hr1G000630	steelblue
Hordeum_vulgare_newGene_9578	steelblue
Hordeum_vulgare_newGene_9902	steelblue
HORVU7Hr1G064170	steelblue
HORVU7Hr1G050510	steelblue
HORVU6Hr1G000680	steelblue
HORVU1Hr1G060110	steelblue
HORVU7Hr1G082040	steelblue
Hordeum_vulgare_newGene_11612	steelblue
Hordeum_vulgare_newGene_3534	steelblue
Hordeum_vulgare_newGene_737	steelblue
HORVU5Hr1G077810	steelblue
HORVU3Hr1G074000	steelblue
HORVU5Hr1G010330	steelblue
HORVU5Hr1G092150	steelblue
HORVU3Hr1G056710	steelblue
HORVU5Hr1G109290	steelblue
HORVU6Hr1G000490	steelblue
HORVU4Hr1G001450	steelblue
Hordeum_vulgare_newGene_13934	steelblue
HORVU3Hr1G080110	steelblue
HORVU3Hr1G068390	steelblue
HORVU4Hr1G021720	steelblue
HORVU7Hr1G108790	steelblue
Hordeum_vulgare_newGene_1286	steelblue
HORVU7Hr1G106230	steelblue

Hordeum_vulgare_newGene_1135	steelblue
HORVU5Hr1G051280	steelblue
HORVU1Hr1G079150	steelblue
Hordeum_vulgare_newGene_7034	steelblue
HORVU3Hr1G039700	steelblue
Hordeum_vulgare_newGene_2015	steelblue
HORVU2Hr1G108580	steelblue
HORVU5Hr1G073140	steelblue
Hordeum_vulgare_newGene_13757	steelblue
HORVU2Hr1G075930	steelblue
HORVU5Hr1G103420	steelblue
Hordeum_vulgare_newGene_4247	steelblue
HORVU5Hr1G012710	steelblue
HORVU1Hr1G083660	steelblue
HORVU6Hr1G089570	steelblue
HORVU3Hr1G057810	steelblue
Hordeum_vulgare_newGene_8303	steelblue
Hordeum_vulgare_newGene_8302	steelblue
HORVU7Hr1G078960	steelblue
Hordeum_vulgare_newGene_9822	steelblue
HORVU4Hr1G012060	steelblue
Hordeum_vulgare_newGene_16158	steelblue
Hordeum_vulgare_newGene_12435	steelblue
HORVU4Hr1G052450	steelblue
Hordeum_vulgare_newGene_5832	steelblue
HORVU5Hr1G006290	steelblue
HORVU2Hr1G073730	steelblue
Hordeum_vulgare_newGene_14920	steelblue
HORVU3Hr1G084420	steelblue
HORVU1Hr1G015620	steelblue
HORVU7Hr1G071230	steelblue
HORVU3Hr1G099770	steelblue
Hordeum_vulgare_newGene_12080	steelblue
HORVU3Hr1G011110	steelblue
HORVU3Hr1G043210	steelblue
HORVU5Hr1G098280	steelblue
HORVU3Hr1G011350	steelblue
Hordeum_vulgare_newGene_4401	steelblue
Hordeum_vulgare_newGene_6045	steelblue
HORVU1Hr1G059920	steelblue
HORVU6Hr1G030080	steelblue
Hordeum_vulgare_newGene_1625	steelblue
Hordeum_vulgare_newGene_1628	steelblue
HORVU4Hr1G051780	steelblue
Hordeum_vulgare_newGene_16220	steelblue
HORVU2Hr1G009830	steelblue
HORVU3Hr1G117370	steelblue

HORVU7Hr1G027870	steelblue
HORVU5Hr1G072160	steelblue
HORVU2Hr1G009580	steelblue
Hordeum_vulgare_newGene_2575	steelblue
HORVU5Hr1G094680	steelblue
Hordeum_vulgare_newGene_4130	steelblue
Hordeum_vulgare_newGene_7443	steelblue
HORVU1Hr1G011930	steelblue
HORVU7Hr1G032040	steelblue
Hordeum_vulgare_newGene_6228	steelblue
HORVU4Hr1G059280	steelblue
HORVU3Hr1G052500	steelblue
Hordeum_vulgare_newGene_858	steelblue
HORVU3Hr1G058570	steelblue
HORVU1Hr1G004200	steelblue
HORVU7Hr1G058810	steelblue
HORVU5Hr1G092100	steelblue
HORVU2Hr1G030660	steelblue
Hordeum_vulgare_newGene_8751	steelblue
HORVU4Hr1G090460	steelblue
Hordeum_vulgare_newGene_10620	steelblue
Hordeum_vulgare_newGene_13626	steelblue
Hordeum_vulgare_newGene_1103	steelblue
Hordeum_vulgare_newGene_4558	steelblue
Hordeum_vulgare_newGene_9383	steelblue
HORVU5Hr1G118530	steelblue
HORVU3Hr1G108100	steelblue
HORVU4Hr1G058560	steelblue
HORVU7Hr1G026860	steelblue
HORVU6Hr1G025430	steelblue
HORVU6Hr1G076530	steelblue
HORVU0Hr1G000500	steelblue
HORVU1Hr1G076500	steelblue
HORVU5Hr1G081060	steelblue
HORVU6Hr1G038050	steelblue
HORVU2Hr1G065960	steelblue
HORVU3Hr1G026120	steelblue
HORVU3Hr1G004300	steelblue
HORVU0Hr1G027480	steelblue
HORVU2Hr1G105760	steelblue
HORVU3Hr1G113080	steelblue
HORVU1Hr1G087130	steelblue
HORVU3Hr1G016230	steelblue
HORVU5Hr1G118010	steelblue
HORVU5Hr1G053020	steelblue
HORVU1Hr1G082820	steelblue
HORVU3Hr1G097860	steelblue

Hordeum_vulgare_newGene_3756	black
Hordeum_vulgare_newGene_14186	black
Hordeum_vulgare_newGene_14180	black
Hordeum_vulgare_newGene_5759	black
Hordeum_vulgare_newGene_1185	black
HORVU5Hr1G029410	black
HORVU1Hr1G056560	black
Hordeum_vulgare_newGene_6333	black
HORVU1Hr1G089780	black
HORVU3Hr1G009510	black
HORVU6Hr1G085320	black
HORVU7Hr1G085450	black
HORVU6Hr1G016710	black
HORVU4Hr1G009340	black
HORVU5Hr1G006910	black
HORVU5Hr1G087930	black
Hordeum_vulgare_newGene_2303	black
HORVU3Hr1G050770	black
Hordeum_vulgare_newGene_9372	black
HORVU4Hr1G073730	black
HORVU7Hr1G040720	black
HORVU1Hr1G018710	black
HORVU2Hr1G116150	black
Hordeum_vulgare_newGene_5495	black
HORVU2Hr1G014140	black
HORVU4Hr1G074620	black
HORVU5Hr1G012500	black
HORVU3Hr1G003060	black
HORVU3Hr1G019590	black
HORVU2Hr1G009160	black
HORVU4Hr1G025330	black
HORVU3Hr1G038220	black
HORVU7Hr1G029620	black
HORVU2Hr1G060010	black
HORVU7Hr1G002200	black
Hordeum_vulgare_newGene_2481	black
Hordeum_vulgare_newGene_8076	black
Hordeum_vulgare_newGene_14232	black
HORVU7Hr1G056310	black
HORVU3Hr1G113210	black
Hordeum_vulgare_newGene_2817	black
HORVU5Hr1G062410	black
HORVU2Hr1G085280	black
HORVU4Hr1G082040	black
HORVU4Hr1G031220	black
HORVU3Hr1G079180	black
HORVU3Hr1G029200	black

HORVU2Hr1G038940	black
HORVU2Hr1G103130	black
Hordeum_vulgare_newGene_13780	black
Hordeum_vulgare_newGene_6863	black
HORVU4Hr1G046620	black
HORVU2Hr1G008440	black
HORVU6Hr1G023070	black
HORVU5Hr1G013290	black
HORVU3Hr1G034040	black
HORVU7Hr1G046040	black
HORVU5Hr1G059070	black
HORVU4Hr1G069990	black
HORVU7Hr1G084420	black
HORVU7Hr1G006600	black
HORVU2Hr1G010660	black
HORVU5Hr1G098780	black
HORVU3Hr1G108620	black
HORVU6Hr1G090280	black
HORVU5Hr1G068800	black
Hordeum_vulgare_newGene_14439	black
HORVU1Hr1G081430	black
HORVU3Hr1G036970	black
HORVU1Hr1G088870	black
HORVU5Hr1G104050	black
Hordeum_vulgare_newGene_7663	black
HORVU4Hr1G069380	black
HORVU7Hr1G092070	black
HORVU6Hr1G001160	black
Hordeum_vulgare_newGene_9034	black
Hordeum_vulgare_newGene_9037	black
Hordeum_vulgare_newGene_4661	black
HORVU7Hr1G079830	black
Hordeum_vulgare_newGene_2233	black
HORVU7Hr1G054530	black
HORVU2Hr1G004790	black
HORVU4Hr1G002050	black
HORVU7Hr1G053260	black
HORVU5Hr1G124880	black
HORVU7Hr1G120660	black
HORVU2Hr1G101710	black
HORVU7Hr1G078490	black
HORVU5Hr1G115820	black
HORVU2Hr1G116390	black
Hordeum_vulgare_newGene_14837	black
HORVU3Hr1G079920	black
HORVU1Hr1G083160	black
HORVU5Hr1G054010	black

HORVU5Hr1G058740	black
HORVU2Hr1G002690	black
Hordeum_vulgare_newGene_11183	black
HORVU7Hr1G069770	black
HORVU6Hr1G095070	black
HORVU5Hr1G000800	black
Hordeum_vulgare_newGene_711	black
HORVU2Hr1G021870	black
Hordeum_vulgare_newGene_4979	black
HORVU6Hr1G008640	black
Hordeum_vulgare_newGene_6534	black
Hordeum_vulgare_newGene_15931	black
HORVU3Hr1G041850	black
Hordeum_vulgare_newGene_2090	black
HORVU2Hr1G066680	black
HORVU2Hr1G014030	black
HORVU6Hr1G076040	black
HORVU3Hr1G069830	black
HORVU7Hr1G063030	black
HORVU2Hr1G011870	black
HORVU5Hr1G072770	black
HORVU4Hr1G074320	black
HORVU2Hr1G119820	black
HORVU5Hr1G078950	black
HORVU5Hr1G122960	black
HORVU5Hr1G011100	black
HORVU4Hr1G012220	black
Hordeum_vulgare_newGene_4638	black
Hordeum_vulgare_newGene_2036	black
HORVU1Hr1G030200	black
Hordeum_vulgare_newGene_2376	black
HORVU5Hr1G097370	black
HORVU2Hr1G009900	black
Hordeum_vulgare_newGene_4263	black
HORVU7Hr1G018780	black
Hordeum_vulgare_newGene_11101	black
HORVU6Hr1G033600	black
HORVU7Hr1G076290	black
HORVU7Hr1G050030	black
HORVU7Hr1G028840	black
Hordeum_vulgare_newGene_9495	black
HORVU1Hr1G073720	black
HORVU3Hr1G079800	black
HORVU5Hr1G114540	black
HORVU4Hr1G011170	black
HORVU0Hr1G013180	black
HORVU3Hr1G079340	black

HORVU7Hr1G016770	black
HORVU6Hr1G022370	black
HORVU5Hr1G042180	black
HORVU4Hr1G076940	black
HORVU5Hr1G007890	black
HORVU0Hr1G033170	black
Hordeum_vulgare_newGene_1557	black
Hordeum_vulgare_newGene_14426	black
HORVU5Hr1G122540	black
HORVU1Hr1G061230	black
HORVU6Hr1G085370	black
HORVU7Hr1G001020	black
HORVU2Hr1G099100	black
HORVU5Hr1G074340	black
Hordeum_vulgare_newGene_780	black
Hordeum_vulgare_newGene_1446	black
HORVU6Hr1G011970	black
HORVU6Hr1G071500	black
Hordeum_vulgare_newGene_3603	black
HORVU1Hr1G029180	black
HORVU5Hr1G058240	black
HORVU7Hr1G055080	black
HORVU7Hr1G040080	black
HORVU5Hr1G122080	black
HORVU3Hr1G071300	black
HORVU4Hr1G054200	black
HORVU3Hr1G096500	black
HORVU3Hr1G074470	black
HORVU4Hr1G077310	black
HORVU4Hr1G032980	black
HORVU7Hr1G101860	black
HORVU3Hr1G017000	black
HORVU0Hr1G005570	black
HORVU5Hr1G069910	black
HORVU1Hr1G094180	black
HORVU3Hr1G092520	black
HORVU2Hr1G063510	black
Hordeum_vulgare_newGene_13984	black
Hordeum_vulgare_newGene_13980	black
HORVU7Hr1G081770	black
HORVU7Hr1G030810	black
Hordeum_vulgare_newGene_14615	black
HORVU3Hr1G068300	black
HORVU7Hr1G037780	black
HORVU3Hr1G083680	black
HORVU3Hr1G011990	black
HORVU2Hr1G125600	black

HORVU3Hr1G078680	black
HORVU7Hr1G042150	black
HORVU3Hr1G089210	black
Hordeum_vulgare_newGene_663	black
HORVU4Hr1G015930	black
Hordeum_vulgare_newGene_13976	black
HORVU1Hr1G000090	black
Hordeum_vulgare_newGene_2945	black
Hordeum_vulgare_newGene_2947	black
HORVU3Hr1G001940	black
HORVU3Hr1G033790	black
HORVU1Hr1G023220	black
HORVU1Hr1G066340	black
HORVU3Hr1G099690	black
Hordeum_vulgare_newGene_10264	black
HORVU4Hr1G025410	black
HORVU2Hr1G031410	black
HORVU2Hr1G108180	black
HORVU3Hr1G103630	black
Hordeum_vulgare_newGene_1833	black
HORVU5Hr1G071080	black
Hordeum_vulgare_newGene_13479	black
Hordeum_vulgare_newGene_8786	black
HORVU3Hr1G000970	black
HORVU6Hr1G016070	black
HORVU1Hr1G083170	black
HORVU4Hr1G057330	black
HORVU3Hr1G052250	black
HORVU1Hr1G049760	black
Hordeum_vulgare_newGene_4576	black
Hordeum_vulgare_newGene_7004	black
HORVU3Hr1G043160	black
HORVU5Hr1G122070	black
HORVU0Hr1G016150	black
HORVU3Hr1G025690	black
HORVU5Hr1G039560	black
HORVU4Hr1G003580	black
HORVU5Hr1G016850	black
HORVU4Hr1G005320	black
HORVU2Hr1G017860	black
HORVU1Hr1G067330	black
Hordeum_vulgare_newGene_13891	black
HORVU3Hr1G108540	black
HORVU3Hr1G062010	black
Hordeum_vulgare_newGene_741	black
HORVU6Hr1G012290	black
Hordeum_vulgare_newGene_14661	black

Hordeum_vulgare_newGene_9199	black
HORVU4Hr1G088080	black
HORVU7Hr1G080950	black
Hordeum_vulgare_newGene_4922	black
HORVU0Hr1G039090	black
Hordeum_vulgare_newGene_15962	black
HORVU7Hr1G031140	black
HORVU1Hr1G023070	black
HORVU5Hr1G063620	black
HORVU7Hr1G037090	black
HORVU1Hr1G082330	black
HORVU7Hr1G091780	black
HORVU4Hr1G055210	black
HORVU6Hr1G041610	black
HORVU4Hr1G085100	black
Hordeum_vulgare_newGene_11494	black
Hordeum_vulgare_newGene_11492	black
Hordeum_vulgare_newGene_16149	black
HORVU2Hr1G021530	black
HORVU5Hr1G121450	black
Hordeum_vulgare_newGene_14166	black
Hordeum_vulgare_newGene_3773	black
HORVU6Hr1G072050	black
Hordeum_vulgare_newGene_11824	black
Hordeum_vulgare_newGene_11820	black
HORVU7Hr1G039080	black
HORVU0Hr1G005640	black
HORVU6Hr1G094780	black
HORVU5Hr1G006930	black
Hordeum_vulgare_newGene_7244	black
Hordeum_vulgare_newGene_12094	black
Hordeum_vulgare_newGene_12091	black
Hordeum_vulgare_newGene_3903	black
Hordeum_vulgare_newGene_3635	black
Hordeum_vulgare_newGene_1006	black
HORVU6Hr1G082880	black
HORVU1Hr1G085480	black
HORVU6Hr1G083250	black
HORVU0Hr1G000910	black
HORVU6Hr1G008320	black
HORVU2Hr1G002660	black
Hordeum_vulgare_newGene_3923	black
HORVU2Hr1G077970	black
HORVU3Hr1G079720	black
HORVU3Hr1G006940	black
HORVU3Hr1G108360	black
HORVU0Hr1G007360	black

HORVU3Hr1G074100	black
HORVU3Hr1G104090	black
Hordeum_vulgare_newGene_5470	black
Hordeum_vulgare_newGene_10567	black
HORVU3Hr1G018680	black
HORVU7Hr1G082330	black
HORVU2Hr1G025630	black
Hordeum_vulgare_newGene_7615	black
Hordeum_vulgare_newGene_12683	black
HORVU6Hr1G072740	black
HORVU5Hr1G039730	black
HORVU1Hr1G054250	black
HORVU7Hr1G038900	black
HORVU2Hr1G054890	black
HORVU3Hr1G095780	black
HORVU2Hr1G019010	black
HORVU1Hr1G093390	black
HORVU5Hr1G099170	black
HORVU1Hr1G003960	black
Hordeum_vulgare_newGene_5158	black
HORVU7Hr1G037830	black
Hordeum_vulgare_newGene_8054	black
Hordeum_vulgare_newGene_4607	black
Hordeum_vulgare_newGene_4603	black
HORVU2Hr1G043960	black
HORVU4Hr1G048110	black
HORVU6Hr1G057500	black
Hordeum_vulgare_newGene_634	black
Hordeum_vulgare_newGene_7455	black
Hordeum_vulgare_newGene_4819	black
HORVU6Hr1G087310	black
HORVU1Hr1G048670	black
HORVU3Hr1G077980	black
HORVU2Hr1G100660	black
HORVU3Hr1G099390	black
Hordeum_vulgare_newGene_11653	black
HORVU4Hr1G063240	black
HORVU5Hr1G005740	black
Hordeum_vulgare_newGene_12352	black
HORVU2Hr1G092800	black
Hordeum_vulgare_newGene_13401	black
HORVU3Hr1G048950	black
HORVU3Hr1G110550	black
Hordeum_vulgare_newGene_15763	black
Hordeum_vulgare_newGene_6920	black
Hordeum_vulgare_newGene_8565	black
HORVU6Hr1G032080	black

Hordeum_vulgare_newGene_12439	black
HORVU7Hr1G106960	black
HORVU3Hr1G002840	black
HORVU2Hr1G033320	black
HORVU5Hr1G095990	black
HORVU7Hr1G003860	black
Hordeum_vulgare_newGene_5226	black
HORVU3Hr1G075960	black
Hordeum_vulgare_newGene_11283	black
HORVU2Hr1G115710	black
Hordeum_vulgare_newGene_196	black
HORVU4Hr1G073650	black
HORVU6Hr1G061270	black
HORVU7Hr1G066630	black
HORVU1Hr1G058470	black
HORVU7Hr1G039930	black
HORVU5Hr1G044830	black
Hordeum_vulgare_newGene_2058	black
HORVU4Hr1G000280	black
HORVU3Hr1G040310	black
HORVU6Hr1G087420	black
HORVU1Hr1G011660	black
HORVU2Hr1G117050	black
HORVU3Hr1G032490	black
Hordeum_vulgare_newGene_4698	black
Hordeum_vulgare_newGene_11167	black
HORVU3Hr1G108530	black
HORVU7Hr1G045580	black
HORVU6Hr1G078110	black
Hordeum_vulgare_newGene_779	black
HORVU1Hr1G051160	black
HORVU5Hr1G017640	black
HORVU2Hr1G118190	black
HORVU3Hr1G007210	black
HORVU5Hr1G064130	black
HORVU4Hr1G061680	black
HORVU5Hr1G057590	black
HORVU5Hr1G044430	black
HORVU4Hr1G021820	black
HORVU1Hr1G000120	black
HORVU6Hr1G077770	black
Hordeum_vulgare_newGene_11464	black
HORVU5Hr1G071480	black
HORVU5Hr1G037200	black
Hordeum_vulgare_newGene_13565	black
Hordeum_vulgare_newGene_7914	black
HORVU7Hr1G038120	black

HORVU3Hr1G052780	black
Hordeum_vulgare_newGene_3747	black
HORVU0Hr1G009120	black
HORVU2Hr1G032360	black
HORVU6Hr1G083200	black
HORVU2Hr1G000280	black
HORVU0Hr1G001280	black
HORVU3Hr1G009560	black
HORVU3Hr1G067910	black
HORVU6Hr1G012270	black
HORVU2Hr1G020920	black
Hordeum_vulgare_newGene_1996	black
HORVU1Hr1G087570	black
Hordeum_vulgare_newGene_796	black
HORVU4Hr1G073460	black
HORVU4Hr1G011320	black
HORVU6Hr1G081080	black
Hordeum_vulgare_newGene_4202	black
HORVU5Hr1G060980	black
HORVU5Hr1G082990	black
Hordeum_vulgare_newGene_2155	black
HORVU3Hr1G061130	black
HORVU2Hr1G121250	black
HORVU2Hr1G102280	black
Hordeum_vulgare_newGene_4991	black
Hordeum_vulgare_newGene_14481	black
HORVU0Hr1G008590	black
HORVU7Hr1G037510	black
HORVU7Hr1G017670	black
HORVU3Hr1G030850	black
HORVU3Hr1G042330	black
HORVU4Hr1G073070	black
HORVU2Hr1G101360	black
HORVU4Hr1G023580	black
HORVU1Hr1G012770	black
HORVU3Hr1G077850	black
HORVU3Hr1G018110	black
HORVU2Hr1G114340	black
HORVU5Hr1G100670	black
HORVU7Hr1G044270	black
Hordeum_vulgare_newGene_11099	black
Hordeum_vulgare_newGene_11545	black
HORVU3Hr1G026900	black
Hordeum_vulgare_newGene_7817	black
HORVU2Hr1G063740	black
HORVU7Hr1G028670	black
Hordeum_vulgare_newGene_4283	black

HORVU3Hr1G047670	black
HORVU1Hr1G021390	black
HORVU4Hr1G074250	black
HORVU0Hr1G018100	black
HORVU5Hr1G012170	black
HORVU5Hr1G029060	black
HORVU2Hr1G038570	black
Hordeum_vulgare_newGene_8615	black
HORVU2Hr1G048680	black
Hordeum_vulgare_newGene_1898	black
HORVU3Hr1G038290	black
HORVU1Hr1G055320	black
HORVU7Hr1G048020	black
Hordeum_vulgare_newGene_2747	black
HORVU3Hr1G042440	black
Hordeum_vulgare_newGene_3825	black
HORVU7Hr1G101140	black
Hordeum_vulgare_newGene_4655	black
HORVU6Hr1G061450	black
HORVU1Hr1G078740	black
HORVU7Hr1G043920	black
HORVU3Hr1G109530	black
HORVU1Hr1G056120	black
Hordeum_vulgare_newGene_5002	black
HORVU4Hr1G089910	black
HORVU7Hr1G106510	black
Hordeum_vulgare_newGene_1786	black
HORVU1Hr1G024550	black
HORVU2Hr1G004890	black
HORVU7Hr1G052030	black
HORVU1Hr1G082090	black
HORVU4Hr1G032660	black
HORVU5Hr1G050180	black
HORVU2Hr1G108420	black
HORVU3Hr1G041440	black
HORVU3Hr1G099360	black
Hordeum_vulgare_newGene_10249	black
HORVU1Hr1G004080	black
Hordeum_vulgare_newGene_2670	black
HORVU4Hr1G077360	black
HORVU6Hr1G051070	black
HORVU3Hr1G010980	black
HORVU7Hr1G109380	black
HORVU1Hr1G056500	black
HORVU5Hr1G122180	black
HORVU6Hr1G073080	black
HORVU7Hr1G071470	black

HORVU2Hr1G111690	black
HORVU2Hr1G072870	black
Hordeum_vulgare_newGene_6030	black
HORVU3Hr1G044720	black
HORVU5Hr1G062240	black
HORVU1Hr1G054380	black
HORVU1Hr1G073300	black
HORVU7Hr1G121830	black
HORVU7Hr1G025750	black
HORVU3Hr1G077530	black
Hordeum_vulgare_newGene_8106	black
Hordeum_vulgare_newGene_14537	black
Hordeum_vulgare_newGene_14534	black
Hordeum_vulgare_newGene_15373	black
HORVU0Hr1G013010	black
HORVU0Hr1G039000	black
HORVU7Hr1G002940	black
HORVU7Hr1G087750	black
HORVU6Hr1G070860	black
HORVU3Hr1G055420	black
HORVU6Hr1G071380	black
HORVU3Hr1G087010	black
HORVU1Hr1G066240	black
HORVU7Hr1G097760	black
HORVU5Hr1G080580	black
HORVU3Hr1G078420	black
HORVU4Hr1G067340	black
HORVU7Hr1G105620	black
HORVU4Hr1G076970	black
HORVU5Hr1G125120	black
HORVU5Hr1G105790	black
HORVU6Hr1G085360	black
Hordeum_vulgare_newGene_11937	black
HORVU0Hr1G014170	black
HORVU2Hr1G097180	black
HORVU4Hr1G020950	black
HORVU0Hr1G005060	black
HORVU5Hr1G096010	black
HORVU4Hr1G072130	black
HORVU0Hr1G004060	black
HORVU4Hr1G016280	black
HORVU4Hr1G089060	black
HORVU5Hr1G109040	black
HORVU4Hr1G009210	black
HORVU6Hr1G074830	black
HORVU5Hr1G095910	black
Hordeum_vulgare_newGene_2105	black

HORVU2Hr1G116880	black
HORVU2Hr1G035020	black
Hordeum_vulgare_newGene_569	black
Hordeum_vulgare_newGene_15782	black
Hordeum_vulgare_newGene_15783	black
HORVU3Hr1G032540	black
HORVU7Hr1G087860	black
HORVU5Hr1G105360	black
HORVU3Hr1G006220	black
HORVU1Hr1G021600	black
HORVU5Hr1G016210	black
HORVU5Hr1G112630	black
HORVU7Hr1G040680	black
HORVU6Hr1G089750	black
Hordeum_vulgare_newGene_8036	black
HORVU1Hr1G063250	black
HORVU3Hr1G067110	black
HORVU1Hr1G003300	black
HORVU6Hr1G080230	black
HORVU3Hr1G009370	black
HORVU6Hr1G019350	black
HORVU2Hr1G097670	black
HORVU3Hr1G073630	black
HORVU6Hr1G090250	black
HORVU3Hr1G030150	black
HORVU3Hr1G026950	black
HORVU7Hr1G038270	black
HORVU2Hr1G084750	black
Hordeum_vulgare_newGene_10073	black
HORVU6Hr1G075900	black
HORVU6Hr1G077410	black
Hordeum_vulgare_newGene_4872	black
HORVU6Hr1G029640	black
HORVU6Hr1G078770	black
HORVU3Hr1G116650	black
HORVU4Hr1G017370	black
HORVU4Hr1G015330	black
HORVU1Hr1G058000	black
HORVU5Hr1G066490	black
Hordeum_vulgare_newGene_13898	black
HORVU4Hr1G052060	black
Hordeum_vulgare_newGene_10279	black
HORVU7Hr1G001600	black
Hordeum_vulgare_newGene_13462	black
Hordeum_vulgare_newGene_13466	black
Hordeum_vulgare_newGene_3555	black
HORVU5Hr1G115360	black

HORVU3Hr1G059610	black
HORVU6Hr1G073620	black
HORVU5Hr1G002110	black
HORVU3Hr1G082380	black
HORVU1Hr1G020240	black
HORVU7Hr1G042390	black
HORVU6Hr1G021460	black
HORVU2Hr1G118210	black
HORVU5Hr1G117610	black
HORVU3Hr1G075210	black
Hordeum_vulgare_newGene_7357	black
HORVU7Hr1G108110	black
Hordeum_vulgare_newGene_2273	black
Hordeum_vulgare_newGene_13637	black
Hordeum_vulgare_newGene_949	black
HORVU4Hr1G089410	black
HORVU7Hr1G107780	black
HORVU7Hr1G025700	black
HORVU1Hr1G052960	black
HORVU1Hr1G063620	black
HORVU3Hr1G031940	black
HORVU5Hr1G116850	black
HORVU3Hr1G035650	black
HORVU6Hr1G033160	black
HORVU2Hr1G037700	black
HORVU2Hr1G063460	black
HORVU6Hr1G062320	black
HORVU5Hr1G014170	black
HORVU7Hr1G052770	black
HORVU2Hr1G124800	black
HORVU4Hr1G087590	black
HORVU6Hr1G000620	black
HORVU4Hr1G015820	black
HORVU3Hr1G070200	black
HORVU2Hr1G122280	black
HORVU2Hr1G105980	black
HORVU0Hr1G017000	black
HORVU4Hr1G090810	black
HORVU6Hr1G095380	black
Hordeum_vulgare_newGene_34	black
Hordeum_vulgare_newGene_32	black
HORVU2Hr1G000140	black
Hordeum_vulgare_newGene_14750	black
HORVU2Hr1G085000	black
HORVU1Hr1G052890	black
Hordeum_vulgare_newGene_14193	black
HORVU4Hr1G024950	black

HORVU5Hr1G076400	black
Hordeum_vulgare_newGene_7097	black
HORVU1Hr1G011730	black
HORVU5Hr1G017660	black
Hordeum_vulgare_newGene_4270	black
HORVU2Hr1G023170	black
HORVU5Hr1G077680	black
HORVU2Hr1G025810	black
HORVU1Hr1G042870	black
Hordeum_vulgare_newGene_7775	black
Hordeum_vulgare_newGene_6121	black
HORVU5Hr1G074220	black
HORVU3Hr1G114920	black
HORVU7Hr1G006520	black
Hordeum_vulgare_newGene_4229	black
HORVU6Hr1G085600	black
HORVU5Hr1G117990	black
Hordeum_vulgare_newGene_2139	black
HORVU2Hr1G019120	black
Hordeum_vulgare_newGene_13348	black
HORVU6Hr1G064070	black
HORVU2Hr1G005530	black
HORVU2Hr1G085990	black
Hordeum_vulgare_newGene_1600	black
HORVU7Hr1G024270	black
Hordeum_vulgare_newGene_11449	black
HORVU5Hr1G077010	black
HORVU3Hr1G038230	black
Hordeum_vulgare_newGene_14112	black
Hordeum_vulgare_newGene_5809	black
Hordeum_vulgare_newGene_5149	black
HORVU3Hr1G089830	black
HORVU7Hr1G021820	black
Hordeum_vulgare_newGene_3843	black
HORVU3Hr1G034640	black
HORVU3Hr1G097810	black
HORVU6Hr1G081800	black
HORVU7Hr1G012380	black
HORVU2Hr1G096550	black
HORVU7Hr1G098460	black
HORVU7Hr1G099650	black
Hordeum_vulgare_newGene_3648	black
Hordeum_vulgare_newGene_14325	black
HORVU6Hr1G072300	black
HORVU7Hr1G096610	black
HORVU6Hr1G085270	black
HORVU4Hr1G080190	black

HORVU5Hr1G115980	black
Hordeum_vulgare_newGene_1875	black
Hordeum_vulgare_newGene_12347	black
HORVU2Hr1G092810	black
HORVU4Hr1G046270	black
HORVU3Hr1G081760	black
Hordeum_vulgare_newGene_2692	black
Hordeum_vulgare_newGene_15776	black
Hordeum_vulgare_newGene_15774	black
Hordeum_vulgare_newGene_783	black
Hordeum_vulgare_newGene_784	black
Hordeum_vulgare_newGene_8572	black
HORVU4Hr1G028110	black
HORVU0Hr1G006640	black
HORVU6Hr1G081400	black
HORVU4Hr1G082910	black
Hordeum_vulgare_newGene_2767	black
Hordeum_vulgare_newGene_2769	black
HORVU1Hr1G040130	black
Hordeum_vulgare_newGene_1444	black
HORVU4Hr1G029420	black
HORVU4Hr1G058340	black
HORVU0Hr1G031830	black
HORVU3Hr1G096910	black
HORVU7Hr1G048570	black
HORVU1Hr1G021920	black
HORVU4Hr1G080720	black
Hordeum_vulgare_newGene_1166	black
HORVU5Hr1G096480	black
HORVU1Hr1G056580	black
HORVU0Hr1G001180	black
HORVU5Hr1G116800	black
HORVU5Hr1G008670	black
HORVU5Hr1G111870	black
HORVU6Hr1G093840	black
HORVU4Hr1G079710	black
HORVU4Hr1G034860	black
Hordeum_vulgare_newGene_4734	black
HORVU6Hr1G053810	black
HORVU6Hr1G074820	black
HORVU2Hr1G024950	black
HORVU2Hr1G099480	black
HORVU7Hr1G091800	black
HORVU3Hr1G052560	black
HORVU7Hr1G025800	black
HORVU0Hr1G022670	black
HORVU1Hr1G009920	black

HORVU2Hr1G020900	black
Hordeum_vulgare_newGene_4961	black
HORVU7Hr1G032340	black
HORVU7Hr1G052190	black
Hordeum_vulgare_newGene_9879	black
HORVU6Hr1G072250	black
HORVU2Hr1G056710	black
HORVU5Hr1G069310	black
HORVU3Hr1G071220	black
HORVU1Hr1G065250	black
Hordeum_vulgare_newGene_160	black
HORVU3Hr1G081610	black
HORVU4Hr1G043680	black
HORVU5Hr1G113580	black
Hordeum_vulgare_newGene_12487	black
Hordeum_vulgare_newGene_3486	black
Hordeum_vulgare_newGene_6053	black
Hordeum_vulgare_newGene_5881	black
HORVU2Hr1G051180	black
Hordeum_vulgare_newGene_7592	black
HORVU4Hr1G007260	black
HORVU7Hr1G000260	black
HORVU5Hr1G109100	black
HORVU3Hr1G028540	black
Hordeum_vulgare_newGene_13739	black
HORVU1Hr1G040920	black
HORVU0Hr1G016250	black
HORVU3Hr1G069650	black
HORVU1Hr1G020370	black
Hordeum_vulgare_newGene_1047	black
HORVU1Hr1G058630	black
HORVU5Hr1G112710	black
HORVU6Hr1G084690	black
HORVU6Hr1G089730	black
Hordeum_vulgare_newGene_8689	black
HORVU2Hr1G011570	black
Hordeum_vulgare_newGene_3971	black
Hordeum_vulgare_newGene_12645	black
HORVU6Hr1G030720	black
HORVU4Hr1G037290	black
HORVU7Hr1G018470	black
HORVU5Hr1G075510	black
Hordeum_vulgare_newGene_15456	black
HORVU3Hr1G030840	black
HORVU3Hr1G046780	black
HORVU2Hr1G079020	black
HORVU1Hr1G051290	black

HORVU1Hr1G055970	black
HORVU5Hr1G117040	black
Hordeum_vulgare_newGene_3402	black
HORVU7Hr1G109340	black
HORVU5Hr1G063420	black
Hordeum_vulgare_newGene_5150	black
HORVU0Hr1G033160	black
HORVU5Hr1G015050	black
HORVU4Hr1G048210	black
HORVU5Hr1G060340	black
HORVU3Hr1G084880	black
HORVU1Hr1G089760	black
HORVU6Hr1G074910	black
HORVU1Hr1G080080	black
HORVU1Hr1G049420	black
HORVU6Hr1G076660	black
HORVU2Hr1G116660	black
Hordeum_vulgare_newGene_10245	black
Hordeum_vulgare_newGene_8443	black
HORVU0Hr1G005820	black
HORVU5Hr1G095710	black
HORVU3Hr1G099180	black
HORVU1Hr1G067080	black
HORVU6Hr1G010580	black
HORVU7Hr1G056990	black
HORVU6Hr1G010840	black
HORVU3Hr1G061850	black
Hordeum_vulgare_newGene_12310	black
HORVU4Hr1G022020	black
Hordeum_vulgare_newGene_14410	black
HORVU5Hr1G103730	black
HORVU3Hr1G013790	black
HORVU3Hr1G082320	black
Hordeum_vulgare_newGene_15215	black
HORVU5Hr1G073370	black
HORVU2Hr1G097010	black
Hordeum_vulgare_newGene_5193	black
Hordeum_vulgare_newGene_5192	black
Hordeum_vulgare_newGene_10194	black
Hordeum_vulgare_newGene_14992	black
Hordeum_vulgare_newGene_14994	black
HORVU3Hr1G081180	black
Hordeum_vulgare_newGene_12128	black
HORVU7Hr1G097550	black
HORVU5Hr1G109760	black
HORVU7Hr1G043930	black
Hordeum_vulgare_newGene_11000	black

HORVU3Hr1G026970	black
Hordeum_vulgare_newGene_3367	black
HORVU5Hr1G007980	black
HORVU4Hr1G005910	black
HORVU3Hr1G099680	black
HORVU0Hr1G001500	black
HORVU7Hr1G111010	black
HORVU4Hr1G066070	black
HORVU3Hr1G090560	black
HORVU7Hr1G038080	black
Hordeum_vulgare_newGene_7606	black
Hordeum_vulgare_newGene_1553	black
Hordeum_vulgare_newGene_6551	black
HORVU1Hr1G070960	black
HORVU5Hr1G113560	black
HORVU4Hr1G052470	black
HORVU5Hr1G115630	black
HORVU5Hr1G011160	black
HORVU7Hr1G021560	black
Hordeum_vulgare_newGene_153	black
Hordeum_vulgare_newGene_157	black
HORVU2Hr1G110130	black
HORVU6Hr1G016880	black
Hordeum_vulgare_newGene_7378	black
HORVU2Hr1G032970	black
Hordeum_vulgare_newGene_5700	black
Hordeum_vulgare_newGene_963	black
HORVU6Hr1G064460	black
Hordeum_vulgare_newGene_1283	black
HORVU7Hr1G090280	black
HORVU7Hr1G000250	black
Hordeum_vulgare_newGene_5093	black
HORVU4Hr1G083940	black
HORVU2Hr1G072510	black
HORVU5Hr1G080700	black
HORVU7Hr1G045830	black
Hordeum_vulgare_newGene_14521	black
Hordeum_vulgare_newGene_2358	black
HORVU5Hr1G079700	black
HORVU7Hr1G030120	black
HORVU4Hr1G015570	black
HORVU2Hr1G108110	black
HORVU5Hr1G122680	black
HORVU1Hr1G016770	black
HORVU7Hr1G046320	black
HORVU7Hr1G026680	black
HORVU2Hr1G102240	black

HORVU7Hr1G032330	black
HORVU6Hr1G068910	black
HORVU4Hr1G073580	black
HORVU6Hr1G087250	black
HORVU7Hr1G078030	black
HORVU4Hr1G048060	black
HORVU0Hr1G001420	black
HORVU2Hr1G063800	black
HORVU5Hr1G121440	black
Hordeum_vulgare_newGene_14172	black
Hordeum_vulgare_newGene_5836	black
HORVU5Hr1G032980	black
HORVU7Hr1G073040	black
Hordeum_vulgare_newGene_7521	black
HORVU7Hr1G099250	black
HORVU3Hr1G009520	black
HORVU2Hr1G065430	black
HORVU3Hr1G088000	black
HORVU3Hr1G006600	black
HORVU4Hr1G059050	black
HORVU3Hr1G073930	black
HORVU0Hr1G003020	black
HORVU4Hr1G043860	black
HORVU6Hr1G081270	black
HORVU6Hr1G009620	black
HORVU1Hr1G053000	black
Hordeum_vulgare_newGene_8655	black
HORVU7Hr1G012550	black
HORVU7Hr1G020760	black
Hordeum_vulgare_newGene_10576	black
HORVU3Hr1G054050	black
HORVU2Hr1G021280	black
HORVU3Hr1G095340	black
HORVU2Hr1G087730	black
Hordeum_vulgare_newGene_13824	black
HORVU3Hr1G028550	black
HORVU7Hr1G040690	black
HORVU0Hr1G013900	black
HORVU5Hr1G109240	black
HORVU3Hr1G104100	black
HORVU2Hr1G112230	black
Hordeum_vulgare_newGene_3130	black
Hordeum_vulgare_newGene_14635	black
HORVU5Hr1G064100	black
HORVU3Hr1G012010	black
HORVU0Hr1G012190	black
HORVU3Hr1G047030	black

Hordeum_vulgare_newGene_10324	black
HORVU4Hr1G056000	black
HORVU7Hr1G043960	black
Hordeum_vulgare_newGene_11056	black
Hordeum_vulgare_newGene_11058	black
HORVU3Hr1G095600	black
HORVU3Hr1G014890	black
Hordeum_vulgare_newGene_11587	black
HORVU6Hr1G048170	black
HORVU6Hr1G024930	black
Hordeum_vulgare_newGene_647	black
Hordeum_vulgare_newGene_14341	black
Hordeum_vulgare_newGene_14340	black
Hordeum_vulgare_newGene_14342	black
HORVU1Hr1G070480	black
HORVU7Hr1G096630	black
Hordeum_vulgare_newGene_10040	black
HORVU3Hr1G026550	black
HORVU6Hr1G073180	black
HORVU7Hr1G102030	black
HORVU5Hr1G043090	black
HORVU5Hr1G030330	black
HORVU5Hr1G104980	black
HORVU5Hr1G089740	black
HORVU7Hr1G020410	black
HORVU5Hr1G021610	black
HORVU6Hr1G085250	black
HORVU3Hr1G064340	black
HORVU2Hr1G096700	black
HORVU3Hr1G089510	black
HORVU3Hr1G059620	black
HORVU3Hr1G075870	black
HORVU4Hr1G016770	black
Hordeum_vulgare_newGene_2093	black
Hordeum_vulgare_newGene_2095	black
Hordeum_vulgare_newGene_9009	black
HORVU2Hr1G097350	black
HORVU1Hr1G036060	black
Hordeum_vulgare_newGene_1705	black
HORVU4Hr1G073990	black
HORVU2Hr1G114960	black
Hordeum_vulgare_newGene_7340	black
HORVU7Hr1G064800	black
Hordeum_vulgare_newGene_9576	black
HORVU2Hr1G094660	black
HORVU7Hr1G096250	black
HORVU5Hr1G091840	black

HORVU7Hr1G028260	black
HORVU5Hr1G094460	black
Hordeum_vulgare_newGene_3338	black
HORVU2Hr1G076180	black
HORVU5Hr1G087970	black
HORVU6Hr1G020600	black
HORVU0Hr1G022380	black
HORVU3Hr1G102720	black
HORVU0Hr1G005750	black
HORVU7Hr1G064100	black
HORVU7Hr1G088770	black
HORVU4Hr1G010000	black
HORVU2Hr1G005600	black
HORVU5Hr1G101990	black
HORVU4Hr1G009190	black
HORVU3Hr1G086870	black
HORVU1Hr1G001140	black
HORVU4Hr1G085820	black
HORVU2Hr1G119480	black
Hordeum_vulgare_newGene_14266	black
Hordeum_vulgare_newGene_5944	black
HORVU2Hr1G019400	black
HORVU5Hr1G015560	black
HORVU3Hr1G034280	black
HORVU5Hr1G062510	black
HORVU4Hr1G089330	black
HORVU3Hr1G009490	black
HORVU1Hr1G065270	black
HORVU5Hr1G000170	black
HORVU5Hr1G107410	black
HORVU2Hr1G004720	darkolivegreen
HORVU5Hr1G068060	darkolivegreen
HORVU6Hr1G004120	darkolivegreen
HORVU5Hr1G115720	darkolivegreen
HORVU2Hr1G122580	darkolivegreen
HORVU3Hr1G011870	darkolivegreen
HORVU2Hr1G004550	darkolivegreen
HORVU7Hr1G024090	darkolivegreen
HORVU2Hr1G102720	darkolivegreen
HORVU3Hr1G014880	darkolivegreen
HORVU1Hr1G089830	darkolivegreen
HORVU7Hr1G009310	darkolivegreen
HORVU3Hr1G096280	darkolivegreen
HORVU5Hr1G073640	darkolivegreen
HORVU5Hr1G073700	darkolivegreen
HORVU5Hr1G059890	darkolivegreen
HORVU1Hr1G020410	darkolivegreen

HORVU3Hr1G096360	darkolivegreen
HORVU2Hr1G004610	darkolivegreen
HORVU5Hr1G005210	darkolivegreen
HORVU6Hr1G088440	darkolivegreen
HORVU2Hr1G101920	darkolivegreen
HORVU1Hr1G090930	darkolivegreen
HORVU5Hr1G067760	darkolivegreen
HORVU0Hr1G002250	darkolivegreen
HORVU5Hr1G005290	darkolivegreen
HORVU5Hr1G057730	darkolivegreen
HORVU0Hr1G000840	darkolivegreen
HORVU2Hr1G098670	darkolivegreen
HORVU5Hr1G052150	darkolivegreen
HORVU7Hr1G118770	darkolivegreen
HORVU5Hr1G006330	darkolivegreen
HORVU7Hr1G116650	darkolivegreen
Hordeum_vulgare_newGene_1529	darkolivegreen
HORVU5Hr1G124650	darkolivegreen
HORVU1Hr1G018370	darkolivegreen
HORVU2Hr1G109590	darkolivegreen
HORVU5Hr1G075540	darkolivegreen
HORVU5Hr1G106850	darkolivegreen
HORVU5Hr1G068350	darkolivegreen
HORVU2Hr1G004620	darkolivegreen
HORVU6Hr1G070740	darkolivegreen
HORVU5Hr1G023730	darkolivegreen
HORVU1Hr1G019740	darkolivegreen
Hordeum_vulgare_newGene_13474	darkolivegreen
HORVU5Hr1G117080	darkolivegreen
HORVU3Hr1G068380	darkolivegreen
HORVU4Hr1G079600	darkolivegreen
HORVU5Hr1G115870	darkolivegreen
HORVU2Hr1G004530	darkolivegreen
HORVU3Hr1G028620	darkolivegreen
HORVU4Hr1G071370	darkolivegreen
HORVU2Hr1G012980	darkolivegreen
HORVU1Hr1G049280	darkolivegreen
HORVU1Hr1G015090	darkolivegreen
HORVU6Hr1G076620	darkolivegreen
HORVU6Hr1G020540	darkolivegreen
HORVU4Hr1G072480	darkolivegreen
HORVU1Hr1G093480	darkolivegreen
Hordeum_vulgare_newGene_2942	darkolivegreen
HORVU5Hr1G103770	darkolivegreen
HORVU4Hr1G079620	darkolivegreen
HORVU7Hr1G002740	darkolivegreen
HORVU2Hr1G027480	darkolivegreen

HORVU2Hr1G114390	darkolivegreen
HORVU3Hr1G074960	darkolivegreen
HORVU1Hr1G009490	darkolivegreen
HORVU6Hr1G053710	darkolivegreen
HORVU7Hr1G096700	darkolivegreen
HORVU3Hr1G095760	darkolivegreen
HORVU5Hr1G085710	darkolivegreen
HORVU5Hr1G062290	darkolivegreen
HORVU2Hr1G095460	darkolivegreen
HORVU2Hr1G034290	darkolivegreen
HORVU6Hr1G005400	darkolivegreen
HORVU3Hr1G071470	darkolivegreen
HORVU4Hr1G061120	darkolivegreen
HORVU4Hr1G056500	darkolivegreen
HORVU7Hr1G051430	darkolivegreen
HORVU2Hr1G004600	darkolivegreen
HORVU5Hr1G099470	darkolivegreen
HORVU7Hr1G033530	darkolivegreen
HORVU5Hr1G087800	darkolivegreen
HORVU5Hr1G066130	darkolivegreen
HORVU6Hr1G032810	darkolivegreen
HORVU1Hr1G081240	darkolivegreen
HORVU1Hr1G090860	darkolivegreen
HORVU5Hr1G074740	darkolivegreen
HORVU7Hr1G091860	darkolivegreen
HORVU2Hr1G116800	darkolivegreen
HORVU3Hr1G100190	darkolivegreen
HORVU3Hr1G095730	darkolivegreen
HORVU7Hr1G121090	darkolivegreen
HORVU3Hr1G117390	darkolivegreen
HORVU3Hr1G096860	darkolivegreen
HORVU5Hr1G113790	darkolivegreen
HORVU3Hr1G033740	darkolivegreen
HORVU5Hr1G034180	darkolivegreen
HORVU4Hr1G010890	darkolivegreen
HORVU4Hr1G008520	darkolivegreen
HORVU3Hr1G074390	darkolivegreen
HORVU2Hr1G114910	darkolivegreen
HORVU1Hr1G083380	darkolivegreen
HORVU7Hr1G019390	darkolivegreen
HORVU2Hr1G113300	darkolivegreen
HORVU1Hr1G049230	darkolivegreen
HORVU2Hr1G064160	darkolivegreen
Hordeum_vulgare_newGene_8283	darkolivegreen
HORVU7Hr1G074660	darkolivegreen
HORVU7Hr1G108670	darkolivegreen
HORVU7Hr1G036070	darkolivegreen

HORVU4Hr1G065800	darkolivegreen
HORVU7Hr1G116810	darkolivegreen
Hordeum_vulgare_newGene_6840	darkolivegreen
HORVU4Hr1G020230	darkolivegreen
HORVU2Hr1G004540	darkolivegreen
HORVU5Hr1G066720	darkolivegreen
HORVU3Hr1G065320	darkolivegreen
HORVU2Hr1G119500	darkolivegreen
Hordeum_vulgare_newGene_554	darkolivegreen
HORVU7Hr1G030660	darkolivegreen
HORVU3Hr1G016860	darkolivegreen
HORVU5Hr1G062300	darkolivegreen
HORVU3Hr1G020140	darkolivegreen
HORVU4Hr1G071020	darkolivegreen
HORVU4Hr1G067450	darkolivegreen
HORVU5Hr1G073710	darkolivegreen
HORVU3Hr1G035680	darkolivegreen
Hordeum_vulgare_newGene_14259	darkolivegreen
HORVU3Hr1G098340	darkolivegreen
Hordeum_vulgare_newGene_10564	darkolivegreen
HORVU2Hr1G003210	darkolivegreen
HORVU5Hr1G065620	darkolivegreen
HORVU1Hr1G080680	darkolivegreen
HORVU4Hr1G080560	darkolivegreen
HORVU6Hr1G014600	darkolivegreen
HORVU3Hr1G011850	darkolivegreen
HORVU2Hr1G001780	darkolivegreen
HORVU3Hr1G092940	darkolivegreen
HORVU7Hr1G040850	darkolivegreen
HORVU5Hr1G118800	darkolivegreen
Hordeum_vulgare_newGene_13356	darkolivegreen
HORVU2Hr1G026890	darkolivegreen
HORVU5Hr1G023720	darkolivegreen
HORVU1Hr1G019750	darkolivegreen
HORVU7Hr1G096740	darkolivegreen
HORVU2Hr1G004480	darkolivegreen
HORVU4Hr1G066200	darkolivegreen
HORVU7Hr1G095230	darkolivegreen
HORVU1Hr1G049210	darkolivegreen
HORVU6Hr1G010890	darkolivegreen
Hordeum_vulgare_newGene_4072	darkolivegreen
HORVU2Hr1G004230	darkolivegreen
HORVU3Hr1G090410	darkolivegreen
HORVU5Hr1G008270	darkolivegreen
Hordeum_vulgare_newGene_10571	darkolivegreen
HORVU2Hr1G102710	darkolivegreen
HORVU5Hr1G023690	darkolivegreen

HORVU0Hr1G000780	darkolivegreen
HORVU2Hr1G004640	darkolivegreen
HORVU3Hr1G002000	darkolivegreen
HORVU4Hr1G052320	darkolivegreen
HORVU1Hr1G027700	darkolivegreen
HORVU7Hr1G100850	darkolivegreen
HORVU5Hr1G111240	darkolivegreen
HORVU2Hr1G112060	darkolivegreen
HORVU7Hr1G037910	darkolivegreen
HORVU4Hr1G060280	darkolivegreen
HORVU1Hr1G065670	darkolivegreen
HORVU5Hr1G120810	darkolivegreen
HORVU6Hr1G084740	darkolivegreen
HORVU5Hr1G122410	darkolivegreen
HORVU4Hr1G066240	darkolivegreen
HORVU3Hr1G098360	darkolivegreen
HORVU7Hr1G094320	darkolivegreen
Hordeum_vulgare_newGene_10011	darkolivegreen
HORVU6Hr1G089460	darkolivegreen
HORVU1Hr1G048900	darkolivegreen
HORVU1Hr1G010120	darkolivegreen
HORVU3Hr1G094340	darkolivegreen
HORVU7Hr1G105780	darkolivegreen
HORVU1Hr1G027350	orangered4
HORVU3Hr1G086620	orangered4
HORVU5Hr1G046880	orangered4
Hordeum_vulgare_newGene_11745	orangered4
HORVU0Hr1G008670	orangered4
HORVU0Hr1G023990	orangered4
Hordeum_vulgare_newGene_14296	orangered4
HORVU0Hr1G037040	orangered4
HORVU6Hr1G057770	orangered4
HORVU0Hr1G029340	orangered4
HORVU5Hr1G109130	orangered4
HORVU2Hr1G032180	orangered4
HORVU3Hr1G046320	orangered4
Hordeum_vulgare_newGene_254	orangered4
HORVU1Hr1G074600	orangered4
HORVU6Hr1G062190	orangered4
HORVU2Hr1G030380	orangered4
Hordeum_vulgare_newGene_13215	orangered4
HORVU6Hr1G076450	orangered4
HORVU3Hr1G092980	orangered4
HORVU4Hr1G022770	orangered4
HORVU1Hr1G073480	orangered4
HORVU6Hr1G057780	orangered4
HORVU5Hr1G093710	orangered4

HORVU2Hr1G104300	orangered4
HORVU7Hr1G040380	orangered4
HORVU1Hr1G050050	orangered4
HORVU1Hr1G093620	orangered4
HORVU3Hr1G080410	orangered4
HORVU7Hr1G035730	orangered4
HORVU5Hr1G039810	orangered4
HORVU2Hr1G032240	orangered4
HORVU6Hr1G004910	orangered4
HORVU4Hr1G088670	orangered4
HORVU1Hr1G056000	orangered4
HORVU3Hr1G115180	orangered4
HORVU4Hr1G040510	orangered4
Hordeum_vulgare_newGene_11966	orangered4
HORVU5Hr1G057600	orangered4
HORVU7Hr1G121440	orangered4
HORVU3Hr1G034070	orangered4
HORVU4Hr1G058070	orangered4
HORVU2Hr1G084700	orangered4
HORVU3Hr1G068650	orangered4
HORVU2Hr1G032220	orangered4
HORVU7Hr1G045370	orangered4
HORVU5Hr1G075660	orangered4
HORVU6Hr1G054000	orangered4
HORVU1Hr1G027340	orangered4
HORVU4Hr1G001420	orangered4
HORVU3Hr1G064120	orangered4
HORVU2Hr1G072950	orangered4
Hordeum_vulgare_newGene_2600	orangered4
HORVU2Hr1G077410	orangered4
Hordeum_vulgare_newGene_5928	orangered4
HORVU5Hr1G009540	orangered4
HORVU5Hr1G104750	orangered4
HORVU3Hr1G034060	orangered4
HORVU6Hr1G053140	orangered4
Hordeum_vulgare_newGene_10860	orangered4
HORVU3Hr1G088080	orangered4
Hordeum_vulgare_newGene_15	orangered4
HORVU6Hr1G082990	orangered4
Hordeum_vulgare_newGene_10121	orangered4
HORVU0Hr1G003660	orangered4
Hordeum_vulgare_newGene_951	orangered4
Hordeum_vulgare_newGene_4551	orangered4
HORVU0Hr1G022610	orangered4
HORVU3Hr1G057840	orangered4
HORVU4Hr1G054970	lightgreen
HORVU7Hr1G057650	lightgreen

Hordeum_vulgare_newGene_1822	lightgreen
HORVU3Hr1G074780	lightgreen
Hordeum_vulgare_newGene_2385	lightgreen
HORVU4Hr1G070530	lightgreen
Hordeum_vulgare_newGene_9031	lightgreen
HORVU1Hr1G000530	lightgreen
HORVU1Hr1G000700	lightgreen
Hordeum_vulgare_newGene_9418	lightgreen
HORVU2Hr1G014380	lightgreen
HORVU1Hr1G064110	lightgreen
Hordeum_vulgare_newGene_5899	lightgreen
HORVU2Hr1G069440	lightgreen
Hordeum_vulgare_newGene_8817	lightgreen
HORVU2Hr1G037480	lightgreen
Hordeum_vulgare_newGene_14713	lightgreen
Hordeum_vulgare_newGene_12411	lightgreen
Hordeum_vulgare_newGene_10101	lightgreen
HORVU4Hr1G074400	lightgreen
HORVU7Hr1G007790	lightgreen
HORVU3Hr1G056440	lightgreen
HORVU7Hr1G055330	lightgreen
Hordeum_vulgare_newGene_14469	lightgreen
Hordeum_vulgare_newGene_13161	lightgreen
HORVU1Hr1G026100	lightgreen
Hordeum_vulgare_newGene_11326	lightgreen
Hordeum_vulgare_newGene_15013	lightgreen
Hordeum_vulgare_newGene_13649	lightgreen
HORVU6Hr1G082310	lightgreen
HORVU7Hr1G118570	lightgreen
HORVU5Hr1G061710	lightgreen
Hordeum_vulgare_newGene_14007	lightgreen
Hordeum_vulgare_newGene_11525	lightgreen
HORVU6Hr1G038250	lightgreen
HORVU2Hr1G032420	lightgreen
HORVU7Hr1G006570	lightgreen
HORVU6Hr1G076070	lightgreen
HORVU5Hr1G054420	lightgreen
HORVU4Hr1G067280	lightgreen
Hordeum_vulgare_newGene_13768	lightgreen
HORVU4Hr1G018150	lightgreen
HORVU6Hr1G089560	lightgreen
HORVU6Hr1G055440	lightgreen
HORVU2Hr1G014410	lightgreen
Hordeum_vulgare_newGene_3094	lightgreen
Hordeum_vulgare_newGene_4233	lightgreen
HORVU5Hr1G034820	lightgreen
Hordeum_vulgare_newGene_13836	lightgreen

HORVU1Hr1G075750	lightgreen
Hordeum_vulgare_newGene_8059	lightgreen
HORVU7Hr1G116220	lightgreen
HORVU1Hr1G082220	lightgreen
Hordeum_vulgare_newGene_14177	lightgreen
Hordeum_vulgare_newGene_223	lightgreen
HORVU1Hr1G059810	lightgreen
HORVU1Hr1G005460	lightgreen
HORVU2Hr1G021730	lightgreen
HORVU0Hr1G010340	lightgreen
Hordeum_vulgare_newGene_4912	lightgreen
Hordeum_vulgare_newGene_57	lightgreen
HORVU7Hr1G011050	lightgreen
HORVU7Hr1G091580	lightgreen
Hordeum_vulgare_newGene_10405	lightgreen
Hordeum_vulgare_newGene_10401	lightgreen
HORVU5Hr1G022940	lightgreen
HORVU1Hr1G085150	lightgreen
HORVU3Hr1G031680	lightgreen
HORVU7Hr1G003940	lightgreen
Hordeum_vulgare_newGene_9361	lightgreen
HORVU1Hr1G043710	lightgreen
HORVU4Hr1G088880	lightgreen
Hordeum_vulgare_newGene_14730	lightgreen
HORVU3Hr1G016800	lightgreen
HORVU0Hr1G017220	lightgreen
Hordeum_vulgare_newGene_3017	lightgreen
HORVU2Hr1G082340	lightgreen
HORVU1Hr1G010210	lightgreen
HORVU6Hr1G063510	lightgreen
HORVU5Hr1G125570	lightgreen
HORVU5Hr1G011730	lightgreen
HORVU1Hr1G000680	lightgreen
Hordeum_vulgare_newGene_8797	lightgreen
HORVU1Hr1G000520	lightgreen
Hordeum_vulgare_newGene_6549	lightgreen
HORVU5Hr1G100890	lightgreen
HORVU0Hr1G014040	lightgreen
HORVU1Hr1G001420	lightgreen
Hordeum_vulgare_newGene_10478	lightgreen
Hordeum_vulgare_newGene_5796	lightgreen
HORVU7Hr1G095840	lightgreen
Hordeum_vulgare_newGene_3611	lightgreen
Hordeum_vulgare_newGene_9135	lightgreen
Hordeum_vulgare_newGene_5459	lightgreen
HORVU4Hr1G089510	lightgreen
HORVU4Hr1G018180	lightgreen

HORVU0Hr1G009860	lightgreen
Hordeum_vulgare_newGene_16045	lightgreen
Hordeum_vulgare_newGene_4120	lightgreen
HORVU2Hr1G054010	lightgreen
HORVU7Hr1G027960	lightgreen
Hordeum_vulgare_newGene_13913	lightgreen
HORVU5Hr1G009510	lightgreen
HORVU1Hr1G013680	lightgreen
HORVU0Hr1G007410	lightgreen
HORVU3Hr1G005350	lightgreen
HORVU1Hr1G056820	lightgreen
HORVU1Hr1G092960	lightgreen
Hordeum_vulgare_newGene_13117	lightgreen
Hordeum_vulgare_newGene_15124	lightgreen
HORVU3Hr1G014100	lightgreen
Hordeum_vulgare_newGene_12611	lightgreen
HORVU2Hr1G023590	lightgreen
HORVU1Hr1G000660	lightgreen
Hordeum_vulgare_newGene_14940	lightgreen
HORVU5Hr1G096260	lightgreen
HORVU1Hr1G058310	lightgreen
HORVU3Hr1G063680	lightgreen
HORVU0Hr1G014570	lightgreen
Hordeum_vulgare_newGene_16202	lightgreen
Hordeum_vulgare_newGene_14435	lightgreen
Hordeum_vulgare_newGene_6499	lightgreen
HORVU3Hr1G037580	lightgreen
HORVU1Hr1G005030	lightgreen
Hordeum_vulgare_newGene_15771	lightgreen
HORVU2Hr1G079580	lightgreen
Hordeum_vulgare_newGene_10492	lightgreen
HORVU7Hr1G080510	lightgreen
Hordeum_vulgare_newGene_10022	lightgreen
Hordeum_vulgare_newGene_14594	lightgreen
Hordeum_vulgare_newGene_14595	lightgreen
Hordeum_vulgare_newGene_14597	lightgreen
HORVU7Hr1G119250	lightgreen
HORVU7Hr1G045620	lightgreen
HORVU1Hr1G015560	lightgreen
Hordeum_vulgare_newGene_13684	lightgreen
Hordeum_vulgare_newGene_11863	lightgreen
HORVU5Hr1G027110	lightgreen
HORVU5Hr1G109980	lightgreen
HORVU2Hr1G037040	lightgreen
HORVU1Hr1G087380	lightgreen
HORVU4Hr1G011670	lightgreen
HORVU3Hr1G046900	lightgreen

HORVU3Hr1G059130	lightgreen
HORVU5Hr1G112610	lightgreen
Hordeum_vulgare_newGene_10018	lightgreen
Hordeum_vulgare_newGene_4852	lightgreen
HORVU5Hr1G012180	lightgreen
HORVU7Hr1G116060	lightgreen
HORVU7Hr1G082980	lightgreen
HORVU1Hr1G004120	lightgreen
HORVU7Hr1G033760	lightgreen
HORVU6Hr1G015390	lightgreen
HORVU4Hr1G056260	lightgreen
HORVU1Hr1G057170	lightgreen
Hordeum_vulgare_newGene_14922	lightgreen
HORVU2Hr1G045120	lightgreen
HORVU2Hr1G104500	lightgreen
HORVU5Hr1G104230	lightgreen
HORVU7Hr1G043530	lightgreen
Hordeum_vulgare_newGene_15793	lightgreen
HORVU7Hr1G110900	lightgreen
Hordeum_vulgare_newGene_9084	lightgreen
HORVU4Hr1G007610	lightgreen
Hordeum_vulgare_newGene_11620	lightgreen
HORVU1Hr1G068930	lightgreen
HORVU2Hr1G093670	lightgreen
HORVU6Hr1G079600	lightgreen
Hordeum_vulgare_newGene_8549	lightgreen
HORVU1Hr1G013690	lightgreen
Hordeum_vulgare_newGene_13743	lightgreen
HORVU5Hr1G060790	lightgreen
Hordeum_vulgare_newGene_13963	lightgreen
HORVU5Hr1G106110	brown4
HORVU5Hr1G085250	brown4
HORVU3Hr1G089420	brown4
HORVU2Hr1G027150	brown4
Hordeum_vulgare_newGene_3687	brown4
HORVU5Hr1G087730	brown4
Hordeum_vulgare_newGene_7710	brown4
HORVU5Hr1G084510	brown4
HORVU5Hr1G111810	brown4
HORVU6Hr1G065740	brown4
HORVU1Hr1G079970	brown4
Hordeum_vulgare_newGene_14646	brown4
HORVU7Hr1G038630	brown4
Hordeum_vulgare_newGene_3044	brown4
HORVU1Hr1G078050	brown4
Hordeum_vulgare_newGene_10219	brown4
HORVU0Hr1G002480	brown4

HORVU2Hr1G090980	brown4
HORVU7Hr1G010750	brown4
HORVU5Hr1G103450	brown4
Hordeum_vulgare_newGene_14678	brown4
HORVU5Hr1G057520	brown4
HORVU5Hr1G080170	brown4
Hordeum_vulgare_newGene_2116	brown4
HORVU3Hr1G004420	brown4
EPlHVUG00000039881	brown4
Hordeum_vulgare_newGene_1497	brown4
HORVU2Hr1G080370	brown4
HORVU1Hr1G078310	brown4
HORVU1Hr1G087040	brown4
HORVU5Hr1G098290	brown4
HORVU2Hr1G112860	brown4
HORVU6Hr1G026840	brown4
HORVU6Hr1G072650	brown4
Hordeum_vulgare_newGene_12937	brown4
Hordeum_vulgare_newGene_3924	brown4
HORVU6Hr1G087400	brown4
HORVU1Hr1G067850	brown4
Hordeum_vulgare_newGene_10147	brown4
HORVU7Hr1G092680	brown4
HORVU7Hr1G096880	brown4
HORVU5Hr1G113740	brown4
HORVU5Hr1G112390	brown4
HORVU2Hr1G027230	brown4
HORVU3Hr1G099490	brown4
HORVU2Hr1G099610	brown4
HORVU7Hr1G012850	brown4
HORVU4Hr1G084240	brown4
HORVU3Hr1G027200	brown4
HORVU6Hr1G091860	brown4
Hordeum_vulgare_newGene_12218	brown4
HORVU7Hr1G010890	brown4
HORVU6Hr1G077730	brown4
HORVU3Hr1G114240	brown4
HORVU2Hr1G099660	brown4
HORVU3Hr1G088850	brown4
Hordeum_vulgare_newGene_15136	brown4
HORVU7Hr1G033470	brown4
HORVU5Hr1G021300	brown4
Hordeum_vulgare_newGene_13029	brown4
Hordeum_vulgare_newGene_2305	lightcyan1
HORVU6Hr1G012340	lightcyan1
HORVU4Hr1G001570	lightcyan1
HORVU0Hr1G007340	lightcyan1

Hordeum_vulgare_newGene_12896	lightcyan1
HORVU5Hr1G041400	lightcyan1
HORVU3Hr1G117580	lightcyan1
HORVU3Hr1G117630	lightcyan1
HORVU1Hr1G075040	lightcyan1
HORVU5Hr1G056030	lightcyan1
HORVU4Hr1G051400	lightcyan1
HORVU2Hr1G117120	lightcyan1
Hordeum_vulgare_newGene_10827	lightcyan1
Hordeum_vulgare_newGene_10828	lightcyan1
Hordeum_vulgare_newGene_8910	lightcyan1
HORVU2Hr1G076060	lightcyan1
HORVU1Hr1G079450	lightcyan1
HORVU2Hr1G028670	lightcyan1
HORVU3Hr1G057690	lightcyan1
HORVU3Hr1G111580	lightcyan1
Hordeum_vulgare_newGene_12915	lightcyan1
HORVU1Hr1G043890	lightcyan1
HORVU3Hr1G087100	lightcyan1
HORVU2Hr1G045340	lightcyan1
HORVU3Hr1G081570	lightcyan1
HORVU3Hr1G071370	lightcyan1
HORVU7Hr1G035010	lightcyan1
Hordeum_vulgare_newGene_13423	lightcyan1
HORVU6Hr1G011860	lightcyan1
HORVU5Hr1G023740	lightcyan1
HORVU5Hr1G125280	lightcyan1
HORVU1Hr1G089970	lightcyan1
HORVU7Hr1G112900	lightcyan1
HORVU2Hr1G109100	lightcyan1
Hordeum_vulgare_newGene_11346	lightcyan1
HORVU7Hr1G024610	lightcyan1
HORVU5Hr1G014130	lightcyan1
HORVU6Hr1G043980	lightcyan1
Hordeum_vulgare_newGene_10914	lightcyan1
HORVU1Hr1G025530	lightcyan1
HORVU1Hr1G043470	lightcyan1
HORVU4Hr1G076840	lightcyan1
Hordeum_vulgare_newGene_15200	lightcyan1
HORVU6Hr1G078670	lightcyan1
HORVU7Hr1G118010	lightcyan1
HORVU3Hr1G062920	lightcyan1
HORVU2Hr1G036930	lightcyan1
Hordeum_vulgare_newGene_179	lightcyan1
Hordeum_vulgare_newGene_178	lightcyan1
HORVU5Hr1G012010	lightcyan1
HORVU3Hr1G026610	lightcyan1

HORVU3Hr1G006310	lightcyan1
HORVU4Hr1G050510	lightcyan1
HORVU4Hr1G078310	lightcyan1
Hordeum_vulgare_newGene_10327	lightcyan1
Hordeum_vulgare_newGene_8818	lightcyan1
HORVU3Hr1G013910	lightcyan1
Hordeum_vulgare_newGene_13868	lightcyan1
Hordeum_vulgare_newGene_2528	lightcyan1
HORVU0Hr1G000760	lightcyan1
HORVU3Hr1G087020	lightcyan1
HORVU3Hr1G016280	lightcyan1
HORVU3Hr1G016820	lightcyan1
HORVU5Hr1G066930	lightcyan1
HORVU7Hr1G009340	lightcyan1
HORVU6Hr1G092780	lightcyan1
Hordeum_vulgare_newGene_14157	lightcyan1
HORVU1Hr1G008360	lightcyan1
HORVU2Hr1G018420	lightcyan1
Hordeum_vulgare_newGene_1486	lightcyan1
Hordeum_vulgare_newGene_1489	lightcyan1
Hordeum_vulgare_newGene_4050	lightcyan1
HORVU6Hr1G004900	lightcyan1
HORVU5Hr1G115750	lightcyan1
HORVU7Hr1G009680	lightcyan1
HORVU2Hr1G020430	lightcyan1
Hordeum_vulgare_newGene_11953	lightcyan1
HORVU0Hr1G013470	lightcyan1
HORVU2Hr1G108730	lightcyan1
HORVU7Hr1G121860	lightcyan1
HORVU1Hr1G073940	lightcyan1
HORVU3Hr1G094010	lightcyan1
Hordeum_vulgare_newGene_14797	lightcyan1
HORVU7Hr1G113020	lightcyan1
HORVU2Hr1G080190	lightcyan1
HORVU7Hr1G007690	lightcyan1
HORVU4Hr1G051450	lightcyan1
HORVU3Hr1G023230	lightcyan1
HORVU7Hr1G072620	lightcyan1
Hordeum_vulgare_newGene_5188	lightcyan1
Hordeum_vulgare_newGene_14988	lightcyan1
Hordeum_vulgare_newGene_1408	lightcyan1
Hordeum_vulgare_newGene_13642	lightcyan1
HORVU1Hr1G092680	lightcyan1
Hordeum_vulgare_newGene_5684	lightcyan1
HORVU6Hr1G080690	lightcyan1
HORVU5Hr1G012190	lightcyan1
HORVU5Hr1G051970	lightcyan1

HORVU5Hr1G066640	lightcyan1
Hordeum_vulgare_newGene_6560	lightcyan1
HORVU6Hr1G073630	lightcyan1
HORVU6Hr1G000480	lightcyan1
HORVU5Hr1G120110	lightcyan1
Hordeum_vulgare_newGene_6016	lightcyan1
HORVU6Hr1G062430	lightcyan1
HORVU5Hr1G001750	lightcyan1
Hordeum_vulgare_newGene_1293	lightcyan1
HORVU5Hr1G115780	lightcyan1
HORVU7Hr1G039180	lightcyan1
HORVU4Hr1G002180	lightcyan1
HORVU1Hr1G078860	lightcyan1
HORVU2Hr1G094160	lightcyan1
HORVU2Hr1G070620	lightcyan1
HORVU0Hr1G012850	lightcyan1
HORVU0Hr1G029570	lightcyan1
Hordeum_vulgare_newGene_12601	lightcyan1
HORVU2Hr1G006850	lightcyan1
HORVU1Hr1G081410	lightcyan1
HORVU5Hr1G057740	lightcyan1
HORVU7Hr1G009370	lightcyan1
Hordeum_vulgare_newGene_128	lightcyan1
HORVU2Hr1G100500	lightcyan1
HORVU1Hr1G000650	lightcyan1
HORVU2Hr1G075370	lightcyan1
HORVU1Hr1G005760	lightcyan1
HORVU1Hr1G090870	lightcyan1
HORVU2Hr1G084130	lightcyan1
HORVU5Hr1G005340	lightcyan1
HORVU2Hr1G016720	lightcyan1
HORVU1Hr1G082100	lightcyan1
HORVU3Hr1G099760	lightcyan1
HORVU2Hr1G043890	lightcyan1
Hordeum_vulgare_newGene_6808	lightcyan1
HORVU4Hr1G005450	lightcyan1
Hordeum_vulgare_newGene_7760	lightcyan1
HORVU1Hr1G079680	lightcyan1
HORVU7Hr1G121850	lightcyan1
HORVU3Hr1G039470	lightcyan1
HORVU1Hr1G070310	lightcyan1
HORVU4Hr1G002730	lightcyan1
HORVU1Hr1G062380	lightcyan1
HORVU4Hr1G072360	lightcyan1
HORVU6Hr1G034760	lightcyan1
HORVU2Hr1G065000	lightcyan1
Hordeum_vulgare_newGene_2718	lightcyan1

Hordeum_vulgare_newGene_5842	lightcyan1
HORVU2Hr1G076320	lightcyan1
HORVU3Hr1G088040	lightcyan1
Hordeum_vulgare_newGene_3124	lightcyan1
Hordeum_vulgare_newGene_14642	lightcyan1
HORVU1Hr1G000590	lightcyan1
HORVU3Hr1G018800	lightcyan1
HORVU3Hr1G034020	lightcyan1
HORVU2Hr1G092390	lightcyan1
Hordeum_vulgare_newGene_15496	lightcyan1
HORVU2Hr1G088640	lightcyan1
HORVU3Hr1G053060	lightcyan1
Hordeum_vulgare_newGene_3136	lightcyan1
Hordeum_vulgare_newGene_198	lightcyan1
HORVU2Hr1G096890	lightcyan1
HORVU1Hr1G095430	lightcyan1
HORVU0Hr1G007730	lightcyan1
HORVU7Hr1G054890	lightcyan1
HORVU4Hr1G055610	lightcyan1
Hordeum_vulgare_newGene_11989	lightcyan1
HORVU7Hr1G089270	lightcyan1
HORVU4Hr1G077850	lightcyan1
HORVU1Hr1G039720	lightcyan1
HORVU7Hr1G046830	lightcyan1
HORVU3Hr1G081590	lightcyan1
HORVU5Hr1G104580	lightcyan1
HORVU7Hr1G045630	lightcyan1
HORVU2Hr1G035870	lightcyan1
Hordeum_vulgare_newGene_778	lightcyan1
HORVU6Hr1G073990	lightcyan1
HORVU3Hr1G032260	lightcyan1
HORVU6Hr1G021060	lightcyan1
HORVU3Hr1G065630	lightcyan1
Hordeum_vulgare_newGene_11461	lightcyan1
Hordeum_vulgare_newGene_2297	lightcyan1
Hordeum_vulgare_newGene_5742	lightcyan1
HORVU1Hr1G068490	lightcyan1
HORVU1Hr1G056570	lightcyan1
HORVU2Hr1G093700	lightcyan1
HORVU7Hr1G027010	lightcyan1
HORVU2Hr1G044360	lightcyan1
HORVU3Hr1G097180	lightcyan1
HORVU3Hr1G019510	lightcyan1
HORVU3Hr1G117590	lightcyan1
HORVU1Hr1G012090	lightcyan1
HORVU1Hr1G084980	lightcyan1
HORVU2Hr1G110860	lightcyan1

HORVU7Hr1G122680	lightcyan1
HORVU5Hr1G069380	lightcyan1
HORVU0Hr1G023760	lightcyan1
Hordeum_vulgare_newGene_3940	lightcyan1
HORVU3Hr1G091680	lightcyan1
HORVU7Hr1G100130	lightcyan1
HORVU1Hr1G072720	lightcyan1
HORVU7Hr1G118610	lightcyan1
HORVU5Hr1G108690	lightcyan1
HORVU5Hr1G058190	lightcyan1
HORVU1Hr1G062600	lightcyan1
HORVU1Hr1G065150	lightcyan1
HORVU1Hr1G009800	lightcyan1
HORVU1Hr1G004980	lightcyan1
Hordeum_vulgare_newGene_3678	lightcyan1
HORVU6Hr1G074690	lightcyan1
HORVU2Hr1G017420	lightcyan1
Hordeum_vulgare_newGene_11543	lightcyan1
HORVU5Hr1G081840	lightcyan1
HORVU2Hr1G028660	lightcyan1
HORVU3Hr1G024500	lightcyan1
HORVU5Hr1G071140	lightcyan1
Hordeum_vulgare_newGene_5626	lightcyan1
HORVU3Hr1G049730	lightcyan1
HORVU5Hr1G088130	lightcyan1
HORVU1Hr1G067670	lightcyan1
HORVU5Hr1G123520	lightcyan1
Hordeum_vulgare_newGene_2957	lightcyan1
HORVU5Hr1G065350	lightcyan1
HORVU1Hr1G057410	lightcyan1
Hordeum_vulgare_newGene_10936	lightcyan1
HORVU6Hr1G080260	lightcyan1
HORVU3Hr1G100350	lightcyan1
HORVU7Hr1G051120	lightcyan1
HORVU5Hr1G083330	lightcyan1
HORVU5Hr1G104240	lightcyan1
Hordeum_vulgare_newGene_2929	lightcyan1
HORVU4Hr1G002650	lightcyan1
HORVU7Hr1G088350	lightcyan1
HORVU3Hr1G028020	lightcyan1
Hordeum_vulgare_newGene_3503	lightcyan1
HORVU7Hr1G027410	lightcyan1
HORVU2Hr1G061910	lightcyan1
HORVU4Hr1G063780	lightcyan1
HORVU7Hr1G088780	lightcyan1
Hordeum_vulgare_newGene_914	lightcyan1
HORVU7Hr1G113290	lightcyan1

HORVU6Hr1G092840	lightcyan1
Hordeum_vulgare_newGene_12951	lightcyan1
HORVU7Hr1G006180	lightcyan1
HORVU2Hr1G121090	lightcyan1
HORVU1Hr1G062590	lightcyan1
Hordeum_vulgare_newGene_14531	lightcyan1
HORVU5Hr1G096930	lightcyan1
HORVU4Hr1G079940	lightcyan1
HORVU2Hr1G070310	lightcyan1
HORVU5Hr1G083320	lightcyan1
HORVU2Hr1G098140	lightcyan1
HORVU4Hr1G069860	lightcyan1
HORVU5Hr1G072780	lightcyan1
HORVU2Hr1G018680	lightcyan1
HORVU2Hr1G059320	lightcyan1
HORVU1Hr1G065600	lightcyan1
HORVU1Hr1G018140	lightcyan1
HORVU6Hr1G017390	lightcyan1
HORVU7Hr1G118560	lightcyan1
HORVU2Hr1G043900	lightcyan1
HORVU3Hr1G113120	lightcyan1
Hordeum_vulgare_newGene_10333	lightcyan1
HORVU6Hr1G094880	lightcyan1
Hordeum_vulgare_newGene_16042	lightcyan1
HORVU1Hr1G038130	lightcyan1
HORVU6Hr1G088540	lightcyan1
Hordeum_vulgare_newGene_13639	lightcyan1
HORVU1Hr1G044780	lightcyan1
HORVU7Hr1G107670	lightcyan1
HORVU7Hr1G098250	lightcyan1
HORVU2Hr1G017530	lightcyan1
HORVU5Hr1G042370	lightcyan1
HORVU5Hr1G099670	lightcyan1
HORVU3Hr1G100360	lightcyan1
HORVU5Hr1G097270	lightcyan1
HORVU3Hr1G117550	lightcyan1
HORVU5Hr1G069360	lightcyan1
HORVU1Hr1G090670	lightcyan1
Hordeum_vulgare_newGene_8741	lightcyan1
HORVU3Hr1G010190	lightcyan1
Hordeum_vulgare_newGene_6000	lightcyan1
Hordeum_vulgare_newGene_6009	lightcyan1
HORVU2Hr1G046550	lightcyan1
HORVU5Hr1G065920	lightcyan1
HORVU6Hr1G091560	lightcyan1
HORVU6Hr1G074560	lightcyan1
HORVU7Hr1G038330	lightcyan1

Hordeum_vulgare_newGene_14095	lightcyan1
HORVU1Hr1G065030	lightcyan1
HORVU0Hr1G030280	lightcyan1
HORVU6Hr1G088310	lightcyan1
Hordeum_vulgare_newGene_5953	lightcyan1
HORVU0Hr1G039080	lightcyan1
HORVU2Hr1G041520	lightcyan1
HORVU0Hr1G003980	lightcyan1
HORVU2Hr1G098800	lightcyan1
HORVU3Hr1G016830	lightcyan1
HORVU7Hr1G050670	lightcyan1
HORVU1Hr1G040700	lightcyan1
HORVU6Hr1G092740	lightcyan1
HORVU2Hr1G066200	lightcyan1
HORVU5Hr1G056420	lightcyan1
HORVU3Hr1G078360	lightcyan1
HORVU5Hr1G027980	lightcyan1
HORVU5Hr1G107220	lightcyan1
HORVU6Hr1G011050	lightcyan1
HORVU7Hr1G089160	lightcyan1
HORVU7Hr1G035870	lightcyan1
HORVU2Hr1G015140	lightcyan1
HORVU5Hr1G107230	lightcyan1
HORVU1Hr1G068020	lightcyan1
HORVU2Hr1G070700	lightcyan1
HORVU2Hr1G036570	lightcyan1
HORVU6Hr1G004770	lightcyan1
Hordeum_vulgare_newGene_12888	lightcyan1
HORVU3Hr1G005540	lightcyan1
HORVU7Hr1G080780	lightcyan1
HORVU5Hr1G094280	lightcyan1
HORVU2Hr1G101990	lightcyan1
HORVU3Hr1G086690	lightcyan1
Hordeum_vulgare_newGene_13547	lightcyan1
HORVU1Hr1G069550	lightcyan1
HORVU5Hr1G095180	lightcyan1
HORVU3Hr1G077790	lightcyan1
Hordeum_vulgare_newGene_274	lightcyan1
HORVU7Hr1G084920	lightcyan1
HORVU2Hr1G033610	lightcyan1
Hordeum_vulgare_newGene_4112	lightcyan1
HORVU1Hr1G063740	lightcyan1
HORVU7Hr1G101590	lightcyan1
HORVU1Hr1G079610	lightcyan1
HORVU2Hr1G087880	lightcyan1
HORVU1Hr1G066300	lightcyan1
HORVU7Hr1G093020	lightcyan1

Hordeum_vulgare_newGene_14257	lightcyan1
HORVU4Hr1G011740	lightcyan1
HORVU7Hr1G027810	lightcyan1
HORVU3Hr1G017940	lightcyan1
HORVU6Hr1G078390	lightcyan1
HORVU7Hr1G088560	lightcyan1
Hordeum_vulgare_newGene_2048	lightcyan1
HORVU2Hr1G048290	lightcyan1
Hordeum_vulgare_newGene_857	lightcyan1
HORVU0Hr1G010470	lightcyan1
HORVU2Hr1G005620	lightcyan1
HORVU5Hr1G109610	lightcyan1
HORVU5Hr1G112900	lightcyan1
Hordeum_vulgare_newGene_10225	lightcyan1
HORVU1Hr1G083610	lightcyan1
HORVU2Hr1G083020	lightcyan1
Hordeum_vulgare_newGene_1350	lightcyan1
Hordeum_vulgare_newGene_45	lightcyan1
HORVU5Hr1G125300	lightcyan1
HORVU2Hr1G012790	lightcyan1
HORVU3Hr1G058300	lightcyan1
Hordeum_vulgare_newGene_4022	lightcyan1
HORVU0Hr1G000850	lightcyan1
Hordeum_vulgare_newGene_10166	lightcyan1
HORVU1Hr1G091010	lightcyan1
HORVU7Hr1G100090	lightcyan1
Hordeum_vulgare_newGene_2986	lightcyan1
HORVU7Hr1G079160	lightcyan1
Hordeum_vulgare_newGene_9310	lightcyan1
HORVU4Hr1G067850	lightcyan1
HORVU7Hr1G040740	lightcyan1
Hordeum_vulgare_newGene_2165	lightcyan1
Hordeum_vulgare_newGene_15150	lightcyan1
Hordeum_vulgare_newGene_1533	lightcyan1
HORVU3Hr1G113020	lightcyan1
Hordeum_vulgare_newGene_13118	lightcyan1
HORVU4Hr1G050210	lightcyan1
HORVU7Hr1G001750	lightcyan1
HORVU1Hr1G063100	lightcyan1
HORVU2Hr1G088110	lightcyan1
HORVU1Hr1G024770	lightcyan1
HORVU4Hr1G072830	lightcyan1
Hordeum_vulgare_newGene_490	lightcyan1
HORVU3Hr1G086220	lightcyan1
HORVU6Hr1G059500	lightcyan1
Hordeum_vulgare_newGene_11942	lightcyan1
HORVU4Hr1G010580	lightcyan1

HORVU3Hr1G022780	lightcyan1
HORVU6Hr1G064500	lightcyan1
HORVU5Hr1G114700	lightcyan1
HORVU0Hr1G014580	lightcyan1
HORVU3Hr1G065390	lightcyan1
HORVU1Hr1G025380	lightcyan1
HORVU2Hr1G097980	lightcyan1
Hordeum_vulgare_newGene_5387	lightcyan1
Hordeum_vulgare_newGene_14412	lightcyan1
HORVU4Hr1G060810	lightcyan1
HORVU2Hr1G113940	lightcyan1
HORVU3Hr1G094720	lightcyan1
Hordeum_vulgare_newGene_13603	lightcyan1
HORVU7Hr1G036970	lightcyan1
Hordeum_vulgare_newGene_4857	lightcyan1
HORVU4Hr1G017350	lightcyan1
HORVU5Hr1G102140	lightcyan1
HORVU3Hr1G021610	lightcyan1
Hordeum_vulgare_newGene_1823	lightcyan1
HORVU6Hr1G088530	lightcyan1
HORVU1Hr1G043450	lightcyan1
HORVU3Hr1G078840	lightcyan1
HORVU1Hr1G057460	lightcyan1
HORVU6Hr1G091980	lightcyan1
Hordeum_vulgare_newGene_960	lightcyan1
HORVU3Hr1G107350	lightcyan1
Hordeum_vulgare_newGene_1282	lightcyan1
HORVU1Hr1G067980	lightcyan1
HORVU2Hr1G058390	lightcyan1
HORVU2Hr1G123270	lightcyan1
HORVU3Hr1G056100	lightcyan1
HORVU5Hr1G083340	lightcyan1
HORVU3Hr1G094800	lightcyan1
HORVU3Hr1G096890	lightcyan1
HORVU1Hr1G021160	lightcyan1
HORVU6Hr1G093260	lightcyan1
HORVU5Hr1G051980	lightcyan1
HORVU7Hr1G000040	lightcyan1
HORVU1Hr1G076710	lightcyan1
HORVU0Hr1G019300	lightcyan1
HORVU1Hr1G045360	lightcyan1
HORVU4Hr1G025360	lightcyan1
HORVU1Hr1G081300	lightcyan1
HORVU7Hr1G035480	lightcyan1
HORVU5Hr1G010560	lightcyan1
HORVU7Hr1G038400	lightcyan1
HORVU4Hr1G051010	lightcyan1

HORVU5Hr1G066230	lightcyan1
HORVU4Hr1G009380	lightcyan1
HORVU2Hr1G067350	lightcyan1
HORVU5Hr1G014090	lightcyan1
HORVU5Hr1G075150	lightcyan1
Hordeum_vulgare_newGene_6107	lightcyan1
Hordeum_vulgare_newGene_11974	lightcyan1
Hordeum_vulgare_newGene_14609	lightcyan1
HORVU6Hr1G070450	lightcyan1
HORVU5Hr1G046550	lightcyan1
HORVU3Hr1G056660	lightcyan1
HORVU6Hr1G055230	lightcyan1
HORVU7Hr1G094730	lightcyan1
HORVU2Hr1G036590	lightcyan1
HORVU2Hr1G109440	lightcyan1
HORVU1Hr1G091990	lightcyan1
HORVU5Hr1G058060	lightcyan1
Hordeum_vulgare_newGene_9085	lightcyan1
HORVU5Hr1G063430	lightcyan1
HORVU2Hr1G118570	lightcyan1
HORVU5Hr1G082620	lightcyan1
HORVU5Hr1G084700	lightcyan1
HORVU1Hr1G055470	lightcyan1
HORVU1Hr1G044000	lightcyan1
HORVU4Hr1G011250	lightcyan1
HORVU5Hr1G047510	lightcyan1
Hordeum_vulgare_newGene_16052	lightcyan1
HORVU2Hr1G062940	lightcyan1
HORVU7Hr1G042790	lightcyan1
Hordeum_vulgare_newGene_14343	lightcyan1
Hordeum_vulgare_newGene_14344	lightcyan1
HORVU7Hr1G103380	lightcyan1
Hordeum_vulgare_newGene_4796	lightcyan1
HORVU5Hr1G066460	lightcyan1
HORVU2Hr1G028780	lightcyan1
HORVU2Hr1G071270	lightcyan1
HORVU5Hr1G074360	lightcyan1
HORVU2Hr1G102380	lightcyan1
HORVU6Hr1G011580	lightcyan1
HORVU6Hr1G088470	lightcyan1
HORVU3Hr1G070850	lightcyan1
HORVU4Hr1G064790	lightcyan1
HORVU0Hr1G012930	lightcyan1
HORVU2Hr1G083490	lightcyan1
HORVU5Hr1G115100	lightcyan1
Hordeum_vulgare_newGene_13025	lightcyan1
HORVU0Hr1G011720	lightcyan1

HORVU1Hr1G005800	lightcyan1
HORVU3Hr1G098580	lightcyan1
HORVU7Hr1G044990	lightcyan1
Hordeum_vulgare_newGene_3271	lightcyan1
HORVU6Hr1G074200	lightcyan1
HORVU6Hr1G078640	lightcyan1
HORVU0Hr1G000730	lightcyan1
HORVU3Hr1G032270	lightcyan1
HORVU7Hr1G050660	lightcyan1
HORVU1Hr1G061980	lightcyan1
HORVU7Hr1G068080	lightcyan1
Hordeum_vulgare_newGene_103	lightcyan1
HORVU0Hr1G025850	lightcyan1
HORVU4Hr1G057360	skyblue3
HORVU2Hr1G073210	skyblue3
HORVU7Hr1G108460	skyblue3
HORVU4Hr1G016460	skyblue3
HORVU4Hr1G012680	skyblue3
Hordeum_vulgare_newGene_10504	skyblue3
HORVU4Hr1G012330	skyblue3
HORVU5Hr1G106020	skyblue3
HORVU6Hr1G010400	skyblue3
Hordeum_vulgare_newGene_719	skyblue3
HORVU2Hr1G007570	skyblue3
HORVU3Hr1G006800	skyblue3
HORVU2Hr1G102920	skyblue3
HORVU4Hr1G063790	skyblue3
Hordeum_vulgare_newGene_3797	skyblue3
HORVU2Hr1G103000	skyblue3
HORVU1Hr1G001080	skyblue3
HORVU5Hr1G086650	skyblue3
HORVU5Hr1G062720	skyblue3
HORVU3Hr1G093140	skyblue3
HORVU1Hr1G085720	skyblue3
HORVU6Hr1G064650	skyblue3
Hordeum_vulgare_newGene_12941	skyblue3
HORVU0Hr1G036340	skyblue3
Hordeum_vulgare_newGene_3689	skyblue3
HORVU2Hr1G112620	skyblue3
HORVU6Hr1G063240	skyblue3
HORVU1Hr1G051300	skyblue3
HORVU6Hr1G012210	skyblue3
HORVU7Hr1G094870	skyblue3
HORVU5Hr1G041930	skyblue3
HORVU0Hr1G030830	skyblue3
HORVU1Hr1G079140	skyblue3
HORVU4Hr1G053250	skyblue3

HORVU3Hr1G031950	skyblue3
HORVU0Hr1G003340	skyblue3
HORVU4Hr1G016880	skyblue3
HORVU3Hr1G083330	skyblue3
HORVU5Hr1G093460	skyblue3
HORVU6Hr1G073090	skyblue3
HORVU3Hr1G013380	skyblue3
HORVU1Hr1G003320	skyblue3
HORVU5Hr1G080630	skyblue3
HORVU1Hr1G030060	skyblue3
HORVU4Hr1G008950	skyblue3
HORVU3Hr1G003880	skyblue3
HORVU1Hr1G081140	skyblue3
HORVU2Hr1G095880	skyblue3
Hordeum_vulgare_newGene_476	skyblue3
HORVU2Hr1G007490	skyblue3
HORVU7Hr1G007220	skyblue3
HORVU7Hr1G058090	skyblue3
Hordeum_vulgare_newGene_6495	skyblue3
HORVU7Hr1G006370	skyblue3
HORVU1Hr1G052530	skyblue3
HORVU2Hr1G071070	skyblue3
HORVU1Hr1G071500	skyblue3
Hordeum_vulgare_newGene_6069	skyblue3
HORVU6Hr1G075850	skyblue3
Hordeum_vulgare_newGene_15745	skyblue3
HORVU3Hr1G006640	skyblue3
HORVU1Hr1G046370	skyblue3
Hordeum_vulgare_newGene_15993	skyblue3
HORVU5Hr1G046160	skyblue3
HORVU2Hr1G102950	skyblue3
HORVU7Hr1G056320	skyblue3
HORVU4Hr1G019490	skyblue3
HORVU2Hr1G100450	skyblue3
HORVU5Hr1G101710	skyblue3
HORVU4Hr1G000830	skyblue3
HORVU1Hr1G065630	skyblue3
Hordeum_vulgare_newGene_1668	skyblue3
HORVU2Hr1G115240	skyblue3
HORVU2Hr1G094180	skyblue3
HORVU3Hr1G044150	skyblue3
HORVU4Hr1G000050	skyblue3
HORVU4Hr1G085250	skyblue3
HORVU1Hr1G000440	skyblue3
HORVU1Hr1G000340	skyblue3
HORVU0Hr1G031170	skyblue3
Hordeum_vulgare_newGene_10545	skyblue3

HORVU2Hr1G067330	skyblue3
HORVU2Hr1G017470	skyblue3
HORVU7Hr1G110380	skyblue3
HORVU1Hr1G085500	skyblue3
HORVU3Hr1G018820	skyblue3
HORVU5Hr1G097460	skyblue3
HORVU5Hr1G086610	skyblue3
HORVU7Hr1G040170	skyblue3
HORVU2Hr1G105430	skyblue3
HORVU2Hr1G081540	skyblue3
HORVU3Hr1G081640	skyblue3
HORVU0Hr1G000960	skyblue3
HORVU7Hr1G040730	skyblue3
HORVU7Hr1G028290	skyblue3
HORVU1Hr1G000450	skyblue3
HORVU0Hr1G019800	skyblue3
HORVU5Hr1G013510	skyblue3
HORVU1Hr1G054200	skyblue3
HORVU3Hr1G099990	skyblue3
HORVU5Hr1G009460	skyblue3
Hordeum_vulgare_newGene_10490	skyblue3
HORVU5Hr1G095580	skyblue3
HORVU2Hr1G020090	skyblue3
HORVU2Hr1G117940	skyblue3
HORVU4Hr1G060670	skyblue3
HORVU3Hr1G029470	skyblue3
HORVU2Hr1G109330	skyblue3
Hordeum_vulgare_newGene_13724	skyblue3
HORVU1Hr1G086310	skyblue3
HORVU2Hr1G027680	skyblue3
HORVU3Hr1G083450	skyblue3
Hordeum_vulgare_newGene_1534	skyblue3
Hordeum_vulgare_newGene_6150	skyblue3
HORVU0Hr1G019020	skyblue3
HORVU6Hr1G088610	skyblue3
HORVU4Hr1G082710	skyblue3
Hordeum_vulgare_newGene_15028	skyblue3
HORVU4Hr1G060560	skyblue3
HORVU7Hr1G013470	skyblue3
Hordeum_vulgare_newGene_8728	skyblue3
Hordeum_vulgare_newGene_11427	skyblue3
Hordeum_vulgare_newGene_1136	skyblue3
HORVU6Hr1G062850	skyblue3
HORVU2Hr1G082700	skyblue3
Hordeum_vulgare_newGene_9642	skyblue3
HORVU5Hr1G106010	skyblue3
Hordeum_vulgare_newGene_13654	skyblue3

HORVU3Hr1G105560	skyblue3
HORVU4Hr1G070260	skyblue3
HORVU7Hr1G112710	skyblue3
HORVU7Hr1G049070	skyblue3
HORVU5Hr1G097930	skyblue3
HORVU3Hr1G090860	skyblue3
HORVU1Hr1G074580	skyblue3
HORVU5Hr1G023710	skyblue3
HORVU7Hr1G007480	skyblue3
Hordeum_vulgare_newGene_643	skyblue3
HORVU3Hr1G073290	skyblue3
HORVU5Hr1G042130	skyblue3
HORVU2Hr1G118120	skyblue3
HORVU4Hr1G008390	darkorange2
HORVU3Hr1G093100	darkorange2
HORVU1Hr1G078140	darkorange2
HORVU5Hr1G076250	darkorange2
HORVU2Hr1G068880	darkorange2
HORVU1Hr1G065950	darkorange2
HORVU2Hr1G103710	darkorange2
HORVU3Hr1G026690	darkorange2
HORVU5Hr1G034460	darkorange2
HORVU6Hr1G093210	darkorange2
Hordeum_vulgare_newGene_15095	darkorange2
Hordeum_vulgare_newGene_13089	darkorange2
HORVU4Hr1G005710	darkorange2
HORVU7Hr1G096270	darkorange2
HORVU0Hr1G000810	darkorange2
HORVU6Hr1G002290	darkorange2
HORVU1Hr1G067070	darkorange2
Hordeum_vulgare_newGene_5963	darkorange2
Hordeum_vulgare_newGene_561	darkorange2
HORVU1Hr1G075910	darkorange2
HORVU3Hr1G084220	darkorange2
Hordeum_vulgare_newGene_8933	darkorange2
HORVU2Hr1G080260	darkorange2
HORVU7Hr1G012910	darkorange2
HORVU6Hr1G066000	darkorange2
HORVU3Hr1G058990	darkorange2
HORVU0Hr1G020590	darkorange2
Hordeum_vulgare_newGene_5747	darkorange2
HORVU7Hr1G060530	darkorange2
HORVU0Hr1G027230	darkorange2
HORVU1Hr1G058750	darkorange2
Hordeum_vulgare_newGene_5294	darkorange2
HORVU7Hr1G051770	darkorange2
Hordeum_vulgare_newGene_4908	darkorange2

HORVU1Hr1G054110	darkorange2
HORVU7Hr1G056700	darkorange2
HORVU4Hr1G057450	darkorange2
HORVU2Hr1G023540	darkorange2
HORVU6Hr1G013350	darkorange2
HORVU7Hr1G036210	darkorange2
Hordeum_vulgare_newGene_9101	darkorange2
HORVU7Hr1G082990	darkorange2
HORVU2Hr1G006830	darkorange2
HORVU7Hr1G090100	darkorange2
HORVU1Hr1G031250	darkorange2
HORVU2Hr1G071940	darkorange2
HORVU5Hr1G084900	darkorange2
HORVU1Hr1G094420	darkorange2
HORVU7Hr1G042120	darkorange2
Hordeum_vulgare_newGene_9484	darkorange2
Hordeum_vulgare_newGene_14214	darkorange2
HORVU1Hr1G071560	darkorange2
Hordeum_vulgare_newGene_12022	darkorange2
HORVU7Hr1G106660	darkorange2
HORVU5Hr1G051590	darkorange2
HORVU6Hr1G030260	darkorange2
Hordeum_vulgare_newGene_12093	darkorange2
HORVU7Hr1G026030	darkorange2
HORVU2Hr1G118060	darkorange2
HORVU2Hr1G117560	darkorange2
HORVU6Hr1G053680	darkorange2
Hordeum_vulgare_newGene_12883	darkorange2
HORVU5Hr1G084860	darkorange2
HORVU7Hr1G001030	darkorange2
HORVU6Hr1G034140	darkorange2
HORVU5Hr1G045650	darkorange2
HORVU7Hr1G000630	darkorange2
HORVU7Hr1G022330	darkorange2
HORVU5Hr1G022140	darkorange2
Hordeum_vulgare_newGene_545	darkorange2
HORVU4Hr1G003270	darkorange2
HORVU5Hr1G064230	darkorange2
HORVU5Hr1G056670	darkorange2
HORVU3Hr1G098640	darkorange2
HORVU5Hr1G041590	darkorange2
HORVU7Hr1G079410	darkorange2
HORVU5Hr1G109880	darkorange2
Hordeum_vulgare_newGene_16187	darkorange2
HORVU2Hr1G079620	darkorange2
HORVU5Hr1G122530	darkorange2
Hordeum_vulgare_newGene_6568	darkorange2

HORVU4Hr1G013580	darkorange2
HORVU7Hr1G048310	darkorange2
HORVU6Hr1G089980	darkorange2
HORVU1Hr1G094400	darkorange2
HORVU5Hr1G018480	darkorange2
HORVU3Hr1G112010	darkorange2
HORVU4Hr1G055910	darkorange2
HORVU7Hr1G041850	darkorange2
Hordeum_vulgare_newGene_7528	darkorange2
HORVU7Hr1G014870	darkorange2
HORVU1Hr1G094380	darkorange2
HORVU4Hr1G071000	darkorange2
HORVU5Hr1G063570	darkorange2
HORVU1Hr1G085540	darkorange2
HORVU5Hr1G122490	darkorange2
Hordeum_vulgare_newGene_181	darkorange2
HORVU5Hr1G121530	darkorange2
Hordeum_vulgare_newGene_5943	darkorange2
HORVU7Hr1G054980	darkmagenta
HORVU5Hr1G105650	darkmagenta
HORVU0Hr1G040540	darkmagenta
Hordeum_vulgare_newGene_527	darkmagenta
HORVU5Hr1G097940	darkmagenta
HORVU4Hr1G020130	darkmagenta
Hordeum_vulgare_newGene_11024	darkmagenta
HORVU3Hr1G051610	darkmagenta
HORVU5Hr1G122990	darkmagenta
HORVU7Hr1G042930	darkmagenta
HORVU1Hr1G069830	darkmagenta
Hordeum_vulgare_newGene_5798	darkmagenta
HORVU7Hr1G034120	darkmagenta
HORVU6Hr1G036760	darkmagenta
HORVU6Hr1G073980	darkmagenta
HORVU7Hr1G101740	darkmagenta
HORVU6Hr1G040040	darkmagenta
HORVU3Hr1G021550	darkmagenta
Hordeum_vulgare_newGene_14466	darkmagenta
HORVU4Hr1G008180	darkmagenta
HORVU3Hr1G003860	darkmagenta
Hordeum_vulgare_newGene_12265	darkmagenta
Hordeum_vulgare_newGene_3016	darkmagenta
HORVU5Hr1G063340	darkmagenta
HORVU5Hr1G081610	darkmagenta
HORVU6Hr1G012640	darkmagenta
Hordeum_vulgare_newGene_5479	darkmagenta
HORVU2Hr1G040880	darkmagenta
Hordeum_vulgare_newGene_3879	darkmagenta

HORVU3Hr1G110760	darkmagenta
Hordeum_vulgare_newGene_9687	darkmagenta
HORVU2Hr1G015870	darkmagenta
Hordeum_vulgare_newGene_228	darkmagenta
HORVU7Hr1G108280	darkmagenta
Hordeum_vulgare_newGene_11835	darkmagenta
HORVU3Hr1G012000	darkmagenta
Hordeum_vulgare_newGene_4913	darkmagenta
HORVU1Hr1G082340	darkmagenta
Hordeum_vulgare_newGene_12908	darkmagenta
HORVU4Hr1G005480	darkmagenta
HORVU4Hr1G070520	darkmagenta
HORVU7Hr1G098670	darkmagenta
Hordeum_vulgare_newGene_3994	darkmagenta
HORVU4Hr1G064040	darkmagenta
HORVU4Hr1G012400	darkmagenta
HORVU4Hr1G022280	darkmagenta
HORVU7Hr1G096760	darkmagenta
HORVU7Hr1G111310	darkmagenta
Hordeum_vulgare_newGene_8377	darkmagenta
HORVU7Hr1G030800	darkmagenta
Hordeum_vulgare_newGene_14353	darkmagenta
Hordeum_vulgare_newGene_10276	darkmagenta
HORVU2Hr1G108380	darkmagenta
HORVU1Hr1G082320	darkmagenta
HORVU0Hr1G018320	darkmagenta
HORVU2Hr1G026450	darkmagenta
HORVU1Hr1G004720	darkmagenta
HORVU4Hr1G083930	darkmagenta
HORVU3Hr1G057860	darkmagenta
HORVU6Hr1G032760	darkmagenta
HORVU1Hr1G060030	darkmagenta
HORVU4Hr1G083020	darkmagenta
HORVU3Hr1G018350	darkmagenta
Hordeum_vulgare_newGene_11618	darkmagenta
Hordeum_vulgare_newGene_11617	darkmagenta
HORVU1Hr1G089380	darkmagenta
Hordeum_vulgare_newGene_5644	darkmagenta
HORVU7Hr1G087810	darkmagenta
Hordeum_vulgare_newGene_9771	darkmagenta
HORVU6Hr1G074410	darkmagenta
HORVU5Hr1G096420	darkmagenta
HORVU3Hr1G107980	darkmagenta
HORVU3Hr1G025630	darkmagenta
HORVU1Hr1G088620	bisque4
HORVU1Hr1G088010	bisque4
Hordeum_vulgare_newGene_1938	bisque4

HORVU1Hr1G088360	bisque4
HORVU5Hr1G011200	bisque4
HORVU3Hr1G109170	bisque4
HORVU5Hr1G006720	bisque4
HORVU5Hr1G030250	bisque4
HORVU1Hr1G088590	bisque4
Hordeum_vulgare_newGene_12568	bisque4
HORVU1Hr1G088270	bisque4
HORVU1Hr1G087970	bisque4
HORVU1Hr1G088600	bisque4
HORVU1Hr1G088810	bisque4
HORVU0Hr1G002270	bisque4
HORVU1Hr1G087880	bisque4
HORVU1Hr1G088110	bisque4
HORVU1Hr1G087870	bisque4
Hordeum_vulgare_newGene_1249	bisque4
HORVU1Hr1G088440	bisque4
HORVU1Hr1G088140	bisque4
HORVU7Hr1G098440	bisque4
HORVU1Hr1G064020	bisque4
HORVU1Hr1G088060	bisque4
Hordeum_vulgare_newGene_14024	bisque4
Hordeum_vulgare_newGene_14026	bisque4
Hordeum_vulgare_newGene_14020	bisque4
Hordeum_vulgare_newGene_14021	bisque4
Hordeum_vulgare_newGene_14028	bisque4
Hordeum_vulgare_newGene_13927	bisque4
HORVU3Hr1G114990	bisque4
HORVU1Hr1G087990	bisque4
HORVU1Hr1G088580	bisque4
HORVU3Hr1G027340	bisque4
HORVU1Hr1G088280	bisque4
HORVU0Hr1G005350	bisque4
HORVU1Hr1G087940	bisque4
HORVU1Hr1G088460	bisque4
HORVU1Hr1G009980	bisque4
HORVU6Hr1G060020	bisque4
HORVU7Hr1G100740	bisque4
Hordeum_vulgare_newGene_14042	bisque4
Hordeum_vulgare_newGene_14041	bisque4
HORVU1Hr1G088370	bisque4
HORVU1Hr1G088550	bisque4
HORVU6Hr1G069180	bisque4
HORVU1Hr1G088070	bisque4
Hordeum_vulgare_newGene_14037	bisque4
Hordeum_vulgare_newGene_14036	bisque4
Hordeum_vulgare_newGene_14035	bisque4

HORVU1Hr1G088340	bisque4
HORVU1Hr1G088560	bisque4
HORVU1Hr1G088300	bisque4
HORVU1Hr1G087950	bisque4
HORVU1Hr1G002000	saddlebrown
HORVU2Hr1G006130	saddlebrown
HORVU3Hr1G112860	saddlebrown
HORVU5Hr1G079040	saddlebrown
Hordeum_vulgare_newGene_4504	saddlebrown
HORVU1Hr1G002790	saddlebrown
Hordeum_vulgare_newGene_9593	saddlebrown
Hordeum_vulgare_newGene_15209	saddlebrown
HORVU1Hr1G071010	saddlebrown
HORVU1Hr1G003140	saddlebrown
Hordeum_vulgare_newGene_12305	saddlebrown
HORVU1Hr1G089310	saddlebrown
HORVU1Hr1G002950	saddlebrown
HORVU7Hr1G047790	saddlebrown
HORVU5Hr1G125450	saddlebrown
HORVU1Hr1G001400	saddlebrown
HORVU1Hr1G089280	saddlebrown
HORVU1Hr1G002920	saddlebrown
Hordeum_vulgare_newGene_12445	saddlebrown
Hordeum_vulgare_newGene_12446	saddlebrown
Hordeum_vulgare_newGene_846	saddlebrown
HORVU1Hr1G088680	saddlebrown
HORVU1Hr1G088920	saddlebrown
HORVU1Hr1G001150	saddlebrown
HORVU1Hr1G000720	saddlebrown
HORVU5Hr1G111860	saddlebrown
HORVU7Hr1G035440	saddlebrown
Hordeum_vulgare_newGene_8180	saddlebrown
HORVU4Hr1G016600	saddlebrown
HORVU3Hr1G078620	saddlebrown
HORVU1Hr1G002540	saddlebrown
HORVU1Hr1G002090	saddlebrown
Hordeum_vulgare_newGene_14492	saddlebrown
Hordeum_vulgare_newGene_5214	saddlebrown
Hordeum_vulgare_newGene_14027	saddlebrown
Hordeum_vulgare_newGene_7922	saddlebrown
HORVU2Hr1G006170	saddlebrown
Hordeum_vulgare_newGene_9657	saddlebrown
Hordeum_vulgare_newGene_9592	saddlebrown
HORVU1Hr1G088190	saddlebrown
HORVU1Hr1G001710	saddlebrown
HORVU1Hr1G089240	saddlebrown
HORVU4Hr1G069100	saddlebrown

HORVU4Hr1G013910	saddlebrown
HORVU6Hr1G042780	saddlebrown
HORVU2Hr1G018830	saddlebrown
HORVU3Hr1G003600	saddlebrown
HORVU1Hr1G001040	saddlebrown
HORVU7Hr1G056530	saddlebrown
HORVU1Hr1G001490	saddlebrown
HORVU2Hr1G115090	saddlebrown
Hordeum_vulgare_newGene_12714	saddlebrown
Hordeum_vulgare_newGene_14043	saddlebrown
Hordeum_vulgare_newGene_8133	saddlebrown
HORVU1Hr1G002440	saddlebrown
HORVU1Hr1G089180	saddlebrown
HORVU1Hr1G001670	saddlebrown
HORVU1Hr1G002680	saddlebrown
Hordeum_vulgare_newGene_15158	saddlebrown
Hordeum_vulgare_newGene_7987	saddlebrown
HORVU3Hr1G028440	saddlebrown
HORVU6Hr1G065710	saddlebrown
HORVU5Hr1G094570	saddlebrown
Hordeum_vulgare_newGene_14379	saddlebrown
HORVU3Hr1G082000	saddlebrown
HORVU1Hr1G001090	saddlebrown
HORVU1Hr1G003100	saddlebrown
HORVU3Hr1G002980	saddlebrown
HORVU3Hr1G099630	saddlebrown
HORVU3Hr1G105360	saddlebrown
Hordeum_vulgare_newGene_13307	saddlebrown
Hordeum_vulgare_newGene_12178	saddlebrown
Hordeum_vulgare_newGene_12779	saddlebrown
HORVU5Hr1G094750	saddlebrown
Hordeum_vulgare_newGene_237	saddlebrown
Hordeum_vulgare_newGene_954	saddlebrown
HORVU2Hr1G006160	saddlebrown
HORVU4Hr1G074740	saddlebrown
HORVU1Hr1G088930	saddlebrown
HORVU1Hr1G001850	saddlebrown
HORVU1Hr1G003280	saddlebrown
HORVU1Hr1G001750	darkslateblue
HORVU1Hr1G003130	darkslateblue
HORVU1Hr1G001950	darkslateblue
HORVU1Hr1G001540	darkslateblue
Hordeum_vulgare_newGene_2896	darkslateblue
HORVU1Hr1G002820	darkslateblue
Hordeum_vulgare_newGene_6048	darkslateblue
HORVU1Hr1G001660	darkslateblue
HORVU1Hr1G002850	darkslateblue

HORVU1Hr1G001630	darkslateblue
HORVU1Hr1G002400	darkslateblue
Hordeum_vulgare_newGene_4232	darkslateblue
HORVU1Hr1G001770	darkslateblue
HORVU1Hr1G003020	darkslateblue
HORVU1Hr1G003270	darkslateblue
Hordeum_vulgare_newGene_1777	darkslateblue
HORVU1Hr1G002830	darkslateblue
HORVU1Hr1G003070	darkslateblue
HORVU1Hr1G001810	darkslateblue
HORVU1Hr1G002460	darkslateblue
HORVU1Hr1G002160	darkslateblue
HORVU1Hr1G001890	darkslateblue
HORVU1Hr1G001220	darkslateblue
HORVU1Hr1G002590	darkslateblue
Hordeum_vulgare_newGene_4414	darkslateblue
HORVU1Hr1G001990	darkslateblue
Hordeum_vulgare_newGene_4871	darkslateblue
HORVU1Hr1G002510	darkslateblue
HORVU1Hr1G002410	darkslateblue
HORVU1Hr1G002990	darkslateblue
Hordeum_vulgare_newGene_4198	darkslateblue
HORVU1Hr1G002890	darkslateblue
HORVU1Hr1G003260	darkslateblue
HORVU1Hr1G003050	darkslateblue
HORVU1Hr1G002600	darkslateblue
HORVU1Hr1G003150	darkslateblue
Hordeum_vulgare_newGene_4472	darkslateblue
HORVU1Hr1G001200	darkslateblue
HORVU1Hr1G001970	darkslateblue
HORVU1Hr1G003080	darkslateblue
HORVU1Hr1G002960	darkslateblue
HORVU1Hr1G002340	darkslateblue
HORVU1Hr1G003060	darkslateblue
HORVU1Hr1G001350	darkslateblue
Hordeum_vulgare_newGene_6214	darkslateblue
HORVU1Hr1G003230	darkslateblue
HORVU1Hr1G002470	darkslateblue
HORVU1Hr1G002150	darkslateblue
HORVU1Hr1G003250	darkslateblue
HORVU1Hr1G002620	darkslateblue
HORVU1Hr1G003170	darkslateblue
HORVU1Hr1G002740	darkslateblue
HORVU1Hr1G001590	darkslateblue
HORVU1Hr1G001460	darkslateblue
HORVU6Hr1G036510	maroon
HORVU0Hr1G021280	maroon

HORVU3Hr1G088190	maroon
HORVU1Hr1G048610	maroon
HORVU6Hr1G043490	maroon
HORVU6Hr1G021620	maroon
HORVU6Hr1G085120	maroon
HORVU7Hr1G095760	maroon
HORVU7Hr1G027740	maroon
Hordeum_vulgare_newGene_14464	maroon
HORVU7Hr1G001790	maroon
HORVU2Hr1G090330	maroon
HORVU3Hr1G019280	maroon
HORVU4Hr1G078400	maroon
HORVU0Hr1G028930	maroon
Hordeum_vulgare_newGene_14270	maroon
HORVU5Hr1G062030	maroon
Hordeum_vulgare_newGene_14409	maroon
HORVU7Hr1G052850	maroon
Hordeum_vulgare_newGene_10335	maroon
HORVU2Hr1G015850	maroon
HORVU7Hr1G023940	maroon
Hordeum_vulgare_newGene_6989	maroon
HORVU6Hr1G077750	maroon
Hordeum_vulgare_newGene_11381	maroon
Hordeum_vulgare_newGene_1876	maroon
HORVU6Hr1G067470	maroon
HORVU3Hr1G103960	maroon
Hordeum_vulgare_newGene_15956	maroon
HORVU3Hr1G016150	maroon
HORVU2Hr1G037620	maroon
Hordeum_vulgare_newGene_11831	maroon
HORVU6Hr1G015770	maroon
HORVU6Hr1G000660	salmon4
HORVU1Hr1G075610	salmon4
Hordeum_vulgare_newGene_15164	salmon4
HORVU2Hr1G123800	salmon4
HORVU3Hr1G062430	salmon4
Hordeum_vulgare_newGene_13756	salmon4
Hordeum_vulgare_newGene_1317	salmon4
HORVU6Hr1G002530	salmon4
HORVU3Hr1G105420	salmon4
HORVU3Hr1G069030	salmon4
HORVU1Hr1G055710	salmon4
HORVU3Hr1G113100	salmon4
HORVU1Hr1G016980	salmon4
Hordeum_vulgare_newGene_11367	salmon4
HORVU7Hr1G027440	salmon4
Hordeum_vulgare_newGene_16119	salmon4

Hordeum_vulgare_newGene_13693	salmon4
HORVU7Hr1G031480	salmon4
HORVU5Hr1G063670	salmon4
Hordeum_vulgare_newGene_15867	salmon4
HORVU5Hr1G010120	salmon4
HORVU0Hr1G005300	salmon4
HORVU4Hr1G063590	salmon4
HORVU2Hr1G008640	salmon4
HORVU4Hr1G053980	salmon4
HORVU3Hr1G105290	salmon4
HORVU6Hr1G081750	salmon4
HORVU1Hr1G057270	salmon4
Hordeum_vulgare_newGene_15486	salmon4
HORVU0Hr1G005720	salmon4
HORVU0Hr1G015950	salmon4
HORVU7Hr1G051310	salmon4
HORVU1Hr1G069800	salmon4
HORVU3Hr1G089090	salmon4
HORVU2Hr1G093230	salmon4
HORVU3Hr1G059480	salmon4
HORVU5Hr1G010130	salmon4
HORVU4Hr1G008700	salmon4
HORVU7Hr1G043620	salmon4
HORVU0Hr1G032870	salmon4
Hordeum_vulgare_newGene_1953	salmon4
HORVU2Hr1G044640	salmon4
HORVU3Hr1G030980	lightpink4
HORVU5Hr1G114780	lightpink4
HORVU4Hr1G072880	lightpink4
HORVU3Hr1G039020	lightpink4
HORVU7Hr1G074330	lightpink4
HORVU3Hr1G095790	lightpink4
Hordeum_vulgare_newGene_8310	lightpink4
HORVU3Hr1G021080	lightpink4
HORVU6Hr1G054240	lightpink4
Hordeum_vulgare_newGene_10530	lightpink4
HORVU3Hr1G001020	lightpink4
HORVU4Hr1G087110	lightpink4
HORVU6Hr1G003750	lightpink4
HORVU2Hr1G034580	lightpink4
HORVU3Hr1G087090	lightpink4
HORVU6Hr1G008730	lightpink4
HORVU3Hr1G034310	lightpink4
HORVU5Hr1G106100	lightpink4
HORVU3Hr1G116300	lightpink4
HORVU6Hr1G006140	lightpink4
HORVU2Hr1G023840	lightpink4

HORVU2Hr1G071570	lightpink4
HORVU2Hr1G036680	lightpink4
HORVU2Hr1G099710	lightpink4
HORVU7Hr1G119060	lightpink4
HORVU0Hr1G006420	lightpink4
HORVU6Hr1G033060	lightpink4
HORVU5Hr1G122760	lightpink4
HORVU6Hr1G024190	lightpink4
HORVU3Hr1G001060	lightpink4
Hordeum_vulgare_newGene_6717	lightpink4
