# Supplementary material for: Molecular mechanisms of how black barley accumulates higher anthocyanins than blue barley following transcriptomic evaluation and expression analysis of key genes in anthocyanins biosynthesis pathway
Source: Front Plant Sci. 2025 Aug 29;16:1650803. doi: 10.3389/fpls.2025.1650803 (PMC12427265; doi:10.3389/fpls.2025.1650803)
Supplement: Supplementary file 1 [file Supplementaryfile1.zip › Supplementary Material/Data Sheet 2.PDF]

Supplementary Table 1. Description of the primers used in quantitative real-time PCR experiment.

Gene name	Primer ID	Primer Sequences
	HORVU5Hr1G094280-F	AGATCCCCGTTGTTGACGTG
ANS1	HORVU5Hr1G094280-R	CGATGAGGTCCTCCGGGAT
	HORVU5Hr1G065620-F	CCTCATCGATCTTACCCGGC
LDOX1	HORVU5Hr1G065620-R	GGAAGAACTCCCTCGACACG
	HORVU0Hr1G019590-F	TACCCATTGTTCCTCGCACC
LDOX2	HORVU0Hr1G019590-R	TCGGGGTCAACCGAGTAGAA
	HORVU7Hr1G009310-F	GGAGGACGAGCGATGTATGG
LDOX3	HORVU7Hr1G009310-R	GCTGGTCCACGAAGTAGTCC
	LOC123446308 actin7-F	ATACACGAAGCGACATACA
	LOC123446308 actin7-R	AGAACCACCACTGAGAAC
