# Supplementary material for: Molecular mechanisms of how black barley accumulates higher anthocyanins than blue barley following transcriptomic evaluation and expression analysis of key genes in anthocyanins biosynthesis pathway
Source: Front Plant Sci. 2025 Aug 29;16:1650803. doi: 10.3389/fpls.2025.1650803 (PMC12427265; doi:10.3389/fpls.2025.1650803)
Supplement: Supplementary file 1 [file Supplementaryfile1.zip › Supplementary Material/Data Sheet 3.pdf]

Supplementary Table 2a. Properties of Hordeum_vulgare_newGene_711 that significantly interacted with the anthocyanin biosynthesis pathway during analyzing protein-protein interaction.

Gene Name	Chr Start End Strand
Hordeum_vulgare_newGene_711	Chr2H; 713276864--713278076 -
	**Annotations**
COG_class_annotation	Cell wall/membrane/envelope biogenesis
GO_annotation	Molecular Function: catalytic activity (GO:0003824);;
KEGG_annotation	K08695|5.0e-96|ats:109778519|K08695 anthocyanidin reductase [EC:1.3.1.77] | (RefSeq) LOC109778519; anthocyanidin reductase ((2S)-flavan-3-ol-forming)-like
KOG_class_annotation	Defense mechanisms
Pfam_annotation	NAD dependent epimerase/dehydratase family
Swiss_Prot_annotation	Anthocyanidin reductase ((2S)-flavan-3-ol-forming) OS=Vitis vinifera OX=29760 GN=ANR PE=1 SV=1
eggNOG_class_annotation	Defense mechanisms
NR_annotation	unnamed protein product [Triticum turgidum subsp. durum]
KEGG_pathway_annotation	Flavonoid biosynthesis (ko00941)

Protein Sequence

MRSCVRAGTVKRVVLTSSAAAVSSRPLQGDGHVLDEESWSDVEFLRSRKTGPWAYPVSKVLLE
KAACAFALEHGISLVTVCPVVTVGAAPAAKANTSVPDILSLLSGDDARVRKLEFIERRTGSIPMV
HIDDLCRAEVFVAEEEAASGRYNCGSVNTTVVELARFLAAKYPQYNVKTDRFAGLTEKPRVCIS
SAKLVGEGFEFRYKNLDQIYDDVVEYGRGLGILPY

CDS Sequence

AGAAAGACGTGATCGAGCCGGCCATCCACGGAACCCTCAACGTGATGAGGTCGTGCGTGAG
AGCGGGCACGGTGAAGCGCGTGGTCCTGACATCGTCGGCGGCCGCCGTCTCCAGCCGGCCG
CTGCAAGGGGATGGCCATGTCCTGGACGAGGAGTCCTGGTCCGACGTCGAGTTCCTCAGATC
GAGAAAGACCGGTCCCTGGGCGTACCCTGTCTCCAAGGTGCTTCTGGAGAAGGCGGCGTGC
GCGTTCGCCCTGGAGCACGGCATCAGCCTGGTCACCGTGTGCCCCGTCGTCACCGTCGGCGC
GGCGCCGGCGGCCAAGGCCAACACCAGCGTGCCCGACATCCTCTCCCTGCTCTCCGGCGATG
ATGCGAGGGTGAGGAAACTTGAATTCATCGAGAGGAGGACCGGCTCGATCCCGATGGTCCA
CATCGACGACCTCTGCCGCGCCGAGGTGTTCGTCGCCGAGGAGGAGGCCGCGTCGGGGCGG
TACAACTGCGGCAGCGTCAACACCACCGTCGTGGAGCTCGCCCGCTTCCTGGCGGCCAAGTA
CCCGCAGTACAACGTCAAGACCGACCGGTTCGCCGGTCTCACCGAGAAGCCGAGAGTCTGC

ATTTCGTCGGCGAAGCTCGTCGGGGAAGGGTTTGAGTTCAGGTACAAGAACCTGGACCAGA
TATACGACGACGTCGTCGAGTACGGGAGGGGGTTGGGAATCCTTCCGTACTAGTATGATTGA
TTGATCTTTGAACCAAACGATGCGCTTGCAAGCAAAATAGGGTTAACCTCCTTGTTTTGTTGG
TTAATAAGCTTATAACCTCCTAAAGTACATATATGATGAAATGTGGTTGTTTAGCTCATATGA
AAAATGATTTGTAAAACATACTTGTATGAAAAGTGGTATATACCATTGTGAACATTCTCGGA
ACAATGGTTGTGATCTGC

Supplementary Table 2b. Properties of Hordeum_vulgare_newGene_8522 that significantly interacted with the anthocyanin biosynthesis pathway during analyzing protein-protein interaction.

Gene Name	Chr Start End Strand
Hordeum_vulgare_newGene_8522	Chr1H; 427165649--427169354 +
	**Annotations**
COG_class_annotation	Carbohydrate transport and metabolism
GO_annotation	Molecular Function: hydrolase activity, hydrolyzing O-glycosyl compounds (GO:0004553);; Biological Process: carbohydrate metabolic process (GO:0005975);;
KEGG_annotation	K01188|1.2e-284|ats:109771504|K01188 beta-glucosidase [EC:3.2.1.21] | (RefSeq) LOC109771504; beta-glucosidase 22-like isoform X1
KOG_class_annotation	Carbohydrate transport and metabolism
Pfam_annotation	Glycosyl hydrolase family 1
Swiss_Prot_annotation	Beta-glucosidase 22 OS=Oryza sativa subsp. japonica OX=39947 GN=BGLU22 PE=2 SV=1
eggNOG_class_annotation	Carbohydrate transport and metabolism
NR_annotation	predicted protein [Hordeum vulgare subsp. vulgare]
KEGG_pathway_annotation	Cyanoamino acid metabolism (ko00460);; Starch and sucrose metabolism (ko00500);; Phenylpropanoid biosynthesis (ko00940)
GO_second_level_annotation	molecular function: catalytic activity (GO:0003824);; biological process: metabolic process (GO:0008152

Protein Sequence

MRPTTHIGGPRGCPQDIEASLLSERRREMRATPCAVPLMQLLPAVLLLAGGAAVAGALNFTRA
DFPGAFVFGAGTSAYQYEGATDEGGRSPSIWDTFTHAGRMPDKSTGDLGADGYHRYKEDVEL

MVDTGLEAYRFSISWSRLIPRGRGPVNPKGLEYYNNLINELTKRGIQIHVTLYHLDFPQILEDEY
HGWLSPRVVDDFTAFADACFREFGDRVRHWTTMDEPNVIAIAAYDSGAFPPCRCSAPYGVNCT
TGDSTVEPYTVAHHSILAHASAVRLYRDKYQATQGGLVGINIYTFWNYPFSHSPADVAATQRSL
DFMVGWILDPLVKGDYPEIMKKKAGPRIPSFTKQQSELIRGCIDFVGINHYTSVYVSDGKSSADA
SLRDYNADMSATFRMSRNDSGSGQFIPINMPNDPQGLQCMLRYLTDTYQNVPIYVQENGYGQF
FVDSVNDHNRVEYLSGYIGSTLAALRNGANVKGYFVWSFLDVFELMAGYYLRYGLHYIDFQD
PDLPRQPKLSAKWYSKFLKSEIGINIENILSPDTRSDAQQ

CDS Sequence

CTGATGAATAAGACATGCGGCCAACAACACATATAGGAGGACCCAGAGGTTGCCCACAAG
ATATCGAGGCGTCACTTTTGAGCGAGCGACGGAGAGAGATGAGGGCCACACCCTGCGCCGT
GCCGCTCATGCAGCTGCTACCGGCGGTGCTGCTGCTCGCCGGCGGCGCGGCGGTGGCCGGA
GCTCTCAACTTCACGAGGGCGGACTTCCCCGGGGCCTTCGTCTTCGGGGCCGGCACGTCGG
CCTACCAGTACGAGGGCGCGACCGATGAAGGCGGGAGGAGCCCTAGCATTTGGGACACTTT
TACTCATGCAGGGAGGATGCCAGACAAGAGCACCGGCGATTTGGGCGCGGACGGCTACCA
CAGATACAAGGAGGATGTAGAGTTGATGGTGGACACTGGCCTGGAGGCGTACCGGTTCTCC
ATTTCCTGGTCGAGGCTCATTCCAAGAGGAAGAGGGCCCGTGAATCCGAAAGGGCTGGAGT
ACTACAACAACCTTATAAACGAGCTGACAAAACGAGGAATCCAGATACATGTGACCCTGTA
CCACCTCGACTTCCCCCAGATCCTTGAAGACGAGTACCACGGCTGGCTCAGCCCCAGGGTC
GTGGATGACTTCACGGCGTTCGCGGACGCGTGCTTCCGGGAGTTCGGGGACCGGGTGCGGC
ACTGGACCACCATGGACGAGCCCAACGTGATCGCCATCGCCGCCTACGACAGCGGCGCCTT
CCCGCCGTGCCGCTGCTCTGCGCCCTACGGGGTGAACTGCACCACGGGGGACTCCACCGTG
GAGCCCTACACCGTGGCGCACCACTCCATCCTGGCGCACGCCTCCGCCGTCAGGCTCTACC
GGGACAAGTACCAGGCCACGCAGGGGGGCCTCGTCGGCATCAACATCTACACCTTCTGGAA
CTACCCCTTCTCCCACAGCCCCGCCGACGTCGCCGCCACGCAGAGGTCGCTCGACTTCATGG
TCGGCTGGATCTTGGACCCCTTGGTGAAAGGTGACTACCCTGAGATCATGAAGAAGAAAGC
CGGGCCACGGATTCCGTCCTTCACCAAACAACAGTCCGAGCTGATCCGAGGGTGCATCGAC
TTCGTCGGCATCAACCACTACACGTCGGTGTATGTCAGCGATGGCAAGAGCAGCGCCGACG
CGAGCCTCCGGGACTACAACGCGGACATGTCTGCTACATTCAGAATGTCAAGGAATGATAG
CGGGTCTGGTCAGTTCATCCCTATCAACATGCCGAATGATCCACAAGGGCTGCAATGCATG
CTCCGGTACCTCACAGACACATACCAGAACGTCCCTATCTATGTCCAAGAAAATGGTTATG
GGCAGTTCTTCGTCGACTCGGTCAATGATCATAACAGAGTGGAGTACTTAAGTGGCTACAT
CGGCAGCACACTCGCCGCGCTCAGGAACGGAGCCAACGTGAAGGGCTACTTTGTTTGGTCA
TTCCTGGATGTGTTCGAGCTAATGGCGGGATATTATTTGCGATACGGACTGCACTACATTGA
TTTTCAGGATCCTGACTTGCCAAGGCAGCCAAAGCTCTCTGCCAAGTGGTACTCCAAGTTCT
TAAAGAGTGAAATCGGGATAAACATTGAGAACATACTTAGTCCTGACACAAGGTCAGATGC
TCAACAATGAGGTTAACTAGCAGTGCATATATAGACACATTCAGGTTGGAGGGGCCGGAGA
AACAAGAAAAAAACTAGATGGTTGCTTATAATTTTCTTACTCGATTTGCAAAGATGAAATTT
TATTCGTCTTCTTATATAGTTGATGGTGGCTCGCCGTCAGGTGTTTTTCTGATGAGAAATAG
GTGTATACCCTTATGCTCTTGGTGTCATAACTAATTACATTTCGGTAATCAGTTATAAATAA
AGCAG
