# Supplementary material for: Molecular mechanisms of how black barley accumulates higher anthocyanins than blue barley following transcriptomic evaluation and expression analysis of key genes in anthocyanins biosynthesis pathway
Source: Front Plant Sci. 2025 Aug 29;16:1650803. doi: 10.3389/fpls.2025.1650803 (PMC12427265; doi:10.3389/fpls.2025.1650803)
Supplement: Supplementary file 1 [file Supplementaryfile1.zip › Supplementary Material/Data Sheet 4.pdf]

Supplementary Table 3. KEGG (Kyoto Encyclopedia of Genes and Genomes) classification of differentially (

KEGG_A_	KEGG_B_	**ID**	**Pathway**	GeneRatio	BgRatio	enrich_fac	pvalue	**qvalue**	geneID
Metabolisı	Energy me	ko00196	Photosyntl	1.20%	0.27%	4.38	2.93E-12	2.81E-10	HORVU1I
Metabolisı	Carbohydr	ko00500	Starch and	6.23%	3.92%	1.59	2.93E-08	1.40E-06	HORVU0I
Metabolisı	Amino aci	ko00250	Alanine, as	1.65%	0.65%	2.54	9.53E-08	3.04E-06	HORVU0I
Metabolisı	Carbohydr	ko00630	Glyoxylate	2.54%	1.24%	2.05	1.62E-07	3.87E-06	HORVU0I
Metabolisı	Amino aci	ko00350	Tyrosine n	1.74%	0.77%	2.26	1.12E-06	2.14E-05	HORVU0I
Metabolisı	Global anc	ko01200	Carbon me	5.03%	3.31%	1.52	5.98E-06	9.55E-05	HORVU0I
Metabolisı	Carbohydr	ko00010	Glycolysis	3.24%	1.93%	1.68	1.10E-05	0.000151	HORVU0I
Metabolisı	Carbohydr	ko00052	Galactose	2.14%	1.19%	1.81	4.92E-05	0.000589	HORVU0I
Metabolisı	Biosynthes	ko00941	Flavonoid	2.24%	1.28%	1.75	7.46E-05	0.000732	HORVU0I
Metabolisı	Amino aci	ko00220	Arginine b	1%	0.41%	2.41	7.64E-05	0.000732	HORVU0I
Metabolisı	Carbohydr	ko00030	Pentose ph	1.35%	0.66%	2.05	0.000122	0.000966	HORVU0I
Metabolisı	Lipid meta	ko00071	Fatty acid	1.55%	0.80%	1.94	0.000129	0.000966	HORVU0I
Metabolisı	Energy me	ko00710	Carbon fix	1.94%	1.08%	1.79	0.000131	0.000966	HORVU0I
Metabolisı	Biosynthes	ko00950	Isoquinolir	1.20%	0.60%	1.99	0.000476	0.003254	HORVU0I
Metabolisı	Metabolisı	ko00904	Diterpenoi	1.50%	0.82%	1.83	0.000536	0.003425	HORVU0I
Metabolisı	Nucleotide	ko00230	Purine met	2.14%	1.36%	1.58	0.001195	0.007153	HORVU0I
Metabolisı	Glycan bic	ko00603	Glycosphir	0.40%	0.12%	3.2	0.001397	0.00787	HORVU0I
Metabolisı	Global anc	ko01230	Biosynthes	4.04%	2.98%	1.36	0.002106	0.011206	HORVU0I
Metabolisı	Metabolisı	ko00906	Carotenoic	1.10%	0.59%	1.85	0.002346	0.011306	HORVU0I
Metabolisı	Amino aci	ko00360	Phenylalar	1.35%	0.78%	1.73	0.002468	0.011306	HORVU0I
Metabolisı	Metabolisı	ko00480	Glutathion	2.34%	1.57%	1.49	0.002479	0.011306	HORVU0I
Metabolisı	Metabolisı	ko00460	Cyanoamir	1.74%	1.09%	1.6	0.002652	0.011547	HORVU0I
Metabolisı	Biosynthes	ko00944	Flavone ar	0.45%	0.17%	2.61	0.003943	0.01642	HORVU1I
Metabolisı	Lipid meta	ko00073	Cutin, sub	1.74%	1.15%	1.51	0.006847	0.027329	HORVU0I
Metabolisı	Carbohydr	ko00520	Amino sug	3.14%	2.34%	1.34	0.007685	0.028455	HORVU0I
Metabolisı	Metabolisı	ko00730	Thiamine r	0.65%	0.32%	2.03	0.007723	0.028455	HORVU1I
Metabolisı	Biosynthes	ko00940	Phenylpro	5.33%	4.29%	1.24	0.008257	0.029295	HORVU0I
Metabolisı	Energy me	ko00910	Nitrogen n	0.85%	0.47%	1.81	0.008827	0.030197	HORVU1I
Metabolisı	Metabolisı	ko00903	Limonene	0.30%	0.10%	2.95	0.00925	0.030553	HORVU2I
Metabolisı	Amino aci	ko00280	Valine, leu	1.25%	0.79%	1.58	0.011407	0.036421	HORVU1I
Metabolisı	Glycan bic	ko00511	Other glyc	1.40%	0.91%	1.53	0.012339	0.038128	HORVU0I
Metabolisı	Lipid meta	ko00561	Glycerolip	1.94%	1.38%	1.41	0.014898	0.044597	HORVU0I
Environme	Signal tran	ko04070	Phosphatic	1.89%	1.35%	1.4	0.016872	0.048974	HORVU0I
Metabolisı	Amino aci	ko00330	Arginine a	1.15%	0.74%	1.55	0.01917	0.052869	HORVU0I
Environme	Membrane	ko02010	ABC trans	1.94%	1.40%	1.38	0.019358	0.052869	HORVU1I
Metabolisı	Biosynthes	ko00960	Tropane, p	0.60%	0.32%	1.87	0.01987	0.052869	HORVU0I
Metabolisı	Metabolisı	ko00410	beta-Alani	0.90%	0.55%	1.62	0.022924	0.059347	HORVU2I
Metabolisı	Lipid meta	ko00061	Fatty acid	0.90%	0.56%	1.6	0.026235	0.066132	HORVU1I
Metabolisı	Metabolisı	ko00900	Terpenoid	0.90%	0.57%	1.58	0.029894	0.073423	HORVU1I
Metabolisı	Carbohydr	ko00620	Pyruvate n	1.60%	1.15%	1.38	0.032622	0.078122	HORVU0I
Metabolisı	Metabolisı	ko00430	Taurine an	0.20%	0.07%	2.84	0.038967	0.091039	HORVU2I
Metabolisı	Global anc	ko01212	Fatty acid	1.45%	1.06%	1.36	0.047387	0.108076	HORVU1I
Metabolisı	Lipid meta	ko00592	alpha-Linc	1.50%	1.12%	1.34	0.053451	0.116837	HORVU0I
Metabolisı	Lipid meta	ko00590	Arachidon	0.65%	0.41%	1.6	0.053668	0.116837	HORVU2I
Genetic In	Replicatioı	ko03440	Homologo	1.84%	1.43%	1.29	0.056884	0.121086	HORVU1I

Metabolisı	Carbohydr	ko00640	Propanoate	0.80%	0.54%	1.48	0.064193	0.133674	HORVU1I
Metabolisı	Glycan bic	ko00604	Glycosphi	0.40%	0.23%	1.76	0.071718	0.146166	HORVU1I
Metabolisı	Amino aci	ko00400	Phenylalar	0.85%	0.59%	1.43	0.07669	0.15136	HORVU0I
Cellular Pı	Transport :	ko04146	Peroxisom	1.79%	1.42%	1.26	0.077427	0.15136	HORVU0I
Metabolisı	Metabolisı	ko00860	Porphyrin	1%	0.73%	1.37	0.082011	0.153564	HORVU0I
Metabolisı	Carbohydr	ko00650	Butanoate	0.55%	0.35%	1.56	0.082764	0.153564	HORVU0I
Metabolisı	Metabolisı	ko00902	Monoterpe	0.35%	0.19%	1.79	0.083398	0.153564	HORVU1I
Metabolisı	Glycan bic	ko00514	Other type	0.40%	0.23%	1.7	0.084967	0.153564	HORVU1I
Genetic In	Folding, sc	ko03018	RNA degra	2.34%	1.94%	1.21	0.094085	0.166896	HORVU0I
Metabolisı	Amino aci	ko00260	Glycine, se	1.10%	0.83%	1.31	0.104367	0.17981	HORVU1I
Metabolisı	Carbohydr	ko00562	Inositol ph	1.65%	1.33%	1.24	0.106875	0.17981	HORVU1I
Metabolisı	Biosynthes	ko00402	Benzoxazi	0.80%	0.58%	1.38	0.106997	0.17981	HORVU0I
Metabolisı	Biosynthes	ko00965	Betalain bi	0.55%	0.37%	1.46	0.119477	0.197321	HORVU1I
Metabolisı	Global anc	ko01210	2-Oxocarb	1.05%	0.81%	1.29	0.12732	0.20671	HORVU0I
Genetic In	Replicatioı	ko03450	Non-homo	0.20%	0.10%	1.97	0.13275	0.211935	HORVU1I
Metabolisı	Carbohydr	ko00051	Fructose aı	1.20%	0.95%	1.26	0.13551	0.212794	HORVU0I
Metabolisı	Metabolisı	ko00130	Ubiquinon	1.10%	0.87%	1.26	0.149558	0.231065	HORVU0I
Metabolisı	Carbohydr	ko00053	Ascorbate	1.15%	0.92%	1.25	0.151981	0.231082	HORVU2I
Metabolisı	Glycan bic	ko00531	Glycosami	0.50%	0.36%	1.39	0.172464	0.256939	HORVU1I
Metabolisı	Amino aci	ko00270	Cysteine a	1.69%	1.44%	1.17	0.175523	0.256939	HORVU0I
Metabolisı	Lipid meta	ko00100	Steroid bic	0.65%	0.49%	1.32	0.177033	0.256939	HORVU1I
Metabolisı	Lipid meta	ko00591	Linoleic ac	0.45%	0.32%	1.4	0.181524	0.259524	HORVU2I
Metabolisı	Metabolisı	ko00450	Selenocom	0.40%	0.30%	1.35	0.235149	0.331248	HORVU2I
Metabolisı	Glycan bic	ko00601	Glycosphi	0.10%	0.05%	2.13	0.238737	0.331428	HORVU0I
Metabolisı	Metabolisı	ko00770	Pantothena	0.55%	0.44%	1.26	0.252828	0.345975	HORVU1I
Metabolisı	Metabolisı	ko00908	Zeatin bios	0.90%	0.76%	1.17	0.266005	0.35888	HORVU1I
--	--	ko00998	Biosynthes	0.05%	0.02%	3.2	0.288433	0.383735	HORVU2I
Metabolisı	Glycan bic	ko00513	Various ty	0.60%	0.50%	1.2	0.294075	0.385881	HORVU1I
Metabolisı	Metabolisı	ko00780	Biotin met	0.35%	0.27%	1.28	0.302293	0.391303	HORVU1I
Metabolisı	Lipid meta	ko00564	Glyceroph	2.04%	1.89%	1.08	0.31326	0.395497	HORVU0I
Metabolisı	Amino aci	ko00310	Lysine deg	0.70%	0.60%	1.16	0.31379	0.395497	HORVU1I
Metabolisı	Amino aci	ko00340	Histidine r	0.35%	0.28%	1.24	0.329127	0.405085	HORVU2I
Metabolisı	Biosynthes	ko00945	Stilbenoid,	0.80%	0.70%	1.14	0.329855	0.405085	HORVU1I
Metabolisı	Biosynthes	ko00261	Monobacta	0.20%	0.15%	1.35	0.345492	0.418918	HORVU2I
Genetic In	Replicatioı	ko03030	DNA repli	1.20%	1.11%	1.08	0.373794	0.447569	HORVU0I
Metabolisı	Biosynthes	ko00232	Caffeine n	0.05%	0.02%	2.13	0.399775	0.472769	HORVU1I
Metabolisı	Carbohydr	ko00020	Citrate cyc	0.70%	0.64%	1.09	0.406087	0.474376	HORVU0I
Metabolisı	Metabolisı	ko00670	One carboı	0.20%	0.17%	1.16	0.458882	0.529591	HORVU2I
Metabolisı	Lipid meta	ko00072	Synthesis a	0.10%	0.08%	1.28	0.479267	0.538622	HORVU4I
Metabolisı	Lipid meta	ko00600	Sphingolip	0.70%	0.67%	1.04	0.481078	0.538622	HORVU0I
Metabolisı	Amino aci	ko00290	Valine, leu	0.25%	0.23%	1.1	0.483576	0.538622	HORVU1I
Metabolisı	Carbohydr	ko00660	C5-Branch	0.15%	0.13%	1.13	0.51043	0.561998	HORVU2I
Metabolisı	Amino aci	ko00300	Lysine bio	0.20%	0.19%	1.07	0.530666	0.577638	HORVU3I
Metabolisı	Lipid meta	ko01040	Biosynthes	0.45%	0.44%	1.01	0.544177	0.58569	HORVU1I
Metabolisı	Metabolisı	ko00740	Riboflavin	0.30%	0.32%	0.94	0.636746	0.677706	HORVU2I
Metabolisı	Amino aci	ko00380	Tryptopha	1.55%	1.63%	0.95	0.656945	0.68804	HORVU1I
Metabolisı	Metabolisı	ko00790	Folate bios	0.30%	0.33%	0.91	0.660821	0.68804	HORVU1I

Metabolisı	Lipid meta	ko00062	Fatty acid	0.70%	0.76%	0.92	0.671689	0.691836	HORVU1I
Metabolisı	Biosynthes	ko00942	Anthocyan	0.05%	0.05%	0.91	0.69616	0.709413	HORVU2I
--	--	ko04016	MAPK sig	4.54%	4.80%	0.95	0.740039	0.720222	HORVU0I
Metabolisı	Metabolisı	ko00909	Sesquiterp	0.40%	0.48%	0.84	0.758627	0.720222	HORVU2I
Metabolisı	Metabolisı	ko00905	Brassinost	0.20%	0.25%	0.8	0.759943	0.720222	HORVU2I
Organisma	Environme	ko04626	Plant-path	9.07%	9.53%	0.95	0.787795	0.720222	HORVU0I
Genetic In	Translatioı	ko03008	Ribosome	1.20%	1.36%	0.88	0.789527	0.720222	HORVU1I
--	--	ko00515	Mannose t	0.25%	0.32%	0.78	0.790524	0.720222	HORVU3I
Cellular Pı	Transport	ko04145	Phagosom	0.85%	0.99%	0.86	0.793447	0.720222	HORVU1I
Metabolisı	Lipid meta	ko00565	Ether lipid	0.40%	0.50%	0.8	0.804395	0.720222	HORVU1I
Metabolisı	Energy me	ko00920	Sulfur met	0.40%	0.51%	0.77	0.831053	0.720222	HORVU2I
--	--	ko04136	Autophagy	0.30%	0.41%	0.74	0.84409	0.720222	HORVU2I
Genetic In	Replicatioı	ko03420	Nucleotide	0.95%	1.15%	0.83	0.848417	0.720222	HORVU1I
Organisma	Environme	ko04712	Circadian	0.80%	0.99%	0.81	0.859559	0.720222	HORVU0I
Metabolisı	Biosynthes	ko00901	Indole alka	0.10%	0.16%	0.61	0.862791	0.720222	HORVU3I
Genetic In	Folding, sc	ko04141	Protein prc	3.04%	3.44%	0.88	0.872383	0.720222	HORVU0I
Environme	Signal tran	ko04075	Plant horm	6.48%	7.06%	0.92	0.875021	0.720222	HORVU0I
Metabolisı	Metabolisı	ko00760	Nicotinate	0.25%	0.37%	0.68	0.878782	0.720222	HORVU1I
Genetic In	Replicatioı	ko03410	Base excis	0.55%	0.73%	0.76	0.879979	0.720222	HORVU1I
Metabolisı	Glycan bic	ko00563	Glycosylpl	0.20%	0.31%	0.64	0.891503	0.720222	HORVU1I
Metabolisı	Carbohydr	ko00040	Pentose an	1.15%	1.45%	0.79	0.913581	0.720222	HORVU1I
Cellular Pı	Transport	ko04144	Endocytos	2.14%	2.56%	0.84	0.915166	0.720222	HORVU0I
Metabolisı	Biosynthes	ko00943	Isoflavono	0.10%	0.19%	0.51	0.920072	0.720222	HORVU2I
Metabolisı	Metabolisı	ko00750	Vitamin B	0.05%	0.12%	0.43	0.922196	0.720222	HORVU2I
Metabolisı	Nucleotide	ko00240	Pyrimidine	0.85%	1.13%	0.75	0.926981	0.720222	HORVU1I
Metabolisı	Glycan bic	ko00510	N-Glycan	0.35%	0.54%	0.65	0.930384	0.720222	HORVU1I
Genetic In	Translatioı	ko00970	Aminoacy	1%	1.45%	0.69	0.978761	0.720222	HORVU1I
Genetic In	Folding, sc	ko04130	SNARE in	0.20%	0.43%	0.46	0.980366	0.720222	HORVU3I
Metabolisı	Biosynthes	ko00966	Glucosinol	0.05%	0.19%	0.27	0.983215	0.720222	HORVU5I
Genetic In	Folding, sc	ko03060	Protein exp	0.40%	0.76%	0.53	0.989568	0.720222	HORVU3I
Genetic In	Translatioı	ko03015	mRNA sur	1%	1.53%	0.65	0.989765	0.720222	HORVU0I
Genetic In	Translatioı	ko03013	RNA trans	1.94%	2.69%	0.72	0.992	0.720222	HORVU1I
Genetic In	Replicatioı	ko03430	Mismatch	0.65%	1.15%	0.56	0.995169	0.720222	HORVU1I
Metabolisı	Energy me	ko00195	Photosynt	1.74%	2.67%	0.65	0.998698	0.720222	HORVU0I
Genetic In	Transcripti	ko03020	RNA poly	0.45%	0.99%	0.45	0.998879	0.720222	HORVU2I
Genetic In	Folding, sc	ko04120	Ubiquitin	1.99%	2.99%	0.67	0.99895	0.720222	HORVU1I
Genetic In	Transcripti	ko03022	Basal trans	0.20%	0.64%	0.31	0.99941	0.720222	HORVU3I
Genetic In	Folding, sc	ko03050	Proteasom	0.40%	0.97%	0.41	0.999466	0.720222	HORVU0I
Genetic In	Transcripti	ko03040	Spliceosor	1.74%	3.12%	0.56	0.999987	0.720222	HORVU0I
Metabolisı	Energy me	ko00190	Oxidative	0.85%	3.94%	0.22	1	0.720222	HORVU1I
Genetic In	Translatioı	ko03010	Ribosome	2.39%	6.07%	0.39	1	0.720222	HORVU0I

expressed g

Hr1G078380;HORVU1Hr1G088870;HORVU1Hr1G088880;HORVU1Hr1G088920;HORVU1Hr1G089180;H
Hr1G000910;HORVU0Hr1G013220;HORVU0Hr1G014150;HORVU0Hr1G019020;HORVU0Hr1G022780;H
Hr1G017370;HORVU1Hr1G038060;HORVU1Hr1G070220;HORVU1Hr1G080320;HORVU2Hr1G033320;H
Hr1G013900;HORVU0Hr1G014980;HORVU0Hr1G024560;HORVU0Hr1G030280;HORVU1Hr1G013740;H
Hr1G008870;HORVU0Hr1G010220;HORVU0Hr1G017370;HORVU1Hr1G010120;HORVU1Hr1G015090;H
Hr1G004830;HORVU0Hr1G013220;HORVU0Hr1G013900;HORVU0Hr1G014980;HORVU0Hr1G024560;H
Hr1G008870;HORVU0Hr1G010220;HORVU0Hr1G013220;HORVU0Hr1G014980;HORVU1Hr1G013740;H
Hr1G007690;HORVU1Hr1G043820;HORVU1Hr1G075550;HORVU1Hr1G085230;HORVU1Hr1G095430;H
Hr1G019590;HORVU1Hr1G070960;HORVU1Hr1G081430;HORVU1Hr1G082020;HORVU1Hr1G094880;H
Hr1G017370;HORVU1Hr1G015560;HORVU1Hr1G038060;HORVU2Hr1G002600;HORVU2Hr1G033320;H
Hr1G013220;HORVU1Hr1G075550;HORVU1Hr1G085230;HORVU2Hr1G012790;HORVU2Hr1G032970;H
Hr1G008870;HORVU0Hr1G010220;HORVU1Hr1G016200;HORVU1Hr1G038330;HORVU1Hr1G082330;H
Hr1G004830;HORVU0Hr1G024560;HORVU1Hr1G041250;HORVU1Hr1G061160;HORVU1Hr1G067300;H
Hr1G017370;HORVU1Hr1G010120;HORVU1Hr1G015090;HORVU2Hr1G033320;HORVU2Hr1G103040;H
Hr1G017000;HORVU0Hr1G021720;HORVU0Hr1G021760;HORVU1Hr1G061830;HORVU2Hr1G004480;H
Hr1G000280;HORVU1Hr1G027570;HORVU1Hr1G040670;HORVU1Hr1G049690;HORVU1Hr1G054380;H
Hr1G007690;HORVU1Hr1G043820;HORVU1Hr1G064700;HORVU2Hr1G017880;HORVU2Hr1G037480;H
Hr1G000490;HORVU0Hr1G017370;HORVU0Hr1G030280;HORVU1Hr1G015560;HORVU1Hr1G038060;H
Hr1G017560;HORVU0Hr1G017690;HORVU1Hr1G053080;HORVU2Hr1G015140;HORVU2Hr1G021320;H
Hr1G017370;HORVU1Hr1G010120;HORVU1Hr1G015090;HORVU2Hr1G033320;HORVU2Hr1G089440;H
Hr1G017540;HORVU0Hr1G019300;HORVU1Hr1G021160;HORVU1Hr1G022430;HORVU1Hr1G043470;H
Hr1G019840;HORVU0Hr1G022360;HORVU0Hr1G031760;HORVU1Hr1G009200;HORVU2Hr1G000280;H
Hr1G094880;HORVU2Hr1G019680;HORVU2Hr1G090170;HORVU2Hr1G090200;HORVU3Hr1G023230;H
Hr1G005180;HORVU1Hr1G012170;HORVU1Hr1G039830;HORVU1Hr1G084980;HORVU1Hr1G094400;H
Hr1G002090;HORVU0Hr1G002120;HORVU0Hr1G013220;HORVU0Hr1G014150;HORVU1Hr1G064700;H
Hr1G058420;HORVU1Hr1G060610;HORVU1Hr1G084900;HORVU2Hr1G126150;HORVU3Hr1G090190;H
Hr1G002720;HORVU0Hr1G004850;HORVU0Hr1G005060;HORVU0Hr1G012860;HORVU0Hr1G012940;H
Hr1G038060;HORVU2Hr1G022920;HORVU2Hr1G044560;HORVU3Hr1G057090;HORVU3Hr1G063050;H
Hr1G092530;HORVU2Hr1G092540;HORVU5Hr1G037200;HORVU6Hr1G001270;HORVU6Hr1G061220;H
Hr1G013740;HORVU1Hr1G021600;HORVU1Hr1G038330;HORVU1Hr1G070220;HORVU1Hr1G091230;H
Hr1G031170;HORVU1Hr1G010670;HORVU1Hr1G019530;HORVU1Hr1G029460;HORVU1Hr1G064700;H
Hr1G002530;HORVU0Hr1G007690;HORVU1Hr1G032150;HORVU1Hr1G043820;HORVU1Hr1G057940;H
Hr1G001280;HORVU0Hr1G002530;HORVU1Hr1G051700;HORVU1Hr1G057940;HORVU1Hr1G077420;H
Hr1G017370;HORVU0Hr1G025870;HORVU1Hr1G068850;HORVU1Hr1G076870;HORVU1Hr1G080320;H
Hr1G003300;HORVU1Hr1G009920;HORVU1Hr1G030200;HORVU1Hr1G056210;HORVU2Hr1G009580;H
Hr1G017370;HORVU1Hr1G015090;HORVU2Hr1G033320;HORVU3Hr1G097180;HORVU4Hr1G031510;H
Hr1G092530;HORVU2Hr1G092540;HORVU3Hr1G003050;HORVU4Hr1G008700;HORVU4Hr1G064790;H
Hr1G016200;HORVU1Hr1G018140;HORVU2Hr1G010440;HORVU2Hr1G023390;HORVU2Hr1G068270;H
Hr1G060610;HORVU1Hr1G084900;HORVU2Hr1G036800;HORVU2Hr1G111540;HORVU2Hr1G118670;H
Hr1G014980;HORVU1Hr1G013740;HORVU1Hr1G041250;HORVU1Hr1G054380;HORVU1Hr1G061160;H
Hr1G079960;HORVU3Hr1G003050;HORVU4Hr1G008700;HORVU4Hr1G064790
Hr1G016200;HORVU1Hr1G018140;HORVU1Hr1G038330;HORVU1Hr1G055600;HORVU2Hr1G010440;H
Hr1G000810;HORVU0Hr1G008870;HORVU0Hr1G010220;HORVU1Hr1G038330;HORVU1Hr1G063250;H
Hr1G079960;HORVU3Hr1G073710;HORVU3Hr1G103960;HORVU4Hr1G002230;HORVU4Hr1G035380;H
Hr1G005760;HORVU1Hr1G062970;HORVU1Hr1G077590;HORVU2Hr1G015760;HORVU2Hr1G075870;H

Hr1G013740;HORVU1Hr1G021600;HORVU2Hr1G010440;HORVU2Hr1G012440;HORVU3Hr1G105880;H
Hr1G064700;HORVU2Hr1G037480;HORVU3Hr1G050450;HORVU3Hr1G081960;HORVU3Hr1G090160;H
Hr1G000490;HORVU0Hr1G017370;HORVU1Hr1G015090;HORVU1Hr1G050970;HORVU1Hr1G055370;H
Hr1G010800;HORVU0Hr1G013900;HORVU1Hr1G003300;HORVU1Hr1G016200;HORVU1Hr1G038330;H
Hr1G007360;HORVU0Hr1G040200;HORVU1Hr1G006780;HORVU1Hr1G011520;HORVU1Hr1G044680;H
Hr1G014980;HORVU2Hr1G119600;HORVU3Hr1G003050;HORVU3Hr1G049900;HORVU4Hr1G008700;H
Hr1G089830;HORVU2Hr1G017120;HORVU2Hr1G114340;HORVU6Hr1G012270;HORVU6Hr1G088610;H
Hr1G004980;HORVU1Hr1G048690;HORVU1Hr1G048700;HORVU2Hr1G038030;HORVU3Hr1G094370;H
Hr1G025460;HORVU1Hr1G013990;HORVU1Hr1G019000;HORVU1Hr1G075550;HORVU1Hr1G085230;H
Hr1G013740;HORVU1Hr1G056180;HORVU1Hr1G070220;HORVU1Hr1G093480;HORVU2Hr1G005140;H
Hr1G015620;HORVU1Hr1G051700;HORVU1Hr1G068860;HORVU1Hr1G077420;HORVU1Hr1G080460;H
Hr1G007370;HORVU1Hr1G018600;HORVU1Hr1G078740;HORVU2Hr1G005670;HORVU2Hr1G006830;H
Hr1G010120;HORVU2Hr1G075950;HORVU2Hr1G114390;HORVU2Hr1G114450;HORVU3Hr1G081590;H
Hr1G017370;HORVU0Hr1G030280;HORVU1Hr1G015560;HORVU1Hr1G090490;HORVU2Hr1G033320;H
Hr1G031270;HORVU2Hr1G100690;HORVU2Hr1G116540;HORVU7Hr1G085450
Hr1G017220;HORVU1Hr1G024400;HORVU1Hr1G075550;HORVU1Hr1G085230;HORVU3Hr1G019580;H
Hr1G002720;HORVU0Hr1G018190;HORVU1Hr1G015090;HORVU1Hr1G036200;HORVU1Hr1G051190;H
Hr1G009940;HORVU2Hr1G092530;HORVU2Hr1G092540;HORVU2Hr1G096250;HORVU2Hr1G101730;H
Hr1G064700;HORVU2Hr1G037480;HORVU3Hr1G050450;HORVU3Hr1G081960;HORVU3Hr1G090160;H
Hr1G017370;HORVU0Hr1G039040;HORVU1Hr1G015090;HORVU1Hr1G020410;HORVU1Hr1G041250;H
Hr1G052620;HORVU1Hr1G052890;HORVU2Hr1G003210;HORVU3Hr1G028620;HORVU3Hr1G073710;H
Hr1G115960;HORVU3Hr1G073710;HORVU5Hr1G001180;HORVU5Hr1G093700;HORVU6Hr1G033600;H
Hr1G004890;HORVU2Hr1G023170;HORVU3Hr1G069210;HORVU3Hr1G104600;HORVU4Hr1G069130;H
Hr1G013010;HORVU1Hr1G083700
Hr1G090490;HORVU2Hr1G092530;HORVU2Hr1G092540;HORVU2Hr1G119600;HORVU5Hr1G037200;H
Hr1G025830;HORVU1Hr1G042360;HORVU2Hr1G004720;HORVU2Hr1G004790;HORVU2Hr1G066790;H
Hr1G014240
Hr1G064700;HORVU2Hr1G037480;HORVU3Hr1G090160;HORVU4Hr1G014070;HORVU4Hr1G075560;H
Hr1G018140;HORVU2Hr1G023390;HORVU2Hr1G068270;HORVU2Hr1G077970;HORVU2Hr1G089160;H
Hr1G000810;HORVU0Hr1G002530;HORVU1Hr1G015620;HORVU1Hr1G032150;HORVU1Hr1G057940;H
Hr1G013740;HORVU2Hr1G092530;HORVU2Hr1G092540;HORVU3Hr1G007830;HORVU4Hr1G006720;H
Hr1G092530;HORVU2Hr1G092540;HORVU4Hr1G074400;HORVU5Hr1G037200;HORVU6Hr1G001270;H
Hr1G081430;HORVU1Hr1G089520;HORVU2Hr1G086380;HORVU2Hr1G109590;HORVU2Hr1G115890;H
Hr1G004890;HORVU3Hr1G096500;HORVU4Hr1G063600;HORVU5Hr1G094300
Hr1G017330;HORVU1Hr1G029770;HORVU1Hr1G050450;HORVU1Hr1G059180;HORVU1Hr1G063700;H
Hr1G049690
Hr1G030280;HORVU1Hr1G013740;HORVU1Hr1G041250;HORVU2Hr1G001600;HORVU2Hr1G061040;H
Hr1G103130;HORVU4Hr1G011500;HORVU5Hr1G056400;HORVU7Hr1G049510
Hr1G085740;HORVU5Hr1G052090
Hr1G007690;HORVU1Hr1G043820;HORVU2Hr1G017880;HORVU2Hr1G028610;HORVU3Hr1G050450;H
Hr1G090490;HORVU2Hr1G119600;HORVU3Hr1G069300;HORVU7Hr1G059460;HORVU7Hr1G066450
Hr1G119600;HORVU3Hr1G069300;HORVU7Hr1G066450
Hr1G096500;HORVU4Hr1G063600;HORVU4Hr1G071300;HORVU5Hr1G045450
Hr1G038330;HORVU3Hr1G013790;HORVU5Hr1G006930;HORVU5Hr1G039730;HORVU6Hr1G020600;H
Hr1G086080;HORVU4Hr1G000510;HORVU4Hr1G012400;HORVU5Hr1G087800;HORVU7Hr1G082590;H
Hr1G010120;HORVU1Hr1G013740;HORVU1Hr1G022530;HORVU1Hr1G074200;HORVU1Hr1G090870;H
Hr1G076470;HORVU2Hr1G110680;HORVU5Hr1G006850;HORVU5Hr1G011960;HORVU5Hr1G017830;H

Hr1G055600;HORVU1Hr1G091540;HORVU2Hr1G112860;HORVU3Hr1G013790;HORVU3Hr1G059880;H
Hr1G102380
Hr1G001280;HORVU0Hr1G004060;HORVU1Hr1G020370;HORVU1Hr1G025250;HORVU1Hr1G055340;H
Hr1G024120;HORVU5Hr1G094080;HORVU6Hr1G035730;Hordeum_vulgare_newGene_14177;Hordeum_vu
Hr1G081650;HORVU3Hr1G023910;HORVU3Hr1G085400;HORVU7Hr1G084390
Hr1G001280;HORVU0Hr1G001780;HORVU0Hr1G008580;HORVU1Hr1G001490;HORVU1Hr1G004080;H
Hr1G076500;HORVU1Hr1G078310;HORVU2Hr1G020690;HORVU2Hr1G065960;HORVU2Hr1G083090;H
Hr1G002840;HORVU3Hr1G003040;HORVU5Hr1G006290;HORVU6Hr1G016330;HORVU7Hr1G048570
Hr1G064870;HORVU3Hr1G029880;HORVU3Hr1G052560;HORVU3Hr1G089830;HORVU3Hr1G114200;H
Hr1G015620;HORVU1Hr1G079150;HORVU1Hr1G085090;HORVU2Hr1G027010;HORVU3Hr1G024530;H
Hr1G000280;HORVU2Hr1G004890;HORVU4Hr1G079940;HORVU4Hr1G081310;HORVU5Hr1G034810;H
Hr1G049500;HORVU2Hr1G057610;HORVU2Hr1G103010;HORVU3Hr1G054070;HORVU6Hr1G084430;H
Hr1G050450;HORVU3Hr1G034860;HORVU3Hr1G072410;HORVU3Hr1G072460;HORVU4Hr1G067940;H
Hr1G031850;HORVU1Hr1G079450;HORVU2Hr1G013400;HORVU2Hr1G116390;HORVU3Hr1G024220;H
Hr1G074780;HORVU5Hr1G043200
Hr1G013830;HORVU0Hr1G020420;HORVU1Hr1G004200;HORVU1Hr1G057330;HORVU1Hr1G073130;H
Hr1G000450;HORVU0Hr1G021630;HORVU0Hr1G022070;HORVU0Hr1G040070;HORVU1Hr1G000660;H
Hr1G056560;HORVU2Hr1G027680;HORVU3Hr1G109880;HORVU5Hr1G056620;HORVU6Hr1G090080
Hr1G007730;HORVU1Hr1G073540;HORVU1Hr1G077820;HORVU3Hr1G003060;HORVU3Hr1G078490;H
Hr1G012420;HORVU1Hr1G074530;HORVU5Hr1G009620;HORVU5Hr1G121120
Hr1G038220;HORVU1Hr1G067840;HORVU1Hr1G070310;HORVU1Hr1G075860;HORVU1Hr1G079130;H
Hr1G013830;HORVU0Hr1G015420;HORVU0Hr1G027130;HORVU1Hr1G004200;HORVU1Hr1G074130;H
Hr1G063220;HORVU5Hr1G096930
Hr1G060730
Hr1G025380;HORVU1Hr1G072750;HORVU1Hr1G087380;HORVU2Hr1G029350;HORVU3Hr1G026180;H
Hr1G057330;HORVU4Hr1G014070;HORVU4Hr1G075560;HORVU5Hr1G062720;Hordeum_vulgare_newGe
Hr1G046630;HORVU1Hr1G057910;HORVU1Hr1G069900;HORVU1Hr1G090770;HORVU2Hr1G028920;H
Hr1G066580;HORVU3Hr1G112450;HORVU6Hr1G011180;HORVU6Hr1G081270
Hr1G074170
Hr1G031940;HORVU3Hr1G110160;HORVU4Hr1G021950;HORVU5Hr1G014470;HORVU5Hr1G049980;H
Hr1G035930;HORVU1Hr1G057650;HORVU1Hr1G057880;HORVU1Hr1G084450;HORVU2Hr1G105930;H
Hr1G058470;HORVU1Hr1G076500;HORVU1Hr1G088780;HORVU2Hr1G015870;HORVU2Hr1G045250;H
Hr1G050450;HORVU3Hr1G034860;HORVU3Hr1G072410;HORVU3Hr1G072460;HORVU4Hr1G067940;H
Hr1G001490;HORVU0Hr1G003270;HORVU1Hr1G078140;HORVU2Hr1G019820;HORVU2Hr1G038940;H
Hr1G084270;HORVU3Hr1G077450;HORVU4Hr1G007530;HORVU5Hr1G023090;HORVU5Hr1G117770;H
Hr1G008940;HORVU1Hr1G038130;HORVU1Hr1G040800;HORVU1Hr1G065030;HORVU2Hr1G022590;H
Hr1G023590;HORVU4Hr1G043930;HORVU4Hr1G046620;HORVU7Hr1G071800
Hr1G001180;HORVU1Hr1G058180;HORVU1Hr1G068490;HORVU1Hr1G081810;HORVU2Hr1G096510;H
Hr1G003340;HORVU0Hr1G013830;HORVU1Hr1G000720;HORVU1Hr1G004200;HORVU1Hr1G020000;H
Hr1G006780;HORVU1Hr1G025060;HORVU2Hr1G061040;HORVU2Hr1G061080;HORVU3Hr1G029880;H
Hr1G006020;HORVU0Hr1G009120;HORVU0Hr1G010170;HORVU0Hr1G038820;HORVU1Hr1G000040;H

ORVU2Hr1G036960;HORVU2Hr1G040780;HORVU2Hr1G041270;HORVU2Hr1G079920;HORVU4Hr1G01

ORVU0Hr1G028350;HORVU0Hr1G031760;HORVU1Hr1G000810;HORVU1Hr1G011100;HORVU1Hr1G01

ORVU2Hr1G063740;HORVU2Hr1G113260;HORVU3Hr1G003050;HORVU3Hr1G013910;HORVU3Hr1G0

ORVU1Hr1G038060;HORVU1Hr1G041250;HORVU2Hr1G005140;HORVU2Hr1G010630;HORVU2Hr1G01

ORVU2Hr1G020900;HORVU2Hr1G033320;HORVU2Hr1G103040;HORVU2Hr1G114390;HORVU2Hr1G11

ORVU0Hr1G030280;HORVU1Hr1G013740;HORVU1Hr1G041250;HORVU1Hr1G054380;HORVU1Hr1G0

ORVU1Hr1G054380;HORVU1Hr1G061160;HORVU1Hr1G067300;HORVU1Hr1G070310;HORVU1Hr1G0

ORVU2Hr1G017880;HORVU2Hr1G020920;HORVU2Hr1G073210;HORVU2Hr1G080100;HORVU2Hr1G0

ORVU2Hr1G019680;HORVU2Hr1G033610;HORVU2Hr1G038260;HORVU2Hr1G086380;HORVU2Hr1G1

ORVU2Hr1G063740;HORVU3Hr1G103540;HORVU4Hr1G007610;HORVU4Hr1G058840;HORVU4Hr1G0

ORVU2Hr1G036110;HORVU2Hr1G070400;HORVU3Hr1G019580;HORVU3Hr1G070300;HORVU3Hr1G11

ORVU2Hr1G020900;HORVU2Hr1G092530;HORVU2Hr1G092540;HORVU3Hr1G013880;HORVU3Hr1G0

ORVU2Hr1G010630;HORVU2Hr1G010670;HORVU2Hr1G010690;HORVU2Hr1G012790;HORVU2Hr1G0

ORVU2Hr1G114390;HORVU2Hr1G114450;HORVU3Hr1G077790;HORVU4Hr1G031510;HORVU4Hr1G0

ORVU2Hr1G004510;HORVU2Hr1G004530;HORVU2Hr1G004540;HORVU2Hr1G004600;HORVU2Hr1G0

ORVU1Hr1G059810;HORVU1Hr1G078310;HORVU1Hr1G087380;HORVU2Hr1G004890;HORVU2Hr1G0

ORVU3Hr1G090160;HORVU6Hr1G075240;Hordeum_vulgare_newGene_9576

ORVU1Hr1G050970;HORVU1Hr1G054380;HORVU1Hr1G055370;HORVU1Hr1G056160;HORVU1Hr1G0

ORVU2Hr1G068610;HORVU2Hr1G094160;HORVU3Hr1G070660;HORVU4Hr1G054980;HORVU4Hr1G0

ORVU2Hr1G114390;HORVU2Hr1G114450;HORVU2Hr1G126540;HORVU4Hr1G031510;HORVU4Hr1G0

ORVU1Hr1G049090;HORVU1Hr1G049210;HORVU1Hr1G049230;HORVU1Hr1G049250;HORVU1Hr1G0

ORVU2Hr1G023590;HORVU2Hr1G079960;HORVU2Hr1G082170;HORVU2Hr1G088980;HORVU2Hr1G11

ORVU4Hr1G063780;HORVU6Hr1G087250;HORVU6Hr1G087310;HORVU7Hr1G007580

ORVU1Hr1G094420;HORVU2Hr1G001150;HORVU2Hr1G028780;HORVU2Hr1G088540;HORVU2Hr1G0

ORVU1Hr1G078050;HORVU1Hr1G082350;HORVU1Hr1G095430;HORVU2Hr1G006130;HORVU2Hr1G0

ORVU3Hr1G099770;HORVU4Hr1G003580;HORVU6Hr1G014480;HORVU7Hr1G043620;HORVU7Hr1G0

ORVU0Hr1G018190;HORVU0Hr1G031760;HORVU1Hr1G016770;HORVU1Hr1G016980;HORVU1Hr1G0

ORVU4Hr1G007610;HORVU4Hr1G066860;HORVU4Hr1G076970;HORVU6Hr1G012290;HORVU6Hr1G0

ORVU6Hr1G061270

ORVU2Hr1G012440;HORVU2Hr1G092530;HORVU2Hr1G092540;HORVU3Hr1G000350;HORVU3Hr1G0

ORVU2Hr1G025810;HORVU2Hr1G037480;HORVU3Hr1G028020;HORVU3Hr1G050450;HORVU3Hr1G0

ORVU1Hr1G070310;HORVU2Hr1G017880;HORVU2Hr1G022160;HORVU2Hr1G040170;HORVU2Hr1G0

ORVU1Hr1G080460;HORVU1Hr1G094160;HORVU1Hr1G094230;HORVU2Hr1G013730;HORVU2Hr1G01

ORVU2Hr1G033320;HORVU2Hr1G092530;HORVU2Hr1G092540;HORVU2Hr1G126540;HORVU3Hr1G0

ORVU2Hr1G009830;HORVU2Hr1G018920;HORVU2Hr1G019010;HORVU2Hr1G090960;HORVU3Hr1G0

ORVU4Hr1G074530;HORVU4Hr1G074620;HORVU5Hr1G083170;HORVU5Hr1G122860;HORVU7Hr1G01

ORVU4Hr1G074530;HORVU4Hr1G074620;HORVU5Hr1G006930;HORVU5Hr1G037200;HORVU5Hr1G0

ORVU2Hr1G077970;HORVU2Hr1G089160;HORVU2Hr1G114920;HORVU3Hr1G105880;HORVU4Hr1G01

ORVU2Hr1G126150;HORVU3Hr1G078420;HORVU3Hr1G090560;HORVU4Hr1G071070;HORVU4Hr1G0

ORVU2Hr1G001600;HORVU2Hr1G010440;HORVU2Hr1G084130;HORVU2Hr1G092530;HORVU2Hr1G0

ORVU2Hr1G023390;HORVU2Hr1G068270;HORVU2Hr1G077970;HORVU2Hr1G089160;HORVU2Hr1G11

ORVU1Hr1G090670;HORVU2Hr1G004230;HORVU2Hr1G020330;HORVU2Hr1G020900;HORVU2Hr1G0

ORVU5Hr1G011960;HORVU5Hr1G115720;HORVU5Hr1G115780;HORVU6Hr1G005400;HORVU6Hr1G0

ORVU2Hr1G116540;HORVU3Hr1G015880;HORVU3Hr1G017990;HORVU3Hr1G034860;HORVU3Hr1G0

ORVU5Hr1G006930;HORVU5Hr1G034820;HORVU5Hr1G052090;HORVU5Hr1G109390;HORVU5Hr1G11
ORVU5Hr1G045820;HORVU7Hr1G086690;Hordeum_vulgare_newGene_15051
ORVU1Hr1G056160;HORVU1Hr1G093480;HORVU2Hr1G033320;HORVU3Hr1G052780;HORVU4Hr1G03
ORVU1Hr1G049690;HORVU1Hr1G094400;HORVU2Hr1G031910;HORVU2Hr1G060010;HORVU2Hr1G06
ORVU2Hr1G023540;HORVU2Hr1G023560;HORVU3Hr1G039930;HORVU4Hr1G077730;HORVU5Hr1G00
ORVU4Hr1G064790;HORVU4Hr1G081290;HORVU4Hr1G085740;HORVU5Hr1G052090;HORVU6Hr1G03
ORVU7Hr1G024270;Hordeum_vulgare_newGene_11543
ORVU7Hr1G027410;HORVU7Hr1G029400;Hordeum_vulgare_newGene_15087
ORVU2Hr1G007840;HORVU2Hr1G011960;HORVU2Hr1G056650;HORVU2Hr1G056710;HORVU2Hr1G07
ORVU2Hr1G063740;HORVU2Hr1G097910;HORVU2Hr1G103130;HORVU3Hr1G067110;HORVU3Hr1G09
ORVU1Hr1G085090;HORVU2Hr1G013730;HORVU2Hr1G019950;HORVU2Hr1G027010;HORVU2Hr1G04
ORVU2Hr1G006850;HORVU3Hr1G011850;HORVU3Hr1G011870;HORVU3Hr1G099530;HORVU4Hr1G01
ORVU5Hr1G105360;HORVU6Hr1G002390;HORVU7Hr1G010750;HORVU7Hr1G010850;HORVU7Hr1G01
ORVU2Hr1G063740;HORVU2Hr1G119600;HORVU3Hr1G069300;HORVU3Hr1G096500;HORVU3Hr1G10

ORVU3Hr1G070300;HORVU3Hr1G071210;HORVU3Hr1G117540;HORVU4Hr1G019570;HORVU4Hr1G06
ORVU1Hr1G066240;HORVU3Hr1G013400;HORVU3Hr1G077250;HORVU3Hr1G089000;HORVU4Hr1G00
ORVU3Hr1G081530;HORVU3Hr1G099760;HORVU4Hr1G008270;HORVU4Hr1G057170;HORVU4Hr1G05
ORVU5Hr1G045820;HORVU6Hr1G087000;HORVU7Hr1G086690;Hordeum_vulgare_newGene_15051;Hord
ORVU1Hr1G056180;HORVU1Hr1G070220;HORVU1Hr1G083380;HORVU2Hr1G000280;HORVU2Hr1G03
ORVU4Hr1G018150;HORVU4Hr1G018180;HORVU4Hr1G023430;HORVU4Hr1G084000;HORVU4Hr1G09
ORVU7Hr1G050660;HORVU7Hr1G050670;HORVU7Hr1G050680;Hordeum_vulgare_newGene_10698
ORVU5Hr1G094300;HORVU5Hr1G105250;HORVU7Hr1G028540

ORVU6Hr1G001270;HORVU6Hr1G017390;HORVU6Hr1G061220;HORVU6Hr1G061270;HORVU6Hr1G07
ORVU2Hr1G096890;HORVU2Hr1G096910;HORVU3Hr1G027430;HORVU3Hr1G027460;HORVU3Hr1G07

ORVU5Hr1G062720;HORVU5Hr1G063330;HORVU6Hr1G059800;Hordeum_vulgare_newGene_1699;Horde
ORVU5Hr1G053010;HORVU7Hr1G030660
ORVU1Hr1G063250;HORVU1Hr1G067480;HORVU1Hr1G078350;HORVU1Hr1G079150;HORVU1Hr1G08
ORVU5Hr1G037200;HORVU5Hr1G041830;HORVU5Hr1G052090;HORVU5Hr1G074840;HORVU6Hr1G00
ORVU6Hr1G061220;HORVU6Hr1G061270
ORVU3Hr1G026150;HORVU3Hr1G110170;HORVU5Hr1G064280;HORVU6Hr1G003020;HORVU6Hr1G00

ORVU1Hr1G070110;HORVU2Hr1G069780;HORVU2Hr1G105420;HORVU3Hr1G034860;HORVU3Hr1G04

ORVU2Hr1G061080;HORVU4Hr1G062440;HORVU5Hr1G099910;HORVU5Hr1G112850;HORVU7Hr1G04

ORVU3Hr1G081960;HORVU5Hr1G045820;HORVU5Hr1G057320;HORVU6Hr1G075240;HORVU7Hr1G07

ORVU7Hr1G029110;HORVU7Hr1G044990;Hordeum_vulgare_newGene_11133;Hordeum_vulgare_newGene
ordeum_vulgare_newGene_449
ORVU2Hr1G092530;HORVU2Hr1G092540;HORVU2Hr1G114390;HORVU2Hr1G114450;HORVU2Hr1G12
ORVU5Hr1G089780

ƆRVU4Hr1G063420;HORVU4Hr1G067340;HORVU4Hr1G072960;HORVU4Hr1G076940;HORVU6Hr1G08

ƆRVU1Hr1G070480;HORVU1Hr1G078050;HORVU1Hr1G078710;HORVU1Hr1G078860;HORVU1Hr1G09
lgare_newGene_14454;Hordeum_vulgare_newGene_14455;Hordeum_vulgare_newGene_14704;Hordeum_vul

ƆRVU1Hr1G011720;HORVU1Hr1G012190;HORVU1Hr1G025250;HORVU1Hr1G057410;HORVU1Hr1G06
ƆRVU2Hr1G113320;HORVU3Hr1G067910;HORVU3Hr1G099400;HORVU4Hr1G013530;HORVU4Hr1G04

ƆRVU4Hr1G002530;HORVU4Hr1G009520;HORVU4Hr1G087740;HORVU5Hr1G049980;HORVU5Hr1G09
ƆRVU3Hr1G073710;HORVU5Hr1G060030;HORVU5Hr1G084740
ƆRVU5Hr1G094300;HORVU7Hr1G021700;Hordeum_vulgare_newGene_15962
ɔrdeum_vulgare_newGene_5234
ƆRVU4Hr1G090160;HORVU4Hr1G090210;HORVU5Hr1G044830;HORVU5Hr1G080050;HORVU6Hr1G05
ƆRVU3Hr1G087100;HORVU4Hr1G008610;HORVU4Hr1G019380;HORVU5Hr1G035980;HORVU5Hr1G08

ƆRVU1Hr1G074130;HORVU2Hr1G011690;HORVU2Hr1G048680;HORVU2Hr1G077710;HORVU2Hr1G08
ƆRVU1Hr1G015210;HORVU1Hr1G020370;HORVU1Hr1G047390;HORVU1Hr1G052350;HORVU1Hr1G05

ƆRVU5Hr1G080050;HORVU5Hr1G083000;HORVU7Hr1G039710;HORVU7Hr1G096270;Hordeum_vulgare

ƆRVU2Hr1G023820;HORVU2Hr1G032220;HORVU2Hr1G086910;HORVU2Hr1G096130;HORVU3Hr1G05
ƆRVU1Hr1G079150;HORVU2Hr1G045100;HORVU3Hr1G012080;HORVU3Hr1G013390;HORVU3Hr1G01

ƆRVU3Hr1G108360;HORVU4Hr1G087230;HORVU5Hr1G002320;HORVU5Hr1G057410;HORVU6Hr1G06
ne_1699;Hordeum_vulgare_newGene_3447;Hordeum_vulgare_newGene_4026
ƆRVU3Hr1G055980;HORVU3Hr1G065830;HORVU3Hr1G108620;HORVU4Hr1G018650;HORVU4Hr1G06

ƆRVU5Hr1G067800;HORVU7Hr1G098810;HORVU7Hr1G109250
ƆRVU2Hr1G122920;HORVU2Hr1G123110;HORVU3Hr1G104360;HORVU4Hr1G009190;HORVU4Hr1G00
ƆRVU2Hr1G058390;HORVU2Hr1G065960;HORVU2Hr1G105930;HORVU2Hr1G122920;HORVU2Hr1G12
ƆRVU5Hr1G044830;HORVU5Hr1G080050;HORVU6Hr1G059440;HORVU6Hr1G081140;HORVU6Hr1G09
ƆRVU2Hr1G041610;HORVU2Hr1G043960;HORVU2Hr1G057700;HORVU2Hr1G060480;HORVU2Hr1G06
ƆRVU7Hr1G080710;HORVU7Hr1G081980;Hordeum_vulgare_newGene_14757;Hordeum_vulgare_newGene
ƆRVU2Hr1G076180;HORVU2Hr1G077230;HORVU2Hr1G118840;HORVU2Hr1G124270;HORVU3Hr1G00

ƆRVU5Hr1G086500;HORVU6Hr1G010420;HORVU7Hr1G018660
ƆRVU1Hr1G074130;HORVU1Hr1G075040;HORVU1Hr1G078320;HORVU1Hr1G081570;HORVU2Hr1G12
ƆRVU3Hr1G116580;HORVU3Hr1G116650;HORVU4Hr1G004820;HORVU4Hr1G058970;HORVU4Hr1G07
ƆRVU1Hr1G042450;HORVU1Hr1G052180;HORVU1Hr1G056510;HORVU1Hr1G066630;HORVU1Hr1G08

15260;HORVU5Hr1G062240;HORVU5Hr1G066280;HORVU5Hr1G082420;HORVU5Hr1G087250;HORVU
11260;HORVU1Hr1G015610;HORVU1Hr1G019500;HORVU1Hr1G021590;HORVU1Hr1G093950;HORVU
53050;HORVU3Hr1G082280;HORVU4Hr1G005910;HORVU4Hr1G007610;HORVU4Hr1G008700;HORVU
10670;HORVU2Hr1G010690;HORVU2Hr1G022920;HORVU2Hr1G044560;HORVU2Hr1G045340;HORVU
14450;HORVU3Hr1G034820;HORVU3Hr1G077790;HORVU4Hr1G013370;HORVU4Hr1G016770;HORVU
56180;HORVU1Hr1G061160;HORVU1Hr1G067300;HORVU1Hr1G075550;HORVU1Hr1G085230;HORVU
75550;HORVU1Hr1G085230;HORVU2Hr1G001600;HORVU2Hr1G020900;HORVU2Hr1G036110;HORVU
30190;HORVU2Hr1G109080;HORVU2Hr1G109120;HORVU3Hr1G005350;HORVU3Hr1G019580;HORVU
08110;HORVU2Hr1G108180;HORVU2Hr1G108260;HORVU2Hr1G109590;HORVU2Hr1G110130;HORVU
56860;HORVU5Hr1G014730;HORVU5Hr1G062990;HORVU5Hr1G114090;HORVU6Hr1G012290;HORVU
17540;HORVU4Hr1G007620;HORVU4Hr1G019570;HORVU4Hr1G060630;HORVU4Hr1G090310;HORVU
34820;HORVU4Hr1G016770;HORVU4Hr1G016810;HORVU4Hr1G019780;HORVU4Hr1G074840;HORVU
33320;HORVU2Hr1G063740;HORVU2Hr1G070400;HORVU3Hr1G029200;HORVU3Hr1G029210;HORVU
52730;HORVU4Hr1G074530;HORVU4Hr1G074620;HORVU4Hr1G080610;HORVU5Hr1G083170;HORVU
04610;HORVU2Hr1G004620;HORVU2Hr1G004640;HORVU2Hr1G090030;HORVU2Hr1G099440;HORVU
22360;HORVU3Hr1G050350;HORVU3Hr1G059470;HORVU3Hr1G082280;HORVU4Hr1G005910;HORVU

56180;HORVU1Hr1G067300;HORVU1Hr1G075550;HORVU1Hr1G085230;HORVU1Hr1G090490;HORVU
72360;HORVU5Hr1G005740;HORVU5Hr1G054970;HORVU5Hr1G088130;HORVU6Hr1G068720;HORVU
74530;HORVU4Hr1G074620;HORVU5Hr1G047530;HORVU5Hr1G083170;HORVU5Hr1G087780;HORVU
49280;HORVU1Hr1G062210;HORVU2Hr1G009940;HORVU2Hr1G026810;HORVU2Hr1G045200;HORVU
13260;HORVU3Hr1G079720;HORVU3Hr1G096910;HORVU3Hr1G097010;HORVU3Hr1G098340;HORVU

38640;HORVU2Hr1G088660;HORVU2Hr1G088760;HORVU3Hr1G002050;HORVU3Hr1G014080;HORVU
06170;HORVU2Hr1G019180;HORVU2Hr1G037480;HORVU2Hr1G043900;HORVU2Hr1G044640;HORVU
77220;HORVU7Hr1G090100;HORVU7Hr1G090950;Hordeum_vulgare_newGene_8655
23750;HORVU1Hr1G047220;HORVU1Hr1G051190;HORVU1Hr1G058820;HORVU1Hr1G068020;HORVU
74030;HORVU6Hr1G080750;HORVU6Hr1G087300;HORVU7Hr1G067110;Hordeum_vulgare_newGene_131

57440;HORVU3Hr1G069070;HORVU4Hr1G085740;HORVU5Hr1G034820;HORVU5Hr1G037200;HORVU
58440;HORVU3Hr1G081960;HORVU3Hr1G090160;HORVU4Hr1G085460;HORVU5Hr1G045820;HORVU
73850;HORVU2Hr1G092530;HORVU2Hr1G092540;HORVU3Hr1G056830;HORVU3Hr1G073710;HORVU
19950;HORVU2Hr1G022160;HORVU2Hr1G045100;HORVU2Hr1G114960;HORVU3Hr1G077030;HORVU
24150;HORVU5Hr1G037200;HORVU5Hr1G057520;HORVU5Hr1G064040;HORVU5Hr1G087780;HORVU
22800;HORVU3Hr1G065240;HORVU3Hr1G065320;HORVU3Hr1G065390;HORVU3Hr1G068650;HORVU
10790;HORVU7Hr1G082450;HORVU7Hr1G089290
33170;HORVU6Hr1G001270;HORVU6Hr1G020600;HORVU6Hr1G061220;HORVU6Hr1G061270;HORVU
19780;HORVU4Hr1G055240;HORVU5Hr1G036460;HORVU5Hr1G039730;HORVU5Hr1G053010;HORVU
35740;HORVU5Hr1G052090;HORVU5Hr1G103730;HORVU5Hr1G122530;HORVU6Hr1G064460;HORVU
92540;HORVU3Hr1G029200;HORVU3Hr1G029210;HORVU3Hr1G059470;HORVU3Hr1G070850;HORVU

14920;HORVU3Hr1G013790;HORVU3Hr1G013880;HORVU3Hr1G105880;HORVU4Hr1G019780;HORVU
77210;HORVU2Hr1G098800;HORVU3Hr1G013880;HORVU3Hr1G034820;HORVU3Hr1G073710;HORVU
92740;HORVU7Hr1G030810;HORVU7Hr1G045620;HORVU7Hr1G045640
54100;HORVU3Hr1G072410;HORVU3Hr1G072460;HORVU3Hr1G077800;HORVU3Hr1G105820;HORVU

6770;HORVU6Hr1G020600;HORVU7Hr1G029110;HORVU7Hr1G085310;Hordeum_vulgare_newGene_15

1510;HORVU4Hr1G061120;HORVU5Hr1G052150;HORVU7Hr1G010790;HORVU7Hr1G037910;HORVU

0070;HORVU2Hr1G118670;HORVU3Hr1G103960;HORVU4Hr1G001450;HORVU4Hr1G019780;HORVU

9900;HORVU5Hr1G011100;HORVU5Hr1G081500;HORVU5Hr1G081610;HORVU6Hr1G075900;HORVU

1480;HORVU7Hr1G082330

2520;HORVU2Hr1G077410;HORVU2Hr1G079610;HORVU2Hr1G081140;HORVU2Hr1G081440;HORVU

6500;HORVU4Hr1G011500;HORVU4Hr1G051010;HORVU4Hr1G074530;HORVU4Hr1G074620;HORVU

5100;HORVU2Hr1G114960;HORVU3Hr1G088190;HORVU3Hr1G099390;HORVU4Hr1G002820;HORVU

1760;HORVU4Hr1G089410;HORVU5Hr1G114700;HORVU7Hr1G019520;HORVU7Hr1G100850;HORVU

0990;HORVU7Hr1G091860

3540;HORVU4Hr1G058840;HORVU5Hr1G014730;HORVU5Hr1G062990;HORVU5Hr1G074170;HORVU

0630;HORVU4Hr1G069230;HORVU5Hr1G019030;HORVU5Hr1G032980;HORVU5Hr1G046370;HORVU

2050;HORVU4Hr1G031510;HORVU4Hr1G072130;HORVU4Hr1G072150;HORVU4Hr1G076420;HORVU

7210;HORVU4Hr1G089060;HORVU5Hr1G013840;HORVU5Hr1G031870;HORVU5Hr1G037200;HORVU

eum_vulgare_newGene_3468

3320;HORVU2Hr1G058800;HORVU3Hr1G065230;HORVU3Hr1G075690;HORVU3Hr1G096500;HORVU

0810;HORVU5Hr1G065920;HORVU7Hr1G118180;Hordeum_vulgare_newGene_4409

4200;HORVU7Hr1G059460

8860;HORVU5Hr1G096260;HORVU6Hr1G059500;HORVU6Hr1G078110;HORVU7Hr1G036890;HORVU

um_vulgare_newGene_3447;Hordeum_vulgare_newGene_4026;Hordeum_vulgare_newGene_6242

5090;HORVU1Hr1G090670;HORVU2Hr1G012200;HORVU2Hr1G020330;HORVU2Hr1G022160;HORVU

1270;HORVU6Hr1G061220;HORVU6Hr1G061270;Hordeum_vulgare_newGene_15022;Hordeum_vulgare_

3160;HORVU6Hr1G062190;HORVU6Hr1G088530;HORVU7Hr1G026680;HORVU7Hr1G030670;HORVU

7230;HORVU3Hr1G072410;HORVU3Hr1G072460;HORVU4Hr1G067940;HORVU5Hr1G028260;HORVU

5470;HORVU7Hr1G074890;HORVU7Hr1G095680;Hordeum_vulgare_newGene_15022;Hordeum_vulgare_

6680;HORVU7Hr1G078770;HORVU7Hr1G086690;Hordeum_vulgare_newGene_15051;Hordeum_vulgare_

_7775

6540;HORVU4Hr1G082040;HORVU5Hr1G037200;HORVU5Hr1G052090;HORVU5Hr1G087780;HORVU

39730;HORVU6Hr1G089750;HORVU7Hr1G044990;HORVU7Hr1G084610;Hordeum_vulgare_newGene_111

00360;HORVU1Hr1G094160;HORVU1Hr1G094230;HORVU2Hr1G000090;HORVU2Hr1G050260;HORVU2
gare_newGene_5668

54110;HORVU1Hr1G065990;HORVU1Hr1G066020;HORVU1Hr1G068660;HORVU1Hr1G070480;HORVU1
41810;HORVU4Hr1G049150;HORVU4Hr1G054880;HORVU5Hr1G084510;HORVU5Hr1G092800;HORVU5

98960;HORVU5Hr1G116590;HORVU6Hr1G027620;HORVU7Hr1G069660;HORVU7Hr1G077710;HORVU7

59440;HORVU6Hr1G081140;HORVU6Hr1G094080;HORVU7Hr1G043410;HORVU7Hr1G064170;HORVU7
30210;HORVU5Hr1G109880;HORVU5Hr1G117990;HORVU6Hr1G058740;HORVU6Hr1G075650;Hordeum

30770;HORVU2Hr1G086170;HORVU2Hr1G091220;HORVU3Hr1G006940;HORVU3Hr1G012080;HORVU3
52420;HORVU1Hr1G055340;HORVU1Hr1G056920;HORVU1Hr1G065120;HORVU1Hr1G066340;HORVU1

e_newGene_10320;Hordeum_vulgare_newGene_184

54810;HORVU3Hr1G056440;HORVU3Hr1G081530;HORVU3Hr1G087030;HORVU4Hr1G008270;HORVU4
14580;HORVU3Hr1G024530;HORVU3Hr1G069650;HORVU3Hr1G070240;HORVU3Hr1G086380;HORVU3

59280;HORVU6Hr1G074200;HORVU6Hr1G087960;HORVU7Hr1G027430;HORVU7Hr1G041710;HORVU7

50200;HORVU4Hr1G069130;HORVU5Hr1G026140;HORVU5Hr1G092240;HORVU5Hr1G125110;HORVU6

09210;HORVU4Hr1G009310;HORVU5Hr1G053230;HORVU5Hr1G121860;HORVU5Hr1G122080;HORVU6
23110;HORVU3Hr1G047040;HORVU3Hr1G056400;HORVU3Hr1G077960;HORVU3Hr1G083990;HORVU3
94080;HORVU7Hr1G064170;Hordeum_vulgare_newGene_10320;Hordeum_vulgare_newGene_184
50880;HORVU2Hr1G072880;HORVU2Hr1G073370;HORVU2Hr1G080260;HORVU2Hr1G090100;HORVU2
_196
04520;HORVU3Hr1G016010;HORVU3Hr1G031950;HORVU3Hr1G038430;HORVU3Hr1G062370;HORVU3

23070;HORVU3Hr1G012080;HORVU3Hr1G013390;HORVU3Hr1G014960;HORVU3Hr1G039480;HORVU3
77430;HORVU6Hr1G014540;HORVU6Hr1G084740;HORVU7Hr1G005040;HORVU7Hr1G074890;Hordeum
31790;HORVU1Hr1G084690;HORVU1Hr1G089760;HORVU2Hr1G080340;HORVU3Hr1G056580;HORVU3

5Hr1G109250;HORVU5Hr1G124160;HORVU6Hr1G016880;HORVU6Hr1G016890;HORVU6Hr1G091650;
2Hr1G002750;HORVU2Hr1G006100;HORVU2Hr1G020920;HORVU2Hr1G023590;HORVU2Hr1G030870;
4Hr1G055970;HORVU4Hr1G056240;HORVU4Hr1G064790;HORVU4Hr1G066860;HORVU5Hr1G014730;
2Hr1G060010;HORVU2Hr1G060070;HORVU2Hr1G063740;HORVU2Hr1G084130;HORVU2Hr1G084750;
4Hr1G016810;HORVU4Hr1G031510;HORVU4Hr1G074530;HORVU4Hr1G074620;HORVU4Hr1G078460;
2Hr1G000280;HORVU2Hr1G001600;HORVU2Hr1G005140;HORVU2Hr1G010630;HORVU2Hr1G010670;
2Hr1G080100;HORVU2Hr1G080190;HORVU2Hr1G092530;HORVU2Hr1G092540;HORVU2Hr1G101360;
3Hr1G020780;HORVU3Hr1G050450;HORVU3Hr1G070300;HORVU3Hr1G081530;HORVU3Hr1G081960;
2Hr1G115890;HORVU2Hr1G116390;HORVU3Hr1G056560;HORVU3Hr1G069830;HORVU3Hr1G088040;
6Hr1G074030;HORVU7Hr1G010790;HORVU7Hr1G089290;Hordeum_vulgare_newGene_13117;Hordeum_v
5Hr1G019030;HORVU5Hr1G032980;HORVU5Hr1G069850;HORVU5Hr1G069880;HORVU5Hr1G069910;
4Hr1G078460;HORVU5Hr1G006930;HORVU5Hr1G036460;HORVU5Hr1G037200;HORVU5Hr1G052090;
3Hr1G070850;HORVU3Hr1G085270;HORVU3Hr1G102340;HORVU3Hr1G117540;HORVU4Hr1G019570;
5Hr1G105360;HORVU7Hr1G010750;HORVU7Hr1G010790;HORVU7Hr1G010850;HORVU7Hr1G010990;
2Hr1G099480;HORVU2Hr1G114980;HORVU2Hr1G118350;HORVU3Hr1G029010;HORVU3Hr1G029040;
4Hr1G007620;HORVU4Hr1G023210;HORVU4Hr1G052090;HORVU4Hr1G087230;HORVU4Hr1G090310;

1Hr1G093480;HORVU2Hr1G000280;HORVU2Hr1G002600;HORVU2Hr1G012790;HORVU2Hr1G033320;
7Hr1G076030;HORVU7Hr1G096970;HORVU7Hr1G114000;HORVU7Hr1G114020;HORVU7Hr1G114050;
5Hr1G105360;HORVU6Hr1G027650;HORVU6Hr1G058820;HORVU6Hr1G073990;HORVU6Hr1G078210;
2Hr1G079960;HORVU2Hr1G095460;HORVU2Hr1G101730;HORVU2Hr1G111840;HORVU3Hr1G024500;
3Hr1G098360;HORVU3Hr1G098400;HORVU4Hr1G006480;HORVU4Hr1G006530;HORVU4Hr1G011500;

3Hr1G035730;HORVU3Hr1G062900;HORVU3Hr1G096360;HORVU4Hr1G001450;HORVU4Hr1G074840;
2Hr1G085270;HORVU2Hr1G085280;HORVU2Hr1G091270;HORVU2Hr1G126740;HORVU3Hr1G023370;

1Hr1G081430;HORVU1Hr1G089700;HORVU1Hr1G092680;HORVU2Hr1G023590;HORVU2Hr1G026450;
17;Hordeum_vulgare_newGene_13684;Hordeum_vulgare_newGene_13743;Hordeum_vulgare_newGene_160

5Hr1G052090;HORVU6Hr1G001270;HORVU6Hr1G061220;HORVU6Hr1G061270;HORVU6Hr1G062320;
5Hr1G080110;HORVU5Hr1G086390;HORVU5Hr1G113880;HORVU6Hr1G079720;HORVU7Hr1G024220;
3Hr1G077030;HORVU3Hr1G084990;HORVU3Hr1G087020;HORVU4Hr1G008550;HORVU4Hr1G074470;
3Hr1G088190;HORVU3Hr1G099390;HORVU4Hr1G018430;HORVU4Hr1G053130;HORVU4Hr1G064440;
5Hr1G001270;HORVU6Hr1G061220;HORVU6Hr1G061270;HORVU6Hr1G073990;HORVU6Hr1G078210;
3Hr1G071470;HORVU3Hr1G085890;HORVU3Hr1G092650;HORVU3Hr1G098580;HORVU3Hr1G104290;

6Hr1G074200;HORVU7Hr1G029110;HORVU7Hr1G085310;Hordeum_vulgare_newGene_7775
5Hr1G099350;HORVU5Hr1G116770;HORVU7Hr1G019830;HORVU7Hr1G030660
6Hr1G075900;HORVU7Hr1G106230;Hordeum_vulgare_newGene_15240;Hordeum_vulgare_newGene_5668
3Hr1G102340;HORVU3Hr1G105880;HORVU4Hr1G055030;HORVU4Hr1G062440;HORVU4Hr1G081290;

4Hr1G055240;HORVU5Hr1G006930;HORVU5Hr1G036460;HORVU5Hr1G039730;HORVU5Hr1G052090;
3Hr1G110330;HORVU4Hr1G016770;HORVU4Hr1G016810;HORVU5Hr1G001180;HORVU5Hr1G006930;

3Hr1G106210;HORVU4Hr1G002650;HORVU4Hr1G010810;HORVU4Hr1G063730;HORVU4Hr1G067940;

022;Hordeum_vulgare_newGene_15200;Hordeum_vulgare_newGene_7775

7Hr1G073460;HORVU7Hr1G082450;HORVU7Hr1G089290
4Hr1G066370;HORVU4Hr1G082040;HORVU4Hr1G087110;HORVU5Hr1G006930;HORVU5Hr1G036460;
7Hr1G012690;HORVU7Hr1G054360;HORVU7Hr1G096250;Hordeum_vulgare_newGene_11449;Hordeum_v

2Hr1G105930;HORVU2Hr1G119500;HORVU2Hr1G122920;HORVU2Hr1G123110;HORVU2Hr1G123480;
5Hr1G045450;HORVU5Hr1G074840;HORVU5Hr1G083170;HORVU6Hr1G020310;HORVU6Hr1G076880;
4Hr1G018430;HORVU4Hr1G064440;HORVU4Hr1G071730;HORVU5Hr1G034810;HORVU5Hr1G075330;
7Hr1G107280;Hordeum_vulgare_newGene_719

5Hr1G099910;HORVU7Hr1G010790;HORVU7Hr1G045470;HORVU7Hr1G059460;HORVU7Hr1G066450;

5Hr1G069880;HORVU5Hr1G069910;HORVU5Hr1G069960;HORVU5Hr1G104050;HORVU5Hr1G115310;
5Hr1G118710;HORVU6Hr1G027650;HORVU6Hr1G048850;HORVU6Hr1G068410;HORVU7Hr1G073860;
5Hr1G096370;HORVU5Hr1G104120;HORVU6Hr1G001270;HORVU6Hr1G029240;HORVU6Hr1G061220;

4Hr1G031510;HORVU4Hr1G067280;HORVU4Hr1G081310;HORVU5Hr1G013290;HORVU5Hr1G045450;

7Hr1G038510;HORVU7Hr1G040760;HORVU7Hr1G096070;HORVU7Hr1G118130

2Hr1G027010;HORVU2Hr1G040170;HORVU2Hr1G070700;HORVU2Hr1G081920;HORVU2Hr1G098800;
newGene_8164

7Hr1G118090;Hordeum_vulgare_newGene_7380

5Hr1G044830;HORVU5Hr1G080050;HORVU6Hr1G059440;HORVU6Hr1G081140;HORVU6Hr1G094080;

newGene_963

newGene_9576

5Hr1G103890;HORVU5Hr1G105360;HORVU6Hr1G001270;HORVU6Hr1G008640;HORVU6Hr1G061220;

33

2Hr1G075030;HORVU2Hr1G079900;HORVU2Hr1G097010;HORVU2Hr1G099570;HORVU2Hr1G110230;

1Hr1G087050;HORVU1Hr1G091540;HORVU1Hr1G092920;HORVU1Hr1G094160;HORVU1Hr1G094230;
5Hr1G109880;HORVU6Hr1G007020;HORVU6Hr1G072650;HORVU6Hr1G077410;HORVU6Hr1G091860;

7Hr1G086010;HORVU7Hr1G101140;Hordeum_vulgare_newGene_8906

7Hr1G071800;Hordeum_vulgare_newGene_10320;Hordeum_vulgare_newGene_1279;Hordeum_vulgare_new
_vulgare_newGene_13547;Hordeum_vulgare_newGene_13659

3Hr1G013390;HORVU3Hr1G039700;HORVU3Hr1G063680;HORVU3Hr1G086500;HORVU3Hr1G089490;
1Hr1G068760;HORVU1Hr1G069510;HORVU1Hr1G070480;HORVU1Hr1G073230;HORVU1Hr1G073490;

4Hr1G058450;HORVU5Hr1G010860;HORVU5Hr1G096370;HORVU6Hr1G015390;HORVU6Hr1G061300;
3Hr1G086500;HORVU3Hr1G095300;HORVU3Hr1G099390;HORVU3Hr1G115020;HORVU4Hr1G059260;

7Hr1G077620;HORVU7Hr1G100090;HORVU7Hr1G119870

5Hr1G071500;HORVU7Hr1G011750;HORVU7Hr1G040390;HORVU7Hr1G048880;Hordeum_vulgare_new

5Hr1G075070;HORVU7Hr1G078670;Hordeum_vulgare_newGene_10448;Hordeum_vulgare_newGene_1369
3Hr1G086430;HORVU3Hr1G099400;HORVU3Hr1G114920;HORVU4Hr1G010940;HORVU4Hr1G055910;

3Hr1G009210;HORVU3Hr1G075870;HORVU3Hr1G082730;HORVU3Hr1G082740;HORVU3Hr1G086190;

3Hr1G062430;HORVU3Hr1G068780;HORVU3Hr1G115170;HORVU4Hr1G043860;HORVU4Hr1G056260;

3Hr1G086500;HORVU3Hr1G115020;HORVU4Hr1G011060;HORVU4Hr1G052730;HORVU4Hr1G059260;
_vulgare_newGene_10040;Hordeum_vulgare_newGene_1067;Hordeum_vulgare_newGene_12352
3Hr1G059810;HORVU3Hr1G062490;HORVU3Hr1G071530;HORVU3Hr1G078780;HORVU3Hr1G095330;

HORVU6Hr1G091660;HORVU7Hr1G046320;HORVU7Hr1G058120;Hordeum_vulgare_newGene_15172;Ho
HORVU2Hr1G033470;HORVU2Hr1G036980;HORVU2Hr1G047410;HORVU2Hr1G072500;HORVU2Hr1G
HORVU5Hr1G048100;HORVU5Hr1G062990;HORVU5Hr1G105840;HORVU6Hr1G012290;HORVU6Hr1G
HORVU2Hr1G103130;HORVU3Hr1G018610;HORVU3Hr1G018690;HORVU3Hr1G049900;HORVU3Hr1G
HORVU5Hr1G012160;HORVU5Hr1G047530;HORVU5Hr1G057090;HORVU5Hr1G083170;HORVU5Hr1G
HORVU2Hr1G010690;HORVU2Hr1G012790;HORVU2Hr1G032970;HORVU2Hr1G033320;HORVU2Hr1G
HORVU3Hr1G018550;HORVU3Hr1G018650;HORVU3Hr1G019580;HORVU3Hr1G034820;HORVU3Hr1G
HORVU3Hr1G094300;HORVU4Hr1G007620;HORVU4Hr1G052450;HORVU4Hr1G086300;HORVU5Hr1G
HORVU4Hr1G056500;HORVU4Hr1G061990;HORVU4Hr1G063780;HORVU5Hr1G046480;HORVU5Hr1G
ulgare_newGene_13684;Hordeum_vulgare_newGene_13743
HORVU5Hr1G069960;HORVU5Hr1G104050;HORVU6Hr1G003210;HORVU7Hr1G071730;HORVU7Hr1G
HORVU5Hr1G057090;HORVU5Hr1G099350;HORVU6Hr1G001270;HORVU6Hr1G020600;HORVU6Hr1G
HORVU4Hr1G055030;HORVU4Hr1G060630;HORVU4Hr1G062440;HORVU4Hr1G082700;HORVU5Hr1G
HORVU7Hr1G028850;HORVU7Hr1G082450;HORVU7Hr1G086570;HORVU7Hr1G089290;HORVU7Hr1G
HORVU5Hr1G013880;HORVU5Hr1G067360;HORVU5Hr1G088060;HORVU5Hr1G122540;HORVU7Hr1G
HORVU5Hr1G057410;HORVU5Hr1G061710;HORVU5Hr1G069800;HORVU5Hr1G087800;HORVU5Hr1G

HORVU2Hr1G063740;HORVU2Hr1G070400;HORVU2Hr1G097910;HORVU2Hr1G119600;HORVU3Hr1G
HORVU7Hr1G120660;Hordeum_vulgare_newGene_14635;Hordeum_vulgare_newGene_3338
HORVU7Hr1G010750;HORVU7Hr1G010790;HORVU7Hr1G010850;HORVU7Hr1G010990;HORVU7Hr1G
HORVU3Hr1G064320;HORVU3Hr1G074000;HORVU3Hr1G107350;HORVU3Hr1G117370;HORVU3Hr1G
HORVU4Hr1G055970;HORVU5Hr1G013040;HORVU5Hr1G073370;HORVU5Hr1G074170;HORVU5Hr1G

HORVU4Hr1G083930;HORVU4Hr1G083940;HORVU4Hr1G087110;HORVU5Hr1G057590;HORVU6Hr1G
HORVU3Hr1G059480;HORVU3Hr1G081530;HORVU3Hr1G084220;HORVU3Hr1G089420;HORVU3Hr1G

HORVU2Hr1G026810;HORVU2Hr1G063460;HORVU2Hr1G082170;HORVU2Hr1G085990;HORVU2Hr1G
99

HORVU6Hr1G077770;HORVU6Hr1G077790;HORVU7Hr1G041850;HORVU7Hr1G085310;Hordeum_vulga
HORVU7Hr1G024240;HORVU7Hr1G024250;HORVU7Hr1G063430;HORVU7Hr1G076680;HORVU7Hr1G
HORVU5Hr1G037200;HORVU5Hr1G049370;HORVU5Hr1G057210;HORVU6Hr1G001270;HORVU6Hr1G
HORVU5Hr1G034810;HORVU5Hr1G057210;HORVU5Hr1G075330;HORVU6Hr1G003620;HORVU6Hr1G
HORVU7Hr1G010790;HORVU7Hr1G055560;HORVU7Hr1G089290;Hordeum_vulgare_newGene_7586
HORVU4Hr1G072620;HORVU4Hr1G077060;HORVU4Hr1G079940;HORVU5Hr1G049490;HORVU5Hr1G

HORVU5Hr1G037200;HORVU5Hr1G052090;HORVU5Hr1G109390;HORVU5Hr1G112850;HORVU5Hr1G

HORVU5Hr1G053010;HORVU5Hr1G099350;HORVU5Hr1G116770;HORVU6Hr1G020600;HORVU7Hr1G
HORVU5Hr1G057090;HORVU5Hr1G075540;HORVU6Hr1G005250;HORVU6Hr1G020600;HORVU6Hr1G

HORVU4Hr1G074230;HORVU5Hr1G005180;HORVU5Hr1G009830;HORVU5Hr1G021560;HORVU5Hr1G

HORVU5Hr1G066230;HORVU5Hr1G066930;HORVU5Hr1G074840;HORVU5Hr1G089280;HORVU5Hr1G[illegible]
ulgare_newGene_12594;Hordeum_vulgare_newGene_8216

HORVU3Hr1G012280;HORVU3Hr1G019070;HORVU3Hr1G019580;HORVU3Hr1G061730;HORVU3Hr1G[illegible]
HORVU7Hr1G073460;Hordeum_vulgare_newGene_15022;Hordeum_vulgare_newGene_2358
HORVU5Hr1G104620;HORVU5Hr1G112350;HORVU6Hr1G003620;HORVU6Hr1G073100;HORVU7Hr1G[illegible]

HORVU7Hr1G089290;Hordeum_vulgare_newGene_963

HORVU6Hr1G009620;HORVU7Hr1G038710;HORVU7Hr1G056770;HORVU7Hr1G076150;Hordeum_vulga
HORVU7Hr1G082450;Hordeum_vulgare_newGene_13381;Hordeum_vulgare_newGene_1676
HORVU6Hr1G061270;HORVU7Hr1G038770;HORVU7Hr1G075760;HORVU7Hr1G088260

HORVU5Hr1G064040;HORVU5Hr1G067530;HORVU5Hr1G067880;HORVU6Hr1G079600;HORVU6Hr1G[illegible]

HORVU3Hr1G021080;HORVU3Hr1G024530;HORVU3Hr1G056830;HORVU3Hr1G068380;HORVU3Hr1G[illegible]

HORVU7Hr1G064170;HORVU7Hr1G102300;Hordeum_vulgare_newGene_10320;Hordeum_vulgare_newGen

HORVU6Hr1G061270;HORVU6Hr1G073990;HORVU6Hr1G078210;HORVU7Hr1G010750;HORVU7Hr1G[illegible]

HORVU2Hr1G126740;HORVU3Hr1G002080;HORVU3Hr1G013380;HORVU3Hr1G061410;HORVU3Hr1G(

HORVU2Hr1G002690;HORVU2Hr1G012010;HORVU2Hr1G036930;HORVU2Hr1G040600;HORVU2Hr1G(
HORVU7Hr1G024890;HORVU7Hr1G033470;HORVU7Hr1G082980;HORVU7Hr1G084420;Hordeum_vulga

Gene_1284;Hordeum_vulgare_newGene_184

HORVU3Hr1G089830;HORVU3Hr1G114200;HORVU3Hr1G115020;HORVU4Hr1G012220;HORVU4Hr1G(
HORVU1Hr1G076690;HORVU1Hr1G079450;HORVU1Hr1G090360;HORVU2Hr1G000090;HORVU2Hr1G(

HORVU7Hr1G032330;HORVU7Hr1G056770;HORVU7Hr1G078960;HORVU7Hr1G095080
HORVU4Hr1G062110;HORVU4Hr1G063980;HORVU5Hr1G032650;HORVU5Hr1G052010;HORVU5Hr1G(

Gene_5796;Hordeum_vulgare_newGene_7340

4;Hordeum_vulgare_newGene_13736;Hordeum_vulgare_newGene_255
HORVU5Hr1G008250;HORVU5Hr1G045070;HORVU5Hr1G053230;HORVU5Hr1G084510;HORVU5Hr1G

HORVU4Hr1G058970;HORVU5Hr1G071920;HORVU5Hr1G100140;HORVU5Hr1G109710;HORVU5Hr1G

HORVU4Hr1G060670;HORVU4Hr1G061900;HORVU4Hr1G064670;HORVU4Hr1G066070;HORVU5Hr1G(

HORVU4Hr1G060670;HORVU5Hr1G032650;HORVU5Hr1G044640;HORVU5Hr1G080870;HORVU5Hr1G

HORVU4Hr1G013640;HORVU4Hr1G016190;HORVU4Hr1G038570;HORVU4Hr1G052470;HORVU4Hr1G(

rdeum_vulgare_newGene_8689
72890;HORVU2Hr1G073210;HORVU2Hr1G077120;HORVU2Hr1G082170;HORVU2Hr1G085800;HORV
31480;HORVU6Hr1G074030;HORVU7Hr1G010790;HORVU7Hr1G082330;HORVU7Hr1G089290;Hordeu
67110;HORVU4Hr1G007610;HORVU4Hr1G011500;HORVU4Hr1G066860;HORVU4Hr1G081290;HORV
105360;HORVU6Hr1G027650;HORVU7Hr1G010750;HORVU7Hr1G010790;HORVU7Hr1G010850;HORV
36110;HORVU2Hr1G045340;HORVU2Hr1G060010;HORVU2Hr1G060070;HORVU2Hr1G061040;HORV
59470;HORVU3Hr1G070300;HORVU3Hr1G117540;HORVU4Hr1G007620;HORVU4Hr1G016770;HORV
45820;HORVU5Hr1G055920;HORVU5Hr1G069880;HORVU5Hr1G069910;HORVU5Hr1G069960;HORV
46490;HORVU5Hr1G064280;HORVU5Hr1G065620;HORVU5Hr1G094280;HORVU6Hr1G062190;HORV

71760;Hordeum_vulgare_newGene_13116;Hordeum_vulgare_newGene_13685;Hordeum_vulgare_newGene_
61220;HORVU6Hr1G061270;HORVU6Hr1G081800;HORVU6Hr1G092740;HORVU7Hr1G029110;Hordeu
14730;HORVU5Hr1G032980;HORVU5Hr1G033540;HORVU5Hr1G038630;HORVU5Hr1G050990;HORV
91860
03090;HORVU7Hr1G116060;Hordeum_vulgare_newGene_14259;Hordeum_vulgare_newGene_1948;Horde
94300;HORVU5Hr1G100910;HORVU5Hr1G114090;HORVU6Hr1G014480;HORVU6Hr1G056910;HORV

13910;HORVU3Hr1G019580;HORVU3Hr1G052780;HORVU3Hr1G059470;HORVU3Hr1G063050;HORV

82450;HORVU7Hr1G089290;HORVU7Hr1G091860;Hordeum_vulgare_newGene_7586
117390;HORVU4Hr1G035380;HORVU4Hr1G057170;HORVU4Hr1G057210;HORVU4Hr1G057910;HORV
77910;HORVU5Hr1G095080;HORVU6Hr1G087300;HORVU7Hr1G028290;HORVU7Hr1G084230;Hordeu

13350;HORVU6Hr1G053810;HORVU6Hr1G058000;HORVU6Hr1G081800;HORVU6Hr1G089980;HORV
90160;HORVU4Hr1G007620;HORVU4Hr1G008270;HORVU4Hr1G026150;HORVU4Hr1G058500;HORV

86380;HORVU2Hr1G088980;HORVU2Hr1G089440;HORVU2Hr1G090120;HORVU2Hr1G109440;HORV

re_newGene_15022;Hordeum_vulgare_newGene_15200
86690;HORVU7Hr1G092450;Hordeum_vulgare_newGene_1243;Hordeum_vulgare_newGene_15051;Horde
61220;HORVU6Hr1G061270;HORVU6Hr1G075240;HORVU6Hr1G083620;HORVU6Hr1G090370;HORV
73100;HORVU6Hr1G078670;HORVU6Hr1G083620;HORVU7Hr1G021700;HORVU7Hr1G027740;HORV

95990;HORVU5Hr1G106850;HORVU5Hr1G124650;HORVU5Hr1G125010;HORVU6Hr1G027440;HORV

116770;HORVU6Hr1G001270;HORVU6Hr1G061220;HORVU6Hr1G061270;HORVU7Hr1G060260;HORV

19830;HORVU7Hr1G029110;HORVU7Hr1G030660;HORVU7Hr1G044990;Hordeum_vulgare_newGene_1
33600;HORVU7Hr1G029110;HORVU7Hr1G050660;HORVU7Hr1G050670;HORVU7Hr1G050680;HORV

44830;HORVU5Hr1G080050;HORVU5Hr1G093270;HORVU5Hr1G105080;HORVU5Hr1G113990;HORV

)99350;HORVU5Hr1G115720;HORVU5Hr1G115780;HORVU6Hr1G005400;HORVU6Hr1G008640;HORVU

)61750;HORVU3Hr1G070300;HORVU3Hr1G074660;HORVU3Hr1G074770;HORVU4Hr1G021570;HORVU

)21700;HORVU7Hr1G058500;HORVU7Hr1G069660;HORVU7Hr1G077710;HORVU7Hr1G088260;Hordeu

re_newGene_8302

)79640;HORVU6Hr1G089250;HORVU7Hr1G010790;HORVU7Hr1G028540;HORVU7Hr1G028840;HORVU

)68390;HORVU3Hr1G073710;HORVU3Hr1G077030;HORVU3Hr1G079900;HORVU3Hr1G084990;HORVU

ıe_184;Hordeum_vulgare_newGene_6379

)10850;HORVU7Hr1G010990;HORVU7Hr1G017620;HORVU7Hr1G091860;Hordeum_vulgare_newGene_1

)61450;HORVU3Hr1G065610;HORVU3Hr1G065630;HORVU3Hr1G079230;HORVU3Hr1G085930;HORVU

)60560;HORVU2Hr1G065060;HORVU2Hr1G066680;HORVU2Hr1G075030;HORVU2Hr1G096270;HORVU
re_newGene_13231

)43910;HORVU4Hr1G059260;HORVU4Hr1G060720;HORVU4Hr1G066900;HORVU5Hr1G006670;HORVU
)17650;HORVU2Hr1G025290;HORVU2Hr1G036280;HORVU2Hr1G050260;HORVU2Hr1G075030;HORVU

)52030;HORVU5Hr1G058330;HORVU5Hr1G060030;HORVU5Hr1G080870;HORVU5Hr1G080990;HORVU

100200;HORVU5Hr1G100700;HORVU6Hr1G081400;HORVU6Hr1G085320;HORVU6Hr1G085370;HORVU

113900;HORVU6Hr1G000620;HORVU6Hr1G085170;HORVU7Hr1G000900;HORVU7Hr1G002210;HORVU

)38440;HORVU5Hr1G053810;HORVU6Hr1G003170;HORVU6Hr1G044080;HORVU6Hr1G068080;HORVU

118010;HORVU6Hr1G078060;HORVU7Hr1G052140;HORVU7Hr1G093450;HORVU7Hr1G097760;Hordeu

)57450;HORVU4Hr1G058360;HORVU4Hr1G060970;HORVU4Hr1G061900;HORVU4Hr1G084240;HORVU

J2Hr1G088980;HORVU2Hr1G090980;HORVU2Hr1G091360;HORVU2Hr1G109080;HORVU2Hr1G109120
m_vulgare_newGene_13117;Hordeum_vulgare_newGene_1350;Hordeum_vulgare_newGene_13684;Hordeum
J4Hr1G082040;HORVU5Hr1G033540;HORVU5Hr1G038630;HORVU5Hr1G050990;HORVU5Hr1G051010
J7Hr1G010990;HORVU7Hr1G082450;HORVU7Hr1G089290;HORVU7Hr1G091860;HORVU7Hr1G107350
J2Hr1G061080;HORVU2Hr1G063740;HORVU2Hr1G070400;HORVU2Hr1G084130;HORVU2Hr1G084750
J4Hr1G016810;HORVU4Hr1G019570;HORVU4Hr1G051010;HORVU4Hr1G056050;HORVU4Hr1G060630
J5Hr1G104050;HORVU6Hr1G075240;HORVU7Hr1G034070;HORVU7Hr1G048710;HORVU7Hr1G086690
J6Hr1G078640;HORVU6Hr1G087250;HORVU6Hr1G087310;HORVU6Hr1G088440;HORVU6Hr1G088470

_13742
m_vulgare_newGene_14172;Hordeum_vulgare_newGene_14180;Hordeum_vulgare_newGene_7775
J5Hr1G051010;HORVU5Hr1G062990;HORVU6Hr1G003210;HORVU6Hr1G067660;HORVU7Hr1G001570

ım_vulgare_newGene_2048;Hordeum_vulgare_newGene_3900;Hordeum_vulgare_newGene_9451
J6Hr1G087960;HORVU7Hr1G027430;HORVU7Hr1G041710;HORVU7Hr1G049510;HORVU7Hr1G056990

J3Hr1G069300;HORVU3Hr1G070300;HORVU3Hr1G096500;HORVU3Hr1G103540;HORVU4Hr1G007610

J5Hr1G057410;HORVU5Hr1G074000;HORVU5Hr1G085070;HORVU5Hr1G103420;HORVU5Hr1G103430
m_vulgare_newGene_10096;Hordeum_vulgare_newGene_13111;Hordeum_vulgare_newGene_13112;Hordeur

J7Hr1G068080;HORVU7Hr1G112710;Hordeum_vulgare_newGene_11464;Hordeum_vulgare_newGene_197;
J4Hr1G064890;HORVU4Hr1G069230;HORVU5Hr1G007050;HORVU5Hr1G023640;HORVU5Hr1G045150

J2Hr1G109590;HORVU2Hr1G115890;HORVU2Hr1G122590;HORVU3Hr1G038290;HORVU3Hr1G074960

ım_vulgare_newGene_181
J6Hr1G090390;HORVU7Hr1G021310;HORVU7Hr1G021620;HORVU7Hr1G021630;HORVU7Hr1G046970
J7Hr1G027770;HORVU7Hr1G058500;HORVU7Hr1G069660;HORVU7Hr1G077710;HORVU7Hr1G079830

J6Hr1G067170;HORVU6Hr1G067200;HORVU7Hr1G009310;HORVU7Hr1G030540;HORVU7Hr1G063020

J7Hr1G080510;HORVU7Hr1G101220;Hordeum_vulgare_newGene_15022;Hordeum_vulgare_newGene_903'

1133;Hordeum_vulgare_newGene_7775
J7Hr1G118010;Hordeum_vulgare_newGene_7775

J6Hr1G059440;HORVU6Hr1G074560;HORVU6Hr1G081140;HORVU6Hr1G094080;HORVU7Hr1G064170

J6Hr1G020600;HORVU7Hr1G029110;HORVU7Hr1G045620;HORVU7Hr1G045640;HORVU7Hr1G060130

J4Hr1G060840;HORVU4Hr1G072850;HORVU5Hr1G053230;HORVU5Hr1G069880;HORVU5Hr1G069910

m_vulgare_newGene_11620;Hordeum_vulgare_newGene_12548;Hordeum_vulgare_newGene_13513;Hordeur

J7Hr1G055560;HORVU7Hr1G082450;HORVU7Hr1G089290;Hordeum_vulgare_newGene_11161;Hordeum_

J3Hr1G098160;HORVU3Hr1G104790;HORVU3Hr1G110330;HORVU4Hr1G032960;HORVU4Hr1G074470

5022;Hordeum_vulgare_newGene_1574;Hordeum_vulgare_newGene_4025;Hordeum_vulgare_newGene_758(

J3Hr1G088000;HORVU3Hr1G089250;HORVU3Hr1G089420;HORVU3Hr1G099590;HORVU4Hr1G003340

J2Hr1G107970;HORVU2Hr1G109330;HORVU2Hr1G112860;HORVU2Hr1G114240;HORVU2Hr1G119780

J5Hr1G007340;HORVU5Hr1G011730;HORVU5Hr1G014470;HORVU5Hr1G032650;HORVU5Hr1G049980

J2Hr1G077000;HORVU2Hr1G079900;HORVU2Hr1G089670;HORVU2Hr1G094360;HORVU2Hr1G099570

J5Hr1G084740;HORVU5Hr1G093260;HORVU5Hr1G120210;HORVU6Hr1G025460;HORVU6Hr1G032310

J6Hr1G088840;HORVU6Hr1G088870;HORVU6Hr1G089470;HORVU7Hr1G016130;HORVU7Hr1G084420

J7Hr1G005040;HORVU7Hr1G077740;HORVU7Hr1G077750;Hordeum_vulgare_newGene_10040;Hordeum_

J6Hr1G089560;HORVU6Hr1G089590;HORVU7Hr1G039760;HORVU7Hr1G099950;HORVU7Hr1G103380

m_vulgare_newGene_10448;Hordeum_vulgare_newGene_12063;Hordeum_vulgare_newGene_14325;Hordeur

J4Hr1G084410;HORVU4Hr1G084830;HORVU5Hr1G098340;HORVU5Hr1G111640;HORVU5Hr1G112390

;HORVU3Hr1G005350;HORVU3Hr1G023370;HORVU3Hr1G029470;HORVU3Hr1G033620;HORVU3Hr1(
_vulgare_newGene_13743;Hordeum_vulgare_newGene_3923
;HORVU5Hr1G052090;HORVU5Hr1G099910;HORVU5Hr1G109390;HORVU6Hr1G008640;HORVU6Hr1(
;Hordeum_vulgare_newGene_14172;Hordeum_vulgare_newGene_14180
;HORVU2Hr1G103130;HORVU3Hr1G019580;HORVU3Hr1G029200;HORVU3Hr1G029210;HORVU3Hr1(
;HORVU4Hr1G060840;HORVU4Hr1G062440;HORVU4Hr1G078460;HORVU4Hr1G081290;HORVU5Hr1(
;HORVU7Hr1G106540;Hordeum_vulgare_newGene_10828;Hordeum_vulgare_newGene_12819;Hordeum_vu
;HORVU6Hr1G088530;HORVU6Hr1G088540;HORVU7Hr1G007580;HORVU7Hr1G026680;HORVU7Hr1(

;HORVU7Hr1G010790;HORVU7Hr1G060260;HORVU7Hr1G080510;HORVU7Hr1G089290;HORVU7Hr1(

;HORVU7Hr1G077220;HORVU7Hr1G077620;HORVU7Hr1G090950;HORVU7Hr1G100090;Hordeum_vulg

;HORVU4Hr1G011500;HORVU4Hr1G019570;HORVU4Hr1G051010;HORVU4Hr1G056240;HORVU4Hr1(

;HORVU5Hr1G104120;HORVU5Hr1G118800;HORVU6Hr1G012320;HORVU6Hr1G087960;HORVU6Hr1(
n_vulgare_newGene_13702;Hordeum_vulgare_newGene_13766;Hordeum_vulgare_newGene_8522;Hordeum_

;Hordeum_vulgare_newGene_198;Hordeum_vulgare_newGene_4326;Hordeum_vulgare_newGene_5798
;HORVU5Hr1G045700;HORVU5Hr1G055920;HORVU5Hr1G069850;HORVU5Hr1G069860;HORVU5Hr1(

;HORVU3Hr1G079720;HORVU3Hr1G089000;HORVU3Hr1G096910;HORVU3Hr1G097010;HORVU3Hr1(

;HORVU7Hr1G079830;Hordeum_vulgare_newGene_1293;Hordeum_vulgare_newGene_1317;Hordeum_vulg
;HORVU7Hr1G099560;Hordeum_vulgare_newGene_11620;Hordeum_vulgare_newGene_2974;Hordeum_vul

;HORVU7Hr1G080320;HORVU7Hr1G081250;HORVU7Hr1G088210;HORVU7Hr1G098490;HORVU7Hr1(

7

;HORVU7Hr1G085450;HORVU7Hr1G099650;HORVU7Hr1G105200;Hordeum_vulgare_newGene_12461

;Hordeum_vulgare_newGene_11464;Hordeum_vulgare_newGene_577;Hordeum_vulgare_newGene_7775

;HORVU5Hr1G069960;HORVU5Hr1G077990;HORVU5Hr1G078050;HORVU5Hr1G088210;HORVU5Hr1(

n_vulgare_newGene_7281;Hordeum_vulgare_newGene_9891

_vulgare_newGene_2116;Hordeum_vulgare_newGene_9383

;HORVU5Hr1G057210;HORVU5Hr1G060030;HORVU5Hr1G084740;HORVU6Hr1G052420;HORVU6Hr1(

5

;HORVU4Hr1G026160;HORVU4Hr1G053130;HORVU4Hr1G060370;HORVU4Hr1G081670;HORVU4Hr1

;HORVU2Hr1G122810;HORVU3Hr1G001570;HORVU3Hr1G002080;HORVU3Hr1G002150;HORVU3Hr1

;HORVU5Hr1G053810;HORVU5Hr1G060790;HORVU5Hr1G065400;HORVU5Hr1G070720;HORVU5Hr1
;HORVU2Hr1G100360;HORVU2Hr1G103890;HORVU2Hr1G104040;HORVU2Hr1G109650;HORVU2Hr1

;HORVU7Hr1G037090;HORVU7Hr1G044270;HORVU7Hr1G052140;HORVU7Hr1G074660;HORVU7Hr1

;HORVU7Hr1G088780;Hordeum_vulgare_newGene_10207;Hordeum_vulgare_newGene_10347;Hordeum_vu

_vulgare_newGene_14743;Hordeum_vulgare_newGene_4819;Hordeum_vulgare_newGene_8353

;HORVU7Hr1G103550;HORVU7Hr1G107010;Hordeum_vulgare_newGene_1279;Hordeum_vulgare_newGe

n_vulgare_newGene_3091;Hordeum_vulgare_newGene_3124;Hordeum_vulgare_newGene_7182;Hordeum_vu

;HORVU5Hr1G115340;HORVU5Hr1G120250;HORVU5Hr1G120650;HORVU6Hr1G018830;HORVU6Hr1

G067620;HORVU3Hr1G074010;HORVU3Hr1G079720;HORVU3Hr1G093830;HORVU3Hr1G094300;HORV

G012290;HORVU6Hr1G053090;HORVU6Hr1G074030;HORVU6Hr1G076880;HORVU6Hr1G087300;HORV

G049900;HORVU3Hr1G059470;HORVU3Hr1G067110;HORVU3Hr1G070300;HORVU3Hr1G070850;HORV
G019030;HORVU5Hr1G032980;HORVU5Hr1G037200;HORVU5Hr1G056390;HORVU5Hr1G057090;HORV
ılgare_newGene_13116;Hordeum_vulgare_newGene_13654;Hordeum_vulgare_newGene_13685;Hordeum_vu
G030670;HORVU7Hr1G082350;HORVU7Hr1G101860;HORVU7Hr1G118090;Hordeum_vulgare_newGene_

G097550;Hordeum_vulgare_newGene_9037

gare_newGene_13116;Hordeum_vulgare_newGene_13685;Hordeum_vulgare_newGene_13742;Hordeum_vulg

G058840;HORVU4Hr1G060840;HORVU4Hr1G061120;HORVU4Hr1G063600;HORVU4Hr1G066860;HORV

G089780;HORVU6Hr1G090560;HORVU7Hr1G027430;HORVU7Hr1G030810;HORVU7Hr1G038770;HORV
_vulgare_newGene_9407

G074340;HORVU5Hr1G077110;HORVU5Hr1G081520;HORVU5Hr1G096370;HORVU6Hr1G062820;HORV

G111970;HORVU4Hr1G006480;HORVU4Hr1G006530;HORVU4Hr1G016940;HORVU4Hr1G022280;HORV

are_newGene_13405;Hordeum_vulgare_newGene_2974;Hordeum_vulgare_newGene_9576
gare_newGene_7281;Hordeum_vulgare_newGene_9891

G108110;Hordeum_vulgare_newGene_3503

G104050;HORVU6Hr1G090250;HORVU7Hr1G017640;HORVU7Hr1G043930;HORVU7Hr1G043960;HORV

G083620;HORVU7Hr1G054980;HORVU7Hr1G079830;Hordeum_vulgare_newGene_2974

G082040;HORVU4Hr1G083000;HORVU4Hr1G083020;HORVU4Hr1G084850;HORVU4Hr1G086500;HORV

G010310;HORVU3Hr1G010980;HORVU3Hr1G010990;HORVU3Hr1G012620;HORVU3Hr1G012800;HORV

G078080;HORVU5Hr1G080870;HORVU5Hr1G116590;HORVU5Hr1G121610;HORVU6Hr1G044080;HORV
G110230;HORVU2Hr1G125360;HORVU3Hr1G002080;HORVU3Hr1G019750;HORVU3Hr1G022060;HORV

G086010;HORVU7Hr1G101140;Hordeum_vulgare_newGene_13976;Hordeum_vulgare_newGene_14325;Hor

ulgare_newGene_10448;Hordeum_vulgare_newGene_5213;Hordeum_vulgare_newGene_9147

ne_1284;Hordeum_vulgare_newGene_13022;Hordeum_vulgare_newGene_15135;Hordeum_vulgare_newGene

ulgare_newGene_9892

G041020;HORVU6Hr1G056610;HORVU6Hr1G062040;HORVU6Hr1G078290;HORVU6Hr1G093030;HORV

/U3Hr1G096910;HORVU3Hr1G097010;HORVU3Hr1G105190;HORVU3Hr1G105290;HORVU3Hr1G1053[illegible]

/U7Hr1G019390;HORVU7Hr1G045470;Hordeum_vulgare_newGene_10225;Hordeum_vulgare_newGene_13

/U3Hr1G085270;HORVU3Hr1G102340;HORVU3Hr1G117540;HORVU4Hr1G011500;HORVU4Hr1G0195[illegible]
/U5Hr1G069850;HORVU5Hr1G069880;HORVU5Hr1G069910;HORVU5Hr1G069960;HORVU5Hr1G1040[illegible]
lgare_newGene_13742;Hordeum_vulgare_newGene_15051;Hordeum_vulgare_newGene_3648;Hordeum_vulg
14296;Hordeum_vulgare_newGene_2824;Hordeum_vulgare_newGene_711;Hordeum_vulgare_newGene_738[illegible]

are_newGene_3364;Hordeum_vulgare_newGene_3923;Hordeum_vulgare_newGene_8655

/U4Hr1G071300;HORVU4Hr1G074400;HORVU4Hr1G081310;HORVU4Hr1G090310;HORVU5Hr1G0132[illegible]

/U7Hr1G039140;HORVU7Hr1G041710;HORVU7Hr1G055560;Hordeum_vulgare_newGene_1286;Hordeum_

/U6Hr1G065690;HORVU6Hr1G082160;HORVU7Hr1G016770;HORVU7Hr1G038200;HORVU7Hr1G03871

/U4Hr1G027260;HORVU4Hr1G052060;HORVU4Hr1G072130;HORVU4Hr1G072150;HORVU4Hr1G08491

VU7Hr1G052190;Hordeum_vulgare_newGene_11142;Hordeum_vulgare_newGene_1637;Hordeum_vulgare_n

/U4Hr1G087590;HORVU5Hr1G002090;HORVU5Hr1G024550;HORVU5Hr1G041590;HORVU5Hr1G04741

/U3Hr1G013380;HORVU3Hr1G014890;HORVU3Hr1G018390;HORVU3Hr1G018430;HORVU3Hr1G0191[illegible]

/U6Hr1G091250;HORVU7Hr1G026240;HORVU7Hr1G026250;HORVU7Hr1G036540;HORVU7Hr1G0365[illegible]
/U3Hr1G029670;HORVU3Hr1G031460;HORVU3Hr1G033790;HORVU3Hr1G080740;HORVU3Hr1G0847[illegible]

deum_vulgare_newGene_2463;Hordeum_vulgare_newGene_7519

_3940

/U7Hr1G003940;HORVU7Hr1G052800;HORVU7Hr1G091910;HORVU7Hr1G114170;HORVU7Hr1G1150[illegible]

)0;HORVU3Hr1G105420;HORVU3Hr1G105600;HORVU3Hr1G105790;HORVU4Hr1G000520;HORVU4Hr

117;Hordeum_vulgare_newGene_13684;Hordeum_vulgare_newGene_13743;Hordeum_vulgare_newGene_15(

'0;HORVU4Hr1G051010;HORVU4Hr1G055030;HORVU4Hr1G060630;HORVU4Hr1G060840;HORVU4Hr
;0;HORVU5Hr1G109390;HORVU5Hr1G112350;HORVU5Hr1G112850;HORVU6Hr1G001270;HORVU6Hr
;are_newGene_9576
)

)0;HORVU5Hr1G014730;HORVU5Hr1G032980;HORVU5Hr1G045450;HORVU5Hr1G048100;HORVU5Hr

_vulgare_newGene_3594

.0;HORVU7Hr1G039260;HORVU7Hr1G053260;HORVU7Hr1G067620;HORVU7Hr1G076150;HORVU7Hr

.0;HORVU4Hr1G085100;HORVU5Hr1G013040;HORVU5Hr1G023460;HORVU5Hr1G062030;HORVU5Hr

ewGene_4852;Hordeum_vulgare_newGene_9484

0;HORVU5Hr1G062490;HORVU5Hr1G068070;HORVU5Hr1G074340;HORVU5Hr1G078630;HORVU5Hr

40;HORVU3Hr1G027200;HORVU3Hr1G033740;HORVU3Hr1G059250;HORVU3Hr1G059880;HORVU3Hr

70;HORVU7Hr1G039930;HORVU7Hr1G040080;HORVU7Hr1G052140;HORVU7Hr1G092320;HORVU7Hr
50;HORVU3Hr1G085210;HORVU3Hr1G085930;HORVU3Hr1G088000;HORVU3Hr1G089250;HORVU3Hr

40;Hordeum_vulgare_newGene_5150

1G006480;HORVU4Hr1G006530;HORVU4Hr1G007620;HORVU4Hr1G008950;HORVU4Hr1G011160;HOF

)22;Hordeum_vulgare_newGene_2358;Hordeum_vulgare_newGene_577

1G062440;HORVU4Hr1G078460;HORVU4Hr1G081290;HORVU4Hr1G081310;HORVU4Hr1G082040;HOF
1G020310;HORVU6Hr1G061220;HORVU6Hr1G061270;HORVU7Hr1G001570;HORVU7Hr1G017640;HOF

1G052150;HORVU5Hr1G062990;HORVU5Hr1G069880;HORVU5Hr1G069910;HORVU5Hr1G069960;HOF

1G099320;HORVU7Hr1G113270;HORVU7Hr1G121850;Hordeum_vulgare_newGene_12935;Hordeum_vulga

1G064280;HORVU5Hr1G065330;HORVU5Hr1G073370;HORVU5Hr1G073870;HORVU5Hr1G073960;HOF

1G080140;HORVU5Hr1G082940;HORVU5Hr1G096800;HORVU5Hr1G119550;HORVU5Hr1G121440;HOR

1G061410;HORVU3Hr1G061450;HORVU3Hr1G076940;HORVU3Hr1G079230;HORVU3Hr1G081030;HOR

1G098810;HORVU7Hr1G099950;HORVU7Hr1G117000;Hordeum_vulgare_newGene_14325;Hordeum_vulga
1G093530;HORVU3Hr1G099590;HORVU3Hr1G106880;HORVU3Hr1G108540;HORVU3Hr1G114970;HOR

RVU4Hr1G015570;HORVU4Hr1G016880;HORVU4Hr1G049500;HORVU4Hr1G063240;HORV

RVU4Hr1G082700;HORVU4Hr1G090310;HORVU5Hr1G006930;HORVU5Hr1G014730;HORV
RVU7Hr1G034070;HORVU7Hr1G097550;Hordeum_vulgare_newGene_13116;Hordeum_

RVU5Hr1G099910;HORVU5Hr1G104050;HORVU5Hr1G105840;HORVU6Hr1G003210;HORV

are_newGene_13116;Hordeum_vulgare_newGene_13685;Hordeum_vulgare_

RVU5Hr1G077910;HORVU5Hr1G082610;HORVU5Hr1G094450;HORVU5Hr1G095010;HORV

:VU5Hr1G125270;HORVU6Hr1G008640;HORVU6Hr1G013290;HORVU6Hr1G054770;HORV

:VU3Hr1G088000;HORVU3Hr1G089090;HORVU3Hr1G089260;HORVU3Hr1G093310;HORV

are_newGene_15135;Hordeum_vulgare_newGene_1699;Hordeum_vulgare_n
:VU4Hr1G001570;HORVU4Hr1G001680;HORVU4Hr1G003340;HORVU4Hr1G009340;HORV
