# Supplementary material for: Molecular mechanisms of how black barley accumulates higher anthocyanins than blue barley following transcriptomic evaluation and expression analysis of key genes in anthocyanins biosynthesis pathway
Source: Front Plant Sci. 2025 Aug 29;16:1650803. doi: 10.3389/fpls.2025.1650803 (PMC12427265; doi:10.3389/fpls.2025.1650803)
Supplement: Supplementary file 1 [file Supplementaryfile1.zip › Supplementary Material/Data Sheet 6.PDF]

Supplementary Table 5 Number of 12 types alternative splicing events.

**Types**	**GB-1**	**GB-2**	**GB-3**	**GH-1**	**GH-2**	**GH-3**
TSS	26,079	26,322	27,164	27,215	27,689	28,245
TTS	22,136	22,245	22,878	22,851	23,296	23,657
IR	9,992	10,486	10,966	11,174	1,1440	11,879
AE	6,859	7,289	7,821	7,390	7,703	7,667
XIR	6,768	7,244	7,623	7,768	7,974	8,662
XAE	4,304	4,607	4,863	4,829	5,022	5,270
SKIP	1,841	1,836	2,118	1641	1,731	1,723
MIR	1,642	1,727	1,767	1,821	1,851	1,927
XMIR	1,120	1,244	1,298	1,312	1,276	1,418
XSKIP	758	741	872	700	800	798
MSKIP	345	304	382	298	294	320
XMSKIP	93	72	109	73	96	93

Note: Here, GB and GH represent black and blue barley respectively whereas, 1-3 represents three independent biological replicates.
